# H9 Consensus Hemagglutinin Subunit Vaccine with Adjuvants Induces Robust Mucosal and Systemic Immune Responses in Mice by Intranasal Administration

**DOI:** 10.3390/microorganisms12112294

**Published:** 2024-11-12

**Authors:** Liming Lin, Shunfan Zhu, Beibei Yang, Xin Zhang, Huimin Wu, Shixiang Wu, Li Wu, Jianhong Shu, Yulong He, Huapeng Feng

**Affiliations:** 1 Department of Biopharmacy, College of Life Sciences and Medicine, Zhejiang Sci-Tech University, Hangzhou 310018, China; aminggo@yeah.net (L.L.); zhushunfan_2022@foxmail.com (S.Z.); yangbeibeiybb@foxmail.com (B.Y.); zhangxin20000112@gmail.com (X.Z.); whmtse@foxmail.com (H.W.); shixiang_wu@foxmail.com (S.W.); shujianhong@zstu.edu.cn (J.S.); heyl79@zstu.edu.cn (Y.H.); 2 Department of Biology, College of Life Sciences, China Jiliang University, Hangzhou 310018, China; 19a0902131@cjlu.edu.cn

**Keywords:** H9 HA, subunit vaccine, adjuvant, H9N2 subtype avian influenza virus, baculovirus system

## Abstract

The H9N2 subtype avian influenza viruses mainly cause respiratory symptoms, reduce the egg production and fertility of poultry, and result in secondary infections, posing a great threat to the poultry industry and human health. Currently, all H9N2 avian influenza commercial vaccines are inactivated vaccines, which provide protection for immunized animals but cannot inhibit the spread of the virus and make it difficult to distinguish between the infected animals and vaccinated animals. In this study, a trimeric consensus H9 hemagglutinin (HA) subunit vaccine for the H9N2 subtype avian influenza virus based on a baculovirus expression system was first generated, and then the effects of three molecular adjuvants on the H9 HA subunit vaccine, Cholera toxin subunit B (CTB), flagellin, and granulocyte-macrophage colony-stimulating factor (GM-CSF) fused with H9 HA, and one synthetic compound, a polyinosinic–polycytidylic acid (PolyI:C) adjuvant, were evaluated in mice by intranasal administration. The results showed that these four adjuvants enhanced the immunogenicity of the H9 HA subunit vaccine for avian influenza viruses, and that GM-CSF and PolyI:C present better mucosal adjuvant activity for the H9 HA subunit vaccine. These results demonstrate that we have developed a potential universal H9 HA mucosal subunit vaccine with adjuvants in a baculovirus system that would be helpful for the prevention and control of H9N2 subtype avian influenza viruses.

## 1. Introduction

H9N2 avian influenza viruses (AIVs) are highly infectious diseases that cause respiratory symptoms, reduce the laying rate and fertilization rate of poultry, and cause secondary infections, resulting in huge economic losses [1,2]. In 1966, Hommee et al. isolated a strain of H9N2 subtype AIV-A/Turkey/Wisconsin/1/66 from turkeys suffering from mild respiratory illness, which was the first reported case of H9N2 AIV worldwide [3]. It was not until 1990 that H9N2 AIV was first isolated from domestic poultry in China, followed by a large outbreak of the virus, which rapidly spread to most parts of Asia, the Middle East, North Africa, and West Africa, where it was prevalent in domestic poultry [4]. A study showed that the positive rate of H9N2 AIV was still 78.18% from 2013 to 2018 in Southern China in poultry [5]. Based on the surveillance, it was found that H9N2 AIVs have infected multiple species, including pigs, dogs, minks, horses, rabbits, and bats [6,7,8]. AIVs are prone to antigen drift and shift, and the ability of H9N2 AIV to bind the mammalian upper respiratory tract cells has gradually strengthened; as a result, humans have become the host of the H9N2 virus [9]. As of 19 July 2024, nearly 130 cases of human infection with the H9N2 AIV had been reported to the World Health Organization (WHO) since 2003, including six that were severe or fatal [10]. Therefore, the prevention and control of H9N2 AIVs is good for the health of both poultry and humans.

In addition, large-scale H9N2 infection provides an extensive gene pool for other subtypes of AIVs. It has been shown that H9N2 viruses can extensively recombine with the H1N1 and H3N2 AIVs [11,12,13]. Replacing the polymerase acidic (PA) or Nucleoprotein (NP) genes of the H5N1 virus with those of the H9N2 AIV can significantly increase its pathogenicity in mice [14]. There is evidence that previously isolated H5N6, H7N7, H10N8, and H7N9 AIVs have internal genes derived from the H9N2 virus, and the more highly pathogenic recombinant H5N2 virus has seven segments derived from the H9N2 virus [15]. Since early 2013, the H7N9 avian influenza virus has infected 1568 humans and resulted in 616 fatal cases, and the mortality is up to 39%. The six internal genes of these H7N9 AIVs are derived from circulating H9N2 viruses [16,17,18,19]. Therefore, it is reasonable to predict that the H9N2 AIV is a major potential threat to the health of humans and poultry.

Vaccination is the best strategy to prevent and control avian influenza viruses. Currently, all commercial vaccines against avian influenza viruses in China are whole-virus-inactivated vaccines. Although the generation of this kind of vaccine is simple and the protection effect is good, there are still some problems, such as the long-term preparation and production cycle and excessive dependence on chicken embryos. The supply of chicken embryos cannot be guaranteed when the epidemic is spreading on a large scale, and the disposal of used chicken embryos also causes some problems with environmental safety [20,21]. In addition, the application of whole-virus-inactivated vaccines interferes with immunological monitoring and epidemiological investigation, making it impossible to distinguish the infected animals from the vaccinated animals [22]. With the antigenic drift and the continuous emergence of immune escape mutants in the HA protein of circulating H9N2 subtype AIVs, the efficacy of the current vaccine is constantly being challenged. Therefore, the development of novel vaccine candidates is urgently needed.

With a high safety profile and the ability to effectively distinguish between the infected and vaccinated animals, subunit vaccines are continuously being applied in the development of various novel vaccines against infectious diseases. Previous studies have shown that TAT-fused NP protein can be used as a broad-spectrum subunit vaccine. After intranasal immunization, high levels of IgA and IgG antibodies were induced in mice, which protected against the challenge of H3N2 and H9N2 viruses [23]. Chowdhury et al. developed a subunit vaccine combining the fusion peptide of the M2 protein and HA2 protein, which was prepared by adding a mucosal adjuvant. The vaccine can effectively induce an immune response and defense against viral challenges by intranasal administration [24]. Song et al. used plants to express a trimeric HA protein and formulated a vaccine that induced a strong immune response in vaccinated animals without adjuvants [25].

In this study, we expressed the trimetric consensus HA of H9N2 AIVs with or without the molecular adjuvants Cholera toxin subunit B (CTB), flagellin, and granulocyte-macrophage colony-stimulating factor (GM-CSF) in the baculovirus expression system. The mucosal immunogenicity of different types of H9 HA subunit vaccines was evaluated in mice by intranasal administration.

## 2. Materials and Methods

### 2.1. Cells

Insect cell Sf9 (Cat. No. 11496015, Thermo Fisher Scientific, Waltham, MA, USA) was suspended and cultured in Sf-900™ II SFM (Cat. No. 10902088, Thermo Fisher Scientific, Waltham, MA, USA) at 28 °C in the cell incubator.

### 2.2. Optimization and Synthesis of H9 HA and Molecular Adjuvants

We used the computationally optimized broadly reactive antigen (COBRA) approach to design antigens through multiple rounds of consensus building based on thousands of circulating strains of influenza virus over a period of time. All the HA sequences of the H9N2 subtype avian influenza viruses published recently were downloaded from the NCBI (Supplementary Materials). After alignment by MEGA11 software (Version 11.0.13), a consensus H9 HA sequence was generated (Supplementary Materials). The transmembrane region of the consensus H9 HA was predicted by the online software (https://dtu.biolib.com/DeepTMHMM, accessed on 16 June 2022). Then, the H9 HA gene without the transmembrane region, including the signal peptide (amino acid position, 1-506I, H9 numbering, see Supplementary Materials, blue highlight), added the T4 foldon for trimerization and the 6xHis tag for purification at the C-terminus, and the modified gene sequence was *Spodoptera frugiperda* codon-optimized and synthesized in the GenScript (Nanjing, China) Biotech Corporation.

**Figure 1:**
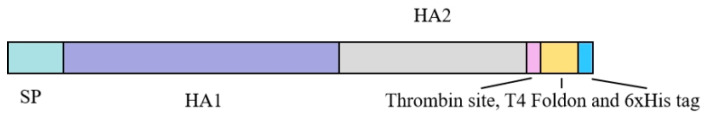
Schematic representation of the structure of HA.

Cholera toxin subunit B (CTB) (accession number: KP037243.1), flagellin (FliC) (accession number: WP_000079805.1), and granulocyte-macrophage colony-stimulating factor (GM-CSF) (accession number: AAI16879.1) without their signal peptides via the use of the HA signal peptide instead and synthesized after codon optimization were fused to the amino-terminal of the consensus H9 HA, and (GGGGS) _3_ linkers were used to link the molecular adjuvants and the H9 HA. The chimeric HA genes are shown in Figure 1.

### 2.3. Construction of Recombinant Transfer Plasmids and Bacmids

The fragments of CTB-HA, FliC-HA, GM-CSF-HA, and HA were amplified and constructed by overlapping PCR with the primers in Table 1, and then they were cloned into pFastBac by digestion with *EcoR*I and *Pst*I double restriction enzymes. The recombinant plasmids were termed pFastBac-HA, pFastBac-CTB-HA, pFastBac-FliC-HA, and pFastBac-GM-CSF-HA. The plasmids were verified by the digestion of double restriction enzymes and Sanger sequencing. Then, these four plasmids were transformed into DH10Bac-competent *E. coli* cells to generate recombinant bacmids by site-specific recombination. *E. coli* with recombinant bacmids were selected by blue–white screening and were identified by PCR with M13-F/M13-R. The recombinant bacmids were confirmed by sequencing to avoid unwanted mutations.

### 2.4. Expression and Purification of H9 HA and Molecular Adjuvant Fusion HAs in Bac-to-Bac Expression System

Sf9 insect cells were cultured in suspension at 28 °C in Sf-900 II serum-free medium supplemented with 1% penicillin and streptomycin (Cat. No. 15140122, Gibco, Grand Island, NY, USA). To rescue the recombinant baculoviruses for H9 HA expression, the recombinant bacmids were transfected into sf9 cells with Lipofectamine^TM^ LTX reagent (Cat. NO. 15338100, Thermo Fisher Scientific) according to the manufacturer’s instructions. The transfected cells were then incubated at 28 °C for 120 h with daily monitoring for the cytopathic effect (CPE). After identification with Western blotting, the recombinant baculoviruses (rBVs) were amplified from passage 1 to passage 3 (P3). Then, the P3 rBVs were used to infect the sf9 cells with 2% (*v*/*v*), and the mixture containing the supernatants and the infected cells was collected. After three rounds of the freezing and thawing treatment, the supernatant was collected and combined with Ni Sepharose at 4 °C for 4 h. After washing the column with washing buffer (20 mM Tris, 25 mM Imidazole, 300 mM NaCl, pH 8.0), bound protein was eluted with elution buffer (250 mM imidazole in 20 mM Tris, 300 mM NaCl), and the purified protein was concentrated with an ultrafiltration concentration tube to remove the imidazole. The purity of the recombinant protein was evaluated by SDS-PAGE with Coomassie blue staining and Western blotting. According to the manufacturer’s instructions (Cat. No. P0009 Beyotime), the concentration of the purified protein was determined with a BCA protein assay [26].

### 2.5. Western Blotting

Lysates prepared from the cells infected with recombinant baculovirus or purified recombinant proteins were subjected to 12.5% SDS-PAGE gel and isolated at 120 V for 60 min. Proteins were then transferred to polyvinylidene difluoride (PVDF) membranes, and then the transferred PVDF was blocked with 5% non-fat milk in PBS with 0.1% Tween 20 (PBST). The membrane was incubated with HRP-conjugated goat anti-mouse His-tag antibody (Cat. No. HRP-66005, Proteintech, Wuhan, China) at a 1:2000 dilution for 1.5 h at 37 °C and was then washed three times in PBST. The reaction was detected by ECL reagent (Cat. No. SQ201, Epizyme Biotech, Cambridge, MA, USA) according to the manufacturer’s instructions.

### 2.6. Indirect Immunofluorescence Assay

Sf9 cells were seeded at a concentration of 3 × 10^5^ cells/mL in six-well plates. The cells were infected with four different recombinant baculovirus suspensions for 48 h. Then, the cells were fixed using methanol for 10 min. After washing three times, the H9N2-positive chicken serum (generated in our lab) was added (1:200) and incubated for 1 h at 37 °C. Then, the cells were incubated with fluorescein isothiocyanate (FITC)-conjugated goat anti-chicken IgY diluted in 1:400 for 1 h at 37 °C. After washing, the cells were observed under a fluorescence microscope.

### 2.7. Animal Immunization

**Figure 2:**
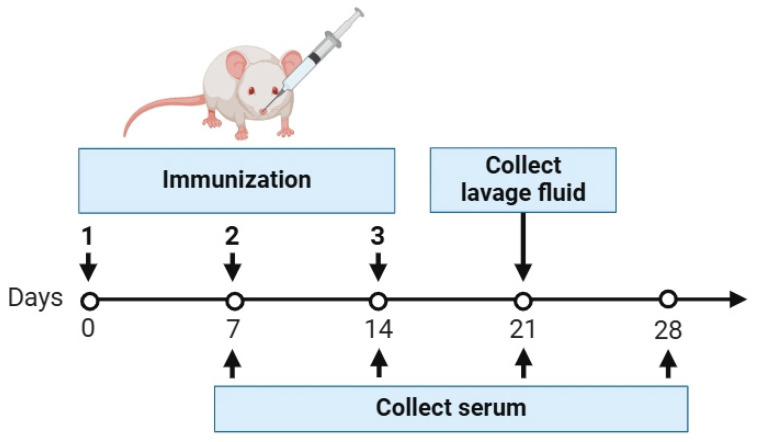
Immunization schedule and sample collection time points.

A total of 56 6-week-old female mice were randomly divided into the following seven groups: the HA group, CTB-HA group, FliC-HA group, GM-CSF-HA group, HA plus PolyI:C group, FliC-HA + PolyI:C group, and PBS group (Table 2). At day 0, day 7, and day 14, the mice were anesthetized with dry ice, and the H9 HA or fused H9 HA (5 µg per dose) or HA plus PolyI:C (10 µg per dose) mice were subjected to immunization by intranasal administration at the one-week interval, which was performed three times in total. The immunization schedule is shown in Figure 2.

On day 7, day 14, day 21, and day 28 after the first immunization, the sera were collected from each mouse. Three mice per group were killed on day 21 after the first immunization, nasal washes (NWs) were obtained from the nasopharynx by flushing the nasal passages with 1 mL of PBS, and bronchoalveolar lavage fluid (BALF) was collected by washing the organs first with 0.7 mL of PBS and then with 0.5 mL of PBS, as described previously [27]. The collected NWs and BALFs were stored at −80 °C until use.

### 2.8. Enzyme-Linked Immunosorbent Assay

To detect the HA-specific IgA and IgG antibodies, ELISA plates were coated with 100 µL of recombinant HA protein (2 µg/mL) at 4 °C overnight and blocked with 1% BSA for 2 h at 37 °C. Then, 100 µL NWs, BALFs, or diluted mouse sera (1: 200) was added, and the samples were incubated for 1 h at 37 °C. After washing three times, HRP-conjugated goat anti-mouse IgA or IgG was added and incubated for 1 h at 37 °C. The plates were incubated with the tetramethylbenzidine (TMB) substrate for 15 min at 37 °C after washing three times, and the reaction was terminated with 2 M H_2_SO_4_. The optical density (OD) was measured at a wavelength of 405 nm.

### 2.9. Statistical Analysis

Values are presented as means ± standard deviations (SDs). For comparing the significant differences between the two groups, we used the two-way ANOVA performed by using GraphPad Prism Version 6.07 (GraphPad Software Inc., San Diego, CA, USA); *p* < 0.05 indicated statistically significant differences.

## 3. Results

### 3.1. Identification of Recombinant Transfer Plasmids and Recombinant Bacmids

**Figure 3:**
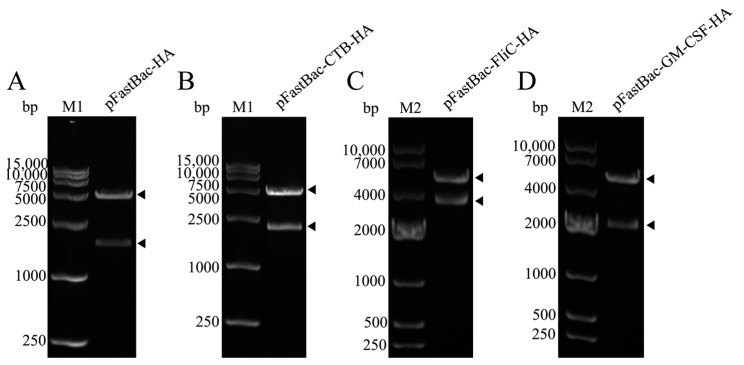
Double-digestion identification of recombinant transfer plasmids. (**A**) pFastBac-HA, M1: DL15000 DNA marker; (**B**) pFastBac-CTB-HA; (**C**) pFastBac-FliC-HA, M2: DL10000 DNA marker; (**D**) pFastBac-GM-CSF-HA.

The recombinant plasmids pFastBac-HA, pFastBac-CTB-HA, pFastBac-FliC-HA, and pFastBac-GM-CSF-HA were identified by double digestion with the *EcoR*I and *Pst*I restriction enzymes (Figure 3).

**Figure 4:**
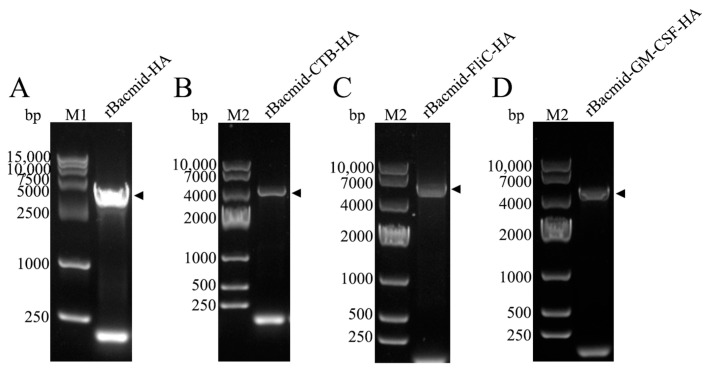
Identification of recombinant bacmids by PCR. (**A**) rBacmid-HA, M1: DL15000 DNA marker; (**B**) rBacmid-CTB-HA, M2: DL10000 DNA marker; (**C**) rBacmid-FliC-HA; (**D**) rBacmid-GM-CSF-HA.

The bands after the double enzyme digestion of recombinant plasmid were consistent with the expectations, among which the two bands of pFastBac-HA were 4714 bp and 1699 bp and the two bands of pFastBac-CTB-HA were 4714 bp and 2059 bp, respectively. The two bands of pFastBac-FliC-HA were 4714 bp and 3229 bp and the two bands of pFastBac-GM-CSF-HA were 4714 bp and 2116 bp, respectively. The recombinant Bacmid-HA, Bacmid-ctxB-HA, Bacmid-FliC-HA, and Bacmid-GM-CSF-HA were verified by PCR with M13 primers (Figure 4).

The amplifications of the Bacmid-HA, Bacmid-CTB-HA, Bacmid-FliC-HA, and Bacmid-GM-CSF-HA were 3983 bp, 3983 bp, 4343 bp, 5513 bp and 4400 bp, respectively, which were consistent with the expected size. This indicates that the target genes were recombined to the correct sites.

### 3.2. Identification of Recombinant Protein Expression in rBV-Infected sf9 Cells by Western Blotting and IFA

**Figure 5:**
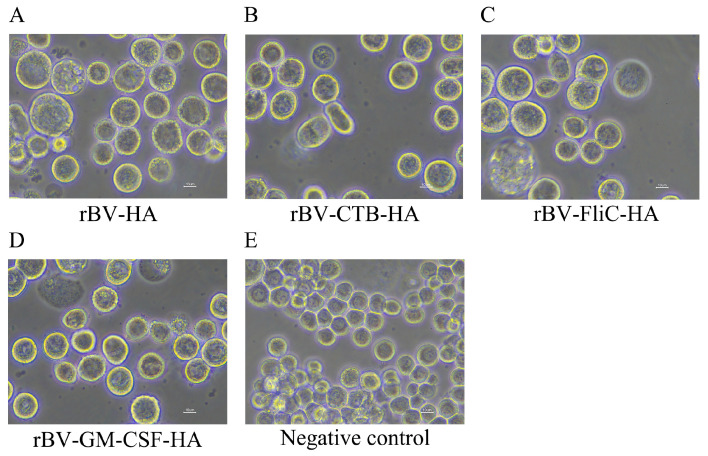
Observation of cytopathic effect in Sf9 cells transfected with recombinant bacmids. (**A**) rBV-HA; (**B**) rBV-CTB-HA; (**C**) rBV-FliC-HA; (**D**) rBV-GM-CSF-HA; (**E**) negative control. All images were magnified at 400×.

Sf9 cells were infected with recombinant baculovirus (rBV) for 48 h at a concentration of 3 × 10^5^ cells/mL, and the cytopathic effect of the infected cells could be significantly observed. Compared with the control group, the infected Sf9 cells were larger and there were less of them (Figure 5).

**Figure 6:**
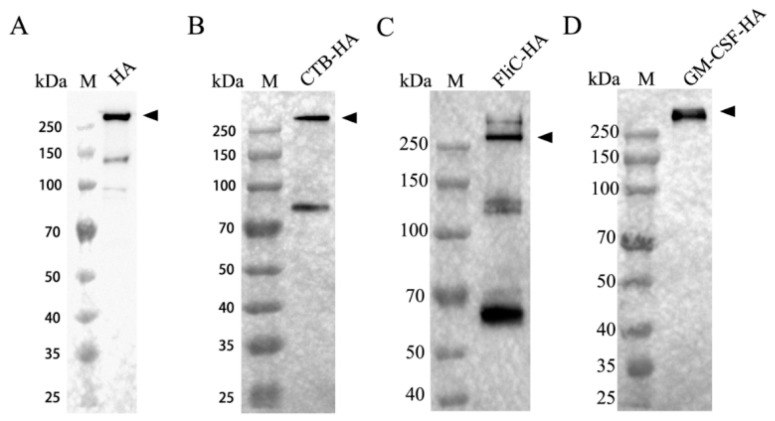
Identification of trimer formation of recombinant proteins by Western blotting. (**A**) HA; (**B**) CTB-HA; (**C**) FliC-HA; (**D**) GM-CSF-HA.

To identify the expression of the H9 HA and molecular adjuvant-fused HAs, the transfected cell supernatant was collected and mixed with reducing agent-free sample buffer (5×) and subjected to analysis with SDS-PAGE and Western blotting. The results showed that most of the HA proteins were trimerized except a small amount of monomers, and the molecular weight of these HA proteins was in the range of 300–400 kDa, which was consistent with the expected size of the trimer protein after glycosylation (Figure 6).

**Figure 7:**
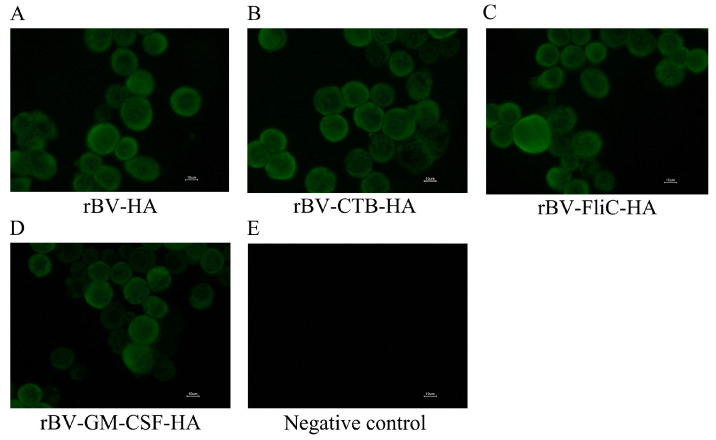
Identification of HA expression with the recombinant baculoviruses by indirect immunofluorescence assay. (**A**) rBV-HA; (**B**) rBV-CTB-HA; (**C**) rBV-FliC-HA; (**D**) rBV-GM-CSF-HA; (**E**) negative control. All images were magnified at 400×.

To further confirm the expression and distribution of the H9 HAs, Sf9 cells were infected with rBVs at 1% for 48 h. Except for the control group, the other infected Sf9 cells exhibited obvious green fluorescence after staining with anti-H9N2 chicken-positive sera, indicating that HA proteins could be expressed in these cells and mainly distributed at the membranes in the infected cells (Figure 7).

### 3.3. Identification of the Purified HAs by SDS-PAGE and Western Blotting

**Figure 8:**
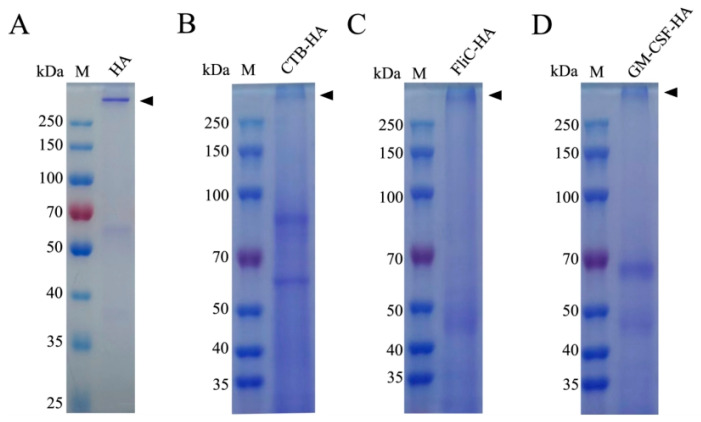
Identification of the purified proteins by SDS-PAGE. (**A**) HA; (**B**) CTB-HA; (**C**) FliC-HA; (**D**) GM-CSF-HA.

**Figure 9:**
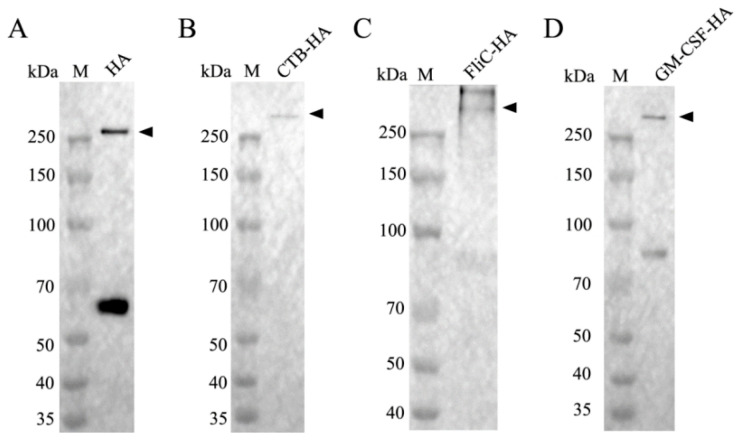
Identification of the purified HA proteins by Western blotting. (**A**) HA; (**B**) CTB-HA; (**C**) FliC-HA; (**D**) GM-CSF-HA.

The purity of the recombinant protein was evaluated by SDS-PAGE with Coomassie blue staining and Western blotting. The results showed that the HA, CTB-HA, FliC-HA, and GM-CSF-HA proteins were mostly expressed in trimer form, with molecular weights above 250 kDa (Figure 8 and Figure 9).

### 3.4. HA-Specific IgA and IgG Antibodies Were Induced by rHA Proteins and Molecular Adjuvant Fused-HAs with Intranasal Administration in Mice

**Figure 10:**
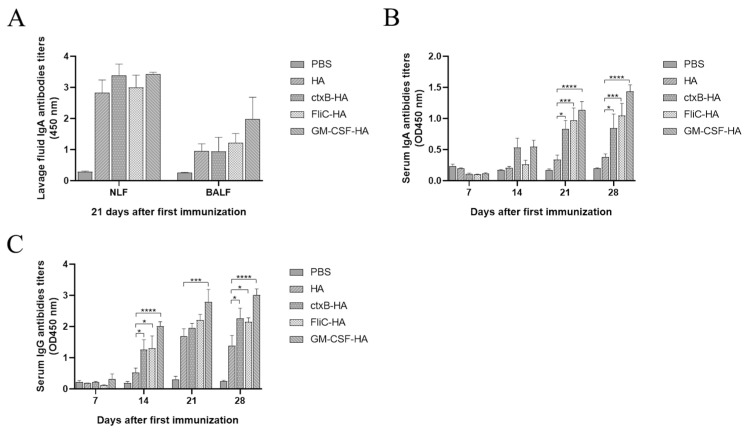
Specific IgA and IgG antibodies were induced in NW, BALF, and sera by HA and three molecular adjuvant-fused HAs through intranasal immunization. (**A**) IgA antibody titers in NLF and BALF, (**B**) IgA antibody titers in sera, (**C**) IgG antibody titers in NLF and BALF, * *p* < 0.05, *** *p* < 0.001, **** *p* < 0.0001; Two-way ANOVA was used for significant analysis.

The levels of H9 HA-specific IgA and IgG antibodies were evaluated in sera at day 7, day 14, day 21, and day 28 after the first immunization, as well as the levels of specific IgA antibodies in the NW and BALF at day 21 after the initial immunization. The results showed that the HA and three molecular adjuvants (CTB, FliC, and GM-CSF) fused HAs to induce high levels of IgA antibodies in NWs, and GM-CSF-HA induced slightly higher levels of IgA antibodies in BALFs, indicating that these antigens mainly induced the mucosal immune response in the upper respiratory tract (Figure 10A). Three molecular adjuvants significantly enhanced the IgA and IgG antibody titers, especially on day 28 after the first immunization (on day 7 after the third immunization), and GM-CSF-HA induced the highest level of IgG antibodies in sera (Figure 10B,C). The IgG levels in the sera of the CTB-HA, FliC-HA, and GM-CSF-HA groups were significantly higher than those in the HA vaccine group on day 14 after the first immunization. On day 21 after the first immunization, the IgA levels in the lavage fluid of the CTB-HA, FliC-HA, and GM-CSF-HA groups were a little higher than those of the HA group, but there was no significant difference. The IgG level in the sera of the GM-CSF-HA group was significantly higher than that of the HA group. The levels of IgA and IgG in the sera of the CTB-HA, FliC-HA and GM-CSF-HA groups were significantly higher than those of the H9 HA group on day 28 after the first immunization (Figure 10B,C). These results demonstrate that HA and molecular adjuvant-fused recombinant HAs possess good immunogenicity and GM-CSF-fused HAs have the best immunogenicity among them by intranasal administration.

### 3.5. PolyI:C Significantly Enhanced the Mucosal and Systematic Immune Responses of Recombinant H9 HA Proteins with Intranasal Administration in Mice

**Figure 11:**
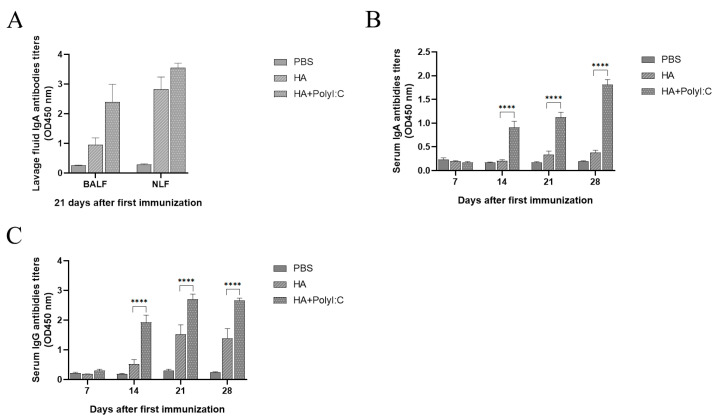
Poly I:C significantly enhanced the induction of IgA and IgG antibodies by intranasal administration. (**A**) IgA antibody titers in NW and BALF; (**B**) IgA antibody titers in sera; (**C**) IgG antibody titers in sera.; **** *p* < 0.0001; two-way ANOVA was used for significant analysis.

To investigate the mucosal adjuvant effect of PolyI:C on H9 HA recombinant subunit vaccines, an ELISA was used to compare the differences in the IgA and IgG antibody levels in the lavage fluids and sera in the groups with or without PolyI:C. The results showed that the antibody levels in the NW and BALF in the HA + PolyI:C group were a little higher than those in the HA group on day 21 after the first immunization (Figure 11A). The IgA antibody levels in the sera in the HA + PolyI:C group were significantly higher than those in the HA group on days 21 and 28 after the first immunization (Figure 11B). The IgG antibody levels in the sera in the HA + PolyI:C group were significantly higher than those in the HA group on day 14, day 21, and day 28 after the first immunization (Figure 11C). These results demonstrate that PolyI:C could significantly enhance the mucosal immune response and humoral immune response induced with the recombinant H9 HA subunit influenza vaccine.

## 4. Discussion

H9N2 subtype avian influenza viruses have been circulating in China for nearly 30 years and have not only caused huge economic losses but also serve as gene reservoirs of other subtype avian influenza viruses, bringing great risks for the next outbreak of influenza [17,28,29,30]. Traditional whole-virus-inactivated vaccines against the H9N2 avian influenza virus cannot induce cellular immunity, and they also cannot induce enough cross-protection against the H9N2 mutant viruses. In addition, the conventional inactivated H9N2 avian influenza vaccines are commonly administered by intramuscular injection, and this route induces no or a very weak mucosal immune response [31]. So, the development of novel influenza vaccines is urgently needed. In this study, we first aligned the 5934 H9N2 HA sequences isolated in the past five years and reintegrated them into one representative consensus H9 HA. We removed the transmembrane domain and cytoplasmic tail and added the T4 foldon to facilitate the trimerization of the expressed H9 HA. Then, we synthesized the modified H9 HA after codon optimization. We generated the recombinant consensus H9 HA protein in a Bac-to-Bac baculovirus expression system and the H9 HA presented in the trimers (Figure 6). We also generated molecular adjuvant-fused recombinant HA proteins, including CTB-H9 HA, FliC-H9 HA, and GM-CSF-H9 HA. We found that the expressed H9 HA, CTB-H9 HA, FliC-H9 HA, and GM-CSF-H9 HA induced the production of IgA in the NW, BALF, and sera and the production of IgG in the sera by intranasal administration. GM-CSF showed the best adjuvant activity among these three molecular adjuvants. PolyI:C significantly enhanced the immunogenicity in the mucosal immune response and humoral immune response combined with the rH9 HA. In sum, we developed potential mucosal consensus H9 HA subunit vaccines by a baculovirus expression system and found that GM-CSF or PolyI:C is a good mucosal adjuvant for consensus H9 HA subunit vaccines.

The COBRA methodology is an excellent strategy to develop next-generation influenza vaccines. COBRA-based H1 and H3 HA vaccines induced broad-spectrum reactive antibodies and elicited long-term protective immunity in mammalian models [32,33,34]. In addition, COBRA-based H2, H5, and H7 influenza vaccines increased the serological breadth against drifted variants [35,36]. Until now, the COBRA-based H9 HA influenza vaccine has not yet been reported. In this study, we aligned the H9 HA sequences published in the NCBI between 2017 and 2022 and found one H9 HA consensus sequence. Then, we expressed the consensus H9 HA trimer in a baculovirus system (Figure 8 and Figure 9). H9 HA exhibits good immunogenicity and induces the production of IgA and IgG antibodies (Figure 10 and Figure 11). Therefore, the COBRA-based H9 HA generated in this study could be a potential next-generation H9N2 influenza vaccine.

Influenza HA proteins have been generated in various expression systems, including the prokaryotic expression system, yeast expression system, baculovirus expression system, and mammalian expression system (CHO, 293F) [37,38,39,40]. The HA proteins expressed in *E. coli* usually present less immunogenicity due to a lack of glycosylation modification and the high cost of HA proteins expressed in mammals. Therefore, we selected a baculovirus expression system to express the COBRA H9 HA in this study, and the HA generated in insect cells possesses good glycosylation and immunogenicity in animals and humans. The seasonal influenza HA vaccines produced in insect cells, called Flublock, have been approved by the FDA and are mainly used for adults and the elderly with high doses [41,42]. In this study, the expressed consensus H9 HA in insect cells presented trimers, which is consistent with the previous studies [39,43].

Adjuvants could further improve the immunogenicity of antigens, and adding adjuvants is one effective strategy to improve the effect of vaccines [44,45]. In this study, we tried two kinds of strategies for adding the adjuvants. One was to fuse three molecular mucosal adjuvants, including CTB, FliC, and GM-CSF, with the consensus H9 HA and its co-expression. The results demonstrate that GM-CSF possesses the strongest mucosal adjuvant activity among them (Figure 10). A previous study indicates that GM-CSF is an effective adjuvant for influenza DNA vaccines by epidermis administration [46]. GM-CSF released by airway epithelial cells was the effector of the adjuvant activity of the TLR5 agonist flagellin [47,48]. We reported at first that the GM-CSF fused with the HA protein could improve the effect of the H9 HA subunit vaccine by intranasal administration. The other strategy is to add the synthesized chemical compound. The TLR3 agonist PolyI:C showed strong mucosal adjuvant activity for the H1N1, H3N2, and H5N1 split influenza vaccine [46,49]. We demonstrated that PolyI:C significantly enhanced the mucosal immune response of an insect cell-based consensus H9 HA subunit vaccine by intranasal administration.

Currently, all the H9N2 inactivated avian influenza vaccines are whole-viral-particle vaccines and are inoculated by intramuscular injection [50,51]. These types of vaccines cannot induce the mucosal immune response and cannot distinguish between the infected and vaccinated animals [52]. The mucosal subunit vaccines have several advantages. First, they can induce the mucosal immune responses and the production of IgA, which could inhibit the invasion of the viruses at the first defense line. Moreover, the subunit vaccines can distinguish between the infected and vaccinated animals, since they only contain the main viral surface antigens [53]. In addition, intranasal vaccines do not need to be injected and are more friendly for humans and animals.

## 5. Conclusions

In this study, we generated a consensus H9 HA by aligning the 5934 H9 HA sequences between 2017 and 2022, and we then expressed the CORBA-based H9 HA in trimers in a baculovirus system. Subsequently, we evaluated the immunogenicity of the COBRA H9 HA subunit vaccine in mice and demonstrated that this antigen induces the production of IgA and IgG by intranasal administration. Finally, we found that the molecular adjuvant GM-CSF further improves the mucosal immune response by fusing with consensus H9 HA. Adding the TLR3 agonist PolyI:C is the other effective strategy to improve the effect of the mucosal COBRA H9 HA vaccine. It is worth mentioning that although the H9 consensus HA subunit vaccine presented good immunogenicity in a murine model, the evaluation of the vaccine efficacy needs to be conducted within avian models to confirm the effectiveness in the target species. Above all, we developed a potential COBRA-based mucosal H9 HA subunit vaccine fused with molecular adjuvants or combined with PolyI:C that will be helpful in the prevention and control of the circulation of H9N2 influenza viruses in humans and animals.

## 6. Patents

This work has been filed for patent application by Zhejiang Sci-Tech University.

## Figures and Tables

**Table 1:** The primers used for the PCR in this study.

Gene	Primer	Sequence (5′-3′)
CTB	P1	GGAATTCGCCACCATGGAAACCAC
	P2	CGATGCAGATCTTGTCAGAGCCGCCACCT
FliC	P3	GGAATTCGCCACCATGGAAACCAC
	P4	CGATGCAGATCTTGTCAGAGCCGCCACCT
GM-CSF	P5	GGAATTCGCCACCATGGAAACCAC
	P6	CGATGCAGATCTTGTCAGAGCCGCCACCT
HA	P7	AGGAGGTGGCGGCTCTGACAAGATCTGCA
	P8	AGACTGCAGCTAGTGATGGTGATG
M13-F	P9	CCCAGTCACGACGTTGTAAAACG
M13-R	P10	AGCGGATAACAATTTCACACAGG

Note: The underlined parts are the nucleotide sequences recognized by restriction endonucleases.

**Table 2:** Groups of immunized mice.

Group	Immunogen	Dose (Per Mouse)
1	H9 HA	5 µg
2	CTB-HA	5 µg
3	FliC-HA	5 µg
4	GM-CSF-HA	5 µg
5	HA + PolyI:C	5 µg + 10 µg
6	FliC-HA + PolyI:C	5 µg + 10 µg
7	PBS	20 µL

## Data Availability

Data are contained within the article and Supplementary Materials.

## Supplementary Materials

The following supporting information can be downloaded at https://www.mdpi.com/article/10.3390/microorganisms12112294/s1.
