# Supplementary material for: H9 Consensus Hemagglutinin Subunit Vaccine with Adjuvants Induces Robust Mucosal and Systemic Immune Responses in Mice by Intranasal Administration
Source: Microorganisms. 2024 Nov 12;12(11):2294. doi: 10.3390/microorganisms12112294 (PMC11596065; doi:10.3390/microorganisms12112294)
Supplement: Supplementary file 1 [file microorganisms-12-02294-s001.zip › microorganisms-3277010-supplementary.pdf]

Supplementary information

1. H9 HA consensuses sequence

METTSLITILLVVTVSNADKICIGYQSTNSTETVDTLTENNVPVTHAKELLLTEHNGMLCATSLGQPLILDTCTIEGLIYGN
PSCDLLLEGREWSYIVERPSAVNGLCYPGNVENLEELRSLFSSARSYQRIQIFPDTIWNVSYDGTSTACSGSFYRSMRWL
TRKNGNYPIQDAQYTNNQGKNILFMWGINHPPTDTTQTNLYTRTDTTTSVATEEINRIFKPLIGPRPLVNGLMGRINYYW
SVLKPGQTLRIKSDGNLIAPWYGHILSGESHGRILKTDLKRGSCTVQCQTEKGGLNTTLPFQNVSKYAFGNCSKYIGIKS
LKLAVGLRNVPSRSSRGLFGAIAGFIEGGWSGLVAGWYGFQHSNDQGVGMAADRDSTQKAIDKITSKVNNIVDKMNK
QYEIIDHEFSEVETRLNMINNKIDDQIQDIWAYNAELLVLLENQKTLDEHDANVNNLYNKVKRALGSNAVEDGKGCFE
LYHKCDDQCMETIRNGTYNRRKYQEESKLERQKIEGVKLESEGTYKILTIYSTVASSLVIAMGFAAFLFWAMSNGSCRC
NICI

2. All H9N2 HA sequences used for alignment in this study

>QCU81269.1 hemagglutinin [Influenza A virus]

METVSLITILLVATVSKADKICIGYQSTNSTETVDTLTENNVPVTHVKELLHTEHNGMLCATSLGHPLIL

DTCTIEGLIYGNPSCDLLLGGREWSYIVERPSAVNGLCYPGNVENLEELRSLFSSARSYQRIQIFPDTIW

NVSYSGTSKACSDSFYRSMRWLTQKNNAYPIQDAQYTNNQEKNILFMWGINHPPTDTVQTNLYTRTDTTT

SVATEEINRIFKPLIGPRPLVNGLMGRIDYYWSVLKPGQTLRIRSDGNLIAPWYGHILSGESHGRILKTD

LKRGSCTVQCQTEKGGLNTTLPFQNVSKYAFGNCSKYIGIKSLKLAVGLRNVPSRSSRGLFGAIAGFIEG

GWSGLVAGWYGFQHSNDQGVGMAADRDSTQKAIDKITSKVNNIVDKMNKQYEIIDHEFSEVETRLNMINN

KIDDQIQDIWAYNAELLVLLENQKTLDEHDANVNNLYNKVKRVLGSNAVEDGKGCFELYHKCDDQCMETI

RNGTYNRRKYQEESKLERQKIEGVKLESEGTYKILTIYSTVASSLVIAMGFAAFLFWAMSNGSCRCNICI

>QCU81257.1 hemagglutinin [Influenza A virus]

METVSLITILLVATVSNADKICIGYQSTNSTETVDTLTENNVPVTHAKELLHTEHNGMLCATSLGHPLIL

DTCTIEGLIYGNPSCDLLLGGREWSYIVERPSAVNGLCYPGNVENLEELRSLFSSARSYQRIQIFPDTIW

NVSYSGTSKACSDSFYRSMRWLTQKNNAYPIQDAQYTNNQEKNILFMWGINHPPTDTAQTNLYTRTDTTT

SVATEEINRIFKPLIGPRPLVNGLMGRIDYYWSVLKPGQTLRIRSNGNLISPWYGHILSGESHGRILKTD

LKRGSCTVQCQTEKGGLNTTLPFQNVSKYAFGNCSKYIGIKSLKLAVGLRNVPSRSSRGLFGAIAGFIEG

GWSGLVAGWYGFQHSNDQGVGMAADRDSTQKAIDKITSKVNNIVDKMNKQYEIIDHEFSEVETRLNMINN

KIDDQIQDIWAYNAELLVLLENQKTLDEHDANVNNLYKKVKRALGSNAVEDGKGCFELYHKCDDQCMETI

RNGTYNRRKYQEESKLERQKIEGVKLESEGTYKILTIYSTVASSLVIAMGFAAFLFWAMSNGSCRCNICI

>QCU81245.1 hemagglutinin [Influenza A virus]

METASLITILLVVTVINADKICIGYQSTNSTETVDTLTENNVPVTHAKELLHTEHNGMLCATSLGHPLIL

DTCTIEGLIYGNPSCDLLLGGREWSYIVERPSAVNGLCYPGNVENLEELRSLFSSVRSYQRIQIFPDTIW
NVSYSGTSKACSDSFYRSMRWLTQKNNAYPIQDAQYTNNQEKNILFMWGINHPPTDTVQTNLYTRTDTTT
SVATEEINRTFKPLIGPRPLVNGLMGRIDYYWSVLKPGQTLRIRSNGNLIAPWYGHILSGESHGRILKTD
LKRGSCTVQCQTEKGGLNTTLPFQNVSKYAFGNCSKYIGIKSLKLAVGLRNVPSRSSRGLFGAIAGFIEG
GWSGLVAGWYGFQHSNDQGVGMAADRDSTQKAIDKITSKVNNIVDKMNKQYEIIDHEFSEVETRLNMINN
KIDDQIQDIWAYNAELLVLLENQKTLDEHDANVNNLYNKVKRALGSNAVEDGKGCFELYHKCDDQCMETI
RNGTYNRRKYQEESKLEKQKIEGVKLESEGTYKILTIYSTVASSLVIAMGFAAFLFWAMSNGSCRCNICI

>QCU81233.1 hemagglutinin [Influenza A virus]
MEVASLITILLVVTVSNADKICIGYQSTNSTETVDTLTENNVPVTHAKELLHTEHNGMLCATSLGHPLIL
DTCTIEGLIYGNPSCDLLLGGREWSYIVERPSAVNGLCYPGNVENLEELRSLFSSARSYQRIQIFPDTIW
NVSYSGTSKACSDSFYRSMRWLTQKNNAYPIQDAQYTNNQEKNILFMWGINHPPTDTTQTNLYTRTDTTT
SVATEEINRTFKPLIGPRPLVNGLMGRIDYYWSVLKPGQTLRIRSNGNLIAPWYGHILSGESHGRILKTD
LKRGSCTVQCQTEKGGLNTTLPFQNVSKYAFGNCSKYIGIKSLKLAVGLRNVPSRSSRGLFGAIAGFIEG
GWSGLVAGWYGFQHSNDQGVGMAADRDSTQKAIDKITSKVNNIVDKMNKQYEIIDHEFSEVETRLNMINN
KIDDQIQDIWAYNAELLVLLENQKTLDEHDANVNNLYNKVKRALGSNAVEDGKGCFELYHKCDDQCMETI
RNGTYNRRKYQEESKLERQKIEGVKLESEGTYKILTIYSTVASSLVIAMGFAAFLFWAMSNGSCRCNICI

>QCU81221.1 hemagglutinin [Influenza A virus]
METVSLITILLVAAVSNADKICIGYQSTNSTETVDTLTENNVPVTHAKELLHTEHNGMLCATSLGHPLIL
DTCTIEGLIYGNPSCDLLLGGREWSYIVERPSAVNGLCYPGNVENLEELRSLFSSARSYQRIQIFPDTIW
NVSYSGTSKACSDSFYRSMRWLTQKDNAYPIQDAQYTNNQEKNILFMWGINHPPTDTTQTNLYTRTDTTT
SVATEEINRIFKPLIGPRPLVNGLMGRIDYYWSVLKPGQTLRIRSNGNLIAPWYGHILSGESHGRILKTD
LKRGSCTVQCQTEKGGLNTTLPFQNVSKYAFGNCSKYIGIKSLKLAVGLRNVPSRSSRGLFGAIAGFIEG
GWSGLVAGWYGFQHSNDQGVGMAADRDSTQKAIDKITSKVNNIVDKMNKQYEIIDHEFSEVETRLNMINN
KIDDQIQDIWAYNAELLVLLENQKTLDEHDANVNNLYNKVKRALGFNAVEDGKGCFELYHKCDDQCMETI
RNGTYNRRKYQEESKLERQKVEGVKLESEGTYKILTIYSTVASSLVIAMGFAAFLFWAMSNGSCRCNICI

>QCU81209.1 hemagglutinin [Influenza A virus]
MEIVSLITILLVATVSNADKICIGYQSTNSTETVDTLTENNIPVTHAKELLHTEHNGMLCATNLGQPLIL
DTCTIEGLIYGNPSCELLLDGREWSYIVERPSAVNGLCYPGNVENLEELRSLFSSARSYQRIQIFPVTIW
NVSYNGTSKACSGSFYRSMRWLTQKNSNYPIQDAQYTNNQGKNILFMWGINHPPTDTTQRDLYMRTDTTT
SVATEEINRVFKPLIGPRPLVNGLMGRIDYYWSVLKPGQTLRIKSDGNLIAPWYGYILSGESHGRILKTD
LKRGSCTVQCQTEKGGLNTTLPFQNVSKYAFGNCSKYIGIKSLKLAVGLRNVPSRSSRGLFGAIAGFIEG

GWSGLVAGWYGFQHSNDQGVGMAADRDSTQKAIDKITSKVNNIVDKMNKQYEIIDHEFSEVETRLNMINN
KIDDQIQDIWAYNAELLVLLENQKTLDEHDANVNNLYDKVKRALGSNAVEDGKGCFELYHKCDDQCMETI
RNGTYNRRKYQEESKLVRQKIEGVKLESEGTYKILTIYSTVASSLVIAMGFAAFLFWAMSNGSCRCNICI

>QCU81197.1 hemagglutinin [Influenza A virus]
METVSLITILLVATVSKADKICIGYQSTNSTETVDTLTENNVPVTHVKELLHTEHNGMLCATSLGHPLIL
DTCTIEGLIYGNPSCDLLLGGREWSYIVERPSAVNGLCYPGNVENLEELRSLFSSARSYQRIQIFPDTIW
NVSYSGTSKACSDSFYRSMRWLTQKNNAYPIQDAQYTNNQEKNILFMWGINHPPTDTVQTNLYTRTDTTT
SVATEEINRIFKPLIGPRPLVNGLMGRIDYYWSVLKPGQTLRIRSDGNLIAPWYGHILSGESHGRILKTD
LKRGSCTVQCQTEKGGLNTTLPFQNVSKYAFGNCSKYIGIKSLKLAVGLRNVPSRSSRGLFGAIAGFIEG
GWSGLVAGWYGFQHSNDQGVGMAADRDSTQKAIDKITSKVNNIVDKMNKQYEIIDHEFSEVETRLNMINN
KIDDQIQDIWAYNAELLVLLENQKTLDEHDANVNNLYNKVKRALGSNAVEDGKGCFELYHKCDDQCMETI
RNGTYNRRKYQEESKLERQKIEGVKLESEGTYKILTIYSTVASSLVIAMGFAAFLFWAMSNGSCRCNICI

>QCU81185.1 hemagglutinin [Influenza A virus]
METVSLITILLVATVSNADKICIGYQSTNSTETVDTLTENNVPVTHAKELLHTEHNGMLCATSLGHPLIL
DTCTIEGLIYGNPSCDLLLGGREWSYIVERPSAVNGLCYPGNVENLEELRSLFSSARSYQRIQIFPDTIW
NVSYSGTSKACSDSFYRSMRWLTQKDNAYPIQDAQYTNNQEKNILFMWGINHPPTDTTQTNLYTRTDTTT
SVATEEINRIFKPLIGPRPLVNGLMGRIDYYWSVLKPGQTLRIRSNGNLIAPWYGHILSGESHGRILKTD
LKRGSCTVQCQTEKGGLNTTLPFQNVSKYAFGNCSKYIGIKSLKLAVGLRNVPSRSSRGLFGAIAGFIEG
GWSGLVAGWYGFQHSNDQGVGMAADRDSTQKAIDKITSKVNNIVDKMNKQYEIIDHEFSEVETRLNMINN
KIDDQIQDIWAYNAELLVLLENQKTLDEHDANVNNLYNKVKRALGFNAVEDGKGCFELYHKCDDQCMETI
RNGTYNRRKYQEESKLERQKVEGVKLESEGTYKILTIYSTVASSLVIAMGFAAFLFWAMSNGSCRCNICI

>QCU81173.1 hemagglutinin [Influenza A virus]
METVSLITILLVATVSNADKICIGYQSTNSTETVDTLTENNVPVTHAKELLHTEHNGMLCATSLGHPLIL
DTCTIEGLIYGNPSCDLLLGGREWSYIVERPSAVNGLCYPGNVENLEELRSLFSSARSYQRIQIXPDTIW
NVSYSGTSKACSDSFYRSMRWLTQKNNAYPIQDAQYTNNQEKNILFMWGINHPPTDTAQTNLYTRTDTTT
SVATEEINRIFKPLIGPRPLVNGLMGRIDYYWSVLKPGQTLRIKSNGNLIAPWYGHLLSGESHGRILKTD
LKKGSCTVQCQTEKGGLNTTLPFQNVSKYAFGNCSKYIGIKSLKLAVGLRNVPSRSSRGLFGAIAGFIEG
GWSGLVAGWYGFQHSNDQGVGIAADRDSTQKAIDKITSKVNNIVDKMNKQYEIIDHEFSEVETRLNMINN
KIDDQIQDIWAYNAELLVLLENQKTLDEHDANVNNLYNKVKRALGSNAVEDGKGCFELYHKCDDQCMETI
RNGTYNRRKYQEESKLERQKIEGVKLESEGTYKILTIYSTVASSLVIAMGFAAFLFWAMSNGSCRCNICI

>QCU81161.1 hemagglutinin [Influenza A virus]
METVSLITILLVATVSNADKICIGYQSTNSTETVDTLTENNVPVTHAKELLHTEHNGMLCATSLGHPLIL
DTCTIEGLIYGNPSCDLLLGGREWSYIVERPSAVNGLCYPGNVENLEELRSLFSSARSYXRIQIFPDTIW
NVSYSGTSKACSDSFYRSMRWLTQKNNAYPIQDAQYTNNQEKNILFMWGINHPPTDTAQTNLYTRTDTTT
SVATEEINRIFKPLIGPRPLVNGLMGRIDYYWSVLKPGQTLRIKSNGNLIAPWYGHILSGESHGRILKTD
LKKGSCTVQCQTEKGGLNTTLPFQNVSKYAFGNCSKYIGIKSLKLAVGLRNVPSRSSRGLFGAIAGFIEG
GWSGLVAGWYGFQHSNDQGVGIAADRDSTQKAIDKITSKVNNIVDKMNKQYEIIDHEFSEVETRLNMINN
KIDDQIQDIWAYNAELLVLLENQKTLDEHDANVNNLYNKVKRALGSNAVEDGKGCFELYHKCDDQCMETI
RNGTYNRRKYQEESKLERQKIEGVKLESEGTYKILTIYSTVASSLVIAMGFAAFLFWAMSNGSCRCNICI

>AWK91180.1 hemagglutinin [Influenza A virus]
MKTISLITILLVVTTSNADKICIGHQSTNSTETVDTLTETNVPVTHAKELLHTEHNGMLCATNLGHPLIL
DTCTIEGLIYGNPSCDLLLGGREWSYIVERPSAVNGTCYPGNVENLEELRTLFSSSSSYQRIQIFPDTIW
NVTYTGTSKSCSDSFYRNMRWLTQKNGLYPVQDAQYTNNRGKDILFLWGIHHPPTDTAQTNLYTRTDTTT
SVTTENLDRTFKPLIGPRPLVNGLIGRINYYWSVLKPGQTLRVRSNGNLIAPWYGHVLSGESHGRILKTD
LNSGNCVVQCQTEKGGLNSTLPFHNISKYAFGTCPKYIGVKSLKLAIGLRNVPARSSRGLFGAIAGFIEG
GWPGLVAGWYGFQHSNDQGVGMAADRDSTQKAIDKITSKVNNIVDKMNKQYEIIDHEFSEVETRLNMINN
KIDDQIQDVWAYNAELLVLLENQKTLDEHDANVNNLYNKVKRALGSNAMEDGKGCFELYHKCDDQCMETI
RNGTYNRRKYKEEVRLERQKIEGVKLESEGTYKILTIYSTVASSLVLAMGFAAFLFWAMSNGSCRCNICI

>QWE52403.2 hemagglutinin [Influenza A virus]
MEAISLMIILLVVTTSNADKICIGHQSTNSTETVDTLTESNIPVTQAKELLHTEHNGMLCATNLGRPLIL
DTCTVEGLVYGNPSCDLLLGGREWSYIVERPSAVNGTCYPGNVENLEELRMLFSSASSYQRIQIFPDAIW
NVTYDGTSKSCSNSFYRNMRWLTQKNGNYPIQDAQYTNNRGEDILFIWGIHHPPTDTAQTNLYTRTDTTT
SVTTENLDRTFKPLIGPRPLVNGLIGRINYYWSVLKPGQTLRVRSNGNLIAPWYGHVLSGESHGRILKTD
LKSGNCVVQCQTEKGGLNSTLPFHNISKYAFGTCPKYIGVKSLKLAIGLRNVPAKSSRGLFGAIAGFIEG
GWPGLVAGWYGFQHSNDQGVGMAADRSSTQKAVDKITSKVNNIIDKMNRQYEIIDHEFSEIETRLNMINN
KIDDQIQDVWAYNAELLVLLENQKTLDEHDANVNNLYNKVKRALGSNAMEDGKGCFELYHKCDDQCMETI
RNGTYNRRKYTEESRLERQKIEGVKLESEGTYKILTIYSTAASSLVLAMGVAAFLFWAMSNGSCRCNICI

>QQD48004.1 hemagglutinin [Influenza A virus]
METVSLMTILVVATVSNADKICIGYQSTNSTETVDTLTENNVPVTHAKELLHTEHNGMLCATSLGHPLVL
DTCTIEGLIYGNPSCDPLLGGREWSYIVERPSAVNGLCYPGNVENLEELRSLFSSSRSYQRIQIFPDTIW
NVSYSGTSKACSDSFYRSMRWLTQKNNAYPTQDAQYTNNQGKNILFMWGINHPPTDTAQTNLYTRTDTTT

SVATEEMNRIFKPLIGPRPLVNGLMGRINYYWSVLKPGQTLRIKSDGNLIAPWYGHILSGESHGRILKTD
LKRGSCTVQCQTEKGGLNTTLPFQNVSKYAFGNCSKYIGVKSLKLAVGLRNVPSKSSRGLFGAIAGFIEG
GWSGLVAGWYGFQHSNDQGVGMAADRDSTQKAIDKITSKVNNIVDKMNKQYEIIDHEFSEVETRLNMINN
KVDDQIQDIWAYNAELLVLLENQKTLDEHDANVNNLYNKVKRALGSNAVEDGKGCFELYHKCDDHCMETI
RNGTYNRRKYQEESKLERQKIEGVKLESEETYKILTIYSTVASSLVIAMGFAAFLFWAMSNGSCRCNICI

>QQD47992.1 hemagglutinin [Influenza A virus]
METVSLITILVVATVSNADKICIGYQSTNSTETVDTLTENNVPVTHAKELLHTEHNGMLCATSLGHPLIL
DTCTIEGLIYGNPSCDPLLGGREWSYIVERPSAVNGLCYPGNVENLEELRSLFSSSRSYQRIQIFPDTIW
NVSYSGTSKACSDSFYRSMRWLTQKNNAYPTQDAQYTNNQGKNILFMWGINHPPTDTVQTNLYTRTDTTT
SVATEEMNRIFKPLIGPRPLVNGLMGRIKYYWSVLKPGQTLRIKSDGNLIAPWYGHILSGESHGRILKTD
LKRGSCTVQCQTEKGGLNTTLPFQNVSKYAFGNCSKYIGVKSLKLAVGLRNVPSRSSRGLFGAIAGFIEG
GWSGLVAGWYGFQHSNDQGVGMAADRDSTQKAIDKITSKVNNIVDKMNKQYEIIDHEFSEVETRLNMINN
KVDDQIQDIWAYNAELLVLLENQKTLDEHDANVNNLYNKVKRALGSNAVEDGKGCFELYHKCDDHCMETI
RNGTYNRRKYQEESKLERQKIEGVKLESEETYKILTIYSTVASSLVIAMGFAAFLFWAMSNGSCRCNICI

>QQD47980.1 hemagglutinin [Influenza A virus]
METVSLITILVVATVSNADKICIGYQSTNSTETVDTLTENNVPVTHAKELLHTEHNGMLCATSLGHPLIL
DTCTIEGLIYGNPSCDPLLGGREWSYIVERPSAVNGLCYPGNVENLEELRSLFSSSRSYQRIQIFPDTIW
NVSYSGTSKACSDSFYRSMRWLTQKNNAYPTQDAQYTNNQGKNILFMWGINHPPTDTAQTNLYTRTDTTT
SVATEEMNRIFKPLIGPRPLVNGLMGRINYYWSVLKPGQTLRIKSDGNLIAPWYGHILSGESHGRILKTD
LKRGSCTVQCQTEKGGLNTTLPFQNVSKYAFGNCSKYIGVKSLKLAVGLRNVPSRSRRGLFGAIAGFIEG
GWSGLVAGWYGFQHSNDQGVGMAADRDSTQKAIDKITSKVNNIVDKMNKQYEIIDHEFSEVETRLNMINN
KVDDQIQDIWAYNAELLVLLENQKTLDEHDANVNNLYNKVRRALGSNAVEDGKGCFELYHKCDDHCMETI
RNGTYNRRKYQEESKLERQKIEGVKLESEETYKILTIYSTVASSLVIAMGFAAFLFWAMSNGSCRCNICI

>QCA40742.1 hemagglutinin, partial [Influenza A virus]
MTILLVVTTSNADKICIGHQSTNSTETVDTLTETNVPVTQAKELLHTEHNGMLCATNLGRPLILDTCTIE
GLIYGNPSCDMLLGGREWSYIVERPSAVNGTCYPGNVENLEELRTLFSSSSSYQRIQLFPDSIWNVTYTG
TSKSCSDSFYRNMRWLTQKNGGYPIQDAQYTNNRGKDILFVWGIHHPPTDATQTNLYTRADTTTSVTTET
LDRTFKPLIGPRPLVNGLIGRINYYWSVLKPGQTLRVRSNGNLIAPWFGHVLSGESHGRILRTDLSSGNC
VVQCQTEKGGLNSTMPFHNISKYAFGTCPKYIGVKSLKLAIGLRNVHARSSRGLFGAIAGFIEGGWPGLV
AGWYGFQHSNDQGVGMAADRVSTQKAVDKITSKVNNIVDKMNKQYEIIDHEFSEVETRLNMINNKIDDQI
QDIWAYNAELLVLLENQKTLDEHDANVNNLYNKVKRALGSNAMEDGKGCFELYHKCDDQCMETIRNGTYN

RRKYTEESRLERQKIEGVKLEAEGTYKILSIYSTVASSLVLAMGFAAFLFWAMSNGSCRCNICI

>QCA40741.1 hemagglutinin, partial [Influenza A virus]
MTILLVVTTSNADKICIGHQSTNSTETVDTLTETNVPVTQAKELLHTEHNGMLCATNLGRPLILDTCTIE
GLIYGNPSCDMLLGGREWSYIVERPSAVNGTCYPGNVENLEELRTLFSSSSSYQRIQLFPDSIWNVTYTG
TSKSCSDSFYRNMRWLTQKNGGYPIQDAQYTNNRGKDILFVWGIHHPPTDATQTNLYTRADTTTSVTTET
LDRTFKPLIGPRPLVNGLIGRINYYWSVLKPGQTLRVRSNGNLIAPWFGHVLSGESHGRILRTDLSSGNC
VVQCQTEKGGLNSTMPFHNISKYAFGTCPKYIGVKSLKLAIGLRNVHARSSRGLFGAIAGFIEGGWPGLV
AGWYGFQHSNDQGVGMAADRVSTQKAVDKITSKVNNIVDKMNKQYEIIDHEFSEVETRLNMINNKIDDQI
QDIWAYNAELLVLLENQKTLDEHDANVNNLYNKVKRALGSNAMEDGKGCFELYHKCDDQCMETIRNGTYN
RRKYTEESRLERQKIEGVKLEAEGTYKILSIYSTVASSLVLAMGFAAFLFWAMSNGSCRCNICI

>QCA40740.1 hemagglutinin, partial [Influenza A virus]
MTILLVVTTSNADKICIGHQSTNSTETVDTLTETNVPVTQAKELLHTEHNGMLCATNLGRPLILDTCTIE
GLIYGNPSCDMLLGGREWSYIVERPSAVNGTCYPGNVENLEELRTLFSSSSSYQRIQLFPDSIWNVTYTG
TSKSCSDSFYRNMRWLTQKNGGYPIQDAQYTNNRGKDILFVWGIHHPPTNATQTNLYTRADTTTSVTTET
LDRTFKPLIGPRPLVNGLIGRINYYWSVLKPGQTLRVRSNGNLIAPWFGHVLSGESHGRILRTDLSSGNC
VVQCQTEKGGLNSTMPFHNISKYAFGTCPKYIGVKSLKLAIGLRNVHARSSRGLFGAIAGFIEGGWPGLV
AGWYGFQHSNDQGVGMAADRVSTQKAVDKITSKVNNIVDKMNKQYEIIDHEFSEVETRLNMINNKIDDQI
QDIWAYNAELLVLLENQKTLDEHDANVNNLYNKVKRALGSNAMEDGKGCFELYHKCDDQCMETIRNGTYN
RRKYTEESRLERQKIEGVKLEAEGTYKILSIYSTVASSLVLAMGFAAFLFWAMSNGSCRCNICI

>QCA40739.1 hemagglutinin, partial [Influenza A virus]
MTILLVVTTSNADKICIGHQSTNSTETVDTLTETNVPVTQAKELLHTEHNGMLCATNLGRPLILDTCTIE
GLIYGNPSCDMLLGGREWSYIVERPSAVNGTCYPGNVENLEELRTLFSSSSSYQRIQLFPDSIWNVTYTG
TSKSCSDSFYRNMRWLTQKNGGYPIQDAQYTNNRGKDILFVWGIHHPPTDATQTNLYTRADTTTSVTTET
LDRTFKPLIGPRPLVNGLIGRINYYWSVLKPGQTLRVRSNGNLIAPWFGHVLSGESHGRILRTDLSSGNC
VVQCQTEKGGLNSTMPFHNISKYAFGTCPKYIGVKSLKLAIGLRNVHARSSRGLFGAIAGFIEGGWPGLV
AGWYGFQHSNDQGVGMAADRVSTQKAVDKITSKVNNIVDKMNKQYEIIDHEFSEVETRLNMINNKIDDQI
QDIWAYNAELLVLLENQKTLDEHDANVNNLYNKVKRALGSNAMEDGKGCFELYHKCDDQCMETIRNGTYN
RRKYTEESRLERQKIEGVKLEAEGTYKILSIYSTVASSLVLAMGFAAFLFWAMSNGSCRCNICI

>QCA40738.1 hemagglutinin, partial [Influenza A virus]
MTILLVVTTSNADKICIGHQSTNSTETVDTLTETNVPVTQAKELLHTEHNGMLCATNLGRPLILDTCTIE

GLIYGNPSCDMLLGGREWSYIVERPSAVNGTCYPGNVENLEELRTLFSSSSSYQRIQLFPDSIWNVTYTG
TSKSCSDSFYRNMRWLTQKNGGYPIQDAQYTNNRGKDILFVWGIHHPPTNATQTNLYTRADTTTSVTTET
LDRTFKPLIGPRPLVNGLIGRINYYWSVLKPGQTLRVRSNGNLIAPWFGHVLSGESHGRILRTDLSSGNC
VVQCQTEKGGLNSTMPFQNISKYAFGTCPKYIGVKSLKLAIGLRNVHARSSRGLFGAIAGFIEGGWPGLV
AGWYGFQHSNDQGVGMAADRVSTQKAVDKITSKVNNIVDKMNKQYEIIDHEFSEVETRLNMINNKIDDQI
QDIWAYNAELLVLLENQKTLDEHDANVNNLYNKVKRALGSNAMEDGKGCFELYHKCDDQCMETIRNGTYN
RRKYTEESRLERQKIEGVKLEAEGTYKILSIYSTVASSLVLAMGFAAFLFWAMSNGSCRCNICI

>QCA40737.1 hemagglutinin, partial [Influenza A virus]
MTILLVVTTSNADKICIGHQSTNSTETVDTLTETNVPVTQAKELLHTEHNGMLCATNLGRPLILDTCTIE
GLIYGNPSCDMLLGGREWSYIVERPSAVNGTCYPGNVENLEELRTLFSSSSSYQRIQLFPDSIWNVTYTG
TSKSCSDSFYRNMRWLTQKNGGYPIQDAQYTNNRGKDILFVWGIHHPPTDATQTNLYTRADTTTSVTTET
LDRTFKPLIGPRPLVNGLIGRINYYWSVLKPGQTLRVRSNGNLIAPWFGHVLSGESHGRILRTDLSSGNC
VVQCQTEKGGLNSTMPFHNISKYAFGTCPKYIGVKSLKLAIGLRNVHARSSRGLFGAIAGFIEGGWPGLV
AGWYGFQHSNDQGVGMAADRVSTQKAVDKITSKVNNIVDKMNKQYEIIDHEFSEVETRLNMINNKIDDQI
QDIWAYNAELLVLLENQKTLDEHDANVNNLYNKVKRALGSNAMEDGKGCFELYHKCDDQCMETIRNGTYN
RRKYTEESRLERQKIEGVKLEAEGTYKILSIYSTVASSLVLAMGFAAFLFWAMSNGSCRCNICI

>QCA40736.1 hemagglutinin, partial [Influenza A virus]
MTILLVVTTSNADKICIGHQSTNSTETVDTLTETNVPVTQAKELLHTEHNGMLCATNLGRPLILDTCTIE
GLIYGNPSCDMLLGGREWSYIVERPSAVNGTCYPGNVENLEELRTLFSSSSSYQRIQLFPDSIWNVTYTG
TSKSCSDSFYRNMRWLTQKNGGYPIQDAQYTNNRGKDILFVWGIHHPPTDATQTNLYTRADTTTSVTTET
LDRTFKPLIGPRPLVNGLIGRINYYWSVLKPGQTLRVRSNGNLIAPWFGHVLSGESHGRILRTDLSSGNC
VVQCQTEKGGLNSTMPFHNISKYAFGTCPKYIGVKSLKLAIGLRNVHARSSRGLFGAIAGFIEGGWPGLV
AGWYGFQHSNDQGVGMAADRVSTQKAVDKITSKVNNIVDKMNKQYEIIDHEFSEVETRLNMINNKIDDQI
QDIWAYNAELLVLLENQKTLDEHDANVNNLYNKVKRALGSNAMEDGKGCFELYHKCDDQCMETIRNGTYN
RRKYTEESRLERQKIEGVKLEAEGTYKILSIYSTVASSLVLAMGFAAFLFWAMSNGSCRCNICI

>QCA40735.1 hemagglutinin, partial [Influenza A virus]
MTILLVVTTSNADKICIGHQSTNSTETVDTLTETNVPVTQAKELLHTEHNGMLCATNLGRPLILDTCTIE
GLIYGNPSCDMLLGGREWSYIVERPSAVNGTCYPGNVENLEELRTLFSSSSSYQRIQLFPDSIWNVTYTG
TSKSCSDSFYRNMRWLTQKNGGYPIQDAQYTNNRGKDILFVWGIHHPPTDATQTNLYTRADTTTSVTTET
LDRTFKPLIGPRPLVNGLIGRINYYWSVLKPGQTLRVRSNGNLIAPWFGHVLSGESHGRILRTDLSSGNC
VVQCQTEKGGLNSTMPFHNISKYAFGTCPKYIGVKSLKLAIGLRNVHARSSRGLFGAIAGFIEGGWPGLV

AGWYGFQHSNDQGVGMAADRVSTQKAVDKITSKVNNIVDKMNKQYEIIDHEFSEVETRLNMINNKIDDQI
QDIWAYNAELLVLLENQKTLDEHDANVNNLYNKVKRALGSNAMEDGKGCFELYHKCDDQCMETIRNGTYN
RRKYTEESRLERQKIEGVKLEAEGTYKILSIYSTVASSLVLAMGFAAFLFWAMSNGSCRCNICI

>QCA40734.1 hemagglutinin, partial [Influenza A virus]
MTILLVVTTSNADKICIGHQSTNSTETVDTLTETNVPVTQAKELLHTEHNGMLCATNLGRPLILDTCTIE
GLIYGNPSCDMLLGGREWSYIVERPSAVNGTCYPGNVENLEELRTLFSSSSSYQRIQLFPDSIWNVTYTG
TSKSCSDSFYRNMRWLTQKNGGYPIQDAQYTNNRGKDILFVWGIHHPPTDATQTNLYTRADTTTSVTTET
LDRTFKPLIGPRPLVNGLIGRINYYWSVLKPGQTLRVRSNGNLIAPWFGHVLSGESHGRILRTDLSSGNC
VVQCQTEKGGLNSTMPFHNISKYAFGTCPKYIGVKSLKLAIGLRNVHARSSRGLFGAIAGFIEGGWPGLV
AGWYGFQHSNDQGVGMAADRVSTQKAVDKITSKVNNIVDKMNKQYEIIDHEFSEVETRLNMINNKIDDQI
QDIWAYNAELLVLLENQKTLDEHDANVNNLYNKVKRALGSNAMEDGKGCFELYHKCDDQCMETIRNGTYN
RRKYTEESRLERQKIEGVKLEAEGTYKILSIYSTVASSLVLAMGFAAFLFWAMSNGSCRCNICI

>QCA40733.1 hemagglutinin, partial [Influenza A virus]
MTILLVVTTSNADKICIGHQSTNSTETVDTLTETNVPVTQAKELLHTEHNGMLCATNLGRPLILDTCTIE
GLIYGNPSCDMLLEGREWSYIVERPSAVNGTCYPGNVENLEELRTLFSSSSSYQRVQLFPDSIWNVTYTG
TSKSCSDSFYRNMRWLTQKNGGYPIQDAQYTNNRGKDILFVWGIHHPPTDTTQTNLYTRTDTTTSVTTET
LDRTFKPLIGPRPLVNGLIGRINYYWSVLKPGQTLRVRSNGNLIAPWFGHVLSGESHGRILRTDLSSGNC
VVQCQTEKGGLNSTMPFHNISKYAFGTCPKYIGVKSLKLAIGLRNVHARSSRGLFGAIAGFIEGGWPGLV
AGWYGFQHSNDQGVGMAADRVSTQKAVDKITSKVNNIVDKMNKQYEIIDHEFSEVETRLNMINNKIDDQI
QDIWAYNAELLVLLENQKTLDEHDANVNNLYNKVKRALGSNAMEDGKGCFELYHKCDDQCMETIRNGTYN
RRKYTEESRLERQKIEGVKLEAEGTYKILSIYSTVASSLVLAMGFAAFLFWAMSNGSCRCNICI

>QMY23001.1 hemagglutinin [Influenza A virus]
METISLITILLAATVIYADKICIGYQSTNSTETVDTLTENNVPVTHAKELLHTEHNGMLCATSLGQPLIL
DTCTIEGLIYGNPSCDLSLEGKEWSYIVERPSAVNGLCYPGNVENLEELRSLFSSARSFQRVQIFPDTIW
NVSYDGTSTACSGSFYRSMRWLTRKDGNYPTQDAQYTNNQGKNILFMWGINHPPTDDTQRSLYTKTDTTT
SVATEEINRIFKPLIGPRPLVNGLMGRIDYYWSVLKPGQTLRIKSDGNLIAPWYGYVLSGESHGRILKTD
LKRGSCTVQCQTEKGGLNTTLPFQNVSKYAFGNCSKYIGIKSLKLAVGLRNVPSRSSRGLFGAIAGFIEG
GWSGLVAGWYGFQHSNDQGVGMAADRDSTQKAIDKITSKVNNIVDKMNKQYEIIDHEFSEVETRLNMINN
KIDDQIQDIWAYNAELLVLLENQKTLDEHDANVNNLYNKVKRALGSNAVEDGKGCFELYHKCDDQCMETI
RNGTYNRRKYQEESKLERQKIEGVKLESEGTYKILTIYSTVASSLVIAMGFAAFLFWAMSNGSCRCNICI

>QDQ72376.1 hemagglutinin, partial [Influenza A virus]

METVSLITILLIATVSNADKICIGYQSTNSTETVDTLTENNVPVTHAKELLHTEHNGMLCATSLGHPLIL

DTCTIEGLIYGNPSCDPLLGGREWSYIVERPSAVNGLCYPGNVENLEELRSLFSSSRSYQRIQIFPDTIW

NVSYSGTSKACSDSFYRSMRWLTQKNNAYPTQDAQYTNNQEKNILFMWGINHPPTDTVQTNLYTRTDTTT

SVATEEMNRIFKPLIGPRPLVNGLMGRINYYWSVLKPGQTLRIKSDGNLIAPWYGHILSGESHGRILKTD

LKRGSCTVQCQTEKGGLNTTLPFQNVSKYAFGNCSKYIGVKSLKLAVGLRNVPSRSSRGLFGAIAGFIEG

GWSGLVAGWYGFQHSNDQGVGIAADRDSTQKAIDKITSKVNNIVDKMNKQYEIIDHEFSEVETRLNMINN

KVDDQIQDIWAYNAELLVLLENQKTLDEHDANVNNLYNKVKRALGSNAVEDGKGCFELYHKCDDHCMETI

RNGTYNRRKYQEESKLERQKIEGVKLESEETYKILTIYSTVASSLVIAMGFAAFLFWAMSNGSCRCNICI

>QCT26977.1 hemagglutinin [Influenza A virus]

METASLMTILLVVTTSNADKICIGYQSTNSTETVDTLTENNVPVTHAKELLHTEHNGMLCATSLGHPLIL

DTCTIEGLIYGNPSCDLLLGGREWSYIVERPSAVNGLCYPGXXXXXXXXXXXXXXXXXXXXXXXXXXXXX

XXXXXXXXXXXXXXXXXXXXXXXXXXXXXXXXXXXXXXXXXXXXXXXXXXXXXXXXXXXXXXXXXXXXXX

XXXXXXXXXXXXXXXXXXXXXXXXXXXXXXXXXXXXXXXXXXXXXXXXXXXXXXXXXXXXXXXXXXXXXX

XXXXXXXXXXXXXXXXXXXXXXXXXXXXXXXXXXXXXXXXXXXXXXXXXXXXXXXXXXXXXXXXAGFIEG

GWSGLVAGWYGFQHSNDQGVGMAADRDSTQKAIDKITSKVNNIVDKMNKQYEIIDHEFSEVETRLNMINS

KIDDQIQDIWAYNAELLVLLENQKTLDEHDANVNNLYNKVKRALGSNAVEDGKGCFELYHKCDDQCMETI

RNGTYNRRKYQEESKLERQKIEGVKLESEGTYKILTIYSTVASSIVIAMGFAAFLFWAMSNGSCRCNICI

>QCT26765.1 hemagglutinin [Influenza A virus]

METASLMTILLVVTASNADKICIGYQSTNSTETVDTLTENNVPVTHAKELLHTEHNGMLCATSLGHPLIL

DTCTIEGLIYGNPSCDLLLGGREWSYIVERPSAVNGLCYPXXXXXXXXXXXXXXXXXXXXXXXXXXXXXX

XXXXXXXXXXXXXXXXXXXXXXXXXXXXXXXXXXXXXXXXXXXXXXXXXXXXXXXXXXXXXXXXXXXXXX

XXXXXXXXXXXXXXXXXXXXXXXXXXXXXXXXXXXXXXXXXXXXXXXXXXXXXXXXXXXXXXXXXXXXXX

XXXXXXXXXXXXXXXXXXXXXXXXXXXXXXXXXXXXXXXXXXXXXXXXXXXVPSRSSRGLFGAKAGFIEG

GWSGLVAGWYGFQHSNDQGVGMAADRDSTQKAIDKITSKVNNIVDKMNKQYEIIDHEFSEVETRLNMINS

KIDDQIQDIWAYNAELLVLLENQKTLDEHDANVNNLYNKVKRALGSNAVEDGKGCFELYHKCDDQCMETI

RNGTYNRRKYQEESKLERQKIEGVKLESEGTYKILTIYSTVASSIVIAMGFAAFLFWAMSNGSCRCNICI

>QCT26728.1 hemagglutinin [Influenza A virus]

METASLMTILLVVTASNADKICIGYQSTNSTETVDTLTENNVPVTHAKELLHTEHNGMLCATSLGHPLIL

DTCTIEGLIYGNPSCDLLLGGREWSYIVEXXXXXXXXXXXXXXXXXXXXXXXXXXXXXXXXXXXXXXXXX

XXXXXXXXXXXXXXXXXXXXXXXXXXXXXXXXXXXXXXXXXXXXXXXXXXXXXXXXXXXXXXXXXXXXXX

XXXXXXXXXXXXXXXXXXXXXXXXXXXXXXXXXXXXXXXXXXXXXXXXXXXXXXXXXXXXXXXXXXXXXX
XXXXXXXXXXXXXXXXXXXXXXXXXXXXXXXXXXXXXXXXXXXXXXXXXXXXXXXXXXXXXXXXAGFIEG
GWSGLVAGWYGFQHSNDQGVGMAADRDSTQKAIDKITSKVNNIVDKMNKQYEIIDHEFSEVETRLNMINS
KIDDQIQDIWAYNAELLVLLENQKTLDEHDANVNNLYNKVKRALGSNAVEDGKGCFELYHKCDDQCMETI
RNGTYNRRKYQEESKLERQKIEGVKLESEGTYKILTIYSTVASSIVIAMGFAAFLFWAMSNGSCRCNICI

>QCT25812.1 hemagglutinin [Influenza A virus]
METASLMTILLVVTASNADKICIGYQSTNSTETVDTLTENNVPVTHAKELLHTEHNGMLCATSLGHPLIL
DTCTIEGLIYGNPSCDLLLGGREWSYIVERPSXXXXXXXXXXXXXXXXXXXXXXXXXXXXXXXXXXXXXX
XXXXXXXXXXXXXXXXXXXXXXXXXXXXXXXXXXXXXXXXXXXXXXXXXXXXXXXXXXXXXXXXXXXXXX
XXXXXXXXXXXXXXXXXXXXXXXXXXXXXXXXXXXXXXXXXXXXXXXXXXXXXXXXXXXXXXXXXXXXXX
XXXXXXXXXXXXXXXXXXXXXXXXXXXXXXXXXXXXXXXXXXXXXXXXXXXXXXXXXXXXXXXXAGFIEG
GWSGLVAGWYGFQHSNDQGVGMAADRDSTQKAIDKITSKVNNIVDKMNKQYEIIDHEFSEVETRLNMINS
KIDDQIQDIWAYNAELLVLLENQKTLDEHDANVNNLYNKVKRALGSNAVEDGKGCFELYHKCDDQCMETI
RNGTYNRRKYQEESKLERQKIEGVKLESEGTYKILTIYSTVASSIVIAMGFAAFLFWAMSNGSCRCNICI

>AYW17103.1 hemagglutinin [Influenza A virus]
METVSLITILLVATVSYADKICIGYQSTNSTETVDTLTENNVPVTHAKELLHTEHNGMLCATSLGQPLIL
DTCTIEGLIYGNPSCDLSLEGREWSYIVERPSAVNGLCYPGNVENLEELRSLFSSARSYQRVQIFPDTIW
NVSYDGTSTACSGSFYRSMRWLTRKDGNYPTQDAQYTNNQGKNILFMWGINHPPTDDTQRSLYTRTDTTT
SVATEEINRIFKPLIGPRPLVNGLMGRIDYYWSVLKPGQTLRIKSDGNLIAPWYGYILSGESHGRILKTD
LKRGSCTVQCQTEKGGLNTTLPFQNVSKYAFGNCSKYIGIKSLKLAVGLRNVPSRSSRGLFGAIAGFIEG
GWSGLVAGWYGFQHSNDQGVGMAADRDSTQKAIDKITSKVNNIVDKMNKQYEIIDHEFSEVETRLNMINN
KIDDQIQDIWAYNAELLVLLENQKTLDEHDANVNNLYNKVKRALGSNAVEDGKGCFELYHKCNDQCMETI
RNGTYNKRKYQEESKLERQKIEGVKLESEGTYKILTIYSTVASSLVIAMGFAAFLFWAMSNGSCRCNICI

>AYW17101.1 hemagglutinin [Influenza A virus]
METISLIAILLVVTVSNADKICIGYQSTNSTETVDTLTENNVPVTHAKELLHTEHNGMLCATNLGHPLIL
NTCTIEGLIYGNPSCDQLLGGGKWSYIVERPSAVNGMCYPGNVENLEELRSLFSSASSYQRIQIFPDTIW
NVSYNGTSNACSDSFYRSMRWLTQKNNAYPIQDAQYTNNRGKSILFMWGINHPPTDTVQTNLYTRTDTTT
SVTTEDITRTFKPMIGPRPLVNGQQGRIDYYWSVLKPGQTLRIRSNGNLIAPWYGHILSGESHGRILKTD
LNSGNCVVQCQTERGGLNTTLPFHNVSKYAFGNCPKYVGVKSLKLAVGLRNVPARSSRGLFGAIAGFIEG
GWSGLVAGWYGFQHSNDQGVGMAADRESTQRAIDKITSKVNNIVDKMNKQYEIIDHEFSEVETRLNMINN
KIDDQIQDIWAYNAELLVLLENQKTLDEHDANVNNLYNKVKRALGSNAMEDGKGCFELYHKCDDQCMETI

RNGTYNRRKYKEEAKLERQKIEGVKLESEGTYKILTIYSTVASSLVIAMGFAAFLFWAMSNGSCRCNICI

>AYW17099.1 hemagglutinin [Influenza A virus]
METVSLITILLVATVSNADKICIGYQSTNSTETVDTLTENNVPVTHAKELLHTEHNGMLCATSLGQPLIL
DTCTIEGLIYGNPSCDLSLEGREWSYIVERPSAVNGLCYPGNVENLEELRSLFSSARSYQRLQIFPDTIW
NVSYDGTSTACSGSFYRSMRWLTRKNGEYPTQDVQYTNNQGKNILFMWGIHHPPTDTTQRDLYTRTDTTT
SVATEEINRIFKPLIGPRPLVNGLMGRIDYYWSILKPGQTLRIKSDGNLIAPWYGHILSGESHGRILKTD
LKKGSCTVQCQTEKGGLDTTLPFQNVSKYAFGNCSKYIGIKSLKLAVGLRNVPSRSSRGLFGAIAGFIEG
GWSGLVAGWYGFQHSNDQGVGMAADRDSTQKAIDKITSKVNNIVDKMNKQYEIIDHEFSEVETRLNMINN
KIDDQIQDIWAYNAELLVLLENQKTLDEHDANVNNLYNKVKRALGSNAVEDGKGCFELYHKCDNQCMETI
RNGTYNRRKYQEESKLERQKIEGVKLESEGTYKILTIYSTVASSLVIAMGFAAFLFWAMSNGSCRCNICI

>QXI62278.1 hemagglutinin [Influenza A virus]
METVSLITILLVATVSYADKICIGYQSTNSTETVDTLTENNVPVTHAKELLHTEHNGMLCATSLGQPLIL
DTCTIEGLIYGNPSCDLSLEGREWSYIVERPSAVNGLCYPGNVENLEELRSLFSSARSYQRVQIFPDTIW
NVSYDGTSTACSGSFYRSMRWLTRKDGNYPTQDAQYTNNQGKNILFMWGINHPPTDDTQRSLYTRTDTTT
SVATEEINRIFKPLIGPRPLVNGLMGRIDYYWSVLKPGQTLRIKSDGNLIAPWYGYILSGESHGRILKTD
LKRGSCTVQCQTEKGGLNTTLPFQNVSKYAFGNCSKYIGIKSLKLAVGLRNVPSRSSRGLFGAIAGFIEG
GWSGLVAGWYGFQHSNDQGVGMAADRDSTQKAIDKITSKVNNIVDKMNKQYEIIDHEFSEVETRLNMINN
KIDDQIQDIWAYNAELLVLLENQKTLDEHDANVNNLYNKVKRALGSNAAEDGKGCFELYHKCNDQCMETI
RNGTYNRKKYQEESKLERQKIEGVKLESEGTYKILTIYSTVASSLVIAMGFAAFLFWAMSNGSCRCNICI

>QPG87084.1 hemagglutinin [Influenza A virus]
MEAISLMILLIVVTTSNADKICIGHQSTNSTETVDTLTESNIPVTQAKELLHTEHNGMLCATNLGRPLIL
DTCTVEGLIYGNPSCDLLLGGREWSYIVERPSAVNGTCYPGNVENLEELRMLFSSASSYQRVQIFPDAIW
NVTYDGTSKSCSNSFYRNMRWLTQKNGNYPIQDAQYINNRGKDILFIWGIHHPPTDTAQTNLYTRTDTTT
SVTTEDLDRTFKPLIGPRPLVNGLIGRINYYWSVLKPGQTLRVRSNGNLIAPWYGHVLSGESHGRILKTD
LKSGNCVVQCQTEKGGLNSTLPFHNISKYAFGTCPKYIGVKSLKLAIGLRNVPAKSSRGLFGAIAGFIEG
GWPGLVAGWYGFQHSNDQGVGMAADRSSTQKAVDKITSKVNNIIDKMNRQYEIIDHEFSEIETRLNMINN
KIDDQIQDVWAYNAELLVLLENQKTLDEHDANVNNLYNKVKRALGSNAMEDGKGCFELYHKCDDQCMETI
RNGTYNRRKYTEESRLERQKIEGVKLESEGTYKILTIYSTAASSLVLAMGVAAFLFWAMSNGSCRCNICI

>QLI63414.1 hemagglutinin [Influenza A virus]
METVSLITILLVATVSNADKICIGYQSTNSTETVDTLTENNVPVTHAKELLHTEHNGMLCATSLGQPLIL

DTCTIEGLIYGNPSCDLSLEGREWSYIVERPSAVNGLCYPGNVENLEELRSLFSSARSYQRIQIFPDTIW
NVSYDGTSTACSGSFYRSMRWLTRKNGDYPTQDAQYTNNQGKNILFMWGINHPPTDDTQRNLYTRTDTTT
SVATEEINRIFKPLIGPRPLVNGLMGRIDYYWSVLKPGQTLRIKSDGNLIAPWYGHILSGESHGRILKTD
LKRGSCTVQCQTEKGGLNTTLPFQNVSKYAFGNCSKYIGIKSLKLAVGLRNVPSRSSRGLFGAIAGFIEG
GWSGLVAGWYGFQHSNDQGVGMAADRDSTQKAIDKITSKVNNIVDKMNKQYEIIDHEFSEVETRLNMINN
KIDDQIQDIWAYNAELLVLLENQKTLDEHDANVNNLYNKVKRALGSNAVEDGKGCFELYHKCNDQCMETI
RNGTYNRRKYQEESKLERQKIEGVKLESEGTYKILTIYSTVASSLVIAMGFAAFLFWAMSNGSCRCNICI

>QTD79673.1 hemagglutinin [Influenza A virus]
METVSLITILLVATVSNADKICIGYQSTNSTETVNTLTENNVPVTHAKELLHTEHNGMLCATSLGQPLIL
DTCTIEGLIYGNPSCDLSLEGREWSYIVERPSAINGLCYPGNVENLEELRSLFSSARSYQRIQIFPDTIW
NVSYDGTSTACSNSFYRSMRWLTRKDGNYPTQDAQYTNNQGKNILFMWGINHPPTDETQRNLYTRTDTTT
SVATEEINRIFKPLIGPRPLVNGLMGRIDYYWSVLKPGQTLRIKSDGNLIAPWYGHILSGESHGRILKTD
LKRGSCTVQCQTEKGGLNTTLPFQNVSKYAFGNCSKYIGIKSLKLAVGLRNVPSRSSRGLFGAIAGFIEG
GWSGLVAGWYGFQHSNDQGVGMAADRDSTQKAIDKITSKVNNIVDKMNKQYEIIDHEFSEVETRLNMINN
KIDDQIQDIWAYNAELLVLLENQKTLDEHDANVNNLYNKVKRALGSNAVEDGKGCFELYHKCNDQCMETI
RNGTYNRKKYQEESKLERQRIDGVKLESEGTYKILTIYSTVASSLVIAMGFAAFLFWAMSNGSCRCNICI

>QQX99376.1 hemagglutinin [Influenza A virus]
MEAISLMTILLAVTTSNADKICIGHQSTNSTETVDTLTETNVPVTQAKELLHTEHNGMLCATNLGRPLIL
DTCTIEGLIYGNPSCDMLLGGREWAYIVERPSAVNGTCYPGNVENLEELRTFFSSSSSYQRVQLFPDSIW
NVTYTGTSKSCSGSFYRNMRWLTQKDGSYPIQDAQYTNNRGKDILFVWGIHHPPTDTAQTNLYTRTDTTT
SVTTETLDRTFKPLIGPRPLVNGLIGRINYYWSVLKPGQTLRVRSNGNLIAPWFGHVLSGESHGRILRTD
LSSGNCVVQCQTEKGGLNSTMPFHNISKYAFGTCPKYIGVKSLKLAIGLRNVHAKSSRGLFGAIAGFIEG
GWPGLVAGWYGFQHSNDQGVGMAADRVSTQKAVDKITSKVNNIVDKMNKQYEIIDHEFSEVENRLNMINN
KIDDQIQDIWAYNAELLVLLENQKTLDEHDANVNNLYNKVKRALGSNAMEDGKGCFELYHKCDDQCMETI
RNGTYNRRKYTEESRLEKQRIEGVKLEAEGTYKILSIYSTVASSLVLAMGFAAFLFWAMSNGSCRCNICI

>QQX99364.1 hemagglutinin [Influenza A virus]
MEAISLMTILLAVTTSNADKICIGHQSTNSTETVDTLTETNVPVTQAKELLHTEHNGMLCATNLGRPLIL
DTCTIEGLIYGNPSCDMLLGGREWAYIVERPSAVNGTCYPGNVENLEELRTFFSSSSSYQRVQLFPDSIW
NVTYTGTSKSCSGSFYRNMRWLTQKDGSYPIQDAQYTNNRGKDILFVWGIHHPPTDTAQTNLYTRTDTTT
SVTTETLDRTFKPLIGPRPLVNGLIGRINYYWSVLKPGQTLRVRSNGNLIAPWFGHVLSGESHGRILRTD
LSSGNCVVQCQTEKGGLNSTMPFHNISKYAFGTCPKYIGVKSLKLAIGLRNVHAKSSRGLFGAIAGFIEG

GWPGLVAGWYGFQHSNDQGVGMAADRVSTQKAVDKITSKVNNIVDKMNKQYEIIDHEFSEVENRLNMINN
KIDDQIQDIWAYNAELLVLLENQKTLDEHDANVNNLYNKVKRALGSNAMEDGKGCFELYHKCDDQCMETI
RNGTYNRRKYTEESRLEKQRIEGVKLEAEGTYKILSIYSTVASSLVLAMGFAAFLFWAMSNGSCRCNICI

>QQX99352.1 hemagglutinin [Influenza A virus]
MEAISLMTILLVVTTSNADKICIGHQSTNSTETVDTLTETNVPVTQAKELLHTEHNGMLCATNLGRPLIL
DTCTIEGLIYGNPSCDMLLGGREWAYIVERPSAVNGTCYPGNVENLEELRTFFSSSSSYQRVQLFPDSIW
NVTYTGTSKSCSGSFYRNMRWLTQKDGSYPIQDAQYTNNRGKDILFVWGIHHPPTDTAQTNLYTRTDTTT
SVTTETLDRTFKPLIGPRPLVNGLIGRINYYWSVLKPGQTLRVRSNGNLIAPWFGHVLSGESHGRILRTD
LSSGNCVVQCQTEKGGLNSTMPFHNISKYAFGTCPKYIGVKSLKLAIGLRNVHAKSSRGLFGAIAGFIEG
GWPGLVAGWYGFQHSNDQGVGMAADRVSTQKAVDKITSKVNNIVDKMNKQYEIIDHEFSEVENRLNMINN
KIDDQIQDIWAYNAELLVLLENQKTLDEHDANVNNLYNKVKRALGSNAMEDGKGCFELYHKCDDQCMETI
RNGTYNRRKYTEESRLEKQRIEGVKLEAEGTYKILSIYSTVASSLVLAMGFAAFLFWAMSNGSCRCNICI

>QQX99340.1 hemagglutinin [Influenza A virus]
MEAISLMTILLAVTTSNADKICIGHQSTNSTETVDTLTETNVPVTQAKELLHTEHNGMLCATNLGRPLIL
DTCTIEGLIYGNPSCDMLLGGREWAYIVERPSAVNGTCYPGNVENLEELRTFFSSSSSYQRVQLFPDSIW
NVTYTGTSKSCSGSFYRNMRWLTQKDGSYPIQDAQYTNNRGKDILFVWGIHHPPTDTAQTNLYTRTDTTT
SVTTETLDRTFKPLIGPRPLVNGLIGRINYYWSVLKPGQTLRVRSNGNLIAPWFGHVLSGESHGRILRTD
LSSGNCVVQCQTEKGGLNSTMPFHNISKYAFGTCPKYIGVKSLKLAIGLRNVHAKSSRGLFGAIAGFIEG
GWPGLVAGWYGFQHSNDQGVGMAADRVSTQKAVDKITSKVNNIVDKMNKQYEIIDHEFSEIENRLNMINN
KIDDQIQDIWAYNAELLVLLENQKTLDEHDANVNNLYNKVKRALGSNAMEDGKGCFELYHKCDDQCMETI
RNGTYNRRKYTEESRLEKQRIEGVKLEAEGTYKILSIYSTVASSLVLAMGFAAFLFWAMSNGSCRCNICI

>QQX99328.1 hemagglutinin [Influenza A virus]
MEAISLMTILLAVTTSNADKICIGHQSTNSTETVDTLTETNVPVTQAKELLHTEHNGMLCATNLGRPLIL
DTCTIEGLIYGNPSCDMLLGGREWAYIVERPSAVNGTCYPGNVENLEELRTFFSSSSSYQRVQLFPDSIW
NVTYTGTSKSCSGSFYRNMRWLTQKDGSYPIQDAQYTNNRGKDILFVWGIHHPPTDTAQTNLYTRTDTTT
SVTTETLDRTFKPLIGPRPLVNGLIGRINYYWSVLKPGQTLRVRSNGNLIAPWFGHVLSGESHGRILRTD
LSSGNCVVQCQTEKGGLNSTMPFHNISKYAFGTCPKYIGVKSLKLAIGLRNVHAKSSRGLFGAIAGFIEG
GWPGLVAGWYGFQHSNDQGVGMAADRVSTQKAVDKITSKVNXIVDKMNKQYEIIDHEFSEVENRLNMINN
KIDDQIQDIWAYNAELLVLLENQKTLDEHDANVNNLYNKVKRALGSNAMEDGXGCFELYHKCDDQCMETI
RNGTYNRRKYTEESRLXKQRIEGVKLEAEGTYKILSIYSTVASSLVLAMGFAAFLFWAMSNGSCRCNICI

>QQX99316.1 hemagglutinin [Influenza A virus]

MEAISLMTILLVVTTSDADKICIGHQSTNSTETVDTLTETNVPVTQAKELLHTEHNGMLCATKLGRPLIL

DTCTIEGLIYGNPSCDMLLGGREWAYIVERPSATNGTCYPGNVENLEELRTFFSSSSSYQRVQLFPDSIW

NVTYTGTSKSCSDSFYRNMRWLTQKNGNYPIQDAQYTNNRGKDILFVWGIHHPPTDTAQTNLYTRTDTTT

SVTTETLDRTFKPLIGPRPLVNGLIGRINYYWSVLKPGQTLRVRSNGNLIAPWFGHVLSGESHGRILRTN

LSSGNCVVQCQTEKGGLNSTMPFHNISKYAFGTCPKYIGVKSLKLAIGLRNVHARTSRGLFGAIAGFIEG

GWPGLVAGWYGFQHSNDQGVGMAADRVSTQKAVDKITSKVNNIVDKMNKQYEIIDHEFSEIETRLNMINN

KIDDQIQDIWAYNAELLVLLENQKTLDEHDANVNNLYNKVKRALGSNAMEDGKGCFELYHKCDDQCMETI

RNGTYNRRKYTEESRLEKQKIEGVKLEAEGTYKILSIYSTVASSLVLAMGFAAFLFWAMSNGSCRCNICI

>QQX99304.1 hemagglutinin [Influenza A virus]

MEAISLMTILLVVTTSDADKICIGHQSTNSTETVDTLTETNVPVTQAKELLHTXHNGMLCATKLGRPLIL

DTCTIEGLIYGNPSCDMLLGGREWAYIVERPSATNGTCYPGNVENLEELRTFFSSSSSYQRVQLFPDSIW

NVTYTGTSKSCSDSFYRNMRWLTQKNGNYPIQDAQYTNNRGKDILFVWGIHHPPTDTAQTNLYTRTDTTT

SVTTETLDRTFKPLIGPRPLVNGLIGRINYYWSVLKPGQTLRVRSNGNLIAPWFGHVLSGESHGRILRTN

LSSGNCVVQCQTEKGGLNSTMPFHNISKYAFGTCPKYIGVKSLKLAIGLRNVHARTSRGLFGAIAGFIEG

GWPGLVAGWYGFQHSNDQGVGMAADRVSTQKAVDKITSKVNNIVDKMNKQYEIIDHEFSEIETRLNMINN

KIDDQIQDIWAYNAELLVLLENQKTLDEHDANVNNLYNKVKRALGSNAMEDGKGCFELYHKCDDQCMETI

RNGTYNRRKYTEESRLEXQKIEGVKLEAEGTYKILSIYSTVASSLVLAMGFAAFLFWAMSNGSCRCNICI

>QQX99292.1 hemagglutinin [Influenza A virus]

MEAISLMTILLVVTTSDADKICIGHQSTNSTETVDTLTETNVPVTQAKELLHTXHNGMLCATKLGRPLIL

DTCTIEGLIYGNPSCDMLLGGREWAYIVERPSATNGTCYPGNVENLEELRTFFSSSSSYQRVQLFPDSIW

NVTYTGTSKSCSDSFYRNMRWLTQKNGNYPIQDAQYTNNRGKDILFVWGIHHPPTDTAQTNLYTRTDTTT

SVTTETLDRTFKPLIGPRPLVNGLIGRINYYWSVLKPGQTLRVRSNGNLIAPWFGHVLSGESHGRILRTN

LSSGNCVVQCQTEKGGLNSTMPFHNISKYAFGTCPKYIGVKSLKLAIGLRNVHARTSRGLFGAIAGFIEG

GWPGLVAGWYGFQHSNDQGVGMAADRVSTQKAVDKITSKVNNIVDKMNKQYEIIDHEFSEIETRLNMINN

KIDDQIQDIWAYNAELLVLLENQKTLDEHDANVNNLYNKVKRALGSNAMEDGKGCFELYHKCDDQCMETI

RNGTYNRRKYTEESRLEXQKIEGVKLEAEGTYKILSIYSTVASSLVLAMGFAAFLFWAMSNGSCRCNICI

>QQX99280.1 hemagglutinin [Influenza A virus]

MEAISLMTILLVVTTTSNADKICIGHQSTNSTETVDTLTETNVPVTQAKELLHTEHNGMLCATKLGRPLI

LDTCTIEGLIYGNPSCDMLLGGREWAYIVERPSAVNGTCYPGNVENLEELRTFFSSSSSYQRIQLFPDSI

WNVTYTGTSKSCSDSFYRNMRWLTQKNGNYPIQDAQYTNNRGKDILFVWGIHHPPTDTAQTNLYTRTDTT

TSVTTETLDRTFKPLIGPRPLVNGLIGRINYYWSVLKPGQTLRVRSNGNLIAPWFGHVLSGESHGRILRT
NLSSGNCVVQCQTEKGGLNSTMPFHNISKYAFGTCPKYIGVKSLKLAIGLRNVHARSSRGLFGAIAGFIE
GGWPGLVAGWYGFQHSNDQGVGMAADRVSTQKAVDKITSKVNNIVDKMNKQYEIIDHEFSEIETRLNMIN
NKIDDQIQDIWAYNAELLVLLENQKTLDEHDANVNNLYNKVKRALGSNAMEDGKGCFELYHKCDDQCMET
IRNGTYNRRKYTEESRLERQRIEGVKLEAEGTYKILSIYSTVASSLVLAMGFAAFLFWAMSNGSCRCNIC
I

>QQX99268.1 hemagglutinin [Influenza A virus]
MEAISLMTILLVVTTTSNADKICIGHQSTNSTETVDTLTETNVPVTQAKELLHTEHNGMLCATKLGRPLI
LDTCTIEGLIYGNPSCDMLLGGREWAYIVERPSAVNGTCYPGNVENLEELRTFFSSSSSYQRIQLFPDSI
WNVTYTGTSKSCSDSFYRNMRWLTQKNGNYPIQDAQYTNNRGKDILFVWGIHHPPTDTAQTNLYTRTDTT
TSVTTETLDRTFKPLIGPRPLVNGLIGRINYYWSVLKPGQTLRVRSNGNLIAPWFGHVLSGESHGRILRT
NLSSGNCVVQCQTEKGGLNSTMPFHNISKYAFGTCPKYIGVKSLKLAIGLRNVHARSSRGLFGAIAGFIE
GGWPGLVAGWYGFQHSNDQGVGMAADRVSTQKAVDKITSKVNNIVDKMNKQYEIIDHEFSEIETRLNMIN
NKIDDQIQDIWAYNAELLVLLENQKTLDEHDANVNNLYNKVKRALGSNAMEDGKGCFELYHKCDDQCMET
IRNGTYNRRKYTEESRLERQRIEGVKLEAEGTYKILSIYSTVASSLVLAMGFAAFLFWAMSNGSCRCNIC
I

>QQX99256.1 hemagglutinin [Influenza A virus]
MEAISLMTILLVVTTTSNADKICIGHQSTNSTETVDTLTETNVPVTQAKELLHTEHNGMLCATKLGRPLI
LDTCTIEGLIYGNPSCDMLLGGREWAYIVERPSAVNGTCYPGNVENLEELRTFFSSSSSYQRIQLFPDSI
WNVTYTGTSKSCSDSFYRNMRWLTQKNGNYPIQDAQYTNNRGKDILFVWGIHHPPTDTAQTNLYTRTDTT
TSVTTETLDRTFKPLIGPRPLVNGLIGRINYYWSVLKPGQTLRVRSNGNLIAPWFGHVLSGESHGRILRT
NLSSGNCVVQCQTEKGGLNSTMPFHNISKYAFGTCPKYIGVKSLKLAIGLRNVHARSSRGLFGAIAGFIE
GGWPGLVAGWYGFQHSNDQGVGMAADRVSTQKAVDKITSKVNNIVDKMNKQYEIIDHEFSEIETRLNMIN
NKIDDQIQDIWAYNAELLVLLENQKTLDEHDANVNNLYNKVKRALGSNAMEDGKGCFELYHKCDDQCMET
IRNGTYNRRKYTEESRLERQRIEGVKLEAEGTYKILSIYSTVASSLVLAMGFAAFLFWAMSNGSCRCNIC
I

>QQX99244.1 hemagglutinin [Influenza A virus]
MEAISLMTILLVVTTSDADKICIGHQSTNSTETVDTLTETNVPVTQAKELLHTXHNGMLCATKLGRPLIL
DTCTIEGLIYGNPSCDMLLGGREWAYIVERPSATNGTCYPGNVENLEELRTFFSSSSSYQRVQLFPDSIW
NVTYTGTSKSCSDSFYRNMRWLTQKNGNYPIQDAQYTNNRGKDILFVWGIHHPPTDTAQTNLYTRTDTTT
SVTTETLDRTFKPLIGPRPLVNGLIGRINYYWSVLKPGQTLRVRSNGNLIAPWFGHVLSGESHGRILRTN

LSSGNCVVQCQTEKGGLNSTMPFHNISKYAFGTCPKYIGVKSLKLAIGLRNVHARTSRGLFGAIAGFIEG
GWPGLVAGWYGFQHSNDQGVGMAADRVSTQKAVDKITSKVNNIVDKMNKQYEIIDHEFSEIETRLNMINN
KIDDQIQDIWAYNAELLVLLENQKTLDEHDANVNNLYNKVKRALGSNAMEDGKGCFELYHKCDDQCMETI
RNGTYNRRKYTEESRLEKQKIEGVKLEAEGTYKILSIYSTVASSLVLAMGFAAFLFWAMSNGSCRCNICI

>QAU21889.1 hemagglutinin [Influenza A virus]
METVSLITILLVATVSNADKICIGYQSTNSTETVDTLTENNVPVTHAKELLHTEHNGMLCATSLGQPLIL
DTCTIEGLIYGNPSCDLSLEGREWSYIVERPSAVHGLCYPGKVEDLEELRSLFSSARSYQRIQIFPDTIW
NVSYDGTSTACSGSFYRSMRWLTRKNGDYPIQDAQYTNNQGKNILFMWGINHPPTDETQRGLYTRTDTTT
SVATEEINRIFKPLIGPRPLVNGLMGRINYYWSVLKPGQTLRIKSDGNLIAPWYGHILSGESHGRILKTD
LKKGSCTVQCQTEKGGLNTTLPFQNVSKYAFGNCSKYIGIKSLKLAVGLRNVPSRSSRGLFGAIAGFIEG
GWSGLVAGWYGFQHSNDQGVGMAADRDSTQKAIDKITSKVNNIVDKMNKQYEIIDHEFSEVETRLNMINN
KIDDQIQDIWAYNAELLVLLENQKTLDEHDANVNNLYNKVKRALGSNAVEDGKGCFELYHKCDDQCMETI
RNGTYNRREYQEESKLERQKIEGVKLESEGTYKILTIYSTVASSLVIAMGFAAFLFWAMSNGSCRCNICI

>QAU21888.1 hemagglutinin [Influenza A virus]
METVSLITILLVATVSNADKICIGYQSTNSTETVDTLTENNVPVTHAKELLHTEHNGMLCATSLGHPLIL
DTCTIEGLIYGNPSCDPLLGGREWSYIVERPSAVNGLCYPGNVENLEELRSLFSSSRSYQRIQIFPDTIW
NVSYNGTSKACSDSFYRSMRWLTQKNNAYPTQDAQYTNNQGKNILFMWGINHPPTDTAQTNLYTRTDTTT
SVATEEINRIFKPLIGPRPLVNGLMGRINYYWSVLKPGQTLRIKSDGNLIAPWYGHILSGESHGRILKTD
LKRGSCTVQCQTEKGGLNTTLPFQNVSKYAFGNCSKYIGVKSLKLAVGLRNVPSRSSRGLFGAIAGFIEG
GWSGLVAGWYGFQHSNDQGVGMAADRDSTQKAIDKITSKVNNIVDKMNKQYEIIDHEFSEVETRLNMINN
KVDDQIQDIWAYNAELLVLLENQKTLDEHDANVNNLYNKVKRALGSNAVEDGKGCFELYHKCDDHCMETI
RNGTYNRRKYQEESKLERQKIEGVKLESEETYKILTIYSTVASSLVIAMGFAAFLFWAMSNGSCRCNICI

>QAU21887.1 hemagglutinin [Influenza A virus]
METVSLITILLVATVSNADKICIGYQSTNSTETVDTLTENNVPVTHAKELLHTEHNGMLCATSLGQPLIL
DTCTIEGLIYGNPSCDLSLEGREWSYIVERPSAVHGLCYPGKVEDLEELRSLFSSARSYQRIQIFPDTIW
NVSYDGTSTACSGSFYRSMRWLTRKNGDYPIQDAQYTNNQGKNILFMWGINHPPTDETQRGLYTRTDTTT
SVATEEINRIFKPLIGPRPLVNGLMGRINYYWSVLKPGQTLRIKSDGNLIAPWYGHILSGESHGRILKTD
LKKGSCTVQCQTEKGGLNTTLPFQNVSKYAFGNCSKYIGIKSLKLAVGLRNVPSRSSRGLFGAIAGFIEG
GWSGLVAGWYGFQHSNDQGVGMAADRDSTQKAIDKITSKVNNIVDKMNKQYEIIDHEFSEVETRLNMINN
KIDDQIQDIWAYNAELLVLLENQKTLDEHDANVNNLYNKVKRALGSNAVEDGKGCFELYHKCDDQCMETI
RNGTYNRREYQEESKLERQKIEGVKLESEGTYKILTIYSTVASSLVIAMGFAAFLFWAMSNGSCRCNICI

>QAU21886.1 hemagglutinin [Influenza A virus]

METVSLITILLVATVSNADKICIGYQSTNSTETVDTLTESNVPVTHAKELLHTEHNGMLCATSLGHPLIL

DTCTIEGLIYGNPSCDPLLEGREWSYIVERPSAVNGLCYPGNVENLEELRSLFSSSRSYQRIQIFPDTIW

NVSYNGTSKACSDSFYRSMRWLTQKNNDYPTQDAQYTNNQGKNILFMWGINHPPTDTAQTNLYTRTDTTT

SVATEEMNRIFKPLIGPRPLVNGLMGRINYYWSVLKPGQTLRIKSDGNLIAPWYGHILSGESHGRILKTD

LKRGSCTVQCQTEKGGLNTTLPFQNVSKYAFGNCSKYIGVKSLKLAVGLRNVPSRSSRGLFGAIAGFIEG

GWSGLVAGWYGFQHSNDQGVGMAADRDSTQKAIDKITSKVNNIVDKMNKQYEIIDHEFSEVETRLNMINN

KVDDQIQDIWAYNAELLVLLENQKTLDEHDANVNNLYNKVKRALGSNAVEDGKGCFELYHKCDDHCMETI

RNGTYNRRKYQEESKLERQKIEGVKLESEETYKILTIYSTVASSLVIAMGFAAFLFWAMSNGSCRCNICI

>QAU21885.1 hemagglutinin [Influenza A virus]

METVSLITILLVATVSNADKICIGYQSTNSTETVDTLTENNVPVTHAKELLHTEHNGMLCATSLGQPLIL

DTCTIEGLIYGNPSCDLSLEGREWSYIVERPSAVHGLCYPGKVEDLEELRSLFSSARSYQRIQIFPDTIW

NVSYDGTSTACSGSFYRSMRWLTRKNGNYPIQDAQYTNNQGKNILFMWGINHPPTDETQRGLYTRTDTTT

SVATEEINRIFKPLIGPRPLVNGLMGRINYYWSVLKPGQTLRIKSDGNLIAPWYGHILSGESHGRILKTD

LKKGSCTVQCQTEKGGLNTTLPFQNVSKYAFGNCSKYIGIKSLKLAVGLRNVPSRSSRGLFGAIAGFIEG

GWSGLVAGWYGFQHSNDQGVGMAADRDSTQKAIDKITSKVNNIVDKMNKQYEIIDHEFSEVETRLNMINN

KIDDQIQDIWAYNAELLVLLENQKTLDEHDANVNNLYNKVKRALGSNAVEDGKGCFELYHKCDDQCMETI

RNGTYNRRKYQEESKLERQKIEGVKQESEGTYKILTIYSTVASSLVIAMGFAAFLFWAMSNGSCRCNICI

>QAU21884.1 hemagglutinin [Influenza A virus]

METVSLITILLVATVSNADKICIGYQSTNSTETVDTLTENNVPVTHAKELLHTEHNGMLCATSLGQPLIL

DTCTIEGLIYGNPSCDLSLEGREWSYIVERPSAVHGLCYPGKVEDLEELRSLFSSARSYQRIQIFPDTIW

NVSYDGTSTACSGSFYRSMRWLTRKNGDYPIQDAQYTNNQGKNILFMWGINHPPTDETQRGLYTRTDTTT

SVATEEINRIFKPLIGPRPLVNGLMGRINYYWSVLKPGQTLRIKSDGNLIAPWYGHILSGESHGRILKTD

LKKGSCTVQCQTEKGGLNTTLPFQNVSKYAFGNCSKYIGIKSLKLAVGLRNVPSRSSRGLFGAIAGFIEG

GWSGLVAGWYGFQHSNDQGVGMAADRDSTQKAIDKITSKVNNIVDKMNKQYEIIDHEFSEVETRLNMINN

KIDDQIQDIWAYNAELLVLLENQKTLDEHDANVNNLYNKVKRALGSNAVEDGKGCFELYHKCDDQCMETI

RNGTYNRREYQEESKLERQKIEGVKLESEGTYKILTIYSTVASSLVIAMGFAAFLFWAMSNGSCRCNICI

>QAU21883.1 hemagglutinin [Influenza A virus]

METVSLITILLVATVSNADKICIGYQSTNSTETVDTLTENNVPVTHAKELLHTEHNGMLCATSLGQPLIL

DTCTIEGLIYGNPSCDLSLEGREWSYIVERPSAVHGLCYPGKVEDLEELRSLFSSARSYQRIQIFPDTIW

NVSYDGTSTACSGSFYRSMRWLTRKNGNYPIQDAQYTNNQGKNILFMWGINHPPTDETQRGLYTRTDTTT
SVATEEINRIFKPLIGPRPLVNGLMGRINYYWSVLKPGQTLRIKSDGNLIAPWYGHILSGESHGRILKTD
LKKGSCTVQCQTEKGGLNTTLPFQNVSKYAFGNCSKYIGIKSLKLAVGLRNVPSRSSRGLFGAIAGFIEG
GWSGLVAGWYGFQHSNDQGVGMAADRDSTQKAIDKITSKVNNIVDKMNKQYEIIDHEFSEVETRLNMINN
KIDDQIQDIWAYNAELLVLLENQKTLDEHDANVNNLYNKVKRALGSNAVEDGKGCFELYHKCDDQCMETI
RNGTYNRRKYQEESKLERQKIEGVKLESEGTYKILTIYSTVASSLVIAMGFAAFLFWAMSNGSCRCNICI

>QAU21882.1 hemagglutinin [Influenza A virus]
METVSLITILLVATVSNADKICIGYQSTNSTETVDTLTENNVPVTHAKELLHTEHNGMLCATSLGHPLIL
DTCTIEGLIYGNPSCDPLLGGREWSYIVERPSAVNGLCYPGNVENLEELRSLFSSSRSYQRIQIFPDTIW
NVSYSGTSRACSDSFYRSMRWLTQKNNAYPTQDAQYTNNQEKNILFMWGINHPPTDTAQTNLYTRTDTTT
SVATEEINRIFKPLIGPRPLVNGLMGRINYYWSVLKPGQTLRIKSDGNLIAPWYGHILSGESHGRILKTD
LKRGSCTVQCQTEKGGLNTTLPFQNVSKYAFGNCSKYIGVKSLKLAVGLRNVPSRSSRGLFGAIAGFIEG
GWSGLVAGWYGFQHSNDQGVGMAADRDSTQKAIDKITSKVNNIVDKMNKQYEIIDHEFSEVETRLNMINN
KVDDQIQDIWAYNAELLVLLENQKTLDEHDANVNNLYNKVKRALGSNAVEDGKGCFELYHKCDDHCMETI
RNGTYNRRKYQEESKLERQKIEGVKLESEETYKILTIYSTVASSLVIAMGFAAFLFWAMSNGSCRCNICI

>QAU21881.1 hemagglutinin [Influenza A virus]
METVSLITILLVATVSNADKICIGYQSTNSTETVDTLTENNVPVTHAKELLHTEHNGMLCATSLGQPLIL
DTCTIEGLIYGSPSCDLPLEGREWSYIVERPSAVNGLCYPGNVENLEELRSLFSSARSYQRIQIFPDTIW
NVSYSGTSKACSDSFYRSMRWLTQKNGNYPTQDAQYTNNQGKNILFMWGINHPPTDDTQRNLYTRTDTTT
SVATEEINRVFKPLIGPRPLVNGLMGRIDYYWSVLKPGQTLRIKSDGNLIAPWYGHILSGESHGRILKTD
LKRGSCTVQCQTEKGGLNTTLPFQNVSKYAFGNCSKYIGIKSLKLAVGLRNVPSRSSRGLFGAIAGFIEG
GWSGLVAGWYGFQHSNDQGVGMAADRDSTQKAIDKITSKVNNIVDKMNKQYEIIDHEFSEVETRLNMINN
KIDDQIQDIWAYNAELLVLLENQKTLDEHDANVNNLYNKVKRALGSNAMEDGKGCFELYHKCNDQCMETI
RNGTYNRRKYQEESKLERQKIEGVKLESEGTYKILTIYSTVASSLVIAMGFAAFLFWAMSNGSCRCNICI

>QAU21880.1 hemagglutinin [Influenza A virus]
METVSLITILLVATVSNADKICIGYQSTNSTETVDTLTENNVPVTHAKELLHTEHNGMLCATSLGQPLIL
DTCTIEGLIYGNPSCDLSLEGREWSYIVERPSAVHGLCYPGKVEDLEELRSLFSSARSYQRIQIFPDTIW
NVSYDGTSTACSGSFYRSMRWLTRKNGNYPIQDAQYTNNQGKNILFMWGINHPPTDETQRGLYTRTDTTT
SVATEEINRIFKPLIGPRPLVNGLMGRINYYWSVLKPGQTLRIKSDGNLIAPWYGHILSGESHGRILKTD
LKKGSCTVQCQTEKGGLNTTLPFQNVSKYAFGNCSKYIGIKSLKLAVGLRNVPSRSSRGLFGAIAGFIEG
GWSGLVAGWYGFQHSNDQGVGMAADRDSTQKAIDKITSKVNNIVDKMNKQYEIIDHEFSEVETRLNMINN

KIDDQIQDIWAYNAELLVLLENQKTLDEHDANVNNLYNKVKRALGSNAVEDGKGCFELYHKCDDQCMETI
RNGTYNRRKYQEESKLERQKIEGVKQESEGTYKILTIYSTVASSLVIAMGFAAFLFWAMSNGSCRCNICI

>QAU21879.1 hemagglutinin [Influenza A virus]
METVSLITILVVATVSNADKICIGYQSTNSTETVDTLTENNVPVTHAKELLHTEHNGMLCATSLGHPLIL
DTCTIEGLIYGNPSCDPLLGGREWSYIVERPSAVNGLCYPGNVENLEELRSLFSSSRSYQRIQIFPDTIW
NVSYSGTSKACSDSFYRSMRWLTQKNNAYPTQDAQYTNNQGKNILFMWGINHPPTDTAQTNLYTRTDTTT
SVATEEMNRIFKPLIGPRPLVNGLMGRINYYWSVLKPGQTLRIKSDGNLIAPWYGHILSGESHGRILKTD
LKRGSCTVQCQTEKGGLNTTLPFQNVSKYAFGNCSKYIGVNSLKLAVGLRNVPSRSSRGLFGAIAGFIEG
GWSGLVAGWYGFQHSNDQGVGMAADRDSTQKAIDKITSKVNNIVDKMNKQYEIIDHEFSEVETRLNMINN
KVDDQIQDIWAYNAELLVLLENQKTLDEHDANVNNLYNKVKRALGSNAVEDGKGCFELYHKCDDHCMETI
RNGTYNRRKYQEESKLERQKIEGVKLESEETYKILTIYSTVASSLVIAMGFAAFLFWAMSNGSCRCNICI

>QAU21878.1 hemagglutinin [Influenza A virus]
METVSLITILLVATVSNADKICIGYQSTNSTETVDTLTENNVPVTHAKELLHTEHNGMLCATSLGQPLIL
DTCTIEGLIYGSPSCDLPLEGREWSYIVERPSAVNGLCYPGNVENLEELRSLFSSARSYQRIQIFPDTIW
NVSYSGTSKACSDSFYRSMRWLTQKNGNYPTQDAQYTNNQGKNILFMWGINHPPTDDTQRNLYTRTDTTT
SVATEEINRVFKPLIGPRPLVNGLMGRIDYYWSVLKPGQTLRIKSDGNLIAPWYGHILSGESHGRILKTD
LKRGSCTVQCQTEKGGLNTTLPFQNVSKYAFGNCSKYIGIKSLKLAVGLRNVPSRSSRGLFGAIAGFIEG
GWSGLVAGWYGFQHSNDQGVGMAADRDSTQKAIDKITSKVNNIVDKMNKQYEIIDHEFSEVETRLNMINN
KIDDQIQDIWAYNAELLVLLENQKTLDEHDANVNNLYNKVKRALGSNAMEDGKGCFELYHKCNDQCMETI
RNGTYNRRKYQEESKLERQKIEGVKLESEGTYKILTIYSTVASSLVIAMGFAAFLFWAMSNGSCRCNICI

>QAU21877.1 hemagglutinin [Influenza A virus]
METVSLITILLVATVSNADKICIGYQSTNSTETVDTLTENNVPVTHAKELIHTEHNGMLCATSLGQPLIL
DTCTIEGLIYGNPSCDLMLEGREWSYIVERPSAVNGLCYPGHVENLEELRSLFSSARSYQRVQIFPDTIW
NVSYDGTSTACSGSFYRSMRWLTRKNGNYPIQDAQYTNNQGKSILFMWGINHPPSDTTQRDLYTRTDTTT
SVATEEINRIFKPLIGPRPLVNGLMGRIDYYWSVLKPGQTLRIKSDGNLIAPWYGHILSGESHGRILKTD
LKRGSCTVQCQTEKGGLNTTLPFQNVSKYAFGNCSKYIGMKSLKLAVGLRNVPSRSSRGLFGAIAGFIEG
GWSGLVAGWYGFQHSNDQGVGMAADRDSTQKAIDKITSKVNNIVDKMNKQYEIIDHEFSEVETRLNMINN
KIDDQIQDIWAYNAELLVLLENQKTLDEHDANVNNLYNKVKRALGSNAVEDGKGCFDLYHKCDDQCMETI
RNGTYNRRKYQEESKLERQKIEGVKLESEGTYKILTIYSTVASSLVIAMGFAAFLFWAMSNGSCRCNICI

>QAU21876.1 hemagglutinin [Influenza A virus]

METVSLITILLVATVSNADKICIGYQSTNSTETVDTLTENNVPVTHAKELIHTEHNGMLCATSLGQPLIL
DTCTIEGLIYGNPSCDLMLEGREWSYIVERPSAVNGLCYPGHVENLEELRSLFSSARSYQRVQIFPDTIW
NVSYDGTSTACSGSFYRSMRWLTRKNGNYPIQDAQYTNNQGKSILFMWGINHPPADTTQRDLYTRTDTTT
SVATEEINRIFKPLIGPRPLVNGLMGRIDYYWSVLKPGQTLRIKSDGNLIAPWYGHILSGESHGRILKTD
LKRGSCTVQCQTEKGGLNTTLPFQNVSKYAFGNCSKYIGMKSLKLAVGLRNVPSRSSRGLFGAIAGFIEG
GWSGLVAGWYGFQHSNDQGVGMAADRDSTQKAIDKITSKVNNIVDKMNKQYEIIDHEFSEVETRLNMINN
KIDDQIQDIWAYNAELLVLLENQKTLDEHDANVNNLYNKVKRALGSNAVEDGKGCFDLYHKCDDQCMETI
RNGTYNRRKYQEESKLERQKIEGVKLESEGTYKILTIYSTVASSLVIAMGFAAFLFWAMSNGSCRCNICI

>QAU21875.1 hemagglutinin [Influenza A virus]
METVSLITILLVATVSNADKICIGYQSTNSTETVDTLTENNVPVTHAKELIHTEHNGMLCATSLGQPLIL
DTCTIEGLIYGNPSCDLMLEGREWSYIVERPSAVNGLCYPGHVENLEELRSLFSSARSYQRVQIFPDTIW
NVSYDGTSTACSGSFYRSMRWLTRKNGNYPIQDAQYTNNQGKSILFMWGINHPPSDTTQRDLYTRTDTTT
SVATEEINRIFKPLIGPRPLVNGLMGRIDYYWSVLKPGQTLRIKSDGNLIAPWYGHILSGESHGRILKTD
LKRGSCTVQCQTEKGGLNTTLPFQNVSKYAFGNCSKYIGMKSLKLAVGLRNVPSRSSRGLFGAIAGFIEG
GWSGLVAGWYGFQHSNDQGVGMAADRDSTQKAIDKITSKVNNIVDKMNKQYEIIDHEFSEVETRLNMINN
KIDDQIQDIWAYNAELLVLLENQKTLDEHDANVNNLYNKVKRALGSNAVEDGKGCFDLYHKCDDQCMETI
RNGTYNRRKYQEESKLERQKIEGVKLESEGTYKILTIYSTVASSLVIAMGFAAFLFWAMSNGSCRCNICI

>QAU21874.1 hemagglutinin [Influenza A virus]
METVSLITILLVATVSNADKICIGYQSTNSTETVDTLTENNVPVTHAKELIHTEHNGMLCATSLGQPLIL
DTCTIEGLIYGNPSCDLMLEGREWSYIVERPSAVNGLCYPGHVENLEELRSLFSSARSYQRVQIFPDTIW
NVSYDGTSTACSGSFYRSMRWLTRKNGNYPIQDAQYTNNQGKSILFMWGINHPPADTTQRDLYTRTDTTT
SVATEEINRIFKPLIGPRPLVNGLMGRIDYYWSVLKPGQTLRIKSDGNLIAPWYGHILSGESHGRILKTD
LKRGSCTVQCQTEKGGLNTTLPFQNVSKYAFGNCSKYIGMKSLKLAVGLRNVPSRSSRGLFGAIAGFIEG
GWSGLVAGWYGFQHSNDQGVGMAADRDSTQKAIDKITSKVNNIVDKMNKQYEIIDHEFSEVETRLNMINN
KIDDQIQDIWAYNAELLVLLENQKTLDEHDANVNNLYNKVKRALGSNAVEDGKGCFDLYHKCDDQCMETI
RNGTYNRRKYQEESKLERQKIEGVKLESEGTYKILTIYSTVASSLVIAMGFAAFLFWAMSNGSCRCNICI

>QAU21873.1 hemagglutinin [Influenza A virus]
METVSLITILLVATVSNADKICIGYQSTNSTETVDTLTENNVPVTHAKELIHTEHNGMLCATSLGQPLIL
DTCTIEGLIYGNPSCDLMLEGREWSYIVERPSAVNGLCYPGHVENLEELRSLFSSARSYQRVQIFPDTIW
NVSYDGTSTACSGSFYRSMRWLTRKNGNYPIQDAQYTNNQGKSILFMWGINHPPADTTQRDLYTRTDTTT
SVATEEINRIFKPLIGPRPLVNGLMGRIDYYWSVLKPGQTLRIKSDGNLIAPWYGHILSGESHGRILKTD

LKRGSCTVQCQTEKGGLNTTLPFQNVSKYAFGNCSKYIGMKSLKLAVGLRNVPSRSSRGLFGAIAGFIEG
GWSGLVAGWYGFQHSNDQGVGMAADRDSTQKAIDKITSKVNNIVDKMNKQYEIIDHEFSEVETRLNMINN
KIDDQIQDIWAYNAELLVLLENQKTLDEHDANVNNLYNKVKRALGSNAVEDGKGCFDLYHKCDDQCMETI
RNGTYNRRKYQEESKLERQKIEGVKLESEGTYKILTIYSTVASSLVIAMGFAAFLFWAMSNGSCRCNICI

>QAU21872.1 hemagglutinin [Influenza A virus]
METVSLITILLVATVSNADKICIGYQSTNSTETVDTLTENNVPVTHAKELIHTEHNGMLCATSLGQPLIL
DTCTIEGLIYGNPSCDLMLEGREWSYIVERPSAVNGLCYPGHVENLEELRSLFSSARSYQRVQIFPDTIW
NVSYDGTSTACSGSFYRSMRWLTRKNGNYPIQDAQYTNNQGKSILFMWGINHPPADTTQRDLYTRTDTTT
SVATEEINRIFKPLIGPRPLVNGLMGRIDYYWSVLKPGQTLRIKSDGNLIAPWYGHILSGESHGRILKTD
LKRGSCTVQCQTEKGGLNTTLPFQNVSKYAFGNCSKYIGMKSLKLAVGLRNVPSRSSRGLFGAIAGFIEG
GWSGLVAGWYGFQHSNDQGVGMAADRDSTQKAIDKITSKVNNIVDKMNKQYEIIDHEFSEVETRLNMINN
KIDDQIQDIWAYNAELLVLLENQKTLDEHDANVNNLYNKVKRALGSNAVEDGKGCFDLYHKCDDQCMETI
RNGTYNRRKYQEESKLERQKIEGVKLESEGTYKILTIYSTVASSLVIAMGFAAFLFWAMSNGSCRCNICI

>QAU21871.1 hemagglutinin [Influenza A virus]
METVSLITILLVATVSNADKICIGYQSTNSTETVDTLTENNVPVTHAKELIHTEHNGMLCATSLGQPLIL
DTCTIEGLIYGNPSCDLMLEGREWSYIVERPSAVNGLCYPGHVENLEELRSLFSSARSYQRVQIFPDTIW
NVSYDGTSTACSGSFYRSMRWLTRKNGNYPIQDAQYTNNQGKSILFMWGINHPPADTTQRDLYTRTDTTT
SVATEEINRIFKPLIGPRPLVNGLMGRIDYYWSVLKPGQTLRIKSDGNLIAPWYGHILSGESHGRILKTD
LKRGSCTVQCQTEKGGLNTTLPFQNVSEYAFGNCSKYIGMKSLKLAVGLRNVPSRSSRGLFGAIAGFIEG
GWSGLVAGWYGFQHSNDQGVGMAADRDSTQKAIDKITSKVNNIVDKMNKQYEIIDHEFSEVETRLNMINN
KIDDQIQDIWAYNAELLVLLENQKTLDEHDANVNNLYNKVKRALGSNAVEDGKGCFDLYHKCDDQCMETI
RNGTYNRRKYQEESKLERQKIEGVKLESEGTYKILTIYSTVASSLVIAMGFAAFLFWAMSNGSCRCNICI

>QAU21870.1 hemagglutinin [Influenza A virus]
METVSLITILLVATVSNADKICIGYQSTNSTETVDTLTENNVPVTHAKELIHTEHNGMLCATSLGQPLIL
DTCTIEGLIYGNPXCDLMLEGREWSYIVERPSAVNGLCYPGHVENLEELRSLFSSARSYQRVQIFPDTIW
NVSYDGTSTACSGSFYRSMRWLTRKNGNYPIQDAQYTNNQGKSILFMWGINHPPADTTQRDLYTRTDTTT
SVATEEINRIFKPLIGPRPLVNGLMGRIDYYWSVLKPGQTLRIKSDGNLIAPWYGHILSGESHGRILKTD
LKRGSCTVQCQTEKGGLNTTLPFQNVSKYAFGNCSKYIGMKSLKLAVGLRNVPSRSSRGLFGAIAGFIEG
GWSGLVAGWYGFQHSNDQGVGMAADRDSTQKAIDKITSKVNNIVDKMNKQYEIIDHEFSEVETRLNMINN
KIDDQIQDIWAYNAELLVLLENQKTLDEHDANVNNLYNKVKRALGSNAVEDGKGCFDLYHKCDDQCMETI
RNGTYNRRKYQEESKLERQKIEGVKLESEGTYKILTIYSTVASSLVIAMGFAAFLFWAMSNGSCRCNICI

>QAU21869.1 hemagglutinin [Influenza A virus]

METVSLITILLVATVSNADKICIGYQSTNSTETVDTLTENNVPVTHAKELIHTEHNGMLCATSLGQPLIL

DTCTIEGLIYGNPSCDLMLEGREWSYIVERPSAVNGLCYPGHVENLEELRSLFSSARSYQRVQIFPDTIW

NVSYDGTSTACSGSFYRSMRWLTRKNGNYPIQDAQYTNNQGKSILFMWGINHPPADTTQRDLYTRTDTTT

SVATEEINRIFKPLIGPRPLVNGLMGRIDYYWSVLKPGQTLRIKSDGNLIAPWYGHILSGESHGRILKTD

LKRGSCTVQCQTEKGGLNTTLPFQNVSKYAFGNCSKYIGMKSLKLAVGLRNVPSRSSRGLFGAIAGFIEG

GWSGLVAGWYGFQHSNDQGVGMAADRDSTQKAIDKITSKVNNIVDKMNKQYEIIDHEFSEVETRLNMINN

KIDDQIQDIWAYNAELLVLLENQKTLDEHDANVNNLYNKVKRALGSNAVEDGKGCFDLYHKCDDQCMETI

RNGTYNRRKYQEESKLERQKIEGVKLESEGTYKILTIYSTVASSLVIAMGFAAFLFWAMSNGSCRCNICI

>QAU21868.1 hemagglutinin [Influenza A virus]

METVSLITILLVAAVSNADKICIGYQSTNSTETVDTLTENNVPVTHAKELLHTEHNGMLCATSLGQPLIL

DTCTIEGLIYGNPYCDLSLEGREWSYIVERPSAVNGLCYPGNVENLEELRSLFSSARSYQRIQIFPDTIW

NVSYDGTSAACAGSFYKSMRWLTRTNGNYPIQDAQYTNNQGKNILFMWGINHPPTDTTQRDLYTRTDTTT

SVATEEINRVFKPLIGPRPLVNGLMGRIDYYWSVLKPGQTLRIKSDGNLIAPWFGHILSGESHGRILKTD

LKRGSCTVQCQTEKGGLNTTLPFQNISKYAFGNCSKYIGIKSLKLAVGLRNVPSRSSRGLFGAIAGFIEG

GWSGLVAGWYGFQHSNDQGVGMAADRDSTQKAIDKITSKVNNIVDKMNKQYEIIDHEFNEVETRLNMINN

KIDDQIQDIWAYNAELLVLLENQKTLDEHDANVNNLYNKVKRALGSNAVEDGKGCFELYHKCDDQCMETI

RNGTYNRRKYREESKLERQKIEGVKLESEGTYKILTIYSTVASSLVIAMGFAAFLFWAMSNGSCRCNICI

>QAU21867.1 hemagglutinin [Influenza A virus]

METVSLITILLVAAVSNADKICIGYQSTNSTETVDTLTENNVPVTHAKELLHTEHNGMLCATSLGQPLIL

DTCTIEGLIYGNPYCDLSLEGREWSYIVERPSAVNGLCYPGNVENLEELRSLFSSARSYQRIQIFPDTIW

NVSYDGTSAACSGSFYKSMRWLTRKNGDYPIQDAQYTNNQGKNILFMWGINHPPTDTTQRDLYTRTDTTT

SVATEEINRVFKPLIGPRPLVNGLMGRIDYYWSVLKPGQTLRIKSDGNLIAPWFGHILSGESHGRILKTD

LKRGSCTVQCQTEKGGLNTTLPFQNISKYAFGNCSKYIGIKSLKLAVGLRNVPSRSSRGLFGAIAGFIEG

GWSGLVAGWYGFQHSNDQGVGMAADRDSTQKAIDKITSKVNNIVDKMNKQYEIIDHEFSEVETRLNMINN

KIDDQIQDIWAYNAELLVLLENQKTLDEHDANVNNLYNKVKRALGSNAVEDGKGCFELYHKCDDQCMETI

RNGTYNRRKYREESRLERQKIEGVKLESEGTYKILTIYSTVASSLVIAMGFAAFLFWAMSNGSCRCNICI

>QAU21866.1 hemagglutinin [Influenza A virus]

METVSLITILLVATVSNADKICIGYQSTNSTETVDTLTENNVPVTHVKELLHTEHNGMLCATSLGHPLIL

DTCTIEGLIYGNPSCDLLLGGREWSYIVERPSAVNGLCYPGNVENLEELRSLFSSARSYQRIQIFPDTIW

NVSYSGTSKACSDSFYRSMRWLTQKNNAYPIQDAQYTNNQEKNILFMWGINHPPTDTTQTNLYTRTDTTT
SVATEEINRIFKPLIGPRPLVNGLMGRIDYYWSVLKPGQTLRIRSDGNLIAPWYGHILSGESHGRILKTD
LKRGSCTVQCQTEKGGLNTTLPFQNVSKYAFGNCSKYIGIKSLKLAVGLRNVPSRSSRGLFGAIAGFIEG
GWSGLVAGWYGFQHSNDQGVGMAADRDSTQKAIDKITSKVNNIVDKMNKQYEIIDHEFSEVETRLNMINN
KIDDQIQDIWAYNAELLVLLENQKTLDEHDANVNNLYNKVKRALGSNAVEDGKGCFELYHKCDDQCMETI
RNGTYNRRKYQEESKLERQKIEGVKLESEGTYKILTIYSTVASSLVIAMGFTAFLFWAMSNGSCRCNICI

>QAU21865.1 hemagglutinin [Influenza A virus]
METVSLITILLVAAVSNADKICIGYQSTNSTETVDTLTENNVPVTHAKELLHTEHNGMLCATSLGQPLIL
DTCTIEGLIYGNPYCDLSLEGREWSYIVERPSAVNGLCYPGNVENLEELRSLFSSARSYQRIQIFPDTIW
NVSYDGTSAACAGSFYKSMRWLTRKNGDYPIQDAQYTNNQGKNILFMWGINHPPTDTTQRDLYTRTDTTT
SVATEEINRVFKPLIGPRPLVNGLMGRIDYYWSVLKPGQTLRIKSDGNLIAPWFGHILSGESHGRILKTD
LKRGSCTVQCQTEKGGLNTTLPFQNISKYAFGNCSKYIGIKSLKLAVGLRNVPSRSSRGLFGAIAGFIEG
GWSGLVAGWYGFQHSNDQGVGMAADRDSTQKAIDKITSKVNNIVDKMNKQYEIIDHEFNEVETRLNMINN
KIDDQIQDIWAYNAELLVLLENQKTLDEHDANVNNLYNKVKRALGSNAVEDGKGCFELYHKCDDQCMETI
RNGTYNRRKYREESKLERQKIEGVKLESEGTYKILTIYSTVASSLVIAMGFAAFLFWAMSNGSCRCNICI

>QAU21864.1 hemagglutinin [Influenza A virus]
METVSLITILLVAAVSNADKICIGYQSTNSTETVDTLTENNVPVTHAKELLHTEHNGMLCATSLGQPLIL
DTCTIEGLIYGNPYCDLSLEGREWSYIVERPSAVNGLCYPGNVENLEELRSLFSSARSYQRIQIFPDTIW
NVSYDGTSAACAGSFYKSMRWLTRKNGDYPIQDAQYTNNQGKNILFMWGINHPPTDTTQRDLYTRTDTTT
SVATEEINRVFKPLIGPRPLVNGLMGRIDYYWSVLKPGQTLRIKSDGNLIAPWFGHILSGESHGRILKTD
LKRGSCTVQCQTEKGGLNTTLPFQNISKYAFGNCSKYIGIKSLKLAVGLRNVPSRSSRGLFGAIAGFIEG
GWSGLVAGWYGFQHSNDQGVGMAADRDSTQKAIDKITSKVNNIVDKMNKQYEIIDHEFNEVETRLNMINN
KIDDQIQDIWAYNAELLVLLENQKTLDEHDANVNNLYNKVKRALGSNAVEDGKGCFELYHKCDDQCMETI
RNGTYNRRKYREESKLERQKIEGVKLESEGTYKILTIYSTVASSLVIAMGFAAFLFWAMSNGSCRCNICI

>QAU21863.1 hemagglutinin [Influenza A virus]
METVSLITILLVAAVSNADKICIGYQSTNSTETVDTLTENNVPVTHAKELLHTEHNGMLCATSLGQPLIL
DTCTIEGLIYGNPYCDLSLEGREWSYIVERPSAVNGLCYPGNVENLEELRSLFSSARSYQRIQIFPDTIW
NVSYDGTSAACSGSFYKSMRWLTRKNGDYPIQDAQYTNNQGKNILFMWGINHPPTDTTQRDLYTRTDTTT
SVATEEINRVFKPLIGPRPLVNGLMGRIDYYWSVLKPGQTLRIKSDGNLIAPWFGHILSGESHGRILKTD
LKRGSCTVQCQTEKGGLNTTLPFQNISKYAFGNCSKYIGIKSLKLAVGLRNVPSRSSRGLFGAIAGFIEG
GWSGLVAGWYGFQHSNDQGVGMAADRDSTQKAIDKITSKVNNIVDKMNKQYEIIDHEFSEVETRLNMINN

KIDDQIQDIWAYNAELLVLLENQKTLDEHDANVNNLYNKVKRALGSNAVEDGKGCFELYHKCDDQCMETI
RNGTYNRRKYREESRLERQKIEGVKLESEGTYKILTIYSTVASSLVIAMGFAAFLFWAMSNGSCRCNICI

>QAU21862.1 hemagglutinin [Influenza A virus]
METVSLITILLVATVSNADKICIGYQSTNSTETVDTLTENNVPVTHAKELLQTEHNGMLCATSLGQPLIL
DTCTIEGLIYGNPSCDLSLEGREWSYIVERPSAVNGLCYPGNVENLEELRSLFSSARSYQRIQIFPDTIW
NVSYDGTSTACSGSFYKSMRWLTRKNGDYPIQDAQYTNNQGKNILFMWGINHPPTDTTQRDLYTRTDTTT
SVATEEINRVFKPLIGPRPLVNGLMGRIDYYWSVLKPGQTLRIKSDGNLIAPWFGHILSGESHGRILKTD
LKRGSCTVQCQTEKGGLNTTLPFQNVSKYAFGNCSKYIGIKSLKLAVGLRNVPSRSSRGLFGAIAGFIEG
GWSGLVAGWYGFQHSNDQGVGMAADRDSTQKAIDKITSKVNNIVDKMNKQYEIIDHEFSEVETRLNMINN
KIDDQIQDIWAYNAELLVLLENQKTLDEHDANVNNLYNKVKRALGSNAVEDGKGCFELYHKCDDQCMETI
RNGTYNRRKYQEESKLERQKIEGVKLESEGTYKILTIYSTVASSLVIAMGFAAFLFWAMSNGSCRCNICI

>QAU21861.1 hemagglutinin [Influenza A virus]
METVSLITILVVATVSNADKICIGYQSTNSTETVDTLTENNVPVTHAKELLHTEHNGMLCATSLGHPLIL
DTCTIEGLIYGNPSCDPLLGGREWSYIVERPSAVNGLCYPGNVENLEELRSLFSSSRSYQRIQIFPDTIW
NVSYSGTSKACSDSFYRSMRWLTQKNNAYPTQDAQYTNNQGKNILFMWGINHPPTDTVQTNLYTRTDTTT
SVATEEMNRIFKPLIGPRPLVNGLMGRINYYWSVLKPGQTLRIKSDGNLIAPWYGHILSGESHGRILKTD
LKRGSCTVQCQTEKGGLNTTLPFQNVSKYAFGNCSKYIGVKSLKLAVGLRNVPSRSSRGLFGAIAGFIEG
GWSGLVAGWYGFQHSNDQGVGMAADRDSTQKAIDKITSKVNNIVDKMNKQYEIIDHEFSEVETRLNMINN
KVDDQIQDIWAYNAELLVLLENQKTLDEHDANVNNLYNKVKRALGSNAVEDGKGCFELYHKCDDHCMETI
RNGTYNRRKYQEESKLERQKIEGVKLESEETYKILTIYSTVASSLVIAMGFAAFLFWAMSNGSCRCNICI

>QAU21860.1 hemagglutinin [Influenza A virus]
METVSLITILVVATVSNADKICIGYQSTNSTETVDTLTENNVPVTHAKELLHTEHNGMLCATSLGHPLIL
DTCTIEGLIYGNPSCDPLLGGREWSYIVERPSAVNGLCYPGNVENLEELRSLFSSSRSYQRIQIFPDTIW
NVSYSGTSKACSDSFYRSMRWLTQKNNAYPTQDAQYTNNQGKNILFMWGINHPPTDTVQTNLYTRTDTTT
SVATEEMNRIFKPLIGPRPLVNGLMGRINYYWSVLKPGQTLRIKSDGNLIAPWYGHILSGESHGRILKTD
LKRGSCTVQCQTEKGGLNTTLPFQNVSKYAFGNCSKYIGVKSLKLAVGLRNVPSRSSRGLFGAIAGFIEG
GWSGLVAGWYGFQHSNDQGVGMAADRDSTQKAIDKITSKVNNIVDKMNKQYEIIDHEFSEVETRLNMINN
KVDDQIQDIWAYNAELLVLLENQKTLDEHDANVNNLYNKVKRALGSNAVEDGKGCFELYHKCDDHCMETI
RNGTYNRRKYQEESKLERQKIEGVKLESEETYKILTIYSTVASSLVIAMGFAAFLFWAMSNGSCRCNICI

>QAU21859.1 hemagglutinin [Influenza A virus]

METVSLITILVVATVSNADKICIGYQSTNSTETVDTLTENNVPVTHAKELLHTEHNGMLCATSLGHPLIL
DTCTIEGLIYGNPSCDPLLGGREWSYIVERPSAVNGLCYPGNVENLEELRSLFSSSRSYQRIQIFPDTIW
NVSYSGTSKACSDSFYRSMRWLTQKNNAYPTQDAQYTNNQGKNILFMWGINHPPTDTVQTNLYTRTDTTT
SVATEEMNRIFKPLIGPRPLVNGLMGRINYYWSVLKPGQTLRIKSDGNLIAPWYGHILSGESHGRILKTD
LKRGSCTVQCQTEKGGLNTTLPFQNVSKYAFGNCSKYIGVKSLKLAVGLRNVPSRSSRGLFGAIAGFIEG
GWSGLVAGWYGFQHSNDQGVGMAADRDSTQKAIDKITSKVNNIVDKMNKQYEIIDHEFSEVETRLNMINN
KVDDQIQDIWAYNAELLVLLENQKTLDEHDANVNNLYNKVKRALGSNAVEDGKGCFELYHKCDDHCMETI
RNGTYNRRKYQEESKLERQKIEGVKLESEETYKILTIYSTVASSLVIAMGFAAFLFWAMSNGSCRCNICI

>QAU21858.1 hemagglutinin [Influenza A virus]
METVSLITILLVATVSNADKICIGYQSTNSTETVDTLTENNVPVTHAKELLHTEHNGMLCATSLGQPLIL
DTCTIEGLIYGNPSCDLSLEGREWSYIVERPSAVHGLCYPGNVEDLEELRSLFSSARSYQRIQIFPDTIW
NVSYDGTSTACSGSFYRSMRWLTRKNGEYPVQDAQYTNNQGKDLLFMWGINHPPTDDTQRGLYTRTDTTT
SVATEEINRIFKPLIGPRPLVNGLMGRINYYWSVLKPGQTLRIKSDGNLIAPWYGHILSGESHGRILKTD
LKRGSCTVQCQTEKGGLNTTLPFQNVSKYAFGNCSKYIGIKSLKLAVGLRNVPSRSSRGLFGAIAGFIEG
GWSGLVAGWYGFQHSNDQGIGMAADRDSTQKAIDKITSKVNNIVDKMNKQYEIIDHEFSEVETRLNMINN
KIDDQIQDIWAYNAELLVLLENQKTLDEHDANVNNLYNKVKRALGSNAVEDGKGCFELYHKCDDQCMETI
RNGTYNRRKYQEESKLERQKIEGVKLESEGTYKILTIYSTVASSLVIAMGFAAFLFWAMSNGSCRCNICI

>QAU21857.1 hemagglutinin [Influenza A virus]
METVSLITILLVATVSNADKICIGYQSTNSTETVDTLTENNVPVTHAKELLHTEHNGMLCATSLGQPLIL
DTCTIEGLIYGNPSCDLSLEGREWSYIVERPSAVHGLCYPGNVENLEELRSLFSSARSYQRIQIFPDTIW
NVSYDGTSTACSGSFYRSMRWLTRKNGEYPVQDAQYTNNQGKDILFMWGINHPPTDDTQRGLYTRTDTTT
SVATEEINRIFKPLIGPRPLVNGLMGRINYYWSVLKPGQTLRIKSDGNLIAPWYGHILSGESHGRILKTD
LKRGNCTVQCQTEKGGLNTTLPFQNVSKYAFGDCSKYIGIKSLKLAVGLRNVPSRSSRGLFGAIAGFIEG
GWSGLVAGWYGFQHSNDQGVGMAADRDSTQKAIDKITSKVNNIVDKMNKQYEIIDHEFSEVETRLNMINN
KIDDQIQDIWAYNAELLVLLENQKTLDELDANVNNLYNKVKRALGSNAVEDGKGCFELYHKCDDQCMETI
RNGTYNRRKYQEESKLERQKIEGVKLESEGTYKILTIYSTVASSLVIAMGFAAFLFWAMSNGSCRCNICI

>QAU21856.1 hemagglutinin [Influenza A virus]
METVSLITILLVATVSNADKICIGYQSTNSTETVDTLTENNVPVTHAKELLHTEHNGMLCATSLGQPLIL
DTCTIEGLIYGNPSCDLSLEGKEWSYIVERPSAVNGLCYPGNVENLEELRSLFSSARSYQRIQIFPDTIW
NVSYDGTSTACSGSFYRSMRWLTRKDGNYPTQDAQYTNNQGKNILFMWGINHPPTDDTQRNLYTRTDTTT
SVATEEINRIFKPLIGPRPLVNGLMGRIDYYWSVLKPGQTLRIKSDGNLIAPWYGHILSGESHGRILKTD

LKRGSCTVQCQTEKGGLNTTLPFQNVSKYAFGNCSKYIGIKSLKLAVGLRNVPSRSSRGLFGAIAGFIEG
GWSGLVAGWYGFQHSNDQGVGMAADRDSTQKAIDKITSKVNNIVDKMNKQYEIIDHEFSEVETRLNMINN
KIDDQIQDIWAYNAELLVLLENQKTLDEHDANVNNLYNKVKRALGSNAVEDGKGCFELYHKCNDQCMETI
RNGTYNRRKYQEESKLERQKIEGVKLESEGTYKILTIYSTVASSLVIAMGFAAFLFWAMSNGSCRCNICI

>QAU21855.1 hemagglutinin [Influenza A virus]
METVSLITILLVATVSNADKICIGYQSTNSTETVDTLTENNVPVTHAKELLHTEHNGMLCATSLGQPLIL
DTCTIEGLIYGNPSCDLSLEGREWSYIVERPSAVNGLCYPGNVENLEELRSLFSSARSYQRIQIFPDTIW
NVSYDGTSTACSGSFYRSMRWLTRKDGNYPTQDAQYTNNQGKNILFMWGINHPPTDDTQRNLYTRTDTTT
SVATEEINRIFKPLIGPRPLVNGLMGRIDYYWSVLKPGQTLRIKSDGNLIAPWYGHILSGESHGRILKTD
LKRGSCTVQCQTEKGGLNTTLPFQNVSKYAFGNCSKYIGIKSLKLAVGLRNVPSRSSRGLFGAIAGFIEG
GWSGLVAGWYGFQHSNDQGVGMAADRDSTQKAIDKITSKVNNIVDKMNKQYEIIDHEFSEVETRLNMINN
KIDDQIQDIWAYNAELLVLLENQKTLDEHDANVNNLYNKVKRALGSNAVEDGKGCFELYHKCNDQCMETI
RNGTYNRRKYQEESKLERQKIEGVKLESEGTYKILTIYSTVASSLVIAMGFAAFLFWAMSNGSCRCNICI

>QAU21854.1 hemagglutinin [Influenza A virus]
METVSLITILLVATVSNADKICIGYQSTNSTETVDTLTENNVPVTHAKELLHTEHNGMLCATSLGQPLIL
DTCTIEGLIYGNPSCDLSLEGREWSYIVERPSAVNGLCYPGNVENLEELRSLFSSARSYQRIQIFPDTIW
NVSYDGTSTACSGSFYRSMRWLTRKDGNYPTQDAQYTNNQGKNILFMWGINHPPTDDTQRNLYTRTDTTT
SVATEEINRIFKPLIGPRPLVNGLMGRIDYYWSVLKPGQTLRIKSDGNLIAPWYGHILSGESHGRILKTD
LKRGSCTVQCQTEKGGLNTTLPFQNVSKYAFGNCSKYIGIKSLKLAVGLRNVPSRSSRGLFGAIAGFIEG
GWSGLVAGWYGFQHSNDQGVGMAADRDSTQKAVDKITSKVNTIVDKMNKQYEIIDHEFSEVETRLNMINN
KIDDQIQDIWAYNAELLVLLENQKTLDEHDANVNNLYNKVKRALGSNAVEDGKGCFELYHKCDDQCMETI
RNGTYNRRKYQEESKLERQKIEGVKLESEGTYKILTIYSTVASSLVIAMGFAAFLFWAMSNGSCRCNICI

>QAU21853.1 hemagglutinin [Influenza A virus]
METVSLITILLVATVSNADKICIGYQSTNSTETVDTLTENNVPVTHAKELLHTEHNGMLCATSLGQPLIL
DTCTIEGLIYGNPSCDLSLEGREWSYIVERPSAVNGLCYPGNVENLEELRSLFSSARSYQRIQIFPDTIW
NVSYDGTSTACSGSFYRSMRWLTRKDGNYPTQDAQYTNNQGKNILFMWGINHPPTDDTQRNLYTRTDTTT
SVATEEINRIFKPLIGPRPLVNGLMGRIDYYWSVLKPGQTLRIKSDGNLIAPWYGHILSGESHGRILKTD
LKRGSCTVQCQTEKGGLNTTLPFQNVSKYAFGNCSKYIGIKSLKLAVGLRNVPSRSSRGLFGAIAGFIEG
GWSGLVAGWYGFQHSNDQGVGMAADRDSTQKAIDKITSKVNNIVDKMNKQYEIIDHEFSEVETRLNMINN
KIDDQIQDIWAYNAELLVLLENQKTLDEHDANVNNLYNKVKRALGSNAVEDGKGCFELYHKCNDQCMETI
RNGTYNRRKYQEESKLERQKIEGVKLESEGTYKILTIYSTVASSLVIAMGFAAFLFWAMSNGSCRCNICI

>QAU21852.1 hemagglutinin [Influenza A virus]

METVSLITILLVATVSNADKICIGYQSTNSTETVDTLTENNVPVTHAKELLHTEHNGMLCATSLGQPLIL
DTCTIEGLIYGNPSCDLSLEGREWSYIVERPSAVNGLCYPGNVENLEELRSLFSSARSYQRIQIFPDTIW
NVSYDGTSTACSGSFYRSMRWLTRKDGNYPTQDAQYTNNQGKNILFMWGINHPPTDDTQRNLYTRTDTTT
SVATEEINRIFKPLIGPRPLVNGLMGRIDYYWSVLKPGQTLRIKSDGNLIAPWYGHILSGESHGRILKTD
LKRGSCTVQCQTEKGGLNTTLPFQNVSKYAFGNCSKYIGIKSLKLAVGLRNVPSRSSRGLFGAIAGFIEG
GWSGLVAGWYGFQHSNDQGVGMAADRDSTQKAIDKITSKVNNIVDKMNKQYEIIDHEFSEVETRLNMINN
KIDDQIQDIWAYNAELLVLLENQKTLDEHDANVNNLYNKVKRALGSNAVEDGKGCFELYHKCNDQCMETI
RNGTYNRRKYQEESKLERQKIEGVKLESEGTYKILTIYSTVASSLVIAMGFAAFLFWAMSNGSCRCNICI

>QAU21851.1 hemagglutinin [Influenza A virus]

METVSLITILLVATVSNADKICIGYQSTNSTETVDTLTENNVPVTHAKELLHTEHNGMLCATSLGQPLIL
DTCTIEGLIYGNPSCDLSLEGREWSYIVERPSAVNGLCYPGNVENLEELRSLFSSARSYQRIQIFPDTIW
NVSYDGTSTACSGSFYRSMRWLTRKDGNYPTQDAQYTNNQGKNILFMWGINHPPTDDTQRNLYTRTDTTT
SVATEEINRIFKPLIGPRPLVNGLMGRIDYYWSVLKPGQTLRIKSDGNLIAPWYGHILSGESHGRILKTD
LKRGSCTVQCQTEKGGLNTTLPFQNVSKYAFGNCSKYIGIKSLKLAVGLRNVPSRSSRGLFGAIAGFIEG
GWSGLVAGWYGFQHSNDQGVGMAADRDSTQKAIDKITSKVNNIVDKMNKQYEIIDHEFSEVETRLNMINN
KIDDQIQDIWAYNAELLVLLENQKTLDEHDANVNNLYNKVKRALGSNAVEDGKGCFELYHKCNDQCMETI
RNGTYNRRKYQEESKLERQKIEGVKLESEGTYKILTIYSTVASSLVIAMGFAAFLFWAMSNGSCRCNICI

>QAU21850.1 hemagglutinin [Influenza A virus]

METVSLITILLVATVSNADKICIGYQSTNSTETVDTLTENNVPVTHAKELLHTEHNGMLCATSLGQPLIL
DTCTIEGLIYGNPSCDLSLEGREWSYIVERPSAVNGLCYPGNVENLEELRSLFSSARSYQRIQIFPDTIW
NVSYDGTSTACSGSFYRSMRWLTRKDGNYPTQDAQYTNNQGKNILFMWGINHPPTDDTQRNLYTRTDTTT
SVATEEINRIFKPLIGPRPLVNGLMGRIDYYWSVLKPGQTLRIKSDGNLIAPWYGHILSGESHGRILKTD
LKRGSCTVQCQTEKGGLNTTLPFQNVSKYAFGNCSKYIGIKSLKLAVGLRNVPSRSSRGLFGAIAGFIEG
GWSGLVAGWYGFQHSNDQGVGMAADRDSTQKAIDKITSKVNNIVDKMNKQYEIIDHEFSEVETRLNMINN
KIDDQIQDIWAYNAELLVLLENQKTLDEHDANVNNLYNKVKRALGSNAVEDGKGCFELYHKCNDQCMETI
RNGTYNRRKYQEESKLERQKIEGVKLESEGTYKILTIYSTVASSLVIAMGFAAFLFWAMSNGSCRCNICI

>QAU21849.1 hemagglutinin [Influenza A virus]

METVSLITILLVATVSNADKICIGYQSTNSTETVDTLTENNVPVTHAKELLHTEHNGMLCATSLGQPLIL
DTCTIEGLIYGNPSCDLSLEGREWSYIVERPSAVNGLCYPGNVENLEELRSLFSSARSYQRIQIFPDTIW

NVSYDGTSTACSGSFYRSMRWLTRKDGNYPTQDAQYTNNQGKNILFMWGINHPPTDDTQRNLYTRTDTTT
SVATEEINRIFKPLIGPRPLVNGLMGRIDYYWSVLKPGQTLRIKSDGNLIAPWYGHILSGESHGRILKTD
LKRGSCTVQCQTEKGGLNTTLPFQNVSKYAFGNCSKYIGIKSLKLAVGLRNVPSRSSRGLFGAIAGFIEG
GWSGLVAGWYGFQHSNDQGVGMAADRDSTQKAIDKITSKVNNIVDKMNKQYEIIDHEFSEVETRLNMINN
KIDDQIQDIWAYNAELLVLLENQKTLDEHDANVNNLYNKVKRALGSNAVEDGKGCFELYHKCNDQCMETI
RNGTYNRRKYQEESKLERQKIEGVKLESEGTYKILTIYSTVASSLVIAMGFAAFLFWAMSNGSCRCNICI

>QAU21848.1 hemagglutinin [Influenza A virus]
METVSLITILLIATVSNADKICIGYQSTNSTETVDTLTENNVPVTHAKELLHTEHNGMLCATSLGQPLIL
DTCTIEGLIYGNPSCDLSLEGREWSYIVERPSAVNGLCYPGNVENLEELRSLFSSARSYQRIQIFPDTIW
NVSYDGTSTACSGSFYRSMRWLTRKDGNYPTQDAQYTNNQGKNILFMWGINHPPTDDTQRNLYTRTDTTT
SVATEEINRIFKPLIGPRPLVNGLMGRIDYYWSVLKPGQTLRIKSDGNLIAPWYGHILSGESHGRILKTD
LKRGSCTVQCQTEKGGLNTTLPFQNVSKYAFGNCSKYIGIKSLKLAVGLRNVPSRSSRGLFGAIAGFIEG
GWSGLVAGWYGFQHSNDQGVGMAADRDSTQKAIDKITSKVNNIVDRMNKQYEIIDHEFSEVETRLNMINN
KIDDQIQDIWAYNAELLVLLENQKTLDEHDANVNNLYNKVKRALGSNAVEDGKGCFELYHKCNDQCMETI
RNGTYNRRKYQEESKLERQKIEGVKLESEGTYKILTIYSTVASSLVIAMGFAAFLFWAMSNGSCRCNICI

>QAU21847.1 hemagglutinin [Influenza A virus]
METVSLITILLVATVSNADKICIGYQSTNSTETVDTLTENNVPVTHAKELLHTEHNGMLCATSLGQPLIL
DTCTIEGLIYGNPSCDLSLEGREWSYIVERPSAVNGLCYPGNVENLEELRSLFSSARSYQRIQIFPDTIW
NVSYDGTSTACSGSFYRSMRWLTRKDGNYPTQDAQYTNNQGKNILFMWGINHPPTDDTQRNLYTRTDTTT
SVATEEINRIFKPLIGPRPLVNGLMGRIDYYWSVLKPGQTLRIKSDGNLIAPWYGHILSGESHGRILKTD
LKRGSCTVQCQTEKGGLNTTLPFQNVSKYAFGNCSKYIGIKSLKLAVGLRNVPSRSSRGLFGAIAGFIEG
GWSGLVAGWYGFQHSNDQGVGMAADRDSTQKAIDKITSKVNNIVDRMNKQYEIIDHEFSEVETRLNMINN
KIDDQIQDIWAYNAELLVLLENQKTLDEHDANVNNLYNKVKRALGSNAVEDGKGCFELYHKCNDQCMETI
RNGTYNRRKYQEESKLERQKIEGVKLESEGTYKILTIYSTVASSLVIAMGFAAFLFWAMSNGSCRCNICI

>QAU21846.1 hemagglutinin [Influenza A virus]
METVSLITILLVATVSNADKICIGYQSTNSTETVDTLTENNVPVTHAKELLHTEHNGMLCATSLGQPLIL
DTCTIEGLIYGNPSCDLSLEGREWSYIVERPSAVNGLCYPGNVENLEELRSLFSSARSYQRIQIFPDTIW
NVSYDGTSTACSGSFYRSMRWLTRKDGNYPTQDAQYTNNQGKNILFMWGINHPPTDDTQRNLYTRTDTTT
SVATEEINRIFKPLIGPRPLVNGLMGRIDYYWSVLKPGQTLRIKSDGNLIAPWYGHILSGESHGRILKTD
LKRGSCTVQCQTEKGGLNTTLPFQNVSKYAFGNCSKYIGIKSLKLAVGLRNVPSRSSRGLFGAIAGFIEG
GWSGLVAGWYGFQHSNDQGVGMAADRDSTQKAIDKITSKVNNIVDKMNKQYEIIDHEFSEVETRLNMINN

KIDDQIQDIWAYNAELLVLLENQKTLDEHDANVNNLYNKVKRALGSNAVEDGKGCFELYHKCNDQCMETI
RNGTYNRRKYQEESKLERQKIEGVKLESEGTYKILTIYSTVASSLVIAMGFAAFLFWAMSNGSCRCNICI

>QAU21845.1 hemagglutinin [Influenza A virus]
METVSLITILLIATVSNADKICIGYQSTNSTETVDTLTENNVPVTHAKELLHTEHNGMLCATSLGQPLIL
DTCTIEGLIYGNPSCDLSLEGREWSYIVERPSAVNGLCYPGNVENLEELRSLFSSARSYQRIQIFPDTIW
NVSYDGTSTACSGSFYRSMRWLTRKDGNYPTQDAQYTNNQGKNILFMWGINHPPTDDTQRNLYTRTDTTT
SVATEEINRIFKPLIGPRPLVNGLMGRIDYYWSVLKPGQTLRIKSDGNLIAPWYGHILSGESHGRILKTD
LKRGSCTVQCQTEKGGLNTTLPFQNVSKYAFGNCSKYIGIKSLKLAVGLRNVPSRSSRGLFGAIAGFIEG
GWSGLVAGWYGFQHSNDQGVGMAADRDSTQKAIDKITSKVNNIVDRMNKQYEIIDHEFSEVETRLNMINN
KIDDQIQDIWAYNAELLVLLENQKTLDEHDANVNNLYNKVKRALGSNAVEDGKGCFELYHKCNDQCMETI
RNGTYNRRKYQEESKLERQKIEGVKLESEGTYKILTIYSTVASSLVIAMGFAAFLFWAMSNGSCRCNICI

>QAU21844.1 hemagglutinin [Influenza A virus]
METVSLITILLIATVSNADKICIGYQSTNSTETVDTLTENNVPVTHAKELLHTEHNGMLCATSLGQPLIL
DTCTIEGLIYGNPSCDLSLEGREWSYIVERPSAVNGLCYPGNVENLEELRSLFSSARSYQRIQIFPDTIW
NVSYDGTSTACSGSFYRSMRWLTRKDGNYPTQDAQYTNNQGKNILFMWGINHPPTDDTQRNLYTRTDTTT
SVATEEINRIFKPLIGPRPLVNGLMGRIDYYWSVLKPGQTLRIKSDGNLIAPWYGHILSGESHGRILKTD
LKRGSCTVQCQTEKGGLNTTLPFQNVSKYAFGNCSKYIGIKSLKLAVGLRNVPSRSSRGLFGAIAGFIEG
GWSGLVAGWYGFQHSNDQGVGMAADRDSTQKAIDKITSKVNNIVDRMNKQYEIIDHEFSEVETRLNMINN
KIDDQIQDIWAYNAELLVLLENQKTLDEHDANVNNLYNKVKRALGSNAVEDGKGCFELYHKCNDQCMETI
RNGTYNRRKYQEESKLERQKIEGVKLESEGTYKILTIYSTVASSLVIAMGFAAFLFWAMSNGSCRCNICI

>QAU21843.1 hemagglutinin [Influenza A virus]
METVSLITILLVATVSNADKICIGYQSTNSTETVDTLTENNVPVTHAKELLHTEHNGMLCATSLGQPLIL
DTCTIEGLIYGNPSCDLSLEGREWSYIVERPSAVNGLCYPGNVENLEELRSLFSSARSYQRIQIFPDTIW
NVSYDGTSTACSGSFYRSMRWLTRKDGNYPTQDAQYTNNQGKNILFMWGINHPPTDDTQRNLYTRTDTTT
SVATEEINRIFKPLIGPRPLVNGLMGRIDYYWSVLKPGQTLRIKSDGNLIAPWYGHILSGESHGRILKTD
LKRGSCTVQCQTEKGGLNTTLPFQNVSKYAFGNCSKYIGIKSLKLAVGLRNVPSRSSRGLFGAIAGFIEG
GWSGLVAGWYGFQHSNDQGVGMAADRDSTQKAIDKITSKVNNIVDKMNKQYEIIDHEFSEVETRLNMINN
KIDDQIQDIWAYNAELLVLLENQKTLDEHDANVNNLYNKVKRALGSNAVEDGKGCFELYHKCNDQCMETI
RNGTYNRRKYQEESKLERQKIEGVKLESEGTYKILTIYSTVASSLVIAMGFAAFLFWAMSNGSCRCNICI

>QAU21842.1 hemagglutinin [Influenza A virus]

METVSLITILLLATVSNADKICIGYQSTNSTETVDTLTENNVPVTHAKELLHTEHNGMLCATSLGQPLIL
DTCTIEGLIYGNPSCDLSLEGKEWSYIVERPSAVNGLCYPGNVENLEELRSLFSSARSYQRIQIFPDTIW
NVSYDGTSTACSGSFYKSMRWLTRKNGDYPTQDAQYTNNQGKNILFMWGINHPPTDDTQRNLYTRTDTTT
SVATEEINRIFKPLIGPRPLVNGLMGRIDYYWSVLKPGQTLRIKSDGNLIAPWYGHILSGESHGRILKTD
LKRGSCTVQCQTEKGGLNTTLPFQNVSKYAFGNCSKYIGLKSLKLAVGLRNVPSRSSRGLFGAIAGFIEG
GWSGLVAGWYGFQHSNDQGVGMAADRDSTQKAIDKITSKVNNIVDKMNKQYEIIDHEFSEVETRLNMINN
KIDDQIQDIWAYNAELLVLLENQKTLDEHDANVNNLYNKVKRALGSNAVEDGKGCFELYHKCNDQCMETI
RNGTYNRRKYQEESKLERQKIEGVKLESEGTYKILTIYSTVASSLVIAMGFAAFLFWAMSNGSCRCNICI

>QAU21841.1 hemagglutinin [Influenza A virus]
METVSLITILLVATVSNADKICIGYQSTNSTETVDTLTENNVPVTHAKELLHTEHNGMLCATSLGQPLIL
DTCTIEGLIYGNPSCDLSLEGREWSYIVERPSAVNGLCYPGNVENLEELRSLFSSARSYQRIQIFPDTIW
NVSYDGTSTACSGSFYRSMRWLTRKDGNYPTQDAQYTNNQGKNILFMWGINHPPTDDTQRNLYTRTDTTT
SVATEEINRIFKPLIGPRPLVNGLMGRIDYYWSVLKPGQTLRIKSDGNLIAPWYGHILSGESHGRILKTD
LKRGSCTVQCQTEKGGLNTTLPFQNVSKYAFGNCSKYIGIKSLKLAVGLRNVPSRSSRGLFGAIAGFIEG
GWSGLVAGWYGFQHSNDQGVGMAADRDSTQKAIDKITSKVNNIVDKMNKQYEIIDHEFSEVETRLNMINN
KIDDQIQDIWAYNAELLVLLENQKTLDEHDANVNNLYNKVKRALGSNAVEDGKGCFELYHKCNDQCMETI
RNGTYNRRKYQEESKLERQKIEGVKLESEGTYKILTIYSTVASSLVIAMGFAAFLFWAMSNGSCRCNICI

>QAU21840.1 hemagglutinin [Influenza A virus]
METVSLITILLVAAVSNADKICIGYQSTNSTETVDTLTENNVPVTHAKELLHTEHNGMLCATSLGQPIIL
DTCTIEGLIYGNPSCDLSLEGREWSYIVERPSAVNGLCYPGNVENLEELRSLFSSARSYQRIQIFPDTIW
NVSYDGTSTACSGSFYRNMRWLTRKNGEYPTQDAQYTNNQGKNILFMWGINHPPADTTQRNLYTRTDTTT
SVATEEINRIFKPLIGPRPLVNGLMGRIDYYWSVLKPGQTLRIKSDGNLIAPWYGHILSGESHGRILKTD
LKRGSCTVQCQTEKGGLNTTLPFQNVSKYAFGNCSKYIGIKSLKLAVGLRNVPSRSSRGLFGAIAGFIEG
GWSGLVAGWYGFQHSNDQGVGMAADRDSTQKAVDKITSKVNTIVDKMNKQYEIIDHEFSEVETRLNMINN
KIDDQIQDIWAYNAELLVLLENQKTLDEHDANVNNLYNKVKRALGSNAVEDGKGCFELYHKCDDQCMETI
RNGTYNRRKYQEESKLERQKIEGVKLESEGTYKILTIYSTVASSLVIAMGFAAFLFWAMSNGSCRCNICI

>QAU21839.1 hemagglutinin [Influenza A virus]
METVSLITILLVATVSNADKICIGYQSTNSTETVDTLTENNVPVTHAKELLHTEHNGMLCATSLGQPLIL
DTCTIEGLIYGNPSCDLSLEGREWSYIVERPSAVNGLCYPGNVENLEELRSLFSSARSYQRIQIFPDTIW
NVSYDGTSTACSGSFYRSMRWLTRKDGNYPTQDAQYTNNQGKNILFMWGINHPPTDDTQRNLYTRTDTTT
SVATEEINRIFKPLIGPRPLVNGLMGRIDYYWSVLKPGQTLRIKSDGNLIAPWYGHILSGESHGRILKTD

LKRGSCTVQCQTEKGGLNTTLPFQNVSKYAFGNCSKYIGIKSLKLAVGLRNVPSRSSRGLFGAIAGFIEG
GWSGLVAGWYGFQHSNDQGVGMAADRDSTQKAIDKITSKVNNIVDKMNKQYEIIDHEFSEVETRLNMINN
KIDDQIQDIWAYNAELLVLLENQKTLDEHDANVNNLYNKVKRALGSNAVEDGKGCFELYHKCNDQCMETI
RNGTYNRRKYQEESKLERQKIEGVKLESEGTYKILTIYSTVASSLVIAMGFAAFLFWAMSNGSCRCNICI

>QAU21838.1 hemagglutinin [Influenza A virus]
METVSLITILLVATVSNADKICIGYQSTNSTETVDTLTENNVPVTHAKELLHTEHNGMLCATSLGQPLIL
DTCTIEGLIYGNPSCDLSLEGREWSYIVERPSAVNGLCYPGNVENLEELRSLFSSARSYQRIQIFPDTIW
NVSYDGTSTACSNSFYRSMRWLTRKDGNYPTQDAQYTNNQGKNILFMWGINHPPTDDTQRNLYTRTDTTT
SVATEEINRIFKPLIGPRPLVNGLMGRIDYYWSVLKPGQTLRIKSDGNLIAPWYGHILSGESHGRILKTD
LKRGSCTVQCQTEKGGLNTTLPFQNVSKYAFGNCSKYIGIKSLKLAVGLRNVPSRSSRGLFGAIAGFIEG
GWSGLVAGWYGFQHSNDQGVGMAADRDSTQKAIDKITSKVNNIVDKMNKQYEIIDHEFSEVETRLNMINN
KIDDQIQDIWAYNAELLVLLENQKTLDEHDANVNNLYNKVKRALGSNAVEDGKGCFELYHKCNDQCMETI
RNGTYNRRKYQEESKLERQKIEGVKLESEGTYKILTIYSTVASSLVIAMGFAAFLFWAMSNGSCRCNICI

>QAU21837.1 hemagglutinin [Influenza A virus]
METVSLITILLVATVSNADKICIGYQSTNSTETVDTLTENNVPVTHAKELLHTEHNGMLCATSLGQPLIL
DTCTIEGLIYGNPSCDLSLEGREWSYIVERPSAVNGLCYPGNVENLEELRSLFSSARSYQRIQIFPDTIW
NVSYDGTSTACSNSFYRSMRWLTRKDGNYPTQDAQYTNNQGKNILFMWGINHPPTDDTQRNLYTRTDTTT
SVATEEINRIFKPLIGPRPLVNGLMGRIDYYWSVLKPGQTLRIKSDGNLIAPWYGHILSGESHGRILKTD
LKRGSCTVQCQTEKGGLNTTLPFQNVSKYAFGNCSKYIGIKSLKLAVGLRNVPSRSSRGLFGAIAGFIEG
GWSGLVAGWYGFQHSNDQGVGMAADRDSTQRAIDKITSKVNNIVDKMNKQYEIIDHEFSEVETRLNMINN
KIDDQIQDIWAYNAELLVLLENQKTLDEHDANVNNLYNKVKRALGSNAVEDGKGCFELYHKCNDQCMETI
RNGTYNRRKYQEESKLERQKIEGVKLESEGTYKILTIYSTVASSLVIAMGFAAFLFWAMSNGSCRCNICI

>QAU21836.1 hemagglutinin [Influenza A virus]
METVSLITILLVATVSNADKICIGYQSTNSTETVDTLTENNVPVTHAKELLHTEHNGMLCATSLGQPLIL
DTCTIEGLIYGNPSCDLSLEGREWSYIVERPSAVNGLCYPGNVENLEELRSLFSSARSYQRIQIFPDTIW
NVSYDGTSTACSNSFYRSMRWLTRKDGNYPTQDAQYTNNQGKNILFMWGINHPPTDDTQRNLYTRTDTTT
SVATEEINRIFKPLIGPRPLVNGLMGRIDYYWSVLKPGQTLRIKSDGNLIAPWYGHILSGESHGRILKTD
LKRGSCTVQCQTEKGGLNTTLPFQNVSKYAFGNCSKYIGIKSLKLAVGLRNVPSRSSRGLFGAIAGFIEG
GWSGLVAGWYGFQHSNDQGVGMAADRDSTQKAIDKITSKVNNIVDKMNKQYEIIDHEFSEVETRLNMINN
KIDDQIQDIWAYNAELLVLLENQKTLDEHDANVNNLYNKVKRALGSNAVEDGKGCFELYHKCNDQCMETI
RNGTYNRRKYQEESKLERQKIEGVKLESEGTYKILTIYSTVASSLVIAMGFAAFLFWAMSNGSCRCNICI

>QAU21835.1 hemagglutinin [Influenza A virus]

METASLITILLAVTVSNADKICIGYQSTNSTETVDTLTENNVPVTHAKELLHTEHNGMLCATSLGHPLIL
DTCTIEGLIYGNPSCDLLLGGREWSYIVERPSAVNGLCYPGNVENLEELRSLFSSARSYQRIQIFPDTIW
NVSYSGTSKACSDSFYRSMRWLTQKDNAYPVQDAQYTNNQEKNILFMWGINHPPTDTAQTNLYTRTDTTT
SVATEEINRTFKPLIGPRPLVNGLMGRINYYWSVLKPGQTLRIKSNGNLIAPWYGHILSGESHGRILKTD
LKRGSCTVQCQTEKGGLNTTLPFQNVSKYAFGNCSKYIGIKSLKLAVGLRNVPSRSSRGLFGAIAGFIEG
GWSGLVAGWYGFQHSNDQGVGMAADRDSTQKAIDKITSKVNNIVDKMNKQYEIIDHEFSEVETRLNMINN
KIDDQIQDIWAYNAELLVLLENQKTLDEHDANVNNLYNKVKRALGSNAVEDGKGCFELYHKCDDQCMETI
RNGTYNRRKYQEESKLERQKIEGVKLESEETYKILTIYSTVASSLVIAMGFAAFLFWAMSNGSCRCNICI

>QAU21834.1 hemagglutinin [Influenza A virus]

METVSLITILLVATVSNADKICIGYQSTNSTETVDTLTENNVPVTHAKELLHTEHNGMLCATSLGHPLIL
DTCTIEGIIYGNPSCDPLLGEREWSYIVERPSAVNGLCYPGNVENLEELRSLFSSSRSYQRIQIFPDTIW
NVSYSGTSKACSDSFYRSMRWLTQKNNAYPTQDAQYTNNQEKNILFMWGINHPPTDTAQTNLYTRTDTTT
SVATEEINRIFKPLIGPRPLVNGLMGRINYYWSVLKPGQTLRIKSNGNLIAPWYGHILSGESHGRILKTD
LKSGSCTVQCQTEKGGLNTTLPFQNVSKYAFGNCSKYIGVKSLKLAVGLRNVPSRSSRGLFGAIAGFIEG
GWSGLVAGWYGFQHSNDQGVGMAADRDSTQKAIDKITSKVNNIVDKMNKQYEIIDHEFSEVETRLNMINN
KVDDQIQDIWAYNAELLVLLENQKTLDEHDANVNNLYNKVKRALGSNAVEDGKGCFELYHKCDDHCMETI
RNGTYNRRKYQEESKLERQKIEGVKLESEETYKILTIYSTVASSLVIAMGFAAFLFWAMSNGSCRCNICI

>QAU21833.1 hemagglutinin [Influenza A virus]

METASLITILLAVTVSNADKICIGYQSTNSTETVDTLTENNVPVTHAKELLHTEHNGMLCATSLGHPLIL
DTCTIEGLIYGNPSCDLLLGGREWSYIVERPSAVNGLCYPGNVENLEELRSLFSSARSYQRIQIFPDTIW
NVSYSGTSKACSDSFYRSMRWLTQKNNAYPVQDAQYTNNQEKNILFMWGINHPPTDTAQTNLYTRTDTTT
SVATEEINRTFKPLIGPRPLVNGLMGRINYYWSVLKPGQTLRIKSNGNLIAPWYGHILSGESHGRILKTD
LKRGSCTVQCQTEKGGLNTTLPFQNVSKYAFGNCSKYIGIKSLKLAVGLRNVPSRSSRGLFGAIAGFIEG
GWSGLVAGWYGFQHSNDQGVGMAADRDSTQKAIDKITSKVNNIVDKMNKQYEIIDHEFSEVETRLNMINN
KIDDQIQDIWAYNAELLVLLENQKTLDEHDANVNNLYNKVKRALGSNAVEDGKGCFELYHKCDDQCMETI
RNGTYNRRKYQEESKLERQKIEGVKLESEETYKILTIYSTVASSLVIAMGFAAFLFWAMSNGSCRCNICI

>QAU21832.1 hemagglutinin [Influenza A virus]

METASLITILLAVTVSNADKICIGYQSTNSTETVDTLTENNVPVTHAKELLHTEHNGMLCATSLGHPLIL
DTCTIEGLIYGNPSCDLLLGGREWSYIVERPSAVNGLCYPGNVENLEELRSLFSSARSYQRIQIFPDTIW

NVSYSGTSKACSDSFYRSMRWLTQKDNAYPVQDAQYTNNQEKNILFMWGINHPPTDTVQTNLYTRTDTTT
SVATEEINRTFKPLIGPRPLVNGLMGRINYYWSVLKPGQTLRIKSNGNLIAPWYGHILSGESHGRILKTD
LKRGSCTVQCQTEKGGLNTTLPFQNVSKYAFGNCSKYIGIKSLKLAVGLRNVPSRSSRGLFGAIAGFIEG
GWSGLVAGWYGFQHSNDQGVGMAADRDSTQKAIDKITSKVNNIVDKMNKQYEIIDHEFSEVETRLNMINN
KIDDQIQDIWAYNAELLVLLENQKTLDEHDANVNNLYNKVKRALGSNAVEDGKGCFELYHKCDDQCMETI
RNGTYNRRKYQEESKLERQKIEGVKLESEETYKILTIYSTVASSLVIAMGFAAFLFWAMSNGSCRCNICI

>QAU21831.1 hemagglutinin [Influenza A virus]
METVSLITILLVATVSNADKICIGYQSTNSTETVDTLTENNVPVTHAKELLHTEHNGMLCATSLGQPLIL
DTCTIEGLIYGNPSCDLSLEGKEWSYIVERPSAVNGLCYPGNVENLEELRSLFSSARSYQRIQIFPDTIW
NVSYDGTSTACSNSFYRSMRWLTRKDGNYPTQDAQYTNNQGKNILFMWGINHPPTDDTQRNLYTRTDTTT
SVATEEINRIFKPLIGPRPLVNGLMGRIDYYWSVLKPGQTLRIKSDGNLIAPWYGHILSGESHGRILKTD
LKRGSCTVQCQTEKGGLNTTLPFQNVSKYAFGNCSKYIGIKSLKLAVGLRNVPSRSSRGLFGAIAGFIEG
GWSGLVAGWYGFQHSNDQGVGMAADRDSTQKAIDKITSKVNNIVDKMNKQYEIIDHEFSEVETRLNMINN
KIDDQIQDIWAYNAELLVLLENQKTLDEHDANVNNLYNKVKRALGSNAVEDGKGCFELYHKCNDQCMETI
RNGTYNRRKYQEESKLERQKIEGVKLESEGTYKILTIYSTVASSLVIAMGFAAFLFWAMSNGSCRCNICI

>QAU21830.1 hemagglutinin [Influenza A virus]
METVSLITILLVATVSNADKICIGYQSTNSTETVDTLTENNVPVTHAKELLHTEHNGMLCATSLGQPLIL
DTCTIEGLIYGNPSCDLSLEGKEWSYIVERPSAVNGLCYPGNVENLEELRSLFSSARSYQRIQIFPDTIW
NVSYDGTSTACSNSFYRSMRWLTRKDGNYPTQDAQYTNNQGKNILFMWGINHPPTDDTQRNLYTRTDTTT
SVATEEINRIFKPLIGPRPLVNGLMGRIDYYWSVLKPGQTLRIKSDGNLIAPWYGHILSGESHGRILKTD
LKRGSCTVQCQTEKGGLNTTLPFQNVSKYAFGNCSKYIGIKSLKLAVGLRNVPSRSSRGLFGAIAGFIEG
GWSGLVAGWYGFQHSNDQGVGMAADRDSTQKAIDKITSKVNNIVDKMNKQYEIIDHEFSEVETRLNMINN
KIDDQIQDIWAYNAELLVLLENQKTLDEHDANVNNLYNKVKRALGSNAVEDGKGCFELYHKCNDQCMETI
RNGTYNRRKYQEESKLERQKIEGVKLESEGTYKILTIYSTVASSLVIAMGFAAFLFWAMSNGSCRCNICI

>QAU21829.1 hemagglutinin [Influenza A virus]
METVSLITILLVATVSNADKICIGYQSTNSTETVDTLTENNVPVTHAKELLHTEHNGMLCATSLGQPLIL
DTCTIEGLIYGNPSCDLSLEGKEWSYIVERPSAVNGLCYPGNVENLEELRSLFSSARSYQRIQIFPDTIW
NVSYDGTSTACSNSFYRSMRWLTRKDGNYPTQDAQYTNNQGKNILFMWGINHPPTDDTQRNLYTRTDTTT
SVATEEINRIFKPLIGPRPLVNGLMGRIDYYWSVLKPGQTLRIKSDGNLIAPWYGHILSGESHGRILKTD
LKRGSCTVQCQTEKGGLNTTLPFQNVSKYAFGNCSKYIGIKSLKLAVGLRNVPSRSSRGLFGAIAGFIEG
GWSGLVAGWYGFQHSNDQGVGMAADRDSTQKAIDKITSKVNNIVDKMNKQYEIIDHEFSEVETRLNMINN

KIDDQIQDIWAYNAELLVLLENQKTLDEHDANVNNLYNKVKRALGSNAVEDGKGCFELYHKCNDQCMETI
RNGTYNRRKYQEESKLERQKIEGVKLESEGTYKILTIYSTVASSLVIAMGFAAFLFWAMSNGSCRCNICI

>QAU21828.1 hemagglutinin [Influenza A virus]
METVSLITILVVATVSNADKICIGYQSTNSTETVDTLTENNVPVTHAKELLHTEHNGMLCATSLGHPLIL
DTCTIEGLIYGNPSCDPLLGGREWSYIVERPSAVNGLCYPGNVENLEELRSLFSSSRSYQRIQIFPDTIW
NVSYSGTSKACSDSFYRSMRWLTQKNNAYPTQDAQYTNNQGKNILFMWGINHPPTDTAQTNLYTRTDTTT
SVATEEMNRIFKPLIGPRPLVNGLMGRINYYWSVLKPGQTLRIKSDGNLIAPWYGHILSGESHGRILKTD
LKRGSCTVQCQTEKGGLNTTLPFQNVSKYAFGNCSKYIGVKSLKLAVGLRNVPSRSSRGLFGAIAGFIEG
GWSGLVAGWYGFQHSNDQGVGMAADRDSTQKAIDKITSKVNNIVDKMNKQYEIIDHEFSEVETRLNMINN
KIDDQIQDIWAYNAELLVLLENQKTLDEHDANVNNLYNKVKRALGSNAVEDGKGCFELYHKCDDHCMETI
RNGTYNRRKYQEESKLERQRIEGVKLESEGTYKILTIYSTVASSLVIAMGFAAFLFWAMSNGSCRCNICI

>QAU21827.1 hemagglutinin [Influenza A virus]
METVSLITILLVATVSNADKICIGYQSTNSTETVDTLTENNVPVTHAKELLHTEHNGMLCATSLGQPLIL
DTCTIEGLIYGNPSCDLSLEGKEWSYIVERPSAVNGLCYPGNVENLEELRSLFSSARSYQRIQIFPDTIW
NVSYDGTSTACSNSFYRSMRWLTRKDGNYPTQDAQYTNNQGKNILFMWGINHPPTDDTQRNLYTRTDTTT
SVATEEINRIFKPLIGPRPLVNGLMGRIDYYWSVLKPGQTLRIKSDGNLIAPWYGHILSGESHGRILKTD
LKRGSCTVQCQTEKGGLNTTLPFQNVSKYAFGNCSKYIGIKSLKLAVGLRNVPSRSSRGLFGAIAGFIEG
GWSGLVAGWYGFQHSNDQGVGMAADRDSTQKAIDKITSKVNNIVDKMNKQYEIIDHEFSEVETRLNMINN
KIDDQIQDIWAYNAELLVLLENQKTLDEHDANVNNLYNKVKRALGSNAVEDGKGCFELYHKCNDQCMETI
RNGTYNRKKYQEESKLERQRIEGVKLESEGTYKILTIYSTVASSLVIAMGFAAFLFWAMSNGSCRCNICI

>QAU21826.1 hemagglutinin [Influenza A virus]
METVSLITILLVATVSNADKICIGYQSTNSTETVDTLTENNVPVTHAKELLHTEHNGMLCATSLGQPLIL
DTCTIEGLIYGNPSCDLSLEGKEWSYIVERPSAVNGLCYPGNVENLEELRSLFSSARSYQRIQIFPDTIW
NVSYDGTSTACSNSFYRSMRWLTRKDGNYPTQDAQYTNNQGKNILFMWGINHPPTDDTQRNLYTRTDTTT
SVATEEINRIFKPLIGPRPLVNGLMGRIDYYWSVLKPGQTLRIKSDGNLIAPWYGHILSGESHGRILKTD
LKRGSCTVQCQTEKGGLNTTLPFQNVSKYAFGNCSKYIGIKSLKLAVGLRNVPSRSSRGLFGAIAGFIEG
GWSGLVAGWYGFQHSNDQGVGMAADRDSTQKAIDKITSKVNNIVDKMNKQYEIIDHEFSEVETRLNMINN
KIDDQIQDIWAYNAELLVLLENQKTLDEHDANVNNLYNKVKRALGSNAVEDGKGCFELYHKCNDQCMETI
RNGTYNRRKYQEESKLERQKIEGVKLESEGTYKILTIYSTVASSLVIAMGFAAFLFWAMSNGSCRCNICI

>QAU21825.1 hemagglutinin [Influenza A virus]

METVSLITILLVATVSNADKICIGYQSTNSTETVDTLTENNVPVTHAKELLHTEHNGMLCATSLGQPLIL
DTCTIEGLIYGNPSCDLSLEGREWSYIVERPSAVHGLCYPGNVEDLEELRSLFSSARSYQRIQIFPDTIW
NVSYDGTSTACSGSFYRSMRWLTRKNGEYPIQDAQYTNNQGKNILFMWGINHPPTDDTQRGLYTRTDTTT
SVATEEINRIFKPLIGPRPLVNGLMGRINYYWSVLKPGQTLRIKSDGNLIAPWYGHILSGESHGRILKTD
LKRGICTVQCQTEKGGLNTTLPFQNVSKYAFGNCSKYIGIKSLKLAVGLRNVPSRSSRGLFGAIAGFIEG
GWSGLVAGWYGFQHSNDQGVGMAADRDSTQKAIDKITSKVNNIVDKMNKQYEIIDHEFSEVETRLNMINN
KIDDQIQDIWAYNAELLVLLENQKTLDEHDANVNNLYNKVKRALGSNAVEDGKGCFELYHKCNDQCMETI
RNGTYNRRKYQEESKLERQKIEGVKLESEGTYKILTIYSTVASSLVIAMGFAAFLFWAMSNGSCRCNICI

>QAU21824.1 hemagglutinin [Influenza A virus]
METVSLITILLVATVSNADKICIGYQSTNSTETVDTLTENNVPVTHAKELLHTEHNGMLCATSLGQPLIL
DTCTIEGLIYGNPSCDLSLEGKEWSYIVERPSAVNGLCYPGNVENLEELRSLFSSARSYQRIQIFPDTIW
NVSYDGTSTACSNSFYRSMRWLTRKDGNYPTQDAQYTNNQGKNILFMWGINHPPTDDTQRNLYTRTDTTT
SVATEEINRIFKPLIGPRPLVNGLMGRIDYYWSVLKPGQTLRIKSDGNLIAPWYGHILSGESHGRILKTD
LKRGSCTVQCQTEKGGLNTTLPFQNVSKYAFGNCSKYIGIKSLKLAVGLRNVPSRSSRGLFGAIAGFIEG
GWSGLVAGWYGFQHSNDQGVGMAADRDSTQKAIDKITSKVNNIVDKMNKQYEIIDHEFSEVETRLNMINN
KIDDQIQDIWAYNAELLVLLENQKTLDEHDANVNNLYNKVKRALGSNAVEDGKGCFELYHKCNDQCMETI
RNGTYNRRKYQEESKLERQKIEGVKLESEGTYKILTIYSTVASSLVIAMGFAAFLFWAMSNGSCRCNICI

>QAU21823.1 hemagglutinin [Influenza A virus]
METVSLITILLVATVSNADKICIGYQSTNSTETVDTLTENNVPVTHAKELLHTEHNGMLCATSLGQPLIL
DTCTIEGLIYGNPSCDLSLEGKEWSYIVERPSAVNGLCYPGNVENLEELRSLFSSARSYQRIQIFPDTIW
NVSYDGTSTACSNSFYRSMRWLTRKDGNYPTQDAQYTNNQGKNILFMWGINHPPTDDTQRNLYTRTDTTT
SVATEEINRIFKPLIGPRPLVNGLMGRIDYYWSVLKPGQTLRIKSDGNLIAPWYGHILSGESHGRILKTD
LKRGSCTVQCQTEKGGLNTTLPFQNVSKYAFGNCSKYIGIKSLKLAVGLRNVPSRSSRGLFGAIAGFIEG
GWSGLVAGWYGFQHSNDQGVGMAADRDSTQKAIDKITSKVNNIVDKMNKQYEIIDHEFSEVETRLNMINN
KIDDQIQDIWAYNAELLVLLENQKTLDEHDANVNNLYNKVKRALGSNAVEDGKGCFELYHKCNDQCMETI
RNGTYNRKKYQEESKLERQRIEGVKLESEGTYKILTIYSTVASSLVIAMGFAAFLFWAMSNGSCRCNICI

>QAU21822.1 hemagglutinin [Influenza A virus]
METISLITILLLATVSNADKICIGYQSTNSTETVDTLTENNVPVTHAKELIHTEHNGMLCATSLGQPLIL
DTCTIEGLIYGNPSCDLSLEGREWSYIVERPSAVNGLCYPGNVENLEELRSLFSSARSYQRIQIFPDTIW
NVSYDGTSTACSGSFYRNMRWLTRKDGNYPTQDAQYTNNQGKNILFMWGINHPPTDTTQSGLYTRTDTTT
SVATEEINRIFKPLIGPRPLVNGLMGRIDYYWSVLKPGQTLRIKSDGNLIAPWFGHILSGESHGRILKTD

LKRGSCTVQCQTEKGGLNTTLPFQNVSKYAFGNCSKYIGIKSLKLAVGLRNVPSRSSRGLFGAIAGFIEG
GWSGLVAGWYGFQHSNDQGVGMAADRDSTQKAIDKITSKVNNIVDKMNKQYEIIDHEFSEVETRLNMINN
KIDDQIQDIWAYNAELLVLLENQKTLDEHDANVNNLYNKVKRALGSNAVEDGKGCFELYHKCDDQCMETI
RNGTYNRRKYQEESKLERQKIEGVKLESEGTYKILTIYSTVASSLVIAMGFAAFLFWAMSNGSCRCNICI

>QAU21821.1 hemagglutinin [Influenza A virus]
METVSLITILLVATVSNADKICIGYQSTNSTETVDTLTENNVPVTHAKELLHTEHNGMLCATSLGQPLIL
DTCTIEGLIYGNPSCDLSLEGKEWSYIVERPSAVNGLCYPGNVENLEELRSLFSSARSYQRIQIFPDTIW
NVSYDGTSTACSNSFYRSMRWLTRKDGNYPTQDAQYTNNQGKNILFMWGINHPPTDDTQRNLYTRTDTTT
SVATEEINRIFKPLIGPRPLVNGLMGRIDYYWSVLKPGQTLRIKSDGNLIAPWYGHILSGESHGRILKTD
LKRGSCTVQCQTEKGGLNTTLPFQNVSKYAFGNCSKYIGIKSLKLAVGLRNVPSRSSRGLFGAIAGFIEG
GWSGLVAGWYGFQHSNDQGVGMAADRDSTQKAIDKITSKVNNIVDKMNKQYEIIDHEFSEVETRLNMINN
KIDDQIQDIWAYNAELLVLLENQKTLDEHDANVNNLYNKVKRALGSNAVEDGKGCFELYHKCNDQCMETI
RNGTYNRRKYQEESKLERQKIEGVKLESEGTYKILTIYSTVASSLVIAMGFAAFLFWAMSNGSCRCNICI

>QAU21820.1 hemagglutinin [Influenza A virus]
METVSLITILLVATVSNADKICIGYQSTNSTETVDTLTENNVPVTHAKELLHTEHNGMLCATSLGQPLIL
DTCTIEGLIYGNPSCDLSLEGREWSYIVERPSAVNGLCYPGNVENLEELRSLFSSARSYQRIQIFPDTIW
NVSYDGTSTACSNSFYRSMRWLTRKDGNYPTQDAQYTNNQGKNILFMWGINHPPTDDTQRNLYTRTDTTT
SVATEEINRIFKPLIGPRPLVNGLMGRIDYYWSVLKPGQTLRIKSDGNLIAPWYGHILSGESHGRILKTD
LKRGSCTVQCQTEKGGLNTTLPFQNVSKYAFGNCSKYIGIKSLKLAVGLRNVPSRSSRGLFGAIAGFIEG
GWSGLVAGWYGFQHSNDQGVGMAADRDSTQKAIDKITSKVNNIVDKMNKQYEIIDHEFSEVETRLNMINN
KIDDQIQDIWAYNAELLVLLENQKTLDEHDANVNNLYNKVKRALGSNAVEDGKGCFELYHKCNDQCMETI
RNGTYNRRKYQEESKLERQKIEGVKLESEGTYKILTIYSTVASSLVIAMGFAAFLFWAMSNGSCRCNICI

>QAU21819.1 hemagglutinin [Influenza A virus]
METASLITILLVATVSNADKICIGYQSTNSTETVDTLTENNVPVTHAKELLHTEHNGMLCATSLGQPLIL
DTCTIEGLIYGNPSCDLSLEGREWSYIVERPSAVNGLCYPGNVENLEELRSLFSSARSYQRIQIFPDTIW
NVSYDGTSTACSNSFYRSMRWLTRKDGNYPTQDAQYTNNQGKNILFMWGINHPPTDETQRNLYTRTDTTT
SVATEEINRIFKPLIGPRPLVNGLMGRIDYYWSVLRPGQTLRIKSDGNLIAPWYGHILSGESHGRILKTD
LKRGSCTVQCQTEKGGLNTTLPFQNVSKYAFGNCSKYIGIKSLKLAVGLRNVPSRSSRGLFGAIAGFIEG
GWSGLVAGWYGFQHSNDQGVGMAADRDSTQKAIDKITSKVNNIVDKMNKQYEIIDHEFSEVETRLNMINN
KIDDQIQDIWAYNAELLVLLENQKTLDEHDANVNNLYNKVKRALGSNAVEDGKGCFELYHKCNDQCMETI
RNGTYNRKKYQEESKLERQRIEGVKLESEGTYKILTIYSTVASSLVIAMGFAAFLFWAMSNGSCRCNICI

>QAU21818.1 hemagglutinin [Influenza A virus]

METVSLITILLVATVSNADKICIGYQSTNSTETVDTLTENNVPVTHAKELLHTEHNGMLCATSLGQPIIL
DTCTIEGLIYGNPSCDLSLEGREWSYIVERPSAVNGLCYPGNVENLEELRSLFSSARSYQRIQIFPDTIW
NVSYDGTSTACSGSFYRNMRWLTRKNGDYPIQDAQYTNNQGKNILFMWGINHPPADTTQISLYTRTDTTT
SVATEEINRIFKPLIGPRPLVNGLMGRIDYYWSILKPGQTLRIKSDGNLIAPWYGHILSGESHGRILKTD
LKRGSCTVQCQTEKGGLNTTLPFQNVSKYAFGNCSKYIGIKSLKLAVGLRNVPSRSSRGLFGAIAGFIEG
GWSGLVAGWYGFQHSNDQGVGMAADRDSTQKAIDKITSKVNNIVDKMNKQYEIIDHEFSEVETRLNMINN
KIDDQIQDIWAYNAELLVLLENQKTLDEHDANVNNLYNKVKRALGSNAVEDGKGCFELYHKCDDQCMETI
RNGTYNRRKYQEESKLERQKIEGVKLESEGTYKILTIYSTVASSLVIAMGFAAFLFWAMSNGSCRCNICI

>QAU21817.1 hemagglutinin [Influenza A virus]

METVSLITILLVAAVSNADKICIGYQSTNSTETVDTLTENNVPVTHAKELLHTEHNGMLCATNLGQPIIL
DTCTIEGLIYGNPSCDLSLEGREWSYIVERPSAVNGLCYPGNVENLEELRSLFSSARSYQRIQIFPDTIW
NVSYDGTSTACSGSFYRNMRWLTRKNGEYPIQDAQYTNNQGKNILFMWGINHPPADTTQRNLYTRTDTTT
SVATEEINRIFKPLIGPRPLVNGLMGRIDYYWSVLKPGQTLRIKSDGNLIAPWYGHILSGESHGRILKTD
LKRGSCTVQCQTEKGGLNTTLPFQNVSKYAFGNCSKYIGIKSLKLAVGLRNVPSRSSRGLFGAIAGFIEG
GWSGLVAGWYGFQHSNDQGVGMAADRDSTQKAVDKITSKVNTIVDKMNKQYEIIDHEFSEVETRLNMINN
KIDDQIQDIWAYNAELLVLLENQKTLDEHDANVNNLYNKVKRALGSNAVEDGKGCFELYHKCDDQCMETI
RNGTYNRRKYQEESKLERQKIEGVKLESEGTYKILTIYSTVASSLVIAMGFAAFLFWAMSNGSCRCNICI

>QAU21816.1 hemagglutinin [Influenza A virus]

METVSLITILLVAAVSNADKICIGYQSTNSTETVDTLTENNVPVTHAKELLHTEHNGMLCATSLGQPIIL
DTCTIEGLIYGNPSCDLSLEGREWSYIVERPSAVNGLCYPGNVENLEELRSLFSSARSYQRIQIFPDTIW
NVSYDGTSTACSGSFYRNMRWLTRKNGEYPIQDAQYTNNQGKNILFMWGINHPPADTTQRNLYTRTDTTT
SVATEEINRIFKPLIGPRPLVNGLMGRIDYYWSVLKPGQTLRIKSDGNLIAPWYGHILSGESHGRILKTD
LKRGSCTVQCQTEKGGLNTTLPFQNVSKYAFGNCSKYIGIKSLKLAVGLRNVPSRSSRGLFGAIAGFIEG
GWSGLVAGWYGFQHSNDQGVGMAADRDSTQKAVDKITSKVNTIVDKMNKQYEIIDHEFSEVETRLNMINN
KIDDQIQDIWAYNAELLVLLENQKTLDEHDANVNNLYNKVKRALGSNAVEDGKGCFELYHKCDDQCMETI
RNGTYNRRKYQEESKLERQKIEGVKLESEGTYKILTIYSTVASSLVIAMGFAAFLFWAMSNGSCRCNICI

>QAU21815.1 hemagglutinin [Influenza A virus]

METVSLITILLVAAVSNADKICIGYQSTNSTETVDTLTENNVPVTHAKELLHTEHNGMLCATSLGQPIIL
DTCTIEGLIYGNPSCDLSLEGREWSYIVERPSAVNGLCYPGNVENLEELRSLFSSARSYQRIQIFPDTIW

NVSYDGTSTACSGSFYRNMRWLTRKNGEYPIQDAQYTNNQGKNILFMWGINHPPADTTQRDLYTRTDTTT
SVATEEINRIFKPLIGPRPLVNGLMGRIDYYWSVLKPGQTLRIKSDGNLIAPWYGHILSGESHGRILKTD
LKRGSCTVQCQTEKGGLNTTLPFQNVSKYAFGNCSKYIGIKSLRLAVGLRNVPSRSSRGLFGAIAGFIEG
GWSGLVAGWYGFQHSNDQGVGMAADRDSTQKAVDKITSKVNTIVDKMNKQYEIIDHEFSEVETRLNMINN
KIDDQIQDIWAYNAELLVLLENQKTLDEHDANVNNLYNKVKRALGSNAVEDGKGCFELYHKCDDQCMETI
RNGTYNRRKYQEESKLERQKIEGVKLESEGTYKILTIYSTVASSLVIAMGFAAFLFWAMSNGSCRCNICI

>QAU21814.1 hemagglutinin [Influenza A virus]
METVSLITILLVAAVSNADKICIGYQSTNSTETVDTLTENNVPVTHAKELLHTEHNGMLCATSLGQPIIL
DTCTIEGLIYGNPSCDLSLEGREWSYIVERPSAVNGLCYPGNVENLEELRSLFSSARSYQRIQIFPDTIW
NVSYDGTSTACSGSFYRNMRWLTRKNGEYPIQDAQYTNNQGKNILFMWGINHPPADTTQRDLYTRTDTTT
SVATEEINRIFKPLIGPRPLVNGLMGRIDYYWSVLKPGQTLRIKSDGNLIAPWYGHILSGESHGRILKTD
LKRGSCTVQCQTEKGGLNTTLPFQNVSKYAFGNCSKYIGIKSLKLAVGLRNVPSRSSRGLFGAIAGFIEG
GWSGLVAGWYGFQHSNDQGVGMAADRDSTQKAIDKITSKVNNIVDKMNKQYEIIDHEFSEVETRLNMINN
KVDDQIQDIWAYNAELLVLLENQKTLDEHDSNVNNLYNKVKRALGSNAVEDGKGCFELYHKCDNQCMETI
RNGTYNRRKYQEESKLERQKIEGVKLESEGTYKILTIYSTVASSLGIAMGFAAFLFWAMSNGSCRCNICI

>QAU21813.1 hemagglutinin [Influenza A virus]
METVSLITILLVAAVSNADKICIGYQSTNSTETVDTLTENNVPVTHAKELLHTEHNGMLCATSLGQPIIL
DTCTIEGLIYGNPSCDLSLEGREWSYIVERPSAVNGLCYPGNVENLEELRSLFSSARSYQRIQIFPDTIW
NVSYDGTSTACSGSFYRNMRWLTRKNGEYPIQDAQYTNNQGKNILFMWGINHPPADTTQRDLYTRTDTTT
SVATEEINRIFKPLIGPRPLVNGLMGRIDYYWSVLKPGQTLRIKSDGNLIAPWYGHILSGESHGRILKTD
LKRGSCTVQCQTEKGGLNTTLPFQNVSKYAFGNCSKYIGIKSLKLAVGLRNVPSRSSRGLFGAIAGFIEG
GWSGLVAGWYGFQHSNDQGVGMAADRDSTQKAVDKITSKVNTIVDKMNKQYEIIDHEFSEVETRLNMINN
KIDDQIQDIWAYNAELLVLLENQKTLDEHDANVNNLYNKVKRALGSNAVEDGKGCFELYHKCDDQCMETI
RNGTYNRRKYQEESKLERQKIEGVKLESEGTYKILTIYSTVASSLVIAMGFAAFLFWAMSNGSCRCNICI

>QAU21812.1 hemagglutinin [Influenza A virus]
METVSLITILLVAAVSNADKICIGYQSTNSTETVDTLTENNVPVTHAKELLHTEHNGMLCATSLGQPIIL
DTCTIEGLIYGNPSCDLSLEGREWSYIVERPSAVNGLCYPGNVENLEELRSLFSSARSYQRIQIFPDTIW
NVSYDGTSTACSGSFYRNMRWLTRKNGEYPIQDAQYTNNQGKNILFMWGINHPPADTTQRDLYTRTDTTT
SVATEEINRIFKPLIGPRPLVNGLMGRIDYYWSVLKPGQTLRIKSDGNLIAPWYGHILSGESHGRILKTD
LKRGSCTVQCQTEKGGLNTTLPFQNVSKYAFGNCSKYIGIKSLKLAVGLRNVPSRSSRGLFGAIAGFIEG
GWSGLVAGWYGFQHSNDQGVGMAADRDSTQKAVDKITSKVNTIVDKMNKQYEIIDHEFSEVETRLNMINN

KIDDQIQDIWAYNAELLVLLENQKTLDEHDANVNNLYNKVKRALGSNAVEDGKGCFELYHKCDDQCMETI
RNGTYNRRKYQEESKLERQKIEGVKLESEGTYKILTIYSTVASSLVIAMGFAAFLFWAMSNGSCRCNICI

>QAU21811.1 hemagglutinin [Influenza A virus]
METVSLITILLVAAVSNADKICIGYQSTNSTETVDTLTENNVPVTHAKELLHTEHNGMLCATSLGQPIIL
DTCTIEGLIYGNPSCDLSLEGREWSYIVERPSAVNGLCYPGNVENLEELRSLFSSARSYQRIQIFPDTIW
NVSYDGTSTACSGSFYRNMRWLTRKNGEYPIQDAQYTNNQGKNILFMWGINHPPADTTQRDLYTRTDTTT
SVATEEINRIFKPLIGPRPLVNGLMGRIDYYWSVLKPGQTLRIKSDGNLIAPWYGHILSGESHGRILKTD
LKRGSCTVQCQTEKGGLNTTLPFQNVSKYAFGNCSKYIGIKSLKLAVGLRNVPSRSSRGLFGAIAGFIEG
GWSGLVAGWYGFQHSNDQGVGMAADRDSTQKAVDKITSKVNTIVDKMNKQYEIIDHEFSEVETRLNMINN
KIDDQIQDIWAYNAELLVLLENQKTLDEHDANVNNLYNKVKRALGSNAVEDGKGCFELYHKCDDQCMETI
RNGTYNRRKYQEESKLERQKIEGVKLESEGTYKILTIYSTVASSLVIAMGFAAFLFWAMSNGSCRCNICI

>QAU21810.1 hemagglutinin [Influenza A virus]
METVSLITILLVAAVSNADKICIGYQSTNSTETVDTLTENNVPVTHAKELLHTEHNGMLCATSLGQPIIL
DTCTIEGLIYGNPSCDLSLEGREWSYIVERPSAVNGLCYPGNVENLEELRSLFSSARSYQRIQIFPDTIW
NVSYDGTSTACSGSFYRNMRWLTRKNGEYPIQDAQYTNNQGKNILFMWGINHPPADTTQRDLYTRTDTTT
SVATEEINRIFKPLIGPRPLVNGLMGRIDYYWSVLKPGQTLRIKSDGNLIAPWYGHILSGESHGRILKTD
LKRGSCTVQCQTEKGGLNTTLPFQNVSKYAFGNCSKYIGIKSLKLAVGLRNVPSRSSRGLFGAIAGFIEG
GWSGLVAGWYGFQHSNDQGVGMAADRDSTQKAVDKITSKVNTIVDKMNKQYEIIDHEFSEVETRLNMINN
KIDDQIQDIWAYNAELLVLLENQKTLDEHDANVNNLYNKVKRALGSNAVEDGKGCFELYHKCDDQCMETI
RNGTYNRRKYQEESKLERQKIEGVKLESEGTYKILTIYSTVASSLVIAMGFAAFLFWAMSNGSCRCNICI

>QAU21809.1 hemagglutinin [Influenza A virus]
METVSLITILLVAAVSNADKICIGYQSTNSTETVDTLTENNVPVTHAKELLHTEHNGMLCATSLGQPIIL
DTCTIEGLIYGNPSCDLSLEGREWSYIVERPSAVNGLCYPGNVENLEELRSLFSSARSYQRIQIFPDTIW
NVSYDGTSTACSGSFYRNMRWLTRRNGEYPTQDAQYTNNQGKNILFMWGINHPPADTTQRDLYTRTDTTT
SVATEEINRIFKPLIGPRPLVNGLMGRIDYYWSVLKPGQTLRIKSDGNLIAPWYGHILSGESHGRILKTD
LKRGSCTVQCQTEKGGLNTTLPFQNVSKYAFGNCSKYIGIKSLKLAVGLRNVPSRSSRGLFGAIAGFIEG
GWSGLVAGWYGFQHSNDQGVGMAADRDSTQKAVDKITSKVNTIVDKMNKQYEIIDHEFSEVETRLNMINN
KIDDQIQDIWAYNAELLVLLENQKTLDEHDANVNNLYNKVKRALGSNAVEDGKGCFELYHKCDDQCMETI
RNGTYNRRKYQEESKLERQKIEGVKLESEGTYKILTIYSTVASSLVIAMGFAAFLFWAMSNGSCRCNICI

>QAU21808.1 hemagglutinin [Influenza A virus]

METVSLITILLVAAVSNADKICIGYQSTNSTETVDTLTENNVPVTHAKELLHTEHNGMLCATSLGQPIIL
DTCTIEGLIYGNPSCDLSLEGREWSYIVERPSAVNGLCYPGNVENLEELRSLFSSARSYQRIQIFPDTIW
NVSYDGTSTACSGSFYRNMRWLTRKNGEYPIQDAQYTNNQGKNILFMWGINHPPADTTQRDLYTRTDTTT
SVATEEINRIFKPLIGPRPLVNGLMGRIDYYWSVLKPGQTLRIKSDGNLIAPWYGHILSGESHGRILKTD
LKRGSCTVQCQTEKGGLNTTLPFQNVSKYAFGNCSKYIGIKSLKLAVGLRNVPSRSSRGLFGAIAGFIEG
GWSGLVAGWYGFQHSNDQGVGMAADRDSTQKAVDKITSKVNTIVDKMNKQYEIIDHEFSEVETRLNMINN
KIDDQIQDIWAYNAELLVLLENQKTLDEHDANVNNLYNKVKRALGSNAVEDGKGCFELYHKCDDQCMETI
RNGTYNRRKYQEESKLERQKIEGVKLESEGTYKILTIYSTVASSLVIAMGFAAFLFWAMSNGSCRCNICI

>QAU21807.1 hemagglutinin [Influenza A virus]
METVSLITILLVAAVSNADKICIGYQSTNSTETVDTLTENNVPVTHAKELLHTEHNGMLCATSLGQPIIL
DTCTIEGLIYGNPSCDLSLEGREWSYIVERPSAVNGLCYPGNVENLEELRSLFSSARSYQRIQIFPDTIW
NVSYDGTSTACSGSFYRNMRWLTRKNGEYPIQDAQYTNNQGKNILFMWGINHPPADTTQRDLYTRTDTTT
SVATEEINRIFKPLIGPRPLVNGLMGRIDYYWSVLKPGQTLRIKSDGNLIAPWYGHILSGESHGRILKTD
LKRGSCTVQCQTEKGGLNTTLPFQNVSKYAFGNCSKYIGIKSLKLAVGLRNVPSRSSRGLFGAIAGFIEG
GWSGLVAGWYGFQHSNDQGVGMAADRDSTQKAVDKITSKVNTIVDKMNKQYEIIDHEFSEVETRLNMINN
KIDDQIQDIWAYNAELLVLLENQKTLDEHDANVNNLYNKVKRALGSNAVEDGKGCFELYHKCDDQCMETI
RNGTYNRRKYQEESKLERQKIEGVKLESEGTYKILTIYSTVASSLVIAMGFAAFLFWAMSNGSCRCNICI

>QAU21806.1 hemagglutinin [Influenza A virus]
METVSLITIILVATVSNADKICIGYQSTNSTETVDTLTENNVPVTHAKELLHTEHNGMLCATSLGHPLIL
DTCTIEGLIYGNPSCDPLLGGREWSYIVERPSAVNGLCYPGNVENLEELRSLFSSSRSYQRIQIFPDTIW
NVSYSGTSRACSDSFYRSMRWLTQKNNAYPTQDAQYTNNQEKNILFMWGINHPPTDTAQTNLYTRTDTTT
SVATEEINRIFKPLIGPRPLVNGLMGRINYYWSVLKPGQTLRIKSDGNLIAPWYGHILSGESHGRILKTD
LKRGSCTVQCQTEKGGLNTTLPFQNVSKYAFGNCSKYIGVKSLKLAVGLRNVPSRSSRGLFGAIAGFIEG
GWSGLVAGWYGFQHSNDQGVGMAADRDSTQKAIDKITSKVNNIVDKMNKQYEIIDHEFSEVETRLNMINN
KVDDQIQDIWAYNAELLVLLENQKTLDEHDANVNNLYNKVKRALGSNAVEDGKGCFELYHKCDDHCMETI
RNGTYNRRKYQEESKLERQKIEGVKLESEETYKILTIYSTVASSLVIAMGFAAFLFWAMSNGSCRCNICI

>QAU21805.1 hemagglutinin [Influenza A virus]
METVSLITILLVATVSNADKICIGYQSTNSTETVDTLTENNVPVTHAKELLHTEHNGMLCATSLGHPLIL
DTCTIEGLIYGNPSCDPLLGGREWSYIVERPSAVNGLCYPGNVENLEELRSLFSSSRSYQRIQIFPDTIW
NVSYSGTSRACSDSFYRSMRWLTQKNNAYPTQDAQYTNNQEKNILFMWGINHPPTDTAQTNLYTRTDTTT
SVATEEINRIFKPLIGPRPLVNGLMGRINYYWSVLKPGQTLRIKSDGNLIAPWYGHILSGESHGRILKTD

LKRGSCTVQCQTEKGGLNTTLPFQNVSKYAFGNCSKYIGVKSLKLAVGLRNVPSRSSRGLFGAIAGFIEG
GWSGLVAGWYGFQHSNDQGVGMAADRDSTQKAIDKITSKVNNIVDKMNKQYEIIDHEFSEVETRLNMINN
KVDDQIQDIWAYNAELLVLLENQKTLDEHDANVNNLYNKVKRALGSNAVEDGKGCFELYHKCDDHCMETI
RNGTYNRRKYQEESKLERQKIEGVKLESEETYKILTIYSTVASSLVIAMGFAAFLFWAMSNGSCRCNICI

>QAU21804.1 hemagglutinin [Influenza A virus]
METVSLMTILLVATVSNADKICIGYQSTNSTETVDTLTENNVPVTHAKELLHTEHNGMLCATSLGNPLIL
DTCTIEGLIYGNPSCDPLLGGREWSYIVERPSAVDGLCYPGSVENLEELRSLFSSAGSYQRIQIFPDTIW
NVSYSGTSKACSDSFYRSMRWLTQKNTAYPIQDAQYTNNQEKNILFMWGINHPPTETAQTNLYTRTDTTT
SVATEEINRIFKPLIGPRPLVNGLMGRINYYWSVLKPGQTLRIKSDGNLIAPWYGHILSGESHGRILKTD
LKRGSCTVQCQTEKGGLNTTLPFQNVSKYAFGNCSKYIGIKSLKLAVGLRNVPSRSSRGLFGAIAGFIEG
GWSGLVAGWYGFQHSNDQGVGMAADRDSTQKAIDKITSKVNNIVDKMNKQYEIIDHEFSEVETRLNMINN
KVDDQIQDIWAYNAELLVLLENQKTLDEHDSNVNNLYNKVKRALGSNAVEDGKGCFELYHKCDNQCMETI
RNGTYNRRKYQEESKLERQKIEGVKLESEGTYKILTIYSTVASSLVIAMGFAAFLFWAMSNGSCRCNICI

>QAU21803.1 hemagglutinin [Influenza A virus]
METASLITILLAVTVSNADKICIGYQSTNSTETVDTLTENNVPVTHAKELLHTEHNGMLCATSLGHPLIL
DTCTIEGLIYGNPSCDLLLGGREWSYIVERPSAVNGLCYPGNVENLEELRSLFSSARSYQRIQIFPDTIW
NVSYSGTSKACSDSFYRSMRWLTQKDNAYPVQDAQYTNNQEKNILFMWGINHPPTDTAQTNLYTRTDTTT
SVATEEINRTFKPLIGPRPLVNGLMGRINYYWSVLKPGQTLRIKSNGNLIAPWYGHILSGESHGRILKTD
LKRGSCTVQCQTEKGGLNTTLPFQNVSKYAFGNCSKYIGIKSLKLAVGLRNVPSRSSRGLFGAIAGFIEG
GWSGLVAGWYGFQHSNDQGVGMAADRDSTQKAIDKITSKVNNIVDKMNKQYEIIDHEFSEVETRLNMINN
KIDDQIQDIWAYNAELLVLLENQKTLDEHDANVNNLYNKVKRALGSNAVEDGKGCFELYHKCDDQCMETI
RNGTYNRKKYQEESKLERQKIEGVKLESEGTYKILTIYSTVASSLVIAMGFAAFLFWAMSNGSCRCNICI

>QAU21802.1 hemagglutinin [Influenza A virus]
METASLITILLAVTVSNADKICIGYQSTNSTETVDTLTENNVPVTHAKELLHTEHNGMLCATSLGHPLIL
DTCTIEGLIYGNPSCDLLLGGREWSYIVERPSAVNGLCYPGNVENLEELRSLFSSARSYQRIQIFPDTIW
NVSYSGTSKACSDSFYRSMRWLTQKDNAYPVQDAQYTNNQEKNILFMWGINHPPTDTAQTNLYTRTDTTT
SVATEEINRTFKPLIGPRPLVNGLMGRINYYWSVLKPGQTLRIKSNGNLIAPWYGHILSGESHGRILKTD
LKRGSCTVQCQTEKGGLNTTLPFQNVSKYAFGNCSKYIGIKSLKLAVGLRNVPSRSSRGLFGAIAGFIEG
GWSGLVAGWYGFQHSNDQGVGMAADRDSTQKAIDKITSKVNNIVDKMNKQYEIIDHEFSEVETRLNMINN
KIDDQIQDIWAYNAELLVLLENQKTLDEHDANVNNLYNKVKRALGSNAVEDGKGCFELYHKCDDQCMETI
RNGTYNRKKYQEESKLERQKIEGVKLESEGTYKILTIYSTVASSLVIAMGFAAFLFWAMSNGSCRCNICI

>QAU21801.1 hemagglutinin [Influenza A virus]

METVSLITILLVATVSNADKICIGYQSTNSTETVDTLTENNVPVTHAKELLHTEHNGMLCATSLGQPLIL
DTCTIEGLIYGNPSCDLSLEGREWSYIVERPSAVNGLCYPGNVENLEELRSLFSSARSYQRIQIFPDTIW
NVSYDGTSTACSDSFYRSMRWLTRKDGNYPTQDAQYTNNQGKNILFMWGINHPPTDDTQRKLYTRTDTTT
SVATEEINRIFKPLIGPRPLVNGLMGRIDYYWSVLKPGQTLRIKSDGNLIAPWYGHILSGESHGRILKTD
LKRGSCTVQCQTEKGGLNTTLPFQNVSKYAFGNCSKYIGIKSLKLAVGLRNVPSRSSRGLFGAIAGFIEG
GWSGLVAGWYGFQHSNDQGVGMAADRDSTQKAINKITSKVNNIVDKMNKQYEIIDHEFSEVETRLNMINN
KIDDQIQDIWAYNAELLVLLENQKTLDEHDANVNNLYNKVKRALGSNAVEDGKGCFELYHKCNDQCMETI
RNGTYNRRKYQEESKLERQKIEGVKLESEGTYKILTIYSTVASSLVIAMGFAAFLFWAMSNGSCRCNICI

>QAU21800.1 hemagglutinin [Influenza A virus]

METVSLITILLVATVSNADKICIGYQSTNSTETVDTLTENNVPVTHAKELLHTEHNGMLCATSLGQPLIL
DTCTIEGLIYGNPSCDLSLEGREWSYIVERPSAVNGLCYPGNVENLEELRSLFSSARSYQRIQIFPDTIW
NVSYDGTSTACSDSFYRSMRWLTRKDGNYPTQDAQYTNNQGKNILFMWGINHPPTDDTQRKLYTRTDTTT
SVATEEINRIFKPLIGPRPLVNGLMGRIDYYWSVLKPGQTLRIKSDGNLIAPWYGHILSGESHGRILKTD
LKRGSCTVQCQTEKGGLNTTLPFQNVSKYAFGNCSKYIGIKSLKLAVGLRNVPSRSSRGLFGAIAGFIEG
GWSGLVAGWYGFQHSNDQGVGMAADRDSTQKAINKITSKVNNIVDKMNKQYEIIDHEFSEVETRLNMINN
KIDDQIQDIWAYNAELLVLLENQKTLDEHDANVNNLYNKVKRALGSNAVEDGKGCFELYHKCNDQCMETI
RNGTYNRRKYQEESKLERQKIEGVKLESEGTYKILTIYSTVASSLVIAMGFAAFLFWAMSNGSCRCNICI

>QAU21799.1 hemagglutinin [Influenza A virus]

METVSLITILLVATVSNADKICIGYQSTNSTETVDTLTENNVPVTHAKELLHTEHNGMLCATSLGQPLIL
DTCTIEGLIYGNPSCDLSLEGREWSYIVERPSAVNGLCYPGNVENLEELRSLFSSARSYQRIQIFPDTIW
NVSYDGTSTACSDSFYRSMRWLTRKDGNYPTQDAQYTNNQGKNILFMWGINHPPTDDTQRKLYTRTDTTT
SVATEEINRIFKPLIGPRPLVNGLMGRIDYYWSVLKPGQTLRIKSDGNLIAPWYGHILSGESHGRILKTD
LKRGSCTVQCQTEKGGLNTTLPFQNVSKYAFGNCSKYIGIKSLKLAVGLRNVPSRSSRGLFGAIAGFIEG
GWSGLVAGWYGFQHSNDQGVGMAADRDSTQKAINKITSKVNNIVDKMNKQYEIIDHEFSEVETRLNMINN
KIDDQIQDIWAYNAELLVLLENQKTLDEHDANVNNLYNKVKRALGSNAVEDGKGCFELYHKCNDQCMETI
RNGTYNRRKYQEESKLERQKIEGVKLESEGTYKILTIYSTVASSLVIAMGFAAFLFWAMSNGSCRCNICI

>QAU21798.1 hemagglutinin [Influenza A virus]

METVSLITILLVATVSNADKICIGYQSTNSTETVDTLTENNVPVTHAKELLHTEHNGMLCATSLGQPLIL
DTCTIEGLIYGNPSCDLSLEGREWSYIVERPSAVNGLCYPGNVENLEELRSLFSSARSYQRIQIFPDTIW

NVSYDGTSTACSDSFYRSMRWLTRKDGNYPTQDAQYTNNQGKNILFMWGINHPPTDDTQRKLYTRTDTTT
SVATEEINRIFKPLIGPRPLVNGLMGRIDYYWSVLKPGQTLRIKSDGNLIAPWYGHILSGESHGRILKTD
LKRGSCTVQCQTEKGGLNTTLPFQNVSKYAFGNCSKYIGIKSLKLAVGLRNVPSRSSRGLFGAIAGFIEG
GWSGLVAGWYGFQHSNDQGVGMAADRDSTQKAINKITSKVNNIVDKMNKQYEIIDHEFSEVETRLNMINN
KIDDQIQDIWAYNAELLVLLENQKTLDEHDANVNNLYNKVKRALGSNAVEDGKGCFELYHKCNDQCMETI
RNGTYNRRKYQEESKLERQKIEGVKLESEGTYKILTIYSTVASSLVIAMGFAAFLFWAMSNGSCRCNICI

>QAU21797.1 hemagglutinin [Influenza A virus]
METVSLITILLVATVSNADKICIGYQSTNSTETVDTLTENNVPVTHAKELLHTEHNGMLCATSLGQPLIL
DTCTIEGLIYGNPSCDLSLEGREWSYIVERPSAVNGLCYPGNVENLEELRSLFSSARSYQRIQIFPDTIW
NVSYDGTSTACSDSFYRSMRWLTRKDGNYPTQDAQYTNNQGKNILFMWGINHPPTDDTQRKLYTRTDTTT
SVATEEINRIFKPLIGPRPLVNGLMGRIDYYWSVLKPGQTLRIKSDGNLIAPWYGHILSGESHGRILKTD
LKRGSCTVQCQTEKGGLNTTLPFQNVSKYAFGNCSKYIGIKSLKLAVGLRNVPSRSSRGLFGAIAGFIEG
GWSGLVAGWYGFQHSNDQGVGMAADRDSTQKAINKITSKVNNIVDKMNKQYEIIDHEFSEVETRLNMINN
KIDDQIQDIWAYNAELLVLLENQKTLDEHDANVNNLYNKVKRALGSNAVEDGKGCFELYHKCNDQCMETI
RNGTYNRRKYQEESKLERQKIEGVKLESEGTYKILTIYSTVASSLVIAMGFAAFLFWAMSNGSCRCNICI

>QAU21796.1 hemagglutinin [Influenza A virus]
METVSLITILLVATVSNADKICIGYQSTNSTETVDTLTENNVPVTHAKELLHTEHNGMLCATSLGQPLIL
DTCTIEGLIYGNPSCDLSLEGREWSYIVERPSAVNGLCYPGNVENLEELRSLFSSARSYQRIQIFPDTIW
NVSYDGTSTACSDSFYRSMRWLTRKDGNYPTQDAQYTNNQGKNILFMWGINHPPTDDTQRKLYTRTDTTT
SVATEEINRIFKPLIGPRPLVNGLMGRIDYYWSVLKPGQTLRIKSDGNLIAPWYGHILSGESHGRILKTD
LKRGSCTVQCQTEKGGLNTTLPFQNVSKYAFGNCSKYIGIKSLKLAVGLRNVPSRSSRGLFGAIAGFIEG
GWSGLVAGWYGFQHSNDQGVGMAADRDSTQKAINKITSKVNNIVDKMNKQYEIIDHEFSEVETRLNMINN
KIDDQIQDIWAYNAELLVLLENQKTLDEHDANVNNLYNKVKRALGSNAVEDGKGCFELYHKCNDQCMETI
RNGTYNRRKYQEESKLERQKIEGVKLESEGTYKILTIYSTVASSLVIAMGFAAFLFWAMSNGSCRCNICI

>QAU21795.1 hemagglutinin [Influenza A virus]
METVSLITILLVATVSNADKICIGYQSTNSTETVDTLTENNVPVTHAKELLHTEHNGMLCATSLGQPLIL
DTCTIEGLIYGNPSCDLSLEGREWSYIVERPSAVNGLCYPGNVENLEELRSLFSSARSYQRIQIFPDTIW
NVSYDGTSTACSDSFYRSMRWLTRKDGNYPTQDAQYTNNQGKNILFMWGINHPPTDDTQRKLYTRTDTTT
SVATEEINRIFKPLIGPRPLVNGLMGRIDYYWSVLKPGQTLRIKSDGNLIAPWYGHILSGESHGRILKTD
LKRGSCTVQCQTEKGGLNTTLPFQNVSKYAFGNCSKYIGIKSLKLAVGLRNVPSRSSRGLFGAIAGFIEG
GWSGLVAGWYGFQHSNDQGVGMAADRDSTQKAINKITSKVNNIVDKMNKQYEIIDHEFSEVETRLNMINN

KIDDQIQDIWAYNAELLVLLENQKTLDEHDANVNNLYNKVKRALGSNAVEDGKGCFELYHKCNDQCMETI
RNGTYNRRKYQEESKLERQKIEGVKLESEGTYKILTIYSTVASSLVIAMGFAAFLFWAMSNGSCRCNICI

>QAU21794.1 hemagglutinin [Influenza A virus]
METVSLITILLVATVSNADKICIGYQSTNSTETVDTLTENNVPVTHAKELLHTEHNGMLCATSLGQPLIL
DTCTIEGLIYGNPSCDLSLEGREWSYIVERPSAVNGLCYPGNVENLEELRSLFSSARSYQRIQIFPDTIW
NVSYDGTSTACSDSFYRSMRWLTRKDGNYPTQDAQYTNNQGKNILFMWGINHPPTDDTQRKLYTRTDTTT
SVATEEINRIFKPLIGPRPLVNGLMGRIDYYWSVLKPGQTLRIKSDGNLIAPWYGHILSGESHGRILKTD
LKRGSCTVQCQTEKGGLNTTLPFQNVSKYAFGNCSKYIGIKSLKLAVGLRNVPSRSSRGLFGAIAGFIEG
GWSGLVAGWYGFQHSNDQGVGMAADRDSTQKAINKITSKVNNIVDKMNKQYEIIDHEFSEVETRLNMINN
KIDDQIQDIWAYNAELLVLLENQKTLDEHDANVNNLYNKVKRALGSNAVEDGKGCFELYHKCNDQCMETI
RNGTYNRRKYQEESKLERQKIEGVKLESEGTYKILTIYSTVASSLVIAMGFAAFLFWAMSNGSCRCNICI

>QAU21793.1 hemagglutinin [Influenza A virus]
METVSLITILLVAAVSNADKICIGYQSTNSTETVDTLTENNVPVTHAKELLHTEHNGMLCATSLGQPLIL
DTCTIEGLIYGNPYCDLSLEGREWSYIVERPSAVNGLCYPGNVENLEELRSLFSSARSYQRIQIFPDTIW
NVSYDGTSAACSGSFYKSMRWLTRKNGDYPIQDAQYTNNQGKNILFMWGINHPPTDTTQRDLYTRTDTTT
SVATEEINRVFKPLIGPRPLVNGLMGRIDYYWSVLKPGQTLRIKSDGNLIAPWFGHILSGESHGRILKTD
LKRGSCTVQCQTEKGGLNTTLPFQNISKYAFGNCSKYIGIKSLKLAVGLRNVPSRSSRGLFGAIAGFIEG
GWSGLVAGWYGFQHSNDQGVGMAADRDSTQKAIDKITSKVNNIVDKMNKQYEIIDHEFSEVETRLNMINN
KLDDQIQDIWAYNAELLVLLENQKTLDEHDANVNNLYNKVKRALGSNAVEDGKGCFELYHKCDDQCMETI
RNGTYNRRKYQEESKLERQKIEGVKLESEGTYKILTIYSTVASSLVIAMGFAAFLFWAMSNGSCRCNICI

>QAU21792.1 hemagglutinin [Influenza A virus]
METVSLITILLVAAVSNADKICIGYQSTNSTETVDTLTENNVPVTHAKELLHTEHNGMLCATSLGQPLIL
DTCTIEGLIYGNPYCDLSLEGREWSYIVERPSAVNGLCYPGNVENLEELRSLFSSARSYQRIQIFPDTIW
NVSYDGTSAACSGSFYKSMRWLTRKNGDYPIQDAQYTNNQGKNILFMWGINHPPTDTTQRDLYTRTDTTT
SVATEEINRVFKPLIGPRPLVNGLMGRIDYYWSVLKPGQTLRIKSDGNLIAPWFGHILSGESHGRILKTD
LKRGSCTVQCQTEKGGLNTTLPFQNISKYAFGNCSKYIGIKSLKLAVGLRNVPSRSSRGLFGAIAGFIEG
GWSGLVAGWYGFQHSNDQGVGMAADRDSTQKAIDKITSKVNNIVDKMNKQYEIIDHEFSEVETRLNMINN
KLDDQIQDIWAYNAELLVLLENQKTLDEHDANVNNLYNKVKRALGSNAVEDGKGCFELYHKCDDQCMETI
RNGTYNRRKYQEESKLERQKIEGVKLESEGTYKILTIYSTVASSLVIAMGFAAFLFWAMSNGSCRCNICI

>QAU21791.1 hemagglutinin [Influenza A virus]

METVSLITILLVAAVSNADKICIGYQSTNSTETVNTLTENNVPVTHAKELLHTEHNGMLCATSLGQPLIL
DTCTIEGLIYGNPYCDLSLEGREWSYIVERPSAVNGLCYPGNVENLEELRSLFSSARSYQRIQIFPDTIW
NVSYDGTSAACSGSFYKSMRWLTRKNGDYPIQDAQYTNNQGKNILFMWGINHPPTDTTQRDLYTRTDTTT
SVATEEINRVFKPLIGPRPLVNGLMGRIDYYWSVLKPGQTLRIKSDGNLIAPWFGHILLGESHGRILKTD
LKRGSCTVQCQTEKGGLNTTLPFQNISKYAFGNCSKYIGIKSLKLAVGLRNVPSRSSRGLFGAIAGFIEG
GWSGLVAGWYGFQHSNDQGVGMAADRDSTQKAIDKITSKVNNIVDKMNKQYEIIDHEFSEVETRLNMINN
KLDDQIQDIWAYNAELLVLLENQKTLDEHDANVNNLYNKVKRALGSNAVEDGKGCFELYHKCDDQCMETI
RNGTYNRRKYQEESKLERQKIEGVKLESEGTYKILTIYSTVASSLVIAMGFAAFLFWAMSNGSCRCNICI

>QAU21790.1 hemagglutinin [Influenza A virus]
METASLITILLAVTVSNADKICIGYQSTNSTETVDTLTENNVPVTHAKELLHTEHNGMLCATSLGHPLIL
DTCTIEGLIYGNPSCDLLLGGREWSYIVERPSAVNGLCYPGNVENLEELRSLFSSARSYQRIQIFPDTIW
NVSYSGTSKACSDSFYRSMRWLTQKDNAYPVQDAQYTNNQEKNILFMWGINHPPTDTAQTNLYTRTDTTT
SVATEEINRTFKPLIGPRPLVNGLMGRINYYWSVLKPGQTLRIKSNGNLIAPWYGHILSGESHGRILKTD
LKRGSCTVQCQTEKGGLNTTLPFQNVSKYAFGNCSKYIGIKSLKLAVGLRNVPSRSSRGLFGAIAGFIEG
GWSGLVAGWYGFQHSNDQGVGMAADRDSTQKAIDKITSKVNNIVDKMNKQYEIIDHEFSEVETRLNMINN
KIDDQIQDIWAYNAELLVLLENQKTLDEHDANVNNLYNKVKRALGSNAVEDGKGCFELYHKCDDQCMETI
RNGTYNRRKYQEESKLERQKIEGVKLESEGTYKILTIYSTVASSLVIAMGFAAFLFWAMSNGSCRCNICI

>QAU21788.1 hemagglutinin [Influenza A virus]
METVSLITILLVATVSNADKICIGYQSTNSTETVDTLTENNVPVTHAKELLHTEHNGMLCATSLGHPLIL
DTCTIEGIIYGNPSCDPLLGGREWSYIVERPSAVNGLCYPGNVENLEELRSLFSSSRSYQRIQIFPDTIW
NVSYSGTSKACSDSFYRSMRWLTQKNNAYPTQDAQYTNNQEKNILFMWGINHPPTDTAQTNLYTRTDTTT
SVATEEINRIFKPLIGPRPLVNGLMGRINYYWSVLKPGQTLRIKSNGNLIAPWYGHILSGESHGRILKTD
LKSGSCTVQCQTEKGGLNTTLPFQNVSKYAFGNCSKYIGVKSLKLAVGLRNVPSRSSRGLFGAIAGFIEG
GWSGLVAGWYGFQHSNDQGVGMAADRDSTQKAIDKITSKVNNIVDKMNKQYEIIDHEFSEVETRLNMINN
KVDDQIQDIWAYNAELLVLLENQKTLDEHDANVNNLYNKVKRALGSNAVEDGKGCFELYHKCDDHCMETI
RNGTYNRRKYQEESKLERQKIEGVKLESEETYKILTIYSTVASSLVIAMGFAAFLFWAMSNGSCRCNICI

>QAU21787.1 hemagglutinin [Influenza A virus]
METVSLIAILLVAAVSNADKICIGYQSTNSTETVDTLTENNVPVTHAKELLHTEHNGMLCATSLGQPLIL
DTCTIEGLIYGNPSCDLSLEGREWSYIVERPSAVNGLCYPGNVENLEELRSLFSSARSYQRIQIFPDTIW
NVSYDGTSAACSGSFYKSMRWLTRKNGDYPIQDAQYTNNQGKNILFMWGINHPPTDTTQRDLYTRTDTTT
SVATEEINRVFKPLIGPRPLVNGLMGRIDYYWSVLKPGQTLRIKSDGNLIAPWFGHILSGESHGRILKTD

LKRGSCTVQCQTEKGGLNTTLPFQNVSKYAFGNCSKYIGIKSLKLAVGLRNVPSRSNRGLFGAIAGFIEG
GWSGLVAGWYGFQHSNDQGVGMAADRDSTQKAIDKITSKVNNIVDKMNKQYEIIDHEFSEVETRLNMINN
KIDDQIQDIWAYNAELLVLLENQKTLDEHDANVNNLYNKVKRALGSNAVEDGKGCFELYHKCDDQCMETI
RNGTYNRRKYQEESKLGRQKIEGVKLESEGTYKILTIYSTVASSLVIAMGFAAFLFWAMSNGSCRCNICI

>QAU21786.1 hemagglutinin [Influenza A virus]
METVSLITTLLVATVSNADKICIGYQSTNSTETVDTLTENNVPVTHAKELLHTEHNGMLCATSLGQPLIL
DTCTIEGLIYGNPSCDLSLEGREWSYIVERPSAVNGLCYPGNVENLEELRSLFSSARSYQRIQIFPDTIW
NVSYDGTSTACSGSFYKSMRWLTRKNGDYPIQDAQYTNNQGKNILFMWGINHPPTDTTQRDLYTRIDTTT
SVATEEINRVFKPLIGPRPRVNGLMGRIDYYWSVLKPGQTLRIKSDGNLIAPWFGHILSGESHGRILKTD
LKRGSCTVQCQTEKGGLNSTLPFQNVSKYAFGNCSKYIGIKSLKLAVGLRNVPSRSSRGLFGAIAGFIEG
GWSGLVAGWYGFQHSNDQGVGMAADRDSTQKAIDKITSKVNNIVDKMNKQYEIIDHEFSEVETRLNMINN
KIDDQIQDIWAYNAELLVLLENQKTLDEHDANVNNLYNKVKRALGSNAVEDGKGCFELYHKCDDQCMETI
RNGTYNRRKYQEESKLERQKIEGVKLESEGTYKILTIYSTVASSLVIAMGFAAFLFWAMSNGSCRCNICI

>QAU21785.1 hemagglutinin [Influenza A virus]
METASLITILLAVTVSNADKICIGYQSTNSTETVDTLTENNVPVTHAKELLHTEHNGMLCATSLGHPLIL
DTCTIEGLIYGNPSCDLLLGGREWSYIVERPSAVNGLCYPGNVENLEELRSLFSSARSYQRIQIFPDTIW
NVSYSGTSKACSDSFYRSMRWLTQKDNAYPVQDAQYTNNQEKNILFMWGINHPPTDTAQTNLYTRTDTTT
SVATEEINRTFKPLIGPRPLVNGLMGRINYYWSVLKPGQTLRIKSNGNLIAPWYGHILSGESHGRILKTD
LKRGSCTVQCQTEKGGLNTTLPFQNVSKYAFGNCSKYIGIKSLKLAVGLRNVPSRSSRGLFGAIAGFIEG
GWSGLVAGWYGFQHSNDQGVGMAADRDSTQKAIDKITSKVNNIVDKMNKQYEIIDHEFSEVETRLNMINN
KIDDQIQDIWAYNAELLVLLENQKTLDEHDANVNNLYNKVKKALGSNAVEDGKGCFELYHKCDDQCMETI
RNGTYNRRKYQEESKLERQKIEGVKLESEGTYKILTIYSTVASSLVIAMGFAAFLFWAMSNGSCRCNICI

>QAU21784.1 hemagglutinin [Influenza A virus]
METVSLIAILLVAAVSNADKICIGYQSTNSTETVDTLTENNVPVTHAKELLHTEHNGMLCATSLGQPLIL
DTCTIEGLIYGNPSCDLSLEGREWSYIVERPSAVNGLCYPGNVENLEELRSLFSSARSYQRIQIFPDTIW
NVSYDGTSAACSGSFYKSMRWLTRKNGDYPIQDAQYTNNQGKNILFMWGINHPPTDTTQRDLYTRTDTTT
SVATEEINRVFKPLIGPRPLVNGLMGRIDYYWSVLKPGQTLRIKSDGNLIAPWFGHILSGESHGRILKTD
LKRGSCTVQCQTEKGGLNTTLPFQNVSKYAFGNCSKYIGIKSLKLAVGLRNVPSRSNRGLFGAIAGFIEG
GWSGLVAGWYGFQHSNDQGVGMAADRDSTQKAIDKITSKVNNIVDKMNKQYEIIDHEFSEVETRLNMINN
KIDDQIQDIWAYNAELLVLLENQKTLDEHDANVNNLYNKVKRALGSNAVEDGKGCFELYHKCDDQCMETI
RNGTYNRRKYQEESKLGRQKIEGVKLESEGTYKILTIYSTVASSLVIAMGFAAFLFWAMSNGSCRCNICI

>QAU21783.1 hemagglutinin [Influenza A virus]

METVSLITILLVATVSSADKICIGYQSTNSTETVDTLTENNVPVTHAKELLHTEHNGMLCATSLGQPLIL
DTCTIEGLIYGNPSCDLSLEGREWSYIVERPSAVNGLCYPGNVENLEELRSLFSSARSYQRIQIFPDTIW
NVSYDGTSTACSGSFYRSMRWLTRKDGNYPTQDAQYTNNQGENILFMWGINHPPTDDTQRNLYTRTDTTT
SVATEEINRIFKPLIGPRPLVNGLMGRIDYYWSVLKPGQTLRIKSDGNLIAPWYGHILSGESHGRILKTD
LKRGSCTVQCQTEKGGLNTTLPFQNVSKYAFGNCSKYIGIKSLKLAVGLRNVPSRSSRGLFGAIAGFIEG
GWSGLVAGWYGFQHSNDQGVGMAADRDSTQKAIDKITSKVNNIVDKMNKQYEIIDHEFSEVETRLNMINN
KIDDQIQDIWAYNAELLVLLENQKTLDEHDANVNNLYNKVKRALGSNAVEDGKGCFELYHKCNDQCMETI
RNGTYNRRKYQEESKLERQKIEGVKLESEGTYKILTIYSTVASSLVIAMGFAAFLFWAMSNGSCRCNICI

>QAU21782.1 hemagglutinin [Influenza A virus]

METASLITILLAVTVSNADKICIGYQSTNSTETVDTLTENNVPVTHAKELLHTEHNGMLCATSLGHPLIL
DTCTIEGLIYGNPSCDLLLGGREWSYIVERPSAVNGLCYPGNVENLEELRSLFSSARSYQRIQIFPDTIW
NVSYSGTSKACSDSFYRSMRWLTQKDNAYPVQDAQYTNNQEKNILFMWGINHPPTDTAQTNLYTRTDTTT
SVATEEINRTFKPLIGPRPLVNGLMGRINYYWSVLKPGQTLRIKSNGNLIAPWYGHILSGESHGRILKTD
LKRGSCTVQCQTEKGGLNTTLPFQNVSKYAFGNCSKYIGIKSLKLAVGLRNVPSRSSRGLFGAIAGFIEG
GWSGLVAGWYGFQHSNDQGVGMAADRDSTQKAIDKITSKVNNIVDKMNKQYEIIDHEFSEVETRLNMINN
KIDDQIQDIWAYNAELLVLLENQKTLDEHDANVNNLYNKVKRALGSNAVEDGKGCFELYHKCDDQCMETI
RNGTYNRRKYQEESKLERQKIEGVKLESEGTYKILTIYSTVASSLVIAMGFAAFLFWAMSNGSCRCNICI

>QAU21781.1 hemagglutinin [Influenza A virus]

METASLITILLAVTVSNADKICIGYQSTNSTETVDTLTENNVPVTHAKELLHTEHNGMLCATSLGHPLIL
DTCTIEGLIYGNPSCDLLLGGREWSYIVERPSAVNGLCYPGNVENLEELRSLFSSARSYQRIQIFPDTIW
NVSYSGTSKACSDSFYRSMRWLTQKDNAYPVQDAQYTNNQEKNILFMWGINHPPTDTVQTNLYTRTDTTT
SVATEEINRTFKPLIGPRPLVNGLMGRINYYWSVLKPGQTLRIKSNGNLIAPWYGHILSGESHGRILKTD
LKRGSCTVQCQTEKGGLNSTLPFQNVSKYAFGNCSKYIGIKSLKLAVGLRNVPSRSSRGLFGAIAGFIEG
GWSGLVAGWYGFQHSNDQGVGMAADRDSTQKAIDKITSKVNNIVDKMNKQYEIIDHEFSEVETRLNMINN
KIDDQIQDIWAYNAELLVLLENQKTLDEHDANVNNLYNKVKRALGSNAVEDGKGCFELYHKCDDQCMETI
RNGTYNRRKYQEESKLERQKIEGVKLESEGTYKILTIYSTVASSLVIAMGFAAFLFWAMSNGSCRCNICI

>QAU21780.1 hemagglutinin [Influenza A virus]

METVSLITILLVAAVSNADKICIGYQSTNSTETVDTLTENNVPVTHAKELLHTEHNGMLCATSLGQPIIL
DTCTIEGLIYGNPSCDLSLEGREWSYIVERPSAVNGLCYPGNVENLEELRSLFSSARSYQRIQIFPDTIW

NVSYDGTSTACSGSFYRNMRWLTRKNGEYPIQDAQYTNNQGKNILFMWGINHPPADTTQRDLYTRTDTTT
SVATEEVNRIFKPLIGPRPLVNGLMGRIDYYWSVLKPGQTLRIKSDGNLIAPWYGHILSGESHGRILKTD
LKRGSCTVQCQTEKGGLNTTLPFQNVSKYAFGNCSKYIGIKSLKLAVGLRNVPSRSSRGLFGAIAGFIEG
GWSGLVAGWYGFQHSNDQGVGMAADRDSTQKAVDKITSKVNTIVDKMNKQYEIIDHEFSEVETRLNMINN
KIDDQIQDIWAYNAELLVLLENQKTLDEHDANVNNLYNKVKRALGSNAVEDGKGCFELYHKCDDQCMETI
RNGTYNRKKYQEESKLERQKIEGVKLESEGTYKILTIYSTVASSLVIAMGFAAFLFWAMSNGSCRCNICI

>QAU21779.1 hemagglutinin [Influenza A virus]
METVSLITILLVATVSNADKICIGYQSTNSTETVDTLTENNVPVTHAKELLHTEHNGMLCATSLGQPLIL
DTCTIEGLIYGNPSCDLSLEGREWSYIVERPSAVNGLCYPGNVENLEELRSLFSSARSYQRIQIFPDTIW
NVSYDGTSTACSGSFYRSMRWLTRKNGDYPIQDAQYTNNQGKNILFMWGINHPPTDTTQRNLYTRIDTTT
SVATEEINRIFKPLIGPRPLVNGLMGRINYYWSVLKPGQTLRIKSDGNLIAPWYGHILSGESHGRILKTD
LKRGSCTVQCQTEKGGLNTTLPFQNVSKYAFGNCSKYIGIKSLKLAVGLRNVPSRSSRGLFGAIAGFIEG
GWSGLVAGWYGFQHSNDQGVGMAADRDSTQKAIDKITSKVNNIVDKMNKQYEIIDHEFSEVETRLNMINN
KIDDQIQDIWAYNAELLVLLENQKTLDEHDANVNNLYNKVKRALGSNAVEDGKGCFELYHKCDDQCMETI
RNGTYNRRKYQEESKLERQKIEGVKLESEGTYKILTIYSTVASSLVIAMGFAAFLFWAMSNGSCRCNICI

>QQO75684.1 hemagglutinin [Influenza A virus]
METVSLITILLVATVSNADKICIGYQSTNSTETVDTLTENNVPVTHAKELLHTEHNGMLCATSLGQPLIL
DTCTIEGLIYGNPSCDLSLEGREWSYIVERPSAVHGLCYPGKVEDLEELRSLFSSARSYQRIQIFPDTIW
NVSYDGTSTACSGSFYRSMRWLTRKNGDYPIQDAQYTNNQGKNILFMWGINHPPTDETQRGLYTRTDTTT
SVATEEINRIFKPLIGPRPLVNGLMGRINYYWSVLKPGQTLRIKSDGNLIAPWYGHILSGESHGRILKTD
LKKGSCTVQCQTEKGGLNTTLPFQNVSKYAFGNCSKYIGIKSLKLAVGLRNVPSRSSRGLFGAIAGFIEG
GWSGLVAGWYGFQHSNDQGVGMAADRDSTQKAIDKITSKVNNIVDKMNKQYEIIDHEFSEVETRLNMINN
KIDDQIQDIWAYNAELLVLLENQKTLDEHDANVNNLYNKVKRALGSNAVEDGKGCFELYHKCDDQCMETI
RNGTYNRRKYQEESKLERQKIEGVKLESEGTYKILTIYSTVASSLVIAMGFAAFLFWAMSNGSCRCNICI

>QQO75683.1 hemagglutinin [Influenza A virus]
METVSLITILLVATVSNADKICIGYQSTNSTETVDTLTENNVPVTHAKELLHTEHNGMLCATSLGQPLIL
DTCTIEGLIYGNPSCDLSLEGREWSYIVERPSAVHGLCYPGKVEDLEELRSLFSSARSYQRIQIFPDTIW
NVSYDGTSTACSGSFYRSMRWLTRKNGDYPIQDAQYTNNQGKNILFMWGINHPPTDETQRGLYTRTDTTT
SVATEEINRIFKPLIGPRPLVNGLMGRINYYWSVLKPGQTLRIKSDGNLIAPWYGHILSGESHGRILKTD
LKKGSCTVQCQTEKGGLNTTLPFQNVSKYAFGNCSKYIGIKSLKLAVGLRNVPSRSSRGLFGAIAGFIEG
GWSGLVAGWYGFQHSNDQGVGMAADRDSTQKAIDKITSKVNNIVDKMNKQYEIIDHEFSEVETRLNMINN

KIDDQIQDIWAYNAELLVLLENQKTLDEHDANVNNLYNKVKRALGSNAVEDGKGCFELYHKCDDQCMETI
RNGTYNRRKYQEESKLERQKIEGVKLESEGTYKILTIYSTVASSLVIAMGFAAFLFWAMSNGSCRCNICI

>QQO75682.1 hemagglutinin [Influenza A virus]
METVSLITILLVATVSNADKICIGYQSTNSTETVDTLTENNVPVTHAKELLHTEHNGMLCATSLGQPLIL
DTCTIEGLIYGNPSCDLSLEGREWSYIVERPSAVHGLCYPGKVEDLEELRSLFSSARSYQRIQIFPDTIW
NVSYDGTSTACSGSFYRSMRWLTRKNGDYPIQDAQYTNNQGKNILFMWGINHPPTDETQRGLYTRTDTTT
SVATEEINRIFKPLIGPRPLVNGLMGRINYYWSVLKPGQTLRIKSDGNLIAPWYGHILSGESHGRILKTD
LKKGSCTVQCQTEKGGLNTTLPFQNVSKYAFGNCSKYIGIKSLKLAVGLRNVPSRSSRGLFGAIAGFIEG
GWSGLVAGWYGFQHSNDQGVGMAADRDSTQKAIDKITSKVNNIVDKMNKQYEIIDHEFSEVETRLNMINN
KIDDQIQDIWAYNAELLVLLENQKTLDEHDANVNNLYNKVKRALGSNAVEDGKGCFELYHKCDDQCMETI
RNGTYNRRKYQEESKLERQKIEGVKLESEGTYKILTIYSTVASSLVIAMGFAAFLFWAMSNGSCRCNICI

>QQO75681.1 hemagglutinin [Influenza A virus]
METVSLITILLVATVSNADKICIGYQSTNSTETVDTLTENNVPVTHAKELLHTEHNGMLCATSLGQPLIL
DTCTIEGLIYGNPSCDLSLEGREWSYIVERPSAVHGLCYPGKVEDLEELRSLFSSARSYQRIQIFPDTIW
NVSYDGTSTACSGSFYRSMRWLTRKNGDYPIQDAQYTNNQGKNILFMWGINHPPTDETQRGLYTRTDTTT
SVATEEINRIFKPLIGPRPLVNGLMGRINYYWSVLKPGQTLRIKSDGNLIAPWYGHILSGESHGRILKTD
LKKGSCTVQCQTEKGGLNTTLPFQNVSKYAFGNCSKYIGIKSLKLAVGLRNVPSRSSRGLFGAIAGFIEG
GWSGLVAGWYGFQHSNDQGVGMAADRDSTQKAIDKITSKVNNIVDKMNKQYEIIDHEFSEVETRLNMINN
KIDDQIQDIWAYNAELLVLLENQKTLDEHDANVNNLYNKVKRALGSNAVEDGKGCFELYHKCDDQCMETI
RNGTYNRRKYQEESKLERQKIEGVKLESEGTYKILTIYSTVASSLVIAMGFAAFLFWAMSNGSCRCNICI

>QQO75680.1 hemagglutinin [Influenza A virus]
METVSLITILLVATVSNADKICIGYQSTNSTETVDTLTENNVPVTHAKELLHTEHNGMLCATSLGQPLIL
DTCTIEGLIYGNPSCDLSLEGREWSYIVERPSAVHGLCYPGKVEDLEELRSLFSSARSYQRIQIFPDTIW
NVSYDGTSTACSGSFYRSMRWLTRKNGDYPIQDAQYTNNQGKNILFMWGINHPPTDETQRGLYTRTDTTT
SVATEEINRIFKPLIGPRPLVNGLMGRINYYWSVLKPGQTLRIKSDGNLIAPWYGHILSGESHGRILKTD
LKKGSCTVQCQTEKGGLNTTLPFQNVSKYAFGNCSKYIGIKSLKLAVGLRNVPSRSSRGLFGAIAGFIEG
GWSGLVAGWYGFQHSNDQGVGMAADRDSTQKAIDKITSKVNNIVDKMNKQYEIIDHEFSEVETRLNMINN
KIDDQIQDIWAYNAELLVLLENQKTLDEHDANVNNLYNKVKRALGSNAVEDGKGCFELYHKCDDQCMETI
RNGTYNRRKYQEESKLERQKIEGVKLESEGTYKILTIYSTVASSLVIAMGFAAFLFWAMSNGSCRCNICI

>QQO75679.1 hemagglutinin [Influenza A virus]

METVSLITILLVATVSNADKICIGYQSTNSTETVDTLTENNVPVTHAKELLHTEHNGMLCATSLGQPLIL
DTCTIEGLIYGNPSCDLSLEGREWSYIVERPSAVHGLCYPGKVEDLEELRSLFSSARSYQRIQIFPDTIW
NVSYDGTSTACSGSFYRSMRWLTRKNGDYPIQDAQYTNNQGKNILFMWGINHPPTDETQRGLYTRTDTTT
SVATEEINRIFKPLIGPRPLVNGLMGRINYYWSVLKPGQTLRIKSDGNLIAPWYGHILSGESHGRILKTD
LKKGSCTVQCQTEKGGLNTTLPFQNVSKYAFGNCSKYIGIKSLKLAVGLRNVPSRSSRGLFGAIAGFIEG
GWSGLVAGWYGFQHSNDQGVGMAADRDSTQKAIDKITSKVNNIVDKMNKQYEIIDHEFSEVETRLNMINN
KIDDQIQDIWAYNAELLVLLENQKTLDEHDANVNNLYNKVKRALGSNAVEDGKGCFELYHKCDDQCMETI
RNGTYNRRKYQEESKLERQKIEGVKLESEGTYKILTIYSTVASSLVIAMGFAAFLFWAMSNGSCRCNICI

>QQO75678.1 hemagglutinin [Influenza A virus]
METVSLITILIVATVSNADKICIGYQSTNSTETVDTLTENNVPVTHAKELLHTEHNGMLCATSLGQPLIL
DTCTIEGLIYGNPSCDLSLEGREWSYIVERPSAINGLCYPGNVENLEELRSLFSSARSYQRIQIFPDTIW
NVSYDGTSTACSNSFYRSMRWLTRKDGNYPTQDAQYTNNQGKNILFMWGINHPPTDDTQRNLYTRTDTTT
SVATEEINRIFKPLIGPRPLVNGLMGRIDYYWSVLKPGQTLRIKSDGNLIAPWYGHILSGESHGRILKTD
LKRGSCTVQCQTEKGGLNTTLPFQNVSKYAFGNCSKYIGIKSLKLAVGLRNVPSRSSRGLFGAIAGFIEG
GWSGLVAGWYGFQHSNDQGVGMAADRDSTQKAIDKITSKVNNIVDKMNKQYEIIDHEFSEVETRLNMINN
KIDDQIQDIWAYNAELLVLLENQKTLDEHDANVNNLYNKVKRALGSNAVEDGKGCFELYHKCNDQCMETI
RNGTYNRRKYQEESKLERQKIEGVKLESEGTYKILTIYSTVASSLVIAMGFAAFLFWAMSNGSCRCNICI

>QQO75677.1 hemagglutinin [Influenza A virus]
METVSLITILLVATVSNADKICIGYQSTNSTETVDTLTENNVPVTHAKELLHTEHNGMLCATSLGQPLIL
DTCTIEGLIYGNPSCDLSLEGREWSYIVERPSAVHGLCYPGKVEDLEELRSLFSSARSYQRIQIFPDTIW
NVSYDGTSTACSGSFYRSMRWLTRKNGDYPIQDAQYTNNQGKNILFMWGINHPPTDETQRGLYTRTDTTT
SVATEEINRIFKPLIGPRPLVNGLMGRINYYWSVLKPGQTLRIKSDGNLIAPWYGHILSGESHGRILKTD
LKKGSCTVQCQTEKGGLNTTLPFQNVSKYAFGNCSKYIGIKSLKLAVGLRNVPSRSSRGLFGAIAGFIEG
GWSGLVAGWYGFQHSNDQGVGMAADRDSTQKAIDKITSKVNNIVDKMNKQYEIIDHEFSEVETRLNMINN
KIDDQIQDIWAYNAELLVLLENQKTLDEHDANVNNLYNKVKRALGSNAVEDGKGCFELYHKCDDQCMETI
RNGTYNRRKYQEESKLERQKIEGVKLESEGTYKILTIYSTVASSLVIAMGFAAFLFWAMSNGSCRCNICI

>QQO75676.1 hemagglutinin [Influenza A virus]
METVSLITILLVATVSNADKICIGYQSTNSTETVDTLTENNVPVTHAKELLHTEHNGMLCATSLGQPLIL
DTCTIEGLIYGNPSCDLSLEGREWSYIVERPSAVHGLCYPGKVEDLEELRSLFSSARSYQRIQIFPDTIW
NVSYDGTSTACSGSFYRSMRWLTRKNGDYPIQDAQYTNNQGKNILFMWGINHPPTDETQRGLYTRTDTTT
SVATEEINRIFKPLIGPRPLVNGLMGRINYYWSVLKPGQTLRIKSDGNLIAPWYGHILSGESHGRILKTD

LKKGSCTVQCQTEKGGLNTTLPFQNVSKYAFGNCSKYIGIKSLKLAVGLRNVPSRSSRGLFGAIAGFIEG
GWSGLVAGWYGFQHSNDQGVGMAADRDSTQKAIDKITSKVNNIVDKMNKQYEIIDHEFSEVETRLNMINN
KIDDQIQDIWAYNAELLVLLENQKTLDEHDANVNNLYNKVKRALGSNAVEDGKGCFELYHKCDDQCMETI
RNGTYNRRKYQEESKLERQKIEGVKLESEGTYKILTIYSTVASSLVIAMGFAAFLFWAMSNGSCRCNICI

>QQO75675.1 hemagglutinin [Influenza A virus]
METVSLITILLVATVSNADKICIGYQSTNSTETVDTLTENNVPVTHAKELLHTEHNGMLCATSLGQPLIL
DTCTIEGLIYGNPSCDLSLEGREWSYIVERPSAVHGLCYPGKVEDLEELRSLFSSARSYQRIQIFPDTIW
NVSYDGTSTACSGSFYRSMRWLTRKNGDYPIQDAQYTNNQGKNILFMWGINHPPTDETQRGLYTRTDTTT
SVATEEINRIFKPLIGPRPLVNGLMGRINYYWSVLKPGQTLRIKSDGNLIAPWYGHILSGESHGRILKTD
LKKGSCTVQCQTEKGGLNTTLPFQNVSKYAFGNCSKYIGIKSLKLAVGLRNVPSRSSRGLFGAIAGFIEG
GWSGLVAGWYGFQHSNDQGVGMAADRDSTQKAIDKITSKVNNIVDKMNKQYEIIDHEFSEVETRLNMINN
KIDDQIQDIWAYNAELLVLLENQKTLDEHDANVNNLYNKVKRALGSNAVEDGKGCFELYHKCDDQCMETI
RNGTYNRRKYQEESKLERQKIEGVKLESEGTYKILTIYSTVASSLVIAMGFAAFLFWAMSNGSCRCNICI

>QQO75674.1 hemagglutinin [Influenza A virus]
METVSLITILLVATVSNADKICIGYQSTNSTETVDTLTENNVPVTHAKELLHTEHNGMLCATSLGQPLIL
DTCTIEGLIYGNPSCDLSLEGREWSYIVERPSAVHGLCYPGKVEDLEELRSLFSSARSYQRIQIFPDTIW
NVSYDGTSTACSGSFYRSMRWLTRKNGDYPIQDAQYTNNQGKNILFMWGINHPPTDETQRGLYTRTDTTT
SVATEEINRIFKPLIGPRPLVNGLMGRINYYWSVLKPGQTLRIKSDGNLIAPWYGHILSGESHGRILKTD
LKKGSCTVQCQTEKGGLNTTLPFQNVSKYAFGNCSKYIGIKSLKLAVGLRNVPSRSSRGLFGAIAGFIEG
GWSGLVAGWYGFQHSNDQGVGMAADRDSTQKAIDKITSKVNNIVDKMNKQYEIIDHEFSEVETRLNMINN
KIDDQIQDIWAYNAELLVLLENQKTLDEHDANVNNLYNKVKRALGSNAVEDGKGCFELYHKCDDQCMETI
RNGTYNRRKYQEESKLERQKIEGVKLESEGTYKILTIYSTVASSLVIAMGFAAFLFWAMSNGSCRCNICI

>QQO75673.1 hemagglutinin [Influenza A virus]
METVSLITILLVATVSNADKICIGYQSTNSTETVDTLTENNVPVTHAKELLHTEHNGMLCATSLGQPLIL
DTCTIEGLIYGNPSCDLSLEGREWSYIVERPSAVHGLCYPGNVEDLEELRSLFSSARSYQRIQIFPDTIW
NVSYDGTSTACSGSFYRSMRWLTRKNGEYPIQDAQYTNNQGKNILFMWGINHPPTDDTQRGLYTRTDTTT
SVATEEINRIFKPLIGPRPLVNGLMGRINYYWSVLKPGQTLRIKSDGNLIAPWYGHILSGESHGRILKTD
LKRGSCTVQCQTEKGGLNTTLPFQNVSKYAFGNCSKYIGIKSLKLAVGLRNVPSRSSRGLFGAIAGFIEG
GWPGLVAGWYGFQHSNDQGVGMAADRDSTQKAIDKITSKVNNIVDKMNKQYEIIDHEFSEVETRLNMINN
KIDDQIQDIWAYNAELLVLLENQKTLDEHDANVNNLYSKVKRALGSNAVEDGKGCFELYHKCDDQCMETI
RNGTYNRRKYQEESKLERQRIEGVKLESEGTYKILTIYSTVASSLVIAMGFAAFLFWAMSNGSCRCNICI

>QQO75672.1 hemagglutinin [Influenza A virus]

METVSLITILLVATVSNADKICIGYQSTNSTETVDTLTENNVPVTHAKELLHTEHNGMLCATSLGQPLIL
DTCTIEGLIYGNPSCDLSLEGREWSYIVERPSAVHGLCYPGNVEDLEELRSLFSSARSYQRIQIFPDTIW
NVSYDGTSTACSGSFYKSMRWLTRKNGEYPTQDAQYTNNQGKNILFMWGINHPPTDDTQRGLYTRTDTTT
SVATEEINRIFKPLIGPRPLVNGLMGRINYYWSVLKPGQTLRIKSDGNLIAPWYGHILSGESHGRILKTD
LKRGSCTVQCQTEKGGLNTTLPFQNVSKYAFGNCSKYIGIKSLKLAVGLRNVPSRSSRGLFGAIAGFIEG
GWSGLVAGWYGFQHSNDQGVGMAADRDSTQKAIDKITSKVNNIVDKMNKQYEIIDHEFSEVETRLNMINN
KIDDQIQDIWAYNAELLVLLENQKTLDEHDANVNNLYNKVKRALGSNAVEDGKGCFELYHKCDDQCMETI
RNGTYNRRKYQEESKLERQRIEGVKLESEGTYKILTIYSTVASSLVIAMGFAAFLFWAMSNGSCRCNICI

>QQO75671.1 hemagglutinin [Influenza A virus]

METVSLITILLVATASNADKICIGYQSTNSTETVDTLTENNVPVTHAKELLHTEHNGMLCATRLGQPLIL
DTCTIEGLIYGNPSCDLSLEGREWSYIVERPSAVHGLCYPGNVEDLEELRSLFSSARSYQRIQIFPDTIW
NVSYDGTSTACSGSFYRSMRWLTRKNGEYPIQDAQYTNNQGKNILFMWGINHPPTDDTQRGLYTRTDTTT
SVATEEINRIFKPLIGPRPRVNGLMGRINYYWSVLKPGQTLRIKSDGNLIAPWYGHILSGESHGRILKTD
LKRGSCTVQCQTEKGGLNTTLPFQNVSKYAFGNCSKYIGIKSLKLAVGLRNVPSRSSRGLFGAIAGFIEG
GWSGLVAGWYGFQHSNDQGVGMAADRDSTQKAIDKITSKVNNIVDKMNKQYEIIDHEFSEVETRLNMINN
KIDDQIQDIWAYNAELLVLLENQKTLDEHDANVNNLYNKVKRALGSNAVEDGKGCFELYHKCDDKCMETI
RNGTYNRRKYQEESKLERQRIEGVKLESEGTYKILTIYSTVASSLVIAMGFAAFLFWAMSNGSCRCNICI

>QQO75670.1 hemagglutinin [Influenza A virus]

METVSLITILIVATVSNADKICIGYQSTNSTETVDTLTENNVPVTHAKELLHTEHNGMLCATSLGQPLIL
DTCTIEGLIYGNPSCDLSLEGREWSYIVERPSAINGLCYPGNVENLEELRSLFSSARSYQRIQIFPDTIW
NVSYDGTSTACSNSFYRSMRWLTRKDGNYPTQDAQYTNNQGKNILFMWGINHPPTDDTQRNLYTRTDTTT
SVATEEINRIFKPLIGPRPLVNGLMGRIDYYWSVLKPGQTLRIKSDGNLIAPWYGHILSGESHGRILKTD
LKRGSCTVQCQTEKGGLNTTLPFQNVSKYAFGNCSKYIGIKSLKLAVGLRNVPSRSSRGLFGAIAGFIEG
GWSGLVAGWYGFQHSNDQGVGMAADRDSTQKAIDKITSKVNNIVDKMNKQYEIIDHEFSEVETRLNMINN
KIDDQIQDIWAYNAELLVLLENQKTLDEHDANVNNLYNKVKRALGSNAVEDGKGCFELYHKCNDQCMETI
RNGTYNRRKYQEESKLERQKIEGVKLESEGTYKILTIYSTVASSLVIAMGFAAFLFWAMSNGSCRCNICI

>QQO75669.1 hemagglutinin [Influenza A virus]

METVSLITILLVATVSNADKICIGYQSTNSTETVDTLTENNVPVTHAKELLHTEHNGMLCATSLGQPLIL
DTCTIEGIIYGNPSCDLSLEGREWSYIVERPSAVNGLCYPGNVENLEELRSLFSSARSYQRVQIFPDTIW

NVSYDGTSTACSDSFYRSMRWLTRKDGNYPTQDAQYTNNQGKNILFMWGINHPPTDDTQRNLYTRTDTTT
SVATEEINRIFKPLIGPRPLVNGLMGRIDYYWSVLKPGQTLRIKSDGNLIAPWYGHILSGESHGRILKTD
LKRGSCTVQCQTEKGGLNTTLPFQNVSKYAFGNCSKYIGIKSLKLAVGLRNVPSRSSRGLFGAIAGFIEG
GWSGLVAGWYGFQHSNDQGVGMAADRDSTQKAIDKITSKVNNIVDKMNKQYEIIDHEFSEVETRLNMINN
KIDDQIQDIWAYNAELLVLLENQKTLDEHDANVNNLYNKVKRALGSNAVEDGKGCFELYHKCNDQCMESI
RNGTYNRRKYQEESKLERQKIEGVKLESEGTYKILTIYSTVASSLVIAMGFAAFLFWAMSNGSCRCNICI

>QQO75668.1 hemagglutinin [Influenza A virus]
METVSLITILLVATVSNADKICIGYQSTNSTETVDTLTENNVPVTHAKELLHTEHNGMLCATSLGQPLIL
DTCTIEGLIYGNPSCDLSLEGREWSYIVERPSAVHGLCYPGNVEDLEELRSLFSSARSYQRIQIFPDTIW
NVSYDGTSTACSGSFYKSMRWLTRKNGEYPTQDAQYTNNQGKNILFMWGINHPPTDDTQRGLYTRTDTTT
SVATEEINRIFKPLIGPRPLVNGLMGRINYYWSVLKPGQTLRIKSDGNLIAPWYGHILSGESHGRILKTD
LKRGSCTVQCQTEKGGLNTTLPFQNVSKYAFGNCSKYIGIKSLKLAVGLRNVPSRSSRGLFGAIAGFIEG
GWSGLVAGWYGFQHSNDQGVGMAADRDSTQKAIDKITSKVNNIVDKMNKQYEIIDHEFSEVETRLNMINN
KIDDQIQDIWAYNAELLVLLENQKTLDEHDANVNNLYNKVKRALGSNAVEDGKGCFELYHKCDDQCMETI
RNGTYNRRKYQEESKLERQRIEGVKLESEGTYKILTIYSTVASSLVIAMGFAAFLFWAMSNGSCRCNICI

>QQO75667.1 hemagglutinin [Influenza A virus]
METVSLITILLVATVSNADKICIGYQSTNSTETVDTLTENNVPVTHAKELLHTEHNGMLCATSLGQPLIL
DTCTIEGLIYGNPSCDLSLEGREWSYIVERPSAVHGLCYPGKVEDLEELRSLFSSARSYQRIQIFPDTIW
NVSYDGTSTACSGSFYRSMRWLTRKNGDYPIQDAQYTNNQGKNILFMWGINHPPTDETQRGLYTRTDTTT
SVATEEINRIFKPLIGPRPLVNGLMGRINYYWSVLKPGQTLRIKSDGNLIAPWYGHILSGESHGRILKTD
LKKGSCTVQCQTEKGGLNTTLPFQNVSKYAFGNCSKYIGIKSLKLAVGLRNVPSRSSRGLFGAIAGFIEG
GWSGLVAGWYGFQHSNDQGVGMAADRDSTQKAIDKITSKVNNIVDKMNKQYEIIDHEFSEVETRLNMINN
KIDDQIQDIWAYNAELLVLLENQKTLDEHDANVNNLYNKVKRALGSNAVEDGKGCFELYHKCDDQCMETI
RNGTYNRRKYQEESKLERQKIEGVKLESEGTYKILTIYSTVASSLVIAMGFAAFLFWAMSNGSCRCNICI

>QQO75666.1 hemagglutinin [Influenza A virus]
METVSLITILLVATVSNADKICIGYQSTNSTETVDTLTENNVPVTHAKELLHTEHNGMLCATSLGQPLIL
DTCTIEGLIYGNPSCDLSLEGREWSYIVERPSAVHGLCYPGKVEDLEELRSLFSSARSYQRIQIFPDTIW
NVSYDGTSTACSGSFYRSMRWLTRKNGDYPIQDAQYTNNQGKNILFMWGINHPPTDETQRGLYTRTDTTT
SVATEEINRIFKPLIGPRPLVNGLMGRINYYWSVLKPGQTLRIKSDGNLIAPWYGHILSGESHGRILKTD
LKKGSCTVQCQTEKGGLNTTLPFQNVSKYAFGNCSKYIGIKSLKLAVGLRNVPSRSSRGLFGAIAGFIEG
GWSGLVAGWYGFQHSNDQGVGMAADRDSTQKAIDKITSKVNNIVDKMNKQYEIIDHEFSEVETRLNMINN

KIDDQIQDIWAYNAELLVLLENQKTLDEHDANVNNLYNKVKRALGSNAVEDGKGCFELYHKCDDQCMETI
RNGTYNRRKYQEESKLERQKIEGVKLESEGTYKILTIYSTVASSLVIAMGFAAFLFWAMSNGSCRCNICI

>QQO75665.1 hemagglutinin [Influenza A virus]
METVSLITILLVATVSNADKICIGYQSTNSTETVDTLTENNVPVTHAKELLHTEHNGMLCATSLGQPLIL
DTCTIEGLIYGNPSCDLSLEGREWSYIVERPSAVHGLCYPGNVEDLEELRSLFSSARSYQRIQIFPDTIW
NVSYDGTSTACSGSFYKSMRWLTRKNGEYPTQDAQYTNNQGKNILFMWGINHPPTDDTQRGLYTRTDTTT
SVATEEINRIFKPLIGPRPLVNGLMGRINYYWSVLKPGQTLRIKSDGNLIAPWYGHILSGESHGRILKTD
LKRGSCTVQCQTEKGGLNTTLPFQNVSKYAFGNCSKYIGIKSLKLAVGLRNVPSRSSRGLFGAIAGFIEG
GWSGLVAGWYGFQHSNDQGVGMAADRDSTQKAIDKITSKVNNIVDKMNKQYEIIDHEFSEVETRLNMINN
KIDDQIQDIWAYNAELLVLLENQKTLDEHDANVNNLYNKVKRALGSNAVEDGKGCFELYHKCDDQCMETI
RNGTYNRRKYQEESKLERQRIEGVKLESEGTYKILTIYSTVASSLVIAMGFAAFLFWAMSNGSCRCNICI

>QQO75664.1 hemagglutinin [Influenza A virus]
METVSLITILIVATVSNADKICIGYQSTNSTETVDTLTENNVPVTHAKELLHTEHNGMLCATSLGQPLIL
DTCTIEGLIYGNPSCDLSLEGREWSYIVERPSAINGLCYPGNVENLEELRSLFSSARSYQRIQIFPDTIW
NVSYDGTSTACSNSFYRSMRWLTRKDGNYPTQDAQYTNNQGKNILFMWGINHPPTDDTQRNLYTRTDTTT
SVATEEINRIFKPLIGPRPLVNGLMGRIDYYWSVLKPGQTLRIKSDGNLIAPWYGHILSGESHGRILKTD
LKRGSCTVQCQTEKGGLNTTLPFQNVSKYAFGNCSKYIGIKSLKLAVGLRNVPSRSSRGLFGAIAGFIEG
GWSGLVAGWYGFQHSNDQGVGMAADRDSTQKAIDKITSKVNNIVDKMNKQYEIIDHEFSEVETRLNMINN
KIDDQIQDIWAYNAELLVLLENQKTLDEHDANVNNLYNKVKRALGSNAMEDGKGCFELYHKCNDQCMETI
RNGTYNRRKYQEESKLERQKIEGVKLESEGTYKILTIYSTVASSLVIAMGFAAFLFWAMSNGSCRCNICI

>QQO75663.1 hemagglutinin [Influenza A virus]
METVSLITILLVATVSNADKICIGYQSTNSTETVDTLTENNVPVTHAKELLHTEHNGMLCATSLGQPLIL
DTCTIEGLIYGNPSCDLSLEGREWSYIVERPSAVHGLCYPGKVEDLEELRSLFSSARSYQRIQIFPDTIW
NVSYDGTSTACSGSFYRSMRWLTRKNGDYPIQDAQYTNNQGKNILFMWGINHPPTDETQRGLYTRTDTTT
SVATEEINRIFKPLIGPRPLVNGLMGRINYYWSVLKPGQTLRIKSDGNLIAPWYGHILSGESHGRILKTD
LKKGSCTVQCQTEKGGLNTTLPFQNVSKYAFGNCSKYIGIKSLKLAVGLRNVPSRSSRGLFGAIAGFIEG
GWSGLVAGWYGFQHSNDQGVGMAADRDSTQKAIDKITSKVNNIVDKMNKQYEIIDHEFSEVETRLNMINN
KIDDQIQDIWAYNAELLVLLENQKTLDEHDANVNNLYNKVKRALGSNAVEDGKGCFELYHKCDDQCMETI
RNGTYNRRKYQEESKLERQKIEGVKLESEGTYKILTIYSTVASSLVIAMGFAAFLFWAMSNGSCRCNICI

>QQO75662.1 hemagglutinin [Influenza A virus]

METVSLITILLVATVSNADKICIGYQSTNSTETVDTLTENNVPVTHAKELLHTEHNGMLCATSLGQPLIL
DTCTIEGLIYGNPSCDLSLEGREWSYIVERPSAVHGLCYPGKVEDLEELRSLFSSARSYQRIQIFPDTIW
NVSYDGTSTACSGSFYRSMRWLTRKNGDYPIQDAQYTNNQGKNILFMWGINHPPTDETQRGLYTRTDTTT
SVATEEINRIFKPLIGPRPLVNGLMGRINYYWSVLKPGQTLRIKSDGNLIAPWYGHILSGESHGRILKTD
LKKGSCTVQCQTEKGGLNTTLPFQNVSKYAFGNCSKYIGIKSLKLAVGLRNVPSRSSRGLFGAIAGFIEG
GWSGLVAGWYGFQHSNDQGVGMAADRDSTQKAIDKITSKVNNIVDKMNKQYEIIDHEFSEVETRLNMINN
KIDDQIQDIWAYNAELLVLLENQKTLDEHDANVNNLYNKVKRALGSNAVEDGKGCFELYHKCDDQCMETI
RNGTYNRRKYQEESKLERQKIEGVKLESEGTYKILTIYSTVASSLVIAMGFAAFLFWAMSNGSCRCNICI

>QQO75661.1 hemagglutinin [Influenza A virus]
METVSLITILLVATVSNADKICIGYQSTNSTETVDTLTENNVPVTHAKELLHTEHNGMLCATSLGQPLIL
DTCTIEGLIYGNPSCDLSLEGREWSYIVERPSAVHGLCYPGNVEDLEELRSLFSSARSYQRIQIFPDTIW
NVSYDGTSTACSGSFYKSMRWLTRKNGEYPTQDAQYTNNQGKNILFMWGINHPPTDDTQRGLYTRTDTTT
SVATEEINRIFKPLIGPRPLVNGLMGRINYYWSVLKPGQTLRIKSDGNLIAPWYGHILSGESHGRILKTD
LKRGSCTVQCQTEKGGLNTTLPFQNVSKYAFGNCSKYIGIKSLKLAVGLRNVPSRSSRGLFGAIAGFIEG
GWSGLVAGWYGFQHSNDQGVGMAADRDSTQKAIDKITSKVNNIVDKMNKQYEIIDHEFSEVETRLNMINN
KIDDQIQDIWAYNAELLVLLENQKTLDEHDANVNNLYNKVKRALGSNAVEDGKGCFELYHKCDDQCMETI
RNGTYNRRKYQEESKLERQRIEGVKLESEGTYKILTIYSTVASSLVIAMGFAAFLFWAMSNGSCRCNICI

>QQO75660.1 hemagglutinin [Influenza A virus]
METVSLITILLVATVSNADKICIGYQSTNSTETVDTLTENNVPVTHAKELLHTEHNGMLCATSLGQPLIL
DTCTIEGLIYGNPSCDLSLEGREWSYIVERPSAVHGLCYPGKVEDLEELRSLFSSARSYQRIQIFPDTIW
NVSYDGTSTACSGSFYRSMRWLTRKNGDYPIQDAQYTNNQGKNILFMWGINHPPTDETQRGLYTRTDTTT
SVATEEINRIFKPLIGPRPLVNGLMGRINYYWSVLKPGQTLRIKSDGNLIAPWYGHILSGESHGRILKTD
LKKGSCTVQCQTEKGGLNTTLPFQNVSKYAFGNCSKYIGIKSLKLAVGLRNVPSRSSRGLFGAIAGFIEG
GWSGLVAGWYGFQHSNDQGVGMAADRDSTQKAIDKITSKVNNIVDKMNKQYEIIDHEFSEVETRLNMINN
KIDDQIQDIWAYNAELLVLLENQKTLDEHDANVNNLYNKVKRALGSNAVEDGKGCFELYHKCDDQCMETI
RNGTYNRRKYQEESKLERQKIEGVKLESEGTYKILTIYSTVASSLVIAMGFAAFLFWAMSNGSCRCNICI

>QQO75659.1 hemagglutinin [Influenza A virus]
METVSLITILIVATVSNADKICIGYQSTNSTETVDTLTENNVPVTHAKELLHTEHNGMLCATSLGQPLIL
DTCTIEGLIYGNPSCDLSLEGREWSYIVERPSAINGLCYPGNVENLEELRSLFSSARSYQRIQIFPDTIW
NVSYDGTSTACSNSFYRSMRWLTRKDGNYPTQDAQYTNNQGKNILFMWGINHPPTDDTQRNLYTRTDTTT
SVATEEINRIFKPLIGPRPLVNGLMGRIDYYWSVLKPGQTLRIKSDGNLIAPWYGHILSGESHGRILKTD

LKRGSCTVQCQTEKGGLNTTLPFQNVSKYAFGNCSKYIGIKSLKLAVGLRNVPSRSSRGLFGAIAGFIEG
GWSGLVAGWYGFQHSNDQGVGMAADRDSTQKAIDKITSKVNNIVDKMNKQYEIIDHEFSEVETRLNMINN
KIDDQIQDIWAYNAELLVLLENQKTLDEHDANVNNLYNKVKRALGSNAVEDGKGCFELYHKCNDQCMETI
RNGTYNRRKYQEESKLERQKIEGVKLESEGTYKILTIYSTVASSLVIAMGFAAFLFWAMSNGSCRCNICI

>QQO75658.1 hemagglutinin [Influenza A virus]
METVSLITILLVATVSNADKICIGYQSTNSTETVDTLTENNVPVTHAKELLHTEHNGMLCATSLGNPLIL
DTCTIEGLIYGNPSCDPLLGGREWSYIVERPSAVNGLCYPGIVENLEELRSLFSSARSYQRIQIFPETIW
NVTYSGTSKACSDSFYRNMRWLTQKNNAYPIQDAQYTNNQEKNILFMWGINHPPTETSQTNLYTRTDTTT
SVATEEINRIFKPLIGPRPLVNGLMGRINYYWSVLKPGQTLRIKSDGNLIAPWYGHILSGESHGRILKTD
LKRGSCTVQCQTEKGGLNTTLPFQNVSKYAFGNCSKYIGIKSLKLAVGLRNVPSRSSRGLFGAIAGFIEG
GWSGLVAGWYGFQHSNDQGVGMAADRDSTQKAIDKITSKVNNIVDKMNKQYEIIDHEFSEVETRLNMINN
KVDDQIQDIWAYNAELLVLLENQKTLDEHDSNVNNLYNKVKRALGSNAVEDGKGCFELYHKCDDQCMETI
RNGTYNRRKYQEESKLERQKIEGVKLESEGTYKILTIYSTVASSLVIAMGFAAFLFWAMSNGSCRCNICI

>QQO75657.1 hemagglutinin [Influenza A virus]
METVSLMTILLVATVSNADKICIGYQSTNSTETVDTLTENNVPVTHAKELLHTEHNGMLCATSLGNPLIL
DTCTIEGLIYGNPSCDPLLGGREWSYIVERPSAVNGLCYPGIVENLEELRSLFSSARSYQRIQIFPETIW
NVTYSGTSKACSDSFYRNMRWLTQKNNAYPIQDAQYTNNQEKNILFMWGINHPPTETLQTNLYTRTDTTT
SVATEEINRIFKPLIGPRPLVNGLMGRINYYWSVLKPGQTLRIKSDGNLIAPWYGHILSGESHGRILKTD
LKRGSCTVQCQTEKGGLNTTLPFQNVSKYAFGNCSKYIGIKSLKLAVGLRNVPSRSSRGLFGAIAGFIEG
GWSGLVAGWYGFQHSNDQGVGMAADRDSTQKAIDKITSKVNNIVDKMNKQYEIIDHEFSEVETRLNMINN
KVDDQIQDIWAYNAELLVLLENQKTLDEHDSNVNNLYNKVKRALGSNAVEDGKGCFELYHKCDDQCMETI
RNGTYNRRKYQEESKLERQKIEGVKLESEGTYKILTIYSTVASSLVIAMGFAAFLFWAMSNGSCRCNICI

>QQO75656.1 hemagglutinin [Influenza A virus]
METVSLITILIVATVSKADKICIGYQSTNSTETVDTLTENNVPVTHAKELLHTEHNGMLCATSLGHPLIL
DTCTIEGLIYGNPSCDPLLGGREWSYIVERPSAVNGLCYPGNVENLEELRSLFSSSRSYQRIQIFPDTIW
NVSYSGTSKACSDSFYRSMRWLTQKNNAYPTQDAQYTNNQGKNILFMWGINHPPTDTAQTNLYTRIDTTT
SVATEEMNRIFKPLIGPRPLVNGLMGRINYYWSVLKPGQTLRIKSDGNLIAPWYGHILSGESHGRILKTD
LKRGSCTVQCQTEKGGLNTTLPFQNVSKYAFGNCSKYIGVKSLKLAVGLRNVPSRSSRGLFGAIAGFIEG
GWSGLVAGWYGFQHSNDQGVGMAADRDSTQKAIDKITSKVNNIVDKMNKQYEIIDHEFSEVETRLNMINN
KVDDQIQDIWAYNAELLVLLENQKTLDEHDANVNNLYNKVKRALGSNAVEDGKGCFELYHKCDDHCMETI
RNGTYNRRKYQEESKLERQKIEGVKLESEETYKILTIYSTVASSLVIAMGFAAFLFWAMSNGSCRCNICI

>QQO75655.1 hemagglutinin [Influenza A virus]

METVSLITILLVATVSNADKICIGYQSTNSTETVDTLTENNVPVTHAKELLHTEHNGMLCATSLGQPLIL
DTCTIEGLIYGNPSCDLSLEGREWSYIVERPSAVHGLCYPGKVEDLEELRSLFSSARSYQRIQIFPDTIW
NVSYDGTSTACSGSFYRSMRWLTRKNGDYPIQDAQYTNNQGKNILFMWGINHPPTDETQRGLYTRTDTTT
SVATEEINRIFKPLIGPRPLVNGLMGRINYYWSVLKPGQTLRIKSDGNLIAPWYGHILSGESHGRILKTD
LKKGSCTVQCQTEKGGLNTTLPFQNVSKYAFGNCSKYIGIKSLKLAVGLRNVPSRSSRGLFGAIAGFIEG
GWSGLVAGWYGFQHSNDQGVGMAADRDSTQKAIDKITSKVNNIVDKMNKQYEIIDHEFSEVETRLNMINN
KIDDQIQDIWAYNAELLVLLENQKTLDEHDANVNNLYNKVKRALGSNAVEDGKGCFELYHKCDDQCMETI
RNGTYNRRKYQEESKLERQKIEGVKLESEGTYKILTIYSTVASSLVIAMGFAAFLFWAMSNGSCRCNICI

>QQO75654.1 hemagglutinin [Influenza A virus]

METVSLITILLVATVSNADKICIGYQSTNSTETVDTLTENNVPVTHAKELLHTEHNGMLCATSLGQPLIL
DTCTIEGLIYGNPSCDLSLEGREWSYIVERPSAVHGLCYPGKVEDLEELRSLFSSARSYQRIQIFPDTIW
NVSYDGTSTACSGSFYRSMRWLTRKNGDYPIQDAQYTNNQGKNILFMWGINHPPTDETQRGLYTRTDTTT
SVATEEINRIFKPLIGPRPLVNGLMGRINYYWSVLKPGQTLRIKSDGNLIAPWYGHILSGESHGRILKTD
LKKGSCTVQCQTEKGGLNTTLPFQNVSKYAFGNCSKYIGIKSLKLAVGLRNVPSRSSRGLFGAIAGFIEG
GWSGLVAGWYGFQHSNDQGVGMAADRDSTQKAIDKITSKVNNIVDKMNKQYEIIDHEFSEVETRLNMINN
KIDDQIQDIWAYNAELLVLLENQKTLDEHDANVNNLYNKVKRALGSNAVEDGKGCFELYHKCDDQCMETI
RNGTYNRRKYQEESKLERQKIEGVKLESEGTYKILTIYSTVASSLVIAMGFAAFLFWAMSNGSCRCNICI

>QQO75653.1 hemagglutinin [Influenza A virus]

METVSLITILIVATVSNADKICIGYQSTNSTETVDTLTENNVPVTHAKELLHTEHNGMLCATSLGQPLIL
DTCTIEGLIYGNPSCDLSLEGREWSYIVERPSAINGLCYPGNVENLEELRSLFSSARSYQRIQIFPDTIW
NVSYDGTSTACSNSFYRSMRWLTRKDGNYPTQDAQYTNNQGKNILFMWGINHPPTDDTQRNLYTRTDTTT
SVATEEINRIFKPLIGPRPLVNGLMGRIDYYWSVLKPGQTLRIKSDGNLIAPWYGHILSGESHGRILKTD
LKRGSCTVQCQTEKGGLNTTLPFQNVSKYAFGNCSKYIGIKSLKLAVGLRNVPSRSSRGLFGAIAGFIEG
GWSGLVAGWYGFQHSNDQGVGMAADRDSTQKAIDKITSKVNNIVDKMNKQYEIIDHEFSEVETRLNMINN
KIDDQIQDIWAYNAELLVLLENQKTLDEHDANVNNLYNKVKRALGSNAVEDGKGCFELYHKCNDQCMETI
RNGTYNRRKYQEESKLERQKIEGVKLESEGTYKILTIYSTVASSLVIAMGFAAFLFWAMSNGSCRCNICI

>QQO75652.1 hemagglutinin [Influenza A virus]

METVSLITILIVATVSKADKICIGYQSTNSTETVDTLTENNVPVTHAKELLHTEHNGMLCATSLGHPLIL
DTCTIEGLIYGNPSCDPLLGGREWSYIVERPSAVNGLCYPGNVENLEELRSLFSSSRSYQRIQIFPDTIW

NVSYSGTSKACSDSFYRSMRWLTQKNNAYPTQDAQYTNNQGKNILFMWGINHPPTDTAQTNLYTRTDTTT
SVATEEMNRIFKPLIGPRPLVNGLMGRINYYWSVLKPGQTLRIKSDGNLIAPWYGHILSGESHGRILKTD
LKRGSCTVQCQTEKGGLNTTLPFQNVSKYAFGNCSKYIGVKSLKLAVGLRNVPSRSSRGLFGAIAGFIEG
GWSGLVAGWYGFQHSNDQGVGMAADRDSTQKAIDKITSKVNNIVDKMNKQYEIIDHEFSEVETRLNMINN
KVDDQIQDIWAYNAELLVLLENQKTLDEHDANVNNLYNKVKRALGSNAVEDGKGCFELYHKCDDHCMETI
RNGTYNRRKYQEESKLERQKIEGVKLESEETYKILTIYSTVASSLVIAMGFAAFLFWAMSNGSCRCNICI

>QQO75651.1 hemagglutinin [Influenza A virus]
METVSLITILLVATVSNADKICIGYQSTNSTETVDTLTENNVPVTHAKELLHTEHNGMLCATSLGQPLIL
DTCTIEGLIYGNPSCDLSLEGREWSYIVERPSAVHGLCYPGNVEDLEELRSLFSSARSYQRIQIFPDTIW
NVSYDGTSTACSGSFYKSMRWLTRKNGEYPTQDAQYTNNQGKNILFMWGINHPPTDDTQRGLYTRTDTTT
SVATEEINRIFKPLIGPRPLVNGLMGRINYYWSVLKPGQTLRIKSDGNLIAPWYGHILSGESHGRILKTD
LKRGSCTVQCQTEKGGLNTTLPFQNVSKYAFGNCSKYIGIKSLKLAVGLRNVPSRSSRGLFGAIAGFIEG
GWSGLVAGWYGFQHSNDQGVGMAADRDSTQKAIDKITSKVNNIVDKMNKQYEIIDHEFSEVETRLNMINN
KIDDQIQDIWAYNAELLVLLENQKTLDEHDANVNNLYNKVKRALGSNAVEDGKGCFELYHKCDDQCMETI
RNGTYNRRKYQAESKLERQRIEGVKLESEGTYKILTIYSTVASSLVIAMGFAAFLFWAMSNGSWRCNICI

>QQO75650.1 hemagglutinin [Influenza A virus]
METVSLITILLVATVSNADKICIGYQSTNSTETVDTLTENNVPVTHAKELLHTEHNGMLCATSLGQPLIL
DTCTIEGLIYGNPSCDLSLEGREWSYIVERPSAVHGLCYPGNVEDLEELRSLFSSARSYQRIQIFPDTIW
NVSYDGTSTACSGSFYKSMRWLTRKNGEYPTQDAQYTNNQGKNILFMWGINHPPTDDTQRGLYTRTDTTT
SVATEEINRIFKPLIGPRPLVNGLMGRINYYWSVLKPGQTLRIKSDGNLIAPWYGHILSGESHGRILKTD
LKRGSCTVQCQTEKGGLNTTLPFQNVSKYAFGNCSKYIGIKSLKLAVGLRNVPSRSSRGLFGAIAGFIEG
GWSGLVAGWYGFQHSNDQGVGMAADRDSTQKAIDKITSKVNNIVDKMNKQYEIIDHEFSEVETRLNMINN
KIDDQIQDIWAYNAELLVLLENQKTLDEHDANVNNLYNKVKRALGSNAVEDGKGCFELYHKCDDQCMETI
RNGTYNRRKYQAESKLERQRIEGVKLESEGTYKILTIYSTVASSLVIAMGFAAFLFWAMSNGSCRCNICI

>QQO75649.1 hemagglutinin [Influenza A virus]
METVSLITILIVATVSNADKICIGYQSTNSTETVDTLTENNVPVTHAKELLHTEHNGMLCATSLGQPLIL
DTCTIEGLIYGNPSCDLSLEGREWSYIVERPSAINGLCYPGNVENLEELRSLFSSARSYQRIQIFPDTIW
NVSYDGTSTACSNSFYRSMRWLTRKDGNYPTQDAQYTNNQGKNILFMWGINHPPTDDTQRNLYTRTDTTT
SVATEEINRIFKPLIGPRPLVNGLMGRIDYYWSVLKPGQTLRIKSDGNLIAPWYGHILSGESHGRILKTD
LKRGSCTVQCQTEKGGLNTTLPFQNVSKYAFGNCSKYIGIKSLKLAVGLRNVPSRSSRGLFGAIAGFIEG
GWSGLVAGWYGFQHSNDQGVGMAADRDSTQKAIDKITSKVNNIVDKMNKQYEIIDHEFSEVETRLNMINN

KIDDQIQDIWAYNAELLVLLENQKTLDEHDANVNNLYNKVKRALGSNAVEDGKGCFELYHKCNDQCMETI
RNGTYNRRKYQEECKLERQKIEGVKLESEGTYKILTIYSTVASSLVIAMGFAAFLFWAMSNGSCRCNICI

>QQO75648.1 hemagglutinin [Influenza A virus]
METVSLITILLVATVSNADKICIGYQSTNSTETVDTLTENNVPVTHAKELLHTEHNGMLCATSLGNPLIL
DTCTIEGLIYGNPSCDPLLGGREWSYIVERPSAVNGLCYPGIVENLEELRSLFSSARSYQRIQIFPETIW
NVTYSGTSKACSDSFYRNMRWLTQKNNAYPIQDAQYTNNQEKNILFMWGINHPPTETSQTNLYTRTDTTT
SVATEEINRIFKPLIGPRPLVNGLMGRINYYWSVLKPGQTLRIKSDGNLIAPWYGHILSGESHGRILKTD
LKRGSCTVQCQTEKGGLNTTLPFQNVSKYAFGNCSKYIGIKSLKLAVGLRNVPSRSSRGLFGAIAGFIEG
GWSGLVAGWYGFQHSNDQGVGMAADRDSTQKAIDKITSKVNNIVDKMNKQYEIIDHEFSEVETRLNMINN
KVDDQIQDIWAYNAELLVLLENQKTLDEHDSNVNNLYNKVKRALGSNAVEDGKGCFELYHKCDDQCMETI
RNGTYNRRKYQEESKLERQKIEGVKLESEGTYKILTIYSTVASSLVIAMGFAAFLFWAMSNGSCRCNICI

>QQO75647.1 hemagglutinin [Influenza A virus]
METVSLITILIVATVSNADKICIGYQSTNSTETVDTLTENNVPVTHAKELLHTEHNGMLCATSLGQPLIL
DTCTIEGLIYGNPSCDLSLEGREWSYIVERPSAINGLCYPGNVENLEELRSLFSSARSYQRIQIFPDTIW
NVSYDGTSTACSNSFYRSMRWLTRKDGNYPTQDAQYTNNQGKNILFMWGINHPPTDDTQRNLYTRTDTTT
SVATEEINRIFKPLIGPRPLVNGLMGRIDYYWSVLKPGQTLRIKSDGNLIAPWYGHILSGESHGRILKTD
LKRGSCTVQCQTEKGGLNTTLPFQNVSKYAFGNCSKYIGIKSLKLAVGLRNVPSRSSRGLFGAIAGFIEG
GWSGLVAGWYGFQHSNDQGVGMAADRDSTQKAIDKITSKVNNIVDKMNKQYEIIDHEFSEVETRLNMINN
KIDDQIQDIWAYNAELLVLLENQKTLDEHDANVNNLYNKVKRALGSNAVEDGKGCFELYHKCNDQCMETI
RNGTYNRRKYQEESKLERQKIEGVKLESEGTYKILTIYSTVASSLVIAMGFAAFLFWAMSNGSCRCNICI

>QQO75646.1 hemagglutinin [Influenza A virus]
METVSLITILIVATVSKADKICIGYQSTNSTETVDTLTENNVPVTHAKELLHTEHNGMLCATSLGHPLIL
DTCTIEGLIYGNPSCDPLLGGREWSYIVERPSAVNGLCYPGNVENLEELRSLFSSSRSYQRIQIFPDTIW
NVSYSGTSKACSDSFYRSMRWLTQKNNAYPTQDAQYTNNQGKNILFMWGINHPPTDTAQTNLYTRTDTTT
SVATEEMNRIFKPLIGPRPLVNGLMGRINYYWSVLKPGQTLRIKSDGNLIAPWYGHILSGESHGRILKTD
LKRGSCTVQCQTEKGGLNTTLPFQNVSKYAFGNCSKYIGVKSLKLAVGLRNVPSRSSRGLFGAIAGFIEG
GWSGLVAGWYGFQHSNDQGVGMAADRDSTQKAIDKITSKVNNIVDKMNKQYEIIDHEFSEVETRLNMINN
KVDDQIQDIWAYNAELLVLLENQKTLDEHDANVNNLYNKVKRALGSNAVEDGKGCFELYHKCDDHCMETI
RNGTYNRRKYQEESKLERQKIEGVKLESEETYKILTIYSTVASSLVIAMGFAAFLFWAMSNGSCRCNICI

>QQO75645.1 hemagglutinin [Influenza A virus]

METVSLITILIVATVSKADKICIGYQSTNSTETVDTLTENNVPVTHAKELLHTEHNGMLCATSLGHPLIL
DTCTIEGLIYGNPSCDPLLGGREWSYIVERPSAVNGLCYPGNVENLEELRSLFSSSRSYQRIQIFPDTIW
NVSYSGTSKACSDSFYRSMRWLTQKNNAYPTQDAQYTNNQGKNILFMWGINHPPTDTAQTNLYTRTDTTT
SVATEEMNRIFKPLIGPRPLVNGLMGRINYYWSVLKPGQTLRIKSDGNLIAPWYGHILSGESHGRILKTD
LKRGSCTVQCQTEKGGLNTTLPFQNVSKYAFGNCSKYIGVKSLKLAVGLRNVPSRSSRGLFGAIAGFIEG
GWSGLVAGWYGFQHSNDQGVGMAADRDSTQKAIDKITSKVNNIVDKMNKQYEIIDHEFSEVETRLNMINN
KVDDQIQDIWAYNAELLVLLENQKTLDEHDANVNNLYNKVKRALGSNAVEDGKGCFEIYHKCDDQCMETI
RNGTYNRRKYQEESKLERQKIEGVKLESEETYKILSIYSTVASSLVIAMGFAAFLFWAMSNGSCRCNICI

>QQO75644.1 hemagglutinin [Influenza A virus]
METVSLMTILLVATVSNADKICIGYQSTNSTETVDTLTENNVPVTHAKELLHTEHNGMLCATSLGNPLIL
DTCTIEGLIYGNPSCDPLLGGREWSYIVERPSAVNGLCYPGIVENLEELRSLFSSARSYQRIQIFPETIW
NVTYSGTSKACSGSFYRNMRWLTQKNNAYPIQDAQYTNNQEKNILFMWGINHPPTETSQTNLYTRTDTTT
SVATEEINRIFKPLIGPRPLVNGLMGRINYYWSVLKPGQTLRIKSDGNLIAPWYGHILSGESHGRILKTD
LKRGSCTVQCQTEKGGLNTTLPFQNVSKYAFGNCSKYIGIKSLKLAVGLRNVPSRSSRGLFGAIAGFIEG
GWSGLVAGWYGFQHSNDQGVGMAADRDSTQKAIDKITSKVNNIVDKMNKQYEIIDHEFSEVETRLNMINN
KVDDQIQDIWAYNAELLVLLENQKTLDEHDSNVNNLYNKVKRALGSNAVEDGKGCFELYHKCDDQCMETI
RNGTYNRRKYQEESKLERQKIEGVKLESEGTYKILTIYSTVASSLVIAMGFAAFLFWAMSNGSCRCNICI

>QQO75643.1 hemagglutinin [Influenza A virus]
METVSLMTILLVATVSNADKICIGYQSTNSTETVDTLTENNVPVTHAKELLHTEHNGMLCATSLGNPLVL
DTCTIEGLIYGNPSCDPLLGGREWSYIVERPSAVNGLCYPGSVENLEELRSLFSSARSYQRIQIFPDTIW
NVSYSGTSKACSDSFYRSMRWLTQKNNAYPIQDAQYTNNQEKNILFMWGINHPPTETAQTNLYTRTDTTT
SVATEEINRIFKPLIGPRPLVNGLMGRINYYWSVLKPGQTLRIKSDGNLIAPWYGHILSGESHGRILKTD
LKRGSCTVQCQTEKGGLNTTLPFQNVSKYAFGNCSKYIGIKSLKLAVGLRNVPSRSSRGLFGAIAGFIEG
GWSGLVAGWYGFQHSNDQGVGMAADRDSTQKAIDKITSKVNNIVDKMNKQYEIIDHEFSEVETRLNMINN
KVDDQIQDIWAYNAELLVLLENQKTLDEHDSNVNNLYNKVKRALGSNAVEDGKGCFELYHKCDNQCMETI
RNGTYNRRKYQEESKLERQRIEGVKLESEGTYKILTIYSTVASSLVIAMGFAAFLFWAMSNGSCRCNICI

>QQO75642.1 hemagglutinin [Influenza A virus]
METVSLITILIVATVSNADKICIGYQSTNSTETVDTLTENNVPVTHAKELLHTEHNGMLCATSLGQPLIL
DTCTIEGLIYGNPSCDLSLEGREWSYIVERPSAINGLCYPGNVENLEELRSLFSSARSYQRIQIFPDTIW
NVSYDGTSTACSNSFYRSMRWLTRKDGNYPTQDAQYTNNQGKNILFMWGINHPPTDDTQRNLYTRTDTTT
SVATEEINRIFKPLIGPRPLVNGLMGRIDYYWSVLKPGQTLRIKSDGNLIAPWYGHILSGESHGRILKTD

LKRGSCTVQCQTEKGGLNTTLPFQNVSKYAFGNCSKYIGIKSLKLAVGLRNVPSRSSRGLFGAIAGFIEG
GWSGLVAGWYGFQHSNDQGVGMAADRDSTQKAIDKITSKVNNIVDKMNKQYEIIDHEFSEVETRLNMINN
KIDDQIQDIWAYNAELLVLLENQKTLDEHDANVNNLYNKVKRALGSNAVEDGKGCFELYHKCNDQCMETI
RNGTYNRRKYQEESKLERQKIEGVKLESEGTYKILTIYSTVASSLVIAMGFAAFLFWAMSNGSCRCNICI

>QQO75641.1 hemagglutinin [Influenza A virus]
METVSLITILIVATVSNADKICIGYQSTNSTETVDTLTENNVPVTHAKELLHTEHNGMLCATSLGQPLIL
DTCTIEGLIYGNPSCDLSLEGREWSYIVERPSAINGLCYPGNVENLEELRSLFSSARSYQRIQIFPDTIW
NVSYDGTSTACSNSFYRSMRWLTRKDGNYPTQDAQYTNNQGKNILFMWGINHPPTDDTQRNLYTRTDTTT
SVATEEINRIFKPLIGPRPLVNGLMGRIDYYWSVLKPGQTLRIKSDGNLIAPWYGHILSGESHGRILKTD
LKRGSCTVQCQTEKGGLNTTLPFQNVSKYAFGNCSKYIGIKSLKLAVGLRNVPSRSSRGLFGAIAGFIEG
GWSGLVAGWYGFQHSNDQGVGMAADRDSTQKAIDKITSKVNNIVDKMNKQYEIIDHEFSEVETRLNMINN
KIDDQIQDIWAYNAELLVLLENQKTLDEHDANVNNLYNKVKRALGSNAVEDGKGCFELYHKCNDQCMETI
RNGTYNRRKYQEESKLERQKIEGVKLESEGTYKILTIYSTVASSLVIAMGFAAFLFWAMSNGSCRCNICI

>QEX14692.1 hemagglutinin, partial [Influenza A virus]
METISLMTILLVVTTSNADKICIGHQSTNSTETVDTLTETNVPVTHAKELLHTEHNGMLCATNLGHPLIL
EACTIEGLIYGNPVCDLLLGGREWSYIVERPSAVNGTCYPGNIENLEELRTLFSSSSSYQRIQIFPDTIW
NVTYTGTSKSCSDSFYRNMRWLTQKNGLYPVQDAQYTNNRGKDILFVWGIHHPPTDTTQTNLYTRTDTTT
SVTTENLDRTFKPLIGPRPLVNGLIGRINYYWSVLKPGQTLRIRSNGNLIAPWFGHVLSGESHGRILKTN
LNSGNCVVQCQTEKGGLNSTLPFHNISKYAFGTCPKYIGVKSLKLAIGLRNVPSRSSRGLFGAIAGFIEG
GWPGLVAGWYGFQHSNDQGVGMAADRDSTQKAVDKITSKVNNIVDKMNKQYEIIDHEFSEVETRLNMINN
KIDDQIQDVWAYNAELLVLLENQKTLDEHDANVNNLYNKVKRALGSNAMEDGKGCFELYHKCDDQCMETI
RNGTYNRRKYTEESRLERQKIEGVKLESEGTYKILTIYSTVASSIVIAMGFSAFLFWAMSNGSCRCNICI

>QEX14691.1 hemagglutinin, partial [Influenza A virus]
METISLMTILLVVTTSNADKICIGHQSTNSTETVDTLTETNVPVTHAKELLHTEHNGMLCATNLGHPLIL
EACTIEGLIYGNPVCDLLLGGREWSYIVERPSAVNGTCYPGNIENLEELRTLFSSSSSYQRIQIFPETIW
NVTYTGTSKSCSDSFYRNMRWLTQKNGLYPVQDAQYTNNRGKDILFVWGIHHPPTDTAQTNLYTRTDTTT
SVTTENLDRTFKPLIGPRPLVNGLIGRINYYWSVLKPGQTLRVRSNGNLIAPWFGHVLSGESHGRILKTN
LNSGNCVVQCQTEKGGLNSTLPFHNISKYAFGTCPKYIGVKSLKLAIGLRNVPARSSRGLFGAIAGFIEG
GWPGLVAGWYGFQHSNDQGVGMAADRDSTQKAVDKITSKVNNIVDKMNKQYEIIDHEFSEVETRLNMINN
KIDDQIQDVWAYNAELLVLLENQKTLDEHDANVNNLYNKVKRALGSNAMEDGKGCFELYHKCDDQCMETI
RNGTYNRRKYTEESRLERQKIEGVKLESEGTYKILTIYSTVASSLVIAMGFAAFLFWAMSNGSCRCNICI

>QEX14690.1 hemagglutinin, partial [Influenza A virus]

METISLMTILLVVTTSNADKICIGHQSTNSTETVDTLTETNVPVTHAKELLHTEHNGMLCATNLGHPLIL
EACTIEGLIYGNPVCDLLLGGREWSYIVERPSAVNGTCYPGNIENLEELRTLFSSSSSYQRIQIFPETIW
NVTYTGTSKSCSDSFYRNMRWLTQKNGLYPVQDAQYTNNRGKDILFVWGIHHPPTDTAQTNLYTRTDTTT
SVTTENLDRTFKPLIGPRPLVNGLIGRINYYWSVLKPGQTLRVRSNGNLIAPWFGHVLSGESHGRILKTN
LNSGNCVVQCQTEKGGLNSTLPFHNISKYAFGTCPKYIGVKSLKLAIGLRNVPARSSRGLFGAIAGFIEG
GWPGLVAGWYGFQHSNDQGVGMAADRDSTQKAVDKITSKVNNIVDKMNKQYEIIDHEFSEVETRLNMINN
KIDDQIQDVWAYNAELLVLLENQKTLDEHDANVNNLYNKVKRALGSNAMEDGKGCFELYHKCDDQCMETI
RNGTYNRRKYTEESRLERQKIEGVKLESEGTYKILTIYSTVASSLVIAMGFAAFLFWAMSNGSCRCNICI

>QEX14689.1 hemagglutinin, partial [Influenza A virus]

METISLMTILLVVTTSNADKICIGHQSTNSTETVDTLTETNVPVTHAKELLHTEHNGMLCATNLGHPLIL
EACTIEGLIYGNPVCDLLLGGREWSYIVERPSAVNGTCYPGNIENLEELRTLFSSSSSYQRIQIFPETIW
NVTYTGTSKSCSDSFYRNMRWLTQKNGLYPVQDAQYTNNRGKDILFVWGIHHPPTDTAQTNLYTRTDTTT
SVTTENLDRTFKPLIGPRPLVNGLIGRINYYWSVLKPGQTLRVRSNGNLIAPWFGHVLSGESHGRILKTN
LNSGNCVVQCQTEKGGLNSTLPFHNISKYAFGTCPKYIGVKSLKLAIGLRNVPARSSRGLFGAIAGFIEG
GWPGLVAGWYGFQHSNDQGVGMAADRDSTQKAVDKITSKVNNIVDKMNKQYEIIDHEFSEVETRLNMINN
KIDDQIQDVWAYNAELLVLLENQKTLDEHDANVNNLYNKVKRALGSNAMEDGKGCFELYHKCDDQCMETI
RNGTYNRRKYTEESRLERQKIEGVKLESEGTYKILTIYSTVASSLVIAMGFAAFLFWAMSNGSCRCNICI

>QEX14688.1 hemagglutinin, partial [Influenza A virus]

METISLMTILLVVTTSNADKICIGHQSTNSTETVDTLTETNVPVTHAKELLHTGHNGMLCATNLGHPLIL
EACTIEGLIYGNPVCDLLLGGREWSYIVERPSAVNGTCYPGNIENLEELRTLFSSSSSYQRIQIFPDTIW
NVTYTGTSKSCSDSFYRNMRWLTQKNGLYPVQDAQYTNNRGKDILFVWGIHHPPTDTAQTNLYTRTDTTT
SVTTENLDRTFKPLIGPRPLVNGLIGRINYYWSVLKPGQTLRVRSNGNLIAPWFGHVLSGESHGRILKTN
LNSGNCVVQCQTEKGGLNSTLPFHNISKYAFGTCPKYIGVKSLKLAIGLRNVPSRSSRGLFGAIAGFIEG
GWPGLVAGWYGFQHSNDQGVGMAADRDSTQKAVDKITSKVNNIVDKMNKQYEIIDHEFSEVETRLNMINN
KIDDQIQDVWAYNAELLVLLENQKTLDEHDANVNNLYNKVKRALGSNAMEDGKGCFELYHKCDDQCMETI
RNGTYNRRKYTEESRLERQKIEGVKLESEGTYKILTIYSTVASSLVIAMGFAAFLFWAMSNGSCRCNICI

>QGA46695.1 hemagglutinin [Influenza A virus]

METVSLITILLVATVSNADKICIGYQSTNSTETVDTLTENNVPVTHAKELLHTEHNGMLCATSLGQPLIL
DTCTIEGLIYGNPSCDLSLEGREWSYIVERPSAVHGLCYPGNVEDLEELRSLFSSARSYQRIQIFPDTIW

NVSYDGTSTACSGSFYKSLRWLTRKNGEYPTQDAQYTNNQGKNILFMWGINHPPTDDTQRGLYTRTDTTT
SVATEEINRIFKPLIGPRPLVNDLMGRINYYWSVLKPGQTLRIKSDGNLIAPWYGHILSGESHGRILKTD
LKRGSCTVQCQTEKGGLNTTLPFQNVSKYAFGNCSKYIGIKSLKLAVGLRNVPSRSSRGLFGAIAGFIEG
GWPGLVAGWYGFQHSNDQGVGMAADRDSTQKAIDKITSKVNNIVDKMNKQYEIIDHEFSEVETRLNMINN
KIDDQIQDIWAYNAELLVLLENQKTLDEHDANVNNLYNKVKRALGSNAVEDGKGCFELYHKCDDQCMETI
RNGTYNRRKYQEESKLERQRIEGVKLESEGTYKILTIYSTVASSLVIAMGFAAFLFWAMSNGSCRCNICI

>QGA46683.1 hemagglutinin [Influenza A virus]
METVSLITILLVATVSNADKICIGYQSTNSTETVDTLTENNVPVTHAKELLHTEHNGMLCATSLGQPLIL
DTCTIEGLIYGNPSCDLSLEGREWSYIVERPSAVHGLCYPGNVEDLEELRSLFSSARSYQRIQIFPDTIW
NVSYDGTSTACSGSFYKSMRWLTRKNGEYPTQDAQYTNNQGKNILFMWGINHPPTDDTQRGLYTRTDTTT
SVATEEINRIFKPLIGPRPLVNGLMGRINYYWSVLKPGQTLRIKSDGNLIAPWYGHILSGESHGRILKTD
LKRGSCTVQCQTEKGGLNTTLPFQNVSKYAFGNCSKYIGIKSLKLAVGLRNVPSRSSRGLFGAIAGFIEG
GWPGLVAGWYGFQHSNDQGVGMAADRDSTQKAIDKITSKVNNIVDKMNKQYEIIDHEFSEVETRLNMINN
KIDDQIQDIWAYNAELLVLLENQKTLDEHDANVNNLYNKVKRALGSNAVEDGKGCFELYHKCDDQCMETI
RNGTYNRRKYQEESKLERQRIEGVKLESEGTYKILTIYSTVASSLVIAMGFAAFLFWAMSNGSCRCNICI

>QGA46671.1 hemagglutinin [Influenza A virus]
METASLITILLVVTVSSADKICIGYQSTNSTETVDTLTENNVPVTHAKELLHTEHNGMLCATSLGHPLIL
DTCTIEGLIYGNPSCDLLLGGREWSYIVERPSAVNGLCYPGNVENLEELRSLFSSARSYQRIQIFPDTIW
NVSYSGTSKACSDSFYRSMRWLTQKDNAYPIQDAQYTNNQEKNILFMWGINHPPTDTVQTNLYTRTDTTT
SVATEEINRTFKPLIGPRPLVNGLMGRINYYWSVLKPGQTLRIKSNGNLIAPWYGHILSGESHGRILKTD
LKRGSCTVQCQTEKGGLNTTLPFQNVSKYAFGNCSKYIGIKSLKLAVGLRNVPSRSSRGLFGAIAGFIEG
GWSGLVAGWYGFQHSNDQGVGMAADRDSTQKAIDKITSKVNNIVDKMNKQYEIIDHEFSEVETRLNMINN
KIDDQIQDIWAYNAELLVLLENQKTLDEHDANVNNLYNKVKRALGSNAVEDGKGCFELYHKCDDQCMETI
RNGTYNRRKYQEESKLERQKIEGVKLESEGTYKILTIYSTVASSLVIAMGFAAFLFWAMSNGSCRCNICI

>QGA46659.1 hemagglutinin [Influenza A virus]
METVSLITILLVATASNADKICIGYQSTNSTETVDTLTENNVPVTHAKELLHTEHNGMLCATSLGQPLIL
DTCTVEGLIYGNPSCDLSLEGREWSYIVERPSAVNGLCYPGNVENLEELRSLFSSARSYQRIQIFPDTIW
NVSYDGTSTACSDSFYRSMRWLTRKDGNYPTQDAQYTNNQGKNILFMWGINHPPTDDAQRNLYTRTDTTT
SVATEEINRIFKPLIGPRPLVNGLMGRINYYWSVLKPGQTLRIKSDGNLIAPWYGHILSGESHGRILKTD
LKRGSCTVQCQTEKGGLNTTLPFQNVSKYAFGNCSKYIGVKSLKLAVGLRNVPSRSSRGLFGAIAGFIEG
GWSGLVAGWYGFQHSNDQGVGMAADRDSTQKAIDKITSKVNNIVDKMNKQYEIIDHEFSEVETRLNMINN

KIDDQIQDIWAYNAELLVLLENQKTLDEHDANVNNLYNKVKRALGSNAVEDGKGCFELYHKCNDQCMETI
RNGTYNRRKYQEESKLERQKIEGVKLESEGTYKILTIYSTVASSLVIAMGFAAFLFWAMSNGSCRCNICI

>QGA46647.1 hemagglutinin [Influenza A virus]
METASLITILLVVTVSSADKICIGYQSTNSTETVDTLTENNVPVTHAKELLHTEHNGMLCATSLGHPLIL
DTCTIEGLIYGNPSCDLLLGGREWSYIVERPSAVNGLCYPGNVENLEELRSLFSSARSYQRIQIFPDTIW
NVSYSGTSKACSDSFYRSMRWLTQKDNAYPIQDAQYTNNQEKNILFMWGINHPPTDTAQTNLYTRTDTTT
SVATEEINRTFKPLIGPRPLVNGLMGRINYYWSVLKPGQTLRIKSNGNLIAPWYGHILSGESHGRILKTD
LKRGSCTVQCQTEKGGLNTTLPFQNVSKYAFGNCSKYIGIKSLKLAVGLRNVPSRSSRGLFGAIAGFIEG
GWSGLVAGWYGFQHSNDQGVGMAADRDSTQKAIDKITSKVNNIVDKMNKQYEIIDHEFSEVETRLNMINN
KIDDQIQDIWAYNAELLVLLENQKTLDEHDANVNNLYNKVKRALGSNAVEDGKGCFELYHKCDDQCMETI
RNGTYNRRKYQEESKLERQKIEGVKLESEGTYKILTIYSTVASSLVIAMGFAAFLFWAMSNGSCRCNICI

>QGA46635.1 hemagglutinin [Influenza A virus]
METVSLITILLVATVSNADKICIGYQSTNSTETVDTLTENNVPVTHAKELLHTEHNGMLCATSLGQPLIL
DTCTIEGLIYGNPSCDLSLEGREWSYIVERPSAVHGLCYPGNVEDLEELRSLFSSARSYQRIQIFPDTIW
NVSYDGTSTACSGSFYKSMRWLTRKNGEYPTQDAQYTNNQGKNILFMWGINHPPTDDTQRGLYTRTDTTT
SVATEEINRIFKPLIGPRPLVNGLMGRINYYWSVLKPGQTLRIKSDGNLIAPWYGHILSGESHGRILKTD
LKRGSCTVQCQTEKGGLNTTLPFQNVSKYAFGNCSKYIGIKSLKLAVGLRNVPSRSSRGLFGAIAGFIEG
GWPGLVAGWYGFQHSNDQGVGMAADRDSTQKAIDKITSKVNNIVDKMNKQYEIIDHEFSEVETRLNMINN
KIDDQIQDIWAYNAELLVLLENQKTLDEHDANVNNLYNKVKRALGSNAVEDGKGCFELYHKCDDQCMETI
RNGTYNRRKYQEESKLERQRIEGVKLESEGTYKILTIYSTVASSLVIAMGFAAFLFWAMSNGSCRCNICI

>QGA46623.1 hemagglutinin [Influenza A virus]
METLSLITILLVATISNADKICIGYQSTNSTETVDTLTENNVPVTHAKELLHTEHNGMLCATSLGQPLIL
DTCTIEGLIYGNPSCDLSLEGREWSYIVERPSAVNGLCYPGNVENLEELRSLFSSARSYQRIQIFPDTIW
NVSYDGTSTACSGSFYRSMRWLTRKNGDYPTQDAQYTNNQGKNILFMWGINHPPTDDTQRNLYTRTDTTT
SVATEEINRIFKPLIGPRPLVNGLMGRIDYYWSVLKPGQTLRIKSDGNLIAPWYGHILSGESHGRILKTD
LKRGSCTVQCQTEKGGLNTTLPFQNVSKYAFGNCSKYIGIKSLKLAVGLRNVPSRSSRGLFGAIAGFIEG
GWSGLVAGWYGFQHSNDQGVGMAADRDSTQKAIDKITSKVNNIVDKMNKQYEIIDHEFSEVETRLNMINN
KIDDQIQDIWAYNAELLVLLENQKTLDEHDANVNNLYNKVKRALGSNAVEDGKGCFELYHKCNDQCMETI
RNGTYNRRKYQEESKLERQKIEGVKLESEGTYKILTIYSTVASSLVIAMGFAAFLFWAMSNGSCRCNICI

>QGA46611.1 hemagglutinin [Influenza A virus]

METVSLITILLVATVSNADKICIGYQSTNSTETVDTLTENNVPVTHAKELLHTEHNGMLCATSLGHPLIL
DTCTIEGLIYGNPSCDPLLGGREWSYIVERPSAVNGLCYPGNVENLEELRSLFSSARSYQRIQIFPDTIW
NVSYSGTSKACSDSFYRSMRWLTQKNNAYPIQDAQYTNNQEKNILFMWGINHPPTDTAQTNLYTRTDTTT
SVATEEINRIFKPLIGPRPLVNGLMGRINYYWSVLKPGQTLRIKSDGNLIAPWYGHILSGESHGRILKTD
LTRGSCTVQCQTEKGGLNTTLPFQNVSKYAFGNCSKYIGIKSLKLAVGLRNVPSRSSRGLFGAIAGFIEG
GWSGLVAGWYGFQHSNDQGVGMAADRDSTQKAIDKITSKVNNIVDKMNKQYEIIDHEFSEVETRLNMINN
KVDDQIQDIWAYNAELLVLLENQKTLDEHDANVNNLYNKVKRALGSNAVEDGKGCFELYHKCDDQCMETI
RNGTYNRRKYQEESKLERQKIEGVKLESEETYKILTIYSTVASSLVIAMGFAAFLFWAMSNGSCRCNICI

>QGA46599.1 hemagglutinin [Influenza A virus]
METVSLITMLLVATVSNADKICIGYQSTNSTETVDTLTENNVPVTHAKELLHTEHNGMLCATSLGQPLIL
DTCTIEGLIYGNPSCDLSLEGREWSYIVERPSAVNGLCYPGNVENLEELRSLFSSARSYQRIQIFPDTIW
NVSYDGTSTACSGSFYRSMRWLTRKNGDYPIQDAQYTNNQGKNILFMWGINHPPTDTTQRDLYTRTDTTT
SVATEEINRIFKPLIGPRPLVNGLMGRIDYYWSVLKPGQTLRIKSDGNLIAPWYGHILSGESHGRILKTD
LKRGSCTVQCQTEKGGLNTTLPFQNVSKYAFGNCSKYIGIKSLKLAVGLRNVPSRSSRGLFGAIAGFIEG
GWSGLVAGWYGFQHSNDQGVGMAADRDSTQKAIDKITSKVNNIVDKMNKQYEIIDHEFSEVETRLNMINN
KIDDQIQDIWAYNAELLVLLENQKTLDEHDANVNNLYNKVKRALGSNAVEDGKGCFELYHKCDDQCMETI
RNGTYNRRKYQEESKLERQKIEGVKLESEGTYKILTIYSTVASSLVIAMGFAAFLFWAMSNGSCRCNICI

>QGA46587.1 hemagglutinin [Influenza A virus]
METASLMTILLVVTVSNADKICIGYQSTNSTETVDTLTENNVPVTHAKELLHTEHNGMLCATSLGHPLIL
DTCTIEGLIYGNPSCDLLLGGREWSYIVERPSAVNGLCYPGNVENLEELRSLFSSARSYQRIQIFPDTIW
NVSYSGTSKACSDSFYRSMRWLTQKDNAYPVQDAQYTNNQEKNILFMWGINHPPTDTAQTNLYTRTDTTT
SVATEEINRIFKPLIGPRPLVNGLMGRINYYWSVLKPGQTLRIKSNGNLIAPWYGHILSGESHGRILKTD
LKRGSCTVQCQTEKGGLNTTLPFQNVSKYAFGNCSKYIGIKSLKLAVGLRNVPSRSSRGLFGAIAGFIEG
GWSGLVAGWYGFQHSNDQGVGMAADRDSTQKAIDKITSKVNNIVDKMNKQYEIIDHEFSEVETRLNMINN
KIDDQIQDIWAYNAELLVLLENQKTLDEHDANVNNLYNKVKRALGSNAVEDGKGCFELYHKCDDQCMETI
RNGTYNRRKYQEESKLERQKIEGVKLESEGTYKILTIYSTVASSLVIAMGFAAFLFWAMSNGSCRCNICI

>QGA46575.1 hemagglutinin [Influenza A virus]
METVSLITILLVATVSNADKICIGYQTTNSTETVDTLTENNVPVTHAKELLHTEHNGMLCATSLGQPLIL
DTCTIEGLIYGNPSCDLSLEGREWSYIVERPSAVNGLCYPGNVEDLEELRSLFSSARSYQRIQIFPDTIW
NVSYDGTSTACSGSFYRSMRWLTRKNGDYPIQDAQYTNNQGKNILFMWGINHPPTDTTQRELYTRTDTTT
SVATEEINRIFKPLIGPRPLVNGLMGRINYYWSVLKPGQTLRIKSDGNLIAPWYGHILSGESHGRILKTD

LKRGSCTVQCQTEKGGLNTTLPFQNVSKYAFGNCSKYIGIKSLKLAVGLRNVPSRSSRGLFGAIAGFIEG
GWSGLVAGWYGFQHSNDQGVGMAADRDSTQKAIDKITSKVNNIVDKMNKQYEIIDHEFSEVETRLNMINN
KIDDQIQDIWAYNAELLVLLENQKTLDEHDANVNNLYNKVKRALGSNAVEDGKGCFELYHKCDDQCMETI
RNGTYNRRKYQEESKLERQKIEGVKLESEGTYKILTIYSTVASSLVIAMGFAAFLFWAMSNGSCRCNICI

>QGA46563.1 hemagglutinin [Influenza A virus]
METVSLITILLVATVSNADKICIGYQSTNSTETVDTLTENNVPVTHAKELLHTEHNGMLCATSLGQPLIL
DTCTIEGLIYGNPSCDLSLEGREWSYIVERPSAVNGLCYPGNVENLEELRSLFSSARSYQRIQIFPDTIW
NVSYDGTSKACSGSFYRSMRWLTRKNGEYPIQDAQYTNNQGKNILFMWGINHPPTDTTQRDLYTRNDTTT
SVATEEINRVFKPLIGPRPLVNGLMGRIDYYWSVLKPGQTLRIKSDGNLIAPWFGHILSGESHGRILKTD
LKRGSCTVQCQTEKGGLNTTLPFQNVSKYAFGNCSKYIGIKSLKLAVGLRNVPSRSSRGLFGAIAGFIEG
GWSGLVAGWYGFQHSNDQGVGMAADRDSTQKAIDKITSKVNNIVDKMNKQYEIINHEFSEVEARLNMINN
KIDDQIQDIWAYNAELLVLLENQKTLDEHDANVNNLYNKVKRALGSNAVEDGKGCFELYHKCDDQCMETI
RNGTYNRRKYQEESKLEKQKIEGVKLESEGTYKILTIYSTVASSLVIAMGFAAFLFWAMSNGSCRCNIYI

>QGA46551.1 hemagglutinin [Influenza A virus]
METASLITILLVATVSNADKICIGYQSTNSTETVDTLTENNVPVTHAKELLHTEHNGMLCATSLGHPLIL
DTCTIEGLIYGNPSCDPLLGGREWSYIVERPSAVNGLCYPGNVENLEELRSLFSSARSYQRIQIFPDTIW
NVSYSGTSKACSDSFYRSMRWLTQKNNAYPIQDAQYTNNQEKNILFMWGINHPPTDTVQTNLYTRTDTTT
SVATEEINRIFKPLIGPRPLVNGLMGRINYYWSVLKPGQTLRIKSDGNLIAPWYGHILSGESHGRILKTD
LTRGSCTVQCQTEKGGLNTTLPFQNVSKYAFGNCSKYIGIKSLKLAVGLRNVPSRSSRGLFGAIAGFIEG
GWSGLVAGWYGFQHSNDQGVGMAADRDSTQKAIDKITSKVNNIVDKMNKQYEIIDHEFSEVETRLNMINN
KVDDQIQDIWAYNAELLVLLENQKTLDEHDANVNNLYNKVKRALGSNAVEDGKGCFELYHKCDDQCMETI
RNGTYNRRKYQEESKLERQKIEGVKLESEETYKILTIYSTVASSLVIAMGFAAFLFWAMSNGSCRCNICI

>QGA46539.1 hemagglutinin [Influenza A virus]
METVSLITMLLVATVSNADKICIGYQSTNSTETVDTLTENNVPVTHAKELLHTEHNGMLCATSLGQPLIL
DTCTIEGLIYGNPSCDLSLEGREWSYIVERPSAVNGLCYPGNVENLEELRSLFSSARSYQRIQIFPDTIW
NVSYDGTSTACSGSFYRSMRWLTRKNGDYPIQDAQYTNNQGKNILFMWGINHPPTDTTQRDLYTRTDTTT
SVATEEINRIFKPLIGPRPLVNGLMGRIDYYWSVLKPGQTLRIKSDGNLIAPWYGHILSGESHGRILKTD
LKRGSCTVQCQTEKGGLNTTLPFQNVSKYAFGNCSKYIGIKSLKLAVGLRNVPSRSSRGLFGAIAGFIEG
GWSGLVAGWYGFQHSNDQGVGMAADRDSTQKAIDKITSKVNNIVDKMNKQYEIIDHEFSEVETRLNMINN
KIDDQIQDIWAYNAELLVLLENQKTLDEHDANVNNLYNKVKRALGSNAVEDGKGCFELYHKCDDQCMETI
RNGTYNRRKYQEESKLERQKIEGVKLESEGTYKILTIYSTVASSLVIAMGFAAFLFWAMSNGSCRCNICI

>QGA46527.1 hemagglutinin [Influenza A virus]

METVSLITILLVATVSNADKICIGYQSTNSTETVDTLTENNVPVTHAKELLHTEHNGMLCATSLGQPLIL

DTCTIEGLIYGNPSCDLSLEGREWSYIVERPSAVNGLCYPGNVENLEELRSLFSSARSYQRIQIFPDTIW

NVSYDGTSTACSGSFYRSMRWLTRKNGDYPIQDAQYTNNQGKNILFMWGINHPPTDTTQRDLYTRTDTTT

SVATEEINRIFKPLIGPRPLVNGLMGRIDYYWSVLKPGQTLRIKSDGNLIAPWYGHILSGESHGRILKTD

LKRGSCTVQCQTEKGGLNTTLPFQNVSKYAFGNCSKYIGIKSLKLAVGLRNVPSRSSRGLFGAIAGFIEG

GWSGLVAGWYGFQHSNDQGVGMAADRDSTQKAIDKITSKVNNIVDKMNKQYEIIDHEFSEVETRLNMINN

KIDDQIQDIWAYNAELLVLLENQKTLDEHDANVNNLYNKVKRALGSNAVEDGKGCFELYHKCDDQCMETI

RNGTYNRRKYQEESKLERQKIEGVKLESEGTYKILTIYSTVASSLVIAMGFAAFLFWAMSNGSCRCNICI

>QGA46515.1 hemagglutinin [Influenza A virus]

METVSLITILLVATVSNADKICIGYQSTNSTETVDTLTENNVPVTHAKELLHTEHNGMLCATSLGQPLIL

DTCTIEGLIYGNPSCDLSLEGREWSYIVERPSAVHGLCYPGNVEDLEELRSLFSSARSYQRIQIFPDTIW

NVSYDGTSTACSGSFYKSLRWLTRKNGEYPTQDAQYTNNQGKNILFMWGINHPPTDDTQRGLYTRTDTTT

SVATEEINRIFKPLIGPRPLVNSLMGRINYYWSVLKPGQTLRIKSDGNLIAPWYGHILSGESHGRILKTD

LKRGSCTVQCQTEKGGLNTTLPFQNVSKYAFGNCSKYIGIKSLKLAVGLRNVPSRSSRGLFGAIAGFIEG

GWPGLVAGWYGFQHSNDQGVGMAADRDSTQKAIDKITSKVNNIVDKMNKQYEIIDHEFSEVETRLNMINN

KIDDQIQDIWAYNAELLVLLENQKTLDEHDANVNNLYNKVKRALGSNAVEDGKGCFELYHKCDDQCMETI

RNGTYNRRKYQEESKLERQRIEGVKLESEGTYKILTIYSTVASSLVIAMGFAAFLFWAMSNGSCRCNICI

>QGA46503.1 hemagglutinin [Influenza A virus]

MEPVSLITILLVATVSNADKICIGYQSTNSTETVDTLTENNVPVTHAKELLHTEHNGMLCATSLGQPLIL

DTCTIEGLIYGNPSCDLSLEGREWSYIVERPSAVNGLCYPGNVEDLEELRSLFSSARSYQRIQIFPDTIW

NVSYDGTSTACSGSFYRSMRWLTRKNGDYPIQDAQYTNNQGKNILFMWGINHPPTDTTQRELYTRTDTTT

SVATEEINRIFKPLIGPRPLVNGLMGRINYYWSVLKPGQTLRIKSDGNLIAPWYGHILSGESHGRILKTD

LKRGSCTVQCQTEKGGLNTTLPFQNVSKYAFGNCSKYIGIKSLKLAVGLRNVPSRSSRGLFGAIAGFIEG

GWSGLVAGWYGFQHSNDQGVGMAADRDSTQKAIDKITSKVNNIVDKMNKQYEIIDHEFSEVETRLNMINN

KIDDQIQDIWAYNAELLVLLENQKTLDEHDANVNNLYNKVKRALGSNAVEDGKGCFELYHKCDDQCMETI

RNGTYNRRKYQEESKLERQKIEGVKLESEGTYKILTIYSTVASSLVIAMGFAAFLFWAMSNGSCRCNICI

>QGA46491.1 hemagglutinin [Influenza A virus]

METVSLITILLVATVSNADKICIGYQSTNSTETVDTLTENNVPVTHAKELLHTEHNGMLCATSLGQPLIL

DTCTIEGLIYGNPSCDLSLEGREWSYIVERPSAVNGLCYPGNVEDLEELRSLFSSARSYQRIQIFPDTIW

NVSYDGTSTACSGSFYRSMRWLTRKNGDYPIQDAQYTNNQGKNILFMWGINNPPTDTTQRELYTRTDTTT
SVATEEINRIFKPLIGPRPLVNGLMGRINYYWSVLKPGQTLRIKSDGNLIAPWYGHILSGESHGRILKTD
LKRGSCTVQCQTEKGGLNTTLPFQNVSKYAFGNCSKYIGIKSLKLAVGLRNVPSRSSRGLFGAIAGFIEG
GWSGLVAGWYGFQHSNDQGVGMAADRDSTQKAIDKITSKVNNIVDKMNKQYEIIDHEFSEVETRLNMINN
KIDDQIQDIWAYNAELLVLLENQKTLDEHDANVNNLYNKVKRALGSNAVEDGKGCFELYHKCDDQCMETI
RNGTYNRRKYQEESKLERQKIEGVKLESEGTYKILTIYSTVASSLVIAMGFAAFLFWAMSNGSCRCNICI

>QGA46479.1 hemagglutinin [Influenza A virus]
METVSLITILLLATVSNADKICIGYQSTNSTETVDTLTENNVPVTHAKELLHTEHNGMLCATSLGQPLIL
DTCTIEGLIYGNPSCDLSLEGREWSYIVERPSAVNGLCYPGNVENLEELRSLFSSARSYQRIQIFPDTIW
NVSYDGTSTACSGSFYRSMRWLTRKNGDYPTQDAQYTNNQGKNILFMWGINHPPTDDTQRNLYTRTDTTT
SVATEEINRIFKPLIGPRPLVNGLMGRIDYYWSVLKPGQTLRIKSDGNLIAPWYGHILSGESHGRILKTD
LKRGNCTVQCQTEKGGLNTTLPFQNVSKYAFGNCSKYIGIKSLKLAVGLRNVPSRSSRGLFGAIAGFIEG
GWSGLVAGWYGFQHSNDQGVGMAADRESTQKAIDKITSKVNNIVDKMNKQYEIIDHEFSEVETRLNMINN
KIDDQIQDIWAYNAELLVLLENQKTLDEHDANVNNLYNKIKRALGSNAVEDGKGCFELYHKCNDQCMETI
RNGTYNRRKYQEESKLERQKIEGVKLESEGTYKILTIYSTVASSLVIAMGFAAFLFWAMSNGSCRCNICI

>QGA46467.1 hemagglutinin [Influenza A virus]
METISLMTILLVATVSNADKICIGYQSTNSTETVDTLTENNVPVTHAKELLHTEHNGMLCATSLGNPLIL
DTCTIEGLIYGNPSCDPLLGGREWSYIVERPSAVNGLCYPGIVENLEELRSLFSSARSYQRIQIFPDTIW
NVSYSGTSKACSDSFYRSMRWLTQKNNAYPIQDAQYTNNQEKNILFMWGINHPPTETAQTNLYTRTDTTT
SVATEEINRIFKPLIGPRPLVNGLMGRINYYWSVLKPGQTLRIKSDGNLIAPWYGHILSGESHGRILKTD
LKRGSCTVQCQTEKGGLNTTLPFQNVSKYAFGNCSKYIGIKSLKLAVGLRNVPSRSSRGLFGAIAGFIEG
GWSGLVAGWYGFQHSNDQGVGMAADRDSTQKAIDKITSKVNNIVDKMNKQYEIIDHEFSEVETRLNMINN
KVDDQIQDIWAYNAELLVLLENQKTLDEHDSNVNNLYNKVKRALGSNAVEDGKGCFELYHKCDDQCMETI
RNGTYNRRKYQEESKLERQKIEGVKLESEGTYKILTIYSTVASSLVIAMGFAAFLFWAMSNGSCRCNICI

>QGA46455.1 hemagglutinin [Influenza A virus]
METLSLITILLVATISNADKICIGYQSTNSTETVDTLTENNVPVTHAKELLHTEHNGMLCATSLGQPLIL
DTCTIEGLIYGNPSCDLSLEGREWSYIVERPSAVNGLCYPGNVENLEELRSLFSSARSYQRIQIFPDTIW
NVSYDGTSTACSGSFYRSMRWLTRKNGDYPTQDAQYTNNQGKNILFMWGINHPPTDDTQRNLYTRTDTTT
SVATEEINRIFKPLIGPRPLVNGLMGRIDYYWSVLKPGQTLRIKSDGNLIAPWYGHILSGESHGRILKTD
LKKGSCTVQCQTEKGGLNTTLPFQNVSKYAFGNCSKYIGIKSLKLAVGLRNVPSRSSRGLFGAIAGFIEG
GWSGLVAGWYGFQHSNDQGVGMAADRDSTQKAIDKITSKVNNIVDKMNKQYEIIDHEFSEVETRLNMINN

KIDDQIQDIWAYNAELLVLLENQKTLDEHDANVNNLYNKVKRALGSNAVEDGKGCFELYHKCNDQCMETI
RNGTYNRRKYQEESKLERQKIEGVKLESEGTYKILTIYSTVASSLVIAMGFAAFLFWAMSNGSCRCNICI

>QGA46443.1 hemagglutinin [Influenza A virus]
MRTVSLITILLVATVSNADKICIGYQSTNSTETVDTLTENNVPVTHAKELLHTEHNGMLCATSLGQPLIL
DTCTIEGLIYGNPSCDLSLEGREWSYIVERPSAVNGLCYPGNVENLEELRSLFSSARSYQRIQIFPDTIW
NVSYDGTSTACSGSFYRSMRWLTRKNGDYPIQDAQYTNNQGKNILFMWGINHPPTDTTQRDLYTRTDTTT
SVATEEINRIFKPLIGPRPLVNGLMGRIDYYWSVLKPGQTLRIKSDGNLIAPWYGHILSGESHGRILKTD
LKRGSCTVQCQTEKGGLNTTLPFQNVSKYAFGNCSKYIGIKSLKLAVGLRNVPSRSSRGLFGAIAGFIEG
GWSGLVAGWYGFQHSNDQGVGMAADRDSTQKAIDKITSKVNNIVDKMNKQYEIIDHEFSEVETRLNMINN
KIDDQIQDIWAYNAELLVLLENQKTLDEHDANVNNLYNKVKRALGSNAVEDGKGCFELYHKCDDQCMETI
RNGTYNRRKYQEESKLERQKIEGVKLESEGTYKILTIYSTVASSLVIAMGFAAFLFWAMSNGSCRCNICI

>QGA46431.1 hemagglutinin [Influenza A virus]
METVSLITILLVATVSTADKICIGYQSTNSTETVDTLTENNVPVTHAKELLHTEHNGMLCATSLGQPLIL
DTCTIEGLIYGNPSCDLSLEGREWSYIVERPSAVNGLCYPGNVENLEELRSLFSSARSYQRIQIFPDTIW
NVSYDGTSTACSGSFYRSMRWLTRKNGDYPIQDAQYTNNQGKNILFMWGINHPPTDTTQRDLYTRTDTTT
SVATEEINRIFKPLIGPRPLVNGLMGRIDYYWSVLKPGQTLRIKSDGNLIAPWYGHILSGESHGRILKTD
LKRGSCTVQCQTEKGGLNTTLPFQNVSKYAFGNCSKYIGIKSLKLAVGLRNVPSRSSRGLFGAIAGFIEG
GWSGLVAGWYGFQHSNDQGVGMAADRDSTQKAIDKITSKVNNIVDKMNKQYEIIDHEFSEVETRLNMINN
KIDDQIQDIWAYNAELLVLLENQKTLDEHDANVNNLYNKVKRALGSNAVEDGKGCFELYHKCDDQCMETI
RNGTYNRRKYQEESKLERQKIEGVKLESEGTYKILTIYSTVASSLVIAMGFAAFLFWAMSNGSCRCNICI

>QGA46419.1 hemagglutinin [Influenza A virus]
METVSLITILLVATVSNADKICIGYQSTNSTETVDTLTENNVPVTHAKELLHTEHNGMLCATSLGQPLIL
DTCTIEGLIYGNPSCDLSLEGREWSYIVERPSAVNGLCYPGNVEDLEELRSLFSSARSYQRVQIFPDTIW
NVSYDGTSTACSGSFYRSMRWLTRKNGEYPIQDAQYTNNQGKNILFMWGINHPPTDTTQRELYTRTDTTT
SVATEEINRIFKPLIGPRPLVNGLMGRINYYWSVLKPGQTLRIKSDGNLIAPWYGHILSGESHGRILKTD
LKRGSCTVQCQTEKGGLNTTLPFQNVSKYAFGNCSKYIGIKSLKLAVGLRNVPSRSSRGLFGAIAGFIEG
GWSGLVAGWYGFQHSNDQGVGMAADRDSTQKAIDKITSKVNNIVDKMNKQYEIIDHEFSEVETRLNMINN
KIDDQIQDIWAYNAELLVLLENQKTLDEHDANVNNLYNKVKRALGSNAVEDGKGCFELYHKCDDQCMETI
RNGTYNRRKYQEESKLERQKIEGVKLESEGTYKILTIYSTVASSLVIAMGFAAFLFWAMSNGSCRCNICI

>QGA46407.1 hemagglutinin [Influenza A virus]

METVSLITILLVATVSNADKICIGYQSTNSTETVDTLTENNVPVTHAKELLHTEHNGMLCATGLGQPLIL
DTCTIEGLIYGNPSCDLSMEGREWSYIVERPSAVNGLCYPGNVENLEELRSLFSSARSYQRIQIFPDTIW
NVSYDGTSTACSGSFYRSMRWLTRKNGEYPIQDAQYTNNQGKNILFMWGINHPPTDTTQRDLYTRTDTTT
SVATEEINRVFKPLIGPRPLVNGLMGRIDYYWSVLKPGQTLRIKSDGNLIAPWYGHILSGESHGIILKTD
LKKGSCTVQCQTEKGGLNTTLPFQNVSKYAFGNCSKYIGIKSLKLAVGLRNVPSRSSRGLFGAIAGFIEG
GWSGLVAGWYGFQHSNDQGVGMAADRDSTQKAIDKITSKVNNIVDKMNKQYEIIDHEFSEVETRLNMINN
KIDDQIQDIWAYNAELLVLLENQKTLDEHDANVNNLYSKVKRALGSNAVEDGKGCFELYHKCDDQCMETI
RNGTYNRRKYQEESKLERQKIEGVKLESEGTYKILTIYSTVASSLVIAMGFAAFLFWAMSNGSCRCNICI

>QGA46395.1 hemagglutinin [Influenza A virus]
METLSLITILLVATISNADKICIGYQSTNSTETVDTLTENNVPVTHAKELLHTEHNGMLCATSLGQPLIL
DTCTIEGLIYGNPSCDLSLEGREWSYIVERPSAVNGLCYPGNVENLEELRSLFSSARSYQRIQIFPDTIW
NVSYDGTSTACSGSFYRSMRWLTRKNGDYPTQDAQYTNNQGKNILFMWGINHPPTDDTQRNLYTRTDTTT
SVATEEINRIFKPLIGPRPLVNGLMGRIDYYWSVLKPGQTLRIKSDGNLIAPWYGHILSGESHGRILKTD
LKRGSCTVQCQTEKGGLNTTLPFQNVSKYAFGNCSKYIGIKSLKLAVGLRNVPSRSSRGLFGAIAGFIEG
GWSGLVAGWYGFQHSNDQGVGMAADRDSTQKAIDKITSKVNNIVDKMNKQYEIIDHEFSEVETRLNMINN
KIDDQIQDIWAYNAELLVLLENQKTLDEHDANVNNLYNKVKRALGSNAVEDGKGCFELYHKCNDQCMETI
RNGTYNRRKYQEESKLERQKIEGVKLESEGTYKILTIYSTVASSLVIAMGFAAFLFWAMSNGSCRCNICI

>QGA46383.1 hemagglutinin [Influenza A virus]
METVSLITMLLVATVSNADKICIGYQSTNSTETVDTLTENNVPVTHAKELLHTEHNGMLCATSLGQPLIL
DTCTIEGLIYGNPSCDLSLEGREWSYIVERPSAVNGLCYPGNVENLEELRSLFSSARSYQRIQIFPDTIW
NVSYDGTSTACSGSFYRSMRWLTRKNGDYPIQDAQYTNNQGKNILFMWGINHPPTDTTQRDLYTRTDTTT
SVATEEINRIFKPLIGPRPLVNGLMGRIDYYWSVLKPGQTLRIKSDGNLIAPWYGHILSGESHGRILKTD
LKRGSCTVQCQTEKGGLNTTLPFQNVSKYAFGNCSKYIGIKSLKLAVGLRNVPSRSSRGLFGAIAGFIEG
GWSGLVAGWYGFQHSNDQGVGMAADRDSTQKAIDKITSKVNNIVDKMNKQYEIIDHEFSEVETRLNMINN
KIDDQIQDIWAYNAELLVLLENQKTLDEHDANVNNLYNKVKRALGSNAVEDGKGCFELYHKCDDQCMETI
RNGTYNRRKYQEESKLERQKIEGVKLESEGTYKILTIYSTVASSLVIAMGFAAFLFWAMSNGSCRCNICI

>QGA46371.1 hemagglutinin [Influenza A virus]
METASLITILLVVTVSNADKICIGYQSTNSTETVDTLTENNVPVTHAKELLHTEHNGMLCATSLGQPLIL
DTCTIEGLIYGNPSCDLLLGGREWSYIVERPSAVNGLCYPGNVENLEELRSLFSSARSYQRIQIFPDTIW
NVSYSGTSKACSDSFYRSMRWLTQKDNAYPIQDAQYTNNQEKNILFMWGINHPPTDTAQTNLYTRTDTTT
SVATEEINRTFKPLIGPRPLVNGLMGRINYYWSVLKPGQTLRIKSNGNLIAPWYGHILSGESHGRILKTD

LKRGSCTVQCQTEKGGLNTTLPFQNVSKYAFGNCSKYIGIKSLKLAVGLRNVPSRSSRGLFGAIAGFIEG
GWSGLVAGWYGFQHSNDQGVGMAADRDSTQKAIDKITSKVNNIVDKMNKQYEIIDHEFSEVETRLNMINN
KIDDQIQDIWAYNAELLVLLENQKTLDEHDANVNNLYNKVKRALGSNAVEDGKGCFELYHKCDDQCMETI
RNGTYNRRKYQEESKLEKQKIEGVKLESEGTYKILTIYSTVASSLVIAMGFAAFLFWAMSNGSCRCNICI

>QGA46359.1 hemagglutinin [Influenza A virus]
METASLITILLVVTVSNADKICIGYQSTNSTETVDTLTENNVPVTHAKELLHTEHNGMLCATSLGQPLIL
DTCTIEGLIYGNPSCDLLLGGREWSYIVERPSAVNGLCYPGNVENLEELRSLFSSARSYQRIQIFPDTIW
NVSYSGTSKACSDSFYRSMRWLTQKDNAYPIQDAQYTNNQEKNILFMWGINHPPTDTAQTNLYTRTDTTT
SVATEEINRTFKPLIGPRPLVNGLMGRINYYWSVLKPGQTLRIKSNGNLIAPWYGHILSGESHGRILKTD
LKRGSCTVQCQTEKGGLNTTLPFQNVSKYAFGNCSKYIGIKSLKLAVGLRNVPSRSSRGLFGAIAGFIEG
GWSGLVAGWYGFQHSNDQGVGMAADRDSTQKAIDKITSKVNNIVDKMNKQYEIIDHEFSEVETRLNMINN
KIDDQIQDIWAYNAELLVLLENQKTLDEHDANVNNLYNKVKRALGSNAVEDGKGCFELYHKCDDQCMETI
RNGTYNRRKYQEESKLEKQKIEGVKLESEGTYKILTIYSTVASSLVIAMGFAAFLFWAMSNGSCRCNICI

>QGA46347.1 hemagglutinin [Influenza A virus]
METVSLITILLVATVSNADKICIGYQSTNSTETVDTLTENNVPVTHAKELLHTEHNGMLCATSLGQPLIL
DTCTIEGLIYGNPSCDLSLEGREWSYIVERPSAVNGLCYPGNVEDLEELRSLFSSARSYQRIQIFPDTIW
NVSYDGTSTACSGSFYRSMRWLTRKNGDYPIQDAQYTNNQGKNILFMWGINHPPTDTTQRELYTRTDTTT
SVATEEINRIFKPLIGPRPLVNGLMGRINYYWSVLKPGQTLRIKSDGNLIAPWYGHILSGESHGRILKTD
LKRGSCTVQCQTEKGGLNTTLPFQNVSKYAFGNCSKYIGIKSLKLAVGLRNVPSRSSRGLFGAIAGFIEG
GWSGLVAGWYGFQHSNDQGVGMAADRDSTQKAIDKITSKVNNIVDKMNKQYEIIDHEFSEVETRLNMINN
KIDDQIQDIWAYNAELLVLLENQKTLDEHDANVNNLYNKVKRALGSNAVEDGKGCFELYHKCDDQCMETI
RNGTYNRRKYQEESKLERQKIEGVKLESEGTYKILTIYSTVASSLVIAMGFAAFLFWAMSNGSCRCNICI

>QGA46335.1 hemagglutinin [Influenza A virus]
METISLMTILLVATVSNADKICIGYQSTNSTETVDTLTENNVPVTHAKELLHTEHNGMLCATSLGNPLIL
DTCTIEGLIYGNPSCDPLLGGREWSYIVERPSAVNGLCYPGIVENLEELRSLFSSARSYQRIQIFPDTIW
NVSYSGTSKACSDSFYRSMRWLTQKNNAYPIQDAQYTNNQEKNILFMWGINHPPTETAQTNLYTRTDTTT
SVATEEINRIFKPLIGPRPLVNGLMGRINYYWSVLKPGQTLRIKSDGNLIAPWYGHILSGESHGRILKTD
LKRGSCTVQCQTEKGGLNTTLPFQNVSKYAFGNCSKYIGIKSLKLAVGLRNVPSRSSRGLFGAIAGFIEG
GWSGLVAGWYGFQHSNDQGVGMAADRDSTQKAIDKITSKVNNIVDKMNKQYEIIDHEFSEVETRLNMINN
KVDDQIQDIWAYNAELLVLLENQKTLDEHDSNVNNLYNKVKRALGSNAVEDGKGCFELYHKCDDQCMETI
RNGTYNRRKYQEESKLERQKIEGVKLESEGTYKILTIYSTVASSLVIAMGFAAFLFWAMSNGSCRCNICI

>QGA46323.1 hemagglutinin [Influenza A virus]

METVSLITILLVATVSNADKICIGYQSTNSTETVDTLTENNVPVTHAKELLHTEHNGMLCATSLGQPLIL

DTCTIEGLIYGNPSCDLSLEGREWSYIVERPSAVNGLCYPGNVEDLEELRSLFSSARSYQRIQIFPDTIW

NVSYDGTSTACSGSFYRSMRWLTRKNGDYPIQDAQYTNNQEKNILFMWGINHPPTDTTQRELYTRTDTTT

SVATEEINRIFKPLIGPRPLVNGLMGRINYYWSVLKPGQTLRIKSDGNLIAPWYGHILSGESHGRILKTD

LKRGSCTVQCQTEKGGLNTTLPFQNVSKYAFGNCSKYIGIKSLKLAVGLRNVPSRSSRGLFGAIAGFIEG

GWSGLVAGWYGFQHSNDQGVGMAADRDSTQKAIDKITSKVNNIVDKMNKQYEIIDHEFSEVETRLNMINN

KIDDQIQDIWAYNAELLVLLENQKTLDEHDANVNNLYNKVKRALGSNAVEDGKGCFELYHKCDDQCMETI

RNGTYNRRKYQEESKLERQKIEGVKLESEGTYKILTIYSTVASSLVIAMGFAAFLFWAMSNGSCRCNICI

>QGA46311.1 hemagglutinin [Influenza A virus]

METVSLITILLVATVSNADKICIGYQSTNSTETVDTLTENNVPVTHAKELLHTEHNGMLCATSLGQPLIL

DTCTIEGLIYGNPSCDLSLEGREWSYIVERPSAVNGLCYPGNVEDLEELRSLFSSARSYQRIQIFPDTIW

NVSYDGTSTACSGSFYRSMRWLTRKNGDYPIQDAQYTNNQGKNILFMWGINHPPTDTTQRELYTRIDTTT

SVATEEINRIFKPLIGPRPLVNGLMGRINYYWSVLKPGQTLRIKSDGNLIAPWYGHILSGESHGRILKTD

LKRGSCTVQCQTEKGGLNTTLPFQNVSKYAFGNCSKYIGIKSLKLAVGLRNVPSRSSRGLFGAIAGFIEG

GWSGLVAGWYGFQHSNDQGVGMAADRDSTQKAIDKITSKVNNIVDKMNKQYEIIDHEFSEVETRLNMINN

KIDDQIQDIWAYNAELLVLLENQKTLDEHDANVNNLYNKVKRALGSNAVEDGKGCFELYHKCDDQCMETI

RNGTYNRRKYQEESKLERQKIEGVKLESEGTYKILTIYSTVASSLVIAMGFAAFLFWAMSNGSCRCNICI

>QGA46299.1 hemagglutinin [Influenza A virus]

METISLMTILLVATVSNADKICIGYQSTNSTETVDTLTENNVPVTHAKELLHTEHNGMLCATSLGNPLIL

DTCTIEGLIYGNPSCDPLLGGREWSYIVERPSAVNGLCYPGSVENLEELRSLFSSARSYQRIQIFPDTIW

NVSYSGTSKACSDSFYRSMRWLTQKNNAYPIQDAQYTNNQEKNILFMWGINHPPTETAQTNLYTRTDTTT

SVATEEINRIFKPLIGPRPLVNGLMGRINYYWSVLKPGQTLRIKSDGNLIAPWYGHILSGESHGRILKTD

LKRGSCTVQCQTEKGGLNTTLPFQNVSKYAFGNCSKYIGIKSLKLAVGLRNVPSRSSRGLFGAIAGFIEG

GWSGLVAGWYGFQHSNDQGVGMAADRDSTQKAIDKITSKVNNIVDKMNKQYEIIDHEFSEVETRLNMINN

KVDDQIQDIWAYNAELLVLLENQKTLDEHDSNVNNLYNKVKRALGSNAVEDGKGCFELYHKCDDQCMETI

RNGTYNRRKYQEESKLERQKIEGVKLESEGTYKILTIYSTVASSLVIAMGFAAFLFWAMSNGSCRCNICI

>QGA46287.1 hemagglutinin [Influenza A virus]

METVSLITILLVATVSNADKICIGYQSTNSTETVDTLTENNVPVTHAKELLHTEHNGMLCATSLGQPLIL

DTCTIEGLIYGNPSCDLSLEGREWSYIVERPSAVNGLCYPGNVENLEELRSLFSSARSYQRIQIFPDTIW

NVSYDGTSTACSGSFYRSMRWLTRKNGDYPIQDAQYTNNQGKNILFMWGINHPPTDTTQRDLYTRTDTTT
SVATEEINRIFKPLIGPRPLVNGLMGRINYYWSVLKPGQTLRIKSDGNLIAPWYGHILSGESHGRILKTD
LKRGSCTVQCQTEKGGLNTTLPFQNVSKYAFGNCSKYIGIKSLKLAVGLRNVPSRSSRGLFGAIAGFIEG
GWSGLVAGWYGFQHSNDQGVGMAADRDSTQKAIDKITSKVNNIVDKMNKQYEIIDHEFSEVETRLNMINN
KIDDQIQDIWAYNAELLVLLENQKTLDEHDANVNNLYNKVKRALGSNAVEDGKGCFELYHKCDDQCMETI
RNGTYNRRKYQEESKLERQKIEGVKLESEGTYKILTIYSTVASSLVIAMGFAAFLFWAMSNGSCRCNICI

>QGA46275.1 hemagglutinin [Influenza A virus]
METVSLITILLVATVSNADKICIGYQSTNSTETVDTLTENNVPVTHAKELLHTEHNGMLCATSLGQPLIL
DTCTIEGLIYGNPSCDLSLEGREWSYIVERPSAVHGLCYPGNVEDLEELRSLFSSARSYQRIQIFPDTIW
NVSYDGTSTACSGSFYRSMRWLTRKNGDYPIQDAQYTNNQGKNILFMWGINHPPTDDTQRGLYTRTDTTT
SVATEEINRIFKPLIGPRPLVNGLMGRINYYWSVLKPGQTLRIKSDGNLIAPWYGHILSGESHGRILKTD
LKRGSCTVQCQTEKGGLNTTLPFQNVSKYAFGNCSKYIGIKSLKLAVGLRNVPSRSSRGLFGAIAGFIEG
GWSGLVAGWYGFQHSNDQGVGMAADRDSTQKAIDKITSKVNNIVDKMNKQYEIIDHEFSEVETRLNMINN
KIDDQIQDIWAYNAELLVLLENQKTLDEHDANVNNLYNKVKRALGSNAVEDGKGCFELYHKCDDQCMETI
RNGTYNRRKYQEESKLERQKIEGVKLESEGTYKILTIYSTVASSLVIAMGFAAFLFWAMSNGSCRCNICI

>QDF63510.1 hemagglutinin [Influenza A virus]
METVSLITILIVATVSNADKICIGYQSTNSTETVDTLTENNVPVTHAKELLHTEHNGMLCATSLGHPLIL
DTCTIEGLIYGNPSCDPLLGGREWSYIVERPSAVNGLCYPGNVENLEELRSLFSSSRSYQRIQIFPDTIW
NVSYSGTSKACSDSFYRSMRWLTQKNNAYPTQDAQYTNNQGKNILFVWGINHPPTDTAQTNLYTRTDTTT
SVATEEMNRIFKPLIGPRPLVNGLMGRINYYWSVLKPGQTLRIKSDGNLIAPWYGHILSGESHGRILKTD
LKRGSCTVQCQTEKGGLNTTLPFQNVSKYAFGNCSKYIGVKSLKLAVGLRNVPSRSSRGLFGAIAGFIEG
GWSGLVAGWYGFQHSNDQGVGMAADRDSTQKAIDKITSKVNNIVDKMNKQYEIIDHEFSEVETRLNMINN
KVDDQIQDIWAYNAELLVLLENQKTLDEHDANVNNLYNKVKRALGSNAVEDGKGCFELYHKCDDHCMETI
RNGTYNRRKYQEESKLERQKIEGVKLESEETYKILTIYSTVASSLVIAMGFAAFLFWAMSNGSCRCNICI

>QDF63509.1 hemagglutinin [Influenza A virus]
METVSLITILLVATVSYADKICIGYQSTNSTETVDTLTESNVPVTHAKELLHTEHNGMLCATSLGQPLIL
DTCTIEGLIYGNPSCDLSLEGREWSYIVERPSAVNGLCYPGNVENLEELRSLFSSARSYQRVQIFPDTIW
NVSYDGTSTACSGSFYRSMRWLTRKDGNYPTQDAQYTNNQGKNILFMWGINHPPTDDTQRSLYTRTDTTT
SVATEEINRIFKPLIGPRPLVNGLMGRIDYYWSVLKPGQTLRIKSDGNLIAPWYGYILSGESHGRILKTD
LKRGSCTVQCQTEKGGLNTTLPFQNVSKYAFGNCSKYIGIKSLKLAVGLRNVPSRSSRGLFGAIAGFIEG
GWSGLVAGWYGFQHSNDQGVGMAADRDSTQKAIDKITSKVNNIVDKMNKQYEIIDHEFSEVETRLNMINN

KIDDQIQDIWAYNAELLVLLENQKTLDEHDANVNNLYNKVKRALGSNAAEDGKGCFELYHKCNDQCMETI
RNGTYNRRKYQEESKLERQKIEGVKLESEGTYKILTIYSTVASSLVIAMGFAAFLFWAMSNGSCRCNICI

>QDF63508.1 hemagglutinin [Influenza A virus]
METVSLITILLAATVSNADKICIGYQSTNSTETVDTLTENNVPVTHAKELLHTEHNGMLCATSLGQPLIL
DTCTIEGLIYGNPSCDPLLEEREWSYIVERPSAVNGLCYPGNVENLEELRSLFSSARSYQRIQIFPDTIW
NVSYDGTSNTCSGSFYRNMRWLTRKNGNYPIQDAQYTNNQGKNILFMWGINHPPTDDTQRNLYTRTDTTT
SVATEEINRIFKPLIGPRPLVNGLMGRINYYWSVLKPGQTLRIKSDGNLIAPWYGYILSGESHGRILRTD
LKRGSCTVQCQTEKGGLNTTLPFQNVSKYAFGNCSKYIGIKSLKLAVGLRNVPSRSSRGLFGAIAGFIEG
GWSGLVAGWYGFQHSNDQGVGMAADRESTQKAIDKITSKVNNIVDKMNKQYEIIDHEFSEVETRLNMINN
KIDDQIQDIWAYNAELLVLLENQKTLDEHDANVNNLYNKIKRALGSNAVEDGKGCFELYHKCDDQCMETI
RNGTYNRRKYQEESKLERQKIEGVKLESEGTYKILTIYSTVASSLVIAMGFAAFLFWAMSNGSCRCNICI

>QDF63507.1 hemagglutinin [Influenza A virus]
METVSLITILIVATVSNADKICIGYQSTNSTETVDTLTENNVPVTHAKELLHTEHNGMLCATSLGHPLIL
DTCTIEGLIYGNPSCDPLLGGREWSYIVERPSAVNGLCYPGNVENLEELRSLFSSSRSYQRIQIFPDTIW
NVSYSGTSKACSDSFYRSMRWLTQKNNAYPTQDAQYTNNQGKNILFVWGINHPPTDTAQTNLYTRTDTTT
SVATEEMNRIFKPLIGPRPLVNGLMGRINYYWSVLKPGQTLRIKSDGNLIAPWYGHILSGESHGRILKTD
LKRGSCTVQCQTEKGGLNTTLPFQNVSKYAFGNCSKYIGVKSLKLAVGLRNVPSRSSRGLFGAIAGFIEG
GWSGLVAGWYGFQHSNDQGVGMAADRDSTQKAIDKITSKVNNIVDKMNKQYEIIDHEFSEVETRLNMINN
KVDDQIQDIWAYNAELLVLLENQKTLDEHDANVNNLYNKVKRALGSNAVEDGKGCFELYHKCDDHCMETI
RNGTYNRRKYQEESKLERQKIEGVKLESEETYKILTIYSTVASSLVIAMGFAAFLFWAMSNGSCRCNICI

>QDF63506.1 hemagglutinin [Influenza A virus]
METVSLITILLVAAVSYADKICIGYQSTNSTETVDTLTENNVPVTHAKELLHTEHNGMLCATSLGQPLIL
DTCTIEGLIYGNPSCDLSLEGREWSYIVERPSAVNGLCYPGNVENLEELRSLFSSARSYQRVQIFPDTIW
NVSYDGTSTACSGSFYRSMRWLTRKDGNYPTQDAQYTNNQGKNILFMWGINHPPTDDTQRSLYTRTDTTT
SVATEEINRIFKPLIGPRPLVNGLMGRIDYYWSVLKPGQTLRIKSDGNLIAPWYGYILSGESHGRILKTD
LKRGSCTVQCQTEKGGLNTTLPFQNVSKYAFGNCSKYIGIKSLKLAVGLRNVPSKSSRGLFGAIAGFIEG
GWSGLVAGWYGFQHSNDQGVGMAADRDSTQKAIDKITSKVNNIVDKMNKQYEIIDHEFSEVETRLNMINN
KIDDQIQDIWAYNAELLVLLENQKTLDEHDANVNNLYNKVKRALGSNAAEDGKGCFELYHKCNDQCMETI
RNGTYNRRKYQEESKLERQKIEGVKLESEGTYKILTIYSTVASSLVIAMGFAAFLFWAMSNGSCRCNICI

>QDF63505.1 hemagglutinin [Influenza A virus]

METVSLMTILIVATVSNADKICIGYQSTNSTETVDTLTENNVPVTHAKELLHTEHNGMLCATSLGHPLIL
DTCTIEGLIYGNPSCDPLLGGREWSYIVERPSAVNGLCYPGNVENLEELRSLFSSSRSYQRIQIFPDTIW
NVSYSGTSKACSDSFYRSMRWLTQKNNAYPTQDAQYTNNQGKNILFVWGINHPPTDTAQTNLYTRTDTTT
SVATEEMNRIFKPLIGPRPLVNGLMGRINYYWSVLKPGQTLRIKSDGNLIAPWYGHILSGESHGRILKTD
LKRGSCTVQCQTEKGGLNTTLPFQNVSKYAFGNCSKYIGVKSLKLAVGLRNVPSRSSRGLFGAIAGFIEG
GWSGLVAGWYGFQHSNDQGVGMAADRDSTQKAIDKITSKVNNIVDKMNKQYEIIDHEFSEVETRLNMINN
KVDDQIQDIWAYNAELLVLLENQKTLDEHDANVNNLYNKVKRALGSNAVEDGKGCFELYHKCDDHCMETI
RNGTYNRRKYQEESKLERQKIEGVKLESEETYKILTIYSTVASSLVIAMGFAAFLFWAMSNGSCRCNICI

>QDF63504.1 hemagglutinin [Influenza A virus]
METVSLITILLVATVSNADKICIGYQSTNSTETVDTLTENNVPVTHAKELLHTEHNGMLCATSLGQPLIL
DTCTIEGLIYGNPSCDLSLEGREWSYIVERPSAVHGLCYPGNVEDLEELRSLFSSARSYQRIQIFPDTIW
NVSYDGTSTACSGSFYRSMRWLTRKNGEYPIQDAQYTNNQGKNILFMWGINHPPTDDTQRGLYTRTDTTT
SVATEEINRIFKPLIGPRPLVNGLMGRINYYWSVLKPGQTLRIKSDGNLIAPWYGHILSGESHGRILKTD
LKRGSCTVQCQTEKGGLNTTLPFQNVSKYAFGNCSKYIGIKSLKLAVGLRNVPSRSSRGLFGAIAGFIEG
GWSGLVAGWYGFQHSNDQGVGMAADRDSTQKAIDKITSKVNNIVDKMNKQYEIIDHEFSEVETRLNMINN
KIDDQIQDIWAYNAELLVLLENQKTLDEHDANVNNLYNKVKRALGSNAVEDGKGCFELYHKCDDQCMETI
RNGTYNRRKYQEESKLERQRIEGVKLESEGTYKILTIYSTVASSLVIAMGFAAFLFWAMSNGSCRCNICI

>QDF63503.1 hemagglutinin [Influenza A virus]
METVSLITILIVATVSNADKICIGYQSTNSTETVDTLTENNVPVTHAKELLHTEHNGMLCATSLGHPLIL
DTCTIEGLIYGNPSCDPLLGGREWSYIVERPSAVNGLCYPGNVENLEELRSLFSSSRSYQRIQIFPDTIW
NVSYGGTSKACSDSFYRSMRWLTQKNNAYPTQDAQYTNNQGKNILFVWGINHPPTDTAQTNLYTRTDTTT
SVATEEMNRIFKPLIGPRPLVNGLMGRINYYWSVLKPGQTLRIKSDGNLIAPWYGHILSGESHGRILKTD
LKRGSCTVQCQTEKGGLNTTLPFQNVSKYAFGNCSKYIGVKSLKLAVGLRNVPSRSSRGLFGAIAGFIEG
GWSGLVAGWYGFQHSNDQGVGMAADRDSTQKAIDKITSKVNNIVDKMNKQYEIIDHEFSEVETRLNMINN
KVDDQIQDIWAYNAELLVLLENQKTLDEHDANVNNLYNKVKRALGSNAVEDGKGCFELYHKCDDHCMETI
RNGTYNRRKYQEESKLERQKIEGVKLESEETYKILTIYSTVASSLVIAMGFAAFLFWAMSNGSCRCNICI

>QDF63502.1 hemagglutinin [Influenza A virus]
METVSLITILLVATVSNADKICIGYQSTNSTETVDTLTENNVPVTHAKELLHTEHNGMLCATSLGQPLIL
DTCTIEGLIYGNPSCDLSLEGREWSYIVERPSAVHGLCYPGNVEDLEELRSLFSSARSYQRIQIFPDTIW
NVSYDGTSTACSGSFYRSMRWLTRKNGEYPIQDAQYTNNQGKNILFMWGINHPPTDDTQRGLYTRTDTTT
SVATEEINRIFKPLIGPRPLVNGLMGRINYYWSVLKPGQTLRIKSDGNLIAPWYGHILSGESHGRILKTD

LKRGSCTVQCQTEKGGLNTTLPFQNVSKYAFGNCSKYIGIKSLKLAVGLRNVPSRSSRGLFGAIAGFIEG
GWSGLVAGWYGFQHSNDQGVGMAADRDSTQKAIDKITSKVNNIVDKMNKQYEIIDHEFSEVETRLNMINN
KIDDQIQDIWAYNAELLVLLENQKTLDEHDANVNNLYNKVKRALGSNAVEDGKGCFELYHKCDDQCMETI
RNGTYNRRKYQEESKLERQRIEGVKLESEGTYKILTIYSTVASSLVIAMGFAAFLFWAMSNGSCRCNICI

>QDF63501.1 hemagglutinin [Influenza A virus]
METISLITILLVATVSNADKICIGYQSTNSTETVDTLTENNVPVTHAKELLHTEHNGMLCATSLGQPLIL
DTCTIEGLIYGNPSCDLSLEGREWSYIVERPSAVHGLCYPGNVEDLEELRSLFSSARSYQRIQIFPDTIW
NVSYDGTSTACSDSFYRSMRWLTRKNGEYPIQDAQYTNNQGKNILFMWGINHPPTDETQRSLYTRTDTTT
SVATEEINRIFKPLIGPRPLVNGLMGRIDYYWSVLKPGQTLRIKSDGNLIAPWYGHILSGESHGRILKTD
LKRGSCTVQCQTEKGGLNTTLPFQNVSKYAFGNCSKYIGIKSLKLAVGLRNVPSRSSRGLFGAIAGFIEG
GWSGLVAGWYGFQHSNDQGVGMAADRDSTQKAIDKITSKVNNIVDKMNKQYEIIDHEFSEVETRLNMINN
KIDDQIQDIWAYNAELLVLLENQKTLDEHDANVNNLYNKVKRALGSNAVEDGKGCFELYHKCNDQCMETI
RNGTYNRKKYQEESKLERQRIEGVKLESEGTYKILTIYSTVASSLVIAMGFAAFLFWAMSNGSCRCNICI

>QDF63500.1 hemagglutinin [Influenza A virus]
METVSLITILLVATVSNADKICIGYQSTNSTETVDTLTENNVPVTHAKELIHTEHNGMLCATSLGQPLIL
DTCTIEGLIYGNPFCGLSLEGREWSYIVERPSAVNGLCYPGNVENLEELRSLFSSARSYQRIQIFPDTIW
NVSYDGTSAACSGSFYRNMRWLTRKDGNYPTQDAQYTNNQGKNILFMWGINHPPTDTTQRDLYTRTDTTT
SVATEEINRIFKPLIGPRPLVNGLMGRIDYYWSVLKPGQTLRIKSDGNLIAPWFGHILSGESHGRILKTD
LKRGSCTVQCQTEKGGLNTTLPFQNVSKYAFGNCSKYIGIKSLKLAVGLRNVPSRSSRGLFGAIAGFIEG
GWSGLIAGWYGFQHSNDQGVGMAADRDSTQKAIDKITSKVNNIVDKMNKQYEIIDHEFSEVETRLNMINN
KIDDQIQDIWAYNAELLVLLENQKTLDEHDANVNNLYNKVKRALGSNAVEDGKGCFELYHKCDDQCMETI
RNGTYNRRKYQEESKLERQKIEGVKLESEGTYKILTIYSTVASSLVIAMGFAAFLFWAMSNGSCRCNICI

>QDF63499.1 hemagglutinin [Influenza A virus]
METVSLITILLVAAVSYADKICIGYQSTNSTETVDTLTENNVPVTHAKELLHTEHNGMLCATSLGQPLIL
DTCTIEGLIYGNPSCDLSLEGREWSYIVERPSAVNGLCYPGNVENLEELRSLFSSARSYQRVQIFPDTIW
NVSYDGTSTACSGSFYRSMRWLTRKDGNYPTQDAQYTNNQGKNILFMWGINHPPTDDTQRSLYTRTDTTT
SVATEEINRIFKPLIGPRPLVNGLMGRIDYYWSVLKPGQTLRIKSDGNLIAPWYGYILSGESHGRILKTD
LKRGSCTVQCQTEKGGLNTTLPFQNVSKYAFGNCSKYIGIKSLKLAVGLRNVPSKSSRGLFGAIAGFIEG
GWSGLVAGWYGFQHSNDQGVGMAADRDSTQKAIDKITSKVNNIVDKMNKQYEIIDHEFSEVETRLNMINN
KIDDQIQDIWAYNAELLVLLENQKTLDEHDANVNNLYNKVKRALGSNAAEDGKGCFELYHKCNDQCMETI
RNGTYNRRKYQEESKLERQKIEGVKLESEGTYKILTIYSTVASSLVIAMGFAAFLFWAMSNGSCRCNICI

>QDF63498.1 hemagglutinin [Influenza A virus]

METISLMTILLVATVSNADKICIGYQSTNSTETVDTLTENNVPVTHAKELLHTEHNGMLCATSLGNPLIL

DTCTIEGLIYGNPSCDPLLGGREWSYIVERPSAVNGLCYPGSVENLEELRSLFSSARSYQRIQIFPDTIW

NVSYSGTSKACSDSFYRSMRWLTQKNNAYPIQDAQYTNNQEKNILFMWGINHPPTETVQTNLYTRTDTTT

SVATEEINRIFKPLIGPRPLVNGLMGRINYYWSVLKPGQTLRIKSDGNLIAPWYGHILSGESHGRILKTD

LKRGSCTVQCQTEKGGLNTTLPFQNVSKYAFGNCSKYIGIKSLKLAVGLRNVPSRSSRGLFGAIAGFIEG

GWSGLVAGWYGFQHSNDQGVGMAADRDSTQKAIDKITSKVNNIVDKMNKQYEIIDHEFSEVETRLNMINN

KVDDQIQDIWAYNAELLVLLENQKTLDEHDSNVNNLYNKVKRALGSNAVEDGKGCFELYHKCDNQCMETI

RNGTYNRRKYQEESKLERQKIEGVKLESEGTYKILTIYSTVASSLVIAMGFAAFLFWAMSNGSCRCNICI

>QDF63497.1 hemagglutinin [Influenza A virus]

METVSLITILIVATVSNADKICIGYQSTNSTETVDTLTENNVPVTHAKELLHTEHNGMLCATSLGQPLIL

DTCTIEGLIYGNPSCDLSLEGREWSYIVERPSAINGLCYPGNVENLEELRSLFSSARSYQRIQIFPDTIW

NVSYDGTSTACSNSFYRSMRWLTRKDGNYPTQDAQYTNNQGKNILFMWGINHPPTDDTQRNLYTRTDTTT

SVATEEINRIFKPLIGPRPLVNGLMGRIDYYWSVLKPGQTLRIKSDGNLIAPWYGHILSGESHGRILKTD

LKRGSCTVQCQTEKGGLNTTLPFQNVSKYAFGNCSKYIGIKSLKLAVGLRNVPSRSSRGLFGAIAGFIEG

GWSGLVAGWYGFQHSNDQGVGMAADRDSTQKAIDKITSKVNNIVDKMNKQYEIIDHEFSEVETRLNMINN

KIDDQIQDIWAYNAELLVLLENQKTLDEHDANVNNLYNKVKRALGSNAVEDGKGCFELYHKCNDQCMETI

RNGTYNRRKYQEESKLERQKIEGVKLESEGTYKILTIYSTVASSLVIAMGFAAFLFWAMSNGSCRCNICI

>QDF63496.1 hemagglutinin [Influenza A virus]

METASLITILLAVTVSNADKICIGYQSTNSTETVDTLTENNVPVTHAKELLHTEHNGMLCATSLGHPLIL

DTCTIEGLIYGNPSCDLLLGGREWSYIVERPSAVNGLCYPGNVENLEELRSLFSSARSYQRIQIFPDTIW

NVSYSGTSKACSDSFYRSMRWLTQKDNAYPVQDAQYTNNQEKNILFMWGINHPPTDTAQTNLYTRTDTTT

SVATEEINRTFKPLIGPRPLVNGLMGRINYYWSVLKPGQTLRIKSNGNLIAPWYGHILSGESHGRILKTD

LKRGSCTVQCQTEKGGLNTTLPFQNVSKYAFGNCSKYIGIKSLKLAVGLRNVPSRSSRGLFGAIAGFIEG

GWSGLVAGWYGFQHSNDQGVGMAADRDSTQKAIDKITSKVNNIVDKMNKQYEIIDHEFNEVETRLNMINN

KIDDQIQDIWAYNAELLVLLENQKTLDEHDANVNNLYNKVKRALGSNAVEDGKGCFELYHKCDDQCMETI

RNGTYNRRKYQEESKLERQKIEGVKLESEGTYKILTIYSTVASSLVIAMGFAAFLFWAMSNGSCRCNICI

>QDF63495.1 hemagglutinin [Influenza A virus]

METVSLITILLVATISNADKICIGYQSTNSTETVDTLTENNVPVTHAKELLHTEHNGMLCATSLGQPLIL

DTCTIEGLIYGNPSCDLSLEGREWSYIVERPSAVNGLCYPGNVENLEELRSLFSSARSYQRIQIFPDTIW

NVSYDGTSTACSGSFYRNMRWLTRKNGDYPTQDAQYTNNQGKNILFMWGINHPPADDTQRTLYTRNDTTT
SVATEEINRIFKPLIGPRPLVNGLMGRIDYYWSVLKPGQTLRIKSDGNLIAPWYGHILSGESHGRILKTD
LKRGSCTVQCQTEKGGLNTTLPFQNVSKYAFGNCSKYIGIKSLKLAVGLRNVPSRSSRGLFGAIAGFIEG
GWSGLVAGWYGFQHSNDQGVGMAADRDSTQKAIDKITSKVNNIVDKMNKQYEIIDHEFSEVETRLNMINN
KIDDQIQDIWAYNAELLVLLENQKTLDEHDANVNNLYNKVKRALGSNAVEDGKGCFELYHKCDDQCMETI
RNGTYNRKKYQEESKLERQKIEGVKLESEGTYKILTIYSTVASSLVIAMGFAAFLVWAMSNGSCRCNICI

>QDF63494.1 hemagglutinin [Influenza A virus]
METVSLITILLVAAVSNADKICIGYQSTNSTETVDTLTENNVPVTHAKELLHTEHNGMLCATSLGQPIIL
DTCTIEGLIYGNPSCDLSLEGREWSYIVERPSAVNGLCYPGNVENLEELRSLFSSARSYQRIQIFPDTIW
NVSYDGTSTACSGSFYRNMRWLTRKNGEYPIQDAQYTNNQGKNILFMWGINHPPADTTQRDLYTRTDTTT
SVATEEINRIFKPLIGPRPLVNGLMGRIDYYWSVLKPGQTLRIKSDGNLIAPWYGHILSGESHGRILKTD
LKRGSCTVQCQTEKGGLNTTLPFQNVSKYAFGNCSKYIGIKSLKLAVGLRNVPSRSSRGLFGAIAGFIEG
GWSGLVAGWYGFQHSNDQGVGMAADRDSTQKAVDKITSKVNTIVDKMNKQYEIIDHEFSEVETRLNMINN
KIDDQIQDIWAYNAELLVLLENQKTLDEHDANVNNLYNKVKRALGSNAVEDGKGCFELYHKCDDQCMETI
RNGTYNRRKYQEESKLERQKIEGVKLESEGTYKILTIYSTVASSLVIAMGFAAFLFWAMSNGSCRCNICI

>QDF63493.1 hemagglutinin [Influenza A virus]
METVSLITILLVATVSNADKICIGYQSTNSTETVDTLTENNVPVTHAKELLHTEHNGMLCATSLGQPLIL
DTCTIEGLIYGNPSCDLSLEGREWSYIVERPSAVNGLCYPGNVENLEELRSLFSSARSYQRIQIFPDTIW
NVSYDGTSTACSNSFYRSMRWLTRKDGNYPTQDAQYTNNQGKNILFMWGINHPPTDDTQRNLYTRTDTTT
SVATEEINRIFKPLIGPRPLVNGLMGRIAYYWSVLKPGQTLRIKSDGNLIAPWYGHILSGESHGRILKTD
LKRGSCTVQCQTEKGGLNTTLPFQNVSKYAFGNCSKYIGIKSLKLAVGLRNVPSRSSRGLFGAIAGFIEG
GWSGLVAGWYGFQHSNDQGVGMAADRDSTQKAIDKITSKVNNIVDKMNKQYEIIDHEFSEVETRLNMINN
KIDDQIQDIWAYNAELLVLLENQKTLDEHDANVNNLYNKVKRALGSNAVEDGKGCFELYHKCNDQCMETI
RNGTYNRRKYQEESKLERQKIEGVKLESEGTYKILTIYSTVASSLVIAMGFAAFLFWAMSNGSCRCNICI

>QDF63492.1 hemagglutinin [Influenza A virus]
METVSLITILLVATVSNADKICIGYQSTNSTETVDTLTENNVPVTHAKELLHTEHNGMLCATSLGQPIIL
DTCTIEGLIYGNPSCDLSLEGREWSYIVERPSAVNGLCYPGNVENLEELRSLFSSARSYQRIQIFPDTIW
NVSYDGTSTACSGSFYKNMRWLTRKNGEYPIQDAQYTNNQGKNILFMWGINHPPADTTQRDLYTRTDTTT
SVATEEINRIFKPLIGPRPLVNGLMGRIDYYWSVLKPGQTLRIKSDGNLIAPWYGHILSGESHGRILKTD
LKRGSCTVQCQTEKGGLNTTLPFQNVSKYAFGNCSKYIGIKSLKLAVGLRNVPSRSSRGLFGAIAGFIEG
GWSGLVAGWYGFQHSNDQGVGMAADRESTQKAVDKITSKVNNIVDKMNKQYEIIDHEFSEVETRLNMINN

KIDDQIQDIWAYNAELLVLLENQKTLDEHDANVNNLYNKVKRALGSNAMEDGKGCFELYHKCDDQCMETI
RNGTYNRRKYQEESKLERQKIEGVKLESEGTYKILTIYSTVASSLVIAMGFAAFLFWAMSNGSCRCNICI

>QDF63491.1 hemagglutinin [Influenza A virus]
METVSLITILLVATVSYADKICIGYQSTNSTETVDTLTENNVPVTHAKELLHTEHNGMLCATSLGQPLIL
DTCTIEGLIYGNPSCDLSLEGREWSYIVERPSAVNGLCYPGNVENLEELRSLFSSARSYQRVQIFPDTIW
NVSYDGTSTACSGSFYRSMRWLTRKNGDYPTQDAQYTNNQGKNILFMWGINHPPTDDTQRTLYTRTDTTT
SVATEEINRIFKPLIGPRPLVNGLMGRIDYYWSVLKPGQTLRIKSDGNLIAPWYGYILSGESHGRILKTD
LKRGSCTVQCQTEKGGLNTTLPFQNVSKYAFGNCSKYIGIKSLKLAVGLRNVPSRSSRGLFGAIAGFIEG
GWSGLVAGWYGFQHSNDQGVGMAADRDSTQKAIDKITSKVNNIVDKMNKQYEIIDHEFSEVETRLNMINN
KIDDQIQDIWAYNAELLVLLENQKTLDEHDANVNNLYNKVKRALGSNAVEDGKGCFELYHKCNDQCMETI
RNGTYNRRKYQEESKLERQKIEGVKLESEGTYKILTIYSTVASSLVIAMGFAAFLFWAMSNGSCRCNICI

>QDF63490.1 hemagglutinin [Influenza A virus]
METVSLITILLVATVSYADKICIGYQSTNSTETVDTLTENNVPVTHAKELLHTEHNGMLCATSLGQPLIL
DTCTIEGLIYGNPSCDLSLEGREWSYIVERPSAVNGLCYPGNVENLEELRSLFSSARSYQRVQIFPDTIW
NVSYDGTSTACSGSFYRSMRWLTRKNDNYPTQDAQYTNNQGKNILFMWGINHPPTDDTQRNLYTRTDTTT
SVATEEINRIFKPLIGPRPLVNGLMGRINYYWSVLKPGQTLRIKSDGNLIAPWYGYILSGESHGRILKTD
LKRGSCTVQCQTEKGGLNTTLPFQNVSKYAFGNCSKYIGIKSLKLAVGLRNVPSRSSRGLFGAIAGFIEG
GWSGLVAGWYGFQHSNDQGVGMAADRDSTQKAIDKITSKVNNIVDKMNKQYEIIDHEFSEVETRLNMINN
KIDDQIQDIWAYNAELLVLLENQKTLDEHDANVNNLYNKVKRSLGSNAVEDGKGCFELYHKCNDQCMETI
RNGTYNRRKYQEESKLERQKIEGVKLESEGTYKILTIYSTVASSLVIAMGFAAFLFWAMSNGSCRCNICI

>QDF63489.1 hemagglutinin [Influenza A virus]
METVSLITILLVATVSNADKICIGYQSTNSTETVDTLTENNVPVTHAKELLHTEHNGMLCATSLGQPLIL
DTCTIEGLIYGNPSCDLSLEGREWSYIVERPSAVNGLCYPGNVENLEELRSLFSSARSYQRIQIFPDTIW
NVSYDGTSTACSNSFYRSMRWLTRKDGNYPTQDAQYTNNQGKNILFMWGINHPPTDDTQRNLYTRTDTTT
SVATEEINRIFKPLIGPRPLVNGLMGRIAYYWSVLKPGQTLRIKSDGNLIAPWYGHILSGESHGRILKTD
LKRGSCTVQCQTEKGGLNTTLPFQNVSKYAFGNCSKYIGIKSLKLAVGLRNVPSRSSRGLFGAIAGFIEG
GWSGLVAGWYGFQHSNDQGVGMAADRDSTQKAIDKITSKVNNIVDKMNKQYEIIDHEFSEVETRLNMINN
KIDDQIQDIWAYNAELLVLLENQKTLDEHDANVNNLYNKVKRALGSNAVEDGKGCFELYHKCNDQCMETI
RNGTYNRRKYQEESKLERQKIEGVKLESEGTYKILTIYSTVASSLVIAMGFAAFLFWAMSNGSCRCNICI

>QDF63488.1 hemagglutinin [Influenza A virus]

METVSLITILLVATVSNADKICIGYQSTNSTETVDTLTENNVPVTHAKELLHTEHNGMLCATSLGQPIIL
DTCTIEGLIYGNPSCDLSLEGREWSYIVERPSAVNGLCYPGNVENLEELRSLFSSARSYQRIQIFPDTIW
NVSYDGTSTACSGSFYRNMRWLTRKNGEYPIQDAQYTNNQEKNILFMWGINHPPADTTQRDLYTRTDTTT
SVATEEINRIFKPLIGPRPLVNGLMGRIDYYWSVLKPGQTLRIKSDGNLIAPWYGHILSGESHGRILKTD
LKRGSCTVQCQTEKGGLNTTLPFQNVSKYAFGNCSKYIGIKSLKLAVGLRNVPSRSSRGLFGAIAGFIEG
GWSGLVAGWYGFQHSNDQGVGMAADRESTQKAVDKITSKVNNIVDKMNKQYEIIDHEFSEVETRLNMINN
KIDDQIQDIWAYNAELLVLLENQKTLDEHDANVNNLYNKVKRALGSNAMEDGKGCFELYHKCDDQCMETI
RNGTYNRRKYQEESKLERQKIEGVKLESEGTYKILTIYSTVASSLVIAMGFAAFLFWAMSNGSCRCNICI

>QDF63487.1 hemagglutinin [Influenza A virus]
METVSLITILLVATVSNADKICIGYQSTNSTETVDTLTENNVPVTHAKELLHTEHNGMLCATSLGQPIIL
DTCTIEGLIYGNPSCDLSLEGREWSYIVERPSAVNGLCYPGNVENLEELRSLFSSARSYQRIQIFPDTIW
NVSYDGTSTACSGSFYRNMRWLTRKNGEYPIQDAQYTNNQGKNILFMWGINHPPADTTQRDLYTRTDTTT
SVATEEINRIFKPLIGPRPLVNGLMGRIDYYWSVLKPGQTLRIKSDGNLIAPWYGHILSGESHGRILKTD
LKRGSCTVQCQTEKGGLNTTLPFQNVSRYAFGNCSKYIGIKSLKLAVGLRNVPSRSSRGLFGAIAGFIEG
GWSGLVAGWYGFQHSNDQGVGMAADRESTQKAVDKITSKVNNIVDKMNKQYEIIDHEFSEVETRLNMINN
KIDDQIQDIWAYNAELLVLLENQKTLDEHDANVNNLYNKVKRALGSNAMEDGKGCFELYHKCDDQCMETI
RNGTYNRRKYQEESKLERQKIEGVKLESEGTYKILTIYSTVASSLVIAMGFAAFLFWAMSNGSCRCNICI

>QDF63486.1 hemagglutinin [Influenza A virus]
METVSLITILLVAAVSNADKICIGYQSTNSTETVDTLTENNVPVTHAKELLHTEHNGMLCATSLGQPIIL
DTCTIEGLIYGNPSCDLSLEGREWSYIVERPSAVNGLCYPGNVENLEELRSLFSSARSYQRIQIFPDTIW
NVSYDGTSTACSGSFYRNMRWLTRKNGEYPIQDAQYTNNQGKNILFMWGINHPPADTTQRDLYTRTDTTT
SVATEEINRIFKPLIGPRPLVNGLMGRIDYYWSVLKPGQTLRIKSDGNLIAPWYGHILSGESHGRILKTD
LKRGSCTVQCQTEKGGLNTTLPFQNVSKYAFGNCSKYIGIKSLKLAVGLRNVPSRSSRGLFGAIAGFIEG
GWSGLVAGWYGFQHSNDQGVGMAADRDSTQKAVDKITSKVNTIVDKMNKQYEIIDHEFSEVETRLNMINN
KIDDQIQDIWAYNAELLVLLENQKTLDEHDANVNNLYNKVKRALGSNAVEDGKGCFELYHKCDDQCMETI
RNGTYNRRKYQEESKLERQKIEGVKLESEGTYKILTIYSTVASSLVIAMGFAAFLFWAMSNGSCRCNICI

>QDF63485.1 hemagglutinin [Influenza A virus]
METISLMTILLVATVSNADKICIGYQSTNSTETVDTLTESNVPVTHAKELLHTEHNGMLCATSLGNPLIL
DTCTIEGLIYGNPSCDPLLGGREWSYIVERPSAVNGLCYPGSVENLEELRSLFSSARSYQRIQIFPDTIW
NVSYSGTSKACSDSFYRSMRWLTQKNNAYPIQDAQYINNQEKNILFMWGINHPPTETVQTNLYTRTDTTT
SVATEEINRIFKPLIGPRPLVNGLMGRINYYWSVLKPGQTLRIKSDGNLIAPWYGHILSGESHGRILKTD

LKRGSCTVQCQTEKGGLNTTLPFQNVSKYAFGNCSKYIGIKSLKLAVGLRNVPSRSSRGLFGAIAGFIEG
GWSGLVAGWYGFQHSNDQGVGMAADRDSTQKAIDKITSKVNNIVDKMNKQYEIIDHEFSEVETRLNMINN
KVDDQIQDIWAYNAELLVLLENQKTLDEHDSNVNNLYNKVKRALGSNAVEDGKGCFELYHKCDNQCMETI
RNGTYNRRKYQEESKLERQKIEGVKLESEGTYKILTIYSTVASSLVIAMGFAAFLFWAMSNGSCRCNICI

>QDF63484.1 hemagglutinin [Influenza A virus]
METISLITILLVATVSNADKICIGYQSTNSTETVDTLTENNVPVTHAKELLHTEHNGMLCATSLGQPLIL
DTCTIEGLIYGNPSCDLSLEGREWSYIVERPSAVNGLCYPGNVENLEELRSLFSSARSYQRVQIFPDTIW
NVSYDGTSTACSGSFYRSMRWLTRKNGDYPTQDAQYTNNQRKNILFMWGINHPPTDDTQRNLYTRTDTTT
SVATEEINRIFKPLIGPRPLVNGLMGRIDYYWSVLKPGQTLRIKSDGNLIAPWYGHILSGESHGRILKTD
LKRGSCTVQCQTEKGGLNTTLPFQNVSKYAFGNCSKYIGIKSLKLAVGLRNVPSRSSRGLFGAIAGFIEG
GWSGLVAGWYGFQHSNDQGVGMAADRDSTQKAIDKITSKVNNIVDKMNKQYEIIDHEFSEVETRLNMINN
KIDDQIQDIWAYNAELLVLLENQKTLDEHDANVNNLYNKVKRALGSNAMEDGKGCFELYHKCNDQCMETI
RNGTYNRRKYQEESKLERQKIEGVKLESEGTYKILTIYSTVASSLVIAMGFAAFLFWAMSNGSCRCNICI

>QDF63483.1 hemagglutinin [Influenza A virus]
METVSLITILLVATVSNADKICIGYQSTNSTETVDTLTENNVPVTHAKELIHTEHNGMLCATSLGQPLIL
DTCTIEGLIYGNPSCDLSLEGREWSYIVERPSAVNGLCYPGNVENLEELRSLFSSARSYQRIQIFPDTIW
NVSYDGTSTACSGSFYRNMRWLTRKNGNYPTQDVQYTNNQGKNILFMWGINHPPTDTTQSGLYTRTDTTT
SVATEEINRIFKPLIGPRPLVNGLMGRIDYYWSVLKPGQTLRIKSDGNLIAPWFGHILSGESHGRILKTD
LKRGSCTVQCQTEKGGLNTTLPFQNVSKYAFGNCSKYIGIKSLKLAVGLRNVPSRSSRGLFGAIAGFIEG
GWSGLVAGWYGFQHSNDQGVGMAADRDSTQKAIDKITSKVNNIVDKMNKQYEIIDHEFSEVETRLNMINN
KIDDQIQDIWAYNAELLVLLENQKTLDEHDANVNNLYNKVKRALGSNAVEDGKGCFELYHKCDDQCMETI
RNGTYNRRKYQEESKLERQKIEGVKLESEGTYKILTIYSTVASSLVIAMGFAAFLFWAMSNGSCRCNICI

>QDF63482.1 hemagglutinin [Influenza A virus]
METISLITILLVATVSNADKICIGYQSTNSTETVDTLTENNVPVTHAKELLHTEHNGMLCATSLGQPLIL
DTCTIEGLIYGNPSCDLSLEGREWSYIVERPSAVNGLCYPGNVENLEELRSLFSSARSYQRIQIFPDTIW
NVSYDGTSTACSGSFYRSMRWLTRKNGDYPTQDAQYTNNQGKNILFMWGINHPPTDDTQRNLYTRTDTTT
SVATEEINRIFKPLIGPRPLVNGLMGRIDYYWSVLKPGQTLRIKSDGNLIAPWYGYILSGESHGRILKTD
LKRGSCTVQCQTEKGGLNTTLPFQNVSKYAFGNCSKYIGIKSLKLAVGLRNVPSRSSRGLFGAIAGFIEG
GWSGLVAGWYGFQHSNDQGVGMAADRDSTQKAIDKITSKVNNIVDKMNKQYEIIDHEFSEVETRLNMINN
KIDDQIQDIWAYNAELLVLLENQKTLDEHDANVNNLYNKVKRALGSNAVEDGKGCFELYHKCNDQCMETI
RNGTYNRRKYQEESKLERQKIEGVKLESEGTYKILTIYSTVASSLVIAMGFAAFLFWAMSNGSCRCNICI

>QDF63481.1 hemagglutinin [Influenza A virus]

METISLITILLVATVSNADKICIGYQSTNSTETVDTLTENNVPVTHAKELLHTEHNGMLCATSLGQPLIL
DTCTIEGLIYGNPSCDLSLEGREWSYIVERPSAVNGLCYPGNVENLEELRSLFSSARSYQRIQIFPDTIW
NVSYDGTSTACSGSFYRSMRWLTRKNGDYPTQDAQYTNNQGKNILFMWGINHPPTDDTQRNLYTRTDTTT
SVATEEINRIFKPLIGPRPLVNGLMGRIDYYWSVLKPGQTLRIKSDGNLIAPWYGYILSGESHGRILKTD
LKRGSCTVQCQTEKGGLNTTLPFQNVSKYAFGNCSKYIGIKSLKLAVGLRNVPSRSSRGLFGAIAGFIEG
GWSGLVAGWYGFQHSNDQGVGMAADRDSTQKAIDKITSKVNNIVDKMNKQYEIIDHEFSEVETRLNMINN
KIDDQIQDIWAYNAELLVLLENQKTLDEHDANVNNLYNKVKRALGSNAVEDGKGCFELYHKCNDQCMETI
RNGTYNRRKYQEESKLERQKIEGVKLESEGTYKILTIYSTVASSLVIAMGFAAFLFWAMSNGSCRCNICI

>QDF63479.1 hemagglutinin [Influenza A virus]

METISLITILLVATVSNADKICIGYQSTNSTETVDTLTENNVPVTHAKELLHTEHNGMLCATGLGQPLIL
DTCTIEGLIYGNPSCDLSVEGREWSYIVERPSAVNGLCYPGNVENLEELRSLFSSARSYQRIQIFPDTIW
NVSYDGTSTACSGSFYRSMRWLTRKNGEYPIQDAQYTNNQGKNILFMWGINHPPTDTTQRDLYTRTDTTT
SVATEEINRVFKPLIGPRPLVNGLMGRIDYYWSVLKPGQTLRIKSDGNLIAPWYGHILSGESHGRILKTD
LKKGSCTVQCQTEKGGLNTTLPFQNVSKYAFGNCSKYIGIKSLKLAVGLRNVPSRSSRGLFGAIAGFIEG
GWSGLVAGWYGFQHSNDQGVGMAADRDSTQKAIDKITSKVNNIVDKMNKQYEIIDHEFSEVETRLNMINN
KIDDQIQDIWAYNAELLVLLENQKTLDEHDANVNNLYNKVKRALGSNAVEDGKGCFELYHKCDDQCMETI
RNGTYNRRKYQEESKLERQKIEGVKLESEGTYKILTIYSTVASSLVIAMGFAAFLFWAMSNGSCRCNICI

>QDF63478.1 hemagglutinin [Influenza A virus]

METISLITILLVATVSNADKICIGYQSTNSTETVDTLTENNVPVTHAKELLHTEHNGMLCATSLGHPLIL
DTCTIEGLIYGNPSCDPLLGGREWSYIVERPSAVNGLCYPGNVENLEELRSLFSSSRSYQRIQIFPDTIW
NVSYSGTSRACSDSFYRSMRWLTQKNNAYPTQDAQYTNNQEKNILFMWGINHPPTDTAQTNLYTRTDTTT
SVATEEINRIFKPLIGPRPLVNGLMGRINYYWSVLKPGQTLRIKSDGNLIAPWYGHILSGESHGRILKTD
LKRGSCTVQCQTEKGGLNTTLPFQNVSKYAFGNCSKYIGVKSLKLAVGLRNVPSRSSRGLFGAIAGFIEG
GWSGLVAGWYGFQHSNDQGVGMAADRDSTQKAIDKITSKVNNIVDKMNKQYEIIDHEFSEVETRLNMINN
KVDDQIQDIWAYNAELLVLLENQKTLDEHDANVNNLYNKVKRALGSNAVEDGKGCFELYHKCDDHCMETI
RNGTYNRRKYQEESKLERQKIEGVKLESEETYKILTIYSTVASSLVIAMGFAAFLFWAMSNGSCRCNICI

>QDF63477.1 hemagglutinin [Influenza A virus]

METVSLITILLVATVSNADKICIGYQSTNSTETVDTLTENNVPVTHAKELLHTEHNGMLCATSLGQPIIL
DTCTIEGLIYGNPSCDLSLEGREWSYIVERPSAVNGLCYPGNVENLEELRSLFSSARSYQRIQIFPDTIW

NVSYDGTSTACSGSFYRNMRWLTRKNGDYPIQDAQYTNNQGKNILFMWGINHPPADTTQRNLYTRTDTTT
SVATEEINRIFKPLIGPRPLVNGLMGRIDYYWSVLKPGQTLRIKSDGNLIAPWYGHILSGESHGRILKTD
LKRGSCTVQCQTEKGGLNTTLPFQNVSKYAFGNCSKYIGIKSLKLAVGLRNVPSRSSRGLFGAIAGFIEG
GWSGLVAGWYGFQHSNDQGVGMAADRDSTQKAVDKITSKVNNIVDKMNKQYEIIDHEFSEVETRLNMINN
KIDDQIQDIWAYNAELLVLLENQKTLDEHDANVNNLYNKVKRALGSNAVEDGKGCFELYHKCDDQCMETI
RNGTYNRRKYQEESKLERQKIEGVKLESEGTYKILTIYSTVASSLVIAMGFAAFLFWAMSNGSCRCNICI

>QDF63476.1 hemagglutinin [Influenza A virus]
METISLITILLVATVSNADKICIGYQSTNSTETVDTLTENNVPVTHAKELLHTEHNGMLCATSLGDPLIL
DTCTIEGLIYGNPSCDSLLGGREWSYIVERPSAVNGLCYPGNVENLEELRSLFSSARSYQRVQIFPDTIW
NVSYSGTSKACSDSFYRSMRWLTQKNNAYPIQDAQYTNNQEKNILFMWGINHPPTETAQTNLYTRTDTTT
SVATEEMNRIFKPLIGPRPLVNGLMGRINYYWSVLKPGQTLRIKSDGNLIAPWYGHILSGESHGRILKTD
LKRGSCIVQCQTEKGGLNTTLPFQNVSKYAFGNCSKYIGIKSLKLAVGLRNVPSRSSRGLFGAIAGFIEG
GWSGLVAGWYGFQHSNDQGVGMVADRDSTQKAIDKITSKVNNIVDKMNKQYEIIDHEFSEVETRLNMINN
KVDDQIQDIWAYNAELLVLLENQKTLDEHDSNVNNLYNKVKRALGSNAVEDGKGCFELYHKCDDQCMETI
RNGTYNRRKYQEESKLERQKIEGVKLESEGTYKILTIYSTVASSLVIAMGFAAFLFWAMSNGSCRCNICI

>QDF63475.1 hemagglutinin [Influenza A virus]
METISLITILLVATVSNADKICIGYQSTNSTETVDTLTENNVPVTHAKELLHTEHNGMLCATSLGHPLIL
DTCTIEGLIYGNPSCDPLLGGREWSYIVERPSAVNGLCYPGNVENLEELRSLFSSARSYQRIQIFPDTIW
NVSYSGTSKACSDSFYRSMRWLTQKNNAYPIQDAQYTNNQEKNILFMWGINHPPTETVQTNLYTRTDTTT
SVATEEINRIFKPLIGPRPLVNGLMGRINYYWSVLKPGQTLRIKSDGNLIAPWYGHILSGESHGRILKTD
LKRGSCTVQCQTEKGGLNTTLPFQNVSKYAFGNCSKYIGIKSLKLAIGLRNVPSRSSRGLFGAIAGFIEG
GWSGLVAGWYGFQHSNDQGVGMAADRDSTQKAIDKITSKVNNIVDKMNRQYEIIDHEFSEVETRLNMINN
KVDDQIQDIWAYNAELLVLLENQKTLDEHDSNVNNLYNKVKRALGSNAVEDGKGCFELYHKCDDQCMETI
RNGTYNRRKYQEESKLERQKIEGVKLESEGTYKILTIYSTVASSLVIAMGFAAFLFWAMSNGSCRCNICI

>QDF63474.1 hemagglutinin [Influenza A virus]
METVSLITILLVATVSNADKICIGYQSTNSTETVDTLTENNVPVTHAKELLHTEHNGMLCATSLGQPLIL
DTCTIEGLIYGNPSCDLSLEGREWSYIVERPSAVNGLCYPGNVENLEELRSLFSSARSYQRIQIFPDTIW
NVSYDGTSTACSGSFYRSMRWLTRKNGDYPTQDAQYTNNQGKNILFMWGINHPPTDDTQRNLYTRTDTTT
SVATEEINRIFKPLIGPRPLVNGLMGRIDYYWSVLKPGQTLRIKSDGNLIAPWYGHILSGESHGRILKTD
LKRGSCTVQCQTEKGGLNTTLPFQNVSKYAFGNCSKYIGIKSLKLAVGLRNVPSRSSRGLFGAIAGFIEG
GWSGLVAGWYGFQHSNDQGVGMAADRDSTQKAIDKITSKVNNIVDKMNKQYEIIDHEFSEVETRLNMINN

KIDDQIQDIWAYNAELLVLLENQKTLDEHDANVNNLYNKVKRALGSNAVEDGKGCFELYHKCNDQCMETI
RNGTYNRRKYQEESKLERQKIEGVKLESEGTYKILTIYSTVASSLVIAMGFAAFLFWAMSNGSCRCNICI

>QDF63473.1 hemagglutinin [Influenza A virus]
METVSLITILLVATASNADKICIGYQSTNSTETVDTLTENNVPVTHAKELLHTEHNGMLCATSLGQPLIL
DTCTIEGLIYGNPSCDLSLEGREWSYIVERPSAVNGLCYPGNVENLEELRSLFSSARSYQRIQIFPDTIW
NVSYDGTSTACSGSFYRSMRWLTRKNGDYPTQDAQYTNNQGKNILFMWGINHPPTDDTQRNLYTRTDTTT
SVATEEINRIFKPLIGPRPLVNGLMGRIDYYWSVLKPGQTLRIKSDGNLIAPWYGHILSGESHGRILKTD
LKRGSCTVQCQTEKGGLNTTLPFQNVSKYAFGNCSKYIGIKSLKLAVGLRNVPSRSSRGLFGAIAGFIEG
GWSGLVAGWYGFQHSNDQGVGMAADRDSTQKAIDKITSKVNNIVDKMNKQYEIIDHEFSEVETRLNMINN
KIDDQIQDIWAYNAELLVLLENQKTLDEHDANVNNLYNKVKRALGSNAVEDGKGCFELYHKCNDQCMETI
RNGTYNRRMYQEESKLERQKIEGVKLESEGTYKILTIYSTVASSLVIAMGFAAFLFWAMSNGSCRCNICI

>QDF63472.1 hemagglutinin [Influenza A virus]
METVSLITILLVATVSNADKICIGYQSTNSTETVDTLTENNVPVTHAKELLHTEHNGMLCATSLGQPLIL
DTCTIEGLIYGNPSCDLSLEGREWSYIVERPSAVNGLCYPGNVENLEELRSLFSSARSYQRIQIFPDTIW
NVSYDGTSTACSGSFYRSMRWLTRKNGDYPTQDAQYTNNQGKNILFMWGINHPPTDDTQRNLYTRTDTTT
SVATEEINRIFKPLIGPRPLVNGLMGRIDYYWSVLKPGQTLRIKSDGNLIAPWYGHILSGESHGRILKTD
LKRGSCTVQCQTEKGGLNTTLPFQNVSKYAFGNCSKYIGIKSLKLAVGLRNVPSRSSRGLFGAIAGFIEG
GWSGLVAGWYGFQHSNDQGVGMAADRDSTQKAIDKITSKVNNIVDKMNKQYEIIDHEFSEVETRLNMINN
KIDDQIQDIWAYNAELLVLLENQKTLDEHDANVNNLYNKVKRALGSNAVEDGKGCFELYHKCNDQCMETI
RNGTYNRRKYQEESKLERQKIEGVKLESEGTYKILTIYSTVASSLVIAMGFAAFLFWAMSNGSCRCNICI

>QDF63471.1 hemagglutinin [Influenza A virus]
METVSLITILLVATVSNADKICIGYQSTNSTETVDTLTENNVPVTHAKELLHTEHNGMLCATSLGQPLIL
DTCTIEGLIYGNPSCDLSLEGREWSYIVERPSAVNGLCYPGNVENLEELRSLFSSARSYQRIQIFPDTIW
NVSYDGTSTACSGSFYRSMRWLTRKNGDYPTQDAQYTNNQGKNILFMWGINHPPTDDTQRNLYTRTDTTT
SVATEEINRIFKPLIGPRPLVNGLMGRIDYYWSVLKPGQTLRIKSDGNLIAPWYGHILSGESHGRILKTD
LKRGSCTVQCQTEKGGLNTTLPFQNVSKYAFGNCSKYIGIKSLKLAVGLRNVPSRSSRGLFGAIAGFIEG
GWSGLVAGWYGFQHSNDQGVGMAADRDSTQKAIDKITSKVNNIVDKMNKQYEIIDHEFSEVETRLNMINN
KIDDQIQDIWAYNAELLVLLENQKTLDEHDANVNNLYNKVKRALGSNAVEDGKGCFELYHKCNDQCMETI
RNGTYNRRKYQEESKLERQKIEGVKLESEGTYKILTIYSTVASSLVIAMGFAAFLFWAMSNGSCRCNICI

>QDF63470.1 hemagglutinin [Influenza A virus]

METISLITILLVATVSNADKICIGYQSTNSTETVDTLTENNVPVTHAKELLHTEHNGMLCATSLGQPLIL
DTCTIEGLIYGNPSCDLSLEGREWSYIVERPSAVNGLCYPGNVENLEELRSLFSSARSYQRIQIFPDTIW
NVSYDGTSTACSGSFYRSMRWLTRKNGDYPTQDAQYTNNQGKNILFMWGINHPPTDDTQRNLYTRTDTTT
SVATEEINRIFKPLIGPRPLVNGLMGRIDYYWSVLKPGQTLRIKSDGNLIAPWYGLILSGESHGRILKTD
LKRGSCTVQCQTEKGGLNTTLPFQNVSKYAFGNCSKYIGIKSLKLAVGLRNVPSRSSRGLFGAIAGFIEG
GWSGLVAGWYGFQHSNDQGVGMAADRDSTQKAIDKITSKVNNIVDKMNKQYEIIDHEFSEVETRLNMINN
KIDDQIQDIWAYNAELLVLLENQKTLDEHDANVNNLYNKVKRALGSNAVEDGKGCFELYHKCNDQCMETI
RNGTYNRRKYQEESKLERQKIEGVKLESEGTYKILTIYSTVASSLVIAMGFAAFLFWAMSNGSCRCNICI

>QDF63468.1 hemagglutinin [Influenza A virus]
METISLITILLVVTVSNADKICIGYQSTNSTETVDTLTENNVPVTHAKELLHTEHNGMLCATNLGHPLIL
DTCTIEGLIYGNPSCNLLLGGREWSYIVERPSAVNGLCYPGNVENLEELRSLFSSASSYQRIQIFPDTIW
NVSYSGTSKACSDSFYRSMRWLTQKNNTYPIQDAQYTNNRGKNILFMWGINHPPTDTVQTNLYTRTDTTT
SVATEDINRTFKPLIGPRPLVNGLQGRIDYYWSVLKPGQTLRVRSNGNLIAPWYGHILSGESHGRILKTD
LNSGNCVVQCQTERGGLNTTLPFHNVSKYAFGNCPKYVGVKSLKLAVGLRNVPARSSRGLFGAIAGFIEG
GWSGLVAGWYGFQHSNDQGVGMAADRDSTQKATDKITSKVNNIVDKMNKQYEIIDHEFSEVETRLNMIND
KIDDQIQDIWAYNAELLVLLENQKTLDEHDANVNNLYNKVKRTLGSNAVEDGKGCFELYHKCDDQCMETI
RNGTYDRRKYKEESRLERQKIEGVKLESEGTYKILTIYSTVASSLVIAMGFAAFLFWAMSNGSCRCNICI

>QDF63466.1 hemagglutinin [Influenza A virus]
METISLITILLVATVSSADKICIGYQSTNSTETVDTLTENNVPVTHAKELLHTEHNGMLCATSLGQPLIL
DTCTIEGIIYGNPSCDLSLEGREWSYIVERPSAVNGLCYPGNVENLEELRSLFSSARSYQRIQIFPDTIW
NVSYDGTSTACSGSFYKSMRWLTRKNGDYPIQDAQYTNNQGKNILFMWGINHPPTDTTQRDLYTRTDTTT
SVATEEINRVFKPLIGPRPLVNGLMGRIDYYWSVLKPGQTLRIKSDGNLIAPWFGHILSGESHGRILKTD
LKRGSCTVQCQTEKGGLNTTLPFQNVSKYAFGNCSKYIGIKSLKLAVGLRNVPSRSSRGLFGAIAGFIEG
GWSGLVAGWYGFQHSNDQGVGMAADRDSTQKAIDKITSKVNNIVDKMNKQYEIIDHEFSEVETRLNMINN
KIDDQIQDIWAYNAELLVLLENQKTLDEHDANVNNLYNKVKRALGSNAVEDGKGCFELYHKCDDQCMETI
RNGTYNRRKYQEESKLERQKIEGVKLESEGTYKILTIYSTVASSLVIAMGFAAFLFWAMSNGSCRCNICI

>QDF63465.1 hemagglutinin [Influenza A virus]
METISLITILLGKQQVSNADKICIGYQSTNSTETVDTLTENNVPVTHAKELLHTEHNGMLCATSLGHPLI
LDTCTIEGLIYGNPSCDPLLGGREWSYIVERPSAVNGLCYPGNVENLEELRSLFSSARSYQRIQIFPDTI
WNVSYDGTSKACSDSFYRSMRWLTQKNNAYPIQDAQYTNNQEKNILFMWGINHPPTETAQTNLYTRTDTT
TSVATEEINRIFKPLIGPRPLVNGLMGRINYYWSVLKPGQTLRIKSDGNLIAPWYGHILSGESHGRILKT

DLKRGSCTVQCQTEKGGLNTTLPFQNVSKYAFGNCSKYIGIKSLKLAIGLRNVPSRSSRGLFGAIAGFIE
GGWSGLVAGWYGFQHSNDQGVGMAADRDSTQKAIDKITSKVNNIVDKMNRQYEIIDHEFSEVETRLNMIN
NKVDDQIQDIWAYNAELLVLLENQKTLDEHDSNVNNLYNKVKRALGSNAVEDGKGCFELYHKCDDQCMET
IRNGTYNRRKYQEESKLERQKIEGVKLESEGTYKILTIYSTVASSLVIAMGFAAFLFWAMSNGSCRCNIC
I

>QDF63463.1 hemagglutinin [Influenza A virus]
METVSLITILLVATVSNADKICIGYQSTNSTETVDTLTENNVPVTHAKELLHTEHNGMLCATSLGHPLIL
DTCTIEGLIYGNPSCDPLLGGREWSYIVERPSAVNGLCYPGNVENLEELRSLFSSARSYQRIQIFPDTIW
NVSYNGTSTACSDSFYRSMRWLTQKNNDYPIQDAQYTNNQGKNILFMWGINHPPTDTAQTNLYTRTDTTT
SVATEEINRIFKPLIGPRPLVNGLMGRINYYWSVLKPGQTLRIKSDGNLIAPWYGHILSGESHGRILKTD
LKRGSCTVQCQTEKGGLNTTLPFQNVSKYAFGNCSKYIGIKSLKLAVGLRNVPSRSSRGLFGAIAGFIEG
GWSGLVAGWYGFQHSNDQGVGIAADRDSTQKAIDKITSKVNNIVDKMNKQYEIIDHEFSEVETRLNMINN
KVDDQIQDIWAYNAELLVLLENQKTLDEHDANVNNLYNKVKRALGSNAVEDGKGCFELYHKCDDQCMETI
RNGTYNRRKYQEESKLERQKIEGVKLESEETYKILTIYSTVASSLVIAMGFAAFLFWAMSNGSCRCNICI

>QDF63462.1 hemagglutinin [Influenza A virus]
METISLITILLVVTVSNADKICIGYQSTNSTETVDTLTENNVPVTHAKELLHTEHNGMLCATGLGHPLIL
DTCTIEGLIYGNPSCDLLLGGREWSYIVERPSAVNGLCYPGNVENLEELRSLFSSASSYQRIQIFPDTIW
NVTYSGTSKACSDSFYRSMRWLTQKNNAYPIQDAQYTNNQEKNILFMWGINHPPTDTVQTNLYTRTDTTT
SVATEEINRTFKPLIGPRPLVNGLQGRIDYYWSVLKPGQTLRIRSNGNLIAPWYGHILSGGSHGRILKTD
LKRGSCTVQCQTEKGGLNTTLPFQNISKYAFGNCSKYIGIKSLKLAVGLRNVPSRSSRGLFGAIAGFIEG
GWSGLVAGWYGFQHSNDQGVGMAADRDSTQKAIDKITSKVNNIVDKMNKQYEIIDHEFSEVEARLNMINN
KIDDQIQDIWAYNAELLVLLENQKTLDEHDANVNNLYNKVKRALGSNAMEDGKGCFELYHKCDNQCMETI
RNGTYNRRKYQEESKLERQKIEGVKLESEGTYKILTIYSTVASSLVIAMGFAAFLFWAMSNGSCRCNICI

>QDF63461.1 hemagglutinin [Influenza A virus]
METVSLITILLVATVSNADKICIGYQSTNSTETVDTLTENNVPVTHAKELLHTEHNGMLCATSLGQPLIL
DTCTIEGLIYGNPSCDLSLEGREWSYIVERPSAVNGLCYPGNVENLEELRSLFSSARSYQRIQIFPDTIW
NVSYDGTSTACSGSFYRSMRWLTRKNGDYPTQDAQYTNNQGKNILFMWGINHPPTDDTQRNLYTRTDTTT
SVATEEINRIFKPLIGPRPLVNGLMGRIDYYWSVLKPGQTLRIKSDGNLIAPWYGHILSGESHGRILKTD
LKRGSCTVQCQTEKGGLNTTLPFQNVSKYAFGNCSKYIGIKSLKLAVGLRNVPSRSSRGLFGAIAGFIEG
GWSGLVAGWYGFQHSNDQGVGMAADRDSTQKAIDKITSKVNNIVDKMNKQYEIIDHEFSEVETRLNMINN
KIDDQIQDIWAYNAELLVLLENQKTLDEHDANVNNLYNKVKRALGSNAVEDGKGCFELYHKCNDQCMETI

RNGTYNRRKYQEESKIERQKIEGVKLESEGTYKILTIYSTVASSLVIAMGFAAFLFWAMSNGSCRCNICI

>QDF63460.1 hemagglutinin [Influenza A virus]
METVSLITILLVATVSNADKICIGYQSTNSTETVDTLTENNVPVTHAKELLHTEHNGMLCATSLGQPLIL
DTCTIEGLIYGNPSCDLSLEGREWSYIVERPSAVNGLCYPGNVENLEELRSLFSSARSYQRIQIFPDTIW
NVSYDGTSTACSGSFYRSMRWLTRKNGDYPTQDAQYTNNQGKNILFMWGINRPPTDDTQRNLYTRTDTTT
SVATEEINRIFKPLIGPRPLVNGLMGRIDYYWSVLKPGQTLRIKSDGNLIAPWYGHILSGESHGRILKTD
LKRGSCTVQCQTEKGGLNTTLPFQNVSKYAFGNCSKYIGIKSLKLAVGLRNVPSRSSRGLFGAIAGFIEG
GWSGLVAGWYGFQHSNDQGVGMAADRDSTQKAIDKITSKVNNIVDKMNKQYEIIDHEFSEVETRLNMINN
KIDDQIQDIWAYNAELLVLLENQKTLDEHDANVNNLYNKVKRALGSNAVEDGKGCFELYHKCNDQCMETI
RNGTYNRRKYQEESKLERQKIEGVKLESEGTYKILTIYSTVASSLVIAMGFAAFLFWAMSNGSCRCNICI

>QDF63459.1 hemagglutinin [Influenza A virus]
METVSLITILLVATVSNADKICIGYQSTNSTETVDTLTENNVPVTHAKELLHTEHNGMLCATSLGQPLIL
DTCTIEGLIYGNPSCDLSLEGREWSYIVERPSAVNGLCYPGNVENLEELRSLFSSARSYQRIQIFPDTIW
NVSYDGTSTACSGSFYRSMRWLTRKNGDYPTQDAQYTNNQGKNILFMWGINHPPTDDTQRNLYTRTDTTT
SVATEEINRIFKPLIGPRPLVNGLMGRIDYYWSVLKPGQTLRIKSDGNLIAPWYGHILSGESHGRILKTD
LKRGSCTVQCQTEKGGLNTTLPFQNVSKYAFGNCSKYIGIKSLKLAVGLRNVPSRSSRGLFGAIAGFIEG
GWSGLVAGWYGFQHSNDQGVGMAADRDSTQKAIDKITSKVNNIVDKMNKQYEIIDHEFSEVETRLNMINN
KIDDQIQDIWAYNAELLVLLENQKTLDEHDANVNNLYNKVKRALGSNAVEDGKGCFELYHKCNDQCMETI
RNGTYNRRKYQEESKLERQKIEGVKLESEGTYKILTIYSTVASSLVIAMGFAAFLFWAMSNGSCRCNICI

>QDF63458.1 hemagglutinin [Influenza A virus]
METVSLITILLVATVSNADKICIGYQSTNSTETVDTLTENNVPVTHAKELLHTEHNGMLCATSLGQPLIL
DTCTIEGLIYGNPSCDLSLEGREWSYIVERPSAVNGLCYPGNVENLEELRSLFSSARSYQRIQIFPDTIW
NVSYDGTSTACSGSFYRSMRWLTRKNGDYPTQDAQYTNNQGKNILFMWGINHPPTDDTQRNLYTRTDTTT
SVATEEINRIFKPLIGPRPLVNGLMGRIDYYWSVLKPGQTLRIKSDGNLIAPWYGHILSGESHGRILKTD
LKRGSCTVQCQTEKGGLNTTLPFQNVSKYAFGNCSKYIGIKSLKLAVGLRNVPSRSSRGLFGAIAGFIEG
GWSGLVAGWYGFQHSNDQGVGMAADRDSTQKAIDKITSKVNNIVDKMNKQYEIIDHEFSEVETRLNMINN
KIDDQIQDIWAYNAELLVLLENQKTLDEHDANVNNLYNKVKRALGSNAVEDGKGCFELYHKCNDQCMETI
RNGTYNRRKYQEESKLERQKIEGVKLESEGTYKILTIYSTVASSLVIAMGFAAFLFWAMSNGSCRCNICI

>QDF63457.1 hemagglutinin [Influenza A virus]
METVSLITILLAATVSNADKICIGYQSTNSTETVDTLTENNVPVTHAKELLHTEHNGMLCATSLGQPLIL

DTCTIEGLIYGNPSCDLSLEGREWSYIVERPSAVNGLCYPGNVENLEELRSLFSSARSYQRIQIFPDTIW
NVSYDGTSNTCSGSFYRNMRWLTRKDGNYPIQDAQYTNNQGKNILFMWGINHPPTDNTQRNLYTRTDTTT
SVATEEINRIFKPLIGPRPLVNGLMGRIDYYWSVLKPGQTLRIKSDGNLIAPWYGYILSGESHGRILKTD
LKRGSCTVQCQTEKGGLNTTLPFQNVSKYAFGNCSKYIGIKSLKLAVGLRNVPSRSSRGLFGAIAGFIEG
GWSGLVAGWYGFQHSNDQGVGMAADRESTQKAIDKITSKVNNIVDKMNKQYEIIDHEFSEVETRLNMINN
KIDDQIQDIWAYNAELLVLLENQKTLDEHDANVNNLYNKVKRALGSNAVEDGKGCFELYHKCDDQCMETI
RNGTYNRRKYQEESKLERQKIEGVKLESEGTYKILTIYSTVASSLVIAVGFAAFLFWAMSNGSCRCNICI

>QDF63456.1 hemagglutinin [Influenza A virus]
METVSLITILLVATVSNADKICIGYQSTNSTETVDTLTENNVPVTHAKELLHTEHNGMLCATSLGQPLIL
DTCTIEGLIYGNPSCDLSLEGREWSYIVERPSAVNGLCYPGNVENLEELRSLFSSARSYQRIQIFPDTIW
NVSYDGTSTACSGSFYRSMRWLTRKNGDYPTQDAQYTNNQGKNILFMWGINHPPTDTTQRDLYTRTDTTT
SVATEEINRIFKPLIGPRPLVNGLMGRIDYYWSVLKPGQTLRIKSDGNLIAPWFGHILSGESHGRILKTD
LKRGSCTVQCQTEKGGLNTTLPFQNVSKYAFGNCSKYIGIKSLKLAVGLRNVPSRSSRGLFGAIAGFIEG
GWSGLVAGWYGFQHSNDQGVGMAADRDSTQKAIDKITSKVNNIVDKMNKQYEIIDHEFSEVETRLNMINN
KIDDQIQDIWAYNAELLVLLENQKTLDEHDANVNNLYNKVKRALGSNAVEDGKGCFELYHKCDDQCMETI
RNGTYNRRKYQEESKLERQKIEGVKLESEGTYKILTIYSTVASSLVIAMGFAAFLFWAMSNGSCRCNICI

>QDF63455.1 hemagglutinin [Influenza A virus]
METVSLITILLVAAVSNADKICIGYQSTNSTETVDTLTENNVPVTHAKELLHTEHNGMLCATSLGQPLIL
DTCTIEGLIYGNPSCDLSLEGREWSYIVERPSAVNGLCYPGNVENLEELRSLFSSARSYQRIQIFPDTIW
NVSYDGTSTACSGSFYRSMRWLTRKNGDYPIQDAQYTNNQGKNILFMWGINHPPTDTTQRDLYTRTDTTT
SVATEEINRIFKPLIGPRPLVNGLMGRINYYWSVLKPGQTLRIKSDGNLIAPWFGHILSGESHGRILKTD
LKRGSCTVQCQTEKGGLNTTLPFQNVSKYAFGNCSKYIGIKSLKLAVGLRNVPSRSSRGLFGAIAGFIEG
GWSGLVAGWYGFQHSNDQGVGMAADRDSTQKAIDKITSKVNNIVDKMNKQYEIIDHEFSEVETRLNMINN
KIDDQIQDIWAYNAELLVLLENQKTLDEHDANVNNLYNKVKRALGSNAVEDGKGCFELYHKCDDQCMETI
RNGTYNRRKYQEESKLERQKIEGVKLESEGTYKILTIYSTVASSLVIAMGFAAFLFWAMSNGSCRCNICI

>QDF63454.1 hemagglutinin [Influenza A virus]
METASLITILLAVTVSNADKICIGYQSTNSTETVDTLTENNVPVTHAKELLHTEHNGMLCATSLGHPLIL
DTCTIEGLIYGNPSCDLLLGGREWSYIVERPSAVNGLCYPGNVENLEELRSLFSSARSYQRIQIFPDTIW
NVSYSGTSKACSDSFYRSMRWLTQKDNAYPVQDAQYTNNQEKNILFMWGINHPPTDTVQTNLYTRTDTTT
SVATEEINRTFKPLIGPRPLVNGLMGRINYYWSVLKPGQTLRIKSNGNLIAPWYGHILSGESHGRILKTD
LKRGSCTVQCQTEKGGLNTTLPFQNVSKYAFGNCSKYIGVKSLKLAVGLRNVPSRSSRGLFGAIAGFIEG

GWSGLVAGWYGFQHSNDQGVGMAADRDSTQKAIDKITSKVNNIVDKMNRQYEIIDHEFSEVETRLNMINN
KIDDQIQDIWAYNAELLVLLENQKTLDEHDANVNNLYNKVKRALGSNAVEDGKGCFELYHKCDDQCMETI
RNGTYNRRKYQEESKLERQKIEGVKLESEGTYKILTIYSTVASSLVIAMGFAAFLFWAMSNGSCRCNICI

>QDF63453.1 hemagglutinin [Influenza A virus]
METVSLITILLVATVSNADKICIGYQSTNSTETVDTLTENNVPVTHAKELLHTEHNGMLCATSLGQPLVL
DTCTIEGLIYGNPSCDLSLEGREWSYIVERPSAVNGLCYPGNVENLEELRSLFSSARSYQRIQIFPDTIW
NVSYDGTSTACSGSFYRSMRWLTRKNGDYPIQDAQYTNNQGKNILFMWGINQPPTDTTQRDLYTRTDTTT
SVATEEINRIFKPLIGPRPLVNGLMGRIDYYWSVLKPGQTLRIKSDGNLIAPWYGHILSGESHGRILKTD
LKRGSCTVQCQTEKGGLNTTLPFQNVSKYAFGNCSKYIGIKSLKLAVGLRNVPSRSSRGLFGAIAGFIEG
GWSGLVAGWYGFQHSNDQGVGMAADRDSTQKAIDKITSKVNNIVDKMNKQYEIIDHEFSEVETRLNMINN
KIDDQIQDIWAYNAELLVLLENQKTLDEHDANVNNLYNKVKRALGSNAVEDGKGCFELYHKCDDQCMETI
RNGTYNRRKYQEESKLERQKIEGVKLESEGTYKILTIYSTVASSLVIAMGFAAFLFWAMSNGSCRCNICI

>QDF63452.1 hemagglutinin [Influenza A virus]
METVSLITILLVATVSNADKICIGYQSTNSTETVDTLTENNVPVTHAKELLHTEHNGMLCATSLGQPLIL
DTCTIEGLIYGNPSCDLSLEGREWSYIVERPSAVNGLCYPGNVENLEELRSLFSSARSYQRIQIFPDTIW
NVSYDGTSTACSGSFYRSMRWLTRKNGDYPIQDAQYTNNQGKNILFMWGINHPPTDTTQRDLYTRTDTTT
SVATEEINRIFKPLIGPRPLVNGLMGRINYYWSVLKPGQTLRIKSDGNLIAPWYGHILSGESHGRILKTD
LKRGSCTVQCQTEKGGLNTTLPFQNVSKYAFGNCSKYIGIKSLKLAVGLRNVPSRSSRGLFGAIAGFIEG
GWSGLVAGWYGFQHSNDQGVGMAADRDSTQKAIDKITSKVNNIVDKMNKQYEIIDHEFSEVETRLNMINN
KIDDQIQDIWAYNAELLVLLENQKTLDEHDANVNNLYNKVKRALGSNAVEDGKGCFELYHKCDDQCMETI
RNGTYNRRKYQEESKLERQKIEGVKLESEGTYKILTIYSTVASSLVIAMGFAAFLFWAMSNGSCRCNICI

>QDF63451.1 hemagglutinin [Influenza A virus]
METVSLITILLVATVSNADKICIGYQSTNSTETVDTLTENNVPVTHAKELLHTEHNGMLCATSLGQPLVL
DTCTIEGLIYGNPSCDLSLEGREWSYIVERPSAVNGLCYPGNVENLEELRSLFSSARSYQRIQIFPDTIW
NVSYDGTSSACSGSFYKSMRWLTRKNGDYPIQDAQYTNNQGKNILFMWGINHPPTDTTQRDLYTRTDTTT
SVATEEINRIFKPLIGPRPLVNGLMGRIDYYWSVLKPGQTLRIKSDGNLIAPWYGHILSGESHGRILKTD
LKRGSCTVQCQTEKGGLNTTLPFQNVSKYAFGNCSKYIGIKSLKLAVGLRNVPSRSSRGLFGAIAGFIEG
GWSGLVAGWYGFQHSNDQGVGMAADRDSTQKAIDKITSKVNNIVDKMNKQYEIIDHEFSEIETRLNMINN
KIDDQIQDIWAYNAELLVLLENQKTLDEHDANVNNLYNKVKRALGSNAVEDGKGCFELYHKCDDQCMETI
RNGTYNRRKYQEESKLERQKIEGVKLESEGTYKILTIYSTVASSLVIAMGFAAFLFWAMSNGSCRCNICI

>QDF63450.1 hemagglutinin [Influenza A virus]

METVSLITILLVATVSNADKICIGYQSTNSTETVDTLTENNVPVTHAKELLHTEHNGMLCATSLGQPLIL
DTCTIEGLIYGNPSCDLSLEGREWSYIVERPSAVNGLCYPGNVENLEELRSLFSSARSYQRIQIFPDTIW
NVSYDGTSTACSGSFYRSMRWLTRKNGEYPIQDAQYTNNQGKNILFMWGINHPPTDTTQRDLYTRTDTTT
SVATEEINRVFKPLIGPRPLVNGLMGRIDYYWSVLKPGQTLRIKSDGNLIAPWFGHILSGESHGRILKTD
LKRGSCTVQCQTEKGGLNTTLPFQNVSKYAFGNCSKYIGIKSLKLAVGLRNVPSRSSRGLFGAIAGFIEG
GWSGLVAGWYGFQHSNDQGVGMAADRDSTQKAIDKITSKVNNIVDKMNKQYEIIDHEFSEVEARLNMINN
KIDDQIQDIWAYNAELLVLLENQKTLDEHDANVNNLYNKVKRALGSNAVEDGKGCFELYHKCDDQCMETI
RNGTYNRRKYQEESKLERQKIEGVKLESEGTYKILTIYSTVASSLVIAMGFAAFLFWAMSNGSCRCNICI

>QDF63447.1 hemagglutinin [Influenza A virus]

METVSLITILLVATVSNADKICIGYQSTNSTETVDTLTENNVPVTHAKELLHTEHNGMLCATSLGHPLIL
DTCTIEGLIYGNPSCDPLLGGREWSYIVERPSAVNGLCYPGNVENLEELRSLFSSARSYQRIQIFPDTIW
NVSYSGTSKACSDSFYRSMRWLTQKNNAYPIQDAQYTNNQEKNILFMWGINHPPTETAQTNLYTRTDTTT
SVATEEINRIFKPLIGPRPLVNGLMGRINYYWSVLKPGQTLRIKSDGNLIAPWYGHILSGESHGRILKTD
LKRGSCTVQCQTEKGGLNTTLPFQNVSKYAFGNCSKYIGIKSLKLAIGLRNVPSRSSRGLFGAIAGFIEG
GWSGLVAGWYGFQHSNDQGVGMAADRDSTQKAIDKITSKVNNIVDKMNRQYEIIDHEFSEVETRLNMINN
KVDDQIQDIWAYNAELLVLLENQKTLDEHDSNVNNLYNKVKRALGSNAVEDGKGCFELYHKCDDQCMETI
RNGTYNRRKYQEESKLERQKIEGVKLESEGTYKILTIYSTVASSLVIAMGFAAFLFWAMSNGSCRCNICI

>QDF63445.1 hemagglutinin [Influenza A virus]

METVSLITILLVATVSNADKICIGYQSTNSTETVDTLTENNVPVTHAKELLHTEHNGMLCATSLGQPIIL
DTCTIEGLIYGNPSCDLSLEGREWSYIVERPSAVNGLCYPGNVENLEELRSLFSSARSYQRIQIFPDTIW
NVSYDGTSTACSGSFYRNMRWLTRKNGDYPIQDAQYTNNQGKNILFMWGINHPPADTTQRDLYTGTDTTT
SVATEEINRIFKPLIGPRPLVNGLMGRIDYYWSVLKPGQTLRIKSDGNLIAPWYGHILSGESHGRILKTD
LKRGSCTVQCQTEKGGLNTTLPFQNVSKYAFGNCSKYIGIKSLKLAVGLRNVPSRSSRGLFGAIAGFIEG
GWSGLVAGWYGFQHSNDQGVGMAADRDSTQKAIDKITSKVNNIVDKMNKQYEIIDHEFSEVETRLNMINN
KIDDQIQDIWAYNAELLVLLENQKTLDEHDANVNNLYNKVKRALGSNAVEDGKGCFELYHKCDDQCMETI
RNGTYNRRKYQEESKLERQKIEGVKLESEGTYKILTIYSTVASSLVIAMGFAAFLFWAMSNGSCRCNICI

>QDF63444.1 hemagglutinin [Influenza A virus]

METVSLITILLVATVSNADKICIGYQSTNSTETVDTLTENNVPVTHAKELLQTEHNGMLCATSLGQPLIL
DTCTIEGLIYGNPSCDLSLEGREWSYIVERPSAVNGLCYPGNVENLEELRSLFSSARSYQRIQIFPDTIW
NVSYDGTSTACSGSFYKSMRWLTRKNGDYPIQDAQYTNNQGKNILFMWGINHPPTDTTQRDLYTRTDTTT

SVATEEINRVFKPLIGPRPPVNGLMGRIDYYWSVLKPGQTLRIKSDGNLIAPWFGHILSGESHGRILKTD
LKRGSCTVQCQTEKGGLNTTLPFQNVSKYAFGNCSKYIGIKSLKLAVGLRNVPSRSSRGLFGAIAGFIEG
GWSGLVAGWYGFQHSNDQGVGMAADRDSTQKAIDKITSKVNNIVDKMNKQYEIIDHEFSEVETRLNMINN
KIDDQIQDIWAYNAELLVLLENQKTLDEHDANVNNLYNKVKRALGSNAVEDGKGCFELYHKCDDQCMETI
RNGTYNRRKYQEESKLERQKIEGVKLESEGTYKILTIYSTVASSLVIAMGFAAFLFWAMSNGSCRCNICI

>QDF63443.1 hemagglutinin [Influenza A virus]
METVSLITILLVATVSNADKICIGYQSTNSTETVDTLTENNVPVTHAKELLHTEHNGMLCATSLGHPLIL
DTCTIEGLIYGNPSCDPLLGGREWSYIVERPSAVNGLCYPGSVENLEELRSLFSSARSYQRIQIFPDTIW
NVSYSGTSKACSDSFYRSMRWLTQKNNAYPIQDAQYTNNQEKNILFMWGINHPPTETAQTNLYTRTDTTT
SVATEEINRIFKPLIGPRPLVNGLMGRINYYWSVLKPGQTLRIKSDGNLIAPWYGHILSGESHGRILKTD
LKRGSCTVQCQTEKGGLNTTLPFQNVSKYAFGNCSKYIGIKSLKLAIGLRNVPSRSSRGLFGAIAGFIEG
GWSGLVAGWYGFQHSNDQGVGMAADRDSTQKAIDKITSKVNNIVDKMNRQYEIIDHEFSEVETRLNMINN
KVDDQIQDIWAYNAELLVLLENQKTLDEHDSNVNNLYNKVKRALGSNAVEDGKGCFELYHKCDDQCMETI
RNGTYNRRKYQEESKLERQKIEGVKLESEGTYKILTIYSTVASSLVIAMGFAAFLFWAMSNGSCRCNICI

>QDF63442.1 hemagglutinin [Influenza A virus]
METVSLITILLVATVSNADKICIGYQSTNSTETVDTLTENNVPVTHAKELLHTEHNGMLCATSLGHPLIL
DTCTIEGLIYGNPSCDPLLGGREWSYIVERPSAVNGLCYPGSVENLEELRSLFSSARSYQRIQIFPDTIW
NVSYSGTSKACSDSFYRSMRWLTQKNNAYPIQDAQYTNNQEKNILFMWGINHPPTETAQTNLYTRTDTTT
SVATEEINRIFKPLIGPRPLVNGLMGRINYYWSVLKPGQTLRIKSDGNLIAPWYGHILSGESHGRILKTD
LKRGSCTVQCQTEKGGLNTTLPFQNVSKYAFGNCSKYIGIKSLKLAIGLRNVPSRSSRGLFGAIAGFIEG
GWSGLVAGWYGFQHSNDQGVGMAADRDSTQKAIDKITSKVNNIVDKMNRQYEIIDHEFSEVETRLNMINN
KVDDQIQDIWAYNAELLVLLENQKTLDEHDSNVNNLYNKVKRALGSNAVEDGKGCFELYHKCDDQCMETI
RNGTYNRRKYQEESKLERQKIEGVKLESEGTYKILTIYSTVASSLVIAMGFAAFLFWAMSNGSCRCNICI

>QDF63441.1 hemagglutinin [Influenza A virus]
METVSLITILLVATVSNADKICIGYQSTNSTETVDTLTENNVPVTHAKELLHTEHNGMLCATSLGQPLIL
DTCTIEGLIYGNPSCDLSLEGREWSYIVERPSAVNGLCYPGNVENLEELRSLFSSARSYQRIQIFPDTIW
NVSYDGTSTACSGSFYRSMRWLTRKNGNYPIQDAQYTNNQGKNILFMWGINHPPTDTTQRDLYTRTDTTT
SVATEEINRIFKPLIGPRPLVNGLMGRIDYYWSVLKPGQTLRIKSDGNLIAPWFGHILSGESHGRILKTD
LKRGSCTVQCQTEKGGLNTTLPFQNVSKYAFGNCSKYIGIKSLKLAVGLRNVPSRSSRGLFGAIAGFIEG
GWSGLVAGWYGFQHSNDQGVGMAADRDSTQKAIDKITSKVNNIVDKMNKQYEIIDHEFSEVETRLNMINN
KIDDQIQDIWAYNAELLVLLENQKTLDEHDANVNNLYNKVKRALGSNAVEDGKGCFELYHKCDDQCMETI

RNGTYNRRKYQEESKLERQKIEGVKLESEGTYKILTIYSTVASSLVIAMGFAAFLFWAMSNGSCRCNICI

>QDF63440.1 hemagglutinin [Influenza A virus]
METVSLITILLVAAVSNADKICIGYQSTNSTETVDTLTENNVPVTHAKELLHTEHNGMLCATSLGQPLIL
DTCTIEGLIYGNPSCDLSLEGREWSYIVERPSAVNGLCYPGNVENLEELRSLFSSARSYQRIQIFPDTIW
NVSYDGTSTACSGSFYKSMRWLTRKNGDYPIQDAQYTNNQGKNILFMWGINHPPTDTTQRDLYTRTDTTT
SVATEEINRVFKPLIGPRPLVNGLMGRIDYYWSVLKPGQTLRIKSDGNLIAPWFGHILSGESHGRILKTD
LKRGSCTVQCQTEKGGLNTTLPFQNVSKYAFGNCSKYIGIKSLKLAVGLRNVPSRSSRGLFGAIAGFIEG
GWSGLVAGWYGFQHSNDQGVGMAADRDSTQKAIDKITSKVNNIVDKMNKQYEIIDHEFSEVETRLNMINN
KIDDQIQDIWAYNAELLVLLENQKTLDEHDANVNNLYNKVKRALGSNAVEDGKGCFELYHKCDDQCMETI
RNGTYNRRKYQEESKLERQKIEGVKLESEGTYKILTIYSTVASSLVIAMGFAAFLFWAMSNGSCRCNICI

>QDF63439.1 hemagglutinin [Influenza A virus]
METVSLITILLVATVSNADKICIGYQSTNSTETVDTLTENNVPVTHAKELLHTEHNGMLCATSLGQPLIL
DTCTIEGIIYGNPSCDLSLEGREWSYIVERPSAVNGLCYPGNVENLEELRSLFSSARSYQRIQIFPDTIW
NVSYDGTSKACSGSFYKSMRWLTRKNGDYPIQDAQYTNNQGKNILFMWGINHPPTDTTQRDLYTRTDTTT
SVATEEINRVFKPLIGPRPLVNGLMGRIDYYWSVLKPGQTLRIKSDGNLIAPWFGHILSGESHGRILKTD
LKRGNCTVQCQTEKGGLNTILPFQNVSKYAFGNCSKYIGIKSLKLAVGLRNVPSRSSRGLFGAIAGFIEG
GWSGLVAGWYGFQHSNDQGVGMAADRDSTQKAIDKITSKVNNIVDKMNKQYEIIDHEFSEVEIRLNMINN
KIDDQIQDIWAYNAELLVLLENQKTLDEHDANVNNLYNKVKRALGSNAVEDGKGCFELYHKCDDQCMETI
RNGTYNRRKYQEESKLERQKIEGVKLESEGTYKILTIYSTVASSLVIAMGFAAFLFWAMSNGSCRCNICI

>QDF63438.1 hemagglutinin [Influenza A virus]
METVSLITILLVATVSNADKICIGYQSTNSTETVDTLTENNVPVTHAKELIHTEHNGMLCATSLGQPLIL
DTCTIEGLIYGNPSCDLMLEGREWSYIVERPSAVNGLCYPGHVENLEELRSLFSSARSYQRVQIFPDTIW
NVSYDGTSTACSGSFYRSMRWLTRKNGDYPIQDAQYTNNQGKNILFMWGINHPPADTTQRDLYTRTDTTT
SVATEEINRIFKPLIGPRPLVNGLMGRIDYYWSVLKPGQTLRIKSDGNLIAPWYGHILSGESHGRILKTD
LKRGSCTVQCQTEKGGLNTTLPFQNVSKYAFGNCSKYIGMKSLKLAVGLRNVPSRSSRGLFGAIAGFIEG
GWSGLVAGWYGFQHSNDQGVGMAADRDSTQKAIDKITSKVNNIVDKMNKQYEIIDHEFSEVETRLNMINN
KIDDQIQDIWAYNAELLVLLENQKTLDEHDANVNNLYNKVKRALGSNAVEDGKGCFDLYHKCDDQCMETI
RNGTYNRRKYQEESKLERQKIEGVKLESEGTYKILTIYSTVASSLVIAMGFAAFLFWAMSNGSCRCNICI

>QDF63437.1 hemagglutinin [Influenza A virus]
METVSLITILLVATVSNADKICIGYQSTNSTETVDTLTENNVPVTHAKELLHTEHNGMLCATSLGQPLIL

DTCTIEGIIYGNPSCDLSLEGREWSYIVERPSAVNGLCYPGNVENLEELRSLFSSARSYQRIQIFPDTIW
NVSYDGTSTACSGSFYKSMRWLTRKNGDYPIQDAQYTNNQGKNILFMWGINHPPTDTTQRDLYTRTDTTT
SVATEEINRVFKPLIGPRPLVNGLMGRIDYYWSVLKPGQTLRIKSDGNLIAPWFGHILSGESHGRILKTD
LKRGSCTVQCQTEKGGLNTTLPFQNVSKYAFGNCSKYIGIKSLKLAVGLRNVPSRSSRGLFGAIAGFIEG
GWSGLVAGWYGFQHSNDQGVGMAADRDSTQKAIDKITSKVNNIVDKMNKQYEIIDHEFSEVEIRLNMINN
KIDDQIQDIWAYNAELLVLLENQKTLDEHDANVNNLYNKVKRALGSNAVEDGKGCFELYHKCDDQCMETI
RNGTYNRRKYQEESKLERQKIEGVKLESEGTYKILTIYSTVASSLVIAMGFAAFLFWAMSNGSCRCNICI

>QDF63435.1 hemagglutinin [Influenza A virus]
METVSLITILLVATVSNADKICIGYQSTNSTETVDTLTENNVPVTHAKELIHTEHNGMLCATSLGQPLIL
DTCTIEGLIYGNPSCDLMLEGREWSYIVERPSAVNGLCYPGHVENLEELRSLFSSARSYQRVQIFPDTIW
NVSYDGTSTACSGSFYRSMRWLTRKNGDYPIQDAQYTNNQGKNILFMWGINHPPADTTQRDLYTRTDTTT
SVATEEINRIFKPLIGPRPLVNGLMGRIDYYWSVLKPGQTLRIKSDGNLIAPWYGHILSGESHGRILKTD
LKRGSCTVQCQTEKGGLNTTLPFQNVSKYAFGNCSKYIGMKSLKLAVGLRNVPSRSSRGLFGAIAGFIEG
GWSGLVAGWYGFQHSNDQGVGMAADRDSTQKAIDKITSKVNNIVDKMNKQYEIIDHEFSEVETRLNMINN
KIDDQIQDIWAYNAELLVLLENQKTLDEHDANVNNLYNKVKRALGSNAVEDGKGCFDLYHKCDDQCMETI
RNGTYNRRKYQEESKLERQKIEGVKLESEGTYKILTIYSTVASSLVIAMGFAAFLFWAMSNGSCRCNICI

>QDF63434.1 hemagglutinin [Influenza A virus]
METVSLITILLVATVSNADKICIGYQSTNSTETVDTLTENNVPVTHAKELLHTEHNGMLCATSLGQPLIL
DTCTIEGLIYGNPSCNLMLEGREWSYIVERPSAVNGLCYPGHVENLEELRSLFSSARSYQRIQIFPDTIW
NVSYDGTSNACSGSFYRSMRWLTRKNGDYPIQDAQYTNNQGKNILFMWGINHPPTDTTQRDLYTRTDTTT
SVATEEINRIFKPLIGPRPLVNGLMGRIDYYWSILKPGQTLRIKSDGNLIAPWYGHILSGESHGRFLKTD
LKRGSCTVQCQTEKGGLNTTLPFQNVSKYAFGNCSKYIGMKSLKLAVGLRNVPSRSSRGLFGAIAGFIEG
GWSGLVAGWYGFQHSNDQGVGMAADRDSTQKAIDKITSKVNNIVDKMNKQYEIIDHEFSEVESRLNMINN
KIDDQIQDIWAYNAELLVLLENQKTLDEHDANVNNLYNKVKRALGSNAVEDGKGCFELYHKCDDQCMETI
RNGTYNRRKYQEESKLERQKIEGVKLESEGTYKILTIYSTVASSLVIAMGFAAFLFWAMSNGSCRCNICI

>QDF63433.1 hemagglutinin [Influenza A virus]
METVSLITMLLVATVSNADKICIGYQSTNSTETVDTLTENNVPVTHAKELLHTEHNGMLCATSLGQPLIL
DTCTIEGLIYGNPSCNLSLEGREWSYIVERPSAVNGLCYPGNVENLEELRSLFSSARSYQRIQIFPDTIW
NVSYDGTSKACSGSFYRSMRWLTRKNGDYPIQDAQYTNNQGKNILFMWGINHPPTDTTQRDLYTRTDTTT
SVATEEINRIFKPLIGPRPLVNGLMGRIDYYWSVLKPGQTLRIKSDGNLIAPWFGHILSGESHGRILKTD
LKRGSCTVQCQTEKGGLNTTLPFQNVSKYAFGNCSKYIGIKSLKLAVGLRNVPSRSSRGLFGAIAGFIEG

GWSGLVAGWYGFQHSNDQGVGMAADRDSTQKAIDKITSKVNNIVDKMNKQYEIIDHEFSEVETRLNMINN
KIDDQIQDIWAYNAELLVLLENQKTLDEHDANVNNLYNKVKRALGSNAVEDGKGCFELYHKCDDQCMETI
RNGTYNRRKYQEESKLERQKIEGVKLESEGTYKILTIYSTVASSLVIAMGFAAFLFWAMSNGSCRCNICI

>QDF63432.1 hemagglutinin [Influenza A virus]
METVSLITILLVATVSNADKICIGYQSTNSTETVDTLTENNVPVTHAKELLHTEHNGMLCATSLGHPLIL
DTCTIEGLIYGNPSCDPLLGGREWSYIVERPSAVNGLCYPGNVENLEELRSLFSSARSYQRIQIFPDTIW
NVSYSGTSRACSDSFYRSMRWLTQKNNAYPIQDAQYTNNQDKNILFMWGINHPPTETTQTNLYTRTDTTT
SVATEEINRIFKPLIGPRPLVNGLMGRINYYWSVLKPGQTLRIKSDGNLIAPWYGHILSGESHGRILKTD
LKRGSCTVQCQTEKGGLNTTLPFQNVSKYAFGNCSKYIGIKSLKLAVGLRNVPSRSSRGLFGAIAGFIEG
GWSGLVAGWYGFQHSNDQGVGMAADRDSTQKAIDKITSKVNNIVDKMNRQYEIIDHEFSEVETRLNMINN
KVDDQIQDIWAYNAELLVLLENQKTLDEHDSNVNNLYNKVKRALGSNAVEDGKGCFELYHKCDDQCMETI
RNGTYNRRKYQEESKLERQKIEGVKLESEGTYKILTIYSTVASSLVIAMGFAAFLFWAMSNGSCRCNICI

>QDF63431.1 hemagglutinin [Influenza A virus]
METVSLITILLVATVSNADKICIGYQSTNSTETVDTLTENNVPVTHAKELLHTEHNGMLCATSLGQPLIL
DTCTIEGLIYGNPSCDLSLEGREWSYIVERPSAVNGLCYPGNVENLEELRSLFSSARSYQRIQIFPDTIW
NVSYDGTSTACSGSFYKSMRWLTRKNGDYPIQDAQYTNNQGKNILFMWGINHPPTDTTQRDLYTRTDTTT
SVATEEINRVFKPLIGPRPLVNGLMGRIDYYWSVLKPGQTLRIKSDGNLIAPWFGHILSGESHGRILKTD
LKRGSCTVQCQTEKGGLNTTLPFQNVSKYAFGNCSKYIGIKSLKLAVGLRNVPSRSSRGLFGAIAGFIEG
GWSGLVAGWYGFQHSNDQGVGMAADRDSTQKAIDKITSKVNNIVDKMNKQYEIIDHEFSEVETRLNMINN
KIDDQIQDIWAYNAELLVLLENQKTLDEHDANVNNLYNKVKRALGSNAVEDGKGCFELYHKCDDQCMETI
RNGTYNRRKYQEESKLERQKIEGVKLESEGTYKILTIYSTVASSLVIAMGFAAFLFWAMSNGSCRCNICI

>QDF63430.1 hemagglutinin [Influenza A virus]
METVSLITILLVATVSNADKICIGYQSTNSTETVDTLTENNVPVTHAKELLHTEHNGMLCATSLGQPIIL
DTCTIEGLIYGNPSCDLSLEGREWSYIVERPSAVNGLCYPGNVENLEELRSLFSSARSYQRIQIFPDTIW
NVSYDGTSTACSGSFYRNMRWLTRKNGDYPIQDAQYTNNQGKNILFMWGINHPPADTTQRDLYTRTDTTT
SVATEEINRIFKPLIGPRPLVNGLMGRIDYYWSVLKPGQTLRIKSDGNLIAPWYGHILSGESHGRILKTD
LKRGSCTVQCQTEKGGLNTTLPFQNVSKYAFGNCSKYIGIKSLKLAVGLRNVPSRSSRGLFGAIAGFIEG
GWSGLVAGWYGFQHSNDQGVGMAADRDSTQKAIDKITSKVNNIVDKMNKQYEIIDHEFSEVETRLNMINN
KIDDQIQDIWAYNAELLVLLENQKTLDEHDANVNNLYNKVKRALGSNAVEDGKGCFELYHKCDDQCMETI
RNGTYNRRKYQEESKLERQKIEGVKLESEGTYKILTIYSTVASSLVIAMGFAAFLFWAMSNGSCRCNICI

>QDF63428.1 hemagglutinin [Influenza A virus]

METVSLITILLVATVSNADKICIGYQSTNSTETVDTLTENNVPVTHAKELLHTEHNGMLCATSLGQPLIL

DTCTIEGLIYGNPSCDLSLEGREWSYIVERPSAVNGLCYPGNVENLEELRSLFSSARSYQRIQIFPDTIW

NVSYDGTSTACSGSFYKSMRWLTRKNGDYPIQDAQYTNNQGKNILFMWGINHPPTDTTQRNLYTRTDTTT

SVATEEINRVFKPLIGPRPLVNGLMGRIDYYWSVLKPGQTLRIKSDGNLIAPWFGHILSGESHGRILKTD

LKRGSCTVQCQTEKGGLNTTLPFQNVSKYAFGNCSKYIGIKSLKLAVGLRNVPSRSSRGLFGAIAGFIEG

GWSGLVAGWYGFQHSNDQGVGMAADRDSTQKAIDKITSKVNNIVDKMNKQYEIIDHEFSEVETRLNMINN

KVDDQIQDIWAYNAELLVLLENQKTLDEHDANVNNLYNKVKRALGSNAVEDGKGCFELYHKCDDQCMETI

RNGTYNRRKYQEESKLERQKIEGVKLESEGTYKILTIYSTVASSLVIAMGFAAFLFWAMSNGSCRCNICI

>QDF63427.1 hemagglutinin [Influenza A virus]

METVSLITILLVATVSNADKICIGYQSTNSTETVDTLTENNVPVTHAKELLHTEHNGMLCATSLGQPIIL

DTCTIEGLIYGNPSCDLSLEGREWSYIVERPSAVNGLCYPGNVENLEELRSLFSSARSYKRIQIFPDTIW

NVSYDGTSTACSGSFYRNMRWLTRKNGDYPIQDAQYTNNQGKNILFMWGINHPPADTTQRDLYTRTDTTT

SVATEEINRIFKPLIGPRPLVNGLMGRIDYYWSVLKPGQTLRIKSDGNLIAPWYGHILSGESHGRILKTD

LKRGSCTVQCQTEKGGLNTTLPFQNVSKYAFGNCSKYIGIKSLKLAVGLRNVPSRSSRGLFGAIAGFIEG

GWSGLVAGWYGFQHSNDQGVGMAADRDSTQKAIDKITSKVNNIVDKMNKQYEIIDHEFSEVETRLNMINN

KIDDQIQDIWAYNAELLVLLENQKTLDEHDANVNNLYNKVKRALGSNAVEDGKGCFELYHKCDDQCMETI

RNGTYNRRKYQEESKLERQKIEGVKLESEGTYKILTIYSTVASSLVIAMGFAAFLFWAMSNGSCRCNICI

>QDF63425.1 hemagglutinin [Influenza A virus]

METASLITILLVATVSNADKICIGYQSTNSTETVDTLTENNVPVTHAKELLHTEHNGMLCATSLGHPLIL

DTCTIEGLIYGNPSCDPLLGGREWSYIVERPSAVNGLCYPGNVENLEELRSLFSSARSYQRIQIFPDTIW

NVSYSGTSKACSDSFYRSMRWLTQKNNAYPIQDAQYTNNQEKNILFMWGINHPPTETVQTNLYTRTDTTT

SVATEEINRVFKPLIGPRPLVNGLMGRINYYWSVLKPGQTLRIKSDGNLIAPWYGHILSGESHGRILKTD

LKRGSCTVQCQTEKGGLNTTLPFQNVSKYAFGNCSKYIGIKSLKLAVGLRNVPSRSSRGLFGAIAGFIEG

GWSGLVAGWYGFQHSNDQGVGMAADRDSTQKAIDKITSKVNNIVDKMNKQYEIIDHEFSEVETRLNMINN

KVDDQIQDIWAYNAELLVLLENQKTLDEHDSNVNNLYNKVKRALGSNAMEDGKGCFELYHKCDDQCMETI

RNGTYNRRKYQEESKLERQKIEGVKLESEGTYKILTIYSTVASSLVIAMGFAAFLFWAMSNGSCRCNICI

>QDF63424.1 hemagglutinin [Influenza A virus]

METVSLITILLVATVSNADKICIGYQSTNSTETVDTLTENNVPVTHAKELLHTEHNGMLCATSLGQPLIL

DTCTIEGLIYGNPSCDLSLEGREWSYIVERPSAVNGLCYPGNVENLEELRSLFSSARSYQRIQIFPDTIW

NVSYDGTSTACSGSFYRSMRWLTRKNGDYPIQDAQYTNNQGKNILFMWGINHPPTDTTQRDLYTRTDTTT

SVATEEINRIFKPLIGPRPLVNGLMGRINYYWSVLKPGQTLRIKSDGNLIAPWYGHILSGESHGRILKTD
LKRGSCTVQCQTEKGGLNTTLPFQNVSKYAFGNCSKYIGIKSLKLAVGLRNVPSRSSRGLFGAIAGFIEG
GWSGLVAGWYGFQHSNDQGVGMAADRDSTQKAIDKITSKVNNIVDKMNKQYEIIDHEFSEVETRLNMINN
KIDDQIQDIWAYNAELLVLLENQKTLDEHDANVNNLYNKVKRALGSNAVEDGKGCFELYHKCDDQCMETI
RNGTYNRRKYQEESKLERQKIEGVKLESEGTYKILTIYSTVASSLVIAMGFAAFLFWAMSNGSCRCNICI

>QDF63423.1 hemagglutinin [Influenza A virus]
METVSLITILLVATVSNADKICIGYQSTNSTETVDTLTENNVPVTHAKELLHTEHNGMLCATSLGQPLIL
DTCTIEGLIYGNPSCDLSLEGREWSYIVERPSAVNGLCYPGNVENLEELRSLFSSARSYQRIQIFPDTIW
NVSYDGTSTACSGSFYRSMRWLTRKNGDYPIQDAQYTNNQGKNILFMWGINHPPTDTTQRDLYTRTDTTT
SVATEEINRIFKPLIGPRPLVNGLMGRIDYYWSVLKPGQTLRIKSDGNLIAPWYGHILSGESHGRILKTD
LKRGSCTVQCQTEKGGLNTTLPFQNVSKYAFGNCSKYIGIKSLKLAVGLRNVPSRSSRGLFGAIAGFIEG
GWSGLVAGWYGFQHSNDQGVGMAADRDSTQKAIDKITSKVNNIVDKMNKQYEIIDHEFSEVETRLNMINN
KIDDQIQDIWAYNAELLVLLENQKTLDEHDANVNNLYNKVKRALGSNAVEDGKGCFELYHKCDDQCMETI
RNGTYNRRKYQEESKLERQKIEGVKLESEGTYKILTIYSTVASSLVIAMGFAAFLFWAMSNGSCRCNICI

>QDF63422.1 hemagglutinin [Influenza A virus]
METVSLITILLVATVSNADKICIGYQSTNSTETVDTLTENNVPVTHAKELLHTEHNGMLCATSLGHPLIL
DTCTIEGLIYGNPSCDLLLGGREWSYIVERPSAVNGLCYPGNVENLEELRSLFSSARSYQRIQIFPDTIW
NVSYSGTSKACSDSFYRSMRWLTQKNNAYPIQDAQYTNNQEKNILFMWGINHPPTDTAQTNLYTRTDTTT
SVATEEINRIFKPLIGPRPLVNGLMGRINYYWSVLKPGQTLRIKSDGNLIAPWYGHILSGESHGRILKTD
LKRGSCTVQCQTEKGGLNTTLPFQNVSKYAFGNCSKYIGIKSLKLAVGLRNVPSRSSRGLFGAIAGFIEG
GWSGLVAGWYGFQHSNDQGVGMAADRDSTQKAIDKITSKVNNIVDKMNKQYEIIDHEFSEVETRLNMINN
KVDDQIQDIWAYNAELLVLLENQKTLDEHDANVNNLYNKVKRALGSNAVEDGKGCFELYHKCDDQCMETI
RNGTYNRRKYQEESKLERQKIEGVKLESEETYKILTIYSTVASSLVIAMGFAAFLFWAMSNGSCRCNICI

>QDF63421.1 hemagglutinin [Influenza A virus]
METVSLITILLVATVSNADKICIGYQSTNSTETVDTLTENNVPVTHAKELIHTEHNGMLCATSLGHPLIL
DTCTIEGLIYGNPSCDPLLGGREWSYIVERPSAVNGLCYPGSVENLEELRSLFSSARSYQRIQIFPDTIW
NVSYSGTSKACSDSFYRSMRWLTQKNNAYPIQDAQYTNNQEKNILFMWGINHPPTETVQTNLYTRTDTTT
SVATEEINRIFKPLIGPRPLVNGLMGRINYYWSVLKPGQTLRIKSDGNLIAPWYGHILSGESHGRILKTD
LKRGSCTVQCQTEKGGLNTTLPFQNVSKYAFGNCSKYIGIKSLKLAIGLRNVPSRSSRGLFGAIAGFIEG
GWSGLVAGWYGFQHSNDQGVGMAADRDSTQKAIDKITSKVNNIVDKMNRQYEIIDHEFSEVETRLNMINN
KVDDQIQDIWAYNAELLVLLENQKTLDEHDSNVNNLYNKVKRALGSNAVEDGKGCFELYHKCDDQCMETI

RNGTYNRRKYQEESKLERQKIEGVKLESEGTYKILTIYSTVASSLVIAMGFAAFLFWAMSNGSCRCNICI

>QDF63420.1 hemagglutinin [Influenza A virus]
METVSLITILLVATVSNADKICIGYQSTNSTETVDTLTENNVPVTHAKELLHTEHNGMLCATSLGQPLIL
DTCTIEGLIYGNPSCDLSLEGREWSYIVERPSAVNGLCYPGNVENLEELRSLFSSARSYQRIQIFPDTIW
NVSYDGTSTACSGSFYRSMRWLTRKNGNYPIQDAQYTNNQGKNILFMWGINHPPTDTTQRDLYTRTDTTT
SVATEEINRIFKPLIGPRPLVNGLMGRIDYYWSVLKPGQTLRIKSDGNLIAPWYGHILSGESHGRILKTD
LKRGSCTVQCQTEKGGLNTTLPFQNVSKYAFGNCSKYIGIKSLKLAVGLRNVPSRSSRGLFGAIAGFIEG
GWSGLVAGWYGFQHSNDQGVGMAADRDSTQKAIDKITSKVNNIVDKMNKQYEIIDHEFSEVETRLNMINN
KIDDQIQDIWAYNAELLVLLENQKTLDEHDANVNNLYNKVKRALGSNAVEDGKGCFELYHKCDDQCMETI
RNGTYNRRKYQEESKLERQKIEGVKLESEGTYKILTIYSTVASSLVIAMGFAAFLFWAMSNGSCRCNICI

>QDF63419.1 hemagglutinin [Influenza A virus]
METVSLITILLVATVSNADKICIGYQSTNSTETVDTLTENNVPVTHAKELLHTEHNGMLCATSLGQPLVL
DTCTIEGLIYGNPSCDLSLEGREWSYIVERPSAVNGLCYPGNVENLEELRSLFSSARSYQRIQIFPDTIW
NVSYDGTSNACSGSFYRSMRWLTRKNGDYPIQDAQYTNNQGKNILFMWGINHPPTDTTQRDLYTRTDTTT
SVATEEINRIFKPLIGPRPLVNGLMGRIDYYWSVLKPGQTLRIKSDGNLIAPWYGHILSGESHGRILKTD
LKRGSCTVQCQTEKGGLNTTLPFQNVSKYAFGNCSKYIGIKSLKLAVGLRNVPSRSSRGLFGAIAGFIEG
GWSGLVAGWYGFQHSNDQGVGMAADRDSTQKAIDKITSKVNNIVDKMNKQYEIIDHEFSEVETRLNMINN
KIDDQIQDIWAYNAELLVLLENQKTLDEHDANVNNLYNKVKRALGSNAVEDGKGCFELYHKCDDQCMETI
RNGTYNRRKYQEESKLERQKIEGVKLESEGTYKILTIYSTVASSLVIAMGFAAFLFWAMSNGSCRCNICI

>QDF63417.1 hemagglutinin [Influenza A virus]
METVSLITILLVATVSNADKICIGYQSTNSTETVDTLTENNVPVTHAKELLHTEHNGMLCATSLGHPLIL
DTCTIEGLIYGNPSCDPLLGGREWSYIVERPSAVNGLCYPGNVENLEELRSLFSSARSYQRIQIFPDTIW
NVSYSGTSKACSDSFYRSMRWLTQKNNAYPIQDAQYTNNQEKNILFMWGINHPPTETAQTNLYTRTDTTT
SVATEEINRIFKPLIGPRPLVNGLMGRINYYWSVLKPGQTLRIKSDGNLIAPWYGHILSGESHGRILKTD
LKRGSCTVQCQTEKGGLNTTLPFQNVSKYAFGNCSKYIGIKSLKLAVGLRNVPSRSSRGLFGAIAGFIEG
GWSGLVAGWYGFQHSNDQGVGMAADRDSTQKAIDKITSKVNNIVDKMNRQYEIIDHEFSEVETRLNMINN
KVDDQIQDIWAYNAELLVLLENQKTLDEHDSNVNNLYNKVKRALGSNAVEDGKGCFELYHKCDDQCMETI
RNGTYNRRKYQEESKLERQKIEGVKLESEGTYKILTIYSTVASSLVIAMGFAAFLFWAMSNGSCRCNICI

>QDF63416.1 hemagglutinin [Influenza A virus]
METVSLITILLVATVSNADKICIGYQSTNSTETVDTLTENNVPVTHAKELLHTEHNGMLCATSLGHPLIL

DTCTIEGLIYGNPSCDLLLGGREWSYIVERPSAVNGLCYPGNVENLEELRSLFSSARSYQRIQIFPDTIW
NVSYSGTSKACSDSFYRSMRWLTQKNNAYPIQDAQYTNNQEKNILFMWGINHPPTDTAQTNLYTRTDTTT
SVATEEINRIFKPLIGPRPLVNGLMGRINYYWSVLKPGQTLRIKSDGNLIAPWYGHILSGESHGRILKTD
LKRGSCTVQCQTEKGGLNTTLPFQNVSKYAFGNCSKYIGIKSLKLAVGLRNVPSRSSRGLFGAIAGFIEG
GWSGLVAGWYGFQHSNDQGVGMAADRDSTQKAIDKITSKVNNIVDKMNKQYEIIDHEFSEVETRLNMINN
KVDDQIQDIWAYNAELLVLLENQKTLDEHDANVNNLYNKVKRALGSNAVEDGKGCFELYHKCDDQCMETI
RNGTYNRRKYQEESKLERQKIEGVKLESEETYKILTIYSTVASSLVIAMGFAAFLFWAMSNGSCRCNICI

>QDF63414.1 hemagglutinin [Influenza A virus]
METVSLITILLVATVSNADKICIGYQSTNSTETVDTLTENNVPVTHAKELLHTEHNGMLCATSLGHPLIL
DTCTIEGLIYGNPSCDPLLGGREWSYIVERPSAVNGLCYPGNVENLEELRSLFSSARSYQRIQIFPDTIW
NVSYSGTSKACSDSFYRSMRWLTQKNNAYPIQDAQYTNNQEKNILFMWGINHPPTETAQTNLYTRTDTTT
SVATEEINRIFKPLIGPRPLVNGLMGRINYYWSVLKPGQTLRIKSDGNLIAPWYGHILSGESHGRILKTD
LKRGSCTVQCQTEKGGLNTTLPFQNVSKYAFGNCSKYIGIKSLKLAVGLRNVPSRSSRGLFGAIAGFIEG
GWSGLVAGWYGFQHSNDQGVGMAADRDSTQKAIDKITSKVNNIVDKMNRQYEIIDHEFSEVETRLNMINN
KVDDQIQDIWAYNAELLVLLENQKTLDEHDSNVNNLYNKVKRALGSNAVEDGKGCFELYHKCDDQCMETI
RNGTYNRRKYQEESKLERQKIEGVKLESEGTYKILTIYSTVASSLVIAMGFAAFLFWAMSNGSCRCNICI

>QDF63413.1 hemagglutinin [Influenza A virus]
METVSLITILLVATVSNADKICIGYQSTNSTETVDTLTENNVPVTHAKELLHTEHNGMLCATSLGQPIIL
DTCTIEGLIYGNPSCDLSLEGREWSYIVERPSAVNGLCYPGNVENLEELRSLFSSARSYQRIQIFPDTIW
NVSYDGTSTACSGSFYRNMRWLTRKNGDYPIQDAQYTNNQGKNILFMWGINHPPADTTQRDLYTRTDTTT
SVATEEINRIFKPLIGPRPLVNGLMGRIDYYWSVLKPGQTLRIRSDGNLIAPWYGHILSGESHGRILKTD
LKRGSCTVQCQTEKGGLNTTLPFQNVSKYAFGNCSKYIGIKSLKLAVGLRNVPSRSSRGLFGAIAGFIEG
GWSGLVAGWYGFQHSNDQGVGMAADRDSTQKAIDKITSKVNNIVDKMNKQYEIIDHEFSEVETRLNMINN
KIDDQIQDIWAYNAELLVLLENQKTLDEHDANVNNLYNKVKRALGSNAVEDGKGCFELYHKCDDQCMETI
RNGTYNRRKYQEESKLERQKIEGVKLESEGTYKILTIYSTVASSLVIAMGFAAFLFWAMSNGSCRCNICI

>QDF63411.1 hemagglutinin [Influenza A virus]
METASLITVLLVVTVSNADKICIGYQSTNSTETVDTLTENNVPVTHAKELLHTEHNGMLCATSLGNPLIL
DTCTIEGLIYGNPSCDLLLGGREWSYIVERPSAVNGLCYPGNVENLEELRSLFSSARSYKRVQIFPDTIW
NVSYNGISKACSDSFYRSMRWLTQKNNAYPTQDAQYTNNQEKNILFMWGINHPPTDTAQTNLYTRTDTTT
SVATEEINRTFKPLIGPRPLVNGLMGRIDYYWSVLKPGQTLRIRSNGNLIAPWYGHILSGESHGRILKTD
LKKGSCTVQCQTEKGGLNTTLPFQNVSKYAFGNCSKYIGIKSLKLAVGLRNVPSRSSRGLFGAIAGFIEG

GWSGLVAGWYGFQHSNDQGVGMAADRDSTQKAIDKITSKVNNIVDKMNKQYEIIDHEFSEVETRINMINN
KIDDQIQDIWAYNAELLVLLENQKTLDEHDANVNNLYNKVKRALGSNAVEDGKGCFELYHKCDDQCMETI
RNGTYNRRKYQEESKLKRQKIEGVKLESEETYKILTIYSTVASSLVIAMGFAAFLFWAMSNGSCRCNICI

>QDF63410.1 hemagglutinin [Influenza A virus]
METASLITVLLVVTVSNADKICIGYQSTNSTETVDTLTENNVPVTHAKELLHTEHNGMLCATSLGNPLIL
DTCTIEGLIYGNPSCDLLLGGREWSYIVERPSAVNGLCYPGNVENLEELRSLFSSARSYKRVQIFPDTIW
NVSYNGISKACSDSFYRSMRWLTQKNNAYPTQDAQYTNNQEKNILFMWGINHPPTDTAQTNLYTRTDTTT
SVATEEINRTFKPLIGPRPLVNGLMGRIDYYWSVLKPGQTLRIRSNGNLIAPWYGHILSGESHGRILKTD
LKKGSCTVQCQTEKGGLNTTLPFQNVSKYAFGNCSKYIGIKSLKLAVGLRNVPSRSSRGLFGAIAGFIEG
GWSGLVAGWYGFQHSNDQGVGMAADRDSTQKAIDKITSKVNNIVDKMNKQYEIIDHEFSEVETRINMINN
KIDDQIQDIWAYNAELLVLLENQKTLDEHDANVNNLYNKVKRALGSNAVEDGKGCFELYHKCDDQCMETI
RNGTYNRRKYQEESKLERQKIEGVKLESEETYKILTIYSTVASSLVIAMGFAAFLFWAMSNGSCRCNICI

>QDF63409.1 hemagglutinin [Influenza A virus]
METVSLITILLVATVSNADKICIGYQSTNSTETVDTLTENNVPVTHAKELLHTEHNGMLCATSLGQPLIL
DTCTIEGLIYGNPSCDLLLEGREWSYIVERPSAVNGLCYPGNVENLEELRSLFSSARSYQRIQIFPDTIW
NVSYDGTSTACSGSFYRSMRWLTRKNGNYPIQDAQYTNNQGKNILFMWGINHPPTDTTQRDLYTRTDTTT
SVATEEINRIFKPLIGPRPLVNGLMGRIDYYWSVLKPGQTLRIKSDGNLIAPWYGHILSGESHGRILKTD
LKRGSCTVQCQTEKGGLNTTLPFQNVSKYAFGNCSKYLGIKSLKLAVGLRNVPSRSSRGLFGAIAGFIEG
GWSGLVAGWYGFQHSNDQGVGMAADRDSTQKAIDKITSKVNNIVDKMNKQYEIIDHEFSEVETRLNMINN
KIDDQIQDIWAYNAELLVLLENQKTLDEHDANVNNLYNKVKRALGSNAVEDGKGCFELYHKCDDQCMETI
RNGTYNRRKYQEESKLERQKIEGVKLESEGTYKILTIYSTVASSLVIAMGFAAFLFWAMSNGSCRCNICI

>QDF63408.1 hemagglutinin [Influenza A virus]
METVSLITILLVATVSNADKICIGYQSTNSTETVDTLTENNVPVTHAKELLHTEHNGMLCATSLGQPLVL
DTCTIEGLIYGNPSCDLSLEGREWSYIVERPSAVNGLCYPGNVENLEELRSLFSSARSYQRIQIFPDTIW
NVSYDGTSNACSGSFYRSMRWLTRKNGDYPIQDAQYTNNQGKNILFMWGINHPPTDTTQRDLYTRTDTTT
SVATEEINRIFKPLIGPRPLVNGLMGRIDYYWSVLKPGQTLRIKSDGNLIAPWYGHILSGESHGRILKTD
LKRGSCTVQCQTEKGGLNTTLPFQNVSKYAFGNCSKYIGIKSLKLAVGLRNVPSRSSRGLFGAIAGFIEG
GWSGLVAGWYGFQHSNDQGVGMAADRDSTQKAIDKITSKVNNIVDKMNKQYEIIDHEFSEVETRLNMINN
KIDDQIQDIWAYNAELLVLLENQKTLDEHDANVNNLYNKVKRALGSNAVEDGKGCFELYHKCDDQCMETI
RNGTYNRRKYQEESKLERQKIEGVKLESEGTYKILTIYSTVASSLVIAMGFAAFLFWAMSNGSCRCSICI

>QDF63407.1 hemagglutinin [Influenza A virus]

METVSLITILLVATVSNADKICIGYQSTNSTETVDTLTENNVPVTHAKELLHTEHNGMLCATSLGQPLIL

DTCTIEGLIYGNPSCDLLLEGREWSYIVERPSAVNGLCYPGHVENLEELRSLFSSARSYQRIQIFPDTIW

NVSYDGTSNACSGSFYRSMRWLTRKNGDYPIQDAQYTNNQGKNILFMWGINHPPTDTTQRDLYTRTDTTT

SVATEEINRIFKPLIGPRPLVNGLMGRIDYYWSVLKPGQTLRIKSDGNLIAPWYGHILSGESHGRILKTD

LKRGSCTVQCQTEKGGLNTTLPFQNVSKYAFGNCSKYIGIKSLKLAVGLRNVPSRSSRGLFGAIAGFIEG

GWSGLVAGWYGFQHSNDQGVGMAADRDSTQKAIDKITSKVNNIVDKMNKQYEIIDHEFSEVETRLNMINN

KIDDQIQDIWAYNAELLVLLENQKTLDEHDANVNNLYNKVKRALGSNAVEDGKGCFELYHKCDDQCMETI

RNGTYNRRKYQEESKLERQKIEGVKLESEGTYKILTIYSTVASSLVIAMGFAAFLFWAMSNGSCRCNICI

>QDF63406.1 hemagglutinin [Influenza A virus]

METVSLITILLVSTVSNADKICIGYQSTNSTETVDTLTENNVPVTHAKELLHTEHNGMLCATSLGQPLIL

DTCTIEGLIYGNPSCDLSLEGREWSYIVERPSAVNGLCYPGNVENLEELRSLFSSARSYQRIQIFPDTIW

NVSYNGISKACSDSFYRSMRWLTQKNSDYPIQDAQYTNNQGKNILFMWGINHPPTDTTQRDLYTRTDTTT

SVATEEINRVFKPLIGPRPLVNGLMGRIDYYWSVLKPGQTLRIKSDGNLIAPWYGHILSGESHGRILKTD

LKRGSCTVQCQTEKGGLNTTLPFQNVSKYAFGNCSKYIGIKSLKLAFGLRNVPSRSSRGLFGAIAGFIEG

GWSGLVAGWYGFQHSNDQGVGMAADRDSTQKAIDKITSKVNNIVDKMNKQYEIIDHEFSEVETRLNMINN

KIDDQIQDIWAYNAELLVLLENQKTLDEHDANVNNLYNKVKRALGSNAVEDGKGCFELYHKCDDQCMETI

RNGTYNRRKYQEESKLERQKIEGVKLESEGTYKILTIYSTVASSLVIAMGFAAFLFWAMSNGSCRCNICI

>QDF63405.1 hemagglutinin [Influenza A virus]

METVSLITILLVATVSNADKICIGYQSTNSTETVDTLTENNVPVTHAKELLHTEHNGMLCATSLGQPLIL

DTCTIEGLIYGNPSCDLLLEGREWSYIVERPSAVNGLCYPGNVENLEELRSLFSSARSYQRIQIFPDTIW

NVSYNGTSKACSGSFYRSMRWLTRKNGDYPIQDAQYTNNQGKNILFMWGINHPPTDTTQRDLYTRTDTTT

SVATEEINRIFKPLIGPRPLVNGLMGRIDYYWSVLKPGQTLRIKSDGNLIAPWYGHILSGESHGRILKTD

LKRGSCTVQCQTEKGGLNTTLPFQNVSKYAFGNCSKYIGIKSLKLAVGLRNVPSRSSRGLFGAIAGFIEG

GWSGLVAGWYGFQHSNDQGVGMAADRDSTQKAIDKITSKVNNIVDKMNKQYEIIDHEFSEVETRLNMINN

KIDDQIQDIWAYNAELLVLLENQKTLDEHDANVNNLYNKVKRALGSNAVEDGKGCFELYHKCDNQCMETI

RNGTYNRRKYQEESKLERQKIEGVKLESEGTYKILTIYSTVASSLVIAMGFAAFLFWAMSNGSCRCNICI

>QDF63404.1 hemagglutinin [Influenza A virus]

METVSLITILLVATVSNADKICIGYQSTNSTETVDTLTENNVPVTHAKELLHTEHNGMLCATSLGQPLIL

DTCTIEGLIYGNPSCDLLLEGREWSYIVERPSAVNGLCYPGNVENLEELRSLFSSARSYQRIQIFPDTIW

NVSYDGTSTACSGSFYRSMRWLTRKNGDYPIQDAQYTNNQGKNILFMWGINHPPTDTTQRDLYTRTDTTT

SVATEEINRIFKPLIGPRPLVNGLMGRIDYYWSVLKPGQTLRIKSDGNLIAPWYGHILSGESHGRILKTD
LKRGSCTVQCQTEKGGLNTTLPFQNVSKYAFGNCSKYIGIKSLKLAVGLRNVPSRSSRGLFGAIAGFIEG
GWSGLVAGWYGFQHSNDQGVGMAADRDSTQKAIDKITSKVNNIVDKMNKQYEIIDHEFSEVETRLNMIHN
KIDDQIQDIWAYNAELLVLLENQKTLDEHDANVNNLYNKVKRALGSNAVEDGKGCFELYHKCDDQCMETI
RNGTYNRRKYQEESKLERQKIEGVKLESEGTYKILTIYSTVASSLVIAMGFAAFLFWAMSNGSCRCNICI

>QDF63403.1 hemagglutinin [Influenza A virus]
METVSLITILLVATVSNADKICIGYQSTNSTETVDTLTENNVPVTHAKELLHTEHNGMLCATSLGQPLIL
DTCTIEGLIYGNPSCELLLEGREWSYIVERPSAVNGLCYPGNVENLEELRSLFSSARSYQRIQIFPVTIW
NVSYNGTSKACSGSFYRSMRWLTRKNGDYPIQDAQYTNNQGKNILFMWGINHPPTDTTQRDLYTRTDTTT
SVATEEINRVFKPLIGPRPLVNGLMGRIDYYWSVLKPGQTLRIKSDGNLIAPWYGYILSGESHGRILKTD
LKRGSCTVQCQTEKGGLNTTLPFQNVSKYAFGNCSKYIGIKSLKLAVGLRNVPSRSSRGLFGAIAGFIEG
GWSGLVAGWYGFQHSNDQGVGMAADRDSTQKAIDKITSKVNNIVDKMNKQYEIIDHEFSEVETRLNMINN
KIDDQIQDIWAYNAELLVLLENQKTLDEHDANVNNLYNKVKRALGSNAVEDGKGCFELYHKCDDQCMETI
RNGTYNRRKYQEESKLERQKIEGVKLESEGTYKILTIYSTVASSLVIAMGFAAFLFWAMSNGSCRCNICI

>QDF63402.1 hemagglutinin [Influenza A virus]
METASLITILLVATVSNADKICIGYQSTNSTETVDTLTENNVPVTHAKELLHTEHNGMLCATSLGQPLIL
DTCTIEGLIYGNPSCDLLLEGREWSYIVERPSAVNGLCYPGNVENLEELRSLFSSARSYQRIQIFPDTIW
NVSYDGTSTACSGSFYRSMRWLTRKNGDYPIQDAQYTNNQGKNILFMWGINHPPTDTTQRDLYTRTDTTT
SVATEEINRVFKPLIGPRPLVNGLMGRIDYYWSVLKPGQTLRIKSDGNLIAPWYGYILSGESHGRILKTD
LKRGSCTVQCQTEKGGLNTTLPFQNVSKYAFGNCSKYIGIKSLKLAVGLRNVPSRSSRGLFGAIAGFIEG
GWSGLVAGWYGFQHSNDQGVGMAADRDSTQKAIDKITSKVNNIVDKMNKQYEIIDHEFSEVETRLNMINN
KIDDQIQDIWAYNAELLVLLENQKTLDEHDANVNNLYNKVKRALGSNAVEDGKGCFELYHKCDDQCMETI
RNGTYNRRKYQEESKLERQKIEGVKLESEGTYKILTIYSTVASSLVIAMGFAAFLFWAMSNGSCRCNICI

>QDF63401.1 hemagglutinin [Influenza A virus]
METVSLITILLVATVSNADKICIGYQSTNSTETVDTLTENNVPVTHAKELLHTEHNGMLCATSLGQPLIL
DTCTIEGLIYGNPSCDLLLEGREWSYIVERPSAVNGLCYPGNVENLEELRSLFSSARSYQRIQIFPNTIW
NVSYNGTSKACSGSFYRSMRWLTQKNGNYPVQDAQYTNNQGKNILFMWGINHPPTDTTQRDLYTRTDTTT
SVATEEINRIFKPLIGPRPLVNGLMGRIDYYWSVLKPGQTLRIKSDGNLIAPWYGHILSGESHGRILKTD
LKRGSCTVQCQTEKGGLNTTLPFQNVSKYAFGNCSKYIGIKSLKLAVGLRNVPSRSSRGLFGAIAGFIEG
GWSGLVAGWYGFQHSNDQGVGMAADRDSTQKAIDKITSKVNNIVDKMNKQYEIIDHEFSEVETRLNMINN
KIDDQIQDIWAYNAELLVLLENQKTLDEHDANVNNLYNKVKRALGSNAMEDGKGCFDLYHKCDDQCMETI

RNGTYNRRKYQEESKLERQKIEGVKLESEGTYKILTIYSTVASSLVIAMGFAAFLFWAMSNGSCRCNICI

>QDF63399.1 hemagglutinin [Influenza A virus]

MEAVTIITILLAITGSNADKICIGYQSTNSTETVDTLTENNVPVTHAKELLHTEHNGMLCATNLGSPLIL
DTCTIEGIIYGNPSCDLPLEGRKWSYIVERPSAVNGVCYPGNVENLEELRSLFSSASSYQRIQIFPDSIW
NVSYSGTSKACSDSFYKSMRWLTQKNNNYPVQDAQYTNNRGKNILFMWGINHPPTDTAQTNLYTRTDTTT
SVATEDINRIFKPLIGPRPLVNGLQGRIDYYWAVLKPGQTLRVRSNGNLIAPWYGHILLGESHGRILKTD
LKSGNCVVQCQTERGGLNTTLPFHNVSKYAFGNCPKYIGVKSLKLAVGLRNVPARSSRGLFGAIAGFIEG
GWSGLVAGWYGFQHSNDQGTGMAADRDSTQKAIDKITSKVNNIVDKMNKQYEIIDHEFSEIETRLNMINN
KIDDQIQDIWAYNAELLVLLENQKTLDEHDANVNNLYNKVKRALGSNAVEDGKGCFDLYHKCDNQCMETI
RNGTYNRRKYQEESRLERQKIEGVKLESEGTYKILTIYSTVASSLVIAMGFAAFLFWAMSNGSCRCNICI

>QDF63398.1 hemagglutinin [Influenza A virus]

MEAVTIITILLAITGSNADKICIGYQSTNSTETVDTLTENNVPVTHAKELLHTEHNGMLCATNLGSPLIL
DTCTIEGIIYGNPSCDLPLEGRKWSYIVERPSAVNGVCYPGNVENLEELRSLFSSASSYQRIQIFPDSIW
NVSYSGTSKACSDSFYKSMRWLTQKNNNYPVQDAQYTNNRGKNILFMWGINHPPTDTAQTNLYTRTDTTT
SVATEDINRIFKPLIGPRPLVNGLQGRIDYYWAVLKPGQTLRVRSNGNLIAPWYGHILLGESHGRILKTD
LKSGNCVVQCQTERGGLNTTLPFHNVSKYAFGNCPKYIGVKSLKLAVGLRNVPARSSRGLFGAIAGFIEG
GWSGLVAGWYGFQHSNDQGTGMAADRDSTQKAIDKITSKVNNIVDKMNKQYEIIDHEFSEIETRLNMINN
KIDDQIQDIWAYNAELLVLLENQKTLDEHDANVNNLYNKVKRALGSNAVEDGKGCFDLYHKCDNQCMETI
RNGTYNRRKYQEESRLERQKIEGVKLESEGTYKILTIYSTVASSLVIAMGFAAFLFWAMSNGSCRCNICI

>QDF63397.1 hemagglutinin [Influenza A virus]

METVSLITILLVATVSNADKICIGYQSTNSTETVDTLTENNVPVTHAKELLHTEHNGMLCATSLGQPLIL
DTCTIEGLIYGNPSCDLLLEGREWSYIVERPSAVNGLCYPGNVENLEELRSLFSSARSYQRIQIFPDTIW
NVSYNGTSKACSGSFYRSMRWLTRKNGDYPIQDAQYTNNQGKNILFMWGINHPPTDTTQRDLYTRTDTTT
SVATEEINRIFKPLIGPRPLVNGLMGRIDYYWSVLKPGQTLRIKSDGNLIAPWYGHILSGESHGRILKTD
LKRGSCTVQCQTEKGGLNTTLPFQNVSKYAFGNCSKYIGIKSLKLAVGLRNVPSRSSRGLFGAIAGFIEG
GWSGLVAGWYGFQHSNDQGVGMAADRDSTQKAIDKITSKVNNIVDKMNKQYEIIDHEFSEVETRLNMINN
KIDDQIQDIWAYNAELLVLLENQKTLDEHDANVNNLYNKVKRALGSNAVEDGKGCFELYHKCDDQCMETI
RNGTYNRRKYQEESKLERQKIEGVKLESEGTYKILTIYSTVASSLVIAMGFAAFLFWAMSNGSCRCNICI

>QDF63396.1 hemagglutinin [Influenza A virus]

METVSLITILLVATVSNADKICIGYQSTNSTETVDTLTENNVPVTHAKELIHTEHNGMLCATSLGQPLIL

DTCTIEGLIYGNPSCDLLLEGREWSYIVERPSAVNGLCYPGNVENLEELRSLFSSARSYQRIQIFPDTIW
NVSYNGTSKACSGSFYRSMRWLTRKNGDYPIQDAQYTNNQGKNILFMWGINHPPTDTTQRDLYTRTDTTT
SVATEEINRVFKPLIGPRPLVNGLMGRIDYYWSVLKPGQTLRIKSDGNLIAPWYGHILSGESHGRILKTD
LKRGSCTVQCQTEKGGLNTTLPFQNVSKYAFGNCSKYIGIKSLKLAVGLRNVPSRSSRGLFGAIAGFIEG
GWSGLVAGWYGFQHSNDQGVGMAADRDSTQKAIDKITSKVNNIVDKMNKQYEIIDHEFSEVETRLNMINN
KIDDQIQDIWAYNAELLVLLENQKTLDEHDANVNNLYNKVKRALGSNAVEDGKGCFELYHKCDDQCMETI
RNGTYNRRKYQEESKLERQKIEGVKLESEGTYKILTIYSTVASSLVIAMGFAAFLFWAMSNGSCRCNICI

>QDF63395.1 hemagglutinin [Influenza A virus]
METASLITILLVVTVSNADKICIGYQSTNSTETVDTLTENNVPVTHAKELLHTEHNGMLCATSLGHPLIL
DTCTIEGLIYGNPSCDLLLGGREWSYIVERPSAVNGLCYPGNVENLEELRSLFSSARSYQRVQIFPDTIW
NVSYSGTSRACSDSFYRSMRWLTQKDNAYPTQDAQYTNNQEKNILFMWGINHPPTDTAQTNLYTRTDTTT
SVATEEINRIFKPLIGPRPLVNGLMGRINYYWSVLKPGQTLRIKSNGNLIAPWYGHILSGESHGRILKTD
LKRGSCTVQCQTEKGGLNTTLPFQNVSKYAFGNCSKYIGIKSLKLAVGLRNVPSRSSRGLFGAIAGFIEG
GWSGLVAGWYGFQHSNDQGVGMAADRDSTQKAIDKITSKVNNIVDKMNKQYEIIDHEFSEVETRLNMINN
KIDDQIQDIWAYNAELLVLLENQKTLDEHDANVNNLYNKVKRALGSNAVEDGKGCFELYHKCDDQCMETI
RNGTYNRRKYQEESKLERQKIEGVKLESEGTYKILTIYSTVASSLVIAMGFAAFLFWAMSNGSCRCNICI

>QDF63394.1 hemagglutinin [Influenza A virus]
METASLITILLVVTVSNADKICIGYQSTNSTETVDTLTENNVPVTHAKELLHTEHNGMLCATSLGQPLIL
DTCTIEGLIYGNPSCDLLLGGREWSYIVERPSAVNGLCYPGNVENLEELRSLFSSARSYQRIQIFPDTIW
NVSYSGTSKACSDSFYRSMRWLTQKDNAYPIQDAQYTNNQEKNILFMWGINHPPTDTAQTNLYTRTDTTT
SVATEEINRTFKPLIGPRPLVNGLMGRINYYWSVLKPGQTLRIKSNGNLIAPWYGHILSGESHGRILKTD
LKRGSCTVQCQTEKGGLNTTLPFQNVSKYAFGNCSKYIGIKSLKLAVGLRNVPSRSSRGLFGAIAGFIEG
GWPGLVAGWYGFQHSNDQGVGMAADRDSTQKAIDKITSKVNNIVDKMNKQYEIIDHEFSEVETRLNMINN
KIDDQIQDIWAYNAELLVLLENQKTLDEHDANVNNLYNKVKRALGSNAVEDGKGCFELYHKCDDQCMETI
RNGTYNRKKYQEESKLEKQKIEGVKLESEGTYKILTIYSTVASSLVIAMGFAAFLFWAMSNGSCRCNICI

>QDF63393.1 hemagglutinin [Influenza A virus]
METASLITILLVVTVSNADKICIGYQSTNSTETVDTLTENNVPVTHAKELLHTEHNGMLCATSLGNPLIL
DTCTIEGLIYGNPSCDLLLGGREWSYIVERPSAVNGLCYPGNVENLEELRSLFSSARSYQRIQIFPDTIW
NVSYSGTSKACSDSFYRSMRWLTQKDNAYPIQDAQYTNNQGKNILFMWGINHPPTDTAQTNLYTRTDTTT
SVATEEINRTFKPLIGPRPLVNGLMGRINYYWSVLKPGQTLRIKSNGNLIAPWYGYILSGESHGRILKTD
LKRGSCTVQCQTEKGGLNTTLPFQNVSKYAFGNCSKYIGMKSLKLAVGLRNVPSRSSRGLFGAIAGFIEG

GWSGLVAGWYGFQHSNDQGVGMAADRDSTQKAIDKITSKVNNIVDKMNKQYEIIDHEFSEVETRLNMINN
KIDDQIQDIWAYNAELLVLLENQKTLDEHDANVNNLYNKVKRALGSNAVEDGKGCFELYHKCDDQCMETI
RNGTYNRRKYQEESKLERQKIEGVKLESEGTYKILTIYSTVASSLVIAMGFAAFLFWAMSNGSCRCNICI

>QDF63392.1 hemagglutinin [Influenza A virus]
METVSLIAILLVATVSNADKICIGYQSTNSTETVDTLTENNVPVTHAKELLHTEHNGMLCATSLGQPLIL
DTCTIEGLIYGNPSCDLLLEGREWSYIVERPSAVNGLCYPGHVENLEELRSLFSSARSYQRIQIFPDTIW
NVSYNGTSKACSGSFYRSMRWLTRKNGDYPIQDAQYTNNQGKNILFMWGINHPPTDTTQRDLYTRTDTTT
SVATEEINRIFKPLIGPRPLVNGLMGRIDYYWSVLKPGQTLRIKSDGNLIAPWYGHILSGESHGRILKTD
LKRGSCTVQCQTEKGGLNTTLPFQNVSKYAFGNCSKYIGIKSLKLAVGLRNVPSRSSRGLFGAIAGFIEG
GWSGLVAGWYGFQHSNDQGVGMAADRESTQKAIDKITSKVNNIVDKMNKQYEIIDHEFSEVETRLNMINN
KIDDQIQDIWAYNAELLVLLENQKTLDEHDANVNNLYNKVKRALGSNAVEDGKGCFELYHKCDDQCMETI
RNGTYNRRKYQEESKLERQKIEGVKLESEGTYKILTIYSTVASSLVIAMGFAAFLFWAMSNGSCRCNICI

>QDF63391.1 hemagglutinin [Influenza A virus]
METASLITILLVVTVSNADKICIGYQSTNSTETVDTLTENNVPVTHAKELLHTEHNGMLCATSLGQPLIL
DTCTIEGLIYGNPSCDLLLGGREWSYIVERPSAVNGLCYPGNVENLEELRSLFSSARSYQRIQIFPDTIW
NVSYSGTSKACSDSFYRSMRWLTQKDNAYPIQDAQYTNNQEKNILFMWGINHPPTDTAQTNLYTRTDTTT
SVATEEINRTFKPLIGPRPLVNGLMGRINYYWSVLKPGQTLRIKSNGNLIAPWYGHILSGESHGRILKTD
LKRGSCTVQCQTEKGGLNTTLPFQNVSKYAFGNCSKYIGIKSLKLAVGLRNVPSRSSRGLFGAIAGFIEG
GWSGLVAGWYGFQHSNDQGVGMAADRDSTQKAIDKITSKVNNIVDKMNKQYEIIDHEFSEVETRLNMINN
KIDDQIQDIWAYNAELLVLLENQKTLDEHDANVNNLYNKVKRALGSNAVEDGKGCFELYHKCDDQCMETI
RNGTYNRRKYQEESKLEKQKIEGVKLESEGTYKILTIYSTVASSLVIAMGFAAFLFWAMSNGSCRCNICI

>QDF63390.1 hemagglutinin [Influenza A virus]
METVSLIAILLVATVSNADKICIGYQSTNSTETVDTLTENNVPVTHAKELLHTEHNGMLCATSLGQPLIL
DTCTIEGLIYGNPSCDLLLEGREWSYIVERPSAVNGLCYPGHVENLEELRSLFSSARSYQRIQIFPDTIW
NVSYNGTSKACSGSFYRSMRWLTRKNGDYPIQDAQYTNNQGKNILFMWGINHPPTDTTQRDLYTRTDTTT
SVATEEINRIFKPLIGPRPLVNGLMGRIDYYWSVLKPGQTLRIKSDGNLIAPWYGHILSGESHGRILKTD
LKRGSCTVQCQTEKGGLNTTLPFQNVSKYAFGNCSKYIGIKSLKLAVGLRNVPSRSSRGLFGAIAGFIEG
GWSGLVAGWYGFQHSNDQGVGMAADRESTQKAIDKITSKVNNIVDKMNKQYEIIDHEFSEVETRLNMINN
KIDDQIQDIWAYNAELLVLLENQKTLDEHDANVNNLYNKVKRALGSNAVEDGKGCFELYHKCDDQCMETI
RNGTYNRRKYQEESKLERQKIEGVKLESEGTYKILTIYSTVASSLVIAMGFAAFLFWAMSNGSCRCNICI

>QDF63389.1 hemagglutinin [Influenza A virus]

METASLITILLVVTVSNADKICIGYQSTNSTETVDTLTENNVPVTHAKELLHTEHNGMLCATSLGQPLIL
DTCTIEGLIYGNPSCDLLLGGREWSYIVERPSAVNGLCYPGNVENLEELRSLFSSARSYQRIQIFPDTIW
NVSYSGTSKACSDSFYRSMRWLTQKNNAYPIQDAQYTNNQEKNILFMWGINHPPTDTAQTNLYTRTDTTT
SVATEEINRTFKPLIGPRPLVNGLMGRINYYWSVLKPGQTLRIKSNGNLIAPWYGHILSGESHGRILKTD
LKRGSCTVQCQTEKGGLNTTLPFQNVSKYAFGNCSKYIGIKSLKLAVGLRNVPSRSSRGLFGAIAGFIEG
GWSGLVAGWYGFQHSNDQGVGMAADRDSTQKAIDKITSKVNNIVDKMNKQYEIIDHEFSEVETRLNMINN
KIDDQIQDIWAYNAELLVLLENQKTLDEHDANVNNLYNKVKRALGSNAVEDGKGCFELYHKCDDQCMETI
RNGTYNRRKYQEESKLEKQKIEGVKLESEGTYKILTIYSTVASSLVIAMGFAAFLFWAMSNGSCRCNICI

>QDF63388.1 hemagglutinin [Influenza A virus]

METASLITILLVVTVSNADKICIGYQSTNSTETVDTLTENNVPVTHAKELLHTEHNGMLCATSLGNPLIL
DTCTIEGLIYGNPSCDLLLGGREWSYIVERPSAVNGLCYPGNVENLEELRSLFSSARSYQRIQIFPDTIW
NVSYSGTSKACSDSFYRSMRWLTQKDNAYPIQDAQYTNNQGKNILFMWGINHPPTDTVQTNLYTRTDTTT
SVATEEINRTFKPLIGPRPLVNGLMGRINYYWSVLKPGQTLRIKSNGNLIAPWYGYILSGESHGRILKTD
LKRGSCTVQCQTEKGGLNTTLPFQNVSKYAFGNCSKYIGMKSLKLAVGLRNVPSRSSRGLFGAIAGFIEG
GWSGLVAGWYGFQHSNDQGVGMAADRDSTQKAIDKITSKVNNIVDKMNKQYEIIDHEFSEVETRLNMINN
KIDDQIQDIWAYNAELLVLLENQKTLDEHDANVNNLYNKVKRALGSNAVEDGKGCFELYHKCDDQCMETI
RNGTYNRRKYQEESKLERQKIEGVKLESEGTYKILTIYSTVASSLVIAMGFAAFLFWAMSNGSCRCNICI

>QDF63387.1 hemagglutinin [Influenza A virus]

METVSLIAILLVATVSNADKICIGYQSTNSTETVDTLTENNVPVTHAKELLHTEHNGMLCATSLGQPLIL
DTCTIEGLIYGNPSCDLLLEGREWSYIVERPSAVNGLCYPGHVENLEELRSLFSSARSYQRIQIFPDTIW
NVSYNGTSKACSGSFYRSMRWLTRKNGDYPIQDAQYTNNQGKNILFMWGINHPPTDTTQRDLYTRTDTTT
SVATEEINRIFKPLIGPRPLVNGLMGRIDYYWSVLKPGQTLRIKSDGNLIAPWYGHILSGESHGRILKTD
LKRGSCTVQCQTEKGGLNTTLPFQNVSKYAFGNCSKYIGIKSLKLAVGLRNVPSRSSRGLFGAIAGFIEG
GWSGLVAGWYGFQHSNDQGVGMAADRESTQKAIDKITSKVNNIIDKMNKQYEIIDHEFSEVETRLNMINN
KIDDQIQDIWAYNAELLVLLENQKTLDEHDANVNNLYNKVKRALGSNAVEDGKGCFELYHKCDDQCMETI
RNGTYNRRKYQEESKLERQKIEGVKLESEGTYKILTIYSTVASSLVIAMGFAAFLFWAMSNGSCRCNICI

>QDF63386.1 hemagglutinin [Influenza A virus]

METVSLITILLVATVSNADKICIGYQSTNSTETVDTLTENNVPVTHAKELIHTEHNGMLCATSLGQPLIL
DTCTIEGLIYGNPSCDLLLEGREWSYIVERPSAVNGLCYPGNVENLEELRSLFSSARSYQRIQIFPDTIW
NVSYNGTSKACSGSFYRSMRWLTRKNGDYPIQDAQYTNNQGKNILFMWGINHPPTDTTQRDLYTRTDTTT

SVATEEINRVFKPLIGPRPLVNGLMGRIDYYWSVLKPGQTLRIKSDGNLIAPWYGHILSGESHGRILKTD
LKRGSCTVQCQTEKGGLNTTLPFQNVSKYAFGNCSKYIGIKSLKLAVGLRNVPSRSSRGLFGAIAGFIEG
GWSGLVAGWYGFQHSNDQGVGMAADRDSTQKAIDKITSKVNNIVDKMNKQYEIIDHEFSEVETRLNMINN
KIDDQIQDIWAYNAELLVLLENQKTLDEHDANVNNLYNKVKRALGSNAVEDGKGCFELYHKCDDQCMETI
RNGTYNRRKYQEESKLERQKIEGVKLESEGTYKILTIYSTVASSLVIAMGFAAFLFWAMSNGSCRCNICI

>QDF63385.1 hemagglutinin [Influenza A virus]
METVSLITILLVATVSNADKICIGYQSTNSTETVDTLTENNVPVTHAKELLHTEHNGMLCATSLGQPLIL
DTCTIEGLIYGNPSCDLLLEGREWSYIVERPSAVNGLCYPGNVENLEELRSLFSSARSYQRIQIFPDTIW
NVSYNGTSKACSGSFYRSMRWLTRKNGDYPIQDAQYTNNQGKNILFMWGINHPPTDTTQRDLYTRTDTTT
SVATEEINRIFKPLIGPRPLVNGLMGRIDYYWSVLKPGQTLRIKSDGNLIAPWYGHILSGESHGRILKTD
LKRGSCTVQCQTEKGGLNTTLPFQNVSKYAFGNCSKYIGIKSLKLAVGLRNVPSRSSRGLFGAIAGFIEG
GWSGLVAGWYGFQHSNDQGVGMAADRDSTQKAIDKITSKVNNIVDKMNKQYEIIDHEFSEVETRLNMINN
KIDDQIQDIWAYNAELLVLLENQKTLDEHDANVNNLYNKVKRALGSNAVEDGKGCFELYHKCDDQCMETI
RNGTYNRRKYQEESKLERQKIEGVKLESEGTYKILTIYSTVASSLVIAMGFAAFLFWAMSNGSCKCNICI

>QDF63384.1 hemagglutinin [Influenza A virus]
METVSLITILLVATVSNADKICIGYQSTNSTETVDTLTENNVPVTHAKELLHTEHNGMLCATSLGQPLIL
DTCTIEGLIYGNPSCDLLLEGREWSYIVERPSAVNGLCYPGNVENLEELRSLFSSARSYQRIQIFPDTIW
NVSYDGTSKACSDSFYRSMRWLTRRNGDYPIQDAQYTNNQGKNILFMWGINHPPTDTTQRDLYTRTDTTT
SVATEEINRIFKPLIGPRPLVNGLMGRIDYYWSVLKPGQTLRIKSDGNLIAPWYGHILSGESHGRILKTD
LKRGSCTVQCQTEKGGLNTTLPFQNVSKYAFGNCSKYIGIKSLKLAVGLRNVPSRSSRGLFGAIAGFIEG
GWSGLVAGWYGFQHSNDQGVGMAADRDSTQKAIDKITSKVNNIVDKMNKQYEIIDHEFSEVETRLNMINN
KIDDQIQDIWAYNAELLVLLENQKTLDEHDANVNNLYNKVKRALGSNAVEDGKGCFELYHKCDDQCMETI
RNGTYNRRKYQEESKLERQKIEGVKLESEGTYKILTIYSTVASSLVIAMGFAAFLFWAMSNGSCRCNICI

>QDF63383.1 hemagglutinin [Influenza A virus]
METISLVTILLVATVSNADKICIGYQSTNSTETVDTLTENNVPVTHAKELLHTEHNGMLCATSLGQPLIL
DTCTIEGLIYGNPSCDLLLEGREWSYIVERPSAVNGLCYPGNVENLEELRSLFSSARSYQRIQIFPVTIW
NVSHNGTSRACSGSFYRSMRWLTQKNSDYPIQDAQYTNNQGKNILFMWGINHPPTDTTQRDLYTRTDTTT
SVATEEINRVFKPLIGPRPLVNGLMGRIDYYWSVLKPGQTLRIKSDGNLIAPWYGYVLSGESHGRILKTD
LKRGSCTVQCQTEKGGLNTTLPFQNVSKYAFGNCSKYIGIKSLKLAVGLRNVPSRSSRGLFGAIAGFIEG
GWSGLVAGWYGFQHSNDQGVGMAADRDSTQKAIDKITSKVNNIVDKMNKQYEIIDHEFSEVETRLNMINN
KIDDQIQDIWAYNAELLVLLENQKTLDEHDANVNNLYNKVKRALGSNAVEDGKGCFELYHKCDDQCMETI

RNGTYNRRKYQEESKLERQKIEGVKLESEGTYKILTIYSTVASSLVIAMGFAAFLFWAMSNGSCRCNICI

>QDF63382.1 hemagglutinin [Influenza A virus]
METASLITILLVVTVSNADKICIGYQSTNSTETVDTLTENNVPVTHAKELLHTEHNGMLCATSLGHPLIL
DTCTVEGLIYGNPSCDLLLGGREWSYIVERPSAVNGLCYPGNVENLEELRSLFSSARSYQRIQIFPDTIW
NVSYSGTSKACSDSFYRSMRWLTQKDNAYPTQDAQYTNNQGKNILFMWGINHPPTDTVQTNLYTRTDTTT
SVATEEINRTFKPLIGPRPLVNGLMGRINYYWSVLKPGQTLRIKSNGNLIAPWYGHILSGESHGRILKTD
LKRGSCTVQCQTEKGGLNTTLPFQNVSKYAFGNCSKYIGIKSLKLAVGLRNVPSRSSRGLFGAIAGFIEG
GWSGLVAGWYGFQHSNDQGVGMAADRDSTQKAIDKITSKVNNIVDKMNKQYEIIDHEFSEVETRLNMINN
KIDDQIQDIWAYNAELLVLLENQKTLDEHDANVNNLYNKVKRSLGSNAVEDGKGCFELYHKCDDQCMETI
RNGTYNRRKYQEESKLERQKIEGVKLESEGTYKILTIYSTVASSLVIAMGFAAFLFWAMSNGSCRCNICI

>QDF63381.1 hemagglutinin [Influenza A virus]
METASLITILLVVTVSNADKICIGYQSTNSTETVDTLTENNVPVTHAKELLHTEHNGMLCATSLGHPLIL
DTCTIEGLIYGNPSCDLLLGGREWSYIVERPSAVNGLCYPGNVENLEELRSLFSSARSYQRIQIFPDTIW
NVSYSGTSKACSDSFYRSMRWLTQKDNAYPTQDAQYTNNQGKNILFMWGINHPPTDTVQTNLYTRTDTTT
SVATEEINRTFKPLIGPRPLVNGLMGRINYYWSVLKPGQTLRIKSDGNLIAPWYGHILSGESHGRILKTD
LKRGSCTVQCQTEKGGLNTTLPFQNVSKYAFGNCSKYIGIKSLKLAVGLRNVPSRSSRGLFGAIAGFIEG
GWSGLVAGWYGFQHSNDQGVGMAADRDSTQKAIDKITSKVNNIVDKMNKQYEIIDHEFSEVETRLNMINN
KIDDQIQDIWAYNAELLVLLENQKTLDEHDANVNNLYNKVKRSLGSNAVEDGKGCFELYHKCDDQCMETI
RNGTYNRRKYQEESKLERQKIEGVKLESEGTYKILTIYSTVASSLVIAMGFAAFLFWAMSNGSCRCNICI

>QDF63380.1 hemagglutinin [Influenza A virus]
METVSLITILLVATVSNADKICIGYQSTNSTETVDTLTENNVPVTHAKELLHTEHNGMLCATSLGQPLIL
DTCTIEGLIYGNPSCDLLLEGREWSYIVERPSAVNGLCYPGNVENLEELRSLFSSARSYQRIQIFPDTIW
NVSYDGTSTACSGSFYRSMRWLTQKDNDYPIQDAQYTNNQGKNILFMWGINHPPTDTAQTNLYTRTDTTT
SVATEEINRTFKPLIGPRPLVNGLMGRINYYWSVLKPGQTLRIKSNGNLIAPWYGHILSGESHGRILKTD
LKRGSCTVQCQTEKGGLNTTLPFQNVSKYAFGNCSKYIGIKSLKLAVGLRNVPSRSSRGLFGAIAGFIEG
GWSGLVAGWYGFQHSNDQGVGMAADRDSTQKAIDKITSKVNNIVDKMNKQYEIIDHEFSEVETRLNMINN
KIDDQIQDIWAYNAELLVLLENQKTLDEHDANVNNLYNKVKRALGSNAVEDGKGCFELYHKCDDQCMETI
RNGTYNRRKYQEESKLERQKIEGVKLESEGTYKILTIYSTVASSLVIAMGFAAFLFWAMSNGSCRCNICI

>QDF63379.1 hemagglutinin [Influenza A virus]
METASLITILLVVTVSNADKICIGYQSTNSTETVDTLTENNVPVTHAKELLHTEHNGMLCATSLGQPLIL

DTCTIEGLIYGNPSCDLLLGGREWSYIVERPSAVNGLCYPGNVENLEELRSLFSSARSYQRIQIFPDTIW
NVSYSGTSKACSDSFYRSMRWLTQKDNAYPIQDAQYTNNQEKNILFMWGINHPPTDTVQTNLYTRTDTTT
SVATEEINRTFKPLIGPRPLVNGLMGRINYYWSVLKPGQTLRIKSNGNLIAPWYGHILSGESHGRILKTD
LKRGSCTVQCQTEKGGLNTTLPFQNVSKYAFGNCSKYIGIKSLKLAVGLRNVPSRSSRGLFGAIAGFIEG
GWSGLVAGWYGFQHSNDQGVGMAADRDSTQKAIDKITSKVNNIVDKMNKQYEIIDHEFSEVETRLNMINN
KIDDQIQDIWAYNAELLVLLENQKTLDEHDANVNNLYNKVKRALGSNAVEDGKGCFELYHKCDDQCMETI
RNGTYNRRKYQEESKLERQKIEGVKLESEGTYKILTIYSTVASSLVIAMGFAAFLFWAMSNGSCRCNICI

>QDF63378.1 hemagglutinin [Influenza A virus]
METVSLITILLVATVSNADKICIGYQSTNSTETVDTLTENNVPVTHAKELLHTEHNGMLCATSLGQPLIL
DTCTIEGLIYGNPSCDLLLEGREWSYIVERPSAVNGLCYPGNVENLEELRSLFSSARSYQRIQIFPDTIW
NVSYDGTSTACSGSFYRSMRWLTRKNGDYPIQDAQYTNNQGKNILFMWGINHPPTDTTQRNLYTRTDTTT
SVATEEINRIFKPLIGPRPLVNGLMGRIDYYWSVLKPGQTLRIKSDGNLIAPWYGHILSGESHGRILKTD
LKRGSCTVQCQTEKGGLNTTLPFQNVSKYAFGNCSKYIGIKSLKLAVGLRNVPSRSSRGLFGAIAGFIEG
GWSGLVAGWYGFQHSNDQGVGMAADRDSTQKAIDKITSKVNNIVDKMNKQYEIIDHEFSEVETRLNMINN
KIDDQIQDIWAYNAELLVLLENQKTLDEHDANVNNLYNKVKRALGSNAVEDGKGCFELYHKCDDQCMETI
RNGTYNRRKYQEESKLERQKIEGVKLESEGTYKILTIYSTVASSLVIAMGFAAFLFWAMSNGSCRCNICI

>QDF63377.1 hemagglutinin [Influenza A virus]
METVSLITILLVATVSNADKICIGYQSTNSTETVDTLTENNVPVTHAKELLHTEHNGMLCATSLGQPLIL
DTCTIEGLIYGNPSCDLSLEGREWSYIVERPSAVNGLCYPGNVENLEELRSLFSSARSYQRIQIFPDTIW
NVSYDGTSKACSDSFYRSMRWLTRKNGDYPIQDAQYTNNQGKNILFMWGINHPPTDTTQRNLYTRNDTTT
SVATEEINRIFKPLIGPRPLVNGLMGRIDYYWSVLKPGQTLRIKSDGNLIAPWYGHILSGESHGRILKTD
LKRGSCTVQCQTEKGGLNTTLPFQNVSKYAFGNCSKYIGIKSLKLAVGLRNVPSRSSRGLFGAIAGFIEG
GWSGLVAGWYGFQHSNDQGVGMAADRDSTQKAIDKITSKVNNIVDKMNKQYEIIDHEFSEVETRLNMINN
KIDDQIQDIWAYNAELLVLLENQKTLDEHDANVNNLYNKVKRALGSNAVEDGKGCFELYHKCDDQCMETI
RNGTYNRRKYQEESKLERQKIEGVKLESEGTYKILTIYSTVASSLVIAMGFAAFLFWAMSNGSCRCNICI

>QKV35093.1 hemagglutinin, partial [Influenza A virus]
DKICIGHQSTNSTETVDTLTETGVPVTHAKELLHTEHNGKLCATNLGNPLILDTCTVEGLIYGNPSCDML
LGGREWSYIVERPSAVNGTCYPGNVENLEELRILFSSSSSYQRIQMFPDTVWNVTYSGTSKSCSDSFYRN
MRWLTQKNGNYPVQDAQYTNTRGKDILFVWGIHHPPTDTAQTNLYTRTDTTTSITTESLDRTFKPLIGPR
PLVNGLIGRINYYWSVLKPGQTLRVRSNGNLIAPWFGHVLSGESHGRILKTDLNSGNCVVQCQTEKGGLN
STLPFHNISKYAFGDCPKYIGVKSLKLAIGLRNVPARSSRGLFGAIAGFIEGGWPGLVAGWYGFQHSNDQ

GVGMAADRDSTQKAIDKITSKVNNIVDKMNKQYEIIDHEFSEVENRLNMINNKIDDQIQDIWAYNAELLV
LLENQKTLDEHDANVNNLYNKVKRALGSNAMEDGKGCFELYHKCDDQCMETIRNGTYNRRKYMGESRLGR
QKIEGVKLESEGT

>QKV35092.1 hemagglutinin, partial [Influenza A virus]
DKICIGHQSTNSTETVDTLTETGVPVTHAKELLHTEHNGRLCSTNLGNPLILDTCTVEGIIYGNPSCDML
LGGREWSYIVERPSAVNGTCYPGNVENLEELRVLFSSSSSYQRIQMFPDTIWNVTYSGTSKSCSDSFYRN
MRWLTQKNGNYPVQDAQYTNTRGKDILFVWGIHHPPTDTAQTNLYTRTDTTTSITTESLDRTFKPLIGPR
PLVNGLIGRINYYWSVLKPGQTLRVRSNGNLIAPWFGHVLSGESHGRILKTDLNSGNCVVQCQTEKGGLN
STLPFHNISKYAFGDCPKYIGVKSLKLAIGLRNVPARSSRGLFGAIAGFIEGGWPGLVAGWYGFQHSNDQ
GVGMAADRDSTQKAVDKITSKVNNIVDKMNKQYEIIDHEFSEVETRLNMINNKIDDQIQDIWAYNAELLV
LLENQKTLDEHDANVNNLYNKVKRALGSNAMEDGKGCFELYHKCDNQCMETIRNGTYNRRKYMEESRLGR
QKIEGVKLESEGT

>QKV35091.1 hemagglutinin, partial [Influenza A virus]
DKICIGHQSTNSTETVDTLTETNVPVTHAKELLHTEHNGKLCATNLGNPLILDTCTIEGLIYGNPSCDML
LGGREWSYIVERPSAVNGTCYPGNVENLEELRILFSSSRSYQRIQMFPDTIWNVTYSGTSKSCSDSFYRN
MRWLTQKNGNYPVQDAQYTNTRGKDILFVWGIHHPPTDTAQTNLYTRTDTTTSITTENLDRTFKPLIGPR
PLVNGLIGRINYYWSVLKPGQTLRVRSNGNLIAPWFGHVLSGESHGRILKTDLNSGNCVVQCQTEKGGLN
STLPFHNISKYAFGDCPKYIGVKSLKLAIGLRNVPARSSRGLFGAIAGFIEGGWPGLVAGWYGFQHSNDQ
GVGMAADRDSTQKAVDKITSKVNNIVDKMNKQYEIIDHEFNEVETRLNMINNKIDDQIQDIWAYNAELLV
LLENQKTLDEHDANVNNLYNKVKRALGSNAMEDGKGCFELYHKCDDQCMETIRNGTYNRRKYMEESRLGR
QKIDGVKLESEGT

>AWM12410.1 hemagglutinin [Influenza A virus]
METVSLITILVVATVSNADKICIGYQSTNSTETVDTLTENNVPVTHAKELLHTEHNGMLCATSLGHPLIL
DTCTIEGLIYGNPSCDPLLGGREWSYIVERPSAVNGLCYPGNVENLEELRSLFSSSRSYQRIQIFPDTIW
NVSYSGTSKACSDSFYRSMRWLTQKNNAYPTQDAQYTNNQGKNILFMWGINHPPTDTAQTNLYTRTDTTT
SVATEEMNRIFKPLIGPRPLVNGLMGRINYYWSVLKPGQTLRIKSDGNLIAPWYGHILSGESHGRILKTD
LKRGSCTVQCQTEKGGLNTTLPFQNVSKYAFGNCSKYIGVKSLKLAVGLRNVPSRSSRGLFGAIAGFIEG
GWSGLVAGWYGFQHSNDQGVGMAADRDSTQKAIDKITSKVNNIVDKMNKQYEIIDHEFSEVETRLNMINN
KVDDQIQDIWAYNAELLVLLENQKTLDEHDANVNNLYNKVKRALGSNAVEDGKGCFELYHKCDDHCMETI
RNGTYNRRKYQEESKLERQKIEGVKLESEETYKILTIYSTVASSLVIAMGFAAFLFWAMSNGSCRRNICI

>AWM12351.1 hemagglutinin [Influenza A virus]

METVSLITILLVATVSNADKICIGYQSTNSTETVDTLTENNVPVTHAKELLHTDHNGMLCATSLGHPLIL
DTCTIEGLIYGNPSCDPLLGGREWSYIVERPSAVNGLCYPGNVENLEELRSLFSSSRSYQRIQIFPDTIW
NVSYSGTSKACSDSFYRSMRWLTQKNNAYPTQDAQYTNNQEKNILFMWGINHPPTDTAQTNLYTRTDTTT
SVATEEINRIFKPLIGPRPLVNGLMGRINYYWSVLRPGQTLRIKSNGNLIAPWYGHILSGESHGRILKTD
LKSGSCTVQCQTERGGLNTTLPFQNESKYAFGNCSKYIGVKSLKLAVGLRNVPSRSSRGLFGAIAGFIEG
GWSGLVAGWYGFQHSNDQGVGMAADRDSTQKAIDKITSKVNNIVDKMNKQYGIIDHEFSEVGTRLNMINN
KVDDQIQDIWAYNAELLVLLENQKTLDEHDANVNNLYNKVKRALGSNAVEDGKGCFELYHKCDDHCMETM
RNGTYNRRKYQEESKLERQKIEGVKLESEETYKILTIYSTVASSLVIAMGFAAFLFWAMSNGSCRCNRWI

>AWM12339.1 hemagglutinin [Influenza A virus]

METVSLITILLVATVSNADKICIGYQSTNSTETVDTLTENNVPVTHAKELLHTEHNGMLCATSLGQPLIL
DTCTIEGLIYGNPSCDLSLEGREWSYIVERPSAVNGLCYPGNVENLEELRSLFSSARSYQRIQIFPDTIW
NVSYDGTSTACSGSFYKSMRWLTRKDGNYPIQDAQYTNNQGKNILFMWGINHPPTDTTQRELYTRIDTTT
SVATEEINRVFKPLIGPRPLVNGLMGRIDYYWSVLKPGQTLRIKSDGNLIAPWFGHILSGESHGRILKTD
LKRGSCTVQCQTERGGLNSTLPFQNVSKYAFGNCSKYIGIKSLKLAVGLRNVPSRSSRGLFGAIAGFIEG
GWSGLVAGWYGFQHSNDQGVGMAADRDSTQKAIDKITSKVNNIVDKMNKQYEIIDHEFSEVEARLNMINN
KIDDQIQDIWAYNAELLVLLENQKTLDEHDANVNNLYNKVKRALGSNAVEDGKGCFELYHKCDDQCMETI
RNGTYNRRKYQEESKLERQKIEGVKLESEGTYKILTIYSTVASSLVIAMGFAAFLFWAMSNGSCRRNICI

>AWM12316.1 hemagglutinin [Influenza A virus]

METAPLITILLVVTVSNADKICIGYQSTNSTETVDTLTENNVPVTHAKELLHTEHNGMLCATSLGQPLIL
DTCTIEGLIYGNPSCDLLLGGREWSYIVERPSAVNGLCYPGNVENLEELRSLFSSARSYQRIQIFPDTIW
NVSYSGTSKACSDSFYRSMRWLTQKDNAYPIQDAQYTNNQEKNILFMWGINHPPTDTVQTNLYTRTDTTT
SVATEEINRTFKPLIGPRPLVNGLMGRINYYWSVLKPGQTLRIKSNGNLIAPWYGHILSGESHGRILKTD
LKRGSCTVQCQTEKGGLNTTLPFQNVSKYAFGNCSKYIGIKSLKLAVGLRNVPSRSSRGLFGAIAGFIEG
GWSGLVAGWYGFQHSNDQGVGMAADRDSTQKAIDKITSKVNNIVDKMNKQYEIIDHEFSEVETRLNMINN
KIDDQIQDIWAYNAELLVLLENQKTLDEHDANVNNLYNKVKRALGSNAVEDGKGCFELYHKCDDQCMETI
RNGTYNRRKYQEESKLERQEIEGVKLESEGTYKILTIYSTVASSLVIAMGFAAFLFWAMSNGSCRRNICI

>AWM12304.1 hemagglutinin [Influenza A virus]

METASLITILLAVTVSNADKICIGYQSTNSTETVDTLTENNVPVTHAKELLHTEHNGMLCATSLGHPLIL
DTCTIEGLIYGNPSCDLLLGGREWSYIVERPSAVNGLCYPGNVENLEELRSLFSSARSYQRIQIFPDTIW
NVSYSGTSKACSDSFYRSMRWLTQKDNAYPVQDAQYTNNQEKNILFMWGINHPPTDTVQTNLYTRTDTTT

SVATEEINRTFKPLIGPRPLVNGLMGRINYYWSVLKPGQTLRIKSNGNLIAPWYGHILSGESHGRILKTD
LKRGSCTVQCQTERGGLNTTLPFQNVSKYAFGNCSKYIGIKSLKLAVGLRNVPSRSSRGLFGAIAGFIEG
GWSGLVAGWYGFQHSNDQGVGMAADRDSTQKAIDKITSKVNNIVDKMNKQYEIIDHEFSEVETRLNMINN
KIDDQIQDIWAYNAELLVLLENQKTLDEHDANVNNLYNKVKRALGSNAVEDGKGCFELYHKCDDQCMETI
RNGTYNRRKYQEESKLERQKIEGVKLEAEGTYKILTIYSTVASSLVIAMGFAAFLFWAMSNGSCRRNICI

>AWM12292.1 hemagglutinin [Influenza A virus]
METASLITILLAVTVSNADKICIGYQSTNSTETVDTLTENNVPVTHAKELLHTEHNGMLCATSLGHPLIL
DTCTIEGLIYGNPSCDLLLGGREWSYIVERPSAVNGLCYPGNVENLEELRSLFSSARSYQRIQIFPDTIW
NVSYSGTSKACSDSFYRSMRWLTQKDNAYPVQDAQYTNNQEKNILFMWGINHPPTDTVQTNLYTRTDTTT
SVATEEINRTFKPLIGPRPLVNGLMGRINYYWSVLKPGQTLRIKSNGNLIAPWYGHILSGESHGRILKTD
LKRGSCTVQCQTEKGGLNTTLPFQNVSKYAFGNCSKYIGIKSLKLAVGLRNVPSRSSRGLFGAIAGFIEG
GWSGLVAGWYGFQHSNDQGVGMAADRDSTQKAIDKITSKVNNIVDKMNKQYEIIDHEFSEVETRLNMINN
KIDDQIQDIWAYNAELLVLFENQKTLDEHDANVNNLYNKVKRALGSNAVEDGKGCFELYHKCDDQCMETI
RNGTYNRRKYQEESKLERQKIEGVKLESEGTYKILTIYSTVASSLVIAMGFAAFLFWAMSNGSCRRNICI

>QDB63642.1 hemagglutinin [Influenza A virus]
METVSLITILLVATVSNADKICIGYQSTNSTETVDTLTENNVPVTHAKELLHTEHNGMLCATSLGHPLIL
DTCTIEGLIYGNPSCDPLLGGREWSYIVERPSAVNGLCYPGNVENLEELRSLFSSARSYQRIQIFPDTIW
NVSYSGTSKACSDSFYRSMRWLTQKNNAYPIQDAQYTNNQEKNILFMWGINHPPTETVQTNLYTRTDTTT
SVATEEINRILKPLIGPRPLVNGLMGRINYYWSVLKPGQTLRIKSDGNLIAPWYGHILSGESHGRILKTD
LKRGSCTVQCQTEKGGLNTTLPFQNVSKYAFGNCSKYIGIKSLKLAIGLRNVPSRSSRGLFGAIAGFIEG
GWSGLVAGWYGFQHSNDQGVGMAADRDSTQKAIDKITSKVNNIVDKMNRQYEIIDHEFSEVETRLNMIND
KVDDQIQDIWAYNAELLVLLENQKTLDEHDSNVNNLYNKVKRALGSNAVEDGKGCFELYHKCDDQCMETI
RNGTYNRRKYQEESKLERQKIEGVKLESEGTYKILTIYSTVASSLVIAMGFAAFLFWAMSNGSCRCNICI

>QDB63641.1 hemagglutinin [Influenza A virus]
METVSLITILLVATVSNADKICIGYQSTNSTETVDTLTENNVPVTHAKELLHTEHNGMLCATSLGQPLIL
DTCTIEGLIYGNPSCDQLLEGREWSYIVERPSAVNGLCYPGSVENLEELRSLFSSARSYQRIQIFPDTIW
NVSYDGTSTACSGSFYRSMRWLTRKNGDYPIQDAQYTNNQGKNILFMWGINHPPTDTTQRDLYTRTDTTT
SVATEEINRIFKPLIGPRPLVNGLMGRINYYWSVLKPGQTLRIKSDGNLIAPWYGHILSGESHGRILKTD
LKKGSCTVQCQTEKGGLNTTLPFQNVSKYAFGNCSKYIGIKSLKLAVGLRNVPSRSSRGLFGAIAGFIEG
GWSGLVAGWYGFQHSNDQGVGMTADRDSTQKAIDKITSKVNNIVDKMNKQYEIIDHEFSEVETRLNMINN
KIDDQIQDIWAYNAELLVLLENQKTLDEHDANVNNLYNKVKRALGSNAVEDGKGCFELYHKCDDQCMETI

RNGTYNRRKYQEESKLERQKIEGVKLESEGTYKILTIYSTVASSLVIAMGFAAFLFWAMSNGSCRCNICI

>BCB92169.1 hemagglutinin [Influenza A virus]
MEIIPLMTILLLVTINNADKICIGHQSTNSTETXDTLTETNVPVTHAKELLHTEHNGKLCATNLGNPLIL
DTCTIEGLIYGNPSCDMLLGGREWSYIVERPSAVNGTCYPGNVENLEELRILFSSSSSYQRIQMFPDTIW
NVTYSGTSKSCSDSFYRNMRWLTQKNGNYPVQDAQYTNTRGKDILFVWGIHHSPTDTAQTNLYTRTDTTT
SVTTENLDRTFKPLIGPRPLVNGLIGRINYYWSVLKPGQTLRVRSNGNLIAPWFGHVLSGESHGRILKTD
LNSGNCVVQCQTEKGGLNSTLPFHNISKYAFGDCPKYIGVKSLKLAIGLRNVPARSSRGLFGAIAGFIEG
GWPGLVAGWYGFQHSNDQGVGMAADRDSTQKAVDKITSKANNIVDKMNKQYEIIDHEFSEVETRLNMINN
KIDDQIQDIWAYNAELLVLLENQKTLDEHDANVNNLYNKVKRALGSNAMEDGKGCFELYHKCDDQCMETI
RNGTYNRRKYKEESRLGRLKIEGVKLESEGTYKILTIYSTVASSLVLAMGFAALLFWAMSNGSCRCNICI

>BCB92168.1 hemagglutinin [Influenza A virus]
MEIIPLMTILLLVTINNADKICIGHQSTNSTETVDTLTETNVPVTHAKELLHTEHNGKLCATNLGNPLIL
DTCTIEGLIYGNPSCDMLLGGREWSYIVERPSAVNGTCYPGNVENLEELRILFSSSSSYQRIQMFPDTIW
NVTYSGTSKSCSDSFYRNMRWLTQKNGNYPVQDAQYTNTRGKDILFVWGIHHPPTDTAQTNLYTRTDTTT
SVTTENLDRTFKPLIGPRPLVNGLIGRINYYWSVLKPGQTLRVRSNGNLIAPWFGHVLSGESHGRILKTD
LNSGNCVVQCQTEKGGLNSTLPFHNISKYAFGDCPKYIGVKSLKLAIGLRNVPARSSRGLFGAIAGFIEG
GWPGLVAGWYGFQHSNDQGVGMAADRDSTQKAVDKITSKVNNIVDKMNKQYEIIDHEFSEVETRLNMINN
KIDDQIQDIWAYNAELLVLLENQKTLDEHDANVNNLYNKVKRALGSNAMEDGKGCFELYHKCDDQCMETI
RNGTYNRRKYKEESRLGRLKIEGVKLESEGTYKILTIYSTVASSLVLAMGFAALLFWAMSNGSCRCNICI

>BCB92167.1 hemagglutinin [Influenza A virus]
MEIIPLMTILLLVTINNADKICIGHQSTNSTETVDTLTETNVPVTHAKELLHTEHNGKLCATNLGNPLIL
DTCTIEGLIYGNPSCDMLLGGREWSYIVERPSAVNGTCYPGNVENLEELRILFSSSSSYQRIQMFPDTIW
NVTYSGTSKSCSDSFYRNMRWLTQKNGNYPVQDAQYTNTRGKDILFVWGIHHPPTDTAQTNLYTRTDTTT
SVTTENLDRTFKPLIGPRPLVNGLIGRINYYWSVLKPGQTLRVRSNGNLIAPWFGHVLSGESHGRILKTD
LNSGNCVVQCQTEKGGLNSTLPFHNISKYAFGDCPKYIGVKSLKLAIGLRNVPARSSRGLFGAIAGFIEG
GWPGLVAGWYGFQHSNDQGVGMAADRDSTQKAVDEITSKVNNIVDKMNKQYEIIDHEFSEVETRLNMINN
KIDDQIQDIWAYNAELLVLLENQKTLDEHDANVNNLYNKVKRALGSNAMEDGKGCFELYHKCDDQCMETI
RNGTYNRRKYKEESRLGRLKIEGVKLESEGTYKILTIYSTVASSLVLAMGFAALLFWAMSNGSCRCNICI

>QIJ97507.1 hemagglutinin, partial [Influenza A virus]
MEAISMMIALVVVSTSSADIICIGHQSTNCTETVDTLTESNIPVTQAKELLHTEHNGMLCATNLGRPLIL

DTCTVEGLIYGNPSCDLLLGGREWSYIVERPSAVNGTCYPGNVENLEELRMLFSSANSYQRIQIFPDAIW
NVTYDGTSKSCSNSFYRNMRWLTQKNGNYPIQDAQYTNNRGKDILFIWGIHHPPTDTAQTNLYTRTDTTT
SVTTENLDRTFKPLIGPRPLVNGLIGRINYYWSVLKPGQTLRVRSNGNLIAPWYGHVISGESHGRILKTD
KSNCVVQCQTEKGGLNSTLPFHNISKYAFGTCPKYIGVKSLKLAIGLRNVPAKSSRGLFGAIAGFIEGGW
PGLVAGWYGFQHSNDQGVGMAADRGSTQKAVDKITSKVNNIIDKMNRQYEIIDHEFSEIETRLNMINNKI
DDQIQDVWAYNAELLVLLENQKTLDEHDANVNNLYNKVKRALGSNAMEDGKGCFELYHKCDDQCMETIRN
GTYNRRKYAEESRLERQKIEGVKLESEENYKILTIYSTVASSLVLAMGAAAFLFWAMFN

>QHB50135.1 hemagglutinin, partial [Influenza A virus]
METASLITILLVVTASNADKICIGYQSTNSTETVDTLTENNVPVTHAKELLHTEHNGMLCATSLGHPLIL
DTCTIEGLIYGNPSCDPLLEGGEWSYIVERPSAVNGLCYPGNVENLEELRSLFSSARSYKRILIFPDTIW
NVSYSGTSKACSDSFYRSMRWLTQKNNAYPIQDAQYTNNQEKNILFMWGINHPPTDTVQTNLYTRTDTTT
SVATEEISRTFKPLIGPRPLVNGLMGRINYYWSVLKPGQTLRIKSNGNLIAPWYGHILSGESHGRILKTD
LKRGSCTVQCQTEKGGLNTTLPFQNVSRYAFGNCSKYIGINSLKLAVGLRNVPSRSSRGLFGAIAGFIEG
GWPGLVAGWYGFQHSNDQGVGIAADRDSTQKAIDKITSKVNNIVNKMNKQYEIIDHEFSEVETRLNMINN
KIDDQIQDIWAYNAELLVLLENQKTLDEHDSNVNNLYNKVKRALGSNAVEDGKGCFELYHKCDDQCMETI
RNGTYNRRKYQEESKLERQKIEGVKLESEGTYKILTIYSTVASSLVIAMGFAA

>QHB50134.1 hemagglutinin, partial [Influenza A virus]
METASLITILLVVTASNADKICIGYQSTNSTETVDTLTENNVPVTHAKELLHTEHNGMLCATSLGHPLIL
DTCTIEGLIYGNPSCDPLLEGGEWSYIVERPSAVNGLCYPGNVENLEELRSLFSSARSYKRILIFPDTIW
NVSYSGTSKACSDSFYRSMRWLTQKNNAYPIQDAQYTNNQEKNILFMWGINHPPTDTVQTNLYTRTDTTT
SVATEEISRTFKPLIGPRPLVNGLMGRINYYWSVLKPGQTLRIKSNGNLIAPWYGHILSGESHGRILKTD
LKRGSCTVQCQTEKGGLNTTLPFQNVSRYAFGNCSKYIGINSLKLAVGLRNVPSRSSRGLFGAIAGFIEG
GWPGLVAGWYGFQHSNDQGVGIAADRDSTQKAIDKITSKVNNIVNKMNKQYEIIDHEFSEVETRLNMINN
KIDDQIQDIWAYNAELLVLLENQKTLDEHDSNVNNLYNKVKRALGSNAVEDGKGCFELYHKCDDQCMETI
RNGTYNRRKYQEESKLERQKIEGVKLESEGTYKILTIYSTVASSLVIAMGFAA

>QHB50133.1 hemagglutinin, partial [Influenza A virus]
METASLITILLVVTASNADKICIGYQSTNSTETVDTLTENNVPVTHAKELLHTEHNGMLCATSLGHPLIL
DTCTIEGLIYGNPSCDPLLEGREWSYIVERPSAVNGLCYPGIVENLEELRSLFSSARSYQRILIFPDTIW
NVSYSGTSKACSDSFYRSMRWLTQKNNAYPIQDTQYTNNQEKNILFMWGINHPPTDTVQTNLYTRTDTTT
SVATEEINRTFKPLIGPRPLVNGLMGRINYYWSLLKPGQTLRIKSNGNLIAPWYGHILSGESHGRILKTD
LKRGSCTVQCQTEKGGLNTTLPFQNVSKYAFGNCSKYIGIKSLKLAVGLRNVPSRSSRGLFGAIAGFIEG

GWSGLVAGWYGFQHSNDQGVGMAADRDSTQKAIDKITSKVNNIVEKMNKQYEIIDHEFSEVETRLNMINN
KIDDQIQDIWAYNAELLVLLENQKTLDEHDANVNNLYNKVKRALGSNAVEDGKGCFELYHKCDDQCMETI
RNGTYNRRKYQEESKLERQKIEGVKLESEGTYKILTIYSTVASSLVIAMGFAA

>QHB50132.1 hemagglutinin, partial [Influenza A virus]
METASLITMLLVVTASNADKICIGYQSTNSTETVDTLTENNVPVTHAKELLHTEHNGMLCATSLGHPLIL
DTCTIEGLIYGNPSCDPLLEGREWSYIVERPSAVNGLCYPGIVENLEELRSLFSSARSYQRILIFPDTIW
NVSYSGTSKACSDSFYRSMRWLTQKNNAYPIQDAQYTNNQEKNILFMWGINHPPTDTAQTNLYTRTDTTT
SVATEEINRTFKPLIGPRPLVNGLMGRINYYWSLLKPGQTLRIKSNGNLIAPWYGHILSGESHGRILKTD
LKRGSCTVQCQTEKGGLNTTLPFQNVSKYAFGNCSKYIGIKSLKLAVGLRNVPSRSSRGLFGAIAGFIEG
GWSGLVAGWYGFQHSNDQGVGMAADRDSTQKAIDKITSKVNNIVEKMNKQYEIIDHEFSEVETRLNMINN
KIDDQIQDIWAYNAELLVLLENQKTLDEHDANVNNLYNKVKRALGSNAVEDGKGCFELYHKCDDQCMETI
RNGTYNRRKYQEESKLERQKIEGVKLESEGTYKILTIYSTVASSLVIAMGFAA

>QHB50131.1 hemagglutinin, partial [Influenza A virus]
METASLITMLLVVTASNADKICIGYQSTNSTETVDTLTENNVPVTHAKELLHTEHNGMLCATSLGHPLIL
DTCTIEGLIYGNPSCDPLLEGGEWSYIVERPSAVNGLCYPGNVENLEELRSLFSSARSYKRILIFPDTIW
NVSYSGTSKACSDSFYRSMRWLTQKNNAYPIQDAQYTNNQEKNILFMWGINHPPTDTAQTNLYTRTDTTT
SVATEEISRTFKPLIGPRPLVNGLMGRINYYWSVLKPGQTLRIKSNGNLIAPWYGHILSGESHGRILKTD
LKRGSCTVQCQTEKGGLNTTLPFQNVSRYAFGNCSKYIGINSLKLAVGLRNVPSRSSRGLFGAIAGFIEG
GWPGLVAGWYGFQHSNDQGVGMAADRDSTQKAIDKITSKVNNIVDKMNKQYEIIDHEFSEVETRLNMINN
KIDDQIQDIWAYNAELLVLLENQKTLDEHDSNVNNLYNKVKRALGSNAVEDGKGCFELYHKCDDQCMETI
RNGTYNRRKYQEESKLERQKIEGVKLESEGTYKILTIYSTVASSLVIAMGFAA

>QHB50130.1 hemagglutinin, partial [Influenza A virus]
METASLITILLVVTASNADKICIGYQSTNSTETVDTLTENNVPVTHAKELLHTEHNGMLCATSLGHPLIL
DTCTIEGLIYGNPSCDPLLEGGEWSYIVERPSAVNGLCYPGNVENLEELRSLFSSARSYKRILIFPDTIW
NVSYSGTSKACSDSFYRSMRWLTQKNNAYPIQDAQYTNNQEKNILFMWGINHPPTDTAQTNLYTRTDTTT
SVATEEISRTFKPLIGPRPLVNGLMGRINYYWSVLKPGQTLRIKSNGNLIAPWYGHILSGESHGRILKTD
LKRGSCTVQCQTEKGGLNTTLPFQNVSRYAFGNCSKYIGINSLKLAVGLRNVPSRSSRGLFGAIAGFIEG
GWPGLVAGWYGFQHSNDQGVGIAADRDSTQKAIDKITSKVNNIVNKMNKQYEIIDHEFSEVETRLNMINN
KIDDQIQDIWAYNAELLVLLENQKTLDEHDSNVNNLYNKVKRALGSNAVEDGKGCFELYHKCDDQCMETI
RNGTYNRRKYQEESKLERQKIEGVKLESEGTYKILTIYSTVASSLVIAMGFAA

>QHB50129.1 hemagglutinin, partial [Influenza A virus]

METASLITILLVVTASNADKICIGYQSTNSTETVDTLTENNVPVTHAKELLHTEHNGMLCATSLGHPLIL
DTCTIEGLIYGNPSCDPLLKGGEWSYIVERPSAVNGLCYPGNVENLEELRSLFSSARSYKRILIFPDTIW
NVSYSGTSKACSDSFYRSMRWLTQKNNAYPIQDAQYTNNQEKNILFMWGINHPPTDTVQTNLYTRTDTTT
SVATEEISRTFKPLIGPRPLVNGLMGRINYYWSVLKPGQTLRIKSNGNLIAPWYGHILSGESHGRILKTD
LKRGSCTVQCQTEKGGLNTTLPFQNVSRYAFGNCSKYIGINSLKLAVGLRNVPSRSSRGLFGAIAGFIEG
GWPGLVAGWYGFQHSNDQGVGIAADRDSTQKAIDKITSKVNNIVNKMNKQYEIIDHEFSEVETRLNMINN
KIDDQIQDIWAYNAELLVLLENQKTLDEHDSNVNNLYNKVKRALGSNAVEDGKGCFELYHKCDDQCMETI
RNGTYNRRKYQEESKLERQKIEGVKLESEGTYKILTIYSTVASSLVIAMGFAA

>QHB50128.1 hemagglutinin, partial [Influenza A virus]

MEIIALIAILVVTGTSDADKICIGYQSTNSTETVDTLVENNVPVTHAKELLHTEHNGMLCATNLGHPLIL
DTCTVEGLVYGNPACDSLLGGKEWSYIVERSSAVNGMCYPGRVENLEELRSFFSSARSYKKLLLFPDRTW
NVTFNGTSRACSGSFYRSMRWLTHKDNSYPIQDAQYTNDWGKNILFVWGIHHPPTDTEQMNLYKKADTTT
SITTEDINRTFKPAVGPRPLVNGQQGRIDYYWSVLKPGQTLRIRSNGNLIAPWYGHILSGESHGRILKTD
LNSGNCTIQCQTEKGGLNTTLPFQNVSKYAFGNCPKYVGVKSLKLAIGLRNVPARSKRGLFGAIAGFIEG
GWPGLVAGWYGFQHSNDNGVGIAADKKSTQEAVDKITSKVNNIIDKMNKQYEIIDHEFSEIEARLNMINN
KIDDQIQDIWAYNAELLVLLENQKTLDDHDANVNNLYNKVKRALGSNAIEDGKGCFELYHKCDDQCMETI
RNGTYDRLKYKEESKLERQKIEGVKLESEETYKILTIYSTVASSLMLAIGIAA

>QHB50127.1 hemagglutinin, partial [Influenza A virus]

MEIIALIAILVVTGTSDADKICIGYQSTNSTETVDTLVENNVPVTHAKELLHTEHNGMLCATNLGHPLIL
DTCTVEGLVYGNPSCDSLLGGKEWSYIVERSSAVNGMCYPGRVENLEELRSFFSSARSYKKLLLFPDRTW
NVTFNGTSRACSGSFYRSMRWLTHKDNSYPIQDAQYTNDWGKNILFVWGIHHPPTDTEQMNLYKKADTTT
SITTEDINRTFKPAVGPRPLVNGQQGRIDYYWSVLKPGQTLRIRSNGNLIAPWYGHILSGESHGRILKTD
LNSGNCTIQCQTEKGGLNTTLPFQNVSKYAFGNCPKYVGVKSLKLAIGLRNVPARSKRGLFGAIAGFIEG
GWPGLVAGWYGFQHSNDNGVGIAADKKSTQEAVDKITSKVNNIIDKMNKQYEIIDHEFSEIEARLNMINN
KIDDQIQDIWAYNAELLVLLENQKTLDDHDANVNNLYNKVKRALGSNAIEDGKGCFELYHKCDDQCMETI
RNGTYDRLKYKEESKLERQKIEGVKLESEETYKILTIYSTVASSLMLAIGIAA

>QHB50126.1 hemagglutinin, partial [Influenza A virus]

MEIIALIAILVVTGTSDADKICIGYQSTNSTETVDTLVENNVPVTHAKELLHTEHNGMLCATNLGHPLIL
DTCTVEGLVYGNPSCDSLLGGKEWSYIVERSSAVNGMCYPGRVENLEELRSFFSSARSYKKLLLFPDRTW
NVTFNGTSRACSGSFYRSMRWLTHKDNSYPIQDAQYTNDWGKNILFVWGIHHPPTDTEQMNLYKKADTTT

SITTEDINRTFKPAVGPRPLVNGQQGRIDYYWSVLKPGQTLRIRSNGNLIAPWYGHILSGESHGRILKTD
LNSGNCTIQCQTEKGGLNTTLPFQNVSKYAFGNCPKYVGVKSLKLAIGLRNVPARSKRGLFGAIAGFIEG
GWPGLVAGWYGFQHSNDNGVGIAADKKSTQEAVDKITSKVNNIIDKMNKQYEIIDHEFSEIEARLNMINN
KIDDQIQDIWAYNAELLVLLENQKTLDDHDANVNNLYNKVKRALGSNAIEDGKGCFELYHKCDDQCMETI
RNGTYDRLKYKEESKLERQKIEGVKLESEETYKILTIYSTVASSLMLAIGIAA

>QHB50125.1 hemagglutinin, partial [Influenza A virus]
MEIIALIAILVVTGTSDADKICIGYQSTNSTETVDTLVENNVPVTHAKELLHTEHNGMLCATNLGHPLIL
DTCTIEGLVYGNPSCDSLLGGREWSYIVERSSAVNGMCYPGRVENLEELRSFFSSARSYKKLLLFPDRTW
NVTFNGTSRACSGSFYRSMRWLTHKDNSYPIQDAQYTNDWGKNILFVWGIHHPPTDTEQMNLYKKADTTT
SITTEDINRTFKPAVGPRPPVNGQQGRIDYYWSVLKPGQTLRIRSNGNLIAPWYGHILSGESHGRILKTD
LNSGNCTIRCQTEKGGLNTTLPFQNVSKYAFGNCPKYVGVKSLKLAIGLRNVPARSKRGLFGAIAGFIEG
GWPGLVAGWYGFQHSNDNGVGIAADKKSTQEAVDKITSKVNNVIDKMNKQYEIIDHEFSEVEARLNMINN
KIDDQIQDIWAYNAELLVLLENQKTLDDHDANVNNLYNKVKRALGSNAIEDGKGCFELYHKCDDQCMETI
RNGTYDRLKYKEESKLERQKIEGVKLESEETYKILTIYSTVASSLMLAIGIAA

>QHD56067.1 hemagglutinin [Influenza A virus]
METISLITILLAATVSYADKICIGYQSTNSTETVDTLTENNVPVTHAKELLHTEHNGMLCATSLGQPLIL
DTCTIEGLIYGNPSCDLSLEEKEWSYIVERPSAVNGLCYPGNVENLEELRSLFSSARSFQRIQIFPDTIW
NVSYDGTSTACSGSFYRNMRWLTRKDGNYPTQDAQYTNNQGKNILFMWGINQPPSDDTQRSLYTKTDTTT
SVATEEINRIFKPLIGPRPLVNGLMGRIDYYWSVLKPGQTLRIKSDGNLIAPWYGYILSGESHGRILKTD
LKRGSCTVQCQTEKGGLNTTLPFQNVSKYAFGNCSKYIGIKSLKLAVGLRNVPSRSSRGLFGAIAGFIEG
GWSGLVAGWYGFQHSNDQGVGMAADRDSTQKAIDKITSKVNNIVDKMNKQYEIIDHEFSEVETRLNMINN
KIDDQIQDIWAYNAELLVLLENQKTLDEHDANVNNLYNKVKRALGSNAAEDGKGCFELYHKCDDQCMETI
RNGTYNRRKYQEESKLERQKIEGVKLESEGTYKILTIYSTVASSLVIAMGFAAFLFWAMSNGSCRCNICI

>QHD56055.1 hemagglutinin [Influenza A virus]
METISLMTILLVATVSNADKICIGYQSTNSTETVDTLTENNVPVTHAKELLHTEHNGMLCATSLGNPLIL
DTCTIEGLIYGNPSCDPLLGGREWSYIVERPSAVNGLCYPGSVENLEELRSLFSSARSYQRIQIFPDTIW
NVSYSGTSKACSDSFYRSMRWLTQKNNAYPTQDAQYTNNQEKNILFMWGINHPPTETAQTNLYTKTDTTT
SVATEEINRIFKPLIGPRPLVNGLMGRINYYWSVLKPGQTLRIKSDGNLIAPWYGHILSGESHGRILKTD
LKRGSCTVQCQTEKGGLNTTLPFQNVSKYAFGNCSKYIGIKSLKLAVGLRNVPSRSSRGLFGAIAGFIEG
GWSGLVAGWYGFQHSNDQGVGMAADRDSTQRAIDKITSKVNNIVDKMNKQYEIIDHEFSEVETRLNMINN
KVDDQIQDIWAYNAELLVLLENQKTLDEHDSNVNNLYNKVKRALGSNAVEDGKGCFELYHKCDNQCMETI

RNGTYNRRKYQEESKLERQKIEGVKLESEGTYKILTIYSTVASSLVIAMGFAAFLFWAMSNGSCRCNICI

>QHD56043.1 hemagglutinin [Influenza A virus]
METVSLITILLAATVSNADKICIGYQSTNSTETVDTLTENNVPVTHAKELLHTEHNGMLCATSLGQPLIL
DTCTIEGLIYGNPSCDPLPEEREWSYIVERPSAVNGLCYPGNVENLEELRSLFSSARSYQRIQIFPDTIW
NVSYDGTSNTCSGSFYRNMRWLTRKDGNYPTQDAQYTNNQGKNILFMWGINNPPTDDTQRNLYTRTDTTT
SVATEEINRIFKPLIGPRPLVNGLMGRINYYWSILKPGQTLRIKSDGNLVAPWYGYILSGESHGRILRTD
LKRGSCTVQCQTEKGGLNTTLPFQNVSKYAFGNCSKYIGIKSLKLAVGLRNVPSRSSRGLFGAIAGFIEG
GWSGLVAGWYGFQHSNDQGVGMAADRESTQKAVDKITSKVNNIVDKMNKQYEIIDHEFSEVETRLNMINN
KIDDQIQDIWAYNAELLVLLENQKTLDEHDANVNNLYNRVKRALGSNAVEDGKGCFELYHKCDDQCMETI
RNGTYNRRKYQEESKLERQKIEGVKLESEGTYKILTIYSTVASSLVIAMGFAAFLFWAMSNGSCRCNICI

>QHD56031.1 hemagglutinin [Influenza A virus]
METVSLITILLAATVSNADKICIGYQSTNSTETVDTLTENNVPVTHAKELLHTEHNGMLCATSLGQPLIL
DTCTIEGLIYGNPSCDPLPEEREWSYIVERPSAVNGLCYPGNVENLEELRSLFSSARSYQRIQIFPDTIW
NVSYDGTSNTCSGSFYRNMRWLTRKDGNYPTQDAQYTNNQGKNILFMWGINNPPTDDTQRNLYTRTDTTT
SVATEEINRIFKPLIGPRPLVNGLMGRINYYWSILKPGQTLRIKSDGNLVAPWYGYILSGESHGRILRTD
LKRGSCTVQCQTEKGGLNTTLPFQNVSKYAFGNCSKYIGIKSLKLAVGLRNVPSRSSRGLFGAIAGFIEG
GWSGLVAGWYGFQHSNDQGVGMAADRESTQKAVDKITSKVNNIVDKMNKQYEIIDHEFSEVETRLNMINN
KIDDQIQDIWAYNAELLVLLENQKTLDEHDANVNNLYNRVKRALGSNAVEDGKGCFELYHKCDDQCMETI
RNGTYNRRKYQEESKLERQKIEGVKLESEGTYKILTIYSTVASSLVIAMGFAAFLFWAMSNGSCRCNICI

>QHB21339.1 hemagglutinin, partial [Influenza A virus]
DKICIGHQSTNSTETVDTLTETGVPVTHAKELLHTEHNGKLCATNLGNPLILDTCTVEGLIYGNPSCDLL
LGGREWSYIVERPSAVNGTCYPGNVENLEELRILFSSSSSYQRIQMFPDTIWNVTYSGTSKSCSDSFYRN
MRWLTQKNGNYPVQDAQYTNTRGKDILFVWGIHHPPTDTAQTNLYTRTDTTTSITTESLDRTFKPLIGPR
PLVNGLIGRINYYWSVLKPGQTLRVRSNGNLIAPWFGHVLSGESHGRILKTDLNSGNCVVQCQTEKGGLN
STLPFHNISKYAFGDCPKYIGVKSLKLAIGLRNVPARSSRGLFGAIAGFIEGGWPGLVAGWYGFQHSNDQ
GVGMAADRDSTQKAIDKITSKVNNIVDKMNKQYEIIDHEFSEVENRLNMINNKVDDQIQDIWAYNAELLV
LLENQKTLDEHDANVNNLYNKVKRALGSNAMEDGKGCFELYHKCDDQCMETIRNGTYNRRKYVEESRLGR
QKIEGVKLESEGT

>QHB21338.1 hemagglutinin, partial [Influenza A virus]
DKICIGHQSTNSTETVDTLTETGVPVTHAKELLHAEHNGMLCATNLGNPLILDTCTVEGLIYGNPSCDML

LGGREWSYIVERPSAVNGTCYPGNVENLEELRVLFSSSSSYQRIQMFPDTIWNVTYSGTSKSCSDSFYRN
MRWLTQKNGNYPVQDAQYTNTQGKDILFVWGIHHPPTDTAQTNLYTRTDTTTSITTESLDRTFKPLIGPR
PLVNGLIGRINYYWSVLKPGQTLRVRSNGNLIAPWFGHILSGVSHGRILKTDLNSGNCVVQCQTERGGLN
STLPFHNISKYAFGDCPKYIGVKSLKLAIGLRNVPARSSRGLFGAIAGFIEGGWPGLVAGWYGFQHSNDQ
GVGMAADRDSTQKAVDKITSKVNNIVDKMNKQYEIINHEFSEVETRLNMINNKIDDQIQDIWAYNAELLV
LLENQKTLDEHDANVNNLYNKVKRALGSNAMEDGKGCFELYHKCDDQCMETIRNGTYNRRKYVEESRLGR
QKIEGVKLESEGT

>QHB21337.1 hemagglutinin, partial [Influenza A virus]
DKICIGHQSTNSTETVDTLTETGVPVTHAKELLHTEHNGKLCATNLGNPLILDTCTVEGIIYGNPSCDML
LGGRKWSYIVERPSAVNGTCYPGNVENLEELRVLFSSSSSYQRIQMFPDTIWNVTYTGTSKSCSDSFYRN
MRWLTQKNGNYPVQDAQYTNTRGKDILFVWGIHHPPTDTAQTNLYTRTDTTTSITTEKLDRTFKPLIGQR
ALVNGLMGRIYYYWSVLKPGQTLRVRSNGNLIAPWFGHILSGESHGRILKTDLNSGNCVVQCQTERGGLN
STLPFHNISKYAFGDCPKYIGVKSLKLAIGLRNVPARSSRGLFGAIAGFIEGGWPGLVAGWYGFQHSNDQ
GVGMAADRDSTQKAVDKITSKVNNIVDKMNKQYEIIDHEFNEVETRLNMINNKIDDQIQDIWAYNAELLV
LLENQKTLDEHDANVNNLYNKVKRALGSNAMEDGKGCFELYHKCDDQCMETIRNGTYNRRKYVEESRLGR
QKIEGVKLESEGT

>QHB21336.1 hemagglutinin, partial [Influenza A virus]
DKICIGHQSTNSTETVDTLTETGVPVTHAKELLHAEHNGMLCATNLGNPLILDTCTVEGLIYGNPSCDML
LGGREWSYIVERPSAVNGTCYPGNVVNLEELRVLFSSSSSYQRIQMFPDTIWNVTYSGTSKSCSDSFYRN
MRWLTQKNGNYPVQDAQYTNTQGKDILFVWGIHHPPTDTVQTNLYTRTDTTTSITTESLDRTFKPLIGPR
PLVNGLIGRINYYWSVLKPGQTLRVRSNGNLIAPWFGHILSGVSHGRILKTDLNSGNCVVQCQTERGGLN
STLPFHNISKYAFGDCPKYIGVKSLKLAIGLRNVPARSSRGLFGAIAGFIEGGWPGLVAGWYGFQHSNDQ
GVGMAADRDSTQKAVDKITSKVNNIVDKMNKQYEIINHEFSEVETRLNMINNKIDDQIQDIWAYNAELLV
LLENQKTLDEHDANVNNLYNKVKRALGSNAMEDGKGCFELYHKCDDQCMETIRNGTYNRRKYVEESRLGR
QKIEGVKLESEGT

>QHB21334.1 hemagglutinin, partial [Influenza A virus]
DKICIGHQSTNSTETVDTLTETGVPVTHAKELLHTEHNGKLCATNLGNPLILDTCTVEGLIYGNPSCDML
LGGREWSYIVERPSAVNGTCYPGNVENLEELRILFSSSSSYQRIQMFPDAIWNVTYSGTSKSCSDSFYRN
MRWLTQKNGNYPVQDAQYTNTRGKDILFVWGIHHPPTDTAQTNLYTRTDTTTSITTESLDRTFKPLIGPR
PLVNGLIGRINYYWSVLKPGQTLRVRSNGNLIAPWFGHLLSGESHGRILKTDLNSGNCVVQCQTEKGGLN
STLPFHNISKYAFGDCPKYIGVKSLKLAIGLRNVPARSSRGLFGAIAGFIEGGWPGLVAGWYGFQHSNDQ

GVGMAADRDSTQKAVDKITSKVNNIVDKMNKQYEIIDHEFSEVENRLNMINNKIDDQIQDIWAYNAELLV
LLENQKTLDEHDANVNNLYNKVKRALGSNAVEDGKGCFELYHKCDDQCMETIRNGTYNRRKYTEESRLGR
QKIEGVKLESEGT

>QHB21333.1 hemagglutinin, partial [Influenza A virus]
DKICIGHQSTNSTETVDTLTETGVPVTHAKELLHTEHNGRLCATNLGNPLILDTCTVEGIIYGNPSCDML
LGGREWSYIVERPSAVNGTCYPGNVENLEELRVLFSSSSSYQRIQMFPDTIWNVTYSGTSKSCSDSFYRN
MRWLTQKSGNYPVQDAQYTNTRGKDILFVWGIHHPPTDTAQTNLYTRTDTTTSITTESLDRTFKPLIGPR
PLVNGLIGRINYYWSVLKPGQTLRVRSNGNLIAPWFGHVLSGESHGRILKTDLNSGNCVVQCQTEKGGLN
STLPFHNISKYAFGDCPKYIGVKSLKLAIGLRNVPARSSRGLFGAIAGFIEGGWPGLVAGWYGFQHSNDQ
GVGIAADRDSTQKAVDKITSKVNNIVDKMNKQYEIIDHEFSEVETRLNMINNKIDDQIQDIWAYNAELLV
LLENQKTLDEHDANVNNLYNKVRRALGSNAMEDGKGCFELYHKCDDQCMETIRNGTYNRRKYMEESRLGR
QKIEGVKLESEGT

>QHB21332.1 hemagglutinin, partial [Influenza A virus]
DKICIGHQSTNSTETVDTLTETGVPVTHAKELLHTEHNGRLCATNLGNPLILDTCTVEGLIYGNPSCDML
LGGREWSYIVERPSAVNGTCYPGNVENLEELRILFSSSSSYQRIQMFPDTIWNVTYSGTSKSCSDSFYRN
MRWLTQKNGNYPVQDAQYTNTQGKDILFVWGIHHPPTDTAQTNLYTRTDTTTSITTESLDRTFKPLIGPR
PLVNGLIGRINYYWSVLKPGQTLRVRSNGNLIAPWFGHVLSGESHGRILKTDLNSGNCVVQCQTEKGGLN
STLPFHNISKYAFGDCPKYIGVKSLKLAIGLRNVPARSSRGLFGAIAGFIEGGWPGLVAGWYGFQHSNDQ
GVGMAADRDSTQKAVDKITSKVNNIVDKMNKQYEIIDHEFSEIENRLNMINNKVDDQIQDIWAYNAELLV
LLENQKTLDEHDANVNNLYNKVKRALGSNAMEDGKGCFELYHKCDDQCMETIRNGTYNRRKYMEESRLGR
QKIEGVKLESEGT

>QHB21331.1 hemagglutinin, partial [Influenza A virus]
DKICIGHQSTNSTETVDTLTETGVPVTHAKELLHTEHNGRLCATNLGNPLILDTCTVEGIIYGNPSCDML
LGGREWSYIVERPSAVNGTCYPGNVENLEELRVLFSSSSSYQRIQMFPDTIWNVTYSGTSKSCSDSFYRN
MRWLTQKNGNYPVQDAQYTNTQGKDILFVWGIHHPPTDTAQTNLYTRTDTTTSITTESLDRTFKPLIGPR
PLVNGLIGRINYYWSVLKPGQTLRVRSNGNLIAPWFGHILSGESHGRILKTDLNSGNCVVQCQTERGGLN
STLPFHNISKYAFGDCPKYIGVKSLKLAIGLRNVPARSSRGLFGAIAGFIEGGWPGLVAGWYGFQHSNDQ
GVGMAADRDSTQKAVDKITSKVNNIVDKMNKQYEIIDHEFSEVETRLNMINNKIDDQIQDIWAYNAELLV
LLENQKTLDEHDANVNNLYNKVKRALGSNAMEDGKGCFELYHKCDDQCMEIIRNGTYNRRKYVEESRLGR
QKIEGVKLESEGT

>QHB21329.1 hemagglutinin, partial [Influenza A virus]
DKICIGHQSTNSTETVDTLTETGVPVTHAKELLHTEHNGRLCSTNLGNPLILDTCTVEGIIYGNPSCDML
LGGREWSYIVERPSAVNGTCYPGNVENLEELRVLFSSSSSYQRIQMFPDTIWNVTYSGTSKSCSDSFYRN
MRWLTQKNGNYPVQDAQYTNTRGKDILFVWGIHHPPTDTAQTNLYTRTDTTTSITTESLDRTFKPLIGPR
PLVNGLIGRINYYWSVLKPGQTLRVRSNGNLIAPWFGHVLSGESHGRILKTDLNSGNCVVQCQTEKGGLN
STLPFHNISKYAFGDCPKYIGVKSLKLAIGLRNVPARSSRGLFGAIAGFIEGGWPGLVAGWYGFQHSNDQ
GVGMAADRDSTQKAVDKITSKVNNIVDKMNKQYEIIDHEFSEVETRLNMINNKIDDQIQDIWAYNAELLV
LLENQKTLDEHDANVNNLYNKVRRALGSNAMEDGKGCFELYHKCDNQCMETIRNGTYNRRKYMEESRLER
QKIEGVKLESEGT

>QHB21328.1 hemagglutinin, partial [Influenza A virus]
DKICIGHQSTNSTETVDTLTETGVPVTHAKELLHTEHNGKLCATNLGNPLILDTCTVEGLIYGNPSCDML
LGGREWSYIVERPSAVNGTCYPGNVENLEELRILFSSSSSYQRIQMFPDAIWNVTYSGTSKSCSDSFYRN
MRWLTQKNGNYPVQDAQYTNTRGKDILFVWGIHHPPTDTTQTNLYTRTDTTTSITTESLDRTFKPLIGPR
PLVNGLIGRINYYWSVLKPGQTLRVRSNGNLIAPWFGHLLSGESHGRILKTDLNSGNCVVQCQTEKGGLN
STLPFHNISKYAFGDCPKYIGVKSLKLAIGLRNVPARSSRGLFGAIAGFIEGGWPGLVAGWYGFQHSNDQ
GVGMAADRDSTQKAVDKITSKVNNIVDKMNKQYEIIDHEFSEVENRLNMINNKIDDQIQDIWAYNAELLV
LLENQKTLDEHDANVNNLYNKVKRALGSNAVEDGKGCFELYHKCDDQCMETIRNGTYNRRKYTEESRLGR
QKIEGVKLESEGT

>QHB21327.1 hemagglutinin, partial [Influenza A virus]
DKICIGHQSTNSTETVDTLTETGVPVTHAKELLHTEHNGRLCATNLGNPLILDTCTVEGIIYGNPSCDTL
LGGREWSYIVERPSAVNGTCYPGNVENLEELRVLFSSSSSYQRIQMFPDTIWNVTYSGTSKSCSDSFYRN
MRWLTQKNGNYPVQDAQYTNTQGKDILFVWGIHHPPTDTAQTNLYTRTDTTTSITTESLDRTFKPLIGPR
PLVNGLIGRINYYWSVLKPGQTLRVRSNGNLIAPWFGHILSGESHGRILKTDLNSGNCVVQCQTERGGLN
STLPFHNISKYAFGDCPKYIGVKSLKLAIGLRNVPARSSRGLFGAIAGFIEGGWPGLVAGWYGFQHSNDQ
GVGMAADRDSTQKAVDKITSKVNNIVDKMNKQYEIIDHEFSEVETRLNMINNKIDDQIQDIWAYNAELLV
LLENQKTLDEHDANVNNLYNKVKRALGSNAMEDGKGCFELYHKCDDQCMETIRNGTYNRRKYVEESRLGR
QKIEGVKLESEGT

>QHB21326.1 hemagglutinin, partial [Influenza A virus]
DKICIGHQSTNSTETVDTLTETGVPVTQAKELLHTEHNRKLCATNLGNPLILDTCTVEGLIYGNPSCDLL
LGGREWSYIVERPSAVNGTCYPGNVENLEELRILFSSSSSYRRIQMFPDTIWNVTYSGTSKSCSDSFYRN
MRWLTQKNGNYPVQDAQYTNTRGKDILFVWGIHHPPTDTTQTNLYTRTDTTTSITTESLDRTFKPLIGPR

PLVNGLIGRINYYWSVLKPGQTLRVRSNGNLIAPWFGHVLSGESHGRILKTDLNSGNCVVQCQTEKGGLN
STLPFHNISKYAFGDCPKYIGVKSLKLAIGLRNVPARSSRGLFGAIAGFIEGGWPGLVAGWYGFQHSNDQ
GVGMAADRDSTQKAVDKITSKVNNIVDKMNKQYEIIDHEFSEVENRLNMINNKVDDQIQDIWAYNAELLV
LLENQKTLDEHDANVNNLYNKVKRALGSNAMEDGKGCFELYHKCDDQCMETIRNGTYNRRKYMEESRLGR
QKIEGVKLESEGT

>QHB21325.1 hemagglutinin, partial [Influenza A virus]
DKICIGHQSTNSTETVDTLTETNVPVTHAKELLHTEHNGKLCATNLGNPLILDTCTIEGLIYGNPSCDML
LGGREWSYIVERPSAVNGTCYPGNVENLEELRILFSSSSSYQRIQMFPDTIWNVTYSGTSKSCSDSFYRN
MRWLTQKNGNYPVQDAQYTNTRGKDILFVWGIHHPPTDTAQTNLYTRTDTTTSITTENLDRTFKPLIGPR
PLVNGLIGRINYYWSVLKPGQTLRVRSNGNLIAPWLGHVLSGESHGRILKTDLNSGNCVVQCQTEKGGLN
STLPFHNISKYAFGDCPKYIGVKSLKLAIGLRNVPARSSRGLFGAIAGFIEGGWPGLVAGWYGFQHSNDQ
GVGMAADRDSTQKAVDKITSKVNNIVDKMNKQYEIIDHEFSEVETRLNMINNKIDDQIQDIWAYNAELLV
LLENQKTLDEHDANVNNLYNKVKRALGSNAMEDGKGCFELYHKCDDQCMETIRNGTYNRRKYMEESRLGR
QKIEGVKLESEGT

>QHB21324.1 hemagglutinin, partial [Influenza A virus]
DKICIGHQSTNSTETVDTLTETGVPVTHAKELLHTEHNGRLCATNLGNPLILDTCTVEGIIYGNPSCDML
LGGREWSYIVERPSAVNGTCYPGNVENLEELRVLFSSSSSYQRIQMFPDTIWNVTYSGTSKSCSDSFYRN
MRWLTQKNGNYPVQDAQYTNTRGKDILFVWGIHHPPTDTAQTNLYTRTDTTTSITTESLDRTFKPLIGPR
PLVNGLIGRINYYWSVLKPGQTLRVRSNGNLIAPWFGHVLSGESHGRILKTDLNSGNCVVQCQTEKGGLN
STLPFHNISKYAFGDCPKYIGVKSLKLAIGLRNVPARSSRGLFGAIAGFIEGGWPGLVAGWYGFQHSNDQ
GVGMAADRDSTQKAVDKITSKVNNIVDKMNKQYEIIDHEFSEIETRLNMINNKIDDQIQDIWAYNAELLV
LLENQKTLDEHDANVNNLYNKVKRALGSNAMEDGKGCFELYHKCDNQCMETIRNGTYNRRKYMEESRLGR
QKIEGVKLESEGT

>QHB21323.1 hemagglutinin, partial [Influenza A virus]
DKICIGHQSTNSTETVDTLTETGVPVTHAKELLHTEHNGKLCATNLGNPLILDTCTVEGLIYGNPSCDLL
LGGREWSYIVERPSAVNGTCYPGNVENLEELRILFSSSSSYQRIQMFPDAIWNVTYSGTSKSCSDSFYRN
MRWLTQKNGNYPVQDAQYTNTRGKDILFVWGIHHPPTDTAQTNLYTRTDTTTSITTESLDRTFKPLIGPR
PLVNGLIGRINYYWSVLKPGQTLRVRSNGNLIAPWFGHVLSGESHGRILKTDLNSGNCVVQCQTEKGGLN
STLPFHNISKYAFGDCPKYIGVKSLKLAIGLRNVPARSSRGLFGAIAGFIEGGWPGLVAGWYGFQHSNDQ
GVGMAADRDSTQKAVDKITSKVNNIVDKMNKQYEIIDHEFSEVENRLNMINNKVDDQIQDIWAYNAELLV
LLENQKTLDEHDANVNNLYNKVKRALGSNAMEDGKGCFKLYHKCDDQCMETIRNGTYNRRKYMEESRLGR

QKIEGVKLESEGT

>QHB21322.1 hemagglutinin, partial [Influenza A virus]
DKICIGHQSTNSTETVDTLTETGVPVTHAKELLHTEHNGKLCATNLGNPLILDTCTVEGLIYGNPSCDML
LGGREWSYIVERPSAVNGTCYPGNVENLEELRILFSSSSSYQRIQMFPDTIWNVTYSGTSKSCSDSFYRN
MRWLTQKNGNYPVQDAQYTNTRGKDILFVWGIHHPPTDTAQTNLYTRTDTTTSITTESLDRTFKPLIGPR
PLVNGLIGRINYYWSVLKPGQTLRVRSNGNLIAPWFGHVLSGESHGRILKTDLNSGNCVVQCQTEKGGLN
STLPFHNISKYAFGDCPKYIGVKSLKLAIGLRNVPARSSRGLFGAIAGFIEGGWPGLVAGWYGFQHSNDQ
GVGMAADRDSTQKAIDKITSKVNNIVDKMNKQYEIIDHEFSEVENRLNMINNKIDDQIQDIWAYNAELLV
LLENQKTLDEHDANVNNLYNKVKRALGSNAMEDGKGCFELYHKCDDQCMETIRNGTYNRRKYMEESRLGR
QKIEGVKLESEGT

>QHB21321.1 hemagglutinin, partial [Influenza A virus]
DKICIGHQSTNSTETVDTLTETGVPVTHAKELLHTEHNGKLCATNLGNPLILDTCTVEGLIYGNPSCDML
LGGREWSYIVERPSAVNGTCYPGNVENLEELRILFSSSSSYQRIQMFPDTIWNVTYSGTSKSCSDSFYRN
MRWLTQKNGNYPVQDAQYTNTRGKDILFVWGIHHPPTDTAQTNLYTRTDTTTSITTESLDRTFKPLIGPR
PLVNGLIGRINYYWSVLKSGQTLRVRSNGNLIAPWFGHVLSGESHGRILKTDLNSGNCVVQCQTEKGGLN
STLPFHNISKYAFGDCPKYIGVKSLKLAIGLRNVPARSSRGLFGAIAGFIEGGWPGLVAGWYGFQHSNDQ
GVGMAADRDSTQKAIDKITSKVNNIVDKMNKQYEIIDHEFSEVENRLNMINNKIDDQIQDIWAYNAELLV
LLENQKTLDEHDANVNNLYNKVKRALGSNAMEDGKGCFELYHKCDDQCMETIRNGTYNRRKYMEESRLGR
QKIEGVKLESEGT

>QHB21320.1 hemagglutinin, partial [Influenza A virus]
DKICIGHQSTNSTETVDTLTETGVPVTHAKELLHTEHNGKLCATNLGNPLILDTCTVEGLIYGNPSCDML
LGGREWSYIVERPSAVNGTCYPGNVENLEELRILFSSSSSYQRIQMFPDTIWNVTYSGTSKSCSDSFYRN
MRWLTQKNGNYPVQDAQYTNTQGKDILFVWGIHHPPTDTTQTNLYTRTDTTTSITTESLDRTFKPLIGPR
PLVNGLIGRINYYWSVLKPGQTLRVRSNGNLIAPWFGHVLSGESHGRILKTDLNSGNCVVQCQTEKGGLN
STLPFHNISKYAFGDCPKYIGVKSLKLAIGLRNVPARSSRGLFGAIAGFIEGGWPGLVAGWYGFQHSNDQ
GVGMAADRDSTQKAVDKITSKVNNIVDKMNKQYEIIDHEFSEVENRLNMINNKIDDQIQDIWAYNAELLV
LLENQKTLDEHDANVNNLYNKVKRALGSNAMEDGKGCFELYHKCDDQCMETIRNGTYNRRKYMEESRLGR
QKIEGVKLESEGT

>QHB21319.1 hemagglutinin, partial [Influenza A virus]
DKICIGHQSTNSTETVDTLTETGVPVTHAKELLHTEHNVRLCATNLGNPLILDTCTVEGIIYGNPSCDML

LGGREWSYIVERPSAVNGTCYPGNVENLEELRVLFSSSSSYQRIQMFPDTIWNVTYSGTSKSCSDSFYRN
MRWLTQKNGNYPVQDAQYTNTRGKDILFVWGIHHPPTDTAQTNLYTRTDTTTSITTESLDRTFKPLIGPR
PLVNGLIGRINYYWSVLKPGQTLRVRSNGNLIAPWFGHVLSGESHGRILKTDLNSGNCVVQCQTEKGGLN
STLPFHNISKYAFGDCPKYIGVKSLKLAIGLRNVPARSSRGLFGAIAGFIEGGWPGLVAGWYGFQHSNDQ
GVGMAADRDSTQKAVDKITSKVNNIVDKMNKQYEIIDHEFSEVETRLNMINNKIDDQIQDIWAYNAELLV
LLENQKTLDEHDANVNNLYNKVKRALGSNAMEDGKGCFELYHKCDDQCMETIRNGTYNRRKYMEESRLGR
QKIEGVKLESEGT

>QHB21318.1 hemagglutinin, partial [Influenza A virus]
DKICIGHQSTNSTETVDTLTETGVPVTYAKELLHTEHNGRLCATNLGDPLILDTCTVEGIIYGNPSCDML
LGGREWSYIVERPSAVNGTCYPGNVENLEELRILFSSSSSYQRIQMFPDTIWNVTYSGTSKSCSDSFYRN
MRWLTQKNGNYPVQDAQYTNTRGKDILFVWGIHHPPTDTAQTNLYTRTDTTTSITTESLDRTFKPLIGPR
PLVNGLIGRINYYWSVLKPGQTLRVRSNGNLIAPWFGHVLSGESHGRILKTDLNSGNCVVQCQTEKGGLN
STLPFHNISKYAFGDCPKYIGVKSLKLAIGLRNVPARSSRGLFGAIAGFIEGGWPGLVAGWYGFQHSNDQ
GVGMAADRDSTQKAVDKITSKVNNIVDKMNKQYEIIDHEFSEVETRLNMINNKIDDQIQDIWAYNAELLV
LLENQKTLDEHDANVNNLYNKVKRALGSNAMEDGKGCFELYHKCDDQCMETIRNGTYNRRKYMEESRLGR
QKIEGVKLESEGT

>QGH80954.1 hemagglutinin [Influenza A virus]
METVSLITILLVVTVSNADKICIGYQSTNSTETVDTLTEDNVPVTHAKELLHTEHNGMLCATSLGHPLIL
DTCTIEGLIYGNPSCDLLLGGREWSYIVERPSAVNGLCYPGNVENLEELRSLFSSASSYQRIQIFPDTIW
NVSYSGTSKACSDSFYRSMRWLTQKNNAYPIQDAQYTNNQEKNILFMWGINHPPTDTVQTNLYTRTDTTT
SVATEEINRTFKPLIGPRPLVNGLQGRIDYYWSVLKPGQTLRIRSNGNLIAPWYGHILSGESHGRILKTD
LKKGSCTVQCQTEKGGLNTTLPFQNVSKYAFGNCSKYIGIKSLKLAVGLRNVPSRSSRGLFGAIAGFIEG
GWSGLVAGWYGFQHSNDQGVGMAADRDSTQKAIDKITSKVNNIVDKMNKQYEIIDHEFSEVETRLNMINN
KIDDQIQDIWAYNAELLVLLENQKTLDEHDANVNNLYNKVKRALGSNAMEDGKGCFELYHKCDDQCMETI
RNGTYNRRKYQEESKLERQKIEGVKLESEGTYKILTIYSTVASSLVIAMGFAAFLFCAMTSGSCRCNICI

>QGH80882.1 hemagglutinin [Influenza A virus]
METVSLITILLVVTVSNADKICIGYQSTNSTETVDTLTENNVPVTHAKELLHTEHNGMLCATSLGHPLIL
DTCTIEGLIYGNPSCDLLLGGREWSYIVERPSAVNGLCYPGNVENLEELRSLFSSARSYQRIQIFPDTIW
NVSYSGTSKACSDSFYRSMRWLTQKNNAYPIQDAQYTNNQEKNILFMWGINHPPTDTVQTNLYTRTDTTT
SVATEEINRTFKPLIGPRPLVNGLMGRIDYYWSVLKPGQTLRIRSNGNLIAPWYGHILSGESHGRILKTD
LKRGSCTVQCQTEKGGLNTTLPFQNVSKYAFGNCSKYIGIKSLKLAVGLRNVPSRSSRGLFGAIAGFIEG

GWSGLVAGWYGFQHSNDQGVGMAADRDSTQKAIDKITSKVNNIVDKMNKQYEIIDHEFSEVETRLNMINN
KIDDQIQDIWAYNAELLVLLENQKTLDEHDANVNNLYNKVKRALGSNAVEDGKGCFELYHKCDDQCMETI
RNGTYNRRKYQEESKLEKQKIEGVKLESEGTYKILTIYSTVASSLVIAMGFAAFLFWAMTNGSCRCNICI

>QGH80861.1 hemagglutinin [Influenza A virus]
METVSLITILLVVTVSNADKICIGYQSTNSTETVDTLTENNVPVTHAKELLHTEHNGMLCATSLGHPLIL
DTCTIEGLIYGNPSCDLLLGGREWSYIVERPSAVNGLCYPGNVENLEELRSLFSSARSYQRIQIFPDTIW
NVSYSGTSKACSDSFYRSMRWLTQKNNAYPIQDAQYTNNQEKNILFMWGINHPPTDTAQTNLYTRTDTTT
SVATEEINRTFKPLIGPRPLVNGLQGRIDYYWSVLKPGQTLRIRSNGNLIAPWYGHILSGESHGRILKTD
LKRGSCTVQCQTEKGGLNTTLPFQNVSKYAFGNCSKYIGIKSLKLAVGLRNVPSRSSRGLFGAIAGFIEG
GWSGLVAGWYGFQHSNDQGVGMAADRDSTQKAIDKITSKVNNIVDKMNKQYEIIDHEFSEVETRLNMINN
KIDDQIQDIWAYNAELLVLLENQKTLDEHDANVNNLYNKVKRALGSNAVEDGKGCFELYHKCDDQCMETI
RNGTYNRRKYQEESKLERQKIEGVKLESEGTYKILTIYSTVASSLVIAMGFAAFLFWAMTNGSCRCNICI

>QGZ20395.1 hemagglutinin [Influenza A virus]
METVSLITILLVATVSNADKICIGYQSTNSTETVDTLTENNVPVTHAKELLHTEHNGMLCATSLGQPLIL
DTCTIEGLIYGNPSCDLSLEGREWSYIVERPSAVHGLCYPGKVEDLEELRSLFSSARSYQRIQIFPDTIW
NVSYDGTSTACSGSFYRSMRWLTRKNGDYPIQDAQYTNNQGKNILFMWGINHPPTDETQRRLYTRTDTTT
SVATEEINRIFKPLIGPRPLVNGLMGRINYYWSVLKPGQTLRIKSDGNLIAPCYGHILSGESHGRILKTD
LKKGSCTVQCQTEKGGLNTTLPFQNVSKYAFGNCSKYIGIKSLKLAVGLRNVPSRSSRGLFGAIAGFIEG
GWSGLVAGWYGFQHSNDQGVGMAADRDSTQKAIDKITSKVNNIVDKMNKQYEIIDHEFSEIETRLNMINN
KIDDQIQDIWAYNAELLVLLENQKTLDEHDANVNNLYNKVKRALGSNAVEDGKGCFELYHKCDDQCMETI
RNGTYNRRKYQEESKLERQKIEGVKLESEGTYKILTIYSTVASSLVIAMGFAAFLFWAMSNGSCRCNICI

>AYG88115.1 hemagglutinin [Influenza A virus]
METVSLITILLVATVSNADKICIGYQSTNSTETVDTLTENNVPVTHAKELLHTEHNGMLCATSLGQPIIL
DTCTIEGLIYGNPSCDLSLEGREWSYIVERPSAVNGLCYPGNVENLEELRSLFSSARSYKRIQIFPDTIW
NVSYDGTSTACSGSFYRNMRWLTRKNGDYPIQDAQYTNNQGKNILFMWGINHPPADTTQRDLYTRTDTTT
SVATEEINRIFKPLIGPRPLVNGLMGRIDYYWSVLKPGQTLRIKSDGNLIAPWYGHILSGESHGRILKTD
LKRGSCTVQCQTEKGGLNTTLPFQNVSKYAFGNCSKYIGIKSLKLAVGLRNVPSRSSRGLFGAIAGFIEG
GWSGLVAGWYGFQHSNDQGVGMAADRDSTQKAIDKITSKVNNIVDKMNKQYEIIDHEFSEVETRLNMINN
KIDDQIQDIWAYNAELLVLLENQKTLDEHDANVNNLYNKVKRALGSNAVEDGKGCFELYHKCDDQCMETI
RNGTYNRRKYQEESKLERQKIEGVKLESEGTYKILTIYSTVASSLVIAMGFAAFLFWAMSNGSCRCNICI

>AYG88114.1 hemagglutinin [Influenza A virus]

METVSLITILLVATVSNADKICIGYQSTNSTETVDTLTENNVPVTHAKELLHTEHNGMLCATSLGHPLIL
DTCTIEGLIYGNPSCDPLLGGREWSYIVERPSAINGLCYPGNVENLEELRSLFSSARSYQRIQIFPDTIW
NVSYSGTSKACSDSFYRSMRWLTQKNNAYPIQDAQYTNNQEKNILFMWGINHPPTETVQTNLYTRTDTTT
SVATEEINRIFKPLIGPRPLVNGLMGRINYYWSVLKPGQTLRIKSDGNLIAPWYGHILSGESHGRILKTD
LKRGSCTVQCQTEKGGLNTTLPFQNVSKYAFGNCSKYIGIKSLKLAVGLRNVPSRSSRGLFGAIAGFIEG
GWSGLVAGWYGFQHSNDQGVGMAADRDSTQKAIDKITSKVNNIVDKMNRQYEIIDHEFSEVETRLNMINN
KVDDQIQDIWAYNAELLVLLENQKTLDEHDSNVNNLYNKVKRALGSNAVEDGKGCFELYHKCDDQCMETI
RNGTYNRRKYQEESKLERQKIEGVKLESEGTYKILTIYSTVASSLVIAMGFAAFLFWAMSNGSCRCNICI

>AYG88113.1 hemagglutinin [Influenza A virus]

METASLITILLVATVSNADKICIGYQSTNSTETVDTLTENNVPVTHAKELLHTEHNGMLCATSLGHPLIL
DTCTIEGLIYGNPSCDPLLGGREWSYIVERPSAVNGLCYPGNVENLEELRSLFSSARSYQRIQIFPDTIW
NVSYSGTSKACSDSFYRSMRWLTQKNNAYPIQDAQYTNNQEKNILFMWGINHPPTETVQTNLYTRTDTTT
SVATEEINRVFKPLIGPRPLVNGLMGRINYYWSVLKPGQTLRIKSDGNLIAPWYGHILSGESHGRILKTD
LKRGSCTVQCQTEKGGLNTTLPFQNVSKYAFGNCSKYIGIKSLKLAVGLRNVPSRSSRGLFGAIAGFIEG
GWSGLVAGWYGFQHSNDQGVGMAADRDSTQKAIDKITSKVNNIVDKMNKQYEIIDHEFSEVETRLNMINN
KVDDQIQDIWAYNAELLVLLENQKTLDEHDSNVNNLYNKVKRALGSNAMEDGKGCFELYHKCDDQCMETI
RNGTYNRRKYQEESKLERQKIEGVKLESEGTYKILTIYSTVASSLVIAMGFAAFLFWAMSNGSCRCNICI

>AYG88112.1 hemagglutinin [Influenza A virus]

METVSLITILLVATVSNADKICIGYQSTNSTETVDTLTENNVPVTHAKELLHTEHNGMLCATSLGQPLIL
DTCTIEGLIYGNPSCDLLLEGREWSYIVERPSAVNGLCYPGNVENLEELRSLFSSARSYQRIQIFPDTIW
NVSYDGTSTACSGSFYRSMRWLTRKNGNYPIQDAQYTNNQGKNILFMWGINHPPTDTTQRDLYTRTDTTT
SVATEEINRIFKPLIGPRPLVNGLMGRIDYYWSVLKPGQTLRIKSDGNLIAPWYGHILSGESHGRILKTD
LKRGSCTVQCQTEKGGLNTTLPFQNVSKYAFGNCSKYLGIKSLKLAVGLRNVPSRSSRGLFGAIAGFIEG
GWSGLVAGWYGFQHSNDQGVGMAADRDSTQKAIDKITSKVNNIVDKMNKQYEIIDHEFSEVETRLNMINN
KIDDQIQDIWAYNAELLVLLENQKTLDEHDANVNNLYNKVKRALGSNAVEDGKGCFELYHKCDDQCMETI
RNGTYNRRKYQEESKLERQKIEGVKLESEGTYKILTIYSTVASSLVIAMGFAAFLFWAMSNGSCRCNICI

>AYG88111.1 hemagglutinin [Influenza A virus]

METVSLITILLVATVSNADKICIGYQSTNSTETVDTLTENNVPVTHAKELLHTEHNGMLCATSLGQPLVL
DTCTIEGLIYGNPSCDLSLEGREWSYIVERPSAVNGLCYPGNVENLEELRSLFSSARSYQRIQIFPDTIW
NVSYDGTSNACSGSFYRSMRWLTRKNGDYPIQDAQYTNNQGKNILFMWGINHPPTDTTQRDLYTRTDTTT

SVATEEINRIFKPLIGPRPLVNGLMGRIDYYWSVLKPGQTLRIKSDGNLIAPWYGHILSGESHGRILKTD
LKRGSCTVQCQTEKGGLNTTLPFQNVSKYAFGNCSKYIGIKSLKLAVGLRNVPSRSSRGLFGAIAGFIEG
GWSGLVAGWYGFQHSNDQGVGMAADRDSTQKAIDKITSKVNNIVDKMNKQYEIIDHEFSEVETRLNMINN
KIDDQIQDIWAYNAELLVLLENQKTLDEHDANVNNLYNKVKRALGSNAVEDGKGCFELYHKCDDQCMETI
RNGTYNRRKYQEESKLERQKIEGVKLESEGTYKILTIYSTVASSLVIAMGFAAFLFWAMSNGSCRCSICI

>AYG88110.1 hemagglutinin [Influenza A virus]
METISLITILLVVTVSNADKICIGYQSTNSTETVDTLTENNVPVTHAKELLHTEHNGMLCATGLGHPLIL
DTCTIEGLIYGNPSCDLLLGGREWSYIVERPSAVNGLCYPGNVENLEELRSLFSSASSYQRIQIFPDTIW
NVTYSGTSKACSDSFYRSMRWLTQKNNAYPIQDAQYTNNQEKNILFMWGINHPPTDTVQTNLYTRTDTTT
SVATEEINRTFKPLIGPRPLVNGLQGRIDYYWSVLKPGQTLRIRSNGNLIAPWYGHILSGGSHGRILKTD
LKRGSCTVQCQTEKGGLNTTLPFQNISKYAFGNCSKYIGIKSLKLAVGLRNVPSRSSRGLFGAIAGFIEG
GWSGLVAGWYGFQHSNDQGVGMAADRDSTQKAIDKITSKVNNIVDKMNKQYEIIDHEFSEVEARLNMINN
KIDDQIQDIWAYNAELLVLLENQKTLDEHDANVNNLYNKVKRALGSNAMEDGKGCFELYHKCDNQCMETI
RNGTYNRRKYQEESKLERQKIEGVKLESEGTYKILTIYSTVASSLVIAMGFAAFLFWAMSNGSCRCNICI

>AYG88109.1 hemagglutinin [Influenza A virus]
METVSLITILLVATVSNADKICIGYQSTNSTETVDTLTENNVPVTHAKELLHTEHNGMLCATSLGQPLIL
DTCTIEGLIYGNPSCDLSLEGREWSYIVERPSAVNGLCYPGNVENLEELRSLFSSARSYQRIQIFPDTIW
NVSYDGTSTACSGSFYRSMRWLTRKNGDYPTQDAQYTNNQGKNILFMWGINHPPTDDTQRNLYTRTDTTT
SVATEEINRIFKPLIGPRPLVNGLMGRIDYYWSVLKPGQTLRIKSDGNLIAPWYGHILSGESHGRILKTD
LKRGSCTVQCQTEKGGLNTTLPFQNVSKYAFGNCSKYIGIKSLKLAVGLRNVPSRSSRGLFGAIAGFIEG
GWSGLVAGWYGFQHSNDQGVGMAADRDSTQKAIDKITSKVNNIVDKMNKQYEIIDHEFSEVETRLNMINN
KIDDQIQDIWAYNAELLVLLENQKTLDEHDANVNNLYNKVKRALGSNAVEDGKGCFELYHKCNDQCMETI
RNGTYNRRKYQEESKIERQKIEGVKLESEGTYKILTIYSTVASSLVIAMGFAAFLFWAMSNGSCRCNICI

>AYG88108.1 hemagglutinin [Influenza A virus]
METVSLITILLVATVSNADKICIGYQSTNSTETVDTLTENNVPVTHAKELLHTEHNGMLCATSLGQPIIL
DTCTIEGLIYGNPSCDLSLEGREWSYIVERPSAVNGLCYPGNVENLEELRSLFSSARSYQRIQIFPDTIW
NVSYDGTSTACSGSFYRNMRWLTRKNGEYPIQDAQYTNNQGKNILFMWGINHPPADTTQRDLYTRTDTTT
SVATEEINRIFKPLIGPRPLVNGLMGRIDYYWSVLKPGQTLRIKSDGNLIAPWYGHILSGESHGRILKTD
LKRGSCTVQCQTEKGGLNTTLPFQNVSRYAFGNCSKYIGIKSLKLAVGLRNVPSRSSRGLFGAIAGFIEG
GWSGLVAGWYGFQHSNDQGVGMAADRESTQKAVDKITSKVNNIVDKMNKQYEIIDHEFSEVETRLNMINN
KIDDQIQDIWAYNAELLVLLENQKTLDEHDANVNNLYNKVKRALGSNAMEDGKGCFELYHKCDDQCMETI

RNGTYNRRKYQEESKLERQKIEGVKLESEGTYKILTIYSTVASSLVIAMGFAAFLFWAMSNGSCRCNICI

>AYG88107.1 hemagglutinin [Influenza A virus]
METVSLITILLVATVSNADKICIGYQSTNSTETVDTLTENNVPVTHAKELLHTEHNGMLCATSLGQPLIL
DTCTIEGLIYGNPSCDLSLEGREWSYIVERPSAVNGLCYPGNVENLEELRSLFSSARSYQRIQIFPDTIW
NVSYDGTSTACSNSFYRSMRWLTRKDGNYPTQDAQYTNNQGKNILFMWGINHPPTDDTQRNLYTRTDTTT
SVATEEINRIFKPLIGPRPLVNGLMGRIAYYWSVLKPGQTLRIKSDGNLIAPWYGHILSGESHGRILKTD
LKRGSCTVQCQTEKGGLNTTLPFQNVSKYAFGNCSKYIGIKSLKLAVGLRNVPSRSSRGLFGAIAGFIEG
GWSGLVAGWYGFQHSNDQGVGMAADRDSTQKAIDKITSKVNNIVDKMNKQYEIIDHEFSEVETRLNMINN
KIDDQIQDIWAYNAELLVLLENQKTLDEHDANVNNLYNKVKRALGSNAVEDGKGCFELYHKCNDQCMETI
RNGTYNRRKYQEESKLERQKIEGVKLESEGTYKILTIYSTVASSLVIAMGFAAFLFWAMSNGSCRCNICI

>AYG88106.1 hemagglutinin [Influenza A virus]
METVSLITILLVATISNADKICIGYQSTNSTETVDTLTENNVPVTHAKELLHTEHNGMLCATSLGQPLIL
DTCTIEGLIYGNPSCDLSLEGREWSYIVERPSAVNGLCYPGNVENLEELRSLFSSARSYQRIQIFPDTIW
NVSYDGTSTACSGSFYRNMRWLTRKNGDYPTQDAQYTNNQGKNILFMWGINHPPADDTQRTLYTRNDTTT
SVATEEINRIFKPLIGPRPLVNGLMGRIDYYWSVLKPGQTLRIKSDGNLIAPWYGHILSGESHGRILKTD
LKRGSCTVQCQTEKGGLNTTLPFQNVSKYAFGNCSKYIGIKSLKLAVGLRNVPSRSSRGLFGAIAGFIEG
GWSGLVAGWYGFQHSNDQGVGMAADRDSTQKAIDKITSKVNNIVDKMNKQYEIIDHEFSEVETRLNMINN
KIDDQIQDIWAYNAELLVLLENQKTLDEHDANVNNLYNKVKRALGSNAVEDGKGCFELYHKCDDQCMETI
RNGTYNRKKYQEESKLERQKIEGVKLESEGTYKILTIYSTVASSLVIAMGFAAFLVWAMSNGSCRCNICI

>QEI48649.1 hemagglutinin [Influenza A virus]
METVSLITILLAATVSNADKICIGYQSTNSTETVDTLTENNVPVTHAKELLHTEHNGMLCATSLGQPLIL
DTCTIEGLIYGNPSCDPLLEEREWSYIVERPSAVNGLCYPGNVENLEELRSLFSSARSYQRIQIFPDTIW
NVSYDGTSNTCSGSFYRNMRWLTRKNGNYPIQDAQYTNNQGKNILFMWGINHPPTDDTQRNLYTRTDTTT
SVATEEINRIFRPLIGPRPLVNGLMGRINYYWSVLKPGQTLRIKSDGNLIAPWYGYILSGESHGRILRTD
LKKGSCTVQCQTEKGGLNTTLPFQNVSKYAFGNCSKYIGIKSLKLAVGLRNVPSRSSRGLFGAIAGFIEG
GWSGLVAGWYGFQHSNDQGVGMAADRESTQKAIDKITSKVNNIVDKMNKQYEIIDHEFSEVETRLNMINN
KIDDQIQDIWAYNAELLVLLENHKTLDEHDANVNNLYNKVKRALGSNAVEDGKGCFELYHKCDDQCMETI
RNGTYNRRKYQEESKLERQKIEGVKLESEGTYKILTIYSTVASSLVIAMGFAAFLFWAMSNGSCRCNICI

>QEI48637.1 hemagglutinin [Influenza A virus]
METVSLMTILLAATVSNADKICIGYQSTNSTETVDTLTENNVPVTHAKELLHTEHNGMLCATSLGQPLIL

DTCTIEGLIYGNPSCDPPPEEREWSYIVERPSAVNGLCYPGNVENLEELRSLFSSARSYQRIQIFPDTIW
NVSYDGTSNTCSGSFYRNMRWLTRKNGNYPIQDAQYTNNQGKNILFMWGINNPSTDDTQRNLYTRTDTTT
SVATEEINRIFKPLIGPRPLVNGLMGRINYYWSVLKPGQTLRIKSDGNLVAPWYGYILSGESHGRILRTD
LKRGSCTVQCQTEKGGLNTTLPFQNVSKYAFGNCSKYIGIKSLKLAVGLRNVPSRSSRGLFGAIAGFIEG
GWSGLVAGWYGFQHSNDQGVGMAADRESTQKAVDKITSKVNNIVDKMNKQYEIIDHEFNEVETRLNMINN
KIDDQIQDIWAYNAELLVLLENQKTLDEHDANVNNLYNKVKRALGSNAVEDGKGCFELYHKCDDQCMETI
RNGTYNRRKYQEESKLERQKIEGVKLESEGTYKILTIYSTVASSLVIAMGFAAFLFWAMSNGSCRCNICI

>QEI48625.1 hemagglutinin [Influenza A virus]
METVSLITILLAATVSNADKICIGYQSTNSTETVDTLTENNVPVTHAKELLHTEHNGMLCATSLGQPLIL
DTCTIEGLIYGNPSCDPLLEEREWSYIVERPSAVNGLCYPGNVENLEELRSLFSSARSYQRIQIFPDTIW
NVSYDGTSNTCSGSFYRNMRWLTRKNGNYPIQDAQYTNNQGKNILFMWGINHPPTDDTQRNLYTRTDTTT
SVATEEINRIFKPLIGPRPLVNGLMGRINYYWSVLKPGQTLRIKSDGNLIAPWYGYILSGESHGRILKTD
LKRGSCTVQCQTEKGGLNTTLPFQNVSKYAFGNCSKYIGIKSLKLAVGLRNVPSRSSRGLFGAIAGFIEG
GWSGLVAGWYGFQHSNDQGVGMAADRESTQKAIDKITSKVNNIVDKMNKQYEIIDHEFSEVETRLNMINN
KIDDQIQDIWAYNAELLVLLENQKTLDEHDANVNNLYNKVKRALGSNAVEDGKGCFELYHKCDDQCMETI
RNGTYNRRKYQEESKLERQKIEGVKLESEGTYKILTIYSTVASSLVIAMGFAAFLYWAMSNGSCRCNICI

>QEI48613.1 hemagglutinin [Influenza A virus]
METVSLITILLAATVSNADKICIGYQSTNSTETVDTLTENNVPVTHAKELLHTEHNGMLCATSLGQPLIL
DTCTIEGLIYGNPSCDPLLEEREWSYIVERPSAVNGLCYPGNVENLEELRSLFSSARSYQRIQIFPDTIW
NVSYDGTSNTCSGSFYRNMRWLTRKNGNYPIQDAQYTNNQGKNILFMWGINHPPTDDTQRNLYTRTDTTT
SVATEEINRIFKPLIGPRPLVNGLMGRINYYWSVLKPGQTLRIKSDGNLIAPWYGYILSGESHGRILKTD
LKRGSCTVQCQTEKGGLNTTLPFQNVSKYAFGNCSKYIGIKSLKLAVGLRNVPSRSSRGLFGAIAGFIEG
GWSGLVAGWYGFQHSNDQGVGMAADRESTQKAIDKITSKVNNIVDKMNKQYEIIDHEFSEVETRLNMINN
KIDDQIQDIWAYNAELLVLLENQKTLDEHDANVNNLYNKVKRALGSNAVEDGKGCFELYHKCDDQCMETI
RNGTYNRRKYQEESKLERQKIEGVKLESEGTYKILTIYSTVASSLVIAMGFAAFLFWAMSNGSCRCNICI

>QEI48601.1 hemagglutinin [Influenza A virus]
METISLMTILLVTTVSNADKICIGYQSTNSTETVDTLTENNVPVTHAKELLHTEHNGMLCATSLGNPLIL
DTCTIEGLIYGNPSCDPLLGGREWSYIVERPSAVNGLCYPGSVENLEELRSLFSSARSYQRIQIFPDTIW
NVSYGGTSKACSDSFYRSMRWLTQKNNAYPIQDAQYTNNQEKNILFMWGINHPPTETAQTNLYTRTDTTT
SVATEEINRIFKPLIGPRPLVNGLMGRINYYWSVLKPGQTLRIKSDGNLIAPWYGHILSGESHGRILKTD
LKRGICTVQCQTEKGGLNTTLPFQNVSKYAFGNCSKYIGIKSLKLAVGLRNVPSRSSRGLFGAIAGFIEG

GWSGLVAGWYGFQHSNDQGVGMAADRDSTQKAIDKITSKVNNIVDKMNKQYEIIDHEFSEIETRLNMINN
KVDDQIQDIWAYNAELLVLLENQKTLDEHDSNVDNLYNKVKRALGSNAVEDGKGCFELYHKCDNQCMETI
WNGTYNRRKYQEESKLERQKIEGVKLESEGTYKILTIYSTVASSLVIAMGFAAFLFWAMSNGSCRCNICI

>QEI48436.1 hemagglutinin [Influenza A virus]
METVSLITILLVATVSNADKICIGYQSTNSTETVDTLTENNVPVTHAKELLHTEHNGMLCATSLGQPLIL
DTCTIEGLIYGNPSCDLSLEGREWSYIVERPSAVHGLCYPGNVEDLEELRSLFSSARSYQRIQIFPDTIW
NVSYDGTSTACSGSFYKSMRWLTRKNGEYPTQDAQYTNNQGKNILFMWGINHPPTDDTQRGLYTRTDTTT
SVATEEINRIFKPLIGPRPLVNGLMGRINYYWSVLKPGQTLRIKSDGNLIAPWYGHILSGESHGRILKTD
LKRGSCTVQCQTEKGGLNTTLPFQNVSKYAFGNCSKYIGVKSLKLAVGLRNVPSRSSRGLFGAIAGFIEG
GWPGLVAGWYGFQHSNDQGVGMAADRDSTQKAIDKITSKVNNIVDKMNKQYEIIDHEFSEVETRLNMINN
KIDDQIQDIWAYNAELLVLLENQKTLDEHDANVNNLYNKVKRALGSNAVEDGKGCFELYHKCDDQCMETI
RNGTYNRRKYQEESKLERQRIEGVKLESEGTYKILTIYSTVASSLVIAMGFAAFLFWAMSNGSCRCNICI

>QEI48226.1 hemagglutinin [Influenza A virus]
MEIASLITILLVATVSNADKICIGYQSTNSTETVDTLTENDVPVTHAKELLHTEHNGMLCATSLGQPLIL
DTCTIEGLIYGNPSCDLSLEGREWSYIVERPSAVNGLCYPGNVENLEELRSLFSSARSYQRVQIFPDTIW
NVSYDGTSTACSGSFYSSMRWLIRKNGDYPTQDAQYTNNQGKNILFVWGINHPPTDTTQRNLYTRTDTTT
SVATEEINRVFRPLIGPRPLVNGLMGRIDYYWSVLKPGQTLRIKSDGNLIAPWFGHILSGESHGRILKTD
LKRGSCTVQCQTEKGGLNTTLPFQNVSKYAFGNCSKYIGIKSLKLAVGLRNVPSRSSRGLFGAIAGFIEG
GWSGLVAGWYGFQHSNDQGVGMAADRDSTQKAIDKITSKVNNIVDKMNKQYEIIDHEFSEVETRLNMINN
KIDDQIQDIWAYNAELLVLLENQKTLDEHDANVNNLYNKVKRALGSNAVEDGKGCFELYHKCDDQCMETI
RNGTYNRRKYQEESKLERQKIEGVKLESEGTYKILTIYSTVASSLVIAMGFAAFLFWAMSNGSCRCNICI

>AXV48291.1 hemagglutinin [Influenza A virus]
MEAISLMTILLIMTTSNADKICIGHQSTNSTETVDTLTESNIPVTQAKELLHTEHNGMLCATNLGRPLIL
DTCTVEGLIYGNPSCDLLLGGREWSYIVERSSAVNGTCYPGSIENSEELRMLFSSASSYQRIQIFPDAIW
NVTYDGTSKSCSNSFYRNMRWLTQKNGNYPIQDAQYTNNRGKDILFIWGIHHPPTDTAQTNLYTRTDTTT
SVTTESLDRTFKPLIGPRPLVNGLIGRINYYWSVLKPGQTLRVRSNGNLIAPWYGHILSGESHGRILKTD
LKSGNCIVQCQTEKGGLNSTLPFHNISKYAFGTCPKYIGVKSLKLAIGLRNVPAKSSRGLFGAIAGFIEG
GRPGLVAGWYGFQHSNDQGVGMAADRSSTQKAVDKITSKVNNIIDKMNRQYEIIDHEFSEIETRLNMINN
KIDDQIQDVWAYNAELLVLLENQKTLDEHDANVNNLYNKVKRALGFNAMEDGKGCFELYHKCDDQCMETI
RNGTYNRRKYTEESRLERQKIEGVKLESEGTYKILTIYSTVASSLVLAMGVAAFLFWAMSNGSCRCNICI

>AXV48284.1 hemagglutinin [Influenza A virus]

MEAISLMTILLIMTTSNADKICIGHQSTNSTETVDTLTESNIPVTQAKELLHTEHNGMLCATNLGRPLIL

DTCTVEGLIYGNPSCDLLLGGREWSYIVERSSAVNGTCYPGSIENSEELRMLFSSASSYQRIQIFPDAIW

NVTYDGTSKSCSNSFYRNMRWLTQKNGNYPIQDAQYTNNRGKDILFIWGIHHPPTDTAQTNLYTRTDTTT

SVTTESLDRTFKPLIGPRPLVNGLIGRINYYWSVLKPGQTLRVRSNGNLIAPWYGHILSGESHGRILKTD

LKSGNCIVQCQTEKGGLNSTLPFHNISKYAFGTCPKYIGVKSLKLAIGLRNVPAKSSRGLFGAIAGFIEG

GRPGLVAGWYGFQHSNDQGVGMAADRSSTQKAVDKITSKVNNIIDKMNRQYEIIDHEFSEIETRLNMINN

KIDDQIQDVWAYNAELLVLLENQKTLDEHDANVNNLYNKVKRALGFNAMEDGKGCFELYHKCDDQCMETI

RNGTYNRRKYTEESRLERQKIEGVKLESEGTYKILTIYSTVASSLVLAMGVAAFLFWAMSNGSCRCNICI

>AWW21765.1 hemagglutinin [Influenza A virus]

METVSLITILLVATVSNADKICIGYQSTNSTETVDTLTENDVPVTHAKELLHTEHNGMLCATSLGQPLIL

DTCTIEGLIYGNPSCDLLLEGREWSYIVERPSAVNGLCYPGNVENLEELRSLFSSARSYQRIQIFPDTIW

NVSYNGTSKACSGSFYRSMRWLTRKNGDYPIQDAQYTNNQGKNILFMWGINHPPTDTTQRDLYTRTDTTT

SVATEEINRIFKPLIGPRPLVNGLMGRIDYYWSVLKPGQTLRIKSDGNLIAPWYGHILSGESHGRILKTD

LKRGSCTVQCQTEKGGLNTTLPFQNVSKYAFGNCSKYIGIKSLKLAVGLRNVPSRSSRGLFGAIAGFIEG

GWSGLVAGWYGFQHSNDQGVGMAADRDSTQKAIDKITSKVNNIVDKMNKQYEIIDHEFSEVETRLNMINN

KIDDQIQDIWAYNAELLVLLENQKTLDEHDANVNNLYNKVKRALGSNAVEDGKGCFELYHKCDDQCMETI

RNGTYNRRKYQEESKLKRQKIEGVKLESEGTYKILTIYSTVASSLVIAMGFAAFLFWAMSNGSCRCNICI

>AVT44463.1 hemagglutinin [Influenza A virus]

MEAISLMIILLVVTTSNADKICIGHQSTNSTETVDTLTESNIPVTQAKELLHTEHNGMLCATNLGRPLIL

DTCTVEGLVYGNPSCDLLLGGREWSYIVERPSAVNGTCYPGNVENLEELRMLFSSASSYQRIQIFPDAIW

NVTYDGTSKSCSNSFYRNMRWLTQKNGNYPIQDAQYTNNRGKDILFIWGIHHPPTDTAQTNLYTRTDTTT

SVTTENLDRTFKPLIGPRPLVNGLIGRINYYWSVLKPGQTLRVRSNGNLIAPWYGHVLSGESHGRILKTD

LKSGNCVVQCQTEKGGLNSTLPFHNISKYAFGTCPKYIGVKSLKLAIGLRNVPAKSSRGLFGAIAGFIEG

GWPGLVAGWYGFQHSNDQGVGMAADRGSTQKAVDKITSKVNNIIDKMNRQYEIIDHEFSEIETRLNMINN

KIDDQIQDVWAYNAELLVLLENQKTLDEHDANVNNLYNKVKRALGSNAMEDGKGCFELYHKCDDQCMETI

RNGTYNRRKYTEESRLERQKIEGVKLESEENYKILTIYSTVASSLVLAMGVAAFLFWAMSNGSCRCNICI

>AVT44462.1 hemagglutinin [Influenza A virus]

MEAISLMIILLVVTTSNADKICIGHQSTNSTETVDTLTESNIPVTQAKELLHTEHNGMLCATNLGRPLIL

DTCTVEGLVYGNPSCDLLLGGREWSYIVERPSAVNGTCYPGNVENLEELRMLFSSASSYQRIQIFPDAIW

NVTYDGTSKSCSNSFYRNMRWLTQKNGNYPIQDAQYTNNRGKDILFIWGIHHPPTDTAQTNLYTRTDTTT

SVTTENLDRTFKPLIGPRPLVNGLIGRINYYWSVLKPGQTLRVRSNGNLIAPWYGHVLSGESHGRILKTD
LKSGNCVVQCQTEKGGLNSTLPFHNISKYAFGTCPKYIGVKSLKLAIGLRNVPAKSSRGLFGAIAGFIEG
GWPGLVAGWYGFQHSNDQGVGMAADRSSTQKAVDKITSKVNNIIDKMNRQYEIIDHEFSEIETRLNMINN
KIDDQIQDVWAYNAELLVLLENQKTLDEHDANVNNLYNKVKRALGSNAMEDGKGCFELYHKCDDQCMETI
RNGTYNRRKYTEESRLERQKIEGVKLESEGTYKILTIYSTVASSLVLAMGVAAFLFWAMSNGSCRCNICI

>AVT44461.1 hemagglutinin [Influenza A virus]
MEAISLMIILLVVTTSNADKICIGHQSTNSTETVDTLTESNIPVTQAKELLHTEHNGMLCATNLGRPLIL
DTCTVEGLIYGNPSCDLLLGGREWSYIVERPSAVNGTCYPGNVENLEELRMLFSSASSYQRIQIFPDAIW
NVTYDGTSKSCSNSFYRNMRWLTQKNGNYPIQDAQYTNNRGKDILFIWGIHHPPTDTAQTNLYTRTDTTT
SVTTENLDRTFKPLIGPRPLVNGLIGRINYYWSVLKPGQTLRVRSNGNLIAPWYGHVLSGESHGRILKTD
LKSGNCVVQCQTEKGGLNSTLPFHNISKYAFGTCPKYIGVKSLKLAIGLRNVPAKSSRGLFGAIAGFIEG
GWPGLVAGWYGFQHSNDQGVGMAADRSSTQKAVDKITSKVNNIIDKMNRQYEIIDHEFSEIETRLNMINN
KIDDQIQDVWAYNAELLVLLENQKTLDEHDANVNNLYNKVKRALGSNAMEDGKGCFELYHKCDDQCMETI
RNGTYNRRKYAEESRLERQKIEGVKLESEGTYKILTIYSTVASSLVLAMGVAAFLFWAMSNGSCRCNICI

>AVT44460.1 hemagglutinin [Influenza A virus]
MEAISLMIILLVVTTSNADKICIGHQSTNSTETVDTLTESNIPVTQAKELLHTEHNGMLCATNLGRPLIL
DTCTVEGLVYGNPSCDLLLGGREWSYIVERPSAVNGTCYPGNVENLEELRMLFSSASSYQRIQIFPDAIW
NVTYDGTSKSCSNSFYRNMRWLTQKNGNYPIQDAQYTNNRGKDILFIWGIHHPPTDTAQTNLYTRTDTTT
SVTTENLDRTFKPLIGPRPLVNGLIGRINYYWSVLKPGQTLRVRSNGNLIAPWYGHVLSGESHGRILKTD
LKSGNCVVQCQTEKGGLNSTLPFHNISKYAFGTCPKYIGVKSLKLAIGLRNVPAKSSRGLFGAIAGFIEG
GWPGLVAGWYGFQHSNDQGVGMAADRSSTQKAVDKITSKVNNIIDKMNRQYEIIDHEFSEIETRLNMINN
KIDDQIQDVWAYNAELLVLLENQKTLDEHDANVNNLYNKVKRALGSNAMEDGKGCFELYHKCDDQCMETI
RNGTYNRRKYAEESRLERQKIEGVKLESEGTYKILTIYSTVASSLVLAMGVAAFLFWAMSNGSCRCNICI

>AVT44459.1 hemagglutinin [Influenza A virus]
MEAISLMIILLVVTTSSADKICIGHQSTNSTETVDTLTESNIPVTQAKELLHTEHNGMLCATNLGRPLLL
DTCTVEGLIYGNPSCDLLLGGREWSYIVERPSAVNGTCYPGNVENLKELRMLFSSASSYQRIQIFPDAIW
NVTYDGTSKSCSNSFYRNMRWLTQKNGNYPIQDAQYTNNRGKDILFIWGIHHPPTDTAQTNLYKRTDTTT
SVTTENLDRTFKPLIGPRPLVNGLIGRINYYWSVLKPGQTLRVRSNGNLIAPWYGHVLSGESHGRILKTD
LKSGNCVVQCQTEKGGLNSTLPFHNISKYAFGTCPKYIGVKSLKLAIGLRNVPAKSSRGLFGAIAGFIEG
GWPGLVAGWYGFQHSNDQGVGMTADRGSTQKAVDKITSKVNNIIDKMNRQYEIIDHEFSEIETRLNMINN
KIDDQIQDVWAYNAELLVLLENQKTLDEHDANVNNLYNKVKRALGSNAMEDGKGCFELYHKCDDQCMETI

RNGTYNRRKYTEESRLERLKIEGVKLESEENYKILTIYSTVASSLVLAMGAAAFLFWAMYNGSCRCNICI

>AVT44458.1 hemagglutinin [Influenza A virus]

MEAISLMIILLVVTASSADKICIGHQSTDSAESVDTLTESNIPVTQAKELLHTEHNGMLCATNLGRPLIL

DTCTVEGLIYGNPSCDLLLGGRKWSYIVERPSAVNGTCYPGNVENLEELRMLFSSASSYQRIQIFPDAIW

NVTYDGTSKSCSNSFYRNMRWLTQKNGNYPIQDAQYTNNRGKDILFIWGIHHPPTDTAQTNLYTRTDTTT

SVTTENMDRTFKPLIGPRPLVNGLIGRINYYWSVLKPGQTLRVRSNGNLIAPWYGHVLSGESHGRILKTD

LKSGNCVVQCQTEKGGLNSTLPFHNISKYAFGTCPKYIGVKSLKLAIGLRNVPAKSSRGLFGAIAGFIEG

GWPGLVAGWYGFQHSNDQGVWMTADRGSTQKAVDKITSKVNNIIDKMNRQYEIIDHEFSEIETRLNMINN

KIDDQIQDVWAYNAELLVLLENQKTLDEHDANVNNLYNKVKRALGSNAMEDGKGCFELYHKCDDQCMETI

RNGTYNRRKYTEESRLERQKIEGVKLESEENYKILTIYSTVASSLVLAMGVAAFLFWAMSNGSCRCNICI

>AVT44457.1 hemagglutinin [Influenza A virus]

MEAISLMIILLVVTTSNADKICIGHQSTNSTETVDTLTESNIPVTQAKELLHTEHNGMLCATNLGRPLIL

DTCTVEGLVYGNPSCDLLLGGREWSYIVERPSAVNGTCYPGNVENLEELRMLFSSASSYQRIQIFPDAIW

NVTYDGTSKSCSNSFYRNMRWLTQKNGNYPIQDAQYTNNRGKDILFIWGIHHPPTDTAQTNLYTRTDTTT

SVTTENLDRTFKPLIGPRPLVNGLIGRINYYWSVLKPGQTLRVRSNGNLIAPWYGHVLSGESPGRILKTD

LKSGNCVVQCQTEKGGLNSTLPFHNISKYAFGTCPKYIGVKSLKLAIGLRNVPAKSSRGLFGAIAGFIEG

GWPGLVAGWYGFQHSNDQGVGMAADRSSTQKAVDKITSKVNNIIDKMNRQYEIIDHEFSEIETRLNMINN

KIDDQIQDVWAYNAELLVLLENQKTLDEHDANVNNLYNKVKRALGSNAMEDGKGCFELYHKCDDQCMETI

RNGTYNRRKYTEESRLERQKIEGVKLESEGTYKILTIYSTVASSLVLAMGVAAFLFWAMSNGSCRCNICI

>QDY98876.1 hemagglutinin [Influenza A virus]

METVSLITILLVATVSYADKICIGYQSTNSTETVDTLTENNVPVTHAKELLHTEHNGMLCATSLGQPLIL

DTCTIEGLIYGNPSCDLSLEGREWSYIVERPSAVNGLCYPGNVENLEELRSLFSSARSYQRVQIFPDTIW

NVSYDGTSTACSGSFYRSMRWLTRKDGNYPTQDAQYTNNQGKNILFMWGINHPPTDDTQRSLYTRTDTTT

SVATEEINRIFKPLIGPRPLVNGLMGRIDYYWSVLKPGQTLRIKSDGNLISPWYGYILSGESHGRILKTD

LKRGSCTVQCQTEKGGLNTTLPFQNVSKYAFGNCSKYIGIKSLKLAVGLRNVPSRSSRGLFGAIAGFIEG

GWSGLVAGWYGFQHSNDQGVGMAADRDSTQKAIDKITSKVNNIVDKMNKQYEIIDHEFNEVETRLNMINN

KIDDQIQDIWAYNAELLVLLENQKTLDEHDANVNNLYNKVKRALGSNAAEDGKGCFELYHKCNDQCMETI

RNGTYNRRKYQEESKLERQKIEGVKLESEGTYKILTIYSTVASSLVIAMGFAAFLFWAMSNGSCRCNICI

>QDY98864.1 hemagglutinin [Influenza A virus]

METVSLITILLVATVSNADKICIGYQSTNSTETVDTLTENNVPVTHAKELLHTEHNGMLCATSLGQPLIL

DTCTIEGLIYGNPSCDLSLEGREWSYIVERPSAVHGLCYPGNVEDLEELRSLFSSARSYQRIQIFPDTIW
NVSYDGTSTACSGSFYKSMRWLTRKNGEYPTQDAQYTNNQGKNILFMWGINHPPTDDTQRGLYTRTDTTT
SVATEEINRIFKPLIGPRPLVNGLMGRINYYWSVLKPGQTLRIKSDGNLIAPWYGHILSGESHGRILKTD
LKRGSCTVQCQTEKGGLNTTLPFQNVSKYAFGNCSKYIGIKSLKLAVGLRNVPSRSSRGLFGAIAGFIEG
GWPGLVAGWYGFQHSNDQGVGMAADRDSTQKAIDKITSKVNNIVDKMNKQYEIIDHEFSEVETRLNMINN
KIDDQIQDIWAYNAELLVLLENQKTLDEHDANVNNLYNKVKRALGSNAVEDGKGCFELYHKCDDQCMETI
RNGTYNRRKYQEESKLERQRIEGVKLESEGTYKILTIYSTVASSLVIAMGFAAFLFWAMSNGSCRCNICI

>AVX19113.1 hemagglutinin [Influenza A virus]
MEAISLMIILLVVTTSNADKICIGHQSTNSTETVDTLTESNIPVTQAKELLHTEHNGMLCATNLGRPLIL
DTCTVEGLIYGNPSCDLLLGGREWSYIVERPSAVNGTCYPGSVENLEELRMLFSSASSYQRIQIFPDAIW
NVTYDGTSKSCSNSFYRNMRWLTQKSGNYPIQDAQYTNNRGKDILFIWGIHHPPTDTAQTNLYTRTDTTT
SVTTENLDRTFKPLIGPRPLVNGLIGRINYYWSVLKPGQTLRVRSNGNLIAPWYGHVLSGESHGRILKTD
LKSGNCVVQCQTEKGGLNSTLPFHNISKYAFGTCPKYIGVKSLKLAIGLRNVPAKSSRGLFGAIAGFIEG
GWPGLVAGWYGFQHSNDQGVGMAADRGSTQKAVDKITSKVNNIIDKMNRQYEIIDHEFSEIETRLNMINN
KIDDQIQDVWAYNAELLVLLENQKTLDEHDANVNNLYNKVKRALGSNAMEDGKGCFDLYHKCDDQCMETI
RNGTYNRRKYTEESRLERQKIEGVKLESEGTYKILTIYSTVASSLVLAMGVAAFLFWAMSNGSCRCNICI

>AVX19112.1 hemagglutinin [Influenza A virus]
MEAISLMITLLVVTTSNADKICIGHQSTNSTETVDTLTESNIPVTQAKELLHTEHNGMLCATNLGRPLIL
DTCTVEGLIYGNPSCDLLLGGREWSYIVERPSAVNGTCYPGNVENLEELRMLFSSASSYQRIQIFPDAIW
NVTYDGTSKSCSNSFYRNMRWLTQKNGNYPIQDAQYTNNRGNDILFIWGIHHPPTDTAQTNLYTRTDTTT
SVTTENLDRTFKPLIGPRPLVNGLIGRINYYWSVLKPGQTLRVRSNGNLIAPWYGHVLSGESHGRILKTD
LKSGNCVVQCQTEKGGLNSTLPFHNISKYAFGTCPKYIGVKSLKLAIGLRNVPAKSSRGLFGAIAGFIEG
GWPGLVAGWYGFQHSNDQGVGMAADRGSTQKAVDKITSKVNNIIDKMNRQYEIIDHEFSEIETRLNMINN
KIDDQIQDVWAYNAELLVLLENQKTLDEHDANVNNLYNKVKRALGSNAMEDGKGCFELYHKCDDQCMETI
RNGTYNRRKYTEESRLERQKIEGVKLESEGTYKILTIYSTVASSLVLAMGVAAFLFWAMSNGSCRCNICI

>AVX19111.1 hemagglutinin [Influenza A virus]
MEAISLMITLLVVTTSNADKICIGHQSTNSTETVDTLTESNIPVTQAKELLHTEHNGMLCATNLGRPLIL
DTCTVEGLIYGNPSCDLLLGGREWSYIVERPSAVNGTCYPGNVENLEELRMLFSSASSYQRIQIFPDAIW
NVTYDGTSKSCSNSFYRNMRWLTQKNGNYPIQDAQYTNNRGKDILFIWGIHHPPTDTAQTNLYTRTDTTT
SVTTENLDRTFKPLIGPRPLVNGLIGRINYYWSVLKPGQTLRVRSNGNLIAPWYGHVLSGESHGRILKTD
LKSGNCVVQCQTEKGGLNSTLPFHNISKYAFGTCPKYIGVKSLKLAIGLRNVPAKSSRGLFGAIAGFIEG

GWPGLVAGWYGFQHSNDQGVGMAADRGSTQKAVDKITSKVNNIIDKMNRQYEIIDHEFSEIETRLNMINN
KIDDQIQDVWAYNAELLVLLENQKTLDEHDANVNNLYNKVKRALGSNAMEDGKGCFELYHKCDDQCMETI
RNGTYNRRKYTEESRLERQKIEGVKLESEGTYKILTIYSTVASSLVLAMGVAAFLFWAMSNGSCRCNICI

>AVX19110.1 hemagglutinin [Influenza A virus]
MEAISLVTILLVMTTSNADKICIGHQSTNSTETVDTLTETNIPVTQAKELLHTEHNGMLCATNLGRPLIL
DTCTVEGLIYGNPSCDLLLGGREWSYIVERPSAVNGTCYPGNVENLEELRILFSSASSYQRIQIFPDAIW
NVTYDGTSKSCSNSFYRNMRWLTQKNGNYPIQDAQYTNNQGKDILFIWGIHHPPTDTAQTNLYTRTDTTT
SVTTENLDRTFKPLIGPRPLVNGLIGRINYYWSVLKPGQTLRVRSNGNLIAPWYGHVLSGESHGRILKTN
LKSGNCVVQCQTEKGGLNSTLPFHNVSKYAFGTCPKYIGVKSLKLAIGLRNVPAKSSRGLFGAIAGFIEG
GWPGLVAGWYGFQHSNDQGVGMAADRGSTQKAVDKITSKVNNIIDKMNRQYEIIDHEFSEIETRLNMINN
KIDDQIQDVWAYNAELLVLLENQKTLDEHDANVNNLYNKVKRALGSNAMEDGKGCFELYHKCDDQCMETI
RNGTYNRRKYTEESRLERQKIEGVKLESEGTYKILTIYSTVASSLVLAMGFAAFLFWAMSNGSCRCNICI

>AVX19109.1 hemagglutinin [Influenza A virus]
MEAISLMITLLVVTTSNADKICIGHQSTNSTETVDTLTESNIPVTQAKELLHTEHNGMLCATNLGRPLIL
DTCTVEGLIYGNPSCDLLLGGREWSYIVERPSAVNGTCYPGNVENLEELRMLFSSASSYQRIQIFPDAIW
NVTYDGTSKSCSNSFYRNMRWLTQKNGNYPIQDAQYTNNRGKDILFIWGIHHPPTDTAQTNLYTRTDTTT
SVTTENLDRTFKPLIGPRPLVNGLIGRINYYWSVLKPGQTLRVRSNGNLIAPWYGHVLSGESHGRILKTD
LKSGNCVVQCQTEKGGLNSTLPFHNISKYAFGTCPKYIGVKSLKLAIGLRNVPAKSSRGLFGAIAGFIEG
GWPGLVAGWYGFQHSNDQGVGMAADRGSTQKAVDKITSKVNNIIDKMNRQYEIIDHEFSEIETRLNMINN
KIDDQIQDVWAYNAELLVLLENQKTLDEHDANVNNLYNKVKRALGSNAMEDGKGCFELYHKCDDQCMETI
RNGTYNRRKYTEESRLERQKIEGVKLESEGTYKILTIYSTVASSLVFAMGVAAFLFWAMSNGSCRCNICI

>AVX19108.1 hemagglutinin [Influenza A virus]
MEAISLMIILLVVTTSNADKICIGHQSTNSTETVDTLTESNIPVTQAKELLHTEHNGMLCATNLGRPLIL
DTCTVEGLIYGNPSCDLLLGGREWSYIVERPSAVNGTCYPGSVENLEELRMLFSSASSYQRIQIFPDAIW
NVTYDGTSKSCSNSFYRNMRWLTQKSGNYPIQDAQYTNNRGKDILFIWGIHHPPTDTAQTNLYTRTDTTT
SVTTENLDRTFKPLIGPRPLVNGLIGRINYYWSVLKPGQTLRVRSNGNLIAPWYGHVLSGESHGRILKTD
LKSGNCVVQCQTEKGGLNSTLPFHNISKYAFGTCPKYIGVKSLKLAIGLRNVPAKSSRGLFGAIAGFIEG
GWPGLVAGWYGFQHSNDQGVGMAADRGSTQKAVDKITSKVNNIIDKMNRQYEIIDHEFSEIETRLNMINN
KIDDQIQDVWAYNAELLVLLENQKTLDEHDANVNNLYNKVKRALGSNAMEDGKGCFDLYHKCDDQCMETI
RNGTYNRRKYTEESRLERQKIEGVKLESEGTYKILTIYSTVASSLVLAMGVAAFLFWAMSNGSCRCNICI

>AVX19107.1 hemagglutinin [Influenza A virus]

MEAISLMITLLVVTTSNADKICIGHQSTNSTETVDTLTESNIPVTQAKELLHTEHNGMLCATNLGRPLIL

DTCTVEGLIYGNPSCDLLLGGREWSYIVERPSAVNGTCYPGNVENLEELRMLFSSASSYQRIQIFPDAIW

NVTYDGTSKSCSNSFYRNMRWLTQKNGNYPIQDAQYTNNRGNDILFIWGIHHPPTDTAQTNLYTRTDTTT

SVTTENLDRTFKPLIGPRPLVNGLIGRINYYWSVLKPGQTLRVRSNGNLIAPWYGHVLSGESHGRILKTD

LKSGNCVVQCQTEKGGLNSTLPFHNISKYAFGTCPKYIGVKSLKLAIGLRNVPAKSSRGLFGAIAGFIEG

GWPGLVAGWYGFQHSNDQGVGMAADRGSTQKAVDKITSKVNNIIDKMNRQYEIIDHEFSEIETRLNMINN

KIDDQIQDVWAYNAELLVLLENQKTLDEHDANVNNLYNKVKRALGSNAMEDGKGCFELYHKCDDQCMETI

RNGTYNRRKYTEESRLERQKIEGIKLESEGTYKILTIYSTVASSLVLAMGVAAFLFWAMSNGSCRCNICI

>AVX19106.1 hemagglutinin [Influenza A virus]

MEAISLMIILLVVTTSNADKICIGHQSTNSTETVDTLTESNIPVTQAKELLHTEHNGMLCATNLGRPLIL

DTCTVEGLIYGNPSCDLLLGGREWSYIVERPSAVNGTCYPGNVENLEELRMLFSSASSYQRIQIFPDAIW

NVTYDGTSKSCSNSFYRNMRWLTQKNGNYPIQDAQYTNNRGKDILFIWGIHHPPTDTAQTNLYTRTDTTT

SVTTENLDRTFKPLIGPRPLVNGLIGRINYYWSVLKPGQTLRVRSNGNLIAPWYGHVLSGESHGRILKTD

LKIGNCVVQCQTEKGGLNSTLPFHNISKYAFGTCPKYIGVKSLKLAIGLRNVPAKSNRGLFGAIAGFIEG

GWPGLIAGWYGFQHSNDQGVGMAADRGSTQKAVDKITSKVNNIIDKMNRQYEIIDHEFSEIETRLNMINN

KIDDQIQDVWAYNAELLVLLENQKTLDEHDANVNNLYNKVKRALGSNAMEDGKGCFELYHKCDDQCMETI

RNGTYNRRKYTEESRLERQKIEGVKLESEGTYKILTIYSTVASSLVLAMGVAAFLFWAMSNGSCRCNICI

>AVX19105.1 hemagglutinin [Influenza A virus]

MEAISLMIILLVVTTSNADKICIGHQSTNSTETVDTLTESNIPVTQAKELLHTEHNGMLCATNLGRPLIL

DTCTVEGLIYGNPSCDLLLGGREWSYIVERPSAVNGTCYPGNVENLEELRMLFSSASSYQRIQIFPDAIW

NVTYDGTSKSCSNSFYRNMRWLTQKNGNYPIQDAQYTNNRGKDILFIWGIHHPPTDTAQTNLYTRTDTTT

SVTTEDLDRTFKPLIGPRPLVNGLIGRINYYWSVLKPGQTLRVRSNGNLIAPWYGHVLSGESHGRILKTD

LKSGNCVVQCQTEKGGLNSTLPFHNISKYAFGTCPKYIGVKSLKLAIGLRNVPAKSSRGLFGAIAGFIEG

GWPGLVAGWYGFQHSNDQGVGMAADRGSTQKAVDKITSKVNNIIDKMNRQYEIIDHEFSEIETRLNMINN

KIDDQIQDVWAYNAELLVLLENQKTLDEHDANVNNLYNKVKRALGSNAMEDGKGCFELYHKCDDQCMETI

RNGTYNRRKYTEESRLERQKIEGVKLESEGTYKILTIYSTVASSLVLAMGVAAFLFWAMSNGSCRCNICI

>AVX19104.1 hemagglutinin [Influenza A virus]

MEAISLMIILLVVTKSSADKICIGHQSTNSTETVDTLTESNIPVTQAKELLHTEHNGMLCATNLGRPLIL

DTCTVEGLIYGNPSCDLLLGGREWSYIVERPSAVNGTCYPGNVENLEELRMLFSSASSYQRIQIFSDAIW

NVTYDGTSKSCSNSFYRNMRWLTQKNGNYPIQDAQYTNNRGKDILFIWGIHHPPTDTAQTNLYTRTDTTT

SVTTENLDRTFKPLIGPRPLVNGLIGRINYYWSVLKPGQTLRVRSNGNLIAPWYGHVLSGESHGRILKTD
LKSGNCVVQCQTEKGGLNSTLPFHNISKYAFGTCPKYVGVKSLKLAIGLRNVPAKSSRGLFGAIAGFIEG
GWPGLVAGWYGFQHSNDQGVGMAADRGSTQKAVDKITSKVNNIIDKMNRQYEIIDHEFNEIETRLNMINN
KIDDQIQDVWAYNAELLVLLENQKTLDEHDANVNNLYNKVKRALGSNAMEDGKGCFELYHKCDDQCMETI
RNGTYNRRKYTEESRLERQKIEGVKLESEGTYKILTIYSTVASSLVLAMGVAAFLFWAMSNGSCRCNICI

>AVX19103.1 hemagglutinin [Influenza A virus]
MEAISLMIILLVVTTSSADKICIGHQSTNSTETVDTLTESNIPVTQAKELLHTEHNGMLCATNLGRPLIL
DTCTVEGLIYGNPSCDLLLGGREWSYIVERPSAVNGTCYPGNVENLEELRMLFSSASSYQRIQIFPDAIW
NVTYDGTSKSCSNSFYRNMRWLTQKNGNYPIQDAQYTNNRGKDILFIWGIHHPPTDTAQTNLYTRTDTTT
SVTTENLDRTFKPLIGPRPLVNGLIGRINYYWSVLKPGQTLRVRSNGNLIAPWYGHVLSGESHGRILKTD
LKSGNCVVQCQTEKGGLNSTLPFHNISKYAFGTCPKYVGVKSLKLAIGLRNVPAKSSRGLFGAIAGFIEG
GWPGLVAGWYGFQHSNDQGVGMAADRGSTQKAVDKITSKVNNIIDKMNRQYEIIDHEFNEIETRLNMINN
KIDDQIQDVWAYNAELLVLLENQKTLDEHDANVNNLYNKVKRALGSNAMEDGKGCFELYHKCDDQCMETI
RNGTYNRRKYTEEARLERQKIEGVKLESEGTYKILTIYSTVASSLVLAMGVAAFLFWAMSNGSCRCNICI

>AVX19102.1 hemagglutinin [Influenza A virus]
MEAISLMITLLVVTTSNADKICIGHQSTNSTETVDTLTESNIPVTQAKELLHTEHNGMLCATNLGRPLIL
DTCTVEGLIYGNPSCDLLLGGREWSYIVERPSAVNGTCYPGNVENLEELRMLFSSASSYQRIQIFPDAIW
NVTYDGTSKSCSNSFYRNMRWLTQKNGNYPIQDAQYTNNRGNDILFIWGIHHPPTDTAQTNLYTRTDTTT
SVTTENLDRTFKPLIGPRPLVNGLIGRINYYWSVLKPGQTLRVRSNGNLIAPWYGHVLSGESHGRILKTD
LKSGNCVVQCQTEKGGLNSTLPFHNISKYAFGTCPKYIGVKSLKLAIGLRNVPAKSSRGLFGAIAGFIEG
GWPGLVAGWYGFQHSNDQGVGMAADRGSTQKAVDKITSKVNNIIDKMNRQYEIIDHEFSEIETRLNMINN
KIDDQIQDVWAYNAELLVLLENQKTLDEHDANVNNLYNKVKRALGSNAMEDGKGCFELYHKCDDQCMETI
RNGTYNRRKYTEESRLERQKIEGIKLESEGTYKILTIYSTVASSLVLAMGVAAFLFWAMSNGSCRCNICI

>AVX19100.1 hemagglutinin [Influenza A virus]
MEAISLMIILLVVTTSNADKICIGHQSTNSTETVDTLTESNIPVTQAKELLHTEHNGMLCATNLGRPLIL
DTCTVEGLIYGNPSCDLLLGGREWSYIVERPSAVNGTCYPGNVENLEELRMLFSSASSYQRIQLFPDAIW
NVTYDGTSKSCSNSFYRNMRWLTQKNGNYPIQDAQYTNNRGKDILFIWGIHHPPTDTAQTNLYTRTDTTT
SVTTENLDRTFKPLIGPRPLVNGLIGRINYYWSVLKPGQTLRVRSNGNLIAPWYGHVLSGESHGRILKTD
LKSGNCVVQCQTEKGGLNSTLPFHNISKYAFGTCPKYIGVKSLKLAIGMRNVPAKSSRGLFGAIAGFIEG
GWPGLVAGWYGFQHSNDQGVGMAADRGSTQKAVDKITSKVNNIIDKMNRQYEIIDHEFSEIETRLNMINN
KIDDQIQDVWAYNAELLVLLENQKTLDEHDANVNNLYNKVKRALGSNAMEDGKGCFELYHKCDDQCMETI

RNGTYNRRKYTEESRLERQKIEGVKLESEGTYKILTIYSTVASSLVLAMGVAAFLFWAMSNGSCRCNICI

>AVX19099.1 hemagglutinin [Influenza A virus]
MEAISLMIILLVVTTSNADKICIGHQSTNSTETVDTLTESNIPVTQAKELLHTEHNGMLCATNLGRPLIL
DTCTVEGLIYGNPSCDLLLGGREWSYIVERPSAVNGTCYPGNVENLEELRMLFSSASSYQRIQIFPDAIW
NVTYDGTSKSCSNSFYRNMRWLTQKNGNYPIQDAQYTNNRGKDILFIWGIHHPPTDTAQTNLYTRTDTTT
SVTTENLDRTFKPLIGPRPLVNGLIGRINYYWSVLKPGQTLRVRSNGNLIAPWYGHVLSGESHGRILKTD
LKSGNCVVQCQTEKGGLNSTLPFHNISKYAFGTCPKYIGVKSLKLAIGLRNVPAKSNRGLFGAIAGFIEG
GWPGLVAGWYGFQHSNDQGVGMAADRGSTQKAVDKITSKVNNIIDKMNRQYEIIDHEFSEIETRLNMINN
KIDDQIQDVWAYNAELLVLLENQKTLDEHDANVNNLYNKVKRALGSNAMEDGKGCFELYHKCDDQCMETI
RNGTYNRRKYTEESRLERQKIEGVKLESEGTYKILTIYSTVASSLVLAMGVAAFLFWAMSNGSCRCNICI

>AVX19098.1 hemagglutinin [Influenza A virus]
MEAISLMIILLVVTTSNADKICIGHQSTNSTETVDTLTESNIPVTQAKELLHTEHNGMLCATNLGRPLIL
DTCTVEGLIYGNPSCDLLLGGREWSYIVERPSAVNGTCYPGNVENLEELRMLFSSASSYQRIQIFPDAIW
NVTYDGTSKSCSNSFYRNMRWLTQKNGNYPIQDAQYTNNREKDILFIWGIHHPPTDTAQTNLYTRTDTTT
SVTTENLDRTFKPLIGPRPLVNGLIGRINYYWSVLKPGQTLRVRSNGNLIAPWYGHVLSGESHGRILKTD
LKSGNCVVQCQTEKGGLNSTLPFHNISKYAFGTCPKYIGVKSLKLAIGLRNVPAKSSRGLFGAIAGFIEG
GWPGLVAGWYGFQHSNDQGVGMAADRSSTQKAVDKITSKVNNIIDKMNRQYEIIDHEFSEIETRLNMINN
KIDDQIQDVWAYNAELLVLLENQKTLDEHDANVNNLYNKVKRALGSNAMEDGKGCFELYHKCDDQCMETI
RNGTYNRRKYTEESRLERQKIEGVKLESEGTYKILTIYSTVASSLVLAMGVAAFLFWAMSNGSCRCNICI

>AVX19097.1 hemagglutinin [Influenza A virus]
MEAISLMIILLVVTTSNADKICIGHQSTNSTETVDTLTESNIPVTQAKELLHTEHNGMLCATNLGRPLIL
DTCTVEGLIYGNPSCDLLLGGREWSYIVERPSAVNGTCYPGNVENLEELRMLFSSASSYQRIQIFPDAIW
NVTYDGTSKSCSNSFYRNMRWLTQKNGNYPIQDAQYTNNRGKDILFIWGIHHPPTDTAQTNLYTRTDTTT
SVTTENLDRTFKPLIGPRPLVNGLIGRINYYWSVLKPGQTLRVRSNGNLIAPWYGHVLSGESHGRILKTD
LKSGNCVVQCQTEKGGLNSTLPFHNISKYAFGTCPKYIGVKSLKLAIGLRNVPAKSNRGLFGAIAGFIEG
GWPGLVAGWYGFQHSNDQGVGMAADRGSTQKAVDKITSKVNNIIDKMNRQYEIIDHEFSEIETRLNMINN
KIDDQIQDVWAYNAELLVLLENQKTLDEHDANVNNLYNKVKRALGSNAMEDGKGCFELYHKCDDQCMETI
RNGTYNRRKYTEESRLERQKIEGVKLESEGTYKILTIYSTVASSLVLAMGVAAFLFWAMSNGSCRCNICI

>AVX19096.1 hemagglutinin [Influenza A virus]
MEAISLMIILLVVTTSNADKICIGHQSTNSTETVDTLTESNIPVTQAKELLHTEHNGMLCATNLGRPLIL

DTCTVEGLIYGNPSCDLLLGGREWSYIVERPSAVNGTCYPGNVENLEELRMLFSSASSYQRIQIFPDAIW
NVTYDGTSKSCSNSFYRNMRWLTQKNGNYPIQDAQYTNNRGKDILFIWGIHHPPTDTAQTNLYTRTDTTT
SVTTENLDRTFKPLIGPRPLVNGLIGRINYYWSVLKPGQTLRVRSNGNLIAPWYGHVLSGESHGRILKTD
LKSGNCVVQCQTEKGGLNSTLPFHNISKYAFGTCPKYIGVKSLKLAIGLRNVPAKSNRGLFGAIAGFIEG
GWPGLVAGWYGFQHSNDQGVGMAADRGSTQKAVDKITSKVNNIIDKMNRQYEIIDHEFSEIETRLNMINN
KIDDQIQDVWAYNAELLVLLENQKTLDEHDANVNNLYNKVKRALGSNAMEDGKGCFELYHKCDDQCMETI
RNGTYNRRKYTEESRLERQKIEGVKLESEGTYKILTIYSTVASSLVLVMGVAAFLFWAMSNGSCRCNICI

>AVX19095.1 hemagglutinin [Influenza A virus]
MEAISLMIILLVVTTSNADKICIGHQSTNSTETVDTLTESNIPVTQAKELLHTEHNGMLCATNLGRPLIL
DTCTVEGLIYGNPSCDLLLGGREWSYIVERPSAVNGTCYPGNVENLEELRMLFSSASSYQRIQIFPDAIW
NVTYDGTSKSCSNSFYRNMRWLTQKNGNYPIQDAQYTNNRGKDILFIWGIHHPPTDTAQTNLYTRTDTTT
SVTTENLDRTFKPLIGPRPLVNGLIGRINYYWSVLKPGQTLRVRSNGNLIAPWYGHVLSGESHGRILKTD
LKSGNCVVQCQTEKGGLNSTLPFHNISKYAFGTCPKYIGVKSLKLAIGLRNVPAKSNRGLFGAIAGFIEG
GWPGLVAGWYGFQHSNDQGVGMAADRGSTQKAVDKITSKVNNIIDKMNRQYEIIDHEFSEIETRLNMINN
KIDDQIQDVWAYNAELLVLLENQKTLDEHDANVNNLYNKVKRALGSNAMEDGKGCFELYHKCDDQCMETI
RNGTYNRRKYTEESRLERQKIEGVKLESEGTYKILTIYSTVASSLVLVMGVAAFLFWAMSNGSCRCNICI

>AVX19094.1 hemagglutinin [Influenza A virus]
MEAISLMIILLVVTTSNADKICIGHQSTNSTETVDTLTESNIPVTQAKELLHTEHNGMLCATNLGRPLIL
DTCTVEGLIYGNPSCDLLLGGREWSYIVERPSAVNGTCYPGNVENLEELRMLFSSASSYQRIQIFPDAIW
NVTYDGTSKSCSNSFYRNMRWLTQKNGNYPIQDAQYTNNRGKDILFIWGIHHPPTDTAQTNLYTRTDTTT
SVTTENLDRTFKPLIGPRPLVNGLIGRINYYWSVLKPGQTLRVRSNGNLIAPWYGHVLSGESHGRILKTD
LKSGNCVVQCQTEKGGLNSTLPFHNISKYAFGTCPKYIGVKSLKLAIGLRNVPAKSNRGLFGAIAGFIEG
GWPGLVAGWYGFQHSNDQGVGMAADRGSTQKAVDKITSKVNNIIDKMNRQYEIIDHEFSEIETRLNMINN
KIDDQIQDVWAYNAELLVLLENQKTLDEHDANVNNLYNKVKRALGSNAMEDGKGCFELYHKCDDQCMETI
RNGTYNRRKYTEESRLERQKIEGVKLESEGTYKILTIYSTVASSLVLAMGVAAFLFWAMSNGSCRCNICI

>AVX19093.1 hemagglutinin [Influenza A virus]
MEAISLMIILLVVTTSNADKICIGHQSTNSTETVDTLTESNIPVTQAKELLHTEHNGMLCATNLGRPLIL
DTCTVEGLIYGNPSCDLLLGGREWSYIVERPSAVNGTCYPGNVENLEELRMLFSSASSYQRIQIFPDAIW
NVTYDGTSKSCSNSFYRNMRWLTQKNGNYPIQDAQYTNNRGKDILFIWGIHHPPTDTAQTNLYTRTDTTT
SVTTENLDRTFKPLIGPRPLVNGLIGRINYYWSVLKPGQTLRVRSNGNLIAPWYGHVLSGESHGRILKTD
LKSGNCVVQCQTEKGGLNSTLPFHNISKYAFGTCPKYIGVKSLKLAIGLRNVPAKSNRGLFGAIAGFIEG

GWPGLVAGWYGFQHSNDQGVGMAADRGSTQKAVDKITSKVNNIIDKMNRQYEIIDHEFSEIETRLNMINN
KIDDQIQDVWAYNAELLVLLENQKTLDEHDANVNNLYNKVKRALGSNAMEDGKGCFELYHKCDDQCMETI
RNGTYNRRKYTEESRLERQKIEGVKLESEGTYKILTIYSTVASSLVLAMGVAAFLFWAMSNGSCRCNICI

>AVX19092.1 hemagglutinin [Influenza A virus]
MEAISLMITLLVVTTSNADKICIGHQSTNSTETVDTLTESNIPVTQAKELLHTEHNGMLCATNLGRPLIL
DTCTVEGLIYGNPSCDLLLGGREWSYIVERPSAVNGTCYPGNVENLEELRMLFSSASSYQRIQIFPDAIW
NVTYDGTSKSCSNSFYRNMRWLTQKNGNYPIQDAQYTNNRGNDILFIWGIHHPPTDTTQTNLYTRTDTTT
SVTTENLDRTFKPLIGPRPLVNGLIGRINYYWSVLKPGQTLRVRSNGNLIAPWYGHVLSGESHGRILKTD
LKSGNCVVQCQTEKGGLNSTLPFHNISKYAFGTCPKYIGVKSLKLAIGLRNVPAKSSRGLFGAIAGFIEG
GWPGLVAGWYGFQHSNDQGVGMAADRGSTQKAVDKITSKVNNIIDKMNRQYEIIDHEFSEIETRLNMINN
KIDDQIQDVWAYNAELLVLLENQKTLDEHDANVNNLYNKVKRALGSNAMEDGKGCFELYHKCDDQCMETI
RNGTYNRRKYTEESRLERQKIEGVKLESEGTYKILTIYSTVASSLVLAMGVAAFLFWAMSNGSCRCNICI

>AVX19091.1 hemagglutinin [Influenza A virus]
MEAISLMIILLVVTTSNADKICIGHQSTNSTETVDTLTESNIPVTQAKELLHTEHNGMLCATNLGRPLIL
DTCTVEGLIYGNPSCDLLLGGREWSYIVERPSAVNGTCYPGNVENLEELRMLFSSASSYQRIQIFPDAIW
NVTYDGTSKSCSNSFYRNMRWLTQKNGNYPIQDAQYTNNRGKDILFIWGIHHPPTDTTQTNLYTRTDTTT
SVTTENLDRTFKPLIGPRPLVNGLIGRINYYWSVLKPGQTLRVRSNGNLIAPWYGHVLSGESHGRILKTD
LKSGNCVVQCQTEKGGLNSTLPFHNISKYAFGTCPKYIGVKSLKLAIGLRNVPAKSNRGLFGAIAGFIEG
GWPGLVAGWYGFQHSNDQGVGMAADRGSTQKAVDKITSKVNNIIDKMNRQYEIIDHEFSEIETRLNMINN
KIDDQIQDVWAYNAELLVLLENQKTLDEHDANVNNLYNKVKRALGSNAMEDGKGCFELYHKCDDQCMETI
RNGTYNRRKYTEESRLERQKIEGVKLESEGTYKILTIYSTVASSLVLAMGVAAFLFWAMSNGSCRCNICI

>AVX19090.1 hemagglutinin [Influenza A virus]
MEAISLMIILLVVTTSNADKICIGHQSTNSTETVDTLTESNIPVTQAKELLHTEHNGMLCATNLGRPLIL
DTCTVEGLIYGNPSCDLLLGGREWSYIVERPSAVNGTCYPGNVENLEELRMLFSSASSYQRIQIFPDAIW
NVTYDGTSKSCSNSFYRNMRWLTQKNGNYPIQDAQYTNNRGKDILFIWGIHHPPTDTAQTNLYTRTDTTT
SVTTENLDRTFKPLIGPRPLVNGLIGRINYYWSVLKPGQTLRVRSNGNLIAPWYGHVLSGESHGRILKTD
LKSGNCVVQCQTEKGGLNSTLPFHNISKYAFGTCPKYIGVKSLKLAIGLRNVPAKSSRGLFGAIAGFIEG
GWPGLVAGWYGFQHSNDQGVGMAADRSSTQKAVDKITSKVNNIIDKMNRQYEIIDHEFSEIETRLNMINN
KIDDQIQDVWAYNAELLVLLENQKTLDEHDANVNNLYNKVKRALGSNAMEDGKGCFELYHKCDDQCMETI
RNGTYNRRKYTEESRLERQKIEGVKLESEGTYKILTIYSTVASSLVLAMGVAAFLLWAMSNGSCRCNICI

>AVX19089.1 hemagglutinin [Influenza A virus]

MEAISLMITLLVVTTSNADKICIGHQSTNSTETVDTLTESNIPVTQAKELLHTEHNGMLCATNLGRPLIL
DTCTVEGLIYGNPSCDLLLGGREWSYIVERPSAVNGTCYPGNVENLEELRMLFSSASSYQRIQIFPDAIW
NVTYDGTSKSCSNSFYRNMRWLTQKNGNYPIQDAQYTNNRGNDILFIWGIHHPPTDTAQTNLYTRTDTTT
SVTTENLDRTFKPLIGPRPLVNGLIGRINYYWSVLKPGQTLRVRSNGNLIAPWYGHVLSGESHGRILKTD
LKSGNCVVQCQTEKGGLNSTLPFHNISKYAFGTCPKYIGVKSLKLAIGLRNVPAKSSRGLFGAIAGFIEG
GWPGLVAGWYGFQHSNDQGVGMAADRGSTQKAVDKITSKVNNIIDKMNRQYEIIDHEFSEIETRLNMINN
KIDDQIQDVWAYNAELLVLLENQKTLDEHDANVNNLYNKVKRALGSNAMEDGKGCFELYHKCDDQCMETI
RNGTYNRRKYTEESRLERQKIEGVKLESEGTYKILTIYSTVASSLVLAMGVAAFLFWAMSNGSCRCNICI

>AVX19088.1 hemagglutinin [Influenza A virus]

MEAISLMIILLVVTTSNADKICIGHQSTNSTETVDTLTESNIPVTQAKELLHTEHNGMLCATNLGRPLIL
DTCTVEGLIYGNPSCDLLLGGREWSYIVERPSAVNGTCYPGSVENLEELRMLFSSASSYQRIQIFPDAIW
NVTYDGTSKSCSNSFYRNMRWLTQKSGNYPIQDAQYTNNRGKDILFIWGIHHPPTDTAQTNLYTRTDTTT
SVTTENLDRTFKPLIGPRPLVNGLIGRINYYWSVLKPGQTLRVRSNGNLIAPWYGHVLSGESHGRILKTD
LKSGNCVVQCQTEKGGLNSTLPFHNISKYAFGTCPKYIGVKSLKLAIGLRNVPAKSSRGLFGAIAGFIEG
GWPGLVAGWYGFQHSNDQGVGMAADRGSTQKAVDKITSKVNNIIDKMNRQYEIIDHEFSEIETRLNMINN
KIDDQIQDVWAYNAELLVLLENQKTLDEHDANVNNLYNKVKRALGSNAMEDGKGCFDLYHKCDDQCMETI
RNGTYNRRKYTEESRLERQKIEGVKLESEGTYKILTIYSTVASSLVLAMGVAAFLFWAMSNGSCRCNICI

>AVX19087.1 hemagglutinin [Influenza A virus]

MEAISLMITLLVVTTSNADKICIGHQSTNSTETVDTLTESNIPVTQAKELLHTEHNGMLCATNLGRPLIL
DTCTVEGLIYGNPSCDLLLGGREWSYIVERPSAVNGTCYPGNVENLEELRMLFSSASSYQRIQIFPDAIW
NVTYDGTSKSCSNSFYRNMRWLTQKNGNYPIQDAQYTNNRGKDILFIWGIHHPPTDTAQTNLYTRTDTTT
SVTTENLDRTFKPLIGPRPLVNGLIGRINYYWSVLKPGQTLRVRSNGNLIAPWYGHVLSGESHGRILKTD
LKSGNCVVQCQTEKGGLNSTLPFHNISKYAFGTCPKYIGVKSLKLAIGLRNVPAKSSRGLFGAIAGFIEG
GWPGLVAGWYGFQHSNDQGVGMAADRSSTQKAVDKITSKVNNIIDKMNRQYEIIDHEFSEIETRLNMINN
KIDDQIQDVWAYNAELLVLLENQKTLDEHDANVNNLYNKVKRALGSNAMEDGKGCFELYHKCDDQCMETI
RNGTYNRRKYTEESRLERQKIEGVKLESEGTYKILTIYSTVASSLVLAMGVAAFLFWAMSNGSCRCNICI

>AVX19086.1 hemagglutinin [Influenza A virus]

MEAISLMITLLVVTTSNADKICIGHQSTNSTETVDTLTESNIPVTQAKELLHTEHNGMLCATNLGRPLIL
DTCTVEGLIYGNPSCDLLLGGREWSYIVERPSAVNGTCYPGNVENLEELRMLFSSASSYQRIQIFPDAIW
NVTYDGTSKSCSNSFYRNMRWLTQKNGNYPIQDAQYTNNRGKDILFIWGIHHPPTDTAQTNLYTRTDTTT

SVTTENLDRTFKPLIGPRPLVNGLIGRINYYWSVLKPGQTLRVRSNGNLIAPWYGHVLSGESHGRILKTD

LKSGNCVVQCQTEKGGLNSTLPFHNISKYAFGTCPKYIGVKSLKLAIGLRNVPAKSSRGLFGAIAGFIEG

GWPGLVAGWYGFQHSNDQGVGMAADRGSTQKAVDKITSKVNNIIDKMNRQYEIIDHEFSEIETRLNMINN

KIDDQIQDVWAYNAELLVLLENQKTLDEHDANVNNLYNKVKRALGSNAMEDGKGCFELYHKCDDQCMETI

RNGTYNRRKYTEESRLERQKIEGVKLESEGTYKILTIYSTVASSLVLAMGVAAFLFWAMSNGSCRCNICI

>AVX19085.1 hemagglutinin [Influenza A virus]

MEAISLMIILLVVTTSNADKICIGHQSTNSTETVDTLTESNIPVTQAKELLHTEHNGMLCATNLGRPPIL

DTCTVEGLIYGNPSCDLLLGGREWSYIVERPSAVNGTCYPGNVENLEELRMLFSSASSYQRIQIFPDAIW

NVTYDGTSKSCSNSFYRNMRWLTQKNGNYPIQDAQYTNNRGKDILFIWGIHHPPTDTAQTNLYTRTDTTT

SVTTENLDRTFKPLIGPRPLVNGLIGRINYYWSVLKPGQTLRVRSNGNLIAPWYGHVLSGESHGRILKTD

LKSGNCVVQCQTEKGGLNSTLPFHNISKYAFGTCPKYIGVKSLKLAIGLRNVPAKSSRGLFGAIAGFIEG

GWPGLVAGWYGFQHSNDQGVGMAADRGSTQKAVDKITSKVNNIIDKMNRQYEIIDHEFSEVETRLNMINN

KIDDQIQDVWAYNAELLVLLENQKTLDEHDANVNNLYNKVKRALGSNAMEDGKGCFELYHKCDDQCMETI

RNGTYNRRKYTEESRLERQKIEGVKLESEGTYKILTIYSTVASSLVLAMGVAAFLFWAMSNGSCRCNICI

>AVX19084.1 hemagglutinin [Influenza A virus]

MEAISLMIILLVVTTSNADKICIGHQSTNSTETVDTLTESNIPVTQAKELLHTEHNGMLCATNLGRPLIL

DTCTVEGLIYGNPSCDLLLGGREWSYIVERPSAVNGTCYPGNVENLEELRMLFSSASSYQRIQIFPDAIW

NVTYDGTSKSCSNSFYRNMRWLTQKNGNYPIQDAQYTNNRGKDILFIWGIHHPPTDTAQTNLYTRTDTTT

SVTTENLDRTFKPLIGPRPLVNGLIGRINYYWSVLKPGQTLRVRSNGNLIAPWYGHVLSGESHGRILKTD

LKSGNCVVQCQTEKGGLNSTLPFHNISKYAFGTCPKYIGVKSLKLAIGLRNVPAKSSRGLFGAIAGFIEG

GWPGLVAGWYGFQHSNDQGVGMAADRGSTQKAVDKITSKVNNIIDKMNRQYEIIDHEFSEIETRLNMINN

KIDDQIQDVWAYNAELLVLLENQKTLDEHDANVNNLYNKVKRALGSNAMEDGKGCFELYHKCDDQCMETI

RNGTYNRRKYTEESRLERQKIEGVKLESEGTYKILTIYSTVASSLVLAMGVAAFLFWAMSNGSCRCNICI

>AVX19083.1 hemagglutinin [Influenza A virus]

MEAISLMIILLVVTTSNADKICIGHQSTNSTETVDTLTESNIPVTQAKELLHTEHNGMLCATNLGRPLIL

DTCTVEGLIYGNPSCDLLLGGREWSYIVERPSAVNGTCYPGNVENLEELRMLFSSASSYQRIQIFPDAIW

NVTYDGTSKSCSNSFYRNMRWLTQKNGNYPIQDAQYTNNREKDILFIWGIHHPPTDTAQTNLYTRTDTTT

SVTTENLDRTFKPLIGPRPLVNGLIGRINYYWSVLKPGQTLRVRSNGNLIAPWYGHVLSGESHGRILKTD

LKSGNCVVQCQTEKGGLNSTLPFHNISKYAFGTCPKYIGVKSLKLAIGLRNVPAKSSRGLFGAIAGFIEG

GWPGLVAGWYGFQHSNDQGVGMAADRSSTQKAVDKITSKVNNIIDKMNRQYEIIDHEFSEIETRLNMINN

KIDDQIQDVWAYNAELLVLLENQKTLDEHDANVNNLYNKVKRALGSNAMEDGKGCFELYHKCDDQCMETI

RNGTYNRRKYTEESRLERQKIEGVKLESEGTYKILTIYSTVASSLVLAMGVAAFLFWAMSNGSCRCNICI

>AVX19082.1 hemagglutinin [Influenza A virus]
MEAISLMIILLVVTTSNADKICIGHQSTNSTETVDTLTESNIPVTQAKELLHTEHNGMLCATNLGRPLIL
DTCTVEGLIYGNPSCDLLLGGREWSYIVERPSAVNGTCYPGNVENLEELRMLFSSASSYQRIQIFPDAIW
NVTYDGTSKSCLNSFYRNMRWLTQKNGNYPIQDAQYTNNRGKDILFIWGIHHPPTDTAQTNLYTRTDTTT
SVTTENLDRTFKPLIGPRPLVNGLIGRINYYWSVLKPGQTLRVRSNGNLIAPWYGHVLSGESHGRILKTD
LKSGNCVVQCQTEKGGLNSTLPFHNISKYAFGTCPKYIGVKSLKLAIGLRNVPAKSSRGLFGAIAGFIEG
GWPGLVAGWYGFQHSNDQGVGMAADRSSTQKAVDKITSKVNNIIDKMNRQYEIIDHEFSEIETRLNMINN
KIDDQIQDVWAYNAELLVLLENQKTLDEHDANVNNLYNKVKRALGSNAMEDGKGCFELYHKCDDQCMETI
RNGTYNRRKYTEESRLERQKIEGVKLESEGTYKILTIYSTVASSLVLAMGVAAFLFWAMSNGSCRCNICI

>AVX19081.1 hemagglutinin [Influenza A virus]
MEAISLMIILLVVTTSNADKICIGHQSTNSTETVDTLTESNIPVTQAKELLHTEHNGMLCATNLGRPLIL
DTCTVEGLIYGNPSCDLLLGGREWSYIVERPSAVNGTCYPGNVENLEELRMLFSSASSYQRIQIFPDAIW
NVTYDGTSKSCSNSFYRNMRWLTQKNGNYPIQDAQYTNNRGKDILFIWGIHHPPTDTAQTNLYTRTDTTT
SVTTENLDRTFKPLIGPRPLVNGLIGRINYYWSVLKPGQTLRVRSNGNLIAPWYGHVLSGESHGRILKTD
LKSGNCVVQCQTEKGGLNSTLPFHNISKYAFGTCPKYIGIKSLKLAIGLRNVPAKSSRGLFGAIAGFIEG
GWPGLVAGWYGFQHSNDQGVGMAADRSSTQKAVDKITSKVNNIIDKMNRQYEIIDHEFSEIETRLNMINN
KIDDQIQDVWAYNAELLVLLENQKTLDEHDANVNNLYNRVKRALGSNAMEDGKGCFELYHKCDDQCMETI
RNGTYNRRKYTEESRLERQKIEGVKLESEGTYKILTIYSTVASSLVLAMGVAAFLFWAMSNGSCRCNICI

>AVX19080.1 hemagglutinin [Influenza A virus]
MEAISLMITLLVVTTSNADKICIGHQSTNSTETVDTLTESNIPVTQAKELLHTEHNGMLCATNLGRPLIL
DTCTVEGLIYGNPSCDLLLGGREWSYIVERPSAVNGTCYPGNVENLEELRMLFSSASSYQRIQIFPDAIW
NVTYDGTSKSCSNSFYRNMRWLTQKNGNYPIQDAQYTNNRGKDILFIWGIHHPPTDTAQTNLYTRTDTTT
SVTTENLDRTFKPLIGPRPLVNGLIGRINYYWSVLKPGQTLRVRSNGNLIAPWYGHVLSGESHGRILKTD
LKSGNCVVQCQTEKGGLNSTLPFHNISKYAFGTCPKYIGVKSLKLAIGLRNVPAKSSRGLFGAIAGFIEG
GWPGLVAGWYGFQHSNDQGVGMAADRGSTQKAVDKITSKVNNIIDKMNRQYEIIDHEFSEIETRLNMINN
KIDDQIQDVWAYNAELLVLLENQKTLDEHDANVNNLYNKVKRALGSNAMEDGKGCFELYHKCDDQCMETI
RNGTYNRRKYTEESRLERQKIEGVKLESEGTYKILTIYSTVASSLVLAMGVAAFLFWAMSNGSCRCNICI

>AVX19079.1 hemagglutinin [Influenza A virus]
MEAISLMIILLVVTTSNADKICIGHQSTNSTETVDTLTESNIPVTQAKELLHTEHNGMLCATNLGRPLIL

DTCTVEGLIYGNPSCDLLLGGREWSYIVERPSAVNGTCYPGNVENLEELRMLFSSASSYQRIQIFPDAIW
NVTYDGTSKSCSNSFYRNMRWLTQKNGNYPIQDVQYTNNRGKDILFIWGIHHPPTDTAQTNLYTRTDTTT
SVTTENLDRTFKPLIGPRPLVNGLIGRINYYWSVLKPGQTLRVRSNGNLIAPWYGHVLSGESHGRILKTD
LKSGNCVVQCQTEKGGLNSTLPFHNISKYAFGTCPKYIGVKSLKLAIGLRNVPAKSSRGLFGAIAGFIEG
GWPGLVAGWYGFQHSNDQGVGMAADRGSTQKAVDKITSKVNSIIDKMNRQYEIIDHEFSEIETRLNMINN
KIDDQIQDVWAYNAELLVLLENQKTLDEHDANVNNLYNKVKRALGSNAMEDGKGCFELYHKCDDQCMETI
RNGTYNRRKYTEESRLKRQKIEGVKLEPEGTYKILTIYSTVASSLVLAMGVAAFLFWAMSNGSCRCNICI

>AVX19078.1 hemagglutinin [Influenza A virus]
MEAISLMIILLVVTTSNADKICIGHQSTNSTETVDTLTESNIPVTQAKELLHTEHNGMLCATNLGRPLIL
DTCTVEGLIYGNPSCDLLLGGREWSYIVERPSAVNGTCYPGNVENLEELRMLFSSASSYQRIQIFPDAIW
NVTYDGTSKSCSNSFYRNMRWLTQKNGNYPIQDAQYTNNRGKDILFIWGIHHPPTDTAQTNLYTRTDTTT
SVTTENLDRTFKPLIGPRPLVNGLIGRINYYWSVLKPGQTLRVRSNGNLIAPWYGHVLSGESHGRILKTD
LKSGNCVVQCQTEKGGLNSTLPFHNISKYAFGTCPKYIGVKSLKLAIGLRNVPAKSSRGLFGAIAGFIEG
GWPGLVAGWYGFQHSNDQGVGMAADRSSTQKAVDKITSKVNNIIDKMNRQYEIIDHEFSEIETRLNMINN
KIDDQIQDVWAYNAELLVLLENQKTLDEHDANVNNLYNKVKRALGSNAMEDGKGCFELYHKCDDQCMETI
RNGTYNRRKYTEESRLERQKIEGVKLESEGTYKILTIYSTVASSLVLAMGVAAFLFWAMSNGSCRCNICI

>AVX19077.1 hemagglutinin [Influenza A virus]
MEAISLMITLLVVTTSNADKICIGHQSTNSTETVDTLTESNIPVTQAKELLHTEHNGMLCATNLGRPLIL
DTCTVEGLIYGNPSCDLLLGGREWSYIVERPSAVNGTCYPGNVENLEELRMLFSSASSYQRIQIFPDAIW
NVTYDGTSKSCSNSFYRNMRWLTQKNGNYPIQDAQYTNNRGKDILFIWGIHHPPTDTAQTNLYTRTDTTT
SVTTENLDRTFKPLIGPRPLVNGLIGRINYYWSVLKPGQTLRVRSNGNLIAPWYGHVLSGESHGRILKTD
LKSGNCVVQCQTEKGGLNSTLPFHNISKYAFGTCPKYIGVKSLKLAIGLRNVPAKSSRGLFGAIAGFIEG
GWPGLVAGWYGFQHSNDQGVGMAADRSSTQKAVDKITSKVNNIIDKMNRQYEIIDHEFSEIETRLNMINN
KIDDQIQDVWAYNAELLVLLENQKTLDEHDANVNNLYNKVKRALGSNAMEDGKGCFELYHKCDDQCMETI
RNGTYNRRKYTEESRLERQKIEGVKLESEGTYKILTIYSTVASSLVLAMGVAAFLFWAMSNGSCRCNICI

>AVX19076.1 hemagglutinin [Influenza A virus]
MEAISLMTILLVMTTSNADKICIGHQSTNSTETVDTLTETNIPVTQAKELLHTEHNGMLCATNLGRPLIL
DTCTVEGLIYGNPSCDLLLGGREWSYIVERPSAVNGTCYPGNVENLEELRILFSSASSYQRIQIFPDAIW
NVTYDGTSKSCSNSFYRNMRWLTQKNGNYPIQDAQYTNNQGKDILFIWGIHHPPTDTAQTNLYTRTDTTT
SVTTENLDRTFKPLIGPRPLVNGLIGRINYYWSVLKPGQTLRVRSNGNLIAPWYGHVLSGESHGRILKTN
LKSGNCVVQCQTEKGGLNSTLPFHNVSKYAFGTCPKYIGVKSLKLAIGLRNVPAKSSRGLFGAIAGFIEG

GWPGLVAGWYGFQHSNDQGVGMAADRGSTQKAVDKITSKVNNIIDKMNRQYEIIDHEFSEIETRLNMINN
KIDDQIQDVWAYNAELLVLLENQKTLDEHDANVNNLYNKVKRALGSNAMEDGKGCFELYHKCDDQCMETI
RNGTYNRRKYTEESRLERQKIEGVKLESEGTYKILTIYSTVASSLVLAMGFAAFLFWAMSNGSCRCNICI

>AVX19075.1 hemagglutinin [Influenza A virus]
MEAISLMIILLVVTTSNADKICIGHQSTNSTETVDTLTESNIPVTQAKELLHTEHNGMLCATNLGRPLIL
DTCTVEGLIYGNPSCDLLLGGREWSYIVERPSAVNGTCYPGNVENLEELRMLFSSASSYQRIQIFPDAIW
NVTYDGTSKSCSNSFYRNMRWLTQKNGNYPIQDAQYTNNRGKDILFIWGIHHPPTDTAQTNLYTRTDTTT
SVTTENLDRTFKPLIGPRPLVNGLIGRINYYWSVLKPGQTLRVRSNGNLIAPWYGHVLSGESHGRILKTD
LKSGNCVVQCQTEKGGLNSTLPFHNISKYAFGTCPKYIGVKSLKLAIGLRNVPAKSSRGLFGAIAGFIEG
GWPGLVAGWYGFQHSNDQGVGMAADRGSTQKAVDKITSKVNNIIDKMNRQYEIIDHEFSEIETRLNMINN
KIDDQIQDVWAYNAELLVLLENQKTLDEHDANVNNLYNKVKRALGSNAMEDGKGCFELYHKCDDQCMETI
RNGTYNRRKYTEESRLERQKIEGVKLESEGTYKILTIYSTVASSLVLAMGVAAFLFWAMSNGSCRCNICI

>AVX19074.1 hemagglutinin [Influenza A virus]
MEAISLMITLLVVTTSNADKICIGHQSTNSTETVDTLTESNIPVTQAKELLHTEHNGMLCATNLGRPLIL
DTCTVEGLIYGNPSCDLLLGGREWSYIVERPSAVNGTCYPGNVENLEELRMLFSSASSYQRIQIFPDAIW
NVTYDGTSKSCSNSFYRNMRWLTQKNGNYPIQDAQYTNNRGKDILFIWGIHHPPTDTAQTNLYTRTDTTT
SVTTENLDRTFKPLIGPRPLVNGLIGRINYYWSVLKPGQTLRVRSNGNLIAPWYGHVLSGESHGRILKTD
LKSGNCVVQCQTEKGGLNSTLPFHNISKYAFGTCPKYIGVKSLKLAIGLRNVPAKSSRGLFGAIAGFIEG
GWPGLVAGWYGFQHSNDQGVGMAADRGSTQKAVDKITSKVNNIIDKMNRQYEIIDHEFSEIETRLNMINN
KIDDQIQDVWAYNAELLVLLENQKTLDEHDANVNNLYNKVKRALGSNAMEDGKGCFELYHKCDDQCMETI
RNGTYNRRKYTEESRLERQKIEGVKLESEGTYKILTIYSTVASSLVLAMGVAAFLFWAMSNGSCRCNICI

>AVX19073.1 hemagglutinin [Influenza A virus]
MEAISLMITLLVVTTSNADKICIGHQSTNSTETVDTLTESNIPVTQAKELLHTEHNGMLCATNLGRPLIL
DTCTVEGLIYGNPSCDLLLGGREWSYIVERPSAVNGTCYPGNVENLEELRMLFSSASSYQRIQIFPDAIW
NVTYDGTSKSCSNSFYRNMRWLTQKNGNYPIQDAQYTNNRGKDILFIWGIHHPPTDTAQTNLYTRTDTTT
SVTTENLDRTFKPLIGPRPLVNGLIGRINYYWSVLKPGQTLRVRSNGNLIAPWYGHVLSGESHGRILKTD
LKSGNCVVQCQTEKGGLNSTLPFHNISKYAFGTCPKYIGVKSLKLAIGLRNVPAKSSRGLFGAIAGFIEG
GWPGLVAGWYGFQHSNDQGVGMAADRGSTQKAVDKITSKVNNIIDKMNRQYEIIDHEFSEIETRLNMINN
KIDDQIQDVWAYNAELLVLLENQKTLDEHDANVNNLYNKVKRALGSNAMEDGKGCFELYHKCDDQCMETI
RNGTYNRRKYTEESRLERQKIEGVKLESEGTYKILTIYSTVASSLVLAMGVAAFLFWAMSNGSCRCNICI

>AVX19072.1 hemagglutinin [Influenza A virus]

MEAISLMIILLVVTTSNADKICIGHQSTNSTETVDTLTESNIPVTQAKELLHTEHNGMLCATNLGRPLIL

DTCTVEGLIYGNPSCDLLLGGREWSYIVERPSAVNGTCYPGNVENLEELRMLFSSASSYQRIQIFPDAIW

NVTYDGTSKSCSNSFYRNMRWLTQKNGNYPIQDAQYTNNRGKDILFIWGIHHPPTDTAQTNLYTRTDTTT

SVTTENLDRTFKPLIGPRPLVNGLIGRINYYWSVLKPGQTLRVRSNGNLIAPWYGHVLSGESHGRILKTD

LKSGNCVVQCQTEKGGLNSTLPFHNISKYAFGTCPKYIGVKSLKLAIGLRNVPAKSSRGLFGAIAGFIEG

GWPGLVAGWYGFQHSNDQGVGMAADRGSTQKAVDKITSKVNNIIDKMNRQYEIIDHEFSEIETRLNMINN

KVDDQIQDVWAYNAELLVLLENQKTLDEHDANVNNLYNKVKRALGSNAMEDGKGCFELYHKCDDQCMETI

RNGTYNRRKYTEESRLERQKIEGVKLESEGTYKILTIYSTVASSLVLAMGVAAFLFWAMSNGSCRCNICI

>AVX19071.1 hemagglutinin [Influenza A virus]

MEAISLMIILLVVTTSNADKICIGHQSTNSTETVDTLTESNIPVTQAKELLHTEHNGMLCATNLGRPLIL

DTCTVEGLIYGNPSCDLLLGGREWSYIVERPSAVNGTCYPGNVENLEELRMLFSSASSYQRIQIFPDAIW

NVTYDGTSKSCSNSFYRNMRWLTQKNGNYPIQDAQYTNNRGKDILFIWGIHHPPTDTTQTNLYTRTDTTT

SVTTENLDRTFKPLIGPRPLVNGLIGRINYYWSVLKPGQTLRVRSNGNLIAPWYGHVLSGESHGRILKTD

LKSGNCVVQCQTEKGGLNSTLPFHNISKYAFGTCPKYIGVKSLKLAIGLRNVPAKSSRGLFGAIAGFIEG

GWPGLVAGWYGFQHSNDQGVGMAADRSSTQKAVDKITSKVNNIIDKMNRQYEIIDHEFSEIETRLNMINN

KIDDQIQDVWAYNAELLVLLENQKTLDEHDANVNNLYNKVKRALGSNAMEDGKGCFELYHKCDDQCMETI

RNGTYNRRKYTEESRLERQKIEGVKLESEGTYKILTIYSTVASSLVLAMGVAAFLFWAMSNGSCRCNICI

>AVX19070.1 hemagglutinin [Influenza A virus]

MEAISLMIILLVVTTSSADKICIGHQSTNSTETVDTLTESNIPVTQAKELLHTEHNGMLCATNLGRPLIL

DTCTVEGLIYGNPSCDLLLGGREWSYIVERPSAVNGTCYPGNVENLEELRMLFSSASSYQRIQIFPDAIW

NVTYDGTSKSCSNSFYRNMRWLTQKNGNYPIQDAQYTNNRGKDILFIWGIHHPPTDTAQTNLYTRTDTTT

SVTTENLDRTFKPLIGPRPLVNGLIGRINYYWSVLKPGQTLRVRSNGNLIAPWYGHVLSGESHGRILKTD

LKSGNCVVQCQTEKGGLNSTLPFHNISKYAFGTCPKYVGVKSLKLAIGLRNVPAKSSRGLFGAIAGFIEG

GWPGLVAGWYGFQHSNDQGVGMAADRGSTQKAVDKITSKVNNIIDKMNRQYEIIDHEFNEIETRLNMINN

KIDDQIQDVWAYNAELLVLLENQKTLDEHDANVNNLYNKVKRALGSNAMEDGKGCFELYHKCDDQCMETI

RNGTYNRRKYTEESRLERQKIEGVKLESEGTYKILTIYSTVASSLVLAMGVAAFLFWAMSNGSCRCNICI

>AVX19069.1 hemagglutinin [Influenza A virus]

MEAISLMIILLVVTTSNADKICIGHQSTNSTETVDTLTESNIPVTQAKELLHTEHNGMLCATNLGRPLIL

DTCTVEGLIYGNPSCDLLLGGREWSYIVERPSAVNGTCYPGNVENLEELRMLFSSASSYQRIQIFPDAIW

NVTYDGTSKSCSNSFYRNMRWLTQKNGNYPIQDAQYTNNRGKDILFIWGIHHPPTDTAQTNLYTRTDTTT

SVTTENLDRTFKPLIGPRPLVNGLIGRINYYWSVLKPGQTLRVRSNGNLIAPWYGHVLSGESHGRILKTD
LKSGNCVVQCQTEKGGLNSTLPFHNISKYAFGTCPKYIGVKSLKLAIGLRNVPAKSSRGLFGAIAGFIEG
GWPGLVAGWYGFQHSNDQGVGMAADRSSTQKAVDKITSKVNNIIDKMNRQYEIIDHEFSEIETRLNMINN
KIDDQIQDVWAYNAELLVLLENQKTLDEHDANVNNLYNKVKRALGSNAMEDGKGCFELYHKCDDQCMETI
RNGTYNRRKYTEESRLERQKIEGVKLESEGTYKILTIYSTVASSLVLAMGVAAFLFWAMSNGSCRCNICI

>AVX19068.1 hemagglutinin [Influenza A virus]
MEAISLMIILLVVTTSNADKICIGHQSTNSTETVDTLTESNIPVTQAKELLHTEHNGMLCATNLGRPLIL
DTCTVEGLIYGNPSCDLLLGGREWSYIVERPSAVNGTCYPGNVENLEELRMLFSSASSYQRIQIFPDAIW
NVTYDGTSKSCSNSFYRNMRWLTQKNGNYPIQDAQYTNNRGKDILFIWGIHHPPTDTAQTNLYTRTDTTT
SVTTEDLDRTFKPLIGPRPLVNGLIGRINYYWSVLKPGQTLRVRSNGNLIAPWYGHVLSGESHGRILKTD
LKSGNCVVQCQTEKGGLNSTLPFHNISKYAFGTCPKYIGVKSLKLAIGLRNVPAKSSRGLFGAIAGFIEG
GWPGLVAGWYGFQHSNDQGVGMAADRGSTQKAVDKITSKVNNIIDKMNRQYEIIDHEFSEIETRLNMINN
KIDDQIQDVWAYNAELLVLLENQKTLDEHDANVNNLYNKVKRALGSNAMEDGKGCFELYHKCDDQCMETI
RNGTYNRRKYTEESRLERQKIEGVKLESEGTYKILTIYSTVASSLVLAMGVAAFLFWAMSNGSCRCNICI

>AVX19067.1 hemagglutinin [Influenza A virus]
MEAISLMIILLVVTTSNADKICIGHQSTNSTETVDTLTESNIPVTQAKELLHTEHNGMLCATNLGRPLIL
DTCTVEGLIYGNPSCDLLLGGREWSYIVERPSAVNGTCYPGNVENLEELRMLFSSASSYQRIQIFPDAIW
NVTYDGTSKSCSNSFYRNMRWLTQKNGNYPIQDAQYTNNRGKDILFIWGIHHPPTDTAQTNLYTRTDTTT
SVTTEDLDRTFKPLIGPRPLVNGLIGRINYYWSVLKPGQTLRVRSNGNLIAPWYGHVLSGESHGRILKTD
LKSGNCVVQCQTEKGGLNSTLPFHNISKYAFGTCPKYIGVKSLKLAIGLRNVPAKSSRGLFGAIAGFIEG
GWPGLVAGWYGFQHSNDQGVGMAADRGSTQKAVDKITSKVNNIIDKMNRQYEIIDHEFSEIETRLNMINN
KIDDQIQDVWAYNAELLVLLENQKTLDEHDANVNNLYNKVKRALGSNAMEDGKGCFELYHKCDDQCMETI
RNGTYNRRKYTEESRLERQKIEGVKLESEGTYKILTIYSTVASSLVLAMGVAAFLFWAMSNGSCRCNICI

>AVX19066.1 hemagglutinin [Influenza A virus]
MEAISLMIILLVVTTSNADKICIGHQSTNSTETVDTLTESNIPVTQAKELLHTEHNGMLCATNLGRPLIL
DTCTVEGLIYGNPSCDLLLGGREWSYIVERPSAVNGTCYPGNVENLEELRMLFSSASSYQRIQIFPDAIW
NVTYDGTSKSCSNSFYRNMRWLTQKNGNYPIQDAQYTNNRGKDILFIWGIHHPPTDTAQTNLYTRTDTTT
SVTTENLDRTFKPLIGPRPLVNGLIGRINYYWSVLKPGQTLRVRSNGNLIAPWYGHVLSGESHGRILKTD
LKSGNCVVQCQTEKGGLNSTLPFHNISKYAFGTCPKYIGVKSLKLAIGLRNVPAKSNRGLFGAIAGFIEG
GWPGLVAGWYGFQHSNDQGVGMAADRGSTQKAVDKITSKVNNIIDKMNRQYEIIDHEFSEIETRLNMINN
KIDDQIQDVWAYNAELLVLLENQKTLDEHDANVNNLYNKVKRALGSNAMEDGKGCFELYHKCDDQCMETI

RNGTYNRRKYTEESRLERQKIEGVKLESEGTYKILTIYSTVASSLVLVMGVAAFLFWAMSNGSCRCNICI

>AVX19065.1 hemagglutinin [Influenza A virus]
MEAISLMIILLVVTTSNADKICIGHQSTNSTETVDTLTESNIPVTQAKELLHTEHNGMLCATNLGRPLIL
DTCTVEGLIYGNPSCDLLLGGREWSYIVERPSAVNGTCYPGNVENLEELRMLFSSASSYQRIQIFPDAIW
NVTYDGTSKSCSNSFYRNMRWLTQKNGNYPIQDAQYTNNRGKDILFIWGIHHPPTDTAQTNLYTRTDTTT
SVTTENLDRTFKPLIGPRPLVNGLIGRINYYWSVLKPGQTLRVRSNGNLIAPWYGHVLSGESHGRILKTD
LKSGNCVVQCQTEKGGLNSTLPFHNISKYAFGTCPKYIGVKSLKLAIGLRNVPAKSSRGLFGAIAGFIEG
GWPGLVAGWYGFQHSNDQGVGMAADRSSTQKAVDKITSKVNNIIDKMNRQYEIIDHEFSEIETRLNMINN
KIDDQIQDVWAYNAELLVLLENQKTLDEHDANVNNLYNKVKRALGSNAMEDGKGCFELYHKCDDQCMETI
RNGTYNRRKYTEESRLERQKIEGVKLESEGTYKILTIYSTVASSLVLAMGVAAFLFWAMSNGSCRCNICI

>AVX19064.1 hemagglutinin [Influenza A virus]
MEAISLMIILLVVTTSNADKICIGHQSTNSTETVDTLTESNIPVTQAKELLHTEHNGMLCATNLGRPLIL
DTCTVEGLIYGNPSCDLLLGGREWSYIVERPSAVNGTCYPGNVENLEELRMLFSSASSYQRIQIFPDAIW
NVTYDGTSKSCSNSFYRNMRWLTQKNGNYPIQDAQYTNNRGKDILFIWGIHHPPTDTAQTNLYTRTDTTT
SVTTENLDRTFKPLIGPRPLVNGLIGRINYYWSVLKPGQTLRVRSNGNLIAPWYGHVLSGESHGRILKTD
LKSGNCVVQCQTEKGGLNSTLPFHNISKYAFGTCPKYIGVKSLKLAIGLRNVPAKSSRGLFGAIAGFIEG
GWPGLVAGWYGFQHSNDQGVGMAADRGSTQKAVDKITSKVNNIIDKMNRQYEIIDHEFSEIETRLNMINN
KIDDQIQDVWAYNAELLVLLENQKTLDEHDANVNNLYNKVKRALGSNAMEDGKGCFELYHKCDDQCMETI
RNGTYNRRKYTEESRLERQKIEGVKLESEGTYKILTIYSTVASSLVLAMGVAAFLFWAMSNGSCRCNICI

>AVX19063.1 hemagglutinin [Influenza A virus]
MEAISLMIILLVVTTSNADKICIGHQSTNSTETVDTLTESNIPVTQAKELLHTEHNGMLCATNLGRPLIL
DTCTVEGLIYGNPSCDLLLGGREWSYIVERPSAVNGTCYPGNVENLEELRMLFSSASSYQRIQIFPDAIW
NVTYDGTSKSCSNSFYRNMRWLTQKNGNYPIQDAQYTNNRGKDILFIWGIHHPPTDTAQTNLYTRTDTTT
SVTTENLDRTFKPLIGPRPLVNGLIGRINYYWSVLKPGQTLRVRSNGNLIAPWYGHVLSGESHGRILKTD
LKSGNCVVQCQTEKGGLNSTLPFHNISKYAFGTCPKYIGVKSLKLAIGLRNVPAKSSRGLFGAIAGFIEG
GWPGLVAGWYGFQHSNDQGVGMAADRSSTQKAVDKITSKVNNIIDKMNRQYEIIDHEFSEIETRLNMINN
KIDDQIQDVWAYNAELLVLLENQKTLDEHDANVNNLYNKVKGALGSNAMEDGKGCFELYHKCDDQCMETI
RNGTYNRRKYTEESRLERQKIEGVKLESEGTYKILTIYSTVASSLVLAMGVAAFLFWAMSNGSCRCNICI

>AVX19062.1 hemagglutinin [Influenza A virus]
MEAISLMTILLVVTTSNADKICIGHQSTNSTETVDTLTESNIPVTQAKELLHTEHNGMLCATNLGRPLIL

DTCTVEGLIYGNPSCDLLLGGREWSYIVERPSAVNGTCYPGNVENLEELRMLFSSASSYQRIQIFPDAIW
NVTYDGTSKSCSNSFYRNMRWLTQKNGNYPIQDAQYTNNRGKDILFIWGIHHPPTDTAQTNLYTRTDTTT
SVTTENLDRTFKPLIGPRPLVNGLIGRINYYWSVLKPGQTLRVRSNGNLIAPWYGHVLSGESHGRILKTD
LKSGNCVVQCQTEKGGLNSTLPFHNISKYAFGTCPKYIGVKSLKLAIGLRNVPAKSSRGLFGAIAGFIEG
GWPGLVAGWYGFQHSNDQGVGMAADRGSTQKAVDKITSKVNNIIDKMNRQYEIIDHEFSEIETRLNMINN
KIDDQIQDVWAYNAELLVLLENQKTLDEHDANVNNLYNKVKRALGSNAMEDGKGCFELYHKCDDQCMETI
RNGAYNRRKYTEESRLERQKIEGIKLESEGTYKILTIYATVASSLVLAMGAAAFLFWAMSNGSCRCNICI

>AVX19059.1 hemagglutinin [Influenza A virus]
MEAISLMIILLVVTTSNADKICIGHQSTNSTETVDTLTESNIPVTQAKELLHTEHNGMLCATNLGRPLIL
DTCTVEGLIYGNPSCDLLLGGREWSYIVERPSAVNGTCYPGNVENLEELRMLFSSASSYQRIQIFPDAIW
NVTYDGTSKSCSNSFYRNMRWLTQKNGNYPIQDAQYTNNRGKDILFIWGIHHPPTDTAQTNLYTRTDTTT
SVTTENLDRTFKPLIGPRPLVNGLIGRINYYWSVLKPGQTLRVRSNGNLIAPWYGHVLSGESHGRILKTD
LKSGNCVVQCQTEKGGLNSTLPFHNISKYAFGTCPKYIGVKSLKLAIGLRNVPAKSSRGLFGAIAGFIEG
GWPGLVAGWYGFQHSNDQGVGMAADRGSTQKAVDKITSKVNNIIDKMNRQYEIIDHEFSEVETRLNMINN
KIDDQIQDVWAYNAELLVLLENQKTLDEHDANVNNLYNKVKRALGSNAMEDGKGCFELYHKCDDQCMETI
RNGTYNRRKYTEESRLERQKIEGVKLESEGTYKILTIYSTVASSLVLAMGVAAFLFWAMSNGSCRCNICI

>QCR64082.1 hemagglutinin [Influenza A virus]
MKAISLMTILLVVTTSDADKICIGHQSTNSTETVDTLTETNVPVTQAKELLHTEHNGMLCATNLGRPLIL
DTCTIEGLIYGNPSCDMLLGGREWSYIVERPSAVNGTCYPGNVENLEELRTLFSSSSSYQRIQLFPDSIW
NVTYSGTSKSCSDSFYRNMRWLTQKNGLYPVQDAQYINNRGKDILFVWGIHHPPTDTAQTNLYTRTDTTT
SVTTENLDRTFKPLIGPRPLVNGLIGRINYYWSVLKPGQTLRIRSNGNLIAPWFGHILSGESHGRILRTD
LSSGNCVVQCQTEKGGLNSTLPFHNISKYAFGTCPKYIGVKSLRLAIGLRNVHARSNRGLFGAIAGFIEG
GWPGLVAGWYGFQHSNDQGVGMAADRVSTQKAVDKITSKVNNIVDKMNKQYEIIDHEFSEVESRLNMINN
KIDDQIQDIWAYNAELLVLLENQKTLDEHDANVNNLYNKVKRALGSNAMEDGKGCFELYHKCDDQCMETI
RNGTYNRRKYTEESRLERQKIEGVKLEAEGTYKILSIYSTVASSLVLAMGFAAFLFWAMSNGSCRCNICI

>QCR64076.1 hemagglutinin [Influenza A virus]
MKAISLMTILLVVTTSDADKICIGHQSTNSTETVDTLTETNVPVTQAKELLHTEHNGMLCATNLGRPLIL
DTCTIEGLIYGNPSCDMLLGGREWSYIVERPSAVNGTCYPGNVENLEELRTLFSSSSSYQRIQLFPDSIW
NVTYSGTSKSCSDSFYRNMRWLTQKNGLYPVQDAQYINNRGKDILFVWGIHHPPTDTAQTNLYTRTDTTT
SVTTENLDRTFKPLIGPRPLVNGLIGRINYYWSVLKPGQTLRIRSNGNLIAPWFGHILSGESHGRILRTD
LSSGNCVVQCQTEKGGLNSTLPFHNISKYAFGTCPKYIGVKSLRLAIGLRNVHARSNRGLFGAIAGFIEG

GWPGLVAGWYGFQHSNDQGVGMAADRVSTQKAVDKITSKVNNIVDKMNKQYEIIDHEFSEVESRLNMINN
KIDDQIQDIWAYNAELLVLLENQKTLDEHDANVNNLYNKVKRALGSNAMEDGKGCFELYHKCDDQCMETI
RNGTYNRRKYTEESRLERQKIEGVKLEAEGTYKILSIYSTVASSLVLAMGFAAFLFWAMSNGSCRCNICI

>QBK46344.1 hemagglutinin [Influenza A virus]
MEAISLMIILLVVTTSSADKICIGHQSTNSTETVDTLTESNIPVTQAKELLHTEHNGMLCATNLGRPLIL
DTCTVEGLIYGNPSCDLLLGGREWSYIVERPSAVNGTCYPGNVENLEELRMLFSSASSYQRIQIFPDAIW
NVTYDGTSKSCSNSFYRNMRWLTQKNGNYPIQDAQYTNNRGKDILFIWGIHHPPTDTAQTNLYTRTDTTT
SVTTENLDRTFKPLIGPRPLVNGLIGRINYYWSVLKPGQTLRVRSNGNLIAPWYGHVLSGESHGRILKTD
LKSGNCVVQCQTEKGGLNSTLPFHNISKYAFGTCPKYIGVKSLKLAIGLRNVPAKSSRGLFGAIAGFIEG
GWPGLVAGWYGFQHSNDQGVGMAADSGSTQKAVDKITSKVNNIIDKMNRQYEIIDHEFSEIETRLNMINN
KIDDQIQDVWAYNAELLVLLENQKTLDEHDANVNNLYNKVKRALGSNAMEDGKGCFELYHKCDDQCMETI
RNGTYNRRKYTEESRLERQKIEGVKLESEGTYKILTIYSTVASSLVLAMGVAAFLFWAMSNGSCRCNICI

>AZL88848.1 hemagglutinin, partial [Influenza A virus]
AISLMTILLVVTTSNADKICIGHQSTNSTETVDTLTETNVPVTQAKELLHTEHNGMLCATNLGRPLILDT
CTIEGLIYGNPSCDMLLGGREWAYIVERPSAVNGTCYPGNVENLEELRTFFSSSSSYQRVQLFPDSIWNV
TYTGTSKSCSDSFYRNMRWLTQKNGGYPIQDAQYTNNRGKDILFVWGIHHPPTDTAQTNLYTRTDTTTSV
TTETLDRTFKPLIGPRPLVNGLIGRINYYWSVLKPGQTLRVRSNGNLIAPWFGHVLSGESHGRILRTNLS
SGNCVVQCQTEKGGLNSTMPFHNISKYAFGTCPKYIGVKSLKLAIGLRNVHARSSRGLFGAIAGFIEGGW
PGLVAGWYGFQHSNDQGVGMAADRVSTQKAVDKITSKVNNIVDKMNKQYEIIDHEFSEIETRLNMINNKI
DDQIQDIWAYNAELLVLLENQKTLDEHDANVNNLYNKVKRALGSNAMEDGKGCFELYHKCDDQCMETIRN
GTYNRRKYTEESRLERQKIEGVKLEAEGTYKILSIYSTVASSLVLAMGFAAFLFWAMSNGSCRCNICI

>AZL88847.1 hemagglutinin, partial [Influenza A virus]
AISLMTILLVVTTSNADKICIGHQSTNSTETVDTLTETNVPVTQAKELLHTEHNGMLCATNLGRPLILDT
CTIEGLIYGNPSCDMLLGGREWAYIVERPSAVNGTCYPGNVENLEELRTFFSSSSSYQRVQLFPDSIWNV
TYTGTSKSCSDSFYRNMRWLTQKNGGYPIQDAQYTNNRGKDILFVWGIHHPPTDTAQTNLYTRTDTTTSV
TTETLDRTFKPLIGPRPLVNGLIGRINYYWSVLKPGQTLRVRSNGNLIAPWFGHVLSGESHGRILRTNLS
SGNCVVQCQTEKGGLNSTMPFHNISKYAFGTCPKYIGVKSLKLAIGLRNVHARSSRGLFGAIAGFIEGGW
PGLVAGWYGFQHSNDQGVGMAADRVSTQKAVDKITSKVNNIVDKMNKQYEIIDHEFSEIETRLNMINNKI
DDQIQDIWAYNAELLVLLENQKTLDEHDANVNNLYNKVKRALGSNAMEDGKGCFELYHKCDDQCMETIRN
GTYNRRKYTEESRLERQKIEGVKLEAEGTYKILSIYSTVASSLVLAMGFAAFLFWAMSNGSCRCNICI

>AZL88846.1 hemagglutinin, partial [Influenza A virus]

AISLMTILLVVTTSNADKICIGHQSTNSTETVDTLTETNVPVTQAKELLHTEHNGMLCATNLGRPLILDT
CTIEGLIYGNPSCDMLLGGREWAYIVERPSAVNGTCYPGNVENLEELRTFFSSSSSYQRVQLFPDSIWNV
TYTGTSKSCSDSFYRNMRWLTQKNGGYPIQDAQYTNNRGKDILFVWGIHHPPTDTAQTNLYTRTDTTTSV
TTETLDRTFKPLIGPRPLVNGLIGRINYYWSVLKPGQTLRVRSNGNLIAPWFGHVLSGESHGRILRTNLS
SGNCVVQCQTEKGGLNSTMPFHNISKYAFGTCPKYIGVKSLKLAIGLRNVHARSSRGLFGAIAGFIEGGW
PGLVAGWYGFQHSNDQGVGMAADRVSTQKAVDKITSKVNNIVDKMNKQYEIIDHEFSEIETRLNMINNKI
DDQIQDIWAYNAELLVLLENQKTLDEHDANVNNLYNKVKRALGSNAMEDGKGCFELYHKCDDQCMETIRN
GTYNRRKYTEESRLERQKIEGVKLEAEGTYKILSIYSTVASSLVLAMGFAAFLFWAMSNGSCRCNICI

>AZL88845.1 hemagglutinin, partial [Influenza A virus]

AISLMTILLVVTTSNADKICIGHQSTNSTETVDTLTETNVPVTQAKELLHTEHNGMLCATNLGRPLILDT
CTIEGLIYGNPSCDMLLGGREWAYIVERPSAVNGTCYPGNVENLEELRTFFSSSSSYQRVQLFPDSIWNV
TYTGTSKSCSDSFYRNMRWLTQKNGGYPIQDAQYTNNRGKDILFVWGIHHPPTDTAQTNLYTRTDTTTSV
TTETLDRTFKPLIGPRPLVNGLIGRINYYWSVLKPGQTLRVRSNGNLIAPWFGHVLSGESHGRILRTNLS
SGNCVVQCQTEKGGLNSTMPFHNISKYAFGTCPKYIGVKSLKLAIGLRNVHARSSRGLFGAIAGFIEGGW
PGLVAGWYGFQHSNDQGVGMAADRVSTQKAVDKITSKVNNIVDKMNKQYEIIDHEFSEIETRLNMINNKI
DDQIQDIWAYNAELLVLLENQKTLDEHDANVNNLYNKVKRALGSNAMEDGKGCFELYHKCDDQCMETIRN
GTYNRRKYTEESRLERQKIEGVKLEAEGTYKILSIYSTVASSLVLAMGFAAFLFWAMSNGSCRCNICI

>AZL88844.1 hemagglutinin [Influenza A virus]

MEAISLMTILLVVTTSNADKICIGHQSTNSTETVDTLTETNVPVTQAKELLHTEHNGMLCATNLGRPLIL
DTCTIEGLIYGNPSCDMLLGGREWSYIVERPSAVNGTCYPGNVENLEELRTFFSSSSSYQRVQLFPDSIW
NVTYTGTSKSCSDSFYRNMRWLTQKNGAYPIQDAQYTNNRGKDILFVWGIHHPPTDTAQTNLYTRTDTTT
SVTTETLDRTFKPLIGPRPLVNGLIGRINYYWSVLKPGQTLRVRSNGNLIAPWFGHVLSGESHGRILRTD
LSSGNCVVQCQTEKGGLNSTMPFHNISKYAFGTCPKYIGVKSLKLAIGLRNVHARSSRGLFGAIAGFIEG
GWPGLVAGWYGFQHSNDQGVGMAADRVSTQKAVDKITSKVNNIVDKMNKQYEIIDHEFSEVETRLNMINN
KIDDQIQDIWAYNAELLVLLENQKTLDEHDANVNNLYNKVKRALGSNAMEDGKGCFELYHKCDDQCMETI
RNGTYNRRKYTEESRLERQKIEGVKLEAEGTYKILSIYSTVASSLVLAMGFAAFLFWAMSNGSCRCNICI

>AZL88843.1 hemagglutinin [Influenza A virus]

MEAISLMTILLVVTTSNADKICIGHQSTNSTETVDTLTETNVPVTQAKELLXTEHNGMLCATNLGRPLIL
DTCTIEGLIYGNPSCDMLLGGREWSYIVERPSAVNGTCYPGNVENLEELRTFFSSSSSYQRVQLFPDSIW
NVTYTGTSKSCSDSFYRNMRWLTQKNGAYPIQDAQYTNNRGKDILFVWGIHHPPTDTXQTNLYTRTDTTT

SVTTETLDRTFKPLIGPRPLVNGLIGRINYYWSVLKPGQTLRVRSNGNLIAPWFGHVLSGESHGRILRTD
LSSGNCVVQCQTEKGGLNSTMPFHNISKYAFGTCPKYIGVKSLKLAIGLRNVHARSSRGLFGAIAGFIEG
GWPGLVAGWYGFQHSNDQGVGMAADRVSTQKAVDKITSKVNNIVDKMNKQYEIIDHEFSEVETRLNMINN
KIDDQIQDIWAYNAELLVLLENQKTLDEHDANVNNLYNKVRRALGSNAMEDGKGCFELYHKCDDQCMETI
RNGTYNRRKYTEESRLERQKIEGVKLEAEGTYKILSIYSTVASSLVLAMGFAAFLFWAMSNGSCRCNICI

>AZL88842.1 hemagglutinin [Influenza A virus]
MEAISLMTILLVVTTSNADKICIGHQSTNSTETVDTLTETNVPVTQAKELLHTEHNGMLCATNLGRPLIL
DTCTIEGLIYGNPSCDMLLGGREWSYIVERPSAVNGTCYPGNVENLEELRTFFSSSSSYQRVQLFPDSIW
NVTYTGTSKSCSDSFYRNMRWLTQKNGAYPIQDAQYTNNRGKDILFVWGIHHPPTDTAQTNLYTRTDTTT
SVTTETLDRTFKPLIGPRPLVNGLIGRINYYWSVLKPGQTLRVRSNGNLIAPWFGHVLSGESHGRILRTD
LSSGNCVVQCQTEKGGLNSTMPFHNISKYAFGTCPKYIGVKSLKLAIGLRNVHARSSRGLFGAIAGFIEG
GWPGLVAGWYGFQHSNDQGVGMAADRVSTQKAVDKITSKVNNIVDKMNKQYEIIDHEFSEVETRLNMINN
KIDDQIQDIWAYNAELLVLLENQKTLDEHDANVNNLYNKVRRALGSNAMEDGKGCFELYHKCDDQCMETI
RNGTYNRRKYTEESRLERQKIEGVKLEAEGTYKILSIYSTVASSLVLAMGFAAFLFWAMSNGSCRCNICI

>QCT27584.1 hemagglutinin [Influenza A virus]
METASLITILLVVTVSNADKICIGYQSTNSTETVDTLTENNVPVTHAKELLHTEHNGMLCATSLGHPLIL
DTCTIEGLIYGNPSCDLLLGGREWSYIVERPSAVNGLCYPGNVENLEELRSLFSSASSYQRIQIFPDTIW
NVSYSGTSKACSDSFYRSMRWLTQKDNAYPTQDAQYTNNQEKNILFMWGINHPPTDTAQTNLYTRTDTTT
SVATEEINRTFKPLIGPRPLVNGLMGRINYYWSVLKPGQTLRIKSNGNLIAPWYGHILSGESHGRILKTD
LKKGSCTVQCQTEKGGLNTTLPFQNVSKYAFGNCSKYIGIKSLKLAVGLRNVPSRSSRGLFGAIAGFIEG
GWSGLVAGWYGFQHSNDQGVGMAADRDSTQKAIDKITSKVNNIVDKMNKQYEIIDHEFSEVETRLNMINN
KIDDQIQDIWAYNAELLVLLENQKTLDEHDANVNNLYNKVKRALGSNAVEDGKGCFELYHKCDDQCMETI
RNGTYNRRKYQEESKLERQKIEGVKLESEGTYKILTIYSTVASSLVIAMGFAAFLFWAMSNGSCRCNICI

>QCT27573.1 hemagglutinin [Influenza A virus]
MDTASLITILLVVTVSNADKICIGYQSTNSTETVDTLTENNVPVTHAKELLHTEHNGMLCATSLGHPLIL
DTCTIEGLIYGNPSCDLLLGGREWSYIVERPSAVNGLCYPGNVENLEELRSLFSSARSYQRIQIFPDTIW
NVSYSGTSKACSDSFYRSMRWLTQKDNAYPIQDAQYTNNQEKNILFMWGINHPPTDTAQTNLYTRTDTTT
SVATEEINRTFKPLIGPRPLVNGLMGRINYYWSVLKPGQTLRIKSNGNLIAPWYGHILSGESHGRILKTD
LKRGSCTVQCQTEKGGLNTTLPFQNVSKYAFGNCSKYIGIKSLKLAVGLRNVPSRSSRGLFGAIAGFIEG
GWSGLVAGWYGFQHSNDQGVGMAADRDSTQKAIDKITSKVNNIVDKMNKQYEIIDHEFSEVETRLNMINN
KIDDQIQDIWAYNAELLVLLENQKTLDEHDANVNNLYNKVKRALGSNAVEDGKGCFELYHKCDDQCMETI

RNGTYNRRKYQEESKLERQKIEGVKLESEGTYKILTIYSTVASSLVIAMGFAAFLFWAMSNGSCRCNICI

>QCT27561.1 hemagglutinin [Influenza A virus]
MDTASLITILLVVTVSNADKICIGYQSTNSTETVDTLTENNVPVTHAKELLHTEHNGMLCATSLGHPLIL
DTCTIEGLIYGNPSCDLLLGGREWSYIVERPSAVNGLCYPGNVENLEELRSLFSSARSYQRIQIFPDTIW
NVSYSGTSKACSDSFYRSMRWLTQKDNAYPIQDAQYTNNQEKNILFMWGINHPPTDTAQTNLYTRTDTTT
SVATEEINRTFKPLIGPRPLVNGLMGRINYYWSVLKPGQTLRIKSNGNLIAPWYGHILSGESHGRILKTD
LKRGSCTVQCQTEKGGLNTTLPFQNVSKYAFGNCSKYIGIKSLKLAVGLRNVPSRSSRGLFGAIAGFIEG
GWSGLVAGWYGFQHSNDQGVGMAADRDSTQKAIDKITSKVNNIVDKMNKQYEIIDHEFSEVETRLNMINN
KIDDQIQDIWAYNAELLVLLENQKTLDEHDANVNNLYNKVKRALGSNAVEDGKGCFELYHKCDDQCMETI
RNGTYNRRKYQEESKLERQKIEGVKLESEGTYKILTIYSTVASSLVIAMGFAAFLFWAMSNGSCRCNICI

>QCT27549.1 hemagglutinin [Influenza A virus]
MDTASLITILLVVTVSNADKICIGYQSTNSTETVDTLTENNVPVTHAKELLHTEHNGMLCATSLGHPLIL
DTCTIEGLIYGNPSCDLLLGGREWSYIVERPSAVNGLCYPGNVENLEELRSLFSSARSYQRIQIFPDTIW
NVSYSGTSKACSDSFYRSMRWLTQKDNAYPIQDAQYTNNQEKNILFMWGINHPPTDTAQTNLYTRTDTTT
SVATEEINRTFKPLIGPRPLVNGLMGRINYYWSVLKPGQTLRIKSNGNLIAPWYGHILSGESHGRILKTD
LKRGSCTVQCQTEKGGLNTTLPFQNVSKYAFGNCSKYIGIKSLKLAVGLRNVPSRSSRGLFGAIAGFIEG
GWSGLVAGWYGFQHSNDQGVGMAADRDSTQKAIDKITSKVNNIVDKMNKQYEIIDHEFSEVETRLNMINN
KIDDQIQDIWAYNAELLVLLENQKTLDEHDANVNNLYNKVKRALGSNAVEDGKGCFELYHKCDDQCMETI
RNGTYNRRKYQEESKLERQKIEGVKLESEGTYKILTIYSTVASSLVIAMGFAAFLFWAMSNGSCRCNICI

>QCT27537.1 hemagglutinin [Influenza A virus]
METASLMTILLVVTASNADKICIGYQSTNSTETVDTLTENNVPVTHAKELLHTEHNGMLCATSLGHPLIL
DTCTIEGLIYGNPSCDLLLGGREWSYIVERPSAVNGLCYPGNVENLEELRSLFSSARSYQRIQIFPDTIW
NVSYSGTSKACSDSFYRSMRWLTQKDNAYPIQDAQYTNNQEKNILFMWGINHPPTDTAQTNLYTRTDTTT
SVATEEINRTFKPLIGPRPLVNGLMGRINYYWSVLKPGQTLRIKSNGNLIAPWYGHILSGESHGRILKTD
LKRGSCTVQCQTEKGGLNTTLPFQNVSKYAFGNCSKYIGIKSLKLAVGLRNVPSRSSRGLFGAIAGFIEG
GWSGLVAGWYGFQHSNDQGVGMAADRDSTQKAIDKITSKVNNIVDKMNKQYEIIDHEFSEVETRLNMINS
KIDDQIQDIWAYNAELLVLLENQKTLDEHDANVNNLYNKVKRALGSNAVEDGKGCFELYHKCDDQCMETI
RNGTYNRRKYQEESKLERQKIEGVKLESEGTYKILTIYSTVASSIVIAMGFAAFLFWAMSNGSCRCSICI

>QCT27525.1 hemagglutinin [Influenza A virus]
METASLMTILLVVTASNADKICIGYQSTNSTETVDTLTENNVPVTHAKELLHTEHNGMLCATSLGHPLIL

DTCTIEGLIYGNPSCDLLLGGREWSYIVERPSAVNGLCYPGNVENLEELRSLFSSARSYQRIQIFPDTIW

NVSYSGTSKACSDSFYRSMRWLTQKDNAYPIQDAQYTNNQEKNILFMWGINHPPTDTAQTNLYTRTDTTT

SVATEEINRTFKPLIGPRPLVNGLMGRINYYWSVLKPGQTLRIKSNGNLIAPWYGHILSGESHGRILKTD

LKRGSCTVQCQTEKGGLNTTLPFQNVSKYAFGNCSKYIGIKSLKLAVGLRNVPSRSSRGLFGAIAGFIEG

GWSGLVAGWYGFQHSNDQGVGMAADRDSTQKAIDKITSKVNNIVDKMNKQYEIIDHEFSEVETRLNMINS

KIDDQIQDIWAYNAELLVLLENQKTLDEHDANVNNLYNKVKRALGSNAVEDGKGCFELYHKCDDQCMETI

RNGTYNRRKYQEESKLERQKIEGVKLESEGTYKILTIYSTVASSIVIAMGFAAFLFWAMSNGSCRCSICI

>QCT27513.1 hemagglutinin [Influenza A virus]

METASLMTILLVVTASNADKICIGYQSTNSTETVDTLTENNVPVTHAKELLHTEHNGMLCATSLGHPLIL

DTCTIEGLIYGNPSCDLLLGGREWSYIVERPSAVNGLCYPGNVENLEELRSLFSSARSYQRIQIFPDTIW

NVSYSGTSKACSDSFYRSMRWLTQKDNAYPIQDAQYTNNQEKNILFMWGINHPPTDTAQTNLYTRTDTTT

SVATEEINRTFKPLIGPRPLVNGLMGRINYYWSVLKPGQTLRIKSNGNLIAPWYGHILSGESHGRILKTD

LKRGSCTVQCQTEKGGLNTTLPFQNVSKYAFGNCSKYIGIKSLKLAVGLRNVPSRSSRGLFGAIAGFIEG

GWSGLVAGWYGFQHSNDQGVGMAADRDSTQKAIDKITSKVNNIVDKMNKQYEIIDHEFSEVETRLNMINS

KIDDQIQDIWAYNAELLVLLENQKTLDEHDANVNNLYNKVKRALGSNAVEDGKGCFELYHKCDDQCMETI

RNGTYNRRKYQEESKLERQKIEGVKLESEGTYKILTIYSTVASSIVIAMGFAAFLFWAMSNGSCRCSICI

>QCT27501.1 hemagglutinin [Influenza A virus]

MDTASLITILLVVTVSNADKICIGYQSTNSTETVDTLTENNVPVTHAKELLHTEHNGMLCATSLGHPLIL

DTCTIEGLIYGNPSCDLLLGGREWSYIVERPSAVNGLCYPGNVENLEELRSLFSSARSYQRIQIFPDTIW

NVSYSGTSKACSDSFYRSMRWLTQKDNAYPIQDAQYTNNQEKNILFMWGINHPPTDTAQTNLYTRTDTTT

SVATEEINRTFKPLIGPRPLVNGLMGRINYYWSVLKPGQTLRIKSNGNLIAPWYGHILSGESHGRILKTD

LKRGSCTVQCQTEKGGLNTTLPFQNVSKYAFGNCSKYIGIKSLKLAVGLRNVPSRSSRGLFGAIAGFIEG

GWSGLVAGWYGFQHSNDQGVGMAADRDSTQKAIDKITSKVNNIVDKMNKQYEIIDHEFSEVETRLNMINN

KIDDQIQDIWAYNAELLVLLENQKTLDEHDANVNNLYNKVKRALGSNAVEDGKGCFELYHKCDDQCMETI

RNGTYNRRKYQEESKLERQKIEGVKLESEGTYKILTIYSTVASSLVIAMGFAAFLFWAMSNGSCRCNICI

>QCT27476.1 hemagglutinin [Influenza A virus]

METASLITILLVVTASYADKICIGYQSTNSTETVDTLTENNVPVTHAKELLHTEHNGMLCATSLGHPLIL

DTCTIEGLIYGNPSCDLLLGGREWSYIVERPTAVNGLCYPGNVENLEELRSLFSSARSYQRIQIFPDTIW

NVSYSGTSKACSDSFYRSMRWLTQKDNAYPIQDAQYTNNQEKNILFMWGINHPPTDTAQTNLYTRTDTTT

SVATEEINRTFKPLIGPRPLVNGLMGRINYYWSVLKPGQTLRIKSNGNLIAPWYGHILSGESHGRILKTD

LKRGSCTVQCQTEKGGLNTTLPFQNVSKYAFGNCSKYIGIKSLKLAVGLRNVPSRSSRGLFGAIAGFIEG

GWSGLVAGWYGFQHSNDQGVGMAADRDSTQKAIDKITSKVNNIVDKMNKQYEIIDHEFSEVETRLNMINS
KIDDQIQDIWAYNAELLVLLENQKTLDEHDANVNNLYNKVKRALGSNAVEDGKGCFELYHKCDDRCMETI
RNGTYNRRKYQEESKLERQKIEGVKLESEGTYKILTIYSTVASSLVIAMGFAAFLFWAMSNGSCRCNICI

>QCT27453.1 hemagglutinin [Influenza A virus]
METASLITILLVVTASNADKICIGYQSTNSTETVDTLTENNVPVTHAKELLHTEHNGMLCATSLGHPLIL
DTCTIEGLIYGNPSCDLLLGGREWSYIVERPTAVNGLCYPGNVENLEELRSLFSSARSYQRIQIFPDTIW
NVSYSGTSKACSDSFYRSMRWLTQKDNAYPIQDAQYTNNQEKNILFMWGINHPPTDTAQTNLYTRTDTTT
SVATEEINRTFKPLIGPRPLVNGLMGRINYYWSVLKPGQTLRIKSNGNLIAPWYGHILSGESHGRILKTD
LKRGSCTVQCQTEKGGLNTTLPFQNVSKYAFGNCSKYIGIKSLKLAVGLRNVPSRSSRGLFGAIAGFIEG
GWSGLVAGWYGFQHSNDQGVGMAADRDSTQKAIDKITSKVNNIVDKMNKQYEIIDHEFSEVETRLNMINS
KIDDQIQDIWAYNAELLVLLENQKTLDEHDANVNNLYNKVKRALGSNAVEDGKGCFELYHKCDDRCMETI
RNGTYNRRKYQEESKLERQKIEGVKLESEGTYKILTIYSTVASSLVIAMGFAAFLFWAMSNGSCRCNICI

>QCT27429.1 hemagglutinin [Influenza A virus]
MEAASLMTILLVVTASNADKICIGYQSTNSTETVDTLTENNVPVTHAKELLHTEHNGMLCATSLGHPLIL
DTCTIEGLIYGNPSCDLLLGGREWSYIVERPSAVNGLCYPGNVENLEELRSLFSSARSYQRIQIFPDTIW
NVSYSGTSKACSDSFYRSMRWLTQKENAYPIQDAQYTNNQEKNILFMWGINHPPTDTAQTNLYTRTDTTT
SVATEEINRTFKPLIGPRPLVNGLMGRINYYWSVLKPGQTLRIKSNGNLIAPWYGHILSGESHGRILKTD
LKRGSCTVQCQTEKGGLNTTLPFQNVSKYAFGNCSKYIGIKSLKLAVGLRNVPSRSSRGLFGAIAGFIEG
GWSGLVAGWYGFQHSNDQGVGMAADRDSTQKAIDKITSKVNNIVDKMNKQYEIIDHEFSEVETRLNMINS
KIDDQIQDIWAYNAELLVLLENQKTLDEHDANVNNLYNKVKRALGSNAVEDGKGCFELYHKCDDQCMETI
RNGTYNRRKYQEESKLERQKIEGVKLESEGTYKILTIYSTVASSIVIAMGFAAFLFWAMSNGSCRCNICI

>QCT27417.1 hemagglutinin [Influenza A virus]
METASLMTILLVVTASNADKICIGYQSTNSTETVDTLTENNVPVTHAKELLHTEHNGMLCATSLGHPLIL
DTCTIEGLIYGNPSCDLLLGGREWSYIVERPSAVNGLCYPGNVENLEELRSLFSSARSYQRIQIFPDTIW
NVSYSGTSKACSDSFYRSMRWLTQKNNAYPIQDAQYTNNQEKNILFMWGINHPPTDTAQTNLYTRTDTTT
SVATEEINRTFKPLIGPRPLVNGLMGRINYYWSVLKPGQTLRIKSNGNLIAPWYGHILSGESHGRILKTD
LKRGSCTVQCQTEKGGLNTTLPFQNVSKYAFGNCSKYIGIKSLKLAVGLRNVPSRSSRGLFGAIAGFIEG
GWSGLVAGWYGFQHSNDQGVGMAADRDSTQKAIDKITSKVNNIVDKMNKQYEIIDHEFSEVETRLNMINS
KIDDQIQDIWAYNAELLVLLENQKTLDEHDANVNNLYNKVKRALGSNAVEDGKGCFELYHKCDDQCMETI
RNGTYNRRKYQEESKLERQKIEGVKLESEGTYKILTIYSTVASSIVIAMGFAAFLFWAMSNGSCRCNICI

>QCT27405.1 hemagglutinin [Influenza A virus]

MESASLMTILLVVTASNADKICIGYQSTNSTETVDTLTENNVPVTHAKELLHTEHNGMLCATSLGHPLIL
DTCTIEGLIYGNPSCDLLLGGREWSYIVERPSAVNGLCYPGNVENLEELRSLFSSARSYQRIQIFPDTIW
NVSYSGTSKACSDSFYRSMRWLTQKDNAYPIQDAQYTNNQEKNILFMWGINHPPTDTAQTNLYTRTDTTT
SVATEEINRTFKPLIGPRPLVNGLMGRINYYWSVLKPGQTLRIKSNGNLIAPWYGHILSGESHGRILKTD
LKRGSCTVQCQTEKGGLNTTLPFQNVSKYAFGNCSKYIGIKSLKLAVGLRNVPSRSSRGLFGAIAGFIEG
GWSGLVAGWYGFQHSNDQGVGMAADRDSTQKAIDKITSKVNNIVDKMNKQYEIIDHEFSEVETRLNMINS
KIDDQIQDIWAYNAELLVLLENQKTLDEHDANVNNLYNKVKRALGSNAVEDGKGCFELYHKCDDQCMETI
RNGTYNRRKYQEESKLERQKIEGVKLESEGTYKILTIYSTVASSIVIAMGFAAFLFWAMSNGSCRCNICI

>QCT27393.1 hemagglutinin [Influenza A virus]

METASLMTILLVVTASNADKICIGYQSTNSTETVDTLTENNVPVTHAKELLHTEHNGMLCATSLGHPLIL
DTCTIEGLIYGNPSCDLLLGGREWSYIVERPSAVNGLCYPGNVENLEELRSLFSSARSYQRIQIFPDTIW
NVSYSGTSKACSDSFYRSMRWLTQKDNAYPIQDAQYTNNQEKNILFMWGINHPPTDTAQTNLYTRTDTTT
SVATEEINRTFKPLIGPRPLVNGLMGRINYYWSVLKPGQTLRIKSNGNLIAPWYGHILSGESHGRILKTD
LKRGSCTVQCQTEKGGLNTTLPFQNVSKYAFGNCSKYIGIKSLKLAVGLRNVPSRSSRGLFGAIAGFIEG
GWSGLVAGWYGFQHSNDQGVGMAADRDSTQKAIDKITSKVNNIVDKMNKQYEIIDHEFSEVETRLNMINS
KIDDQIQDIWAYNAELLVLLENQKTLDEHDANVNNLYNKVKRALGSNAVEDGKGCFELYHKCDDQCMETI
RNGTYNRRKYQEESKLERQKIEGVKLESEGTYKILTIYSTVASSIVIAMGFAAFLFWAMSNGSCRCNICI

>QCT27381.1 hemagglutinin [Influenza A virus]

METASLMTILLVVTASNADKICIGYQSTNSTETVDTLTENNVPVTHAKELLHTEHNGMLCATSLGHPLIL
DTCTIEGLIYGNPSCDLLLGGREWSYIVERPSAVNGLCYPGNVENLEELRSLFSSARSYQRIQIFPDTIW
NVSYSGTSKACSDSFYRSMRWLTQKDNAYPIQDAQYTNNQEKNILFMWGINHPPTDTAQTNLYTRTDTTT
SVATEEINRTFKPLIGPRPLVNGLMGRINYYWSVLKPGQTLRIKSNGNLIAPWYGHILSGESHGRILKTD
LKRGSCTVQCQTEKGGLNTTLPFQNVSKYAFGNCSKYIGIKSLKLAVGLRNVPSRSSRGLFGAIAGFIEG
GWSGLVAGWYGFQHSNDQGVGMAADRDSTQKAIDKITSKVNNIVDKMNKQYEIIDHEFSEVETRLNMINS
KIDDQIQDIWAYNAELLVLLENQKTLDEHDANVNNLYNKVKRALGSNAVEDGKGCFELYHKCDDQCMETI
RNGTYNRRKYQEESKLERQKIEGVKLESEGTYKILTIYSTVASSIVIAMGFAAFLFWAMSNGSCRCNICI

>QCT27355.1 hemagglutinin [Influenza A virus]

METASLMTILLVVTASNADKICIGYQSTNSTETVDTLTENNVPVTHAKELLHTEHNGMLCATSLGHPLIL
DTCTIEGLIYGNPSCDLLLGGREWSYIVERPSAVNGLCYPGNVENLEELRSLFSSARSYQRIQIFPDTIW
NVSYSGTSKACSDSFYRSMRWLTQKDNAYPIQDAQYTNNQEKNILFMWGINHPPTDTAQTNLYTRTDTTT

SVATEEINRTFKPLIGPRPLVNGLMGRINYYWSVLKPGQTLRIKSNGNLIAPWYGHILSGESHGRILKTD
LKRGSCTVQCQTEKGGLNTTLPFQNVSKYAFGNCSKYIGIKSLKLAVGLRNVPSRSSRGLFGAIAGFIEG
GWSGLVAGWYGFQHSNDQGVGMAADRDSTQKAIDKITSKVNNIVDKMNKQYEIIDHEFSEVETRLNMINS
KIDDQIQDIWAYNAELLVLLENQKTLDEHDANVNNLYNKVKRALGSNAVEDGKGCFELYHKCDDQCMETI
RNGTYNRRKYQEESKLERQKIEGVKLESEGTYKILTIYSTVASSIVIAMGFAAFLFWAMSNGSCRCNICI

>QCT27329.1 hemagglutinin [Influenza A virus]
METASLMTILLVVTASNADKICIGYQSTNSTETVDTLTENNVPVTHAKELLHTEHNGMLCATSLGHPLIL
DTCTIEGLIYGNPSCDLLLGGREWSYIVERPSAVNGLCYPGNVENLEELRSLFSSARSYQRIQIFPDTIW
NVSYSGTSKACSDSFYRSMRWLTQKDNAYPIQDAQYTNNQEKNILFMWGINHPPTDTAQTNLYTRTDTTT
SVATEEINRTFKPLIGPRPLVNGLMGRINYYWSVLKPGQTLRIKSNGNLIAPWYGHILSGESHGRILKTD
LKRGSCTVQCQTEKGGLNTTLPFQNVSKYAFGNCSKYIGIKSLKLAVGLRNVPSRSSRGLFGAIAGFIEG
GWSGLVAGWYGFQHSNDQGVGMAADRDSTQKAIDKITSKVNNIVDKMNKQYEIIDHEFSEVETRLNMINS
KIDDQIQDIWAYNAELLVLLENQKTLDEHDANVNNLYNKVKRALGSNAVEDGKGCFELYHKCDDQCMETI
RNGTYNRRKYQEESKLERQKIEGVKLESEGTYKILTIYSTVASSIVIAMGFAAFLFWAMSNGSCRCNICI

>QCT27303.1 hemagglutinin [Influenza A virus]
METASLMTILLVVTASNADKICIGYQSTNSTETVDTLTENNVPVTHAKELLHTEHNGMLCATSLGHPLIL
DTCTIEGLIYGNPSCDLLLGGREWSYIVERPSAVNGLCYPGNVENLEELRSLFSSARSYQRIQIFPDTIW
NVSYSGTSKACSDSFYRSMRWLTQKDNAYPIQDAQYTNNQEKNILFMWGINHPPTDTAQTNLYTRTDTTT
SVATEEINRTFKPLIGPRPLVNGLMGRINYYWSVLKPGQTLRIKSNGNLIAPWYGHILSGESHGRILKTD
LKRGSCTVQCQTEKGGLNTTLPFQNVSKYAFGNCSKYIGIKSLKLAVGLRNVPSRSSRGLFGAIAGFIEG
GWSGLVAGWYGFQHSNDQGVGMAADRDSTQKAIDKITSKVNNIVDKMNKQYEIIDHEFSEVETRLNMINS
KIDDQIQDIWAYNAELLVLLENQKTLDEHDANVNNLYNKVKRALGSNAVEDGKGCFELYHKCDDQCMETI
RNGTYNRRKYQEESKLERQKIEGVKLESEGTYKILTIYSTVASSIVIAMGFAAFLFWAMSNGSCRCNICI

>QCT27291.1 hemagglutinin [Influenza A virus]
METASLMTILLVVTASNADKICIGYQSTNSTETVDTLTENNVPVTHAKELLHTEHNGMLCATSLGHPLIL
DTCTIEGLIYGNPSCDLLLGGREWSYIVERPSAVNGLCYPGNVENLEELRSLFSSARSYQRIQIFPDTIW
NVSYSGTSKACSDSFYRSMRWLTQKDNAYPIQDAQYTNNQEKNILFMWGINHPPTDTAQTNLYTRTDTTT
SVATEEINRTFKPLIGPRPLVNGLMGRINYYWSVLKPGQTLRIKSNGNLIAPWYGHILSGESHGRILKTD
LKRGSCTVQCQTEKGGLNTTLPFQNVSKYAFGNCSKYIGIKSLKLAIGLRNVPSRSSRGLFGAIAGFIEG
GWSGLVAGWYGFQHSNDQGVGMAADRDSTQKAIDKITSKVNNIVDKMNKQYEIIDHEFSEVETRLNMINS
KIDDQIQDIWAYNAELLVLLENQKTLDEHDANVNNLYNKVKRALGSNAVEDGKGCFELYHKCDDQCMETI

RNGTYNRRKYQEESKLERQKIEGVKLESEGTYKILTIYSTVASSIVIAMGFAAFLFWAMSNGSCRCNICI

>QCT27267.1 hemagglutinin [Influenza A virus]
MEAATIIIILLAITGSNADKICIGYQSTNSTETVDTLTENNVPVTHAKELLHTEHNGMLCATNLGRPLIL
DTCTIEGLIYGNPSCDLPLEGREWSYIVERPSAVNGVCYPGNVENLEELRSLFSSASSYQRIQIFPDSIW
NVSYSGTSKACSDSFYRSMRWLTQKNNNYPVQDAQYTNNRGKNILFMWGINHPPTDTAQTNLYTRTETTT
SVATEDINRTFKPLIGPRPLVNGLQGRIDYYWAVLKPGQTLRIRSNGNLIAPWYGHILLGESHGRILKTD
LKSGNCVVQCQTERGGLNTTLPFHNVSKYAFGNCPKYIGVKSLKLAVGLRNVPARSSRGLFGAIAGFIEG
GWSGLVAGWYGFQHSNDQGTGMAADRDSTQKAVDKITSKVNNIVDKMNKQYEIIDHEFSEIETRLNMIND
KIDDQIQDIWAYNAELLVLLENQKTLDEHDANVNNLYNKVKRALGSNAVEDGKGCFDLYHKCNNQCMETI
RNGTYNRRKYQEESRLERQKIEGVKLESEGTYKILTIYSTVASSLVIAMGFAAFLFWAMSNGSCRCNICI

>QCT27241.1 hemagglutinin [Influenza A virus]
MAATTIITILLAITGSNADKICIGYQSTNSTETVDTLTENNVPVTHAKELLHTEHNGMLCATNLGRPLIL
DTCTIEGLIYGNPSCDLPLEGREWSYIVERPSAVNGVCYPGNVENLEELRSLFSSASSYQRIQIFPDSIW
NVSYSGTSKACSDSFYRSMRWLTQKNNNYPVQDAQYTNNRGKNILFMWGINHPPTDTAQTNLYTRTDTTT
SVATEDINRTFKPLIGPRPLVNGLQGRIDYYWAVLKPGQTLRVRSNGNLIAPWYGHILLGESHGRILKTD
LKSGNCVVQCQTERGGLNTTLPFHNVSKYAFGNCPKYIGVKSLKLAVGLRNVPARSSRGLFGAIAGFIEG
GWSGLVAGWYGFQHSNDQGTGMAADRDSTQKAIDKITSKVNNIVDKMNKQYEIIDHEFSEIETRLNMIND
KIDDQIQDIWAYNAELLVLLENQKTLDEHDANVNNLYNKVKRALGSNAVEDGKGCFDLYHKCDNQCMETI
RNGTYNRRKYQEESRLERQKIEGVKLESEGTYKILTIYSTVASSLVIAMGFAAFLFWAMSNGSCRCNICI

>QCT27229.1 hemagglutinin [Influenza A virus]
MEAATTIITILLAITGSNADKICIGYQSTNSTETVDTLTENNVPVTHAKELLHTEHNGMLCATNLGRPLI
LDTCTIEGLIYGNPSCDLPLEGREWSYIVERPSAVNGVCYPGNVENLEELRSLFSSASSYQRTQIFPDSI
WNVSYSGTSKACSDSFYRSMRWLTQKNNNYPVQDAQYTNNRGKNILFMWGINHPPTDTAQTNLYTRTDTT
TSVATEDINRTFKPLIGPRPLVNGLQGRIDYYWAVLKPGQTLRVRSNGNLIAPWYGHILLGESHGRILKT
DLKSGNCVVQCQTERGGLNTTLPFHNVSKYAFGNCPKYIGVKSLKLAVGLRNVPARSSRGLFGAIAGFIE
GGWSGLVAGWYGFQHSNDQGTGMAADRDSTQKAIDKITSKVNNIVDKMNKQYEIIDHEFSEIETRLNMIN
DKIDDQIQDIWAYNAELLVLLENQKTLDEHDANVNNLYNKVKRALGSNAVEDGKGCFDLYHKCDNQCMET
IRNGTYNRRKYQEESRLERQKIEGVKLESEGTYKILTIYSTVASSLVIAMGFAAFLFWAMSNGSCRCNIC
I

>QCT27217.1 hemagglutinin [Influenza A virus]

MEATTIITILLAITGSNADKICIGYQSTNSTETVDTLTENNVPVTHAKELLHTEHNGMLCATNLGRPLIL
DTCTIEGLIYGNPSCDLPLEGREWSYIVERPSAVNGVCYPGNVENLEELRSLFSSASSYQRIQIFPDSIW
NVSYSGTSKACSDSFYRSMRWLTQKNNNYPVQDAQYTNNRGKNILFMWGINHPPTDTAQTNLYTRTDTTT
SVATEDINRTFKPLIGPRPLVNGLQGRIDYYWAVLKPGQTLRVRSNGNLIAPWYGHILLGESHGRILKTD
LKSGNCVVQCQTERGGLNTTLPFHNVSKYAFGNCPKYIGVKSLKLAVGLRNVPARSSRGLFGAIAGFIEG
GWSGLVAGWYGFQHSNDQGTGMAADRDSTQKAIDKITSKVNNIVDKMNKQYEIIDHEFSEIETRLNMIND
KIDDQIQDIWAYNAELLVLLENQKTLDEHDANVNNLYNKVKRALGSNAVEDGKGCFDLYHKCDNQCMETI
RNGTYNRRKYQEESRLERQKIEGVKLESEGTYKILTIYSTVASSLVIAMGFAAFLFWAMSNGSCRCNICI

>QCT27205.1 hemagglutinin [Influenza A virus]
METASLITILLVVTVSNADKICIGYQSTNSTETVDTLTENNVPVTHAKELLHTEHNGMLCATSLGHPLIL
DTCTIEGLIYGNPSCDLLLGGREWSYIVERPSAVNGLCYPGNVENLEELRSLFSSARSYQRIQIFPDTIW
NVSYSGTSKACSDSFYRSMRWLTQKDNAYPIQDAQYTNNQEKNILFMWGINHPPTDTAQTNLYTRTDTTT
SVATEEINRTFKPLIGPRPLVNGLMGRINYYWSVLKPGQTLRIKSNGNLIAPWYGHILSGESHGRILKTD
LKRGSCTVQCQTEKGGLNTTLPFQNVSKYAFGNCSKYIGIKSLKLAVGLRNVPSRSSRGLFGAIAGFIEG
GWSGLVAGWYGFQHSNDQGVGMAADRDSTQKAIDKITSKVNNIVDKMNKQYEIIDHEFSEVETRLNMINN
KIDDQIQDVWAYNAELLVLLENQKTLDEHDANVNNLYNKVKRALGSNAVEDGKGCFELYHKCDDQCMETI
RNGTYNRRKYQEESKLERQKIEGVKLESEGTYKILTIYSTVASSLVIAMGFAAFLFWAMSNGSCRCNICI

>QCT27193.1 hemagglutinin [Influenza A virus]
METASLITILLVVTVSNADKICIGYQSTNSTETVDTLTENNVPVTHAKELLHTEHNGMLCATSLGHPLIL
DTCTIEGLIYGNPSCDLLLGGREWSYIVERPSAVNGLCYPGNVENLEELRSLFSSARSYQRIQIFPDTIW
NVSYSGTSKACSDSFYRSMRWLTQKDNAYPIQDAQYTNNQEKNILFMWGINHPPTDTAQTNLYTRTDTTT
SVATEEINRTFKPLIGPRPLVNGLMGRINYYWSVLKPGQTLRIKSNGNLIAPWYGHILSGESHGRILKTD
LKRGSCTVQCQTEKGGLNTTLPFQNVSKYAFGNCSKYIGIKSLKLAVGLRNVPSRSSRGLFGAIAGFIEG
GWSGLVAGWYGFQHSNDQGVGMAADRDSTQKAIDKITSKVNNIVDKMNKQYEIIDHEFSEVETRLNMINN
KIDDQIQDVWAYNAELLVLLENQKTLDEHDANVNNLYNKVKRALGSNAVEDGKGCFELYHKCDDQCMETI
RNGTYNRRKYQEESKLERQKIEGVKLESEGTYKILTIYSTVASSLVIAMGFAAFLFWAMSNGSCRCNICI

>QCT27181.1 hemagglutinin [Influenza A virus]
METASLITILLVVTASNADKICIGYQSTNSTETVDTLTENNVPVTHAKELLHTEHNGMLCATSLGHPLIL
DTCTIEGLIYGNPSCDPLLGGREWSYIVERPSAVNGLCYPGNVENLEELRSLFSSARSYQRIQIFPDTIW
NVSYSGTSKACSDSFYRSMRWLTQKNNAYPIQDAQYTNNQEKNILFMWGINHPPTDTAQTNLYTRTDTTT
SVATEEINRTFKPLIGPRPLVNGLMGRINYYWSVLKPGQTLRIKSNGNLIAPWYGHILSGESHGRILKTD

LKRGSCTVQCQTEKGGLNTTLPFQNVSKYAFGNCSKYIGIKSLKLAVGLRNVPSRSSRGLFGAIAGFIEG
GWSGLVAGWYGFQHSNDQGVGMAADRDSTQKAIDKITSKVNNIVDKMNKQYEIIDHEFSEVETRLNMINN
KIDDQIQDIWAYNAELLVLLENQKTLDEHDANVNNLYNKVKRALGSNAVEDGKGCFELYHKCDDQCMETI
RNGTYNKRKYQEESKLERQKIDGVKLESEGTYKILTIYSTVASSLVIAMGFAAFLFWAMSNGSCRCNICI

>QCT27169.1 hemagglutinin [Influenza A virus]
METASLITILLVVTASNADKICIGYQSTNSTETVDTLTENNVPVTHAKELLHTEHNGMLCATSLGHPLIL
DTCTIEGLIYGNPSCDPLLGGREWSYIVERPSAVNGLCYPGNVENLEELRSLFSSARSYQRIQIFPDTIW
NVSYSGTSKACSDSFYRSMRWLTQKNNAYPIQDAQYTNNQEKNILFMWGINHPPTDTAQTNLYTRTDTTT
SVATEEINRTFKPLIGPRPLVNGLMGRINYYWSVLKPGQTLRIKSNGNLIAPWYGHILSGESHGRILKTD
LKRGSCTVQCQTEKGGLNTTLPFQNVSKYAFGNCSKYIGIKSLKLAVGLRNVPSRSSRGLFGAIAGFIEG
GWSGLVAGWYGFQHSNDQGVGMAADRDSTQKAIDKITSKVNNIVDKMNKQYEIIDHEFSEVETRLNMINN
KIDDQIQDIWAYNAELLVLLENQKTLDEHDANVNNLYNKVKRALGSNAVEDGKGCFELYHKCDDQCMETI
RNGTYNRRKYQEESKLERQKIEGVKLESEGTYKILTIYSTVASSLVIAMGFAAFLFWAMSNGSCRCNICI

>QCT27157.1 hemagglutinin [Influenza A virus]
METASLITILLVVTASNADKICIGYQSTNSTETVDTLTENNVPVTHAKELLHTEHNGMLCATSLGHPLIL
DTCTIEGLIYGNPSCDPLLGGREWSYIVERPSAVNGLCYPGNVENLEELRSLFSSARSYQRIQIFPDTIW
NVSYSGTSKACSDSFYRSMRWLTQKNNAYPIQDAQYTNNQEKNILFMWGINHPPTDTAQTNLYTRTDTTT
SVATEEINRTFKPLIGPRPLVNGLMGRINYYWSVLKPGQTLRIKSNGNLIAPWYGHILSGESHGRILKTD
LKRGSCTVQCQTEKGGLNTTLPFQNVSKYAFGNCSKYIGIKSLKLAVGLRNVPSRSSRGLFGAIAGFIEG
GWSGLVAGWYGFQHSNDQGVGMAADRDSTQKAIDKITSKVNNIVDKMNKQYEIIDHEFSEVETRLNMINN
KIDDQIQDIWAYNAELLVLLENQKTLDEHDANVNNLYNKVKRALGSNAVEDGKGCFELYHKCDDQCMETI
RNGTYNRRKYQEESKLERQKIEGVKLESEGTYKILTIYSTVASSLVIAMGFAAFLFWAMSNGSCRCNICI

>QCT27145.1 hemagglutinin [Influenza A virus]
METASLITILLVVTASNADKICIGYQSTNSTETVDTLTENNVPVTHAKELLHTEHNGMLCATSLGHPLIL
DTCTIEGLIYGNPSCDPLLGGREWSYIVERPSAVNGLCYPGNVENLEELRSLFSSARSYQRIQIFPDTIW
NVSYSGTSKACSDSFYRSMRWLTQKNNAYPIQDAQYTNNQEKNILFMWGINHPPTDTAQTNLYTRTDTTT
SVATEEINRTFKPLIGPRPLVNGLMGRINYYWSVLKPGQTLRIKSNGNLIAPWYGHILSGESHGRILKTD
LKRGSCTVQCQTEKGGLNTTLPFQNVSKYAFGNCSKYIGIKSLKLAVGLRNVPSRSSRGLFGAIAGFIEG
GWSGLVAGWYGFQHSNDQGVGMAADRDSTQKAIDKITSKVNNIVDKMNKQYEIIDHEFSEVETRLNMINN
KIDDQIQDIWAYNAELLVLLENQKTLDEHDANVNNLYNKVKRALGSNAVEDGKGCFELYHKCDDQCMETI
RNGTYNRRKYQEESKLERQKIEGVKLESEGTYKILTIYSTVASSLVIAMGFAAFLFWAMSNGSCRCNICI

>QCT27110.1 hemagglutinin [Influenza A virus]

METASLITILLVVTASNADKICIGYQSTNSTETVDTLTENNVPVTHAKELLHTEHNGMLCATSLGHPLIL

DTCTIEGLIYGNPSCDLLLGGREWSYIVERPSAVNGLCYPGNVENLEELRSLFSSARSYQRIQIFPDTIW

NVSYSGTSKACSDSFYRSMRWLTQKDNAYPIQDAQYTNNQEKNILFMWGINHPPTDTAQTNLYTRTDTTT

SVATEEINRTFKPLIGPRPLVNGLMGRINYYWSVLKPGQTLRIKSNGNLIAPWYGHILSGESHGRILKTD

LKRGSCTVQCQTEKGGLNTTLPFQNVSKYAFGNCSKYIGIKSLKLAVGLRNVPSRSSRGLFGAIAGFIEG

GWSGLVAGWYGFQHSNDQGVGMAADRDSTQKAIDKITSKVNNIVDKMNKQYEIIDHEFSEVETRLNMINS

KIDDQIQDVWAYNAELLVLLENQKTLDEHDANVNNLYNKVKRALGSNAVEDGKGCFELYHKCDDQCMETI

RNGTYNRRKYQEESKLERQKIEGVKLESEGTYKILTIYSTVASSLVIAMGFAAFLFWAMSNGSCRCNICI

>QCT27098.1 hemagglutinin [Influenza A virus]

METASLITILLVVTASNADKICIGYQSTNSTETVDTLTENNVPVTHAKELLHTEHNGMLCATSLGHPLIL

DTCTIEGLIYGNPSCDLLLGGREWSYIVERPSAVNGLCYPGNVENLEELRSLFSSARSYQRIQIFPDTIW

NVSYSGTSKACSDSFYRSMRWLTQKDNAYPIQDAQYTNNQEKNILFMWGINHPPTDTAQTNLYTRTDTTT

SVATEEINRTFKPLIGPRPLVNGLMGRINYYWSVLKPGQTLRIKSNGNLIAPWYGHILSGESHGRILKTD

LKRGSCTVQCQTEKGGLNTTLPFQNVSKYAFGNCSKYIGIKSLKLAVGLRNVPSRSSRGLFGAIAGFIEG

GWSGLVAGWYGFQHSNDQGVGMAADRDSTQKAIDKITSKVNNIVDKMNKQYEIIDHEFSEVETRLNMINS

KIDDQIQDVWAYNAELLVLLENQKTLDEHDANVNNLYNKVKRALGSNAVEDGKGCFELYHKCDDQCMETI

RNGTYNRRKYQEESKLERQKIEGVKLESEGTYKILTIYSTVASSLVIAMGFAAFLFWAMSNGSCRCNICI

>QCT27086.1 hemagglutinin [Influenza A virus]

METASLITILLVVTASNADKICIGYQSTNSTETVDTLTENNVPVTHAKELLHTEHNGMLCATSLGHPLIL

DTCTIEGLIYGNPSCDLLLGGREWSYIVERPSAVNGLCYPGNVENLEELRSLFSSARSYQRIQIFPDTIW

NVSYSGTSKACSDSFYRSMRWLTQKDNAYPIQDAQYTNNQEKNILFMWGINHPPTDTAQTNLYTRTDTTT

SVATEEINRTFKPLIGPRPLVNGLMGRINYYWSVLKPGQTLRIKSNGNLIAPWYGHILSGESHGRILKTD

LKRGSCTVQCQTEKGGLNTTLPFQNVSKYAFGNCSKYIGIKSLKLAVGLRNVPSRSSRGLFGAIAGFIEG

GWSGLVAGWYGFQHSNDQGVGMAADRDSTQKAIDKITSKVNNIVDKMNKQYEIIDHEFSEVETRLNMINS

KIDDQIQDVWAYNAELLVLLENQKTLDEHDANVNNLYNKVKRALGSNAVEDGKGCFELYHKCDDQCMETI

RNGTYNRRKYQEESKLERQKIEGVKLESEGTYKILTIYSTVASSLVIAMGFAAFLFWAMSNGSCRCNICI

>QCT27074.1 hemagglutinin [Influenza A virus]

METASLITILLVVTASNADKICIGYQSTNSTETVDTLTENNVPVTHAKELLHTEHNGMLCATSLGHPLIL

DTCTIEGLIYGNPSCDLLLGGREWSYIVERPSAVNGLCYPGNVENLEELRSLFSSARSYQRIQIFPDTIW

NVSYSGTSKACSDSFYRSMRWLTQKDNAYPIQDAQYTNNQEKNILFMWGINHPPTDTAQTNLYTRTDTTT
SVATEEINRTFKPLIGPRPLVNGLMGRINYYWSVLKPGQTLRIKSNGNLIAPWYGHILSGESHGRILKTD
LKRGSCTVQCQTEKGGLNTTLPFQNVSKYAFGNCSKYIGIKSLKLAVGLRNVPSRSSRGLFGAIAGFIEG
GWSGLVAGWYGFQHSNDQGVGMAADRDSTQKAIDKITSKVNNIVDKMNKQYEIIDHEFSEVETRLNMINS
KIDDQIQDVWAYNAELLVLLENQKTLDEHDANVNNLYNKVKRALGSNAVEDGKGCFELYHKCDDQCMETI
RNGTYNRRKYQEESKLERQKIEGVKLESEGTYKILTIYSTVASSLVIAMGFAAFLFWAMSNGSCRCNICI

>QCT27062.1 hemagglutinin [Influenza A virus]
METASLITILLVVTASNADKICIGYQSTNSTETVDTLTENNVPVTHAKELLHTEHNGMLCATSLGHPLIL
DTCTIEGLIYGNPSCDLLLGGREWSYIVERPSAVNGLCYPGNVENLEELRSLFSSARSYQRIQIFPDTIW
NVSYSGTSKACSDSFYRSMRWLTQKDNAYPIQDAQYTNNQEKNILFMWGINHPPTDTAQTNLYTRTDTTT
SVATEEINRTFKPLIGPRPLVNGLMGRINYYWSVLKPGQTLRIKSNGNLIAPWYGHILSGESHGRILKTD
LKRGSCTVQCQTEKGGLNTTLPFQNVSKYAFGNCSKYIGIKSLKLAVGLRNVPSRSSRGLFGAIAGFIEG
GWSGLVAGWYGFQHSNDQGVGMAADRDSTQKAIDKITSKVNNIVDKMNKQYEIIDHEFSEVETRLNMINS
KIDDQIQDIWAYNAELLVLLENQKTLDEHDANVNNLYNKVKRALGSNAVEDGKGCFELYHKCDDQCMETI
RNGTYNRRKYQEESKLERQKIEGVKLESEGTYKILTIYSTVASSIVIAMGFAAFLFWAMSNGSCRCNICI

>QCT27050.1 hemagglutinin [Influenza A virus]
METASLITILLVVTASNADKICIGYQSTNSTETVDTLTENNVPVTHAKELLHTEHNGMLCATSLGHPLIL
DTCTIEGLIYGNPSCDLLLGGREWSYIVERPSAVNGLCYPGNVENLEELRSLFSSARSYQRIQIFPDTIW
NVSYSGTSKACSDSFYRSMRWLTQKDNAYPIQDAQYTNNQEKNILFMWGINHPPTDTAQTNLYTRTDTTT
SVATEEINRTFKPLIGPRPLVNGLMGRINYYWSVLKPGQTLRIKSNGNLIAPWYGHILSGESHGRILKTD
LKRGSCTVQCQTEKGGLNTTLPFQNVSKYAFGNCSKYIGIKSLKLAVGLRNVPSRSSRGLFGAIAGFIEG
GWSGLVAGWYGFQHSNDQGVGMAADRDSTQKAIDKITSKVNNIVDKMNKQYEIIDHEFSEVETRLNMINS
KIDDQIQDIWAYNAELLVLLENQKTLDEHDANVNNLYNKVKRALGSNAVEDGKGCFELYHKCDDQCMETI
RNGTYNRRKYQEESKLERQKIEGVKLESEGTYKILTIYSTVASSIVIAMGFAAFLFWAMSNGSCRCNICI

>QCT27038.1 hemagglutinin [Influenza A virus]
METASLITILLVVTASNADKICIGYQSTNSTETVDTLTENNVPVTHAKELLHTEHNGMLCATSLGHPLIL
DTCTIEGLIYGNPSCDLLLGGREWSYIVERPSAINGLCYPGNVENLEELRSLFSSARSYQRIQIFPDTIW
NVSYSGTSKACSDSFYRSMRWLTQKDNAYPIQDAQYTNNQEKNILFMWGINHPPTDTTQTNLYTRTDTTT
SVATEEINRTFKPLIGPRPLVNGLMGRINYYWSVLKPGQTLRIKSNGNLIAPWYGHILSGESHGRILKTD
LKRGSCTVQCQTEKGGLNTTLPFQNVSKYAFGNCSKYIGIKSLKLAVGLRNVPSRSSRGLFGAIAGFIEG
GWSGLVAGWYGFQHSNDQGVGMAADRDSTQKAIDKITSKVNNIVDKMNKQYEIIDHEFSEVETRLNMINS

KIDDQIQDIWAYNAELLVLLENQKTLDEHDANVNNLYNKVKRALGSNAVEDGKGCFELYHKCDDQCMETI
RNGTYNRRKYQEESKLERQKIEGVKLESEGTYKILTIYSTVASSIVIAMGFAAFLFWAMSNGSCRCNICI

>QCT27027.1 hemagglutinin [Influenza A virus]
METASLITILLVVTASNADKICIGYQSTNSTETVDTLTENNVPVTHAKELLHTEHNGMLCATSLGHPLIL
DTCTIEGLIYGNPSCDLLLGGREWSYIVERPSAVNGLCYPGNVENLEELRSLFSSARSYQRIQIFPDTIW
NVSYSGTSKACSDSFYRSMRWLTQKDNAYPIQDAQYTNNQEKNILFMWGINHPPTDTVQTNLYTRTDTTX
XXXXXXXXXXXXXXXXXXXXXXXXXXXXXXXXXXXXXXXXXXXXXXXXXLIAPWYGHILSGESHGRILKTD
LKRGSCTVQCQTEKGGLNTTLPFQNVSKYAFGNCSKYIGIKSLKLAVGLRNVPSRSSRGLFGAIAGFIEG
GWSGLVAGWYGFQHSNDQGVGMAADRDSTQKAIDKITSKVNNIVDKMNKQYEIIDHEFSEVETRLNMINS
KIDDQIQDIWAYNAELLVLLENQKTLDEHDANVNNLYNKVKRALGSNAVEDGKGCFELYHKCDDQCMETI
RNGTYNRRKYQEESKLERQKIEGVKLESEGTYKILTIYSTVASSIVIAMGFAAFLFWAMSNGSCRCNICI

>QCT26969.1 hemagglutinin [Influenza A virus]
MEAATIIIILLAITGSNADKICIGYQSTNSTETVDTLTENNVPVTHAKELLHTEHNGMLCATNLGRPLIL
DTCTIEGLIYGNPSCDLPLEGREWSYIVERPSAVNGVCYPGNVENLEELRSLFSSASSYQRIQIFPDSIW
NVSYSGTSKACSDSFYRSMRWLTQKNNNYPVQDAQYTNNRGKNILFMWGINHPPTDTAQTNLYTRTETTT
SVATEDINRTFKPLIGPRPLVNGLQGRIDYYWAVLKPGQTLRIRSNGNLIAPWYGHILLGESHGRILKTD
LKSGNCVVQCQTERGGLNTTLPFHNVSKYAFGNCPKYIGVKSLKLAVGLRNVPARSSRGLFGAIAGFIEG
GWSGLVAGWYGFQHSNDQGTGMAADRDSTQKAVDKITSKVNNIVDKMNKQYEIIDHEFSEIETRLNMIND
KIDDQIQDIWAYNAELLVLLENQKTLDEHDANVNNLYNKVKRALGSNAVEDGKGCFDLYHKCNNQCMETI
RNGTYNRRKYQEESRLERQKIEGVKLESEGTYKILTIYSTVASSLVIAMGFAAFLFWAMSNGSCRCNICI

>QCT26956.1 hemagglutinin, partial [Influenza A virus]
ATTIITILLAITGSNADKICIGYQSTNSTETVDTLTENNVPVTHAKELLHTEHNGMLCATNLGRPLILDT
CTIEGLIYGNPSCDLPLEGREWSYIVERPSAVNGVCYPGNVENLEELRSLFSSASSYQRIQIFPDSIWNV
SYSGTSKACSDSFYRSMRWLTQKNNNYPVQDAQYTNNRGKNILFMWGINHPPTDTAQTNLYTRTDTTTSV
ATEDINRTFKPLIGPRPLVNGLQGRIDYYWAVLKPGQTLRVRSNGNLIAPWYGHILLGESHGRILKTDLK
SGNCVVQCQTERGGLNTTLPFHNVSKYAFGNCPKYIGVKSLKLAVGLRNVPARSSRGLFGAIAGFIEGGW
SGLVAGWYGFQHSNDQGTGMAADRDSTQKAIDKITSKVNNIVDKMNKQYEIIDHEFSEIETRLNMINDKI
DDQIQDIWAYNAELLVLLENQKTLDEHDTNVNNLYNKVKRALGSNAVEDGKGCFDLYHKCDNQCMETIRN
GTYNRRKYQEESRLERQKIEGVKLESEGTYKILTIYSTVASSLVIAMGFAAFLFWAMSNGSCRCNICI

>QCT26884.1 hemagglutinin [Influenza A virus]

METASLMTILLVVTASNADKICIGYQSTNSTETVDTLTENNVPVTHAKELLHTEHNGMLCATSLGHPLIL
DTCTIEGLIYGNPSCDLLLGGREWSYIVERPSAVNGLCYPGNVENLEELRSLFSSARSYQRIQIFPDTIW
NVSYSGTSKACSDSFYRSMRWLTQKDNAYPIQDAQYTNNQEKNILFMWGINHPPTDTAQTNLYTRTDTTT
SVATEEINRTFKPLIGPRPLVNGLMGRINYYWSVLKPGQTLRIKSNGNLIAPWYGHILSGESHGRILKTD
LKRGSCTVQCQTEKGGLNTTLPFQNVSKYAFGNCSKYIGIKSLKLAVGLRNVPSRSSRGLFGAIAGFIEG
GWSGLVAGWYGFQHSNDQGVGMAADRDSTQKAIDKITSKVNNIVDKMNKQYEIIDHEFSEVETRLNMINS
KIDDQIQDIWAYNAELLVLLENQKTLDEHDANVNNLYNKVKRALGSNAVEDGKGCFELYHKCDDQCMETI
RNGTYNRRKYQEESKLERQKIEGVKLESEGTYKILTIYSTVASSIVIAMGFAAFLFWAMSNGSCRCNICI

>QCT26858.1 hemagglutinin, partial [Influenza A virus]
ATTIITILLAITGSNADKICIGYQSTNSTETVDTLTENNVPVTHAKELLHTEHNGMLCATNLGRPLILDI
CTIEGLIYGNPSCDLPLEGREWSYIVERPSAVNGVCYPGNVENLEELRSLFSSASSYQRIQIFPDSIWNV
SYSGTSKACSDSFYRSMRWLTQKNNNYPVQDAQYTNNRGKNILFMWGINHPPTDTAQTNLYTRTDTTTSV
ATEDINRTFKPLIGPRPLVNGLQGRIDYYWAVLKPGQTLRVRSNGNLIAPWYGHILLGESHGRILKTDLK
SGNCVVQCQTERGGLNTTLPFHNVSKYAFGNCPKYIGVKSLKLAVGLRNVPARSSRGLFGAIAGFIEGGW
SGLVAGWYGFQHSNDQGTGMAADRDSTQKAIDKITSKVNNIVDKMNKQYEIIDHEFSEIETRLNMINDKI
DDQIQDIWAYNAELLVLLENQKTLDEHDANVNNLYNKVKRALGSNAVEDGKGCFDLYHKCDNQCMETIRN
GTYNRRKYQEESRLERQKIEGVKLESEGTYKILTIYSTVASSLVIAMGFAAFLFWAMSNGSCRCNICI

>QCT26789.1 hemagglutinin [Influenza A virus]
METASLMTILLVVTASNADKICIGYQSTNSTETVDTLTENNVPVTHAKELLHTEHNGMLCATSLGHPLIL
DTCTIEGLIYGNPSCDLLLGGREWSYIVERPSAVNGLCYPGNVENLEELRSLFSSARSYQRIQIFPDTIW
NVSYSGTSKACSDSFYRSMRWLTQKDNAYPIQDAQYTNNQEKNILFMWGINHPPTDTAQTNLYTRTDTTT
SVATEEINRTFKPLIGPRPLVNGLMGRINYYWSVLKPGQTLRIKSNGNLIAPWYGHILSGESHGRILKTD
LKRGSCTVQCQTEKGGLNTTLPFQNVSKYAFGNCSKYIGIKSLKLAVGLRNVPSRSSRGLFGAIAGFIEG
GWSGLVAGWYGFQHSNDQGVGMAADRDSTQKAIDKITSKVNNIVDKMNKQYEIIDHEFSEVETRLNMINS
KIDDQIQDIWAYNAELLVLLENQKTLDEHDANVNNLYNKVKRALGSNAVEDGKGCFELYHKCDDQCMETI
RNGTYNRRKYQEESKLERQKIEGVKLESEGTYKILTIYSTVASSIVIAMGFAAFLFWAMSNGSCRCNICI

>QCT26778.1 hemagglutinin [Influenza A virus]
METASLMTILLVVTASNADKICIGYQSTNSTETVDTLTENNVPVTHAKELLHTEHNGMLCATSLGHPLIL
DTCTIEGLIYGNPSCDLLLGGREWSYIVERPSAVNGLCYPGNVENLEELRSLFSSARSYQRIQIFPDTIW
NVSYSGTSKACSDSFYRSMRWLTQKDNAYPIQDAQYTNNQEKNILFMWGINHPPTDTAQTNLYTRTDTTT
SVATEEINRTFKPLIGPRPLVNGLMGRINYYWSVLKPGQTLRIKSNGNLIAPWYGHILSGESHGRILKTD

LKRGSCTVQCQTEKGGLNTTLPFQNVSKYAFGNCSKYIGIKSLKLAVGLRNVPSRSSRGLFGAIAGFIEG
GWSGLVAGWYGFQHSNDQGVGMAADRDSTQKAIDKITSKVNNIVDKMNKQYEIIDHEFSEVETRLNMINS
KIDDQIQDIWAYNAELLVLLENQKTLDEHDANVNNLYNKVKRALGSNAVEDGKGCFELYHKCDDQCMETI
RNGTYNRRKYQEESKLERQKIEGVKLESEGTYKILTIYSTVASSIVIAMGFAAFLFWAMSNGSCRCNICI

>QCT26662.1 hemagglutinin [Influenza A virus]
METASLITILLVVTVSNADKICIGYQSTNSTETVDTLTENNVPVTHAKELLHTEHNGMLCATSLGHPLIL
DTCTIEGLIYGNPSCDLLLGGREWSYIVERPSAVNGLCYPGNVENLEELRSLFSSARSYQRIQIFPDTIW
NVSYSGTSKACSDSFYRSMRWLTQKDNAYPIQDAQYTNNQEKNILFMWGINHPPTDTAQTNLYTRTDTTT
SVATEEINRTFKPLIGPRPLVNGLMGRINYYWSVLKPGQTLRIKSNGNLIAPWYGHILSGESHGRILKTD
LKRGSCTVQCQTEKGGLNTTLPFQNVSKYAFGNCSKYIGIKSLKLAVGLRNVPSRSSRGLFGAIAGFIEG
GWSGLVAGWYGFQHSNDQGVGMAADRDSTQKAIDKITSKVNNIVDKMNKQYEIIDHEFSEVETRLNMINN
KIDDQIQDVWAYNAELLVLLENQKTLDEHDANVNNLYNKVKRALGSNAVEDGKGCFELYHKCDDQCMETI
RNGTYNRRKYQEESKLERQKIEGVKLESEGTYKILTIYSTVASSLVIAMGFAAFLFWAMSNGSCRCNICI

>QCT26650.1 hemagglutinin [Influenza A virus]
METASLITILLVVTVSNADKICIGYQSTNSTETVDTLTENNVPVTHAKELLHTEHNGMLCATSLGHPLIL
DTCTIEGLIYGNPSCDLLLGGREWSYIVERPSAVNGLCYPGNVENLEELRSLFSSARSYQRIQIFPDTIW
NVSYSGTSKACSDSFYRSMRWLTQKDNAYPIQDAQYTNNQEKNILFMWGINHPPTDTAQTNLYTRTDTTT
SVATEEINRTFKPLIGPRPLVNGLMGRINYYWSVLKPGQTLRIKSNGNLIAPWYGHILSGESHGRILKTD
LKRGSCTVQCQTEKGGLNTTLPFQNVSKYAFGNCSKYIGIKSLKLAVGLRNVPSRSSRGLFGAIAGFIEG
GWSGLVAGWYGFQHSNDQGVGMAADRDSTQKAIDKITSKVNNIVDKMNKQYEIIDHEFSEVETRLNMINN
KIDDQIQDVWAYNAELLVLLENQKTLDEHDANVNNLYNKVKRALGSNAVEDGKGCFELYHKCDDQCMETI
RNGTYNRRKYQEESKLERQKIEGVKLESEGTYKILTIYSTVASSLVIAMGFAAFLFWAMSNGSCRCNICI

>QCT26607.1 hemagglutinin [Influenza A virus]
METASLITILLVVTVSNADKICIGYQSTNSTETVDTLTENNVPVTHAKELLHTEHNGMLCATSLGHPLIL
DTCTIEGLIYGNPSCDLLLGGREWSYIVERPSAVNGLCYPGNVENLEELRSLFSSASSYQRIQIFPDTIW
NVSYSGTSKACSDSFYRSMRWLTQKDNAYPTQDAQYTNNQEKNILFMWGINHPPTDTAQTNLYTRTDTTT
SVATEEINRTFKPLIGPRPLVNGLMGRINYYWSVLKPGQTLRIKSNGNLIAPWYGHILSGESHGRILKTD
LKKGSCTVQCQTEKGGLNTTLPFQNVSKYAFGNCSKYIGIKSLKLAVGLRNVPSRSSRGLFGAIAGFIEG
GWSGLVAGWYGFQHSNDQGVGMAADRDSTQKAIDKITSKVNNIVDKMNKQYEIIDHEFSEVETRLNMINN
KIDDQIQDIWAYNAELLVLLENQKTLDEHDANVNNLYNKVKRALGSNAVEDGKGCFELYHKCDDQCMETI
RNGTYNRRKYQEESKLERQKIEGVKLESEGTYKILTIYSTVASSLVIAMGFAAFLFWAMSNGSCRCNICI

>QCT26555.1 hemagglutinin [Influenza A virus]

METASLITILLVVTVSNADKICIGYQSTNSTETVDTLTENNVPVTHAKELLHTEHNGMLCATSLGHPLIL

DTCTIEGLIYGNPSCDLLLGGREWSYIVERPSAVNGLCYPGNVENLEELRSLFSSASSYQRIQIFPDTIW

NVSYSGTSKACSDSFYRSMRWLTQKDNAYPTQDAQYTNNQEKNILFMWGINHPPTDTAQTNLYTRTDTTT

SVATEEINRTFKPLIGPRPLVNGLMGRINYYWSVLKPGQTLRIKSNGNLIAPWYGHILSGESHGRILKTD

LKKGSCTVQCQTEKGGLNTTLPFQNVSKYAFGNCSKYIGIKSLKLAVGLRNVPSRSSRGLFGAIAGFIEG

GWSGLVAGWYGFQHSNDQGVGMAADRDSTQKAIDKITSKVNNIVDKMNKQYEIIDHEFSEVETRLNMINN

KIDDQIQDIWAYNAELLVLLENQKTLDEHDANVNNLYNKVKRALGSNAVEDGKGCFELYHKCDDQCMETI

RNGTYNRRKYQEESKLERQKIEGVKLESEGTYKILTIYSTVASSLVIAMGFAAFLFWAMSNGSCRCNICI

>QCT26516.1 hemagglutinin, partial [Influenza A virus]

ATTIITILLAITGSNADKICIGYQSTNSTETVDTLTENNVPVTHAKELLHTEHNGMLCATNLGRPLILDT

CTIEGLIYGNPSCDLPLEGREWSYIVERPSAVNGVCYPGNVENLEELRSLFSSASSYQRIQIFPDSIWNV

SYSGTSKACSDSFYRSMRWLTQKNNNYPVQDAQYTNNRGKNILFMWGINHPPTETAQTNLYTRTDTTTSV

ATEDINRTFKPLIGPRPLVNGLQGRIDYYWAVLKPGQTLRVRSNGNLIAPWYGHILLGESHGRILKTDLK

SGNCVVQCQTERGGLNTTLPFHNVSKYAFGNCPKYIGVKSLKLAVGLRNVPARSSRGLFGAIAGFIEGGW

SGLVAGWYGFQHSNDQGTGMAADRDSTQKAIDKITSKVNNIVDKMNKQYEIIDHEFSEIETRLNMINDKI

DDQIQDIWAYNAELLVLLENQKTLDEHDANVNNLYNKVKRALGSNAVEDGKGCFDLYHKCDNQCMETIRN

GTYNRRKYQEESRLERQKIEGVKLESEGTYKILTIYSTVASSLVIAMGFAAFLFWAMSNGSCRCNICI

>QCT26492.1 hemagglutinin [Influenza A virus]

METASLITILLVVTASNADKICIGYQSTNSTETVDTLTENNVPVTHAKELLHTEHNGMLCATSLGHPLIL

DTCTIEGLIYGNPSCDPLLGGREWSYIVERPSAVNGLCYPGNVENLEELRSLFSSARSYQRIQIFPDTIW

NVSYSGTSKACSDSFYRSMRWLTQKNNAYPIQDAQYTNNQEKNILFMWGINHPPTDTAQTNLYTRTDTTT

SVATEEINRTFKPLIGPRPLVNGLMGRINYYWSVLKPGQTLRIKSNGNLIAPWYGHILSGESHGRILKTD

LKRGSCTVQCQTEKGGLNTTLPFQNVSKYAFGNCSKYIGIKSLKLAVGLRNVPSRSSRGLFGAIAGFIEG

GWSGLVAGWYGFQHSNDQGVGMAADRDSTQKAIDKITSKVNNIVDKMNKQYEIIDHEFSEVETRLNMINN

KIDDQIQDIWAYNAELLVLLENQKTLDEHDANVNNLYNKVKRALGSNAVEDGKGCFELYHKCDDQCMETI

RNGTYNRRKYQEESKLERQKIEGVKLESEGTYKILTIYSTVASSLVIAMGFAAFLFWAMSNGSCRCNICI

>QCT26469.1 hemagglutinin [Influenza A virus]

METASLMTILLVVTASNADKICIGYQSTNSTETVDTLTENNVPVTHAKELLHTEHNGMLCATSLGHPLIL

DTCTIEGLIYGNPSCDLLLGGREWSYIVERPSAVNGLCYPGNVENLEELRSLFSSARSYQRIQIFPDTIW

NVSYSGTSKACSDSFYRSMRWLTQKDNAYPIQDAQYTNNQEKNILFMWGINHPPTDTAQTNLYTRTDTTT
SVATEEINRTFKPLIGPRPLVNGLMGRINYYWSVLKPGQTLRIKSNGNLIAPWYGHILSGESHGRILKTD
LKRGSCTVQCQTEKGGLNTTLPFQNVSKYAFGNCSKYIGIKSLKLAVGLRNVPSRSSRGLFGAIAGFIEG
GWSGLVAGWYGFQHSNDQGVGMAADRDSTQKAIDKITSKVNNIVDKMNKQYEIIDHEFSEVETRLNMINS
KIDDQIQDIWAYNAELLVLLENQKTLDEHDANVNNLYNKVKRALGSNAVEDGKGCFELYHKCDDQCMETI
RNGTYNRRKYQEESKLERQKIEGVKLESEGTYKILTIYSTVASSIVIAMGFAAFLFWAMSNGSCRCNICI

>QCT26463.1 hemagglutinin [Influenza A virus]
MEAATTVITILLAITGSNADKICIGYQSTNSTETVDTLTENNVPVTHAKELLHTEHNGMLCATNLGRPLI
LDTCTIEGLIYGNPSCDLPLEGREWSYIVERPSAVNGVCYPGNVENLEELRSLFSSASSYQRIQIFPDSI
WNVSYSGTSKACSDSFYRSMRWLTQKNNNYPVQDAQYTNNRGKNILFMWGINQPPTDTAQTNLYTRTDTT
TSVATEDINRTFKPLIGPRPLVNGLQGRIDYYWAVLKPGQTLRVRSNGNLIAPWYGHILLGESHGRILKT
DLKSGNCVVQCQTERGGLNTTLPFHNVSKYAFGNCPKYIGVKSLKLAVGLRNVPARSSRGLFGAIAGFIE
GGWSGLVAGWYGFQHSNDQGTGMAADRDSTQKAIDKITSKVNNIVDKMNKQYEVIDHEFSEIETRLNMIN
DKIDDQIQDIWAYNAELLVLLENQKTLDEHDANVNNLYNKVKRALGSNAVEDGKGCFDLYHKCDNQCMET
IRNGTYNRRKYQEESRLERQKIEGVKLESEGTYKILTIYSTVASSLVIAMGFAAFLFWAMSNGSCRCNIC
I

>QCT26234.1 hemagglutinin [Influenza A virus]
METASLITILLVVTVSNADKICIGYQSTNSTETVDTLTENNVPVTHAKELLHTEHNGMLCATSLGHPLIL
DTCTIEGLIYGNPSCDLLLGGREWSYIVERPTAVNGLCYPGNVENLEELRSLFSSARSYQRIQIFPDTIW
NVSYSGTSKACSDSFYRSMRWLTQKDNAYPIQDAQYTNNQEKNILFMWGINHPPTDTAQTNLYTRTDTTT
SVATEEINRTFKPLIGPRPLVNGLMGRINYYWSVLKPGQTLRIKSNGNLIAPWYGHILSGESHGRILKTD
LKKGSCTVQCQTEKGGLNTTLPFQNVSKYAFGNCSKYIGIKSLKLAVGLRNVPSRSSRGLFGAIAGFIEG
GWSGLVAGWYGFQHSNDQGVGMAADRDSTQKAIDKITSKVNNIVDKMNKQYEIIDHEFSEVETRLNMINN
KIDDQIQDIWAYNAELLVLLENQKTLDEHDANVNNLYNKVKRALGSNAVEDGKGCFELYHKCDDRCMETI
RNGTYNRRKYQEESKLERQKIEGVKLESEGTYKILTIYSTVASSLVIAMGFAAFLFWAMSNGSCRCNICI

>QCT26194.1 hemagglutinin [Influenza A virus]
METASLITILLVVTASNADKICIGYQSTNSTETVDTLTENNVPVTHAKELLHTEHNGMLCATSLGHPLIL
DTCTIEGLIYGNPSCDLLLGGREWSYIVERPSAVNGLCYPGNVENLEELRSLFSSARSYQRIQIFPDTIW
NVSYSGTSKACSDSFYRSMRWLTQKDNAYPIQDAQYTNNQEKNILFMWGINHPPTDTAQTNLYTRTDTTT
SVATEEINRTFKPLIGPRPLVNGLMGRINYYWSVLKPGQTLRIKSNGNLIAPWYGHILSGESHGRILKTD
LKRGSCTVQCQTEKGGLNTTLPFQNVSKYAFGNCSKYIGIKSLKLAVGLRNVPSRSSRGLFGAIAGFIEG

GWSGLVAGWYGFQHSNDQGVGMAADRDSTQKAIDKITSKVNNIVDKMNKQYEIIDHEFSEVETRLNMINS
KIDDQIQDIWAYNAELLVLLENQKTLDEHDANVNNLYNKVKRALGSNAVEDGKGCFELYHKCDDQCMETI
RNGTYNRRKYQEESKLERQKIEGVKLESEGTYKILTIYSTVASSIVIAMGFAAFLFWAMSNGSCRCNICI

>QCT26126.1 hemagglutinin [Influenza A virus]
METASLMTILLVVTTSNADKICIGYQSTNSTETVDTLTENNVPVTHAKELLHTEHNGMLCATSLGHPLIL
DTCTIEGLIYGNPSCDLLLGGREWSYIVERPSAVNGLCYPGNVENLEELRSLFSSARSYQRIQIFPDTIW
NVSYSGTSKACSDSFYRSMRWLTQKDNAYPIQDAQYTNNQEKNILFMWGINHPPTDTAQTNLYTRTDTTT
SVATEEINRTFKPLIGPRPLVNGLMGRINYYWSVLKPGQTLRIKSNGNLIAPWYGHILSGESHGRILKTD
LKRGSCTVQCQTEKGGLNTTLPFQNVSKYAFGNCSKYIGIKSLKLAVGLRNVPSRSSRGLFGAIAGFIEG
GWSGLVAGWYGFQHSNDQGVGMAADRDSTQKAIDKITSKVNNIVDKMNKQYEIIDHEFSEVETRLNMINS
KIDDQIQDIWAYNAELLVLLENQKTLDEHDANVNNLYNKVKRALGSNAVEDGKGCFELYHKCDDQCMETI
RNGTYNRRKYQEESKLERQKIEGVKLESEGTYKILTIYSTVASSIVIAMGFAAFLFWAMSNGSCRCNICI

>QCT26067.1 hemagglutinin [Influenza A virus]
METASLMTILLVVTASNADKICIGYQSTNSTETVDTLTENNVPVTHAKELLHTEHNGMLCATSLGHPLIL
DTCTIEGLIYGNPSCDLLLGGREWSYIVERPSAVNGLCYPGNVENLEELRSLFSSARSYQRIQIFPDTIW
NVSYSGTSKACSDSFYRSMRWLTQKDNAYPIQDAQYTNNQEKNILFMWGINHPPTDTAQTNLYTRTDTTT
SVATEEINRTFKPLIGPRPLVNGLMGRINYYWSVLKPGQTLRIKSNGNLIAPWYGHILSGESHGRILKTD
LKRGSCTVQCQTEKGGLNTTLPFQNVSKYAFGNCSKYIGIKSLKLAVGLRNVPSRSSRGLFGAIAGFIEG
GWSGLVAGWYGFQHSNDQGVGMAADRDSTQKAIDKITSKVNNIVDKMNKQYEIIDHEFSEVETRLNMINS
KIDDQIQDIWAYNAELLVLLENQKTLDEHDANVNNLYNKVKRALGSNAVEDGKGCFELYHKCDDQCMETI
RNGTYNRRKYQEESKLERQKIEGVKLESEGTYKILTIYSTVASSIVIAMGFAAFLFWAMSNGSCRCNICI

>QCT26039.1 hemagglutinin [Influenza A virus]
METASLMTILLVVTASNADKICIGYQSTNSTETVDTLTENNVPVTHAKELLHTEHNGMLCATSLGHPLIL
DTCTIEGLIYGNPSCDLLLGGREWSYIVERPSAVNGLCYPGNVENLEELRSLFSSARSYQRIQIFPDTIW
NVSYSGTSKACSDSFYRSMRWLTQKDNAYPIQDAQYTNNQEKNILFMWGINHPPTDTAQTNLYTRTDTTT
SVATEEINRTFKPLIGPRPLVNGLMGRINYYWSVLKPGQTLRIKSNGNLIAPWYGHILSGESHGRILKTD
LKRGSCTVQCQTEKGGLNTTLPFQNVSKYAFGNCSKYIGIKSLKLAVGLRNVPSRSSRGLFGAIAGFIEG
GWSGLVAGWYGFQHSNDQGVGMAADRDSTQKAIDKITSKVNNIVDKMNKQYEIIDHEFSEVETRLNMINS
KIDDQIQDIWAYNAELLVLLENQKTLDEHDANVNNLYNKVKRALGSNAVEDGKGCFELYHKCDDQCMETI
RNGTYNRRKYQEESKLERQKIEGVKLESEGTYKILTIYSTVASSIVIAMGFAAFLFWAMSNGSCRCNICI

>QCT25978.1 hemagglutinin [Influenza A virus]

METASLITILLVVTVSNADKICIGYQSTNSTETVDTLTENNVPVTHAKELLHTEHNGMLCATSLGHPLIL

DTCTIEGLIYGNPSCDLLLGGREWSYIVERPSAVNGLCYPGNVENLEELRSLFSSASSYQRIQIFPDTIW

NVSYSGTSKACSDSFYRSMRWLTQKDNAYPTQDAQYTNNQEKNILFMWGINHPPTDTAQTNLYTRTDTTT

SVATEEINRTFKPLIGPRPLVNGLMGRINYYWSVLKPGQTLRIKSNGNLIAPWYGHILSGESHGRILKTD

LKKGSCTVQCQTEKGGLNTTLPFQNVSKYAFGNCSKYIGIKSLKLAVGLRNVPSRSSRGLFGAIAGFIEG

GWSGLVAGWYGFQHSNDQGVGMAADRDSTQKAIDKITSKVNNIVDKMNKQYEIIDHEFSEVETRLNMINN

KIDDQIQDIWAYNAELLVLLENQKTLDEHDANVNNLYNKVKRALGSNAVEDGKGCFELYHKCDDQCMETI

RNGTYNRRKYQEESKLERQKIEGVKLESEGTYKILTIYSTVASSLVIAMGFAAFLFWAMSNGSCRCNICI

>QCT25967.1 hemagglutinin, partial [Influenza A virus]

SLMTILLVVTTSNADKICIGYQSTNSTETVDTLTENNVPVTHAKELLHTEHNGMLCATSLGHPLILDTCT

IEGLIYGNPSCDLLLGGREWSYIVERPSAVNGLCYPGNVENLEELRSLFSSARSYQRIQIFPDTIWNVSY

SGTSKACSDSFYRSMRWLTQKDNAYPIQDAQYTNNQEKNILFMWGINHPPTDTAQTNLYTRTDTTTSVAT

EEINRTFKPLIGPRPLVNGLMGRINYYWSVLKPGQTLRIKSNGNLIAPWYGHILSGESHGRILKTDLKRG

SCTVQCQTEKGGLNTTLPFQNVSKYAFGNCSKYIGIKSLKLAIGLRNVPSRSSRGLFGAIAGFIEGGWSG

LVAGWYGFQHSNDQGVGMAADRDSTQKAIDKITSKVNNIVDKMNKQYEIIDHEFSEVETRLNMINSKIDD

QIQDIWAYNAELLVLLENQKTLDEHDANVNNLYNKVKRALGSNAVEDGKGCFELYHKCDDQCMETIRNGT

YNRRKYQEESKLERQKIEGVKLESEGTYKILTIYSTVASSIVIAMGFAAFLFWAMSNGSCRCNICI

>QCT25955.1 hemagglutinin [Influenza A virus]

METASLMTILLVVTASNADKICIGYQSTNSTETVDTLTENNVPVTHAKELLHTEHNGMLCATSLGHPLIL

DTCTIEGLIYGNPSCDLLLGGREWSYIVERPSAVNGLCYPGNVENLEELRSLFSSARSYQRIQIFPDTIW

NVSYSGTSKACSDSFYRSMRWLTQKDNAYPIQDAQYTNNREKNILFMWGINHPPTDTAQTNLYTRTDTTT

SVATEEINRTFKPLIGPRPLVNGLMGRINYYWSVLKPGQTLRIKSXXXXXXXXXXXXXXXXXXXXXXXXX

XXXXXXXXXXXXXXXXXXXXXXXXXXXXXXXXXXXXXXXXXXXXXXXXXXXXXXXXXXXXXXXKAGFIEG

GWSGLVAGWYGFQHSNDQGVGMAADRDSTQKAIDKITSKVNNIVDKMNKQYEIIDHEFSEVETRLNMINS

KIDDQIQDIWAYNAELLVLLENQKTLDEHDANVNNLYNKVKRALGSNAVEDGKGCFELYHKCDDQCMETI

RNGTYNRRKYQEESKLERQKIEGVKLESEGTYKILTIYSTVASSIVIAMGFAAFLFWAMSNGSCRCNICI

>QCT25882.1 hemagglutinin [Influenza A virus]

MEAATIIIILLAITGSNADKICIGYQSTNSTETVDTLTENNVPVTHAKELLHTEHNGMLCATNLGRPLIL

DTCTIEGLIYGNPSCDLPLEGREWSYIVERPSAVNGVCYPGNVENLEELRSLFSSASSYQRIQIFPDSIW

NVSYSGTSKACSDSFYRSMRWLTQKNNNYPVQDAQYTNNRGKNILFMWGINHPPTDTAQTNLYTRTETTT

SVATEDINRTFKPLIGPRPLVNGLQGRIDYYWAVLKPGQTLRIRSNGNLIAPWYGHILLGESHGRILKTD
LKSGNCVVQCQTERGGLNTTLPFHNVSKYAFGNCPKYIGVKSLKLAVGLRNVPARSSRGLFGAIAGFIEG
GWSGLVAGWYGFQHSNDQGTGMAADRDSTQKAVDKITSKVNNIVDKMNKQYEIIDHEFSEIETRLNMIND
KIDDQIQDIWAYNAELLVLLENQKTLDEHDANVNNLYNKVKRALGSNAVEDGKGCFDLYHKCNNQCMETI
RNGTYNRRKYQEESRLERQKIEGVKLESEGTYKILTIYSTVASSLVIAMGFAAFLFWAMSNGSCRCNICI

>QCT25857.1 hemagglutinin [Influenza A virus]
METASLITILLVVTASNADKICIGYQSTNSTETVDTLTENNVPVTHAKELLHTEHNGMLCATSLGHPLIL
DTCTIEGLIYGNPSCDLLLGGREWSYIVERPSAVNGLCYPGNVENLEELRSLFSSARSYQRIQIFPDTIW
NVSYSGTSKACSDSFYRSMRWLTQKDNAYPIQDAQYTNNQEKNILFMWGINHPPTDTAQTNLYTRTDTTT
SVATEEINRTFKPLIGPRPLVNGLMGRINYYWSVLKPGQTLRIKSNGNLIAPWYGHILSGESHGRILKTD
LKRGSCTVQCQTEKGGLNTTLPFQNVSKYAFGNCSKYIGIKSLKLAVGLRNVPSRSSRGLFGAIAGFIEG
GWSGLVAGWYGFQHSNDQGVGMAADRDSTQKAIDKITSKVNNIVDKMNKQYEIIDHEFSEVETRLNMINS
KIDDQIQDVWAYNAELLVLLENQKTLDEHDANVNNLYNKVKRALGSNAVEDGKGCFELYHKCDDQCMETI
RNGTYNRRKYQEESKLERQKIEGVKLESEGTYKILTIYSTVASSLVIAMGFAAFLFWAMSNGSCRCNICI

>QCT25831.1 hemagglutinin [Influenza A virus]
METASLITILLVVTVSNADKICIGYQSTNSTETVDTLTENNVPVTHAKELLHTEHNGMLCATSLGHPLIL
DTCTIEGLIYGNPSCDLLLGGREWSYIVERPSAVNGLCYPGNVENLEELRSLFSSASSYQRIQIFPDTIW
NVSYSGTSKACSDSFYRSMRWLTQKDNAYPTQDAQYTNNQEKNILFMWGINHPPTDTAQTNLYTRTDTTT
SVATEEINRTFKPLIGPRPLVNGLMGRINYYWSVLKPGQTLRIKSNGNLIAPWYGHILSGESHGRILKTD
LKKGSCTVQCQTEKGGLNTTLPFQNVSKYAFGNCSKYIGIKSLKLAVGLRNVPSRSSRGLFGAIAGFIEG
GWSGLVAGWYGFQHSNDQGVGMAADRDSTQKAIDKITSKVNNIVDKMNKQYEIIDHEFSEVETRLNMINN
KIDDQIQDIWAYNAELLVLLENQKTLDEHDANVNNLYNKVKRALGSNAVEDGKGCFELYHKCDDQCMETI
RNGTYNRRKYQEESKLERQKIEGVKLESEGTYKILTIYSTVASSLVIAMGFAAFLFWAMSNGSCRCNICI

>QCT25799.1 hemagglutinin, partial [Influenza A virus]
AATTIITILLAITGSNADKICIGYQSTNSTETVDTLTENNVPVTHAKELLHTEHNGMLCATNLGRPLILD
TCTIEGLIYGNPSCDLPLEGREWSYIVERPSAVNGVCYPGNVENLEELRSLFSSASSYQRIQIFPDSIWN
VSYSGTSKACSDSFYRSMRWLTQKNNNYPVQDAQYTNNRGKNILFMWGINHPPTETAQTNLYTRTDTTTS
VATEDINRTFKPLIGPRPLVNGLQGRIDYYWAVLKPGQTLRVRSNGNLIAPWYGHILLGESHGRILKTDL
KSGNCVVQCQTERGGLNTTLPFHNVSKYAFGNCPKYIGVKSLKLAVGLRNVPARSSRGLFGAIAGFIEGG
WSGLVAGWYGFQHSNDQGTGMAADRDSTQKAIDKITSKVNNIVDKMNKQYEIIDHEFSEIETRLNMINDK
IDDQIQDIWAYNAELLVLLENQKTLDEHDANVNNLYNKVKRALGSNAVEDGKGCFDLYHKCDNQCMETIR

NGTYNRRKYQEESRLERQKIEGVKLESEGTYKILTIYSTVASSLVIAMGFAAFLFWAMSNGSCRCNICI

>QCT25713.1 hemagglutinin [Influenza A virus]
METASLITILLVVTVSNADKICIGYQSTNSTETVDTLTENNVPVTHAKELLHTEHNGMLCATSLGHPLIL
DTCTIEGLIYGNPSCDLLLGGREWSYIVERPSAVNGLCYPGNVENLEELRSLFSSASSYQRIQIFPDTIW
NVSYSGTSKACSDSFYRSMRWLTQKDNAYPTQDAQYTNNQEKNILFMWGINHPPTDTAQTNLYTRTDTTT
SVATEEINRTFKPLIGPRPLVNGLMGRINYYWSVLKPGQTLRIKSNGNLIAPWYGHILSGESHGRILKTD
LKKGSCTVQCQTEKGGLNTTLPFQNVSKYAFGNCSKYIGIKSLKLAVGLRNVPSRSSRGLFGAIAGFIEG
GWSGLVAGWYGFQHSNDQGVGMAADRDSTQKAIDKITSKVNNIVDKMNKQYEIIDHEFSEVETRLNMINN
KIDDQIQDIWAYNAELLVLLENQKTLDEHDANVNNLYNKVKRALGSNAVEDGKGCFELYHKCDDQCMETI
RNGTYNRRKYQEESKLERQKIEGVKLESEGTYKILTIYSTVASSLVIAMGFAAFLFWAMSNGSCRCNICI

>QCT25631.1 hemagglutinin [Influenza A virus]
METASLITILLVVTVSNADKICIGYQSTNSTETVDTLTENNVPVTHAKELLHTEHNGMLCATSLGHPLIL
DTCTIEGLIYGNPSCDLLLGGREWSYIVERPSAVNGLCYPGNVENLEELRSLFSSASSYQRIQIFPDTIW
NVSYSGTSKACSDSFYRSMRWLTQKDNAYPTQDAQYTNNQEKNILFMWGINHPPTDTAQTNLYTRTDTTT
SVATEEINRTFKPLIGPRPLVNGLMGRINYYWSVLKPGQTLRIKSNGNLIAPWYGHILSGESHGRILKTD
LKKGSCTVQCQTEKGGLNTTLPFQNVSKYAFGNCSKYIGIKSLKLAVGLRNVPSRSSRGLFGAIAGFIEG
GWSGLVAGWYGFQHSNDQGVGMAADRDSTQKAIDKITSKVNNIVDKMNKQYEIIDHEFSEVETRLNMINN
KIDDQIQDIWAYNAELLVLLENQKTLDEHDANVNNLYNKVKRALGSNAVEDGKGCFELYHKCDDQCMETI
RNGTYNRRKYQEESKLERQKIEGVKLESEGTYKILTIYSTVASSLVIAMGFAAFLFWAMSNGSCRCNICI

>QCT25613.1 hemagglutinin [Influenza A virus]
MEAATIIIILLAITGSNADKICIGYQSTNSTETVDTLTENNVPVTHAKELLHTEHNGMLCATNLGRPLIL
DTCTIEGLIYGNPSCDLPLEGREWSYIVERPSAVNGVCYPGNVENLEELRSLFSSASSYQRIQIFPDSIW
NVSYSGTSKACSDSFYRSMRWLTQKNNNYPVQDAQYTNNRGKNILFMWGINHPPTDTAQTNLYTRTETTT
SVATEDINRTFKPLIGPRPLVNGLQGRIDYYWAVLKPGQTLRIRSNGNLIAPWYGHILLGESHGRILKTD
LKSGNCVVQCQTERGGLNTTLPFHNVSKYAFGNCPKYIGVKSLKLAVGLRNVPARSSRGLFGAIAGFIEG
GWSGLVAGWYGFQHSNDQGTGMAADRDSTQKAVDKITSKVNNIVDKMNKQYEIIDHEFSEIETRLNMIND
KIDDQIQDIWAYNAELLVLLENQKTLDEHDANVNNLYNKVKRALGSNAVEDGKGCFDLYHKCNNQCMETI
RNGTYNRRKYQEESRLERQKIEGVKLESEGTYKILTIYSTVASSLVIAMGFAAFLFWAMSNGSCRCNICI

>QCR65173.1 hemagglutinin [Influenza A virus]
METASLITILLVVTASKADKICIGYQSTNSTETVDTLTENNVPVTHAKELLHTEHNGMLCATSLGHPLIL

DTCTIEGLIYGNPSCDLLLGGREWSYIVERPSAVNGLCYPGNVENLEELRSLFSSARSYHRIQIFPDTIW
NVSYSGTSKACSDSFYRSMRWLTQKNNAYPIQDAQYTNNQEKNILFMWGINHPPTDTAQTNLYTRTDTTT
SVATEEINRTFKPLIGPRPLVNGLMGRINYYWSVLKPGQTLRIKTNGNLIAPWYGHILSGESHGRILKTD
LKSGSCTVQCQTEKGGLNTTLPFQNVSRYAFGNCSKYIGIKSLKLAVGLRNVPSKSSRGLFGAIAGFIEG
GWPGLVAGWYGFQHSNDQGVGMAADRDSTQKAIDKITSKVNNIVDKMNKQYEIIDHEFSEVETRLNMINS
KIDDQIQDIWAYNAELLVLLENQKTLDEHDANVNNLYNKVKRALGSNAVEDGKGCFELYHKCDDQCMETI
RNGTYNRRKYQEESKLERQKIEGVKLESEGTYKILTIYSTVASSLVIAMGFAAFLFWAMSNGSCRCNICI

>QCR65149.1 hemagglutinin [Influenza A virus]
METASLMTILLVVTASNADKICIGYQSTNSTETVDTLTENNVPVTHAKELLHTEHNGMLCATSLGHPLIL
DTCTIEGLIYGNPSCDPLLGGREWSYIVERPSAVNGLCYPGNVENLEELRSLFSSARSYQRILIFPDTIW
NVSYSGTSKACSDSFYRSMRWLTQKNNAYPIQDAQYTNNQEKNILFMWGINHPPTDTAQTNLYTRTDTTT
SVATEEINRTFKPLIGPRPLVNGLMGRINYYWSVLKPGQTLRIKSNGNLIAPWYGHILSGESHGRILKTD
LKRGSCTVQCQTEKGGLNTTLPFQNVSKYAFGNCSKYIGIKSLKLAVGLRNVPSRSSRGLFGAIAGFIEG
GWSGLVAGWYGFQHSNDQGVGMAADRDSTQKAIDKITSKVNNIVDKMNKQYEIIDHEFSEVETRLNMINN
KIDDQIQDIWAYNAELLVLLENQKTLDEHDANVNNLYNKVKRALGSNAVEDGKGCFELYHKCNDQCMETI
RNGTYNRRKYQEESKLERQKIEGVKLESEGTYKILTIYSTVASSLVIAMGFAAFLFWAMSNGSCRCNICI

>QCR65137.1 hemagglutinin [Influenza A virus]
METASLMTILLVVTASNADKICIGYQSTNSTETVDTLTENNVPVTHAKELLHTEHNGMLCATSLGHPLIL
DTCTIEGLIYGNPSCDPLLGGREWSYIVERPSAVNGLCYPGNVENLEELRSLFSSARSYQRILIFPDTIW
NVSYSGTSKACSDSFYRSMRWLTQKNNAYPIQDAQYTNNQEKNILFMWGINHPPTDTAQTNLYTRTDTTT
SVATEEINRTFKPLIGPRPLVNGLMGRINYYWSVLKPGQTLRIKSNGNLIAPWYGHILSGESHGRILKTD
LKRGSCTVQCQTEKGGLNTTLPFQNVSKYAFGNCSKYIGIKSLKLAVGLRNVPSRSSRGLFGAIAGFIEG
GWSGLVAGWYGFQHSNDQGVGMAADRDSTQKAIDKITSKVNNIVDKMNKQYEIIDHEFSEVETRLNMINN
KIDDQIQDIWAYNAELLVLLENQKTLDEHDANVNNLYNKVKRALGSNAVEDGKGCFELYHKCNDQCMETI
RNGTYNRRKYQEESKLERQKIEGVKLESEGTYKILTIYSTVASSLVIAMGFAAFLFWAMSNGSCRCNICI

>QCR65114.1 hemagglutinin [Influenza A virus]
METASLITILLVVTASKADKICIGYQSTNSTETVDTLTENNVPVTHAKELLHTEHNGMLCATSLGHPLIL
DTCTIEGLIYGNPSCDLLLGGREWSYIVERPSAVNGLCYPGNVENLEELRSLFSSARSYHRIQIFPDTIW
NVSYSGTSKACSDSFYRSMRWLTQKNNAYPIQDAQYTNNQEKNILFMWGINHPPTDTAQTNLYTRTDTTT
SVATEEINRTFKPLIGPRPLVNGLMGRINYYWSVLKPGQTLRIKTNGNLIAPWYGHILSGESHGRILKTD
LKSGSCTVQCQTEKGGLNTTLPFQNVSRYAFGNCSKYIGIKSLKLAVGLRNVPSKSSRGLFGAIAGFIEG

GWPGLVAGWYGFQHSNDQGVGMAADRDSTQKAIDKITSKVNNIVDKMNKQYEIIDHEFSEVETRLNMINS
KIDDQIQDIWAYNAELLVLLENQKTLDEHDANVNNLYNKVKRALGSNAVEDGKGCFELYHKCDDQCMETI
RNGTYNRRKYQEESKLERQKIEGVKLESEGTYKILTIYSTVASSLVIAMGFAAFLFWAMSNGSCRCNICI

>QCR65082.1 hemagglutinin [Influenza A virus]
METASLITILLVVTASNADKICIGYQSTNSTETVDTLTENNVPVTHAKELLHTEHNGMLCATSLGHPLIL
DTCTIEGLIYGNPSCDPLLGGREWSYIVERPSAVNGLCYPGNVENLEELRSLFSSARSYQRILIFPDTIW
NVSYSGTSKACSDSFYRSMRWLTQKDNAYPIQDAQYTNNQEKNILFMWGINHPPTDTAQTNLYTRTDTTT
SVATEEINRTFKPLIGPRPLVNGLMGRINYYWSVLKPGQTLRIKSNGNLIAPWYGHILSGESHGRILKTD
LKRGSCTVQCQTEKGGLNTTLPFQNVSKYAFGNCSKYIGIKSLKLAVGLRNVPSRSSRGLFGAIAGFIEG
GWSGLVAGWYGFQHSNDQGVGMAADRDSTQKAIDKITSKVNNIVDKMNKQYEIIDHEFSEVETRLNMINN
KIDDQIQDIWAYNAELLVLLENQKTLDEHDANVNNLYNKVKRALGSNAVEDGKGCFELYHKCDDQCMETI
RNGTYNRRKYQEESKLERQKIEGVKLESEGTYKILTIYSTVASSLVIAMGFAAFLFWAMSNGSCRCNICI

>QCR64889.1 hemagglutinin [Influenza A virus]
METASLITILLVVTASNADKICIGYQSTNSTETVDTLTENNVPVTHAKELLHTEHNGMLCATSLGHPLIL
DTCTIEGLIYGNPSCDPLLGGREWSYIVERPSAVNGLCYPGNVENLEELRSLFSSARSYQRILIFPDTIW
NVSYSGTSKACSDSFYRSMRWLTQKDNAYPIQDAQYTNNQEKNILFMWGINHPPTDTAQTNLYTRTDTTT
SVATEEINRTFKPLIGPRPLVNGLMGRINYYWSVLKPGQTLRIKSNGNLIAPWYGHILSGESHGRILKTD
LKRGSCTVQCQTEKGGLNTTLPFQNVSKYAFGNCSKYIGIKSLKLAVGLRNVPSRSSRGLFGAIAGFIEG
GWSGLVAGWYGFQHSNDQGVGMAADRDSTQKAIDKITSKVNKIVDKMNKQYEIIDHEFSEVETRLNMINN
KIDDQIQDIWAYNAELLVLLENQKTLDEHDANVNNLYNKVKRALGSNAVEDGKGCFELYHKCDDQCMETI
RNGTYNRRKYQEESKLERQKIEGVKLESEGTYKILTIYSTVASSLVIAMGFAAFLFWAMSNGSCRCNICI

>QCR64766.1 hemagglutinin [Influenza A virus]
METASLITILLVVTASNADKICIGYQSTNSTETVDTLTENNVPVTHAKELLHTEHNGMLCATSLGHPLIL
DTCTIEGLIYGNPSCDPLLGGREWSYIVERPSAVNGLCYPGNVENLEELRSLFSSARSYQRILIFPDTIW
NVSYSGTSKACSDSFYRSMRWLTQKDNAYPIQDAQYTNNQEKNILFMWGINHPPTDTAQTNLYTRTDTTT
SVATEEINRTFKPLIGPRPLVNGLMGRINYYWSVLKPGQTLRIKSNGNLIAPWYGHILSGESHGRILKTD
LKRGSCTVQCQTEKGGLNTTLPFQNVSKYAFGNCSKYIGIKSLKLAVGLRNVPSRSSRGLFGAIAGFIEG
GWSGLVAGWYGFQHSNDQGVGMAADRDSTQKAIDKITSKVNNIVDKMNKQYEIIDHEFSEVETRLNMINN
KIDDQIQDIWAYNAELLVLLENQKTLDEHDANVNNLYNKVKRALGSNAVEDGKGCFELYHKCDDQCMETI
RNGTYNRRKYQEESKLERQKIEGVKLESEGTYKILTIYSTVASSLVIAMGFAAFLFWAMSNGSCRCNICI

>QCR64723.1 hemagglutinin [Influenza A virus]

METASLITILLVVTASKADKICIGYQSTNSTETVDTLTENNVPVTHAKELLHTEHNGMLCATSLGHPLIL

DTCTIEGLIYGNPSCDLLLGGREWSYIVERPSAVNGLCYPGNVENLEELRSLFSSARSYHRIQIFPDTIW

NVSYSGTSKACSDSFYRSMRWLTQKNNAYPIQDAQYTNNQEKNILFMWGINHPPTDTAQTNLYTRTDTTT

SVATEEINRTFKPLIGPRPLVNGLMGRINYYWSVLKPGQTLRIKTNGNLIAPWYGHILSGESHGRILKTD

LKSGSCTVQCQTEKGGLNTTLPFQNVSRYAFGNCSKYIGIKSLKLAVGLRNVPSKSSRGLFGAIAGFIEG

GWPGLVAGWYGFQHSNDQGVGMAADRDSTQKAIDKITSKVNNIVDKMNKQYEIIDHEFSEVETRLNMINS

KIDDQIQDIWAYNAELLVLLENQKTLDEHDANVNNLYNKVKRALGSNAVEDGKGCFELYHKCDDQCMETI

RNGTYNRRKYQEESKLERQKIEGVKLESEGTYKILTIYSTVASSLVIAMGFAAFLFWAMSNGSCRCNICI

>QCR64667.1 hemagglutinin [Influenza A virus]

METASLITILLVVTASNADKICIGYQSTNSTETVDTLTENNVPVTHAKELLHTEHNGMLCATSLGHPLIL

DTCTIEGLIYGNPSCDSLLGGREWSYIVERPSAVNGLCYPGNVENLEELRSLFSSARSYQRILIFPDTIW

NVSYSGTSKACSDSFYRNMRWLTQKDNAYPIQDAQYTNNQEKNILFMWGINHPPTDTAQTNLYTRTDTTT

SVATEEINRTFKPLIGPRPLVNGLMGRINYYWSVLKPGQTLRIKSNGNLIAPWYGHILSGESHGRILKTD

LKRGSCTVQCQTEKGGLNTTLPFQNVSKYAFGNCSKYIGIKSLKLAVGLRNVPSRSSRGLFGAIAGFIEG

GWSGLVAGWYGFQHSNDQGVGMAADRDSTQKAIDKITSKVNNIVNKMNKQYEIIDHEFSEVETRLNMINN

KIDDQIQDIWAYNAELLVLLENQKTLDEHDANVNNLYNKVKRALGSNAVEDGKGCFELYHKCDDQCMETI

RNGTYNRRKYQEESKLERQKIEGVKLESEGTYKILTIYSTVASSLVIAMGFAAFLFWAMSNGSCRCNICI

>QCR64654.1 hemagglutinin, partial [Influenza A virus]

MTILLVVTTSNADKICIGHQSTNSTETVDTLTETNVPVTHAKELLHTEHNGMLCATNLGRPLILEACTIE

GLIYGNPACDMLLGGREWSYIVERPSAVNGTCYPGNVENLEELRTLFSYSSSYQRIQIFPDSIWNVTYTG

TSKSCSDSFYRNMRWLTQKNGLYPIQDAQYTNNRGKDILFVWGIHHPPTDNAQTNLYTRTDTTTSVTTEN

LDRTFKPLIGPRPLVNGQIGRINYFWSVLKPGQTLRIRSNGNLIAPCFGHVLSGESHGRILRTDLNSGNC

VVQCQTEKGGLNSTLPFHNISKYAFGNCPKYIGVKSLKLAIGLRNVHARSSRGLFGAIAGFIEGGWPGLV

AGWYGFQHSNDQGVGMAADRVSTQKAVDKITSKVNNIVDKMNKQYEIIDHEFNEVETRLNMINNKIDDQI

QDIWAYNAELLVLLENQKTLDEHDANVNNLYNKVKRALGSNAMEDGKGCFELYHKCDDQCMETIRNGTHN

RRKYTEESRLERQKIEGVKLEAEGTYKILSIYSTVASSLVLAMGFAAFLFWAMSNGSCRCNICI

>AVM33076.1 hemagglutinin [Influenza A virus]

METISLISILLIVATSNADKICVGHQSTNSTETVDTLTETNVPVTHAKELLHTEHNGMLCATNLGHPLIL

DTCTIEGLIYGNPSCDLLLGGREWSYIVERPSAVNGTCYPGNVENLEELRTLFSSSSSYQRIQIFPDTIW

NVTHTGTSKSCSDSFYRNMRWLTQKNGLYPVQDAQYTNNRGKDILFVWGIHHPPTDTAQTNLYTRTDTTT

SVTTENLDRTFKPLIGPRPLVNGLIGRINYYWSVLKPGQTLRVRSNGNLIAPWYGHVLSGESHGRILKTN
LNSGNCVVQCQTEKGGLNSTLPFHNISKYAFGNCPKYIGVKSLKLAIGLRNVPAKSSRGLFGAIAGFIEG
GWPGLVAGWYGFQHSNDQGVGMAADRDSTQKAIDKITTKVNNIIDKMNNQYEIIDHEFSEVETRLNMINN
KLDDQIQDVWAYNAELLVLLENQKTLDEHDANVNNLYNKVKRALGSNAMEDGKGCFELYHKCDDQCMETI
RNGTYNRRKYKEESRLERQKIEGIKLESEETYKILSIYSTVASSLVLAMGFAAFLFWAMSTGSCRCNICI

>AVK43547.1 hemagglutinin [Influenza A virus]
METVSLITILLVATISNADKICIGYQSTNSTETVDTLTENNVPVTHAKELLHTEHNGMLCATSLGHPLIL
DTCTIEGLIYGNPSCDLLLGGREWSYIVERPSAVNGLCYPGNVENLEELRSLFSSARSYQRIQIFPDTIW
NVSYSGTSKACSDSFYRSMRWLTQKNNAYPIQDAQYTNNQEKNILFMWGINHPPTDTAQTNLYTRTSTTT
SVATEEINRIFKPLIGPRPLVNGLMGRIDYYWSVLKPGQTLRIRSDGNLIAPWYGHILSGESHGRILKTD
LKRGSCTVQCQTEKGGLNTTLPFQNVSKYAFGNCSKYIGIKSLKLAVGLRNVPSRSSRGLFGAIAGFIEG
GWSGLVAGWYGFQHSNDQGVGMAADRDSTQKAIDKITSKVNNIVDKMNKQYEIIDHEFSEVETRLNMINN
KIDDQIQDIWAYNAELLVLLENQKTLDEHDANVNNLYNKVKRALGSNAVEDGKGCFELYHKCNDQCMETI
RNGTYNRKKYQEESKLERQKIEGVKLESEGTYKILTIYSTVASSLVIAMGFAAFLFWAMSNGSCRRNICI

>AVK43546.1 hemagglutinin [Influenza A virus]
METASLITILLVVTVSNADKICIGYQSTNSTETVDTLTENNVPVTHAKELLHTEHNGMLCATSLGHPLIL
DTCTIEGLIYGNPSCDLLLGGREWSYIVERPSAVNGLCYPGNVENLEELRSLFSSARSYQRIQIFPDTIW
NVSYSGTSKACSDSFYRSMRWLTQKNNAYPIQDAQYTNNQEKNILFMWGINHPPTDTTQTNLYTRTDTTT
SVATEEINRTFKPLIGPRPLVNGLMGRIDYYWSVLKPGQTLRIRSNGNLIAPWYGHILSGESHGRILKTD
LKRGSCTVQCQTEKGGLNTTLPFQNVSKYAFGNCSKYIGIKSLKLAVGLRNVPSRSSRGLFGAIAGFIEG
GWSGLVAGWYGFQHSNDQGVGMAADRDSTQKAIDKITSKVNNIVDKMNKQYEIIDHEFSEVETRLNMINN
KIDDQIQDIWAYNAELLVLLENQKTLDEHDANVNNLYNKVKRALGSNAVEDGKGCFELYHKCDDQCMETI
RNGTYNRRKYQEESKLEKQKIEGVKLESEGTYKILTIYSTVASSLVIAMGFAAFLFWAMSNGSCRCNICI

>AVK43545.1 hemagglutinin [Influenza A virus]
METAPLVTMLLVVAVSNADKICIGYQSTNSTETVDTLTENNVPVTHAKELLHTEHNGMLCATSLGHPLIL
DTCTIEGLIYGNPSCDLLLEGREWSYIVERPSAVNGLCYPGNVEGLEELRSLFSSASSYQRIQIFPDTIW
NVSYSGTSKACSESFYRSMRWLTQKNNAYPIQDAQYTNNQEKNILFMWGINHPPTDTVQTNLYTRTDTTT
SVATEEINRTFKPLIGPRPLVNGLQGRIDYYWSVLKPGQTLRIRSNGNLIAPWYGHILSGESHGRILKTD
LKRGSCTVQCQTEKGGLNTTLPFQNVSKYAFGNCSKYIGTKSLKLAVGLRNVPSRSSRGLFGAIAGFIEG
GWSGLVAGWYGFQHSNDQGVGMAADRDSTQKAIDKITSKVNNIVDKMNKQYEIIDHEFSEVETRLNMINN
KIDDQIQDIWAYNAELLVLLENQKTLDEHDANVNNLYNKVKRALGSNAVEDGKGCFELYHKCDDQCMETI

RNGTYNRRKYQEESKLERQKIEGVKLESEGTYKILTIYSTVASSLVIAMGFAAFLFWAMSNESCRRNICI

>AVK43544.1 hemagglutinin [Influenza A virus]
METASLIIMLLITTVSNADKICIGYQSTNSTETVDTLTENNVPVTHAKELLHTEHNGMLCATSLGHPLIL
DTCTIEGLIYGNPSCDPLLGGRKWSYIVERPSAVNGLCYPGNVENLEELRSLFSSASSYQRIQIFPDTIW
NVSYBGTSKACSDSFYRSMRWLTQKNNAYPIQDAQYTNNQEKNILFMWGINHPPTDTTQTNLYTRTDTTT
SVATEEINRTFKPLIGPRPLVNGLQGRIDYYWSVLKPGQTLRIRSNGNLIAPWYGHILSGESHGRILKTD
LKRGSCTVQCQTERGGLNATLPFQNVSKYAFGNCSKYIGIKSLKLAVGLRNVPSRSSRGLFGAIAGFIEG
GWSGLVAGWYGFQHSNDQGVGMAADRDSTQKAIDKITSKVNNIVDKMNKQYEIIDHEFSEVETRLNMINN
KIDDQIQDIWAYNAELLVLLENQKTLDEHDANVNNLYNKVKRALGSNAVEDGKGCFELYHKCDDQCMETI
RNGTYNRRKYQEESKLERQKIEGVKLESEGTYKILTIYSTVASSLVIAMGFAAFLFWAMSNGSCRRNICI

>AVK43543.1 hemagglutinin [Influenza A virus]
MEVVSLITILLVTTVSNADKICIGYQSKNSTETVDTLTENNVLVTHAKELLHTGHNGMLCATNLGHPLIL
DTCTIEGLIYGNPSCDLLLGGREWSYIVERPSAVNGLCYPGNVENLEELRSLFSSASSYQRIQIFPDTIW
NVSYSGTSKACSDSFYRSMRWLTQKNNTYPIQDAQYTNNRGKNILFMWGISHPPTDTVQTNLYTRTDTTT
SVATEDINRTFKPLIGPRPFVNGQQGRIDYYWSILKPGQTLRVRSNGNLIAPWYGHILPGESHGRILKTD
LNNGNCVVQCQTERGGLNTTLPFQNVSKYAFGNCSKYIGIESLKLAVGLRNVPSKSSRGLFGAIAGFIEG
GWSGLVAGWYGFQHSNDQGVGMAADRDSTQKAIDKITSKVNNIVDKMNKQYEIIDHEFSEVETRLNMINN
KIDDQIQDIWAYNAELLVLLENQKTLDEHDANVNNLYNKVKRALGSNAVEDGKGCFELYHKCDDQCMETI
RNGTYNRRKYQEESKLKRQKIEGVKLESEGTYKILTIYSTVASSLVIAMGFAAFLFWGHVQLSCRRNICI

>AVK43542.1 hemagglutinin [Influenza A virus]
MEVVSLITILLVTTVSNADKICIGYQSTNSTETVDTLTENNVPVTHAKELLHTGHNGMLCATNLGHPLIL
DTCTIEGLIYGNPSCDLLLGGREWSYIVERPSAVNGLCYPGNVENLEELRSLFSSASSYQRIQIFPDTIW
NVSYSGTSKACSDSFYRSMRWLTQKNNTYPIQDAQYTNNRGKNILFMWGISHPPTDTVQTNLYTRTDTTT
SVATEDINRTFKPLIGPRPLVNGQQGRIDYYWSILKPGQTLRVRSNGNLIAPWYGHILSGESHGRILKTD
LNNGNCVVQCQTEKGGLNTTLPFQNVSKYAFGNCPKYVGVKSLKLAVGLRNVPARSSRGLFGAIAGFIEG
GWSGLVAGWYGFQHSNDQGVGMAADRDSTQKAIDKITSKVNNIVDKMNRQYEIIDHEFSEVETRLHMINN
KIDDQIQDIWAYNAELLVLLENQKTLDEHDANVNNLYNKVKRALGSNAMEDGKGCFELYHKCDDQCMETI
RNGTYNRRKYKEESKLERQKIEGVKLESEGTYKILTIYSTVASSLVIAMGFAAFLFWAMSNGSCRRNICI

>AVK43541.1 hemagglutinin [Influenza A virus]
MEVVSLITILLVVTVSNADKICIGYQSTNSTETVDTLTENNVPVTHAKELLHTEHNGMLCATNLGHPLIL

DTCTIEGLIYGNPSCDLLLGGREWSYIVERPSAVNGLCYPGNVENLEELRSLFSSASSYQRIQIFPDTIW
NVSYSGTSKACSDSFYRSMRWLTQKNNAYPIQDAQYTNNRGKNILFMWGINHPPTDTTQTDLYTRTDTTT
SVATEDINRTFKPLIGPRPLVNGQQGRIDYYWSVLKPGQTLRVRSNGNLIAPWYGHILSGESHGRILKTD
LNSGNCVVQCQTEKGGLNTTLPFHNVSKYAFGNCPKYVGVKSLKLAVGLRNVPARSSRGLFGAIAGFIEG
GWSGLVAGWYGFQHSNDQGVGMAADRDSTRKAIDKITSKVNNIVDKMNKRYEIIDHEFSEVETRLNMINN
KIDDQIQDIWAYNAELLVLLENQKTLDEHDANVNNLYNKVKRALGSNAVEDGKGCFELYHKCDDQCMETI
RNGTYNRRKYKEESRLERQKIEGVKLESEGTYKILTIYSTVASSLVIAMGFAAFLFWAMSNGSCRRNICI

>AVK43540.1 hemagglutinin [Influenza A virus]
MEAVSLITILLVVTVSNADKICIGYQSTNSTETVDTLTENNVPVTHAKELLHTEHNGMLCATNLGHPLIL
DTCTIEGLIYGNPSCDLLLGGREWSYIVERPSAVNGMCYPGKVENLEELRSLFSSASSYQRIQIFPDTIW
NVSYSGTSKACSDSFYRNMRWLTQKNNAYPIQDAQYTNNREKSILFMWGINHPPTDTAQTNLYTRTDTTT
SVATEDINRTFKPLIGPRPLVNGLQGRIDYYWSVLKPGQTLRIRSNGNLIAPWYGHILSGESHGRILKTD
LKSGNCVVQCQTERGGLNTTLPFHNVSKYAFGNCPKYVGVKSLKLAVGLRNVPARSSRGLFGAIAGFIEG
GWSGLVAGWYGFQHSNDQGVGMAADRDSTQKAIDKITSKVNSIVDKMNRQYEIIDHEFSEVEARLNMINN
KIDDQIQDIWAYNAELLVLLENQKTLDEHDANVNNLYNKVKRALGSNAMEDGKGCFELYHKCDDQCMETI
RNGTYNRRKYKEESRLKRQKIEGVKLESEGTYKILTIYSTVASSLVIAMGFAAFLFWAMSNGSCRRNICI

>AVK43539.1 hemagglutinin [Influenza A virus]
MEVVSLITILLVVTVSNADKICIGYQSTNSTETVDTLTENNVPVTHAKELLHTEHNGMLCATNLGHPLIL
DTCTIEGLIYGNPSCNLLLGGREWSYIVERPSAVNGLCYPGNVENLEELRSLFSSASSYQSIQIFPDTIW
NVSYSGTSKACSDSFYRSMRWLTQKNNAYPIQDAQYTNNRGKNILFMWGINHPPTDTVQTDLYTRTDTTT
SVATEDINRTFKPLIGPRPLVNGLQGRIDYYWSVLKPGQTLRVRSNGNLIAPWYGHILSGESHGRILKTD
LNSGNCVVQCQTERGGLNTTLPFHNVSKYAFGNCPKYVGVKSLKLAVGLRNVPARSSRGLFGAIAGFIEG
GWSGLVAGWYGFQHSNDQGVGMAADRDSTQKAIDKITSKVNNIVDKMNKQYEIIDHEFSEVETRLNMIND
KIDDQIQNIWAYNAELLVLLENQKTLDEHDANVNNLYNKVKRTLGSNAVEDGKGCFELYHKCDDQCMETI
RNGTYNRRKYKEESRLERQKIEGVKLESEGTYKILTIYSTVASSLVIAMGFAAFLFWAMSNGSCRCNICI

>AVK43538.1 hemagglutinin [Influenza A virus]
MEVVSLITILLVVTVSNADKICIGYQSTNSTETVDTLTENNVPVTHAKELLHTEHNGMLCATNLGHPLIL
DTCTIEGLIYGNPSCNLLLGGREWSYIVERPSAVNGLCYPGNVENLEELRSLFSSASSYQRIQIFPDTIW
NVSYSGTSKACSDSFYRSMRWLTQKNNAYPIQDAQYTNNRGKNILFMWGINHPPTDTVQTDLYTRTDTTT
SVATEDINRTFKPLIGPRPLVNGLQGRIDYYWSVLKPGQTLRVRSNGNLIAPWYGHILSGESHGRILKTD
LNSGNCVVQCQTEKGGLNTTLPFHNVSKYAFGNCPKYVGAKSLKLAVGLRNVPARSSRGLFGAIAGFIEG

GWSGLVAGWYGFQHSNDQGVGMAADRDSTQKAIDKITSKVNNIVDKMNKQYEIIDHEFSEVETRLNMINN
KIDDQIQDIWAYNAELLVLLENQKTLDEHDANVNNLYNKVKRTLGSNAVEDGKGCFELYHKCDDQCMETI
RSGTYNRRKYKEESRLERQKIEGVKLESEGTYKILTIYSTVASSLVIAMGFAAFLFWAMSNGSCRRNICI

>AVK43537.1 hemagglutinin [Influenza A virus]
MEAVSLITILLVVTVSNADKICIGYQSTNSTETVDTLTENNVPVTHAKELLHTEHNGMLCATNLGHPLIL
DTCNIEGLIYGNPSCDLLLGGREWSYIVERPSAVNGLCYPGNVENLEELRSLFSSASSYQRIQIFPDAIW
NVSYNGTSKACSVSFYRSMRWLTQKSNAYPIQDAQYTNNRGKNILFMWGINHPPTDTVQTNLYTRTDTTT
SVATEDINRTFKPLIGPRPPVNGQQGRIDYYWSILKPGQTLRIRSNGNLIAPWYGHVLSGESHGRILKTD
LNSGNCVVQCQTERGGLNTTLPFQNVSKYAFGNCPKYVSVKSLKLAVGLRNVPARSSRGLFGAIAGFIEG
GWSGLVAGWYGFQHSNDQGVGMAADRDSTQKAIDKITSKVNNIVDKMNKQYEIIDHEFSEVETRLNMINN
KIDDQIQDIWAYNAELLVLLENQKTLDEHDANVNNLYNKVKRALGSNAVEDGKGCFELYHKCDDQCMETI
RNGTYNRRKYKEESKLERQKIEGVKLESEGTYKILTIYSTVASSLVIAMGFAAFLFWAMSNVSCRRNICI

>AYK28683.1 hemagglutinin [Influenza A virus]
METVSLITILLVATVSHADKICIGYQSTNSTETVDTLTENNVPVTHAKELLHTEHNGMLCATSLGQPLIL
DTCTIEGLIYGNPSCDLSLEEREWSYIVERPSAVNGLCYPGNVENLEELRSLFSSARSYQRIQIFPDTIW
NVSYDGTSTACSNSFYRSMRWLTRNDGNYPTQDAQYTNNQGKNILFMWGINHPPTDETQRNLYTEKNPTT
SVATEEINRIFKPLIGPRPLVNGLMGRIDYYWSVLKPGQTLRIKSDGNLIAPWYGHILSGESHGRILKTD
LKKGSCTVQCQTEKGGLNTTLPFQNVSKYAFGNCSKYIGIKSLKLAVGLRNVPSRSSRGLFGAIAGFIEG
GWSGLVAGWYGFQHSNDQGVGMAADRDSTQKAIDKITSKVNNIVDKMNKQYEIIDHEFSEVETRLNMINN
KIDDQIQDIWAYNAELLVLLENQKTLDEHDANVNNLYNKVKRALGSNAVEDGKGCFELYHKCNDQCMETI
RNGTYNRKKYQEESKLERQKIEGVKLESEGTYKILTIYSTVASSLVIAMGFAAFLFWAMSNGSCRCNICI

>AYK28671.1 hemagglutinin [Influenza A virus]
METISLITILLVATVSNADKICIGYQSTNSTETVDTLTENNVPVTHAKELLHTEHNGMLCATSLGNPLIL
DTCTIEGLIYGNPSCDPLLGGREWSYIVERPSAVNGLCYPGSVENLEELRSLFSSARSYQRIQIFPDTIW
NVSYSGTSKACSDSFYRSMRWLTQKNNAYPTQDAQYTNNQEKNILFMWGINHPPTETAQTNLYTKTDTTT
SVATEEINRIFKPLIGPRPLVNGLMGRIDYYWSVLKPGQTLRIKSDGNLIAPWYGYILSGESHGRILKTD
LKRGSCTVQCQTEKGGLNTTLPFQNVSKYAFGNCSKYIGIKSLKLAVGLRNVPSRSSRGLFGAIAGFIEG
GWSGLVAGWYGFQHSNDQGVGMAADRDSTQKAIDKITSKVNNIVDKMNKQYEIIDHEFSEVETRLNMINN
KIDDQIQDIWAYNAELLVLLENQKTLDEHDANVNNLYNKVKRALGSNAVEDGKGCFELYHKCNDQCMETI
RNGTYNRRKYQEESKLERQKIEGVKLESEGTYKILTIYSTVASSLVIAMGFAASLFWPMSNGSCRCNICI

>AYK28659.1 hemagglutinin [Influenza A virus]

METVSLITILLVATVSYADKICIGYQSTNSTETVDTLTENNVPVTHAKELLQTEHNGMLCATSLGQPLIL
DTCTIEGLIYGNPSCDLSLEGREWSYIVERPSAVNGLCYPGNVENLEELRSLFSSARSYQRVQIFPDTIW
NVSYDGTSTACSGSFYRSMRWLTRKDGNYPTQDAQYTNNQGKNILFMWGINHPPTDDTQRSRFTRTDATT
SVATEEINRIFKPLIGPRPLVNGLMGRIDYYWSVLKPGQTLRIKSDGNLIAPWYGYILSGESHGRILKTD
LKRGSCTVQCQTEKGGLNTTLPFQNVSKYAFGNCSKYIGIKSLKLAVGLRNVPSRSSRGLFGAIAGFIEG
GWSGLVAGWYGFQHSNDQGVGMAADRDSTQKAIDKITSKVNNIVDKMNKQYEIIDHEFSEVETRLNMINN
KIDDQIQDIWAYNAELLVLLENQKTLDEHDANVNNLYNKVKRALGSNAVEDGKGCFELYHKCNDQCMETI
RNGTYNRRKYQEESKLERQKIEGVKLESEGTYKILTIYSTVASSLVIAMGFAAFLFWAMSNGSCRCNICI

>AYK28647.1 hemagglutinin [Influenza A virus]

METVSLITILLVATVSYADKICIGYQSTNSTETVDTLTENNVPVTHAKELLHTEHNGMLCATSLGQPLIL
DTCTIEGLIYGNPSCDLSLEGKEWSYIVERPSAVNGLCYPGNVENLEELRSLFSSARSFQRVQIFPDTIW
NVSYDGTSTACSGSFYRSMRWLTRKDGNYPTQDAQYTNNQGKNILFMWGINHPPTDDTQRSLYTKTDTTT
SVATEEINRIFKPLIGPRPLVNGLMGRIDYYWSVLKPGQTLRIKSDGNLIAPWYGYILSGESHGRILKTD
LKRGSCTVQCQTEKGGLNTTLPFQNVSKYAFGNCSKYIGIKSLKLAVGLRNVPSRSSRGLFGAIAGFIEG
GWSGLVAGWYGFQHSNDQGVGMAADRDSTQKAIDKITSKVNNIVDKMNKQYEIIDHEFSEVETRLNMINN
KIDDQIQDIWAYNAELLVLLENQKTLDEHDANVNNLYNKVKRALGSNAAEDGKGCFELYHKCDDQCMETI
RNGTYNRRKYQEESKLERQKIEGVKLESEGTYKILTIYSTVASSLVIAMGFAAFLFWAMSNGSCRCNICI

>AYK28635.1 hemagglutinin [Influenza A virus]

METVSLITILLVATVSYADKICIGYQSTNSTETVDTLTENNVPVTHAKELLHTEHNGMLCATSLGQPLIL
DTCTIEGLIYGNPSCDLSLEGKEWSYIVERPSAVNGLCYPGNVENLEELRSLFSSARSFQRVQIFPDTIW
NVSYDGTSTACSGSFYRSMRWLTRKDGNYPTQDAQYTNNQGKNILFMWGINHPPTDDTQRSLYTKTDTTT
SVATEEINRIFKPLIGPRPLVNGLMGRIDYYWSVLKPGQTLRIKSDGNLIAPWYGYILSGESHGRILKTD
LKRGSCTVQCQTEKGGLNTTLPFQNVSKYAFGNCSKYIGIKSLKLAVGLRNVPSRSSRGLFGAIAGFIEG
GWSGLVAGWYGFQHSNDQGVGMAADRDSTQKAIDKITSKVNNIVDKMNKQYEIIDHEFSEVETRLNMINN
KIDDQIQDIWAYNAELLVLLENQKTLDEHDANVNNLYNKVKRALGSNAAEDGKGCFELYHKCDDQCMETI
RNGTYNRRKYQEESKLERQKLEGVKLESEGTYKILTIYSTVASSLVIAMGFAAFLFWAMSNGSCTCTIGI

>AYK28623.1 hemagglutinin [Influenza A virus]

METVSLITILLVATVSHADKICIGYQSTNSTETVDTLTENNVPVTHAKELLHTEHNGMLCATSLGQPLIL
DTCTIEGLIYGNPSCDLSLEGREWSYIVERSSAVNGLCYPGNVENLEELRSLFSSARSYQRIQIFPDTIW
NVSYDGTSTACSNSFYRSMRWLTRKDGNYPTQDAQYTNNQGKNILFMWGINHPPTDETQRNRFLRKNATT

SVATEEINRIFKPLIGPRPLVNGLMGRIDYYWSVLKPGQTLRIKSDGNLIAPWYGHILSGESHGRILKTD
LKRGSCTVQCQTEKGGLNTTLPFQNVSKYAFGNCSKYIGIKSLKLAVGLRNVPSRSSRGLFGAIAGFIEG
GWSGLVAGWYGFQHSNDQGVGMAADRDSTQKAIDKITSKVNNIVDKMNKQYEIIDHEFSEVETRLNMINN
KIDDQIQDIWAYNAELLVLLENQKTLDEHDANVNNLYNKIKRALGSNAVEDGKGCFELYHKCNDQCMETI
RNGTYNRKKYQEESKLERQRIEGVKLESEGTYKILTIYSTVASSLVIAMGFAAFLFWAMSNGSCRCNICI

>AYW17109.1 hemagglutinin [Influenza A virus]
MEVISLITILLVATVSNADKICIGYQSTNSTETVDTLTENNVPVTHAKELLHTEHNGMLCATSLGHPLIL
DTCTIEGLIYGNPSCDLLLGGKEWSYIVERPSPVNGLCYPGNVENLEKLRSLFSSARSYQRIQIFPHTIW
NVSYSGTSKACSHSFYRSMRWLTQKNNAYPIQDAQYTNNQEKNILFMWGINHPPTDTVQTDLYTRTDTTT
SVATEEIDRIFKPLIGPRPLVNGLMGRINYYWSVLKPGQTLRIKSDGNLIAPWYGHILSGESHGRILKTD
LKRGSCTVQCQTEKGGLNTTLPFQNVSKYAFGNRSKYIGIKSLKLAVGLRNVPSRSSRGLFGAIAGFIEG
GWSGLVAGWYGFQHSNDQGVGMAADRDSTQKAIDKITSKVNNIVDKMNKQYEIIDHEFSEVETRLNMINN
KVDDQIQDIWAYNAELLVLLENQKTLDEHDSNVNNLYNKVKRALGSNAVEDGKGCFELYHKCDDQCMETI
RNGTYNRRKYQEESKLERQKIEGVKLESEGTYKILTIYSTVASSLVIAMGFAAFLFWAMSNGSCRCNICI

>AYW17107.1 hemagglutinin [Influenza A virus]
MEVVSLMTILLVVTVSNADKICIGYQSTNSTETVDTLTENNVPVTHAKELLHTEHNGMLCATSLGDPLIL
DTCTIEGLIYGNPSCDLLLGGREWSYIVERPSAVNGLCYPGNVENLEKLRSLFSSARSYQRVQIFPDTIW
NVSYSGTSKACSDSFYRSMRWLTQKDNAYPIQDAQYTNNQGKNILFMWGINHPPTDTAQTNLYTRTDTTT
SVATEEINRTFKPLIGPRPLVNGLMGRINYYWSVLKPGQTLRIKSNGNLIAPWYGHILSGESHGRILKTD
IHRGSCTVQCQTEKGGLNTTLPFQNVSKYAFGNSSKYIGMKSVELAVSMRNVRSRYSRGLLGAIAGFIEG
GWSGLVDGWYGFQHSNDHGVGITADSDSTQKAIDKITSKVNNIVDKMNKPYEIIDHEFSEVETRINMINN
MVDDQIQDIGAFNAELLVLLENQQTLDEHDANTNNSNNKVKRASGSNAGKDGKGCFELYHKCDDQSMETI
RNGTYNWRKYQEESKLERQKIEGVKLETEGTYKILTIYSTVASSLVIAMGSAAFLFWAMHKGYSRCVICI

>BBD75311.1 hemagglutinin [Influenza A virus]
MEIIPLMTVLLLVTTSNADKICIGHQSTNSTETVDTLTETNVPVTHAKELLHTEHNGMLCATNLGNPLIL
DTCTIEGLIYGNPSCDMLLGGREWSYIVERPTAVNGTCYPGNVENLEELRILFSSSSSYQRIQMFPDTIW
NVTYSGTSKSCSDSFYRNMRWLTQKNGNYPVQDAQYTNTRGKDILFVWGIHHPPTDTAQTNLYTRTDTTT
SVTTENLDRTFKPLIGPRSLVNGLIGRINYYWSVLKPGQTLRVRSNGNLIAPWFGHVLSGESHGRILKTD
LNSGNCVVQCQTEKGGLNSTLPFHNISKYAFGDCPKYIGVKSLKLAIGLRNVPARSSRGLFGAIAGFIEG
GWPGLVAGWYGFQHSNDQGVGMAADRDSTQKAVGKITAKVNNIVDKMNKQYEIIDHEFSEVETRLNMINN
KIDDQIQNVWAYNAELLVLLENQKTLDEHDANVNNLYNKVKRALGSNAIEDGKGCFELYHKCDDQCMETI

RNGTYNRRKYKEESRLERQKIEGVKLESEGTYKILTIYSTVASSLVLAMGFAAFLFWAMSNGSCRCNICI

>AYM84940.1 hemagglutinin [Influenza A virus]

METASLITILLVVTVSNADKICIGYQSTNSTETVDTLTENNVPVTHAKELLHTEHNGMLCATSLGQPLIL
DTCTIEGLIYGNPSCDLLLGGREWSYIVERPSAVNGLCYPGNVENLEELRSLFSSARSYQRIQIFPDTIW
NVSYSGTSKACSDSFYRSMRWLTQKDNAYPIQDAQYTNNQEKNILFMWGINHPPTDTVQTNLYTRTNTTT
SVATEEINRTFKPLIGPRPLVNGLMGRINYYWSVLKPGQTLRIKSNGNLIAPWYGHILSGESHGRILKTD
LKRGSCTVQCQTEKGGLNTTLPFQNVSKYAFGNCSKYIGIKSLKLAVGLRNVPSRSSRGLFGAIAGFIEG
GWSGLVAGWYGFQHSNDQGVGMAADRDSTQKAIDKITSKVNNIVDKMNKQYEIIDHEFSEVETRLNMINN
KIDDQIQDIWAYNAELLVLLENQKTLDEHDANVNNLYNKVKRALGSNAVEDGKGCFELYHKCDDQCMETI
RNGTYNRRKYQEESKLERQKIEGVKLESEGTYKILTIYSTVASSLVIAMGFAAFLFWAMSNGSCRCNICI

>AYA50666.1 hemagglutinin [Influenza A virus]

MEAISLMIILLVVTTSNADKICIGHQSTNSTETVDTLTESNIPVTQAKELLHTEHNGMLCATNLGRPLIL
DTCTVEGLIYGNPSCDLLLGGREWSYIVERPSAVNGTCYPGNVENLEELRMLFSSASSYQRIQIFPDAIW
NVTYDGTSKSCSNSFYRNMRWLTQKNGNYPIQDAQYTNNRGKDILFIWGIHHPPTDTAQTNLYTRTDTTT
SVTTENLDRTFKPLIGPRPLVNGLIGRINYYWSVLKPGQTLRVRSNGNLIAPWYGHVLSGESHGRILKTD
LKSGNCVVQCQTEKGGLNSTLPFHNISKYAFGTCPKYIGVKSLKLAIGLRNVPAKSSRGLFGAIAGFIEG
GWPGLVAGWYGFQHSNDQGVGMAADRSSTQKAVDKITSKVNNIIDKMNRQYEIIDHEFSEIETRLNMINN
KIDDQIQDVWAYNAELLVLLENQKTLDEHDANVNNLYNKVKRALGSNAMEDGKGCFELYHKCDDQCMETI
RNGTYNRRKYTEESRLERQKIEGVKLESEGTYKILTIYSTVASSLVLAMGVAAFLFWAMSNGSCRCNICI

>AYA50665.1 hemagglutinin [Influenza A virus]

MEAISLMIILLVVTTSNADKICIGHQSTNSTETVDTLTESNIPVTQAKELLHTEHNGMLCATNLGRPLIL
DTCTVEGLIYGNPSCDLLLGGREWSYIVERPSAVNGTCYPGNVENLEELRMLFSSASSYQRIQLFPDAIW
NVTYDGTSKSCSNSFYRNMRWLTQKNGNYPIQDAQYTNNRGKDILFIWGIHHPPTDTAQTNLYTRTDTTT
SVTTENLDRTFKPLIGPRPLVNGLIGRINYYWSVLKPGQTLRVRSNGNLIAPWYGHVLSGESHGRILKTD
LKSGNCVVQCQTEKGGLNSTLPFHNISKYAFGTCPKYIGVKSLKLAIGMRNVPAKSSRGLFGAIAGFIEG
GWPGLVAGWYGFQHSNDQGVGMAADRGSTQKAVDKITSKVNNIIDKMNRQYEIIDHEFSEIETRLNMINN
KIDDQIQDVWAYNAELLVLLENQKTLDEHDANVNNLYNKVKRALGSNAMEDGKGCFELYHKCDDQCMETI
RNGTYNRRKYTEESRLERQKIEGVKLESEGTYKILTIYSTVASSLVLAMGVAAFLFWAMSNGSCRCNICI

>AYA50664.1 hemagglutinin [Influenza A virus]

MEAISLMIILLVVTTSNADKICIGHQSTNSTETVDTLTESNIPVTQAKELLHTEHNGMLCATNLGRPLIL

DTCTVEGLIYGNPSCDLLLGGREWSYIVERPSAVNGTCYPGNVENLEELRMLFSSASSYQRIQIFPDAIW
NVTYDGTSKSCSNSFYRNMRWLTQKNGNYPIQDAQYTNNRGKDILFIWGIHHPPTDTAQTNLYTRTDTTT
SVTTENLDRTFKPLIGPRPLVNGLIGRINYYWSVLKPGQTLRVRSNGNLIAPWYGHVLSGESHGRILKTD
LKSGNCVVQCQTEKGGLNSTLPFHNISKYAFGTCPKYIGVKSLKLAIGLRNVPAKSSRGLFGAIAGFIEG
GWPGLVAGWYGFQHSNDQGVGMAADRSSTQKAVDKITSKVNNIIDKMNRQYEIIDHEFSEIETRLNMINN
KIDDQIQDVWAYNAELLVLLENQKTLDEHDANVNNLYNKVKRALGSNAMEDGKGCFELYHKCDDQCMETI
RNGTYNRRKYTEESRLERQKIEGVKLESEGTYKILTIYSTVASSLVLAMGVAAFLFWAMSNGSCRCNICI

>AYA50663.1 hemagglutinin [Influenza A virus]
MEAISLMIILLVVTTSNADKICIGHQSTNSTETVDTLTESNIPVTQAKELLHTEHNGMLCATNLGRPLIL
DTCTVEGLIYGNPSCDLLLGGREWSYIVERPSAVNGTCYPGNVENLEELRMLFSSASSYQRIQIFPDAIW
NVTYDGTSKSCSNSFYRNMRWLTQKNGNYPIQDAQYTNNRGKDILFIWGIHHPPTDTAQTNLYTRTDTTT
SVTTENLDRTFKPLIGPRPLVNGLIGRINYYWSVLKPGQTLRVRSNGNLIAPWYGHVLSGESHGRILKTD
LKSGNCVVQCQTEKGGLNSTLPFHNISKYAFGTCPKYIGVKSLKLAIGLRNVPAKSSRGLFGAIAGFIEG
GWPGLVAGWYGFQHSNDQGVGMAADRSSTQKAVDKITSKVNNIIDKMNRQYEIIDHEFSEIETRLNMINN
KIDDQIQDVWAYNAELLVLLENQKTLDEHDANVNNLYNKVKRALGSNAMEDGKGCFELYHKCDDQCMETI
RNGTYNRRKYTEESRLERQKIEGVKLESEGTYKILTIYSTVASSLVLAMGVAAFLFWAMSNGSCRCNICI

>AYA50662.1 hemagglutinin [Influenza A virus]
MEAISLMIILLVVTTSNADKICIGHQSTNSTETVDTLTESNIPVTQAKELLHTEHNGMLCATNLGRPLIL
DTCTVEGLIYGNPSCDLLLGGREWSYIVERPSAVNGTCYPGNVENLEELRMLFSSASSYQRIQIFPDAIW
NVTYDGTSKSCSNSFYRNMRWLTQKNGNYPIQDAQYTNNREKDILFIWGIHHPPTDTAQTNLYTRTDTTT
SVTTENLDRTFKPLIGPRPLVNGLIGRINYYWSVLKPGQTLRVRSNGNLIAPWYGHVLSGESHGRILKTD
LKSGNCVVQCQTEKGGLNSTLPFHNISKYAFGTCPKYIGVKSLKLAIGLRNVPAKSSRGLFGAIAGFIEG
GWPGLVAGWYGFQHSNDQGVGMAADRSSTQKAVDKITSKVNNIIDKMNRQYEIIDHEFSEIETRLNMINN
KIDDQIQDVWAYNAELLVLLENQKTLDEHDANVNNLYNKVKRALGSNAMEDGKGCFELYHKCDDQCMETI
RNGTYNRRKYTEESRLERQKIEGVKLESEGTYKILTIYSTVASSLVLAMGVAAFLFWAMSNGSCRCNICI

>AYA50661.1 hemagglutinin [Influenza A virus]
MEAISLMIILLVVTTSNADKICIGHQSTNSTETVDTLTESNIPVTQAKELLHTEHNGMLCATNLGRPLIL
DTCTVEGLIYGNPSCDLLLGGREWSYIVERPSAVNGTCYPGNVENLEELRMLFSSASSYQRIQIFPDAIW
NVTYDGTSKSCSNSFYRNMRWLTQKNGNYPIQDAQYTNNRGEDILFIWGIHHPPTDTAQTNLYTRTDTTT
SVTTENLDRTFKPLIGPRPLVNGLIGRINYYWSVLKPGQTLRVRSNGNLIAPWYGHVLSGESHGRILKTD
LKSGNCVVQCQTEKGGLNSTLPFHNVSKYAFGTCPKYIGVKSLKLAIGLRNVPAKSSRGLFGAIAGFIEG

GWPGLVAGWYGFQHSNDQGVGMAADRSSTQKAVDKITSKVNNIIDKMNRQYEIIDHEFSEIETRLNMINN
KIDDQIQDVWAYNAELLVLLENQKTLDEHDANVNNLYNKVKRALGSNAMEDGKGCFELYHKCDDQCMETI
RNGTYNRRKYTEESRLERQKIEGVKLESEGTYKILTIYSTVASSLVLAMGVAAFLFWAMSNGSCRCNICI

>AYA50660.1 hemagglutinin [Influenza A virus]
MEAISLMIILLVVTTSNADKICIGHQSTNSTETVDTLTETNIPVTQAKELLHTEHNGMLCATNLGRPLIL
DTCTVEGLIYGNPSCDLLLGGREWSYIVERPSAVNGTCYPGNVENLEELRILFSSASSYQRIQIFPDAIW
NVTYDGTSKSCSNSFYRNMRWLTQKNGNYPIQDAQYTNNQGKDILFIWGIHHPPTDTAQTNLYTRTDTTT
SVTTENLDRTFKPLIGPRPLVNGLIGRINYYWSVLKPGQTLRVRSNGNLIAPWYGHVLSGESHGRILKTN
LKSGNCVVQCQTEKGGLNSTLPFHNVSKYAFGTCPKYIGVKSLKLAIGLRNVPAKSSRGLFGAIAGFIEG
GWPGLVAGWYGFQHSNDQGVGMAADRGSTQKAVDKITSKVNNIIDKMNRQYEIIDHEFSEIETRLNMINN
KIDDQIQDVWAYNAELLVLLENQKTLDEHDANVNNLYNKVKRALGSNAMEDGKGCFELYHKCDDQCMETI
RNGTYNRRKYTEESRLERQKIEGVKLESEGTYKILTIYSTVASSLVLAMGFAAFLFWAMSNGSCRCNICI

>AYA50659.1 hemagglutinin [Influenza A virus]
MEAISLMIILLVVTTSNADKICIGHQSTNSTETVDTLTESNIPVTQAKELLHTEHNGMLCATNLGRPLIL
DTCTVEGLIYGNPSCDLLLGGREWSYIVERPSAVNGTCYPGNVENLEELRMLFSSASSYQRIQIFPDAIW
NVTYDGTSKSCSNSFYRNMRWLTQKNGNYPIQDAQYTNNRGKDILFIWGIHHPPTDTAQTNLYTRTDTTT
SVTTENLDRTFKPLIGPRPLVNGLIGRINYYWSVLKPGQTLRVRSNGNLIAPWYGHVLSGESHGRILKTD
LKSGNCVVQCQTEKGGLNSTLPFHNISKYAFGTCPKYIGVKSLKLAIGLRNVPAKSSRGLFGAIAGFIEG
GWPGLVAGWYGFQHSNDQGVGMAADRSSTQKAVDKITSKVNNIIDKMNRQYEIIDHEFSEIETRLNMINN
KIDDQIQDVWAYNAELLVLLENQKTLDEHDANVNNLYNKVKRALGSNAMEDGKGCFELYHKCDDQCMETI
RNGTYNRRKYTEESRLERQKIEGVKLESEGTYKILTIYSTVASSLVLAMGVAAFLLWAMSNGSCRCNICI

>AXU22768.1 hemagglutinin, partial [Influenza A virus]
VTTSNADKICIGHQSTNSTETVDTLTESNIPVTQAKELLHTEHNGMLCATNLGRPLILDTCTVEGLIYGN
PSCDLLLGGREWSYIVERPSAVNGTCYPGNVENLEELRMLFSSASSYQRIQIFPDAIWNVTYDGTSKSCS
NSFYRNMRWLTQKNGNYPIQDAQYTNNRGKDILFIWGIHHPPTDTAQTNLYTRTDTTTSVTTENLDRTFK
PLIGPRPLVNGLIGRINYYWSVLKPGQTLRVRSNGNLIAPWYGHVLSGESHGRILKTDLKSGNCVVQCQT
EKGGLNSTLPFHNISKYAFGTCPKYIGVKSLKLAIGLRNVPAKSSRGLFGAIAGFIEGGWPGLVAGWYGF
QHSNDQGVGMAADRGSTQKAVDKITSKVNNIIDKMNRQYEIIDHEFSEIETRLNMINNKIDDQIQDVWAY
NAELLVLLENQKTLDEHDANVNNLYNKVKRALGSNAMEDGKGCFELYHKCDDQCMETIRNGTYNRRKYTE
ESRLERQKIEGVKLESEGTYKILTIYSTVASSLVFAMGVAAFLFWAM

>AXU22767.1 hemagglutinin, partial [Influenza A virus]
VTTSNADKICIGHQSTNSTETVDTLTESNIPVTQAKELLHTEHNGMLCATNLGRPLILDTCTVEGLIYGN
PSCDLLLGGREWSYIVERPSAVNGTCYPGSVENLEELRMLFSSASSYQRIQIFPDAIWNVTYDGTSKSCS
NSFYRNMRWLTQKSGNYPIQDAQYTNNRGKDILFIWGIHHPPTDTAQTNLYTRTDTTTSVTTENLDRTFK
PLIGPRPLVNGLIGRINYYWSVLKPGQTLRVRSNGNLIAPWYGHVLSGESHGRILKTDLKSGNCVVQCQT
EKGGLNSTLPFHNISKYAFGTCPKYIGVKSLKLAIGLRNVPAKSSRGLFGAIAGFIEGGWPGLVAGWYGF
QHSNDQGVGMAADRGSTQKAVDKITSKVNNIIDKMNRQYEIIDHEFSEIETRLNMINNKIDDQIQDVWAY
NAELLVLLENQKTLDEHDANVNNLYNKVKRALGSNAMEDGKGCFDLYHKCDDQCMETIRNGTYNRRKYTE
ESRLERQKIEGVKLESEGTYKILTIYSTVASSLVLAMGVAAFLFWAM

>AXU22766.1 hemagglutinin, partial [Influenza A virus]
VTTSNADKICIGHQSTNSTETVDTLTESNIPVTQAKELLHTEHNGMLCATNLGRPLILDTCTVEGLIYGN
PSCDLLLGGREWSYIVERPSAVNGTCYPGNVENLEELRMLFSSASSYQRIQIFPDAIWNVTYDGTSKSCS
NSFYRNMRWLTQKNGNYPIQDAQYTNNRGNDILFIWGIHHPPTDTAQTNLYTRTDTTTSVTTENLDRTFK
PLIGPRPLVNGLIGRINYYWSVLKPGQTLRVRSNGNLIAPWYGHVLSGESHGRILKTDLKSGNCVVQCQT
EKGGLNSTLPFHNISKYAFGTCPKYIGVKSLKLAIGLRNVPAKSSRGLFGAIAGFIEGGWPGLVAGWYGF
QHSNDQGVGMAADRGSTQKAVDKITSKVNNIIDKMNRQYEIIDHEFSEIETRLNMINNKIDDQIQDVWAY
NAELLVLLENQKTLDEHDANVNNLYNKVKRALGSNAMEDGKGCFELYHKCDDQCMETIRNGTYNRRKYTE
ESRLERQKIEGVKLESEGTYKILTIYSTVASSLVLAMGVAAFLFWAM

>AXU22765.1 hemagglutinin, partial [Influenza A virus]
VTTSNADKICIGHQSTNSTETVDTLTESNIPVTQAKELLHTEHNGMLCATNLGRPLILDTCTVEGLIYGN
PSCDLLLGGREWSYIVERPSAVNGTCYPGNVENLEELRMLFSSASSYQRIQIFPDAIWNVTYDGTSKSCS
NSFYRNMRWLTQKNGNYPIQDAQYTNNRGKDILFIWGIHHPPTDTAQTNLYTRTDTTTSVTTENLDRTFK
PLIGPRPLVNGLIGRINYYWSVLKPGQTLRVRSNGNLIAPWYGHVLSGESHGRILKTDLKIGNCVVQCQT
EKGGLNSTLPFHNISKYAFGTCPKYIGVKSLKLAIGLRNVPAKSNRGLFGAIAGFIEGGWPGLIAGWYGF
QHSNDQGVGMAADRGSTQKAVDKITSKVNNIIDKMNRQYEIIDHEFSEIETRLNMINNKIDDQIQDVWAY
NAELLVLLENQKTLDEHDANVNNLYNKVKRALGSNAMEDGKGCFELYHKCDDQCMETIRNGTYNRRKYTE
ESRLERQKIEGVKLESEGTYKILTIYSTVASSLVLAMGVAAFLFWAM

>AXU22764.1 hemagglutinin, partial [Influenza A virus]
VTTSNADKICIGHQSTNSTETVDTLTESNIPVTQAKELLHTEHNGMLCATNLGRPLILDTCTVEGLIYGN
PSCDLLLGGREWSYIVERPSAVNGTCYPGNVENLEELRMLFSSASSYQRIQIFPDAIWNVTYDGTSKSCS
NSFYRNMRWLTQKNGNYPIQDAQYTNNRGKDILFIWGIHHPPTDTAQTNLYTRTDTTTSVTTEDLDRTFK

PLIGPRPLVNGLIGRINYYWSVLKPGQTLRVRSNGNLIAPWYGHVLSGESHGRILKTDLKSGNCVVQCQT
EKGGLNSTLPFHNISKYAFGTCPKYIGVKSLKLAIGLRNVPAKSSRGLFGAIAGFIEGGWPGLVAGWYGF
QHSNDQGVGMAADRGSTQKAVDKITSKVNNIIDKMNRQYEIIDHEFSEIETRLNMINNKIDDQIQDVWAY
NAELLVLLENQKTLDEHDANVNNLYNKVKRALGSNAMEDGKGCFELYHKCDDQCMETIRNGTYNRRKYTE
ESRLERQKIEGVKLESEGTYKILTIYSTVASSLVLAMGVAAFLFWAM

>AXO10677.1 hemagglutinin, partial [Influenza A virus]
DKICIGHQSTNSTETVDTLTETGVPVTHAKELLHTEHNGKLCATNMGNPLILDTCTVEGLIYGNPSCDML
LGGREWSYIVERPSAVNGTCYPGNVENLEELRILFSSSSSYQRIQMFPDTIWNVTYSGTSKSCSDSFYRN
MRWLTQKNGNYPVQDAQYTNTRGKDILFVWGIHHPPTDTSQTNLYTRTDTTTSITTESLDRTFKPLIGPR
PLVNGLIGRINYYWSVLKPGQTLRVRSNGNLIAPWFGHVLSGESHGRILKTDLNSGNCVVKCQTEKGGLN
STLPFHNISKYAFGDCPKYIGVKSLKLAIGLRNVPARSSRGLFGAIAGFIEGGWPGLVAGWYGFQHSNDQ
GVGMAADRDSTQKAVDKITSKVNNIVDKMNKQYEIIDHEFNEVENRLNMINNKIDDQIQDIWAYNAELLV
LLENQKTLDEHDANVNNLYNKVKRALGSNAMEDGKGCFELYHKCDDQCMETIRNGTYNRRKYMEESRLGR
QKIEGVKLESEGT

>AXO10676.1 hemagglutinin, partial [Influenza A virus]
DKICIGHQSTNSTETVDTLTETGVPVTHAKELLHTEHNGRLCATNLGNPLILDTCTVEGIIYGNPSCDML
LGGREWSYIVERPSAVNGTCYPGNVENLEELRILFSSSSSYQRIQMFPDTIWNVTYSGTSKSCSDSFYRN
MRWLTQKNGNYPVQDAQYTNTRGKDILFVWGIHHPATDTAQTNLYTRTDTTTSITTESLDRTFKPLIGPR
PLVNGLIGRINYYWSVLKPGQTLRVRSNGNLIAPWFGHVLSGESHGRILKTDLNSGNCVVQCQTEKGGLN
STLPFHNISKYAFGDCPKYIGVKSLKLAIGLRNVPARSSRGLFGAIAGFIEGGWPGLVAGWYGFQHSNDQ
GVGMAADRDSTQKAVDKITSKVNNIVDKVNKQYEIIDHEFSEVETRLNMINNKIDDQIQDIWAYNAELLV
LLENQKTLDEHDANVNNLYNKVKRALGSNAMEDGKGCFELYHKCDDQCMETIRNGTYNRRKYMEESRLGR
QKIEGVKLESEGT

>AXO10675.1 hemagglutinin, partial [Influenza A virus]
DKICIGHQSTNSTETVDTLTETNVPVTHAKELLHTEHNGKLCATNLGNPLILDTCTIEGLIYGNPSCDML
LGGREWSYIVERPSAVNGTCYPGNVENLEELRILFSSSSSYQRIQMFPDSIWNVTYSGTSKSCSDSFYRN
MRWLTQKNGNYPVQDAQYTNTRGKDILFVWGIHHPPTDTAQTNLYTRTDTTTSITTENLDRTFKPLIGPR
PLVNGLIGRINYYWSVLKPGQTLRVRSNGNLIAPWFGHVLSGESHGRILKTDLNSGNCVVQCQTEKGGLN
STLPFHNISKYAFGDCPKYIGVKSLKLAIGLRNVPARSSRGLFGAIAGFIEGGWPGLVAGWYGFQHSNDQ
GVGMAADRDSTQKAVDKITSKVNNIVDKMNKQYEIIDHEFSEVETRLNMINNKIDDQIQDIWAYNAELLV
LLENQKTLDEHDANVNNLYNKVKRALGSNAMEDGKGCFELYHKCDDQCMETIRNGTYNRRKYMEESRLGR

QKIEGVKLESEGT

>AXO10673.1 hemagglutinin, partial [Influenza A virus]
DKICIGHQSTNSTETVDTLTETNVPVTHAKELLHTEHNGKLCATNLGNPLILDTCTIEGLIYGNPSCDML
LGGREWSYIVERPSAVNGTCYPGNVENLEELRILFSSSSSYQRIQMFPDTIWNVTYSGTSKSCSDSFYRN
MRWLTQKNGNYPVQDAQYTNTRGKDILFVWGIHHPPTDTAQTNLYTRTDTTTSITTENLDRTFKPLIGPR
PLVNGLIGRINYYWSVLKPGQTLRVRSNGNLIAPWFGHVLSGESHGRILKTDLNSGNCAVQCQTEKGGLN
STLPFHNISKYAFGDCPKYIGVKSLKLAIGLRNVPARSSRGLFGAIAGFIEGGWPGLVAGWYGFQHSNDQ
GVGMAADRDSTQKAVDKIHSKVNNIGCKMNKHMEIIDMEFSEVETRPNMTINKIDDQIPGVGAYNAELLV
LLENQKTPEGCEANVNNLYNKVKRGLGSNAMEDGKGCFELYHKCDDQCMETIRNGTYNRRKYMEESRLGR
QKIEGVKLESEGT

>AXO10672.1 hemagglutinin, partial [Influenza A virus]
DKICIGHQSTNSTETVDTLTETNVPVTHAKELLHTEHNGKLCATNLGNPLILDTCTIEGLIYGNPSCDML
LGGREWSYIVERPSAVNGTCYPGNVENLEELRILFSSSSSYQRIQMFPDTIWNVTYSGTSKSCSDSFYRN
MRWLTQRNGNYPVPDAQYTNTRGKDILFVGGIHHPPTDTAQTNLYTRTDTTTSITTENLDRTFKPLIGPR
PLVNGLIGRINYYWSVLKPGQTLRVRSNGNLIAPWFGHVLSGESHGRILKTDLNSGNCVVQCQTEKGGLN
STLPFHNISKYAFGDCPKYIGVKSLKLAIGLRNVPARSSRGLFGAIAGFIEGGWPGLVAGWYGFQHSNDQ
GVGMAADRDSTQKAVDKITSKVNNIVDKMNKQYEIIDHEFSEVETRLNMINNKIDDQIQDIWAYNAELLV
LLENQKTLDEHDANVNNLYNKVKRALGSNAMKDGKGCFELYHKCDDQXMEXIRNGTYNRRKYMEESRLGR
QKIEGVKLESEGT

>AXO10671.1 hemagglutinin, partial [Influenza A virus]
DKICIGHQSTNSTETVDTLTETNVPVTHAKELLHTEHNGKLCATNLGNPLILDTCTIEGLIYGNPSCDML
LGGREWSYIVERPSAVNGTCYPGNVENLEELRILFSSSSSYQRIQMFPDTIWNVTYSGTSKSCSDSFYRN
MRWLTQKNGNYPVQDAQYTNTRGKDILFVWGIHHPPTDTAQTNLYTRTDTTTSITTENLDRTFKPLIGPR
PLVNGLIGRINYYWSVLKPGQTLRVRSNGNLIAPWFGHVLSGESHGRILKTDLNSGNCVVQCQTEKGGLN
STLPFHNISKYAFGDCPKYIGVKSLKLAIGLRNVPARSSRGLFGAIAGFIEGGWPGLVAGWYGFQHSNDQ
GVGMAADRDSTQKAVDKITSKVNNIVDKMNKQYEIIDHEFSEVETRLNMINNKIDDQIQDIWAYNAELLV
LLENQKTLDEHDANVNNLYNKVKRALGSNAMEDGKGCFELYHKCDDQCMETIRNGTYNRRKYMEESRLGR
QRIEGVKLESEGT

>AXO10670.1 hemagglutinin, partial [Influenza A virus]
DKICIGHQSTNSTETVDTLTETNVPVTHAKELLHTEHNGKLCATNLGNPLILDTCTIEGLIYGNPSCDML

LGGREWSYIVERPSAVNGTCYPGNVENLEELRILFSSSSSYQRIQMFPDTIWNVTYSGTSKSCSDSFYRN
MRWLTQKNGNYPVQDAQYTNTRGKDILFVWGIHHPPTDTAQTNLYTRTDTTTSVTTENLDRTFKPLIGPR
PLVNGLIGRINYYWSVLKPGQTLRVRSNGNLIAPWFGHVLSGESHGRILKTDLNSGNCVVQCQTEKGGLN
STLPFHNISKYAFGDCPKYIGVKSLKLAIGLRNVPARSSRGLFGAIAGFIEGGWPGLVAGWYGFQHSNDQ
GVGMAADRDSTQKAVDKITSKVNNIVDKMNKQYEIIDHEFSEVETRLNMINNKIDDQIQDIWAYNAELLV
LLENQKTLDEHDANVNNLYNKVKRALGSNAMEDGKGCFELYHKCDDQCMETIRNGTYNRRKYKEESRLGR
QKIEGVKLESEGT

>AXO10669.1 hemagglutinin, partial [Influenza A virus]
DKICIGHQSTNSTETVDTLTETNVPVTHAKELLHTEHNGKLCATNLGNPLILDTCTIEGLIYGNPSCDML
LGGREWSYIVERPSAVNGTCYPGNVENLEELRILFSSSSSYQRIQMFPDTIWNVTYSGTSKSCSDSFYRN
MRWLTQKNGNYPVQDAQYTNTRGKDILFVWGIHHPPTDTAQTNLYTRTDTTTSVTTENLDRTFKPLIGPR
PLVNGLIGRINYYWSVLKPGQTLRVRSNGNLIAPWFGHVLSGESHGRILKTDLNSGNCVVQCQTEKGGLN
STLPFHNISKYAFGDCPKYIGVKSLKLAIGLRNVPARSSRGLFGAIAGFIEGGWPGLVAGWYGFQHSNDQ
GVGMAADRDSTQKAVDKITSKVNNIVDKMNKQYEIIDHEFSEVETRLNMINNKIDDQIQDIWAYNAELLV
LLENQKTLDEHDANVNNLYNKVKRALGSNAMEDGKGCFELYHKCDDQCMETIRNGTYNRRKYKEESRLGR
QKIEGVKLESEGT

>AXO10668.1 hemagglutinin, partial [Influenza A virus]
DKICIGHQSTNSTETVDTLTETNVPVTHAKELLHTEHNGKLCATNLGNPLILDTCTIEGLIYGNPSCDML
LGGREWSYIVERPSAVNGTCYPGNVENLEELRILFSSSSSYQRIQMFPDTIWNVTYSGTSKSCSDSFYRN
MRWLTQKNGNYPVQDAQYTNTRGKDILFVWGIHHPPTDTAQTNLYTRTDTTTSITTENLDRTFKPLIGPR
PLVNGLIGRINYYWSVLKPGQTLRVRSNGNLIAPWFGHVLSGESHGRILKTDLNSGNCVVQCQTEKGGLN
STLPFHNISKYAFGDCPKYIGVKSLKLAIGLRNVPARSSRGLFGAIAGFIEGGWPGLVAGWYGFQHSNDQ
GVGMAADRDSTQKAVDKITSKVNNIVDKMNKQYEIIDHEFSEVETRLNMINNKIDDQIQDIWAYNAELLV
LLENQKTLDEHDANVNNLYNKVKRALGSNAMEDGKGCFELYHKCDDQCMETIRNGTYNRRKYMEESRLGR
QKIEGVKLESEGT

>AXO10667.1 hemagglutinin, partial [Influenza A virus]
DKICIGHQSTNSTETVDTLTETNVPVTHAKELLHTEHNGKLCATNLGNPLILDTCTIEGLIYGNPSCDML
LGGREWSYIVERPSAVNGTCYPGNVENLEELRILFSSSSSYQRIQMFPDTIWNVTYSGTSKSCSDSFYRN
MRWLTQKNGNYPVQDAQYTNTRGKDILFVWGIHHPPTDTVQTNLYTRTDTTTSITTENLDRTFKPLIGPR
PLVNGLIGRINYYWSVLKPGQTLRVRSNGNLIAPWFGHVLSGESHGRILKTDLNSGNCVVQCQTEKGGLN
STLPFHNISKYAFGDCPKYIGVKSLKLAIGLRNVPARSSRGLFGAIAGFIEGGWPGLVAGWYGFQHSNDQ

GVGMAADRDSTQKAVDKITSKVNNIVDKMNKQYEIIDHEFSEVETRLNMINNKIDDQIQDIWAYNAELLV
LLENQKTLDEHDANVNNLYNKVKRALGSNAMEDGKGCFELYHKCDDQCMETIRNGTYNRRKYMEESRLGR
QKIEGVKLESEGT

>AXO10666.1 hemagglutinin, partial [Influenza A virus]
DKICIGHQSTNSTETVDTLTESNVPVTHAKELLHTEHNGKLCATNLGNPLILDTCTIEGLIYGNPSCDML
LGGREWSYIVERPSAVNGTCYPGNVENLEELRILFSSSSSYQRIQMFPDTIWNVTYSGTSKSCSDSFYRN
MRWLTQKNGNYPVQDAQYTNTRGKDILFVWGIHHPPTDTAQTNLYTRTDTTTSITTEXLDRTFKPLIGPR
PLVNGLIGRINYYWSVLKPGQTLRVRSNGNLIAPWFGHVLSGESHGRVLKTDLNSGNCVVQCQTEKGGLN
STLPFHNISKYAFGDCPKYIGVKSLKLAIGLRNVPARSSRGLFGAIAGFIEGGWPGLVAGWYGFQHSNDQ
GVGMAADRDSTQKAVDKITSKVNNIVDKMNKQYEIIDHEFSEVETRLNMINNKIDDQIQDIWAYNAELLV
LLENQKTLDEHDANVNNLYNKVKRALGSNAMEDGKGCFELYHKCDDQCMETIRNGTYNRRKYMEESRLGR
QKIEGVKLESEGT

>AXO10665.1 hemagglutinin, partial [Influenza A virus]
DKICIGHQSTNSTETVDTLTETNVPVTHAKELLHTEHNGKLCATNLGNPLILDTCTIEGLIYGNPSCDML
LGGREWSYIVERPSAVNGTCYPGNVENLEELRILFSSSSSYQRIQMFPDTIWNVTYSGTSKSCSDSFYRN
MRWLTQKNGNYPVQDAQYTNTRGKDILFVWGIHHPPTDTAQTNLYTRTDTTTSITTENLDRTFKPLIGPR
PLVNGLIGRINYYWSVLKPGQTLRVRSNGNLIAPWFGHVLSGESHGRILKTDLNSGNCVVQCQTEKGGLN
STLPFHNISKYAFGDCPKYIGVKSLKLAIGLRNVPARSSRGLFGAIAGFIEGGWPGLVAGWYGFQHSNDQ
GVGMAADRDSTQQAVDKITSKVNNIVDKMNKQYEIIDHEFSEVETRLNMINNKIDDQIQDIWAYNAELLV
LLENQKTLDEHDANVNNLYNKVKRALGSNAMEDGKGCFELYHKCDDQCMETIRNGTYNRRKYMEESRLGR
QKIEGVKLESEGT

>AXO10664.1 hemagglutinin, partial [Influenza A virus]
DRICIGHQSTNSTETVDTLTETNVPVTHAKELLHTEHNGKLCATNLGNPLILDTCTIEGLIYGNPSCDML
LGGREWSYIVERPSAVNGTCYPGNVENLEELRILFSSSSSYQRIQMFPDTIWNVTYSGTSKSCSDSFYRN
MRWLTQKNGNYPVQDAQYTNTRGKDILFVWGIHHPPTDTAQTNLYTRTDTTTSVTTENLDRTFKPLIGPR
PLVNGLIGRINYYWSVLKPGQTLRVRSNGNLIAPWFGHVLSGESHGRILKTDLNSGNCVVQCQTEKGGLN
STLPFHNISKYAFGNCPKYIGVKSLKLAIGLRNVPARSSRGLFGAIAGFIEGGWPGLVAGWYGFQHSNDQ
GVGMAADRDSTQKAVDKITSKVNNIVDKMNKQYEIIDHEFSEVETRLNMINNKIDDQIQDVWAYNAELLV
LLENQKTLDEHDANVNNLYNKVKRALGSNAMEDGKGCFELYHKCDDQCMETIRNGTYNRRKYKEESRLGR
QKIEGVKLESEGT

>AXO10663.1 hemagglutinin, partial [Influenza A virus]

DKICIGHQSTNSTETVDTLTETNVPVTHAKELLHTEHNGKLCATNLGNPLILDTCTIEGLIYGNPSCDML
LGGREWSYIVERPSAVNGTCYPGTVENLEELRILFSSSSSYQRIQMFPDTIWNVTYSGTSKSCSDSFYRN
MRWLTQKNGNYPVQDAQYTNTRGKDILFVWGIHHPPTDTAQTNLYTRTDTTTSITTENLDRTFKPLIGPR
PLVNGLIGRINYYWSVLKPGQTLRVRSNGNLIAPWFGHVLSGESHGRILKTDLNSGNCVVQCQTEKGGLN
STLPFHNISKYAFGDCPKYIGVKSLKLAIGLRNVPARSSRGLFGAIAGFIEGGWPGLVAGWYGFQHSNDQ
GVGMAADRDSTQKAVDKITSKVNNIVDKMNKQYEIIDHEFSEVETRLNMINNKIDDQIQDIWAYNAELLV
LLENQKTLDEHDANVNNLYNKVKRALGSNAMEDGKGCFELYHKCDDQCMETIRNGTYNRRKYMEESRLGR
QKIEGVKLESEGA

>AXO10662.1 hemagglutinin, partial [Influenza A virus]

DKICIGHQSTNSTETVDTLTETNVPVTHAQELLHTEHNGKLCATNLGNPLILDTCTIEGLIYGNPSCDML
LGGREWSYIVERPSAVNGTCYPGNVENLEELRILFSSSSSYQRIQMFPDTIWNVTYSGTSKSCSDSFYRN
MRWLTQKNGNYPVQDAQYTNTRGKDILFVWGIHHPPTDTAQTNLYTRTDTTTSITTENLDRTFKPLIGPR
PLVNGLIGRINYYWSVLKPGQTLRVRSNGNLIAPWFGHVLSGESHGRILKTDLNSGNCVVQCQTEKGGLN
STLPFHNISKYAFGDCPKYIGVKSLKLAIGLRNVPARSSRGLFGAIAGFIEGGWPGLVAGWYGFQHSNDQ
GVGMAADRDSTQKAVDKITSKVNNIVDKMNKQYEIIDHEFSEVETRLNMINNKIDDQIQDIWAYNAELLV
LLENQKTLDEHDANVNNLYNKVKRALGSNAMEDGKGCFELYHKCDDQCMETIRNGTYNRRKYMEESRLGR
QKIEGVKLESEGT

>AXO10660.1 hemagglutinin, partial [Influenza A virus]

DRICIGHQSTNSTETVDTLTETNVPVTHAKELLHTEHNGKLCATNLGNPLILDTCTIEGLIYGNPSCDML
LGGREWSYIVERPSAVNGTCYPGNVENLEELRMLFSSSSSYQRIQMFPDTIWNVTYSGTSKSCSDSFYRN
MRWLTQKNGNYPVQVAQYTNTRGKDILFVWGIHHPPTDTAQTNLYTRTDTTTSVTTENLDRTFKPLIGPR
PLVNGLIGRINYYWSVLKPGQTLRVRSNGNLIAPWFGHVLSGESHGRILKTDLNSGNCVVQCQTEKGGLN
STLPFHNISKYAFGNCPKYIGVKSLKLAIGLRNVPARSSRGLFGAIAGFIEGGWPGLVAGWYGFQHSNDQ
GVGMAADRDSTQKAVDKITSKVNNIVDKMNKQYEIIDHEFSEVETRLNMINNKIDDQIQDVWAYNAELLV
LLENQKTLDEHDANVNNLYNKVKRALGSNAMEDGKGCFELYHKCDDQCMETIRNGTYNRRKYKEESRLGR
QKIEGVKLESEGT

>AXO10659.1 hemagglutinin, partial [Influenza A virus]

DKICIGHQSTNSTETVDTLTETNVPVTHAKELLHTEHNGKLCATNLGNPLILDTCTIEGLIYGNPSCDML
LGGREWSYIVERPSAVNGTCYPGYVENLEKLRILFSSSISYHQIQIFPGPIWNGTYSGTSNPCSDPFYRN
MRWLTQKNGNYPVQDAPYTNTRGKDILFVWGIHHPPTDTAQTNLYTRTDTTTSITTENLDRTFKPLIGPR

PLVNGLIGRINYYWSVLKPGQTLRVRSNGNLIAPWFGHVLSGESHGRILKTDLNSGNCVVQCQTEKGGLN
STLPFHNISKYAFGDCPKYIGVKSLKLAIGLRNVPARSSRGLFGAIAGFIEGGWPGLVAGWYGFQHSNDQ
GVGMAADRDSTQKAVDKITSKVNNIVDKMNKQYEIIDHEFSEVETRLNMINNKIDDQIQDIWAYNAELLV
LLENQKTLDEHDANVNNLYNKVKRALGSNAMEDGKGCFELYHKCDDQCMETIRNGTYNRRKYMEESRLGR
QKIEGVKLESEGT

>AXO10658.1 hemagglutinin, partial [Influenza A virus]
DKICVGHQSTNSTETVDTLTETNVPVTHAKELLHTEHNGKLCATNLGNPLILDTCTIEGLIYGNPSCDML
LGGREWSYIVERPSAVNGTCYPGNVENLEELRILFSSSSSYQRIQMFPDTIWNVTYSGTSKSCSDSFYRN
MRWLTQKNGNYPVQDAQYTNTRGKDILFVWGIHHPPTDTAQTNLYTRTDTTTSITTENLDRTFKPLIGPR
PLVNGLIGRINYYWSVLKPGQTLRVRSNGNLIAPWFGHVLSGESHGRILKTDLNNGNCVVQCQTEKGGLN
STLPFHNISKYAFGDCPKYIGVTSLKLAIGLRNVPARSSRGLFGAIAGFIEGGWPGLVAGWYGFQHSNDQ
GVGMAADRDSTQKAVDKITSKVNNIVDKMNKQYEIIDHEFSEVETRLNMINNKIDDQIQDVWAYNAELLV
LLENQKTLDEHDANVNNLYNKVKRALGSNAMEDGKGCFELYHKCDDQCMETIRNGTYNRRKYMEESRLGR
QKIEGVKLESEGT

>AXO10657.1 hemagglutinin, partial [Influenza A virus]
DKICIGHQSTNSTETVDTLTETNIPVTHAKELLHTEHNGKLCATNLGNPLILDTCTIEGLIYGNPSCDML
LGGREWSYIVERPSAVNGTCYPGNVENLEELRILFSSSSSYQRIQMFPDTIWNVTYSGTSKSCSDSFYRN
MRWLTQKNGNYPVQDAQYTNTRGKDILFVWGIHHPPTDTAQTNLYTRTDTTTSITTENLDRTFKPLIGPR
PLVNGLIGRINYYWSVLKPGQTLRVRSNGNLIAPWFGHVLSGESHGRILKTDLNSGDCAVQCQTEKGGLN
STLPFHNISKYAFGDCPKYIGVKSLKLAIGLRNVPARSSRGLFGAIAGFIEGGWPGLVAGWYGFQHSNDQ
GVGMAADRDSTQKAVDKITSKVNNIVDKMNKQYEIIDHEFSEVETRLNMINNKIDDQIQDVWAYNAELLV
LLENQKTLDEHDANVNNLYNKVKRALGSNAMEDGKGCFELYHKCDDQCMETIRNGTYNRRKYMEESRLGR
QKIEGVKLESEGT

>AXO10656.1 hemagglutinin, partial [Influenza A virus]
DKICIGHQSTNSTETVDTLAETNVPVTHAKELLHTEHNGKLCATNLGNPLILDTCTIEGLIYGNPSCDML
LGGREWSYIVERPSAVNGTCYPGNVENLEELRILFSSSSSYQRIQMFPDTIWNVTYSGTSKSCSDSFYRN
MRWLTQKNGNYPVQDAQYTNTRGKDILFVWGIHHPPTDTAQTNLYTRTDTTTSITTENLDRTFKPLIGPR
PLVNGLIGRINYYWSVLKPGQTLRVRSNGNLIAPWFGHILSGESHGRILKTDLNSGNCVVQCQTEKGGLN
STLPFHNISKYAFGDCPKYIGVKSLKLAIGLRNVPARSSRGLFGAIAGFIEGGWPGLVAGWYGFQHSNDQ
GVGMAADRDSTQKAVDKITSKVNNIVDKMNKQYEIIDHEFSEVETRLNMINNKIDDQIQDIWAYNAELLV
LLENQKTLDEHDANVNNLYNKVKRALGSNAMEDGKGCFELYHKCDDQCMETIRNGTYNRRKYMEESRLGR

QRIEGVKLESEGT

>AXO10655.1 hemagglutinin, partial [Influenza A virus]

DKICIGHQSTNSTETVDTLTETNVPVTHAKELLHTEHNGKLCATNLGNPLILDTCTIEGLIYGNPSCDML
LGGREWSYIVERPSAVNGTCYPGNVENLEELRILFSSSSSYQRIQMFPDTIWNVTYSGTSKSCSDSFYRN
MRWLTQKNGNYPVQDAQYTNTRGKDILFVWGIHHPPTDTTQTNLYTRTDTTTSITTENLDRTFKPLIGPR
PLVNGLIGRINYYWSVLKPGQTLRVRSNGNLIAPWFGHVLSGESHGRILKTDLNSGNCVVQCQTEKGGLN
STLPFHNISKYAFGDCPKYIGVKSLKLAIGLRNVPARSGRGLFGAIAGFIEGGWPGLVAGWYGFQHSNDQ
GVGMAADRDSTQKAVDKITSKVNNIVDKMNKQYEIIDHEFSEVETRLNMINNKIDDQIQDIWAYNAELLV
LLENQKTLDEHDANVNNLYNKVKRALGSNAMEDGKGCFELYHKCNDQCMETIRNGTYNRRKYMEESRLGR
QKIEGVKLESEGT

>AXO10654.1 hemagglutinin, partial [Influenza A virus]

DKICIGHQSTNSTETVDTLTETNVPVTHAKELLHTEHNGKLCATNLGNPLILDTCTIEGLIYGNPSCDML
LGGREWSYIVERPSAVNGTCYPGNVENLEELRILFSSSSSYQRIQMFPDTIWNVSYSGTSKSCSDSFYRN
MRWLTQKNGNYPVQDAQYTNTRGKDILFVWGIHHPPTDTAQTNLYTRTDTTTSITTENLDRTFKPLIGPR
PLVNGLIGRINYYWSVLKPGQTLRVRSNGNLIAPWFGHVLSGESHGRILKTDLNNGNCVVQCQTEKGGLN
STLPFHNISKYAFGDCPKYIGVKSLKLAIGLRNVPARSSRGLFGAIAGFIEGGWPGLVAGWYGFQHSNDQ
GVGMAADRDSTQKAVDKITSKVNNIVDKMNKQYEIIDHEFSEVETRLNMINNKIDDQIQDIWAYNAELLV
LLENQKTLDEHDANVNNLYNKVKRALGSNAMEDGKGCFELYHKCDDQCMETIRNGTYNRRKYMEESRLGR
QKIEGVKLESEGT

>AXO10653.1 hemagglutinin, partial [Influenza A virus]

DKICIGHQSRNSTETVDTLTETNVPVTHAKELLHTEHNGMLCATNLGNPLILDTCTIEGLIYGNPSCDML
LGGREWSYIVERPSAVNGTCYPGNVENLEELRILFSSSSSYQRIQMFPDTIWNVTYSGTSKSCSDSFYRN
MRWLTQKNGNYPVQDAQYTNTRGKDILFVWGIHHPPTDTAQTNLYTRTDTTTSVTTENLDRTFKPLIGPR
PLVNGQIGRINYYWSVLKPGQTLRVRSNGNLIAPWFGHVLSGESHGRILKTDLNSGNCVVQCQTEKGGLN
STLPFHNISKYAFGDCPKYIGVKSLKLAIGLRNVPARSSRGLFGAIAGFIEGGWPGLVSGWYGFQHSNDQ
GVGMAADRDSTQKAVDKITSKVNNIVGKMNKQYEIIDHEFSEVETRLNMINNKIDDQIQDVWAYNAELLV
LLENQKTLDEHDANVNNLYNKVKRALGSNAMEDGKGCFELYHKCDDQCMETIRNGTYNRRKYKEESRLER
LKIEGVKLESEGT

>AXO10651.1 hemagglutinin, partial [Influenza A virus]

DKICIGHQSTNSTETVDTLTETNVPVTHAKELLHTEHNGKLCATNLGNPLILDTCTIEGLIYGNPSCDML

LGGREWSYIVERPSAVNGTCYPGNVENLEELRILFSSSSSYQRIQMFPDTIWNVTYSGTSKSCSDSFYRN
MRWLTQKNGNYPVQDAQYTNTRGKDILFVWGIHHPPTDTAQTNLYTRTDTTTSVTTENLDRTFKPLIGPR
PLVNGLIGRINYYWSVLKPGQTLRVRSNGNLIAPWFGHVLSGESHGRILKTDLNSGNCVVQCQTEKGGLN
STLPFHNISKYAFGDCPKYIGVKSLKLAIGLRNVPARSSRGLFGAIAGFIEGGWPGLVAGWYGFQHSNDQ
GVGMAADRDSTQKAVDKITSKVNNIIDKMNKQYEIIDHEFSEVETRLNMINNKIDDQIQDIWAYNAELLV
LLENQKTLDEHDANVNNLYNKVKRALGSNAMEDGKGCFELYHKCDDQCMETIRNGTYNRRKYKEESRLGR
QKIEGVKLESEGT

>AXO10650.1 hemagglutinin, partial [Influenza A virus]
DKICIGHQSTNSTETVDTLTETNVPVTHAKELLHTEHNGKLCATNLGNPLILDTCTIEGLIYGNPSCDVL
LGGREWSYIVERPSAVNGTCYPGNVENLEELRILFSSSSSYQRIQMFPDTIWNVTYSGTSKSCSDSFYRN
MRWLTQKNGNYPVQDAQYTNTRGKDILFVWGIHHPPTETAQTNLYTRTDTTTSITTENLDRTFKPLIGPR
PLVNGLIGRINYYWSVLKPGQTLRVRSNGNLIAPWFGHVLSGESHGRILKTDLNSGNCVVQCQTEKGGLN
STLPFHNISKYAFGDCPKYIGVKSLKLAIGLRNVPARSSRGLFGAIAGFIEGGWPGLVAGWYGFQHSNDQ
GVGMAADRDSTQKAVDKITSKVNSIVDKMNKQYEIIDHEFSEVETRLNMINNKIDDQIQDIWAYNAELLV
LLENQRTLDEHDANVNNLYNKVKRALGSNAREDGKGCFELYHKCDDQCMETIRNGTYNRRKYMEESRLGR
QKIEGVKLESEGT

>AXO10649.1 hemagglutinin, partial [Influenza A virus]
DKICIGHQSTNSTETVDTLTETKVPVTHAKELLHTEHNGKLCATNLGNPLILDTCTIEGLIYGNPSCDML
LGGREWSYIVERPSAVNGTCYPGNVENLEELRILFSSSSSYQRIQMFPDTIWNVTYSGTSKSCSDSFYRN
MRWLTQKNGNYPVQDAQYTNTRGKDILFVWGIHHPPTDTAQTNLYTRTDTTTSITTENLDRTFKPLIGPR
PLVNGLIGRINYYWSVLKPGQTLRVRSNGNLIAPWFGHVLSGESHGRILKTDLNSGSCVVQCQTEKGGLN
STLPFHNISKYAFGDCPKYIGVKSLKLAIGLRNVPARSSRGLFGAIAGFIEGGWPGLVAGWYGFQHSNDQ
GVGMAADRDSTQKAVDKITSKVNNIVDKMNKQYEIIDHEFSEVETRLNMINNKIDDQIQDIWAYNAELLV
LLENQKTLDEHDANVNNLYNKVKRALGSNAMEDGKGCFELYHKCDDQCMETIRNGTYNRRKYMEESRLGR
QKIEGVKLESEGT

>AXO10648.1 hemagglutinin, partial [Influenza A virus]
DKICIGHQSTNSTETVDTLTETNVPVTHAKELLHTEHNGKLCATNLGNPLILDTCTIEGLIYGNPSCDML
LGGRKWSYIVERPSAVNGTCYPGNVENLEELRILFSSSSSYQRIQMFPDTIWNVTYSGTSKSCSDSFYRN
MRWLTQKNGNYPVQDAQYTNTRGKDILFVWGIHHPPTDTAQTNLYTRTDTTTSITTENLDRTFKPLIGPR
PLVNGLIGRINYYWSVLKPGQTLRVRSNGNLIAPWFGHVLSGESHGRILKTDLNSGNCVVQCQTEKGGLN
STLPFHNISKYAFGDCPKYIGVKSLKLAIGLRNVPARSSRGLFGAIAGFIEGGWPGLVAGWYGFQHSNDQ

GVGMAADRDSTQKAVDKITSKVNNIVDKMNKQYEIIDHEFSEVETRLNMINNKIDDQIQDIWAYNAELLV
LLENQKTLDEHDANVNNLYNKVKRALGSNAMEDGKGCFELYHKCDDQCMETIRNGTYNRRKYMEESRLGR
QKIEGVKLESEGT

>AXO10647.1 hemagglutinin, partial [Influenza A virus]
DRICIGHQSTNSTETVDTLTETNVPVTHAKELLHTEHNGKLCATNLGNPLILDTCTIEGLIYGNPSCDML
LGGREWSYIVERPSAVNGTCYPGNVENLEELRILFSSSSSYQRIQMFPDTIWNVTYSGTSKSCSDSFYRN
MRWLTQKNGNYPVQDAQYTNTRGKDILFVWGIHHPPTDTAQTNLYTRTDTTTSVTTENLDRTFKPLIGPR
PLVNGLIGRINYYWSVLKPGQTLRVRSNGNLIAPWFGHVLSGESHGRILKTDLNSGNCVVQCQTEKGGLN
STLPFHNISKYAFGNCPKYIGVKSLKLAIGLRNVPARSSRGLFGAIAGFIEGGWPGLVAGWYGFQHSNDQ
GVGMAADRDSTQKAVDKITSKVNNIVDKMNKQYEIIDHEFSEVETRLNMINNKIDDQIQDVWAYNAELLV
LLENQKTLDEHDANVNNLYNKVKRALGSNAMEDGKGCFELYHKCDDQCMETIRNGTYNRRKYKEESRLGR
QKIEGVKLESEGT

>AXO10646.1 hemagglutinin, partial [Influenza A virus]
DKICIGHQSTNSTETVDTLTETNVPVTHAKELLHTEHNGKLCATNLGNPLILDTCTIEGLIYGNPSCDML
LGGREWSYIVERPSAVNGTCYPGNVENLEELRILFSSSSSYQRIQMFPDTIWNVTYSGTSKSCSDSFYRN
MRWLTQKNGNYPVQDAQYTNTRGKDILFVWGIHHPPTDTAQTNLYTRTDTTTSITTENLDRTFKPLIGPR
PLVNGLIGRINYYWSVLKPGQTLRVRSNGNLIAPWFGHVLSGESHGRILKTDLNSGNCVVQCQTEKGGLN
STLPFHNISKYAFGDCPKYIGVKSLKLAIGLRNVPARSSRGLFGAIAGFIEGGWPGLVAGWYGFQHSNDQ
GVGIAADRDSTQKAVDKITSKVNNIVDKMNKQYEIIDHEFSEVETRLNMINNKIDDQIQDIWAYNAELLV
LLENQKTLDEHDANVNNLYNKVKRALGSNAMEDGKGCFELYHKCDDQCMETIRNGTYNRRKYMEESRLGR
QKIEGVKLESEGT

>AXO10645.1 hemagglutinin, partial [Influenza A virus]
DKICIGHQSTNSTETVDTLTETNVPVTHAKELLHTEHNGKLCATNLGNPLILDTCTIEGLIYGNPSCDML
LGGREWSYIVERPSAVNGTCYPGNVENLEELRILFSSSSSYQRIQMFPDTIWNVTYSGTSKSCSDSFYRN
MRWLTQKNGNYPVQDAQYTNTRGKDILFVWGIHHPPTDTAQTNLYTRTDTTTSVTTENLDRTFKPLIGPR
PLVNGLIGRINYYWSVLKPGQTLRVRSNGNLIAPWFGHVLSGESHGRILKTDLNSGNCVVQCQTEKGGLN
STLPFHNISKYAFGDCPKYIGVKSLKLAIGLRNVPARSSRGLFGAIAGFIEGGWPGLVAGWYGFQHSNDQ
GVGMAADRDSTQKAVDKITSKVNNIVDKMNKQYEIIDHEFSEVETRLNMINNKIDDQIQDIWAYNAELLV
LLENQKTLDEHDANVNNLYNKVKRALGSNAMEDGKGCFELYHKCDDQCMETIRNGTYNRRKYKEESRLGR
QKIEGVKLESEGT

>AXO10644.1 hemagglutinin, partial [Influenza A virus]

DKICIGHQSTNSTETVDTLTETNVPVTQAKELLHTEHNGKLCATNLGNPLILDTCTIEGLIYGNPSCDML
LGGREWSYIVERPSAVNGTCYPGNVENLEELRILFSSSSSYQRIQMFPDTIWNVTYSGTSKSCSDSFYRN
MRWLTQKNGNYPVQDAQYTNTRGKDILFVWGIHHPPTDTAQTNLYTRTDTTTSITTENLDRTFKPLIGPR
PLVNGLIGRINYYWSVLKPGQTLRVRSNGNLIAPWFGHVLSGESHGRILKTDLNSGNCVVQCQTEKGGLN
STLPFHNISKYAFGDCPKYIGVKSLKLAIGLRNVPARSSRGLFGAIAGFIEGGWPGLVAGWYGFQHSNDQ
GVGMAADRDSTQRAVDKITSKVNNIVDKMNKQYEIIDHEFSEVETRLNMINNKIDDQIQDIWAYNAELLV
LLENQKTLDEHDANVNNLYNKVRRALGSNAMEDGRGCFELYHKCDDQCMETIRNGTYNRRKYKEESRLGR
QKIEGVKLESEGT

>AXL94634.1 hemagglutinin, partial [Influenza A virus]

DKICIGHQSTNSTETVDTLTETNVPVTHAKELLHTEHNGKLCATNLGNPLILDTCTIEGLIYGNPSCDML
LGGREWSYIVERPSAVNGTCYPGNVENLEELRILFSSSSSYQRIQMFPDTIWNVTYSGTSKSCSDSFYRN
MRWLTQKNGNYPVQDAQYTNTRGKDILFVWGIHHPPTDTAQTNLYTRTDTTTSITTENLDRTFKPLIGPR
PLVNGLIGRINYYWSVLKPGQTLRVRSNGNLIAPWFGHVLSGESHGRILKTDLNSGNCVVQCQTEKGGLN
STLPFHNISKYAFGDCPKYIGVKSLKLAIGLRNVPARSSRGLFGAIAGFIEGGWPGLVAGWYGFQHSNDQ
GVGMAADRDSTQKAVDKITSKVNNIVDKMNKQYEIIDHEFSEVETRLNMINNKIDDQIQDIWAYNAELLV
LLENQKTLDEHDANVNNLYNKVKRALGSNAMEDGKGCFELYHKCDDQCMETIRNGTYNRRKYMEESRLGR
QKIEGVKLESEGT

>AXL94633.1 hemagglutinin, partial [Influenza A virus]

DKICIGHQSTNSTETVDTLTETNVPVTHAKELLHTEHNGKLCATNLGNPLILDTCTIEGLIYGNPSCDML
LGGREWSYIVERPSAVNGTCYPGNVENLEELRILFSSSSSYQRIQMFPDTIWNVTYSGTSKSCSDSFYRN
MRWLTQKNGNYPVQDAQYTNTRGKDILFVWGIHHPPTDTAQTNLYTRTDTTTSITTENLDRTFKPLIGPR
PLANGLIGRINYYWSVLKPGQTLRVRSNGNLIAPWFGHVLSGESHGRILKTDLNSGNCVVQCQTEKGGLN
STLPFHNISKYAFGDCPKYIGVKSLKLAIGLRNVPARSSRGLFGAIAGFIEGGWPGLVAGWYGFQHSNDQ
GVGMAADRDSTQKAVDKITSKVNNIVDKMNKQYEIIDHEFSEVETRLNMINNKIDDQIQDIWAYNAELLV
LLENQKTLDEHDANVNNLYNKVKRALGSNAMEDGKGCFELYHKCDDQCMETIRNGTYNRRKYMEESRLGR
QKIEGVKLESEGT

>AXL94632.1 hemagglutinin, partial [Influenza A virus]

DKICIGHQSTNSTETVDTLTETNVPVTHAKELLHTEHNGKLCATNLGNPLILDTCTIEGLIYGNPSCDML
LGGREWSYIVERPSAVNGTCYPGNVENLEELRILFSSSSSYQRIQMFPDTIWNVTYSGTSKSCSDSFYRN
MRWLTQKNGNYPVQDAQYTNTRGKDILFVWGIHHPPTDTTQTNLYTRTDTTTSITTENLDRTFKPLIGPR

PLVNGLIGRINYYWSVLKPGQTLRVRSNGNLIAPWFGHVLSGESHGRILKTDLNRGNCVVQCQTEKGGLN
STLPFHNISKYAFGDCPKYIGVKSLKLAIGLRNVPARSSRGLFGAIAGFIEGGWPGLVAGWYGFQHSNDQ
GVGMAADRDSTQKAVDKITSKVNNIVDKMNKQYEIIDHEFSEVETRLNMINNKIDDQIQDIWAYNAELLV
LLENQKTLDEHDANVNNLYNKVKRALGSNAMEDGKGCFELYHKCDDQCMETIRNGTYNRRKYMEESRLGR
QKIEGFKLESEGT

>AWH12385.1 hemagglutinin [Influenza A virus]
METSSLITILLVVTASNADKICIGYQSTNSTETVDTLTENNVPVTHAKELLHTEHNGMLCATNLGHPLIL
DTCTIEGLIYGNPSCDLLLGGREWSYIVERPSAVNGLCYPGNVENLEELRSLFSSARSYQRIQIFPDTIW
NVSYSGTSKACSDSFYRSMRWLTQKDNAYPIQDAQYTNNQEKNILFMWGINHPPTDTAQTNLYTRTDTTT
SVATEEINRTFKPLIGPRPLVNGLMGRINYYWSVLKPGQTLRIKSNGNLIAPWYGHILSGESHGRILKTD
LKSGSCTVQCQTEKGGLNTTLPFQNVSKYAFGNCSKYIGIKSLKLAVGLRNVPSRSSRGLFGAIAGFIEG
GWSGLVAGWYGFQHSNDQGVGMAADRDSTQKAIDKITSKVNNIVDKMNKQYEIIDHEFSEVETRLNMINS
KIDDQIQDIWAYNAELLVLLENQKTLDEHDANVNNLYNKVKRALGSNAVEDGKGCFELYHKCDDQCMETI
RNGTYNRRKYQEESKLERQKIEGVKLESEGTYKILTIYSTVASSLVIAMGFAAFLFWAMSNGSCRCNICI

>AWH12373.1 hemagglutinin [Influenza A virus]
METASLITILLVVTASKADKICIGYQSTNSTETVDTLTENNVPVTHAKELLHTEHNGMLCATSLGHPLIL
DTCTIEGLIYGNPSCDLLLGGREWSYIVERPSAVNGLCYPGNVENLEELRSLFSSARSYQRIQIFPDTIW
NVSYSGTSKACSDSFYRSMRWLTQKDNAYPIQDAQYTNNQEKNILFMWGINHPPTDTAQTNLYTRTDTTT
SVATEEINRTFKPLIGPRPLVNGLMGRINYYWSVLKPGQTLRIKSNGNLIAPWYGHILSGESHGRILKTD
LKSGSCTVQCQTEKGGLNTTLPFQNVSRYAFGNCSKYIGIKSLKLAVGLRNVPSKSSRGLFGAIAGFIEG
GWSGLVAGWYGFQHSNDQGVGMAADRDSTQKAIDKITSKVNNIVDKMNKQYEIIDHEFSEVETRLNMINS
KIDDQIQDIWAYNAELLVLLENQKTLDEHDANVNNLYNKVKRALGSNAVEDGKGCFELYHKCDDQCMETI
RNGTYNRRKYQEESKLERQKIEGVKLESEDTYKILTIYSTVASSLVIAMGFAAFLFWAMSNGSCRCNICI

>AXG75386.1 hemagglutinin [Influenza A virus]
METTSLITILLVVTASNADKICIGYQSTNSTETVDTLTENNVPVTHAKELLHTEHNGMLCATSLGHPLIL
DTCTIEGLIYGNPSCDPLLGEREWSYIVERPSAVNGLCYPGNVENLEELRSLFSSARSYQRILIFPDTIW
NVSYSGTSKACSDSFYRSMRWLTQKNNAYPIQDAQYTNNQEKNILFMWGINHPPTDTVQTNLYTRTDTTT
SVATEEINRTFKPLIGPRPLVNGLMGRINYYWSVLKPGQTLRIKSNGNLIAPWYGHILSGKSHGRILKTD
LKRGSCTVQCQTEKGGLNTTLPFQNVSKYAFGNCSKYIGIKSLKLAVGLRNVPSRSSRGLFGAIAGFIEG
GWSGLVAGWYGFQHSNDQGVGMAADRDSTQKAIDKITSKVNNIVDKMNKQYEIIDHEFSEVETRLNMINN
KIDDQIQDIWAYNAELLVLLENQKTLDEHDANVNNLYNKVKRALGSNAAEDGKGCFELYHKCDDQCMETI

RNGTYNRRKYQEESKLERQKIEGVKLESEGTYKILTIYSTVASSLVIAMGFAAFLFWAMSNGSCRCNICI

>AXG75384.1 hemagglutinin [Influenza A virus]
METASLITILLVVTASNADKICIGYQSTNSTETVDTLTENNVPVTHAKELLHTEHNGMLCATSLGHPLIL
DTCTIEGLIYGNPSCDQLLGGREWSYIVERPSAVNGLCYPGNVENLEELRSLFSSARSYQRILIFPDTIW
NVSYSGTSRACSDSFYRSMRWLTQKDNAYPIQDAQYTNNQEKNILFMWGINHPPTDTAQTNLYTRTDTTT
SVATEEINRTFKPLIGPRPLVNGLMGRINYYWSVLKPGQTLRIKSNGNLIAPWYGHILSGESHGRILKTD
LKRGSCTVQCQTEKGGLNTTLPFQNVSKYAFGNCSKYIGIKSLKLAVGLRNVPSRSSRGLFGAIAGFIEG
GWSGLVAGWYGFQHSNDQGVGMAADRDSTQKAIDKITSKVNNIVDKMNKQYEIINHEFSEVETRLNMINN
KIDDQIQDIWAYNAELLVLLENQKTLDEHDANVNNLYNKVKRALGSNAVEDGKGCFELYHKCDDQCMETI
RNGTYNRRKYQEESKLERQKIEGVKLESEGTYKILTIYSTVASSLVIVMGFAAFLFWAMSNGSCRCNICI

>AXG75383.1 hemagglutinin [Influenza A virus]
METASLITILLVVTASNADKICIGYQSTNSTETVDTLTENNVPVTHAKELLHTEHNGMLCATSLGHPLIL
DTCTIEGLIYGNPSCDPLLGGREWSYIVERPSAVNGLCYPGNVENLEELRSLFSSARSYQRILIFPDTIW
NVSYSGTSKACSDSFYRSMRWLTQKDNAYPIQDAQYTNNQEKNILFMWGINHPPTDTAQTNLYTRTDTTT
SVATEEINRTFKPLIGPRPLVNGLMGRINYYWSVLKPGQTLRIKSNGNLIAPWYGHILSGESHGRILKTD
LKRGSCTVQCQTEKGGLNTTLPFQNVSKYAFGNCSKYIGIKSLKLAVGLRNVPSRSSRGLFGAIAGFIEG
GWSGLVAGWYGFQHSNDQGVGMAADRDSTQKAIDKITSKVNNIVDKMNKQYEIIDHEFSEVETRLNMINN
KIDDQIQDIWAYNAELLVLLENQKTLDEHDANVNNLYNKVKRALGSNAVEDGKGCFELYHKCDDQCMETI
RNGTYNRRKYQEESKLERQKIEGVKLESEGTYKILTIYSTVASSLVIAMGFAAFLFWAMSNGSCRCNICI

>AXG75382.1 hemagglutinin [Influenza A virus]
METASLITILLVVTASNADKICIGYQSTNSTETVDTLTENNVPVTHAKELLHTEHNGMLCATSLGHPLIL
DTCTIEGLIYGNPSCDPLLGGREWSYIVERPSAVNGLCYPGNVENLEELRSLFSSARSYQRILIFPDTIW
NVSYSGTSKACSDSFYRSMRWLTQKDNAYPIQDAQYTNNQEKNILFMWGINHPPTDTAQTNLYTRTDTTT
SVATEEINRTFKPLIGPRPLVNGLMGRINYYWSVLKPGQTLRIKSNGNLIAPWYGHILSGESHGRILKTD
LKRGSCTVQCQTEKGGLNTTLPFQNVSKYAFGNCSKYIGIKSLKLAVGLRNVPSRSSRGLFGAIAGFIEG
GWSGLVAGWYGFQHSNDQGVGMAADRDSTQKAIDKITSKVNNIVDKMNKQYEIIDHEFSEVETRLNMINN
KIDDQIQDIWAYNAELLVLLENQKTLDEHDANVNNLYNKVKRALGSNAVEDGKGCFELYHKCDDQCMETI
RNGTYNRRKYQEESKLERQKIEGVKLESEGTYKILTIYSTVASSLVIAMGFAAFLFWAMSNGSCRCNICI

>AXG75381.1 hemagglutinin [Influenza A virus]
METASLITILLVVTVSNADKICIGYQSTNSTETVDTLTENNVPVTHAKELLHTEHNGMLCATSLGHPLIL

DTCTIEGLIYGNPSCDPLLGGREWSYIVERPSAVNGLCYPGNVENLEELRSLFSSARSYQRIQIFPDTIW
NVSYSGTSKACSDSFYRSMRWLTQKNNAYPIQDAQYTNNQEKNILFMWGINHPPTDTAQTNLYTRTDTTT
SVATEEINRTFKPLIGPRPLVNGLMGRINYYWSVLKPGQTLRIKSNGNLIAPWYGHILSGESHGRILKTD
LKRGSCTVQCQTEKGGLNTTLPFQNVSKYAFGNCSKYIGKKSLKLAVGLRNVPSRSSRGLFGAIAGFIEG
GWSGLVAGWYGFQHSNDQGVGMAADRDSTQRAIDKITSKVNNIVDKMNKQYEIIDHEFSEVETRLNMINN
KIDDQIQDIWAYNAELLVLLENQKTLDEHDANVNNLYNKVKRALGSNAVEDGKGCFELYHKCDDQCMETI
RNGTYNRRKYQEESKLERQKIEGVKLESEGTYKILTIYSTVASSLVIAMGFAAFLFWAMSNGSCRCNICI

>AXG75380.1 hemagglutinin [Influenza A virus]
METVSLITILLVATVSNADKICIGYQSTNSTETVDTLTENNVPVTHAKELLHTEHNGMLCATSLGQPLIL
DTCTIEGLIYGNPSCDPLLDGREWSYIVERPSAVNGLCYPGHVENLEELRSLFSSARSYQRIQIFPDTIW
NVSYDGTSNACSGSFYRSMRWLTRKNGDYPIQDAQYTNNQGKNILFMWGINHPPTDTTQRELYTRTDTTT
SVATEEINRIFKPLIGPRPLVNGLMGRIDYYWSVLRPGQTLRIKSDGNLIAPWYGHILSGESHGRILKTD
LKRGSCTVQCQTEKGGLNTTLPFQNVSKYAFGNCSKYIGIKSLKLAVGLRNVPSRSSRGLFGAIAGFIEG
GWSGLVAGWYGFQHSNDQGVGMAADRDSTQKAIDKITSKVNNIVDKMNKQYEIIDHEFSEVETRLNMINN
KIDDQIQDIWAYNAELLVLLENQKTLDEHDANVNNLYNKVKRALGSNAVEDGKGCFELYHKCDDQCMETI
RNGTYNRRKYQEESKLERQKIEGVKLESEGTYKILTIYSTVASSLVIAMGFAAFLFWAMSNGSCRCNICI

>AXG75379.1 hemagglutinin [Influenza A virus]
METVSLITILLVATVSNADKICIGYQSTNSTETVDTLTENNVPVTHAKELLHTEHNGMLCATSLGQPLIL
DTCTIEGLIYGNPSCDPLLDGREWSYIVERPSAVNGLCYPGHVENLEELRSLFSSARSYQRIQIFPDTIW
NVSYDGTSNACSGSFYRSMRWLTRKNGDYPIQDAQYTNNQGKNILFMWGINHPPTDTTQRELYTRTDTTT
SVATEEINRIFKPLIGPRPLVNGLMGRIDYYWSVLRPGQTLRIKSDGNLIAPWYGHILSGESHGRILKTD
LKRGSCTVQCQTEKGGLNTTLPFQNVSKYAFGNCSKYIGIKSLKLAVGLRNVPSRSSRGLFGAIAGFIEG
GWSGLVAGWYGFQHSNDQGVGMAADRDSTQKAIDKITSKVNNIVDKMNKQYEIIDHEFSEVETRLNMINN
KIDDQIQDIWAYNAELLVLLENQKTLDEHDANVNNLYNKVKRALGSNAVEDGKGCFELYHKCDDQCMETI
RNGTYNRRKYQEESKLERQKIEGVKLESEGTYKILTIYSTVASSLVIAMGFAAFLFWAMSNGSCRCNICI

>AXG75378.1 hemagglutinin [Influenza A virus]
METASLITILLVVTASNADKICIGYQSTNSTETVDTLTENNVPVTHAKELLHTEHNGMLCATSLGHPLIL
DTCTIEGLIYGNPSCDPLLGGREWSYIVERPSAVNGLCYPGNVENLEELRSLFSSARSYQRILIFPDTIW
NVSYSGTSKACSDSFYRSMRWLTQKDNAYPIQDAQYTNNQEKNILFMWGINHPPTDTAQTNLYTRTDTTT
SVATEEINRTFKPLIGPRPLVNGLMGRINYYWSVLKPGQTLRIKSNGNLIAPWYGHILSGESHGRILKTD
LKRGSCTVQCQTEKGGLNTTLPFQNVSKYAFGNCSKYIGIKSLKLAVGLRNVPSRSSRGLFGAIAGFIEG

GWPGLVAGWYGFQHSNDQGVGMAADRDSTQKAIDKITSKVNNIVDKMNKQYEIIDHEFSEVETRLNMINN
KIDDQIQDIWAYNAELLVLLENQKTLDEHDANVNNLYNKVKRALGSNAVEDGKGCFELYHKCDDQCMETI
RNGTYNRRKYQEESKLERQKIEGVKLESEGTYKILTIYSTVASSLVIAMGFAAFLFWAMSNGSCRCNICI

>AXG75377.1 hemagglutinin [Influenza A virus]
METAPLITILLVVTVSNADKICIGYQSTNSTETVDTLTENNVPVTHAKELLHTEHNGMLCATSLGHPLIL
DTCTIEGLIYGNPSCDPLLEGREWSYIVERPSAVNGLCYPGNVENLEELRSLFSSARSYQRIQIFPDTIW
NVSYSGTSKACSDSFYRSMRWLTQKNNAYPIQDAQYTNNQEKNILFMWGINHPPTDTAQTNLYTRTDTTT
SVATEEINRTFKPLIGPRPLVNGLMGRINYYWSVLKPGQTLRIKSNGNLIAPWYGHILSGESHGRILKTD
LKRGSCTVQCQTEKGGLNTTLPFQNVSKYAFGNCSKYIGIKSLKLAIGLRNVPSRSSRGLFGAIAGFIEG
GWSGLVAGWYGFQHSNDQGVGMAADRDSTQKAIDKITSKVNNIVDKMNKQYEIIDHEFSEVETRLNMINN
KIDDQIQDIWAYNAELLVLLENQKTLDEHDANVNNLYNKVKRALGSNAVEDGKGCFELYHKCDDQCMETI
RNGTYNRRKYQEESKLERQKIEGVKLESEGTYKILTIYSTVASSLVIAMGFAAFLFWAMSNGSCRCNICI

>AXG75376.1 hemagglutinin [Influenza A virus]
METAPLITILLVVTVSNADKICIGYQSTNSTETVDTLTENNVPVTHAKELLHTEHNGMLCATSLGHPLIL
DTCTIEGLIYGNPSCDPLLEGREWSYIVERPSAVNGLCYPGNVENLEELRSLFSSARSYQRIQIFPDTIW
NVSYSGTSKACSDSFYRSMRWLTQKNNAYPIQDAQYTNNQEKNILFMWGINHPPTDTAQTNLYTRTDTTT
SVATEEINRTFKPLIGPRPLVNGLMGRINYYWSVLKPGQTLRIKSNGNLIAPWYGHILSGESHGRILKTD
LKRGSCTVQCQTEKGGLNTTLPFQNVSKYAFGNCSKYIGIKSLKLAVGLRNVPSRSSRGLFGAIAGFIEG
GWSGLVAGWYGFQHSNDQGVGMAADRDSTQKAIDKITSKVNNIVDKMNKQYEIIDHEFSEVETRLNMINN
KIDDQIQDIWAYNAELLVLLENQKTLDEHDANVNNLYNKVKRALGSNAVEDGKGCFELYHKCDDQCMETI
RNGTYNRRKYQEESKLERQKIEGVKLESEGTYKILTIYSTVASSLVIAMGFAAFLFWAMSNGSCRCNICI

>AXG75375.1 hemagglutinin [Influenza A virus]
METVSLITILLVATVSNADKICIGYQSTNSTETVDTLTENNVPVTHAKELLHTEHNGMLCATSLGQPLIL
DTCTIEGLIYGNPSCDPLLDGREWSYIVERPSAVNGLCYPGHVENLEELRSLFSSARSYQRIQIFPDTIW
NVSYDGTSSACSGSFYRSMRWLTRKNGDYPIQDAQYTNNQGKNILFMWGINHPPTDTTQRELYTRTDTTT
SVATEEINRIFKPLIGPRPLVNGLMGRIDYYWSVLRPGQTLRIKSDGNLIAPWYGHILSGESHGRILKTD
LKRGSCTVQCQTEKGGLNTTLPFQNVSKYAFGNCSKYIGIKSLKLAVGLRNVPSRSSRGLFGAIAGFIEG
GWSGLVAGWYGFQHSNDQGVGMAADRDSTQKAIDKITSKVNNIVDKMNKQYEIIDHEFSEVETRLNMINN
KIDDQIQDIWAYNAELLVLLENQKTLDEHDANVNNLYNKVKRALGSNAVEDGKGCFELYHKCDDQCMETI
RNGTYNRRKYQEESKLERQKIEGVKLESEGTYKILTIYSTVASSLVVAMGFAAFLFWAMSNGSCRCNICI

>AXG75374.1 hemagglutinin [Influenza A virus]

METASLITILLVVTVSNADKICIGYQSTNSTETVDTLTENNVPVTHAKELLHTEHNGMLCATSLGHPLIL
DTCTIEGLIYGNPSCDPLLGGREWSYIVERPSAVNGLCYPGNVENLEELRSLFSSARSYQRIQIFPDTIW
NVSYSGTSKACSDSFYRSMRWLTQKNNAYPIQDAQYTNNQEKNILFMWGINHPPTDTAQTNLYTRTDTTT
SVATEEINRTFKPLIGPRPLVNGLMGRINYYWSVLKPGQTLRIKSNGNLIAPWYGHILSGESHGRILKTD
LKRGSCTVQCQTEKGGLNTTLPFQNVSKYAFGNCSKYIGKKSLKLAVGLRNVPSRSSRGLFGAIAGFIEG
GWSGLVAGWYGFQHSNDQGVGMAADRDSTQRAIDKITSKVNNIVDKMNKQYEIIDHEFSEVETRLNMINN
KIDDQIQDIWAYNAELLVLLENQKTLDEHDANVNNLYNKVKRALGSNAVEDGKGCFELYHKCDDQCMETI
RNGTYNRRKYQEESKLERQKIEGVKLESEGTYKILTIYSTVASSLVIAMGFAAFLFWAMSNGSCRCNICI

>AXG75373.1 hemagglutinin [Influenza A virus]

METVSLITILLVATVSNADKICIGYQSTNSTETVDTLTENNVPVTHAKELLHTEHNGMLCATSLGQPLIL
DTCTIEGLIYGNPSCDPLLDGREWSYIVERPSAVNGLCYPGHVENLEELRSLFSSARSYQRIQIFPDTIW
NVSYDGTSNACSGSFYRSMRWLTRKNGDYPIQDAQYTNNQGKNILFMWGINHPPTDTTQRELYTRTDTTT
SVATEEINRIFKPLIGPRPLVNGLMGRIDYYWSVLRPGQTLRIKSDGNLIAPWYGHILSGESHGRILKTD
LKKGSCTVQCQTEKGGLNTTLPFQNVSKYAFGNCSKYIGIKSLKLAVGLRNVPSRSSRGLFGAIAGFIEG
GWSGLVAGWYGFQHSNDQGVGMAADRDSTQKAIDKITSKVNNIVDKMNKQYEIIDHEFSEVETRLNMINN
KIDDQIQDIWAYNAELLVLLENQKTLDEHDANVNNLYNKVKRALGSNAVEDGKGCFELYHKCDDQCMETI
RNGTYNRRKYQEESKLERQKIEGVKLESEGTYKILTIYSTVASSLVIAMGFAAFLFWAMSNGSCRCNICI

>AXG75372.1 hemagglutinin [Influenza A virus]

METASLITILLVVTASNADKICIGYQSTNSTETVDTLTENNVPVTHAKELLHTEHNGMLCATSLGHPLIL
DTCTIEGLIYGNPSCDPLLGGREWSYIVERPSAVNGLCYPGNVENLEELRSLFSSARSYQRIQIFPDTIW
NVSYSGTSKACSDSFYRSMRWLTQKNNAYPIQDAQYTNNQEKNILFMWGINHPPTDTAQTNLYTRTDTTT
SVATEEINRTFKPLIGPRPLVNGLMGRINYYWSVLKPGQTLRIKSNGNLIAPWYGHILSGESHGRILKTD
LKRGSCTVQCQTEKGGLNTTLPFQNVSKYAFGNCSKYIGIKSLKLAVGLRNVPSRSSRGLFGAIAGFIEG
GWSGLVAGWYGFQHSNDQGVGMAADRDSTQKAIDKITSKVNNIVDKMNKQYEIIDHEFSEVETRLNMINN
KIDDQIQDIWAYNAELLVLLENQKTLDEHDANVNNLYNKVKRALGSNAVEDGKGCFELYHKCDDQCMETI
RNGTYNKRKYQEESKLERQKIEGVKLESEGTYKILTIYSTVASSLVIAMGFAAFLFWAMSNGSCRCNICI

>AXG75371.1 hemagglutinin [Influenza A virus]

METASLITILLVVTVSNADKICIGYQSTNSTETVDTLTENNVPVTHAKELLHTEHNGMLCATSLGHPLIL
DTCTIEGLIYGNPSCDPLLGGREWSYIVERPSAVNGLCYPGNVENLEELRSLFSSARSYQRIQIFPDTIW
NVSYSGTSKACSDSFYRSMRWLTQKNNAYPIQDAQYTNNQEKNILFMWGINHPPTDTAQTNLYTRTDTTT

SVATEEINRTFKPLIGPRPLVNGLMGRINYYWSVLKPGQTLRIKSNGNLIAPWYGHILSGESHGRILKTD
LKRGSCTVQCQTEKGGLNTTLPFQNVSKYAFGNCSKYIGKKSLKLAVGLRNVPSRSSRGLFGAIAGFIEG
GWSGLVAGWYGFQHSNDQGVGMAADRDSTQRAIDKITSKVNNIVDKMNKQYEIIDHEFSEVETRLNMINN
KIDDQIQDIWAYNAELLVLLENQKTLDEHDANVNNLYNKVKRALGSNAVEDGKGCFELYHKCDDQCMETI
RNGTYNRRKYQEESKLERQKIEGVKLESEGTYKILTIYSTVASSLVIAMGFAAFLFWAMSNGSCRCNICI

>AXG75370.1 hemagglutinin [Influenza A virus]
METASLITILLVVTVSNADKICIGYQSTNSTETVDTLTENNVPVTHAKELLHTEHNGMLCATSLGHPLIL
DTCTIEGLIYGNPSCDPLLGGREWSYIVERPSAVNGLCYPGNVENLEELRSLFSSARSYQRIQIFPDTIW
NVSYSGTSKACSDSFYRSMRWLTQKNNAYPIQDAQYTNNQEKNILFMWGINHPPTDTAQTNLYTRTDTTT
SVATEEINRTFKPLIGPRPLVNGLMGRINYYWSVLKPGQTLRIKSNGNLIAPWYGHILSGESHGRILKTD
LKRGSCTVQCQTEKGGLNTTLPFQNVSKYAFGNCSKYIGKKSLKLAVGLRNVPSRSSRGLFGAIAGFIEG
GWSGLVAGWYGFQHSNDQGVGMAADRDSTQRAIDKITSKVNNIVDKMNKQYEIIDHEFSEVETRLNMINN
KIDDQIQDIWAYNAELLVLLENQKTLDEHDANVNNLYNKVKRALGSNAVEDGKGCFELYHKCDDQCMETI
RNGTYNRRKYQEESKLERQKIEGVKLESEGTYKILTIYSTVASSLVIAMGFAAFLFWAMSNGSCRCNICI

>AXG75369.1 hemagglutinin [Influenza A virus]
METASLITILLVVTVSNADKICIGYQSTNSTETVDTLTENNVPVTHAKELLHTEHNGMLCATSLGHPLIL
DTCTIEGLIYGNPSCDPLLGGREWSYIVERPSAVNGLCYPGNVENLEELRSLFSSARSYQRIQIFPDTIW
NVSYSGTSKACSDSFYRSMRWLTQKNNAYPIQDAQYTNNQEKNILFMWGINHPPTDTAQTNLYTRTDTTT
SVATEEINRTFKPLIGPRPLVNGLMGRINYYWSVLKPGQTLRIKSNGNLIAPWYGHILSGESHGRILKTD
LKRGSCTVQCQTEKGGLNTTLPFQNVSKYAFGNCSKYIGKKSLKLAVGLRNVPSRSSRGLFGAIAGFIEG
GWSGLVAGWYGFQHSNDQGVGMAADRDSTQRAIDKITSKVNNIVDKMNKQYEIIDHEFSEVETRLNMINN
KIDDQIQDIWAYNAELLVLLENQKTLDEHDANVNNLYNKVKRALGSNAVEDGKGCFELYHKCDDQCMETI
RNGTYNRRKYQEESKLERQKIEGVKLESEGTYKILTIYSTVASSLVIAMGFAAFLFWAMSNGSCRCNICI

>AXG75368.1 hemagglutinin, partial [Influenza A virus]
VSLITILLVATVSNADKICIGYQSTNSTETVDTLTENNVPVTHAKELLQTEHNGMLCATSLGQPLILDTC
TIEGLIYGNPSCDPLLDGREWSYIVERPSAVNGLCYPGHVENLEELRSLFSSARSYQRIQIFPDTIWNVS
YDGTSNACSGSFYRSMRWLTRKNGDYPIQDAQYTNNQGKNILFMWGINHPPTDTTQRELYTRTDTTTSVA
TEEINRIFKPLIGPRPLVNGLMGRIDYYWSVLRPGQTLRIKSDGNLIAPWYGHILSGESHGRILKTDLKR
GSCTVQCQTEKGGLNTTLPFQNVSKYAFGNCSKYIGIKSLKLAVGLRNVPSRSSRGLFGAIAGFIEGGWS
GLVAGWYGFQHSNDQGVGMAADRDSTQKAIDKITSKVNNIVDKMNKQYEIIDHEFSEVETRLNMINNKID
DQIQDIWAYNAELLVLLENQKTLDEHDANVNNLYNKVKRALGSNAVEDGKGCFELYHKCDDQCMETIRNG

TYNRRKYQEESKLERQKIEGVKLESEGTYKILTIYSTVASSLVIAMGFAAFLFWAMSNGSCRCNICI

>AXF50182.1 hemagglutinin [Influenza A virus]
METISLITILLVATASNADKICIGYQSTNSTETVDTLTENNVPVTHAKELLHTEHNGMLCATSLGQPLIL
DTCTVEGLIYGNPSCDLSLEGREWSYIVERPSAVNGLCYPGNVENLEELRSLFSSARSYQRIQIFPDTIW
NVSYDGTSTACSDSFYRSMRWLTRKDGNYPTQDAQYTNNQGKNILFMWGINHPPTDDTQRNLYTRTDTTT
SVATEEINRIFKPLIGPRPLVNGLMGRINYYWSVLKPGQTLRIKSDGNLIAPWYGHILSGESHGRILKTD
LKRGSCTVQCQTEKGGLNTTLPFQNVSKYAFGNCSKYIGIKSLKLAVGLRNVPSRSSRGLFGAIAGFIEG
GWSGLVAGWYGFQHSNDQGVGMAADRDSTQKAIDKITSKVNNIVDKMNKQYEIIDHEFNEVETRLNMINN
KIDDQIQDIWAYNAELLVLLENQKTLDEHDANVNNLYNKVKRALGSNAVEDGKGCFELYHKCNDQCMETI
RNGTYNRKKYQEESKLERQKIEGVKLESEETYKILTIYSTVASSLVIAMGFAAFLFWAMSNGSCRCNICI

>AXF50170.1 hemagglutinin [Influenza A virus]
METISLITILVVATVSNADKICIGYQSTNSTETVDTLTENNVPVTHAKELLHTEHNGMLCATSLGHPLIL
DTCTIEGLIYGNPSCDPLLGGREWSYIVERPSAVNGLCYPGNVENLEELRSLFSSSRSYQRIQIFPDTIW
NVSYSGTSKACSDSFYRSMRWLTQKNNAYPTQDAQYTNNQGKNILFMWGINHPPTDTAQTNLYTRTDTTT
SVATEEMNRIFKPLIGPRPLVNGLMGRINYYWSVLKPGQTLRIKSDGNLIAPWYGHILSGESHGRILKTD
LKRGSCTVQCQTEKGGLNTTLPFQNVSKYAFGNCSKYIGVKSLKLAVGLRNVPSRSSRGLFGAIPGFIEG
GWSGLVAGWYGFQHSNDQGVGMAADRDSTQKAIDKITSKVNNIVDKMNKQYEIIDHEFSEVETRLNMINN
KVDDQIQDIWAYNAELLVLLENQKTLDEHDANVNNLYNKVKRALGSNAVEDGKGCFELYHTCDDHCMETI
RNGTYNPRKYHEESKLEDPKLEGFKLESEKTYKILTIYSTVASSLVIAMGFAAFLFWARYTGSCRCNICI

>AXF50158.1 hemagglutinin [Influenza A virus]
METISLITILLVATVSNADKICIGYQSTNSTETVDTLTENNVPVTHAKELLHTEHNGMLCATSLGQPIIL
DTCTIEGLIYGNPSCDLSLEGREWSYIVERPSAVNGLCYPGNVENLEELRSLFSSARSYQRIQIFPDTIW
NVSYDGTSTACSGSFYKNMRWLTRKSGEYPIQDAQYTNNQGKNILFMWGINHPPADTTQINLYTRTNTTT
SVATEEINRIFKPLIGPRPLVNGLMGRINYYWSVLKPGQTLRIRSDGNLIAPWYGHILSGESHGRILKTD
LKRGSCTVQCQTEKGGLNTTLPFQNVSKYAFGNCSKYIGIKSLKLAVGLRNVPSRSSRGLFGAIAGFIEG
GWSGLVAGWYGFQHSNDQGVGMAADRESTQKAVDKITSKVNNIVDKMNKQYEIIDHEFSEVETRLNMINN
KIDDQIQDIWAYNAELLVLLENQKTLDEHDANVNNLYNKVKRALGSNAMEDGKGCFELYHKCDDQCMETI
RNGTYNRTKYQEESKLERQKIEGVKLESERTYKILTIYSTVASSLVIAMGFAAFLFWAMSNGSCRCNICI

>AXF50146.1 hemagglutinin [Influenza A virus]
METISLITILLVATISNADKICIGYQSTNSTETVDTLTENNVPVTHAKELLHTEHNGMLCATSLGQPLIL

DTCTIEGLIYGNPSCDLSLEGREWSYIVERPSAVNGLCYPGNVENLEELRSLFSSARSYQRIQIFPDTIW
NVSYDGTSKACSGSFYRSMRWLTQKNGNYPTQDAQYTNNQGKNILFMWGINHPPADDTQRNLYTRTDTTT
SVATEEINRIFKPLIGPRPLVNGLMGRIDYYWSVLKPGQTLRIKSDGNLVAPWYGHILSGESHGRILKTD
LKRGSCTVQCQTEKGGLNTTLPFQNISKYAFGNCSKYIGIKSLKLAVGLRNVPSRSSRGLFGAIAGFIEG
GWSGLVAGWYGFQHSNDQGVGMAADRDSTQKAIDKVTSKVNNIIDKMNKQYEIIDHEFSEVETRLNMINN
KIDDQIQDIWAYNAELLVLLENQKTLDEHDANVNNLYNKVKRALGSNAVEDGKGCFELYHKCNDQCMETI
RNGTYNRRKYHEESKLERQKIEGVKLESEGTYKILTIYSTVASSLVIAMGFAAFLFWAMSNGSCRCNICI

>AXF50134.1 hemagglutinin [Influenza A virus]
METISLITILLVATVSNADKICIGYQSTNSTETVDTLTENNVPVTHAKELLHTEHNGMLCATSLGNPLIL
DTCTIEGLIYGNPSCDPLLGGRKWSYIVERPSAVNGLCYPGSVENLEELRSLFSSARSYQRIQIFPDTIW
NVSYSGTSKACSDSFYRSMRWLTQKNNAYPIQDAQYTNNQEKNILFMWGINHPPTETVQTNLYTRTDTTT
SVATEEINRIFKPLIGPRPLVNGLMGRINYYWSVLKPGQTLRIKSDGNLIAPWYGHILSGESHGRILKTD
LKRGSCTVQCQTEKGGLNTTLPFQNVSKYAFGNCSKYIGIKSLKLAVGLRNVPSRSSRGLFGAIAGFIEG
GWSGLVAGWYGFQHSNDQGVGMAADRDSTQKAIDKITSKVNNIVDKMNKQYEIIDHEFSEVETRLNMINN
KVDDQIQDIWAYNAELLVLLENQKTLDEHDSNVNNLYNKVKRALGSNAVEDGKGCFELYHKCNDQCMETI
RNGTYNRRKYQEESKLERQKIEGVKLGSEGTYKILTIYSTAASSLVIAMGFAAFLFWAMSNGSCRCNICI

>AXF50122.1 hemagglutinin [Influenza A virus]
METISLITILVVATVSNADKICIGYQSTNSTETVDTLTENNVPVTHAKELLHTEHNGMLCATSLGHPLIL
DTCTIEGLIYGNPSCDPLLGGREWSYIVERPSAVNGLCYPGNVENLEELRSLFSSSRSYQRIQIFPDTIW
NVSYSGTSKACSDSFYRSMRWLTQKNNAYPTQDAQYTNNQGKNILFMWGINHPPTDTTQTNLYTRTDTTT
SVATEEMNRIFKPLIGPRPLVNGLMGRINYYWSVLKPGQTLRIKSDGNLIAPWYGHILSGESHGRILKTD
LKRGSCTVQCQTEKGGLNTTLPFQNVSKYAFGNCSKYIGVKSLKLAVGLRNVPSRSSRGLFGAIAGFIEG
GWSGLVAGWYGFQHSNDQGVGMAADRDSTQKAIDKITSKVNNIVDKMNKQYEIIDHEFSEVETRLNMINN
KVDDQIQDIWAYNAELLVLLENQKTLDEHDANVNNLYNKVKRALGSNAVEDGKGCFELYHKCDDHCMETI
RNGTYNRRKYQEESKLERQKIEGVKLESEETYKILTIYSTVASSLVIAMGFAAFLFWAMSNGSCRCNICI

>AXF50110.1 hemagglutinin [Influenza A virus]
METISLITILLIAAVSNADKICIGYQSTNSTETVDTLTENNVPVTHAKELLHTEHNGMLCATSLGQPIIL
DTCTIEGLIYGNPSCDLSLEGREWSYIVERPSAVNGLCYPGNVENLEELRSLFSSARSYQRIQIFPDTIW
NVSYDGTSTACSGSFYRNMRWLTRKNGEYPIQDAQYTNNQGKNILFMWGINHPPADTTQRDLYTRTDTTT
SVATEEINRIFKPLIGPRPLVNGLMGRIDYYWSVLKPGQTLRIKSDGNLIAPWYGHILSGESHGRILKTD
LKRGSCTVQCQTEKGGLNTTLPFQNVSKYAFGNCSKYIGIKSLKLAVGLRNVPSRSSRGLFGAIAGFIEG

GWSGLVAGWYGFQHSNDQGVGMAADRDSTQKAVDKITSKVNTIVDKMNKQYEIIDHEFSEVETRLNMINN
KIDDQIQDIWAYNAELLVLLENQKTLDEHDANVNNLYNKVKRALGSNAVEDGKGCFELYHKCDDQCMETI
RNGTYNRRKYQEESKLERQKIEGVKLESEGTYKILTIYSTVASSLVIAMGFAAFLFWAMSNGSCRCNICI

>AXF50098.1 hemagglutinin [Influenza A virus]
METISLITILVVATVSNADKICIGYQSTNSTETVDTLTENNVPVTHAKELLHTEHNGMLCATSLGHPLIL
DTCTIEGLIYGNPSCDPLLGGREWSYIVERPSAVNGLCYPGNVENLEELRSLFSSSRSYQRIQIFPDTIW
NVSYSGTSKACSDSFYRSMRWLTQKNNAYPTQDAQYTNNQGKNILFMWGINHPPTDTAQTNLYTRTDTTT
SVATEEMNRIFKPLIGPRPLVNGLMGRINYYWSVLKPGQTLRIKSDGNLIAPWYGHILSGESHGRILKTD
LKRGSCTVQCQTEKGGLNTTLPFQNVSKYAFGNCSKYIGVKSLKLAVGLRNVPSRSSRGLFGAIAGFIEG
GWSGLVAGWYGFQHSNDQGVGMAADRDSTQKAIDKITSKVNNIVDKMNKQYEIIDHEFSEVETRLNMINN
KVDDQIQDIWAYNAELLVLLENQKTLDEHDANVNNLYNKVKRALGSNAVEDGKGCFELYHKCDDHCMETI
RNGTYNRRKYQEESKLERQENRGVKLESEETYKILTIYSTVASSLVIAMGFAAFLFWAMSNGSCRCNICI

>AXF50086.1 hemagglutinin [Influenza A virus]
METISLITILVVATVSNADKICIGYQSTNSTETVDTLTENNVPVTHAKELLHTEHNGMLCATSLGHPLIL
DTCTIEGLIYGNPSCDPLLGGREWSYIVERPSAVNGLCYPGNVENLEELRSLFSSSRSYQRIQIFPDTIW
NVSYSGTSKACSDSFYRSMRWLTQKNNAYPTQDAQYTNNQGKNILFMWGINHPPTDTAQTNLYTRTDTTT
SVATEEMNRIFKPLIGPRPLVNGLMGRINYYWSVLKPGQTLRIKSDGNLIAPWYGHILSGESHGRILKTD
LKRGSCTVQCQTEKGGLNTTLPFQNVSKYAFGNCSKYIGVKSLKLAVGLRNVPSRSSRGLFGAIAGFIEG
GWSGLVAGWYGFQHSNDQGVGMAADRDSTQKAIDKITSKVNNIVDKMNKQYEIIDHEFSEVETRLNMINN
KVDDQIQDIWAYNAELLVLLENQKTLDEHDANVNNLYNKVKRALGSNAVEDGKGCFELYHKCDDHCMETI
RNVTYNRRKYQEESKLQRQEIEGVKLESEETYKILIIYSTVASSLVIAMGFAAFLFWAMSHASCRCNICI

>AXF50074.1 hemagglutinin [Influenza A virus]
METISLITILLVATVSYADKICIGYQSTNSTETVDTLTENNVPVTHAKELLHTEHNGMLCATSLGQPLIL
DTCTIEGLIYGNPSCDLSLEGREWSYIVERPSAVNGLCYPGNVENLEELRSLFSSARSYQRVQIFPDTIW
NVSYDGTSTACSGSFYRSMRWLTRKNGNYPTQDAQYTNNQGKNILFMWGINHPPTDDTQRNLYTRNDTTT
SVATEEINRIFKPLIGPRPLVNGLMGRIDYYWSVLKPGQTLRIKSDGNLIAPWYGYILSGESHGRILKTD
LKRGSCTVQCQTEKGGLNTTLPFQNVSKYAFGNCSKYIGIKSLKLAVGLRNVPSRSSRGLFGAIAGFIEG
GWSGLVAGWYGFQHSNDQGVGMAADRDSTQKAIDKITSKVNNIVDKMNKQYEIIDHEFSEVETRLNMINN
KIDDQIQDIWAYNAELLVLLENQKTLDEHDANVNNLYNKVKRALGSNAVEDGKGCFELYHKCNDQCMETI
RNGTYNRRKYQEESKLERQKIEGVKLESEGTYKILTIYSTVASSLVIAMGFAAFLFWAMSNGSCRCNICI

>AWY10923.1 hemagglutinin [Influenza A virus]

MEAISLMTILLVVTTSNADKICIGHQSTNSTETVDTLTETNVPVTQAKELLHTEHNGMLCATNLGRPLIL

DTCTIEGLIYGNPSCDMLLGGREWSYIVERPSAVNGTCYPGNVENLEELRTLFSSSSSYQRIQLFPDSIW

NVTYSGTSKSCSDSFYRNMRWLTQKNGLYPVQDAQYINNRGKDILFVWGIHHPPTDTAQTNLYTRTDTTT

SVTTENLDRTFKPLIGPRPLVNGLIGRINYYWSVLKPGQTLRIRSNGNLIAPWFGHILSGESHGRILRTD

LSSGNCVVQCQTEKGGLNSTLPFHNISKYAFGTCPKYIGVKSLKLAIGLRNVHARSNRGLFGAIAGFIEG

GWPGLVAGWYGFQHSNDQGVGMAADRVSTQKAVDKITSKVNNIVDKMNKQYEIIDHEFSEVESRLNMINN

KIDDQIQDIWAYNAELLVLLENQKTLDEHDANVNNLYNKVKRALGSNAMEDGKGCFELYHKCDDQCMETI

RNGTYNRRKYTEESRLERQKIEGVKLEAEGTYKILSIYSTVASSLVLAMGFAAFLFWAMSNGSCRCNICI

>AWY10922.1 hemagglutinin [Influenza A virus]

MKAISLMTILLVVTTSDADKICIGHQSTNSTETVDTLTETNVPVTQAKELLHTEHNGMLCATNLGRPLIL

DTCTIEGLIYGNPSCDMLLGGREWSYIVERPSAVNGTCYPGNVENLEELRTLFSSSSSYQRIQLFPDSIW

NVTYSGTSKSCSESFYRNMRWLTQKNGLYPVQDAQYINNRGKDILFVWGIHHPPTDTAQTNLYTRTDTTT

SVTTENLDRTFKPLIGPRPLVNGLIGRINYYWSVLKPGQTLRIRSNGNLIAPWFGHILSGESHGRILRTD

LSSGNCVVQCQTEKGGLNSTLPFHNISKYAFGTCPKYIGVKSLRLAIGLRNVHARSNRGLFGAIAGFIEG

GWPGLVAGWYGFQHSNDQGVGMAADRVSTQKAVDKITSKVNNIVDKMNKQYEIIDHEFSEVESRLNMINN

KIDDQIQDIWAYNAELLVLLENQKTLDEHDANVNNLYNKVKRALGSNAMEDGKGCFELYHKCDDQCMETI

RNGTYNRRKYTEESRLERQKIEGVKLEAEGTYKILSIYSTVASSLVLAMGFAAFLFWAMSNGSCRCNICI

>AWY10921.1 hemagglutinin [Influenza A virus]

MXAISLITIXLVVTISNADKICIGHQSTNSTETVDTLTEANVPVTQAKELLHTEHNGMLCATNLGRPLIL

DTCTIEGLIYGNPSCDMLLGEREWSYIVERPSAVNGTCYPGNVENLEELRTLFSSSSSSQRIQLFPDSIW

NVTYTGTSKSCSDSFYRNMRWLTQKNGAYPVQDAQYLNNQGKDILFVWGIHHPPSDTTQTDLYTRTDTTT

SITTENLDRTFKPLIGPRPLVNGLIGRINYYWSVLKPGQTLRVRSNGNLIAPWFGHVLSGESHGRILRTD

LNSGNCVVQCQTEKGGLNSTLPFHNISKYAFGTCPKYIGVKSLKLAIGLRNVHTRSSRGLFGAIAGFIEG

GWPGLVAGWYGFQHSNDQGVGMAADRVSTQKAVDKITSKVNNIVDKMNKQYEIIDHEFSEVESRLNMINN

KIDDQVQDIWAYNAELLVLLENQKTLDEHDANVNNLYNKVRRALGSNAMEDGKGCFELYHKCDDQCMETI

RNGTYNRRKYTKESRLERQKIEGVKLETEGTYKILSIYSTVASSLMLAIGFAAFLFWAMSNGSCRCNVCI

>AWY10920.1 hemagglutinin [Influenza A virus]

MEAISLITILLVVTISNADKICIGHQSTNSTETVDTLTEANVPVTQAKELLHTEHNGMLCATNLGRPLIL

DTCTIEGLIYGNPSCDMLLGEREWSYIVERPSAVNGTCYPGNVENLEELRTLFSSSSSSQRIQLFPDSIW

NVTYTGTSKSCSDSFYRNMRWLTQKNGAYPVQDAQYLNNQGKDILFVWGIHHPPSDTTQTDLYTRTDTTT

SITTENLDRTFKPLIGPRPLVNGLIGRINYYWSVLKPGQTLRVRSNGNLIAPWFGHVLSGESHGRILRTD
LNSGNCVVQCQTEKGGLNSTLPFHNISKYAFGTCPKYIGVKSLKLAIGLRNVHARSSRGLFGAIAGFIEG
GWPGLVAGWYGFQHSNDQGVGMAADRVSTQKAVDKITSKVNNIVDKMNKQYEIIDHEFSEVESRLNMINN
KIDDQVQDIWAYNAELLVLLENQKTLDEHDANVNNLYNKVRRALGSNAMEDGKGCFELYHKCDDQCMETI
RNGTYNRRKYTKESRLERQKIEGVKLETEGTYKILSIYSTVASSLMLAIGFAAFLFWAMSNGSCRCNVCI

>AWY10919.1 hemagglutinin [Influenza A virus]
MEVIPLMTMLLLVTTNNADKICIGHQSTNSTETVDTLTETGVPVTHAKELLHTEHNGRLCATNLGNPLIL
DTCTVEGIIYGNPSCDMLLGGREWSYIVERPSAVNGTCYPGNVENLEELRVLFSSSSSYQRIQMFPDTIW
NVTYSGTSKSCSDSFYRNMRWLTQKNGNYPVQDAQYTNTRGKDILFVWGIHHPPTDTAQTNLYTRTDTTT
SITTESLDRTFKPLIGPRPLVNGLIGRINYYWSVLKPGQTLRVRSNGNLIAPWFGHVLSGESHGRILKTD
LNSGNCVVQCQTEKGGLNSTLPFHNISKYAFGDCPKYIGVKSLKLAIGLRNVPARSSRGLFGAIAGFIEG
GWPGLVAGWYGFQHSNDQGVGMAADRDSTQKAVDKITSKVNNIVDKMNKQYEIIDHEFSEVETRLNMINN
KIDDQIQDIWAYNAELLVLLENQKTLDEHDANVNNLYNKVKRALGSNAMEDGKGCFELYHKCDDQCMETI
RNGTYNRRKYMEESRLGRQKIEGVKLESEGTYKILTIYSTVASSLVLAMGFAAFLFWAMSNGSCRCNICI

>AWY10918.1 hemagglutinin [Influenza A virus]
MKAISLMTILLVVTTSDADKICIGHQSTNSTETVDTLTETNVPVTQAKELLHTEHNGMLCATNLGRPLIL
DTCTIEGLIYGNPSCDMLLGGREWSYIVERPSAVNGTCYPGNVENLEELRTLFSSSSSYQRIQLFPDSIW
NVTYSGTSKSCSDSFYRNMRWLTQKNGLYPVQDAQYINNRGKDILFVWGIHHPPTDTAQTNLYTRTDTTT
SVTTENLDRTFKPLIGPRPLVNGLIGRINYYWSVLKPGQTLRIRSNGNLIAPWFGHILSGESHGRILRTD
LSSGNCVVQCQTEKGGLNSTLPFHNISKYAFGTCPKYIGVKSLRLAIGLRNVHARSNRGLFGAIAGFIEG
GWPGLVAGWYGFQHSNDQGVGMAADRVSTQKAVDKITSKVNNIVDKMNKQYEIIDHEFSEVESRLNMINN
KIDDQIQDIWAYNAELLVLLENQKTLDEHDANVNNLYNKVKRALGSNAMEDGKGCFELYHKCDDQCMETI
RNGTYNRRKYTEESRLERQKIEGVKLEAEGTYKILSIYSTVASSLVLAMGFAAFLFWAMSNGSCRCNICI

>AWY10917.1 hemagglutinin [Influenza A virus]
MXAISLITILLVVTISNADKICIGHQSTNSTETVDTLTEANVPVTQAKELLHTEHNGMLCATNLGRPLIL
DTCTIEGLIYGNPSCDMLLGEREWSYIVERPSAVNGTCYPGNVENLEELRTLFSSSSSSQRIQLFPDSIW
NVTYTGTSKSCSDSFYRNMRWLTQKNGAYPVQDAQYLNNQGKDILFVWGIHHPPSDTTQTDLYTRTDTTT
SITTENLDRTFKPLIGPRPLVNGLIGRINYYWSVLKPGQTLRVRSNGNLIAPWFGHVLSGESHGRILRTD
LNSGNCVVQCQTEKGGLNSTLPFHNISKYAFGTCPKYIGVKSLKLAIGLRNVHARSSRGLFGAIAGFIEG
GWPGLVAGWYGFQHSNDQGVGMAADRVSTQKAVDKITSKVNNIVDKMNKQYEIIDHEFSEVESRLNMINN
KIDDQVQDIWAYNAELLVLLENQKTLDEHDANVNNLYNKVRRALGSNAMEDGKGCFELYHKCDDQCMETI

RNGTYNRRKYTKESRLERQKIEGVKLETEGTYKILSIYSTVASSLMLAIGFAAFLFWAMSNGSCRCNVCI

>AWY10916.1 hemagglutinin [Influenza A virus]

MEAISLITILLVVTISNADKICIGHQSTNSTETVDTLTEANVPVTQAKELLHTEHNGMLCATNLGRPLIL

DTCTIEGLIYGNPSCDMQLGEREWSYIVERPSAVNGTCYPGNVENLEELRTLFSSSSSSQRIQLFPDSIW

NVTYTGTSKSCSDSFYRNMRWLTQKNGAYPVQDAQYLNNQGKDILFVWGIHHPPSDTTQTDLYTRTDTTT

SITTENLDRTFKPLIGPRPLVNGLIGRINYYWSVLKPGQTLRVRSNGNLIAPWFGHVLSGESHGRILRTD

LNSGNCVVQCQTEKGGLNSTLPFHNISKYAFGTCPKYIGVKSLKLAIGLRNVHARSSRGLFGAIAGFIEG

GWPGLVAGWYGFQHSNDQGVGMAADRVSTQKAVDKITSKVNNIVDKMNKQYEIIDHEFSEVESRLNMINN

KIDDQVQDIWAYNAELLVLLENQKTLDEHDANVNNLYNKVRRALGSNAMEDGKGCFELYHKCDDQCMETI

RNGTYNRRKYTKESRLERQKIEGVKLETEGTYKILSIYSTVASSLMLAIGFAAFLFWAMSNGSCRCNVCI

>AWY10915.1 hemagglutinin [Influenza A virus]

MEIIPLMTMLLLVTTNNADKICIGHQSTNSTETVDTLTETNVPVTHAKELLHTGHNGKLCATNLGNPLIL

DTCTIEGLIYGNPSCDMLLGGREWSYIVERPSAVNGTCYPGNVENLEELRILFSSSSSYQRIQMFPDTIW

NVTYSGTSKSCSNSFYRNMRWLTQKNGNYPVQDAQYTNTQGKDILFVWGIHHPPTNTAQTNLYTRTDTTT

SITTENLDRTFKPLIGPRPLVNGLIGRINYYWSVLKPGQTLRVRSNGNLIAPWFGHVLSGESHGRILKTD

LKSGNCVVQCQTEKGGLNSTLPFHNISKYAFGDCPKYIGVKSLKLAIGLRNVPARSSRGLFGAIAGFIEG

GWPGLVAGWYGFQHSNDQGVGMAADRDSTQKAVDKITSKVNNIVDKMNKQYEIIDHEFSEVETRLNMINN

KIDDQIQDIWAYNAELLVLLENQKTLDEHDANVNNLYNKVKRALGSNAMEDGKGCFELYHKCDDQCMETI

RNGTYNRRKYMEESRLGRQRIEGVKLESEGTYKILTIYSTVASSLVLAMGFAAFLFWAMSNGSCRCNICI

>AWY10914.1 hemagglutinin [Influenza A virus]

MEAISLITILLVVTISNADKICIGHQSTNSTETVDTLTEANVPVTQAKELLHTEHNGMLCATNLGRPLIL

DTCTIEGLIYGNPSCDMLLGEREWSYIVERPSAVNGTCYPGNVENLEELRTLFSSSSSSQRIQLFPDSIW

NVTYTGTSKSCSDSFYRNMRWLTQKNGAYPVQDAQYLNNQGKDILFVWGIHHPPSDTTQTDLYTRTDTTT

SITTENLDRTFKPLIGPRPLVNGLIGRINYYWSVLKPGQTLRVRSNGNLIAPWFGHVLSGESHGRILRTD

LNSGNCVVQCQTEKGGLNSTLPFHNISKYAFGTCPKYIGVKSLKLAIGLRNVHARSSRGLFGAIAGFIEG

GWPGLVAGWYGFQHSNDQGVGMAADRVSTQKAVDKITSKVNNIVDKMNKQYEIIDHEFSEVESRLNMINN

KIDDQVQDIWAYNAELLVLLENQKTLDEHDANVNNLYNKVRRALGSNAMEDGKGCFELYHKCDDQCMETI

RNGTYNRRKYTKESRLERQKIEGVKLETEGTYKILSIYSTVASSLMLAIGFAAFLFWAMSNGSCRCNVCI

>AWW21773.1 hemagglutinin [Influenza A virus]

METASLITILLAVTVSNADKICIGYQSTNSTETVDTLTENNVPVTHAKELLHTEHNGMLCATSLGHPLIL

DTCTIEGLIYGNPSCDLLLGGREWSYIVERPSAVNGLCYPGNVENLEELRSLFSSARSYQRIQIFPDTIW
NVSYSGTSKACSDSFYRSMRWLTQKDNAYPVQDAQYTNNQEKNILFMWGINHPPTDTVQTNLYTRTDTTT
SVATEEINRTFKPLIGPRPLVNGLMGRINYYWSVLKPGQTLRIKSNGNLIAPWYGHILSGESHGRILKTD
LKRGSCTVQCQTEKGGLNTTLPFQNVSKYAFGNCSKYIGIKSLKLAVGLRNVPSRSSRGLFGAIAGFIEG
GWSGLVAGWYGFQHSNDQGVGMAADRDSTQKAIDKITSKVNNIVDKMNKQYEIIDHEFSEVETRLNMINN
KIDDQIQDIWAYNAELLVLLENQKTLDEHDANVNNLYNKVKRALGSNAVEDGKGCFELYHKCDDQCMETI
RNGTYNRRKYQEESKLERQKIEGVKLESEGTYKILTIYSTVASSLVIAMGFAAFLFWAMSNGSCRCNICI

>AWC68529.1 hemagglutinin [Influenza A virus]
MEAISLMTILLVVTTSNADKICIGHQSTNSTETVDTLTETNVPVTQAKELLHTEHNGMLCATNLGRPLIL
DTCTIEGLIYGNPSCDMLLGGREWSYIVERPSAVNGTCYPGNVENLEELRTLFSSSSSYQRIQLFPDSIW
NVTYTGTSKSCSDSFYRNMRWLTQKNGVYPVQDAQYINNRGKDILFVWGIHHPPTDTEQTNLYTRTDTTT
SVTTENLDRTFKPLIGPRPLVNGRIGRINYYWSVLKPGQTLRVRSNGNLIAPWFGHVLSGESHGRILRTD
LSSGNCVVQCQTEKGGLNSTLPFHNISKYAFGTCPKYIGVKSLKLAIGLRNVHARSSRGLFGAIAGFIEG
GWPGLVAGWYGFQHSNDQGVGMAADRVSTQKAVDKITSKVNNIVDKMNKQYEIIDHEFSEVESRLNMINN
KIDDQIQDIWAYNAELLVLLENQKTLDEHDANVNNLYNKVKRALGSNAMEDGKGCFELYHKCDDQCMETI
RNGTYNRRKYTEESRLERQKIEGVKLETEGTYKILSIYSTVASSLVLAMGFAAFLFWAMSNGSCRCNICI

>AWB36461.1 hemagglutinin, partial [Influenza A virus]
DKICIGHQSTNSTETVDTLTETNVPVTHAKELLHTEHNGKLCATNLGNPLILDTCTIEGLIYGNPSCDML
LGGREWSYIVERPSAVNGTCYPGNVENLEELRILFSSSSSYQRIQMFPDTIWNVTYSGTSKSCSDSFYRN
MRWLTQKNGNYPVQDAQYTNTRGKDILFVWGIHHPPTDTAQTNLYTRTDTTTSITTENLDRTFKPLIGPR
PLVNGLIGRINYYWSVLKPGQTLRVRSNGNLIAPWFGHVLSGESHGRILKTDLNSGNCVVQCQTEKGGLN
STLPFHNISKYAFGNCPKYIGVKSLKLAIGLRNVPARSSRGLFGAIAGFIEGGWPGLVAGWYGFQHSNDQ
GVGMAADRDSTQKAVDKITSKVNNIVDKMNKQYEIIDHEFSEVETRLNMINNKIDDQIQDVWAYNAELLV
LLENQKTLDEHDANVNNLYNKVKRALGSNAMEDGKGCFELYHKCDDQCMETIRNGTYNRRKYKEESRLGR
QKIEGVKLESEGT

>AVX28224.1 hemagglutinin, partial [Influenza A virus]
LVTTNNADKICIGHQSTNSTETVDTLTETNVPVTHAKELLHTEHNGKLCATNLGNPLILDTCTIEGLIYG
NPSCDMLLGGREWSYIVERPSAVNGTCYPGNVENLEELRILFSSSSSYQRIQMFPDTIWNVTYSGTSKSC
SDSFYRNMRWLTQKNGNYPVQDAQYTNTWGKDILFVWGIHHPPTDTAQTNLYTRTDTTTSITTENLDRTF
KPLIGPRPLVNGLIGRINYYWSVLKPGQTLRVRSNGNLIAPWFGHVLSGESHGRILKTDLNSGNCVVQCQ
TEKGGLNSTLPFHNISKYAFGDCPKYIGVKSLKLAIGLRNVPARSSRGLFGAIAGFIEGGWPGLVAGWYG

FQHSNDQGVGMAADRDSTQKAVDKITSKVNNIVDKMNKQYEIIDHEFSEVETRLNMINNKIDDQIQDIWA
YNAELLVLLENQKTLDEHDANVNNLYNKVKRALGSNAMEDGKGCFELYHKCDDQCMETIRNGTYNRRKYM
EESRLGRQRIEGVKLESEGTYKILTIYSTVASSLVLAMGFAAFLFWA

>AVX28223.1 hemagglutinin, partial [Influenza A virus]
MLLLVTTNNADKICIGHQSTNSTETVDTLTETNVPVTHAKELLHTEHNGKLCATNLGNPLILDTCTIEGL
IYGNPSCDMLLGGREWSYIVERPSAVNGTCYPGNVENLEELRILFSSSSSYQRIQMFPDTIWNVTYSGTS
KSCSDSFYRNMRWLTQKNGNYPVQDAQYTNTRGKDILFVWGIHHPPTDTTQTNLYTRTDTTTSITTENLD
RTFKPSIGPRPLVNGLIGRINYYWSVLKPGQTLRVRSNGNLIAPWFGHVLSGESHGRILKTDLNNGNCLV
QCQTEKGGLNSTLPFHNISKYAFGDCPKYIGVKSLKLAIGLRNVPARSSRGLFGAIAGFIEGGWPGLVAG
WYGFQHSNDQGVGMAADRDSTQKAVDKITSKVNNIVDKMNKQYEIIDHEFSEVETRLNMINNKIDDQIQD
IWAYNAELLVLLENQKTLDEHDANVNNLYNKVKRALGSNAMEDGKGCFELYHKCDDQCMETIRNGTYNRR
KYMEESRLGRQKIEGVKLESEGTYKILTIYSTVAS

>AVX28222.1 hemagglutinin, partial [Influenza A virus]
TMLLLVTTNNADKICIGHQSTNSTETVDTLTETNVPVTHAKELLHTEHNGKLCATNLGNPLILDTCNIEG
LIYGNPSCDMLLGGREWSYIVERPSAVNGTCYPGNVENLEELRILFSSSSSYQRIQMFPDTIWNVTYSGT
SKSCSDSFYRNMRWLTQKNGNYPVQDAQYTNTRGKDILFVWGIHHPPTDTAQTNLYTRIDTTTSITTENL
DRTFKPLIGPRPLVNGLIGRINYYWSVLKPGQTLRVRSNGNLIAPWFGHVLSGESHGRILKTDLNSGNCV
VQCQTEKGGLNSTLPFHNISKYAFGDCPKYIGVKSLKLAIGLRNVPAKSSRGLFGAIAGFIEGGWPGLVA
GWYGFQHSNDQGVGIAADRDSTQKAVDKITSKVNNIVDKMNKQYEIIDHEFSEVETRLNMINNKIDDQIQ
DIWAYNAELLVLLENQKTLDEHDANVNNLYNKVKKALGSNAMEDGKGCFELYHKCDDQCMETIRNGTYNR
RKYVEESRLGRQKIEGVKLESEGTYKILTIY

>AVX28221.1 hemagglutinin, partial [Influenza A virus]
TMLLLVTTNNADKICIGHQSTNSTETVDTLTETNVPVTHAKELLHTEHNGKLCATNLGNPLILDTCTIEG
LIYGNPSCDMLLGGREWSYIVERPSAVNGTCYPGNVENLEELRILFSSSSSYQRIQMFPDTIWNVTYSGT
SKSCSDSFYRNMRWLTQKNGNYPVQDAQYTNTWGKDILFVWGIHHPPTDTAQTNLYTRTDTTTSITTENL
DRTFKPLIGPRPLVNGLIGRINYYWSVLKPGQTLRVRSNGNLIAPWFGHVLSGESHGRILKTDLNSGNCV
VQCQTEKGGLNSTLPFHNISKYAFGDCPKYIGVKSLKLAIGLRNVPARSSRGLFGAIAGFIEGGWPGLVA
GWYGFQHSNDQGVGMAADRDSTQKAVDKITSKVNNIVDKMNKQYEIIDHEFSEVETRLNMINNKIDDQIQ
DIWAYNAELLVLLENQKTLDEHDANVNNLYNKVKRALGSNAMEDGKGCFELYHKCDDQCMETIRNGTYNR
RKYMEESRLGRQRIEGVKLESEGTYKILTIYSTVASSLVLAMGFAAFLFWA

>AVX28220.1 hemagglutinin, partial [Influenza A virus]

TILLLVTTNNADKICIGHQSTNSTETVDTLTETNVPVTHAKELLHTEHNGKLCATNLGNPLILDTCTIEG

LIYGNPSCDMLLGGREWSYIVERPSAVNGTCYPGNVENLEELRILFSSSSSYQRIQMFPDTIWNVTYSGT

SKSCSDSFYRNMRWLTQKNGNYPVQDAQYTNTRGKDILFVWGIHHPPTDTAQTNLYTRTDTTTSITTENL

DRTFKPLIGPRPLVNGLIGRINYYWSVLKPGQTLRVRSNGNLIAPWFGHVLSGESHGRILKTDLNSGNCV

VQCQTEKGGLNSTLPFHNISKYAFGDCPKYIGVKSLKLAIGLRNVPARSSRGLFGAIAGFIEGGWPGLVA

GWYGFQHSNDQGVGMAADRDSTQKAVDKITSKVNNIVDKMNKQYEIIDHEFSEVETRLNMINNKIDDQIQ

DVWAYNAELLVLLENQKTLDEHDANVNNLYNKVKRALGSNAMEDGKGCFELYHKCDDQCMETIRNGTYNR

RKYMEESRLGRQKIEGVKLESEGTYKILTIYSTVASSLVLAMGFAAFLFWA

>AVX28219.1 hemagglutinin, partial [Influenza A virus]

MEIIPLMTVLLLVTTSNADKICIGHQSTNSTETVDTLTETNVPVTHAKELLHTEHNGMLCATNLGNPLIL

DTCTIEGLIYGNPSCDMLLGGREWSYIVERPTAVNGTCYPGNVENLEELRILFSSSSSYQRIQMFPDTIW

NVTYSGTSKSCSDSFYRNMRWLTQKNGNYPVQDAQYTNTQGKDILFVWGIHHPPTDTTQTNLYTRTDTTT

SVTTENLDRTFKPLIGPRPLVNGQIGRINYYWSVLKPGQTLRVRSNGNLIAPWFGHVLSGESHGRILKTD

LNSGNCVVQCQTEKGGLNSTLPFHNISKYAFGDCPKYIGVKSLKLAIGLRNVPARSSRGLFGAIAGFIEG

GWPGLVAGWYGFQHSNDQGVGMAADRDSTQKAVDKITAKVNNIVDKMNKQYEIIDHEFSEVETRLNMINN

KIDDQIQNVWAYNAELLVLLENQKTLDEHDANVNNLYNKVKRALGSNAMEDGKGCFELYHKCDDQCMETI

RNGTYNRIKYKEESRLERQKIEGVKLESEGTYKILTIYSTVASSLVLAMGFAAFLFWA

>AVX28218.1 hemagglutinin, partial [Influenza A virus]

MEIIPLMTILLLVTTNNADKICIGHQSTNSTETVDTLTETNVPVTHAKELLHTEHNGKLCATNLGNPLIL

DTCTIEGLIYGNPSCDMLLGGREWSYIVERPSAVNGTCYPGNVENLEELRILFSSSSSYQRIQMFPDTIW

NVTYSGTSKSCSDSFYRNMRWLTQKNGNYPVQDAQYTNTRGKDILFVWGIHHPPTDTAQTNLYTRTDTTT

SVTTENLDRTFKPLVGPRPLVNGLIGRINYYWSVLKPGQTLRVRSNGNLIAPWFGHVLSGESHGRILKTD

LNSGNCVVQCQTEKGGLNSTLPFHNISKYAFGNCPKYIGVKSLKLAIGLRNVPARSSRGLFGAIAGFIEG

GWPGLVAGWYGFQHSNDQGVGMAADRDSTQKAVDKITSKVNNIVDKMNKQYEIIDHEFSEVETRLNMINN

KIDDQIQDVWAYNAELLVLLENQKTLDEHDANVNNLYNKVKRALGSNAMEDGKGCFELYHKCDDQCMETI

RNGTYNRRKYKEESRLGRQKIEGVKLESEGTYRILTIYSTVASSLVLAMGFAAFLFWA

>AVX28217.1 hemagglutinin, partial [Influenza A virus]

EIIPLMTILLLVTTNNADKICIGHQSTNSTETVDTLTETNVPVTHAKELLHTEHNGKLCATNLGNPLILD

TCTIEGLIYGNPSCDMLLGGREWSYIVERPSAVNGTCYPGNVENLEELRILFSSSSSYQRIQMFPDTIWN

VTYSGTSKSCSDSFYRNMRWLTQKNGNYPVQDAQYTNTQGKDILFVWGIHHPPTDTAQTNLYTRTDTTTS

VTTENLDRTFKPLVGPRPLVNGLIGRINYYWSVLKPGQTLRVRSNGNLIAPWFGHVLSGESHGRILKTDL
NSGNCVVQCQTEKGGLNSTLPFHNISKYAFGNCPKYIGVKSLKLAIGLRNVPARSSRGLFGAIAGFIEGG
WPGLVAGWYGFQHSNDQGVGMAADRDSTQKAVDKITSKVNNIVDKMNKQYEIIDHEFSEVETRLNMINNK
IDDQIQDVWAYNAELLVLLENQKTLDEHDANVNNLYNKVKRALGSNAMEDGKGCFELYHKCDDQCMETIR
NGTYNRRKYKEESRLGRQKIEGVKLESEGTYRILTIYSTVASSLVLAMGFAAFLFWA

>AVX28216.1 hemagglutinin, partial [Influenza A virus]
MEIIPLMTILLLVTTNNADKICIGHQSTNSTETVDTLTETNVPVTHAKELLHTEHNGKLCATNLGNPLIL
DTCTIEGLIYGNPSCDMLLGGREWSYIVERPSAVNGTCYPGNVENLEELRILFSSSSSYQRIQMFPDTIW
NVTYSGTSKSCSDSFYRNMRWLTQKNGNYPVQDAQYTNTQGKDILFVWGIHHPPTDTAQTNLYTRTDTTT
SVTTENLDRTFKPLVGPRPLVNGLIGRINYYWSVLKPGQTLRVRSNGNLIAPWFGHVLSGESHGRILKTD
LNSGNCVVQCQTEKGGLNSTLPFHNISKYAFGNCPKYIGVKSLKLAIGLRNVPARSSRGLFGAIAGFIEG
GWPGLVAGWYGFQHSNDQGVGMAADRDSTQKAVDKITSKVNNIVDKMNKQYEIIDHEFSEVETRLNMINN
KIDDQIQDVWAYNAELLVLLENQKTLDEHDANVNNLYNKVKRALGSNAMEDGKGCFELYHKCDDQCMETI
RNGTYNRRKYKEESRLGRQKIEGVKLESEGTYRILTIYSTVASSLVLAMGFAAFLFWA

>AVX28215.1 hemagglutinin, partial [Influenza A virus]
MEIIPLMTILLLVTTNNADKICIGHQSTNSTETVDTLTETNVPVTHAKELLHTEHNGKLCATNLGNPLIL
DTCTIEGLIYGNPSCDMLLGGREWSYIVERPSAVNGTCYPGNVENLEELRILFSSSSSYQRIQMFPDTIW
NVTYSGTSKSCSDSFYRNMRWLTQKNGNYPVQDAQYTNTQGKDILFVWGIHHPPTDTAQTNLYTRTDTTT
SVTTENLDRTFKPLVGPRPLVNGLIGRINYYWSVLKPGQTLRVRSNGNLIAPWFGHVLSGESHGRILKTD
LNSGNCVVQCQTEKGGLNSTLPFHNISKYAFGNCPKYIGVKSLKLAIGLRNVPARSSRGLFGAIAGFIEG
GWPGLVAGWYGFQHSNDQGVGMAADRDSTQKAVDKITSKVNNIVDKMNKQYEIIDHEFSEVETRLNMINN
KIDDQIQDVWAYNAELLVLLENQKTLDEHDANVNNLYNKVKRALGSNAMEDGKGCFELYHKCDDQCMETI
RNGTYNRRKYKEESRLGRQKIEGVKLESEGTYRILTIYSTVASSLVLAMGFAAFLFWA

>AVV61746.1 hemagglutinin [Influenza A virus]
METISLIAILLVVTVSNADKICIGYQSTNSTETVDTLTENNVPVTHAKELLHTEHNGMLCATNLGHPLIL
NTCTIEGLIYGNPSCDQLLGGGKWSYIVERPSAVNGMCYPGNVENLEELRSLFSSASSYQRIQIFPDTIW
NVSYNGTSKACSDSFYRSMRWLTQKNNAYPIQDAQYTNNRGKSILFMWGINHPPTDTVQTNLYTRTDTTT
SVTTEDITRTFKPMIGPRPLVNGQQGRIDYYWSVLKPGQTLRIRSNGNLIAPWYGHILSGESHGRILKTD
LNSGNCVVQCQTERGGLNTTLPFHNVSKYAFGNCPKYVGVKSLKLAVGLRNVPARSSRGLFGAIAGFIEG
GWSGLVAGWYGFQHSNDQGVGMAADRESTQRAIDKITSKVNNIVDKMNKQYEIIDHEFSEVETRLNMINN
KIDDQIQDIWAYNAELLVLLENQKTLDEHDANVNNLYNKVKRALGSNAMEDGKGCFELYHKCDDQCMETI

RNGTYNRRKYKEEAKLERQKIEGVKLESEGTYKILTIYSTVASSLVIAMGFAAFLFWAMSNGSCRCNICI

>AVR58643.1 hemagglutinin [Influenza A virus]
MEAISLMTILLVVTTSNADKICIGHQSTNSTETVDTLTETNVPVTQAKELLHTEHNGMLCATNLGRPLIL
DTCTIEGLIYGNPSCDMLLGGREWSYIVERPSAVNGTCYPGNVENLEELRTLFSSSSSYQRVQLFPDSIW
NVTYTGTSKSCSDSFYRNMRWLTQKNGGYPIQDAQYTNNRGKDILFVWGIHHPPTDTAQTNLYTRTDTTT
SVTTETLDRTFKPLIGPRPLVNGLIGRINYYWSVLKPGQTLRVRSNGNLIAPWFGHVLSGESHGRILRTD
LSSGNCVVQCQTEKGGLNSTMPFHNISKYAFGTCPKYIGVKSLKLAIGLRNVHARSSRGLFGAIAGFIEG
GWPGLVAGWYGFQHSNDQGVGMAADRVSTQKAVDKITSKVNNIVDKMNKQYEIIDHEFSEVETRLNMINN
KIDDQIQDIWAYNAELLVLLENQKTLDEHDANVNNLYNKVKRALGSNAMEDGKGCFELYHKCDDQCMETI
RNGTYNRRKYTEESRLERQKIEGVKLEAEGTYKILSIYSTVASSLVLAMGFAAFLFWAMSNGSCRCNICI

>AVR58631.1 hemagglutinin [Influenza A virus]
MEAISLMTILLVVTTSNADKICIGHQSTNSTETVDTLTETNVPVTQAKELLHTEHNGMLCATNLGRPLIL
DTCTIEGLIYGNPSCDMLLGGREWSYIVERPSAVNGTCYPGNVENLEELRTLFSSSSSYQRVQLFPDSIW
NVTYTGTSKSCSDSFYRNMRWLTQKNGGYPIQDAQYTNNRGKDILFVWGIHHPPTDTAQTNLYTRTDTTT
SVTTETLDRTFKPLIGPRPLVNGLIGRINYYWSVLKPGQTLRVRSNGNLIAPWFGHVLSGESHGRILRTD
LSSGNCVVQCQTEKGGLNSTMPFHNISKYAFGTCPKYIGVKSLKLAIGLRNVHARSSRGLFGAIAGFIEG
GWPGLVAGWYGFQHSNDQGVGMAADRVSTQKAVDKITSKVNNIVDKMNKQYEIIDHEFSEVETRLNMINN
KIDDQIQDIWAYNAELLVLLENQKTLDEHDANVNNLYNKVKRALGSNAMEDGKGCFELYHKCDDQCMETI
RNGTYNRRKYTEESRLERQKIEGVKLEAEGTYKILSIYSTVASSLVLAMGFAAFLFWAMSNGSCRCNICI

>UVB12133.1 hemagglutinin, partial [Influenza A virus]
DKICIGYQSTNSTETVDTLTENNVPVTHAKELLHTEHNGMLCATSLGQPLVLDTCTIEGLIYGNPSCDPL
LGGREWSYIVERPSAVNGLCYPGNVENLEELRSLFSSARSYQRVLIFPDTIWNVSYSGTSKACSDSFYRS
MRWLTQKGNAYPVQDAQYTNNQEKNILFMWGINHPPTDTAQTNLYTRTDTTTSVATEEINRTFKPLIGPR
PLVNGLMGRINYYWSVLKPGQTLRIKSNGNLIAPWYGHILSGESHGRILKTDLKRGSCTVQCQTEKGGLN
TTLPFQNVSKYAFGNCSKYIGIKSLKLAVGLRNVPSRSSRGLFGAIAGFIEGGWSGLVAGWYGFQHSNDQ
GVGMAADRDSTQKAIDKITSKVNNIVDKMNKQYEIINHEFSEVETRLNMINNKIDDQIQDIWAYNAELLV
LLENQKTLDEHDANVNNLYNKVKRALGSNAMEDGKGCFELYHKCDDQCMETIRNGTYNRRKYQEESKLER
QKIEGVKLESEETYKILTIYSTVASSLVIAMGFAAFLFWAMSNGSCRCNICI

>UVB12132.1 hemagglutinin, partial [Influenza A virus]
DKICIGYQSTNSTETVDTLTENNVPVTHAKELLHTEHNGMLCATSLGQPLILDTCTIEGLIYGNPSCDPL

PEEREWSYIVERPSAVNGLCYPGNVENLEELRSLFSSARSYQRVQIFPDTIWNVSYDGTSNTCSGSFYRN
MRWLTRKDGNYPIQDAQYTNNQGKNILFMWGINNPPTDDTQRNLYTRTDTTTSVATEEINRIFKPLIGPR
PLVNGLMGRINYYWSVLKPGQTLRIKSDGNLVAPWYGYILSGESHGRILRTDLKRGSCTVQCQTEKGGLN
TTLPFQNVSKYAFGNCSKYIGIKSLKLAVGLRNVPSRSSRGLFGAIAGFIEGGWSGLVAGWYGFQYSNDQ
GVGMAADRESTQKAVDKITSKVNNIVDKMNKQYEIIDHEFSEIETRLNMINNKIDDQIQDIWAYNAELLV
LLENQKTLDEHDANVNNLYNKVKRALGTNAVEDGKGCFELYHKCDDQCMETIRNGTYNRRKYQEESKLER
QKIEGVKLESEGTYKILTIYSTVASSLVIAMGFAAFLFWAMSNGSCRCNICI

>UVB12131.1 hemagglutinin, partial [Influenza A virus]
DKICIGYQSTNSTETVDTLTENNVPVTHAKELLHTEHNGMLCATSLGQPLILDTCTIEGLIYGNPSCDPL
PDEREWSYIVERPSAVNGLCYPGNVENLEELRSLFSSARSYQRIQIFPDTIWNVSYDGTSNSCSGSFYRN
MRWLTRKDGNYPTQDAQYTNNQGKNILFMWGINNPPTDDTQRNLYTRTDTTTSVATEEMNRIFKPLIGPR
PLVNGLMGRINYYWSVLKPGQTLRIKSDGNLVAPWYGYILLGESHGRILRTDLKRGSCTVQCQTEKGGLN
TTLPFQNVSKYAFGNCSKYIGIKSLKLAVGLRNVPSRSSRGLFGAIAGFIEGGWSGLVAGWYGFQHSNGQ
GVGMAADRESTQKAIDKITSKVNNIVDKMNKQYEIIDHEFSEVETRLNMINNKIDDQIQDIWAYNAELLV
LLENQKTLDEHDANVNNLYNKVKRALGSNAVEDGKGCFELYHKCDDQCMETIRNGTYNRRKYQEESKLER
QKIEGVKLESEGTYKILTIYSTVASSLVIAMGFAAFLFWAMSNGSCRCNICI

>UVB12130.1 hemagglutinin, partial [Influenza A virus]
DKICIGYQSTNSTETVDTLTENNVPVTHAKELLHTEHNGMLCATNLGQPLILDTCTIEGLIYGNPSCDPL
LGGREWSYIVERPSAVNGLCYPGNVENLEELRSLFSSARSYQRVLIFPDTIWNVSYSGTSKACSDSFYRS
MRWLTQKDNAYPVQDAQYTNNQEKNILFMWGINHPPTDTAQTNLYTRTDTTTSVATEEINRTFKPLIGPR
PLVNGLMGRINYYWSVLKPGQTLRIKSNGNLIAPWYGHILSGESHGRILKTDLKRGSCTVQCQTEKGGLN
TTLPFQNVSKYAFGNCSKYIGIKSLKLAVGLRNVPSRSSRGLFGAIAGFIEGGWSGLVAGWYGFQHSNDQ
GVGMAADRDSTQKAIDKITSKVNNIVDKMNKQYEIIDHEFSEVETRLNMINNKIDDQIQDIWAYNAELLV
LLENQKTLDEHDANVNNLYNKVKRALGSNAVEDGKGCFELYHKCDDQCMETIRNGTYNRRKYQEESKLER
QKIEGVKLESEGTYKILTIYSTVASSLVIAMGFAAFLFWAMSNGSCRCNICI

>UVB12129.1 hemagglutinin, partial [Influenza A virus]
DKICIGYQSTNSTETVDTLTENNVPVTHAKELLHTEHNGMLCATSLGQPLILDTCTIEGLIYGNPSCDPL
LGGREWSYIVERPSAVNGLCYPGNVENLEELRSLFSSARSYQRVLIFPDTIWNVSYSGTSKACSDSFYRS
MRWLTQKDNTYPVQDAQYTNNQEKNILFMWGINHPPTDTTQTNLYTRTDTTTSVATEEINRTFKPLIGPR
PLVNGLMGRINYYWSVLKPGQTLRIKSNGNLIAPWYGHILSGESHGRILKTDLKRGSCTVQCQTEKGGLN
TTLPFQNVSKYAFGNCSKYIGIKSLKLAVGLRNVPSRSSRGLFGAIAGFIEGGWSGLVAGWYGFQHSNDQ

GVGMAADRDSTQKAIDKITSKVNNIVDKMNKQYEIIDHEFSEVETRLNMINNKIDDQIQDIWAYNAELLV
LLENQKTLDEHDANVNNLYNKVKRALGSNAVEDGKGCFELYHKCDDQCMETIRNGTYNRRKYQEESKLER
QKIEGVKLESEGTYKILTIYSTVASSLVIAMGFAAFLFWAMSNGSCRCNICI

>UVB12128.1 hemagglutinin, partial [Influenza A virus]
DKICIGYQSTNSTETVDTLTENNVPVTHAKELLHTEHNGMLCATSLGHPLILDTCTIEGLIYGNPSCDLL
LGGREWSYIVERPSAVNGLCYPGNVENLEELRSLFSSARSYQRIQIFPDTIWNVSYSGTSKACSDSFYRS
MRWLTQKNNAYPIQDAQYTNNQGKNILFMWGINHPPTDTAQTNLYTRTDTTTSVATEEINRTFKPLIGPR
PLVNGLMGRINYYWSVLKPGQTLRIKSDGNIIAPWYGHILSGESHGRILKTDLKSGSCTVQCQTEKGGLN
TTLPFQNVSKYAFGNCSKYIGVKSLKLAVGLRNVPSKSSRGLFGAIAGFIEGGWSGLVAGWYGFQHSNDQ
GVGMAADRDSTQKAIDKITSKVNNIVDKMNKQYEIIDHEFSEVETRLNMINNKIDDQIQDIWAYNAELLV
LLENQKTLDEHDANVNNLYNKVKRALGSNAVEDGKGCFELYHKCDNQCMETIRNGTYNRRKYQEESKLER
QKIEGVKLESEGTYKILTIYSTVASSLVIAMGFAAFLFWAMSNGSCRCNICI

>UVB12127.1 hemagglutinin, partial [Influenza A virus]
DKICIGYQSTNSTETVDTLTENNVPVTHAKELLHTEHNGMLCATSLGHPLILDTCTIEGLIYGNPSCDPL
LGGREWSYIVERPSAVNGLCYPGNVENLEELRSLFSSARSYQRIQIFPDTIWNVSYSGTSKACSDSFYRS
MRWLTQKNNAYPIQDAQYTNNQGKNILFMWGINHPPTDTVQTNLYTRTDTTTSVATEEINRTFKPLIGPR
PLVNGLMGRINYYWSVLKPGQTLRIKSNGNLIAPWYGHILTGESHGRILKTDLKSGSCTVQCQTEKGGLN
TTLPFQNVSRYAFGNCSKYIGIKSLKLAVGLRNVPSKSSRGLFGAIAGFIEGGWSGLVAGWYGFQHSNDQ
GVGMAADRDSTQKAIDKITSKVNNIVDKMNKQYEIIDHEFSEVETRLNMINNKIDDQIQDIWAYNAELLV
LLENQKTLDEHDANVNNLYNKVKRALGSNAVEDGKGCFELYHKCDDQCMETIRNGTYNRRKYQEESKLER
QKIEGVKLESEGTYKILTIYSTVASSLVIAMGFAAFLFWAMSNGSCRCNICI

>UVB12126.1 hemagglutinin, partial [Influenza A virus]
DKICIGYQSTNSTETVDTLTENNVPVTHAKELLHTEHNGMLCATSLGQPLILDTCTIEGLIYGNPSCDPL
PDEREWSYIVERPSAINGLCYPGNVENLEELRSLFSSARSYQRIQIFPDTIWNVSYDGTSNTCSGSFYRN
MRWLTRKDGNYPTQDAQYTNNQGKNILFMWGINNPPTDDTQRNLYTRTDTTTSVATEEMNRIFKPLIGPR
PLVNGLMGRINYYWSVLKPGQTLRIKSDGNLVAPWYGYILLGESHGRILRTDLKRGSCTVQCQTEKGGLN
TTLPFQNVSKYAFGNCSKYIGIKSLKLAVGLRNVPSRSSRGLFGAIAGFIEGGWSGLVAGWYGFQHSNDQ
GVGMAADRESTQKAIDKITSKVNNIVDKMNKQYEIIDHEFSEVETRLNMINNKIDDQIQDIWAYNAELLV
LLENQKTLDEHDANVNNLYNKVKRALGSNAVEDGKGCFELYHKCDDQCMETIRNGTYNRRKYQEESKLER
QKIEGVKLESEGTYKILTIYSTVASSLVIAMGFAAFLFWAMSNGSCRCNIC

>UVB12125.1 hemagglutinin, partial [Influenza A virus]

DKICIGYQSTNSTETVDTLTENNVPVTHAKELLHTEHNGMLCATSLGQPLILDTCTIEGLIYGNPSCDPL
PDEREWSYIVERPSAVNGLCYPGNVENLEELRSLFSSARSYQRIQIFPDTIWNVSYDGTSNACSGSFYRN
MRWLTRKDGNYPTQDAQYTNNQGKNILFMWGINNPPTDDTQRNLYTRTDTTTSVATEEMNRIFKPLIGPR
PLVNGLMGRINYYWSVLKPGQTLRIKSDGNLVAPWYGYILLGESHGRILRTDLKRGSCTVQCQTEKGGLN
TTLPFQNVSKYAFGNCSKYIGIKSLKLAVGLRNVPSRSSRGLFGAIAGFIEGGWSGLVAGWYGFQHSNDQ
GVGMAADRESTQKAIDKITSKVNNIVDKMNKQYEIIDHEFSEVETRLNMINNKIDDQIQDIWAYNAELLV
LLENQKTLDEHDANVNNLYNKVKRALGSNAVEDGKGCFELYHKCDDQCMETIRNGTYNRRKYQEESKLER
QKIEGVKLESEGTYKILTIYSTVASSLVIAMGFAAFLFWAMSNGSCRCNICI

>UVB12124.1 hemagglutinin, partial [Influenza A virus]

DKICIGYQSTNSTETVDTLTENNVPVTHAKELLQTEHNGMLCATSLGQPLILDTCTIEGLIYGNPSCDPL
LGGREWSYIVERPSAVNGLCYPGNVENLEELRSLFSSARSYQRVLIFPDTIWNVSYSGTSKACSDSFYRS
MRWLTQKNNAYPVQDAQYTNNQEKNILFMWGINHPPTDTTQTNLYTRTDTTTSVATEEINRTFRPLIGPR
PLVNGLMGRINYYWSVLKPGQTLRIKSNGNLIAPWYGHILSGESHGRILKTDLKRGSCTVQCQTEKGGLN
TTLPFQNVSKYAFGNCSKYIGIKSLKLAVGLRNVPSRSSRGLFGAIAGFIEGGWSGLVAGWYGFQHSNDQ
GVGMAADRDSTQKAIDKITSKVNNIVDKMNKQYEIIDHEFSEVETRLNMINNKIDDQIQDIWAYNAELLV
LLENQKTLDEHDANVNNLYNKVKRALGSNAVEDGKGCFELYHKCDDQCMETIRNGTYNRRKYQEESRLER
QKIEGVTLESEGTYKILTIYSTVASSIVIAMGFAAFLFWAMSNGSCRCNICI

>UVB12123.1 hemagglutinin, partial [Influenza A virus]

DKICIGYQSTNSTETVDTLTENNVPVTHAKELLHTEHNGMLCATSLGQPLILDTCTIEGLIYGNPSCDPL
PDEREWSYIVERPSAVNGLCYPGNVENLEELRSLFSSARSYQRIQIFPDTIWNVSYDGTSNTCSGSFYRN
MRWLTRKDGNYPTQDAQYTNNQGKNILFMWGINNPPTDDTQRNLYTRTDTTTSVATEEMNRIFKPLIGPR
PLVNGLMGRINYYWSVLKPGQTLRIKSDGNLVAPWYGYILLGESHGRILRTGLKRGSCTVQCQTEKGGLN
TTLPFQNVSKYAFGNCSKYIGIKSLKLAVGLRNVPSRSSRGLFGAIAGFIEGGWSGLVAGWYGFQHSNDQ
GVGMAADRETTQKAIDKITSKVNNIVDKMNKQYEIIDHEFSEVETRLNMINNKIDDQIQDIWAYNAELLV
LLENQKTLDEHDANVNNLYNKVKRALGSNAVEDGKGCFELYHKCDDQCMETIRNGTYNRRKYQEESKLER
QKIEGVKLESEGTYKILTIYSTVASSLVIAMGFAAFLFWAMSNGSCRCNICI

>UVB12122.1 hemagglutinin, partial [Influenza A virus]

DKICIGYQSTNSTETVDTLTENNVPVTHAKELLHTEHNGMLCATSLGQPLVLDTCTIEGLIYGNPSCDPL
PEEREWSYIVERPSAVNGLCYPGNVENLEELRSLFSSARSYQRIQIFPDTIWNVSYDGTSNTCSGSFYRN
MRWLTRKDGNYPVQDAQYTNNQGKNILFMWGINNPPTDDTQRNLYTRTDTTTSVATEEINRIFKPLIGPR

PLVNGLMGRINYYWSVLKPGQTLRIKSDGNLVAPWYGYILSGESHGRILRTDLKRGSCTVQCQTEKGGLN
TTLPFQNVSKYAFGNCSKYIGIKSLKLAVGLRNVPSRSSRGLFGAIAGFIEGGWSGLVAGWYGFQHSNDQ
GVGMAADRESTQKAVDKITSKVNNIVDKMNKQYEIIDHEFSEVETRLNMINNKIDDQIQDIWAYNAELLV
LLENQKTLDEHDANVNNLYNKVKRALGSNAVEDGKGCFELYHKCDDQCMETIRNGTYNRRKYQEESKLER
QKIEGVKLESEGTYKILTIYSTVASSLVIAMGFAAFLFWAMSNGSCRCNICI

>UVB12121.1 hemagglutinin, partial [Influenza A virus]
DKICIGYQSTNSTETVDTLTENNVPVTHAKELLHTEHNGMLCATSLGHPLILETCTIEGLIYGNPSCDLL
LGGREWSYIVERPSAVNGLCYPGNVENLEELRSLFSSARSYKRIQIFPDTIWNVSYSGTSKACSDSFYRS
MRWLTQKNNDYPIQDAQYTNNQGKNILFLWGINHPPTDTVQTNLYTRTDTTTSVATEEINRTFKPLIGPR
PLVNGLMGRINYYWSVLKPGQTLRIKSNGNLIAPWYGHILSGESHGRILKTDLKSGSCTVQCQTEKGGLN
TTLPFQNVSKYAFGNCSKYIGIKSLKLAVGLRNVPSRSSRGLFGAIAGFIEGGWSGLVAGWYGFQHSNAQ
GVGMAADRDSTQKAIDKITSKVNNIVDKMNKQYEIIDHEFSEVETRLNMINSKIDDQIQDIWAYNAELLV
LLENQKTLDEHDANVNNLYNKVKRALGSNAVEDGRGCFELYHKCDDQCMETIRNGTYNRRKYQEESKLER
QKIEGVKLESEGTYKILTIYSTVASSLVIAMGFAAFLFWAMSNGSCRCNICI

>UVB12120.1 hemagglutinin, partial [Influenza A virus]
DKICIGYQSTNSTETVDTLTENNVPVTHAKELLHTKHNGMLCATSLGHPLILDTCTIEGLIYGNPSCDPL
LGGREWSYIVERPSAVNGLCYPGNVENLEELRSLFSSARSYQRIQIFPDTIWNVSYSGTSKACSDSFYRS
MRWLTQKNNAYPIQDAQYTNNQGKNILFMWGINHPPTDTAQTNLYTRTDTTTSVATEEINRTFKPLIGPR
PLVNGLMGRINYYWSVLKPGQTLRIKSNGNLIAPWYGHILTGESHGRILKTDLKSGSCTVQCQTEKGGLN
TTLPFQNVSRYAFGNCSKYIGVKSLKLAVGLRNVPSKSSRGLFGAIAGFIEGGWSGLVAGWYGFQHSNDQ
GVGMAADRDSTQKAIDKITSKVNNIVDKMNKQYEIIDHEFSEVETRLNMINNKIDDQIQDIWAYNAELLV
LLENQKTLDEHDANVNNLYNKVKRALGSNAVEDGKGCFELYHKCDDQCMETIRNGTYNRRKYQEESKLER
QKIEGVKLESEGTYKILTIYSTVASSLVIAMGFAAFLFWAMSNGSCRCNIC

>UVB12119.1 hemagglutinin, partial [Influenza A virus]
DKICIGYQSTNSTETVDTLTENNVPVTHAKELLHTEHNGMLCATSLGHPLILDTCTIEGLIYGNPSCDPL
LGGREWSYIVERPSAVNGLCYPGNVENLEELRSLFSSARSYQRIQIFPDTIWNVSYSGTSKACSDSFYRS
MRWLTQKNNAYPIQDAQYTNNQGKNILFMWGINHPPTDTAQTNLYTRTDTTTSVATEEINRTFKPLIGPR
PLVNGLMGRINYYWSVLKPGQTLRIKSNGNLIAPWYGHILTGESHGRILKTDLKSGSCTVQCQTEKGGLN
TTLPFQNVSRYAFGNCSKYIGIKSLKLAVGLRNVPSKSSRGLFGAIAGFIEGGWSGLVAGWYGFQHSNDQ
GVGIAADRDSTQKAIDKITSKVNNIVDKMNKQYEIIDHEFSEVETRLNMINNKIDDQIQDIWAYNAELLV
LLENQKTLDEHDANVNNLYNKVKRALGSNAVEDGKGCFELYHKCDDQCMETIRNGTYNRRKYQEESKLER

QKIEGVKLESEGTYKILTIYSTVASSLVIAMGFAAFLFWAMSNGSCRCNICI

>UVB12118.1 hemagglutinin, partial [Influenza A virus]
DKICIGYQSTNSTETVDTLTENNVPVTHAKELLHTEHNGMLCATSLGHPLILETCTIEGLIYGNPSCDLL
LGGREWSYIVERPSAVNGLCYPGNVENLEELRSLFSSARSYQRIQIFPDTIWNVSYSGTSKACSDSFYRS
MRWLTQKNNAYPIQDAQYTNNQGKNILFLWGINHPPTDTVQTNLYTRTDTTTSVATEEINRTFKPLIGPR
PLVNGLMGRINYYWSVLKPGQTLRIKSNGNLIAPWYGHILSGESHGRILKTDLKSGSCTVQCQTEKGGLN
TTLPFQNVSKYAFGNCSKYIGIKSLKLAVGLRNVPSRSSRGLFGAIAGFIEGGWSGLVAGWYGFQHSNAQ
GVGMAADRDSTQKAIDKITSKVNNIVDKMNKQYEIIDHEFSEVETRLNMINSKIDDQIQDIWAYNAELLV
LLENQKTLDEHDANVNNLYNKVKRALGSNAVEDGRGCFELYHKCDDQCMETIRNGTYNRRKYQEESKLER
QKIEGVKLESEGTYKILTIYSTVASSLVIAIGFAAFLFWAMSNGSCRCNICI

>UVB12117.1 hemagglutinin, partial [Influenza A virus]
DKICIGYQSTNSTETVDTLTENNVPVTHAKELLHTEHNGMLCATSLGHPLILETCTIEGLIYGNPSCDLL
LGGREWSYIVERPSAVNGLCYPGNVENLEELRSLFSSARSYQRIQIFPDTIWNVSYSGTSKACSDSFYRS
MRWLTQKNNAYPIQDAQYTNNQGKNILFLWGINHPPTDTVQTNLYTRTDTTTSVATEEINRTFKPLIGPR
PLVNGLMGRINYYWSVLKPGQTLRIKSNGNLIAPWYGHILSGESHGRILKTDLKSGSCTVQCQTEKGGLN
TTLPFQNVSKYAFGNCSKYIGIKSLKLAVGLRNVPSRSSRGLFGAIAGFIEGGWSGLVAGWYGFQHSNAQ
GVGMAADRDSTQKAIDKITSKVNNIVDKMNKQYEIIDHEFSEVETRLNMINSKIDDQIQDIWAYNAELLV
LLENQKTLDEHDANVNNLYNKVKRALGSNAVEDGRGCFELYHKCDDQCMETIRNGTYNRRKYQEESKLER
QKIEGVKLESEGTYKILTIYSTVASSLVIAMGFAAFLFWAMSNGSCRCNICI

>UVB12116.1 hemagglutinin, partial [Influenza A virus]
DKICIGYQSTNSTETVDTLTENNVPVTHAKELLHTEHNGMLCATSLGQPLILDTCTIEGLIYGNPSCGPL
PDEREWSYIVERPSAVNGLCYPGNVENLEELRSLFSSARSYQRIQIFPDTIWNVSYDGTSNTCSGSFYRN
MRWLTRKDGNYPTQDAQYTNNQRKNILFMWGINNPPTDDTQRNLYTRTDTTTSVATEEMNRIFKPLIGPR
PLVNGLMGRINYYWSVLRPGQTLRIKSDGNLIAPWYGYILLGESHGRILRTDLKRGSCTVQCQTEKGGLN
TTLPFQNVSKYAFGNCSKYIGIKSLKLAVGLRNVPSRSSRGLFGAIAGFIEGGWSGLVAGWYGFQHSNDQ
GVGMAADRESTQKAIDKITSKVNNIVDKMNKQYEIIDHEFSEVETRLNMINNKIDDQIQDIWAYNAELLI
LLENQKTLDEHDANVNNLYNKVKRALGSNAVEDGKGCFELYHKCDDQCMETIRNGTYNRRKYQEESKLER
QKIEGVKLESEGTYKILTIYSTVASSLVIAMGFAAFLFWAMSNGSCRCNICI

>URN67695.1 hemagglutinin [Influenza A virus]
MEAISLMTILMVVTTSNADKICIGHQSTNSTETVDTLTETNVPVTQAKELLHTDHNGMLCATNLGRPLIL

EACTIEGLIYGNPTCDMLLGGREWSYIVERPSAVNGTCYPGNVENLEELRTLFSSSSSYQRIQLFPDSIW
NVTYTGTSKSCSDSFYRNMRWLTQKNGLYPIQDAQYTNNRGKDILYVWGIHHPPTDTAQTNLYTRTDTTT
SVTTENLDRTFKPLIGPRPLVNGLIGRINYYWSVLKPGQTLRIRSNGNLIAPWFGHVLSGESHGRILKTD
LNSGNCVVQCQTEKGGLNSTLPFHNISKYAFGNCPKYIGVKSLKLAIGLRNVHARSSRGLFGAIAGFIEG
GWPGLVAGWYGFQHSNDQGVGMAADRVSTQKAVDKITSKVNNIVDKMNKQYEIIDHEFNEVETRLNMINN
KIDDQIQDIWAYNAELLVLLENQKTLDEHDANVNNLYNKVKRALGSNAVEDGKGCFELYHKCDDQCMETI
RNGTYNRRKYTEESRLERQKIEGVKLEAEGTYKILSIYSTVASSLVLAMGFAAFIFWAMSNGSCRCNICI

>URN67694.1 hemagglutinin [Influenza A virus]
MEAISLMTILMVVTTSNADKICIGHQSTNSTETVDTLTETNVPVTQAKELLHTDHNGMLCATNLGRPLIL
EACTIEGLIYGNPTCDMLLGGREWSYIVERPSAVNGTCYPGNVENLEELRTLFSSSSSYQRIQLFPDSIW
NVTYTGTSKSCSDSFYRNMRWLTQKNGLYPIQDAQYTNNRGKDILYVWGIHHPPTDTAQTNLYTRTDTTT
SVTTENLDRTFKPLIGPRPLVNGLIGRINYYWSVLKPGQTLRIRSNGNLIAPWFGHVLSGESHGRILRTD
LNSGNCVVQCQTEKGGLNSTLPFHNISKYAFGNCPKYIGVKSLKLAIGLRNVHARSSRGLFGAIAGFIEG
GWPGLVAGWYGFQHSNDQGVGMAADRVSTQKAVDKITSKVNNIVDKMNKQYEIIDHEFNEVETRLNMINN
KIDDQIQDIWAYNAELLVLLENQKTLDEHDANVNNLYNKVKRALGSNAVEDGKGCFELYHKCDDQCMETI
RNGTYNRRKYTEESRLERQKIEGVKLEAEGTYKILSIYSTVASSLVLAMGFAAFIFWAMSNGSCRCNICI

>URN67693.1 hemagglutinin [Influenza A virus]
MEAISLMTILMVVTTSNADKICIGHQSTNSTETVDTLTETNVPVTQAKELLHTDHNGMLCATNLGRPLIL
EACTIEGLIYGNPTCDMLLGGREWSYIVERPSAVNGTCYPGNVENLEELRTLFSSSSSYQRIQIFPDSIW
NVTYTGTSKSCSDSFYRNMRWLTQKNGLYPIQDAQYTNNRGKDILYVWGIHHPPTDTAQTNLYTRTDTTT
SVTTENLDRTFKPLIGPRPIVNGLIGRINYYWSVLKPGQTLRIRSNGNLIAPWFGHVLSGESHGRILRTD
LNSGNCVVQCQTEKGGLNSTLPFHNISKYAFGNCPKYIGVKSLKLAIGLRNVHARSSRGLFGAIAGFIEG
GWPGLVAGWYGFQHSNDQGVGMAADRVSTQKAVDKITSKVNNIVDKMNKQYEIIDHEFNEVETRLNMINN
KIDDQIQDIWAYNAELLVLLENQKTLDEHDANVNNLYNKVKRALGSNAVEDGKGCFELYHKCDDQCMETI
RNGTYNRRKYTEESRLERQKIEGVKLEAEGTYKILSIYSTVASSLVLAMGFAAFIFWAMSNGSCRCNICI

>URN67692.1 hemagglutinin [Influenza A virus]
MEAISLMTILMVVTTSNADKICIGHQSTNSTETVDTLTETNVPVTQAKELLHTDHNGMLCATNLGRPLIL
EACTIEGLIYGNPTCDMLLGGREWSYIVERPSAVNGTCYPGNVENLEELRTLFSSSSSYQRIQLFPDSIW
NVTYTGTSKSCSDSFYRNMRWLTQKNGLYPIQDAQYTNNRGKDILYVWGIHHPPTDTVQTNLYTRTDTTT
SVTTENLDRTFKPLIGPRPLVNGLIGRINYYWSVLKPGQTLRIRSNGNLIAPWFGHVLSGESHGRILRTD
LNSGNCVVQCQTEKGGLNSTLPFHNISKYAFGNCPKYIGVKSLKLAIGLRNVHARSSRGLFGAIAGFIEG

GWPGLVAGWYGFQHSNDQGVGMAADRVSTQKAVDKITSKVNNIVDKMNKQYEIIDHEFNEVETRLNMINN
KIDDQIQDIWAYNAELLVLLENQKTLDEHDANVNNLYNKVKRALGSNAVEDGKGCFELYHKCDDQCMETI
RNGTYNRRKYTEESRLERQKIEGVKLEAEGTYKILSIYSTVASSLVLAMGFAAFIFWAMSNGSCRCNICI

>ULM88427.1 hemagglutinin [Influenza A virus]
MEAVSLITILVVATVSNADKICIGYQSTNSTETVDTLTENNVPVTHAKELLHTEHNGMLCATSLGHPLIL
DTCTIEGLIYGNPSCDPLLGGREWSYIVERPSAVNGLCYPGNVENLEELRSLFSSSRSYQRIQIFPDTIW
NVSYSGTSKACSDSFYRSMRWLTQKNNAYPTQDAQYTNNQGKNILFMWGINHPPTDAAQTNLYTRTDTTT
SVATEEMNRVFKPLIGPRPLVNGLMGRINYYWSVLKPGQTLRIKSDGNLIAPWYGHILSGESHGRILKTD
LKMGSCTVQCQTEKGGLNTTLPFQNVSKYAFGNCSKYIGVKSLKLAVGLRNVPSRSSRGLFGAIAGFIEG
GWSGLVAGWYGFQHSNDQGVGMAADRDSTQKAVDKITSKVNNIVDKMNKQYEIIDHEFSEVETRLNMIND
KVDDQIQDIWAYNAELLVLLENQKTLDEHDANVNNLYNKVKRALGSNAVEDGRGCFELYHKCDNHCMETI
RNGTYNRRKYQEESKLERQKIEGVKLESEETYKILTIYSTVASSLVIAMGFAAFLFWAMSNGSCRCNICI

>QRG27120.1 hemagglutinin, partial [Influenza A virus]
TILVVATVSNADKICIGYQSTNSTETVDTLTENNVPVTHAKELLHTEHNGMLCATSLGHPLILDTCTIEG
LIYGNPSCDPLLGGREWSYIVERPSAVNGLCYPGNVENLEELRSLFSSSRSYQRIQIFPDTIWNVSYSGT
SNACSDSFYRSMRWLTQKNNAYPTQDAQYTNNQGKNILFMWGINHPPTDAAQTNLYTRTDTTTSVATEEM
NRVFKPLIGPRPLVNGLMGRINYYWSVLKPGQTLRIKSDGNLIAPWYGHILSGESHGRILKTDLKMGSCT
VQCQTEKGGLNTTLPFQNVSKYAFGNCSKYIGVKSLKLAVGLRNVPSRSSRGLFGAIAGFIEGGWSGLIA
GWYGFQHSNDQGVGMAADRDSTQKAVDKITSKVNNIVDKMNKQYEIIDHEFSEVETRLNMINDKVDDQIQ
DIWAYNAELLVLLENQKTLDEHDANVNNLYNKVKRALGSNAVEDGRGCFELYHKCDNHCMETIRNGTYNR
RKYQEESKLERQKIEGVKLESEENYKILTIYSTVASSLVIA

>QRG27119.1 hemagglutinin, partial [Influenza A virus]
TILVVATVSNADKICIGYQSTNSTETVDTLTENNVPVTNAKELLHTEHNGMLCATSLGHPLILDTCTIEG
LIYGNPSCDPLLGGREWSYIVERPSAVNGLCYPGNVENLEELRSLFSSSRSYQRIQIFPDTIWNVSYSGT
SKACSDSFYRSMRWLTQKNNAYPTQDAQYTNNQGKNILFMWGINHPPTDDVQTNLYTRTDTTTSVATEEM
NRVFKPLIGPRPLVNGLMGRINYYWSVLKPGQTLRIKSDGNLIAPWYGHILSGESHGRILKTDLKMGNCT
VQCQTEKGGLNTTLPFQNVSKYAFGNCSKYIGVKSLKLAVGLRNVPSRSSRGLFGAIAGFIEGGWSGLVA
GWYGFQHSNDQGVGMAADRDSTQKAVDKITSKVNNIVDKMNKQYEIIDHEFSEVETRLNMINDKVDDQIQ
DIWAYNAELLVLLENQKTLDEHDANVNNLYNKVKRALGSNAVEDGRGCFELYHKCDNHCMETIRNGTYNR
RKYQEESKLERQKIEGVKLESEETYKILTIYSTVASSLVIA

>QRG27118.1 hemagglutinin, partial [Influenza A virus]

TILVVATVSNADKICIGYQSTNSTETVDTLTENNVPVTNAKELLHTEHNGMLCATSLGHPLILDTCTIEG
LIYGNPSCDPLLGGREWSYIVERPSAVNGLCYPGNVENLEELRSLFSSSRSYQRIQIFPDTIWNVSYSGT
SKACSDSFYRSMRWLTQKNNAYPTQDAQYTNNQGKNILFMWGINHPPTDDVQTNLYTRTDTTTSVATEEM
NRVFKPLIGPRPLVNGLMGRINYYWSVLKPGQTLRIKSDGNLIAPWYGHILSGESHGRILKTDLKMGTCT
VQCQTEKGGLNTTLPFQNVSKYAFGNCSKYIGVKSLKLAVGLRNVPSRSSRGLFGAIAGFIEGGWSGLVA
GWYGFQHSNDQGVGMAADRDSTQKAVDKITSKVNNIVDKMNKQYEIIDHEFSEVETRLNMINDKVDDQIQ
DIWAYNAELLVLLENQKTLDEHDANVNNLYNKVKRALGSNAVEDGRGCFELYHKCDNHCMETIRNGTYNR
RKYQEESKLERQKIEGVKLESEETYKILTIYSTVASSLVIA

>QRG27117.1 hemagglutinin, partial [Influenza A virus]

TILVVATVSNADKICIGYQSTNSTETVDTLTENNVPVTHAKELLHTEHNGMLCATSLGHPLILDTCTIEG
LIYGNPSCDPLLGGREWSYIVERPSAVNGLCYPGNVENLEELRSLFSSSRSYQRIQIFPDTIWNVSYSGT
SKACSDSFYRSMRWLTQKNNAYPTQDAQYTNNQGKNVLFMWGINHPPTDAAQTNLYTRTDTTTSVATEEM
NRVFKPLIGPRPLVNGLMGRINYYWSVLKPGQTLRIKSDGNLIAPWYGHILSGESHGRILKTDLKMGSCT
VQCQTEKGGLNTTLPFQNVSKYAFGNCSKYIGVKSLKLAVGLRNVPSRSSRGLFGAIAGFIEGGWSGLIA
GWYGFQHSNDQGVGMAADRDSTQKAVDKITSKVNNIVDKMNKQYEIIDHEFSEVETRLNMINDKVDDQIQ
DIWAYNAELLVLLENQKTLDEHDANVNNLYNKVKRALGSNAVEDGRGCFELYHKCDNHCMETIRNGTYNR
RKYQEESKLERQKIEGVKLESEETYKILTIYSTVASSLVIA

>QRG27116.1 hemagglutinin, partial [Influenza A virus]

TILVVATVSNADKICIGYQSTNSTETVDTLTENNVPVTNAKELLHTEHNGMLCATSLGHPLILDTCTIEG
LIYGNPSCDPLLGGREWSYIVERPSAVNGLCYPGNVENLEELRSLFSSSRSYQRIQIFPDTIWNVSYSGT
SKACSDSFYRSMRWLTQKNNAYPTQDAQYTNNQGKNILFMWGINHPPTDDVQTNLYTRTDTTTSVATEEM
NRVFKPLIGPRPLVNGLMGRINYYWSVLKPGQTLRIKSDGNLIAPWYGHILSGESHGRILKTDLKMGNCT
VQCQTEKGGLNTTLPFQNVSKYSFGNCSKYIGVKSLKLAVGLRNVPSRSSRGLFGAIAGFIEGGWSGLVA
GWYGFQHSNDQGVGMAADRDSTQKAVDKITSKVNNIVDKMNKQYEIIDHEFSEVETRLNMINDKVDDQIQ
DIWAYNAELLVLLENQKTLDEHDANVNNLYNKVKRALGSNAVEDGRGCFELYHKCDNHCMETIRNGTYNR
RKYQEESKLERQKIEGVKLESEETYKILTIYSTVASSLVIA

>QRG27115.1 hemagglutinin, partial [Influenza A virus]

TILVVATVSNADKICIGYQSTNSTETVDTLTENNVPVTHAKELLHTEHNGMLCATSLGHPLILDTCTIEG
LIYGNPSCDPLLGGREWSYIVERPSAVNGLCYPGNVENLEELRSLFSSSRSYQRIQIFPDTIWNVSYNGT
SKACSDSFYRSMRWLTQKNNAYPTQDAQYTNNQGKNILFMWGINHPPTDAVQTNLYTRTDTTTSVATEEM

NRVFKPLIGPRPLVNGLMGRINYYWSVLKPGQTLRIKSDGNLIAPWYGHILSGESHGRILKTDLKMGSCT
VQCQTEKGGLNTTLPFQNVSKYAFGNCSKYIGVKSLKLAVGLRNVPSRSSRGLFGAIAGFIEGGWSGLIA
GWYGFQHSNDQGVGMAADRDSTQKAVDKITSKVNNIVDKMNKQYEIIDHEFSEVETRLNMINDKVDDQIQ
DIWAYNAELLVLLENQKTLDEHDANVNNLYNKVKRALGSNAVEDGRGCFELYHKCDNHCMETIRNGTYNR
RKYQEESKLERQKIEGVKLESEETYKILTIYSTVASSLVIA

>QRG27114.1 hemagglutinin, partial [Influenza A virus]
TILVVATVSNADKICIGYQSTNSTETVDTLTENNVPVTHAKELLHTEHNGMLCATSLGHPLILDTCTIEG
LIYGNPSCDPLLGGREWSYIVERPSAVNGLCYPGNVENLEELRSLFSSSRSYQRIQIFPDTIWNVSYSGT
SKACSDSFYRSMRWLTQKNNAYPTQDAQYTNNQGKNILFMWGINHPPTDDVQTNLYTRTDTTTSVATEEM
NRVFKPLIGPRPLVNGLMGRINYYWSVLKPGQTLRIKSDGNLIAPWYGHILSGESHGRILKTDLKMGNCT
VQCQTEKGGLNTTLPFQNVSKYAFGNCSKYIGVKSLKLAVGLRNVPSRSSRGLFGAIAGFIEGGWSGLVA
GWYGFQHSNDQGVGMAADRDSTQKAVDKITSKVNNIVDKMNKQYEIIDHEFSEVETRLNMINDKVDDQIQ
DIWAYNAELLVLLENQKTLDEHDANVNNLYNKVKRALGSNAVEDGRGCFELYHKCDNHCMETIRNGTYNR
RKYQEESKLERQKIEGVKLESEETYKILTIYSTVASSLVIA

>QRG27113.1 hemagglutinin, partial [Influenza A virus]
TILVVATVSNADKICIGYQSTNSTETVDTLTENNVPVTHAKELLHTEHNGMLCATSLGHPLILDTCTIEG
LIYGNPSCDPLLGGREWSYIVERPSAVNGLCYPGNVENLEELRSLFSSSRSYQRIQIFPDTIWNVSYSGT
SKACSDSFYRSMRWLTQKNNAYPTQDAQYTNNQGKNILFMWGINHPPTDDVQTNLYTRTDTTTSVATEEM
NRVFKPLIGPRPLVNGLMGRINYYWSVLKPGQTLRIKSDGNLIAPWYGHILSGESHGRILKTDLKMGSCT
VQCQTEKGGLNTTLPFQNVSKYAFGNCSKYIGVKSLKLAVGLRNVPSRSSRGLFGAIAGFIEGGWSGLVA
GWYGFQHSNDQGVGMAADRDSTQKAVDKITSKVNNIVDKMNKQYEIIDHEFSEVETRLNMINDKVDDQIQ
DIWAYNAELLVLLENQKTLDEHDANVNNLYNKVKRALGSNAVEDGRGCFELYHKCDNHCMETIRNGTYNR
RKYQEESKLERQKIEGVKLESEETYKILTIYSTVASSLVIA

>QZX86308.1 hemagglutinin, partial [Influenza A virus]
SLIIILLVATVSNADKICIGYQSTNSTETVDTLTENNVPVTHAKELLHTEHNGMLCATSLGHPLILDTCT
IEGLIYGNPSCDLLWEGKEWSYIVERPSAVNGLCYPGNVENLEELRSLFSSARSYRRIQIFPDTIWNVSY
SGTSKACSNSFYRSMRWLTKKDNAYPIQDAQYTNNQEKNILFMWGINHPPTDTVQTDLYARADTTTSVAT
EEINRIFKPLIGPRPLVNGLMGRIDYYWSVLKPGQTLRIRSDGNLIAPWYGHVLSGESHGRILKTDLKRG
SCTVQCQTEKGGLNTTLPFQNVSKYAFGNCSKYIGIKSLKLAVGLRNVPSRSSRGLFGAIAGFIEGGWSG
LVAGWYGFQHSNDQGVGMAADRDSTQKAIDKITSKVNNIVDKMNKQYEIIDHEFSEVETRLNMINSKIDD
QIQDIWAYNAELLVLLENQKTLDEHDANVNNLYNKVKRALGSNAVEDGKGCFELYHKCDDQCMETIRNGT

YNRRKYQEESKLERQKIEGVKLESEGTYKILTIYSTVASSLVIAMGFAAFLFWAMSNGSCRCNICI

>UDE32127.1 hemagglutinin [Influenza A virus]
METVSLITILLVATVSNADKICIGYQSTNSTETVDTLTENNVPVTHAKELLHTEHNGMLCATSLGQPLIL
DTCTIEGLIYGNPSCDLSLEGREWSYIVERPSAVNGLCYPGNVENLEELRSLFSSARSYQRIQIFPDTIW
NVSYDGTSTACSGSFYRSMRWLTRKNGDYPTQDAQYTNNQGKNILFMWGINHPPTDDTQRNLYTRTDTTT
SVATEEINRIFKPLIGPRPLVNGLMGRIDYYWSVLKPGQTLRIKSDGNLIAPWYGHILSGESHGRILKTD
LKRGSCTVQCQTEKGGLNTTLPFQNVSKYAFGNCSKYIGIKSLKLAVGLRNVPSRSSRGLFGAIAGFIEG
GWSGLVAGWYGFQHSNDQGVGMAADRDSTQKAIDKITSKVNNIVDKMNKQYEIIDHEFSEVETRLNMINN
KIDDQIQDIWAYNAELLVLLENQKTLDEHDANVNNLYNKVKRALGSNAVEDGKGCFELYHKCNDQCMETI
RNGTYNRRKYQEESKLERQKIEGVKLESEGTYKILTIYSTVASSLVIAMGFAAFLFWAMSNGSCRCNICI

>UDE32126.1 hemagglutinin [Influenza A virus]
METVSLITILLVATVSNADKICIGYQSTNSTETVDTLTENNVPVTHAKELLHTEHNGMLCATSLGQPLIL
DTCTIEGLIYGNPSCDLSLEGREWSYIVERPSAVNGLCYPGNVENLEELRSLFSSARSYQRIQIFPDTIW
NVSYDGTSTACSGSFYRSMRWLTRKNGDYPTQDAQYTNNQGKNILFMWGINHPPTDDTQRNLYTRTDTTT
SVATEEINRIFKPLIGPRPLVNGLMGRIDYYWSVLKPGQTLRIKSDGNLIAPWYGHILSGESHGRILKTD
LKRGSCTVQCQTEKGGLNTTLPFQNVSKYAFGNCSKYIGIKSLKLAVGLRNVPSRSSRGLFGAIAGFIEG
GWSGLVAGWYGFQHSNDQGVGMAADRDSTQKAIDKITSKVNNIVDKMNKQYEIIDHEFSEVETRLNMINN
KIDDQIQDIWAYNAELLVLLENQKTLDEHDANVNNLYNKVKRALGSNAVEDGKGCFELYHKCNDQCMETI
RNGTYNRRKYQEESKIERQKIEGVKLESEGTYKILTIYSTVASSLVIAMGFAAFLFWAMSNGSCRCNICI

>UDE32125.1 hemagglutinin [Influenza A virus]
METVSLITILLVATVSNADKICIGYQSTNSTETVDTLTENNVPVTHAKELLHTEHNGMLCATSLGQPLIL
DTCTIEGLIYGNPSCDLLLEGREWSYIVERPSAVNGLCYPGNVENLEELRSLFSSARSYQRIQIFPDTIW
NVSYDGTSTACSGSFYRSMRWLTRKNGDYPIQDAQYTNNQGKNILFMWGINHPPTDTXQXDLYTRTDTTT
SVATEEINRIFKPLIGPRPLVNGLMGRIDYYWSVLKPGQTLRIKSDGNLIAPWYGHILSGESHGRILKTD
LKRGSCTVQCQTEKGGLNTTLPFQNVSKYAFGNCSKYIGIKSLKLAVGLRNVPSRSSRGLFGAIAGFIEG
GWSGLVAGWYGFQHSNDQGVGMAADRDSTQKAIDKITSKVNNIVDKMNKQYEIIDHEFSEVETRLNMIHN
KIDDQIQDIWAYNAELLVLLENQKTLDEHDANVNNLYNKVKRALGSNAVEDGKGCFELYHKCDDQCMETI
RNGTYNRRKYQEESKLERQKIEGVKLESEGTYKILTIYSTVASSLVIAMGFAAFLFWAMSNGSCRCNICI

>UDE32124.1 hemagglutinin [Influenza A virus]
METVSLITILLVATVSNADKICIGYQSTNSTETVDTLTENNVPVTHAKELLHTEHNGMLCATSLGQPLIL

DTCTIEGLIYGNPSCDLSLEGREWSYIVERPSAVNGLCYPGNVENLEELRSLFSSARSYQRIQIFPDTIW
NVSYDGTSTACSGSFYRSMRWLTRKNGDYPTQDAQYTNNQGKNILFMWGINRPPTDDTQRNLYTRTDTTT
SVATEEINRIFKPLIGPRPLVNGLMGRIDYYWSVLKPGQTLRIKSDGNLIAPWYGHILSGESHGRILKTD
LKRGSCTVQCQTEKGGLNTTLPFQNVSKYAFGNCSKYIGIKSLKLAVGLRNVPSRSSRGLFGAIAGFIEG
GWSGLVAGWYGFQHSNDQGVGMAADRDSTQKAIDKITSKVNNIVDKMNKQYEIIDHEFSEVETRLNMINN
KIDDQIQDIWAYNAELLVLLENQKTLDEHDANVNNLYNKVKRALGSNAVEDGKGCFELYHKCNDQCMETI
RNGTYNRRKYQEESKLERQKIEGVKLESEGTYKILTIYSTVASSLVIAMGFAAFLFWAMSNGSCRCNICI

>UDE32123.1 hemagglutinin [Influenza A virus]
METVSLITILLVATVSNADKICIGYQSTNSTETVDTLTENNVPVTHAKELLHTEHNGMLCATSLGQPLIL
DTCTIEGLIYGNPSCDLSLEGREWSYIVERPSAVNGLCYPGNVENLEELRSLFSSARSYQRIQIFPDTIW
NVSYDGTSTACSGSFYRSMRWLTRKNGDYPTQDAQYTNNQGKNILFMWGINHPPTDDTQRNLYTRTDTTT
SVATEEINRIFKPLIGPRPLVNGLMGRIDYYWSVLKPGQTLRIKSDGNLIAPWYGHILSGESHGRILKTD
LKRGSCTVQCQTEKGGLNTTLPFQNVSKYAFGNCSKYIGIKSLKLAVGLRNVPSRSSRGLFGAIAGFIEG
GWSGLVAGWYGFQHSNDQGVGMAADRDSTQKAIDKITSKVNNIVDKMNKQYEIIDHEFSEVETRLNMINN
KIDDQIQDIWAYNAELLVXLENQKTLDEHDANVNNLYNKVKRALGSNAVEDGKGCFELYHKCNDQCMETI
RNGTYNRRKYQEESKLERQKIEGVKLESEGTYKILTIYSTVASSLVIAMGFAAFLFWAMSNGSCRCNICI

>UDE32122.1 hemagglutinin [Influenza A virus]
METVSLITILLVATVSNADKICIGYQSTNSTETVDTLTENNVPVTHAKELLHTEHNGMLCATSLGQPIIL
DTCTIEGLIYGNPSCDLSLEGREWSYIVERPSAVNGLCYPGNVENLEELRSLFSSARSYQRIQIFPDTIW
NVSYDGTSTACSGSFYRNMRWLTRKNGDYPIQDAQYTNNQGKNILFMWGINHPPADTTQRDLYTRTDTTT
SVATEEINRIFKPLIGPRPLVNGLMGRIDYYWSVLKPGQTLRIKSDGNLIAPWYGHILSGESHGRILKTD
LKRGSCTVQCQTEKGGLNTTLPFQNVSKYAFGNCSKYIGIKSLKLAVGLRNVPSRSSRGLFGAIAGFIEG
GWSGLVAGWYGFQHSNDQGVGMAADRDSTQKAIDKITSKVNNIVDKMNKQYEIIDHEFSEVETRLNMINN
KIDDQIQDIWAYNAELLVLLENQKTLDEHDANVNNLYNKVKRALGSNAVEDGKGCFELYHKCDDQCMETI
RNGTYNRRKYQEESKLERQKIEGVKLESEGTYKILTIYSTVASSLVIAMGFAAFLFWAMSNGSCRCNICI

>UDE32121.1 hemagglutinin [Influenza A virus]
METVSLITILLAATVSNADKICIGYQSTNSTETVDTLTENNVPVTHAKELLHTEHNGMLCATSLGQPLIL
DTCTIEGLIYGNPSCDLSLEGREWSYIVERPSAVNGLCYPGNVENLEELRSLFSSARSYQRIQIFPDTIW
NVSYDGTSNTCSGSFYRNMRWLTRKDGNYPIQDAQYTNNQGKNILFMWGINHPPTDNTQRNLYTRTDTTT
SVATEEINRIFKPLIGPRPLVNGLMGRIDYYWSVLKPGQTLRIKSDGNLIAPWYGYILSGESHGRILKTD
LKRGSCTVQCQTEKGGLNTTLPFQNVSKYAFGNCSKYIGIKSLKLAVGLRNVPSRSSRGLFGAIAGFIEG

GWSGLVAGWYGFQHSNDQGVGMAADRESTQKAIDKITSKVNNIVDKMNKQYEIIDHEFSEVETRLNMINN
KIDDQIQDIWAYNAELLVLLENQKTLDEHDANVNNLYNKVKRALGSNAVEDGKGCFELYHKCDDQCMETI
RNGTYNRRKYQEESKLERQKIEGVKLESEGTYKILTIYSTVASSLVIAVGFAAFLFWAMSNGSCRCNICI

>UDE32120.1 hemagglutinin [Influenza A virus]
METVSLITILLVSTVSNADKICIGYQSTNSTETVDTLTENNVPVTHAKELLHTEHNGMLCATSLGQPLIL
DTCTIEGLIYGNPSCDLSLEGREWSYIVERPSAVNGLCYPGNVENLEELRSLFSSARSYQRIQIFPDTIW
NVSYNGISKACSDSFYRSMRWLTQKNSDYPIQDAQYTNNQGKNILFMWGINHPPTDTTQRDLYTRTDTTT
SVATEEINRVFKPLIGPRPLVNGLMGRIDYYWSVLKPGQTLRIKSDGNLIAPWYGHILSGESHGRILKTD
LKRGSCTVQCQTEKGGLNTTLPFQNVSKYAFGNCSKYIGIKSLKLAFGLRNVPSRSSRGLFGAIAGFIEG
GWSGLVAGWYGFQHSNDQGVGMAADRDSTQKAIDKITSKVNNIVDKMNKQYEIIDHEFSEVETRLNMINN
KIDDQIQDIWAYNAELLVLLENQKTLDEHDANVNNLYNKVKRALGSNAVEDGKGCFELYHKCDDQCMETI
RNGTYNRRKYQEESKLERQKIEGVKLESEGTYKILTIYSTVASSLVIAMGFAAFLFWAMSNGSCRCNICI

>UDE32119.1 hemagglutinin [Influenza A virus]
METVSLITILLVATVSNADKICIGYQSTNSTETVDTLTENNVPVTHAKELLHTEHNGMLCATSLGQPLIL
DTCTIEGLIYGNPSCDLLLEGREWSYIVERPSAVNGLCYPGNVENLEELRSLFSSARSYQRIQIFPDTIW
NVSYNGTSKACSGSFYRSMRWLTRKNGDYPIQDAQYTNNQGKNILFMWGINHPPTDTTQRDLYTRTDTTT
SVATEEINRIFKPLIGPRPLVNGLMGRIDYYWSVLKPGQTLRIKSDGNLIAPWYGHILSGESHGRILKTD
LKRGSCTVQCQTEKGGLNTTLPFQNVSKYAFGNCSKYIGIKSLKLAVGLRNVPSRSSRGLFGAIAGFIEG
GWSGLVAGWYGFQHSNDQGVGMAADRDSTQKAIDKITSKVNNIVDKMNKQYEIIDHEFSEVETRLNMINN
KIDDQIQDIWAYNAELLVLLENQKTLDEHDANVNNLYNKVKRALGSNAVEDGKGCFELYHKCDNQCMETI
RNGTYNRRKYQEESKLERQKIEGVKLESEGTYKILTIYSTVASSLVIAMGFAAFLFWAMSNGSCRCNICI

>UDE32118.1 hemagglutinin [Influenza A virus]
METASLITILLVATVSNADKICIGYQSTNSTETVDTLTENNVPVTHAKELLHTEHNGMLCATSLGQPLIL
DTCTIEGLIYGNPSCDLLLEGREWSYIVERPSAVNGLCYPGNVENLEELRSLFSSARSYQRIQIFPDTIW
NVSYDGTSTACSGXFYRSMRWLTRKNGDYPIQDAQYTNNQGKNILFMWGINHPPTDTTQRDLYTRTDTTT
SVATEEINRVFKPLIGPRPLVNGLMGRIDYYWSVLKPGQTLRIKSDGNLIAPWYGYILSGESHGRILKTD
LKRGSCTVQCQTEKGGLNTTLPFQNVSKYAFGNCSKYIGIKSLKLAVGLRNVPSRSSRGLFGAIAGFIEG
GWSGLVAGWYGFQHSNDQGVGMAADRDSTQKAIDKITSKVNNIVDKMNKQYEIIDHEFSEVETRLNMINN
KIDDQIQDIWAYNAELLVLLENQKTLDEHDANVNNLYNKVKRALGSNAVEDGKGCFELYHKCDDQCMETI
RNGTYNRRKYQEESKLERQKIEGVKLESEGTYKILTIYSTVASSLVIAMGFAAFLFWAMSNGSCRCNICI

>UDE32117.1 hemagglutinin [Influenza A virus]

METVSLITILLVATVSNADKICIGYQSTNSTETVDTLTENNVPVTHAKELLHTEHNGMLCATSLGQPLIL

DTCTIEGLIYGNPSCDLLLEGREWSYIVERPSAVNGLCYPGNVENLEELRSLFSSARSYQRIQIFPNTIW

NVSYNGTSKACSGSFYRSMRWLTQKNGNYPVQDAQYTNNQGKNILFMWGINHPPTDTTQRDLYTRTDTTT

SVATEEINRIFKPLIGPRPLVNGLMGRIDYYWSVLKPGQTLRIKSDGNLIAPWYGHILSGESHGRILKTD

LKRGSCTVQCQTEKGGLNTTLPFQNVSKYAFGNCSKYIGIKSLKLAVGLRNVPSRSSRGLFGAIAGFIEG

GWSGLVAGWYGFQHSNDQGVGMAADRDSTQKAIDKITSKVNNIVDKMNKQYEIIDHEFSEVETRLNMINN

KIDDQIQDIWAYNAELLVLLENQKTLDEHDANVNNLYNKVKRALGSNAMEDGKGCFDLYHKCDDQCMETI

RNGTYNRRKYQEESKLERQKIEGVKLESEGTYKILTIYSTVASSLVIAMGFAAFLFWAMSNGSCRCNICI

>UDE32115.1 hemagglutinin [Influenza A virus]

MEAVTIITILLAITGSNADKICIGYQSTNSTETVDTLTENNVPVTHAKELLHTEHNGMLCATNLGSPLIL

DTCTIEGIIYGNPSCDLPLEGRKWSYIVERPSAVNGVCYPGNVENLEELRSLFSSASSYQRIQIFPDSIW

NVSYSGTSKACSDSFYKSMRWLTQKNNNYPVQDAQYTNNRGKNILFMWGINHPPTDTAQTNLYTRTDTTT

SVATEDINRIFKPLIGPRPLVNGLQGRIDYYWAVLKPGQTLRVRSNGNLIAPWYGHILLGESHGRILKTD

LKSGNCVVQCQTERGGLNTTLPFHNVSKYAFGNCPKYIGVKSLKLAVGLRNVPARSSRGLFGAIAGFIEG

GWSGLVAGWYGFQHSNDQGTGMAADRDSTQKAIDKITSKVNNIVDKMNKQYEIIDHEFSEIETRLNMINN

KIDDQIQDIWAYNAELLVLLENQKTLDEHDANVNNLYNKVKRALGSNAVEDGKGCFDLYHKCDNQCMETI

RNGTYNRRKYQEESRLERQKIEGVKLESEGTYKILTIYSTVASSLVIAMGFAAFLFWAMSNGSCRCNICI

>UDE32114.1 hemagglutinin [Influenza A virus]

METVSLITILLVATVSNADKICIGYQSTNSTETVDTLTENNVPVTHAKELLHTEHNGMLCATSLGQPLIL

DTCTIEGLIYGNPSCDLLLEGREWSYIVERPSAVNGLCYPGNVENLEELRSLFSSARSYQRIQIFPDTIW

NVSYNGTSKACSGSFYRSMRWLTRKNGDYPIQDAQYTNNQGKNILFMWGINHPPTDTTQRDLYTRTDTTT

SVATEEINRIFKPLIGPRPLVNGLMGRIDYYWSVLKPGQTLRIKSDGNLIAPWYGHILSGESHGRILKTD

LKRGSCTVQCQTEKGGLNTTLPFQNVSKYAFGNCSKYIGIKSLKLAVGLRNVPSRSSRGLFGAIAGFIEG

GWSGLVAGWYGFQHSNDQGVGMAADRDSTQKAIDKITSKVNNIVDKMNKQYEIIDHEFSEVETRLNMINN

KIDDQIQDIWAYNAELLVLLENQKTLDEHDANVNNLYNKVKRALGSNAVEDGKGCFELYHKCDDQCMETI

RNGTYNRRKYQEESKLERQKIEGVKLESEGTYKILTIYSTVASSLVIAMGFAAFLFWAMSNGSCRCNICI

>UDE32113.1 hemagglutinin [Influenza A virus]

METASLITILLVVTVSNADKICIGYQSTNSTETVDTLTENNVPVTHAKELLHTEHNGMLCATSLGHPLIL

DTCTIEGLIYGNPSCDLLLGGREWSYIVERPSAVNGLCYPGNVENLEELRSLFSSARSYQRVQIFPDTIW

NVSYSGTSRACSDSFYRSMRWLTQKDNAYPTQDAQYTNNQEKNILFMWGINHPPTDTAQTNLYTRTDTTT

SVATEEINRIFKPLIGPRPLVNGLMGRINYYWSVLKPGQTLRIKSNGNLIAPWYGHILSGESHGRILKTD
LKRGSCTVQCQTEKGGLNTTLPFQNVSKYAFGNCSKYIGIKSLKLAVGLRNVPSRSSRGLFGAIAGFIEG
GWSGLVAGWYGFQHSNDQGVGMAADRDSTQKAIDKITSKVNNIVDKMNKQYEIIDHEFSEVETRLNMINN
KIDDQIQDIWAYNAELLVLLENQKTLDEHDANVNNLYNKVKRALGSNAVEDGKGCFELYHKCDDQCMETI
RNGTYNRRKYQEESKLERQKIEGVKLESEGTYKILTIYSTVASSLVIAMGFAAFLFWAMSNGSCRCNICI

>UDE32112.1 hemagglutinin [Influenza A virus]
METVSLITILLVATVSNADKICIGYQSTNSTETVDTLTENNVPVTHAKELLHTEHNGMLCATSLGQPLIL
DTCTIEGLIYGNPSCDLLLEGREWSYIVERPSAVNGLCYPGNVENLEELRSLFSSARSYQRIQIFPDTIW
NVSYNGTSKACSGSFYRSMRWLTRKNGDYPIQDAQYTNNQGKNILFMWGINHPPTDTTQRDLYTRTDTTT
SVATEEINRIFKPLIGPRPLVNGLMGRIDYYWSVLKPGQTLRIKSDGNLIAPWYGHILSGESHGRILKTD
LKRGSCTVQCQTEKGGLNTTLPFQNVSKYAFGNCSKYIGIKSLKLAVGLRNVPSRSSRGLFGAIAGFIEG
GWSGLVAGWYGFQHSNDQGVGMAADRDSTQKAIDKITSKVNNIVDKMNKQYEIIDHEFSEVETRLNMINN
KIDDQIQDIWAYNAELLVLLENQKTLDEHDANVNNLYNKVKRALGSNAVEDGKGCFELYHKCDDQCMETI
RNGTYNRRKYQEESKLERQKIEGVKLESEGTYKILTIYSTVASSLVIAMGFAAFLFWAMSNGSCKCNICI

>UDE32111.1 hemagglutinin [Influenza A virus]
METVSLITILLVATVSNADKICIGYQSTNSTETVDTLTENNVPVTHAKELLHTEHNGMLCATSLGQPLIL
DTCTIEGLIYGNPSCDLLLEGREWSYIVERPSAVNGLCYPGNVENLEELRSLFSSARSYQRIQIFPDTIW
NVSYDGTSKACSDSFYRSMRWLTRRNGDYPIQDAQYTNNQGKNILFMWGINHPPTDTTQRDLYTRTDTTT
SVATEEINRIFKPLIGPRPLVNGLMGRIDYYWSVLKPGQTLRIKSDGNLIAPWYGHILSGESHGRILKTD
LKRGSCTVQCQTEKGGLNTTLPFQNVSKYAFGNCSKYIGIKSLKLAVGLRNVPSRSSRGLFGAIAGFIEG
GWSGLVAGWYGFQHSNDQGVGMAADRDSTQKAIDKITSKVNNIVDKMNKQYEIIDHEFSEVETRLNMINN
KIDDQIQDIWAYNAELLVLLENQKTLDEHDANVNNLYNKVKRALGSNAVEDGKGCFELYHKCDDQCMETI
RNGTYNRRKYQEESKLERQKIEGVKLESEGTYKILTIYSTVASSLVIAMGFAAFLFWAMSNGSCRCNICI

>UDE32110.1 hemagglutinin [Influenza A virus]
METVSLITILLVATVSNADKICIGYQSTNSTETVDTLTENNVPVTHAKELLHTEHNGMLCATSLGQPLIL
DTCTIEGLIYGNPSCDLLLEGREWSYIVERPSAVNGLCYPGHVENLEELRSLFSSARSYQRIQIFPDTIW
NVSYDGTSNACSGSFYRSMRWLTRKNGDYPIQDAQYTNNQGKNILFMWGINHPPTDTTQRDLYTRTDTTT
SVATEEINRIFKPLIGPRPLVNGLMGRIDYYWSVLKPGQTLRIKSDGNLIAPWYGHILSGESHGRILKTD
LKRGSCTVQCQTEKGGLNTTLPFQNVSKYAFGNCSKYIGIKSLKLAVGLRNVPSRSSRGLFGAIAGFIEG
GWSGLVAGWYGFQHSNDQGVGMAADRDSTQKAIDKITSKVNNIVDKMNKQYEIIDHEFSEVETRLNMINN
KIDDQIQDIWAYNAELLVLLENQKTLDEHDANVNNLYNKVKRALGSNAVEDGKGCFELYHKCDDQCMETI

RNGTYNRRKYQEESKLERQKIEGVKLESEGTYKILTIYSTVASSLVIAMGFAAFLFWAMSNGSCRCNICI

>UDE32109.1 hemagglutinin [Influenza A virus]

METASLITILLVVTVSNADKICIGYQSTNSTETVDTLTENNVPVTHAKELLHTEHNGMLCATSLGHPLIL
DTCTIEGLIYGNPSCDLLLGGREWSYIVERPSAVNGLCYPGNVENLEELRSLFSSARSYQRIQIFPDTIW
NVSYSGTSKACSDSFYRSMRWLTQKDNAYPTQDAQYTNNQGKNILFMWGINHPPTDTVQTNLYTRTDTTT
SVATEEINRTFKPLIGPRPLVNGLMGRINYYWSVLKPGQTLRIKSDGNLIAPWYGHILSGESHGRILKTD
LKRGSCTVQCQTEKGGLNTTLPFQNVSKYAFGNCSKYIGIKSLKLAVGLRNVPSRSSRGLFGAIAGFIEG
GWSGLVAGWYGFQHSNDQGVGMAADRDSTQKAIDKITSKVNNIVDKMNKQYEIIDHEFSEVETRLNMINN
KIDDQIQDIWAYNAELLVLLENQKTLDEHDANVNNLYNKVKRSLGSNAVEDGKGCFELYHKCDDQCMETI
RNGTYNRRKYQEESKLERQKIEGVKLESEGTYKILTIYSTVASSLVIAMGFAAFLFWAMSNGSCRCNICI

>UDE32108.1 hemagglutinin [Influenza A virus]

METVSLITILLVATVSNADKICIGYQSTNSTETVDTLTENNVPVTHAKELLHTEHNGMLCATSLGQPLIL
DTCTIEGLIYGNPSCDLLLEGREWSYIVERPSAVNGLCYPGNVENLEELRSLFSSARSYQRIQIFPDTIW
NVSYDGTSTACSGSFYRSMRWLTXKDXXYPIQDAQYTNNQXKNILFMWGINHPPTDTAQTNLYTRTDTTT
SVATEEINRTFKPLIGPRPLVNGLMGRINYYWSVLKPGQTLRIKSNGNLIAPWYGHILSGESHGRILKTD
LKRGSCTVQCQTEKGGLNTTLPFQNVSKYAFGNCSKYIGIKSLKLAVGLRNVPSRSSRGLFGAIAGFIEG
GWSGLVAGWYGFQHSNDQGVGMAADRDSTQKAIDKITSKVNNIVDKMNKQYEIIDHEFSEVETRLNMINN
KIDDQIQDIWAYNAELLVLLENQKTLDEHDANVNNLYNKVKRALGSNAVEDGKGCFELYHKCDDQCMETI
RNGTYNRRKYQEESKLERQKIEGVKLESEGTYKILTIYSTVASSLVIAMGFAAFLFWAMSNGSCRCNICI

>UDE32107.1 hemagglutinin [Influenza A virus]

METASLITILLVVTVSNADKICIGYQSTNSTETVDTLTENNVPVTHAKELLHTEHNGMLCATSLGQPLIL
DTCTIEGLIYGNPSCDLLLGGREWSYIVERPSAVNGLCYPGNVENLEELRSLFSSARSYQRIQIFPDTIW
NVSYSGTSKACSDSFYRSMRWLTQKDNAYPIQDAQYTNNQEKNILFMWGINHPPTDTVQTNLYTRTDTTT
SVATEEINRTFKPLIGPRPLVNGLMGRINYYWSVLKPGQTLRIKSNGNLIAPWYGHILSGESHGRILKTD
LKRGSCTVQCQTEKGGLNTTLPFQNVSKYAFGNCSKYIGIKSLKLAVGLRNVPSRSSRGLFGAIAGFIEG
GWSGLVAGWYGFQHSNDQGVGMAADRDSTQKAIDKITSKVNNIVDKMNKQYEIIDHEFSEVETRLNMINN
KIDDQIQDIWAYNAELLVLLENQKTLDEHDANVNNLYNKVKRALGSNAVEDGKGCFELYHKCDDQCMETI
RNGTYNRRKYQEESKLERQKIEGVKLESEGTYKILTIYSTVASSLVIAMGFAAFLFWAMSNGSCRCNICI

>UDE32106.1 hemagglutinin [Influenza A virus]

METVSLITILLVATVSNADKICIGYQSTNSTETVDTLTENNVPVTHAKELLHTEHNGMLCATSLGQPLIL

DTCTIEGLIYGNPSCDLSLEGREWSYIVERPSAVNGLCYPGNVENLEELRSLFSSARSYQRIQIFPDTIW
NVSYDGTSKACSDSFYRSMRWLTRKNGDYPIQDAQYTNNQGKNILFMWGINHPPTDTTQRNLYTRNDTTT
SVATEEINRIFKPLIGPRPLVNGLMGRIDYYWSVLKPGQTLRIKSDGNLIAPWYGHILSGESHGRILKTD
LKRGSCTVQCQTEKGGLNTTLPFQNVSKYAFGNCSKYIGIKSLKLAVGLRNVPSRSSRGLFGAIAGFIEG
GWSGLVAGWYGFQHSNDQGVGMAADRDSTQKAIDKITSKVNNIVDKMNKQYEIIDHEFSEVETRLNMINN
KIDDQIQDIWAYNAELLVLLENQKTLDEHDANVNNLYNKVKRALGSNAVEDGKGCFELYHKCDDQCMETI
RNGTYNRRKYQEESKLERQKIEGVKLESEGTYKILTIYSTVASSLVIAMGFAAFLFWAMSNGSCRCNICI

>UDE32105.1 hemagglutinin [Influenza A virus]
METVSLITILLVATVSNADKICIGYQSTNSTETVDTLTENNVPVTHAKELLHTEHNGMLCATSLGQPLIL
DTCTIEGLIYGNPSCDLLLEGREWSYIVERPSAVNGLCYPGNVENLEELRSLFSSARSYQRIQIFPDTIW
NVSYDGTSTACSGSFYRSMRWLTRKNGDYPIQDAQYTNNQGKNILFMWGINHPPTDTTQRNLYTRTDTTT
SVATEEINRIFKPLIGPRPLVNGLMGRIDYYWSVLKPGQTLRIKSDGNLIAPWYGHILSGESHGRILKTD
LKRGSCTVQCQTEKGGLNTTLPFQNVSKYAFGNCSKYIGIKSLKLAVGLRNVPSRSSRGLFGAIAGFIEG
GWSGLVAGWYGFQHSNDQGVGMAADRDSTQKAIDKITSKVNNIVDKMNKQYEIIDHEFSEVETRLNMINN
KIDDQIQDIWAYNAELLVLLENQKTLDEHDANVNNLYNKVKRALGSNAVEDGKGCFELYHKCDDQCMETI
RNGTYNRRKYQEESKLERQKIEGVKLESEGTYKILTIYSTVASSLVIAMGFAAFLFWAMSNGSCRCNICI

>UDE32104.1 hemagglutinin [Influenza A virus]
METVSLITILLVATVSNADKICIGYQSTNSTETVDTLTENNVPVTHAKELLHTEHNGMLCATSLGQPLIL
DTCTIEGLIYGNPSCELLLEGREWSYIVERPSAVNGLCYPGNVENLEELRSLFSSARSYQRIQIFPVTIW
NVSYNGTSKACSGSFYRSMRWLTRKNGDYPIQDAQYTNNQGKNILFMWGINHPPTDTTQRDLYTRTDTTT
SVATEEINRVFKPLIGPRPLVNGLMGRIDYYWSVLKPGQTLRIKSDGNLIAPWYGYILSGESHGRILKTD
LKRGSCTVQCQTEKGGLNTTLPFQNVSKYAFGNCSKYIGIKSLKLAVGLRNVPSRSSRGLFGAIAGFIEG
GWSGLVAGWYGFQHSNDQGVGMAADRDSTQKAIDKITSKVNNIVDKMNKQYEIIDHEFSEVETRLNMINN
KIDDQIQDIWAYNAELLVLLENQKTLDEHDANVNNLYNKVKRALGSNAVEDGKGCFELYHKCDDQCMETI
RNGTYNRRKYQEESKLERQKIEGVKLESEGTYKILTIYSTVASSLVIAMGFAAFLFWAMSNGSCRCNICI

>UDE32103.1 hemagglutinin [Influenza A virus]
MEAVTIITILLAITGSNADKICIGYQSTNSTETVDTLTENNVPVTHAKELLHTEHNGMLCATNLGSPLIL
DTCTIEGIIYGNPSCDLPLEGRKWSYIVERPSAVNGVCYPGNVENLEELRSLFSSASSYQRIQIFPDSIW
NVSYSGTSKACSDSFYKSMRWLTQKNNNYPVQDAQYTNNRGKNILFMWGINHPPTDTAQTNLYTRTDTTT
SVATEDINRIFKPLIGPRPLVNGLQGRIDYYWAVLKPGQTLRVRSNGNLIAPWYGHILLGESHGRILKTD
LKSGNCVVQCQTERGGLNTTLPFHNVSKYAFGNCPKYIGVKSLKLAVGLRNVPARSSRGLFGAIAGFIEG

GWSGLVAGWYGFQHSNDQGTGMAADRDSTQKAIDKITSKVNNIVDKMNKQYEIIDHEFSEIETRLNMINN
KIDDQIQDIWAYNAELLVLLENQKTLDEHDANVNNLYNKVKRALGSNAVEDGKGCFDLYHKCDNQCMETI
RNGTYNRRKYQEESRLERQKIEGVKLESEGTYKILTIYSTVASSLVIAMGFAAFLFWAMSNGSCRCNICI

>UDE32102.1 hemagglutinin [Influenza A virus]
METVSLITILLVATVSNADKICIGYQSTNSTETVDTLTENNVPVTHAKELIHTEHNGMLCATSLGQPLIL
DTCTIEGLIYGNPSCDLLLEGREWSYIVERPSAVNGLCYPGNVENLEELRSLFSSARSYQRIQIFPDTIW
NVSYNGTSKACSGSFYRSMRWLTRKNGDYPIQDAQYTNNQGKNILFMWGINHPPTDTTQRDLYTRTDTTT
SVATEEINRVFKPLIGPRPLVNGLMGRIDYYWSVLKPGQTLRIKSDGNLIAPWYGHILSGESHGRILKTD
LKRGSCTVQCQTEKGGLNTTLPFQNVSKYAFGNCSKYIGIKSLKLAVGLRNVPSRSSRGLFGAIAGFIEG
GWSGLVAGWYGFQHSNDQGVGMAADRDSTQKAIDKITSKVNNIVDKMNKQYEIIDHEFSEVETRLNMINN
KIDDQIQDIWAYNAELLVLLENQKTLDEHDANVNNLYNKVKRALGSNAVEDGKGCFELYHKCDDQCMETI
RNGTYNRRKYQEESKLERQKIEGVKLESEGTYKILTIYSTVASSLVIAMGFAAFLFWAMSNGSCRCNICI

>UDE32101.1 hemagglutinin [Influenza A virus]
METASLITILLVVTVSNADKICIGYQSTNSTETVDTLTENNVPVTHAKELLHTEHNGMLCATSLGHPLIL
DTCTVEGLIYGNPSCDLLLGGREWSYIVERPSAVNGLCYPGNVENLEELRSLFSSARSYQRIQIFPDTIW
NVSYSGTSKACSDSFYRSMRWLTQKDNAYPTQDAQYTNNQGKNILFMWGINHPPTDTVQTNLYTRTDTTT
SVATEEINRTFKPLIGPRPLVNGLMGRINYYWSVLKPGQTLRIKSNGNLIAPWYGHILSGESHGRILKTD
LKRGSCTVQCQTEKGGLNTTLPFQNVSKYAFGNCSKYIGIKSLKLAVGLRNVPSRSSRGLFGAIAGFIEG
GWSGLVAGWYGFQHSNDQGVGMAADRDSTQKAIDKITSKVNNIVDKMNKQYEIIDHEFSEVETRLNMINN
KIDDQIQDIWAYNAELLVLLENQKTLDEHDANVNNLYNKVKRSLGSNAVEDGKGCFELYHKCDDQCMETI
RNGTYNRRKYQEESKLERQKIEGVKLESEGTYKILTIYSTVASSLVIAMGFAAFLFWAMSNGSCRCNICI

>UDE32100.1 hemagglutinin [Influenza A virus]
METVSLITILLVATVSNADKICIGYQSTNSTETVDTLTENNVPVTHAKELLHTEHNGMLCATSLGQPLIL
DTCTIEGLIYGNPSCDLSLEGREWSYIVERPSAVNGLCYPGNVENLEELRSLFSSARSYQRIQIFPDTIW
NVSYDGTSTACSGSFYRSMRWLTRKNGDYPTQDAQYTNNQGKNILFMWGINHPPTDTTQRDLYTRTDTTT
SVATEEINRIFKPLIGPRPLVNGLMGRIDYYWSVLKPGQTLRIKSDGNLIAPWFGHILSGESHGRILKTD
LKRGSCTVQCQTEKGGLNTTLPFQNVSKYAFGNCSKYIGIKSLKLAVGLRNVPSRSSRGLFGAIAGFIEG
GWSGLVAGWYGFQHSNDQGVGMAADRDSTQKAIDKITSKVNNIVDKMNKQYEIIDHEFSEVETRLNMINN
KIDDQIQDIWAYNAELLVLLENQKTLDEHDANVNNLYNKVKRALGSNAVEDGKGCFELYHKCDDQCMETI
RNGTYNRRKYQEESKLERQKIEGVKLESEGTYKILTIYSTVASSLVIAMGFAAFLFWAMSNGSCRCNICI

>UDE32099.1 hemagglutinin [Influenza A virus]

METVSLITILLVAAVSNADKICIGYQSTNSTETVDTLTENNVPVTHAKELLHTEHNGMLCATSLGQPLIL
DTCTIEGLIYGNPSCDLSLEGREWSYIVERPSAVNGLCYPGNVENLEELRSLFSSARSYQRIQIFPDTIW
NVSYDGTSTACSGSFYRSMRWLTRKNGDYPIQDAQYTNNQGKNILFMWGINHPPTDTTQRDLYTRTDTTT
SVATEEINRIFKPLIGPRPLVNGLMGRINYYWSVLKPGQTLRIKSDGNLIAPWFGHILSGESHGRILKTD
LKRGSCTVQCQTEKGGLNTTLPFQNVSKYAFGNCSKYIGIKSLKLAVGLRNVPSRSSRGLFGAIAGFIEG
GWSGLVAGWYGFQHSNDQGVGMAADRDSTQKAIDKITSKVNNIVDKMNKQYEIIDHEFSEVETRLNMINN
KIDDQIQDIWAYNAELLVLLENQKTLDEHDANVNNLYNKVKRALGSNAVEDGKGCFELYHKCDDQCMETI
RNGTYNRRKYQEESKLERQKIEGVKLESEGTYKILTIYSTVASSLVIAMGFAAFLFWAMSNGSCRCNICI

>UDE32098.1 hemagglutinin [Influenza A virus]

METVSLITILLVATVSNADKICIGYQSTNSTETVDTLTENNVPVTHAKELLHTEHNGMLCATSLGQPLVL
DTCTIEGLIYGNPSCDLSLEGREWSYIVERPSAVNGLCYPGNVENLEELRSLFSSARSYQRIQIFPDTIW
NVSYDGTSTACSGSFYRSMRWLTRKNGDYPIQDAQYTNNQGKNILFMWGINQPPTDTTQRDLYTRTDTTT
SVATEEINRIFKPLIGPRPLVNGLMGRIDYYWSVLKPGQTLRIKSDGNLIAPWYGHILSGESHGRILKTD
LKRGSCTVQCQTEKGGLNTTLPFQNVSKYAFGNCSKYIGIKSLKLAVGLRNVPSRSSRGLFGAIAGFIEG
GWSGLVAGWYGFQHSNDQGVGMAADRDSTQKAIDKITSKVNNIVDKMNKQYEIIDHEFSEVETRLNMINN
KIDDQIQDIWAYNAELLVLLENQKTLDEHDANVNNLYNKVKRALGSNAVEDGKGCFELYHKCDDQCMETI
RNGTYNRRKYQEESKLERQKIEGVKLESEGTYKILTIYSTVASSLVIAMGFAAFLFWAMSNGSCRCNICI

>UDE32097.1 hemagglutinin [Influenza A virus]

METVSLITILLVATVSNADKICIGYQSTNSTETVDTLTENNVPVTHAKELLHTEHNGMLCATSLGQPLIL
DTCTIEGLIYGNPSCDLSLEGREWSYIVERPSAVNGLCYPGNVENLEELRSLFSSARSYQRIQIFPDTIW
NVSYDGTSTACSGSFYRSMRWLTRKNGDYPIQDAQYTNNQGKNILFMWGINHPPTDTTQRDLYTRTDTTT
SVATEEINRIFKPLIGPRPLVNGLMGRINYYWSVLKPGQTLRIKSDGNLIAPWYGHILSGESHGRILKTD
LKRGSCTVQCQTEKGGLNTTLPFQNVSKYAFGNCSKYIGIKSLKLAVGLRNVPSRSSRGLFGAIAGFIEG
GWSGLVAGWYGFQHSNDQGVGMAADRDSTQKAIDKITSKVNNIVDKMNKQYEIIDHEFSEVETRLNMINN
KIDDQIQDIWAYNAELLVLLENQKTLDEHDANVNNLYNKVKRALGSNAVEDGKGCFELYHKCDDQCMETI
RNGTYNRRKYQEESKLERQKIEGVKLESEGTYKILTIYSTVASSLVIAMGFAAFLFWAMSNGSCRCNICI

>UDE32096.1 hemagglutinin [Influenza A virus]

METVSLITILLVATVSNADKICIGYQSTNSTETVDTLTENNVPVTHAKELLHTEHNGMLCATSLGQPLVL
DTCTIEGLIYGNPSCDLSLEGREWSYIVERPSAVNGLCYPGNVENLEELRSLFSSARSYQRIQIFPDTIW
NVSYDGTSSACSGSFYKSMRWLTRKNGDYPIQDAQYTNNQGKNILFMWGINHPPTDTTQRDLYTRTDTTT

SVATEEINRIFKPLIGPRPLVNGLMGRIDYYWSVLKPGQTLRIKSDGNLIAPWYGHILSGESHGRILKTD
LKRGSCTVQCQTEKGGLNTTLPFQNVSKYAFGNCSKYIGIKSLKLAVGLRNVPSRSSRGLFGAIAGFIEG
GWSGLVAGWYGFQHSNDQGVGMAADRDSTQKAIDKITSKVNNIVDKMNKQYEIIDHEFSEIETRLNMINN
KIDDQIQDIWAYNAELLVLLENQKTLDEHDANVNNLYNKVKRALGSNAVEDGKGCFELYHKCDDQCMETI
RNGTYNRRKYQEESKLERQKIEGVKLESEGTYKILTIYSTVASSLVIAMGFAAFLFWAMSNGSCRCNICI

>UDE32095.1 hemagglutinin [Influenza A virus]
METVSLITILLVATVSNADKICIGYQSTNSTETVDTLTENNVPVTHAKELLHTEHNGMLCATSLGQPLIL
DTCTIEGLIYGNPSCDLSLEGREWSYIVERPSAVNGLCYPGNVENLEELRSLFSSARSYQRIQIFPDTIW
NVSYDGTSTACSGSFYRSMRWLTRKNGEYPIQDAQYTNNQGKNILFMWGINHPPTDTTQRDLYTRTDTTT
SVATEEINRVFKPLIGPRPLVNGLMGRIDYYWSVLKPGQTLRIKSDGNLIAPWFGHILSGESHGRILKTD
LKRGSCTVQCQTEKGGLNTTLPFQNVSKYAFGNCSKYIGIKSLKLAVGLRNVPSRSSRGLFGAIAGFIEG
GWSGLVAGWYGFQHSNDQGVGMAADRDSTQKAIDKITSKVNNIVDKMNKQYEIIDHEFSEVEARLNMINN
KIDDQIQDIWAYNAELLVLLENQKTLDEHDANVNNLYNKVKRALGSNAVEDGKGCFELYHKCDDQCMETI
RNGTYNRRKYQEESKLERQKIEGVKLESEGTYKILTIYSTVASSLVIAMGFAAFLFWAMSNGSCRCNICI

>UDE32092.1 hemagglutinin [Influenza A virus]
METVSLITILLVATVSNADKICIGYQSTNSTETVDTLTENNVPVTHAKELLHTEHNGMLCATSLGHPLIL
DTCTIEGLIYGNPSCDPLLGGREWSYIVERPSAVNGLCYPGNVENLEELRSLFSSARSYQRIQIFPDTIW
NVSYSGTSKACSDSFYRSMRWLTQKNNAYPIQDAQYTNNQEKNILFMWGINHPPTETAQTNLYTRTDTTT
SVATEEINRIFKPLIGPRPLVNGLMGRINYYWSVLKPGQTLRIKSDGNLIAPWYGHILSGESHGRILKTD
LKRGSCTVQCQTEKGGLNTTLPFQNVSKYAFGNCSKYIGIKSLKLAIGLRNVPSRSSRGLFGAIAGFIEG
GWSGLVAGWYGFQHSNDQGVGMAADRDSTQKAIDKITSKVNNIVDKMNRQYEIIDHEFSEVETRLNMINN
KVDDQIQDIWAYNAELLVLLENQKTLDEHDSNVNNLYNKVKRALGSNAVEDGKGCFELYHKCDDQCMETI
RNGTYNRRKYQEESKLERQKIEGVKLESEGTYKILTIYSTVASSLVIAMGFAAFLFWAMSNGSCRCNICI

>UDE32090.1 hemagglutinin [Influenza A virus]
METVSLITILLVATVSNADKICIGYQSTNSTETVDTLTENNVPVTHAKELLQTEHNGMLCATSLGQPLIL
DTCTIEGLIYGNPSCDLSLEGREWSYIVERPSAVNGLCYPGNVENLEELRSLFSSARSYQRIQIFPDTIW
NVSYDGTSTACSGSFYKSMRWLTRKNGDYPIQDAQYTNNQGKNILFMWGINHPPTDTTQRDLYTRTDTTT
SVATEEINRVFKPLIGPRPPVNGLMGRIDYYWSVLKPGQTLRIKSDGNLIAPWFGHILSGESHGRILKTD
LKRGSCTVQCQTEKGGLNTTLPFQNVSKYAFGNCSKYIGIKSLKLAVGLRNVPSRSSRGLFGAIAGFIEG
GWSGLVAGWYGFQHSNDQGVGMAADRDSTQKAIDKITSKVNNIVDKMNKQYEIIDHEFSEVETRLNMINN
KIDDQIQDIWAYNAELLVLLENQKTLDEHDANVNNLYNKVKRALGSNAVEDGKGCFELYHKCDDQCMETI

RNGTYNRRKYQEESKLERQKIEGVKLESEGTYKILTIYSTVASSLVIAMGFAAFLFWAMSNGSCRCNICI

>UDE32089.1 hemagglutinin [Influenza A virus]
METVSLITILLVATVSNADKICIGYQSTNSTETVDTLTENNVPVTHAKELLHTEHNGMLCATSLGHPLIL
DTCTIEGLIYGNPSCDPLLGGREWSYIVERPSAVNGLCYPGSVENLEELRSLFSSARSYQRIQIFPDTIW
NVSYSGTSKACSDSFYRSMRWLTQKNNAYPIQDAQYTNNQEKNILFMWGINHPPTETAQTNLYTRTDTTT
SVATEEINRIFKPLIGPRPLVNGLMGRINYYWSVLKPGQTLRIKSDGNLIAPWYGHILSGESHGRILKTD
LKRGSCTVQCQTEKGGLNTTLPFQNVSKYAFGNCSKYIGIKSLKLAIGLRNVPSRSSRGLFGAIAGFIEG
GWSGLVAGWYGFQHSNDQGVGMAADRDSTQKAIDKITSKVNNIVDKMNRQYEIIDHEFSEVETRLNMINN
KVDDQIQDIWAYNAELLVLLENQKTLDEHDSNVNNLYNKVKRALGSNAVEDGKGCFELYHKCDDQCMETI
RNGTYNRRKYQEESKLERQKIEGVKLESEGTYKILTIYSTVASSLVIAMGFAAFLFWAMSNGSCRCNICI

>UDE32088.1 hemagglutinin [Influenza A virus]
METVSLITILLVATVSNADKICIGYQSTNSTETVDTLTENNVPVTHAKELLHTEHNGMLCATSLGQPLIL
DTCTIEGIIYGNPSCDLSLEGREWSYIVERPSAVNGLCYPGNVENLEELRSLFSSARSYQRIQIFPDTIW
NVSYDGTSKACSGSFYKSMRWLTRKNGDYPIQDAQYTNNQGKNILFMWGINHPPTDTTQRDLYTRTDTTT
SVATEEINRVFKPLIGPRPLVNGLMGRIDYYWSVLKPGQTLRIKSDGNLIAPWFGHILSGESHGRILKTD
LKRGNCTVQCQTEKGGLNTILPFQNVSKYAFGNCSKYIGIKSLKLAVGLRNVPSRSSRGLFGAIAGFIEG
GWSGLVAGWYGFQHSNDQGVGMAADRDSTQKAIDKITSKVNNIVDKMNKQYEIIDHEFSEVEIRLNMINN
KIDDQIQDIWAYNAELLVLLENQKTLDEHDANVNNLYNKVKRALGSNAVEDGKGCFELYHKCDDQCMETI
RNGTYNRRKYQEESKLERQKIEGVKLESEGTYKILTIYSTVASSLVIAMGFAAFLFWAMSNGSCRCNICI

>UDE32087.1 hemagglutinin [Influenza A virus]
METVSLITILLVAAVSNADKICIGYQSTNSTETVDTLTENNVPVTHAKELLHTEHNGMLCATSLGQPLIL
DTCTIEGLIYGNPSCDLSLEGREWSYIVERPSAVNGLCYPGNVENLEELRSLFSSARSYQRIQIFPDTIW
NVSYDGTSTACSGSFYKSMRWLTRKNGDYPIQDAQYTNNQGKNILFMWGINHPPTDTTQRDLYTRTDTTT
SVATEEINRVFKPLIGPRPLVNGLMGRIDYYWSVLKPGQTLRIKSDGNLIAPWFGHILSGESHGRILKTD
LKRGSCTVQCQTEKGGLNTTLPFQNVSKYAFGNCSKYIGIKSLKLAVGLRNVPSRSSRGLFGAIAGFIEG
GWSGLVAGWYGFQHSNDQGVGMAADRDSTQKAIDKITSKVNNIVDKMNKQYEIIDHEFSEVETRLNMINN
KIDDQIQDIWAYNAELLVLLENQKTLDEHDANVNNLYNKVKRALGSNAVEDGKGCFELYHKCDDQCMETI
RNGTYNRRKYQEESKLERQKIEGVKLESEGTYKILTIYSTVASSLVIAMGFAAFLFWAMSNGSCRCNICI

>UDE32086.1 hemagglutinin [Influenza A virus]
METVSLITILLVATVSNADKICIGYQSTNSTETVDTLTENNVPVTHAKELIHTEHNGMLCATSLGQPLIL

DTCTIEGLIYGNPSCDLMLEGREWSYIVERPSAVNGLCYPGHVENLEELRSLFSSARSYQRVQIFPDTIW
NVSYDGTSTACSGSFYRSMRWLTRKNGDYPIQDAQYTNNQGKNILFMWGINHPPADTTQRDLYTRTDTTT
SVATEEINRIFKPLIGPRPLVNGLMGRIDYYWSVLKPGQTLRIKSDGNLIAPWYGHILSGESHGRILKTD
LKRGSCTVQCQTEKGGLNTTLPFQNVSKYAFGNCSKYIGMKSLKLAVGLRNVPSRSSRGLFGAIAGFIEG
GWSGLVAGWYGFQHSNDQGVGMAADRDSTQKAIDKITSKVNNIVDKMNKQYEIIDHEFSEVETRLNMINN
KIDDQIQDIWAYNAELLVLLENQKTLDEHDANVNNLYNKVKRALGSNAVEDGKGCFDLYHKCDDQCMETI
RNGTYNRRKYQEESKLERQKIEGVKLESEGTYKILTIYSTVASSLVIAMGFAAFLFWAMSNGSCRCNICI

>UDE32085.1 hemagglutinin [Influenza A virus]
METVSLITILLVATVSNADKICIGYQSTNSTETVDTLTENNVPVTHAKELLHTEHNGMLCATSLGQPLIL
DTCTIEGIIYGNPSCDLSLEGREWSYIVERPSAVNGLCYPGNVENLEELRSLFSSARSYQRIQIFPDTIW
NVSYDGTSTACSGSFYKSMRWLTRKNGDYPIQDAQYTNNQGKNILFMWGINHPPTDTTQRDLYTRTDTTT
SVATEEINRVFKPLIGPRPLVNGLMGRIDYYWSVLKPGQTLRIKSDGNLIAPWFGHILSGESHGRILKTD
LKRGSCTVQCQTEKGGLNTTLPFQNVSKYAFGNCSKYIGIKSLKLAVGLRNVPSRSSRGLFGAIAGFIEG
GWSGLVAGWYGFQHSNDQGVGMAADRDSTQKAIDKITSKVNNIVDKMNKQYEIIDHEFSEVEIRLNMINN
KIDDQIQDIWAYNAELLVLLENQKTLDEHDANVNNLYNKVKRALGSNAVEDGKGCFELYHKCDDQCMETI
RNGTYNRRKYQEESKLERQKIEGVKLESEGTYKILTIYSTVASSLVIAMGFAAFLFWAMSNGSCRCNICI

>UDE32084.1 hemagglutinin [Influenza A virus]
METVSLITILLVATVSNADKICIGYQSTNSTETVDTLTENNVPVTHAKELLHTEHNGMLCATSLGQPLIL
DTCTIEGLIYGNPSCDLMLEGREWSYIVERPSAVNGLCYPGHVENLEELRSLFSSARSYQRIQIFPDTIW
NVSYDGTSNACSGSFYRSMRWLTRKNGDYPIQDAQYTNNQGKNILFMWGINHPPTDTTQRDLYTRTDTTT
SVATEEINRIFKPLIGPRPLVNGLMGRIDYYWSILKPGQTLRIKSDGNLIAPWYGHILSGESHGRILKTD
LKRGSCTVQCQTEKGGLNTTLPFQNVSKYAFGNCSKYIGMKSLKLAVGLRNVPSRSSRGLFGAIAGFIEG
GWSGLVAGWYGFQHSNDQGVGMAADRDSTQKAIDKITSKVNNIVDKMNKQYEIIDHEFSEVESRLNMINN
KIDDQIQDIWAYNAELLVLLENQKTLDEHDANVNNLYNKVKRALGSNAVEDGKGCFELYHKCDDQCMETI
RNGTYNRRKYQEESKLERQKIEGVKLESEGTYKILTIYSTVASSLVIAMGFAAFLFWAMSNGSCRCNICI

>UDE32083.1 hemagglutinin [Influenza A virus]
METVSLITILLVATVSNADKICIGYQSTNSTETVDTLTENNVPVTHAKELLHTEHNGMLCATSLGQPLIL
DTCTIEGLIYGNPSCNLMLEGREWSYIVERPSAVNGLCYPGHVENLEELRSLFSSARSYQRIQIFPDTIW
NVSYDGTSNACSGSFYRSMRWLTRKNGDYPIQDAQYTNNQGKNILFMWGINHPPTDTTQRDLYTRTDTTT
SVATEEINRIFKPLIGPRPLVNGLMGRIDYYWSILKPGQTLRIKSDGNLIAPWYGHILSGESHGRFLKTD
LKRGSCTVQCQTEKGGLNTTLPFQNVSKYAFGNCSKYIGMKSLKLAVGLRNVPSRSSRGLFGAIAGFIEG

GWSGLVAGWYGFQHSNDQGVGMAADRDSTQKAIDKITSKVNNIVDKMNKQYEIIDHEFSEVESRLNMINN
KIDDQIQDIWAYNAELLVLLENQKTLDEHDANVNNLYNKVKRALGSNAVEDGKGCFELYHKCDDQCMETI
RNGTYNRRKYQEESKLERQKIEGVKLESEGTYKILTIYSTVASSLVIAMGFAAFLFWAMSNGSCRCNICI

>UDE32082.1 hemagglutinin [Influenza A virus]
METVSLITMLLVATVSNADKICIGYQSTNSTETVDTLTENNVPVTHAKELLHTEHNGMLCATSLGQPLIL
DTCTIEGLIYGNPSCNLSLEGREWSYIVERPSAVNGLCYPGNVENLEELRSLFSSARSYQRIQIFPDTIW
NVSYDGTSKACSGSFYRSMRWLTRKNGDYPIQDAQYTNNQGKNILFMWGINHPPTDTTQRDLYTRTDTTT
SVATEEINRIFKPLIGPRPLVNGLMGRIDYYWSVLKPGQTLRIKSDGNLIAPWFGHILSGESHGRILKTD
LKRGSCTVQCQTEKGGLNTTLPFQNVSKYAFGNCSKYIGIKSLKLAVGLRNVPSRSSRGLFGAIAGFIEG
GWSGLVAGWYGFQHSNDQGVGMAADRDSTQKAIDKITSKVNNIVDKMNKQYEIIDHEFSEVETRLNMINN
KIDDQIQDIWAYNAELLVLLENQKTLDEHDANVNNLYNKVKRALGSNAVEDGKGCFELYHKCDDQCMETI
RNGTYNRRKYQEESKLERQKIEGVKLESEGTYKILTIYSTVASSLVIAMGFAAFLFWAMSNGSCRCNICI

>UDE32081.1 hemagglutinin [Influenza A virus]
METVSLITILLVATVSNADKICIGYQSTNSTETVDTLTENNVPVTHAKELLHTEHNGMLCATSLGQPLIL
DTCTIEGLIYGNPSCDLSLEGREWSYIVERPSAVNGLCYPGNVENLEELRSLFSSARSYQRIQIFPDTIW
NVSYDGTSTACSGSFYKSMRWLTRKNGDYPIQDAQYTNNQGKNILFMWGINHPPTDTTQRDLYTRTDTTT
SVATEEINRVFKPLIGPRPLVNGLMGRIDYYWSVLKPGQTLRIKSDGNLIAPWFGHILSGESHGRILKTD
LKRGSCTVQCQTEKGGLNTTLPFQNVSKYAFGNCSKYIGIKSLKLAVGLRNVPSRSSRGLFGAIAGFIEG
GWSGLVAGWYGFQHSNDQGVGMAADRDSTQKAIDKITSKVNNIVDKMNKQYEIIDHEFSEVETRLNMINN
KIDDQIQDIWAYNAELLVLLENQKTLDEHDANVNNLYNKVKRALGSNAVEDGKGCFELYHKCDDQCMETI
RNGTYNRRKYQEESKLERQKIEGVKLESEGTYKILTIYSTVASSLVIAMGFAAFLFWAMSNGSCRCNICI

>UDE32079.1 hemagglutinin [Influenza A virus]
METVSLITILLVATVSNADKICIGYQSTNSTETVDTLTENNVPVTHAKELLHTEHNGMLCATSLGQPIIL
DTCTIEGLIYGNPSCDLSLEGREWSYIVERPSAVNGLCYPGNVENLEELRSLFSSARSYKRIQIFPDTIW
NVSYDGTSTACSGSFYRNMRWLTRKNGDYPIQDAQYTNNQGKNILFMWGINHPPADTTQRDLYTRTDTTT
SVATEEINRIFKPLIGPRPLVNGLMGRIDYYWSVLKPGQTLRIKSDGNLIAPWYGHILSGESHGRILKTD
LKRGSCTVQCQTEKGGLNTTLPFQNVSKYAFGNCSKYIGIKSLKLAVGLRNVPSRSSRGLFGAIAGFIEG
GWSGLVAGWYGFQHSNDQGVGMAADRDSTQKAIDKITSKVNNIVDKMNKQYEIIDHEFSEVETRLNMINN
KIDDQIQDIWAYNAELLVLLENQKTLDEHDANVNNLYNKVKRALGSNAVEDGKGCFELYHKCDDQCMETI
RNGTYNRRKYQEESKLERQKIEGVKLESEGTYKILTIYSTVASSLVIAMGFAAFLFWAMSNGSCRCNICI

>UDE32077.1 hemagglutinin [Influenza A virus]

METASLITILLVATVSNADKICIGYQSTNSTETVDTLTENNVPVTHAKELLHTEHNGMLCATSLGHPLIL
DTCTIEGLIYGNPSCDPLLGGREWSYIVERPSAVNGLCYPGNVENLEELRSLFSSARSYQRIQIFPDTIW
NVSYSGTSKACSDSFYRSMRWLTQKNNAYPIQDAQYTNNQEKNILFMWGINHPPTETVQTNLYTRTDTTT
SVATEEINRVFKPLIGPRPLVNGLMGRINYYWSVLKPGQTLRIKSDGNLIAPWYGHILSGESHGRILKTD
LKRGSCTVQCQTEKGGLNTTLPFQNVSKYAFGNCSKYIGIKSLKLAVGLRNVPSRSSRGLFGAIAGFIEG
GWSGLVAGWYGFQHSNDQGVGMAADRDSTQKAIDKITSKVNNIVDKMNKQYEIIDHEFSEVETRLNMINN
KVDDQIQDIWAYNAELLVLLENQKTLDEHDSNVNNLYNKVKRALGSNAMEDGKGCFELYHKCDDQCMETI
RNGTYNRRKYQEESKLERQKIEGVKLESEGTYKILTIYSTVASSLVIAMGFAAFLFWAMSNGSCRCNICI

>UDE32076.1 hemagglutinin [Influenza A virus]

METVSLITILLVATVSNADKICIGYQSTNSTETVDTLTENNVPVTHAKELLHTEHNGMLCATSLGHPLIL
DTCTIEGLIYGNPSCDLLLGGREWSYIVERPSAVNGLCYPGNVENLEELRSLFSSARSYQRIQIFPDTIW
NVSYSGTSKACSDSFYRSMRWLTQKNNAYPIQDAQYTNNQEKNILFMWGINHPPTDTAQTNLYTRTDTTT
SVATEEINRIFKPLIGPRPLVNGLMGRINYYWSVLKPGQTLRIKSDGNLIAPWYGHILSGESHGRILKTD
LKRGSCTVQCQTEKGGLNTTLPFQNVSKYAFGNCSKYIGIKSLKLAVGLRNVPSRSSRGLFGAIAGFIEG
GWSGLVAGWYGFQHSNDQGVGMAADRDSTQKAIDKITSKVNNIVDKMNKQYEIIDHEFSEVETRLNMINN
KVDDQIQDIWAYNAELLVLLENQKTLDEHDANVNNLYNKVKRALGSNAVEDGKGCFELYHKCDDQCMETI
RNGTYNRRKYQEESKLERQKIEGVKLESEETYKILTIYSTVASSLVIAMGFAAFLFWAMSNGSCRCNICI

>UDE32075.1 hemagglutinin [Influenza A virus]

METVSLITILLVATVSNADKICIGYQSTNSTETVDTLTENNVPVTHAKELIHTEHNGMLCATSLGHPLIL
DTCTIEGLIYGNPSCDPLLGGREWSYIVERPSAVNGLCYPGSVENLEELRSLFSSARSYQRIQIFPDTIW
NVSYSGTSKACSDSFYRSMRWLTQKNNAYPIQDAQYTNNQEKNILFMWGINHPPTETVQTNLYTRTDTTT
SVATEEINRIFKPLIGPRPLVNGLMGRINYYWSVLKPGQTLRIKSDGNLIAPWYGHILSGESHGRILKTD
LKRGSCTVQCQTEKGGLNTTLPFQNVSKYAFGNCSKYIGIKSLKLAIGLRNVPSRSSRGLFGAIAGFIEG
GWSGLVAGWYGFQHSNDQGVGMAADRDSTQKAIDKITSKVNNIVDKMNRQYEIIDHEFSEVETRLNMINN
KVDDQIQDIWAYNAELLVLLENQKTLDEHDSNVNNLYNKVKRALGSNAVEDGKGCFELYHKCDDQCMETI
RNGTYNRRKYQEESKLERQKIEGVKLESEGTYKILTIYSTVASSLVIAMGFAAFLFWAMSNGSCRCNICI

>UDE32074.1 hemagglutinin [Influenza A virus]

METVSLITILLVATVSNADKICIGYQSTNSTETVDTLTENNVPVTHAKELLHTEHNGMLCATSLGQPLIL
DTCTIEGLIYGNPSCDLSLEGREWSYIVERPSAVNGLCYPGNVENLEELRSLFSSARSYQRIQIFPDTIW
NVSYDGTSTACSGSFYRSMRWLTRKNGNYPIQDAQYTNNQGKNILFMWGINHPPTDTTQRDLYTRTDTTT

SVATEEINRIFKPLIGPRPLVNGLMGRIDYYWSVLKPGQTLRIKSDGNLIAPWYGHILSGESHGRILKTD
LKRGSCTVQCQTEKGGLNTTLPFQNVSKYAFGNCSKYIGIKSLKLAVGLRNVPSRSSRGLFGAIAGFIEG
GWSGLVAGWYGFQHSNDQGVGMAADRDSTQKAIDKITSKVNNIVDKMNKQYEIIDHEFSEVETRLNMINN
KIDDQIQDIWAYNAELLVLLENQKTLDEHDANVNNLYNKVKRALGSNAVEDGKGCFELYHKCDDQCMETI
RNGTYNRRKYQEESKLERQKIEGVKLESEGTYKILTIYSTVASSLVIAMGFAAFLFWAMSNGSCRCNICI

>UDE32073.1 hemagglutinin [Influenza A virus]
METVSLITILLVATVSNADKICIGYQSTNSTETVDTLTENNVPVTHAKELLHTEHNGMLCATSLGQPLVL
DTCTIEGLIYGNPSCDLSLEGREWSYIVERPSAVNGLCYPGNVENLEELRSLFSSARSYQRIQIFPDTIW
NVSYDGTSNACSGSFYRSMRWLTRKNGDYPIQDAQYTNNQGKNILFMWGINHPPTDTTQRDLYTRTDTTT
SVATEEINRIFKPLIGPRPLVNGLMGRIDYYWSVLKPGQTLRIKSDGNLIAPWYGHILSGESHGRILKTD
LKRGSCTVQCQTEKGGLNTTLPFQNVSKYAFGNCSKYIGIKSLKLAVGLRNVPSRSSRGLFGAIAGFIEG
GWSGLVAGWYGFQHSNDQGVGMAADRDSTQKAIDKITSKVNNIVDKMNKQYEIIDHEFSEVETRLNMINN
KIDDQIQDIWAYNAELLVLLENQKTLDEHDANVNNLYNKVKRALGSNAVEDGKGCFELYXKCDDQCMETI
RNGTYNRRKYQEESKLERQKIEGVKLESEGTYKILTIYSTVASSLVIAMGFAAFLFWAMSNGSCRCNICI

>UDE32071.1 hemagglutinin [Influenza A virus]
METVSLITILLVATVSNADKICIGYQSTNSTETVDTLTENNVPVTHAKELLHTEHNGMLCATSLGHPLIL
DTCTIEGLIYGNPSCDPLLGGREWSYIVERPSAVNGLCYPGNVENLEELRSLFSSARSYQRIQIFPDTIW
NVSYSGTSKACSDSFYRSMRWLTQKNNAYPIQDAQYTNNQEKNILFMWGINHPPTETAQTNLYTRTDTTT
SVATEEINRIFKPLIGPRPLVNGLMGRINYYWSVLKPGQTLRIKSDGNLIAPWYGHILSGESHGRILKTD
LKRGSCTVQCQTEKGGLNTTLPFQNVSKYAFGNCSKYIGIKSLKLAVGLRNVPSRSSRGLFGAIAGFIEG
GWSGLVAGWYGFQHSNDQGVGMAADRDSTQKAIDKITSKVNNIVDKMNRQYEIIDHEFSEVETRLNMINN
KVDDQIQDIWAYNAELLVLLENQKTLDEHDSNVNNLYNKVKRALGSNAVEDGKGCFELYHKCDDQCMETI
RNGTYNRRKYQEESKLERQKIEGVKLESEGTYKILTIYSTVASSLVIAMGFAAFLFWAMSNGSCRCNICI

>UDE32070.1 hemagglutinin [Influenza A virus]
METVSLITILLVATVSNADKICIGYQSTNSTETVDTLTENNVPVTHAKELLHTEHNGMLCATSLGHPLIL
DTCTIEGLIYGNPSCDLLLGGREWSYIVERPSAVNGLCYPGNVENLEELRSLFSSARSYQRIQIFPDTIW
NVSYSGTSKACSDSFYRSMRWLTQKNNAYPIQDAQYTNNQEKNILFMWGINHPPTDTAQTNLYTRTDTTT
SVATEEINRIFKPLIGPRPLVNGLMGRINYYWSVLKPGQTLRIKSDGNLIAPWYGHILSGESHGRILKTD
LKRGSCTVQCQTEKGGLNTTLPFQNVSKYAFGNCSKYIGIKSLKLAVGLRNVPSRSSRGLFGAIAGFIEG
GWSGLVAGWYGFQHSNDQGVGMAADRDSTQKAIDKITSKVNNIVDKMNKQYEIIDHEFSEVETRLNMINN
KVDDQIQDIWAYNAELLVLLENQKTLDEHDANVNNLYNKVKRALGSNAVEDGKGCFELYHKCDDQCMETI

RNGTYNRRKYQEESKLERQKIEGVKLESEETYKILTIYSTVASSLVIAMGFAAFLFWAMSNGSCRCNICI

>UDE32069.1 hemagglutinin [Influenza A virus]

METVSLITILLVATVSNADKICIGYQSTNSTETVDTLTENNVPVTHAKELLHTEHNGMLCATSLGHPLIL

DTCTIEGLIYGNPSCDPLLGGREWSYIVERPSAVNGLCYPGNVENLEELRSLFSSARSYQRIQIFPDTIW

NVSYSGTSKACSDSFYRSMRWLTQKNNAYPIQDAQYTNNQEKNILFMWGINHPPTETAQTNLYTRTDTTT

SVATEEINRIFKPLIGPRPLVNGLMGRINYYWSVLKPGQTLRIKSDGNLIAPWYGHILSGESHGRILKTD

LKRGSCTVQCQTEKGGLNTTLPFQNVSKYAFGNCSKYIGIKSLKLAVGLRNVPSRSSRGLFGAIAGFIEG

GWSGLVAGWYGFQHSNDQGVGMAADRDSTQKAIDKITSKVNNIVDKMNRQYEIIDHEFSEVETRLNMINN

KVDDQIQDIWAYNAELLVLLENQKTLDEHDSNVNNLYNKVKRALGSNAVEDGKGCFELYHKCDDQCMETI

RNGTYNRRKYQEESKLERQKIEGVKLESEGTYKILTIYSTVASSLVIAMGFAAFLFWAMSNGSCRCNICI

>UDE32068.1 hemagglutinin [Influenza A virus]

METVSLITILLVATVSNADKICIGYQSTNSTETVDTLTENNVPVTHAKELLHTEHNGMLCATSLGQPIIL

DTCTIEGLIYGNPSCDLSLEGREWSYIVERPSAVNGLCYPGNVENLEELRSLFSSARSYQRIQIFPDTIW

NVSYDGTSTACSGSFYRNMRWLTRKNGDYPIQDAQYTNNQGKNILFMWGINHPPADTTQRDLYTRTDTTT

SVATEEINRIFKPLIGPRPLVNGLMGRIDYYWSVLKPGQTLRIRSDGNLIAPWYGHILSGESHGRILKTD

LKRGSCTVQCQTEKGGLNTTLPFQNVSKYAFGNCSKYIGIKSLKLAVGLRNVPSRSSRGLFGAIAGFIEG

GWSGLVAGWYGFQHSNDQGVGMAADRDSTQKAIDKITSKVNNIVDKMNKQYEIIDHEFSEVETRLNMINN

KIDDQIQDIWAYNAELLVLLENQKTLDEHDANVNNLYNKVKRALGSNAVEDGKGCFELYHKCDDQCMETI

RNGTYNRRKYQEESKLERQKIEGVKLESEGTYKILTIYSTVASSLVIAMGFAAFLFWAMSNGSCRCNICI

>UDE32067.1 hemagglutinin [Influenza A virus]

METVSLITILLVATVSNADKICIGYQSTNSTETVDTLTENNVPVTHAKELLHTEHNGMLCATSLGQPLIL

DTCTIEGLIYGNPSCDLLLEGREWSYIVERPSAVNGLCYPGNVENLEELRSLFSSARSYQRIQIFPDTIW

NVSYDGTSTACSGSFYRSMRWLTRKNGNYPIQDAQYTNNQGKNILFMWGINHPPTDTTQRDLYTRTDTTT

SVATEEINRIFKPLIGPRPLVNGLMGRIDYYWSVLKPGQTLRIKSDGNLIAPWYGHILSGESHGRILKTD

LKRGSCTVQCQTEKGGLNTTLPFQNVSKYAFGNCSKYLGIKSLKLAVGLRNVPSRSSRGLFGAIAGFIEG

GWSGLVAGWYGFQHSNDQGVGMAADRDSTQKAIDKITSKVNNIVDKMNKQYEIIDHEFSEVETRLNMINN

KIDDQIQDIWAYNAELLVLLENQKTLDEHDANVNNLYNKVKRALGSNAVEDGKGCFELYHKCDDQCMETI

RNGTYNRRKYQEESKLERQKIEGVKLESEGTYKILTIYSTVASSLVIAMGFAAFLFWAMSNGSCRCNICI

>UDE32066.1 hemagglutinin [Influenza A virus]

METVSLITILLVATVSNADKICIGYQSTNSTETVDTLTENNVPVTHAKELLHTEHNGMLCATSLGQPLVL

DTCTIEGLIYGNPSCDLSLEGREWSYIVERPSAVNGLCYPGNVENLEELRSLFSSARSYQRIQIFPDTIW
NVSYDGTSNACSGSFYRSMRWLTRKNGDYPIQDAQYTNNQGKNILFMWGINHPPTDTTQRDLYTRTDTTT
SVATEEINRIFKPLIGPRPLVNGLMGRIDYYWSVLKPGQTLRIKSDGNLIAPWYGHILSGESHGRILKTD
LKRGSCTVQCQTEKGGLNTTLPFQNVSKYAFGNCSKYIGIKSLKLAVGLRNVPSRSSRGLFGAIAGFIEG
GWSGLVAGWYGFQHSNDQGVGMAADRDSTQKAIDKITSKVNNIVDKMNKQYEIIDHEFSEVETRLNMINN
KIDDQIQDIWAYNAELLVLLENQKTLDEHDANVNNLYNKVKRALGSNAVEDGKGCFELYHKCDDQCMETI
RNGTYNRRKYQEESKLERQKIEGVKLESEGTYKILTIYSTVASSLVIAMGFAAFLFWAMSNGSCRCSICI

>UDE32065.1 hemagglutinin [Influenza A virus]
METVSLITILLVATVSNADKICIGYQSTNSTETVDTLTENNVPVTHAKELLHTEHNGMLCATSLGHPLIL
DTCTIEGLIYGNPSCDPLLGGREWSYIVERPSAVNGLCYPGNVENLEELRSLFSSARSYQRIQIFPDTIW
NVSYSGTSRACSDSFYRSMRWLTQKNNAYPIQDAQYTNNQDKNILFMWGINHPPTETTQTNLYTRTDTTT
SVATEEINRIFKPLIGPRPLVNGLMGRINYYWSVLKPGQTLRIKSDGNLIAPWYGHILSGESHGRILKTD
LKRGSCTVQCQTEKGGLNTTLPFQNVSKYAFGNCSKYIGIKSLKLAVGLRNVPSRSSRGLFGAIAGFIEG
GWSGLVAGWYGFQHSNDQGVGMAADRDSTQKAIDKITSKVNNIVDKMNRQYEIIDHEFSEVETRLNMINN
KVDDQIQDIWAYNAELLVLLENQKTLDEHDSNVNNLYNKVKRALGSNAVEDGKGCFELYHKCDDQCMETI
RNGTYNRRKYQEESKLERQKIEGVKLESEGTYKILTIYSTVASSLVIAMGFAAFLFWAMSNGSCRCNICI

>UDE32063.1 hemagglutinin [Influenza A virus]
METVSLITILLVATVSNADKICIGYQSTNSTETVDTLTENNVPVTHAKELLHTEHNGMLCATSLGQPLIL
DTCTIEGLIYGNPSCDLSLEGREWSYIVERPSAVNGLCYPGNVENLEELRSLFSSARSYQRIQIFXDTIW
NVSYDGTSTACSGSFYRSMRWLTRKNGNYPIQDAQYTNNQGKNILFMWGINHPPTDTTQRDLYTRTDTTT
SVATEEINRIFKPLIGPRPLVNGLMGRIDYYWSVLKPGQTLRIKSDGNLIAPWFGHILSGESHGRILKTD
LKRGSCTVQCQTEKGGLNTTLPFQNVSKYAFGNCSKYIGIKSLKLAVGLRNVPSRSSRGLFGAIAGFIEG
GWSGLVAGWYGFQHSNDQGVGMAADRDSTQKAIDKITSKVNNIVDKMNKQYEIIDHEFSEVETRLNMINN
KIDDQIQDIWAYNAELLVLLENQKTLDEHDANVNNLYNKVKRALGSNAVEDGKGCFELYHKCDDQCMETI
RNGTYNRRKYQEESKLERQKIEGVKLESEGTYKILTIYSTVASSLVIAMGFAAFLFWAMSNGSCRCNICI

>UDE32061.1 hemagglutinin [Influenza A virus]
METVSLITILLVATVSNADKICIGYQSTNSTETVDTLTENNVPVTHAKELLHTEHNGMLCATSLGHPLIL
DTCTIEGLIYGNPSCDPLLGGREWSYIVERPSAVNGLCYPGSVENLEELRSLFSSARSYQRIQIFPDTIW
NVSYSGTSKACSDSFYRSMRWLTQKNNAYPIQDAQYTNNQEKNILFMWGINHPPTETAQTNLYTRTDTTT
SVATEEINRIFKPLIGPRPLVNGLMGRINYYWSVLKPGQTLRIKSDGNLIAPWYGHILSGESHGRILKTD
LKRGSCTVQCQTEKGGLNTTLPFQNVSKYAFGNCSKYIGIKSLKLAIGLRNVPSRSSRGLFGAIAGFIEG

GWSGLVAGWYGFQHSNDQGVGMAADRDSTQKAIDKITSKVNNIVDKMNRQYEIIDHEFSEVETRLNMINN
KVDDQIQDIWAYNAELLVLLENQKTLDEHDSNVNNLYNKVKRALGSNAVEDGKGCFELYHKCDDQCMETI
RNGTYNRRKYQEESKLERQKIEGVKLESEGTYKILTIYSTVASSLVIAMGFAAFLFWAMSNGSCRCNICI

>UDE32060.1 hemagglutinin [Influenza A virus]
METVSLITILLVATVSNADKICIGYQSTNSTETVDTLTENNVPVTHAKELIHTEHNGMLCATSLGQPLIL
DTCTIEGLIYGNPSCDLSLEGREWSYIVERPSAVNGLCYPGNVENLEELRSLFSSARSYQRIQIFPDTIW
NVSYDGTSTACSGSFYRNMRWLTRKNGNYPTQDVQYTNNQGKNILFMWGINHPPTDTTQSGLYTRTDTTT
SVATEEINRIFKPLIGPRPLVNGLMGRIDYYWSVLKPGQTLRIKSDGNLIAPWFGHILSGESHGRILKTD
LKRGSCTVQCQTEKGGLNTTLPFQNVSKYAFGNCSKYIGIKSLKLAVGLRNVPSRSSRGLFGAIAGFIEG
GWSGLVAGWYGFQHSNDQGVGMAADRDSTQKAIDKITSKVNNIVDKMNKQYEIIDHEFSEVETRLNMINN
KIDDQIQDIWAYNAELLVLLENQKTLDEHDANVNNLYNKVKRALGSNAVEDGKGCFELYHKCDDQCMETI
RNGTYNRRKYQEESKLERQKIEGVKLESEGTYKILTIYSTVASSLVIAMGFAAFLFWAMSNGSCRCNICI

>UDE32059.1 hemagglutinin [Influenza A virus]
METISLITILLVATVSNADKICIGYQSTNSTETVDTLTENNVPVTHAKELLHTEHNGMLCATSLGQPLIL
DTCTIEGLIYGNPSCDLSLEGREWSYIVERPSAVNGLCYPGNVENLEELRSLFSSARSYQRIQIFPDTIW
NVSYDGTSTACSGSFYRSMRWLTRKNGDYPTQDAQYTNNQGKNILFMWGINHPPTDDTQRNLYTRTDTTT
SVATEEINRIFKPLIGPRPLVNGLMGRIDYYWSVLKPGQTLRIKSDGNLIAPWYGYILSGESHGRILKTD
LKRGSCTVQCQTEKGGLNTTLPFQNVSKYAFGNCSKYIGIKSLKLAVGLRNVPSRSSRGLFGAIAGFIEG
GWSGLVAGWYGFQHSNDQGVGMAADRDSTQKAIDKITSKVNNIVDKMNKQYEIIDHEFSEVETRLNMINN
KIDDQIQDIWAYNAELLVLLENQKTLDEHDANVNNLYNKVKRALGSNAVEDGKGCFELYHKCNDQCMETI
RNGTYNRRKYQEESKLERQKIEGVKLESEGTYKILTIYSTVASSLVIAMGFAAFLFWAMSNGSCRCNICI

>UDE32058.1 hemagglutinin [Influenza A virus]
METXSLITILLVATVSNADKICIGYQSTNSTETVDTLTENNVPVTHAKELLHTEHNGMLCATSLGQPLIL
DTCTIEGLIYGNPSCDLSLEGREWSYIVERPSAVNGLCYPGNVENLEELRSLFSSARSYQRIQIFPDTIW
NVSYDGTSTACSGSFYRSMRWLTRKNGDYPTQDAQYTNNQGKNILFMWGINHPPTDDTQRNLYTRTDTTT
SVATEEINRIFKPLIGPRPLVNGLMGRIDYYWSVLKPGQTLRIKSDGNLIAPWYGYILSGESHGRILKTD
LKRGSCTVQCQTXKGGLNTTLPFQNVSKYAFGNCSKYIGIKSLKLAVGLRNVPSRSSRGLFGAIAGFIEG
GWSGLVAGWYGFQHSNDQGVGMAADRDSTQKAIDKITSKVNNIVDKMNKQYEIIDHEFSEVETRLNMINN
KIDDQIQDIWAYNAELLVLLENQKTLDEHDANVNNLYNKVKRALGSNAVEDGKGCFELYHKCNDQCMETI
RNGTYNRRKYQEESKLERQKIEGVKLESEGTYKILTIYSTVASSLVIAMGFAAFLFWAMSNGSCRCNICI

>UDE32056.1 hemagglutinin [Influenza A virus]

METISLITILLVATVSNADKICIGYQSTNSTETVDTLTENNVPVTHAKELLHTEHNGMLCATGLGQPLIL
DTCTIEGLIYGNPSCDLSVEGREWSYIVERPSAVNGLCYPGNVENLEELRSLFSSARSYQRIQIFPDTIW
NVSYDGTSTACSGSFYRSMRWLTRKNGEYPIQDAQYTNNQGKNILFMWGINHPPTDTTQRDLYTRTDTTT
SVATEEINRVFKPLIGPRPLVNGLMGRIDYYXSVLKPGQTLRIKSDGNLIAPWYGHILSGESHGRILKTD
LKKGSCTVQCQTEKGGLNTTLPFQNVSKYAFGNCSKYIGIKSLKLAVGLRNVPSRSSRGLFGAIAGFIEG
GWSGLVAGWYGFQHSNDQGVGMAADRDSTQKAIDKITSKVNNIVDKMNKQYEIIDHEFSEVETRLNMINN
KIDDQIQDIWAYNAELLVLLENQKTLDEHDANVNNLYNKVKRALGSNAVEDGKGCFELYHKCDDQCMETI
RNGTYNRRKYQEESKLERQKIEGVKLESEGTYKILTIYSTVASSLVIAMGFAAFLFWAMSNGSCRCNICI

>UDE32055.1 hemagglutinin [Influenza A virus]

METVSLITILLVATASNADKICIGYQSTNSTETVDTLTENNVPVTHAKELLHTEHNGMLCATSLGQPLIL
DTCTIEGLIYGNPSCDLSLEGREWSYIVERPSAVNGLCYPGNVENLEELRSLFSSARSYQRIQIFPDTIW
NVSYDGTSTACSGSFYRSMRWLTRKNGDYPTQDAQYTNNQGKNILFMWGINHPPTDDTQRNLYTRTDTTT
SVATEEINRIFKPLIGPRPLVNGLMGRIDYYWSVLKPGQTLRIKSDGNLIAPWYGHILSGESHGRILKTD
LKRGSCTVQCQTEKGGLNTTLPFQNVSKYAFGNCSKYIGIKSLKLAVGLRNVPSRSSRGLFGAIAGFIEG
GWSGLVAGWYGFQHSNDQGVGMAADRDSTQKAIDKITSKVNNIVDKMNKQYEIIDHEFSEVETRLNMINN
KIDDQIQDIWAYNAELLVLLENQKTLDEHDANVNNLYNKVKRALGSNAVEDGKGCFELYHKCNDQCMETI
RNGTYNRRMYQEESKLERQKIEGVKLESEGTYKILTIYSTVASSLVIAMGFAAFLFWAMSNGSCRCNICI

>UDE32054.1 hemagglutinin [Influenza A virus]

METVSLITILLVATVSNADKICIGYQSTNSTETVDTLTENNVPVTHAKELLHTEHNGMLCATSLGQPLIL
DTCTIEGLIYGNPSCDLSLEGREWSYIVERPSAVNGLCYPGNVENLEELRSLFSSARSYQRIQIFPDTIW
NVSYDGTSTACSGSFYRSMRWLTRKNGDYPTQDAQYTNNQGKNILFMWGINHPPTDDTQRNLYTRTDTTT
SVATEEINRIFKPLIGPRPLVNGLMGRIDYYWSVLKPGQTLRIKSDGNLIAPWYGHILSGESHGRILKTD
LKRGSCTVQCQTEKGGLNTTLPFQNVSKYAFGNCSKYIGIKSLKLAVGLRNVPSRSSRGLFGAIAGFIEG
GWSGLVAGWYGFQHSNDQGVGMAADRDSTQKAIDKITSKVNNIVDKMNKQYEIIDHEFSEVETRLNMINN
KIDDQIQDIWAYNAELLVLLENQKTLDEHDANVNNLYNKVKRALGSNAVEDGKGCFELYHKCNDQCMETI
RNGTYNRRKYQEESKLERQKIEGVKLESEGTYKILTIYSTVASSLVIAMGFAAFLFWAMSNGSCRCNICI

>UDE32053.1 hemagglutinin [Influenza A virus]

METISLITILLVATVSNADKICIGYQSTNSTETVDTLTENNVPVTHAKELLHTEHNGMLCATSLGQPLIL
DTCTIEGLIYGNPSCDLSLEGREWSYIVERPSAVNGLCYPGNVENLEELRSLFSSARSYQRIQIFPDTIW
NVSYDGTSTACSGSFYRSMRWLTRKNGDYPTQDAQYTNNQGKNILFMWGINHPPTDDTQRNLYTRTDTTT

SVATEEINRIFKPLIGPRPLVNGLMGRIDYYWSVLKPGQTLRIKSDGNLIAPWYGLILSGESHGRILKTD
LKRGSCTVQCQTEKGGLNTTLPFQNVSKYAFGNCSKYIGIKSLKLAVGLRNVPSRSSRGLFGAIAGFIEG
GWSGLVAGWYGFQHSNDQGVGMAADRDSTQKAIDKITSKVNNIVDKMNKQYEIIDHEFSEVETRLNMINN
KIDDQIQDIWAYNAELLVLLENQKTLDEHDANVNNLYNKVKRALGSNAVEDGKGCFELYHKCNDQCMETI
RNGTYNRRKYQEESKLERQKIEGVKLESEGTYKILTIYSTVASSLVIAMGFAAFLFWAMSNGSCRCNICI

>UDE32051.1 hemagglutinin [Influenza A virus]
METISLITILLVVTVSNADKICIGYQSTNSTETVDTLTENNVPVTHAKELLHTEHNGMLCATNLGHPLIL
DTCTIEGLIYGNPSCNLLLGGREWSYIVERPSAVNGLCYPGNVENLEELRSLFSSASSYQRIQIFPDTIW
NVSYSGTSKACSDSFYRSMRWLTQKNNTYPIQDAQYTNNRGKNILFMWGINHPPTDTVQTNLYTRTDTTT
SVATEDINRTFKPLIGPRPLVNGLQGRIDYYWSVLKPGQTLRVRSNGNLIAPWYGHILSGESHGRILKTD
LNSGNCVVQCQTERGGLNTTLPFHNVSKYAFGNCPKYVGVKSLKLAVGLRNVPARSSRGLFGAIAGFIEG
GWSGLVAGWYGFQHSNDQGVGMAADRDSTQKATDKITSKVNNIVDKMNKQYEIIDHEFSEVETRLNMIND
KIDDQIQDIWAYNAELLVLLENQKTLDEHDANVNNLYNKVKRTLGSNAVEDGKGCFELYHKCDDQCMETI
RNGTYDRRKYKEESRLERQKIEGVKLESEGTYKILTIYSTVASSLVIAMGFAAFLFWAMSNGSCRCNICI

>UDE32050.1 hemagglutinin [Influenza A virus]
METISLITILLVATVSSADKICIGYQSTNSTETVDTLTENNVPVTHAKELLHTEHNGMLCATSLGQPLIL
DTCTIEGIIYGNPSCDLSLEGREWSYIVERPSAVNGLCYPGNVENLEELRSLFSSARSYQRIQIFPDTIW
NVSYDGTSTACSGSFYKSMRWLTRKNGDYPIQDAQYTNNQGKNILFMWGINHPPTDTTQRDLYTRTDTTT
SVATEEINRVFKPLIGPRPLVNGLMGRIDYYWSVLKPGQTLRIKSDGNLIAPWFGHILSGESHGRILKTD
LKRGSCTVQCQTEKGGLNTTLPFQNVSKYAFGNCSKYIGIKSLKLAVGLRNVPSRSSRGLFGAIAGFIEG
GWSGLVAGWYGFQHSNDQGVGMAADRDSTQKAIDKITSKVNNIVDKMNKQYEIIDHEFSEVETRLNMINN
KIDDQIQDIWAYNAELLVLLENQKTLDEHDANVNNLYNKVKRALGSNAVEDGKGCFELYHKCDDQCMETI
RNGTYNRRKYQEESKLERQKIEGVKLESEGTYKILTIYSTVASSLVIAMGFAAFLFWAMSNGSCRCNICI

>UDE32049.1 hemagglutinin [Influenza A virus]
METISLITILLGKQQVSNADKICIGYQSTNSTETVDTLTENNVPVTHAKELLHTEHNGMLCATSLGHPLI
LDTCTIEGLIYGNPSCDPLLGGREWSYIVERPSAVNGLCYPGNVENLEELRSLFSSARSYQRIQIFPDTI
WNVSYDGTSKACSDSFYRSMRWLTQKNNAYPIQDAQYTNNQEKNILFMWGINHPPTETAQTNLYTRTDTT
TSVATEEINRIFKPLIGPRPLVNGLMGRINYYWSVLKPGQTLRIKSDGNLIAPWYGHILSGESHGRILKT
DLKRGSCTVQCQTEKGGLNTTLPFQNVSKYAFGNCSKYIGIKSLKLAIGLRNVPSRSSRGLFGAIAGFIE
GGWSGLVAGWYGFQHSNDQGVGMAADRDSTQKAIDKITSKVNNIVDKMNRQYEIIDHEFSEVETRLNMIN
NKVDDQIQDIWAYNAELLVLLENQKTLDEHDSNVNNLYNKVKRALGSNAVEDGKGCFELYHKCDDQCMET

IRNGTYNRRKYQEESKLERQKIEGVKLESEGTYKILTIYSTVASSLVIAMGFAAFLFWAMSNGSCRCNIC
I

>UDE32047.1 hemagglutinin, partial [Influenza A virus]
SLITILLVATVSNADKICIGYQSTNSTETVDTLTENNVPVTHAKELLHTEHNGMLCATSLGHPLILDTCT
IEGLIYGNPSCDPLLGGREWSYIVERPSAVNGLCYPGNVENLEELRSLFSSARSYQRIQIFPDTIWNVSY
NGTSTACSDSFYRSMRWLTQKNNXYPIQDAQYTNNQGKNILFMWGINHPPTDTAQTNLYTRTDTTTSVAT
EEINRIFKPLIGPRPLVNGLMGRINYYWSVLKPGQTLRIKSDGNLIAPWYGHILSGESHGRILKTDLKRG
SCTVQCQTEKGGLNXTLPFQNVSKYAFGNCSKYIGIKSLKLAVGLRNVPSRSSRGLFGAIAGFIEGGWSG
LVAGWYGFQHSNDQGVGIAADRDSTQKAIDKITSKVNNIVDKMNKQYEIIDHEFSEVETRLNMINNKVDD
QIQDIWAYNAELLVLLENQKTLDEHDANVNNLYNKVKRALGSNAVEDGKGCFELYHKCDDQCMETIRNGT
YNRRKYQEESKLERQKIEGVKLESEETYKILTIYSTVASSLVIAMGFAAFLFWAMSNGSCRCNICI

>UDE32046.1 hemagglutinin [Influenza A virus]
METISLITILLVATVSNADKICIGYQSTNSTETVDTLTENNVPVTHAKELLHTEHNGMLCATSLGHPLIL
DTCTIEGLIYGNPSCDPLLGGREWSYIVERPSAVNGLCYPGNVENLEELRSLFSSARSYQRIQIFPDTIW
NVSYSGTSKACSDSFYRSMRWLTQKNNAYPIQDAQYTNNQEKNILFMWGINHPPTETVQTNLYTRTDTTT
SVATEEINRIFKPLIGPRPLVNGLMGRINYYWSVLKPGQTLRIKSDGNLIAPWYGHILSGESHGRILKTD
LKRGSCTVQCQTEKGGLNTTLPFQNVSKYAFGNCSKYIGIKSLKLAIGLRNVPSRSSRGLFGAIAGFIEG
GWSGLVAGWYGFQHSNDQGVGMAADRDSTQKAIDKITSKVNNIVDKMNRQYEIIDHEFSEVETRLNMINN
KVDDQIQDIWAYNAELLVLLENQKTLDEHDSNVNNLYNKVKRALGSNAVEDGKGCFELYHKCDDQCMETI
RNGTYNRRKYQEESKLERQKIEGVKLESEGTYKILTIYSTVASSLVIAMGFAAFLFWAMSNGSCRCNICI

>UDE32045.1 hemagglutinin [Influenza A virus]
METVSLITILLVATVSNADKICIGYQSTNSTETVDTLTENNVPVTHAKELLHTEHNGMLCATSLGQPLIL
DTCTIEGLIYGNPSCDLSLEGREWSYIVERPSAVNGLCYPGNVENLEELRSLFSSARSYQRIQIFPDTIW
NVSYDGTSTACSGSFYRSMRWLTRKNGDYPTQDAQYTNNQGKNILFMWGINHPPTDDTQRNLYTRTDTTT
SVATEEINRIFKPLIGPRPLVNGLMGRIDYYWSVLKPGQTLRIKSDGNLIAPWYGHILSGESHGRILKTD
LKRGSCTVQCQTEKGGLNTTLPFQNVSKYAFGNCSKYIGIKSLKLAVGLRNVPSRSSRGLFGAIAGFIEG
GWSGLVAGWYGFQHSNDQGVGMAADRDSTQKAIDKITSKVNNIVDKMNKQYEIIDHEFSEVETRLNMINN
KIDDQIQDIWAYNAELLVLLENQKTLDEHDANVNNLYNKVKRALGSNAVEDGKGCFELYHKCNDQCMETI
RNGTYNRRKYQEESKLERQKIEGVKLESEGTYKILTIYSTVASSLVIAMGFAAFLFWAMSNGSCRCNICI

>UDE32044.1 hemagglutinin [Influenza A virus]

METISLITILLVVTVSNADKICIGYQSTNSTETVDTLTENNVPVTHAKELLHTEHNGMLCATGLGHPLIL
DTCTIEGLIYGNPSCDLLLGGREWSYIVERPSAVNGLCYPGNVENLEELRSLFSSASSYQRIQIFPDTIW
NVTYSGTSKACSDSFYRSMRWLTQKNNAYPIQDAQYTNNQEKNILFMWGINHPPTDTVQTNLYTRTDTTT
SVATEEINRTFKPLIGPRPLVNGLQGRIDYYWSVLKPGQTLRIRSNGNLIAPWYGHILSGGSHGRILKTD
LKRGSCTVQCQTEKGGLNTTLPFQNISKYAFGNCSKYIGIKSLKLAVGLRNVPSRSSRGLFGAIAGFIEG
GWSGLVAGWYGFQHSNDQGVGMAADRDSTQKAIDKITSKVNNIVDKMNKQYEIIDHEFSEVEARLNMINN
KIDDQIQDIWAYNAELLVLLENQKTLDEHDANVNNLYNKVKRALGSNAMEDGKGCFELYHKCDNQCMETI
RNGTYNRRKYQEESKLERQKIEGVKLESEGTYKILTIYSTVASSLVIAMGFAAFLFWAMSNGSCRCNICI

>UDE32043.1 hemagglutinin [Influenza A virus]
METVSLITILLVATVSNADKICIGYQSTNSTETVDTLTENNVPVTHAKELLHTEHNGMLCATSLGQPLIL
DTCTIEGLIYGNPSCDLSLEGREWSYIVERPSAVNGLCYPGNVENLEELRSLFSSARSYQRIQIFPDTIW
NVSYDGTSTACSGSFYRSMRWLTRKNGDYPTQDAQYTNNQGKNILFMWGINHPPTDDTQRNLYTRTDTTT
SVATEEINRIFKPLIGPRPLVNGLMGRIDYYWSVLKPGQTLRIKSDGNLIAPWYGHILSGESHGRILKTD
LKRGSCTVQCQTEKGGLNTTLPFQNVSKYAFGNCSKYIGIKSLKLAVGLRNVPSRSSRGLFGAIAGFIEG
GWSGLVAGWYGFQHSNDQGVGMAADRDSTQKAIDKITSKVNNIVDKMNKQYEIIDHEFSEVETRLNMINN
KIDDQIQDIWAYNAELLVLLENQKTLDEHDANVNNLYNKVKRALGSNAVEDGKGCFELYHKCNDQCMETI
RNGTYNRRKYQEESKLERQKIEGVKLESEGTYKILTIYSTVASSLVIAMGFAAFLFWAMSNGSCRCNICI

>UDE32042.1 hemagglutinin [Influenza A virus]
METVSLITILLVATVSNADKICIGYQSTNSTETVDTLTENNVPVTHAKELLHTEHNGMLCATSLGQPIIL
DTCTIEGLIYGNPSCDLSLEGREWSYIVERPSAVNGLCYPGNVENLEELRSLFSSARSYQRIQIFPDTIW
NVSYDGTSTACSGSFYRNMRWLTRKNGDYPIQDAQYTNNQGKNILFMWGINHPPADTTQRNLYTRTDTTT
SVATEEINRIFKPLIGPRPLVNGLMGRIDYYWSVLKPGQTLRIKSDGNLIAPWYGHILSGESHGRILKTD
LKRGSCTVQCQTEKGGLNTTLPFQNVSKYAFGNCSKYIGIKSLKLAVGLRNVPSRSSRGLFGAIAGFIEG
GWSGLVAGWYGFQHSNDQGVGMAADRDSTQKAVDKITSKVNNIVDKMNKQYEIIDHEFSEVETRLNMINN
KIDDQIQDIWAYNAELLVLLENQKTLDEHDANVNNLYNKVKRALGSNAVEDGKGCFELYHKCDDQCMETI
RNGTYNRRKYQEESKLERQKIEGVKLESEGTYKILTIYSTVASSLVIAMGFAAFLFWAMSNGSCRCNICI

>UDE32041.1 hemagglutinin [Influenza A virus]
METVSLITILLVATVSYADKICIGYQSTNSTETVDTLTENNVPVTHAKELLHTEHNGMLCATSLGQPLIL
DTCTIEGLIYGNPSCDLSLEGREWSYIVERPSAVNGLCYPGNVENLEELRSLFSSARSYQRVQIFPDTIW
NVSYDGTSTACSGSFYRSMRWLTRKNDNYPTQDAQYTNNQGKNILFMWGINHPPTDDTQRNLYTRTDTTT
SVATEEINRIFKPLIGPRPLVNGLMGRINYYWSVLKPGQTLRIKSDGNLIAPWYGYILSGESHGRILKTD

LKRGSCTVQCQTEKGGLNTTLPFQNVSKYAFGNCSKYIGIKSLKLAVGLRNVPSRSSRGLFGAIAGFIEG
GWSGLVAGWYGFQHSNDQGVGMAADRDSTQKAIDKITSKVNNIVDKMNKQYEIIDHEFSEVETRLNMINN
KIDDQIQDIWAYNAELLVLLENQKTLDEHDANVNNLYNKVKRSLGSNAVEDGKGCFELYHKCNDQCMETI
RNGTYNRRKYQEESKLERQKIEGVKLESEGTYKILTIYSTVASSLVIAMGFAAFLFWAMSNGSCRCNICI

>UDE32040.1 hemagglutinin [Influenza A virus]
METVSLITILLVATVSNADKICIGYQSTNSTETVDTLTENNVPVTHAKELLHTEHNGMLCATSLGQPLIL
DTCTIEGLIYGNPSCDLSLEGREWSYIVERPSAVHGLCYPGNVEDLEELRSLFSSARSYQRIQIFPDTIW
NVSYDGTSTACSGSFYRSMRWLTRKNGEYPIQDAQYTNNQGKNILFMWGINHPPTDVTQRGLYTRTDTTT
SVATEEINRIFKPLIGPRPLVNGLMGRINYYWSVLKPGQTLRIKSDGNLIAPWYGHILSGESHGRILKTD
LKRGSCTVQCQTEKGGLNTTLPFQNVSKYAFGNCSKYIGIKSLKLAVGLRNVPSRSSRGLFGAIAGFIEG
GWSGLVAGWYGFQHSNDQGVGMAADRGSTQKAIDKITSKVNNIVDKMNKQYEIIDHEFSEVETRLNMINN
KIDDQIQDIWAYNAELLVLLENQKTLDEHDANVNNLYNKVKRALGSNAVEDGKGCFELYHKCDDQCMETI
RNGTYNRRKYQEESKLERQRIEGVKLESEGTYKILTIYSTVASSLVIAMGFAAFLFWAMSNGSCRCNICI

>UDE32039.1 hemagglutinin [Influenza A virus]
METVSLITILIVATVSNADKICIGYQSTNSTETVDTLTENNVPVTHAKELLHTEHNGMLCATSLGQPLIL
DTCTIEGLIYGNPSCDLSLEGREWSYIVERPSAINGLCYPGNVENLEELRSLFSSARSYQRIQIFPDTIW
NVSYDGTSTACSNSFYRSMRWLTRKDGNYPTQDAQYTNNQGKNILFMWGINHPPTDDTQRNLYTRTDTTT
SVATEEINRIFKPLIGPRPLVNGLMGRIDYYWSVLKPGQTLRIKSDGNLIAPWYGHILSGESHGRILKTD
LKRGSCTVQCQTEKGGLNTTLPFQNVSKYAFGNCSKYIGIKSLKLAVGLRNVPSRSSRGLFGAIAGFIEG
GWSGLVAGWYGFQHSNDQGVGMAADRDSTQKAIDKITSKVNNIVDKMNKQYEIIDHEFSEVETRLNMINN
KIDDQIQDIWAYNAELLVLLENQKTLDEHDANVNNLYNKVKRALGSNAVEDGKGCFELYHKCNDQCMETI
RNGTYNRRKYQEESKLERQKIEGVKLESEGTYKILTIYSTVASSLVIAMGFAAFLFWAMSNGSCRCNICI

>UDE32038.1 hemagglutinin [Influenza A virus]
METISLMTILLVATVSNADKICIGYQSTNSTETVDTLTESNVPVTHAKELLHTEHNGMLCATSLGNPLIL
DTCTIEGLIYGNPSCDPLLGGREWSYIVERPSAVNGLCYPGSVENLEELRSLFSSARSYQRIQIFPDTIW
NVSYSGTSKACSDSFYRSMRWLTQKNNAYPIQDAQYINNQEKNILFMWGINHPPTETVQTNLYTRTDTTT
SVATEEINRIFKPLIGPRPLVNGLMGRINYYWSVLKPGQTLRIKSDGNLIAPWYGHILSGESHGRILKTD
LKRGSCTVQCQTEKGGLNTTLPFQNVSKYAFGNCSKYIGIKSLKLAVGLRNVPSRSSRGLFGAIAGFIEG
GWSGLVAGWYGFQHSNDQGVGMAADRDSTQKAIDKITSKVNNIVDKMNKQYEIIDHEFSEVETRLNMINN
KVDDQIQDIWAYNAELLVLLENQKTLDEHDSNVNNLYNKVKRALGSNAVEDGKGCFELYHKCDNQCMETI
RNGTYNRRKYQEESKLERQKIEGVKLESEGTYKILTIYSTVASSLVIAMGFAAFLFWAMSNGSCRCNICI

>UDE32037.1 hemagglutinin [Influenza A virus]

METVSLITILLVATVSNADKICIGYQSTNSTETVDTLTENNVPVTHAKELIHTEHNGMLCATSLGQPLIL

DTCTIEGLIYGNPSCDLSLEGREWSYVVERPSAVNGLCYPGNVENLEELRSLFSSARSYQRVQIFPDTIW

NVSYDGTSIACSGSFYRNMRWLTRKDGNYPTQDAQYTNNQGKNILFMWGINHPPTDTTQSGLYTRTDTTT

SVATEEINRIFKPLIGPRPLVNGLMGRIDYYWSVLKPGQTLRIKSDGNLIAPWFGHILSGESHGRILKTD

LKRGSCTVQCQTEKGGLNTTLPFQNVSKYAFGNCSKYIGIKSLKLAVGLRNVPSRSSRGLFGAIAGFIEG

GWSGLVAGWYGFQHSNDQGVGMAADRDSTQKAIDKITSKVNNIVDKMNKQYEIIDHEFSEVETRLNMINN

KIDDQIQDIWAYNAELLVLLENQKTLDEHDANVNNLYNKVKRALGSNAVEDGKGCFELYHKCDDQCMETI

RNGTYNRRKYQEESKLERQKIEGVKLESEGTYKILTIYSTVASSLVIAMGFAAFLFWAMSNGSCRCNICI

>UDE32036.1 hemagglutinin [Influenza A virus]

METVSLITILLVATVGNADKICIGYQSTNSTETVDTLTENNVPVTHAKELLHTEHNGMLCATSLGQPLIL

DTCTIEGLIYGNPSCDLSLEGREWSYIVERPSAVHGLCYPGNVEDLEELRSLFSSARSYQRIQIFPDTIW

NVSYDGTSTACSGSFYRSMRWLTRKNGEYPIQDAQYTNNQGKNILFMWGINHPPTDDTQRGLYTRTDTTT

SVATEEINRIFKPLIGPRPLVNGLMGRINYYWSVLKPGQTLRIKSDGNLIAPWYGHILSGESHGRILKTD

LKRGSCTVQCQTEKGGLNTTLPFQNVSKYAFGNCSKYIGIKSLKLAVGLRNVPSRSSRGLFGAIAGFIEG

GWPGLVAGWYGFQHSNDQGVGMAADRDSTQKAIDKITSKVNNIVDKMNKQYEIIDHEFSEVETRLNMINN

KIDDQIQDIWAYNAELLVLLENQKTLDEHDANVNNLYSKVKRALGSNAVEDGKGCFELYHKCDDQCMETI

RNGTYNRRKYQEESKLERQRIEGVKLESEGTYKILTIYSTVASSLVIAMGFAAFLFWAMSNGSCRCNICI

>UDE32035.1 hemagglutinin [Influenza A virus]

METVSLITILLVAAVSNADKICIGYQSTNSTETVDTLTENNVPVTHAKELLHTEHNGMLCATSLGQPIIL

DTCTIEGLIYGNPSCDLSLEGREWSYIVERPSAVNGLCYPGNVENLEELRSLFSSARSYQRIQIFPDTIW

NVSYDGTSTACSGSFYRNMRWLTRKNGEYPIQDAQYTNNQGKNILFMWGINHPPADTTQRDLYTRTDTTT

SVATEEINRIFKPLIGPRPRVNGLMGRIDYYWSVLKPGQTLRIKSDGNLIAPWYGHILSGESHGRILKTD

LKRGSCTVQCQTEKGGLNTTLPFQNVSKYAFGNCSKYIGIKSLKLAVGLRNVPSRSSRGLFGAIAGFIEG

GWSGLVAGWYGFQHSNDQGVGMAADRDSTQKAVDKITSKVNTIVDKMNKQYEIIDHEFSEVETRLNMINN

KIDDQIQDIWAYNAELLVLLENQKTLDEHDANVNNLYNKVKRALGSNAVEDGKGCFELYHKCDDQCMETI

RNGTYNRRKYQEESKLERQKIEGVKLESEGTYKILTIYSTVASSLVIAMGFAAFLFWAMSNGSCRCNICI

>UDE32034.1 hemagglutinin [Influenza A virus]

METVSLITILLAATVSNADKICIGYQSTNSTETVDTLTENNVPVTHAKELLHTEHNGMLCATSLGQPLIL

DTCTIEGLIYGNPSCDPLLEEREWSYIVERPSAVNGLCYPGNVENLEELRSLFSSARSYQRIQIFPDTIW

NVSYDGTSNTCSGSFYRNMRWLTRKNGNYPIQDAQYTNNQGKNILFMWGINHPPTDDTQRNLYTRTDTTT
SVATEEINRIFKPLIGPRPLVNGLMGRINYYWSVLKPGQTLRIKSDGNLIAPWYGYILSGESHGRILRTD
LKRGSCTVQCQTEKGGLNTTLPFQNVSKYAFGNCSKYIGIKSLKLAVGLRNVPSRSSRGLFGAIAGFIEG
GWSGLVAGWYGFQHSNDQGVGMAADRESTQKAIDKITSKVNNIVDKMNKQYEIIDHEFSEVETRLNMINN
KIDDQIQDIWAYNAELLVLLENQKTLDEHDANVNNLYNKIKRALGSNAVEDGKGCFELYHKCDDQCMETI
RNGTYNRRKYQEESKLERQKIEGVKLESEGTYKILTIYSTVASSLVIAMGFAAFLFWAMSNGSCRCNICI

>UDE32033.1 hemagglutinin [Influenza A virus]
METVSLITILLVATVSNADKICIGYQSTNSTETVDTLTENNVPVTHAKELIHTEHNGMLCATSLGQPLIL
DTCTIEGLIYGNPFCGLSLEGREWSYIVERPSAVNGLCYPGNVENLEELRSLFSSARSYQRIQIFPDTIW
NVSYDGTSAACSGSFYRNMRWLTRKDGNYPTQDAQYTNNQGKNILFMWGINHPPTDTTQRDLYTRTDTTT
SVATEEINRIFKPLIGPRPLVNGLMGRIDYYWSVLKPGQTLRIKSDGNLIAPWFGHILSGESHGRILKTD
LKRGSCTVQCQTEKGGLNTTLPFQNVSKYAFGNCSKYIGIKSLKLAVGLRNVPSRSSRGLFGAIAGFIEG
GWSGLIAGWYGFQHSNDQGVGMAADRDSTQKAIDKITSKVNNIVDKMNKQYEIIDHEFSEVETRLNMINN
KIDDQIQDIWAYNAELLVLLENQKTLDEHDANVNNLYNKVKRALGSNAVEDGKGCFELYHKCDDQCMETI
RNGTYNRRKYQEESKLERQKIEGVKLESEGTYKILTIYSTVASSLVIAMGFAAFLFWAMSNGSCRCNICI

>UDE32032.1 hemagglutinin [Influenza A virus]
METVSLITILLVATVSYADKICIGYQSTNSTETVDTLTESNVPVTHAKELLHTEHNGMLCATSLGQPLIL
DTCTIEGLIYGNPSCDLSLEGREWSYIVERPSAVNGLCYPGNVENLEELRSLFSSARSYQRVQIFPDTIW
NVSYDGTSTACSGSFYRSMRWLTRKDGNYPTQDAQYTNNQGKNILFMWGINHPPTDDTQRSLYTRTDTTT
SVATEEINRIFKPLIGPRPLVNGLMGRIDYYWSVLKPGQTLRIKSDGNLIAPWYGYILSGESHGRILKTD
LKRGSCTVQCQTEKGGLNTTLPFQNVSKYAFGNCSKYIGIKSLKLAVGLRNVPSRSSRGLFGAIAGFIEG
GWSGLVAGWYGFQHSNDQGVGMAADRDSTQKAIDKITSKVNNIVDKMNKQYEIIDHEFSEVETRLNMINN
KIDDQIQDIWAYNAELLVLLENQKTLDEHDANVNNLYNKVKRALGSNAAEDGKGCFELYHKCNDQCMETI
RNGTYNRRKYQEESKLERQKIEGVKLESEGTYKILTIYSTVASSLVIAMGFAAFLFWAMSNGSCRCNICI

>UDE32031.1 hemagglutinin [Influenza A virus]
METISLMTILLVATVSNADKICIGYQSTNSTETVDTLTENNVPVTHAKELLHTEHNGMLCATSLGNPLIL
DTCTIEGLIYGNPSCDPLLGGREWSYIVERPSAVNGLCYPGSVENLEELRSLFSSARSYQRIQIFPDTIW
NVSYSGTSKACSDSFYRSMRWLTQKNNAYPIQDAQYTNNQEKNILFMWGINHPPTETVQTNLYTRTDTTT
SVATEEINRIFKPLIGPRPLVNGLMGRINYYWSVLKPGQTLRIKSDGNLIAPWYGHILSGESHGRILKTD
LKRGSCTVQCQTEKGGLNTTLPFQNVSKYAFGNCSKYIGIKSLKLAVGLRNVPSRSSRGLFGAIAGFIEG
GWSGLVAGWYGFQHSNDQGVGMAADRDSTQKAIDKITSKVNNIVDKMNKQYEIIDHEFSEVETRLNMINN

KVDDQIQDIWAYNAELLVLLENQKTLDEHDSNVNNLYNKVKRALGSNAVEDGKGCFELYHKCDNQCMETI
RNGTYNRRKYQEESKLERQKIEGVKLESEGTYKILTIYSTVASSLVIAMGFAAFLFWAMSNGSCRCNICI

>UDE32030.1 hemagglutinin [Influenza A virus]
METVSLITILIVATVSNADKICIGYQSTNSTETVDTLTENNVPVTHAKELLHTEHNGMLCATSLGHPLIL
DTCTIEGLIYGNPSCDPLLGGREWSYIVERPSAVNGLCYPGNVENLEELRSLFSSSRSYQRIQIFPDTIW
NVSYSGTSKACSDSFYRSMRWLTQKNNAYPTQDAQYTNNQGKNILFVWGINHPPTDTVQTNLYTRTDTTT
SVATEEMNRIFKPLIGPRPLVNGLMGRINYYWSVLKPGQTLRIKSDGNLIAPWYGHILSGESHGRILKTD
LKRGSCTVQCQTEKGGLNTTLPFQNVSKYAFGNCSKYIGVKSLKLAVGLRNVPSRSSRGLFGAIAGFIEG
GWSGLVAGWYGFQHSNDQGVGMAADRDSTQKAIGKITSKVNNIVDKMNKQYEIIDHEFSEVETRLNMINN
KVDDQIQDIWAYNAELLVLLENQKTLDEHDANVNNLYNKVKRALGSNAVEDGKGCFELYHKCDDHCMETI
RNGTYNRRKYQEESKLERQKIEGVKLESEETYKILTIYSTVASSLVIAMGFAAFLFWAMSNGSCRCNICI

>UDE32029.1 hemagglutinin [Influenza A virus]
METVSLITILLVATVSNADKICIGYQSTNSTETVDTLTENNVPVTHAKELLHTEHNGMLCATSLGQPLIL
DTCTIEGLIYGNPSCDLSLEGREWSYIVERPSAVHGLCYPGNVEDLEELRSLFSSARSYQRIQIFPDTIW
NVSYDGTSTACSGSFYRSMRWLTRKNGEYPIQDAQYTNNQGKNILFMWGINHPPTDDTQRGLYTRTDTTT
SVATEEINRIFKPLIGPRPLVTGLMGRINYYWSVLKPGQTLRIKSDGNLIAPWYGHILSGESHGRILKTD
LKRGSCTVQCQTEKGGLNTTLPFQNVSKYAFGNCSKYIGIKSLKLAVGLRNVPSRSSRGLFGAIAGFIEG
GWSGLVAGWYGFQHSNDQGVGMAADRDSTQKAIDKITSKVNNIVDKMNKQYEIIDHEFSEVETRLNMINN
KIDDQIQDIWAYNAELLVLLENQKTLDEHDANVNNLYNKVKRALGSNAVEDGKGCFELYHKCDDQCMETI
RNGTYNRRKYQEESKLERQRIEGVKLESEGTYKILTIYSTVASSLVIAMGFAAFLFWAMSNGSCRCNICI

>UDE32028.1 hemagglutinin [Influenza A virus]
METISLMTILLVATVSNADKICIGYQSTNSTETVDTLTENNVPVTHAKELLHTEHNGMLCATSLGNPLIL
DTCTIEGLIYGNPSCDPLLGGREWSYIVERPSAVNGLCYPGSVENLEELRSLFSSARSYQRIQIFPDTIW
NVSYSGTSKACSDSFYRSMRWLTQKNNAYPIQDAQYTNNQEKNILFMWGINHPPTETAQTNLYTRTDTTT
SVATEEINRIFKPLIGPRPLVNGLMGRINYYWSVLKPGQTLRIKSDGNLIAPWYGHILSGESHGRILKTD
LKRGSCTVQCQTEKGGLNTTLPFQNVSKYAFGNCSKYIGIKSLKLAVGLRNVPSRSSRGLFGAIAGFIEG
GWSGLVAGWYGFQHSNDQGVGMAADRDSTQKAIDKITSKVNNIVDKMNKQYEIIDHEFSEVETRLNMINN
KVDDQIQDIWAYNAELLVLLENQKTLDEHDSNVNNLYNKVKRALGSNAVEDGKGCFELYHKCDNQCMETI
RNGTYNRRKYQEESKLERQKIEGVKLESEGTYKILTIYSTVASSLVIAMGFAAFLFWAMSNGSCRCNICI

>UDE32027.1 hemagglutinin [Influenza A virus]

METVSLITILIVATVSNADKICIGYQSTNSTETVDTLTENNVPVTHAKELLHTEHNGMLCATSLGHPLIL
DTCTIEGLIYGNPSCDPLLGGREWSYIVERPSAVNGLCYPGNVENLEELRSLFSSSRSYQRIQIFPDTIW
NVSYSGTSKACSDSFYRSMRWLTQKNNAYPTQDAQYTNNQGKNILFVWGINHPPTDTAQTNLYTRTDTTT
SVATEEMNRIFKPLIGPRPLVNGLMGRINYYWSVLKPGQTLRIKSDGNLIAPWYGHILSGESHGRILKTD
LKRGSCTVQCQTEKGGLNTTLPFQNVSKYAFGNCSKYIGVKSLKLAVGLRNVPSRSSRGLFGAIAGFIEG
GWSGLVAGWYGFQHSNDQGVGMAADRDSTQKAIDKITSKVNNIVDKMNKQYEIIDHEFSEVETRLNMINN
KVDDQIQDIWAYNAELLVLLENQKTLDEHDANVNNLYNKVKRALGSNAVEDGKGCFELYHKCDDHCMETI
RNGTYNRRKYQEESKLERQKIEGVKLESEETYKILTIYSTVASSLVIAMGFAAFLFWAMSNGSCRCNICI

>UDE32026.1 hemagglutinin [Influenza A virus]
METVSLITILIVATVSNADKICIGYQSTNSTETVDTLTENNVPVTHAKELLHTEHNGMLCATSLGHPLIL
DTCTIEGLIYGNPSCDPLLGGREWSYIVERPSAVNGLCYPGNVENLEELRSLFSSSRSYQRIQIFPDTIW
NVSYSGTSKACSDSFYRSMRWLTQKNNAYPTQDAQYTNNQGKNILFVWGINHPPTDTAQTNLYTRTDTTT
SVATEEMNRIFKPLIGPRPLVNGLMGRINYYWSVLKPGQTLRIKSDGNLIAPWYGHILSGESHGRILKTD
LKRGSCTVQCQTEKGGLNTTLPFQNVSKYAFGNCSKYIGVKSLKLAVGLRNVPSRSSRGLFGAIAGFIEG
GWSGLVAGWYGFQHSNDQGVGMAADRDSTQKAIDKITSKVNNIVDKMNKQYEIIDHEFSEVETRLNMINN
KVDDQIQDIWAYNAELLVLLENQKTLDEHDANVNNLYNKVKRALGSNAVEDGKGCFELYHKCDDHCMETI
RNGTYNRRKYQEESKLERQKIEGVKLESEETYKILTIYSTVASSLVIAMGFAAFLFWAMSNGSCRCNICI

>UDE32025.1 hemagglutinin [Influenza A virus]
METVSLITILLVATVSYADKICIGYQSTNSTETVDTLTESNVPVTHAKELLHTEHNGMLCATSLGQPLIL
DTCTIEGLIYGNPSCDLSLEGREWSYIVERPSAVNGLCYPGNVENLEELRSLFSSARSYQRVQIFPDTIW
NVSYDGTSTACSGSFYRSMRWLTRKDGNYPTQDAQYTNNQGKNILFMWGINHPPTDDTQRSLYTRTDTTT
SVATEEINRIFKPLIGPRPLVNGLMGRIDYYWSVLKPGQTLRIKSDGNLIAPWYGYILSGESHGRILKTD
LKRGSCTVQCQTEKGGLNTTLPFQNVSKYAFGNCSKYIGIKSLKLAVGLRNVPSRSSRGLFGAIAGFIEG
GWSGLVAGWYGFQHSNDQGVGMAADRDSTQKAIDKITSKVNNIVDKMNKQYEIIDHEFSEVETRLNMINN
KIDDQIQDIWAYNAELLVLLENQKTLDEHDANVNNLYNKVKRALGSNAAEDGKGCFELYHKCNDQCMETI
RNGTYNRRKYQEESKLERQKIEGVKLESEGTYKILTIYSTVASSLVIAMGFAAFLFWAMSNGSCRCNICI

>UDE32024.1 hemagglutinin [Influenza A virus]
METVSLITILLVATASNADKICIGYQSTNSTETVDTLTENNVPVTHAKELLHTEHNGMLCATSLGQPLIL
DTCTVEGLIYGNPSCDLSLEGREWSYIVERPSAVNGLCYPGNVENLEELRSLFSSARSYQRIQIFPDTIW
NVSYDGTSTACSNSFYRSMRWLTRKDGNYPTQDAQYTNNQGKNILFMWGINHPPTDDTQRNLYTRTDTTT
SVATEEINRIFKPLIGPRPRVNGLMGRIDYYWSVLKPGQTLRIKSDGNLIAPWYGHILSGESHGRILKTD

LKRGSCTVQCQTEKGGLNTTLPFQNVSKYAFGNCSKYIGIKSLKLAVGLRNVPSRSSRGLFGAIAGFIEG
GWSGLVAGWYGFQHSNDQGVGMAADRDSTQKAIDKITSKVNNIVDKMNKQYEIIDHEFSEVETRLNMINN
KIDDQIQDIWAYNAELLVLLENQKTLDEHDANVNNLYNKVKRALGSNAVEDGKGCFELYHKCNDQCMETI
RNGTYNRRTYQEESKLERQKIEGVKLESEGTYKILTIYSTVASSLVIAMGFAAFLFWAMSNGSCRCNICI

>UDE32023.1 hemagglutinin [Influenza A virus]
MDTVSLITILLVATVSNADKICIGYQSTNSTETVDTLTENNVPVTHAKELLHTEHNGMLCATSLGQPLIL
DTCTIEGLIYGNPSCDLSLEGREWSYIVERPSAVHGLCYPGNVEDLEELRSLFSSARSYQRIQIFPDTIW
NVSYDGTSTACSGSFYKSMRWLTRKNGEYPTQDAQYTNNQGKNILFMWGINHPPTDDTQRGLYTRTDTTT
SVATEEINRIFKPLIGPRPLVNGLMGRINYYWSVLKPGQTLRIKSDGNLIAPWYGHILSGESHGRILKTD
LKRGSCTVQCQTEKGGLNTTLPFQNVSKYAFGNCSKYIGIKSLKLAVGLRNVPSRSSRGLFGAIAGFIEG
GWSGLVAGWYGFQHSNDQGVGMAADRDSTQKAIDKITSKVNNIVDKMNKQYEIIDHEFSEVETRLNMINN
KIDDQIQDIWAYNAELLVLLENQKTLDEHDANVNNLYNKVKRALGSNAVEDGKGCFELYHKCDDQCMETI
RNGTYNRRKYQEESKLERQRIEGVKLESEGTYKILTIYSTVASSLVIAMGFAAFLFWAMSNGSCRCNICI

>UDE32022.1 hemagglutinin [Influenza A virus]
METVSLITILLVATVSNADKICIGYQSTNSTETVDTLTENNVPVTHAKELLHTEHNGMLCATSLGQPLIL
DTCTIEGLIYGNPSCDLSLEGREWSYIVERPSAVHGLCYPGNVEDLEELRSLFSSARSYQRIQIFPDTIW
NVSYDGTSTACSGSFYKSMRWLTRKNGEYPTQDAQYTNNQGKNILFMWGINHPPTDETQRRLYTRTDTTT
SVATEEINRIFKPLIGPRPLVNGLMGRINYYWSVLKPGQTLRIKSDGNLIAPWYGHILSGESHGRILKTD
LKRGSCTVQCQTEKGGLNTTLPFQNVSKYAFGNCSKYIGIKSLKLAVGLRNVPSRSSRGLFGAIAGFIEG
GWSGLVAGWYGFQHSNDQGVGMAADRDSTQKAIDKITSKVNNIVDKMNKQYEIIDHEFSEVETRLNMINN
KIDDQIQDIWAYNAELLVLLENQKTLDEHDANVNNLYNKVKRALGSNAVEDGKGCFELYHKCDDQCMETI
RNGTYNRRKYQEESKLERQRIEGVKLESEGTYKILTIYSTVASSLVIAMGFAAFLFWAMSNGSCRCNICI

>UDE32021.1 hemagglutinin [Influenza A virus]
METVSLMTILLVATVSNADKICIGYQSTNSTETVDTLTENDVPVTHAKELIHTEHNGMLCATSLGQPLIL
DTCTIEGLIYGNPSCDLSLEGREWSYIVERPSAVNGLCYPGNVENLEELRSLFSSARSYQRIQIFPDTIW
NVSYDGTSTACSGSFYKNMRWLTRKDGNYPTQDAQYTNNQGKNILFMWGINHPPADTTQTGLYTRTDTTT
SVATEEINRIFKPLIGPRPLVNGLMGRIDYYWSVLKPGQTLRIKSDGNLIAPWFGHILSGESHGRILKTD
LKRGSCTVQCQTEKGGLNTTLPFQNVSKYAFGNCSKYIGIKSLKLAVGLRNVPSRSSRGLFGAIAGFIEG
GWSGLVAGWYGFQHSNDQGVGMAADRDSTQKAIDKITSKVNNIVDKMNKQYEIIDHEFSEVETRLNMINN
KIDDQIQDIWAYNAELLVLLENQKTLDEHDANVNNLYNKVKRALGSNAVEDGKGCFELYHKCDDQCMETI
RNGTYNRRKYQEESKLERQKIEGVKLESEGTYKILTIYSTVASSLVIAMGFAAFLFWAMSNGSCRCNICI

>UDE32020.1 hemagglutinin [Influenza A virus]

METVSLITILLTATVSNADKICIGYQSTNSTETVDTLTENNVPVTHAKELLHTEHNGMLCATNLGQPLIL
DTCTIEGLIYGNPSCDPLPEEREWSYIVERPSAVNGLCYPGNVENLEELRSLFSSARSYQRVQIFPDTIW
NVSYDGTSNTCSGSFYRNMRWLTRKDGNYPIQDAQYTNNQGKNILFMWGINNPPTDNTQRNLYTRTDTTT
SVATEEINRIFKPLIGPRPLVNGLMGRINYYWSVLKPGQTLRIKSDGNLVAPWYGYILSGESHGRILRTD
LKRGSCTVQCQTEKGGLNTTLPFQNVSKYAFGNCSKYIGIKSLKLAVGLRNVPSRSSRGLFGAIAGFIEG
GWSGLVAGWYGFQHSNDQGVGMAADRESTQKAVDKITSKVNNIVDKMNKQYEIIDHEFSEVETRLNMINN
KIDDQIQDIWAYNAELLVLLENQKTLDEHDANVNNLYNKVKRALGSNAVEDGKGCFELYHKCDDQCMETI
RNGTYNRRKYQEESKLERQKIEGVKLESEGTYKILTIYSTVASSLVIAMGFAAFLFWAMSNGSCRCNICI

>UDE32019.1 hemagglutinin [Influenza A virus]

METVSLITILLVATVSNADKICIGYQSSNSTETVDTLTENNVPVTHAKELLHTEHNGMLCATSLGQPLIL
DTCTIEGLIYGNPYCDLSLEGREWSYIVERPSAVHGLCYPGNVEDLEELRSLFSSARSYQRIQIFPDTIW
NVSYDGTSTACSGSFYKSMRWLTRKNGEYPTQDAQYTNNQGKNILFMWGINHPPTDDTQRGLYTRTDTTT
SVATEEINRIFKPLIGPRPLVNGLMGRINYYWSVLKPGQTLRIKSDGNLIAPWYGHILSGESHGRILKTD
LKRGSCTVQCQTEKGGLNTTLPFQNVSKYAFGNCSKYIGVMSLKLAVGLRNVPSRSSRGLFGAIAGFIEG
GWPGLVAGWYGFQHSNDQGVGMAADRDSTQKAIDKITSKVNNIVDKMNKQYEIIDHEFSEVETRLNMINN
KIDDQIQDIWAYNAELLVLLENQKTLDEHDANVNNLYNKVKRALGSNAVEDGKGCFELYHKCDDQCMETI
RNGTYNRRKYQEESKLERQRIEGVKLESEGTYKILTIYSTVASSLVIAMGFAAFLFWAMSNGSCRCNVCI

>UDE32018.1 hemagglutinin [Influenza A virus]

METVSLITILLAATVSNADKICIGYQSTNSTETVDTLTENNVPVTHAKELLHTEHNGMLCATSLGQPLIL
DTCTIEGLIYGNPSCDPLLEEREWSYIVERPSAVNGLCYPGNVENLEELRSLFSSARSYQRIQIFPDTIW
NVSYDGTSNACSGSFYRNMRWLTRKNGNYPIQDAQYTNNQGKNILFMWGINHPPTDDTQRNLYTRTDTTT
SVATEEINRIFRPLIGPRPLVNGLMGRINYYWSVLKPGQTLRIKSDGNLIAPWYGYILSGESHGRILRTD
LKRGSCTVKCQTEKGGLNTTLPFQNVSKYAFGNCSKYIGIKSLKLAVGLRNVPSRSSRGLFGAIAGFIEG
GWSGLVAGWYGFQHSNDQGVGMAADRESTQKAIDKITSKVNNIVDKMNKQYEIIDHEFSEVETRLNMINN
KIDDQIQDIWAYNAELLVLLENQKTLDEHDANVNNLYNKVKRALGSNAVEDGKGCFELYHKCDDQCMETI
RNGTYNRRKYQEESKLERQKIEGVKLESEGTYKILTIYSTVASSLVIAMGFAAFLFWAMSNGSCRCNICI

>UDE32017.1 hemagglutinin [Influenza A virus]

MDTVSLITILLVATVSNADKICIGYQSTNSTETVDTLTENNVPVTHAKELLHTEHNGMLCATSLGQPLIL
DTCTIEGLIYGNPSCDLSLEGREWSYIVERPSAVHGLCYPGNVEDLEELRSLFSSARSYQRIQIFPDTIW

NVSYDGTSTACSGSFYKSMRWLTRKNGEYPTQDAQYTNNQGKNILFMWGINHPPTDDTQRGLYTRTDTTT
SVATEEINRIFKPLIGPRPLVNGLMGRINYYWSVLKPGQTLRIKSDGNLIAPWYGHILSGESHGRILKTD
LKRGSCTVQCQTEKGGLNTTLPFQNVSKYAFGNCSKYIGIKSLKLAVGLRNVPSRSSRGLFGAIAGFIEG
GWSGLVAGWYGFQHSNDQGVGMAADRDSTQKAIDKITSKVNNIVDKMNKQYEIIDHEFSEVETRLNMINN
KIDDQIQDIWAYNAELLVLLENQKTLDEHDANVNNLYNKVKRALGSNAVEDGKGCFELYHKCDDQCMETI
RNGTYNRRKYQEESKLERQRIEGVKLESEGTYKILTIYSTVASSLVIAMGFAAFLFWAMSNGSCRCNICI

>UDE32016.1 hemagglutinin [Influenza A virus]
METVSLITILLVATVSNADKICIGYQSTNSTETVDTLTENNVPVTHAKELLHTEHNGMLCATSLGQPLIL
DTCTIEGLIYGNPSCDLSLEGREWSYIVERPSAVHGLCYPGNVEDLEELRSLFSSARSYQRIQIFPDTIW
NVSYDGTSTACSGSFYKSMRWLTRKNGEYPTQDAQYTNNQGKNILFMWGINHPPTDETQRRLYTRTDTTT
SVATEEINRIFKPLIGPRPLVNGLMGRINYYWSVLKPGQTLRIKSDGNLIAPWYGHILSGESHGRILKTD
LKRGSCTVQCQTEKGGLNTTLPFQNVSKYAFGNCSKYIGIKSLKLAVGLRNVPSRSSRGLFGAIAGFIEG
GWSGLVAGWYGFQHSNDQGVGMAADRDSTQKAIDKITSKVNNIVDKMNKQYEIIDHEFSEVETRLNMINN
KIDDQIQDIWAYNAELLVLLENQKTLDEHDANVNNLYNKVKRALGSNAVEDGKGCFELYHKCDDQCMETI
RNGTYNRRKYQEESKLERQRIEGVKLESEGTYKILTIYSTVASSLVIAMGFAAFLFWAMSNGSCRCNICI

>UDE32015.1 hemagglutinin [Influenza A virus]
METVSLITILLIATVSNADKICIGYQSTNSTETVDTLTENNVPVTHAKELLHTEHNGMLCATSLGQPLIL
DTCTIEGLIYGNPSCDLSLEGREWSYIVERPSAVHGLCYPGNVEDLEELRSLFSSARSYQRIQIFPDTIW
NVSYDGTSTACSGSFYKSMRWLTRKNGEYPTQDAQYTNNQGKNILFMWGINHPPTDDTQRGLYTRTDTTT
SVATEEINRIFKPLIGPRPLVNGLMGRINYYWSILKPGQTLRIKSDGNLIAPWYGHILSGESHGRILKTD
LTRGSCTVQCQTEKGGLNTTLPFQNVSKYAFGNCSKYIGVKSLKLAVGLRNVPSRSSRGLFGAIAGFIEG
GWPGLVAGWYGFQHSNDQGVGMAADRDSTQKAIDKITSKVNNIVDKMNKQYEIIDHEFSEVETRLNMINN
KIDDQIQDIWAYNAELLVLLENQKTLDEHDANVNNLYNKVKRALGSNAVEDGKGCFELYHKCDDQCMETI
RNGTYNRRKYQEESKLERQRIEGVKLESEGTYKILTIYSTVASSLVIAMGFAAFLFWAMSNGSCRCNICI

>QUX07003.1 hemagglutinin [Influenza A virus]
MEAISLMTILLVVTTINADKICIGHQSTNSTETVDTLTETNVPVTQVKELLHTEHNGMLCATKLGRPLIL
DTCTIEGLIYGNPSCDMLLGGREWAYIVERPSAANGTCYPGNVENLEELRTFFSSSSSYQRVQLFPDSIW
NVTYTGTSKSCSDSFYRNMRWLTQKNGNYPIQDAQYTNNRGKDILFVWGIHHPPTDTAQTNLYTRTDTTT
SVTTETLDRTFKPLIGPRPLVNGLIGRINYYWSVLKPGQTLRVRSNGNLIAPWFGHVLSGESHGRILRTN
LSSGNCVVQCQTEKGGLNSTMPFHNISKYAFGTCPKYIGVKSLKLAIGLRNVHARSSRGLFGAIAGFIEG
GWPGLVAGWYGFQHSNDQGVGMAADRVSTQKAVDKITSKVNNIVDKMNKQYEIIDHEFSEIETRLNMINN

KIDDQIQDIWAYNAELLVLLENQKTLDEHDANVNNLYNKVKRALGSNAMEDGKGCFELYHKCDDQCMETI
RNGTYNRRKYTEESRLEKQKIEGVKLEAEGTYKILSIYSTVASSLVLAMGFAAFLFWAMSNGSCRCNICI

>QUX06980.1 hemagglutinin [Influenza A virus]
MEAISLMTILLVVTTINADKICIGHQSTNSTETVDTLTETNVPVTQVKELLHTEHNGMLCATKLGRPLIL
DTCTIEGLIYGNPSCDMLLGGREWAYIVERPSAANGTCYPGNVENLEELRTFFSSSSSYQRVQLFPDSIW
NVTYTGTSKSCSDSFYRNMRWLTQKNGNYPIQDAQYTNNRGKDILFVWGIHHPPTDTAQTNLYTRTDTTT
SVTTETLDRTFKPLIGPRPLVNGLIGRINYYWSVLKPGQTLRVRSNGNLIAPWFGHVLSGESHGRILRTN
LSSGNCVVQCQTEKGGLNSTMPFHNISKYAFGTCPKYIGVKSLKLAIGLRNVHARSSRGLFGAIAGFIEG
GWPGLVAGWYGFQHSNDQGVGMAADRVSTQKAVDKITSKVNNIVDKMNKQYEIIDHEFSEIETRLNMINN
KIDDQIQDIWAYNAELLVLLENQKTLDEHDANVNNLYNKVKRALGSNAMEDGKGCFELYHKCDDQCMETI
RNGTYNRRKYTEESRLEKQKIEGVKLEAEGTYKILSIYSTVASSLVLAMGFAAFLFWAMSNGSCRCNICI

>QUX06979.1 hemagglutinin [Influenza A virus]
MEAISLMTILLVVTTINADKICIGHQSTNSTETVDTLTETNVPVTQVKELLHTEHNGMLCATKLGRPLIL
DTCTIEGLIYGNPSCDMLLGGREWAYIVERPSAANGTCYPGNVENLEELRTFFSSSSSYQRVQLFPDSIW
NVTYTGTSKSCSDSFYRNMRWLTQKNGXYPIQDAQYTNNRGKDILFVWGIHHPPTDTXQTNLYTRTDTTT
SVTTETLDRTFKPLIGPRPLVNGLIGRINYYWSVLKPGQTLRVRSNGNLIAPWFGHVLSGESHGRILRTN
LSSGNCVVQCQTEKGGLNSTMPFHNISKYAFGTCPKYIGVKSLKLAIGLRNVHARSSRGLFGAIAGFIEG
GWPGLVAGWYGFQHSNDQGVGMAADRVSTQKAVDKITSKVNNIVDKMNKQYEIIDHEFSEIETRLNMINN
KIDDQIQDIWAYNAELLVLLENQKTLDEHDANVNNLYNKVKRALGSNAMEDGKGCFELYHKCDDQCMETI
RNGTYNRRKYTEESRLEKQKIEGVKLEAEGTYKILSIYSTVASSLVLAMGFAAFLFWAMSNGSCRCNICI

>QUX06967.1 hemagglutinin [Influenza A virus]
MEAKSLMTILLVVTTSNADKICIGHQSTNSTETVDTLTETNVPVTQAKELLHTEHNGMLCATKLGRPLIL
DTCTIEGLIYGNPSCDMLLGGREWAYIVERPSAANGTCYPGNVENLEELRTFFSSSSSYQRVQLFPDSIW
NVTYTGTSKSCSDSFYRNMRWLTQKNGNYPIQDAQYTNNRGKDILFVWGIHHPPTDTAQTNLYTRTDTTT
SVTTETLDRTFKPLIGPRPLVNGLIGRINYYWSVLKPGQTLRVRSNGNLIAPWFGHVLSGESHGRILRTN
LSSGNCVVQCQTEKGGLNSTMPFHNISKYAFGTCPKYIGVKSLKLAIGLRNVHARSSRGLFGAIAGFIEG
GWPGLVAGWYGFQHSNDQGVGIAADRVSTQRAVDKITSKVNNIVDKMNKQYEIIDHEFSEIETRLNMINN
KIDDQIQDIWAYNAELLVLLENQKTLDEHDANVNNLYNKVKRALGSNAMEDGKGCFELYHKCDDQCMETI
RNGTYNKRKYTEESRLEKQKIEGVKLEAEGTYKILSIYSTVASSLVLAMGFAAFLFWAMSNGSCRCNICI

>QUX06955.1 hemagglutinin [Influenza A virus]

MEAKSLMTILLVVTTSNADKICIGHQSTNSTETVDTLTETNVPVTQAKELLHTEHNGMLCATXLGRPLIL
DTCTIEGLIYGNPSCDMLLGGREWAYIVERPSAANGTCYPGNVENLEELRTFFSSSSSYQRVQLFPDSIW
NVTYTGTSKSCSDSFYRNMRWLTQKNGNYPIQDAQYTNNRGKDILFVWGIHHPPTDTAQTNLYTRTDTTT
SVTTETLDRTFKPLIGPRPLVNGLIGRINYYWSVLKPGQTLRVRSNGNLIAPWFGHVLSGESHGRILRTN
LSSGNCVVQCQTEKGGLNSTMPFHNISKYAFGTCPKYIGVKSLKLAIGLRNVHARSSRGLFGAIAGFIEG
GWPGLVAGWYGFQHSNDQGVGIAADRVSTQRAVDKITSKVNNIVDKMNKQYEIIDHEFSEIETRLNMINN
KIDDQIQDIWAYNAELLVLLENQKTLDEHDANVNNLYNKVKRALGSNAMEDGKGCFELYHKCDDQCMETI
RNGTYNRRKYTEESRLEKQKIEGVKLEAEGTYKILSIYSTVASSLVLAMGFAAFLFWAMSNGSCRCNICI

>QUX06943.1 hemagglutinin [Influenza A virus]
MEAISLMTILLVVTTSNADKICIGHQSTNSTETVDTLTETNVPVTQAKELLHTEHNGMLCATKLGRPLIL
DTCTIEGLIYGNPSCDMLLGGREWAYIVERPSAANGTCYPGNVENLEELRTFFSSSSSYQRVQLFPDSIW
NVTYTGTSKSCSDSFYRNMRWLTQKNGNYPIQDAQYTNNRGKDILFVWGIHHPPTDTAQTNLYTRTDTTT
SVTTETLDRTFKPLIGPRPLVNGLIGRINYYWSVLKPGQTLRVRSNGNLIAPWFGHXLSGESHGRILRTN
LSSGNCVVQCQTEKGGLNSTMPFHNISKYAFGTCPKYIGVKSLKLAIGLRNVHARSSRGLFGAIAGFIEG
GWPGLVAGWYGFQHSNDQGVGXAADRVSTQXAVDKITSKVNNIVDKMNKQYEIIDHEFSEIETRLNMINN
KIDDQIQDIWAYNAELLVLLENQKTLDEHDANVNNLYNKVKRXLGSNAMEDGKGCFELYHKCDDQCMETI
RNGTYNXRKYTEESRLEKQKIEGVKLEAEGTYKILSIYSTVASSLVLAMGFAAFLFWAMSNGSCRCNICI

>QUX06931.1 hemagglutinin [Influenza A virus]
MEAISLMTILLVVTTSNADKICIGHQSTNSTETVDTLTETNVPVTQAKELLHTEHNGMLCATKLGRPLIL
DTCTIEGLIYGNPSCDMLLGGREWAYIVERPSATNGTCYPGNVENLEELRTFFSSSSSYQRVQLFPDSIW
NVTYTGTSKSCSDSFYRNMRWLTQKNGNYPIQDAQYTNNRGKDILFVWGIHHPPTDTAQTNLYTRTDTTT
SVTTETLDRTFKPLIGPRPLVNGLIGRINYYWSVLKPGQTLRVRSNGNLIAPWFGHVLSGESHGRILRTN
LSSGNCVVQCQTEKGGLNSTMPFHNISKYAFGTCPKYIGVKSLKLAIGLRNVHARSSRGLFGAIAGFIEG
GWPGLIAGWYGFQHSNDQGVGMAADRVSTQKAVDKITSKVNNIVDKMNKQYEIIDHEFSEIETRLNMINN
KIDDQIQDIWAYNAELLVLLENQKTLDEHDANVNNLYNKVKRALGSNAMEDGKGCFELYHKCDDQCMETI
RNGTYNRRKYTEESRLEKQKIEGVKLEAEGTYKILSIYSTVASSLVLAMGFAAFLFWAMSNGSCRCNICI

>QUX06919.1 hemagglutinin [Influenza A virus]
MEAISLMTILLVVTTSNADKICIGHQSTNSTETVDTLTETNVPVTQAKELLHTEHNGMLCATKLGRPLIL
DTCTIEGLIYGNPSCDMLLGGREWAYIVERPSATNGTCYPGNVENLEELRTFFSSSSSYQRVQLFPDSIW
NVTYTGTSKSCSDSFYRNMRWLTQKNGNYPIQDAQYTNNRGKDILFVWGIHHPPTDTAQTNLYTRTDTTT
SVTTETLDRTFKPLIGPRPLVNGLIGRINYYWSVLKPGQTLRVRSNGNLIAPWFGHVLSGESHGRILRTN

LSSGNCVVQCQTEKGGLNSTMPFHNISKYAFGTCPKYIGVKSLKLAIGLRNVHARSSRGLFGAIAGFIEG
GWPGLIAGWYGFQHSNDQGVGMAADRVSTQKAVDKITSKVNNIVDKMNKQYEIIDHEFSEIETRLNMINN
KIDDQIQDIWAYNAELLVLLENQKTLDEHDANVNNLYNKVKRALGSNAMEDGKGCFELYHKCDDQCMETI
RNGTYNRRKYTEESRLEKQKIEGVKLEAEGTYKILSIYSTVASSLVLAMGFAAFLFWAMSNGSCRCNICI

>QUX06907.1 hemagglutinin [Influenza A virus]
MEAISLMTILLVVTTSNADKICIGHQSTNSTETVDTLTETNVPVTQAKELLHTEHNGMLCATKLGRPLIL
DTCTIEGLIYGNPSCDMLLGGREWAYIVERPSATNGTCYPGNVENLEELRTFFSSSSSYQRVQLFPDSIW
NVTYTGTSKSCSDSFYRNMRWLTQKNGNYPIQDAQYTNNRGKDILFVWGIHHPPTDTAQTNLYTRTDTTT
SVTTETLDRTFKPLIGPRPLVNGLIGRINYYWSVLKPGQTLRVRSNGNLIAPWFGHVLSGESHGRILRTN
LSSGNCVVQCQTEKGGLNSTMPFHNISKYAFGTCPKYIGVKSLKLAIGLRNVHARSSRGLFGAIAGFIEG
GWPGLVAGWYGFQHSNDQGVGMAADRVSTQKAVDKITSKVNNIVDKMNKQYEIIDHEFSEIETRLNMINN
KIDDQIQDIWAYNAELLVLLENQKTLDEHDANVNNLYNKVKRALGSNAMEDGKGCFELYHKCDDQCMETI
RNGTYNRRKYTEESRLEKQKIEGVKLEAEGTYKILSIYSTVASSLVLAMGFAAFLFWAMSNGSCRCNICI

>QUX06895.1 hemagglutinin [Influenza A virus]
MEAIPLMTILLVVTTSNADKICIGHQSTNSTETVDTLTETNVPVTQAKELLHTEHNGMLCATNLGRPLIL
DTCTIEGLIYGNPSCDMLLGGREWAYIVERPSAVNGTCYPGNVENLEELRTFFSSSSSYQRVQLFPDSIW
NVTYTGTSKSCSGSFYRNMRWLTQKDGSYPIQDAQYTNNRGKDILFVWGIHHPPTDTAQTNLYTRTDTTT
SVTTETLDRTFKPLIGPRPLVNGLIGRINYYWSVLKPGQTLRVRSNGNLIAPWFGHVLSGESHGRILRTD
LSSGNCVVQCQTEKGGLNSTMPFHNISKYAFGTCPKYIGVKSLKLAIGLRNVHAKSSRGLFGAIAGFIEG
GWPGLVAGWYGFQHSNDQGVGMAADRVSTQKAVDKITSKVNNIVDKMNKQYEIIDHEFSEVENRLNMINN
KIDDQIQDIWAYNAELLVLLENQKTLDEHDANVNNLYNKVKRALGSNAMEDGKGCFELYHKCDDQCMETI
RNGTYNRRKYTEESRLEKQRIEGVKLEAEGTYKILSIYSTVASSLVLAMGFAAFLFWAMSNGSCRCNICI

>QUX06883.1 hemagglutinin [Influenza A virus]
MEAKSLMTILLVVTTSNADKICIGHQSTNSTETVDTLTETNVPVTQAKELLHTEHNGMLCATKLGXPLIL
DTCTIEGLIYGNPSCDMLLGGREWAYIVERPSAXNGTCYPGNVENLEELRTFFSSSSSYQRVQLFPDSIW
NVTYTGTSKSCSDSFYRNMRWLTQKNGNYPIQDAQYTNNRGKDILFVWGIHHPPTDTAQTNLYTRTDTTT
SVTTETLDRTFKPLIGPRPLVNGLIGRINYYWSVLKPGQTLRVRSNGNLIAPWFGHVLSGESHGRILRTN
LSSGNCVVQCQTEKGGLNSTMPFHNISKYAFGTCPKYIGVKSLKLAIGLRNVHARSSRGLFGAIAGFIEG
GWPGLVAGWYGFQHSNDQGVGMAADRVSTQKAVDKITSKVNNIVDKMNKQYEIIDHEFSEIETRLNMINN
KIDDQIQDIWAYNAELLVLLENQKTLDEHDANVNNLYNKVKRALGSNAMEDGKGCFELYHKCDDQCMETI
RNGTYNRRKYTEESRLEKQKIEGVKLEAEGAYKILSIYSTVASSLVLAMGFAAFLFWAMSNGSCRCNICI

>QUX06871.1 hemagglutinin [Influenza A virus]

MEAISLMTILLVVTTSNADKICIGHQSTNSTETVDTLTETNVPVTQAKELLHTEHNGMLCATKLGRPLIL
DTCTIEGLIYGNPSCDMLLGGREWAYIVERPSAANGTCYPGNVENLEELRTFFSSSSSYQRVQLFPDSIW
NVTYTGTSKSCSDSFYRNMRWLTQKNGNYPIQDAQYTNNRGKDILFVWGIHHPPTDTAQTNLYTRTDTTT
SVTTETLDRTFKPLIGPRPLVNGLIGRINYYWSVLKPGQTLRVRSNGNLIAPWFGHVLSGESHGRILRTN
LSSGNCVVQCQTEKGGLNSTMPFHNISKYAFGTCPKYIGVKSLKLAIGLRNVHARSSRGLFGAIAGFIEG
GWPGLVAGWYGFQHSNDQGVGMAADRVSTQKAVDKITSKVNNIVDKMNKQYEIIDHEFSEIETRLNMINN
KIDDQIQDIWAYNAELLVLLENQKTLDEHDANVNNLYNKVKRALGSNAMEDGKGCFELYHKCDDQCMETI
RNGTYNRRKYTEESRLEKQKIEGVKLEAEGTYKILSIYSTVASSLVLAMGFAAFLFWAMSNGSCRCNICI

>QUX06859.1 hemagglutinin [Influenza A virus]

MEAKSLMTILLVVTTSNADKICIGHQSTNSTETVDTLTETNVPVTQAKELLHTEHNGMLCATKLGRPLIL
DTCTIEGLIYGNPSCDMLLGGREWAYIVERPSAANGTCYPGNVENLEELRTFFSSSSSYQRVQLFPDSIW
NVTYTGTSKSCSDSFYRNMRWLTQKNGNYPXQDAQYTNNRGKDILFVWGIHHPPTDTAQTNLYTRTDTTT
SVTTETLDRTFKPLIGPRPLVNGLIGRINYYWSVLKPGQTLRVRSNGNLIAPWFGHVLSGESHGRILRTN
LSSGNCVVQCQTEKGGLNSTMPFHNISKYAFGTCPKYIGVKSLKLAIGLRNVHARSSRGLFGAIAGFIEG
GWPGLVAGWYGFQHSNDQGVGMAADRVSTQKAVDKITSKVNNIVDKMNKQYEIIDHEFSEIETRLNMINN
KIDDQIQDIWAYNAELLVLLENQKTLDEHDANVNNLYNKVKRALGSNAMEDGKGCFELYHKCDDQCMETI
RNGTYNRRKYTEESRLEXQKIEGVKLEAEGTYKILSIYSTVASSLVLAMGFAAFLFWAMSNGSCRCNICI

>QUX06847.1 hemagglutinin [Influenza A virus]

MEAKSLMTILLVVTTSNADKICIGHQSTNSTETVDTLTETNVPVTQAKELLHTEHNGMLCATKLGRPLIL
DTCTIEGLIYGNPSCDMLLGGREWAYIVERPSAANGTCYPGNVENLEELRTFFSSSSSYQRVQLFPDSIW
NVTYTGTSKSCSDSFYRNMRWLTQKNGNYPIQDAQYTNNRGKDILFVWGIHHPPTDTAQTNLYTRTDTTT
SVTTETLDRTFKPLIGPRPLVNGLIGRINYYWSVLKPGQTLRVRSNGNLIAPWFGHVLSGESHGRILRTN
LSSGNCVVQCQTEKGGLNSTMPFHNISKYAFGTCPKYIGVKSLKLAIGLRNVHARSSRGLFGAIAGFIEG
GWPGLVAGWYGFQHSNDQGVGIAADRVSTQRAVDKITSKVNNIVDKMNKQYEIIDHEFSEIETRLNMINN
KIDDQIQDIWAYNAELLVLLENQKTLDEHDANVNNLYNKVKRALGSNAMEDGKGCFELYHKCDDQCMETI
RNGTYNRRKYTEESRLEKQKIEGVKLEAEGTYKILSIYSTVASSLVLAMGFAAFLFWAMSNGSCRCNICI

>QUX06835.1 hemagglutinin [Influenza A virus]

MEAKSLMTILLVVTTSNADKICIGHQSTNSTETVDTLTETNVPVTQAKELLHTEHNGMLCATKLGRPLIL
DTCTIEGLIYGNPSCDMLLGGREWAYIVERPSAANGTCYPGNVENLEELRTFFSSSSSYQRVQLFPDSIW

NVTYTGTSKSCSDSFYRNMRWLTQKNGNYPIQDAQYTNNRGKDILFVWGIHHPPTDTAQTNLYTRTDTTT
SVTTETLDRTFKPLIGPRPLVNGLIGRINYYWSVLKPGQTLRVRSNGNLIAPWFGHVLSGESHGRILRTN
LSSGNCVVQCQTEKGGLNSTMPFHNISKYAFGTCPKYIGVKSLKLAIGLRNVHARSSRGLFGAIAGFIEG
GWPGLVAGWYGFQHSNDQGVGMAADRVSTQKAIDKITSKVNNIVDKMNKQYEIIDHEFSEIETRLNMINN
KIDDQIQDIWAYNAELLVLLENQKTLDEHDANVNNLYNKVKRALGSNAMEDGKGCFELYHKCDDQCMETI
RNGTYNRRKYTEESRLERQKIEGVKLEAEGTYKILSIYSTVASSLVLAMGFAAFLFWAMSNGSCRCNICI

>UKJ62205.1 hemagglutinin [Influenza A virus]
MEAXSLMTILLVVTXXTAXKICIGHQSTNSTETVDTLTETNVPVTQAKELLHTEHNGMLCATSLGRPLIL
DTCTIEGLIYGNPSCDMLLGGRKWSYIVERPSAVNGTCYPGNVENLEELRTLFSSSSSYQRVQLFPDSIW
NVTYTGTSKSCSDSFYRNMRWLTQKNGGYPIQDAQYTNNRGKDILFVWGIHHPPTDTAQTNLYARTDTTT
SVTTETLDRTFKPLVGPRPLVNGLIGRINYYWSVLKPGQTLRVRSNGNLIAPWFGHVLSGESHGRILRTD
LSSSNCVVQCQTEKGGLNSTMPFHNISKYAFGNCPKYIGVKSLKLAIGMRNVHARSSRGLFGAIAGFIEG
GWPGLVAGWYGFQHSNDQGVGMAADRVSTQKAVDKITSKVNNIVDKMNKQYEIIDHEFSEVETRLNMINN
KIDDQIQDIWAYNAELLVLLENQKTLDEHDANVNNLYNKVKRALGSNAMEDGKGCFELYHKCDDQCMETI
RNGTYNRIKYIEESRLERQKIEGVKLEAEGTYKILSIYSTVASSLVLAMGFAAFLFWAMSNGSCRCNICI

>UKJ62204.1 hemagglutinin [Influenza A virus]
MEAISLMTILLVVTTSTADKICIGHQSTNSTETVDTLTETNVPVTQAKELLHTEHNGMLCATSLGRPLIL
DTCTIEGLIYGNPSCDMLLGGREWSYIVERPSAVNGTCYPGNVENLEELRTLFSSSSSYQRVQLFPDSIW
NVTYTGTSKSCSDSFYRNMRWLTQKNGGYPIQDAQYTNNRGKDILFVWGIHHPPTDTVQTNLYTRTDTTT
SVTTETLDRTFKPLIGPRPLVNGLIGRINYYWSVLKPGQTLRVRSNGNLIAPWFGHVLSGESHGRILRTD
LSSSNCVVQCQTEKGGLNSTMPFHNISKYAFGNCPKYIGVKSLKLAIGMRNVHARSSRGLFGAIAGFIEG
GWPGLVAGWYGFQHSNDQGVGMAADRVSTQKAVDKITSKVNNIVDKMNKQYEIIDHEFSEVETRLNMINN
KIDDQIQDIWAYNAELLVLLENQKTLDEHDANVNNLYNKVKRALGSNAMEDGKGCFELYHKCDDQCMETI
RNGTYNRRKYIEESRLERQKIEGVKLEAEGTYKILSIYSTVASSLVLAMGFAAFLFWAMSNGSCRCNICI

>UKJ62203.1 hemagglutinin [Influenza A virus]
MEAISLMTILLVVTTSTADKICIGHQSTNSTETVDTLTETNVPVTQAKELLHTEHNGMLCATSLGRPLIL
DTCTIEGLIYGNPSCDMLLGGREWSYIVERPSAVNGTCYPGNVENLEELRTLFSSSSSYQRVQLFPDSIW
NVTYTGTSKSCSDSFYRNMRWLTQKNGGYPIQDAQYTNNRGKDILFVWGIHHPPTDTAQTNLYTRTDTTT
SVTTETLDRTFKPLIGPRPLVNGLIGRINYYWSVLKPGQTLRVRSNGNLIAPCFGHVLSGESHGRILRTD
LSSSNCVVQCQTEKGGLNSTMPFHNISKYAFGNCPKYIGVKSLKLAIGMRNVHARSSRGLFGAIAGFIEG
GWPGLVAGWYGFQHSNDQGVGMAADRVSTQKAVDKITSKVNNIVDKMNKQYEIIDHEFSEVETRLNMINN

KIDDQIQDIWAYNAELLVLLENQKTLDEHDANVNNLYNKVKRALGSNAMEDGKGCFELYHKCDDQCMETI
RNGTYNRRKYIEESRLERQKIEGVKLEAEGTYKILSIYSTVASSLVLAMGFAAFLFWAMSNGSCRCNICI

>UKJ62202.1 hemagglutinin [Influenza A virus]
MEAISLMTILLVVTTSTADKICIGHQSTNSTETVDTLTETNVPVTQAKELLHTEHNGMLCATSLGRPLIL
DTCTIEGLIYGNPSCDMLLGGREWSYIVERPSAVNGTCYPGNVENLEELRTLFSSSSSYQRVQLFPDSIW
NVTYTGTSKSCSDSFYRNMRWLTQKNGGYPIQDAQYTNNRGKDILFVWGIHHPPTDTAQTNLYTRTDTTT
SVTTETLDRTFKPLIGPRPLVNGLIGRINYYWSVLKPGQTLRVRSNGNLIAPWFGHVLSGESHGRILRTD
LSSSNCVVQCQTEKGGLNSTMPFHNISKYAFGNCPKYIGVKSLKLAIGMRNVHARSSRGLFGAIAGFIEG
GWPGLVAGWYGFQHSNDQGVGMAADRVSTQKAVDKITSKVNNIVDKMNKQYEIIDHEFSEVETRLNMINN
KIDDQIQDIWAYNAELLVLLENQKTLDEHDANVNNLYNKVKRALGSNAMEDGKGCFELYHKCDDQCMETI
RNGTYNRRKYIEESRLERQKIEGVKLEAEGTYKILSIYSTVASSLVLAMGFAAFLFWAMSNGSCRCNICI

>UKJ62201.1 hemagglutinin [Influenza A virus]
MXAXSLMTILLVVTTSTADKICIGHQSTNSTETVDTLTETNVPVTQAKELLHTEHNGMLCATSLGRPLIL
DTCTIEGLIYGNPSCDMLLGGREWSYIVERPSAVNGTCYPGNVENLEELRTLFSSSSSYQRVQLFPDSIW
NVTYTGTSKSCSDSFYRNMRWLTQKNGGYPIQDAQYTNNRGKDILFVWGIHHPPTDTAQTNLYTRTDTTT
SVTTETLDRTFKPLIGPRPLVNGLIGRINYYWSVLRPGQTLRVRSNGNLIAPWFGHVLSGESHGRILRTD
LSSSNCVVQCQTEKGGLNSTMPFHNISKYAFGNCPKYXXXXSLKLAIGMRNVHARSSRGLFGAIAGFIEG
GWPGLVAGWYGFQHSNDQGVGMAADRVSTQKAVDKITSKVNNIVDKMNKQYEIIDHEFSEVETRLNMINN
KIDDQIQDIWAYNAELLVLLENQKTLDEHDANVNNLYNKVKRALGSNAMEDGKGCFELYHKCDDQCMETI
RNGTYNRRKYIEESRLERQKIEGVKLEAEGTYKILSIYSTVASSLVLAMGFAAFLFWAMSNGSCRCNICI

>UKJ62200.1 hemagglutinin [Influenza A virus]
MEAISLMTILLVVTTSTADKICIGHQSTNSTETVDTLTETNVPVTQAKELLHTEHNGMLCATSLGRPLIL
DTCTIEGLIYGNPSCDMLLGGRKWSYIVERPSAVNGTCYPGNVENLEELRTLFSSSSSYQRVQLFPDSIW
NVTYTGTSKSCSDSFYRNMRWLTQKNGGYPIQDAQYTNNRGKDILFVWGIHHPPTDTAQTNLYARTDTTT
SVTTETLDRTFKPLVGPRPLVNGLIGRINYYWSVLKPGQTLRVRSNGNLIAPWFGHVLSGESHGRILRTD
LSSSNCVVQCQTEKGGLNSTMPFHNISKYAFGNCPKYIGVKSLKLAIGMRNVHARSSRGLFGAIAGFIEG
GWPGLVAGWYGFQHSNDQGVGMAADRVSTQKAVDKITSKVNNIVDKMNKQYEIIDHEFSEVETRLNMINN
KIDDQIQDIWAYNAELLVLLENQKTLDEHDANVNNLYNKVKRALGSNAMEDGKGCFELYHKCDDQCMETI
RNGTYNRIKYIEESRLERQKIEGVKLEAEGTYKILSIYSTVASSLVLAMXFAXFLFWAMSXGSCRCXXCI

>UKJ62199.1 hemagglutinin [Influenza A virus]

MXXISLMTILLVVTTSTADKICIGHQSTNSTETVDTLTETNVPVTQAKELLHTEHNGMLCATSLGRPLIL
DTCTVEGLIYGNPSCDMLLGGREWSYIVERPSAVNGTCYPGNVENLEELRTLFSSSSSYQRVQLFPDSIW
NVTYTGTSKSCSDSFYRNMRWLTQKNGGYPIQDAQYTNNRGKDILFVWGIHHPPTDTAQTNLYTRTDTTT
SVTTETLDRTFKPLIGPRPLVNGLIGRINYYWSVLKPGQTLRVRSNGNLIAPWFGHVLSGESHGRILRTD
LSSSNCVVQCQTEKGGLNSTMPFHNISKYAFGNCPKYIGVKSLKLAIGMRNVHARSSRGLFGAIAGFIEG
GWPGLVAGWYGFQHSNDQGVGMAADRVSTQKAVDKITSKVNNIVDKMNKQYEIIDHEFSEVETRLNMINN
KIDDQIQDIWAYNAELLVLLENQKTLDEHDANVNNLYNKVKRALGSNAMEDGKGCFELYHKCDDQCMETI
RNGTYNRRKYIEESRLERQKIEGVKLEAEGTYKILSIYSTVASSLVLAMGFAAFLFWAMSNGSCRCNICI

>UKJ62198.1 hemagglutinin [Influenza A virus]
MXXISLMTILLVVTTSTADKICIGHQSTNSTETVDTLTETNVPVTQAKELLHTEHNGMLCATSLGRPLIL
DTCTIEGLIYGNPSCDMLLGGREWSYIVERPSAVNGTCYPGNVENLEELRTLFSSSSSYQRVQLFPDSIW
NVTYTGTSKSCSDSFYRNMRWLTQKNGGYPIQDAQYTNNRGKDILFVWGIHHPPTDTAQTNLYTRTDTTT
SVTTETLDRTFKPLIGPRPLVNGLIGRINYYWSVLKPGQTLRVRSNGNLIAPWFGHVLSGESHGRILRTD
LSSSNCVVQCQTEKXGLNSTMPFHNISKYAFGNCPKYXGVKSLKLAIGMRNVHARSSRGLFGAIAGFIEG
GWPGLVAGWYGFQHSNDQGVGMAADRVSTQKAVDKITSKVNNIVDKMNKQYEIIDHEFSEVETRLNMINN
KIDDQIQDIWAYNAELLVLLENQKTLDEHDANVNNLYNKVKRALGSNAMEDGKGCFELYHKCDDQCMETI
RNGTYNRRKYIEESRLERQKIEGVKLEAEGTYKILSIYSTVASSLVLAMGFAAFLFWAMSNGSCRCNICI

>UKJ62197.1 hemagglutinin [Influenza A virus]
MEXISLMTILLVVTTSNADKICIGHQSTNSTETVDTLTETNVPVTQAKELLHTEHNGMLCATNLGRPLIL
DTCTIEGLIYGNPSCDMLLGGREWSYIVERPSAVNGTCYPGNVENLEELRTLFSSSSSYQRVQLFPDSIW
NVTYTGTSKSCSDSFYRNMRWLTQKNGGYPIQDAQYTNNRGKDILFVWGIHHPPTDTAQTNLYTRTDTTT
SVTTETLDRTFKPLIGPRPLVNGLIGRINYYWSVLKPGQTLRVRSNGNLIAPWFGHVLSGESHGRILRTD
LSSSNCVVQCQTEKGGLNSTMPFHNISKYAFGNCPKYIGVKSLKLAIGMRNVHARSSRGLFGAIAGFIEG
GWPGLVAGWYGFQHSNDQGVGMAADRVSTQKAVDKITSKVNNIVDKMNKQYEIIDHEFSEVETRLNMINN
KIDDQIQDIWAYNAELLVLLENQKTLDEHDANVNNLYNKVKRALGSNAMEDGKGCFELYHKCDDQCMETI
RNGTYNRRKYIEESRLKRQKIEGVKLEAEGTYKILSIYSTVASSLVLAMGFAAFLFWAMSNGSCRCNICI

>UKJ62196.1 hemagglutinin [Influenza A virus]
MEVVPLMTMLLLVTTNNADKICIGHQSTNSTETVDTLTETGVPVTHAKELLHTEHNGKLCATNLGNPLIL
DTCTVEGIIYGNPSCDMLLGGREWSYIVERPSAVNGTCYPGNVENLEELRVLFSSSSSYQRIQMFPDTIW
NVTYSGTSKSCSDSFYRNMRWLTQKNGNYPVQDAQYTNTRGKDILFVWGIHHPPTDTAQTNLYTRTDTTT
SITTESLDRTFKPLIGPRPLVNGLIGRINYYWSVLKPGQTLRVRSSGNLIAPWFGHVLSGESHGRILKTD

LNSGNCVVQCQTEKGGLNSTLPFHNISKYAFGDCPKYIGVKSLKLAIGLRNVPARSSRGLFGAIAGFIEG
GWPGLVAGWYGFQHSNDQGVGMAADRDSTQKAVDKITSKVNNIVDKMNKQYEIIDHEFSEIETRLNMINN
KIDDQIQDIWAYNAELLVLLENQKTLDEHDANVNNLYNKVRRALGSNAMEDGKGCFELYHKCDDQCMETI
RNGTYNRRKYVEESRLGRQKIEGVKLESEGTYKILTIYSTVASSLVIAMGFAAFLFWAMSNGSCRCNICI

>UKJ62195.1 hemagglutinin [Influenza A virus]
MEVISLMTMLLLITTNNADKICIGHQSTNSTETVDTLTETGVPVTHAKELLHTEHNGKLCATNLGNPLIL
DTCTVEGLIYGNPSCDLLLGGREWSYIVERPSAVNGTCYPGNVENLEELRILFSSSSSYQRIQMFPDTIW
NVTYSGTSKSCSDSFYRNMRWLTQKNGNYPVQDAQYTNTRGKNILFVWGIHHPPTDTTQTNLYTRTDTTT
SITTESLDRTFKPLIGPRPLVNGLIGRINYYWSVLKPGQTLRVRSNGNLIAPWFGHVLSGESHGRILKTD
LNSGNCVVQCQTEKGGLNSTLPFHNISKYAFGNCPKYIGVKSLKLAIGLRNVPARSSRGLFGAIAGFIEG
GWPGLVAGWYGFQHSNDQGVGMAADRDSTQKAVDKITSKVNNIVDKMNKQYEIIDHEFSEVENRLNMINN
KVDDQIQDIWAYNAELLVLLENQKTLDEHDANVNNLYNKVKRALGSNAMEDGKGCFELYHKCDDQCMETI
RNGTYNRRKYMEESRLGRQKIEGVKLESEGTYKILTIYSTVASSLVLAMGFAAFLFWAMSNGSCRCNVCI

>UKJ62194.1 hemagglutinin [Influenza A virus]
MXVVPLMTMLLLVTTNNADKICIGHQSTNSTETVDTLTETGVPVTHAKELLHTEHNGKLCATNLGNPLIL
DTCTVEGIIYGNPSCDMLLGGREWSYIVERPSAVNGTCYPGNVENLEELRVLFSSSSSYQRIQMFPDTIW
NVTYSGTSKSCSDSFYRNMRWLTQKNGNYPVQDAQYTNTRGKDILFVWGIHHPPTDTTQTNLYTRTDTTT
SITTESLDRTFKPLIGPRPLVNGLIGRINYYWSVLKPGQTLRVRSSGNLIAPWFGHVLSGESHGRILKTD
LNSGNCVVQCQTEKGGLNSTLPFHNISKYAFGDCPKYIGVKSLKLAIGLRNVPARSSRGLFGAIAGFIEG
GWPGLVAGWYGFQHSNDQGVGMAADRDSTQKAVDKITSKVNNIVDKMNKQYEIIDHEFSEIETRLNMINN
KIDDQIQDIWAYNAELLVLLENQKTLDEHDANVNNLYNKVRRALGSNAMEDGKGCFELYHKCDDQCMETI
RNGTYNRRKYVEESRLGRQKIEGVKLESEGTYKILTIYSTVASSLVIAMGFAAFLFWAMSNGSCRCNICI

>UKJ62193.1 hemagglutinin [Influenza A virus]
MXXIPLMTMMLXSTTXNAXKXCXXHQSTNSTETVDTLTETGVPVTHAKELLHTEHNGKLCATNLGNPLIL
DTCTVEGLIYGNPSCDLLLGGREWSYIVERPSAANGTCYPGNVENLEELRILFSSSSSYQRIQMFPDTIW
NVTYSGTSKSCSDSFYRNMRWLTQKNGNYPVQDAQYTNTRGKDILFVWGIHHPPTDTAQTNLYTRTDTTT
SITTESLDRTFKPLIGPRPLVNGLIGRINYYWSVLKPGQTLRVRSNGNLIAPWFGHMLSGESHGRILKTD
LNSGNCVVQCQTEKGGLNSTLPFHNISKYAFGNCPKYIRVKSLKLAIGLRNVPARSSRGLFGAIAGFIEG
GWPGLVAGWYGFQHSNDQGVGMAADRDSTQKAVDKITSKVNNIVDKMNKQYEIIDHEFSEVENRLNMINN
KVDDQIQDIWAYNAELLVLLENQKTLDEHDANVNNLYNKVKRALGSNALEDGKGFFXLYHKCDDQCMETI
RNGTYNRRKYMEESRLGRQEIEGVKLEPEGTYKILTIYSTVASSLVLAMGFAAFLFWAMSNGSCRCNICI

>UKJ62192.1 hemagglutinin [Influenza A virus]

MEIIPLVTVLLLATTNNADKICIGHQSTNSTETVDTLTETGVPVTHAKELLHTEHNGKLCATNLGNPLIL
DTCTVEGLIYGNPSCDMLLGGREWSYIVERPSAVNGTCYPGSVENLEELRILFSSSSSYQRVQMFPDAIW
NVTYSGTSKSCSDSFYRNMRWLTQKNGNYPVQDAQYTNTRGKDILFVWGIHHPPTDTAQTNLYTRTDTTT
SITTESLDRAFKPLIGPRPLVNGLIGRINYYWSVLKPGQTLRVRSNGNLIAPWFGHLLSGESHGRILKTD
LKSGNCVVQCQTEKGGLNSTLPFHNISKYAFGNCPKYIGVKSLKLAIGLRNVPARSSRGLFGAIAGFIEG
GWPGLVAGWYGFQHSNDQGVGMAADRDSTQRAVDKITSKVNNIVDKMNKQYEIIDHEFSEVENRLNMINN
KIDDQIQDIWAYNAELLVLLENQKTLDEHDANVNNLYNKVKRALGSNAVEDGKGCFELYHKCDDQCMETI
RNGTYNRRKYTEESRLGRQKIEGVKLESEGTYKILTIYSTVASSLVLAMGFAAFLFWAMSNGSCRCNICI

>UKJ62191.1 hemagglutinin [Influenza A virus]

MEVXPLMAMLLLVTTNNADKICIGHQSTNSTETVDTLTETGVPVTHAKELLHTEHNGKLCATNLGNPLIL
DTCTVEGIIYGNPSCDMLLGGREWSYIVERPSAVNGTCYPGNVENLEELRVLFSSSSSYQRIQMFPDTIW
NVTYSGTSKSCSDSFYRNMRWLTQKNGNYPVQDAQYTNTRGKDILFVWGIHHPPTDTAQTNLYTRTDTMT
SITTESLDRTFKPLIGPRPLVNGLIGRINYYWSVLKPGQTLRVRSSGNLIAPWFGHVLSGESHGRILKTD
LNSGNCVVQCQTEKGGLNSTLPFHNISKYAFGDCPKYIGVKSLKLAIGLRNVPARSSRGLFGAIAGFIEG
GWPGLVAGWYGFQHSNDQGVGMAADRDSTQKAVDKITSKVNNIVDKMNKQYEIIDHEFSEIETRLNMINN
KIDDQIQDIWAYNAELLVLLENQKTLDEHDANVNNLYNKVRRALGSNAMEDGKGCFELYHKCDDQCMETI
RNGTYNRRKYVEESRLGRQKIEGVKLESEGTYKILTIYSTVASSLVIAMGFAAFLFWAMSNGSCRCNICI

>UKJ62190.1 hemagglutinin [Influenza A virus]

MEVIPLMTMLLLVTTNNADKICIGHQSTNSTETVDTLTETGVPVTHAKELLHTEHNGKLCATNLGNPLIL
DTCTVEGIIYGNPSCDMLLGGREWSYIVERPSAVNGTCYPGNVENLEELRVLFSSSSSYQRIQMFPDTIW
NVTYSGTSKSCSDSFYRNMRWLTQKNGNYPVQDAQYTNTRGKDILFVWGIHHPPTDTAQTNLYTRTDTTT
SITTESLDRTFKPLIGPRPLVNGLIGRINYYWSVLKPGQTLRVRSSGNLIAPWFGHVLSGESHGRILKTD
LNSGNCVVQCQTEKGGLNSTLPFHNISKYAFGDCPKYIGVKSLKLAIGLRNVPARSSRGLFGAIAGFIEG
GWPGLVAGWYGFQHSNDQGVGMAADRVSTQKAVDKITSKVNNIVDKMNKQYEIIDHEFSEVETRLNMINN
KIDDQIQDIWAYNAELLVLLENQKTLDEHDANVNNLYNKVKRALGSNAMEDGKGCFELYHKCDDQCMETI
RNGTYNRIKYMEESRLERQKIEGVKLXAEGTYKILSIYSTVASSLVIAMGFAAFLFWAMSNGSCXCNICI

>UKF78684.1 hemagglutinin [Influenza A virus]

METTSLMIILLAVVTTINADKICIGHQSTNSTETVDTLTENNVPVTHAKELLHTEHNGMLCATNLGRPLI
LDTCTIEGLIYGNPSCDLLLGGKEWSYIVERPSAVNGTCYPGHVENLEELRTLFSSSNSYQRIQIFPDTI

WNVTYTGTSKSCSDSFYRNMRWLTQKNGVYTVQDAQFTNNRGKDILFVWGIHHPPSDTAQTNLYTRTDTT
TSVTTENLDRTFKPLIGPRPLVNGLIGRINYYWSVLKPGQTLRVRSNGNLIAPWYGHVLSGESHGRILKT
DLKSGDCLVQCQTEKGGLNSTLPFHNISKYAFGNCPKYIGVKSFKLAIGLRNVPAKSSRGLFGAIAGFIE
GGWPGLVAGWYGFQHSNDQGVGIAADRVSTQQAVDKITSKVNNIVDKMNKQYEIIDHEFSEVESRLNMIN
NKIDDQIQDVWAYNAELLVLLENQKTLDEHDANVNNLYNKVKRALGSNAQEDGKGCFELYHKCDDQCMET
IRNGTYNRRKYTEESRLERQKIEGVKLESEGTYKILTIYSTVASSLVLAMGFAAFLFWAMSNGSCRCNIC
I

>QAR21027.1 hemagglutinin [Influenza A virus]
METVSLITILLVAAISNADKICIGYQSTNSTETVDTLTENNVPVTHAKELLHTEHNGMLCATSLGQPLIL
DTCTIEGLIYGNPYCDLSLEGREWSYIVERPSAVNGLCYPGNVENLEELRSLFSSARSYQRIQIFPDTIW
NVSYDGTSAACSGSFYKSMRWLFRKNGNYPIQDAQYTNNQGKNILFMWGINHPPTDTTQIDLYTRTDTTT
SVATEEINRVFKPLIGPRPLVNGLMGRIDYYWSVLKPGQTLRIKSDGNLIAPWFGHILSGESHGRILKTD
LKRGSCTVQCQTEKGGLNTTLPFQNISKYAFGNCSKYIGIKSLKLAVGLRNVPSRSSRGLFGAIAGFIEG
GWSGLVAGWYGFQHSNDQGVGMAADRDSTQKAIDKITSKVNNIVDKMNKQYEIIDHEFSEVETRLNMINN
KIDDQIQDIWAYNAELLVLLENQKTLDEHDANVNNLYNKVKRALGSNAVEDGKGCFELYHKCDDQCMETI
RNGTYNRRKYQEESKLERQKIEGVKLESEGTYKILTIYSTVASSLVIAMGFAAFLFWAMSNGSCRCNICI

>QAR21026.1 hemagglutinin [Influenza A virus]
METVSLITILLVAAISNADKICIGYQSTNSTETVDTLTENNVPVTHAKELLHTEHNGMLCATSLGQPLIL
DTCTIEGLIYGNPYCDLSLEGREWSYIVERPSAVNGLCYPGNVENLEELRSLFSSARSYQRIQIFPDTIW
NVSYDGTSAACSGSFYKSMRWLIRKNGNYPIQDAQYTNNQGKNILFMWGINHPPTDTTQIDLYTRTDTTT
SVATEEINRVFKPLIGPRPLVNGLMGRIDYYWSVLKPGQTLRIKSDGNLIAPWFGHILSGESHGRILKTD
LKRGSCTVQCQTEKGGLNTTLPFQNISKYAFGNCSKYIGIKSLKLAVGLRNVPSRSSRGLFGAIAGFIEG
GWSGLVAGWYGFQHSNDQGVGMAADRDSTQKAIDKITSKVNNIVDKMNKQYEIIDHEFSEVETRLNMINN
KIDDQIQDIWAYNAELLVLLENQKTLDEHDANVNNLYNKVKRALGSNAVEDGKGCFELYHKCDDQCMETI
RNGTYNRRKYQEESKLERQKIEGVKLESEGTYKILTIYSTVASSLVIAMGFAAFLFWAMSNGSCRCNICI

>QHA34395.1 hemagglutinin [Influenza A virus]
METASLITILLVVTVSNADKICIGYQSTNSTETVDTLTENNVPVTHAKELLHTEHNGMLCATSLGHPLIL
DTCTIEGLIYGNPSCDLLLGGREWSYIVERPSAANGLCYPGNVENLEELRSLFSSARSYQRIQIFPDTIW
NVSYSGTSKACSDSFYRSMRWLTQKDNAYPIQDAQYTNNQGKNILFMWGINHPPTDSVQTNLYTRTDTTT
SVATEEINRTFKPLIGPRPLVNGLMGRINYYWSVLKPGQTLRIKSNGNLIAPWYGHILSGESHGRILKTD
LKRGSCTVQCQTEKGGLNTTLPFQNVSKYAFGNCSKYIGIKSLKLAVGLRNVPSRSSRGLFGAIAGFIEG

GWSGLVAGWYGFQHSNNQGVGMAADRDSTQKAIDKITSKVNNIVDKMNKQYEIIDHEFNEVETRLNMINN
KIDDQIQDIWAYNAELLVLLENQKTLDEHDANVNNLYNKVKRALGSNAVEDGKGCFELYHKCDDQCMETI
RNGTYNRRKYQEESKLERQKIEGVKLESEGTYKILTIYSTVASSLVIAMGFAAFLFWAMSNGSCRCNICI

>QHA34383.1 hemagglutinin [Influenza A virus]
METISLITILLVVTVSNADKICIGYQSTNSTETVDTLTENNVPVTHAKELLHTEHNGMLCATGLGHPLIL
DTCTIEGLIYGNPSCDLLLGGREWSYIVERPSAVNGLCYPGNVENLEELRSLFSSASSYQRIQIFPDTIW
NVTYSGTSKACSDSFYRSMRWLTQKNNAYPIQDAQYTNNQEKNILFMWGINHPPTDTAQTNLYTRTDTTT
SVATEEINRTFKPLIGPRPLVNGLQGRIDYYWSVLKPGQTLRIRSNGNLIAPWYGHILSGESHGRILKTD
LKRGSCTVQCQTEKGGLNTTLPFQNVSKYAFGNCSKYIGIKSLKLAVGLRNVPSRSSRGLFGAIAGFIEG
GWSGLVAGWYGFQHSNDQGVGMAADRDSTQKAIDKITSKVNSIVDKMNKQYEIIDHEFSEVETRLNMINN
KIDDQIQDIWAYNAELLVLLENQKTLDEHDANVNNLYNKVKRALGSNAMEDGKGCFELYHKCDNQCMETI
RNGTYNRRKYQEESKLERQKIEGVKLESEGTYKILTIYSTVASSLVIAMGFAAFLFWAMSNGSCRCHICI

>QHA34371.1 hemagglutinin [Influenza A virus]
METASLITILLVATVSNADKVCIGYQSTNSTETVDTLTENNVPVTHAKELLHTEHNGMLCATSLGHPLIL
DTCTIEGLIYGNPSCDPLLGGREWSYIVERPSAVNGLCYPGNVENLEELRSLFSSARSYQRIQIFPDTIW
NVSYSGTSKACSDSFYRSMRWLTQKDNAYPIQDAQYTNNQEKNILFMWGINHPPTDTVQTNLYTRTDTTT
SVATEEINRTFKPLIGPRPLVNGLMGRINYYWSVLKPGQTLRIKSNGNLIAPWYGHILSGESHGRILKTD
LKRGSCTVQCQTEKGGLNTTLPFQNVSKYAFGNCSKYIGIKSLKLAVGLRNVPSKSSRGLFGAIAGFIEG
GWPGLVAGWYGFQHSNDQGVGMAADRDSTQKAIDKITSKVNNIIDKMNKQYEIIDHEFSEVETRLNMINN
KVDDQIQDIWAYNAELLVLLENQKTLDEHDANVNNLYNKVKRALGSNAVEDGKGCFELYHKCDDQCMETI
RNGTYNRRKYQEESKLERQKIEGVKLESEGTYKILTIYSTVASSLVIAMGFAAFLFWAMSNGSCRCNICI

>QHA24409.1 hemagglutinin [Influenza A virus]
METASLITILLVVTVSNADKICIGYQSTNSTETVDTLTENNVPVTHAKELLHTEHNGMLCATSLGHPLIL
DTCTIEGLIYGNPSCDLLLGGREWSYIVERPSAVNGLCYPGNVENLEELRSLFSSARSYQRIQIFPDTIW
NVSYSGTSKACSDSFYRSMRWLTQKDNAYPIQDAQYTNNQEKNILFMWGINHPPTDTAQTNLYTRTDTTT
SVATEEINRTFKPLIGPRPLVNGLMGRINYYWSVLKPGQTLRIKSNGNLIAPWYGHILSGESHGRILKTD
LKRGSCTVQCQTEKGGLNTTLPFQNVSKYAFGNCSKYIGIKSLKLAVGLRNVPSRSSRGLFGAIAGFIEG
GWSGLVAGWYGFQHSNDQGVGMAADRDSTQKAIDKITSKVNNIVDKMNKQYEIIDHEFSEVETRLNMINN
KIDDQIQDIWAYNAELLVLLENQKTLDEHDANVNNLYNKVKRALGSNAVEDGKGCFELYHKCDDQCMETI
RNGTYNRRKYQEESKLERQKIEGVKLESEGTYKILTIYSTVASSLVIAMGFAAFLFWAMSNGSCRCNICI

>QHA24397.1 hemagglutinin [Influenza A virus]

METVSLITMLLVITMSNADKICIGYQSTNSTETVDTLTENSVPVTHAKELLHTEHNGMLCATTLGQPLIL
DTCTIEGLIYGNPSCDLLLGGREWSYIVERPSAVNGLCYPGNVENLEELRSLFSSASSYQRIQIFPDTIW
NVSYSGTSKACSDSFYRSMRWLTQKNNAYPVQDAQYTNNQGKNILFMWGINHPPTDTVQTNLYTRTDTTT
SVATEEINRTFKPLIGPRPLVNGLQGRIDYYWSVLKPGQTLRIRSNGNLIAPWYGHILSGESHGRILKTD
LKRGSCTVQCQTEKGGLNTTLPFQNVSKYAFGNCSKYIGVKSLKLAVGLRNVPSRSSRGLFGAIAGFIEG
GWSGLVAGWYGFQHSNDQGVGMAADRDSTQKAIDKITSKVNNIVDKMNKQYEIIDHEFSEIETRLNMINN
KIDDQIQDIWAYNAELLVLLENQKTLDEHDANVNNLYNKVKRVLGSNAIEDGKGCFELYHKCDNQCMETI
RNGTYNRRRYQEESKLERQKIEGVKLESEGTYKILTIYSTVASSLVIAMGFAAFLFWAMSNGSCRCNICI

>QHA24337.1 hemagglutinin [Influenza A virus]

METASLITILLLVTVSNADKICIGYQSTNSTETVDTLTENNVPVTHAKGLLHTEHNGMLCATSLGHPLIL
DTCTIEGLIYGNPSCDLLLGGREWSYIVERPSAVNGLCYPGNVENLEELRSLFSSARSYQRIQIFPDTIW
NVSYSGTSKACSDSFYRSMRWLTQKDNAYPIQDAQYTNNQEKNILFMWGINHPPTDTVQTNLYTRTDTTT
SVATEEINRIFKPLIGPRPLVNGLMGRINYYWSVLKPGQTLRIKSNGNLIAPWYGHILSGESHGRILKTD
LKRGSCTVQCQTEKGGLNTTLPFQNVSKYAFGNCSKYIGIKSLKLAVGLRNVPSRSSRGLFGAIAGFIEG
GWSGLVAGWYGFQHSNDQGVGMAADRDSTQKAIDKITSKVNNIVDKMNKQYEIIDHEFSEVETRLNMINN
KIDDQIQDIWAYNAELLVLLENQKTLDEHDANVNNLYNKVKRALGSNAVEDGKGCFELYHKCDDQCMETI
RNGTYNRRKYQEESKLERQKIEGVKLESEGTYKILTIYSTVASSLVIAMGFAAFLFWAMSNGSCRCNICI

>QHA24314.1 hemagglutinin [Influenza A virus]

METATIITILLAITGSNADKICIGYQSTNSTETVDTLTENNVPVTHAKELLHTEHNGMLCATNLGRPLIL
DTCTIEGIIYGNPSCDLPLEGREWSYIVERPSAINGVCYPGNVENLEELRSLFSSASSHQRIQIFPDSIW
NVSYSGTSKACSDSFYRNMRWLTQKNNNYPVQDAQYTNNRGKNILFMWGINHPPTDTAQTNLYTRTDTTT
SVATEDLTRIFEPLIGPRPLVNGLQGRIDYYWAVLKPGQTLRVRSNGNLIAPWYGHILLGESHGRILKTD
LKSGNCVVQCQTERGGLNTTLPFHNVSKYAFGNCPKYIGVKSLKLAVGLRNVPARSSRGLFGAIAGFIEG
GWSGLVAGWYGFQHSNDQGTGMAADRDSTQKAIDKITSKVNNIVDKMNKQYEIIDHEFSEIEARLNMIND
KIDDQIQDIWAYNAELLVLLENQKTLDEHDANVNNLYNKVKRALGANAVEDGKGCFDLYHKCDNQCMETI
RNGTYNRRKYQEESRLERQKIGGVKLESEGTYKILTIYSTVASSLVIAMGFAAFLFWAMSNGSCRCNICI

>QHA24302.1 hemagglutinin [Influenza A virus]

MEAATTIITILLAITGSNADKICIGYQSTNSTETVDTLTENNVPVTHAKELLHTEHNGVLCATNLGRPLI
LDTCTIEGLIYGNPSCDLPLEGREWSYIVERPSAVNGVCYPGNVENLEELRSLFSSASSYQRIQIFPDSI
WNVSYSGTSKACSDSFYRNMRWLTQKNNNYPVQDAQYTNNRGKNILFMWGINHPPTDTAQTNLYTRTDTT

TSVATEDINRTFKPLIGPRPLVNGLQGRIDYYWAVLKPGQTLRVRSNGNLIAPWYGHILLGESHGRILKT
DLKSGNCVVQCQTERGGLNTTLPFHNVSKYAFGNCPKYIGVKSLKLAVGLRNVPARSSRGLFGAIAGFIE
GGWSGLVAGWYGFQHSNDQGTGMAADRDSTQKAIDKITSKINNVVDKMNKQYEIIDHEFSEIETRLNMIN
DKIDDQIQDIWAYNAELLVLLENQKTLDEHDANVNNLYNKVKRALGSNAVEDGKGCFDLYHKCDNQCMET
IRNGTYNRRKYQEESRLERQKIEGVKLESEGTYKILTIYSAVASSLVIAMGFAAFLFWAMSNGSCRCNIC
I

>QGZ07232.1 hemagglutinin [Influenza A virus]
MEAATIITILLAITGSNADKICIGYQSTNSTETVDTLTENNVPVTHAKELLHTEHNGMLCATNLGRPLIL
DTCTIEGLIYGNPSCDLPLEGREWSYIVERPSAVNGVCYPGNVENLEELRSLFSSASSYQRIQIFPDSIW
NVSYSGTSKACSDSFYRSMRWLTQKNNNYPVQDAQYTNNRGKNILFMWGINHPPTDTAQTNLYTRTDTTT
SVATEDINRTFKPLIGPRPLVNGLQGRIDYYWAVLKPGQTLRIRSNGNLIAPWYGHILLGESHGRILKTD
LKSGNCVVQCQTERGGLNTTLPFHNVSKYAFGNCPKYIGVKSLKLAVGLRNVPARSSRGLFGAIAGFIEG
GWSGLVAGWYGFQHSNDQGTGMAADRDSTQKAIGKITSKVNNIVDKMNKQYEIIDHEFSEIETRLNMIND
KIDDQIQDIWAYNAELLVLLENQKTLDEHDANVNNLYNKVKRALGSNAMEDGKGCFDLYHKCDNQCMETI
RNGTYNRRKYQEESRLERQKIEGVKLESEGTYKILTIYSTVASSLVIAMGFAAFLFWAMSNGSCRCNICI

>QPC52359.1 hemagglutinin [Influenza A virus]
METVSLITILLVAAVSNADKICIGYQSTNSTETVDTLTENNVPVTHAKELLHTEHNGMLCATSLGQPIIL
DTCTIEGLIYGNPSCDLSLEGREWSYIVERPSAVNGLCYPGNVENLEELRSLFSSARSYQRIQIFPDTIW
NVSYDGTSTACSGSFYRNMRWLTRKNGEYPIQDAQYTNNQGKNILFMWGINHPPADTTQRDLYTRTDTTT
SVATEEINRIFKPLIGPRPLVNGLMGRIDYYWSVLKPGQTLRIKSDGNLIAPWYGHILSGESHGRILKTD
LKRGSCTVQCQTEKGGLNTTLPFQNVSKYAFGNCSKYIGIKSLKLAVGLRNVPSRSSRGLFGAIAGFIEG
GWSGLVAGWYGFQHSNDQGVGMAADRDSTQKAVDKITSKVNTIVDKMNKQYEIIDHEFSEVETRLNMINN
KIDDQIQDIWAYNAELLVLLENQKTLDEHDANVNNLYNKVKRALGSNAVEDGKGCFELYHKCDDQCMETI
RNGTYNRRKYQEESKLERQKIEGVKLESEGTYKILTIYSTVASSLVIAMGFAAFLFWAMSNGSCRCNICI

>UAU42822.1 hemagglutinin, partial [Influenza A virus]
NADKICIGHQSTNSTETVDTLTETGVPVTHAKELLHTEHNGKLCATNLGNPLILDTCTVEGLIYGNPSCD
LLLGGREWSYIVERPSAVNGTCYPGNVENLEELRILFSSSSSYQRIQMFPDTIWNVTYSGTSKSCSDSFY
RNMRWLTQKNGNYPVQDAQYTNTRGKNILFVWGIHHPPTDTAQTNLYTRTDTTTSITTESLDRTFKPLIG
PRPLVNGLIGRINYYWSVLKPGQTLRVRSNGNLIAPWFGHVLSGESHGRILKTDLNSGNCVVQCQTEKGG
LNSTLPFHNISKYAFGNCPKYIGVKSLKLAIGLRNVPARSSRGLFGAIAGFIEGGWPGLVAGWYGFQHSN
DQGVGMAADRDSTQKAVDKITSKVNNIVDKMNKQYEIIDHEFSEVENRLNMINNKVDDQIQDIWAYNAEL

LVLLENQKTLDEHDANVNNLYNKVKRALGSNAMEDGKGCFELYHKCDDQCMETIRNGTYNRRKYMEESRL
GRQKIEGVKLESEGTYKILTIYSTVASSLVLAMG

>QXI62312.1 hemagglutinin [Influenza A virus]
METISLITILLVATVSNADKICIGYQSTNSTETVDTLTENNVPVTHAKELLHTEHNGMLCATSLGQPLIL
DTCTIEGLIYGNPSCDLSLEGREWSYIVERPSAVNGLCYPGNVENLEELRSLFSSAISYQRIQIFPDTIW
NVSYDGTSTACSNSFYRSMRWLTRKDGNYPTQDAQYTNNQGKNILFMWGINHPPTDDTQRNLYTRTDTTT
SVATEEINRIFKPLIGPRPLVNGLMGRIDYYWSVLKPGQTLRIKSDGNLIAPWYGYILSGETHGRILKTD
LKRGSCTVQCQTEKGGLNTTLPFQNVSKYAFGNCSKYIGIKSLKLAVGLRNVPSRSSRGLFGAIAGFIEG
GWSGLVAGWYGFQHSNDQGVGMAADRDSTQKAIDKITSKVNNIVDKMNKQYEIIDHEFSEVETRLNMINN
KIDDQIQDIWAYNAELLVLLENQKTLDEHDANVNNLYNKVKRALGSNAVEDGKGCFELYHKCNDQCMETI
RNGTYNKRKYQEESKLERQKIEGVKLESEGTYKILTIYSTVASSLVIAMGFAAFLFWAMSNGSCRCNICI

>QXI62289.1 hemagglutinin [Influenza A virus]
METVSLITILVVATVSNADKICIGYQSTNSTETVDTLTENNVPVTHAKELLHTEHNGMLCATSLGHPLIL
DTCTIEGLIYGNPSCDPLLGGREWSYIVERPSAVNGLCYPGNVENLEELRSLFSSSRSYQRIQIFPDTIW
NVSYSGTSKACSDSFYRSMRWLTQKNNAYPTQDAQYTNNQGKNILFMWGINHPPTDTAQTNLYTRTDTTT
SVATEEMNRIFKPLIGPRPLVNGLMGRINYYWSVLKPGQTLRIKSDGNLIAPWYGHILSGESHGRILKTD
LKRGSCTVQCQTEKGGLNTTLPFQNVSKYAFGNCSKYIGVKSLKLAVGLRNVPSKSSRGLFGAIAGFIEG
GWSGLVAGWYGFQHSNDQGVGMAADRDSTQKAIDKITSKVNNIVDKMNKQYEIIDHEFSEVETRLNMINN
KFDDQIQDIWAYNAELLVLLENQKTLDEHDANVNNLYNKVKRALGSNAVEDGKGCFELYHKCDDHCMETI
RNGTYNRRKYQEESKLERQKIEGVKLESEETYKILTIYSTVASSLVIAMGFAAFLFWAMSNGSCRCNICI

>QXM00737.1 hemagglutinin [Influenza A virus]
MEAIPLLTLLLVVTTSSADKICVGHQSTNSTETVDTLTETNVPVTQAKELLHTEHNGMLCATNLGRPLIL
NTCTIEGLIYGNPSCDMLLGGREWSYIVERPSAVNGTCYPGNIENLEELRTLFSSSSSYQRIQLFPDTIW
NVTYTGTSKSCSDSFYRNMRWLTQKNGLYPIQDAQYTNNRGKDILFVWGIHHPPTDTAQTNLYTRTDTTT
SVTTENLDRTFKPLIGPRPLVNGLIGRINYYWSVLKPGQTLRVRSNGNLVAPWFGHVLSGESHGRILKTD
LSSGNCVVQCQTEKGGLNSTLPFHNISKYAFGTCPKYIGVKSLKLAVGLRNVHARSSRGLFGAIAGFIEG
GWPGLVAGWYGFQHSNDQGVGMAADRDSTQKAVDKITSKVNNIVDKMNKQYEIIDHEFSEVETRLNMINS
KIDDQIQDIWAYNAELLVLLENQKTLDEHDANVNNLYNKVKRALGSNAMEDGKGCFELYHKCDDQCMETI
RNGTYNRRKYTEESRLERQKIEGVKLESEGTYKILSIYSTVASSLVLAMGFAAFLFWAMSNGSCRCNICI

>QXM00761.1 hemagglutinin [Influenza A virus]

MEAIPLLTLLLVVTTSSADKICVGHQSTNSTETVDTLTETNVPVTQAKELLHTEHNGMLCATNLGRPLIL
NTCTIEGLIYGNPSCDMLLGGREWSYIVERPSAVNGTCYPGNIENLEELRTLFSSSSSYQRIQLFPDTIW
NVTYTGTSKSCSDSFYRNMRWLTQKNGLYPIQDAQYTNNRGKDILFVWGIHHPPTDTAQTNLYTRTDTTT
SVTTENLDRTFKPLIGPRPLVNGLIGRINYYWSVLKPGQTLRVRSNGNLVAPWFGHVLSGESHGRILKTD
LSSGNCVVQCQTEKGGLNSTLPFHNISKYAFGTCPKYIGVKSLKLAVGLRNVHARSSRGLFGAIAGFIEG
GWPGLVAGWYGFQHSNDQGVGMAADRDSTQKAVDKITSKVNNIVDKMNKQYEIIDHEFSEVETRLNMINS
KIDDQIQDIWAYNAELLVLLENQKTLDEHDANVNNLYNKVKRALGSNAMEDGKGCFELYHKCDDQCMETI
RNGTYNRRKYTEESRLERQKIEGVKLESEGTYKILSIYSTVASSLVLAMGFAAFLFWAMSNGSCRCNICI

>QXM00725.1 hemagglutinin [Influenza A virus]
MEAIPLLTLLLVVTTSSADKICVGHQSTNSTETVDTLTETNVPVTQAKELLHTEHNGMLCATNLGRPLIL
NTCTIEGLIYGNPSCDMLLGGREWSYIVERPSAVNGTCYPGNIENLEELRTLFSSSSSYQRIQLFPDTIW
NVTYTGTSKSCSDSFYRNMRWLTQKNGLYPIQDAQYTNNRGKDILFVWGIHHPPTDTAQTNLYTRTDTTT
SVTTENLDRTFKPLIGPRPLVNGLIGRINYYWSVLKPGQTLRVRSNGNLVAPWFGHVLSGESHGRILKTD
LSSGNCVVQCQTEKGGLNSTLPFHNISKYAFGTCPKYIGVKSLKLAVGLRNVHARSSRGLFGAIAGFIEG
GWPGLVAGWYGFQHSNDQGVGMAADRDSTQKAVDKITSKVNNIVDKMNKQYEIIDHEFSEVETRLNMINS
KIDDQIQDIWAYNAELLVLLENQKTLDEHDANVNNLYNKVKRALGSNAMEDGKGCFELYHKCDDQCMETI
RNGTYNRRKYTEESRLERQKIEGVKLESEGTYKILSIYSTVASSLVLAMGFAAFLFWAMSNGSCRCNICI

>QXM00713.1 hemagglutinin [Influenza A virus]
MEAIPLLTLLLVVTTSSADKICVGHQSTNSTETVDTLTETNVPVTQAKELLHTEHNGMLCATNLGRPLIL
NTCTIEGLIYGNPSCDMLLGGREWSYIVERPSAVNGTCYPGNIENLEELRTLFSSSSSYQRIQLFPDTIW
NVTYTGTSKSCSDSFYRNMRWLTQKNGLYPIQDAQYTNNRGKDILFVWGIHHPPTDTAQTNLYTRTDTTT
SVTTENLDRTFKPLIGPRPLVNGLIGRINYYWSVLKPGQTLRVRSNGNLIAPWFGHVLSGESHGRILKTD
LSSGNCVVQCQTEKGGLNSTLPFHNISKYAFGTCPKYIGVKSLKLAVGLRNVHARSSRGLFGAIAGFIEG
GWPGLVAGWYGFQHSNDQGVGMAADRDSTQKAVDKITSKVNNIVDKMNKQYEIIDHEFSEVETRLNMINS
KIDDQIQDIWAYNAELLVLLENQKTLDEHDANVNNLYNKVKRALGSNAMEDGKGCFELYHKCDDQCMETI
RNGTYNRRKYTEESRLERQKIEGVKLESEGTYKILSIYSTVASSLVLAMGFAAFLFWAMSNGSCRCNICI

>QXM00677.1 hemagglutinin [Influenza A virus]
MEAIPLLTLLLVVTTSSADKICVGHQSTNSTETVDTLTETNVPVTQAKELLHTEHNGMLCATNLGRPLIL
NTCTIEGLIYGNPSCDMLLGGREWSYIVERPSAVNGTCYPGNIENLEELRTLFSSSSSYQRIQLFPDTIW
NVTYTGTSKSCSDSFYRNMRWLTQKNGLYPIQDAQYTNNRGKDILFVWGIHHPPTDTAQTNLYTRTDTTT
SVTTENLDRTFKPLIGPRPLVNGLIGRINYYWSVLKPGQTLRVRSNGNLVAPWFGHVLSGESHGRILKTD

LSSGNCVVQCQTEKGGLNSTLPFHNISKYAFGTCPKYIGVKSLKLAVGLRNVHARSSRGLFGAIAGFIEG
GWPGLVAGWYGFQHSNDQGVGMAADRDSTQKAVDKITSKVNNIVDKMNKQYEIIDHEFSEVETRLNMINS
KIDDQIQDIWAYNAELLVLLENQKTLDEHDANVNNLYNKVKRALGSNAMEDGKGCFELYHKCDDQCMETI
RNGTYNRRKYTEESRLERQKIEGVKLESEGTYKILSIYSTVASSLVLAMGFAAFLFWAMSNGSCRCNICI

>QXM00605.1 hemagglutinin [Influenza A virus]
MEAIPLLTLLLVVTTSSADKICVGHQSTNSTETVDTLTETNVPVTQAKELLHTEHNGMLCATNLGRPLIL
NTCTIEGLIYGNPSCDMLLGGREWSYIVERPSAVNGTCYPGNIENLEELRTLFSSSSSYQRIQLFPDTIW
NVTYTGTSKSCSDSFYRNMRWLTQKNGLYPIQDAQYTNNRGKDILFVWGIHHPPTDTAQTNLYTRTDTTT
SVTTENLDRTFKPLIGPRPLVNGLIGRINYYWSVLKPGQTLRVRSNGNLVAPWFGHVLSGESHGRILKTD
LSSGNCVVQCQTEKGGLNSTLPFHNISKYAFGTCPKYIGVKSLKLAVGLRNVHARSSRGLFGAIAGFIEG
GWPGLVAGWYGFQHSNDQGVGMAADRDSTQKAVDKITSKVNNIVDKMNKQYEIIDHEFSEVETRLNMINS
KIDDQIQDIWAYNAELLVLLENQKTLDEHDANVNNLYNKVKRALGSNAMEDGKGCFELYHKCDDQCMETI
RNGTYNRRKYTEESRLERQKIEGVKLESEGTYKILSIYSTVASSLVLAMGFAAFLFWAMSNGSCRCNICI

>QXM00583.1 hemagglutinin [Influenza A virus]
MEAIPLLTLLLVVTTSSADKICVGHQSTNSTETVDTLTETNVPVTQAKELLHTEHNGMLCATNLGRPLIL
NTCTIEGLIYGNPSCDMLLGGREWSYIVERPSAVNGTCYPGNIENLEELRTLFSSSSSYQRIQLFPDTIW
NVTYTGTSKSCSDSFYRNMRWLTQKNGLYPIQDAQYTNNRGKDILFVWGIHHPPTDTAQTNLYTRTDTTT
SVTTENLDRTFKPLIGPRPLVNGLIGRINYYWSVLKPGQTLRVRSNGNLVAPWFGHVLSGESHGRILKTD
LSSGNCVVQCQTEKGGLNSTLPFHNISKYAFGTCPKYIGVKSLKLAVGLRNVHARSSRGLFGAIAGFIEG
GWPGLVAGWYGFQHSNDQGVGMAADRDSTQKAVDKITSKVNNIVDKMNKQYEIIDHEFSEVETRLNMINS
KIDDQIQDIWAYNAELLVLLENQKTLDEHDANVNNLYNKVKRALGSNAMEDGKGCFELYHKCDDQCMETI
RNGTYNRRKYTEESRLERQKIEGVKLESEGTYKILSIYSTVASSLVLAMGFAAFLFWAMSNGSCRCNICI

>QXM00571.1 hemagglutinin [Influenza A virus]
MEAIPLLTLLLVVTTSSADKICVGHQSTNSTETVDTLTETNVPVTQAKELLHTEHNGMLCATNLGRPLIL
NTCTIEGLIYGNPSCDMLLGGREWSYIVERPSAVNGTCYPGNIENLEELRTLFSSSSSYQRIQLFPDTIW
NVTYTGTSKSCSDSFYRNMRWLTQKNGLYPIQDAQYTNNRGKDILFVWGIHHPPTDTAQTNLYTRTDTTT
SVTTENLDRTFKPLIGPRPLVNGLIGRINYYWSVLKPGQTLRVRSNGNLVAPWFGHVLSGESHGRILKTD
LSSGNCVVQCQTEKGGLNSTLPFHNISKYAFGTCPKYIGVKSLKLAVGLRNVHARSSRGLFGAIAGFIEG
GWPGLVAGWYGFQHSNDQGVGMAADRDSTQKAVDKITSKVNNIVDKMNKQYEIIDHEFSEVETRLNMINS
KIDDQIQDIWAYNAELLVLLENQKTLDEHDANVNNLYNKVKRALGSNAMEDGKGCFELYHKCDDQCMETI
RNGTYNRRKYTEESRLERQKIEGVKLESEGTYKILSIYSTVASSLVLAMGFAAFLFWAMSNGSCRCNICI

>QXM00547.1 hemagglutinin [Influenza A virus]

MEAIPLLTLLLVVTTSSADKICVGHQSTNSTETVDTLTETNVPVTQAKELLHTEHNGMLCATNLGRPLIL
NTCTIEGLIYGNPSCDMLLGGREWSYIVERPSAVNGTCYPGNIENLEELRTLFSSSSSYQRIQLFPDTIW
NVTYTGTSKSCSDSFYRNMRWLTQKNGLYPIQDAQYTNNRGKDILFVWGIHHPPTDTAQTNLYTRTDTTT
SVTTENLDRTFKPLIGPRPLVNGLIGRINYYWSVLKPGQTLRVRSNGNLVAPWFGHVLSGESHGRILKTD
LSSGNCVVQCQTEKGGLNSTLPFHNISKYAFGTCPKYIGVKSLKLAVGLRNVHARSSRGLFGAIAGFIEG
GWPGLVAGWYGFQHSNDQGVGMAADRDSTQKAVDKITSKVNNIVDKMNKQYEIIDHEFSEVETRLNMINS
KIDDQIQDIWAYNAELLVLLENQKTLDEHDANVNNLYNKVKRALGSNAMEDGKGCFELYHKCDDQCMETI
RNGTYNRRKYTEESRLERQKIEGVKLESEGTYKILSIYSTVASSLVLAMGFAAFLFWAMSNGSCRCNICI

>QXM00511.1 hemagglutinin [Influenza A virus]

MEAIPLLTLLLVVTTSSADKICVGHQSTNSTETVDTLTETNVPVTQAKELLHTEHNGMLCATNLGRPLIL
NTCTIEGLIYGNPSCDMLLGGREWSYIVERPSAVNGTCYPGNIENLEELRTLFSSSSSYQRIQLFPDTIW
NVTYTGTSKSCSDSFYRNMRWLTQKNGLYPIQDAQYTNNRGKDILFVWGIHHPPTDTAQTNLYTRTDTTT
SVTTENLDRTFKPLIGPRPLVNGLIGRINYYWSVLKPGQTLRVRSNGNLVAPWFGHVLSGESHGRILKTD
LSSGNCVVQCQTEKGGLNSTLPFHNISKYAFGTCPKYIGVKSLKLAVGLRNVHARSSRGLFGAIAGFIEG
GWPGLVAGWYGFQHSNDQGVGMAADRDSTQKAVDKITSKVNNIVDKMNKQYEIIDHEFSEVETRLNMINS
KIDDQIQDIWAYNAELLVLLENQKTLDEHDANVNNLYNKVKRALGSNAMEDGKGCFELYHKCDDQCMETI
RNGTYNRRKYTEESRLERQKIEGVKLESEGTYKILSIYSTVASSLVLAMGFAAFLFWAMSNGSCRCNICI

>QXM00499.1 hemagglutinin [Influenza A virus]

MEAIPLLTLLLVVTTSSADKICVGHQSTNSTETVDTLTETNVPVTQAKELLHTEHNGMLCATNLGRPLIL
NTCTIEGLIYGNPSCDMLLGGREWSYIVERPSAVNGTCYPGNIENLEELRTLFSSSSSYQRIQLFPDTIW
NVTYTGTSKSCSDSFYRNMRWLTQKNGLYPIQDAQYTNNRGKDILFVWGIHHPPTDTAQTNLYTRTDTTT
SVTTENLDRTFKPLIGPRPLVNGLIGRINYYWSVLKPGQTLRVRSNGNLVAPWFGHVLSGESHGRILKTD
LSSGNCVVQCQTEKGGLNSTLPFHNISKYAFGTCPKYIGVKSLKLAVGLRNVHARSSRGLFGAIAGFIEG
GWPGLVAGWYGFQHSNDQGVGMAADRDSTQKAVDKITSKVNNIVDKMNKQYEIIDHEFSEVETRLNMINS
KIDDQIQDIWAYNAELLVLLENQKTLDEHDANVNNLYNKVKRALGSNAMEDGKGCFELYHKCDDQCMETI
RNGTYNRRKYTEESRLERQKIEGVKLESEGTYKILSIYSTVASSLVLAMGFAAFLFWAMSNGSCRCNICI

>QXM00463.1 hemagglutinin [Influenza A virus]

MEAIPLLTLLLVVTTSSADKICVGHQSTNSTETVDTLTETNVPVTQAKELLHTEHNGMLCATNLGRPLIL
NTCTIEGLIYGNPSCDMLLGGREWSYIVERPSAVNGTCYPGNIENLEELRTLFSSSSSYQRIQLFPDTIW

NVTYTGTSKSCSDSFYRNMRWLTQKNGLYPIQDAQYTNNRGKDILFVWGIHHPPTDTAQTNLYTRTDTTT
SVTTENLDRTFKPLIGPRPLVNGLIGRINYYWSVLKPGQTLRVRSNGNLVAPWFGHVLSGESHGRILKTD
LSSGNCVVQCQTEKGGLNSTLPFHNISKYAFGTCPKYIGVKSLKLAVGLRNVHARSSRGLFGAIAGFIEG
GWPGLVAGWYGFQHSNDQGVGMAADRDSTQKAVDKITSKVNNIVDKMNKQYEIIDHEFSEVETRLNMINS
KIDDQIQDIWAYNAELLVLLENQKTLDEHDANVNNLYNKVKRALGSNAMEDGKGCFELYHKCDDQCMETI
RNGTYNRRKYTEESRLERQKIEGVKLESEGTYKILSIYSTVASSLVLAMGFAAFLFWAMSNGSCRCNICI

>QXM00451.1 hemagglutinin [Influenza A virus]
MEAIPLLTLLLVVTTSSADKICVGHQSTNSTETVDTLTETNVPVTQAKELLHTEHNGMLCATNLGRPLIL
NTCTIEGLIYGNPSCDMLLGGREWSYIVERPSAVNGTCYPGNIENLEELRTLFSSSSSYQRIQLFPDTIW
NVTYTGTSKSCSDSFYRNMRWLTQKNGLYPIQDAQYTNNRGKDILFVWGIHHPPTDTAQTNLYTRTDTTT
SVTTENLDRTFKPLIGPRPLVNGLIGRINYYWSVLKPGQTLRVRSNGNLVAPWFGHVLSGESHGRILKTD
LSSGNCVVQCQTEKGGLNSTLPFHNISKYAFGTCPKYIGVKSLKLAVGLRNVHARSSRGLFGAIAGFIEG
GWPGLVAGWYGFQHSNDQGVGMAADRDSTQKAVDKITSKVNNIVDKMNKQYEIIDHEFSEVETRLNMINS
KIDDQIQDIWAYNAELLVLLENQKTLDEHDANVNNLYNKVKRALGSNAMEDGKGCFELYHKCDDQCMETI
RNGTYNRRKYTEESRLERQKIEGVKLESEGTYKILSIYSTVASSLVLAMGFAAFLFWAMSNGSCRCNICI

>QXM00439.1 hemagglutinin [Influenza A virus]
MEAIPLLTLLLVVTTSSADKICVGHQSTNSTETVDTLTETNVPVTQAKELLHTEHNGMLCATNLGRPLIL
NTCTIEGLIYGNPSCDMLLGGREWSYIVERPSAVNGTCYPGNIENLEELRTLFSSSSSYQRIQLFPDTIW
NVTYTGTSKSCSDSFYRNMRWLTQKNGLYPIQDAQYTNNRGKDILFVWGIHHPPTDTAQTNLYTRTDTTT
SVTTENLDRTFKPLIGPRPLVNGLIGRINYYWSVLKPGQTLRVRSNGNLVAPWFGHVLSGESHGRILKTD
LSSGNCVVQCQTEKGGLNSTLPFHNISKYAFGTCPKYIGVKSLKLAVGLRNVHARSSRGLFGAIAGFIEG
GWPGLVAGWYGFQHSNDQGVGMAADRDSTQKAVDKITSKVNNIVDKMNKQYEIIDHEFSEVETRLNMINS
KIDDQIQDIWAYNAELLVLLENQKTLDEHDANVNNLYNKVKRALGSNAMEDGKGCFELYHKCDDQCMETI
RNGTYNRRKYTEESRLERQKIEGVKLESEGTYKILSIYSTVASSLVLAMGFAAFLFWAMSNGSCRCNICI

>QXM00415.1 hemagglutinin [Influenza A virus]
MEAIPLLTLLLVVTTSSADKICVGHQSTNSTETVDTLTETNVPVTQAKELLHTEHNGMLCATNLGRPLIL
NTCTIEGLIYGNPSCDMLLGGREWSYIVERPSAVNGTCYPGNIENLEELRTLFSSSSSYQRIQLFPDTIW
NVTYTGTSKSCSDSFYRNMRWLTQKNGLYPIQDAQYTNNRGKDILFVWGIHHPPTDTAQTNLYTRTDTTT
SVTTENLDRTFKPLIGPRPLVNGLIGRINYYWSVLKPGQTLRVRSNGNLVAPWFGHVLSGESHGRILKTD
LSSGNCVVQCQTEKGGLNSTLPFHNISKYAFGTCPKYIGVKSLKLAVGLRNVHARSSRGLFGAIAGFIEG
GWPGLVAGWYGFQHSNDQGVGMAADRDSTQKAVDKITSKVNNIVDKMNKQYEIIDHEFSEVETRLNMINS

KIDDQIQDIWAYNAELLVLLENQKTLDEHDANVNNLYNKVKRALGSNAMEDGKGCFELYHKCDDQCMETI
RNGTYNRRKYTEESRLERQKIEGVKLESEGTYKILSIYSTVASSLVLAMGFAAFLFWAMSNGSCRCNICI

>QXM00379.1 hemagglutinin [Influenza A virus]
MEAIPLLTLLLVVTTSSADKICVGHQSTNSTETVDTLTETNVPVTQAKELLHTEHNGMLCATNLGRPLIL
NTCTIEGLIYGNPSCDMLLGGREWSYIVERPSAVNGTCYPGNIENLEELRTLFSSSSSYQRIQLFPDTIW
NVTYTGTSKSCSDSFYRNMRWLTQKNGLYPIQDAQYTNNRGKDILFVWGIHHPPTDTAQTNLYTRTDTTT
SVTTENLDRTFKPLIGPRPLVNGLIGRINYYWSVLKPGQTLRVRSNGNLVAPWFGHVLSGESHGRILKTD
LSSGNCVVQCQTEKGGLNSTLPFHNISKYAFGTCPKYIGVKSLKLAVGLRNVHARSSRGLFGAIAGFIEG
GWPGLVAGWYGFQHSNDQGVGMAADRDSTQKAVDKITSKVNNIVDKMNKQYEIIDHEFSEVETRLNMINS
KIDDQIQDIWAYNAELLVLLENQKTLDEHDANVNNLYNKVKRALGSNAMEDGKGCFELYHKCDDQCMETI
RNGTYNRRKYTEESRLERQKIEGVKLESEGTYKILSIYSTVASSLVLAMGFAAFLFWAMSNGSCRCNICI

>QXM00333.1 hemagglutinin [Influenza A virus]
MEAIPLLTLLLVVTTSSADKICVGHQSTNSTETVDTLTETNVPVTQAKELLHTEHNGMLCATNLGRPLIL
NTCTIEGLIYGNPSCDMLLGGREWSYIVERPSAVNGTCYPGNIENLEELRTLFSSSSSYQRIQLFPDTIW
NVTYTGTSKSCSDSFYRNMRWLTQKNGLYPIQDAQYTNNRGKDILFVWGIHHPPTDTAQTNLYTRTDTTT
SVTTENLDRTFKPLIGPRPLVNGLIGRINYYWSVLKPGQTLRVRSNGNLVAPWFGHVLSGESHGRILKTD
LSSGNCVVQCQTEKGGLNSTLPFHNISKYAFGTCPKYIGVKSLKLAVGLRNVHARSSRGLFGAIAGFIEG
GWPGLVAGWYGFQHSNDQGVGMAADRDSTQKAVDKITSKVNNIVDKMNKQYEIIDHEFSEVETRLNMINS
KIDDQIQDIWAYNAELLVLLENQKTLDEHDANVNNLYNKVKRALGSNAMEDGKGCFELYHKCDDQCMETI
RNGTYNRRKYTEESRLERQKIEGVKLESEGTYKILSIYSTVASSLVLAMGFAAFLFWAMSNGSCRCNICI

>QXM00273.1 hemagglutinin [Influenza A virus]
MEAIPLLTLLLVVTTSSADKICVGHQSTNSTETVDTLTETNVPVTQAKELLHTEHNGMLCATNLGRPLIL
NTCTIEGLIYGNPSCDMLLGGREWSYIVERPSAVNGTCYPGNIENLEELRTLFSSSSSYQRIQLFPDTIW
NVTYTGTSKSCSDSFYRNMRWLTQKNGLYPIQDAQYTNNRGKDILFVWGIHHPPTDTAQTNLYTRTDTTT
SVTTENLDRTFKPLIGPRPLVNGLIGRINYYWSVLKPGQTLRVRSNGNLVAPWFGHVLSGESHGRILKTD
LSSGNCVVQCQTEKGGLNSTLPFHNISKYAFGTCPKYIGVKSLKLAVGLRNVHARSSRGLFGAIAGFIEG
GWPGLVAGWYGFQHSNDQGVGMAADRDSTQKAVDKITSKVNNIVDKMNKQYEIIDHEFSEVETRLNMINS
KIDDQIQDIWAYNAELLVLLENQKTLDEHDANVNNLYNKVKRALGSNAMEDGKGCFELYHKCDDQCMETI
RNGTYNRRKYTEESRLERQKIEGVKLESEGTYKILSIYSTVASSLVLAMGFAAFLFWAMSNGSCRCNICI

>QXM00309.1 hemagglutinin [Influenza A virus]

MEAIPLLTLLLVVTTSSADKICVGHQSTNSTETVDTLTETNVPVTQAKELLHTEHNGMLCATNLGRPLIL
NTCTIEGLIYGNPSCDMLLGGREWSYIVERPSAVNGTCYPGNIENLEELRTLFSSSSSYQRIQLFPDTIW
NVTYTGTSKSCSDSFYRNMRWLTQKNGLYPIQDAQYTNNRGKDILFVWGIHHPPTDTAQTNLYTRTDTTT
SVTTENLDRTFKPLIGPRPLVNGLIGRINYYWSVLKPGQTLRVRSNGNLVAPWFGHVLSGESHGRILKTD
LSSGNCVVQCQTEKGGLNSTLPFHNISKYAFGTCPKYIGVKSLKLAVGLRNVHARSSRGLFGAIAGFIEG
GWPGLVAGWYGFQHSNDQGVGMAADRDSTQKAVDKITSKVNNIVDKMNKQYEIIDHEFSEVETRLNMINS
KIDDQIQDIWAYNAELLVLLENQKTLDEHDANVNNLYNKVKRALGSNAMEDGKGCFELYHKCDDQCMETI
RNGTYNRRKYTEESRLERQKIEGVKLESEGTYKILSIYSTVASSLVLAMGFAAFLFWAMSNGSCRCNICI

>QXM00145.1 hemagglutinin [Influenza A virus]
MEAIPLLTLLLVVTTSSADKICVGHQSTNSTETVDTLTETNVPVTQAKELLHTEHNGMLCATNLGRPLIL
NTCTIEGLIYGNPSCDMLLGGREWSYIVERPSAVNGTCYPGNIENLEELRTLFSSSSSYQRIQLFPDTIW
NVTYTGTSKSCSDSFYRNMRWLTQKNGLYPIQDAQYTNNRGKDILFVWGIHHPPTDTAQTNLYTRTDTTT
SVTTENLDRTFKPLIGPRPLVNGLIGRINYYWSVLKPGQTLRVRSNGNLVAPWFGHVLSGESHGRILKTD
LSSGNCVVQCQTEKGGLNSTLPFHNISKYAFGTCPKYIGVKSLKLAVGLRNVHARSSRGLFGAIAGFIEG
GWPGLVAGWYGFQHSNDQGVGMAADRDSTQKAVDKITSKVNNIVDKMNKQYEIIDHEFSEVETRLNMINS
KIDDQIQDIWAYNAELLVLLENQKTLDEHDANVNNLYNKVKRALGSNAMEDGKGCFELYHKCDDQCMETI
RNGTYNRRKYTEESRLERQKIEGVKLESEGTYKILSIYSTVASSLVLAMGFAAFLFWAMSNGSCRCNICI

>QXM00121.1 hemagglutinin [Influenza A virus]
MEAIPLLTLLLVVTTSSADKICVGHQSTNSTETVDTLTETNVPVTQAKELLHTEHNGMLCATNLGRPLIL
NTCTIEGLIYGNPSCDMLLGGREWSYIVERPSAVNGTCYPGNIENLEELRTLFSSSSSYQRIQLFPDTIW
NVTYTGTSKSCSDSFYRNMRWLTQKNGLYPIQDAQYTNNRGKDILFVWGIHHPPTDTAQTNLYTRTDTTT
SVTTENLDRTFKPLIGPRPLVNGLIGRINYYWSVLKPGQTLRVRSNGNLVAPWFGHVLSGESHGRILKTD
LSSGNCVVQCQTEKGGLNSTLPFHNISKYAFGTCPKYIGVKSLKLAVGLRNVHARSSRGLFGAIAGFIEG
GWPGLVAGWYGFQHSNDQGVGMAADRDSTQKAVDKITSKVNNIVDKMNKQYEIIDHEFSEVETRLNMINS
KIDDQIQDIWAYNAELLVLLENQKTLDEHDANVNNLYNKVKRALGSNAMEDGKGCFELYHKCDDQCMETI
RNGTYNRRKYTEESRLERQKIEGVKLESEGTYKILSIYSTVASSLVLAMGFAAFLFWAMSNGSCRCNICI

>QXM00085.1 hemagglutinin [Influenza A virus]
MEAIPLLTLLLVVTTSSADKICVGHQSTNSTETVDTLTETNVPVTQAKELLHTEHNGMLCATNLGRPLIL
NTCTIEGLIYGNPSCDMLLGGREWSYIVERPSAVNGTCYPGNIENLEELRTLFSSSSSYQRIQLFPDTIW
NVTYTGTSKSCSDSFYRNMRWLTQKNGLYPIQDAQYTNNRGKDILFVWGIHHPPTDTAQTNLYTRTDTTT
SVTTENLDRTFKPLIGPRPLVNGLIGRINYYWSVLKPGQTLRVRSNGNLVAPWFGHVLSGESHGRILKTD

LSSGNCVVQCQTEKGGLNSTLPFHNISKYAFGTCPKYIGVKSLKLAVGLRNVHARSSRGLFGAIAGFIEG
GWPGLVAGWYGFQHSNDQGVGMAADRDSTQKAVDKITSKVNNIVDKMNKQYEIIDHEFSEVETRLNMINS
KIDDQIQDIWAYNAELLVLLENQKTLDEHDANVNNLYNKVKRALGSNAMEDGKGCFELYHKCDDQCMETI
RNGTYNRRKYTEESRLERQKIEGVKLESEGTYKILSIYSTVASSLVLAMGFAAFLFWAMSNGSCRCNICI

>QVV58232.1 hemagglutinin, partial [Influenza A virus]
MEVIPLMTMLLLITTNNADKICIGHQSTNSTETVDTLTETGVPVTHAKELLHTEHNGRLCATNLGNPLIL
DTCTVEGIVYGNPSCDMLLGGREWSYIVERPSAVNGTCYPGNVENLEELRVLFSSSSSYQRIQMFPDTIW
NVTYSGTSKSCSDSFYRNMRWLTQKNGNYPVQDAQYTNTRGKDILFVWGIHHPPTETAQTNLYTRTDTTT
SITTESLDRTFKPLIGPRPLVNGLIGRINYYWSVLKPGQTLRVRSNGNLIAPWFGHILSGESHGRILKTD
LNSGNCVVQCQTEKGGLNSTLPFHNISKYAFGDCPKYIGVKSLKLAIGLRNVPARSSRGLFGAIAGFIEG
GWPGLVAGWYGFQHSNDQGVGMAADRDSTQKAVDKITSKVNNIVDKMNKQYEIIDHEFSEVENRLNMINN
KIDDQIQDIWAYNAELLVLLENQKTLDEHDANVNNLYNKVKRALGSNAMEDGKGCFELYHKCDDQCMETI
RNGTYNRKKYMEESRLGRQKIEGVKLESEGTYKILTIYSTVASSIVLAMGFAAFLLWAMSNGSC

>QVV58231.1 hemagglutinin [Influenza A virus]
MEVIPLVTMLLLVTTNNADKICIGHQSTNSTETVDTLTETGVPVTHAKELLHTEHNGRLCATNLGNPLIL
DTCTVEGIIYGNPSCDMLLGGREWSYIVERPSAVNGTCYPGNVENLEELRVLFSSSNSYQRIQMFPDTIW
NVTYSGTSKSCSDSFYRNMRWLTQKNGNYPVQDAQYTNTRGKDILFVWGIHHPPTETAQTNLYTRTDTTT
SITTESLDRTFKPLIGPRPLVNGLIGRINYYWSVLKPGQTLRVRSNGNLIAPWFGHVLSGESHGRILKTD
LNSGNCVVQCQTEKGGLNSTLPFHNISKYAFGDCPKYIGVKSLKLAIGLRNVPARSSRGLFGAIAGFIEG
GWPGLVSGWYGFQHSNDQGVGMAADRDSTQKAVDKITSKVNSIVDKMNKQYEIIDHEFSEVENRLNMINN
KIDDQIQDIWAYNAELLVLLENQKTLDEHDANVNNLYNKVKRALGSNAMEDGKGCFELYHKCDDQCMETI
RNGTYNRGKYMEESRLGRQKIEGVKLESEGTYKILTIYSTVASSLVLAMGFAAFLFWAMSNGSCRCNICI

>QVV58230.1 hemagglutinin [Influenza A virus]
MEVIPLMTMLLLVTTNNADKICIGHQSTNSTETVDTLTETGVPVTHAKELLHTEHNGRLCATNLGNPLIL
DTCTVEGIIYGNPSCDMLLGGREWSYIVERPSAVNGTCYPGNVENLEELRVLFSSSSSYQRIQMFPDTIW
NVTYSGTSKSCSDSFYRNMRWLTQKNGNYPVQDAQYTNTRGKDILFVWGIHHPPTETTQTNLYTRTDTTT
SITTENLDRTFKPLIGPRPLVNGLIGRINYYWSVLKPGQTLRVRSNGNLIAPWFGHVLSGKSHGRILKTD
LNSGNCVVQCQTEKGGLNSTLPFHNISKYAFGDCPKYIGVKSLKLAIGLRNVPARSSRGLFGAIAGFIEG
GWPGLVAGWYGFQHSNDQGVGMAADRDSTQKAVDKITSKVNNIVDKMNKQYEIIDHEFSEVENRLNMINN
KIDDQIQDIWAYNAELLVLLENQKTLDEHDANVNNLYNKVKRALGSNAMEDGKGCFELYHKCDDQCMETI
RNGTYNRRKYIEESRLGRQKIEGVKLESEGTYKILTIYSTVASSLVLAMGFAAFLFWAMSNGSCRCNICI

>QVV58229.1 hemagglutinin [Influenza A virus]

MEVIPLMXMLLLVTTNNADKICIGHQSTNSTETVDTLTETGVPVTHAKELLHTEHNGRLCATNLGNPLIL
DTCTVEGIIYGNPSCDMLLGGREWSYIVERPSAVNGTCYPGNVENLEELRVLFSSSSSYQRIQMFPDTIW
NVTYSGTSKSCSDSFYRNMRWLTQKNGNYPVQDAQYTNTRGKDILFVWGIHHPPTETTQTNLYTRTDTTT
SITTENLDRTFKPLIGPRPLVNGLIGRINYYWSVLKPGQTLRVRSNGNLIAPWFGHVLSGKSHGRILKTD
LNSGNCVVQCQTEKGGLNSTLPFHNISKYAFGDCPKYIGVKSLKLAIGLRNVPARSSRGLFGAIAGFIEG
GWPGLVAGWYGFQHSNDQGVGMAADRDSTQKAVDKITSKVNNIVDKMNKQYEIIDHEFSEVENRLNMINN
KIDDQIQDIWAYNAELLVLLENQKTLDEHDANVNNLYNKVKRALGSNAMEDGKGCFELYHKCDDQCMETI
RNGTYNRRKYIEESRLGRQKIEGVKLESEGTYKILTIYSTVASSLVLAMGFAAFLFWAMSNGSCRCNICI

>QVV58228.1 hemagglutinin [Influenza A virus]

MEVIPLMTMLLLVTTNNADKICIGHQSTNSTETVDTLTETGVPVTHAKELLHTEHNGRLCATNLGNPLIL
DTCTVEGIIYGNPSCDMLLGGREWSYIVERPSAVNGTCYPGNVENLEELRVLFSSSNSYQRIQMFPDTIW
NVTYSGTSKSCSDSFYRNMRWLTQKNGNYPVQDAQYTNTRGKDILFVWGIHHPPTETAQTNLYTRTDTTT
SITTESLDRTFKPLIGPRPLVNGLIGRINYYWSVLKPGQTLRVRSNGNLIAPWFGHVLSGESHGRILKTD
LNSGNCVVQCQTEKGGLNSTLPFHNISKYAFGDCPKYIGVKSLKLAIGLRNVPARSSRGLFGAIAGFIEG
GWPGLVSGWYGFQHSNDQGVGMAADRDSTQKAVDKITSKVNSIVDKMNKQYEIIDHEFSEVENRLNMINN
KIDDQIQDIWAYNAELLVLLENQKTLDEHDANVNNLYNKVKRALGSNAMEDGKGCFELYHKCDDQCMETI
RNGTYNRGKYMEESRLGRQKIEGVKLESEGTYKILTIYSTVASSLVLAMGFAAFLFWAMSNGSCRCNICI

>QVV58227.1 hemagglutinin [Influenza A virus]

MEVIPLMTMLLLVTTNNADKICIGHQSTNSTETVDTLTETGVPVTHAKELLHTEHNGRLCATNLGNPLIL
DTCTVEGIIYGNPSCDMLLGGREWSYIVERPSAVNGTCYPGNVENLEELRVLFSSSNSYQRIQMFPDTIW
NVTYSGTSKSCSDSFYRNMRWLTQKNGNYPVQDAQYTNTRGKDILFVWGIHHPPTETAQTNLYTRTDTTT
SITTESLDRTFKPLIGPRPLVNGLIGRINYYWSVLKPGQTLRVRSNGNLIAPWFGHVLSGESHGRILKTD
LNSGNCVVQCQTEKGGLNSTLPFHNISKYAFGDCPKYIGVKSLKLAIGLRNVPARSSRGLFGAIAGFIEG
GWPGLVSGWYGFQHSNDQGVGMAADRDSTQKAVDKITSKVNSIVDKMNKQYEIIDHEFSEVENRLNMINN
KIDDQIQDIWAYNAELLVLLENQKTLDEHDANVNNLYNKVKRALGSNAMEDGKGCFELYHKCDDQCMETI
RNGTYNRGKYMEESRLGRQKIEGVKLESEGTYKILTIYSTAASSLVLAMGFAAFLFWAMSNGSCXCNICI

>QVV58225.1 hemagglutinin [Influenza A virus]

MEVXPLMTMLLXVTTNNADKICIGHQSTNSTETVDTLTETGVPVTHAKELLHTEHNGRLCATNLGNPXIL
DTCTVEGIIYGNPSCDMLLGGREWSYIVERPSAVNGTCYPGNVENLEELRVLFSSSNSYQRIQMFPDTIW

NVTYSGTSKSCSDSFYRNMRWLTQKNGNYPVQDAQYTNTRGKDILFVWGIHHPPTETAQTNLYTRTDTTT
SITTESLDRTFKPLIGPRPLVNGQIGRINYYWSVLKPGQTLRVRSNGNLIAPWFGHVLSGESHGRILKTD
LNSGNCVVQCQTEKGGLNSTLPFHNISKYAFGDCPKYIGVKSLKLAIGLRNVPARSSRGLFGAIAGFIEG
GWPGLVSGWYGFQHSNDQGVGMAADRDSTQKAVDKITSKVNSIVDKMNKQYEIIDHEFSEVENRLNMINN
KIDDQIQDIWAYNAELLVLLENQKTLDEHDANVNNLYNKVKRALGSNAMEDGKGCFELYHKCDDQCMETI
RNGTYNRGKYMEESRLGRQKIEGVKLESEGTYKILTIYSTVASSLVLAMGFAAFLFWAMSNGSCRCNICI

>QVV58224.1 hemagglutinin [Influenza A virus]
MEVIPLMTMLLLVTTNNADKICIGHQSTNSTETVDTLTETGVPVTHAKELLHTEHNGRLCATNLGNPLIL
DTCTVEGIIYGNPSCDMLLGGREWSYIVERPSAVNGTCYPGNVENLEELRVLFSSSNSYQRIQMFPDTIW
NVTYSGTSKSCSDSFYRNMRWLTQKNGNYPVQDAQYTNTRGKDILFVWGIHHPPTETAQTNLYTRTDTTT
SITTESLDRTFKPLIGPRPLVNGLIGRINYYWSVLKPGQTLRVRSNGNLIAPWFGHVLSGESHGRILKTD
LNSGNCVVQCQTEKGGLNSTLPFHNISKYAFGDCPKYIGVKSLKLAIGLRNVPARSSRGLFGAIAGFIEG
GWPGLVSGWYGFQHSNDQGVGMAADRDSTQKAVDKITSKVNSIVDKMNKQYEIIDHEFSEVENRLNMINN
KIDDQIQDIWAYNAELLVLLENQKTLDEHDANVNNLYNKVKRALGSNAMEDGKGCFELYHKCDDQCMETI
RNGTYNRGKYMEESRLGRQKIEGVKLESEGTYKILTIYSTAASSLVLAMGFAAFLFWAMSNGSCXCNICI

>QVV58223.1 hemagglutinin [Influenza A virus]
MEIIPLMTMLLLVTTNNADKICIGHQSTNSTETVDTLTEANVPVTHAKELLHTEHNGKLCATDLGNPLIL
DTCTIEGLIYGNPSCDMLLGGREWSYIVERPSAVNGTCYPGNVENLEELRILFSSSSSYQRIQMFPDTIW
NVTYSGTSKSCSNSFYRNMRWLTQKNGNYPVQDAQYTNTQGKDILFVWGIHHPPTDTTQTDLYTRTDTTT
SITTENLDRTFKPLIGPRPLVNGLIGRINYYWSVLKPGQTLRVRSNGNLIAPWFGHVLSGESHGRILKTD
LKSGNCVVQCQTEKGGLNSTLPFHNISKYAFGDCPKYIGVKSLKLAIGLRNVPARSSRGLFGAIAGFIEG
GWPGLVAGWYGFQHSNDQGVGMAADRDSTQKAVDKITSKVNNIVDKMNKQYEIIDHEFSEVETRLNMINN
KIDDQIQDIWAYNAELLVLLENQKTLDEHDANVNNLYNKVKRALGSNAMEDGKGCFELYHKCDDQCMETI
RNGTYNRRKYMEESRLGRQRIEGVKLESEGTYKILTIYSTVASSLVLAMGFAAFLFWAMSNGSCRCNICI

>QVV58222.1 hemagglutinin, partial [Influenza A virus]
MXXIPLMIMLLLVTTNNADKICIGHQSTNSTETVDTLTETGVPVTHAKELLHAEHNGMLCATNLGNPLIL
DTCTVEGLIYGNPSCDMLLGGRKWSYIVERPSAVNGTCYPGNVENLEELRVLFSSSSSYQRIQMFPDTIW
NVTYSGTSKSCSDSFYRNMRWLTQKNGNYPVQDAQYKNTQGKDILFVWGIHHPPTDTAQTNLYTRTDTTT
SITTESLDRTFKPLIGPRPLVNGLIGRINYYWSVLKPGQTLRVRSNGNLIAPWFGHILSGVSHGRILKTD
LNSGNCVVQCQTDRGGLNSTLPFHNISKYAFGDCPKYIGVKSLKLAIGLRNVPARSSRGLFGAIAGFIEG
GWPGLVAGWYGFQHSNDQGVGMAADRDSTQKAVDKITSKVNNIVDKMNKQYEIINHEFSEVETRLNMINN

KIDDQIQDIWAYNAELLVLLENQKTLDEHDANVNNLYNKVKRALGSNAIEDGRGCFELYHKCDDQCMETI
RNGTYNRRKYVEESRLGRQKIEGVKLESEGTYKILTIYSTVASSLVLAMGLAAFLFWAMSNGSCRC

>QUE40578.1 hemagglutinin [Influenza A virus]
METISLMAILLVVTTSNADKICIGHQSTNSTETVDTLTETNVPVTHAKELLHTEHNGMLCATNLGRPLIL
DTCTIEGLIYGNPSCDLLLGGREWSYIVERSSAVNGTCYPGNVENLEELRTLFSSSSSYQRIQIFPDTIW
NVTYTGTSKSCSDSFYRNMRWLTQKNGLYPIQDAQYTNNRGKDILFVWGIHHPPTDTTQTNLYTRTDTTT
SVTTENLDRTFKPLIGPRPLVNGLIGRINYYWSILKPGQTLRVRSNGNLIAPWFGHVLSGESHGRILKTD
LNSGNCVVQCQTEKGGLNSTLPFHNISKFAFGTCPKYIGVKSLKLAIGLRNVPAKSSRGLFGAIAGFIEG
GWPGLVAGWYGFQHSNDQGVGMAADRDSTQKAVDKITSKVNTIVDKMNKQYEIIDHEFSEVETRLNMINN
KIDDQIQDVWAYNAELLVLLENQKTLDEHDANVNNLYNKVKRALGSNAMEDGKGCFELYHKCDDQCMETI
RNGTYNRRKYKEESRLERQKIEGVKLESEGTYKILTIYSTVASSLVLAMGFAAFLFWAMSNGSCRCNICI

>QTY73219.1 hemagglutinin [Influenza A virus]
METVSLITILLVATVSYANEICIGYQSTNSTETVDTLTENNVPVTHAKELLHTEHNGMLCATSLGQPLIL
DTCTIEGLIYGNPSCDLSLEGREWSYIVERPSAVNGLCYPGNVENLEELRSLFSSARSYQRVQIFPDTIW
NVSYDGTSTACSGSFYRSMRWLTRKNGDYPTQDAQYTNNQGKNILFMWGINHPPTDDTQRNLYTRTDTTT
SVATEEINRIFKPLIGPRPLVNGLMGRIDYYWSVLNPDQTLRIKSDGNLIAPWYGYILSGESHGRILKTD
LKRGSCTVQCQTEKGGLNTTLPFQNVSKYAFGNCSKYIGIKSLKLAVGLRNVPSRSSRGLFGAIAGFIEG
GWSGLVAGWYGFQHSNDQGVGMAADRDSTQKAIDKITSKVNNIVDKMNKQYEIIDHEFSEVETRLNMINN
KIDDQIQDIWAYNAELLVLLENQKTLDEHDANVNNLYNKVKRALGSNAVEDGKGCFELYHKCDDQCMETI
RNGTYNRRKYQEESKLERQKIEGVKLESEGTYKILTIYSTVASSLVIAMGLAAFLFWAMSNGSCRCNICI

>QTY73207.1 hemagglutinin [Influenza A virus]
METVSLITILLVATVSNADKICIGYQSTNSTETVDTLTENNVPVTHAKELLHTEHNGMLCATSLGQPLVL
DTCTIEGLIYGNPSCDLSLEGREWSYIVERPSAVNGLCYPGNVENLEELRSLFSSARSYQRVQIFPDTIW
NVSYDGTSSACSGSFYKSMRWLTRKNGDYPTQDAQYTNNQGKNILFMWGINHPPTDTAQRNLYTRTDTTT
SVATEEINRIFKPLIGPRPLVNGLMGRIDYYWSVLKPGQTLRIKSDGNLIAPWYGHILSGESHGRILKTD
LKRGSCTVQCQTEKGGLNTTLPFQNVSKYAFGNCSKYIGIKSLKLAVGLRNVPSRSSRGLFGAIAGFIEG
GWSGLVAGWYGFQHSNDQGVGMAADRDSTQKAIDKITSKVNNIVDKMNKQYEIIDHEFSEVETRLNMINN
KIDDQIQDIWAYNAELLVLLENQKTLDEHDANVNNLYNKVKRALGSNAMEDGKGCFELYHKCNDQCMETI
RNGTYNRRKYQEESKLERQKIEGVKLESEGTYKILTIYSTVASSLVIAMGFAAFLFWAMSNGSCRCNICI

>QTY73195.1 hemagglutinin [Influenza A virus]

METVSLITILLVATVSNADKICIGYQSTNSTETVDTLTENNVPVTHAKELLHTEHNGMLCATSLGQPLIL
DTCTIEGLIYGNPSCDLSLEGREWSYIVERPSAVNGLCYPGNVENLEELRSLFSSARSYQRLQIFPDTIW
NVSYDGTSTACSGSFYRSMRWLTRKNGEYPTQDAQYTNNQGKNILFMWGINHPPTDTTQRNLYTRTDTTT
SVATEEINRIFKPLIGPRPLVNGLMGRIDYYWSILKPGQTLRIKSDGNLIAPWYGYILSGESHGRILKTD
LKRGSCTVQCQTEKGGLNTTLPFQNVSKYAFGNCSKYIGIKSLKLAVGLRNVPSRSSRGLFGAIAGFIEG
GWSGLVAGWYGFQHSNDQGVGMAADRDSTQKAIDKITSKVNNIVDKMNKQYEIIDHEFSEVETRLNMINN
KIDDQIQDIWAYNAELLVLLENQKTLDEHDANVNNLYNKVKRALGSNAVEDGKGCFELYHKCDNQCMETI
RNGTYNRRKYQEESKLERQKIEGVKLESEGAYKILTIYSTVASSLVIAMGFAAFLFWAMSNGSCRCNICI

>QTY73183.1 hemagglutinin [Influenza A virus]
METVPLITILLVATVSNADKICIGYQSTNSTETVDTLTENNVPVTHAKELLHTEHNGMLCATSLGQPIIL
DTCTIEGLIYGNPSCDLSLEGREWSYIVERPSAVNGLCYPGNVENLEELRSLFSSARSYQRIQIFPDTIW
NVSYDGTSTACSGSFYRNMRWLTRKNGEYPIQDAQYTNNQGKNILFMWGINHPPADTTQRDLYTRTDTTT
SVATEEINRIFKPLVGPRPLVNGLMGRINYYWSVLKPGQTLRIKSDGNLIAPWYGHILSGESHGRILKTD
LKRGSCTVQCQTEKGGLNTTLPFQNVSKYAFGNCSKYIGIKSLKLAVGLRNVPSRSSRGLFGAIAGFIEG
GWSGLVAGWYGFQHSNDQGVGMAADRESTQKAVDKITSKVNNIVDKMNKQYEIIDHEFSEVETRLNMINN
KIDDQIQDIWAYNAELLVLLENQKTLDEHDANVNNLYNKVKRALGSNAVEDGKGCFELYHKCDDQCMETI
RNGTYNRRKYQEESKLERQKIEGVKLESEGTYKILTIYSTVASSLVIAMGFAAFLFWAMSNGSCRCNICI

>QTY73172.1 hemagglutinin [Influenza A virus]
METVSLITILLVATVSNADKICIGYQSTNSTETVDTLTENNVPVTHAKELLHTEHNGMLCATSLGQPIIL
DTCTIEGLIYGNPSCDLSLEGREWSYIVERPSAVNGLCYPGNVENLEELRSLFSSARSYQRIQIFPDTIW
NVSYDGTSTACSGSFYRNMRWLTRKNGEYPIQDAQYTNNQGKNILFMWGINHPPSDTTQRDLYTRTDTTT
SVATEEINRIFKPLIGPRPLVNGLMGRIDYYWSVLKPGQTLRIKSDGNLIAPWYGHILSGESHGRILKTD
LKRGSCTVQCQTEKGGLNTTLPFQNVSKYAFGNCSKYIGIKSLKLAVGLRNVPSRSSRGLFGAIAGFIEG
GWSGLVAGWYGFQHSNDQGVGMAADRDSTQKAVDKITSKVNTIVDKMNKQYEIIDHEFSEVETRLNMINN
KIDDQIQDIWAYNAELLVLLENQKTLDEHDANVNNLYNKVKRALGSNAVEDGKGCFELYHKCDDQCMETI
RNGTYNRRKYQEESKLERQKIEGVKLESEGTYKILTIYSTVASSLVIAMGFAAFLFWAMSNGSCRCNICI

>QTY73159.1 hemagglutinin [Influenza A virus]
METVSLITILLVATGSNADKICIGYQSTNSTETVDTLTENNVPVTHAKELLHTEHNGMLCATSLGQPIIL
DTCTIEGLIYGNPSCDLSLEGREWSYIVERPSAVNGLCYPGNVENLEELRSLFSSARSYQRIQIFPDTIW
NVSYDGTSTACSGSFYRNMRWLTRKNGEYPIQDAQYTNNQGKNILFMWGINHPPADTTQRDLYTRTDTTT
SVATEEINRIFKPLIGPRPLVNGLMGRIDYYWSVLKPGQTLRIKSDGNLIAPWYGHILSGESHGRILKTD

LKRGSCTVQCQTEKGGLNTTLPFQNVSKYAFGNCSKYIGIKSLKLAVGLRNVPSRSSRGLFGAIAGFIEG
GWSGLVAGWYGFQHSNDQGVGMAADRESTQKAVDKITSKVNNIVDKMNKQYEIIDHEFSEVETRLNMINN
KIDDQIQDIWAYNAELLVLLENQKTLDEHDANVNNLYNKVKRALGSNAMEDGKGCFELYHKCDDQCMETI
RNGTYNRRKYQEESKLERQKIEGVKLESEGTYKILTIYSTVASSLVIAMGFAAFLFWAMSNGSCRCNICI

>QTC30510.1 hemagglutinin [Influenza A virus]
MEAISLMIILLVVTTSNADKICIGHQSTNSTETVDTLTESNIPVTQAKELLHTEHNGMLCATNLGRPLIL
DTCTVEGLVYGNPSCDLLLGGREWSYIVERPSAVNGTCYPGNVENLEELRMLFSSASSYQRIQIFPDAIW
NVTYDGTSKSCSNSFYRNMRWLTQKNGNYPIQDAQYTNNRGEDILFIWGIHHPPTDTAQTNLYTRTDTTT
SVTTENLDRTFKPLIGPRPLVNGLIGRINYYWSVLKPGQTLRVRSNGNLIAPWYGHVLSGESHGRILKTD
LKSGNCVVPCQTGKGGLNSTLPFHNISKYAFGTCPKYIGVKSLMLAIGLRNVPAKSSRGLFGSIAGFIEG
GWPGLVAGWYGFQHSNDQGVGMAADRSSTQKAVDKITSKVNNIIDKMNRQYEIIDHEFSEIETRLNMINN
KIDDQIQDVWAYNAELLVLLENQKTLDEHDANVNNLYNKVKRALGSNAMEDGKGCFELYHKCDDQCMETI
RNGTYNRRKYTEESRLERQKIEGVKLESEGTYKILTIYSTAASSLVLAMGVAAFLFWAMSNGSCRCNICI

>QTC11221.1 hemagglutinin [Influenza A virus]
MEAKSLMITLLVVTTSSADKICIGHQSTNSTETVDTLTESNIPVTQAKELLHTEHNGMLCATNLGRPLIL
DTCTVEGLIYGNPSCDLLLGGREWSYIVERPSAVNGTCYPGNVENLEELRMLFSSASSYQRIQIFADAIW
NVTYDGTSKSCSNSFYRNMRWLTQKNGNYPIQDAQYTNNLGKDILFIWGIHHPPTDTAQTNLYTRTDTTT
SVTTENLDRTFKPLIGPRPLVNGLIGRINYYWSVLKPGQTLRVRSNGNLIAPWYGHVLSGESHGRILKTD
LKSGNCVVQCQTEICGSNSTLQFHNISKYAFGTCPKYIGVKSLKLAIGLRNVPAKSSRGLFGAIAGFIEG
GWPGLVAGWYGFQHSNDQGVGMAADRGSTQKAVDKITSKVNNIIDKMNRQYEIIDHEFSEIETRLNMINN
KIDDQIQDVWAYNAELLVLLENQKTLDEHDANVNNLYNKVKSALGSYAMEDGKGFFELYHKCDDQCMETI
RNGTYNRRKYTEESRLERQKIEGVKLESEGTYKILTIYSTVASSLVLAMGVAAFLFWAMSNGSCRCNICI

>QTB11586.1 hemagglutinin [Influenza A virus]
MEAISLMIILLVVTTSNADKICIGHQSTNSTETVDTLTESNIPVTQAKELLHTEHNGMLCATNLGRPLIL
DTCTVEGLVYGNPSCDLLLGGREWSYIVERPSAVNGTCYPGNVENLEELRMLFSSASSYQRIQIFPDAIW
NVTYDGTSKSCSNSFYRNMRWLTQKNGNYPIQDAQYTNNRGEDILFIWGIHHPPTDTAQTNLYTRTDTTT
SVTTENLDRTFKPLIGPRPLVNGLIGRINYYWSVLKPGQTLRVRSNGNLIAPWYGHVLSGESHGRILKTD
LKSGNCVVQCQTEKGGLNSTLPFHNISKYAFGTCPKYIGVKSLKLAIGLRNVPAKSSRGLFGAIAGFIEG
GWPGLVAGWYGFQHLNDQGVGMAADRSSTQKAVDKITSKVNNIIDKMNRQYEIIDHEFSEIETRLNMINN
KIDDQIQDVWAYNAELLVLLENQKTLDEHDANVNNLYNKVKRALGSNAMEDGKGCFELYHKCDDQCMETI
RNGTYNRRKYTEESRLERQKIEGVKLESEETYKILTIYSTVASSLVLAMGVAAFLFWAMSNGSCRCNICI

>QSH74822.1 hemagglutinin, partial [Influenza A virus]

DKICIGHQSTNSTETVDTLTETGVPVTHAKELLHTEHNGKLCATNLGNPLILDTCTVEGLIYGNPSCDLL
LGGREWSYIVERPSAVNGTCYPGNVENLEELRILFSSSSSYQRIQMFPDTIWNVTYSGTSKSCSDSFYRN
MRWLTQKNGNYPVQDAQYTNTRGKDILFVWGIHHPPTDTAQTNLYTRTDTTTSITTESLDRTFKPLIGPR
PLVNGLIGRINYYWSVLKPGQTLRVRSNGNLIAPWFGHMLSGESHGRILKTDLNSGNCVVQCQTEKGGLN
STLPFHNISKYAFGNCPKYIGVKSLKLAIGLRNVPARSSRGLFGAIAGFIEGGWPGLVAGWYGFQHSNDQ
GVGMAADRDSTQKAVDKITSKVNNIVDKMNKQYEIIDHEFSEVENRLNMINNKVDDQIQDIWAYNAELLV
LLENQKTLDEHDANVNNLYNKVKRALGSNAMEDGKGCFELYHKCDDQCMETIRNGTYNRRKYMEESRLGR
QKIEGVKLEPEGTYKILTIYSTVASSLVLAMGFAA

>QSH74821.1 hemagglutinin, partial [Influenza A virus]

DKICIGHQSTNSTETVDTLTETGVPVTHAKELLHTEHNGRLCATNLGDPLILDACTVEGIIYGNPSCDMF
LGGRKWSYIVERPSAVNGTCYPGNVENLEELRVLFSSSSSYQRIQMFPDTIWNVTYSGTSKSCSNSFYRN
MRWLTQKNGNYPVQDTQYTNNQGKDILFVWGIHHPPTDTAQTNLYTRTDTTTSITTESLDRTFKPLIGPR
PLVNGLIGRINYYWSVLKPGQTLRVRSNGNLIAPWFGHVLSGESHGRILKTDLNSGNCVVQCQTEKGGLN
STLPFHNISKYAFGDCPKYIGVKSLKLAIGLRNVPARSSRGLFGAIAGFIEGGWPGLVAGWYGFQHSNDQ
GVGMAADRDSTQKAVDKITSKVNNIVDKMNKQYEIIDHEFSEVETRLNMINNKIDDQIQDIWAYNAELLV
LLENQKTLDEHDANVNNLYNKVRRTLGSNAMEDGKGCFELYHKCDDQCMETIRNGTYNRRKYMEESRLGR
QKIEGVKLESEGTYKILTIYSTVASSLVIAMGFAA

>QSH74819.1 hemagglutinin, partial [Influenza A virus]

DKICIGHQSTNSTETVDTLTETGVPVTHAKELLHTEHNGKLCATNLGNPLILDTCTVEGLIYGNPSCDLL
LGGREWSYIVERPSAVNGTCYPGNVENLEELRILFSSSSSYQRIQMFPDTIWNVTYSGTSKSCSDSFYRN
MRWLTQKNGNYPVQDAQYTNTRGKNILFVWGIHHPPTDTAQTNLYTRTDTTTSITTESLDRTFKPLIGPR
PLVNGLIGRINYYWSVLKPGQTLRVRSNGNLIAPWFGHVLSGESHGRILKTDLNSGNCVVQCQTEKGGLN
STLPFHNISKYAFGNCPKYIGVKSLKLAIGLRNVPARSSRGLFGAIAGFIEGGWPGLVAGWYGFQHSNDQ
GVGMAADRDSTQKAVDKITSKVNNIVDKMNKQYEIIDHEFSEVENRLNMINNKVDDQIQDIWAYNAELLV
LLENQKTLDEHDANVNNLYNKVKRALGSNAMEDGKGCFELYHKCDDQCMETIRNGTYNRRKYMEESRLGR
QKIEGVKLESGGTYKILTIYSTVASSLVLAMGFAAFLFWAMSN

>QSH74818.1 hemagglutinin, partial [Influenza A virus]

DKICIGHQSTNSTETVDTLTETGVPVTQAKELLHTEHNGRLCATNLGDPLILDACTVEGIIYGNPSCDMF
LGGRKWSYIVERPSAVNGTCYPGNVENLEELRVLFSSSSSYQRIQMFPDTIWNVTYSGTSKSCSDSFYRN

MRWLTQKNGNYPVQDTQYTNNQGKDILFVWGIHHPPTDTAQTNLYTRTDTTTSITTESLDRTFKPLIGPR
PLVNGLIGRINYYWSVLKPGQTLRVRSNGNLIAPWFGHLLSGESHGRILKTDLRSGNCVVQCQTEKGGLN
STLPFHNISKYAFGNCPKYIGVKSLKLAIGLRNVPARSSRGLFGAIAGFIEGGWPGLVAGWYGFQHSNDQ
GVGMAADRDSTQKAVDKITSKVNNIVDKMNKQYEIIDHEFSEVENRLNMINNKIDDQIQDIWAYNAGLLV
LLENQKTLDEHDANVNNLYNKVKRALGSNAVEDGKGCFELYHKCDDQCMETIRNGTYNRRKYTEESRLGR
QKIEGVKLESEGTYKILTIYSTVASSLVLAMGFAAFLFWA

>QSH74816.1 hemagglutinin, partial [Influenza A virus]
QSTNSTETVDTLTETGVPVTHAKELLHTEHNGKLCATNLGNPLILDTCTVEGLIYGNPSCDLLLGGREWS
YIVERPSAVNGTCYPGNVENLEELRILFSSSSSYQRIQMFPDTIWNVTYSGTSKSCSDSFYRNMRWLTQK
NGNYPVQDAQYTNTRGKNILFVWGIHHPPTDTTQTNLYTRTDTTTSITTESLDRTFKPLIGPRPLVNGLI
GRINYYWSVLKPGQTLRVRSNGNLIAPWFGHVLSGESHGRILKTDLNSGNCVVQCQTEKGGLNSTLPFHN
ISKYAFGNCPKYIGVKSLKLAIGLRNVPARSSRGLFGAIAGFIEGGWPGLVAGWYGFQHSNDQGVGMAAD
RDSTQKAVDKITSKVNNIVDKMNKQYEIIDHEFSEVENRLNMINNKVDDQIQDIWAYNAELLVLLENQKT
LDEHDANVNNLYNKVKRALGSNAMEDGKGCFELYHKCDDQCMETIRNGTYNRRKYMEESRLGRQKIEGVK
LESGGTYKILTIFSTVASSLVLAMGFAAFLFWAMSNGSCRCNVCI

>QSH74815.1 hemagglutinin, partial [Influenza A virus]
DKICIGHQSTNSTETVDTLTETGVPVTHAKELLHTEHNGKLCATNLGNPLILDTCTVEGLIYGNPSCDLL
LGGREWSYIVERPSAVNGTCYPGNVENLEELRILFSSSSSYQRIQMFPDTIWNVTYSGTSKSCSDSFYRN
MRWLTQKNGNYPVQDAQYTNTRGKNILFVWGIHHPPTDTAQTNLYTRTDTTTSITTESLDRTFKPLIGPR
PLVNGLIGRINYYWSVLKPGQTLRVRSNGNLIAPWFGHVLSGESHGRILKTDLNSGNCVVQCQTEKGGLN
STLPFHNISKYAFGNCPKYIGVKSLKLAIGLRNVPARSSRGLFGAIAGFIEGGWPGLVAGWYGFQHSNDQ
GVGMAADRDSTQKAVDKITSKVNNIVDKMNKQYEIIDHEFSEVENRLNMINNKVDDQIQDIWAYNAELLV
LLENQKTLDEHDANVNNLYNKVKRALGSNAMEDGKGCFELYHKCDDQCMETIRNGTYNRRKYMEESRLGR
QKIEGVKLESGGTYKILTIYSTVASSLVLAMGFAAFLFWAMSNGSCRCNVCI

>QSH74814.1 hemagglutinin, partial [Influenza A virus]
DKICIGHQSTNSTETVDTLTETGVPVTHAKELLHTEHNGKLCATNLGNPLILDTCTVEGLIYGNPSCDLL
LGGREWSYIVERPSAVNGTCYPGNVENLEELRILFSSSSSYQRIQMFPDTIWNVTYSGTSKSCSDSFYRN
MRWLTQKNGNYPVQDAQYTNTRGKNILFVWGIHHPPTDTAQTNLYTRTDTTTSITTESLDRTFKPLIGPR
PLVNGLIGRINYYWSVLKPGQTLRVRSNGNLIAPWFGHVLSGESHGRILKTDLNSGNCVVQCQTEKGGLN
STLPFHNISKYAFGNCPKYIGVKSLKLAIGLRNVPARSSRGLFGAIAGFIEGGWPGLVAGWYGFQHSNDQ
GVGMAADRDSTQKAVDKITSKVNNIVDKMNKQYEIIDHEFSEVENRLNMINNKVDDQIQDIWAYNAELLV

LLENQKTLDEHDANVNNLYNKVKRALGSNAMEDGKGCFELYHKCDDQCMETIRNGTYNRRKYMEESRLGR
QKIEGVKLESEGTYKILTIYSTVASSLVLAMGFAAFLFWAMSNGSCRCNVCI

>QSH74813.1 hemagglutinin, partial [Influenza A virus]
DKICIGHQSTNSTETVDTLTETGVPVTHARELLHTEHNGRLCATNLGNPLILDTCTVEGIIYGNPSCDML
LGGRKWSYIVERPSAVNGTCYPGNVENLEELRVLFSSSSSYQRIQMFPDTIWNVTYTGTSKSCSDSFYRN
MRWLTQKNGNYPVQDAQYTNTQGKDILFVWGIHHPPTDTAQTNLYTRTDTTTSISTESLDRNFKPLIGPR
PLVNGLIGRINYYWSVLKPGQTLRVRSNGNLIAPWFGHILSGESHGRILKTDLNSGNCVVQCQTERGGLN
STLPFHNISKYAFGDCPKYIGVKSLKLAIGLRNVPARSSRGLFGAIAGFIEGGWPGLVAGWYGFQHSNDQ
GVGMAADRDSTQKAVDKITSKVNNIVDKMNKQYEIIDHEFNEVETRLNMINNKIDDQIQDIWAYNAELLV
LLENQKTLDEHDANVNNLYNKVKRALGSNAMEDGKGCFELYHKCDDQCMETIRNGTYNRRKYVEESRLGR
QKIEGVKLESEGTYKILTIYSTVASSLVLAMGFAAFLFWAMSNGSCRCNICI

>QSH74812.1 hemagglutinin, partial [Influenza A virus]
DKICIGHQSTNSTETVDTLTETGVPVTHAKELLHTEHNGKLCATNLGNPLILDTCTVEGLIYGNPSCDLL
LGGREWSYIVERPSAVNGTCYPGNVENLEELRILFSSSSSYQRIQMFPDAIWNVTYSGTSKSCSDSFYRN
MRWLTQKNGNYPVQDAQYTNTQGKDILFVWGIHHPPTDTAQTNLYTRTDTTTSITTESLDRTFKPLIGPR
PLVNGLIGRINYYWSVLKPGQTLRVRSNGNLIAPWFGHVLSGESHGRILKTDLNNGNCVVQCQTEKGGLN
STLPFHNISKYAFGDCPKYIGVKSLKLAIGLRNVPARSSRGLFGAIAGFIEGGWPGLVAGWYGFQHSNDQ
GVGMAADRDSTQKAVDKITSKVNNIVDKMNKQYEIIDHEFSEVENRLNMINNKVDDQIQDIWAYNAELLV
LLENQKTLDEHDANVNNLYNKVKRALGSNAMEDGKGCFELYHKCDDQCMETIRNGTYNRRKYMEESRLGR
QKIEGVKLESEGTYKILTIYSTVASSLVLAMGFAAFLFWAMSNG

>QSH74811.1 hemagglutinin, partial [Influenza A virus]
DKICIGHQSTNSTETVDTLTETGVPVTHAKELLHTEHNGKLCATNLGNPLILDTCTVEGLIYGNPSCDLL
LGGREWSYIVERPSAVNGTCYPGNVENLEELRILFSSSSSYQRIQMFPDAIWNVTYSGTSKSCSDSFYRN
MRWLTQKNGNYPVQDAQYTNTQGKDILFVWGIHHPPTDTAQTNLYTRTDTTTSITTESLDRTFKPLIGPR
PLVNGLIGRINYYWSVLKPGQTLRVRSNGNLIAPWFGHVLSGESHGRILKTDLNNGNCVVQCQTEKGGLN
STLPFHNISKYAFGDCPKYIGVKSLKLAIGLRNVPARSSRGLFGAIAGFIEGGWPGLVAGWYGFQHSNDQ
GVGMAADRDSTQKAVDKITSKVNNIVDKMNKQYEIIDHEFSEVENRLNMINNKVDDQIQDIWAYNAELLV
LLENQKTLDEHDANVNNLYNKVKRALGSNAMEDGKGCFELYHKCDDQCMETIRNGTYNRRKYMEESRLGR
QKIEGVKLESEGTYKILTIYSTVASSLVLAMGFAAFLFWAMSNGSCRCNICI

>QSH74810.1 hemagglutinin, partial [Influenza A virus]

DKICIGHQSTNSTETVDTLTETGVPVTHAKELLHTEHNGKLCATNLGNPLILDTCTVEGLIYGNPSCDLL
LGGREWSYIVERPSAVNGTCYPGNVENLEELRILFSSSSSYQRIQMFPDTIWNVTYSGTSKSCSDSFYRN
MRWLTQKNGNYPVQDAQYTNTKGKDILFVWGIHHPPTDTAQTNLYTRTDTTTSITTESLDRTFKPLIGPR
PLVNGLIGRINYYWSVLKPGQTLRVRSNGNLIAPWFGHVLSGESHGRILKTDLNSGNCVVQCQTEKGGLN
STLPFHNISKYAFGNCPKYIGVKSLKLAIGLRNVPARSSRGLFGAIAGFIEGGWPGLVAGWYGFQHSNDQ
GVGMAADRDSTQKAVDKITSKVNNIVDKMNKQYEIIDHEFSEVENRLNMINNKVDDQIQDIWAYNAELLV
LLENQKTLDEHDANVNNLYNKVKRALGSNAMEDGKGCFELYHKCDDQCMETIRNGTYNRRKYMEESRLGR
QKIEGVKLESEGTYKILTIYSTVASSLVLAMGFAAF

>QSH74809.1 hemagglutinin, partial [Influenza A virus]
DKICIGHQATNSTETVDTLTETGVPVTHAKELLHTEHNGRLCSTNLGNPLILDTCTVEGIIYGNPSCDML
LGGREWSYIVERPSAVNGTCYPGNVENLEELRLLFSSSSSYQRIQMFPDTIWNVTYSGTSKSCSDSFYRN
MRWLTQKNGNYPVQDAQYTNTRGKDILFVWGIHHPPTDTAQTNLYTRTDTTTSITTESLDRTFKPLIGPR
PLVNGLIGRINYYWSVLKPGQTLRVRSNGNLIAPWFGHVLSGESHGRILKTDLNSGNCVVQCQTEKGGLN
STLPFHNISKYAFGDCPKYIGVKSLKLAIGLRNVPARSSRGLFGAIAGFIEGGWPGLVAGWYGFQHSNDQ
GVGMAADRDSTQKAVDKITSKVKNIVDKMNKQYEIIDHEFSEVETRLNMINNKIDDQIQDIWAYNAELLV
LLENQKTLDEHDANVNNLYNKVRRALGSNAMEDGKGCFELYHKCDNQCMETIRNGTYNRRTYMEESRLGR
QKIEGVKLESEGTYKILTIYSTVASSLVIAMGFAAFLFWA

>QSH74808.1 hemagglutinin, partial [Influenza A virus]
DKICIGHQSTNSTETVDTLTETGVPVTHAKELLHTGHNGMLCATNLGNPLILDTCTVEGLIYGNPSCDVL
LGGREWSYIVERPSAVNGTCYPGNVENLEELRVLFSSSSSYQRIQMFPDTIWNVTYSGTSKSCSDSFYRN
MRWLTQKNGNYPVQDAQYTNTQGKDILFVWGIHHPPTDTAQTNLYTRTDTTTSITTESLDRTFKPLIGPR
PLVNGLIGRINYYWSVLKPGQTLRVRSNGNLIAPWFGHILSGVSHGRILKTDLNSGNCVVQCQTERGGLN
STLPFHNISKYAFGDCPKYIGVKSLKLAIGLRNVPARSSRGLFGAIAGFIEGGWPGLVAGWYGFQHSNDQ
GVGMAADRDSTQKAVDKITSKVNNIVDKMNKQYEIINHEFSEVETRLNMINNKIDDQIQDIWAYNAELLV
LLENQKTLDEHDANVNNLYNKVKRALGSNAMEDGKGCFELYHKCDDQCMETIRNGTYNRRKYVEESRLGR
QKIEGVKLESEGTYKVLTIYSTVASSLVLAMGLAAFLFWAMSK

>QOJ79819.1 hemagglutinin, partial [Influenza A virus]
VIVLLVVTTSNADKICIGHQSTNSTETVDTLTESNVPVTHAKELLHTDHNGMLCATNLGHPLILDTCTIE
GLIYGNPSCDLLLGGREWSYIVERPSAVNGKCYPGNIENLEELRTLFSSSSSYQRVQIFPDTIWNVTYTG
TSKSCSGSFYRNMRWLTQKNGLYPVQDAQYTNNRQKDILFVGGIHHPSSDDQQTNLYTRTDTTTSVTTEN
LDRTFKPLIGPRPLVNGLIGRINYYWSILKPGQTLRVRSNGNLIAPWYGHVLSGESHGRILKTNLNNGNC

VVQCQTEKGGLNSTLPFHNVSKYAFGNCPKYIGVKSLKLAIGLRNVPARSSRGLFGAIAGFIEGGWPGLV
AGWYGFQHSNDQGVGIAADRVSTQKAVDKITSKVNNIVDKMNKQYEIIDHEFSEVEARLNMINNKIDDQI
QDIWAYNAELLVLLENQKTLDEHDANVNNLYNKVKRALGLNAMEDGKGCFELYHKCDDQCMETIRNGTYN
RRKYEEESRLERQKIEGVKLESEGTYKILTIYSTVASSLVLAIGFAAFLFWAMSNGSCRCNICI

>QRA19072.1 hemagglutinin [Influenza A virus]
METVSLITILLIATVSNADKICIGYQSTNSTETVDTLTENNVPVTHAKELLHTEHNGMLCATSLGQPLIL
DTCTIEGLIYGNPSCDLSLEGREWSYIVERPSAVHGLCYPGNVEDLEELRSLFSSARSYQRIQIFPDTIW
NVSYDGTSTACSGSFYKSMRWLTRKNGEYPTQDAQYTNNQGKNILFMWGINHPPTDETQRGLYTRTDTTT
SVATEEINRIFKPLIGPRPLVNGLMGRINYYWSVLKPGQTLRIKSDGNLIAPWYGHILSGESHGRILKTD
LKRGSCTVQCQTEKGGLNTTLPFQNVSKYAFGNCSKYIGVKSLKLAVGLRNVPSRSSRGLFGAIAGFIEG
GWPGLVAGWYGFQHSNDQGVGMAADRDSTQKAVDKITSKVNNIVDKMNKQYEIIDHEFSEVETRLNMINN
KIDDQIQDIWAYNAELLVLLENQKTLDEHDANVNNLYNKVKRALGSNAVEDGKGCFELYHKCDDQCMETI
RNGTYNRRKYQEESKLERQRIEGVKLESEGTYKILTIYSTVASSLVLAMGFAAFLFWAMSNGSCRCNYCI

>QQS81631.1 hemagglutinin [Influenza A virus]
METVSLITILLVATVSNADKICIGYQSTNSTETVDTLTENNVPVTHAKELLHTEHNGMLCATSLGQPIIL
DTCTIEGLIYGNPSCDLSLEGREWSYIVERPSAVNGLCYPGNVENLEELRSLFSSARSYQRIQIFPDTIW
NVSYDGTSTACSGSFYRNMRWLTRKNGDYPIQDAQYTNNRGKNILFMWGIHHPPADTTQRDLYTRIDTTT
SVATEEINRIFKPLIGPRPLVNGLGRIDYYWSVLKPGQTLRIKSDGNLIAPWYGHILSGESHGRILKTDL
KRGSCTVQCQTEKGGLNTTLPFQNVSKYAFGNCSKYIGIKSLKLAVGLRNVPSRSSRGLFGAIAGFIEGG
WSGLVAGWYGFQHSNDQGVGMAADRDSTQKAIDKITSKVNNIVDKMNKQYEIIDHEFSEVETRLNMINNK
IDDQIQDIWAYNAELLVLLENQKTLDEHDANVNNLYNKVKRALGSNAVEDGKGCFELYHKCDDQCMETIR
NGTYSRRKYQEESKLERQKIEGVKLESEGTYKILTIYSTVASSLVIAMGFAAFLFWAMSNGSCRCNICI

>QQO74793.1 hemagglutinin [Influenza A virus]
METITLMTTLLLTTTSLADKICIGHQSTNSTETVDTLTETNVPVTHAKELLHTDHNGMLCATNLGHPLIL
DKCTVEGLIYGNPSCDSLLGGREWSYIVERSSAVNGTCYPGNVENLEELRTLFSSSSSYQRIQIFPDTIW
NVTYTGTSKSCSDSFYRNMRWLTQKNSLYPVQDAQFTNNRGKDILFVWGIHHPPTDTAQTNLYTRTDTTT
SVTTEDLDRTFKPLIGPRPLVNGLIGRINYYWSILKPGQTLRVRSNGNLIAPWYGHVLSGESHGRILKTD
LNGGNCVVQCQTEKGGLNSTLPFHNISKYAFGNCPKYIGVKSLKLAIGLRNVPAKSKRGLFGAIAGFIEG
GWPGLVAGWYGFQHSNDQGVGIAADKISTQKAVDKITSKVNNIVDKMNKQYEIIDHEFSEVETRLSMINN
KIDDQIQDVWAYNAELLVLLENQKTLDEHDANVNNLYNKVRRALGSNAMEDGKGCFELYHKCDDQCMETI
RNGTYNRKRYQEESRLERQRIEGVKLESEGTYKILTIYSTVASSLVLAMGFAAFLFWAMSNGSCRCNICI

>QQO74787.1 hemagglutinin [Influenza A virus]

METITLMTTLLLITTSLADKICVGHQSTNSTETVDTLTETNVPVTHAKELLHTDHNGMLCATNLGRPLIL
DKCNVEGLIYGNPSCDSLLGGREWSYIVERPSAVNGTCYPGNVENLEELRTLFSSSSSYQRIQIFPDTIW
NVTYTGTSKSCSDSFYRNMRWLTQKNSLYPVQDAQFTNNRGKDILFVWGIHHPPTDTAQTNLYTRTDTTT
SVTTEDLDRTFKPLIGPRPLVNGLIGRINYYWSILKPGQTLRVRSNGNLIAPWYGHVLSGESHGRILKTD
LNSGNCVVQCQTEKGGLNSTLPFHNISKYAFGNCPKYIGVKSLKLAIGLRNVPAKSKRGLFGAIAGFIEG
GWPGLVAGWYGFQHSNDQGVGIAADKISTQKAVDKITSKVNNIVDKMNKQYEIIDHEFSEVETRIYMINN
KIDDQIQDVWAYNAELLVLLENQKTLDEHDANVNNLYNKVRRALGSNAMEDGKGCFELYHKCDDQCMETI
RNGTYNREKYQEESRLERQRIEGVKLESEGTYKILTIYSTVASSLVLAMGFAAFLFWAMSNGSCRCNICI

>QHU98666.1 hemagglutinin [Influenza A virus]

METVSLITILLAATASNADKICIGYQSTNSTETVDTLTENNVPVTHAKELLHTEHNGMLCATSLGQPLIL
DTCTIEGLIYGNPSCDPLLEEREWSYIVERPSAVNGLCYPGNVENLEELRSLFSSARSYQRIQIFPDTIW
NVSYDGTSNTCSGSFYRNMRWLTRKNGNYPIQDAQYTNNQGKNILFMWGINHPPTDDTQRNLYTRTDTTT
SVATEEINRIFKPLIGPRPRVNGLMGRINYYWSVLKPGQTLRIKSDGNLIAPWYGYILSRESHGRILRTD
LKRGSCSVQCQTEKGGLNTTLPFQNVSKYAFGNCSKYIGIKSLKLAVGLRNVPSRSSRGLFGAIAGFIEG
GWSGLVAGWYGFQHSNDQGVGMAADRESTQKAIDKITSKVNNIVDKMNKQYEIIDHEFSEVETRLNMINN
KIDDQIQDIWAYNAELLVLLENQKTLDEHDANVNNLYNKVKRALGSNAVEDGKGCFELYHKCDDQCMETI
RNGTYNRRKYQEESKLERQKIEGVKLESEGTYKILTIYSTVASSLVIAMGFAAFLFWAMSNGSCRCNICI

>QQD80211.1 hemagglutinin [Influenza A virus]

METIPLMTILLMVTAINADKICIGYQSTNSTETVDTLTKTNVPVTQAKELLHTEHNGMLCATNLGHPLIL
DTCTIEGLIYGNPSCDLLLGGREWSYIVERPSAVNGMCYPGNVENLEELRLLFSSASSYQRVQIFPDTIW
NVTYSGTSSACSNSFYRSMRWLTQKDNTYPVQDAQYTNNRGKSILFMWGINHPPTDTVQTNLYTRTDTTT
SVTTEDINRAFKPVIGPRPLVNGLQGRIDYYWSVLKPGQTLRVRSNGNLIAPWYGHILSGESHGRILKSD
LNSGNCVVQCQTERGGLNTTLPFHNVSKYAFGNCPKYVGVKSLKLAVGMRNVPARSSRGLFGAIAGFIEG
GWPGLVAGWYGFQHSNDQGVGMAADRDSTQKAIDKITSKVNNIVDKMNKQYEIIDHEFSEIETRLNMINN
KIDDQIQDIWAYNAELLVLLENQKTLDEHDANVNNLYNKVKRALGSNAMEDGKGCFELYHKCDDRCMETI
RNGTYNRGKYKEESRLERQKIEGVKLESEGTYKILTIYSTVASSLVIAMGFAAFLFWAMSNGSCRCNICI

>QQD80199.1 hemagglutinin [Influenza A virus]

METIPLMTILLMVTAINADKICIGYQSTNSTETVDTLTKTNVPVTQAKELLHTEHNGMLCATNLGHPLIL
DTCTIEGLIYGNPSCDLLLGGREWSYIVERPSAVNGMCYPGNVENLEELRLLFSSASSYQRVQIFPDTIW

NVTYSGTSSACSNSFYRSMRWLTQKDNTYPVQDAQYTNNRGKSILFMWGINHPPTDTVQTNLYTRTDTTT
SVTTEDINKAFKPVIGPRPLVNGQQGRIDYYWSVLKPGQTLRVRSNGNLIAPWYGHILSGESHGRILKSD
LNSGNCVVQCQTERGGLNTTLPFHNVSKYAFGNCPKYVGVKSLKLAVGMRNVPARSSRGLFGAIAGFIEG
GWPGLVAGWYGFQHSNDQGVGMAADRDSTQKAIDKITSKVNNIVDKMNKQYEIIDHEFSEIETRLNMINN
KIDDQIQDIWAYNAELLVLLENQKTLDEHDANVNNLYNKVKRALGSNAMEDGKGCFELYHKCDDRCMETI
RNGTYNRGKYKEESRLERQKIEGVKLESEGTYKILTIYSTVASSLVIAMGFAAFLFWAMSNGSCRCNICI

>QNQ07348.1 hemagglutinin [Influenza A virus]
METISLISILLVVATSNADKICIGHQSTNSTETVDTLTENNVPVTHAKELLHTEHNGMLCATNLGRPLIL
DTCTIEGLIYGNPSCDPLLGGREWSYIVERPSAVNGTCYPGNVENLEELRTLFSSSSSYQRIQIFPDTVW
NVTHTGTSKSCSDSFYRNMRWLTQKNGLYPIQDAQYTNNRGKDILFVWGIHHPPTDTAQTNLYTRTDTTT
SVTTESLDRTFKPLIGPRPLVNGLIGRINYYWSVLKPGQTLRVRSNGNLIAPWYGHVLSGESHGRILKTN
LNGGNCVVQCQTEKGGLNSTLPFHNVSKYAFGNCPKYIGVKSLKLAIGLRNIPAKSKRGLFGAIAGFIEG
GWPGLVAGWYGFQHSNDQGVGIAADRDSTQKAIDKITTKVNNIIDKMNNQYEIIDHEFSEIETRLNMINN
KIDDQIQDVWAYNAELLVLLENQKTLDEHDANVNNLYNKVKRALGSNAMEDGKGCFELYHKCDDQCMETI
RNGTYNRRKYTQESRLERQKIEGIKLESEETYKILSIYSTVASSLVLAIGFAAFLFWAMSNGSCRCNICI

>QNN30851.1 hemagglutinin [Influenza A virus]
METISLISILLVVATSNADKICIGHQSTNSTETVDTLTETNVPVTHAKELLHTEHNGMLCATNLGRPLIL
DTCTIEGLIYGNPSCDPLLGGREWSYIVERPSAVNGTCYPGNVENLEELRTLFSSSSSYQRIQIFPDTIW
NVTHTGTSKSCSDSFYRNMRWLTQKNGLYPIQDAQYTNNRGKDILFVWGIHHPPTDTAQTNLYTRTNTTT
SVTTESLDRTFKPLIGPRPLVNGQIGRINYYWSVLKPGQTLRVRSNGNLIAPWYGHVLSGESHGRILKTN
LNGGNCVVQCQTEKGGLNSTLPFHNVSKYAFGNCPKYIGVKSLKLAIGLRNVPAKSKRGLFGAIAGFIEG
GWPGLVAGWYGFQHSNDQGVGIAADRDSTQKAIDKITTKVNNIIDKMNNQYEIIDHEFSEIETRLNMINN
KIDDQIQDVWAYNAELLVLLENQKTLDEHDANVNNLYNKVKRALGSNAMEDGKGCFELYHKCDDQCMETI
RNGTYNRRKYTQESRLERQKIEGIKLESEETYKILSIYSTVASSLVLAIGFAAFLFWAMSNGSCRCNICI

>QED95697.1 hemagglutinin [Influenza A virus]
MEAISLMIILIVVTTSSADKICIGHQSTNSTETVDTLTESNIPVTQAKELLHTEHNGMLCATNLGRPLIL
DTCTVEGLIYGNPSCDLLLGGREWSYIVERPSAVNGTCYPGSVENLEELRMLFSSASSYQRIQIFPDAIW
NVTYDGTSKSCSNSFYRNMRWLTQKNGNYPIQDAQYTNNRGKDILFIWGIRHPPTDTAQTNLYTRTDTTT
SVTTENLDRTFKPLIGPRPLVNGLIGRINYYWSVLKPGQTLRVRSNGNLIAPWYGHVLSGESHGRILKTD
LKSGNCVVQCQTEKGGLNSTLPFHNISKYAFGTCPKYIGVKSLKLAIGLRNVPAKSSRGLFGAIAGFIEG
GWPGLVAGWYGFQHSNDQGVGIAADRGSTQKAVDKITSKVNNIIDKMNRQYEIIDHEFSEIETRLNMINN

KIDDQIQDVWAYNAELLVLLENQKTLDEHDANVNNLYNKVKRALGSNAMEDGKGCFELYHKCDDQCMETI
RNGTYNRRKYTEESRLERQKIEGVKLESEGTYKILTIYSTVASSLVLAMGVAAFLFWAMSNGSCRCNICI

>QED95709.1 hemagglutinin [Influenza A virus]
METVSLITILLVVTASNADKICIGHQSTNSTETVDTLTETNVPVTHAKELLHTEHNGMLCATNLGHPLIL
DTCTIEGLIYGNPSCDLLLGGREWSYIVERSSAVNGTCYPGNVENLEELRTLFSSASSYQRIQIFPDTIW
NVTYTGTSKACSGSFYRSMRWLTQKNGSYPVQDAQYTNNRGKSILFVWGIHHPPTDTEQTSLYIRTDTTT
SVTTEDLNRIFKPMIGPRPLVNGQQGRINYYWSVLKPGQTLRVRSNGNLIAPWYGHVLSGGSHGRILKTD
LNSGNCVVQCQTEKGGLNSTLPFHNISKYAFGNCPKYVRVKSLKLAVGLRNVPARSSRGLFGAIAGFIEG
GWPGLVAGWYGFQHSNDQGVGMAADRDSTQKAIDKITSKVNNIVDKMNKQYEIIDHEFSEVETRLNMINN
KIDDQIQDVWAYNAELLVLLENQKTLDEHDANVNNLYNKVKRALGSNAMEDGKGCFELYHKCDDQCMETI
RNGTYNRRKYKEESRLERQKIEGVKLESEGTYKILTIYSTVASSLVLAMGFAAFLFWAMSNGSCRCNISI

>QED95708.1 hemagglutinin [Influenza A virus]
MEAISLMIILLVVTIGNADKICIGHQSTNSTETVDTLTESNIPVTQAKELLHTEHNGMLCATNLGRPLIL
DTCTVEGLIYGNPSCDLLLGGREWSYIVERPSAVNGTCYPGNVENLEELRMLFSSASSYQRIQIFPDAVW
NVTYDGTSKSCSNSFYRNMRWLTQKNGNYPIQDAQYTNNRGKDILFIWGIHHPPTDTAQTNLYTRTDTTT
SVTTESLDRTFKPLIGPRPLVNGLIGRINYYWSVLKPGQTLRVRSNGNLIAPWYGHVLSGESHGRILKTD
LKSGNCIVQCQTEKGGLNSTLPFHNISKYAFGTCPKYVGVRSLKLAVGLRNVPAKSSRGLFGAIAGFIEG
GWPGLVAGWYGFQHSNDQGVGMAADRSSTQKAVDKITSKVNNIIDKMNRQYEIIDHEFSEIETRLNMINN
KIDDQIQDVWAYNAELLVLLENQKTLDEHDANVNNLYNKVKRALGSNAMEDGKGCFELYHKCDDQCMETI
RNGTYNRRKYTEESRLERQKIEGVKLESEGTYKILTIYSTVASSLVLAMGIAAFLFWAMSNGSCRCNICI

>QED95707.1 hemagglutinin [Influenza A virus]
MESISLMIILLVVTISNADKICIGHQSTNSTETVDTLTESNIPVTQAKELLHTEHNGMLCATNLGRPLIL
DTCTVEGLIYGNPSCDLLLGGREWSYIVERPSAVNGTCYPGNVENLEELRMLFSSASSYQRIQIFPDAVW
NVTYDGTSKSCSNSFYRNMRWLTQKNGNYPIQDAQYTNNRGKDILFIWGIHHPPTDTAQTNLYTRTDTTT
SVTTESLDRTFKPLIGPRPLVNGLIGRINYYWSVLKPGQTLRVRSNGNLIAPWYGHVLSGESHGRILKTD
LKSGNCVVQCQTEKGGLNSTLPFHNISKYAFGTCPKYVGVKSLKLAVGLRNVPAKSSRGLFGAIAGFIEG
GWPGLVAGWYGFQHSNDQGVGMAADRSSTQKAVDKITSKVNNIIDKMNRQYEIIDHEFSEIETRLNMINN
KIDDQIQDVWAYNAELLVLLENQKTLDEHDANVNNLYNKVKRALGSNAMEDGKGCFELYHKCDDQCMETI
RNGTYNRRKYTEESRLERQKIEGVKLESEGTYKILTIYSTVASSLVLAMGIAAFLFWAMSNGSRVNNICI

>QED95706.1 hemagglutinin [Influenza A virus]

MEAISLMIILLVVTISSADKICIGHQSTNSTETVDTLTESNIPVTQAKELLHTEHNGMLCATNLGSPLIL
DTCTVEGLIYGNPSCDLLLGGREWSYIVERPSAVNGTCYPGNVENLEELRMLFSSASSYQRIQIFPDAVW
NVTYDGTSKSCSNSFYRNMRWLTQKNGNYPIQDAQYTNNRGKDILFIWGIHHPPTDTAQTNLYTRTDTTT
SVTTESLDRTFKPLIGPRPLVNGLIGRINYYWSVLKPGQTLRVRSNGNLIAPWYGHVLSGESHGRILKTD
LKSGNCVVQCQTEKGGLNSTLPFHNISKYAFGTCPKYVGVKSLKLAVGLRNVPAKSSRGLFGAIAGFIEG
GWPGLVAGWYGFQHSNDQGVGMAADRSSTQKAVDKITSKVNNIIDKMNRQYEIIDHEFSEIETRLNMINN
KIDDQIQDVWAYNAELLVLLENQKTLDEHDANVNNLYNKVKRALGSNAMEDGKGCFELYHKCDDQCMETI
RNGTYNRRKYTEESRLERQKIEGVKLESEGTYKILTIYSTVASSLVLAMGIAAFLFWAMSNGSCRCNICI

>QED95705.1 hemagglutinin [Influenza A virus]
MEAISLMIILLVVTISNADKICIGHQSTNSTETVDTLTESNIPVTQAKELLHTEHNGMLCATNLGRPLIL
DTCTVEGLIYGNPSCDLLLGGREWSYIVERPSAVNGTCYPGNVENLEELRMLFSSASSYQRIQIFPDAVW
NVTYDGTSKSCSNSFYRNMRWLTQKNGNYPIQDAQYTNNRGKDILFIWGIHHPPTDTAQTNLYTRTDTTT
SVTTESLDRTFKPLIGPRPLVNGLIGRINYYWSVLKPGQTLRVRSNGNLIAPWYGHVLSGESHGRILKTD
LKSGNCVVQCQTEKGGLNSTLPFHNISKYAFGTCPKYVRVKSLKLAVGLRNVPAKSSRGLFGAIAGFIEG
GWPGLVAGWYGFQHSNDQGVGMAADRSSTQKAIDKITSKVNNIIDKMNRQYEIIDHEFSEIETRLNMINN
KIDDQIQDVWAYNAELLVLLENQKTLDEHDANVNNLYNKVKRALGSNAMEDGKGCFELYHKCDDQCMETI
RNGTYNRRKYTEESRLERQKIEGVKLESEGTYKILTIYSTVASSLVLAMGIAAFLFWAMSNGSCRCNICI

>QED95704.1 hemagglutinin [Influenza A virus]
MEAISLMIILLVVTISNADKICIGHQSTNSTETVDTLTESNIPVTQAKELLHTEHNGMLCATNLGRPLIL
DTCTVEGLIYGNPSCDLLLGGREWSYIVERPSAVNGTCYPGNVENLEELRMLFSSASSYQRIQIFPDAVW
NVTYDGTSKSCSNSFYRNMRWLTQKNGNYPIQDAQYTNNQGKDILFIWGIHHPPTDTTQTNLYTRTDTTT
SVTTESLDRTFKPLIGPRPLVNGLIGRINYYWSVLKPGQTLRVRSNGNLIAPWYGHVLSGESHGRILKTD
LKSGNCVVQCQTEKGGLNSTLPFHNISKYAFGTCPKYVGVKSLKLAVGLRNVPAKSSRGLFGAIAGFIEG
GWPGLVAGWYGFQHSNDQGVGMAADRSSTQKAVDKITSKVNNIIDKMNRQYEIIDHEFSEIETRLNMINN
KIDDQIQDVWAYNAELLVLLENQKTLDEHDANVNNLYNKVKRALGSNAMEDGKGCFELYHKCDDQCMETI
RNGTYNRRKYTEESRLGRQKIEGVKLESEGTYKILTIYSTVASSLVLAMGIAAFLFWAMSNGSCRCNICI

>QED95703.1 hemagglutinin [Influenza A virus]
MEAISLMIILLVVTISNADKICIGHQSTNSTETVDTLTESNIPVTQAKELLHTEHNGMLCATNLGRPLIL
DTCTVEGLIYGNPSCDLLLGGREWSYIVERPSAVNGTCYPGNVENLEELRMLFSSASSYQRIQIFPDAVW
NVTYDGTSKSCSNSFYRNMRWLTQKNGNYPIQDAQYTNNRGKDILFIWGIHHPPTDTAQTNLYTRTDTTT
SVTTESLDRTFKPLIGPRPLVNGLIGRINYYWSVLKPGQTLRVRSNGNLIAPWYGHVLSGESHGRILKTD

LKSGNCVVQCQTEKGGLNSTLPFHNISKYAFGTCPKYVGVKSLKLAVGLRNVPAKSSRGLFGAIAGFIEG
GWPGLVAGWYGFQHSNDQGVGMAADRSSTQKAVDKITSKVNNIIDKMNRQYEIIDHEFSEIETRLNMINN
KIDDQIQDVWAYNAELLVLLENQKTLDEHDANVNNLYNKVKRALGSNAMEDGKGCFELYHKCDDQCMETI
RNGTYNRRKYTEESRLERQKIEGVKLESEGTYKILTIYSTVASSLVLAMGIAAFLFWAMSNGSCRCNICI

>QED95702.1 hemagglutinin [Influenza A virus]
MEAISLMIILLVVTISNADKICIGHQSTNSTETVDTLTESNIPVTQAKELLHTEHNGMLCATNLGRPLIL
DTCTVEGLIYGNPSCDLLLGGREWSYIVERPSAVNGTCYPGNVENLEELRMLFSSASSYQRIQIFPDAVW
NVTYDGTSKSCSNSFYRNMRWLTQKNGNYPIQDAQYTNNRGKDILFIWGIHHPPTDTAQTNLYTRTDTTT
SVTTESLDRTFKPLIGPRPLVNGLIGRINYYWSVLKPGQTLRVRSNGNLIAPWYGHVLSGESHGRILKTD
LKSGNCVVQCQTEKGGLNSTLPFHNISKYAFGTCPKYVGVRSLKLAVGLRNVPAKSSRGLFGAIAGFIEG
GWPGLVAGWYGFQHSNDQGVGMAADRSSTQKAVDKITSKVNNIIDKMNRQYEIIDHEFSEIETRLNMINN
KIDDQIQDVWAYNAELLVLLENQKTLDEHDANVNNLYNKVKRALGSNAMEDGKGCFELYHKCDDQCMETI
RNGTYNRRKYTEESRLERQKIEGVKLESEGTYKILTIYSTVASSLVLAMGIAAFLFWAMSNGSCRCNICI

>QED95701.1 hemagglutinin [Influenza A virus]
MEAISLMIILLVVTISNADKICIGHQSTNSTETVDTLTESNIPVTQAKELLHTEHNGMLCATNLGRPLIL
DTCTVEGLIYGNPSCDLLLGGREWSYIVERPSAVNGTCYPGNVENLEELRMLFSSASSYQRIQIFPDAVW
NVTYDGTSKSCSNSFYRNMRWLTQKNGNYPIQDAQYTNNRGKDILFIWGIHHPPTDTAQTNLYTRTDTTT
SVTTESLDRTFKPLIGPRPLVNGLIGRINYYWSVLKPGQTLRVRSNGNLIAPWYGHVLSGESHGRILKTD
LKSGNCVVQCQTEKGGLNSTLPFHNISKYAFGTCPKYVGVKSLKLAVGLRNVPAKSSRGLFGAIAGFIEG
GWPGLVAGWYGFQHSNDQGVGMAADRSSTQKAVDKITSKVNNIIDKMNRQYEIIDHEFSEIETRLNMINN
KIDDQIQDVWAYNAELLVLLENQKTLDEHDANVNNLYNKVKRALGSNAMEDGKGCFELYHKCDDQCMETI
RNGTYNRRKYTEESRLERQKIEGVKLESEGNYKILTIYSTVASSLVLAMGIAAFLFWAMSNGSCRCNICI

>QED95700.1 hemagglutinin [Influenza A virus]
MEAIPLMIILLVVTISNADKICIGHQSTNSTETVDTLTESNIPVTQAKELLHTEHNGMLCATNLGRPLIL
DTCTVEGLIYGNPSCDLLLGGREWSYIVERPSAVNGTCYPGNVENLEELRMLFSSASSYQRIQIFPDAIW
NVTYDGTSKSCSNSFYRNMRWLTQKNGNYPIQDAQYTNNRGEDILFIWGIHHPPTDTAQTNLYTRTDTTT
SVTTESLDRTFKPLIGPRPLVNGLIGRINYYWSVLKPGQTLRVRSNGNLIAPWYGHVLSGESHGRILKTD
LKSGNCVVQCQTEKGGLNSTLPFHNISKYAFGTCPKYVGVKSLKLAVGLRNVPAKSSRGLFGAIAGFIEG
GWPGLVAGWYGFQHSNDQGVGMAADRSSTQKAVDKITSKVNNIIDKMNRQYEIIDHEFSEIETRLNMINN
KIDDQIQDVWAYNAELLVLLENQKTLDEHDANVNNLYNKVKRALGSNAMEDGKGCFELYHKCDDQCMETI
RNGTYNRRKYTEESRLERQKIEGVKLESEGTYKILTIYSTVASSLVLTMGIAAFLFWAMSNGSCRCNICI

>QED95699.1 hemagglutinin [Influenza A virus]

MEAISLMIILLVVTISNADKICIGHQSTNSTETVDTLTESNIPVTQAKELLHTEHNGMLCATDLGRPLIL

DTCTVEGLIYGNPSCDLLLGGREWSYIVERPSAVNGTCYPGNVENLEELRMLFSSASSYQRIQIFPDAIW

NVTYNGTSKSCSNSFYRNMRWLTQKNGNYPIQDAQYTNSQGKDILFIWGIHHPPTDTTQTNLYTRTDTTT

SVTTESLDRTFKPLIGPRPLVNGLIGRINYYWSVLKPGQTLRVRSNGNLIAPWYGHVLSGESHGRILKTD

LKSGNCVVQCQTEKGGLNSTLPFHNISKYAFGMCPKYVGVKSLKLAVGLRNVPAKSSRGLFGAIAGFIEG

GWPGLVAGWYGFQHSNDQGVGMAADRSSTQKAVDKITSKVNNIIDKMNRQYEIIDHEFSEIETRLNMINN

KIDDQIQDVWAYNAELLVLLENQKTLDEHDANVNNLYNKVKRALGSNAMEDGKGCFELYHKCDDQCMETI

RNGTYNRRKYTEESRLERQKIEGVKLESEGTYKILTIYSTVASSLVLAMGIAAFLFWAMSTGSCRCNICI

>QED95698.1 hemagglutinin [Influenza A virus]

MEAISLMIILLVVTISNADKICIGHQSTNSTETVDTLTESNIPVTQAKELLHTEHNGMLCATNLGRPLIL

DTCTVEGLIYGNPSCDLLLGGREWSYIVERPSAVNGTCYPGNVENLEELRMLFSSASSYQRIQIFPDAVW

NVTYDGTSKSCSNSFYRNMRWLTQKNGNYPIQDAQYTNNRGKDIFFIWGIHHPATDTAQTNLYTRTDTTT

SVTTESLDRTFKPLIGPRPLVNGLIGRINYYWSVLKPGQTLRVRSNGNLIAPWYGHVLSGESHGRILKTD

LKSGNCVVQCQTEKGGLNSTLPFHNISKYAFGTCPKYVGVKSLKLAVGLRNVPAKSSRGLFGAIAGFIEG

GWPGLVAGWYGFQHSNDQGVGMAADRSSTQKAVDKITSKVNNIIDKMNRQYEIIDHEFSEIETRLNMINN

KIDDQIQDVWAYNAELLVLLENQKTLDEHDANVNNLYNKVKRALGSNAMEDGKGCFELYHKCDDQCMETI

RNGTYNRRKYTEESRLERQKIEGVKLESEGTYKILTIYSTVASSLVLAMGIAAFLFWAMSNGSCRCNICI

>QED95696.1 hemagglutinin [Influenza A virus]

MEAISLMIILIVVTTSSADKICIGHQSTNSTETVDTLTESNIPVTQAKELLHTEHNGMLCATNLGRPLIL

DTCTVEGLIYGNPSCDLLLGGREWSYIVERPSAVNGTCYPGSVENLEELRMLFSSASSYQRIQIFPDAIW

NVTYDGTSKSCSNSFYRNMRWLTQKNGNYPIQDAQYTNNRGKDILFIWGIHHPPTDTAQTNLYTRTDTTT

SVTTENLDRTFKPLIGPRPLVNGLIGRINYYWSVLKPGQTLRVRSNGNLIAPWYGHVLSGESHGRILKTD

LKSGNCVVQCQTEKGGLNSTLPFHNISKYAFGTCPKYIGVKSLKLAIGLRNVPAKSSRGLFGAIAGFIEG

GWPGLVAGWYGFQHSNDQGVGIAADRGSTQKAVDKITSKVNNIIDKMNRQYEIIDHEFSEIETRLNMINN

KIDDQIQDVWAYNAELLVLLENQKTLDEHDANVNNLYNKVKRALGSNAMEDGKGCFELYHKCDDQCMETI

RNGTYNRRKYTEESRLERQKIEGVKLESEGTYKILTIYSTVASSLVLAMGVAAFLFWAMSNGSCRCNICI

>QED95695.1 hemagglutinin [Influenza A virus]

METIPLMAILLVVTTSNADKICIGHQSTNSTETVDTLTETNVPVTHAKELLHTEHNGMLCATNLGRPLIL

DTCTIEGLIYGNPSCDLLLGGREWSYIVERSSAVNGTCYPGNVENLEELRTLFSSSSSYQRIQIFPDTIW

NVTYTGTSKSCSDSFYRNMRWLTQKNGLYPIQDAQYTNNRGKDILFVWGIHHPPTDTTQTNLYTRTDTTT
SVTTENLDRTFKPLIGPNGLIGRINYYWSILKPGQTLRVRSNGNLIAPWFGHVLSGESHGRILKTDLNSG
NCVVQCQTEKGGLNSTLPFHNISKFAFGTCPKYIGVKSLKLAIGLRNVPAKSSRGLFGAIAGFIEGGWPG
LVAGWYGFQHSNDQGVGMVADRDSTQKAVDKITSKVNTIVDKMNKQYEIIDHEFSEVETRLNMINNKIDD
QIQDVWAYNAELLVLLENQKTLDEHDANVNNLYNKVKRALGSNAMEDGKGCFELYHKCDDQCMETIRNGT
YNRRKYKEESRLERQKIEGVKLESEGTYKILTIYSTVASSLVLAMGFAAFLFWAMSNGSCRCNICI

>QOW78228.1 hemagglutinin [Influenza A virus]
MEAIPLLTLLLVVTTSSADKICVGHQSTNSTETVDTLTETNVPVTQAKELLHTEHNGMLCATNLGRPLIL
NTCTIEGLIYGNPSCDMLLGGREWSYIVERPSAVNGTCYPGNIENLEELRTLFSSSSSYQRIQLFPDTIW
NVTYTGTSKSCSDSFYRNMRWLTQKNGLYPIQDAQYTNNRGKDILFVWGIHHPPTDTAQTNLYTRTDTTT
SVTTENLDRTFKPLIGPRPLVNGLIGRINYYWSVLKPGQTLRVRSNGNLVAPWFGHVLSGESHGRILKTD
LSSGNCVVQCQTEKGGLNSTLPFHNISKYAFGTCPKYIGVKSLKLAVGLRNVHARSSRGLFGAIAGFIEG
GWPGLVAGWYGFQHSNDQGVGMAADRDSTQKAVDKITSKVNNIVDKMNKQYEIIDHEFSEVETRLNMINS
KIDDQIQDIWAYNAELLVLLENQKTLDEHDANVNNLYNKVKRALGSNAMEDGKGCFELYHKCDDQCMETI
RNGTYNRRKYTEESRLERQKIEGVKLESEGTYKILSIYSTVASSLVLAMGFAAFLFWAMSNGSCRCNICI

>QOW78210.1 hemagglutinin [Influenza A virus]
MEAIPLLTLLLVVTTSNADKICVGHQSTNSTETVDTLTETNVPVTQAKELLHTEHNGMLCATNLGRPLIL
NTCTIEGLIYGNPSCDMLLGGREWSYIVERPSAVNGTCYPGNIENLEELRTLFSSSSSYQRIQLFPDTIW
NVTYTGTSKSCSDSFYRNMRWLTQKNGLYPIQDAQYTNNRGKDILFVWGIHHPPTDTAQTNLYTRTDTTT
SVTTENLDRTFKPLIGPRPLVNGLIGRINYYWSVLKPGQTLRVRSNGNLIAPWFGHVLSGESHGRILRTD
LSSGNCVVQCQTEKGGLNSTLPFHNISKYAFGTCPKYIGVKSLKLAVGLRNVHARSSRGLFGAIAGFIEG
GWPGLVAGWYGFQHSNDQGVGMAADRDSTQKAVDKITSKVNNIIDKMNKQYEIIDHEFSEVETRLNMINS
KIDDQIQDIWAYNAELLVLLENQKTLDEHDANVNNLYNKVKRALGSNAMEDGKGCFELYHKCDDQCMETI
RNGTYNRRKYTEESRLERQKIEGVKLESEGTYKILSIYSTVASSLVLAMGFAAFLFWAMSNGSCRCNICI

>QOW78199.1 hemagglutinin [Influenza A virus]
MEAIPLLTLLLVVTTSNADKICVGHQSTNSTETVNTLTETNVPVTQAKELLHTEHNGMLCATNLGRPLIL
NTCTIEGLIYGNPSCDMLLGGREWSYIVERPSAVNGTCYPGNIENLEELRTLFSSSSSYQRIQLFPDTIW
NVTYTGTSKSCSDSFYRNMRWLTQKNGLYPIQDAQYTNNRGKDILFVWGIHHPPTDTAQTNLYTRTDTTT
SVTTENLDRTFKPLIGPRPLVNGLIGRINYYWSVLKPGQTLRVRSNGNLIAPWFGHVLSGESHGRILRTD
LSSGNCVVQCQTEKGGLNSTLPFHNISKYAFGTCPKYIGVKSLKLAVGLRNVHARSSRGLFGAIAGFIEG
GWPGLVAGWYGFQHSNDQGVGMAADRDSTQKAVDKITSKVNNIVDKMNKQYEIIDHEFSEVETRLNMINS

KIDDQIQDIWAYNAELLVLLENQKTLDEHDANVNNLYNKVKRALGSNAMEDGKGCFELYHKCDDQCMETI
RNGTYNRRKYTEESRLERQKIEGVKLESEGTYKILSIYSTVASSLVLAMGFAAFLFWAMSNGSCRCNICI

>QOW78193.1 hemagglutinin [Influenza A virus]
MEAIPLLTLLLVVTTSNADKICVGHQSTNSTETVDTLTETNVPVTQAKELLHTEHNGMLCATNLGRPLIL
NTCTIEGLIYGNPSCDMLLGGREWSYIVERPSAVNGTCYPGNIENLEELRTLFSSSSSYQRIQLFPDTIW
NVTYTGTSKSCSGSFYRNMRWLTQKNGLYPIQDAQYTNNRGKDILFVWGIHHPPTDTAQTNLYTRTDTTT
SVTTENLDRTFKPLIGPRPLVNGLIGRINYYWSVLKPGQTLRVRSNGNLIAPWFGHALSGESHGRILRTD
LSSGNCVVQCQTEKGGLNSTLPFHNISKYAFGTCPKYIGVKSLKLAVGLRNVHARSSRGLFGAIAGFIEG
GWPGLVAGWYGFQHSNDQGIGMAADRDSTQKAVDKITSKVNNIVDKMNKQYEIIDHEFSEVETRLNMINS
KIDDQIQDIWAYNAELLVLLENQKTLDEHDANVNNLYNKVKRALGSNAMEDGKGCFELYHKCDDQCMETI
RNGTYNRRKYTEESRLERQKIEGVKLESEGTYKILSIYSTVASSLVLAMGFAAFLVWAMSNGSCRCNICI

>QOW78148.1 hemagglutinin [Influenza A virus]
MEAIPLLTLLLVVTTSNADKICVGHQSTNSTETVDTLTETNVPVTQAKELLHTEHNGMLCATNLGRPLIL
NTCTIEGLIYGNPSCDMLLGGREWSYIVERPSAVNGTCYPGNIENLEELRTLFSSSSSYQRIQLFPDTIW
NVTYTGTSKSCSDSFYRNMRWLTQKNGLYPIQDAQYTNNRGKDILFVWGIHHPPTDTAQTNLYTRTDTTT
SVTTENLDRTFKPLIGPRPLVNGLIGRINYYWSVLKPGQTLRVRSNGNLIAPWFGHILSGESHGRILRTD
LSSGNCVVQCQTEKGGLNSTLPFHNISKYAFGTCPKYIGVKSLKLAVGLRNVHARSSRGLFGAIAGFIEG
GWPGLVAGWYGFQHSNDQGVGMAADRDSTQKAVDKITSKVNNIVDKMNKQYEIIDHEFSEVETRLNMINS
KIDDQIQDIWAYNAELLVLLENQKTLDEHDANVNNLYNKVKRALGSNAMEDGKGCFELYHKCDDQCMETI
RNGTYNRRKYTEESRLERQKIEGVKLESEGTYKILSIYSTVASSLVLAMGFAAFLFWAMSNGSCRCNICI

>QOW78162.1 hemagglutinin [Influenza A virus]
MEAIPLLTLLLVVTTSNADKICVGHQSTNSTETVDTLTETNVPVTQAKELLHTEHNGMLCATNLGRPLIL
NTCTIEGLIYGNPSCDMLLEGREWSYIVERPSAVNGTCYPGNIENLEELRTLFSSSSSYQRIQLFPDTIW
NVTYTGTSKSCSDSFYRNMRWLTQKNGLYPIQDAQYTNNRGKDILFVWGIHHPPTDTAQTNLYTRTDTTT
SVTTENLDRTFKPLIGPRPLVNGLIGRINYYWSVLKPGQTLRVRSNGNLIAPWFGHVLSGESHGRILRTD
LSSGNCVVQCQTEKGGLNSTLPFHNISKYAFGTCPKYIGVKSLKLAVGLRNVHARSSRGLFGAIAGFIEG
GWPGLVAGWYGFQHSNDQGVGMAADRDSTQKAVDKITSKVNNIVDKMNKQYEIIDHEFSEVETRLNMINS
KIDDQIQDIWAYNAELLVLLENQKTLDEHDANVNNLYNKVKRALGSNAMEDGKGCFELYHKCDDQCMETI
RNGTYNRRKYTEESRLERQKIEGVKLESEGTYKILSIYSTVASSLVLAMGFAAFLFWAMSNGSCRCNICI

>QOW78181.1 hemagglutinin [Influenza A virus]

MEAIPLLTLLLVVTTSNADKICVGHQSTNSTETVDTLTETNVPVTQAKELLHTEHNGMLCATNLGRPLIL
NTCTIEGLIYGNPSCDMLLGGREWSYIVERPSAVNGTCYPGNIENLEELRTLFSSSSSYQRIQLFPDTIW
NVTYTGTSKSCSDSFYRNMRWLTQKNGLYPIQDAQYTNNRGKDILFVWGIHHPPTDTAQTNLYTRTDTTT
SVTTENLDRTFKPLIGPRPLVNGLIGRINYYWSVLKPGQTLRVRSNGNLIAPWFGHILSGESHGRILRTD
LSSGNCVVQCQTEKGGLNSTLPFHNISKYAFGTCPKYIGVKSLKLAVGLRNVHARSSRGLFGAIAGFIEG
GWPGLVAGWYGFQHSNDQGVGMAADRDSTQKAVDKITSKVNNIVDKMNKQYEIIDHEFSEVETRLNMINS
KIDDQIQDIWAYNAELLVLLENQKTLDEHDANVNNLYNKVKRALGSNAMEDGKGCFELYHKCDDQCMETI
RNGTYNRRKYTEESRLERQKIEGVKLESEGTYKILSIYSTVASSLVLAMGFAAFLFWAMSNGSCRCNICI

>QOW78144.1 hemagglutinin [Influenza A virus]
MEAIPLLTLLLVVTTSNADKICVGHQSTNSTETVDTLTETNVPVTQAKELLHTEHNGMLCATNLGRPLIL
NTCTIEGLIYGNPSCDMLLGGREWSYIVERPSAVNGTCYPGNIENLEELRTLFSSSSSYQRIQLFPDTIW
NVTYTGTSKSCSDSFYRNMRWLTQKNGLYPIQDAQYTNSRGKDILFVWGIHHPPTDTAQTNLYTRTDTTT
SVTTENLDRTFKPLIGPRPLVNGLIGRINYYWSVLKPGQTLRVRSNGNLIAPWFGHILSGESHGRILRTD
LSSGNCVVQCQTEKGGLNSTLPFHNISKYAFGTCPKYIGVKSLKLAVGLRNVHARSSRGLFGAIAGFIEG
GWPGLVAGWYGFQHSNDQGVGMAADRDSTQKAVDKITSKVNNIVDKMNKQYEIIDHEFSEVETRLNMINS
KIDDQIQDIWAYNAELLVLLENQKTLDEHDANVNNLYNKVKRALGSNAMEDGKGCFELYHKCDDQCMETI
RNGTYNRRKYTEESRLERQKIEGVKLESEGTYKILSIYSTVASSLVLAMGFAAFLFWAMSNGSCRCNICI

>QOW78125.1 hemagglutinin [Influenza A virus]
MEAIPLLTLLLVVTTGNADKICVGHQSTNSTETVDTLTETNVPVTQAKELLHTEHNGKLCATNLGRPLIL
NTCTIEGLIYGNPSCDMLLGGREWSYIVERPSAVNGTCYPGNIENLEELRTLFSSSSSYQRIQLFPDTIW
NVTYTGTSKSCSDSFYRNMRWLTQKNGLYPIQDAQYTNSRGKDILFVWGIHHPPTDTAQTNLYTRTDTTT
SVTTENLDRTFKPLIGPRPLVNGLIGRINYYWSVLKPGQTLRVRSNGNLIAPWFGHILSGESHGRILRTD
LSSGNCVVQCQTEKGGLNSTLPFHNISKYAFGTCPKYIGVKSLKLAVGLRNVHARSSRGLFGAIAGFIEG
GWPGLVAGWYGFQHSNDQGVGMAADRDSTQKAVDKITSKVNNIVDKMNKQYEIIDHEFSEVETRLNMINS
KIDDQIQDIWAYNAELLVLLENQKTLDEHDANVNNLYNKVKRALGSNAMEDGKGCFELYHKCDDQCMETI
RNGTYNRRKYTEESRLERQKIEGVKLESEGTYKILSIYSTVASSLVLAMGFAAFLFWAMSNGSCRCNICI

>QOW78117.1 hemagglutinin [Influenza A virus]
MEAIPLLTLLLVVTTSNADKICVGHQSTNSTETVDTLTETNVPVTQAKELLHTEHNGMLCATNLGRPLIL
NTCTIEGLIYGNPSCDMLLGGREWSYIVERPSAVNGTCYPGNIENLEELRTLFSSSSSYQRIQLFPDTIW
NVTYTGTSKSCSDSFYRNMRWLTQKNGLYPIQDAQYTNNRGKDILFVWGIHHPPTDTAQTNLYTRTDTTT
SVTTENLDRTFKPLIGPRPLVNGLIGRINYYWSVLKPGQTLRVRSNGNLIAPWFGHVLSGESHGRILRTD

LSSGNCVVQCQTEKGGLNSTLPFHNISKYAFGTCPKYIGVKSLKLAVGLRNVHARSSRGLFGAIAGFIEG
GWPGLVAGWYGFQHSNDQGVGMAADRDSTQKAVDKITSKVNNIVDKMNKQYEIIDHEFSEVETRLNMINS
KIDDQIQDIWAYNAELLVLLENQKTLDEHDANVNNLYNKVKRALGSNAMEDGKGCFELYHKCDDQCMETI
RNGTYNRRKYTEESRLERQKIEGVKLESEGTYKILSIYSTVASSLVLAMGFAAFLVWAMSNGSCRCNICI

>QOW78104.1 hemagglutinin [Influenza A virus]
MKAIPLLTILLVVTTSNADKICVGHQSTNSTETVDTLTETNVPVTQAKELLHTEHNGMLCATNLGRPLIL
NTCTIEGLIYGNPSCDMLLGGREWSYIVERPSAVNGTCYPGNIENLEELRTLFSSSSSYQRIQLFPDTIW
NVTYTGTSKSCSDSFYRNMRWLTQKNGLYPIQDAQYTNNRGKDILFVWGIHHPPTDTAQTNLYTRTDTTT
SVTTENLDRTFKPLIGPRPLVNGLIGRINYYWSVLKPGQTLRVRSNGNLIAPWFGHVLSGESHGRILKTD
LSSGNCVVQCQTERGGLNSTLPFHNISKYAFGTCPKYIGVKSLKLAVGLRNVRARSSRGLFGAIAGFIEG
GWPGLVAGWYGFQHSNDQGIGMAADRDSTQKAVDKITSKVNNIVDKMNKQYEIIDHEFSEVETRLNMINS
KIDDQIQDIWAYNAELLVLLENQKTLDEHDANVNNLYNKVKRALGSNAMEDGKGCFELYHKCDDQCMETI
RNGTYNRRKYTEESRLERLKIEGVKLESEGTYKILSIYSTVASSLVLAMGFAAFLFWAMSNGSCRCNICI

>QOW78098.1 hemagglutinin [Influenza A virus]
MEAIPLLTLLLMVTTSNADKICVGHQSTNSTETVDTLTETNVPVTQAKELLHTEHNGMLCATNLGRPLIL
NTCTIEGLIYGNPSCDMLLGGREWSYIVERPSAVNGTCYPGNIENLEELRTLFSSSSSYQRIQLFPDTIW
NVTYTGTSKSCSDSFYRNMRWLTQKNGLYPIQDAQYTNNRGKDILFVWGIHHPPTDTAQTNLYTRTDTTT
SVTTENLDRTFKPLIGPRPLVNGLIGRINYYWSVLKPGQTLRVRSNGNLIAPWFGHVLSGESHGRILRTD
LSSGNCVVQCQTEKGGLNSTLPFHNISKYAFGTCPKYIGVKSLKLAVGLRNVHARSSRGLFGAIAGFIEG
GWPGLVAGWYGFQHSNDQGVGMAADRDSTQKAVDKITSKVNNIVDKMNKQYEIIDHEFSEVETRLNMINS
KIDDQIQDIWAYNAELLVLLENQKTLDEHDANVNNLYNKVKRALGSNAMEDGKGCFELYHKCDDQCMETI
RNGTYNRRKYTEESKLERQKIEGVKLESEGTYKILSIYSTVASSLVLAMGFAAFLFWAMSNGSCRCNICI

>QOW78077.1 hemagglutinin [Influenza A virus]
MEAISLLTLLLVVTTSNADKICVGHQSTNSTETVDTLTETNVPVTQAKELLHTEHNGMLCATNLGRPLIL
DTCTIEGLIYGNPSCDVLLGGREWSYIVERPSAVNGTCYPGNIENLEELRTLFSSSSSYQRIQLFPDTIW
NVTYTGTSKSCSGSFYRNMRWLTQKNGLYPIQDAQYTNSRGKDILFVWGIHHPPTDTAQTNLYTRTDTTT
SVTTENLDRTFKPLIGPRPLVNGLIGRINYYWSVLKPGQTLRVRSNGNLIAPWFGHILSGESHGRILRTD
LSSGNCVVQCQTEKGGLNSTLPFHNISKYAFGTCPKYIGVKSLKLAVGLRNVHARSSRGLFGAIAGFIEG
GWPGLVAGWYGFQHSNDQGVGMAADRDSTQKAVDKITSKVNNIVDKMNKQYEIIDHEFSEVETRLNMINS
KIDDQIQDIWAYNAELLVLLENQKTLDEHDANVNNLYNKVKRALGSNAMEDGKGCFELYHKCDDQCMETI
RNGTYNRRKYTEESRLERQKIEGVKLESEGTYKILSIYSTVASSLVLAMGFAAFLFWAMSNGSCRCNICI

>QOW78064.1 hemagglutinin [Influenza A virus]

MEAIPLLTLLLVVTTSNADKICVGHQSTNSTETVDTLTETNVPVTQAKELLHTEHNGMLCATNLGRPLIL

NTCTIEGLIYGNPSCDMLLGGREWSYIVERPSAVNGTCYPGNIENLEELRTLFSSSSSYQRIQLFPDTIW

NVTYTGTSKSCSDSFYRNMRWLTQKNGLYPIQDAQYTNNRGKDILFVWGIHHPPTDTAQTNLYTRTDTTT

SVTTENLDRTFKPLIGPRPLVNGLIGRINYYWSVLKPGQTLRVRSNGNLIAPWFGHVLSGESHGRILRTD

LSSGNCVVQCQTEKGGLNSTLPFHNISKYAFGTCPKYIGVKSLKLAVGLRNVHARSSRGLFGAIAGFIEG

GWPGLVAGWYGFQHSNDQGVGMAADRDSTQKAVDKITSKVNNIVDKMNKQYEIIDHEFSEVETRLNMINS

KIDDQIQDIWAYNAELLVLLENQKTLDEHDANVNNLYNKVKRALGSNAMEDGKGCFELYHKCDDQCMETI

RNGTYNRRKYTEESRLERQKIEGVKLESEGTYKILSIYSTVASSLVLAMGFAAFLFWAMSNGSCRCNICI

>QOW78058.1 hemagglutinin [Influenza A virus]

MEAIPLLTLLLMVTTSNADKICVGHQSTNSTETVDTLTETNVPVTQAKELLHTEHNGMLCATNLGRPLIL

NTCTIEGLIYGNPSCDMLLGGREWSYIVERPSAVNGTCYPGNIENLEELRTLFSSSSSYQRIQLFPDTIW

NVTYTGTSKSCSDSFYRNMRWLTQKNGLYPIQDAQYTNNRGKDILFVWGIHHPPTDTTQTNLYTRTDTTT

SVTTENLDRTFKPLIGPRPLVNGLIGRINYYWSVLKPGQTLRVRSNGNLIAPWFGHVLSGESHGRILRTD

LSSGNCVVQCQTEKGGLNSTLPFHNISKYAFGTCPKYIGVKSLKLAVGLRNVHARSSRGLFGAIAGFIEG

GWPGLVAGWYGFQHSNDQGVGMAADRDSTQKAVDKITSKVNNIVDKMNKQYEIIDHEFSEVETRLNMINS

KIDDQIQDIWAYNAELLVLLENQKTLDEHDANVNNLYNKVKRALGSNAMEDGKGCFELYHKCDDQCMETI

RNGTYNRRKYTEESKLERQKIEGVKLESEGTYKILSIYSTVASSLVLAMGFAAFLFWAMSNGSCRCNICI

>QOW78051.1 hemagglutinin [Influenza A virus]

MEAIPLLTLLLVVTTSSADKICVGHQSTNSTETVDTLTETNVPVTQAKELLHTEHNGMLCATNLGRPLIL

NTCTIEGLIYGNPSCDMLLGGREWSYIVERPSAVNGTCYPGNIENLEELRTLFSSSSSYQRIQLFPDTIW

NVTYTGTSKSCSDSFYRNMRWLTQKNGLYPIQDAQYTNNRGKDILFVWGIHHPPTDTAQTNLYTRTDTTT

SVTTENLDRTFKPLIGPRPLVNGLIGRINYYWSVLKPGQTLRVRSNGNLVAPWFGHVLSGESHGRILKTD

LSSGNCVVQCQTEKGGLNSTLPFHNISKYAFGTCPKYIGVKSLKLAVGLRNVHARSSRGLFGAIAGFIEG

GWPGLVAGWYGFQHSNDQGVGMAADRDSTQKAVDKITSKVNNIVDKMNKQYEIIDHEFSEVETRLNMINS

KIDDQIQDIWAYNAELLVLLENQKTLDEHDANVNNLYNKVKRALGSNAMEDGKGCFELYHKCDDQCMETI

RNGTYNRRKYTEESRLERQKIEGVKLESEGTYKILSIYSTVASSLVLAMGFAAFLFWAMSNGSCRCNICI

>QOW78016.1 hemagglutinin [Influenza A virus]

MKAIPLLTILLVVTTSNADKICVGHQSTNSTETVDTLTETNVPVTQAKELLHTEHNGMLCATNLGRPLIL

NTCTIEGLIYGNPSCDMLLGGREWSYIVERPSAVNGTCYPGNIENLEELRTLFSSSSSYQRIQLFPDTIW

NVTYTGTSKSCSDSFYRNMRWLTQKNGLYPIQDAQYTNNRGKDILFVWGIHHPPTDTAQTNLYTRTDTTT
SVTTENLDRTFKPLIGPRPLVNGLIGRINYYWSVLKPGQTLRVRSNGNLIAPWFGHVLSGESHGRILKTD
LSSGNCVVQCQTERGGLNSTLPFHNISKYAFGTCPKYIGVKSLKLAVGLRNVRARSSRGLFGAIAGFIEG
GWPGLVAGWYGFQHSNDQGIGMAADRDSTQKAVDKITSKVNNIVDKMNKQYEIIDHEFSEVETRLNMINS
KIDDQLQDIWAYNAELLVLLENQKTLDEHDANVNNLYNKVKRALGSNAMEDGKGCFELYHKCDDQCMETI
RNGTYNRRKYTEESRLERLKIEGVKLESEGTYKILSIYSTVASSLVLAMGFAAFLFWAMSNGSCRCNICI

>QOW78037.1 hemagglutinin [Influenza A virus]
MKAIPLLTILLVVTTSNADKICVGHQSTNSTETVDTLTETNVPVTQAKELLHTEHNGMLCATNLGRPLIL
NTCTIEGLIYGNPSCDMLLGGREWSYIVERPSAVNGTCYPGNIENLEELRTLFSSSSSYQRIQLFPDTIW
NVTYTGTSKSCSDSFYRNMRWLTQKNGLYPIQDAQYTNNRGKDILFVWGIHHPPTDTAQTNLYTRTDTTT
SVTTENLDRTFKPLIGPRPLVNGLIGRINYYWSVLKPGQTLRVRSNGNLIAPWFGHVLSGESHGRILKTD
LSSGNCVVQCQTERGGLNSTLPFHNISKYAFGTCPKYIGVKSLKLAVGLRNVRARSSRGLFGAIAGFIEG
GWPGLVAGWYGFQHSNDQGIGMAADRDSTQKAVDKITSKVNNIVDKMNKQYEIIDHEFSEVETRLNMINS
KIDDQIQDIWAYNAELLVLLENQKTLDEHDANVNNLYNKVKRALGSNAMEDGKGCFELYHKCDDQCMETI
RNGTYNRRKYTEESRLERLKIEGVKLESEGTYKILSIYSTVASSLVLAMGFAAFLFWAMSNGSCRCNICI

>QOW78015.1 hemagglutinin [Influenza A virus]
MEAIPLLTLLLVVTTSSADKICVGHQSTNSTETVDTLTETNVPVTQAKELLHTEHNGMLCATNLGRPLIL
NTCTIEGLIYGNPSCDMLLGGREWSYIVERPSAVNGTCYPGNIENLEELRTLFSSSSSYQRIQLFPDTIW
NVTYTGTSKSCSDSFYRNMRWLTQKNGLYPIQDAQYTNNRGKDILFVWGIHHPPTDTVQTNLYTRTDTTT
SVTTENLDRTFKPLIGPRPLVNGLIGRINYYWSVLKPGQTLRVRSNGNLVAPWFGHVLSGESHGRILKTD
LSSGNCVVQCQTEKGGLNSTLPFHNISKYAFGTCPKYIGVKSLKLAVGLRNVHARSSRGLFGAIAGFIEG
GWPGLVAGWYGFQHSNDQGVGMAADRDSTQKAVDKITSKVNNIVDKMNKQYEIIDHEFSEVETRLNMINS
KIDDQIQDIWAYNAELLVLLENQKTLDEHDANVNNLYNKVKRALGSNAMEDGKGCFELYHKCDDQCMETI
RNGTYNRRKYTEESRLERQKIEGVKLESEGNYKILSIYSTVASSLVLAMGFAAFLFWAMSNGSCRCNICI

>QOW77994.1 hemagglutinin [Influenza A virus]
MEAIPLLTLLLVVTTSNADKICVGHQSTNSTETVDTLTETNVPVTQAKELLHTEHNGMLCATNLGRPLIL
NTCTIEGLIYGNPSCDMLLGGREWSYIVERPSAVNGTCYPGNIENLEELRTLFSSSSSYQRIQLFPDTIW
NVTYTGTSKSCSDSFYRNMRWLTQKNGLYPIQDAQYTNSRGKDILFVWGIHHPPTDTAQTNLYTRTDTTT
SVTTENLDRTFKPLIGPRPLVNGLIGRINYYWSVLKPGQTLRVRSNGNLIAPWFGHILSGESHGRILRTD
LSSGNCVVQCQTEKGGLNSTLPFHNISKYAFGTCPKYIGVKSLKLAVGLRNVHARSSRGLFGAIAGFIEG
GWPGLVAGWYGFQHSNDQGVGMAADRDSTQKAVDKITSKVNNIVDKMNKQYEIIDHEFSEVETRLNMINS

KIDDQIQDIWAYNAELLVLLENQKTLDEHDANVNNLYNKVKRALGSNAMEDGKGCFELYHKCDDQCMETI
RNGTYNRRKYTEESRLERQKIEGVKLESEGTYKILSIYSTVASSLVLAMGFAAFLFWAMSNGSCRCNICI

>QOW77987.1 hemagglutinin [Influenza A virus]
MEAIPLLTLLLVVTTSSADKICVGHQSTNSTETVDTLTETNVPVTQAKELLHTEHNGMLCATNLGRPLIL
NTCTIEGLIYGNPSCDMLLGGREWSYIVERPSAVNGTCYPGNIENLEELRTLFSSSSSYQRIQLFPDTIW
NVTYTGTSKSCSDSFYRNMRWLTQKNGLYPIQDAQYTNNRGKDILFVWGIHHPPTDTAQTNLYTRTDTTT
SVTTENLDRTFKPLIGPRPLVNGLIGRINYYWSVLKPGQTLRVRSNGNLVAPWFGHVLSGESHGRILKTD
LSSGNCVVQCQTEKGGLNSTLPFHNISKYAFGTCPKYIGVKSLKLAVGLRNVHARSSRGLFGAIAGFIEG
GWPGLVAGWYGFQHSNDQGVGMAADRDSTQKAVDKITSKVNNIVDKMNKQYEIIDHEFSEVETRLNMINS
KIDDQIQDIWAYNAELLVLLENQKTLDEHDANVNNLYNKVKRALGSNAMEDGKGCFELYHKCDDQCMETI
RNGTYNRRKYTEESRLERQKIEGVKLESEGTYKILSIYSTVASSLVLAMGFAAFLFWAMSNGSCRCNICI

>QOW77978.1 hemagglutinin [Influenza A virus]
MEAIPLLTLLLVVTTSSADKICVGHQSTNSTETVDTLTETNVPVTQAKELLHTEHNGMLCATNLGRPLIL
NTCTIEGLIYGNPSCDMLLGGREWSYIVERPSAVNGTCYPGNIENLEELRTLFSSSSSYQRIQLFPDTIW
NVTYTGTSKSCSDSFYRNMRWLTQKNGLYPIQDAQYTNNRGKDILFVWGIHHPPTDTAQTNLYTRTDTTT
SVTTENLDRTFKPLIGPRPLVNGLIGRINYYWSVLKPGQTLRVRSNGNLIAPWFGHVLSGESHGRILKTD
LSSGNCVVQCQTEKGGLNSTLPFHNISKYAFGTCPKYIGVKSLKLAVGLRNVHARSSRGLFGAIAGFIEG
GWPGLVAGWYGFQHSNDQGVGMAADRDSTQKAVDKITSKVNNIVDKMNKQYEIIDHEFSEVETRLNMINS
KIDDQIQDIWAYNAELLVLLENQKTLDEHDANVNNLYNKVKRALGSNAMEDGKGCFELYHKCDDQCMETI
RNGTYNRRKYTEESRLERQKIEGVKLESEGTYKILSIYSTVASSLVLAMGFAAFLFWAMSNGSCRCNICI

>QOW77959.1 hemagglutinin [Influenza A virus]
MEAIPLLTLLLVVTTSNADKICVGHQSTNSTETVDTLTETNVPVTQAKELLHTEHNGMLCATNLGRPLIL
NTCTIEGLIYGNPSCDMLLGGREWSYIVERPSAVNGTCYPGNIENLEELRTLFSSSSSYQRIQLFPDTIW
NVTYTGTSKSCSDSFYRNMRWLTQKNGLYPIQDAQYTNNRGKDILFVWGIHHPPTDTAQTNLYTRTDTTT
SVTTENLDRTFKPLIGPRPLVNGLIGRINYYWSVLKPGQTLRVRSNGNLIAPWFGHVLSGESHGRILRTD
LSSGNCVVQCQTEKGGLNSTLPFHNISKYAFGTCPKYIGVKSLKLAVGLRNVHARSSRGLFGAIAGFIEG
GWPGLVAGWYGFQHSNDQGVGMAADRDSTQKAVDKITSKVNNIVDKMNKQYEIIDHEFSEVETRLNMINS
KIDDQIQDIWAYNAELLVLLENQKTLDEHDANVNNLYNKVKRALGSNAMEDGKGCFELYHKCDDQCMETI
RNGTYNRRKYTEESRLERQKIEGVKLESEGTYKILSIYSTVASSLVLAMGFAAFLVWAMSNGSCRCNICI

>QNL29317.1 hemagglutinin [Influenza A virus]

METVSLITMLLVVTVSNADKICIGYQSTNSTETVDTLTEDNVPVTHAKELLHTEHNGMLCATSLGHPLIL
DTCTIEGLIYGNPSCDLLLGGREWSYIVERPSAVNGLCYPGNVENLEELRSLFSSASSYQRIQIFPDTIW
NVSYSGTSKACSDSFYRSMRWLTQKNNAYPIQDAQYTNNQEKNILFMWGINHPPTDTVQTNLYTRTDTTT
SVATEEINRTFKPLIGPRPLVNGMQGRIDYYWSVLKPGQTLRIISNGNLIAPWYGHILSRESHGRILKTD
LKKGSCTVQCQTEKGGLNTTLPFQNVSKYAFGNCSKYIGIKSLKLAVGLRNVPSRSSRGLFGAIAGFIEG
GWSGLVAGWYGFQHSNDQGVGMAADRDSTQKAIDKITSKVNNIVDKMNKQYEIIDHEFSEVETRLNMINN
KIDDQIQDIWAYNAELLVLLENQKTLDEHDANVNNLYNKVKRALGSNAMEDGKGCFELYHKCDDQCMETI
RNGTYNRRKYQEESKLERQKIEGVKLESEGTYKILTIYSTVASSLVIAMGFAAFLFWAMSNGSCRCNICI

>QOQ50962.1 hemagglutinin [Influenza A virus]
MEAISLMTILLVVTTSNADKICIGHQSTNSTETVDTLTETNVPVTQAKELLHTEHNGMLCATNLGRPLIL
DTCTIEGLIYGNPSCDMLLGGREWSYIVERPSAVNGTCYPGNVENLEELRTLFSSSSSYQRVQLFPDSIW
NVTYTGTSKSCSDSFYRNMRWLTQKNGGYPIQDAQYTNNRGKDILFVWGIHHPPTDTTQTNLYTRTDTTT
SVTTETLDRTFKPLIGPRPLVNGLIGRINYYWSVLKPGQTLRVRSNGNLIAPWFGHVLSGESHGRILRTD
LSSGNCVVQCQTEKGGLNSTMPFHNISKYAFGTCPKYIGVKSLKLAIGLRNVHARSSRGLFGAIAGFIEG
GWPGLVAGWYGFQHSNDQGVGIAADRVSTQKAVDKITSKVNNIVDKMNKQYEIIDHEFSEVETRLNMINN
KIDDQIQDIWAYNAELLVLLENQKTLDEHDANVNNLYNKVKRALGSNAMEDGKGCFELYHKCDDQCMETI
RNGTYNRRKYTEESRLERQKIEGVKLEAEGTYKILSIYSTVASSLVLAMGFAAFLFWAMSNGSCRCNICI

>QOQ50949.1 hemagglutinin [Influenza A virus]
MEAISLMTILLVVTTSNADKICIGHQSTNSTETVDTLTETNVPVTQAKELLHTEHNGMLCATNLGRPLIL
DTCTIEGLIYGNPSCDMLLGGREWSYIVERPSAVNGTCYPGNVENLEELRTLFSSSSSYQRVQLFPDSIW
NVTYNGTSKSCSDSFYRNMRWLTQKNGGYPIQDAQYTNNRGKDILFVWGIHHPPTDTVQTNLYTRTDTTT
SVTTETLDRTFKPLIGPRPIVNGRIGRINYYWSVLKPGQTLRVRSNGNLIAPWFGHVLSGESHGRILRTD
LSSGNCVVQCQTEKGGLNSTMPFHNISKYAFGTCPKYIGVKSLKLAIGLRNVHARSSRGLFGAIAGFIEG
GWPGLVAGWYGFQHSNDQGVGMAADRVSTQKAVDKITSKVNNIVDKMNKQYEIIDHEFSEVETRLNMINN
KIDDQIQDIWAYNAELLVLLENQKTLDEHDANVNNLYNKVKRALGSNAMEDGKGCFELYHKCDDQCMETI
RNGTYNRRKYTEESRLERQKIEGVKLEAEGTYKILSIYSTVASSLVLAMGFAAFLFWAMSNGSCRCNICI

>QOQ50939.1 hemagglutinin [Influenza A virus]
MEAISLMTILLVVTTSNADKICIGHQSTNSTETVDTLTETNVPVTQAKELLHTEHNGMLCATNLGRPLIL
DTCTIEGLIYGNPSCDMLLGGREWSYIVERPSAVNGTCYPGNVENLEELRTLFSSSSSYQRVQLFPDSIW
NVTYTGTSKSCSDSFYRNMRWLTQKNGGYPIQDAQYTNNRGKDILFVWGIHHPPTDTAQTNLYTRTDTTT
SVTTETLDRTFKPLIGPRPLVNGLIGRINYYWSVLKPGQTLRVRSNGNLIAPWFGHVLSGESHGRILRTD

LSSGNCVVQCQTEKGGLNSTMPFHNVSKYAFGTCPKYIGVKSLKLAIGLRNVHARSSRGLFGAIAGFIEG
GWPGLVAGWYGFQHSNDQGVGMAADRVSTQKAIDKITSKVNNIVDKMNKQYEIIDHEFSEVETRLSMINN
KIDDQIQDIWAYNAELLVLLENQKTLDEHDANVNNLYNKVKRALGSNAMEDGKGCFELYHKCDDQCMETI
RNGTYNRRKYTEESRLERQKIEGVKLEAEGTYKILSIYSTVASSLVLAMGFAAFLFWAMSNGSCRCNICI

>QOQ50928.1 hemagglutinin [Influenza A virus]
MEAISLMTILLVVTTGNADKICIGHQSTNSTETVNTLTETNVPVTQAKELLHTEHNGMLCATNLGRPLIL
DTCTIEGLIYGNPSCDMLLEGREWAYIVERPSAVNGTCYPGNVENLEELRTFFSSSSSYQRVQLFPDSIW
NVTYTGTSKSCSDSFYRNMRWLTQKSGSYPIQDAQYTNNRGKDILFVWGIHHPPTDTAQTNLYTRTDTTT
SVTTETLDRTFKPLIGPRPLVNGLIGRINYYWSVLKPGQTLRVRSNGNLIAPWFGHVLSGESHGRILRTN
LRSGNCVVQCQTEKGGLNSTMPFHNISKYAFGTCPKYIGVKSLKLAIGLRNVHARSSRGLFGAIAGFIEG
GWPGLVAGWYGFQHSNDQGVGMAADRVSTQKAVDKITSKVNNIVDKMNKQYEIIDHEFSEIETRLNMINN
KIDDQIQDIWAYNAELLVLLENQKTLDEHDANVNNLYNKVKRALGSNAMEDGKGCFELYHKCDDQCMETI
RNGTYNRRKYTEESRIERQKIEGVKLEAEGTYKILSIYSTVASSLVLAMGFAAFLFWAMSNGSCRCNICI

>QOQ50918.1 hemagglutinin [Influenza A virus]
MEAISLMTILLVVTTSNADKICIGHQSTNSTETVDTLTETNVPVTQAKELLHTEHNGMLCATNLGRPLIL
DTCTIEGLIYGNPSCDMLLGGREWSYIVERPSAVNGTCYPGNVENLEELRTLFSSSSSYQRVQLFPDSIW
NVTYTGTSKSCSDSFYRNMRWLTQKNGGYPIQDAQYTNNRGKDILFVWGIHHPPTDTVQTNLYTRTDTTT
SVTTETLDRTFKPLIGPRPLVNGLIGRINYYWSVLKPGQTLRVRSNGNLIAPWFGHVLSGESHGRILRTD
LSSGNCVVQCQTEKGGLNSTMPFHNISKYAFGTCPKYIGVKSLKLAIGLRNVHARSSRGLFGAIAGFIEG
GWPGLVAGWYGFQHSNDQGVGMAADRVSTQKAVDKITSKVNNIVDKMNKQYEIINHEFSEVETRLNMINN
KIDDQIQDIWAYNAELLVLLENQKTLDEHDANVNNLYNKVKRALGSNAMEDGKGCFELYHKCDDQCMETI
RNGTYNRRKYTEESRLERQKIEGVKLEAEGTYKILSIYSTVASSLVLAMGFAAFLFWAMSNGSCRCNICI

>QOQ50902.1 hemagglutinin [Influenza A virus]
MEAISLMTILLVVTTSNADKICIGHQSTNSTETVDTLTETNVPVTQAKELLHTEHNGMLCATNLGRPLIL
DTCTIEGLIYGNPSCDMLLGGREWSYIVERPSAVNGTCYPGNVENLEELRTLFSSSSSYQRVQLFPDSIW
NVTYTGTSKSCSDSFYRNMRWLTQKNGGYPIQDAQYTNNRGKDILFVWGIHHPPTNTAQTNLYTRTDTTT
SVTTETLDRTFKPLIGPRPLVNGQIGRINYYWSVLKPGQTLRVRSNGNLIAPWFGHVLSGESHGRILRTD
LSSGNCVVQCQTEKGGLNSTMPFHNVSKYAFGTCPKYIGVKSLKLAIGLRNVHARSSRGLFGAIAGFIEG
GWPGLVAGWYGFQHSNDQGVGMAADRVSTQKAVDKITSKVNNIVDKMNKQYEIIDHEFSEVETRLNMINN
KIDDQIQDIWAYNAELLVLLENQKTLDEHDANVNNLYNKVKRALGSNAMEDGKGCFELYHKCDDQCMETI
RNGTYNRRKYTEESRLERQKIEGVKLEAEGTYKILSIYSTVASSLVLAMGFAAFLFWAMSNGSCRCNICI

>QOQ50895.1 hemagglutinin [Influenza A virus]

MEAISLMTILLVVTTSNADKICIGHQSTNSTETVDTLTETNVPVTQAKELLHTEHNGMLCATNLGRPLIL
DTCTIEGLIYGNPSCDMLLGGREWSYIVERPSAVNGTCYPGNVENLEELRTLFSSSSSYQRVQLFPDSIW
NVTYTGTSKSCSDSFYRNMRWLTQKNGGYPIQDAQYTNNRGKDILFVWGIHHPPTDTAQTNLYTRTDTTT
SVTTETLDRTFKPLIGPRPLVNGLIGRINYYWSVLKPGQTLRVRSNGNLIAPWFGHVLSGESHGRILRTD
LSSGNCVVQCQTEKGGLNSTMPFHNVSKYAFGTCPKYIGVKSLKLAIGLRNVHARSSRGLFGAIAGFIEG
GWPGLVAGWYGFQHSNDQGVGMAADRVSTQKAVDKITSKVNNIVDKMNKQYEIIDHEFSEVETRLNMINN
KIDDQIQDIWAYNAELLVLLENQKTLDEHDANVNNLYNKVKRALGSNAMEDGKGCFELYHKCDDQCMETI
RNGTYNRRKYTEESRLERQKIEGVKLEAEGTYKILSIYSTVASSLVLAMGFAAFLFWAMSNGSCRCNICI

>QOQ50872.1 hemagglutinin [Influenza A virus]

MEAISLMTILLVVTTSNADKICIGHQSTNSTETVDTLTETNVPVTQAKELLHTEHNGMLCATNLGRPLIL
DTCTIEGLIYGNPSCDMLLGGREWSYIVERPSAVNGTCYPGNVENLEELRTLFSSSSSYQRVQLFPDSIW
NVTYTGTSKSCSDSFYRNMRWLTQKNGGYPIQDAQYTNNRGKDILFVWGIHHPPTDTAQTNLYTRTDTTT
SVTTETLDRTFKPLIGPRPLVNGLIGRINYYWSVLKPGQTLRVRSNGNLIAPWFGHVLSGESHGRILRTD
LSSGNCVVQCQTEKGGLNSTMPFHNVSKYAFGTCPKYIGVKSLKLAIGLRNVHARSSRGLFGAIAGFIEG
GWPGLVAGWYGFQHSNDQGVGMAADRVSTQKAVDKITSKVNNIVDKMNKQYEIIDHEFSDVETRLNMINN
KIDDQIQDIWAYNAELLVLLENQKTLDEHDANVNNLYNKVKRALGSNAMEDGKGCFELYHKCDDQCMETI
RNGTYNRRKYTEESRLERQKIEGVKLEAEGTYKILSIYSTVASSLVLAMGFAAFLFWAMSNGSCRCNICI

>QOQ50882.1 hemagglutinin [Influenza A virus]

MEAISLMTILLVVTTSNADKICIGHQSTNSTETVDTLTETNVPVTQAKELLHTEHNGMLCATNLGRPLIL
DTCTIEGLIYGNPSCDMLLGGREWSYIVERPSAVNGTCYPGNVENLEELRTLFSSSSSYQRVQLFPDSIW
NVTYTGTSKSCSDSFYRNMRWLTQKNGGYPIQDAQYTNNRGKDILFVWGIHHPPTDTAQTNLYTRTDTTT
SVTTETLDRTFKPLIGPRPLVNGLIGRINYYWSVLKPGQTLRVRSNGNLIAPWFGHVLSGESHGRILRTD
LSSGNCVVQCQTEKGGLNSTMPFHNISKYAFGTCPKYIGVKSLKLAIGLRNVHARSSRGLFGAIAGFIEG
GWPGLVAGWYGFQHSNDQGVGMAADRVSTQKAVDKITSKVNNIVDKMNKQYEIIDHEFSEVETRLNMINN
KIDDQIQDIWAYNAELLVLLENQKTLDEHDANVNNLYNKVKRALGSNAMEDGKGCFELYHKCDDQCMETI
RNGTYNRRKYTEESRLERQKIEGVKLEAEGTYKILSIYSTVASSLVLAMGFAAFLFWAMSNGSCRCNICI

>QOQ50863.1 hemagglutinin [Influenza A virus]

MEAISLMTILLVVTTSNADKICIGHQSTNSTETVDTLTETNVPVTQAKELLHTEHNGMLCATNLGRPLIL
DTCTIEGLIYGNPSCDMLLGGREWSYIVERPSAVNGTCYPGNVENLEELRTLFSSSSSYQRVQLFPDSIW

NVTYTGTSKSCSDSFYRNMRWLTQKNGGYPIQDAQYTNNRGKDILFVWGIHHPPTDTAQTNLYTRTDTTT
SVTTETLDRTFKPLIGPRPLVNGLIGRINYYWSVLKPGQTLRVRSNGNLIAPWFGHVLSGESHGRILRTD
LSSGNCVVQCQTEKGGLNSTMPFHNISKYAFGTCPKYIGVKSLKLAIGLRNVQARSSRGLFGAIAGFIEG
GWPGLVAGWYGFQHSNDQGVGMAADRVSTQKAVDKITSKVNNIVDKMNKQYEIIDHEFSEVETRLNMINN
KIDDQIQDIWAYNAELLVLLENQKTLDEHDANVNNLYNKVKRALGSNAMEDGKGCFELYHKCDDQCMETI
RNGTYNRRKYIEESRLERQKIEGVKLEAEGTYKILSIYSTVASSLVLAMGFAAFLFWAMSNGSCRCNICI

>QOQ30275.1 hemagglutinin [Influenza A virus]
MEAISLMTILLVVTTSNADKICIGHQSTNSTETVDTLTETNVPVTQAKELLHTEHNGMLCATNLGRPLIL
DTCTIEGLIYGNPSCDMLLGGREWAYIVERPSAVNGTCYPGNVENLEELRTFFSSSSSYQRVQLFPDSIW
NVTYTGTSKSCSGSFYRNMRWLTQKDGSYPIQDAQYTNNRGKDILFVWGIHHPPTDTAQTNLYTRTDTTT
SVTTETLDRTFKPLIGPRPLVNGLIGRINYYWSVLKPGQTLRVRSNGNLIAPWFGHVLSGESHGRILRTD
LSSGNCVVQCQTEKGGLNSTMPFHNISKYAFGTCPKYIGVKSLKLAIGLRNVHAKSSRGLFGAIAGFIEG
GWPGLVAGWYGFQHSNDQGVGMAADRVSTQKAVDKITSKVNNIVDKMNKQYEIIDHEFSEVENRLNMINN
KIDDQIQDIWAYNAELLVLLENQKTLDEHDANVNNLYNKVKRALGSNAMEDGKGCFELYHKCDDQCMETI
RNGTYNRRKYTEESRLEKQRIEGVKLEAEGTYKILSIYSTVASSLVLAMGFAAFLFWAMSNGSCRCNICI

>QOQ30260.1 hemagglutinin [Influenza A virus]
MEAISLMTILLVVTTSNADKICIGHQSTNSTETVDTLTETNVPVTQAKELLHTEHNGMLCATNLGRPLIL
DTCTIEGLIYGNPSCDMLLGGREWAYIVERPSAVNGTCYPGNVENLEELRTFFSSSSSYQRVQLFPDSIW
NVTYTGTSKSCSGSFYRNMRWLTQKDGSYPIQDAQYTNNRGKDILFVWGIHHPPTDTAQTNLYTRTDTTT
SVTTETLDRTFKPLIGPRPLVNGLIGRINYYWSVLKPGQTLRVRSNGNLIAPWFGHVLSGESHGRILRTD
LSSGNCVVQCQTEKGGLNSTMPFHNISKYAFGTCPKYIGVKSLKLAIGLRNVHAKSSRGLFGAIAGFIEG
GWPGLVAGWYGFQHSNDQGVGMAADRVSTQKAVDKITSKVNNIVDKMNKQYEIIDHEFSEVENRLNMINN
KIDDQIQDIWAYNAELLVLLENQKTLDEHDANVNNLYNKVKRALGSNAMEDGKGCFELYHKCDDQCMETI
RNGTYNRRKYTEESRLEKQRIEGVKLEAEGTYKILSIYSTVASSLVLAMGFAAFLFWAMSNGSCRCNICI

>QOQ30244.1 hemagglutinin [Influenza A virus]
MEAISLMTILLVVTTSNADKICIGHQSTNSTETVDTLTETNVPVTQAKELLHTEHNGMLCATNLGRPLIL
DTCTIEGLIYGNPSCDMLLGGREWAYIVERPSAVNGTCYPGNVENLEELRTFFSSSSSYQRVQLFPDSIW
NVTYTGTSKSCSGSFYRNMRWLTQKDGSYPIQDAQYTNNRGKDILFVWGIHHPPTDTAQTNLYTRTDTTT
SVTTETLDRTFKPLIGPRPLVNGLIGRINYYWSVLKPGQTLRVRSNGNLIAPWFGHVLSGESHGRILRTD
LSSGNCVVQCQTEKGGLNSTMPFHNISKYAFGTCPKYIGVKSLKLAIGLRNVHAKSSRGLFGAIAGFIEG
GWPGLVAGWYGFQHSNDQGVGMAADRVSTQKAVDKITSKVNNIVDKMNKQYEIIDHEFSEVENRLNMINN

KIDDQIQDIWAYNAELLVLLENQKTLDEHDANVNNLYNKVKRALGSNAMEDGKGCFELYHKCDDQCMETI
RNGTYNRRKYTEESRLEKQRIEGVKLEAEGTYKILSIYSTVASSLVLAMGFAAFLFWAMSNGSCRCNICI

>QOQ30237.1 hemagglutinin [Influenza A virus]
MEAISLMTILLVVTTSNADKICIGHQSTNSTETVDTLTETNVPVTQAKELLHTEHNGMLCATNLGRPLIL
DTCTIEGLIYGNPSCDMLLGGREWAYIVERPSAVNGTCYPGNVENLEELRTFFSSSSSYQRVQLFPDSIW
NVTYTGTSKSCSGSFYRNMRWLTQKDGSYPIQDAQYTNNRGKDILFVWGIHHPPTDTAQTNLYTRTDTTT
SVTTETLDRTFKPLIGPRPLVNGLIGRINYYWSVLKPGQTLRVRSNGNLIAPWFGHVLSGESHGRILRTD
LSSGNCVVQCQTEKGGLNSTMPFHNISKYAFGTCPKYIGVKSLKLAIGLRNVHAKSSRGLFGAIAGFIEG
GWPGLVAGWYGFQHSNDQGVGMAADRVSTQKAVDKITSKVNNIVDKMNKQYEIIDHEFSEVENRLNMINN
KIDDQIQDIWAYNAELLVLLENQKTLDKHDANVNNLYNKVKRALGSNAMEDGKGCFELYHKCDDQCMETI
RNGTYNRRKYTEESRLEKQRIEGVKLEAEGTYKILSIYSTVASSLVLAMGFAAFLFWAMSNGSCRCNICI

>QOQ30222.1 hemagglutinin [Influenza A virus]
MEAISLMTILLVVTTSNADKICIGHQSTNSTETVDTLTETNVPVTQAKELLHTEHNGMLCATNLGRPLIL
DTCTIEGLIYGNPSCDMLLGGREWAYIVERPSAVNGTCYPGNVENLEELRTFFSSSSSYQRVQLFPDSIW
NVTYTGTSKSCSGSFYRNMRWLTQKDGSYPIQDAQYTNNRGKDILFVWGIHHPPTDTAQTNLYTRTDTTT
SVTTETLDRTFKPLIGPRPLVNGLIGRINYYWSVLKPGQTLRVRSNGNLIAPWFGHVLSGESHGRILRTD
LSSGNCVVQCQTEKGGLNSTMPFHNISKYAFGTCPKYIGVKSLKLAIGLRNVHAKSSRGLFGAIAGFIEG
GWPGLVAGWYGFQHSNDQGVGMAADRVSTQKAVDKITSKVNNIVDKMNKQYEIIDHEFSEVENRLNMINN
KIDDQIQDIWAYNAELLVLLENQKTLDEHDANVNNLYNKVKRALGSNAMEDGKGCFELYHKCDDQCMETI
RNGTYNRRKYTEESRLEKQRIEGVKLEAEGTYKILSIYSTVASSLVLAMGFAAFLFWAMSNGSCRCNICI

>QOQ30194.1 hemagglutinin [Influenza A virus]
MEAISLMTILLVVTTSNADKICIGHQSTNSTETVDTLTETNVPVTQAKELLHTEHNGMLCATNLGRPLIL
DTCTIEGLIYGNPSCDMLLGGREWAYIVERPSAVNGTCYPGNVENLEELRTFFSSSSSYQRVQLFPDSIW
NVTYTGTSKSCSGSFYRNMRWLTQKDGSYPIQDAQYTNNRGKDILFVWGIHHPPTDTAQTNLYTRTDTTT
SVTTETLDRTFKPLIGPRPLVNGLIGRINYYWSVLKPGQTLRVRSNGNLIAPWFGHVLSGESHGRILRTD
LSSGNCVVQCQTEKGGLNSTMPFHNISKYAFGTCPKYIGVKSLKLAIGLRNVHAKSSRGLFGAIAGFIEG
GWPGLVAGWYGFQHSNDQGVGMAADRVSTQKAVDKITSKVNNIVDKMNKQYEIIDHEFSEVENRLNMINN
KIDDQIQDIWAYNAELLVLLENQKTLDEHDANVNNLYNKVKRALGSNAMEDGKGCFELYHKCDDQCMETI
RNGTYNRRKYTEESRLEKQRIEGVKLEAEGTYKILSIYSTVASSLVLAMGFAAFLFWAMSNGSCRCNICI

>QOQ30211.1 hemagglutinin [Influenza A virus]

MEAISLMTILLVVTTSNADKICIGHQSTNSTETVDTLTETNVPVTQAKELLHTEHNGMLCATNLGRPLIL
DTCTIEGLIYGNPSCDMLLGGREWAYIVERPSAVNGTCYPGNVENLEELRTFFSSSSSYQRVQLFPDSIW
NVTYTGTSKSCSGSFYRNMRWLTQKDGSYPIQDAQYTNNRGKDILFVWGIHHPPTDTAQTNLYTRTDTTT
SVTTETLDRTFKPLIGPRPLVNGLIGRINYYWSVLKPGQTLRVRSNGNLIAPWFGHVLSGESHGRILRTD
LSSGNCVVQCQTEKGGLNSTMPFHNISKYAFGTCPKYIGVKSLKLAIGLRNVHAKSSRGLFGAIAGFIEG
GWPGLVAGWYGFQHSNDQGVGMAADRVSTQKAVDKITSKVNNIVDKMNKQYEIIDHEFSEVENRLNMINN
KIDDQIQDIWAYNAELLVLLENQKTLDEHDANVNNLYNKVKRALGSNAMEDGKGCFELYHKCDDQCMETI
RNGTYNRRKYTEESRLEKQRIEGVKLEAEGTYKILSIYSTVASSLVLAMGFAAFLFWAMSNGSCRCNICI

>QOQ30162.1 hemagglutinin [Influenza A virus]
MEAISLMTILLVVTTSNADKICIGHQSTNSTETVDTLTETNVPVTQAKELLHTEHNGMLCATNLGRPLIL
DTCTIEGLIYGNPSCDMLLGGREWAYIVERPSAVNGTCYPGNVENLEELRTFFSSSSSYQRVQLFPDSIW
NVTYTGTSKSCSGSFYRNMRWLTQKDGSYPIQDAQYTNNRGKDILFVWGIHHPPTDTAQTNLYTRTDTTT
SVTTETLDRTFKPLIGPRPLVNGLIGRINYYWSVLKPGQTLRVRSNGNLIAPWFGHVLSGESHGRILRTD
LSSGNCVVQCQTEKGGLNSTMPFHNISKYAFGTCPKYIGVKSLKLAIGLRNVHAKSSRGLFGAIAGFIEG
GWPGLVAGWYGFQHSNDQGVGMAADRVSTQKAVDKITSKVNNIVDKMNKQYEIIDHEFSEVENRLNMINN
KIDDQIQDIWAYNAELLVLLENQKTLDEHDANVNNLYNKVKRALGSNAMEDGKGCFELYHKCDDQCMETI
RNGTYNRRKYTEESRLEKQRIEGVKLEAEGTYKILSIYSTVASSLVLAMGFAAFLFWAMSNGSCRCNICI

>QOQ30191.1 hemagglutinin [Influenza A virus]
MEAISLMTILLVVTTSNADKICIGHQSTNSTETVDTLTETNVPVTQAKELLHTEHNGMLCATNLGRPLIL
DTCTIEGLIYGNPSCDMLLGGREWAYIVERPSAVNGTCYPGNVENLEELRTFFSSSSSYQRVQLFPDSIW
NVTYTGTSKSCSGSFYRNMRWLTQKDGSYPIQDAQYTNNRGKDILFVWGIHHPPTDTAQTNLYTRTDTTT
SVTTETLDRTFKPLIGPRPLVNGLIGRINYYWSVLKPGQTLRVRSNGNLIAPWFGHVLSGESHGRILRTD
LSSGNCVVQCQTEKGGLNSTMPFHNISKYAFGTCPKYIGVKSLKLAIGLRNVHAKSSRGLFGAIAGFIEG
GWPGLVAGWYGFQHSNDQGVGMAADRVSTQKAVDKITSKVNNIVDKMNKQYEIIDHEFSEVENRLNMINN
KIDDQIQDIWAYNAELLVLLENQKTLDEHDANVNNLYNKVKRALGSNAMEDGKGCFELYHKCDDQCMETI
RNGTYNRRKYTEESRLEKQRIEGVKLEAEGTYKILSIYSTVASSLVLAMGFAAFLFWAMSNGSCRCNICI

>QOQ30172.1 hemagglutinin [Influenza A virus]
MEAISLMTILLVVTTSNADKICIGHQSTNSTETVDTLTETNVPVTQAKELLHTEHNGMLCATNLGRPLIL
DTCTIEGLIYGNPSCDMLLGGREWAYIVERPSAVNGTCYPGNVENLEELRTFFSSSSSYQRVQLFPDSIW
NVTYTGTSKSCSGSFYRNMRWLTQKDGSYPIQDAQYTNNRGKDILFVWGIHHPPTDTAQTNLYTRTDTTT
SVTTETLDRTFKPLIGPRPLVNGLIGRINYYWSVLKPGQTLRVRSNGNLIAPWFGHVLSGESHGRILRTD

LSSGNCVVQCQTEKGGLNSTMPFHNISKYAFGTCPKYIGVKSLKLAIGLRNVHAKSSRGLFGAIAGFIEG
GWPGLVAGWYGFQHSNDQGVGMAADRVSTQKAVDKITSKVNNIVDKMNKQYEIIDHEFSEVENRLNMINN
KIDDQIQDIWAYNAELLVLLENQKTLDEHDANVNNLYNKVKRALGSNAMEDGKGCFELYHKCDDQCMETI
RNGTYNRRKYTEESRLEKQRIEGVKLEAEGTYKILSIYSTVASSLVLAMGFAAFLFWAMSNGSCRCNICI

>QOQ30142.1 hemagglutinin [Influenza A virus]
MEAISLMTILLVVTTSNADKICIGHQSTNSTETVDTLTETNVPVTQAKELLHTEHNGMLCATNLGRPLIL
DTCTIEGLIYGNPSCDMLLGGREWAYIVERPSAVNGTCYPGNVENLEELRTFFSSSSSYQRVQLFPDSIW
NVTYTGTSKSCSGSFYRNMRWLTQKDGSYPIQDAQYTNNRGKDILFVWGIHHPPTDTAQTNLYTRTDTTT
SVTTETLDRTFKPLIGPRPLVNGLIGRINYYWSVLKPGQTLRVRSNGNLIAPWFGHVLSGESHGRILRTD
LSSGNCVVQCQTEKGGLNSTMPFHNISKYAFGTCPKYIGVKSLKLAIGLRNVHAKSSRGLFGAIAGFIEG
GWPGLVAGWYGFQHSNDQGVGMAADRVSTQKAVDKITSKVNNIVDKMNKQYEIIDHEFSEVENRLNMINN
KIDDQIQDIWAYNAELLVLLENQKTLDEHDANVNNLYNKVKRALGSNAMEDGKGCFELYHKCDDQCMETI
RNGTYNRRKYTEESRLEKQRIEGVKLEAEGTYKILSIYSTVASSLVLAMGFAAFLFWAMSNGSCRCNICI

>QOQ30149.1 hemagglutinin [Influenza A virus]
MEAISLMTILLVVTTSNADKICIGHQSTNSTETVDTLTETNVPVTQAKELLHTEHNGMLCATNLGRPLIL
DTCTIEGLIYGNPSCDMLLGGREWAYIVERPSAVNGTCYPGNVENLEELRTFFSSSSSYQRVQLFPDSIW
NVTYTGTSKSCSGSFYRNMRWLTQKDGSYPIQDAQYTNNRGKDILFVWGIHHPPTDTAQTNLYTRTDTTT
SVTTETLDRTFKPLIGPRPLVNGLIGRINYYWSVLKPGQTLRVRSNGNLIAPWFGHVLSGESHGRILRTD
LSSGNCVVQCQTEKGGLNSTMPFHNISKYAFGTCPKYIGVKSLKLAIGLRNVHAKSSRGLFGAIAGFIEG
GWPGLVAGWYGFQHSNDQGVGMAADRVSTQKAVDKITSKVNNIVDKMNKQYEIIDHEFSEVENRLNMINN
KIDDQIQDIWAYNAELLVLLENQKTLDEHDANVNNLYNKVKRALGSNAMEDGKGCFELYHKCDDQCMETI
RNGTYNRRKYTEESRLEKQRIEGVKLEAEGTYKILSIYSTVASSLVLAMGFAAFLFWAMSNGSCRCNICI

>QOQ30122.1 hemagglutinin [Influenza A virus]
MEAISLMTILLVVTTSNADKICIGHQSTNSTETVDTLTETNVPVTQAKELLHTEHNGMLCATNLGRPLIL
DTCTIEGLIYGNPSCDMLLGGREWAYIVERPSAVNGTCYPGNVENLEELRTFFSSSSSYQRVQLFPDSIW
NVTYTGTSKSCSGSFYRNMRWLTQKDGSYPIQDAQYTNNRGKDILFVWGIHHPPTDTAQTNLYTRTDTTT
SVTTETLDRTFKPLIGPRPLVNGLIGRINYYWSVLKPGQTLRVRSNGNLIAPWFGHVLSGESHGRILRTD
LSSGNCVVQCQTEKGGLNSTMPFHNISKYAFGTCPKYIGVKSLKLAIGLRNVHAKSSRGLFGAIAGFIEG
GWPGLVAGWYGFQHSNDQGVGMAADRVSTQKAVDKITSKVNNIVDKMNKQYEIIDHEFSEVENRLNMINN
KIDDQIQDIWAYNAELLVLLENQKTLDEHDANVNNLYNKVKRALGSNAMEDGKGCFELYHKCDDQCMETI
RNGTYNRRKYTEESRLEKQRIEGVKLEAEGTYKILSIYSTVASSLVLAMGFAAFLFWAMSNGSCRCNICI

>QOQ30115.1 hemagglutinin [Influenza A virus]

MEAISLMTILLVVTTSNADKICIGHQSTNSTETVDTLTETNVPVTQAKELLHTEHNGMLCATNLGRPLIL

DTCTIEGLIYGNPSCDMLLGGREWAYIVERPSAVNGTCYPGNVENLEELRTFFSSSSSYQRVQLFPDSIW

NVTYTGTSKSCSGSFYRNMRWLTQKDGSYPIQDAQYTNNRGKDILFVWGIHHPPTDTAQTNLYTRTDTTT

SVTTETLDRTFKPLIGPRPLVNGLIGRINYYWSVLKPGQTLRVRSNGNLIAPWFGHVLSGESHGRILRTD

LSSGNCVVQCQTEKGGLNSTMPFHNISKYAFGTCPKYIGVKSLKLAIGLRNVHAKSSRGLFGAIAGFIEG

GWPGLVAGWYGFQHSNDQGVGMAADRVSTQKAVDKITSKVNNIVDKMNKQYEIIDHEFSEVENRLNMINN

KIDDQIQDIWAYNAELLVLLENQKTLDEHDANVNNLYNKVKRALGSNAMEDGKGCFELYHKCDDQCMETI

RNGTYNRRKYTEESRLEKQRIEGVKLEAEGTYKILSIYSTVASSLVLAMGFAAFLFWAMSNGSCRCNICI

>QOQ30083.1 hemagglutinin [Influenza A virus]

MEAISLMTILLVVTTSNADKICIGHQSTNSTETVDTLTETNVPVTQAKELLHTEHNGMLCATNLGRPLIL

DTCTIEGLIYGNPSCDMLLGGREWAYIVERPSAVNGTCYPGNVENLEELRTFFSSSSSYQRVQLFPDSIW

NVTYTGTSKSCSGSFYRNMRWLTQKDGSYPIQDAQYTNNRGKDILFVWGIHHPPTDTAQTNLYTRTDTTT

SVTTETLDRTFKPLIGPRPLVNGLIGRINYYWSVLKPGQTLRVRSNGNLIAPWFGHVLSGESHGRILRTD

LSSGNCVVQCQTEKGGLNSTMPFHNISKYAFGTCPKYIGVKSLKLAIGLRNVHAKSSRGLFGAIAGFIEG

GWPGLVAGWYGFQHSNDQGVGMAADRVSTQKAVDKITSKVNNIVDKMNKQYEIIDHEFSEVENRLNMINN

KIDDQIQDIWAYNAELLVLLENQKTLDEHDANVNNLYNKVKRALGSNAMEDGKGCFELYHKCDDQCMETI

RNGTYNRRKYTEESRLEKQRIEGVKLEAEGTYKILSIYSTVASSLVLAMGFAAFLFWAMSNGSCRCNICI

>QOQ30104.1 hemagglutinin [Influenza A virus]

MEAISLMTILLVVTTSNADKICIGHQSTNSTETVDTLTETNVPVTQAKELLHTEHNGMLCATNLGRPLIL

DTCTIEGLIYGNPSCDMLLGGREWAYIVERPSAVNGTCYPGNVENLEELRTFFSSSSSYQRVQLFPDSIW

NVTYTGTSKSCSGSFYRNMRWLTQKDGSYPIQDAQYTNNRGKDILFVWGIHHPPTDTAQTNLYTRTDTTT

SVTTETLDRTFKPLIGPRPLVNGLIGRINYYWSVLKPGQTLRVRSNGNLIAPWFGHVLSGESHGRILRTD

LSSGNCVVQCQTEKGGLNSTMPFHNISKYAFGTCPKYIGVKSLKLAIGLRNVHAKSSRGLFGAIAGFIEG

GWPGLVAGWYGFQHSNDQGVGMAADRVSTQKAVDKITSKVNNIVDKMNKQYEIIDHEFSEVENRLNMINN

KIDDQIQDIWAYNAELLVLLENQKTLDEHDANVNNLYNKVKRALGSNAMEDGKGCFELYHKCDDQCMETI

RNGTYNRRKYTEESRLEKQRIEGVKLEAEGTYKILSIYSTVASSLVLAMGFAAFLFWAMSNGSCRCNICI

>QOQ30091.1 hemagglutinin [Influenza A virus]

MEAISLMTILLVVTTSNADKICIGHQSTNSTETVDTLTETNVPVTQAKELLHTEHNGMLCATNLGRPLIL

DTCTIEGLIYGNPSCDMLLGGREWAYIVERPSAVNGTCYPGNVENLEELRTFFSSSSSYQRVQLFPDSIW

NVTYTGTSKSCSGSFYRNMRWLTQKDGSYPIQDAQYTNNRGKDILFVWGIHHPPTDTAQTNLYTRTDTTT
SVTTETLDRTFKPLIGPRPLVNGLIGRINYYWSVLKPGQTLRVRSNGNLIAPWFGHVLSGESHGRILRTD
LSSGNCVVQCQTEKGGLNSTMPFHNISKYAFGTCPKYIGVKSLKLAIGLRNVHAKSSRGLFGAIAGFIEG
GWPGLVAGWYGFQHSNDQGVGMAADRVSTQKAVDKITSKVNNIVDKMNKQYEIIDHEFSEVENRLNMINN
KIDDQIQDIWAYNAELLVLLENQKTLDEHDANVNNLYNKVKRALGSNAMEDGKGCFELYHKCDDQCMETI
RNGTYNRRKYTEESRLEKQRIEGVKLEAEGTYKILSIYSTVASSLVLAMGFAAFLFWAMSNGSCRCNICI

>QOQ30056.1 hemagglutinin [Influenza A virus]
MEAISLMTILLVVTTSNADKICIGHQSTNSTETVDTLTETNVPVTQAKELLHTEHNGMLCATNLGRPLIL
DTCTIEGLIYGNPSCDMLLGGREWAYIVERPSAVNGTCYPGNVENLEELRTFFSSSSSYQRVQLFPDSIW
NVTYTGTSKSCSGSFYRNMRWLTQKDGSYPIQDAQYTNNRGKDILFVWGIHHPPTDTAQTNLYTRTDTTT
SVTTETLDRTFKPLIGPRPLVNGLIGRINYYWSVLKPGQTLRVRSNGNLIAPWFGHVLSGESHGRILRTD
LSSGNCVVQCQTEKGGLNSTMPFHNISKYAFGTCPKYIGVKSLKLAIGLRNVHAKSSRGLFGAIAGFIEG
GWPGLVAGWYGFQHSNDQGVGMAADRVSTQKAVDKITSKVNNIVDKMNKQYEIIDHEFSEVENRLNMINN
KIDDQIQDIWAYNAELLVLLENQKTLDEHDANVNNLYNKVKRALGSNAMEDGKGCFELYHKCDDQCMETI
RNGTYNRRKYTEESRLEKQRIEGVKLEAEGTYKILSIYSTVASSLVLAMGFAAFLFWAMSNGSCRCNICI

>QOQ30062.1 hemagglutinin [Influenza A virus]
MEAISLMTILLVVTTSNADKICIGHQSTNSTETVDTLTETNVPVTQAKELLHTEHNGMLCATNLGRPLIL
DTCTIEGLIYGNPSCDMLLGGREWAYIVERPSAVNGTCYPGNVENLEELRTFFSSSSSYQRVQLFPDSIW
NVTYTGTSKSCSGSFYRNMRWLTQKDGSYPIQDAQYTNNRGKDILFVWGIHHPPTDTAQTNLYTRTDTTT
SVTTETLDRTFKPLIGPRPLVNGLIGRINYYWSVLKPGQTLRVRSNGNLIAPWFGHVLSGESHGRILRTD
LSSGNCVVQCQTEKGGLNSTMPFHNISKYAFGTCPKYIGVKSLKLAIGLRNVHAKSSRGLFGAIAGFIEG
GWPGLVAGWYGFQHSNDQGVGMAADRVSTQKAVDKITSKVNNIVDKMNKQYEIIDHEFSEVENRLNMINN
KIDDQIQDIWAYNAELLVLLENQKTLDEHDANVNNLYNKVKRALGSNAMEDGKGCFELYHKCDDQCMETI
RNGTYNRRKYTEESRLEKQRIEGVKLEAEGTYKILSIYSTVASSLVLAMGFAAFLFWAMSNGSCRCNICI

>QOQ30049.1 hemagglutinin [Influenza A virus]
MEAISLMTILLVVTTSNADKICIGHQSTNSTETVDTLTETNVPVTQAKELLHTEHNGMLCATNLGRPLIL
DTCTIEGLIYGNPSCDMLLGGREWAYIVERPSAVNGTCYPGNVENLEELRTFFSSSSSYQRVQLFPDSIW
NVTYTGTSKSCSGSFYRNMRWLTQKDGSYPIQDAQYTNNRGKDILFVWGIHHPPTDTAQTNLYTRTDTTT
SVTTETLDRTFKPLIGPRPLVNGLIGRINYYWSVLKPGQTLRVRSNGNLIAPWFGHVLSGESHGRILRTD
LSSGNCVVQCQTEKGGLNSTMPFHNISKYAFGTCPKYIGVKSLKLAIGLRNVHAKSSRGLFGAIAGFIEG
GWPGLVAGWYGFQHSNDQGVGMAADRVSTQKAVDKITSKVNNIVDKMNKQYEIIDHEFSEVENRLNMINN

KIDDQIQDIWAYNAELLVLLENQKTLDEHDANVNNLYNKVKRALGSNAMEDGKGCFELYHKCDDQCMETI
RNGTYNRRKYTEESRLEKQRIEGVKLEAEGTYKILSIYSTVASSLVLAMGFAAFLFWAMSNGSCRCNICI

>QOQ30026.1 hemagglutinin [Influenza A virus]
MEAISLMTILLVVTTSNADKICIGHQSTNSTETVDTLTETNVPVTQAKELLHTEHNGMLCATNLGRPLIL
DTCTIEGLIYGNPSCDMLLGGREWAYIVERPSAVNGTCYPGNVENLEELRTFFSSSSSYQRVQLFPDSIW
NVTYTGTSKSCSGSFYRNMRWLTQKDGSYPIQDAQYTNNRGKDILFVWGIHHPPTDTAQTNLYTRTDTTT
SVTTETLDRTFKPLIGPRPLVNGLIGRINYYWSVLKPGQTLRVRSNGNLIAPWFGHVLSGESHGRILRTD
LSSGNCVVQCQTEKGGLNSTMPFHNISKYAFGTCPKYIGVKSLKLAIGLRNVHAKSSRGLFGAIAGFIEG
GWPGLVAGWYGFQHSNDQGVGMAADRVSTQKAVDKITSKVNNIVDKMNKQYEIIDHEFSEVENRLNMINN
KIDDQIQDIWAYNAELLVLLENQKTLDEHDANVNNLYNKVKRALGSNAMEDGKGCFELYHKCDDQCMETI
RNGTYNRRKYTEESRLEKQRIEGVKLEAEGTYKILSIYSTVASSLVLAMGFAAFLFWAMSNGSCRCNICI

>QOQ30018.1 hemagglutinin [Influenza A virus]
MEAISLMTILLVVTTGNADKICIGHQSTNSSETVDTLTETNVPVTQAKELLHTEHNGMLCATNLGRPLIL
DTCTIEGLIYGNPSCDMLLEGREWAYIVERPSAVNGTCYPGNVENLEELRTFFSSSSSYQRVQLFPDSIW
NVTYTGTSKSCSDSFYRNMRWLTQKSGSYPIQDAQYTNNRGKDILFVWGIHHPPTDTAQTNLYTRTDTTT
SVTTETLDRTFKPLIGPRPLVNGLIGRINYYWSVLKPGQTLRVRSNGNLIAPWFGHVLSGESHGRILRTN
LRSGNCVVQCQTEKGGLNSTMPFHNISKYAFGTCPKYIGVKSLKLAIGLRNVHARSSRGLFGAIAGFIEG
GWPGLVAGWYGFQHSNDQGVGMAADRVSTQKAVDKITSKVNNIVDKMNKQYEIIDHEFSEIETRLNMINN
KIDDQIQDIWAYNAELLVLLENQKTLDEHDANVNNLYNKVKRALGSNAMEDGKGCFELYHKCDDQCMETI
RNGTYNRRKYTEESRLERQKIEGVKLEAEGTYKILSIYSTVASSLVLAMGFAAFLFWAMSNGSCRCNICI

>QOQ30013.1 hemagglutinin [Influenza A virus]
MEAISLMTILLVVTTSNADKICIGHQSTNSTETVDTLTETNVPVTQAKELLHTEHNGMLCATNLGRPLIL
DTCTIEGLIYGNPSCDMLLGGREWAYIVERPSAVNGTCYPGNVENLEELRTFFSSSSSYQRVQLFPDSIW
NVTYTGTSKSCSGSFYRNMRWLTQKDGSYPIQDAQYTNNRGKDILFVWGIHHPPTDTAQTNLYTRTDTTT
SVTTETLDRTFKPLIGPRPLVNGLIGRINYYWSVLKPGQTLRVRSNGNLIAPWFGHVLSGESHGRILRTD
LSSGNCVVQCQTEKGGLNSTMPFHNISKYAFGTCPKYIGVKSLKLAIGLRNVHAKSSRGLFGAIAGFIEG
GWPGLVAGWYGFQHSNDQGVGMAADRVSTQKAVDKITSKVNNIVDKMNKQYEIIDHEFSEVENRLNMINN
KIDDQIQDIWAYNAELLVLLENQKTLDEHDANVNNLYNKVKRALGSNAMEDGKGCFELYHKCDDQCMETI
RNGTYNRRKYTEESRLEKQRIEGVKLEAEGTYKILSIYSTVASSLVLAMGFAAFLFWAMSNGSCRCNICI

>QOQ29994.1 hemagglutinin [Influenza A virus]

MEAISLMTILLVVTTGNADKICIGHQSTNSTETVDTLTETNVPVTQAKELLHTEHNGMLCATNLGRPLIL
DTCTIEGLIYGNPSCDMLLGGREWAYIVERPSAVNGTCYPGNVENLEELRTFFSSSSSYQRVQLFPDSIW
NVTYTGTSKSCSDSFYRNMRWLTQKSGSYPIQDAQYTNNRGKDILFVWGIHHPPTDTAQTNLYTRTDTTT
SVTTETLDRTFKPLIGPRPLVNGLIGRINYYWSVLKPGQTLRVRSNGNLIAPWFGHVLSGESHGRILRTN
LRSGNCVVQCQTEKGGLNSTMPFHNISKYAFGTCPKYIGVKSLKLAIGLRNVHARSSRGLFGAIAGFIEG
GWPGLVAGWYGFQHSNDQGVGMAADRVSTQKAVDKITSKVNKIVDKMNKQYEIIDHEFSEIETRLNMINN
KIDDQIQDIWAYNAELLVLLENQKTLDEHDANVNNLYNKVKRALGSNAMEDGKGCFELYHKCDDQCMETI
RNGTYNRRKYTEESRLERQKIEGVKLEAEGTYKILSIYSTVASSLVLAMGFTAFLFWAMSNGSCRCNICI

>QOK12803.1 hemagglutinin [Influenza A virus]
METVSLITILLVATVSNADKICIGYQSTNSTETVDTLTENNVPVTHAKELIHTEHNGMLCATSLGQPLIL
DTCTIEGLIYGNPSCDLSLEGREWSYIVERPSAVNGLCYPGNVENLEELRSLFSSARSYQRIQIFPDTIW
NVSYDGTSTACSGSFYRNMRWLTRKDGNYPTQDAQYTNNQGKNILFMWGINHPPTDTTQSGLYTRTDTTT
SVATEEINRIFKPLIGPRPLVNGLMGRIDYYWSVLKPGQTLRIKSDGNLIAPWFGHILSGESHGRILKTD
LKRGSCTVQCQTEKGGLNTTLPFQNVSKYAFGNCSKYIGIKSLKLAVGLRNVPSRSSRGLFGAIAGFIEG
GWSGLVAGWYGFQHSNDQGVGMAADRDSTQKAIDKITSKVNNIVDKMNKQYEIIDHEFSEVETRLNMINN
KIDDQIQDIWAYNAELLVLLENQKTLDEHDANVNNLYNKVKRALGSNAVEDGKGCFELYHKCDDQCMETI
RNGTYNRRKYQEESKLERQKIEGVKLESEGTYKILTIYSTVASSLVIAMGFAAFLFWAMSNGSCRCNICI

>QOK12780.1 hemagglutinin [Influenza A virus]
METVSLITILLVATVSNADKICIGYQSTNSTETVDTLTENNVPVTHAKELIHTEHNGMLCATSLGQPLIL
DTCTIEGLIYGNPSCDLSLEGREWSYIVERPSAVNGLCYPGNVENLEELRSLFSSARSYQRIQIFPDTIW
NVSYDGTSTACSGSFYRNMRWLTRKDGNYPTQDAQYTNNQGKNILFMWGINHPPTDTTQSGLYTRTDTTT
SVATEEINRIFKPLIGPRPLVNGLMGRIDYYWSVLKPGQTLRIKSDGNLIAPWFGHILSGESHGRILKTD
LKRGSCTVQCQTEKGGLNTTLPFQNVSKYAFGNCSKYIGIKSLKLAVGLRNVPSRSSRGLFGAIAGFIEG
GWSGLXAGWYGFQHSNDQGVGMAADRDSTQKAIDKITSKVNNIVDKMNKQYEIIDHEFSEVETRLNMINN
KIDDQIQDIWAYNAELLVLLENQKTLDEHDANVNNLYNKVKRALGSNAVEDGKGCFELYHKCDDQCMETI
RNGTYNRRKYQEESKLERQKIEGVKLESEGTYKILTIYSTVASSLVIAMGFAAFLFWAMSNGSCRCNICI

>QOK12756.1 hemagglutinin [Influenza A virus]
METVSLITILLVATVSNADKICIGYQSTNSTETVDTLTENNVPVTHAKELIHTEHNGMLCATSLGQPLIL
DTCTIEGLIYGNPSCDLSLEGREWSYIVERPSAVNGLCYPGNVENLEELRSLFSSARSYQRIQIFPDTIW
NVSYDGTSTACSGSFYRNMRWLTRKDGNYPTQDAQYTNNQGKNILFMWGINHPPTDTTQSGLYTRTDTTT
SVATEEINRVFKPLIGPRPLVNGLMGRIDYYWSVLKPGQTLRIKSDGNLIAPWFGHILSGESHGRILKTD

LKRGSCTVQCQTEKGGLNTTLPFQNVSKYAFGNCSKYIGIKSLKLAVGLRNVPSRSSRGLFGAIAGFIEG
GWSGLVAGWYGFQHSNDQGVGMAADRDSTQKAIDKITSKVNNIVDKMNKQYEIIDHEFSEVETRLNMINN
KIDDQIQDIWAYNAELLVLLENQKTLDEHDANVNNLYNKVKRALGSNAVEDGKGCFELYHKCDDQCMETI
RNGTYNRRKYQEESKLERQKIEGVKLESEGTYKILTIYSTVASSLVIAMGFAAFLFWAMSNGSCRCNICI

>QOK12720.1 hemagglutinin [Influenza A virus]
METVSLITILLVATVSNADKICIGYQSTNSTETVDTLTENNVPVTHAKELIHTEHNGMLCATSLGQPLIL
DTCTIEGLIYGNPSCDLSLEGREWSYIVERPSAVNGLCYPGNVENLEELRSLFSSARSYQRIQIFPDTIW
NVSYDGTSTACSGSFYRNMRWLTRKDGNYPTQDAQYTNNQGKNILFMWGINHPPTDTTQSGLYTRTDTTT
SVATEEINRVFKPLIGPRPLVNGLMGRIDYYWSVLKPGQTLRIKSDGNLIAPWFGHILSGESHGRILKTD
LKRGSCTVQCQTEKGGLNTTLPFQNVSKYAFGNCSKYIGMKGLKLAVGLRNVPSRSSRGLFGAIAGFIEG
GWSGLVAGWYGFQHSNDQGVGMAADRDSTQKAIDKITSKVNNIVDKMNKQYEIIDHEFSEVETRLNMINN
KIDDQIQDIWAYNAELLVLLENQKTLDEHDANVNNLYNKVKRALGSNAVEDGKGCFELYHKCDDQCMETI
RNGTYNRRKYQEESKLERQKIEGVKLESEGTYKILTIYSTVASSLVIAMGFAAFLFWAMSNGSCRCNICI

>QOK12592.1 hemagglutinin [Influenza A virus]
METVSLITILLVATVSNADKICIGYQSTNSTETVDTLTENNVPVTHAKELLHTEHNGMLCATSLGQPLIL
DTCTIEGLIYGNPSCDLSLEGREWSYIVERPSAVNGLCYPGNVENLEELRSLFSSARSYQRIQIFPDTIW
NVSYDGTSTACSNSFYRSMRWLTRKDGNYPTQDAQYTNNQGKNILFMWGINHPPTDDTQRNLYTRTDTTT
SVATEEINRIFKPLIGPRPLVNGLMGRIDYYWSVLKPGQTLRIKSDGNLIAPWYGHILSGESHGRILKTD
LKRGSCTVQCQTEKGGFNTTLPFQNVSKYAFGNCSKYIGIKSLKLAVGLRNVPSRSSRGLFGAIAGFIEG
GWSGLVAGWYGFQHSNDQGVGMAADRDSTQKAIDKITSKVNNIVDKMNKQYEIIDHEFSEVETRLNMINN
KIDDQIQDIWAYNAELLVLLENQKTLDEHDANVNNLYNKVKRALGSNAVEDGKGCFELYHKCNDQCMDTI
RNGTYNRRKYQEESKLERQKIEGVKLESEGTYKILTIYSTVASSLVIAMGFAAFLFWAMSNGSCRCNIXI

>QOK12568.1 hemagglutinin [Influenza A virus]
METVSLITILLVATVSNADKICIGYQSTNSTETVDTLTENNVPVTHAKELLHTEHNGMLCATSLGQPLIL
DTCTIEGLIYGNPSCDLSLEGREWSYIVERPSAVNGLCYPGNVENLEELRSLFSSARSYQRIQIFPDTIW
NVSYDGTSTACSNSFYRSMRWLTRKDGNYPTQDAQYTNNQGKNILFMWGINHPPTDDTQRNLYTRTDTTT
SVATEEINRIFKPLIGPRPLVNGLMGRIDYYWSVLKPGQTLRIKSDGNLIAPWYGHILSGESHGRILKTD
LKRGSCTVQCQTEKGGFNTTLPFQNISKYAFGNCSKYIGIKSLKLAVGLRNVPSRSSRGLFGAIAGFIEG
GWSGLVAGWYGFQHSNDQGVGMAADRDSTQKAIDKITSKVNNIVDKMNKQYEIIDHEFSEVETRLNMINN
KIDDQIQDIWAYNAELLVLLENQKTLDEHDANVNNLYNKVKRALGSNAVEDGKGCFELYHKCNDQCMDTI
RNGTYNRRKYQEESKLERQKIEGVKLESEGTYKILTIYSTVASSLVIAMGFAAFLFWAMSNGSCRCNICI

>QOK12556.1 hemagglutinin [Influenza A virus]

METVSLITILVVATVSNADKICIGYQSTNSTETVDTLTENNVPVTHAKELLHTEHNGMLCATSLGHPLIL
DICTIEGLIYGNPSCDPLLGGREWSYIVERPSAVNGLCYPGNVENLEELRSLFSSSRSYQRIQIFPDTIW
NVSYSGTSKACSDSFYRSMRWLTQKNNAYPTQDAQYTNNQGKNILFMWGINHPPTDTAQTNLYTRTDTTT
SVATEEMNRIFKPLIGPRPLVNGLMGRINYYWSVLKPGQTLRIKSDGNLIAPWYGHILSGESHGRILKTD
LKMGSCTVQCQTEKGGLNTTLPFQNVSKYAFGNCSKYIGVKSLKLAVGLRNVPSRSSRGLFGAIAGFIEG
GWSGLVAGWYGFQHSNDQGVGMAADRDSTQKAIDKITSKVNNIVDKMNKQYEIIDHEFSEVETRLNMIND
KVDDQIQDIWAYNAELLVLLENQKTLDEHDANVNNLYNKVKRALGSNAVEDGRGCFELYHKCDDHCMETI
RNGTYNRRKYQEESKLERQKIEGVKLESEETYKILTIYSTVASSLVIAMGFAAFLFWAMSNGSCRCNICI

>QOK12544.1 hemagglutinin [Influenza A virus]

METVSLITILLVATVSNADKICIGYQSTNSTETVDTLTENNVPVTHAKELLHTEHNGMLCATSLGQPLIL
DTCTIEGLIYGNPSCDLSLEGREWSYIVERPSAVHGLCYPGNVENLEELRSLFSSARSYQRIQIFPDTIW
NVSYDGTSTACSGSFYRSMRWLTRKNGDYPIQDAQYTNNQGKNILFMWGINHPPTDETQRGLYTRTDTTT
SVATEEINRIFKPLIGPRPLVNGLMGRINYYWSVLKPGQTLRIKSDGNLIAPWYGHILSGESHGRILKTD
LKRGSCTVQCQTEKGGLNTTLPFQNVSKYAFGNCSKYIGIKSLKLAVGLRNVPSRSSRGLFGAIAGFIEG
GWSGLVAGWYGFQHSNDQGVGMAADRDSTQKAIDKITSKVNNIVDKMNKQYEIIDHEFSEVETRINMINN
KIDDQIQDIWTYNAELLVLLENQKTLDEHDANVNNLYNKVKRALGSNAVEDGKGCFELYHKCDDQCMETI
RNGTYNRRKYQEESKLERQKIEGVKLESEGTYKILTIYSTVASSLVIAMGFAAFLFWAMSNGSCRCNICI

>QOK12532.1 hemagglutinin [Influenza A virus]

METVSLITILLVATVSNADKICIGYQSTNSTETVDTLTENNVPVTHAKELLHTEHNGMLCATSLGQPLIL
DTCTIEGLIYGNPSCDLSLEGREWSYIVERPSAVHGLCYPGNVEDLEELRSLFSSARSYQRIQIFPDTIW
NVSYDGTSTACSGSFYRSMRWLTRKNGEYPIQDAQYTNNQGKNILFMWGINHPPTDDTQRGLYTRTDTTT
SVATEEINRIFKPLIGPRPLVNGLMGRINYYWSVLKPGQTLRIKSDGNLIAPWYGHILSGESHGRILKTD
LKRGSCTVQCQTEKGGLNTTLPFQNVSKYAFGNCSKYIGIKSLKLAVGLRNVPSRSSRGLFGAIAGFIEG
GWSGLVAGWYGFQHSNDQGVGMAADRDSTQKAIDKITSKVNNIVDKMNKQYEIIDHEFSEVETRLNMINN
KIDDQIQDIWAYNAELLVLLENQKTLDEHDANVNNLYNKVKRALGSNAVEDGKGCFELYHKCDDQCMETI
RNGTYNRRKYQEESKLERQKIEGVKLESEGTYKILTIYSTVASSLVIAMGFAAFLFWAMSNGSCRCNICI

>QOK12508.1 hemagglutinin [Influenza A virus]

METVSLITILLVATVSNADKICIGYQSTNSTETVDTLTENNVPVTHAKELLHTEHNGMLCATSLGQPLIL
DTCTIEGLIYGNPSCDLSLEGREWSYIVERPSAVHGLCYPGNVEDLEELRSLFSSARSYQRIQIFPDTIW

NVSYDGTSTACSGSFYRSMRWLTRKNGEYPIQDAQYTNNQGKNILFMWGINHPPTDDTQRGLYTRTDTTT
SVATEEINRIFKPLIGPRPLVNGLMGRINYYWSVLKPGQTLRIKSDGNLIAPWYGHILSGESHGRILKTD
LKRGSCTVQCQTEKGGLNTTLPFQNVSKYAFGNCSKYIGIKSLKLAVGLRNVPSRSSRGLFGAIAGFIEG
GWSGLVAGWYGFQHSNDQGVGMAADRDSTQKAIDKITSKVNNIVDKMNKQYEIIDHEFSEVETRLNMINN
KIDDQIQDIWAYNAELLVLLENQKTLDEHDANVNNLYNKVKRALGSNAVEDGKGCFELYHKCDDQCMETI
RNGTYNRRKYQEESKLERQKIEGVKLESEGTYKILTIYSTVASSLVIAMGFAAFLFWAMSNGSCRCNICI

>QOK12496.1 hemagglutinin [Influenza A virus]
METVSLITILLVATVSNADKICIGYQSTNSTETVDTLTENNVPVTHAKELLHTEHNGMLCATSLGQPLIL
DTCTIEGLIYGNPSCDLSLEGREWSYIVERPSAVHGLCYPGNVENLEELRSLFSSARSYQRIQIFPDTIW
NVSYDGTSTACSGSFYRSMRWLTRKNGDYPIQDAQYTNNQGKNILFMWGINHPPTDETQRGLYTRTDTTT
SVATEEINRIFKPLIGPRPLVNGLMGRINYYWSVLKPGQTLRIKSDGNLIAPWYGHILSGESHGRILKTD
LKRGSCTVQCQTEKGGLNTTLPFQNVSKYAFGNCSKYIGIKSLKLAVGLRNVPSRSSRGLFGAIAGFIEG
GWSGLVAGWYGFQHSNDQGVGMAADRDSTQKAIDKITSKVNNIVDKMNKQYEIIDHEFSEVETRLNMINN
KIDDQIQDIWAYNAELLVLLENQKTLDEHDANVNNLYNKVKRALGSNAVEDGKGCFELYHKCDDQCMETI
RNGTYNRRKYQEESKLERQKIEGVKLESEGTYKILTIYSTVASSLVIAMGFAAFLFWAMSNGSCRCNICI

>QOK12484.1 hemagglutinin [Influenza A virus]
METVSLITILLVATVSNADKICIGYQSTNSTETVDTLTENNVPVTHAKELLHTEHNGMLCATSLGQPLIL
DTCTIEGLIYGNPSCDLSLEGREWSYIVERPSAVHGLCYPGNVENLEELRSLFSSARSYQRIQIFPDTIW
NVSYDGTSTACSGSFYRSMRWLTRKNGDYPIQDAQYTNNQGKNILFMWGINHPPTDETQRGLYTRTDTTT
SVATEEINRIFKPLIGPRPLVNGLMGRINYYWSVLKPGQTLRIKSDGNLIAPWHGHILSGESHGRILKTD
LKRGSCTVQCQTEKGGLNTTLPFQNVSKYAFGNCSKYIGIKSLKLAVGLRNVPSRSSRGLFGAIAGFIEG
GWSGLVAGWYGFQHSNDQGVGMAADRDSTQKAIDKITSKVNNIVDKMNKQYEIIDHEFSEVETRINMINN
KIDDQIQDIWAYNAELLVLLENQKTLDEHDANVNNLYNKVKRALGSNAVEDGKGCFELYHKCDDQCMETI
RNGTYNRRKYQEESKLERQKIEGVKLESEGTYKILTIYSTVASSLVIAMGFAAFLFWAMSNGSCRCNICI

>QOK12472.1 hemagglutinin [Influenza A virus]
METVSLITILVVATVSNADKICIGYQSTNSTETVDTLTENNVPVTHAKELLHTEHNGMLCATSLGHPLIL
DTCTIEGLIYGNPSCDPLLGGREWSYIVERPSAVNGLCYPGNVENLEELRSLFSSSRSYQRIQIFPDTIW
NVSYSGTSKACSDSFYRSMRWLTQKNNAYPTQDAQYTNNQGKNILFMWGINHPPTDTAQTNLYTRTDTTT
SVATEEMNRIFKPLIGPRPLVNGLMGRINYYWSVLKPGQTLRIKSDGNLIAPWYGHILSGESHGRILKTD
LKMGSCTVQCQTEKGGLNTTLPFQNVSKYAFGNCSKYIGVKSLKLAVGLRNVPSRSSRGLFGAIAGFIEG
GWSGLVAGWYGFQHSNDQGVGMAADRDSTQKAIDKITSKVNNIVDKMNKQYEIIDHEFSEVETRLNMIND

KVDDQIQDIWAYNAELLVLLENQKTLDEHDANVNNLYNKVKRALGSNAVEDGRGCFELYHKCDDHCMETI
RNGTYNRRKYQEESKLERQKIEGVKLESEETYKILTIYSTVASSLVIAMGFAAFLFWAMSNGSCRCNICI

>QOK12460.1 hemagglutinin [Influenza A virus]
METVSLITILLVATVSNADKICIGYQSTNSTETVDTLTENNVPVTHAKELLHTEHNGMLCATSLGQPLIL
DTCTIEGLIYGNPSCDLSLEGREWSYIVERPSAVHGLCYPGNVEDLEELRSLFSSARSYQRIQLFPDTIW
NVSYDGTSTACSGSFYRSMRWLTRKNGEYPIQDAQYTNNQGKNILFMWGINHPPTDDTQRGLYTRTDTTT
SVATEEINRIFKPLIGPRPLVNGLMGRINYYWSVLKPGQTLRIKSDGNLIAPWYGHILSGESHGRILKTD
LKRGSCTVQCQTEKGGLNTTLPFQNVSKYAFGNCSKYIGIKSLKLAVGLRNVPSRSSRGLFGAIAGFIEG
GWSGLVAGWYGFQHSNDQGVGMAADRDSTQKAIDKITSKVNNIVDKMNKQYEIIDHEFSEVETRLNMINN
KIDDQIQDIWAYNAELLVLLENQKTLDEHDANVNNLYNKVKRALGSNAVEDGKGCFELYHKCDDQCMETI
RNGTYNRRKYQEESKLERQKIEGVKLESEGTYKILTIYSTVASSLVIAMGFAAFLFWAMSNGSCRCNICI

>QOK12448.1 hemagglutinin [Influenza A virus]
METVSLITILVVATVSNADKICIGYQSTNSTETVDTLTENNVPVTHAKELLHTEHNGMLCATSLGHPLIL
DTCTIEGLIYGNPSCDPLLGGREWSYIVERPSAVNGLCYPGNVEHLEELRSLFSSSRSYQRIQIFPDAIW
NVSYSGTSKACSDSFYRSMRWLTQKNNAYPTQDAQYTNNQGKNILFMWGINHPPTDTAQTNLYTRTDTTT
SVATEEMNRIFKPLIGPRPLVNGLMGRINYYWSVLKPGQTLRIKSDGNLIAPWYGHILSGESHGRILKTD
LKRGSCTVQCQTEKGGLNTTLPFQNVSKYAFGNCSKYIGVKSLKLAVGLRNVPSRSSRGLFGAIAGFIEG
GWSGLVAGWYGFQHSNDQGVGIAADRDSTQKAIDKITSKVNNIVDKMNKQYEIIDHEFSEIETRLNMINN
KVDDQIQDIWAYNAELLVLLENQKTLDEHDANVNNLYNKVKRALGSNAVEDGKGCFELYHKCDDHCMETI
RNGTYNRRKYQEESKLERQKIEGVKLESEETYKILTIYSTVASSLVIAVGFAAFLFWAMSNGSCRCNICI

>QOK12436.1 hemagglutinin [Influenza A virus]
METISLITILVVATVSNADKICIGYQSTNSTETVDTLTENNVPVTHAKELLHTEHNGMLCATSLGHPLIL
DTCTIEGLIYGNPSCDPLLGGREWSYIVERPSAVNGLCYPGNVEHLEELRSLFSSSRSYQRIQIFPDAIW
NVSYSGTSKACSDSFYRSMRWLTQKNNAYPTQDAQYTNNQGKNILFMWGINHPPTDTAQTNLYTRTDTTT
SVATEEMNRIFKPLIGPRPLVNGLMGRINYYWSVLKPGQTLRIKSDGNLIAPWYGHILSGESHGRILKTD
LKRGSCTVQCQTEKGGLNTTLPFQNVSKYAFGNCSKYIGVKSLKLAVGLRNVPSRSSRGLFGAIAGFIEG
GWSGLVAGWYGFQHSNDQGVGIAADRDSTQKAIDKITSKVNNIVDKMNKQYEIIDHEFSEIETRLNMINN
KVDDQIQDIWAYNAELLVLLENQKTLDEHDANVNNLYNKVKRALGSNAVEDGKGCFELYHKCDDHCMETI
RNGTYNRRKYQEESKLERQKIEGVKLESEETYKILTIYSTVASSLVIAVGFAAFLFWAMSNGSCRCNICI

>QOK12424.1 hemagglutinin [Influenza A virus]

METVSLITILLVATVSNADKICIGYQSTNSTETVDTLTENNVPVTHAKELLHTEHNGMLCATSLGQPLIL
DTCTIEGLIYGNPSCDLSLEGREWSYIVERPSAVHGLCYPGNVEDLEELRSLFSSARSYQRIQIFPDTIW
NVSYDGTSTACSGSFYRSMRWLTRKNGEYPIQDAQYTNNQGKNILFMWGINHPPTDDTQRGLYTRTDTTT
SVATEEINRIFKPLIGPRPLVNGLMGRINYYWSVLKPGQTLRIKSDGNLIAPWYGHILSGESHGRILKTD
LKRGSCTVQCQTEKGGLNTTLPFQNVSKYAFGNCSKYIGIKSLKLAVGLRNVPSRSSRGLFGAIAGFIEG
GWSGLVAGWYGFQHSNDQGVGMAADRDSTQKAIDKITSKVNNIVDKMNKQYEIIDHEFSEVETRLNMINN
KIDDQVQDIWAYNAELLVLLENQKTLDEHDANVNNLYNKVKRALGSNAVEDGKGCFELYHKCDDQCMETI
RNGTYNRRKYQEESKLERQKIEGVKLESEGTYKILTIYSTVASSLVIAMGFAAFLFWAMSNGSCRCNICI

>QOK12412.1 hemagglutinin [Influenza A virus]
METVSLITILVVATVSNADKICIGYQSTNSTETVDTLTENNVPVTHAKELLHTEHNGMLCATSLGHPLIL
DTCTIEGLIYGNPSCDPLLGGREWSYIVERPSAVNGLCYPGNVEHLEELRSLFSSSRSYQRIQIFPDAIW
NVSYSGTSKACSDSFYRSMRWLTQKNNAYPTQDAQYTNNQGKNILFMWGINHPPTDTAQTNLYTRTDTTT
SVATEEMNRIFKPLIGPRPLVNGLMGRINYYWSVLKPGQTLRIKSDGNLIAPWYGHILSGESHGRILKTD
LKRGSCTVQCQTEKGGLNTTLPFQNVSKYAFGNCSKYIGVKSLKLAVGLRNVPSRSSRGLFGAIAGFIEG
GWSGLVAGWYGFQHSNDQGVGIAADRDSTQKAIDKITSKVNNIVDKMNKQYEIIDHEFSEIETRLNMIND
KVDDQIQDIWAYNAELLVLLENQKTLDEHDANVNNLYNKVKRALGSNAVEDGKGCFELYHKCDDHCMETI
RNGTYNRRKYQEESKLERQKIEGVKLESEETYKILTIYSTVASSLVIAVGFAAFLFWAMSNGSCRCNICI

>QOK12400.1 hemagglutinin [Influenza A virus]
METVSLITILVVATVSNADKICIGYQSTNSTETVDTLTENNVPVTHAKELLHTEHNGMLCATSLGHPLIL
DTCTIEGLIYGNPSCDPLLGGREWSYIVERPSAVNGLCYPGNVENLEELRSLFSSSRSYQRIQIFPDTIW
NVSYSGTSKACSDSFYRSMRWLTQKNNAYPTQDAQYTNNQEKNILFMWGINHPPTDTAQTNLYTRTDTTT
SVATEEMNRIFKPLIGPRPLVNGLMGRINYYWSVLKPGQTLRIKSDGNLIAPWYGHILSGESHGRILKTD
LKRGSCTVQCQTEKGGLNTTLPFQNVSKYAFGNCSKYIGVKSLKLAVGLRNVPSRSSRGLFGAIAGFIEG
GWSGLIAGWYGFQHSNDQGVGMAADRDSTQKAIDKITSKVNNIVDKMNKQYEIIDHEFSEVETRLNMIND
KVDDQIQDIWAYNAELLVLLENQKTLDEHDANVNNLYNKVKRALGSNAVEDGKGCFELYHKCDDHCMETI
RNGTYNRRKYQEESKLERQKIEGVKLESEETYKILTIYSTVASSLVIAMGFAAFLFWAMSNGSCRCNICI

>QOK12388.1 hemagglutinin [Influenza A virus]
METVSLITILVVATVSNADKICIGYQSTNSTETVDTLTENNVPVTHAKELLHTEHNGMLCATSLGHPLIL
DTCTIEGLIYGNPSCDPLLGGREWSYIVERPSAVNGLCYPGNVENLEELRSLFSSSRSYQRIQIFPDTIW
NVSYSGTSKACSDSFYRSMRWLTQKNNAYPTQDAQYTNNQEKNILFMWGINHPPTDTAQTNLYTRTDTTT
SVATEEMNRIFKPLIGPRPLVNGLMGRINYYWSVLKPGQTLRIKSDGNLIAPWYGHILSGESHGRILKTD

LKRGSCTVQCQTEKGGLNTTLPFQNVSKYAFGNCSKYIGVKSLKLAVGLRNVPSRSSRGLFGAIAGFIEG
GWSGLIAGWYGFQHSNDQGVGMAADRDSTQKAIDKITSKVNNIVDKMNKQYEIIDHEFSEVETRLNMIND
KVDDQIQDIWAYNAELLVLLENQKTLDEHDANVNNLYNKVKRALGSNAVEDGKGCFELYHKCDDHCMETI
RNGTYNRRKYQEESKLERQKIEGVKLESEETYKILTIYSTVASSLVIAMGFAAFLFWAMSNGSCRCNICI

>QOK12376.1 hemagglutinin [Influenza A virus]
METVSLITILVVATVSNADKICIGYQSTNSTETVDTLTENNVPVTHAKELLHTEHNGMLCATSLGHPLIL
DTCTIEGLIYGNPSCDPLLGGREWSYIVERPSAVNGLCYPGNVENLEELRSLFSSSRSYQRIQIFPDTIW
NVSYSGTSKACSDSFYRSMRWLTQKNNAYPTQDAQYTNNQEKNILFMWGINHPPTDTAQTNLYTRTDTTT
SVATEEMNRIFKPLIGPRPLVNGLMGRINYYWSVLKPGQTLRIKSDGNLIAPWYGHILSGESHGRILKTD
LKRGSCTVQCQTEKGGLNTTLPFQNVSKYAFGNCSKYIGVKSLKLAVGLRNVPSRSSRGLFGAIAGFIEG
GWSGLIAGWYGFQHSNDQGVGMAADRDSTQKAIDKITSKVNNIVDKMNKQYEIIDHEFSEVETRLNMIND
KVDDQIQDIWAYNAELLVLLENQKTLDEHDANVNNLYNKVKRALGSNAVEDGKGCFELYHKCDDHCMETI
RNGTYNRRKYQEESKLERQKIEGVKLESEETYKILTIYSTVASSLVIAMGFAAFLFWAMSNGSCRCNICI

>QOK12364.1 hemagglutinin [Influenza A virus]
METVSLITILLVATVSNADKICIGYQSTNSTETVDTLTENNVPVTHAKELLHTEHNGMLCATSLGQPLIL
DTCTIEGLIYGNPSCDLSLEGREWSYIVERPSAVHGLCYPGNVENLEELRSLFSSARSYQRIQIFPDTIW
NVSYDGTSTACSGSFYRSMRWLTRKNGDYPIQDAQYTNNQGKNILFMWGINHPPTDETQRGLYTRTDTTT
SVATEEINRIFKPLIGPRPLVNGLMGRINYYWSVLKPGQTLRIKSDGNLIAPWYGHILSGESHGRILKTD
LKRGSCTVQCQTEKGGLNTTLPFQNVSKYAFGNCSKYIGIKSLKLAVGLRNVPSRSNRGLFGAIAGFIEG
GWSGLVAGWYGFQHSNDQGVGMAADRDSTQKAIDKITSKVNNIVDKMNKQYEIIDHEFSEVETRLNMINN
KIDDQIQDIWAYNAELLVLLENQKTLDEHDANVNNLYNKVKRALGSNAVEDGKGCFELYHKCDDQCMETI
RNGTYNRRKYQEESKLERQKIEGVKLESEGTYKILTIYSTVASSLVIAMGFAAFLFWAMSNGSCRCNICI

>QOK12352.1 hemagglutinin [Influenza A virus]
METVSLITILLVATVSNADKICIGYQSTNSTETVDTLTENNVPVTHAKELLHTEHNGMLCATSLGQPLIL
DTCTIEGLIYGNPSCDLSLEGREWSYIVERPSAVNGLCYPGNVENLEELRSLFSSARSYQRIQIFPDTIW
NVSYDGTSTACSNSFYRSMRWLTRKDGNYPTQDAQYTNNQGKNILFMWGINHPPTDETQRNLYTRTDTTT
SVATEEINRIFKPLIGPRPLVNGLMGRIDYYWSVLKPGQTLRIKSDGNLIAPWYGHILSGESHGRILKTD
LKKGSCTVQCQTEKGGLNTTLPFQNVSKYAFGNCSKYIGIKSLKLAVGLRNVPSRSSRGLFGAIAGFIEG
GWSGLVAGWYGFQHSNDQGVGMAADRDSTQKAIDKITSKVNNIVDKMNKQYEIIDHEFSEVETRLNMINN
KIDDQIQDIWAYNAELLVLLENQKTLDEHDANVNNLYNKVKRALGSNAMEDGKGCFELYHKCNDQCMETI
RNGTYNRKKYQEESKLERQRVEGVKLESEGTYKILTIYSTVASSLVIAMGFAAFLFWAMSNGSCRCNICI

>QOK12340.1 hemagglutinin [Influenza A virus]

METVSLITILLVATVSNADKICIGYQSTNSTETVDTLTENNVPVTHAKELLHTEHNGMLCATSLGQPLIL

DTCTIEGLIYGNPSCDLSLEGREWSYIVERPSAVNGLCYPGNVENLEELRSLFSSARSYQRIQIFPDTIW

NVSYDGTSTACSNSFYRSMRWLTRKDGNYPTQDAQYTNNQGKNILFMWGINHPPTDETQRNLYTRTDTTT

SVATEEINRIFKPLIGPRPLVNGLMGRIDYYWSVLKPGQTLRIKSDGNLIAPWYGHILSGESHGRILKTD

LKKGSCTVQCQTEKGGLNTTLPFQNVSKYAFGNCSKYIGIKSLKLAVGLRNVPSRSSRGLFGAIAGFIEG

GWSGLVAGWYGFQHSNDQGVGMAADRDSTQKAIDKITSKVNNIVDKMNKQYEIIDHEFSEVETRLNMINN

KIDDQIQDIWAYNAELLVLLENQKTLDEHDANVNNLYNKVKRALGSNAMEDGKGCFELYHKCNDQCMETI

RNGTYNRKKYQEESKLERQRVEGVKLESEGTYKILTIYSTVASSLVIAMGFAAFLFWAMSNGSCRCNICI

>QOK12304.1 hemagglutinin [Influenza A virus]

METISLITILLVATVSNADKICIGYQSTNSTETVNTLTENNVPVTHAKELLHTEHNGMLCATSLGQPLIL

DTCTIEGLIYGNPSCDLSLEGREWSYIVERPSAVHGLCYPGNVEDLEELRSLFSSARSYQRIQIFPDTIW

NVSYDGTSTACSGSFYRSMRWLTRKNGEYPIQDAQYTNNQGKNILFMWGINHPPTDDTQRGLYTRTDTTT

SVATEEINRIFKPLIGPRPLVNGLMGRINYYWAVLKPGQTLRIKSDGNLIAPWYGHILSGESHGRILKTD

LKRGSCTVQCQTEKGGLNTTLPFQNVSKYAFGNCSKYIGIKSLKLAVGLRNVPSRSSRGLFGAIAGFIEG

GWSGLVAGWYGFQHSNDQGVGMAADRDSTQKAIDKITSKVNNIVDKMNKQYEIIDHEFSEVETRLNMINN

KIDDQIQDIWAYNAELLVLLENQKTLDEHDANVNNLYNKVKRALGSNAVEDGKGCFELYHKCDDQCMETI

RNGTYNRRKYQEESKLERQKIEGVKLESEGTYKILTIYSTVASSLVIAMGFAAFLFWAMSNGSCRCNICI

>QOK12292.1 hemagglutinin [Influenza A virus]

METISLITILLVATVSNADKICIGYQSTNSTETVNTLTENNVPVTHAKELLHTEHNGMLCATSLGQPLIL

DTCTIEGLIYGNPSCDLSLEGREWSYIVERPSAVHGLCYPGNVEDLEELRSLFSSARSYQRIQIFPDTIW

NVSYDGTSTACSGSFYRSMRWLTRKNGEYPIQDAQYTNNQGKNILFMWGINHPPTDDTQRGLYTRTDTTT

SVATEEINRIFKPLIGPRPLVNGLMGRINYYWAVLKPGQTLRIKSDGNLIAPWYGHILSGESHGRILKTD

LKRGSCTVQCQTEKGGLNTTLPFQNVSKYAFGNCSKYIGIKSLKLAVGLRNVPSRSSRGLFGAIAGFIEG

GWSGLVAGWYGFQHSNDQGVGMAADRDSTQKAIDKITSKVNNIVDKMNKQYEIIDHEFSEVETRLNMINN

KIDDQIQDIWAYNAELLVLLENQKTLDEHDANVNNLYNKVKRALGSNAVEDGKGCFELYHKCDDQCMETI

RNGTYNRRKYQEESKLERQKIEGVKLESEGTYKILTIYSTVASSLVIAMGFAAFLFWAMSNGSCRCNICI

>QOK12221.1 hemagglutinin [Influenza A virus]

METVSLITILLVATVSNADKICIGYQSTNSTETVDTLTENNVPVTHAKELLHTEHNGMLCATSLGQPLIL

DTCTIEGLIYGNPSCDLSLEGREWSYIVERPSAVNGLCYPGNVENLEELRSLFSSARSYQRIQIFPDTIW

NVSYDGTSTACSNSFYRSMRWLTRKDGNYPTQDAQYTNNQGKNILFMWGINHPPTDETQRNLYTRTDTTT
SVATEEINRIFKPLIGPRPLVNGLMGRIDYYWSVLKPGQTLRIKSDGNLIAPWYGHILSGESHGRILKTD
LKKGSCTVQCQTEKGGLNTTLPFQNVSKYAFGNCSKYIGIKSLKLAVGLRNVPSRSSRGLFGAIAGFIEG
GWSGLVAGWYGFQHSNDQGVGMAADRDSTQKAIDKITSKVNNIVDKMNKQYEIIDHEFSEVETRLNMINN
KIDDQIQDIWAYNAELLVLLENQKTLDEHDANVNNLYNKVKRALGSNAMEDGKGCFELYHKCNDQCMETI
RNGTYNRKKYQEESKLERQRVEGVKLESEGTYKILTIYSTVASSLVIAMGFAAFLFWAMSNGSCRCNICI

>QOK12185.1 hemagglutinin [Influenza A virus]
METVSLITILLVATVSNADKICIGYQSTNSTETVDTLTENNVPVTHAKELLHTEHNGMLCATSLGQPLIL
DTCTIEGLIYGNPSCDLSLEGREWSYIVERPSAVNGLCYPGNVENLEELRSLFSSARSYQRIQIFPDTIW
NVSYDGTSTACSNSFYRSMRWLTRKDGNYPTQDAQYTNNQGKNILFMWGINHPPTDETQRNLYTRTDTTT
SVATEEINRIFKPLIGPRPLVNGLMGRIDYYWSVLKPGQTLRIKSDGNLIAPWYGHILSGESHGRILKTD
LKKGSCTVQCQTEKGGLNTTLPFQNVSKYAFGNCSKYIGIKSLKLAVGLRNVPSRSSRGLFGAIAGFIEG
GWSGLVAGWYGFQHSNDQGVGMAADRDSTQKAIDKITSKVNNIVDKMNKQYEIIDHEFSEVETRLNMINN
KIDDQIQDIWAYNAELLVLLENQKTLDEHDANVNNLYNKVKRALGSNAMEDGKGCFELYHKCNDQCMETI
RNGTYNRKKYQEESKLERQRVEGVKLESEGTYKILTIYSTVASSLVIAVGFAAFLFWAMSNGSCRCNICI

>QOK12104.1 hemagglutinin [Influenza A virus]
METVSLITILLVATVSNADKICIGYQSTNSTETVDTLTENNVPVTHAKELLHTEHNGMLCATSLGQPLIL
DTCTIEGLIYGNPSCDLSLEGREWSYIVERPSAVNGLCYPGNVENLEELRSLFSSARSYQRIQIFPDTIW
NVSYDGTSTACSGSFYKSMRWLTRKNGDYPIQDAQYTNNQGKNILFMWGINHPPTDTTQRDLYTRIDTTT
SVATEEINRVFKPLIGPRPLVNGLGRIDYYWSVLKPGQTLRIKSDGNLIAPWFGHILSGESHGRILKTDL
KKGSCTVQCQTEKGGLNSTLPFQNVSKYAFGNCSKYIGIKSLKLAVGLRNVPSRSSRGLFGAIAGFIEGG
WSGLVAGWYGFQHSNDQGVGMAADRDSTQKAIDKITSKVNNIVDKMNKQYEIIDHEFSEVETRLNMINNK
IDDQIQDIWAYNAELLVLLENQKTLDEHDANVNNLYNKVKRALGSNAVEDGKGCFELYHKCDDQCMETIR
NGTYNRRKYQEESKLERQKIEGVKLESEGTYKILTIYSTVASSLVIAMGFAAFLFWAMSNGSCRCNICI

>QOK12093.1 hemagglutinin [Influenza A virus]
METVSLITILLVATVSNADKICIGYQSTNSTETVDTLTENNVPVTHAKELLHTEHNGMLCATSLGQPLIL
DTCTIEGLIYGNPSCDLSLEGREWSYIVERPSAVHGLCYPGNVEDLEELRSLFSSARSYQRIQIFPDTIW
NVSYDGTSTACSGSFYRSMRWLTRKNGDYPIQDAQYTNNQGKNILFMWGINHPPTDTTQTNLYTRIDTTT
SVATEEINRIFKPLIGPRPLVNGLMGRINYYWSVLKPGQTLRIKSDGNLIAPWYGHILSGESHGRILKTD
LKRGSCTVQCQTEKGGLNTTLPFQNVSKYAFGNCSKYVGIKSLKLAVGLRNVPSRSSRGLFGAIAGFIEG
GWSGLVAGWYGFQHSNDQGVGMAADRDSTQKAIDKITSKVNNIVDKMNKQYEIIDHEFSEVETRLNMINN

KIDDQIQDIWAYNAELLVLLENQKTLDEHDANVNNLYNKVKRALGSNAVEDGKGCFELYHKCDDQCMETI
RNGTYNRRKYQEESKLERQKIEGVKLESEGTYKILTIYSTVASSLVIAMGFAAFLFWAMSNGSCRCNICI

>QOK12082.1 hemagglutinin [Influenza A virus]
METISLMTILLVATVSNADKICIGYQSTNSTETVDTLTENNVPVTHAKELLHTEHNGMLCATSLGNPLIL
DTCTIEGLIYGNPSCDPLLGGREWSYIVERPSAVNGLCYPGSVENLEELRSLFSSARSYQRIQIFPDTIW
NVSYSGTSKACSDSFYRSMRWLTQKNNAYPIQDAQYTNNQEKNILFMWGINHPPTETTQTNLYTRTDTTT
SVATEEINRIFKPLIGPRPLVNGLMGRINYYWSVLKPGQTLRIKSDGNLIAPWYGHILSGESHGRILKTD
LKRGSCTVQCQTEKGGLNTTLPFQNVSKYAFGNCSKYIGIKSLKLAVGLRNVPSRSSRGLFGAIAGFIEG
GWSGLVAGWYGFQHSNDQGVGMAADRDSTQKAIDKITSKVNNIVDKMNKQYEIIDHEFSEVETRLNMINN
KVDDQIQDIWAYNAELLVLLENQKTLDEHDSNVNNLYNKVKRALGSNAVEDGKGCFELYHKCDNQCMETI
RNGTYNRRKYQEESKLERQKIEGVKLESEGTYKILTIYSTVASSLVIAMGFAAFLFWAMSNGSCRCNICI

>QOK12071.1 hemagglutinin [Influenza A virus]
METISLMTILLVATVSNADKICIGYQSTNSTETVDTLTENNVPVTHAKELLHTEHNGMLCATSLGNPLIL
DTCTIEGLIYGNPSCDPLLGGREWSYIVERPSAVNGLCYPGSVENLEELRSLFSSARSYQRIQIFPDTIW
NVSYSGTSKACSDSFYRSMRWLTQKNNAYPIQDAQYTNNQEKNILFMWGINHPPTETAQTNLYTRTDTTT
SVATEEINRIFKPLIGPRPLVNGLMGRINYYWSVLKPGQTLRIKSDGNLIAPWYGHILSGESHGRILKTD
LKRGSCTVQCQTEKGGLNTTLPFQNVSKYAFGNCSKYIGIKSLKLAVGLRNVPSRSSRGLFGAIAGFIEG
GWSGLVAGWYGFQHSNDQGVGMAADRDSTQKAIDKITSKVNNIVDKMNKQYEIIDHEFSEVETRLNMINN
KVDDQIQDIWAYNAELLVLLENQKTLDEHDSNVNNLYNKVKRALGSNAVEDGKGCFELYHKCDNQCMETI
RNGTYNRRKYQEESKLERQKIEGVKLESEGTYKILTIYSTVASSLVIAMGFAAFLFWAMSNGSCRCNICI

>QOK12060.1 hemagglutinin [Influenza A virus]
METISLMTILLVATVSNADKICIGYQSTNSTETVDTLTENNVPVTHAKELLHTEHNGMLCATSLGNPLIL
DTCTIEGLIYGNPSCDPLLGGREWSYIVERPSAVNGLCYPGSVENLEELRSLFSSARSYQRIQIFPDTIW
NVSYSGTSKACSDSFYRSMRWLTQKNNAYPIQDAQYTNNQEKNILFMWGINHPPTETAQTNLYTRTDTTT
SVATEEINRIFKPLIGPRPLVNGLMGRINYYWSVLKPGQTLRIKSDGNLIAPWYGHILSGESHGRILKTD
LKRGSCTVQCQTEKGGLNTTLPFQNVSKYAFGNCSKYIGIKSLKLAVGLRNVPSRSSRGLFGAIAGFIEG
GWSGLVAGWYGFQHSNDQGVGMAADRDSTQKAIDKITSKVNNIVDKMNKQYEIIDHEFSEVETRLNMINN
KVDDQIQDIWAYNAELLVLLENQKTLDEHDSNVNNLYNKVKRALGSNAVEDGKGCFELYHKCDNQCMETI
RNGTYNRRKYQEESKLERQKIEGVKLESEGTYKILTIYSTVASSLVIAMGFAAFLFWAMSNGSCRCNICI

>QOK12025.1 hemagglutinin [Influenza A virus]

METVSLITILLVATVSNADKICIGYQSTNSTETVDTLTENNVPVTHAKELLHTEHNGMLCATSLGQPLIL
DTCTIEGLIYGNPSCDLSLEGREWSYIVERPSAVHGLCYPGNVEDLEELRSLFSSARSYQRIQIFPDTIW
NVSYDGTSTACSGSFYKSMRWLTRKNGEYPTQDAQYTNNQGKNILFMWGINHPPTDDTQRGLYTRTDTTT
SVATEEINRIFKPLIGPRPLVNGLMGRINYYWSVLKPGQTLRIKSDGNLIAPWYGHILSGESHGRILKTD
LKRGSCTVQCQTEKGGLNTTLPFQNVSKYAFGNCSKYIGIKSLKLAVGLRNVPSRSSRGLFGAIAGFIEG
GWPGLVAGWYGFQHSNDQGVGMAADRDSTQKAIDKITSKVNNIVDKMNKQYEIIDHEFSEIETRLNMINN
KIDDQIQDIWAYNAELLVLLENQKTLDEHDANVNNLYNKVKRALGSNAVEDGKGCFELYHKCDDQCMETI
RNGTYNRRKYQEESKLERQRIEGVKLESEGTYKILTIYSTVASSLVIAMGFAAFLFWAMSNGSCRCNICI

>QOK11978.1 hemagglutinin [Influenza A virus]
METVSLITILLXATVSNADKICIGYQSTNSTETVDTLTENNVPVTHAKELLHTEHNGMLCATSLGQPLIL
DTCTIEGLIYGNPSCDLSLEGREWSYIVERPSAVHGLCYPGNVEDLEELRSLFSSARSYQRIQIFPDTIW
NVSYDGTSTACSGSFYKSMRWLTRKNGEYPTQDAQYTNNQGKNILFMWGINHPPTDDTQRGLYTRTDTTT
SVATEEINRIFKPLIGPRPLVNGLMGRINYYWSVLKPGQTLRIKSDGNLIAPWYGHILSGESHGRILKTD
LKRGSCTVQCQTEKGGLNTTLPFQNVSKYAFGNCSKYIGIKSLKLAVGLRNVPSRSSRGLFGAIAGFIEG
GWXGLVAGWYGFQHSNDQGVGMAADRXSTQKAIDKITSKVNNIVDKMNKQYEIIDHEFSEXETRLNMINN
KIDDQIQDIWAYNAELLVLLENQKTLDEHDANVNNLYNKVKRALGSNAVEDGKGCFELYHKCDDQCMETI
RNGTYNRRKYQEESKLERQXIEGVKLESEGTYKILTIYSTVASSLVIAMGFAAFLFWAMSNGSCRCNICI

>QOK11954.1 hemagglutinin [Influenza A virus]
METVSLITILLAATVSNADKICIGYQSTNSTETVDTLTENNVPVTHAKELLHTEHNGMLCATSLGQPLIL
DTCTIEGLIYGNPSCDPLLEEREWSYIVERPSAVNGLCYPGNVENLEELRSFFSSARSYQRIQIFPDTIW
NVSYDGTSNTCSGSFYRNMRWLTRKNGNYPIQDAQYTNNQGKSILFMWGINHPPTDDTQRNLYTRTDTTT
SVATEEINRIFKPLIGPRPLVNGLMGRINYYWSVLKPGQTLRIKSDGNLIAPWYGYILSGESHGRILRTD
LTKGSCTVQCQTEKGGLNTTLPFQNVSKYAFGNCSKYIGIKSLKLAVGLRNVPSRSSRGLFGAIAGFIEG
GWSGLVAGWYGFQHSNDQGVGMAADRESTQKAIDKITSKVNNIVDKMNKQYEIIDHEFSEVETRLNMINN
KIDDQIQDIWAYNAELLVLLENQKTLDEHDANVNNLYNKVKRALGSNAVEDGKGCFELYHKCDDQCMETI
RNGTYNRRKYQEESKLERQKIEGVKLESEGTYKILTIYSTVASSLVIAMGFAAFLFWAMSNGSCRCNICI

>QOK11942.1 hemagglutinin [Influenza A virus]
METVSLITILIVATVSNADKICIGYQSTNSTETVDTLTENNVPVTHAKELLHTEHNGMLCATSLGHPLIL
DTCTIEGLIYGNPSCDPLLGGREWSYIVERPSAVNGLCYPGNVENLEELRSLFSSSRSYQRIQIFPDTIW
NVSYSGTSKACSDSFYRSMRWLTQKNNAYPTQDAQYTNNQGKNILFVWGINHPPTDTAQTNLYTRTDTTT
SVATEEMNRIFKPLIGPRPLVNGLMGRINYYWSVLKPGQTLRIKSDGNLIAPWYGHILSGESHGRILKTD

LKRGSCTVQCQTEKGGLNTTLPFQNVSKYAFGNCSKYIGVKSLKLAVGLRNVPSRSSRGLFGAIAGFIEG
GWSGLVAGWYGFQHSNDQGVGMAADRDSTQKAIDKITSKVNNIVDKMNKQYEIIDHEFSEVETRLNMINN
KVDDQIQDIWAYNAELLVLLENQKTLDEHDANVNNLYNKVKRALGSNAVEDGKGCFELYHKCDDHCMETI
RNGTYNRRKYQEESKLERQKIEGVKLESEETYKILTIYSTVASSLVIAMGFAAFLFWAMSNGSCRCNICI

>QOK11930.1 hemagglutinin [Influenza A virus]
METVSLITILIVATVSNADKICIGYQSTNSTETVDTLTENNVPVTHAKELLHTEHNGMLCATSLGHPLIL
DTCTIEGLIYGNPSCDPLLGGREWSYIVERPSAVNGLCYPGNVENLEELRSLFSSSRSYQRIQIFPDTIW
NVSYSGTSKACSDSFYRSMRWLTQKNNAYPTQDAQYTNNQGKNILFVWGINHPPTDTAQTNLYTRTDTTT
SVATEEMNRIFKPLIGPRPLVNGLMGRINYYWSVLKPGQTLRIKSDGNLIAPWYGHILSGESHGRILKTD
LKRGSCTVQCQTEKGGLNTTLPFQNVSKYAFGNCSKYIGVKSLKLAVGLRNVPSRSSRGLFGAIAGFIEG
GWSGLVAGWYGFQHSNDQGVGMAADRDSTQKAIDKITSKVNNIVDKMNKQYEIIDHEFSEVETRLNMINN
KVDDQIQDIWAYNAELLVLLENQKTLDEHDANVNNLYNKVKRALGSNAVEDGKGCFELYHKCDDHCMETI
RNGTYNRRKYQEESKLERQKIEGVKLESEETYKILTIYSTVASSLVIAMGFAAFLFWAMSNGSCRCNICI

>QOK11918.1 hemagglutinin [Influenza A virus]
METVSLITILVVATVSNADKICIGYQSTNSTETVDTLTENNVPVTHAKELLHTEHNGMLCATSLGHPLIL
DTCTIEGLIYGNPSCDPLLGGREWSYIVERPSAVNGLCYPGNVENLEELRSLFSSSRSYQRIQIFPDTIW
NVSYSGTSKACSDSFYRSMRWLTQKNNAYPTQDAQYTNNQGKNILFMWGINHPPTDTAQTNLYTRTDTTT
SVATEEMNRIFKPLIGPRPLVNGLMGRINYYWSVLKPGQTLRIKSDGNLIAPWYGHILSGESHGRILKTD
LKRGSCTVQCQTEKGGLNTTLPFQNVSKYAFGNCSKYIGVKSLKLAVGLRNVPSRSSRGLFGAIAGFIEG
GWSGLVAGWYGFQHSNDQGVGMAADRDSTQKAIDKITSKVNNIVDKMNKQYEIIDHEFSEVETRLNMINN
KVDDQIQDIWAYNAELLVLLENQKTLDEHDANVNNLYNKVKRALGSNAVEDGKGCFELYHKCDDHCMETI
RNGTYNRRKYQEESKLERQKIEGVKLESEETYKILTIYSTVASSLVIAMGFAAFLFWAMSNGSCRCNICI

>QOK11894.1 hemagglutinin [Influenza A virus]
METVSLITILVVATVSNADKICIGYQSTNSTETVDTLTENNVPVTHAKELLHTEHNGMLCATSLGHPLIL
DTCTIEGLIYGNPSCDPLLGGREWSYIVERPSAVNGLCYPGNVENLEELRSLFSSSRSYQRIQIFPDTIW
NVSYSGTSKACSDSFYRSMRWLTQKNNAYPTQDAQYTNNQGKNILFMWGINHPPTDTAQTNLYTRTDTTT
SVATEEMNRIFKPLIGPRPLVNGLMGRINYYWSVLKPGQTLRIKSDGNLIAPWYGHILSGESHGRILKTD
LKRGSCTVQCQTEKGGLNTTLPFQNVSKYAFGNCSKYIGVKSLKLAVGLRNVPSRSSRGLFGAIAGFIEG
GWSGLVAGWYGFQHSNEQGVGMAADRDSTQKAIDKITSKVNNIVDKMNKQYEIIDHEFSEVETRLNMINN
KVDDQIQDIWAYNAELLVLLENQKTLDEHDANVNNLYNKVKRALGSNAVEDGKGCFELYHKCDDHCMETI
RNGTYNRRKYQEESKLERQKIEGVKLESEETYKILTIYSTVASSLVIAMGFAAFLFWAMSNGSCRCNICI

>QOK11848.1 hemagglutinin [Influenza A virus]

METVSLITILLAATVSNADKICIGYQSTNSTETVDTLTENNVPVTHAKELLHTEHNGMLCATSLGQPLIL
DTCTIEGLIYGNPSCDPLLEEREWSYIVERPSAVNGLCYPGNVENLEELRSFFSSARSYQRIQIFPDTIW
NVSYDGTSNTCSGSFYRNMRWLTRKNGNYPIQDAQYTNNQGKSILFMWGINHPPTDDTQRNLYTRTDTTT
SVATEEINRIFKPLIGPRPLVNGLMGRINYYWSVLKPGQTLRIKSDGNLIAPWYGYILSGESHGRILRTD
LTKGSCTVQCQTEKGGLNTTLPFQNVSKYAFGNCSKYIGIKSLKLAVGLRNVPSRSSRGLFGAIAGFIEG
GWSGLVAGWYGFQHSNDQGVGMAADRESTQKAIDKITSKVNNIVDKMNKQYEIIDHEFSEVETRLNMINN
KIDDQIQDIWAYNAELLVLLENQKTLDEHDANVNNLYNKVKRALGSNAVEDGKGCFELYHKCDDQCMETI
RBGTYNRRKYQEESKLERQKIEGVKLESEGTYKILTIYSTVASSLVIAMGFAAFLFWAMSNGSCRCNICI

>QOK11836.1 hemagglutinin [Influenza A virus]

METVSLITILIVATVSNADKICIGYQSTNSTETVDTLTENNVPVTHAKELLHTEHNGMLCATSLGHPLIL
DTCTIEGLIYGNPSCDPLLGGREWSYIVERPSAVNGLCYPGNVENLEELRSLFSSSRSYQRIQIFPDTIW
NVSYSGTSKACSDSFYRSMRWLTQKNNAYPTQDAQYTNNQGKNILFVWGINHPPTDTAQTNLYTRTDTTT
SVATEEMNRIFKPLIGPRPLVNGLMGRINYYWSVLKPGQTLRIKSDGNLIAPWYGHILSGESHGRILKTD
LKRGSCTVQCQTEKGGLNTTLPFQNVSKYAFGNCSKYIGVKSLKLAVGLRNVPSRSSRGLFGAIAGFIEG
GWSGLVAGWYGFQHSNDQGVGMAADRDSTQKAIDKITSKVNNIVDKMNKQYEIIDHEFSEVETRLNMINN
KVDDQIQDIWAYNAELLVLLENQKTLDEHDANVNNLYNKVKRALGSNAVEDGKGCFELYHKCDDHCMETI
RNGTYNRRKYQEESKLERQKIEGVKLESEETYKILTIYSTVASSLVIAMGFAAFLFWAMSNGSCRCNICI

>QOK11824.1 hemagglutinin [Influenza A virus]

METVSLITILVVATVSNADKICIGYQSTNSTETVDTLTENNVPVTHAKELLHTEHNGMLCATSLGHPLIL
DTCTIEGLIYGNPSCDPLLGGREWSYIVERPSAVNGLCYPGNVENLEELRSLFSSSRSYQRIQIFPDTIW
NVSYSGTSKACSDSFYRSMRWLTQKNNAYPTQDAQYTNNQGKNILFMWGINHPPTDTAQTNLYTRTDTTT
SVATEEMNRIFKPLIGPRPLVNGLMGRINYYWSVLKPGQTLRIKSDGNLIAPWYGHILSGESHGRILKTD
LKRGSCTVQCQTEKGGLNTTLPFQNVSKYAFGNCSKYIGVKSLKLAVGLRNVPSRSSRGLFGAIAGFIEG
GWSGLVAGWYGFQHSNDQGVGMAADRDSTQKAIDKITSKVNNIVDKMNKQYEIIDHEFSEVETRLNMINN
KVDDQIQDIWAYNAELLVLLENQKTLDEHDANVNNLYNKVKRALGSNAVEDGKGCFELYHKCDDHCMETI
RNGTYNRRKYQEESKLERQKIEGVKLESEETYKILTIYSTVASSLVIAMGFAAFLFWAMSNGSCRCNICI

>QOK11812.1 hemagglutinin [Influenza A virus]

METVSLITILVVATVSNADKICIGYQSTNSTETVDTLTENNVPVTHAKELLHTEHNGMLCATSLGHPLIL
DTCTIEGLIYGNPSCDPLLGGREWSYIVERPSAVNGLCYPGNVENLEELRSLFSSSRSYQRIQIFPDTIW

NVSYSGTSKACSDSFYRSMRWLTQKNNAYPTQDAQYTNNQGKNILFMWGINHPPTDTAQTNLYTRTDTTT
SVATEEMNRIFKPLIGPRPLVNGLMGRINYYWSVLKPGQTLRIKSDGNLIAPWYGHILSGESHGRILKTD
LKRGSCTVQCQTEKGGLNTTLPFQNVSKYAFGNCSKYIGVKSLKLAVGLRNVPSRSSRGLFGAIAGFIEG
GWSGLVAGWYGFQHSNDQGVGMAADRDSTQKAIDKITSKVNNIVDKMNKQYEIIDHEFSEVETRLNMINN
KVDDQIQDIWAYNAELLVLLENQKTLDEHDANVNNLYNKVKRALGSNAVEDGKGCFELYHKCDDHCMETI
RNGTYNRRKYQEESKLERQKIEGVKLESEETYKILTIYSTVASSLVIAMGFAAFLFWAMSNGSCRCNICI

>QOK11800.1 hemagglutinin [Influenza A virus]
METVSLITILVVATVSNADKICIGYQSTNSTETVDTLTENNVPVTHAKELLHTEHNGMLCATSLGHPLIL
DTCTIEGLIYGNPSCDPLLGGREWSYIVERPSAVNGLCYPGNVENLEELRSLFSSSRSYQRIQIFPDTIW
NVSYSGTSKACSDSFYRSMRWLTQKNNAYPTQDAQYTNNQGKNILFMWGINHPPTDTAQTNLYTRTDTTT
SVATEEMNRIFKPLIGPRPLVNGLMGRINYYWSVLKPGQTLRIKSDGNLIAPWYGHILSGESHGRILKTD
LKRGSCTVQCQTEKGGLNTTLPFQNVSKYAFGNCSKYIGVKSLKLAVGLRNVPSRSSRGLFGAIAGFIEG
GWSGLVAGWYGFQHSNDQGVGMAADRDSTQKAIDKITSKVNNIVDKMNKQYEIIDHEFSEVETRLNMINN
KVDDQIQDIWAYNAELLVLLENQKTLDEHDANVNNLYNKVKRALGSNAVEDGKGCFELYHKCDDHCMETI
RNGTYNRRKYQEESKLERQKIEGVKLESEETYKILTIYSTVASSLVIAMGFAAFLFWAMSNGSCRCNICI

>QOK11788.1 hemagglutinin [Influenza A virus]
METVSLITILVVATVSNADKICIGYQSTNSTETVDTLTENNVPVTHAKELLHTEHNGMLCATSLGHPLIL
DTCTIEGLIYGNPSCDPLLGGREWSYIVERPSAVNGLCYPGNVENLEELRSLFSSSRSYQRIQIFPDTIW
NVSYSGTSKACSDSFYRSMRWLTQKNNAYPTQDAQYTNNQGKNILFMWGINHPPTDTAQTNLYTRTDTTT
SVATEEMNRIFKPLIGPRPLVNGLMGRINYYWSVLKPGQTLRIKSDGNLIAPWYGHILSGESHGRILKTD
LKRGSCTVQCQTEKGGLNTTLPFQNVSKYAFGNCSKYIGVKSLKLAVGLRNVPSRSSRGLFGAIAGFIEG
GWSGLVAGWYGFQHSNDQGVGMAADRDSTQKAIDKITSKVNNIVDKMNKQYEIIDHEFSEVETRLNMINN
KVDDQIQDIWAYNAELLVLLENQKTLDEHDANVNNLYNKVKRALGSNAVEDGKGCFELYHKCDDHCMETI
RNGTYNRRKYQEESKLERQKIEGVKLESEETYKILTIYSTVASSLVIAMGFAAFLFWAMSNGSCRCNICI

>QOK11776.1 hemagglutinin [Influenza A virus]
METVSLITILVVATVSNADKICIGYQSTNSTETVDTLTENNVPVTHAKELLHTEHNGMLCATSLGHPLIL
DTCTIEGLIYGNPSCDPLLGGREWSYIVERPSAVNGLCYPGNVENLEELRSLFSSSRSYQRIQIFPDTIW
NVSYSGTSKACSDSFYRSMRWLTQKNNAYPTQDAQYTNNQGKNILFMWGINHPPTDTAQTNLYTRTDTTT
SVATEEMNRIFKPLIGPRPLVNGLMGRINYYWSVLKPGQTLRIKSDGNLIAPWYGHILSGESHGRILKTD
LKRGSCTVQCQTEKGGLNTTLPFQNVSKYAFGNCSKYIGVKSLKLAVGLRNVPSRSSRGLFGAIAGFIEG
GWSGLVAGWYGFQHSNDQGVGMAADRDSTQKAIDKITSKVNNIVDKMNKQYEIIDHEFSEVETRLNMINN

KVDDQIQDIWAYNAELLVLLENQKTLDEHDANVNNLYNKVKRALGSNAVEDGKGCFELYHKCDDHCMETI
RNGTYNRRKYQEESKLERQKIEGVKLESEETYKILTIYSTVASSLVIAMGFAAFLFWAMSNGSCRCNICI

>QOK11764.1 hemagglutinin [Influenza A virus]
METVSLITILVVATVSNADKICIGYQSTNSTETVDTLTENNVPVTHAKELLHTEHNGMLCATSLGHPLIL
DTCTIEGLIYGNPSCDPLLGGREWSYIVERPSAVNGLCYPGNVENLEELRSLFSSSRSYQRIQIFPDTIW
NVSYSGTSKACSDSFYRSMRWLTQKNNAYPTQDAQYTNNQGKNILFMWGINHPPTDTAQTNLYTRTDTTT
SVATEEMNRIFKPLIGPRPLVNGLMGRINYYWSVLKPGQTLRIKSDGNLIAPWYGHILSGESHGRILKTD
LKRGSCTVQCQTEKGGLNTTLPFQNVSKYAFGNCSKYIGVKSLKLAVGLRNVPSRSSRGLFGAIAGFIEG
GWSGLVAGWYGFQHSNDQGVGMAADRDSTQKAIDKITSKVNNIVDKMNKQYEIIDHEFSEVETRLNMINN
KVDDQIQDIWAYNAELLVLLENQKTLDEHDANVNNLYNKVKRALGSNAVEDGKGCFELYHKCDDHCMETI
RNGTYNRRKYQEESKLERQKIEGVKLESEETYKILTIYSTVASSLVIAMGFAAFLFWAMSNGSCRCNICI

>QOK11752.1 hemagglutinin [Influenza A virus]
METVSLITILVVATVSNADKICIGYQSTNSTETVDTLTENNVPVTHAKELLHTEHNGMLCATSLGHPLIL
DTCTIEGLIYGNPSCDPLLGGREWSYIVERPSAVNGLCYPGNVENLEELRSLFSSSRSYQRIQIFPDTIW
NVSYSGTSKACSDSFYRSMRWLTQKNNAYPTQDAQYTNNQGKNILFMWGINHPPTDTAQTNLYTRTDTTT
SVATEEMNRIFKPLIGPRPLVNGLMGRINYYWSVLKPGQTLRIKSDGNLIAPWYGHILSGESHGRILKTD
LKRGSCTVQCQTEKGGLNTTLPFQNVSKYAFGNCSKYIGVKSLKLAVGLRNVPSRSSRGLFGAIAGFIEG
GWSGLVAGWYGFQHSNDQGVGMAADRDSTQKAIDKITSKVNNIVDKMNKQYEIIDHEFSEVETRLNMINN
KVDDQIQDIWAYNAELLVLLENQKTLDEHDANVNNLYNKVKRALGSNAVEDGKGCFELYHKCDDHCMETI
RNGTYNRRKYQEESKLERQKIEGVKLESEETYKILTIYSTVASSLVIAMGFAAFLFWAMSNGSCRCNICI

>QOK11740.1 hemagglutinin [Influenza A virus]
METVSLITILVVATVSNADKICIGYQSTNSTETVDTLTENNVPVTHAKELLHTEHNGMLCATSLGHPLIL
DTCTIEGLIYGNPSCDPLLGGREWSYIVERPSAVNGLCYPGNVENLEELRSLFSSSRSYQRIQIFPDTIW
NVSYSGTSKACSDSFYRSMRWLTQKNNAYPTQDAQYTNNQGKNILFMWGINHPPTDTVQTNLYTRTDTTT
SVATEEMNRIFKPLIGPRPLVNGLMGRINYYWSVLKPGQTLRIKSDGNLIAPWYGHILSGESHGRILKTD
LKRGSCTVQCQTEKGGLNTTLPFQNVSKYAFGNCSKYIGVKSLKLAVGLRNVPSRSSRGLFGAIAGFIEG
GWSGLVAGWYGFQHSNDQGVGMAADRDSTQKAIDKITSKVNNIVDKMNKQYEIIDHEFSEVETRLNMINN
KVDDQIQDIWAYNAELLVLLENQKTLDEHDANVNNLYNKVKRALGSNAVEDGKGCFELYHKCDDHCMETI
RNGTYNRRKYQEESKLERQKIEGVKLESEETYKILTIYSTVASSLVIAMGFAAFLFWAMSNGSCRCNICI

>QOK11728.1 hemagglutinin [Influenza A virus]

METVSLITILVVATVSNADKICIGYQSTNSTETVDTLTENNVPVTHAKELLHTEHNGMLCATSLGHPLIL
DTCTIEGLIYGNPSCDPLLGGREWSYIVERPSAVNGLCYPGNVENLEELRSLFSSSRSYQRIQIFPDTIW
NVSYSGTSKACSDSFYRSMRWLTQKNNAYPTQDAQYTNNQGKNILFMWGINHPPTDTAQTNLYTRTDTTT
SVATEEMNRIFKPLIGPRPLVNGLMGRINYYWSVLKPGQTLRIKSDGNLIAPWYGHILSGESHGRILKTD
LKRGSCTVQCQTEKGGLNTTLPFQNVSKYAFGNCSKYIGVKSLKLAVGLRNVPSRSSRGLFGAIAGFIEG
GWSGLVAGWYGFQHSNDQGVGMAADRDSTQKAIDKITSKVNNIVDKMNKQYEIIDHEFSEVETRLNMINN
KVDDQIQDIWAYNAELLVLLENQKTLDEHDANVNNLYNKVKRALGSNAVEDGKGCFELYHKCDDHCMETI
RNGTYNRRKYQEESKLERQKIEGVKLESEETYKILTIYSTVASSLVIAMGFAAFLFWAMSNGSCRCNICI

>QOK11716.1 hemagglutinin [Influenza A virus]
METVSLITILVVATVSNADKICIGYQSTNSTETVDTLTENNVPVTHAKELLHTEHNGMLCATSLGHPLIL
DTCTIEGLIYGNPSCDPLLGGREWSYIVERPSAVNGLCYPGNVENLEELRSLFSSSRSYQRIQIFPDTIW
NVSYSGTSKACSDSFYRSMRWLTQKNNAYPTQDAQYTNNQGKNILFMWGINHPPTDTAQTNLYTRTDTTT
SVATEEMNRIFKPLIGPRPLVNGLMGRINYYWSVLKPGQTLRIKSDGNLIAPWYGHILSGESHGRILKTD
LRRGSCTVQCQTEKGGLNTTLPFQNVSKYAFGNCSKYIGVKSLKLAVGLRNVPSRSSRGLFGAIAGFIEG
GWSGLVAGWYGFQHSNDQGVGMAADRDSTQKAIDKITSKVNNIVDKMNKQYEIIDHEFSEVETRLNMINN
KVDDQIQDIWAYNAELLVLLENQKTLDEHDANVNNLYNKVKRALGSNAVEDGKGCFELYHKCDDHCMETI
RNGTYNRRKYQEESKLERQKIEGVKLESEETYKILTIYSTVASSLVIAMGFAAFLFWAMSNGSCRCNICI

>QOK11704.1 hemagglutinin [Influenza A virus]
METVSLITILVVATVSNADKICIGYQSTNSTETVDTLTENNVPVTHAKELLHTEHNGMLCATSLGHPLIL
DTCTIEGLIYGNPSCDPLLGGREWSYIVERPSAVNGLCYPGNVENLEELRSLFSSSRSYQRIQILGERMW
NVCYSGRRKGWWDSLYRRMRWLSEKKKGYGREDGEYRNNEGKKIVLMWGINEGGRDSGETNLYTRTDTTT
SVATEEMNRIFKPLIGPRPLVNGLMGRINYYWSVLKPGQTLRIKSDGNLIAPWYGHILSGESHGRILKTD
LKRGSCTVQCQTEKGGLNTTLPFQNVSKYAFGNCSKYIGVKSLKLAVGLRNVPSRSSRGLFGAIAGFIEG
GWSGLVAGWYGFQHSNDQGVGMAADRDSTQKAIDKITSKVNNIVDKMNKQYEIIDHEFSEVETRLNMINN
KVDDQIQDIWAYNAELLVLLENQKTLDEHDANVNNLYNKVKRALGSNAVEDGKGCFELYHKCDDHCMETI
RNGTYNRRKYQEESKLERQKIEGVKLESEETYKILTIYSTVASSLVIAMGFAAFLFWAMSNGSCRCNICI

>QOK11692.1 hemagglutinin [Influenza A virus]
METVSLITILIVATVSNADKICIGYQSTNSTETVDTLTENNVPVTHAKELLHTEHNGMLCATSLGHPLIL
DTCTIEGLIYGNPSCDPLLGGREWSYIVERPSAVNGLCYPGNVENLEELRSLFSSSRSYQRIQIFPDTIW
NVSYSGTSKACSDSFYRSMRWLTQKNNAYPTQDAQYTNNQGKNILFVWGINHPPTDTAQTNLYTRTDTTT
SVATEEMNRIFKPLIGPRPLVNGLMGRINYYWSVLKPGQTLRIKSDGNLIAPWYGHILSGESHGRILKTD

LKRGSCTVQCQTEKGGLNTTLPFQNVSKYAFGNCSKYIGVKSLKLAVGLRNVPSRSSRGLFGAIAGFIEG
GWSGLVAGWYGFQHSNDQGVGMAADRDSTQKAIDKITSKVNNIVDKMNKQYEIIDHEFSEVETRLNMINN
KVDDQIQDIWAYNAELLVLLENQKTLDEHDANVNNLYNKVKRALGSNAVEDGKGCFELYHKCDDHCMETI
RNGTYNRRKYQEESKLERQKIEGVKLESEETYKILTIYSTVASSLVIAMGFAAFLFWAMSNGSCRCNICI

>QOK11680.1 hemagglutinin [Influenza A virus]
METVSLITILVVATVSNADKICIGYQSTNSTETVDTLTENNVPVTHAKELLHTEHNGMLCATSLGHPLIL
DTCTIEGLIYGNPSCDPLLGGREWSYIVERPSAVNGLCYPGNVENLEELRSLFSSSRSYQRIQIFPDTIW
NVSYSGTSKACSDSFYRSMRWLTQKNNAYPTQDAQYTNNQGKNILFMWGINHPPTDTAQTNLYTRTDTTT
SVATEEMNRIFKPLIGPRPLVNGLMGRINYYWSVLKPGQTLRIKSDGNLIAPWYGHILSGESHGRILKTD
LKRGSCTVQCQTEKGGLNTTLPFQNVSKYAFGNCSKYIGVKSLKLAVGLRNVPSRSSRGLFGAIAGFIEG
GWSGLVAGWYGFQHSNDQGVGMAADRDSTQKAIDKITSKVNNIVDKMNKQYEIIDHEFSEVETRLNMINN
KVDDQIQDIWAYNAELLVLLENQKTLDEHDANVNNLYNKVKRALGSNAVEDGKGCFELYHKCDDHCMETI
RNGTYNRRKYQEESKLERQKIEGVKLESEETYKILTIYSTVASSLVIAMGFAAFLFWAMSNGSCRCNICI

>QOK11668.1 hemagglutinin [Influenza A virus]
METVSLITILVVATVSNADKICIGYQSTNSTETVDTLTENNVPVTHAKELLHTEHNGMLCATSLGHPLIL
DTCTIEGLIYGNPSCDPLLGGREWSYIVERPSAVNGLCYPGNVENLEELRSLFSSSRSYQRIQIFPDTIW
NVSYSGTSKACSDSFYRSMRWLTQKNNAYPTQDAQYTNNQGKNILFMWGINHPPTDTAQTNLYTRTDTTT
SVATEEMNRIFKPLIGPRPLVNGLMGRINYYWSVLKPGQTLRIKSDGNLIAPWYGHILSGESHGRILKTD
LKRGSCTVQCQTEKGGLNTTLPFQNVSKYAFGNCSKYIGVKSLKLAVGLRNVPSRSSRGLFGAIAGFIEG
GWSGLVAGWYGFQHSNDQGVGMAADRDSTQKAIDKITSKVNNIVDKMNKQYEIIDHEFSEVETRLNMINN
KVDDQIQDIWAYNAELLVLLENQKTLDEHDANVNNLYNKVKRALGSNAVEDGKGCFELYHKCDDHCMETI
RNGTYNRRKYQEESKLERQKIEGVKLESEETYKILTIYSTVASSLVIAMGFAAFLFWAMSNGSCRCNICI

>QOK11656.1 hemagglutinin [Influenza A virus]
METVSLITILIVATVSNADKICIGYQSTNSTETVDTLTENNVPVTHAKELLHTEHNGMLCATSLGHPLIL
DTCTIEGLIYGNPSCDPLLGGREWSYIVERPSAVNGLCYPGNVENLEELRSLFSSSRSYQRIQIFPDTIW
NVSYSGTSKACSDSFYRSMRWLTQKNNAYPTQDAQYTNNQGKNILFVWGINHPPTDTAQTNLYTRTDTTT
SVATEEMNRIFKPLIGPRPLVNGLMGRINYYWSVLKPGQTLRIKSDGNLIAPWYGHILSGESHGRILKTD
LKRGSCTVQCQTEKGGLNTTLPFQNVSKYAFGNCSKYIGVKSLKLAVGLRNVPSRSSRGLFGAIAGFIEG
GWSGLVAGWYGFQHSNDQGVGMAADRDSTQKAIDKITSKVNNIVDKMNKQYEIIDHEFSEVETRLNMINN
KVDDQIQDIWAYNAELLVLLENQKTLDEHDANVNNLYNKVKRALGSNAVEDGKGCFELYHKCDDHCMETI
RNGTYNRRKYQEESKLERQKIEGVKLESEETYKILTIYSTVASSLVIAMGFAAFLFWAMSNGSCRCNICI

>QOK11644.1 hemagglutinin [Influenza A virus]

METVSLITILVVATVSNADKICIGYQSTNSTETVDTLTENNVPVTHAKELLHTEHNGMLCATSLGHPLIL
DTCTIEGLIYGNPSCDPLLGGREWSYIVERPSAVNGLCYPGNVENLEELRSLFSSSRSYQRIQIFPDTIW
NVSYSGTSKACSDSFYRSMRWLTQKNNAYPTQDAQYTNNQGKNILFMWGINHPPTDTAQTNLYTRTDTTT
SVATEEMNRIFKPLIGPRPLVNGLMGRINYYWSVLKPGQTLRIKSDGNLIAPWYGHILSGESHGRILKTD
LKRGSCTVQCQTEKGGLNTTLPFQNVSKYAFGNCSKYIGVKSLKLAVGLRNVPSRSSRGLFGAIAGFIEG
GWSGLVAGWYGFQHSNDQGVGMAADRDSTQKAIDKITSKVNNIVDKMNKQYEIIDHEFSEVETRLNMINN
KVDDQIQDIWAYNAELLVLLENQKTLDEHDANVNNLYNKVKRALGSNAVEDGKGCFELYHKCDDHCMETI
RNGTYNRRKYQEESKLERQKIEGVKLESEETYKILTIYSTVASSLVIAMGFAAFLFWAMSNGSCRCNICI

>QOK11632.1 hemagglutinin [Influenza A virus]

METVSLITILVVATVSNADKICIGYQSTNSTETVDTLTENNVPVTHAKELLHTEHNGMLCATSLGHPLIL
DTCTIEGLIYGNPSCDPLLGGREWSYIVERPSAVNGLCYPGNVENLEELRSLFSSSRSYQRIQIFPDTIW
NVSYSGTSKACSDSFYRSMRWLTQKNNAYPTQDAQYTNNQGKNILFMWGINHPPTDTAQTNLYTRTDTTT
SVATEEMNRIFKPLIGPRPLVNGLMGRINYYWSVLKPGQTLRIKSDGNLIAPWYGHILSGESHGRILKTD
LKRGSCTVQCQTEKGGLNTTLPFQNVSKYAFGNCSKYIGVKSLKLAVGLRNVPSRSSRGLFGAIAGFIEG
GWSGLVAGWYGFQHSNDQGVGMAADRDSTQKAIDKITSKVNNIVDKMNKQYEIIDHEFSEVETRLNMINN
KVDDQIQDIWAYNAELLVLLENQKTLDEHDANVNNLYNKVKRALGSNAVEDGKGCFELYHKCDDHCMETI
RNGTYNRRKYQEESKLERQKIEGVKLESEETYKILTIYSTVASSLVIAMGFAAFLFWAMSNGSCRCNICI

>QOK11620.1 hemagglutinin [Influenza A virus]

METVSLITILVVATVSNADKICIGYQSTNSTETVDTLTENNVPVTHAKELLHTEHNGMLCATSLGHPLIL
DTCTIEGLIYGNPSCDPLLGGREWSYIVERPSAVNGLCYPGNVENLEELRSLFSSSRSYQRIQIFPDTIW
NVSYSGTSKACSDSFYRSMRWLTQKNNAYPTQDAQYTNNQGKNILFMWGINHPPTDTAQTNLYTRTDTTT
SVATEEMNRIFKPLIGPRPLVNGLMGRINYYWSVLKPGQTLRIKSDGNLIAPWYGHILSGESHGRILKTD
LKRGSCTVQCQTEKGGLNTTLPFQNVSKYAFGNCSKYIGVKSLKLAVGLRNVPSRSSRGLFGAIAGFIEG
GWSGLVAGWYGFQHSNEQGVGMAADRDSTQKAIDKITSKVNNIVDKMNKQYEIIDHEFSEVETRLNMINN
KVDDQIQDIWAYNAELLVLLENQKTLDEHDANVNNLYNKVKRALGSNAVEDGKGCFELYHKCDDHCMETI
RNGTYNRRKYQEESKLERQKIEGVKLESEETYKILTIYSTVASSLVIAMGFAAFLFWAMSNGSCRCNICI

>QOK11608.1 hemagglutinin [Influenza A virus]

METVSLITILIVATVSNADKICIGYQSTNSTETVDTLTENNVPVTHAKELLHTEHNGMLCATSLGHPLIL
DTCTIEGLIYGNPSCDPLLGGREWSYIVERPSAVNGLCYPGNVENLEELRSLFSSSRSYQRIQIFPDTIW

NVSYSGTSKACSDSFYRSMRWLTQKNNAYPTQDAQYTNNQGKNILFVWGINHPPTDTAQTNLYTRTDTTT
SVATEEMNRIFKPLIGPRPLVNGLMGRINYYWSVLKPGQTLRIKSDGNLIAPWYGHILSGESHGRILKTD
LKRGSCTVQCQTEKGGLNTTLPFQNVSKYAFGNCSKYIGVKSLKLAVGLRNVPSRSSRGLFGAIAGFIEG
GWSGLVAGWYGFQHSNDQGVGMAADRDSTQKAIDKITSKVNNIVDKMNKQYEIIDHEFSEVETRLNMINN
KVDDQIQDIWAYNAELLVLLENQKTLDEHDANVNNLYNKVKRALGSNAVEDGKGCFELYHKCDDHCMETI
RNGTYNRRKYQEESKLERQKIEGVKLESEETYKILTIYSTVASSLVIAMGFAAFLFWAMSNGSCRCNICI

>QOK11596.1 hemagglutinin [Influenza A virus]
METVSLITILVVATVSNADKICIGYQSTNSTETVDTLTENNVPVTHAKELLHTEHNGMLCATSLGHPLIL
DTCTIEGLIYGNPSCDPLLGGREWSYIVERPSAVNGLCYPGNVENLEELRSLFSSSRSYQRIQIFPDTIW
NVSYSGTSKACSDSFYRSMRWLTQKNNAYPTQDAQYTNNQGKNILFMWGINHPPTDTAQTNLYTRTDTTT
SVATEEMNRIFKPLIGPRPLVNGLMGRINYYWSVLKPGQTLRIKSDGNLIAPWYGHILSGESHGRILKTD
LKRGSCTVQCQTEKGGLNTTLPFQNVSKYAFGNCSKYIGVKSLKLAVGLRNVPSRSSRGLFGAIAGFIEG
GWSGLVAGWYGFQHSNDQGVGMAADRDSTQKAIDKITSKVNNIVDKMNKQYEIIDHEFSEVETRLNMINN
KVDDQIQDIWAYNAELLVLLENQKTLDEHDANVNNLYNKVKRALGSNAVEDGKGCFELYHKCDDHCMETI
RNGTYNRRKYQEESKLERQKIEGVKLESEETYKILTIYSTVASSLVIAMGFAAFLFWAMSNGSCRCNICI

>QOK11584.1 hemagglutinin [Influenza A virus]
METVSLITILIVATVSNADKICIGYQSTNSTETVDTLTENNVPVTHAKELLHTEHNGMLCATSLGHPLIL
DTCTIEGLIYGNPSCDPLLGGREWSYIVERPSAVNGLCYPGNVENLEELRSLFSSSRSYQRIQIFPDTIW
NVSYSGTSKACSDSFYRSMRWLTQKNNAYPTQDAQYTNNQGKNILFVWGINHPPTDTAQTNLYTRTDTTT
SVATEEMNRIFKPLIGPRPLVNGLMGRINYYWSVLKPGQTLRIKSDGNLIAPWYGHILSGESHGRILKTD
LKRGSCTVQCQTEKGGLNTTLPFQNVSKYAFGNCSKYIGVKSLKLAVGLRNVPSRSSRGLFGAIAGFIEG
GWSGLVAGWYGFQHSNDQGVGMAADRDSTQKAIDKITSKVNNIVDKMNKQYEIIDHEFSEVETRLNMINN
KVDDQIQDIWAYNAELLVLLENQKTLDEHDANVNNLYNKVKRALGSNAVEDGKGCFELYHKCDDHCMETI
RNGTYNRRKYQEESKLERQKIEGVKLESEETYKILTIYSTVASSLVIAMGFAAFLFWAMSNGSCRCNICI

>QOK11572.1 hemagglutinin [Influenza A virus]
METVSLITILVVATVSNADKICIGYQSTNSTETVDTLTENNVPVTHAKELLHTEHNGMLCATSLGHPLIL
DTCTIEGLIYGNPSCDPLLGGREWSYIVERPSAVNGLCYPGNVENLEELRSLFSSSRSYQRIQIFPDTIW
NVSYSGTSKACSDSFYRSMRWLTQKNNAYPTQDAQYTNNQGKNILFMWGINHPPTDTAQTNLYTRTDTTT
SVATEEMNRIFKPLIGPRPLVNGLMGRINYYWSVLKPGQTLRIKSDGNLIAPWYGHILSGESHGRILKTD
LKRGSCTVQCQTEKGGLNTTLPFQNVSKYAFGNCSKYIGVKSLKLAVGLRNVPSRSSRGLFGAIAGFIEG
GWSGLVAGWYGFQHSNDQGVGMAADRDSTQKAIDKITSKVNNIVDKMNKQYEIIDHEFSEVETRLNMINN

KVDDQIQDIWAYNAELLVLLENQKTLDEHDANVNNLYNKVKRALGSNAVEDGKGCFELYHKCDDHCMETI
RNGTYNRRKYQEESKLERQKIEGVKLESEETYKILTIYSTVASSLVIAMGFAAFLFWAMSNGSCRCNICI

>QOK11560.1 hemagglutinin [Influenza A virus]
METVSLITILIVATVSNADKICIGYQSTNSTETVDTLTENNVPVTHAKELLHTEHNGMLCATSLGHPLIL
DTCTIEGLIYGNPSCDPLLGGREWSYIVERPSAVNGLCYPGNVENLEELRSLFSSSRSYQRIQIFPDTIW
NVSYSGTSKACSDSFYRSMRWLTQKNNAYPTQDAQYTNNQGKNILFVWGINHPPTDTAQTNLYTRTDTTT
SVATEEMNRIFKPLIGPRPLVNGLMGRINYYWSVLKPGQTLRIKSDGNLIAPWYGHILSGESHGRILKTD
LKRGSCTVQCQTEKGGLNTTLPFQNVSKYAFGNCSKYIGVKSLKLAVGLRNVPSRSSRGLFGAIAGFIEG
GWSGLVAGWYGFQHSNDQGVGMAADRDSTQKAIDKITSKVNNIVDKMNKQYEIIDHEFSEVETRLNMINN
KVDDQIQDIWAYNAELLVLLENQKTLDEHDANVNNLYNKVKRALGSNAVEDGKGCFELYHKCDDHCMETI
RNGTYNRRKYQEESKLERQKIEGVKLESEETYKILTIYSTVASSLVIAMGFAAFLFWAMSNGSCRCNICI

>QOK11548.1 hemagglutinin [Influenza A virus]
METVSLITILVVATVSNADKICIGYQSTNSTETVDTLTENNVPVTHAKELLHTEHNGMLCATSLGHPLIL
DTCTIEGLIYGNPSCDPLLGGREWSYIVERPSAVNGLCYPGNVENLEELRSLFSSSRSYQRIQIFPDTIW
NVSYSGTSKACSDSFYRSMRWLTQKNNAYPTQDAQYTNNQGKNILFMWGINHPPTDTAQTNLYTRTDTTT
SVATEEMNRIFKPLIGPRPLVNGLMGRINYYWSVLKPGQTLRIKSDGNLIAPWYGHILSGESHGRILKTD
LKRGSCTVQCQTEKGGLNTTLPFQNVSKYAFGNCSKYIGVKSLKLAVGLRNVPSRSSRGLFGAIAGFIEG
GWSGLVAGWYGFQHSNDQGVGMAADRDSTQKAIDKITSKVNNIVDKMNKQYEIIDHEFSEVETRLNMINN
KVDDQIQDIWAYNAELLVLLENQKTLDEHDANVNNLYNKVKRALGSNAVEDGKGCFELYHKCDDHCMETI
RNGTYNRRKYQEESKLERQKIEGVKLESEETYKILTIYSTVASSLVIAMGFAAFLFWAMSNGSCRCNICI

>QOK11536.1 hemagglutinin [Influenza A virus]
METVSLITILIVATVSNADKICIGYQSTNSTETVDTLTENNVPVTHAKELLHTEHNGMLCATSLGHPLIL
DTCTIEGLIYGNPSCDPLLGGREWSYIVERPSAVNGLCYPGNVENLEELRSLFSSSRSYQRIQIFPDTIW
NVSYSGTSKACSDSFYRSMRWLTQKNNAYPTQDAQYTNNQGKNILFVWGINHPPTDTAQTNLYTRTDTTT
SVATEEMNRIFKPLIGPRPLVNGLMGRINYYWSVLKPGQTLRIKSDGNLIAPWYGHILSGESHGRILKTD
LKRGSCTVQCQTEKGGLNTTLPFQNVSKYAFGNCSKYIGVKSLKLAVGLRNVPSRSSRGLFGAIAGFIEG
GWSGLVAGWYGFQHSNDQGVGMAADRDSTQKAIDKITSKVNNIVDKMNKQYEIIDHEFSEVETRLNMINN
KVDDQIQDIWAYNAELLVLLENQKTLDEHDANVNNLYNKVKRALGSNAVEDGKGCFELYHKCDDHCMETI
RNGTYNRRKYQEESKLERQKIEGVKLESEETYKILTIYSTVASSLVIAMGFAAFLFWAMSNGSCRCNICI

>QOK11512.1 hemagglutinin [Influenza A virus]

METVSLITILVVATVSNADKICIGYQSTNSTETVDTLTENNVPVTHAKELLHTEHNGMLCATSLGHPLIL
DTCTIEGLIYGNPSCDPLLGGREWSYIVERPSAVNGLCYPGNVENLEELRSLFSSSRSYQRIQIFPDTIW
NVSYSGTSKACSDSFYRSMRWLTQKNNAYPTQDAQYTNNQGKNILFMWGINHPPTDTAQTNLYTRTDTTT
SVATEEMNRIFKPLIGPRPLVNGLMGRINYYWSVLKPGQTLRIKSDGNLIAPWYGHILSGESHGRILKTD
LKRGSCTVQCQTEKGGLNTTLPFQNVSKYAFGNCSKYIGVKSLKLAVGLRNVPSRSSRGLFGAIAGFIEG
GWSGLVAGWYGFQHSNDQGVGMAADRDSTQKAIDKITSKVNNIVDKMNKQYEIIDHEFSEVETRLNMINN
KVDDQIQDIWAYNAELLVLLENQKTLDEHDANVNNLYNKVKRALGSNAVEDGKGCFELYHKCDDHCMETI
RNGTYNRRKYQEESKLERQKIEGVKLESEETYKILTIYSTVASSLVVAMGFAAFLFWAMSNGSCRCNICI

>QOK11500.1 hemagglutinin [Influenza A virus]
METVSLITILVVATVSNADKICIGYQSTNSTETVDTLTENNVPVTHAKELLHTEHNGMLCATSLGHPLIL
DTCTIEGLIYGNPSCDPLLGGREWSYIVERPSAVNGLCYPGNVENLEELRSLFSSSRSYQRIQIFPDTIW
NVSYSGTSKACSDSFYRSMRWLTQKNNAYPTQDAQYTNNQGKNILFMWGINHPPTDTVQTNLYTRTDTTT
SVATEEMNRIFKPLIGPRPLVNGLMGRINYYWSVLKPGQTLRIKSDGNLIAPWYGHILSGESHGRILKTD
LKRGSCTVQCQTEKGGLNTTLPFQNVSKYAFGNCSKYIGVKSLKLAVGLRNVPSRSSRGLFGAIAGFIEG
GWSGLVAGWYGFQHSNDQGVGMAADRDSTQKAIDKITSKVNNIVDKMNKQYEIIDHEFSEVETRLNMINN
KVDDQIQDIWAYNAELLVLLENQKTLDEHDANVNNLYNKVKRALGSNAVEDGKGCFELYHKCDDHCMETI
RNGTYNRRKYQEESKLERQKIEGVKLESEETYKILTIYSTVASSLVIAMGFAAFLFWAMSNGSCRCNICI

>QOK11476.1 hemagglutinin [Influenza A virus]
METVSLITILLVATTSNADKICIGYQSTNSTETVDTLTENNVPVTHAKELLHTEHNGMLCATSLGQPLIL
DTCTVEGLIYGNPSCDLSLEGREWSYIVERPSAVNGLCYPGNVENLEELRSLFSSARSYQRIQIFPDTIW
NVSYDGTSTACSDSFYRSMRWLTRKDGNYPTQDAQYTNNQGKNILFMWGINHPPTDDTQRKLYTRTDTTT
SVATEEINRIFKPLIGPRPLVNGLMGRIDYYWSVLKPGQTLRIKSDGNLIAPWYGHILSGESHGRILKTD
LKRGSCTVQCQTEKGGLNTTLPFQNVSKYAFGNCSKYIGIKSLKLAVGLRNVPSRSSRGLFGAIAGFIEG
GWSGLVAGWYGFQHSNDQGVGMAADRDSTQKAIDKITSKVNNIVDKMNKQYEIIDHEFSEVETRLNMINN
KIDDQIQDIWAYNAELLVLLENQKTLDEHDANVNNLYNKVKRALGSNAVEDGKGCFELYHKCNDQCMETI
RNGTYNRRKYQEESKLERQKIEGVKLESEGTYKILTIYSTVASSLVIAMGFAAFLFWAMSNGSCRCNICI

>QOK11440.1 hemagglutinin [Influenza A virus]
METVSLITILIVATVSNADKICIGYQSTNSTETVDTLTENNVPVTHAKELLHTEHNGMLCATSLGHPLIL
DTCTIEGLIYGNPSCDPLLGGREWSYIVERPSAVNGLCYPGNVENLEELRSLFSSSRSYQRIQIFPDTIW
NVSYSGTSKACSDSFYRSMRWLTQKNNAYPTQDAQYTNNQGKNILFVWGINHPPTDTAQTNLYTRTDTTT
SVATEEMNRIFKPLIGPRPLVNGLMGRINYYWSVLKPGQTLRIKSDGNLIAPWYGHILSGESHGRILKTD

LKRGSCTVQCQTEKGGLNTTLPFQNVSKYAFGNCSKYIGVKSLKLAVGLRNVPSRSSRGLFGAIAGFIEG
GWSGLVAGWYGFQHSNDQGVGMAADRDSTQKAIDKITSKVNNIVDKMNKQYEIIDHEFSEVETRLNMINN
KVDDQIQDIWAYNAELLVLLENQKTLDEHDANVNNLYNKVKRALGSNAVEDGKGCFELYHKCDDHCMETI
RNGTYNRRKYQEESKLERQKIEGVKLESEETYKILTIYSTVASSLVIAMGFAAFLFWAMSNGSCRCNICI

>QOK11392.1 hemagglutinin [Influenza A virus]
METISLITILIVTTVSNADKICIGYQSTNSTETVDTLTENNVPVTHAKELLHTEHNGMLCATSLGQPLIL
ETCTIEGLIYGNPSCDLSLEGREWSYIVERPSAVNGLCYPGNVENLEELRSLFSSARSYQRIQIFPDTIW
NVSYDGTSTACSNSFYRSMRWLTRKNDNYPTQDAQYTNNQGKNILFMWGINHPPTDDTQRNLYTRTDTTT
SVATEEINRIFKPLIGPRPLVNGLMGRIDYYWSVLKPGQTLRIKSDGNLIAPWYGHILSGESHGRILKTD
LKRGSCTVQCQTEKGGLNTTLPFQNVSKYAFGNCSKYIGIKSLKLAVGLRNVPSRSSRGLFGAIAGFIEG
GWSGLVAGWYGFQHSNDQGVGMAADRDSTQKAIDKITSKVNNIVDKMNKQYEIIDHEFSEVETRLNMINN
KIDDQIQDIWAYNAELLVLLENQKTLDEHDANVNNLYNKVKRALGSNAVEDGKGCFELYHKCNDQCMETI
RNGTYNRKKYQEESKLERQKIEGVKLESEGTYKILTIYSTVASSLVIAMGFAAFLFWAMSNGSCRCNICI

>QOK11369.1 hemagglutinin [Influenza A virus]
METISLITILIVTTVSNADKICIGYQSTNSTETVDTLTENNVPVTHAKELLHTEHNGMLCATSLGQPLIL
ETCTIEGLIYGNPSCDLSLEGREWSYIVERPSAVNGLCYPGNVENLEELRSLFSSARSYQRIQIFPDTIW
NVSYDGTSTACSNSFYRSMRWLTRKNDNYPTQDAQYTNNQGKNILFMWGINHPPTDDTQRNLYTRTDTTT
SVATEEINRIFKPLIGPRPLVNGLMGRIDYYWSVLKPGQTLRIKSDGNLIAPWYGHILSGESHGRILKTD
LKRGSCTVQCQTEKGGLNTTLPFQNVSKYAFGNCSKYIGIKSLKLAVGLRNVPSRSSRGLFGAIAGFIEG
GWSGLVAGWYGFQHSNDQGVGMAADRDSTQKAIDKITSKVNNIVDKMNKQYEIIDHEFSEVETRLNMINN
KIDDQIQDIWAYNAELLVLLENQKTLDEHDANVNNLYNKVKRALGSNAVEDGKGCFELYHKCNDQCMETI
RNGTYNRKKYQEESKLERQKIEGVKLESEGTYKILTIYSTVASSLVIAMGFAAFLFWAMSNGSCRCNICI

>QOK11357.1 hemagglutinin [Influenza A virus]
METVSLITILIVTTVSNADKICIGYQSTNSTETVDTLTENNVPVTHAKELLHTEHNGMLCATSLGQPLIL
ETCTIEGLIYGNPSCDLSLEGREWSYIVERPSAVNGLCYPGNVENLEELRSLFSSARSYQRIQIFPDTIW
NVSYDGTSTACSNSFYRSMRWLTRKNGNYPTQDAQYTNNQGKNILFMWGINHPPTDDTQRNLYTRTDTTT
SVATEEINRIFKPLIGPRPLVNGLMGRIDYYWSVLKPGQTLRIKSDGNLIAPWYGHILSGESHGRILKTD
LKRGSCTVQCQTEKGGLNTTLPFQNVSKYAFGNCSKYIGIKSLKLAVGLRNVPSRSSRGLFGAIAGFIEG
GWSGLVAGWYGFQHSNDQGVGMAADRDSTQKAIDKITSKVNNIVDKMNKQYEIIDHEFSEVETRLNMINN
KIDDQIQDIWAYNAELLVLLENQKTLDEHDANVNNLYNKVKRALGSNAVEDGKGCFELYHKCNDQCMETI
RNGTYNRKKYQEESKLERQKIEGVKLESEGTYKILTIYSTVASSLVIAMGFAAFLFWAMSNGSCRCNICI

>QOK11346.1 hemagglutinin [Influenza A virus]

METISLITILIVTTVSNADKICIGYQSTNSTETVDTLTENNVPVTHAKELLHTEHNGMLCATSLGQPLIL

ETCTIEGLIYGNPSCDLSLEGREWSYIVERPSAVNGLCYPGNVENLEELRSLFSSARSYQRIQIFPDTIW

NVSYDGTSTACSNSFYRSMRWLTRKNDNYPTQDAQYTNNQGKNILFMWGINHPPTDDTQRNLYTRTDTTT

SVATEEINRIFKPLIGPRPLVNGLMGRIDYYWSVLKPGQTLRIKSDGNLIAPWYGHILSGESHGRILKTD

LKRGSCTVQCQTEKGGLNTTLPFQNVSKYAFGNCSKYIGIKSLKLAVGLRNVPSRSSRGLFGAIAGFIEG

GWSGLVAGWYGFQHSNDQGVGMAADRDSTQKAIDKITSKVNNIVDKMNKQYEIIDHEFSEVETRLNMINN

KIDDQIQDIWAYNAELLVLLENQKTLDEHDANVNNLYNKVKRALGSNAVEDGKGCFELYHKCNDQCMETI

RNGTYNRKKYQEESKLERQKIEGVKLESEGTYKILTIYSTVASSLVIAMGFAAFLFWAMSNGSCRCNICI

>QOK11335.1 hemagglutinin [Influenza A virus]

METISLMTILLVATVSNADKICIGYQSTNSTETVDTLTENNVPVTHAKELLHTEHNGMLCATSLGNPLIL

DTCTIEGLIYGNPSCDPLLGGREWSYIVERPSAVNGLCYPGSVENLEELRSLFSSARSYQRIQIFPDTIW

NVSYSGTSNACSDSFYRSMRWLTQKNNNYPIQDAQYTNNQEKNILFMWGINHPPTETTQTNLYTRTDTTT

SVATEEINRIFKPLIGPRPLVNGLMGRINYYWSVLKPGQTLRIKSNGNLIAPWYGHILSGESHGRILKTD

LKSGSCTVQCQTEKGGLNTTLPFQNVSKYAFGNCSKYIGIKSLKLAVGLRNVPSRSSRGLFGAIAGFIEG

GWSGLVAGWYGFQHSNDQGVGMAADRDSTQKAIDKITSKVNNIIDKVNKQYEIIDHEFSEVETRLNMINR

KIDDQIQDIWAYNAELLVLLENQKTLDEHDSNVNNLYNKVKRALGSNAVEDGKGCFELYHKCDNQCMETI

RNGTYNRRKYQEESKLERQKIEGVKLESEGTYKILTIYSTVASSLVIAMGFAAFLFWAMSNGSCRCNICI

>QOK11323.1 hemagglutinin [Influenza A virus]

METVSLITILLVATTSNADKICIGYQSTNSTETVDTLTENNVPVTHAKELLHTEHNGMLCATSLGQPLIL

DTCTVEGLIYGNPSCDLSLEGREWSYIVERPSAVNGLCYPGNVENLEELRSLFSSARSYQRIQIFPDTIW

NVSYDGTSTACSDSFYRSMRWLTRKDGNYPTQDAQYTNNQGKNILFMWGINHPPTDDTQRKLYTRTDTTT

SVATEEINRIFKPLIGPRPLVNGLMGRIDYYWSVLKPGQTLRIKSDGNLIAPWYGHILSGESHGRILKTD

LKRGSCTVQCQTEKGGLNTTLPFQNVSKYAFGNCSKYIGIKSLKLAVGLRNVPSRSSRGLFGAIAGFIEG

GWSGLVAGWYGFQHSNDQGVGMAADRDSTQKAIDKITSKVNNIVDKMNKQYEIIDHEFSEVETRLNMINN

KIDDQIQDIWAYNAELLVLLENQKTLDEHDANVNNLYNKVKRALGSNAVEDGKGCFELYHKCNDQCMETI

RNGTYNRRKYQEESKLERQKIEGVKLESEGTYKILTIYSTVASSLVIAMGFAAFLFWAMSNGSCRCNICI

>QOK11311.1 hemagglutinin [Influenza A virus]

METISLITILIVTTVSNADKICIGYQSTNSTETVDTLTENNVPVTHAKELLHTEHNGMLCATSLGQPLIL

ETCTIEGLIYGNPSCDLSLEGREWSYIVERPSAVNGLCYPGNVENLEELRSLFSSARSYQRIQIFPDTIW

NVSYDGTSTACSNSFYRSMRWLTRKNDNYPTQDAQYTNNQGKNILFMWGINHPPTDDTQRNLYTRTDTTT
SVATEEINRIFKPLIGPRPLVNGLMGRIDYYWSVLKPGQTLRIKSDGNLIAPWYGHILSGESHGRILKTD
LKRGSCTVQCQTEKGGLNTTLPFQNVSKYAFGNCSKYIGIKSLKLAVGLRNVPSRSSRGLFGAIAGFIEG
GWSGLVAGWYGFQHSNDQGVGMAADRDSTQKAIDKITSKVNNIVDKMNKQYEIIDHEFSEVETRLNMINN
KIDDQIQDIWAYNAELLVLLENQKTLDEHDANVNNLYNKVKRALGSNAVEDGKGCFELYHKCNDQCMETI
RNGTYNRKKYQEESKLERQKIEGVKLESEGTYKILTIYSTVASSLVIAMGFAAFLFWAMSNGSCRCNICI

>QOK11288.1 hemagglutinin [Influenza A virus]
METISLMTILLVATVSNADKICIGYQSTNSTETVDTLTENNVPVTHAKELLHTEHNGMLCATSLGNPLIL
DTCTIEGLIYGNPSCDPLLGGREWSYIVERPSAVNGLCYPGSVENLEELRSLFSSARSYQRIQIFPDTIW
NVSYSGTSNACSDSFYRSMRWLTQKNNNYPIQDAQYTNNQEKNILFMWGINHPPTETTQTNLYTRTDTTT
SVATEEINRIFKPLIGPRPLVNGLMGRINYYWSVLKPGQTLRIKSNGNLIAPWYGHILSGESHGRILKTD
LKSGSCTVQCQTEKGGLNTTLPFQNVSKYAFGNCSKYIGIKSLKLAVGLRNVPSRSSRGLFGAIAGFIEG
GWSGLVAGWYGFQHSNDQGVGMAADRDSTQKAIDKITSKVNNIIDKVNKQYEIIDHEFSEVETRLNMINR
KIDDQIQDIWAYNAELLVLLENQKTLDEHDSNVNNLYNKVKRALGSNAVEDGKGCFELYHKCDNQCMETI
RNGTYNRRKYQEESKLERQKIEGVKLESEGTYKILTIYSTVASSLVIAMGFAAFLFWAMSNGSCRCNICI

>QOK11228.1 hemagglutinin [Influenza A virus]
METISLMTILLVATVSNADKICIGYQSTNSTETVDTLTENNVPVTHAKELLHTEHNGMLCATSLGNPLIL
DTCTIEGLIYGNPSCDPLLGGREWSYIVERPSAVNGLCYPGSVENLEELRSLFSSARSYQRIQIFPDTIW
NVSYSGTSNACSDSFYRSMRWLTQKNNNYPIQDAQYTNNQEKNILFMWGINHPPTETTQTNLYTRTDTTT
SVATEEINRIFKPLIGPRPLVNGLMGRINYYWSVLKPGQTLRIKSNGNLIAPWYGHILSGESHGRILKTD
LKSGSCTVQCQTEKGGLNTTLPFQNVSKYAFGNCSKYIGIKSLKLAVGLRNVPSRSSRGLFGAIAGFIEG
GWSGLVAGWYGFQHSNDQGVGMAADRDSTQKAIDKITSKVNNIIDKVNKQYEIIDHEFSEVETRLNMINR
KIDDQIQDIWAYNAELLVLLENQKTLDEHDSNVNNLYNKVKRALGSNAVEDGKGCFELYHKCDNQCMETI
RNGTYNRRKYQEESKLERQKIEGVKLESEGTYKILTIYSTVASSLVIAMGFAAFLFWAMSNGSCRCNICI

>QOK11216.1 hemagglutinin [Influenza A virus]
METVSLITILIVATVSNADKICIGYQSTNSTETVDTLTENNVPVTHAKELLHTEHNGMLCATSLGHPLIL
DTCTIEGLIYGNPSCDPLLGGREWSYIVERPSAVNGLCYPGNVENLEELRSLFSSSRSYQRIQIFPDTIW
NVSYSGTSKACSDSFYRSMRWLTQKNNAYPTQDAQYTNNQGKNILFVWGINHPPTDTAQTNLYTRTDTTT
SVATEEMNRIFKPLIGPRPLVNGLMGRINYYWSVLKPGQTLRIKSDGNLIAPWYGHILSGESHGRILKTD
LKRGSCTVQCQTEKGGLNTTLPFQNVSKYAFGNCSKYIGVKSLKLAVGLRNVPSRSSRGLFGAIAGFIEG
GWSGLVAGWYGFQHSNDQGVGMAADRDSTQKAIDKITSKVNNIVDKMNKQYEIIDHEFSEVETRLNMINN

KVDDQIQDIWAYNAELLVLLENQKTLDEHDANVNNLYNKVKRALGSNAVEDGKGCFELYHKCDDHCMETI
RNGTYNRRKYQEESKLERQKIEGVKLESEETYKILTIYSTVASSLVIAMGFAAFLFWAMSNGSCRCNICI

>QOK11146.1 hemagglutinin [Influenza A virus]
METISLITILLAATVSNADKICIGYQSTNSTETVDTLTENNVPVTHAKELIHTEHNGMLCATSLGQPLIL
DTCTIEGLIYGNPSCDLSLEGREWSYIVERPSAVNGLCYPGNVENLEELRSLFSSARSYQRIQIFPDTIW
NVSYDGTSTACSGSFYRNMRWLTRKDGNYPTQDAQYTNNQGKNILFMWGINHPPTDTTQSGLYTRIETTT
SVATEEINRIFKPLIGPRPLVNGLMGRIDYYWSVLKPGQTLRIKSDGNLIAPWFGHILSGESHGRILKTD
LKRGSCTVQCQTEKGGLNTTLPFQNVSKYAFGNCSKYIGIKSLKLAVGLRNVPSRSNRGLFGAIAGFIEG
GWSGLVAGWYGFQHSNDQGVGMAADRDSTQKAIDKITSKVNNIVDKMNKQYEIIDHEFSEVETRLNMINN
KIDDQIQDIWAYNAELLVLLENQKTLDEHDANVNNLYNKVKRALGSNAVEDGKGCFELYHKCNDQCMETI
RNGTYNRRKYQEESKLERQKIEGVKLESEGTYKILTIYSTVASSLVIAMGFAAFLFWAMSNGSCRCNICI

>QOK11134.1 hemagglutinin [Influenza A virus]
METISLITILLAATVSNADKICIGYQSTNSTETVDTLTENNVPVTHAKELIHTEHNGMLCATSLGQPLIL
DTCTIEGLIYGNPSCDLSLEGREWSYIVERPSAVNGLCYPGNVENLEELRSLFSSARSYQRIQIFPDTIW
NVSYDGTSTACSGSFYRNMRWLTRKDGNYPTQDAQYTNNQGKNILFMWGINHPPTDTTQSGLYTRIETTT
SVATEEINRIFKPLIGPRPLVNGLMGRIDYYWSVLKPGQTLRIKSDGNLIAPWFGHILSGESHGRILKTD
LKRGSCTVQCQTEKGGLNTTLPFQNVSKYAFGNCSKYIGIKSLKLAVGLRNVPSRSNRGLFGAIAGFIEG
GWSGLVAGWYGFQHSNDQGVGMAADRDSTQKAIDKITSKVNNIVDKMNKQYEIIDHEFSEVETRLNMINN
KIDDQIQDIWAYNAELLVLLENQKTLDEHDANVNNLYNKVKRALGSNAVEDGKGCFELYHKCDDQCMETI
RNGTYNRRKYQEESKLERQKIEGVKLESEGTYKILTIYSTVASSLVIAMGFAAFLFWAMSNGSCRCNICI

>QOK11122.1 hemagglutinin [Influenza A virus]
METISLITILLAATVSNADKICIGYQSTNSTETVDTLTENNVPVTHAKELIHTEHNGMLCATSLGQPLIL
DTCTIEGLIYGNPSCDLSLEGREWSYIVERPSAVNGLCYPGNVENLEELRSLFSSARSYQRIQIFPDTIW
NVSYDGTSTACSGSFYRNMRWLTRKDGNYPTQDAQYTNNQGKNILFMWGINHPPTDTTQSGLYTRIETTT
SVATEEINRIFKPLIGPRPLVNGLMGRIDYYWSVLKPGQTLRIKSDGNLIAPWFGHILSGESHGRILKTD
LKRGSCTVQCQTEKGGLNTTLPFQNVSKYAFGNCSKYIGIKSLKLAVGLRNVPSRSNRGLFGAIAGFIEG
GWSGLVAGWYGFQHSNDQGVGMAADRDSTQKAIDKITSKVNNIVDKMNKQYEIIDHEFSEVETRLNMINN
KIDDQIQDIWAYNAELLVLLENQKTLDEHDANVNNLYNKVKRALGSNAVEDGKGCFELYHKCDDQCMETI
RNGTYNRRKYQEESKLERQKIEGVKLESEGTYKILTIYSTVASSLVIAMGFAAFLFWAMSNGSCRCNICI

>QOK11062.1 hemagglutinin [Influenza A virus]

METVSLITILIVATVSNADKICIGYQSTNSTETVDTLTENNVPVTHAKELLHTEHNGMLCATSLGQPLIL
DTCTIEGLIYGNPSCDLSLEGREWSYIVERPSAINGLCYPGNVENLEELRSLFSSARSYQRVQIFPDTIW
NVSYDGTSTACSNSFYRSMRWLTRKDGNYPTQDAQYTNNQGKNILFMWGINHPPTDDTQRNLYTRTDTTT
SVATEEINRIFKPLIGPRPLVNGLMGRIDYYWSILKPGQTLRIKSDGNLIAPWYGHILSGESHGRILKTD
LKRGSCTVQCQTEKGGLNTTLPFQNVSKYAFGNCSKYIGIKSLKLAVGLRNVPSRSSRGLFGAIAGFIEG
GWSGLVAGWYGFQHSNDQGVGMAADRDSTQKAIDKITSKVNNIVDKMNKQYEIIDHEFSAVETRLNMINN
KIDDQIQDIWAYNAELLVLLENQKTLDEHDANVNNLYNKVKRALGSNAVEDGKGCFELYHKCNDQCMETI
RNGTYNRRKYQEESKLERQKIEGVKLESEGTYKILTIYSTVASSLVIAMGFAAFLFWAMSNGSCRCNICI

>QOK11038.1 hemagglutinin [Influenza A virus]
METVSLITILIVATVSNADKICIGYQSTNSTETVDTLTENNVPVTHAKELLHTEHNGMLCATGLGQPLIL
DTCTIEGLIYGNPSCDLSLEGREWSYIVERPSAINGLCYPGNVENLEELRSLFSSARSYQRIQIFPDTIW
NVSYDGTSTACSNSFYRSMRWLTRKDGNYPTQDAQYTNNQGKNILFMWGINHPPTDDTQRNLYTRTDTTT
SVATEEINRIFKPLIGPRPLVNGLMGRIDYYWSILKPGQTLRIKSDGNLIAPWYGHILSGESHGRILKTD
LKRGSCTVQCQTEKGGLNTTLPFQNVSKYAFGNCSKYIGIKSLKLAVGLRNVPSRSSRGLFGAIAGFIEG
GWSGLVAGWYGFQHSNDQGVGMAADRDSTQKAIDKITSKVNNIVDKMNKQYEIIDHEFSEVETRLNMINN
KIDDQIQDIWAYNAELLVLLENQKTLDEHDANVNNLYNKVKRALGSNAVEDGKGCFELYHKCNDQCMETI
RNGTYNRRKYQEESKLERQKIEGVKLESEGTYKILTIYSTVASSLVIAMGFAAFLFWAMSNGSCRCNICI

>QOK11026.1 hemagglutinin [Influenza A virus]
METVSLITILIVATVSNADKICIGYQSTNSTETVDTLTENNVPVTHAKELLHTEHNGMLCATSLGQPLIL
DTCTIEGLIYGNPSCDLSLEGREWSYIVERPSAVNGLCYPGNVENLEELRSLFSSARSYQRIQIFPDTIW
NVSYDGTSTACSNSFYKSMRWLTRKNGDYPTQDAQYTNNQGKNILFMWGINHPPTDDTQRNLYTRTDTTT
SVATEEINRIFKPLIGPRPLVNGLMGRIDYYWSVLKPGQTLRIKSDGNLIAPWYGHILSGESHGRILKTD
LKRGSCTVQCQTEKGGLNTTLPFQNVSKYAFGNCSKYIGIKSLKLAVGLRNVPSRSSRGLFGAIAGFIEG
GWSGLVAGWYGFQHSNDQGVGMAADRDSTQKAIDKITSKVNNIVDKMNKQYEIIDHEFSEVETRLNMINN
KIDDQIQDIWAYNAELLVLLENQKTLDEHDANVNNLYNKVKRALGSNAVEDGKGCFELYHKCNDQCMETI
RNGTYNRRKYQEESKLERQKIEGVKLESEGTYKILTIYSTVASSLVIAMGFAAFLFWAMSNGSCRCNICI

>QOK11014.1 hemagglutinin [Influenza A virus]
METVSLITILIVATVSNADKICIGYQSTNSTETVDTLTENNVPVTHAKELLHTEHNGMLCATSLGQPLIL
DTCTIEGLIYGNPSCDLSLEGREWSYIVERPSAINGLCYPGNVENLEELRSLFSSARSYQRIQIFPDTIW
NVSYDGTSTACSNSFYRSMRWLTRKDGNYPTQDAQYTNNQGKNILFMWGINHPPTDDTQRNLYTRTDTTT
SVATEEINRIFKPLIGPRPLVNGLMGRIDYYWSILKPGQTLRIKSDGNLIAPWYGHILSGESHGRILKTD

LKRGSCTVQCQTEKGGLNTTLPFQNVSKYAFGNCSKYIGIKSLKLAVGLRNVPSRSSRGLFGAIAGFIEG
GWSGLVAGWYGFQHSNDQGVGMAADRDSTQKAIDKITSKVNNIVDKMNKQYEIIDHEFSEVETRLNMINN
KIDDQIQDIWAYNAELLVLLENQKTLDEHDANVNNLYNKVKRALGSNAVEDGKGCFELYHKCNDQCMETI
RNGTYNRRKYQEESKLERQKIEGVKLESEGTYKILTIYSTVASSLVIAMGFAAFLFWAMSNGSCRCNICI

>QOK10954.1 hemagglutinin [Influenza A virus]
METVSLITILLVATVSSADKICIGYQSTNSTETVDTLTENNVPVTHAKELLHTEHNGMLCATSLGQPLIL
DTCTIEGLIYGNPSCDLSLEGREWSYIVERPSAVNGLCYPGNVENLEELRSLFSSARSYQRIQIFPDTIW
NVSYDGTSTACSGSFYGSMRWLTQKNGNYPTQDAQYTNNQGKNILFMWGINHPPTDDTQRNLYTRTDTTT
SVATEEMNRIFKPLIGPRPLVNGLMGRINYYWSVLKPGQTLRIKSDGNLIAPWYGHILSGESHGRILKTD
LKRGSCTVQCQTEKGGLNTTLPFQNVSKYAFGNCSKYIGIKSLKLAVGLRNVPSRSSRGLFGAIAGFIEG
GWSGLVAGWYGFQHSNDQGVGMAADRDSTQKAIDKITSKVNNIVDKMNKQYEIIDHEFSEVETRLNMINN
KIDDQIQDIWAYNAELLVLLENQKTLDEHDANVNNLYNKVKRALGSNAVEDGKGCFELYHKCNDQCMETI
RNGTYNRRKYQEESKLERQKIEGVKLESEGTYKILTIYSTVASSLVIAMGFAAFLFWAMSNGSCRCNICI

>QOK10942.1 hemagglutinin [Influenza A virus]
METVSLITILIVATVSNADKICIGYQSTNSTETVDTLTENNVPVTHAKELLHTEHNGMLCATSLGQPLIL
DTCTIEGLIYGNPSCDLSLEGREWSYIVERPSAINGLCYPGNVENLEELRSLFSSARSYQRIQIFPDTIW
NVSYDGTSTACSNSFYRSMRWLTRKDGNYPTQDAQYTNNQGKNILFMWGINHPPTDDTQRNLYTRTDTTT
SVATEEINRIFKPLIGPRPLVNGLMGRIDYYWSVLKPGQTLRIKSDGNLIAPWYGHILSGESHGRILKTD
LKRGSCTVQCQTEKGGLNTTLPFQNVSKYAFGNCSKYIGIKSLKLAVGLRNVPSRSSRGLFGAIAGFIEG
GWSGLVAGWYGFQHSNDQGVGMAADRDSTQKAIDKITSKVNNIVDKMNKQYEIIDHEFSEVETRLNMINN
KIDDQIQDIWAYNAELLVLLENQKTLDEHDANVNNLYNKVKRALGSNAVEDGKGCFELYHKCNDQCMETI
RNGTYNRRKYQEESKLERQKIEGVKLESEGTYKILTIYSTVASSLVIAMGFAAFLFWAMSNGSCRCNICI

>QOK10906.1 hemagglutinin [Influenza A virus]
METVSLITILLAATVSNADKICIGYQSTNSTETVDTLTENNVPVTHAKELLHTEHNGMLCATSLGQPLIL
DTCTIEGLIYGNPSCDPLLEEREWSYIVERPSAVNGLCYPGNVENLEELRSFFSSARSYQRIQIFPDTIW
NVSYDGTSNTCSGSFYRNMRWLTRKNGNYPIQDALYTNNQGKSILFMWGINHPPTDDTQRNLYTRTDTTT
SVATEEINRIFKPLIGPRPLVNGLMGRINYYWSVLKPGQTLRIKSDGNLIAPWYGYILSGESHGRILRTD
LTKGRCTVQCQTEKGGLNTTLPFQNVSKYAFGNCSKYIGIKSLKLAVGLRNVPSRSSRGLFGAIAGFIEG
GWSGLVAGWYGFQHSNDQGVGMAADRESTQKAIDKITSKVNNIVDKMNKQYEIIDHEFSEVETRLNMINN
KIDDQIQDIWAYNAELLVLLENQKTLDEHDANVNNLYNKVKRALGSNAMEDGKGCFELYHKCDDQCMETI
RNGTYNRRKYQEESKLERQKIEGVKLESEGTYKILTIYSTVASSLVIAMGFAAFLFWAMSNGSCRCNICI

>QOK10894.1 hemagglutinin [Influenza A virus]

METVSLITILLAATVSNADKICIGYQSTNSTETVDTLTENNVPVTHAKELLHTEHNGMLCATSLGQPLIL
DTCTIEGLIYGNPSCDPLLEEREWSYIVERPSAVNGLCYPGNVENLEELRSFFSSARSYQRIQIFPDTIW
NVSYDGTSNTCSGSFYRNMRWLTRKNGNYPIQDAQYTNNQGKSILFMWGINHPPTDDTQRNLYTRTDTTT
SVATEEINRIFKPLIGPRPLVNGLMGRINYYWSVLKPGQTLRIKSDGNLIAPWYGYILSGESHGRILRTD
LTKGSCTVQCQTEKGGLNTTLPFQNVSKYAFGNCSKYIGIKSLKLAVGLRNVPSRSSRGLFGAIAGFIEG
GWSGLVAGWYGFQHSNDQGVGMAADRESTQKAIDKITSKVNNIVDKMNKQYEIIDHEFSEVETRLNMINN
KIDDQIQDIWAYNAELLVLLENQKTLDEHDANVNNLYNKVKRALGSNAVEDGKGCFELYHKCDDQCMETI
RNGTYNRRKYQEESKLERQKIEGVKLESEGTYKILTIYSTVASSLVIAMGFAAFLFWAMSNGSCRCNICI

>QOK10882.1 hemagglutinin [Influenza A virus]

METVSLITILLAATVSNADKICIGYQSTNSTETVDTLTENNVPVTHAKELLHTEHNGMLCATSLGQPLIL
DTCTIEGLIYGNPSCDPLLEEREWSYIVERPSAVNGLCYPGNVENLEELRSFFSSARSYQRIQIFPDTIW
NVSYDGTSNTCSGSFYRNMRWLTRKNGNYPIQDAQYTNNQGKSILFMWGINHPPTDDTQRNLYTRTDTTT
SVATEEINRIFKPLIGPRPLVNGLMGRINYYWSVLKPGQTLRIKSDGNLIAPWYGYILSGESHGRILRTD
LTKGSCTVQCQTEKGGLNTTLPFQNVSKYAFGNCSKYIGIKSLKLAVGLRNVPSRSSRGLFGAIAGFIEG
GWSGLVAGWYGFQHSNDQGVGMAADRESTQKAIDKITSKVNNIVDKMNKQYEIIDHEFSEVETRLNMINN
KIDDQIQDIWAYNAELLVLLENQKTLDEHDANVNNLYNKVKRALGSNAVEDGKGCFELYHKCDDQCMETI
RNGTYNRRKYQEESKLERQKIEGVKLESEGTYKILTIYSTVASSLVIAMGFAAFLFWAMSNGSCRCNICI

>QOK10870.1 hemagglutinin [Influenza A virus]

METVSLITILLAATVSNADKICIGYQSTNSTETVDTLTENNVPVTHAKELLHTEHNGMLCATSLGQPLIL
DTCTIEGLIYGNPSCDPLLEEREWSYIVERPSAVNGLCYPGNVENLEELRSFFSSARSYQRIQIFPDTIW
NVSYDGTSNTCSGSFYRNMRWLTRKNGNYPIQDAQYTNNQGKSILFMWGINHPPTDDTQRNLYTRTDTTT
SVATEEINRIFKPLIGPRPLVNGLMGRINYYWSVLKPGQTLRIKSDGNLIAPWYGYILSGESHGRILRTD
LTKGSCTVQCQTEKGGLNTTLPFQNVSKYAFGNCSKYIGIKSLKLAVGLRNVPSRSSRGLFGAIAGFIEG
GWSGLVAGWYGFQHSNDQGVGMAADRESTQKAIDKITSKVNNIVDKMNKQYEIIDHEFSEVETRLNMINN
KIDDQIQDIWAYNAELLVLLENQKTLDEHDANVNNLYNKVKRALGSNAVEDGKGCFELYHKCDDQCMETI
RNGTYNRRKYQEESKLERQKIEGVKLESEGTYKILTIYSTVASSLVIAMGFAAFLFWAMSNGSCRCNICI

>QOK10846.1 hemagglutinin [Influenza A virus]

METVSLITILLAATVSNADKICIGYQSTNSTETVDTLTENNVPVTHAKELLHTEHNGMLCATSLGQPLIL
DTCTIEGLIYGNPSCDPLLEEREWSYIVERPSAVNGLCYPGNVENLEELRSFFSSARSYQRIQIFPDTIW

NVSYDGTSNTCSGSFYRNMRWLTRKNGNYPIQDAQYTNNQGKSILFMWGINHPPTDDTQRNLYTRTDTTT
SVATEEINRIFKPLIGPRPLVNGLMGRINYYWSVLKPGQTLRIKSDGNLIAPWYGYILSGESHGRILRTD
LTKGSCTVQCQTEKGGLNTTLPFQNVSKYAFGNCSKYIGIKSLKLAVGLRNVPSRSSRGLFGAIAGFIEG
GWSGLVAGWYGFQHSNDQGVGMAADRESTQKAIDKITSKVNNIVDKMNKQYEIIDHEFSEVETRLNMINN
KIDDQIQDIWAYNAELLVLLENQKTLDEHDANVNNLYNKVKRALGSNAVEDGKGCFELYHKCDDQCMETI
RNGTYNRRKYQEESKLERQKIEGVKLESEGTYKILTIYSTVASSLVIAMGFAAFLFWAMSNGSCRCNICI

>QOK10834.1 hemagglutinin [Influenza A virus]
METVSLITILLAATVSNADKICIGYQSTNSTETVDTLTENNVPVTHAKELLHTEHNGMLCATSLGQPLIL
DTCTIEGLIYGNPSCDPLLEEREWSYIVERPSAVNGLCYPGNVENLEELRSFFSSARSYQRIQIFPDTIW
NVSYDGTSNTCSGSFYRNMRWLTRKNGNYPIQDAQYTNNQGKSILFMWGINHPPTDDTQRNLYTRTDTTT
SVATEEINRIFKPLIGPRPLVNGLMGRINYYWSVLKPGQTLRIKSDGNLIAPWYGYILSGESHGRILRTD
LTKGSCTVQCQTEKGGLNTTLPFQNVSKYAFGNCSKYIGIKSLKLAVGLRNVPSRSSRGLFGAIAGFIEG
GWSGLVAGWYGFQHSNDQGVGMAADRESTQKAIDKITSKVNNIVDKMNKQYEIIDHEFSEVETRLNMINN
KIDDQIQDIWAYNAELLVLLENQKTLDEHDANVNNLYNKVKRALGSNAVEDGKGCFELYHKCDDQCMETI
RNGTYNRRKYQEESKLERQKIEGVKLESEGTYKILTIYSTVASSLVIAMGFAAFLFWAMSNGSCRCNICI

>QOK10822.1 hemagglutinin [Influenza A virus]
METVSLITILLAATVSNADKICIGYQSTNSTETVDTLTENNVPVTHAKELLHTEHNGMLCATSLGQPLIL
DTCTIEGLIYGNPSCDPLLEEREWSYIVERPSAVNGLCYPGNVENLEELRSFFSSARSYQRIQIFPDTIW
NVSYDGTSNTCSGSFYRNMRWLTRKNGNYPIQDAQYTNNQGKSILFMWGINHPPTDDTQRNLYTRTDTTT
SVATEEINRIFKPLIGPRPLVNGLMGRINYYWSVLKPGQTLRIKSDGNLIAPWYGYILSGESHGRILRTD
LTKGSCTVQCQTEKGGLNTTLPFQNVSKYAFGNCSKYIGIKSLKLAVGLRNVPSRSSRGLFGAIAGFIEG
GWSGLVAGWYGFQHSNDQGVGMAADRESTQKAIDKITSKVNNIVDKMNKQYEIIDHEFSEVETRLNMINN
KIDDQIQDIWAYNAELLVLLENQKTLDEHDANVNNLYNKVKRALGSNAVEDGKGCFELYHKCDDQCMETI
RNGTYNRRKYQEESKLERQKIEGVKLESEGTYKILTIYSTVASSLVIAMGFAAFLFWAMSNGSCRCNICI

>QOK10810.1 hemagglutinin [Influenza A virus]
METVSLITILLAATVSNADKICIGYQSTNSTETVDTLTENNVPVTHAKELLHTEHNGMLCATSLGQPLIL
DTCTIEGLIYGNPSCDPLLEEREWSYIVERPSAVNGLCYPGNVENLEELRSFFSSARSYQRIQIFPDTIW
NVSYDGTSNTCSGSFYRNMRWLTRKDGNYPIQDAQYTNNQGKSILFMWGINHPPTDDTQRNLYTRTDTTT
SVATEEINRIFKPLIGPRPLVNGLMGRINYYWSVLKPGQTLRIKSDGNLIAPWYGYILSGESHGRILRTD
LTKGSCTVQCQTEKGGLNTTLPFQNVSKYAFGNCSKYIGIKSLKLAVGLRNVPSRSSRGLFGAIAGFIEG
GWSGLVAGWYGFQHSNDQGVGMAADRESTQKAIDKITSKVNNIVDKMNKQYEIIDHEFSEVETRLNMINN

KIDDQIQDIWAYNAELLVLLENQKTLDEHDANVNNLYNKVKRALGSNAVEDGKGCFELYHKCDDQCMETI
RNGTYNRRKYQEESKLERQKIEGVKLESEGTYKILTIYSTVASSLVIAMGFAAFLFWAMSNGSCRCNICI

>QOK10798.1 hemagglutinin [Influenza A virus]
METVSLITILLAATVSNADKICIGYQSTNSTETVDTLTENNVPVTHAKELLHTEHNGMLCATSLGQPLIL
DTCTIEGLIYGNPSCDPLLEEREWSYIVERPSAVNGLCYPGNVENLEELRSFFSSARSYQRIQIFPDTIW
NVSYDGTSNTCSGSFYRNMRWLTRKNGNYPIQDAQYTNNQGKSILFMWGINHPPTDDTQRNLYTRTDTTT
SVATEEINRIFKPLIGPRPLVNGLMGRINYYWSVLKPGQTLRIKSDGNLIAPWYGYILSGESHGRILRTD
LTKGSCTVQCQTEKGGLNTTLPFQNVSKYAFGNCSKYIGIKSLKLAVGLRNVPSRSSRGLFGAKAGFIEG
GWSGLVAGWYGFQHSNDQGVGMAADRESTQKAIDKITSKVNNIVDKMNKQYEIIDHEFSEVETRLNMINN
KIDDQIQDIWAYNAELLVLLENQKTLDEHDANVNNLYNKVKRALGSNAVEDGKGCFELYHKCDDQCMETI
RNGTYNRRKYQEESKLERQKIEGVKLESEGTYKILTIYSTVASSLVIAMGFAAFLFWAMSNGSCRCNICI

>QOK10786.1 hemagglutinin [Influenza A virus]
METVSLITILLAATVSNADKICIGYQSTNSTETVDTLTENNVPVTHAKELLHTEHNGMLCATSLGQPLIL
DTCTIEGLIYGNPSCDPLLEEREWSYIVERPSAVNGLCYPGNVENLEELRSFFSSARSYQRIQIFPDTIW
NVSYDGTSNTCSGSFYRNMRWLTRKNGNYPIQDAQYTNNQGKSILFMWGINHPPTDDTQRNLYTRTDTTT
SVATEEINRIFKPLIGPRPLVNGLMGRINYYWSVLKPGQTLRIKSDGNLIAPWYGYILSGESHGRILRTD
LTKGSCTVQCQTEKGGLNTTLPFQNVSKYAFGNCSKYIGIKSLKLAVGLRNVPSRSSRGLFGAIAGFIEG
GWSGLVAGWYGFQHSNDQGVGMAADRESTQKAIDKITSKVNNIVDKMNKQYEIIDHEFSEVETRLNMINN
KIDDQIQDIWAYNAELLVLLENQKTLDEHDANVNNLYNKVKRALGSNAVEDGKGCFELYHKCDDQCMETI
RNGTYNRRKYQEESKLERQKIEGVKLESEGTYKILTIYSTVASSLVIAMGFAAFLFWAMSNGSCRCNICI

>QOK10774.1 hemagglutinin [Influenza A virus]
METVSLITILLAATVSNADKICIGYQSTNSTETVDTLTENNVPVTHAKELLHTEHNGMLCATSLGQPLIL
DTCTIEGLIYGNPSCDPLLEEREWSYIVERPSAVNGLCYPGNVENLEELRSFFSSARSYQRIQIFPDTIW
NVSYDGTSNTCSGSFYRNMRWLTRKNGNYPIQDAQYTNNQGKSILFMWGINHPPTDDTQRNLYTRTDTTT
SVATEEINRIFKPLIGPRPLVNGLMGRINYYWSVLKPGQTLRIKSDGNLIAPWYGYILSGESHGRILRTD
LTKGSCTVQCQTEKGGLNTTLPFQNVSKYAFGNCSKYIGIKSLKLAVGLRNVPSRSSRGLFGAIAGFIEG
GWSGLVAGWYGFQHSNDQGVGMAADRESTQKAIDKITSKVNNIVDKMNKQYEIIDHEFSEVETRLNMINN
KIDDQIQDIWAYNAELLVLLENQKTLDEHDANVNNLYNKVKRALGSNAVEDGKGCFELYHKCDDQCMETI
RNGTYNRRKYQEESKLERQKIEGVKLESEGTYKILTIYSTVASSLVIAMGFAAFLFWAMSNGSCRCNICI

>QOK10750.1 hemagglutinin [Influenza A virus]

METVSLITILLAATVSNADKICIGYQSTNSTETVDTLTENNVPVTHAKELLHTEHNGMLCATSLGQPLIL
DTCTIEGLIYGNPSCDPLLEEREWSYIVERPSAVNGLCYPGNVENLEELRSFFSSARSYQRIQIFPDTIW
NVSYDGTSNTCSGSFYRNMRWLTRKDGNYPIQDAQYTNNQGKSILFMWGINHPPTDNTQRNLYTRTDTTT
SVATEEINRIFKPLIGPRPLVNGLMGRINYYWSVLKPGQTLRIKSDGNLIAPWYGYILSGESHGRILRTD
LTKGSCTVQCQTEKGGLNTTLPFQNVSKYAFGNCSKYIGIKSLKLAVGLRNVPSRSSRGLFGAIAGFIEG
GWSGLVAGWYGFQHSNDQGVGMAADRESTQKAIDKITSKVNNIVDKMNKQYEIIDHEFSEVETRLNMINN
KIDDQIQDIWAYNAELLVLLENQKTLDEHDANVNNLYNKVKRALGSNAVEDGKGCFELYHKCDDQCMETI
RNGTYNRRKYQEESKLERQKIEGVKLESEGTYKILTIYSTVASSLVIAMGFAAFLFWAMSNGSCRCNICI

>QOK10726.1 hemagglutinin [Influenza A virus]
METVSLITILLAATVSNADKICIGYQSTNSTETVDTLTENNVPVTHAKELLHTEHNGMLCATSLGQPLIL
DTCTIEGLIYGNPSCDPLLEEREWSYIVERPSAINGLCYPGNVENLEELRSLFSSARSYQRIQIFPDTIW
NVSYDGTSNTCSGSFYRNMRWLNRKNGNYPIQDAQYTNNQGKSILFMWGINHPPTDDTQRNLYTRTDTTT
SVATEEINRIFKPLIGPRPLVNGLMGRINYYWSVLKPGQTLRIKSDGNLIAPWYGYILSGESHGRILRTD
LTKGSCTVQCQTEKGGLNTTLPFQNVSKYAFGNCSKYIGIKSLKLAVGLRNVPSRSSRGLFGAIAGFIEG
GWSGLVAGWYGFQHSNDQGVGMAADRESTQKAIDKITSKVNNIVDKMNKQYEIIDHEFSEVETRLNMINN
KIDDQIQDIWAYNAELLVLLENQKTLDEHDANVNNLYNKVKRALGSNAVEDGKGCFELYHKCDDQCMETI
RNGTYNRRKYQEESKLERQKIEGVKLESEGTYKILTIYSTVASSLVIAMGFAAFLFWAMSNGSCRCNICI

>QOK10714.1 hemagglutinin [Influenza A virus]
METVSLITILLAATVSNADKICIGYQSTNSTETVDTLTENNVPVTHAKELLHTEHNGMLCATSLGQPLIL
DTCTIEGLIYGNPSCDPLLEEREWSYIVERPSAVNGLCYPGNVENLEELRSFFSSARSYQRIQIFPDTIW
NVSYDGTSNTCSGSFYRNMRWLTRKNGNYPIQDAQYTNNQGKSILFMWGINHPPTDDTQRNLYTRTDTTT
SVATEEINRIFKPLIGPRPLVNGLMGRINYYWSVLKPGQTLRIKSDGNLIAPWYGYILSGESHGRILRTD
LTKGSCTVQCQTEKGGLNTTLPFQNVSKYAFGNCSKYIGIKSLKLAVGLRNVPSRSSRGLFGAIAGFIEG
GWSGLVAGWYGFQHSNDQGVGMAADRESTQKAIDKITSKVNNIVDKMNKQYEIIDHEFSEVETRLNMINN
KIDDQIQDIWAYNAELLVLLENQKTLDEHDANVNNLYNKVKRALGSNAVEDGKGCFELYHKCDDQCMETI
RNGTYNRRKYQEESKLERQKIEGVKLESEGTYKILTIYSTVASSLVIAMGFAAFLFWAMSNGSCRCNICI

>QOK10702.1 hemagglutinin [Influenza A virus]
METVSLITILLAATVSNADKICIGYQSTNSTETVDTLTENNVPVTHAKELLHTEHNGMLCATSLGQPLIL
DTCTIEGLIYGNPSCDPLLEEREWSYIVERPSAVNGLCYPGNVENLEELRSFFSSARSYQRIQIFPDTIW
NVSYDGTSNACSGSFYRNMRWLTRKNGNYPIQDAQYTNNQGKSILFMWGINHPPTDDTQRNLYTRTDTTT
SVATEEINRIFKPLIGPRPLVNGLMGRINYYWSVLKPGQTLRIKSDGNLIAPWYGYILSGESHGRILRTD

LTKGSCTVQCQTEKGGLNTTLPFQNVSKYAFGNCSKYIGIKSLKLAVGLRNVPSRSSRGLFGAIAGFIEG
GWSGLVAGWYGFQHSNDQGVGMAADRESTQKAIDKITSKVNNIVDKMNKQYEIIDHEFSEVETRLNMINN
KIDDQIQDIWAYNAELLVLLENQKTLDEHDANVNNLYNKVKRALGSNAVEDGKGCFELYHKCDDQCMETI
RNGTYNRRKYQEESKLERQKIEGVKLESEGTYKILTIYSTVASSLVIAMGFAAFLFWAMSNGSCRCNICI

>QOK10690.1 hemagglutinin [Influenza A virus]
METVSLITILLAATVSNADKICIGYQSTNSTETVDTLTENNVPVTHAKELLHTEHNGMLCATSLGQPLIL
DTCTIEGLIYGNPSCDPLLEEREWSYIVERPSAVNGLCYPGNVENLEELRSFFSSARSYQRIQIFPDTIW
NVSYDGTSNTCSGSFYRNMRWLTRKNGNYPIQDAQYTNNQGKSILFMWGINHPPTDDTQRNLYTRTDTTT
SVATEEINRIFKPLIGPRPLVNGLMGRINYYWSVLKPGQTLRIKSDGNLIAPWYGYILSGESHGRILRTD
LTKGSCTVQCQTEKGGLNTTLPFQNVSKYAFGNCSKYIGIKSLKLAVGLRNVPSRSSRGLFGAIAGFIEG
GWSGLVAGWYGFQHSNDQGVGMAADRESTQKAIDKITSKVNNIVDKMNKQYEIIDHEFSEVETRLNMINN
KIDDQIQDIWAYNAELLVLLENQKTLDEHDANVNNLYNKVKRALGSNAVEDGKGCFELYHKCDDQCMETI
RNGTYNRRKYQEESKLERQKIEGVKLESEGTYKILTIYSTVASSLVIAMGFAAFLFWAMSNGSCRCNICI

>QOK10678.1 hemagglutinin [Influenza A virus]
METVSLITILLAATVSNADKICIGYQSTNSTETVDTLTENNVPVTHAKELLHTEHNGMLCATSLGQPLIL
DTCTIEGLIYGNPSCDPLLEEREWSYIVERPSAVNGLCYPGNVENLEELRSFFSSARSYQRIQIFPDTIW
NVSYDGTSNTCSGSFYRNMRWLTRKNGNYPIQDAQYTNNQGKSILFMWGINHPPTDDTQRNLYTRTDTTT
SVATEEINRIFKPLIGPRPLVNGLMGRINYYWSVLKPGQTLRIKSDGNLIAPWYGYILSGESHGRILRTD
LTKGSCTVQCQTEKGGLNTTLPFQNVSKYAFGNCSKYIGIKSLKLAVGLRNVPSRSSRGLFGAIAGFIEG
GWSGLVAGWYGFQHSNDQGVGMAADRESTQKAIDKITSKVNNIVDKMNKQYEIIDHEFSEVETRLNMINN
KIDDQIQDIWAYNAELLVLLENQKTLDEHDANVNNLYNKVKRALGSNAVEDGKGCFELYHKCDDQCMETI
RNGTYNRRKYQEESKLERQKIEGVKLESEGTYKILTIYSTVASSLVIAMGFAAFLFWAMSNGSCRCNICI

>QOK10666.1 hemagglutinin [Influenza A virus]
METVSLITILLAATVSNADKICIGYQSTNSTETVDTLTENNVPVTHAKELLHTEHNGMLCATSLGQPLIL
DTCTIEGLIYGNPSCDPLLEEREWSYIVERPSAVNGLCYPGNVENLEELRSFFSSARSYQRIQIFPDTIW
NVSYDGTSNTCSGSFYRNMRWLTRKDGNYPIQDAQYTNNQGKSILFMWGINHPPTDDTQRNLYTRTDTTT
SVATEEINRIFKPLIGPRPLVNGLMGRINYYWSVLKPGQTLRIKSDGNLIAPWYGYILSGESHGRILRTD
LTKGSCTVQCQTEKGGLNTTLPFQNVSKYAFGNCSKYIGIKSLKLAVGLRNVPSRSSRGLFGAIAGFIEG
GWSGLVAGWYGFQHSNDQGVGMAADRESTQKAIDKITSKVNNIVDKMNKQYEIIDHEFSEVETRLNMINN
KIDDQIQDIWAYNAELLVLLENQKTLDEHDANVNNLYNKVKRALGSNAVEDGKGCFELYHKCDDQCMETI
RNGTYNRRKYQEESKLERQKIEGVKLESEGTYKILTIYSTVASSLVIAMGFAAFLFWAMSNGSCRCNICI

>QOK10654.1 hemagglutinin [Influenza A virus]

METVSLITILLAATVSNADKICIGYQSTNSTETVDTLTENNVPVTHAKELLHTEHNGMLCATSLGQPLIL
DTCTIEGLIYGNPSCDPLLEEREWSYIVERPSAVNGLCYPGNVENLEELRSFFSSARSYQRIQIFPDTIW
NVSYDGTSNTCSGSFYRNMRWLTRKDGNYPIQDAQYTNNQGKSILFMWGINHPPTDDTQRNLYTRTDTTT
SVATEEINRIFKPLIGPRPLVNGLMGRINYYWSVLKPGQTLRIKSDGNLIAPWYGYILSGESHGRILRTD
LTKGSCTVQCQTEKGGLNTTLPFQNVSKYAFGNCSKYIGIKSLKLAVGLRNVPSRSSRGLFGAIAGFIEG
GWSGLVAGWYGFQHSNDQGVGMAADRESTQKAIDKITSKVNNIVDKMNKQYEIIDHEFSEVETRLNMINN
KIDDQIQDIWAYNAELLVLLENQKTLDEHDANVNNLYNKVKRALGSNAVEDGKGCFELYHKCDDQCMETI
RNGTYNRRKYQEESKLERQKIEGVKLESEGTYKILTIYSTVASSLVIAMGFAAFLFWAMSNGSCRCNICI

>QOK10642.1 hemagglutinin [Influenza A virus]

METVSLITILLAATVSNADKICIGYQSTNSTETVDTLTENNVPVTHAKELLHTEHNGMLCATSLGQPLIL
DTCTIEGLIYGNPSCDPLLEEREWSYIVERPSAVNGLCYPGNVENLEELRSFFSSARSYQRIQIFPDTIW
NVSYDGTSNTCSGSFYRNMRWLTRKNGNYPIQDAQYTNNQGKSILFMWGINHPPTDDTQRNLYTRTDTTT
SVATEEINRIFKPLIGPRPLVNGLMGRINYYWSVLKPGQTLRIKSDGNLIAPWYGYILSGESHGRILRTD
LTKGSCTVQCQTEKGGLNTTLPFQNVSKYAFGNCSKYIGIKSLKLAVGLRNVPSRSSRGLFGAIAGFIEG
GWSGLVAGWYGFQHSNDQGVGMAADRESTQKAIDKITSKVNNIVDKMNKQYEIIDHEFSEVETRLNMINN
KIDDQIQDIWAYNAELLVLLENQKTLDEHDANVNNLYNKVKRALGSNAVEDGKGCFELYHKCDDQCMETI
RNGTYNRRKYQEESKLERQKIEGVKLESEGTYKILTIYSTVASSLVIAMGFAAFLFWAMSNGSCRCNICI

>QOK10630.1 hemagglutinin [Influenza A virus]

METVSLITILLAATVSNADKICIGYQSTNSTETVDTLTENNVPVTHAKELLHTEHNGMLCATSLGQPLIL
DTCTIEGLIYGNPSCDPLLEEREWSYIVERPSAVNGLCYPGNVENLEELRSFFSSARSYQRIQIFPDTIW
NVSYDGTSNTCSGSFYRNMRWLTRKNGNYPIQDAQYTNNQGKSILFMWGINHPPTDDTQRNLYTRTDTTT
SVATEEINRIFKPLIGPRPLVNGLMGRINYYWSVLKPGQTLRIKSDGNLIAPWYGYILSGESHGRILRTD
LTKGSCTVQCQTEKGGLNTTLPFQNVSKYAFGNCSKYIGIKSLKLAVGLRNVPSRSSRGLFGAIAGFIEG
GWSGLVAGWYGFQHSNDQGVGMAADRESTQKAIDKITSKVNNIVDKMNKQYEIIDHEFSEVETRLNMINN
KIDDQIQDIWAYNAELLVLLENQKTLDEHDANVNNLYNKVKRALGSNAVEDGKGCFELYHKCDDQCMETI
RNGTYNRRKYQEESKLERQKIEGVKLESEGTYKILTIYSTVASSLVIAMGFAAFLFWAMSNGSCRCNICI

>QOK10618.1 hemagglutinin [Influenza A virus]

METVSLITILLAVTVSNADKICIGYQSTNSTETVDTLTENNVPVTHAKELLHTEHNGMLCATSLGQPLIL
DTCTIEGLIYGNPSCDPLLEEREWSYIVERPSAVNGLCYPGNVENLEELRSLFSSARSYQRVQIFPDTIW

NVSYDGTSNACSGSFYRNMRWLTQKNGNYPIQDTQYTNNQGKNILFMWGINHPPTDDTQRNLYTRTDTTT
SVATEEINRIFKPLIGPRPLVNGLMGRINYYWSVLKPGQTLRIKSDGNLIAPSYGYILSGESHGRILRTD
LKRGSCTVQCQTEKGGLNTTLPFQNVSKYAFGNCSKYIGIKSLKLAVGLRNVPSRSSRGLFGAIAGFIEG
GWSGLVAGWYGFQHSNDQGVGMAADRESTQKAIDKITSKVNNIVDKMNKQYEIIDHEFSEVEARLNMINN
KIDDQIQDIWAYNAELLVLLENQKTLDEHDANVNNLYNKVKRALGSNAVEDGKGCFELYHKCDDQCMETI
RNGTYNRRKYQEESKLERQKIEGVKLESEGTYKILTIYSTVASSLVIAMGFAAFLFWAMSNGSCRCNICI

>QOK10606.1 hemagglutinin [Influenza A virus]
METVSLITILLAATVSNADKICIGYQSTNSTETVDTLTENNVPVTHAKELLHTEHNGMLCATSLGQPLIL
DTCTIEGLIYGNPSCDPLLEEREWSYIVERPSAVNGLCYPGNVENLEELRSFFSSARSYQRIQIFPDTIW
NVSYDGTSNTCSGSFYRNMRWLTRKDGNYPIQDAQYTNNQGKSILFMWGINHPPTDDTQRNLYTRTDTTT
SVATEEINRIFKPLIGPRPLVNGLMGRINYYWSVLKPGQTLRIKSDGNLIAPWYGYILSGESHGRILRTD
LTKGSCTVQCQTEKGGLNTTLPFQNVSKYAFGNCSKYIGIKSLKLAVGLRNVPSRSSRGLFGAIAGFIEG
GWSGLVAGWYGFQHSNDQGVGMAADRESTQKAIDKITSKVNNIVDKMNKQYEIIDHEFSEVETRLNMINN
KIDDQIQDIWAYNAELLVLLENQKTLDEHDANVNNLYNKVKRALGSNAVEDGKGCFELYHKCDDQCMETI
RNGTYNRRKYQEESKLERQKIEGVKLESEGTYKILTIYSTVASSLVIAMGFAAFLFWAMSNGSCRCNICI

>QOK10594.1 hemagglutinin [Influenza A virus]
METVSLITILLAATVSNADKICIGYQSTNSTETVDTLTENNVPVTHAKELLHTEHNGMLCATSLGQPLIL
DTCTIEGLIYGNPSCDPLLEEREWSYIVERPSAVNGLCYPGNVENLEELRSFFSSARSYQRIQIFPDTIW
NVSYDGTSNTCSGSFYRNMRWLTRKNGNYPIQDAQYTNNQGKSILFMWGINHPPTDDTQRNLYTRTDTTT
SVATEEINRIFKPLIGPRPLVNGLMGRINYYWSVLKPGQTLRIKSDGNLIAPWYGYILSGESHGRILRTD
LTKGSCTVQCQTEKGGLNTTLPFQNVSKYAFGNCSKYIGIKSLKLAVGLRNVPSRSSRGLFGAIAGFIEG
GWSGLVAGWYGFQHSNDQGVGMAADRESTQKAIDKITSKVNNIVDKMNKQYEIIDHEFSEVETRLNMINN
KIDDQIQDIWAYNAELLVLLENQKTLDEHDANVNNLYNKVKRALGSNAVEDGKGCFELYHKCDDQCMETI
RNGTYNRRKYQEESKLERQKIEGVKLESEGTYKILTIYSTVASSLVIAMGFAAFLFWAMSNGSCRCNICI

>QOK10582.1 hemagglutinin [Influenza A virus]
METVSLITILLAATVSNADKICIGYQSTNSTETVDTLTENNVPVTHAKELLHTEHNGMLCATSLGQPLIL
DTCTIEGLIYGNPSCDPLLEEREWSYIVERPSAVNGLCYPGNVENLEELRSFFSSARSYQRIQIFPDTIW
NVSYDGTSNTCSGSFYRNMRWLTRKNGNYPIQDAQYTNNQGKSILFMWGINHPPTDDTQRNLYTRIDTTT
SVATEEINRIFKPLIGPRPLVNGLMGRINYYWSVLKPGQTLRIKSDGNLIAPWYGYILSGESHGRILRTD
LTKGSCTVQCQTEKGGLNTTLPFQNVSKYAFGNCSKYIGIKSLKLAVGLRNVPSRSSRGLFGAIAGFIEG
GWSGLVAGWYGFQHSNDQGVGMAADRESTQKAIDKITSKVNNIVDKMNKQYEIIDHEFSEVETRLNMINN

KIDDQIQDIWAYNAELLVLLENQKTLDEHDANVNNLYNKVKRALGSNAVEDGKGCFELYHKCDDQCMETI
RNGTYNRRKYQEESKLERQKIEGVKLESEGTYKILTIYSTVASSLVIAMGFAAFLFWAMSNGSCRCNICI

>QOK10570.1 hemagglutinin [Influenza A virus]
METVSLITILLAATVSNADKICIGYQSTNSTETVDTLTENNVPVTHAKELLHTEHNGMLCATSLGQPLIL
DTCTIEGLIYGNPSCDPLLEEREWSYIVERPSAVNGLCYPGNVENLEELRSFFSSARSYQRIQIFPDTIW
NVSYDGTSNTCSGSFYRNMRWLTRKNGNYPIQDAQYTNNQGKSILFMWGINHPPTDDTQRNLYTRTDTTT
SVATEEINRIFKPLIGPRPLVNGLMGRINYYWSVLKPGQTLRIKSDGNLIAPWYGYILSGESHGRILRTD
LTKGSCTVQCQTEKGGLNTTLPFQNVSKYAFGNCSKYIGIKSLKLAVGLRNVPSRSSRGLFGAIAGFIEG
GWSGLVAGWYGFQHSNDQGVGMAADRESTQKAIDKITSKVNNIVDKMNKQYEIIDHEFSEVETRLNMINN
KIDDQIQDIWAYNAELLVLLENQKTLDEHDANVNNLYNKVKRALGSNAVEDGKGCFELYHKCDDQCMETI
RNGTYNRRKYQEESKLERQKIEGVKLESEGTYKILTIYSTVASSLVIAMGFAAFLFWAMSNGSCRCNICI

>QOK10558.1 hemagglutinin [Influenza A virus]
METVSLITILLAATVSNADKICIGYQSTNSTETVDTLTENNVPVTHAKELLHTEHNGMLCATSLGQPLIL
DTCTIEGLIYGNPSCDPLLEEREWSYIVERPSAVNGLCYPGNVENLEELRSFFSSARSYQRIQIFPDTIW
NVSYDGTSNTCSGSFYRNMRWLTRKDGNYPIQDAQYTNNQGKSILFMWGINHPPTDDTQRNLYTRTDTTT
SVATEEINRIFKPLIGPRPLVNGLMGRINYYWSVLKPGQTLRIKSDGNLIAPWYGYILSGESHGRILRTD
LTKGSCTVQCQTEKGGLNTTLPFQNVSKYAFGNCSKYIGIKSLKLAVGLRNVPSRSSRGLFGAIAGFIEG
GWSGLVAGWYGFQHSNDQGVGMAADRESTQKAIDKITSKVNNIVDKMNKQYEIIDHEFSEVETRLNMINN
KIDDQIQDIWAYNAELLVLLENQKTLDEHDANVNNLYNKVKRALGSNAVEDGKGCFELYHKCDDQCMETI
RNGTYNRRKYQEESKLERQKIEGVKLESEGTYKILTIYSTVASSLVIAMGFAAFLFWAMSNGSCRCNICI

>QOK10546.1 hemagglutinin [Influenza A virus]
METVSLMTILLAATVSNADKICIGYQSTNSTETVDTLTENNVPVTHAKELLHTEHNGMLCATSLGQPLIL
DTCTIEGLIYGNPSCDPLLEEREWSYIVERPSAVNGLCYPGNVENLEELRSFFSSARSYQRIQIFPDTIW
NVSYDGTSNTCSGSFYRNMRWLTRKNGNYPIQDAQYTNNQGKSILFMWGINHPPTDDTQRNLYTRTDTTT
SVATEEINRIFKPLIGPRPLVNGLMGRINYYWSVLKPGQTLRIKSDGNLIAPWYGYILSGESHGRILRTD
LTKGSCTVQCQTEKGGLNTTLPFQNVSKYAFGNCSKYIGIKSLKLAVGLRNVPSRSSRGLFGAIAGFIEG
GWSGLVAGWYGFQHSNDQGVGMAADRESTQKAIDKITSKVNNIVDKMNKQYEIIDHEFSEVETRLNMINN
KIDDQIQDIWAYNAELLVLLENQKTLDEHDANVNNLYNKVKRALGSNAVEDGKGCFELYHKCDDQCMETI
RNGTYNRRKYQEESKLERQKIEGVKLESEGTYKILTIYSTVASSLVIAMGFAAFLFWAMSNGSCRCNICI

>QOK10534.1 hemagglutinin [Influenza A virus]

METVSLITILLAATVSNADKICIGYQSTNSTETVDTLTENNVPVTHAKELLHTEHNGMLCATSLGQPLIL
DTCTIEGLIYGNPSCDPLLEEREWSYIVERPSAVNGLCYPGNVENLEELRSFFSSARSYQRIQIFPDTIW
NVSYDGTSNTCSGSFYRNMRWLTRKNGNYPIQDAQYTNNQGKSILFMWGINHPPTDDTQRNLYTRTDTTT
SVATEEINRIFKPLIGPRPLVNGLMGRINYYWSVLKPGQTLRIKSDGNLIAPWYGYILSGESHGRILRTD
LTKGSCTVQCQTEKGGLNTTLPFQNVSKYAFGNCSKYIGIKSLKLAVGLRNVPSRSSRGLFGAIAGFIEG
GWSGLVAGWYGFQHSNDQGVGMAADRESTQKAIDKITSKVNNIVDKMNKQYEIIDHEFSEVETRLNMINN
KIDDQIQDIWAYNAELLVLLENQKTLDEHDANVNNLYNKVKRALGSNAVEDGKGCFELYHKCDDQCMETI
RNGTYNRRKYQEESKLERQKIEGVKLESEGTYKILTIYSTVASSLVIAMGFAAFLFWAMSNGSCRCNICI

>QOK10522.1 hemagglutinin [Influenza A virus]
METVSLITILLVATVSNADKICIGYQSTNSTETVDTLTENNVPVTHAKELLHTEHNGMLCATNLGQPLIL
DTCTIEGLIYGNPSCDLSLEGREWSYIVERPSAVHGLCYPGNVEDLEELRSLFSSARSYQRIQIFPDTIW
NVSYDGTSNTCSGSFYRNMRWLTRKNGNYPIQDAQYTNNQGKNILFMWGINHPPTDDTQRNLYTRTDTTT
SVATEEINRIFKPLIGPRPLVNGLMGRINYYWSVLKPGQTLRIKSDGNLIAPWYGHILSGESHGRILRTD
LTKGSCTVQCQTEKGGLNTTLPFQNVSKYAFGNCSKYIGIKSLKLAVGLRNVPSRSSRGLFGAIAGFIEG
GWPGLVAGWYGFQHSNDQGVGMAADRDSTQKAIDKITSKVNNIVDKMNKQYEIIDHEFSEVETRLNMINN
KIDDQIQDIWAYNAELLVLLENQKTLDEHDANVNNLYNKVKRALGSNAVEDGKGCFELYHKCDDQCMETI
RNGTYNRRKYQEESKLERQKIEGVKLESEGTYKILTIYSTVASSLVIAMGFAAFLFWAMSNGSCRCNICI

>QOK10510.1 hemagglutinin [Influenza A virus]
METVSLITILLAATVSNADKICIGYQSTNSTETVDTLTENNVPVTHAKELLHTEHNGMLCATSLGQPLIL
DTCTIEGLIYGNPSCDPLLEEREWSYIVERPSAVNGLCYPGNVENLEELRSFFSSARSYQRIQIFPDTIW
NVSYDGTSNTCSGSFYRNMRWLTRKNGNYPIQDAQYTNNQGKSILFMWGINHPPTDDTQRNLYTRIDTTT
SVATEEINRIFKPLIGPRPLVNGLMGRINYYWSVLKPGQTLRIKSDGNLIAPWYGYILSGESHGRILRTD
LTKGSCTVQCQTEKGGLNTTLPFQNVSKYAFGNCSKYIGIKSLKLAVGLRNVPSRSSRGLFGAIAGFIEG
GWSGLVAGWYGFQHSNDQGVGMAADRESTQKAIDKITSKVNNIVDKMNKQYEIIDHEFSEVETRLNMINN
KIDDQIQDIWAYNAELLVLLENQKTLDEHDANVNNLYNKVKRALGSNAVEDGKGCFELYHKCDDQCMETI
RNGTYNRRKYQEESKLERQKIEGVKLESEGTYKILTIYSTVASSLVIAMGFAAFLFWAMSNGSCRCNICI

>QOK10498.1 hemagglutinin [Influenza A virus]
METVSLITILLAATVSNADKICIGYQSTNSTETVDTLTENNVPVTHAKELLHTEHNGMLCATSLGQPLIL
DTCTIEGLIYGNPSCDPLLEEREWSYIVERPSAVNGLCYPGNVENLEELRSFFSSARSYQRIQIFPDTIW
NVSYDGTSNTCSGSFYRNMRWLTRKNGNYPIQDAQYTNNQGKSILFMWGINHPPTDDTQRNLYTRTDTTT
SVATEEINRIFKPLIGPRPLVNGLMGRINYYWSVLKPGQTLRIKSDGNLIAPWYGYILSGESHGRILRTD

LTKGSCTVQCQTEKGGLNTTLPFQNVSKYAFGNCSKYIGIKSLKLAVGLRNVPSRSSRGLFGAIAGFIEG
GWSGLVAGWYGFQHSNDQGVGMAADRESTQKAIDKITSKVNNIVDKMNKQYEIIDHEFSEVETRLNMINN
KIDDQIQDIWAYNAELLVLLENQKTLDEHDANVNNLYNKVKRALGSNAVEDGKGCFELYHKCDDQCMETI
RNGTYNRRKYQEESKLERQKIEGVKLESEGTYKILTIYSTVASSLVIAMGFAAFLFWAMSNGSCRCNICI

>QOK10486.1 hemagglutinin [Influenza A virus]
METVSLITILLAATVSNADKICIGYQSTNSTETVDTLTENNVPVTHAKELLHTEHNGMLCATSLGQPLIL
DTCTIEGLIYGNPSCDPLLEEREWSYIVERPSAVNGLCYPGNVENLEELRSFFSSARSYQRIQIFPDTIW
NVSYDGTSNTCSGSFYRNMRWLTRKNGNYPIQDAQYTNNQGKSILFMWGINHPPTDDTQRNLYTRTDTTT
SVATEEINRIFKPLIGPRPLVNGLMGRINYYWSVLKPGQTLRIKSDGNLIAPWYGYILSGESHGRILRTD
LTKGSCTVQCQTEKGGLNTTLPFQNVSKYAFGNCSKYIGIKSLKLAVGLRNVPSRSSRGLFGAIAGFIEG
GWSGLVAGWYGFQHSNDQGVGMAADRESTQKAIDKITSKVNNIVDKMNKQYEIIDHEFSEVETRLNMINN
KIDDQIQDIWAYNAELLVLLENQKTLDEHDANVNNLYNKVKRALGSNAVEDGKGCFELYHKCDDQCMETI
RNGTYNRRKYQEESKLERQKIEGVKLESEGTYKILTIYSTVASSLVIAMGFAAFLFWAMSNGSCRCNICI

>QOK10474.1 hemagglutinin [Influenza A virus]
METVSLITILLAATVSNADKICIGYQSTNSTETVDTLTENNVPVTHAKELLHTEHNGMLCATSLGQPLIL
DTCTIEGLIYGNPSCDPLLEEREWSYIVERPSAVNGLCYPGNVENLEELRSFFSSARSYQRIQIFPDTIW
NVSYDGTSNTCSGSFYRNMRWLTRKNGNYPIQDAQYTNNQGKSILFMWGINHPPTDDTQRNLYTRIDTTT
SVATEEINRIFKPLIGPRPLVNGLMGRINYYWSVLKPGQTLRIKSDGNLIAPWYGYILSGESHGRILRTD
LTKGSCTVQCQTEKGGLNTTLPFQNVSKYAFGNCSKYIGIKSLKLAVGLRNVPSRSSRGLFGAIAGFIEG
GWSGLVAGWYGFQHSNDQGVGMAADRESTQKAIDKITSKVNNIVDKMNKQYEIIDHEFSEVETRLNMINN
KIDDQIQDIWAYNAELLVLLENQKTLDEHDANVNNLYNKVKRALGSNAVEDGKGCFELYHKCDDQCMETI
RNGTYNRRKYQEESKLERQKIEGVKLESEGTYKILTIYSTVASSLVIAMGFAAFLFWAMSNGSCRCNICI

>QOK10462.1 hemagglutinin [Influenza A virus]
METVSLITILLAATVSNADKICIGYQSTNSTETVDTLTENNVPVTHAKELLHTEHNGMLCATSLGQPLIL
DTCTIEGLIYGNPSCDPLLEEREWSYIVERPSAVNGLCYPGNVENLEELRSFFSSARSYQRIQIFPDTIW
NVSYDGTSNTCSGSFYRNMRWLTRKNGNYPIQDAQYTNNQGKSILFMWGINHPPTDDTQRNLYTRTDTTT
SVATEEINRIFKPLIGPRPLVNGLMGRINYYWSVLKPGQTLRIKSDGNLIAPWYGYILSGESHGRILRTD
LTKGSCTVQCQTEKGGLNTTLPFQNVSKYAFGNCSKYIGIKSLKLAVGLRNVPSRSSRGLFGAIAGFIEG
GWSGLVAGWYGFQHSNDQGVGMAADRESTQKAIDKITSKVNNIVDKMNKQYEIIDHEFSEVETRLNMINN
KIDDQIQDIWAYNAELLVLLENQKTLDEHDANVNNLYNKVKRALGSNAVEDGKGCFELYHKCDDQCMETI
RNGTYNRRKYQEESKLERQKIEGVKLESEGTYKILTIYSTVASSLVIAMGFAAFLFWAMSNGSCRCNICI

>QOK10438.1 hemagglutinin [Influenza A virus]

METVSLMTILLVAAVSNADKICIGYQSTNSTETVDTLTENNVPVTHAKELLHTEHNGMLCATSLGQPIIL

DTCTIEGLIYGNPSCDLSLEGREWSYIVERPSAVNGLCYPGNVENLEELRSLFSSARSYQRIQIFPDTIW

NVSYDGTSTACSGSFYRNMRWLTRKNGEYPIQDAQYTNNQGKNILFMWGINHPPADTTQRDLYTRTDTTT

SVATEEINRIFKPLIGPRPLVNGLMGRIDYYWSVLKPGQTLRIKSDGNLIAPWYGHILSGESHGRILKTD

LKRGSCTVQCQTEKGGLNTTLPFQNVSKYAFGNCSKYIGIKSLKLAVGLRNVPSRSSRGLFGAIAGFIEG

GWSGLVAGWYGFQHSNDQGVGMAADRDSTQKAVDKITSKVNTIVEKMNKQYEIIDHEFSEVETRLNMINN

KIDDQIQDIWAYNAELLVLLENQKTLDEHDANVNNLYNKVKRALGSNAVEDGKGCFELYHKCDDQCMETI

RNGTYNRRKYQEESKLERQKIEGVKLESEGTYKILTIYSTVASSLVIAMGFAAFLFWAMSNGSCRCNICI

>QOK10426.1 hemagglutinin [Influenza A virus]

METVSLITILIVATVSKADKICIGYQSTNSTETVDTLTENNVPVTHAKELLHTEHNGMLCATSLGHPLIL

DTCTIEGLIYGNPSCDPLLGGREWSYIVERPSAVNGLCYPGNVENLEELRSLFSSSRSYQRIQIFPDTIW

NVSYSGTSKACSDSFYRSMRWLTQKNNAYPTQDAQYTNNQGKNILFMWGINHPPTDTAQTNLYTRTDTTT

SVATEEMNRIFKPLIGPRPLVNGLMGRINYYWSVLKPGQTLRIKSDGNLIAPWYGHILSGESHGRILKTD

LKRGSCTVQCQTEKGGLNTTLPFQNVSKYAFGNCSKYIGVKSLKLAVGLRNVPSRSSRGLFGAIAGFIEG

GWSGLVAGWYGFQHSNDQGVGMAADRDSTQKAIDKITSKVNNIVDKMNKQYEIIDHEFSEVETRLNMINN

KVDDQIQDIWAYNAELLVLLENQKTLDEHDANVNNLYNKVKRALGSNAVEDGKGCFELYHKCDDHCMETI

RNGTYNRRKYQEESKLERQKIEGVKLESEETYKILTIYSTVASSLVIAMGFAAFLFWAMSNGSCRCNICI

>QOK10390.1 hemagglutinin [Influenza A virus]

METVSLITILIVATVSKADKICIGYQSTNSTETVDTLTENNVPVTHAKELLHTEHNGMLCATSLGHPLIL

DTCTIEGLIYGNPSCDPLLGGREWSYIVERPSAVNGLCYPGNVENLEELRSLFSSSRSYQRIQIFPDTIW

NVSYSGTSKACSDSFYRSMRWLTQKNNAYPTQDAQYTNNQGKNILFMWGINHPPTDTAQTNLYTRTDTTT

SVATEEMNRIFKPLIGPRPLVNGLMGRINYYWSVLKPGQTLRIKSDGNLIAPWYGHILSGESHGRILKTD

LKRGSCTVQCQTEKGGLNTTLPFQNVSKYAFGNCSKYIGVKSLKLAVGLRNVPSRSSRGLFGAIAGFIEG

GWSGLVAGWYGFQHSNDQGVGMAADRDSTQKAIDKITSKVNNIVDKMNKQYEIIDHEFSEVETRLNMINN

KVDDQIQDIWAYNAELLVLLENQKTLDEHDANVNNLYNKVKRALGSNAVEDGKGCFELYHKCDDHCMETI

RNGTYNRRKYQEESKLERQKIEGVKLESEETYKILTIYSTVASSLVIAMGFAAFLFWAMSNGSCRCNICI

>QOK10378.1 hemagglutinin [Influenza A virus]

METVSLITILIVATVSKADKICIGYQSTNSTETVDTLTENNVPVTHAKELLHTEHNGMLCATSLGHPLIL

DTCTIEGLIYGNPSCDPLLGGREWSYIVERPSAVNGLCYPGNVENLEELRSLFSSSRSYQRIQIFPDTIW

NVSYSGTSKACSDSFYRSMRWLTQKNNAYPTQDAQYTNNQGKNILFMWGINHPPTDTVQTNLYTRTDTTT
SVATEEMNRIFKPLIGPRPLVNGLMGRINYYWSVLKPGQTLRIKSDGNLIAPWYGHILSGESHGRILKTD
LKRGSCTVQCQTEKGGLNTTLPFQNVSKYAFGNCSKYIGVKSLKLAVGLRNVPSRSSRGLFGAIAGFIEG
GWSGLVAGWYGFQHSNDQGVGMAADRDSTQKAIDKITSKVNNIVDKMNKQYEIIDHEFSEVETRLNMINN
KVDDQIQDIWAYNAELLVLLENQKTLDEHDANVNNLYNKVKRALGSNAVEDGKGCFELYHKCDDHCMETI
RNGTYNRRKYQEESKLERQKIEGVKLESEETYKILTIYSTVASSLVIAMGFAAFLFWAMSNGSCRCNICI

>QOK10318.1 hemagglutinin [Influenza A virus]
METVSLITILIVATVSNADKICIGYQSTNSTETVDTLTENNVPVTHAKELLHTEHNGMLCATSLGQPLIL
DTCTIEGLIYGNPSCDLSLEGREWSYIVERPSAINGLCYPGNVENLEELRSLFSSARSYQRIQIFPDTIW
NVSYDGTSTACSNSFYRSMRWLTRKDGNYPTQDAQYTNNQGKNILFMWGINHPPTDDTQRNLYTRTDTTT
SVATEEINRIFKPLIGPRPLVNGLMGRIDYYWSVLKPGQTLRIKSDGNLIAPWYGHILSGESHGRILKTD
LKRGSCTVQCQTEKGGLNTTLPFQNVSKYAFGNCSKYIGIKSLKLAVGLRNVPSRSSRGLFGAIAGFIEG
GWSGLVAGWYGFQHSNDQGVGMAADRDSTQKAIDKITSKVNNIVDKMNKQYEIIDHEFSEVETRLNMINN
KIDDQIQDIWAYNAELLVLLENQKTLDEHDANVNNLYNKVKRALGSNAVEDGKGCFELYHKCNDQCMETI
RNGTYNRRKYQEESKLERQKIEGVKLESEGTYKILTIYSTVASSLVIAMGFAAFLFWAMSNGSCRCNICI

>QOK10222.1 hemagglutinin [Influenza A virus]
METVSLITILIVATVSKADKICIGYQSTNSTETVDTLTENNVPVTHAKELLHTEHNGMLCATSLGHPLIL
DTCTIEGLIYGNPSCDPLLGGREWSYIVERPSAVNGLCYPGNVENLEELRSLFSSSRSYQRIQIFPDTIW
NVSYSGTSKACSDSFYRSMRWLTQKNNAYPTQDAQYTNNQGKNILFMWGINHPPTDTAQTNLYTRTDTTT
SVATEEMNRIFKPLIGPRPLVNGLMGRINYYWSVLKPGQTLRIKSDGNLIAPWYGHILSGESHGRILKTD
LKRGSCTVQCQTEKGGLNTTLPFQNVSKYAFGNCSKYIGVKSLKLAVGLRNVPSRSSRGLFGAIAGFIEG
GWSGLVAGWYGFQHSNDQGVGMAADRDSTQKAIDKITSKVNNIVDKMNKQYEIIDHEFSEVETRLNMINN
KVDDQIQDIWAYNAELLVLLENQKTLDEHDANVNNLYNKVKRALGSNAVEDGKGCFELYHKCDDHCMETI
RNGTYNRRKYQEESKLERQKIEGVKLESEETYKILTIYSTVASSLVIAMGFAAFLFWAMSNGSCRCNICI

>QOK10186.1 hemagglutinin [Influenza A virus]
METVSLMTILLVAAVSNADKICIGYQSTNSTETVDTLTENNVPVTHAKELLHTEHNGMLCATSLGQPIIL
DTCTIEGLIYGNPSCDLSLEGREWSYIVERPSAVNGLCYPGNVENLEELRSLFSSARSYQRIQIFPDTIW
NVSYDGTSTACSGSFYRNMRWLTRKNGEYPIQDAQYTNNQGKNILFMWGINHPPADTTQRNLYTRTDTTT
SVATEEINRIFKPLIGPRPLVNGLMGRIDYYWSVLKPGQTLRIKSDGNLIAPWYGHILSGESHGRILKTD
LKRGSCTVQCQTEKGGLNTTLPFQNVSKYAFGNCSKYIGIKSLKLAVGLRNVPSRSSRGLFGAIAGFIEG
GWSGLVAGWYGFQHSNDQGVGMAADRDSTQKAVDKITSKVNTIVEKMNKQYEIIDHEFSEVETRLNMINN

KIDDQIQDIWAYNAELLVLLENQKTLDEHDANVNNLYNKVKRALGSNAVEDGKGCFELYHKCDDQCMETI
RNGTYNRRKYQEESKLERQKIEGVKLESEGTYKILTIYSTVASSLVIAMGFAAFLFWAMSNGSCRCNICI

>QOK10150.1 hemagglutinin [Influenza A virus]
METVSLITILIVATVSKADKICIGYQSTNSTETVDTLTENNVPVTHAKELLHTEHNGMLCATSLGHPLIL
DTCTIEGLIYGNPSCDPLLGGREWSYIVERPSAVNGLCYPGNVENLEELRSLFSSSRSYQRIQIFPDTIW
NVSYSGTSKACSDSFYRSMRWLTQKNNAYPTQDAQYTNNQGKNILFMWGINHPPTDTAQTNLYTRTDTTT
SVATEEMNRIFKPLIGPRPLVNGLMGRINYYWSVLKPGQTLRIKSDGNLIAPWYGHILSGESHGRILKTD
LKRGSCTVQCQTEKGGLNTTLPFQNVSKYAFGNCSKYIGVKSLKLAVGLRNVPSRSSRGLFGAIAGFIEG
GWSGLVAGWYGFQHSNDQGVGMAADRDSTQKAIDKITSKVNNIVDKMNKQYEIIDHEFSEVETRLNMINN
KVDDQIQDIWAYNAELLVLLENQKTLDEHDANVNNLYNKVKRALGSNAVEDGKGCFELYHKCDDHCMETI
RNGTYNRRKYQEESKLERQKIEGVKLESEETYKILTIYSTVASSLVIAMGFAAFLFWAMSNGSCRCNICI

>QOK10138.1 hemagglutinin [Influenza A virus]
METVSLITILIVATVSKADKICIGYQSTNSTETVDTLTENNVPVTHAKELLHTEHNGMLCATSLGHPLIL
DTCTIEGLIYGNPSCDPLLGGREWSYIVERPSAVNGLCYPGNVENLEELRSLFSSSRSYQRIQIFPDTIW
NVSYSGTSKACSDSFYRSMRWLTQKNNAYPTQDAQYTNNQGKNILFMWGINHPPTDTAQTNLYTRTDTTT
SVATEEMNRIFKPLIGPRPLVNGLMGRINYYWSVLKPGQTLRIKSDGNLIAPWYGHILSGESHGRILKTD
LKRGSCTVQCQTEKGGLNTTLPFQNVSKYAFGNCSKYIGVKSLKLAVGLRNVPSRSSRGLFGAIAGFIEG
GWSGLVAGWYGFQHSNDQGVGMAADRDSTQKAIDKITSKVNNIVDKMNKQYEIIDHEFSEVETRLNMINN
KVDDQIQDIWAYNAELLVLLENQKTLDEHDANVNNLYNKVKRALGSNAVEDGKGCFELYHKCDDHCMETI
RNGTYNRRKYQEESKLERQKIEGVKLESEETYKILTIYSTVASSLVIAMGFAAFLFWAMSNGSCRCNICI

>QOK10126.1 hemagglutinin [Influenza A virus]
METVSLITILIVATVSKADKICIGYQSTNSTETVDTLTENNVPVTHAKELLHTEHNGMLCATSLGHPLIL
DTCTIEGLIYGNPSCDPLLGGREWSYIVERPSAVNGLCYPGNVENLEELRSLFSSSRSYQRIQIFPDTIW
NVSYSGTSKACSDSFYRSMRWLTQKNNAYPTQDAQYTNNQGKNILFMWGINHPPTDTAQTNLYTRTDTTT
SVATEEMNRIFKPLIGPRPLVNGLMGRINYYWSVLKPGQTLRIKSDGNLIAPWYGHILSGESHGRILKTD
LKRGSCTVQCQTEKGGLNTTLPFQNVSKYAFGNCSKYIGVKSLKLAVGLRNVPSRSSRGLFGAIAGFIEG
GWSGLVAGWYGFQHSNDQGVGMAADRDSTQKAIDKITSKVNNIVDKMNKQYEIIDHEFSEVETRLNMINN
KVDDQIQDIWAYNAELLVLLENQKTLDEHDANVNNLYNKVKRALGSNAVEDGKGCFELYHKCDDHCMETI
RNGTYNRRKYQEESKLERQKIEGVKLESEETYKILTIYSTVASSLVIAMGFAAFLFWAMSNGSCRCNICI

>QOK10114.1 hemagglutinin [Influenza A virus]

METVSLITILIVATVSNADKICIGYQSTNSTETVDTLTENNVPVTHAKELLHTEHNGMLCATSLGQPLIL
DTCTIEGLIYGNPSCDLSLEGREWSYIVERPSAINGLCYPGNVENLEELRSLFSSARSYQRIQIFPDTIW
NVSYDGTSTACSNSFYRSMRWLTRKDGNYPTQDAQYTNNQGKNILFMWGINHPPTDDTQRNLYTRTDTTT
SVATEEINRIFKPLIGPRPLVNGLMGRIDYYWSILKPGQTLRIKSDGNLIAPWYGHILSGESHGRILKTD
LKRGSCTVQCQTEKGGLNTTLPFQNVSKYAFGNCSKYIGIKSLKLAVGLRNVPSRSSRGLFGAIAGFIEG
GWSGLVAGWYGFQHSNDQGVGMAADRDSTQKAIDKITSKVNNIVDKMNKQYEIIDHEFSEVETRLNMINN
KIDDQIQDIWAYNAELLVLLENQKTLDEHDANVNNLYNKVKRALGSNAVEDGKGCFELYHKCNDQCMETI
RNGTYNRRKYQEESKLERQKIEGVKLESEGTYKILTIYSTVASSLVIAMGFAAFLFWAMSNGSCRCNICI

>QOK10102.1 hemagglutinin [Influenza A virus]
METVSLITILIVATVSNADKICIGYQSTNSTETVDTLTENNVPVTHAKELLHTEHNGMLCATSLGQPLIL
DTCTIEGLIYGNPSCDLSLEGREWSYIVERPSAINGLCYPGNVENLEELRSLFSSARSYQRIQIFPDTIW
NVSYDGTSTACSNSFYRSMRWLTRKDGNYPTQDAQYTNNQGKNILFMWGINHPPTDDTQRNLYTRTDTTT
SVATEEINRIFKPLIGPRPLVNGLMGRIDYYWSILKPGQTLRIKSDGNLIAPWYGHILSGESHGRILKTD
LKRGSCTVQCQTEKGGLNTTLPFQNVSKYAFGNCSKYIGIKSLKLAVGLRNVPSRSSRGLFGAIAGFIEG
GWSGLVAGWYGFQHSNDQGVGMAADRDSTQKAIDKITSKVNNIVDKMNKQYEIIDHEFSEVETRLNMINN
KIDDQIQDIWAYNAELLVLLENQKTLDEHDANVNNLYNKVKRALGSNAVEDGKGCFELYHKCNDQCMETI
RNGTYNRRKYQEESKLERQKIEGVKLESEGTYKILTIYSTVASSLVIAMGFAAFLFWAMSNGSCRCNICI

>QOK10078.1 hemagglutinin [Influenza A virus]
METISLMTILLVTTVSNADKICIGYQSTNSTETVDTLTENNVPVTHAKELLHTEHNGMLCATSLGNPLIL
DTCTIEGLIYGNPSCDPLLGGREWSYIVERPSAVNGLCYPGNVENLEELRSLFSSARSYQRIQIFPDTIW
NVSYSGTSKACSDSFYRSMRWLTQKNNAYPIQDAQYTNNQEKNILFMWGINHPPTETAQTNLYTRTDTTT
SVATEEINRIFKPLIGPRPLVNGLMGRINYYWSVLKPGQTLRIKSDGNLIAPWYGHILSGESHGRILKTD
LKRGSCTVQCQTEKGGLNTTLPFQNVSKYAFGNCSKYIGIKSLKLAVGLRNVPSRSSRGLFGAIAGFIEG
GWSGLVAGWYGFQHSNDQGVGMAADRDSTQKAIDKITSKVNNIVDKMNKQYEIIDHEFSEVETRLNMINN
KVDDQIQDIWAYNAELLVLLENQKTLDEHDSNVNNLYNKVKRALGSNAVEDGKGCFELYHKCDNQCMETI
RNGTYNRRKYQEESKLERQKIEGVKLESEGTYKILTIYSTVASSLVIAMGFAAFLFWAMSNGSCRCNICI

>QOK10066.1 hemagglutinin [Influenza A virus]
METVSLITILIVATVSNADKICIGYQSTNSTETVDTLTENNVPVTHAKELLHTEHNGMLCATSLGQPLIL
DTCTIEGLIYGNPSCDLSLEGREWSYIVERPSAINGLCYPGNVENLEELRSLFSSARSYQRIQIFPDTIW
NVSYDGTSTACSNSFYRSMRWLTRKDGNYPTQDAQYTNNQGKNILFMWGINHPPTDDTQRNLYTRTDTTT
SVATEEINRIFKPLIGPRPLVNGLMGRIDYYWSVLKPGQTLRIKSDGNLIAPWYGHILSGESHGRILKTD

LKRGSCTVQCQTEKGGLNTTLPFQNVSKYAFGNCSKYIGIKSLKLAVGLRNVPSRSSRGLFGAIAGFIEG
GWSGLVAGWYGFQHSNDQGVGMAADRDSTQKAIDKITSKVNNIVDKMNKQYEIIDHEFSEVETRLNMINN
KIDDQIQDIWAYNAELLVLLENQKTLDEHDANVNNLYNKVKRALGSNAVEDGKGCFELYHKCNDQCMETI
RNGTYNRRKYQEESKLERQKIEGVKLESEGTYKILTIYSTVASSLVIAMGFAAFLFWAMSNGSCRCNICI

>QOK10054.1 hemagglutinin [Influenza A virus]
METVSLITILIVATVSNADKICIGYQSTNSTETVDTLTENNVPVTHAKELLHTEHNGMLCATSLGQPLIL
DTCTIEGLIYGNPSCDLSLEGREWSYIVERPSAINGLCYPGNVENLEELRSLFSSARSYQRIQIFPDTIW
NVSYDGTSTACSNSFYRSMRWLTRKDGNYPTQDAQYTNNQGKNILFMWGINHPPTDDTQRNLYTRTDTTT
SVATEEINRIFKPLIGPRPLVNGLMGRIDYYWSVLKPGQTLRIKSDGNLIAPWYGHILSGESHGRILKTD
LKRGSCTVQCQTEKGGLNTTLPFQNVSKYAFGNCSKYIGIKSLKLAVGLRNVPSRSSRGLFGAIAGFIEG
GWSGLVAGWYGFQHSNDQGVGMAADRDSTQKAIDKITSKVNNIVDKMNKQYEIIDHEFSEVETRLNMINN
KIDDQIQDIWAYNAELLVLLENQKTLDEHDANVNNLYNKVKRALGSNAVEDGKGCFELYHKCNDQCMETI
RNGTYNRRKYQEESKLERQKIEGVKLESEGTYKILTIYSTVASSLVIAMGFAAFLFWAMSNGSCRCNICI

>QOK09994.1 hemagglutinin [Influenza A virus]
METVSLITILLVATVSNADKICIGYQTTNSTETVDTLTENNVPVTHAKELLHTEHNGMLCATSLGQPLIL
DTCTIEGLIYGNPSCDLSLEGREWSYIVERPSAVHGLCYPGNVEDLEELRSLFSSARSYQRIQIFPDTIW
NVSYDGTSTACSGSFYKSMRWLTRKNGEYPTQDAQYTNNQGKNILFMWGINHPPXDDTQRGLYTRTDTTT
SVATEEINRIFKPLIGPRPLVNGLMGRINYYWSVLKPGQTLRIKSDGNLIAPWYGHILSGESHGRILKTD
LKRGSCTVQCQTEKGGLNTTLPFQNVSKYAFGNCSKYIGVKSLKLAVGLRNVPSRSSRGLFGAIAGFIEG
GWPGLVAGWYGFQHSNDQGVGMAADRDSTQKAIDKITSKVNNIVDKMNKQYEIIDHEFSEVETRLNMINN
KIDDQIQDIWAYNAELLVLLENQKTLDEHDANVNNLYNKVKRALGSNAVEDGKGCFELYHKCDDQCMETI
RNGTYNRRKYQEESKLERQRIEGVKLESEGTYKILTIYSTVASSLVIAMGFAAFLFWAMSNGSCRCNICI

>QOK09970.1 hemagglutinin [Influenza A virus]
METVSLITILLVATVSNADKICIGYQTTNSTETVDTLTENNVPVTHAKELLHTEHNGMLCATSLGQPLIL
DTCTIEGLIYGNPSCDLSLEGREWSYIVERPSAVHGLCYPGNVEDLEELRSLFSSARSYQRIQIFPDTIW
NVSYDGTSTACSGSFYKSMRWLTRKNGEYPTQDAQYTNNQGKNILFMWGINHPPTDDTQRGLYTRTDTTT
SVATEEINRIFKPLIGPRPLVNGLMGRINYYWSVLKPGQTLRIKSDGNLIAPWYGHILSGESHGRILKTD
LKRGSCTVQCQTEKGGLNTTLPFQNVSKYAFGNCSKYIGVKSLKLAVGLRNVPSRSSRGLFGAIAGFIEG
GWPGLVAGWYGFQHSNDQGVGMAADRDSTQKAIDKITSKVNNIVDKMNKQYEIIDHEFSEVETRLNMINN
KIDDQIQDIWAYNAELLVLLENQKTLDEHDANVNNLYNKVKRALGSNAVEDGKGCFELYHKCDDQCMETI
RNGTYNRRKYQEESKLERQRIEGVKLESEGTYKILTIYSTVASSLVIAMGFAAFLFWAMSNGSCRCNICI

>QOK09958.1 hemagglutinin [Influenza A virus]
METVSLITILLVATVSNADKICIGYQSTNSTETVDTLTESNVPVTHAKELLHTEHNGMLCATSLGQPLIL
DTCTIEGLIYGNPSCDLSLEGREWSYIVERPSAVHGLCYPGNVEDLEELRSLFSSARSYQRIQIFPDTIW
NVSYDGTSTACSGSFYRSMRWLTRKNGEYPIQDAQYTNNQGKNILFMWGINHPPTDDTQRGLYTRTDTTT
SVATEEINRIFKPLIGPRPLVNGLMGRINYYWSVLKPGQTLRIKSDGNLIAPWYGHILSGESHGRILKTD
LKRGSCTVQCQTEKGGLNTTLPFQNVSKYAFGNCSKYIGIKSLKLAVGLRNVPSRSSRGLFGAIAGFIEG
GWPGLVAGWYGFQHSNDQGVGMAADRDSTQKAIDKITSKVNNIVDKMNKQYEIIDHEFSEVETRLNMINN
KIDDQIQDIWAYNAELLVLLENQKTLDEHDANVNNLYSKVKRALGSNAVEDGKGCFELYHKCDDQCMETI
RNGTYNRRKYQEESKLERQRIEGVKLESEGTYKILTIYSTVASSLVIAMGFAAFLFWAMSNGSCRCNICI

>QOK09946.1 hemagglutinin [Influenza A virus]
METVSLITILVVATVSNADKICIGYQSTNSTETVDTLTENNVPVTHAKELLHTEHNGMLCATSLGHPLIL
DTCTIEGLIYGNPSCDPLLGGREWSYIVERPSAVNGLCYPGNVENLEELRSLFSSSRSYQRIQIFPDTIW
NVSYSGTSKACSDSFYRSMRWLTQKNNAYPTQDAQYTNNQGKNILFMWGINHPPTDTAQTNLYTRTDTTT
SVATEEMNRIFKPLIGPRPLVNGLMGRINYYWSVLKPGQTLRIKSDGNLIAPWYGHILSGESHGRILKTD
LKRGSCTVQCQTEKGGLNTTLPFQNVSKYAFGNCSKYIGVKSLKLAVGLRNVPSRSSRGLFGAIAGFIEG
GWSGLVAGWYGFQHSNDQGVGMAADRDSTQKAIDKITSKVNNIVDKMNKQYEIIDHEFSEVETRLNMINN
KVDDQIQDIWAYNAELLVLLENQKTLDEHDANVNNLYNKVKRALGSNAVEDGKGCFELYHKCDDHCMETI
RNGTYNRRKYQEESKLERQKIEGVKLESEETYKILTIYSTVASSLVIAMGFAAFLFWAMSNGSCRCNICI

>QOK09934.1 hemagglutinin [Influenza A virus]
METVSLITILVVATVSNADKICIGYQSTNSTETVDTLTENNVPVTHAKELLHTEHNGMLCATSLGHPLIL
DTCTIEGLIYGNPSCDPLLGGREWSYIVERPSAVNGLCYPGNVENLEELRSLFSSSRSYQRIQIFPDTIW
NVSYSGTSKACSDSFYRSMRWLTQKNNAYPTQDAQYTNNQGKNILFMWGINHPPTDTTQTNLYTRTDTTT
SVATEEMNRIFKPLIGPRPLVNGLMGRINYYWSVLKPGQTLRIKSDGNLIAPWYGHILSGESHGRILKTD
LKRGSCTVQCQTEKGGLNTTLPFQNVSKYAFGNCSKYIGVKSLKLAVGLRNVPSRSSRGLFGAIAGFIEG
GWSGLVAGWYGFQHSNDQGVGMAADRDSTQKAIDKITSKVNNIVDKMNKQYEIIDHEFSEVETRLNMINN
KVDDQIQDIWAYNAELLVLLENQKTLDEHDANVNNLYNKVKRALGSNAVEDGKGCFELYHKCDDHCMETI
RNGTYNRRKYQEESKLERQKIEGVKLESEETYKILTIYSTVASSLVIAMGFAAFLFWAMSNGSCRCNICI

>QOK09683.1 hemagglutinin [Influenza A virus]
METVSLITILLVATVSNADKICIGYQSTNSTETVDTLTENNVPVTHAKELLHTEHNGMLCATSLGQPLIL
DTCTIEGLIYGNPSCDLSLEGREWSYIVERPSAVHGLCYPGNVEDLEELRSLFSSARSYQRIQIFPDTIW

NVSYDGTSTACSGSFYRSMRWLTRKNGEYPIQDAQYTNNQGKNILFMWGINHPPTDDTQRGLYTRTDTTT
SVATEEINRIFKPLIGPRPLVNGLMGRINYYWSVLKPGQTLRIKSDGNLIAPWYGHILSGESHGRILKTD
LKRGSCTVQCQTEKGGLNTTLPFQNVSKYAFGNCSKYIGIKSLKLAVGLRNVPSRSSRGLFGAIAGFIEG
GWSGLVAGWYGFQHSNDQGVGMAADRDSTQKAIDKITSKVNNIVDKMNKQYEIIDHEFSEVETRLNMINN
KIDDQIQDIWAYNAELLVLLENQKTLDEHDANVNNLYNKVKRALGSNAVEDGKGCFELYHKCDDQCMETI
RNGTYNRRKYQEESKLERQRIEGVKLESEGTYKILTIYSTVASSLVIAMGFAAFLFWAMSNGSCRCNICI

>QOK09672.1 hemagglutinin [Influenza A virus]
METVSLITILLVATVSNADKICIGYQSTNSTETVDTLTENNVPVTHAKELLHTEHNGMLCATSLGQPLIL
DTCTIEGLIYGNPSCDLSLEGREWSYIVERPSAVHGLCYPGNVEDLEELRSLFSSARSYQRIQIFPDTIW
NVSYDGTSTACSGSFYRSMRWLTRKNGEYPIQDAQYTNNQGKNILFMWGINHPPTDDTQRGLYTRTDTTT
SVATEEINRIFKPLIGPRPLVNGLMGRINYYWSVLKPGQTLRIKSDGNLIAPWYGHILSGESHGRILKTD
LKRGSCTVQCQTEKGGLNTTLPFQNVSKYAFGNCSKYIGIKSLKLAVGLRNVPSRSSRGLFGAIAGFIEG
GWSGLVAGWYGFQHSNDQGVGMAADRDSTQKAIDKITSKVNNIVDKMNKQYEIIDHEFSEVETRLNMINN
KIDDQIQDIWAYNAELLVLLENQKTLDEHDANVNNLYNKVKRALGSNAVEDGKGCFELYHKCDDQCMETI
RNGTYNRRKYQEESKLERQRIEGVKLESEGTYKILTIYSTVASSLVIAMGFAAFLFWAMSNGSCRCNICI

>QOK09649.1 hemagglutinin [Influenza A virus]
METVSLITILLVATVSNADKICIGYQSTNSTETVDTLTENNVPVTHAKELLHTEHNGMLCATSLGQPLIL
DTCTIEGLIYGNPSCDLSLEGREWSYIVERPSAVHGLCYPGNVEDLEELRSLFSSARSYQRIQIFPDTIW
NVSYDGTSTACSGSFYRSMRWLTRKNGEYPIQDAQYTNNQGKNILFMWGINHPPTDDTQRGLYTRTDTTT
SVATEEINRIFKPLIGPRPLVNGLMGRINYYWSVLKPGQTLRIKSDGNLIAPWYGHILSGESHGRILKTD
LKRGSCTVQCQTEKGGLNTTLPFQNVSKYAFGNCSKYIGIKSLKLAVGLRNVPSRSSRGLFGAIAGFIEG
GWSGLVAGWYGFQHSNDQGVGMAADRDSTQKAIDKITSKVNNIVDKMNKQYEIIDHEFSEVETRLNMINN
KIDDQIQDIWAYNAELLVLLENQKTLDEHDANVNNLYNKVKRALGSNAVEDGKGCFELYHKCDDQCMETI
RNGTYNRRKYQEESKLERQRIEGVKLESEGTYKILTIYSTVASSLVIAMGFAAFLFWAMSNGSCRCNICI

>QOK09637.1 hemagglutinin [Influenza A virus]
METVSLITILLVATVSNADKICIGYQSTNSTETVDTLTENNVPVTHAKELLHTEHNGMLCATSLGQPLIL
DTCTIEGLIYGNPSCDLSLEGREWSYIVERPSAVHGLCYPGNVEDLEELRSLFSSARSYQRIQIFPDTIW
NVSYDGTSTACSGSFYRSMRWLTRKNGEYPIQDAQYTNNQGKNILFMWGINHPPTDDTQRGLYTRTDTTT
SVATEEINRIFKPLIGPRPLVNGLMGRINYYWSVLKPGQTLRIKSDGNLIAPWYGHILSGESHGRILKTD
LKRGSCTVQCQTEKGGLNTTLPFQNVSKYAFGNCSKYIGIKSLKLAVGLRNVPSRSSRGLFGAIAGFIEG
GWSGLVAGWYGFQHSNDQGVGMAADRDSTQKAIDKITSKVNNIVDKMNKQYEIIDHEFSEVETRLNMINN

KIDDQIQDIWAYNAELLVLLENQKTLDEHDANVNNLYNKVKRALGSNAVEDGKGCFELYHKCDDQCMETI
RNGTYNRRKYQEESKLERQRIEGVKLESEGTYKILTIYSTVASSLVIAMGFAAFLFWAMSNGSCRCNICI

>QOK09625.1 hemagglutinin [Influenza A virus]
METVSLITILLVATVSNADKICIGYQSTNSTETVDTLTENNVPVTHAKELLHTEHNGMLCATSLGQPLIL
DTCTIEGLLYGNPSCDLSLEGREWSYIVERPSAVNGLCYPGNVENLEELRSLFSSARSYQRIQIFPDTIW
NVSYDGTSTACSNSFYRSMRWLTRKDGNYPTQDAQYTNNQGKNILFMWGINHPPTDDTQRNLYTRTDTTT
SVATEEINRIFKPLIGPRPLVNGLMGRIDYYWSVLKPGQTLRIKSDGNLIAPWYGHILTGESHGRILKTD
LKRGSCTVQCQTEKGGFNTTLPFQNVSKYAFGNCSKYIGIKSLKLAVGLRNVPSRSSRGLFGAIAGFIEG
GWSGLVAGWYGFQHSNDQGVGMAADRDSTQKAIDKITSKVNNIVDKMNKQYEIIDHEFSEVETRLNMINN
KIDDQIQDIWAYNAELLVLLENQKTLDEHDANVNNLYNKVKRALGSNAVEDGKGCFELYHKCNDQCMDTI
RNGTYNRRKYQEESKLERQKIEGVKLEPEGTYKILTIYSTVASSLVIAMGFAAFLFWAMSNGSCRCNICI

>QOK09613.1 hemagglutinin [Influenza A virus]
METVSLITILLIATVSNADKICIGYQSTNSTETVDTLTENNVPVTHAKELLHTEHNGMLCATSLGQPIIL
DTCTIEGLIYGNPSCDLSLEGREWSYIVERPSAVNGLCYPGNVENLEELRSLFSSARSYQRIQIFPDTIW
NVSYDGTSTACSGSFYRNMRWLTRKNGEYPIQDAQYTNNQGKNILFMWGINHPPADTTQRDLYTRTDTTT
SVATEEINRIFKPLIGPRPLVNGLMGRIDYYWSVLKPGQTLRIKSDGNLIAPWYGHILSGESHGRILKTD
LKRGSCTVQCQTEKGGLNTTLPFQNVSKYAFGNCSKYIGIKSLKLAVGLRNVPSRSSRGLFGAIAGFIEG
GWSGLVAGWYGFQHSNDQGVGMAADRESTQKAVDKITSKVNNIVDKMNKQYEIIDHEFSEVETRLNMINN
KIDDQIQDIWAYNAELLVLLENQKTLDEHDANVNNLYNKVKRALGSNAMEDGKGCFELYHKCDDQCMETI
RNGTYNRRKYQEESKLERQKIEGVKLESEGTYKILTIYSTVASSLVIAMGFAAFLFWAMSNGSCRCNICI

>QOK09601.1 hemagglutinin [Influenza A virus]
METVSLITILLVATVSNADKICIGYQSTNSTETVDTLTENNVPVTHAKELLHTEHNGMLCATSLGQPLIL
DTCTIEGLIYGNPSCDLSLEGREWSYIVERPSAVHGLCYPGNVEDLEELRSLFSSARSYQRIQIFPDTIW
NVSYDGTSTACSGSFYRSMRWLTRKNGEYPIQDAQYTNNQGKNILFMWGINHPPTDDTQRGLYTRTDTTT
SVATEEINRIFKPLIGPRPLVNGLMGRINYYWSVLKPGQTLRIKSDGNLIAPWYGHILSGESHGRILKTD
LKRGSCTVQCQTEKGGLNTTLPFQNVSKYAFGNCSKYIGIKSLKLAVGLRNVPSRSSRGLFGAIAGFIEG
GWSGLVAGWYGFQHSNDQGVGMAADRDSTQKAIDKITSKVNNIVDKMNKQYEIIDHEFSEVETRLNMINN
KIDDQIQDIWAYNAELLVLLENQKTLDEHDANVNNLYNKVKRALGSNAVEDGKGCFELYHKCDDQCMETI
RNGTYNRRKYQEESKLERQRIEGVKLESEGTYKILTIYSTVASSLVIAMGFAAFLFWAMSNGSCRCNICI

>QOK09578.1 hemagglutinin [Influenza A virus]

METVSLITILLVATVSNADKICIGYQSTNSTETVDTLTENNVPVTHAKELLHTEHNGMLCATSLGQPLIL
DTCTIEGLIYGNPSCDLSLEGREWSYIVERPSAVHGLCYPGNVEDLEELRSLFSSARSYQRIQIFPDTIW
NVSYDGTSTACSGSFYRSMRWLTRKNGEYPIQDAQYTNNQGKNILFMWGINHPPTDDTQRGLYTRTDTTT
SVATEEINRIFKPLIGPRPLVNGLMGRINYYWSVLKPGQTLRIKSDGNLIAPWYGHILSGESHGRILKTD
LKRGSCTVQCQTEKGGLNTTLPFQNVSKYAFGNCSKYIGIKSLKLAVGLRNVPSRSSRGLFGAIAGFIEG
GWSGLVAGWYGFQHSNDQGVGMAADRDSTQKAIDKITSKVNNIVDKMNKQYEIIDHEFSEVETRLNMINN
KIDDQIQDIWAYNAELLVLLENQKTLDEHDANVNNLYNKVKRALGSNAVEDGKGCFELYHKCDDQCMETI
RNGTYNRRKYQEESKLERQRIEGVKLESEGTYKILTIYSTVASSLVIAMGFAAFLFWAMSNGSCRCNICI

>QOK09556.1 hemagglutinin [Influenza A virus]
METVSLITILLVATVSNADKICIGYQSTNSTETVDTLTENNVPVTHAKELLHTEHNGMLCATSLGQPLIL
DTCTIEGLIYGNPSCDLSLEGREWSYIVERPSAVHGLCYPGNVEDLEELRSLFSSARSYQRIQIFPDTIW
NVSYDGTSTACSGSFYRSMRWLTRKNGDYPIQDAQYTNNQGKNILFMWGINHPPTDETQRGLYTRIDTTT
SVATEEINRIFKPLIGPRPLVNGLMGRINYYWSVLKPGQTLRIKSDGNLIAPWYGHILSGESHGRILKTD
LKRGSCTVQCQTEKGGLNTTLPFQNVSKYAFGNCSKYIGIKSLKLAVGLRNVPSRSSRGLFGAIAGFIEG
GWSGLVAGWYGFQHSNDQGVGMAADRDSTQKAIDKITSKVNNIVDKMNKQYEIIDHEFSEVETRLNMINN
KIDDQIQDIWAYNAELLVLLENQKTLDEHDANVNNLYNKVKRALGSNAVEDGKGCFELYHKCDDQCMETI
RNGTYNRRKYQEESKLERQKIEGVKLESEGTYKILTIYSTVASSLVIAMGFAAFLFWAMSNGSCRCNICI

>QOK09532.1 hemagglutinin [Influenza A virus]
METISLITILLVATVSNADKICIGYQSTNSTETVDTLTENNVPVTHAKELLHTEHNGMLCATSLGQPLIL
DTCTIEGLIYGNPSCDLSLEGREWSYIVERPSAVNGLCYPGNVENLEELRSLFSSARSYQRIQIFPDTIW
NVSYDGTSTACSNSFYRSMRWLTRKDGNYPTQDAQYTNNQGKNILFMWGINHPPTDETQRNLYTRTDTTT
SVATEEINRIFKPLIGPRPLVNGLMGRIDYYWSVLKPGQTLRIKSDGNLIAPWYGHILSGESHGRILKTD
LKRGSCTVQCQTEKGGLNTTLPFQNVSKYAFGNCSKYIGIKSLKLAVGLRNVPSRSSRGLFGAIAGFIEG
GWSGLVAGWYGFQHSNDQGVGMAADRDSTQKAIDKITSKVNNIVDKMNKQYEIIDHEFSEVETRLNMINN
KIDDQIQDIWAYNAELLVLLENQKTLDEHDANVNNLYNKVKRALGSNAVEDGKGCFELYHKCNDQCMETI
RNGTYNRKKYQEESKLERQRIEGVKLESEGTYKILTIYSTVASSLVIAMGFAAFLFWAMSNGSCRCNICI

>QOK09520.1 hemagglutinin [Influenza A virus]
METVPLITILLVAAVSNADKICIGYQSTNSTETVDTLTENNVPVTHAKELLHTEHNGMLCATSLGQPIIL
DTCTIEGLIYGNPSCDLSLEGREWSYIVERPSAVNGLCYPGNVENLEELRSLFSSARSYQRIQIFPDTIW
NVSYDGTSTACSGSFYRNMRWLTRKNGEYPIQDAQYTNNQGKNILFMWGINHPPADTTQRDLYTRTDTTT
SVATEEINRIFKPLIGPRPLVNGLMGRIDYYWSVLKPGQTLRIKSDGNLIAPWYGHILSGESHGRILKTD

LKRGSCTVQCQTEKGGLNTTLPFQNVSKYAFGNCSKYIGIKSLKLAVGLRNVPSRSSRGLFGAIAGFIEG
GWSGLVAGWYGFQHSNDQGVGMAADRDSTQKAVDKITSKVNTIVDKMNKQYEIIDHEFSEVETRLNMINN
KIDDQIQDIWAYNAELLVLLENQKTLDEHDANVNNLYNRVKRALGSNAVEDGKGCFELYHKCDDQCMETI
RNGTYNRRKYQEESKLERQKIEGVKLESEGTYKILTIYSTVASSLVIAMGFAAFLFWAMSNGSCRCNICI

>QOK09508.1 hemagglutinin [Influenza A virus]
METVPLITILLVAAVSNADKICIGYQSTNSTETVDTLTENNVPVTHAKELLHTEHNGMLCATSLGQPIIL
DTCTIEGLIYGNPSCDLSLEGREWSYIVERPSAVNGLCYPGNVENLEELRSLFSSARSYQRIQIFPDTIW
NVSYDGTSTACSGSFYRNMRWLTRKNGEYPIQDAQYTNNQGKNILFMWGINHPPADTTQRDLYTRTDTTT
SVATEEINRIFKPLIGPRPLVNGLMGRIDYYWSVLKPGQTLRIKSDGNLIAPWYGHILSGESHGRILKTD
LKRGSCTVQCQTEKGGLNTTLPFQNVSKYAFGNCSKYIGIKSLKLAVGLRNVPSRSSRGLFGAIAGFIEG
GWSGLVAGWYGFQHSNDQGVGMAADRDSTQKAVDKITSKVNTIVDKMNKQYEIIDHEFSEVETRLNMINN
KIDDQIQDIWAYNAELLVLLENQKTLDEHDANVNNLYNRVKRALGSNAVEDGKGCFELYHKCDDQCMETI
RNGTYNRRKYQEESKLERQKIEGVKLESEGTYKILTIYSTVASSLVIAMGFAAFLFWAMSNGSCRCNICI

>QOK09496.1 hemagglutinin [Influenza A virus]
METVSLITILLVATVSNADKICIGYQSTNSTETVDTLTENNVPVTHAKELLHTEHNGMLCATSLGQPLIL
DTCTIEGLIYGNPSCDLSLEGREWSYIVERPSAVHGLCYPGNVEDLEELRSLFSSARSYQRIQIFPDTIW
NVSYDGTSTACSGSFYRSMRWLTRKNGEYPIQDAQYTNNQGKNILFMWGINHPPTDDTQRGLYTRTDTTT
SVATEEINRIFKPLIGPRPLVNGLMGRINYYWSVLKPGQTLRIKSDGNLIAPWYGHILSGESHGRILKTD
LKRGSCTVQCQTEKGGLNTTLPFQNVSKYAFGNCSKYIGIKSLKLAVGLRNVPSRSSRGLFGAIAGFIEG
GWSGLVAGWYGFQHSNDQGVGMAADRDSTQKAIDKITSKVNNIVDKMNKQYEIIDHEFSEVETRLNMINN
KIDDQIQDIWAYNAELLVLLENQKTLDEHDANVNNLYNKVKRALGSNAVEDGKGCFELYHKCDDQCMETI
RNGTYNRRKYQEESKLERQRIEGVKLESEGTYKILTIYSTVASSLVIAMGFAAFLFWAMSNGSCRCNICI

>QOK09485.1 hemagglutinin [Influenza A virus]
METVSLITILLVATVSNADKICIGYQSTNSTETVDTLTENNVPVTHAKELLHTEHNGMLCATSLGQPLIL
DTCTIEGLIYGNPSCDLSLEGREWSYIVERPSAVNGMCYPGNVENLEELRSLFSSARSYQRIQIFPDTIW
NVSYDGTSTACSGSFYRSMRWLTRKNGEYPIQDAQYTNNQGKNILFMWGINHPPTDTTQRDLYTRTDTTT
SVATEEINRVFKPLIGPRPLVNGLMGRIDYYWSVLKPGQTLRIKSDGNLIAPWFGHILSGESHGRILKTD
LKRGSCTVQCQTEKGGLNTTLPFQNVSKYAFGNCSKYIGIKSLKLAVGLRNVPSRSSRGLFGAIAGFIEG
GWSGLVAGWYGFQHSNDQGVGMAADRDSTQKAIDKITSKVNNIVDKMNKQYEIIDHEFSEVEARLNMINN
KIDDQIQDIWAYNAELLVLLENQKTLDEHDANVNNLYNKVKRALGSNAVEDGKGCFELYHKCDDQCMETI
RNGTYNRRKYQEESKLERQKIEGVKLESEGTYKILTIYSTVASSLVIAMGFAAFLFWAMSNGSCRCNICI

>QOK09473.1 hemagglutinin [Influenza A virus]

METVSLITILLVATVSNADKICIGYQSTNSTETVDTLTENNVPVTHAKELLHTEHNGMLCATSLGQPLIL

DTCTIEGLIYGNPSCDLSLEGREWSYIVERPSAVHGLCYPGNVEDLEELRSLFSSARSYQRIQIFPDTIW

NVSYDGTSTACSGSFYRSMRWLTRKNGDYPIQDAQYTNNQGKNILFMWGINHPPTDETQRGLYTRTDTTT

SVATEEINRIFKPLIGPRPLVNGLMGRINYYWSVLKPGQTLRIKSDGNLIAPWYGHILSGESHGRILKTD

LKRGSCTVQCQTEKGGLNTTLPFQNVSKYAFGNCSKYIGIKSLKLAVGLRNVPSRSSRGLFGAIAGFIEG

GWSGLVAGWYGFQHSNDQGVGMAADRDSTQKAIDKITSKVNNIVDKMNKQYEIIDHEFSEVETRLNMINN

KIDDQIQDIWAYNAELLVLLENQKTLDEHDANVNNLYNKVKRALGSNAVEDGKGCFELYHKCDDQCMETI

RNGTYNRRKYQEESKLERQKIEGVKLESEGTYKILTIYSTVASSLVIAMGFAAFLFWAMSNGSCRCNICI

>QOK09461.1 hemagglutinin [Influenza A virus]

METVSLITILLVATVSNADKICIGYQSTNSTETVNTLTENNVPVTHAKELLHTEHNGMLCATSLGQPLIL

DTCTIEGLIYGNPSCDLSLEGREWSYIVERPSAINGLCYPGNVENLEELRSLFSSARSYQRIQIFPDTIW

NVSYDGTSTACSNSFYRSMRWLTRKDGNYPTQDAQYTNNQGKNILFMWGINHPPTDETQRNLYTRTDTTT

SVATEEINRIFKPLIGPRPLVNGLMGRIDYYWSVLKPGQTLRIKSDGNLIAPWYGHILSGESHGRILKTD

LKRGSCTVQCQTEKGGLNTTLPFQNVSKYAFGNCSKYIGIKSLKLAVGLRNVPSRSSRGLFGAIAGFIEG

GWSGLVSGWYGFQHSNDQGVGMAADRDSTQKAIDKITSKVNNIVDKMNKQYEIIDHEFSEVETRLNMINN

KIDDQIQDIWAYNADLLVLLENQKTLDEHDANVNNLYNKVKRALGSNAVEDGKGCFELYHKCNDQCMETI

RNGTYNRKKYQEESKLERQRIDGVKLESEGTYKILTIYSTVASSLVIAMGFAAFLFWAMSNGSCRCNICI

>QOK09344.1 hemagglutinin [Influenza A virus]

METVSLITILLVATVSNADKICIGYQSTNSTETVDTLTENNVPVTHAKELLHTEHNGMLCATSLGQPLIL

DTCTIEGLIYGNPSCDLSLEGREWSYIVERPSAVHGLCYPGNVEDLEELRSLFSSARSYQRIQIFPDTIW

NVSYDGTSTACSGSFYRSMRWLTRKNGEYPIQDAQYTNNQGKNILFMWGINHPPTDDTQRGLYTRTDTTT

SVATEEINRIFKPLIGPRPLVNGLMGRINYYWSVLKPGQTLRIKSDGNLIAPWYGHILSGESHGRILKTD

LKRGSCTVQCQTEKGGLNTTLPFQNVSKYAFGNCSKYIGIKSLKLAVGLRNVPSRSSRGLFGAIAGFIEG

GWSGLVAGWYGFQHSNDQGVGMAADRDSTQKAIDKITSKVNNIVDKMNKQYEIIDHEFSEVETRLNMINN

KIDDQIQDIWAYNAELLVLLENQKTLDEHDANVNNLYNKVKRALGSNAVEDGKGCFELYHKCDDQCMETI

RNGTYNRRKYQEESKLERQKIEGVKLESEGTYKILTIYSTVASSLVIAMGFAAFLFWAMSNGSCRCNICI

>QOK09332.1 hemagglutinin [Influenza A virus]

METVSLITILLVATVSNADKICIGYQSTNSTETVDTLTENNVPVTHAKELLHTEHNGMLCATSLGQPLIL

DTCTIEGLIYGNPSCDLSLEGREWSYIVERPSAVHGLCYPGNVEDLEELRSLFSSARSYQRIQIFPDTIW

NVSYDGTSTACSGSFYRSMRWLTRKNGEYPIQDAQYTNNQGKNILFMWGINHPPTDDTQRGLYTRTDTTT
SVATEEINRIFKPLIGPRPLVNGLMGRINYYWSVLKPGQTLRIKSDGNLIAPWYGHILSGESHGRILKTD
LKRGSCTVQCQTEKGGLNTTLPFQNVSKYAFGNCSKYIGIKSLKLAVGLRNVPSRSSRGLFGAIAGFIEG
GWSGLVAGWYGFQHSNDQGVGMAADRDSTQKAIDKITSKVNNIVDKMNKQYEIIDHEFSEVETRLNMINN
KIDDQIQDIWAYNAELLVLLENQKTLDEHDANVNNLYNKVKRALGSNAVEDGKGCFELYHKCDDQCMETI
RNGTYNRRKYQEESKLERQKIEGVKLESEGTYKILTIYSTVASSLVIAMGFAAFLFWAMSNGSCRCNICI

>QOK09320.1 hemagglutinin [Influenza A virus]
METVSLITILLVATVSNADKICIGYQSTNSTETVDTLTENNVPVTHAKELLHTEHNGMLCATSLGQPLIL
DTCTIEGLIYGNPSCDLSLEGREWSYIVERPSAVHGLCYPGNVEDLEELRSLFSSARSYQRIQIFPDTIW
NVSYDGTSTACSGSFYRSMRWLTRKNGEYPIQDAQYTNNQGKNILFMWGINHPPTDDTQRGLYTRTDTTT
SVATEEINRIFKPLIGPRPLVNGLMGRINYYWSVLKPGQTLRIKSDGNLIAPWYGHILSGESHGRILKTD
LKRGSCTVQCQTEKGGLNTTLPFQNVSKYAFGNCSKYIGIKSLKLAVGLRNVPSRSSRGLFGAIAGFIEG
GWSGLVAGWYGFQHSNDQGVGMAADRDSTQKAIDKITSKVNNIVDKMNKQYEIIDHEFSEVETRLNMINN
KIDDQIQDIWAYNAELLVLLENQKTLDEHDANVNNLYNKVKRALGSNAVEDGKGCFELYHKCDDQCMETI
RNGTYNRRKYQEESKLERQKIEGVKLESEGTYKILTIYSTVASSLVIAMGFAAFLFWAMSNGSCRCNICI

>QOK09308.1 hemagglutinin [Influenza A virus]
METVSLITILLVATVSNADKICIGYQSTNSTETVDTLTENNVPVTHAKELLHTEHNGMLCATSLGQPLIL
DTCTIEGLIYGNPSCDLSLEGREWSYIVERPSAVHGLCYPGNVEDLEELRSLFSSARSYQRIQIFPDTIW
NVSYDGTSTACSGSFYRSMRWLTRKNGEYPIQDAQYTNNQGKNILFMWGINHPPTDDTQRGLYTRTDTTT
SVATEEINRIFKPLIGPRPLVNGLMGRINYYWSVLKPGQTLRIKSDGNLIAPWYGHILSGESHGRILKTD
LKRGSCTVQCQTEKGGLNTTLPFQNVSKYAFGNCSKYIGIKSLKLAVGLRNVPSRSSRGLFGAIAGFIEG
GWSGLVAGWYGFQHSNDQGVGMAADRDSTQKAIDKITSKVNNIVDKMNKQYEIIDHEFSEVETRLNMINN
KIDDQIQDIWAYNAELLVLLENQKTLDEHDANVNNLYNKVKRALGSNAVEDGKGCFELYHKCDDQCMETI
RNGTYNRRKYQEESKLERQKIEGVKLESEGTYKILTIYSTVASSLVIAMGFAAFLFWAMSNGSCRCNICI

>QOK09296.1 hemagglutinin [Influenza A virus]
METVSLITILLVATVSNADKICIGYQSTNSTETVDTLTENNVPVTHAKELLHTEHNGMLCATSLGQPLIL
DTCTIEGLIYGNPSCDLSLEGREWSYIVERPSAVHGLCYPGNVEDLEELRSLFSSARSYQRIQIFPDTIW
NVSYDGTSTACSGSFYRSMRWLTRKNGEYPIQDAQYTNNQGKNILFMWGINHPPTDDTQRGLYTRTDTTT
SVATEEINRIFKPLIGPRPLVNGLMGRINYYWSVLKPGQTLRIKSDGNLIAPWYGHILSGESHGRILKTD
LKRGSCTVQCQTEKGGLNTTLPFQNVSKYAFGNCSKYIGIKSLKLAVGLRNVPSRSSRGLFGAIAGFIEG
GWSGLVAGWYGFQHSNDQGVGMAADRDSTQKAIDKITSKVNNIVDKMNKQYEIIDHEFSEVETRLNMINN

KIDDQIQDIWAYNAELLVLLENQKTLDEHDANVNNLYNKVKRALGSNAVEDGKGCFELYHKCDDQCMETI
RNGTYNRRKYQEESKLERQKIEGVKLESEGTYKILTIYSTVASSLVIAMGFAAFLFWAMSNGSCRCNICI

>QOK09284.1 hemagglutinin [Influenza A virus]
METVSLITILLVATVSNADKICIGYQSTNSTETVDTLTENNVPVTHAKELLHTEHNGMLCATSLGQPLIL
DTCTIEGLIYGNPSCDLSLEGREWSYIVERPSAVHGLCYPGNVEDLEELRSLFSSARSYQRIQIFPDTIW
NVSYDGTSTACSGSFYRSMRWLTRKNGEYPIQDAQYTNNQGKNILFMWGINHPPTDDTQRGLYTRTDTTT
SVATEEINRIFKPLIGPRPLVNGLMGRINYYWSVLKPGQTLRIKSDGNLIAPWYGHILSGESHGRILKTD
LKRGSCTVQCQTEKGGLNTTLPFQNVSKYAFGNCSKYIGIKSLKLAVGLRNVPSRSSRGLFGAIAGFIEG
GWSGLVAGWYGFQHSNDQGVGMAADRDSTQKAIDKITSKVNNIVDKMNKQYEIIDHEFSEVETRLNMINN
KIDDQIQDIWAYNAELLVLLENQKTLDEHDANVNNLYNKVKRALGSNAVEDGKGCFELYHKCDDQCMETI
RNGTYNRRKYQEESKLERQKIEGVKLESEGTYKILTIYSTVASSLVIAMGFAAFLFWAMSNGSCRCNICI

>QOK09272.1 hemagglutinin [Influenza A virus]
METVSLITILLVATVSNADKICIGYQSTNSTETVDTLTENNVPVTHAKELLHTEHNGMLCATSLGQPLIL
DTCTIEGLIYGNPSCDLSLEGREWSYIVERPSAVHGLCYPGNVEDLEELRSLFSSARSYQRIQIFPDTIW
NVSYDGTSTACSGSFYRSMRWLTRKNGEYPIQDAQYTNNQGKNILFMWGINHPPTDDTQRGLYTRTDTTT
SVATEEINRIFKPLIGPRPLVNGLMGRINYYWSVLKPGQTLRIKSDGNLIAPWYGHILSGESHGRILKTD
LKRGSCTVQCQTEKGGLNTTLPFQNVSKYAFGNCSKYIGIKSLKLAVGLRNVPSRSSRGLFGAIAGFIEG
GWSGLVAGWYGFQHSNDQGVGMAADRDSTQKAIDKITSKVNNIVDKMNKQYEIIDHEFSEVETRLNMINN
KIDDQIQDIWAYNAELLVLLENQKTLDEHDANVNNLYNKVKRALGSNAVEDGKGCFELYHKCDDQCMETI
RNGTYNRRKYQEESKLERQKIEGVKLESEGTYKILTIYSTVASSLVIAMGFAAFLFWAMSNGSCRCNICI

>QOK09260.1 hemagglutinin [Influenza A virus]
METVSLITILLVATVSNADKICIGYQSTNSTETVDTLTENNVPVTHAKELLHTEHNGMLCATSLGQPLIL
DTCTIEGLIYGNPSCDLSLEGREWSYIVERPSAVHGLCYPGNVEDLEELRSLFSSARSYQRIQIFPDTIW
NVSYDGTSTACSGSFYRSMRWLTRKNGEYPIQDAQYTNNQGKNILFMWGINHPPTDDTQRGLYTRTDTTT
SVATEEINRIFKPLIGPRPLVNGLMGRINYYWSVLKPGQTLRIKSDGNLIAPWYGHILSGESHGRILKTD
LKRGSCTVQCQTEKGGLNTTLPFQNVSKYAFGNCSKYIGIKSLKLAVGLRNVPSRSSRGLFGAIAGFIEG
GWSGLVAGWYGFQHSNDQGVGMAADRDSTQKAIDKITSKVNNIVDKMNKQYEIIDHEFSEVETRLNMINN
KIDDQIQDIWAYNAELLVLLENQKTLDEHDANVNNLYNKVKRALGSNAVEDGKGCFELYHKCDDQCMETI
RNGTYNRRKYQEESKLERQKIEGVKLESEGTYKILTIYSTVASSLVIAMGFAAFLFWAMSNGSCRCNICI

>QOK09248.1 hemagglutinin [Influenza A virus]

METVSLITILLVATVSNADKICIGYQSTNSTETVDTLTENNVPVTHAKELLHTEHNGMLCATSLGQPLIL
DTCTIEGLIYGNPSCDLSLEGREWSYIVERPSAVHGLCYPGNVEDLEELRSLFSSARSYQRIQIFPDTIW
NVSYDGTSTACSGSFYRSMRWLTRKNGEYPIQDAQYTNNQGKNILFMWGINHPPTDDTQRGLYTRTDTTT
SVATEEINRIFKPLIGPRPLVNGLMGRINYYWSVLKPGQTLRIKSDGNLIAPWYGHILSGESHGRILKTD
LKRGSCTVQCQTEKGGLNTTLPFQNVSKYAFGNCSKYIGIKSLKLAVGLRNVPSRSSRGLFGAIAGFIEG
GWSGLVAGWYGFQHSNDQGVGMAADRDSTQKAIDKITSKVNNIVDKMNKQYEIIDHEFSEVETRLNMINN
KIDDQIQDIWAYNAELLVLLENQKTLDEHDANVNNLYNKVKRALGSNAVEDGKGCFELYHKCDDQCMETI
RNGTYNRRKYQEESKLERQKIEGVKLESEGTYKILTIYSTVASSLVIAMGFAAFLFWAMSNGSCRCNICI

>QOK09236.1 hemagglutinin [Influenza A virus]
METVSLITILLVATVSNADKICIGYQSTNSTETVDTLTENNVPVTHAKELLHTEHNGMLCATSLGQPLIL
DTCTIEGLIYGNPSCDLSLEGREWSYIVERPSAVHGLCYPGNVEDLEELRSLFSSARSYQRIQIFPDTIW
NVSYDGTSTACSGSFYRSMRWLTRKNGEYPIQDAQYTNNQGKNILFMWGINHPPTDDTQRGLYTRTDTTT
SVATEEINRIFKPLIGPRPLVNGLMGRINYYWSVLKPGQTLRIKSDGNLIAPWYGHILSGESHGRILKTD
LKRGSCTVQCQTEKGGLNTTLPFQNVSKYAFGNCSKYIGIKSLKLAVGLRNVPSRSSRGLFGAIAGFIEG
GWSGLVAGWYGFQHSNDQGVGMAADRDSTQKAIDKITSKVNNIVDKMNKQYEIIDHEFSEVETRLNMINN
KIDDQIQDIWAYNAELLVLLENQKTLDEHDANVNNLYNKVKRALGSNAVEDGKGCFELYHKCDDQCMETI
RNGTYNRRKYQEESKLERQKIEGVKLESEGTYKILTIYSTVASSLVIAMGFAAFLFWAMSNGSCRCNICI

>QOK09224.1 hemagglutinin [Influenza A virus]
METISLITILLVATVSNADKICIGYQSTNSTETVNTLTENNVPVTHAKELLHTEHNGMLCATSLGQPLIL
DTCTIEGLIYGNPSCDLSLEGREWSYIVERPSAVHGLCYPGNVEDLEELRSLFSSARSYQRIQIFPDTIW
NVSYDGTSTACSGSFYRSMRWLTRKNGEYPIQDAQYTNNQGKNILFMWGINHPPTDDTQRGLYTRTDTTT
SVATEEINRIFKPLIGPRPLVNGLMGRINYYWAVLKPGQTLRIKSDGNLIAPWYGHILSGESHGRILKTD
LKRGSCTVQCQTEKGGLNTTLPFQNVSKYAFGNCSKYIGIKSLKLAVGLRNVPSRSSRGLFGAIAGFIEG
GWSGLVAGWYGFQHSNDQGVGMAADRDSTQKAIDKITSKVNNIVDKMNKQYEIIDHEFSEVETRLNMINN
KIDDQIQDIWAYNAELLVLLENQKTLDEHDANVNNLYNKVKRALGSNAVEDGKGCFELYHKCDDQCMETI
RNGTYNRRKYQEESKLERQKIEGVKLESEGTYKILTIYSTVASSLVIAMGFAAFLFWAMSNGSCRCNICI

>QOK09212.1 hemagglutinin [Influenza A virus]
METISLITILLVATVSNADKICIGYQSTNSTETVNTLTENNVPVTHAKELLHTEHNGMLCATSLGQPLIL
DTCTIEGLIYGNPSCDLSLEGREWSYIVERPSAVHGLCYPGNVEDLEELRSLFSSARSYQRIQIFPDTIW
NVSYDGTSTACSGSFYRSMRWLTRKNGEYPIQDAQYTNNQGKNILFMWGINHPPTDDTQRGLYTRTDTTT
SVATEEINRIFKPLIGPRPLVNGLMGRINYYWAVLKPGQTLRIKSDGNLIAPWYGHILSGESHGRILKTD

LKRGSCTVQCQTEKGGLNTTLPFQNVSKYAFGNCSKYIGIKSLKLAVGLRNVPSRSSRGLFGAIAGFIEG
GWSGLVAGWYGFQHSNDQGVGMAADRDSTQKAIDKITSKVNNIVDKMNKQYEIIDHEFSEVETRLNMINN
KIDDQIQDIWAYNAELLVLLENQKTLDEHDANVNNLYNKVKRALGSNAVEDGKGCFELYHKCDDQCMETI
RNGTYNRRKYQEESKLERQKIEGVKLESEGTYKILTIYSTVASSLVIAMGFAAFLFWAMSNGSCRCNICI

>QOK09200.1 hemagglutinin [Influenza A virus]
METVSLITILLVATVSNADKICIGYQSTNSTETVDTLTENNVPVTHAKELLHTEHNGMLCATSLGQPLIL
DTCTIEGLIYGNPSCDLSLEGREWSYIVERPSAVHGLCYPGNVEDLEELRSLFSSARSYQRIQIFPDTIW
NVSYDGTSTACSGSFYKSMRWLTRKNGEYPTQDAQYTNNQGKNILFMWGINHPPTDDTQRGLYTRTDTTT
SVATEEINRIFKPLIGPRPLVNGLMGRINYYWSVLKPGQTLRIKSDGNLIAPWYGHILSGESHGRILKTD
LKRGSCTVQCQTEKGGLNTTLPFQNVSKYAFGNCSKYIGIKSLKLAVGLRNVPSRSSRGLFGAIAGFIEG
GWSGLVAGWYGFQHSNDQGVGMAADRDSTQKAIDKITSKVNNIVDKMNKQYEIIDHEFSEVETRLNMINN
KIDDQIQDIWAYNAELLILLENQKTLDEHDANVNNLYNKVKRALGSNAVEDGKGCFELYHKCDDQCMETI
RNGTYNRRKYQEESKLERQRIEGVKLESEGTYKILTIYSTVASSLVIAMGFAAFLFWAMSNGSCRCNICI

>QOK09188.1 hemagglutinin [Influenza A virus]
METVSLITILLVATVSNADKICIGYQSTNSTETVDTLTENNVPVTHAKELLHTEHNGMLCATSLGQPLIL
DTCTIEGLIYGNPSCDLSLEGREWSYIVERPSAVHGLCYPGNVEDLEELRSLFSSARSYQRIQIFPDTIW
NVSYDGTSTACSGSFYRSMRWLTRKNGEYPIQDAQYTNNQGKNILFMWGINHPPTDDTQRGLYTRTDTTT
SVATEEINRIFKPLIGPRPLVNGLMGRINYYWSVLKPGQTLRIKSDGNLIAPWYGHILSGESHGRILKTD
LKRGSCTVQCQTEKGGLNTTLPFQNVSKYAFGNCSKYIGIKSLKLAVGLRNVPSRSSRGLFGAIAGFIEG
GWSGLVAGWYGFQHSNDQGVGMAADRDSTQKAIDKITSKVNNIVDKMNKQYEIIDHEFSEVETRLNMINN
KIDDQIQDIWAYNAELLVLLENQKTLDEHDANVNNLYNKVKRALGSNAVEDGKGCFELYHKCDDQCMETI
RNGTYNRRKYQEESKLERQKIEGVKLESEGTYKILTIYSTVASSLVIAMGFAAFLFWAMSNGSCRCNICI

>QOK09176.1 hemagglutinin [Influenza A virus]
METVSLITILLVATVSNADKICIGYQSTNSTETVDTLTENNVPVTHAKELLHTEHNGMLCATSLGQPLIL
DTCTIEGLIYGNPSCDLSLEGREWSYIVERPSAVHGLCYPGNVEDLEELRSLFSSARSYQRIQIFPDTIW
NVSYDGTSTACSGSFYRSMRWLTRKNGEYPIQDAQYTNNQGKNILFMWGINHPPTDDTQRGLYTRTDTTT
SVATEEINRIFKPLIGPRPLVNGLMGRINYYWSVLKPGQTLRIKSDGNLIAPWYGHILSGESHGRILKTD
LKRGSCTVQCQTEKGGLNTTLPFQNVSKYAFGNCSKYIGIKSLKLAVGLRNVPSRSSRGLFGAIAGFIEG
GWSGLVAGWYGFQHSNDQGVGMAADRDSTQKAIDKITSKVNNIVDKMNKQYEIIDHEFSEVETRLNMINN
KIDDQIQDIWAYNAELLVLLENQKTLDEHDANVNNLYNKVKRALGSNAVEDGKGCFELYHKCDDQCMETI
RNGTYNRRKYQEESKLERQKIEGVKLESEGTYKILTIYSTVASSLVIAMGFAAFLFWAMSNGSCRCNICI

>QOK09164.1 hemagglutinin [Influenza A virus]
METVSLITILLVATVSNADKICIGYQSTNSTETVDTLTENNVPVTHAKELLHTEHNGMLCATSLGQPLIL
DTCTIEGLIYGNPSCDLSLEGREWSYIVERPSAVHGLCYPGNVEDLEELRSLFSSARSYQRIQIFPDTIW
NVSYDGTSTACSGSFYRSMRWLTRKNGEYPIQDAQYTNNQGKNILFMWGINHPPTDDTQRGLYTRTDTTT
SVATEEINRIFKPLIGPRPLVNGLMGRINYYWSVLKPGQTLRIKSDGNLIAPWYGHILSGESHGRILKTD
LKRGSCTVQCQTEKGGLNTTLPFQNVSKYAFGNCSKYIGIKSLKLAVGLRNVPSRSSRGLFGAIAGFIEG
GWSGLVAGWYGFQHSNDQGVGMAADRDSTQKAIDKITSKVNNIVDKMNKQYEIIDHEFSEVETRLNMINN
KIDDQIQDIWAYNAELLVLLENQKTLDEHDANVNNLYNKVKRALGSNAVEDGKGCFELYHKCDDRCMETI
RNGTYNRRKYQEESKLERQKIEGVKLESEGTYKILTIYSTVASSLVIAMGFAAFLFWAMSNGSCRCNICI

>QOK09152.1 hemagglutinin [Influenza A virus]
METVSLITILLVATVSNADKICIGYQSTNSTETVDTLTENNVPVTHAKELLHTEHNGMLCATSLGQPLIL
DTCTIEGLIYGNPSCDLSLEGREWSYIVERPSAVHGLCYPGNVEDLEELRSLFSSARSYQRIQIFPDTIW
NVSYDGTSTACSGSFYRSMRWLTRKNGEYPIQDAQYTNNQGKNILFMWGINHPPTDDTQRGLYTRTDTTT
SVATEEINRIFKPLIGPRPLVNGLMGRINYYWSVLKPGQTLRIKSDGNLIAPWYGHILSGESHGRILKTD
LKRGSCTVQCQTEKGGLNTTLPFQNVSKYAFGNCSKYIGIKSLKLAVGLRNVPSRSSRGLFGAIAGFIEG
GWSGLVAGWYGFQHSNDQGVGMAADRDSTQKAIDKITSKVNNIVDKMNKQYEIIDHEFSEVETRLNMINN
KIDDQIQDIWAYNAELLVLLENQKTLDEHDANVNNLYNKVKRALGSNAVEDGKGCFELYHKCDDQCMETI
RNGTYNRRKYQEESKLERQKIEGVKLESEGTYKILTIYSTVASSLVIAMGFAAFLFWAMSNGSCRCNICI

>QOK09140.1 hemagglutinin [Influenza A virus]
METVSLITILLVATVSNADKICIGYQSTNSTETVDTLTENNVPVTHAKELLHTEHNGMLCATSLGQPLIL
DTCTIEGLIYGNPSCDLSLEGREWSYIVERPSAVHGLCYPGNVEDLEELRSLFSSARSYQRIQIFPDTIW
NVSYDGTSTACSGSFYRSMRWLTRKNGEYPIQDAQYTNNQGKNILFMWGINHPPTDDTQRGLYTRTDTTT
SVATEEINRIFKPLIGPRPLVNGLMGRINYYWSVLKPGQTLRIKSDGNLIAPWYGHILSGESHGRILKTD
LKRGSCTVQCQTEKGGLNTTLPFQNVSKYAFGNCSKYIGIKSLKLAVGLRNVPSRSSRGLFGAIAGFIEG
GWSGLVAGWYGFQHSNDQGVGMAADRDSTQKAIDKITSKVNNIVDKMNKQYEIIDHEFSEVETRLNMINN
KIDDQIQDIWAYNAELLVLLENQKTLDEHDANVNNLYNKVKRALGSNAVEDGKGCFELYHKCDDQCMETI
RNGTYNRRKYQEESKLERQKIEGVKLESEGTYKILTIYSTVASSLVIAMGFAAFLFWAMSNGSCRCNICI

>QOK09128.1 hemagglutinin [Influenza A virus]
METVSLITILLVATVSNADKICIGYQSTNSTETVDTLTENNVPVTHAKELLHTEHNGMLCATSLGQPLIL
DTCTIEGLIYGNPSCDLSLEGREWSYIVERPSAVHGLCYPGNVEDLEELRSLFSSARSYQRIQIFPDTIW

NVSYDGTSTACSGSFYRSMRWLTRKNGEYPIQDAQYTNNQGKNILFMWGINHPPTDDTQRGLYTRTDTTT
SVATEEINRIFKPLIGPRPLVNGLMGRINYYWSVLKPGQTLRIKSDGNLIAPWYGHILSGESHGRILKTD
LKRGSCTVQCQTEKGGLNTTLPFQNVSKYAFGNCSKYIGIKSLKLAVGLRNVPSRSSRGLFGAIAGFIEG
GWSGLVAGWYGFQHSNDQGVGMAADRDSTQKAIDKITSKVNNIVDKMNKQYEIIDHEFSEVETRLNMINN
KIDDQIQDIWAYNAELLVLLENQKTLDEHDANVNNLYNKVKRALGSNAVEDGKGCFELYHKCDDQCMETI
RNGTYNRRKYQEESKLERQKIEGVKLESEGTYKILTIYSTVASSLVIAMGFAAFLFWAMSNGSCRCNICI

>QOK09116.1 hemagglutinin [Influenza A virus]
METVSLITILLVATVSNADKICIGYQSTNSTETVDTLTENNVPVTHAKELLHTEHNGMLCATSLGQPLIL
DTCTIEGLIYGNPSCDLSLEGREWSYIVERPSAVHGLCYPGNVEDLEELRSLFSSARSYQRIQIFPDTIW
NVSYDGTSTACSGSFYRSMRWLTRKNGEYPIQDAQYTNNQGKNILFMWGINHPPTDDTQRGLYTRTDTTT
SVATEEINRIFKPLIGPRPLVNGLMGRINYYWSVLKPGQTLRIKSDGNLIAPWYGHILSGESHGRILKTD
LKRGSCTVQCQTEKGGLNTTLPFQNVSKYAFGNCSKYIGIKSLKLAVGLRNVPSRSSRGLFGAIAGFIEG
GWSGLVAGWYGFQHSNDQGVGMAADRDSTQKAIDKITSKVNNIVDKMNKQYEIIDHEFSEVETRLNMINN
KIDDQIQDIWAYNAELLVLLENQKTLDEHDANVNNLYNKVKRALGSNAVEDGKGCFELYHKCDDQCMETI
RNGTYNRRKYQEESKLERQKIEGVKLESEGTYKILTIYSTVASSLVIAMGFAAFLFWAMSNGSCRCNICI

>QOK09104.1 hemagglutinin [Influenza A virus]
METVSLITILLVATVSNADKICIGYQSTNSTETVDTLTENNVPVTHAKELLHTEHNGMLCATSLGQPLIL
DTCTIEGLIYGNPSCDLSLEGREWSYIVERPSAVHGLCYPGNVEDLEELRSLFSSARSYQRIQIFPDTIW
NVSYDGTSTACSGSFYRSMRWLTRKNGEYPIQDAQYTNNQGKNILFMWGINHPPTDDTQRGLYTRTDTTT
SVATEEINRIFKPLIGPRPLVNGLMGRINYYWSVLKPGQTLRIKSDGNLIAPWYGHILSGESHGRILKTD
LKRGSCTVQCQTEKGGLNTTLPFQNVSKYAFGNCSKYIGIKSLKLAVGLRNVPSRSSRGLFGAIAGFIEG
GWSGLVAGWYGFQHSNDQGVGMAADRDSTQKAIDKITSKVNNIVDKMNKQYEIIDHEFSEVETRLNMINN
KIDDQIQDIWAYNAELLVLLENQKTLDEHDANVNNLYNKVKRALGSNAVEDGKGCFELYHKCDDQCMETI
RNGTYNRRKYQEESKLERQKIEGVKLESEGTYKILTIYSTVASSLVIAMGFAAFLFWAMSNGSCRCNICI

>QOK09092.1 hemagglutinin [Influenza A virus]
METVSLITILLVATVSNADKICIGYQSTNSTETVDTLTENNVPVTHAKELLHTEHNGMLCATSLGQPLIL
DTCTIEGLIYGNPSCDLSLEGREWSYIVERPSAVHGLCYPGNVEDLEELRSLFSSARSYQRIQIFPDTIW
NVSYDGTSTACSGSFYRSMRWLTRKNGEYPIQDAQYTNNQGKNILFMWGINHPPTDDTQRGLYTRTDTTT
SVATEEINRIFKPLIGPRPLVNGLMGRINYYWSVLKPGQTLRIKSDGNLIAPWYGHILSGESHGRILKTD
LKRGSCTVQCQTEKGGLNTTLPFQNVSKYAFGNCSKYIGIKSLKLAVGLRNVPSRSSRGLFGAIAGFIEG
GWSGLVAGWYGFQHSNDQGVGMAADRDSTQKAIDKITSKVNNIVDKMNKQYEIIDHEFSEVETRLNMINN

KIDDQIQDIWAYNAELLVLLENQKTLDEHDANVNNLYNKVKRALGSNAVEDGKGCFELYHKCDDQCMETI
RNGTYNRRKYQEESKLERQKIEGVKLESEGTYKILTIYSTVASSLVIAMGFAAFLFWAMSNGSCRCNICI

>QOK09080.1 hemagglutinin [Influenza A virus]
METVSLITILLVATVSNADKICIGYQSTNSTETVDTLTENNVPVTHAKELLHTEHNGMLCATSLGQPLIL
DTCTIEGLIYGNPSCDLSLEGREWSYIVERPSAVHGLCYPGNVEDLEELRSLFSSARSYQRIQIFPDTIW
NVSYDGTSTACSGSFYRSMRWLTRKNGEYPIQDAQYTNNQGKNILFMWGINHPPTDDTQRGLYTRTDTTT
SVATEEINRIFKPLIGPRPLVNGLMGRINYYWSVLKPGQTLRIKSDGNLIAPWYGHILSGESHGRILKTD
LKRGSCTVQCQTEKGGLNTTLPFQNVSKYAFGNCSKYIGIKSLKLAVGLRNVPSRSSRGLFGAIAGFIEG
GWSGLVAGWYGFQHSNDQGVGMAADRDSTQKAIDKITSKVNNIVDKMNKQYEIIDHEFSEVETRLNMINN
KIDDQIQDIWAYNAELLVLLENQKTLDEHDANVNNLYNKVKRALGSNAVEDGKGCFELYHKCDDQCMETI
RNGTYNRRKYQEESKLERQKIEGVKLESEGTYKILTIYSTVASSLVIAMGFAAFLFWAMSNGSCRCNICI

>QOK09068.1 hemagglutinin [Influenza A virus]
METVSLITILLVATVSNADKICIGYQSTNSTETVDTLTENNVPVTHAKELLHTEHNGMLCATSLGQPLIL
DTCTIEGLIYGNPSCDLSLEGREWSYIVERPSAVHGLCYPGNVEDLEELRSLFSSARSYQRIQIFPDTIW
NVSYDGTSTACSGSFYRSMRWLTRKNGEYPIQDAQYTNNQGKNILFMWGINHPPTDDTQRGLYTRTDTTT
SVATEEINRIFKPLIGPRPLVNGLMGRINYYWSVLKPGQTLRIKSDGNLIAPWYGHILSGESHGRILKTD
LKRGSCTVQCQTEKGGLNTTLPFQNVSKYAFGNCSKYIGIKSLKLAVGLRNVPSRSSRGLFGAIAGFIEG
GWSGLVAGWYGFQHSNDQGVGMAADRDSTQKAIDKITSKVNNIVDKMNKQYEIIDHEFSEVETRLNMINN
KIDDQIQDIWAYNAELLVLLENQKTLDEHDANVNNLYNKVKRALGSNAVEDGKGCFELYHKCDDQCMETI
RNGTYNRRKYQEESKLERQKIEGVKLESEGTYKILTIYSTVASSLVIAMGFAAFLFWAMSNGSCRCNICI

>QOK09056.1 hemagglutinin [Influenza A virus]
METVSLITILLVATVSNADKICIGYQSTNSTETVDTLTENNVPVTHAKELLHTEHNGMLCATSLGQPLIL
DTCTIEGLIYGNPSCDLSLEGREWSYIVERPSAVHGLCYPGNVEDLEELRSLFSSARSYQRIQIFPDTIW
NVSYDGTSTACSGSFYRSMRWLTRKNGEYPIQDAQYTNNQGKSILFMWGINHPPTDDTQRGLYTRTDTTT
SVATEEINRIFKPLIGPRPLVNGLMGRINYYWSVLKPGQTLRIKSDGNLIAPWYGHILSGESHGRILKTD
LKRGSCTVQCQTEKGGLNTTLPFQNVSKYAFGNCSKYIGIKSLKLAVGLRNVPSRSSRGLFGAIAGFIEG
GWSGLVAGWYGFQHSNDQGVGMAADRDSTQKAIDKITSKVNNIVDKMNKQYEIIDHEFSEVETRLNMINN
KIDDQIQDIWAYNAELLVLLENQKTLDEHDANVNNLYNKVKRALGSNAVEDGKGCFELYHKCDDQCMETI
RNGTYNRRKYQEESKLERQKIEGVKLESEGTYKILTIYSTVASSLVIAMGFAAFLFWAMSNGSCRCNICI

>QOK09044.1 hemagglutinin [Influenza A virus]

METVSLITILLVATVSNADKICIGYQSTNSTETVDTLTENNVPVTHAKELLHTEHNGMLCATSLGQPLIL
DTCTIEGLIYGNPSCDLSLEGREWSYIVERPSAVHGLCYPGNVEDLEELRSLFSSARSYQRIQIFPDTIW
NVSYDGTSTACSGSFYRSMRWLTRKNGEYPIQDAQYTNNQGKNILFMWGINHPPTDDTQRGLYTRTDTTT
SVATEEINRIFKPLIGPRPLVNGLMGRINYYWSVLKPGQTLRIKSDGNLIAPWYGHILSGESHGRILKTD
LKRGSCTVQCQTEKGGLNTTLPFQNVSKYAFGNCSKYIGIKSLKLAVGLRNVPSRSSRGLFGAIAGFIEG
GWSGLVAGWYGFQHSNDQGVGMAADRDSTQKAIDKITSKVNNIVDKMNKQYEIIDHEFSEVETRLNMINN
KIDDQIQDIWAYNAELLVLLENQKTLDEHDANVNNLYNKVKRALGSNAVEDGKGCFELYHKCDDQCMETI
RNGTYNRRKYQEESKLERQKIEGVKLESEGTYKILTIYSTVASSLVIAMGFAAFLFWAMSNGSCRCNICI

>QOK09032.1 hemagglutinin [Influenza A virus]
METVSLITILLVATVSNADKICIGYQSTNSTETVDTLTENNVPVTHAKELLHTEHNGMLCATSLGQPLIL
DTCTIEGLIYGNPSCDLSLEGREWSYIVERPSAVHGLCYPGNVENLEELRSLFSSARSYQRIQIFPDTIW
NVSYDGTSTACSGSFYRSMRWLTRKNGDYPTQDAQYTNNQGKNILFMWGINHPPTDTTQSGLYTRTDTTT
SVATEEINRIFKPLIGPRPLVNGLMGRIDYYWSVLKPGQTLRIKSDGNLIAPWYGHILSGESHGRILKTD
LKRGSCTVQCQTEKGGLNTTLPFQNVSKYAFGNCSKYIGIKSLKLAVGLRNVPSRSSRGLFGAIAGFIEG
GWSGLVAGWYGFQHSNDQGVGMAADRDSTQKAIDKITSKVNNIVDKMNKQYEIIDHEFSEVETRLNMINN
KIDDQIQDIWAYNAELLVLLENQKTLDEHDANVNNLYNKVKRALGSNAVEDGKGCFELYHKCDDQCMETI
RNGTYNRRKYQEESKLERQKIEGVKLESEGTYKILTIYSTVASSLVIAMGFAAFLFWAMSNGSCRCNICI

>QOK09020.1 hemagglutinin [Influenza A virus]
METVSLITILLVATVSNADKICIGYQSTNSTETVDTLTENNVPVTHAKELLHTEHNGMLCATSLGQPLIL
DTCTIEGLIYGNPSCDLSLEGREWSYIVERPSAVHGLCYPGNVEDLEELRSLFSSARSYQRIQIFPDTIW
NVSYDGTSTACSGSFYRSMRWLTRKNGEYPIQDAQYTNNQGKSILFMWGINHPPTDDTQRGLYTRTDTTT
SVATEEINRIFKPLIGPRPLVNGLMGRINYYWSVLKPGQTLRIKSDGNLIAPWYGHILSGESHGRILKTD
LKRGSCTVQCQTEKGGLNTTLPFQNVSKYAFGNCSKYIGIKSLKLAVGLRNVPSRSSRGLFGAIAGFIEG
GWSGLVAGWYGFQHSNDQGVGMAADRDSTQKAIDKITSKVNNIVDKMNKQYEIIDHEFSEVETRLNMINN
KIDDQIQDIWAYNAELLVLLENQKTLDEHDANVNNLYNKVKRALGSNAVEDGKGCFELYHKCDDQCMETI
RNGTYNRRKYQEESKLERQKIEGVKLESEGTYKILTIYSTVASSLVIAMGFAAFLFWAMSNGSCRCNICI

>QOK09008.1 hemagglutinin [Influenza A virus]
METVSLITILLVATVSNADKICIGYQSTNSTETVDTLTENNVPVTHAKELLHTEHNGMLCATSLGQPLIL
DTCTIEGLIYGNPSCDLSLEGREWSYIVERPSAVHGLCYPGNVEDLEELRSLFSSARSYQRIQIFPDTIW
NVSYDGTSTACSGSFYRSMRWLTRKNGEYPIQDAQYTNNQGKNILFMWGINHPPTDDTQRGLYTRTDTTT
SVATEEINRIFKPLIGPRPLVNGLMGRINYYWSVLKPGQTLRIKSDGNLIAPWYGHILSGESHGRILKTD

LKRGSCTVQCQTEKGGLNTTLPFQNVSKYAFGNCSKYIGIKSLKLAVGLRNVPSRSSRGLFGAIAGFIEG
GWSGLVAGWYGFQHSNDQGVGMAADRDSTQKAIDKITSKVNNIVDKMNKQYEIIDHEFSEVETRLNMINN
KIDDQIQDIWAYNAELLVLLENQKTLDEHDANVNNLYNKVKRALGSNAVEDGKGCFELYHKCDDQCMETI
RNGTYNRRKYQEESKLERQKIEGVKLESEGTYKILTIYSTVASSLVIAMGFAAFLFWAMSNGSCRCNICI

>QOK08996.1 hemagglutinin [Influenza A virus]
METVSLITILLVATVSNADKICIGYQSTNSTETVDTLTENNVPVTHAKELLHTEHNGMLCATSLGQPLIL
DTCTIEGLIYGNPSCDLSLEGREWSYIVERPSAVHGLCYPGNVEDLEELRSLFSSARSYQRIQIFPDTIW
NVSYDGTSTACSGSFYRSMRWLTRKNGEYPIQDAQYTNNQGKNILFMWGINHPPTDDTQRGLYTRTDTTT
SVATEEINRIFKPLIGPRPLVNGLMGRINYYWSVLKPGQTLRIKSDGNLIAPWYGHILSGESHGRILKTD
LKRGSCTVQCQTEKGGLNTTLPFQNVSKYAFGNCSKYIGIKSLKLAVGLRNVPSRSSRGLFGAIAGFIEG
GWSGLVAGWYGFQHSNDQGVGMAADRDSTQKAIDKITSKVNNIVDKMNKQYEIIDHEFSEVETRLNMINN
KIDDQIQDIWAYNAELLVLLENQKTLDEHDANVNNLYNKVKRALGSNAVEDGKGCFELYHKCDDQCMETI
RNGTYNRRKYQEESKLERQKIEGVKLESEGTYKILTIYSTVASSLVIAMGFAAFLFWAMSNGSCRCNICI

>QOK08972.1 hemagglutinin [Influenza A virus]
METVSLITILLVATVSNADKICIGYQSTNSTETVDTLTENNVPVTHAKELLHTEHNGMLCATSLGQPLIL
DTCTIEGLIYGNPSCDLSLEGREWSYIVERPSAVHGLCYPGNVEDLEELRSLFSSARSYQRIQIFPDTIW
NVSYDGTSTACSGSFYRSMRWLTRKNGEYPIQDAQYTNNQGKNILFMWGINHPPTDDTQRRLYTRTDTTT
SVATEEINRIFKPLIGPRPLVNGLMGRINYYWSVLKPGQTLRIKSDGNLIAPWYGHILSGESHGRILKTD
LKRGSCTVQCQTEKGGLNTTLPFQNVSKYAFGNCSKYIGIKSLKLAVGLRNVPSRSSRGLFGAIAGFIEG
GWSGLVAGWYGFQHSNDQGVGMAADRDSTQKAIDKITSKVNNIVDKMNKQYEIIDHEFSEVETRLNMINN
KIDDQIQDIWAYNAELLVLLENQKTLDEHDANVNNLYNKVKRALGSNAVEDGKGCFELYHKCDDQCMETI
RNGTYNRRKYQEESKLERQKIEGVKLESEGTYKILTIYSTVASSLVIAMGFAAFLFWAMSNGSCRCNICI

>QOK08924.1 hemagglutinin [Influenza A virus]
METVSLITILLVATVSNADKICIGYQSTNSTETVDTLTENNVPVTHAKELLHTEHNGMLCATSLGQPLIL
DTCTIEGLIYGNPSCDLSLEGREWSYIVERPSAVHGLCYPGNVEDLEELRSLFSSARSYQRIQIFPDTIW
NVSYDGTSTACSGSFYKSMRWLTRKNGEYPTQDAQYTNNQGKNILFMWGINHPPTDDTQRGLYTRTDTTT
SVATEEINRIFKPLIGPRPLVNGLMGRINYYWSVLKPGQTLRIKSDGNLIAPWYGHILSGESHGRILKTD
LKRGSCTVQCQTEKGGLNTTLPFQNVSKYAFGNCSKYIGIKSLKLAVGLRNVPSKSSRGLFGAIAGFIEG
GWSGLVAGWYGFQHSNDQGVGIAADRDSTQKAIDKITSKVNNIVDKMNKQYEIIDHEFSEVETRLNMINN
KIDDQIQDIWAYNAELLVLLENQKTLDEHDANVNNLYNKVKRALGSNAVEDGKGCFELYHKCDDQCMETI
RNGTYNRRKYQEESKLERQRIEGVKLESEETYKILTIYSTVASSLVFAMGFAALLFWAMSNGSCRCNICI

>QOK08912.1 hemagglutinin [Influenza A virus]
METVSLITILLVATVSNADKICIGYQSTNSTETVDTLTENNVPVTHAKELLHTEHNGMLCATSLGQPLIL
DTCTIEGLIYGNPSCDLSLEGREWSYIVERPSAVHGLCYPGNVEDLEELRSLFSSARSYQRIQIFPDTIW
NVSYDGTSTACSGSFYKSMRWLTRKNGEYPTQDAQYTNNQGKNILFMWGINHPPTDDTQRGLYTRTDTTT
SVATEEINRIFKPLIGPRPLVNGLMGRINYYWSVLKPGQTLRIKSDGNLIAPWYGHILSGESHGRILKTD
LKRGSCTVQCQTEKGGLNTTLPFQNVSKYAFGNCSKYIGIKSLKLAVGLRNVPSKSSRGLFGAIAGFIEG
GWSGLVAGWYGFQHSNDQGVGIAADRDSTQKAIDKITSKVNNIVDKMNKQYEIIDHEFSEVETRLNMINN
KIDDQIQDIWAYNAELLVLLENQKTLDEHDANVNNLYNKVKRALGSNAVEDGKGCFELYHKCDDQCMETI
RNGTYNRRKYQEESKLERQRIEGVKLESEETYKILTIYSTVASSLVFAMGFAALLFWAMSNGSCRCNICI

>QOK08900.1 hemagglutinin [Influenza A virus]
METVSLITILLVATVSNADKICIGYQSTNSTETVDTLTENNVPVTHAKELLHTEHNGMLCATSLGQPLIL
DTCTIEGLIYGNPSCDLSLEGREWSYIVERPSAVHGLCYPGNVEDLEELRSLFSSARSYQRIQIFPDTIW
NVSYDGTSTACSGSFYKSMRWLTRKNGEYPTQDAQYTNNQGKNILFMWGINHPPTDDTQRGLYTRTDTTT
SVATEEINRIFKPLIGPRPLVNGLMGRINYYWSVLKPGQTLRIKSDGNLIAPWYGHILSGESHGRILKTD
LKRGSCTVQCQTEKGGLNTTLPFQNVSKYAFGNCSKYIGIKSLKLAVGLRNVPSKSSRGLFGAIAGFIEG
GWSGLVAGWYGFQHSNDQGVGIAADRDSTQKAIDKITSKVNNIVDKMNKQYEIIDHEFSEVETRLNMINN
KIDDQIQDIWAYNAELLVLLENQKTLDEHDANVNNLYNKVKRALGSNAVEDGKGCFELYHKCDDQCMETI
RNGTYNRRKYQEESKLERQRIEGVKLESEETYKILTIYSTVASSLVFAMGFAALLFWAMSNGSCRCNICI

>QOK08888.1 hemagglutinin [Influenza A virus]
METVSLITILLVATVSNADKICIGYQSTNSTETVDTLTENNVPVTHAKELLHTEHNGMLCATSLGQPLIL
DTCTIEGLIYGNPSCDLSLEGREWSYIVERPSAVHGLCYPGNVEDLEELRSLFSSARSYQRIQIFPDTIW
NVSYDGTSTACSGSFYKSMRWLTRKNGEYPTQDAQYTNNQGKNILFMWGINHPPTDDTQRGLYTRTDTTT
SVATEEINRIFKPLIGPRPLVNGLMGRINYYWSVLKPGQTLRIKSDGNLIAPWYGHILSGESHGRILKTD
LKRGSCTVQCQTEKGGLNTTLPFQNVSKYAFGNCSKYIGIKSLKLAVGLRNVPSKSSRGLFGAIAGFIEG
GWSGLVAGWYGFQHSNDQGVGIAADRDSTQKAIDKITSKVNNIVDKMNKQYEIIDHEFSEVETRLNMINN
KIDDQIQDIWAYNAELLVLLENQKTLDEHDANVNNLYNKVKRALGSNAVEDGKGCFELYHKCDDQCMETI
RNGTYNRRKYQEESKLERQRIEGVKLESEETYKILTIYSTVASSLVFAMGFAALLFWAMSNGSCRCNICI

>QOK08876.1 hemagglutinin [Influenza A virus]
METVSLITILLVATVSNADKICIGYQSTNSTETVDTLTENNVPVTHAKELLHTEHNGMLCATSLGQPLIL
DTCTIEGLIYGNPSCDLSLEGREWSYIVERPSAVHGLCYPGNVEDLEELRSLFSSARSYQRIQIFPDTIW

NVSYDGTSTACSGSFYKSMRWLTRKNGEYPTQDAQYTNNQGKNILFMWGINHPPTDDTQRGLYTRTDTTT
SVATEEINRIFKPLIGPRPLVNGLMGRINYYWSVLKPGQTLRIKSDGNLIAPWYGHILSGESHGRILKTD
LKRGSCTVQCQTEKGGLNTTLPFQNVSKYAFGNCSKYIGIKSLKLAVGLRNVPSKSSRGLFGAIAGFIEG
GWSGLVAGWYGFQHSNDQGVGIAADRDSTQKAIDKITSKVNNIVDKMNKQYEIIDHEFSEVETRLNMINN
KIDDQIQDIWAYNAELLVLLENQKTLDEHDANVNNLYNKVKRALGSNAVEDGKGCFELYHKCDDQCMETI
RNGTYNRRKYQEESKLERQRIEGVKLESEETYKILTIYSTVASSLVFAMGFAALLFWAMSNGSCRCNICI

>QOK08864.1 hemagglutinin [Influenza A virus]
METVSLITILLVATVSNADKICIGYQSTNSTETVDTLTENNVPVTHAKELLHTEHNGMLCATSLGQPLIL
DTCTIEGLIYGNPSCDLSLEGREWSYIVERPSAVHGLCYPGNVEDLEELRSLFSSARSYQRIQIFPDTIW
NVSYDGTSTACSGSFYKSMRWLTRKNGEYPTQDAQYTNNQGKNILFMWGINHPPTDDTQRGLYTRTDTTT
SVATEEINRIFKPLIGPRPLVNGLMGRINYYWSVLKPGQTLRIKSDGNLIAPWYGHILSGESHGRILKTD
LKRGSCTVQCQTEKGGLNTTLPFQNVSKYAFGNCSKYIGIKSLKLAVGLRNVPSKSSRGLFGAIAGFIEG
GWSGLVAGWYGFQHSNDQGVGIAADRDSTQKAIDKITSKVNNIVDKMNKQYEIIDHEFSEVETRLNMINN
KIDDQIQDIWAYNAELLVLLENQKTLDEHDANVNNLYNKVKRALGSNAVEDGKGCFELYHKCDDQCMETI
RNGTYNRRKYQEESKLERQRIEGVKLESEETYKILTIYSTVASSLVFAMGFAALLFWAMSNGSCRCNICI

>QOK08852.1 hemagglutinin [Influenza A virus]
METVSLITILLVATVSNADKICIGYQSTNSTETVDTLTENNVPVTHAKELLHTEHNGMLCATSLGQPLIL
DTCTIEGLIYGNPSCDLSLEGREWSYIVERPSAVNGLCYPGSVENLEELRSLFSSARSYQRIQIFPDTIW
NVSYDGTSTACSGSFYRSMRWLTRKNGVYPIQDAQYTNNQGKNILFIWGINHPPTDDTQTGLYTRTDTTT
SVATEEINRIFKPLIGPRPLVNGLMGRINYYWSVLKPGQTLRIKSDGNLIAPWYGHILSGESHGRILKTD
LKRGSCTVQCQTEKGGLNTTLPFQNVSKYAFGNCSKYIGIKSLKLAVGLRNVPSRSSRGLFGAIAGFIEG
GWSGLVAGWYGFQHSNDQGVGMAADRDSTQKAIDKITSKVNNIVDKMNKQYEIIDHEFSEVEARLNMINN
KIDDQIQDIWAYNAELLVLLENQKTLDEHDANVNNLYNKVKRALGSNAVEDGKGCFELYHKCDDQCMETI
RNGTYNRRKYQEESKLARQKIEGVKLESEGTYKILTIYSTVASSLVIAMGFAAFLFWAMSNGSCRCNICI

>QOK08840.1 hemagglutinin [Influenza A virus]
METVSLITILLVATVSNADKICIGYQSTNSTETVDTLTENNVPVTHAKELLHTEHNGMLCATSLGQPLIL
DTCTIEGLIYGNPSCDLSLEGREWSYIVERPSAVHGLCYPGKVEDLEELRSLFSSARSYQRIQIFPDTIW
NVSYDGTSTACSGSFYKSMRWLTRKNGEYPTQDAQYTNNQGKNILFMWGINHPPTDNTQRELYTRTDTTT
SVATEEINRIFKPLIGPRPLVNGLMGRISYYWSVLKPGQTLRIKSDGNLIAPWYGHILSGESHGRILKTD
LKRGSCTVQCQTEKGGLNTTLPFQNVSKYAFGNCSKYIGIKSLKLAVGLRNVPSKSSRGLFGAIAGFIEG
GWSGLVAGWYGFQHSNDQGVGMAADRDSTQKAIDKITSKVNNIVDKMNKQYEIIDHEFSEVETRLNMINN

KIDDQIQDIWAYNAELLVLLENQKTLDEHDANVNNLYNKVKRALGSNAVEDGKGCFELYHKCDDQCMETI
RNGTYNRRKYQEESKLERQRIEGVKLESEETYKILTIYSTVASSLVFAMGFAALLFWAMSNGSCRCNICI

>QOK08828.1 hemagglutinin [Influenza A virus]
METVSLITILLVATVSNADKICIGYQSTNSTETVDTLTENNVPVTHAKELLHTEHNGMLCATSLGQPLIL
DTCTIEGLIYGNPSCDLSLEGREWSYIVERPSAVHGLCYPGNVEDLEELRSLFSSARSYQRIQIFPDTIW
NVSYDGTSTACSGSFYKSMRWLTRKNGEYPTQDAQYTNNQGKNILFMWGINHPPTDDTQRGLYTRTDTTT
SVATEEINRIFKPLIGPRPLVNGLMGRINYYWSVLKPGQTLRIKSDGNLIAPWYGHILSGESHGRILKTD
LKRGSCTVQCQTEKGGLNTTLPFQNVSKYAFGNCSKYIGIKSLKLAVGLRNVPSKSSRGLFGAIAGFIEG
GWSGLVAGWYGFQHSNDQGVGIAADRDSTQKAIDKITSKVNNIVDKMNKQYEIIDHEFSEVETRLNMINN
KIDDQIQDIWAYNAELLVLLENQKTLDEHDANVNNLYNKVKRALGSNAVEDGKGCFELYHKCDDQCMETI
RNGTYNRRKYQEESKLERQRIEGVKLESEETYKILTIYSTVASSLVFAMGFAALLFWAMSNGSCRCNICI

>QOK08816.1 hemagglutinin [Influenza A virus]
METVSLITILLVATVSNADKICIGYQSTNSTETVDTLTENNVPVTHAKELLHTEHNGMLCATSLGQPLIL
DTCTIEGLIYGNPSCNLSLEGREWSYIVERPSAVHGLCYPGKIEDLEELRSLFSSARSYQRIQIFPDTIW
NVSYDGTSTACSGSFYRSMRWLTRKDGNYPIQDAQYTNNQGKNILFMWGINHPPTDETQRGLYTRTDTTT
SVATEEINRIFKPLIGPRPLVNGLMGRINYYWSVLKPGQTLRIKSDGNLIAPWYGHILSGESHGRILKTD
LKKGSCTVQCQTEKGGLNTTLPFQNVSKYAFGNCSKYIGIKSLKLAVGLRNVPSRSSRGLFGAIAGFIEG
GWPGLVAGWYGFQHSNDQGVGMAADRDSTQKAIDKITSKVNNIVDKMNKQYEIIDHEFSEVETRLNMINN
KIDDQIQDIWAYNAELLVLLENQKTLDEHDANVNNLYNKVKRALGSNAVEDGKGCFELYHKCDDQCMETI
RNGTYNRRKYQEESKLERQKIEGVKLESEGTYKILTIYSTVASSLVIAMGFAAFLFWAMSNGSCRCNICI

>QOK08804.1 hemagglutinin [Influenza A virus]
METVSLITILVVATVSNADKICIGYQSTNSTETVDTLTENNVPVTHAKELLHTEHNGMLCATSLGHPLIL
DTCTIEGLIYGNPSCDPLLGGREWSYIVERPSAVNGLCYPGNVENLEELRSLFSSSRSYQRIQIFPDTIW
NVSYSGTSKACSDSFYRSMRWLTQKNNAYPTQDAQYTNNQGKNILFMWGINHPPTDTAQTNLYTRTDTTT
SVATEEMNRIFKPLIGPRPLVNGLMGRINYYWSVLKPGQTLRIKSDGNLIAPWYGHILSGESHGRILKTD
LKRGSCTVQCQTEKGGLNTTLPFQNVSKYAFGNCSKYIGVKSLKLAVGLRNVPSRSSRGLFGAIAGFIEG
GWSGLVAGWYGFQHSNDQGVGMAADRDSTQKAIDKITSKVNNIVDKMNKQYEIIDHEFSEVETRLNMINN
KVDDQIQDIWAYNAELLVLLENQKTLDEHDANVNNLYNKVKRALGSNAVEDGKGCFELYHKCDDHCMETI
RNGTYNRRKYQEESKLERQKIEGVKLESEETYKILTIYSTVASSLVIAMGFAAFLFWAMSNGSCRCNICI

>QOK08792.1 hemagglutinin [Influenza A virus]

METVSLITILVVATVSNADKICIGYQSTNSTETVDTLTENNVPVTHAKELLHTEHNGMLCATSLGHPLIL
DTCTIEGLIYGNPSCDPLLGGREWSYIVERPSAVNGLCYPGNVENLEELRSLFSSSRSYQRIQIFPDTIW
NVSYSGTSKACSDSFYRSMRWLTQKNNAYPTQDAQYTNNQGKNILFMWGINHPPTDTAQTNLYTRTDTTT
SVATEEMNRIFKPLIGPRPLVNGLMGRINYYWSVLKPGQTLRIKSDGNLIAPWYGHILSGESHGRILKTD
LKRGSCTVQCQTEKGGLNTTLPFQNVSKYAFGNCSKYIGVKSLKLAVGLRNVPSRSSRGLFGAIAGFIEG
GWSGLVAGWYGFQHSNDQGVGMAADRDSTQKAIDKITSKVNNIVDKMNKQYEIIDHEFSEVETRLNMINN
KVDDQIQDIWAYNAELLVLLENQKTLDEHDANVNNLYNKVKRALGSNAVEDGKGCFELYHKCDDHCMETI
RNGTYNRRKYQEESKLERQKIEGVKLESEETYKILTIYSTVASSLVIAMGFAAFLFWAMSNGSCRCNICI

>QOK08780.1 hemagglutinin [Influenza A virus]
METVSLITILVVATVSNADKICIGYQSTNSTETVDTLTENNVPVTHAKELLHTEHNGMLCATSLGHPLIL
DTCTIEGLIYGNPSCDPLLGGREWSYIVERPSAVNGLCYPGNVENLEELRSLFSSSRSYQRIQIFPDTIW
NVSYSGTSKACSDSFYRSMRWLTQKNNAYPTQDAQYTNNQGKNILFMWGINHPPTDTAQTNLYTRTDTTT
SVATEEMNRIFKPLIGPRPLVNGLMGRINYYWSVLKPGQTLRIKSDGNLIAPWYGHILSGESHGRILKTD
LKRGSCTVQCQTEKGGLNTTLPFQNVSKYAFGNCSKYIGVKSLKLAVGLRNVPSRSSRGLFGAIAGFIEG
GWSGLVAGWYGFQHSNDQGVGMAADRDSTQKAIDKITSKVNNIVDKMNKQYEIIDHEFSEVETRLNMINN
KVDDQIQDIWAYNAELLVLLENQKTLDEHDANVNNLYNKVKRALGSNAVEDGKGCFELYHKCDDHCMETI
RNGTYNRRKYQEESKLERQKIEGVKLESEETYKILTIYSTVASSLVIAMGFAAFLFWAMSNGSCRCNICI

>QOK08768.1 hemagglutinin [Influenza A virus]
METVSLITILVVATVSNADKICIGYQSTNSTETVDTLTENNVPVTHAKELLHTEHNGMLCATSLGHPLIL
DTCTIEGLIYGNPSCDPLLGGREWSYIVERPSAVNGLCYPGNVENLEELRSLFSSSRSYQRIQIFPDTIW
NVSYSGTSKACSDSFYRSMRWLTQKNNAYPTQDAQYTNNQGKNILFMWGINHPPTDTAQTNLYTRTDTTT
SVATEEMNRIFKPLIGPRPLVNGLMGRINYYWSVLKPGQTLRIKSDGNLIAPWYGHILSGESHGRILKTD
LKRGSCTVQCQTEKGGLNTTLPFQNVSKYAFGNCSKYIGVKSLKLAVGLRNVPSRSSRGLFGAIAGFIEG
GWSGLVAGWYGFQHSNDQGVGMAADRDSTQKAIDKITSKVNNIVDKMNKQYEIIDHEFSEVETRLNMINN
KVDDQIQDIWAYNAELLVLLENQKTLDEHDANVNNLYNKVKRALGSNAVEDGKGCFELYHKCDDHCMETI
RNGTYNRRKYQEESKLERQKIEGVKLESEETYKILTIYSTVASSLVIAMGFAAFLFWAMSNGSCRCNICI

>QOK08744.1 hemagglutinin [Influenza A virus]
METVSLITILLAATVSNADKICIGYQSTNSTETVDTLTENNVPVTHAKELLHTEHNGMLCATSLGQPLIL
DTCTIEGLIYGNPSCDPLPEEREWSYIVERPSAVNGLCYPGNVENLEELRSLFSSARSYQRIQIFPDTIW
NVSYDGTSNTCSGSFYRNMRWLTRKDGNYPIQDAQYTNNQGKNILFMWGINNPPTDDTQRNLYTRTDTTT
SVATEEINRIFKPLIGPRPLVNGLMGRINYYWSVLKPGQTLRIKSDGNLVAPWYGYILSGESHGRILRTD

LKRGSCTVQCQTEKGGLNTTLPFQNVSKYAFGNCSKYIGIKSLKLAVGLRNVPSRSSRGLFGAIAGFIEG
GWSGLVAGWYGFQHSNDQGVGMAADRESTQKAVDKITSKVNNIVDKMNKQYEIIDHEFSEVETRLNMINN
KIDDQIQDIWAYNAELLVLLENQKTLDEHDANVNNLYNKVKRALGSNAVEDGKGCFELYHKCDDQCMETI
RNGTYNRRKYQEESKLERQKIEGVKLESEGTYKILTIYSTVASSLVIAMGFAAFLFWAMSNGSCRCNICI

>QOK08733.1 hemagglutinin [Influenza A virus]
METVSLITILVVATVSNADKICIGYQSTNSTETVDTLTENNVPVTHAKELLHTEHNGMLCATSLGHPLIL
DTCTIEGLIYGNPSCDPLLGGREWSYIVERPSAVNGLCYPGNVENLEELRSLFSSSRSYQRIQIFPDTIW
NVSYSGTSKACSDSFYRSMRWLTQKNNAYPTQDAQYTNNQGKNILFMWGINHPPTDTAQTNLYTRTDTTT
SVATEEMNRIFKPLIGPRPLVNGLMGRINYYWSVLKPGQTLRIKSDGNLIAPWYGHILSGESHGRILKTD
LKRGSCTVQCQTEKGGLNTTLPFQNVSKYAFGNCSKYIGVKSLKLAVGLRNVPSRSSRGLFGAIAGFIEG
GWSGLVAGWYGFQHSNDQGVGMAADRDSTQKAIDKITSKVNNIVDKMNKQYEIIDHEFSEVETRLNMINN
KVDDQIQDIWAYNAELLVLLENQKTLDEHDANVNNLYNKVKRALGSNAVEDGKGCFELYHKCDDHCMETI
RNGTYNRRKYQEESKLERQKIEGVKLESEETYKILTIYSTVASSLVIAMGFAAFLFWAMSNGSCRCNICI

>QOK08721.1 hemagglutinin [Influenza A virus]
METVSLITILVVATVSNADKICIGYQSTNSTETVDTLTENNVPVTHAKELLHTEHNGMLCATSLGHPLIL
DTCTIEGLIYGNPSCDPLLGGREWSYIVERPSAVNGLCYPGNVENLEELRSLFSSSRSYQRIQIFPDTIW
NVSYSGTSKACSDSFYRSMRWLTQKNNAYPTQDAQYTNNQGKNILFMWGINHPPTDTAQTNLYTRTDTTT
SVATEEMNRIFKPLIGPRPLVNGLMGRINYYWSVLKPGQTLRIKSDGNLIAPWYGHILSGESHGRILKTD
LKRGSCTVQCQTEKGGLNTTLPFQNVSKYAFGNCSKYIGVKSLKLAVGLRNVPSRSSRGLFGAIAGFIEG
GWSGLVAGWYGFQHSNDQGVGMAADRDSTQKAIDKITSKVNNIVDKMNKQYEIIDHEFSEVETRLNMINN
KVDDQIQDIWAYNAELLVLLENQKTLDEHDANVNNLYNKVKRALGSNAVEDGKGCFELYHKCDDHCMETI
RNGTYNRRKYQEESKLERQKIEGVKLESEETYKILTIYSTVASSLVIAMGFAAFLFWAMSNGSCRCNICI

>QOK08709.1 hemagglutinin [Influenza A virus]
METVSLITILVVATVSNADKICIGYQSTNSTETVDTLTENNVPVTHAKELLHTEHNGMLCATSLGHPLIL
DTCTIEGLIYGNPSCDPLLGGREWSYIVERPSAVNGLCYPGNVENLEELRSLFSSSRSYQRIQIFPDTIW
NVSYSGTSKACSDSFYRSMRWLTQKNNAYPTQDAQYTNNQGKNILFMWGINHPPTDTAQTNLYTRTDTTT
SVATEEMNRIFKPLIGPRPLVNGLMGRINYYWSVLKPGQTLRIKSDGNLIAPWYGHILSGESHGRILKTD
LKRGSCTVQCQTEKGGLNTTLPFQNVSKYAFGNCSKYIGVKSLKLAVGLRNVPSRSSRGLFGAIAGFIEG
GWSGLVAGWYGFQHSNDQGVGMAADRDSTQKAIDKITSKVNNIVDKMNKQYEIIDHEFSEVETRLNMINN
KVDDQIQDIWAYNAELLVLLENQKTLDEHDANVNNLYNKVKRALGSNAVEDGKGCFELYHKCDDHCMETI
RNGTYNRRKYQEESKLERQKIEGVKLESEETYKILTIYSTVASSLVIAMGFAAFLFWAMSNGSCRCNICI

>QOK08697.1 hemagglutinin [Influenza A virus]

METVSLITILLAATVSNADKICIGYQSTNSTETVDTLTENNVPVTHAKELLHTEHNGMLCATSLGQPLIL

DTCTIEGLIYGNPSCDPLLEEREWSYIVERPSAVNGLCYPGNVENLEELRSFFSSARSYQRIQIFPDTIW

NVSYDGTSNTCSGSFYRNMRWLTRKNGNYPIQDAQYTNNQGKSILFMWGINHPPTDDTQRNLYTRTDTTT

SVATEEINRIFKPLIGPRPLVNGLMGRINYYWSVLKPGQTLRIKSDGNLIAPWYGYILSGESHGRILRTD

LTKGSCTVQCQTEKGGLNTTLPFQNVSKYAFGNCSKYIGIKSLKLAVGLRNVPSRSSRGLFGAIAGFIEG

GWSGLVAGWYGFQHSNDQGVGMAADRESTQKAIDKITSKVNNIVDKMNKQYEIIDHEFSEVETRLNMINN

KIDDQIQDIWAYNAELLVLLENQKTLDEHDANVNNLYNKVKRALGSNAVEDGKGCFELYHKCDDQCMETI

RNGTYNRRKYQEESKLERQKIEGVKLESEGTYKILTIYSTVASSLVIAMGFAAFLFWAMSNGSCRCNICI

>QOK08685.1 hemagglutinin [Influenza A virus]

METVSLITILVVATVSNADKICIGYQSTNSTETVDTLTENNVPVTHAKELLHTEHNGMLCATSLGHPLIL

DTCTIEGLIYGNPSCDPLLGGREWSYIVERPSAVNGLCYPGNVENLEELRSLFSSSRSYQRIQIFPDTIW

NVSYSGTSKACSDSFYRSMRWLTQKNNAYPTQDAQYTNNQGKNILFMWGINHPPTDTAQTNLYTRTDTTT

SVATEEMNRIFKPLIGPRPLVNGLMGRINYYWSVLKPGQTLRIKSDGNLIAPWYGHILSGESHGRILKTD

LKRGSCTVQCQTEKGGLNTTLPFQNVSKYAFGNCSKYIGVKSLKLAVGLRNVPSRSSRGLFGAIAGFIEG

GWSGLVAGWYGFQHSNDQGVGMAADRDSTQKAIDKITSKVNNIVDKMNKQYEIIDHEFSEVETRLNMINN

KVDDQIQDIWAYNAELLVLLENQKTLDEHDANVNNLYNKVKRALGSNAVEDGKGCFELYHKCDDHCMETI

RNGTYNRRKYQEESKLERQKIEGVKLESEETYKILTIYSTVASSLVIAMGFAAFLFWAMSNGSCRCNICI

>QOK08673.1 hemagglutinin [Influenza A virus]

METVSLITILLAATVSNADKICIGYQSTNSTETVDTLTENNVPVTHAKELLHTEHNGMLCATSLGQPLIL

DTCTIEGLIYGNPSCDPLLEEREWSYIVERPSAVNGLCYPGNVENLEELRSFFSSARSYQRIQIFPDTIW

NVSYDGTSNTCSGSFYRNMRWLTRKNGNYPIQDAQYTNNQGKSILFMWGINHPPTDDTQRNLYTRTDTTT

SVATEEINRIFKPLIGPRPLVNGLMGRINYYWSVLKPGQTLRIKSDGNLIAPWYGYILSGESHGRILRTD

LTKGSCTVQCQTEKGGLNTTLPFQNVSKYAFGNCSKYIGIKSLKLAVGLRNVPSRSSRGLFGAIAGFIEG

GWSGLVAGWYGFQHSNDQGVGMAADRESTQKAIDKITSKVNNIVDKMNKQYEIIDHEFSEVETRLNMINN

KIDDQIQDIWAYNAELLVLLENQKTLDEHDANVNNLYNKVKRALGSNAVEDGKGCFELYHKCDDQCMETI

RNGTYNRRKYQEESKLERQKIEGVKLESEGTYKILTIYSTVASSLVIAMGFAAFLFWAMSNGSCRCNICI

>QOK08662.1 hemagglutinin [Influenza A virus]

METVSLITILVVATVSNADKICIGYQSTNSTETVDTLTENNVPVTHAKELLHTEHNGMLCATSLGHPLIL

DTCTIEGLIYGNPSCDPLLGGREWSYIVERPSAVNGLCYPGNVENLEELRSLFSSSRSYQRIQIFPDTIW

NVSYSGTSKACSDSFYRSMRWLTQKNNAYPTQDAQYTNNQGKNILFMWGINHPPTDTAQTNLYTRTDTTT
SVATEEMNRIFKPLIGPRPLVNGLMGRINYYWSVLKPGQTLRIKSDGNLIAPWYGHILSGESHGRILKTD
LKRGSCTVQCQTEKGGLNTTLPFQNVSKYAFGNCSKYIGVKSLKLAVGLRNVPSRSSRGLFGAIAGFIEG
GWSGLVAGWYGFQHSNDQGVGMAADRDSTQKAIDKITSKVNNIVDKMNKQYEIIDHEFSEVETRLNMINN
KVDDQIQDIWAYNAELLVLLENQKTLDEHDANVNNLYNKVKRALGSNAVEDGKGCFELYHKCDDHCMETI
RNGTYNRRKYQEESKLERQKIEGVKLESEETYKILTIYSTVASSLVIAMGFAAFLFWAMSNGSCRCNICI

>QOK08650.1 hemagglutinin [Influenza A virus]
METVSLIIILLVATVSNADKICIGYQSTNSTETVDTLTENNVPVTHAKELLHTEHNGMLCATSLGQPLIL
DTCTIEGLIYGNPSCDLSLEGREWSYIVERPSAVNGLCYPGTVENLEELRSLFSSARSYQRIQIFPDTIW
NVSYDGTSTACSGSFYRSMRWLTRKNGEYPIQDAQYTNSQGKNILFMWGINHPPTDTTQETLYTRTDTTT
SVATEEINRIFKPLIGPRPLVNGLMGRINYYWSVLKPGQTLRIKSDGNLIAPWYGHILSGESHGRILKTD
LKRGSCTVQCQTEKGGLNTTLPFQNVSRYAFGNCSKYIGIKSLKLAVGLRNVPSRSSRGLFGAIAGFIEG
GWPGLVAGWYGFQHSNDQGVGMAADRDSTQKAIDKITSKVNNIVDKMNKQYEIIDHEFSEVETRLNMINN
KIDDQIQDIWAYNAELLVLLENQKTLDEHDANVNNLYNKVKRALGSNAVEDGKGCFELYHKCDDQCMDTI
RNGTYNRKKYQEESKLERQKIEGVKLESEGTYKILTIYSTVASSLVIAMGFAAFLFWAMSNGSCRCNICI

>QOK08639.1 hemagglutinin [Influenza A virus]
METVSLITILVVATVSNADKICIGYQSTNSTETVDTLTENNVPVTHAKELLHTEHNGMLCATSLGHPLIL
DTCTIEGLIYGNPSCDPLLGGREWSYIVERPSAVNGLCYPGNVENLEELRSLFSSSRSYQRIQIFPDTIW
NVSYSGTSKACSDSFYRSMRWLTQKNNAYPTQDAQYTNNQGKNILFMWGINHPPTDTAQTNLYTRTDTTT
SVATEEMNRIFKPLIGPRPLVNGLMGRINYYWSVLKPGQTLRIKSDGNLIAPWYGHILSGESHGRILKTD
LKRGSCTVQCQTEKGGLNTTLPFQNVSKYAFGNCSKYIGVKSLKLAVGLRNVPSRSSRGLFGAIAGFIEG
GWSGLVAGWYGFQHSNDQGVGMAADRDSTQKAIDKITSKVNNIVDKMNKQYEIIDHEFSEVETRLNMINN
KVDDQIQDIWAYNAELLVLLENQKTLDEHDANVNNLYNKVKRALGSNAVEDGKGCFELYHKCDDHCMETI
RNGTYNRRKYQEESKLERQKIEGVKLESEETYKILTIYSTVASSLVIAMGFAAFLFWAMSNGSCRCNICI

>QOK08627.1 hemagglutinin [Influenza A virus]
METVSLITILVVATVSNADKICIGYQSTNSTETVDTLTENNVPVTHAKELLHTEHNGMLCATSLGHPLIL
DTCTIEGLIYGNPSCDPLLGGREWSYIVERPSAVNGLCYPGNVENLEELRSLFSSSRSYQRIQIFPDTIW
NVSYSGTSKACSDSFYRSMRWLTQKNNAYPTQDAQYTNNQGKNILFMWGINHPPTDTAQTNLYTRTDTTT
SVATEEMNRIFKPLIGPRPLVNGLMGRINYYWSVLKPGQTLRIKSDGNLIAPWYGHILSGESHGRILKTD
LKRGSCTVQCQTEKGGLNTTLPFQNVSKYAFGNCSKYIGVKSLKLAVGLRNVPSRSSRGLFGAIAGFIEG
GWSGLVAGWYGFQHSNDQGVGMAADRDSTQKAIDKITSKVNNIVDKMNKQYEIIDHEFSEVETRLNMINN

KVDDQIQDIWAYNAELLVLLENQKTLDEHDANVNNLYNKVKRALGSNAVEDGKGCFELYHKCDDHCMETI
RNGTYNRRKYQEESKLERQKIEGVKLESEETYKILTIYSTVASSLVIAMGFAAFLFWAMSNGSCRCNICI

>QOK08615.1 hemagglutinin [Influenza A virus]
METVSLITILVVATVSNADKICIGYQSTNSTETVDTLTENNVPVTHAKELLHTEHNGMLCATSLGHPLIL
DTCTIEGLIYGNPSCDPLLGGREWSYIVERPSAVNGLCYPGNVENLEELRSLFSSSRSYQRIQIFPDTIW
NVSYSGTSKACSDSFYRSMRWLTQKNNAYPTQDAQYTNNQGKNILFMWGINHPPTDTAQTNLYTRTDTTT
SVATEEMNRIFKPLIGPRPLVNGLMGRINYYWSVLKPGQTLRIKSDGNLIAPWYGHILSGESHGRILKTD
LKRGSCTVQCQTEKGGLNTTLPFQNVSKYAFGNCSKYIGVKSLKLAVGLRNVPSRSSRGLFGAIAGFIEG
GWSGLVAGWYGFQHSNDQGVGMAADRDSTQKAIDKITSKVNNIVDKMNKQYEIIDHEFSEVETRLNMINN
KVDDQIQDIWAYNAELLVLLENQKTLDEHDSNVNNLYNKVKRALGSNAVEDGKGCFELYHKCDDHCMETI
RNGTYNRRKYQEESKLERQKIEGVKLESEETYKILTIYSTVASSLVIAMGFAAFLFWAMSNGSCRCNICI

>QOK08603.1 hemagglutinin [Influenza A virus]
METVSLITILVVATVSNADKICIGYQSTNSTETVDTLTENNVPVTHAKELLHTEHNGMLCATSLGHPLIL
DTCTIEGLIYGNPSCDPLLGGREWSYIVERPSAVNGLCYPGNVENLEELRSLFSSSRSYQRIQIFPDTIW
NVSYSGTSKACSDSFYRSMRWLTQKNNAYPTQDAQYTNNQGKNILFMWGINHPPTDTAQTNLYTRTDTTT
SVATEEMNRIFKPLIGPRPLVNGLMGRINYYWSVLKPGQTLRIKSDGNLIAPWYGHILSGESHGRILKTD
LKRGSCTVQCQTEKGGLNTTLPFQNVSKYAFGNCSKYIGVKSLKLAVGLRNVPSRSSRGLFGAIAGFIEG
GWSGLVAGWYGFQHSNDQGVGMAADRDSTQKAIDKITSKVNNIVDKMNKQYEIIDHEFSEVETRLNMINN
KVDDQIQDIWAYNAELLVLLENQKTLDEHDANVNNLYNKVKRALGSNAVEDGKGCFELYHKCDDHCMETI
RNGTYNRRKYQEESKLERQKIEGVKLESEETYKILTIYSTVASSLVIAMGFAAFLFWAMSNGSCRCNICI

>QOK08591.1 hemagglutinin [Influenza A virus]
METVSLITILVVATVSNADKICIGYQSTNSTETVDTLTENNVPVTHAKELLHTEHNGMLCATSLGHPLIL
DTCTIEGLIYGNPSCDPLLGGREWSYIVERPSAVNGLCYPGNVENLEELRSLFSSSRSYQRIQIFPDTIW
NVSYSGTSKACSDSFYRSMRWLTQKNNAYPTQDAQYTNNQGKNILFMWGINHPPTDTAQTNLYTRTDTTT
SVATEEMNRIFKPLIGPRPLVNGLMGRINYYWSVLKPGQTLRIKSDGNLIAPWYGHILSGESHGRILKTD
LKRGSCTVQCQTEKGGLNTTLPFQNVSKYAFGNCSKYIGVKSLKLAVGLRNVPSRSSRGLFGAIAGFIEG
GWSGLVAGWYGFQHSNDQGVGMAADRDSTQKAIDKITSKVNNIVDKMNKQYEIIDHEFSEVETRLNMINN
KVDDQIQDIWAYNAELLVLLENQKTLDEHDANVNNLYNKVKRALGSNAVEDGKGCFELYHKCDDHCMETI
RNGTYNRRKYQEESKLERQKIEGVKLESEETYKILTIYSTVASSLVIAMGFAAFLFWAMSNGSCRCNICI

>QOK08579.1 hemagglutinin [Influenza A virus]

METVSLITILVVATVSNADKICIGYQSTNSTETVDTLTENNVPVTHAKELLHTEHNGMLCATSLGHPLIL
DTCTIEGLIYGNPSCDPLLGGREWSYIVERPSAVNGLCYPGNVENLEELRSLFSSSRSYQRIQIFPDTIW
NVSYSGTSKACSDSFYRSMRWLTQKNNAYPTQDAQYTNNQGKNILFMWGINHPPTDTAQTNLYTRTDTTT
SVATEEMNRIFKPLIGPRPLVNGLMGRINYYWSVLKPGQTLRIKSDGNLIAPWYGHILSGESHGRILKTD
LKRGSCTVQCQTEKGGLNTTLPFQNVSKYAFGNCSKYIGVKSLKLAVGLRNVPSRSSRGLFGAIAGFIEG
GWSGLIAGWYGFQHSNDQGVGMAADRDSTQKAIDKITSKVNNIVDKMNKQYEIIDHEFSEVETRLNMINN
KVDDQIQDIWAYNAELLVLLENQKTLDEHDANVNNLYNKVKRALGSNAVEDGKGCFELYHKCDDHCMETI
RNGTYNRRKYQEESKLERQKIEGVKLESEETYKILTIYSTVASSLVIAMGFAAFLFWAMSNGSCRCNICI

>QOK08567.1 hemagglutinin [Influenza A virus]
METVSLITILLAATVSNADKICIGYQSTNSTETVDTLTENNVPVTHAKELLHTEHNGMLCATSLGQPLIL
DTCTIEGLIYGNPSCDPLLEEREWSYIVERPSAVNGLCYPGNVENLEELRSLFSSARSYQRIQIFPDTIW
NVSYDGTSNTCSGSFYRNMRWLTRKNGNYPIQDAQYTNNQGKDILFMWGINHPPTNDTQRNLYTRTDTTT
SVATEEINRIFKPLIGPRPLVNGLMGRINYHWSVLKPGQTLRIKSDGNLIAPWYGYILSGESHRRILRTD
LKRGSCTVQCQTEKGGLNTTLPFQNVSKYAFGNCSKYIGIKSLKLAVGLRNVPSRSSRGLFGAIAGFIEG
GWSGLVAGWYGFQHSNDQGVGMAADRESTQKAIDKITSKVNNIVDKMNKQYEIIDHEFSEVETRLNMINN
KIDDQIQDIWAYNAELLVLLENQKTLDEHDANVNNLYNKVKRALGSNAVEDGKGCFELYHKCDDQCMETI
RNGTYNRRKYQEESKLERQKIXXVKLESEGTYRILTIYSTVASSLVIAMGFAAFLFWAMSNGSCRCNICI

>QOK08543.1 hemagglutinin [Influenza A virus]
METVPLITILLAATVSNADKICIGYQSTNSTETVDTLTENNVPVTHAKELLHTEHNGMLCATSLGQPLIL
DTCTIEGLIYGNPSCDPLPEEREWSYIVERPSAVNGLCYPGNVENLEELRSLFSSAGSYQRIQIFPDTIW
NVSYDGTSNXCSGSFYRNMRWLTRKDGNYPTQDAQYTNNQGKNILFMWGINNPPTDDTQRNLYTRTDTTT
SVATEEINRIFKPLIGPRPLVNGLMGRINYYWSVLKPGQTLRIKSDGNLVAPWYGYILSGESHGRILRTD
LKRGSCTVQCQTEKGGLNTTLPFQNVSKYAFGNCSKYIGIKSLKLAVGLRNVPSRSSRGLFGAIAGFIEG
GWSGLVAGWYGFQHSNDQGVGMAADRESTQKAVDKITSKVNNIVDKMNKQYEIIDHEFSEVETRLNMINN
KIDDQIQDIWAYNAELLVLLENQKTLDEHDANVNNLYNKVKRALGSNAVEDGKGCFELYHKCDDQCMETI
RNGTYNRRKYQEESKLERQKIEGVKLESEGTYKILTIYSTVASSLVIAMGFAAFLFWAMSNGSCRCNICI

>QOK08531.1 hemagglutinin [Influenza A virus]
METVPLITILLAATVSNADKICIGYQSTNSTETVDTLTENNVPVTHAKELLHTEHNGMLCATSLGQPLIL
DTCTIEGLIYGNPSCDPLPEEREWSYIVERPSAVNGLCYPGNVENLEELRSLFSSAGSYQRIQIFPDTIW
NVSYDGTSNTCSGSFYRNMRWLTRKDGNYPTQDAQYTNNQGKNILFMWGINNPPTDDTQRNLYTRTDTTT
SVATEEINRIFKPLIGPRPLVNGLMGRINYYWSVLKPGQTLRIKSDGNLVAPWYGYILSGESHGRILRTD

LKRGSCAVQCQTEKGGLNTTLPFQNVSKYAFGNCSKYIGIKSLKLAVGLRNVPSRSSRGLFGAIAGFIEG
GWSGLVAGWYGFQHSNDQGVGMAADRESTQKAVDKITSKVNNIVDKMNKQYEIIDHEFSEVETRLNMINN
KIDDQIQDIWAYNAELLVLLENQKTLDEHDANVNNLYNKVKRALGSNAVEDGKGCFELYHKCDDQCMETI
RNGTYNRRKYQEESKLERQKIEGVKLESEGTYKILTIYSTVASSLVIAMGFAAFLFWAMSNGSCRCNICI

>QOK08519.1 hemagglutinin [Influenza A virus]
METVPLITILLAATVSNADKICIGYQSTNSTETVDTLTENNVPVTHAKELLHTEHNGMLCATSLGQPLIL
DTCTIEGLIYGNPSCDPLPEEREWSYIVERPSAVNGLCYPGNVENLEELRSLFSSAGSYQRIQIFPDTIW
NVSYDGTSNTCSGSFYRNMRWLTRKDGNYPTQDAQYTNNQGKNILFMWGINNPPTDDTQRNLYTRTDTTT
SVATEEINRIFKPLIGPRPRVNGLMGRINYYWSVLKPGQTLRIKSDGNLVAPWYGYILSGESHGRILRTD
LKRGSCTVQCQTEKGGLNTTLPFQNVSKYAFGNCSKYIGIKSLKLAVGLRNVPSRSSRGLFGAIAGFIEG
GWSGLVAGWYGFQHSNDQGVGMAADRESTQKAVDKITSKVNNIVDKMNKQYEIIDHEFSEVETRLNMINN
KIDDQIQDIWAYNAELLVLLENQKTLDEHDANVNNLYNKVKRALGSNAVEDGKGCFELYHKCDDQCMETI
RNGTYNRRKYQEESKLERQKIEGVKLESEGTYKILTIYSTVASSLVIAMGFAAFLFWAMSNGSCRCNICI

>QOK08507.1 hemagglutinin [Influenza A virus]
METVSLITILLAATVSNADKICIGYQSTNSTETVDTLTENNVPVTHAKELLHTEHNGMLCATSLGQPLIL
DTCTIEGLIYGNPSCDPLLEEREWSYIVERPSAVNGLCYPGNVENLEELRSLFSSARSYQRIQIFPDTIW
NVSYDGTSNTCSGSFYRNMRWLTRKNGNYPIQDAQYTNNQGKNILFMWGINHPPTDDTQRNLYTRTDTTT
SVATEEINRIFKPLIGPRPLVNGLMGRINYYWSVLKPGQTLRIKSDGNLIAPWYGYILSGESHGRILKTD
LKRGSCTVQCQTEKGGLNTTLPFQNVSKYAFGNCSKYIGIKSLKLAVGLRNVPSRSSRGLFGAIAGFIEG
GWSGLVAGWYGFQHSNDQGVGMAADRESTQKAIDKITSKVNNIVNKMNKQYEIIDHEFSEVETRLNMINN
KIDDQIQDIWAYNAELLVLLENQKTLDEHDANVNNLYNKVKRALGSNAVEDGKGCFELYHKCDDQCMETI
RNGTYNRRKYQEESKLERQKIEGVKLESEETYKILTIYSTVASSLVIAMGFAAFLFWAMSNGSCRCNICI

>QOK08495.1 hemagglutinin [Influenza A virus]
METVSLITILLAATVSNADKICIGYQSTNSTETVDTLTENNVPVTHAKELLHTEHNGMLCATSLGQPLIL
DTCTIEGLIYGNPSCDPLLEEREWSYIVERPSAVNGLCYPGNVENLEELRSLFSSARSYQRIQIFPDTIW
NVSYDGTSNTCSGSFYRNMRWLTRKNGNYPIQDAQYTNNQGKNILFMWGINHPPTDDTQRNLYTRTDTTT
SVATEEMNRIFRPLIGPRPLVNGLMGRINYYWSVLKPGQTLRIKSDGNLIAPWYGYILSGESHGRILRTD
LKRGSCTVQCQTEKGGLNTTLPFQNVSKYAFGNCSKYIGIKSLKLAVGLRNVPSRSSRGLFGAIAGFIEG
GWSGLVAGWYGFQHSNDQGVGMAADRESTQKAIDKITSKVNNIVDKMNKQYEIIDHEFSEVETRLNMINN
KIDDQIQDIWAYNAELLVLLENQKTLDEHDANVNNLYNKVKRALGSNAVEDGKGCFELYHKCDDQCMETI
RNGTYNRRKYQEESKLERQKIEGVKLESEGTYKILTIYSTVASSLVIAMGFAAFLFWAMSNGSCRCNICI

>QOK08483.1 hemagglutinin [Influenza A virus]

METVSLITILLAATVSNADKICIGYQSTNSTETVDTLTENNVPVTHAKELLHTEHNGMLCATSLGQPLIL
DTCTIEGLIYGNPSCDPLLEEREWSYIVERPSAVNGLCYPGNVENLEELRSLFSSARSYQRIQIFPDTIW
NVSYDGTSNTCSGSFYRNMRWLTRKNGNYPIQDAQYTNNQGKNILFMWGINHPPTDDTQRNLYTRTDTTT
SVATEEINRIFKPLIGPRPLVNGLMGRINYYWSVLKPGQTLRIKSDGNLIAPWYGYILSGESHGRILRTD
LKRGSCTVQCQTEKGGLNTTLPFQNVSKYAFGNCSKYIGIKSLKLAVGLRNVPSRSSRGLFGAIAGFIEG
GWSGLVAGWYGFQHSNDQGVGMAADRESTQKAIDKITSKVNNIVDKMNKQYEIIDHEFSEVETRLNMINN
KIDDQIQDIWAYNAELLVLLENQKTLDEHDANVNNLYNKVKRALGSNAVEDGKGCFELYHKCDDQCMETI
RNGTYNRRKYQEESKLERQKIEGVKLESEGTYKILTIYSTVASSLVIAMGFAAFLFWAMSNGSCRCNICI

>QOK08471.1 hemagglutinin [Influenza A virus]

METVSLITILLAATVSNADKICIGYQSTNSTETVDTLTENNVPVTHAKELLHTEHNGMLCATSLGQPLIL
DTCTIEGLIYGNPSCDPLLEEREWSYIVERPSAVNGLCYPGNVENLEELRSLFSSARSYQRIQIFPDTIW
NVSYDGTSNTCSGSFYRNMRWLTRKNGNYPIQDAQYTNNQGKNILFMWGINHPPTDDTQRNLYTRTDTTT
SVATEEINRIFKPLIGPRPLVNGLMGRINYYWSVLKPGQTLRIKSDGNLIAPWYGYILSGESHGRILKTD
LKRGSCTVQCQTEKGGLNTTLPFQNVSKYAFGNCSKYIGIKSLKLAVGLRNVPSRSSRGLFGAIAGFIEG
GWSGLVAGWYGFQHSNDQGVGMAADRESTQKAIDKITSKVNNIVNKMNKQYEIIDHEFSEVETRLNMINN
KIDDQIQDIWAYNAELLVLLENQKTLDEHDANVNNLYNKVKRALGSNAVEDGKGCFELYHKCDDQCMETI
RNGTYNRRKYQEESKLERQKIEGVKLESEETYKILTIYSTVASSLVIAMGFAAFLFWAMSNGSCRCNICI

>QOK08459.1 hemagglutinin [Influenza A virus]

METVSLITILLAATVSNADKICIGYQSTNSTETVDTLTENNVPVTHAKELLHTEHNGMLCATSLGQPLIL
DTCTIEGLIYGNPSCDPLLEEREWSYIVERPSAVNGLCYPGNVENLEELRSLFSSARSYQRIQIFPDTIW
NVSYDGTSNTCSGSFYRNMRWLTRKNGNYPIQDAQYTNNQGKNILFMWGINHPPTDDTQRNLYTRTDTTT
SVATEEINRIFKPLIGPRPLVNGLMGRINYYWSVLKPGQTLRIKSDGNLIAPWYGYILSGESHGRILRTD
LNRGSCTVQCQTEKGGLNTTLPFQNVSKYAFGNCSKYIGIKSLKLAVGLRNVPSRSSRGLFGAIAGFIEG
GWSGLVAGWYGFQHSNDQGVGMAADRESTQKAIDKITSKVNNIVDKMNKQYEIIDHEFSEVETRLNMINN
KIDDQIQDIWAYNAELLVLLENQKTLDEHDANVNNLYNKVKRALGSNAVEDGKGCFELYHKCDDQCMETI
RNGTYNRRKYQEESKLERQKIEGVKLESEGTYKILTIYSTVASSLVIAMGFAAFLFWAMSNGSCRCNICI

>QOK08447.1 hemagglutinin [Influenza A virus]

METVSLITILVVATVSNADKICIGYQSTNSTETVDTLTENNVPVTHAKELLHTEHNGMLCATGLGHPLIL
DTCTIEGLIYGNPSCDPLLGGREWSYIVERPSAVNGLCYPGNVENLEELRSLFSSSRSYQRIQIFPDTIW

NVSYSGTSKACSDSFYRSMRWLTQKNNAYPTQDAQYTNNQGKNILFMWGINHPPTDTVQTNLYTRTDTTT
SVATEEMNRIFKPLIGPRPLVNGLMGRINYYWSVLKPGQTLRIKSDGNLIAPWYGHILSGESHGRILKTD
LKRGSCIVQCQTEKGGLNTTLPFQNVSKYAFGNCSKYIGVKSLKLAVGLRNVPSRSSRGLFGAIAGFIEG
GWSGLVAGWYGFQHSNDQGVGMAADRDSTQKAIDKITSKVNNIVDKMNKQYEIIDHEFSEVETRLNMINN
KVDDQIQDIWAYNAELLVLLENQKTLDEHDANVNNLYNKVKRALGSNAVEDGKGCFELYHKCDDHCMETI
RNGTYNRRKYQEESKLERQKIEGVKLESEETYKILTIYSTVASSLVIAMGFAAFLFWAMSNGSCRCNICI

>QOK08435.1 hemagglutinin [Influenza A virus]
METVSLITILLVATVSNADKICIGYQSTNSTETVDTLTENNVPVTHAKELIHTEHNGMLCATSLGQPLIL
ETCTIEGLIYGNPSCDLSLEGREWSYIVERPSAVNGLCYPGNVENLEELRSLFSSARSYQRIQIFPDTIW
NVSYDGTSTACSGSFYRNMRWLTRKDGNYPTQDAQYTNNQGKNILFMWGINHPPSDTTQSGLYTRTDTTT
SVATEEINRIFKPLIGPRPLVNGLMGRIDYYWSVLKPGQTLRIKSDGNLIAPWFGHILSGESHGRILKTD
LKRGSCTVQCQTEKGGLNTTLPFQNVSKYAFGNCSKYIGIKSLKLAVGLRNVPSRSSRGLFGAKAGFIEG
GWSGLVAGWYGFQHSNDQGVGMAADRDSTQKAIDKITSKVNNIVDKMNKQYEIIDHEFSEVETRLNMINN
KIDDQIQDIWAYNAELLVLLENQKTLDEHDANVNNLYNKVKRALGSNAVEDGKGCFELYHKCDDQCMETI
RNGTYNRRKYQEESKLERQKIEGVKLESEGTYKILTIYSTVASSLVIAMGFAAFLFWAMSNGSCRCNICI

>QOK08423.1 hemagglutinin [Influenza A virus]
METVSLITILVVATVSNADKICIGYQSTNSTETVDTLTENNVPVTHAKELLHTEHNGMLCATSLGHPLIL
DTCTIEGLIYGNPSCDPLLGGREWSYIVERPSAVNGLCYPGNVENLEELRSLFSSSRSYQRIQIFPDTIW
NVSYSGTSKACSDSFYRSMRWLTQKNNAYPTQDAQYTNNQGKNILFMWGINHPPTDTAQTNLYTRTDTTT
SVATEEMNRIFKPLIGPRPLVNGLMGRINYYWSVLKPGQTLRIKSDGNLIAPWYGHILSGESHGRILKTD
LKMGSCTVQCQTEKGGLNTTLPFQNVSKYAFGNCSKYIGVKSLKLAVGLRNVPSRSSRGLFGAIAGFIEG
GWSGLVAGWYGFQHSNDQGVGMAADRESTQKAIDKITSKVNNIVDKMNKQYEIIDHEFSEVETRLNMIND
KVDDQIQDIWAYNAELLVLLENQKTLDEHDANVNNLYNKVKRALGSNAVEDGRGCFELYHKCDDHCMETI
RNGTYNRRKYQEESKLERQKIEGVKLESEETYKILTIYSTVASSLVIAMGFAAFLFWAMSNGSCRCNICI

>QOK08411.1 hemagglutinin [Influenza A virus]
METVSLITILLVATVSNADKICIGYQSTNSTETVDTLTENNVPVTHAKELIHTEHNGMLCATSLGQPLIL
ETCTIEGLIYGNPSCDLSLEGREWSYIVERPSAVNGLCYPGNVENLEELRSLFSSARSYQRIQIFPDTIW
NVSYDGTSTACSGSFYRNMRWLTRKDGNYPTQDAQYTNNQGKNILFMWGINHPPSDTTQSGLYTRTDTTT
SVATEEINRIFKPLIGPRPLVNGLMGRIDYYWSVLKPGQTLRIKSDGNLIAPWFGHILSGESHGRILKTD
LKRGSCTVQCQTEKGGLNTTLPFQNVSKYAFGNCSKYIGIKSLKLAVGLRNVPSRSSRGLFGAIAGFIEG
GWSGLVAGWYGFQHSNDQGVGMAADRDSTQKAIDKITSKVNNIVDKMNKQYEIIDHEFSEVETRLNMINN

KIDDQIQDIWAYNAELLVLLENQKTLDEHDANVNNLYNKVKRALGSNAVEDGKGCFELYHKCDDQCMETI
RNGTYNRRKYQEESKLERQKIEGVKLESEGTYKILTIYSTVASSLVIAMGFAAFLFWAMSNGSCRCNICI

>QOK08399.1 hemagglutinin [Influenza A virus]
METVSLITILLVATVSNADKICIGYQSTNSTETVDTLTENNVPVTHAKELIHTEHNGMLCATSLGQPLIL
ETCTIEGLIYGNPSCDLSLEGREWSYIVERPSAVNGLCYPGNVENLEELRSLFSSARSYQRIQIFPDTIW
NVSYDGTSTACSGSFYRNMRWLTRKDGNYPTQDAQYTNNQGKNILFMWGINHPPSDTTQSGLYTRTDTTT
SVATEEINRIFKPLIGPRPLVNGLMGRIDYYWSVLKPGQTLRIKSDGNLIAPWFGHILSGESHGRILKTD
LKRGSCTVQCQTEKGGLNTTLPFQNVSKYAFGNCSKYIGIKSLKLAVGLRNVPSRSSRGLFGAIAGFIEG
GWSGLVAGWYGFQHSNDQGVGMAADRDSTQKAIDKITSKVNNIVDKMNKQYEIIDHEFSEVETRLNMINN
KIDDQIQDIWAYNAELLVLLENQKTLDEHDANVNNLYNKVKRALGSNAVEDGKGCFELYHKCDDQCMETI
RNGTYNRRKYQEESKLERQKIEGVKLESEGTYKILTIYSTVASSLVIAMGFAAFLFWAMSNGSCRCNICI

>QOK08387.1 hemagglutinin [Influenza A virus]
METVSLITILLVATVSNADKICIGYQSTNSTETVDTLTENNVPVTHAKELIHTEHNGMLCATSLGQPLIL
ETCTIEGLIYGNPSCDLSLEGREWSYIVERPSAVNGLCYPGNVENLEELRSLFSSARSYQRIQIFPDTIW
NVSYDGTSTACSGSFYRNMRWLTRKDGNYPTQDAQYTNNQGKNILFMWGINHPPSDTTQSGLYTRTDTTT
SVATEEINRIFKPLIGPRPLVNGLMGRIDYYWSVLKPGQTLRIKSDGNLIAPWFGHILSGESHGRILKTD
LKRGSCTVQCQTEKGGLNTTLPFQNVSKYAFGNCSKYIGIKSLKLAVGLRNVPSRSSRGLFGAIAGFIEG
GWSGLVAGWYGFQHSNDQGVGMAADRDSTQKAIDKITSKVNNIVDKMNKQYEIIDHEFSEVETRLNMINN
KIDDQIQDIWAYNAELLVLLENQKTLDEHDANVNNLYNKVKRALGSNAVEDGKGCFELYHKCDDQCMETI
RNGTYNRRKYQEESKLERQKIEGVKLESEGTYKILTIYSTVASSLVIAMGFAAFLFWAMSNGSCRCNICI

>QOK08375.1 hemagglutinin [Influenza A virus]
METVSLITILLAATVSNADKICIGYQSTNSTETVDTLTENNVPVTHAKELLHTEHNGMLCATSLGQPLIL
DTCTIEGLIYGNPSCDPVLEEREWSYIVERPSAVNGLCYPGNVENLEELRSLFSSARFYQRIQIFPDTIW
NVSYDGTSNTCSGSFYRNMRWLTRKDGNYPIQDAQYTNNQGKNILFMWGINHPPTDDTQRNLYTRTDTTT
SVATEEINRIFKPLIGPRPLVNGLMGRINYYWSVLKPGQTLRIKSDGNLIAPWYGYILSGESHGRILRTD
LKRGSCTVQCQTEKGGLNTTLPFQNVSKYAFGNCSKYIGIKSLKLAVGLRNVPSRSSRGLFGAIAGFIEG
GWSGLVAGWYGFQHSNDQGVGMAADRESTQKAIDKITSKVNNIVDKMNKQYEIIDHEFSEVETRLNMINN
KIDDQIQDIWAYNAELLVLLENQKTLDEHDANVNNLYNKVKRALGSNAVEDGKGCFELYHKCDDQCMETI
RNGTYNRRKYQEESKLERQKIEGVKLESEGTYKILTIYSTVASSLVIAMGFAAFLFWAMSNGSCRCNICI

>QOK08363.1 hemagglutinin [Influenza A virus]

METVSLITILLAATVSNADKICIGYQSTNSTETVDTLTENNVPVTHAKELLHTEHNGMLCATSLGQPLIL
DTCTIEGLIYGNPSCDPVLEEREWSYIVERPSAVNGLCYPGNVENLEELRSLFSSARFYQRIQIFPDTIW
NVSYDGTSNTCSGSFYRNMRWLTRKDGNYPIQDAQYTNNQGKNILFMWGINHPPTDDTQRNLYTRTDTTT
SVATEEINRIFKPLIGPRPLVNGLMGRINYYWSVLKPGQTLRIKSDGNLIAPWYGYILSGESHGRILRTD
LKRGSCTVQCQTEKGGLNTTLPFQNVSKYAFGNCSKYIGIKSLKLAVGLRNVPSRSSRGLFGAIAGFIEG
GWSGLVAGWYGFQHSNDQGVGMAADRESTQKAIDKITSKVNNIVDKMNKQYEIIDHEFSEVETRLNMINN
KIDDQIQDIWAYNAELLVLLENQKTLDEHDANVNNLYNKVKRALGSNAVEDGKGCFELYHKCDDQCMETI
RNGTYNRRKYQEESKLERQKIEGVKLESEGTYKILTIYSTVASSLVIAMGFAAFLFWAMSNGSCRCNICI

>QOK08351.1 hemagglutinin [Influenza A virus]
METVSLITILLVATVSNADKICIGYQSTNSTETVDTLTENNVPVTHAKELIHTEHNGMLCATSLGQPLIL
ETCTIEGLIYGNPSCDLSLEGREWSYIVERPSAVNGLCYPGNVENLEELRSLFSSARSYQRIQIFPDTIW
NVSYDGTSTACSGSFYRNMRWLTRKDGNYPTQDAQYTNNQGKNILFMWGINHPPSDTTQSGLYTRTDTTT
SVATEEINRIFKPLIGPRPLVNGLMGRIDYYWSVLKPGQTLRIKSDGNLIAPWFGHILSGESHGRILKTD
LKRGSCTVQCQTEKGGLNTTLPFQNVSKYAFGNCSKYIGIKSLKLAVGLRNVPSRSSRGLFGAIAGFIEG
GWSGLVAGWYGFQHSNDQGVGMAADRDSTQKAIDKITSKVNNIVDKMNKQYEIIDHEFSEVETRLNMINN
KIDDQIQDIWAYNAELLVLLENQKTLDEHDANVNNLYNKVKRALGSNAVEDGKGCFELYHKCDDQCMETI
RNGTYNRRKYQEESKLERQKIEGVKLESEGTYKILTIYSTVASSLVIAMGFAAFLFWAMSNGSCRCNICI

>QOK08327.1 hemagglutinin [Influenza A virus]
METVSLITILLAATVSNADKICIGYQSTNSTETVDTLTENNVPVTHAKELLHTEHNGMLCATSLGQPLIL
DTCTIEGLIYGNPSCDPLLEEREWSYIVERPSAVNGLCYPGNVENLEELRSLFSSARSYQRIQIFPDTIW
NVSYDGTSNTCSGSFYRNMRWLTRKNGNYPIQDAQYTNNQGKNILFMWGINHPPTDDTQRNLYTRTDTTT
SVATEEINRIFKPLIGPRPLVNGLMGRINYYWSVLKPGQTLRIKSDGNLIAPWYGYILSGVSHGRILRTD
LKRGSCTVQCQTEKGGLNTTLPFQNVSKYAFGNCSKYIRIKSLKLAVGLRNVPSRSSRGLFGAIAGFIEG
GWSGLVAGWYGFQHSNDQGVGMAADRESTQKAIDKITSKVNNIVDKMNKQYEIIDHEFSEVETRLNMINN
KIDDQIQDIWAYNAELLVLLENQKTLDEHDANVNNLYNKVKRALGSNAVEDGKGCFELYHKCDDQCMETI
RNGTYNRRKYQEESKLERQKIEGVKLESEGTYKILTIYSTVASSLVIAMGFAAFLFWAMSNGSCRCNICI

>QOK08304.1 hemagglutinin [Influenza A virus]
METVSLITILLTATISNADKICIGYQSTNSTETVDTLTENNVPVTHAKELLHTEHNGMLCATSLGQPLIL
DTCTIEGLIYGNPSCDPLPEEREWSYIVERPSAVNGLCYPGNVENLEELRSLFSSARSYQRIQIFPDTIW
NVSYDGTSNTCSGSFYRNMRWLTRKDGNYPIQDAQYTNNQGKNILFMWGINNPPTDDTQRNLYTRTDTTT
SVATEEINRIFKPLIGPRPLVNGLMGRINYYWSVLKPGQTLRIKSDGNLVAPWYGYILSGESHGRILRTG

LKRGSCTVQCQTEKGGLNTTLPFQNVSKYAFGNCSKYIGIKSLKLAVGLRNVPSRSSRGLFGAIAGFIEG
GWSGLVAGWYGFQHSNDQGVGMAADKESTQKAVDKITSKVNNIVDKMNKQYEIIDHEFSEVETRLNMINN
KIDDQIQDIWAYNAELLVLLENQKTLDEHDANVNNLYNKVKRALGSNAVEDGKGCFELYHKCDDRCMETI
RNGTYNRRKYQEESKLERQKIEGVKLESEGTYKILTIYSTVASSLVIAMGFAAFLFWAMSNGSCRCNICI

>QOK08292.1 hemagglutinin [Influenza A virus]
METVSLITILLTATISNADKICIGYQSTNSTETVDTLTENNVPVTHAKELLHTEHNGMLCATSLGQPLIL
DTCTIEGLIYGNPSCDPLPEEREWSYIVERPSAVNGLCYPGNVENLEELRSLFSSARSYQRIQIFPDTIW
NVSYDGTSNTCSGSFYRNMRWLTRKDGNYPIQDAQYTNNQGKNILFMWGINNPPTDDTQRNLYTRTDTTT
SVATEEINRIFKPLIGPRPLVNGLMGRINYYWSVLKPGQTLRIKSDGNLVAPWYGYILSGESHGRILRTD
LKRGSCTVQCQTEKGGLNTTLPFQNVSKYAFGNCSKYIGIKSLKLAVGLRNVPSRSSRGLFGAIAGFIEG
GWSGLVAGWYGFQHSNDQGVGMAADKESTQKAVDKITSKVNNIVDKMNKQYEIIDHEFSEVETRLNMINN
KIDDQIQDIWAYNAELLVLLENQKTLDEHDANVNNLYNKVKRALGSNAVEDGKGCFELYHKCDDRCMETI
RNGTYNRRKYQEESKLERQKIEGVKLESEGTYKILTIYSTVASSLVIAMGFAAFLFWAMSNGSCRCNICI

>QOK08280.1 hemagglutinin [Influenza A virus]
METVSLITILLTATISNADKICIGYQSTNSTETVDTLTENNVPVTHAKELLHTEHNGMLCATSLGQPLIL
DTCTIEGLIYGNPSCDPLPEEREWSYIVERPSAVNGLCYPGNVENLEELRSLFSSARSYQRIQIFPDTIW
NVSYDGTSNTCSGSFYRNMRWLTRKDGNYPIQDAQYTNNQGKNILFMWGINNPPTDDTQRNLYTRTDTTT
SVATEEINRIFKPLIGPRPLVNGLMGRINYYWSVLKPGQTLRIKSDGNLVAPWYGYILSGESHGRILRTD
LKRGSCTVQCQTEKGGLNTTLPFQNVSKYAFGNCSKYIGIKSLKLAVGLRNVPSRSSRGLFGAIAGFIEG
GWSGLVAGWYGFQHSNDQGVGMAADKESTQKAVDKITSKVNNIVDKMNKQYEIIDHEFSEVETRLNMINN
KIDDQIQDIWAYNAELLVLLENQKTLDEHDANVNNLYNKVKRALGSNAVEDGKGCFELYHKCDDRCMETI
RNGTYNRRKYQEESKLERQKIEGVKLESEGTYKILTIYSTVASSLVIAMGFAAFLFWAMSNGSCRCNICI

>QOK08268.1 hemagglutinin [Influenza A virus]
METVSLITILLTATISNADKICIGYQSTNSTETVDTLTENNVPVTHAKELLHTEHNGMLCATSLGQPLIL
DTCTIEGLIYGNPSCDPLPEEREWSYIVERPSAVNGLCYPGNVENLEELRSLFSSARSYQRIQIFPDTIW
NVSYDGTSNTCSGSFYRNMRWLTRKDGNYPIQDAQYTNNQGKNILFMWGINNPPTDDTQRNLYTRTDTTT
SVATEEINRIFKPLIGPRPLVNGLMGRINYYWSVLKPGQTLRIKSDGNLVAPWYGYILSGESHGRILRTD
LKRGSCTVQCQTEKGGLNTTLPFQNVSKYAFGNCSKYIGIKSLKLAVGLRNVPSRSSRGLFGAIAGFIEG
GWSGLVAGWYGFQHSNDQGVGMAADKESTQKAVDKITSKVNNIVDKMNKQYEIIDHEFSEVETRLNMINN
KIDDQIQDIWAYNAELLVLLENQKTLDEHDANVNNLYNKVKRALGSNAVEDGKGCFELYHKCDDRCMETI
RNGTYNRRKYQEESKLERQKIEGVKLESEGTYKILTIYSTVASSLVIAMGFAAFLFWAMSNGSCRCNICI

>QOK08256.1 hemagglutinin [Influenza A virus]

METVSLITILLTATISNADKICIGYQSTNSTETVDTLTENNVPVTHAKELLHTEHNGMLCATSLGQPLIL
DTCTIEGLIYGNPSCDPLPEEREWSYIVERPSAVNGLCYPGNVENLEELRSLFSSARSYQRIQIFPDTIW
NVSYDGTSNTCSGSFYRNMRWLTRKDGNYPIQDAQYTNNQGKNILFMWGINNPPTDDTQRNLYTRTDTTT
SVATEEINRIFKPLIGPRPLVNGLMGRINYYWSVLKPGQTLRIKSDGNLVAPWYGYILSGESHGRILRTD
LKRGSCTVQCQTEKGGLNTTLPFQNVSKYAFGNCSKYIGIKSLKLAVGLRNVPSRSSRGLFGAIAGFIEG
GWSGLVAGWYGFQHSNDQGVGMAADKESTQKAVDKITSKVNNIVDKMNKQYEIIDHEFSEVETRLNMINN
KIDDQIQDIWAYNAELLVLLENQKTLDEHDANVNNLYNKVKRALGSNAVEDGKGCFELYHKCDDRCMETI
RNGTYNRRKYQEESKLERQKIEGVKLESEGTYKILTIYSTVASSLVIAMGFAAFLFWAMSNGSCRCNICI

>QOK08244.1 hemagglutinin [Influenza A virus]

METVSLITILLTATISNADKICIGYQSTNSTETVDTLTENNVPVTHAKELLHTEHNGMLCATSLGQPLIL
DTCTIEGLIYGNPSCDPLPEEREWSYIVERPSAVNGLCYPGNVENLEELRSLFSSARSYQRIQIFPDTIW
NVSYDGTSNTCSGSFYRNMRWLTRKDGNYPIQDAQYTNNQGKNILFMWGINNPPTDDTQRNLYTRTDTTT
SVATEEINRIFKPLIGPRPLVNGLMGRINYYWSVLKPGQTLRIKSDGNLVAPWYGYILSGESHGRILRTD
LKRGSCTVQCQTEKGGLNTTLPFQNVSKYAFGNCSKYIGIKSLKLAVGLRNVPSRSSRGLFGAIAGFIEG
GWSGLVAGWYGFQHSNDQGVGMAADKESTQKAVDKITSKVNNIVDKMNKQYEIIDHEFSEVETRLNMINN
KIDDQIQDIWAYNAELLVLLENQKTLDEHDANVNNLYNKVKRALGSNAVEDGKGCFELYHKCDDRCMETI
RNGTYNRRKYQEESKLERQKIEGVKLESEGTYKILTIYSTVASSLVIAMGFAAFLFWAMSNGSCRCNICI

>QOK08232.1 hemagglutinin [Influenza A virus]

METVSLITILLTATISNADKICIGYQSTNSTETVDTLTENNVPVTHAKELLHTEHNGMLCATSLGQPLIL
DTCTIEGLIYGNPSCDPLPEEREWSYIVERPSAVNGLCYPGNVENLEELRSLFSSARSYQRIQIFPDTIW
NVSYDGTSNTCSGSFYRNMRWLTRKDGNYPIQDAQYTNNQGKNILFMWGINNPPTDDTQRNLYTRTDTTT
SVATEEINRIFKPLIGPRPLVNGLMGRINYYWSVLKPGQTLRIKSDGNLVAPWYGYILSGESHGRILRTD
LKRGSCTVQCQTEKGGLNTTLPFQNVSKYAFGNCSKYIGIKSLKLAVGLRNVPSRSSRGLFGAIAGFIEG
GWSGLVAGWYGFQHSNDQGVGMAADKESTQKAVDKITSKVNNIVDKMNKQYEIIDHEFSEVETRLNMINN
KIDDQIQDIWAYNAELLVLLENQKTLDEHDANVNNLYNKVKRALGSNAVEDGKGCFELYHKCDDRCMETI
RNGTYNRRKYQEESKLERQKIEGVKLESEGTYKILTIYSTVASSLVIAMGFAAFLFWAMSNGSCRCNICI

>QOK08220.1 hemagglutinin [Influenza A virus]

METVSLITILLTATISNADKICIGYQSTNSTETVDTLTENNVPVTHAKELLHTEHNGMLCATSLGQPLIL
DTCTIEGLIYGNPSCDPLPEEREWSYIVERPSAVNGLCYPGNVENLEELRSLFSSARSYQRIQIFPDTIW

NVSYDGTSNTCSGSFYRNMRWLTRKDGNYPIQDAQYTNNQGKNILFMWGINNPPTDDTQRNLYTRTDTTT
SVATEEINRIFKPLIGPRPLVNGLMGRINYYWSVLKPGQTLRIKSDGNLVAPWYGYILSGESHGRILRTD
LKRGSCTVQCQTEKGGLNTTLPFQNVSKYAFGNCSKYIGIKSLKLAVGLRNVPSRSSRGLFGAIAGFIEG
GWSGLVAGWYGFQHSNDQGVGMAADKESTQKAVDKITSKVNNIVDKMNKQYEIIDHEFSEVETRLNMINN
KIDDQIQDIWAYNAELLVLLENQKTLDEHDANVNNLYNKVKRALGSNAVEDGKGCFELYHKCDDRCMETI
RNGTYNRRKYQEESKLERQKIEGVKLESEGTYKILTIYSTVASSLVIAMGFAAFLFWAMSNGSCRCNICI

>QOK08208.1 hemagglutinin [Influenza A virus]
METVSLITILLTATISNADKICIGYQSTNSTETVDTLTENNVPVTHAKELLHTEHNGMLCATSLGQPLIL
DTCTIEGLIYGNPSCDPLPEEREWSYIVERPSAVNGLCYPGNVENLEELRSLFSSARSYQRIQIFPDTIW
NVSYDGTSNTCSGSFYRNMRWLTRKDGNYPIQDAQYTNNQGKNILFMWGINNPPTDDTQRNLYTRTDTTT
SVATEEINRIFKPLIGPRPLVNGLMGRINYYWSVLKPGQTLRIKSDGNLVAPWYGYILSGESHGRILRTD
LKRGSCTVQCQTEKGGLNTTLPFQNVSKYAFGNCSKYIGIKSLKLAVGLRNVPSRSSRGLFGAIAGFIEG
GWSGLVAGWYGFQHSNDQGVGMAADKESTQKAVDKITSKVNNIVDKMNKQYEIIDHEFSEVETRLNMINN
KIDDQIQDIWAYNAELLVLLENQKTLDEHDANVNNLYNKVKRALGSNAVEDGKGCFELYHKCDDRCMETI
RNGTYNRRKYQEESKLERQKIEGVKLESEGTYKILTIYSTVASSLVIAMGFAAFLFWAMSNGSCRCNICI

>QOK08196.1 hemagglutinin [Influenza A virus]
METVSLITILLTATISNADKICIGYQSTNSTETVDTLTENNVPVTHAKELLHTEHNGMLCATSLGQPLIL
DTCTIEGLIYGNPSCDPLPEEREWSYIVERPSAVNGLCYPGNVENLEELRSLFSSARSYQRIQIFPDTIW
NVSYDGTSNTCSGSFYRNMRWLTRKDGNYPIQDAQYTNNQGKNILFMWGINNPPTDDTQRNLYTRTDTTT
SVATEEINRIFKPLIGPRPLVNGLMGRINYYWSVLKPGQTLRIKSDGNLVAPWYGYILSGESHGRILRTD
LKRGSCTVQCQTEKGGLNTTLPFQNVSKYAFGNCSKYIGIKSLKLAVGLRNVPSRSSRGLFGAIAGFIEG
GWSGLVAGWYGFQHSNDQGVGMAADKESTQKAVDKITSKVNNIVDKMNKQYEIIDHEFSEVETRLNMINN
KIDDQIQDIWAYNAELLVLLENQKTLDEHDANVNNLYNKVKRALGSNAVEDGKGCFELYHKCDDRCMETI
RNGTYNRRKYQEESKLERQKIEGVKLESEGTYKILTIYSTVASSLVIAMGFAAFLFWAMSNGSCRCNICI

>QOK08184.1 hemagglutinin [Influenza A virus]
METVSLITILLAATVSNADKICIGYQSTNSTETVDTLTENNVPVTHAKELLHTEHNGMLCATSLGQPLIL
DTCTIEGLIYGNPSCDPLLEEREWSYIVERPSAVNGLCYPGNVENLEELRSLFSSARSYQRIQIFPDTIW
NVSYDGTSNTCSGSFYRNMRWLTRKNGNYPIQDAQYTNNQGKNILFMWGINHPPTNDTQRNLYTRTDTTT
SVATEEINRIFKPLIGPRPLVNGLMGRINYHWSVLKPGQTLRIKSDGNLIAPWYGYILSGESHRRILRTD
LKRGSCTVQCQTEKGGLNTTLPFQNVSKYAFGNCSKYIGIKSLKLAVGLRNVPSRSSRGLFGAIAGFIEG
GWSGLVAGWYGFQHSNDQGVGMAADRESTQKAIDKITSKVNNIVDKMNKQYEIIDHEFSEVETRLNMINN

KIDDQIQDIWAYNAELLVLLENQKTLDEHDANVNNLYNKVKRALGSNAVEDGKGCFELYHKCDDQCMETI
RNGTYNRRKYQEESKLERQKIEGVKLESEGTYRILTIYSTVASSLVIAMGFAAFLFWAMSNGSCRCNICI

>QOK08172.1 hemagglutinin [Influenza A virus]
METVSLITILLTATISNADKICIGYQSTNSTETVDTLTENNVPVTHAKELLHTEHNGMLCATSLGQPLIL
DTCTIEGLIYGNPSCDPLPEEREWSYIVERPSAVNGLCYPGNVENLEELRSLFSSARSYQRIQIFPDTIW
NVSYDGTSNTCSGSFYRNMRWLTRKDGNYPIQDAQYTNNQGKNILFMWGINNPPTDDTQRNLYTRTDTTT
SVATEEINRIFKPLIGPRPLVNGLMGRINYYWSVLKPGQTLRIKSDGNLVAPWYGYILSGESHGRILRTD
LKRGSCTVQCQTEKGGLNTTLPFQNVSKYAFGNCSKYIGIKSLKLAVGLRNVPSRSSRGLFGAIAGFIEG
GWSGLVAGWYGFQHSNDQGVGMAADKESTQKAVDKITSKVNNIVDKMNKQYEIIDHEFSEVETRLNMINN
KIDDQIQDIWAYNAELLVLLENQKTLDEHDANVNNLYNKVKRALGSNAVEDGKGCFELYHKCDDRCMETI
RNGTYNRRKYQEESKLERQKIEGVKLESEGTYKILTIYSTVASSLVIAMGFAAFLFWAMSNGSCRCNICI

>QOK08160.1 hemagglutinin [Influenza A virus]
METVSLITILLTATISNADKICIGYQSTNSTETVDTLTENNVPVTHAKELLHTEHNGMLCATSLGQPLIL
DTCTIEGLIYGNPSCDPLPEEREWSYIVERPSAVNGLCYPGNVENLEELRSLFSSARSYQRIQIFPDTIW
NVSYDGTSNTCSGSFYRNMRWLTRKDGNYPIQDAQYTNNQGKNILFMWGINNPPTDDTQRNLYTRTDTTT
SVATEEINRIFKPLIGPRPLVNGLMGRINYYWSVLKPGQTLRIKSDGNLVAPWYGYILSGESHGRILRTG
LKRGSCTVQCQTEKGGLNTTLPFQNVSKYAFGNCSKYIGIKSLKLAVGLRNVPSRSSRGLFGAIAGFIEG
GWSGLVAGWYGFQHSNDQGVGMAADKESTQKAVDKITSKVNNIVDKMNKQYEIIDHEFSEVETRLNMINN
KIDDQIQDIWAYNAELLVLLENQKTLDEHDANVNNLYNKVKRALGSNAVEDGKGCFELYHKCDDRCMETI
RNGTYNRRKYQEESKLERQKIEGVKLESEGTYKILTIYSTVASSLVIAMGFAAFLFWAMSNGSCRCNICI

>QOK08148.1 hemagglutinin [Influenza A virus]
METVSLITILLTATISNADKICIGYQSTNSTETVDTLTENNVPVTHAKELLHTEHNGMLCATSLGQPLIL
DTCTIEGLIYGNPSCDPLPEEREWSYIVERPSAVNGLCYPGNVENLEELRSLFSSARSYQRIQIFPDTIW
NVSYDGTSNTCSGSFYRNMRWLTRKDGNYPIQDAQYTNNQGKNILFMWGINNPPTDDTQRNLYTRTDTTT
SVATEEINRIFKPLIGPRPLVNGLMGRINYYWSVLKPGQTLRIKSDGNLVAPWYGYILSGESHGRILRTD
LKRGSCTVQCQTEKGGLNTTLPFQNVSKYAFGNCSKYIGIKSLKLAVGLRNVPSRSSRGLFGAIAGFIEG
GWSGLVAGWYGFQHSNDQGVGMAADKESTQKAVDKITSKVNNIVDKMNKQYEIIDHEFSEVETRLNMINN
KIDDQIQDIWAYNAELLVLLENQKTLDEHDANVNNLYNKVKRALGSNAVEDGKGCFELYHKCDDRCMETI
RNGTYNRRKYQEESKLERQKIEGVKLESEGTYKILTIYSTVASSLVIAMGFAAFLFWAMSNGSCRCNICI

>QOK08136.1 hemagglutinin [Influenza A virus]

METVSLITILLTATISNADKICIGYQSTNSTETVDTLTENNVPVTHAKELLHTEHNGMLCATSLGQPLIL
DTCTIEGLIYGNPSCDPLPEEREWSYIVERPSAVNGLCYPGNVENLEELRSLFSSARSYQRIQIFPDTIW
NVSYDGTSNTCSGSFYRNMRWLTRKDGNYPIQDAQYTNNQGKNILFMWGINNPPTDDTQRNLYTRTDTTT
SVATEEINRIFKPLIGPRPLVNGLMGRINYYWSVLKPGQTLRIKSDGNLVAPWYGYILSGESHGRILRTD
LKRGSCTVQCQTEKGGLNTTLPFQNVSKYAFGNCSKYIGIKSLKLAVGLRNVPSRSSRGLFGAIAGFIEG
GWSGLVAGWYGFQHSNDQGVGMAADKESTQKAVDKITSKVNNIVDKMNKQYEIIDHEFSEVETRLNMINN
KIDDQIQDIWAYNAELLVLLENQKTLDEHDANVNNLYNKVKRALGSNAVEDGKGCFELYHKCDDRCMETI
RNGTYNRRKYQEESKLERQKIEGVKLESEGTYKILTIYSTVASSLVIAMGFAAFLFWAMSNGSCRCNICI

>QOK08124.1 hemagglutinin [Influenza A virus]
METVSLITILLTATISNADKICIGYQSTNSTETVDTLTENNVPVTHAKELLHTEHNGMLCATSLGQPLIL
DTCTIEGLIYGNPSCDPLPEEREWSYIVERPSAVNGLCYPGNVENLEELRSLFSSARSYQRIQIFPDTIW
NVSYDGTSNTCSGSFYRNMRWLTRKDGNYPIQDAQYTNNQGKNILFMWGINNPPTDDTQRNLYTRTDTTT
SVATEEINRIFKPLIGPRPLVNGLMGRINYYWSVLKPGQTLRIKSDGNLVAPWYGYILSGESHGRILRTD
LKRGSCTVQCQTEKGGLNTTLPFQNVSKYAFGNCSKYIGIKSLKLAVGLRNVPSRSSRGLFGAIAGFIEG
GWSGLVAGWYGFQHSNDQGVGMAADKESTQKAVDKITSKVNNIVDKMNKQYEIIDHEFSEVETRLNMINN
KIDDQIQDIWAYNAELLVLLENQKTLDEHDANVNNLYNKVKRALGSNAVEDGKGCFELYHKCDDRCMETI
RNGTYNRRKYQEESKLERQKIEGVKLESEGTYKILTIYSTVASSLVIAMGFAAFLFWAMSNGSCRCNICI

>QOK08112.1 hemagglutinin [Influenza A virus]
METVSLITILLTATISNADKICIGYQSTNSTETVDTLTENNVPVTHAKELLHTEHNGMLCATSLGQPLIL
DTCTIEGLIYGNPSCDPLPEEREWSYIVERPSAVNGLCYPGNVENLEELRSLFSSARSYQRIQIFPDTIW
NVSYDGTSNTCSGSFYRNMRWLTRKDGNYPIQDAQYTNNQGKNILFMWGINNPPTDDTQRNLYTRTDTTT
SVATEEINRIFKPLIGPRPLVNGLMGRINYYWSVLKPGQTLRIKSDGNLVAPWYGYILSGESHGRILRTD
LKRGSCTVQCQTEKGGLNTTLPFQNVSKYAFGNCSKYIGIKSLKLAVGLRNVPSRSSRGLFGAIAGFIEG
GWSGLVAGWYGFQHSNDQGVGMAADKESTQKAVDKITSKVNNIVDKMNKQYEIIDHEFSEVETRLNMINN
KIDDQIQDIWAYNAELLVLLENQKTLDEHDANVNNLYNKVKRALGSNAVEDGKGCFELYHKCDDRCMETI
RNGTYNRRKYQEESKLERQKIEGVKLESEGTYKILTIYSTVASSLVIAMGFAAFLFWAMSNGSCRCNICI

>QOK08100.1 hemagglutinin [Influenza A virus]
METVSLITILLTATISNADKICIGYQSTNSTETVDTLTENNVPVTHAKELLHTEHNGMLCATSLGQPLIL
DTCTIEGLIYGNPSCDPLPEEREWSYIVERPSAVNGLCYPGNVENLEELRSLFSSARSYQRIQIFPDTIW
NVSYDGTSNTCSGSFYRNMRWLTRKDGNYPIQDAQYTNNQGKNILFMWGINNPPTDDTQRNLYTRTDTTT
SVATEEINRIFKPLIGPRPLVNGLMGRINYYWSVLKPGQTLRIKSDGNLVAPWYGYILSGESHGRILRTD

LKRGSCTVQCQTEKGGLNTTLPFQNVSKYAFGNCSKYIGIKSLKLAVGLRNVPSRSSRGLFGAIAGFIEG
GWSGLVAGWYGFQHSNDQGVGMAADKESTQKAVDKITSKVNNIVDKMNKQYEIIDHEFSEVETRLNMINN
KIDDQIQDIWAYNAELLVLLENQKTLDEHDANVNNLYNKVKRALGSNAVEDGKGCFELYHKCDDRCMETI
RNGTYNRRKYQEESKLERQKIEGVKLESEGTYKILTIYSTVASSLVIAMGFAAFLFWAMSNGSCRCNICI

>QOK08088.1 hemagglutinin [Influenza A virus]
METVSLITILLTATISNADKICIGYQSTNSTETVDTLTENNVPVTHAKELLHTEHNGMLCATSLGQPLIL
DTCTIEGLIYGNPSCDPLPEEREWSYIVERPSAVNGLCYPGNVENLEELRSLFSSARSYQRIQIFPDTIW
NVSYDGTSNTCSGSFYRNMRWLTRKDGNYPIQDAQYTNNQGKNILFMWGINNPPTDDTQRNLYTRTDTTT
SVATEEINRIFKPLIGPRPLVNGLMGRINYYWSVLKPGQTLRIKSDGNLVAPWYGYILSGESHGRILRTD
LKRGSCTVQCQTEKGGLNTTLPFQNVSKYAFGNCSKYIGIKSLKLAVGLRNVPSRSSRGLFGAIAGFIEG
GWSGLVAGWYGFQHSNDQGVGMAADKESTQKAVDKITSKVNNIVDKMNKQYEIIDHEFSEVETRLNMINN
KIDDQIQDIWAYNAELLVLLENQKTLDEHDANVNNLYNKVKRALGSNAVEDGKGCFELYHKCDDRCMETI
RNGTYNRRKYQEESKLERQKIEGVKLESEGTYKILTIYSTVASSLVIAMGFAAFLFWAMSNGSCRCNICI

>QOK08076.1 hemagglutinin [Influenza A virus]
METVSLITILLVATVSNADKICIGYQSTNSTETVDTLTENNVPVTHAKELLHTEHNGMLCATSLGQPLIL
DTCTIEGLIYGNPSCDLSLEGREWSYIVERPSAVHGLCYPGNVEDLEELRSLFSSARSYQRIQIFPDTIW
NVSYDGTSTACSGSFYKSMRWLTRKNGEYPTQDAQYTNNQGKNILFMWGINHPPTDDTQRGLYTRTDTTT
SVATEEINRIFKPLIGPRPLVNGLMGRINYYWSVLKPGQTLRIKSDGNLIAPWYGHILSGESHGRILKTD
LKRGSCTVQCQTEKGGLNTTLPFQNVSKYAFGNCSKYIGIKSLKLAVGLRNVPSRSSRGLFGAIAGFIEG
GWSGLVAGWYGFQHSNDQGVGMAADRDSTQKAIDKITSKVNNIVDKMNKQYEIIDHEFSEVETRLNMINN
KIDDQIQDIWAYNAELLVLLENQKTLDEHDANVNNLYNKVKRALGSNAVEDGKGCFELYHKCDDQCMETI
RNGTYNRRKYQEESKLERQRIEGVKLESEGTYKILTIYSTVASSLVIAMGFAAFLFWAMSNGSCRCNICI

>QOK08064.1 hemagglutinin [Influenza A virus]
METVSLITILLVATVSNADKICIGYQSTNSTETVDTLTENNVPVTHAKELLHTEHNGMLCATSLGQPLIL
DTCTIEGLIYGNPSCDLSLEGREWSYIVERPSAVHGLCYPGNVEDLEELRSLFSSARSYQRIQIFPDTIW
NVSYDGTSTACSGSFYKSMRWLTRKNGEYPTQDAQYTNNQGKNILFMWGINHPPTDDTQRGLYTRTDTTT
SVATEEINRIFKPLIGPRPLVNGLMGRINYYWSVLKPGQTLRIKSDGNLIAPWYGHILSGESHGRILKTD
LKRGSCTVQCQTEKGGLNTTLPFQNVSKYAFGNCSKYIGIKSLKLAVGLRNVPSRSSRGLFGAIAGFIEG
GWSGLVAGWYGFQHSNDQGVGMAADRDSTQKAIDKITSKVNNIVDKMNKQYEIIDHEFSEVETRLNMINN
KIDDQIQDIWAYNAELLVLLENQKTLDEHDANVNNLYNKVKRALGSNAVEDGKGCFELYHKCDDQCMETI
RNGTYNRRKYQEESKLERQRIEGVKLESEGTYKILTIYSTVASSLVIAMGFAAFLFWAMSNGSCRCNICI

>QOK08052.1 hemagglutinin [Influenza A virus]
METVSLITILLAATVSNADKICIGYQSTNSTETVDTLTENNVPVTHAKELLHTEHNGMLCATSLGQPLIL
DTCTIEGLIYGNPSCDPLLEEREWSYIVERPSAVNGLCYPGNVENLEELRSLFSSARSYQRIQIFPDTIW
NVSYDGTSNTCSSSFYRNMRWLTRKNGNYPIQDAQYTNNQGKNILFMWGINHPPTDDTQRNLYTRTDTTT
SVATEEINRIFKPLIGPRPLVNGLMGRINYYWSVLKPGQTLRIKSDGNLIAPWYGYILSGESHGRILKTD
LKRGSCTVQCQTEKGGLNTTLPFQNVSKYAFGNCSKYIGIKSLKLAVGLRNVPSRSSRGLFGAIAGFIEG
GWSGLVAGWYGFQHSNDQGVGMAADRESTQKAIDKITSKVNNIVDKMNKQYEIIDHEFSEVETRLNMINN
KIDDQIQDIWAYNAELLVLLENQKTLDEHDANVNNLYNKVKRALGSNAVEDGKGCFELYHKCDDQCMETI
RNGTYNRRKYQEESKLERQKIEGVKLESEGTYKILTIYSTVASSLVIAMGFAAFLFWAMSNGSCRCNICI

>QOK08040.1 hemagglutinin [Influenza A virus]
METVSLITILLAATVSNADKICIGYQSTNSTETVDTLTENNVPVTHAKELLHTEHNGMLCATSLGQPLIL
DTCTIEGLIYGNPSCDPLLEEREWSYIVERPSAVNGLCYPGNVENLEELRSLFSSARSYQRIQIFPDTIW
NVSYDGTSNTCSSSFYRNMRWLTRKNGNYPIQDAQYTNNQGKNILFMWGINHPPTDDTQRNLYTRTDTTT
SVATEEINRIFKPLIGPRPLVNGLMGRINYYWSVLKPGQTLRIKSDGNLIAPWYGYILSGESHGRILKTD
LKRGSCTVQCQTEKGGLNTTLPFQNVSKYAFGNCSKYIGIKSLKLAVGLRNVPSRSSRGLFGAIAGFIEG
GWSGLVAGWYGFQHSNDQGVGMAADRESTQKAIDKITSKVNNIVDKMNKQYEIIDHEFSEVETRLNMINN
KIDDQIQDIWAYNAELLVLLENQKTLDEHDANVNNLYNKVKRALGSNAVEDGKGCFELYHKCDDQCMETI
RNGTYNRRKYQEESKLERQKIEGVKLESEGTYKILTIYSTVASSLVIAMGFAAFLFWAMSNGSCRCNICI

>QOK08028.1 hemagglutinin [Influenza A virus]
METVSLITILLAATVSNADKICIGYQSTNSTETVDTLTENNVPVTHAKELLHTEHNGMLCATSLGQPLIL
DTCTIEGLIYGNPSCDPLLEEREWSYIVERPSAVNGLCYPGNVENLEELRSLFSSARSYQRIQIFPDTIW
NVSYDGTSNTCSSSFYRNMRWLTRKNGNYPIQDAQYTNNQGKNILFMWGINHPPTDDTQRNLYTRTDTTT
SVATEEINRIFKPLIGPRPLVNGLMGRINYYWSVLKPGQTLRIKSDGNLIAPWYGYILSGESHGRILKTD
LKRGSCTVQCQTEKGGLNTTLPFQNVSKYAFGNCSKYIGIKSLKLAVGLRNVPSRSSRGLFGAIAGFIEG
GWSGLVAGWYGFQHSNDQGVGMAADRESTQKAIDKITSKVNNIVDKMNKQYEIIDHEFSEVETRLNMINN
KIDDQIQDIWAYNAELLVLLENQKTLDEHDANVNNLYNKVKRALGSNAVEDGKGCFELYHKCDDQCMETI
RNGTYNRRKYQEESKLERQKIEGVKLESEGTYKILTIYSTVASSLVIAMGFAAFLFWAMSNGSCRCNICI

>QOK08016.1 hemagglutinin [Influenza A virus]
METVSLITILLAATVSNADKICIGYQSTNSTETVDTLTENNVPVTHAKELLHTEHNGMLCATSLGQPLIL
DTCTIEGLIYGNPSCDPLLEEREWSYIVERPSAVNGLCYPGNVENLEELRSLFSSARSYQRIQIFPDTIW

NVSYDGTSNTCSSSFYRNMRWLTRKNGNYPIQDAQYTNNQGKNILFMWGINHPPTDDTQRNLYTRTDTTT

SVATEEINRIFKPLIGPRPLVNGLMGRINYYWSVLKPGQTLRIKSDGNLIAPWYGYILSGESHGRILKTD

LKRGSCTVQCQTEKGGLNTTLPFQNVSKYAFGNCSKYIGIKSLKLAVGLRNVPSRSSRGLFGAIAGFIEG

GWSGLVAGWYGFQHSNDQGVGMAADRESTQKAIDKITSKVNNIVDKMNKQYEIIDHEFSEVETRLNMINN

KIDDQIQDIWAYNAELLVLLENQKTLDEHDANVNNLYNKVKRALGSNAVEDGKGCFELYHKCDDQCMETI

RNGTYNRRKYQEESKLERQKIEGVKLESEGTYKILTIYSTVASSLVIAMGFAAFLFWAMSNGSCRCNICI

>QOK08004.1 hemagglutinin [Influenza A virus]

METVSLITILLAATVSNADKICIGYQSTNSTETVDTLTENNVPVTHAKELLHTEHNGMLCATSLGQPLIL

DTCTIEGLIYGNPSCDPLLEEREWSYIVERPSAVNGLCYPGNVENLEELRSLFSSARSYQRIQIFPDTIW

NVSYDGTSNTCSSSFYRNMRWLTRKNGNYPIQDAQYTNNQGKNILFMWGINHPPTDDTQRNLYTRTDTTT

SVATEEINRIFKPLIGPRPLVNGLMGRINYYWSVLKPGQTLRIKSDGNLIAPWYGYILSGESHGRILKTD

LKRGSCTVQCQTEKGGLNTTLPFQNVSKYAFGNCSKYIGIKSLKLAVGLRNVPSRSSRGLFGAIAGFIEG

GWSGLVAGWYGFQHSNDQGVGMAADRESTQKAIDKITSKVNNIVDKMNKQYEIIDHEFSEVETRLNMINN

KIDDQIQDIWAYNAELLVLLENQKTLDEHDANVNNLYNKVKRALGSNAVEDGKGCFELYHKCDDQCMETI

RNGTYNRRKYQEESKLERQKIEGVKLESEGTYKILTIYSTVASSLMIAMGFAAFLFWAMSNGSCRCNICI

>QOK07992.1 hemagglutinin [Influenza A virus]

METVSLITILLAATVSNADKICIGYQSTNSTETVDTLTENNVPVTHAKELLHTEHNGMLCATSLGQPLIL

DTCTIEGLIYGNPSCDPLLEEREWSYIVERPSAVNGLCYPGNVENLEELRSLFSSARSYQRIQIFPDTIW

NVSYDGTSNTCSSSFYRNMRWLTRKNGNYPIQDAQYTNNQGKNILFMWGINHPPTDDTQRNLYTRTDTTT

SVATEEINRIFKPLIGPRPLVNGLMGRINYYWSVLKPGQTLRIKSDGNLIAPWYGYILSGESHGRILKTD

LKRGSCTVQCQTEKGGLNTTLPFQNVSKYAFGNCSKYIGIKSLKLAVGLRNVPSRSSRGLFGAIAGFIEG

GWSGLVAGWYGFQHSNDQGVGMAADRESTQKAIDKITSKVNNIVDKMNKQYEIIDHEFSEVETRLNMINN

KIDDQIQDIWAYNAELLVLLENQKTLDEHDANVNNLYNKVKRALGSNAVEDGKGCFELYHKCDDQCMETI

RNGTYNRRKYQEESKLERQKIEGVKLESEGTYKILTIYSTVASSLVIAMGFAAFLFWAMSNGSCRCNICI

>QOK07980.1 hemagglutinin [Influenza A virus]

METVSLITILLAATVSNADKICIGYQSTNSTETVDTLTENNVPVTHAKELLHTEHNGMLCATSLGQPLIL

DTCTIEGLIYGNPSCDPLLEEREWSYIVERPSAVNGLCYPGNVENLEELRSLFSSARSYQRIQIFPDTIW

NVSYDGTSNTCSSSFYRNMRWLTRKNGNYPIQDAQYTNNQGKNILFMWGINHPPTDDTQRNLYTRTDTTT

SVATEEINRIFKPLIGPRPLVNGLMGRINYYWSVLKPGQTLRIKSDGNLIAPWYGYILSGESHGRILKTD

LKRGSCTVQCQTEKGGLNTTLPFQNVSKYAFGNCSKYIGIKSLKLAVGLRNVPSRSSRGLFGAIAGFIEG

GWSGLVAGWYGFQHSNDQGVGMAADRESTQKAIDKITSKVNNIVDKMNKQYEIIDHEFSEVETRLNMINN

KIDDQIQDIWAYNAELLVLLENQKTLDEHDANVNNLYNKVKRALGSNAVEDGKGCFELYHKCDDQCMETI
RNGTYNRRKYQEESKLERQKIEGVKLESEGTYKILTIYSTVASSLVIAMGFAAFLFWAMSNGSCRCNICI

>QOK07968.1 hemagglutinin [Influenza A virus]
METVSLITILLAATVSNADKICIGYQSTNSTETVDTLTENNVPVTHAKELLHTEHNGMLCATSLGQPLIL
DTCTIEGLIYGNPSCDPLLEEREWSYIVERPSAVNGLCYPGNVENLEELRSLFSSARSYQRIQIFPDTIW
NVSYDGTSNTCSSSFYRNMRWLTRKNGNYPIQDAQYTNNQGKNILFMWGINHPPTDDTQRNLYTRTDTTT
SVATEEINRIFKPLIGPRPLVNGLMGRINYYWSVLKPGQTLRIKSDGNLIAPWYGYILSGESHGRILKTD
LKRGSCTVQCQTEKGGLNTTLPFQNVSKYAFGNCSKYIGIKSLKLAVGLRNVPSRSSRGLFGAIAGFIEG
GWSGLVAGWYGFQHSNDQGVGMAADRESTQKAIDKITSKVNNIVDKMNKQYEIIDHEFSEVETRLNMINN
KIDDQIQDIWAYNAELLVLLENQKTLDEHDANVNNLYNKVKRALGSNAVEDGKGCFELYHKCDDQCMETI
RNGTYNRRKYQEESKLERQKIEGVKLESEGTYKILTIYSTVASSLVIAMGFAAFLFWAMSNGSCRCNICI

>QOK07956.1 hemagglutinin [Influenza A virus]
METVSLITILLAATVSNADKICIGYQSTNSTETVDTLTENNVPVTHAKELLHTEHNGMLCATSLGQPLIL
DTCTIEGLIYGNPSCDPLLEEREWSYIVERPSAVNGLCYPGNVENLEELRSLFSSARSYQRIQIFPDTIW
NVSYDGTSNTCSSSFYRNMRWLTRKNGNYPIQDAQYTNNQGKNILFMWGINHPPTDDTQRNLYTRTDTTT
SVATEEINRIFKPLIGPRPLVNGLMGRINYYWSVLKPGQTLRIKSDGNLIAPWYGYILSGESHGRILKTD
LKRGSCTVQCQTEKGGLNTTLPFQNVSKYAFGNCSKYIGIKSLKLAVGLRNVPSRSSRGLFGAIAGFIEG
GWSGLVAGWYGFQHSNDQGVGMAADRESTQKAIDKITSKVNNIVDKMNKQYEIIDHEFSEVETRLNMINN
KIDDQIQDIWAYNAELLVLLENQKTLDEHDANVNNLYNKVKRALGSNAVEDGKGCFELYHKCDDQCMETI
RNGTYNRRKYQEESKLERQKIEGVKLESEGTYKILTIYSTVASSLVIAMGFAAFLFWAMSNGSCRCNICI

>QOK07944.1 hemagglutinin [Influenza A virus]
METVSLITILLAATVSNADKICIGYQSTNSTETVDTLTENNVPVTHAKELLHTEHNGMLCATSLGQPLIL
DTCTIEGLIYGNPSCDPLLEEREWSYIVERPSAVNGLCYPGNVENLEELRSLFSSARSYQRIQIFPDTIW
NVSYDGTSNTCSSSFYRNMRWLTRKNGNYPIQDAQYTNNQGKNILFMWGINHPPTDDTQRNLYTRTDTTT
SVATEEINRIFKPLIGPRPLVNGLMGRINYYWSVLKPGQTLRIKSDGNLIAPWYGYILSGESHGRILKTD
LKRGSCTVQCQTEKGGLNTTLPFQNVSKYAFGNCSKYIGIKSLKLAVGLRNVPSRSSRGLFGAIAGFIEG
GWSGLVAGWYGFQHSNDQGVGMAADRESTQKAIDKITSKVNNIVDKMNKQYEIIDHEFSEVETRLNMINN
KIDDQIQDIWAYNAELLVLLENQKTLDEHDANVNNLYNKVKRALGSNAVEDGKGCFELYHKCDDQCMETI
RNGTYNRRKYQEESKLERQKIEGVKLESEGTYKILTIYSTVASSLVIAMGFAAFLFWAMSNGSCRCNICI

>QOK07932.1 hemagglutinin [Influenza A virus]

METVSLITILLAATVSNADKICIGYQSTNSTETVDTLTENNVPVTHAKELLHTEHNGMLCATSLGQPLIL
DTCTIEGLIYGNPSCDPLLEEREWSYIVERPSAVNGLCYPGNVENLEELRSLFSSARSYQRIQIFPDTIW
NVSYDGTSNTCSSSFYRNMRWLTRKNGNYPIQDAQYTNNQGKNILFMWGINHPPTDDTQRNLYTRTDTTT
SVATEEINRIFKPLIGPRPLVNGLMGRINYYWSVLKPGQTLRIKSDGNLIAPWYGYILSGESHGRILKTD
LKRGSCTVQCQTEKGGLNTTLPFQNVSKYAFGNCSKYIGIKSLKLAVGLRNVPSRSSRGLFGAIAGFIEG
GWSGLVAGWYGFQHSNDQGVGMAADRESTQKAIDKITSKVNNIVDKMNKQYEIIDHEFSEVETRLNMINN
KIDDQIQDIWAYNAELLVLLENQKTLDEHDANVNNLYNKVKRALGSNAVEDGKGCFELYHKCDDQCMETI
RNGTYNRRKYQEESKLERQKIEGVKLESEGTYKILTIYSTVASSLVIAMGFAAFLFWAMSNGSCRCNICI

>QOK07920.1 hemagglutinin [Influenza A virus]
METVSLITILLAATVSNADKICIGYQSTNSTETVDTLTENNVPVTHAKELLHTEHNGMLCATSLGQPLIL
DTCTIEGLIYGNPSCDPLLEEREWSYIVERPSAVNGLCYPGNVENLEELRSLFSSARSYQRIQIFPDTIW
NVSYDGTSNTCSSSFYRNMRWLTRKNGNYPIQDAQYTNNQGKNILFMWGINHPPTDDTQRNLYTRTDTTT
SVATEEINRIFKPLIGPRPLVNGLMGRINYYWSVLKPGQTLRIKSDGNLIAPWYGYILSGESHGRILKTD
LKRGSCTVQCQTEKGGLNTTLPFQNVSKYAFGNCSKYIGIKSLKLAVGLRNVPSRSSRGLFGAIAGFIEG
GWSGLVAGWYGFQHSNDQGVGMAADRESTQKAIDKITSKVNNIVDKMNKQYEIIDHEFSEVETRLNMINN
KIDDQIQDIWAYNAELLVLLENQKTLDEHDANVNNLYNKVKRALGSNAVEDGKGCFELYHKCDDQCMETI
RNGTYNRRKYQEESKLERQKIEGVKLESEGTYKILTIYSTVASSLVIAMGFAAFLFWAMSNGSCRCNICI

>QOK07908.1 hemagglutinin [Influenza A virus]
METVSLITILLVATVSNADKICIGYQSTNSTETVDTLTENNVPVTHAKELLHTEHNGMLCATSLGQPLIL
DTCTIEGLIYGNPSCDLSLEGREWSYIVERPSAVHGLCYPGNVEDLEELRSLFSSARSYQRIQIFPDTIW
NVSYDGTSTACSGSFYKSMRWLTRKNGEYPTQDAQYTNNQGKNILFMWGINHPPTDDTQRGLYTRTDTTT
SVATEEINRIFKPLIGPRPLVNGLMGRINYYWSVLKPGQTLRIKSDGNLIAPWYGHILSGESHGRILKTD
LKRGSCTVQCQTEKGGLNTTLPFQNVSKYAFGNCSKYIGIKSLKLAVGLRNVPSRSSRGLFGAIAGFIEG
GWSGLVAGWYGFQHSNDQGVGMAADRDSTQKAIDKITSKVNNIVDKMNKQYEIIDHEFSEVETRLNMINN
KIDDQIQDIWAYNAELLVLLENQKTLDEHDANVNNLYNKVKRALGSNAVEDGKGCFELYHKCDDQCMETI
RNGTYNRRKYQEESKLERQRIEGVKLESEGTYKILTIYSTVASSLVIAMGFAAFLFWAMSNGSCRCNICI

>QOK07896.1 hemagglutinin [Influenza A virus]
METVSLITILLVATVSNADKICIGYQSTNSTETVDTLTENNVPVTHAKELLHTEHNGMLCATSLGQPLIL
DTCTIEGLIYGNPSCDLSLEGREWSYIVERPSAVHGLCYPGNVEDLEELRSLFSSARSYQRIQIFPDTIW
NVSYDGTSTACSGSFYKSMRWLTRKNGEYPTQDAQYTNNQGKNILFMWGINHPPTDDTQRGLYTRTDTTT
SVATEEINRIFKPLIGPRPLVNGLMGRINYYWSVLKPGQTLRIKSDGNLIAPWYGHILSGESHGRILKTD

LKRGSCTVQCQTEKGGLNTTLPFQNVSKYAFGNCSKYIGIKSLKLAVGLRNVPSRSSRGLFGAIAGFIEG
GWSGLVAGWYGFQHSNDQGVGMAADRDSTQKAIDKITSKVNNIVDKMNKQYEIIDHEFSEVETRLNMINN
KIDDQIQDIWAYNAELLVLLENQKTLDEHDANVNNLYNKVKRALGSNAVEDGKGCFELYHKCDDQCMETI
RNGTYNRRKYQEESKLERQRIEGVKLESEGTYKILTIYSTVASSLVIAMGFAAFLFWAMSNGSCRCNICI

>QOK07884.1 hemagglutinin [Influenza A virus]
METVSLITILLAATVSNADKICIGYQSTNSTETVDTLTENNVPVTHAKELLHTEHNGMLCATSLGQPLIL
DTCTIEGLIYGNPSCDPLLEEREWSYIVERPSAVNGLCYPGNVENLEELRSLFSSARSYQRIQIFPDTIW
NVSYDGTSNTCSSSFYRNMRWLTRKNGNYPIQDAQYTNNQGKNILFMWGINHPPTDDTQRNLYTRTDTTT
SVATEEINRIFKPLIGPRPLVNGLMGRINYYWSVLKPGQTLRIKSDGNLIAPWYGYILSGESHGRILKTD
LKRGSCTVQCQTEKGGLNTTLPFQNVSKYAFGNCSKYIGIKSLKLAVGLRNVPSRSSRGLFGAIAGFIEG
GWSGLVAGWYGFQHSNDQGVGMAADRESTQKAIDKITSKVNNIVDKMNKQYEIIDHEFSEVETRLNMINN
KIDDQIQDIWAYNAELLVLLENQKTLDEHDANVNNLYNKVKRALGSNAVEDGKGCFELYHKCDDQCMETI
RNGTYNRRKYQEESKLERQKIEGVKLESEGTYKILTIYSTVASSLVIAMGFAAFLFWAMSNGSCRCNICI

>QOK07872.1 hemagglutinin [Influenza A virus]
METVSLITILLAATVSNADKICIGYQSTNSTETVDTLTENNVPVTHAKELLHTEHNGMLCATSLGQPLIL
DTCTIEGLIYGNPSCDPLLEEREWSYIVERPSAVNGLCYPGNVENLEELRSLFSSARSYQRIQIFPDTIW
NVSYDGTSNTCSSSFYRNMRWLTRKNGNYPIQDAQYTNNQGKNILFMWGINHPPTDDTQRNLYTRTDTTT
SVATEEINRIFKPLIGPRPLVNGLMGRINYYWSVLKPGQTLRIKSDGNLIAPWYGYILSGESHGRILKTD
LKRGSCTVQCQTEKGGLNTTLPFQNVSKYAFGNCSKYIGIKSLKLAVGLRNVPSRSSRGLFGAIAGFIEG
GWSGLVAGWYGFQHSNDQGVGMAADRESTQKAIDKITSKVNNIVDKMNKQYEIIDHEFSEVETRLNMINN
KIDDQIQDIWAYNAELLVLLENQKTLDEHDANVNNLYNKVKRALGSNAVEDGKGCFELYHKCDDQCMETI
RNGTYNRRKYQEESKLERQKIEGVKLESEGTYKILTIYSTVASSLVIAMGFAAFLFWAMSNGSCRCNICI

>QOK07860.1 hemagglutinin [Influenza A virus]
METVSLITILLAATVSNADKICIGYQSTNSTETVDTLTENNVPVTHAKELLHTEHNGMLCATSLGQPLIL
DTCTIEGLIYGNPSCDPLLEEREWSYIVERPSAVNGLCYPGNVENLEELRSLFSSARSYQRIQIFPDTIW
NVSYDGTSNTCSSSFYRNMRWLTRKNGNYPIQDAQYTNNQGKNILFMWGINHPPTDDTQRNLYTRTDTTT
SVATEEINRIFKPLIGPRPLVNGLMGRINYYWSVLKPGQTLRIKSDGNLIAPWYGYILSGESHGRILKTD
LKRGSCTVQCQTEKGGLNTTLPFQNVSKYAFGNCSKYIGIKSLKLAVGLRNVPSRSSRGLFGAIAGFIEG
GWSGLVAGWYGFQHSNDQGVGMAADRESTQKAIDKITSKVNNIVDKMNKQYEIIDHEFSEVETRLNMINN
KIDDQIQDIWAYNAELLVLLENQKTLDEHDANVNNLYNKVKRALGSNAVEDGKGCFELYHKCDDQCMETI
RNGTYNRRKYQEESKLERQKIEGVKLESEGTYKILTIYSTVASSLVIAMGFAAFLFWAMSNGSCRCNICI

>QOK07848.1 hemagglutinin [Influenza A virus]

METVSLITILLAATVSNADKICIGYQSTNSTETVDTLTENNVPVTHAKELLHTEHNGMLCATSLGQPLIL
DTCTIEGLIYGNPSCDPLLEEREWSYIVERPSAVNGLCYPGNVENLEELRSLFSSARSYQRIQIFPDTIW
NVSYDGTSNTCSSSFYRNMRWLTRKNGNYPIQDAQYTNNQGKNILFMWGINHPPTDDTQRNLYTRTDTTT
SVATEEINRIFKPLIGPRPLVNGLMGRINYYWSVLKPGQTLRIKSDGNLIAPWYGYILSGESHGRILKTD
LKRGSCTVQCQTEKGGLNTTLPFQNVSKYAFGNCSKYIGIKSLKLAVGLRNVPSRSSRGLFGAIAGFIEG
GWSGLVAGWYGFQHSNDQGVGMAADRESTQKAIDKITSKVNNIVDKMNKQYEIIDHEFSEVETRLNMINN
KIDDQIQDIWAYNAELLVLLENQKTLDEHDANVNNLYNKVKRALGSNAVEDGKGCFELYHKCDDQCMETI
RNGTYNRRKYQEESKLERQKIEGVKLESEGTYKILTIYSTVASSLVIAMGFAAFLFWAMSNGSCRCNICI

>QOK07836.1 hemagglutinin [Influenza A virus]

METVSLITILLAATVSNADKICIGYQSTNSTETVDTLTENNVPVTHAKELLHTEHNGMLCATSLGQPLIL
DTCTIEGLIYGNPSCDPLLEEREWSYIVERPSAVNGLCYPGNVENLEELRSLFSSARSYQRIQIFPDTIW
NVSYDGTSNTCSSSFYRNMRWLTRKNGNYPIQDAQYTNNQGKNILFMWGINHPPTDDTQRNLYTRTDTTT
SVATEEINRIFKPLIGPRPLVNGLMGRINYYWSVLKPGQTLRIKSDGNLIAPWYGYILSGESHGRILKTD
LKRGSCTVQCQTEKGGLNTTLPFQNVSKYAFGNCSKYIGIKSLKLAVGLRNVPSRSSRGLFGAIAGFIEG
GWSGLVAGWYGFQHSNDQGVGMAADRESTQKAIDKITSKVNNIVDKMNKQYEIIDHEFSEVETRLNMINN
KIDDQIQDIWAYNAELLVLLENQKTLDEHDANVNNLYNKVKRALGSNAVEDGKGCFELYHKCDDQCMETI
RNGTYNRRKYQEESKLERQKIEGVKLESEGTYKILTIYSTVASSLVIAMGFAAFLFWAMSNGSCRCNICI

>QOK07824.1 hemagglutinin [Influenza A virus]

METVSLITILLAATVSNADKICIGYQSTNSTETVDTLTENNVPVTHAKELLHTEHNGMLCATSLGQPLIL
DTCTIEGLIYGNPSCDPLLEEREWSYIVERPSAVNGLCYPGNVENLEELRSLFSSARSYQRIQIFPDTIW
NVSYDGTSNTCSSSFYRNMRWLTRKNGNYPIQDAQYTNNQGKNILFMWGINHPPTDDTQRNLYTRTDTTT
SVATEEINRIFKPLIGPRPLVNGLMGRINYYWSVLKPGQTLRIKSDGNLIAPWYGYILSGESHGRILKTD
LKRGSCTVQCQTEKGGLNTTLPFQNVSKYAFGNCSKYIGIKSLKLAVGLRNVPSRSSRGLFGAIAGFIEG
GWSGLVAGWYGFQHSNDQGVGMAADRESTQKAIDKITSKVNNIVDKMNKQYEIIDHEFSEVETRLNMINN
KIDDQIQDIWAYNAELLVLLENQKTLDEHDANVNNLYNKVKRALGSNAVEDGKGCFELYHKCDDQCMETI
RNGTYNRRKYQEESKLERQKIEGVKLESEGTYKILTIYSTVASSLVIAMGFAAFLFWAMSNGSCRCNICI

>QOK07812.1 hemagglutinin [Influenza A virus]

METVSLITILLAATVSNADKICIGYQSTNSTETVDTLTENNVPVTHAKELLHTEHNGMLCATSLGQPLIL
DTCTIEGLIYGNPSCDPLLEEREWSYIVERPSAVNGLCYPGNVENLEELRSLFSSARSYQRIQIFPDTIW

NVSYDGTSNTCSSSFYRNMRWLTRKNGNYPIQDAQYTNNQGKNILFMWGINHPPTDDTQRNLYTRTDTTT
SVATEEINRIFKPLIGPRPLVNGLMGRINYYWSVLKPGQTLRIKSDGNLIAPWYGYILSGESHGRILKTD
LKRGSCTVQCQTEKGGLNTTLPFQNVSKYAFGNCSKYIGIKSLKLAVGLRNVPSRSSRGLFGAIAGFIEG
GWSGLVAGWYGFQHSNDQGVGMAADRESTQKAIDKITSKVNNIVDKMNKQYEIIDHEFSEVETRLNMINN
KIDDQIQDIWAYNAELLVLLENQKTLDEHDANVNNLYNKVKRALGSNAVEDGKGCFELYHKCDDQCMETI
RNGTYNRRKYQEESKLERQKIEGVKLESEGTYKILTIYSTVASSLVIAMGFAAFLFWAMSNGSCRCNICI

>QOK07800.1 hemagglutinin [Influenza A virus]
METVSLITILLAATVSNADKICIGYQSTNSTETVDTLTENNVPVTHAKELLHTEHNGMLCATSLGQPLIL
DTCTIEGLIYGNPSCDPLLEEREWSYIVERPSAVNGLCYPGNVENLEELRSLFSSARSCQRIQIFPDTIW
NVSYDGTSNTCSSSFYRNMRWLTRKNGNYPIQDAQYTNNQGKNILFMWGINHPPTDDTQRNLYTRTDTTT
SVATEEINRIFKPLIGPRPLVNGLMGRINYYWSVLKPGQTLRIKSDGNLIAPWYGYILSGESHGRILKTD
LKRGSCTVQCQTEKGGLNTTLPFQNVSKYAFGNCSKYIGIKSLKLAVGLRNVPSRSSRGLFGAIAGFIEG
GWSGLVAGWYGFQHSNDQGVGMAADRESTQKAIDKITSKVNNIVDKMNKQYEIIDHEFSEVETRLNMINN
KIDDQIQDIWAYNAELLVLLENQKTLDEHDANVNNLYNKVKRALGSNAVEDGKGCFELYHKCDDQCMETI
RNGTYNRRKYQEESKLERQKIEGVKLESEGTYKILTIYSTVASSLVIAMGFAAFLFWAMSNGSCRCNICI

>QOK07788.1 hemagglutinin [Influenza A virus]
METVSLITILLAATVSNADKICIGYQSTNSTETVDTLTENNVPVTHAKELLHTEHNGMLCATSLGQPLIL
DTCTIEGLIYGNPSCDPLLEEREWSYIVERPSAVNGLCYPGNVENLEELRSLFSSARSYQRIQIFPDTIW
NVSYDGTSNTCSSSFYRNMRWLTRKNGNYPIQDAQYTNNQGKNILFMWGINHPPTDDTQRNLYTRTDTTT
SVATEEINRIFKPLIGPRPLVNGLMGRINYYWSVLKPGQTLRIKSDGNLIAPWYGYILSGESHGRILKTD
LKRGSCTVQCQTEKGGLNTTLPFQNVSKYAFGNCSKYIGIKSLKLAVGLRNVPSRSSRGLFGAIAGFIEG
GWSGLVAGWYGFQHSNDQGVGMAADRESTQKAIDKITSKVNNIVDKMNKQYEIIDHEFSEVETRLNMINN
KIDDQIQDIWAYNAELLVLLENQKTLDEHDANVNNLYNKVKRALGSNAVEDGKGCFELYHKCDDQCMETI
RNGTYNRRKYQEESKLERQKIEGVKLESEGTYKILTIYSTVASSLVIAMGFAAFLFWAMSNGSCRCNICI

>QOK07776.1 hemagglutinin [Influenza A virus]
METVSLITILLAATVSNADKICIGYQSTNSTETVDTLTENNVPVTHAKELLHTEHNGMLCATSLGQPLIL
DTCTIEGLIYGNPSCDPLLEEREWSYIVERPSAVNRLCYPGNVENLEELRSLFSSARSYQRIQIFPDTIW
NVSYDGTSNTCSGSFYRNMRWLTRKNGNYPIQDAQYTNNQGKNILFMWGINHPPTDDTQRNLYTRTDTTT
SVATEEINRIFKPLIGPRPLVNGLMGRINYYWSVLKPGQTLRIKSDGNLIAPWYGYILSGESHGRILRTD
LKRGSCTVQCQTEKGGLNTTLPFQNVSKYAFGNCSKYIGTKSLKLAVGLRNVPSRSSRGLFGAIAGFIEG
GWSGLVAGWYGFQHSNDQGVGMAADRESTQKAIDKITSKVNNIVDKMNKQYEIIDHEFSEVETRLNMINN

KIDDQIQDIWAYNAELLVLLENQKTLDEHDANVNNLYNKVKRALGSNAVEDGKGCFELYHKCDDQCMETI
RNGTYNRRKYQEESKLERQKIEGVKLESEGTYKILTIYSTVASSLVIAMGFAAFLFWAMSNGSCRCNICI

>QOK07764.1 hemagglutinin [Influenza A virus]
METVSLITILLAATVSNADKICIGYQSTNSTETVDTLTENNVPVTHAKELLHTEHNGMLCATSLGQPLIL
DTCTIEGLIYGNPSCDPLLEEREWSYIVERPSAVNGLCYPGNVENLEELRSLFSSARSYQRIQIFPDTIW
NVSYDGTSNTCSGSFYRNMRWLTRKNGNYPIQDAQYTNNQGKNILFMWGINHPPTDDTQRNLYTRTDTTT
SVATEEINRIFKPLIGPRPLVNGLMGRINYYWSVLKPGQTLRIKSDGNLIAPWYGYILSGESHGRILRTD
LKRGSCTVQCQTEKGGLNTTLPFQNVSKYAFGNCSKYIGIKSLKLAVGLRNVPSRSSRGLFGAIAGFIEG
GWSGLVAGWYGFQHSNDQGVGMAADRESTQKAIDKITSKVNNIVDKMNKQYEIIDHEFSEVETRLNMINN
KIDDQIQDIWAYNAELLVLLENQKTLDEHDANVNNLYNKVKRALGSNAVEDGKGCFELYHKCDDQCMETI
RNGTYNRRKYQEESKLERQKIEGVKLESEGTYKILTIYSTVASSLLIAMGFAAFLFWAMSNGSCRCNICI

>QOK07752.1 hemagglutinin [Influenza A virus]
METVSLITILLAATVSNADKICIGYQSTNSTETVDTLTENNVPVTHAKELLHTEHNGMLCATSLGQPLIL
DTCTIEGLIYGNPSCDPLLEEREWSYIVERPSAVNGLCYPGNVENLEELRSLFSSARSYQRIQIFPDTIW
NVSYDGTSNTCSGSFYRNMRWLTRKNGNYPIQDAQYTNNQGKNILFMWGINHPPTDDTQRNLYTRTDTTT
SVATEEINRIFKPLIGPRPLVNGLMGRINYYWSVLKPGQTLRIKSDGNLIAPWYGYILSGESHGRILRTD
LKRGSCTVQCQTEKGGLNTTLPFQNVSKYAFGNCSKYIGIKSLKLAVGLRNVPSRSSRGLFGAIAGFIEG
GWSGLVAGWYGFQHSNDQGVGMAADRESTQKAIDKITSKVNNIVDKMNKQYEIIDHEFSEVETRLNMINN
KIDDQIQDIWAYNAELLVLLENQKTLDEHDANVNNLYNKVKRALGSNAVEDGKGCFELYHKCDDQCMETI
RNGTYNRRKYQEESKLERQKIEGVKLESEGTYKILTIYSTVASSLLIAMGFAAFLFWAMSNGSCRCNICI

>QOK07740.1 hemagglutinin [Influenza A virus]
METLSLITILLVATISNADKICIGYQSTNSTETVDTLTENNVPVTHAKELLHTEHNGMLCATSLGQPLIL
DTCTIEGLIYGNPSCDLSLEGREWSYIVERPSAVNGLCYPGNVENLEELRSLFSSARSYQRIQIFPDTIW
NVSYDGTSTACSGSFYRSMRWLTRKNGDYPTQDAQYTNNQGKNILFMWGINHPPTDDTQRNLYTRTDTTT
SVATEEINRIFKPLIGPRPLVNGLMGRIDYYWSVLKPGQTLRIKSDGNLIAPWYGHILSGESHGRILKTD
LKRGSCTVQCQTEKGGLNTTLPFQNVSKYAFGNCSKYIGMKSLKLAVGLRNVPSKSSRGLFGAIAGFIEG
GWSGLVAGWYGFQHSNDQGVGMAADRDSTQKAIDKITSKVNNIVDKMNKQYEIIDHEFSEVETRLNLINN
KIDDQIQDIWAYNAELLVLLENQKTLDEHDANVNNLYNKVKRALGSNAVEDGKGCFELYHKCDDQCMETI
RNGTYNRRKYQEESKLERQKIEGVKLESEGTYKILTIYSTVASSLVIAMGFAAFLFWAMSNGSCRCNICI

>QOK07728.1 hemagglutinin [Influenza A virus]

METLSLITILLVATISNADKICIGYQSTNSTETVDTLTENNVPVTHAKELLHTEHNGMLCATSLGQPLIL
DTCTIEGLIYGNPSCDLSLEGREWSYIVERPSAVNGLCYPGNVENLEELRSLFSSARSYQRIQIFPDTIW
NVSYDGTSTACSGSFYRSMRWLTRKNGDYPTQDAQYTNNQGKNILFMWGINHPPTDDTQRNLYTRTDTTT
SVATEEINRIFKPLIGPRPLVNGLMGRIDYYWSVLKPGQTLRIKSDGNLIAPWYGHILSGESHGRILKTD
LKRGSCTVQCQTEKGGLNTTLPFQNVSKYAFGNCSKYIGMKSLKLAVGLRNVPSKSSRGLFGAIAGFIEG
GWSGLVAGWYGFQHSNDQGVGMAADRDSTQKAIDKITSKVNNIVDKMNKQYEIIDHEFSEVETRLNLINN
KIDDQIQDIWAYNAELLVLLENQKTLDEHDANVNNLYNKVKRALGSNAVEDGKGCFELYHKCDDQCMETI
RNGTYNRRKYQEESKLERQKIEGVKLESEGTYKILTIYSTVASSLVIAMGFAAFLFWAMSNGSCRCNICI

>QOK07716.1 hemagglutinin [Influenza A virus]
METVSLITILLVATVSNADKICIGYQSTNSTETVDTLTENNVPVTHAKELIHTEHNGMLCATSLGQPLIL
DTCTIEGLIYGNPSCDLSLEGREWSYIVERPSAVNGLCYPGNVENLEELRSLFSSARSYQRIQIFPDTIW
NVSYDGTSTACSGSFYRNMRWLTRKDGNYPTQDAQYTNNQGKNILFMWGINHPPTDTTQSGLYTRTDTTT
SVATEEINRIFKPLIGPRPLVNGLMGRIDYYWSVLKPGQTLRIKSDGNLIAPSFGHILSGESHGRILKTD
LKRGSCTVQCQTEKGGLNTTLPFQNVSKYAFGNCSKYIGIKSLKLAVGLRNVPSRSSRGLFGAIAGFIEG
GWSGLVAGWYGFQHSNDQGVGMAADRDSTQKAIDKITSKVNNIVDKMNKQYEIIDHEFSEVETRLNMINN
KIDDQIQDIWAYNAELLVLLENQKTLDEHDANVNNLYNKVKRALGSNAVEDGKGCFELYHKCDDQCMETI
RNGTYNRRKYQEESKLERQKIEGVKLESEGTYKILTIYSTVASSLVIAMGFAAFLFWAMSNGSCRCNICI

>QOK07692.1 hemagglutinin [Influenza A virus]
METASLITILLVATVSNADKICIGYQSTNSTETVDTLTESNVPVTHAKELLHTEHNGMLCATSLGQPLIL
DTCTIEGLIYGNPSCDLSLEGREWSYIVERPSAVNGLCYPGNVENLEELRSLFSSARSYQRIQIFPDTIW
NVSYDGTSTACSGSFYRSMRWLTRKNGDYPTQDAQYTNNQGKNILFMWGINHPPTDDTQRNLYTRTDTTT
SVATEEINRIFKPLIGPRPLVNGLMGRIDYYWSVLKPGQTLRIKSDGNLIAPWYGHILSGESHGRILKTD
LKRGSCTVQCQTEKGGLNTTLPFQNVSKYAFGNCSKYIGIKSLKLAVGLRNVPSRSSRGLFGAIAGFIEG
GWPGLVAGWYGFQHSNDQGVGMAADRDSTQKAIDKITSKVNNIVDKMNKQYEIIDHEFSEVETRLNMINN
KIDDQIQDIWAYNAELLVLLENQKTLDEHDANVNNLYNKVKRALGSNAVEDGKGCFELYHKCNDQCMETI
RNGTYNRRKYQEESKLERQKIEGVKLESEGTYKILTIYSTVASSLVIAMGFAAFLFWAMSNGSCRCNICI

>QOK07680.1 hemagglutinin [Influenza A virus]
METVSLITILLAATVSNADKICIGYQSTNSTETVDTLTENNVPVTHAKELLHTEHNGMLCATSLGQPLIL
DTCTIEGLIYGNPSCDPLLEEREWSYIVERPSAVNGLCYPGNVENLEELRSLFSSARSYQRIQIFPDTIW
NVSYDGTSNTCSGSFYRNMRWLTRKNGNYPIQDAQYTNNQGKNILFMWGINHPPTDDTQRNLYTRTDTTT
SVATEEINRIFKPLIGPRPLVNGLMGRINYYWSVLKPGQTLRIKSDGNLIAPWYGYILSGESHGRILKTD

LKRGSCTVQCQTEKGGLNTTLPFQNVSKFAFGNCSKYIGIKSLKLAVGLRNVPSRSSRGLFGAIAGFIEG

GWSGLVAGWYGFQHSNDQGVGMAADRESTQKAIDKITSKVNNIVDKMNKQYEIIDHEFSEVETRLNMINN

KIDDQIQDIWAYNAELLVLLENQKTLDEHDANVNNLYNKVKRALGSNAVEDGKGCFELYHKCDDQCMETI

RNGTYNRRKYQEESKLERQKIEGVKLESEGTYKILTIYSTVASSLVIAMGFAAFLFWAMSNGSCRCNICI

>QOK07668.1 hemagglutinin [Influenza A virus]

METVSLITILLAATVSNADKICIGYQSTNSTETVDTLTENNVPVTHAKELLHTEHNGMLCATSLGQPLIL

DTCTIEGLIYGNPSCDPLLEEREWSYIVERPSAVNGLCYPGNVENLEELRSLFSSARSYQRIQIFPDTIW

NVSYDGTSNTCSGSFYRNMRWLTQKNGNYPIQDAQYTNNQGKNILFMWGINHPPTDDTQRNLYTRTDTTT

SVATEEINRIFRPLIGPRPLVNGLMGRINYHWSVLKPGQTLRIRSDGNLIAPWYGYILSGESHGRILRTD

LKRGSCTVQCQTEKGGLNTTLPFQNVSKYAFGNCSKYIGIKSLKLAVGLRNVPSRSSRGLFGAIAGFIEG

GWSGLVAGWYGFQHSNDQGVGMAADRESTQKAIDKITSKVNNIVDKMNKQYEIIEHEFSEVETRLNMINN

KIDDQIQDIWAYNAELLVLLENQKTLDEHDANVNNLYNKVKRALGSNAVEDGKGCFELYHKCDDQCMETI

RNGTYNRRKYQEESKLERQKIEGVKLESEGTYKILTIYSTVASSLVIAMGFAAFLFWAMSNGSCRCNICI

>QOK07656.1 hemagglutinin [Influenza A virus]

METVSLITILLAATVSNADKICIGYQSTNSTETVDTLTENNVPVTHAKELLHTEHNGMLCATSLGQPLIL

DTCTIEGLIYGNPSCDPLLEEREWSYIVERPSAVNGLCYPGNVENLEELRSLFSSARSYQRIQIFPDTIW

NVSYDGTSNTCSGSFYRNMRWLTQKNGNYPIQDAQYTNNQGKNILFMWGINHPPTDDTQRNLYTRTDTTT

SVATEEINRIFRPLIGPRPLVNGLMGRINYHWSVLKPGQTLRIRSDGNLIAPWYGYILSGESHGRILRTD

LKRGSCTVQCQTEKGGLNTTLPFQNVSKYAFGNCSKYIGIKSLKLAVGLRNVPSRSSRGLFGAIAGFIEG

GWSGLVAGWYGFQHSNDQGVGMAADRESTQKAIDKITSKVNNIVDKMNKQYEIIEHEFSEVETRLNMINN

KIDDQIQDIWAYNAELLVLLENQKTLDEHDANVNNLYNKVKRALGSNAVEDGKGCFELYHKCDDQCMETI

RNGTYNRRKYQEESKLERQKIEGVKLESEGTYKILTIYSTVASSLVIAMGFAAFLFWAMSNGSCRCNICI

>QOK07644.1 hemagglutinin [Influenza A virus]

METVSLITILLAATVSNADKICIGYQSTNSTETVDTLTENNVPVTHAKELLHTEHNGMLCATSLGQPLIL

DTCTIEGLIYGNPSCDPLLEEREWSYIVERPSAVNGLCYPGNVENLEELRSLFSSARSYQRIQIFPDTIW

NVSYDGTSNTCSGSFYRNMRWLTQKNGNYPIQDAQYTNNQGKNILFMWGINHPPTDDTQRNLYTRTDTTT

SVATEEINRIFRPLIGPRPLVNGLMGRINYHWSVLKPGQTLRIRSDGNLIAPWYGYILSGESHGRILRTD

LKRGSCTVQCQTEKGGLNTTLPFQNVSKYAFGNCSKYIGIKSLKLAVGLRNVPSRSSRGLFGAIAGFIEG

GWSGLVAGWYGFQHSNDQGVGMAADRESTQKAIDKITSKVNNIVDKMNKQYEIIEHEFSEVETRLNMINN

KIDDQIQDIWAYNAELLVLLENQKTLDEHDANVNNLYNKVKRALGSNAVEDGKGCFELYHKCDDQCMETI

RNGTYNRRKYQEESKLERQKIEGVKLESEGTYKILTIYSTVASSLVIAMGFAAFLFWAMSNGSCRCNICI

>QOK07632.1 hemagglutinin [Influenza A virus]

METVSLITILLAATVSNADKICIGYQSTNSTETVDTLTENNVPVTHAKELLHTEHNGMLCATSLGQPLIL

DTCTIEGLIYGNPSCDPLLEEREWSYIVERPSAVNGLCYPGNVENLEELRSLFSSARSYQRIQIFPDTIW

NVSYDGTSNTCSGSFYRNMRWLTQKNGNYPIQDAQYTNNQGKNILFMWGINHPPTDDTQRNLYTRTDTTT

SVATEEINRIFRPLIGPRPLVNGLMGRINYHWSVLKPGQTLRIRSDGNLIAPWYGYILSGESHGRILRTD

LKRGSCTVQCQTEKGGLNTTLPFQNVSKYAFGNCSKYIGIKSLKLAVGLRNVPSRSSRGLFGAIAGFIEG

GWSGLVAGWYGFQHSNDQGVGMAADRESTQKAIDKITSKVNNIVDKMNKQYEIIEHEFSEVETRLNMINN

KIDDQIQDIWAYNAELLVLLENQKTLDEHDANVNNLYNKVKRALGSNAVEDGKGCFELYHKCDDQCMETI

RNGTYNRRKYQEESKLERQKIEGVKLESEGTYKILTIYSTVASSLVIAMGFAAFLFWAMSNGSCRCNICI

>QOK07620.1 hemagglutinin [Influenza A virus]

METVSLITILLAATVSNADKICIGYQSTNSTETVDTLTENNVPVTHAKELLHTEHNGMLCATSLGQPLIL

DTCTIEGLIYGNPSCDPLLEEREWSYIVERPSAVNGLCYPGNVENLEELRSLFSSARSYQRIQIFPDTIW

NVSYDGTSNTCSGSFYRNMRWLTQKNGNYPIQDAQYTNNQGKNILFMWGINHPPTDDTQRNLYTRTDTTT

SVATEEINRIFRPLIGPRPLVNGLMGRINYHWSVLKPGQTLRIRSDGNLIAPWYGYILSGESHGRILRTD

LKRGSCTVQCQTEKGGLNTTLPFQNVSKYAFGNCSKYIGIKSLKLAVGLRNVPSRSSRGLFGAIAGFIEG

GWSGLVAGWYGFQHSNDQGVGMAADRESTQKAIDKITSKVNNIVDKMNKQYEIIEHEFSEVETRLNMINN

KIDDQIQDIWAYNAELLVLLENQKTLDEHDANVNNLYNKVKRALGSNAVEDGKGCFELYHKCDDQCMETI

RNGTYNRRKYQEESKLERQKIEGVKLESEGTYKILTIYSTVASSLVIAMGFAAFLFWAMSNGSCRCNICI

>QOK07608.1 hemagglutinin [Influenza A virus]

METVSLITILLAATVSNADKICIGYQSTNSTETVDTLTENNVPVTHAKELLHTEHNGMLCATSLGQPLIL

DTCTIEGLIYGNPSCDPLLEEREWSYIVERPSAVNGLCYPGNVENLEELRSLFSSARSYQRIQIFPDTIW

NVSYDGTSNTCSGSFYRNMRWLTRKNGNYPIQDAQYTNNQGKNILFMWGINHPPTDDTQRNLYTRTDTTT

SVATEEINRIFKPLIGPRPLVNGLMGRINYYWSVLKPGQTLRIKSDGNLIAPWYGYILSGESHGRILRTD

LNRGSCTVQCQTEKGGLNTTLPFQNVSKYAFGNCSKYIGIKSLKLAVGLRNVPSRSSRGLFGAIAGFIEG

GWSGLVAGWYGFQHSNDQGVGMAADRESTQKAIDKITSKVNNIVDKMNKQYEIIDHEFSEVETRLNMINN

KIDDQIQDIWAYNAELLVLLENQKTLDEHDANVNNLYNKVKRALGSNAVEDGKGCFELYHKCDDQCMETI

RNGTYNRRKYQEESKLERQKIEGVKLESEGTYKILTIYSTVASSLVIAMGFAAFLFWAMSNGSCRCNICI

>QOK07596.1 hemagglutinin [Influenza A virus]

METVSLITILLAATVSNADKICIGYQSTNSTETVDTLTENNVPVTHAKELLHTEHNGMLCATSLGQPLIL

DTCTIEGLIYGNPSCDPLLEEREWSYIVERPSAVNGLCYPGNVENLEELRSLFSSARSYQRIQIFPDTIW

NVSYDGTSNTCSGSFYRNMRWLTRKNGNYPIQDAQYTNNQGKNILFMWGINHPPTDDTQRNLYTRTDTTT
SVATEEINRIFKPLIGPRPLVNGLMGRINYYWSVLKPGQTLRIKSDGNLIAPWYGYILSGESHGRILRTD
LNRGSCTVQCQTEKGGLNTTLPFQNVSKYAFGNCSKYIGIKSLKLAVGLRNVPSRSSRGLFGAIAGFIEG
GWSGLVAGWYGFQHSNDQGVGMAADRESTQKAIDKITSKVNNIVDKMNKQYEIIDHEFSEVETRLNMINN
KIDDQIQDIWAYNAELLVLLENQKTLDEHDANVNNLYNKVKRALGSNAVEDGKGCFELYHKCDDQCMETI
RNGTYNRRKYQEESKLERQKIEGVKLESEGTYKILTIYSTVASSLVIAMGFAAFLFWAMSNGSCRCNICI

>QOK07584.1 hemagglutinin [Influenza A virus]
METISLITILLVATVSNADKICIGYQSTNSTETVDTLTENNVPVTHAKELIHTEHNGMLCATSLGQPLIL
DTCTIEGLIYGNPSCDLSLEGREWSYIVERPSAVNGLCYPGNVENLEELRSLFSSARSYQRIQIFPDTIW
NVSYDGTSTACSGSFYRNMRWLTRKDGNYPTQDAQYTNNQGKNILFMWGINHPPTDTTQSGLYTRTDTTT
SVATEEINRIFKPLIGPRPLVNGLMGRIDYYWSVLKPGQTLRIKSDGNLIAPWFGHILSGESHGRILKTD
LKRGSCTVQCQTEKGGLNTTLPFQNVSKYAFGNCSKYIGIKSLKLAVGLRNVPSRSSRGLFGAIAGFIEG
GWSGLVAGWYGFQHSNDQGVGMAADRDSTQKAIDKITSKVNNIVDKMNKQYEIIDHEFSEVETRLNMINN
KIDDQIQDIWAYNAELLVLLENQKTLDEHDANVNNLYNKVKRALGSNAVEDGKGCFELYHKCDDQCMETI
RNGTYNRRKYQEESKLERQKIEGVKLESEGTYKILTIYSTVASSLVIAMGFAAFLFWAMSNGSCRCNICI

>QOK07572.1 hemagglutinin [Influenza A virus]
METVSLITILLVATVSNADKICIGYQSTNSTETVDTLTENNVPVTHAKELIHTEHNGMLCATSLGQPLIL
DTCTIEGLIYGNPSCDLSLEGREWSYIVERPSAVNGLCYPGNVENLEELRSLFSSARSYQRTQIFPDTIW
NVSYDGTSTACSGSFYRNMRWLTRKDGNYPTQDAQYTNNQGKNILFMWGINHPPTDTTQSGLYTRTDTTT
SVATEEINRIFKPLIGPRPLVNGLMGRIDYYWSVLKPGQTLRIKSDGNLIAPSFGHILSGESHGRILKTD
LKRGSCTVQCQTEKGGLNTTLPFQNVSKYAFGNCSKYIGIKSLKLAVGLRNVPSRSSRGLFGAIAGFIEG
GWSGLVAGWYGFQHSNDQGVGMAADRDSTQKAIDKITSKVNNIVDKMNKQYEIIDHEFSEVETRLNMINN
KIDDQIQDIWAYNAELLVLLENQKTLDEHDANVNNLYNKVKRALGSNAVEDGKGCFELYHKCDDQCMETI
RNGTYNRRKYQEESKLERQKIEGVKLESEGTYKILTIYSTVASSLVIAMGFAAFLFWAMSNGSCRCNICI

>QOK07560.1 hemagglutinin [Influenza A virus]
METVSLITILLVATVSNADKICIGYQSTNSTETVDTLTENNVPVTHAKELIHTEHNGMLCATSLGQPLIL
DTCTIEGLIYGNPSCDLSLEGREWSYIVERPSAVNGLCYPGNVENLEELRSLFSSARSYQRIQIFPDTIW
NVSYDGTSTACSGSFYRNMRWLTRKDGNYPTQDAQYTNNQGKNILFMWGINHPPTDTTQSGLYTRTDTTT
SVATEEINRIFKPLIGPRPLVNGLMGRIDYYWSVLKPGQTLRIKSDGNLIAPSFGHILSGESHGRILKTD
LKRGSCTVQCQTEKGGLNTTLPFQNVSKYAFGNCSKYIGIKSLKLAVGLRNVPSRSSRGLFGAIAGFIEG
GWSGLVAGWYGFQHSNDQGVGMAADRDSTQKAIDKITSKVNNIVDKMNKQYEIIDHEFSEVETRLNMINN

KIDDQIQDIWAYNAELLVLLENQKTLDEHDANVNNLYNKVKRALGSNAVEDGKGCFELYHKCDDQCMETI
RNGTYNRRKYQEESKLERQKIEGVKLESEGTYKILTIYSTVASSLVIAMGFAAFLFWAMSNGSCRCNICI

>QOK07548.1 hemagglutinin [Influenza A virus]
METVSLITILLVATVSNADKICIGYQSTNSTETVDTLTENNVPVTHAKELIHTEHNGMLCATSLGQPLIL
DTCTIEGLIYGNPSCDLSLEGREWSYIVERPSAVNGLCYPGNVENLEELRSLFSSARSYQRIQIFPDTIW
NVSYDGTSTACSGSFYRNMRWLTRKDGNYPTQDAQYTNNQGKNILFIWGINHPPTDTTQSGLYTRTDTTT
SVATEEINRIFKPLIGPRPLVNGLMGRIDYYWSVLKPGQTLRIKSDGNLIAPWFGHILSGESHGRILKTD
LKRGSCTVQCQTEKGGLNTTLPFQNVSKYAFGNCSKYIGIKSLKLAVGLRNVPSRSSRGLFGAIAGFIEG
GWSGLVAGWYGFQHSNDQGVGMAADRDSTQKAIDKITSKVNNIVDKMNKQYEIIDHEFSEVETRLNMINN
KIDDQIQDIWAYNAELLVLLENQKTLDEHDANVNNLYNKVKRALGSNAVEDGKGCFELYHKCDDQCMETI
RNGTYNRRKYQEESKLERQKIEGVKLESEGTYKILTIYSTVASSLVIAMGFAAFLFWAMSNGSCRCNICI

>QOK07536.1 hemagglutinin [Influenza A virus]
METVSLITILLVATVSNADKICIGYQSTNSTETVDTLTENNVPVTHAKELIHTEHNGMLCATSLGQPLIL
ETCTIEGLIYGNPSCDLSLEGREWSYIVERPSAVNGLCYPGNVENLEELRSLFSSARSYQRIQIFPDTIW
NVSYDGTSTACSGSFYRNMRWLTRKDGNYPTQDAQYTNNQGKNILFMWGINHPPSDTTQSGLYTRTDTTT
SVATEEINRIFKPLIGPRPLVNGLMGRIDYYWSVLKPGQTLRIKSDGNLIAPWFGHILSGESHGRILKTD
LKRGSCTVQCQTEKGGLNTTLPFQNVSKYAFGNCSKYIGIKSLKLAVGLRNVPSRSSRGLFGAIAGFIEG
GWSGLVAGWYGFQHSNDQGVGMAADRDSTQKAIDKITSKVNNIVDKMNKQYEIIDHEFSEVETRLNMINN
KIDDQIQDIWAYNAELLVLLENQKTLDEHDANVNNLYNKVKRALGSNAVEDGKGCFELYHKCDDQCMETI
RNGTYNRRKYQEESKLERQKIEGVKLESEGTYKILTIYSTVASSLVIAMGFAAFLFWAMSNGSCRCNICI

>QOK07524.1 hemagglutinin [Influenza A virus]
METVSLITILLAATVSNADKICIGYQSTNSTETVDTLTENNVPVTHAKELLHTEHNGMLCATNLGQPLIL
DTCTIEGLIYGNPSCDPLLEEREWSYIVERPSAVNGLCYPGNVENLEELRSLFSSARSYQRIQIFPDTIW
NVSYDGTSNTCSGSFYRNMRWLTRKNGNYPIQDAQYTNNQGKNILFMWGINHPPTDDTQRNLYTRTDTTT
SVATEEINRIFRPLIGPRPLVNGLMGRINYYWSVLKPGQTLRIKSDGNLIAPWYGYILSGESHGRILRTD
LKRGSCTVQCQTEKGGLNTTLPFQNVSKYAFGNCSKYIGIKSLKLAVGLRNVPSRSSRGLFGAIAGFIEG
GWSGLVAGWYGFQHSNDQGVGMAADRESTQKAIDKITSKVNNIVDKMNKQYEIIDHEFSEVETRLNMINN
KIDDQIQDIWAYNAELLVLLENQKTLDEHDANVNNLYNKVKRALGSNAVEDGKGCFELYHKCDDQCMETI
RNGTYNRRKYQEESKLERQKIEGVKLESEGTYKILTIYSTVASSLVIAMGFAAFLFWAMSNGSCRCNICI

>QOK07512.1 hemagglutinin [Influenza A virus]

METVSLITILLAATVSNADKICIGYQSTNSTETVDTLTENNVPVTHAKELLHTEHNGMLCATNLGQPLIL
DTCTIEGLIYGNPSCDPLLEEREWSYIVERPSAVNGLCYPGNVENLEELRSLFSSARSYQRIQIFPDTIW
NVSYDGTSNTCSGSFYRNMRWLTRKNGNYPIQDAQYTNNQGKNILFMWGINHPPTDDTQRNLYTRTDTTT
SVATEEINRIFRPLIGPRPLVNGLMGRINYYWSVLKPGQTLRIKSDGNLIAPWYGYILSGESHGRILRTD
LKRGSCTVQCQTEKGGLNTTLPFQNVSKYAFGNCSKYIGIKSLKLAVGLRNVPSRSSRGLFGAIAGFIEG
GWSGLVAGWYGFQHSNDQGVGMAADRESTQKAIDKITSKVNNIVDKMNKQYEIIDHEFSEVETRLNMINN
KIDDQIQDIWAYNAELLVLLENQKTLDEHDANVNNLYNKVKRALGSNAVEDGKGCFELYHKCDDQCMETI
RNGTYNRRKYQEESKLERQKIEGVKLESEGTYKILTIYSTVASSLVIAMGFAAFLFWAMSNGSCRCNICI

>QOK07500.1 hemagglutinin [Influenza A virus]
METVSLITILLAATVSNADKICIGYQSTNSTETVDTLTENNVPVTHAKELLHTEHNGMLCATSLGQPLIL
DTCTIEGLIYGNPSCDPLLEEREWSYIVERPSAVNGLCYPGNVENLEELRSLFSSARSYQRIQIFPDTIW
NVSYDGTSNTCSGSFYRNMRWLTRKNGNYPIQDAQYTNNQGKNILFMWGINHPPTDDTQRNLYTRTDTTT
SVATEEINRIFKPLIGPRPLVNGLMGRINYYWSVLKPGQTLRIKSDGNLIAPWYGYILSGESHGRILRTD
LKRGSCTVQCQTEKGGLNTTLPFQNVSKYAFGNCSKYIGIKSLKLAVGLRNVPSRSSRGLFGAIAGFIEG
GWSGLVAGWYGFQHSNDQGVGMAADRESTQKAIDKITSKVNNIVDKMNKQYEIIDHEFSEVETRLNMINN
KIDDQIQDIWAYNAELLVLLENQKTLDEHDANVNNLYNKVKRALGSNAVEDGKGCFELYHKCDDQCMETI
RNGTYNRRKYQEESKLERQKIEGVKLESEGTYKILTIYSTVASSLLIAMGFAAFLFWAMSNGSCRCNICI

>QOK07476.1 hemagglutinin [Influenza A virus]
METVSLITILLVATVSNADKICIGYQSTNSTETVDTLTENNVPVTHAKELIHTEHNGMLCATSLGQPLIL
DTCTIEGLIYGNPSCGLSLEGREWSYIVERPSAVNGLCYPGNVENLEELRSLFSSARSYQRVQIFPDTIW
NVSYDGTSTACSGSFYRNMRWLTRKDGNYPTQDAQYTNNQGKNILFMWGINHPPTDTTQSGLYTRTDTTT
SVATEEINRIFKPLIGPRPLVNGLMGRIDYYWSVLKPGQTLRIKSDGNLIAPWFGHILSGESHGRILKTD
LKRGSCTVQCQTEKGGLNTTLPFQNVSKYAFGNCSKYIGIKSLKLAVGLRNVPSRSSRGLFGAIAGFIEG
GWSGLVAGWYGFQHSNDQGVGMAADRDSTQKAIDKITSKVNNIVDKMNKQYEIIDHEFSEVETRLNMINN
KIDDQIQDIWAYNAELLVLLENQKTLDEHDANVNNLYNKVKRALGSNAVEDGKGCFELYHKCDDQCMETI
RNGTYNRRKYQEESKLERQKIEGVKLESEGTYKILTIYSTVASSLVIAMGFAAFLFWAMSNGSCRCNICI

>QOK07464.1 hemagglutinin [Influenza A virus]
METVSLITILLVATVSNADKICIGYQSTNSTETVDTLTENNVPVTHAKELIHTEHNGMLCATSLGQPLIL
DTCTIEGLIYGNPSCGLSLEGREWSYIVERPSAVNGLCYPGNVENLEELRSLFSSARSYQRVQIFPDTIW
NVSYDGTSTACSGSFYRNMRWLTRKDGNYPTQDAQYTNNQGKNILFMWGINHPPTDTTQSGLYTRTDTTT
SVATEEINRIFKPLIGPRPLVNGLMGRIDYYWSVLKPGQTLRIKSDGNLIAPWFGHILSGESHGRILKTD

LKRGSCTVQCQTEKGGLNTTLPFQNVSKYAFGNCSKYIGIKSLKLAVGLRNVPSRSSRGLFGAIAGFIEG
GWSGLVAGWYGFQHSNDQGVGMAADRDSTQKAIDKITSKVNNIVDKMNKQYEIIDHEFSEVETRLNMINN
KIDDQIQDIWAYNAELLVLLENQKTLDEHDANVNNLYNKVKRALGSNAVEDGKGCFELYHKCDDQCMETI
RNGTYNRRKYQEESKLERQKIEGVKLESEGTYKILTIYSTVASSLVIAMGFAAFLFWAMSNGSCRCNICI

>QOK07200.1 hemagglutinin [Influenza A virus]
METVSLMTILLVATVSNADKICIGYQSTNSTETVDTLTENDVPVTHAKELIHTEHNGMLCATSLGQPLIL
DTCTIEGLIYGNPSCDLSLEGREWSYIVERPSAVNGLCYPGNVENLEELRSLFSSARSYQRIQIFPDTIW
NVSYDGTSTACSGSFYKNMRWLTRKDGNYPTQDAQYTNNQGKNILFMWGINHPPADTTQTGLYTRTDTTT
SVATEEINRIFKPLIGPRPLVNGLMGRIDYYWSVLKPGQTLRIKSDGNLIAPWFGHILSGESHGRILKTD
LKRGSCTVQCQTEKGGLNTTLPFQNVSKYAFGNCSKYIGIKSLKLAVGLRNVPSRSSRGLFGAIAGFIEG
GWSGLVAGWYGFQHSNDQGVGMAADRDSTQKAIDKITSKVNNIVDKMNKQYEIIDHEFSEVETRLNMINN
KIDDQIQDIWAYNAELLVLLENQKTLDEHDANVNNLYNKVKRALGSNAVEDGKGCFELYHKCDDQCMETI
RNGTYNRRKYQEESKLERQKIEGVKLESEGTYKILTIYSTVASSLVIAMGFAAFLFWAMSNGSCRCNICI

>QOK07164.1 hemagglutinin [Influenza A virus]
METVSLMTILLVATVSNADKICIGYQSTNSTETVDTLTENDVPVTHAKELIHTEHNGMLCATSLGQPLIL
DTCTIEGLIYGNPSCDLSLEGREWSYIVERPSAVNGLCYPGNVENLEELRSLFSSARSYQRIQIFPDTIW
NVSYDGTSTACSGSFYKNMRWLTRKDGNYPTQDAQYTNNQGKNILFMWGINHPPADTTQTGLYTRTDTTT
SVATEEINRIFKPLIGPRPLVNGLMGRIDYYWSVLKPGQTLRIKSDGNLIAPWFGHILSGESHGRILKTD
LKRGSCTVQCQTEKGGLNTTLPFQNVSKYAFGNCSKYIGIKSLKLAVGLRNVPSRSSRGLFGAIAGFIEG
GWSGLVAGWYGFQHSNDQGVGMAADRDSTQKAIDKITSKVNNIVDKMNKQYEIIDHEFSEVETRLNMINN
KIDDQIQDIWAYNAELLVLLENQKTLDEHDANVNNLYNKVKRALGSNAVEDGKGCFELYHKCDDQCMETI
RNGTYNRRKYQEESKLERQKIEGVKLESEGTYKILTIYSTVASSLVIAMGFAAFLFWAMSNGSCRCNICI

>QOK07152.1 hemagglutinin [Influenza A virus]
METVSLMTILLVATVSNADKICIGYQSTNSTETVDTLTENDVPVTHAKELIHTEHNGMLCATSLGQPLIL
DTCTIEGLIYGNPSCDLSLEGREWSYIVERPSAVNGLCYPGNVENLEELRSLFSSARSYQRIQIFPDTIW
NVSYDGTSTACSGSFYKNMRWLTRKDGNYPTQDAQYTNNQGKNILFMWGINHPPADTTQTGLYTRTDTTT
SVATEEINRIFKPLIGPRPLVNGLMGRIDYYWSVLKPGQTLRIKSDGNLIAPWFGHILSGESHGRILKTD
LKRGSCTVQCQTEKGGLNTTLPFQNVSKYAFGNCSKYIGIKSLKLAVGLRNVPSRSSRGLFGAIAGFIEG
GWSGLVAGWYGFQHSNDQGVGMAADRDSTQKAIDKITSKVNNIVDKMNKQYEIIDHEFSEVETRLNMINN
KIDDQIQDIWAYNAELLVLLENQKTLDEHDANVNNLYNKVKRALGSNAVEDGKGCFELYHKCDDQCMETI
RNGTYNRRKYQEESKLERQKIEGVKLESEGTYKILTIYSTVASSLVIAMGFAAFLFWAMSNGSCRCNICI

>QOK07128.1 hemagglutinin [Influenza A virus]

METVSLMTILLVATVSNADKICIGYQSTNSTETVDTLTENDVPVTHAKELIHTEHNGMLCATSLGQPLIL

DTCTIEGLIYGNPSCDLSLEGREWSYIVERPSAVNGLCYPGNVENLEELRSLFSSARSYQRIQIFPDTIW

NVSYDGTSTACSGSFYKNMRWLTRKDGNYPTQDAQYTNNQGKNILFMWGINHPPADTTQTGLYTRTDTTT

SVATEEINRIFKPLIGPRPLVNGLMGRIDYYWSVLKPGQTLRIKSDGNLIAPWFGHILSGESHGRILKTD

LKRGSCTVQCQTEKGGLNTTLPFQNVSKYAFGNCSKYIGIKSLKLAVGLRNVPSRSSRGLFGAIAGFIEG

GWSGLVAGWYGFQHSNDQGVGMAADRDSTQKAIDKITSKVNNIVDKMNKQYEIIDHEFSEVETRLNMINN

KIDDQIQDIWAYNAELLVLLENQKTLDEHDANVNNLYNKVKRALGSNAVEDGKGCFELYHKCDDQCMETI

RNGTYNRRKYQEESKLERQKIEGVKLESEGTYKILTIYSTVASSLVIAMGFAAFLFWAMSNGSCRCNICI

>QOK07080.1 hemagglutinin [Influenza A virus]

METISLITILLVATVSYADKICIGYQSTNSTETVDTLTENNVPVTHAKELLHTEHNGMLCATSLGQPLIL

DTCTIEGLIYGNPSCDLSLEGKEWSYIVERPSAVNGLCYPGNVENLEELRSLFSSARSYQRVQIFPDTIW

NVSYDGTSKACSGSFYRSMRWLNRKDGNYPTQDAQYTNNQGKNILFMWGINHPPTDDTQRSLYTKTDTTT

SVATEEINRIFKPLIGPRPLVNGLMGRIDYYWSVLKPGQTLRIKSDGNLIAPWYGYILSGESHGRILKTD

LKRGSCTVQCQTEKGGLNTTLPFQNVSKYAFGNCSKYIGIKSLKLAVGLRNVPSRSSRGLFGAIAGFIEG

GWSGLVAGWYGFQHSNDQGVGMAADRDSTQKAIDKITSKVNNIVDKMNKQYEIIDHEFSEVETRLNMINN

KIDDQIQDIWAYNAELLVLLENQKTLDEHDANVNNLYNKVKRALGSNAAEDGKGCFELYHKCNDQCMETI

RNGTYNRRKYQEESKLERQKIEGVKLESEGTYKILTIYSTVASSLVIAMGFAAFLFWAMSNGSCRCNICI

>QOK07068.1 hemagglutinin [Influenza A virus]

METVSLITILLVATVSNADKICIGYQSTNSTETVDTLTENNVPVTHAKELLHTEHNGMLCATSLGQPIIL

DTCTIEGLIYGNPSCDLSLEGREWSYIVERPSAVNGLCYPGNVENLEELRSLFSSARSYQRIQIFPDTIW

NVSYDGTSTACSGSFYKNMRWLTRKNGEYPIQDAQYTNNQGKNILFMWGINHPPADTTQRDLYTRTDTTT

SVATEEINRIFKPLIGPRPLVNGLMGRIDYYWSVLKPGQTLRIKSDGNLIAPWYGHILSGESHGRILKTD

LKRGSCTVQCQTEKGGLNTTLPFQNVSKYAFGNCSKYIGIKSLKLAVGLRNVPSRSSRGLFGAIAGFIEG

GWSGLVAGWYGFQHSNDQGVGMAADRESTQKAVDKITSKVNNIVDKMNKQYEIIDHEFSEVETRLNMINN

KIDDQIQDIWAYNAELLVLLENQKTLDEHDANVNNLYNKVKRALGSNAMEDGKGCFELYHKCDDQCMETI

RNGTYNRRKYQEESKLERQKIEGVKLESEGTYKILTIYSTVASSLVIAMGFAAFLFWAMSNGSCRCNICI

>QOK07056.1 hemagglutinin [Influenza A virus]

METVSLITILLVATVSYADKICIGYQSTNSTETVDTLTENNVPVTHAKELLHTEHNGMLCATSLGQPLIL

DTCTIEGLIYGNPSCDLSLEGREWSYIVERPSAVNGLCYPGNVENLEELRSLFSSARSYQRVQIFPDTIW

NVSYDGTSTACSGSFYRSMRWLTRKDGNYPTQDAQYTNNQGKNILFMWGINHPPTDDTQRSLYTRTDTTT
SVATEEINRIFKPLIGPRPLVNGLMGRIDYYWSVLKPGQTLRIKSDGNLIAPWYGYILSGESHGRILKTD
LKRGSCTVQCQTEKGGLNTTLPFQNVSKYAFGNCSKYIGIKSLKLAVGLRNVPSRSSRGLFGAIAGFIEG
GWSGLVAGWYGFQHSNDQGVGMAADRDSTQKAIDKITSKVNNIVDKMNKQYEIIDHEFSEVETRLNMINN
KIDDQIQDIWAYNAELLVLLENQKTLDEHDANVNNLYNKVKRALGSNAAEDGKGCFELYHKCNDQCMETI
RNGTYNRKKYQEESKLERQKIEGVKLESEGTYKILTIYSTVASSLVIAMGFAAFLFWAMSNGSCRCNICI

>QOK07044.1 hemagglutinin [Influenza A virus]
METVSLITILLVATVSYADKICIGYQSTNSTETVDTLTENNVPVTHAKELLHTEHNGMLCATSLGQPLIL
DTCTIEGLIYGNPSCDLSLEGREWSYIVERPSAVNGLCYPGNVENLEELRSLFSSARSYQRVQIFPDTIW
NVSYDGTSTACSGSFYRSMRWLTRKDGNYPTQDAQYTNNQGKNILFMWGINHPPTDDTQRSLYTRTDTTT
SVATEEINRIFKPLIGGRGLVNGLMGRIDYYWSVLKPGQTLRIKSDGNLIAPWYGYILSGESHGRILKTD
LKRGSCTVQCQTEKGGLNTTLPFQNVSKYAFGNCSKYIGIKSLKLAVGLRNVPSRSSRGLFGAIAGFIEG
GWSGLVAGWYGFQHSNDQGVGMAADRDSTQKAIDKITSKVNNIVDKMNKQYEIIDHEFSEVETRLNMINN
KIDDQIQDIWAYNAELLVLLENQKTLDEHDANVNNLYNKVKRALGSNAAEDGKGCFELYHKCNDQCMETI
RNGTYNRKKYQEESKLERQKIEGVKLESEGTYKILTIYSTVASSLVIAMGFAAFLFWAMSNGSCRCNICI

>QOK07032.1 hemagglutinin [Influenza A virus]
METVSLITILLVATVSYADKICIGYQSTNSTETVDTLTENNVPVTHAKELLHTEHNGMLCATSLGQPLIL
DTCTIEGLIYGNPSCDLSLEGREWSYIVERPSAVNGLCYPGNVENLEELRSLFSSARSYQRVQIFPDTIW
NVSYDGTSTACSGSFYRSMRWLTRKDGNYPTQDAQYTNNQGKNILFMWGINHPPTDDTQRSLYTRTDTTT
SVATEEINRIFKPLIGPRPLVNGLMGRIDYYWSVLKPGQTLRIKSDGNLIAPWYGYILSGESHGRILKTD
LKRGSCTVQCQTEKGGLNTTLPFQNVSKYAFGNCSKYIGIKSLKLAVGLRNVPSRSSRGLFGAIAGFIEG
GWSGLVAGWYGFQHSNDQGVGMAADRDSTQKAIDKITSKVNNIVDKMNKQYEIIDHEFSEVETRLNMINN
KIDDQIQDIWAYNAELLVLLENQKTLDEHDANVNNLYNKVKRALGSNAAEDGKGCFELYHKCNDQCMETI
RNGTYNRKKYQEESKLERQKIEGVKLESEGTYKILTIYSTVASSLVIAMGFAAFLFWAMSNGSCRCNICI

>QOK07008.1 hemagglutinin [Influenza A virus]
METVSLITILLVATVSYADKICIGYQSTNSTETVDTLTENNVPVTHAKELLHTEHNGMLCATSLGQPLIL
DTCTIEGLIYGNPSCDLSLEGREWSYIVERPSAVNGLCYPGNVENLEELRSLFSSARSYQRVQIFPDTIW
NVSYDGTSTACSGSFYRSMRWLTRKDGNYPTQDAQYTNNQGKNILFMWGINHPPTDDTQRSLYTRTDTTT
SVATEEINRIFKPLIGPRPLVNGLMGRIDYYWSVLKPGQTLRIKSDGNLIAPWYGYILSGESHGRILKTD
LKRGSCTVQCQTEKGGLNTTLPFQNVSKYAFGNCSKYIGIKSLKLAVGLRNVPSRSSRGLFGAIAGFIEG
GWSGLVAGWYGFQHSNDQGVGMAADRDSTQKAIDKITSKVNNIVDKMNKQYEIIDHEFSEVETRLNMINN

KIDDQIQDIWAYNAELLVLLENQKTLDEHDANVNNLYNKVKRALGSNAAEDGKGCFELYHKCNDQCMETI
RNGTYNRKKYQEESKLERQKIEGVKLESEGTYKILTIYSTVASSLVIAMGFAAFLFWAMSNGSCRCNICI

>QOK06996.1 hemagglutinin [Influenza A virus]
METVSLITILLVATVSYADKICIGYQSTNSTETVDTLTENNVPVTHAKELLHTEHNGMLCATSLGQPLIL
DTCTIEGLIYGNPSCDLSLEGREWSYIVERPSAVNGLCYPGNVENLEELRSLFSSARSYQRVQIFPDTIW
NVSYDGTSTACSGSFYRSMRWLTRKDGNYPTQDAQYTNNQGKNILFMWGINHPPTDDTQRSLYTRTDTTT
SVATEEINRIFKPLIGPRPLVNGLMGRIDYYWSVLKPGQTLRIKSDGNLIAPWYGYILSGESHGRILKTD
LKRGSCTVQCQTEKGGLNTTLPFQNVSKYAFGNCSKYIGIKSLKLAVGLRNVPSRSSRGLFGAIAGFIEG
GWSGLVAGWYGFQHSNDQGVGMAADRDSTQKAIDKITSKVNNIVDKMNKQYEIIDHEFSEVETRLNMINN
KIDDQIQDIWAYNAELLVLLENQKTLDEHDANVNNLYNKVKRALGSNAAEDGKGCFELYHKCNDQCMETI
RNGTYNRKKYQEESKLERQKIEGVKLESEGTYKILTIYSTVASSLVIAMGFAAFLFWAMSNGSCRCNICI

>QOK06984.1 hemagglutinin [Influenza A virus]
METVSLITILLVATVSNADKICIGYQSTNSTETVDTLTENNVPVTHAKELLHTEHNGMLCATSLGQPIIL
DTCTIEGLIYGNPSCDLSLEGREWSYIVERPSAVNGLCYPGNVENLEELRSLFSSARSYQRIQIFPDTIW
NVSYDGTSTACSGSFYKNMRWLTRKNGEYPIQDAQYTNNQGKNILFMWGINHPPADTTQRDLYTRTDTTT
SVATEEINRIFKPLIGPRPLVNGLMGRIDYYWSVLKPGQTLRIKSDGNLIAPWYGHILSGESHGRILKTD
LKRGSCTVQCQTEKGGLNTTLPFQNVSKYAFGNCSKYIGIKSLKLAVGLRNVPSRSSRGLFGAIAGFIEG
GWSGLVAGWYGFQHSNDQGVGMAADRESTQKAVDKITSKVNNIVDKMNKQYEIIDHEFSEVETRLNMINN
KIDDQIQDIWAYNAELLVLLENQKTLDEHDANVNNLYNKVKRALGSNAMEDGKGCFELYHKCDDQCMETI
RNGTYNRRKYQEESKLERQKIEGVKLESEGTYKILTIYSTVASSLVIAMGFAAFLFWAMSNGSCRCNICI

>QOK06960.1 hemagglutinin [Influenza A virus]
METVSLITILLVATVSYADKICIGYQSTNSTETVDTLTENNVPVTHAKELLHTEHNGMLCATSLGQPLIL
DTCTIEGLIYGNPSCDLSLEGREWSYIVERPSAVNGLCYPGNVENLEELRSLFSSARSYQRVQIFPDTIW
NVSYDGTSTACSGSFYRSMRWLTRKDGNYPTQDAQYTNNQGKNILFMWGINHPPTDDTQRSLYTRTDTTT
SVATEEINRIFKPLIGPRPLVNGLMGRIDYYWSVLKPGQTLRIKSDGNLIAPWYGYILSGESHGRILKTD
LKRGSCTVQCQTEKGGLNTTLPFQNVSKYAFGNCSKYIGIKSLKLAVGLRNVPSRSSRGLFGAIAGFIEG
GWSGLVAGWYGFQHSNDQGVGMAADRDSTQKAIDKITSKVNNIVDKMNKQYEIIDHEFSEVETRLNMINN
KIDDQIQDIWAYNAELLVLLENQKTLDEHDANVNNLYNKVKRALGSNAAEDGKGCFELYHKCNDQCMETI
RNGTYNRKKYQEESKLERQKIEGVKLESEGTYKILTIYSTVASSLVIAMGFAAFLFWAMSNGSCRCNICI

>QOK06948.1 hemagglutinin [Influenza A virus]

METVSLITILLVATVSNADKICIGYQSTNSTETVDTLTENNVPVTHAKELLHTEHNGMLCATSLGQPIIL
DTCTIEGLIYGNPSCDLSLEGREWSYIVERPSAVNGLCYPGNVENLEELRSLFSSARSYQRIQIFPDTIW
NVSYDGTSTACSGSFYKNMRWLTRKNGEYPIQDAQYTNNQGKNILFMWGINHPPADTTQRDLYTRTDTTT
SVATEEINRIFKPLIGPRPLVNGLMGRIDYYWSVLKPGQTLRIKSDGNLIAPWYGHILSGESHGRILKTD
LKRGSCTVQCQTEKGGLNTTLPFQNVSKYAFGNCSKYIGIKSLKLAVGLRNVPSRSSRGLFGAIAGFIEG
GWSGLVAGWYGFQHSNDQGVGMAADRESTQKAVDKITSKVNNIVDKMNKQYEIIDHEFSEVETRLNMINN
KIDDQIQDIWAYNAELLVLLENQKTLDEHDANVNNLYNKVKRALGSNAMEDGKGCFELYHKCDDQCMETI
RNGTYNRRKYQEESKLERQKIEGVKLESEGTYKILTIYSTVASSLVIAMGFAAFLFWAMSNGSCRCNICI

>QOK06936.1 hemagglutinin [Influenza A virus]
METVSLITILLVATVSYADKICIGYQSTNSTETVDTLTENNVPVTHAKELLHTEHNGMLCATSLGQPLIL
DTCTIEGLIYGNPSCDLSLEGREWSYIVERPSAVNGLCYPGNVENLEELRSLFSSARSYQRVQIFPDTIW
NVSYDGTSTACSGSFYRSMRWLTRKDGNYPTQDAQYTNNQGKNILFMWGINHPPTDDTQRSLYTRTDTTT
SVATEEINRIFKPLIGPRPLVNGLMGRIDYYWSVLKPGQTLRIKSDGNLIAPWYGYILSGESHGRILKTD
LKRGSCTVQCQTEKGGLNTTLPFQNVSKYAFGNCSKYIGIKSLKLAVGLRNVPSRSSRGLFGAIAGFIEG
GWSGLVAGWYGFQHSNDQGVGMAADRDSTQKAIDKITSKVNNIVDKMNKQYEIIDHEFSEVETRLNMINN
KIDDQIQDIWAYNAELLVLLENQKTLDEHDANVNNLYNKVKRALGSNAAEDGKGCFELYHKCNDQCMETI
RNGTYNRKKYQEESKLERQKIEGVKLESEGTYKILTIYSTVASSLVIAMGFAAFLFWAMSNGSCRCNICI

>QOK06924.1 hemagglutinin [Influenza A virus]
METVSLITILLVATVSYADKICIGYQSTNSTETVDTLTENNVPVTHAKELLHTEHNGMLCATSLGQPLIL
DTCTIEGLIYGNPSCDLSLEGREWSYIVERPSAVNGLCYPGNVENLEELRSLFSSARSYQRVQIFPDTIW
NVSYDGTSTACSGSFYRSMRWLTRKDGNYPTQDAQYTNNQGKNILFMWGINHPPTDDTQRSLYTRTDTTT
SVATEEINRIFKPLIGPRPLVNGLMGRIDYYWSVLKPGQTLRIKSDGNLIAPWYGYILSGESHGRILKTD
LKRGSCTVQCQTEKGGLNTTLPFQNVSKYAFGNCSKYIGIKSLKLAVGLRNVPSRSSRGLFGAIAGFIEG
GWSGLVAGWYGFQHSNDQGVGMAADRDSTQKAIDKITSKVNNIVDKMNKQYEIIDHEFSEVETRLNMINN
KIDDQIQDIWAYNAELLVLLENQKTLDEHDANVNNLYNKVKRALGSNAAEDGKGCFELYHKCNDQCMETI
RNGTYNRKKYQEESKLERQKIEGVKLESEGTYKILTIYSTVASSLVIAMGFAAFLFWAMSNGSCRCNICI

>QOK06912.1 hemagglutinin [Influenza A virus]
METVSLITILLVATVSYADKICIGYQSTNSTETVDTLTENNVPVTHAKELLHTEHNGMLCATSLGQPLIL
DTCTIEGLIYGNPSCDLSLEGREWSYIVERPSAVNGLCYPGNVENLEELRSLFSSARSYQRVQIFPDTIW
NVSYDGTSTACSGSFYRSMRWLTRKDGNYPTQDAQYTNNQGKNILFMWGINHPPTDDTQRSLYTRTDTTT
SVATEEINRIFKPLIGPRPLVNGLMGRIDYYWSVLKPGQTLRIKSDGNLIAPWYGYILSGESHGRILKTD

LKRGSCTVQCQTEKGGLNTTLPFQNVSKYAFGNCSKYIGIKSLKLAVGLRNVPSRSSRGLFGAIAGFIEG
GWSGLVAGWYGFQHSNDQGVGMAADRDSTQKAIDKITSKVNNIVDKMNKQYEIIDHEFSEVETRLNMINN
KIDDQIQDIWAYNAELLVLLENQKTLDEHDANVNNLYNKVKRALGSNAAEDGKGCFELYHKCNDQCMETI
RNGTYNRKKYQEESKLERQKIEGVKLESEGTYKILTIYSTVASSLVIAMGFAAFLFWAMSNGSCRCNICI

>QOK06900.1 hemagglutinin [Influenza A virus]
METVSLITILLVATVSYADKICIGYQSTNSTETVDTLTENNVPVTHAKELLHTEHNGMLCATSLGQPLIL
DTCTIEGLIYGNPSCDLSLEGREWSYIVERPSAVNGLCYPGNVENLEELRSLFSSARSYQRVQIFPDTIW
NVSYDGTSTACSGSFYRSMRWLTRKDGNYPTQDAQYTNNQGKNILFMWGINHPPTDDTQRSLYTRTDTTT
SVATEEINRIFKPLIGPRPLVNGLMGRIDYYWSVLKPGQTLRIKSDGNLIAPWYGYILSGESHGRILKTD
LKRGSCTVQCQTEKGGLNTTLPFQNVSKYAFGNCSKYIGIKSLKLAVGLRNVPSRSSRGLFGAIAGFIEG
GWSGLVAGWYGFQHSNDQGVGMAADRDSTQKAIDKITSKVNNIVDKMNKQYEIIDHEFSEVETRLNMINN
KIDDQIQDIWAYNAELLVLLENQKTLDEHDANVNNLYNKVKRALGSNAAEDGKGCFELYHKCNDQCMETI
RNGTYNRKKYQEESKLERQKIEGVKLESEGTYKILTIYSTVASSLVIAMGFAAFLFWAMSNGSCRCNICI

>QOK06888.1 hemagglutinin [Influenza A virus]
METVSLITILLVATVSYADKICIGYQSTNSTETVDTLTENNVPVTHAKELLHTEHNGMLCATSLGQPLIL
DTCTIEGLIYGNPSCDLSLEGREWSYIVERPSAVNGLCYPGNVENLEELRSLFSSARSYQRVQIFPDTIW
NVSYDGTSTACSGSFYRSMRWLTRKDGNYPTQDAQYTNNQGKNILFMWGINHPPTDDTQRSLYTRTDTTT
SVATEEINRIFKPLIGPRPLVNGLMGRIDYYWSVLKPGQTLRIKSDGNLIAPWYGYILSGESHGRILKTD
LKRGSCTVQCQTEKGGLNTTLPFQNVSKYAFGNCSKYIGIKSLKLAVGLRNVPSRSSRGLFGAIAGFIEG
GWSGLVAGWYGFQHSNDQGVGMAADRDSTQKAIDKITSKVNNIVDKMNKQYEIIDHEFSEVETRLNMINN
KIDDQIQDIWAYNAELLVLLENQKTLDEHDANVNNLYNKVKRALGSNAAEDGKGCFELYHKCNDQCMETI
RNGTYNRKKYQEESKLERQKIEGVKLESEGTYKILTIYSTVASSLVIAMGFAAFLFWAMSNGSCRCNICI

>QOK06876.1 hemagglutinin [Influenza A virus]
METVSLITILLVATVSYADKICIGYQSTNSTETVDTLTENNVPVTHAKELLHTEHNGMLCATSLGQPLIL
DTCTIEGLIYGNPSCDLSLEGREWSYIVERPSAVNGLCYPGNVENLEELRSLFSSARSYQRVQIFPDTIW
NVSYDGTSTACSGSFYRSMRWLTRKDGNYPTQDAQYTNNQGKNILFMWGINHPPTDDTQRSLYTRTDTTT
SVATEEINRIFKPLIGPRPLVNGLMGRIDYYWSVLKPGQTLRIKSDGNLIAPWYGYILSGESHGRILKTD
LKRGSCTVQCQTEKGGLNTTLPFQNVSKYAFGNCSKYIGIKSLKLAVGLRNVPSRSSRGLFGAIAGFIEG
GWSGLVAGWYGFQHSNDQGVGMAADRDSTQKAIDKITSKVNNIVDKMNKQYEIIDHEFSEVETRLNMINN
KIDDQIQDIWAYNAELLVLLENQKTLDEHDANVNNLYNKVKRALGSNAAEDGKGCFELYHKCNDQCMETI
RNGTYNRKKYQEESKLERQKIEGVKLESEGTYKILTIYSTVASSLVIAMGFAAFLFWAMSNGSCRCNICI

>QOK06864.1 hemagglutinin [Influenza A virus]

METVSLITILLVATVSYADKICIGYQSTNSTETVDTLTENNVPVTHAKELLHTEHNGMLCATSLGQPLIL

DTCTIEGLIYGNPSCDLSLEGREWSYIVERPSAVNGLCYPGNVENLEELRSLFSSARSYQRVQIFPDTIW

NVSYDGTSTACSGSFYRSMRWLTRKDGNYPTQDAQYTNNQGKNILFMWGINHPPTDDTQRSLYTRTDTTT

SVATEEINRIFKPLIGPRPLVNGLMGRIDYYWSVLKPGQTLRIKSDGNLIAPWYGYILSGESHGRILKTD

LKRGSCTVQCQTEKGGLNTTLPFQNVSKYAFGNCSKYIGIKSLKLAVGLRNVPSRSSRGLFGAIAGFIEG

GWSGLVAGWYGFQHSNDQGVGMAADRDSTQKAIDKITSKVNNIVDKMNKQYEIIDHEFSEVETRLNMINN

KIDDQIQDIWAYNAELLVLLENQKTLDEHDANVNNLYNKVKRALGSNAAEDGKGCFEVYEKCNEEWMERI

GKGRYNRKKYQEESKLERQKIEGVKLESEGTYKILTIYSTVASSLVIAMGFAAFLFWAMSNGSCRCNICI

>QOK06852.1 hemagglutinin [Influenza A virus]

METVSLITILLVATVSYADKICIGYQSTNSTETVDTLTENNVPVTHAKELLHTEHNGMLCATSLGQPLIL

DTCTIEGLIYGNPSCDLSLEGREWSYIVERPSAVNGLCYPGNVENLEELRSLFSSARSYQRVQIFPDTIW

NVSYDGTSTACSGSFYRSMRWLTRKDGNYPTQDAQYTNNQGKNILFMWGINHPPTDDTQRSLYTRTDTTT

SVATEEINRIFKPLIGPRPLVNGLMGRIDYYWSVLKPGQTLRIKSDGNLIAPWYGYILSGESHGRILKTD

LKRGSCTVQCQTEKGGLNTTLPFQNVSKYAFGNCSKYIGIKSLKLAVGLRNVPSRSSRGLFGAIAGFIEG

GWSGLVAGWYGFQHSNDQGVGMAADRDSTQKAIDKITSKVNNIVDKMNKQYEIIDHEFSEVETRLNMINN

KIDDQIQDIWAYNAELLVLLENQKTLDEHDANVNNLYNKVKRALGSNAAEDGKGCFELYHKCNDQCMETI

RNGTYNRKKYQEESKLERQKIEGVKLESEGTYKILTIYSTVASSLVIAMGFAAFLFWAMSNGSCRCNICI

>QOK06840.1 hemagglutinin [Influenza A virus]

METVSLITILLVATVSYADKICIGYQSTNSTETVDTLTENNVPVTHAKELLHTEHNGMLCATSLGQPLIL

DTCTIEGLIYGNPSCDLSLEGREWSYIVERPSAVNGLCYPGNVENLEELRSLFSSARSYQRVQIFPDTIW

NVSYDGTSTACSGSFYRSMRWLTRKDGNYPTQDAQYTNNQGKNILFMWGINHPPTDDTQRSLYTRTDTTT

SVATEEINRIFKPLIGPRPLVNGLMGRIDYYWSVLKPGQTLRIKSDGNLIAPWYGYILSGESHGRILKTD

LKRGSCTVQCQTEKGGLNTTLPFQNVSKYAFGNCSKYIGIKSLKLAVGLRNVPSRSSRGLFGAIAGFIEG

GWSGLVAGWYGFQHSNDQGVGMAADRDSTQKAIDKITSKVNNIVDKMNKQYEIIDHEFSEVETRLNMINN

KIDDQIQDIWAYNAELLVLLENQKTLDEHDANVNNLYNKVKRALGSNAAEDGKGCFELYHKCNDQCMETI

RNGTYNRKKYQEESKLERQKIEGVKLESEGTYKILTIYSTVASSLVIAMGFAAFLFWAMSNGSCRCNICI

>QOK06828.1 hemagglutinin [Influenza A virus]

METVSLITILLVATVSYADKICIGYQSTNSTETVDTLTENNVPVTHAKELLHTEHNGMLCATSLGQPLIL

DTCTIEGLIYGNPSCDLSLEGREWSYIVERPSAVNGLCYPGNVENLEELRSLFSSARSYQRVQIFPDTIW

NVSYDGTSTACSGSFYRSMRWLTRKDGNYPTQDAQYTNNQGKNILFMWGINHPPTDDTQRSLYTRTDTTT
SVATEEINRIFKPLIGPRPLVNGLMGRIDYYWSVLKPGQTLRIKSDGNLIAPWYGYILSGESHGRILKTD
LKRGSCTVQCQTEKGGLNTTLPFQNVSKYAFGNCSKYIGIKSLKLAVGLRNVPSRSSRGLFGAIAGFIEG
GWSGLVAGWYGFQHSNDQGVGMAADRDSTQKAIDKITSKVNNIVDKMNKQYEIIDHEFSEVETRLNMINN
KIDDQIQDIWAYNAELLVLLENQKTLDEHDANVNNLYNKVKRALGSNAAEDGKGCFELYHKCNDQCMETI
RNGTYNRKKYQEESKLERQKIEGVKLESEGTYKILTIYSTVASSLVIAMGFAAFLFWAMSNGSCRCNICI

>QOK06816.1 hemagglutinin [Influenza A virus]
METVSLITILLVATVSYADKICIGYQSTNSTETVDTLTENNVPVTHAKELLHTEHNGMLCATSLGQPLIL
DTCTIEGLIYGNPSCDLSLEGREWSYIVERPSAVNGLCYPGNVENLEELRSLFSSARSYQRVQIFPDTIW
NVSYDGTSTACSGSFYRSMRWLTRKDGNYPTQDAQYTNNQGKNILFMWGINHPPTDDTQRSLYTRTDTTT
SVATEEINRIFKPLIGPRPLVNGLMGRIDYYWSVLKPGQTLRIKSDGNLIAPWYGYILSGESHGRILKTD
LKRGSCTVQCQTEKGGLNTTLPFQNVSKYAFGNCSKYIGIKSLKLAVGLRNVPSRSSRGLFGAIAGFIEG
GWSGLVAGWYGFQHSNDQGVGMAADRDSTQKAIDKITSKVNNIVDKMNKQYEIIDHEFSEVETRLNMINN
KIDDQIQDIWAYNAELLVLLENQKTLDEHDANVNNLYNKVKRALGSNAAEDGKGCFELYHKCNDQCMETI
RNGTYNRKKYQEESKLERQKIEGVKLESEGTYKILTIYSTVASSLVIAMGFAAFLFWAMSNGSCRCNICI

>QOK06804.1 hemagglutinin [Influenza A virus]
METVSLITILLVATVSYADKICIGYQSTNSTETVDTLTENNVPVTHAKELLHTEHNGMLCATSLGQPLIL
DTCTIEGLIYGNPSCDLSLEGREWSYIVERPSAVNGLCYPGNVENLEELRSLFSSARSYQRVQIFPDTIW
NVSYDGTSTACSGSFYRSMRWLTRKDGNYPTQDAQYTNNQGKNILFMWGINHPPTDDTQRSLYTRTDTTT
SVATEEINRIFKPLIGPRPLVNGLMGRIDYYWSVLKPGQTLRIKSDGNLIAPWYGYILSGESHGRILKTD
LKRGSCTVQCQTEKGGLNTTLPFQNVSKYAFGNCSKYIGIKSLKLAVGLRNVPSRSSRGLFGAIAGFIEG
GWSGLVAGWYGFQHSNDQGVGMAADRDSTQKAIDKITSKVNNIVDKMNKQYEIIDHEFSEVETRLNMINN
KIDDQIQDIWAYNAELLVLLENQKTLDEHDANVNNLYNKVKRALGSNAAEDGKGCFELYHKCNDQCMETI
RNGTYNRKKYQEESKLERQKIEGVKLESEGTYKILTIYSTVASSLVIAMGFAAFLFWAMSNGSCRCNICI

>QOK06792.1 hemagglutinin [Influenza A virus]
METVSLITILLVATVSYADKICIGYQSTNSTETVDTLTENNVPVTHAKELLHTEHNGMLCATSLGQPLIL
DTCTIEGLIYGNPSCDLSLEGREWSYIVERPSAVNGLCYPGNVENLEELRSLFSSARSYQRVQIFPDTIW
NVSYDGTSTACSGSFYRSMRWLTRKDGNYPTQDAQYTNNQGKNILFMWGINHPPTDDTQRSLYTRTDTTT
SVATEEINRIFKPLIGPRPLVNGLMGRIDYYWSVLKPGQTLRIKSDGNLIAPWYGYILSGESHGRILKTD
LKRGSCTVQCQTEKGGLNTTLPFQNVSKYAFGNCSKYIGIKSLKLAVGLRNVPSRSSRGLFGAIAGFIEG
GWSGLVAGWYGFQHSNDQGVGMAADRDSTQKAIDKITSKVNNIVDKMNKQYEIIDHEFSEVETRLNMINN

KIDDQIQDIWAYNAELLVLLENQKTLDEHDANVNNLYNKVKRALGSNAAEDGKGCFELYHKCNDQCMETI
RNGTYNRKKYQEESKLERQKIEGVKLESEGTYKILTIYSTVASSLVIAMGFAAFLFWAMSNGSCRCNICI

>QOK06780.1 hemagglutinin [Influenza A virus]
METVSLITILLVATVSYADKICIGYQSTNSTETVDTLTENNVPVTHAKELLHTEHNGMLCATSLGQPLIL
DTCTIEGLIYGNPSCDLSLEGREWSYIVERPSAVNGLCYPGNVENLEELRSLFSSARSYQRVQIFPDTIW
NVSYDGTSTACSGSFYRSMRWLTRKDGNYPTQDAQYTNNQGKNILFMWGINHPPTDDTQRSLYTRTDTTT
SVATEEINRIFKPLIGPRPLVNGLMGRIDYYWSVLKPGQTLRIKSDGNLIAPWYGYILSGESHGRILKTD
LKRGSCTVQCQTEKGGLNTTLPFQNVSKYAFGNCSKYIGIKSLKLAVGLRNVPSRSSRGLFGAIAGFIEG
GWSGLVAGWYGFQHSNDQGVGMAADRDSTQKAIDKITSKVNNIVDKMNKQYEIIDHEFSEVETRLNMINN
KIDDQIQDIWAYNAELLVLLENQKTLDEHDANVNNLYNKVKRALGSNAAEDGKGCFELYHKCNDQCMETI
RNGTYNRKKYQEESKLERQKIEGVKLESEGTYKILTIYSTVASSLVIAMGFAAFLFWAMSNGSCRCNICI

>QOK06768.1 hemagglutinin [Influenza A virus]
METVSLITILLVATVSYADKICIGYQSTNSTETVDTLTENNVPVTHAKELLHTEHNGMLCATSLGQPLIL
DTCTIEGLIYGNPSCDLSLEGREWSYIVERPSAVNGLCYPGNVENLEELRSLFSSARSYQRVQIFPDTIW
NVSYDGTSTACSGSFYRSMRWLTRKDGNYPTQDAQYTNNQGKNILFMWGINHPPTDDTQRSLYTRTDTTT
SVATEEINRIFKPLIGPRPLVNGLMGRIDYYWSVLKPGQTLRIKSDGNLIAPWYGYILSGESHGRILKTD
LKRGSCTVQCQTEKGGLNTTLPFQNVSKYAFGNCSKYIGIKSLKLAVGLRNVPSRSSRGLFGAIAGFIEG
GWSGLVAGWYGFQHSNDQGVGMAADRDSTQKAIDKITSKVNNIVDKMNKQYEIIDHEFSEVETRLNMINN
KIDDQIQDIWAYNAELLVLLENQKTLDEHDANVNNLYNKVKRALGSNAAEDGKGCFELYHKCNDQCMETI
RNGTYNRKKYQEESKLERQKIEGVKLESEGTYKILTIYSTVASSLVIAMGFAAFLFWAMSNGSCRCNICI

>QOK06756.1 hemagglutinin [Influenza A virus]
METVSLITILLVATVSYADKICIGYQSTNSTETVDTLTENNVPVTHAKELLHTEHNGMLCATSLGQPLIL
DTCTIEGLIYGNPSCDLSLEGREWSYIVERPSAVNGLCYPGNAENPEEPRSLFSSARSYQRVQIFPDTIW
NVSYDGTSTACSGSFYRSMRWLTRKDGNYPTQDAQYTNNQGKNILFMWGINHPPTDDTQRSLYTRTDTTT
SVATEEINRIFKPLIGPRPLVNGLMGRIDYYWSVLKPGQTLRIKSDGNLIAPWYGYILSGESHGRILKTD
LKRGSCTVQCQTEKGGLNTTLPFQNVSKYAFGNCSKYIGIKSLKLAVGLRNVPSRSSRGLFGAIAGFIEG
GWSGLVAGWYGFQHSNDQGVGMAADRDSTQKAIDKITSKVNNIVDKMNKQYEIIDHEFSEVETRLNMINN
KIDDQIQDIWAYNAELLVLLENQKTLDEHDANVNNLYNKVKRALGSNAAEDGKGCFELYHKCNDQCMETI
RNGTYNRKKYQEESKLERQKIEGVKLESEGTYKILTIYSTVASSLVIAMGFAAFLFWAMSNGSCRCNICI

>QOK06744.1 hemagglutinin [Influenza A virus]

METVSLITILLVATVSYADKICIGYQSTNSTETVDTLTENNVPVTHAKELLHTEHNGMLCATSLGQPLIL
DTCTIEGLIYGNPSCDLSLEGREWSYIVERPSAVNGLCYPGNVENLEELRSLFSSARSYQRVQIFPDTIW
NVSYDGTSTACSGSFYRSMRWLTRKDGNYPTQDAQYTNNQGKNILFMWGINHPPTDDTQRSLYTRTDTTT
SVATEEINRIFKPLIGPRPLVNGLMGRIDYYWSVLKPGQTLRIKSDGNLIAPWYGYILSGESHGRILKTD
LKRGSCTVQCQTEKGGLNTTLPFQNVSKYAFGNCSKYIGIKSLKLAVGLRNVPSRSSRGLFGAIAGFIEG
GWSGLVAGWYGFQHSNDQGVGMAADRDSTQKAIDKITSKVNNIVDKMNKQYEIIDHEFSEVETRLNMINN
KIDDQIQDIWAYNAELLVLLENQKTLDEHDANVNNLYNKVKRALGSNAAEDGKGCFELYHKCNDQCMETI
RNGTYNRKKYQEESKLERQKIEGVKLESEGTYKILTIYSTVASSLVIAMGFAAFLFWAMSNGSCRCNICI

>QOK06732.1 hemagglutinin [Influenza A virus]
METVSLITILLVATVSYADKICIGYQSTNSTETVDTLTENNVPVTHAKELLHTEHNGMLCATSLGQPLIL
DTCTIEGLIYGNPSCDLSLEGREWSYIVERPSAVNGLCYPGNVENLEELRSLFSSARSYQRVQIFPDTIW
NVSYDGTSTACSGSFYRSMRWLTRKDGNYPTQDAQYTNNQGKNILFMWGINHPPTDDTQRSLYTRTDTTT
SVATEEINRIFKPLIGPRPLVNGLMGRIDYYWSVLKPGQTLRIKSDGNLIAPWYGYILSGESHGRILKTD
LKRGSCTVQCQTEKGGLNTTLPFQNVSKYAFGNCSKYIGIKSLKLAVGLRNVPSRSSRGLFGAIAGFIEG
GWSGLVAGWYGFQHSNDQGVGMAADRDSTQKAIDKITSKVNNIVDKMNKQYEIIDHEFSEVETRLNMINN
KIDDQIQDIWAYNAELLVLLENQKTLDEHDANVNNLYNKVKRALGSNAAEDGKGCFELYHKCNDQCMETI
RNGTYNRKKYQEESKLERQKIEGVKLESEGTYKILTIYSTVASSLVIAMGFAAFLFWAMSNGSCRCNICI

>QOK06720.1 hemagglutinin [Influenza A virus]
METVSLITILLVATVSYADKICIGYQSTNSTETVDTLTENNVPVTHAKELLHTEHNGMLCATSLGQPLIL
DTCTIEGLIYGNPSCDLSLEGREWSYIVERPSAVNGLCYPGNVENLEELRSLFSSARSYQRVQIFPDTIW
NVSYDGTSTACSGSFYRSMRWLTRKDGNYPTQDAQYTNNQGKNILFMWGINHPPTDDTQRSLYTRTDTTT
SVATEEINRIFKPLIGPRPLVNGLMGRIDYYWSVLKPGQTLRIKSDGNLIAPWYGYILSGESHGRILKTD
LKRGSCTVQCQTEKGGLNTTLPFQNVSKYAFGNCSKYIGIKSLKLAVGLRNVPSRSSRGLFGAIAGFIEG
GWSGLVAGWYGFQHSNDQGVGMAADRDSTQKAIDKITSKVNNIVDKMNKQYEIIDHEFSEVETRLNMINN
KIDDQIQDIWAYNAELLVLLENQKTLDEHDANVNNLYNKVKRALGSNAAEDGKGCFELYHKCNDQCMETI
RNGTYNRKKYQEESKLERQKIEGVKLESEGTYKILTIYSTVASSLVIAMGFAAFLFWAMSNGSCRCNICI

>QOK06708.1 hemagglutinin [Influenza A virus]
METVSLITILLVATVSYADKICIGYQSTNSTETVDTLTENNVPVTHAKELLHTEHNGMLCATSLGQPLIL
DTCTIEGLIYGNPSCDLSLEGREWSYIVERPSAVNGLCYPGNVENLEELRSLFSSARSYQRVQIFPDTIW
NVSYDGTSTACSGSFYRSMRWLTRKDGNYPTQDAQYTNNQGKNILFMWGINHPPTDDTQRSLYTRTDTTT
SVATEEINRIFKPLIGPRPLVNGLMGRIDYYWSVLKPGQTLRIKSDGNLIAPWYGYILSGESHGRILKTD

LKRGSCTVQCQTEKGGLNTTLPFQNVSKYAFGNCSKYIGIKSLKLAVGLRNVPSRSSRGLFGAIAGFIEG
GWSGLVAGWYGFQHSNDQGVGMAADRDSTQKAIDKITSKVNNIVDKMNKQYEIIDHEFSEVETRLNMINN
KIDDQIQDIWAYNAELLVLLENQKTLDEHDANVNNLYNKVKRALGSNAAEDGKGCFELYHKCNDQCMETI
RNGTYNRKKYQEESKLERQKIEGVKLESEGTYKILTIYSTVASSLVIAMGFAAFLFWAMSNGSCRCNICI

>QOK06684.1 hemagglutinin [Influenza A virus]
METVSLITILLVATVSYADKICIGYQSTNSTETVDTLTENNVPVTHAKELLHTEHNGMLCATSLGQPLIL
DTCTIEGLIYGNPSCDLSLEGREWSYIVERPSAVNGLCYPGNVENLEELRSLFSSARSYQRVQIFPDTIW
NVSYDGTSTACSGSFYRSMRWLTRKDGNYPTQDAQYTNNQGKNILFMWGINHPPTDDTQRSLYTRTDTTT
SVATEEINRIFKPLIGPRPLVNGLMGRIDYYWSVLKPGQTLRIKSDGNLIAPWYGYILSGESHGRILKTD
LKRGSCTVQCQTEKGGLNTTLPFQNVSKYAFGNCSKYIGIKSLKLAVGLRNVPSRSSRGLFGAIAGFIEG
GWSGLVAGWYGFQHSNDQGVGMAADRDSTQKAIDKITSKVNNIVDKMNKQYEIIDHEFSEVETRLNMINN
KIDDQIQDIWAYNAELLVLLENQKTLDEHDANVNNLYNKVKRALGSNAAEDGKGCFELYHKCNDQCMETI
RNGTYNRKKYQEESKLERQKIEGVKLESEGTYKILTIYSTVASSLVIAMGFAAFLFWAMSNGSCRCNICI

>QOK06672.1 hemagglutinin [Influenza A virus]
METVSLITILLVATVSYADKICIGYQSTNSTETVDTLTENNVPVTHAKELLHTEHNGMLCATSLGQPLIL
DTCTIEGLIYGNPSCDLSLEGREWSYIVERPSAVNGLCYPGNVENLEELRSLFSSARSYQRVQIFPDTIW
NVSYDGTSTACSGSFYRSMRWLTRKDGNYPTQDAQYTNNQGKNILFMWGINHPPTDDTQRSLYTRTDTTT
SVATEEINRIFKPLIGPRPLVNGLMGRIDYYWSVLKPGQTLRIKSDGNLIAPWYGYILSGESHGRILKTD
LKRGSCTVQCQTEKGGLNTTLPFQNVSKYAFGNCSKYIGIKSLKLAVGLRNVPSRSSRGLFGAIAGFIEG
GWSGLVAGWYGFQHSNDQGVGMAADRDSTQKAIDKITSKVNNIVDKMNKQYEIIDHEFSEVETRLNMINN
KIDDQIQDIWAYNAELLVLLENQKTLDEHDANVNNLYNKVKRALGSNAAEDGKGCFELYHKCNDQCMETI
RNGTYNRKKYQEESKLERQKIEGVKLESEGTYKILTIYSTVASSLVIAMGFAAFLFWAMSNGSCRCNICI

>QOK06648.1 hemagglutinin [Influenza A virus]
METVSLITILLVATVSYADKICIGYQSTNSTETVDTLTENNVPVTHAKELLHTEHNGMLCATSLGQPLIL
DTCTIEGLIYGNPSCDLSLEGREWSYIVERPSAVNGLCYPGNVENLEELRSLFSSARSYQRVQIFPDTIW
NVSYDGTSTACSGSFYRSMRWLTRKDGNYPTQDAQYTNNQGKNILFMWGINHPPTDDTQRSLYTRTDTTT
SVATEEINRIFKPLIGPRPLVNGLMGRIDYYWSVLKPGQTLRIKSDGNLIAPWYGYILSGESHGRILKTD
LKRGSCTVQCQTEKGGLNTTLPFQNVSKYAFGNCSKYIGIKSLKLAVGLRNVPSRSSRGLFGAIAGFIEG
GWSGLVAGWYGFQHSNDQGVGMAADRDSTQKAIDKITSKVNNIVDKMNKQYEIIDHEFSEVETRLNMINN
KIDDQIQDIWAYNAELLVLLENQKTLDEHDANVNNLYNKVKRALGSNAAEDGKGCFELYHKCNDQCMETI
RNGTYNRKKYQEESKLERQKIEGVKLESEGTYKILTIYSTVASSLVIAMGFAAFLFWAMSNGSCRCNICI

>QOK06636.1 hemagglutinin [Influenza A virus]
METVSLITILLVATVSYADKICIGYQSTNSTETVDTLTENNVPVTHAKELLHTEHNGMLCATSLGQPLIL
DTCTIEGLIYGNPSCDLSLEGREWSYIVERPSAVNGLCYPGNVENLEELRSLFSSARSYQRVQIFPDTIW
NVSYDGTSTACSGSFYRSMRWLTRKDGNYPTQDAQYTNNQGKNILFMWGINHPPTDDTQRSLYTRTDTTT
SVATEEINRIFKPLIGPRPLVNGLMGRIDYYWSVLKPGQTLRIKSDGNLIAPWYGYILSGESHGRILKTD
LKRGSCTVQCQTEKGGLNTTLPFQNVSKYAFGNCSKYIGIKSLKLAVGLRNVPSRSSRGLFGAIAGFIEG
GWSGLVAGWYGFQHSNDQGVGMAADRDSTQKAIDKITSKVNNIVDKMNKQYEIIDHEFSEVETRLNMINN
KIDDQIQDIWAYNAELLVLLENQKTLDEHDANVNNLYNKVKRALGSNAAEDGKGCFELYHKCNDQCMETI
RNGTYNRKKYQEESKLERQKIEGVKLESEGTYKILTIYSTVASSLVIAMGFAAFLFWAMSNGSCRCNICI

>QOK06588.1 hemagglutinin [Influenza A virus]
METVSLMTILLVATVSNADKICIGYQSTNSTETVDTLTENNVPVTHAKELIHTEHNGMLCATSLGQPLIL
DTCTIEGLIYGNPSCDLSLEGREWSYIVERPSAVNGLCYPGNVENLEELRSLFSSARSYQRIQIFPDTIW
NVSYDGTSTACSGSFYKNMRWLTRKDGNYPPQDAQYTNNQGKNILFMWGINHPPADTTQTGLYTRTDTTT
SVATEEINRIFKPLIGPRPLVNGLMGRIDYYWSVLKPGQTLRIKSDGNLIAPWFGHILSGESHGRILKTD
LKRGSCTVQCQTEKGGLNTTLPFQNVSKYAFGNCSKYIGIKSLKLAVGLRNVPSRSSRGLFGAIAGFIEG
GWSGLVAGWYGFQHSNDQGVGMAADRDSTQKAIDKITSKVNNIVDKMNKQYEIIDHEFSEVETRLNMINN
KIDDQIQDIWAYNAELLVLLENQKTLDEHDANVNNLYNKVKRALGSNAVEDGKGCFELYHKCDDQCMETI
RNGTYNRRKYQEESKLERQKIEGVKLESEGTYKILTIYSTVASSLVIAMGFAAFLFWAMSNGSCRCNICI

>QOK06577.1 hemagglutinin [Influenza A virus]
METVSLMTILLVATVSNADKICIGYQSTNSTETVDTLTENNVPVTHAKELIHTEHNGMLCATSLGQPLIL
DTCTIEGLIYGNPSCDLSLEGREWSYIVERPSAVNGLCYPGNVENLEELRSLFSSARSYQRIQIFPDTIW
NVSYDGTSTACSGSFYKNMRWLTRKDGNYPTQDAQYTNNQGKNILFMWGINHPPADTTQTGLYTRTDTTT
SVATEEINRIFKPLIGPRPLVNGLMGRIDYYWSVLKPGQTLRIKSDGNLIAPWFGHILSGESHGRILKTD
LKRGSCTVQCQTEKGGLNTTLPFQNVSKYAFGNCSKYIGIKSLKLAVGLRNVPSRSSRGLFGAIAGFIEG
GWSGLVAGWYGFQHSNDQGVGMAADRDSTQKAIDKITSKVNNIVDKMNKQYEIIDHEFSEVETRLNMINN
KIDDQIQDIWAYNAELLVLLENQKTLDEHDANVNNLYNKVKRALGSNAVEDGKGCFELYHKCDDQCMETI
RNGTYNRRKYQEESKLERQKIEGVKLESEGTYKILTIYSTVASSLVIAMGFAAFLFWAMSNGSCRCNICI

>QOK06566.1 hemagglutinin [Influenza A virus]
METVSLMTILLVATVSNADKICIGYQSTNSTETVDTLTENNVPVTHAKELIHTEHNGMLCATSLGQPLIL
DTCTIEGLIYGNPSCDLSLEGREWSYIVERPSAVNGLCYPGNVENLEELRSLFSSARSYQRIQIFPDTIW

NVSYDGTSTACSGSFYKNMRWLTRKDGNYPTQDAQYTNNQGKNILFMWGINHPPADTTQTGLYTRTDTTT
SVATEEINRIFKPLIGPRPLVNGLMGRIDYYWSVLKPGQTLRIKSDGNLIAPWFGHILSGESHGRILKTD
LKRGSCTVQCQTEKGGLNTTLPFQNVSKYAFGNCSKYIGIKSLKLAVGLRNVPSRSSRGLFGAIAGFIEG
GWSGLVAGWYGFQHSNDQGVGMAADRDSTQKAIDKITSKVNNIVDKMNKQYEIIDHEFSEVETRLNMINN
KIDDQIQDIWAYNAELLVLLENQKTLDEHDANVNNLYNKVKRALGSNAVEDGKGCFELYHKCDDQCMETI
RNGTYNRRKYQEESKLERQKIEGVKLESEGTYKILTIYSTVASSLVIAMGFAAFLFWAMSNGSCRCNICI

>QOK06555.1 hemagglutinin [Influenza A virus]
METVSLMTILLVATVSNADKICIGYQSTNSTETVDTLTENNVPVTHAKELIHTEHNGMLCATSLGQPLIL
DTCTIEGLIYGNPSCDLSLEGREWSYIVERPSAVNGLCYPGNVENLEELRSLFSSARSYQRIQIFPDTIW
NVSYDGTSTACSGSFYKNMRWLTRKDGNYPTQDAQYTNNQGKNILFMWGINHPPADTTQTGLYTRTDTTT
SVATEEINRIFKPLIGPRPLVNGLMGRIDYYWSVLKPGQTLRIKSDGNLIAPWFGHILSGESHGRILKTD
LKRGSCTVQCQTEKGGLNTTLPFQNVSKYAFGNCSKYIGIKSLKLAVGLRNVPSRSSRGLFGAIAGFIEG
GWSGLVAGWYGFQHSNDQGVGMAADRDSTQKAIDKITSKVNNIVDKMNKQYEIIDHEFSEVETRLNMINN
KIDDQIQDIWAYNAELLVLLENQKTLDEHDANVNNLYNKVKRALGSNAVEDGKGCFELYHKCDDQCMETI
RNGTYNRRKYQEESKLERQKIEGVKLESEGTYKILTIYSTVASSLVIAMGFAAFLFWAMSNGSCRCNICI

>QOK06544.1 hemagglutinin [Influenza A virus]
METASLITILLVATVSYADKICIGYQSTNSTETVDTLTENNVPVTHAKELLHTEHNGMLCATSLGQPLIL
DTCTIEGLIYGNPSCDLSLEGREWSYIVERPSAVNGLCYPGNVENLEELRSLFSSARSYQRVQIFPDTIW
NVSYDGTSTACSGSFYRSMRWLTRKNGDYPTQDAQYTNNQGKNILFMWGINHPPTDDTQRNLYTRTDTTT
SVATEEINRIFKPLIGPRPLVNGLMGRIDYYWSVLKPGQTLRIKSDGNLIAPWYGYILSGESHGRILKTD
LKRGSCTVQCQTEKGGLNTTLPFQNVSKYAFGNCSKYIGIRSLKLAVGLRNVPSRSSRGLFGAIAGFIEG
GWSGLVAGWYGFQHSNDQGVGMAADRDSTQKAIDKITSKVNNIVDKMNKQYEIIDHEFSEVETRLNMINN
KIDDQIQDIWAYNAELLVLLENQKTLDEHDANVNNLYNKVKRALGSNAVEDGKGCFELYHKCNDQCMETI
RNGTYNRRKYQEESKLERQKIEGVKLESEGTYKILTIYSTVASSLVIAMGFAAFLFWAMSNGSCRCNICI

>QOK06508.1 hemagglutinin [Influenza A virus]
METVSLITILLVAAVSYADKICIGYQSTNSTETVDTLTENNVPVTHAKELLHTEHNGMLCATSLGQPLIL
DTCTIEGLIYGNPSCDLSLEGREWSYIVERPSAVNGLCYPGNVENLEELRSLFSSARSYQRVQIFPDTIW
NVSYDGTSTACSGSFYRSMRWLTRKDGNYPTQDAQYTNNQGKNILFMWGINHPPTDDTQRSLYTRTDTTT
SVATEEINRIFKPLIGPRPLVNGLMGRIDYYWSVLKPGQTLRIKSDGNLIAPWYGYILSGESHGRILKTD
LKRGSCTVQCQTEKGGLNTTLPFQNVSKYAFGNCSKYIGIKSLKLAVGLRNVPSKSSRGLFGAIAGFIEG
GWSGLVAGWYGFQHSNDQGVGMAADRDSTQKAIDKITSKVNNIVDKMNKQYEIIDHEFSEVETRLNMINN

KIDDQIQDIWAYNAELLVLLENQKTLDEHDANVNNLYNKVKRALGSNAAEDGKGCFELYHKCNDQCMETI
RNGTYNRRKYQEESKLERQKIEGVKLESEGTYKILTIYSTVASSLVIAMGFAAFLFWAMSNGSCRCNICI

>QOK06485.1 hemagglutinin [Influenza A virus]
METVSLXTILLVATVSNADKICIGYQSTNSTETVDTLTENBVPVTHAKELIHTEHNGMLCATSLGQPLIL
DTCTIEGLIYGNPSCDLSLEGREWSYIVERPSAVNGLCYPGXVENLEELRSLFSSARSYQRIQIFPDTIW
NVSYDGTSTACSGSFYKNMRWLTRKDGNYPTQDAQYTNNQGKNILFMWGINHPPXDTTQTXLYTRTDTTT
SVATEEINRIFKPLIGPRPLVNGLMGRIDYYWSVLKPGQTLRIKSDGNLIAPWFGHILSGESHGRILKTD
LKRGSCTVQCQTEKGGLNTTLPFQNVSKYAFGNCSKYIGIKSLKLAVGXRNVPSRSSRGLFGAIAGFIEG
GWSGLVAGWYGFQHSNDQGVGMAADRXSTQKAIDKITSKVNNIVDKMNKQYEIIDHEFSEVETRLNMINN
KIDDQIQDIWAYNAELLVLLENQKTLDEHDANVNNLYNKVKRALGSNAXEDGKGCFELYHKCDDQCMETI
RNGTYNRRKYQEESKLERQKIEGVKLESEGTYKILTIYSTVASSLVIAMGFAAFLFWAMSNGSCRCNICI

>QOK06473.1 hemagglutinin [Influenza A virus]
METVSLMTILLVATVSNADKICIGYQSTNSTETVDTLTENDVPVTHAKELIHTEHNGMLCATSLGQPLIL
DTCTIEGLIYGNPSCDLSLEGREWSYIVERPSAVNGLCYPGKVENLEELRSLFSSARSYQRIQIFPDTIW
NVSYDGTSTACSGSFYKNMRWLTRKDGNYPTQDAQYTNNQGKNILFMWGINHPPADTTQTGLYTRTDTTT
SVATEEINRIFKPLIGPRPLVNGLMGRIDYYWSVLKPGQTLRIKSDGNLIAPWFGHILSGESHGRILKTD
LKRGSCTVQCQTEKGGLNTTLPFQNVSKYAFGNCSKYIGIKSLKLAVGLRNVPSRSSRGLFGAIAGFIEG
GWSGLVAGWYGFQHSNDQGVGMAADRDSTQKAIDKITSKVNNIVDKMNKQYEIIDHEFSEVETRLNMINN
KIDDQIQDIWAYNAELLVLLENQKTLDEHDANVNNLYNKVKRALGSNAMEDGKGCFELYHKCDDQCMETI
RNGTYNRRKYQEESKLERQKIEGVKLESEGTYKILTIYSTVASSLVIAMGFAAFLFWAMSNGSCRCNICI

>QOK06461.1 hemagglutinin [Influenza A virus]
METVSLITILVVATVSNADKICIGYQSTNSTETVDTLTENNVPVTHAKELLHTEHNGMLCATSLGQPLIL
DTCTIEGLIYGNPSCDPLLGGREWSYIVERPSAVNGLCYPGNVENLEELRSLFSSSRSYQRIQIFPDTIW
NVSYSGTSKACSDSFYRSMRWLTQKNNAYPTQDAQYTNNQGKNILFMWGINHPPTDTTQRNLYTRTDTTT
SVATEEINRIFRPLIGPRPLVNGLMGRINYYWSVLKPGQTLRIKSDGNLIAPWYGYILSGESHGRILKTD
LKRGSCTVQCQTEKGGLNTTLPFQNVSKYAFGNCSKYIGIKSLKLAVGLRNVPSRSSRGLFGAIAGFIEG
GWSGLVAGWYGFQHSNDQGVGMAADRESTQKAIDKITSKVNNIVDKMNKQYEIIDHEFSEVETRLNMINN
KIDDQIQDIWAYNAELLVLLENQKTLDEHDANVNNLYNKVKRALGSNAVEDGKGCFELYHKCDDQCMETI
RNGTYNRRKYQEESKLERQKIEGVKLESEGTYKILTIYSTVASSLVIAMGFAAFLFWAMSNGSCRCNICI

>QOK06401.1 hemagglutinin [Influenza A virus]

METVSLITILLVATVSYADKICIGYQSTNSTETVDTLTENNVPVTHAKELLHTEHNGMLCATSLGQPLIL
DTCTIEGLIYGNPSCDLSLEGREWSYIVERPSAVNGLCYPGNVENLEELRSLFSSARSYQRVQIFPDTIW
NVSYDGTSTACSGSFYRSMRWLTRKNGDYPTQDAQYTNNQGKNILFMWGINHPPTDDTQRNLYTRTDTTT
SVATEEINRIFKPLIGPRPLVNGLMGRIDYYWSVLKPGQTLRIKSDGNLIAPWYGYILSGESHGRILKTD
LKRGSCTVQCQTEKGGLNTTLPFQNVSKYAFGNCSKYIGIRSLKLAVGLRNVPSRSSRGLFGAIAGFIEG
GWSGLVAGWYGFQHSNDQGVGMAADRDSTQKAIDKITSKVNNIVDKMNKQYEIIDHEFSEVETRLNMINN
KIDDQIQDIWAYNAELLVLLENQKTLDEHDANVNNLYNKVKRALGSNAVEDGKGCFELYHKCNDQCMETI
RNGTYNRRKYQEESKLERQKIEGVKLESEGTYKILTIYSTVASSLVIAMGFAAFLFWAMSNGSCRCNICI

>QOK06389.1 hemagglutinin [Influenza A virus]
METVSLITILLVATVSYADKICIGYQSTNSTETVDTLTENNVPVTHAKELLHTEHNGMLCATSLGQPLIL
DTCTIEGLIYGNPSCDLSLEGREWSYIVERPSAVNGLCYPGNVENLEELRSLFSSARSYQRVQIFPDTIW
NVSYDGTSTACSGSFYRSMRWLTRKNGDYPTQDAQYTNNQGKNILFMWGINHPPTDDTQRNLYTRTDTTT
SVATEEINRIFKPLIGPRPLVNGLMGRIDYYWSVLKPGQTLRIKSDGNLIAPWYGYILSGESHGRILKTD
LKRGSCTVQCQTEKGGLNTTLPFQNVSKYAFGNCSKYIGIRSLKLAVGLRNVPSRSSRGLFGAIAGFIEG
GWSGLVAGWYGFQHSNDQGVGMAADRDSTQKAIDKITSKVNNIVDKMNKQYEIIDHEFSEVETRLNMINN
KIDDQIQDIWAYNAELLVLLENQKTLDEHDANVNNLYNKVKRALGSNAVEDGKGCFELYHKCNDQCMETI
RNGTYNRRKYQEESKLERQKIEGVKLESEGTYKILTIYSTVASSLVIAMGFAAFLFWAMSNGSCRCNICI

>QOK06377.1 hemagglutinin [Influenza A virus]
METVSLITILLVATVSYADKICIGYQSTNSTETVDTLTENNVPVTHAKELLHTEHNGMLCATSLGQPLIL
DTCTIEGLIYGNPSCDLSLEGREWSYIVERPSAVNGLCYPGNVENLEELRSLFSSARSYQRVQIFPDTIW
NVSYDGTSTACSGSFYRSMRWLTRKNGDYPTQDAQYTNNQGKNILFMWGINHPPTDDTQRNLYTRTDTTT
SVATEEINRIFKPLIGPRPLVNGLMGRIDYYWSVLKPGQTLRIKSDGNLIAPWYGYILSGESHGRILKTD
LKRGSCTVQCQTEKGGLNTTLPFQNVSKYAFGNCSKYIGIRSLKLAVGLRNVPSRSSRGLFGAIAGFIEG
GWSGLVAGWYGFQHSNDQGVGMAADRDSTQKAIDKITSKVNNIVDKMNKQYEIIDHEFSEVETRLNMINN
KIDDQIQDIWAYNAELLVLLENQKTLDEHDANVNNLYNKVKRALGSNAVEDGKGCFELYHKCNDQCMETI
RNGTYNRRKYQEESKLERQKIEGVKLESEGTYKILTIYSTVASSLVIAMGFAAFLFWAMSNGSCRCNICI

>QOK06365.1 hemagglutinin [Influenza A virus]
METVSLITILLVATVSYADKICIGYQSTNSTETVDTLTENNVPVTHAKELLHTEHNGMLCATSLGQPLIL
DTCTIEGLIYGNPSCDLSLEGREWSYIVERPSAVNGLCYPGNVENLEELRSLFSSARSYQRVQIFPDTIW
NVSYDGTSTACSGSFYRSMRWLTRKNGDYPTQDAQYTNNQGKNILFMWGINHPPTDDTQRNLYTRTDTTT
SVATEEINRIFKPLIGPRPLVNGLMGRIDYYWSVLKPGQTLRIKSDGNLIAPWYGYILSGESHGRILKTD

LKRGSCTVQCQTEKGGLNTTLPFQNVSKYAFGNCSKYIGIRSLKLAVGLRNVPSRSSRGLFGAIAGFIEG
GWSGLVAGWYGFQHSNDQGVGMAADRDSTQKAIDKITSKVNNIVDKMNKQYEIIDHEFSEVETRLNMINN
KIDDQIQDIWAYNAELLVLLENQKTLDEHDANVNNLYNKVKRALGSNAVEDGKGCFELYHKCNDQCMETI
RNGTYNRRKYQEESKLERQKIEGVKLESEGTYKILTIYSTVASSLVIAMGFAAFLFWAMSNGSCRCNICI

>QOK06353.1 hemagglutinin [Influenza A virus]
METVSLITILLVATVSYADKICIGYQSTNSTETVDTLTENNVPVTHAKELLHTEHNGMLCATSLGQPLIL
DTCTIEGLIYGNPSCDLSLEGREWSYIVERPSAVNGLCYPGNVENLEELRSLFSSARSYQRVQIFPDTIW
NVSYDGTSTACSGSFYRSMRWLTRKNGDYPTQDAQYTNNQGKNILFMWGINHPPTDDTQRNLYTRTDTTT
SVATEEINRIFKPLIGPRPLVNGLMGRIDYYWSVLKPGQTLRIKSDGNLIAPWYGYILSGESHGRILKTD
LKRGSCTVQCQTEKGGLNTTLPFQNVSKYAFGNCSKYIGIRSLKLAVGLRNVPSRSSRGLFGAIAGFIEG
GWSGLVAGWYGFQHSNDQGVGMAADRDSTQKAIDKITSKVNNIVDKMNKQYEIIDHEFSEVETRLNMINN
KIDDQIQDIWAYNAELLVLLENQKTLDEHDANVNNLYNKVKRALGSNAVEDGKGCFELYHKCNDQCMETI
RNGTYNRRKYQEESKLERQKIEGVKLESEGTYKILTIYSTVASSLVIAMGFAAFLFWAMSNGSCRCNICI

>QOK06341.1 hemagglutinin [Influenza A virus]
METVSLITILLVATVSYADKICIGYQSTNSTETVDTLTENNVPVTHAKELLHTEHNGMLCATSLGQPLIL
DTCTIEGLIYGNPSCDLSLEGREWSYIVERPSAVNGLCYPGNVENLEELRSLFSSARSYQRVQIFPDTIW
NVSYDGTSTACSGSFYRSMRWLTRKDGNYPTQDAQYTNNQGKNILFMWGINHPPTDDTQRSLYTRTDTTT
SVATEEINRIFKPLIGPRPLVNGLMGRIDYYWSVLKPGQTLRIKSDGNLIAPWYGYILSGESHGRILKTD
LKRGSCTVQCQTEKGGLNTTLPFQNVSKYAFGNCSKYIGIKSLKLAVGLRNVPSRSSRGLFGAIAGFIEG
GWSGLVAGWYGFQHSNDQGVGMAADRDSTQKAIDKITSKVNNIVDKMNKQYEIIDHEFSEVETRLNMINN
KIDDQIQDIWAYNAELLVLLENQKTLDEHDANVNNLYNKVKRALGSNAAEDGKGCFELYHKCNDQCMETI
RNGTYNRKKYQEESKLERQKIEGVKLESEGTYKILTIYSTVASSLVIAMGFAAFLFWAMSNGSCRCNICI

>QOK06329.1 hemagglutinin [Influenza A virus]
METVSLITILLVATVSYADKICIGYQSTNSTETVDTLTENNVPVTHAKELLHTEHNGMLCATSLGQPLIL
DTCTIEGLIYGNPSCDLSLEGREWSYIVERPSAVNGLCYPGNVENLEELRSLFSSARSYQRVQIFPDTIW
NVSYDGTSTACSGSFYRSMRWLTRKDGNYPTQDAQYTNNQGKNILFMWGINHPPTDDTQRSLYTRTDTTT
SVATEEINRIFKPLIGPRPLVNGLMGRIDYYWSVLKPGQTLRIKSDGNLIAPWYGYILSGESHGRILKTD
LKRGSCTVQCQTEKGGLNTTLPFQNVSKYAFGNCSKYIGIKSLKLAVGLRNVPSRSSRGLFGAIAGFIEG
GWSGLVAGWYGFQHSNDQGVGMAADRDSTQKAIDKITSKVNNIVDKMNKQYEIIDHEFSEVETRLNMINN
KIDDQIQDIWAYNAELLVLLENQKTLDEHDANVNNLYNKVKRALGSNAAEDGKGCFELYHKCNDQCMETI
RNGTYNRKKYQEESKLERQKIEGVKLESEGTYKILTIYSTVASSLVIAMGFAAFLFWAMSNGSCRCNICI

>QOK06317.1 hemagglutinin [Influenza A virus]

METVSLITILLVATVSYADKICIGYQSTNSTETVDTLTENNVPVTHAKELLHTEHNGMLCATSLGQPLIL

DTCTIEGLIYGNPSCDLSLEGREWSYIVERPSAVNGLCYPGNVENLEELRSLFSSARSYQRVQIFPDTIW

NVSYDGTSTACSGSFYRSMRWFTPNHPNYPTQHPQYTNNQPNNILFMWGINHPPTDDTQRSLYTRTDTTT

SVATEEINRIFKPLIGPRPLVNGLMGRIDYYWSVLKPGQTLRIKSDGNLIAPWYGYILSGESHGRILKTD

LKRGSCTVQCQTEKGGLNTTLPFQNVSKYAFGNCSKYIGIKSLKLAVGLRNVPSRSSRGLFGAIAGFIEG

GWSGLVAGWYGFQHSNDQGVGMAADRDSTQKAIDKITSKVNNIVDKMNKQYEIIDHEFSEVETRLNMINN

KIDDQIQDIWAYNAELLVLLENQKTLDEHDANVNNLYNKVKRALGSNAAEDGKGCFELYHKCNDQCMETI

RNGTYNRKKYQEESKLERQKIEGVKLESEGTYKILTIYSTVASSLVIAMGFAAFLFWAMSNGSCRCNICI

>QOK06305.1 hemagglutinin [Influenza A virus]

METVSLITILLVATVSYADKICIGYQSTNSTETVDTLTENNVPVTHAKELLHTEHNGMLCATSLGQPLVL

DTCTIEGLIYGNPSCDLSLEGREWSYIVERPSAVNGLCYPGNVENLEELRSLFSSARSYQRVQIFPDTIW

NVSYDGTSTACSGSFYRSMRWLTRKDGNYPTQDAQYTNNQGKNILFMWGINHPPTDDTQRSLYTRTDTTT

SVATEEINRIFKPLIGPRPLVNGLMGRIDYYWSVLKPGQTLRIKSDGNLIAPWYGYILSGESHGRILKTD

LKRGSCTVQCQTEKGGLNTTLPFQNVSKYAFGNCSKYIGIKSLKLAVGLRNVPSRSSRGLFGAIAGFIEG

GWSGLVAGWYGFQHSNDQGVGMAADRDSTQKAIDKITSKVNNIVDKMNKQYEIIDHEFSEVETRLNMINN

KIDDQIQDIWAYNAELLVLLENQKTLDEHDANVNNLYNKVKRALGSNAAEDGKGCFELYHKCNDQCMETI

RNGTYNRKKYQEESKLERQKIEGVKLESEGTYKILTIYSTVASSLVIAMGFAAFLFWAMSNGSCRCNICI

>QOK06293.1 hemagglutinin [Influenza A virus]

METVSLITILLVATVSYADKICIGYQSTNSTETVDTLTENNVPVTHAKELLHTEHNGMLCATSLGQPLIL

DTCTIEGLIYGNPSCDLSLEGREWSYIVERPSAVNGLCYPGNVENLEELRSLFSSARSYQRVQIFPDTIW

NVSYDGTSTACSGSFYRSMRWLTRKDGNYPTQDAQYTNNQGKNILFMWGINHPPTDDTQRSLYTRTDTTT

SVATEEINRIFKPLIGPRPLVNGLMGRIDYYWSVLKPGQTLRIKSDGNLIAPWYGYILSGESHGRILKTD

LKRGSCTVQCQTEKGGLNTTLPFQNVSKYAFGNCSKYIGIKSLKLAVGLRNVPSRSSRGLFGAIAGFIEG

GWSGLVAGWYGFQHSNDQGVGMAADRDSTQKAIDKITSKVNNIVDKMNKQYEIIDHEFSEVETRLNMINN

KIDDQIQDIWAYNAELLVLLENQKTLDEHDANVNNLYNKVKRALGSNAAEDGKGCFELYHKCNDQCMETI

RNGTYNRKKYQEESKLERQKIEGVKLESEGTYKILTIYSTVASSLVIAMGFAAFLFWAMSNGSCRCNICI

>QOK06281.1 hemagglutinin [Influenza A virus]

METVSLITILLVATVSYADKICIGYQSTNSTETVDTLTENNVPVTHAKELLHTEHNGMLCATSLGQPLIL

DTCTIEGLIYGNPSCDLSLEGREWSYIVERPSAVNGLCYPGNVENLEELRSLFSSARSYQRVQIFPDTIW

NVSYDGTSTACSGSFYRSMRWLTRKDGNYPTQDAQYTNNQGKNILFMWGINHPPTDDTQRSLYTRTDTTT
SVATEEINRIFKPLIGPRPLVNGLMGRIDYYWSVLKPGQTLRIKSDGNLIAPWYGYILSGESHGRILKTD
LKRGSCTVQCQTEKGGLNTTLPFQNVSKYAFGNCSKYIGIKSLKLAVGLRNVPSRSSRGLFGAIAGFIEG
GWSGLVAGWYGFQHSNDQGVGMAADRDSTQKAIDKITSKVNNIVDKMNKQYEIIDHEFSEVETRLNMINN
KIDDQIQDIWAYNAELLVLLENQKTLDEHDANVNNLYNKVKRALGSNAAEDGKGCFELYHKCNDQCMETI
RNGTYNRKKYQEESKLERQKIEGVKLESEGTYKILTIYSTVASSLVIAMGFAAFLFWAMSNGSCRCNICI

>QOK06269.1 hemagglutinin [Influenza A virus]
METVSLITILLVATVSYADKICIGYQSTNSTETVDTLTENNVPVTHAKELLHTEHNGMLCATSLGQPLIL
DTCTIEGLIYGNPSCDLSLEGREWSYIVERPSAVNGLCYPGNVENLEELRSLFSSARSYQRVQIFPDTIW
NVSYDGTSTACSGSFYRSMRWLTRKDGNYPTQDAQYTNNQGKNILFMWGINHPPTDDTQRSLYTRTDTTT
SVATEEINRIFKPLIGPRPLVNGLMGRIDYYWSVLKPGQTLRIKSDGNLIAPWYGYILSGESHGRILKTD
LKRGSCTVQCQTEKGGLNTTLPFQNVSKYAFGNCSKYIGIKSLKLAVGLRNVPSRSSRGLFGAIAGFIEG
GWSGLVAGWYGFQHSNDQGVGMAADRDSTQKAIDKITSKVNNIVDKMNKQYEIIDHEFSEVETRLNMINN
KIDDQIQDIWAYNAELLVLLENQKTLDEHDANVNNLYNKVKRALGSNAAEDGKGCFELYHKCNDQCMETI
RNGTYNRKKYQEESKLERQKIEGVKLESEGTYKILTIYSTVASSLVIAMGFAAFLFWAMSNGSCRCNICI

>QOK06257.1 hemagglutinin [Influenza A virus]
METVSLITILLVATVSYADKICIGYQSTNSTETVDTLTENNVPVTHAKELLHTEHNGMLCATSLGQPLVL
DTCTIEGLIYGNPSCDLSLEGREWSYIVERPSAVNGLCYPGNVENLEELRSLFSSARSYQRVQIFPDTIW
NVSYDGTSTACSGSFYRSMRWLTRKDGNYPTQDAQYTNNQGKNILFMWGINHPPTDDTQRSLYTRTDTTT
SVATEEINRIFKPLIGPRPLVNGLMGRIDYYWSVLKPGQTLRIKSDGNLIAPWYGYILSGESHGRILKTD
LKRGSCTVQCQTEKGGLNTTLPFQNVSKYAFGNCSKYIGIKSLKLAVGLRNVPSRSSRGLFGAIAGFIEG
GWSGLVAGWYGFQHSNDQGVGMAADRDSTQKAIDKITSKVNNIVDKMNKQYEIIDHEFSEVETRLNMINN
KIDDQIQDIWAYNAELLVLLENQKTLDEHDANVNNLYNKVKRALGSNAAEDGKGCFELYHKCNDQCMETI
RNGTYNRKKYQEESKLERQKIEGVKLESEGTYKILTIYSTVASSLVIAMGFAAFLFWAMSNGSCRCNICI

>QOK06245.1 hemagglutinin [Influenza A virus]
METVSLITILLVATVSYADKICIGYQSTNSTETVDTLTENNVPVTHAKELLHTEHNGMLCATSLGQPLIL
DTCTIEGLIYGNPSCDLSLEGREWSYIVERPSAVNGLCYPGNVENLEELRSLFSSARSYQRVQIFPDTIW
NVSYDGTSTACSGSFYRSMRWLTRKDGNYPTQDAQYTNNQGKNILFMWGINHPPTDDTQRSLYTRTDTTT
SVATEEINRIFKPLIGPRPLVNGLMGRIDYYWSVLKPGQTLRIKSDGNLIAPWYGYILSGESHGRILKTD
LKRGSCTVQCQTEKGGLNTTLPFQNVSKYAFGNCSKYIGIKSLKLAVGLRNVPSRSSRGLFGAIAGFIEG
GWSGLVAGWYGFQHSNDQGVGMAADRDSTQKAIDKITSKVNNIVDKMNKQYEIIDHEFSEVETRLNMINN

KIDDQIQDIWAYNAELLVLLENQKTLDEHDANVNNLYNKVKRALGSNAAEDGKGCFELYHKCNDQCMETI
RNGTYNRKKYQEESKLERQKIEGVKLESEGTYKILTIYSTVASSLVIAMGFAAFLFWAMSNGSCRCNICI

>QOK06233.1 hemagglutinin [Influenza A virus]
METVSLMTILLVATVSNADKICIGYQSTNSTETVDTLTENNVPVTHAKELIHTEHNGMLCATSLGQPLIL
DTCTIEGLIYGNPSCDLSLEGREWSYIVERPSAVNGLCYPGNVENLEELRSLFSSARSYQRIQIFPDTIW
NVSYDGTSTACSGSFYKNMRWLTRKDGNYPTQDAQYTNNQGKNILFMWGINHPPADTTQTGLYTRTDTTT
SVATEEINRIFKPLIGPRPLVNGLMGRIDYYWSVLKPGQTLRIKSDGNLIAPWFGHILSGESHGRILKTD
LKRGSCTVQCQTEKGGLNTTLPFQNVSKYAFGNCSKYIGIKSLKLAVGLRNVPSRSSRGLFGAIAGFIEG
GWSGLVAGWYGFQHSNDQGVGMAADRDSTQKAIDKITSKVNNIVDKMNKQYEIIDHEFSEVETRLNMINN
KIDDQIQDIWAYNAELLVLLENQKTLDEHDANVNNLYNKVKRALGSNAVEDGKGCFELYHKCDDQCMETI
RNGTYNRRKYQEESKLERQKIEGVKLESEGTYKILTIYSTVASSLVIAMGFAAFLFWAMSNGSCRCNICI

>QOK06210.1 hemagglutinin [Influenza A virus]
METVSLITILLAAAVSNADKICIGYQSTNSTETVDTLTENNVPVTHAKELLHTEHNGMLCATSLGQPLIL
DTCTIEGLIYGNPSCDPLPEEREWSYIVERPSAVNGLCYPGNVENLEELRSLFSSARSYQRIQIFPDTIW
NVSYDGTSNTCSGSFYRNMRWLTRKDGNYPIQDAQYTNKQGKNILFMWGINNPPTDDTQRNLYTRTDTTT
SVATEEINRIFKPLIGPRPLVNGLMGRINYYWSVLKPGQTLRIKSDGNLVAPWYGYILSGESHGRILRTN
LKRGSCTVQCQTEKGGLNTTLPFQNVSKYAFGNCSKYIGIKSLKLAVGLRNVPSRSSRGLFGAIAGFIEG
GWSGLVAGWYGFQHSNDQGVGMAADRESTQKAVDKITSKVNNIVDKMNKQYEIIDHEFSEVETRLNMINN
KIDDQIQDIWAYNAELLVLLENQKTLDEHDANVNNLYNKVKRALGSNAVEDGKGCFELYHKCDDQCMETI
RNGTYNRRKYQEESKLERQKIEGVKLESEGTYKILTIYSTVASSLVIAMGFAAFLFWAMSNGSCRCNICI

>QOK06198.1 hemagglutinin [Influenza A virus]
METVSLITILLAAAVSNADKICIGYQSTNSTETVDTLTENNVPVTHAKELLHTEHNGMLCATSLGQPLIL
DTCTIEGLIYGNPSCDPLPEEREWSYIVERPSAVNGLCYPGNVENLEELRSLFSSARSYQRIQIFPDTIW
NVSYDGTSNTCSGSFYRNMRWLTRKDGNYPIQDAQYTNNQGKNILFMWGINNPPTDDTQRNLYTRTDTTT
SVATEEINRIFKPLIGPRPLVNGLMGRINYYWSVLKPGQTLRIKSDGNLVAPWYGYILSGESHGRILRTN
LKRGSCTVQCQTEKGGLNTTLPFQNVSKYAFGNCSKYIGIKSLKLAVGLRNVPSRSSRGLFGAIAGFIEG
GWSGLVAGWYGFQHSNDQGVGMAADRESTQKAVDKIXSKVNNIVDKMNKQYEIIDHEFSEVETRLNMINN
KIDDQIQDIWAYNAELLVLLENQKTLDEHDANVNNLYNKVKRALGSNAVEDGKGCFELYHKCDDQCMETI
RNGTYNRRKYQEESKLERQKIEGVKLESEGTYKILTIYSTVASSLVIAMGFAAFLFWAMSNGSCRCNICI

>QOK06186.1 hemagglutinin [Influenza A virus]

METVSLITILLAAAVSNADKICIGYQSTNSTETVDTLTENNVPVTHAKELLHTEHNGMLCATSLGQPLIL
DTCTIEGLIYGNPSCDPLPEEREWSYIVERPSAVNGLCYPGNVENLEELRSLFSSARSYQRIQIFPDTIW
NVSYDGTSNTCSGSFYRNMRWLTRKDGNYPIQDAQYTNNQGKNILFMWGINNPPTDDTQRNLYTRTDTTT
SVATEEINRIFKPLIGPRPLVNGLMGRINYYWSVLKPGQTLRIKSDGNLVAPWYGYILSGESHGRILRTN
LKRGSCTVQCQTEKGGLNTTLPFQNVSKYAFGNCSKYIGIKSLKLAVGLRNVPSRSSRGLFGAIAGFIEG
GWSGLVAGWYGFQHSNDQGVGMAADRESTQKAVDKITSKVNNIVDKMNKQYEIIDHEFSEVETRLNMINN
KIDDQIQDIWAYNAELLVLLENQKTLDEHDANVNNLYNKVKRALGSNAVEDGKGCFELYHKCDDQCMETI
RNGTYNRRKYQEESKLERQKIEGVKLESEGTYKILTIYSTVASSLVIAMGFAAFLFWAMSNGSCRCNICI

>QOK06174.1 hemagglutinin [Influenza A virus]
METVSLITILLAAAVSNADKICIGYQSTNSTETVDTLTENNVPVTHAKELLHTEHNGMLCATSLGQPLIL
DTCTIEGLIYGNPSCDPLPEEGEWSYIVERPSAVNGLCYPGSVENLEELRSLFSSARSYQRIQIFPDTIW
NVSYDGTSNTCSGSFYRNMRWLTRKDGNYPIQDAQYTNKQGKNILFMWGINNPPTDDTQRNLYTRTDTTT
SVATEEINRIFKPLIGPRPLVNGLMGRINYYWSVLKPGQTLRIKSDGNLVAPWYGYILSGESHGRILRTN
LKRGSCTVQCQTEKGGLNTTLPFQNVSKYAFGNCSKYIGIKSLKLAVGLRNVPSRSSRGLFGAIAGFIEG
GWSGLVAGWYGFQHSNDQGVGMAADRESTQKAVDKITSKVNNIVDKMNKQYEIIDHEFSEVETRLNMINN
KIDDQIQDIWAYNAELLVLLENQKTLDEHDANVNNLYNKVKRALGSNAVEDGKGCFELYHKCDDQCMETI
RNGTYNRRKYQEESKLERQKIEGVKLESEGTYKILTIYSTVASSLVIAMGFAAFLFWAMSNGSCRCNICI

>QOK06162.1 hemagglutinin [Influenza A virus]
METVSLITILLAAAVSNADKICIGYQSTNSTETVDTLTENNVPVTHAKELLHTEHNGMLCATSLGQPLIL
DTCTIEGLIYGNPSCDPLPEEXEWSYIVERPSAVNGLCYPGNVENLEELRSLFSSARSYQRIQIFPDTIW
NVSYDGTSNTCSGSFYRNMRWLTRKDGNYPIQDAQYTNNQGKNILFMWGINNPPTDDTQRNLYTRTDTTT
SVATEEINRIFKPLIGPRPLVNGLMGRINYYWSVLKPGQTLRIKSDGNLVAPWYGYILSGESHGRILRTB
LKRGSCTVQCQTEKGGLNTTLPFQNVSKYAFGNCSKYIGIKSLKLAVGLRNVPSRSSRGLFGAIAGFIEG
GWSGLVAGWYGFQHSNDQGVGMAADRESTQKAVDKITSKVNNIVDKMNKQYEIIDHEFSEVETRLNMINN
KIDDQIQDIWAYNAELLVLLENQKTLDEHDANVNNLYNKVKRALGSNAVEDGKGCFELYHKCDDQCMETI
RNGTYNXRKYQEESKLERQKIEGVKLESEGTYKILTIYSTVASSLVIAMGFAAFLFWAMSNGSCRCNICI

>QOK06150.1 hemagglutinin [Influenza A virus]
METVSLITILLAAAVSNADKICIGYQSTNSTETVDTLTENNVPVTHAKELLHTEHNGMLCATSLGQPLIL
DTCTIEGLIYGNPSCDPLPEEREWSYIVERPSAVNGLCYPGNVENLEELRSLFSSARSYQRIQIFPDTIW
NVSYDGTSNTCSGSFYRNMRWLTRKDGNYPIQDAQYTNNQGKNILFMWGINNPPTDDTQRNLYTRTDTTT
SVATEEINRIFKPLIGPRPLVNGLMGRINYYWSVLKPGQTLRIKSDGNLVAPWYGYILSGESHGRILRTN

LKRGSCTVQCQTEKGGLNTTLPFQNVSKYAFGNCSKYIGIKSLKLAVGLRNVPSRSSRGLFGAIAGFIEG
GWSGLVAGWYGFQHSNDQGVGMAADRESTQKAVDKITSKVNNIVDKMNKQYEIIDHEFSEVETRLNMINN
KIDDQIQDIWAYNAELLVLLENQKTLDEHDANVNNLYNKVKRALGSNAVEDGKGCFELYHKCDDQCMETI
RNGTYNRRKYQEESKLERQKIEGVKLESEGTYKILTIYSTVASSLVIAMGFAAFLFWAMSNGSCRCNICI

>QOK06138.1 hemagglutinin [Influenza A virus]
METVSLITILLAAAVSNADKICIGYQSTNSTETVDTLTENNVPVTHAKELLHTEHNGMLCATSLGQPLIL
DTCTIEGLIYGNPSCDPLPEEREWSYIVERPSAVNGLCYPGNVENLEELRSLFSSARSYQRIQIFPDTIW
NVSYDGTSNTCSGSFYRNMRWLTRKDGNYPIQDAQYTNNQGKNILFMWGINNPPTDDTQRNLYTRTDTTT
SVATEEINRIFKPLIGPRPLVNGLMGRINYYWSVLKPGQTLRIKSDGNLVAPWYGYILSGESHGRILRTD
LKRGSCTVQCQTEKGGLNTTLPFQNVSKYAFGNCSKYIGIKSLKLAVGLRNVPSRSSRGLFGAIAGFIEG
GWSGLVAGWYGFQHSNDQGVGMAADRESTQKAVDKITSKVNNIVDKMNKQYEIIDHEFSEVETRLNMINN
KIDDQIQDIWAYNAELLVLLENQKTLDEHDANVNNLYNKVKRALGSNAVEDGKGCFELYHKCDDQCMETI
RNGTYNRRKYQEESKLERQKIEGVKLESEGTYKILTIYSTVASSLVIAMGFAAFLFWAMSNGSCRCNICI

>QOK06126.1 hemagglutinin [Influenza A virus]
METVSLITILLAAAVSNADKICIGYQSTNSTETVDTLTENNVPVTHAKELLHTEHNGMLCATSLGQPLIL
DTCTIEGLIYGNPSCDXXXEXREWSYIVERPSAVNGLCYPGNVENLEELRSLFSSARSYQRIQIFPDTIW
NVSYDGTSNTCSGSFYRNMRWLTRKDGNYPIQDAQYTNNQGKNILFMWGINNPPTDDTQRNLYTRTDTTT
SVATEEINRIFKPLIGPRPLVNGLMGRINYYWSVLKPGQTLRIKSDGNLVAPWYGYILSGESHGRILRTD
LKRGSCTVQCQTEKGGLNTTLPFQNVSKYAFGNCSKYIGIKSLKLAVGLRNVPSRSSRGLFGAIAGFIEG
GWSGLVAGWYGFQHSNDQGVGMAADRESTQKAVDKITSKVNNIVDKMNKQYEIIDHEFSEVETRLNMINN
KIDDQIQDIWAYNAELLVLLENQKTLDEHDANVNNLYNKVKRALGSNAVEDGKGCFELYHKCDDQCMETI
RNGTYNRRKYQEESKLERQKIEGVKLESEGTYKILTIYSTVASSLVIAMGFAAFLFWAMSNGSCRCNICI

>QOK06114.1 hemagglutinin [Influenza A virus]
METVSLITILLAAAVSNADKICIGYQSTNSTETVDTLTENNVPVTHAKELLHTEHNGMLCATSLGQPLIL
DTCTIEGLIYGNPSCDPLPEEREWSYIVERPSAVNGLCYPGNVENLEELRSLFSSARSYQRIQIFPDTIW
NVSYDGTSNTCSGSFYRNMRWLTRKDGNYPIQDAQYTNNQGKNILFMWGINNPPTDDTQRNLYTRTDTTT
SVATEEINRIFKPLIGPRPLVNGLMGRINYYWSVLKPGQTLRIKSDGNLVAPWYGYILSGESHGRILRTD
LKRGSCTVQCQTEKGGLNTTLPFQNVSKYAFGNCSKYIGIKSLKLAVGLRNVPSRSSRGLFGAIAGFIEG
GWSGLVAGWYGFQHSNDQGVGMAADRESTQKAVDKITSKVNNIVDKMNKQYEIIDHEFSEVETRLNMINN
KIDDQIQDIWAYNAELLVLLENQKTLDEHDANVNNLYNKVKRALGSNAVEDGKGCFELYHKCDDQCMETI
RNGTYNRRKYQEESKLERQKIEGVKLESEGTYKILTIYSTVASSLVIAMGFAAFLFWAMSNGSCRCNICI

>QOK06102.1 hemagglutinin [Influenza A virus]

METVSLITILLAAAVSNADKICIGYQSTNSTETVDTLTENNVPVTHAKELLHTEHNGMLCATSLGQPLIL

DTCTIEGLIYGNPSCDPLPEEREWSYIVERPSAVNGLCYPGNVENLEELRSLFSSARSYQRIQIFPDTIW

NVSYDGTSNTCSGSFYRNMRWLTRKDGNYPIQDAQYTNNQGKNILFMWGINNPPTDDTQRNLYTRTDTTT

SVATEEINRIFKPLIGPRPLVNGLMGRINYYWSVLKPGQTLRIKSDGNLVAPWYGYILSGESHGRILRTN

LKRGSCTVQCQTEKGGLNTTLPFQNVSKYAFGNCSKYIGIKSLKLAVGLRNVPSRSSRGLFGAIAGFIEG

GWSGLVAGWYGFQHSNDQGVGMAADRESTQKAVDKITSKVNNIVDKMNKQYEIIDHEFSEVETRLNMINN

KIDDQIQDIWAYNAELLVLLENQKTLDEHDANVNNLYNKVKRALGSNAVEDGKGCFELYHKCDDQCMETI

RNGTYNRRKYQEESKLERQKIEGVKLESEGTYKILTIYSTVASSLVIAMGFAAFLFWAMSNGSCRCNICI

>QOK06090.1 hemagglutinin [Influenza A virus]

METVSLITILLAAAVSNADKICIGYQSTNSTETVDTLTENNVPVTHAKELLHTEHNGMLCATSLGQPLIL

DTCTIEGLIYGNPSCDPLPEEREWSYIVERPSAVNGLCYPGNVENLEELRSLFSSARSYQRIQIFPDTIW

NVSYDGTSNTCSGSFYRNMRWLTRKDGNYPIQDAQYTNNQGKNILFMWGINNPPTDDTQRNLYTRTDTTT

SVATEEINRIFKPLIGPRPLVNGLMGRINYYWSVLKPGQTLRIKSDGNLVAPWYGYILSGESHGRILRTN

LKRGSCTVQCQTEKGGLNTTLPFQNVSKYAFGNCSKYIGIKSLKLAVGLRNVPSRSSRGLFGAIAGFIEG

GWSGLVAGWYGFQHSNDQGVGMAADRESTQKAVDKITSKVNNIVDKMNKQYEIIDHEFSEVETRLNMINN

KIDDQIQDIWAYNAELLVLLENQKTLDEHDANVNNLYNKVKRALGSNAVEDGKGCFELYHKCDDQCMETI

RNGTYNRRKYQEESKLERQKIEGVKLESEGTYKILTIYSTVASSLVIAMGFAAFLFWAMSNGSCRCNICI

>QOK06078.1 hemagglutinin [Influenza A virus]

METVSLITILLAAAVSNADKICIGYQSTNSTETVDTLTENNVPVTHAKELLHTEHNGMLCATSLGQPLIL

DTCTIEGLIYGNPSCDPLPEEREWSYIVERPSAVNGLCYPGNVENLEELRSLFSSARSYQRIQIFPDTIW

NVSYDGTSNTCSGSFYRNMRWLTRKDGNYPIQDAQYTNNQGKNILFMWGINNPPTDDTQRNLYTRTDTTT

SVATEEINRIFKPLIGPRPLVNGLMGRINYYWSVLKPGQTLRIKSDGNLVAPWYGYILSGESHGRILRTB

LKRGSCTVQCQTEKGGLNTTLPFQNVSKYAFGNCSKYIGIKSLKLAVGLRNVPSRSSRGLFGAIAGFIEG

GWSGLVAGWYGFQHSNDQGVGMAADRESTQKAVDKITSKVNNIVDKMNKQYEIIDHEFSEVETRLNMINN

KIDDQIQDIWAYNAELLVLLENQKTLDEHDANVNNLYNKVKRALGSNAVEDGKGCFELYHKCDDQCMETI

RNGTYNXRKYQEESKLERQKIEGVKLESEGTYKILTIYSTVASSLVIAMGFAAFLFWAMSNGSCRCNICI

>QOK06066.1 hemagglutinin [Influenza A virus]

METVSLITILLAAAVSNADKICIGYQSTNSTETVDTLTENNVPVTHAKELLHTEHNGMLCATSLGQPLIL

DTCTIEGLIYGNPSCDPLPEEREWSYIVERPSAVNGLCYPGNVENLEELRSLFSSARSYQRIQIFPDTIW

NVSYDGTSNTCSGSFYRNMRWLTRKDGNYPIQDAQYTNNQGKNILFMWGINNPPTDDTQRNLYTRTDTTT
SVATEEINRIFKPLIGPRPLVNGLMGRINYYWSVLKPGQTLRIKSDGNLVAPWYGYILSGESHGRILRTN
LKRGSCTVQCQTEKGGLNTTLPFQNVSKYAFGNCSKYIGIKSLKLAVGLRNVPSRSSRGLFGAIAGFIEG
GWSGLVAGWYGFQHSNDQGVGMAADRESTQKAVDKITSKVNNIVDKMNKQYEIIDHEFSEVETRLNMINN
KIDDQIQDIWAYNAELLVLLENQKTLDEHDANVNNLYNKVKRALGSNAVEDGKGCFELYHKCDDQCMETI
RNGTYNRRKYQEESKLERQKIEGVKLESEGTYKILTIYSTVASSLVIAMGFAAFLFWAMSNGSCRCNICI

>QOK05943.1 hemagglutinin [Influenza A virus]
METVSLITILLAATVSNADKICIGYQSTNSTETVDTLTENNVPVTHAKELLHTEHNGMLCATSLGQPLIL
DTCTIEGLIYGNPSCDPLLEEREWSYIVERPSAVNGLCYPGNVENLEELRSFFSSARSYQRIQIFPDTIW
NVSYDGTSXXCSGSFYRNMRWLTRKBGNYPXQDAQYTNNQXKXILFMWGINHPPTDDTQRNLYTRTDTTT
SVATEEINRIFKPLIGPRPLVNGLMGRINYYWSVLKPGQTLRIKSDGNLIAPWYGYILSGESHGRILXTD
LTKGSCTVQCQTEKGGLNTTLPFQNVSKYAFGNCSKYIGIKSLKLAVGLRNVPSRSSRGLFGAIAGFIEG
GWSGLVAGWYGFQHSNDQGVGMAADRESTQKAIDKITSKVNNIVDKMNKQYEIIDHEFSEVETRLNMINN
KIDDQIQDIWAYNAELLVLLENQKTLDEHDANVNNLYNKVKRALGSNAVEDGKGCFELYHKCDDQCMETI
RNGTYNRXKYQEESKLERQKIEGVKLESEGTYKILTIYSTVASSLVIAMGFAAFLFWAMSNGSCRCNICI

>QOK05931.1 hemagglutinin [Influenza A virus]
METVSLITILLVATVSNADKICIGYQSSNSTETVDTLTENNVPVTHAKELLHTEHNGMLCATSLGQPLIL
DTCTIEGLIYGNPSCDLSLEGREWSYIVERPSAVHGLCYPGNVEDLEELRSLFSSARSYQRIQIFPDTIW
NVSYDGTSTACSGSFYKSMRWLTRKNGEYPTQDAQYTNNQGKNILFMWGINHPPTDDTQRGLYTRTDTTT
SVATEEINRIFKPLIGPRPLVNGLMGRINYYWSVLKPGQTLRIKSDGNLIAPWYGHILSGESHGRILKTD
LKRGSCTVQCQTEKGGLNTTLPFQNVSKYAFGNCSKYIGVKSLKLAVGLRNVPSRSSRGLFGAIAGFIEG
GWPGLVAGWYGFQHSNDQGVGMAADRDSTQKAIDKITSKVNNIVDKMNKQYEIIDHEFSEVETRLNMINN
KIDDQIQDIWAYNAELLVLLENQKTLDEHDANVNNLYNKVKRALGSNAVEDGKGCFELYHKCDDQCMETI
RNGTYNRRKYQEESKLERQRIEGVKLESEGTYKILTIYSTVASSLVIAMGFAAFLFWAMSNGSCRCNICI

>QOK05919.1 hemagglutinin [Influenza A virus]
METVSLITILLVATVSNADKICIGYQSSNSTETVDTLTENNVPVTHAKELLHTEHNGMLCATSLGQPLIL
DTCTIEGLIYGNPSCDLSLEGREWSYIVERPSAVHGLCYPGNVEDLEELRSLFSSARSYQRIQIFPDTIW
NVSYDGTSTACSGSFYKSMRWLTRKNGEYPTQDAQYTNNQGKNILFMWGINHPPTDDTQRGLYTRTDTTT
SVATEEINRIFKPLIGPRPLVNGLMGRINYYWSVLKPGQTLRIKSDGNLIAPWYGHILSGESHGRILKTD
LKRGSCTVQCQTEKGGLNTTLPFQNVSKYAFGNCSKYIGVKSLKLAVGLRNVPSRSSRGLFGAIAGFIEG
GWPGLVAGWYGFQHSNDQGVGMAADRDSTQKAIDKITSKVNNIVDKMNKQYEIIDHEFSEVETRLNMINN

KIDDQIQDIWAYNAELLVLLENQKTLDEHDANVNNLYNKVKRALGSNAVEDGKGCFELYHKCDDQCMETI
RNGTYNRRKYQEESKLERQRIEGVKLESEGTYKILTIYSTVASSLVIAMGFAAFLFWAMSNGSCRCNICI

>QOK05896.1 hemagglutinin [Influenza A virus]
METVSLITILLXATVSNADKICIGYQSTNSTETVDTLTENNVPVTHAKELLHTEHNGMLCATSLGQPLIL
DTCTIEGLIYGNPSCDPLLEEREWSYIVERPSAVNGLCYPGNVENLEELRSFFSSARSYQRIQIFPDTIW
NVSYDGTSNTCSGSFYRNMRWLTRKNGNYPIQDAQYTNNQGKSILFMWGINHPPTDDTQRNLYTRTDTTT
SVATEEINRIFKPLIGPRPLVNGLMGRINYYWSVLKPGQTLRIKSDGNLIAPWYGYILSGESHGRILRTD
LTKGSCTVQCQTEKGGLNTTLPFQNVSKYAFGNCSKYIGIKSLKLAVGLRNVPSRSSRGLFGAIAGFIEG
GWSGLVAGWYGFQHSNDQGVGMAADRESTQKAIDKITSKVNNIVDKMNKQYEIIDHEFSEVETRLNMINN
KIDDQIQDIWAYNAELLVLLENQKTLDEHDANVNNLYNKVKRALGSNAVEDGKGCFELYHKCDDQCMETI
RNGTYNRRKYQEESKLERQKIEGVKLESEGTYKILTIYSTVASSLVIAMGFAAFLFWAMSNGSCRCNICI

>QOJ99979.1 hemagglutinin, partial [Influenza A virus]
ETVSLITILVVATVSNADKICIGYQSTNSTETVDTLTENNVPVTHAKELLHTEHNGMLCATSLGHPLILD
TCTIEGLIYGNPSCDPLLGGREWSYIVERPSAVNGLCYPGNVENLEELRSLFSSSRSYQRIQIFPDTIWN
VSYSGTSKACSDSFYRSMRWLTQKNNAYPTQDAQYTNNQGKNILFMWGINHPPTDTAQTNLYTRTDTTTS
VATEEMNRIFKPLIGPRPLVNGLMGRINYYWSVLKPGQTLRIKSDGNLIAPWYGHILSGESHGRILKTDL
KMGSCTVQCQTEKGGLNTTLPFQNVSKYAFGNCSKYIGXKSLKLAVGLRNVPSRSSRGLFGAIAGFIEGG
WSGLVAGWYGFQHSNDQGVGMAADRDSTQKAIDKITSKVNNIVXKMNKQYEIIDHEFSEVETRLNMINBK
XDDQIQDIWAYNAELLVLLENQKTLDEHDANVNNLYNKVKRALGSNAVEDGRGCFELYHKCDDHCMETIR
NGTYNRRKYQEESKLERQKIEGVKLESEETYKILTIYSTVASSLVIAMGFAAFLFWAMSNGSCRCNICI

>QOJ99969.1 hemagglutinin, partial [Influenza A virus]
ETVSLMTILLVATVSNADKICIGYQSTNSTETVDTLTENNVPVTHAKELLHTEHNGMLCATXLGXPLILD
TCTIEGLIYGNPSCDPLLGGREWSYIVERPSAVNGLCYPGNVENLEELRSLFSSSRSYQRIQIFPDTIWN
VSYSGTSKACSDSFYRSMRWLTQKNNAYPTQDAQYTNNQGKNILFMWGINHPPTDTAQTNLYTRTDTTTS
VATEEINRIFKPLIGPRPLVNGLMGRINYYWSVLKPGQTLRIKSDGNLIAPWYGHILSGESHGRILKTDL
KRGSCTVQCQTEKGGLNTTLPFQNVSKYAFGNCSKYIGVKSLKLAVGLRNVPSRSSRGLFGAIAGFIEGG
WXGLVAGWYGFQHSNDQGVGMAADXDSTQKAIDKITSKVNNIVDKMNKQYEIIDHEFSEVETRLNMINNK
XDDQIQDIWAYNAELLVLLENQKTLDEHDANVNNLYNKVKRALGSNAVEDGKGCFELYHKCDDXCMETIR
NGTYNRRKYQEESKLERQXXEGVKLESEXTYKILTIYSTVASSLVIAMGFAAFLFWAMSNGSCRCNICI

>QOJ99952.1 hemagglutinin, partial [Influenza A virus]

METVSLITILVVATVSNADKICIGYQSTNSTETVDTLTENNVPVTHAKELLHTEHNGMLCATDLGHPLIL
DTCTIEGLIYGNPSCDPLLGGREWSYIVERPSAVNGLCYPGNVENLEELRSLFSSSRSYQRIQIFPDTIW
NVSYSGTSKACSDSFYRSMRWLTQKNNAYPTQDAQYTNNQGKNILFMWGINHPPTDTAQTNLYTRTDTTT
SVATEEINRIFKPLIGPRPLVNGLMGRINYYWSVLKPGQTLRIKSDGNLIAPWYGHILSGESHGRILKTD
LKRGSCTVQCQTEKGGLNTTLPFQNVSKYAFGNCSKYIGVKSLKLAVGLRNVPSRSSRGLFGAIAGFIEG
GWPGLVAGWYGFQHSNDQGVGMAADRDSTQKAIDKITSKVNNIVDKMNKQYEIIDHEFSEVETRLNMINN
KVDDQIQDIWAYNAELLVLLENQKTLDEHDANVNNLYNKVKRALGSNAVEDGKGCFELYHKCDDHCMETI
RNGTYNRRKYQEESKLERQKIEGVKLESEETYKILTIYSTVASSLVIAM

>QOJ99951.1 hemagglutinin, partial [Influenza A virus]
METVSLITILVVATVSNADKICIGYQSTNSTETVDTLTENNVPVTHAKELLHTEHNGMLCATDLGHPLIL
DTCTIEGLIYGNPSCDPLLGGREWSYIVERPSAVNGLCYPGNVENLEELRSLFSSSRSYQRIQIFPDTIW
NVSYSGTSKACSDSFYRSMRWLTQKNNAYPTQDAQYTNNQGKNILFMWGINHPPTDTAQTNLYTRTDTTT
SVATEEINRIFKPLIGPRPLVNGLMGRINYYWSVLKPGQTLRIKSDGNLIAPWYGHILSGESHGRILKTD
LKRGSCTVQCQTEKGGLNTTLPFQNVSKYAFGNCSKYIGVKSLKLAVGLRNVPSRSSRGLFGAIAGFIEG
GWPGLVAGWYGFQHSNDQGVGMAADRDSTQKAIDKITSKVNNIVDKMNKQYEIIDHEFSEVETRLNMINN
KVDDQIQDIWAYNAELLVLLENQKTLDEHDANVNNLYNKVKRALGSNAVEDGKGCFELYHKCDDHCMETI
RNGTYNRRKYQEESKLERQKIEGVKLESEETYKILTIYSTVASSLVIAM

>QOJ99950.1 hemagglutinin, partial [Influenza A virus]
METVSLITILVVATVSNADKICIGYQSTNSTETVDTLTENNVPVTHAKELLHTEHNGMLCATDLGHPLIL
DTCTIEGLIYGNPSCDPLLGGREWSYIVERPSAVNGLCYPGNVENLEELRSLFSSSRSYQRIQIFPDTIW
NVSYSGTSKACSDSFYRSMRWLTQKNNAYPTQDAQYTNNQGKNILFMWGINHPPTDTAQTNLYTRTDTTT
SVATEEINRIFKPLIGPRPLVNGLMGRINYYWSVLKPGQTLRIKSDGNLIAPWYGHILSGESHGRILKTD
LKRGSCTVQCQTEKGGLNTTLPFQNVSKYAFGNCSKYIGVKSLKLAVGLRNVPSRSSRGLFGAIAGFIEG
GWPGLVAGWYGFQHSNDQGVGMAADRDSTQKAIDKITSKVNNIVDKMNKQYEIIDHEFSEVETRLNMINN
KVDDQIQDIWAYNAELLVLLENQKTLDEHDANVNNLYNKVKRALGSNAVEDGKGCFELYHKCDDHCMETI
RNGTYNRRKYQEESKLERQKIEGVKLESEETYKILTIYSTVASSLVIAM

>QOJ99949.1 hemagglutinin, partial [Influenza A virus]
METVSLITILVVATVSNADKICIGYQSTNSTETVDTLTENNVPVTHAKELLHTEHNGMLCATGLGHPLIL
DTCTIEGLIYGNPSCDPLLGGREWSYIVERPSAVNGLCYPGNVENLEELRSLFSSSRSYQRIQIFPDTIW
NVSYSGTSKACSDSFYRSMRWLTQKNNAYPTQDAQYTNNQGKNILFMWGINHPPTDTAQTNLYTRTDTTT
SVATEEINRIFKPLIGPRPLVNGLMGRINYYWSVLKPGQTLRIKSDGNLIAPWYGHILSGESHGRILKTD

LKRGSCTVQCQTEKGGLNTTLPFQNVSKYAFGNCSKYIGVKSLKLAVGLRNVPSRSSRGLFGAIAGFIEG
GWPGLVAGWYGFQHSNDQGVGMAADRDSTQKAIDKITSKVNNIVDKMNKQYEIIDHEFSEVETRLNMINN
KVDDQIQDIWAYNAELLVLLENQKTLDEHDANVNNLYNKVKRALGSNAVEDGKGCFELYHKCDDHCMETI
RNGTYNRRKYQEESKLERQKIEGVKLESEETYKILTIYSTVASSLVIAM

>QOJ99948.1 hemagglutinin, partial [Influenza A virus]
METVSLITILVVATVSNADKICIGYQSTNSTETVDTLTENNVPVTHAKELLHTEHNGMLCATGLGHPLIL
DTCTIEGLIYGNPSCDPLLGGREWSYIVERPSAVNGLCYPGNVENLEELRSLFSSSRSYQRIQIFPDTIW
NVSYSGTSKACSDSFYRSMRWLTQKNNAYPTQDAQYTNNQGKNILFMWGINHPPTDTAQTNLYTRTDTTT
SVATEEINRIFKPLIGPRPLVNGLMGRINYYWSVLKPGQTLRIKSDGNLIAPWYGYILSGESHGRILKTD
LKRGSCTVQCQTEKGGLNTTLPFQNVSKYAFGNCSKYIGVKSLKLAVGLRNVPSRSSRGLFGAIAGFIEG
GWPGLVAGWYGFQHSNDQGVGMAADRDSTQKAIDKITSKVNNIVDKMNKQYEIIDHEFSEVETRLNMINN
KVDDQIQDIWAYNAELLVLLENQKTLDEHDANVNNLYNKVKRALGSNAVEDGKGCFELYHKCDDHCMETI
RNGTYNRRKYQEESKLERQKIEGVKLESEETYKILTIYSTVASSLVIAM

>QOJ99947.1 hemagglutinin, partial [Influenza A virus]
METVSLITILVVATVSNADKICIGYQSTNSTETVDTLTENNVPVTHAKELLHTEHNGMLCATDLGHPLIL
DTCTIEGLIYGNPSCDPLLGGREWSYIVERPSAVNGLCYPGNVENLEELRSLFSSSRSYQRIQIFPDTIW
NVSYSGTSKACSDSFYRSMRWLTQKNNAYPTQDAQYTNNQGKNILFMWGINHPPTDTAQTNLYTRTDTTT
SVATEEINRIFKPLIGPRPLVNGLMGRINYYWSVLKPGQTLRIKSDGNLIAPWYGHILSGESHGRILKTD
LKRGSCTVQCQTEKGGLNTTLPFQNVSKYAFGNCSKYIGVKSLKLAVGLRNVPSRSSRGLFGAIAGFIEG
GWPGLVAGWYGFQHSNDQGVGMAADRDSTQKAIDKITSKVNNIVDKMNKQYEIIDHEFSEVETRLNMINN
KVDDQIQDIWAYNAELLVLLENQKTLDEHDANVNNLYNKVKRALGSNAVEDGKGCFELYHKCDDHCMETI
RNGTYNRRKYQEESKLERQKVEGVKLESEETYKILTIYSTVASSLVIAM

>QOJ99946.1 hemagglutinin, partial [Influenza A virus]
METVSLITILVVATVSNADKICIGYQSTNSTETVDTLTENNVPVTHAKELLHTEHNGMLCATGLGHPLIL
DTCTIEGLIYGNPSCDPLLGGREWSYIVERPSAVNGLCYPGNVENLEELRSLFSSSRSYQRIQIFPDTIW
NVSYSGTSKACSDSFYRSMRWLTQKNNAYPTQDAQYTNNQGKNILFMWGINHPPTDTVQTNLYTRTDTTT
SVATEEINRIFKPLIGPRPLVNGLMGRINYYWSVLKPGQTLRIKSDGNLIAPWYGHILSGESHGRILKTD
LKRGSCTVQCQTEKGGLNTTLPFQNVSKYAFGNCSKYIGVKSLKLAVGLRNVPSRSSRGLFGAIAGFIEG
GWPGLVAGWYGFQHSNDQGVGMAADRDSTQKAIDKITSKVNNIVDKMNKQYEIIDHEFSEVETRLNMINN
KVDDQIQDIWAYNAELLVLLENQKTLDEHDANVNNLYNKVKRALGSNAVEDGKGCFELYHKCDDHCMETI
RNGTYNRRKYQEESKLERQKIEGVKLESEETYKILTIYSTVASSLVIAMGFAAFLFWAMSNGSC

>QOJ99945.1 hemagglutinin, partial [Influenza A virus]

METVSLITILVVATVSNADKICIGYQSTNSTETVDTLTENNVPVTHAKELLHTEHNGMLCATDLGHPLIL
DTCTIEGLIYGNPSCDPLLGGREWSYIVERPSAVNGLCYPGNVENLEELRSLFSSSRSYQRIQIFPDTIW
NVSYSGTSKACSDSFYRSMRWLTQKNNAYPTQDAQYTNNQGKNILFMWGINHPPTDTAQTNLYTRTDTTT
SVATEEINRIFKPLIGPRPLVNGLMGRINYYWSVLKPGQTLRIKSDGNLIAPWYGHILSGESHGRILKTD
LKRGSCTVQCQTEKGGLNTTLPFQNVSKYAFGNCSKYIGVKSLKLAVGLRNVPSRSSRGLFGAIAGFIEG
GWPGLVAGWYGFQHSNDQGVGMAADRDSTQKAIDKITSKVNNIVDKMNKQYEIIDHEFSEVETRLNMINN
KVDDQIQDIWAYNAELLVLLENQKTLDEHDANVNNLYNKVKRALGSNAVEDGKGCFELYHKCDDHCMETI
RNGTYNRRKYQEESKLERQKIEGVKLESEETYKILTIYSTVASSLVIAM

>QOJ99944.1 hemagglutinin, partial [Influenza A virus]

METVSLITILVVATVSNADKICIGYQSTNSTETVDTLTENNVPVTHAKELLHTEHNGMLCATDLGHPLIL
DTCTIEGLIYGNPSCDPLLGGREWSYIVERPSAVNGLCYPGNVENLEELRSLFSSSRSYQRIQIFPDTIW
NVSYSGTSKACSDSFYRSMRWLTQKNNAYPTQDAQYTNNQGKNILFMWGINHPPTDTAQTNLYTRTDTTT
SVATEEINRIFKPLIGPRPLVNGLMGRINYYWSVLKPGQTLRIKSDGNLIAPWYGHILSGESHGRILKTD
LKRGSCTVQCQTEKGGLNTTLPFQNVSKYAFGNCSKYIGVKSLKLAVGLRNVPSRSSRGLFGAIAGFIEG
GWPGLVAGWYGFQHSNDQGVGMAADRDSTQKAIDKITSKVNNIVDKMNKQYEIIDHEFSEVETRLNMINN
KVDDQIQDIWAYNAELLVLLENQKTLDEHDANVNNLYNKVKRALGSNAVEDGKGCFELYHKCDDHCMETI
RNGTYNRRKYQEESKLERQKIEGVKLESEETYKILTIYSTVASSLVIAM

>QOJ99943.1 hemagglutinin, partial [Influenza A virus]

METVSLITILVVATVSNADKICIGYQSTNSTETVDTLTENNVPVTHAKELLHTEHNGMLCATXLGHPLIL
DTCTIEGLIYGNPSCDPLLGGREWSYIVERPSAVNGLCYPGNVENLEELRSLFSSSRSYQRIQIFPDTIW
NVSYSGTSKACSDSFYRSMRWLTQKNNAYPTQDAQYTNNQGKNILFMWGINHPPTDTXQTNLYTRTDTTT
SVATEEINRIFKPLIGPRPLVNGLMGRINYYWSVLKPGQTLRIKSDGNLIAPWYGXILSGESHGRILKTD
LKRGSCTVQCQTEKGGLNTTLPFQNVSKYAFGNCSKYIGVKSLKLAVGLRNVPSRSSRGLFGAIAGFIEG
GWPGLVAGWYGFQHSNDQGVGMAADRDSTQKAIDKITSKVNNIVDKMNKQYEIIDHEFSEVETRLNMINN
KVDDQIQDIWAYNAELLVLLENQKTLDEHDANVNNLYNKVKRALGSNAVEDGKGCFELYHKCDDHCMETI
RNGTYNRRKYQEESKLERQKIEGVKLESEETYKILTIYSTVASSLVIAMGFAAFLFWAMSNGSC

>QOJ99942.1 hemagglutinin, partial [Influenza A virus]

METVSLITILVVATVSNADKICIGYQSTNSTETVDTLTENNVPVTHAKELLHTEHNGMLCATGLGHPLIL
DTCTIEGLIYGNPSCDPLLGGREWSYIVERPSAVNGLCYPGNVENLEELRSLFSSSRSYQRIQIFPDTIW

NVSYSGTSKACSDSFYRSMRWLTQKNNAYPTQDAQYTNNQGKNILFMWGINHPPTDTVQTNLYTRTDTTT
SVATEEINRIFKPLIGPRPLVNGLMGRINYYWSVLKPGQTLRIKSDGNLIAPWYGYILSGESHGRILKTD
LKRGSCTVQCQTEKGGLNTTLPFQNVSKYAFGNCSKYIGVKSLKLAVGLRNVPSRSSRGLFGAIAGFIEG
GWPGLVAGWYGFQHSNDQGVGMAADRDSTQKAIDKITSKVNNIVDKMNKQYEIIDHEFSEVETRLNMINN
KVDDQIQDIWAYNAELLVLLENQKTLDEHDANVNNLYNKVKRALGSNAVEDGKGCFELYHKCDDHCMETI
RNGTYNRRKYQEESKLERQKIEGVKLESEETYKILTIYSTVASSLVIAMGFAAFLFWAMSNGSC

>QOJ99941.1 hemagglutinin, partial [Influenza A virus]
METVSLITILVVATVSNADKICIGYQSTNSTETVDTLTENNVPVTHAKELLHTEHNGMLCATDLGHPLIL
DTCTIEGLIYGNPSCDPLLGGREWSYIVERPSAVNGLCYPGNVENLEELRSLFSSSRSYQRIQIFPDTIW
NVSYSGTSKACSDSFYRSMRWLTQKNNAYPTQDAQYTNNQGKNILFMWGINHPPTDTAQTNLYTRTDTTT
SVATEEINRIFKPLIGPRPLVNGLMGRINYYWSVLKPGQTLRIKSDGNLIAPWYGHILSGESHGRILKTD
LKRGSCTVQCQTEKGGLNTTLPFQNVSKYAFGNCSKYIGVKSLKLAVGLRNVPSRSSRGLFGAIAGFIEG
GWPGLVAGWYGFQHSNDQGVGMAADRDSTQKAIDKITSKVNNIVDKMNKQYEIIDHEFSEVETRLNMINN
KVDDQIQDIWAYNAELLVLLENQKTLDEHDANVNNLYNKVKRALGSNAVEDGKGCFELYHKCDDHCMETI
RNGTYNRRKYQEESKLERQKIEGVKLESEETYKILTIYSTVASSLVIAM

>QOJ99940.1 hemagglutinin, partial [Influenza A virus]
METVSLITILVVATVSNADKICIGYQSTNSTETVDTLTENNVPVTHAKELLHTEHNGMLCATDLGHPLIL
DTCTIEGLIYGNPSCDPLLGGREWSYIVERPSAVNGLCYPGNVENLEELRSLFSSSRSYQRIQIFPDTIW
NVSYSGTSKACSDSFYRSMRWLTQKNNAYPTQDAQYTNNQGKNILFMWGINHPPTDTAQTNLYTRTDTTT
SVATEEINRIFKPLIGPRPLVNGLMGRINYYWSVLKPGQTLRIKSDGNLIAPWYGHILSGESHGRILKTD
LKRGSCTVQCQTEKGGLNTTLPFQNVSKYAFGNCSKYIGVKSLKLAVGLRNVPSRSSRGLFGAIAGFIEG
GWPGLVAGWYGFQHSNDQGVGMAADRDSTQKAIDKITSKVNNIVDKMNKQYEIIDHEFSEVETRLNMINN
KVDDQIQDIWAYNAELLVLLENQKTLDEHDANVNNLYNKVKRALGSNAVEDGKGCFELYHKCDDHCMETI
RNGTYNRRKYQEESKLERQKVEGVKLESEETYKILTIYSTVASSLVIAM

>QOJ99938.1 hemagglutinin, partial [Influenza A virus]
METVSLITILVVATVSNADKICIGYQSTNSTETVDTLTENNVPVTHAKELLHTEHNGMLCATXLGHPLIL
DTCTIEGLIYGNPSCDPLLGGREWSYIVERPSAVNGLCYPGNVENLEELRSLFSSSRSYQRIQIFPDTIW
NVSYSGTSKACSDSFYRSMRWLTQKNNAYPTQDAQYTNNQGKNILFMWGINHPPTDTAQTNLYTRTDTTT
SVATEEINRIFKPLIGPRPLVNGLMGRINYYWSVLKPGQTLRIKSDGNLIAPWYGHILSGESHGRILKTD
LKRGSCTVQCQTEKGGLNTTLPFQNVSKYAFGNCSKYIGVKSLKLAVGLRNVPSRSSRGLFGAIAGFIEG
GWPGLVAGWYGFQHSNDQGVGMAADRDSTQKAIDKITSKVNNIVDKMNKQYEIIDHEFSEVETRLNMINN

KVDDQIQDIWAYNAELLVLLENQKTLDEHDANVNNLYNKVKRALGSNAVEDGKGCFELYHKCDDHCMETI
RNGTYNRRKYQEESKLERQKIEGVKLESEETYKILTIYSTVASSLVIAM

>QOJ99937.1 hemagglutinin, partial [Influenza A virus]
METVXLITILVVATVSNADKICIGYQSTNSTETVDTLTENNVPVTHAKELLHTEHNGMLCATDLGHPLIL
DTCTIEGLIYGNPSCDPLLGGREWSYIVERPSAVNGLCYPGNVENLEELRSLFSSSRSYQRIQIFPDTIW
NVSYSGTSKACSDSFYRSMRWLTQKNNAYPTQDAQYTNNQGKNILFMWGINHPPTDTAQTNLYTRTDTTT
SVATEEINRIFKPLIGPRPLVNGLMGRINYYWSVLKPGQTLRIKSDGNLIAPWYGHILSGESHGRILKTD
LKRGSCTVQCQTEKGGLNTTLPFQNVSKYAFGNCSKYIGVKSLKLAVGLRNVPSRSSRGLFGAIAGFIEG
GWPGLVAGWYGFQHSNDQGVGMAADRDSTQKAIDKITSKVNNIVDKMNKQYEIIDHEFSEVETRLNMINN
KVDDQIQDIWAYNAELLVLLENQKTLDEHDANVNNLYNKVKRALGSNAVEDGKGCFELYHKCDDHCMETI
RNGTYNRRKYQEESKLERQKIEGVKLESEETYKILTIYSTVASSLVIAM

>QOJ99936.1 hemagglutinin, partial [Influenza A virus]
METVXLITILVVATVSNADKICIGYQSTNSTETVDTLTENNVPVTHAKELLHTEHNGMLCATDLGHPLIL
DTCTIEGLIYGNPSCDPLLGGREWSYIVERPSAVNGLCYPGNVENLEELRSLFSSSRSYQRIQIFPDTIW
NVSYSGTSKACSDSFYRSMRWLTQKNNAYPTQDAQYTNNQGKNILFMWGINHPPTDTAQTNLYTRTDTTT
SVATEEINRIFKPLIGPRPLVNGLMGRINYYWSVLKPGQTLRIKSDGNLIAPWYGHILSGESHGRILKTD
LKRGSCTVQCQTEKGGLNTTLPFQNVSKYAFGNCSKYIGVKSLKLAVGLRNVPSRSSRGLFGAIAGFIEG
GWPGLVAGWYGFQHSNDQGVGMAADRDSTQKAIDKITSKVNNIVDKMNKQYEIIDHEFSEVETRLNMINN
KVDDQIQDIWAYNAELLVLLENQKTLDEHDANVNNLYNKVKRALGSNAVEDGKGCFELYHKCDDHCMETI
RNGTYNRRKYQEESKLERQKIEGVKLESEETYKILTIYSTVASSLVIAM

>QOJ99935.1 hemagglutinin, partial [Influenza A virus]
METVSLITILVVATVSNADKICIGYQSTNSTETVDTLTENNVPVTHAKELLHTEHNGMLCATDLGHPLIL
DTCTIEGLIYGNPSCDPLLGGREWSYIVERPSAVNGLCYPGNVENLEELRSLFSSSRSYQRIQIFPDTIW
NVSYSGTSKACSDSFYRSMRWLTQKNNAYPTQDAQYTNNQGKNILFMWGINHPPTDTAQTNLYTRTDTTT
SVATEEINRIFKPLIGPRPLVNGLMGRINYYWSVLKPGQTLRIKSDGNLIAPWYGHILSGESHGRILKTD
LKRGSCTVQCQTEKGGLNTTLPFQNVSKYAFGNCSKYIGVKSLKLAVGLRNVPSRSSRGLFGAIAGFIEG
GWPGLVAGWYGFQHSNDQGVGMAADRDSTQKAIDKITSKVNNIVDKMNKQYEIIDHEFSEVETRLNMINN
KVDDQIQDIWAYNAELLVLLENQKTLDEHDANVNNLYNKVKRALGSNAVEDGKGCFELYHKCDDHCMETI
RNGTYNRRKYQEESKLERQKVEGVKLESEETYKILTIYSTVASSLVIAM

>QOJ99934.1 hemagglutinin, partial [Influenza A virus]

METVSLITILVVATVSNADKICIGYQSTNSTETVDTLTENNVPVTHAKELLHTEHNGMLCATDLGHPLIL
DTCTIEGLIYGNPSCDPLLGGREWSYIVERPSAVNGLCYPGNVENLEELRSLFSSSRSYQRIQIFPDTIW
NVSYSGTSKACSDSFYRSMRWLTQKNNAYPTQDAQYTNNQGKNILFMWGINHPPTDTAQTNLYTRTDTTT
SVATEEINRIFKPLIGPRPLVNGLMGRINYYWSVLKPGQTLRIKSDGNLIAPWYGHILSGESHGRILKTD
LKRGSCTVQCQTEKGGLNTTLPFQNVSKYAFGNCSKYIGVKSLKLAVGLRNVPSRSSRGLFGAIAGFIEG
GWPGLVAGWYGFQHSNDQGVGMAADRDSTQKAIDKITSKVNNIVDKMNKQYEIIDHEFSEVETRLNMINN
KVDDQIQDIWAYNAELLVLLENQKTLDEHDANVNNLYNKVKRALGSNAVEDGKGCFELYHKCDDHCMETI
RNGTYNRRKYQEESKLERQKXEGVKLESEETYKILTIYSTVASSLVIAM

>QOJ99816.1 hemagglutinin [Influenza A virus]
METVSLITILLVATVSNADKICIGYQTTNSTETVDTLTEXNVPVTHAKELLHTEHNGMLCATSLGQPLIL
DTCTIEGLIYGNPSCDLSLEGREWSYIVERPSAVHGLCYPGNVEDLEELRSLFSSARSYQRIQIFPDTIW
NVSYDGTSTACSGSFYKSMRWLTRKNGEYPTQDAQYTNNQGKNILFMWGINHPPTDDTQRGLYTRTDTTT
SVATEEINRIFKPLIGPRPLVNGLMGRINYYWSVLKPGQTLRIKSDGNLIAPWYGHILSGESHGRILKTD
LKRGSCTVQCQTEKGGLNTTLPFQNVSKYAFGNCSKYIGVKSLKLAVGLRNVPSRSSRGLFGAIAGFIEG
GWXGLVAGWYGFQHSNDQGVGMAADRDSTQKAIDKITSKVNNIVDKMNKQYEIIDHEFSEVETRLNMINN
KXDDQIQDIWAYNAELLVLLENQKTLDEHDANVNNLYNKVKRALGSNAVEDGKGCFELYHKCDDQCMETI
RNGTYNRRKYQEESKLERQXIEGVKLESEXTYKILTIYSTVASSLVIAMGFAAFLFWAMSNGSCRCNICI

>QOJ99799.1 hemagglutinin [Influenza A virus]
METVSLITILXXATVSNADKICIGYQSTNSTETVDTLTENNVPVTHAKELLHTEHNGMLCATSLGQPLIL
DTCTIEGLIYGNPSCDPLLEXREWSYIVERPSAVNGLCYPGNVENLEELRSFFSSARSYQRIQIFPDTIW
NVSYDGTSNTCSGSFYRNMRWLTRKNGNYPIQDAQYTNNQGKSILFMWGINHPPTDDTQRNLYTRTDTTT
SVATEEINRIFKPLIGPRPLVNGLMGRINYYWSVLKPGQTLRIKSDGNLIAPWYGXILSGESHGRILXTD
LXXGSCTVQCQTEKGGLNTTLPFQNVSKYAFGNCSKYIGIKSLKLAVGLRNVPSRSSRGLFGAIAGFIEG
GWSGLVAGWYGFQHSNDQGVGMAADRESTQKAIDKITSKVNNIVDKMNKQYEIIDHEFSEVETRLNMINN
KIDDQIQDIWAYNAELLVLLENQKTLDEHDANVNNLYNKVKRALGSNAVEDGKGCFELYHKCDDQCMETI
RBGTYNRRKYQEESKLERQKIEGVKLESEGTYKILTIYSTVASSLVIAMGFAAFLFWAMSNGSCRCNICI

>QOJ99796.1 hemagglutinin, partial [Influenza A virus]
SLMTILLVATVSNADKICIGYQSTNSTETVDTLTENNVPVTHAKELLHTEHNGMLCATSLGHPLILDTCT
IEGLIYGNPSCDPLLGGREWSYIVERPSAVNGLCYPGNVENLEELRSLFSSSRSYQRIQIFPDTIWNVSY
SGTSKACSDSFYRSMRWLTQKNNAYPTQDAQYTNNQGKNILFMWGINHPPTDTAQTNLYTRTDTTTSVAT
EEMNRIFKPLIGPRPLVNGLMGRINYYWSVLKPGQTLRIKSDGNLIAPWYGHILSGESHGRILKTDLKRG

SCTVQCQTEKGGLNTTLPFQNVSKYAFGNCSKYIGVKSLKLAVGLRNVPSRSSRGLFGAIAGFIEGGWSG
LVAGWYGFQHSNDQGVGMAADRDSTQKAIDKITSKVNNIVDKMNKQYEIIDHEFSEVETRLNMINNKXDD
QIQDIWAYNAELLVLLENQKTLDEHDANVNNLYNKVKRALGSNAVEDGKGCFELYHKCDDXCMETIRNGT
YNRRKYQEESKLERQKIEGVKLESEXTYKILTIYSTVASSLVIAMGFAAFLXWAMSNGSCRCNICI

>QOJ99790.1 hemagglutinin, partial [Influenza A virus]
METVSLITILVVATVSNADKICIGYQSTNSTETVDTLTENNVPVTHAKELLHTEHNGMLCATSLGHPLIL
DTCTIEGLIYGNPSCDPLLGGREWSYIVERPSAVNGLCYPGNVENLEELRSLFSSSRSYQRIQIFPDTIW
NVSYSGTSKACSDSFYRSMRWLTQKNNAYPTQDAQYTNNQGKNILFMWGINHPPTDTAQTNLYIRTDTTT
SVATEEMNRIFKPLIGPRPLVNGLMGRINYYWSVLKPGQTLRIKSDGNLIAPWYGHILSGESHGRILKTD
LKMGSCTVQCQTEKGGLNTTLPFQNVSKYAFGNCSKYIGVKSLKLAVGLRNVPSRSSRGLFGAIAGFIEG
GWSGLVAGWYGFQHSNDQGVGMAADRDSTQKAIDKITSKVNNIVDKMNKQYEIIDHEFSEVETRLNMIND
KVDDQIQDIWAYNAELLVLLENQKTLDEHDANVNNLYNKVKRALGSNAVEDGRGCFELYHKCDDHCMETI
RNGTYNRRKYQEESKLERQKIEGVKLESEETYKILTIYSTVASSLVIAM

>QOJ99785.1 hemagglutinin [Influenza A virus]
METVSLITILLVATVSNADKICIGYQTTNSTETVDTLTENNVPVTHAKELLHTEHNGMLCATSLGQPLIL
DTCTIEGLIYGNPSCDLSLEGREWSYIVERPSAVHGLCYPGNVEDLEELRSLFSSARSYQRIQIFPDTIW
NVSYDGTSTACSGSFYKSMRWLTRKNGEYPTQDAQYTNNQGKNILFMWGINHPPTDDTQRGLYTRTDTTT
SVATEEINRIFKPLIGPRPLVNGLMGRINYYWSVLKPGQTLRIKSDGNLIAPWYGHILSGESHGRILKTD
LKRGSCTVQCQTEKGGLNTTLPFQNVSKYAFGNCSKYIGVKSLKLAVGLRNVPSRSSRGLFGAIAGFIEG
GWPGLVAGWYGFQHSNDQGVGMAADRDSTQKAIDKITSKVNNIVDKMNKQYEIIDHEFSEVETRLNMINN
KIDDQIQDIWAYNAELLVLLENQKTLDEHDANVNNLYNKVKRALGSNAVEDGKGCFELYHKCDDQCMETI
RNGTYNRRKYQEESKLERQRIEGVKLESEGTYKILTIYSTVASSLVIAMGFAAFLFWAMSNGSCRCNICI

>QOJ99784.1 hemagglutinin [Influenza A virus]
METVSLITILLVATVSNADKICIGYQTTNSTETVDTLTENNVPVTHAKELLHTEHNGMLCATSLGQPLIL
DTCTIEGLIYGNPSCDLSLEGKEWSYIVERPSAVHGLCYPGNVEDLEELRSLFSSARSYQRIQIFPDTIW
NVSYDGTSTACSGSFYKSMRWLTRKNGEYPTQDAQYTNNQGKNILFMWGINHPPTDDTQRGLYTRTDTTT
SVATEEINRIFKPLIGPRPLVNGLMGRINYYWSVLKPGQTLRIKSDGNLIAPWYGHILSGESHGRILKTD
LKRGSCTVQCQTEKGGLNTTLPFQNVSKYAFGNCSKYIGVKSLKLAVGLRNVPSRSSRGLFGAIAGFIEG
GWPGLVAGWYGFQHSNDQGVGMAADRDSTQKAIDKITSKVNNIVDKMNKQYEIIDHEFSEVETRLNMINN
KIDDQIQDIWAYNAELLVLLENQKTLDEHDANVNNLYNKVKRALGSNAVEDGKGCFELYHKCDDQCMETI
RNGTYNRRKYQEESKLERQRIEGVKLESEGTYKILTIYSTVASSLVIAMGFAAFLFWAMSNGSCRCNICI

>QOJ99783.1 hemagglutinin, partial [Influenza A virus]

METVSLITILVVATVSNADKICIGYQSTNSTETVDTLTENNVPVTHAKELLHTEHNGMLCATSLGHPLIL
DTCTIEGLIYGNPSCDPLLGGREWSYIVERPSAVNGLCYPGNVENLEELRSLFSSSRSYQRIQIFPDTIW
NVSYSGTSKACSDSFYRSMRWLTQKNNAYPTQDAQYTNNQGKNILFMWGINHPPTDTAQTNLYTRTDTTT
SVATEEMNRIFKPLIGPRPLVNGLMGRINYYWSVLKPGQTLRIKSDGNLIAPWYGHILSGESHGRILKTD
LKMGSCTVQCQTEKGGLNTTLPFQNVSKYAFGNCSKYIGVKSLKLAVGLRNVPSRSSRGLFGAIAGFIEG
GWSGLVAGWYGFQHSNDQGVGMAADRDSTQKAIDKITSKVNNIVDKMNKQYEIIDHEFSEVETRLNMIND
KVDDQIQDIWAYNAELLVLLENQKTLDEHDANVNNLYNKVKRALGSNAVEDGRGCFELYHKCDDHCMETI
RNGTYNRRKYQEESKLERQKIEGVKLESEETYKILTIYSTVASSLVIAM

>QOJ99782.1 hemagglutinin, partial [Influenza A virus]

ETVSLITILLVATVSNADKICIGYQTTNSTETVDTLTENNVPVTHAKELLHTEHNGMLCATSLGQPLILD
TCTIEGLIYGNPSCDLSLEGREWSYIVERPSAVHGLCYPGNVEDLEELRSLFSSARSYQRIQIFPDTIWN
VSYDGTSTACSGSFYKSMRWLTRKNGEYPTQDAQYTNNQGKNILFMWGINHPPTDDTQRGLYTRTDTTTS
VATEEINRIFKPLIGPRPLVNGLMGRINYYWSVLKPGQTLRIKSDGNLIAPWYGHILSGESHGRILKTDL
KRGSCTVQCQTEKGGLNTTLPFQNVSKYAFGNCSKYIGVKSLKLAVGLRNVPSRSSRGLFGAIAGFIEGG
WPGLVAGWYGFQHSNDQGVGMAADRDSTQKAIDKITSKVNNIVDKMNKQYEIIDHEFSEVETRLNMINNK
IDDQIQDIWAYNAELLVLLENQKTLDEHDANVNNLYNKVKRALGSNAVEDGKGCFELYHKCDDQCMETIR
NGTYNRRKYQEESKLERQRIEGVKLESEGTYKILTIYSTVASSLVIAMGFAAFLFWAMSNGSC

>QOJ99781.1 hemagglutinin, partial [Influenza A virus]

ETVSLITILLVATVSNADKICIGYQTTNSTETVDTLTENNVPVTHAKELLHTEHNGMLCATSLGQPLILD
TCTIEGLIYGNPSCDLSLEGREWSYIVERPSAVHGLCYPGNVEDLEELRSLFSSARSYQRIQIFPDTIWN
VSYDGTSTACSGSFYKSMRWLTRKNGEYPTQDAQYTNNQGKNILFMWGINHPPTDDTQRGLYTRTDTTTS
VATEEINRIFKPLIGPRPLVNGLMGRINYYWSVLKPGQTLRIKSDGNLIAPWYGHILSGESHGRILKTDL
KRGSCTVQCQTEKGGLNTTLPFQNVSKYAFGNCSKYIGVKSLKLAVGLRNVPSRSSRGLFGAIAGFIEGG
WPGLVAGWYGFQHSNDQGVGMAADRDSTQKAIDKITSKVNNIVDKMNKQYEIIDHEFSEVETRLNMINNK
IDDQIQDIWAYNAELLVLLENQKTLDEHDANVNNLYNKVKRALGSNAVEDGKGCFELYHKCDDQCMETIR
NGTYNRRKYQEESKLERQRIEGVKLESEGTYKILTIYSTVASSLVIAMGFAAFLFWAMSNGSC

>QOJ99777.1 hemagglutinin, partial [Influenza A virus]

ETVSLITILLVATVSNADKICIGYQTTNSTETVDTLTENNVPVTHAKELLHTEHNGMLCATSLGQPLILD
TCTIEGLIYGNPSCDLSLEGREWSYIVERPSAVHGLCYPGNVEDLEELRSLFSSARSYQRIQIFPDTIWN

VSYDGTSTACSGSFYKSMRWLTRKNGEYPTQDAQYTNNQGKNILFMWGINHPPTDDTQRGLYTRTDTTTS
VATEEINRIFKPLIGPRPLVNGLMGRINYYWSVLKPGQTLRIKSDGNLIAPWYGHILSGESHGRILKTDL
KRGSCTVQCQTEKGGLNTTLPFQNVSKYAFGNCSKYIGVKSLKLAVGLRNVPSRSSRGLFGAIAGFIEGG
WPGLVAGWYGFQHSNDQGVGMAADRDSTQKAIDKITSKVNNIVDKMNKQYEIIDHEFSEVETRLNMINNK
IDDQIQDIWAYNAELLVLLENQKTLDEHDANVNNLYNKVKRALGSNAVEDGKGCFELYHKCDDQCMETIR
NGTYNRRKYQEESKLERQRIEGVKLESEGTYKILTIYSTVASSLVIAMGFAAFLFWAMSNGSC

>QOJ99775.1 hemagglutinin [Influenza A virus]
METVSLITILLVATVSNADKICIGYQTTNSTETVDTLTENNVPVTHAKELLHTEHNGMLCATSLGQPLIL
DTCTIEGLIYGNPSCDLSLEGREWSYIVERPSAVHGLCYPGNVEDLEELRSLFSSARSYQRIQIFPDTIW
NVSYDGTSTACSGSFYKSMRWLTRKNGEYPTQDAQYTNNQGKNILFMWGINHPPTDDTQRGLYTRTDTTT
SVATEEINRIFKPLIGPRPLVNGLMGRINYYWSVLKPGQTLRIKSDGNLIAPWYGHILSGESHGRILKTD
LKRGSCTVQCQTEKGGLNTTLPFQNVSKYAFGNCSKYIGVKSLKLAVGLRNVPSRSSRGLFGAIAGFIEG
GWPGLVAGWYGFQHSNDQGVGMAADRDSTQKAIDKITSKVNNIVDKMNKQYEIIDHEFSEVETRLNMINN
KIDDQIQDIWAYNAELLVLLENQKTLDEHDANVNNLYNKVKRALGSNAXEDGKGCFELYHKCDDQCMETI
RNGTYNRRKYQEESKLERQRIEGVKLESEGTYKILTIYSTVASSLVIAMGFAAFLFWAMSNGSCRCNICI

>QOJ99772.1 hemagglutinin [Influenza A virus]
METVSLITILLVATVSNADKICIGYQTTNSTETVDTLTENNVPVTHAKELLHTEHNGMLCATSLGQPLIL
DTCTIEGLIYGNPSCDLSLEGREWSYIVERPSAVHGLCYPGNVEDLEELRSLFSSARSYQRIQIFPDTIW
NVSYDGTSTACSGSFYKSMRWLTRKNGEYPTQDAQYTNNQGKNILFMWGINHPPTDDTQRRLYTRTDTTT
SVATEEINRIFKPLIGPRPLVNGLMGRINYYWSVLKPGQTLRIKSDGNLIAPWYGHILSGESHGRILKTD
LKRGSCTVQCQTEKGGLNTTLPFQNVSKYAFGNCSKYIGVKSLKLAVGLRNVPSRSSRGLFGAIAGFIEG
GWPGLVAGWYGFQHSNDQGVGMAADRDSTQKAIDKITSKVNNIVDKMNKQYEIIDHEFSEVETRLNMINN
KIDDQIQDIWAYNAELLVLLENQKTLDEHDANVNNLYNKVKRALGSNAVEDGKGCFELYHKCDDQCMETI
RNGTYNRRKYQEESKLERQRIEGVKLESEGTYKILTIYSTVASSLVIAMGFAAFLFWAMSNGSCRCNICI

>QOJ99771.1 hemagglutinin, partial [Influenza A virus]
METVSLITILVVATVSNADKICIGYQSTNSTETVDTLTENNVPVTHAKELLHTEHNGMLCATSLGHPLIL
DTCTIEGLIYGNPSCDPLLGGREWSYIVERPSAVNGLCYPGSVENLEELRSLFSSSRSYQRIQIFPDTIW
NVSYSGTSKACSDSFYRSMRWLTQKNNAYPTQDAQYTNNQGKNILFMWGINHPPTDTAQTNLYTRTDTTT
SVATEEMNRIFKPLIGPRPLVNGLMGRINYYWSVLKPGQTLRIKSDGNLIAPWYGHILSGESHGRILKTD
LKRGSCTVQCQTEKGGLNTTLPFQNVSKYAFGNCSKYIGVKSLKLAVGLRNVPSRSSRGLFGAIAGFIEG
GWSGLVAGWYGFQHSNDQGVGMAADRDSTQKAXDKITSKVNNIVDKMNKQYEIIDHEFSEVETRLNMINN

KVDDQIQDIWAYNAELLVLLENQKTLDEHDANVNNLYNKVKRALGSNAVEDGKGCFELYHKCDDHCMETI
RNGTYNRRKYQEESKLERQKIEGVKLESEETYKILTIYSTVASSLVIAM

>QOJ99753.1 hemagglutinin [Influenza A virus]
METVSLITILLVATVSNADKICIGYQSTNSTETVDTLTENNVPVTHAKELLHTEHNGMLCATSLGQPLIL
DTCTIEGLIYGNPSCDLSLEGREWSYIVERPSAVHGLCYPGNVEDLEELRSLFSSARSYQRIQIFPDTIW
NVSYDGTSTACSGSFYKSMRWLTRKNGEYPTQDAQYTNNQGKNILFMWGINHPPTDDTQRGLYTRTDTTT
SVATEEINRIFKPLIGPRPLVNGLMGRINYYWSVLKPGQTLRIKSDGNLIAPWYGHILSGESHGRILKTD
LKRGSCTVQCQTEKGGLNTTLPFQNVSKYAFGNCSKYIGIKSLKLAVGLRNVPSRSSRGLFGAIAGFIEG
GWPGLVAGWYGFQHSNDQGVGMAADRDSTQKAIDKITSKVNNIVDKMNKQYEIIDHEFSEIETRLNMINN
KIDDQIQDIWAYNAELLVLLENQKTLDEHDANVNNLYNKVKRALGSNAVEDGKGCFELYHKCDDQCMETI
RNGTYNRRKYQEESKLERQRIEGVKLESEGTYKILTIYSTVASSLVIAMGFAAFLFWAMSNGSCRCNICI

>QOJ99751.1 hemagglutinin [Influenza A virus]
METVSLITILLAATVSNADKICIGYQSTNSTETVDTLTENNVPVTHAKELLHTEHNGMLCATSLGQPLIL
DTCTIEGLIYGNPSCDPLLEEREWSYIVERPSAVNGLCYPGNVENLEELRSFFSSARSYQRIQIFPDTIW
NVSYDGTSNTCSGSFYRNMRWLTRKNGNYPIQDAQYTNNQGKSILFMWGINHPPTDDTQRNLYTRTDTTT
SVATEEINRIFKPLIGPRPLVNGLMGRINYYWSVLKPGQTLRIKSDGNLIAPWYGYILSGESHGRILRTD
LTKGSCTVQCQTEKGGLNTTLPFQNVSKYAFGNCSKYIGIKSLKLAVGLRNVPSRSSRGLFGAIAGFIEG
GWSGLVAGWYGFQHSNDQGVGMAADRESTQKAIDKITSKVNNIVDKMNKQYEIIDHEFSEVETRLNMINN
KIDDQIQDIWAYNAELLVLLENQKTLDEHDANVNNLYNKVKRALGSNAVEDGKGCFELYHKCDDQCMETI
RNGTYNRRKYQEESKLERQKIEGVKLESEGTYKILTIYSTVASSLVIAMGFAAFLFWAMSNGSCRCNICI

>QOJ99750.1 hemagglutinin, partial [Influenza A virus]
ETVSLITILLVATVSNADKICIGYQSTNSTETVDTLTENNVPVTHAKELLHTEHNGMLCATSLGQPLILD
TCTIEGLIYGNPSCDLSLEGREWSYIVERPSAVHGLCYPGNVEDLEELRSLFSSARSYQRIQIFPDTIWN
VSYDGTSTACSGSFYKSMRWLTRKNGEYPTQDAQYTNNQGKNILFMWGINHPPTDDTQRGLYTRTDTTTS
VATEEINRIFKPLIGPRPLVNGLMGRINYYWSVLKPGQTLRIKSDGNLIAPWYGHILSGESHGRILKTDL
KRGSCTVQCQTEKGGLNTTLPFQNVSKYAFGNCSKYIGIKSLKLAVGLRNVPSRSSRGLFGAIAGFIEGG
WPGLVAGWYGFQHSNDQGVGMAADRDSTQKAIDKITSKVNNIVDKMNKQYEIIDHEFSEIETRLNMINNK
IDDQIQDIWAYNAELLVLLENQKTLDEHDANVNNLYNKVKRALGSNAVEDGKGCFELYHKCDDQCMETIR
NGTYNRRKYQEESKLERQRIEGVKLESEGTYKILTIYSTVASSLVIAMGFAAFLFWAMSNGSC

>QOJ99748.1 hemagglutinin, partial [Influenza A virus]

ETVSLITILLVATVSNADKICIGYQSTNSTETVDTLTENNVPVTHAKELLHTEHNGMLCATSLGQPLILD
TCTIEGLIYGNPSCDLSLEGREWSYIVERPSAVHGLCYPGNVEDLEELRSLFSSARSYQRIQIFPDTIWN
VSYDGTSTACSGSFYKSMRWLTRKNGEYPTQDAQYTNNQGKNILFMWGINHPPTDDTQRGLYTRTDTTTS
VATEEINRIFKPLIGPRPLVNGLMGRINYYWSVLKPGQTLRIKSDGNLIAPWYGHILSGESHGRILKTDL
KRGSCTVQCQTEKGGLNTTLPFQNVSKYAFGNCSKYIGIKSLKLAVGLRNVPSRSSRGLFGAIAGFIEGG
WXGLVAGWYGFQHSNDQGVGMAADRXSTQKAIDKITSKVNNIVDKMNKQYEIIDHEFSEXETRLNMINNK
IDDQIQDIWAYNAELLVLLENQKTLDEHDANVNNLYNKVKRALGSNAVEDGKGCFELYHKCDDQCMETIR
NGTYNRRKYQEESKLERQXIEGVKLESEGTYKILTIYSTVASSLVIAMGFAAFLFWAMSNGSC

>QOJ99747.1 hemagglutinin, partial [Influenza A virus]
ETVSLITILLVATVSNADKICIGYQSTNSTETVDTLTENNVPVTHAKELLHTEHNGMLCATSLGQPLILD
TCTIEGLIYGNPSCDLSLEGREWSYIVERPSAVHGLCYPGNVEDLEELRSLFSSARSYQRIQIFPDTIWN
VSYDGTSTACSGSFYKSMRWLTRKNGEYPTQDAQYTNNQGKNILFMWGINHPPTDDTQRGLYTRTDTTTS
VATEEINRIFKPLIGPRPLVNGLMGRINYYWSVLKPGQTLRIKSDGNLIAPWYGHILSGESHGRILKTDL
KRGSCTVQCQTEKGGLNTTLPFQNVSKYAFGNCSKYIGIKSLKLAVGLRNVPSRSSRGLFGAIAGFIEGG
WPGLVAGWYGFQHSNDQGVGMAADRDSTQKAIDKITSKVNNIVDKMNKQYEIIDHEFSEIETRLNMINNK
IDDQIQDIWAYNAELLVLLENQKTLDEHDANVNNLYNKVKRALGSNAVEDGKGCFELYHKCDDQCMETIR
NGTYNRRKYQEESKLERQRIEGVKLESEGTYKILTIYSTVASSLVIAMGFAAFLFWAMSNGSC

>QOJ99743.1 hemagglutinin, partial [Influenza A virus]
METVSLITILVVATVSNADKICIGYQSTNSTETVDTLTENNVPVTHAKELLHTEHNGMLCATSLGHPLIL
DTCTIEGLIYGNPSCDPLLXGREWSYIVERPSAVNGLCYPGNVENLEELRSLFSSSRSYQRIQIFPDTIW
NVSYSGTSKACSDSFYRSMRWLTQKNNAYPTQDAQYTNNQGKNILFMWGINHPPTDTAQTNLYTRTDTTT
SXATEEMNRIFKPLIGPRPLVNGLMGRINYYWSVLKPGQTLRIKSDGNLIAPWYGHILSGESHGRILKTD
LKRGSCTVQCQTEKGGLNTTLPFQNVSXYAFGNCSKYIGVKSLKLAVGLRNVPSRSSRGLFGAIAGFIEG
GWSGLVAGWYGFQHSNDQGVGMAADRDSTQKAIDKITSKVNNIVDKMNKQYEIIDHEFSEVETRLNMINN
KVDDQIQDIWAYNAELLVLLENQKTLDEHDANVNNLYNKVKRALGSNAVEDGKGCFELYHKCDDHCMETI
RNGTYNRRKYQEESKLERQKIEGVKLESEETYKILTIYSTVASSLVIAMGFAAFLFWAMSNGSC

>QOJ99733.1 hemagglutinin [Influenza A virus]
METVSLITILLXATVSNADKICIGYQSTNSTETVDTLTENNVPVTHAKELLHTEHNGMLCATSLGXPLIL
DTCTIEGLIYGNPSCDPLLEEREWSYIVERPSAVNGLCYPGNVENLEELRSLFSSARSYQRIQIFPDTIW
NVSYDGTSNTCSGSFYRNMRWLTRKBGNYPXQDAQYTNNQGKNILFMWGINHPPTDDTQRNLYTRTDTTT
SVATEEVNRIFKPLIGPRPLVNGLMGRIBYYWSVLKPGQTLRXKSDGNLIAPWYGXILSGESHGRILXTD

LKRGSCTVQCQTEKGGLNTTLPFQNXSKYAFGNCSKYIGIKSLKLAVGLRNVPSRSSRGLFGAIAGFIEG
GWSGLVAGWYGFQHSNDQGVGMAADRESTQKAIDKITSKVNNIVDKMNKQYEIIDHEFSEVETRLNMINN
KIDDQIQDIWAYNAELLVLLENQKTLDEHDANVNNLYNKVKRALGXNAVEDGKGCFELYHKCDDQCMETI
RNGTYNRRKYQEESKLERQKIEGVKLESEGTYKILTIYSTVASSLVIAMGFAAFLFWAMSNGSCRCNICI

>QOJ99694.1 hemagglutinin, partial [Influenza A virus]
ETVSLITXLLVATVSNADKICIGYQSTNSTETVDTLTENNVPVTHAKELJHTEHNGMLCATXLGQPLILD
TCTIEGLIYGNPSCDLSLEGREWSYIVERXSAVXGLCYPGNVENLEELRSLFSSARSYQRXQIFPDTIWN
VSYDGTSTACSGSFYRXMRWLTRKBGXYPXQDAQYTNNQGKNILFMWGINHPPTDTTQXGLYTRTDTTTS
VATEEINRXFKPLIGPRPLVNGLMGRIBYYWSVLKPGQTLRIKSDGNLIAPWXGHILSGESHGRILKTDL
KRGSCTVQCQTEKGGLNTTLPFQNVSKYAFGNCSKYIGIKSLKLAVGLRNVPSRSSRGLFGAXAGFIEGG
WSGLVAGWYGFQHSNDQGVGMAADRDSTQKAIDKITSKVNNIVDKMNKQYEIIDHEFSEVEXRLNMINNK
IDDQIQDIWAYNAELLVLLENQKTLDEHDANVNNLYXKVKRALGSNAVEDGKGCFELYHKCDDQCMETIR
NGTYNRRKYQEESKLERQXIEGVKLESEGTYKILTIYSTVASSLVIAMGFAAFLFWAMSNGSCRCNICI

>QOJ99693.1 hemagglutinin [Influenza A virus]
METISLITVLLVATVSXADKICIGYQSTNSTETVDTLTENNVPVTHAKELJXTEHNGMLCATXLGQPLIL
DTCTIEGLIYGNPSCDLSLEGREWSYIVERPSAVNGLCYPGNVENLEELRSLFSSARSYQRXQIFPDTIW
NVSYDGTSTACSGSFYRNMRWLTRKDGNYPTQDAQYTNNQGKNILFMWGINHPPTDXTQSGLYTRTDTTT
SVATEEINRXFKPLIGPRPLVNGLMGRIDYYWSVLKPGQTLRIKSDGNLIAPWXGXILSGESHGRILKTD
LKRGSCTVQCQTEKGGLNTTLPFQNVSKYAFGNCSKYIGIKSLKLAVGLRNVPSRSSRGLFGAXAGFIEG
GWSGLVAGWYGFQHSNDQGVGMAADRDSTQKAIDKITSKVNNIVDKMNKQYEIIDHEFSEVETRLNMINN
KIDDQIQDIWAYNAELLVLLENQKTLDEHDANVNNLYNKVKRALGSNAVEXGKGCFELYHKCDDQCMETI
RNGTYNRXKYQEESKLERQXIEGVKLESEGTYKILTIYSTVASSLVIAMGFAAFLFWAMSNGSCRCNICI

>QOJ99555.1 hemagglutinin [Influenza A virus]
METVSLITILLVATVSNADKICIGYQSTNSTETVDTLTENNVPVTHAKELLHTEHNGMLCATSLGQPLIL
DTCTIEGLIYGNPSCDLSLEGREWSYIVERPSAVHGLCYPGNVEDLEELRSLFSSARSYQRIQIFPDTIW
NVSYDGTSTACSGSFYKSMRWLTRKNGEYPTQDAQYTNNQGKNILFMWGINHPPTDDTQRGLYTRTDTTT
SVATEEINRIFKPLIGPRPLVNGLMGRINYYWSVLKPGQTLRIKSDGNLIAPWYGHILSGESHGRILKTD
LKRGSCTVQCQTEKGGLNTTLPFQNVSKYAFGNCSKYIGIKSLKLAVGLRNVPSRSSRGLFGAIAGFIEG
GWSGLVAGWYGFQHSNDQGVGMAADRDSTQKAIDKITSKVNNIVDKMNKQYEIIDHEFSEVETRLNMINN
KIDDQIQDIWAYNAELLVLLENQKTLDEHDANVNNLYNKVKRALGSNAVEDGKGCFELYHKCDDQCMETI
RNGTYNRRKYQEESKLERQRIEGVKLESEGTYKILTIYSTVASSLVIAMGFAAFLFWAMSNGSCRCNICI

>QOJ99550.1 hemagglutinin [Influenza A virus]

METVSLITILLVATVSNADKICIGYQSTNSTETVDTLTENNVPVTHAKELLHTEHNGMLCATSLGQPLIL

DTCTIEGLIYGNPSCDLSLEGREWSYIVERPSAVHGLCYPGNVEDLEELRSLFSSARSYQRIQIFPDTIW

NVSYDGTSTACSGSFYKSMRWLTRKNGEYPTQDAQYTNNQGKNILFMWGINHPPTDDTQRGLYTRTDTTT

SVATEEINRIFKPLIGPRPLVNGLMGRINYYWSVLKPGQTLRIKSDGNLIAPWYGHILSGESHGRILKTD

LKRGSCTVQCQTEKGGLNTTLPFQNVSKYAFGNCSKYIGIKSLKLAVGLRNVPSRSSRGLFGAIAGFIEG

GWSGLVAGWYGFQHSNDQGVGMAADRDSTQKAIDKITSKVNNIVDKMNKQYEIIDHEFSEVETRLNMINN

KIDDQIQDIWAYNAELLVLLENQKTLDEHDANVNNLYNKVKRALGSNAVEDGKGCFELYHKCDDQCMETI

RNGTYNRRKYQEESKLERQRIEGVKLESEGTYKILTIYSTVASSLVIAMGFAAFLFWAMSNGSCRCNICI

>QOJ99548.1 hemagglutinin [Influenza A virus]

METVSLITILLVATVSNADKICIGYQSTNSTETVDTLTENNVPVTHAKELLHTEHNGMLCATSLGQPLIL

DTCTIEGLIYGNPSCDLSLEGREWSYIVERPSAVNGLCYPGNVENLEELRSLFSSARSYQRIQXFPDTIW

NVSYDGTSTACSNSFYRSMRWLTRKDGNYPTQDAQYTNNQGKNILFMWGINHPPTDETQRNLYTRTDTTT

SVATEEINRIFKPLIGPRPLVNGLMGRIDYYWSVLKPGQTLRIKSDGNLIAPWYGHILSGESHGRILKTD

LKKGSCTVQCQTEKGGLNTTLPFQNVSKYAFGNCSKYIGIKSLKLAVGLRNVPSRSSRGLFGAIAGFIEG

GWSGLVAGWYGFQHSNDQGVGMAADRDSTQKAIDKITSKVNNIVDKMNKQYEIIDHEFSEVETRLNMINN

KIDDQIQDIWAYNAELLVLLENQKTLDEHDANVNNLYNKVKRALGSNAMEDGKGCFELYHKCNDQCMETI

RNGTYNRKKYQEESKLERQRVEGVKLESEGTYKILTIYSTVASSLVIAMGFAAFLFWAMSNGSCRCNICI

>QOJ99543.1 hemagglutinin, partial [Influenza A virus]

METVSLITILVVATVSSADKICIGYQSTNSTETVDTLTENNVPVTHAKELLHTEHNGMLCATSLGHPLIL

DTCTIEGLIYGNPSCDPLLGGREWSYIVERPSAVNGLCYPGNVENLEELRSLFSSSRSYQRIQIFPDTIW

NVSYSGTSKACSDSFYRSMRWLTQKNNAYPTQDAQYTNNQGKNILFMWGINHPPTDTAQTNLYTRTDTTT

SVATEEMNRIFKPLIGPRPLVNGLMGRINYYWSVLKPGQTLRIKSDGNLIAPWYGHILSGESHRRILKTD

LKRGSCTVQCQTEKGGLNTTLPFQNISKYAFGNCSKYIGVKSLKLAVGLRNVPARSSRGLFGAIAGFIEG

GWSGLVAGWYGFQHSNDQGVGIAADRDSTQKAIDKITSKVNNIVDKMNKQYEIIDHEFSEVETRLNMINN

KVDDQIQDIWAYNAELLVLLENQKTLDEHDANVNNLYNKVKRALGSNAVEDGKGCFELYHKCDDHCMETI

RNGTYNRRKYQEESKLERQKIEGVKLESEETYKILTIYSTVASSLVIAM

>QOJ99542.1 hemagglutinin, partial [Influenza A virus]

ETVSLITILLVATVSNADKICIGYQSTNSTETVDTLTENNVPVTHAKELLHTEHNGMLCATSLGQPLILD

TCTIEGLIYGNPSCDLSLEGREWSYIVERPSAVHGLCYPGNVEDLEELRSLFSSARSYQRIQIFPDTIWN

VSYDGTSTACSGSFYKSMRWLTRKNGEYPTQDAQYTNNQGKNILFMWGINHPPTDDTQRGLYTRTDTTTS
VATEEINRIFKPLIGPRPLVNGLMGRINYYWSVLKPGQTLRIKSDGNLIAPWYGHILSGESHGRILKTDL
KRGSCTVQCQTEKGGLNTTLPFQNVSKYAFGNCSKYIGIKSLKLAVGLRNVPSRSSRGLFGAIAGFIEGG
WSGLVAGWYGFQHSNDQGVGMAADRDSTQKAIDKITSKVNNIVDKMNKQYEIIDHEFSEVETRLNMINNK
IDDQIQDIWAYNAELLVLLENQKTLDEHDANVNNLYNKIKRALGSNAVEDGKGCFELYHKCDDQCMETIR
NGTYNRRKYQEESKLERQRIEGVKLESEGTYKILTIYSTVASSLVIAMGFAAFLFWAMSNGSC

>QOJ99541.1 hemagglutinin [Influenza A virus]
METVSLITILLVATVSNADKICIGYQSTNSTETVDTLTENNVPVTHAKELLHTEHNGMLCATSLGQPLIL
DTCTIEGLIYGNPSCDLSLEGREWSYIVERPSAVHGLCYPGNVEDLEELRSLFSSARSYQRIQIFPDTIW
NVSYDGTSTACSGSFYKSMRWLTRKNGEYPTQDAQYTNNQGKNILFMWGINHPPTDDTQRGLYTRTDTTT
SVATEEINRIFKPLIGPRPLVNGLMGRINYYWSVLKPGQTLRIKSDGNLIAPWYGHILSGESHGRILKTD
LKRGSCTVQCQTEKGGLNTTLPFQNVSKYAFGNCSKYIGIKSLKLAVGLRNVPSRSSRGLFGAIAGFIEG
GWSGLVAGWYGFQHSNDQGVGMAADRDSTQKAIDKITSKVNNIVDKMNKQYEIIDHEFSEVETRLNMINN
KIDDQIQDIWAYNAELLVLLENQKTLDEHDANVNNLYNKVKRALGSNAVEDGKGCFELYHKCDDQCMETI
RNGTYNRRKYQEESKLERQRIEGVKLESEGTYKILTIYSTVASSLVIAMGFAAFLFWAMSNGSCRCNICI

>QOJ99538.1 hemagglutinin [Influenza A virus]
METVSLITILLVATVSNADKICIGYQSTNSTETVDTLTENNVPVTHAKELLHTEHNGMLCATSLGQPLIL
DTCTIEGLIYGNPSCDLSLEGREWSYIVERPSAVHGLCYPGNVEDLEELRSLFSSARSYQRIQIFPDTIW
NVSYDGTSTACSGSFYKSMRWLTRKNGEYPTQDAQYTNNQGKNILFMWGINHPPTDDTQRGLYTRTDTTT
SVATEEINRIFKPLIGPRPLVNGLMGRINYYWSVLKPGQTLRIKSDGNLIAPWYGHILSGESHGRILKTD
LKRGSCTVQCQTEKGGLNTTLPFQNVSKYAFGNCSKYIGIKSLKLAVGLRNVPSRSSRGLFGAIAGFIEG
GWSGLVAGWYGFQHSNDQGVGMAADRDSTQKAIDKITSKVNNIVDKMNKQYEIIDHEFSEVETRLNMINN
KIDDQIQDIWAYNAELLVLLENQKTLDEHDANVNNLYNKVKRALGSNAVEDGKGCFELYHKCDDQCMETI
RNGTYNRRKYQEESKLERQRIEGVKLESEGTYKILTIYSTVASSLVIAMGFAAFLFWAMSNGSCRCNICI

>QOJ99537.1 hemagglutinin, partial [Influenza A virus]
ETVSLITILLVATASNADKICIGYQSTNSTETVDTLTENNVPVTHAKELLHTEHNGMLCATSLGQPLILD
TCTIEGLIYGNPSCDLSLEGREWSYIVERPSAVNGLCYPGNVENLEELRSLFSSARSYQRIQIFPDTIWN
VSYDGTSTACSNSFYRSMRWLTRKDGNYPTQDAQYTNNQGKNILFMWGINHPPTDETQRTLYTRTDTTTS
VATEEINRIFKPLIGPRPLVNGLMGRIDYYWSVLKPGQTLRIKSDGNLIAPWYGHILSGESHGRILKTDL
KKGSCTVQCQTEKGGLNTTLPFQNVSKYAFGNCSKYIGIKSLKLAVGLRNVPSRSSRGLFGAIAGFIEGG
WSGLVAGWYGFQHSNDQGVGMAADRDSTQKAIDKITSKVNNIVDKMNKQYEIIDHEFSEVETRLNMINNK

IDDQIQDIWAYNAELLVLLENQKTLDEHDANVNNLYNKVKRALGSNAAEDGKGCFELYHKCNDQCMETIR
NGTYNRKKYQEESKLERQRIEGVKLESEGTYKILTIYSTVASSLVIAMGFAAFLFWAMSNGSC

>QOJ99536.1 hemagglutinin [Influenza A virus]
METVSLITILLVATVSNADKICIGYQSTNSTETVDTLTENNVPVTHAKELLQTEHNGMLCATSLGQPLIL
DTCTIEGLIYGNPSCDLSLEGREWSYIVERPSAVHGLCYPGNVEDLEELRSLFSSARSYQRIQIFPDTIW
NVSYDGTSTACSGSFYKSMRWLTRKNGEYPTQDAQYTNNQGKNILFMWGINHPPTDDTQRGLYTRTDTTT
SVATEEINRIFKPLIGPRPLVNGLMGRINYYWSVLKPGQTLRIKSDGNLIAPWYGHILSGESHGRILKTD
LKRGSCTVQCQTEKGGLNTTLPFQNVSKYAFGNCSKYIGIKSLKLAVGLRNVPSRSSRGLFGAIAGFIEG
GWSGLVAGWYGFQHSNDQGVGMAADRDSTQKAIDKITSKVNNIVDKMNKQYEIIDHEFSEVETRLNMINN
KIDDQIQDIWAYNAELLVLLENQKTLDEHDANVNNLYNKVKRALGSNAVEDGKGCFELYHKCDDQCMETI
RNGTYNRRKYQEESKLERQRIEGVKLESEGTYKILTIYSTVASSLVIAMGFAAFLFWAMSNGSCRCNICI

>QOJ99535.1 hemagglutinin, partial [Influenza A virus]
ETVSLITILLVATVSNADKICIGYQSTNSTETVDTLTENNVPVTHAKELLQTEHNGMLCATSLGQPLILD
TCTIEGLIYGNPSCDLSLEGREWSYIVERPSAVHGLCYPGNVEDLEELRSLFSSARSYQRIQIFPDTIWN
VSYDGTSTACSGSFYKSMRWLTRKNGEYPTQDAQYTNNQGKNILFMWGINHPPTDDTQRGLYTRTDTTTS
VATEEINRIFKPLIGPRPLVNGLMGRINYYWSVLKPGQTLRIKSDGNLIAPWYGHILSGESHGRILKTDL
KRGSCTVQCQTEKGGLNTTLPFQNVSKYAFGNCSKYIGIKSLKLAVGLRNVPSRSSRGLFGAIAGFIEGG
WSGLVAGWYGFQHSNDQGVGMAADRDSTQKAIDKITSKVNNIVDKMNKQYEIIDHEFSEVETRLNMINNK
IDDQIQDIWAYNAELLVLLENQKTLDEHDANVNNLYNKVKRALGSNAVEDGKGCFELYHKCDDQCMETIR
NGTYNRRKYQEESKLERQRIEGVKLESEGTYKILTIYSTVASSLVIAMGFAAFLFWAMSNGSC

>QOJ99534.1 hemagglutinin, partial [Influenza A virus]
METVSLITILVVATVSNADKICIGYQSTNSTETVDTLTENNVPVTHAKELLHTEHNGMLCATSLGHPLIL
DTCTIEGLIYGNPSCDPLLGGREWSYIVERPSAVNGLCYPGNVENLEELRSLFSSSRSYQRIQIFPDTIW
NVSYSGTSKACSDSFYRSMRWLTQKNNAYPTQDAQYTNNQGKNILFMWGINHPPTDTTQTNLYTRTDTTT
SVATEEMNRIFKPLIGPRPLVNGLMGRINYYWSVLKPGQTLRIKSDGNLIAPWYGHILSGESHGRILKTD
LKRGSCTVQCQTEKGGLNTTLPFQNVSKYAFGNCSKYIGVKSLKLAVGLRNVPSRSSRGLFGAIAGFIEG
GWSGLVAGWYGFQHSNDQGVGMAADRDSTQKAIDKITSKVNNIVDKMNKQYEIIDHEFSEVETRLNMINN
KVDDQIQDIWAYNAELLVLLENQKTLDEHDANVNNLYNKVKRALGSNAVEDGKGCFELYHKCDDHCMETI
RNGTYNRRKYQEESKLERQKIEGVKLESEETYKILTIYSTVASSLVIAMGFAAFLFWAMSNGSC

>QOJ99533.1 hemagglutinin, partial [Influenza A virus]

ETVSLITILLVATASNADKICIGYQSTNSTETVDTLTENNVPVTHAKELLHTEHNGMLCATSLGQPLILD
TCTIEGLIYGNPSCDLSLEGREWSYIVERPSAVNGLCYPGNVENLEELRSLFSSARSYQRIQIFPDTIWN
VSYDGTSTACSNSFYRSMRWLTRKDGNYPTQDAQYTNNQGKNILFMWGINHPPTDETQRTLYTRTDTTTS
VATEEINRIFKPLIGPRPLVNGLMGRIDYYWSVLKPGQTLRIKSDGNLIAPWYGHILSGESHGRILKTDL
KKGSCTVQCQTEKGGLNTTLPFQNVSKYAFGNCSKYIGIKSLKLAVGLRNVPSRSSRGLFGAIAGFIEGG
WSGLVAGWYGFQHSNDQGVGMAADRDSTQKAIDKITSKVNNIVDKMNKQYEIIDHEFSEVETRLNMINNK
IDDQIQDIWAYNAELLVLLENQKTLDEHDANVNNLYNKVKRALGSNAAEDGKGCFELYHKCNDQCMETIR
NGTYNRKKYQEESKLERQRIEGVKLESEGTYKILTIYSTVASSLVIAMGFAAFLFWAMSNGSC

>QOJ99532.1 hemagglutinin, partial [Influenza A virus]
ETVSLITILLVATASNADKICIGYQSTNSTETVDTLTENNVPVTHAKELLHTEHNGMLCATSLGQPLILD
TCTIEGLIYGNPSCDLSLEGREWSYIVERPSAVNGLCYPGNVENLEELRSLFSSARSYQRIQIFPDTIWN
VSYDGTSTACSNSFYRSMRWLTRKDGNYPTQDAQYTNNQGKNILFMWGINHPPTDETQRXLYTRTDTTTS
VATEEINRIFKPLIGPRPLVNGLMGRIDYYWSVLKPGQTLRIKSDGNLIAPWYGHILSGESHGRILKTDL
KKGSCTVQCQTEKGGLNTTLPFQNVSKYAFGNCSKYIGIKSLKLAVGLRNVPSRSSRGLFGAIAGFIEGG
WSGLVAGWYGFQHSNDQGVGMAADRDSTQKAIDKITSKVNNIVDKMNKQYEIIDHEFSEVETRLNMINNK
IDDQIQDIWAYNAELLVLLENQKTLDEHDANVNNLYNKVKRALGSNAXEDGKGCFELYHKCBDQCMETIR
NGTYNRXKYQEESKLERQRIEGVKLESEGTYKILTIYSTVASSLVIAMGFAAFLFWAMSNGSC

>QOJ99531.1 hemagglutinin, partial [Influenza A virus]
ETVSLITILLVATASNADKICIGYQSTNSTETVDTLTENNVPVTHAKELLHTEHNGMLCATSLGQPLILD
TCTIEGLIYGNPSCDLSLEGREWSYIVERPSAVXGLCYPGNVENLEELRSLFSSARSYQRIQXFPDTIWN
VSYDGTSTACSNSFYRSMRWLTRKDGNYPTQDAQYTNNQGKNILFMWGINHPPTDETQRXLYTRTDTTTS
VATEEINRIFKPLIGPRPLVNGLMGRIDYYWSVLKPGQTLRIKSDGNLIAPWYGHILSGESHGRILKTDL
KKGSCTVQCQTEKGGLNTTLPFQNVSKYAFGNCSKYIGIKSLKLAVGLRNVPSRSSRGLFGAIAGFIEGG
WSGLVAGWYGFQHSNDQGVGMAADRDSTQKAIDKITSKVNNIVDKMNKQYEIIDHEFSEVETRLNMINNK
IDDQIQDIWAYNAELLVLLENQKTLDEHDANVNNLYNKVKRALGSNAXEDGKGCFELYHKCBDQCMETIR
NGTYNRXKYQEESKLERQRIEGVKLESEGTYKILTIYSTVASSLVIAMGFAAFLFWAMSNGSC

>QOJ99530.1 hemagglutinin, partial [Influenza A virus]
METVSLITILVVATVSNADKICIGYQSTNSTETVDTLTENNVPVTHAKELLHTEHNGMLCATSLGHPLIL
DTCTIEGLIYGNPSCDPLLGGREWSYIVERPSAVNGLCYPGNVENLEELRSLFSSSRSYQRIQIFPDTIW
NVSYSGTSKACSDSFYRSMRWLTQKNNAYPTQDAQYTNNQGKNILFMWGINHPPTDTAQTNLYTRTDTTT
SVATEEMNRIFKPLIGPRPLVNGLMGRINYYWSVLKPGQTLRIKSDGNLIAPWYGHILSGESHGRILKTD

LKMGSCTVQCQTEKGGLNTTLPFQNVSKYAFGNCSKYIGVKSLKLAVGLRNVPSRSSRGLFGAIAGFIEG
GWSGLVAGWYGFQHSNDQGVGMAADRDSTQKAIDKITSKVNNIVDKMNKQYEIIDHEFSEVETRLNMIND
KVDDQIQDIWAYNAELLVLLENQKTLDEHDANVNNLYNKVKRALGSNAVEDGRGCFELYHKCDDHCMETI
RNGTYNRRKYQEESKLERQKIEGVKLESEETYKILTIYSTVASSLVIAM

>QOJ99529.1 hemagglutinin, partial [Influenza A virus]
METVSLITILVVATVSNADKICIGYQSTNSTETVDTLTENNVPVTHAKELLHTEHNGMLCATSLGHPLIL
DTCTIEGLIYGNPSCDPLLGGREWSYIVERPSAVNGLCYPGNVENLEELRSLFSSSRSYQRIQIFPDTIW
NVSYSGTSKACSDSFYRSMRWLTQKNNAYPTQDAQYTNNQGKNILFMWGINHPPTDTAQTNLYTRTDTTT
SVATEEMNRIFKPLIGPRPLVNGLMGRINYYWSVLKPGQTLRIKSDGNLIAPWYGHILSGESHGRILKTD
LKMGSCTVQCQTEKGGLNTTLPFQNVSKYAFGNCSKYIGVKSLKLAVGLRNVPSRSSRGLFGAIAGFIEG
GWSGLVAGWYGFQHSNDQGVGMAADRDSTQKAIDKITSKVNNIVDKMNKQYEIIDHEFSEVETRLNMIND
KVDDQIQDIWAYNAELLVLLENQKTLDEHDANVNNLYNKVKRALGSNAVEDGRGCFELYHKCDDHCMETI
RNGTYNRRKYQEESKLERQKIEGVKLESEETYKILTIYSTVASSLVIAM

>QOJ99528.1 hemagglutinin, partial [Influenza A virus]
ETVSLITILLVATXSNADKICIGYQSTNSTETVDTLTENNVPVTHAKELLHTEHNGMLCATSLGQPLILD
TCTIEGLIYGNPSCDLSLEGREWSYIVERPSAVNGLCYPGNVENLEELRSLFSSARSYQRIQXFPDTIWN
VSYDGTSTACSNSFYRSMRWLTRKDGNYPTQDAQYTNNQGKNILFMWGINHPPTDETQRXLYTRTDTTTS
VATEEINRIFKPLIGPRPLVNGLMGRIDYYWSVLKPGQTLRIKSDGNLIAPWYGHILSGESHGRILKTDL
KKGSCTVQCQTEKGGLNTTLPFQNVSKYAFGNCSKYIGIKSLKLAVGLRNVPSRSSRGLFGAIAGFIEGG
WSGLVAGWYGFQHSNDQGVGMAADRDSTQKAIDKITSKVNNIVDKMNKQYEIIDHEFSEVETRLNMINNK
IDDQIQDIWAYNAELLVLLENQKTLDEHDANVNNLYNKVKRALGSNAXEDGKGCFELYHKCBDQCMETIR
NGTYNRXKYQEESKLERQRIEGVKLESEGTYKILTIYSTVASSLVIAMGFAAFLFWAMSNGSC

>QOJ99527.1 hemagglutinin, partial [Influenza A virus]
ETVSLITILLVATXSNADKICIGYQSTNSTETVDTLTENNVPVTHAKELLHTEHNGMLCATSLGQPLILD
TCTIEGLIYGNPSCDLSLEGREWSYIVERPSAVXGLCYPGNVEBLEELRSLFSSARSYQRIQXFPDTIWN
VSYDGTSTACSNSFYRSMRWLTRKDGNYPTQDAQYTNNQGKNILFMWGINHPPTDETQRXLYTRTDTTTS
VATEEINRIFKPLIGPRPLVNGLMGRIDYYWSVLKPGQTLRIKSDGNLIAPWYGHILSGESHGRILKTDL
KXGSCTVQCQTEKGGLNTTLPFQNVSKYAFGNCSKYIGIKSLKLAVGLRNVPSRSSRGLFGAIAGFIEGG
WSGLVAGWYGFQHSNDQGVGMAADRDSTQKAIDKITSKVNNIVDKMNKQYEIIDHEFSEVETRLNMINNK
IDDQIQDIWAYNAELLVLLENQKTLDEHDANVNNLYNKVKRALGSNAXEDGKGCFELYHKCBDQCMETIR
NGTYNRXKYQEESKLERQRIEGVKLESEGTYKILTIYSTVASSLVIAMGFAAFLFWAMSNGSC

>QOJ99526.1 hemagglutinin, partial [Influenza A virus]

METVSLITILVVATVSNADKICIGYQSTNSTETVDTLTENNVPVTHAKELLHTEHNGMLCATSLGHPLIL
DTCTIEGLIYGNPSCDPLLGGREWSYIVERPSAVNGLCYPGNVENLEELRSLFSSSRSYQRIQIFPDTIW
NVSYSGTSKACSDSFYRSMRWLTQKNNAYPTQDAQYTNNQGKNILFMWGINHPPTDTAQTNLYTRTDTTT
SVATEEMNRIFKPLIGPRPLVNGLMGRINYYWSVLKPGQTLRIKSDGNLIAPWYGHILSGESHGRILKTD
LKMGSCTVQCQTEKGGLNTTLPFQNVSKYAFGNCSKYIGVKSLKLAVGLRNVPSRSSRGLFGAIAGFIEG
GWSGLVAGWYGFQHSNDQGVGMAADRDSTQKAIDKITSKVNNIVDKMNKQYEIIDHEFSEVETRLNMIND
KVDDQIQDIWAYNAELLVLLENQKTLDEHDANVNNLYNKVKRALGSNAVEDGRGCFELYHKCDDHCMETI
RNGTYNRRKYQEESKLERQKIEGVKLESEETYKILTIYSTVASSLVIAM

>QOJ99525.1 hemagglutinin, partial [Influenza A virus]

METVSLITILVVATVSNADKICIGYQSTNSTETVDTLTENNVPVTHAKELLHTEHNGMLCATSLGHPLIL
DTCTIEGLIYGNPSCDPLLGGREWSYIVERPSAVNGLCYPGNVENLEELRSLFSSSRSYQRIQIFPDTIW
NVSYSGTSKACSDSFYRSMRWLTQKNNAYPTQDAQYTNNQGKNILFMWGINHPPTDTTQTNLYTRTDTTT
SVATEEMNRIFKPLIGPRPLVNGLMGRINYYWSVLKPGQTLRIKSDGNLIAPWYGHILSGESHGRILKTD
LKRGSCTVQCQTEKGGLNTTLPFQNVSKYAFGNCSKYIGVKSLKLAVGLRNVPSRSSRGLFGAIAGFIEG
GWSGLVAGWYGFQHSNDQGVGMAADRDSTQKAIDKITSKVNNIVDKMNKQYEIIDHEFSEVETRLNMINN
KVDDQIQDIWAYNAELLVLLENQKTLDEHDANVNNLYNKVKRALGSNAVEDGKGCFELYHKCDDHCMETI
RNGTYNRRKYQEESKLERQKIEGVKLESEETYKILTIYSTVASSLVIAMGFAAFLFWAMSNGSC

>QOJ99524.1 hemagglutinin, partial [Influenza A virus]

METVSLITILVVATVSNADKICIGYQSTNSTETVDTLTENNVPVTHAKELLHTEHNGMLCATSLGHPLIL
DTCTIEGLIYGNPSCDPLLGGREWSYIVERPSAVNGLCYPGNVENLEELRSLFSSSRSYQRIQIFPDTIW
NVSYSGTSKACSDSFYRSMRWLTQKNNAYPTQDAQYTNNQGKNILFMWGINHPPTDTTQTNLYTRTDTTT
SVATEEMNRIFKPLIGPRPLVNGLMGRINYYWSVLKPGQTLRIKSDGNLIAPWYGHILSGESHGRILKTD
LKRGSCTVQCQTEKGGLNTTLPFQNVSKYAFGNCSKYIGVKSLKLAVGLRNVPSRSSRGLFGAIAGFIEG
GWSGLVAGWYGFQHSNDQGVGMAADRDSTQKAIDKITSKVNNIVDKMNKQYEIIDHEFSEVETRLNMINN
KVDDQIQDIWAYNAELLVLLENQKTLDEHDANVNNLYNKVKRALGSNAVEDGKGCFELYHKCDDHCMETI
RNGTYNRRKYQEESKLERQKIEGVKLESEETYKILTIYSTVASSLVIAMGFAAFLFWAMSNGSC

>QOJ99523.1 hemagglutinin, partial [Influenza A virus]

METVSLITILVVATVSNADKICIGYQSTNSTETVDTLTENNVPVTHAKELLHTEHNGMLCATSLGHPLIL
DTCTIEGLIYGNPSCDPLLGGREWSYIVERPSAVNGLCYPGNVENLEELRSLFSSSRSYQRIQIFPDTIW

NVSYSGTSKACSDSFYRSMRWLTQKNNAYPTQDAQYTNNQGKNILFMWGINHPPTDTTQTNLYTRTDTTT
SVATEEMNRIFKPLIGPRPLVNGLMGRINYYWSVLKPGQTLRIKSDGNLIAPWYGHILSGESHGRILKTD
LKRGSCTVQCQTEKGGLNTTLPFQNVSKYAFGNCSKYIGVKSLKLAVGLRNVPSRSSRGLFGAIAGFIEG
GWSGLVAGWYGFQHSNDQGVGMAADRDSTQKAIDKITSKVNNIVDKMNKQYEIIDHEFSEVETRLNMINN
KVDDQIQDIWAYNAELLVLLENQKTLDEHDANVNNLYNKVKRALGSNAVEDGKGCFELYHKCDDHCMETI
RNGTYNRRKYQEESKLERQKIEGVKLESEETYKILTIYSTVASSLVIAMGFAAFLFWAMSNGSC

>QOJ99522.1 hemagglutinin, partial [Influenza A virus]
METVSLITILVVATVSNADKICIGYQSTNSTETVDTLTENNVPVTHAKELLHTEHNGMLCATSLGHPLIL
DTCTIEGLIYGNPSCDPLLGGREWSYIVERPSAVNGLCYPGNVENLEELRSLFSSSRSYQRIQIFPDTIW
NVSYSGTSKACSDSFYRSMRWLTQKNNAYPTQDAQYTNNQGKNILFMWGINHPPTDTTQTNLYTRTDTTT
SVATEEMNRIFKPLIGPRPLVNGLMGRINYYWSVLKPGQTLRIKSDGNLIAPWYGHILSGESHGRILKTD
LKRGSCTVQCQTEKGGLNTTLPFQNVSKYAFGNCSKYIGVKSLKLAVGLRNVPSRSSRGLFGAIAGFIEG
GWSGLVAGWYGFQHSNDQGVGMAADRDSTQKAIDKITSKVNNIVDKMNKQYEIIDHEFSEVETRLNMINN
KVDDQIQDIWAYNAELLVLLENQKTLDEHDANVNNLYNKVKRALGSNAVEDGKGCFELYHKCDDHCMETI
RNGTYNRRKYQEESKLERQKIEGVKLESEETYKILTIYSTVASSLVIAMGFAAFLFWAMSNGSC

>QOJ99521.1 hemagglutinin, partial [Influenza A virus]
METVSLITILVVATVSNADKICIGYQSTNSTETVDTLTENNVPVTHAKELLHTEHNGMLCATSLGHPLIL
DTCTIEGLIYGNPSCDPLLGGREWSYIVERPSAVNGLCYPGNVENLEELRSLFSSSRSYQRIQIFPDTIW
NVSYSGTSKACSDSFYRSMRWLTQKNNAYPTQDAQYTNNQGKNILFMWGINHPPTDTXQTNLYTRTDTTT
SVATEEMNRIFKPLIGPRPLVNGLMGRINYYWSVLKPGQTLRIKSDGNLIAPWYGHILSGESHGRILKTD
LKXGSCTVQCQTEKGGLNTTLPFQNVSKYAFGNCSKYIGVKSLKLAVGLRNVPSRSSRGLFGAIAGFIEG
GWSGLVAGWYGFQHSNDQGVGMAADRXSTQKAIDKITSKVNNIVDKMNKQYEIIDHEFSEVETRLNMINB
KVDDQIQDIWAYNAELLVLLENQKTLDEHDANVNNLYNKVKRALGSNAVEDGXGCFELYHKCDDHCMETI
RNGTYNRRKYQEESKLERQKIEGVKLESEETYKILTIYSTVASSLVIAMGFAAFLFWAMSNGSC

>QOJ99520.1 hemagglutinin, partial [Influenza A virus]
METVSLITILVVATVSNADKICIGYQSTNSTETVDTLTENNVPVTHAKELLHTEHNGMLCATSLGHPLIL
DTCTIEGLIYGNPSCDXLLGGREWSYIVERPSAVNGLCYPGNVENLEELRSLFSSSRSYQRIQIFPDTIW
NVSYSGTSKACSDSFYRSMRWLTQKNNAYPTQDAQYTNNQGKNILFMWGINHPPTDTXQTNLYTRTDTTT
SVATEEMNRIFKPLIGPRPLVNGLMGRINYYWSVLKPGQTLRIKSDGNLIAPWYGHIJSGESHGRILKTD
LKXGSCTVQCQTEKGGLNTTLPFQNVSKYAFGNCSKYIGVKSLKLAVGLRNVPSRSSRGLFGAIAGFIEG
GWSGLVAGWYGFQHSNDQGVGMAADRXSTQKAIDKITSKVNNIVDKMNKQYEIIDHEFSEVETRLNMINB

KVDDQIQDIWAYNAELLVLLENQKTLDEHDANVNNLYNKVKRALGSNAVEDGXGCFELYHKCDDHCMETI
RNGTYNRRKYQEESKLERQKIEGVKLESEETYKILTIYSTVASSLVIAMGFAAFLFWAMSNGSC

>QOJ99519.1 hemagglutinin, partial [Influenza A virus]
METVSLITILVVATVSNADKICIGYQSTNSTETVDTLTENNVPVTHAKELLHTEHNGMLCATSLGHPLIL
DTCTIEGLIYGNPSCDXLLGGREWSYIVERPSAVNGLCYPGNVENLEELRSLFSSSRSYQRIQIFPDTIW
NVSYSGTSKACSDSFYRSMRWLTQKNNAYPTQDAQYTNNQGKNILFMWGINHPPTDTXQTNLYTRTDTTT
SVATEEMNRIFKPLIGPRPLVNGLMGRINYYWSVLKPGQTLRIKSDGNLIAPWYGHIJSGESHGRILKTD
LKXGSCTVQCQTEKGGLNTTLPFQNVSKYAFGNCSKYIGVKSLKLAVGLRNVPSRSSRGLFGAIAGFIEG
GWSGLVAGWYGFQHSNDQGVGMAADRXSTQKAIDKITSKVNNIVDKMNKQYEIIDHEFSEVETRLNMINB
KVDDQIQDIWAYNAELLVLLENQKTLDEHDANVNNLYNKVKRALGSNAVEDGRGCFELYHKCDDHCMETI
RNGTYNRRKYQEESKLERQKIEGVKLESEETYKILTIYSTVASSLVIAMGFAAFLFWAMSNGSC

>QOJ99518.1 hemagglutinin, partial [Influenza A virus]
METVSLITILVVATVSNADKICIGYQSTNSTETVDTLTENNVPVTHAKELLHTEHNGMLCATSLGHPLIL
DTCTIEGLIYGNPSCDXLLGGREWSYIVERPSAVNGLCYPGNVENLEELRSLFSSSRSYQRIQIFPDTIW
NVSYSGTSKACSDSFYRSMRWLTQKNNAYPTQDAQYTNNQGKNILFMWGINHPPTDTXQTNLYTRTDTTT
SVATEEMNRIFKPLIGPRPLVNGLMGRINYYWSVLKPGQTLRIKSDGNLIAPWYGHIJSGESHGRILKTD
LKXGSCTVQCQTEKGGLNTTLPFQNVSKYAFGNCSKYIGVKSLKLAVGLRNVPSRSSRGLFGAIAGFIEG
GWSGLVAGWYGFQHSNDQGVGMAADRXSTQKAIDKITSKVNNIVDKMNKQYEIIDHEFSEVETRLNMINB
KVDDQIQDIWAYNAELLVLLENQKTLDEHDANVNNLYNKVKRALGSNAVEDGXGCFELYHKCDDHCMETI
RNGTYNRRKYQEESKLERQKIEGVKLESEETYKILTIYSTVASSLVIAMGFAAFLFWAMSNGSC

>QOJ99517.1 hemagglutinin, partial [Influenza A virus]
METVSLITILVVATVSNADKICIGYQSTNSTETVDTLTENNVPVTHAKELLHTEHNGMLCATSLGHPLIL
DTCTIEGLIYGNPSCDPLLGGREWSYIVERPSAVNGLCYPGNVENLEELRSLFSSSRSYQRIQIFPDTIW
NVSYSGTSKACSDSFYRSMRWLTQKNNAYPTQDAQYTNNQGKNILFMWGINHPPTDTAQTNLYTRTDTTT
SVATEEMNRIFKPLIGPRPLVNGLMGRINYYWSVLKPGQTLRIKSDGNLIAPWYGHILSGESHGRILKTD
LKMGSCTVQCQTEKGGLNTTLPFQNVSKYAFGNCSKYIGVKSLKLAVGLRNVPSRSSRGLFGAIAGFIEG
GWSGLVAGWYGFQHSNDQGVGMAADRDSTQKAIDKITSKVNNIVDKMNKQYEIIDHEFSEVETRLNMIND
KVDDQIQDIWAYNAELLVLLENQKTLDEHDANVNNLYNKVKRALGSNAVEDGRGCFELYHKCDDHCMETI
RNGTYNRRKYQEESKLERQKIEGVKLESEETYKILTIYSTVASSLVIAM

>QOJ99516.1 hemagglutinin, partial [Influenza A virus]

METVSLITILVVATVSNADKICIGYQSTNSTETVDTLTENNVPVTHAKELLHTEHNGMLCATSLGHPLIL
DTCTIEGLIYGNPSCDPLLGGREWSYIVERPSAVNGLCYPGNVENLEELRSLFSSSRSYQRIQIFPDTIW
NVSYSGTSKACSDSFYRSMRWLTQKNNAYPTQDAQYTNNQGKNILFMWGINHPPTDTTQTNLYTRTDTTT
SVATEEMNRIFKPLIGPRPLVNGLMGRINYYWSVLKPGQTLRIKSDGNLIAPWYGHILSGESHGRILKTD
LKRGSCTVQCQTEKGGLNTTLPFQNVSKYAFGNCSKYIGVKSLKLAVGLRNVPSRSSRGLFGAIAGFIEG
GWSGLVAGWYGFQHSNDQGVGMAADRDSTQKAIDKITSKVNNIVDKMNKQYEIIDHEFSEVETRLNMINN
KVDDQIQDIWAYNAELLVLLENQKTLDEHDANVNNLYNKVKRALGSNAVEDGKGCFELYHKCDDHCMETI
RNGTYNRRKYQEESKLERQKIEGVKLESEETYKILTIYSTVASSLVIAMGFAAFLFWAMSNGSC

>QOJ99515.1 hemagglutinin, partial [Influenza A virus]
METVSLITILVVATVSNADKICIGYQSTNSTETVDTLTENNVPVTHAKELLHTEHNGMLCATSLGHPLIL
DTCTIEGLIYGNPSCDPLLGGREWSYIVERPSAVNGLCYPGNVENLEELRSLFSSSRSYQRIQIFPDTIW
NVSYSGTSKACSDSFYRSMRWLTQKNNAYPTQDAQYTNNQGKNILFMWGINHPPTDTTQTNLYTRTDTTT
SVATEEMNRIFKPLIGPRPLVNGLMGRINYYWSVLKPGQTLRIKSDGNLIAPWYGHILSGESHGRILKTD
LKRGSCTVQCQTEKGGLNTTLPFQNVSKYAFGNCSKYIGVKSLKLAVGLRNVPSRSSRGLFGAIAGFIEG
GWSGLVAGWYGFQHSNDQGVGMAADRDSTQKAIDKITSKVNNIVDKMNKQYEIIDHEFSEVETRLNMINN
KVDDQIQDIWAYNAELLVLLENQKTLDEHDANVNNLYNKVKRALGSNAVEDGKGCFELYHKCDDHCMETI
RNGTYNRRKYQEESKLERQKIEGVKLESEETYKILTIYSTVASSLVIAMGFAAFLFWAMSNGSC

>QOJ99511.1 hemagglutinin, partial [Influenza A virus]
METVSLITILVVATLSNADKICIGYQSTNSTETVDTLTENNVPVTHAKELLHTEHNGMLCATSLGHPLIL
DTCTVEGLIYGNPSCDPLLGGREWSYIVERPSAVNGLCYPGNVENLEELRSLFSSSRSYQRIQIFPDTIW
NVSYSGTSKACSDSFYRSMRWLTQKNNAYPTQDAQYTNNQGKNILFMWGINHPPTDTAQTNLYTRTDTTT
SVATEEMNRIFKPLIGPRPLVNGLMGRINYYWSVLKPGQTLRIKSDGNLIAPWYGHILSGESHGRILKTD
LKMGSCTVQCQTEKGGLNTTLPFQNVSKYAFGNCSKYIGVKSLKLAVGLRNVPSRSSRGLFGAIAGFIEG
GWSGLVAGWYGFQHSNDQGVGMAADRDSTQKAIDKITSKVNNIVDKMNKQYEIIDHEFSEVETRLNMIND
KIDDQIQDIWAYNAELLVLLENQKTLDEHDANVNNLYNKVKRALGSNAVEDGRGCFELYHKCDDHCMETI
RNGTYNRRKYQEESKLERQKIEGVKLESEETYKILTIYSTVASSLVIAM

>QOJ99510.1 hemagglutinin, partial [Influenza A virus]
METVSLITILVVATVSNADKICIGYQSTNSTETVDTLTENNVPVTHAKELLHTEHNGMLCATSLGHPLIL
DTCTIEGLIYGNPSCDPLLGGREWSYIVERPSAVNGLCYPGNVENLEELRSLFSSSRSYQRIQIFPDTIW
NVSYSGTSKACSDSFYRSMRWLTQKNNAYPTQDAQYTNNQGKNILFMWGINHPPTDTAQTNLYTRTDTTT
SVATEEINRIFKPLIGPRPLVNGLMGRINYYWSVLKPGQTLRIKSDGNLIAPWYGHILSGESHGRILKTD

LKMGSCTVQCQTEKGGLNTTLPFQNVSKYAFGNCSKYIGVKSLKLAVGLRNVPSRSSRGLFGAIAGFIEG
GWSGLVAGWYGFQHSNDQGVGMAADRDSTQKAIDKITSKVNNIVDKMNKQYEIIDHEFSEVETRLNMIND
KVDDQIQDIWAYNAELLVLLENQKTLDEHDANVNNLYNKVKRALGSNAVEDGRGCFELYHKCDDHCMETI
RNGTYNRRKYQEESKLERQKIEGVKLESEETYKILTIYSTVASSLVIAMGFAAFLFWAMSNGSC

>QOJ99509.1 hemagglutinin, partial [Influenza A virus]
METVSLITILVVATLSNADKICIGYQSTNSTETVDTLTENNVPVTHAKELLHTEHNGMLCATSLGHPLIL
DTCTVEGLIYGNPSCDPLLGGREWSYIVERPSAVNGLCYPGNVENLEELRSLFSSSRSYQRIQIFPDTIW
NVSYSGTSKACSDSFYRSMRWLTQKNNAYPTQDAQYTNNQGKNILFMWGINHPPTDTAQTNLYTRTDTTT
SVATEEMNRIFKPLIGPRPLVNGLMGRINYYWSVLKPGQTLRIKSDGNLIAPWYGHILSGESHGRILKTD
LKMGSCTVQCQTEKGGLNKTLPFQNVSKYAFGNCSKYIGVKSLKLAVGLRNVPSRSSRGLFGAIAGFIEG
GWSGLVAGWYGFQHSNDQGVGMAADRDSTQKAIDKITSKVNNIVDKMNKQYEIIDHEFSEVETRLNMIND
KIDDQIQDIWAYNAELLVLLENQKTLDEHDANVNNLYNKVKRALGSNAVEDGRGCFELYHKCDDHCMETI
RNGTYNRRKYQEESKLERQKIEGVKLESEETYKILTIYSTVASSLVIAM

>QOJ99508.1 hemagglutinin [Influenza A virus]
METVSLITILLAATVSNADKICIGYQSTNSTETVDTLTENNVPVTHAKELLHTEHNGMLCATSLGQPLIL
DTCTIEGLIYGNPSCDPLLEEREWSYIVERPSAVNGLCYPGNVENLEELRSFFSSARSYQRIQIFPDTIW
NVSYDGTSNTCSGSFYRNMRWLTRKNGNYPIQDAQYTNNQGKSILFXWGINHPPTDDTQRNLYTRTDTTT
SVATEEINRIFKPLIGPRPLVNGLMGRINYYWSVLKPGQTLRIKSDGNLIAPWYGYILSGESHGRILRTD
LKRGSCTVQCQTEKGGLNTTLPFQNISKYAFGNCSKYIGIKSLKLAVGLRNVPSRSSRGLFGAIAGFIEG
GWSGLVAGWYGFQHSNDQGVGMAADRESTQKAIDKITSKVNNIVDKMNKQYEIIDHEFSEVETRLNMINN
KIDDQIQDIWAYNAELLVLLENQKTLDEHDANVNNLYNKVKRALGSNAVEDGKGCFELYHKCDDQCMETI
RNGTYNRRKYQEESKLERQKIEGVKLESEGTYKILTIYSTVASSLVIAMGFAAFLFWAMSNGSCRCNICI

>QOJ99507.1 hemagglutinin, partial [Influenza A virus]
ETVSLITILXVATVSNADKICIGYQSTNSTETVDTLTENNVPVTHAKELLHTEHNGMLCATXLGHPLILD
TCTIEGLIYGNPSCDPLLGGREWSYIVERPSAVNGLCYPGNVENLEELRSLFSSSRSYQRIQIFPDTIWN
VSYSGTSKACSDSFYRSMRWLTQKNNAYPTQDAQYTNNQGKNILFMWGINHPPTDTAQTNLYTRTDTTTS
VATEEXNRIFKPLIGPRPLVNGLMGRINYYWSVLKPGQTLRIKSDGNLIAPWYGHILSGESHGRILKTDL
KXGSCTVQCQTEXGGLNTTLPFQNVSKYAFGNCSKYIGVKSLKLAVGLRNVPSRSSRGLFGAIAGFIEGG
WXGLVAGWYGFQHSNDQGVGMAADRDSTQKAIDKITSKVNNIVXKMNKQYEIIDHEFSEVETRLNMINBK
VDDQIQDIWAYNAELLVLLENQKTLDEHDANVNNLYNKVKRALGSNAVEDGXGCFELYHKCDDHCMETIR
NGTYNRRKYQEESKLERQKIEGVKLESEETYKILTIYSTVASSLVIAMGFAAFLFWAMSNGSCRCNICI

>QOJ99506.1 hemagglutinin [Influenza A virus]

METVSLITILXVATVSNADKICIGYQSTNSTETVDTLTENNVPVTHAKELLHTEHNGMLCATXLGHPLIL
DTCTIEGLIYGNPSCDPLLGGREWSYIVERPSAVNGLCYPGNVENLEELRSLFSSSRSYQRIQIFPDTIW
NVSYSGTSKACSDSFYRSMRWLTQKNNAYPTQDAQYTNNQGKNILFMWGINHPPTDTAQTNLYTRTDTTT
SVATEEXNRIFKPLIGPRPLVNGLMGRINYYWSVLKPGQTLRIKSDGNLIAPWYGHILSGESHGRILKTD
LKXGSCTVQCQTEKGGLNTTLPFQNVSKYAFGNCSKYIGXKSLKLAVGLRNVPSRSSRGLFGAIAGFIEG
GWXGLVAGWYGFQHSNDQGVGMAADRDSTQKAIDKITSKVNNIVXKMNKQYEIIDHEFSEVETRLNMINB
KXDDQIQDIWAYNAELLVLLENQKTLDEHDANVNNLYNKVKRALGSNAVEDGXGCFELYHKCDDHCMETI
RNGTYNRRKYQEESKLERQKIEGVKLESEETYKILTIYSTVASSLVIAMGFAAFLFWAMSNGSCRCNICI

>QOJ99505.1 hemagglutinin [Influenza A virus]

METVSLITILXVATVSNADKICIGYQSTNSTETVDTLTENNVPVTHAKELLHTEHNGMLCATXLGHPLIL
DTCTIEGLIYGNPSCDPLLGGREWSYIVERPSAVNGLCYPGNVENLEELRSLFSSSRSYQRIQIFPDTIW
NVSYSGTSKACSDSFYRSMRWLTQKNNAYPTQDAQYTNNQGKNILFMWGINHPPTDTAQTNLYTRTDTTT
SVATEEXNRIFKPLIGPRPLVNGLMGRINYYWSVLKPGQTLRIKSDGNLIAPWYGHILSGESHGRILKTD
LKXGSCTVQCQTEKGGLNTTLPFQNVSKYAFGNCSKYIGXKSLKLAVGLRNVPSRSSRGLFGAIAGFIEG
GWXGLVAGWYGFQHSNDQGVGMAADRDSTQKAIDKITSKVNNIVXKMNKQYEIIDHEFSEVETRLNMINB
KXDDQIQDIWAYNAELLVLLENQKTLDEHDANVNNLYNKVKRALGSNAVEDGXGCFELYHKCDDHCMETI
RNGTYNRRKYQEESKLERQKIEGVKLESEXTYKILTIYSTVASSLVIAMGFAAFLFWAMSNGSCRCNICI

>QOJ99504.1 hemagglutinin [Influenza A virus]

METVSLITILVVATVSNADKICIGYQSTNSTETVDTLTENNVPVTHAKELLHTEHNGMLCATSLGHPLIL
DTCTIEGLIYGNPSCDPLLGGREWSYIVERPSAVNGLCYPGNVENLEELRSLFSSSRSYQRIQIFPDTIW
NVSYSGTSKACSDSFYRSMRWLTQKNNAYPTQDAQYTNNQGKNILFMWGINHPPTDTAQTNLYTRTDTTT
SVATEEXNRIFKPLIGPRPLVNGLMGRINYYWSVLKPGQTLRIKSDGNLIAPWYGHILSGESHGRILKTD
LKXGSCTVQCQTEKGGLNXTLPFQNVSKYAFGNCSKYIGVKSLKLAVGLRNVPSRSSRGLXGAIAGFIEG
GWSGLVAGWYGFQHSNDQGVGMAADRDSTQKAIDKITSKVNNIVDKMNKQYEIIDHEFSEVETRLNMINB
KVDDQIQDIWAYNAELLVLLENQKTLDEHDANVNNLYNKVKRALGSNAVEDGXGCFELYHKCDDHCMETI
RNGTYNRRKYQEESKLERQKIEGVKLESEETYKILTIYSTVASSLVIAMGFAAFLFWAMSNGSCRCNICI

>QOJ99502.1 hemagglutinin [Influenza A virus]

METVSLITILXXATVSNADKICIGYQSTNSTETVDTLTENNVPVTHAKELLHTEHNGMLCATXLGXPLIL
DTCTIEGLIYGNPSCDLSLEGREWSYIVERPSAVXGLCYPGNVEBLEELRSLFSSARSYQRIQIFPDTXW

NVSYDGTSTACSGSFYXSMRWLTRKNGEYPXQDAQYTNNQGKNILFMWGINHPPTDDTQRGLYTRTDTTT
SVATEEINRIFKPLIGPRPLVNGLMGRINYYWSXLKPGQTLRIKSDGNLIAPWYGHILSGESHGRILKTD
LKRGSCTVQCQTEKGGLNTTLPFQNVSKYAFGNCSKYIGXKSLKLAVGLRNVPSRSSRGLFGAIAGFIEG
GWXGLVAGWYGFQHSNDQGVGMAADRDSTQKAIDKITSKVNNIVXKMNKQYEIIDHEFSEVETRLNMINB
KXDDQIQDIWAYNAELLVLLENQKTLDEHDANVNNLYNKVKRALGSNAVEDGKGCFELYHKCDDXCMETI
RNGTYNRRXYQEESKLERQXIEGVKLESEXTYKILTIYSTVASSLVIAMGFAAFLFWAMSNGSCRCNICI

>QOJ99488.1 hemagglutinin [Influenza A virus]
METVSLITILLVATVSNADKICIGYQSSNSTETVDTLTENNVPVTHAKELLHTEHNGMLCATSLGQPLIL
DTCTIEGLIYGNPSCDLSLEGREWSYIVERPSAVHGLCYPGNVEDLEELRSLFSSARSYQRIQIFPDTIW
NVSYDGTSTACSGSFYKSMRWLTRKNGEYPTQDAQYTNNQGKNILFMWGINHPPTDDTQRGLYTRTDTTT
SVATEEINRIFKPLIGPRPLVNGLMGRINYYWSVLKPGQTLRIKSDGNLIAPWYGHILSGESHGRILKTD
LKRGSCTVQCQTEKGGLNTTLPFQNVSKYAFGNCSKYIGVKSLKLAVGLRNVPSRSSRGLFGAIAGFIEG
GWPGLVAGWYGFQHSNDQGVGMAADRDSTQKAIDKITSKVNNIVDKMNKQYEIIDHEFSEVETRLNMINN
KIDDQIQDIWAYNAELLVLLENQKTLDEHDANVNNLYNKVKRALGSNAVEDGKGCFELYHKCDDQCMETI
RNGTYNRRKYQEESKLERQRIEGVKLESEGTYKILTIYSTVASSLVIAMGFAAFLFWAMSNGSCRCNICI

>QOJ99487.1 hemagglutinin, partial [Influenza A virus]
METVSLITILVVATLSNADKICIGYQSTNSTETVDTLTENNVPVTHAKELLHTEHNGMLCATSLGHPLIL
DTCTIEGLIYGNPSCDPLLGGREWSYIVERPSAVNGLCYPGNVENLEELRSLFSSSRSYQRIQIFPDTIW
NVSYSGTSKACSDSFYRSMRWLTQKNNAYPTQDAQYTNNQGKNILFMWGINHPPTDTAQTNLYTRTDTTT
SVATEEMNRIFKPLIGPRPLVNGLMGRINYYWSVLKPGQTLRIKSDGNLIAPWYGHILSGESHGRILKTD
LKMGSCTVQCQTEKGGLNTTLPFQNVSKYAFGNCSKYIGVKSLKLAVGLRNVPSRSSRGLFGAIAGFIEG
GWSGLVAGWYGFQHSNDQGVGMAADRDSTQKAIDKITSKVNNIVDKMNKQYEIIDHEFSEVETRLNMIND
KIDDQIQDIWAYNAELLVLLENQKTLDEHDANVNNLYNKVKRALGSNAVEDGRGCFELYHKCDDHCMETI
RNGTYNRRKYQEESKLERQKIEGVKLESEETYKILTIYSTVASSLVIAM

>QOJ99486.1 hemagglutinin, partial [Influenza A virus]
METVSLITILVVATLSNADKICIGYQSTNSTETVDTLTENNVPVTHAKELLHTEHNGMLCATSLGHPLIL
DTCTIEGLIYGNPSCDPLLGGREWSYIVERPSAVNGLCYPGNVENLEELRSLFSSSRSYQRIQIFPDTIW
NVSYSGTSKACSDSFYRSMRWLTQKNNAYPTQDAQYTNNQGKNILFMWGINHPPTDTAQTNLYTRTDTTT
SVATEEMNRIFKPLIGPRPLVNGLMGRINYYWSVLKPGQTLRIKSDGNLIAPWYGHILSGESHGRILKTD
LKMGSCTVQCQTEKGGLNTTLPFQNVSKYAFGNCSKYIGVKSLKLAVGLRNVPSRSSRGLFGAIAGFIEG
GWSGLVAGWYGFQHSNDQGVGMAADRDSTQKAIDKITSKVNNIVDKMNKQYEIIDHEFSEVETRLNMIND

KIDDQIQDIWAYNAELLVLLENQKTLDEHDANVNNLYNKVKRALGSNAVEDGRGCFELYHKCDDHCMETI
RNGTYNRRKYQEESKLERQKIEGVKLESEETYKILTIYSTVASSLVIAM

>QOJ99485.1 hemagglutinin, partial [Influenza A virus]
METVSLITILVVATVSNADKICIGYQSTNSTETVDTLTENNVPVTHAKELLHTEHNGMLCATSLGHPLIL
DTCTIEGLIYGNPSCDPLLGGREWSYIVERPSAVNGLCYPGNVENLEELRSLFSSSRSYQRIQIFPDTIW
NVSYSGTSKACSDSFYRSMRWLXQKNNXYPTQDAQYTNNQGKNILFMWGINHPPTDTAQTNLYTRTDTTT
SVATEEMNRIFKPLIGPRXXXXXXXXXXXXXXXXXXXXXXXXXXXXXXXXXXXXXGHILSGESHGRILKTD
LKRGSCTVQCQTEKGGLNTTLPFQNVSKYAFGNCSKYIGXKSLKLAVGLRNVPSRSSRGLFGAIAGFIEG
GWPGLVAGWYGFRHSNDQGVGMAADRDSTQKAIDKITSKVNNIVDKMNKQYEIIDHEFSEVETRLNMINN
KVDDQIQDIWAYNAELLVLLENQKTLDEHDANVNNLYNKVKRALGSNAVEDGKGCFELYHKCDDHCMETI
RNGTYNRRKYQEESKLERQKIEGVKLESEETYKILTIYSTVASSLVIAMGFAAFLFWAMSNGSC

>QOJ99484.1 hemagglutinin, partial [Influenza A virus]
ETVSLITILLVATASNADKICIGYQSTNSTETVDTLTENNVPVTHAKELLHTEHNGMLCATSLGQPLILD
TCTIEGLIYGNPSCDLSLEGREWSYIVERPSAVNGLCYPGNVENLEELRSLFSSARSYQRIQIFPDTIWN
VSYDGTSTACSNSFYRSMRWLTRKDGNYPTQDAQYTNNQGKNILFMWGINHPPTDETQRNLYTRTDTTTS
VATEEINRIFKPLIGPRPLVNGLMGRIDYYWSVLKPGQTLRIKSDGNLIAPWYGHILSGESHGRILKTDL
KKGSCTVQCQTEKGGLNTTLPFQNVSKYAFGNCSKYIGXKSLKLAVGLRNVPSRSSRGLFGAIAGFIEGG
WSGLVAGWYGFQHSNDQGVGMAADRDSTQKAIDKITSKVNNIVDKMNKQYEIIDHEFSEVETRLNMINNK
IDDQIQDIWAYNAELLVLLENQKTLDEHDANVNNLYNKVKRALGSNAVEDGKGCFELYHKCBDQCMETIR
NGTYNRKKYQEESKLERQXIEGVKLESEGTYKILTIYSTVASSLVIAMGXAAFLFWAMSN

>QOJ99483.1 hemagglutinin, partial [Influenza A virus]
ETVSLITILLVATASNADKICIGYQSTNSTETVDTLTENNVPVTHAKELLHTEHNGMLCATSLGQPLILD
TCTIEGLIYGNPSCDLSLEGREWSYIVERPSAVXGLCYPGNVEBLEELRSLFSSARSYQRIQIFPDTIWN
VSYDGTSTACSDSFYRSMRWLTRKDXXXXXXXXXXXXXXXXKNILFMWGINHPPTDDTQRSLYTRTDTTTS
VATEEINRIFKPLIGPRPLVNGLMGRIBYYWSVLKPGQTLRIKSDGNLIAPWYGYILSGESHGRILXTDL
KRGSCTVQCQTEKGGLNTTXPFQNVSKYAFGNCSKYIGIKSLKLAVGLRNVPSRSSRGLXGAIAGFIEGG
WSGLVAGWYGFQHSNDQGVGMAADRDSTQKAIDKITSKVNNIVDKMNKQYEIIDHEFSEVETRLNMINNK
IDDQIQDIWAYNAELLVLLENQKTLDEHDANVNNLYNKVKRALGSNAVEDGKGCFELYHKCDDQCMETIR
NGTYNRXKYQEESKLERQRIEGVKLESEGTYKILTIYSTVASSLVIAMGFAAFLFWAMSNG

>QOJ99482.1 hemagglutinin, partial [Influenza A virus]

METVSLITILVVATVSNADKICIGYQSTNSTETVDTLTENNVPVTHAKELLHTEHNGMLCATSLGHPLIL
DTCTIEGLIYGNPSCDPLLRGREWSYIVERPSAVNGLCYPGNVENLEELRSLFSSSRSYQRIQIFPDTIW
NVSYSGTSKACSDSFYRSMRWLTQKNNAYPTQDAQYTNNQGKNILFMWGINHPPTDTAQTNLYTRTDTTT
SVATEEMNRIFKPLIGPRPLVNGLMGRINYYWSVLKPGQTXRIKSDGNLIAPWYGHILSGESHGRILKTD
LKRGSCTVQCQTEKGGLNTTLPFQNVSKYAFGNCSKYIGVKSLKLAVGLRNVPSRSSRGLFGAIAGFIEG
GWSGLVAGWYGFQHSNDQGVGMAADXDSTQKAIDKITSKVNNIVDKMNKQYEIIDHEFSEVETRLNMINN
KXDDQIQDIWAYNAELLVLLENQKTLDEHDANVNNLYNKVKRALGSNAVEDGKGCFELYHKCDDHCMETI
RNGTYNRRKYQEESKLERQKIEGVKLESEETYKILTIYSTVASSLVIAMGFAAFLFWAMSNGSC

>QOJ99480.1 hemagglutinin, partial [Influenza A virus]
METVSLITILLVATVSXADKICIGYQSTNSTETVDTLTENNVPVTHAKELLXTEHNGMLCATXLGQPLIL
DTCTIEGLIYGNPSCDLSLEGREWSYIVERPSAVNGLCYPGNVENLEELRSLFSSARSYQRVQIFPDTTW
NVSYDGTSTACSGSFYRSMRWLTRKDGNYPTQDAQYTNNQGKNILFMWGINNPPTDDTQRSLYTRTDTTT
SVATEEINRIFKPLIGPRPLVNGLMGRIDYYWSVLKPGQTLRIKSDGNLXAPWYGYILSGESHGRILXTX
LKRGSCTVQCQTEKGGLNTTLPFQNVSKYAFGNCSKYIGIKSLKLAVGLRNVPSRSSXGLFGAIAGFIEG
GWSGLVAGWYGFQHSNDQGVGMAADRXSTQKAXDKITSKVNNIVDKMNKQYEIIDHEFSEVETRLNMINN
KIDDQIQDIWAYNAELLVLLENQKTLDEHDANVNNLYNKVKRALGSNAVEDGKGCFELYHKCDDQCMETI
RNGTYNRKKYQEESKLERQKIEGVKLESEGTYKILTIYSTVASSLVIAM

>QOJ99479.1 hemagglutinin, partial [Influenza A virus]
METVSLITILLVATVSNADKICIGYQSTNSTETVDTLTENNVPVTHAKELLHTEHNGMLCATSLGHPLIL
DTCTIEGLIYGNPSCDPLLGGREWSYIVERPSAVNGLCYPGNVENLEELRSLFSSSRSYQRIQIFPDTIW
NVSYSGTSKACSDSFYRSMRWLTQKNNAYPTQDAQYTNNQGKNILFMWGINHPPTXTTQTNLYTRTDTTT
SVATEEMNRIFKPLIGPRPLVNGLMGRINYYWSVLXXXXXXXXXXXXXXXXXXXXXXXXXXXXXXXXXX
XXXXXXXXXXXXXXXXXXXXXXXXXXXXXXXXXXXXXXXXXXXXXXXXXXXXXVPSRSSRGLFGAIAGFIEG
GWSGLVAGWYGFQHSNDQGVGMAADRDSTQKAIDKITSKVNNIVDKMNKQYEXIDHEFSEVETRLNMINN
KVDDQIQDIWAYNAELLVLLENQKTLDEHDANVNNLYNKVKRALGSNAVEDGKGCFELYHKCDDXCMETI
RNGTYNRRKYQEESKLERQXIEGVKLESEXTYKILTIYSTVASSLVIAMGF

>QOJ99478.1 hemagglutinin [Influenza A virus]
METVSLITILLVATVSNADKICIGYQSSNSTETVDTLTENNVPVTHAKELLHTEHNGMLCATSLGQPLIL
DTCTIEGLIYGNPSCDLSLEGREWSYIVERPSAVHGLCYPGNVEDLEELRSLFSSARSYQRIQIFPDTIW
NVSYDGTSTACSGSFYKSMRWLTRKNGEYPTQDAQYTNNQGKNILFMWGINHPPTDDTQRGLYTRTDTTT
SVATEEINRIFKPLIGPRPLVNGLMGRINYYWSVLKPGQTLRIKSDGNLIAPWYGHILSGESHGRILKTD

LKRGSCTVQCQTEKGGLNTTLPFQNVSKYAFGNCSKYIGVKSLKLAVGLRNVPSRSSRGLFGAIAGFIEG
GWXGLVAGWYGFQHSNDQGVGMAADRDSTQKAIDKITSKVNNIVDKMNKQYEIIDHEFSEVETRLNMINN
KIDDQIQDIWAYNAELLVLLENQKTLDEHDANVNNLYNKVKRALGSNAVEDGKGCFELYHKCDDQCMETI
RNGTYNRRKYQEESKLERQRIEGVKLESEGTYKILTIYSTVASSLVIAMGFAAFLFWAMSNGSCRCNICI

>QOJ99477.1 hemagglutinin, partial [Influenza A virus]
ETVSLITILLVATVSNADKICIGYQSTNSTETVDTLTENNVPVTHAKELLHTEHNGMLCATSLGQPLILD
TCTIEGLIYGNPSCDLSLEGREWSYIVERPSAVNGLCYPGNVENLEELRSLFSSARSYQRIQIFPDTIWN
VSYDGTSTACSNSFYRSMRWLTRKDGNYPTQDAQYTNNQGKNILFMWGINHPPTDETQRNLYTRTDTTTS
VATEEINRIFKPLIGPRPLVNGLMGRIDYYWSVLKPGQTLRIKSDGNLIAPWYGHILSGESHGRILKTDL
KKGSCTVQCQTEKGGLNTTLPFQNVSKYAFGNCSKYIGXKSLKLAVGLRNVPSRSSRGLFGAIAGFIEGG
WSGLVAGWYGFQHSNDQGVGMAADRDSTQKAIDKITSKVNNIVDKMNKQYEIIDHEFSEVETRLNMINNK
XDDQIQDIWAYNAELLVLLENQKTLDEHDANVNNLYNKVKRALGSNAVEDGKGCFELYHKCNDQCMETIR
NGTYNRKKYQEESKLERQRIEGVKLESEGTYKILTIYSTVASSLVIAMGXAAFLFWAMSNGSC

>QOJ99476.1 hemagglutinin, partial [Influenza A virus]
ETVSLITILLVATVSNADKICIGYQSTNSTETVDTLTENNVPVTHAKELLHTEHNGMLCATSLGQPLILD
TCTIEGLIYGNPSCDLSLEGREWSYIVERPSAVNGLCYPGNVENLEELRSLFSSARSYQRIQIFPDTIWN
VSYDGTSTACSNSFYRSMRWLTRKDGNYPTQDAQYTNNQGKNILFMWGINHPPTDETQRNLYTRTDTTTS
VATEEINRIFKPLIGPRPLVNGLMGRIDYYWSVLKPGQTLRIKSDGNLIAPWYGHILSGESHGRILKTDL
KKGSCTVQCQTEKGGLNTTLPFQNVSKYAFGNCSKYIGIKSLKLAVGLRNVPSRSSRGLFGAIAGFIEGG
WSGLVAGWYGFQHSNDQGVGMAADRDSTQKAIDKITSKVNNIVDKMNKQYEIIDHEFSEVETRLNMINNK
IDDQIQDIWAYNAELLVLLENQKTLDEHDANVNNLYNKVKRALGSNAVEDGKGCFELYHKCNDQCMETIR
NGTYNRKKYQEESKLERQRIEGVKLESEGTYKILTIYSTVASSLVIAMGLAAFLFWAMSNGSC

>QOJ99475.1 hemagglutinin, partial [Influenza A virus]
ETVSLITILLVATVSNADKICIGYQSTNSTETVDTLTENNVPVTHAKELLHTEHNGMLCATSLGQPLILD
TCTIEGLIYGNPSCDLSLEGREWSYIVERPSAVNGLCYPGNVENLEELRSLFSSARSYQRIQIFPDTIWN
VSYDGTSTACSNSFYRSMRWLTRKDGNYPTQDAQYTNNQGKNILFMWGINHPPTDETQRNLYTRTDTTTS
VATEEINRIFKPLIGPRPLVNGLMGRIDYYWSVLKPGQTLRIKSDGNLIAPWYGHILSGESHGRILKTDL
KKGSCTVQCQTEKGGLNTTLPFQNVSKYAFGNCSKYIGIKSLKLAVGLRNVPSRSSRGLFGAIAGFIEGG
WSGLVAGWYGFQHSNDQGVGMAADRDSTQKAIDKITSKVNNIVDKMNKQYEIIDHEFSEVETRLNMINNK
IDDQIQDIWAYNAELLVLLENQKTLDEHDANVNNLYNKVKRALGSNAVEDGKGCFELYHKCNDQCMETIR
NGTYNRKKYQEESKLERQRIEGVKLESEGTYKILTIYSTVASSLVIAMGLAAFLFWAMSNGSC

>QOJ99474.1 hemagglutinin, partial [Influenza A virus]
ETVSLITILLVATVSNADKICIGYQSTNSTETVDTLTENNVPVTHAKELLHTEHNGMLCATSLGQPLILD
TCTIEGLIYGNPSCDLSLEGREWSYIVERPSAVNGLCYPGNVENLEELRSLFSSARSYQRIQIFPDTIWN
VSYDGTSTACSNSFYRSMRWLTRKDGNYPTQDAQYTNNQGKNILFMWGINHPPTDETQRNLYTRTDTTTS
VATEEINRIFKPLIGPRPLVNGLMGRIDYYWSVLKPGQTLRIKSDGNLIAPWYGHILSGESHGRILKTDL
KKGSCTVQCQTEKGGLNTTLPFQNVSKYAFGNCSKYIGIKSLKLAVGLRNVPSRSSRGLFGAIAGFIEGG
WSGLVAGWYGFQHSNDQGVGMAADRDSTQKAIDKITSKVNNIVDKMNKQYEIIDHEFSEVETRLNMINNK
IDDQIQDIWAYNAELLVLLENQKTLDEHDANVNNLYNKVKRALGSNAVEDGKGCFELYHKCNDQCMETIR
NGTYNRKKYQEESKLERQRIEGVKLESEGTYKILTIYSTVASSLVIAMGLAAFLFWAMSNGSC

>QOJ99473.1 hemagglutinin, partial [Influenza A virus]
ETVSLITILLVATVSNADKICIGYQSTNSTETVDTLTENNVPVTHAKELLHTEHNGMLCATSLGQPLILD
TCTIEGLIYGNPSCDLSLEGREWSYIVERPSAVNGLCYPGNVENLEELRSLFSSARSYQRIQIFPDTIWN
VSYDGTSTACSNSFYRSMRWLTRKDGNYPTQDAQYTNNQGKNILFMWGINHPPTDETQRNLYTRTDTTTS
VATEEINRIFKPLIGPRPLVNGLMGRIDYYWSVLKPGQTLRIKSDGNLIAPWYGHILSGESHGRILKTDL
KKGSCTVQCQTEKGGLNTTLPFQNVSKYAFGNCSKYIGIKSLKLAVGLRNVPSRSSRGLFGAIAGFIEGG
WSGLVAGWYGFQHSNDQGVGMAADRDSTQKAIDKITSKVNNIVDKMNKQYEIIDHEFSEVETRLNMINNK
IDDQIQDIWAYNAELLVLLENQKTLDEHDANVNNLYNKVKRALGSNAVEDGKGCFELYHKCNDQCMETIR
NGTYNRKKYQEESKLERQRIEGVKLESEGTYKILTIYSTVASSLVIAMGLAAFLFWAMSNGSC

>QOJ99472.1 hemagglutinin, partial [Influenza A virus]
ETVSLITILIVATVSNADKICIGYQSTNSTETVDTLTENNVPVTHAKELLHTEHNGMLCATSLGQPLILD
TCTIEGLIYGNPSCDLSLEGREWSYIVERPSAVNGLCYPGNVENLEELRSLFSSARSYQRIQIFPDTIWN
VSYDGTSTACSNSFYRSMRWLTRKDGNYPTQDAQYTNNQGKNILFMWGINHPPTDETQRNLYTRTDTTTS
VATEEINRIFKPLIGPRPLVNGLMGRIDYYWSVLKPGQTLRIKSDGNLIAPWYGHILSGESHGRILKTDL
KKGSCTVQCQTEKGGLNTTLPFQNVSKYAFGNCSKYIGIKSLKLAVGLRNVPSRSSRGLFGAIAGFIEGG
WSGLVAGWYGFQHSNDQGVGMAADRDSTQKAIDKITSKVNNIVDKMNKQYEIIDHEFSEVETRLNMINNK
IDDQIQDIWAYNAELLVLLENQKTLDEHDANVNNLYNKVKRALGSNAVEDGKGCFELYHKCNDQCMETIR
NGTYNRKKYQEESKLERQRIEGVKLESEGTYKILTIYSTVASSLVIAMGFAAFLFWAMSNGSC

>QOJ99471.1 hemagglutinin, partial [Influenza A virus]
METVSLITILLVATVSXADKICIGYQSTNSTETVDTLTENNVPVTHAKELLXTEHNGMLCATXLGQPLIL
DTCTIEGLIYGNPSCDLSLEGREWSYIVERPSAVXGLCYPGNVENLEELRSLFSSARSYQRVQIFPDTTW

NVSYDGTSTACSGSFYRSMRWLTRKDGNYPTQDAQYTNNQGKNILFMWGINNPPTDDTQRSLYTRTDTTT
SVATEEINRIFKPLIGPRPLVNGLMGRIDYYWSVLKPGQTLRIKSDGNLIAPWYGYILSGESHGRILKTD
LKRGSCTVQCQTEKGGLNTTLPFQNVSKYAFGNCSKYIGIKSLKLAVGLRNVPSRSSRGLFGAIAGFIEG
GWSGLVAGWYGFQHSNDQGVGMAADRDSTQKAIDKITSKVNNIVDKMNKQYEIIDHEFSEVETRLNMINN
KIDDQIQDIWAYNAELLXLLENQKTLDEHDANVNNLYNKVKRALGSNAVEDGKGCFELYHKCDDQCMETI
RNGTYNRXKYQEESKLERQXIEGVKLESEGTYKILTIYSTVASSLVIAM

>QOJ99470.1 hemagglutinin, partial [Influenza A virus]
METVSLITILLVATVSXADKICIGXQSTNSTETVDTLTENNVPVTHAKELLXTEHNGMLCATXLGQPLIL
DTCTIEGLIYGNPSCDLSLEGREWSYIVERPSAVXGLCYPGNVENLEELRSLFSSARSYQRVQIFPDTTW
NVSYDGTSTACSGSFYRSMRWLTRKDGNYPTQDAQYTNNQGKNILFMWGINNPPTDDTQRSLYTRTDTTT
SVATEEINRIFKPLIGPRPLVNGLMGRIDYYWSVLKPGQTLRIKSDGNLIAPWYGYILSGESHGRILKTD
LKRGSCTVQCQTEKGGLNTTLPFQNVSKYAFGNCSKYIGIKSLKLAVGLRNVPSRSSRGLFGAIAGFIEG
GWSGLVAGWYGFQHSNDQGVGMAADRDSTQKAIDKITSKVNNIVDKMNKQYEIIDHEFSEVETRLNMINN
KIDDQIQDIWAYNAELLXLLENQKTLDEHDANVNNLYNKVKRALGSNAVEDGKGCFELYHKCDDQCMETI
RNGTYNRXKYQEESKLERQXIEGVKLESEGTYKILTIYSTVASSLVIAM

>QOJ99468.1 hemagglutinin, partial [Influenza A virus]
METVSLITILVVATVSNADKICIGYQSTNSTETVDTLTENNVPVTHAKELLHTEHNGMLCATSLGHPLIL
DTCTIEGLIYGNPSCDPLLRGREWSYIVERPSAVNGLCYPGNVENLEELRSLFSSSRSYQRIQIFPDTIW
NVSYSGTSKACSDSFYRSMRWLTQKNNAYPTQDAQYTNNQGKNILFMWGINHPPTDTAQTNLYTRTDTTT
SVATEEMNRIFKPLIGPRPLVNGLMGRINYYWSVLKPGQTLRIKSDGNLIAPWYGHILSGESHGRILKTD
LKRGSCTVQCQTEKGGLNTTLPFQNVSKYAFGNCSKYIGVKSLKLAVGLRNVPSRSSRGLFGAIAGFIEG
GWSGLVAGWYGFQHSNDQGVGMAADRDSTQKAIDKITSKVNNIVDKMNKQYEIIDHEFSEVETRLNMINN
KVDDQIQDIWAYNAELLVLLENQKTLDEHDANVNNLYNKVKRALGSNAVEDGKGCFELYHKCDDHCMETI
RNGTYNRRKYQEESKLERQKIEGVKLESEETYKILTIYSTVASSLVIAMGFAAFLFWAMSNGSC

>QOJ99467.1 hemagglutinin, partial [Influenza A virus]
ETVSLITILLXATVSNADKICIGYQSXNSTETVDTLTENNVPVTHAKELLHTEHNGMLCATSLGQPLILD
TCTIEGLIYGNPSCDLSLEGREWSYIVERPSAVHGLCYPGNVEDLEELRSLFSSARSYQRIQIFPDTIWN
VSYDGTSTACSGSFYKSMRWLTRKNGEYPTQDAQYTNNQGKNILFMWGINHPPTDDTQRGLYTRTDTTTS
VATEEINRIFKPLIGPRPLVNGLMGRINYYWSVLKPGQTLRIKSDGNLIAPWYGHILSGESHGRILKTDL
KRGSCTVQCQTEKGGLNTTLPFQNVSKYAFGNCSKYIGVKSLKLAVGLRNVPSRSXRGLFGAIAGFIEGG
WXGLVAGWYGFQHSNDQGVGMAADRXSTQKAIDKITSKVNNIVDKMNKQYEIIDHEFSEVETRLNMINNK

IDDQIQDIWAYNAELLVLLENQKTLDEHDANVNNLYNKVKRALGSNAVEDGKGCFELYHKCDDQCMETIR
NGTYNRRKYQEESKLERQXIEGVKLESEGTYKILTIYSTVASSLVIAMGFAAFLFWAMSNGSC

>QOJ99466.1 hemagglutinin, partial [Influenza A virus]
METVSLITILVVATVSNADKICIGYQSTNSTETVDTLTENNVPVTHAKELLHTEHNGMLCATSLGHPLIL
DTCTIEGLIYGNPSCDPLLGGREWSYIVERPSAVNGLCYPGNVENLEELRSLFSSSRSYQRIQIFPDTIW
NVSYSGTSKACSDSFYRSMRWLTQKNNAYPTQDAQYTNNQGKNILFMWGINHPPTDTAQTNLYTRTDTTT
SVATEEMNRIFKPLIGPRPLVNGLMGRINYYWSVLKPGQTLRIKSDGNLIAPWYGHILSGESHGRILKTD
LKRGSCTVQCQTEKGGLNTTLPFQNVSKYAFGNCSKYIGVKSLKLAVGLRNVPSRSSRGLFGAIAGFIEG
GWSGLVAGWYGFQHSNDQGVGMAADRDSTQKAIDKITSKVNNIVDKMNKQYEIIDHEFSEVETRLNMINN
KVDDQIQDIWAYNAELLVLLENQKTLDEHDANVNNLYNKVKRALGSNAVEDGKGCFELYHKCDDHCMETI
RNGTYNRRKYQEESKLERQKIEGVKLESEETYKILTIYSTVASSLVIAMGFAAFLFWAMSNGSC

>QOJ99465.1 hemagglutinin, partial [Influenza A virus]
METVSLITILVVATVSNADKICIGYQSTNSTETVDTLTENNVPVTHAKELLHTEHNGMLCATGLGHPLIL
DTCTIEGLIYGNPSCDPFLGGREWSYIVERPSAVNGLCYPGNVENLEELRSLFSSSRSYQRIQIFPDTIW
NVSYSGTSKACSDSFYRSMRWLTQKNNAYPTQDAQYTNNQGKNILFMWGINHPPTDTAQTNLYTRTDTTT
SVATEEMNRIFKPLIGPRPLVNGLMGRINYYWSVLKPGQTLRIKSDGNLIAPWYGHILSGESHGRILKTD
LKRGSCTVQCQTEKGGLNTTLPFQNVSKYAFGNCSKYIGVKSLKLAVGLRNVPSRSSRGLFGAIAGFIEG
GWSGLVAGWYGFQHSNDQGVGMAADRDSTQKAIDKITSKVNNIVDKMNKQYEIIDHEFSEVETRLNMINN
KVDDQIQDIWAYNAELLVLLENQKTLDEHDANVNNLYNKVKRALGSNAVEDGKGCFELYHKCDDHCMETI
RNGTYNRRKYQEESKLERQKIEGVKLESEETYKILTIYSTVASSLVIAMGFAAFLFWAMSNGSC

>QOJ99464.1 hemagglutinin, partial [Influenza A virus]
METVSLITILVVATVSNADKICIGYQSTNSTETVDTLTENNVPVTHAKELLHTEHNGMLCATGLGHPLIL
DTCTIEGLIYGNPSCDPFLGGREWSYIVERPSAVNGLCYPGNVENLEELRSLFSSSRSYQRIQIFPDTIW
NVSYSGTSKACSDSFYRSMRWLTQKNNAYPTQDAQYTNNQGKNILFMWGINHPPTDTAQTNLYTRTDTTT
SVATEEMNRIFKPLIGPRPLVNGLMGRINYYWSVLKPGQTLRIKSDGNLIAPWYGHILSGESHGRILKTD
LKRGSCTVQCQTEKGGLNTTLPFQNVSKYAFGNCSKYIGVKSLKLAVGLRNVPSRSSRGLFGAIAGFIEG
GWSGLVAGWYGFQHSNDQGVGMAADRDSTQKAIDKITSKVNNIVDKMNKQYEIIDHEFSEVETRLNMINN
KVDDQIQDIWAYNAELLVLLENQKTLDEHDANVNNLYNKVKRALGSNAVEDGKGCFELYHKCDDHCMETI
RNGTYNRRKYQEESKLERQKIEGVKLESEETYKILTIYSTVASSLVIAMGFAAFLFWAMSNGSC

>QOJ99463.1 hemagglutinin, partial [Influenza A virus]

METVSLITILVVATVSNADKICIGYQSTNSTETVDTLTENNVPVTHAKELLHTEHNGMLCATGLGHPLIL
DTCTIEGLIYGNPSCDPFLGGREWSYIVERPSAVNGLCYPGNVENLEELRSLFSSSRSYQRIQIFPDTIW
NVSYSGTSKACSDSFYRSMRWLTQKNNAYPTQDAQYTNNQGKNILFMWGINHPPTDTAQTNLYTRTDTTT
SVATEEMNRIFKPLIGPRPLVNGLMGRINYYWSVLKPGQTLRIKSDGNLIAPWYGHILSGESHGRILKTD
LKRGSCTVQCQTEKGGLNTTLPFQNVSKYAFGNCSKYIGVKSLKLAVGLRNVPSRSSRGLFGAIAGFIEG
GWSGLVAGWYGFQHSNDQGVGMAADRDSTQKAIDKITSKVNNIVDKMNKQYEIIDHEFSEVETRLNMINN
KVDDQIQDIWAYNAELLVLLENQKTLDEHDANVNNLYNKVKRALGSNAVEDGKGCFELYHKCDDHCMETI
RNGTYNRRKYQEESKLERQKIEGVKLESEETYKILTIYSTVASSLVIAMGFAAFLFWAMSNGSC

>QOJ99462.1 hemagglutinin, partial [Influenza A virus]
METVSLITILVVATVSNADKICIGYQSTNSTETVDTLTENNVPVTHAKELLHTEHNGMLCATGLGHPLIL
DTCTIEGLIYGNPSCDPFLGGREWSYIVERPSAVNGLCYPGNVENLEELRSLFSSSRSYQRIQIFPDTIW
NVSYSGTSKACSDSFYRSMRWLTQKNNAYPTQDAQYTNNQGKNILFMWGINHPPTDTAQTNLYTRTDTTT
SVATEEMNRIFKPLIGPRPLVNGLMGRINYYWSVLKPGQTLRIKSDGNLIAPWYGHILSGESHGRILKTD
LKRGSCTVQCQTEKGGLNTTLPFQNVSKYAFGNCSKYIGVKSLKLAVGLRNVPSRSSRGLFGAIAGFIEG
GWSGLVAGWYGFQHSNDQGVGMAADRDSTQKAIDKITSKVNNIVDKMNKQYEIIDHEFSEVETRLNMINN
KVDDQIQDIWAYNAELLVLLENQKTLDEHDANVNNLYNKVKRALGSNAVEDGKGCFELYHKCDDHCMETI
RNGTYNRRKYQEESKLERQKIEGVKLESEETYKILTIYSTVASSLVIAMGFAAFLFWAMSNGSC

>QOJ99461.1 hemagglutinin, partial [Influenza A virus]
METVSLITILVVATVSNADKICIGYQSTNSTETVDTLTENNVPVTHAKELLHTEHNGMLCATSLGHPLIL
DTCTIEGLIYGNPSCDPLLGGREWSYIVERPSAVNGLCYPGNVENLEELRSLFSSSRSYQRIQIFPDTIW
NVSYSGTSKACSDSFYRSMRWLTQKNNAYPTQDAQYTNNQGKNILFMWGINHPPTDTAQTNLYTRTDTTT
SVATEEMNRIFKPLIGPRPLVNGLMGRINYYWSVLKPGQTLRIKSDGNLIAPWYGHILSGESHGRILKTD
LKRGSCTVQCQTEKGGLNTTLPFQNVSKYAFGNCSKYIGVKSLKLAVGLRNVPSRSSRGLFGAIAGFIEG
GWSGLVAGWYGFQHSNDQGVGMAADRDSTQKAIDKITSKVNNIVDKMNKQYEIIDHEFSEVETRLNMINN
KVDDQIQDIWAYNAELLVLLENQKTLDEHDANVNNLYNKVKRALGSNAVEDGKGCFELYHKCDDHCMETI
RNGTYNRRKYQEESKLERQKIEGVKLESEETYKILTIYSTVASSLVIAMGFAAFLFWAMSNGSC

>QOJ99460.1 hemagglutinin, partial [Influenza A virus]
METVSLITILVVATVSNADKICIGYQSTNSTETVDTLTENNVPVTHAKELLHTEHNGMLCATSLGHPLIL
DTCTIEGLIYGNPSCDPLLGGREWSYIVERPSAVNGLCYPGNVENLEELRSLFSSSRSYQRIQIFPDTIW
NVSYSGTSKACSDSFYRSMRWLTQKNNAYPTQDAQYTNNQGKNILFMWGINHPPTDTAQTNLYTRTDTTT
SVATEEMNRIFKPLIGPRPLVNGLMGRINYYWSVLKPGQTLRIKSDGNLIAPWYGHILSGESHGRILKTD

LKRGSCTVQCQTEKGGLNTTLPFQNVSKYAFGNCSKYIGVKSLKLAVGLRNVPSRSSRGLFGAIAGFIEG
GWSGLVAGWYGFQHSNDQGVGMAADRDSTQKAIDKITSKVNNIVDKMNKQYEIIDHEFSEVETRLNMINN
KVDDQIQDIWAYNAELLVLLENQKTLDEHDANVNNLYNKVKRALGSNAVEDGKGCFELYHKCDDHCMETI
RNGTYNRRKYQEESKLERQKIEGVKLESEETYKILTIYSTVASSLVIAMGFAAFLFWAMSNGSC

>QOJ99459.1 hemagglutinin, partial [Influenza A virus]
METVSLITILVVATVSNADKICIGYQSTNSTETVDTLTENNVPVTHAKELLHTEHNGMLCATSLGHPLIL
DTCTIEGLIYGNPSCDPLLGGREWSYIVERPSAVNGLCYPGNVENLEELRSLFSSSRSYQRIQIFPDTIW
NVSYSGTSKACSDSFYRSMRWLTQKNNAYPTQDAQYTNNQGKNILFMWGINHPPTDTAQTNLYTRTDTTT
SVATEEMNRIFKPLIGPRPLVNGLMGRINYYWSVLKPGQTLRIKSDGNLIAPWYGHILSGESHGRILKTD
LKRGSCTVQCQTEKGGLNTTLPFQNVSKYAFGNCSKYIGVKSLKLAVGLRNVPSRSSRGLFGAIAGFIEG
GWSGLVAGWYGFQHSNDQGVGMAADRDSTQKAIDKITSKVNNIVDKMNKQYEIIDHEFSEVETRLNMINN
KVDDQIQDIWAYNAELLVLLENQKTLDEHDANVNNLYNKVKRALGSNAVEDGKGCFELYHKCDDHCMETI
RNGTYNRRKYQEESKLERQKIEGVKLESEETYKILTIYSTVASSLVIAMGFAAFLFWAMSNGSC

>QOJ99458.1 hemagglutinin, partial [Influenza A virus]
METVSLITILVVATVSNADKICIGYQSTNSTETVDTLTENNVPVTHAKELLHTEHNGMLCATSLGHPLIL
DTCTIEGLIYGNPSCDPLLGGREWSYIVERPSAVNGLCYPGNVENLEELRSLFSSSRSYQRIQIFPDTIW
NVSYSGTSKACSDSFYRSMRWLTQKNNAYPTQDAQYTNNQGKNILFMWGINHPPTDTAQTNLYTRTDTTT
SVATEEMNRIFKPLIGPRPLVNGLMGRINYYWSVLKPGQTLRIKSDGNLIAPWYGHILSGESHGRILKTD
LKRGSCTVQCQTEKGGLNTTLPFQNVSKYAFGNCSKYIGVKSLKLAVGLRNVPSRSSRGLFGAIAGFIEG
GWSGLVAGWYGFQHSNDQGVGMAADRDSTQKAIDKITSKVNNIVDKMNKQYEIIDHEFSEVETRLNMINN
KVDDQIQDIWAYNAELLVLLENQKTLDEHDANVNNLYNKVKRALGSNAVEDGKGCFELYHKCDDHCMETI
RNGTYNRRKYQEESKLERQKIEGVKLESEETYKILTIYSTVASSLVIAMGFAAFLFWAMSNGSC

>QOJ99457.1 hemagglutinin, partial [Influenza A virus]
METVSLITILLVATVSXADKICIGYQSTNSTETVDTLTENNVPVTHAKELLXTEHNGMLCATGLGQPLIL
DTCTIEGLIYGNPSCDLSLEGREWSYIVERPSAVNGLCYPGNVENLEELRSLFSSARSYQRVQIFPDTTW
NVSYDGTSTACSGSFYRSMRWLTRKDGNYPTQDAQYTNNQGKNILFMWGINNPPTDDTQRSLYTRTDTTT
SVATEEINRIFKPLIGPRPLVNGLMGRIDYYWSVLKPGQTLRIKSDGNLIAPWYGYILSGESHGRILKTD
LKRGSCTVQCQTEKGGLNTTLPFQNVSKYAFGNCSKYIGIKSLKLAVGLRNVPSRSSRGLFGAIAGFIEG
GWSGLVAGWYGFQHSNDQGVGMAADRDSTQKAIDKITSKVNNIVDKMNKQYEIIDHEFSEVETRLNMINN
KIDDQIQDIWAYNAELLVLLENQKTLDEHDANVNNLYNKVKRALGSNAVEDGKGCFELYHKCDDQCMETI
RNGTYNRXKYQEESKLERQXIEGVKLESEGTYKILTIYSTVASSLVIAM

>QOJ99456.1 hemagglutinin, partial [Influenza A virus]

METVSLITILLVATVSYADKICIGYQSTNSTETVDTLTENNVPVTHAKELLQTEHNGMLCATGLGQPLIL
DTCTIEGLIYGNPSCDLSLEGREWSYIVERPSAVNGLCYPGNVENLEELRSLFSSARSYQRVQIFPDTTW
NVSYDGTSTACSGSFYRSMRWLTRKDGNYPTQDAQYTNNQGKNILFMWGINNPPTDDTQRSLYTRTDTTT
SVATEEINRIFKPLIGPRPLVNGLMGRIDYYWSVLKPGQTLRIKSDGNLIAPWYGYILSGESHGRILKTD
LKRGSCTVQCQTEKGGLNTTLPFQNVSKYAFGNCSKYIGIKSLKLAVGLRNVPSRSSRGLFGAIAGFIEG
GWSGLVAGWYGFQHSNDQGVGMAADRDSTQKAIDKITSKVNNIVDKMNKQYEIIDHEFSEVETRLNMINN
KIDDQIQDIWAYNAELLVLLENQKTLDEHDANVNNLYNKVKRALGSNAVEDGKGCFELYHKCDDQCMETI
RNGTYNRKKYQEESKLERQKIEGVKLESEGTYKILTIYSTVASSLVIAM

>QOJ99455.1 hemagglutinin, partial [Influenza A virus]

METVSLITILLVATVSYADKICIGYQSTNSTETVDTLTENNVPVTHAKELLQTEHNGMLCATGLGQPLIL
DTCTIEGLIYGNPSCDLSLEGREWSYIVERPSAVNGLCYPGNVENLEELRSLFSSARSYQRVQIFPDTTW
NVSYDGTSTACSGSFYRSMRWLTRKDGNYPTQDAQYTNNQGKNILFMWGINNPPTDDTQRSLYTRTDTTT
SVATEEINRIFKPLIGPRPLVNGLMGRIDYYWSVLKPGQTLRIKSDGNLIAPWYGYILSGESHGRILKTD
LKRGSCTVQCQTEKGGLNTTLPFQNVSKYAFGNCSKYIGIKSLKLAVGLRNVPSRSSRGLFGAIAGFIEG
GWSGLVAGWYGFQHSNDQGVGMAADRDSTQKAIDKITSKVNNIIDKMNKQYEIIDHEFSEVETRLNMINN
KIDDQIQDIWAYNAELLVLLENQKTLDEHDANVNNLYNKVKRALGSNAVEDGKGCFELYHKCDDQCMETI
RNGTYNRKKYQEESKLERQKIEGVKLESEGTYKILTIYSTVASSLVIAM

>QOJ99454.1 hemagglutinin, partial [Influenza A virus]

METVSLITILLVATVSYADKICIGYQSTNSTETVDTLTENNVPVTHAKELLXTEHNGMLCATGLGQPLIL
DTCTIEGLIYGNPSCDLSLEGREWSYIVERPSAVXGLCYPGNVENLEELRSLFSSARSYQRVQIFPDTTW
NVSYDGTSTACSGSFYRSMRWLTRKDGNYPTQDAQYTNNQGKNILFMWGINNPPTDDTQRSLYTRTDTTT
SVATEEINRIFKPLIGPRPLVNGLMGRIDYYWSVLKPGQTLRIKSDGNLIAPWYGYILSGESHGRILKTD
LKXGSCTVQCQTEKGGLNTTLPFQNVSKYAFGNCSKYIGIKSLKLAVGLRNVPSRSSRGLFGAIAGFIEG
GWSGLVAGWYGFQHSNDQGVGMAADRDSTQKAIDKITSKVNNIVDKMNKQYEIIDHEFSEVETRLNMINN
KIDDQIQDIWAYNAELLVLLENQKTLDEHDANVNNLYNKVKRALGSNAVEDGKGCFELYHKCDDQCMETI
RNGTYNRKKYQEESKLERQXIEGVKLESEGTYKILTIYSTVASSLVIAM

>QOJ99453.1 hemagglutinin, partial [Influenza A virus]

METVSLITILLVATVSYADKICIGYQSTNSTETVDTLTENNVPVTHAKELLQTEHNGMLCATGLGQPLIL
DTCTIEGLIYGNPSCDLSLEGREWSYIVERPSAVNGLCYPGNVENLEELRSLFSSARSYQRVQIFPDTTW

NVSYDGTSTACSGSFYRSMRWLTRKDGNYPTQDAQYTNNQGKNILFMWGINNPPTDDTQRSLYTRTDTTT
SVATEEINRIFKPLIGPRPLVNGLMGRIDYYWSVLKPGQTLRIKSDGNLIAPWYGYILSGESHGRILKTD
LKRGSCTVQCQTEKGGLNTTLPFQNVSKYAFGNCSKYIGIKSLKLAVGLRNVPSRSSRGLFGAIAGFIEG
GWSGLVAGWYGFQHSNDQGVGMAADRDSTQKAIDKITSKVNNIIDKMNKQYEIIDHEFSEVETRLNMINN
KIDDQIQDIWAYNAELLVLLENQKTLDEHDANVNNLYNKVKRALGSNAVEDGKGCFELYHKCDDQCMETI
RNGTYNRKKYQEESKLERQKIEGVKLESEGTYKILTIYSTVASSLVIAM

>QOJ99452.1 hemagglutinin [Influenza A virus]
METVSLITILLVATVSNADKICIGYQSTNSTETVDTLTENNVPVTHAKELLHTEHNGMLCATSLGQPLIL
DTCTIEGLIYGNPSCDLSLEGREWSYIVERPSAVHGLCYPGNVEDLEELRSLFSSARSYQRIQIFPDTIW
NVSYDGTSTACSGSFYKSMRWLTRKNGEYPTQDAQYTNNQGKNILFMWGINHPPTDDTQRGLYTRTDTTT
SVATEEINRIFKPLIGPRPLVNGLMGRINYYWSVLKPGQTLRIKSDGNLIAPWYGHILSGESHGRILKTD
LKRGSCTVQCQTEKGGLNTTLPFQNVSKYAFGNCSKYIGIKSLKLAVGLRNVPSRSSRGLFGAIAGFIEG
GWSGLVAGWYGFQHSNDQGVGMAADRDSTQKAIDKITSKVNNIVDKMNKQYEIIDHEFSEVETRLNMINN
KIDDQIQDIWAYNAELLILLENQKTLDEHDANVNNLYNKVKRALGSNAVEDGKGCFELYHKCDDQCMETI
RNGTYNRRKYQEESKLERQRIEGVKLESEGTYKILTIYSTVASSLVIAMGFAAFLFWAMSNGSCRCNICI

>QOJ99451.1 hemagglutinin [Influenza A virus]
METVSLITILLVATVSNADKICIGYQSTNSTETVDTLTENNVPVTHAKELLHTEHNGMLCATSLGQPLIL
DTCTIEGLIYGNPSCDLSLEGREWSYIVERPSAVHGLCYPGNVEDLEELRSLFSSARSYQRIQIFPDTIW
NVSYDGTSTACSGSFYKSMRWLTRKNGEYPTQDAQYTNNQGKNILFMWGINHPPTDDTQRGLYTRTDTTT
SVATEEINRIFKPLIGPRPLVNGLMGRINYYWSVLKPGQTLRIKSDGNLIAPWYGHILSGESHGRILKTD
LKRGSCTVQCQTEKGGLNTTLPFQNVSKYAFGNCSKYIGIKSLKLAVGLRNVPSRSSRGLFGAIAGFIEG
GWSGLVAGWYGFQHSNDQGVGMAADRDSTQKAIDKITSKVNNIVDKMNKQYEIIDHEFSEVETRLNMINN
KIDDQIQDIWAYNAELLILLENQKTLDEHDANVNNLYNKVKRALGSNAVEDGKGCFELYHKCDDQCMETI
RNGTYNRRKYQEESKLERQRIEGVKLESEGTYKILTIYSTVASSLVIAMGFAAFLFWAMSNGSCRCNICI

>QOJ99450.1 hemagglutinin, partial [Influenza A virus]
METVSLITILLVATVSYADKICIGYQSTNSTETVDTLTENNVPVTHAKELLQTEHNGMLCATGLGQPLIL
DTCTIEGLIYGNPSCDLSLEGREWSYIVERPSAVNGLCYPGNVENLEELRSLFSSARSYQRVQIFPDTTW
NVSYDGTSTACSGSFYRSMRWLTRKDGNYPTQDAQYTNNQGKNILFMWGINNPPTDDTQRSLYTRTDTTT
SVATEEINRIFKPLIGPRPLVNGLMGRIDYYWSVLKPGQTLRIKSDGNLIAPWYGYILSGESHGRILKTD
LKRGSCTVQCQTEKGGLNTTLPFQNVSKYAFGNCSKYIGIKSLKLAVGLRNVPSRSSRGLFGAIAGFIEG
GWSGLVAGWYGFQHSNDQGVGMAADRDSTQKAIDKITSKVNNIVDKMNKQYEIIDHEFSEVETRLNMINN

KIDDQIQDIWAYNAELLVLLENQKTLDEHDANVNNLYNKVKRALGSNAVEDGKGCFELYHKCDDQCMETI
RNGTYNRKKYQEESKLERQKIEGVKLESEGTYKILTIYSTVASSLVIAM

>QOJ99449.1 hemagglutinin, partial [Influenza A virus]
METVSLITILLVATVSYADKICIGYQSTNSTETVDTLTENNVPVTHAKELLQTEHNGMLCATGLGQPLIL
DTCTIEGLIYGNPSCDLSLEGREWSYIVERPSAVNGLCYPGNVENLEELRSLFSSARSYQRVQIFPDTTW
NVSYDGTSTACSGSFYRSMRWLTRKDGNYPTQDAQYTNNQGKNILFMWGINNPPTDDTQRSLYTRTDTTT
SVATEEINRIFKPLIGPRPLVNGLMGRIDYYWSVLKPGQTLRIKSDGNLIAPWYGYILSGESHGRILKTD
LKRGSCTVQCQTEKGGLNTTLPFQNVSKYAFGNCSKYIGIKSLKLAVGLRNVPSRSSRGLFGAIAGFIEG
GWSGLVAGWYGFQHSNDQGVGMAADRDSTQKAIDKITSKVNNIVDKMNKQYEIIDHEFSEVETRLNMINN
KIDDQIQDIWAYNAELLVLLENQKTLDEHDANVNNLYNKVKRALGSNAVEDGKGCFELYHKCDDQCMETI
RNGTYNRKKYQEESKLERQKIEGVKLESEGTYKILTIYSTVASSLVIAM

>QOJ99448.1 hemagglutinin, partial [Influenza A virus]
METVSLITILLVATVSYADKICIGYQSTNSTETVDTLTENNVPVTHAKELLQTEHNGMLCATGLGQPLIL
DTCTIEGLIYGNPSCDLSLEGREWSYIVERPSAVNGLCYPGNVENLEELRSLFSSARSYQRVQIFPDTTW
NVSYDGTSTACSGSFYRSMRWLTRKDGNYPTQDAQYTNNQGKNILFMWGINNPPTDDTQRSLYTRTDTTT
SVATEEINRIFKPLIGPRPLVNGLMGRIDYYWSVLKPGQTLRIKSDGNLIAPWYGYILSGESHGRILKTD
LKRGSCTVQCQTEKGGLNTTLPFQNVSKYAFGNCSKYIGIKSLKLAVGLRNVPSRSSRGLFGAIAGFIEG
GWSGLVAGWYGFQHSNDQGVGMAADRDSTQKAIDKITSKVNNIIDKMNKQYEIIDHEFSEVETRLNMINN
KIDDQIQDIWAYNAELLVLLENQKTLDEHDANVNNLYNKVKRALGSNAVEDGKGCFELYHKCDDQCMETI
RNGTYNRKKYQEESKLERQKIEGVKLESEGTYKILTIYSTVASSLVIAM

>QOJ99447.1 hemagglutinin [Influenza A virus]
METVSLITILLVATVSNADKICIGYQSSNSTETVDTLTENNVPVTHAKELLHTEHNGMLCATSLGQPLIL
DTCTIEGLIYGNPSCDLSLEGREWSYIVERPSAVHGLCYPGNVEDLEELRSLFSSARSYQRIQIFPDTIW
NVSYDGTSTACSGSFYKSMRWLTRKNGEYPTQDAQYTNNQGKNILFMWGINHPPTDDTQRGLYTRTDTTT
SVATEEINRIFKPLIGPRPLVNGLMGRINYYWSVLKPGQTLRIKSDGNLIAPWYGHILSGESHGRILKTD
LKRGSCTVQCQTEKGGLNTTLPFQNVSKYAFGNCSKYIGVKSLKLAVGLRNVPSRSSRGLFGAIAGFIEG
GWPGLVAGWYGFQHSNDQGVGMAADRDSTQKAIDKITSKVNNIVDKMNKQYEIIDHEFSEVETRLNMINN
KIDDQIQDIWAYNAELLVLLENQKTLDEHDANVNNLYNKVKRALGSNAVEDGKGCFELYHKCDDQCMETI
RNGTYNRRKYQEESKLERQRIEGVKLESEGTYKILTIYSTVASSLVIAMGFAAFLFWAMSNGSCRCNICI

>QOJ99446.1 hemagglutinin, partial [Influenza A virus]

METVSLITILLVATVSYADKICIGYQSTNSTETVDTLTENNVPVTHAKELLQTEHNGMLCATGLGQPLIL
DTCTIEGLIYGNPSCDLSLEGREWSYIVERPSAVNGLCYPGNVENLEELRSLFSSARSYQRVQIFPDTTW
NVSYDGTSTACSGSFYRSMRWLTRKDGNYPTQDAQYTNNQGKNILFMWGINNPPTDDTQRSLYTRTDTTT
SVATEEINRIFKPLIGPRPLVNGLMGRIDYYWSVLKPGQTLRIKSDGNLIAPWYGYILSGESHGRILKTD
LKRGSCTVQCQTEKGGLNTTLPFQNVSKYAFGNCSKYIGIKSLKLAVGLRNVPSRSSRGLFGAIAGFIEG
GWSGLVAGWYGFQHSNDQGVGMAADRDSTQKAIDKITSKVNNIVDKMNKQYEIIDHEFSEVETRLNMINN
KIDDQIQDIWAYNAELLVLLENQKTLDEHDANVNNLYNKVKRALGSNAVEDGKGCFELYHKCDDQCMETI
RNGTYNRKKYQEESKLERQKIEGVKLESEGTYKILTIYSTVASSLVIAM

>QOJ99445.1 hemagglutinin [Influenza A virus]
METVSLITILLAAAVSNADKICIGYQSTNSTETVDTLTENNVPVTHAKELLHTEHNGMLCATSLGQPLIL
DTCTIEGLIYGNPSCDPLPEEREWSYIVERPSAVNGLCYPGNVENLEELRSLFSSARSYQRIQIFPDTIW
NVSYDGTSNTCSGSFYRNMRWLTRKDGNYPIQDAQYTNNQGKNILFMWGINNPPTDDTQRNLYTRTDTTT
SVATEEINRIFKPLIGPRPLVNGLMGRINYYWSVLKPGQTLRIKSDGNLVAPWYGYILSGESHGRILRTD
LKRGSCTVQCQTEKGGLNTTLPFQNVSKYAFGNCSKYIGIKSLKLAVGLRNVPSRSSRGLFGAIAGFIEG
GWSGLVAGWYGFQHSNDQGVGMAADRESTQKAVDKITSKVNNIVDKMNKQYEIIDHEFSEVETRLNMINN
KIDDQIQDIWAYNAELLVLLENQKTLDEHDANVNNLYNKVKRALGSNAVEDGKGCFELYHKCDDQCMETI
RNGTYNRRKYQEESKLERQKIEGVKLESEGTYKILTIYSTVASSLVIAMGFAAFLFWAMSNGSCRCNICI

>QOJ99444.1 hemagglutinin [Influenza A virus]
METVSLITILLAAAVSNADKICIGYQSTNSTETVDTLTENNVPVTHAKELLHTEHNGMLCATSLGQPLIL
DTCTIEGLIYGNPSCDPLPEEREWSYIVERPSAVNGLCYPGNVENLEELRSLFSSARSYQRIQIFPDTIW
NVSYDGTSNTCSGSFYRNMRWLTRKDGNYPIQDAQYTNNQGKNILFMWGINNPPTDDTQRNLYTRTDTTT
SVATEEINRIFKPLIGPRPLVNGLMGRINYYWSVLKPGQTLRIKSDGNLVAPWYGYILSGESHGRILRTN
LKRGSCTVQCQTEKGGLNTTLPFQNVSKYAFGNCSKYIGIKSLKLAVGLRNVPSRSSRGLFGAIAGFIEG
GWSGLVAGWYGFQHSNDQGVGMAADRESTQKAVDKITSKVNNIVDKMNKQYEIIDHEFSEVETRLNMINN
KIDDQIQDIWAYNAELLVLLENQKTLDEHDANVNNLYNKVKRALGSNAVEDGKGCFELYHKCDDQCMETI
RNGTYNRRKYQEESKLERQKIEGVKLESEGTYKILTIYSTVASSLVIAMGFAAFLFWAMSNGSCRCNICI

>QOJ99443.1 hemagglutinin, partial [Influenza A virus]
METVSLITILVVATVSNADKICIGYQSTNSTETVDTLTENNVPVTHAKELLHTEHNGMLCATGLGHPLIL
DTCTIEGLIYGNPSCDPLLGGREWSYIVERPSAVNGLCYPGNVENLEELRSLFSSSRSYQRIQIFPDTIW
NVSYSGTSKACSDSFYRSMRWLTQKNNAYPTQDAQYTNNQGKNILFMWGINHPPTDTAQTNLYTRTDTTT
SVATEEXNRIFKPLIGPRPLVNGLMGRINYYWSVLKPGQTLRIKSDGNLIAPWYGHILSGESHGRILKTD

LKRGSCTVQCQTEKGGLNTTLPFQNVSKYAFGNCSKYIGVKSLKLAVGLRNVPSRSSRGLFGAIAGFIEG
GWXGLVAGWYGFQHSNDQGVGMAADRDSTQKAIDKITSKVNNIVDKMNKQYEIIDHEFSEVETRLNMINN
KVDDQIQDIWAYNAELLVLLENQKTLDEHDANVNNLYNKVKRALGSNAVEDGKGCFELYHKCDDHCMETI
RNGTYNRRKYQEESKLERQKIEGVKLESEETYKILTIYSTVASSLVIAM

>QOJ99442.1 hemagglutinin, partial [Influenza A virus]
ETVSLITILLVATVSNADKICIGYQSTNSTETVDTLTENNVPVTHAKELLHTEHNGMLCATSLGQPLILD
TCTIEGLIYGNPSCDLSLEGREWSYIVERPSAVNGLCYPGNVENLEELRSLFSSARSYQRIQIFPDTIWN
VSYDGTSTACSNSFYRSMRWLTRKDGNYPTQDAQYTNNQGKNILFMWGINHPPTDETQRNLYTRTDTTTS
VATEEINRIFKPLIGPRPLVNGLMGRIDYYWSVLKPGQTLRIKSDGNLIAPWYGHILSGESHGRILKTDL
KKGSCTVQCQTEKGGLNTTLPFQNVSKYAFGNCSKYIGIKSLKLAVGLRNVPSRSSRGLFGAIAGFIEGG
WSGLVAGWYGFQHSNDQGVGMAADRDSTQKAIDKITSKVNNIVDKMNKQYEIIDHEFSEVETRLNMINNK
IDDQIQDIWAYNAELLVLLENQKTLDEHDANVNNLYNKVKRALGSNAVEDGKGCFELYHKCNDQCMETIR
NGTYNRKKYQEESKLERQRIEGVKLESEGTYKILTIYSTVASSLVIAMGLAAFLFWAMSNGSC

>QOJ99441.1 hemagglutinin, partial [Influenza A virus]
ETVSLITILLVATVSNADKICIGYQSTNSTETVDTLTENNVPVTHAKELLHTEHNGMLCATSLGQPLILD
TCTIEGLIYGNPSCDLSLEGREWSYIVERPSAVNGLCYPGNVENLEELRSLFSSARSYQRIQIFPDTIWN
VSYDGTSTACSNSFYRSMRWLTRKDGNYPTQDAQYTNNQGKNILFMWGINHPPTDETQRNLYTRTDTTTS
VATEEINRIFKPLIGPRPLVNGLMGRIDYYWSVLKPGQTLRIKSDGNLIAPWYGHILSGESHGRILKTDL
KKGSCTVQCQTEKGGLNTTLPFQNVSKYAFGNCSKYIGIKSLKLAVGLRNVPSRSSRGLFGAIAGFIEGG
WSGLVAGWYGFQHSNDQGVGMAADRDSTQKAIDKITSKVNNIVDKMNKQYEIIDHEFSEVETRLNMINNK
IDDQIQDIWAYNAELLVLLENQKTLDEHDANVNNLYNKVKRALGSNAVEDGKGCFELYHKCNDQCMETIR
NGTYNRKKYQEESKLERQRIEGVKLESEGTYKILTIYSTVASSLVIAMGLAAFLFWAMSNGSC

>QOJ99440.1 hemagglutinin, partial [Influenza A virus]
ETVSLITILLVATVSNADKICIGYQSTNSTETVDTLTENNVPVTHAKELLHTEHNGMLCATSLGQPLILD
TCTIEGLIYGNPSCDLSLEGREWSYIVERPSAVNGLCYPGNVENLEELRSLFSSARSYQRIQIFPDTIWN
VSYDGTSTACSNSFYRSMRWLTRKDGNYPTQDAQYTNNQGKNILFMWGINHPPTDETQRNLYTRTDTTTS
VATEEINRIFKPLIGPRPLVNGLMGRIDYYWSVLKPGQTLRIKSDGNLIAPWYGHILSGESHGRILKTDL
KKGSCTVQCQTEKGGLNTTLPFQNVSKYAFGNCSKYIGIKSLKLAVGLRNVPSRSSRGLFGAIAGFIEGG
WSGLVAGWYGFQHSNDQGVGMAADRDSTQKAIDKITSKVNNIVDKMNKQYEIIDHEFSEVETRLNMINNK
IDDQIQDIWAYNAELLVLLENQKTLDEHDANVNNLYNKVKRALGSNAVEDGKGCFELYHKCNDQCMETIR
NGTYNRKKYQEESKLERQRIEGVKLESEGTYKILTIYSTVASSLVIAMGLAAFLFWAMSNGSC

>QOJ99439.1 hemagglutinin, partial [Influenza A virus]

METVSLITILVVATVSNADKICIGYQSTNSTETVDTLTENNVPVTHAKELLHTEHNGMLCATGLGHPLIL
DTCTIEGLIYGNPSCDPLLGGREWSYIVERPSAVNGLCYPGNVENLEELRSLFSSSRSYQRIQIFPDTIW
NVSYSGTSKACSDSFYRSMRWLTQKNNAYPTQDAQYTNNQGKNILFMWGINHPPTDTVQTNLYTRTDTTT
SVATEEINRIFKPLIGPRPLVNGLMGRINYYWSVLKPGQTLRIKSDGNLIAPWYGHILSGESHGRILKTD
LKRGSCTVQCQTEKGGLNTTLPFQNVSKYAFGNCSKYIGVKSLKLAVGLRNVPSRSSRGLFGAIAGFIEG
GWPGLVAGWYGFQHSNDQGVGMAADRDSTQKAIDKITSKVNNIVDKMNKQYEIIDHEFSEVETRLNMINN
KVDDQIQDIWAYNAELLVLLENQKTLDEHDANVNNLYNKVKRALGSNAVEDGKGCFELYHKCDDHCMETI
RNGTYNRRKYQEESKLERQKIEGVKLESEETYKILTIYSTVASSLVIAMGFAAFLFWAMSNGSC

>QOJ99438.1 hemagglutinin, partial [Influenza A virus]

METVSLITILVVATVSNADKICIGYQSTNSTETVDTLTENNVPVTHAKELLHTEHNGMLCATXLGHPLIL
DTCTIEGLIYGNPSCDPLLGGREWSYIVERPSAVNGLCYPGNVENLEELRSLFSSSRSYQRIQIFPDTIW
NVSYSGTSKACSDSFYRSMRWLTQKNNAYPTQDAQYTNNQGKNILFMWGINHPPTDTAQTNLYTRTDTTT
SVATEEXNRIFKPLIGPRPLVNGLMGRINYYWSVLKPGQTLRIKSDGNLIAPWYGHILSGESHGRILKTD
LKXGSCTVQCQTEKGGLNXTLPFQNVSKYAFGNCSKYIGVKSLKLAVGLRNVPSRSSRGLFGAIAGFIEG
GWXGLVAGWYGFQHSNDQGVGMAADRDSTQKAIDKITSKVNNIVDKMNKQYEIIDHEFSEVETRLNMINB
KVDDQIQDIWAYNAELLVLLENQKTLDEHDANVNNLYNKVKRALGSNAVEDGXGCFELYHKCDDHCMETI
RNGTYNRRKYQEESKLERQKIEGVKLESEETYKILTIYSTVASSLVIAMGFAAFLFWAMSNGSC

>QOJ99437.1 hemagglutinin, partial [Influenza A virus]

METVSLITILVVATVSNADKICIGYQSTNSTETVDTLTENNVPVTHAKELLHTEHNGMLCATSLGHPLIL
DTCTIEGLIYGNPSCDPLLGGREWSYIVERPSAVNGLCYPGNVENLEELRSLFSSSRSYQRIQIFPDTIW
NVSYSGTSKACSDSFYRSMRWLTQKNNAYPTQDAQYTNNQGKNILFMWGINHPPTDTAQTNLYTRTDTTT
SVATEEMNRIFKPLIGPRPLVNGLMGRINYYWSVLKPGQTLRIKSDGNLIAPWYGHILSGESHGRILKTD
LKRGSCTVQCQTEKGGLNTTLPFQNVSKYAFGNCSKYIGVKSLKLAVGLRNVPSRSSRGLFGAIAGFIEG
GWSGLVAGWYGFQHSNDQGVGMAADRDSTQKAIDKITSKVNNIVDKMNKQYEIIDHEFSEVETRLNMINN
KVDDQIQDIWAYNAELLVLLENQKTLDEHDANVNNLYNKVKRALGSNAVEDGKGCFELYHKCDDHCMETI
RNGTYNRRKYQEESKLERQKIEGVKLESEETYKILTIYSTVASSLVIAMGFAAFLFWAMSNGSC

>QOJ99436.1 hemagglutinin, partial [Influenza A virus]

METVSLITILVVATVSNADKICIGYQSTNSTETVDTLTENNVPVTHAKELLHTEHNGMLCATGLGHPLIL
DTCTIEGLIYGNPSCDPLLGGREWSYIVERPSAVNGLCYPGNVENLEELRSLFSSSRSYQRIQIFPDTIW

NVSYSGTSKACSDSFYRSMRWLTQKNNAYPTQDAQYTNNQGKNILFMWGINHPPTDTAQTNLYTRTDTTT
SVATEEXNRIFKPLIGPRPLVNGLMGRINYYWSVLKPGQTLRIKSDGNLIAPWYGHILSGESHGRILKTD
LKRGSCTVQCQTEKGGLNTTLPFQNVSKYAFGNCSKYIGVKSLKLAVGLRNVPSRSSRGLFGAIAGFIEG
GWXGLVAGWYGFQHSNDQGVGMAADRDSTQKAIDKITSKVNNIVDKMNKQYEIIDHEFSEVETRLNMINN
KVDDQIQDIWAYNAELLVLLENQKTLDEHDANVNNLYNKVKRALGSNAVEDGKGCFELYHKCDDHCMETI
RNGTYNRRKYQEESKLERQKIEGVKLESEETYKILTIYSTVASSLVIAM

>QOJ99435.1 hemagglutinin [Influenza A virus]
METVSLITILLAAAVSNADKICIGYQSTNSTETVDTLTENNVPVTHAKELLHTEHNGMLCATSLGQPLIL
DTCTIEGLIYGNPSCDPLPEEREWSYIVERPSAVNGLCYPGNVENLEELRSLFSSARSYQRIQIFPDTIW
NVSYDGTSNTCSGSFYRNMRWLTRKDGNYPIQDAQYTNXQGKNILFMWGINNPPTDDTQRNLYTRTDTTT
SVATEEINRIFKPLIGPRPLVNGLMGRINYYWSVLKPGQTLRIKSDGNLVAPWYGYILSGESHGRILRTN
LKRGSCTVQCQTEKGGLNTTLPFQNVSKYAFGNCSKYIGIKSLKLAVGLRNVPSRSSRGLFGAIAGFIEG
GWSGLVAGWYGFQHSNDQGVGMAADRESTQKAVDKITSKVNNIVDKMNKQYEIIDHEFSEVETRLNMINN
KIDDQIQDIWAYNAELLVLLENQKTLDEHDANVNNLYNKVKRALGSNAVEDGKGCFELYHKCDDQCMETI
RNGTYNRRKYQEESKLERQKIEGVKLESEGTYKILTIYSTVASSLVIAMGFAAFLFWAMSNGSCRCNICI

>QOJ99434.1 hemagglutinin [Influenza A virus]
METVSLITILLAAAVSNADKICIGYQSTNSTETVDTLTENNVPVTHAKELLHTEHNGMLCATSLGQPLIL
DTCTIEGLIYGNPSCDPLPEEXEWSYIVERPSAVNGLCYPGNVENLEELRSLFSSARSYQRIQIFPDTIW
NVSYDGTSNTCSGSFYRNMRWLTRKDGNYPIQDAQYTNNQGKNILFMWGINNPPTDDTQRNLYTRTDTTT
SVATEEINRIFKPLIGPRPLVNGLMGRINYYWSVLKPGQTLRIKSDGNLVAPWYGYILSGESHGRILRTN
LKRGSCTVQCQTEKGGLNTTLPFQNVSKYAFGNCSKYIGIKSLKLAVGLRNVPSRSSRGLFGAIAGFIEG
GWSGLVAGWYGFQHSNDQGVGMAADRESTQKAVDKITSKVNNIVDKMNKQYEIIDHEFSEVETRLNMINN
KIDDQIQDIWAYNAELLVLLENQKTLDEHDANVNNLYNKVKRALGSNAVEDGKGCFELYHKCDDQCMETI
RNGTYNRRKYQEESKLERQKIEGVKLESEGTYKILTIYSTVASSLVIAMGFAAFLFWAMSNGSCRCNICI

>QOJ99433.1 hemagglutinin [Influenza A virus]
METVSLITILLAAAVSNADKICIGYQSTNSTETVDTLTENNVPVTHAKELLHTEHNGMLCATSLGQPLIL
DTCTIEGLIYGNPSCDPLPEEREWSYIVERPSAVNGLCYPGNVENLEELRSLFSSARSYQRIQIFPDTIW
NVSYDGTSNTCSGSFYRNMRWLTRKDGNYPIQDAQYTNXQGKNILFMWGINNPPTDDTQRNLYTRTDTTT
SVATEEINRIFKPLIGPRPLVNGLMGRINYYWSVLKPGQTLRIKSDGNLVAPWYGYILSGESHGRILRTB
LKRGSCTVQCQTEKGGLNTTLPFQNVSKYAFGNCSKYIGIKSLKLAVGLRNVPSRSSRGLFGAIAGFIEG
GWSGLVAGWYGFQHSNDQGVGMAADRESTQKAVDKITSKVNNIVDKMNKQYEIIDHEFSEVETRLNMINN

KIDDQIQDIWAYNAELLVLLENQKTLDEHDANVNNLYNKVKRALGSNAVEDGKGCFELYHKCDDQCMETI
RNGTYNXRKYQEESKLERQKIEGVKLESEGTYKILTIYSTVASSLVIAMGFAAFLFWAMSNGSCRCNICI

>QOJ99432.1 hemagglutinin [Influenza A virus]
METVSLITILLAAAVSNADKICIGYQSTNSTETVDTLTENNVPVTHAKELLHTEHNGMLCATSLGQPLIL
DTCTIEGLIYGNPSCDPLPEEREWSYIVERPSAVNGLCYPGNVENLEELRSLFSSARSYQRIQIFPDTIW
NVSYDGTSNTCSGSFYRNMRWLTRKDGNYPIQDAQYTNKQGKNILFMWGINNPPTDDTQRNLYTRTDTTT
SVATEEINRIFKPLIGPRPLVNGLMGRINYYWSVLKPGQTLRIKSDGNLVAPWYGYILSGESHGRILRTN
LKRGSCTVQCQTEKGGLNTTLPFQNVSKYAFGNCSKYIGIKSLKLAVGLRNVPSRSSRGLFGAIAGFIEG
GWSGLVAGWYGFQHSNDQGVGMAADRESTQKAVDKITSKVNNIVDKMNKQYEIIDHEFSEVETRLNMINN
KIDDQIQDIWAYNAELLVLLENQKTLDEHDANVNNLYNKVKRALGSNAVEDGKGCFELYHKCDDQCMETI
RNGTYNRRKYQEESKLERQKIEGVKLESEGTYKILTIYSTVASSLVIAMGFAAFLFWAMSNGSCRCNICI

>QOJ99431.1 hemagglutinin [Influenza A virus]
METVSLITILLAAAVSNADKICIGYQSTNSTETVDTLTENNVPVTHAKELLHTEHNGMLCATSLGQPLIL
DTCTIEGLIYGNPSCDPLPEEREWSYIVERPSAVNGLCYPGNVENLEELRSLFSSARSYQRIQIFPDTIW
NVSYDGTSNTCSGSFYRNMRWLTRKDGNYPIQDAQYTNNQGKNILFMWGINNPPTDDTQRNLYTRTDTTT
SVATEEINRIFKPLIGPRPLVNGLMGRINYYWSVLKPGQTLRIKSDGNLVAPWYGYILSGESHGRILRTB
LKRGSCTVQCQTEKGGLNTTLPFQNVSKYAFGNCSKYIGIKSLKLAVGLRNVPSRSSRGLFGAIAGFIEG
GWSGLVAGWYGFQHSNDQGVGMAADRESTQKAVDKITSKVNNIVDKMNKQYEIIDHEFSEVETRLNMINN
KIDDQIQDIWAYNAELLVLLENQKTLDEHDANVNNLYNKVKRALGSNAVEDGKGCFELYHKCDDQCMETI
RNGTYNXRKYQEESKLERQKIEGVKLESEGTYKILTIYSTVASSLVIAMGFAAFLFWAMSNGSCRCNICI

>QOJ99430.1 hemagglutinin [Influenza A virus]
METVSLITILLAAAVSNADKICIGYQSTNSTETVDTLTENNVPVTHAKELLHTEHNGMLCATSLGQPLIL
DTCTIEGLIYGNPSCDPLPEEREWSYIVERPSAVNGLCYPGNVENLEELRSLFSSARSYQRIQIFPDTIW
NVSYDGTSNTCSGSFYRNMRWLTRKDGNYPIQDAQYTNXQGKNILFMWGINNPPTDDTQRNLYTRTDTTT
SVATEEINRIFKPLIGPRPLVNGLMGRINYYWSVLKPGQTLRIKSDGNLVAPWYGYILSGESHGRILRTB
LKRGSCTVQCQTEKGGLNTTLPFQNVSKYAFGNCSKYIGIKSLKLAVGLRNVPSRSSRGLFGAIAGFIEG
GWSGLVAGWYGFQHSNDQGVGMAADRESTQKAVDKITSKVNNIVDKMNKQYEIIDHEFSEVETRLNMINN
KIDDQIQDIWAYNAELLVLLENQKTLDEHDANVNNLYNKVKRALGSNAVEDGKGCFELYHKCDDQCMETI
RNGTYNRRKYQEESKLERQKIEGVKLESEGTYKILTIYSTVASSLVIAMGFAAFLFWAMSNGSCRCNICI

>QOJ99429.1 hemagglutinin [Influenza A virus]

METVSLITILLAAAVSNADKICIGYQSTNSTETVDTLTENNVPVTHAKELLHTEHNGMLCATSLGQPLIL
DTCTIEGLIYGNPSCDPLPEEREWSYIVERPSAVNGLCYPGNVENLEELRSLFSSARSYQRIQIFPDTIW
NVSYDGTSNTCSGSFYRNMRWLTRKDGNYPIQDAQYTNNQGKNILFMWGINNPPTDDTQRNLYTRTDTTT
SVATEEINRIFKPLIGPRPLVNGLMGRINYYWSVLKPGQTLRIKSDGNLVAPWYGYILSGESHGRILRTN
LKRGSCTVQCQTEKGGLNTTLPFQNVSKYAFGNCSKYIGIKSLKLAVGLRNVPSRSSRGLFGAIAGFIEG
GWSGLVAGWYGFQHSNDQGVGMAADRESTQKAVDKITSKVNNIVDKMNKQYEIIDHEFSEVETRLNMINN
KIDDQIQDIWAYNAELLVLLENQKTLDEHDANVNNLYNKVKRALGSNAVEDGKGCFELYHKCDDQCMETI
RNGTYNRRKYQEESKLERQKIEGVKLESEGTYKILTIYSTVASSLVIAMGFAAFLFWAMSNGSCRCNICI

>QOJ99412.1 hemagglutinin [Influenza A virus]
METASLITILLVATVSNADKICIGYQSTNSTETVDTLTENNVPVTHAKELLHTEHNGMLCATSLGQPLIL
DTCTIEGLIYGNPSCDLSLEGREWSYIVERPSAVHGLCYPGKVEDLEELRSLFSSARSYQRIQIFPDTIW
NVSYDGTSTACSGSFYRSMRWLTRKDGNYPIQDAQYTNNQGKNILFMWGINHPPTDETQRGLYTRTDTTT
SVATEEINRIFKPLIGPRPLVNGLMGRINYYWSVLKPGQTLRIKSDGNLIAPWYGHILSGESHGRILKTD
LKKGSCTVQCQTEKGGLNTTLPFQNVSKYAFGNCSKYIGIKSLKLAVGLRNVPSRSSRGLFGAIAGFIEG
GWPGLVAGWYGFQHSNDQGVGMAADRDSTQKAIDKITSKVNNIVDKMNKQYEIIDHEFSEVETRLNMINN
KIDDQIQDIWAYNAELLVLLENQKTLDEHDANVNNLYNKVKRALGSNAVEDGKGCFELYHKCDDQCMETI
RNGTYNRRKYQEESKLERQKIEGVKLESEGTYKILTIYSTVASSLVIAMGFAAFLFWAMSNGSCRCNICI

>QOJ99389.1 hemagglutinin [Influenza A virus]
METVSLITILIVATVSKADKICIGYQSTNSTETVDTLTENNVPVTHAKELLHTEHNGMLCATSLGHPLIL
DTCTIEGLIYGNPSCDPLLGGREWSYIVERPSAVNGLCYPGNVENLEELRSLFSSSRSYQRIQIFPDTIW
NVSYSGTSKACSDSFYRSMRWLTQKNNAYPTQDAQYTNNQGKNILFMWGINHPPTDTAQTNLYTRTDTTT
SVATEEMNRIFKPLIGPRPLVNGLMGRINYYWSVLKPGQTLRIKSDGNLIAPWYGHILSGESHGRILKTD
LKRGSCTVQCQTEKGGLNTTLPFQNVSKYAFGNCSKYIGVKSLKLAVGLRNVPSRSSRGLFGAIAGFIEG
GWSGLVAGWYGFQHSNDQGVGMAADRDSTQKAIDKITSKVNNIVDKMNKQYEIIDHEFSEVETRLNMINN
KVDDQIQDIWAYNAELLVLLENQKTLDEHDANVNNLYNKVKRALGSNAVEDGKGCFELYHKCDDHCMETI
RNGTYNRRKYQEESKLERQKIEGVKLESEETYKILTIYSTVASSLVIAMGFAAFLFWAMSNGSCRCNICI

>QOJ99387.1 hemagglutinin [Influenza A virus]
METVSLITILLVATVSNADKICIGYQSTNSTETVDTLTENNVPVTHAKELLQTEHNGMLCATSLGQPLIL
DTCTVEGLIYGNPSCDLSLEGREWSYIVERPSAVNGLCYPGNVENLEELRSLFSSARSYQRIQIFPDTIW
NVSYDGTSTACSGSFYKSMRWLTRKNGDYPIQDAQYTNNQGKNILFMWGINHPPTDTKQRDLYTRTDTTT
SVATEEINRVFKPLIGPRPLVNGLMGRIDYYWSVLKPGQTLRIKSDGNLIAPWFGHILSGESHGRILKTD

LKRGSCTVQCQTEKGGLNTTLPFQNVSKYAFGNCSKYIGIKSLKLAVGLRNVPSRSSRGLFGAIAGFIEG
GWSGLVAGWYGFQHSNDQGVGMAADRDSTQKAIDKITSKVNNIVDKMNKQYEIIDHEFSEVETRLNMINN
KIDDQIQDIWAYNAELLVLLENQKTLDEHDANVNNLYNKVKRALGSNAVEDGKGCFELYHKCDDQCMETI
RNGTYNRRKYQEESKLERQKIEGVKLESEGTYKILTIYSTVASSLVIAMGFAAFLFWAMSNGSCRCNICI

>QOJ99377.1 hemagglutinin [Influenza A virus]
METVSLITILIVATVSKADKICIGYQSTNSTETVDTLTENNVPVTHAKELLHTEHNGMLCATSLGHPLIL
DTCTIEGLIYGNPSCDPLLGGREWSYIVERPSAVNGLCYPGNVENLEELRSLFSSSRSYQRIQIFPDTIW
NVSYSGTSKACSDSFYRSMRWLTQKNNAYPTQDAQYTNNQGKNILFMWGINHPPTDTVQTNLYTRTDTTT
SVATEEMNRIFKPLIGPRPLVNGLMGRINYYWSVLKPGQTLRIKSDGNLIAPWYGHILSGESHGRILKTD
LKRGSCTVQCQTEKGGLNTTLPFQNVSKYAFGNCSKYIGVKSLKLAVGLRNVPSRSSRGLFGAIAGFIEG
GWSGLVAGWYGFQHSNDQGVGMAADRDSTQKAIDKITSKVNNIVDKMNKQYEIIDHEFSEVETRLNMINN
KVDDQIQDIWAYNAELLVLLENQKTLDEHDANVNNLYNKVKRALGSNAVEDGKGCFELYHKCDDHCMETI
RNGTYNRRKYQEESKLERQKIEGVKLESEETYKILTIYSTVASSLVIAMGFAAFLFWAMSNGSCRCNICI

>QOJ99372.1 hemagglutinin [Influenza A virus]
METVSLMTILLVAAVSNADKICIGYQSTNSTETVDTLTENNVPVTHAKELLHTEHNGMLCATSLGQPIIL
DTCTIEGLIYGNPSCDLSLEGREWSYIVERPSAVNGLCYPGNVENLEELRSLFSSARSYQRIQIFPDTIW
NVSYDGTSTACSGSFYRNMRWLTRKNGEYPIQDAQYTNNQGKNILFMWGINHPPADTTQRNLYTRTDTTT
SVATEEINRIFKPLIGPRPLVNGLMGRIDYYWSVLKPGQTLRIKSDGNLIAPWYGHILSGESHGRILKTD
LKRGSCTVQCQTEKGGLNTTLPFQNVSKYAFGNCSKYIGIKSLKLAVGLRNVPSRSSRGLFGAIAGFIEG
GWSGLVAGWYGFQHSNDQGVGMAADRDSTQKAVDKITSKVNTIVEKMNKQYEIIDHEFSEVETRLNMINN
KIDDQIQDIWAYNAELLVLLENQKTLDEHDANVNNLYNKVKRALGSNAVEDGKGCFELYHKCDDQCMETI
RNGTYNRRKYQEESKLERQKIEGVKLESEGTYKILTIYSTVASSLVIAMGFAAFLFWAMSNGSCRCNICI

>QOJ99367.1 hemagglutinin [Influenza A virus]
METVSLITILIAATVSNADKICIGYQSTNSTETVDTLTENNVPVTHAKELLHTEHNGMLCATSLGQPLIL
DTCTIEGLIYGNPSCDLSLEGREWSYIVERPSAINGLCYPGNVENLEELRSLFSSARSYQRIQIFPDTIW
NVSYDGTSTACSNSFYRSMRWLTRKDGNYPTQDAQYTNNQGKNILFMWGINHPPTDDTQRNLYTRTDTTT
SVATEEINRIFKPLIGPRPLVNGLMGRIDYYWSVLKPGQTLRIKSDGNLIAPWYGHILSGESHGRILKTD
LKRGSCTVQCQTEKGGLNTTLPFQNVSKYAFGNCSKYIGIKSLKLAVGLRNVPSRSSRGLFGAIAGFIEG
GWSGLVAGWYGFQHSNDQGVGMAADRDSTQKAIDKITSKVNNIVDKMNKQYEIIDHEFSEVETRLNMINN
KIDDQIQDIWAYNAELLVLLENQKTLDEHDANVNNLYNKVKRALGSNAVEDGKGCFELYHKCNDQCMETI
RNGTYNRRKYQEESKLERQKIEGVKLESEGTYKILTIYSTVASSLVIAMGFAAFLFWAMSNGSCRCNICI

>QOJ99307.1 hemagglutinin [Influenza A virus]

METVSLITILLVATVSNADKICIGYQSTNSTETVDTLTENNVPVTHAKELLHTEHNGMLCATSLGQPLIL
DTCTIEGLIYGNPSCDLSLEGREWSYIVERPSAVNGLCYPGNVENLEELRSLFSSARSYQRXQIFPDTIW
NVSYDGTSTACSXSFYRSMRWLTRKDGNYPTQDAQYTNNQGKNILFMWGINHPPTDETQRNLYTRTDTTT
SVATEEINRIFKPLIGPRPLVNGLMGRIDYYWSVLKPGQTLRIKSDGNLIAPWYGHILSGESHGRILKTD
LKKGSCTVQCQTEKGGLNTTLPFQNVSKYAFGNCSKYIGIKSLKLAVGLRNVPSRSSRGLFGAIAGFIEG
GWSGLVAGWYGFQHSNDQGVGMAADRDSTQKAIDKITSKVNNIVDKMNKQYEIIDHEFSEVETRLNMINN
KIDDQIQDIWAYNAELLVLLENQKTLDEHDANVNNLYNKVKRALGSNAXEDGKGCFELYHKCBDQCMETI
RNGTYNRXKYQEESKLERQXXEGVKLESEGTYKILTIYSTVASSLVIAMGFAAFLFWAMSNGSCRCNICI

>QOJ99299.1 hemagglutinin, partial [Influenza A virus]

ETVSLITILLVAAVSYADKICIGYQSTNSTETVDTLTENNVPVTHAKELLHTEHNGMLCATSLGQPLILD
TCTIEGLIYGNPSCDLSLEGREWSYIVERPSAVNGLCYPGNVENLEELRSLFSSARSYQRVQIFPDTIWN
VSYDGTSTACSGSFYRSMRWLTRKDGNYPTQDAQYTNNQGKNILFMWGINHPPTDDTQRSLYTRTDTTTS
VATEEINRIFKPLIGPRPLVNGLMGRIDYYWSVLKPGQTLRIKSDGNLIAPWYGYILSGESHGRILKTDL
KRGSCTVQCQTEKGGLNTTLPFQNVSKYAFGNCSKYIGIKSLKLAVGLRNVPSKSSRGLFGAIAGFIEGG
WSGLVAGWYGFQHSNDQGVGMAADRDSTQKAIDKITSKVNNIVDKMNKQYEIIDHEFSEVETRLNMINNK
IDDQIQDIWAYNAELLVLLENQKTLDEHDANVNNLYNKVKRALGSNAAEDGKGCFELYHKCNDQCMETIR
NGTYNRRKYQEESKLERQKIEGVKLESEGTYKILTIYSTVASSLVIAMGFAAFLFWAMSNGSCRCNICI

>QOJ99289.1 hemagglutinin [Influenza A virus]

METVSLITILLAATVSNADKICIGYQSTNSTETVDTLTENNVPVTHAKELLHTEHNGMLCATSLGQPLIL
DTCTIEGLIYGNPSCDPLLEEREWSYIVERPSAVNGLCYPGNVENLEELRSLFSSARSYQRIQIFPDTIW
NVSYDGTSNTCSGSFYRNMRWLTRKNGNYPIQDAQYTNNQGKNILFMWGINHPPTDDTQRNLYTRTDTTT
SVATEEINRIFKPLIGPRPLVNGLMGRINYYWSVLKPGQTLRIKSDGNLIAPWYGYILSGESHGRILRTD
LNRGSCTVQCQTEKGGLNTTLPFQNVSKYAFGNCSKYIGIKSLKLAVGLRNVPSRSSRGLFGAIAGFIEG
GWSGLVAGWYGFQHSNDQGVGMAADRESTQKAIDKITSKVNNIVDKMNKQYEIIDHEFSEVETRLNMINN
KIDDQIQDIWAYNAELLVLLENQKTLDEHDANVNNLYNKVKRALGSNAVEDGKGCFELYHKCDDQCMETI
RNGTYNRRKYQEESKLERQKIEGVKLESEGTYKILTIYSTVASSLVIAMGFAAFLFWAMSNGSCRCNICI

>QOJ99288.1 hemagglutinin [Influenza A virus]

METVSLITILLAATVSNADKICIGYQSTNSTETVDTLTENNVPVTHAKELLHTEHNGMLCATSLGQPLIL
DTCTIEGLIYGNPSCDPLLEEREWSYIVERPSAVNGLCYPGNVENLEELRSLFSSARSYQRIQIFPDTIW

NVSYDGTSNTCSGSFYRNMRWLTQKNGNYPIQDAQYTNNQGKNILFMWGINHPPTDDTQRNLYTRTDTTT
SVATEEINRIFRPLIGPRPLVNGLMGRINYHWSVLKPGQTLRIRSDGNLIAPWYGYILSGESHGRILRTD
LKRGSCTVQCQTEKGGLNTTLPFQNVSKYAFGNCSKYIGIKSLKLAVGLRNVPSRSSRGLFGAIAGFIEG
GWSGLVAGWYGFQHSNDQGVGMAADRESTQKAIDKITSKVNNIVDKMNKQYEIIEHEFSEVETRLNMINN
KIDDQIQDIWAYNAELLVLLENQKTLDEHDANVNNLYNKVKRALGSNAVEDGKGCFELYHKCDDQCMETI
RNGTYNRRKYQEESKLERQKIEGVKLESEGTYKILTIYSTVASSLVIAMGFAAFLFWAMSNGSCRCNICI

>QOJ99287.1 hemagglutinin [Influenza A virus]
METVSLITILLAATVSNADKICIGYQSTNSTETVDTLTENNVPVTHAKELIHTEHNGMLCATSLGQPLIL
DTCTIEGLIYGNPSCDPLLEEREWSYIVERPSAVNGLCYPGNVENLEELRSLFSSARSYQRIQIFPDTIW
NVSYDGTSNTCSGSFYRNMRWLTRKNGNYPIQDAQYTNNQGKNILFMWGINHPPTDDTQRNLYTRTDTTT
SVATEEINRIFRPLIGPRPLVNGLMGRINYYWSVLKPDQTLRIKSDGNLIAPWYGYILSGESHGRILRTD
LKRGSCTVQCQTEKGGLNTTLPFQNVSKYAFGNCSKYIGTKSLKLAVGLRNVPSRSSRGLFGAIAGFIEG
GWSGLVAGWYGFQHSNDQGVGMAADRESTQKAIDKITSKVNNIVDKMNKQYEIIDHEFSEVETRLNMINN
KIDDQIQDIWAYNAELLVLLENQKTLDEHDANVNNLYNKVKRALGSNAVEDGKGCFELYHKCDDQCMETI
RNGTYNRRKYQEESKLERQKIEGVKLESEGTYKILTIYSTVASSLVIAMGFAAFLFWAMSNGSCRCNICI

>QOJ99286.1 hemagglutinin, partial [Influenza A virus]
ETVSLITILLAATVSNADKICIGYQSTNSTETVDTLTENNVPVTHAKELLHTEHNGMLCATSLGQPLILD
TCTIEGLIYGNPSCDPLLEEREWSYIVERPSAVNGLCYPGNVENLEELRSLFSSARSYQRIQIFPDTIWN
VSYDGTSNTCSGSFYRNMRWLTRKNGNYPIQDAQYTNNQGKNILFMWGINHPPTDDTQRNLYTRTDTTTS
VATEEINRIFKPLIGPRPLVNGLMGRINYYWSVLKPGQTLRIKSDGNLIAPWYGYILSGESHGRILRTDL
NRGSCTVQCQTEKGGLNTTLPFQNVSKYAFGNCSKYIGIKSLKLAVGLRNVPSRSSRGLFGAIAGFIEGG
WSGLVAGWYGFQHSNDQGVGMAADRESTQKAIDKITSKVNNIVDKMNKQYEIIDHEFSEVETRLNMINNK
IDDQIQDIWAYNAELLVLLENQKTLDEHDANVNNLYNKVKRALGSNAVEDGKGCFELYHKCDDQCMETIR
NGTYNRRKYQDESKLERQKIEGVKLESEGTYKILTIYSTVASSLVIAMGFAAFLFWAMSNGSCRCNICI

>QOJ99285.1 hemagglutinin [Influenza A virus]
METVSLITILLAATVSNADKICIGYQSTNSTETVDTLTENNVPVTHAKELLHTEHNGMLCATSLGQPLIL
DTCTIEGLIYGNPSCDPLLEEREWSYIVERPSAVNGLCYPGNVENLEELRSLFSSARSYQRIQIFPDTIW
NVSYDGTSNTCSGSFYRNMRWLTRKNGNYPIQDAQYTNNQGKNILFMWGINHPPTDDTQRNLYTRTDTTT
SVATEEINRIFKPLIGPRPLVNGLMGRINYYWSVLKPGQTLRIKSDGNLIAPWYGYILSGESHGRILRTD
LNRGSCTVQCQTEKGGLNTTLPFQNVSKYAFGNCSKYIGIKSLKLAVGLRNVPSRSSRGLFGAIAGFIEG
GWSGLVAGWYGFQHSNDQGVGMAADRESTQKAIDKITSKVNNIVDKMNKQYEIIDHEFSEVETRLNMINN

KIDDQIQDIWAYNAELLVLLENQKTLDEHDANVNNLYNKVKRALGSNAVEDGKGCFELYHKCDDQCMETI
RNGTYNRRKYQEESKLERQKIEGVKLESEGTYKILTIYSTVASSLVIAMGFAAFLFWAMSNGSCRCNICI

>QOJ99284.1 hemagglutinin [Influenza A virus]
METVSLITILLAATVSNADKICIGYQSTNSTETVDTLTENNVPVTHAKELLHTEHNGMLCATSLGQPLIL
DTCTIEGLIYGNPSCDPLLEEREWSYIVERPSAVNGLCYPGNVENLEELRSLFSSARSYQRIQIFPDTIW
NVSYDGTSNTCSGSFYRNMRWLTRKNGNYPIQDAQYTNNQGKNILFMWGINHPPTDDTQRNLYTRTDTTT
SVATEEINRIFRPLIGPRPLVNGLMGRINYYWSVLKPGQTLRIRSDGNLIAPWYGYILSGESHGRILRTD
LKRGSCTVQCQTEKGGLNTTLPFQNISKYAFGNCSKYIGIKSLKLAVGLRNVPSRSSRGLFGAIAGFIEG
GWSGLVAGWYGFQHSNDQGVGMAADRESTQKAIDKITSKVNNIVDKMNKQYEIIDHEFSEVETRLNMINN
KIDDQIQDIWAYNAELLVLLENQKTLDEHDANVNNLYNKVKRALGSNAVEDGKGCFELYHKCDDQCMETI
RNGTYNRRKYQEESKLERQKIEGVKLESEGTYKILTIYSTVASSLVIAMGFAAFLFWAMSNGSCRCNICI

>QOJ99283.1 hemagglutinin [Influenza A virus]
METVSLITILLAATVSNADKICIGYQSTNSTETVDTLTENNVPVTHAKELLHTEHNGMLCATSLGQPLIL
DTCTIEGLIYGNPSCDPLLEEREWSYIVERPSAVNGLCYPGNVENLEELRSLFSSARSYQRIQIFPDTIW
NVSYDGTSNTCSGSFYRNMRWLTRKNGNYPIQDAQYTNNQGKNILFMWGINHPPTDDTQRNLYTRTDTTT
SVATEEINRIFRPLIGPRPLVNGLMGRINYYWSVLKPGQTLRIRSDGNLIAPWYGYILSGESHGRILRTD
LKRGSCTVQCQTEKGGLNTTLPFQNISKYAFGNCSKYIGIKSLKLAVGLRNVPSRSSRGLFGAIAGFIEG
GWSGLVAGWYGFQHSNDQGVGMAADRESTQKAIDKITSKVNNIVDKMNKQYEIIDHEFSEVETRLNMINN
KIDDQIQDIWAYNAELLVLLENQKTLDEHDANVNNLYNKVKRALGSNAVEDGKGCFELYHKCDDQCMETI
RNGTYNRRKYQEESKLERQKIEGVKLESEGTYKILTIYSTVASSLVIAMGFAAFLFWAMSNGSCRCNICI

>QOJ99282.1 hemagglutinin [Influenza A virus]
METVSLITILLAATVSNADKICIGYQSTNSTETVDTLTENNVPVTHAKELLHTEHNGMLCATSLGQPLIL
DTCTIEGLIYGNPSCDPLLEEREWSYIVERPSAVNGLCYPGNVENLEELRSLFSSARSYQRIQIFPDTIW
NVSYDGTSNTCSGSFYRNMRWLTRKNGNYPIQDAQYTNNQGKNILFMWGINHPPTDDTQRNLYTRTDTTT
SVATEEINRIFKPLIGPRPLVNGLMGRINYYWSVLKPGQTLRIKSDGNLIAPWYGYILSGESHGRILRTD
LNRGSCTVQCQTEKGGLNTTLPFQNVSKYAFGNCSKYIGIKSLKLAVGLRNVPSRSSRGLFGAIAGFIEG
GWSGLVAGWYGFQHSNDQGVGMAADRESTQKAIDKITSKVNNIVDKMNKQYEIIDHEFSEVETRLNMINN
KIDDQIQDIWAYNAELLVLLENQKTLDEHDANVNNLYNKVKRALGSNAVEDGKGCFELYHKCDDQCMETI
RNGTYNRRKYQEESKLERQKIEGVKLESEGTYKILTIYSTVASSLVIAMGFAAFLFWAMSNGSCRCNICI

>QOJ99281.1 hemagglutinin [Influenza A virus]

METVSLITILLAATVSNADKICIGYQSTNSTETVDTLTENNVPVTHAKELLHTEHNGMLCATSLGQPLIL
DTCTIEGLIYGNPSCDPLLEEREWSYIVERPSAVNGLCYPGNVENLEELRSLFSSARSYQRIQIFPDTIW
NVSYDGTSNTCSGSFYRNMRWLTRKNGNYPIQDAQYTNNQGKNILFMWGINHPPTDDTQRNLYTRTDTTT
SVATEEINRIFKPLIGPRPLVNGLMGRINYYWSVLKPGQTLRIKSDGNLIAPWYGYILSGESHGRILRTD
LKRGSCTVQCQTEKGGLNTTLPFQNVSKYAFGNCSKYIGIKSLKLAVGLRNVPSRSSRGLFGAIAGFIEG
GWSGLVAGWYGFQHSNDQGVGMAADRESTQKAIDKITSKVNNIVDKMNKQYEIIDHEFSEVETRLNMINN
KIDDQIQDIWAYNAELLVLLENQKTLDEHDANVNNLYNKVKRALGSNAVEDGKGCFELYHKCDDQCMETI
RNGTYNRRKYQEESKLERQKIEGVKLESEGTYKILTIYSTVASSLVIAMGFAAFLFWAMSNGSCRCNICI

>QOJ99280.1 hemagglutinin [Influenza A virus]
METVSLITILLAATVSNADKICIGYQSTNSTETVDTLTENNVPVTHAKELLHTEHNGMLCATSLGQPLIL
DTCTIEGLIYGNPSCDPLLEEREWSYIVERPSAVNGLCYPGNVENLEELRSLFSSARSYQRIQIFPDTIW
NVSYDGTSNTCSGSFYRNMRWLTRKNGNYPIQDAQYTNNQGKNILFMWGINHPPTDDTQRNLYTRTDTTT
SVATEEINRIFKPLIGPRPLVNGLMGRINYYWSVLKPGQTLRIKSDGNLIAPWYGYILSGESHGRILKTD
LKRGSCTVQCQTEKGGLNTTLPFQNVSKYAFGNCSKYIGIKSLKLAVGLRNVPSRSSRGLFGAIAGFIEG
GWSGLVAGWYGFQHSNDQGVGMAADRESTQKAIDKITSKVNNIVNKMNKQYEIIDHEFSEVETRLNMINN
KIDDQIQDIWAYNAELLVLLENQKTLDEHDANVNNLYNKVKRALGSNAVEDGKGCFELYHKCDDQCMETI
RNGTYNRRKYQEESKLERQKIEGVKLESEETYKILTIYSTVASSLVIAMGFAAFLFWAMSNGSCRCNICI

>QOJ99279.1 hemagglutinin [Influenza A virus]
METVSLITILLAATVSNADKICIGYQSTNSTETVDTLTENNVPVTHAKELLHTEHNGMLCATSLGQPLIL
DTCTIEGLIYGNPSCDPLLEEREWSYIVERPSAVNGLCYPGNVENLEELRSLFSSARSYQRIQIFPDTIW
NVSYDGTSNTCSGSFYRNMRWLTRKNGNYPIQDAQYTNNQGKNILFMWGINHPPTDDTQRNLYTRTDTTT
SVATEEINRIFKPLIGPRPLVNGLMGRINYYWSVLKPGQTLRIKSDGNLIAPWYGYILSGESHGRILKTD
LKRGSCTVQCQTEKGGLNTTLPFQNVSKYAFGNCSKYIGIKSLKLAVGLRNVPSRSSRGLFGAIAGFIEG
GWSGLVAGWYGFQHSNDQGVGMAADRESTQKAIDKITSKVNNIVNKMNKQYEIIDHEFSEVETRLNMINN
KIDDQIQDIWAYNAELLVLLENQKTLDEHDANVNNLYNKVKRALGSNAVEDGKGCFELYHKCDDQCMETI
RNGTYNRRKYQEESKLERQKIEGVKLESEETYKILTIYSTVASSLVIAMGFAAFLFWAMSNGSCRCNICI

>QOJ99278.1 hemagglutinin [Influenza A virus]
METVSLITILLAATVSNADKICIGYQSTNSTETVDTLTENNVPVTHAKELLHTEHNGMLCATSLGQPLIL
DTCTIEGLIYGNPSCDPLLEEREWSYIVERPSAVNGLCYPGNVENLEELRSLFSSARSYQRIQIFPDTIW
NVSYDGTSNTCSGSFYRNMRWLTRKNGNYPIQDAQYTNNQGKNILFMWGINHPPTDDTQRNLYTRTDTTT
SVATEEINRIFKPLIGPRPLVNGLMGRINYYWSVLKPGQTLRIKSDGNLIAPWYGYILSGESHGRILKTD

LKRGSCTVQCQTEKGGLNTTLPFQNVSKYAFGNCSKYIGIKSLKLAVGLRNVPSRSSRGLFGAIAGFIEG
GWSGLVAGWYGFQHSNDQGVGMAADRESTQKAIDKITSKVNNIVNKMNKQYEIIDHEFSEVETRLNMINN
KIDDQIQDIWAYNAELLVLLENQKTLDEHDANVNNLYNKVKRALGSNAVEDGKGCFELYHKCDDQCMETI
RNGTYNRRKYQEESKLERQKIEGVKLESEETYKILTIYSTVASSLVIAMGFAAFLFWAMSNGSCRCNICI

>QOJ99277.1 hemagglutinin [Influenza A virus]
METVSLITILLAATVSNADKICIGYQSTNSTETVDTLTENNVPVTHAKELLHTEHNGMLCATSLGQPLIL
DTCTIEGLIYGNPSCDPLLEEREWSYIVERPSAVNGLCYPGNVENLEELRSLFSSARSYQRIQIFPDTIW
NVSYDGTSNTCSGSFYRNMRWLTRKNGNYPIQDAQYTNNQGKNILFMWGINHPPTDDTQRNLYTRTDTTT
SVATEEINRIFKPLIGPRPLVNGLMGRINYYWSVLKPGQTLRIKSDGNLIAPWYGYILSGESHGRILKTD
LKRGSCTVQCQTEKGGLNTTLPFQNVSKYAFGNCSKYIGIKSLKLAVGLRNVPSRSSRGLFGAIAGFIEG
GWSGLVAGWYGFQHSNDQGVGMAADRESTQKAIDKITSKVNNIVNKMNKQYEIIDHEFSEVETRLNMINN
KIDDQIQDIWAYNAELLVLLENQKTLDEHDANVNNLYNKVKRALGSNAVEDGKGCFELYHKCDDQCMETI
RNGTYNRRKYQEESKLERQKIEGVKLESEETYKILTIYSTVASSLVIAMGFAAFLFWAMSNGSCRCNICI

>QOJ99275.1 hemagglutinin [Influenza A virus]
METVSLITILLVATVSYADKICVGYQSTNSTETVDTLTENNVPVTHAKELLHTEHNGMLCATSLGQPLIL
DTCTIEGLIYGNPSCDLSLEGKEWSYIVERPSAVNGLCYPGNVENLEELRSLFSSARSYQRVQIFPDTIW
NVSYDGTSTSCSGSFYRSMRWLTRKNGDYPTQDAQYTNNQGKNILFTWGINHPPTDDTQRNLYTRTDTTT
SVATEEINRIFKPLIGPRPLVNGLMGRIAYYWSVLKPGQTLRIKSDGNLIAPWYGYILSGESHGRILKTD
LKRGSCTVQCQTEKGGLNTTLPFQNVSKYAFGNCSKYIGIKSLKLAVGLRNVPSRSSRGLFGAIAGFIEG
GWSGLVAGWYGFQHSNDQGVGMAADRDSTQKAIDKITSKVNNIVDKMNKQYEIIDHEFSEVETRLNMINN
KIDDQIQDIWAYNAELLVLLENQKTLDEHDANVNNLYNKVKRALGSNAVEDGKGCFELYHKCNDQCMETI
RNGTYNRRKYQEESKLERQKIEGVKLESEGTYKILTIYSTVASSLVIAMGFAAFLFWAMSNGSCRCNICI

>QOJ99274.1 hemagglutinin [Influenza A virus]
METVSLITILLVATVSNADKICIGYQSTNSTETVDTLTENNVPVTHAKELLHTEHNGMLCATSLGQPLIL
DTCTIEGLIYGNPSCDLSLEGREWSYIVERPSAVNGLCYPGNVENLEELRSLFSSARSYQRIQIFPDTIW
NVSYDGTSTACSGSFYKSMRWLTRKNGDYPTQDAQYTNNQGKNILFMWGINHPPTDTTQRELYTRTDTTT
SVATEEINRVFKPLIGPRPLVNGLMGRIDYYWSVLKPGQTLRIKSDGNLIAPWFGHILSGESHGRILKTD
LKRGSCTVQCQTEKGGLNTTLPFQNVSKYAFGNCSKYIGIKSLKLAVGLRNVPSRSSRGLFGAIAGFIEG
GWSGLVAGWYGFQHSNDQGVGMAADRDSTQKAIDKITSKVNNIVDKMNKQYEIIDHEFSEVETRLNMINN
KIDDQIQDIWAYNAELLVLLENQKTLDEHDANVNNLYNKVKRALGSNAVEDGKGCFELYHKCDDQCMETI
RNGTYNRRKYQEESKLERQKIEGVKLESEGTYKILTIYSTVASSLVIAMGFAAFLFWAMSNGSCRCNICI

>QOJ99224.1 hemagglutinin, partial [Influenza A virus]
METVSLITILLVAAVSYADKICIGYQSTNSTETVDTLTENNVPVTHAKELLHTEHNGMLCATSLGQPLIL
DTCTIEGLIYGNPSCDLSLEGREWSYIVERPSAVNGLCYPGNVENLEELRSLFSSARSYQRVQIFPDTIW
NVSYDGTSTACSGSFYRSMRWLTRKDGNYPTQDAQYTNNQGKNILFMWGINHPPTDDTQRSLYTRTDTTT
SVATEEINRIFKPLIGPRPLVNGLMGRIDYYWSVLKPGQTLRIKSDGNLIAPWYGYILSGESHGRILKTD
LKRGSCTVQCQTEKGGLNTTLPFQNVSKYAFGNCSKYIGIKSLKLAVGLRNVPSKSSRGLFGAIAGFIEG
GWSGLVAGWYGFQHSNDQGVGMAADRDSTQKAIDKITSKVNNIVDKMNKQYEIIDHEFSEVETRLNMINN
KIDDQIQDIWAYNAELLVLLENQKTLDEHDANVNNLYNKVKRALGSNAAEDGKGCFELYHKCNDQCMETI
RNGTYNRRKYQEESKLERQKIEGVKLESEGTYKILTIYSTVASSLVIAMGFAAFLFWAMSNGSCRCNICI

>QOJ99223.1 hemagglutinin, partial [Influenza A virus]
ETVSLITILLVAAVSYADKICIGYQSTNSTETVDTLTENNVPVTHAKELLHTEHNGMLCATSLGQPLILD
TCTIEGLIYGNPSCDLSLEGREWSYIVERPSAVNGLCYPGNVENLEELRSLFSSARSYQRVQIFPDTIWN
VSYDGTSTACSGSFYRSMRWLTRKDGNYPTQDAQYTNNQGKNILFMWGINHPPTDDTQRSLYTRTDTTTS
VATEEINRIFKPLIGPRPLVNGLMGRIDYYWSVLKPGQTLRIKSDGNLIAPWYGYILSGESHGRILKTDL
KRGSCTVQCQTEKGGLNTTLPFQNVSKYAFGNCSKYIGIKSLKLAVGLRNVPSKSSRGLFGAIAGFIEGG
WSGLVAGWYGFQHSNDQGVGMAADRDSTQKAIDKITSKVNNIVDKMNKQYEIIDHEFSEVETRLNMINNK
IDDQIQDIWAYNAELLVLLENQKTLDEHDANVNNLYNKVKRALGSNAAEDGKGCFELYHKCNDQCMETIR
NGTYNRRKYQEESKLERQKIEGVKLESEGTYKILTIYSTVASSLVIAMGFAAFLFWAMSNGSCRCNICI

>QOJ99222.1 hemagglutinin [Influenza A virus]
METVSLITILLVAAVSYADKICIGYQSTNSTETVDTLTENNVPVTHAKELLHTEHNGMLCATSLGQPLIL
DTCTIEGLIYGNPSCDLSLEGREWSYIVERPSAVNGLCYPGNVENLEELRSLFSSARSYQRVQIFPDTIW
NVSYDGTSTACSGSFYRNMRWLTRKDGNYPTQDAQYTNNQGKNILFMWGINHPPTDDTQRSLYTRTDTTT
SVATEEINRIFKPLIGPRPLVNGLMGRIDYYWSVLKPGQTLRIKSDGNLIAPWYGYILSGESHGRILKTD
LKRGSCTVQCQTEKGGLNTTLPFQNVSKYAFGNCSKYIGIKSLKLAVGLRNVPSKSSRGLFGAIAGFIEG
GWSGLVAGWYGFQHSNDQGVGMAADRDSTQKAIDKITSKVNNIVDKMNKQYEIIDHEFSEVETRLNMINN
KIDDQIQDIWAYNAELLVLLENQKTLDEHDANVNNLYNKVKRALGSNAAEDGKGCFELYHKCNDQCMETI
RNGTYNRRKYQEESKLERQKIEGVKLESEGTYKILTIYSTVASSLVIAMGFAAFLFWAMSNGSCRCNICI

>QOJ99221.1 hemagglutinin [Influenza A virus]
METVSLITILLVAAVSYADKICIGYQSTNSTETVDTLTENNVPVTHAKELLHTEHNGMLCATSLGQPLIL
DTCTIEGLIYGNPSCDLSLEGREWSYIVERPSAVNGLCYPGNVENLEELRSLFSSARSYQRVQIFPDTIW

NVSYDGTSTACSGSFYRSMRWLTRKDGNYPTQDAQYTNNQGKNILFMWGINHPPTDDTQRSLYTRTDTTT
SVATEEINRIFKPLIGPRPLVNGLMGRIDYYWSVLKPGQTLRIKSDGNLIAPWYGYILSGESHGRILKTD
LKRGSCTVQCQTEKGGLNTTLPFQNVSKYAFGNCSKYIGIKSLKLAVGLRNVPSKSSRGLFGAIAGFIEG
GWSGLVAGWYGFQHSNDQGVGMAADRDSTQKAIDKITSKVNNIVDKMNKQYEIIDHEFSEVETRLNMINN
KIDDQIQDIWAYNAELLVLLENQKTLDEHDANVNNLYNKVKRALGSNAAEDGKGCFELYHKCNDQCMETI
RNGTYNRRKYQEESKLERQKIEGVKLESEGTYKILTIYSTVASSLVIAMGFAAFLFWAMSNGSCRCNICI

>QOJ99220.1 hemagglutinin [Influenza A virus]
METVSLITILLVAAVSYADKICIGYQSTNSTETVDTLTENNVPVTHAKELLHTEHNGMLCATSLGQPLIL
DTCTIEGLIYGNPSCDLSLEGREWSYIVERPSAVNGLCYPGNVENLEELRSLFSSARSYQRVQIFPDTIW
NVSYDGTSTACSGSFYRSMRWLTRKDGNYPTQDAQYTNNQGKNILFMWGINHPPTDDTQRSLYTRTDTTT
SVATEEINRIFKPLIGPRPLVNGLMGRIDYYWSVLKPGQTLRIKSDGNLIAPWYGYILSGESHGRILKTD
LKRGSCTVQCQTEKGGLNTTLPFQNVSKYAFGNCSKYIGIKSLKLAVGLRNVPSKSSRGLFGAIAGFIEG
GWSGLVAGWYGFQHSNDQGVGMAADRDSTQKAIDKITSKVNNIVDKMNKQYEIIDHEFSEVETRLNMINN
KIDDQIQDIWAYNAELLVLLENQKTLDEHDANVNNLYNKVKRALGSNAAEDGKGCFELYHKCNDQCMETI
RNGTYNRRKYQEESKLERQKIEGVKLESEGTYKILTIYSTVASSLVIAMGFAAFLFWAMSNGSCRCNICI

>QOJ99219.1 hemagglutinin, partial [Influenza A virus]
ETVSLITILLVATVSNADKICIGYQSTNSTETVDTLTENNVPVTHAKELLHTEHNGMLCATSLGQPLILD
TCTIEGLIYGNPFCDLSLEGREWSYIVERPSAVHGLCYPGNVEDLEELRSLFSSARSYQRIQIFPDTIWN
VSYDGTSTACSGSFYKSMRWLTRKNGEYPTQDAQYTNNQGKNILFMWGINHPPTDETQRGLYTRTDTTTS
VATEEINRIFKPLIGPRPLVNGLMGRINYYWSVLKPGQTLRIKSDGNLIAPWYGHILSGESHGRILKTDL
KRGSCTVQCQTEKGGLNTTLPFQNVSKYAFGNCSKYIGIKSLKLAVGLRNVPSRSSRGLFGAIAGFIEGG
WSGLVAGWYGFQHSNDQGVGMAADRDSTQKAIDKITSKVNNIVDKMNKQYEIIDHEFSEVETRLNMINNK
IDDQIQDIWAYNAELLVLLENQKTLDEHDANVNNLYNKVKRALGSNAVEDGKGCFELYHKCDDQCMETIR
NGTYNRRKYQEESKLERQRIEGVKLESEGTYKILTIYSTVASSLVIAMGFAAFLFWAMSNGSC

>QOJ99215.1 hemagglutinin, partial [Influenza A virus]
ETVSLITILLVAAVSYADKICIGYQSTNSTETVDTLTENNVPVTHAKELLHTEHNGMLCATSLGQPLILD
TCTIEGLIYGNPSCDLSLEGREWSYIVERPSAVNGLCYPGNVENLEELRSLFSSARSYQRVQIFPDTIWN
VSYDGTSTACSGSFYRSMRWLTRKDGNYPTQDAQYTNNQGKNILFMWGINHPPTDDTQRSLYTRTDTTTS
VATEEINRIFKPLIGPRPLVNGLMGRIDYYWSVLKPGQTLRIKSDGNLIAPWYGYILSGESHGRILKTDL
KRGSCTVQCQTEKGGLNTTLPFQNVSKYAFGNCSKYIGIKSLKLAVGLRNVPSKSSRGLFGAIAGFIEGG
WSGLVAGWYGFQHSNDQGVGMAADRDSTQKAIDKITSKVNNIVDKMNKQYEIIDHEFSEVETRLNMINNK

IDDQIQDIWAYNAELLVLLENQKTLDEHDANVNNLYNKVKRALGSNAAEDGKGCFELYHKCNDQCMETIR
NGTYNRRKYQEESKLERQKIEGVKLESEGTYKILTIYSTVASSLVIAMGFAAFLFWAMSNGSCRCNICI

>QOJ99214.1 hemagglutinin [Influenza A virus]
METISLITILLVATVSYADKICIGYQSTNSTETVDTLTENNVPVTHAKELLHTEHNGMLCATSLGQPLIL
DTCTIEGLIYGNPSCDLSLEGKEWSYIVERPSAVNGLCYPGNVENLEELRSLFSSARSYQRVQIFPDTIW
NVSYDGTSKACSGSFYRSMRWLNRKDGNYPTQDAQYTNNQGKNILFMWGINHPPTDDTQRSLYTKTDTTT
SVATEEINRIFKPLIGPRPLVNGLMGRIDYYWSVLKPGQTLRIKSDGNLIAPWYGYILSGESHGRILKTD
LKRGSCTVQCQTEKGGLNTTLPFQNVSKYAFGNCSKYIGIKSLKLAVGLRNVPSRSSRGLFGAIAGFIEG
GWSGLVAGWYGFQHSNDQGVGMAADRDSTQKAIDKITSKVNNIVDKMNKQYEIIDHEFSEVETRLNMINN
KIDDQIQDIWAYNAELLVLLENQKTLDEHDANVNNLYNKVKRALGSNAAEDGKGCFELYHKCNDQCMETI
RNGTYNRRKYQEESKLERQKIEGVKLESEGTYKILTIYSTVASSLVIAMGFAAFLFWAMSNGSCRCNICI

>QOJ99213.1 hemagglutinin [Influenza A virus]
METVSLITILLVAAVSYADKICIGYQSTNSTETVDTLTENNVPVTHAKELLHTEHNGMLCATSLGQPLIL
DTCTIEGLIYGNPSCDLSLEGREWSYIVERPSAVNGLCYPGNVENLEELRSLFSSARSYQRVQIFPDTIW
NVSYDGTSTACSGSFYRNMRWLTRKDGNYPTQDAQYTNNQGKNILFMWGINHPPTDDTQRSLYTRTDTTT
SVATEEINRIFKPLIGPRPLVNGLMGRIDYYWSVLKPGQTLRIKSDGNLIAPWYGYILSGESHGRILKTD
LKRGSCTVQCQTEKGGLNTTLPFQNVSKYAFGNCSKYIGIKSLKLAVGLRNVPSKSSRGLFGAIAGFIEG
GWSGLVAGWYGFQHSNDQGVGMAADRDSTQKAIDKITSKVNNIVDKMNKQYEIIDHEFSEVETRLNMINN
KIDDQIQDIWAYNAELLVLLENQKTLDEHDANVNNLYNKVKRALGSNAAEDGKGCFELYHKCNDQCMETI
RNGTYNRRKYQEESKLERQKIEGVKLESEGTYKILTIYSTVASSLVIAMGFAAFLFWAMSNGSCRCNICI

>QOJ99212.1 hemagglutinin [Influenza A virus]
METVSLITILLVAAVSYADKICIGYQSTNSTETVDTLTENNVPVTHAKELLHTEHNGMLCATSLGQPLIL
DTCTIEGLIYGNPSCDLSLEGREWSYIVERPSAVNGLCYPGNVENLEELRSLFSSARSYQRVQIFPDTIW
NVSYDGTSTACSGSFYRNMRWLTRKDGNYPTQDAQYTNNQGKNILFMWGINHPPTDDTQRSLYTRTDTTT
SVATEEINRIFKPLIGPRPLVNGLMGRIDYYWSVLKPGQTLRIKSDGNLIAPWYGYILSGESHGRILKTD
LKRGSCTVQCQTEKGGLNTTLPFQNVSKYAFGNCSKYIGIKSLKLAVGLRNVPSKSSRGLFGAIAGFIEG
GWSGLVAGWYGFQHSNDQGVGMAADRDSTQKAIDKITSKVNNIVDKMNKQYEIIDHEFSEVETRLNMINN
KIDDQIQDIWAYNAELLVLLENQKTLDEHDANVNNLYNKVKRALGSNAAEDGKGCFELYHKCNDQCMETI
RNGTYNRRKYQEESKLERQKIEGVKLESEGTYKILTIYSTVASSLVIAMGFAAFLFWAMSNGSCRCNICI

>QOJ99211.1 hemagglutinin [Influenza A virus]

METVSLITILLVAAVSYADKICIGYQSTNSTETVDTLTENNVPVTHAKELLHTEHNGMLCATSLGQPLIL
DTCTIEGLIYGNPSCDLSLEGREWSYIVERPSAVNGLCYPGNVENLEELRSLFSSARSYQRVQIFPDTIW
NVSYDGTSTACSGSFYRNMRWLTRKDGNYPTQDAQYTNNQGKNILFMWGINHPPTDDTQRSLYTRTDTTT
SVATEEINRIFKPLIGPRPLVNGLMGRIDYYWSVLKPGQTLRIKSDGNLIAPWYGYILSGESHGRILKTD
LKRGSCTVQCQTEKGGLNTTLPFQNVSKYAFGNCSKYIGIKSLKLAVGLRNVPSKSSRGLFGAIAGFIEG
GWSGLVAGWYGFQHSNDQGVGMAADRDSTQKAIDKITSKVNNIVDKMNKQYEIIDHEFSEVETRLNMINN
KIDDQIQDIWAYNAELLVLLENQKTLDEHDANVNNLYNKVKRALGSNAAEDGKGCFELYHKCNDQCMETI
RNGTYNRRKYQEESKLERQKIEGVKLESEGTYKILTIYSTVASSLVIAMGFAAFLFWAMSNGSCRCNICI

>QOJ99210.1 hemagglutinin, partial [Influenza A virus]
ETVSLITILLVAAVSYADKICIGYQSTNSTETVDTLTENNVPVTHAKELLHTEHNGMLCATSLGQPLILD
TCTIEGLIYGNPSCDLSLEGREWSYIVERPSAVNGLCYPGNVENLEELRSLFSSARSYQRVQIFPDTIWN
VSYDGTSTACSGSFYRNMRWLTRKDGNYPTQDAQYTNNQGKNILFMWGINHPPTDDTQRSLYTRTDTTTS
VATEEINRIFKPLIGPRPLVNGLMGRIDYYWSVLKPGQTLRIKSDGNLIAPWYGYILSGESHGRILKTDL
KRGSCTVQCQTEKGGLNTTLPFQNVSKYAFGNCSKYIGIKSLKLAVGLRNVPSKSSRGLFGAIAGFIEGG
WSGLVAGWYGFQHSNDQGVGMAADRDSTQKAIDKITSKVNNIVDKMNKQYEIIDHEFSEVETRLNMINNK
IDDQIQDIWAYNAELLVLLENQKTLDEHDANVNNLYNKVKRALGSNAAEDGKGCFELYHKCNDQCMETIR
NGTYNRRKYQEESKLERQKIEGVKLESEGTYKILTIYSTVASSLVIAMGFAAFLFWAMSNGSCRCNICI

>QOJ99209.1 hemagglutinin [Influenza A virus]
METVSLITILLVAAVSYADKICIGYQSTNSTETVDTLTENNVPVTHAKELLHTEHNGMLCATSLGQPLIL
DTCTIEGLIYGNPSCDLSLEGREWSYIVERPSAVNGLCYPGNVENLEELRSLFSSARSYQRVQIFPDTIW
NVSYDGTSTACSGSFYRNMRWLTRKDGNYPTQDAQYTNNQGKNILFMWGINHPPTDDTQRSLYTRTDTTT
SVATEEINRIFKPLIGPRPLVNGLMGRIDYYWSVLKPGQTLRIKSDGNLIAPWYGYILSGESHGRILKTD
LKRGSCTVQCQTEKGGLNTTLPFQNVSKYAFGNCSKYIGIKSLKLAVGLRNVPSKSSRGLFGAIAGFIEG
GWSGLVAGWYGFQHSNDQGVGMAADRDSTQKAIDKITSKVNNIVDKMNKQYEIIDHEFSEVETRLNMINN
KIDDQIQDIWAYNAELLVLLENQKTLDEHDANVNNLYNKVKRALGSNAAEDGKGCFELYHKCNDQCMETI
RNGTYNRRKYQEESKLERQKIEGVKLESEGTYKILTIYSTVASSLVIAMGFAAFLFWAMSNGSCRCNICI

>QOJ99208.1 hemagglutinin [Influenza A virus]
METISLITILLVATVSYADKICIGYQSTNSTETVDTLTENNVPVTHAKELLHTEHNGMLCATSLGQPLIL
DTCTIEGLIYGNPSCDLSLEGKEWSYIVERPSAVNGLCYPGNVENLEELRSLFSSARSYQRVQIFPDTIW
NVSYDGTSKACSGSFYRSMRWLNRKDGNYPTQDAQYTNNQGKNILFMWGINHPPTDDTQRSLYTKTDTTT
SVATEEINRIFKPLIGPRPLVNGLMGRIDYYWSVLKPGQTLRIKSDGNLIAPWYGYILSGESHGRILKTD

LKRGSCTVQCQTEKGGLNTTLPFQNVSKYAFGNCSKYIGIKSLKLAVGLRNVPSRSSRGLFGAIAGFIEG
GWSGLVAGWYGFQHSNDQGVGMAADRDSTQKAIDKITSKVNNIVDKMNKQYEIIDHEFSEVETRLNMINN
KIDDQIQDIWAYNAELLVLLENQKTLDEHDANVNNLYNKVKRALGSNAAEDGKGCFELYHKCNDQCMETI
RNGTYNRRKYQEESKLERQKIEGVKLESEGTYKILTIYSTVASSLVIAMGFAAFLFWAMSNGSCRCNICI

>QOJ99207.1 hemagglutinin, partial [Influenza A virus]
METVSLITILLVAAVSYADKICIGYQSTNSTETVDTLTENNVPVTHAKELLHTEHNGMLCATSLGQPLIL
DTCTIEGLIYGNPSCDLSLEGREWSYIVERPSAVNGLCYPGNVENLEELRSLFSSARSYQRVQIFPDTIW
NVSYDGTSTACSGSFYRSMRWLTRKDGNYPTQDAQYTNNQGKNILFMWGINHPPTDDTQRSLYTRTDTTT
SVATEEINRIFKPLIGPRPLVNGLMGRIDYYWSVLKPGQTLRIKSDGNLIAPWYGYILSGESHGRILKTD
LKRGSCTVQCQTEKGGLNTTLPFQNVSKYAFGNCSKYIGIKSLKLAVGLRNVPSKSSRGLFGAIAGFIEG
GWSGLVAGWYGFQHSNDQGVGMAADRDSTQKAIDKITSKVNNIVDKMNKQYEIIDHEFSEVETRLNMINN
KIDDQIQDIWAYNAELLVLLENQKTLDEHDANVNNLYNKVKRALGSNAAEDGKGCFELYHKCNDQCMETI
RNGTYNRRKYQEESKLERQKIEGVKLESEGTYKILTIYSTVASSLVIAMGFAAFLFWAMSNGSCRCNICI

>QOJ99206.1 hemagglutinin [Influenza A virus]
METVSLITILLVAAVSYADKICIGYQSTNSTETVDTLTENNVPVTHAKELLHTEHNGMLCATSLGQPLIL
DTCTIEGLIYGNPSCDLSLEGREWSYIVERPSAVNGLCYPGNVENLEELRSLFSSARSYQRVQIFPDTIW
NVSYDGTSTACSGSFYRSMRWLTRKDGNYPTQDAQYTNNQGKNILFMWGINHPPTDDTQRSLYTRTDTTT
SVATEEINRIFKPLIGPRPLVNGLMGRIDYYWSVLKPGQTLRIKSDGNLIAPWYGYILSGESHGRILKTD
LKRGSCTVQCQTEKGGLNTTLPFQNVSKYAFGNCSKYIGIKSLKLAVGLRNVPSKSSRGLFGAIAGFIEG
GWSGLVAGWYGFQHSNDQGVGMAADRDSTQKAIDKITSKVNNIVDKMNKQYEIIDHEFSEVETRLNMINN
KIDDQIQDIWAYNAELLVLLENQKTLDEHDANVNNLYNKVKRALGSNAAEDGKGCFELYHKCNDQCMETI
RNGTYNRRKYQEESKLERQKIEGVKLESEGTYKILTIYSTVASSLVIAMGFAAFLFWAMSNGSCRCNICI

>QOJ99205.1 hemagglutinin [Influenza A virus]
METVSLITILLVAAVSYADKICIGYQSTNSTETVDTLTENNVPVTHAKELLHTEHNGMLCATSLGQPLIL
DTCTIEGLIYGNPSCDLSLEGREWSYIVERPSAVNGLCYPGNVENLEELRSLFSSARSYQRVQIFPDTIW
NVSYDGTSTACSGSFYRSMRWLTRKDGNYPTQDAQYTNNQGKNILFMWGINHPPTDDTQRSLYTRTDTTT
SVATEEINRIFKPLIGPRPLVNGLMGRIDYYWSVLKPGQTLRIKSDGNLIAPWYGYILSGESHGRILKTD
LKRGSCTVQCQTEKGGLNTTLPFQNVSKYAFGNCSKYIGIKSLKLAVGLRNVPSKSSRGLFGAIAGFIEG
GWSGLVAGWYGFQHSNDQGVGMAADRDSTQKAIDKITSKVNNIVDKMNKQYEIIDHEFSEVETRLNMINN
KIDDQIQDIWAYNAELLVLLENQKTLDEHDANVNNLYNKVKRALGSNAAEDGKGCFELYHKCNDQCMETI
RNGTYNRRKYQEESKLERQKIEGVKLESEGTYKILTIYSTVASSLVIAMGFAAFLFWAMSNGSCRCNICI

>QOJ99204.1 hemagglutinin [Influenza A virus]

METVSLITILLVAAVSYADKICIGYQSTNSTETVDTLTENNVPVTHAKELLHTEHNGMLCATSLGQPLIL

DTCTIEGLIYGNPSCDLSLEGREWSYIVERPSAVNGLCYPGNVENLEELRSLFSSARSYQRVQIFPDTIW

NVSYDGTSTACSGSFYRSMRWLTRKDGNYPTQDAQYTNNQGKNILFMWGINHPPTDDTQRSLYTRTDTTT

SVATEEINRIFKPLIGPRPLVNGLMGRIDYYWSVLKPGQTLRIKSDGNLIAPWYGYILSGESHGRILKTD

LKRGSCTVQCQTEKGGLNTTLPFQNVSKYAFGNCSKYIGIKSLKLAVGLRNVPSKSSRGLFGAIAGFIEG

GWSGLVAGWYGFQHSNDQGVGMAADRDSTQKAIDKITSKVNNIVDKMNKQYEIIDHEFSEVETRLNMINN

KIDDQIQDIWAYNAELLVLLENQKTLDEHDANVNNLYNKVKRALGSNAAEDGKGCFELYHKCNDQCMETI

RNGTYNRRKYQEESKLERQKIEGVKLESEGTYKILTIYSTVASSLVIAMGFAAFLFWAMSNGSCRCNICI

>QOJ99203.1 hemagglutinin [Influenza A virus]

METVSLITILLVAAVSYADKICIGYQSTNSTETVDTLTENNVPVTHAKELLHTEHNGMLCATSLGQPLIL

DTCTIEGLIYGNPSCDLSLEGREWSYIVERPSAVNGLCYPGNVENLEELRSLFSSARSYQRVQIFPDTIW

NVSYDGTSTACSGSFYRSMRWLTRKDGNYPTQDAQYTNNQGKNILFMWGINHPPTDDTQRSLYTRTDTTT

SVATEEINRIFKPLIGPRPLVNGLMGRIDYYWSVLKPGQTLRIKSDGNLIAPWYGYILSGESHGRILKTD

LKRGSCTVQCQTEKGGLNTTLPFQNVSKYAFGNCSKYIGIKSLKLAVGLRNVPSKSSRGLFGAIAGFIEG

GWSGLVAGWYGFQHSNDQGVGMAADRDSTQKAIDKITSKVNNIVDKMNKQYEIIDHEFSEVETRLNMINN

KIDDQIQDIWAYNAELLVLLENQKTLDEHDANVNNLYNKVKRALGSNAAEDGKGCFELYHKCNDQCMETI

RNGTYNRRKYQEESKLERQKIEGVKLESEGTYKILTIYSTVASSLVIAMGFAAFLFWAMSNGSCRCNICI

>QOJ99202.1 hemagglutinin [Influenza A virus]

METVSLITILLVAAVSYADKICIGYQSTNSTETVDTLTENNVPVTHAKELLHTEHNGMLCATSLGQPLIL

DTCTIEGLIYGNPSCDLSLEGREWSYIVERPSAVNGLCYPGNVENLEELRSLFSSARSYQRVQIFPDTIW

NVSYDGTSTACSGSFYRSMRWLTRKDGNYPTQDAQYTNNQGKNILFMWGINHPPTDDTQRSLYTRTDTTT

SVATEEINRIFKPLIGPRPLVNGLMGRIDYYWSVLKPGQTLRIKSDGNLIAPWYGYILSGESHGRILKTD

LKRGSCTVQCQTEKGGLNTTLPFQNVSKYAFGNCSKYIGIKSLKLAVGLRNVPSKSSRGLFGAIAGFIEG

GWSGLVAGWYGFQHSNDQGVGMAADRDSTQKAIDKITSKVNNIVDKMNKQYEIIDHEFSEVETRLNMINN

KIDDQIQDIWAYNAELLVLLENQKTLDEHDANVNNLYNKVKRALGSNAAEDGKGCFELYHKCNDQCMETI

RNGTYNRRKYQEESKLERQKIEGVKLESEGTYKILTIYSTVASSLVIAMGFAAFLFWAMSNGSCRCNICI

>QOJ99201.1 hemagglutinin [Influenza A virus]

METVSLITILLVAAVSYADKICIGYQSTNSTETVDTLTENNVPVTHAKELLHTEHNGMLCATSLGQPLIL

DTCTIEGLIYGNPSCDLSLEGREWSYIVERPSAVNGLCYPGNVENLEELRSLFSSARSYQRVQIFPDTIW

NVSYDGTSTACSGSFYRSMRWLTRKDGNYPTQDAQYTNNQGKNILFMWGINHPPTDDTQRSLYTRTDTTT
SVATEEINRIFKPLIGPRPLVNGLMGRIDYYWSVLKPGQTLRIKSDGNLIAPWYGYILSGESHGRILKTD
LKRGSCTVQCQTEKGGLNTTLPFQNVSKYAFGNCSKYIGIKSLKLAVGLRNVPSKSSRGLFGAIAGFIEG
GWSGLVAGWYGFQHSNDQGVGMAADRDSTQKAIDKITSKVNNIVDKMNKQYEIIDHEFSEVETRLNMINN
KIDDQIQDIWAYNAELLVLLENQKTLDEHDANVNNLYNKVKRALGSNAAEDGKGCFELYHKCNDQCMETI
RNGTYNRRKYQEESKLERQKIEGVKLESEGTYKILTIYSTVASSLVIAMGFAAFLFWAMSNGSCRCNICI

>QOJ99200.1 hemagglutinin, partial [Influenza A virus]
ETVSLITILLVAAVSYADKICIGYQSTNSTETVDTLTENNVPVTHAKELLHTEHNGMLCATSLGQPLILD
TCTIEGLIYGNPSCDLSLEGREWSYIVERPSAVNGLCYPGNVENLEELRSLFSSARSYQRVQIFPDTIWN
VSYDGTSTACSGSFYRSMRWLTRKDGNYPTQDAQYTNNQGKNILFMWGINHPPTDDTQRSLYTRTDTTTS
VATEEINRIFKPLIGPRPLVNGLMGRIDYYWSVLKPGQTLRIKSDGNLIAPWYGYILSGESHGRILKTDL
KRGSCTVQCQTEKGGLNTTLPFQNVSKYAFGNCSKYIGIKSLKLAVGLRNVPSKSSRGLFGAIAGFIEGG
WSGLVAGWYGFQHSNDQGVGMAADRDSTQKAIDKITSKVNNIVDKMNKQYEIIDHEFSEVETRLNMINNK
IDDQIQDIWAYNAELLVLLENQKTLDEHDANVNNLYNKVKRALGSNAAEDGKGCFELYHKCNDQCMETIR
NGTYNRRKYQEESKLERQKIEGVKLESEGTYKILTIYSTVASSLVIAMGFAAFLFWAMSNGSCRCNICI

>QOJ99199.1 hemagglutinin [Influenza A virus]
METVSLITILLVAAVSYADKICIGYQSTNSTETVDTLTENNVPVTHAKELLHTEHNGMLCATSLGQPLIL
DTCTIEGLIYGNPSCDLSLEGREWSYIVERPSAVNGLCYPGNVENLEELRSLFSSARSYQRVQIFPDTIW
NVSYDGTSTACSGSFYRSMRWLTRKDGNYPTQDAQYTNNQGKNILFMWGINHPPTDDTQRSLYTRTDTTT
SVATEEINRIFKPLIGPRPLVNGLMGRIDYYWSVLKPGQTLRIKSDGNLIAPWYGYILSGESHGRILKTD
LKRGSCTVQCQTEKGGLNTTLPFQNVSKYAFGNCSKYIGIKSLKLAVGLRNVPSKSSRGLFGAIAGFIEG
GWSGLVAGWYGFQHSNDQGVGMAADRDSTQKAIDKITSKVNNIVDKMNKQYEIIDHEFSEVETRLNMINN
KIDDQIQDIWAYNAELLVLLENQKTLDEHDANVNNLYNKVKRALGSNAAEDGKGCFELYHKCNDQCMETI
RNGTYNRRKYQEESKLERQKIEGVKLESEGTYKILTIYSTVASSLVIAMGFAAFLFWAMSNGSCRCNICI

>QOJ99198.1 hemagglutinin, partial [Influenza A virus]
ETVSLITILLVAAVSYADKICIGYQSTNSTETVDTLTENNVPVTHAKELLHTEHNGMLCATSLGQPLILD
TCTIEGLIYGNPSCDLSLEGREWSYIVERPSAVNGLCYPGNVENLEELRSLFSSARSYQRVQIFPDTIWN
VSYDGTSTACSGSFYRSMRWLTRKDGNYPTQDAQYTNNQGKNILFMWGINHPPTDDTQRSLYTRTDTTTS
VATEEINRIFKPLIGPRPLVNGLMGRIDYYWSVLKPGQTLRIKSDGNLIAPWYGYILSGESHGRILKTDL
KRGSCTVQCQTEKGGLNTTLPFQNVSKYAFGNCSKYIGIKSLKLAVGLRNVPSKSSRGLFGAIAGFIEGG
WSGLVAGWYGFQHSNDQGVGMAADRDSTQKAIDKITSKVNNIVDKMNKQYEIIDHEFSEVETRLNMINNK

IDDQIQDIWAYNAELLVLLENQKTLDEHDANVNNLYNKVKRALGSNAAEDGKGCFELYHKCNDQCMETIR
NGTYNRRKYQEESKLERQKIEGVKLESEGTYKILTIYSTVASSLVIAMGFAAFLFWAMSNGSCRCNICI

>QOJ99197.1 hemagglutinin [Influenza A virus]
METVSLITILLVATVSYADKICIGYQSTNSTETVDTLTENNVPVTHAKELLHTEHNGMLCATSLGQPLIL
DTCTIEGLIYGNPSCDLSLEGREWSYIVERPSAVNGLCYPGNVENLEELRSLFSSARSYQRVQIFPDTIW
NVSYDGTSTACSGSFYRSMRWLTRKDGNYPTQDAQYTNNQGKNILFMWGINHPPTDDTQRSLYTRTDTTT
SVATEEINRIFKPLIGPRPLVNGLMGRIDYYWSVLKPGQTLRIKSDGNLIAPWYGYILSGESHGRILKTD
LKRGSCTVQCQTEKGGLNTTLPFQNVSKYAFGNCSKYIGIKSLKLAVGLRNVPSRSSRGLFGAIAGFIEG
GWSGLVAGWYGFQHSNDQGVGMAADRDSTQKAIDKITSKVNNIVDKMNKQYEIIDHEFSEVETRLNMINN
KIDDQIQDIWAYNAELLVLLENQKTLDEHDANVNNLYNKVKRALGSNAAEDGKGCFELYHKCNDQCMETI
RNGTYNRKKYQEESKLERQKIEGVKLESEGTYKILTIYSTVASSLVIAMGFAAFLFWAMSNGSCRCNICI

>QOJ99196.1 hemagglutinin [Influenza A virus]
METVSLITILLVATVSYADKICIGYQSTNSTETVDTLTENNVPVTHAKELLHTEHNGMLCATSLGQPLIL
DTCTIEGLIYGNPSCDLSLEGREWSYIVERPSAVNGLCYPGNVENLEELRSLFSSARSYQRVQIFPDTIW
NVSYDGTSTACSGSFYRSMRWLTRKDGNYPTQDAQYTNNQGKNILFMWGINHPPTDDTQRSLYTRTDTTT
SVATEEINRIFKPLIGPRPLVNGLMGRIDYYWSVLKPGQTLRIKSDGNLIAPWYGYILSGESHGRILKTD
LKRGSCTVQCQTEKGGLNTTLPFQNVSKYAFGNCSKYIGIKSLKLAVGLRNVPSRSSRGLFGAIAGFIEG
GWSGLVAGWYGFQHSNDQGVGMAADRDSTQKAIDKITSKVNNIVDKMNKQYEIIDHEFSEVETRLNMINN
KIDDQIQDIWAYNAELLVLLENQKTLDEHDANVNNLYNKVKRALGSNAAEDGKGCFELYHKCNDQCMETI
RNGTYNRKKYQEESKLERQKIEGVKLESEGTYKILTIYSTVASSLVIAMGFAAFLFWAMSNGSCRCNICI

>QOJ99195.1 hemagglutinin [Influenza A virus]
METVSLITILLVATVSYADKICIGYQSTNSTETVDTLTENNVPVTHAKELLHTEHNGMLCATSLGQPLIL
DTCTIEGLIYGNPSCDLSLEGREWSYIVERPSAVNGLCYPGNVENLEELRSLFSSARSYQRVQIFPDTIW
NVSYDGTSTACSGSFYRSMRWLTRKDGNYPTQDAQYTNNQGKNILFMWGINHPPTDDTQRSLYTRTDTTT
SVATEEINRIFKPLIGPRPLVNGLMGRIDYYWSVLKPGQTLRIKSDGNLIAPWYGYILSGESHGRILKTD
LKRGSCTVQCQTEKGGLNTTLPFQNVSKYAFGNCSKYIGIKSLKVGVGVRNVGCRCSRGVLGAIAGFIEG
GWSGLVAGWYGFQHSNDQGVGMAADRDSTQKAIDKITSKVNNIVDKMNKQYEIIDHEFSEVETRLNMINN
KIDDQIQDIWAYNAELLVLLENQKTLDEHDANVNNLYNKVKRALGSNAAEDGKGCFELYHKCNDQCMETI
RNGTYNRKKYQEESKLERQKIEGVKLESEGTYKILTIYSTVASSLVIAMGFAAFLFWAMSNGSCRCNICI

>QOJ99194.1 hemagglutinin [Influenza A virus]

METVSLMTILLVATVSNADKICIGYQSTNSTETVDTLTENNVPVTHAKELIHTEHNGMLCATSLGQPLIL
DTCTIEGLIYGNPSCDLSLEGREWSYIVERPSAVNGLCYPGNVENLEELRSLFSSARSYQRIQIFPDTIW
NVSYDGTSTACSGSFYKNMRWLTRKDGNYPTQDAQYTNNQGKNILFMWGINHPPADTTQTGLYTRTDTTT
SVATEEINRIFKPLIGPRPLVNGLMGRIDYYWSVLKPGQTLRIKSDGNLIAPWFGHILSGESHGRILKTD
LKRGSCTVQCQTEKGGLNTTLPFQNVSKYAFGNCSKYIGIKSLKLAVGLRNVPSRSSRGLFGAIAGFIEG
GWSGLVAGWYGFQHSNDQGVGMAADRDSTQKAIDKITSKVNNIVDKMNKQYEIIDHEFSEVETRLNMINN
KIDDQIQDIWAYNAELLVLLENQKTLDEHDANVNNLYNKVKRALGSNAVEDGKGCFELYHKCDDQCMETI
RNGTYNRRKYQEESKLERQKIEGVKLESEGTYKILTIYSTVASSLVIAMGFAAFLFWAMSNGSCRCNICI

>QOJ99193.1 hemagglutinin, partial [Influenza A virus]
METVSLITILLVATVSNADKICIGYQSTNSTETVDTLTENNVPVTHAKELLHTEHNGMLCATSLGQPLIL
DTCTIEGLIYGNPSCDPLLGGREWSYIVERPSAVNGLCYPGNVENLEELRSLFSSSRSYQRIQIFPDTIW
NVSYDGTSKACSDSFYRSMRWLTQKNNAYPTQDAQYTNNQGKNILFMWGINHPPTDTAQTNLYTRTDTTT
SVATEEINRIFKPLIGPRPLVNGLMGRINYYWSVLKPGQTLRIKSDGNLIAPWYGYILSGESHGRILKTD
LKRGSCTVQCQTEKGGLNTTLPFQNVSKYAFGNCSKYIGIKSLKLAVGLRNVPSRSSRGLFGAIAGFIEG
GWSGLVAGWYGFQHSNDQGVGMAADRESTQKAIDKITSKVNNIVDKMNKQYEIIDHEFSEVETRLNMINN
KIDDQIQDIWAYNAELLVLLENQKTLDEHDANVNNLYNKVKRALGSNAVEDGKGCFELYHKCDDQCMETI
RNGTYNRRKYQEESKLERQKIEGVKLESEGTYKILTIYSTVASSLVIAMGFAAFLF

>QOJ99192.1 hemagglutinin [Influenza A virus]
METVSLITILLVATVSNADKICIGYQSTNSTETVDTLTENNVPVTHAKELLHTEHNGMLCATSLGQPLIL
DTCTIEGLIYGNPSCDPLLGGREWSYIVERPSAVNGLCYPGNVENLEELRSLFSSARSYQRIQIFPDTIW
NVSYDGTSKACSDSFYRSMRWLTQKNNAYPTQDAQYTNNQGKNILFMWGINHPPTDTAQRNLYTRTDTTT
SVATEEINRIFRPLIGPRPLVNGLMGRINYYWSVLKPGQTMRIKSDGNLIAPWYGYILSGESHGRILKTD
LKRGSCTVQCQTEKGGLNTTLPFQNVSKYAFGNCSKYIGIKSLKLAVGLRNVPSRSSRGLFGAIAGFIEG
GWSGLVAGWYGFQHSNDQGVGMAADRESTQKAIDKITSKVNNIVDKMNKQYEIIDHEFSEVETRLNMINN
KIDDQIQDIWAYNAELLVLLENQKTLDEHDANVNNLYNKVKRALGSNAVEDGKGCFELYHKCDDQCMETI
RNGTYNRRKYQEESKLERQKIEGVKLESEGTYKILTIYSTVASSLVIAMGFAAFLFWAMSNGSCRCNICI

>QOJ99191.1 hemagglutinin, partial [Influenza A virus]
METVSLITILVVATVSNADKICIGYQSTNSTETVDTLTENNVPVTHAKELLHTEHNGMLCATSLGHPLIL
DTCTIEGLIYGNPSCDPLLGGREWSYIVERPSAVNGLCYPGSVENLEELRSLFSSSRSYQRIQIFPDTIW
NVSYSGTSKACSDSFYRSMRWLTQKNNAYPTQDAQYTNNQGKNILFMWGINHPPTDTAQTNLYTRTDTTT
SVATEEMNRIFKPLIGPRPLVNGLMGRINYYWSVLKPGQTLRIKSDGNLIAPWYGHILSGESHGRILKTD

LKRGSCTVQCQTEKGGLNTTLPFQNVSKYAFGNCSKYIGVKSLKLAVGLRNVPSRSSRGLFGAIAGFIEG
GWSGLVAGWYGFQHSNDQGVGMAADRDSTQKAIDKITSKVNNIVDKMNKQYEIIDHEFSEVETRLNMINN
KVDDQIQDIWAYNAELLVLLENQKTLDEHDANVNNLYNKVKRALGSNAVEDGKGCFELYHKCDDHCMETI
RNGTYNRRKYQEESKLERQKIEGVKLESEETYKILTIYSTVASSLVIAMGFAAFLFWAMSNGSC

>QOJ99187.1 hemagglutinin, partial [Influenza A virus]
ETVSLITILLVATVSNADKICIGYQSTNSTETVDTLTENNVPVTHAKELLHTEHNGMLCATSLGQPLILD
TCTIEGLIYGNPXCDLSLEGREWSYIVERPSAVHGLCYPGNVEDLEELRSLFSSARSYQRIQIFPDTIWN
VSYDGTSTACSGSFYKSMRWLTRKNGEYPTQDAQYTNNQGKNILFMWGINHPPTDETQRGLYTRTDTTTS
VATEEINRIFKPLIGPRPLVNGLMGRINYYWSVLKPGQTLRIKSDGNLIAPWYGHILSGESHGRILKTDL
KRGSCTVQCQTEKGGLNTTLPFQNVSKYAFGNCSKYIGIKSLKLAVGLRNVPSRSSRGLFGAIAGFIEGG
WSGLVAGWYGFQHSNDQGVGMAADRDSTQKAIDKITSKVNNIVDKMNKQYEIIDHEFSEVETRLNMINNK
IDDQIQDIWAYNAELLVLLENQKTLDEHDANVNNLYNKVKRALGSNAVEDGKGCFELYHKCDDQCMETIR
NGTYNRRKYQEESKLERQRIEGVKLESEGTYKILTIYSTVASSLVIAMGFAAFLFWAMSNGSC

>QOJ99185.1 hemagglutinin [Influenza A virus]
METVSLITILLVATVSNADKICIGYQSTNSTETVDTLTENNVPVTHAKELLHTEHNGMLCATSLGQPLIL
DTCTIEGLIYGNPSCDPLLGGREWSYIVERPSAVNGLCYPGNVENLEELRSLFSSSRSYQRIQIFPDTIW
NVSYSGTSKACSDSFYRSMRWLTQKNNAYPTQDAQYTNNQGKNILFMWGINHPPTDTAQTNLYTRTDTTT
SVATEEMNRIFKPLIGPRPLVNGLMGRINYYWSVLKPGQTLRIKSDGNLIAPWYGYILSGESHGRILKTD
LKRGSCTVQCQTEKGGLNTTLPFQNVSKYAFGNCSKYIGVKSLKLAVGLRNVPSRSSRGLFGAIAGFIEG
GWSGLVAGWYGFQHSNDQGVGMAADRESTQKAIDKITSKVNNIVDKMNKQYEIIDHEFSEVETRLNMINN
KIDDQIQDIWAYNAELLVLLENQKTLDEHDANVNNLYNKVKRALGSNAVEDGKGCFELYHKCDDQCMETI
RNGTYNRRKYQEESKLERQKIEGVKLESEGTYKILTIYSTVASSLVIAMGFAAFLFWAMSNGSCRCNICI

>QOJ99155.1 hemagglutinin, partial [Influenza A virus]
METVSLITILVVATVSNADKICIGYQSTNSTETVDTLTENNVPVTHAKELLHTEHNGMLCATSLGHPLIL
DTCTIEGLIYGNPSCDPLLGGREWSYIVERPSAVNGLCYPGNVENLEELRSLFSSSRSYQRIQIFPDTIW
NVSYSGTSKACSDSFYRSMRWLTQKNNAYPTQDAQYTNNQGKNILFMWGINHPPTDTAQTNLYTRTDTTT
SVATEEMNRIFKPLIGPRPLVNGLMGRINYYWSVLKPGQTLRIKSNGNLIAPWYGHILSGESHGRILKTD
LKRGSCTVQCQTEKGGLNTTLPFQNVSKYAFGNCSKYIGVKSLKLAVGLRNVPSRSSRGLFGAIAGFIEG
GWSGLVAGWYGFQHSNDQGVGMAADRDSTQKAIDKITSKVNNIVDKMNKQYEIIDHEFSEVETRLNMINN
KVDDQIQDIWAYNAELLVLLENQKTLDEHDANVNNLYNKVKRALGSNAVEDGKGCFELYHKCDDHCMETI
RNGTYNRRKYQEESKLGRQKIEGVKLESEETYKILTIYSTVASSLVIAM

>QOJ99154.1 hemagglutinin, partial [Influenza A virus]

METVSLITILVVATVSNADKICIGYQSTNSTETVDTLTENNVPVTHAKELLHTEHNGMLCATSLGHPLIL
DTCTIEGLIYGNPSCDPLLGGREWSYIVERPSAVNGLCYPGNVENLEELRSLFSSSRSYQRIQIFPDTIW
NVSYSGTSKACSDSFYRSMRWLTQKNNAYPTQDAQYTNNQGKNILFMWGINHPPTDTAQTNLYTRTDTTT
SVATEEMNRIFKPLIGPRPLVNGLMGRINYYWSVLKPGQTLRIKSNGNLIAPWYGHILSGESHGRILKTD
LKRGSCTVQCQTEKGGLNTTLPFQNVSKYAFGNCSKYIGVKSLKLAVGLRNVPSRSSRGLFGAIAGFIEG
GWSGLVAGWYGFQHSNDQGVGMAADRDSTQKAIDKITSKVNNIVDKMNKQYEIIDHEFSEVETRLNMINN
KVDDQIQDIWAYNAELLVLLENQKTLDEHDANVNNLYNKVKRALGSNAVEDGKGCFELYHKCDDHCMETI
RNGTYNRRKYQEESKLGRQKIEGVKLESEETYKILTIYSTVASSLVIAM

>QOJ99153.1 hemagglutinin, partial [Influenza A virus]

METVSLITILVVATVSNADKICIGYQSTNSTETVDTLTENNVPVTHAKELLHTEHNGMLCATSLGHPLIL
DTCTIEGLIYGNPSCDPLLGGREWSYIVERPSAVNGLCYPGNVENLEELRSLFSSSRSYQRIQIFPDTIW
NVSYSGTSKACSDSFYRSMRWLTQKNNAYPTQDAQYTNNQGKNILFMWGINHPPTDTAQTNLYTRTDTTT
SVATEEMNRIFKPLIGPRPLVNGLMGRINYYWSVLKPGQTLRIKSNGNLIAPWYGHILSGESHGRILKTD
LKRGSCTVQCQTEKGGLNTTLPFQNVSKYAFGNCSKYIGVKSLKLAVGLRNVPSRSSRGLFGAIAGFIEG
GWSGLVAGWYGFQHSNDQGVGMAADRDSTQKAIDKITSKVNNIVDKMNKQYEIIDHEFSEVETRLNMINN
KVDDQIQDIWAYNAELLVLLENQKTLDEHDANVNNLYNKVKRALGSNAVEDGKGCFELYHKCDDHCMETI
RNGTYNRRKYQEESKLGRQKIEGVKLESEETYKILTIYSTVASSLVIAM

>QOJ99151.1 hemagglutinin, partial [Influenza A virus]

METVSLITILVVATVSNADKICIGYQSTNSTETVDTLTENNVPVTHAKELLHTEHNGMLCATSLGHPLIL
DTCTIEGLIYGNPSCDPLLGGREWSYIVERPSAVNGLCYPGNVENLEELRSLFSSSRSYQRIQIFPDTIW
NVSYSGTSKACSDSFYRSMRWLTQKNNAYPTQDAQYTNNQGKNILFMWGINHPPTDTAQTNLYTRTDTTT
SVATEEMNRIFKPLIGPRPLVNGLMGRINYYWSVLKPGQTLRIKSNGNLIAPWYGHILSGESHGRILKTD
LKRGSCTVQCQTEKGGLNTTLPFQNVSKYAFGNCSKYIGVKSLKLAVGLRNVPSRSSRGLFGAIAGFIEG
GWSGLVAGWYGFQHSNDQGVGMAADRDSTQKAIDKITSKVNNIVDKMNKQYEIIDHEFSEVETRLNMINN
KVDDQIQDIWAYNAELLVLLENQKTLDEHDANVNNLYNKVKRALGSNAVEDGKGCFELYHKCDDHCMETI
RNGTYNRRKYQEESKLGRQKIEGVKLESEETYKILTIYSTVASSLVIAM

>QOJ99144.1 hemagglutinin, partial [Influenza A virus]

VSLIIILLTATVSNADKICIGYQSTNSTETVDTLTENNVPVTHAKELLHTEHNGMLCATSLGQPLILDTC
TIEGLIYGNPSCDPLPEEREWSYIVERPSAVNGLCYPGNVENLEELRSLFSSARSYQRVQIFPDTIWNVS

YDGTSNTCSGSFYRNMRWLTRKDGNYPIQDAQYTNNQGKNILFMWGINNPPTDDTQRNLYTRTDTTTSVA
TEEINRIFKPLIGPRPLVNGLMGRINYYWSVLKPGQTLRIKSDGNLVAPWYGYILSGESHGRILRTDLKR
GSCTVQCQTEKGGLNTTLPFQNVSKYAFGNCSKYIGIKSLKLAVGLRNVPSRSSRGLFGAIAGFIEGGWS
GLVAGWYGFQHSNDQGVGMAADRESTQKAVDKITSKVNNIVDKMNKQYEIIDHEFSEIETRLNMINNKID
DQIQDIWAYNAELLVLLENQKTLDEHDANVNNLYNKVKRALGTNAVEDGKGCFELYHKCDDQCMETIRNG
TYNRRKYQEESKLERQKIEGVKLESEGTYKILTIYSTVASSLVIAMGFAAFLFWAMSNGSCRCNICI

>QOJ99140.1 hemagglutinin [Influenza A virus]
METVSLITILLAATVSNADKICIGYQSTNSTETVDTLTENNVPVTHAKELLHTEHNGMLCATSLGQPLIL
DTCTIEGLIYGNPSCDPLPEEREWSYIVERPSAVNGLCYPGNVENLEELRSLFSSARSYQRIQIFPDTIW
NVSYDGTSNTCSGSFYRNMRWLTRKDGNYPTQDAQYTNNQGKNILFMWGINNPPTDDTQRNLYTRTDTTT
SVATEEINRIFKPLIGPRPLVNGLMGRINYYWSVLKPGQTLRIKSDGNLVAPWYGYILSGESHGRILRTD
LKRGSCTVQCQTEKGGLNTTLPFQNVSKYAFGNCSKYIGIKSLKLAVGLRNVPSRSSRGLFGAIAGFIEG
GWSGLVAGWYGFQHSNDQGVGMAADRESTQKAVDKITSKVNNIVDKMNKQYEIIDHEFSEVETRLNMINN
KIDDQIQDIWAYNAELLVLLENQKTLDEHDANVNNLYNKVKRALGSNAVEDGKGCFELYHKCDDQCMETI
RNGTYNRRKYQEESKLERQKIEGVKLESEGTYKILTIYSTVASSLVIAMGFAAFLFWAMSNGSCRCNICI

>QOJ99139.1 hemagglutinin [Influenza A virus]
METVSLITILLVATVSNADKICIGYQSTNSTETVDTLTENNVPVTHAKELIHTEHNGMLCATSLGQPLIL
DTCTIEGLIYGNPSCDLSLEGREWSYIVERPSAVNGLCYPGNVENLEELRSLFSSARSYQRIQIFPDTIW
NVSYDGTSIACSGSFYRNMRWLTRKDGNYPTQDAQYTNNQGKNILFMWGINHPPTDTTQSDLYTRTDTKT
SVATEEINRVFKPLIGPRPLVNGLMGRIDYYWSVLKPGQTLRIKSDGNLIAPWFGHILSGESHGRILKTD
LKRGSCTVQCQTEKGGLNTTLPFQNVSKYAFGNCSKYIGIKSLKLAIGLRNVPSRSSRGLFGAIAGFIEG
GWSGLVAGWYGFQHSNDQGVGMAADRDSTQKAIDKITSKVNNIVDKMNKQYEIIDHEFSEVETRLNMINN
KIDDQIQDIWAYNAELLVLLENQKTLDEHDANVNNLYNKVKKALGSNAVEDGKGCFELYHKCDDQCMETI
RNGTYNRRKYQEESKLERQKIEGVKLESEGTYKILTIYSTVASSLVIAMGFAAFLFWAMSNGSCRCNICI

>QOJ99138.1 hemagglutinin [Influenza A virus]
METVSLITVLLVATISNADKICIGYQSTNSTETVDTLTENNVPVTHAKELIHTEHNGMLCATNLGQPLIL
DTCTIEGLIYGNPSCDLSLEGREWSYIVERPSAVNGLCYPGNVENLEELRSLFSSARSYQRVQIFPDTIW
NVSYDGTSTACSGSFYRNMRWLTRKDGNYPTQDAQYTNNQGKNILFMWGINHPPTDTTQSGLYTRTDTTT
SVATEEINRIFKPLIGPRPLVNGLMGRIDYYWSVLKPGQTLRIKSDGNLIAPWFGHILSGESHGRILKTD
LKRGSCTVQCQTEKGGLNTTLPFQNVSKYAFGNCSKYIGIKSLKLAVGLRNVPSRSSRGLFGAIAGFIEG
GWSGLVAGWYGFQHSNDQGVGMAADRDSTQKAIDKITSKVNNIVDKMNKQYEIIDHEFSEVETRLNMINN

KIDDQIQDIWAYNAELLVLLENQKTLDEHDANVNNLYNKVKRALGSNAVEDGKGCFELYHKCDDQCMETI
RNGTYNRRKYQEESKLERQKIEGVKLESEGTYKILTIYSTVASSLVIAMGFAAFLFWAMSNGSCRCNICI

>QOJ99126.1 hemagglutinin, partial [Influenza A virus]
ETVSLITILLVATASNADKICIGYQSTNSTETVDTLTENNVPVTHAKELLHTEHNGMLCATSLGQPLILD
TCTVEGLIYGNPSCDLSLEGREWSYIVERPSAVNGLCYPGNVENLEELRLLFSSARSYQRIQIFPDTIWN
VSYDGTSKACSDSFYRSMRWLTRKDGNYPTQDAQYTNNQGKNILFMWGINHPPTDDTQRNLYTRTDTTTS
VATEEINRIFKPLIGPRPLVNGLMGRIDYYWSVLKPGQTLRIKSNGNLIAPWYGHILTGESHGRILKTDL
KRGSCTVQCQTEKGGLNTTLPFQNVSKYAFGNCSKYIGIKSLKLAVGLRNVPSRSSRGLFGAIAGFIEGG
WSGLVAGWYGFQHSNDQGVGMAADRDSTQKAIDKITSKVNNIVDKMNKQYEIIDHEFSEVETRLNMINNK
IDDQIQDIWAYNAELLVLLENQKTLDEHDANVNNLYNKVKRALGSNAVEDGKGCFELYHKCNDQCMETIR
NGTYNRRKYQEESKLKRQKIEGVKLESEGTYKILTIYSTVASSLVIAMGFAAFLFWAMSNGSCR

>QOJ99125.1 hemagglutinin, partial [Influenza A virus]
ETVSLITILLVATASNADKICIGYQSTNSTETVDTLTENNVPVTHAKELLHTEHNGMLCATSLGQPXXXX
TCTVEGLIYGNPSCDLSLEGREWSYIVERPSAVNGLCYPGNVENLEELRSLFSSARSYQRIQIFPDTIWN
VSYDGTSKACSDSFYRSMRXXXXXXXXXXXXXXXXXXXXXXXXXXXXXXXXXXXXXXXXXXXXXXXXXXX
VATEEINRIFKPLIGPRPLVNGLMGRIDYYWSVLKPGQTLRIKSNGNLIAPWYGHILTGESHGRILKTDL
KRGSCTVQCQTEKGGLNXXXXXXXXXXXXXXXXXXXXXXXXXXXXXXXXXXXXXXXXXXXXAIAGFIEGG
WSGLVAGWYGFQHSNDQGVGMAADRDSTQKAIDKITSKVNNIVDKMNKQYEIIDHEFSEVETRLNMINNK
IDDQIQDIWAYNAELLVLLENQKTLDEHDANVNNLYNKVKRALGSNAVEDGKGCFELYHKCBDQCMETIR
NGTYNRRKYQEESKLXRQKIEGVKLESEGTYKILTIYSTVASSLVIAMGFAAFLFWAMSNGS

>QOJ99124.1 hemagglutinin, partial [Influenza A virus]
METVSLITILVVATVSNADKICIGYQSTNSTETVDTLTENNVPVTHAKELLHTEHNGMLCATGLGHPLIL
DTCTIEGLIYGNPSCDPLLGGREWSYIVERPSAVNGLCYPGNVENLEELRSLFSSSRSYQRIQIFPDTIW
NVSYSGTSKACSDSFYRSMRWLTQKNNAYPTQDAQYTNNQGKNILFMWGINHPPTDTXQTNLYTRTDTTT
SVATEEMNRIFKPLIGPRPLVNGLMGRINYYWSVLKPGQTLRIKSDGNLIAPWYGHILSGESHGRILKTD
LKRGSCTVQCQTEKGGLNTTLPFQNVSKYAFGNCSKYIGVKSLKLAVGLRNVPSRSSRGLFGAIAGFIEG
GWPGLVAGWYGFQHSNDQGVGMAADRDSTQKAIDKITSKVNNIVDKMNKQYEIIDHEFSEVETRLNMINN
KVDDQIQDIWAYNAELLVLLENQKTLDEHDANVNNLYNKVKRALGSNAVEDGKGCFELYHKCDDHCMETI
RNGTYNRRKYQEESKLERQKIEGVKLESEETYKILTIYSTVASSLVIAMGFAAFLFWAMSNGSC

>QOJ99123.1 hemagglutinin, partial [Influenza A virus]

ETVSLITILLVATASNADKICIGYQSTNSTETVDTLTENNVPVTHAKELLHTEHNGMLCATSLGQPLILD
TCTVEGLIYGNPSCDLSLEGREWSYIVERPSAVNGLCYPGNVENLEELRSLFSSARSYQRIQIFPDTIWN
VSYDGTSKACSDSFYRSMRWLTQKDGNYPTQDAQYTNNQGKNILFMWGINHPPTDDTQRNLYTRTDTTTS
VATEEINRIFKPLIGPRPLVNGLMGRIDYYWSVLKPGQTLRIKSNGNLIAPWYGHILTGESHGRILKTXX
XXXXXXXXXXXXKGGLNTTLPFQNVSKYAFGNCSKYIGIKSLKLAVGLRNVPSRSSRGLFGAIAGFIEGG
WSGLVAGWYGFQHSNDQGVGMAADRDSTQKAIDKITSKVNNIVDKMNKQYEIIDHEFSEVETRLNMXNNK
IDDQIQDIWAYNAELLVLLENQKTLDEHDANVNNLYNKVKRALGSNAVEDGKGCFELYHKCNDQCMETIR
NGTYNRRKYQEESKLXRQKIEGVKLESEGTYKILTIYSTVASSLVIAMGFAAFLFWAMSNGSC

>QOJ99122.1 hemagglutinin, partial [Influenza A virus]
TVSNADKICIGYQSTNSTETVDTLTENNVPVTHAKELLHTEHNGMLCATSXXXXXXXXXXXXXXXXXXXX
XXXXXXXXXXXXXXXXXXXXXXXXXXXXXXXXXXXXXXXXXXXXXXXXXXXXXXXXXXXXXXXXXXXXXX
XXXXXXXXXXXXXXXXXXXXXXXXXXXXXXXXXXXXXXXXXXXXXXXXXXXXXXXXXXXXXXXXXXXXXX
XXXXXXXXXXXXXXXXXXXXXXXXXXXXXXXXXXXXXXXXXXXXXXXXXXXXXXXXXXXXXXXXVQCQTE
KGGLNTTLPFQNVSKYAFGNCSKYIGIKSLKLAVGLRNVPSRSSRGLFGAIAGFIEGGWSGLVAGWYGFQ
HSNDQGVGMAADRDSTQKAIDKITSKVNNIVDKMNKQYEIIDHEFSEVETRLNMINNKIDDQIQDIWAYN
AELLVLLXNQKTLDEHDANVNNLYNKVKRALGSNAVEDGKGCFELYHKCBDXCMETIRNGTYNRRKYQEE
SKLXRQKIEGVKLESEXTYKILTIYSTVASSLVIAMGFAAXLFWAMSNGSC

>QOJ99120.1 hemagglutinin, partial [Influenza A virus]
ETVSLITILLVATASNADKICIGYQSTNSTETVDTLTENNVPVTHAKELLHTEHNGMLCATSLGQPLILD
TCTVEGLIYGNPSCDLSLEGREWSYIVERPSAVNGLCYPGNVENLEELRSLFSSARSYQRIQIFPDTIWN
VSYDGTSKACSDSFYRSMRWLTQKDGNYPTQDAQYTNNQGKNILFMWGIXHPPTDDTQRNLYTRTDTTTS
VATEEINRIFKPLIGPRPLVNGLMGRIDYYWSVLKPGQTLRIKSNGNLIAPWYGHILTGESHGRILKTDL
KRGSCTVQCQTEKGGLNTTLPFQNVSKYAFGNCSKYIGIKSLKLAVGLRNVPSRSSRGLFGAIAGFIEGG
WSGLVAGWYGFQHSNDQGVGMAADRDSTQKAIDKITSKVNNIVDKMNKQYEIIDHEFSEVETRLNMINNK
IDDQIQDIWAYNAELLVLLENQKTLDEHDANVNNLYNKVKRALGSNAVEDGKGCFELYHKCNDQCMETIR
NGTYNRRKYQEESKLKRQKIEGVKLESEGTYKILTIYSTVASSLVIAMGFAAFLFWAMSNGSCR

>QOJ99116.1 hemagglutinin [Influenza A virus]
METVSLITILLIATVSNAXKICIGYQSTNSTETVDTLTENNVPVTHAKELLHTEHNGMLCATSLGQPLIL
DTCTIEGLIYGNPSCDLSLEGREWSYIVERPSAVHGLCYPGNVEDLEELRSLFSSARSYQRIQIFPDTVW
NVSYDGTSTACSGSFYRSMRWLTRKNGEYPIQDAQYTNNQGKNILFMWGINHPPTDDKQRGLYTRTDTTT
SVATEEINRIFKPLIGPRPLVNGLMGRINYYWSILKPGQTLRIKSDGNLIAPWYGHILSGESHGRILKTD

LKRGSCTVQCQTEKGGLNTTLPFQNVSKYAFGNCSKYIGIKSLKLAVGLRNVPSRSSRGLFGAIAGFIEG
GWSGLVAGWYGFQHSNDQGVGMAADRESTQKAIDKITSKVNNIVDKMNKQYEIIDHEFSEVETRLNMINN
KIDDQIQDIWAYNAELLVLLENQKTLDEHDANVNNLYNKVKRALGSNAVEDGKGCFELYHKCDDQCMETI
RNGTYNRREYQEESKLERQRIEGVKLESEGTYKILTIYSTVASSLVIAMGFAAFLFWAMSNGSCRCNICI

>QOJ99112.1 hemagglutinin [Influenza A virus]
METVSLITILLIATVSNADKICIGYQSTNSTETVDTLTENNVPVTHAKELLHTEHNGMLCATSLGQPLIL
DTCTIEGLIYGNPSCDLSLEGREWSYIVERPSAVHGLCYPGNVEDLEELRSLFSSARSYQRIQIFPDTVW
NVSYDGTSTACSGSFYRSMRWLTRKNGEYPIQDAQYTNNQGKNILFMWGINHPPTDDTQRGLYTRTDTTT
SVATEEINRIFKPLIGPRPLVNGLMGRINYYWSILKPGQTLRIKSDGNLIAPWYGHILSGESHGRILKTD
LKRGSCTVQCQTEKGGLNTTLPFQNVSKYAFGNCSKYIGIKSLKLAVGLRNVPSRSSRGLFGAIAGFIEG
GWSGLVAGWYGFQHSNDQGVGMAADRESTQKAIDKITSKVNNIVDKMNKQYEIIDHEFSEVETRLNMINN
KIDDQIQDIWAYNAELLVLLENQKTLDEHDANVNNLYNKVKRALGSNAVEDGKGCFELYHKCDDQCMETI
RNGTYNRREYQEESKLERQRIEGVKLESEGTYKILTIYSTVASSLVIAMGFAAFLFWAMSNGSCRCNICI

>QOJ99111.1 hemagglutinin, partial [Influenza A virus]
METVSLITILVVATVSNADKICIGYQSTNSTETVDTLTENNVPVTHAKELLHTEHNGMLCATSLGHPLIL
DTCTIEGLIYGNPSCDPLLGGREWSYIVERPSAVNGLCYPGNVENLEELRSLFSSSRSYQRIQIFPDTIW
NVSYSGTSKACSDSFYRSMRWLTQKNNAYPTQDAQYTNNQGKNILFMWGINHPPTDTAQTNLYTRTDTTT
SVATEEMNRIFKPLIGPRPLVNGLMGRINYYWSVLKPGQTLRIKSDGNLIAPWYGHILSGESHGRILKTD
LKMGSCTVQCQTEKGGLNTTLPFQNVSKYAFGNCSKYIGVKSLKLAVGLRNVPSRSSRGLFGAIAGFIEG
GWSGLVAGWYGFQHSNDQGVGMAADRDSTQKAIDKITSKVNNIVDKMNKQYEIIDHEFSEVETRLNMIND
KVDDQIQDIWAYNAELLVLLENQKTLDEHDANVNNLYNKVKRALGSNAVEDGRGCFELYHKCDDHCMETI
RNGTYNRRKYQEESKLERQKIEGVKLESEETYKILTIYSTVASSLVIAMGFAAFLFWAMSNGSC

>QOJ99110.1 hemagglutinin, partial [Influenza A virus]
METVSLITILVVATVSNADKICIGYQSTNSTETVDTLTENNVPVTHAKELLHTEHNGMLCATGLGHPLIL
DTCTIEGLIYGNPSCDPLLGGREWSYIVERPSAVNGLCYPGNVENLEELRSLFSSSRSYQRIQIFPDTIW
NVSYSGTSKACSDSFYRSMRWLTQKNNAYPTQDAQYTNNQGKNILFMWGINHPPTDTAQTNLYTRTDTTT
SVATEEMNRIFKPLIGPRPLVNGLMGRINYYWSVLKPGQTLRIKSDGNLIAPWYGHILSGESHGRILKTD
LKRGSCTVQCQTEKGGLNTTLPFQNVSKYAFGNCSKYIGVKSLKLAVGLRNVPSRSSRGLFGAIAGFIEG
GWPGLVAGWYGFQHSNDQGVGMAADRDSTQKAIDKITSKVNNIVDKMNKQYEIIDHEFSEVETRLNMINN
KVDDQIQDIWAYNAELLVLLENQKTLDEHDANVNNLYNKVKRALGSNAVEDGKGCFELYHKCDDHCMETI
RNGTYNRRKYQEESKLERQKIEGVKLESEETYKILTIYSTVASSLVIAMGFAAFLFWAMSNGSC

>QOJ99109.1 hemagglutinin, partial [Influenza A virus]

METVSLITILVVATVSNADKICIGYQSTNSTETVDTLTENNVPVTHAKELLHTEHNGMLCATGLGHPLIL
DTCTIEGLIYGNPSCDPLLGGREWSYIVERPSAVNGLCYPGNVENLEELRSLFSSSRSYQRIQIFPDTIW
NVSYSGTSKACSDSFYRSMRWLTQKNNAYPTQDAQYTNNQGKNILFMWGINHPPTDTAQTNLYXRTDTTT
SVATEEMNRIFKPLIGPRPLVNGLMGRINYYWSVLKPGQTLRIKSDGNLIAPWYGHILSGESHGRILKTD
LKRGSCTVQCQTEKGGLNTTLPFQNVSKYAFGNCSKYIGVKSLKLAVGLRNVPSRSSRGLFGAIAGFIEG
GWPGLVAGWYGFQHSNDQGVGMAADRDSTQKAIDKITSKVNNIVDKMNKQYEIIDHEFSEVETRLNMINN
KVDDQIQDIWAYNAELLVLLENQKTLDEHDANVNNLYNKVKRALGSNAVEDGKGCFELYHKCDDHCMETI
RNGTYNRRKYQEESKLERQKIEGVKLESEETYKILTIYSTVASSLVIAMGFAAFLFWAMSNGSC

>QOJ99108.1 hemagglutinin, partial [Influenza A virus]

ETVSLITILLVATVSNADKICIGYQSTNSTETVDTLTENNVPVTHAKELLHTEHNGMLCATSLGQPLILN
TCTIEGLIYGNPSCDLSLEGREWSYIVERPSAVHGLCYPGNVEDLEELRSJFSSARSYQRIQIFPDTIWN
VSYDGTSTACSGSFYRSMRWLTRKNGEYPIQDAQYTNNQGKNILFMWGINHPPTDNXQRELYTRTDTTTS
VATEEINRIFKPLIGPRPLVNGLMGRINYYWSVLKPGQTLRIKSDGNLIAPWYGHILSGESHGRILKTDL
KRGSCTVQCQTEKGGLNTTLPFQNVSKYAFGNCSKYIGIKSLKLAVGLRNVPSRSSRGLFGAIAGFIEGG
WSGLVAGWYGFQHSNDQGVGMAADRDSTQKAIDKITSKVNNIVDKMNKQYEIIDHEFSEVETRLNMINNK
IDDQIQDIWAYNAELLVLLENQKTLDEHDANVNNLYNKVKRALGSNAVEDGKGCFELYHKCDDQCMETIR
NGTYNRRKYQEESKLERQKIEGVKLESEGTYKILTIYSTVASSLVIAMGFAAFLFWAMSNGSC

>QOJ99107.1 hemagglutinin, partial [Influenza A virus]

ETVSLITILLVATASNADKICIGYQSTNSTETVDTLTENNVPVTHAKELLHTEHNGMLCATSLGQPLILD
TCTVEGLIYGNPSCDLSLEGREWSYIVERPSAVNGLCYPGNXENLEELRSLFSSARSYQRIQIFPDTIWN
VSYDGTSKACSXSFYRSMRWLTQKDGNYPTQDAQYTNNQGKNILFMWGINHPPTDDTQRNLYTRTDTTTS
VATEEINRIFKPLIGPRPLVNGLMGRIDYYWSVLKPGQTLRIKSNGNLIAPWYGHILTGESHGRILKTDL
KRGSCTVQCQTEKGGLNTTLPFQNVSKYAFGNCSKYIGIKSLKLAVGLRNVPSRSSRGLFGAIAGFIEGG
WSGLVAGWYGFQHSNDQGVGMAADRDSTQKAIDKITSKVNNIVDKMNKQYEIIDHEFSEVETRLNMINNK
IDDQIQDIWAYNAELLVLLENQKTLDEHDANVNNLYNKVKRALGSNAVEDGKGCFELYHKCNDQCMETIR
NGTYNRRKYQEESKLKRQKIEGVKLESEGTYKILTIYSTVASSLVIAMGFAAFLFWAMSNGSC

>QOJ99106.1 hemagglutinin, partial [Influenza A virus]

ETVSLITILLVATVSNADKICIGYQSTNSTETVDTLTENNVPVTHAKELLHTEHNGMLCATSLGQPLILN
TCTIEGLIYGNPSCDLSLEGREWSYIVERPSAVHGLCYPGNVEDLEELRSLFSSARSYQRIQIFPDTIWN

VSYDGTSTACSGSFYRSMRWLTRKNGEYPIQDAQYTNNQGKNILFMWGINHPPTDNTQRELYTRTDTTTS
VATEEINRIFKPLIGPRPLVNGLMGRINYYWSVLKPGQTLRIKSDGNLIAPWYGHILSGESHGRILKTDL
KRGSCTVQCQTEKGGLNTTLPFQNVSKYAFGNCSKYIGIKSLKLAVGLRNVPSRSSRGLFGAIAGFIEGG
WSGLVAGWYGFQHSNDQGVGMAADRDSTQKAIDKITSKVNNIVDKMNKQYEIIDHEFSEVETRLNMINNK
IDDQIQDIWAYNAELLVLLENQKTLDEHDANVNNLYNKVKRALGSNAVEDGKGCFELYHKCDDQCMETIR
NGTYNRRKYQEESKLERQKIEGVKLESEGTYKILTIYSTVASSLVIAMGFAAFLFWAMSNGSC

>QOJ99105.1 hemagglutinin, partial [Influenza A virus]
ETVSLITILLVATVSNADKICIGYQSTNSTETVDTLTENNVPVTHAKELLHTEHNGMLCATSLGQPLILN
TCTIEGLIYGNPSCDLSLEGREWSYIVERPSAVHGLCYPGNVEDLEELRSLFSSARSYQRIQIFPDTIWN
VSYDGTSTACSGSFYRSMRWLTRKNGEYPIQDAQYTNNQGKNILFMWGINHPPTDNTQRELYTRTDTTTS
VATEEINRIFKPLIGPRPLVNGLMGRINYYWSVLKPGQTLRIKSDGNLIAPWYGHILSGESHGRILKTDL
KRGSCTVQCQTEKGGLNTTLPFQNVSKYAFGNCSKYIGIKSLKLAVGLRNVPSRSSRGLFGAIAGFIEGG
WSGLVAGWYGFQHSNDQGVGMAADRDSTQKAIDKITSKVNNIVDKMNKQYEIIDHEFSEVETRLNMINNK
IDDQIQDIWAYNAELLVLLENQKTLDEHDANVNNLYNKVKRALGSNAVEDGKGCFELYHKCDDQCMETIR
NGTYNRRKYQEESKLERQKIEGVKLESEGTYKILTIYSTVASSLVIAMGFAAFLFWAMSNGSC

>QOJ99104.1 hemagglutinin, partial [Influenza A virus]
ETVSLITILLVATVSNADKICIGYQSTNSTETVDTLTENNVPVTHAKELLHTEHNGMLCATSLGQPLILN
TCTIEGLIYGNPSCDLSLEGREWSYIVERPSAVHGLCYPGNVEDLEELRSLFSSARSYQRIQIFPDTIWN
VSYDGTSTACSGSFYRSMRWLTRKNGEYPIQDAQYTNNQGKNILFMWGINHPPTDNTQRELYTRTDTTTS
VATEEINRIFKPLIGPRPLVNGLMGRINYYWSVLKPGQTLRIKSDGNLIAPWYGHILSGESHGRILKTDL
KRGSCTVQCQTEKGGLNTTLPFQNVSKYAFGNCSKYIGIKSLKLAVGLRNVPSRSSRGLFGAIAGFIEGG
WSGLVAGWYGFQHSNDQGVGMAADRDSTQKAIDKITSKVNNIVDKMNKQYEIIDHEFSEVETRLNMINNK
IDDQIQDIWAYNAELLVLLENQKTLDEHDANVNNLYNKVKRALGSNAVEDGKGCFELYHKCDDQCMETIR
NGTYNRRKYQEESKLERQKIEGVKLESEGTYKILTIYSTVASSLVIAMGFAAFLFWAMSNGSC

>QOJ99103.1 hemagglutinin, partial [Influenza A virus]
METVSLITILVVATVSNADKICIGYQSTNSTETVDTLTENNVPVTHAKELLHTEHNGMLCATGLGHPLIL
DTCTIEGLIYGNPSCDPLLGGREWSYIVERPSAVNGLCYPGNVENLEELRSLFSSSRSYQRIQIFPDTIW
NVSYSGTSKACSDSFYRSMRWLTQKNNAYPTQDAQYTNNQGKNILFMWGINHPPTDTXQTNLYTRTDTTT
SVATEEMNRIFKPLIGPRPLVNGLMGRINYYWSVLKPGQTLRIKSDGNLIAPWYGHILSGESHGRILKTD
LKRGSCTVQCQTEKGGLNTTLPFQNVSKYAFGNCSKYIGVKSLKLAVGLRNVPSRSSRGLFGAIAGFIEG
GWPGLVAGWYGFQHSNDQGVGMAADRDSTQKAIDKITSKVNNIVDKMNKQYEIIDHEFSEVETRLNMINN

KVDDQIQDIWAYNAELLVLLENQKTLDEHDANVNNLYNKVKRALGSNAVEDGKGCFELYHKCDDHCMETI
RNGTYNRRKYQEESKLERQKIEGVKLESEETYKILTIYSTVASSLVIAMGFAAFLFWAMSNGSC

>QOJ99102.1 hemagglutinin, partial [Influenza A virus]
METVSLITILVVATVSNADKICIGYQSTNSTETVDTLTENNVPVTHAKELLHTEHNGMLCATGLGHPLIL
DTCTIEGLIYGNPSCDPLLGGREWSYIVERPSAVNGLCYPGNVENLEELRSLFSSSRSYQRIQIFPDTIW
NVSYSGTSKACSDSFYRSMRWLTQKNNAYPTQDAQYTNNQGKNILFMWGINHPPTDTTQTNLYTRTDTTT
SVATEEMNRIFKPLIGPRPLVNGLMGRINYYWSVLKPGQTLRIKSDGNLIAPWYGHILSGESHGRILKTD
LKRGSCTXQCQTEKGGLNTTLPFQNVSKYAFGNCSKYIGVKSLKLAVGLRNVPSRSSRGLFGAIAGFIEG
GWPGLVAGWYGFQHSNDQGVGMAADRDSTQKAIDKITSKVNNIVDKMNKQYEIIDHEFSEVETRLNMINN
KVDDQIQDIWAYNAELLVLLENQKTLDEHDANVNNLYNKVKRALGSNAVEDGKGCFELYHKCDDHCMETI
RNGTYNRRKYQEESKLERQKIEGVKLESEETYKILTIYSTVASSLVIAMGFAAFLFWAMSNGSC

>QOJ99100.1 hemagglutinin, partial [Influenza A virus]
METISLITILLVATVSYADKICIGYQSTNSTETVDTLTENNVPVTHAKELLHTEHNGMLCATSLGQPLIL
DTCTIEGLIYGNPSCDLSLEGREWSYIVERPSAVNGLCYPGNVENLEELRSLFSSARSYQRIQIFPDTIW
NVSYDGTSTACSGSFYRSMRWLTRKDGNYPTQDAQYTNNQGKNILFMWGINHPPTDDTQRSLYTRTDTTT
SVATEEINRIFKPLIGPRPLVNGLMGRIDYYWSVLKPGQTLRIKSDGNLIAPWYGYILSGESHGRILKTD
LKRGSCTVQCQTEKGGLNTTLPFQNVSKYAFGNCSKYIGIKSLKLAVGLRNVPSRSSRGLFGAIAGFIEG
GWSGLVAGWYGFQHSNDQGVGMAADRDSTQKAIDKITSKVNNIVDKMNKQYEIIDHEFSEVETRLNMINN
KIDDQIQDIWAYNAELLVLLENQKTLDEHDANVNNLYNKVKRALGSNAAEDGKGCFELYHKCNDQCMETI
RNGTYNRRKYQEESKLERQKIEGVKLESEGTYKILTIYSTVASSLVIAMGFAAFLF

>QOJ99099.1 hemagglutinin, partial [Influenza A virus]
METISLITILLVATVSYADKICIGYQSTNSTETVDTLTENNVPVTHAKELLHTEHNGMLCATSLGQPLIL
DTCTIEGLIYGNPSCDLSLEGREWSYIVERPSAVNGLCYPGNVENLEELRSLFSSARSYQRIQIFPDTIW
NVSYDGTSTACSGSFYRSMRWLTRKDGNYPTQDAQYTNNQGKNILFMWGINQPPTDDTQRSLYTRTDTTT
SVATEEINRIFKPLIGPRPLVNGLMGRIDYYWSVLKPGQTLRIKSDGNLIAPWYGYILSGESHGRILKTD
LKRGSCTVQCQTEKGGLNTTLPFQNVSKYAFGNCSKYIGIKSLKLAVGLRNVPSRSSRGLFGAIAGFIEG
GWSGLVAGWYGFQHSNDQGVGMAADRDSTQKAIDKITSKVNNIVDKMNKQYEIIDHEFSEVETRLNMINN
KIDDQIQDIWAYNAELLVLLENQKTLDEHDANVNNLYNKVKRALGSNAAEDGKGCFELYHKCNDQCMETI
RNGTYNRRKYQEESKLERQKIEGVKLESEGTYKILTIYSTVASSLVIAMGFAAFLF

>QOJ99098.1 hemagglutinin, partial [Influenza A virus]

METISLITILLVATVSYADKICIGYQSTNSTETVDTLTENNVPVTHAKELLHTEHNGMLCATSLGQPLIL
DTCTIEGLIYGNPSCDLSLEGREWSYIVERPSAVNGLCYPGNVENLEELRSLFSSARSYQRIQIFPDTIW
NVSYDGTSXACSGSFYRSMRWLTRKDGNYPTQDAQYTNNQGKNILFMWGINHPPTDDTQRSLYTRTDTTT
SVATEEINRIFKPLIGPRPLVNGLMGRIDYYWSVLKPGQTLRIKSDGNLIAPWYGYILSGESHGRILKTD
LKXGSCTVQCQTEKGGLNTTLPFQNVSKYAFGNCSKYIGIKSLKLAVGLRNVPSRSSRGLFGAIAGFIEG
GWSGLVAGWYGFQHSNDQGVGMAADRDSTQKAIDKITSKVNNIVDKMNKQYEIIDHEFSEVETRLNMINN
KIDDQIQDIWAYNAELLVLLENQKTLDEHDANVNNLYNKVKRALGSNAAEDGKGCFELYHKCNDQCMETI
RNGTYNRRKYQEESKLERQKIEGVKLESEGTYKILTIYSTVASSLVIAMGFAAFLF

>QOJ99097.1 hemagglutinin, partial [Influenza A virus]
METISLITILLVATVSYADKICIGYQSTNSTETVDTLTENNVPVTHAKELLHTEHNGMLCATSLGQPLIL
DTCTIEGLIYGNPSCDLSLEGREWSYIVERPSAVNGLCYPGNVENLEELRSLFSSARSYQRIQIFPDTIW
NVSYDGTSTACSGSFYRSMRWLTRKDGNYPTQDAQYTNNQGKNILFMWGINHPPTDDTQRSLYTRTDTTT
SVATEEINRIFKPLIGPRPLVNGLMGRIDYYWSVLKPGQTLRIKSDGNLIAPWYGYILSGESHGRILKTD
LKRGSCTVQCQTEKGGLNTTLPFQNVSKYAFGNCSKYIGIKSLKLAVGLRNVPSRSSRGLFGAIAGFIEG
GWSGLVAGWYGFQHSNDQGVGMAADRDSTQKAIDKITSKVNNIVDKMNKQYEIIDHEFSEVETRLNMINN
KIDDQIQDIWAYNAELLVLLENQKTLDEHDANVNNLYNKVKRALGSNAAEDGKGCFELYHKCNDQCMETI
RNGTYNRRKYQEESKLERQKIEGVKLESEGTYKILTIYSTVASSLVIAMGFAAFLF

>QOJ99096.1 hemagglutinin, partial [Influenza A virus]
METISLITILLVATVSYADKICIGYQSTNSTETVDTLTENNVPVTHAKELLHTEHNGMLCATSLGQPLIL
DTCTIEGLIYGNPSCDLSLEGREWSYIVERPSAVNGLCYPGNVENLEELRSLFSSARSYQRIQIFPDTIW
NVSYDGTSTACSGSFYRSMRWLTRKDGNYPTQDAQYTNNQGKNILFMWGINHPPTDDTQRSLYTRTDTTT
SVATEEINRIFKPLIGPRPLVNGLMGRIDYYWSVLKPGQTLRIKSDGNLIAPWYGYILSGESHGRILKTD
LKRGSCTVQCQTEKGGLNTTLPFQNVSKYAFGNCSKYIGIKSLKLAVGLRNVPSRSSRGLFGAIAGFIEG
GWSGLVAGWYGFQHSNDQGVGMAADRDSTQKAIDKITSKVNNIVDKMNKQYEIIDHEFSEVETRLNMINN
KIDDQIQDIWAYNAELLVLLENQKTLDEHDANVNNLYNKVKRALGSNAAEDGKGCFELYHKCNDQCMETI
RNGTYNRRKYQEESKLERQKIEGVKLESEGTYKILTIYSTVASSLVIAMGFAAFLF

>QOJ99095.1 hemagglutinin, partial [Influenza A virus]
METISLITILLXATVSXADKICIGYQSTNSTETVDTLTENNVPVTHAKELLHTEHNGMLCATSLGXPLIL
DTCTIEGLIYGNPSCDLSLEGREWSYIVERPSAVNGLCYPGNVENLEELRSLFSSARSYQRIQIFPDTIW
NVSYDGTSTACSGSFYRSMRWLTRKDGNYPTQDAQYTNNQGKNILFMWGINHPPTDDTQRSLYTRTDTTT
SVATEEINRIFKPLIGPRPLVNGLMGRIDYYWSVLKPGQTLRIKSDGNLIAPWYGYILSGESHGRILXTD

LKRGSCTVQCQTEKGGLNTTLPFQNXSKYAFGNCSKYIGIKSLKLAVGLRNVPSRSXRGLFGAIAGFIEG
GWSGLVAGWYGFQHSNDQGVGMAADRXSTQKAXDKITSKVNNIVDKMNKQYEIIBHEFSEVETRLNMINN
KIDDQIQDIWAYNAELLVLLENQKTLDEHDANVNNLYNKVKRALGSNAAEDGKGCFELYHKCNDQCMETI
RNGTYNRRKYQEESKLERQKIEGVKLESEGTYKILTIYSTVASSLVIAMGFAAFLF

>QOJ99093.1 hemagglutinin, partial [Influenza A virus]
METISLITILLVATVSYADKICIGYQSTNSTETVDTLTENNVPVTHAKELLHTEHNGMLCATSLGQPLIL
DTCTIEGLIYGNPSCDLSLEGREWSYIVERPSAVNGLCYPGNVENLEELRSLFSSARSYQRIQIFPDTIW
NVSYDGTSTACSGSFYRSMRWLTRKDGNYPTQDAQYTNNQGKNILFMWGINHPPTDDTQRSLYTRTDTTT
SVATEEINRIFKPLIGPRPLVNGLMGRIDYYWSVLKPGQTLRIKSDGNLIAPWYGYILSGESHGRILKTD
LKRGSCTVQCQTEKGGLNTTLPFQNVSKYAFGNCSKYIGIKSLKLAVGLRNVPSRSSRGLFGAIAGFIEG
GWSGLVAGWYGFQHSNDQGVGMAADRDSTQKAIDKITSKVNNIVDKMNKQYEIIDHEFSEVETRLNMINN
KIDDQIQDIWAYNAELLVLLENQKTLDEHDANVNNLYNKVKRALGSNAAEDGKGCFELYHKCNDQCMETI
RNGTYNRRKYQEESKLERQKIEGVKLESEGTYKILTIYSTVASSLVIAMGFAAFLF

>QOJ99092.1 hemagglutinin, partial [Influenza A virus]
METISLITILLVATVSYADKICIGYQSTNSTETVDTLTENNVPVTHAKELLHTEHNGMLCATSLGQPLIL
DTCTIEGLIYGNPSCDLSLEGREWSYIVERPSAVNGLCYPGNVENLEELRSLFSSARSYQRIQIFPDTIW
NVSYDGTSTACSGSFYRSMRWLTRKDGNYPTQDAQYTNNQGKNILFMWGINXPPTDDTQRSLYTRTDTTT
SVATEEINRIFKPLIGPRPLVNGLMGRIDYYWSVLKPGQTLRIKSDGNLIAPWYGYILSGESHGRILKTD
LKRGSCTVQCQTEKGGLNTTLPFQNVSKYAFGNCSKYIGIKSLKLAVGLRNVPSRSSRGLFGAIAGFIEG
GWSGLVAGWYGFQHSNDQGVGMAADRDSTQKAIDKITSKVNNIVDKMNKQYEIIDHEFSEVETRLNMINN
KIDDQIQDIWAYNAELLVLLENQKTLDEHDANVNNLYNKVKRALGSNAAEDGKGCFELYHKCNDQCMETI
RNGTYNRRKYQEESKLERQKIEGVKLESEGTYKILTIYSTVASSLVIAMGFAAFLF

>QOJ99091.1 hemagglutinin, partial [Influenza A virus]
METISLITILLVATVSYADKICIGYQSTNSTETVDTLTENNVPVTHAKELLHTEHNGMLCATSLGQPLIL
DTCTIEGLIYGNPSCDLSLEGREWSYIVERPSAVNGLCYPGNVENLEELRSLFSSARSYQRIQIFPDTIW
NVSYDGTSTACSGSFYRSMRWLTRKDGNYPTQDAQYTNNQGKNILFMWGINXPPTDDTQRSLYTRTDTTT
SVATEEINRIFKPLIGPRPLVNGLMGRIDYYWSVLKPGQTLRIKSDGNLIAPWYGYILSGESHGRILKTD
LKRGSCTVQCQTEKGGLNTTLPFQNVSKYAFGNCSKYIGIKSLKLAVGLRNVPSRSSRGLFGAIAGFIEG
GWSGLVAGWYGFQHSNDQGVGMAADRDSTQKAIDKITSKVNNIVDKMNKQYEIIDHEFSEVETRLNMINN
KIDDQIQDIWAYNAELLVLLENQKTLDEHDANVNNLYNKVKRALGSNAAEDGKGCFELYHKCNDQCMETI
RNGTYNRRKYQEESKLERQKIEGVKLESEGTYKILTIYSTVASSLVIAMGFAAFLF

>QOJ99090.1 hemagglutinin, partial [Influenza A virus]

METISLITILLVATVSYADKICIGYQSTNSTETVDTLTENNVPVTHAKELLHTEHNGMLCATSLGQPLIL
DTCTIEGLIYGNPSCDLSLEGREWSYIVERPSAVNGLCYPGNVENLEELRSLFSSARSYQRIQIFPDTIW
NVSYDGTSTACSGSFYRSMRWLTRKDGNYPTQDAQYTNNQGKNILFMWGINHPPTDDTQRSLYTRTDTTT
SVATEEINRIFKPLIGPRPLVNGLMGRIDYYWSVLKPGQTLRIKSDGNLIAPWYGYILSGESHGRILKTD
LKRGSCTVQCQTEKGGLNTTLPFQNVSKYAFGNCSKYIGIKSLKLAVGLRNVPSRSSRGLFGAIAGFIEG
GWSGLVAGWYGFQHSNDQGVGMAADRDSTQKAIDKITSKVNNIVDKMNKQYEIIDHEFSEVETRLNMINN
KIDDQIQDIWAYNAELLVLLENQKTLDEHDANVNNLYNKVKRALGSNAAEDGKGCFELYHKCNDQCMETI
RNGTYNRRKYQEESKLERQKIEGVKLESEGTYKILTIYSTVASSLVIAMGFAAFLF

>QOJ99089.1 hemagglutinin, partial [Influenza A virus]

METVSLITILLAATVSNADKICIGYQSTNSTETVDTLTENNVPVTHAKELLHTEHNGMLCATSLGKPLIL
DTCTIEGLIYGNPSCDXXXXXREWSYIVERPSAVNGLCYPGNVENLEELRSLFSSARSYQRIQIFPDTIW
NVSYDGTSXXCSGSFYRXMRWLTRKDGNYPXQDAQYTNNQGKNILFMWGINXPPTDDTQRXLYTRTDTTT
SVATEEINRIFKPLIGPRPLVNGLMGRIBYYWSVLKPGQTLRIKSDGNLIAPWYGYILSGESHGRILKTD
LKRGSCTVQCQTEKGGLNTTLPFQNISKYAFGNCSKYIGIKSLKLAVGLRNVPSRSNRGLFGAIAGFIEG
GWSGLVAGWYGFQHSNDQGVGMAADRESTQKAVDKITSKVNNIVDKMNKQYEIIDHEFSEVETRLNMINN
KIDDQIQDIWAYNAELLVLLENQKTLDEHDANVNNLYNKVKRALGSNAVEDGKGCFELYHKCBDQCMETI
RNGTYNRRKYQEESKLERQKIEGVXLESEGTYKILTIYSTVASSLVIAMGVC

>QOJ99088.1 hemagglutinin, partial [Influenza A virus]

METISLITILLVATVSYADKICIGYQSTNSTETVDTLTENNVPVTHAKELLHTEHNGMLCATSLGQPLIL
DTCTIEGLIYGNPSCDLSLEGREWSYIVERPSAVNGLCYPGNVENLEELRSLFSSARSYQRIQIFPDTIW
NVSYDGTSTACSGSFYRSMRWLTRKDGNYPTQDAQYTNNQGKNILFMWGINHPPTDDTQRSLYTRTDTTT
SVATEEINRIFKPLIGPRPLVNGLMGRIDYYWSVLKPGQTLRIKSDGNLIAPWYGYILSGESHGRILKTD
LKRGSCTVQCQTEKGGLNTTLPFQNVSKYAFGNCSKYIGIKSLKLAVGLRNVPSRSSRGLFGAIAGFIEG
GWSGLVAGWYGFQHSNDQGVGMAADRDSTQKAIDKITSKVNNIVDKMNKQYEIIDHEFSEVETRLNMINN
KIDDQIQDIWAYNAELLVLLENQKTLDEHDANVNNLYNKVKRALGSNAAEDGKGCFELYHKCNDQCMETI
RNGTYNRRKYQEESKLERQKIEGVKLESEGTYKILTIYSTVASSLVIAMGFAAFLF

>QOJ99087.1 hemagglutinin, partial [Influenza A virus]

METISLITILLVATVSYADKICIGYQSTNSTETVDTLTENNVPVTHAKELLHTEHNGMLCATSLGQPLIL
DTCTIEGLIYGNPSCDLSLEGREWSYIVERPSAVNGLCYPGNVENLEELRSLFSSARSYQRIQIFPDTIW

NVSYDGTSTACSGSFYRSMRWLTRKDGNYPTQDAQYTNNQGKNILFMWGINHPPTDDTQRSLYTRTDTTT
SVATEEINRIFKPLIGPRPLVNGLMGRIDYYWSVLKPGQTLRIKSDGNLIAPWYGYILSGESHGRILKTD
LKRGSCTVQCQTEKGGLNTTLPFQNVSKYAFGNCSKYIGIKSLKLAVGLRNVPSRSSRGLFGAIAGFIEG
GWSGLVAGWYGFQHSNDQGVGMAADRDSTQKAIDKITSKVNNIVDKMNKQYEIIDHEFSEVETRLNMINN
KIDDQIQDIWAYNAELLVLLENQKTLDEHDANVNNLYNKVKRALGSNAAEDGKGCFELYHKCNDQCMETI
RNGTYNRRKYQEESKLERQKIEGVKLESEGTYKILTIYSTVASSLVIAMGFAAFLF

>QOJ99053.1 hemagglutinin, partial [Influenza A virus]
ETVSLITILLVAAVSNADKICIGYQSTNSTETVDTLTENNVPVTHAKELLHTEHNGMLCATSLGQPIILD
TCTIEGIIYGNPSCDLSLEGKEWSYIVERPSAVNGLCYPGNVENLEELRSLFSSARSYQRIQIFPDTIWN
VSYDGTSTACSGSFYRNMRWLTRKNGEYPIQDAQYTNNQGKNILFMWGINHPPADATQRXLYTRTDTTTS
VATEEINRIFKPLIGPRPLVNGLMGRIDYYWSILKPGQTLRIKSDGNLIAPWYGHILSGESHGRILKTDL
KRGSCTVQCQTEKGGLNTTLPFQNVSKYAFGNCSKYIGIKSLKLAVGLRNVPSRSSRGLFGAIAGFIEGG
WSGLVAGWYGFQHSNDQGVGMAADRDSTQKAVDKITSKVNTIVDKMNKQYEIIDHEFSEVETRLNMINNK
IDDQIQDIWAYNAELLVLLENQKTLDEHDANVNNLYNKVKRALGSNAVEDGKGCFELYHKCDDQCMETIR
NGTYNRRKYQEESKLERQKIEGVRLESEGTYKILTIYSTVASSLVIAMGFAAFLFWAMSNGSC

>QOJ99045.1 hemagglutinin [Influenza A virus]
METXSLITILLXATVSNADKICIGYQSTNSTETVDTLTENNVPVTHAKELLHTEHNGMLCATSLGQPLIL
DTCTIEGLIYGNPSCDXXLEXREWSYIVERPSAVNGLCYPGNVENLEELRSLFSSARSYQRIQIFPDTIW
NVSYDGTSNTCSGSFYRNMRWLTRKNGNYPIQDAQYTNNQGKNILFMWGINHPPTDXTQRNLYTRTDTTT
SVATEEINRIFKPLIGPRPLVNGLMGRIBYYWSVLKPGQTLRIKSDGNLIAPWYGXILSGESHGRILXTD
LKRGSCTVQCQTEKGGLNTTLPFQNVSKYAFGNCSKYIGIKSLKLAVGLRNVPSRSSRGLFGAIAGFIEG
GWSGLVAGWYGFQHSNDQGVGMAADRXSTQKAIDKITSKVNNIVDKMNKQYEIIDHEFSEVETRLNMINN
KIDDQIQDIWAYNAELLVLLENQKTLDEHDANVNNLYNKVKRALGSNAVEDGKGCFELYHKCBDQCMETI
RNGTYNRRKYQEESKLERQKIEGVKLESEGTYKILTIYSTVASSLVIAMGFAAFLFWAMSNGSCRCNICI

>QOJ99044.1 hemagglutinin, partial [Influenza A virus]
ETISLITILLVATVSNADKICIGYQSTNSTETVDTLTENNVPVTHAKELLHTEHNGMLCATSLGQPLILD
TCTIEGLIYGNPSCDLSLEGREWSYIVERPSAVNGLCYPGNVENLEELRSLFSSARSYQRIQIFPDTIWN
VSYDGTSTACSNSFYRSMRWLTRKDGNYPTQDAQYTNNQGKNILFMWGINHPPTDETQRNLYTRTDTTTS
VATEEINRIFKPLIGPRPLVNGLMGRIDYYWSVLKPGQTLRIKSDGNLIAPWYGHILSGESHGRILKTDL
KRGSCTVQCQTEKGGLNTTLPFQNVSKYAFGNCSKYIGIKSLKLAVGLRNVPSRSSRGLFGAIAGFIEGG
WSGLVAGWYGFQHSNDQGVGMAADRDSTQKAIDKITSKVNNIVDKMNKQYEIIDHEFSEVETRLNMINNK

IDDQIQDIWAYNAELLVLLENQKTLDEHDANVNNLYNKVKRALGSNAVEDGKGCFELYHKCNDQCMETIR
NGTYNRKKYQEESKLERQRIEGVKLESEGTYKILTIYSTVASSLVIAMGFAAFLFWAMSNGSC

>QOJ99027.1 hemagglutinin [Influenza A virus]
METVSLMTILLVATVSNADKICIGYQSTNSTETVDTLTENNVPVTHAKELIHTEHNGMLCATSLGQPLIL
DTCTIEGLIYGNPSCDLSLEGREWSYIVERPSAVNGLCYPGNVENLEELRSLFSSARSYQRIQIFPDTIW
NVSYDGTSTACSGSFYKNMRWLTRKDGNYPPQDAQYTNNQGKNILFMWGINHPPADTTQTGLYTRTDTTT
SVATEEINRIFKPLIGPRPLVNGLMGRIDYYWSVLKPGQTLRIKSDGNLIAPWFGHILSGESHGRILKTD
LKRGSCTVQCQTEKGGLNTTLPFQNVSKYAFGNCSKYIGIKSLKLAVGLRNVPSRSSRGLFGAIAGFIEG
GWSGLVAGWYGFQHSNDQGVGMAADRDSTQKAIDKITSKVNNIVDKMNKQYEIIDHEFSEVETRLNMINN
KIDDQIQDIWAYNAELLVLLENQKTLDEHDANVNNLYNKVKRALGSNAVEDGKGCFELYHKCDDQCMETI
RNGTYNRRKYQEESKLERQKIEGVKLESEGTYKILTIYSTVASSLVIAMGFAAFLFWAMSNGSCRCNICI

>QOJ99026.1 hemagglutinin [Influenza A virus]
METVSLMTILLVATVSNADKICIGYQSTNSTETVDTLTENNVPVTHAKELIHTEHNGMLCATSLGQPLIL
DTCTIEGLIYGNPSCDLSLEGREWSYIVERPSAVNGLCYPGNVENLEELRSLFSSARSYQRIQIFPDTIW
NVSYDGTSTACSGSFYKNMRWLTRKDGNYPTQDAQYTNNQGKNILFMWGINHPPADTTQTGLYTRTDTTT
SVATEEINRIFKPLIGPRPLVNGLMGRIDYYWSVLKPGQTLRIKSDGNLIAPWFGHILSGESHGRILKTD
LKRGSCTVQCQTEKGGLNTTLPFQNVSKYAFGNCSKYIGIKSLKLAVGLRNVPSRSSRGLFGAIAGFIEG
GWSGLVAGWYGFQHSNDQGVGMAADRDSTQKAIDKITSKVNNIVDKMNKQYEIIDHEFSEVETRLNMINN
KIDDQIQDIWAYNAELLVLLENQKTLDEHDANVNNLYNKVKRALGSNAVEDGKGCFELYHKCDDQCMETI
RNGTYNRRKYQEESKLERQKIEGVKLESEGTYKILTIYSTVASSLVIAMGFAAFLFWAMSNGSCRCNICI

>QOJ99025.1 hemagglutinin, partial [Influenza A virus]
METVSLITILVVATVSNADKICIGYQSTNSTETVDTLTENNVPVTHAKELLHTEHNGMLCATSLGHPLIL
DTCTIEGLIYGNPSCDPLLGGREWSYIVERPSAVNGLCYPGSVENLEELRSLFSSSRSYQRIQIFPDTIW
NVSYSGTSKACSDSFYRSMRWLTQKNNAYPTQDAQYTNNQGKNILFMWGINHPPTDTAQTNLYTRTDTTT
SVATEEMNRIFKPLIGPRPLVNGLMGRINYYWSVLKPGQTLRIKSDGNLIAPWYGHILSGESHGRILKTD
LKRGSCTVQCQTEKGGLNTTLPFQNVSKYAFGNCSKYIGVKSLKLAVGLRNVPSRSSRGLFGAIAGFIEG
GWSGLVAGWYGFQHSNDQGVGMAADRDSTQKAIDKITSKVNNIVDKMNKQYEIIDHEFSEVETRLNMINN
KVDDQIQDIWAYNAELLVLLENQKTLDEHDANVNNLYNKVKRALGSNAVEDGKGCFELYHKCDDHCMETI
RNGTYNRRKYQEESKLERQKIEGVKLESEETYKILTIYSTVASSLVIAM

>QOJ99024.1 hemagglutinin [Influenza A virus]

METVSLITILLAATVSNADKICIGYQSTNSTETVDTLTENNVPVTHAKELLHTEHNGMLCATSLGQPLIL
DTCTIEGLIYGNPSCDPLLEEREWSYIVERPSAVNGLCYPGNVENLEELRSLFSSARSYQRIQIFPDTIW
NVSYDGTSNTCSGSFYRNMRWLTRKNGNYPIQDAQYTNNQGKNILFMWGINHPPTDDTQRNLYTRTDTTT
SVATEEINRIFRPLIGPRPLVNGLMGRINYYWSVLKPGQTMRIKSDGNLIAPWYGYILSGESHGRILRTD
LKKGSCTVQCQTEKGGLNTTLPFQNVSKYAFGNCSKYIGIKSLKLAVGLRNVPSRSSRGLFGAIAGFIEG
GWSGLVAGWYGFQHSNDQGVGMAADRESTQKAIDKITSKVNNIVDKMNKQYEIIDHEFSEVETRLNMINN
KIDDQIQDIWAYNAELLVLLENQKTLDEHDANVNNLYNKVKRALGSNAVEDGKGCFELYHKCDDQCMETI
RNGTYNRRKYQEESKLERQKIEGVKLESEGTYKILTIYSTVASSLVIAMGFAAFLFWAMSNGSCRCNICI

>QOJ99023.1 hemagglutinin [Influenza A virus]
METVSLITILLAATVSNADKICIGYQSTNSTETVDTLTENNVPVTHAKELLHTEHNGMLCATSLGQPLIL
DTCTIEGLIYGNPSCDPLLEEREWSYIVERPSAVNGLCYPGNVENLEELRSLFSSARSYQRIQIFPDTIW
NVSYDGTSNTCSGSFYRNMRWLTRKNGNYPIQDAQYTNNQGKNILFMWGINHPPTDDTQRNLYTRTDTTT
SVATEEINRIFRPLIGPRPLVNGLMGRINYYWSVLKPGQTMRIKSDGNLIAPWYGYILSGESHGRILRTD
LKKGSCTVQCQTEKGGLNTTLPFQNVSKYAFGNCSKYIGIKSLKLAVGLRNVPSRSSRGLFGAIAGFIEG
GWSGLVAGWYGFQHSNDQGVGMAADRESTQKAIDKITSKVNNIVDKMNKQYEIIDHEFSEVETRLNMINN
KIDDQIQDIWAYNAELLVLLENQKTLDEHDANVNNLYNKVKRALGSNAVEDGKGCFELYHKCDDQCMETI
RNGTYNRRKYQEESKLERQKIEGVKLESEGTYKILTIYSTVASSLVIAMGFAAFLFWAMSNGSCRCNICI

>QOJ99022.1 hemagglutinin, partial [Influenza A virus]
METVSLITILVVATVSNADKICIGYQSTNSTETVDTLTENNVPVTHAKELLHTEHNGMLCATSLGHPLIL
DTCTIEGLIYGNPSCDPLLGGREWSYIVERPSAVNGLCYPGSVENLEELRSLFSSSRSYQRIQIFPDTIW
NVSYSGTSKACSDSFYRSMRWLTQKNNAYPTQDAQYTNNQGKNILFMWGINHPPTDTAQTNLYTRTDTTT
SVATEEMNRIFKPLIGPRPLVNGLMGRINYYWSVLKPGQTLRIKSDGNLIAPWYGHILSGESHGRILKTD
LKRGSCTVQCQTEKGGLNTTLPFQNVSKYAFGNCSKYIGVKSLKLAVGLRNVPSRSSRGLFGAIAGFIEG
GWSGLVAGWYGFQHSNDQGVGMAADRDSTQKAIDKITSKVNNIVDKMNKQYEIIDHEFSEVETRLNMINN
KVDDQIQDIWAYNAELLVLLENQKTLDEHDANVNNLYNKVKRALGSNAVEDGKGCFELYHKCDDHCMETI
RNGTYNRRKYQEESKLERQKIEGVKLESEETYKILTIYSTVASSLVIAMGFAAFLFWAMSNGSC

>QOJ99021.1 hemagglutinin, partial [Influenza A virus]
METVSLITILVVATVSNADKICIGYQSTNSTETVDTLTENNVPVTHAKELLHTEHNGMLCATSLGHPLIL
DTCTIEGLIYGNPSCDPLLGGREWSYIVERPSAVNGLCYPGSVENLEELRSLFSSSRSYQRIQIFPDTIW
NVSYSGTSKACSDSFYRSMRWLTQKNNAYPTQDAQYTNNQGKNILFMWGINHPPTDTAQTNLYTRTDTTT
SVATEEMNRIFKPLIGPRPLVNGLMGRINYYWSVLKPGQTLRIKSDGNLIAPWYGHILSGESHGRILKTD

LKRGSCTVQCQTEKGGLNTTLPFQNVSKYAFGNCSKYIGVKSLKLAVGLRNVPSRSSRGLFGAIAGFIEG
GWSGLVAGWYGFQHSNDQGVGMAADRDSTQKAIDKITSKVNNIVDKMNKQYEIIDHEFSEVETRLNMINN
KVDDQIQDIWAYNAELLVLLENQKTLDEHDANVNNLYNKVKRALGSNAVEDGKGCFELYHKCDDHCMETI
RNGTYNRRKYQEESKLERQKIEGVKLESEETYKILTIYSTVASSLVIAMGFAAFLF

>QOJ99020.1 hemagglutinin [Influenza A virus]
METVSLITILLAATVSNADKICIGYQSTNSTETVDTLTENNVPVTHAKELLHTEHNGMLCATSLGQPLIL
DTCTIEGLIYGNPSCDPLLEEREWSYIVERPSAVNGLCYPGNVENLEELRSFFSSARSYQRIQIFPDTIW
NVSYDGTSNTCSGSFYRNMRWLTRKNGNYPIQDAQYTNNQGKSILFMWGINHPPTDDTQRNLYTRTDTTT
SVATEEINRIFKPLIGPRPLVNGLMGRINYYWSVLKPGQTLRIKSDGNLIAPWYGYILSGESHGRILRTD
LTRGSCTVQCQTEKGGLNTTLPFQNVSKYAFGNCSKYIGIKSLKLAVGLRNVPSRSSRGLFGAIAGFIEG
GWSGLVAGWYGFQHSNDQGVGMAADRESTQKAIDKITSKVNNIVDKMNKQYEIIDHEFSEVETRLNMINN
KIDDQIQDIWAYNAELLVLLENQKTLDEHDANVNNLYNKVKRALGSNAVEDGKGCFELYHKCDDQCMETI
RNGTYNRRKYQEESKLERQKIEGVKLESEGTYKILTIYSTVASSLVIAMGFAAFLFWAMSNGSCRCNICI

>QOJ99019.1 hemagglutinin [Influenza A virus]
METVSLMTILLVATVSNADKICIGYQSTNSTETVDTLTENNVPVTHAKELIHTEHNGMLCATSLGQPLIL
DTCTIEGLIYGNPSCDLSLEGREWSYIVERPSAVNGLCYPGNVENLEELRSLFSSARSYQRIQIFPDTIW
NVSYDGTSTACSGSFYKNMRWLTRKDGNYPTQDAQYTNNQGKNILFMWGINHPPADTTQTGLYTRTDTTT
SVATEEINRIFKPLIGPRPLVNGLMGRIDYYWSVLKPGQTLRIKSDGNLIAPWFGHILSGESHGRILKTD
LKRGSCTVQCQTEKGGLNTTLPFQNVSKYAFGNCSKYIGIKSLKLAVGLRNVPSRSSRGLFGAIAGFIEG
GWSGLVAGWYGFQHSNDQGVGMAADRDSTQKAIDKITSKVNNIVDKMNKQYEIIDHEFSEVETRLNMINN
KIDDQIQDIWAYNAELLVLLENQKTLDEHDANVNNLYNKVKRALGSNAVEDGKGCFELYHKCDDQCMETI
RNGTYNRRKYQEESKLERQKIEGVKLESEGTYKILTIYSTVASSLVIAMGFAAFLFWAMSNGSCRCNICI

>QOJ99018.1 hemagglutinin, partial [Influenza A virus]
METVSLMTILLVATVSNADKICIGYQSTNSTETVDTLTENNVPVTHAKELLHTEHNGMLCATSLGQPLIL
DTCTIEGLIYGNPSCDPLLGGREWSYIVERPSAVNGLCYPGNVENLEELRSLFSSSRSYQRIQIFPDTIW
NVSYSGTSKACSDSFYRSMRWLTQKNNAYPTQDAQYTNNQGKNILFMWGINHPPTDTAQTNLYTRTDTTT
SVATEEINRIFKPLIGPRPLVNGLMGRINYYWSVLKPGQTLRIKSDGNLIAPWFGHILSGESHGRILKTD
LKRGSCTVQCQTEKGGLNTTLPFQNVSKYAFGNCSKYIGVKSLKLAVGLRNVPSRSSRGLFGAIAGFIEG
GWSGLVAGWYGFQHSNDQGVGMAADRDSTQKAIDKITSKVNNIVDKMNKQYEIIDHEFSEVETRLNMINN
KIDDQIQDIWAYNAELLVLLENQKTLDEHDANVNNLYNKVKRALGSNAVEDGKGCFELYHKCDDQCMETI
RNGTYNRRKYQEESKLERQKIEGVKLESEGTYKILTIYSTVASSLVIAMGFAAFLF

>QOJ99017.1 hemagglutinin, partial [Influenza A virus]

METVSLITILVVATVSNADKICIGYQSTNSTETVDTLTENNVPVTHAKELLHTEHNGMLCATSLGQPLIL
DTCTIEGLIYGNPSCDPLLGGREWSYIVERPSAVNGLCYPGSVENLEELRSLFSSSRSYQRIQIFPDTIW
NVSYSGTSKACSDSFYRSMRWLTQKNNAYPTQDAQYTNNQGKNILFMWGINHPPTDTAQTNLYTRTDTTT
SVATEEMNRIFKPLIGPRPLVNGLMGRINYYWSVLKPGQTLRIKSDGNLIAPWYGHILSGESHGRILKTD
LKRGSCTVQCQTEKGGLNTTLPFQNVSKYAFGNCSKYIGVKSLKLAVGLRNVPSRSSRGLFGAIAGFIEG
GWSGLVAGWYGFQHSNDQGVGMAADRESTQKAIDKITSKVNNIVDKMNKQYEIIDHEFSEVETRLNMINN
KVDDQIQDIWAYNAELLVLLENQKTLDEHDANVNNLYNKVKRALGSNAVEDGKGCFELYHKCDDHCMETI
RNGTYNRRKYQEESKLERQKIEGVKLESEGTYKILTIYSTVASSLVIAMGFAAFLFWAMSNGSC

>QOJ99015.1 hemagglutinin, partial [Influenza A virus]

METVSLITILVVATVSNADKICIGYQSTNSTETVDTLTENNVPVTHAKELLHTEHNGMLCATSLGHPLIL
DTCTIEGLIYGNPSCDPLLGGREWSYIVERPSAVNGLCYPGSVENLEELRSLFSSSRSYQRIQIFPDTIW
NVSYSGTSKACSDSFYRSMRWLTQKNNAYPTQDAQYTNNQGKNILFMWGINHPPTDTAQTNLYTRTDTTT
SVATEEMNRIFKPLIGPRPLVNGLMGRINYYWSVLKPGQTLRIKSDGNLIAPWYGHILSGESHGRILKTD
LKRGSCTVQCQTEKGGLNTTLPFQNVSKYAFGNCSKYIGVKSLKLAVGLRNVPSRSSRGLFGAIAGFIEG
GWSGLVAGWYGFQHSNDQGVGMAADRDSTQKAIDKITSKVNNIVDKMNKQYEIIDHEFSEVETRLNMINN
KVDDQIQDIWAYNAELLVLLENQKTLDEHDANVNNLYNKVKRALGSNAVEDGKGCFELYHKCDDHCMETI
RNGTYNRRKYQEESKLERQKIEGVKLESEETYKILTIYSTVASSLVIAM

>QOJ99014.1 hemagglutinin, partial [Influenza A virus]

METVSLITILVVATVSNADKICIGYQSTNSTETVDTLTENNVPVTHAKELLHTEHNGMLCATSLGHPLIL
DTCTIEGLIYGNPSCDPLLGGREWSYIVERPSAVNGLCYPGSVENLEELRSLFSSSRSYQRIQIFPDTIW
NVSYSGTSKACSDSFYRSMRWLTQKNNAYPTQDAQYTNNQGKNILFMWGINHPPTDTAQTNLYTRTDTTT
SVATEEMNRIFKPLIGPRPLVNGLMGRINYYWSVLKPGQTLRIKSDGNLIAPWYGHILSGESHGRILKTD
LKRGSCTVQCQTEKGGLNTTLPFQNVSKYAFGNCSKYIGVKSLKLAVGLRNVPSRSSRGLFGAIAGFIEG
GWSGLVAGWYGFQHSNDQGVGMAADRDSTQKAIDKITSKVNNIVDKMNKQYEIIDHEFSEVETRLNMINN
KVDDQIQDIWAYNAELLVLLENQKTLDEHDANVNNLYNKVKRALGSNAVEDGKGCFELYHKCDDHCMETI
RNGTYNRRKYQEESKLERQKIEGVKLESEETYKILTIYSTVASSLVIAMGFAAFLFWAMSNGSC

>QOJ99013.1 hemagglutinin, partial [Influenza A virus]

METVSLITILLAATVSNADKICIGYQSTNSTETVDTLTENNVPVTHAKELLHTEHNGMLCATSLGQPLIL
DTCTIEGLIYGNPSCDPLLGEREWSYIVERPSAVNGLCYPGNVENLEELRSLFSSSRSYQRIQIFPDTIW

NVSYSGTSKACSDSFYRSMRWLTQKNNAYPTQDAQYTNNQGKNILFMWGINHPPTDTAQRNLYTRTDTTT
SVATEEINRIFRPLIGPRPLVNGLMGRINYYWSVLKPGQTMRIKSDGNLIAPWYGHILSGESHGRILKTD
LKRGSCTVQCQTEKGGLNTTLPFQNVSKYAFGNCSKYIGIKSLKLAVGLRNVPSRSSRGLFGAIAGFIEG
GWSGLVAGWYGFQHSNDQGVGMAADRESTQKAIDKITSKVNNIVDKMNKQYEIIDHEFSEVETRLNMINN
KIDDQIQDIWAYNAELLVLLENQKTLDEHDANVNNLYNKVKRALGSNAVEDGKGCFELYHKCDDQCMETI
RNGTYNRRKYQEESKLERQKIEGVKLESEGTYKILTIYSTVASSLVIAMGFAAFLFWAMSNGSC

>QOJ99012.1 hemagglutinin [Influenza A virus]
METVSLITILVVATVSNADKICIGYQSTNSTETVDTLTENNVPVTHAKELLHTEHNGMLCATSLGHPLIL
DTCTIEGLIYGNPSCDPLLGGREWSYIVERPSAVNGLCYPGSVENLEELRSLFSSSRSYQRIQIFPDTIW
NVSYSGTSKACSDSFYRSMRWLTQKNNAYPTQDAQYTNNQGKNILFMWGINHPPTDTAQTNLYTRTDTTT
SVATEEMNRIFKPLIGPRPLVNGLMGRINYYWSVLKPGQTLRIKSDGNLIAPWYGHILSGESHGRILKTD
LKRGSCTVQCQTEKGGLNTTLPFQNVSKYAFGNCSKYIGVKSLKLAVGLRNVPSRSSRGLFGAIAGFIEG
GWSGLVAGWYGFQHSNDQGVGMAADRDSTQKAIDKITSKVNNIVDKMNKQYEIIDHEFSEVETRLNMINN
KVDDQIQDIWAYNAELLVLLENQKTLDEHDANVNNRYNKVKRALGSNAVEDGKGCFELYHKCDDHCMETI
RNGTYNRRKYQEESKLERQKIEGVKLESEETYKILTIYSTVASSLVIAMGFAAFLFWAMSNGSCRCNICI

>QOJ99011.1 hemagglutinin, partial [Influenza A virus]
METVSLITILLAATVSNADKICIGYQSTNSTETVDTLTENNVPVTHAKELLHTEHNGMLCATSLGQPLIL
DTCTIEGLIYGNPSCDPLLGEREWSYIVERPSAVNGLCYPGNVENLEELRSLFSSSRSYQRIQIFPDTIW
NVSYSGTSKACSDSFYRSMRWLTQKNNAYPTQDAQYTNNQGKNILFMWGINHPPTDTAQTNLYTRTDTTT
SVATEEINRIFRPLIGPRPLVNGLMGRINYYWSVLKPGQTMRIKSDGNLIAPWYGYILSGESHGRILKTD
LKRGSCTVQCQTEKGGLNTTLPFQNVSKYAFGNCSKYIGIKSLKLAVGLRNVPSRSSRGLFGAIAGFIEG
GWSGLVAGWYGFQHSNDQGVGMAADRESTQKAIDKITSKVNNIVDKMNKQYEIIDHEFSEVETRLNMINN
KIDDQIQDIWAYNAELLVLLENQKTLDEHDANVNNLYNKVKRALGSNAVEDGKGCFELYHKCDDQCMETI
RNGTYNRRKYQEESKLERQKIEGVKLESEGTYKILTIYSTVASSLVIAMGFAAFLFWAMSNGSC

>QOJ99010.1 hemagglutinin, partial [Influenza A virus]
METVSLITILVVATVSNADKICIGYQSTNSTETVDTLTENNVPVTHAKELLHTEHNGMLCATSLGHPLIL
DTCTIEGLIYGNPSCDPLLGGREWSYIVERPSAVNGLCYPGSVENLEELRSLFSSSRSYQRIQIFPDTIW
NVSYSGTSKACSDSFYRSMRWLTQKNNAYPTQDAQYTNNQGKNILFMWGINHPPTDTAQTNLYTRTDTTT
SVATEEMNRIFKPLIGPRPLVNGLMGRINYYWSVLKPGQTLRIKSDGNLIAPWYGHILSGESHGRILKTD
LKRGSCTVQCQTEKGGLNTTLPFQNVSKYAFGNCSKYIGVKSLKLAVGLRNVPSRSSRGLFGAIAGFIEG
GWSGLVAGWYGFQHSNDQGVGMAADRDSTQKAIDKITSKVNNIVDKMNKQYEIIDHEFSEVETRLNMINN

KVDDQIQDIWAYNAELLVLLENQKTLDEHDANVNNLYNKVKRALGSNAVEDGKGCFELYHKCDDHCMETI
RNGTYNRRKYQEESKLERQKIEGVKLESEETYKILTIYSTVASSLVIAMGFAAFLFWAMSNGSC

>QOJ99009.1 hemagglutinin, partial [Influenza A virus]
METVSLITILLAATVSNADKICIGYQSTNSTETVDTLTENNVPVTHAKELLHTEHNGMLCATSLGQPLIL
DTCTIEGLIYGNPSCDPLLGGREWSYIVERPSAVNGLCYPGSVENLEELRSLFSSSRSYQRIQIFPDTIW
NVSYSGTSKACSDSFYRSMRWLTQKNNAYPTQDAQYTNNQGKNILFMWGINHPPTDTAQTNLYTRTDTTT
SVATEEMNRIFKPLIGPRPLVNGLMGRINYYWSVLKPGQTLRIKSDGNLIAPWYGYILSGESHGRILKTD
LKRGSCTVQCQTEKGGLNTTLPFQNVSKYAFGNCSKYIGVKSLKLAVGLRNVPSRSSRGLFGAIAGFIEG
GWSGLVAGWYGFQHSNDQGVGMAADRESTQKAIDKITSKVNNIVDKMNKQYEIIDHEFSEVETRLNMINN
KIDDQIQDIWAYNAELLVLLENQKTLDEHDANVNNLYNKVKRALGSNAVEDGKGCFELYHKCDDHCMETI
RNGTYNRRKYQEESKLERQKIEGVKLESEGTYKILTIYSTVASSLVIAMGFAAFLFWAMSNGSC

>QOJ98999.1 hemagglutinin [Influenza A virus]
METVSLITILLVATVSYADKICIGYQSTNSTETVDTLTENNVPVTHAKELLHTEHNGMLCATSLGQPLIL
DTCTIEGLIYGNPSCDLSLEGREWSYIVERPSAVNGLCYPGNVENLEELRSLFSSARSYQRVQIFPDTIW
NVSYDGTSTACSGSFYRSMRWLTRKDGNYPTQDAQYTNNQGKNILFMWGINHPPTDDTQRSLYTRTDTTT
SVATEEINRIFKPLIGPRPLVNGLMGRIDYYWSVLKPGQTLRIKSDGNLIAPWYGYILSGESHGRILKTD
LKRGSCTVQCQTEKGGLNTTLPFQNVSKYAFGNCSKYIGIKSLKLAVGLRNVPSRSSRGLFGAIAGFIEG
GWSGLVAGWYGFQHSNDQGVGMAADRESTQKAVDKITSKVNNIVDKMNKQYEIIDHEFSEVETRLNMINN
KIDDQIQDIWAYNAELLVLLENQKTLDEHDANVNNLYNKVKRALGSNAMEDGKGCFELYHKCDDQCMETI
RNGTYNRRKYQEESKLERQKIEGVKLESEGTYKILTIYSTVASSLVIAMGFAAFLFWAMSNGSCRCNICI

>QOJ98989.1 hemagglutinin [Influenza A virus]
METVSLITILLVATVSYADKICIGYQSTNSTETVDTLTENNVPVTHAKELLHTEHNGMLCATSLGQPLIL
DTCTIEGLIYGNPSCDLSLEGREWSYIVERPSAVNGLCYPGNVENLEELRSLFSSARSYQRVQIFPDTIW
NVSYDGTSTACSGSFYRSMRWLTRKNGDYPTQDAQYTNNQGKNILFTWGINHPPTDYTQRNLYTRTDTTT
SVATEEINRIFKPLIGPRPLVNGLMGRIDYYWSVLKPGQTLRIKSDGNLIAPWYGYILSGESHGRILKTD
LKRGSCAVQCQTEKGGLNTTLPFQNVSKYAFGNCSKYIGIKSLKLAVGLRNVPSRSSRGLFGAIAGFIEG
GWSGLVAGWYGFQHSNDQGVGMAADRDSTQKAIDKITSKVNNIVDKMNKQYEIIDHEFSEVETRLNMINN
KIDDQIQDIWAYNAELLVLLENQKTLDEHDANVNNLYNKVKRALGTNAVEDGKGCFELYHKCNDQCMETI
RNGTYNRRKYQEESKLERQKIEGVKLESEGTYKILTIYSTVASSLVIAMGFAAFLFWAMSNGSCRCNICI

>QOJ98975.1 hemagglutinin, partial [Influenza A virus]

METVSLITILLVATVSNADKICIGYQSTNSTETVDTLTENNVPVTHAKELLHTEHNGMLCATSLGQPLIL
DTCTIEGLIYGNPSCDLSLEGREWSYIVERPSAVNGLCYPGNVENLEELRSLFSSARSYQRIQIFPDTIW
NVSYDGTSTACSNSFYRSMRWLTRKDGNYPTQDAQYTNNQGKNILFMWGINHPPTDDTQRNLYTRTDTTT
SVATEEINRIFKPLIGPRPLVNGLMGRIDYYWSVLKPGQTLRIKSDGNLIAPWYGHILSGESHGRILKTD
LKRGSCTVQCQAEKGGFNTTLPFQNVSKYAFGNCSKYIGIKSLKLAVGLRNVPSRSSRGLFGAIAGFIEG
GWSGLVAGWYGFQHSNDQGVGMAADRDSTQKAIDKITSKVNNIVDKMNKQYEIIDHEFSEVETRLNMINN
KIDDQIQDIWAYNAELLVLLENQKTLDEHDANVNNLYNKVKRALGSNAVEDGKGCFELYHKCNDQCMDTI
RNGTYNRRKYQEESKLERQKIEGVKLESEGTYKILTIYSTVASSLVIAM

>QOJ98974.1 hemagglutinin, partial [Influenza A virus]
METVSLITILLVATVSNADKICIGYQSTNSTETVDTLTENNVPVTHAKELLHTEHNGMLCATSLGQPLIL
DTCTIEGLIYGNPSCDLSLEGREWSYIVERPSAVNGLCYPGNVENLEELRSLFSSARSYQRIQIFPDTIW
NVSYDGTSTACSNSFYRSMRWLTRKDGNYPTQDAQYTNNQGKNILFMWGINHPPTDXTQRNLYTRTDTTT
SVATEEINRIFKPLIGPRPLVNGLMGRIDYYWSVLKPGQTLRIKSDGNLIAPWYGHILSGESHGRILKTD
LKRGSCTVQCQAEKGGFNTTLPFQNVSKYAFGNCSKYIGIKSLKLAVGLRNVPSRSSRGLFGAIAGFIEG
GWSGLVAGWYGFQHSNDQGVGMAADRDSTQKAIDKITSKVNNIVDKMNKQYEIIDHEFSEVETRLNMINN
KIDDQIQDIWAYNAELLVLLENQKTLDEHDANVNNLYNKVKRALGSNAVEDGKGCFELYHKCNDQCMDTI
RNGTYNRRKYQEESKLERQKIEGVKLESEGTYKILTIYSTVASSLVIAM

>QOJ98973.1 hemagglutinin, partial [Influenza A virus]
METVSLITILLVATVSNADKICIGYQSTNSTETVDTLTENNVPVTHAKELLHTEHNGMLCATSLGQPLIL
DTCTIEGLIYGNPSCDLSLEGREWSYIVERPSAVNGLCYPGNVENLEELRSLFSSARSYQRIQIFPDTIW
NVSYDGTSTACSNSFYRSMRWLTRKDGNYPTQDAQYTNNQGKNILFMWGINHPPTDXTQRNLYTRTDTTT
SVATEEINRIFKPLIGPRPLVNGLMGRIDYYWSVLKPGQTLRIKSDGNLIAPWYGHILSGESHGRILKTD
LKRGSCTVQCQAEKGGFNTTLPFQNVSKYAFGNCSKYIGIKSLKLAVGLRNVPSRSSRGLFGAIAGFIEG
GWSGLVAGWYGFQHSNDQGVGMAADRDSTQKAIDKITSKVNNIVDKMNKQYEIIDHEFSEVETRLNMINN
KIDDQIQDIWAYNAELLVLLENQKTLDEHDANVNNLYNKVKRALGSNAVEDGKGCFELYHKCNDQCMDTI
RNGTYNRRKYQEESKLERQKIEGVKLESEGTYKILTIYSTVASSLVIAM

>QOJ98971.1 hemagglutinin [Influenza A virus]
MKTVSLITILLVATISNADKICIGYQSTNSTETVDTLTENNVPVTHAKELLHTEHNGMLCATSLGHPLIL
DTCTIEGLIYGNPSCDPLLGGREWSYIVERPSAVNGLCYPGNVENLEELRSLFSSSRSYQRIQIFPDTIW
NVSYSGTSKACSDSFYRSMRWLTQKNNAYPTQDAQYTNNQEKNILFMWGINHPPTDTVQTNLYTRTDTTT
SVATEEINRIFKPLIGPRPLVNGLMGRINYYWSVLKPGQTLRIKSDGNLIAPWYGHILSGESHGRILKTD

LKRGSCTVQCQTEKGGLNTTLPFQNVSKYAFGNCSKYIGVKSLKLAVGLRNVPSRSSRGLFGAIAGFIEG
GWSGLVAGWYGFQHSNDQGVGMAADRDSTQKAIDKITSKVNNIVDKMNKQYEIIDHEFSEVETRLNMINN
KVDDQIQDIWAYNAELLVLLENQKTLDEHDANVNNLYNKVKRALGSNAVEDGKGCFELYHKCDDHCMETI
RNGTYNRRKYQEESKLERQKIEGVKLESEETYKILTIYSTVASSLVIAMGFAAFLFWAMSNGSCRCNICI

>QOJ98970.1 hemagglutinin [Influenza A virus]
METVSLITILLVATVSNADKICIGYQSTNSTETVDTLTENNVPVTHAKELLHTEHNGMLCATSLGQPLIL
DTCTIEGLIYGNPSCDLSLEGREWSYIVERPSAVNGLCYPGNVENLEELRSLFSSARSYQRIQIFPDTIW
NVSYDGTSTACSNSFYRSMRWLTRKDGNYPTQDAQYTNNQGKNILFMWGINHPPTDDTQRNLYTRTDTTT
SVATEEINRIFKPLIGPRPLVNGLMGRIDYYWSVLKPGQTLRIKSDGNLIAPWYGHILSGESHGRILKTD
LKRGSCTVQCQTEKGGFNTTLPFQNVSKYAFGNCSKYIGIKSLKLAVGLRNVPSRSSRGLFGAIAGFIEG
GWSGLVAGWYGFQHSNDQGVGMAADRDSTQKAIDKITSKVNNIVDKMNKQYEIIDHEFSEVETRLNMINN
KIDDQIQDIWAYNAELLVLLENQKTLDEHDANVNNLYNKVKRALGSNAVEDGKGCFELYHKCNDQCMDTI
RNGTYNRRKYQEESKLERQKIEGVKLESEGTYKILTIYSTVASSLVIAMGFAAFLFWAMSNGSCRCNIXI

>QOJ98969.1 hemagglutinin [Influenza A virus]
METVSLITILLVATVSNADKICIGYQSTNSTETVDTLTENNVPVTHAKELLHTEHNGMLCATSLGQPLIL
DTCTIEGLIYGNPSCDLSLEGREWSYIVERPSAVNGLCYPGNVENLEELRSLFSSARSYQRIQIFPDTIW
NVSYDGTSTACSNSFYRSMRWLTRKDGNYPTQDAQYTNNQGKNILFMWGINHPPTDDTQRNLYTRTDTTT
SVATEEINRIFKPLIGPRPLVNGLMGRIDYYWSVLKPGQTLRIKSDGNLIAPWYGHILSGESHGRILKTD
LKRGSCTVQCQTEKGGFNTTLPFQNVSKYAFGNCSKYIGIKSLKLAVGLRNVPSRSSRGLFGAIAGFIEG
GWSGLVAGWYGFQHSNDQGVGMAADRDSTQKAIDKITSKVNNIVDKMNKQYEIIDHEFSEVETRLNMINN
KIDDQIQDIWAYNAELLVLLENQKTLDEHDANVNNLYNKVKRALGSNAVEDGKGCFELYHKCNDQCMDTI
RNGTYNRRKYQEESKLERQKIEGVKLESEGTYKILTIYSTVASSLVIAMGFAAFLFWAMSNGSCRCNIXI

>QOJ98968.1 hemagglutinin [Influenza A virus]
METVSLITILLVATVSNADKICIGYQSTNSTETVDTLTENNVPVTHAKELLHTEHNGMLCATSLGQPLIL
DTCTIEGLIYGNPSCDLSLEGREWSYIVERPSAVNGLCYPGNVENLEELRSLFSSARSYQRIQIFPDTIW
NVSYDGTSTACSNSFYRSMRWLTRKDGNYPTQDAQYTNNQGKNILFMWGINHPPTDDTQRNLYTRTDTTT
SVATEEINRIFKPLIGPRPLVNGLMGRIDYYWSVLKPGQTLRIKSDGNLIAPWYGHILSGESHGRILKTD
LKRGSCTVQCQTEKGGFNTTLPFQNVSKYAFGNCSKYIGIKSLKLAVGLRNVPSRSSRGLFGAIAGFIEG
GWSGLVAGWYGFQHSNDQGVGMAADRDSTQKAIDKITSKVNNIVDKMNKQYEIIDHEFSEVETRLNMINN
KIDDQIQDIWAYNAELLVLLENQKTLDEHDANVNNLYNKVKRALGSNAVEDGKGCFELYHKCNDQCMDTI
RNGTYNRRKYQEESKLERQKIEGVKLESEGTYKILTIYSTVASSLVIAMGFAAFLFWAMSNGSCRCNICI

>QOJ98964.1 hemagglutinin [Influenza A virus]

METVSLITILLVATVSNADKICIGYQSTNSTETVDTLTENNVPVTHAKELLHTEHNGMLCATSLGQPLIL
DTCTIEGLIYGNPSCDLSLEGREWSYIVERPSAVNGLCYPGSVENLEELRSLFSSARSYQRIQIFPDTXW
NVSYDGTSTACSGSFYRSMRWLTRKNGVYPIQDAQYTNNQGKNILFMWGINHPPTDDTQTGLYTRTDTTT
SVATEEINRIFKPLIGPRPLVNGLMGRINYYWSVLKPGQTLRIKSDGNLIAPWYGHILSGESHGRILKTD
LKRGSCTVQCQTEKGGLNTTLPFQNVSKXAFGNCSKYIGIKSLKLAVGLRNVPSRSSRGLFGAIAGFIEG
GWSGLVAGWYGFQHSNDQGVGMAADRDSTQKAIDKITSKVNNIVXKMNKQYEIIDHEFSEVEARLNMINN
KIDDQIQDIWAYNAELLVLLENQKTLDEHDANVNNLYNKVKRALGSNAVEDGKGCFELYHKCDDQCMETI
RNGTYNRRKYQEESKLXRQKIEGVKLESEGTYKILXIYSTVASSLVIAMGFAAFLFWAMSNGSCRCNICI

>QOJ98961.1 hemagglutinin [Influenza A virus]

METVSLITILLVATVSNADKICIGYQSTNSTETVDTLTENNVPVTHAKELLHTEHNGMLCATSLGQPLIL
DTCTIEGLIYGNPSCDLSLEGREWSYIVERPSAVNGLCYPGNVENLEELRSLFSSARSYQRIQIFPDTIW
NVSYDGTSTACSNSFYRSMRWLTRKDGNYPTQDAQYTNNQGKNILFMWGINHPPTDDTQRNLYTRTDTTT
SVATEEINRIFKPLIGPRPLVNGLMGRIDYYWSVLKPGQTLRIKSDGNLIAPWYGHILSGESHGRILKTD
LKRGSCTVQCQTEKGGFNTTLPFQNISKYAFGNCSKYIGIKSLKLAVGLRNVPSRSSRGLFGAIAGFIEG
GWSGLVAGWYGFQHSNDQGVGMAADRDSTQKAIDKITSKVNNIVDKMNKQYEIIDHEFSEVETRLNMINN
KIDDQIQDIWAYNAELLVLLENQKTLDEHDANVNNLYNKVKRALGSNAVEDGKGCFELYHKCNDQCMDTI
RNGTYNRRKYQEESKLERQKIEGVKLESEGTYKILTIYSTVASSLVIAMGFAAFLFWAMSNGSCRCNICI

>QOJ98960.1 hemagglutinin [Influenza A virus]

METVSLITILLAATVSNADKICIGYQSTNSTETVDTLTENNVPVTHAKELLHTEHNGMLCATSLGQPLIL
DTCTIEGLIYGNPSCDPLLEEREWSYIVERPSAVNGLCYPGNVENLEELRSLFSSARSYQRIQIFPDTIW
NVSYDGTSNTCSGSFYRNMRWLTRKNGNYPIQDAQYTNNQGKNILFMWGINHPPTDDTQRNLYTRTDTTT
SVATEEVNRIFKPLIGPRPLVNGLMGRINYYWSVLKPGQTLRVKSDGNLIAPWYGYILSGESHGRILRTD
LKRGSCTVQCQTEKGGLNTTLPFQNVSKYAFGNCSKYIGIKSLKLAVGLRNVPSRSSRGLFGAIAGFIEG
GWSGLVAGWYGFQHSNDQGVGMAADRESTQKAIDKITSKVNNIVDKMNKQYEIIDHEFSEVETRLNMINN
KIDDQIQDIWAYNAELLVLLENQKTLDEHDANVNNLYNKVKRALGSNAVEDGKGCFELYHKCDDQCMETI
RNGTYNRRKYQEESKLERQKIEGVKLESEGTYKILTIYSTVASSLVIAMGFAAFLFWAMSNGSCRCNICI

>QOJ98959.1 hemagglutinin [Influenza A virus]

METVSLITILLAATVSNADKICIGYQSTNSTETVDTLTENNVPVTHAKELLHTEHNGMLCATSLGQPLIL
DTCTIEGLIYGNPSCDPLLEEREWSYIVERPSAVNGLCYPGNVENLEELRSLFSSARSYQRIQIFPDTIW

NVSYDGTSNTCSGSFYRNMRWLTRKNGNYPIQDAQYTNNQGKNILFMWGINHPPTDDTQRNLYTRTDTTT
SVATEEVNRIFKPLIGPRPLVNGLMGRINYYWSVLKPGQTLRVKSDGNLIAPWYGYILSGESHGRILRTD
LKRGSCTVQCQTEKGGLNTTLPFQNVSKYAFGNCSKYIGIKSLKLAVGLRNVPSRSSRGLFGAIAGFIEG
GWSGLVAGWYGFQHSNDQGVGMAADRESTQKAIDKITSKVNNIVDKMNKQYEIIDHEFSEVETRLNMINN
KIDDQIQDIWAYNAELLVLLENQKTLDEHDANVNNLYNKVKRALGSNAVEDGKGCFELYHKCDDQCMETI
RNGTYNRRKYQEESKLERQKIEGVKLESEGTYKILTIYSTVASSLVIAMGFAAFLFWAMSNGSCRCNICI

>QOJ98957.1 hemagglutinin [Influenza A virus]
METVSLITILLAATVSNADKICIGYQSTNSTETVDTLTENNVPVTHAKELLHTEHNGMLCATSLGQPLIL
DTCTIEGLIYGNPSCDPLLEEREWSYIVERPSAVNGLCYPGNVENLEELRSLFSSARSYQRIQIFPDTIW
NVSYDGTSNTCSGSFYRNMRWLTRKNGNYPIQDAQYTNNQGKNILFMWGINHPPTDDTQRNLYTRTDTTT
SVATEEINRIFKPLIGPRPLVNGLMGRINYYWSVLKPGQTLRIKSDGNLIAPWYGYILSGESHGRILRTD
LKRGSCTVQCQTEKGGLNTTLPFQNVSKYAFGNCSKYIGIKSLKLAVGLRNVPSRSSRGLFGAIAGFIEG
GWSGLVAGWYGFQHSNDQGVGMAADRESTQKAIDKITSKVNNIVDKMNKQYEIIDHEFSEVETRLNMINN
KIDDQIQDIWAYNAELLVLLENQKTLDEHDANVNNLYNKVKRALGSNAVEDGKGCFELYHKCDDQCMETI
RNGTYNRRKYQEESKLERQKIEGVKLESEGTYKILTIYSTVASSLVIAMGFAAFLFWAMSNGSCRCNICI

>QOJ98953.1 hemagglutinin, partial [Influenza A virus]
METVSLITILVVATVSNADKICIGYQSTNSTETVDTLTENNVPVTHAKELLHTEHNGMLCATDLGHPLIL
DTCTIEGLIYGNPSCDPLLGGREWSYIVERPSAVNGLCYPGNVENLEELRSLFSSSRSYQRIQIFPDTIW
NVSYSGTSKACSDSFYRSMRWLTQKNNAYPTQDAQYTNNQGKNILFMWGINHPPTDTAQTNLYTRTDTTT
SVATEEINRIFKPLIGPRPLVNGLMGRINYYWSVLKPGQTLRIKSDGNLIAPWYGHILSGESHGRILKTD
LKRGSCTVQCQTEKGGLNTTLPFQNVSKYAFGNCSKYIGVKSLKLAVGLRNVPSRSSRGLFGAIAGFIEG
GWPGLVAGWYGFQHSNDQGVGMAADRDSTQKAIDKITSKVNNIVDKMNKQYEIIDHEFSEVETRLNMINN
KVDDQIQDIWAYNAELLVLLENQKTLDEHDANVNNLYNKVKRALGSNAVEDGKGCFELYHKCDDHCMETI
RNGTYNRRKYQEESKLERQKIEGVKLESEETYKILTIYSTVASSLVIAM

>QOJ98952.1 hemagglutinin, partial [Influenza A virus]
METVSLITILVVATVSNADKICIGYQSTNSTETVDTLTENNVPVTHAKELLHTEHNGMLCATDLGHPLIL
DTCTIEGLIYGNPSCDPLLGGREWSYIVERPSAVNGLCYPGNVENLEELRSLFSSSRSYQRIQIFPDTIW
NVSYSGTSKACSDSFYRSMRWLTQKNNAYPTQDAQYTNNQGKNILFMWGINHPPTDTAQTNLYTRTDTTT
SVATEEINRIFKPLIGPRPLVNGLMGRINYYWSVLKPGQTLRIKSDGNLIAPWYGHILSGESHGRILKTD
LKRGSCTVQCQTEKGGLNTTLPFQNVSKYAFGNCSKYIGVKSLKLAVGLRNVPSRSSRGLFGAIAGFIEG
GWPGLVAGWYGFQHSNDQGVGMAADRDSTQKAIDKITSKVNNIVDKMNKQYEIIDHEFSEVETRLNMINN

KVDDQIQDIWAYNAELLVLLENQKTLDEHDANVNNLYNKVKRALGSNAVEDGKGCFELYHKCDDHCMETI
RNGTYNRRKYQEESKLERQKIEGVKLESEETYKILTIYSTVASSLVIAM

>QOJ98951.1 hemagglutinin, partial [Influenza A virus]
MGTVSLITILVVATVSNADKICIGYQSTNSTETVDTLTENNVPVTHAKELLHTEHNGMLCATXLGHPLIL
DTCTIEGLIYGNPSCDPLLGGREWSYIVERPSAVNGLCYPGNVENLEELRSLFSSSRSYQRIQIFPDTIW
NVSYSGTSKACSDSFYRSMRWLTQKNNAYPTQDAQYTNNQGKNILFMWGINHPPTDTAQTNLYTRTDTTT
SVATEEINRIFKPLIGPRPLVNGLMGRINYYWSVLKPGQTLRIKSDGNLIAPWYGHILSGESHGRILKTD
LKRGSCTVQCQTEKGGLNTTLPFQNVSKYAFGNCSKYIGVKSLKLAVGLRNVPSRSSRGLFGAIAGFIEG
GWPGLVAGWYGFQHSNDQGVGMAADRDSTQKAIDKITSKVNNIVDKMNKQYEIIDHEFSEVETRLNMINN
KVDDQIQDIWAYNAELLVLLENQKTLDEHDANVNNLYNKVKRALGSNAVEDGKGCFELYHKCDDHCMETI
RNGTYNRRKYQEESKLERQKIEGVKLESEETYKILTIYSTVASSLVIAM

>QOJ98950.1 hemagglutinin, partial [Influenza A virus]
METVSLITILVVATVSNADKICIGYQSTNSTETVDTLTENNVPVTHAKELLHTEHNGMLCATXLGHPLIL
DTCTIEGLIYGNPSCDPLLGGREWSYIVERPSAVNGLCYPGNVENLEELRSLFSSSRSYQRIQIFPDTIW
NVSYSGTSKACSDSFYRSMRWLTQKNNAYPTQDAQYTNNQGKNILFMWGINHPPTDTAQTNLYTRTDTTT
SVATEEINRIFKPLIGPRPLVNGLMGRINYYWSVLKPGQTLRIKSDGNLIAPWYGHILSGESHGRILKTD
LKRGSCTVQCQTEKGGLNTTLPFQNVSKYAFGNCSKYIGVKSLKLAVGLRNVPSRSSRGLFGAIAGFIEG
GWPGLVAGWYGFQHSNDQGVGMAADRDSTQKAIDKITSKVNNIVDKMNKQYEIIDHEFSEVETRLNMINN
KVDDQIQDIWAYNAELLVLLENQKTLDEHDANVNNLYNKVKRALGSNAVEDGKGCFELYHKCDDHCMETI
RNGTYNRRKYQEESKLERQKIEGVKLESEETYKILTIYSTVASSLVIAM

>QOJ98948.1 hemagglutinin, partial [Influenza A virus]
METVSLITILVVATVSNADKICIGYQSTNSTETVDTLTENNVPVTHAKELLHTEHNGMLCATGLGHPLIL
DTCTIEGLIYGNPSCDPLLGGREWSYIVERPSAVNGLCYPGNVENLEELRSLFSSSRSYQRIQIFPDTIW
NVSYSGTSKACSDSFYRSMRWLTQKNNAYPTQDAQYTNNQGKNILFMWGINHPPTDTAQTNLYTRTDTTT
SVATEEINRIFKPLIGPRPLVNGLMGRINYYWSVLKPGQTLRIKSDGNLIAPWYGHILSGESHGRILKTD
LKRGSCTVQCQTEKGGLNTTLPFQNVSKYAFGNCSKYIGVKSLKLAVGLRNVPSRSSRGLFGAIAGFIEG
GWPGLVAGWYGFQHSNDQGVGMAADRDSTQKAIDKITSKVNNIVDKMNKQYEIIDHEFSEVETRLNMINN
KVDDQIQDIWAYNAELLVLLENQKTLDEHDANVNNLYNKVKRALGSNAVEDGKGCFELYHKCDDHCMETI
RNGTYNRRKYQEESKLERQKIEGVKLESEETYKILTIYSTVASSLVIAM

>QOJ98946.1 hemagglutinin, partial [Influenza A virus]

METVSLITILVVATVSNADKICIGYQSTNSTETVDTLTENNVPVTHAKELLHTEHNGMLCATDLGHPLIL
DTCTIEGLIYGNPSCDPLLGGREWSYIVERPSAVNGLCYPGNVENLEELRSLFSSSRSYQRIQIFPDTIW
NVSYSGTSKACSDSFYRSMRWLTQKNNAYPTQDAQYTNNQGKNILFMWGINHPPTDTXQTNLYTRTDTTT
SVATEEINRIFKPLIGPRPLVNGLMGRINYYWSVLKPGQTLRIKSDGNLIAPWYGHILSGESHGRILKTD
LKRGSCTVQCQTEKGGLNTTLPFQNVSKYAFGNCSKYIGVKSLKLAVGLRNVPSRSSRGLFGAIAGFIEG
GWPGLVAGWYGFQHSNDQGVGMAADRDSTQKAIDKITSKVNNIVDKMNKQYEIIDHEFSEVETRLNMINN
KVDDQIQDIWAYNAELLVLLENQKTLDEHDANVNNLYNKVKRALGSNAVEDGKGCFELYHKCDDHCMETI
RNGTYNRRKYQEESKLERQKIEGVKLESEETYKILTIYSTVASSLVIAM

>QOJ98945.1 hemagglutinin, partial [Influenza A virus]
METVSLITILVVATVSNADKICIGYQSTNSTETVDTLTENNVPVTHAKELLHTEHNGMLCATDLGHPLIL
DTCTIEGLIYGNPSCDPLLGGREWSYIVERPSAVNGLCYPGNVENLEELRSLFSSSRSYQRIQIFPDTIW
NVSYSGTSKACSDSFYRSMRWLTQKNNAYPTQDAQYTNNQGKNILFMWGINHPPTDTVQTNLYTRTDTTT
SVATEEINRIFKPLIGPRPLVNGLMGRINYYWSVLKPGQTLRIKSDGNLIAPWYGHILSGESHGRILKTD
LKRGSCTVQCQTEKGGLNTTLPFQNVSKYAFGNCSKYIGVKSLKLAVGLRNVPSRSSRGLFGAIAGFIEG
GWPGLVAGWYGFQHSNDQGVGMAADRDSTQKAIDKITSKVNNIVDKMNKQYEIIDHEFSEVETRLNMINN
KVDDQIQDIWAYNAELLVLLENQKTLDEHDANVNNLYNKVKRALGSNAVEDGKGCFELYHKCDDHCMETI
RNGTYNRRKYQEESKLKRQKIEGVKLESEETYKILTIYSTVASSLVIAMGFAAFLFWAMSNGSC

>QOJ98944.1 hemagglutinin, partial [Influenza A virus]
METVSLITILVVATVSNADKICIGYQSTNSTETVDTLTENNVPVTHAKELLHTEHNGMLCATDLGHPLIL
DTCTIEGLIYGNPSCDPLLGGREWSYIVERPSAVNGLCYPGNVENLEELRSLFSSSRSYQRIQIFPDTIW
NVSYSGTSKACSDSFYRSMRWLTQKNNAYPTQDAQYTNNQGKNILFMWGINHPPTDTXQTNLYTRTDTTT
SVATEEINRIFKPLIGPRPLVNGLMGRINYYWSVLKPGQTLRIKSDGNLIAPWYGHILSGESHGRILKTD
LKRGSCTVQCQTEKGGLNTTLPFQNVSKYAFGNCSKYIGVKSLKLAVGLRNVPSRSSRGLFGAIAGFIEG
GWPGLVAGWYGFQHSNDQGVGMAADRDSTQKAIDKITSKVNNIVDKMNKQYEIIDHEFSEVETRLNMINN
KVDDQIQDIWAYNAELLVLLENQKTLDEHDANVNNLYNKVKRALGSNAVEDGKGCFELYHKCDDHCMETI
RNGTYNRRKYQEESKLERQKVEGVKLESEETYKILTIYSTVASSLVIAMGFAAFLFWAMSNGSC

>QOJ98943.1 hemagglutinin, partial [Influenza A virus]
METVSLITILVVATVSNADKICIGYQSTNSTETVDTLTENNVPVTHAKELLHTEHNGMLCATXLGHPLIL
DTCTIEGLIYGNPSCDPLLGGREWSYIVERPSAVNGLCYPGNVENLEELRSLFSSSRSYQRIQIFPDTIW
NVSYSGTSKACSDSFYRSMRWLTQKNNAYPTQDAQYTNNQGKNILFMWGINHPPTDTAQTNLYTRTDTTT
SVATEEINRIFKPLIGPRPLVNGLMGRINYYWSVLKPGQTLRIKSDGNLIAPWYGHILSGESHGRILKTD

LKRGSCTVQCQTEKGGLNTTLPFQNVSKYAFGNCSKYIGVKSLKLAVGLRNVPSRSSRGLFGAIAGFIEG
GWPGLVAGWYGFQHSNDQGVGMAADRDSTQKAIDKITSKVNNIVDKMNKQYEIIDHEFSEVETRLNMINN
KVDDQIQDIWAYNAELLVLLENQKTLDEHDANVNNLYNKVKRALGSNAVEDGKGCFELYHKCDDHCMETI
RNGTYNRRKYQEESKLERQKIEGVKLESEETYKILTIYSTVASSLVIAM

>QOJ98942.1 hemagglutinin, partial [Influenza A virus]
METVSLITILVVATVSNADKICIGYQSTNSTETVDTLTENNVPVTHAKELLHTEHNGMLCATGLGHPLIL
DTCTIEGLIYGNPSCDPLLGGREWSYIVERPSAVNGLCYPGNVENLEELRSLFSSSRSYQRIQIFPDTIW
NVSYSGTSKACSDSFYRSMRWLTQKNNAYPTQDAQYTNNQGKNILFMWGINHPPTDTXQTNLYTRTDTTT
SVATEEINRIFKPLIGPRPLVNGLMGRINYYWSVLKPGQTLRIKSDGNLIAPWYGYILSGESHGRILKTD
LKRGSCTVQCQTEKGGLNTTLPFQNVSKYAFGNCSKYIGVKSLKLAVGLRNVPSRSSRGLFGAIAGFIEG
GWPGLVAGWYGFQHSNDQGVGMAADRDSTQKAIDKITSKVNNIVDKMNKQYEIIDHEFSEVETRLNMINN
KVDDQIQDIWAYNAELLVLLENQKTLDEHDANVNNLYNKVKRALGSNAVEDGKGCFELYHKCDDHCMETI
RNGTYNRRKYQEESKLERQKIEGVKLESEETYKILTIYSTVASSLVIAMGFAAFLFWAMSNGSC

>QOJ98941.1 hemagglutinin, partial [Influenza A virus]
METVSLITILVVATVSNADKICIGYQSTNSTETVDTLTENNVPVTHAKELLHTEHNGMLCATXLGHPLIL
DTCTIEGLIYGNPSCDPLLGGREWSYIVERPSAVNGLCYPGNVENLEELRSLFSSSRSYQRIQIFPDTIW
NVSYSGTSKACSDSFYRSMRWLTQKNNAYPTQDAQYTNNQGKNILFMWGINHPPTDTXQTNLYTRTDTTT
SVATEEINRIFKPLIGPRPLVNGLMGRINYYWSVLKPGQTLRIKSDGNLIAPWYGHILSGESHGRILKTD
LKRGSCTVQCQTEKGGLNTTLPFQNVSKYAFGNCSKYIGVKSLKLAVGLRNVPSRSSRGLFGAIAGFIEG
GWPGLVAGWYGFQHSNDQGVGMAADRDSTQKAIDKITSKVNNIVDKMNKQYEIIDHEFSEVETRLNMINN
KVDDQIQDIWAYNAELLVLLENQKTLDEHDANVNNLYNKVKRALGSNAVEDGKGCFELYHKCDDHCMETI
RNGTYNRRKYQEESKLERQKXEGVKLESEETYKILTIYSTVASSLVIAMGFAAFLFWAMSNGSC

>QOJ98940.1 hemagglutinin, partial [Influenza A virus]
METVSLITILVVATVSNADKICIGYQSTNSTETVDTLTENNVPVTHAKELLHTEHNGMLCATDLGHPLIL
DTCTIEGLIYGNPSCDPLLGGREWSYIVERPSAVNGLCYPGNVENLEELRSLFSSSRSYQRIQIFPDTIW
NVSYSGTSKACSDSFYRSMRWLTQKNNAYPTQDAQYTNNQGKNILFMWGINHPPTDTAQTNLYTRTDTTT
SVATEEINRIFKPLIGPRPLVNGLMGRINYYWSVLKPGQTLRIKSDGNLIAPWYGHILSGESHGRILKTD
LKRGSCTVQCQTEKGGLNTTLPFQNVSKYAFGNCSKYIGVKSLKLAVGLRNVPSRSSRGLFGAIAGFIEG
GWPGLVAGWYGFQHSNDQGVGMAADRDSTQKAIDKITSKVNNIVDKMNKQYEIIDHEFSEVETRLNMINN
KVDDQIQDIWAYNAELLVLLENQKTLDEHDANVNNLYNKVKRALGSNAVEDGKGCFELYHKCDDHCMETI
RNGTYNRRKYQEESKLERQKIEGVKLESEETYKILTIYSTVASSLVIAM

>QOJ98939.1 hemagglutinin, partial [Influenza A virus]

METVSLITILVVATVSNADKICIGYQSTNSTETVDTLTENNVPVTHAKELLHTEHNGMLCATXLGHPLIL
DTCTIEGLIYGNPSCDPLLGGREWSYIVERPSAVNGLCYPGNVENLEELRSLFSSSRSYQRIQIFPDTIW
NVSYSGTSKACSDSFYRSMRWLTQKNNAYPTQDAQYTNNQGKNILFMWGINHPPTDTAQTNLYTRTDTTT
SVATEEINRIFKPLIGPRPLVNGLMGRINYYWSVLKPGQTLRIKSDGNLIAPWYGHILSGESHGRILKTD
LKRGSCTVQCQTEKGGLNTTLPFQNVSKYAFGNCSKYIGVKSLKLAVGLRNVPSRSSRGLFGAIAGFIEG
GWPGLVAGWYGFQHSNDQGVGMAADRDSTQKAIDKITSKVNNIVDKMNKQYEIIDHEFSEVETRLNMINN
KVDDQIQDIWAYNAELLVLLENQKTLDEHDANVNNLYNKVKRALGSNAVEDGKGCFELYHKCDDHCMETI
RNGTYNRRKYQEESKLERQKIEGVKLESEETYKILTIYSTVASSLVIAM

>QOJ98938.1 hemagglutinin, partial [Influenza A virus]

METVSLITILVVATVSNADKICIGYQSTNSTETVDTLTENNVPVTHAKELLHTEHNGMLCATGLGHPLIL
DTCTIEGLIYGNPSCDPLLGGREWSYIVERPSAVNGLCYPGNVENLEELRSLFSSSRSYQRIQIFPDTIW
NVSYSGTSKACSDSFYRSMRWLTQKNNAYPTQDAQYTNNQGKNILFMWGINHPPTDTAQTNLYTRTDTTT
SVATEEINRIFKPLIGPRPLVNGLMGRINYYWSVLKPGQTLRIKSDGNLIAPWYGXILSGESHGRILKTD
LKRGSCTVQCQTEKGGLNTTLPFQNVSKYAFGNCSKYIGVKSLKLAVGLRNVPSRSSRGLFGAIAGFIEG
GWPGLVAGWYGFQHSNDQGVGMAADRDSTQKAIDKITSKVNNIVDKMNKQYEIIDHEFSEVETRLNMINN
KVDDQIQDIWAYNAELLVLLENQKTLDEHDANVNNLYNKVKRALGSNAVEDGKGCFELYHKCDDHCMETI
RNGTYNRRKYQEESKLERQKIEGVKLESEETYKILTIYSTVASSLVIAM

>QOJ98934.1 hemagglutinin, partial [Influenza A virus]

METVSLITILLVATVSNADKICIGYQSTNSTETVDTLTENNVPVTHAKELLHTEHNGMLCATSLGQPLIL
DTCTIEGLIYGNPSCDLSLDGKEWSYIVERPSAVNGLCYPGNVENLEELRSLFSSARSYQRIQIFPDTIW
NVSYDGTSTACSNSFYRSMRWLTRKDGNYPTQDAQYTNNQGKNILFMWGINHPPTDETQRNLYTRTDTTT
SVATEEINRIFKPLIGPRPLVNGLMGRIDYYWSVLKPGQTLRIKSDGNLIAPWYGHILSGESHGRILKTD
LKKGSCTVQCQTEKGGLNTTLPFQNVSKYAFGNCSKYIGIKSLKLAVGLRNVPSRSSRGLFGAIAGFIEG
GWSGLVAGWYGFQHSNDQGVGMAADRDSTQKAIDKITSKVNNIVDKMNKQYEIIDHEFSEVETRLNMINN
KIDDQIQDIWAYNAELLVLLENQKTLDEHDANVNNLYNKVKRALGSNAVEDGKGCFELYHKCNDQCMETI
RNGTYNRKKYQEESKLERQRIEGVKLESEGTYKILTIYSTVASSLVIAMG

>QOJ98933.1 hemagglutinin, partial [Influenza A virus]

ETVSLITILLVATVSNADKICIGYQSTNSTETVDTLTENNVPVTHAKELLHTEHNGMLCATSLGQPLILD
TCTIEGLIYGNPSCDLSLEGREWSYIVERPSAVNGLCYPGNVENLEELRSLFSSARSYQRIQIFPDTIWN

VSYDGTSTACSNSFYRSMRWLTRKDGNYPTQDAQYTNNQGKNILFMWGINHPPTDETQRNLYTRTDTTTS
VATEEINRIFKPLIGPRPLVNGLMGRIDYYWSVLKPGQTLRIKSDGNLIAPWYGHILSGESHGRILKTDL
KKGSCTVQCQTEKGGLNTTLPFQNVSKYAFGNCSKYIGIKSLKLAXGLRNVPSRSSRGLFGAIAGFIEGG
WSGLVAGWYGFQHSNDQGVGMAADRDSTQKAIDKITSKVNNIVDKMNKQYEIIDHEFSEVETRLNMINNK
IDDQIQDIWAYNAELLVLLENQKTLDEHDANVNNLYNKVKRALGSNAVEDGKGCFELYHKCNDQCMETIR
NGTYNRKKYQEESKLERQRIEGVKLESEGTYKILTIYSTVASSLVIAMGFAAFLFWAMSNGSC

>QOJ98932.1 hemagglutinin, partial [Influenza A virus]
ETVSLITILLVATVSNADKICIGYQSTNSTETVDTLTENNVPVTHAKELLHTEHNGMLCATSLGQPLILD
TCTIEGLIYGNPSCDLSLEGREWSYIVERPSAVNGLCYPGNVENLEELRSLFSSARSYQRIQIFPDTIWN
VSYDGTSTACSNSFYRSMRWLTRKDGNYPTQDAQYTNNQGKNILFMWGINHPPTDETQRNLYTRTDTTTS
VATEEINRIFKPLIGPRPLVNGLMGRIDYYWSVLKPGQTLRIKSDGNLIAPWYGHILSGESHGRILKTDL
KKGSCTVQCQTEKGGLNTTLPFQNVSKYAFGNCSKYIGIKSLKLAIGLRNVPSRSSRGLFGAIAGFIEGG
WSGLVAGWYGFQHSNDQGVGMAADRDSTQKAIDKITSKVNNIVDKMNKQYEIIDHEFSEVETRLNMINNK
IDDQIQDIWAYNAELLVLLENQKTLDEHDANVNNLYNKVKRALGSNAVEDGKGCFELYHKCNDQCMETIR
NGTYNRKKYQEESKLERQRIEGVKLESEGTYKILTIYSTVASSLVIAMGFAAFLFWAMSNGSC

>QOJ98931.1 hemagglutinin, partial [Influenza A virus]
ETVSLITILLVATVSNADKICIGYQSTNSTETVDTLTENNVPVTHAKELLHTEHNGMLCATSLGQPLILD
TCTIEGLIYGNPSCDLSLEGREWSYIVERPSAVNGLCYPGNVENLEELRSLFSSARSYQRIQIFPDTIWN
VSYDGTSTACSNSFYRSMRWLTRKDGNYPTQDAQYTNNQGKNILFMWGINHPPTDETQRNLYTRTDTTTS
VATEEINRIFKPLIGPRPLVNGLMGRIDYYWSVLKPGQTLRIKSDGNLIAPWYGHILSGESHGRILKTDL
KKGSCTVQCQTEKGGLNTTLPFQNVSKYAFGNCSKYIGIKSLKLAIGLRNVPSRSSRGLFGAIAGFIEGG
WSGLVAGWYGFQHSNDQGVGMAADRDSTQKAIDKITSKVNNIVDKMNKQYEIIDHEFSEVETRLNMINNK
IDDQIQDIWAYNAELLVLLENQKTLDEHDANVNNLYNKVKRALGSNAVEDGKGCFELYHKCNDQCMETIR
NGTYNRKKYQEESKLERQRIEGVKLESEGTYKILTIYSTVASSLVIAMGFAAFLFWAMSNGSC

>QOJ98930.1 hemagglutinin [Influenza A virus]
METVSLITILLVATVSNADKICIGYQSTNSTETVDTLTENNVPVTHAKELLHTEHNGMLCATSLGQPLIL
DTCTIEGLIYGNPSCDLSLEGREWSYIVERPSAVNGLCYPGNVENLEELRSLFSSARSYQRIQIFPDTIW
NVSYDGTSTACSNSFYRSMRWLTRKDGNYPTQDAQYTNNQGKNILFMWGINHPPTDETQRNLYTRTDTTT
SVATEEINRIFKPLIGPRPLVNGLMGRIDYYWSVLKPGQTLRIKSDGNLIAPWYGHILSGESHGRILKTD
LKKGSCTVQCQTEKGGLNTTLPFQNVSKYAFGNCSKYIGIKSLKLAVGLRNVPSRSSRGLFGAIAGFIEG
GWSGLVAGWYGFQHSNDQGVGMAADRDSTQKAIDKITSKVNNIVDKMNKQYEIIDHEFSEVETRLNMINN

KIDDQIQDIWAYNAELLVLLENQKTLDEHDANVNNLYNKVKRALGSNAMEDGKGCFELYHKCNDQCMETI
RNGTYNRKKYQEESKLERQRVEGVKLESEGTYKILTIYSTVASSLVIAMGFAAFLFWAMSNGSCRCNICI

>QOJ98924.1 hemagglutinin, partial [Influenza A virus]
METVSLITILLVATVSYADKICIGYQSTNSTETVDTLTENNVPVTHAKELLQTEHNGMLCATGLGQPLIL
DTCTIEGLIYGNPSCDLSLEGREWSYIVERPSAVNGLCYPGNVENLEELRSLFSSARSYQRVQIFPDTTW
NVSYDGTSTACSGSFYRSMRWLTRKDGNYPTQDAQYTNNQGKNILFMWGINNPPTDGTQRSLYTRTDTTT
SVATEEINRIFKPLIGPRPLVNGLMGRIDYYWSVLKPGQTLRIKSDGNLIAPWYGYILSGESHGRILKTD
LKRGSCTVQCQTEKGGLNTTLPFQNVSKYAFGNCSKYIGIKSLKLAVGLRNVPSRSSRGLFGAIAGFIEG
GWSGLVAGWYGFQHSNDQGVGMAADRDSTQKAIDKITSKVNNIVDKMNKQYEIIDHEFSEVETRLNMINN
KXDDQIQDIWAYNAELLVLLENQKTLDEHDANVNNLYNKVKRALGSNAVEDGKGCFELYHKCDDQCMETI
RNGTYNRKKYQEESKLERQKIEGVKLESEGTYKILTIYSTVASSLVIAM

>QOJ98923.1 hemagglutinin, partial [Influenza A virus]
METVSLITILLVATVSYADKICIGYQSTNSTETVDTLTENNVPVTHAKELLQTEHNGMLCATGLGQPLIL
DTCTIEGLIYGNPSCDLSLEGREWSYIVERPSAVNGLCYPGNVENLEELRSLFSSARSYQRVQIFPDTTW
NVSYDGTSTACSGSFYRSMRWLTRKDGNYPTQDAQYTNNQGKNILFMWGINNPPTDDTQRSLYTRTDTTT
SVATEEINRIFKPLIGPRPLVNGLMGRIDYYWSVLKPGQTLRIKSDGNLIAPWYGYILSGESHGRILKTD
LKRGSCTVQCQTEKGGLNTTLPFQNVSKYAFGNCSKYIGIKSLKLAVGLRNVPSRSSRGLFGAIAGFIEG
GWSGLVAGWYGFQHSNDQGVGMAADRDSTQKAIDKITSKVNNIVDKMNKQYEIIDHEFSEVETRLNMINN
KIDDQIQDIWAYNAELLVLLENQKTLDEHDANVNNLYNKVKRALGSNAVEDGKGCFELYHKCDDQCMETI
RNGTYNRKKYQEESKLERQKIEGVKLESEGTYKILTIYSTVASSLVIAM

>QOJ98921.1 hemagglutinin, partial [Influenza A virus]
METVSLITILLVATVSYADKICIGYQSTNSTETVDTLTENNVPVTHAKELLQTEHNGMLCATGLGQPLIL
DTCTIEGLIYGNPSCDLSLEGREWSYIVERPSAVNGLCYPGNVENLEELRSLFSSARSYQRVQIFPDTTW
NVSYDGTSTACSGSFYRSMRWLTRKDGNYPTQDAQYTNNQGKNILFMWGINNPPTDGTQRSLYTRTDTTT
SVATEEINRIFKPLIGPRPLVNGLMGRIDYYWSVLKPGQTLRIKSDGNLIAPWYGYILSGESHGRILKTD
LKRGSCTVQCQTEKGGLNTTLPFQNVSKYAFGNCSKYIGIKSLKLAVGLRNVPSRSSRGLFGAIAGFIEG
GWSGLVAGWYGFQHSNDQGVGMAADRDSTQKAIDKITSKVNNIVDKMNKQYEIIDHEFSEVETRLNMINN
KIDDQIQDIWAYNAELLVLLENQKTLDEHDANVNNLYNKVKRALGSNAVEDGKGCFELYHKCDDQCMETI
RNGTYNRKKYQEESKLERQKIEGVKLESEGTYKILTIYSTVASSLVIAM

>QOJ98920.1 hemagglutinin, partial [Influenza A virus]

METVSLITILLVATVSYADKICIGYQSTNSTETVDTLTENNVPVTHAKELLQTEHNGMLCATGLGQPLIL
DTCTIEGLIYGNPSCDLSLEGREWSYIVERPSAVNGLCYPGNVENLEELRSLFSSARSYQRVQIFPDTTW
NVSYDGTSTACSGSFYRSMRWLTRKDGNYPTQDAQYTNNQGKNILFMWGINNPPTDDTQRSLYTRTDTTT
SVATEEINRIFKPLIGPRPLVNGLMGRIDYYWSVLKPGQTLRIKSDGNLIAPWYGYILSGESHGRILKTD
LKRGSCTVQCQTEKGGLNTTLPFQNVSKYAFGNCSKYIGIKSLKLAVGLRNVPSRSSRGLFGAIAGFIEG
GWSGLVAGWYGFQHSNDQGVGMAADRDSTQKAIDKITSKVNNIVDKMNKQYEIIDHEFSEVETRLNMINN
KIDDQIQDIWAYNAELLVLLENQKTLDEHDANVNNLYNKVKRALGSNAVEDGKGCFELYHKCDDQCMETI
RNGTYNRKKYQEESKLERQKIEGVKLESEGTYKILTIYSTVASSLVIAM

>QOJ98919.1 hemagglutinin, partial [Influenza A virus]
METVSLITILLVATVSYADKICIGYQSTNSTETVDTLTENNVPVTHAKELLQTEHNGMLCATGLGQPLIL
DTCTIEGLIYGNPSCDLSLEGREWSYIVERPSAVNGLCYPGNVENLEELRSLFSSARSYQRVQIFPDTTW
NVSYDGTSTACSGSFYRSMRWLTRKDGNYPTQDAQYTNNQGKNILFMWGINNPPTDDTQRSLYTRTDTTT
SVATEEINRIFKPLIGPRPLVNGLMGRIDYYWSVLKPGQTLRIKSDGNLIAPWYGYILSGESHGRILKTD
LKRGSCTVQCQTEKGGLNTTLPFQNVSKYAFGNCSKYIGIKSLKLAVGLRNVPSRSSRGLFGAIAGFIEG
GWSGLVAGWYGFQHSNDQGVGMAADRDSTQKAIDKITSKVNNIVDKMNKQYEIIDHEFSEVETRLNMINN
KIDDQIQDIWAYNAELLVLLENQKTLDEHDANVNNLYNKVKRALGSNAVEDGKGCFELYHKCDDQCMETI
RNGTYNRKKYQEESKLERQKIEGVKLESEGTYKILTIYSTVASSLVIAM

>QOJ98918.1 hemagglutinin, partial [Influenza A virus]
METVSLITILLVATVSYADKICIGYQSTNSTETVDTLTENNVPVTHAKELLQTEHNGMLCATGLGQPLIL
DTCTIEGLIYGNPSCDLSLEGREWSYIVERPSAVNGLCYPGNVENLEELRSLFSSARSYQRVQIFPDTTW
NVSYDGTSTACSGSFYRSMRWLTRKDGNYPTQDAQYTNNQGKNILFMWGINNPPTDDTQRSLYTRTDTTT
SVATEEINRIFKPLIGPRPLVNGLMGRIDYYWSVLKPGQTLRIKSDGNLIAPWYGYILSGESHGRILKTD
LKRGSCTVQCQTEKGGLNTTLPFQNVSKYAFGNCSKYIGIKSLKLAVGLRNVPSRSSRGLFGAIAGFIEG
GWSGLVAGWYGFQHSNDQGVGMAADRDSTQKAIDKITSKVNNIVDKMNKQYEIIDHEFSEVETRLNMINN
KIDDQIQDIWAYNAELLVLLENQKTLDEHDANVNNLYNKVKRALGSNAVEDGKGCFELYHKCDDQCMETI
RNGTYNRKKYQEESKLERQKIEGVKLESEGTYKILTIYSTVASSLVIAM

>QOJ98917.1 hemagglutinin, partial [Influenza A virus]
METVSLITILLVATVSYADKICIGYQSTNSTETVDTLTENNVPVTHAKELLQTEHNGMLCATGLGQPLIL
DTCTIEGLIYGNPSCDLSLEGREWSYIVERPSAVNGLCYPGNVENLEELRSLFSSARSYQRVQIFPDTTW
NVSYDGTSTACSGSFYRSMRWLTRKDGNYPTQDAQYTNNQGKNILFMWGINNPPTDDTQRSLYTRTDTTT
SVATEEINRIFKPLIGPRPLVNGLMGRIDYYWSVLKPGQTLRIKSDGNLIAPWYGYILSGESHGRILKTD

LKRGSCTVQCQTEKGGLNTTLPFQNVSKYAFGNCSKYIGIKSLKLAVGLRNVPSRSSRGLFGAIAGFIEG
GWSGLVAGWYGFQHSNDQGVGMAADRDSTQKAIDKITSKVNNIVDKMNKQYEIIDHEFSEVETRLNMINN
KIDDQIQDIWAYNAELLVLLENQKTLDEHDANVNNLYNKVKRALGSNAVEDGKGCFELYHKCDDQCMETI
RNGTYNRKKYQEESKLERQKIEGVKLESEGTYKILTIYSTVASSLVIAM

>QOJ98915.1 hemagglutinin, partial [Influenza A virus]
METVSLITILLVATVSYADKICIGYQSTNSTETVDTLTENNVPVTHAKELLQTEHNGMLCATGLGQPLIL
DTCTIEGLIYGNPSCDLSLEGREWSYIVERPSAVNGLCYPGNVENLEELRSLFSSARSYQRVQIFPDTTW
NVSYDGTSTACSGSFYRSMRWLTRKDGNYPTQDAQYTNNQGKNILFMWGINNPPTDDTQRSLYTRTDTTT
SVATEEINRIFKPLIGPRPLVNGLMGRIDYYWSVLKPGQTLRIKSDGNLIAPWYGYILSGESHGRILKTD
LKRGSCTVQCQTEKGGLNTTLPFQNVSKYAFGNCSKYIGIKSLKLAVGLRNVPSRSSRGLFGAIAGFIEG
GWSGLVAGWYGFQHSNDQGVGMAADRDSTQKAIDKITSKVNNIVDKMNKQYEIIDHEFSEVETRLNMINN
KIDDQIQDIWAYNAELLVLLENQKTLDEHDANVNNLYNKVKRALGSNAVEDGKGCFELYHKCDDQCMETI
RNGTYNRKKYQEESKLERQKIEGVKLESEGTYKILTIYSTVASSLVIAM

>QOJ98914.1 hemagglutinin, partial [Influenza A virus]
METVSLITILLVATVSYADKICIGYQSTNSTETVDTLTENNVPVTHAKELLQTEHNGMLCATGLGQPLIL
DTCTIEGLIYGNPSCDLSLEGREWSYIVERPSAVNGLCYPGNVENLEELRSLFSSARSYQRVQIFPDTTW
NVSYDGTSTACSGSFYRSMRWLTRKDGNYPTQDAQYTNNQGKNILFMWGINNPPTDDTQRSLYTRTDTTT
SVATEEINRIFKPLIGPRPLVNGLMGRIDYYWSVLKXGQTLRIKSDGNLIAPWYGYILSGESHGRILKTD
LKRGSCTVQCQTEKGGLNTTLPFQNVSKYAFGNCSKYIGIKSLKLAVGLRNVPSRSSRGLFGAIAGFIEG
GWSGLVAGWYGFQHSNDQGVGMAADRDSTQKAIDKITSKVNNIVDKMNKQYEIIDHEFSEVETRLNMINN
KIDDQIQDIWAYNAELLVLLENQKTLDEHDANVNNLYNKVKRALGSNAVEDGKGCFELYHKCDDQCMETI
RNGTYNRKKYQEESKLERQKIEGVKLESEGTYKILTIYSTVASSLVIAM

>QOJ98913.1 hemagglutinin, partial [Influenza A virus]
METVSLITILLVATVSYADKICIGYQSTNSTETVDTLTENNVPVTHAKELLQTEHNGMLCATGLGQPLIL
DTCTIEGLIYGNPSCDLSLEGREWSYIVERPSAVNGLCYPGNVENLEELRSLFSSARSYQRVQIFPDTTW
NVSYDGTSTACSGSFYRSMRWLTRKDGNYPTQDAQYTNNQGKNILFMWGINNPPTDDTQRSLYTRTDTTT
SVATEEINRIFKPLIGPRPLVNGLMGRIDYYWSVLKPGQTLRIKSDGNLIAPWYGYILSGESHGRILKTD
LKRGSCTVQCQTEKGGLNTTLPFQNVSKYAFGNCSKYIGIKSLKLAVGLRNVPSRSSRGLFGAIAGFIEG
GWSGLVAGWYGFQHSNDQGVGMAADRDSTQKAIDKITSKVNNIVDKMNKQYEIIDHEFSEVETRLNMINN
KIDDQIQDIWAYNAELLVLLENQKTLDEHDANVNNLYNKVKRALGSNAVEDGKGCFELYHKCDDQCMETI
RNGTYNRKKYQEESKLERQKIEGVKLESEGTYKILTIYSTVASSLVIAM

>QOJ98912.1 hemagglutinin, partial [Influenza A virus]

METVSLITILLVATVSYADKICIGYQSTNSTETVDTLTENNVPVTHAKELLQTEHNGMLCATGLGQPLIL
DTCTIEGLIYGNPSCDLSLEGREWSYIVERPSAVNGLCYPGNVENLEELRSLFSSARSYQRVQIFPDTTW
NVSYDGTSTACSGSFYRSMRWLTRKDGNYPTQDAQYTNNQGKNILFMWGINNPPTDDTQRSLYTRTDTTT
SVATEEINRIFKPLIGPRPLVNGLMGRIDYYWSVLKPGQTLRIKSDGNLIAPWYGYILSGESHGRILKTD
LKRGSCTVQCQTEKGGLNTTLPFQNVSKYAFGNCSKYIGIKSLKLAVGLRNVPSRSSRGLFGAIAGFIEG
GWSGLVAGWYGFQHSNDQGVGMAADRDSTQKAIDKITSKVNNIVDKMNKQYEIIDHEFSEVETRLNMINN
KIDDQIQDIWAYNAELLVLLENQKTLDEHDANVNNLYNKVKRALGSNAVEDGKGCFELYHKCDDQCMETI
RNGTYNRKKYQEESKLERQKIEGVKLESEGTYKILTIYSTVASSLVIAM

>QOJ98911.1 hemagglutinin, partial [Influenza A virus]

METVSLITILVVATVSNADKICIGYQSTNSTETVDTLTENNVPVTHAKELLHTEHNGMLCATSLGHPLIL
DTCTIEGLIYGNPSCDPFLGGREWSYIVERPSAVNGLCYPGNVENLEELRSLFSSSRSYQRIQIFPDTIW
NVSYSGTSKACSDSFYRSMRWLTQKNNAYPTQDAQYTNNQGKNILFMWGINHPPTDTAQTNLYTRTDTTT
SVATEEMNRIFKPLIGPRPLVNGLMGRINYYWSVLKPGQTLRIKSDGNLIAPWYGHILSGESHGRILKTD
LKMGSCTVQCQTEKGGLNTTLPFQNVSKYAFGNCSKYIGIKSLKLAVGLRNVPSRSSRGLFGAIAGFIEG
GWSGLVAGWYGFQHSNDQGVGMAADRDSTQKAIDKITSKVNNIVDKMNKQYEIIDHEFSEVETRLNMIND
KVDDQIQDIWAYNAELLVLLENQKTLDAHDANVNNLYNKVKRALGSNAVEDGRGCFELYHKCDDHCMETI
RNGTYNRRKYQEESKLERQKIEGVKLESEETYKILTIYSTVASSLVIAMGFAAFLFWAMSNGSC

>QOJ98909.1 hemagglutinin, partial [Influenza A virus]

METVSLITILLVATVSYADKICIGYQSTNSTETVDTLTENNVPVTHAKELLQTEHNGMLCATGLGQPLIL
DTCTIEGLIYGNPSCDLSLEGREWSYIVERPSAVNGLCYPGNVENLEELRSLFSSARSYQRVQIFPDTTW
NVSYDGTSTACSGSFYRSMRWLTRKDGNYPTQDAQYTNNQGKNILFMWGINNPPTDDTQRSLYTRTDTTT
SVATEEINRIFKPLIGPRPLVNGLMGRIDYYWSVLKPGQTLRIKSDGNLIAPWYGYILSGESHGRILKTD
LKRGSCTVQCQTEKGGLNTTLPFQNVSKYAFGNCSKYIGIKSLKLAVGLRNVPSRSSRGLFGAIAGFIEG
GWSGLVAGWYGFQHSNDQGVGMAADRDSTQKAIDKITSKVNNIVDKMNKQYEIIDHEFSEVETRLNMINN
KIDDQIQDIWAYNAELLVLLENQKTLDEHDANVNNLYNKVKRALGSNAVEDGKGCFELYHKCDDQCMETI
RNGTYNRKKYQEESKLERQKIEGVKLESEGTYKILTIYSTVASSLVIAM

>QOJ98908.1 hemagglutinin, partial [Influenza A virus]

METVSLITILVVATVSNADKICIGYQSTNSTETVDTLTENNVPVTHAKELLHTEHNGMLCATSLGHPLIL
DTCTIEGLIYGNPSCDPLLGGREWSYIVERPSAVNGLCYPGNVENLEELRSLFSSSRSYQRIQIFPDTIW

NVSYSGTSKACSDSFYRSMRWLTQKNNAYPTQDAQYTNNQGKNILFMWGINHPPTDTAQTNLYTRTDTTT
SVATEEMNRIFKPLIGPRPLVNGLMGRINYYWSVLKPGQTLRIKSDGNLIAPWYGHILSGESHGRILKTD
LKMGSCTVQCQTEKGGLNTTLPFQNVSKYAFGNCSKYIGVKSLKLAVGLRNVPSRSSRGLFGAIAGFIEG
GWSGLVAGWYGFQHSNDQGVGMAADRDSTQKAIDKITSKVNNIVDKMNKQYEIIDHEFSEVETRLNMIND
KVDDQIQDIWAYNAELLVLLENQKTLDEHDANVNNLYNKVKRALGSNAVEDGRGCFELYHKCDDHCMETI
RNGTYNRRKYQEESKLERQKIEGVKLESEETYKILTIYSTVASSLVIAMGFAAFLFWAMSNGSC

>QOJ98907.1 hemagglutinin, partial [Influenza A virus]
METVSLITILLVATVSYADKICIGYQSTNSTETVDTLTENNVPVTHAKELLQTEHNGMLCATGLGQPLIL
DTCTIEGLIYGNPSCDLSLEGREWSYIVERPSAVNGLCYPGNVENLEELRSLFSSARSYQRVQIFPDTTW
NVSYDGTSTACSGSFYRSMRWLTRKDGNYPTQDAQYTNNQGKNILFMWGINNPPTDDTQRSLYTRTDTTT
SVATEEINRIFKPLIGPRPLVNGLMGRIDYYWSVLKPGQTLRIKSDGNLIAPWYGYILSGESHGRILKTD
LKRGSCTVQCQTEKGGLNTTLPFQNVSKYAFGNCSKYIGIKSLKLAVGLRNVPSRSSRGLFGAIAGFIEG
GWSGLVAGWYGFQHSNDQGVGMAADRDSTQKAIDKITSKVNNIVDKMNKQYEIIDHEFSEVETRLNMINN
KIDDQIQDIWAYNAELLVLLENQKTLDEHDANVNNLYNKVKRALGSNAVEDGKGCFELYHKCDDQCMETI
RNGTYNRKKYQEESKLERQKIEGVKLESEGTYKILTIYSTVASSLVIAM

>QOJ98906.1 hemagglutinin, partial [Influenza A virus]
METVSLITILLVATVSYADKICIGYQSTNSTETVDTLTENNVPVTHAKELLQTEHNGMLCATGLGQPLIL
DTCTIEGLIYGNPSCDLSLEGREWSYIVERPSAVNGLCYPGNVENLEELRSLFSSARSYQRVQIFPDTTW
NVSYDGTSTACSGSFYRSMRWLTRKDGNYPTQDAQYTNNQGKNILFMWGINNPPTDDTQRSLYTRTDTTT
SVATEEINRIFKPLIGPRPLVNGLMGRIDYYWSVLKPGQTLRIKSDGNLIAPWYGYILSGESHGRILKTD
LKRGSCTVQCQTEKGGLNTTLPFQNVSKYAFGNCSKYIGIKSLKLAVGLRNVPSRSSRGLFGAIAGFIEG
GWSGLVAGWYGFQHSNDQGVGMAADRDSTQKAIDKITSKVNNIVDKMNKQYEIIDHEFSEVETRLNMINN
KIDDQIQDIWAYNAELLVLLENQKTLDEHDANVNNLYNKVKRALGSNAVEDGKGCFELYHKCDDQCMETI
RNGTYNRKKYQEESKLERQKIEGVKLESEGTYKILTIYSTVASSLVIAM

>QOJ98905.1 hemagglutinin, partial [Influenza A virus]
METVSLITILLVATVSYADKICIGYQSTNSTETVDTLTENNVPVTHAKELLQTEHNGMLCATGLGQPLIL
DTCTIEGLIYGNPSCDLSLEGREWSYIVERPSAVNGLCYPGNVENLEELRSLFSSARSYQRVQIFPDTTW
NVSYDGTSTACSGSFYRSMRWLTRKDGNYPTQDAQYTNNQGKNILFMWGINNPPTDDTQRSLYTRTDTTT
SVATEEINRIFKPLIGPRPLVNGLMGRIDYYWSVLKPGQTLRIKSDGNLIAPWYGYILSGESHGRILKTD
LKRGSCTVQCQTEKGGLNTTLPFQNVSKYAFGNCSKYIGIKSLKLAVGLRNVPSRSSRGLFGAIAGFIEG
GWSGLVAGWYGFQHSNDQGVGMAADRDSTQKAIDKITSKVNNIVDKMNKQYEIIDHEFSEVETRLNMINN

KIDDQIQDIWAYNAELLVLLENQKTLDEHDANVNNLYNKVKRALGSNAVEDGKGCFELYHKCDDQCMETI
RNGTYNRKKYQEESKLERQKIEGVKLESEGTYKILTIYSTVASSLVIAM

>QOJ98904.1 hemagglutinin, partial [Influenza A virus]
METVSLITILVVATVSNADKICIGYQSTNSTETVDTLTENNVPVTHAKELLHTEHNGMLCATSLGHPLIL
DTCTIEGLIYGNPSCDPLLGGREWSYIVERPSAVNGLCYPGNVENLEELRSLFSSSRSYQRIQIFPDTIW
NVSYSGTSKACSDSFYRSMRWLTQKNNAYPTQDAQYTNNQGKNILFMWGINHPPTDTAQTNLYTRTDTTT
SVATEEMNRIFKPLIGPRPLVNGLMGRINYYWSVLKPGQTLRIKSDGNLIAPWYGHILSGESHGRILKTD
LKMGSCTVQCQTEKGGLNTTLPFQNVSKYAFGNCSKYIGVKSLKLAVGLRNVPSRSSRGLFGAIAGFIEG
GWSGLVAGWYGFQHSNDQGVGMAADRDSTQKAIDKITSKVNNIVDKMNKQYEIIDHEFSEVETRLNMIND
KVDDQIQDIWAYNAELLVLLENQKTLDEHDANVNNLYNKVKRALGSNAVEDGRGCFELYHKCDDHCMETI
RNGTYNRRKYQEESKLERQKIEGVKLESEETYKILTIYSTVASSLVIAMGFAAFLFWAMSNGSC

>QOJ98903.1 hemagglutinin, partial [Influenza A virus]
METVSLITILLVATVSYADKICIGYQSTNSTETVDTLTENNVPVTHAKELLQTEHNGMLCATGLGQPLIL
DTCTIEGLIYGNPSCDLSLEGREWSYIVERPSAVNGLCYPGNVENLEELRSLFSSARSYQRVQIFPDTTW
NVSYDGTSTACSGSFYRSMRWLTRKDGNYPTQDAQYTNNQGKNILFMWGINNPPTDDTQRSLYTRTDTTT
SVATEEINRIFKPLIGPRPLVNGLMGRIDYYWSVLKPGQTLRIKSDGNLIAPWYGYILSGESHGRILKTD
LKRGSCTVQCQTEKGGLNTTLPFQNVSKYAFGNCSKYIGIKSLKLAVGLRNVPSRSSRGLFGAIAGFIEG
GWSGLVAGWYGFQHSNDQGVGMAADRDSTQKAIDKITSKVNNIVDKMNKQYEIIDHEFSEVETRLNMINN
KIDDQIQDIWAYNAELLVLLENQKTLDEHDANVNNLYNKVKRALGSNAVEDGKGCFELYHKCDDQCMETI
RNGTYNRKKYQEESKLERQKIEGVKLESEGTYKILTIYSTVASSLVIAM

>QOJ98901.1 hemagglutinin, partial [Influenza A virus]
METVSLITILVVATVSNADKICIGYQSTNSTETVDTLTENNVPVTHAKELLHTEHNGMLCATSLGHPLIL
DTCTIEGLIYGNPSCDPLLGGREWSYIVERPSAVNGLCYPGNVENLEELRSLFSSSRSYQRIQIFPDTIW
NVSYSGTSKACSDSFYRSMRWLTQKNNAYPTQDAQYTNNQGKNILFMWGINHPPTDTAQTNLYTRTDTTT
SVATEEMNRIFKPLIGPRPLVNGLMGRINYYWSVLKPGQTLRIKSDGNLIAPWYGHILSGESHGRILKTD
LKMGSCTVQCQTEKGGLNTTLPFQNVSKYAFGNCSKYIGVKSLKLAVGLRNVPSRSSRGLFGAIAGFIEG
GWSGLVAGWYGFQHSNDQGVGMAADRDSTQKAIDKITSKVNNIVEKMNKQYEIIDHEFSEVETRLNMIND
KVDDQIQDIWAYNAELLVLLENQKTLDEHDANVNNLYNKVKRALGSNAVEDGRGCFELYHKCDDHCMETI
RNGTYNRRKYQEESKLERQKIEGVKLESEETYKILTIYSTVASSLVIAM

>QOJ98900.1 hemagglutinin, partial [Influenza A virus]

METVSLITILVVATVSNADKICIGYQSTNSTETVDTLTENNVPVTHAKELLHTEHNGMLCATSLGHPLIL
DTCTIEGLIYGNPSCDPLLGGREWSYIVERPSAVNGLCYPGNVENLEELRSLFSSSRSYQRIQIFPDTIW
NVSYSGTSKACSDSFYRSMRWLTQKNNAYPTQDAQYTNNQGKNILFMWGINHPPTDTAQTNLYTRTDTTT
SVATEEMNRIFKPLIGPRPLVNGLMGRINYYWSVLKPGQTLRIKSDGNLIAPWYGHILSGESHGRILKTD
LKMGSCTVQCQTEKGGLNTTLPFQNVSKYAFGNCSKYIGVKSLKLAVGLRNVPSRSSRGLFGAIAGFIEG
GWSGLVAGWYGFQHSNDQGVGMAADRDSTQKAIDKITSKVNNIVEKMNKQYEIIDHEFSEVETRLNMIND
KVDDQIQDIWAYNAELLVLLENQKTLDEHDANVNNLYNKVKRALGSNAVEDGRGCFELYHKCDDHCMETI
RNGTYNRRKYQEESKLERQKIEGVKLESEETYKILTIYSTVASSLVIAM

>QOJ98899.1 hemagglutinin, partial [Influenza A virus]
METVSLITILVVATVSNADKICIGYQSTNSTETVDTLTENNVPVTHAKELLHTEHNGMLCATSLGHPLIL
DTCTIEGLIYGNPSCDPLLGGREWSYIVERPSAVNGLCYPGNVENLEELRSLFSSSRSYQRIQIFPDTIW
NVSYSGTSKACSDSFYRSMRWLTQKNNAYPTQDAQYTNNQGKNILFMWGINHPPTDTAQTNLYTRTDTTT
SVATEEMNRIFKPLIGPRPLVNGLMGRINYYWSVLKPGQTLRIKSDGNLIAPWYGHILSGESHGRILKTD
LKMGSCTVQCQTEKGGLNTTLPFQNVSKYAFGNCSKYIGVKSLKLAVGLRNVPSRSSRGLFGAIAGFIEG
GWSGLVAGWYGFQHSNDQGVGMAADRDSTQKAIDKITSKVNNIVEKMNKQYEIIDHEFSEVETRLNMIND
KVDDQIQDIWAYNAELLVLLENQKTLDEHDANVNNLYNKVKRALGSNAVEDGRGCFELYHKCDDHCMETI
RNGTYNRRKYQEESKLERQKIEGVKLESEETYKILTIYSTVASSLVIAM

>QOJ98897.1 hemagglutinin [Influenza A virus]
METVSLITILLAATVSNADKICIGYQSTNSTETVDTLTENNVPVTHAKELLHTEHNGMLCATNLGQPLIL
DTCTIEGLIYGNPSCDPLLEEREWSYIVERPSAVNGLCYPGNVENLEELRSLFSSARSYQRIQIFPDTIW
NVSYDGTSNTCSGSFYRNMRWLTRKNGNYPIQDAQYTNNQGKNILFMWGINHPPTDDTQRNLYTRTDTTT
SVATEEINRIFRPLIGPRPLVNGLMGRINYYWSVLKPGQTLRIKSDGNLIAPWYGYILSGESHGRILRTD
LKRGSCTVQCQTEKGGLNTTLPFQNVSKYAFGNCSKYIGIKSLKLAVGLRNVPSRSSRGLFGAIAGFIEG
GWSGLVAGWYGFQHSNDQGVGMAADRESTQKAIDKITSKVNNIVDKMNKQYEIIDHEFSEVETRLNMINN
KIDDQIQDIWAYNAELLVLLENQKTLDEHDANVNNLYNKVKRALGSNAVEDGKGCFELYHKCDDQCMETI
RNGTYNRRKYQEESKLERQKIEGVKLESEGTYKILTIYSTVASSLVIAMGFAAFLFWAMSNGSCRCNICI

>QOJ98896.1 hemagglutinin [Influenza A virus]
METVSLITILLAATVSNADKICIGYQSTNSTETVDTLTENNVPVTHAKELLHTEHNGMLCATSLGQPLIL
DTCTIEGLIYGNPSCDPLLEEREWSYIVERPSAVNGLCYPGNVENLEELRSLFSSARSYQRIQIFPDTIW
NVSYDGTSNTCSGSFYRNMRWLTRKNGNYPIQDAQYTNNQGKNILFMWGINHPPTDDTQRNLYTRTDTTT
SVATEEINRIFKPLIGPRPLVNGLMGRINYYWSVLKPGQTLRIKSDGNLIAPWYGYILSGESHGRILRTD

LKRGSCTVQCQTEKGGLNTTLPFQNVSKYAFGNCSKYIGIKSLKLAVGLRNVPSRSSRGLFGAIAGFIEG
GWSGLVAGWYGFQHSNDQGVGMAADRESTQKAIDKITSKVNNIVDKMNKQYEIIDHEFSEVETRLNMINN
KIDDQIQDIWAYNAELLVLLENQKTLDEHDANVNNLYNKVKRALGSNAVEDGKGCFELYHKCDDQCMETI
RNGTYNRRKYQEESKLERQKIEGVKLESEGTYKILTIYSTVASSLLIAMGFAAFLFWAMSNGSCRCNICI

>QOJ98895.1 hemagglutinin [Influenza A virus]
METVSLITILLAATVSNADKICIGYQSTNSTETVDTLTENNVPVTHAKELLHTEHNGMLCATNLGQPLIL
DTCTIEGLIYGNPSCDPLLEEREWSYIVERPSAVNGLCYPGNVENLEELRSLFSSARSYQRIQIFPDTIW
NVSYDGTSNTCSGSFYRNMRWLTRKNGNYPIQDAQYTNNQGKNILFMWGINHPPTDDTQRNLYTRTDTTT
SVATEEINRIFRPLIGPRPLVNGLMGRINYYWSVLKPGQTLRIKSDGNLIAPWYGYILSGESHGRILRTD
LKRGSCTVQCQTEKGGLNTTLPFQNVSKYAFGNCSKYIGIKSLKLAVGLRNVPSRSSRGLFGAIAGFIEG
GWSGLVAGWYGFQHSNDQGVGMAADRESTQKAIDKITSKVNNIVDKMNKQYEIIDHEFSEVETRLNMINN
KIDDQIQDIWAYNAELLVLLENQKTLDEHDANVNNLYNKVKRALGSNAVEDGKGCFELYHKCDDQCMETI
RNGTYNRRKYQEESKLERQKIEGVKLESEGTYKILTIYSTVASSLVIAMGFAAFLFWAMSNGSCRCNICI

>QOJ98894.1 hemagglutinin [Influenza A virus]
METVSLITILLAATVSNADKICIGYQSTNSTETVDTLTENNVPVTHAKELLHTEHNGMLCATSLGQPLIL
DTCTIEGLIYGNPSCDPLLEEREWSYIVERPSAVNGLCYPGNVENLEELRSLFSSARSYQRIQIFPDTIW
NVSYDGTSNTCSGSFYRNMRWLTRKNGNYPIQDAQYTNNQGKNILFMWGINHPPTDDTQRNLYTRTDTTT
SVATEEINRIFKPLIGPRPLVNGLMGRINYYWSVLKPGQTLRIKSDGNLIAPWYGYILSGESHGRILRTD
LKRGSCTVQCQTEKGGLNTTLPFQNVSKYAFGNCSKYIGIKSLKLAVGLRNVPSRSSRGLFGAIAGFIEG
GWSGLVAGWYGFQHSNDQGVGMAADRESTQKAIDKITSKVNNIVDKMNKQYEIIDHEFSEVETRLNMINN
KIDDQIQDIWAYNAELLVLLENQKTLDEHDANVNNLYNKVKRALGSNAVEDGKGCFELYHKCDDQCMETI
RNGTYNRRKYQEESKLERQKIEGVKLESEGTYKILTIYSTVASSLLIAMGFAAFLFWAMSNGSCRCNICI

>QOJ98893.1 hemagglutinin [Influenza A virus]
METVSLITILLAATVSNADKICIGYQSTNSTETVDTLTENNVPVTHAKELLHTEHNGMLCATSLGQPLIL
DTCTIEGLIYGNPSCDPLLEEREWSYIVERPSAVNGLCYPGNVENLEELRSLFSSARSYQRIQIFPDTIW
NVSYDGTSNTCSGSFYRNMRWLTRKNGNYPIQDAQYTNNQGKNILFMWGINHPPTDDTQRNLYTRTDTTT
SVATEEINRIFKPLIGPRPLVNGLMGRINYYWSVLKPGQTLRIKSDGNLIAPWYGYILSGESHGRILRTD
LKRGSCTVQCQTEKGGLNTTLPFQNVSKYAFGNCSKYIGIKSLKLAVGLRNVPSRSSRGLFGAIAGFIEG
GWSGLVAGWYGFQHSNDQGVGMAADRESTQKAIDKITSKVNNIVDKMNKQYEIIDHEFSEVETRLNMINN
KIDDQIQDIWAYNAELLVLLENQKTLDEHDANVNNLYNKVKRALGSNAVEDGKGCFELYHKCDDQCMETI
RNGTYNRRKYQEESKLERQKIEGVKLESEGTYKILTIYSTVASSLLIAMGFAAFLFWAMSNGSCRCNICI

>QOJ98892.1 hemagglutinin [Influenza A virus]

METVSLITILLAATVSNADKICIGYQSTNSTETVDTLTENNVPVTHAKELLHTEHNGMLCATSLGQPLIL

DTCTIEGLIYGNPSCDPLLEEREWSYIVERPSAVNGLCYPGNVENLEELRSLFSSARSYQRIQIFPDTIW

NVSYDGTSNTCSGSFYKNMRWLTRKNGNYPIQDAQYTNNQGKNILFMWGINHPPTDDTQRNLYTRTDTTT

SVATEEINRIFKPLIGPRPLVNGLMGRINYYWSVLKPGQTLRIKSDGNLIAPWYGYILSGESHGRILRTD

LKRGSCTVQCQTEKGGLNTTLPFQNVSKYAFGNCSKYIGIKSLKLAVGLRNVPSRSSRGLFGAIAGFIEG

GWSGLVAGWYGFQHSNDQGVGMAADRESTQKAIDKITSKVNNIVDKMNKQYEIIDHEFSEVETRLNMINN

KIDDQIQDIWAYNAELLVLLENQKTLDEHDANVNNLYNKVKRALGSNAVEDGKGCFELYHKCDDQCMETI

RNGTYNRRKYQEESKLERQKIEGVKLESEGTYKILTIYSTVASSLLIAMGFAAFLFWAMSNGSCRCNICI

>QOJ98891.1 hemagglutinin [Influenza A virus]

METVSLITILLAATVSNADKICIGYQSTNSTETVDTLTENNVPVTHAKELLHTEHNGMLCATSLGQPLIL

DTCTIEGLIYGNPSCDPLLEEREWSYIVERPSAVNGLCYPGNVENLEELRSLFSSARSYQRIQIFPDTIW

NVSYDGTSNTCSGSFYRNMRWLTRKNGNYPVQDAQYTNNQGKNILFMWGINHPPTDDTQRNLYTRTDTTT

SVATEEINRIFKPLIGPRPLVNGLMGRINYYWSVLKPGQTLRIKSDGNLIAPWYGYILSGESHGRILRTD

LNRGSCTVQCQTEKGGLNTTLPFQNVSKYAFGNCSKYIGIKSLKLAVGLRNVPSRSSRGLFGAIAGFIEG

GWSGLVAGWYGFQHSNDQGVGMAADRESTQKAIDKITSKVNNIVDKMNKQYEIIDHEFSEVETRLNMINN

KIDDQIQDIWAYNAELLVLLENQKTLDEHDANVNNLYNKVKRALGSNAVEDGKGCFELYHKCDDQCMETI

RNGTYNRRKYQEESKLERQKIEGIKLESEGTYKILTIYSTVASSLVIAMGFAAFLFWAMSNGSCRCNICI

>QOJ98890.1 hemagglutinin [Influenza A virus]

METVSLITILLAATVSNADKICIGYQSTNSTETVDTLTENNVPVTHAKELLHTEHNGMLCATSLGQPLIL

DTCTIEGLIYGNPSCDPLLEEREWSYIVERPSAVNGLCYPGNVENLEELRSLFSSARSYQRIQIFPDTIW

NVSYDGTSNTCSGSFYRNMRWLTRKNGNYPVQDAQYTNNQGKNILFMWGINHPPTDDTQRNLYTRTDTTT

SVATEEINRIFKPLIGPRPLVNGLMGRINYYWSVLKPGQTLRIKSDGNLIAPWYGYILSGESHGRILRTD

LNRGSCTVQCQTEKGGLNTTLPFQNVSKYAFGNCSKYIGIKSLKLAVGLRNVPSRSSRGLFGAIAGFIEG

GWSGLVAGWYGFQHSNDQGVGMAADRESTQKAIDKITSKVNNIVDKMNKQYEIIDHEFSEVETRLNMINN

KIDDQIQDIWAYNAELLVLLENQKTLDEHDANVNNLYNKVKRALGSNAVEDGKGCFELYHKCDDQCMETI

RNGTYNRRKYQEESKLERQKIEGVKLESEGTYKILTIYSTVASSLVIAMGFAAFLFWAMSNGSCRCNICI

>QOJ98885.1 hemagglutinin, partial [Influenza A virus]

METVSLITILLVATVSYADKICVGYQSTNSTETVDTLTENNVPVTHAKELLHTEHNGMLCATSLGQPLIL

DTCTIEGLIYGNPSCDLSLEGKEWSYIVERPSAVNGLCYPGNVENLEELRSLFSSARSYQRVQIFPDTIW

NVSYDGTSTSCSGSFYRSMRWLTRKNGDYPTQDAQYTNNQGKNILFTWGINHPPTDDTQRNLYTRTDTTT
SVATEEINRIFKPLIGPRPLVNGLMGRIAYYWSVLKPGQTLRIKSDGNLIAPWYGYILSGESHGRILKTD
LKRGSCTVQCQTEKGGLNTTLPFQNVSKYAFGNCSKYIGIKSLKLAVGLRNVPSRSSRGLFGAIAGFIEG
GWSGLVAGWYGFQHSNDQGVGMAADRDSTQKAIDKITSKVNNIVDKMNKQYEIIDHEFSEVETRLNMINN
KIDDQIQDIWAYNAELLVLLENQKTLDEHDANVNNLYNKVKRALGSNAVEDGKGCFELYHKCNDQCMETI
RNGTYNRRKYQEESKLERQKIEGVKLESEGTYKILTIYSTVASSLVIAM

>QOJ98884.1 hemagglutinin [Influenza A virus]
METVSLITILLAATVSNADKICIGYQSTNSTETVDTLTENNVPVTHAKELLHTEHNGMLCATSLGQPLIL
DTCTIEGLIYGNPSCDPLLEEREWSYIVERPSAVNGLCYPGNVENLEELRSLFSSARSYQRIQIFPDTIW
NVSYDGTSNTCSGSFYRNMRWLTRKNGNYPIQDAQYTNNQGKNILFMWGINHPPTDDTQRNLYTRTDTTT
SVATEEINRIFRPLIGPRPLVNGLMGRINYYWSVLKPGQTLRIKSDGNLIAPWYGYILSGESHGRILRTD
LKRGSCTVQCQTEKGGLNTTLPFQNVSKYAFGNCSKYIGIKSLKLAVGLRNVPSRSSRGLFGAIAGFIEG
GWSGLVAGWYGFQHSNDQGVGMAADRESTQKAIDKITSKVNNIVDKMNKQYEIIDHEFSEVETRLNMINN
KIDDQIQDIWAYNAELLVLLENQKTLDEHDANVNNLYNKVKRALGSNAVEDGKGCFELYHKCDDQCMETI
RNGTYNRRKYQEESKLERQKIEGVKLESEGTYKILTIYSTVASSLVIAMGFAAFLFWAMSNGSCRCNICI

>QOJ98883.1 hemagglutinin [Influenza A virus]
METVSLITILLAATVSNADKICIGYQSTNSTETVDTLTENNVPVTHAKELLHTEHNGMLCATSLGQPLIL
DTCTIEGLIYGNPSCDPLLEEREWSYIVERPSAVNGLCYPGNVENLEELRSLFSSARSYQRIQIFPDTIW
NVSYDGTSNTCSGSFYRNMRWLTRKNGNYPIQDAQYTNNQGKNILFMWGINHPPTDDTQRNLYTRTDTTT
SVATEEINRIFRPLIGPRPLVNGLMGRINYYWSVLKPGQTLRIKSDGNLIAPWYGYILSGESHGRILRTD
LKRGSCTVQCQTEKGGLNTTLPFQNVSKYAFGNCSKYIGIKSLKLAVGLRNVPSRSSRGLFGAIAGFIEG
GWSGLVAGWYGFQHSNDQGVGMAADRESTQKAIDKITSKVNNIVDKMNKQYEIIDHEFSEVETRLNMINN
KIDDQIQDIWAYNAELLVLLENQKTLDEHDANVNNLYNKVKRALGSNAVEDGKGCFELYHKCDDQCMETI
RNGTYNRRKYQEESKLERQKIEGVKLESEGTYKILTIYSTVASSLVIAMGFAAFLFWAMSNGSCRCNICI

>QOJ98882.1 hemagglutinin [Influenza A virus]
METVSLITILLVATVSNADKICIGYQSTNSTETVDTLTENNVPVTHAKELLHTEHNGMLCATSLGQPLIL
DTCTIEGLIYGNPSCDLSLEGREWSYIVERPSAVNGLCYPGNVENLEELRSLFSSARSYQRIQIFPDTIW
NVSYDGTSTACSGSFYKSMRWLTRKNGDYPTQDAQYTNNQGKNILFMWGINHPPTDTTQRELYTRTDTTT
SVATEEINRVFKPLIGPRPLVNGLMGRIDYYWSVLKPGQTLRIKSDGNLIAPWFGHILSGESHGRILKTD
LKRGSCTVQCQTEKGGLNTTLPFQNVSKYAFGNCSKYIGIKSLKLAVGLRNVPSRSSRGLFGAIAGFIEG
GWSGLVAGWYGFQHSNDQGVGMAADRDSTQKAIDKITSKVNNIVDKMNKQYEIIDHEFSEVETRLNMINN

KIDDQIQDIWAYNAELLVLLENQKTLDEHDANVNNLYNKVKRALGSNAVEDGKGCFELYHKCDDQCMETI
RNGTYNRRKYQEESKLERQKIEGVKLESEGTYKILTIYSTVASSLVIAMGFAAFLFWAMSNGSCRCNICI

>QOJ98881.1 hemagglutinin [Influenza A virus]
METISLITILLVATVSNADKICIGYQSTNSTETVNTLTENNVPVTHAKELLHTEHNGMLCATSLGQPLIL
DTCTIEGLIYGNPSCDLSLEGREWSYIVERPSAVNGLCYPGNVENLEELRSLFSSARSYQRIQIFPDTIW
NVSYDGTSAACSGSFYKSMRWLTRKNGDYPTQDAQYTNNQGKNILFMWGINHPPTDTTQRDLYTRTDTTT
SVATEEINRVFKPLIGPRPLVNGLMGRIDYYWSVLKPGQTLRIKSDGNLIAPWFGHILSGESHGRILKTD
LKKGSCTVQCQTEKGGLNTTLPFQNVSRYAFGNCSKYIGIKSLKLAVGLRNVPYRSSRGLFGAIAGFIEG
GWSGLVAGWYGFQHSNDQGVGMAADRDSTQKAIDKVTSKVNNIVDKMNKQYEIIDHEFSEVETRLNMINN
KIDDQIQDIWAYNAELLVLLENQKTLDEHDANVNNLYNKVKRALGSNAVEDGKGCFELYHKCDDQCMETI
RNGTYNRRKYQEESKLERQRIEGVKLESEGTYKILTIYSTVASSLVIAMGFAAFLFWAMSNGSCRCNICI

>QOJ98877.1 hemagglutinin [Influenza A virus]
METVSLITILVVATVSNADKICIGYQSTNSTETVDTLTENNVPVTHAKELLHTEHNGMLCATSLGHPLIL
DTCTIEGLIYGNPSCDPLLGGREWSYIVERPSAVNGLCYPGNVENLEELRSLFSSSRSYQRIQIFPDTIW
NVSYSGTSKACSDSFYRSMRWLTQKNNAYPTQDAQYTNNQGKNILFMWGINHPPTDTAQTNLYTRTDTTT
SVATEEMNRIFKPLIGPRPLVNGLMGRINYYWSVLKPGQTLRIKSDGNLIAPWYGHILSGESHGRILKTD
LKRGSCTVQCQTEKGGLNTTLPFQNVSKYAFGNCSKYIGVKSLKLAVGLRNVPSRSSRGLFGAIAGFIEG
GWSGLVAGWYGFQHSNDQGVGMAADRDSTQKAIDKITSKVNNIVDKMNKQYEIIDHEFSEVETRLNMINN
KVDDQIQDIWAYNAELLVLLENQKTLDEHDANVNNLYNKVKRALGSNAVEDGKGCFELYHKCDDHCMETI
RNGTYNRRKYQEESKLERQKIEGVKLESEETYKILTIYSTVASSLVIAMGFAAFLFWAMSNGSCRCNICI

>QOJ98876.1 hemagglutinin [Influenza A virus]
METVSLITILLAATVSNADKICIGYQSTNSTETVDTLTENNVPVTHAKELLHTEHNGMLCATSLGQPLIL
DTCTIEGLIYGNPSCDPLLEEREWSYIVERPSAVNGLCYPGNVENLEELRSFFSSARSYQRIQIFPDTIW
NVSYDGTSNTCSGSFYRNMRWLTRKNGNYPIQDAQYTNNQGKSILFMWGINHPPTDDTQRNLYTRTDTTT
SVATEEINRIFKPLIGPRPLVNGLMGRINYYWSVLKPGQTLRIKSDGNLIAPWYGYILSGESHGRILRTD
LTKGSCTVQCQTEKGGLNTTLPFQNVSKYAFGNCSKYIGIKSLKLAVGLRNVPSRSSRGLFGAIAGFIEG
GWSGLVAGWYGFQHSNDQGVGMAADRESTQKAIDKITSKVNNIVDKMNKQYEIIDHEFSEVETRLNMINN
KIDDQIQDIWAYNAELLVLLENQKTLDEHDANVNNLYNKVKRALGSNAVEDGKGCFELYHKCDDQCMETI
RNGTYNRRKYQEESKLERQKIEGVKLESEGTYKILTIYSTVASSLVIAMGFAAFLFWAMSNGSCRCNICI

>QOJ98874.1 hemagglutinin [Influenza A virus]

METVSLITILLVATVSNADKICIGYQSTNSTETVDTLTENNVPVTHAKELIHTEHNGMLCATSLGQPLIL
ETCTIEGLIYGNPSCDLSLEGREWSYIVERPSAVNGLCYPGNVENLEELRSLFSSARSYQRIQIFPDTIW
NVSYDGTSTACSGSFYRNMRWLTRKDGNYPTQDAQYTNNQGKNILFMWGINHPPSDTTQSGLYTRTDTTT
SVATEEINRIFKPLIGPRPLVNGLMGRIDYYWSVLKPGQTLRIKSDGNLIAPWFGHILSGESHGRILKTD
LKRGSCTVQCQTEKGGLNTTLPFQNVSKYAFGNCSKYIGVKSLKLAVGLRNVPSRSSRGLFGAIAGFIEG
GWSGLVAGWYGFQHSNDQGVGMAADRDSTQKAIDKITSKVNNIVDKMNKQYEIIDHEFSEVETRLNMINN
KIDDQIQDIWAYNAELLVLLENQKTLDEHDANVNNLYNKVKRALGSNAVEDGKGCFELYHKCDDQCMETI
RNGTYNRRKYQEESKLERQKIEGVKLESEGTYKILTIYSTVASSLVIAMGFAAFLVWAMSNGSCRCNICI

>QOJ98873.1 hemagglutinin [Influenza A virus]
METVSLITILLAATVSNADKICIGYQSTNSTETVDTLTENNVPVTHAKELLHTEHNGMLCATSLGQPLIL
DTCTIEGLIYGNPSCDPVLEEREWSYIVERPSAVNGLCYPGNVENLEELRSLFSSARFYQRIQIFPDTIW
NVSYDGTSNTCSGSFYRNMRWLTRKDGNYPIQDAQYTNNQGKNILFMWGINHPPTDDTQRNLYTRTDTTT
SVATEEINRIFKPLIGPRPLVNGLMGRINYYWSVLKPGQTLRIKSDGNLIAPWYGYILSGESHGRILRTD
LKRGSCTVQCQTEKGGLNTTLPFQNVSKYAFGNCSKYIGIKSLKLAVGLRNVPSRSSRGLFGAIAGFIEG
GWSGLVAGWYGFQHSNDQGVGMAADRESTQKAIDKITSKVNNIVDKMNKQYEIIDHEFSEVETRLNMINN
KIDDQIQDIWAYNAELLVLLENQKTLDEHDANVNNLYNKVKRALGSNAVEDGKGCFELYHKCDDQCMETI
RNGTYNRRKYQEESKLERQKIEGVKLESEGTYKILTIYSTVASSLVIAMGFAAFLFWAMSNGSCRCNICI

>QOJ98872.1 hemagglutinin [Influenza A virus]
METVSLITILLAATVSNADKICIGYQSTNSTETVDTLTENNVPVTHAKELLHTEHNGMLCATSLGQPLIL
DTCTIEGLIYGNPSCDPVLEEREWSYIVERPSAVNGLCYPGNVENLEELRSLFSSARFYQRIQIFPDTIW
NVSYDGTSNTCSGSFYRNMRWLTRKDGNYPIQDAQYTNNQGKNILFMWGINHPPTDDTQRNLYTRTDTTT
SVATEEINRIFKPLIGPRPLVNGLMGRINYYWSVLKPGQTLRIKSDGNLIAPWYGYILSGESHGRILRTD
LKRGSCTVQCQTEKGGLNTTLPFQNVSKYAFGNCSKYIGIKSLKLAVGLRNVPSRSSRGLFGAKAGFIEG
GWSGLVAGWYGFQHSNDQGVGMAADRESTQKAIDKITSKVNNIVDKMNKQYEIIDHEFSEVETRLNMINN
KIDDQIQDIWAYNAELLVLLENQKTLDEHDANVNNLYNKVKRALGSNAVEDGKGCFELYHKCDDQCMETI
RNGTYNRRKYQEESKLERQKIEGVKLESEGTYKILTIYSTVASSLVIAMGFAAFLFWAMSNGSCRCNICI

>QOJ98865.1 hemagglutinin [Influenza A virus]
METVSLITILLAATVSNADKICIGYQSTNSTETVDTLTENNVPVTHAKELLHTEHNGMLCATSLGQPLIL
DTCTIEGLIYGNPSCDPLLEEREWSYIVERPSAVNGLCYPGNVENLEELRSFFSSARSYQRIQIFPDTIW
NVSYDGTSNTCSGSFYRNMRWLTRKNGNYPIQDAQYTNNQGKSILFMWGINHPPTDDTQRNLYTRTDTTT
SVATEEINRIFKPLIGPRPLVNGLMGRINYYWSVLKPGQTLRIKSDGNLIAPWYGYILSGESHGRILRTD

LTKGSCTVQCQTEKGGLNTTLPFQNVSKYAFGNCSKYIGIKSLKLAVGLRNVPSRSSRGLFGAIAGFIEG
GWSGLVAGWYGFQHSNDQGVGMAADRESTQKAIDKITSKVNNIVDKMNKQYEIIDHEFSEVETRLNMINN
KIDDQIQDIWAYNAELLVLLENQKTLDEHDANVNNLYNKVKRALGSNAVEDGKGCFELYHKCDDQCMETI
RNGTYNRRKYQEESKLERQKIEGVKLESEGTYKILTIYSTVASSLVIAMGFAAFLFWAMSNGSCRCNICI

>QOJ98864.1 hemagglutinin [Influenza A virus]
METVSLITILLAATVSNADKICIGYQSTNSTETVDTLTENNVPVTHAKELLHTEHNGMLCATSLGQPLIL
DTCTIEGLIYGNPSCDPLLEEREWSYIVERPSAINGLCYPGNVENLEELRSFFSSARSYQRIQIFPDTIW
NVSYDGTSNTCSGSFYRNMRWLTRKNGNYPIQDAQYTNNQGKSILFMWGINHPPTDDTQRNLYTRTDTTT
SVATEEINRIFKPLIGPRPLVNGLMGRINYYWSVLKPGQTLRIKSDGNLIAPWYGYILSGESHGRILRTD
LTKGSCTVQCQTEKGGLNTTLPFQNVSKYAFGNCSKYIGIKSLKLAVGLRNVPSRSSRGLFGAIAGFIEG
GWSGLVAGWYGFQHSNDQGVGMAADRESTQKAIDKITSKVNNIVDKMNKQYEIIDHEFSEVETRLNMINN
KIDDQIQDIWAYNAELLVLLENQKTLDEHDANVNNLYNKVKRALGSNAVEDGKGCFELYHKCDDQCMETI
RNGTYNRRKYQEESKLERQKIEGVKLESEGTYKILTIYSTVASSLVIAMGFAAFLFWAMSNGSCRCNICI

>QOJ98860.1 hemagglutinin [Influenza A virus]
METVSLITILLAATVSNADKICIGYQSTNSTETVDTLTENNVPVTHAKELLHTEHNGMLCATSLGQPLIL
DTCTIEGLIYGNPSCDPLLEEREWSYIVERPSAVNGLCYPGNVENLEELRSFFSSARSYQRIQIFPDTIW
NVSYDGTSNTCSGSFYRNMRWLTRKNGNYPIQDAQYTNNQGKSILFMWGINHPPTDDTQRNLYTRTDTTT
SVATEEINRIFKPLIGPRPLVNGLMGRINYYWSVLKPGQTLRIKSDGNLIAPWYGYILSGESHGRILRTD
LTKGSCTVQCQTEKGGLNTTLPFQNVSKYAFGNCSKYIGIKSLKLAVGLRNVPSRSSRGLFGAIAGFIEG
GWSGLVAGWYGFQHSNDQGVGMAADRESTQKAIDKITSKVNNIVDKMNKQYEIIDHEFSEVETRLNMINN
KIDDQIQDIWAYNAELLVLLENQKTLDEHDANVNNLYNKVKRALGSNAVEDGKGCFELYHKCDDQCMETI
RNGTYNRRKYQEESKLERQKIEGVKLESEGTYKILTIYSTVASSLVIAMGFAAFLFWAMSNGSCRCNICI

>QOJ98859.1 hemagglutinin [Influenza A virus]
METVSLITILLVATVSNADKICIGYQSTNSTETVDTLTENNVPVTHAKELIHTEHNGMLCATSLGQPLIL
ETCTIEGLIYGNPSCDLSLEGREWSYIVERPSAVNGLCYPGNVENLEELRSLFSSARSYQRIQIFPDTIW
NVSYDGTSTACSGSFYRNMRWLTRKDGNYPTQDAQYTNNQGKNILFMWGINHPPSDTTQSGLYTRTDTTT
SVATEEINRIFKPLIGPRPLVNGLMGRIDYYWSVLKPGQTLRIKSDGNLIAPWFGHILSGESHGRILKTD
LKRGSCTVQCQTEKGGLNTTLPFQNVSKYAFGNCSKYIGVKSLKLAVGLRNVPSRSSRGLFGAIAGFIEG
GWSGLVAGWYGFQHSNDQGVGMAADRDSTQKAIDKITSKVNNIVDKMNKQYEIIDHEFSEVETRLNMINN
KIDDQIQDIWAYNAELLVLLENQKTLDEHDANVNNLYNKVKRALGSNAVEDGKGCFELYHKCDDQCMETI
RNGTYNRRKYQEESKLERQKIEGVKLESEGTYKILTIYSTVASSLVIAMGFAAFLVWAMSNGSCRCNICI

>QOJ98858.1 hemagglutinin [Influenza A virus]

METVSLITILLAATVSYADKICIGYQSTNSTETVDTLTENNVPVTHAKELLHTEHNGMLCATSLGQPLIL
DTCTIEGLIYGNPSCDPVLEEREWSYIVERPSAVNGLCYPGNVENLEELRSLFSSARFYQRIQIFPDTIW
NVSYDGTSNTCSGSFYRNMRWLTRKDGNYPIQDAQYTNNQGKNILFMWGINHPPTDDTQRNLYTRTDTTT
SVATEEINRIFKPLIGPRPLVNGLMGRINYYWSVLKPGQTLRIKSDGNLIAPWYGYILSGESHGRILRTD
LKRGSCTVQCQTEKGGLNTTLPFQNVSKYAFGNCSKYIGIKSLKLAVGLRNVPSRSSRGLFGAIAGFIEG
GWSGLVAGWYGFQHSNDQGVGMAADRESTQKAIDKITSKVNNIVDKMNKQYEIIDHEFSEVETRLNMINN
KIDDQIQDIWAYNAELLVLLENQKTLDEHDANVNNLYNKVKRALGSNAVEDGKGCFELYHKCDDQCMETI
RNGTYNRRKYQEESKLERQKIEGVKLESEGTYKILTIYSTVASSLVIAMGFAAFLFWAMSNGSCRCNICI

>QOJ98857.1 hemagglutinin [Influenza A virus]

METVSLITILLAATVSNADKICIGYQSTNSTETVDTLTENNVPVTHAKELLHTEHNGMLCATSLGQPLIL
DTCTIEGLIYGNPSCDPVLEEREWSYIVERPSAVNGLCYPGNVENLEELRSLFSSARFYQRIQIFPDTIW
NVSYDGTSNTCSGSFYRNMRWLTRKDGNYPIQDAQYTNNQGKNILFMWGINHPPTDDTQRNLYTRTDTTT
SVATEEINRIFKPLIGPRPLVNGLMGRINYYWSVLKPGQTLRIKSDGNLIAPWYGYILSGESHGRILRTD
LKRGSCTVQCQTEKGGLNTTLPFQNVSKYAFGNCSKYIGIKSLKLAVGLRNVPSRSSRGLFGAIAGFIEG
GWSGLVAGWYGFQHSNDQGVGMAADRESTQKAIDKITSKVNNIVDKMNKQYEIIDHEFSEVETRLNMINN
KIDDQIQDIWAYNAELLVLLENQKTLDEHDANVNNLYNKVKRALGSNAVEDGKGCFELYHKCDDQCMETI
RNGTYNRRKYQEESKLERQKIEGVKLESEGTYKILTIYSTVASSLVIAMGFAAFLFWAMSNGSCRCNICI

>QOJ98856.1 hemagglutinin [Influenza A virus]

METVSLITILLAATVSNADKICIGYQSTNSTETVDTLTENNVPVTHAKELLHTEHNGMLCATSLGQPLIL
DTCTIEGLIYGNPSCDPVLEEREWSYIVERPSAVNGLCYPGNVENLEELRSLFSSARFYQRIQIFPDTIW
NVSYDGTSNTCSGSFYRNMRWLTRKDGNYPIQDAQYTNNQGKNILFMWGINHPPTDDTQRNLYTRTDTTT
SVATEEINRIFKPLIGPRPLVNGLMGRINYYWSVLKPGQTLRIKSDGNLIAPWYGYILSGESHGRILRTD
LKRGSCTVQCQTEKGGLNTTLPFQNVSKYAFGNCSKYIGIKSLKLAVGLRNVPSRSSRGLFGAIAGFIEG
GWSGLVAGWYGFQHSNDQGVGMAADRESTQKAIDKITSKVNNIVDKMNKQYEIIDHEFSEVETRLNMINN
KIDDQIQDIWAYNAELLVLLENQKTLDEHDANVNNLYNKVKRALGSNAVEDGKGCFELYHKCDDQCMETI
RNGTYNRRKYQEESKLERQKIEGVKLESEGTYKILTIYSTVASSLVIAMGFAAFLFWAMSNGSCRCNICI

>QOJ98855.1 hemagglutinin [Influenza A virus]

METVSLITILLAATVSNADKICIGYQSTNSTETVDTLTENNVPVTHAKELLHTEHNGMLCATSLGQPLIL
DTCTIEGLIYGNPSCDPVLEEREWSYIVERPSAVNGLCYPGNVENLEELRSLFSSARFYQRIQIFPDTIW

NVSYDGTSNTCSGSFYRNMRWLTRKDGNYPIQDAQYTNNQGKNILFMWGINHPPTDDTQRNLYTRTDTTT
SVATEEINRIFKPLIGPRPLVNGLMGRINYYWSVLKPGQTLRIKSDGNLIAPWYGYILSGESHGRILRTD
LKRGSCTVQCQTEKGGLNTTLPFQNVSKYAFGNCSKYIGIKSLKLAVGLRNVPSRSSRGLFGAIAGFIEG
GWSGLVAGWYGFQHSNDQGVGMAADRESTQKAIDKITSKVNNIVDKMNKQYEIIDHEFSEVETRLNMINN
KIDDQIQDIWAYNAELLVLLENQKTLDEHDANVNNLYNKVKRALGSNAVEDGKGCFELYHKCDDQCMETI
RNGTYNRRKYQEESKLERQKIEGVKLESEGTYKILTIYSTVASSLVIAMGFAAFLFWAMSNGSCRCNICI

>QOJ98851.1 hemagglutinin [Influenza A virus]
METVSLITILLVATVSNADKICIGYQSTNSTETVDTLTENNVPVTHAKELLHTEHNGMLCATSLGQPLIL
DTCTIEGLIYGNPSCDLSLEGREWSYIVERPSAVHGLCYPGNVEDLEELRSLFSSARSYQRIQIFPDTIW
NVSYDGTSTACSGSFYKSMRWLTRKNGEYPTQDAQYTNNQGKNILFMWGINHPPTDDTQRGLYTRTDTTT
SVATEEINRIFKPLIGPRPLVNGLMGRINYYWSVLKPGQTLRIKSDGNLIAPWYGHILSGESHGRILKTD
LKRGSCTVQCQTEKGGLNTTLPFQNVSKYAFGNCSKYIGIKSLKLAVGLRNVPSKSSRGLFGAIAGFIEG
GWSGLVAGWYGFQHSNDQGVGIAADRDSTQKAIDKITSKVNNIVDKMNKQYEIIDHEFSEVETRLNMINN
KIDDQIQDIWAYNAELLVLLENQKTLDEHDANVNNLYNKVKRALGSNAVEDGKGCFELYHKCDDQCMETI
RNGTYNRRKYQEESKLERQRIEGVKLESEETYKILTIYSTVASSLVFAMGFAALLFWAMSNGSCRCNICI

>QOJ98850.1 hemagglutinin [Influenza A virus]
METVSLITILLVATVSNADKICIGYQSTNSTETVDTLTENNVPVTHAKELLHTEHNGMLCATSLGQPLIL
DTCTIEGLIYGNPSCDLSLEGREWSYIVERPSAVHGLCYPGNVEDLEELRSLFSSARSYQRIQIFPDTIW
NVSYDGTSTACSGSFYKSMRWLTRKNGEYPTQDAQYTNNQGKNILFMWGINHPPTDDTQRGLYTRTDTTT
SVATEEINRIFKPLIGPRPLVNGLMGRINYYWSVLKPGQTLRIKSDGNLIAPWYGHILSGESHGRILKTD
LKRGSCTVQCQTEKGGLNTTLPFQNVSKYAFGNCSKYIGIKSLKLAVGLRNVPSKSSRGLFGAIAGFIEG
GWSGLVAGWYGFQHSNDQGVGIAADRDSTQKAIDKITSKVNNIVDKMNKQYEIIDHEFSEVETRLNMINN
KIDDQIQDIWAYNAELLVLLENQKTLDEHDANVNNLYNKVKRALGSNAVEDGKGCFELYHKCDDQCMETI
RNGTYNRRKYQEESKLERQRIEGVKLESEETYKILTIYSTVASSLVFAMGFAALLFWAMSNGSCRCNICI

>QOJ98849.1 hemagglutinin [Influenza A virus]
METVSLITILLVATVSNADKICIGYQSTNSTETVDTLTENNVPVTHAKELLHTEHNGMLCATSLGQPLIL
DTCTIEGLIYGNPSCDLSLEGREWSYIVERPSAVHGLCYPGNVEDLEELRSLFSSARSYQRIQIFPDTIW
NVSYDGTSTACSGSFYKSMRWLTRKNGEYPTQDAQYTNNQGKNILFMWGINHPPTDDTQRGLYTRTDTTT
SVATEEINRIFKPLIGPRPLVNGLMGRINYYWSVLKPGQTLRIKSDGNLIAPWYGHILSGESHGRILKTD
LKRGSCTVQCQTEKGGLNTTLPFQNVSKYAFGNCSKYIGIKSLKLAVGLRNVPSKSSRGLFGAIAGFIEG
GWSGLVAGWYGFQHSNDQGVGIAADRDSTQKAIDKITSKVNNIVDKMNKQYEIIDHEFSEVETRLNMINN

KIDDQIQDIWAYNAELLVLLENQKTLDEHDANVNNLYNKVKRALGSNAVEDGKGCFELYHKCDDQCMETI
RNGTYNRRKYQEESKLERQRIEGVKLESEETYKILTIYSTVASSLVFAMGFAALLFWAMSNGSCRCNICI

>QOJ98845.1 hemagglutinin [Influenza A virus]
METVSLITILLVATVSNADKICIGYQSTNSTETVDTLTENNVPVTHAKELLHTEHNGMLCATSLGQPLIL
DTCTIEGLIYGNPSCDLSLEGREWSYIVERPSAVHGLCYPGNVEDLEELRSLFSSARSYQRIQIFPDTIW
NVSYDGTSTACSGSFYKSMRWLTRKNGEYPTQDAQYTNNQGKNILFMWGINHPPTDDTQRGLYTRTDTTT
SVATEEINRIFKPLIGPRPLVNGLMGRINYYWSVLKPGQTLRIKSDGNLIAPWYGHILSGESHGRILKTD
LKRGSCTVQCQTEKGGLNTTLPFQNVSKYAFGNCSKYIGIKSLKLAVGLRNVPSKSSRGLFGAIAGFIEG
GWSGLVAGWYGFQHSNDQGVGIAADRDSTQKAIDKITSKVNNIVDKMNKQYEIIDHEFSEVETRLNMINN
KIDDQIQDIWAYNAELLVLLENQKTLDEHDANVNNLYNKVKRALGSNAVEDGKGCFELYHKCDDQCMETI
RNGTYNRRKYQEESKLERQRIEGVKLESEETYKILTIYSTVASSLVFAMGFAALLFWAMSNGSCRCNICI

>QOJ98844.1 hemagglutinin [Influenza A virus]
METVSLITILLVATVSNADKICIGYQSTNSTETVDTLTENNVPVTHAKELLHTEHNGMLCATSLGQPLIL
DTCTIEGLIYGNPSCDLSLEGREWSYIVERPSAVNGLCYPGSVENLEELRSLFSSARSYQRIQIFPDTIW
NVSYDGTSTACSGSFYRSMRWLTRKNGVYPIQDAQYTNNQGKNILFMWGINHPPTDDTQTGLYTRTDTTT
SVATEEINRIFKPLIGPRPLVNGLMGRINYYWSVLKPGQTLRIKSDGNLIAPWYGHILSGESHGRILKTD
LKRGSCTVQCQTEKGGLNTTLPFQNVSKYAFGNCSKYIGIKSLKLAVGLRNVPSRSSRGLFGAIAGFIEG
GWSGLVAGWYGFQHSNDQGVGMAADRDSTQKAIDKITSKVNNIVDKMNKQYEIIDHEFSEVEARLNMINN
KIDDQIQDIWAYNAELLVLLENQKTLDEHDANVNNLYNKVKRALGSNAVEDGKGCFELYHKCDDQCMETI
RNGTYNRRKYQEESKLARQKIEGVKLESEGIYKILTIYSTVASSLVIAMGFAAFLFWAMSNGSCRCNICI

>QOJ98843.1 hemagglutinin [Influenza A virus]
METVSLITILVVATVSNADKICIGYQSTNSTETVDTLTENNVPVTHAKELLHTEHNGMLCATSLGHPLIL
DTCTIEGLIYGNPSCDPLLGGREWSYIVERPSAVNGLCYPGNVENLEELRSLFSSSRSYQRIQIFPDTIW
NVSYSGTSKACSDSFYRSMRWLTQKNNAYPTQDAQYTNNQGKNILFMWGINHPPTDTVQTNLYTRTDTTT
SVATEEMNRIFKPLIGPRPLVNGLMGRINYYWSVLKPGQTLRIKSDGNLIAPWYGHILSGESHGRILKTD
LKRGSCTVQCQTEKGGLNTTLPFQNVSKYAFGNCSKYIGVKSLKLAVGLRNVPSRSSRGLFGAIAGFIEG
GWSGLVAGWYGFQHSNDQGVGMAADRDSTQKAIDKITSKVNNIVDKMNKQYEIIDHEFSEVETRLNMINN
KVDDQIQDIWAYNAELLVLLENQKTLDEHDANVNNLYNKVKRALGSNAVEDGKGCFELYHKCDDHCMETI
RNGTYNRRKYQEESKLERQKIEGVKLESEETYKILTIYSTVASSLVIAMGFAAFLFWAMSNGSCRCNICI

>QOJ98841.1 hemagglutinin [Influenza A virus]

METVSLITILVVATVSNADKICIGYQSTNSTETVDTLTENNVPVTHAKELLHTEHNGMLCATSLGHPLIL
DTCTIEGLIYGNPSCDPLLGGREWSYIVERPSAVNGLCYPGNVENLEELRSLFSSSRSYQRIQIFPDTIW
NVSYSGTSKACSDSFYRSMRWLTQKNNAYPTQDAQYTNNQGKNILFMWGINHPPTDTAQTNLYTRTDTTT
SVATEEMNRIFKPLIGPRPLVNGLMGRINYYWSVLKPGQTLRIKSDGNLIAPWYGHILSGESHGRILKTD
LKRGSCTVQCQTEKGGLNTTLPFQNVSKYAFGNCSKYIGVKSLKLAVGLRNVPSRSSRGLFGAIAGFIEG
GWSGLVAGWYGFQHSNDQGVGMAADRDSTQKAIDKITSKVNNIVDKMNKQYEIIDHEFSEVETRLNMINN
KVDDQIQDIWAYNAELLVLLENQKTLDEHDANVNNLYNKVKRALGSNAVEDGKGCFELYHKCDDHCMETI
RNGTYNRRKYQEESKLERQKIEGVKLESEETYKILTIYSTVASSLVIAMGFAAFLFWAMSNGSCRCNICI

>QOJ98839.1 hemagglutinin [Influenza A virus]
METVSLITILVAATVSNADKICIGYQSTNSTETVDTLTENNVPVTHAKELLHTEHNGMLCATSLGHPLIL
DTCTIEGLIYGNPSCDPLLGGREWSYIVERPSAVNGLCYPGNVENLEELRSLFSSSRSYQRIQIFPDTIW
NVSYSGTSKACSDSFYRSMRWLTQKNNAYPTQDAQYTNNQGKNILFMWGINHPPTDTAQTNLYTRTDTTT
SVATEEMNRIFKPLIGPRPLVNGLMGRINYYWSVLKPGQTLRIKSDGNLIAPWYGHILSGESHGRILKTD
LERGSCTVQCQTEKGGLNTTLPFQNVSKYAFGNCSKYIGVKSLKLAVGLRNVPSRSSRGLFGAIAGFIEG
GWSGLVAGWYGFQHSNDQGVGMAADRDSTQKAIDKITSKVNNIVDKMNKQYEIIDHEFSEVETRLNMINN
KVDDQIQDIWAYNAELLVLLENQKTLDEHDANVNNLYNKVKRALGSNAVEDGKGCFELYHKCDDHCMETI
RNGTYNRRKYQEESKLERQKIEGVKLESEETYKILTIYSTVASSLVIAMGFAAFLFWAMSNGSCRCNICI

>QOJ98837.1 hemagglutinin [Influenza A virus]
METVSLITILVVATVSNADKICIGYQSTNSTETVDTLTENNVPVTHAKELLHTEHNGMLCATSLGHPLIL
DTCTIEGLIYGNPSCDPLLGGREWSYIVERPSAVNGLCYPGNVENLEELRSLFSSSRSYQRIQIFPDTIW
NVSYSGTSKACSDSFYRSMRWLTQKNNAYPTQDAQYTNNQGKNILFMWGINHPPTDTAQTNLYTRTDTTT
SVATEEMNRIFKPLIGPRPLVNGLMGRINYYWSVLKPGQTLRIKSDGNLIAPWYGHILSGESHGRILKTD
LKRGSCTVQCQTEKGGLNTTLPFQNVSKYAFGNCSKYIGVKSLKLAVGLRNVPSRSSRGLFGAIAGFIEG
GWSGLVAGWYGFQHSNDQGVGMAADRDSTQKAIDKITSKVNNIVDKMNKQYEIIDHEFSEVETRLNMINN
KVDDQIQDIWAYNAELLVLLENQKTLDEHDANVNNLYNKVKRALGSNAVEDGKGCFELYHKCDDHCMETI
RNGTYNRRKYQEESKLERQKIEGVKLESEETYKILTIYSTVASSLVIAMGFAAFLFWAMSNGSCRCNICI

>QOJ98836.1 hemagglutinin [Influenza A virus]
METVSLITILVVATVSNADKICIGYQSTNSTETVDTLTENNVPVTHAKELLHTEHNGMLCATSLGHPLIL
DTCTIEGLIYGNPSCDPLLGGREWSYIVERPSAVNGLCYPGNVENLEELRSLFSSSRSYQRIQIFPDTIW
NVSYSGTSKACSDSFYRSMRWLTQKNNAYPTQDAQYTNNQGKNILFMWGINHPPTDTAQTNLYTRTDTTT
SVATEEMNRIFKPLIGPRPLVNGLMGRINYYWSVLKPGQTLRIKSDGNLIAPWYGHILSGESHGRILKTD

LKRGSCTVQCQTEKGGLNTTLPFQNVSKYAFGNCSKYIGVKSLKLAVGLRNVPSRSSRGLFGAIAGFIEG
GWSGLVAGWYGFQHSNDQGVGMAADRDSTQKAIDKITSKVNNIVDKMNKQYEIIDHEFSEVETRLNMINN
KVDDQIQDIWAYNAELLVLLENQKTLDEHDANVNNLYNKVKRALGSNAVEDGKGCFELYHKCDDHCMETI
RNGTYNRRKYQEESKLERQKIEGVKLESEETYKILTIYSTVASSLVIAMGFAAFLFWAMSNGSCRCNICI

>QOJ98835.1 hemagglutinin [Influenza A virus]
METVSLITILVVATVSNADKICIGYQSTNSTETVDTLTENNVPVTHAKELLHTEHNGMLCATSLGHPLIL
DTCTIEGLIYGNPSCDPLLGGREWSYIVERPSAVNGLCYPGNVENLEELRSLFSSSRSYQRIQIFPDTIW
NVSYSGTSKACSDSFYRSMRWLTQKNNAYPTQDAQYTNNQGKNILFMWGINHPPTDTAQTNLYTRTDTTT
SVATEEMNRIFKPLIGPRPLVNGLMGRINYYWSVLKPGQTLRIKSDGNLIAPWYGHILSGESHGRILKTD
LKRGSCTVQCQTEKGGLNTTLPFQNVSKYAFGNCSKYIGVKSLKLAVGLRNVPSRSSRGLFGAIAGFIEG
GWSGLVAGWYGFQHSNDQGVGMAADRDSTQKAIDKITSKVNNIVDKMNKQYEIIDHEFSEVETRLNMINN
KVDDQIQDIWAYNAELLVLLENQKTLDEHDANVNNLYNKVKRALGSNAVEDGKGCFELYHKCDDHCMETI
RNGTYNRRKYQEESKLERQKIEGVKLESEETYKILTIYSTVASSLVIAMGFAAFLFWAMSNGSCRCNICI

>QOJ98834.1 hemagglutinin [Influenza A virus]
METVSLITILVVATVSNADKICIGYQSTNSTETVDTLTENNVPVTHAKELLHTEHNGMLCATSLGHPLIL
DTCTIEGLIYGNPSCDPLLGGREWSYIVERPSAVNGLCYPGNVENLEELRSLFSSSRSYQRIQIFPDTIW
NVSYSGTSKACSDSFYRSMRWLTQKNNAYPTQDAQYTNNQGKNILFMWGINHPPTDTAQTNLYTRTDTTT
SVATEEMNRIFKPLIGPRPLVNGLMGRINYYWSVLKPGQTLRIKSDGNLIAPWYGHILSGESHGRILKTD
LKRGSCTVQCQTEKGGLNTTLPFQNVSKYAFGNCSKYIGVKSLKLAVGLRNVPSRSSRGLFGAIAGFIEG
GWSGLVAGWYGFQHSNDQGVGMAADRDSTQKAIDKITSKVNNIVDKMNKQYEIIDHEFSEVETRLNMINN
KVDDQIQDIWAYNAELLVLLENQKTLDEHDANVNNLYNKVKRALGSNAVEDGKGCFELYHKCDDHCMETI
RNGTYNRRKYQEESKLERQKIEGVKLESEETYKILTIYSTVASSLVIAMGFAAFLFWAMSNGSCRCNICI

>QOJ98833.1 hemagglutinin [Influenza A virus]
METVSLITILLAATVSNADKICIGYQSTNSTETVDTLTENNVPVTHAKELLHTEHNGMLCATSLGQPLIL
DTCTIEGLIYGNPSCDPLPEEREWSYIVERPSAVNGLCYPGNVENLEELRSLFSSARSYQRIQIFPDTIW
NVSYDGTSNTCSGSFYRNMRWLTRKDGNYPIQDAQYTNNQGKNILFMWGINNPPTDDTQRNLYTRTDTTT
SVATEEINRIFKPLIGPRPLVNGLMGRINYYWSVLKPGQTLRIKSDGNLVAPWYGYILSGESHGRILRTD
LKRGSCTVQCQTEKGGLNTTLPFQNVSKYAFGNCSKYIGIKSLKLAVGLRNVPSRSSRGLFGAIAGFIEG
GWSGLVAGWYGFQHSNDQGVGMAADRESTQKAVDKITSKVNNIVDKMNKQYEIIDHEFSEVETRLNMINN
KIDDQIQDIWAYNAELLVLLENQKTLDEHDANVNNLYNKVKRALGSNAVEDGKGCFELYHKCDDQCMETI
RNGTYNRRKYQEESKLERQKIEGVKLESEGTYKILTIYSTVASSLVIAMGFAAFLFWAMSNGSCRCNICI

>QOJ98832.1 hemagglutinin [Influenza A virus]
METVSLITILLAATVSNADKICIGYQSTNSTETVDTLTENNVPVTHAKELLHTEHNGMLCATSLGQPLIL
DTCTIEGLIYGNPSCDPLPEEREWSYIVERPSAVNGLCYPGNVENLEELRSLFSSARSYQRIQIFPDTIW
NVSYDGTSNTCSGSFYRNMRWLTRKDGNYPIQDAQYTNNQGKNILFMWGINNPPTDDTQRNLYTRTDTTT
SVATEEINRIFKPLIGPRPLVNGLMGRINYYWSVLKPGQTLRIKSDGNLVAPWYGYILSGESHGRILRTD
LKRGSCTVQCQTEKGGLNTTLPFQNVSKYAFGNCSKYIGIKSLKLAVGLRNVPSRSSRGLFGAIAGFIEG
GWSGLVAGWYGFQHSNDQGVGMAADRESTQKAVDKITSKVNNIVDKMNKQYEIIDHEFSEVETRLNMINN
KIDDQIQDIWAYNAELLVLLENQKTLDEHDANVNNLYNKVKRALGSNAVEDGKGCFELYHKCDDQCMETI
RNGTYNRRKYQEESKLERQKIEGVKLESEGTYKILTIYSTVASSLVIAMGFAAFLFWAMSNGSCRCNICI

>QOJ98831.1 hemagglutinin [Influenza A virus]
METVSLITILLAATVSNADKICIGYQSTNSTETVDTLTENNVPVTHAKELLHTEHNGMLCATSLGQPLIL
DTCTIEGLIYGNPSCDPLPEEREWSYIVERPSAVNGLCYPGNVENLEELRSLFSSARSYQRIQIFPDTIW
NVSYDGTSNTCSGSFYRNMRWLTRKDGNYPIQDAQYTNNQGKNILFMWGINNPPTDDTQRNLYTRTDTTT
SVATEEINRIFKPLIGPRPLVNGLMGRINYYWSVLKPGQTLRIKSDGNLVAPWYGYILSGESHGRILRTD
LKRGSCTVQCQTEKGGLNTTLPFQNVSKYAFGNCSKYIGIKSLKLAVGLRNVPSRSSRGLFGAIAGFIEG
GWSGLVAGWYGFQHSNDQGVGMAADRESTQKAVDKITSKVNNIVDKMNKQYEIIDHEFSEVETRLNMINN
KIDDQIQDIWAYNAELLVLLENQKTLDEHDANVNNLYNKVKRALGSNAVEDGKGCFELYHKCDDQCMETI
RNGTYNRRKYQEESKLERQKIEGVKLESEGTYKILTIYSTVASSLVIAMGFAAFLFWAMSNGSCRCNICI

>QOJ98829.1 hemagglutinin, partial [Influenza A virus]
ETVSLITILLVATVSNADKICIGYQSTNSTETVDTLTENNVPVTHAKELLHTEHNGMLCATSLGQPLILD
TCTIEGLIYGNPSCDLSLEGREWSYIVERPSAVHGLCYPGNVENLEELRSLFSSARSYQRIQIFPDTIWN
VSYDGTSTACSGSFYRSMRWLTRKNGEYPIQDAQYTNNQGKNILFMWGINHPPTDDTQRGLYTRTDTTTS
VATEEINRIFKPLIGPRPLVNGLMGRINYYWSVLKPGQTLRIKSDGNLIAPWYGYILSGESHGRILRTDL
KRGSCTVQCQTEKGGLNTTLPFQNVSKYAFGNCSKYIGIKSLKLAVGLRNVPSRSSRGLFGAIAGFIEGG
WSGLVAGWYGFQHSNDQGVGMAADRDSTQKAIDKITSKVNNIVDKMNKQYEIIDHEFSEVETRLNMINNK
IDDQIQDIWAYNAELLVLLENQKTLDEHDANVNNLYNKVKRALGSNAVEDGKGCFELYHKCDDQCMETIR
NGTYNRRKYQEESKLERQKIEGVKLESEGTYKILTIYSTVASSLVIAMGFAAFLFWAMSNGSC

>QOJ98828.1 hemagglutinin [Influenza A virus]
METVSLITILLVATVSNADKICIGYQSTNSTETVDTLTENNVPVTHAKELLHTEHNGMLCATSLGQPLVL
DTCTIEGLIYGNPSCDLSLEGREWSYIVERPSAVHGLCYPGNVEDLEELRSLFSSARSYQRIQIFPDTIW

NVSYDGTSTACSGSFYRSMRWLTRKNGEYPIQDAQYTNNQGKNILFMWGINHPPTDDTQRGLYTRTDTTT
SVATEEINRIFKPLIGPRPLVNGLMGRINYYWSVLKPGQTLRIKSDGNLIAPWYGHILSGESHGRILKTD
LKRGSCTVQCQTEKGGLNTTLPFQNVSKYAFGNCSKYIGIKSLKLAVGLRNVPSRSSRGLFGAIAGFIEG
GWSGLVAGWYGFQHSNDQGVGMAADRDSTQKAIDKITSKVNNIVDKMNKQYEIIDHEFSEVETRLNMINN
KIDDQIQDIWAYNAELLVLLENQKTLDEHDANVNNLYNKVKRALGSNAVEDGKGCFELYHKCDDQCMETI
RNGTYNRRKYQEESKLERQKIEGVKLESEGTYKILTIYSTVASSLVIAMGFAAFLFWAMSNGSCRCNICI

>QOJ98827.1 hemagglutinin [Influenza A virus]
METVSLITILLAATVSNADKICIGYQSTNSTETVDTLTENNVPVTHAKELLHTEHNGMLCATSLGQPLIL
DTCTIEGLIYGNPSCDPLPEEREWSYIVERPSAVNGLCYPGNVENLEELRSLFSSARSYQRIQIFPDTIW
NVSYDGTSNTCSGSFYRNMRWLTRKDGNYPIQDAQYTNNQGKNILFMWGINNPPTDDTQRNLYTRTDTTT
SVATEEINRIFKPLIGPRPLVNGLMGRINYYWSVLKPGQTLRIKSDGNLVAPWYGYILSGESHGRILRTD
LKRGSCTVQCQTEKGGLNTTLPFQNVSKYAFGNCSKYIGIKSLKLAVGLRNVPSRSSRGLFGAIAGFIEG
GWSGLVAGWYGFQHSNDQGVGMAADRESTQKAVDKITSKVNNIVDKMNKQYEIIDHEFSEVETRLNMINN
KIDDQIQDIWAYNAELLVLLENQKTLDEHDANVNNLYNKVKRALGSNAVEDGKGCFELYHKCDDQCMETI
RNGTYNRRKYQEESKLERQKIEGVKLESEGTYKILTIYSTVASSLVIAMGFAAFLFWAMSNGSCRCNICI

>QOJ98826.1 hemagglutinin [Influenza A virus]
METVSLITILLAATVSNADKICIGYQSTNSTETVDTLTENNVPVTHAKELLHTEHNGMLCATSLGQPLIL
DTCTIEGLIYGNPSCDPLPEEREWSYIVERPSAVNGLCYPGNVENLEELRSLFSSARSYQRIQIFPDTIW
NVSYDGTSNTCSGSFYRNMRWLTRKDGNYPIQDAQYTNNQGKNILFMWGINNPPTDDTQRNLYTRTDTTT
SVATEEINRIFKPLIGPRPLVNGLMGRINYYWSVLKPGQTLRIKSDGNLVAPWYGYILSGESHGRILRTD
LKRGSCTVQCQTEKGGLNTTLPFQNVSKYAFGNCSKYIGIKSLKLAVGLRNVPSRSSRGLFGAIAGFIEG
GWSGLVAGWYGFQHSNDQGVGMAADRESTQKAVDKITSKVNNIVDKMNKQYEIIDHEFSEVETRLNMINN
KIDDQIQDIWAYNAELLVLLENQKTLDEHDANVNNLYNKVKRALGSNAVEDGKGCFELYHKCDDQCMETI
RNGTYNRRKYQEESKLERQKIEGVKLESEGTYKILTIYSTVASSLVIAMGFAAFLFWAMSNGSCRCNICI

>QOJ98825.1 hemagglutinin [Influenza A virus]
METVSLITILLVATVSNADKICIGYQSTNSTETVDTLTENNVPVTHAKELLHTEHNGMLCATSLGQPLIL
DTCTIEGLIYGNPSCDLSLEGREWSYIVERPSAVHGLCYPGNVEDLEELRSLFSSARSYQRIQIFPDTIW
NVSYDGTSTACSGSFYKSMRWLTRKNGEYPTQDAQYTNNQGKNILFMWGINHPPTDDTQRGLYTRTDTTT
SVATEEINRIFKPLIGPRPLVNGLMGRINYYWSVLKPGQTLRIKSDGNLIAPWYGHILSGESHGRILKTD
LKRGSCTVQCQTEKGGLNTTLPFQNVSKYAFGNCSKYIGIKSLKLAVGLRNVPSRSSRGLFGAIAGFIEG
GWSGLVAGWYGFQHSNDQGVGMAADRDSTQKAIDKITSKVNNIVDKMNKQYEIIDHEFSEVETRLNMINN

KIDDQIQDIWAYNAELLVLLENQKTLDEHDANVNNLYNKVKRALGSNAVEDGKGCFELYHKCDDQCMETI
RNGTYNRRKYQEESKLERQRIEGVKLESEGTYKILTIYSTVASSLVIAMGFAAFLFWAMSNGSCRCNICI

>QOJ98824.1 hemagglutinin [Influenza A virus]
METVSLITILLVATVSNADKICIGYQSTNSTETVDTLTENNVPVTHAKELLHTEHNGMLCATSLGQPLIL
DTCTIEGLIYGNPSCDLSLEGREWSYIVERPSAVHGLCYPGNVEDLEELRSLFSSARSYQRIQIFPDTIW
NVSYDGTSTACSGSFYKSMRWLTRKNGEYPTQDAQYTNNQGKNILFMWGINHPPTDDTQRGLYTRTDTTT
SVATEEINRIFKPLIGPRPLVNGLMGRINYYWSVLKPGQTLRIKSDGNLIAPWYGHILSGESHGRILKTD
LKRGSCTVQCQTEKGGLNTTLPFQNVSKYAFGNCSKYIGIKSLKLAVGLRNVPSRSSRGLFGAIAGFIEG
GWSGLVAGWYGFQHSNDQGVGMAADRDSTQKAIDKITSKVNNIVDKMNKQYEIIDHEFSEVETRLNMINN
KIDDQIQDIWAYNAELLVLLENQKTLDEHDANVNNLYNKVKRALGSNAVEDGKGCFELYHKCDDQCMETI
RNGTYNRRKYQEESKLERQRIEGVKLESEGTYKILTIYSTVASSLVIAMGFAAFLFWAMSNGSCRCNICI

>QOJ98823.1 hemagglutinin [Influenza A virus]
METVSLITILLVATVSNADKICIGYQSTNSTETVDTLTENNVPVTHAKELLHTEHNGMLCATSLGQPLIL
DTCTIEGLIYGNPSCDLSLEGREWSYIVERPSAVHGLCYPGNVEDLEELRSLFSSARSYQRIQIFPDTIW
NVSYDGTSTACSGSFYKSMRWLTRKNGEYPTQDAQYTNNQGKNILFMWGINHPPTDDTQRGLYTRTDTTT
SVATEEINRIFKPLIGPRPLVNGLMGRINYYWSVLKPGQTLRIKSDGNLIAPWYGHILSGESHGRILKTD
LKRGSCTVQCQTEKGGLNTTLPFQNVSKYAFGNCSKYIGIKSLKLAVGLRNVPSRSSRGLFGAIAGFIEG
GWSGLVAGWYGFQHSNDQGVGMAADRDSTQKAIDKITSKVNNIVDKMNKQYEIIDHEFSEVETRLNMINN
KIDDQIQDIWAYNAELLVLLENQKTLDEHDANVNNLYNKVKRALGSNAVEDGKGCFELYHKCDDQCMETI
RNGTYNRRKYQEESKLERQRIEGVKLESEGTYKILTIYSTVASSLVIAMGFAAFLFWAMSNGSCRCNICI

>QOJ98821.1 hemagglutinin [Influenza A virus]
METVSLITILLAATVSNADKICIGYQSTNSTETVDTLTENNVPVTHAKELLHTEHNGMLCATSLGQPLIL
DTCTIEGLIYGNPSCDPVLEEREWSYIVERPSAVNGLCYPGNVENLEELRSLFSSARFYQRIQIFPDTIW
NVSYDGTSNTCSGSFYRNMRWLTRKDGNYPIQDAQYTNNQGKNILFMWGINHPPTDDTQRNLYTRTDTTT
SVATEEINRIFKPLIGPRPLVNGLMGRINYYWSVLKPGQTLRIKSDGNLIAPWYGYILSGESHGRILRTD
LKRGSCTVQCQTEKGGLNTTLPFQNVSKYAFGNCSKYIGIKSLKLAVGLRNVPSRSSRGLFGAIAGFIEG
GWSGLVAGWYGFQHSNDQGVGMAADRESTQKAIDKITSKVNNIVDKMNKQYEIIDHEFSEVETRLNMINN
KIDDQIQDIWAYNAELLVLLENQKTLDEHDANVNNLYNKVKRALGSNAVEDGKGCFELYHKCDDQCMETI
RNGTYNRRKYQEESKLERQKIEGVKLESEGTYKILTIYSTVASSLVIAMGFAAFLFWAMSNGSCRCNICI

>QOJ98820.1 hemagglutinin [Influenza A virus]

METVSLITILLAATVSNADKICIGYQSTNSTETVDTLTENNVPVTHAKELLHTEHNGMLCATSLGQPLIL
DTCTIEGLIYGNPSCDPVLEEREWSYIVERPSAVNGLCYPGNVENLEELRSLFSSARFYQRIQIFPDTIW
NVSYDGTSNTCSGSFYRNMRWLTRKDGNYPIQDAQYTNNQGKNILFMWGINHPPTDDTQRNLYTRTDTTT
SVATEEINRIFKPLIGPRPLVNGLMGRINYYWSVLKPGQTLRIKSDGNLIAPWYGYILSGESHGRILRTD
LKRGSCTVQCQTEKGGLNTTLPFQNVSKYAFGNCSKYIGIKSLKLAVGLRNVPSRSSRGLFGAIAGFIEG
GWSGLVAGWYGFQHSNDQGVGMAADRESTQKAIDKITSKVNNIVDKMNKQYEIIDHEFSEVETRLNMINN
KIDDQIQDIWAYNAELLVLLENQKTLDEHDANVNNLYNKVKRALGSNAVEDGKGCFELYHKCDDQCMETI
RNGTYNRRKYQEESKLERQKIEGVKLESEGTYKILTIYSTVASSLVIAMGFAAFLFWAMSNGSCRCNICI

>QOJ98819.1 hemagglutinin [Influenza A virus]
METVSLITILLAATVSNADKICIGYQSTNSTETVDTLTENNVPVTHAKELLHTEHNGMLCATSLGQPLIL
DTCTIEGLIYGNPSCDPLLEEREWSYIVERPSAVNGLCYPGNVENLEELRSLFSSARSYQRIQIFPDTIW
NVSYDGTSNTCSSSFYRNMRWLTRKNGNYPIQDAQYTNNQGKNILFMWGINHPPTDDTQRNLYTRTDTTT
SVATEEINRIFKPLIGPRPLVNGLMGRINYYWSVLKPGQTLRIKSDGNLIAPWYGYILSGESHGRILKTD
LKRGSCTVQCQTEKGGLNTTLPFQNVSKYAFGNCSKYIGIKSLKLAVGLRNVPSRSSRGLFGAIAGFIEG
GWSGLVAGWYGFQHSNDQGVGMAADRESTQKAIDKITSKVNNIVDKMNKQYEIIDHEFSEVETRLNMINN
KIDDQIQDIWAYNAELLVLLENQKTLDEHDANVNNLYNKVKRALGSNAVEDGKGCFELYHKCDDQCMETI
RNGTYNRRKYQEESKLERQKIEGVKLESEGTYKILTIYSTVASSLVIAMGFAAFLFWAMSNGSCRCNICI

>QOJ98818.1 hemagglutinin [Influenza A virus]
METVSLITILLAATVSNADKICIGYQSTNSTETVDTLTENNVPVTHAKELLHTEHNGMLCATSLGQPLIL
DTCTIEGLIYGNPSCDPLLEEREWSYIVERPSAVNGLCYPGNVENLEELRSLFSSARSYQRIQIFPDTIW
NVSYDGTSNTCSSSFYRNMRWLTRKNGNYPIQDAQYTNNQGKNILFMWGINHPPTDDTQRNLYTRTDTTT
SVATEEINRIFKPLIGPRPLVNGLMGRINYYWSVLKPGQTLRIKSDGNLIAPWYGYILSGESHGRILKTD
LKRGSCTVQCQTEKGGLNTTLPFQNVSKYAFGNCSKYIGIKSLKLAVGLRNVPSRSSRGLFGAIAGFIEG
GWSGLVAGWYGFQHSNDQGVGMAADRESTQKAIDKITSKVNNIVDKMNKQYEIIDHEFSEVETRLNMINN
KIDDQIQDIWAYNAELLVLLENQKTLDEHDANVNNLYNKVKRALGSNAVEDGKGCFELYHKCDDQCMETI
RNGTYNRRKYQEESKLERQKIEGVKLESEGTYKILTIYSTVASSLVIAMGFAAFLFWAMSNGSCRCNICI

>QOJ98817.1 hemagglutinin [Influenza A virus]
METVSLITILLAATVSNADKICIGYQSTNSTETVDTLTENNVPVTHAKELLHTEHNGMLCATSLGQPLIL
DTCTIEGLIYGNPSCDPLLEEREWSYIVERPSAVNGLCYPGNVENLEELRSLFSSARSYQRIQIFPDTIW
NVSYDGTSNTCSSSFYRNMRWLTRKNGNYPIQDAQYTNNQGKNILFMWGINHPPTDDTQRNLYTRTDTTT
SVATEEINRIFKPLIGPRPLVNGLMGRINYYWSVLKPGQTLRIKSDGNLIAPWYGYILSGESHGRILKTD

LKRGSCTVQCQTEKGGLNTTLPFQNVSKYAFGNCSKYIGIKSLKLAVGLRNVPSRSSRGLFGAIAGFIEG
GWSGLVAGWYGFQHSNDQGVGMAADRESTQKAIDKITSKVNNIVDKMNKQYEIIDHEFSEVETRLNMINN
KIDDQIQDIWAYNAELLVLLENQKTLDEHDANVNNLYNKVKRALGSNAVEDGKGCFELYHKCDDQCMETI
RNGTYNRRKYQEESKLERQKIEGVKLESEGTYKILTIYSTVASSLVIAMGFAAFLFWAMSNGSCRCNICI

>QOJ98816.1 hemagglutinin [Influenza A virus]
METVSLITILLAATVSNADKICIGYQSTNSTETVDTLTENNVPVTHAKELLHTEHNGMLCATSLGQPLIL
DTCTIEGLIYGNPSCDPLLEEREWSYIVERPSAVNGLCYPGNVENLEELRSLFSSARSYQRIQIFPDTIW
NVSYDGTSNTCSSSFYRNMRWLTRKNGNYPIQDAQYTNNQGKNILFMWGINHPPTDDTQRNLYTRTDTTT
SVATEEINRIFKPLIGPRPLVNGLMGRINYYWSVLKPGQTLRIKSDGNLIAPWYGYILSGESHGRILKTD
LKRGSCTVQCQTEKGGLNTTLPFQNVSKYAFGNCSKYIGIKSLKLAVGLRNVPSRSSRGLFGAIAGFIEG
GWSGLVAGWYGFQHSNDQGVGMAADRESTQKAIDKITSKVNNIVDKMNKQYEIIDHEFSEVETRLNMINN
KIDDQIQDIWAYNAELLVLLENQKTLDEHDANVNNLYNKVKRALGSNAVEDGKGCFELYHKCDDQCMETI
RNGTYNRRKYQEESKLERQKIEGVKLESEGTYKILTIYSTVASSLVIAMGFAAFLFWAMSNGSCRCNICI

>QOJ98815.1 hemagglutinin [Influenza A virus]
METVSLITILLAATVSNADKICIGYQSTNSTETVDTLTENNVPVTHAKELLHTEHNGMLCATSLGQPLIL
DTCTIEGLIYGNPSCDPLLEEREWSYIVERPSAVNGLCYPGNVENLEELRSLFSSARSYQRIQIFPDTIW
NVSYDGTSNTCSSSFYRNMRWLTRKNGNYPIQDAQYTNNQGKNILFMWGINHPPTDNTQRNLYTRTDTTT
SVATEEINRIFKPLIGPRPLVNGLMGRINYYWSVLKPGQTLRIKSDGNLIAPWYGYILSGESHGRILKTD
LKRGSCTVQCQTEKGGLNTTLPFQNVSKYAFGNCSKYIGIKSLKLAVGLRNVPSRSSRGLFGAIAGFIEG
GWSGLVAGWYGFQHSNDQGVGMAADRESTQKAIDKITSKVNNIVDKMNKQYEIIDHEFSEVETRLNMINN
KIDDQIQDIWAYNAELLVLLENQKTLDEHDANVNNLYNKVKRALGSNAVEDGKGCFELYHKCDDQCMETI
RNGTYNRRKYQEESKLERQKIEGVKLESEGTYKILTIYSTVASSLVIAMGFAAFLFWAMSNGSCRCNICI

>QOJ98814.1 hemagglutinin [Influenza A virus]
METVSLITILLAATVSNADKICIGYQSTNSTETVDTLTENNVPVTHAKELLHTEHNGMLCATSLGQPLIL
DTCTIEGLIYGNPSCDPLLEEREWSYIVERPSAVNGLCYPGNVENLEELRSLFSSARSYQRIQIFPDTIW
NVSYDGTSNTCSSSFYRNMRWLTRKNGNYPIQDAQYTNNQGKNILFMWGINHPPTDDTQRNLYTRTDTTT
SVATEEINRIFKPLIGPRPLVNGLMGRINYYWSVLKPGQTLRIKSDGNLIAPWYGYILSGESHGRILKTD
LKRGSCTVQCQTEKGGLNTTLPFQNVSKYAFGNCSKYIGIKSLKLAVGLRNVPSRSSRGLFGAIAGFIEG
GWSGLVAGWYGFQHSNDQGVGMAADRESTQKAIDKITSKVNNIVDKMNKQYEIIDHEFSEVETRLDMINN
KIDDQIQDIWAYNAELLVLLENQKTLDEHDANVNNLYNKVKRALGSNAVEDGKGCFELYHKCDDQCMETI
RNGTYNRRKYQEESKLERQKIEGVKLESEGTYKILTIYSTVASSLVIAMGFAAFLFWAMSNGSCRCNICI

>QOJ98813.1 hemagglutinin [Influenza A virus]
METVSLITILLAATVSNADKICIGYQSTNSTETVDTLTENNVPVTHAKELLHTEHNGMLCATSLGQPLIL
DTCTIEGLIYGNPSCDPLLEEREWSYIVERPSAVNGLCYPGNVENLEELRSLFSSARSYQRIQIFPDTIW
NVSYDGTSNTCSSSFYRNMRWLTRKNGNYPIQDAQYTNNQGKNILFMWGINHPPTDDTQRNLYTRTDTTT
SVATEEINRIFKPLIGPRPLVNGLMGRINYYWSVLKPGQTLRIKSDGNLIAPWYGYILSGESHGRILKTD
LKRGSCTVQCQTEKGGLNTTLPFQNVSKYAFGNCSKYIGIKSLKLAVGLRNVPSRSSRGLFGAIAGFIEG
GWSGLVAGWYGFQHSNDQGVGMAADRESTQKAIDKITSKVNNIVDKMNKQYEIIDHEFSEVETRLNMINN
KIDDQIQDIWAYNAELLVLLENQKTLDEHDANVNNLYNKVKRALGSNAVEDGKGCFELYHKCDDQCMETI
RNGTYNRRKYQEESKLERQKIEGVKLESEGTYKILTIYSTVASSLVIAMGFAAFLFWAMSNGSCRCNICI

>QOJ98812.1 hemagglutinin [Influenza A virus]
METVSLITILLAATVSNADKICIGYQSTNSTETVDTLTENNVPVTHAKELLHTEHNGMLCATSLGQPLIL
DTCTIEGLIYGNPSCDPLLEEREWSYIVERPSAVNGLCYPGNVENLEELRSLFSSARSYQRIQIFPDTIW
NVSYDGTSNTCSSSFYRNMRWLTRKNGNYPIQDAQYTNNQGKNILFMWGINHPPTDNTQRNLYTRTDTTT
SVATEEINRIFKPLIGPRPLVNGLMGRINYYWSVLKPGQTLRIKSDGNLIAPWYGYILSGESHGRILKTD
LKRGSCTVQCQTEKGGLNTTLPFQNVSKYAFGNCSKYIGIKSLKLAVGLRNVPSRSSRGLFGAIAGFIEG
GWSGLVAGWYGFQHSNDQGVGMAADRESTQKAIDKITSKVNNIVDKMNKQYEIIDHEFSEVETRLNMINN
KIDDQIQDIWAYNAELLVLLENQKTLDEHDANVNNLYNKVKRALGSNAVEDGKGCFELYHKCDDQCMETI
RNGTYNRRKYQEESKLERQKIEGVKLESEGTYKILTIYSTVASSLVIAMGFAAFLFWAMSNGSCRCNICI

>QOJ98811.1 hemagglutinin [Influenza A virus]
METVSLITILLAATVSNADKICIGYQSTNSTETVDTLTENNVPVTHAKELLHTEHNGMLCATSLGQPLIL
DTCTIEGLIYGNPSCDPLLEEREWSYIVERPSAVNGLCYPGNVENLEELRSLFSSARSYQRIQIFPDTIW
NVSYDGTSNTCSSSFYRNMRWLTRKNGNYPIQDAQYTNNQGKNILFMWGINHPPTDDTQRNLYTRTDTTT
SVATEEINRIFKPLIGPRPLVNGLMGRINYYWSVLKPGQTLRIKSDGNLIAPWYGYILSGESHGRILKTD
LKRGSCTVQCQTEKGGLNTTLPFQNVSKYAFGNCSKYIGIKSLKLAVGLRNVPSRSSRGLFGAIAGFIEG
GWSGLVAGWYGFQHSNDQGVGMAADRESTQKAIDKITSKVNNIVDKMNKQYEIIDHEFSEVETRLNMINN
KIDDQIQDIWAYNAELLVLLENQKTLDEHDANVNNLYNKVKRALGSNAVEDGKGCFELYHKCDDQCMETI
RNGTYNRRKYQEESKLERQKIEGVKLESEGTYKILTIYSTVASSLVIAMGFAAFLFWAMSNGSCRCNICI

>QOJ98810.1 hemagglutinin [Influenza A virus]
METVSLITILLAATVSNADKICIGYQSTNSTETVDTLTENNVPVTHAKELLHTEHNGMLCATSLGQPLIL
DTCTIEGLIYGNPSCDPLLEEREWSYIVERPSAVNGLCYPGNVENLEELRSLFSSARSYQRIQIFPDTIW

NVSYDGTSNTCSSSFYRNMRWLTRKNGNYPIQDAQYTNNQGKNILFMWGINHPPTDDTQRNLYTRTDTTT
SVATEEINRIFKPLIGPRPLVNGLMGRINYYWSVLKPGQTLRIKSDGNLIAPWYGYILSGESHGRILKTD
LKRGSCTVQCQTEKGGLNTTLPFQNVSKYAFGNCSKYIGIKSLKLAVGLRNVPSRSSRGLFGAIAGFIEG
GWSGLVAGWYGFQHSNDQGVGMAADRESTQKAIDKITSKVNNIVDKMNKQYEIIDHEFSEVETRLNMINN
KIDDQIQDIWAYNAELLVLLENQKTLDEHDANVNNLYNKVKRALGSNAVEDGKGCFELYHKCDDQCMETI
RNGTYNRRKYQEESKLERQKIEGVKLESEGTYKILTIYSTVASSLVIAMGFAAFLFWAMSNGSCRCNICI

>QOJ98809.1 hemagglutinin [Influenza A virus]
METVSLITILLAATVSNADKICIGYQSTNSTETVDTLTENNVPVTHAKELLHTEHNGMLCATSLGQPLIL
DTCTIEGLIYGNPSCDPLLEEREWSYIVERPSAVNGLCYPGNVENLEELRSLFSSARSYQRIQIFPDTIW
NVSYDGTSNTCSGSFYRNMRWLTRKNGNYPIQDAQYTNNQGKNILFMWGINHPPTDDTQRNLYTRTDTTT
SVATEEINRIFKPLIGPRPLVNGLMGRINYYWSVLKPGQTLRIKSDGNLIAPWYGYILSGESHGRILRTD
LKRGSCTVQCQTEKGGLNTTLPFQNVSKYAFGNCSKYIGIKSLKLAVGLRNVPSRSSRGLFGAIAGFIEG
GWSGLVAGWYGFQHSNDQGVGMAADRESTQKAIDKITSKVNNIVDKMNKQYEIIDHEFSEVETRLNMINN
KIDDQIQDIWAYNAELLVLLENQKTLDEHDANVNNLYNKVKRALGSNAVEDGKGCFELYHKCDDQCMETI
RNGTYNRRKYQEESKLERQKIEGVKLESEGTYKILTIYSTVASSLLIAMGFAAFLFWAMSNGSCRCNICI

>QOJ98808.1 hemagglutinin [Influenza A virus]
METVSLITILLAATVSNADKICIGYQSTNSTETVDTLTENNVPVTHAKELLHTEHNGMLCATSLGQPLIL
DTCTIEGLIYGNPSCDPLLEEREWSYIVERPSAVNGLCYPGNVENLEELRSLFSSARSYQRIQIFPDTIW
NVSYDGTSNTCSGSFYRNMRWLTRKNGNYPIQDAQYTNNQGKNILFMWGINHPPTDDTQRNLYTRTDTTT
SVATEEINRIFKPLIGPRPLVNGLMGRINYYWSVLKPGQTLRIKSDGNLIAPWYGYILSGESHGRILRTD
LKRGSCTVQCQTEKGGLNTTLPFQNVSKYAFGNCSKYIGIKSLKLAVGLRNVPSRSSRGLFGAIAGFIEG
GWSGLVAGWYGFQHSNDQGVGMAADRESTQKAIDKITSKVNNIVDKMNKQYEIIDHEFSEVETRLNMINN
KIDDQIQDIWAYNAELLVLLENQKTLDEHDANVNNLYNKVKRALGSNAVEDGKGCFELYHKCDDQCMETI
RNGTYNRRKYQEESKLERQKIEGVKLESEGTYKILTIYSTVASSLLIAMGFAAFLFWAMSNGSCRCNICI

>QOJ98807.1 hemagglutinin [Influenza A virus]
METVSLITILLAATVSNADKICIGYQSTNSTETVDTLTENNVPVTHAKELLHTEHNGMLCATSLGQPLIL
DTCTIEGLIYGNPSCDPLLEEREWSYIVERPSAVNGLCYPGNVENLEELRSLFSSARSYQRIQIFPDTIW
NVSYDGTSNTCSGSFYRNMRWLTRKNGNYPIQDAQYTNNQGKNILFMWGINHPPTDDTQRNLYTRTDTTT
SVATEEINRIFKPLIGPRPLVNGLMGRINYYWSVLKPGQTLRIKSDGNLIAPWYGYILSGESHGRILRTD
LKRGSCTVQCQTEKGGLNTTLPFQNVSKYAFGNCSKYIGIKSLKLAVGLRNVPSRSSRGLFGAIAGFIEG
GWSGLVAGWYGFQHSNDQGVGMAADRESTQKAIDKITSKVNNIVDKMNKQYEIIDHEFSEVETRLNMINN

KIDDQIQDIWAYNAELLVLLENQKTLDEHDANVNNLYNKVKRALGSNAVEDGKGCFELYHKCDDQCMETI
RNGTYNRRKYQEESKLERQKIEGVKLESEGTYKILTIYSTVASSLLIAMGFAAFLFWAMSNGSCRCNICI

>QOJ98806.1 hemagglutinin [Influenza A virus]
METVSLITILLAATVSNADKICIGYQSTNSTETVDTLTENNVPVTHAKELLHTEHNGMLCATSLGQPLIL
DTCTIEGLIYGNPSCDPLLEEREWSYIVERPSAVNGLCYPGNVENLEELRSLFSSARSYQRIQIFPDTIW
NVSYDGTSNTCSGSFYRNMRWLTRKNGNYPIQDAQYTNNQGKNILFMWGINHPPTDDTQRNLYTRTDTTT
SVATEEINRIFKPLIGPRPLVNGLMGRINYYWSVLKPGQTLRIKSDGNLIAPWYGYILSGESHGRILRTD
LKRGSCTVQCQTEKGGLNTTLPFQNVSKYAFGNCSKYIGIKSLKLAVGLRNVPSRSSRGLFGAIAGFIEG
GWSGLVAGWYGFQHSNDQGVGMAADRESTQKAIDKITSKVNNIVDKMNKQYEIIDHEFSEVETRLNMINN
KIDDQIQDIWAYNAELLVLLENQKTLDEHDANVNNLYNKVKRALGSNAVEDGKGCFELYHKCDDQCMETI
RNGTYNRRKYQEESKLERQKIEGVKLESEGTYKILTIYSTVASSLLIAMGFAAFLFWAMSNGSCRCNICI

>QOJ98805.1 hemagglutinin [Influenza A virus]
METVSLITILLAATVSNADKICIGYQSTNSTETVDTLTENNVPVTHAKELLHTEHNGMLCATSLGQPLIL
DTCTIEGLIYGNPSCDPLLEEREWSYIVERPSAVNGLCYPGNVENLEELRSLFSSARSYQRIQIFPDTIW
NVSYDGTSNTCSGSFYRNMRWLTRKNGNYPIQDAQYTNNQGKNILFMWGINHPPTDDTQRNLYTRTDTTT
SVATEEINRIFKPLIGPRPLVNGLMGRINYYWSVLKPGQTLRIKSDGNLIAPWYGYILSGESHGRILRTD
LKRGSCTVQCQTEKGGLNTTLPFQNVSKYAFGNCSKYIGIKSLKLAVGLRNVPSRSSRGLFGAIAGFIEG
GWSGLVAGWYGFQHSNDQGVGMAADRESTQKAIDKITSKVNNIVDKMNKQYGIIDHEFSEVETRLNMINN
KIDDQIQDIWAYNAELLVLLENQKTLDEHDANVNNLYNKVKRALGSNAVEDGKGCFELYHKCDDQCMETI
RNGTYNRRKYQEESKLERQKIEGVKLESEGTYKILTIYSTVASSLVIAMGFAAFLFWAMSNGSCRCNICI

>QOJ98797.1 hemagglutinin [Influenza A virus]
METVSLITILLVATVSNADKICIGYQSTNSTETVDTLTENNVPVTHAKELLHTEHNGMLCATSLGQPLIL
DTCTIEGLIYGNPSCDLSLEGREWSYIVERPSAVHGLCYPGNVEDLEELRSLFSSARSYQRIQIFPDTIW
NVSYDGTSTACSGSFYRSMRWLTRKNGDYPIQDAQYTNNQGKNILFMWGINHPPTDETQRGLYTRTDTTT
SVATEEINRIFKPLIGPRPLVNGLMGRINYYWSVLKPGQTLRIKSDGNLIAPWYGHILSGESHGRILKTD
LKKGSCTVQCQTEKGGLNTTLPFQNVSKYAFGNCSKYIGIKSLKLAVGLRNVPSRSSRGLFGAIAGFIEG
GWPGLVAGWYGFQHSNDQGVGMAADRDSTQKAIDKITSKVNNIVDKMNKQYEIIDHEFSEVETRLNMINN
KIDDQIQDIWAYNAELLVLLENQKTLDEHDANVNNLYNKVKRALGSNAVEDGKGCFELYHKCDDQCMETI
RNGTYNRRKYQEESKLERQKIEGVKLESEGTYKILTIYSTVASSLVIAMGFAAFLFWAMSNGSCRCNICI

>QOJ98795.1 hemagglutinin [Influenza A virus]

METASLITILLAVTVSNADKICIGYQSTNSTETVDTLTENNVPVTHAKELLHTEHNGMLCATSLGHPLIL
DTCTIEGLIYGNPSCDLLLGGREWSYIVERPSAVNGLCYPGNVENLEELRSLFSSARSYQRIQIFPDTIW
NVSYSGTSKACSDSFYRSMRWLTQKDNAYPVQDAQYTNNQEKNILFMWGINHPPTDTVQTNLYTRTDTTT
SVATEEINRTFKPLIGPRPLVNGLMGRINYYWSVLKPGQTLRIKSNGNLIAPWYGHILSGESHGRILKTD
LKRGSCTVQCQTEKGGLNTTLPFQNVSKYAFGNCSKYIGIKSLKLAVGLRNVPSRSSRGLFGAIAGFIEG
GWSGLVAGWYGFQHSNDQGVGMAADRDSTQKAIDKITSKVNNIVDKMNKQYEIIDHEFSEVETRLNMINN
KIDDQIQDIWAYNAELLVLLENQKTLDEHDANVNNLYNKVKRALGSNAVEDGKGCFELYHKCDDQCMETI
RNGTYNRRKYQEESKLERQKIEGVKLESEGTYKILTIYSTVASSLVIAMGFAAFLFWAMSNGSCRCNICI

>QOJ98794.1 hemagglutinin [Influenza A virus]
METVSLITILIVATVSNADKICIGYQSTNSTETVDTLTENNVPVTHAKELLHTEHNGMLCATSLGQPLIL
DTCTIEGLIYGNPSCDLSLEGREWSYIVERPSAINGLCYPGNVENLEELRSLFSSARSYQRIQIFPDTIW
NVSYDGTSTACSNSFYRSMRWLTRKDGNYPTQDAQYTNNQGKNILFMWGINHPPTDDTQRNLYTRTDTTT
SVATEEINRIFKPLIGPRPLVNGLMGRIDYYWSVLKPGQTLRIKSDGNLIAPWYGHILSGESHGRILKTD
LKRGSCTVQCQTEKGGLNTTLPFQNVSKYAFGNCSKYIGIKSLKLAVGLRNVPSRSSRGLFGAIAGFIEG
GWSGLVAGWYGFQHSNDQGVGMAADRDSTQKAIDKITSKVNNIVDKMNKQYEIIDHEFSEVETRLNMINN
KIDDQIQDIWAYNAELLVLLENQKTLDEHDANVNNLYNKVKRALGSNAVEDGKGCFELYHKCNDQCMETI
RNGTYNRRKYQEESKLERQKIEGVKLESEGTYKILTIYSTVASSLVIAMGFAAFLFWAMSNGSCRCNICI

>QOJ98793.1 hemagglutinin, partial [Influenza A virus]
ETVSLITILLVATVSNADKICIGYQSTNSTETVDTLTENNVPVTHAKELLHTEHNGMLCATSLGQPLILD
TCTIEGLIYGNPSCDLSLEGREWSYIVERPSAVHGLCYPGNVEDLEELRSLFSSARSYQRIQIFPDTIWN
VSYDGTSTACSGSFYRSMRWLTRKNGEYPIQDAQYTNNQGKNILFMWGINHPPTDDTQRGLYTRTDTTTS
VATEEINRIFKPLIGPRPLVNGLMGRINYYWSVLKPGQTLRIKSDGNLIAPWYGHILSGESHGRILKTDL
KRGSCTVQCQTEKGGLNTTLPFQNVSKYAFGNCSKYIGIKSLKLAVGLRNVPSRSSRGLFGAIAGFIEGG
WSGLVAGWYGFQHSNDQGVGMAADRDSTQKAIDKITSKVNNIVDKMNKQYEIIDHEFSEVETRLNMINNK
IDDQIQDIWAYNAELLVLLENQKTLDEHDANVNNLYSKVKRALGSNAVEDGKGCFELYHKCDDQCMETIR
NGTYNRRKYQEESKLERQRIEGVKLESEGTYKILTIYSTVASSLVIAMGFAAFLFWAMSNGSC

>QOJ98792.1 hemagglutinin, partial [Influenza A virus]
ETVSLITILLVATISNADKICIGYQSTNSTETVDTLTENNVPVTHAKELLHTEHNGMLCATSLGQPLILD
TCTIEGLIYGNPSCDLSLEGREWSYIVERPSAVHGLCYPGNVEDLEELRSLFSSARSYQRIQIFPDTIWN
VSYDGTSTACSGSFYRSMRWLTRKNGEYPIQDAQYTNNQGKNILFMWGINHPPTDDTQRGLYTRTDTTTS
VATEEINRIFKPLIGPRPLVNGLMGRINYYWSVLKPGQTLRIKSDGNLIAPWYGHILSGESHGRILKTDL

KRGSCTVQCQTEKGGLNTTLPFQNVSKYAFGNCSKYIGIKSLKLAVGLRNVPSRSSRGLFGAIAGFIEGG
WSGLVAGWYGFQHSNDQGVGMAADRDSTQKAIDKITSKVNNIVDKMNKQYEIIDHEFSEVETRLNMINNK
IDDQIQDIWAYNAELLVLLENQKTLDEHDANVNNLYSKVKRALGSNAVEDGKGCFELYHKCDDQCMETIR
NGTYNRRKYQEESKLERQRIEGVKLESEGTYKILTIYSTVASSLVIAMGFAAFLFWAMSNGSC

>QOJ98791.1 hemagglutinin, partial [Influenza A virus]
ETVSLITILLVATVSNADKICIGYQSTNSTETVDTLTENNVPVTHAKELLHTEHNGMLCATSLGQPLILD
TCTIEGLIYGNPSCDLSLEGREWSYIVERPSAVHGLCYPGNVEDLEELRSLFSSARSYQRIQIFPDTIWN
VSYDGTSTACSGSFYRSMRWLTRKNGEYPIQDAQYTNNQGKNILFMWGINHPPTDDTQRGLYTRTDTTTS
VATEEINRIFKPLIGPRPLVNGLMGRINYYWSVLKPGQTLRIKSDGNLIAPWYGHILSGESHGRILKTDL
KRGSCTVQCQTEKGGLNTTLPFQNVSKYAFGNCSKYIGIKSLKLAVGLRNVPSRSSRGLFGAIAGFIEGG
WSGLVAGWYGFQHSNDQGVGMAADRDSTQKAIDKITSKVNNIVDKMNKQYEIIDHEFSEVETRLNMINNK
IDDQIQDIWAYNAELLVLLENQKTLDEHDANVNNLYNKVKRALGSNAVEDGKGCFELYHKCDDQCMETIR
NGTYNRRKYQEESKLERQRIEGVKLESEGTYKILTIYSTVASSLVIAMGFAAFLFWAMSNGSC

>QOJ98790.1 hemagglutinin [Influenza A virus]
METVSLITILLVATVSSADKICIGYQSTNSTETVDTLTENNVPVTHAKELLHTEHNGMLCATSLGQPLIL
DTCTIEGLIYGNPSCDLSLEGREWSYIVERPSAVNGLCYPGNVENLEELRSLFSSARSYQRIQIFPDTIW
NVSYDGTSTACSGSFYGSMRWLTQKNGNYPTQDAQYTNNQGKNILFMWGINHPPTDDTQRNLYTRTDTTT
SVATEEMNRIFKPLIGPRPLVNGLMGRINYYWSVLKPGQTLRIKSDGNLIAPWYGHILSGESHGRILKTD
LKRGSCTVQCQTEKGGLNTTLPFQNVSKYAFGNCSKYIGIKSLKLAVGLRNVPSRSSRGLFGAIAGFIEG
GWSGLVAGWYGFQHSNDQGVGMAADRDSTQKAIDKITSKVNNIVDKMNKQYEIIDHEFSEVETRLNMINN
KIDDQILDIWAYNAELLVLLENQKTLDEHDANVNNLYNKVKRALGSNAVEDGKGCFELYHKCNDQCMETI
RNGTYNRRKYQEESKLERQKIEGVKLESEGTYKILTIYSTVASSLVIAMGFAAFLFWAMSNGSCRCNICI

>QOJ98789.1 hemagglutinin, partial [Influenza A virus]
METVSLITILVVATVSNADKICIGYQSTNSTETVDTLTENNVPVTHAKELLHTEHNGMLCATSLGHPLIL
DTCTIEGLIYGNPSCDPLLGGREWSYIVERPSAVNGLCYPGNVENLEELRSLFSSSRSYQRIQIFPDTIW
NVSYSGTSKACSDSFYRSMRWLTQKNNAYPTQDAQYTNNQRKNILFMWGINHPPTDTAQTNLYTRTDTTT
SVATEEMNRIFKPLIGPRPLVNGLMGRINYYWSVLKPGQTLRIKSDGNLIAPWYGHILSGESHGRILKTD
LKRGSCTVQCQTEKGGLNTTLPFQNVSKYAFGNCSKYIGVKSLKLAVGLRNVPSRSSRGLFGAIAGFIEG
GWSGLVAGWYGFQHSNDQGVGMAADRDSTQKAIDKITSKVNNIVDKMNKQYEIIDHEFSEVETRLNMINN
KVDDQIQDIWAYNAELLVLLENQKTLDEHDANVNNLYNKVKRALGSNAVEDGKGCFELYHKCDDQCMETI
RNGTYNRRKYQEESKLERQKIEGVKLESEETYKILTIYSTVASSLVIAMGFAAFLFWAMSNGSC

>QOJ98788.1 hemagglutinin, partial [Influenza A virus]

METVSLMTILIVATVSNADKICIGYQSTNSTETVDTLTENNVPVTHAKELLHTEHNGMLCATSLGHPLIL
DTCTIEGLIYGNPSCDPLLGGREWSYIVERPSAVNGLCYPGNVENLEELRSLFSSSRSYQRIQIFPDTIW
NVSYSGTSKVCSDSFYRSMRWLTQKNNAYPTQDAQYTNNQGKNILFVWGINHPPTDTVQTNLYTRTDTTT
SVATEEMNRIFKPLIGPRPLVNGLMGRINYYWSVLKPGQTLRIKSDGNLIAPWYGHILSGESHGRILKTD
LKRGSCTVQCQTEKGGLNTTLPFQNVSKYAFGNCSKYIGVKSLKLAVGLRNVPSRSSRGLFGAIAGFIEG
GWSGLVAGWYGFQHSNDQGVGMAADRDSTQKAIDKITSKVNNIVDKMNKQYEIIDHEFSEVETRLNMINN
KVDDQIQDIWAYNAELLVLLENQKTLDEHDANVNNLYNKVKRALGSNAVEDGKGCFELYHKCDDHCMETI
RNGTYNRRKYQEESKLERQKIEGVKLESEETYKILTIYSTVASSLVIAMGFAAFLFWAMSNGSC

>QOJ98787.1 hemagglutinin, partial [Influenza A virus]

ETVSLITILLVATASNADKICIGYQSTNSTETVDTLTENNVPVTHAKELLHTEHNGMLCATSLGQPLILD
TCTVEGLIYGNPSCDLSLEGREWSYIVERPSAVNGLCYPGNVENLEELRSLFSSARSYQRIQIFPDTIWN
VSYDGTSTACSDSFYRSMRWLTRKDGNYPTQDAQYTNNQGKNILFMWGINHPPTDDTQRNLYTRTDTTTS
VATEEINRIFKPLIGPRPLVNGLMGRIDYYWSVLKPGQTLRIKSDGNLIAPWYGHILSGESHGRILKTDL
KKGSCTVQCQTEKGGLNTTLPFQNVSKYAFGNCSKYIGIKSLKLAVGLRNVPSRSSRGLFGAIAGFIEGG
WSGLVAGWYGFQHSNDQGVGMAADRDSTQKAIDKITSKVNNIVDKMNKQYEIIDHEFSEVETRLNMINNK
IDDQIQDIWAYNAELLVLLENQKTLDEHDANVNNLYNKVKRALGSNAVEDGKGCFELYHKCNDQCMETIR
NGTYNRRKYQEESKLERQKIEGVKLESEGTYKILTIYSTVASSLVIAMGFAAFLFWAMSNGSCR

>QOJ98778.1 hemagglutinin, partial [Influenza A virus]

ETVSLITILLVATVSNADKICIGYQSTNSTETVDTLTENNVPVTHAKELLHTEHNGMLCATSLGQPLILD
TCTIEGLIYGNPSCDLSLEGREWSYIVERPSAVHGLCYPGNVEDLEELRSLFSSARSYQRIQIFPDTIWN
VSYDGTSTACSGSFYKSMRWLTRKNGEYPTQDAQYTNNQGKNILFMWGINHPPTDDTQRGLYTRTDTTTS
VATEEINRIFKPLIGPRPLVNGLMGRINYYWSVLKPGQTLRIKSDGNLIAPWYGHILSGESHGRILKTDL
KRGSCTVQCQTEKGGLNTTLPFQNISKYAFGNCSKYIGIKSLKLAVGLRNVPSRSSRGLFGAIAGFIEGG
WSGLVAGWYGFQHSNDQGVGMAADRDSTQKAIDKITSKVNNIVDKMNKQYEIIDHEFSEVETRLNMINNK
IDDQIQDIWAYNAELLVLLENQKTLDEHDANVNNLYNKVKRALGSNAVEDGKGCFELYHKCDDQCMETIR
NGTYNRRKYQEESKLERQRIEGVKLESEGTYKILTIYSTVASSLVIAMGFAAFLFWAMSNGSC

>QOJ98773.1 hemagglutinin [Influenza A virus]

METASLITILLVATVSNADKICIGYQSTNSTETVDTLTENNVPVTHAKELLHTEHNGMLCATSLGQPLIL
DTCTIEGLIYGNPSCDLSLEGREWSYIVERPSAVHGLCYPGKVEDLEELRSLFSSARSYQRIQIFPDTIW

NVSYDGTSTACSGSFYRSMRWLTRKDGNYPIQDAQYTNNQGKNILFMWGINHPPTDETQRGLYTRTDTTT
SVATEEINRIFKPLIGPRPLVNGLMGRINYYWSVLKPGQTLRIKSDGNLIAPWYGHILSGESHGRILKTD
LKKGSCTVQCQTEKGGLNTTLPFQNVSKYAFGNCSKYIGIKSLKLAVGLRNVPSRSSRGLFGAIAGFIEG
GWPGLVAGWYGFQHSNDQGVGMAADRDSTQKAIDKITSKVNNIVDKMNKQYEIIDHEFSEVETRLNMINN
KIDDQIQDIWAYNAELLVLLENQKTLDEHDANVNNLYNKVKRALGSNAVEDGKGCFELYHKCDDQCMETI
RNGTYNRRKYQEESKLERQKIEGVKLESEGTYKILTIYSTVASSLVIAMGFAAFLFWAMSNGSCRCNICI

>QOJ98765.1 hemagglutinin, partial [Influenza A virus]
METVSLMTILIVATVSNADKICIGYQSTNSTETVDTLTENNVPVTHAKELLHTEHNGMLCATSLGHPLIL
DTCTIEGLIYGNPSCDPLLGGREWSYIVERPSAVNGLCYPGNVENLEELRSLFSSSRSYQRIQIFPDTIW
NVSYSGTSKVCSDSFYRSMRWLTQKNNAYPTQDAQYTNNQGKNILFVWGINHPPTDTVQTNLYTRTDTTT
SVATEEMNRIFKPLIGPRPLVNGLMGRINYYWSVLKPGQTLRIKSDGNLIAPWYGHILSGESHGRILKTD
LKRGSCTVQCQTEKGGLNTTLPFQNVSKYAFGNCSKYIGVKSLKLAVGLRNVPSRSSRGLFGAIAGFIEG
GWSGLVAGWYGFQHSNDQGVGMAADRDSTQKAIDKITSKVNNIVDKMNKQYEIIDHEFSEVETRLNMINN
KVDDQIQDIWAYNAELLVLLENQKTLDEHDANVNNLYNKVKRALGSNAVEDGKGCFELYHKCDDHCMETI
RNGTYNRRKYQEESKLERQKIEGVKLESEETYKILTIYSTVASSLVIAMGFAAFLFWAMSNGSC

>QOJ98764.1 hemagglutinin, partial [Influenza A virus]
METVSLITILIVATVSNADKICIGYQSTNSTETVDTLTENNVPVTHAKELLHTEHNGMLCATSLGHPLIL
DTCTIEGLIYGNPSCDPLLGGREWSYIVERPSAVNGLCYPGNVENLEELRSLFSSSRSYQRIQIFPDTIW
NVSYSGTSKACSDSFYRSMRWLTQKNNAYPTQDAQYTNNQGKNILFVWGINHPPTDTAQTNLYTRTDTTT
SVATEEMNRIFKPLIGPRPLVNGLMGRINYYWSVLKPGQTLRIKSDGNLIAPWYGHILSGESHGRILKTD
LKRGSCTVQCQTEKGGLNTTLPFQNVSKYAFGNCSKYIGVKSLKLAVGLRNVPSRSSRGLFGAIAGFIEG
GWSGLVAGWYGFQHSNDQGVGMAADRDSTQKAIDKITSKVNNIVDKMNKQYEIIDHEFSEVETRLNMINN
KVDDQIQDIWAYNAELLVLLENQKTLDEHDANVNNLYNKVKRALGSNAVEDGKGCFELYHKCDDHCMETI
RNGTYNRRKYQEESKLERQKIEGVKLESEETYKILTIYSTVASSLVIAMGFAAFLFWAMSNGSC

>QOJ98762.1 hemagglutinin [Influenza A virus]
METASLITILLVATVSNADKICIGYQSTNSTETVDTLTENNVPVTHAKELLHTEHNGMLCATSLGQPLIL
DTCTIEGLIYGNPSCDLSLEGREWSYIVERPSAVHGLCYPGKVEDLEELRSLFSSARSYHRIQIFPDTIW
NVSYDGTSTACSGSFYRSMRWLTRKDGNYPIQDAQYTNNQGKNILFMWGINHPPTDETQRGLYTRTDTTT
SVATEEINRIFKPLIGPRPLVNGLMGRINYYWSVLKPGQTLRIKSDGNLIAPWYGHILSGESHGRILKTD
LKKGSCTVQCQTEKGGLNTTLPFQNVSKYAFGNCSKYIGIKSLKLAVGLRNVPSRSSRGLFGAIAGFIEG
GWPGLVAGWYGFQHSNDQGVGMAADRDSTQKAIDKITSKVNNIVDKMNKQYEIIDHEFSEVETRLNMINN

KIDDQIQDIWAYNAELLVLLENQKTLDEHDANVNNLYNKVKRALGSNAVEDGKGCFELYHKCDDQCMETI
RNGTYNRRKYQEESKLERQKIEGVKLESEGTYKILTIYSTVASSLVIAMGFAAFLFWAMSNGSCRCNICI

>QOJ98758.1 hemagglutinin, partial [Influenza A virus]
METVSLITILIVATVSNADKICIGYQSTNSTETVDTLTENNVPVTHAKELLHTEHNGMLCATSLGHPLIL
DTCTIEGLIYGNPSCDPLLGGREWSYIVERPSAVNGLCYPGNVENLEELRSLFSSSRSYQRIQIFPDTIW
NVSYSGTSKACSDSFYRSMRWLTQKNNAYPTQDAQYTNNQGKNILFVWGINHPPTDTAQTNLYTRTDTTT
SVATEEMNRIFKPLIGPRPLVNGLMGRINYYWSVLKPGQTLRIKSDGNLIAPWYGHILSGESHGRILKTD
LKRGSCTVQCQTEKGGLNTTLPFQNVSKYAFGNCSKYIGVKSLKLAVGLRNVPSRSSRGLFGAIAGFIEG
GWSGLVAGWYGFQHSNDQGVGMAADRDSTQRAIDKITSKVNNIVDKMNKQYEIIDHEFSEVETRLNMINN
KVDDQIQDIWAYNAELLVLLENQKTLDEHDANVNNLYNKVKRALGSNAVEDGKGCFELYHKCDDHCMETI
RNGTYNRKKYQEESKLERQKIEGVKLESEETYKILTIYSTVASSLVIAMGFAAFLFWAMSNGSC

>QOJ98757.1 hemagglutinin, partial [Influenza A virus]
ETVSLITILLIATVSNADKICIGYQSTNSTETVDTLTENNVPVTHAKELLHTEHNGMLCATSLGQPLILD
TCTIEGLIYGNPSCDLSLEGREWSYIVERPSAVNGLCYPGNVENLEELRSLFSSARSYQRIQIFPDTIWN
VSYDGTSTACSNSFYRSMRWLTRKDGNYPTQDAQYTNNQGKNILFMWGINHPPTDETQRNLYTRTDTTTS
VATEEINRIFKPLIGPRPLVNGLMGRIDYYWSVLKPGQTLRIKSDGNLIAPWYGHILSGESHGRILKTDL
KKGSCTVQCQTEKGGLNTTLPFQNVSKYAFGNCSKYIGIKSLKLAVGLRNVPSKSSRGLFGAIAGFIEGG
WSGLVAGWYGFQHSNDQGVGMAADRDSTQKAIDKITSKVNNIVDKMNKQYEIIDHEFSEVETRLNMINNK
IDDQIQDIWAYNAELLVLLENQKTLDEHDANVNNLYNKVKRALGSNAVEDGKGCFELYHKCNDQCMETIR
NGTYNRKKYQEESKLERQRIEGVKLESEGTYKILTIYSTVASSLVIAMGFAAFLFWAMSNGSC

>QOJ98756.1 hemagglutinin, partial [Influenza A virus]
METVSLMTILIVATVSNADKICIGYQSTNSTETVDTLTENNVPVTHAKELLHTEHNGMLCATSLGNPLIL
DTCTIEGLIYGNPSCDPLLGGREWSYIVERPSAVNGLCYPGNVENLEELRSLFSSSRSYQRIQIFPDTIW
NVSYSGTSKACSDSFYRSMRWLTQKNNAYPTQDAQYTNNQGKNILFVWGINHPPTDTAQTNLYTRTDTTT
SVATEEMNRIFKPLIGPRPLVNGLMGRINYYWSVLKPGQTLRIKSDGNLIAPWYGHILSGESHGRILKTD
LKRGSCTVQCQTEKGGLNTTLPFQNVSKYAFGNCSKYIGVKSLKLAVGLRNVPSRSSRGLFGAIAGFIEG
GWSGLVAGWYGFQHSNDQGVGMAADRDSTQKAIDKITSKVNNIVDKMNKQYEIIDHEFSEVETRLNMINN
KVDDQIQDIWAYNAELLVLLENQKTLDEHDANVNNLYNKVKRALGSNAVEDGKGCFELYHKCDDHCMETI
RNGTYNRRKYQEESKLERQKIEGVKLESEETYKILTIYSTVASSLVIAMGFAAFLFWAMSNGSC

>QOJ98754.1 hemagglutinin, partial [Influenza A virus]

METVSLITILIVATVSNADKICIGYQSTNSTETVDTLTENNVPVTHAKELLHTEHNGMLCATSLGHPLIL
DTCTIEGLIYGNPSCDPLLGGREWSYIVERPSAVNGLCYPGNVENLEELRSLFSSSRSYQRIQIFPDTIW
NVSYSGTSKACSDSFYRSMRWLTQKNNAYPTQDAQYTNNQGKNILFVWGINHPPTDTAQTNLYTRTDTTT
SVATEEMNRIFKPLIGPRPLVNGLMGRINYYWSVLKPGQTLRIKSDGNLIAPWYGHILSGESHGRILKTD
LKRGSCTVQCQTEKGGLNTTLPFQNVSKYAFGNCSKYIGVKSLKLAVGLRNVPSRSSRGLFGAIAGFIEG
GWSGLVAGWYGFQHSNDQGVGMAADRDSTQKAIDKITSKVNNIVDKMNKQYEIIDHEFSEVETRLNMINN
KVDDQIQDIWAYNAELLVLLENQKTLDEHDANVNNLYNKVKRALGSNAVEDGKGCFELYHKCDDHCMETI
RNGTYNRRKYQEESKLERQKIEGVKLESEETYKILTIYSTVASSLVIAMGFAAFLFWAMSNGSC

>QOJ98753.1 hemagglutinin, partial [Influenza A virus]
METVSLITILIVATVSNADKICIGYQSTNSTETVDTLTENNVPVTHAKELLHTEHNGMLCATSLGHPLIL
DTCTIEGLIYGNPSCDPLLGGREWSYIVERPSAVNGLCYPGNVENLEELRSLFSSSRSYQRIQIFPDTIW
NVSYSGTSKACSDSFYRSMRWLTQKNNAYPTQDAQYTNNQGKNILFVWGINHPPTDTAQTNLYTRTDTTT
SVATEEMNRIFKPLIGPRPLVNGLMGRINYYWSVLKPGQTLRIKSDGNLIAPWYGHILSGESHGRILKTD
LKRGSCTVQCQTEKGGLNTTLPFQNVSKYAFGNCSKYIGVKSLKLAVGLRNVPSRSSRGLFGAIAGFIEG
GWSGLVAGWYGFQHSNDQGVGMAADRDSTQKAIDKITSKVNNIVDKMNKQYEIIDHEFSEVETRLNMINN
KVDDQIQDIWAYNAELLVLLENQKTLDEHDANVNNLYNKVKRALGSNAVEDGKGCFELYHKCDDHCMETI
RNGTYNRRKYQEESKLERQKIEGVKLESEETYKILTIYSTVASSLVIAMGFAAFLFWAMSNGSC

>QOJ98752.1 hemagglutinin, partial [Influenza A virus]
ETVSLITILLVATVSNADKICIGYQSTNSTETVDTLTENNVPVTHAKELLHTEHNGMLCATSLGQPLILD
TCTIEGLIYGNPSCDLSLEGREWSYIVERPSAVHGLCYPGNVEDLEELRSLFSSARSYQRIQIFPDTIWN
VSYDGTSTACSGSFYKSMRWLTRKNGEYPTQDAQYTNNQGKNILFMWGINHPPTDDTQRGLYTRTDTTTS
VATEEINRIFKPLIGPRPLVNGLMGRINYYWSVLKPGQTLRIKSDGNLIAPWYGHILSGESHGRILKTDL
KRGSCTVQCQTEKGGLNTTLPFQNVSKYAFGNCSKYIGIKSLKLAVGLRNVPSRSSRGLFGAIAGFIEGG
WSGLVAGWYGFQHSNDQGVGMAADRDSTQKAIDKITSKVNNIVDKMNKQYEIIDHEFSEVETRLNMINNK
IDDQIQDIWAYNAELLVLLENQKTLDEHDANVNNLYNKVKRALGSNAVEDGKGCFELYHKCDDQCMETIR
NGTYNRRKYQEESKLERQRIEGVKLESEGTYKILTIYSTVASSLVIAMGFAAFLFWAMSNGSC

>QOJ98751.1 hemagglutinin, partial [Influenza A virus]
ETVSLITILLVATVSNADKICIGYQSTNSTETVDTLTENNVPVTHAKELLHTEHNGMLCATSLGQPLILD
TCTIEGLIYGNPSCDLSLEGREWSYIVERPSAVHGLCYPGNVEDLEELRSLFSSARSYQRIQIFPDTIWN
VSYDGTSTACSGSFYKSMRWLTRKNGEYPTQDAQYTNNQGKNILFMWGINHPPTDDTQRGLYTRTDTTTS
VATEEINRIFKPLIGPRPLVNGLMGRINYYWAVLKPGQTLRIKSDGNLIAPWYGHILSGESHGRILKTDL

KRGSCTVQCQTEKGGLNTTLPFQNVSKYAFGNCSKYIGIKSLKLAVGLRNVPSRSSRGLFGAIAGFIEGG
WSGLVAGWYGFQHSNDQGVGMAADRDSTQKAIDKITSKVNNIVDKMNKQYEIIDHEFSEVETRLNMINNK
IDDQIQDIWAYNAELLVLLENQKTLDEHDANVNNLYNKVKRALGSNAVEDGKGCFELYHKCDDQCMETIR
NGTYNRRKYQEESKLERQRIEGVKLESEGTYKILTIYSTVASSLVIAMGFAAFLFWAMSNGSC

>QOJ98750.1 hemagglutinin, partial [Influenza A virus]
ETVSLITILLVATVSNADKICIGYQSTNSTETVDTLTENNVPVTHAKELLHTEHNGMLCATSLGQPLILD
TCTIEGLIYGNPSCDLSLEGREWSYIVERPSAVHGLCYPGNVEDLEELRSLFSSARSYQRIQIFPDTIWN
VSYDGTSTACSGSFYRSMRWLTRKNGEYPIQDAQYTNNQGKNILFMWGINHPPTDDTQRGLYTRTDTTTS
VATEEINRIFKPLIGPRPLVNGLMGRINYYWSVLKPGQTLRIKSDGNLIAPWYGHILSGESHGRILKTDL
KRGSCTVQCQTEKGGLNTTLPFQNVSKYAFGNCSKYIGIKSLKLAVGLRNVPSRSSRGLFGAIAGFIEGG
WPGLVAGWYGFQHSNDQGVGMAADRDSTQKAIDKITSKVNNIVDKMNKQYEIIDHEFSEVETRLNMINNK
IDDQIQDIWAYNAELLVLLENQKTLDEHDANVNNLYSKVKRALGSNAVEDGKGCFELYHKCDDQCMETIR
NGTYNRRKYQEESKLERQRIEGVKLESEGTYKILTIYSTVASSLVIAMGFAAFLFWAMSNGSC

>QOJ98749.1 hemagglutinin, partial [Influenza A virus]
KTVSLITILLVAKVSNADKICIGYQSTNSTETVDTLTENNVPVTHAKELLHTEHNGMLCATSLGQPLILD
TCTVEGLIYGNPSCDLSLEGREWSYIVERPSAVNGLCYPGNVENLEELRSLFSSARSYQRIQIFPDTIWN
VSYDGTSTACSGSFYRSMRWLTRKDGNYPTQDAQYTNNQGKNILFMWGINHPPTDDTQRNLYTRTDTTTS
VATEEINRIFKPLIGPRPLVNGLMGRIDYYWSVLKPGQTLRIKSDGNLIAPWYGHILSGESHGRILKTDL
KRGSCTVQCQTEKGGLNTTLPFQNVSKYAFGNCSKYIGIKSLKLAVGLRNVPSRSSRGLFGAIAGFIEGG
WSGLVAGWYGFQHSNDQGVGMAADRDSTQKAIDKITSKVNNIVDKMNKQYEIIDHEFSEVETRLNMINNK
IDDQIQDIWAYNAELLVLLENQKTLDEHDANVNNLYNKVKRALGSNAVEDGKGCFELYHKCNDQCMETIR
NGTYNRRKYQEESKLERQKIEGVKLESEGTYKILTIYSTVASSLVIAMGFAAFLFWAMSNGSCR

>QOJ98748.1 hemagglutinin [Influenza A virus]
METISLMTILLVATVSNADKICIGYQSTNSTETVDTLTENNVPVTHAKELLHTEHNGMLCATSLGNPLIL
DTCTIEGLIYGNPSCDPLLGGREWSYIVERPSAVNGLCYPGIVENLEELRSLFSSARSYQRIQIFPDTIW
NVTYSGTSKACSDSFYRNMRWLTQKNNAYPIQDAQYTNNQEKNILFMWGINHPPTETSQTNLYTRTDTTT
SVATEEINRIFKPLIGPRPLVNGLMGRINYYWSVLKPGQTLRIKSDGNLIAPWYGHILSGESHGRILKTD
LKRGSCTVQCQTEKGGLNTTLPFQNVSKYAFGNCSKYIGIKSLKLAVGLRNVPSRSSRGLFGAIAGFIEG
GWSGLVAGWYGFQHSNDQGVGMAADRDSTQKAIDKITSKVNNIVDKMNKQYEIIDHEFSEVETRLNMINN
KIDDQIQDIWAYNAELLVLLENQKTLDEHDSNVNNLYNKVKRALGSNAVEDGKGCFELYHKCDNQCMETI
RNGTYNRRKYQEESKLERQRIEGVKLESEGTYKILTIYSTVASSLVIAMGFAAFLFWAMSNGSCRCNICI

>QOJ98747.1 hemagglutinin, partial [Influenza A virus]

METVSLITILVVATVSNADKICIGYQSTNSTETVDTLTENNVPVTHAKELLHTEHNGMLCATSLGHPLIL
DTCTIEGLIYGNPSCDPLLGGREWSYIVERPSAVNGLCYPGNVENLEELRSLFSSSRSYQRIQIFPDTIW
NVSYSGTSKACSDSFYRSMRWLTQKNNAYPTQDAQYTNNQGKNILFMWGINHPPTDTAQTNLYTRTDTTT
SVATEEMNRIFKPLIGPRPLVNGLMGRINYYWSVLKPGQTLRIKSDGNLIAPWYGHILSGESHGRILKTD
LKRGSCTVQCQTEKGGLNTTLPFQNVSKYAFGNCSKYIGVKSLKLAVGLRNVPSRSSRGLFGAIAGFIEG
GWSGLVAGWYGFQHSNDQGVGMAADRDSTQKAIDKITSKVNNIVDKMNKQYEIIDHEFSEVETRLNMINN
KVDDQIQDIWAYNAELLVLLENQKTLDEHDANVNNLYNKVKRALGSNAVEDGKGCFELYHKCDDHCMETI
RNGTYNRRKYQEESKLERQKIEGVKLESEETYKILTIYSTVASSLVIAMGFAAFLFWAMSNGSC

>QOJ98745.1 hemagglutinin, partial [Influenza A virus]

METVSLITILIVATVSNADKICIGYQSTNSTETVDTLTENNVPVTHAKELLHTEHNGMLCATSLGHPLIL
DTCTIEGLIYGNPSCDPLLGGREWSYIVERPSAVNGLCYPGNVENLEELRSLFSSSRSYQRIQIFPDTIW
NVSYSGTSKACSDSFYRSMRWLTQKNNAYPTQDAQYTNNQGKNILFMWGINHPPTDTAQTNLYTRTDTTT
SVATEEMNRIFKPLIGPRPLVNGLMGRINYYWSVLKPGQTLRIKSDGNLIAPWYGHILSGESHGRILKTD
LKRGSCTVQCQTEKGGLNTTLPFQNVSKYAFGNCSKYIGVKSLKLAVGLRNVPSRSSRGLFGAIAGFIEG
GWSGLVAGWYGFQHSNDQGVGMAADRDSTQKAIDKITSKVNNIVDKMNKQYEIIDHEFSEVETRLNMINN
KVDDQIQDIWAYNAELLVLLENQKTLDEHDANVNNLYNKVKRALGSNAVEDGKGCFELYHKCDDHCMETI
RNGTYNRRKYQEESKLERQKIEGVKLESEETYKILTIYSTVASSLVIAMGFAAFLFWAMSNGSC

>QOJ98744.1 hemagglutinin, partial [Influenza A virus]

METVSLITILIVATVSNADKICIGYQSTNSTETVDTLTENNVPVTHAKELLHTEHNGMLCATSLGHPLIL
DTCTIEGLIYGNPSCDPLLGGREWSYIVERPSAVNGLCYPGNVENLEELRSLFSSSRSYQRIQIFPDTIW
NVSYSGTSKACSDSFYRSMRWLTQKNNAYPTQDAQYTNNQGKNILFVWGINHPPTDTAQTNLYTRTDTTT
SVATEEMNRIFKPLIGPRPLVNGLMGRINYYWSVLKPGQTLRIKSDGNLIAPWYGHILSGESHGRILKTD
LKRGSCTVQCQTEKGGLNTTLPFQNVSKYAFGNCSKYIGVKSLKLAVGLRNVPSRSSRGLFGAIAGFIEG
GWSGLVAGWYGFQHSNDQGVGMAADRDSTQKAIDKITSKVNNIVDKMNKQYEIIDHEFSEVETRLNMINN
KVDDQIQDIWAYNAELLVLLENQKTLDEHDANVNNLYNKVKRALGSNAVEDGKGCFELYHKCDDHCMETI
RNGTYNRRKYQEESKLERQKIEGVKLESEETYKILTIYSTVASSLVIAMGFAAFLFWAMSNGSC

>QOJ98742.1 hemagglutinin, partial [Influenza A virus]

ETVSLITILLVATVSNADKICIGYQSTNSTETVDTLTENNVPVTHAKELLHTEHNGMLCATSLGQPLILD
TCTIEGLIYGNPSCDLSLEGREWSYIVERPSAVHGLCYPGNVEDLEELRSLFSSARSYQRIQIFPDTIWN

VSYDGTSTACSGSFYKSMRWLTRKNGEYPTQDAQYTNNQGKNILFMWGINHPPTDDTQRGLYTRTDTTTS
VATEEINRIFKPLIGPRPLVNGLMGRINYYWSVLKPGQTLRIKSDGNLIAPWYGHILSGESHGRILKTDL
KRGSCTVQCQTEKGGLNTTLPFQNVSKYAFGNCSKYIGIKSLKLAVGLRNVPSRSSRGLFGAIAGFIEGG
WSGLVAGWYGFQHSNDQGVGMAADRDSTQKAIDKITSKVNNIVDKMNKQYEIIDHEFSEVETRLNMINNK
IDDQIQDIWAYNAELLVLLENQKTLDEHDANVNNLYNKVKRALGSNAVEDGKGCFELYHKCDDQCMETIR
NGTYNRRKYQEESKLERQRIEGVKLESEGTYKILTIYSTVASSLVIAMGFAAFLFWAMSNGSC

>QOJ98741.1 hemagglutinin, partial [Influenza A virus]
METVSLITILVVATVSNADKICIGYQSTNSTETVDTLTENNVPVTHAKELLHTEHNGMLCATSLGHPLIL
DTCTIEGLIYGNPSCDPLLGGREWSYIVERPSAVNGLCYPGNVENLEELRSLFSSSRSYQRIQIFPDTIW
NVSYSGTSKACSGSFYRSMRWLTQKNNAYPTQDAQYTNNQGKNILFMWGINHPPTDTAQTNLYTRTDTTT
SVATEEMNRIFKPLIGPRPLVNGLMGRINYYWSVLKPGQTLRIKSDGNLIAPWYGHILSGESHGRILKTD
LKRGSCTVQCQTEKGGLNTTLPFQNVSKYAFGNCSKYIGVKSLKLAVGLRNVPSRSRRGLFGAIAGFIEG
GWSGLVAGWYGFQHSNDQGVGMAADRDSTQKAIDKITSKVNNIVDKMNKQYEIIDHEFSEVETRLNMINN
KVDDQIQDIWAYNAELLVLLENQKTLDEHDANVNNLYNKVRRALGSNAVEDGKGCFELYHKCDDHCMETI
RNGTYNRRKYQEESKLERQKIEGVKLESEETYKILTIYSTVASSLVIAMGFAAFLFWAMSNGSC

>QOJ98737.1 hemagglutinin, partial [Influenza A virus]
METVSLITILIVATVSNADKICIGYQSTNSTETVDTLTENNVPVTHAKELLHTEHNGMLCATSLGHPLIL
DTCTIEGLIYGNPSCDPLLGGREWSYIVERPSAVNGLCYPGNVENLEELRSLFSSSRSYQRIQIFPDTIW
NVSYSGTSKACSDSFYRSMRWLTQKNNAYPTQDAQYTNNQGKNILFVWGINHPPTDTAQTNLYTRTDTTT
SVATEEMNRIFKPLIGPRPLVNGLMGRINYYWSVLKPGQTLRIKSDGNLIAPWYGHILSGESHGRILKTD
LKRGSCTVQCQTEKGGLNTTLPFQNVSKYAFGNCSKYIGVKSLKLAVGLRNVPSRSSRGLFGAIAGFIEG
GWSGLVAGWYGFQHSNDQGVGMAADRDSTQKAIDKITSKVNNIVDKMNKQYEIIDHEFSEVETRLNMINN
KVDDQIQDIWAYNAELLVLLENQKTLDEHDANVNNLYNKVKRALGSNAVEDGKGCFELYHKCDDHCMETI
RNGTYNRRKYQEESKLERQKIEGVKLESEETYKILTIYSTVASSLVIAMGFAAFLFWAMSNGSC

>QOJ98736.1 hemagglutinin, partial [Influenza A virus]
ETVSLITILLVATVSNADKICIGYQSTNSTETVDTLTENNVPVTHAKELLHTEHNGMLCATSLGQPLILD
TCTIEGLIYGNPSCDLSLEGREWSYIVERPSAVHGLCYPGNVEDLEELRSLFSSARSYQRIQIFPDTIWN
VSYDGTSTACSGSFYKSMRWLTRKNGEYPTQDAQYTNNQGKNILFMWGINHPPTDDTQRGLYTRTDTTTS
VATEEINRIFKPLIGPRPLVNGLMGRINYYWSVLKPGQTLRIKSDGNLIAPWYGHILSGESHGRILKTDL
KRGSCTVQCQTEKGGLNTTLPFQNVSKYAFGNCSKYIGIKSLKLAVGLRNVPSRSSRGLFGAIAGFIEGG
WSGLVAGWYGFQHSNDQGVGMAADRDSTQKAIDKITSKVNNIVDKMNKQYEIIDHEFSEVETRLNMINNK

IDDQIQDIWAYNAELLVLLENQKTLDEHDANVNNLYNKVKRALGSNAVEDGKGCFELYHKCDDQCMETIR
NGTYNRRKYQEESKLERQRIEGVKLESEGTYKILTIYSTVASSLVIAMGFAAFLFWAMSNGSC

>QOJ98735.1 hemagglutinin, partial [Influenza A virus]
METVSLITILIVATVSNADKICIGYQSTNSTETVDTLTENNVPVTHAKELLHTEHNGMLCATSLGHPLIL
DTCTIEGLIYGNPSCDPLLGGREWSYIVERPSAVNGLCYPGNVENLEELRSLFSSSRSYQRIQIFPDTIW
NVSYSGTSKACSDSFYRSMRWLTQKNNAYPTQDAQYTNNQGKNILFMWGINHPPTDTAQTNLYTRTDTTT
SVATEEMNRIFKPLIGPRPLVNGLMGRINYYWSVLKPGQTLRIKSDGNLIAPWYGHILSGESHGRILKTD
LKRGSCTVQCQTEKGGLNTTLPFQNVSKYAFGNCSKYIGVKSLKLAVGLRNVPSRSSRGLFGAIAGFIEG
GWSGLVAGWYGFQHSNDQGVGMAADRDSTQKAIDKITSKVNNIVDKMNKQYEIIDHEFSEVETRLNMINN
KVDDQIQDIWAYNAELLVLLENQKTLDEHDANVNNLYNKVKRALGSNAVEDGKGCFELYHKCDDHCMETI
RNGTYNRRKYQEESKLERQKIEGVKLESEETYKILTIYSTVASSLVIAMGFAAFLFWAMSNGSC

>QOJ98733.1 hemagglutinin [Influenza A virus]
METVSLITILLVATVSNADKICIGYQSTNSTETVDTLTENNVPVTHAKELLHTEHNGMLCATSLGQPLIL
DTCTIEGLIYGNPSCDLSLEGREWSYIVERPSAVNGLCYPGNVENLEELRSLFSSARSYQRIQIFPDTIW
NVSYDGTSTACSGSFYRSMRWLTRKNGEYPIQDAQYTNNQGKNILFMWGINHPPTDTTQRDLYTRTDTTT
SVATEEINRVFKPLIGPRPLVNGLMGRIDYYWSVLKPGQTLRIKSDGNLIAPWFGHILSGESHGRILKTD
LKRGSCTVQCQTEKGGLNTTLPFQNVSKYAFGNCSKYIGIKSLKLAVGLRNVPSRSSRGLFGAIAGFIEG
GWSGLVAGWYGFQHSNDQGVGMAADRDSTQKAIDKITSKVNNIVDKMNKQYEIIDHEFSEVEARLNMINN
KIDDQIQDIWAYNAELLVLLENQKTLDEHDANVNNLYNKVKRALGSNAVEDGKGCFELYHKCDDQCMETI
RNGTYNRRKYQEESKLERQKIEGVKLEPEGTYKILTIYSTVASSLVIAMGFAAFLFWAMSNGSCRCNICI

>QOJ98732.1 hemagglutinin [Influenza A virus]
METASLITILLVATVSNADKICIGYQSTNSTETVDTLTENNVPVTHAKELLHTEHNGMLCATSLGQPLIL
DTCTIEGLIYGNPSCDLSLEGREWSYIVERPSAVHGLCYPGKVEDLEELRSLFSSARSYQRIQIFPDTIW
NVSYDGTSTACSGSFYRSMRWLTRKDGNYPIQDAQYTNNQGKNILFMWGINHPPTDETQRGLYTRTDTTT
SVATEEINRIFKPLIGPRPLVNGLMGRINYYWSVLKPGQTLRIKSDGNLIAPWYGHILSGESHGRILKTD
LKKGSCTVQCQTEKGGLNTTLPFQNVSKYAFGNCSKYIGIKSLKLAVGLRNVPSRSSRGLFGAIAGFIEG
GWPGLVAGWYGFQHSNDQGVGMAADRDSTQKAIDKITSKVNNIVDKMNKQYEIIDHEFSEVETRLNMINN
KIDDQIQDIWAYNAELLVLLENQKTLDEHDANVNNLYNKVKRALGSNAVEDGKGCFELYHKCDDQCMETI
RNGTYNRRKYQEESKLERQKIEGVKLESEGTYKILTIYSTVASSLVIAMGFAAFLFWAMSNGSCRCNICI

>QOJ98731.1 hemagglutinin, partial [Influenza A virus]

METVSLITILIVATVSNADKICIGYQSTNSTETVDTLTENNVPVTHAKELLHTEHNGMLCATSLGHPLIL
DTCTIEGLIYGNPSCDPLLGGREWSYIVERPSAVNGLCYPGNVENLEELRSLFSSSRSYQRIQIFPDTIW
NVSYSGTSKACSDSFYRSMRWLTQKNNAYPTQDAQYTNNQGKNILFVWGINHPPTDTAQTNLYTRTDTTT
SVATEEMNRIFKPLIGPRPLVNGLMGRINYYWSVLKPGQTLRIKSDGNLIAPWYGHILSGESHGRILKTD
LKRGSCTVQCQTEKGGLNTTLPFQNVSKYAFGNCSKYIGVKSLKLAVGLRNVPSRSSRGLFGAIAGFIEG
GWSGLVAGWYGFQHSNDQGVGMAADRDSTQKAIDKITSKVNNIVDKMNKQYEIIDHEFSEVETRLNMINN
KVDDQIQDIWAYNAELLVLLENQKTLDEHDANVNNLYNKVKRALGSNAVEDGKGCFELYHKCDDHCMETI
RNGTYNRRKYQEESKLERQKIEGVKLESEETYKILTIYSTVASSLVIAMGFAAFLFWAMSNGSC

>QOJ98729.1 hemagglutinin, partial [Influenza A virus]
METVSLMTILIVATVSNADKICIGYQSTNSTETVDTLTENNVPVTHAKELLHTEHNGMLCATSLGNPLIL
DTCTIEGLIYGNPSCDPLLGGREWSYIVERPSAVNGLCYPGNVENLEELRSLFSSSRSYQRIQIFPDTIW
NVSYSGTSKACSDSFYRSMRWLTQKNNAYPTQDAQYTNNQGKNILFVWGINHPPTDTAQTNLYTRTDTTT
SVATEEMNRIFKPLIGPRPLVNGLMGRINYYWSVLKPGQTLRIKSDGNLIAPWYGHILSGESHGRILKTD
LKRGSCTVQCQTEKGGLNTTLPFQNVSKYAFGNCSKYIGVKSLKLAVGLRNVPSRSSRGLFGAIAGFIEG
GWSGLVAGWYGFQHSNDQGVGMAADRDSTQKAIDKITSKVNNIVDKMNKQYEIIDHEFSEVETRLNMINN
KVDDQIQDIWAYNAELLVLLENQKTLDEHDANVNNLYNKVKRALGSNAVEDGKGCFELYHKCDDHCMETI
RNGTYNRRKYQEESKLERQKIEGVKLESEETYKILTIYSTVASSLVIAMGFAAFLFWAMSNGSC

>QOJ98727.1 hemagglutinin [Influenza A virus]
METVSLITILLVATVSNADKICIGYQSTNSTETVDTLTENNVPVTHAKELLHTEHNGMLCATSLGQPLIL
DTCTIEGLIYGNPSCDLSLEGREWSYIVERPSAVHGLCYPGNVEDLEELRSLFSSARSYQRIQIFPDTIW
NVSYDGTSTACSGSFYRSMRWLTRKNGDYPIQDAQYTNNQGKNILFMWGINHPPTDETQRGLYTRTDTTT
SVATEEINRIFKPLIGPRPLVNGLMGRINYYWSVLKPGQTLRIKSDGNLIAPWYGHILSGESHGRILKTD
LKRGSCTVQCQTEKGGLNTTLPFQNVSKYAFGNCSKYIGIKSLKLAVGLRNVPSRSSRGLFGAIAGFIEG
GWSGLVAGWYGFQHSNDQGVGMAADRDSTQKAIDKITSKVNNIVDKMNKQYEIIDHEFSEVETRLNMINN
KIDDQIQDVWAYNAELLVLLENQKTLDEHDANVNNLYNKVKRALGSNAVEDGKGCFELYHKCDDQCMETI
RNGTYNRRKYQEESKLERQKIEGVKLESEGTYKILTIYSTVASSLVIAMGFAAFLFWAMSNGSCRCNICI

>QOJ98725.1 hemagglutinin [Influenza A virus]
METASLITILLVATVSNADKICIGYQSTNSTETVDTLTENNVPVTHAKELLHTEHNGMLCATSLGQPLIL
DTCTIEGLIYGNPSCDLSLEGREWSYIVERPSAVHGLCYPGKVEDLEELRSLFSSARSYQRIQIFPDTIW
NVSYDGTSTACSGSFYRSMRWLTRKDGNYPIQDAQYTNNQGKNILFMWGINHPPTDETQRGLYTRTDTTT
SVATEEINRIFKPLIGPRPLVNGLMGRINYYWSVLKPGQTLRIKSDGNLIAPWYGHILSGESHGRILKTD

LKKGSCTVQCQTEKGGLNTTLPFQNVSKYAFGNCSKYIGIKSLKLAVGLRNVPSRSSRGLFGAIAGFIEG
GWPGLVAGWYGFQHSNDQGVGMAADRDSTQKAIDKITSKVNNIVDKMNKQYEIIDHEFSEVETRLNMINN
KIDDQIQDIWAYNAELLVLLENQKTLDEHDANVNNLYNKVKRALGSNAVEDGKGCFELYHKCDDQCMETI
RNGTYNRRKYQEESKLERQKIEGVKLESEGTYKILTIYSTVASSLVIAMGFAAFLFWAMSNGSCRCNICI

>QOJ98723.1 hemagglutinin, partial [Influenza A virus]
METVSLITILIVATVSNADKICIGYQSTNSTETVDTLTENNVPVTHAKELLHTEHNGMLCATSLGHPLIL
DTCTIEGLIYGNPSCDPLLGGREWSYIVERPSAVNGLCYPGNVENLEELRSLFSSSRSYQRIQIFPDTIW
NVSYSGTSKACSDSFYRSMRWLTQKNNAYPTQDAQYTNNQGKNILFVWGINHPPTDTAQTNLYTRTDTTT
SVATEEMNRIFKPLIGPRPLVNGLMGRINYYWSVLKPGQTLRIKSDGNLIAPWYGHILSGESHGRILKTD
LKRGSCTVQCQTERGGLNTTLPFQNVSKYAFGNCSKYIGVKSLKLAVGLRNVPSRSSRGLFGAIAGFIEG
GWSGLVAGWYGFQHSNDQGVGMAADRDSTQKAIDKITSKVNNIVDKMNKQYEIIDHEFSEVETRLNMINN
KVDDQIQDIWAYNAELLVLLENQKTLDEHDANVNNLYNKVKRALGSNAVEDGKGCFELYHKCDDHCMETI
RNGTYNRRKYQEESKLERQKIEGVKLESEETYKILTIYSTVASSLVIAMGFAAFLFWAMSNGSC

>QOJ98722.1 hemagglutinin [Influenza A virus]
METVSLITILLVATVSNADKICIGYQSTNSTETVDTLTENNVPVTHAKELLHTEHNGMLCATSLGQPLIL
DTCTIEGLIYGNPSCDLSLEGREWSYIVERPSAVHGLCYPGNVEDLEELRSLFSSARSYQRIQIFPDTIW
NVSYDGTSTACSGSFYRSMRWLTRKNGDYPIQDAQYTNNQGKNILFMWGINHPPTDETQRGLYTRTDTTT
SVATEEINRIFKPLIGPRPLVNGLMGRINYYWSVLKPGQTLRIKSDGNLIAPWYGHILSGESHGRILKTD
LKRGSCTVQCQTEKGGLNTTLPFQNVSKYAFGNCSKYIGIKSLKLAVGLRNVPSRSSRGLFGAIAGFIEG
GWSGLVAGWYGFQHSNDQGVGMAADRDSTQKAIDKITSKVNNIVDKMNKQYEIIDHEFSEVETRLNMINN
KIDDQIQDVWAYNAELLVLLENQKTLDEHDANVNNLYNKVKRALGSNAVEDGKGCFELYHKCDDQCMETI
RNGTYNRRKYQEESKLERQKIEGVKLESEGTYKILTIYSTVASSLVIAMGFAAFLFWAMSNGSCRCNICI

>QOJ98720.1 hemagglutinin, partial [Influenza A virus]
ETVSLITILLVATVSNADKICIGYQSTNSTETVDTLTENNVPVTHAKELLHTEHNGMLCATSLGQPLILD
TCTIEGLIYGNPSCDLSLEGREWSYIVERPSAVHGLCYPGNVEDLEELRSLFSSARSYQRIQIFPDTIWN
VSYDGTSTACSGSFYKSMRWLTRKNGEYPTQDAQYTNNQGKNILFMWGINHPPTDDTQRGLYTRTDTTTS
VATEEINRIFKPLIGPRPLVNGLMGRINYYWSVLKPGQTLRIKSDGNLIAPWYGHILSGESHGRILKTDL
KRGSCTVQCQTEKGGLNTTLPFQNVSKYAFGNCSKYIGIKSLKLAVGLRNVPSRSSRGLFGAIAGFIEGG
WSGLVAGWYGFQHSNDQGVGMAADRDSTQKAIDKITSKVNNIVDKMNKQYEIIDHEFSEVETRLNMINNK
IDDQIQDIWAYNAELLVLLENQKTLDEHDANVNNLYNKVKRALGSNAVEDGKGCFELYHKCDDQCMETIR
NGTYNRRKYQEESKLERQRIEGVKLESEGTYKILTIYSTVASSLVIAMGFAAFLFWAMSNGSC

>QOJ98719.1 hemagglutinin, partial [Influenza A virus]
METVSLITILIVATVSNADKICIGYQSTNSTETVDTLTENNVPVTHAKELLHTEHNGMLCATSLGHPLIL
DTCTIEGLIYGNPSCDPLLGGREWSYIVERPSAVNGLCYPGNVENLEELRSLFSSSRSYQRIQIFPDTIW
NVSYSGTSKACSDSFYRSMRWLTQKNNAYPTQDAQYTNNQGKNILFVWGINHPPTDTAQTNLYTRTDTTT
SVATEEMNRIFKPLIGPRPLVNGLMGRINYYWSVLKPGQTLRIKSDGNLIAPWYGHILSGESHGRILKTD
LKRGSCTVQCQTEKGGLNTTLPFQNVSKYAFGNCSKYIGVKSLKLAVGLRNVPSRSSRGLFGAIAGFIEG
GWSGLVAGWYGFQHSNDQGVGMAADRDSTQKAIDKITSKVNNIVDKMNKQYEIIDHEFSEVETRLNMINN
KVDDQIQDIWAYNAELLVLLENQKTLDEHDANVNNLYNKVKRALGSNAVEDGKGCFELYHKCDDHCMETI
RNGTYNRRKYQEESKLERQKIEGVKLESEETYKILTIYSTVASSLVIAMGFAAFLFWAMSNGSC

>QOJ98718.1 hemagglutinin, partial [Influenza A virus]
ETVSLITILLVATVSNADKICIGYQSTNSTETVDTLTENNVPVTHAKELLHTEHNGMLCATSLGQPLILD
TCTIEGLIYGNPSCDLSLEGREWSYIVERPSAVHGLCYPGNVEDLEELRSLFSSARSYQRIQIFPDTIWN
VSYDGTSTACSGSFYKSMRWLTRKNGEYPTQDAQYTNNQGKNILFMWGINHPPTDDTQRGLYTRTDTTTS
VATEEINRIFKPLIGPRPLVNGLMGRINYYWAVLKPGQTLRIKSDGNLIAPWYGHILSGESHGRILKTDL
KRGSCTVQCQTEKGGLNTTLPFQNVSKYAFGNCSKYIGIKSLKLAVGLRNVPSRSSRGLFGAIAGFIEGG
WSGLVAGWYGFQHSNDQGVGMAADRDSTQKAIDKITSKVNNIVDKMNKQYEIIDHEFSEVETRLNMINNK
IDDQIQDIWAYNAELLVLLENQKTLDEHDANVNNLYNKVKRALGSNAVEDGKGCFELYHKCDDQCMETIR
NGTYNRRKYQEESKLERQRIEGVKLESEGTYKILTIYSTVASSLVIAMGFAAFLFWAMSNGSC

>QOJ98717.1 hemagglutinin [Influenza A virus]
METVSLITILLVATVSNADKICIGYQSTNSTETVDTLTENNVPVTHAKELLHTEHNGMLCATSLGQPLIL
DTCTIEGLIYGNPSCDLSLEGREWSYIVERPSAVHGLCYPGNVEDLEELRSLFSSARSYQRIQIFPDTIW
NVSYDGTSTACSGSFYRSMRWLTRKNGDYPIQDAQYTNNQGKNILFMWGINHPPTDETQRGLYTRTDTTT
SVATEEINRIFKPLIGPRPLVNGLMGRINYYWSVLKPGQTLRIKSDGNLIAPWYGHILSGESHGRILKTD
LKRGSCTVQCQTEKGGLNTTLPFQNVSKYAFGNCSKYIGIKSLKLAVGLRNVPSRSSRGLFGAIAGFIEG
GWSGLVAGWYGFQHSNDQGVGMAADRDSTQKAIDKITSKVNNIVDKMNKQYEIIDHEFSEVETRLNMINN
KIDDQIQDVWAYNAELLVLLENQKTLDEHDANVNNLYNKVKRALGSNAVEDGKGCFELYHKCDDQCMETI
RNGTYNRRKYQEESKLERQKIEGVKLESEGTYKILTIYSTVASSLVIAMGFAAFLFWAMSNGSCRCNICI

>QOJ98716.1 hemagglutinin [Influenza A virus]
METVSLITILLAATVSNADKICIGYQSTNSTETVDTLTENNVPVTHAKELLHTEHNGMLCATSLGQPLIL
DTCTIEGLIYGNPSCDPLLEEREWSYIVERPSAVNGLCYPGNVENLEELRSLFSSARSYQRIQIFPDTIW

NVSYDGTSNTCSGSFYRNMRWLTRKNGNYPIQDAQYTNNQGKNILFMWGINHPPTDDTQRNLYTRTDTTT
SVATEEINRIFRPLIGPRPLVNGLMGRINYYWSVLKPGQTLRIKSDGNLIAPWYGYILSGESHGRILRTD
LKRGSCTVQCQTEKGGLNTTLPFQNVSKYAFGNCSKYIGIKSLKLAVGLRNVPSRSSRGLFGAIAGFIEG
GWSGLVAGWYGFQHSNDQGVGMAADRESTQKAIDKITSKVNNIVDKMNKQYEIIDHEFSEVETRLNMINN
KIDDQIQDIWAYNAELLVLLENQKTLDEHDANVNNLYNKVKRALGSNAVEDGKGCFELYHKCDDQCMETI
RNGTYNRRKYQEESKLERQKIEGVKLEPEGTYKILTIYSTVASSLVIAMGFAAFLFWAMSNGSCRCNICI

>QOJ98714.1 hemagglutinin, partial [Influenza A virus]
ETVSLITILLVATVSNADKICIGYQSTNSTETVDTLTENNVPVTHAKELLHTEHNGMLCATSLGQPLILD
TCTIEGLIYGNPSCDLSLEGREWSYIVERPSAVHGLCYPGSVEDLEELRSLFSSARSYQRIQIFPDTIWN
VSYDGTSTACSGSFYKSMRWLTRKNGEYPTQDAQYTNNQGKNILFMWGINHPPTDDTQRGLYTRTDTTTS
VATEEINRIFKPLIGPRPLVNGLMGRINYYWSVLKPGQTLRIKSDGNLIAPWYGHILSGESHGRILKTDL
KRGSCTVQCQTEKGGLNTTLPFQNVSKYAFGNCSKYIGIKSLKLAVGLRNVPSRSSRGLFGAIAGFIEGG
WSGLVAGWYGFQHSNDQGVGMAADRDSTQKAIDKITSKVNNIVDKMNKQYEIIDHEFSEVETRLNMINNK
IDDQIQDIWAYNAELLVLLENQKTLDEHDANVNNLYNKVKRALGSNAVEDGKGCFELYHKCDDQCMETIR
NGTYNRRKYQEESKLERQRIEGVKLESEGTYKILTIYSTVASSLVIAMGFAAFLFWAMSNGSC

>QOJ98711.1 hemagglutinin, partial [Influenza A virus]
METVSLITILIVATVSNADKICIGYQSTNSTETVDTLTENNVPVTHAKELLHTEHNGMLCATSLGHPLIL
DTCTIEGLIYGNPSCDPLLGGREWSYIVERPSAVNGLCYPGNVENLEELRSLFSSSRSYQRIQIFPDTIW
NVSYSGTSKACSDSFYRSMRWLTQKNNAYPTQDAQYTNNQGKNILFVWGINHPPTDTAQTNLYTRTDTTT
SVATEEMNRIFKPLIGPRPLVNGLMGRINYYWSVLKPGQTLRIKSDGNLIAPWYGHILSGESHGRILKTD
LKRGSCTVQCQTEKGGLNTTLPFQNVSKYAFGNCSKYIGVKSLKLAVGLRNVPSRSSRGLFGAIAGFIEG
GWSGLVAGWYGFQHSNDQGVGMAADRDSTQKAIDKITSKVNNIVDKMNKQYEIIDHEFSEVETRLNMINN
KVDDQIQDIWAYNAELLVLLENQKTLDEHDANVNNLYNKVKRALGSNAVEDGKGCFELYHKCDDHCMETI
RNGTYNRRKYQEESKLERQKIEGVKLESEETYKILTIYSTVASSLVIAMGFAAFLFWAMSNGSC

>QOJ98709.1 hemagglutinin [Influenza A virus]
METISLMTILLVATVSNADKICIGYQSTNSTETVDTLTENNVPVTHAKELLHTEHNGMLCATSLGNPLIL
DTCTIEGLIYGNPSCDPLLGGREWSYIVERPSAVNGLCYPGSVENLEELRSLFSSARSYQRIQIFPDTIW
NVSYSGTSKACSDSFYRSMRWLTQKNNNYPIQDAQYTNNQEKNILFMWGINHPPTETAQTNLYTRTDTTT
SVATEEINRIFKPLIGPRPLVNGLMGRINYYWSVLKPGQTLRIKSDGNLIAPWYGHILSGESHGRILKTD
LKRGSCTVQCQTEKGGLNTTLPFQNVSKYAFGNCSKYIGIKSLKLAVGLRNVPSRSSRGLFGAIAGFIEG
GWSGLVAGWYGFQHSNDQGVGMAADRDSTQKAIDKITSKVNNIVDKMNKQYEIIDHEFSEVETRLNMINN

KVDDQIQDIWAYNAELLVLLENQKTLDEHDSNVNNLYNKVKRALGSNAVEDGKGCFELYHKCDNQCMETI
RNGTYNRRKYQEESKLERQKIEGVKLESEGTYKILTIYSTVASSLVIAMGFAAFLFWAMSNGSCRCNICI

>QOJ98708.1 hemagglutinin, partial [Influenza A virus]
ETVSLITILLVATVSNADKICIGYQSTNSTETVDTLTENNVPVTHAKELLHTEHNGMLCATSLGQPLILD
TCTIEGLIYGNPSCDLSLEGREWSYIVERPSAVHGLCYPGNVEDLEELRSLFSSARSYQRIQIFPDTIWN
VSYDGTSTACSGSFYKSMRWLTRKNGEYPTQDAQYTNNQGKNILFMWGINHPPTDDTQRGLYTRTDTTTS
VATEEINRIFKPLIGPRPLVNGLMGRINYYWSVLKPGQTLRIKSDGNLIAPWYGHILSGESHGRILKTDL
KRGSCTVQCQTEKGGLNTTLPFQNVSKYAFGNCSKYIGIKSLKLAVGLRNVPSRSSRGLFGAIAGFIEGG
WSGLVAGWYGFQHSNDQGVGMAADRDSTQKAIDKITSKVNNIVDKMNKQYEIIDHEFSEVETRLNMINNK
IDDQIQDIWAYNAELLVLLENQKTLDEHDANVNNLYNKVKRALGSNAVEDGKGCFELYHKCDDQCMETIR
NGTYNRRKYQEESKLERQRIEGVKLESEGTYKILTIYSTVASSLVIAMGFAAFLFWAMSNGSC

>QOJ98707.1 hemagglutinin, partial [Influenza A virus]
METVSLITILIVATVSNADKICIGYQSTNSTETVDTLTENNVPVTHAKELLHTEHNGMLCATSLGHPLIL
DTCTIEGLIYGNPSCDPLLGGREWSYIVERPSAVNGLCYPGNVENLEELRSLFSSSRSYQRIQIFPDTIW
NVSYSGTSKACSDSFYRSMRWLTQKNNAYPTQDAQYTNNQGKNILFVWGINHPPTDTAQTNLYTRTDTTT
SVATEEMNRIFKPLIGPRPLVNGLMGRINYYWSVLKPGQTLRIKSDGNLIAPWYGHILSGESHGRILKTD
LKRGSCTVQCQTEKGGLNTTLPFQNVSKYAFGNCSKYIGVKSLKLAVGLRNVPSRSSRGLFGAIAGFIEG
GWSGLVAGWYGFQHSNDQGVGMAADRDSTQKAIDKITSKVNNIVDKMNKQYEIIDHEFSEVETRLNMINN
KVDDQIQDIWAYNAELLVLLENQKTLDEHDANVNNLYNKVKRALGSNAVEDGKGCFELYHKCDDHCMETI
RNGTYNRRKYQEESKLERQKIEGVKLESEETYKILTIYSTVASSLVIAMGFAAFLFWAMSNGSC

>QOJ98706.1 hemagglutinin, partial [Influenza A virus]
ETVSLITILLVATVSNADKICIGYQSTNSTETVDTLTENNVPVTHAKELLHTEHNGMLCATSLGQPLILD
TCTIEGLIYGNPSCDLSLEGREWSYIVERPSAVHGLCYPGNVEDLEELRSLFSSARSYQRIQIFPDTIWN
VSYDGTSTACSGSFYKSMRWLTRKNGEYPTQDAQYTNNQGKNILFMWGINHPPTDDTQRGLYTRTDTTTS
VATEEINRIFKPLIGPRPLVNGLMGRINYYWSVLKPGQTLRIKSDGNLIAPWYGHILSGESHGRILKTDL
KRGSCTVQCQTEKGGLNTTLPFQNVSKYAFGNCSKYIGIKSLKLAVGLRNVPSRSSRGLFGAIAGFIEGG
WSGLVAGWYGFQHSNDQGVGMAADRDSTQKAIDKITSKVNNIVDKMNKQYEIIDHEFSEVETRLNMINNK
IDDQIQDIWAYNAELLVLLENQKTLDEHDANVNNLYNKVKRALGSNAVEDGKGCFELYHKCDDQCMETIR
NGTYNRRKYQEESKLERQRIEGVKLESEGTYKILTIYSTVASSLVIAMGFAAFLFWAMSNGSC

>QOJ98705.1 hemagglutinin, partial [Influenza A virus]

METVSLMTILIVATVSNADKICIGYQSTNSTETVDTLTENNVPVTHAKELLHTEHNGMLCATSLGHPLIL
DTCTIEGLIYGNPSCDPLLGEREWSYIVERPSAVNGLCYPGNVENLEELRSLFSSSRSYQRIQIFPDTIW
NVSYSGTSKVCSDSFYRSMRWLTQKNNAYPTQDAQYTNNQGKNILFVWGINHPPTDTVQTNLYTRTDTTT
SVATEEMNRIFKPLIGPRPLVNGLMGRINYYWSVLKPGQTLRIKSDGNLIAPWYGHILSGESHGRILKTD
LKRGSCTVQCQTEKGGLNTTLPFQNVSKYAFGNCSKYIGVKSLKLAVGLRNVPSRSSRGLFGAIAGFIEG
GWSGLVAGWYGFQHSNDQGVGMAADRDSTQKAIDKITSKVNNIVDKMNKQYEIIDHEFSEVETRLNMINN
KVDDQIQDIWAYNAELLVLLENQKTLDEHDANVNNLYNKVKRALGSNAVEDGKGCFELYHKCDDHCMETI
RNGTYNRRKYQEESKLERQKIEGVKLESEETYKILTIYSTVASSLVIAMGFAAFLFWAMSNGSC

>QOJ98704.1 hemagglutinin, partial [Influenza A virus]
ETVSLITILLVATVSNADKICIGYQSTNSTETVDTLTENNVPVTHAKELLHTEHNGMLCATSLGQPLILD
TCTIEGLIYGNPSCDLSLEGREWSYIVERPSAVHGLCYPGNVEDLEELRSLFSSARSYQRIQIFPDTIWN
VSYDGTSTACSGSFYKSMRWLTRKNGEYPTQDAQYTNNQGKNILFMWGINHPPTDDTQRGLYTRTDTTTS
VATEEINRIFKPLIGPRPLVNGLMGRINYYWSVLKPGQTLRIKSDGNLIAPWYGHILSGESHGRILKTDL
KRGSCTVQCQTEKGGLNTTLPFQNVSKYAFGNCSKYIGIKSLKLAVGLRNVPSRSNRGLFGAIAGFIEGG
WSGLVAGWYGFQHSNDQGVGMAADRDSTQKAIDKITSKVNNIVDKMNKQYEIIDHEFSEVETRLNMINNK
IDDQIQDIWAYNAELLVLLENQKTLDEHDANVNNLYNKVKRALGSNAVEDGKGCFELYHKCDDQCMETIR
NGTYNRRKYQEESKLERQRIEGVKLESEGTYKILTIYSTVASSLVIAMGFAAFLFWAMSNGSC

>QOJ98703.1 hemagglutinin, partial [Influenza A virus]
ETVSLITILLVATVSNADKICIGYQSTNSTETVDTLTENNVPVTHAKELLHTEHNGMLCATSLGQPLILD
TCTIEGLIYGNPSCDLSLEGREWSYIVERPSAVHGLCYPGNVEDLEELRSLFSSARSYQRIQIFPDTIWN
VSYDGTSTACSGSFYKSMRWLTRKNGEYPTQDAQYTNNQGKNILFMWGINHPPTDDTQRGLYTRTDTTTS
VATEEINRIFKPLIGPRPLVNGLMGRINYYWSVLKPGQTLRIKSDGNLIAPWYGHILSGESHGRILKTDL
KRGSCTVQCQTEKGGLNTTLPFQNVSKYAFGNCSKYIGIKSLKLAVGLRNVPSRSSRGLFGAIAGFIEGG
WSGLVAGWYGFQHSNDQGVGMAADRDSTQKAIDKITSKVNNIVDKMNKQYEIIDHEFSEVETRLNMINNK
IDDQIQDIWAYNAELLVLLENQKTLDEHDANVNNLYNKVKRALGSNAVEDGKGCFELYHKCDDQCMETIR
NGTYNRRKYQEESKLERQRIEGVKLESEGTYKILTIYSTVASSLVIAMGFAAFLFWAMSNGSC

>QOJ98702.1 hemagglutinin, partial [Influenza A virus]
ETVSLITILLVATVSNADKICIGYQSTNSTETVDTLTENNVPVTHAKELLHTEHNGMLCATSLGQPLILD
TCTIEGLIYGNPSCDLSLEGREWSYIVERPSAVHGLCYPGNVEDLEELRSLFSSARSYQRIQIFPDTIWN
VSYDGTSTACSGSFYKSMRWLTRKNGEYPTQDAQYTNNQGKNILFMWGINHPPTDDTQRGLYTRTDTTTS
VATEEINRIFKPLIGPRPLVNGLMGRINYYWSVLKPGQTLRIKSDGNLIAPWYGHILSGESHGRILKTDL

KRGSCTVQCQTEKGGLNTTLPFQNVSKYAFGNCSKYIGIKSLKLAVGLRNVPSRSSRGLFGAIAGFIEGG
WSGLVAGWYGFQHSNDQGVGMAADRDSTQKAIDKITSKVNNIVDKMNKQYEIIDHEFSEVETRLNMINNK
IDDQIQDIWAYNAELLVLLENQKTLDEHDANVNNLYNKVKRALGSNAVEDGKGCFELYHKCDDQCMETIR
NGTYNRRKYQEESKLERQRIEGVKLESEGTYKILTIYSTVASSLVIAMGFAAFLFWAMSNGSC

>QOJ98701.1 hemagglutinin, partial [Influenza A virus]
METVSLITILIVATVSNADKICIGYQSTNSTETVDTLTENNVPVTHAKELLHTEHNGMLCATSLGHPLIL
DTCTIEGLIYGNPSCDPLLGGREWSYIVERPSAVNGLCYPGNVENLEELRSLFSSSRSYQRIQIFPDTIW
NVSYSGTSKACSDSFYRSMRWLTQKNNAYPTQDAQYTNNQGKNILFVWGINHPPTDTAQTNLYTRTDTTT
SVATEEMNRIFKPLIGPRPLVNGLMGRINYYWSVLKPGQTLRIKSDGNLIAPWYGHILSGESHGRILKTD
LKRGSCTVQCQTEKGGLNTTLPFQNVSKYAFGNCSKYIGVKSLKLAVGLRNVPSRSSRGLFGAIAGFIEG
GWSGLVAGWYGFQHSNDQGVGMAADRDSTQRAIDKITSKVNNIVDKMNKQYEIIDHEFSEVETRLNMINN
KVDDQIQDIWAYNAELLVLLENQKTLDEHDANVNNLYNKVKRALGSNAVEDGKGCFELYHKCDDHCMETI
RNGTYNRKKYQEESKLERQKIEGVKLESEETYKILTIYSTVASSLVIAMGFAAFLFWAMSNGSC

>QOJ98700.1 hemagglutinin, partial [Influenza A virus]
ETVSLITILLVATVSNADKICIGYQSTNSTETVDTLTENNVPVTHAKELLHTEHNGMLCATSLGQPLILD
TCTIEGLIYGNPSCDLSLEGREWSYIVERPSAVHGLCYPGNVEDLEELRSLFSSARSYQRIQIFPDTIWN
VSYDGTSTACSGSFYKSMRWLTRKNGEYPTQDAQYTNNQGKNILFMWGINHPPTDDTQRGLYTRTDTTTS
VATEEINRIFKPLIGPRPLVNGLMGRINYYWSVLKPGQTLRIKSDGNLIAPWYGHILSGESHGRILKTDL
KRGSCTVQCQTEKGGLNTTLPFQNVSKYAFGNCSKYIGIKSLKLAVGLRNVPSRSNRGLFGAIAGFIEGG
WSGLVAGWYGFQHSNDQGVGMAADRDSTQKAIDKITSKVNNIVDKMNKQYEIIDHEFSEVETRLNMINNK
IDDQIQDIWAYNAELLVLLENQKTLDEHDANVNNLYNKVKRALGSNAVEDGKGCFELYHKCDDQCMETIR
NGTYNRRKYQEESKLERQRIEGVKLESEGTYKILTIYSTVASSLVIAMGFAAFLFWAMSNGSC

>QOJ98697.1 hemagglutinin [Influenza A virus]
METVSLITILLVATVSNADKICIGYQSTNSTETVNTLTENNVPVTHAKELLHTEHNGMLCATSLGQPLIL
DTCTIEGLIYGNPSCDLSLEGREWSYIVERPSAINGLCYPGNVENLEELRSLFSSARSYQRIQIFPDTIW
NVSYDGTSTACSNSFYRSMRWLTRKDGNYPTQDAQYTNNQGKNILFMWGINHPPTDETQRNLYTRTDTTT
SVATEEINRIFKPLIGPRPLVNGLMGRIDYYWSVLKPGQTLRIKSDGNLIAPWYGHILSGESHGRILKTD
LKRGSCTVQCQTEKGGLNTTLPFQNVSKYAFGNCSKYIGIKSLKLAVGLRNVPSRSSRGLFGAIAGFIEG
GWSGLVSGWYGFQHSNDQGVGMAADRDSTQKAIDKITSKVNNIVDKMNKQYEIIDHEFSEVETRLNMINN
KIDDQIQDIWAYNADLLVLLENQKTLDEHDANVNNLYNKVKRALGSNAVEDGKGCFELYHKCNDQCMETI
RNGTYNRKKYQEESKLERQRIDGVKLESEGTYKILTIYSTVASSLVIAMGFAAFLFWAMSNGSCRCNICI

>QOJ98695.1 hemagglutinin [Influenza A virus]

METVSLITILLVATVSNADKICIGYQSTNSTETVNTLTENNVPVTHAKELLHTEHNGMLCATSLGQPLIL
DTCTIEGLIYGNPSCDLSLEGREWSYIVERPSAINGLCYPGNVENLEELRSLFSSARSYQRIQIFPDTIW
NVSYDGTSTACSNSFYRSMRWLTRKDGNYPTQDAQYTNNQGKNILFMWGINHPPTDETQRNLYTRTDTTT
SVATEEINRIFKPLIGPRPLVNGLMGRIDYYWSVLKPGQTLRIKSDGNLIAPWYGHILSGESHGRILKTD
LKRGSCTVQCQTEKGGLNTTLPFQNVSKYAFGNCSKYIGIKSLKLAVGLRNVPSRSSRGLFGAIAGFIEG
GWSGLVSGWYGFQHSNDQGVGMAADRDSTQKAIDKITSKVNNIVDKMNKQYEIIDHEFSEVETRLNMINN
KIDDQIQDIWAYNADLLVLLENQKTLDEHDANVNNLYNKVKRALGSNAVEDGKGCFELYHKCNDQCMETI
RNGTYNRKKYQEESKLERQRIDGVKLESEGTYKILTIYSTVASSLVIAMGFAAFLFWAMSNGSCRCNICI

>QOJ98693.1 hemagglutinin, partial [Influenza A virus]

ETVSLITILLVATVSNADKICIGYQSTNSTETVDTLTENNVPVTHAKELLHTEHNGMLCATSLGQPLILD
TCTIEGLIYGNPSCDLSLEGREWSYIVERPSAVHGLCYPGNVEDLEELRSLFSSARSYQRIQIFPDTIWN
VSYDGTSTACSGSFYKSMRWLTRKNGEYPTQDAQYTNNQGKNILFMWGINHPPTDDTQRGLYTRTDTTTS
VATEEINRIFKPLIGPRPLVNGLMGRINYYWSVLKPGQTLRIKSDGNLIAPWYGHILSGESHGRILKTDL
ERGSCTVQCQTEKGGLNTTLPFQNVSKYAFGNCSKYIGLKSLKLAVGLRNVPSRSSRGLFGAIAGFIEGG
WSGLVAGWYGFQHSNDQGVGMAADRDSTQKAIDKITSKVNNIVDKMNKQYEIIDHEFSEVETRLNMINNK
IDDQIQDIWAYNAELLVLLENQKTLDEHDANVNNLYNKVKRALGSNAVEDGKGCFELYHKCDDQCMETIR
NGTYNRRKYQEESKLERQRIEGVKLESEGTYKILTIYSTVASSLVIAMGFAAFLFWAMSNGSC

>QOJ98691.1 hemagglutinin, partial [Influenza A virus]

ETVSLITILLVATVSNADKICIGYQSTNSTETVDTLTENNVPVTHAKELLHTEHNGMLCATSLGQPLILD
TCTIEGLIYGNPSCDLSLEGREWSYIVERPSAVHGLCYPGNVEDLEELRSLFSSARSYQRIQIFPDTIWN
VSYDGTSTACSGSFYKSMRWLTRKNGEYPTQDAQYTNNQGKNILFMWGINHPPTDDTQRGLYTRTDTTTS
VATEEINRIFKPLIGPRPLVNGLMGRINYYWSVLKPGQTLRIKSDGNLIAPWYGHILSGESHGRILKTDL
KRGSCTVQCQTEKGGLNTTLPFQNISKYAFGNCSKYIGIKSLKLAVGLRNVPSRSSRGLFGAIAGFIEGG
WSGLVAGWYGFQHSNDQGVGMAADRDSTQKAIDKITSKVNNIVDKMNKQYEIIDHEFSEVETRLNMINNK
IDDQIQDIWAYNAELLVLLENQKTLDEHDANVNNLYNKVKRALGSNAVEDGKGCFELYHKCDDQCMETIR
NGTYNRRKYQEESKLERQRIEGVKLESEGTYKILTIYSTVASSLVIAMGFAAFLFWAMSNGSC

>QOJ98690.1 hemagglutinin, partial [Influenza A virus]

ETVSLITILLVATVSNADKICIGYQSTNSTETVDTLTENNVPVTHAKELLHTEHNGMLCATSLGQPLILD
TCTIEGLIYGNPSCDLSLEGREWSYIVERPSAVHGLCYPGSVEDLEELRSLFSSARSYQRIQIFPDTIWN

VSYDGTSTACSGSFYKSMRWLTRKNGEYPTQDAQYTNNQGKNILFMWGINHPPTDDTQRGLYTRTDTTTS
VATEEINRIFKPLIGPRPLVNGLMGRINYYWSVLKPGQTLRIKSDGNLIAPWYGHILSGESHGRILKTDL
KRGSCTVQCQTEKGGLNTTLPFQNVSKYAFGNCSKYIGIKSLKLAVGLRNVPSRSSRGLFGAIAGFIEGG
WSGLVAGWYGFQHSNDQGVGMAADRDSTQKAIDKITSKVNNIVDKMNKQYEIIDHEFSEVETRLNMINNK
IDDQIQDIWAYNAELLVLLENQKTLDEHDANVNNLYNKVKRALGSNAVEDGKGCFELYHKCDDQCMETIR
NGTYNRRKYQEESKLERQRIEGVKLESEGTYKILTIYSTVASSLVIAMGFAAFLFWAMSNGSC

>QOJ98687.1 hemagglutinin, partial [Influenza A virus]
METVSLITILVVATVSNADKICIGYQSTNSTETVDTLTENNVPVTHAKELLHTEHNGMLCATSLGHPLIL
DTCTIEGLIYGNPSCDPLLGGREWSYIVERPSAVNGLCYPGNVENLEELRSLFSSSRSYQRIQIFPDTIW
NVSYSGTSKACSDSFYRSMRWLTQKNNAYPTQDAQYTNNQGKNILFMWGINHPPTDTVQTNLYTRTDTTT
SVATEEMNRIFKPLIGPRPLVNGLMGRINYYWSVLKPGQTLRIKSDGNLIAPWYGHILSGESHGRILKTD
LKRGSCTVQCQTEKGGLNTTLPFQNVSKYAFGNCSKYIGVKSLKLAVGLRNVPSRSSRGLFGAIAGFIEG
GWSGLVAGWYGFQHSNEQGVGMAADRDSTQKAIDKITSKVNNIVDKMNKQYEIIDHEFSEVETRLNMINN
KVDDQIQDIWAYNAELLVLLENQKTLDEHDANVNNLYNKVKRALGSNAVEDGKGCFELYHKCDDHCMETI
RNGTYNRRKYQEESKLERQKIEGVKLESEETYKILTIYSTVASSLVIAMGFAAFLFWAMSNGSC

>QOJ98685.1 hemagglutinin, partial [Influenza A virus]
ETVSLITILLVATVSNADKICIGYQSTNSTETVDTLTENNVPVTHAKELLHTEHNGMLCATSLGQPLILD
TCTIEGLIYGNPSCDLSLEGREWSYIVERPSAVHGLCYPGNVEDLEELRSLFSSARSYQRIQIFPDTIWN
VSYDGTSTACSGSFYKSMRWLTRKNGEYPTQDAQYTNNQGKNILFMWGINHPPTDDTQRGLYTRTDTTTS
VATEEINRIFKPLIGPRPLVNGLMGRINYYWSVLKPGQTLRIKSDGNLIAPWYGHILSGESHGRILKTDL
KRGSCTVQCQTEKGGLNTTLPFQNVSKYAFGNCSKYIGIKSLKLAVGLRNVPSRSSRGLFGAIAGFIEGG
WSGLVAGWYGFQHSNDQGVGMAADRDSTQKAIDKITSKVNNIVDKMNKQYEIIDHEFSEVETRLNMINNK
IDDQIQDIWAYNAELLVLLENQKTLDEHDANVNNLYNKVKRALGSNAVEDGKGCFELYHKCDDQCMETIR
NGTYNRRKYQEESKLERQRIEGVKLESEGTYKILTIYSTVASSLVIAMGFAAFLFWAMSNGSC

>QOJ98684.1 hemagglutinin, partial [Influenza A virus]
METVSLITILIVATVSNADKICIGYQSTNSTETVDTLTENNVPVTHAKELLHTEHNGMLCATSLGHPLIL
DTCTIEGLIYGNPSCDPLLGGREWSYIVERPSAVNGLCYPGNVENLEELRSLFSSSRSYQRIQIFPDTIW
NVSYSGTSKACSDSFYRSMRWLTQKNNAYPTQDAQYTNNQGKNILFVWGINHPPTDTAQTNLYTRTDTTT
SVATEEMNRIFKPLIGPRPLVNGLMGRINYYWSVLKPGQTLRIKSDGNLIAPWYGHILSGESHGRILKTD
LKRGSCTVQCQTEKGGLNTTLPFQNVSKYAFGNCSKYIGVKSLKLAVGLRNVPSRSSRGLFGAIAGFIEG
GWSGLVAGWYGFQHSNDQGVGMAADRDSTQKAIDKITSKVNNIVDKMNKQYEIIDHEFSEVETRLNMINN

KVDDQIQDIWAYNAELLVLLENQKTLDEHDANVNNLYNKVKRALGSNAVEDGKGCFELYHKCDDHCMETI
RNGTYNRRKYQEESKLERQKIEGVKLESEETYKILTIYSTVASSLVIAMGFAAFLFWAMSNGSC

>QOJ98683.1 hemagglutinin, partial [Influenza A virus]
METVSLMTILIVATVSNADKICIGYQSTNSTETVDTLTENNVPVTHAKELLHTEHNGMLCATSLGNPLIL
DTCTIEGLIYGNPSCDPLLGGREWSYIVERPSAVNGLCYPGNVENLEELRSLFSSSRSYQRIQIFPDTIW
NVSYSGTSKACSDSFYRSMRWLTQKNNAYPTQDAQYTNNQGKNILFVWGINHPPTDTAQTNLYTRTDTTT
SVATEEMNRIFKPLIGPRPLVNGLMGRINYYWSVLKPGQTLRIKSDGNLIAPWYGHILSGESHGRILKTD
LKRGSCTVQCQTEKGGLNTTLPFQNVSKYAFGNCSKYIGVKSLKLAVGLRNVPSRSSRGLFGAIAGFIEG
GWSGLVAGWYGFQHSNDQGVGMAADRDSTQKAIDKITSKVNNIVDKMNKQYEIIDHEFSEVETRLNMINN
KVDDQIQDIWAYNAELLVLLENQKTLDEHDANVNNLYNKVKRALGSNAVEDGKGCFELYHKCDDHCMETI
RNGTYNRRKYQEESKLERQKIEGVKLESEETYKILTIYSTVASSLVIAMGFAAFLFWAMSNGSC

>QOJ98682.1 hemagglutinin, partial [Influenza A virus]
ETVSLITILLVATVSNADKICIGYQSTNSTETVDTLTENNVPVTHAKELLHTEHNGMLCATSLGQPLILD
TCTIEGLIYGNPSCDLSLEGREWSYIVERPSAVHGLCYPGNVEDLEELRSLFSSARSYQRIQIFPDTIWN
VSYDGTSTACSGSFYKSMRWLTRKNGEYPTQDAQYTNNQGKNILFMWGINHPPTDDTQRGLYTRTDTTTS
VATEEINRIFKPLIGPRPLVNGLMGRINYYWSVLKPGQTLRIKSDGNLIAPWYGHILSGESHGRILKTDL
KRGSCTVQCQTEKGGLNTTLPFQNVSKYAFGNCSKYIGIKSLKLAVGLRNVPSRSSRGLFGAIAGFIEGG
WSGLVAGWYGFQHSNDQGVGMAADRDSTQKAIDKITSKVNNIVDKMNKQYEIIDHEFSEVETRLNMINNK
IDDQIQDIWAYNAELLVLLENQKTLDEHDANVNNLYNKVKRALGSNAVEDGKGCFELYHKCDDQCMETIR
NGTYNRRKYQEESKLERQRIEGVKLESEGTYKILTIYSTVASSLVIAMGFAAFLFWAMSNGSC

>QOJ98681.1 hemagglutinin, partial [Influenza A virus]
ETVSLITILLVATVSNADKICIGYQSTNSTETVDTLTENNVPVTHAKELLHTEHNGMLCATSLGQPLILD
TCTIEGLIYGNPSCDLSLEGREWSYIVERPSAVHGLCYPGNVEDLEELRSLFSSARSYQRIQIFPDTIWN
VSYDGTSTACSGSFYKSMRWLTRKNGEYPTQDAQYTNNQGKNILFMWGINHPPTDGTQRGLYTRTDTTTS
VATEEINRIFKPLIGPRPLVNGLMGRINYYWSVLKPGQTLRIKSDGNLIAPWYGHILSGESHGRILKTDL
KRGSCTVQCQTEKGGLNTTLPFQNVSKYAFGNCSKYIGIKSLKLAVGLRNVPSRSNRGLFGAIAGFIEGG
WSGLVAGWYGFQHSNDQGVGMAADRDSTQKAIDKITSKVNNIVDKMNKQYEIIDHEFSEVETRLNMINNK
IDDQIQDIWAYNAELLVLLENQKTLDEHDANVNNLYNKVKRALGSNAVEDGKGCFELYHKCDDQCMETIR
NGTYNRRKYQEESKLERQRIEGVKLESEGTYKILTIYSTVASSLVIAMGFAAFLFWAMSNGSC

>QOJ98680.1 hemagglutinin, partial [Influenza A virus]

METVSLITILIVATVSNADKICIGYQSTNSTETVDTLTENNVPVTHAKELLHTEHNGMLCATSLGHPLIL
DTCTIEGLIYGNPSCDPLLGGREWSYIVERPSAVNGLCYPGNVENLEELRSLFSSSRSYQRIQIFPDTIW
NVSYSGTSKACSDSFYRSMRWLTQKNNAYPTQDAQYTNNQGKNILFVWGINHPPTDTAQTNLYTRTDTTT
SVATEEMNRIFKPLIGPRPLVNGLMGRINYYWSVLKPGQTLRIKSDGNLIAPWYGHILSGESHGRILKTD
LKRGSCTVQCQTEKGGLNTTLPFQNVSKYAFGNCSKYIGVKSLKLAVGLRNVPSRSSRGLFGAIAGFIEG
GWSGLVAGWYGFQHSNDQGVGMAADRDSTQKAIDKITSKVNNIVDKMNKQYEIIDHEFSEVETRLNMINN
KVDDQIQDIWAYNAELLVLLENQKTLDEHDANVNNLYNKVKRALGSNAVEDGKGCFELYHKCDDHCMETI
RNGTYNRRKYQEESKLERQKIEGVKLESEETYKILTIYSTVASSLVIAMGFAAFLFWAMSNGSC

>QOJ98676.1 hemagglutinin, partial [Influenza A virus]
ETVSLITILLVATVSNADKICIGYQSTNSTETVDTLTENNVPVTHAKELLHTEHNGMLCATSLGQPLILN
TCTIEGLIYGNPSCDLSLEGREWSYIVERPSAVHGLCYPGNVEDLEELRSLFSSARSYQRIQIFPDTIWN
VSYDGTSTACSGSFYKSMRWLTRKNGEYPTQDAQYTNNQGKNILFMWGINHPPTDDTQRGLYTRTDTTTS
VATEEINRIFKPLIGPRPLVNGLMGRINYYWSVLKPGQTLRIKSDGNLIAPWYGHILSGESHGRILKTDL
KRGSCTVQCQTEKGGLNTTLPFQNVSKYAFGNCSKYIGIKSLKLAVGLRNVPSRSNRGLFGAIAGFIEGG
WSGLVAGWYGFQHSNDQGVGMAADRDSTQKAIDKITSKVNNIVDKMNKQYEIIDHEFSEVETRLNMINNK
IDDQIQDIWAYNAELLVLLENQKTLDEHDANVNNLYNKVKRALGSNAVEDGKGCFELYHKCDDQCMETIR
NGTYNRRKYQEESKLERQRIEGVKLESEGTYKILTIYSTVASSLVIAMGFAAFLFWAMSNGSC

>QOJ98675.1 hemagglutinin, partial [Influenza A virus]
METVSLMTILIVATVSNADKICIGYQSTNSTETVDTLTENNVPVTHAKELLHTEHNGMLCATSLGHPLIL
DTCTIEGLIYGNPSCDPLLGGREWSYIVERPSAVNGLCYPGNVENLEELRSLFSSSRSYQRIQIFPDTIW
NVSYSGTSKVCSDSFYRSMRWLTQKNNAYPTQDAQYTNNQGKNILFVWGINHPPTDTAQTNLYTRTDTTT
SVATEEMNRIFKPLIGPRPLVNGLMGRINYYWSVLKPGQTLRIKSDGNLIAPWYGHILSGESHGRILKTD
LKRGSCTVQCQTEKGGLNTTLPFQNVSKYAFGNCSKYIGVKSLKLAVGLRNVPSRSSRGLFGAIAGFIEG
GWSGLVAGWYGFQHSNDQGVGMAADRDSTQKAIDKITSKVNNIVDKMNKQYEIIDHEFSEVETRLNMINN
KVDDQIQDIWAYNAELLVLLENQKTLDEHDANVNNLYNKVKRALGSNAVEDGKGCFELYHKCDDHCMETI
RNGTYNRRKYQEESKLERQKIEGVKLESEETYKILTIYSTVASSLVIAMGFAAFLFWAMSNGSC

>QOJ98674.1 hemagglutinin, partial [Influenza A virus]
ETVSLITILLVATVSNADKICIGYQSTNSTETVDTLTENNVPVTHAKELLHTEHNGMLCATSLGQPLILD
TCTIEGLIYGNPSCDLSLEGREWSYIVERPSAVHGLCYPGNVEDLEELRSLFSSARSYQRIQIFPDTIWN
VSYDGTSTACSGSFYKSMRWLTRKNGEYPTQDAQYTNNQGKNILFMWGINHPPTDDTQRGLYTRTDTTTS
VATEEINRIFKPLIGPRPLVNGLMGRINYYWSVLKPGQTLRIKSDGNLIAPWYGHILSGESHGRILKTDL

KRGSCTVQCQTEKGGLNTTLPFQNVSKYAFGNCSKYIGIKSLKLAVGLRNVPSRSSRGLFGAIAGFIEGG
WSGLVAGWYGFQHSNDQGVGMAADRDSTQKAIDKITSKVNNIVDKMNKQYEIIDHEFSEVETRLNMINNK
IDDQIQDIWAYNAELLVLLENQKTLDEHDANVNNLYNKVKRALGSNAVEDGKGCFELYHKCDDQCMETIR
NGTYNRRKYQEESKLERQRIEGVKLESEGTYKILTIYSTVASSLVIAMGFAAFLFWAMSNGSC

>QOJ98673.1 hemagglutinin, partial [Influenza A virus]
METVSLITILIVATVSNADKICIGYQSTNSTETVDTLTENNVPVTHAKELLHTEHNGMLCATSLGHPLIL
DTCTIEGLIYGNPSCDPLLGGREWSYIVERPSAVNGLCYPGNVENLEELRSLFSSSRSYQRIQIFPDTIW
NVSYSGTSKACSDSFYRSMRWLTQKNNAYPTQDAQYTNNQGKNILFVWGINHPPTDTAQTNLYTRTDTTT
SVATEEMNRIFKPLIGPRPLVNGLMGRINYYWSVLKPGQTLRIKSDGNLIAPWYGHILSGESHGRILKTD
LKRGSCTVQCQTEKGGLNTTLPFQNVSKYAFGNCSKYIGVKSLKLAVGLRNVPSRSSRGLFGAIAGFIEG
GWSGLVAGWYGFQHSNDQGVGMAADRDSTQKAIDKITSKVNNIVDKMNKQYEIIDHEFSEVETRLNMINN
KVDDQIQDIWAYNAELLVLLENQKTLDEHDANVNNLYNKVKRALGSNAVEDGKGCFELYHKCDDHCMETI
RNGTYNRRKYQEESKLERQKIEGVKLESEETYKILTIYSTVASSLVIAMGFAAFLFWAMSNGSC

>QOJ98672.1 hemagglutinin, partial [Influenza A virus]
ETVSLITILLVATVSNADKICIGYQSTNSTETVDTLTENNVPVTHAKELLHTEHNGMLCATSLGQPLILD
TCTIEGLIYGNPSCDLSLEGREWSYIVERPSAVHGLCYPGNVEDLEELRSLFSSARSYQRIQIFPDTIWN
VSYDGTSTACSGSFYKSMRWLTRKNGEYPTQDAQYTNNQGKNILFMWGINHPPTDDTQRGLYTRTDTTTS
VATEEINRIFKPLIGPRPLVNGLMGRINYYWSVLKPGQTLRIKSDGNLIAPWYGHILSGESHGRILKTDL
KRGSCTVQCQTEKGGLNTTLPFQNVSKYAFGNCSKYIGIKSLKLAVGLRNVPSRSSRGLFGAIAGFIEGG
WSGLVAGWYGFQHSNDQGVGMAADRDSTQKAIDKITSKVNNIVDKMNKQYEIIDHEFSEVETRLNMINNK
IDDQIQDIWAYNAELLVLLENQKTLDEHDANVNNLYNKVKRALGSNAVEDGKGCFELYHKCDDQCMETIR
NGTYNRRKYQEESKLERQRIEGVKLESEGTYKILTIYSTVASSLVIAMGFAAFLFWAMSNGSC

>QOJ98671.1 hemagglutinin, partial [Influenza A virus]
ETVSLITILLVATVSNADKICIGYQSTNSTETVDTLTESNVPVTHAKELLHTEHNGMLCATSLGQPLILD
TCTIEGLIYGNPSCDLSLEGREWSYIVERPSAVHGLCYPGNVEDLEELRSLFSSARSYQRIQIFPDTIWN
VSYDGTSTACSGSFYRSMRWLTRKNGEYPIQDAQYTNNQGKNIFFMWGINHPPTDDTQRGLYTRTDTTTS
VATEEINRIFKPLIGPRPLVNGLMGRINYYWSVLKPGQTLRIKSDGNLIAPWYGHILSGESHGRILKTDL
KRGSCTVQCQTEKGGLNTTLPFQNVSKYAFGNCSKYIGIKSLKLAVGLRNVPSRSSRGLFGAIAGFIEGG
WPGLVAGWYGFQHSNDQGVGMAADRDSTQKAIDKITSKVNNIVDKMNKQYEIIDHEFSEVETRLNMINNK
IDDQIQDIWAYNAELLVLLENQKTLDEHDANVNNLYSKVKRALGSNAVEDGKGCFELYHKCDDQCMETIR
NGTYNRRKYQEESKLERQRIEGVKLESEGTYKILTIYSTVASSLVIAMGFAAFLFWAMSNGSC

>QOJ98670.1 hemagglutinin, partial [Influenza A virus]

METVSLMTILIVATVSNADKICIGYQSTNSTETVDTLTENNVPVTHAKELLHTEHNGMLCATSLGHPLIL
DTCTIEGLIYGNPSCDPLLGGREWSYIVERPSAVNGLCYPGNVENLEELRSLFSSSRSYQRIQIFPDTIW
NVSYSGTSKVCSDSFYRSMRWLTQKNNAYPTQDAQYTNNQGKNILFVWGINHPPTDTVQTNLYTRTDTTT
SVATEEMNRIFKPLIGPRPLVNGLMGRINYYWSVLKPGQTLRIKSDGNLIAPWYGHILSGESHGRILKTD
LKRGSCTVQCQTEKGGLNTTLPFQNVSKYAFGNCSKYIGVKSLKLAVGLRNVPSRSSRGLFGAIAGFIEG
GWSGLVAGWYGFQHSNDQGVGMAADRDSTQKAIDKITSKVNNIVDKMNKQYEIIDHEFSEVETRLNMINN
KVDDQIQDIWAYNAELLVLLENQKTLDEHDANVNNLYNKVKRALGSNAVEDGKGCFELYHKCDDHCMETI
RNGTYNRRKYQEESKLERQKIEGVKLESEETYKILTIYSTVASSLVIAMGFAAFLFWAMSNGSC

>QOJ98668.1 hemagglutinin, partial [Influenza A virus]

ETVSLITILLVATVSNADKICIGYQSTNSTETVDTLTENNVPVTHAKELLHTEHNGMLCATSLGQPLILD
TCTIEGLIYGNPSCDLSLEGREWSYIVERPSAVHGLCYPGNVEDLEELRSLFSSARSYQRIQIFPDTIWN
VSYDGTSTACSGSFYKSMRWLTRKNGEYPTQDAQYTNNQGKNILFMWGINHPPTDDTQRGLYTRTDTTTS
VATEEINRIFKPLIGPRPLVNGLMGRINYYWAVLKPGQTLRIKSDGNLIAPWYGHILSGESHGRILKTDL
KRGSCTVQCQTEKGGLNTTLPFQNVSKYAFGNCSKYIGIKSLKLAVGLRNVPSRSSRGLFGAIAGFIEGG
WSGLVAGWYGFQHSNDQGVGMAADRDSTQKAIDKITSKVNNIVDKMNKQYEIIDHEFSEVETRLNMINNK
IDDQIQDIWAYNAELLVLLENQKTLDEHDANVNNLYNKVKRALGSNAVEDGKGCFELYHKCDDQCMETIR
NGTYNRRKYQEESKLERQRIEGVKLESEGTYKILTIYSTVASSLVIAMGFAAFLFWAMSNGSC

>QOJ98667.1 hemagglutinin, partial [Influenza A virus]

ETVSLITILLVATVSNADKICIGYQSTNSTETVDTLTENNVPVTHAKELLHTEHNGMLCATSLGQPLILD
TCTIEGLIYGNPSCDLSLEGREWSYIVERPSAVHGLCYPGNVEDLEELRSLFSSARSYQRIQIFPDTIWN
VSYDGTSTACSGSFYKSMRWLTRKNGEYPTQDAQYTNNQGKNILFMWGINHPPTDDTQRGLYTRTDTTTS
VATEEINRIFKPLIGPRPLVNGLMGRINYYWSVLKPGQTLRIKSDGNLIAPWYGHILSGESHGRILKTDL
KRGSCTVQCQTEKGGLNTTLPFQNVSKYAFGNCSKYIGIKSLKLAVGLRNVPSRSSRGLFGAIAGFIEGG
WSGLVAGWYGFQHSNDQGVGMAADRDSTQKAIDKITSKVNNIVDKMNKQYEIIDHEFSEVETRLNMINNK
IDDQIQDIWAYNAELLVLLENQKTLDEHDANVNNLYNKVKRALGSNAVEDGKGCFELYHKCDDQCMETIR
NGTYNRRKYQEESKLERQRIEGVKLESEGTYKILTIYSTVASSLVIAMGFAAFLFWAMSNGSC

>QOJ98666.1 hemagglutinin, partial [Influenza A virus]

METASLITILIVATVSNADKICIGYQSTNSTETVDTLTENNVPVTHAKELLHTEHNGMLCATSLGHPLIL
DTCTIEGLIYGNPSCDPLLGGREWSYIVERPSAVNGLCYPGNVENLEELRSLFSSSRSYQRIQIFPDTIW

NVSYSGTSKACSDSFYRSMRWLTQKNNAYPTQDAQYTNNQGKNILFMWGINHPPTDTAQTNLYTRTDTTT
SVATEEMNRIFKPLIGPRPLVNGLMGRINYYWSVLRPGQTLRIKSDGNLIAPWYGHILSGESHGRILKTD
LKRGSCTVQCQTEKGGLNTTLPFQNVSKYAFGNCSKYIGVKSLKLAVGLRNVPSRSSRGLFGAIAGFIEG
GWSGLVAGWYGFQHSNDQGVGMAADRDSTQKAIDKITSKVNNIVDKMNKQYEIIDHEFSEVETRLNMINN
KVDDQIQDIWAYNAELLVLLENQKTLDEHDANVNNLYNKVKRALGSNAVEDGKGCFELYHKCDDHCMETI
RNGTYNRRKYQEESKLERQKIEGVKLESEETYKILTIYSTVASSLVIAMGFAAFLFWAMSNGSC

>QOJ98665.1 hemagglutinin, partial [Influenza A virus]
ETVSLITILLVATASNADKICIGYQSTNSTETVDTLTENNVPVTHAKELLHTEHNGMLCATSLGQPLILD
TCTVEGLIYGNPSCDLSLEGREWSYIVERPSAVNGLCYPGNVENLEELRSLFSSARSYQRIQIFPDTIWN
VSYDGTSIACSDSFYRSMRWLTRKDGNYPTQDAQYTNNQGKNILFMWGINHPPTDDTQRNLYTRTDTTTS
VATEEINRIFKPLIGPRPLVNGLMGRIDYYWSVLKPGQTLRIKSDGNLIAPWYGHILSGESHGRILKTDL
KRGSCTVQCQTEKGGLNTTLPFQNVSKYAFGNCSKYIGIKSLKLAVGLRNVPSRSSRGLFGAIAGFIEGG
WSGLVAGWYGFQHSNDQGVGMAADRDSTQKAIDKITSKVNNIVDKMNKQYEIIDHEFSEVETRLNMINNK
IDDQIQDIWAYNAELLVLLENQKTLDEHDANVNNLYNKVKRALGSNAVEDGKGCFELYHKCNDQCMETIR
NGTYNRRKYQEESKLERQKIEGVKLESEGTYKILTIYSTVASSLVIAMGFAAFLFWAMSNGSCR

>QOJ98664.1 hemagglutinin, partial [Influenza A virus]
ETVSLITILLVATVSNADKICIGYQSTNSTETVDTLTENNVPVTHAKELLHTEHNGMLCATSLGQPLILD
TCTIEGLIYGNPSCDLSLEGREWSYIVERPSAVHGLCYPGNVEDLEELRSLFSSARSYQRIQIFPDTIWN
VSYDGTSTACSGSFYKSMRWLTRKNGEYPTQDAQYTNNQGKNILFMWGINHPPTDDTQRGLYTRTDTTTS
VATEEINRIFKPLIGPRPLVNGLMGRINYYWSVLKPGQTLRIKSDGNLIAPWYGHILSGESHGRILKTDL
KRGSCTVQCQTEKGGLNTTLPFQNVSKYAFGNCSKYIGIKSLKLAVGLRNVPSRSSRGLFGAIAGFIEGG
WSGLVAGWYGFQHSNDQGVGMAADRDSTQKAIDKITSKVNNIVDKMNKQYEIIDHEFSEVETRLNMINNK
IDDQIQDIWAYNAELLVLLENQKTLDEHDANVNNLYNKVKRALGSNAVEDGKGCFELYHKCDDQCMETIR
NGTYNRRKYQEESKLERQRIEGVKLESEGTYKILTIYSTVASSLVIAMGFAAFLFWAMSNGSC

>QOJ98663.1 hemagglutinin, partial [Influenza A virus]
ETVSLITILLVATASNADKICIGYQSTNSTETVDTLTENNVPVTHAKELLHTEHNGMLCATSLGQPLILD
TCTVEGLIYGNPSCDLSLEGREWSYIVERPSAVNGLCYPGNVENLEELRSLFSSARSYQRIQIFPDTIWN
VSYDGTSTACSDSFYRSMRWLTRKDGNYPTQDAQYTNNQGKNILFMWGINHPPTDDTQRNLYTRTDTTTS
VATEEINRIFKPLIGPRPLVNGLMGRIDYYWSVLKPGQTLRIKSDGNLIAPWYGHILSGESHGRILKTDL
KRGSCTVQCQTEKGGLNTTLPFQNVSKYAFGNCSKYIGIKSLKLAVGLRNVPSRSSRGLFGAIAGFIEGG
WSGLVAGWYGFQHSNDQGVGMAADRDSTQKAIDKITSKVNNIVDKMNKQYEIIDHEFSEVETRLNMINNK

IDDQIQDIWAYNAELLVLLENQKTLDEHDANVNNLYNKVKRALGSNAVEDGKGCFELYHKCNDQCMETIR
NGTYNRRKYQEESKLERQKIEGVKLESEGTYKILTIYSTVASSLVIAMGFAAFLFWAMSNGSC

>QOJ98655.1 hemagglutinin [Influenza A virus]
METVSLMTILLVAAVSNADKICIGYQSTNSTETVDTLTENNVPVTHAKELLHTEHNGMLCATSLGQPIIL
DTCTIEGLIYGNPSCDLSLEGREWSYIVERPSAVNGLCYPGNVENLEELRSLFSSARSYQRIQIFPDTIW
NVSYDGTSTACSGSFYRNMRWLTRKNGEYPIQDAQYTNNQGKNILFMWGINHPPADTTQRDLYTRTDTTT
SVATEEINRIFKPLIGPRPLVNGLMGRIDYYWSVLKPGQTLRIKSDGNLIAPWYGHILSGESHGRILKTD
LKRGSCTVQCQTEKGGLNTTLPFQNVSKYAFGNCSKYIGIKSLKLAVGLRNVPSRSSRGLFGAIAGFIEG
GWSGLVAGWYGFQHSNDQGVGMAADRDSTQKAVDKITSKVNTIVEKMNKQYEIIDHEFSEVETRLNMINN
KIDDQIQDIWAYNAELLVLLENQKTLDEHDANVNNLYNKVKRALGSNAVEDGKGCFELYHKCDDQCMETI
RNGTYNRRKYQEESKLERQKIEGVKLESEGTYKILTIYSTVASSLVIAMGFAAFLFWAMSNGSCRCNICI

>QOJ98654.1 hemagglutinin [Influenza A virus]
METVSLMTILLVAAVSNADKICIGYQSTNSTETVDTLTENNVPVTHAKELLHTEHNGMLCATSLGQPIIL
DTCTIEGLIYGNPSCDLSLEGREWSYIVERPSAVNGLCYPGNVENLEELRSLFSSARSYQRIQIFPDTIW
NVSYDGTSTACSGSFYRNMRWLTRKNGEYPIQDAQYTNNQGKNILFMWGINHPPADTTQRDLYTRTDTTT
SVATEEINRIFKPLIGPRPLVNGLMGRIDYYWSVLKPGQTLRIKSNGNLIAPWYGHILSGESHGRILKTD
LKRGSCTVQCQTEKGGLNTTLPFQNVSKYAFGNCSKYIGIKSLKLAVGLRNVPSRSSRGLFGAIAGFIEG
GWSGLVAGWYGFQHSNDQGVGMAADRDSTQKAVDKITSKVNTIVEKMNKQYEIIDHEFSEVETRLNMINN
KIDDQIQDIWAYNAELLVLLENQKTLDEHDANVNNLYNKVKRALGSNAVEDGKGCFELYHKCDDQCMETI
RNGTYNRRKYQEESKLERQKIEGVKLESEGTYKILTIYSTVASSLVIAMGFAAFLFWAMSNGSCRCNICI

>QOJ98647.1 hemagglutinin [Influenza A virus]
METVSLITILVVATVSNADKICIGYQSTNSTETVDTLTENNVPVTHAKELLHTEHNGMLCATSLGHPLIL
DTCTIEGLIYGNPSCDPLLGGREWSYIVERPSAVNGLCYPGNVENLEELRSLFSSSRSYQRIQIFPDTIW
NVSYSGTSKACSDSFYRSMRWLTQKNNAYPTQDAQYTNNQGKNILFMWGINHPPTDTAQTNLYTRTDTTT
SVATEEMNRIFKPLIGPRPLVNGLMGRINYYWSVLKPGQTLRIKSDGNLIAPWYGHILSGESHGRILKTD
LKRGSCTVQCQTEKGGLNTTLPFQNVSKYAFGNCSKYIGVKSLKLAVGLRNVPSRSSRGLFGAIAGFIEG
GWSGLVAGWYGFQHSNDQGVGMAADRDSTQKAIDKITSKVNNIVDKMNKQYEIIDHEFSEVETRLNMINN
KVDDQIQDIWAYNAELLVLLENQKTLDEHDANVNNLYNKVKRALGSNAVEDGKGCFELYHKCDDHCMETI
RNGTYNRRKYQEESKLERQKIEGVKLESEETYKILTIYSTVASSLVIAMGFAAFLFWAMSNGSCRCNICI

>QOJ98644.1 hemagglutinin [Influenza A virus]

METVSLITILIVATVSNADKICIGYQSTNSTETVDTLTENNVPVTHAKELLHTEHNGMLCATSLGHPLIL
DTCTIEGLIYGNPSCDPLLGGREWSYIVERPSAVNGLCYPGNVENLEELRSLFSSSRSYQRIQIFPDTIW
NVSYSGTSKACSDSFYRSMRWLTQKNNAYPTQDAQYTNNQGKNILFVWGINHPPTDTVQTNLYTRTDTTT
SVATEEMNRIFKPLIGPRPLVNGLMGRINYYWSVLKPGQTLRIKSDGNLIAPWYGHILSGESHGRILKTD
LKRGSCTVQCQTEKGGLNTTLPFQNVSKYAFGNCSKYIGVKSLKLAVGLRNVPSRSSRGLFGAIAGFIEG
GWSGLVAGWYGFQHSNDQGVGMAADRDSTQKAIDKITSKVNNIVDKMNKQYEIIDHEFSEVETRLNMINN
KVDDQIQDIWAYNAELLVLLENQKTLDEHDANVNNLYNKVKRALGSNAVEDGKGCFELYHKCDDHCMETI
RNGTYNRRKYQEESKLERQKIEGVKLESEETYKILTIYSTVASSLVIAMGFAAFLFWAMSNGSCRCNICI

>QOJ98638.1 hemagglutinin [Influenza A virus]
METVSLITILVVATVSNADKICIGYQSTNSTETVDTLTENNVPVTHAKELLHTEHNGMLCATSLGHPLIL
DTCTIEGLIYGNPSCDPLLGGREWSYIVERPSAVNGLCYPGNVENLEELRSLFSSSRSYQRIQIFPDTIW
NVSYSGTSKACSDSFYRSMRWLTQKNNAYPTQDAQYTNNQGKNILFMWGINHPPTDTAQTNLYTRTDTTT
SVATEEMNRIFKPLIGPRPLVNGLMGRINYYWSVLKPGQTLRIKSDGNLIAPWYGHILSGESHGRILKTD
LKRGSCTVQCQTEKGGLNTTLPFQNVSKYAFGNCSKYIGVKSLKLAVGLRNVPSRSSRGLFGAIAGFIEG
GWSGLVAGWYGFQHSNDQGVGMAADRDSTQKAIDKITSKVNNIVDKMNKQYEIIDHEFSEVETRLNMINN
KVDDQIQDIWAYNAELLVLLENQKTLDEHDANVNNLYNKVKRALGSNAVEDGKGCFELYHKCDDHCMETI
RNGTYNRRKYQEESKLERQKIEGVKLESEETYKILTIYSTVASSLVIAMGFAAFLFWAMSNGSCRCNICI

>QOJ98637.1 hemagglutinin [Influenza A virus]
METVSLITILVVATVSNADKICIGYQSTNSTETVDTLTENNVPVTHAKELLHTEHNGMLCATSLGHPLIL
DTCTIEGLIYGNPSCDPLLGGREWSYIVERPSAVNGLCYPGNVENLEELRSLFSSSRSYQRIQIFPDTIW
NVSYSGTSKACSDSFYRSMRWLTQKNNAYPTQDAQYTNNQGKNILFMWGINHPPTDTAQTNLYTRTDTTT
SVATEEMNRIFKPLIGPRPLVNGLMGRINYYWSVLKPGQTLRIKSDGNLIAPWYGHILSGESHGRILKTD
LKRGSCTVQCQTEKGGLNTTLPFQNVSKYAFGNCSKYIGVKSLKLAVGLRNVPSRSSRGLFGAIAGFIEG
GWSGLVAGWYGFQHSNDQGVGMAADRDSTQKAIDKITSKVNNIVDKMNKQYEIIDHEFSEVETRLNMINN
KVDDQIQDIWAYNAELLVLLENQKTLDEHDANVNNLYNKVKRALGSNAVEDGKGCFELYHKCDDHCMETI
RNGTYNRRKYQEESKLERQKIEGVKLESEETYKILTIYSTVASSLVIAMGFAAFLFWAMSNGSCRCNICI

>QOJ98635.1 hemagglutinin [Influenza A virus]
METVSLITILIVATVSNADKICIGYQSTNSTETVDTLTENNVPVTHAKELLHTEHNGMLCATSLGHPLIL
DTCTIEGLIYGNPSCDPLLGGREWSYIVERPSAVNGLCYPGNVENLEELRSLFSSSRSYQRIQIFPDTIW
NVSYSGTSKACSDSFYRSMRWLTQKNNAYPTQDAQYTNNQGKNILFVWGINHPPTDTAQTNLYTRTDTTT
SVATEEMNRIFKPLIGPRPLVNGLMGRINYYWSVLKPGQTLRIKSDGNLIAPWYGHILSGESHGRILKTD

LKRGSCTVQCQTEKGGLNTTLPFQNVSKYAFGNCSKYIGVKSLKLAVGLRNVPSRSSRGLFGAIAGFIEG
GWSGLVAGWYGFQHSNDQGVGMAADRDSTQKAIDKITSKVNNIVDKMNKQYEIIDHEFSEVETRLNMINN
KVDDQIQDIWAYNAELLVLLENQKTLDEHDANVNNLYNKVKRALGSNAVEDGKGCFELYHKCDDHCMETI
RNGTYNRRKYQEESKLERQKIEGVKLESEETYKILTIYSTVASSLVIAMGFAAFLFWAMSNGSCRCNICI

>QOJ98633.1 hemagglutinin [Influenza A virus]
METVSLITILVVATVSNADKICIGYQSTNSTETVDTLTENNVPVTHAKELLHTEHNGMLCATSLGHPLIL
DTCTIEGLIYGNPSCDPLLGGREWSYIVERPSAVNGLCYPGNVENLEELRSLFSSSRSYQRIQIFPDTIW
NVSYSGTSKACSDSFYRSMRWLTQKNNAYPTQDAQYTNNQGKNILFMWGINHPPTDTAQTNLYTRTDTTT
SVATEEMNRIFKPLIGPRPLVNGLMGRINYYWSVLKPGQTLRIKSDGNLIAPWYGHILSGESHGRILKTD
LKRGSCTVQCQTEKGGLNTTLPFQNVSKYAFGNCSKYIGVKSLKLAVGLRNVPSRSSRGLFGAIAGFIEG
GWSGLVAGWYGFQHSNDQGVGMAADRDSTQKAIDKITSKVNNIVDKMNKQYEIIDHEFSEVETRLNMINN
KVDDQIQDIWAYNAELLVLLENQKTLDEHDANVNNLYNKVKRALGSNAVEDGKGCFELYHKCDDHCMETI
RNGTYNRRKYQEESKLERQKIEGVKLESEETYKILTIYSTVASSLVIAMGFAAFLFWAMSNGSCRCNICI

>QOJ98632.1 hemagglutinin [Influenza A virus]
METISLITILIVTTVSNADKICIGYQSTNSTETVDTLTENNVPVTHAKELLHTEHNGMLCATSLGQPLIL
ETCTIEGLIYGNPSCDLSLEGREWSYIVERPSAVNGLCYPGNVENLEELRSLFSSARSYQRIQIFPDTIW
NVSYDGTSIACSNSFYRSMRWLTRKNDNYPTQDAQYTNNQGKNILFMWGINHPPTDDTQRNLYTRTDTTT
SVATEEINRIFKPLIGPRPLVNGLMGRIDYYWSVLKPGQTLRIKSDGNLIAPWYGHILSGESHGRILKTD
LKRGSCTVQCQTEKGGLNTTLPFQNVSKYAFGNCSKYIGIKSLKLAVGLRNVPSRSSRGLFGAIAGFIEG
GWSGLVAGWYGFQHSNDQGVGMAADRDSTQKAIDKITSKVNNIVDKMNKQYEIIDHEFSEVETRLNMINN
KIDDQIQDIWAYNAELLVLLENQKTLDEHDANVNNLYNKVKRALGSNAVEDGKGCFELYHKCNDQCMETI
RNGTYNRKKYQEESKLERQKIEGVKLESEGTYKILTIYSTVASSLVIAMGFAAFLFWAMSNGSCRCNICI

>QOJ98631.1 hemagglutinin [Influenza A virus]
METVSLITILVVATVSNADKICIGYQSTNSTETVDTLTENNVPVTHAKELLHTEHNGMLCATSLGHPLIL
DTCTIEGLIYGNPSCDPLLGGREWSYIVERPSAVNGLCYPGNVENLEELRSLFSSSRSYQRIQIFPDTIW
NVSYSGTSKACSDSFYRSMRWLTQKNNAYPTQDAQYTNNQGKNILFMWGINHPPTDTAQTNLYTRTDTTT
SVATEEMNRIFKPLIGPRPLVNGLMGRINYYWSVLKPGQTLRIKSDGNLIAPWYGHILSGESHGRILKTD
LKRGSCTVQCQTEKGGLNTTLPFQNVSKYAFGNCSKYIGVKSLKLAVGLRNVPSRSSRGLFGAIAGFIEG
GWSGLVAGWYGFQHSNDQGVGMAADRDSTQKAIDKITSKVNNIVDKMNKQYEIIDHEFSEVETRLNMINN
KVDDQIQDIWAYNAELLVLLENQKTLDEHDANVNNLYNKVKRALGSNAVEDGKGCFELYHKCDDHCMETI
RNGTYNRRKYQEESKLERQKIEGVKLESEETYKILTIYSTVASSLVIAMGFAAFLFWAMSNGSCRCNICI

>QOJ98629.1 hemagglutinin [Influenza A virus]

METVSLITILVVVTVSNADKICIGYQSTNSTETVDTLTENNVPVTHAKELLHTEHNGMLCATSLGHPLIL
DTCTIEGLIYGNPSCDPLLGGREWSYIVERPSAVNGLCYPGNVENLEELRSLFSSSRSYQRIQIFPDTIW
NVSYSGTSKACSDSFYRSMRWLTQKNNAYPTQDAQYTNNQGKNILFMWGINHPPTDTAQTNLYTRTDTTT
SVATEEMNRIFKPLIGPRPLVNGLMGRINYYWSVLKPGQTLRIKSDGNLIAPWYGHILSGESHGRILKTD
LKRGSCTVQCQTEKGGLNTTLPFQNVSKYAFGNCSKYIGVKSLKLAVGLRNVPSRSSRGLFGAIAGFIEG
GWSGLVAGWYGFQHSNDQGVGMAADRDSTQKAIDKITSKVNNIVDKMNKQYEIIDHEFSEVETRLNMINN
KVDDQIQDIWAYNAELLVLLENQKTLDEHDANVNNLYNKVKRALGSNAVEDGKGCFELYHKCDDHCMETI
RNGTYNRRKYQEESKLERQKIEGVKLESEETYKILTIYSTVASSLVIAMGFAAFLFWAMSNGSCRCNICI

>QOJ98628.1 hemagglutinin [Influenza A virus]

METVSLITILVVATVSNADKICIGYQSTNSTETVDTLTENNVPVTHAKELLHTEHNGMLCATSLGHPLIL
DTCTIEGLIYGNPSCDPLLGGREWSYIVERPSAVNGLCYPGNVENLEELRSLFSSSRSYQRIQIFPDTIW
NVSYSGTSKACSDSFYRSMRWLTQKNNAYPTQDAQYTNNQGKNILFMWGINHPPTDTAQTNLYTRTDTTT
SVATEEMNRIFKPLIGPRPLVNGLMGRINYYWSVLKPGQTLRIKSDGNLIAPWYGHILSGESHGRILKTD
LKRGSCTVQCQTEKGGLNTTLPFQNVSKYAFGNCSKYIGVKSLKLAVGLRNVPSRSSRGLFGAIAGFIEG
GWSGLVAGWYGFQHSNDQGVGMAADRDSTQKAIDKITSKVNNIVDKMNKQYEIIDHEFSEVETRLNMINN
KVDDQIQDIWAYNAELLVLLENQKTLDEHDANVNNLYNKVKRALGSNAVEDGKGCFELYHKCDDHCMETI
RNGTYNRRKYQEESKLERQKIEGVKLESEETYKILTIYSTVASSLVIAMGFAAFLFWAMSNGSCRCNICI

>QOJ98626.1 hemagglutinin [Influenza A virus]

METVSLITILVVATVSNADKICIGYQSTNSTETVDTLTENNVPVTHAKELLHTEHNGMLCATSLGHPLIL
DTCTIEGLIYGNPSCDPLLGGREWSYIVERPSAVNGLCYPGNVENLEELRSLFSSSRSYQRIQIFPDTIW
NVSYSGTSKACSDSFYRSMRWLTQKNNAYPTQDAQYTNNQGKNILFMWGINHPPTDTAQTNLYTRTDTTT
SVATEEMNRIFKPLIGPRPLVNGLMGRINYYWSVLKPGQTLRIKSDGNLIAPWYGHILSGESHGRILKTD
LKRGSCTVQCQTEKGGLNTTLPFQNVSKYAFGNCSKYIGVKSLKLAVGLRNVPSRSSRGLFGAIAGFIEG
GWSGLVAGWYGFQHSNDQGVGMAADRDSTQKAIDKITSKVNNIVDKMNKQYEIIDHEFSEVETRLNMINN
KVDDQIQDIWAYNAELLVLLENQKTLDEHDANVNNLYNKVKRALGSNAVEDGKGCFELYHKCDDHCMETI
RNGTYNRRKYQEESKLERQKIEGVKLESEETYKILTIYSTVASSLVIAMGFAAFLFWAMSNGSCRCNICI

>QOJ98623.1 hemagglutinin, partial [Influenza A virus]

NADKICIGYQSTNSTETVDTLTENNVPVTHAKELLHTEHNGMLCATSLGHPLILDTCTIEGLIYGNPSCD
PLLGGREWSYIVERPSAVNGLCYPGNVENLEELRSLFSSSRSYQRIQIFPDTIWNVSYSGTSKACSDSFY

RSMRWLTQKNNAYPTQDAQYTNNQGKNILFMWGINHPPTDTAQTNLYTRTDTTTSVATEEMNRIFKPLIG
PRPLVNGLMGRINYYWSVLKPGQTLRIKSDGNLIAPWYGHILSGESHGRILKTDLKRGSCTVQCQTEKGG
LNTTLPFQNVSKYAFGNCSKYIGVKSLKLAVGLRNVPSRSSRGLFGAIAGFIEGGWSGLVAGWYGFQHSN
DQGVGMAADRDSTQKAIDKITSKVNNIVDKMNKQYEIIDHEFSEVETRLNMINNKVDDQIQDIWAYNAEL
LVLLENQKTLDEHDANVNNLYNKVKRALGSNAVEDGKGCFELYHKCDDHCMETIRNGTYNRRKYQEESKL
ERQKIEGVKLESEETYKILTIYSTVASSLVIAMGFAAFLFWAMSNGSCRCNICI

>QOJ98621.1 hemagglutinin, partial [Influenza A virus]
ETVSLITILLVATASNADKICIGYQSTNSTETVDTLTENNVPVTHAKELLHTEHNGMLCATSLGQPLILD
TCTVEGLIYGNPSCDLSLEGREWSYIVERPSAVNGLCYPGNVENLEELRSLFSSARSYQRIQIFPDTIWN
VSYDGTSKACSDSFYRSMRWLTQKDGNYPTQDAQYTNNQGKNILFMWGINHPPTDDTQRNLYTRTDTTTS
VATEEINRIFKPLIGPRPLVNGLMGRIDYYWSVLKPGQTLRIKSNGNLIAPWYGHILTGESHGRILKTDL
KRGSCTVQCQTEKGGLNTTLPFQNVSKYAFGNCSKYIGIKSLKLAVGLRNVPSRSSRGLFGAIAGFIEGG
WSGLVAGWYGFQHSNDQGVGMAADRDSTQKAIDKITSKVNNIVDKMNKQYEIIDHEFSEVETRLNMINNK
IDDQIQDIWAYNAELLVLLENQKTLDEHDANVNNLYNKVKRALGSNAVEDGKGCFELYHKCNDQCMETIR
NGTYNRRKYQEESKLKRQKIEGVKLESEGTYKILTIYSTVASSLVIAMGFAAFLFWAMSNGSC

>QOJ98620.1 hemagglutinin, partial [Influenza A virus]
ETVSLITILLVATASNADKICIGYQSTNSTETVDTLTENNVPVTHAKELLHTEHNGMLCATSLGQPLILD
TCTVEGLIYGNPSCDLSLEGREWSYIVERPSAVNGLCYPGNVENLEELRSLFSSARSYQRIQIFPDTIWN
VSYDGTSKACSDSFYRSMRWLTQKDGNYPTQDAQYTNNQGKNILFMWGINHPPTDDTQRNLYTRTDTTTS
VATEEINRIFKPLIGPRPLVNGLMGRIDYYWSVLKPGQTLRIKSNGNLIAPWYGHILTGESHGRILKTDL
KRGSCTVQCQTEKGGLNTTLPFQNVSKYAFGNCSKYIGIKSLKLAVGLRNVPSRSSRGLFGAIAGFIEGG
WSGLVAGWYGFQHSNDQGVGMAADRDSTQKAIDKITSKVNNIVDKMNKQYEIIDHEFSEVETRLNMINNK
IDDQIQDIWAYNAELLVLLENQKTLDEHDANVNNLYNKVKRALGSNAVEDGKGCFELYHKCNDQCMETIR
NGTYNRRKYQEESKLKRQKIEGVKLESEGTYKILTIYSTVASSLVIAMGFAAFLFWAMSNGSC

>QOJ98609.1 hemagglutinin, partial [Influenza A virus]
METVSLITILVVATVSNADKICIGYQSTNSTETVDTLTENNVPVTHAKELLHTEHNGMLCATGLGHPLIL
DTCTIEGLIYGNPSCDPLLGGREWSYIVERPSAVNGLCYPGNVENLEELRSLFSSSRSYQRIQIFPDTIW
NVSYSGTSKACSDSFYRSMRWLTQKNNAYPTQDAQYTNNQGKNILFMWGINHPPTDTVQTNLYTRTDTTT
SVATEEINRIFKPLIGPRPLVNGLMGRINYYWSVLKPGQTLRIKSDGNLIAPWYGHILSGESHGRILKTD
LKRGSCTVQCQTEKGGLNTTLPFQNVSKYAFGNCSKYIGVKSLKLAVGLRNVPSRSSRGLFGAIAGFIEG
GWPGLVAGWYGFQHSNDQGVGMAADRDSTQKAIDKITSKVNNIVDKMNKQYEIIDHEFSEVETRLNMINN

KVDDQIQDIWAYNAELLVLLENQKTLDEHDANVNNLYNKVKRALGSNAVEDGKGCFELYHKCDDHCMETI
RNGTYNRRKYQEESKLERQKIEGVKLESEETYKILTIYSTVASSLVIAMGFAAFLFWAMSNGSC

>QOJ98607.1 hemagglutinin, partial [Influenza A virus]
ETVSLITILLVATASNADKICIGYQSTNSTETVDTLTENNVPVTHAKELLHTEHNGMLCATSLGQPLILD
TCTVEGLIYGNPSCDLSLEGREWSYIVERPSAVNGLCYPGNVENLEELRSLFSSARSYQRIQIFPDTIWN
VSYDGTSKACSDSFYRSIRWLTQRDGNYPTQDAQYTNNQGKNILFMWGINHPPTDDTQRNLYTRTDTTTS
VATEEINRIFKPLIGPRPLVNGLMGRIDYYWSVLKPGQTLRIKSNGNLIAPWYGHILTGESHGRILKTDL
KRGSCTVQCQTEKGGLNTTLPFQNVSKYAFGNCSKYIGIKSLKLAVGLRNVPSRSSRGLFGAIAGFIEGG
WSGLVAGWYGFQHSNDQGVGMAADRDSTQKAIDKITSKVNNIVDKMNKQYEIIDHEFSEIETRLNMINNK
IDDQIQDIWAYNAELLVLLENQKTLDEHDANVNNLYNKVKRALGSNAVEDGKGCFELYHKCDDQCMETIR
NGTYNRRKYQEESKLKRQKIEGVKLESEGTYKILTIYSTVASSLVIAMGFAAFLFWAMSNGSC

>QOJ98606.1 hemagglutinin, partial [Influenza A virus]
ETVSLITILLVATVSNADKICIGYQSTNSTETVDTLTENNVPVTHAKELLHTEHNGMLCATSLGQPLILN
TCTIEGLIYGNPSCDLSLEGREWSYIVERPSAVHGLCYPGNVEDLEELRSLFSSARSYQRIQIFPDTIWN
VSYDGTSTACSGSFYRSMRWLTRKNGEYPIQDAQYTNNQGKNILFMWGINHPPTDNTQRELYTRTDTTTS
VATEEINRIFKPLIGPRPLVNGLMGRINYYWSVLKPGQTLRIKSDGNLIAPWYGHILSGESHGRILKTDL
KRGSCTVQCQTEKGGLNTTLPFQNVSKYAFGNCSKYIGIKSLKLAVGLRNVPSRSSRGLFGAIAGFIEGG
WSGLVAGWYGFQHSNDQGVGMAADRDSTQKAIDKITSKVNNIVDKMNKQYEIIDHEFSEVETRLNMINNK
IDDQIQDIWAYNAELLVLLENQKTLDEHDANVNNLYNKVKRALGSNAVEDGKGCFELYHKCDDQCMETIR
NGTYNRRKYQEESKLERQKIEGVKLESEGTYKILTIYSTVASSLVIAMGFAAFLFWAMSNGSC

>QOJ98605.1 hemagglutinin, partial [Influenza A virus]
ETVSLITILLVATASNADKICIGYQSTNSTETVDTLTENNVPVTHAKELLHTEHNGMLCATSLGQPLILD
TCTVEGLIYGNPSCDLSLEGREWSYIVERPSAVNGLCYPGNVENLEELRSLFSSARSYQRIQIFPDTIWN
VSYDGTSKACSDSFYRSMRWLTQKDGNYPTQDAQYTNNQGKNILLMWGINHPPTDDTQRNLYTRTDTTTS
VATEEINRIFKPLIGPRPLVNGLMGRIDYYWSVLKPGQTLRIKSNGNLIAPWYGHILTGESHGRILKTDL
KRGSCTVQCQTEKGGLNTTLPFQNVSKYAFGNCSKYIGIKSLKLAVGLRNVPSRSSRGLFGAIAGFIEGG
WSGLVAGWYGFQHSNDQGVGMAADRDSTQKAIDKITSKVNNIVDKMNKQYEIIDHEFSEVETRLNMINNK
IDDQIQDIWAYNAELLVLLENQKTLDEHDANVNNLYNKVKRALGSNAVEDGKGCFELYHKCNDQCMETIR
NGTYNRRKYQEESKLKRQKIEGVKLESEGTYKILTIYSTVASSLVIAMGFAAFLFWAMSNGSC

>QOJ98604.1 hemagglutinin, partial [Influenza A virus]

ETVSLITILLVATASNADKICIGYQSTNSTETVDTLTENNVPVTHAKELLHTEHNGMLCATSLGQPLILD
TCTVEGLIYGNPSCDLSLEGREWSYIVERPSAVNGLCYPGNVENLEELRLLFSSARSYQRIQIFPDTIWN
VSYDGTSKACSDSFYRSMRWLTRKDGNYPTQDAQYTNNQGKNILFMWGINHPPTDDTQRNLYTRTDTTTS
VATEEINRIFKPLIGPRPLVNGLMGRIDYYWSVLKPGQTLRIKSNGNLIAPWYGHILTGESHGRILKTDL
KRGSCTVQCQTEKGGLNTTLPFQNVSKYAFGNCSKYIGIKSLKLAVGLRNVPSRSSRGLFGAIAGFIEGG
WSGLVAGWYGFQHSNDQGVGMAADRDSTQKAIDKITSKVNNIVDKMNKQYEIIDHEFSEVETRLNMINNK
IDDQIQDIWAYNAELLVLLENQKTLDEHDANVNNLYNKVKRALGSNAVEDGKGCFELYHKCNDQCMETIR
NGTYNRRKYQEESKLKRQKIEGVKLESEGTYKILTIYSTVASSLVIAMGFAAFLFWAMSNGSC

>QOJ98603.1 hemagglutinin, partial [Influenza A virus]
ETVSLITILLVATASNADKICIGYQSTNSTETVDTLTENNVPVTHAKELLHTEHNGMLCATSLGQPLILD
TCTVEGLIYGNPSCDLSLEGREWSYIVERPSAVNGLCYPGNVENLEELRSLFSSARSYQRIQIFPDTIWN
VSYDGTSKACSDSFYRSMRWLTQKGGNYPTQDAQYTNNQGKNILFMWGINHPPTDDTQRNLYTRTDTTTS
VATEEINRIFKPLIGPRPLVNGLMGRIDYYWSVLKPGQTLRIKSNGNLIAPWYGHILTGESHGRILKTDL
KRGSCTVQCQTEKGGLNTTLPFQNVSKYAFGNCSKYIGIKSLKLAVGLRNVPSRSSRGLFGAIAGFIEGG
WSGLVAGWYGFQHSNDQGVGMAADRDSTQKAIDKITSKVNNIVDKMNKQYEIIDHEFSEVETRLNMINNK
IDDQIQDIWAYNAELLVLLENQKTLDEHDANVNNLYNKVKRALGSNAVEDGKGCFELYHKCNDQCMETIR
NGTYNRRKYQEESKLKRQKIEGVKLESEGTYKILTIYSTVASSLVIAMGFAAFLFWAMSNGSC

>QOJ98602.1 hemagglutinin, partial [Influenza A virus]
ETVSLITILLVATASNADKICIGYQSTNSTETVDTLTENNVPVTHAKELLHTEHNGMLCATSLGQPLILD
TCTVEGLIYGNPSCDLSLEGREWSYIVERPSAVNGLCYPGNVENLEELRSLFSSARSYQRIQIFPDTIWN
VSYDGTSKACSDSFYRSMRWLTQKDGNYPTQDAQYTNNQGKNILFMWGINHPPTDDTQRNLYTRTDTTTS
VATEEINRIFKPLIGPRPLVNGLMGRIDYYWSVLKPGQTLRIKSNGNLIAPWYGHILTGESHGRILKTDL
KRGSCTVQCQTEKGGLNTTLPFQNVSKYAFGNCSKYIGIKSLKLAVGLRNVPSRSSRGLFGAIAGFIEGG
WSGLVAGWYGFQHSNDQGVGMAADRDSTQKAIDKITSKVNNIVDKMNKQYEIIDHEFSEVETRLNMINNK
IDDQIQDIWAYNAELLVLLENQKTLDEHDANVNNLYNKVKRALGSNAVEDGKGCFELYHKCNDQCMETIR
NGTYNRRKYQEESKLKRQKIEGVKLESEGTYKILTIYSTVASSLVIAMGFAAFLFWAMSNGSC

>QOJ98601.1 hemagglutinin [Influenza A virus]
METVSLITILLVATVSNADKICIGYQSTNSTETVDTLTENNVPVTHAKELLHTEHNGMLCATSLGQPLIL
DTCTIEGLIYGNPSCDLSLEGREWSYIVERPSAVHGLCYPGNVENLEELRSLFSSARSYQRIQIFPDTIW
NVSYDGTSTACSGSFYRSMRWLTRKNGDYPIQDAQYTNNQGKNILFMWGINHPPTDETQRGLYTRTDTTT
SVATEEINRIFKPLIGPRPLVNGLMGRINYYWSVLKPGQTLRIKSDGNLIAPWYGHILSGESHGRILKTD

LKRGSCTVQCQTEKGGLNTTLPFQNVSKYAFGNCSKYIGIKSLKLAVGLRNVPSRSSRGLFGAIAGFIEG
GWSGLVAGWYGFQHSNDQGVGMAADRDSTQKAIDKITSKVNNIVDKMNKQYEIIDHEFSEVETRINMINN
KIDDQIQDIWAYNAELLVLLENQKTLDEHDANVNNLYNKVKRALGSNAVEDGKGCFELYHKCDDQCMETI
RNGTYNRRKYQEESKLERQKIEGVKLESEGTYKILTIYSTVASSLVIAMGFAAFLFWAMSNGSCRCNICI

>QOJ98595.1 hemagglutinin [Influenza A virus]
METISLMTILLVATVSNADKICIGYQSTNSTETVDTLTENNVPVTHAKELLHTEHNGMLCATSLGNPLIL
DTCTIEGLIYGNPSCDPLLGGREWSYIVERPSAVNGLCYPGSVENLEELRSLFSSARSYQRIQIFPDTIW
KLSYSGTSKACSDSFYRSMRWLTQKNNAYPTQDAQYTNNQGKNILFMWGINHPPTDTAQTNLYTRTDTTT
SVATEEINRIFKPLIGPRPLVNGLMGRIDYYWSVLKPGQTLRIKSDGNLIAPWYGHILSGESHGRILKTD
LKRGSCTVQCQTEKGGLNTTLPFQNVSKYAFGNCSKYIGIKSLKLAVGLRNVPSRSSRGLFGAIAGFIEG
GWSGLVAGWYGFQHSNDQGVGMAADRDSTQKAIDKITSKVNNIVDKMNKQYEIIDHEFSEVETRLNMINN
KVDDQIQDIWAYNAELLVLLENQKTLDEHDSNVNNLYNKVKRALGSNAVEDGKGCFELYHKCDNQCMETI
RNGTYNRRKYQEESKLERQKIEGVKLESEGTYKILTIYSTVASSLVIAMGFAAFLFWAMSNGSCRCNICI

>QOJ98585.1 hemagglutinin [Influenza A virus]
METVSLITILLVATVSNADKICIGYQSTNSTETVDTLTENNVPVTHAKELLHTEHNGMLCATSLGQPLIL
DTCTIEGLIYGNPSCDLSLEGREWSYIVERPSAVHGLCYPGNVEDLEELRSLFSSARSYQRIQIFPDTIW
NVSYDGTSTACSGSFYRSMRWLTRKNGEYPIQDAQYTNNQGKNILFMWGINHPPTDDTQRGLYTRTDTTT
SVATEEINRIFKPLIGPRPLVNGLMGRINYYWSVLKPGQTLRIKSDGNLIAPWYGHILSGESHGRILKTD
LKRGSCTVQCQTEKGGLNTTLPFQNVSKYAFGNCSKYIGIKSLKLAVGLRNVPSRSSRGLFGAIAGFIEG
GWSGLVAGWYGFQHSNDQGVGMAADRDSTQKAIDKITSKVNNIVDKMNKQYEIIDHEFSEVETRLNMINN
KIDDQIQDIWAYNAELLVLLENQKTLDEHDANVNNLYNKVKRALGSNAVEDGKGCFELYHKCDDQCMETI
RNGTYNRRKYQEESKLERQKIEGVKLESEGTYKILTIYSTVASSLVIAMGFAAFLFWAMSNGSCRCNICI

>QOJ98583.1 hemagglutinin [Influenza A virus]
METVSLITILLVATVSNADKICIGYQSTNSTETVDTLTENNVPVTHAKELLHTEHNGMLCATSLGQPLIL
DTCTIEGLIYGNPSCDLSLEGREWSYIVERPSAVHGLCYPGNVEDLEELRSLFSSARSYQRIQIFPDTIW
NVSYDGTSTACSGSFYRSMRWLTRKNGEYPIQDAQYTNNQGKNILFMWGINHPPTDDTQRGLYTRTDTTT
SVATEEINRIFKPLIGPRPLVNGLMGRINYYWSVLKPGQTLRIKSDGNLIAPWYGHILSGESHGRILKTD
LKRGSCTVQCQTEKGGLNTTLPFQNVSKYAFGNCSKYIGIKSLKLAVGLRNVPSRSSRGLFGAIAGFIEG
GWSGLVAGWYGFQHSNDQGVGMAADRDSTQKAIDKITSKVNNIVDKMNKQYEIIDHEFSEVETRLNMINN
KIDDQIQDIWAYNAELLVLLENQKTLDEHDANVNNLYNKVKRALGSNAVEDGKGCFELYHKCDDQCMETI
RNGTYNRRKYQEESKLERQKIEGVKLESEGTYKILTIYSTVASSLVIAMGFAAFLFWAMSNGSCRCNICI

>QOJ98582.1 hemagglutinin [Influenza A virus]

METVSLITILLVATVSNADKICIGYQSTNSTETVDTLTESNVPVTHAKELLHTEHNGMLCATSLGQPLIL

DTCTIEGLIYGNPSCDLSLEGREWSYIVERPSAVHGLCYPGNVEDLEELRSLFSSARSYQRIQIFPDTIW

NVSYDGTSTACSGSFYRSMRWLTRKNGEYPIQDAQYTNNQGKNILFMWGINHPPTDDTQRKLYTRTDTTT

SVATEEINRIFKPLIGPRPLVNGLMGRINYYWSVLKPGQTLRIKSDGNLIAPWYGHILSGESHGRILKTD

LKRGSCTVQCQTEKGGLNTTLPFQNVSKYAFGNCSKYIGIKSLKLAVGLRNVPSRSSRGLFGAIAGFIEG

GWSGLVAGWYGFQHSNDQGVGMAADRDSTQKAIDKITSKVNNIVDKMNKQYEIIDHEFSEVETRLNMINN

KIDDQIQDIWAYNAELLVLLENQKTLDEHDANVNNLYNKVKRALGSNAVEDGKGCFELYHKCDDQCMETI

RNGTYNRRKYQEESKLERQKIEGVKLESEGTYKILTIYSTVASSLVIAMGFAAFLFWAMSNGSCRCNICI

>QOJ98579.1 hemagglutinin [Influenza A virus]

METVSLITILLIATVSNADKICIGYQSTNSTETVDTLTENNVPVTHAKELLHTEHNGMLCATSLGQPLIL

DTCTIEGLIYGNPSCDLSLEGREWSYIVERPSAVHGLCYPGNVEDLEELRSLFSSARSYQRIQIFPDTVW

NVSYDGTSTACSGSFYRSMRWLTRKNGEYPIQDAQYTNNQGKNILFMWGINHPPTDDTQRGLYTRTDTTT

SVATEEINRIFKPLIGPRPLVNGLMGRINYYWSILKPGQTLRIKSDGNLIAPWYGHILSGESHGRILKTD

LKRGSCTVQCQTEKGGLNTTLPFQNVSKYAFGNCSKYIGIKSLKLAVGLRNVPSRSSRGLFGAIAGFIEG

GWSGLVAGWYGFQHSNDQGVGMAADRESTQKAIDKITSKVNNIVDKMNKQYEIIDHEFSEVETRLNMINN

KIDDQIQDIWAYNAELLVLLENQKTLDEHDANVNNLYNKVKRALGSNAVEDGKGCFELYHKCDDQCMETI

RNGTYNRREYQEESKLERQRIEGVKLESEGTYKILTIYSTVASSLVIAMGFAAFLFWAMSNGSCRCNICI

>QOJ98578.1 hemagglutinin [Influenza A virus]

METVSLITILLIATVSNADKICIGYQSTNSTETVDTLTENNVPVTHAKELLHTEHNGMLCATSLGQPLIL

DTCTIEGLIYGNPSCDLSLEGREWSYIVERPSAVHGLCYPGNVEDLEELRSLFSSARSYQRIQIFPDTVW

NVSYDGTSTACSGSFYRSMRWLTRKNGEYPIQDAQYTNNQGKNILFMWGINHPPTDDXQRGLYTRTDTTT

SVATEEINRIFKPLIGPRPLVNGLMGRINYYWSILKPGQTLRIKSDGNLIAPWYGHILSGESHGRILKTD

LKRGSCTVQCQTEKGGLNTTLPFQNVSKYAFGNCSKYIGIKSLKLAVGLRNVPSRSSRGLFGAIAGFIEG

GWSGLVAGWYGFQHSNDQGVGMAADRESTQKAIDKITSKVNNIVDKMNKQYEIIDHEFSEVETRLNMINN

KIDDQIQDIWAYNAELLVLLENQKTLDEHDANVNNLYNKVKRALGSNAVEDGKGCFELYHKCDDQCMETI

RNGTYNRREYQEESKLERQRIEGVKLESEGTYKILTIYSTVASSLVIAMGFAAFLFWAMSNGSCRCNICI

>AXY36264.1 hemagglutinin [Influenza A virus]

METVSLITILVVATVSNADKICIGYQSTNSTETVDTLTENNVPVTHAKELLHTEHNGMLCATSLGHPLIL

DTCTIEGLIYGNPSCDPLLGGREWSYIVERPSAVNGLCYPGNVENLEELRSLFSSSRSYQRIQIFPDTIW

NVSYSGTSKACSDSFYRSMRWLTQKNSAYPTQDAQYTNNQGKNILFMWGINHPPTDTAQTNLYTRTDTTT
SVATEEMNRIFKPLIGPRPLVNGLMGRINYYWSVLKPGQTLRIKSDGNLIAPWYGHILSGESHGRILKTD
LKRGSCTVQCQTERGGLNTTLPFQNVSKYAFGNCSKYIGVKSLKLAVGLRNVPSRSSRGLFGAIAGFIEG
GWSGLVAGWYGFQHSNDQGVGMAADRDSTQKAIDKITSKVNNIVDKMNKQYEIIDHEFSEVETRLNMINN
KVDDQIQDIWAYNAELLVLLENQKTLDEHDANVNNLYNKVKRALGSNAVEDGKGCFELYHKCDDHCMETI
RNGTYNRRKYQEESKLERQKIEGVKLESEETYKILTIYSTVASSLVIAMGFAAFLFWAMSNGSCRRNICI

>AXY36252.1 hemagglutinin [Influenza A virus]
METVSLITILVVATVSNADKICIGYQSTNSTETVDTLTENNVPVTHAKELLHTEHNGMLCATSLGHPLIL
DTCTIEGLIYGNPSCDPLLGGREWSYIVERPSAVNGLCYPGNVENLEELRSLFSSSRSYQRIQIFPDTIW
NVSYGGTSKACSDSFYRSMRWLTQKNNAYPTQDAQYTNNQGKNILFMWGINHPPTDTVQTNLYTRTDTTT
SVATEEINRIFKPLIGPRPLVNGLMGRINYYWSVLKPGQTLRIKSDGNLIAPWYGHILSGESHGRILKTD
LKRGSCTVQCQTERGGLNTTLPFQNISKYAFGNCSKYIGVKSLKLAVGLRNVPSRSSRGLFGAIAGFIEG
GWSGLVAGWYGFQHSNDQGVGMAADRDSTQKAIDKITSKVNNIVDKMNKQYEIIDHEFNEVETRLNMINN
KADDQIQDIWAYNAELLVLLENQKTLDEHDANVNNLYNKVKRALGSNAVEDGKGCFELYHKCDDHCMETI
RNGTYNRRKYQEESKLERQKIEGVKLESEETYKILTIYSTVASSLVIAMGFAAFLFWAMSNGSCRCNICI

>AXY36240.1 hemagglutinin [Influenza A virus]
METVSLITILLVATVSNADKICIGYQSTNSTETVDTLTENNVPVTHAKELIHTEHNGMLCATSLGQPLIL
DTCTIEGLIYGNPSCDLSLEGREWSYIVERPSAVNGLCYPGNVENLEELRSLFSSARSYQRIQIFPDTIW
NVSYDGTSTACSGSFYRSMRWLTRKNSEYPIQDAQYTNSQGKNILFMWGINHPPTDTTQKALYTRTDTTT
SVATEEINRVFKPLIGPRPLVNGLMGRIDYYWSVLKPGQTLRIKSDGNLIAPWYGHILSGESHGRILKTD
LKKGSCTVQCQTEKGGLNTTLPFQNVSKYAFGNCSKYIGIKSLKLAVGLRNVPSRSSRGLFGAIAGFIEG
GWPGLVAGWYGFQHSNDQGVGMAADRDSTQKAIDKITSKVNNIVDKVNKQYEIIDHEFSEVETRLNMINN
KIDDQIQDIWAYNAELLVLLENQKTLDEHDANVNNLYNKVKRALGSNAVEDGEGCFELYHKCDDQCMDTI
RNGTYNRKKYQEESKLERQKIEGVKLESEGTYKILTIYSTVASSLVIAMGFAAFLFWAMSNGSCRRNICI

>AXY36229.1 hemagglutinin [Influenza A virus]
METVSPITILVVATVSNADKICIGYQSTNSTETVDTLTENNVPVTHAKELLHTEHNGMLCATSLGHPLIL
DTCTIEGLIYGNPSCDPLLGGREWSYIVERPSAVNGLCYPGNVENLEELRSLFSSSRSYQRIQIFPDTIW
NVSYSGTSKACSDSFYRSMRWLTQKNNAYPTQDAQYTNNQGKSILFMWGINHPPTDTTQTNLYTRTDTTT
SVATEEMNRIFKPLIGPRPLVNGLMGRINYYWSVLKPGQTLRIKSDGNLIAPWYGHILSGESHGRILKTD
LKRGSCTVQCQTERGGLNTTLPFQNVSKYAFGNCSKYIGVKSLKLAVGLRNVPSRSSRGLFGAIAGFIER
GWSGLVAGWYGFQHSNDQGVGMAADRDSTQKAIDKITSKVNNIVDKMNKQYEIIDHEFSEVETRLNMINN

KVDDQIQDIWAYNAELLVLLENQKTLDEHDANVNNLYNKVKRALGSNAVEDGKGCFELYHKCDDRCMETI
RNGTYNRRKYQEESKLERQKIEGVKLESEETYKILTIYSTVASSLVIAMGFAAFLFWAMSNGSCRRNICI

>BCK74093.1 hemagglutinin [Influenza A virus]
METASLMTILLVVTVSKADKICIGYQSTNSTETVDTLTENNVPVTHAKELLHTEHNGMLCATSLGHPLIL
DTCTIEGLIYGNPSCDLLLGGREWSYIVERPSAVNGLCYPGNVENLEELRSLFSSARSYQRIQIFPDTIW
NVSYSGTSKACSDSFYRSMRWLTQKNNAYPIQDAQYTNNQGKNILFMWGINHPPTDTAQTNLYTRTDTTT
SVATEEINRTFKPLIGPRPLVNGLMGRINYYWSVLKPGQTLRIKSDGNLIAPWYGHILSGESHGRILKTD
LKSGICTVQCQTEKGGLNTTLPFQNVSKYAFGNCSKYIGVKSLKLAVGLRNVPSKSSRGLFGAIAGFIEG
GWSGLVAGWYGFQHSNDQGVGMAADRDSTQKAIDKITSKVNNIVDKMNKQYEIIDHEFSEVETRLNMINN
KIDDQIQDIWAYNAELLVLLENQKTLDEHDANVNNLYNKVKRALGSNAVEDGKGCFELYHKCDNQCMETI
RNGTYNRRKYQEESKLERQKIEGVKLESEGTYKILTIYSTVASSLVIAIGFAAFLFWAMSNGSCRCNICI

>QIC52675.1 hemagglutinin [Influenza A virus]
METVTLMTILLLVTTSNADKICIGHQSTNSTETVDTLTETNVPVTHAKELLHTDHNGVLCATNLGPPLIL
DTCNVEGLIYGNPSCDLLLGGREWSYIVERPSAVNGTCYPGNVENLEELRTLFSSSSSYQRIQIFPDTIW
NVTYTGTSKSCSDSFYRNMRWLTQKNGQYPVQDAQFTNNRGKDILFVWGIHHPPSDTAQTNLYTRTDTTT
SVTTENLDRTFKPLIGPRPLVNGLIGRINYYWSVLKPGQTLRVRSNGNLIAPWYGHVLSGESHGRILKTN
LNSGNCVVQCQTEKGGLNSTLPFHNISKYAFGNCPKYIGVKSLKLAIGLRNVPAKSKRGLFGAIAGFIEG
GWPGLVAGWYGFQHSNDQGVGIAADKVSTQKAVDKITSKVNNIVDKMNKQYEIIDHEFNEVETRLNMINN
KIDDQIQDVWAYNAELLVLLENQKTLDEHDANVNNLYNKVKRALGSNALEDGKGCFELYHKCDDQCMETI
RNGTYNRRKYQEESRLERQKIEGIKLESEGTYKILTIYSTVASSLVLAMGFAAFLFWAMSNGSCRCNICI

>QIC52604.1 hemagglutinin [Influenza A virus]
MKTITLMTILLLVTTSNADKICIGHQSTNSTETVDTLTETNVPVTHAKELLHTDHNGVLCATNLGPPLIL
DTCNVEGLIYGNPSCDLLLGGREWSYIVERPSAVNGTCYPGNVENLEELRTLFSSSSSYQRIQIFPDTIW
NVTYTGTSKSCSDSFYRNMRWLTQKNGQYPVQDAQFTNNRGKDILFVWGIHHPPSDTAQTNLYTRTDTTT
SVTTENLDRTFKPLIGPRPLVNGLIGRINYYWSVLKPGQTLRVRSNGNLIAPWYGHVLSGESHGRILKTN
LNSGNCVVQCQTEKGGLNSTLPFHNISKYAFGNCPKYIGVKSLKLAIGLRNVPAKSKRGLFGAIAGFIEG
GWPGLVAGWYGFQHSNDQGVGIAADKVSTQKAVDKITSKVNNIVDKMNKQYEIIDHEFNEVETRLNMINN
KIDDQIQDVWAYNAELLVLLENQKTLDEHDANVNNLYNKVKRALGSNALEDGKGCFELYHKCDDQCMETI
RNGTYNRRKYQEESRLERQKIEGIKLESEGTYKILTIYSTVASSLVLAMGFAAFLFWAMSNGSCRCNICI

>QIC52479.1 hemagglutinin [Influenza A virus]

METVTLLSILLLVTTSDADKICVGHQSTNSTETVDTLTETNVPVTHAKELLHTDHNGMLCATNLGHPLIL
DACNVEGIIYGNPSCDLLLGGREWSYIVERPSAVNGTCYPGNVENLEELRTLFSSSSSYQRIQIFPDTIW
NVTYTGTSKSCSDSFYRNMRWLTQKNGLYPVQDAQFTNNRGKNILFVWGIHHPPTDTAQTNLYTRTDTTT
SVTTENLDRTFKPLIGPRPLVNGLIGRINYYWSILKPGQTLRVRSNGNLIAPWYGHVLSGESHGRILKTN
LKSGNCVVQCQTEKGGLNSTLPFHNISKYAFGNCPKYIGVKSLKLAIGLRNVPAKSKRGLFGAIAGFIEG
GWPGLVAGWYGFQHSNDQGVGIAADRVSTQQAVDKITSKVNNIVDKMNKQYEIIDHEFSEVETRLNMINN
KIDDQIQDVWAYNAELLVLLENQKTLDEHDANVNNLYNKVKRALGTNALEDGKGCFELYHKCDDQCMETI
RNGTYNRRKYQEESRLERQKIEGVKLESEGTYKILTIYSTAASSLVLAMGFAAFLFWAMSNGSCRCNICI

>QIC52486.1 hemagglutinin [Influenza A virus]
METVTLMTILLLITTSLADKICIGHQSTNSTETVDTLTETNVPVTHAKELLHTDHNGMLCSTNLGHPLIL
DTCNVEGLIYGNPSCDSLLGGREWSYIVERPSAVNGTCYPGNVENLEELRTLFSSSSSYQRILIFPDTIW
NVTYTGTSKSCSDSFYRNMRWLTQKNSLYPVQDAQFTNNRGKDILFVWGIHHPPTDTAQTNLYTRTDTTT
SVTTETLDRTFKPLIGPRPLVNGLIGRINYYWSVLKPGQTLRVRSNGNLIAPWYGHVLSGESHGRILKTN
LNSGNCVVQCQTERGGLNSTLPFHNISKYAFGNCPKYIGVKSLKLAIGLRNVPAKSKRGLFGAIAGFIEG
GWPGLVAGWYGFQHSNDQGVGIAAGKVSTQNAVDKITSKVNNIVDKMNKQYEIIDHEFSEVETRLNMINN
KLDDQIQDVWAYNAELLVLLENQKTLDEHDANVNNLYNKVRRALGSNALEDGKGCFELYHKCDDQCMETI
RNGTYNRRKYQEESRLERQKIEGVKLESEGTYKILTIYSTVASSLVLAMGFAAFLFWAMSNGSCRCNICI

>QIC52456.1 hemagglutinin [Influenza A virus]
METTTLMTILLLVTTSNADKICIGHQSTNSTETVDTLTETNVPVTHAKELLHTYHNGMLCATNLGHPLIL
DTCNVEGLIYGNPSCDLLLGGREWSYIVERPSAVNGTCYPGNVENLEELRTLFSSSSSYQRIQIFPDTIW
NVTYTGTSKSCSDSFYRNMRWLTQKNGHYPVQDAQFTNNRGKDILFVWGIHHPPTDTAQTNLYTRTDTTT
SVTTENLDRTFKPLIGPRPLVNGLIGRINYYWSILKPGQTLRVRSNGNLIAPWYGHVLSGVSHGRILKTN
LNSGNCVVQCQTEKGGLNSTLPFHNISKYAFGNCPKYIGVKSLKLAIGLRNVPAKSKRGLFGAIAGFIEG
GWPGLVAGWYGFQHSNDQGVGIAADKVSTQEAVDKITSKVNNIVDKMNKQYEIIDHEFNEVETRLNMINN
KIDDQIQDVWAYNAELLVLLENQKTLDEHDANVNNLYNKVKRALGSNALEDGKGCFELYHKCDDQCMETI
RNGTYNRRKYQEESRLERQKIEGVKLESEGTYKILTIYSTVASSLVLAIGFAAFLFWAMSNGSCRCNICI

>QIC52356.1 hemagglutinin [Influenza A virus]
METITLMTTLLLITTSLADKICIGHQSTNSTETVDTLTETNVPVTHAKELLHTDHNGMLCATNLGHPLIL
DTCNVEGLIYGNPSCDSLLEGREWSYIVERPSAVNGTCYPGNVENLEELRTLFSSSSSYQRIQIFPDTIW
NVTYTGTSKSCSDSFYRNMRWLTQKNSLFPVQDAQFTNNRGKDILFVWGIHHPPSDTAQTNLYTRTDTTT
SVTTEDLDRTFNPLIGPRPLVNGLIGRINYYWSILKPGQTLRVRSNGNLIAPWYGHVLSGGSHGRILKTD

LKSGNCVVQCQTEKGGLNSTLPFHNISKYAFGNCPKYIGVKSLKLAIGLRNVPAKSKRGLFGAIAGFIEG
GWPGLVAGWYGFQHSNDQGVGIAADKISTQKAVDKITSKVNNIVDKMNKQYEIIDHEFSEVETRLNMINN
KIDDQIQDVWAYNAELLVLLENQKTLDEHDANVNNLYNKVRRALGSNAMEDGKGCFELYHKCDDQCMETI
RNGTYNRKKYQEESRLERQRIEGVKLESEGTYKILTIYSTVASSLVLAMGFAAFLFWAMSNGSCRCNICI

>QIC52326.1 hemagglutinin [Influenza A virus]
METTTLMTILLLVTTSNADKICIGHQSTNSTETVDTLTETNVPVTHAKELLHTDHNGMLCATNLGHPLIL
DTCNVEGLIYGNPSCDLLLGGREWSYIVERPSAVNGTCYPGNVENLEELRTLFSSSSSYQRIPIFPDTIW
NVTYTGTSKSCSDSFYRNMRWLTQKNGQYPVQDAQFTNNRGKDILFVWGIHHPPTDTAQTNLYTRTDTTT
SVTTENLDRTFKPLIGPRPLVNGLIGRINYYWSILKPGQTLRVRSNGNLIAPWYGHVLSGVSHGRILKTN
LNSGNCVVQCQTEKGGLNSTLPFHNISKYAFGNCPKYIGVKSLKLAIGLRNVPAKSKRGLFGAIAGFIEG
GWPGLVAGWYGFQHSNDQGVGIAADKVSTQEAVDKITSKVNNIVDKMNKQYEIIDHEFNEVETRLNMINN
KIDDQIQDVWAYNAELLVLLENQKTLDEHDANVNNLYNKVKRALGSNALEDGKGCFELYHKCDDQCMETI
RNGTYNRRKYQEESRLERQKIEGVKLESEGTYKILTIYSTVASSLVLAMGFAAFLFWAMSNGSCRCNICI

>QIC52244.1 hemagglutinin [Influenza A virus]
METVTLTTILLLITTSLADKICIGHQSTNSTETVDTLTETNVPVTHAKELLHTDHNGMLCSTNLGHPLIL
DTCNVEGLIYGNPSCDSLLGGREWSYIVERPSAVNGTCYPGSVENLEELRTLFSSSSSYQRIQLFPDTIW
NVTYTGTSKSCSDSFYRNMRWLTQKNSLYPVQDAQFTNNRGKDILFVWGIHHPPTDTAQTNLYTRTDTTT
SVTTENLDRTFKPLIGPRPLVNGLIGRINYYWSVLKPGQTLRVRSNGNLIAPWYGHVLSGESHGRILKTN
LNSGNCVVQCQTEKGGLNSTLPFHNVSKYAFGNCPKYIGVKSLKLAIGLRNVPAKSKRGLFGAIAGFIEG
GWPGLVAGWYGFQHSNDQGVGIAADKVSTQNAVDKITSKVNNIVDKMNKQYEIIDHEFSEVETRLNMINN
KIDDQIQDVWAYNAELLVLLENQKTLDEHDANVNNLYNKVRRALGSNAMEDGKGCFELYHKCDDQCMETI
RNGTYNRRKYQEESRLERQKIEGVKLESEGTYKILTIYSTVASSLVLAMGFAAFLFWAMSNGSCRCNICI

>QIC52202.1 hemagglutinin [Influenza A virus]
METVTLLTILLLATTSNADKICIGHQSTNSTETVDTLTETNVPVTHAKELLHTDHNGMLCATNLGRPLIL
DACNVEGIIYGNPSCDLLLGGREWSYIVERPSAVNGTCYPGNVENLEELRTLFSSSSSYQRIQIFPDTIW
NVTYTGTSKSCSDSFYRNMRWLTQKNGLYPVQDAQFTNNRGKDILFVWGIHHPPTDTAQTNLYTRTDTTT
SVTTENLDRTFKPLIGPRPLVNGLIGRINYYWSVLKPGQTLRVRSNGNLIAPWYGHVLSGESHGRILKTN
LKSGNCVVQCQTEKGGLNSTLPFHNVSKYAFGNCPKYIGVKSLKLAIGLRNVPAKSKRGLFGAIAGFIEG
GWPGLVAGWYGFQHSNDQGVGIAADRVSTQQAVDKITSKVNNIVDKMNKQYEIIDHEFSEVETRLNMINN
KIDDQIQDVWAYNAELLVLLENQKTLDEHDANVNNLYNKVKRALGSNALEDGKGCFELYHKCDDQCMETI
RNGTYNRRKYQEESRLERQKIEGVKLESEGTYKILTIYSTVASSLVLAMGFAAFLFWAMSNGSCRCNICI

>QIC52215.1 hemagglutinin [Influenza A virus]

METITLMTTLLLTTTSLADKICIGHQSTNSTETVDTLTETNVPVTHAKELLHTDHNGMLCATNLGHPLIL
DKCNIEGLIYGNPSCDSLLGGREWSYIVERPSAVNGTCYPGNVENLEELRTLFSSSGSYQRIQIFPDTIW
NVTYTGTSKSCSDSFYRNMRWLTQKNSLYPIQDAQFTNNRGKDILFVWGIHHPPTDTAQTNLYTRTDTTT
SVTTEDLDRTFKPLIGPRPLVNGLIGRINYYWSILKPGQTLRVRSNGNLIAPWYGHVLSGESHGRILKTD
LNSGNCVVQCQTEKGGLNSTLPFHNISKYAFGNCPKYIGVKSLKLAIGLRNVPAKSKRGLFGAIAGFIEG
GWPGLVAGWYGFQHSNDQGVGIAADKISTQKAVDKITSKVNNIVDKMNKQYEIIDHEFSEVETRLNMINN
KIDDQIQDVWAYNAELLVLLENQKTLDEHDANVNNLYNKVRRALGSNAMEDGKGCFELYHKCDDQCMETI
RNGTYNRKRYQEESRLERQRIEGVKLESEGTYKILTIYSTVASSLVLAMGFAAFLFWAMSNGSCRCNICI

>QDP69167.1 hemagglutinin [Influenza A virus]

METVTLLTILLLATTSSADKICVGHQSTNSTETVDTLTETNVPVTHAKELLHTDHNGMLCATNLGRPLIL
DACNVEGIIYGNPSCDLLLGGREWSYIVERPSAVNGTCYPGNVENLEELRTLFSSSSSYQRIQIFPDTIW
NVTYTGTSKSCSDSFYRNMRWLTQKNGLYPVQDAQFTNNRGKDILFVWGIHHPPTDTAQTNLYTRTDTTT
SVTTENLDRTFKPLIGPRPLVNGLVGRINYYWSVLKPGQTLRVRSNGNLIAPWYGHVLSGESHGRILKTN
LKSGNCVVQCQTEKGGLNSTLPFHNISKYAFGNCPKYIGVKSLKLAIGLRNVPAKSKRGLFGAIAGFIEG
GWPGLVAGWYGFQHSNDQGVGIAADRVSTQQAVDKITSKVNNIVDKMNKQYEIIDHEFSEVETRLNMINN
KIDDQMQDIWAYNAELLVLLENQKTLDEHDANVNNLYNKVKRALGTNALEDGKGCFELYHKCDDQCMETI
RNGTYNRRKYQEESRLERQKIEGVNLESEGTYKILTIYSTVASSLVLAMGFAAFLFWAMSNGSCRCNICI

>QDP69149.1 hemagglutinin [Influenza A virus]

METVIMMIILLLVATSNADKICIGHQSTNSTETVDTLTETNVPVTHAKELLHTDHNGMLCATNLGHPLIL
DTCNVEGLIYGNPSCDRLLEGREWSYIVERPSAVNGTCYPGNVENLEELRTLFSSSSSYQRIQIFPDTIW
NVTYTGTSKSCSDSFYRNMRWLTQKNSLYPVQDAQFTNNRGKDILFVWGIHHPPTDTAQTNLYTRTDTTT
SVTTENLDRTFKPLIGPRPLVNGLIGRINYYWSVLKPGQTLRVRSNGNLIAPSYGHVLSGESHGRILKTN
LRNGNCVVQCQTEKGGLNSTLPFHNISKYAFGNCPKYIGVKSLKLAIGLRNVPAKSKRGLFGAIAGFIEG
GWPGLVAGWYGFQHSNDQGVGIAADKVSTQKAVDKITSKVNNIVDKMNKQYEIIDHEFNEVETRLNMINN
KIDDQIQDVWAYNAELLVLLENQKTLDEHDANVNNLYNKVKRALGSNALEDGKGCFELYHKCDDQCMETI
RNGTYNRRKYQEESRLERQKIEGIKLESEGTYKILTIYSTAASSLVLAMGFAAFLFWAMSNGSCRCNICI

>QDP69096.1 hemagglutinin [Influenza A virus]

METVTLMTILLLVTAINADKICIGHQSTNSTETVDTLTETNVPVTHAKELLHTDHNGMLCATNLGHPLIL
DTCNVEGLIYGNPSCDLLLGGREWSYIVERPSAVNGTCYPGNVENLEELRTLFSSSSSYQRIQIFPDTIW

NVTYTGTSKSCSDSFYRNMRWLTQKNGQYPVQDAQFTNNRGKDILFVWGIHHPPTDTAQTNLYTRTDTTT
SVTTENLDRTFKPLIGPRPLVNGLIGRINYYWSVLKPGQTLRVRSNGNLIAPWYGHVLSGESHGRILKTN
LNRGNCVVQCQTEKGGLNSTLPFHNISKYAFGNCPKYIGVKSLKLAIGLRNVPAKSKRGLFGAIAGFIEG
GWPGLVAGWYGFQHSNDQGVGIAADKVSTQKAVDKITSKVNNIVDKMNKQYEIIDHEFNEVETRLNMINN
KIDDQIQDVWAYNAELLVLLENQKTLDEHDANVNNLYNKVKRALGSNALEDGKGCFELYHKCDDQCMETI
RNGTYNRRKYQEESRLERQKIEGIKLESEGTYKILTIYSTVASSLVLAMGFAAFLFWAMSNGSCRCTICI

>QDP69063.1 hemagglutinin [Influenza A virus]
METVTLLTILLLATTSSADKICVGHQSTNSTETVDTLTETNVPVTHAKELLHTDHNGMLCATNLGRPLIL
DACNVEGIIYGNPSCDLLLGGREWSYIVERPSAVNGTCYPGNVENLEELRTLFSSSSSYQRIQIFPDTIW
NVTYTGTSKSCSDSFYRNMRWLTQKNGLYPVQDAQFTNNRGKDILFVWGIHHPPTDTAQTNLYTRTDTTT
SVTTENLDRTFKPLIGPRPLVNGLIGRINYYWSVLKPGQTLRVRSNGNLIAPWYGHVLSGESHGRILKTN
LKSGNCVVQCQTEKGGLNSTLPFHNISKYAFGNCPKYIGVKSLKLAIGLRNVPAKSKRGLFGAIAGFIEG
GWPGLVAGWYGFQHSNDQGVGIAADRVSTQQAVDKITSKVNNIVDKMNKQYEIIDHEFSEVETRLNMINN
KIDDQIQDIWAYNAELLVLLENQKTLDEHDANVNNLYNKVKRALGTNALEDGKGCFELYHKCDDQCMETI
RNGTYNRRKYQEESRLERQKIEGVKLESEGTYKILTIYSTVASSLVLAMGFAAFLFWAMSNGSCRCNICI

>QDP69039.1 hemagglutinin [Influenza A virus]
METVIMMIILLLVATSNADKICIGHQSTNSTETVDTLTETNVPVTHAKELLHTDHNGMLCATNLGHPLIL
DTCNVEGLIYGNPSCDRLLEGREWSYIVERPSAVNGTCYPGNVENLEELRTLFSSSSSYQRIQIFPDTIW
NVTYTGTSKSCSDSFYRNMRWLTQKNSLYPVQDAQFTNNRGKDILFVWGIHHPPTDTAQTNLYTRTDTTT
SVTTENLDRTFKPLIGPRPLVNGLIGRINYYWSVLKPGQTLRVRSNGNLIAPSYGHVLSGESHGRILKTN
LRNGNCVVQCQTEKGGLNSTLPFHNISKYAFGNCPKYIGVKSLKLAIGLRNVPAKSKRGLFGAIAGFIEG
GWPGLVAGWYGFQHSNDQGVGIAADKVSTQKAVDKITSKVNNIVDKMNKQYEIIDHEFNEVETRLNMINN
KIDDQIQDVWAYNAELLVLLENQKTLDEHDANVNNLYNKVKRALGSNALEDGKGCFELYHKCDDQCMETI
RNGTYNRRKYQEESRLERQKIEGIKLESEGTYKILTIYSTAASSLVLAMGFAAFLFWAMSNGSCRCNICI

>QDP68910.1 hemagglutinin [Influenza A virus]
METVILMIILLLVATSNADKICIGHQSTNSTETVDTLTETNVPVTHAKELLHADHNGMLCATNLGHPLIL
DTCNVEGLIYGNPSCDRLLEGREWSYIVERPSAVNGTCYPGNVENLEELRTLFSSSSSYQRIQIFPDTIW
NVTYTGTSKSCSDSFYRNMRWLTQKNSLYPVQDAQFTNNRGKDILFVWGIHHPPTDTAQTNLYTRTDTTT
SVTTENLDRTFKPLIGPRPLVNGLIGRINYYWSVLKPGQTLRVRSNGNLIAPSYGHVLSGESHGRILKTN
LRNGDCVVQCQTEKGGLNSTLPFHNISKYAFGNCPKYIGVKSLKLAIGLRNVPAKSKRGLFGAIAGFIEG
GWPGLVAGWYGFQHSNDQGVGIAADKVSTQKAVDKITSKVNNIVDKMNKQYEIIDHEFNEVETRLNMINN

KIDDQIQDVWAYNAELLVLLENQKTLDEHDANVNNLYNKVKRALGSNALEDGKGCFELYHKCDDQCMETI
RNGTYNRRKYQEESRLERQKIEGIKLESEGTYKILTIYSTAASSLVLAMGFAAFLFWAMSNGSCRCNICI

>QDP68836.1 hemagglutinin [Influenza A virus]
METVTLMTILLLVTTSLADKICIGHQSTNSTETVDTLTETNVPVTHAKELLHTDHNGMLCSTNLGHPLIL
DTCNVEGLIYGNPSCDSLLGGREWSYIVERPSAVNGTCYPGNVENLEELRTLFSSSSSYQRIQLFPDTIW
NVTYTGTSKSCSDSFYRNMRWLTQKNSLYPVQDAQFTNNRGKDILFVWGIHHPPTDTAQTNLYTRTDTTT
SVTTEDLDRTFKPLIGPRPLVNGLIGRINYYWSVLKPGQTLRVRSNGNLIAPWYGHVLSGESHGRILKTN
LNSGNCVVQCQTEKGGLNSTLPFHNISKYAFGNCPKYIGIKSLKLAIGLRNVPAKSKRGLFGAIAGFIEG
GWPGLVAGWYGFQHSNDQGVGIAADKVSTQNAVDKITSKVNNIVDKMNKQYEIIDHEFSEVETRLNMINN
KIDDQIQDVWAYNAELLVLLENQKTLDEHDANVNNLYNKVRRALGSNAMEDGKGCFELYHKCDDQCMETI
RNGTYNRRKYQEESRLERQKIEGVKLESEGTYKILTIYSTVASSLVLAMGFAAFLFWAMSNGSCRCNICI

>QDP68758.1 hemagglutinin [Influenza A virus]
METITLMTTLLLITTSLADKICIGHQSTNSTETVDTLTETNVPVTHAKELLHTDHNGMLCATNLGHPLIL
DTCNVEGLIYGNPSCDSLLGGREWSYIVERPSAVNGTCYPGNVENLEELRTLFSSSRSYQRIQIFPDIIW
NVTYTGTSKSCSDSFYRNMRWLTQKNSLYPVQDAQFTNNRGKDILFVWGIHHPPTDTAQTNLYTRTDTTT
SVTTEDLDRTFKPLIGPRPLVNGLIGRINYYWSILKPGQTLRVRSNGNLIAPWYGHVLSGESHGRILKTD
LNSGNCVVQCQTEKGGLNSTLPFHNISKYAFGNCPKYIGVKSLKLAIGLRNVPAKSKRGLFGAIAGFIEG
GWPGLVAGWYGFQHSNDQGVGIAADKVSTQKAVDKITSKVNNIVDKMNKQYEIIDHEFSEVETRLNMINN
KIDDQIQDVWAYNAELLVLLENQKTLDEHDANVNNLYNKVRRALGSNALEDGKGCFELYHKCDDQCMETI
RNGTYNRKKYQEESRLERQRIEGVKLESEGTYKILTIYSTVASSLVLAMGFAAFLFWAMSNGSCRCNICI

>QDP68697.1 hemagglutinin [Influenza A virus]
METITLMTTLLLTTTSLADKICIGHQSTNSTETVDTLTETNVPVTHAKELLHTDHNGMLCATNLGHPLIL
DKCNVEGLIYGNPSCDSLLGGREWSYIVERPSAVNGTCYPGNVENLEELRTLFSSSSSYQRIQIFPDTIW
NVTYTGTSKSCSDSFYRNMRWLTQKNSLYPVQDAQFTNNRGKDILFVWGIHHPPTDTAQTNLYTRTDTTT
SVTTEDLDRTFKPLIGPRPLVNGLIGRINYYWSILKPGQTLRVRSNGNLIAPWYGHVLSGESHGRILKTD
LNSGNCVVQCQTEKGGLNSTLPFHNISKYAFGNCPKYIGVKSLKLAIGLRNVPAKSKRGLFGAIAGFIEG
GWPGLVAGWYGFQHSNDQGVGIAADKISTQKAVDKITSKVNNIVDKMNKQYEIIDHEFSEVETRLNMINN
KIDDQIQDVWAYNAELLVLLENQKTLDEHDANVNNLYNKVRRALGSNAMEDGKGCFELYHKCDDQCMETI
RNGTYNRKRYQEESRLERQRIEGVKLESEGTYKILTIYSTVASSLVLAMGFAAFLFWAMSNGSCRCNICI

>QDP68691.1 hemagglutinin [Influenza A virus]

METVTLMTIILLITTSLADKICIGHQSTNSTETVDTLTETNVPVTHAKELLHTDHNGMLCSTNLGHPLIL
DTCNVEGLIYGNPSCDSLLGGREWSYIVERPSAVNGTCYPGNVENLEELRTLFSSSSSYQRILIFPDTIW
NVTYTGTSKSCSDSFYRNMRWLTQKNSLYPVQDAQFTNNRGKDILFVWGIHHPPTDTAQTNLYTRTDTTT
SVTTETLDRTFKPLIGPRPLVNGLIGRINYYWSVLKPGQTLRVRSNGNLIAPWYGHVLSGESHGRILKTN
LNSGNCVVQCQTERGGLNSTLPFHNISKYAFGNCPKYIGVKSLKLAIGLRNVPAKSKRGLFGAIAGFIEG
GWPGLVAGWYGFQHSNDQGVGIAADKVSTQNAVDKITSKVNNIVDKMNKQYEIIDHEFSEVETRLNMINN
KLDDQIQDVWAYNAELLVLLENQKTLDEHDANVNNLYNKVRRALGSNALEDGKGCFELYHKCDDQCMETI
RNGTYNRRKYQEESRLERQKIEGVKLESEGTYKILTIYSTVASSLVLAMGFAAFLFWAMSNGSCRCNICI

>QKM77423.1 hemagglutinin [Influenza A virus]
MEIISLMTILLVVTTSNADKICIGHQSTNSTETVDTLTETNVPVTHAKELLHTEHNGMLCATNLGNPLIL
DTCTIEGLIYGNPSCDMLLGGREWSYIVERPSAVNGTCYPGNVENLEELRTLFSSSSSYQRIQIFPDTIW
NVTYTGTSKSCSDSFYRNMRWLTQKNGGYPVQDAQYTNNRGKDILFVWGIHHPPTDTAQTNLYTRTDTTT
SVTTENLDRTFKPLIGPRPLVNGLIGRINYYWSVLKPGQTLRVRSNGNLIAPWFGHVLSGESHGRILKTD
LNSGNCVVQCQTEKGGLNSTLPFHNISKYAFGNCPKYIGVKSLKLAIGLRNVPARSSRGLFGAIAGFIEG
GWPGLVAGWYGFQHSNDQGVGMAADRDSTQKAIDKITSKVNNIVDKMNKQYEIIDHEFSEVETRLNMINN
KIDDQIQDVWAYNAELLVLLENQKTLDEHDANVNNLYNKVKRALGSNAMEDGKGCFELYHKCDDQCMETI
RNGTYNRRKYKEESRLERQKIEGVKLESEGIYKILTIYSTVASSLVLAMGFAAFLFWAMSNGSCRCNICI

>QGR10816.1 hemagglutinin [Influenza A virus]
METVSLITILVVATVSNADKICIGYQSTNSTETVDTLTENNVPVTHAKELLHTEHNGMLCATSLGHPLIL
DTCTIEGLIYGNPSCDPLLGGREWSYIVERPSAVNGLCYPGNVENLEELRSLFSSSRSYQRIQIFPDTIW
NVSYSGTSKACSDSFYRSMRWLTQKNNAYPTQDAQYTNNQGKNILFMWGINHPPTDTVQTNLYTRTDTTT
SVATEEMNRIFKPLIGPRPLVNGLMGRINYYWSILKPGQTLRIKSDGNLIAPWYGHILSGKSHGRILKTD
LKMGSCTVQCQTEKGGLNTTLPFQNVSKYAFGNCSKYIGVKSLKLAVGLRNVPSRSSRGLFGAIAGFIEG
GWSGLVAGWYGFQHSNDQGVGMAADRDSTQKAIDKITSKVNNIVDKMNKQYEIIDHEFSEVETRLNIIND
KVDDQIQDIWAYNAELLVLLENQKTLDEHDANVNNLYNKVKRALGSNAVEDGRGCFELYHKCDDHCMETI
RNGTYNRRKYQEESKLERQKIEGVKLESEETYKILTIYSTVASSLVIAMGFAAFLFWAMSNGSCRRNICI

>QGR10805.1 hemagglutinin [Influenza A virus]
METVSLITILLAATVSNADKICIGYQSTNSTETVDTLTENNVPVTHAKELLHTEHNGMLCATSLGHPLIL
DTCTIEGLIYGNPSCDLLLGGREWSYIVERPSAVNGLCYPGNVENLEELRSLFSSARSYQRIQIFPDTIW
NVSYSGTSKACSDSFYRSMRWLTQKNNTYPIQDAQYTNNQEKNILFMWGINHPPTDTVQTNLYTRTDTTT
SVATEEINRIFKPLIGPRPLVNGLMGRINYYWSVLKPGQTLRIKSDGNLIAPWYGHILSGESHGRILKTD

LKRGSCTVQCQTEKGGLNTTLPFQNVSKYAFGNCSKYVGIKSLKLAVGLRNVPSRSSRGLFGAIAGFIEG
GWSGLVAGWYGFQHSNDQGVGMAADRDSTQKAIDKITSKVNNIVDKMNKQYEIIDHEFSEVETRLNMINN
KVDDQIQDIWAYNAELLVLLENQKTLDEHDANVNNLYNKVKRALGSNAVEDGKGCFELYHKCDDQCMETI
RNGTYNRRKYQEESKLERQKIEGVKLESEGTYKILTIYSTVASSLVIAMGFAAFLFWAMSNESCRRNKSL

>QGR10793.1 hemagglutinin [Influenza A virus]
METVSLITILVVATVSNADKICIGYQSTNSTETVDTLTENNVPVTHAKELLHTEHNGMLCATSLGHPLIL
DTCTIEGLIYGNPSCDPLLGGREWSYIVERPSAVNGLCYPGNVENLEELRLLFSSSRSYQRIQIFPDTIW
NVSYSGTSKACSDSFYRSMRWLTQKNNAYPTQDAQYTNNQGKNILFMWGINHPPTDTVQTNLYTRTDTTT
SVATEEMNRIFKPLIGPRPLVNGLMGRINYYWSVLKPGQTLRIKSDGNLIAPWYGHILSGESHGRILKTD
LKMGSCTVQCQTERGGLNTTLPFQNVSKYAFGNCSKYIGVKSLKLAVGLRNVPSRSSRGLFGAIAGFIEG
GWSGLVAGWYGFQHSNDQGVGMAADRDSTQKAIDKITSKVNNIVDKMNKQYEIIDHEFSEVETRLNMIND
KVDDQIQDIWAYNAELLVLLENQKTLDEHDANVNNLYNKVKRALGSNAVEDGRGCFELYHKCDDHCMETI
RNGTYNRRKYQEESKLERQKIEGVKLESEETYKILTIYSTVASSLVIAMGFAAFLFWAMSNGSCRRNICI

>QGR10782.1 hemagglutinin [Influenza A virus]
METASLITVLLVVTVSNADKICIGYQSTNSTETVDTLTENNVPVTHAKELLHTEHNGMLCATSLGNPLIL
DTCTIEGLIYGNPSCDLLLGGREWSYIVERPSAVNGLCYPGNVENLEELRSLFSSARSYQRIQIFPDTIW
NVSYSGTSKACSDSFYRSMRWLTQKNNAYPTQDAQYTNNQEKNILFMWGINHPPTDTTQTNLYTRTDTTT
SVATEEINRTFKPLIGPRPLVNGLMGRIDYYWSVLKPGQTLRIRSNGNLIAPWYGHILSGESHGRILKTD
LKRGSCTVQCQTEKGGLNTTLPFQNVSKYAFGNCSKYIGIKSLKLAVGLRNLPSRSSRGLFGAIAGFIEG
GWSGLVAGWYGFQHSNDQGVGMAADRDSTQKAIDKITSKVNNIVDKMNKQYEIIDHEFSEVETRLNMINN
KIDDQIQDIWAYNAELLVLLENQKTLDEHDANVNNLYNKVKRALGSNAVEDGKGCFELYHKCDDQCMETI
RNGTYNRRKYQEESKLERQKIEGVKLESEGTYKILTIYSTVASSLVIAMGFAAFLFWAMPNGSCRCNICI

>QGR10770.1 hemagglutinin [Influenza A virus]
METVSLITILLVATVSNADKICIGYQSTDSTETVDTLTENNVPVTHAKELLHTEHNGMLCATSLGQPLIL
DTCTIEGLIYGNPSCDLSLEGREWSYIVERPSAVHGLCYPGNVEDLEELRSLFSSARSYQRIQIFPDTIW
NVSYDGTSTACSGSFYKSMRWLTRKNGEYPTQDAQYTNNQGKNILFMWGINHPPTDDTQKGLYTRTDTTT
SVATEEINRIFKPLIGPRPRVNGLMGRINYYWSVLKPGQTLRIKSDGNLIAPWYGHILSGESHGRILKTD
LKRGSCTVQCQTEKGGLNTTLPFQNVSKYAFGNCSKYIGIKSLKLAVGLRNVPSRSSRGLFGAIAGFIEG
GWSGLVAGWYGFQHSNDQGVGMAADRDSTQKAIDKITSKVNNIVDKMNKQYEIIDHEFSEVETRLNMINN
KIDDQIQDIWAYNAELLVLLENQKTLDEHDANVNNLYNKVKRALGSNAVEDGKGCFELYHKCDDQCMETI
RNGTYNRRKYQEESKLERQRIEGVKLESEGTYKILTIYSTVASSLMIAMGFAAFLFWAMSNGSCRRNICI

>QGR10758.1 hemagglutinin [Influenza A virus]
METVSLITILVVATVSNADKICIGYQSTNSTETVDTLTENNVPVTHAKELLHTEHNGMLCATSLGHPLIL
DTCTIEGLIYGNPSCDPLLGGREWSYIVERPSAVNGLCYPGNIENLEELRSLFSSSRSYQRIQIFPDTIW
NVSYSGTSKACSDSFYRSMRWLTQKNNAYPTQDAQYTNNQGKNILFMWGINHPPTDTVQTNLYTRTDTTT
SVATEEMNRIFKPLIGPRPLVNGLMGRINYYWSILKPGQTLRIKSDGNLIAPWYGHILSGKSHGRILKTD
LKMGSCTVQCQTERGGLNTTLPFQNVSKYAFGNCSKYIGVKSLKLAVGLRNVPSRSSRGLFGAIAGFIEG
GWSGLVAGWYGFQHSNDQGVGMAADRDSTQKAIDKITSKVNNIVDKMNKQYEIIDHEFSEVETRLNMIND
KVDDQIQDIWAYNAELLVLLENQKTLDEHDANVNNLYNKVKRALGSNAVEDGRGCFELYHKCDDHCMETI
RNGTYNRRKYQEESKLERQKIEGVKLESEETYKILTIYSTVASSLVIAMGFAAFLFWAMSNGSCRCNICI

>QGR10747.1 hemagglutinin [Influenza A virus]
METVSLITILVVATVSNADKICIGYQSTNSTETVDTLTENNVPVTHAKELLHTEHNGMLCATSLGHPLIL
DTCTIEGLIYGNPSCDPLLGGREWSYIVERPSAVNGLCCPGNIENLEELRSLFSSSRSYQRIQIFPDTIW
NVSYSGTSKACSDSFYRSMRWLTQKNNAYPTQDAQYTNNQGKNILFMWGINHPPTDTVQTSLYTRTDTTT
SVATEEMNRIFKPLIGPRPLVNGLMGRINYYWSILKPGQTLRIKSDGNLIAPWYGHILSGKSHGRILKTD
LKMGSCTAQCQTEKGGLNTTLPFQNVSKYAFGNCSKYIGVKSLKLAVGLRNVPSRSSRGLFGAIAGFIEG
GWSGLVAGWYGFQHSNDQGVGMAADRDSTQKAIDKITSKVNNIVDKMNKQYEIIDHEFSEVETRLNMIND
KVDDQIQDIWAYNAELLVLLENQKTLDEHDANVNNLYNKVKRALGSNAVEDGRGCFELYHKCDDHCMETI
RNGTYNRRKYQEESKLERQKIEGVKLESEETYKILTIYSTVASSLVIAMGFAAFLFWAMSNGSCRRNICI

>QGR10736.1 hemagglutinin [Influenza A virus]
METVSLITILVVATVSNADKICIGYQSTNSTETVDTLTENNVPVTHAKELLHTEHNGMLCATSLGHPLIL
DTCTIEGLIYGNPSCDPLLGGREWSYIVERPSAVNGLCYPGNVENLEELRSLFSSSRSYQRIQIFPDTIW
NVSYSGTSKACSDSFYRSMRWLTQKNNAYPTQDAQYTNNQGKNILFMWGINHPPTDAVQTNLYTRTDTTT
SVATEEMNRVFKPLIGPRPLVNGLMGRINYYWSVLKPGQTLRIKSDGNLIAPWYGHILSGESHGRILKTD
LKMGSCTVQCQTEKGGLNTTLPFQNVSKYAFGNCSKYIGVKSLELAVGLRNVPSRSSRGLFGAIAGFIEG
GWSGLVAGWYGFQHSNDQGVGMAADRDSTQKAVDKITSKVNNIVDKMNKQYEIIDHEFSEVETRLNMIND
KVDDQIQDIWAYNAELLVLLENQKTLDEHDANINNLYNKVKRALGSNAVEDGRGCFELYHKCDDHCMETI
RNGTYNRRKYQEESKLERQKIEGVKLESEETYKILTIYSTVASSLVIAMGFAAFLFWAMSNGSCRRNICI

>QGR10724.1 hemagglutinin [Influenza A virus]
METVSLITILVVATVSNADKICIGYQSTNSTETVDTLTENNVPVTHAKELLHTEHNGMLCATSLGHPLIL
DTCTIEGLIYGNPSCDPLLGGREWSYIVERPSAVNGLCYPGNVENLEELRSLFSSSRSYQRIQIFPDTIW

NVSYNGTSKACSDSFYRSMRWLTQKNNAYPTQDAQYTNNQGKNILFMWGINHPPTDTVQTNLYTRTDTTT
SVATEEMNRIFKPLIGPRPLVNGLMGRINYYWSILKPGQTLRIKSDGNLIAPWYGHILSGKSHGRILKTD
LKMGSCTVQCQTEKGGLNTTLPFQNVSKYAFGNCSKYIGVKSLKLAVGLRNVPSRSSRGLFGAIAGFIEG
GWSGLVAGWYGFQHSNDQGVGMAADRDSTQKAIDKITSKVNNIVDKMNKQYEIIDHEFSEVETRLNMIND
KVDDQIQDIWAYNAELLVLLENQKTLDEHDANVNNLYNKVKRALGSNAVEDGRGCFELYHKCDDHCMETI
RNGTYNRRKYQEESKLERQKIEGVKLESEETYKILTIYSTVASSLVIAMGFAAFLFWAMSNGSCRRNICI

>QGR10712.1 hemagglutinin [Influenza A virus]
METISLMTILLVVTVSNADKICIGYQSTNSTETVDTLTENNVPVTHAKELLHTEHNGMLCATSLGNPLIL
DTCTIEGLIYGNPSCDPLLGGREWSYIVERPSAVNGLCYPGSVENLEELRSLFSSARSYQRIQIFPDTIW
NVSYSGTSKACSDSFYRSMRWLTQKNNAYPTQDAQYTNNQEKNILFMWGINHPPTETVQTNLYTKTDTTT
SVATEEINRIFKPLIGPRPLVNGLMGRINYYWSVLKPGQTLRIKSDGNLIAPWYGHILSGESHGRILKTD
LKKGSCTVQCQTEKGGLNTTLPFQNVSKYAFGNCSKYIGIKSLKLAVGLRNVPSRSSRGLFGAIAGFIEG
GWSGLVAGWYGFQHSNDQGVGMAADRDSTQKAIDKITSKVNNIVDKINKQYEIIDHEFSEVETRLNMINN
KVDDQIQDIWAYNAELLVLLENQKTLDEHDSNVNNLYNKVKRALGSNAVEDGKGCFELYHKCDNQCMETI
RNGTYNRKKYQEESKLERQKIEGVKLESEGTYKILTIYSTVASSLVIAMGFAAFLFWAMSNGSCRRNICI

>QGR10700.1 hemagglutinin [Influenza A virus]
METVSLITILVVATVSNADKICIGYQSTNSTETVDTLTENNVPVTHAKELLHTEHNGMLCATSLGHPLIL
DTCTIEGLIYGNPSCDPLLGGREWSYIVERPSAVNGLCYPGNVENLEELRSLFSSSRSYQRIQIFPDTIW
NVSYSGTSKACSDSFYRSMRWLTQKNNAYPTQDAQYTNNQGKNILFMWGINHPPTDTVQTNLYTRTDTTT
SVATEEMNRIFKPLIGPRPLVNGLMGRINYYWSILKPGQTLRIKSDGNLIAPWYGHILSGKSHGRILKTD
LKMGSCTVQCQTEKGGLNTTLPFQNVSKYAFGNCSKYIGVKSLKLAVGLRNVPSRSSRGLFGAIAGFIEG
GWSGLVAGWYGFQHSNDQGVGMAADRDSTQKAIDKITSKVNNIVDKMNKQYEIIDHEFSEVETRLNIIND
KVDDQIQDIWAYNAELLVLLENQKTLDEHDANVNNLYNKVKRALGSNAVEDGRGCFELYHKCDDHCMETI
RNGTYNRRKYQEESKLERQKIEGVKLESEETYKILTIYSTVASSLVIAMGFAAFLFWAMSNGSCRRNICI

>QGR10689.1 hemagglutinin [Influenza A virus]
METVSLITILLAATVSNADKICIGYQSTNSTETVDTLTENNVPVTHAKELLHTEHNGMLCATSLGHPLIL
DTCTIEGLIYGNPSCDLLLGGREWSYIVERPSAVNGLCYPGNVENLEELRSLFSSARSYQRIQIFPDTIW
NVSYSGTSKACSDSFYRSMRWLTQKNNTYPIQDAQYTNNQEKNILFMWGINHPPTDTVQTNLYTRTDTTT
SVATEEINRIFKPLIGPRPLVNGLMGRINYYWSVLKPGQTLRIKSDGNLIAPWYGHILSGESHGRILKTD
LKRGSCTVQCQTEKGGLNTTLPFQNVSKYAFGNCSKYVGIKSLKLAVGLRNVPSRSSRGLFGAIAGFIEG
GWSGLVAGWYGFQHSNDQGVGMAADRDSTQKAIDKITSKVNNIVDKMNKQYEIIDHEFSEVETRLNMINN

KVDDQIQDIWAYNAELLVLLENQKTLDEHDANVNNLYNKVKRALGSNAVEDGKGCFELYHKCDDQCMETI
RNGTYNRRKYQEESKLERQKIEGVKLESEGTYKILTIYSTVASSLVIAMGFAAFLFWAMSNESCRRNKSL

>QGR10677.1 hemagglutinin [Influenza A virus]
METVSLITILVVATVSNADKICIGYQSTNSTETVDTLTENNVPVTHAKELLHTEHNGMLCATSLGHPLIL
DTCTIEGLIYGNPSCDPLLGGREWSYIVERPSAVNGLCYPGNVENLEELRLLFSSSRSYQRIQIFPDTIW
NVSYSGTSKACSDSFYRSMRWLTQKNNAYPTQDAQYTNNQGKNILFMWGINHPPTDTVQTNLYTRTDTTT
SVATEEMNRIFKPLIGPRPLVNGLMGRINYYWSVLKPGQTLRIKSDGNLIAPWYGHILSGESHGRILKTD
LKMGSCTVQCQTERGGLNTTLPFQNVSKYAFGNCSKYIGVKSLKLAVGLRNVPSRSSRGLFGAIAGFIEG
GWSGLVAGWYGFQHSNDQGVGMAADRDSTQKAIDKITSKVNNIVDKMNKQYEIIDHEFSEVETRLNMIND
KVDDQIQDIWAYNAELLVLLENQKTLDEHDANVNNLYNKVKRALGSNAVEDGRGCFELYHKCDDHCMETI
RNGTYNRRKYQEESKLERQKIEGVKLESEETYKILTIYSTVASSLVIAMGFAAFLFWAMSNGSCRRNICI

>QGR10663.1 hemagglutinin [Influenza A virus]
METASLITVLLVVTVSNADKICIGYQSTNSTETVDTLTENNVPVTHAKELLHTEHNGMLCATSLGNPLIL
DTCTIEGLIYGNPSCDLLLGGREWSYIVERPSAVNGLCYPGNVENLEELRSLFSSARSYQRIQIFPDTIW
NVSYSGTSKACSDSFYRSMRWLTQKNNAYPTQDAQYTNNQEKNILFMWGINHPPTDTTQTNLYTRTDTTT
SVATEEINRTFKPLIGPRPLVNGLMGRIDYYWSVLKPGQTLRIRSNGNLIAPWYGHILSGESHGRILKTD
LKRGSCTVQCQTEKGGLNTTLPFQNVSKYAFGNCSKYIGIKSLKLAVGLRNLPSRSSRGLFGAIAGFIEG
GWSGLVAGWYGFQHSNDQGVGMAADRDSTQKAIDKITSKVNNIVDKMNKQYEIIDHEFSEVETRLNMINN
KIDDQIQDIWAYNAELLVLLENQKTLDEHDANVNNLYNKVKRALGSNAVEDGKGCFELYHKCDDQCMETI
RNGTYNRRKYQEESKLERQKIEGVKLESEGTYKILTIYSTVASSLVIAMGFAAFLFWAMPNGSCRCNICI

>QGR10088.1 hemagglutinin [Influenza A virus]
METASLITVLLVVTVSNADKICIGYQSTNSTETVDTLTENNVPVTHAKELLHTEHNGMLCATSLGNPLIL
DTCTIEGLIYGNPSCDLLLGGREWSYIVERPSAVNGLCYPGNVENLEELRSLFSSARSYQRIQIFPDTIW
NVSYSGTSKACSDSFYRSMRWLTQKNNAYPTQDAQYTNNQEKNILFMWGINHPPTDTTQTNLYTRTDTTT
SVATEEINRTFKPLIGPRPLVNGLMGRIDYYWSVLKPGQTLRIRSNGNLIAPWYGHILSGESHGRILKTD
LKRGSCTVQCQTEKGGLNTTLPFQNVSKYAFGNCSKYIGIKSLKLAVGLRNLPSRSSRGLFGAIAGFIEG
GWSGLVAGWYGFQHSNDQGVGMAADRDSTQKAIDKITSKVNNIVDKMNKQYEIIDHEFSEVETRLNMINN
KIDDQIQDIWAYNAELLVLLENQKTLDEHDANVNNLYNKVKRALGSNAVEDGKGCFELYHKCDDQCMETI
RNGTYNRRKYQEESKLERQKIEGVKLESEGTYKILTIYSTVASSLVIAMGFAAFLFWAMPNGSCRCNICI

>QGQ73130.1 hemagglutinin [Influenza A virus]

METVSLITILLVATVSNADKICIGYQSTNSTETVDTLTENNVPVTHAKELLHTEHNGMLCATSLGQPLIL
DTCTIEGLIYGNPSCDLSLEGREWSYIVERPSAVNGLCYPGNVENLEELRSLFSSARSYQRIQIFPDTIW
NVSYDGTSTACSNSFYRSMRWLTRKDGNYPTQNAQYTNNQGKNILFMWGINHPPTDETQRNLYTRTDTTT
SVATEEINRIFKPLIGPRPLVNGLMGRIDYYWSVLKSGQTLRIKSDGNLIAPWYGHILSGESHGRILKTD
LKKGSCTVQCQTEKGGLNTTLPFQNVSKYAFGNCSKYIGIKSLKLAVGLRNVPSRSSRGLFGAIAGFIEG
GWSGLVAGWYGFQHSNDQGVGMAADRDSTQKAIDKITSKVNNIVDKMNKQYEIIDHEFSEVETRLNMINN
KIDDQIQDIWAYNAELLVLLENQKTLDEHDANVNNLYNKVKRALGSNAVEDGKGCFELYHKCNDQCMETI
RNGTYNRKKYQEESKLERQRIEGVKLESEGTYKILTIYSTVASSLVIAMGFAAFLFWAMSNGSCRRNICI

>QEX14695.1 hemagglutinin [Influenza A virus]
METASLITILLIVTASNADKICIGYQSTNSTETVDTLTENNVPVTHAKELLHTEHNGMLCATSLGHPLIL
DTCTIEGLIYGNPSCDLLLGGREWSYIVERPSAVNGLCYPGNVENLEELRSLFSSARSYQRIQIFPDTIW
NVSYSGTSRACSDSFYRSMRWLTQKDNAYPTQDAQYTNNQEKNILFMWGINHPPTDTVQTNLYTRTDTTT
SVATEEINRIFKPLIGPRPLVNGLMGRINYYWSVLKPGQTLRIKSNGNLIAPWYGHILSGESHGRILKTD
LKRGSCTVQCQTEKGGLNTTLPFQNVSKYAFGNCSKYIGTKSLKLAVGLRNVPSRSSRGLFGAIAGFIEG
GWSGLVAGWYGFQHSNDQGVGMAADRDSTQKAIDKITSKVNNIVDKMNKQYEIINHEFSEIETRLNMINN
KVDDQIQDIWAYNAELLVLLENQKTLDEHDANVNNLYNKVKRALGSNAVEDGKGCFELYHKCDDQCMETI
RNGTYNRRKYQEESRLERQKIEGVKLESEGTYKILTIYSTVASSLVIAMGFAAFLFWAMSNGSCRCNICI

>QEX14694.1 hemagglutinin [Influenza A virus]
METVSLIIILLTATVSNADKICIGYQSTNSTETVDTLTENNVPVTHAKELLHTEHNGMLCATRLGQPLIL
DTCTIEGLIYGNPSCDPLPEEREWSYIVERPSAVNGLCYPGNVENLEELRSLFSSARSYQRVQIFSDTIW
NVSYDGTSNACSGSFYRNMRWLTRKDGNYPIQDAQYTNNQGKNILFMWGINNPPTDDTQRNLYTRTDTTT
SVATEEINRIFKPLIGPRPLVNGLMGRINYYWSVLKPGQTLRIKSDGNLVAPWYGYILSGESHGRILRTD
LKRGSCTVQCQTEKGGLNTTLPFQNVSKYAFGNCSKYIGIKSLKLAVGLRNVPSRSSRGLFGAIAGFIEG
GWSGLVAGWYGFQHSNDQGVGMAADRESTQKAVDKITSKVNNIVDKMNKQYEIIDHEFSEIETRLNMINN
KIDDQIQDIWAYNAELLVLLENQKTLDEHDANVNNLYNKVKRALGTNAVEDGKGCFELYHKCDDQCMETI
RNGTYNRRKYQEESKLERQKIEGVKLESEGTYKILTIYSTVASSLVIAMGFAAFLFWAMSNGSCRCNICI

>QEX14693.1 hemagglutinin [Influenza A virus]
METVSLITILLVATVSNADKICIGYQSTNSTETVDTLTENNVPVTHAKELLHTEHNGMLCATSLGQPLVL
DTCTIEGLIYGNPSCDLMLEGREWSYIVERPSAVNGLCYPGHVENLEELRSLFSSARSYQRVQIFPDTIW
NVSYDGTSNACSGSFYRSMRWLTRKNGDYPIQDAQYTNNQGKNILFMWGINHPPTDKTQRDLYTRTDTTT
SVATEEINRIFKPLIGPRPLVNGLMGRIDYYWSVLKPGQTLRIKSDGNLIAPWYGHILSGESHGRILKTD

LKRGSCTVQCQTEKGGLNTTLPFQNVSKYAFGNCSKYIGMKSLKLAVGLRNVPSRSSRGLFGAIAGFIEG
GWSGLVAGWYGFQHSNDQGVGMAADRDSTQKAIDKITSKVNNIVDKMNKQYEIIDHEFSEVETRLNMINN
KIDDQIQDIWAYNAELLVLLENQKTLDEHDANVNNLYNKVKRALGSNAVEDGKGCFELYHKCDDQCMETI
RNGTYNRRKYQEESKLERQKIEGVKLESEGTYKILTIYSTVASSLVIAMGFAAFLFWAMSNGSCRCNICI

>QGQ73142.1 hemagglutinin [Influenza A virus]
METVSLITILVVAIVSNADKICIGYQLTNSTETVDTLIENNVPVTHAKELLHTEHNGMLCATSLGHPLIL
DTCTIEGLIYGNPSCDSLLGGREWSYIVERPSAVNGLCYPGNIENLEELRSLFSSSRSYQRIQIFPDTIW
NVSYSGISKACLDSFYRSMRWLTQKNNAYPTQDAQYTNNQGKNILFMWGINHPPTDTAQTNLYTRTDTTM
SVAIEEMNRVFKLLIGPRPLVNGLMGRINYYWSVLKPGQTLRIKSDGNLIAPWYGHILSGESHGRILKTD
LKMGSCTVQCQTEKGGLNTTLPFQNVSKYAFGNCSKYIGIKSLKLAVGLRNVPSRSSRGLFGAIAGFIEG
GWSGLVAGWYGFQHSNDQGVGMAADRDSTQKAIDKITSKVNNIVDKMNKQYEIIDHEFSEVETRLNMINN
KVDDQIQDIWAYNAELLVLLENQKTLDEHDSNVNNLYNKVKRALGSNAVEDGKGCFELYHKCDNQCMETI
RNGTYNRKKYQEESKLERQKIEGVKLESEGTYKILTIYSTVASSLVIAMGFAAFLFWAMSNGSCRRNICI

>QCY50876.1 hemagglutinin [Influenza A virus]
MEIIPLMTMLLLVTTNNADKICIGHQSTNSTETVDTLTETGVPVTHAKELLHTEHNGRLCATNLGNPLIL
DTCTVEGIIYGNPSCDMLLGGREWSYIVERPSAVNGTCYPGNVENLEELRVLFSSSSSYQRIQMFPDTIW
NVTYSGTSKSCSDSFYRNMRWLTQKNGNYPVQDAQYTNTRGKDILFVWGIHHPPTDTAQTNLYTRTDTTT
SITTESLDRTFKPLIGPRPLVNGLIGRINYYWSVLKPGQTLRVRSNGNLIAPWFGHVLSGESHGRILKTD
LNSGNCVVQCQTEKGGLNSTLPFHNVSKYAFGDCPKYIGVKSLKLAIGLRNVPARSSRGLFGAIAGFIEG
GWPGLVAGWYGFQHSNDQGVGMAADRDSTQKAVDKITSKVNNIVDKMNKQYEIIDHEFSEVETRLNMINN
KIDDQIQDIWAYNAELLVLLENQKTLDEHDANVNNLYNKVKRALGSNAMEDGKGCFELYHKCDDQCMETI
RNGTYNRRRYMEESRLGRQKIEGVKLESEGTYKILTIYSTVASSLVLAMGSAAFLFWAMSNGSCRCNICI

>QCY50874.1 hemagglutinin [Influenza A virus]
MEVIPLMAMLLLVTTNNADKICIGHQSTNSTETVDTLTETGVPVTHAKELLHTEHNGKLCATNLGNPLIL
DTCTVEGLIYGNPSCDMLLGGREWSYIVERPSAVNGTCYPGNVENLEELRILFSSSSSYQRIQMFPDTVW
NVTYSGTSKSCSDSFYRNMRWLTQKNGNYPVQDAQYTNTRGKDILFVWGIHHPPTDTAQTNLYTRTDTTT
SITTESLDRTFKPLIGPRPLVNGLIGRINYYWSVLKPGQTLRVRSNGNLIAPWFGHVLSGKSHGRILKTD
LNSGNCVVQCQTEKGGLNSTLPFHNISKYAFGDCPKYIGVKSLKLAIGLRNVPARSSRGLFGAIAGFIEG
GWPGLVAGWYGFQHSNDQGVGMAADRDSTQKAIDKITSKVNNIVDKMNKQYEIIDHEFSEVENRLNMINN
KIDDQIQDIWAYNAELLVLLENQKTLDEHDANVNNLYNKVKRALGSNAMEDGKGCFELYHKCDDQCMETI
RNGTYNRRKYMEESRLGRQKIEGVKLESEGTYKILTIYSTVASSLVLAMGFAAFLFWAMSNGSCRCNICI

>BCD57630.1 hemagglutinin [Influenza A virus]

METASLMTILLVVTTSNADKICIGYQSTNSTETVDTLTENNVPVTHAKELLHTEHNGMLCATSLGQPLIL

DTCTIEGLIYGNPSCDPLLGGREWSYIVERPSAVNGLCYPGNVENLEELRSLFSSARSYQRVLIFPDTIW

NVSYSGTSKACSDSFYRSMRWLTQKDNAYPVQDAQYTNNQEKNILFMWGINHPPTDTVQTNLYTRTDTTT

SVATEEINRTFKPLIGPRPLVNGLMGRINYYWSVLKPGQTLRIKSNGNLIAPWYGHILSGESHGRILKTD

LKRGSCTVQCQTEKGGLNTTLPFQNVSKYTFGNCSKYIGIKSLKLAVGLRNVPSRSSRGLFGAIAGFIEG

GWSGLVAGWYGFQHSNDQGVGMAADRDSTQKAIDKITSKVNNIVDKMNKQYEIIDHEFSEVETRLNMINN

KIDDQIQDIWAYNAELLVLLENQKTLDEHDANVNNLYNKVKRALGSNAVEDGKGCFELYHKCDDQCMETI

RNGTYNRRKYQEESKLERQKIEGVKLESEGTYKILTIYSTVASSLVIAMGFAAFLFWAMSNGSCRCNICI

>QHW19169.1 hemagglutinin [Influenza A virus]

METASLITILLVATVSNADKICIGYQSTNSTETVDTLTENNVPVTHAKELLHTEHNGMLCATSLGQPLIL

DTCTIEGLIYGNPSCDLSLEGREWSYIVERPSAVNGLCYPGNVENLEELRSLFSSARSYQRIQIFPDTIW

NVSYDGTSTACSNSFYRSMRWLTRKDGNYPTQDAQYTNNQGKNILFMWGINHPPTDETQRNLYTRTDTTT

SVATEEINRIFKPLIGPRPRVNGLMGRIDYYWSVLRPGQTLRIKSDGNLIAPWYGHILSGESHGRILKTD

LKRGSCTVQCQTEKGGLNTTLPFQNVSKYAFGNCSKYIGIKSLKLAVGLRNVPSRSSRGLFGAIAGFIEG

GWSGLVAGWYGFQHSNDQGVGMAADRDSTQKAIDKITSKVNNIVDKMNKQYEIIDHEFSEVETRLNMINN

KIDDQIQDIWAYNAELLVLLENQKTLDEHDANVNNLYNKVKRALGSNAVEDGKGCFELYHKCNDQCMETI

RNGTYNRKKYQEESKLERQRIEGVKLESEGTYKILTIYSTVASSLVIAMGFAAFLFWAMSNGSCRCNICI

>QHQ71708.1 hemagglutinin [Influenza A virus]

METVTLLTILLLATTSNADKICVGHQSTNSTETVDTLTETNVPVTHAKELLHTDHNGMLCATNLGRPLIL

DACNVEGIIYGNPSCDLLLGGREWSYIVERPSAVNGTCYPGNVENLEELRTLFSSSSSYQRIQIFPDTIW

NVTYTGTSKSCSDSFYRNMRWLTQKNGLYPVQDAQFTNNRGKDILFVWGIHHPPTDTAQTNLYTRTDTTT

SVTTENLDRTFKPLIGPRPLVNGLIGRINYYWSVLKPGQTLRVRSNGNLIAPWYGHVLSGESHGRILKTN

LKSGNCVVQCQTEKGGLNSTLPFHNISKYAFGNCPKYIGVKSLKLAIGLRNVPAKSKRGLFGAIAGFIEG

GWPGLVAGWYGFQHSNDQGVGIAADRVSTQQAVDKITSKVNNIVDKMNKQYEIIDHEFSEVETRLNMINN

KIDDQIQDVWAYNAELLVLLENQKTLDEHDANVNNLYNKVKRALGTNALEDGKGCFELYHKCDDQCMETI

RNGTYNRRKYQEESRLERQKIEGVKLESEGTYKILTIYSTVASSLVLAMGFAAFLFWAMSNGSCRCNICI

>QHY92949.1 hemagglutinin [Influenza A virus]

MEALSLITILLVVTASNADKICIGYQSTNSTETVDTLTENNVPVTHAKELLHTEHNGMLCATSLGQPLIL

DTCTIEGLIYGNPSCDLLLGGREWSYIVERPSAVNGLCYPGNVENLEELRSLFSSARSYQRIQIFPDTIW

NVSYSGTSKACSDSFYRSMRWLTQKDNAYPIQDAQYTNNQEKNILFMWGINHPPTDTVQTNLYTRTDTTT
SVATEEINRTFKPLIGPRPLVNGLMGRINYYWSVLKPGQTLRIKSNGNLIAPWYGHILSGESHGRILKTD
LKRGSCTVQCQTEKGGLNTTLPFQNVSKYAFGNCSKYIGIKSLKLAVGLRNVPSRSSRGLFGAIAGFIEG
GWSGLVAGWYGFQHSNDQGVGMAADRDSTQKAIDKITSKVNNIVDKMNKQYEIIDHEFSEVETRLNMINN
KIDDQIQDIWAYNAELLVLLENQKTLDEHDANVNNLYHKVKRALGSNAVEDGKGCFELYHKCDDQCMETI
RNGTYNRRKYQEESKLERQKIEGVKLESEGTYKILTIYSTVASSLVIAMGFAAFLFWAMSNGSCRCNICI

>QCC70743.1 hemagglutinin, partial [Influenza A virus]
METVSLITILLVATVSYADKICIGYQSTNSTETVDTLTENNVPVTHAKELLHTEHNGMLCATSLGQPLIL
DTCTIEGLIYGNPSCDLSLEGREWSYIVERPSAVNGLCYPGNVENLEELRSLFSSARSYQRVQIFPDTIW
NVSYDGTSTACSGSFYRSMRWLTRKDGNYPNQDAQYTNNQGKNILFMWGINHPPTDDTQRSLYTRIDTTT
SVATEEINRIFKPLIGPRPLVNGLMGRIDYYWSVLKPGQTLRIKSDGNLIAPWYGYILSGESHGRILKTD
LKRGSCTVQCQTEKGGLNTTLPFQNVSKYAFGNCSKYIGINSLKLAVGLRNVPSRSSRGLFGAIAGFIEG
GWSGLVAGWYGFQHSNDQGVGMAADRDSTQKAIDKITSKVNNIVDKMNKQYEIIDHEFSEVETRLNMINN
KIDDQIQDIWAYNAELLVLLENQKTLDEHDANVNNLYNKVKRALGSNAVEDGKGCFELYHKCNDQCMETI
RNGTYNRRKYQEESKLERQKIEGVKLESEGTYKILTIYSTVASSLVIAMGFAAFLFWAMSNGSCRC

>QCC70742.1 hemagglutinin, partial [Influenza A virus]
METISLITILVVATVSNADKICIGYQSTNSTETVDTLTENNVPVTHAKELLHTEHNGMLCATSLGHPLIL
DTCTIEGLIYGNPSCDPLLRGREWSYIVERPSAVNGLCYPGNVENLEELRSLFSSSRSYQRIQIFPDTIW
NVSYSGTSKACSDSFYRSMRWLTQKNNAYPTQDAQYTNNQGKNILFMWGINHPPTDTVQTNLYTRTDTTT
SVATEEMNRIFKPLIGPRPLVNGLMGRINYYWSVLKPGQTLRIKSDGNLIAPWYGHILSGESHGRILKTD
LKMGSCTVRCQTEKGGLNTTLPFQNVSKYAFGNCSKYIGVKSLKLAVGLRNVPSRSSRGLFGAIAGFIEG
GWSGLVAGWYGFQHSNDQGVGMAADRDSTQKAIDKITSKVNNIVDKMNKQYEIIDHEFSEVETRLNMIND
KVDDQIQDIWAYNAELLVLLENQKTLDEHDANVNNLYNKVKRALGSNAVEDGRGCFELYHKCDDHCMETI
RNGTYNRRKYQEESKLERQKIEGIKLESEETYKILTIYSTVASSLVIAMGFAAFLFWAMSNGSCRC

>QCC70741.1 hemagglutinin, partial [Influenza A virus]
METVSLITILVVATVSNADKICIGYQSTNSTETVDTLTENNVPVTHAKELLHTEHNGMLCATSLGHPLIL
DTCTIEGLXXGNPSXDPLLGGREWSYIVERPSAVNGLCYPGNVENLEELRSLFSSSRSYQRVQIFPDTIW
NVSYSGTSEACSDSFYRSMRWLTQKNNAYPTQDAQYTNNQGKNILFMWGINHPPTDTAQTNLYTRIDTTT
SVATEEMNRIFKPLIGPRPLVNGLMGRINYYWSVLKPGQTLRIKSDGNLIAPWYGHILSGESHGRILKTD
LKRGSCTVQCQTEKGGLNTTLPFXNVSKYAFGNCSKYIGVKSLKLAVGLRNVPSRSSRGLFGAIAGXIEG
GWSRLVAGWYGFQHSNDQGVGMAADRDSTQKAIDKITSKVNNIIDKMNKQYEIIDHEFSEVETRLNMINN

KVDDQIQDIWAYNAELLVXLENQKTLDEHDANVNNLYNKVKRALGSNAVEDGXGCFELYHKCDDHCMEPI
RNGTYNRRKYQQESKLERQKIEGVKLESEETYKILTIYSTVASSLVIAMGFAAFLFWAMSNGSCRC

>QCC70740.1 hemagglutinin, partial [Influenza A virus]
METISLMTILLVATVSNADKICIGYQSTNSTETVDTLTENNVPVTHAKELLHTEHNGMLCATSLGNPLIL
DTCTIEGLIYGNPSCDPLLGGREWSYIVERPSAVNGLCYPGSVENLEELRSLFSSARSYQRIQIFPDTIW
NVSYSGTSKACSDSFYRSMRWLTQKNNAYPIQDAQYTNNQEKNILFMWGINHPPTETTQTNLYTRTDTTT
SVATEEINRIFKPXIGPRPLVNGLMGRINYYWSVLKPGQTLRIKSDGNLIAPWYGHILSGESHGRILKTD
LKRGSCTVQCQTEXGGLNTTLPFQNVSKYAFGNCSKYIGIKSLKLAVGLRNVPSRSSRGLFGAIAGFIEG
GWSGLVAGWYGFQHSNDQGVGMAADRDSTQKAIDKITSKVNNIVDKMNKQYEIIDHEFSEVETRLNMINN
KVDDQIQDIWAYNAELLVLLENQKTLDEHDSNVNNLYNKVKRALGSNAVEDGKGCFELYHKCDNQCMETI
RNGTYNRRKYQEESKLERQKIEGVKLESEGTYKILTIYSTVASSLVIAMGFAAFLFWAMSNGSCRC

>QCC70739.1 hemagglutinin, partial [Influenza A virus]
METVSLITILPVATVSNADKICIGYQSTNSTETVDTLTENNVPVTHAKELLHTEHNGMLCATSLGQPLIL
DTCTIEGLIYGNPSCDLSLEGREWSYIVERPSAVNGLCYPGNVENLEELRSLFSSARSYQRIQIFPDTIW
NVSYDGTSTACSNSFYRSMRWLTRKNGDYPIQDAQYTNNQGKNILFMWGINHPPTDDTQRNLYTRTDTTT
SVATEEINRIFKPLIGPRPLVNGLMGRINYYWSVLKPGQTLRIKSDGNLIAPWYGHILSGESHGRILKTD
LKRGSCTVQCQTEKGGLNTTLPFQNVSKYAFGNCSKYIGIKSLKLAVGLRNVPSRSSRGLFGAIAGFIEG
GWSGLVAGWYGFQHSNDQGVGMAADRDSTQKAIDKITSKVNNTVDKMNKQYEIIDHEFSEVETRLNMINN
KIDDQIQDVWAYNAELLVLLENQKTLDEHDANVNNLYNKVKKALGSNAVEDGKGCFELYHKCNDQCMETI
RNGTYNRRKYQKESKLERQKIEGVKLESEGTYKILTIYSTVASSLVIAMGFAAFLFWA

>QCC70738.1 hemagglutinin, partial [Influenza A virus]
METISLITILLVATVSNADKICIGYQSTNSTETVDTLTESNVPVTHVKELLHTEHNGMLCATSLGQPIIL
DTCTIEGLIYGNPSCDLSLEGREWSYIVERPSAVNGLCYPGNVENLEELRSLFSSARSYQRIQIFPDAIW
NVSYDGTGTACSGSFYRNMRWLTLKNGDYPIQDAQYTNNQGKNILFMWGINHPPSDTTQRNLYTRTDTTT
SVATEEINRIFKPLIGPRPLVNGLMGRIDYYWSVLKPGQTLRIKSDGNLIAPWYGHILSGESHGRILKTD
LKRGSCTVQCQTEKGGLNTTLPFQNVSRYAFGNCSKYIGIKSLKLAVGLRNVPSRSSRGLFGAIAGFIEG
GWSGLVAGWYGFQHSNDQGVGMAADRDSTQKAVDKITSKVNNIIGKMNKQYEIIDHEFSEVETRLNMINN
KIDDQIQDIWAYNAELLVLLENQKTLDEHDANVNNLYNKVKRALGSNAVEDGKGCFELYHKCDDQCMETI
RNGTYNRRKYQEESKLERQKIEGVKLESEGTYKILTIYSTVASSLVIAMGFAAFLFWA

>QCC70737.1 hemagglutinin, partial [Influenza A virus]

METVSLITILVVATVSNADKICIGYQSTNSTETVDTLTENNVPVTHAKELLHTEHNGMLCATSLGHPLIL
DTCTIEGLIYGNPSCDPLLGGREWSYIVERPSAVNGLCYPGNVEDLEELRSLFSSSRSYQRIQIFPDTIW
NVSYSGTSKACSDSFYRSMRWLTQKNNAYPTQDAQYTNNQGKNILFMWGINHPPTDTAQTNLYTRTDTTT
SVATEEMNRIFKPLIGPRPLVNGLMGRINYYWSVLKPGQTLRIKSDGNLIAPWYGHILSGESHGRILKTD
LKMGSCTVQCQTEKGGLNTTLPFQNVSKYAFGNCSKYIGVKSLKLAVGLRNVPSRSSRGLFGAIAGFIEG
GWSGLVAGWYGFQHSNDQGVGMAADRDSTQKAIDKITSKVNNIVDKMNKQYEIIDHEFSEVETRLNMIND
KVDDQIQDIWAYNAELLVLLENQKTLDEHDANVNNLYNKVKRALGSNAVEDGRGCFELYHKCDDHCMETI
RNGTYNRRKYQEESKLERQKIEGVKLESEETYKILTIYSTVASSLVIAMGFAAFLFWAMSNGSCRC

>QCC70736.1 hemagglutinin, partial [Influenza A virus]
METVSLITILVVATVSNADKICIGYQSTNSTETVDTLTENNVPVTHAKELLHTEHNGMLCATSLGHPLIL
DTCTIEGLIYGNPSCDPLLGGREWSYIVERPSAVNGLCYPGNVENLEELRSLFSSSRSYQRVQIFPDTIW
NVSYSGTSKACSDSFYRSMRWLTQKNNAYPTQDAQYTNNQGKNILFMWGINHPPTDTVQTNLYTRTDTTT
SVATEEMNRIFKPLIGPRPLVNGLMGRISYYWSVLKPGQTLRIKSDGNLIAPWYGHILSGESHGRILKTD
LKRGSCTVQCQTEKGGLNTTLPFQNVSKYAFGNCSKYIGVKSLKLAVGLRNVPSRSSRGLFGAIAGFIEG
GWSGLVAGWYGFQHSNDQGVGMAADRDSTQKAIDKITSKVNNIVDKMNKQYEIIDHEFSEVETRLNMINN
KVDDQIQDIWAYNAELLVLLENQKTLDEHDANVNNLYNKVKRALGSNAVEDGKGCFELYHKCDDHCMETI
RNETYNRRKYQQESKLERQKIEGVKLESEETYKILTIYSTVASSLVIAMGFAAFLFWAMSNGSCRC

>QCC70735.1 hemagglutinin, partial [Influenza A virus]
METVSLITILVVATVSSADKICIGYQSTNSTETVDTLTENNVPVTHAKELLHTEHNGMLCATSLGHPLIL
DTCTIEGLIYGNPSCDPLLGGREWSYIVERPSAINGLCYPGIVENLEELRSLFSSSRSYQRIQIFPDTIW
NVSYSGTSKACSDSFYRSMRWLTQKNNAYPTQDAQYTNNQGKNILFMWGINHPPTDTAQTNLYTRTDTTT
SVATEEMNRIFKPLIGPRPLVNGLMGRINYYWSVLKPGQTLRIKSDGNLIAPWYGHILSGESHGRILKTD
LKRGSCTVQCQTEKGGLNTTLPFQNVSKYAFGNCSKYIGVKSLKLAVGLRSVPSRSSRGLFGAIAGFIEG
GWSGLVAGWYGFQHSNDQGVGMAADRDSTQKAIDKITSKVNNIVDKMNKQYEIIDHEFSEVETRLNMINN
KVDDQIQDIWAYNAELLVLLENQKTLDEHDANVNNLYNKVKRALGSNAVEDGKGCFELYHKCDDHCMETI
RNGTYNRRKYQEESKLERQKIEGVKLESEETYKILTIYSTVASSLVIAMGFAAFLFWAMSNGSCRC

>QCC70734.1 hemagglutinin, partial [Influenza A virus]
METVSLITILVVATVSSADKICIGYQSTNSTETVDTLTENNVPVTHAKELLHTEHNGMLCATSLGHPLIL
DTCTIEGLIYGNPSCDPLLGGREWSYIVERPSAINGLCYPGIVENLEELRSLFSSSRSYQRIQIFPDTIW
NVSYSGTSKACSDSFYRSMRWLTQKNNAYPTQDAQYTNNQGKNILFMWGINHPPTDTAQTNLYTRTDTTT
SVATEEMNRIFKPLIGPRPLVNGLMGRINYYWSVLKPGQTLRIKSDGNLIAPWYGHILSGESHGRILKTD

LKRGSCTVQCQTEKGGLNTTLPFQNVSKYAFGNCSKYIGVKSLKLAVGLRSVPSRSSRGLFGAIAGFIEG
GWSGLVAGWYGFQHSNDQGVGMAADRDSTQKAIDKITSKVNNIVDKMNKQYEIIDHEFSEVETRLNMINN
KVDDQIQDIWAYNAELLVLLENQKTLDEHDANVNNLYNKVKRALGSNAVEDGKGCFELYHKCDDHCMETI
RNGTYNRRKYQEESKLERQKIEGVKLESEETYKILTIYSTVASSLVIAMGFAAFLFWAMSNGSCRC

>QCC70733.1 hemagglutinin, partial [Influenza A virus]
METVSLITILVVATVSSADKICIGYQSTNSTETVDTLTENNVPVTHAKELLHTEHNGMPCATSLGHPLIL
DTCTIEGLIYGNPSCDPLLGGREWSYIVERPSAINGLCYPGIVENLEELRSLFSSSRSYQRIQIFPDTIW
NVSYSGTSKACSDSFYRSMRWLTQKNNAYPTQDAQYTNNQGKNILFMWGINHPPTDTAQTNLYTRTDTTT
SVATEEMNRIFKPLIGPRPLVNGLMGRINYYWSVLKPGQTLRIKSDGNLIAPGYGHILSGESHGRILKTD
LKRGSCTVQCQTEKGGLNTTLPFQNVSKYASGNCSKYIGVKSLKLAVGLRNVPSRSSRGLFGAIAGFIEG
GWSGLVAGWYGFQHSNDQGVGMAADRDSTQKAIDKITSKVNNIVDKMNKQYEIIDHEFSEVETRLNMINN
KVDDQIQDIWAYNAELLVLLENQKTLDEHDANVNNLYNKVKSALGSNAVEDGKGCFELYHKCDDHCMETI
RNGTYNRRKYQEESKLERQKIEGVKLESEETYKILTIYSTVASSLVIAMGFAAFLFWAMSNGSCRC

>QCC70732.1 hemagglutinin, partial [Influenza A virus]
METISLITTLLVATVSYADKICIGYQSTNSTETVDTLTENNVPVTHAKELLHTEHNGMLCATSLGQPLIL
DTCTIEGLIYGNPSCDLSLEGREWSYIVERPSAVNGLCYPGNVENLEELRSLFSSARSYQRIQIFPDTIW
NVSYDGTSTACSGSFYRSMRWLTRKDGNYPTQDAQYTNNQGKNILFMWGINHPPTDDTQRSLYTRTDTTT
SVATEEINRIFKPLIGPRPLVNGLMGRIDYYWSVLKPGQTLRIKSDGNLIAPWYGYILSGESHGRILKTD
LKRGSCTVQCQTEKGGLNTTLPFQNVSKYAFGNCSKYIGIKSLKLAVGLRNVPSRSSRGLFGAIAGFIEG
GWSGLVAGWYGFQHSNDQGVGMAADRDSTQKAIDKTTSKVNNIVDKMNKQYEIIDHEFSEVETRLNMINN
KIDDQIQDIWAYNAELLVLLENQKTLDEHDANVNNLYNKVKRALGSNAAEDGKGCFELYHKCNDQCMETI
RNGTYNRRKYQEESKLERQKIEGVKLESEGTYKILTIYSTVASSLVIAMGFAAFLFWAMSNGSCRC

>QCC70731.1 hemagglutinin, partial [Influenza A virus]
METISLMTILLVATVSNADKICIGYQSTNSTETVDTLTENNVPVTHAKELLHTEHNGMLCATSLGNPLIL
DTCTIEGLIYGNPSCDPLLGGREWSYIVERPSAVNGLCYPGSVENLEELRSLFSSARSYQRIQIFPDTIW
NVSYSGTSKACSDSFYRSMRWLTQKNNAYPIQDAQYTNNQEKNILFMWGINHPPTETAQTNLYTRTDTTT
SVATEEINRIFKPLIGPRPLVNGLMGRINYYWSVLKPGQTLRIKSDGNLIAPWYGHILSGESHGRILKTD
LKRGSXTVQCQTEKGGLNTTLPFQNVSKYAFGNCSKYIGIKSLKLAVGLRNVPSRSSRGLFGAIAGFIEG
GWSGLVAGWYGFQHSNDQGVGMAADRGSTQKAIDKITSKVNNIVDKMNKQYEIIDHEFSEVETRLNMINN
KVDDQIQDIWAYNAELLVLLENQKTLDEHDSNVNNLYNKVKRALGSNAVEDGKGCFELYHKCDNQCMETI
RNGTYNRRKYQEESKLERQKIEGVKLESEGTYKILTIYSTVASSLVIAMGFAAFLFWAMSNGSCRC

>QCC70730.1 hemagglutinin, partial [Influenza A virus]

METISLMTILLVATVSNADKICIGYQSTNSTETVDTLTENNVPVTHAKELLHTEHNGMLCATSLGNPLIL

DTCTIEGLIYGNPSCDPLLGGREWSYIVERPSAVNGLCYPGSVENLEELRSLFSSARSYQRIQIFPDTIW

NVSYSGTSKACSDSFYRSMRWLTQKNNAYPIQDAQYTNNQEKNILFMWGINHPPTETVQTNLYTRTDTTT

SVATEEINRIFKPLIGPRPLVNGLMGRINYYWSVLKPGQTLRIKSDGNLIAPWYGHILSGESHGRILKTD

LKRGSCTVQCQTEKGGLNTTLPFQNVSKYAFGNCSKYIGIKSLKLAVGLRNVPSRSSRGLFGAIAGFIEG

GWSGLVAGWYGFQHSNDQGVGMAADRGSTQKAIDKITSKVNNIVDKMNKQYEIIDHEFSEVETRLNMINN

KVDDQIQDIWAYNAELLVLLENQKTLDEHDSNVNNLYNKVKRALGSNAVEDGKGCFELYHKCDNQCMETI

RNGTYNRRKYQEESKLERQKIEGVKLESEGTYKILTIYSTVASSLVIAMGFAAFLFWAMSNGSCRC

>QCC70729.1 hemagglutinin, partial [Influenza A virus]

METVSLITILVVATVSNADKICIGYQSTNSTETVDTLTENNVPVTHAKELLHTEHNGMLCATSLGHPLIL

DTCTIEGLIYGNPSCDPLLGGREWSYIVERPSAVNGLCYPGNVENLEELRSLFSSSRSYQRIQIFPDTVW

NVSYSGTSKACSDSFYRSMRWLTQKNNAYPTQDAQYTNNQGKNILFMWGINHPPTDTTQTNLYTRTDTTT

SVATEEMNRIFKPLIGPRPLVNGLMGRINYYWSVLKPGQTLRIKSDGNLIAPWYGHILSGESHGRILKTD

LKRGSCTVQCQTEKGGLNTTLPFXNXXKYAFGNCSGYIGVKSLKLAVGLRNVPSRSSRGLFGAIAGFIEG

GWSGLVAGWYGFQHSNDQGIGMAADRDSTQKAIDKITSKVNNIVDKMNKQYEIIDHEFSEVETRLNMINN

KVDDQIQDIWAYNAELLVLLENQKTLDEHDANVNNLYNKVKRALGSNAVEDGKGCFELYHKCDDHCMETI

RNGTYNRRKYQEESKLERQKIEGVKLESEETYKILTIYSTVASSLVIAMGFAAFLFWAMSNGSCRC

>QCC70728.1 hemagglutinin, partial [Influenza A virus]

METISLMTILLVATVSNADKICIGYQSTNSTETVDTLTENNVPVTHAKELLHTEHNGMLCATSLGNPLIL

DTCTIEGLIYGNPSCDPLLGGREWSYIVERPSAVNGLCYPGSVENLEELRSLFSSARSYQRIQIFPDTIW

NVSHSGTSKACSDSFYRSMRWLTQKNNAYPIQDAQYTNNQEKNILFMWGINHPPTETVQTNLYTRTDTTT

SVATEEINRIFKPXIGPRPLVNGLMGRINYYWSVLKPGQTLRIKSDGNLIAPWYGHILSGESHGRILKTD

LKRGSCTVQCQTEKGGLNTTLPFXNXSKYAFGNCSKYIGIKSLKLAVGLRNVPSRSSRGLFGAIAGFIEG

GWSGLVAGWYGFQHSNDQGVGMAADRESTQKAIDKITSKVNNIVDKMNKQYEIIDHEFSEVETRLNMINN

KVDDQIQDIWAYNAELLVLLENQKTLDEHDSNVNNLYNKVKRALGSNAMEDGKGCFELYHKCDNQCMETI

RNGTYNRRKYQEESKLERQKIEGVKLESEGTYKILTIYSTVASSLVIAMGFAAFLFWAMSNGSCRC

>QCC70727.1 hemagglutinin, partial [Influenza A virus]

METISLMTILLVATVSNADKICIGYQSTNSTETVDTLTENNVPVTHAKELLHTEHNGMLCATSLGNPLIL

DTCTIEGLIYGNPSCDPLLGGREWSYIVERPSAVNGLCYPGSVENLEELRSLFSSARSYQRIQIFPDTIW

NVSYSGTSKACSDSFYRSMRWLTQKNNAYPIQDAQYTNNQEKNILFMWGINHPPTETAQTNLYTRTDTTT
SVATEEINRIFKPLIGPRPLVNGLMGRINYYWSVLKPGQTLRIKSDGNLIAPWYGHILSGESHGRILKTD
LKRGSCTVQCQTEKGGLNTTLPFQNVSKYAFGNCSKYIGIKSLKLAVGLRNVPSRSSRGLFGAIAGFIEG
GWSGLVAGWYGFQHSNDQGVGMAADRESTQKAIDKITSKVNNIVDKMNKQYEIIDHEFSEVETRLNMINN
KVDDQIQDIWAYNAELLVLLENQKTLDEHDSNVNNLYNKVKRALGSNAVEDGKGCFELYHKCDNQCMETI
RNGTYNRRKYQEESKLERQKIEGVKLESEGTYKILTIYSTVASSLVIAMGFAAFLFWAMSNGSCRC

>QCC70726.1 hemagglutinin, partial [Influenza A virus]
METISLMTILLVATVSNADKICIGYQSTNSTETVDTLTENNVPVTHAKELLHTEHNGMLCATSLGNPLIL
DTCTIEGLIYGNPSCDPLLGGREWSYIVERPSAVNGLCYPGSVENLEELRSLFSSARSYQRIQIFPDTIW
NVSHSGTSKACSDSFYRSMRWLTQKNNAYPIQDAQYTNNQEKNILFMWGINHPPTETAQTNLYTRTDTTT
SVATEEINRIFKPLIGPRPLVNGLMGRINYYWSVLKPGQTLRIKSDGNLIAPWYGHILSGESHGRILKTD
LKRGSCTVQCQTEKGGLNTTLPFQNVSKYAFGNCSKYIGIKSLKLAVGLRNVPSRSSRGLFGAIAGFIEG
GWSGLVAGWYGFQHSNDQGVGMAADRESTQKAIDKITSKVNNIVDKMNKQYEIIDHEFSEVETRLNMINN
KVDDQIQDIWAYNAELLVLLENQKTLDEHDSNVNNLYNKVKRALGSNAMEDGKGCFELYHKCDNQCMETI
RNGTYNRRKYQEESKLERQKIEGVKLESEGTYKILTIYSTVASSLVIAMGFAAFLFWAMSNGSCRC

>QCC70725.1 hemagglutinin, partial [Influenza A virus]
MGTISLMTILLVATVSNADKICIGYQSTNSTETVDTLTENNVPVTHAKELLHTEHNGMLCATSLGNPLIL
DTCTIEGLIYGNPSCDPLLGGREWSYIVERPSAVNGLCYPGSVENLEELRSLFSSARSYQRIQIFPDTIW
NVSHSGTSKACSDSFYRSMRWLTQKNNAYPIQDAQYTNNQEKNILFMWGINHPPTETAQTNLYTRTDTTT
SVATEEINRIFKPLIGPRPLVNGLMGRINYYWSVLKPGQTLRIKSDGNLIAPWYGHILSGESHGRILKTD
LKRGSXTVQCQTEKGGLNTTLPFQNXSKYAFGNCSKYIGIKSLKLAVGLRNVPSRSSRGLFGAIAGFIEG
GWSGLVAGWYGFQHSNDQGVGMAADRESTQKAIDKITSKVNNIVDKMNKQYEIIDHEFSEVETRLNMINN
KVDDQIQDIWAYNAELLVLLENQKTLDEHDSNVNNLYNKVKRALGSNAMEDGKGCFEPYHKCDNQCMETI
RNGTYNRRKYQEESKLERQKIEGVKLESEGTYKILTIYSTVASSLVIAMGFAAFLFWAMSNGSCRC

>QCC70724.1 hemagglutinin, partial [Influenza A virus]
METVSLITILVVATVSNADKICIGYQSTNSTETVDTLTENNVPVTHAKELLHTEHNGMLCATSLGHPLIL
DTCTIEGLIYGNPSCDPLLGGREWSYIVERPSAVNGLCYPGNVENLEELRSLFSSSRSYQRVQIFPDTIW
NVSYSGTSKACSDSFYRSMRWLTQKNNAYPTQDAQYTNNQGKNILFMWGINHPPTDTAQTNLYTRIDTTT
SVATEEMNRIFKPXIGPRPLVNGLMGRINYYWSVLKPGQTLRIKSDGNLIAPWYGHILSGESHGRILKTD
LKRGSCTVQCQTEKGGLNTTLPFQNXSKYAFGNCSKYIGVKSLKLAVGLRNVPSRSSRGLFGAIAGFIEG
GWSGLVAGWYGFQHSNDQGVGMAADRDSTQKAIDKITSKVNNIIDKMNKQYEVIDHEFSEVETRLNMINN

KVDDQIQDIWAYNAELLVLLENQKTLDEHDANVNNLYNKVKRALGSNAVEDGKGCFELYHKCDDHCMETI
RNGTYNRRKYQQESKLERQKIEGVKLESEETYKILTIYSTVASSLVIAMGFAAFLFWAMSNGSCRC

>QCC70723.1 hemagglutinin, partial [Influenza A virus]
ATVSNADKICIGYQSTNSTETVDTLTENNVPVTHAKELLHTEHNGMLCATSLGQPLILDTCTIEGLIYGN
PSCDLSLEGKEWSYIVERPSAVNGLCYPGNVENLEELRSLFSSARSYQRVQIFPDTIWNVSYDGTSTACS
GSFYRSMRWLTRKDGNYPTQDAQYTNNQGKNILFMWGINHPPTDDTQRSLYTKTDTTTSVATEEINRIFK
PLIGPRPLVNGLMGRIDYYWSVLKPGQTLRIKSDGNLIAPWYGYILSGESHGRILKTDLKRGSCTVQCQT
EKGGINTTLPFQNVSKYAFGNCSKYIGIKSLKLAVGLRNVPSRSSRGLFGAIAGFIEGGWSGLVAGWYGF
QHSNDQGVGIAADRDSTQKAIDKITSKVNNIVDKMNKQYEIIDHEFSEVETRLNMINNKIDDQIQDIWAY
NAELLVLLENQKTLDEHGANVNNLYSKVKRALGSNAVEDGKGCFDLYHKCDDQCMETIRNGTYNRRKYQE
ESKLERQKIEGVKLESEGTYKILTIYSTVASSLVIAMGFAAFLFWAMSNGSCRC

>QCC70722.1 hemagglutinin, partial [Influenza A virus]
METISLMTILLVATVSNADKICIGYQSTNSTETVDTLTENNVPVTHAKELLHTEHNGMLCATSLGNPLIL
DTCTIEGLIYGNPSCDPLLGGREWSYIVERPSAVNGLCYPGSVENLEELRSLFSSARSYQRIQIFPDTIW
NVSYSGTSKACSDSFYRSMRWLTRKNNAYPIQDAQYTNNQEKNILFMWGINHPPTETAQTNLYTRTDTTT
SVATEEINRIFKPLIGPRPLVNGLMGRINYYWSVLKPGQTLRIKSDGNLIAPWYGHILSGESHGRILKTN
LKRGSCTVQCQTEKGGLNTTLPFQNVSKYAFGNCSKYIGIKSLKLAVGLRNVPSRSSRGLFGAIAGFIEG
GWSGLVAGWYGFQHSNDQGVGMAADRESTQKAIDKITSKVNNIVDKMNKQYEIIDHEFSEVETRLNMINN
KVDDQIQDIWAYNAELLVLLENQKTLDEHDSNVNNLYNKVKRALGSNAVEDGKGCFELYHKCDNQCMETI
RNGTYNRRRYQEESKLERQKIEGVKLESEGTYKILTIYSTVASSLVIAMGFAAFLFWAMSNGSCRC

>QCC70721.1 hemagglutinin, partial [Influenza A virus]
ATVSNADKICIGYQSTNSTETVDTLTENNVPVTHAKELLHTEHNGMLCATSLGQPLILNTCTIGGLIYGN
PSCDPSLEGREWSYIVERPSAVHGLCYPGNVEDLEELRSIFSSARSYQRIQIFPDTIWNVSYDGTSTACS
GSFYRSMRWLTRKNGEYPIQDAQYTNNQGKNILFMWGINHPPTDNTQRELYTRTDTTTSVATEEINRIFK
PLIGPRPLVNGLMGRINYYWSVLKPGQTLRIKSDGNLIAPWYGHILSGESHGRILKTDLKRGSCTVQCQT
EKGGLNTTLPFQNVSKYAFGNCSKYIGIKSLKLAVGLRNVPSRSSRGLFGAIAGFIEGGWSGLVAGWYGF
QHSNDQGVGMAADRDSTQKAIDKRTSKVNNIVDKMNKQYEIIDHEFSEVETRLNMINNKIDDQIQDIWAY
NAELLVLLENQKTLDEHDANVNNLYNKVKRALGSNAVEDGKGCFELYHKCDDQCMETIRNGTYNRRKYQE
ESKLERQKIEGVKLESEGTYKILTIYSTVASSLVIAMGFAAFLFWAMSNGSCRC

>QCC70720.1 hemagglutinin, partial [Influenza A virus]

ATVSNADKICIGYQSTNSTETVDTLTENNVPVTHAKELLHTEHNGMLCATSLGQPLILNTCTIEGLIYGN
PSCDLSLEGREWSYIVERPSAVHGLCYPGNVEDLEELRSIFSSARSYQRIQIFPDTIWNVSYDGTSTACS
GSFYRSMRWLTRKNGEYPIQDAQYTNNQGKNILFMWGINHPPTDNTQRELYTRTDTTTSVATEEINRIFK
PLIGPRPLVNGLMGRINYYWSVLKPGQTLRIKSDGNLIAPWYGHILSGESHGRILKTDLKRGSCTVQCQT
EKGGLNTTLPFQNVSKYAFGNCSKYIGIKSLKLAVGLRNVPSRSSRGLFGAIAGFIEGGWSGLVAGWYGF
QHSNDQGVGMAADRDSTQKAIDKITSKVNNIVDKMNKQYEIIDHEFSEVETRLNMINNKIDDQIQDIWAY
NAELLVLLENQKTLDEHDANVNNLYNKVKRALGSNAVEDGKGCFELYHKCDDQCMETIRNGTYNRRKYQE
ESKLERQKIEGVKLESEGTYKILTIYSTVASSLVIAMGFAAFLFWAMSNGSCRC

>QCC70719.1 hemagglutinin, partial [Influenza A virus]
METVSLITILLVATVSYADKICIGYQSTNSTETVDTLTENNVPVTHAKELLHTEHNGMLCATSLGQPLIL
DTCTIEGLIYGNPSCDLSLEGREWSYIVERPSAVNGLCYPGNVENLEELRSLFSSARSYQRVQIFPDTIW
NVSYDGTSTACSGSFYRSMRWLTRKDGNYPNQDAQYTNNQGKNILFMWGINHPPTDDTQRSLYTRIDTTT
SVATEEINRIFKPLIGPRPLVNGLMGRIDYYWSVLKPGQTLRIKSDGNLIAPWYGYILSGESHGRILKTD
LKRGSCTVQCQTEKGGLNTTLPFQNVSKYAFGNCSKYIGINSLKLAVGLRNVPSRSSRGLFGAIAGFIEG
GWSGLVAGWYGFQHSNDQGVGMAADRDSTQKAIDKITSKVNNIVDKMNKQYEIIDHEFSEVETRLNMINN
KIDDQIQDIWAYNAELLVLLENQKTLDEHDANVNNLYNKVKRALGSNAVEDGKGCFELYHKCNDQCMETI
RNGTYNRRKYQEESKLERQKIEGVKLESEGTYKILTIYSTVASSLVIAMGFAAFLFWAMSNGSCRC

>QCC70718.1 hemagglutinin, partial [Influenza A virus]
ATVSNADKICIGYQSTNSTETVDTLTENNVPVTHAKELLHTEHNGMLCATSLGQPLILDTCTIEGLIYGN
PSCDLSLEGREWSYIVERPSAVNGLCYPGNVENLEELRSLFSSARSYQRVQIFPDTIWNVSYDGTSTACS
GSFYRSMRWLTRKDGNYPTQDAQYTNDQGKNILFMWGINHPPTDYTQRSLYTRTDTTTSVATEEINRIFK
PLIGPRPLVNGLMGRIDYYWSVLKQGQTLRIKSDGNLIAPWYGYILPGESHGRILKTDLKRGSCTVQCQT
EKGGLNTTLPFQNVSKYAFGNCSKYIGIKSLKLAVGLRNVPSRSSRGLFGAIAGFIEGGWSGLVAGWYGF
QHSNDQGVGMAADRDSTQKAIDKITSKVNNIVDKMNKQYEIIDHEFSEVETRLNMINNKIDDQIQDIWAY
NAELLVLLENQKTLDEHDANVNNLYNKVKRALGSNAAEDGKGCFELYHKCNDQCMETIRNGTYNRRKYQE
ESKLERQKIEGVKLESEGTYKILTIYSTVASSLVIAMGFAAFLFWAMSNGSCRC

>QCC70717.1 hemagglutinin, partial [Influenza A virus]
METISLMTILLVATVSNADKICIGYQSTNSTETVDTLTENNVPVTHAKELLHTEHNGMLCATSLGNPLIL
DTCTIEGLIYGNPSCDPLLGGREWSYIVERPSAVNGLCYPGSVENLEELRSLFSSARSYQRIQIFPDTIW
NVSYSGTSKACSDSFYRSMRWLTQKNNAYPIQDAQYTNNQEKNILFMWGINHPPTETTQTNLYTRTDTTT
SVATEEINRIFKPXIGPRPLVNGLMGRINYYWSVLKPGQTLRIKSDGNLIAPWYGHILSGESHGRILKTD

LKRGSCTVQCQTEKGGLNTTLPFQNXSKYAFGNCSKYIGIKSLKLAVGLRNVPSRSSRGLFGAIAGFIEG
GWSGLVAGWYGFQHSNDQGVGMAADRDSTQKAIDKITSKVNNIVDKMNKQYEIIDHEFSEVETRLNMINN
KVDDQIQDIWAYNAELLVLLENQKTLDEHDSNVNNLYNKVKRALGSNAVEDGKGCFELYHKCDNQCMETI
RNGTYNRRKYQEESKLERQKIEGVKLESEGTYKILTIYSTVASSLVIAMGFAAFLFWAMSNGSCRC

>QCC70713.1 hemagglutinin, partial [Influenza A virus]
METISLITILLVATVSYADKICIGYQSTNSIETVDTLTENNVPVTHAKELLHTEHNGMLCATSLGQPLIL
DTCTIEGLIYGNPSCDLSLEGREWSYIVERPSAVNGLCYPGNVENLEELRSLFSSARSYQRVQIFPDTIW
NVSYDGTSKACSGSFYKSMRWLTRKDGNYPTQDAQYTNNQGKNIFFMWGINHPPTDNTQRSLYTKTDTTT
SVATEEINRIFKPLIGPRPLVNGLMGRIDYYWSVLKPGQTLRIKSDGNLIAPWYGYILSGESHGRILKTD
LKRGSCTVQCQTEKGGLNTTLPFQNVSKYAFGNCSKYIGIKSLKLAVGLRNVPSRSSRGLFGAIAGFIEG
GWSGLVAGWYGFQHSNDQGVGMAADRDSTQKAIDKITSKVNNIVDKMNKQYEIIDHEFSEVETRLNMINN
KIDDQIQDIWAYNAELLVLLENQKTLDEHDANVNNLYNKVKRALGSNAAEDGKGCFELYHKCNDQCMETI
RNGTYNRRKYQEESKLERQKIEGVKLESEGTYKILTIYSTVASSLVIAMGFAAFLFWAMSNGSCRC

>QCC70712.1 hemagglutinin, partial [Influenza A virus]
METISLMTILLVATVSNADKICIGYQSTNSTETVDTLTENNVPVTHAKELLHTEHNGMLCATSLGNPLIL
DTCTIEGLIYGNPSCDPLLGGREWSYIVERPSAVNGLCYPGSVENLEELRSLFSSARSYQRIQIFPDTIW
NVSYSGTSKACSDSFYRSMRWLTQKNNAYPIQDAQYTNNQEKNILFMWGINHPPTETTQTNLYTRTDTTT
SVATEEINRIFKPLIGPRPLVNGLMGRINYYWSVLKPGQTLRIKSDGNLIAPWYGHILSGESHGRILKTD
LKRGSCTVQCQTEKGGLNTTLPFQNVSKYAFGNCSKYIGIKSLKLAVGLRNVPSRSSRGLFGAIAGFIEG
GWSGLVAGWYGFQHSNDQGVGMAADRDSTQKAIDKITSKVNNIVDKMNKQYEIIDHEFSEVETRLNMINN
KVDDQIQDIWAYNAELLVLLENQKTLDEHDSNVNNLYNKVKRALGSNAVEDGKGCFELYHKCDNQCMETI
RNGTYNRRKYQEESKLERQKIEGVKLESEGTYKILTIYSTVASSLVIAMGFAAFLFWAMSNGSCRC

>QCC70711.1 hemagglutinin, partial [Influenza A virus]
METISLITILLVATVSYADKICIGYQSTNSTETVDTLTENNVPVTHAKELLHTEHNGMLCATSLGQPLIL
DTCTIEGLIYGNPSCDLSLEEKEWSYIVERPSAVNGLCYPGNVENLEELRSLFSSARSFQRVQIFPDTIW
NVSYDGTSKACSGSFYRSMRWLTLKDGNYPTQDAQYTNNQGKNILFMWGINHPPTDNTQRSLYTKTDTTT
SVATEEINRIFKPLIGPRPLVNGLMGRIDYYWSVLKPGQTLRIKSDGNLIAPWYGYILSGESHGRILKTD
LKRGSCTVQCQTEKGGLNTTLPFQNVSKYAFGNCSKYIGIKSLKLAVGLRNVPSRSSRGLFGAIAGFIEG
GWSGLVAGWYGFQHSNDQGVGMAADRDSTQKAIDKITSKVNNIVDKMNKQYEIIDHEFSEVETRLNMINN
KIDDQIQDIWAYNAELLVLLENQKTLDEHDANVNNLYNKVKRALGSNAAEDGKGCFELYHKCNDQCMETI
RNGTYNRRKYQEESKLERQKIEGVKLESEGTYKILTIYSTVASSLVIAMGFAAFLFWAMSNGSCRC

>QCC70710.1 hemagglutinin, partial [Influenza A virus]

METISLMTILLVTTVSNADKICIGYQSTNSTETVDTLTENNVPVTHAKELLHTEHNGMLCATSLGNPLIL
DTCTIEGLIYGNPSCDPLLGGREWSYIVERPSAVNGLCYPGSVENLEELRSLFSSARSYQRIQIFPDTIW
NVSYSGTSKACSDSFYRSMRWLTQKNNAYPIQDAQYTNNQEKNILFMWGINHPPTETVQTNLYTRTDTTT
SVATEEINRIFKPLIGPRPLVNGLMGRINYYWSVLKPGQTLRIKSDGNLIAPWFGYILSGESHGRILKTD
LKRGSCTVQCQTEKGGLNTTLPFQNVSKYAFGNCSKYIGIKSLKLAVGLRNVPSRSSRGLFGAIAGFIEG
GWSGLVAGWYGFQHSNDQGVGMAADRDSTQKAIDKITSKVNNIVDKMNKQYEIIDHEFSEVETRLNMINN
KVDDQIQDIWAYNAELLVLLENQKTLDEHDSNVNNLYNKVKRALGSNAVEDGKGCFELYHKCDNQCMETI
RNGTYNRRKYQEESKLERQKIEGVKLESEGTYKILTIYSTVASSLVIAMGFAAFLFWAMSNGSCRC

>QCC70709.1 hemagglutinin, partial [Influenza A virus]

METISLITILLVATVSYADKICIGYQSTNSTETVDTLTENNVPVTHAKELLHTEHNGMLCATSLGQPLIL
DTCTIEGLIYGNPSCDLSLEGKEWSYIVERPSAVNGLCYPGNVENLEELRSLFSSARSYQRVQIFPDTIW
NVSYNGTSKACSGSFYRSMRWLTRKDGNYPTQDAQYTNNQGKNILFMWGINHPPTDDKQRSLYTKTDTTT
SVATEEINRIFKPLIGPRPLVNGLMGRIDYYWSVLKPGQTLRIKSDGNLIAPWYGYILSGESHGRILRTD
LKRGSCTVQCQTEKGGLNTTLPFQNVSKYAFGNCSKYIGIKSLKLAVGLRNVPSRSSRGLFGAIAGFIEG
GWSGLVAGWYGFQHSNDQGVGMAADRDSTQKAIDKITSKVNNIVDKMNKQYEIIDHEFSEVETRLNMINN
KIDDQIQDIWAYNAELLVLLENQKTLDEHDANVNNLYNKVKRALGSNAAEDGKGCFELYHKCNDQCMETI
RNGTYNRRKYQEESKLERQKIEGVKLESEGTYKILTIYSTVASSLVIAMGFAAFLFWAMSNGSCRC

>QCC70707.1 hemagglutinin, partial [Influenza A virus]

METISLITILLVATVSYADKICIGYQSTNSTETVDTLTENNVPVTHAKELLHTEHNGMLCATSLGQPLIL
DTCTIEGLIYGNPSCDLSLEGREWSYIVERPSAVNGLCYPGNVENLEELRSLFSSARSYQRVQIFPDTIW
NVSYDGTSTACSGSFYRSMRWLTRKDGNYPTQDAQYTNNQGKNILFMWGINHPPTDDTQRSLYTKTDTTT
SVATEEINRIFKPLIGPRPLVNGLMGRIDYYWSVLKPGQTLRIKSDGNLIAPWYGYILSGESHGRILKTD
LKRGSCTVQCQTEKGGLNTTLPFQNVSKYAFGNCSKYIGIKSLKLAVGLRNVPSRSSRGLFGAIAGFIEG
GWSGLVAGWYGFQHSNDQGVGMAADRDSTQKAIDKITSKVNNIVDKMNKQYEIIDHEFSEVETRLNMINN
KIDDQIQDIWAYNAELLVLLENQKTLDEHDANVNNLYNKVKRALGSNAAEDGKGCFELYHKCNDQCMETI
RNGTYNRRKYQEESKLERQKIEGVKLESEGTYKILTIYSTVASSLVIAMGFAAFLFWAMSNGSCRC

>QCC70706.1 hemagglutinin, partial [Influenza A virus]

METISLMTILLVATVSNADKICIGYQSTNSTETVDTLTENNVPVTHAKELLHTEHNGMLCATSLGDPLIL
DTCTIEGLIYGNPSCDPLLGGREWSYIVERPSAVNGLCYPGSVENLEELRSLFSSARSYQRIQIFPDTIW

NVSYSGTSKACSDSFYRSMRWLTQKNNAYPIQDAQYTNNQEKNILFMWGINHPPTETVQTNLYTRTDTTT
SVATEEINRIFKPLIGPRPLVNGLMGRINYYWSVLKPGQTLRIKSDGNLIAPWYGHILSGESHGRILKTD
LKRGSCTVQCQTEKGGLNTTLPFQNVSKYAFGNCSKYIGIKSLKLAVGLRNVPSRSSRGLFGAIAGFIEG
GWSGLVAGWYGFQHSNDQGVGMAADRDSTQKAIDKITSKVNNIVDKMNKQYEIIDHEFSEVETRLNMINN
KVDDQIQDIWAYNAELLVLLENQKTLDEHDSNVNNLYNKVKRALGSNAVEDGKGCFELYHKCDNQCMETI
RNGTYNRRKYQEESKLERQKIEGVKLESEGTYKILTIYSTVASSLVIAMGFAAVLFWAMSNGSCRC

>QCC70704.1 hemagglutinin, partial [Influenza A virus]
METVSLITILIVATVSNADKICIGYQSTNSTETVDTLTENNVPVTHAKELLHTEHNGMLCATSLGQPLIL
DTCTIEGLIYGNPSCDLSLEGREWSYIVERPSAVNGLCYPGNVENLEELRSLFSSARSYQRIQIFPDTIW
NVSYNGTSTACSNSFYRSMRWLTRKNGDYPTQDAQYTNNQGKNILFMWGISHPPTDDTQRNLYTRTDTTT
SVATEEINRNFKPLIGPRPLVNGLMGRIDYYWSVLKPGQTLRIKSDGNLIAPWYGHILSGESHGRILKTD
LKRGSCTVQCQTEKGGLNTTLPFRNVSKYAFGNCSKYIGIKSLKLAVGLRNVPSRSSRGLFGAIAGFIEG
GWSGLVAGWYGFQHSNDQGVGMAADRDSTQKAIDKITSKVNNIVDKMNKQYEIIDHEFSEVETRLNMINN
KIDDQIQDIWAYNAELLVLLENQKTLDEHDANVNNLYNKVKRALGSNAVEDGKGCFELYHKCDDQCMETI
RNGTYNRKKYQEESKLERQKIEGVKLESEGTYKILTIYSTVASSLVIAMGFAAFLFWA

>QCC70703.1 hemagglutinin, partial [Influenza A virus]
METVSLITILLVATVSNADKICIGYQSTNSTETVDTLTENNVPVTHAKELLHTEHNGMLCATSLGHPLIL
DTCTIEGLIYGNPSCDLSLEGREWSYIVERPSAVNGLCYPGNVENLEELRSLFSSARSYQRLQIFPDTIW
NVSYDGTSTACSGSFYRSMRWLTRKNGEYPTQDAQYTNNQGKNILFMWGINHPPTDTTQRDLYTRIDTTT
SVATEEINRIFKPLIGPRPLVNGLMGRIDYYWSILKPGQTLRIKSDGNLIAPWHGHILSGESHGRILKTD
LKRGSCTVQCQTEKGGLNTTLPFQNVSKYAFGNCSKYIGIKSLKLAVGLRNVPSRSSRGLFGAIAGFIEG
GWSGLVAGWYGFQHSNDQGVGMAADRDSTQKAIDKITSKVNNIVDKMNKQYEIIDHEFSEVETRLNMINN
KIDDQIQDIWAYNAELLVLLENQKTLDEHDANVNNLYNKVKRALGSNAVEDGKGCFELYHKCDNQCMETI
RNGTYNRRKYQEESKIERQKIEGVKLESEGTYKILTIYSTVASSLVIAMGFAAFLFWAMSNGSCRC

>QCC70701.1 hemagglutinin, partial [Influenza A virus]
METVSLITILLVATVSNADKICIGYQSTNSTETVDTLTENNVPVTHAKELLHTEHNGMLCATSLGQPLIL
DTCTIEGLIYGNPSCDLSLEGREWSYIVERPSAVNGLCYPGNVENLEELRSLFSSARSYQRIQIFPDTIW
NVSYDGTSTACSNSFYRSMRWLTRKNGDYPTQDAQYTNNQGKNILFMWGINHPPTDDTQRNLYTRTDTTT
SVATEEINRIFKPLIGPRPLVNGLMGRINYYWSVLKPGQTLRIKSDGNLIAPWYGHILSGESHGRILKTD
LKRGSCTVQCQTEKGGLNTTLPFQNVSKYAFGNCSKYIGIKSLKLAVGLRNVPSRSSRGLFGAIAGFIEG
GWSGLVAGWYGFQHSNDQGVGMAADRDSTQKAIDKITSKVNNIVDKMNKQYEIIDHEFSEVETRLNMINN

KIDDQIQDVWAYNAELLVLLENQKTLDEHDANVNNLYNKVKKALGSNAVEDGKGCFELYHKCNDQCMETI
RNGTYNRRKYQEESKLERQKIEGVKLESEGTYKILTIYSTVASSLVIAMGFAAFLFWA

>QCC70699.1 hemagglutinin, partial [Influenza A virus]
METVSLITILLVAAVSNADKICIGYQSTNSTETVDTLTENNVPVTHAKELLHTKHNGMLCATSLGQPIIL
DTCTIEGLIYGNPSCDLSLEGREWSYIVERPSAVNGLCYPGNVENLEELRSLFSSARSYQRIQIFPDTIW
NVSYDGTSTACSGSFYRNMRWLTRKNGEYPIQDAQYTNNKGKNILFMWGINHPPTDTTQRNLYTRTDTTT
SVATEEINRIFKPLIGPRPLVNGLMGRIDYYWSVLKPGQTLRIKSDGNLISPWYGHILSGESHGRILKTD
LKRSSCTVQCQTEKGGLNTTLPFQNVSKYAFGNCSKYIGIKSLKLAVGLRNVPSRSSRGLFGAIAGFIEG
GWSGLVAGWYGFQHSNDQGVGMAADRDSTQKAVDRITSKVNTIVDKMNKQYEIIDHEFSEVETRLNMINN
KIDDQIQDIWAYNAELLVLLENQKTLDEHDANVNNLYNKVKRALGSNAVEDGRGCFELYHKCDDQCMETI
RNGTYNRRKYQEESKLERQKIEGVKLESEGTYKILTIYSTVASSLVIAMGFAAFLFWAMSNGSCRC

>QCC70698.1 hemagglutinin, partial [Influenza A virus]
METVSLITILLVATVSNADKICIGYQSTNSTETVDTLTENNVPVTHAKELLHTEHNGMLCATSLGQPLIL
DTCTIEGLIYGNPSCDLSLEGREWSYIVERPSAVNGLCYPGNVENLEELRSLFSSARSYQRIQIFPDTIW
NVSYNGTSIACSNSFYRSMRWLTRKNGDYPTQDAQYTNNQGKNILFMWGINHPPTDDTQRNLYTRTDTTT
SVATEEINRIFKPLIGPRPLVNGLMGRIDYYWSVLKPGQTLRIKSDGNLIAPWYGYILSGESHGRILKTD
LKRGSCTVQCQTEKGGLNTTLPFQNVSKYAFGNCSKYIGIKSLKLAVGLRNVPSRSSRGLFGAIAGFIEG
GWSGLVAGWYGFQHSNDQGVGMAADRDSTQKAIDKITSKVNNIVDKMNKQYEIIDHEFSEVETRLNMINN
KIDDQIQDIWAYDAELLVLLENQKTLDEHDANVNNLYNKVKRALGSNAVEDGKGCFELYHKCNDQCMETI
RNGTYNRRKYQEESKLERQKIEGVKLESEGTYKILTIYSTVASSLVIAMGFAAFLFWAMSNGSCRC

>QCC70697.1 hemagglutinin, partial [Influenza A virus]
METVSLITILLVATVSNADKICIGYQSTNSTETVDTLTENNVPVTHAKELLHTEHNGMLCATSLGQPLIL
DTCTIEGLIYGNPSCDLSLEGREWSYIVERPSAVNGLCYPGNVENLEELRSLFSSARSYQGLQIFPDTIW
NVSYNGTSTACSGSFYRSMRWLTRKNGKYPTQDAQYTNNQGKNILFMWGINHPPTDTTQRDLYTRTDTTT
SVATEEINRIFKPLIGPRPLVNGLMGRIDYYWSILKPGQTLRIKSDGNLIAPWYGHILSGESHGRILKTD
LKRGSCTVQCQTEKGGLNTTLPFQNVSKYAFGNCSKYIGIKSLKLAVGLRNVPSRSSRGLFGAIAGFIEG
GWSGLVAGWYGFQHSNDQGVGMAADRDSTQKAIDKITSKVNNIVDKMNKQYEIIDHEFSEVETRLNMINN
KIDDQIQDIWAYNAELLVLLENQKTLDEHDANVNNLYNKVKRALGSNAVEDGKGCFELYHKCDNQCMETI
RNGTYNRRKYQEESKIERQKIEGVKLESEGTYKILTIYSTVASSLVIAMGFAAFLFWA

>QCC70696.1 hemagglutinin, partial [Influenza A virus]

METASLITILLAVTVSNADKICIGYQSTNSTETVDTLTENNVPVTHAKELLHTEHNGMLCATSLGHPLIL
DTCTIEGLIYGNPSCDLLLGGREWSYIVERPSAVNGLCYPGNVENLEELRSLFSSARSYQRIQIFPDTIW
NVSYSGTSKACSDSFYRSMRWLTQKDNAYPVQDAQYTNNQEKNILFMWGINHPPTDTVQTNLYTRTDTTT
SVATEEINRTFKPLIGPRPLVNGLMGRINYYWSVLKPGQTLRIKSNGNLIAPWYGHILSGESHGRILKTD
LKRGSCTVQCQTEKGGLNTTLPFQNVSKYAFGNCSKYIGIKSLKLAVGLRNVPSRSSRGLFGAIAGFIEG
GWSGLVAGWYGFQHSNDQGVGMAADRDSTQKAIDKITSKVNNIVDKMNKQYEIIDHEFSEVETRLNMINN
KIDDQIQDIWAYNAELLVLLENQKTLDEHDANVNNLYNKVKRALGSNAVEDGKGCFELYHKCDDQCMETI
RNGTYNRRKYQEESKLERQKIEGVKLESEGTYKILTIYSTVASSLVIAMGFAAFLFWA

>QCC70695.1 hemagglutinin, partial [Influenza A virus]
METASLITILLAVTVSNADKICIGYQSTNSTETVDTLTENNVPVTHAKELLHTEHNGMLCATSLGHPLIL
DTCTIEGLIYGNPSCDLLLGGREWSYVVERPSAVNGSCYPGNVENLEELRSLFSSARSYQRIQIFPDTIW
NVSYSGTSKACSDSFYRSMRWLTQKDNAYPVQDAQYTNNQEKNILFMWGINHPPTDTAQTNLYTRTDTTT
SVATEEINRTFKPLIGPRPLVNGLMGRINYYWSVLKPGQTLRIKSNGNLIAPWYGHILSGESHGRILKTD
LKRGSCTVQCQTEKGGLNTTLPFQNVSKYAFGNCSKYIGIKSLKLAVGLRNVPSRSSSGLFGAIAGFIEG
GWSGLVAGWYGFQHSNDQGVGMAADRDSTQKAIDKITSKVNNIVDKMNKQDEIIDHEFSEVETRLNMINN
KIDDQIQDVWAYNAELLVLLENQKTLDEHDANVNNLYNKVKRALGSNAVEDGKGCFELYHKCDDQCMETI
RNGTYNRRKYQEESKLERQKIEGVKLESEGTYKILTIYSTVASSLVIAMGFAAFLFWA

>QCC70694.1 hemagglutinin, partial [Influenza A virus]
METVSLITILLVATVSNADKICIGYQSTNSTETVDTLTENNVPVTHAKELLHTEHNGMLCATSLGQPLIL
DTCTIEGLIYGNPSCDLSLEGREWSYIVERPSAVNGLCYPGNVENLEELRSLFSSARSYQRIQIFPDTIW
NVSYDGTSTACSGSFYKSMRWLTRKNGDYPIQDAQYTNNQGKNILFMWGINHPPTDTTQRNLYTRIDTTT
SVATEEINRVFKPLIGPRPLVNGLMGRIDYYWSVLKPGQTLRIKSDGNLIAPWYGHILSGESHGRILKTD
LKRGSCTVQCQTEKGGLNTTLPFQNVSKYAFGNCSKYIGIKSLKLAVGLRNVPSRSSRGLFGAIAGFIEG
GWSGLVAGWYGFQHSNDQGVGMAADRDSTQKAIDKITSKVNNIVDKMNKQYEIIDHEFSEVETRLNMINN
KIDDQIQDIWAYNAELLVLLENQKTLDEHDANVNNLYNKVKRALGSNAVEDGKGCFELYHKCNDQCMETI
RNGTYNRRKYQEESKLERQKIEGVKLESEGTYKILTIYSTVASSLVIAMGFAAFLFWA

>QCC70692.1 hemagglutinin, partial [Influenza A virus]
METVSLITILLVATVSNADKICIGYQSTNSTETVDTPTENNVPVTHAKELLHTEHNGMLCATSLGQPLIL
DTCTIEGLIYGNPSCDLSLEGREWSYIVERPSAVNGLCYPGNVENLEELRSLFSSARSYQRIQIFPDTIW
NVSYDGTSTACSGSFYKSMRWLTRKNGDYPIQDAQYTNNQGKNILFMWGINHPPTDTTQRNLYTRIDTTT
SVATEEINRVFKPLIGPRPLVNGLMGRIDYYWSVLKPGQTLRIKSDGNLIAPWFGHILSGESHGRILKTD

LKRGSCTVQCQTEKGGLNSTLPFQNVSKYAFGNCSKYIGIKSLKLAVGLRNVPSRSSRGLFGAIAGFIEG
GWSGLVAGWYGFQHSNDQGVGMAADRDSTQKAIDKITSKVNNIVDKMNKQYEIIDHEFSEVETRLNMINN
KIDDQIQDIWAYNAELLVLLENQKTLDEHDANVNNLYNKVKRALGSNAVEDGKGCFELYHKCDDQCMETI
RNGTYNRRKYQEESKLERQKIEGVKLESEGTYKILTIYSTVASSLVIAMGFAAFLFWA

>QCC70691.1 hemagglutinin, partial [Influenza A virus]
METVSLITILLVATVGNADKICIGYQSTNSTETVDTLTENNVPVTHAKELLHTEHNGMLCATSLGQPIIL
DTCTIEGLIYGNPSCDLSLEGREWSYIVERPSAVNGLCYPGNVENLEELRSLFSSARSYQRVQIFPDTIW
NVSYDGTSTACSGSFYRNMRWLTRKNGDYPIQDAQYTNNQGKNIPFMWGINHPLADTTQRDLYTRTDTTT
SVATEEINRIFKPLIGPRPLVNGLMGRIDYYWSVLKPGQTLRIKSDGNLIAPWYGHILSGESHGRILKTD
LKRGSCTVQCQTEKGGLNTTLPFQNVSKYAFGNCSKYIGIKSLKLAVGLRNVPSRSSRGLFGAIAGFIEG
GWSGLVAGWYGFQHSNDQGVGMAADRDSTQKAVDKITSKVNNIVDKMNKQYEIIDHEFSEVETRLNMINN
KIDDQIQDIWAYNAELLVLLENQKTLDEHDANVNNLYNKVKRALGSNAVEDGKGCFELYHKCDDQCMETI
RNGTYNRRKYQEESKLERQKIEGVKLESEGTYKILTIYSTVASSLVIAMGFAAFLFWAMSNGSCRC

>AXK37912.1 hemagglutinin [Influenza A virus]
METVSLITILLVATVSNADKICIGYQSTNSTETVDTLTENNVPVTHAKELLHTEHNGMLCATSLGQPLIL
DTCTIEGLIYGNPSCDLSLEGREWSYIVERPSAVNGLCYPGNVENLEELRSLFSSARSYQRLQIFPDTIW
NVSYDGTSTACSGSFYRSMRWLTRKNGEYPTQDAQYTNNQGKNILFMWGINHPPTDTTQRELYTRTDTTT
SVATEEINRIFKPLIGPRPLVNGLMGRIDYYWSILKPGQTLRIKSDGNLIAPWYGHILSGESHGRILKTD
LKRGSCTVQCQTEKGGLNTTLPFQNVSKYAFGNCSKYIGIKSLKLAVGLRNVPSRSSRGLFGAIAGFIEG
GWSGLVAGWYGFQHSNDQGVGMAADRDSTQKAIDKITSKVNNIVDKMNKQYEIIDHEFSEVETRLNMINN
KIDDQIQDIWAYNAELLVLLENQKTLDEHDANVNNLYNKVKRALGSNAVEDGKGCFELYHKCDNQCMETI
RNGTYNRRKYQEESKLERQKIEGVKLESEGTYKILTIYSTVASSLVIAMGFAAFLFWAMSNGSCRCNICI

>AXF80662.1 hemagglutinin [Influenza A virus]
METVSPITILVVATVSNADKICIGYQSTNSTETVDTLTENNVPVTHAKELLHTEHNGMLCATSLGHPLIL
DTCTIEGLIYGNPSCDPLLGGREWSYIVERPSAVNGLCYPGNVENLEELRSLFSSSRSYQRIQIFPDTIW
NVSYSGTSKACSDSFYRSMRWLTQKNNAYPTQDAQYTNNQGKSILFMWGINHPPTDTTQTNLYTRTDTTT
SVATEEMNRIFKPLIGPRPLVNGLMGRINYYWSVLKPGQTLRIKSDGNLIAPWYGHILSGESHGRILKTD
LKRGSCTVQCQTERGGLNTTLPFQNVSKYAFGNCSKYIGVKSLKLAVGLRNVPSRSSRGLFGAIAGFIER
GWSGLVAGWYGFQHSNDQGVGMAADRDSTQKAIDKITSKVNNIVDKMNKQYEIIDHEFSEVETRLNMINN
KVDDQIQDIWAYNAELLVLLENQKTLDEHDANVNNLYNKVKRALGSNAVEDGKGCFELYHKCDDRCMETI
RNGTYNRRKYQEESKLERQKIEGVKLESEETYKILTIYSTVASSLVIAMGFAAFLFWAMSNGSCRRNICI

>AXF80650.1 hemagglutinin [Influenza A virus]

METVSLITILLVATVSNADKICIGYQSTNSTETVDTLTENNVPVTHAKELLHTEHNGMLCATSLGQPLIL
DTCTIEGLIYGNPSCDLSLEGREWSYIVERPSAVNGLCYPGNVENLEELRSLFSSARSYQRLQIFPDTIW
NVSYDGTSTACSGSFYRSMRWLTRKNGEYPTQDAQYTNNQGKNILFMWGINHPPTDTKQRDLYTRTDTTT
SVATEEINRIFKPLIGPRPLVNGLMGRIDYYWSILKPGQTLRIKSDGNLIAPWYGHILSGESHGRILKTD
LKRGSCTVQCQTEKGGLNTTLPFQNVSKYAFGNCSKYIGIKSLKLAVGLRNVPSRSSRGLFGAIAGFIEG
GWSGLVAGWYGFQHSNDQGVGMAADRDSTQKAIDKITSKVNNIVDKMNKQYEIIDHEFSEVETRLNMINN
KIDDQIQDIWAYNAELLVLLENQKTLDEHDANVNNLYNKVKRALGSNAVEDGKGCFELYHKCDNQCMETI
RNGTYNRRKYQEESKLERQKIEGVKLESEGTYKILTIYSTVASSLVIAMGFAAFLFWAMSNGSCRRNICI

>AXF80638.1 hemagglutinin [Influenza A virus]

METVSLITILLVAAVSNADKICIGYQSTNSTETVDTLTENNVPVTHAKELLHTEHNGMLCATSLGQPLIL
DTCTIEGLIYGNPSCDLSLEGREWSYIVERPSAVNGLCYPGNVENLEELRSLFSSARSYQRIQIFPDTIW
NVSYDGTSAACSGSFYKSMRWLTRKNGDYPIQDAQYTNNQGKNILFMWGINHPPTDTTQRDLYTRTDTTT
SVATEEVNRVFKPLIGPRPLVNGLMGRIDYYWSVLKPGQTLRIKSDGNLIAPWFGHILSGESHGRILKTG
LKRGSCTVQCQTERGGLNTTLPFQNVSKYAFGNCSKYIGIKSLKLAVGLRNVPSRSSRGLFGAIAGFIEG
GWSGLVAGWYGFQHSNDRGVGMAADRDSTQKAIDKITSKVNNIVDKMNKQYEIIDHEFSEVETRLNMINN
KIDDQIQDIWAYNAELLVLLESQKTLDEHDANVNNLYNKVKRALGSNAVEDGKGCFELYHKCDDQCMETI
RNGTYNRRKYQEESKLERQKIEGVKLESEGTYKILTIYSTVASSLVIAMGFAAFLFWAMSNGSCRRNICI

>AXF80626.1 hemagglutinin [Influenza A virus]

METVSLITILVVATVSNADKICIGYQSTNSTETVDTLTENNVPVTHAKELLHTEHNGMLCATSLGHPLIL
DTCTIEGLIYGNPSCDPLLGGREWSYIVERPSAVNGLCYPGNVENLEELRSLFSSSRSYQRIQIFPDTIW
NVSYSGTSKACSDSFYRSMRWLTQKNSAYPTQDAQYANNQGKNILFMWGINHPPTDTAQTNLYTRTDTTT
SVATEEMNRIFKPLIGPRPLVNGLMGRINYYWSVLKPGQTLRIKSDGNLIAPWYGHILSGESHGRILKTD
LKRGSCTVQCQTERGGLNTTLPFQNVSKYAFGNCSKYIGVKSLKLAVGLRNVPSRSSRGLFGAIAGFIEG
GWSGLVAGWYGFQHSNDQGVGMAADRDSTQKAIDKITSKVNNIVDKMNKQYEIIDHEFSEVETRLNMINN
KVDDQIQDIWAYNAELLVLLENQKTLDEHDANVNNLYNKVKRALGSNAVEDGKGCFELYHKCDDHCMETI
RNGTYNRRKYQEESKLERQKIEGVKLESEETYKILTIYSTVASSLVIAMGFAAFLFWAMSNGSCRRNICI

>AXF54277.1 hemagglutinin [Influenza A virus]

METVSLITILLVATVSNADKICIGYQSTNSTETVDTLTENNVPVTHAKELIHTEHNGMLCATSLGQPLIL
DTCTIEGLIYGNPSCDLSLEGREWSYIVERPSAVNGLCYPGNVENLEELRSLFSSARSYQRIQIFPDTIW

NVSYDGTSTACSGSFYRSMRWLTRKNSEYPIQDAQYTNSQGKNILFMWGINHPPTDTTQKALYTRTDTTT
SVATEEINRVFKPLIGPRPLVNGLMGRIDYYWSVLKPGQTLRIKSDGNLIAPWYGHILSGESHGRILKTD
LKKGSCTVQCQTEKGGLNTTLPFQNVSKYAFGNCSKYIGIKSLKLAVGLRNVPSRSSRGLFGAIAGFIEG
GWPGLVAGWDGFQHSNDQGVGMAADRDSTQKAIDKITSKVNNIVDKVNKQYEIIDHEFSEVETRLNMINN
KIDDQIQDIWAYNAELLVLLENQKTLDEHDANVNNLYNKVKRALGSNAVEDGKGCFELYHKCDDQCMDTI
RNGTYNRKKYQEESKLERQKIEGVKLESEGTYKILTIYSTVASSLVIAMGFAAFLFWAMSNGSCRRNICI

>AXF54266.1 hemagglutinin [Influenza A virus]
METVSLITILLVATVSNADKICIGYQSTNSTETVDTLTENNVPVTHAKELIHTEHNGMLCATSLGQPLIL
DTCTIEGLIYGNPSCDLSLEGREWSYIVERPSAVNGLCYPGNVENLEELRSLFSSARSYQRIQIFPDTIW
NVSYDGTSTACSGSFYRSMRWLTRKNSEYPIQDAQYTNSQGKNILFMWGINHPPTDTTQKALYTRTDTTT
SVATEEINRVFKPLIGPRPLVNGLMGRIDYYWSVLKPGQTLRIKSDGNLIAPWYGHILSGESHGRILKTD
LKKGSCTVQCQTERGGLNTTLPFQNVSKYAFGNCSKYIGIKSLKLAVGLRNVPSRSSRGLFGAIAGFIEG
GWPGLVAGWYGFQHSNDQGVGMAADRDSTQKAIDKITSKVNNIVDKVNKQYEIIDHEFSEVETRLNMINN
KIDDQIQDIWAYNAELVVLLENQKTLDEHDANVNNLYNKVKRALGSNAVEDGKGCFELYHKCDDQRMDTI
RNGTYNRKKYQEESKLERQKIEGVKLESEGTYKILTIYSTVASSLVIAMGFAAFLFWAMSNGSCRRNICI

>AZQ05099.1 hemagglutinin [Influenza A virus]
METVSLITILLVATVSNADKICIGYQSTNSTETVDTLTENNVPVTHAKELIHTEHNGMLCATSLGQPLIL
DTCTIEGLIYGNPSCDLSLEGREWSYVVERPSAVNGLCYPGNVENLEELRSLFSSARSYQRVQIFPDTIW
NVSYDGTSIACSGSFYRNMRWLTRKDGNYPTQDAQYTNNQGKNILFMWGINHPPTDTTQSGLYTRTDTTT
SVATEEINRIFKPLIGPRPLVNGLMGRIDYYWSVLKPGQTLRIKSDGNLIAPWFGHILSGESHGRILKTD
LKRGSCTVQCQTEKGGLNTTLPFQNVSKYAFGNCSKYIGIKSLKLAVGLRNVPSRSSRGLFGAIAGFIEG
GWSGLVAGWYGFQHSNDQGVGMAADRDSTQKAIDKITSKVNNIVDKMNKQYEIIDHEFSEVETRLNMINN
KIDDQIQDIWAYNAELLVLLENQKTLDEHDANVNNLYNKVKRALGSNAVEDGKGCFELYHKCDDQCMETI
RNGTYNRRKYQEESKLERQKIEGVKLESEGTYKILTIYSTVASSLVIAMGFAAFLFWAMSNGCRCNICI

>AZQ05098.1 hemagglutinin [Influenza A virus]
METVSLITILLVATVSNADKICIGYQSTNSTETVDTLTENNVPVTHAKELIHTEHNGMLCATSLGQPLIL
DTCTIEGLIYGNPSCDLSLEGREWSYIVERPSAVNGLCYPGNVENLEELRSLFSSARSYQRVQIFPDTIW
NVSYDGTSTACSGSFYRNMRWLTRKDGNYPTQDAQYTNNQGKNILFMWGINHPPTDTTQSGLYTRTDTTT
SVATEEINRIFKPLIGPRPLVNGLMGRIDYYWSVLKPGQTLRIKSDGNLIAPWFGHILSGESHGRILKTD
LKRGSCTVQCQTEKGGLNTTLPFQNVSKYAFGNCSKYIGIKSLKLAVGLRNVPSRSSRGLFGAIAGFIEG
GWSGLVAGWYGFQHSNDQGVGMAADRDSTQKAIDKITSKVNNIVDKMNKQYEIIDHEFSEVETRLNMINN

KIDDQIQDIWAYNAELLVLLENQKTLDEHDANVNNLYNKVKRALGSNAVEDGKGCFELYHKCDDQCMETI
RNGTYNRRKYQEESKLERQKIEGVKLESEGTYKILTIYSTVASSLVIAMGFAAFLFWAMSNGSCRCNICI

>AZQ05097.1 hemagglutinin [Influenza A virus]
METVSLITILIVATVSNADKICIGYQSTNSTETVDTLTENNVPVTHAKELLHTEHNGMLCATSLGHPLIL
DTCTIEGLIYGNPSCDPLLGGREWSYIVERPSAVNGLCYPGNVENLEELRSLFSSSRSYQRIQIFPDTIW
NVSYSGTSKACSDSFYRSMRWLTQKNNAYPTQDAQYTNNQGKNILFMWGINHPPTDTAQTNLYTRTDTTT
SVATEEMNRIFKPLIGPRPLVNGLMGRINYYWSVLKPGQTLRIKSDGNLIAPWYGHILSGESHGRILKTD
LKRGSCTVQCQTEKGGLNTTLPFQNVSKYAFGNCSKYIGVKSLKLAVGLRNVPSRSSRGLFGAIAGFIEG
GWSGLVAGWYGFQHSNDQGVGMAADRDSTQKAIDKITSKVNNIVDKMNKQYEIIDHEFSEVETRLNMINN
KVDDQIQDIWAYNAELLVLLENQKTLDEHDANVNNLYNKVKRALGSNAVEDGKGCFELYHKCDDHCMETI
RNGTYNRRKYQEESKLERQKIEGVKLESEETYKILTIYSTVASSLVIAMGFAAFLFWAMSNGSCRCNICI

>AZQ05096.1 hemagglutinin [Influenza A virus]
METVSLITILIVATVSNADKICIGYQSTNSTETVDTLTENNVPVTHAKELLHTEHNGMLCATSLGQPLIL
DTCTIEGLIYGNPSCDLSLEGREWSYIVERPSAINGLCYPGNVENLEELRSLFSSARSYQRIQIFPDTIW
NVSYDGTSTACSNSFYRSMRWLTRKDGNYPTQDAQYTNNQGKNILFMWGINHPPTDDTQRNLYTRTDTTT
SVATEEINRIFKPLIGPRPLVNGLMGRIDYYWSVLKPGQTLRIKSDGNLIAPWYGHILSGESHGRILKTD
LKRGSCTVQCQTEKGGLNTTLPFQNVSKYAFGNCSKYIGIKSLKLAVGLRNVPSRSSRGLFGAIAGFIEG
GWSGLVAGWYGFQHSNDQGVGMAADRDSTQKAIDKITSKVNNIVDKMNKQYEIIDHEFSEVETRLNMINN
KIDDQIQDIWAYNAELLVLLENQKTLDEHDANVNNLYNKVKRALGSNAVEDGKGCFELYHKCNDQCMETI
RNGTYNRRKYQEESKLERQKIEGVKLESEGTYKILTIYSTVASSLVIAMGFAAFLFWAMSNGSCRCNICI

>AZQ05095.1 hemagglutinin [Influenza A virus]
METVSLITILIVATVSNADKICIGYQSTNSTETVDTLTENNVPVTHAKELLHTEHNGMLCATSLGQPLIL
DTCTIEGLIYGNPSCDLSLEGREWSYIVERPSAINGLCYPGNVENLEELRTLFSSARSYQRIQIFPDTIW
NVSYDGTSTACSNSFYRSMRWLTRKDGNYPTQDAQYTNNQGKNILFMWGINHPPTDDTQRNLYTRTDTTT
SVATEEINRIFKPLIGPRPLVNGLMGRIDYYWSVLKPGQTLRIKSDGNLIAPWYGHILSGESHGRILKTD
LKRGSCTVQCQTEKGGLNTTLPFQNVSKYAFGNCSKYIGIKSLKLAVGLRNVPSRSSRGLFGAIAGFIEG
GWSGLVAGWYGFQHSNDQGVGMAADRDSTQKAIDKITSKVNNIVDKMNKQYEIIDHEFSEVETRLNMINN
KIDDQIQDIWAYNAELLVLLENQKTLDEHDANVNNLYNKVKRALGSNAVEDGKGCFELYHKCNDQCMETI
RNGTYNRRKYQEESKLERQKIEGVKLESEGTYKILTIYSTVASSLVIAMGFAAFLFWAMSNGSCRCNICI

>AZQ05094.1 hemagglutinin [Influenza A virus]

METVSLITILLVATVSNADKICIGYQSTNSTETVDTLTENNVPVTHAKELLHTEHNGMLCATSLGQPLIL
DTCTIEGLIYGNPSCDLSLEGREWSYIVERPSAVHGLCYPGNVEDLEELRSLFSSARSYQRIQIFPDTIW
NVSYDGTSTACSGSFYRSMRWLTRKNGDYPIQDAQYTNNQGKNILFMWGINHPPTDETQRGLYTRTDTTT
SVATEEINRIFKPLIGPRPLVNGLMGRINYYWSVLKPGQTLRIKSDGNLIAPWYGHILSGESHGRILKTD
LKRGICTVQCQTEKGGLNTTLPFQNVSKYAFGNCSKYIGIKSLKLAVGLRNVPSRSSRGLFGAIAGFIEG
GWSGLVAGWYGFQHSNDQGVGMAADRDSTQKAIDKITSKVNNIVDKMNKQYEIIDHEFSEVETRLNMINN
KIDDQIQDVWAYNAELLVLLENQKTLDEHDANVNNLYNKVKRALGSNAVEDGKGCFELYHKCDDQCMETI
RNGTYNRRKYQEESKLERQKIEGVKLESEGTYKILTIYSTVASSLVIAMGFAAFLFWAMSNGSCRCNICI

>AZQ05093.1 hemagglutinin [Influenza A virus]
METVSLITILIVATVSNADKICIGYQSTNSTETVDTLTENNVPVTHAKELLHTEHNGMLCATSLGQPLIL
DTCTIEGLIYGNPSCDLSLEGREWSYIVERPSAINGLCYPGNVENLEELRSLFSSARSYQRIQIFPDTIW
NVSYDGTSTACSNSFYRSMRWLTRKDGNYPTQDAQYTNNQGKNILFMWGINHPPTDDTQRNLYTRTDTTT
SVATEEINRIFKPLIGPRPLVNGLMGRIDYYWSVLKPGQTLRIKSDGNLIAPWYGHILSGESHGRILKTD
LKRGSCTVQCQTEKGGLNTTLPFQNVSKYAFGNCSKYIGIKSLKLAVGLRNVPSRSSRGLFGAIAGFIEG
GWSGLVAGWYGFQHSNDQGVGMAADRDSTQKAIDKITSKVNNIVDKMNKQYEIIDHEFSEVETRLNMINN
KIDDQIQDIWAYNAELLVLLENQKTLDEHDANVNNLYNKVKRALGSNAVEDGKGCFELYHKCNDQCMETI
RNGTYNRRKYQEESKLERQKIEGVKLESEGTYKILTIYSTVASSLVIAMGFAAFLFWAMSNGSCRCNICI

>AZQ05092.1 hemagglutinin [Influenza A virus]
METVSLITILIVATVSNADKICIGYQSTNSTETVDTLTENNVPVTHAKELLHTEHNGMLCATSLGQPLIL
DTCTIEGLIYGNPSCDLSLEGREWSYIVERPSAINGLCYPGNVENLEELRSLFSSARSYQRIQIFPDTIW
NVSYDGTSTACSNSFYRSMRWLTRKDGNYPTQDAQYTNNQGKNILFMWGINHPPTDDTQRNLYTRTDTTT
SVATEEINRIFKPLIGPRPLVNGLMGRIDYYWSVLKPGQTLRIKSDGNLIAPWYGHILSGESHGRILKTD
LKRGSCTVQCQTEKGGLNTTLPFQNVSKYAFGNCSKYIGIKSLKLAVGLRNVPSRSSRGLFGAIAGFIEG
GWSGLVAGWYGFQHSNDQGVGMAADRDSTQKAIDKITSKVNNIVDKMNKQYEIIDHEFSEVETRLNMINN
KIDDQIQDIWAYNAELLVLLENQKTLDEHDANVNNLYNKVKRALGSNAVEDGKGCFELYHKCNDQCMETI
RNGTYNRRKYQEESKLERQKIEGVKLESEGTYKILTIYSTVASSLVIAMGFAAFLFWAMSNGSCRCNICI

>AZQ05091.1 hemagglutinin [Influenza A virus]
METASLITILLVATVSNADKICIGYQSTNSTETVDTLTENNVPVTHAKELLHTEHNGMLCATSLGQPLIL
DTCTIEGLIYGNPSCDLSLEGREWSYIVERPSAVHGLCYPGNVEDLEELRSLFSSARSYQRIQIFPDTIW
NVSYDGTSTACSGSFYRSMRWLTRKNGEYPIQDAQYTNNQGKNILFMWGINHPPTDDTQRGLYTRTDTTT
SVATEEINRIFKPLIGPRPLVNGLMGRINYYWSVLKPGQTLRIKSDGNLIAPWYGHILSGESHGRILKTD

LKRGSCTVQCQTEKGGLNTTLPFQNVSKYAFGNCSKYIGIKSLKLAVGLRNVPSRSSRGLFGAIAGFIEG
GWPGLVAGWYGFQHSNDQGVGMAADRDSTQKAIDKITSKVNNIVDKMNKQYEIIDHEFSEVETRLNMINN
KIDDQIQDIWAYNAELLVLLENQKTLDEHDANVNNLYSKVKRALGSNAVEDGKGCFELYHKCDDQCMETI
RNGTYNRRKYQEESKLERQRIEGVKLESEGTYKILTIYSTVASSLVIAMGFAAFLFWAMSNGSCRCNICI

>AZQ05090.1 hemagglutinin [Influenza A virus]
METVSLITILLVATVSNADKICIGYQSTNSTETVDTLTENNVPVTHAKELIHTEHNGMLCATSLGQPLIL
DTCTIEGLIYGNPSCDLSLEGREWSYIVERPSAVNGLCYPGNVENLEELRSLFSSARSYQRIQIFPDTIW
NVSYDGTSTACSGSFYRNMRWLTRKDGNYPTQDAQYTNNQGKNILFMWGINHPPTDTTQSSLYTRTDTTT
SVATEEINRIFKPLIGPRPLVNGLMGRIDYYWSVLKPGQTLRIKSDGNLIAPWFGHILSGESHGRILKTD
LKRGSCTVQCQTEKGGLNTTLPFQNVSKYAFGNCSKYIGIKSLKLAVGLRNVPSRSSRGLFGAIAGFIEG
GWSGLVAGWYGFQHSNDQGVGMAADRDSTQKAIDKITSKVNNIVDKMNKQYEIIDHEFSEVETRLNMINN
KIDDQIQDIWAYNAELLVLLENQKTLDEHDANVNNLYNKVKRALGSNAVEDGKGCFELYHKCDDQCMETI
RNGTYNRRKYQEESKLERQKIEGVKLESEGTYKILTIYSTVASSLVIAMGFAAFLFWAMSNGSCRCNICI

>AZQ05089.1 hemagglutinin [Influenza A virus]
METVSLITILLVATVSNADKICIGYQSTNSTETVDTLTENNVPVTHAKELIHTEHNGMLCATSLGQPLIL
DTCTIEGLIYGNPSCDLSLEGREWSYIVERPSAVNGLCYPGNVENLEELRSLFSSARSYQRVQIFPDTIW
NVSYDGTSTACSGSFYRNMRWLTRKDGNYPTQDAQYTNNQGKNILFMWGINHPPTDTTQSGLYTRTDTTT
SVATEEINRIFKPLIGPRPLVNGLMGRIDYYWSVLKPGQTLRIKSDGNLIAPWFGHILSGESHGRILKTD
LKRGSCTVQCQTEKGGLNTTLPFQNVSKYAFGNCSKYIGIKSLKLAVGLRNVPSRSSRGLFGAIAGFIEG
GWSGLVAGWYGFQHSNDQGVGMAADRDSTQKAIDKITSKVNNIVDKMNKQYEIIDHEFSEVETRLNMINN
KIDDQIQDIWAYNAELLVLLENQKTLDEHDANVNNLYNKVKRALGSNAVEDGKGCFELYHKCDDQCMETI
RNGTYNRRKYQEESKLERQKIEGVKLESEGTYKILTIYSTVASSLVIAMGFAAFLFWAMSNGSCRCNICI

>AZQ05088.1 hemagglutinin [Influenza A virus]
METVSLITILLVATVSSADKICIGYQSTNSTETVDTLTENNVPVTHAKELIHTEHNGMLCATSLGQPLIL
DTCTIEGLIYGNPSCDLSLEGREWSYIVERPSAVNGLCYPGNVENLEELRSLFSSARSYQRIQIFPDTIW
NVSYDGTSTACSGSFYRNMRWLTRKDGNYPTQDAQYTNNQGKNILFMWGINHPPTETTQSGLYTRTDTTT
SVATEEINRIFKPLIGPRPLVNGLMGRIDYYWSVLKPGQTLRIKSDGNLIAPWFGHILSGESHGRILKTD
LKRGSCTVQCQTEKGGLNTTLPFQNVSKYAFGNCSKYIGIKSLKLAVGLRNVPSRSSRGLFGAIAGFIEG
GWSGLVAGWYGFQHSNDQGVGMAADRDSTQKAIDKITSKVNNIVDKMNKQYEIIDHEFSEVETRLNMINN
KIDDQIQDIWAYNAELLVLLENQKTLDEHDANVNNLYNKVKRALGSNAVEDGKGCFELYHKCDDQCMETI
RNGTYNRRKYQEESKLERQKIEGVKLESEGTYKILTIYSTVASSLVIAMGFAAFLFWAMSNGSCRCNICI

>AZQ05087.1 hemagglutinin [Influenza A virus]

METVSLITILLVATVSSADKICIGYQSTNSTETVDTLTENNVPVTHAKELIHTEHNGMLCATSLGQPLIL

DTCTIEGLIYGNPSCDLSLEGREWSYIVERPSAVNGLCYPGNVENLEELRSLFSSARSYQRIQIFPDTIW

NVSYDGTSTACSGSFYRNMRWLTRKDGNYPTQDAQYTNNQGKNILFMWGINHPPTETTQSGLYTRTDTTT

SVATEEINRIFKPLIGPRPLVNGLMGRIDYYWSVLKPGQTLRIKSDGNLIAPWFGHILSGESHGRILKTD

LKRGSCTVQCQTEKGGLNTTLPFQNVSKYAFGNCSKYIGIKSLKLAVGLRNVPSRSSRGLFGAIAGFIEG

GWSGLVAGWYGFQHSNDQGVGMAADRDSTQKAIDKITSKVNNIVDKMNKQYEIIDHEFSEVETRLNMINN

KIDDQIQDIWAYNAELLVMLENQKTLDEHDANVNNLYNKVKRALGSNAVEDGKGCFELYHKCDDQCMETI

RNGTYNRRKYQEESKLERQKIEGVKLESEGTYKILTIYSTVASSLVIAMGFAAFLFWAMSNGSCRCNICI

>BBN70708.1 hemagglutinin [Influenza A virus]

METASLITILLVVTASNADKICIGYQSTNSTETVDTLTESNVPVTHAKELLHTEHNGMLCATNLGHPLIL

DTCTIEGLIYGNPSCDLLLGGREWSYIVERPSAVNGLCYPGNVENLEELRSLFSSARSYQRVLIFPDTIW

NVSYSGTSKACSDSFYRNMRWLTQKNNAYPIQDAQYTNNQEKNILFMWGINHPPTDTAQTNLYTRTDTTT

SVATEEINRTFKPLIGPRPLVNGLMGRINYYWSVLKPGQTLRIKSNGNLIAPWYGHILLGESHGRILKTD

LKSGSCTVQCQTEKGGLNTTLPFQNVSKYAFGNCSKYIGIKSLKLAVGLRNVPSRSSRGIFGAIAGFIEG

GWSGLVAGWYGFQHSNDQGVGMAADRDSTQKAIDKITSKVNKIVDKMNKQYEIIDHEFSEVETRLNMINS

KIDDQIQDIWAYNAELLVLLENQKTLDEHDANVNNLYNKVKRALGSNAVEDGKGCFELYHKCDDQCMETI

RNGTYNRKKYQEESKLERQKIEGVKLESEGTYKVLTIYSTVASSLVIAMGFAAFLFWAMSNGSCRCNICI

>BBN70638.1 hemagglutinin [Influenza A virus]

METVSLMTILLVATASKADKICIGYQSTNSTETVDTLTENNVPVTHAKELLHTEHNGMLCATSLGHPLIL

DTCTIEGLIYGNPSCDPLLGGREWSYIVERPSAVNGLCYPGNVENLEELRSLFSSARSYQRIQIFPDTIW

NVSYSGTSKACSDSFYRSMRWLTQKNNAYPIQDAQYTNNQGKNILFMWGINHPPTDTVQTNLYTRTDTTT

SVATEEINRTFKPLIGPRPLVNGLMGRINYYWSVLKPGQTLRIKSDGNLIAPWYGHILTGESHGRILKTD

LKSGSCTVQCQTEKGGLNTTLPFQNVSRYAFGNCSKYIGIKSLKLAVGLRNVPSKSSRGLFGAIAGFIEG

GWSGLVAGWYGFQHSNDQGVGMAADRDSTQKAIDKITSKVNNIVDKMNKQYEIIDHEFSEVETRLNMINS

KIDDQIQDIWAYNAELLVLLENQKTLDEHDANVNNLYNKVKRALGSNAVEDGKGCFELYHKCDDQCMETI

RNGTYNRRKYQEESKLERQKIEGVKLESEGTYKILTIYSTVASSLVIAMGFAAFLFWAMSNGSCRCNICI

>BBN23825.1 hemagglutinin [Influenza A virus]

METASLITILLVVTASNADKICIGYQSTNSTETVDTLTESNVPVTHAKELLHTEHNGMLCATNLGHPLIL

DTCTIEGLIYGNPSCDLLLGGREWSYIVERPSAVNGLCYPGNVENLEELRSLFSSARSYQRVLIFPDTIW

NVSYSGTSKACSDSFYRNMRWLTQKNNAYPIQDAQYTNNQEKNILFMWGINHPPTDTAQTNLYTRTDTTT
SVATEEINRTFKPLIGPRPLVNGLMGRINYYWSVLKPGQTLRIKSNGNLIAPWYGHILLGESHGRILKTD
LKSGSCTVQCQTEKGGLNTTLPFQNVSKYAFGNCSKYIGIKSLKLAVGLRNVPSRSSRGIFGAIAGFIEG
GWSGLVAGWYGFQHSNDQGVGMAADRDSTQKAIDKITSKVNKIVDKMNKQYEIIDHEFSEVETRLNMINS
KIDDQIQDIWAYNAELLVLLENQKTLDEHDANVNNLYNKVKRALGSNAVEDGKGCFELYHKCDDQCMETI
RNGTYNRKKYQEESKLERQKIEGVKLESEGTYKVLTIYSTVASSLVIAMGFAAFLFWAMSNGSCRCNICI

>BBN23765.1 hemagglutinin [Influenza A virus]
METASLMTILLVVTTSNADKICIGYQSTNSTETVDTLTENNVPVTHAKELLHTEHNGMLCATSLGQPLIL
DTCTIEGLIYGNPSCDPLLGGREWSYIVERPSAVNGLCYPGNVENLEELRSLFSSARSYQRVLIFPDTIW
NVSYSGTSKACSDSFYRSMRWLTQKDNAYPVQDAQYTNNQEKNILFMWGINHPPTDTVQTNLYTRTDTTT
SVATEEINRTFKPLIGPRPLVNGLMGRINYYWSVLKPGQTLRIKSNGNLIAPWYGHILSGESHGRILKTD
LKRGSCTVQCQTEKGGLNTTLPFQNVSKYAFGNCSKYIGTKSLKLAVGLRNVPSRSSRGLFGAIAGFIEG
GWSGLVAGWYGFQHSNDQGVGMAADRDSTQKAIDKITSKVNNIVDKMNKQYEIIDHEFSEVETRLNMINN
KIDDQIQDIWAYNAELLVLLENQKTLDEHDANVNNLYNKVKRALGSNAVEDGKGCFELYHKCDDQCMETI
RNGTYNRRKYQEESKLERQKIEGVKLESEGTYKILTIYSTVASSLVIAMGFAAFLFWAMSNGSCRCNICI

>BBN23745.1 hemagglutinin [Influenza A virus]
METASLITILLVVTASKADKICIGYQSTNSTETVDTLTENNVPVTHAKELLHTEHNGMLCATSLGNPLIL
DTCTIEGLIYGNPSCDPLLGGREWSYIVERPSAVNGLCYPGNVENLEELRSLFGSARSYQRTLIFPDTIW
NVSYSGTSKACSDSFYRNMRWLTQKNNAYPIQDAQYTNNQEKNILFMWGINHPPTDTAQTNLYTRTDTTT
SVATEEINRTFKPLIGPRPLVNGLMGRINYYWSVLKPGQTLRIKSNGNLIAPWYGHILSGESHGRILKTD
LKMGSCTVQCQTEKGGLNTTLPFQNVSKYAFGNCSKYIGIKSLKLAVGLRNVPSRSSRGLFGAIAGFIEG
GWSGLVAGWYGFQHSNDQGVGMAADRDSTQKAIDKITSKVNNIVDKMNKQYEIINHEFSEVETRLNMINN
KIDDQIQDIWAYNAELLVLLENQKTLDEHDANVNNLYNKVKRALGSNAVEDGKGCFELYHKCNDQCMETI
RNGTYNRSKYQEESKLERQKIEGVKLESEGTYKILTIYSTVASSLVIAMGFAAFLFWAMSNGSCRCNICI

>BBN23735.1 hemagglutinin [Influenza A virus]
METVSLITILLAATVSNADKICIGYQSTNSTETVDTLTENNVPVTHAKELLHTEHNGMLCATSLGQPLIL
DTCTIEGLIYGNPSCDPLPEEREWSYIVERPSAVNGLCYPGNVENLEELRSLFSSARSYQRIQIFPDTIW
NVSYDGTSNTCSGSFYRNMRWLTRKDGNYPTQDAQYTNNQGKNILFMWGINNPPTDDTQRNLYTRTDTTT
SVATEEINRIFKPLIGPRPLVNGLMGRINYYWSVLKPGQTLRIKSDGNLVAPWYGYILLGESHGRILRTD
LKRGSCTVQCQTEKGGLNTTLPFQNVSKYAFGNCSKYIGIKSLKLAVGLRNVPSRSSRGLFGAIAGFIEG
GWSGLVAGWYGFQHSNDQGVGMAADRESTQKAVDKITSKVNNIVDKMNKQYEIIDHEFSEVETRLNMINN

KIDDQIQDIWAYNAELLVLLENQKTLDEHDANVNNLYNKVKRALGSNAVEDGKGCFELYHKCDDQCMETI
RNGTYNRRKYQEESKLERQKIEGVKLESEGTYKILTIYSTVASSLVIAMGFAAFLFWAMSNGSCRCNICI

>BBN23725.1 hemagglutinin [Influenza A virus]
METASLMTILLVVTTSNADKICIGYQSTNSTETVDTLTENNVPVTHAKELLHTEHNGMLCATSLGQPLIL
DTCTIEGLIYGNPSCDPLLGGREWSYIVERPSAVNGLCYPGNVENLEELRSLFSSARSYQRVLIFPDTIW
NVSYSGTSKACSDSFYRSMRWLTQKDNAYPVQDAQYTNNQEKNILFMWGINHPPTDTVQTNLYTRTDTTT
SVATEEINRTFKPLIGPRPLVNGLMGRINYYWSVLKPGQTLRIKSNGNLIAPWYGHILSGESHGRILKTD
LKRGSCTVQCQTEKGGLNTTLPFQNVSKYAFGNCSKYIGIKSLKLAVGLRNVPSRSSRGLFGAIAGFIEG
GWSGLVAGWYGFQHSNDQGVGMAADRDSTQKAIDKITSKVNNIVDKMNKQYEIIDHEFSEVETRLNMINN
KIDDQIQDIWAYNAELLVLLENQKTLDEHDANVNNLYNKVKRALGSNAVEDGKGCFELYHKCDDQCMETI
RNGTYNRRKYQEESKLERQKIEGVKLESEGTYKILTIYSTVASSLVIAMGFAAFLFWAMSNGSCRCNICI

>BBN23704.1 hemagglutinin [Influenza A virus]
METVSLITILLAATVSNADKICIGYQSTNSTETVDTLTENNVPVTHAKELLHTEHNGMLCATSLGQPLIL
DTCTIEGLIYGNPSCDPLQEEREWSYIVERPSAVNGLCYPGNVENLEELRSLFSSARSYQRIQIFPDTIW
NVSYDGTSNTCSGSFYRNMRWLTRKDGNYPTQDAQYTNNQGKNILFMWGINNPPTDDTQRNLYTRTDTTT
SVATEEINRIFKPLIGPRPLVNGLMGRINYYWSVLKPGQTLRIKSDGNLVAPWYGYILSGESHGRILRTD
LKRGSCTVQCQTEKGGLNTTLPFQNVSKYAFGNCSKYIGIKSLKLAVGLRNVPSRSSRGLFGAIAGFIEG
GWSGLVAGWYGFQHSNDQGVGMAADRESTQKAVDKITSKVNNIVDKMNKQYEIIDHEFSEVETRLNMINN
KIDDQIQDIWAYNAELLVLLENQKTLDEHDANVNNLYNKVKRALGSNAVEDGKGCFELYHKCDDQCMETI
RNGTYNRRKYQEESKLERQKIEGVKLESEGTYKILTIYSTVASSLVIAMGFAAFLFWAMSNGSCRCNICI

>BBN23674.1 hemagglutinin [Influenza A virus]
METVSLITILLAATVSNADKICIGYQSTNSTETVDTLTENNVPVTHAKELLHTEHNGMLCATSLGQPLIL
DTCTIEGLIYGNPSCDPLPEEREWSYIVERPSAVNGLCYPGNVENLEELRSLFSSARSYQRIQIFPDTIW
NVSYDGTSNTCSGSFYRNMRWLTRKDGNYPTQDAQYTNNQGKNILFMWGINNPPTDDTQRNLYTRTDTTT
TVATEEINRIFKPLIGPRPLVNGLMGRINYYWSVLKPGQTLRIKSDGNLVAPWYGYILLGESHGRILRTD
LKRGSCTVQCQTEKGGLNTTLPFQNVSKYAFGNCSKYIGIKSLKLAVGLRNVPSRSSRGLFGAIAGFIEG
GWSGLVAGWYGFQHSNDQGVGMAADRESTQKAVDKITSKVNNIVDKMNKQYEIIDHEFSEVETRLNMINN
KIDDQIQDIWAYNAELLVLLENQKTLDEHDANVNNLYNKVKRALGSNAVEDGKGCFELYHKCDDQCMETI
RNGTYNRRKYQEESKLERQKIEGVKLESEGTYKILTIYSTVASSLVIAMGFAAFLFWAMSNGSCRCNICI

>BBN23642.1 hemagglutinin [Influenza A virus]

METVSLITILLIATVSNADKICIGYQSTNSTETVDTLTENNVPVTHAKELLHTEHNGMLCATSLGQPLIL
DTCTIEGLIYGNPSCDPLLDGREWSYIVERPSAVNGLCYPGHVENLEELRSLFSSARSYQRIQIFPDTVW
NVSYDGTSSACSGSFYRSMRWLTRKNGEYPIQDAQYTNNQGKNILFMWGINHPPTDTTQRELYTRTDTTT
SVATEEINRIFKPLIGPRPLVNGLMGRINYYWSVLRPGQTLRIKSDGNLVAPWYGHILSGESHGRILKTD
LKRGSCTVQCQTEKGGLNTTLPFQNVSKYAFGNCSKYIGIKSLKLAVGLRNVPSRSSRGLFGAIAGFIEG
GWSGLVAGWYGFQHSNDQGVGMAADRDSTQKAIDKITSKVNNIVDKMNKQYEIIDHEFSEVETRLNMINN
KIDDQIQDIWAYNAELLVLLENQKTLDEHDANVNNLYNKVKRALGSNAVEDGRGCFELYHKCDNQCMETI
RNGTYSRRKYQEESKLERQKIEGVKLESEGTYKILTIYSTVASSLVIAMGFAAFLFWAMSNGSCRCNICI

>QDW64889.1 hemagglutinin [Influenza A virus]
MEAIPLLTLLLVVTTSNADKICVGHQSTNSTETVDTLTETNVPVTQAKELLHTEHNGMLCATNLGRPLIL
DTCTIEGLIYGNPSCDMLLGGREWSYIVERPSAVNGTCYPGNVENLEELRTLFSSSSSYQRIQLFPDTIW
NVTYTGTSKSCSDSFYRNMRWLTQKNGLYPIQDAQYTNNRGKDILFVWGIHHPPTDTAQTSLYTRTDTTT
SVTTENLDRTFKPLIGPRPLVNGLIGRINYYWSVLKPGQTLRVRSNGNLIAPWFGHILSGESHGRILRTD
LSSGNCVVQCQTEKGGLNSTLPFHNISKYAFGICPKYIGVKSLKLAVGLRNVHARSSRGLFGAIAGFIEG
GWPGLVAGWYGFQHSNDQGVGMAADRDSTQKAVDKITSKVNNIVDKMNKQYEIIDHEFSEIETRLNMINS
KIDDQIQDIWAYNAELLVLLENQKTLDEHDANVNNLYNKVKRALGSNAMEDGKGCFELYHKCDDQCMETI
RNGTYNRRKYTEESRLERQKIEGVKLESEGTYKILSIYSTVASSLVLAMGFAAFLFWAMSNGSCRCNICI

>QDW64879.1 hemagglutinin, partial [Influenza A virus]
MEAIPLLTLLLVVTTSNADKICVGHQSTNSTETVDTLTETNVPVTQAKELLHTEHNGMLCATNLGRPLIL
DTCTIEGLIYGNPSCDMLLGGREWSYIVERPSAVNGTCYPGNVENLEELRTLFSSSSSYQRIQLFPDTIW
NVTYTGTSKSCSDSFYRNMRWLTQKNGLYPIQDAQYTNNRGKDILFVWGIHHPPTDTTQTSLYTRTDTTT
SVTTENLDRTFKPLIGPRPLVNGLIGRINYYWSVLKPGQTLRVRSNGNLIAPWFGHILSGESHGRILRTD
LSSGNCVVQCQTEKGGLNSTLPFHNISKYAFGICPKYIGVKSLKLAVGLRNVHARSSRGLFGAIAGFIEG
GWPGLVAGWYGFQHSNDQGVGMAADRDSTQKAVDKITSKVNNIVDKMNKQYEIIDHEFSEIETRLNMINS
KIDDQIQDIWAYNAELLVLLENQKTLDEHDANVNNLYNKVKRALGSNAMEDGKGCFELYHKCDDQCMETI
RNGTYNRRKYTEESRLERQKIVGVKLESEGTYKILSIYSTVASSLVLAMGFAAFLFWAMSNGSCRC

>QDW64868.1 hemagglutinin [Influenza A virus]
MEAIPLLTLLLVVTTSNADKICVGHQSTNSTETVDTLTETNVPVTQAKELLHTEHNGMLCATNLGRPLIL
DTCTIEGLIYGNPSCDMLLGGREWSYIVERPSAVNGTCYPGNVENLEELRTLFSSSSSYQRIQLFPDTIW
NVTYTGTSKSCSDSFYRNMRWLTQKNGLYPIQDAQYTNNRGKDILFVWGIHHPPTDTAQTSLYTRTDTTT
SVTTENLDRTFKPLIGPRPLVNGLIGRINYYWSVLKPGQTLRVRSNGNLIAPWFGHILSGESHGRILRTD

LSSGNCVVQCQTEKGGLNSTLPFHNISKYAFGICPKYIGVKSLKLAVGLRNVHARSSRGLFGAIAGFIEG
GWPGLVAGWYGFQHSNDQGVGMAADRDSTQKAVDKITSKVNNIVDKMNKQYEIIDHEFSEIETRLNMINS
KIDDQIQDIWAYNAELLVLLENQKTLDEHDANVNNLYNKVKRALGSNAMEDGKGCFELYHKCDDQCMETI
RNGTYNRRKYTEESRLERQKIEGVKLESEGTYKILSIYSTVASSLVLAMGFAAFLFWAMSNGSCRCNICI

>QDW64857.1 hemagglutinin [Influenza A virus]
MEAIPLLTLLLLVVTTSNADKICVGHQSTNSTETVDTLTETNVPVTQAKELLHTEHNGMLCSTNLGRPLI
LDTCTIEGLIYGNPSCDMLLGGREWSYIVERPSAVNGTCYPGNVENLEELRTLFSSSSSYQRIQLFPDTI
WNVTYTGTSKSCSDSFYRNMRWLTQKNGLYPIQDAQYTNNRGKDILFVWGIHHPPTDTTQTSLYTRTDTT
TSVTTENLDRTFKPLIGPRPLVNGLIGRINYYWSVLKPGQTLRVRSNGNLIAPWFGHILSGESHGRILRT
DLSSGNCVVQCQTEKGGLNSTLPFHNISKYAFGICPKYIGVKSLKLAVGLRNVHARSSRGLFGAIAGFIE
GGWPGLVAGWYGFQHSNDQGVGMAADRDSTQKAVDKITSKVNNIVDKMNKQYEIIDHEFSEVETRLNMIN
SKIDDQIQDIWAYNAELLVLLENQKTLDEHDANVNNLYNKVKRALGSNAMEDGKGCFELYHKCDDQCMET
IRNGTYNRRKYTEESRLERQKIEGVKLESEGTYKILSIYSTVASSLVLAMGFAAFLFWAMSNGSCRCNIC
I

>QDW64854.1 hemagglutinin, partial [Influenza A virus]
MEAIPLLTLLLVVTTSNADKICVGHQSTNSTETVDTLTETNVPVTQAKELLHTEHNGMLCATNLGRPLIL
DTCTIEGLIYGNPSCDMLLGGREWSYIVERPSAVNGTCYPGNVENLEELRTLFSSSSSYQRIQLFPDTIW
NVTYTGTSKSCSDSFYRNMRWLTQKNGLYPIQDAQYTNNRGKDILFVWGIHHPPTDTTQTSLYTRTDTTT
SVTTENLDRTFKPLIGPRPLVNGLIGRINYYWSVLKPGQTLRVRSNGNLIAPWFGHILSGESHGRILRTD
LSSGNCVVQCQTEKGGLNSTLPFHNISKYAFGICPKYIGVKSLKLAVGLRNVHARSSRGLFGAIAGFIEG
GWPGLVAGWYGFQHSNDQGVGMAADRDSTQKAVDKITSKVNNIVDKMNKQYEIIDHEFSEIETRLNMINS
KIDDQIQDIWAYNAELLVLLENQKTLDEHDANVNNLYNKVKRALGSNAMEDGKGCFELYHKCDDQCMETI
RNGTYNRRKYTEESRLERQKIVGVKLESEGTYKILSIYSTVASSLVLAMGFAAFLFWAM

>QDM13001.1 hemagglutinin [Influenza A virus]
METASLITILLVVTASKADKICIGYQSTNSTETVDTLTENNVPVTHAKELLHTEHNGMLCATSLGNPLIL
DTCTIEGLIYGNPSCDPLLGGREWSYIVERPSAVNGLCYPGNVENLEELRSLFSSARSYQRIQIFPDTVW
NVSYDGTSSACSGSFYRSMRWLTRKNGEYPIQDAQYTNNQEKNILFMWGINHPPTDTAQRELYTRTDTTT
SVATEEINRTFKPLIGPRPLVNGLMGRINYYWSVLKPGQTLRIKSNGNLIAPWYGHILSGESHGRILKTD
LKSGSCTVQCQTEKGGLNTTLPFQNVSKYAFGNCSKYIGIKSLKLAVGLRNVPSRSSRGLFGAIAGFIEG
GWSGLVAGWYGFQHSNDQGVGMAADRDSTQKAIDKITSKVNNIVDKMNKQYEIIDHEFSEVETRLNMINN
KIDDQIQDIWAYNAELLVLLENQKTLDEHDANVNNLYNKVKRALGSNAVEDGKGCFELYHKCNDQCMETI

RNGTYNRSKYQEESKLERQKIEGVKLESEGTYKILTIYSTVASSLVIAMGFAAFLFWAMSNGSCRCNICI

>QDM13000.1 hemagglutinin [Influenza A virus]
METVSLITILLIAAVSNADKICIGYQSTNSTETVDTLTENNVPVTHAKELLHTEHNGMLCATSLGQPLIL
DTCTIEGLIYGNPSCDPLLDGREWSYIVERPSAVNGLCYPGHVENLEELRSLFSSARSYQRIQIFPDTIW
NVSYDGTSSACSGSFYRSMRWLTRKDGEYPIQDAQYTNNQGKNILFMWGINQPPTDTTQRELYTRTDTTT
SVATEEINRIFKPLIGPRPLVNGLMGRIDYYWSVLRPGQTLRIKSDGNLIAPWYGHILSGESHGRILKTD
LKRGSCTVQCQTEKGGLNTTLPFQNVSKYAFGNCSKYIGIKSLKLAVGLRNVPSRSSRGLFGAIAGFIEG
GWSGLVAGWYGFQHSNDQGVGMAADKDSTQKAIDKITFKVNNIIDKMNKQYEIIDHEFSEVENRLNMINN
KIDDQIQDIWAYNAELLVLLENQKTLDEHDANVNNLYNKVKRALGSNAVEDGRGCFELYHKCDDQCMETI
RNGTYNRRKYQEESKLERQKIEGVKLESEGTYKILTIYSTVASSLVIAMGFAAFLFWAMSNGSCRCNICI

>QDM12999.1 hemagglutinin [Influenza A virus]
METASLITILLVVTASKADKICIGYQSTNSTETVDTLTENNVPVTHAKELLHTEHNGMLCATSLGNPLIL
DTCTIEGLIYGNPSCDPLLGGREWSYIVERPSAVNGLCYPGHVENLEELRSLFSSARSYQRIQIFPDTVW
NVSYDGTSSACSGSFYRSMRWLTRKNGEYPIQDAQYTNNQEKNILFMWGINHPPTDTTQRELYTRTDTTT
SVATEEINRTFKPLIGPRPLVNGLMGRINYYWSVLKPGQTLRIKSNGNLIAPWYGHILSGESHGRILKTD
LKRGSCTVQCQTEKGGLNTTLPFQNVSKYAFGNCSKYIGIKSLKLAVGLRNVPSRSSRGLFGAIAGFIEG
GWSGLVAGWYGFQHSNDQGVGMAADRDSTQKAIDKITSKVNNIVDKMNKQYEIIDHEFSEVETRLNMINN
KIDDQIQDIWAYNAELLVLLENQKTLDEHDANVNNLYNKVKRALGSNAVEDGKGCFELYHKCNDQCMETI
RNGTYNRSKYQEESKLERQKIEGVKLESEGTYKILTIYSTVASSLVIAMGFAAFLFWAMSNGSCRCNICI

>QDM12998.1 hemagglutinin [Influenza A virus]
METASLITILLVVTASKADKICIGYQSTNSTETVDTLTENNVPVTHAKELLHTEHNGMLCATSLGNPLIL
DTCTIEGLIYGNPSCDPLLGGREWSYIVERPSAVNGLCYPGNVENLEELRSLFSSARSYQRVQIFPDTIW
NVSYDGTSTACSGSFYRNMRWLTRKDGNYPIQDAQYTNNQEKNILFMWGINHPPTDTTQSGLYTRTDTTT
SVATEEINRTFKPLIGPRPLVNGLMGRINYYWSVLKPGQTLRIKSNGNLIAPWYGHILSGESHGRILKTD
LKRGSCTVQCQTEKGGLNTTLPFQNVSKYAFGNCSKYIGIKSLKLAVGLRNVPSRSSRGLFGAIAGFIEG
GWSGLVAGWYGFQHSNDQGVGMAADRDSTQKAIDKITSKVNNIVDKMNKQYEIIDHEFSEVETRLNMINN
KIDDQIQDIWAYNAELLVLLENQKTLDEHDANVNNLYNKVKRALGSNAVEDGKGCFELYHKCNDQCMETI
RNGTYNRSKYQEESKLERQKIEGVKLESEGTYKILTIYSTVASSLVIAMGFAAFLFWAMSNGSCRCNICI

>QDM12997.1 hemagglutinin [Influenza A virus]
METVSLITILLVATVSNADKICIGYQSTNSTETVDTLTENNVPVTHAKELLHTEHNGMLCATSLGQPLIL

DTCTIEGLIYGNPSCDLSLEGREWSYIVERPSAVNGLCYPGNVENLEELRSLFSSARSYQRVQIFPDTIW
NVSYDGTSTACSGSFYRNMRWLTRKDGNYPTQDAQYTNNQGKNILFMWGINHPPTDTTQSGLYTRTDTTT
SVATEEINRIFKPLIGPRPLVNGLMGRIDYYWSVLKPGQTLRIKSDGNLIAPWFGHILSGESHGRILKTD
LKRGSCTVQCQTEKGGLNTTLPFQNVSKYAFGNCSKYIGIKSLKLAVGLRNVPSRSSRGLFGAIAGFIEG
GWSGLVAGWYGFQHSNGQGVGMAADRDSTQKAIDKITSKVNNIVDKMNKQYEIIDHEFSEVETRLNMINN
KIDDQIQDIWAYNAELLVLLENQKTLDEHDANVNNLYNKVKRALGSNAVEDGKGCFELYHKCDDQCMETI
RNGTYNRRKYQEESKLERQKIEGVKLESEGTYKILTIYSTVASSLVIAMGFAAFLFWAMSNGSCRCNICI

>QDM12996.1 hemagglutinin [Influenza A virus]
METVSLITILLIATVSNADKICIGYQSTNSTETVDTLTENNVPVTHAKELLHTEHNGMLCATSLGQPLIL
DTCTIEGLIYGNPSCDPLLDGREWSYIVERPSAVNGLCYPGHVENLEELRSLFSSARSYQRIQIFPDTVW
NVSYDGTSSACSGSFYRSMRWLTRKNGEYPIQDAQYTNNQGKNILFMWGINHPPTDTTQRELYTRTDTTT
SVATEEINRIFKPLIGPRPLVNGLMGRIDYYWSVLRPGQTLRIKSDGNLVAPWYGHILSGESHGRILKTD
LKRGSCTVQCQTEKGGLNTTLPFQNVSKYAFGNCSKYIGIKSLKLAVGLRNVPSRSSRGLFGAIAGFIEG
GWSGLVAGWYGFQHSNDQGVGMAADRDSTQKAIDKITSKVNNIVDKMNKQYEIIDHEFSEVETRLNMINN
KIDDQIQDIWAYNAELLVLLENQKTLDEHDANVNNLYNKVKRALGSNAVEDGRGCFELYHKCDNQCMETI
RNGTYNRRKYQEESKLERQKIEGVKLESEGTYKILTIYSTVASSLVIAMGFAAFLFWAMSNGSCRCNICI

>QDM12995.1 hemagglutinin [Influenza A virus]
METVSLITILLIAAVSNADKICIGYQSTNSTETVDTLTENNVPVTHAKELLHTEHNGMLCATSLGQPLIL
DTCTIEGLIYGNPSCDPLLDGREWSYIVERPSAVNGLCYPGHVENLEELRSLFSSARSYQRIQIFPDTIW
NVSYDGTSSACSGSFYRSMRWLTRKDGEYPIQDAQYTNNQGKNILFMWGINQPPTDTTQRELYTRTDTTT
SVATEEINRIFKPLIGPRPLVNGLMGRIDYYWSVLRPGQTLRIKSDGNLIAPWYGHILSGESHGRILKTD
LKRGSCTVQCQTEKGGLNTTLPFQNVSKYAFGNCSKYIGIKSLKLAVGLRNVPSRSSRGLFGAIAGFIEG
GWSGLVAGWYGFQHSNDQGVGMAADKDSTQKAIDKITFKVNNIIDKMNKQYEIIDHEFSEVENRLNMINN
KIDDQIQDIWAYNAELLVLLENQKTLDEHDANVNNLYNKVKRALGSNAVEDGRGCFELYHKCDDQCMETI
RNGTYNRRKYQEESKLERQKIEGVKLESEGTYKILTIYSTVASSLVIAMGFAAFLFWAMSNGSCRCNICI

>QDM12994.1 hemagglutinin [Influenza A virus]
METVSLITILLIATVSNADKICIGYQSTNSTETVDTLTENNVPVTHAKELLHTEHNGMLCATSLGQPLIL
DTCTIEGLIYGNPSCDPLLDGREWSYIVERPSAVNGLCYPGHVENLEELRSLFSSARSYQRIQIFPDTVW
NVSYDGTSSACSGSFYRSMRWLTRKNGEYPIQDAQYTNNQGKNILFMWGINHPPTDTTQRELYTRTDTTT
SVATEEINRIFKPLIGPRPLVNGLMGRIDYYWSVLRPGQTLRIKSDGNLVAPWYGHILSGESHGRILKTD
LKRGSCTVQCQTEKGGLNTTLPFQNVSKYAFGNCSKYIGIKSLKLAVGLRNVPSRSSRGLFGAIAGFIEG

GWSGLVAGWYGFQHSNDQGVGMAADRDSTQKAIDKITSKVNNIVDKMNKQYEIIDHEFSEVETRLNMINN
KIDDQIQDIWAYNAELLVLLENQKTLDEHDANVNNLYNKVKRALGSNAVEDGRGCFELYHKCDNQCMETI
RNGTYNRRKYQEESKLERQKIEGVKLESEGTYKILTIYSTVASSLVIAMGFAAFLFWAMSNGSCRCNICI

>QDM12993.1 hemagglutinin [Influenza A virus]
METASLITILLVVTASSADKICIGYQSTNSTETVDTLTENNVPVTHAKELLHTEHNGMLCATSLGHPLIL
DTCTIEGLIYGNPSCDPLLGGREWSYIVERPSAVNGLCYPGXVENLEELRSLFSSARSYQRIXIFPDTXW
NVSYDGTSSACSXSFYRSMRWLTXKBGEYPIQDAQYTNNQEKNILFMWGINHPPTDTAXRXLYTRTDTTT
SVATEEINRTFKPLIGPRPLVNGLMGRINYYWSVLKPGQTLRIKSNGNLIAPWYGHILSGESHGRILKTD
LKRGSCTVQCQTEKGGLNTTLPFQNVSKYAFGNCSKYIGIKSLKLAVGLRNVPSRSSRGLFGAIAGFIEG
GWSGLVAGWYGFQHSNAQGVGMAADRDSTQKAIDKITSKVNNIVDKMNKQYEIIDHEFSEVETRLNMINN
KIDDQIQDIWAYNAELLVLLENQKTLDEHDANVNNLYNKVKRALGSNAVEDGKGCFELYHKCDNQCMETI
RNGTYNRRKYQEESKLERQKIEGVKLESEGTYKILTIYSTVASSLVIAMGFAAFLFWAMSNGSCRCNICI

>QDM12992.1 hemagglutinin [Influenza A virus]
METASLITILLVVTASKADKICIGYQSTNSTETVDTLTENNVPVTHAKELLHTEHNGMLCATSLGNPLIL
DTCTIEGLIYGNPSCDPLLGGREWSYIVERPSAVNGLCYPGHVENLEELRSLFSSARSYQRIQIFPDTVW
NVSYDGTSSACSGSFYRSMRWLTRKNGEYPIQDAQYTNNQEKNILFMWGINHPPTDTTQRELYTRTDTTT
SVATEEINRTFKPLIGPRPLVNGLMGRINYYWSVLKPGQTLRIKSNGNLIAPWYGHILSGESHGRILKTD
LKRGSCTVQCQTEKGGLNTTLPFQNVSKYAFGNCSKYIGIKSLKLAVGLRNVPSRSSRGLFGAIAGFIEG
GWSGLVAGWYGFQHSNDQGVGMAADRDSTQKAIDKITSKVNNIVDKMNKQYEIIDHEFSEVETRLNMINN
KIDDQIQDIWAYNAELLVLLENQKTLDEHDANVNNLYNKVKRALGSNAVEDGKGCFELYHKCNDQCMETI
RNGTYNRSKYQEESKLERQKIEGVKLESEGTYKILTIYSTVASSLVIAMGFAAFLFWAMSNGSCRCNICI

>QDM12991.1 hemagglutinin [Influenza A virus]
METVSLITILLIATVSYADKICIGYQSTNSTETVDTLTENNVPVTHAKELLHTEHNGMLCATSLGQPLIL
DTCTIEGLIYGNPSCDPLLDGREWSYIVERPSAVNGLCYPGHVENLEELRSLFSSARSYQRIQIFPDTVW
NVSYDGTSSACSGSFYRSMRWLTRKNGEYPIQDAQYTNNQGKNILFMWGINHPPTDTTQRELYTRTDTTT
SVATEEINRIFKPLIGPRPLVNGLMGRIDYYWSVLRPGQTLRIKSDGNIVAPWYGHILSGESHGRILKTD
LKRGSCTVQCQTEKGGLNTTLPFQNVSKYAFGNCSKYIGIKSLKLAVGLRNVPSRSSRGLFGAIAGFIEG
GWPGLVAGWYGFQHSNDQGVGMAADRDSTQKAIDKITSKVNNIVDKMNKQYEIIDHEFSEVETRLNMINN
KIDDQIQDIWAYNAELLVLLENQKTLDEHDANVNNLYNKVKRALGSNAVEDGRGCFELYHKCDNQCMETI
RNGTYNRRKYQEESKLERQKIEGVKLESEGTYKILTIYSTVASSLVIAMGFAAFLFWAMSNGSCRCNICI

>QDM12990.1 hemagglutinin [Influenza A virus]
METVSLITILLIAAVSNADKICIGYQSTNSTETVDTLTENNVPVTHAKELLHTEHNGMLCATSLGQPLIL
DTCTIEGLIYGNPSCDPLLDGREWSYIVERPSAVNGLCYPGHVENLEELRSLFSSARSYQRIQIFPDTIW
NVSYDGTSSACSGSFYRSMRWLTRKDGEYPIQDAQYTNNQGKNILFMWGINQPPTDTTQRELYTRTDTTT
SVATEEINRIFKPLIGPRPLVNGLMGRIDYYWSVLRPGQTLRIKSDGNLIAPWYGHILSGESHGRILKTD
LKRGSCTVQCQTEKGGLNTTLPFQNVSKYAFGNCSKYIGIKSLKLAVGLRNVPSRSSRGLFGAIAGFIEG
GWSGLVAGWYGFQHSNDQGVGMAADKDSTQKAIDKITFKVNNIIDKMNKQYEIIDHEFSEVENRLNMINN
KIDDQIQDIWAYNAELLVLLENQKTLDEHDANVNNLYNKVKRALGSNAVEDGRGCFELYHKCDDQCMETI
RNGTYNRRKYQEESKLERQKIEGVKLESEGTYKILTIYSTVASSLVIAMGFAAFLFWAMSNGSCRCNICI

>QDM12989.1 hemagglutinin [Influenza A virus]
METVSLITILLIATVSYADKICIGYQSTNSTETVDTLTENNVPVTHAKELLHTEHNGMLCATSLGQPLIL
DTCTIEGLIYGNPSCDPLLDGREWSYIVERPSAVNGLCYPGHVENLEELRSLFSSARSYQRIQIFPDTVW
NVSYDGTSSACSGSFYRSMRWLTRKNGEYPIQDAQYTNNQGKNILFMWGINHPPTDTTQRELYTRTDTTT
SVATEEINRIFKPLIGPRPLVNGLMGRIDYYWSVLRPGQTLRIKSDGNIVAPWYGHILSGESHGRILKTD
LKRGSCTVQCQTEKGGLNTTLPFQNVSKYAFGNCSKYIGIKSLKLAVGLRNVPSRSSRGLFGAIAGFIEG
GWSGLVAGWYGFQHSNDQGVGMAADRDSTQKAIDKITSKVNNIVDKMNKQYEIIDHEFSEVETRLNMINN
KIDDQIQDIWAYNAELLVLLENQKTLDEHDANVNNLYNKVKRALGSNAVEDGRGCFELYHKCDNQCMETI
RNGTYNRRKYQEESKLERQKIEGVKLESEGTYKILTIYSTVASSLVIAMGFAAFLFWAMSNGSCRCNICI

>QDM12988.1 hemagglutinin [Influenza A virus]
METVSLITILLAVTASNADKICIGYQSTNSTETVDTLTESNVPVTHAKELLHTEHNGMLCSTSLGHPLIL
DTCTIEGLIYGNPSCDLLLGGREWSYIVERPSAVNGLCYPGNVENLEELRSLFSSARSYQRVQIFPDTIW
NVSYDGTSTACSGSFYRNMRWLTRKDGNYPIQDAQYTNNQEKNILFMWGINHPPTDTAQSGLYTRTDTTT
SVATEEINRIFKPLIGPRPLVNGLMGRIDYYWSVLKPGQTLRIKSDGNLIAPWYGHILSGESHGRILKTD
LKSGSCTVQCQTEKGGLNTTLPFQNVSKYAFGNCSKYIGIKSLKLAVGLRNVPSRSSRGIFGAIAGFIEG
GWSGLVAGWYGFQHSNDQGVGMAADRDSTQKAIDKITSKVNNIVDKMNKQYEIIDHEFSEVETRLNMINS
KIDDQIQDIWAYNAELLVLLENQKTLDEHDANVNNLYNKVKRALGSNAVEDGKGCFELYHKCDDQCMETI
RNGTYNRRKYQEESKLERQKIEGVKLESEGTYKILTIYSTVASSLVIAMGFAAFLFWAMSNGSCRCNICI

>QDM12987.1 hemagglutinin [Influenza A virus]
METVSLITILLIATVSYADKICIGYQSTNSTETVDTLTENNVPVTHAKELLHTEHNGMLCATSLGQPLIL
DTCTIEGLIYGNPSCDPLLDGREWSYIVERPSAVNGLCYPGHIENLEELRSLFSSARSYQRIQIFPDTVW
NVSYDGTSSACSGSFYRSMRWLTRKNGEYPIQDAQYTNNQGKNILFMWGINHPPTDTTQRELYTRTDTTT

SVATEEINRIFKPLIGPRPLVNGLMGRIDYYWSVLRPGQTLRIKSDGNLVAPWYGHILSGESHGRILKTD
LKRGSCTVQCQTEKGGLNTTLPFQNVSKYAFGNCSKYIGIKSLKLAVGLRNVPSRSSRGLFGAIAGFIEG
GWSGLVAGWYGFQHSNDQGVGMAADRDSTQKAIDKITSKVNNIVDKMNKQYEIIDHEFSEVETRLNMINN
KIDDQIQDIWAYNAELLVLLENQKTLDEHDANVNNLYNKVKRALGSNAVEDGRGCFELYHKCDNQCMETI
RNGTYNRRKYQEESKLERQKIEGVKLESEGTYKILTIYSTVASSLVIAMGFAAFLFWAMSNGSCRCNICI

>QDM12986.1 hemagglutinin [Influenza A virus]
METASLITILLVVTASKADKICIGYQSTNSTETVDTLTENNVPVTHAKELLHTEHNGMLCATSLGNPLIL
DICTIEGLIYGNPSCDPLLGGREWSYIVERPSAVNGLCYPGHVENLEELRSLFSSARSYQRIQIFPDTVW
NVSYDGTSSACSDSFYRSMRWLTQKNGEYPIQDAQYTNNQEKNILFMWGINHPPTDTAQRDLYTRTDTTT
SVATEEINRTFKPLIGPRPLVNGLMGRINYYWSVLKPGQTLRIKSNGNLIAPWYGHILSGESHGRILKTD
LKRGSCTVQCQTEKGGLNTTLPFQNVSKYAFGNCSKYIGIKSLKLAVGLRNVPSRSSRGLFGAIAGFIEG
GWSGLVAGWYGFQHSNDQGVGMAADRDSTQKAIDKITSKVNNIVDKMNKQYEIIDHEFSEVETRLNMINN
KIDDQIQDIWAYNAELLVLLENQKTLDEHDANVNNLYNKVKRALGSNAVEDGKGCFELYHKCNDQCMETI
RNGTYNRSKYQEESKLERQKIEGVKLEPEGTYKILTIYSTVASSLVIAMGFAAFLFWAMSNGSCRCNICI

>QDH82733.1 hemagglutinin [Influenza A virus]
METVSLITILLVATVSNADKICIGYQSTNSTETVDTLTENNVPVTHAKELLHTEHNGMLCATSLGHPLIL
DTCTIEGLIYGNPSCDPLLGGREWSYIVERPSAVNGLCYPGNVENLEELRSLFSSSRSYQRIQIFPDTIW
NVSYSGTSRACSDSFYRSMRWLTQKNNTYPTQDAQYTNNQEKNILFMWGINHPPTDTVQTNLYTRTDTTT
SVATEEINRIFKPLIGPRPLVNGLMGRINYYWSVLKPGQTLRIKSDGNLIAPWYGHILSGESHGRILKTD
LKRGSCTVQCQTEKGGLNTTLPFQNVSKYAFGNCSKYIGVKSLKLAVGLRNVPSRSSRGLFGAIAGFIEG
GWSGLVAGWYGFQHSNDQGVGMAADRDSTQKAIDKITSKVNNIVDKMNKQYEIIDHEFSEVETRLNMINN
KVDDQIQDIWAYNAELLVLLENQKTLDEHDANVNNLYNKVKRALGSNAVEDGKGCFELYHKCDDHCMETI
RNGTYNRRKYQEESKLKRQKIEGVKLESEETYKILTIYSTVASSLVIAMGFAAFLFWAMSNGSCRCNMVW

>QDH82721.1 hemagglutinin [Influenza A virus]
METVSLITTLLVATVSNADKICIGYQSTNSTETVDTLTENNVPVTHAKELLHTEHNGMLCATGLGQPLIL
DTCTIEGLIYGNPSCDLSLEGREWSYIVERPSAVNGLCYPGNVENLEELRSLFSSARSYQRIQIFPDTIW
NVSYDGTSTACSGSFYRSMRWLTRKNGDYPIQDAQYTNNQGKNILFMWGINHPPTDTTQRDLYTRTDTTT
SVATEEINRIFKPLIGPRPLVNGLMGRIDYYWSVLKPGQTLRIKSDGNLIAPWYGHILSGESHGRILKTD
LKRGSCTVQCQTEKGGLNTTLPFQNVSKYAFGNCSKYIGIKSLKLAVGLRNVPSRSSRGLFGAIAGFIEG
GWSGLVAGWYGFQHSNDQGVGMAADRDSTQKAIDKITSKVNNIVDKMNKQYEIIDHEFSEVETRLNMINN
KIDDQIQDIWAYNAELLVLLENQKTLDEHDANVNNLYNKVKRALGSNAVEDGKGCFELYHKCDDQCMETI

RNGTYNRRKYQEESKLERQKIEGVKLESEGTYKILTIYSTVASSLVIAMGFAAFLFWAMSNGSCRCNICI

>QDH82709.1 hemagglutinin [Influenza A virus]
METVSLITILLVAAVSNADKICIGYQSTNSTEAVDTLTENNVPVTHAKELLHTEHNGMLCATSLGQPIIL
DTCTIEGLIYGNPSCDLSLEGREWSYIVERPSAVNGLCYPGNVENLEELRSLFSSARSYQRIQIFPDTIW
NVSYDGTSTACSGSFYRNMRWLTRKNGEYPIQDAQYTNNKGKNILFMWGINHPPTDTTQRDLYTRTDTTT
SVATEEINRIFKPLIGPRPLVNGLMGRIDYYWSVLKPGQTLRIKSDGNLISPWYGHILSGESHGRILKTD
LKRGSCTVQCQTEKGGLNTTLPFQNVSKYAFGNCSKYIGIKSLKLAVGLRNVPSRSSRGLFGAIAGFIEG
GWSGLVAGWYGFQHSNDQGVGMAADRDSTQKAVDKITSKVNTIVDKMNKQYEIIDHEFSEVETRLNMINN
KIDDQIQDIWAYNAELLVLLENQKTLDEHDANVNNLYNKVKRALGSNAVEDGRGCFELYHKCDDQCMETI
RNGTYNRRKYQEESKLERQKIEGVKLESEGTYKILTIYSTVASSLVIAMGFAAFLFWAMSNGSCRCNICI

>QDH82697.1 hemagglutinin [Influenza A virus]
MEIVSLITILLVAAVSNADKICIGYQSTNSTETVDTLTENNVPVTHAKELLHTEHNGMLCATSLGQPIIL
DTCTIEGLIYGNPSCDLSLEGREWSYIVERPSAVNGLCYPGNVENLEELRSLFSSARSYQRIQIFPDTIW
NVSYDGTSTACSGSFYRNMRWLTRKNGEYPIQDAQYTNNQGKNILFMWGINHPPTDTTQRDLYTRTDTTT
SVATEEINRIFKPLIGPRPLVNGLMGRIDYYWSVLKPGQTLRIKSDGNLISPWYGHILSGESHGRILKTD
LKRGSCTVQCQTEKGGLNTTLPFQNVSKYAFGNCSKYIGIKSLKLAVGLRNVPSRSSRGLFGAIAGFIEG
GWSGLVAGWYGFQHSNDQGVGMAADRDSTQKAVDKITSKVNAIVDKMNKQYEIIDHEFSEVETRLNMINN
KIDDQIQDIWAYNAELLVLLENQKTLDEHDANVNNLYNKVKRALGSNAVEDGRGCFELYHKCDDQCMETI
RNGTYNRRKYQEESKLERQKIEGVKLESEGTYKILTIYSTVASSLVIAMGFAAFLFWAMSNGSCRCNIFL

>QDH82685.1 hemagglutinin [Influenza A virus]
METVSLITILLVATVSNADKICIGYQSTNSTETVDTLTENNVPVTHAKELLHTEHNGMLCATGLGQPLIL
DTCTIEGLIYGNPSCDLSLEGREWSYIVERPSAVNGLCYPGNVENLEELRSLFSSARSYQRIQIFPDTIW
NVSHDGTSTACSGSFYRSMRWLTQKSGDYPIQDAQYTNNQGKNILFMWGINHPPTDTTQRNLYTRTDTTT
SVATEEINRIFKPLIGPRPLVNGLMGRIDYYWSVLKPGQTLRIKSDGNLIAPWYGHILSGESHGRILKTD
LKRGSCTVQCQTEKGGLNTTLPFQNVSKYAFGNCSKYIGIKSLKLAVGLRNVPSRSSRGLFGAIAGFIEG
GWSGLVAGWYGFQHSNDQGVGMAADRDSTQKAIDKITSKVNNIVDKMNKQYEIIDHEFSEVETRLNMINN
KIDDQIQDIWAYNAELLVLLENQKTLDEHDANVNNLYNKVKRALGSNAVEDGKGCFELYHKCNDQCMETI
RNGTYNRRKYQEESKLERQKIEGVKLESEGTYKILTIYSTVASSLVIAMGFAAFLFWAMSNGSCRCNICI

>QDH82673.1 hemagglutinin [Influenza A virus]
METVSLITILLVATVSNADKICIGYQSTNSTETVDTLTENNVPVTHAKELLHTEHNGMLCATSLGQPIIL

DTCTIEGLIYGNPSCDLSLEGREWSYIVERPSAVNGLCYPGNVENLEELRSLFSSARSYQRIQIFPDTIW
NVSYDGTSTACSGSFYRNMRWLTRKNGEYPIQDAQYTNNQGKNILFMWGINHPPADTTQRDLYTRTDTTT
SVATEEINRIFKPLIGPRPRVNGLMGRIDYYWSVLKPGQTLRIKSDGNLIAPWYGHILSGESHGRILKTD
LKRGSCTVQCQTEKGGLNTTLPFQNVSKYAFGNCSKYIGIKSLKLAVGLRNVPSRSSRGLFGAIAGFIEG
GWSGLVAGWYGFQHSNDQGVGMAADRESTQKAVDKITSKVNNIVDKMNKQYEIIDHEFSEVETRLNMINN
KIDDQIQDIWAYNAELLVLLENQKTLDEHDANVNNLYNKVKRALGSNAMEDGKGCFELYHKCDDQCMETI
RNGTYNRRKYQEESKLERQKIEGVKLESEGTYKILTIYSTVASSLVIAMGFAAFLFWAMSNGSCRCNICI

>QDH82661.1 hemagglutinin [Influenza A virus]
METVSLITILLVATVSNADKICIGYQSTNSTETVDTLTENNVPVTHAKELLHTEHNGMLCATSLGQPLIL
DTCTIEGLIYGNPSCDLSLEGREWSYIVERPSAVNGLCYPGNVENLEELRSLFSSARSYQRIQIFPDTIW
NVSYNGTSTACSGSFYRSMRWLTRKNGDYPTQDAQYTNNQGKNILFMWGINHPPTDTTQRNLYTRTDTTT
SVATEEINRIFKPLIGPRPRVNGLMGRIDYYWSVLKPGQTLRIKSDGNLIAPWYGHILSGESHGRILKTD
LKRGSCTVQCQTEKGGLNTTLPFQNVSKYAFGNCSKYIGVKSLKLAVGLRNVPSRSSRGLFGAIAGFIEG
GWSGLVAGWYGFQHSNDQGVGMAADRDSTQKAIDKITSKVNNIVDKMNKQYEIIDHEFSEVETRLNMINN
KIDDQIQDIWAYNAELLVLLENQKTLDEHDANVNNLYNKVKRALGSNAVEDGKGCFELYHKCNDQCMETI
RNGTYNRRKYQEESKLERQKIEGVKLESEGTYKILTIYSTVASSLVIAMGFAAFLFWAMSNGSCRRNICM

>QDH82650.1 hemagglutinin [Influenza A virus]
METVSLITILLVAAVSNADKICIGYQSTNSTETVDTLTENNVPVTHAKELLHTEHNGMLCATSLGQPIIL
DTCTIEGLIYGNPSCDLSLEGREWSYIVERPSAVNGLCYPGNVENLEELRSLFSSARSYQRIQIFPDTIW
NVSYDGTSTACSGSFYRNMRWLTRKNGEYPIQDAQYTNNQGKNILFMWGINHPPADTTQRDLYTRTDTTT
SVATEEINRIFKPLIGPRPLVNGLMGRIDYYWSVLKPGQTLRIKSDGNLIAPWYGHILSGESHGRILKTD
LKRGSCTVQCQTEKGGLNTTLPFQNVSKYAFGNCSKYIGIKSLKLAVGLRNVPSRSSRGLFGAIAGFIEG
GWSGLVAGWYGFQHSNDQGVGMAADRDSTQKAVDKITSKVNTIVDKMNKQYEIIDHEFSEVETRLNMINN
KIDDQIQDIWAYNAELLVLLENQKTLDEHDANVNNLYNKVKRALGSNAVEDGKGCFELYHKCDDQCMETI
RNGTYNRRKYQEESKLERQKIEGVKLESEGTYKILTIYSTVASSLVIAMGFAAFLFWAMSNGSCRCNICL

>QDH82638.1 hemagglutinin [Influenza A virus]
METVSLITILLVATVSNADKICIGYQSTNSTETVDTLTENNVPVTHAKELLHTEHNGMLCATGLGQPLIL
DTCTIEGLIYGNPSCDLSLEGREWSYIVERPSAVNGLCYPGNVENLEELRSLFSSARSYQRIQIFPDTIW
NVSYDGTSTACSGSFYRSMRWLTQKNGDYPIQDAQYTNNQGKNILFMWGINHPPTDTTQRNLYTRTDTTT
SVATEEINRIFKPLIGPRPLVNGLMGRIDYYWSVLKPGQTLRIKSDGNLIAPWYGHILSGESHGRILKTD
LKRGSCTVQCQTEKGGLNTTLPFQNVSKYAFGNCSKYIGIKSLKLAVGLRNVPSRSSRGLFGAIAGFIEG

GWSGLVAGWYGFQHSNDQGVGMAADRDSTQKAIDKITSKVNNIVDKMNKQYEIIDHEFSEVETRLNMINN
KIDDQIQDIWAYNAELLVLLENQKTLDEHDANVNNLYNKVKRALGSNAVEDGKGCFELYHKCNDQCMETI
RNGTYNRRKYQEESKLERQKIEGVKLESEGTYKILTIYSTVASSLVIAMGFAAFLFWAMSNGSCRCNICI

>QDH82626.1 hemagglutinin [Influenza A virus]
METVSLITILLVATVSNADKICIGYQSTNSTETVDTLTENNVPVTHAKELLHTEHNGMLCATSLGQPLIL
DTCTIEGIIYGNPSCDLSLEGREWSYIVERPSAVNGLCYPGNVENLEELRSLFSSARSYQRIQIFPDTIW
NVSYDGTSTACSGSFYKSMRWLTRKNGNYPIQDAQYTNNQGKNILFMWGINHPPTDTTQRDLYTRTDTTT
SVATEEINRVFKPLIGPRPLVNGLMGRIDYYWSVLKPGQTLRIKSDGNLIAPWFGHILSGESHGRILKTD
LKRGSCTVQCQTEKGGLNTTLPFQNVSKYAFGNCSKYIGIKSLKLAVGLRNVPSRSSRGLFGAIAGFIEG
GWSGLVAGWYGFQHSNDQGVGMAADRDSTQKAIDKITSKVNNIVDKMNKQYEIIDHEFSEVETRLNMINN
KIDDQIQDIWAYNAELLVLLENQKTLDEHDANVNNLYNKVKRALGSNAVEDGKGCFELYHKCDDQCMETI
RNGTYNRRKYQEESKLERQKIEGVKLESEGTYKILTIYSTVASSLVIAMGFAAFLFWAMSNGSCRCNICI

>QCR64132.1 hemagglutinin [Influenza A virus]
MEVIPLMTMLLLVTTNNADKICIGHQSTNSTETVDTLTETGVPVTHAKELLHTEHNGRLCATNLGKPLIL
DTCTVEGIIYGNPSCDMLLGGREWSYIVERPSAVNGTCYPGNVENLEELRVLFSSSSSYQRIQMFPDTIW
NVTYSGTSKSCSDSFYRNMRWLTQKNGNYPVQDAQYTNTRGKDILFVWGMHHPPTETTQTNLYTRIDTTT
SITTESLDRTFKPLIGPRPLVNGLIGRINYYWSVLKPGQTLRVRSNGNLIAPWFGYVLSGESHGRIFETD
LNSGNCVVQCQTEKGGLNSTLPFHNISKYAFGDCPKYIGVKSLKLAIGLRNVPARSSRGLFGAIAGFIEG
GWPGLVAGWYGFQHSNDQGVGMAADRDSTQKAVDKITSKVNNIVDKMNKQYEIIDHEFSEVENRLNMINN
KIDDQIQDIWAYNAELLVLLENQKTLDEHDANVNNLYNKVKRALGSNAMEDGKGCFELYHKCDDQCMETI
RNGTYNRRKYMEESRLGRQKIEGVKLESEGTYKILTIYSTVASSLVLAMGFAAFLFWAMSNGSCRCNICI

>QCR64131.1 hemagglutinin [Influenza A virus]
MEVIXLMXMLLXVTTNNADKICIGHQSTNSTETVDTLTETGVPVTHAKELLHTEHNGRLCATNLGKPLIL
DTCTVEGIIYGNPSCDMLLGGREWSYIVERPSAVNGTCYPGNVENLEELRVLFSSSSSYQRIQMFPDTIW
NVTYSGTSKSCSDSFYRNMRWLTQKNGNYPVQDAQYTNTRGKDILFVWGMHHPPTETTQTNLYTRIDTTT
SITTESLDRTFKPLIGPRPLVNGLIGRINYYWSVLKPGQTLRVRSNGNLIAPWFGYVLSGESHGRILKTD
LNSGNCVVQCQTEKGGLNSTLPFHNISKYAFGDCPKYIGVKSLKLAIGLRNVPARSSRGLFGAIAGFIEG
GWPGLVAGWYGFQHSNDQGVGMAADRDSTQKAVDKITSKVNNIVDKMNKQYEIIDHEFSEVENRLNMINN
KIDDQIQDIWAYNAELLVLLENQKTLDEHDANVNNLYNKVKRALGSNAMEDGKGCFELYHKCDDQCMETI
RNGTYNRRKYMEESRLGRQKIEGVKLESEGTYKILTIYSTVASSLVLAMGFAAFLFWAMSNGSCRCNICI

>QCR64130.1 hemagglutinin [Influenza A virus]

MEVIPXMTMLLLVTTNNADKICIGHQSTNSTETVDTLTETGVPVTHAKELLHTEHNGRLCATNLGKPLIL
DTCTVEGIIYGNPSCDMLLGGREWSYIVERPSAVNGTCYPGNVENLEELRVLFSSSSSYQRIQMFPDTIW
NVTYSGTSKSCSDSFYRNMRWLTQKNGNYPVQDAQYTNTRGKDILFVWGMHHPPTETTQTNLYTRIDTTT
SITTESLDRTFKPLIGPRPLVNGLIGRINYYWSVLKPGQTLRVRSNGNLIAPWFGYVLSGESHGRILKTD
LNSGNCVVQCQTEKGGLNSTLPFHNISKYAFGDCPKYIGVKSLKLAIGLRNVPARSSRGLFGAIAGFIEG
GWPGLVAGWYGFQHSNDQGVGMAADRDSTQKAVDKITSKVNNIVDKMNKQYEIIDHEFSEVENRLNMINN
KIDDQIQDIWAYNAELLVLLENQKTLDEHDANVNNLYNKVKRALGSNAMEDGKGCFELYHKCDDQCMETI
RNGTYNRRKYMEESRLGRQKIEGVKLESEGTYKILTIYSTVASSLVLAMGFAAFLFWAMSNGSCRCNICI

>QCR64129.1 hemagglutinin [Influenza A virus]

MEVIPXMTMLLLVTTNNADKICIGHQSTNSTETVDTLTETGVPVTHAKELLHTEHNGRLCATNLGKPLIL
DTCTVEGIIYGNPSCDMLLGGREWSYIVERPSAVNGTCYPGNVENLEELRVLFSSSSSYQRIQMFPDTIW
NVTYSGTSKSCSDSFYRNMRWLTQKNGNYPVQDAQYTNTRGKDILFVWGMHHPPTETTQTNLYTRIDTTT
SITTESLDRTFKPLIGPRPLVNGLIGRINYYWSVLKPGQTLRVRSNGNLIAPWFGYVLSGESHGRILKTD
LNSGNCVVQCQTEKGGLNSTLPFHNISKYAFGDCPKYIGVKSLKLAIGLRNVPARSSRGLFGAIAGFIEG
GWPGLVAGWYGFQHSNDQGVGMAADRDSTQKAVDKITSKVNNIVDKMNKQYEIIDHEFSEVENRLNMINN
KIDDQIQDIWAYNAELLVLLENQKTLDEHDANVNNLYNKVKRALGSNAMEDGKGCFELYHKCDDQCMETI
RNGTYNRRKYMEESRLGRQKIEGVKLESEGTYKILTIYSTVASSLVLAMGFAAFLFWAMSNGSCRCNICI

>QCR64128.1 hemagglutinin [Influenza A virus]

MKAISXMTILLVVTISDADKICIGHQSTNSTETVDTLTETNVPVTQAKELLHTEHNGMLCATNLGRPLIL
DTCTIEGLIYGNPSCDMLLGGREWSYIVERPSAVNGTCYPGNVENLEELRTLFSSSSSYQRIQLFPDSIW
NVTYSGTSKSCSDSFYRNMRWLTQKNGLYPVQDAQYINNRGKDILFVWGIHHPPTDTTQTNLYTRTDTTT
SVTTENLDRTFKPLIGPRPLVNGLIGRINYYWSVLKPGQTLRIRSNGNLIAPWFGHILSGESHGRILRTD
LSSGNCVVQCQTEKGGLNSTLPFHNISKYAFGTCPKYIGVKSLRLAIGLRNVHARSNRGLFGAIAGFIEG
GWPGLVAGWYGFQHSNDQGVGMAADRVSTQKAVDKITSKVNNIVDKMNKQYEIIDHEFSEVESRLNMINN
KIDDQIQDIWAYNAELLVLLENQKTLDEHDANVNNLYNKVKRALGSNAIEDGKGCFELYHKCDDQCMETI
RNGTYNRRKYTKESRLERQKIEGVKLEAEGTYKILSIYSTVASSLVLAMGFAAFLFWAMSNGSCRCNICI

>QCR64127.1 hemagglutinin [Influenza A virus]

MEXIPXXXMLLLVTTNNADKICIGHQSTNFTETVDTLTETGVPVTHAKELLHTEHNGRLCATNLGKPLIL
DTCTVEGIIYGNPSCDMLLGGREWSYIVERPSAVNGTCYPGNVENLEELRVLFSSSSSYQRIQMFPDTIW
NVTYSGTSKSCSDSFYRNMRWLTQKNGNYPVQDAQYTNTRGKDILFVWGMHHPPTETTQTNLYTRIDTTT

SITTESLDRTFKPLIGPRPLVNGLIGRINYYWSVLKPGQTLRVRSNGNLIAPWFGYVLSGESHGRILKTD
LNSGNCVVQCQTEKGGLNSTLPFHNISKYAFGDCPKYIGVKSLKLAIGLRNVPARSSRGLFGAIAGFIEG
GWPGLVAGWYGFQHSNDQGVGMAADRDSTQKAVDKITSKVNNIVDKMNKQYEIIDHEFSEVENRLNMINN
KIDDQIQDIWAYNAELLVLLENQKTLDEHDANVNNLYNKVKRALGSNAMEDGKGCFELYHKCDDQCMETI
RNGTYNRRKYMEESRLGRQKIEGVKLESEGTYKILTIYSTVASSLVLAMGFAAFLFWAMSNGSCRCNICI

>QCR64126.1 hemagglutinin [Influenza A virus]
MEVIPLMTMLLLVTTNNADKICIGHQSTNSTETVDTLTETGVPVTHAKELLHTEHNGRLCATNLGKPLIL
DTCTVEGIIYGNPSCDMLLGGREWSYIVERPSAVNGTCYPGNVENLEELRVLFSSSSSYQRIQMFPDTIW
NVTYSGTSKSCSDSFYRNMRWLTQKNGNYPVQDAQYTNTRGKDILFVWGMHHPPTETTQTNLYTRIDTTT
SITTESLDRTFKPLIGPRPLVNGLIGRINYYWSVLKPGQTLRVRSNGNLIAPWFGYVLSGESHGRILKTD
LNSGNCVVQCQTEKGGLNSTLPFHNISKYAFGDCPKYIGVKSLKLAIGLRNVPARSSRGLFGAIAGFIEG
GWPGLVAGWYGFQHSNDQGVGMAADRDSTQKAVDKITSKVNNIVDKMNKQYEIIDHEFSEVENRLNMINN
KIDDQIQDIWAYNAELLVLLENQKTLDEHDANVNNLYNKVKRALGSNAMEDGKGCFELYHKCDDQCMETI
RNGTYNRRKYMEESRLGRQKIEGVKLESEGTYKILTIYSTVASSLVLAMGFAAFLFWAMSNGSCRCNICI

>QCR64125.1 hemagglutinin [Influenza A virus]
MEVIPLMTMLLLVTTNNADKICIGHQSTNSTETVDTLTETAVPVTHAKELLHTEHNGRLCATNLGNPLIL
DTCTVEGIIYGNPSCDMLLGGREWSYIVERPSAVNGTCYPGNVENLEELRVLFSSSSSYQRIQMFPDTIW
NVTYNGTSKSCSDSFYRNMRWLTQKNGNYPVQDAQYTNTRGKDILFVWGIHHPPTETTQTNLYTRTDTTT
SITTESLDRTFKPLIGPRPLVNGLIGRINYYWSVLKPGQTLRVRSNGNLIAPWFGHVLSGESHGRILKTD
LNSGNCVVQCQTEKGGLNSTLPFHNISKYAFGDCPKYIGVKSLKLAIGLRNVPARSSRGLFGAIAGFIEG
GWPGLVAGWYGFQHSNDQGVGMAADRDSTQKAVDKITSKVNNIVDKMNKQYEIIDHEFSEVENRLNMINN
KIDDQIQDIWAYNAELLVLLENQKTLDEHDANVNNLYNKVKRALGSNAMEDGKGCFELYHKCDDQCMETI
RNGTYNRRKYMEESRLGRQKIEGVKLESEGTYKILTIYSTVASSLVLAMGFAAFLFWAMSNGSCRCNICI

>QCR64124.1 hemagglutinin [Influenza A virus]
MEVXPXMTMLLLVTTNNADKICIGHQSTNSTETVDTLTETGVPVTHAKELLHTEHNGRLCATNLGKPLIL
DTCTVEGIIYGNPSCDMLLGGREWSYIVERPSAVNGTCYPGNVENLEELRVLFSSSSSYQRIQMFPDTIW
NVTYSGTSKSCSDSFYRNMRWLTQKNGNYPVQDAQYTNTRGKDILFVWGMHHPPTETTQTNLYTRIDTTT
SITTESLDRTFKPLIGPRPLVNGLIGRINYYWSVLKPGQTLRVRSNGNLIAPWFGYVLSGESHGRILKTD
LNSGNCVVQCQTEKGGLNSTLPFHNISKYAFGDCPKYIGVKSLKLAIGLRNVPARSSRGLFGAIAGFIEG
GWPGLVAGWYGFQHSNDQGVGMAADRDSTQKAVDKITSKVNNIVDKMNKQYEIIDHEFSEVENRLNMINN
KIDDQIQDIWAYNAELLVLLENQKTLDEHDANVNNLYNKVKRALGSNAMEDGKGCFELYHKCDDQCMETI

RNGTYNRRKYMEESRLGRQKIEGVKLESEGTYKILTIYSTVASSLVLAMGFAAFLFWAMSNGSCRCNICI

>QCR64123.1 hemagglutinin [Influenza A virus]
MEVIPLMTMLLLVTTNNADKICIGHQSTNSTETVDTLTETGVPVTHAKELLHTEHNGRLCATNLGKPLIL
DTCTVEGIIYGNPSCDMLLGGREWSYIVERPSAVNGTCYPGNVENLEELRVLFSSSSSYQRIQMFPDTIW
NVTYSGTSKSCSDSFYRNMRWLTQKNGNYPVQDAQYTNTRGKDILFVWGMHHPPTETTQTNLYTRIDTTT
SITTESLDRTFKPLIGPRPLVNGLIGRINYYWSVLKPGQTLRVRSNGNLIAPWFGYVLSGESHGRILKTD
LNSGNCVVQCQTEKGGLNSTLPFHNISKYAFGDCPKYIGVKSLKLAIGLRNVPARSSRGLFGAIAGFIEG
GWPGLVAGWYGFQHSNDQGVGMAADRDSTQKAVDKITSKVNNIVDKMNKQYEIIDHEFSEVENRLNMINN
KIDDQIQDIWAYNAELLVLLENQKTLDEHDANVNNLYNKVKRALGSNAMEDGKGCFELYHKCDDQCMETI
RNGTYNRRKYMEESRLGRQKIEGVKLESEGTYKILTIYSTVASSLVLAMGFAAFLFWAMSNGSCRCNVCI

>QCR64122.1 hemagglutinin [Influenza A virus]
MEVIPXMTMLLLVTTNNADKICIGHQSTNSTETVDTLTETGVPVTHAKELLHTEHNGRLCATNLGKPLIL
DTCTVEGIIYGNPSCDMLLGGREWSYIVERPSAVNGTCYPGNVENLEELRVLFSSSSSYQRIQMFPDTIW
NVTYSGTSKSCSDSFYRNMRWLTQKNGNYPVQDAQYTNTRGKDILFVWGMHHPPTETTQTNLYTRIDTTT
SITTESLDRTFKPLIGPRPLVNGLIGRINYYWSVLKPGQTLRVRSNGNLIAPWFGYVLSGESHGRILKTD
LNSGNCVVQCQTEKGGLNSTLPFHNISKYAFGDCPKYIGVKSLKLAIGLRNVPARSSRGLFGAIAGFIEG
GWPGLVAGWYGFQHSNDQGVGMAADRDSTQKAVDKITSKVNNIVDKMNKQYEIIDHEFSEVENRLNMINN
KIDDQIQDIWAYNAELLVLLENQKTLDEHDANVNNLYNKVKRALGSNAMEDGKGCFELYHKCDDQCMETI
RNGTYNRRKYMEESRLGRQKIEGVKLESEGTYKILTIYSTVASSLVLAMGFAAFLFWAMSNGSCRCNICI

>QCR64121.1 hemagglutinin [Influenza A virus]
MEVIPLMTMLLLVTTNNADKICIGHQSTNSTETVDTLTETGVPVTHAKELLHTEHNGKLCATNLGKPLIL
DTCTVEGIIYGNPSCDMLLGGREWSYIVERPSAVNGTCYPGNVENLEELRVLFSSSSSYQRIQMFPDTIW
NVTYSGTSKSCSDSFYRNMRWLTQKNGNYPVQDAQYTNTRGKDILFVWGMHHPPTETTQTNLYTRIDTTT
SITTESLDRTFKPLIGPRPLVNGLIGRINYYWSVLKPGQTLRVRSNGNLIAPWFGYVLSGESHGRILKTD
LNSGNCVVQCQTEKGGLNSTLPXXNISKYAFGDCPKYIGVKSLKLAIGLRNVPARSSRGLFGAIAGFIEG
GWPGLVAGWYGFQHSNDQGVGMAADRDSTQKAVDKITSKVNNIVDKMNKQYEIIDHEFSEVENRLNMINN
KIDDQIQDIWAYNAELLVLLENQKTLDEHDANVNNLYNKVKRALGSNAMEDGKGCFELYHKCDDQCMETI
RNGTYNRRKYMEESRLGRQKIEGVKLESEGTYKILTIYSTVASSLVLAMGFAAFLFWAMSNGSCRCNICI

>QCR64120.1 hemagglutinin [Influenza A virus]
MKAISLMTILLVVTISGADKICIGHQSTNSTETVDTLTETNVPVTQAKELLHTEHNGMLCATNLGRPLIL

DTCTIEGLIYGNPSCDMLLGGREWSYIVERPSAVNGTCYPGNVENLEELRTLFSSSSSYQRIQLFPDSIW
NVTYSGTSKSCSDSFYRNMRWLTQKNGLYPVQDAQYINNRGKDILFVWGIHHPPTDTTQTNLYTRTDTTT
SVTTENLDRTFKPLIGPRPLVNGLIGRINYYWSVLKPGQTLRIRSNGNLIAPWFGHILSGESHGRILRTD
LSSGNCVVQCQTEKGGLNSTLPFHNISKYAFGTCPKYIGVKSLRLAIGLRNVHARSNRGLFGAIAGFIEG
GWPGLVAGWYGFQHSNDQGVGMAADRVSTQKAVDKITSKVNNIVDKMNKQYEIIDHEFSEVESRLNMINN
KIDDQIQDIWAYNAELLVLLENQKTLDEHDANVNNLYNKVKRALGSNAIEDGKGCFELYHKCDDQCMETI
RNGTYNRRKYTKESRLERQKIEGVKLEAEGTYKILSIYSTVASSLVLAMGFAAFLFWAMSNGSCRCNICI

>QCR64119.1 hemagglutinin [Influenza A virus]
MEVIPXMTMLLLVTTNNADKICIGHQSTNSTETVDTLTETGVPVTHAKELLHTEHNGRLCATNLGKPLIL
DTCTVEGIIYGNPSCDMLLGGREWSYIVERPSAVNGTCYPGNVENLEELRVLFSSSSSYQRIQMFPDTIW
NVTYSGTSKSCSDSFYRNMRWLTQKNGNYPVQDAQYTNTRGKDILFVWGMHHPPTETTQTNLYTRIDTTT
SITTESLDRTFKPLIGPRPLVNGLIGRINYYWSVLKPGQTLRVRSNGNLIAPWFGYVLSGESHGRILKTD
LNSGNCVVQCQTEKGGLNSTLPFHNISKYAFGDCPKYIGVKSLKLAIGLRNVPARSSRGLFGAIAGFIEG
GWPGLVAGWYGFQHSNDQGVGMAADRDSTQKAVDKITSKVNNIVDKMNKQYEIIDHEFSEVENRLNMINN
KIDDQIQDIWAYNAELLVLLENQKTLDEHDANVNNLYNKVKRALGSNAMEDGKGCFELYHKCDDQCMETI
RNGTYNRRKYMEESRLGRQKIEGVKLESEGTYKILTIYSTVASSLVLAMGFAAFLFWAMSNGSCRCNICI

>QCR64118.1 hemagglutinin [Influenza A virus]
MKXISLMTILLVVTISDADKICIGHQSTNSTETVDTLTETNVPVTQAKELLHTEHNGMLCATNLGRPLIL
DTCTIEGLIYGNPSCDMLLGGREWSYIVERPSAVNGTCYPGNVENLEELRTLFSSSSSYQRIQLFPDSIW
NVTYSGTSKSCSDSFYRNMRWLTQKNGLYPVQDAQYINNRGKDILFVWGIHHPPTDTTQTNLYTRTDTTT
SVTTENLDRTFKPLIGPRPLVNGLIGRINYYWSVLKPGQTLRIRSNGNLIAPWFGHILSGESHGRILRTD
LSSGNCVVQCQTEKGGLNSTLPFHNISKYAFGTCPKYIGVKSLRLAIGLRNVHARSNRGLFGAIAGFIEG
GWPGLVAGWYGFQHSNDQGVGMAADRVSTQKAVDKITSKVNNIVDKMNKQYEIIDHEFSEVESRLNMINN
KIDDQIQDIWAYNAELLVLLENQKTLDEHDANVNNLYNKVKRALGSNAIEDGKGCFELYHKCDDQCMETI
RNGTYNRRKYTKESRLERQKIEGVKLEAEGTYKILSIYSTVASSLVLAMGFAAFLFWAMSNGSCRCNICI

>QCR64117.1 hemagglutinin, partial [Influenza A virus]
TILLVVTISDADKICIGHQSTNSTETVDTLTETNVPVTQAKELLHTEHNGMLCATNLGRPLILDTCTIEG
LIYGNPSCDMLLGGREWSYIVERPSAVNGTCYPGNVENLEELRTLFSSSSSYQRIQLFPDSIWNVTYSGT
SKSCSDSFYRNMRWLTQKNGLYPVQDAQYINNRGKDILFVWGIHHPPTDTAQTNLYTRTDTTTSVTTENL
DRTFKPLIGPRPLVNGLIGRINYYWSVLKPGQTLRIRSNGNLIAPWFGHILSGESHGRILRTDLSSGNCV
VQCQTEKGGLNSTLPFHNISKYAFGTCPKYIGVKSLRLAIGLRNVHARSNRGLFGAIAGFIEGGWPGLVA

GWYGFQHSNDQGVGMAADRVSTQKAVDKITSKVNNIVDKMNKQYEIIDHEFSEVESRLNMINNKIDDQIQ
DIWAYNAELLVLLENQKTLDEHDANVNNLYNKVKRALGSNAIEDGKGCFELYHKCDDQCMETIRNGTYNR
RKYTKESRLERQKIEGVKLEAEGTYKILSIYSTVASSLVLAMGFAAFLFWAMSNGSCRCNICI

>QCR64116.1 hemagglutinin, partial [Influenza A virus]
LSDADKMCIGHQSTNSTETVDTLTETNVPVTQAKELLHTEHNGMLCATNLGRPLILDTCTIEGLIYGNPS
CDMLLGGREWSYIVERPSAVNGTCYPGNVENLEELRTLFSSSSSYQRIQLFPDSIWNVTYSGTSKSCSDS
FYRNMRWLTQKNGLYPVQDAQYINNRGKDILFVWGIHHPPTDTTQTNLYTRTDTTTSVTTENLDRTFKPL
IGPRPLVNGLIGRINYYWSVLKPGQTLRIRSNGNLIAPWFGHILSGESHGRILRTDLSSGNCVVQCQTEK
GGLNSTLPFHNISKYAFGTCPKYIGVKSLRLAIGLRNVHARSNRGLFGAIAGFIEGGWPGLVAGWYGFQH
SNDQGVGMAADRVSTQKAVDKITSKVNNIVDKMNKQYEIIDHEFSEVESRLNMINNKIDDQIQDIWAYNA
ELLVLLENQKTLDEHDANVNNLYNKVKRALGSNAIEDGKGCFELYHKCDDQCMETIRNGTYNRRKYTKES
RLERQKIEGVKLEAEGTYKILSIYSTVASSLVLAMGFAAFLFWAMSNGSCRCNICI

>QCR64115.1 hemagglutinin [Influenza A virus]
MKAISLMTILLVVTISDADKICIGHQSTNSTETVDTLTETNVPVTQAKELLHTEHNGMLCATNLGRPLIL
DTCTIEGLIYGNPSCDMLLGGREWSYIVERPSAVNGTCYPGNVENLEELRTLFSSSSSYQRIQLFPDSIW
NVTYSGTSKSCSDSFYRNMRWLTQKNGLYPVQDAQYINNRGKDILFVWGIHHPPTDTTQTNLYTRTDTTT
SVTTENLDRTFKPLIGPRPLVNGLIGRINYYWSVLKPGQTLRIRSNGNLIAPWFGHILSGESHGRILRTD
LSSGNCVVQCQTEKGGLNSTLPFHNISKYAFGTCPKYIGVKSLRLAIGLRNVHARSNRGLFGAIAGFIEG
GWPGLVAGWYGFQHSNDQGVGMAADRVSTQKAVDKITSKVNNIVDKMNKQYEIIDHEFSEVESRLNMINN
KIDDQIQDIWAYNAELLVLLENQKTLDEHDANVNNLYNKVKRALGSNAIEDGKGCFELYHKCDDQCMETI
RNGTYNRRKYTKESRLERQKIEGVKLEAEGTYKILSIYSTVASSLVLAMGFAAFLFWAMSXGSCRCNXXX

>QCR64114.1 hemagglutinin [Influenza A virus]
MKAISLMTILLVVTISDADKICIGHQSTNSTETVDTLTETNVPVTQAKELLHTEHNGMLCATNLGRPLIL
DTCTIEGLIYGNPSCDMLLGGREWSYIVERPSAVNGTCYPGNVENLEELRTLFSSSSSYQRIQLFPDSIW
NVTYSGTSKSCSDSFYRNMRWLTQKNGLYPVQDAQYINNRGKDILFVWGIHHPPTDTTQTNLYTRTDTTT
SVTTENLDRTFKPLIGPRPLVNGLIGRINYYWSVLKPGQTLRIRSNGNLIAPWFGHILSGESHGRILRTD
LSSGNCVVQCQTEKXGLNSTLPFHNISKYAFGTCPKYIGVKSLRLAIGLRNVHARSNRGLFGAIAGFIEG
GWPGLVAGWYGFQHSNDQGVGMAADRVSTQKAVDKITSKVNNIVDKMNKQYEIIDHEFSEVESRLNMINN
KIDDQIQDIWAYNAELLVLLENQKTLDEHDANVNNLYNKVKRALGSNAMEDGKGCFELYHKCDDQCMETI
RNGTYNRRKYTKESRLERQKIEGVKLEAEGTYKILSIYSTVASSLVLAMGFAAFLFWAMSNGSCRCNICI

>QCR64113.1 hemagglutinin [Influenza A virus]

MKAISLMTILLVVTISDADKICIGHQSTNSTETVDTLTETNVPVTQAKELLHTEHNGMLCATNLGRPLIL

DTCTIEGLIYGNPSCDMLLGGREWSYIVERPSAVNGTCYPGNVENLEELRTLFSSSSSYQRIQLFPDSIW

NVTYSGTSKSCSDSFYRNMRWLTQKNGLYPVQDAQYINNRGKDILFVWGIHHPPTDTTQTNLYTRTDTTT

SVTTENLDRTFKPLIGPRPLVNGLIGRINYYWSVLKPGQTLRIRSNGNLIAPWFGHILSGESHGRILRTD

LSSGNCVVQCQTEKGGLNSTLPFHNISKYAFGTCPKYIGVKSLRLAIGLRNVHARSNRGLFGAIAGFIEG

GWPGLVAGWYGFQHSNDQGVGMAADRVSTQKAVDKITSKVNNIVDKMNKQYEIIDHEFSEVESRLNMINN

KIDDQIQDIWAYNAELLVLLENQKTLDEHDANVNNLYNKVKRALGSNAMEDGKGCFELYHKCDDQCMETI

RNGTYNRRKYTKESRLERQKIEGVKLEAEGTYKILSIYSTVASSLVLAMGFAAFLFWAMSNGSCRCNICI

>QCR64112.1 hemagglutinin [Influenza A virus]

MEVITLMTMLLLVTTNNADKICMGHQSTNSTETVDTLTETGVPVTHAKELLHTEHNGRLCATNLGNPLIL

DTCTVEGIIYGNPSCDMLLGRREWSYIVERPSAVNGTCYPGNVENLEELRVLFSSSSSYQRIQMFPDTIW

NVTYSGTSKSCSDSFYKNMRWLTQKNGNYPVQDAQYTNTRGKDILFVWGIHHPPTETVQTDLYTRTDTTT

SITTESLDRTFKPLIGPRPLVNGLIGRINYYWSVLKPGQTLRVRSNGNLIAPWFGHVLSGESHGRILKTD

LNSGNCVVQCQTEKGGLNSTLPFHNISKYAFGDCPKYIGVKSLKLAIGLRNVPARSSRGLFGAIAGFIEG

GWPGLVAGWYGFQHSNDQGVGMAADRDSTQKAVDKITSKVNNIVDKMNKQYEIIDHEFSEVENRLNMINN

KIDDQIQDIWAYNAELLVLLENQKTLDEHDANVNNLYNKVKRALGSNAMEDGKGCFELYHKCDDQCMETI

RNGTYNRRKYMEESRLGRQKIEGVKLESEGTYKILTIYSTVASSLVLAMGFAAFLFWAMSNGSCRCNICI

>QCR64111.1 hemagglutinin [Influenza A virus]

MKAISLMTILLVVTISDADKICIGHQSTNSTETVDTLTETNVPVTQAKELLHTEHNGMLCATNLGRPLIL

DTCTIEGLIYGNPSCDMLLGGREWSYIVERPSAVNGTCYPGNVENLEELRTLFSSSSSYQRIQLFPDSIW

NVTYSGTSKSCSDSFYRNMRWLTQKNGLYPVQDAQYINNRGKDILFVWGIHHPPTDTTQTNLYTRTDTTT

SVTTENLDRTFKPLIGPRPLVNGLIGRINYYWSVLKPGQTLRIRSNGNLIAPWFGHILSGESHGRILRTD

LSSGNCVVQCQTEKGGLNSTLPFHNISKYAFGTCPKYIGVKSLRLAIGLRNVHARSNRGLFGAIAGFIEG

GWPGLVAGWYGFQHSNDQGVGMAADRVSTQKAVDKITSKVNNIVDKMNKQYEIIDHEFSEVESRLNMINN

KIDDQIQDIWAYNAELLVLLENQKTLDEHDANVNNLYNKVKRALGSNAMEDGKGCFELYHKCDDQCMETI

RNGTYNRRKYTKESRLERQKIEGVKLEAEGTYKILSIYSTVASSLVLAMGFAAFLFWAMSNGSCRCNICI

>QCR64110.1 hemagglutinin [Influenza A virus]

MKAISLMTILLVVTTSDADKICIGHQSTNSTETVDTLTETNVPVTQAKELLHTEHNGKLCATNLGRPLIL

DTCTIEGLIYGNPSCDMLLGGREWSYIVERPSAVNGTCYPGNVENLEELRTLFSSSSSYQRIQLFPDSIW

NVTYSGTSKSCSDSFYRNMRWLTQKNGLYPVQDAQYINNRGKDILFVWGIHHPPTDTAQTNLYTRTDTTT

SVTTENLDRTFKPLIGPRPLVNGLIGRINYYWSVLKPGQTLRIRSNGNLIAPWFGHILSGESHGRILRTD
LSSGNCVVQCQTEKGGLNSTLPFHNISKYAFGTCPKYIGVKSLRLAIGLRNVHARSNRGLFGAIAGFIEG
GWPGLVAGWYGFQHSNDQGVGMAADRVSTQKAVDKITSKVNNIVDKMNKQYEIIDHEFSEVESRLNMIND
KIDDQIQDIWAYNAELLVLLENQKTLDEHDANVNNLYNKVKRALGSNAMEDGKGCFELYHKCDDQCMETI
RNGTYNRRKYTEESRLERQKIEGVKLEAEGTYKILSIYSTVASSLVLAMGFAAFLFWAMSNGSCRCNICI

>QCR64109.1 hemagglutinin, partial [Influenza A virus]
SLMTILLVVTTSDADKICIGHQSTNSTETVDTLTETNVPVTQAKELLHTEHNGMLCATNLGRPLILDTCT
IEGLIYGNPSCDMLLGGREWSYIVERPSAVNGTCYPGNVENLEELRTLFSSSSSYQRIQLFPDSIWNVTY
SGTSKSCSDSFYRNMRWLTQKNGLYPVQDAQYINNRGKDILFVWGIHHPPTDTVQTDLYTRTDTTTSVTT
ENLDRSFKPLIGPRPLVNGLIGRINYYWSVLKPGQTLRIRSNGNLIAPWFGHILSGESHGRILRTDLSSG
NCVVQCQTEKGGLNSTLPFHNISKYAFGTCPKYIGVKSLRLAIGLRNVHARSNRGLFGAIAGFIEGGWPG
LVAGWYGFQHSNDQGVGMAADRVSTQKAVDKITSKVNNIVDKMNKQYEVIDHEFSEVESRLNMINNKIDD
QIQDIWAYNAELLVLLENQKTLDEHDANVNNLYNKVKRALGSNAMEDGKGCFELYHKCDDQCMETIRNGT
YNRRKYTEESRLERQKIEGVKLEAEGTYKILSIYSTVASSLVLAMGFAAFLFWAMSNGSCRCNICI

>QCR64108.1 hemagglutinin, partial [Influenza A virus]
LLVVTISDADKICIGHQSTNSTETVDTLTETNVPVTQAKELLHTEHNGMLCATNLGRPLILDTCTIEGLI
YGNPSCDMLLGGREWSYIVERPSAVNGTCYPGNVENLEELRTLFSSSSSYQRIQLFPDSIWNVTYSGTSK
SCSDSFYRNMRWLTQKNGLYPVQDAQYINNRGKDILFVWGIHHPPTDTTQTNLYTRTDTTTSVTTENLDR
TFKPLIGPRPLVNGLIGRINYYWSVLKPGQTLRIRSNGNLIAPWFGHILSGESHGRILRTDLSSGNCVVQ
CQTEKGGLNSTLPFHNISKYAFGICPKYIGVKSLRLAIGLRNVHARSNRGLFGAIAGFIEGGWPGLVAGW
YGFQHSNDQGVGMAADRVSTQKAVDKITSKVNNIVDKMNKQYEIIDHEFSEVESRLNMINNKIDDQIQDI
WAYNAELLVLLENQKTLDEHDANVNNLYNKVKRALGSNAMEDGKGCFELYHKCDDQCMETIRNGTYNRRK
YTKESRLKRQKIEGVKLEAEGTYKILSIYSTVASSLVLAMGFAAFLFWAMSNGSCTCNICI

>QCR64107.1 hemagglutinin [Influenza A virus]
MKAISLMTILLVVTISDADKICIGHQSTNSTETVDTLTETNVPVTQAKELLHTEHNGMLCATNLGRPLIL
DTCTIEGLIYGNPSCDMLLGGREWSYIVERPSAVNGTCYPGNVENLEELRTLFSSSSSYQRIQLFPDSIW
NVTYSGTSKSCSDSFYRNMRWLTQKDGLYPVQDAQYINNRGKDILFVWGIHHPPTDTTQTNLYTRTDTTT
SVTTENLDRTFKPLIGPRPLVNGLIGRINYYWSVLKPGQTLRIRSNGNLIAPWFGHILSGESHGRILRTD
LSSGNCVVQCQTXKGGLNSTLPFHNISKYAFGTCPKYIGVKSLRLAIGLRNVHARSNRGLFGAIAGFIEG
GWPGLVAGWYGFQHSNDQGVGMAADRVSTQKAVDKITSKVNNIVDKMNKQYEIIDHEFSEVESRLNMINN
KIDDQIQDIWAYNAELLVLLENQKTLDEHDANVNNLYNKVKRALGSNAMEDGKGCFELYHKCDDQCMETI

RNGTYNRRKYTKESRLERQKIEGVKLEAEGTYKILSIYSTVASSLVLAMGFAAFLFWAMSNGSCMCNICI

>QCR64106.1 hemagglutinin [Influenza A virus]
MEVIPLMTMLLLVTTNNADKICIGHQSTNSTETVDTLTETGVPVTHAKELLHTEHNGRLCATNLGNPLIL
DTCTVEGIIYGNPSCDMLLGGREWSYIVERPSAVNGTCYPGNVENLEELRVLFSSSSSYQRIQMFPDTIW
NVTYSGTSKSCSDSFYRNMRWLTQKNGNYPVQDAQYTNTRGKDILFVWGIHHPPTETAQTNLYTRTDTTT
SITTESLDRTFKPLIGPRPLVNGLIGRINYYWSVLKPGQTLRVRSNGNLIAPWFGHVLSGESHGRILKTD
LNSGNCVVQCQTEKGGLNSTLPFHNISKYAFGDCPKYIGVKSLKLAIGLRNVPARSSRGLFGAIAGFIEG
GWPGLVAGWYGFQHSNDQGVGMAADRDSTQKAVDKITSKVNNIVDKMNKQYEIIDHEFSEVENRLNMINN
KIDDQIQDIWAYNAELLVLLENQKTLDEHDANVNNLYNKVKRALGSNAMEDGKGCFELYHKCDDQCMETI
RNGTYNRRKYMEESRLGRQKIEGVKLESEGTYKILTIYSTVASSLVLAMGFAAFLFWAMSNGSCRCNICI

>QCR64105.1 hemagglutinin, partial [Influenza A virus]
LMTMLLLVTTNNADKICIGHQSTNSTETVDTLTETGVPVTHAKELLHTEHNGRLCATNLGNPLILDTCTV
EGIIYGNPSCDMLLGGREWSYIVERPSAVNGTCYPGNVENLEELRVLFSSSSSYQRIQMFPDTIWNVTYN
GTSKSCSGSFYRNMRWLTQKNGNYPVQDAQYTNTRGKDILFVWGMHHPPTETAQTNLYTRTDTTTSITTE
SLDRTFKPLIGPRPLVNGLIGRINYYWSVLKPGQTLRVRSNGNLIAPWFGHVLSGESHGRILKTDLNSGN
CVVQCQTEKGGLNSTLPFHNISKYAFGDCPKYIGVKSLKLAIGLRNVPARSSRGLFGAIAGFIEGGWPGL
VAGWYGFQHSNDQGVGMAADRDSTQKAVDKITSKVNNIVDKMNKQYEIIDHEFSEVENRLNMINNKIDDQ
IQDIWAYNAELLVLLENQKTLDEHDANVNNLYNKVKRALGSNAMEDGKGCFELYHKCDDQCMETIRNGTY
NRRKYMEESRLGRQKIEGVKLESEGTYKILTIYSTVASSLVLAMGFAAFLFWAMSNGSCRCNICI

>QCR64104.1 hemagglutinin [Influenza A virus]
MKAISXMTILLVVTISDADKICIGHQSTNSTETVDTLTETNVPVTQAKELLHTEHNGMLCATNLGRPLIL
DTCTIEGLIYGNPSCDMLLGGREWSYIVERPSAVNGTCYPGNVENLEELRTLFSSSSSYQRIQLFPDSIW
NVTYNGTSKSCSDSFYRNMRWLTQKNGLYPVQDAQYINNRGKDILFVWGIHHPPTDTTQTNLYTRTDTTT
SVTTENLDRTFKPLIGPRPLVNGLIGRINYYWSVLKPGQTLRIRSNGNLIAPWFGHILSGESHGRILRTD
LSSGNCVVQCQTGKGGLNSTLPFHNISKYAFGTCPKYIGVKSLRLAIGLRNVHARSNRGLFGAIAGFIEG
GWPGLVAGWYGFQHSNDQGVGMAADRVSTQKAVDKITSKVNNIVDKMNKQYEIIDHEFSEVESRLNMINN
KIDDQIQDIWAYNAELLVLLENQKTLDEHDANVNNLYNKVKRALGSNAMEDGKGCFELYHKCDDQCMETI
RNGTYNRRKYTKESRLERQKIEGVKLEAEGTYKILSIYSTVASSLVLAMGFAAFLFWAMSNGSCRCNICI

>QCR64103.1 hemagglutinin, partial [Influenza A virus]
TILLVVTISDADKICIGHQSTNSTETVDTLTETNVPVTQAKELLHTEHNGMLCATNLGRPLILDTCTIEG

LIYGNPSCDMLLGGREWSYIVERPSAVNGTCYPGNVENLEELRTLFSSSSSYQRIQLFPDSIWNVTYSGT
SKSCSDSFYRNMRWLTQKNGLYPVQDAQYINNRGKDILFVWGIHHPPTDTTQTNLYTRTDTTTSVTTENL
DRTFKPLIGPRPLVNGLIGRINYYWSVLKPGQTLRIRSNGNLIAPWFGHILSGESHGRILRTDLSSGNCV
VQCQTEKGGLNSTLPFHNISKYAFGTCPKYIGVKSLRLAIGLRNVHARSNRGLFGAIAGFIEGGWPGLVA
GWYGFQHSNDQGVGMAADRVSTQKAVDKITSKVNNIVDKMNKQYEIIDHEFSEVESRLNMINNKIDDQIQ
DIWAYNAELLVLLENQKTLDEHDANVNNLYNKVKRALGSNAMEDGKGCFELYHKCDDQCMETIRNGTYNR
RKYTKESRLERQKIEGVKLEAEGTYKILSIYSTVASSLVLAMGFAAFLFWAMSNGSCRCNICI

>QCR64102.1 hemagglutinin [Influenza A virus]
MKAISXMTILLVVTISDADKICIGHQSTNSTETVDTLTETNVPVTQAKELLHTEHNGMLCATNLGRPLIL
DTCTIEGLIYGNPSCDMLLGGREWSYIVERPSAVNGTCYPGNVENLEELRTLFSSSSSYQRIQLFPDSIW
NVTYSGTSKSCSDSFYRNMRWLTQKNGLYPVQDAQYINNRGKDILFVWGIHHPPTDTTQTNLYTRTDTTT
SVTTENLDRTFKPLIGPRPLVNGLIGRINYYWSVLKPGQTLRIRSNGNLIAPWFGHILSGESHGRILRTD
LSSGNCVVQCQTEKGGLNSTLPFHNISKYAFGTCPKYIGVKSLRLAIGLRNVHARSNRGLFGAIAGFIEG
GWPGLVAGWYGFQHSNDQGVGMAADRVSTQKAVDKITSKVNNIVDKMNKQYEIIDHEFSEVESRLNMINN
KIDDQIQDIWAYNAELLVLLENQKTLDEHDANVNNLYNKVKRALGSNAMEDGKGCFELYHKCDDQCMETI
RNGTYNRRKYTKESRLERQKIEGVKLEAEGTYKILSIYSTVASSLVLAMGFAAFLFWAMSNGSCRCNICI

>QCR64101.1 hemagglutinin [Influenza A virus]
MEVIPLMTMLLLVTTNNADKICIGHQSTNSTETVDTLTETGVPVTHAKELLHTEHNGRLCATNLGNPLIL
DTCTVEGIIYGNPSCDMLLGGREWSYIVERPSAVNGTCYPGNVENLEELRVLFSSSSSYQRIQMFPDTIW
NVTYSGTSKSCSDSFYRNMRWLTQKNGNYPVQDAQYTNTRGKDILFVWGIHHPATETAQTNLYTRTDTTT
SITTESLDRTFKPLIGPRPLVNGLIGRINYYWSVLKPGQTLRVRSNGNLIAPWFGHVLSGESHGRILKTD
LNSGNCVVQCQTEKGGLNSTLPFHNISKYAFGDCPKYIGVKSLKLAIGLRNVPARSSRGLFGAIAGFIEG
GWPGLVAGWYGFQHSNDQGVGMAADRDSTQKAVDKITSKVNNIVDKMNKQYEIIDHEFSEVENRLNMINN
KIDDQIQDIWAYNAELLVLLENQKTLDEHDANVNNLYNKVKRALGSNAMEDGKGCFELYHKCDDQCMETI
RNGTYNRRKYMEESRLGRQKIEGVKLESEGTYKILTIYSTVASSLVLAMGFAAFLFWAMSNGSCRCNICI

>QCR64100.1 hemagglutinin [Influenza A virus]
MEVIXLMTMLLLVTTNNADKICIGHQSTNSTETVDTLTETGVPVTHAKELLHTEHNGRLCATNLGNPLIL
DTCTVEGIIYGNPSCDMLLGGRKWSYIVERPSAVNGTCYPGNVENLEELRVLFSSSSSYQRIQMFPDTIW
NVTYSGTSKSCSDSFYRNMRWLTQKNGNYPVQDAQYTNTRGKDILFVWGIHHPPTETAQTNLYTRTDTTT
SITTESLDRTFKPLIGPRPLVNGLIGRINYYWSVLKPGQTLRVRSNGNLIAPWFGHVLSGESHGRILKTD
LNSGNCVVQCQTEKGGLNSTLPFHNISKYAFGDCPKYIGVKSLKLAIGLRNVPARSSRGLFGAIAGFIEG

GWPGLVAGWYGFQHSNDQGVGMAADRDSTQKAVDKITSKVNNIVDKMNKQYEIIDHEFSEVENRLNMINN
KIDDQIQDIWAYNAELLVLLENQKTLDEHDANVNNLYNKVKRALGSNAMEDGKGCFELYHKCDNQCMETI
RNGTYNRRKYMEESRLGRQKIEGVKLESEGTYKILTIYSTVASSLVLAMGFAAFLFWAMSNGSCRCNICI

>QCR64099.1 hemagglutinin [Influenza A virus]
MKAISLMTILLVVTTSDADKICIGHQSTNSTETVDTLTETNVPVTQAKELLHTEHNGMLCATNLGRPLIL
DTCTIEGLIYGNPSCDMLLGGREWSYIVERPSAVNGTCYPGNVENLEELRTLFSSSSSYQRIQLFPDSIW
NVTYSGTSKSCSDSFYRNMRWLTQKNGLYPVQDAQYINNRGKDILFVWGIHHPPTDTVQTNLYTRTDTTT
SVTTENLDRSFKPLIGPRPLVNGLIGRINYYWSVLKPGQTLRIRSNGNLIAPWFGHILSGESHGRILRTD
LSSGNCVVQCQTEKGGLNSTLPFHNISKYAFGTCPKYIGVKSLRLAIGLRNVHARSNRGLFGAIAGFIEG
GWPGLVAGWYGFQHSNDQGVGMAADRVSTQKAVDKITSKVNNIVDKMNKQYEIIDHEFSEVESRLNMINN
KIDDQIQDIWAYNAELLVLLENQKTLDEHDANVNNLYNKVKRALGSNAMEDGKGCFELYHKCDDQCMETI
RNGTYNRRKYTEESRLERQKIEGVKLEAEGTYKILSIYSTVASSLVLAMGFAAFLFWAMSNGSCRCNICX

>QCR64098.1 hemagglutinin [Influenza A virus]
MEVIPXMTMLLLVTTNNADKICIGHQSTNSTETVDTLTETGVPVTHAKELLHTEHNGRLCATNLGNPLIL
DTCTVEGIIYGNPSCDMLLGGREWSYIVERPSAVNGTCYPGNVENLEELRVLFSSSSSYQRIQMFPDTIW
NVTYSGTSKSCSDSFYRNMRWLTQKNGNYPVQDAQYTNTRGKDILFVWGIHHPPTETAQTNLYTRTDTTT
SITTESLDRTFKPLIGPRPLVNGLIGRINYYWSVLKPGQTLRVRSNGNLIAPWFGHVLSGESHGRILKTD
LNSGNCVVQCQTEKGGLNSTLPFHNISKYAFGNCPKYIGVKSLKLAIGLRNVPARSSRGLFGAIAGFIEG
GWPGLVAGWYGFQHSNDQGVGMAADRDSTQKAVDKITSKVNNIVDKMNKQYEIIDHEFSEVENRLNMINN
KIDDQIQDIWAYNAELLVLLENQKTLDEHDANVNNLYNKVKRALGSNAMEDGKGCFELYHKCDDQCMETI
RNGTYNRRKYMEESRLGRQKIEGVKLESEGTYKILTIYSTVASSLVLAMGFAAFLFWAMSNGSCRCNICI

>QCR64097.1 hemagglutinin [Influenza A virus]
MKAISLMTILLVVTTSDADKICIGHQSTNSTETVDTLTETNVPVTQAKELLHTEHNGMLCATNLGRPLIL
DTCTIEGLIYGNPSCDMLLGGREWSYIVERPSAVNGTCYPGNVENLEELRTLFSSSSSYQRIQLFPDSIW
NVTYSGTSKSCSDSFYRNMRWLTQKNGLYPVQDAQYINNRGKDILFVWGIHHPPTDTVQTNLYTRTDTTT
SVTTENLDRSFKPLIGPRPLVNGLIGRINYYWSVLKPGQTLRIRSNGNLIAPWFGHILSGESHGRILRTD
LSSGNCVVQCQTEKGGLNSTXPFHNISKYAFGTCPKYIGVKSLRLAIGLRNVHARSNRGLFGAIAGFIEG
GWPGLVAGWYGFQHSNDQGVGMAADRVSTQKAVDKITSKVNNIVDKMNKQYEIIDHEFSEVESRLNMINN
KIDDQIQDIWAYNAELLVLLENQKTLDEHDANVNNLYNKVKRALGSNAMEDGKGCFELYHKCDDQCMETI
RNGTYNRRKYTEESRLERQKIEGVKLEAEGTYKILSIYSTVASSLVLAMGFAAFLFWAMSNGSCRCNICI

>QCR64096.1 hemagglutinin, partial [Influenza A virus]

MTMLLXVTTNNADKICIGHQSTNSTETVDTLTETGVPVTHAKELLHTEHNGRLCATNLGNPLILDTCTVE
GIIYGNPSCDMLLGGREWSYIVERPSAVNGTCYPGNVENLEELRVLFSSSSSYQRIQMFPDTIWNVTYSG
TSKSCSDSFYRNMRWLTQKNGNYPVQDAQYTNTRGKDILFVWGIHHPPTETAQTNLYTRTDTTTSITTES
LDRTFKPLIGPRPLVNGLIGRINYYWSVLKPGQTLRVRSNGNLIAPWFGHVLSGESHGRILKTDLNSGNC
VVQCQTEKGGLNSTLPFHNISKYAFGDCPKYIGVKSLKLAIGLRNVPARSSRGLFGAIAGFIEGGWPGLV
AGWYGFQHSNDQGVGMAADRDSTQKAVDKITSKVNNIVDKMNKQYEIIDHEFSEVENRLNMINNKIDDQI
QDIWAYNAELLVLLENQKTLDEHDANVNNLYNKVKRALGSNAMEDGKGCFELYHKCDDQCMETIRNGTYN
RRKYMEESRLGRQKIEGVKLESEGTYKILTIYSTVASSLVLAMGFAAFLFWAMSNGSCRCNICI

>QCR64095.1 hemagglutinin [Influenza A virus]

MEAILLMTILLVVTTSNADKICIGHQSTNSTETVDTLTETNVPVTQAKELLHTEHNGMLCATNLGRPLIL
DTCTIEGLIYGNPSCDMLLGGREWSYIVERPSAVNGTCYPGNVENLEELRTLFSSSSSYQRIQLFPDSIW
NVTYSGTSKSCSDSFYRNMRWLTQKNGLYPVQDAQYINNRGKDILFVWGIHHPPTDTAQTNLYTRTDTTT
SVTTENLDRTFKPLIGPRPLVNGLIGRINYYWSVLKPGQTLRIRSNGNLIAPWFGHILSGESHGRILRTD
LSSGNCVVQCQTEKGGLNSTLPFHNISKYAFGTCPKYIGVKSLRLAIGLRNVHARSNRGLFGAIAGFIEG
GWPGLVAGWYGFQHSNDQGVGMAADRVSTQKAVDKITSKVNNIVDKMNKQYEIIDHEFSEVESRLNMINN
KIDDQIQDIWAYNAELLVLLENQKTLDEHDANVNNLYNKVKRALGSNAMEDGKGCFELYHKCDDQCMETI
RNGTYNRRKYTEESRLERQKIEGVKLEAEGTYKILSIYSTVASSLVLAMGFAAFLFWAMSXGSCRCNXXX

>QCR64094.1 hemagglutinin [Influenza A virus]

MEVIPLMTMLLLVTTNNADKICIGHQSTNSTETVDTLTETGVPVTHAKELLHTEHNGRLCATNLGNPLIL
DTCTVEGIIYGNPSCDMLLGGREWSYIVERPSAVNGTCYPGNVENLEELRVLFSSSSSYQRIQMFPDAIW
NVTYSGTSKSCSDSFYRNMRWLTQKNGNYPVQDAQYTNTRGKDILFVWGIHHPPTETAQTNLYTRTDTTT
SITTESLDRTFKPLIGPRPLVNGLIGRINYYWSVLKPGQTLRVRSNGNLIAPWFGHVLSGESHGRILKTD
LNSGNCVVQCQTEKGGLNSTLPFHNISKYAFGDCPKYIGVKSLKLAIGLRNVPARSSRGLFGAIAGFIEG
GWPGLVAGWYGFQHSNDQGVGMAADRDSTQKAVDKITSKVNNIVDKMNKQYEIIDHEFSEVENRLNMINN
KIDDQIQDIWAYNAELLVLLENQKTLDEHDANVNNLYNKVKRALGSNAMEDGKGCFELYHKCDDQCMETI
RNGTYNRRKYMEESRLGRQKIEGVKLESEGTYKILTIYSTVASSLVLAMGFAAFLFWAMSNGSCRCNIXX

>QCR64093.1 hemagglutinin, partial [Influenza A virus]

TMLLLVTTNNADKICIGHQSTNSTETVDTLTETGVPVTHAKELLHTEHNGRLCATNLGNPLILDTCTVEG
IIYGNPSCDMLLGGREWSYIVERPSAVNGTCYPGNVENLEELRVLFSSSSSYQRIQMFPDTIWNVTYSGT
SKSCSDSFYRNMRWLTQKNGNYPVQDAQYTNTRGKDILFVWGIHHPPTETAQTNLYTRTDTTTSITTESL

DRTFKPLIGPRPLVNGLIGRINYYWSVLKPGQTLRVRSNGNLIAPWFGHVLSGESHGRILKTDLNSGNCV
VQCQTEKGGLNSTLPFHNISKYAFGDCPKYIGVKSLKLAIGLRNVPARSSRGLFGAIAGFIEGGWPGLVA
GWYGFQHSNDQGVGMAADRDSTQKAVDKITSKVNNIVDKMNKQYEIIDHEFSEVENRLNMINNKIDDQIQ
DIWAYNAELLVLLENQKTLDEHDANVNNLYNKVKRALGSNAMEDGKGCFELYHKCDDQCMETIRNGTYNR
RKYMEESRLGRQKIEGVKLESEGTYKILTIYSTVASSLVLAMGFAAFLFWAMSNGSCRCNICI

>QCR64092.1 hemagglutinin, partial [Influenza A virus]
MTILLVVTTSDADKICIGHQSTNSTETVDTLTETNVPVTQAKELLHTEHNGMLCATNLGRPLILDTCTIE
GLIYGNPSCDMLLGGRKWSYIVERPSAVNGTCYPGNVENLEELRTLFSSSSSYQRIQLFPDSIWNVTYSG
TSKSCSDSFYRNMRWLTQKNGLYPVQDAQYINNRGKDILFVWGIHHPPTDTAQTNLYTRTDTTTSVTTEN
LDRSFKPLIGPRPLVNGLIGRINYYWSVLKPGQTLRIRSNGNLIAPWFGHILSGESHGRILRTDLSSGNC
VVQCQTEKGGLNSTLPFHNISKYAFGTCPKYIGVKSLRLAIGLRNVHARSNRGLFGAIAGFIEGGWPGLV
AGWYGFQHSNDQGVGMAADRVSTQKAVDKITSKVNNIVDKVNKQYEIIDHEFSEVESRLNMINNKIDDQI
QDIWAYNAELLVLLENQKTLDEHDANVNNLYNKVKRALGSNAMEDGKGCFELYHKCDDQCMETIRNGTYN
RRKYTEESRLERQKIEGVKLEAEGTYKILSIYSTVASSLVLAMGFAAFLFWAMSNGSCRCNICI

>QCR64091.1 hemagglutinin [Influenza A virus]
MXVIPLMTMLLLVTTNNADKICIGHQSTNSTETVDTLTETGVPVTHAKELLHTEHNGRLCATNLGNPLIL
DTCTVEGIIYGNPSCDMLLGGREWSYIVERPSAVNGTCYPGNVENLEELRVLFSSSSSYQRIQMFPDTIW
NVTYSGTSKSCSDSFYRNMRWLTQKNGNYPVQDAQYTNTRGKDILFVWGIHHPPTETAQTNLYTRTDTTT
SITTESLDRTFKPLIGPRPLVNGLIGRINYYWSVLKPGQTLRVRSNGNLIAPWFGHVLSGESHGRILKTD
LNSGNCVVQCQTEKGGLNSTLPFHNISKYAFGDCPKYIGVKSLKLAIGLRNVPARSSRGLFGAIAGFIEG
GWPGLVAGWYGFQHSNDQGVGMAADRDSTQKAVDKITSKVNNIVDKMNKQYEIIDHEFSEVENRLNMINN
KIDDQIQDIWAYNAELLVLLENQKTLDEHDANVNNLYNKVKRALGSNAMEDGKGCFELYHKCDDQCMETI
RNGTYNRRKYMEESRLGRQKIEGVKLESEGTYKILTIYSTVASSLVLAMGFAAFLFWAMSNGSCRCNICX

>QCR64090.1 hemagglutinin, partial [Influenza A virus]
VTTSDADKICIGHQSTNSTETVDTLTETNVPVTQAKELLHTEHNGMLCATNLGCPLILDTCTIEGLIYGN
PSCDMLLGGREWSYIVERPSAVNGTCYPGNVENLEELRTLFSSSSSYQRIQLFPDSIWNVTYSGTSKSCS
DSFYRNMRWLTQKNGLYPVQDAQYINNRGKDILFVWGIHHPPTDTAQTNLYTRTDTTTSVTTENLDRTFK
PLIGPRPLVNGLIGRINYYWSVLKPGQTLRIRSNGNLIAPWFGHILSGESHGRILRTDLSSGNCVVQCQT
EKGGLNSTLPFHNISKYAFGTCPKYIGVKSLRLAIGLRNVHARSNRGLFGAIAGFIEGGWPGLVAGWYGF
QHSNDQGVGMAADRVSTQKAVDKITSKVNNIVDKMNKQYEIIDHEFSEVESRLNMINNKIDDQIQDIWAY
NAELLVLLENQKTLDEHDANVNNLYNKVKRALGSNAMEDGKGCFELYHKCDDQCMETIRNGTYNRRKYTE

ESRLERQKIEGVKLEAEGTYKILSIYSTVASSLVLAMGFAAFLFWAMSNGSCRCNICI

>QCR64089.1 hemagglutinin [Influenza A virus]

MEVIPLMTMLLLVTTNNADKICIGHQSTNSTETVDTLTETGVPVTHAKELLHTEHNGRLCATNLGNPLIL
DTCTVEGIIYGNPSCDMLLGGREWSYIVERPSAVNGTCYPGNVENLEELRVLFSSSSSYQRIQMFPDTIW
NVTYSGTSKSCSDSFYRNMRWLTQKNGNYPVQDAQYTNTRGKDILFVWGIHHPPTETAQTNLYTRTDTTT
SITTESLDRTFKPLIGPRPLVNGLIGRINYYWSVLKPGQTLRVRSNGNLIAPWFGHVLSGESHGRILKTD
LNSGNCVVQCQTEKGGLNSTLPFHNISKYAFGDCPKYIGVKSLKLAIGLRNVPARSSRGLFGAIAGFIEG
GWPGLVAGWYGFQHSNDQGVGMAADRDSTQKAVDKITSKVNNIVDKMNKQYEIIDHEFSEVENRLNMINN
KIDDQIQDIWAYNAELLVLLENQKTLDEHDANVNNLYNKVKRALGSNAMEDGKGCFELYHKCDDQCMETI
RNGTYNRRKYMEESRLGRQKIEGVKLESEGTYKILTIYSTVASSLVLAMGFAAFLFWAMSNGSCRCNXXX

>QCR64088.1 hemagglutinin [Influenza A virus]

MEVXXLMXXXXXVTTNNADKICIGHQSTNXTETVDTLTETGVPVTHAKELLHTEHNGRLCATNLGNPLIL
DTCTVEGIIYGNPSCDMLLGGREWSYIVERPSAVNGTCYPGNVENLEELRVLFSSSSSYQRIQMFPDTIW
NVTYSGTSKSCSDSFYRNMRWLTQKNGNYPVQDAQYTNTRGKDILFVWGIHHPPTETAQTNLYTRTDTTT
SITTESLDRTFKPLIGPRPLVNGLIGRINYYWSVLKPGQTLRVRSNGNLIAPWFGHVLSGESHGRILKTD
LKSGNCVVQCQTEKGGLNSTLPFHNISKYAFGDCPKYIGVKSLKLAIGLRNVPARSSRGLFGAIAGFIEG
GWPGLVAGWYGFQHSNDQGVGMAADRDSTQKAVDKITSKVNNIVDKMNKQYEIIDHEFSEVENRLNMINN
KIDDQIQDIWAYNAELLVLLENQKTLDEHDANVNNLYNKVKRALGSNAMEDGKGCFELYHKCDDQCMETI
RNGTYNRRKYMEESRLGRQKIEGVKLESEGTYKILTIYSTVASSLVLAMGFAAFLFWAMSNGSCRCNIXX

>QCR64087.1 hemagglutinin, partial [Influenza A virus]

MKAISLMTILLVVTTSDADKICIGHQSTNSTETVDTLTETNVPVTQAKELLHTEHNGMLCATNLGRPLIL
DTCTIEGLIYGNPSCDMLLGGREWSYIVERPSAVNGTCYPGNVENLEELRTLFSSSSSYQRIQLFPDSIW
NVTYSGTSKSCSDSFYRNMRWLTQKNGLYPVQDAQYINNRGKDILFVWGIHHPPTDTAQTNLYTRTDTTT
SVTTENLDRSFKPLIGPRPLVNGLIGRINYYWSVLKPGQTLRIRSNGNLIAPWFGHILSGESHGRILRTD
LSSGNCVVQCQTEKGGLNSTLPFHNISKYAFGTCPKYIGVKSLRLAIGLRNVHARSNRGLFGAIAGFIEG
GWPGLVAGWYGFQHSNDQGVGMAADRVSTQKAVDKITSKVNNIVDKVNKQYEIIDHEFSEVESRLNMINN
KIDDQIQDIWAYNAELLVLLENQKTLDEHDANVNNLYNKVKRALGSNAMEDGKGCFELYHKCDDQCMETI
RNGTYNRRKYTEESRLERQKIEGVKLEAEGTYKILSIYSTVASSLVLAMGFAAFLFWAMS

>QCR64086.1 hemagglutinin, partial [Influenza A virus]

LVVTTSDADKICIGHQSTNSTETVDTLTETNVPVTQAKELLHTEHNGMLCATNLGRPLILDTCTIEGLIY

GNPSCDMLLGGREWSYIVERPSAVNGTCYPGNVENLEELRTLFSSSSSYQRIQLFPDSIWNVTYSGTSKS
CSDSFYRNMRWLTQKNGLYPVQDAQYINNRGKDILFVWGIHHPPTDTAQTNLYTRTDTTTSVTTENLDRS
FKPLIGPRPLVNGLIGRINYYWSVLKPGQTLRIRSNGNLIAPWFGHILSGESHGRILRTDLSSGNCVVQC
QTEKGGLNSTLPFHNISKYAFGTCPKYIGVKSLRLAIGLRNVHARSNRGLFGAIAGFIEGGWPGLVAGWY
GFQHSNDQGVGMAADRVSTQKAVDKITSKVNNIVDKMNKQYEIIDHEFSEVESRLNMINNKIDDQIQDIW
AYNAELLVLLENQKTLDEHDANVNNLYNKVKRALGSNAMEDGKGCFELYHKCDDQCMETIRNGTYNRRKY
TEESRLERQKIEGVKLEAEGTYKILSIYSTVASSLVLAMGFAAFLFWAMSNGSCRCNIXI

>QCR64085.1 hemagglutinin, partial [Influenza A virus]
VTTSDADKXCXGHQSXNXTEXXDTLTETNVPVTQXKELLHTEHNGMLCATNXGRPLILDTCTIEGLIYGN
PSCDMLLGGREWSYIVERPSAVNGTCYPGNVENLEELRTLFSSSSSYQRIQLFPDSIWNVTYSGTSKSCS
DSFYRNMRWLTQKNGLYPVQDAQYINNRGKDILFVWGIHHPPTDTAQTNLYTRTDTTTSVTTENLDRTFK
PLIGPRPLVNGLIGRINYYWSVLKPGQTLRIRSNGNLIAPWFGHILSGESHGRILRTDLSSGNCVVQCQT
EKGGLNSTLPFHNISKYAFGTCPKYIGVKSLRLAIGLRNVHARSNRGLFGAIAGFIEGGWPGLVAGWYGF
QHSNDQGVGMAADRVSTQKAVDKITSKVNNIVDKMNKQYEIIDHEFSEVESRLNMINNKIDDQIQDIWAY
NAELLVLLENQKTLDEHDANVNNLYNKVKRALGSNAMEDGKGCFELYHKCDDQCMETIRNGTYNRRKYTE
ESRLERQKIEGVKLEAEGTYKILSIYSTVASSLVLAMGFAAFLFWAMSNGSCRCNICI

>QCR64084.1 hemagglutinin, partial [Influenza A virus]
SLMTILLVVTTSDADKICIGHQSTNSTETVDTLTETNVPVTRAKELLHTEHNGMLCATNLGRPLILDTCT
IEGLIYGNPSCDMLLGGREWSYIVERPSAVNGTCYPGNVENLEELRTLFSSSSSYQRIQLFPDSIWNVTY
SGTSKSCSDSFYRNMRWLTQKNGLYPVQDAQYINNRGKDILFVWGIHHPPTDTAQTNLYTRTDTTTSVTT
ENLDRTFKPLIGPRPLVNGLIGRINYYWSVLKPGQTLRIRSNGNLIAPWFGHILSGESHGRILRTDLSSG
NCVVQCQTEKGGLNSTLPFHNISKYAFGTCPKYIGVKSLRLAIGLRNVHARSNRGLFGAIAGFIEGGWPG
LVAGWYGFQHSNDQGVGMAADRVSTQKAVDKITSKVNNIVDKMNKQYEIIDHEFSEVESRLNMINNKIDD
QIQDIWAYNAELLVLLENQKTLDEHDANVNNLYNKVKRALGSNAMEDGKGCFELYHKCDDQCMETIRNGT
YNRRKYTEESRLERQKIEGVKLEAEGTYKILSIYSTVASSLVLAMGFAAFLFWAMSNGSCRCNICI

>QCR64083.1 hemagglutinin, partial [Influenza A virus]
LLVTTNNADKICIGHQSTNSTETVDTLTETGVPVTHAKELLHTEHNGRLCATNLGNPLILDTCTVEGIIY
GNPSCDMLLGGREWSYIVERPSAVNGTCYPGNVENLEELRVLFSSSSSYQRIQMFPDTIWNVTYSGTSKS
CSDSFYRNMRWLTQKNGNYPVQDAQYTNTRGKDILFVWGIHHPPTETTQTNLYTRTDTTTSITTESLDRT
FKPLIGPRPLVNGLIGRINYYWSVLKPGQTLRVRSNGNLIAPWFGHVLSGESHGRILKTDLNSGNCVVQC
QTEKGGLNSTLPFHNISKYAFGDCPKYIGVKSLKLAIGLRNVPARSSRGLFGAIAGFIEGGWPGLVAGWY

GFQHSNDQGVGMAADRDSTQKAVDKITSKVNNIVDKMNKQYEIIDHEFSEVENRLNMINNKIDDQIQDIW
AYNAELLVLLENQKTLDEHDANVNNLYNKVKRALGSNAMEDGKGCFELYHKCDDQCMETIRNGTYNRRKY
MEESRLGRQKIEGVKLESEGTYKILTIYSTVASSLVLAMGFAAFLFWAMSNGSCRCNICI

>QCR64081.1 hemagglutinin [Influenza A virus]
MKAISLMTILLVVTTSDADKICIGHQSTNSTETVDTLTETNVPVTQAKELLHTEHNGMLCATNLGRPLIL
DTCTIEGLIYGNPSCDMLLGGREWSYIVERPSAVNGTCYPGNVENLEELRTLFSSSSSYQRIQLFPDSIW
NVTYSGTSKSCSDSFYRNMRWLTQKNGLYPVQDVQYINNRGKDILFVWGIHHPPTDTAQTNLYTRTDTTT
SVTTENLDRTFKPLIGPRPLVNGQIGRIDYYWSVLKPGQTLRIRSNGNLIAPWFGHILSGESHGRILRTD
LSSGNCVVQCQTEKGGLNSTLPFHNISKYAFGTCPKYIGVKSLRLAIGLRNVHARSNRGLFGAIAGFIEG
GWPGLVAGWYGFQHSNDQGVGMAADRVSTQKAVDKITSKVNNIVDKMNKQYEIIDHEFSEVESRLNMINN
KIDDQIQDIWAYNAELLVLLENQKTLDEHDANVNNLYNKVKRALGSNAMEDGKGCFELYHKCDDQCMETI
RNGTYNRRKYTEESRLERQKIEGVKLEAEGTYKILSIYSTVASSLVLAMGFAAFLFWAMSNGSCRCNICI

>QCR64080.1 hemagglutinin [Influenza A virus]
MEVIPLMTMLLLVTTNNADKICIGHQSTNSTETVDTLTETGVPVTHAKELLHTEHNGRLCATNLGNPLIL
DTCTVEGIIYGNPSCDMLLGGREWSYIVERPSAVNGTCYPGNVENLEELRVLFSSSSSYQRIQMFPDTIW
NVTYSGTSKSCSDSFYRNMRWLTQKNGNYPVQDAQYTNTRGKDILFVWGIHHPPTETAQTNLYTRTDTTT
SITTESLDRTFKPLIGPRPLVNGLIGRINYYWSVLKPGQTLRVRSNGNLIAPWFGHVLSGESHGRILKTD
LNSGNCVVQCQTEKGGLNSTLPFHNISKYAFGDCPKYIGVKSLKLAIGLRNVPARSSRGLFGAIAGFIEG
GWPGLVAGWYGFQHSNDQGVGMAADRDSTQKAVDKITSKVNNIVDKMNKQYEIIDHEFSEVENRLNMINN
KIDDQIQDIWAYNAELLVLLENQKTLDEHDANVNNLYNKVKRALGSNAMEDGKGCFELYHKCDDQCMETI
RNGTYNRRKYMEESRLGRQKIEGVKLESEGTYKILTIYSTVASSLVLAMGFAAFLFWAMSNGSCRCNICI

>QCR64079.1 hemagglutinin [Influenza A virus]
MKQXFXMTXXMVVTTXDADKICXGHQSTNSTETVDTLTETNVPVTQAKELLHTEHNGMLCATNLGRPLIL
DTCTIEGLIYGNPSCDMLLGGREWSYIVERPSAVNGTCYPGNVENLEELRTLFSSSSSYQRIQLFPDSIW
NVTYSGTSKSCSDSFYRNMRWLTQKNGLYPVQDAQYINNRGKDILFVWGIHHPPTDTAQTNLYTRTDTTT
SVTTENLDRTFKPLIGPRPLVNGLIGRINYYWSVLKPGQTLRIRSNGNLIAPWFGHILSGESHGRILRTD
LSSGNCVVQCQTEKGGLNSTLPFHNISKYAFGTCPKYIGVKSLRLAIGLRNVHARSNRGLFGAIAGFIEG
GWPGLVAGWYGFQHSNDQGVGMAADRVSTQKAVDKITSKVNNIVDKMNKQYEIIDHEFSEVESRLNMINN
KIDDQIQDIWAYNAELLVLLENQKTLDEHDANVNNLYNKVKRALGSNAMEDGKGCFELYHKCDDQCMETI
RNGTYNRRKYTEESRLERQKIEGVKLEAEGTYKILSIYSTVASSLVLAMGFAAFLFWAMSNGSCRCNICI

>QCR64078.1 hemagglutinin [Influenza A virus]

MEAISLITILLVVTISNADKICIGHQSTNSTETVDTLTEANVPVTQAKELLHTEHNGMLCATNLGRPLIL
DTCTIEGLIYGNPSCDMLLGEREWSYIVERPSAVNGTCYPGNVENLEELRTLFSSSSSSQRIQLFPDSIW
NVTYTGTSKSCSDSFYRNMRWLTQKNGAYPVQDAQYLNNQGKDILFVWGIHHPPSDTTQTDLYTRTDTTT
SITTENLDRTFKPLIGPRPLVNGLIGRINYYWSVLKPGQMLRVRSNGNLIAPWFGHVLSGESHGRILRTD
LNSGNCVVQCQTEKGGLNSTLPFHNISKYAFGTCPKYIGVKSLKLAIGLRNVHTRSSRGLFGAIAGFIEG
GWPGLVAGWYGFQHSNDQGVGMAADRVSTQKAVDKITSKVNNIVDKMNKQYEIIDHEFSEVESRLNMINN
KIDDQVQDIWAYNAELLVLLENQKTLDEHDANVNNLYNKVRRALGSNAMEDGKGCFELYHKCDDQCMETI
RNGTYNRRKYTKESRLERQKIEGVKLETEGTYKILSIYSTVASSLMLAIGFAAFLFWAMSNGSCRCNVCI

>QCR64077.1 hemagglutinin [Influenza A virus]

MKAISLMTILLVVTTSDADKICIGHQSTNSTETVDTLTETNVPVTQAKELLHTEHNGMLCATNLGRPLIL
DTCTIEGLIYGNPSCDMLLGGREWSYIVERPSAVNGTCYPGNVENLEELRTLFSSSSSYQRIQLFPDSIW
NVTYSGTSKSCSDSFYRNMRWLTQKNGLYPVQDAQYINNRGKDILFVWGIHHPPTDTAQTNLYTRTDTTT
SVTTENLDRTFKPLIGPRPLVNGLIGRINYYWSVLKPGQTLRIRSNGNLIAPWFGHILSGESHGRILRTD
LSSGNCVVQCQTEKGGLNSTLPFHNISKYAFGTCPKYIGVKSLRLAIGVRNVHARSNRGLFGAIAGFIEG
GWPGLVAGWYGFQHSNDQGVGMAADRVSTQKAVDKITSKVNNIVDKMNKQYEIIDHEFSEVESRLNMINN
KIDDQIQDIWAYNAELLVLLENQKTLDEHDANVNNLYNKVKRALGSNAMEDGKGCFELYHKCDDQCMETI
RNGTYNRRKYTEESRLERQKIEGVKLEAEGTYKILSIYSTVASSLVLAMGFAAFLFWAMSNGSCRCNICI

>QCR64075.1 hemagglutinin, partial [Influenza A virus]

VVTTSDADKICIGHQSTNSTETVDTLTETNVPVTQAKELLHTEHNGMLCATNLGRPLILDTCTIEGLIYG
NPSCDMLLGGREWSYIVERPSAVNGTCYPGNVENLEELRTLFSSSSSYQRIQLFPDSIWNVTYSGTSKSC
SDSFYRNMRWLTQKNGLYPVQDAQYINNRGKDILFVWGIHHPPTDTAQTNLYTRTDTTTSVTTENLDRTF
KPLIGPRPLVNGLIGRINYYWSVLKPGQTLRIRSNGNLIAPWFGHILSGESHGRILRTDLSSGNCVVQCQ
TEKGGLNSTLPFHNISKYAFGTCPKYIGVKSLRLAIGLRNVHARSNRGLFGAIAGFIEGGWPGLVAGWYG
FQHSNDQGVGMAADRVSTQKAVDKITSKVNNIVDKMNKQYEIIDHEFSEVESRLNMINNKIDDQIQDIWA
YNAELLVLLENQKTLDEHDANVNNLYNKVKRALGSNAMEDGKGCFELYHKCDDQCMETIRNGTYNRRKYT
EESRLERQKIEGVKLEAEGTYKILSIYSTVASSLVLAMGFAAFLFWAMSNGSCRCNICI

>QCR64074.1 hemagglutinin [Influenza A virus]

MKAISLMTILLVVTTSDADKICIGHQSTNSTETVDTLTETNVPVTQAKELLHTEHNGMLCATNLGRPLIL
DTCTIEGLIYGNPSCDMLLGGREWSYIVERPSAVNGTCYPGNVENLEELRTLFSSSSSYQRIQLFPDSIW
NVTYSGTSKSCSDSFYRNMRWLTQKNGLYPVQDAQYINNRGKDILFVWGIHHPPTDTTQTNLYTRTDTTT

SVTTENLDRTFKPLIGPRPLVNGLIGRINYYWSVLKPGQTLRIRSNGNLIAPWFGHILSGESHGRILRTD
LSSGNCVVQCQTEKGGLNSTLPFHNISKYAFGTCPKYIGVKSLRLAIGLRNVHARSNRGLFGAIAGFIEG
GWPGLVAGWYGFQHSNDQGVGMAADRVSTQKAVDKITSKVNNIVDKMNKQYEIIDHEFSEVESRLNMINN
KIDDQIQDIWAYNAELLVLLENQKTLDEHDANVNNLYNKVKRALGSNAMEDGKGCFELYHKCDDQCMETI
RNGTYNRRKYTEESRLERQKIEGVKLEAEGTYKILSIYSTVASSLVLAMGFAAFLFWAMSNGSCRCNICI

>QCR64073.1 hemagglutinin [Influenza A virus]
MKAISLXXIILVVTTSDADKICIGHQSTNSTETVDTLTETNVPVTQAKELLHTEHNGMLCATNLGRPLIL
DTCTIEGLIYGNPSCDMLLGGREWSYIVERPSAVNGTCYPGNVENLEELRTLFSSSSSYQRIQLFPDSIW
NVTYSGTSKSCSDSFYRNMRWLTQKNGLYPVQDAQYINNRGKDILFVWGIHHPPTDTVQTNLYTRTDTTT
SVTTENLDRTFKPLIGPRPLVNGLIGRINYYWSVLKPGQTLRIRSNGNLIAPWFGHILSGESHGRILRTD
LSSGNCVVQCQTEKGGLNSTLPFHNISKYAFGTCPKYIGVKSLRLAIGLRNVHARSNRGLFGAIAGFIEG
GWPGLVAGWYGFQHSNDQGVGMAADRVSTQKAVDKITSKVNNIVDKMNKQYEIIDHEFSEVESRLNMINN
KIDDQIQDIWAYNAELLVLLENQKTLDEHDANVNNLYNKVKRALGSNAMEDGKGCFELYHKCDDQCMETI
RNGTYNRRKYTEESRLERQKIEGVKLEAEGTYKILSIYSTVASSLVLAMGFAAFLFWAMSNGSCRCNXXX

>QCR64072.1 hemagglutinin [Influenza A virus]
MKAISLMTILLVVTTSDADKICIGHQSTNSTETVDTLTETNVPVTQAKELLHTEHNGMLCATNLGRPLIL
DTCTIEGLIYGNPSCDMLLGGREWSYIVERPSAVNGTCYPGNVENLEELRTLFSSSSSYQRIQLFPDSIW
NVTYSGTSKSCSDSFYRNMRWLTQKNGLYPVQDAQYINNRGKDILFVWGIHHPPTDTAQTNLYTRTDTTT
SVTTENLDRTFKPLIGPRPLVNGLIGRINYYWSVLKPGQTLRIRSNGNLIAPWFGHILSGESHGRILRTD
LNSGNCVVQCQTEKGGLNSTLPFHNISKYAFGTCPKYIGVKSLRLAIGLRNVHARSNRGLFGAIAGFIEG
GWPGLVAGWYGFQHSNDQGVGMAADRVSTQKAVDKITSKVNNIVDKMNKQYEIIDHEFSEVESRLNMINN
KIDDQIQDIWAYNAELLVLLENQKTLDEHDANVNNLYNKVKRALGSNAMEDGKGCFELYHKCDDQCMETI
RNGTYNRRKYTEESRLERQKIEGVKLEAEGTYKILSIYSTVASSLVLTMGFAAFLFWAMSNGSCRCNXXX

>QCU81149.1 hemagglutinin [Influenza A virus]
METASLITILLVATASNADKICIGYQSTNSTETVDTLTENNVPVTHAKELLHTEHNGMLCATGLGHPLIL
DTCTIEGLIYGNPSCDLLLGGREWSYIVERPSAVNGLCYPGNVENLEELRSLFSSARSYQRIQIFPDTIW
NVSYSGTSKACSDSFYRSMRWLTQKDNAYPIQDAQYTNNQEKNILFMWGINHPPTDTAQTNLYTRTDTTT
SVATEEINRTFKPLIGPRPLVNGLMGRINYYWSVLKPGQTLRIKSNGNLIAPWYGHILSGESHGRILKTD
LKSGSCTVQCQTEKGGLNTTLPFQNVSKYAFGNCSKYIGIKSLKLAVGLRNVPSRSSRGLFGAIAGFIEG
GWSGLVAGWYGFQHSNDQGVGMAADRDSTQKAIDKITSKVNNIVDKMNKQYEIIDHEFSEVETRLNMINS
KIDDQIQDIWAYNAELLVLLENQKTLDEHDANVNNLYNKVKRALGSNAVEDGKGCFELYHKCDDQCMETI

RNGTYNRRKYQEESKLERQKIEGVKLESEGTYKILTIYSTVASSLVIAMGFAAFLFWAMSNGSCRCNICI

>QCU81113.1 hemagglutinin [Influenza A virus]
METASLITILLVVTASNADKICIGYQSTNSTETVDTLTENNVPVTHAKELLHTEHNGMLCATSLGHPLIL
DTCTIEGLIYGNPSCDLLLGGREWSYIVERPSAVNGLCYPGNVENLEELRSLFSSARSYQRIQIFPDTIW
NVSYSGTSKACSDSFYRSMRWLTQKDNAYPIQDAQYTNNQEKNILFMWGINHPPTDTAQTNLYTRTDTTT
SVATEEINRTFKPLIGPRPLVNGLMGRINYYWSVLKPGQTLRIKSNGNLIAPWYGHILSGESHGRILKTD
LKSGSCTVQCQTEKGGLNTTLPFQNVSKYAFGNCSKYIGIKSLKLAVGLRNVPSRSSRGLFGAIAGFIEG
GWSGLVAGWYGFQHSNDQGVGMAADRDSTQKAIDKITSKVNNIVDKMNKQYEIIDHEFSEVETRLNMINS
KIDDQIQDIWAYNAELLVLLENQKTLDEHDANVNNLYNKVKRALGSNAVEDGKGCFELYHKCDDQCMETI
RNGTYNRRKYQEESKLERQKIEGVKLESEGTYKILTIYSTVASSLVIAMGFAAFLFWAMSNGSCRCNICI

>AZS93486.1 hemagglutinin [Influenza A virus]
METVSLITILLVATVSNADKICIGYQSTNSTETVDTLTENNVPVTHAKELLHTEHNGMLCATSLGQPLIL
DTCTIEGLIYGNPSCDLSLEGREWSYIVERPSAVNGLCYPGNVENLEELRSLFSSARSYQRIQIFPDTIW
NVSHDGTSTACSNSFYRSMRWLTRKDGNYPTQDAQYTNNQGKNILFMWGINHPPTDETQRSLYTRTDTTT
SVATEEINRIFKPLIGPRPRVNGLMGRIDYYWSVLKPGQTLRIKSDGNLIAPWYGHILSGESHGRILKTD
LKKGSCTVQCQTEKGGLNTTLPFQNVSKYAFGNCSKYIGIKSLKLAVGLRNVPSRSSRGLFGAIAGFIEG
GWSGLVAGWYGFQHSNDQGVGMAADRDSTQKAIDKITSKVNNIVDKMNKQYEIIDHEFSEVETRLNMINN
KIDDQIQDIWAYNAELLVLLENQKTLDEHDANVNNLYNKVKRALGSNAVEDGKGCFELYHKCNDQCMETI
RNGTYNRKKYQEESKLERQRIEGVKLESEGTYKILTIYSTVASSLVIAMGFAAFLFWAMSNGSCRRNICI

>AZS93474.1 hemagglutinin [Influenza A virus]
METASLITVLLVVTVSNADKICIGYQSTNSTETVDTLTENNVPVTHAKELLHTEHNGMLCATSLGNPLIL
DTCTIEGLIYGNPSCDLLLGGREWSYIVERPSAVNGLCYPGNVENLEELRSLFSSARSYQRVQIFPDTIW
NVSYSGTSKACSDSFYRSMRWLTQKNNAYPTQDAQYTNNQEKNILFMWGINHPPTDATQTNLYTRTDTTT
SVATEEINRTFKPLIGPRPLVNGLMGRIDYYWSVLKPGQTLRIRSNGNLIAPWYGHILSGESHGRILKTD
LKRGSCTVQCQTEKGGLNTTLPFQNVSKYAFGNCSKYIGIKSLKLAVGLRNLPSRSSRGLFGAIAGFIEG
GWSGLVAGWYGFQHSNDQGVGMAADRDSTQKAIDKITSKVNNIVDKMNKQYEIIDHEFSEVETRLNMINN
KIDDQIQDIWAYNAELLVLLENQKTLDEHDANVNNLYNKVKRALGSNAVEDGKGCFELYHKCDDQCMETI
RNGTYNRRKYQEESKLERQKIEGVKLESEGTYKILTIYSTVASSLVIAMGFAAFLFWAMSNGSCRCNICI

>AZS93462.1 hemagglutinin [Influenza A virus]
METASLITVLLVVTVSNADKICIGYQSTNSTETVDTLTENNVPVTHAKELLHTEHNGMLCATSLGNPLIL

DTCTIEGLIYGNPSCDLLLGGREWSYIVERPSAVNGLCCPGNVENLEELRSLFSSARSYQRVQIFPDTIW
NVSYSGTSKACSDSFYRSMRWLTQKNNAYPTQDAQYTNNQEKNILFMWGINHPPTDATQTNLYTRTDTTT
SVATEEINRTFKPLIGPRPLVNGLMGRIDYYWSVLKPGQTLRIRSNGNLIAPWYGHILSGESHGRILKTD
LKRGSCTVQCQTEKGGLNTTLPFQNVSKYAFGNCSKYIGIKSLKLAVGLRNVPSRSSRGLFGAIAGFIEG
GWSGLVAGWYGFQHSNDQGVGMAADRESTQKAIDKITSKVNNIVDKMNKQYEIIDHEFSEVETRLNMINN
KVDDQIQDIWAYNAELLVLLENQKTLDEHNSNVNNLYNKVKRALGSNAVEEGKGCFELYHKCDNHCMETI
RNGTYNRRKYQEESKLERQKIEGVKLESEGTYKILTIYSTVASSLVIAMGFAAFLFWAMSNGSCRCNICI

>AZS93450.1 hemagglutinin [Influenza A virus]
METVSLITILLVATVSYADKICIGYQSTNSTETVDTLTENNVPVTHAKELLHTEHNGMLCATSLGQPLIL
DTCTIEGLIYGNPSCDLSLEGREWSYIVERPSAVNGLCYPGNVENLEELRSLFSSARSYQRVQIFPDTIW
NVSYDGTSTACSGSFYRSMRWLTRKDGNYPTQDAQYTNNQGKNILFMWGINQPPTDDTQRSLYTRTDTTT
SVATEEINRIFKPLIGPRPLVNGLMGRIDYYWSVLKPGQTLRIKSDGNLIAPWHGYILSGESHGRILKTD
LKRGSCTVQCQTERGGLNTTLPFQNVSKYAFGNCSKYIGIKSLKLAVGLRNVPSRSSRGLFGAIAGFIEG
GWSGLVAGWYGFQHSNDQGVGMAADRDSTQKAIDKITSKVNNIVDKMNKQYEIIDHEFSEVETRLNMINN
KIDDQIQDIWAYNAELLVLLENQKTLDEHDANVNNLYNKVKRALGSNAAEDGKGCFELYHKCNDQCMETI
RNGTYNRRKYQEESKLERQKIEGVKLESEGTYKILTIYSTVASSLVIAMGFAAFLFWAMSNGSCRRNICI

>AZS93438.1 hemagglutinin [Influenza A virus]
METISLMTILLMAAVSNADKICIGYQSTNSTETVDTLTENNVPVTHAKELLHTEHNGMLCATSLGNPLIL
DTCTIEGLIYGNPSCDPLLGGREWSYIVERPSAVNGLCYPGSVENLEELRSLFSSARSYQRIQIFPDTIW
NVSYSGTSKACSDSFYRSMRWLTQKNNNYPIQDAQYTNNQEKNILFMWGINHPPTETAQTNLYTRTDTTT
SVATEEINRIFKPLIGPRPLVNGLMGRINYYWSVLKPGQTLRIKSDGNLIAPWYGHILSGESHGRILKTD
LKRGSCTVQCQTEKGGLNTTLPFQNVSKYAFGNCSKYIGIKSLKLAVGLRNVPSRSSRGLFGAIAGFIEG
GWSGLVAGWYGFQHSNDQGVGMAADRDSTQKAIDKITSKVNNIVDKMNKQYEIIDHEFSEVETRLNMINN
KIDDQIQDIWAYNAELLVLLENQKTLDEHDANVNNLYNKVKRALGSNAVEDGKGCFELYHKCNDQCMETI
RNGTYNRKKYQEESKLERQRIEGVKLESEGTYKILTIYSTVASSLVIAMGFAAFLFWAMSNGSCRRNICI

>AZS93426.1 hemagglutinin [Influenza A virus]
METISLMTILLMAAVSNADKICIGYQSTNSTETVDTLTENNVPVTHAKELLHTEHNGMLCATSLGNPLIL
DTCTIEGLIYGNPSCDPLLGGREWSYIVERPSAVNGLCYPGSVENLEELRSLFSSARSYQRIQIFPDTIW
NVSYSGTSKACSDSFYRSMRWLTQKNNNYPIQDAQYTNNQEKNILFMWGINHPPTETAQTNLYTRTDTTT
SVATEEINRIFKPLIGPRPLVNGLMGRINYYWSVLKPGQTLRIKSDGNLIAPWYGHILSGESHGRILKTD
LKRGSCTVQCQTEKGGLNTTLPFQNVSKYAFGNCSKYIGIKSLKLAVGLRNVPSRSSRGLFGAIAGFIEG

GWSGLVAGWYGFQHSNDQGVGMAADRDSTQKAIDKITSKVNNIVDKMNKQYEIIDHEFSEVETRLNMINN
KIDDQIQDIWAYNAELLVLLENQKTLDEHDANVNNLYNKVKRALGSNAVEDGKGCFELYHKCNDQCMETI
RNGTYNRKKYQEESKLERQRIEGVKLESEGTYKILTIYSTVASSLVIAMGFAAFLFWAMSNGSCRRNICI

>AZS93414.1 hemagglutinin [Influenza A virus]
METASLITVLLVVTVSNADKICIGYQSTNSTETVDTLTENNVPVTHAKELLHTEHNGMLCATSLGNPLIL
DTCTIEGLIYGNPSCDLLLGGREWSYIVERPSAVNGLCCPGNVENLEELRSLFSSARSYQRVQIFPDTIW
NVSYSGTSKACSDSFYRSMRWLTQKNNAYPTQDAQYTNNQEKNILFMWGINHPPTDATQTNLYTRTDTTT
SVATEEINRTFKPLIGPRPLVNGLMGRIDYYWSVLKPGQTLRIRSNGNLIAPWYGHILSGESHGRILKTD
LKRGSCTVQCQTEKGGLNTTLPFQNVSKYAFGNCSKYIGIKSLKLAVGLRNVPSRSSRGLFGAIAGFIEG
GWSGLVAGWYGFQHSNDQGVGMAADRESTQKAIDKITSKVNNIVDKMNKQYEIIDHEFSEVETRLNMINN
KVDDQIQDIWAYNAELLVLLENQKTLDEHNSNVNNLYNKVKRALGSNAVEEGKGCFELYHKCDNHCMETI
RNGTYNRRKYQEESKLERQKIEGVKLESEGTYKILTIYSTVASSLVIAMGFAAFLFWAMSNGSCRCNICI

>AZS93402.1 hemagglutinin [Influenza A virus]
METVSLITILLVATVSYADKICIGYQSTNSTETVDTLTENNVPVTHAKELLHTEHNGMLCATSLGQPLIL
DTCTIEGLIYGNPSCDLSLEGREWSYIVERPSAVNGLCYPGNVENLEELRSLFSSARSYQRVQIFPDTIW
NVSYDGTSTACSGSFYRSMRWLTRKDGNYPTQDAQYTNNQGKNILFMWGINQPPTDDTQRSLYTRTDTTT
SVATEEINRIFKPLIGPRPLVNGLMGRIDYYWSVLKPGQTLRIKSDGNLIAPWHGYILSGESHGRILKTD
LKRGSCTVQCQTERGGLNTTLPFQNVSKYAFGNCSKYIGIKSLKLAVGLRNVPSRSSRGLFGAIAGFIEG
GWSGLVAGWYGFQHSNDQGVGMAADRDSTQKAIDKITSKVNNIVDKMNKQYEIIDHEFSEVETRLNMINN
KIDDQIQDIWAYNAELLVLLENQKTLDEHDANVNNLYNKVKRALGSNAAEDGKGCFELYHKCNDQCMETI
RNGTYNRRKYQEESKLERQKIEGVKLESEGTYKILTIYSTVASSLVIAMGFAAFLFWAMSNGSCRRNICI

>AZS93389.1 hemagglutinin [Influenza A virus]
METVSLITILLVATVSNADKICIGYQSTNSTETVDTLTENNVPVTHAKELLHTEHNGMLCATSLGQPLIL
DTCTIEGLIYGNPSCDLSLEGREWSYIVERPSAVNGLCYPGNVENLEELRSLFSSARSYQRIQIFPDTIW
NVSHDGTSTACSNSFYRSMRWLTRKDGNYPTQDAQYTNNQGKNILFMWGINHPPTDETQRSLYTRTDTTT
SVATEEINRIFKPLIGPRPRVNGLMGRIDYYWSVLKPGQTLRIKSDGNLIAPWYGHILSGESHGRILKTD
LKKGSCTVQCQTEKGGLNTTLPFQNVSKYAFGNCSKYIGIKSLKLAVGLRNVPSRSSRGLFGAIAGFIEG
GWSGLVAGWYGFQHSNDQGVGMAADRDSTQKAIDKITSKVNNIVDKMNKQYEIIDHEFSEVETRLNMINN
KIDDQIQDIWAYNAELLVLLENQKTLDEHDANVNNLYNKVKRALGSNAVEDGKGCFELYHKCNDQCMETI
RNGTYNRKKYQEESKLERQRIEGVKLESEGTYKILTIYSTVASSLVIAMGFAAFLFWAMSNGSCRRNICI

>AZS69587.1 hemagglutinin [Influenza A virus]
METASLITVLLVVTVSNADKICIGYQSTNSTETVDTLTENNVPVTHAKELLHTEHNGMLCATSLGNPLIL
DTCTIEGLIYGNPSCDLLLGGREWSYIVERPSAVNGLCYPGNVENLEELRSLFSSARSYQRVQIFPDTIW
NVSYSGTSKACSDSFYRSMRWLTQKNNAYPTQDAQYTNNQEKNILFMWGINHPPTDATQTNLYTRTDTTT
SVATEEINRTFKPLIGPRPLVNGLMGRIDYYWSVLKPGQTLRIRSNGNLIAPWYGHILSGESHGRILKTD
LKRGSCTVQCQTEKGGLNTTLPFQNVSKYAFGNCSKYIGIKSLKLAVGLRNLPSRSSRGLFGAIAGFIEG
GWSGLVAGWYGFQHSNDQGVGMAADRDSTQKAIDKITSKVNNIVDKMNKQYEIIDHEFSEVETRLNMINN
KIDDQIQDIWAYNAELLVLLENQKTLDEHDANVNNLYNKVKRALGSNAVEDGKGCFELYHKCDDQCMETI
RNGTYNRRKYQEESKLERQKIEGVKLESEGTYKILTIYSTVASSLVIAMGFAAFLFWAMSNGSCRCNICI

>QBK20966.1 hemagglutinin [Influenza A virus]
METVSLITILLVATVSNADKICIGYQSTNSTETVDTLTENNVPVTHAKELIHTEHNGMLCATSLGQPLIL
DTCTIEGLIYGNPSCDLSLEGREWSYIVERPSAVNGLCYPGNVENLEELRSLFSSARSYQRIQIFPDTIW
NVSYDGTSTACSGSFYRNMRWLTRKDGNYPTQDAQYTNNQGKNILFMWGINHPPTDTTQSGLYTRTDTTT
SVATEEINRIFKPLIGPRPLVNGLMGRIDYYWSVLKPGQTLRIKSDGNLIAPWFGHILSGESHGRILKTD
LKRGSCTVQCQTEKGGLNTTLPFQNVSKYAFGNCSKYIGIKSLKLAVGLRNVPSRSSRGLFGAIAGFIEG
GWSGLVAGWYGFQHSNDQGVGMAADRDSTQKAIDKITSKVNNIVDKMNKQYEIIDHEFSEVETRLNMINN
KIDDQIQDIWAYNAELLVLLENQKTLDEHDANVNNLYNKVKRALGSNAVEDGKGCFELYHKCDDQCMETI
RNGTYNRRKYQEESKLERQKIEGVKLESEGTYKILTIYSTVASSLVIAMGFAAFLFWAMSNGSCRCNICI

>QBK20965.1 hemagglutinin [Influenza A virus]
METVSLITILLVAAVSNADKICIGYQSTNSTETVDTLTENNVPVTHAKELLHTEHNGMLCATSLGQPIIL
DTCTIEGLIYGNPSCDLSLEGREWSYIVERPSAVNGLCYPGNVENLEELRSLFSSARSYQRIQIFPDTIW
NVSYDGTSTACSGSFYRNMRWLTRKNGEYPIQDAQYTNNQGKNILFMWGINHPPADTTQRDLYTRTDTTT
SVATEEINRIFKPLIGPRPLVNGLMGRIDYYWSVLKPGQTLRIKSDGNLIAPWYGHILSGESHGRILKTD
LKRGSCTVQCQTEKGGLNTTLPFQNVSKYAFGNCSKYIGIKSLKLAVGLRNVPSRSSRGLFGAIAGFIEG
GWSGLVAGWYGFQHSNDQGVGMAADRDSTQKAVDKITSKVNTIVDKMNKQYEIIDHEFSEVETRLNMINN
KIDDQIQDIWAYNAELLVLLENQKTLDEHDANVNNLYNKVKRALGSNAVEDGKGCFELYHKCDDQCMETI
RNGTYNRRKYQEESKLERQKIEGVKLESEGTYKILTIYSTVASSLVIAMGFAAFLFWAMSNGSCRCNICI

>QBK20964.1 hemagglutinin [Influenza A virus]
METVSLITILLVAAVSNADKICIGYQSTNSTETVDTLTENNVPVTHAKELLHTEHNGMLCATSLGQPIIL
DTCTIEGLIYGNPSCDLSLEGREWSYIVERPSAVNGLCYPGNVENLEELRSLFSSARSYQRIQIFPDTIW
NVSYDGTSTACSGSFYRNMRWLTRKNGEYPIQDAQYTNNQGKNILFMWGINHPPADTTQRDLYTRTDTTT

SVATEEINRIFKPLIGPRPLVNGLMGRIDYYWSVLKPGQTLRIKSDGNLIAPWYGHILSGESHGRILKTD
LKRGSCTVQCQTEKGGLNTTLPFQNVSKYAFGNCSKYIGIKSLKLAVGLRNVPSRSSRGLFGAIAGFIEG
GWSGLVAGWYGFQHSNDQGVGMAADRDSTQKAVDKITSKVNTIVDKMNKQYEIIDHEFSEVETRLNMINN
KIDDQIQDIWAYNAELLVLLENQKTLDEHDANVNNLYNKVKRALGSNAVEDGKGCFELYHKCDDQCMETI
RNGTYNRRKYQEESKLERQKIEGVKLESEGTYKILTIYSTVASSLVIAMGFAAFLFWAMSNGSCRCNICI

>QBK20963.1 hemagglutinin [Influenza A virus]
METVSLITILLVATVSNADKICIGYQSTNSTETVDTLTENNVPVTHAKELIHTEHNGMLCATSLGQPLIL
DTCTIEGLIYGNPSCDLSLEGREWSYIVERPSAVNGLCYPGNVENLEELRSLFSSARSYQRIQIFPDTIW
NVSYDGTSTACSGSFYRNMRWLTRKDGNYPTQDAQYTNNQGKNILFMWGINHPPTDTTQSGLYTRTDTTT
SVATEEINRIFKPLIGPRPLVNGLMGRIDYYWSVLKPGQTLRIKSDGNLIAPWFGHILSGESHGRILKTD
LKRGSCTVQCQTEKGGLNTTLPFQNVSKYAFGNCSKYIGIKSLKLAVGLRNVPSRSSRGLFGAIAGFIEG
GWSGLVAGWYGFQHSNDQGVGMAADRDSTQKAIDKITSKVNNIVDKMNKQYEIIDHEFSEVETRLNMINN
KIDDQIQDIWAYNAELLVLLENQKTLDEHDANVNNLYNKVKRALGSNAVEDGKGCFELYHKCDDQCMETI
RNGTYNRRKYQEESKLERQKIEGVKLESEGTYKILTIYSTVASSLVIAMGFAAFLFWAMSNGSCRCNICI

>QBK20962.1 hemagglutinin [Influenza A virus]
METVSLITILLVATVSNADKICIGYQSTNSTETVDTLTENNVPVTHAKELIHTEHNGMLCATSLGQPLIL
DTCTIEGLIYGNPSCDLSLEGREWSYIVERPSAVNGLCYPGNVENLEELRSLFSSARSYQRIQIFPDTIW
NVSYDGTSTACSGSFYRNMRWLTRKDGNYPTQDAQYTNNQGKNILFMWGINHPPTDTTQSGLYTRTDTTT
SVATEEINRIFKPLIGPRPLVNGLMGRIDYYWSVLKPGQTLRIKSDGNLIAPWFGHILSGESHGRILKTD
LKRGSCTVQCQTEKGGLNTTLPFQNVSKYAFGNCSKYIGIKSLKLAVGLRNVPSRSSRGLFGAIAGFIEG
GWSGLVAGWYGFQHSNDQGVGMAADRDSTQKAIDKITSKVNNIVDKMNKQYEIIDHEFSEVETRLNMINN
KIDDQIQDIWAYNAELLVLLENQKTLDEHDANVNNLYNKVKRALGSNAVEDGKGCFELYHKCDDQCMETI
RNGTYNRRTYQEESKLERQKIEGVKLESEGTYKILTIYSTVASSLVIAMGFAAFLFWAMSNGSCRCNICI

>QBK20961.1 hemagglutinin [Influenza A virus]
METASLITILLAVTVSNADKICIGYQSTNSTETVDTLTENNVPVTHAKELLHTEHNGMLCATSLGHPLIL
NTCTIEGLIYGNPSCDLLLGGREWSYIVERPSAVNGLCYPGNVENLEELRSLFSSARSYQRIQIFPDTIW
NVSYSGTSKACSDSFYRSMRWLTQKDNAYPVQDAQYTNNQEKNILFMWGINHPPTDTAQTNLYTRTDTTT
SVATEEINRTFKPLIGPRPLVNGLMGRINYYWSVLKPGQTLRIKSNGNLIAPWYGHILSGESHGRILKTD
LKRGSCTVQCQTEKGGLNTTLPFQNVSKYAFGNCSKYIGIKSLKLAVGLRNVPSRSSRGLFGAIAGFIEG
GWSGLVAGWYGFQHSNDQGVGMAADRDSTQKAIDKITSKVNNIVDKMNKQYEIIDHEFSEVETRLNMINN
KIDDQIQDIWAYNAELLVLLENQKTLDEHDANVNNLYNKVKRALGSNAVEDGKGCFELYHKCDDQCMETI

RNGTYNRRKYQEESKLERQKIEGVKLESEETYKILTIYSTVASSLVIAMGFAAFLFWAMSNGSCRCNICI

>QBK20960.1 hemagglutinin [Influenza A virus]

MNTVSLMTILLLATVSNAHKICIGYQSTNSTETVDTLTENNVPVTHAKELLHTEHNGMLCATSLGNPLIL

DTCTIEGLIYGNPSCDPLLGGREWSYIVERPSAVNGLCYPGSVENLEELRSLFSSARSYQRIQIFPDTIW

NVSYSGTSKACSDSFYRSMRWLTQKNNAYPIQDAQYTNNQEKNILFMWGINHPPTETAQTNLYTRTDTTT

SVATEEINRIFKPLIGPRPLVNGLMGRINYYWSVLKPGQTLRIKSDGNLIAPWYGHILSGESHGRILKTD

LKRGSCTVQCQTEKGGLNTTLPFQNVSKYAFGNCSKYIGIKSLKLAVGLRNVPSRSSRGLFGAIAGFIEG

GWSGLVAGWYGFQHSNDQGVGMAADRDSTQKAIDKITSKVNNIVDKMNKQYEIIDHEFSEVETRLNMINN

KVDDQIQDIWAYNAELLVLLENQKTLDEHDSNVNNLYNKVKRALGSNAVEDGKGCFELYHKCDNQCMETI

RNGTYNRRKYQEESKLERQKIEGVKLESEGTYKILTIYSTVASSLVIAMGFAAFLFWAMSNGSCRCNIGI

>QBK20959.1 hemagglutinin [Influenza A virus]

METVSLITILLVATVSNADKICIGYQSTNSTETVDTLTENNVPVTHAKELIHTEHNGMLCATSLGQPLIL

DTCTIEGLIYGNPSCDLSLEGREWSYIVERPSAVNGLCYPGNVENLEELRSLFSSARSYQRIQIFPDTIW

NVSYDGTSTACSGSFYKNMRWLTRKDGNYPTQDAQYTNNQGKNILFMWGINHPPTDTTQSGLYTRTDTTT

SVATEEINRIFKPLIGPRPLVNGLMGRIDYYWSVLKPGQTLRIKSDGNLIAPWFGHILSGESHGRILKTD

LKRGSCTVQCQTEKGGLNTTLPFQNVSKYAFGNCSKYIGIKSLKLAVGLRNVPSRSSRGLFGAIAGFIEG

GWSGLVAGWYGFQHSNDQGVGMAADRDSTQKAIDKITSKVNNIVDKMNKQYEIIDHEFSEVETRLNMINN

KIDDQIQDIWAYNAELLVLLENQKTLDEHDANVNNLYNKVKRALGSNAVEDGKGCFELYHKCDDQCMETI

RNGTYNRRKYQEESKLERQKIEGVKLESEGTYKILSIYSTVASSLVIAMGFAAFLFWAMSNGSCRCNICI

>QBK20957.1 hemagglutinin [Influenza A virus]

METVSLITILLVATVSNADKICIGYQSTNSTETVDTLTENNVPVTHAKELIHTEHNGMLCATSLGQPLIL

DTCTIEGLIYGNPSCDLSLEGREWSYIVERPSAVNGLCYPGNVENLEELRSLFSSARSYQRIQIFPDTIW

NVSYDGTSTACSGSFYKNMRWLTRKDGNYPTQDAQYTNNQGKNILFMWGINHPPTDTTQSGLYTRTDTTT

SVATEEINRIFKPLIGPRPLVNGLMGRIDYYWSVLKPGQTLRIKSDGNLIAPWFGHILSGESHGRILKTD

LKRGSCTVQCQTEKGGLNTTLPFQNVSKYAFGNCSKYIGIKSLKLAVGLRNVPSRSSRGLFGAIAGFIEG

GWSGLVAGWYGFQHSNDQGVGMAADRDSTQKAIDKITSKVNNIVDKMNKQYEIIDHEFSEVETRLNMINN

KIDDQIQDIWAYNAELLVLLENQKTLDEHDANVNNLYNKVKRALGSNAVEDGKGCFELYHKCDDQCMETI

RNGTYNRRKYQEESKLERQKIEGVKLESEGTYKILTIYSTVASSLVIAMGFAAFLFWAMSNGSCRCNICI

>QBK20956.1 hemagglutinin [Influenza A virus]

METVSLMTILLVATVSNADKICIGYQSTNSTETVDTLTENNVPVTHAKELLHTEHNGMLCATSLGNPLIL

DTCTIEGLIYGNPSCDPLLGGREWSYIVERPSAVNGLCYPGSVENLEELRSLFSSARSYQRIQIFPDTIW
NVSYSGTSKACSDSFYRSMRWLTQKNNAYPIQDAQYTNNQEKNILFMWGINHPPTETAQTNLYTRTDTTT
SVATEEINRIFKPLIGPRPLVNGLMGRISYYWSVLKPGQTLRIKSDGNLIAPWYGHILSGESHGRILKTD
LKRGSCTVQCQTEKGGLNTTLPFQNVSKYAFGNCSKYIGIKSLKLAVGLRNVPSRSSRGLFGAIAGFIEG
GWSGLVAGWYGFQHSNDQGVGMAADRDSTQKAIDKITSKVNNIVDKMNKQYEIIDHEFSEVETRLNMINN
KVDDQIQDIWAYNAELLVLLENQKTLDEHDSNVNNLYNKVKRALGSNAVEDGKGCFELYHKCDNQCMETI
RNGTYNRRKYQEESKLERQKIEGVKLESEGTYKILTIYSTVASSLVIAMGFAAFLFWAMSNGSCRCNICI

>QBK20955.1 hemagglutinin [Influenza A virus]
METVSLMTILLVATVSNADKICIGYQSTNSTETVDTLTENNVPVTHAKELLHTEHNGMLCATSLGNPLIL
DTCTIEGLIYGNPSCDPLLGGREWSYIVERPSAVNGLCYPGSVENLEELRSLFSSARSYQRIQIFPDTIW
NVSYSGTSKACSDSFYRSMRWLTQKNNAYPIQDAQYTNNQEKNILFMWGINHPPTETAQTNLYTRTDTTT
SVATEEINRIFKPLIGPRPLVNGLMGRINYYWSVLKPGQTLRIKSDGNLIAPWYGHILSGESHGRILKTD
LKRGSCTVQCQTEKGGLNTTLPFQNVSKYAFGNCSKYIGIKSLKLAVGLRNVPSRSSRGLFGAIAGFIEG
GWSGLVAGWYGFQHSNDQGVGMAADRDSTQKAIDKITSKVNNIVDKMNKQYEIIDHEFSEVETRLNMINN
KVDDQIQDIWAYNAELLVLLENQKTLDEHDSNVNNLYNKVKRALGSNAVEDGKGCFELYHKCDNQCMETI
RNGTYNRRKYQEESKLERQKIEGVKLESEGTYKILTIYSTVASSLVIAMGFAAFLFWAMSNGSCRCNICI

>QBK20954.1 hemagglutinin [Influenza A virus]
METVSLMTILLVAAVSNADKICIGYQSTNSTETVDTLTENNVPVTHAKELLHTEHNGMLCATSLGQPIIL
DTCTIEGLIYGNPSCDLSLEGREWSYIVERPSAVNGLCYPGNVENLEELRSLFSSARSYQRIQIFPDTIW
NVSYDGTSTACSGSFYRNMRWLTRKNGEYPIQDAQYTNNQGKNILFMWGINHPPADTTQRDLYTRTDTTT
SVATEEINRIFKPLIGPRPLVNGLMGRIDYYWSVLKPGQTLRIKSDGNLIAPWYGHILSGESHGRILKTD
LKRGSCTVQCQTEKGGLNTTLPFQNVSKYAFGNCSKYIGIKSLKLAVGLRNVPSRSSRGLFGAIAGFIEG
GWSGLVAGWYGFQHSNDQGVGMAADRDSTQKAVDKITSKVNTIVDKMNKQYEIIDHEFSEVETRLNMINN
KIDDQIQDIWAYNAELLVLLENQKTLDEHDANVNNLYNKVKRALGSNAVEDGKGCFELYHKCDDQCMETI
RNGTYNRRKYQEESKLERQKIEGVKLESEGTYKILTIYSTVASSLVIAMGFAAFLFWAMSNGSCRCNICI

>QBK20953.1 hemagglutinin [Influenza A virus]
METVSLITILLVAAVSNADKICIGYQSTNSTETVDTLTENNVPVTHAKELLHTEHNGMLCATSLGQPIIL
DTCTIEGLIYGNPSCDLSLEGREWSYIVERPSAVNGLCYPGNVENLEELRSLFSSARSYQRIQIFPDTIW
NVSYDGTSTACSGSFYRNMRWLTRKNGEYPIQDAQYTNNQGKNILFMWGINHPPADTTQRDLYTRTDTTT
SVATEEINRIFKPLIGPRPLVNGLMGRIDYYWSVLKPGQTLRIKSDGNLIAPWYGHILSGESHGRILKTD
LKRGSCTVQCQTEKGGLNTTLPFQNVSKYAFGNCSKYIGIKSLKLAVGLRNVPSRSSRGLFGAIAGFIEG

GWSGLVAGWYGFQHSNDQGVGMAADRDSTQKAVDKITSKVNTIVDKMNKQYEIIDHEFSEVETRLNMINN
KIDDQIQDIWAYNAELLVLLENQKTLDEHDANVNNLYNKVKRALGSNAVEDGKGCFELYHKCDDQCMETI
RNGTYNRRKYQEESKLERQKIEGVKLESEGTYKILTIYSTVASSLVIAMGFAAFLFWAMSNGSCRCNICI

>QBK20952.1 hemagglutinin [Influenza A virus]
METVSLITILLVAAVSNADKICIGYQSTNSTETVDTLTENNVPVTHAKELLHTEHNGMLCATSLGQPIIL
DTCTIEGLIYGNPSCDLSLEGREWSYIVERPSAVNGLCYPGNVENLEELRSLFSSARSYQRIQIFPDTIW
NVSYDGTSTACSGSFYRNMRWLTRKNGEYPIQDAQYTNNQGKNILFMWGINHPPADTTQRDLYTRTDTTT
SVATEEINRIFKPLIGPRPLVNGLMGRIDYYWSVLKPGQTLRIKSDGNLIAPWYGHILSGESHGRILKTD
LKRGSCTVQCQTEKGGLNTTLPFQNVSKYAFGNCSKYIGIKSLKLAVGLRNVPSRSSRGLFGAIAGFIEG
GWSGLVAGWYGFQHSNDQGVGMAADRDSTQKAVDKITSKVNTIVDKMNKQYEIIDHEFSEVETRLNMINN
KIDDQIQDIWAYNAELLVLLENQKTLDEHDANVNNLYNKVKRALGSNAVEDGKGCFELYHKCDDQCMETI
RNGTYNRRKYQEESKLERQKIEGVKLESEGTYKILTIYSTVASSLVIAMGFAAFLFWAMSNGSCRCNICI

>QBK20951.1 hemagglutinin [Influenza A virus]
MDSVSLLTILLLSAVSNAHKICIGYQSTNSTETVDTLTENNVPVTHAKELLHTEHNGMLCATSLGQPIIL
DTCTIEGLIYGNPSCDLSLEGREWSYIVERPSAVNGLCYPGNVENLEELRSLFSSARSYQRIQIFPDTIW
NVSYDGTSTACSGSFYRNMRWLTRKNGEYPIQDAQYTNNQGKNILFMWGINHPPADTTQRDLYTRTDTTT
SVATEEINRVFKPLIGPRPLVNGLMGRIDYYWSVLKPGQTLRIKSDGNLIAPWYGHILSGESHGRILKTD
LKRGSCTVQCQTEKGGLNTTLPFQNVSKYAFGNCSKYIGIKSLKLAVGLRNVPSRSSRGLFGAIAGFIEG
GWSGLVAGWYGFQHSNDQGVGMAADRDSTQKAVDKITSKVNTIVDKMNKQYEIIDHEFSEVETRLNMINS
KIDDQIQDIWAYNAELLVLLENQKTLDEHDANVNNLYNKVKRALGSNAVEDGKGCFELYHKCDDQCMETI
RNGTYNRRKYQEESKLERQKIEGVKLESEGTYKILTIYSTVASSLVIAMGFAAFLFWAMSNGSCRCNICI

>QBK20950.1 hemagglutinin [Influenza A virus]
METVSLITILLVATVSNADKICIGYQSTNSTETVDTLTENNVPVTHAKELLHTEHNGMLCATSLGQPLIL
DTCTIEGLIYGNPSCDLSLEGREWSYIVERPSAVNGLCYPGNVENLEELRSLFSSARSYQRIQIFPDTIW
NVSYDGTSTACSDSFYRSMRWLTRKDGNYPTQDAQYTNNQGKNILFMWGINHPPTDDTQRNLYTRTDTTT
SVATEEINRIFKPLIGPRPLVNGLMGRIDYYWSVLKPGQTLRIKSDGNLIAPWYGHILSGESHGRILKTD
LKRGSCTVQCQTEKGGLNTTLPFQNVSKYAFGNCSKYIGIKSLKLAVGLRNVPSRSSRGLFGAIAGFIEG
GWSGLVAGWYGFQHSNDQGVGMAADRDSTQKAIDKITSKVNNIVDKMNKQYEIIDHEFSEVETRLNMINN
KIDDQIQDIWAYNAELLVLLENQKTLDEHDANVNNLYNKVKRALGSNAVEDGKGCFELYHKCNDQCMETI
RNGTYNRRKYQEESKLERQKIEGVKLESEGTYKILTIYSTVASSLVIAMGFAAFLFWAMSNGSCRCNICI

>QBK20949.1 hemagglutinin [Influenza A virus]

METVSLITILLVATVSNADKICIGYQSTNSTETVDTLTENNVPVTHAKELLHTEHNGMLCATSLGQPLIL

DTCTIEGLIYGNPSCDLSLEGREWSYIVERPSAVNGLCYPGNVENLEELRSLFSSARSYQRIQIFPDTIW

NVSYDGTSTACSDSFYRSMRWLTRKDGNYPTQDAQYTNNQGKNILFMWGINHPPTDDTQRNLYTRTDTTT

SVATEEINRIFKPLIGPRPLVNGLMGRIDYYWSVLKPGQTLRIKSDGNLIAPWYGHILSGESHGRILKTD

LKRGSCTVQCQTEKGGLNTTLPFQNVSKYAFGNCSKYIGIKSLKLAVGLRNVPSRSSRGLFGAIAGFIEG

GWSGLVAGWYGFQHSNDQGVGMAADRDSTQKAIDKITSKVNNIVDKMNKQYEIIDHEFSEVETRLNMINN

KIDDQIQDIWAYNAELLVLLENQKTLDEHDANVNNLYNKVKRALGSNAVEDGKGCFELYHKCNDQCMETI

RNGTYNRRKYQEESKLERQKIEGVKLESEGTYKILTIYSTVASSLVIAMGFAAFLFWAMSNGSCRCNICI

>QBK20948.1 hemagglutinin [Influenza A virus]

METVSLITILLVATVSNADKICIGYQSTNSTETVDTLTENNVPVTHAKELLHTEHNGMLCATSLGQPLIL

DTCTIEGLIYGNPSCDLSLEGREWSYIVERPSAVNGLCYPGNVENLEELRSLFSSARSYQRIQIFPDTIW

NVSYDGTSTACSDSFYRSMRWLTRKDGNYPTQDAQYTNNQGKNILFMWGINHPPTDDTQRNLYTRTDTTT

SVATEEINRIFKPLIGPRPLVNGLMGRIDYYWSVLKPGQTLRIKSDGNLIAPWYGHILSGESHGRILKTD

LKRGSCTVQCQTEKGGLNTTLPFQNVSKYAFGNCSKYIGIKSLKLAVGLRNVPSRSSRGLFGAIAGFIEG

GWSGLVAGWYGFQHSNDQGVGMAADRDSTQKAIDKITSKVNNIVDKMNKQYEIIDHEFSEVETRLNMINN

KIDDQIQDIWAYNAELLVLLENQKTLDEHDANVNNLYNKVKRALGSNAVEDGKGCFELYHKCNDQCMETI

RNGTYNRRKYQEESKLERQKIEGVKLESEGTYKILTIYSTVASSLVIAMGFAAFLFWAMSNGSCRCNICI

>QBK20947.1 hemagglutinin [Influenza A virus]

METVSLITILLVATVSNADKICIGYQSTNSTETVDTLTENNVPVTHAKELLHTEHNGMLCATSLGQPLIL

DTCTIEGLIYGNPSCDLSLEGREWSYIVERPSAVNGLCYPGNVENLEELRSLFSSARSYQRIQIFPDTIW

NVSYDGTSTACSDSFYRSMRWLTRKDGNYPTQDAQYTNNQGKNILFMWGINHPPTDDTQRNLYTRTDTTT

SVATEEINRIFKPLIGPRPLVNGLMGRIDYYWSVLKPGQTLRIKSDGNLIAPWYGHILSGESHGRILKTD

LKRGSCTVQCQTEKGGLNTTLPFQNVSKYAFGNCSKYIGIKSLKLAVGLRNVPSRSSRGLFGAIAGFIEG

GWSGLVAGWYGFQHSNDQGVGMAADRDSTQKAIDKITSKVNNIVDKMNKQYEIIDHEFSEVETRLNMINN

KIDDQIQDIWAYNAELLVLLENQKTLDEHDANVNNLYNKVKRALGSNAVEDGKGCFELYHKCNDQCMETI

RNGTYNRRKYQEESKLERQKIEGVKLESEGTYKILTIYSTVASSLVIAMGFAAFLFWAMSNGSCRCNICI

>QBK20946.1 hemagglutinin [Influenza A virus]

METVSLITILLVATVSNADKICIGYQSTNSTETVDTLTENNVPVTHAKELLHTEHNGMLCATSLGQPLIL

DTCTIEGLIYGNPSCDLSLEGREWSYIVERPSAVNGLCYPGNVENLEELRSLFSSARSYQRLQIFPDTIW

NVSYDGTSTACSGSFYRSMRWLTRKNGEYPTQDAQYTNNQGKNILFMWGINHPPTDTTQRDLYTRIDTTT

SVATEEINRIFKPLIGPRPLVNGLMGRIDYYWSILKPGQTLRIKSDGNLIAPWYGHILSGESHGRILKTD
LKRGSCTVQCQTEKGGLNTTLPFQNVSKYAFGNCSKYIGIKSLKLAVGLRNVPSRSSRGLFGAIAGFIEG
GWSGLVAGWYGFQHSNDQGVGMAADRDSTQKAIDKITSKVNNIVDKMNKQYEIIDHEFSEVETRLNMINN
KIDDQIQDIWAYNAELLVLLENQKTLDEHDANVNNLYNKVKRALGSNAVEDGKGCFELYHKCDNQCMETI
RNGTYNRRKYQEESKIERQKIEGVKLESEGTYKILTIYSTVASSLVIAMGFAAFLFWAMSNGSCRCNICI

>QBK20944.1 hemagglutinin [Influenza A virus]
METVSLITILLVAAVSNADKICIGYQSTNSTETVDTLTENNVPVTHAKELLHTEHNGMLCATSLGQPIIL
DTCTIEGLIYGNPSCDLSLEGREWSYIVERPSAVNGLCYPGNVENLEELRSLFSSARSYQRIQIFPDTIW
NVSYDGTSTACSGSFYRNMRWLTRKNGEYPIQDAQYTNNQGKNILFMWGINHPPADTTQRDLYTRTDTTT
SVATEEINRIFKPLIGPRPLVNGLMGRIDYYWSVLKPGQTLRIKSDGNLIAPWYGHILSGESHGRILKTD
LKRGSCTVQCQTEKGGLNTTLPFQNVSKYAFGNCSKYIGIKSLKLALGVRNVRFRYSRGLFGAIAGFIEG
GWSGLVAGWYGFQHSNDQGVGMAADRDSTQKAVDKITSKVNTIVDKMNKQYEITDHEFSEVETRVNTINN
KIDDQIQDIWAYNAEMLVLLENQKTLEEHEAKVNNIYNKVKRGLGFNAVEEGKGCFEIYHKCNDHCTGTL
RNGAYTRGKCHEESKIERHKKEGVKRECEGTYKILTTYSSVASSLVIAKGFAALLFWATSNGCCRCNICI

>QBK20943.1 hemagglutinin [Influenza A virus]
METVSLIAILLVATVGNADKICIGYQSTNSTETVDTLTESNVPVTHAKELLHTEHNGMLCATSLGQPIIL
DTCTIEGLIYGNPSCDQSLEGREWSYIVERPSAVNGLCYPGNVENLEELRSLFSSARSYQRIQIFPDAIW
NVSYDGTSTACSGSFYRNMRWLTRKNGDYPIQDAQYTNNQGKNILFMWGINHPPSDTTQRDLYTRTDTTT
SVATEEINRIFKPLIGPRPLVNGLMGRIDYYWSVLKPGQTLRIKSDGNLIAPWYGHILSGESHGRILKTD
LKRGSCTVQCQTEKGGLNTTLPFQNVSKYAFGNCSKYIGIKSLKLAVGLRNVPSRSSRGLFGAIAGFIEG
GWSGLVAGWYGFQHSNDQGVGMAADRDSTQKAVDKITSKVNNIIDKMNKQYEIINHEFSEVETRLNMINN
KIDDQIQDIWAYNAELLVLLENQKTLDEHDANVNNLYNKVKRALGSNAVEDGKGCFELYHKCDDQCMETI
RNGTYNRRKYQEESKLERQKIEGVKLESEGTYKILTIYSTVASSLVIAMGFAAFLFWAMSNGSCRCNICI

>QBK20942.1 hemagglutinin [Influenza A virus]
METVSLITILLVATVSNADKICIGYQSTNSTETVDTLTENNVPVTHAKELLHTEHNGMLCATSLGQPIIL
DTCTIEGLIYGNPSCDLSLEGREWSYIVERPSAVNGLCYPGNVENLEELRSLFSSAKSYQRIQIFPDTIW
NVSYDGTSTACSGSFYKNMRWLTRKNGEYPIQDAQYTNNQGKNILFMWGINHPPADTTQRDLYTRTDTTT
SVATEEINRIFKPLIGPRPLVNGLMGRIDYYWSVLKPGQTLRIKSDGNLIAPWYGHILSGESHGRILKTD
LKRGSCTVQCQTEKGGLNTTLPFQNVSKYAFGNCSKYIGIKSLKLAVGLRNVPSRSSRGLFGAIAGFIEG
GWSGLVAGWYGFQHSNDQGVGMAADRESTQKAVDKITSKVNNIVDKMNKQYEIIDHEFSEVETRLNMINN
KIDDQIQDIWAYNAELLVLLENQKTLDEHDANVNNLYNKVKRALGSNAMEDGKGCFELYHKCDDQCMETI

RNGTYNRRKYQEESKLERQKIEGVKLESEGTYKILTIYSTVASSLVIAMGFAAFLFWAMSNGSCRCNICI

>QBK20941.1 hemagglutinin [Influenza A virus]
METVSLITILLVAAVSNADKICIGYQSTNSTETVDTLTENNVPVTHAKELLHTEHNGMLCATSLGQPIIL
DTCTIEGLIYGNPSCDLSLEGREWSYIVERPSAVNGLCYPGNVENLEELRSLFSSARSYQRIQIFPDTIW
NVSYDGTSTACSGSFYRNMRWLTRKNGEYPIQDAQYTNNQGKNILFMWGINHPPADTTQRDLYTRTDTTT
SVATEEINRIFKPLIGPRPLVNGLMGRIDYYWSVLKPGQTLRIKSDGNLIAPWYGHILSGESHGRILKTD
LKRGSCTVQCQTEKGGLNTTLPFQNVSRYAFGNCSKYIGIKSLKLAVGLRNVPSRSSRGLFGAIAGFIEG
GWSGLVAGWYGFQHSNDQGVGMAADRDSTQKAVDKITSKVNTIVDKMNKQYEIIDHEFSEVETRLNMINN
KIDDQIQDIWAYNAELLVLLENQKTLDEHDANVNNLYNKVKRALGSNAVEDGKGCFDLYHKCNDQCMETI
RNGTYNRRKYQEESKLERQKIEGVKLESEGTYKILTIYSTVASSLVIAMGFAAFLFWAMSNGSCRCNICI

>QBK20940.1 hemagglutinin [Influenza A virus]
METVSLITILLVAAVSNADKICIGYQSTNSTETVDTLTENNVPVTHAKELLHTEHNGMLCATSLGQPIIL
DTCTIEGLIYGNPSCDLSLEGREWSYIVERPSAVNGLCYPGNVENLEELRSLFSSARSYQRIQIFPDTIW
NVSYDGTSTACSGSFYRNMRWLTRKNGEYPIQDAQYTNNQGKNILFMWGINHPPADTTQRDLYTRTDTTT
SVATEEINRIFKPLIGPRPLVNGLMGRIDYYWSVLKPGQTLRIKSDGNLIAPWYGHILSGESHGRILKTD
LKRGSCTVQCQTEKGGLNTTLPFQNVSRYAFGNCSKYIGIKSLKLAVGLRNVRSRYSRGVFGAIAGFIEG
GWSGVVAGGYGFQHSNDHGVGMAADRDSTQKALDKITSKVNTIVDKMNTQYEITDHEFSEVETRLNTINN
KIDDQIQDIWAHNAEMLVLRENPKTLDEHDANVNNLYNKVKRALGSNAVEDGKGCFELYHKCNDQCTETI
RNGTHNRRKYLEESKLERQKIEGVKLESEGTYKILTIYSTVASSLVIAKGFAAFLFWATCNGCCRCHICI

>QBK20939.1 hemagglutinin [Influenza A virus]
METVSLITILLVATVSNADKICIGYQSTNSTETVDTLTENNVPVTHAKELIHTEHNGMLCATSLGQPLIL
DTCTIEGLIYGNPSCDLSLEGREWSYIVERPSAVNGLCYPGNVENLEELRSLFSSARSYQRIQIFPDTIW
NVSYDGTSTACSGSFYRNMRWLTRKDGNYPTQDAQYTNNQGKNILFMWGINHPPTDTTQSGLYTRTDTTT
SVATEEINRIFKPLIGPRPLVNGLMGRIDYYWSVLKPGQTLRIKSDGNLIAPWFGHILSGESHGRILKTD
LKRGSCTVQCQTEKGGLNTTLPFQNVSKYAFGNCSKYIGIKSLKLAVGLRNVPSRSSRGLFGAIAGFIEG
GWSGLVAGWYGFQHSNDQGVGMAADRDSTQKAIDKITSKVNNIVDKMNKQYEIIDHEFSEVETRLNMINN
KIDDQIQDIWAYNAELLVLLENQKTLDEHDANVNNLYNKVKRALGSNAVEDGKGCFELYHKCDDQCMETI
RNGTYNRRKYQEESKLERQKIEGVKLESEGTYKILTIYSTVASSLVIAMGFAAFLFWAMSNGSCRCNICI

>QBK20938.1 hemagglutinin [Influenza A virus]
METVSLITILLVATVSNADKICIGYQSTNSTETVDTLTENNVPVTHAKELIHTEHNGMLCATSLGQPLIL

DTCTIEGLIYGNPSCDLSLEGREWSYIVERPSAVNGLCYPGNVENLEELRSLFSSARSYQRIQIFPDTIW
NVSYDGTSTACSGSFYRNMRWLTRKDGNYPTQDAQYTNNQGKNILFMWGINHPPTDTTQSGLYTRTDTTT
SVATEEINRIFKPLIGPRPLVNGLMGRIDYYWSVLKPGQTLRIKSDGNLIAPWFGHILSGESHGRILKTD
LKRGSCTVQCQTEKGGLNTTLPFQNVSKYAFGNCSKYIGIKSLKLAVGLRNVPSRSSRGLFGAIAGFIEG
GWSGLVAGWYGFQHSNDQGVGMAADRDSTQKAIDKITSKVNNIVDKMNKQYEIIDHEFSEVETRLNMINN
KIDDQIQDIWAYNAELLVLLENQKTLDEHDANVNNLYNKVKRALGSNAVEDGKGCFELYHKCDDQCMETI
RNGTYNRRKYQEESKLERQKIEGVKLESEGTYKILTIYSTVASSLVIAMGFAAFLFWAMSNGSCRCNICI

>QBK20936.1 hemagglutinin [Influenza A virus]
METVSLITILLVATVSNADKICIGYQSTNSTETVDTLTENNVPVTHAKELIHTEHNGMLCATSLGQPLIL
DTCTIEGLIYGNPSCDLSLEGREWSYIVERPSAVNGLCYPGNVENLEELRSLFSSARSYQRIQIFPDAIW
NVSYDGTSTACSGSFYRNMRWLTRKDGNYPTQDAQYTNNQGKNILFMWGINHPPTDTTQSGLYTRTDTTT
SVATEEINRIFKPLIGPRPLVNGLMGRIDYYWSVLKPGQTLRIKSDGNLIAPWFGHILSGESHGRILKTD
LKRGSCTVQCQTEKGGLNTTLPFQNVSKYAFGNCSKYIGIKSLKLAVGLRNVPSRSSRGLFGAIAGFIEG
GWSGLVAGWYGFQHSNDQGVGMAADRDSTQKAIDKITSKVNNIVDKMNKQYEIIDHEFSEVETRLNMINN
KIDDQIQDIWAYNAELLVLLENQKTLDEHDANVNNLYNKVKRALGSNAVEDGKGCFELYHKCDDQCMETI
RNGTYNRRKYQEESKLERQKIEGVKLESEGTYKILTIYSTVASSLVIAMGFAAFLFWAMSNGSCRCNICI

>QBK20935.1 hemagglutinin [Influenza A virus]
MKTVSLITILLVATVSNADKICIGYQSTNSTETVDTLTENNVPVTHAKELIHTEHNGMLCATSLGQPLIL
DTCTIEGLIYGNPSCDLSLEGREWSYIVERPSAVNGLCYPGNVENLEELRSLFSSARSYQRIQIFPDTIW
NVSYDGTSTACSGSFYRNMRWLTRKDGNYPTQDAQYTNNQGKNILFMWGINHPPTDTTQSGLYTRTDTTT
SVATEEINRIFKPLIGPRPLVNGLMGRIDYYWSVLKPGQTLRIKSDGNLIAPWFGHILSGESHGRILKTD
LKRGSCTVQCQTEKGGLNTTLPFQNVSKYAFGNCSKYIGIKSLKLAVGLRNVPSRSSRGLFGAIAGFIEG
GWSGLVAGWYGFQHSNDQGVGMAADRDSTQKAIDKITSKVNNIVDKMNKQYEVIDHEFSEVETRLNMINN
KIDDQIQDIWAYNAELLVLLENQKTLDEHDANVNNLYNKVKRALGSNAMEDGKGCFELYHKCDDQCMETI
RNGTYNRRKYQEESKLERQKIEGVKLESEGTYKILTIYSTVASSLVIAMGFAAFLFWAMSNGSCRCNICI

>QBK20934.1 hemagglutinin [Influenza A virus]
METVSLITILLVATVSNADKICIGYQSTNSTETVDTLTENNVPVTHAKELIHTEHNGMLCATSLGQPLIL
DTCTIEGLIYGNPSCDLSLEGREWSYIVERPSAVNGLCYPGNVENLEELRSLFSSARSYQRIQIFPDTIW
NVSYDGTSTACSGSFYRNMRWLTRKDGNYPTQDAQYTNNQGKNILFMWGINHPPTDTTQSGLYTRTDTTT
SVATEEINRIFKPLIGPRPLVNGLMGRIDYYWSVLKPGQTLRIKSDGNLIAPWFGHILSGESHGRILKTD
LKRGSCTVQCQTEKGGLNTTLPFQNVSKYAFGNCSKYIGIKSLKLAVGLRNVPSRSSRGLFGAIAGFIEG

GWSGLVAGWYGFQHSNDQGVGMAADRDSTQKAIDKITSKVNNIVDKMNKQYEIIDHEFSEVETRLNMINN
KIDDQIQDIWAYNAELLVLLENQKTLDEHDANVNNLYNKVKRALGSNAVEDGKGCFELYHKCDDQCMETI
RNGTYNRRKYQEESKLERQKIEGVKLESEGTYKILTIYSTVASSLVIAMGFAAFLFWAMSNGSCRCNICI

>QBK20933.1 hemagglutinin [Influenza A virus]
METVSLITILLVATVSNADKICIGYQSTNSTETVDTLTENNVPVTHAKELIHTEHNGMLCATSLGQPLIL
DTCTIEGLIYGNPSCDLSLEGREWSYIVERPSAVNGLCYPGNVENLEELRSLFSSARSYQRIQIFPDTIW
NVSYDGTSTACSGSFYKNMRWLTRKDGNYPTQDAQYTNNQGKNILFMWGINHPPTDTTQSGLYTRTDTTT
SVATEEINRIFKPLIGPRPLVNGLMGRIDYYWSVLKPGQTLRIKSDGNLIAPWFGHILSGESHGRILKTD
LKRGSCTVQCQTEKGGLNTTLPFQNVSKYAFGNCSKYIGIKSLKLAVGLRNVPSRSSRGLFGAIAGFIEG
GWSGLVAGWYGFQHSNDQGVGMAADRDSTQKAIDKITSKVNNIVDKMNKQYEIIDHEFSEVETRLNMINN
KIDDQIQDIWAYNAELLVLLENQKTLDEHDANVNNLYNKVKRALGSNAVEDGKGCFELYHKCDDQCMETI
RNGTYNRRKYQEESKLERQKIEGVKLESEGTYKILTIYSTVASSLVIAMGFAAFLFWAMSNGSCRCNICI

>QBK20932.1 hemagglutinin [Influenza A virus]
METVSLITILLVATVSNADKICIGYQSTNSTETVDTLTENNVPVTHAKELIHTEHNGMLCATSLGQPLIL
DTCTIEGLIYGNPSCDLSLEGREWSYIVERPSAVNGLCYPGNVENLEELRSLFSSARSYQRIQIFPDTVW
NVSYDGTSTACSGSFYRNMRWLTRKDGNYPTQDAQYTNNQGKNILFMWGINHPPTDTTQSGLYTRTDTTT
SVATEEINRIFKPLIGPRPLVNGLMGRIDYYWSVLKPGQTLRIKSDGNLIAPWFGHILSGESHGRILKTD
LKRGSCTVQCQTEKGGLNTTLPFQNVSKYAFGNCSKYIGIKSLKLAVGLRNVPSRSSRGLFGAIAGFIEG
GWSGLVAGWYGFQHSNDQGVGMAADRDSTQKAIDKITSKVNNIVDKMNKQYEIIDHEFSEVETRLNMINN
KIDDQIQDIWAYNAELLVLLENQKTLDEHDANVNNLYNKVKRALGSNAVEDGKGCFELYHKCDDQCMETI
RNGTYNRRKYQEESKLERQKIEGVKLESEGTYKILTIYSTVASSLVIAMGFAAFLFWAMSNGSCRCNICI

>QBK20931.1 hemagglutinin [Influenza A virus]
METVSLITILLVATVSNADKICIGYQSTNSTETVDTLTENNVPVTHAKELIHTEHNGMLCATSLGQPLIL
DTCTIEGLIYGNPSCDLSLEGREWSYIVERPSAVNGLCYPGNVENLEELRSLFSSARSYQRIQIFPDTIW
NVSYDGTSTACSGSFYRNMRWLTRKDGNYPTQDAQYTNNQGKNILFMWGINHPPTDTTQSGLYTRTDTTT
SVATEEINRIFKPLIGPRPLVNGLMGRIDYYWSVLKPGQTLRIKSDGNLIAPWFGHILSGESHGRILKTD
LKRGSCTVQCQTEKGGLNTTLPFQNVSKYAFGNCSKYIGIKSLKLAVGLRNVPSRSSRGLFGAIAGFIEG
GWSGLVAGWYGFQHSNDQGVGMAADRDSTQKAIDKITSKVNNIVDKMNKQYEIIDHEFSEVETRLNMINN
KIDDQIQDIWAYNAELLVLLENQKTLDEHDANVNNLYNKVKRALGSNAVEDGKGCFELYHKCDDQCMETI
RNGTYNRRKYQEESKLERQKIEGVKLESEGTYKILTIYSTVASSLVIAMGFAAFLFWAMSNGSCRCNICI

>QBK20930.1 hemagglutinin [Influenza A virus]

METVSLITILLVATVSNADKICIGYQSTNSTETVDTLTENNVPVTHAKELIHTEHNGMLCATSLGQPLIL
DTCTIEGLIYGNPSCDLPLEGREWSYIVERPSAVNGLCYPGNVENLEELRSLFSSARSYQRIQIFPDTIW
NVSYDGTSTACSGSFYRNMRWLTRKDGNYPTQDAQYTNNQGKNILFMWGINHPPTDTTQSGLYTRTDTTT
SVATEEINRIFKPLIGPRPLVNGLMGRIDYYWSVLKPGQTLRIKSDGNLIAPWFGHILSGESHGRILKTD
LKRGSCTVQCQTEKGGLNTTLPFQNVSKYAFGNCSKYIGIKSLKLAVGLRNVPSRSSRGLFGAIAGFIEG
GWSGLVAGWYGFQHSNDQGVGMAADRDSTQKAIDKITSKVNNIVDKMNKQYEIIDHEFSEVETRLNMINN
KIDDQIQDIWAYNAELLVLLENQKTLDEHDANVNNLYNKVKRALGSNAVEDGKGCFELYHKCDDQCMETI
RNGTYNRRKYQEESKLERQKIEGVKLESEGTYKILTIYSTVASSLVIAMGFAAFLFWAMSNGSCRCNICI

>QBK20929.1 hemagglutinin [Influenza A virus]

METAPLITILLMATVSNADKICIGYQSTNSTETVDTLTENNVPVTHAKELLHTEHNGMLCATSLGQPLIL
DTCTIEGLIYGNPSCDLSLEGREWSYIVERPSAVNGLCYPGSVENLEELRSLFSSARSYQRIQIFPDTIW
NVSYDGTSTACSGSFYRSMRWLTRKNGDYPVQDAQYTNNQGKNILFMWGINHPPTDTTQRDLYTRIDTTT
SVATEEMNRIFKPLIGPRPLVNGLMGRINYYWSVLKPGQTLRIKSDGNLIAPWYGHILSGESHGRILKTD
LKRGSCIVQCQTEKGGLNTTLPFQNVSKYAFGNCSKYIGIKSLKLAVGLRNVPSRSSRGLFGAIAGFIEG
GWSGLVAGWYGFQHSNDQGVGMAADRDSTQKAIDKITSKVNNIVDKMNKQYEIIDHEFSEVETRLNMINN
KIDDQIQDIWAYNAELLVLLENQKTLDEHDANVNNLYNKVKRALGSNAVEDGKGCFELYHKCDDQCMETI
RNGTYNRRKYQEESKLERQKIEGVKLESEGTYKILTIYSTVASSLVIAMGFAAFLFWAMSNGSCRCNICI

>QBK20928.1 hemagglutinin [Influenza A virus]

METVSLITILLMATVSNADKICIGYQSTNSTETVDTLTENNVPVTHAKELLHTEHNGMLCATSLGQPLIL
DTCTIEGLIYGNPSCDPSLEGREWSYIVERPSAVNGLCYPGSVENLEELRSLFSSARSYQRIQIFPDTIW
NVSYDGTSTACSGSFYRSMRWLTRKNGDYPVQDAQYTNNQGKNILFMWGINHPPTDTTQRDLYTRIDTTT
SVATEEMNRIFKPLIGPRPLVNGLMGRINYYWSVLKPGQTLRIKSDGNLIAPWYGHILSGESHGRILKTD
LKRGSCTVQCQTEKGGLNTTLPFQNVSKYAFGNCSKYIGIKSLKLAVGLRNVPSRSSRGLFGAIAGFIEG
GWSGLVAGWYGFQHSNDQGVGMAADRDSTQKAIDKITSKVNNIVDKMNKQYEIIDHEFSEVETRLNMINN
KIDDQIQDIWAYNAELLVLLENQKTLDEHDANVNNLYNKVKRALGSNAVEDGKGCFELYHKCDDQCMETI
RNGTYNRKKYQEESKLERQKIEGVKLESEGTYKILSIYSTVASSLVIAMGFAAFLFWAMSNGSCRCNICI

>QBK20927.1 hemagglutinin [Influenza A virus]

METVSLITILLMATVSNADKICIGYQSTNSTETVDTLTENNVPVTHAKELLHTEHNGMLCATSLGQPLIL
DTCTIEGLIYGNPSCGLSLEGREWSYIVERPSAVNGLCYPGSVENLEELRSLFSSARSYQRIQIFPDTIW
NVSYDGTSTACSGSFYRSMRWLTRKNGNYPVQDAQYTNNQGKNILFMWGINHPPTDTTQRDLYTRIDTTT

SVATEEMNRIFKPLIGPRPLVNGLMGRINYYWSVLKPGQTLRIKSDGNLIAPWYGHILSGESHGRILKTD
LKRGSCTVQCQTEKGGLNTTLPFQNVSKYAFGNCSKYIGIKSLKLAVGLRNVPSRSSRGLFGAIAGFIEG
GWSGLVAGWYGFQHSNDQGVGMAADRDSTQKAIDKITSKVNNIVDKMNKQYEIIDHEFSEVETRLNMINN
KIDDQIQDIWAYNAELLVLLENQKTLDEHDANVNNLYNKVKRALGSNAVEDGKGCFELYHKCDDQCMETI
RNGTYNRRKYQEESKLERQKIEGVKLESEGTYKILTIYSTVASSLVIAMGFAAFLFWAMSNGSCRCNICI

>QBK20926.1 hemagglutinin [Influenza A virus]
METVSLITILLVATVSNADKICIGYQSTNSTETVDTLTENNVPVTHAKELIHTEHNGMLCATSLGQPLIL
DTCTIEGLIYGNPSCDLSLEGREWSYIVERPSAVNGLCYPGNVENLEELRSLFSSARSYQRIQIFPDTIW
NVSYDGTSTACSGSFYRNMRWLTRKDGNYPTQDAQYTNNQGKNILFMWGINHPPTDTTQSGLYTRTDTTT
SVATEEINRIFKPLIGPRPLVNGLMGRIDYYWSVLKPGQTLRIKSDGNLIAPWFGHILSGESHGRILKTD
LKRGSCTVQCQTEKGGLNTTLPFQNVSKYAFGNCSKYVGIKSLKLAVGLRNVPSRSSRGLFGAIAGFIEG
GWSGLVAGWYGFQHSNDQGVGMAADRDSTQKAIDKITSKVNNIVDKMNKQYEIIDHEFSEVETRLNMINN
KIDDQIQDIWAYNAELLVLLENQKTLDEHDANVNNLYNKVKRALGSNAVEDGKGCFELYHKCDDQCMETI
RNGTYNRRKYQEESKLERQKIEGVKLESEGTYKILTIYSTVASSLVIAMGFAAFLFWAMSNGSCRCNICI

>QBK20925.1 hemagglutinin [Influenza A virus]
METVSLITILLVATVSNADKICIGYQSTNSTETVDTLTENNVPVTHAKELIHTEHNGMLCATSLGQPLIL
DTCTIEGLIYGNPSCDLSLEGREWSYIVERPSAVNGLCYPGNVENLEELRSLFSSARSYQRIQIFPDTIW
NVSYDGTSTACSGSFYRNMRWLTRKDGNYPTQDAQYTNNQGKNILFMWGINHPPTDTTQSGLYTRTDTTT
SVATEEINRIFKPLIGPRPLVNGLMGRIDYYWSVLKPGQTLRIKSDGNLIAPWFGHILLGESHGRILKTD
LKRGSCTVQCQTEKGGLNTTLPFQNVSKYAFGNCSKYIGIKSLKLAVGLRNVPSRSSRGLFGAIAGFIEG
GWSGLVAGWYGFQHSNDQGVGMAADRDSTQKAIDKITSKVNNIVDKMNKQYEIIDHEFSEVETRLNMINN
KIDDQIQDIWAYNAELLVLLENQKTLDEHDANVNNLYNKVKRALGSNAVEDGKGCFELYHKCDDQCMETI
RNGTYNRRKYQEESKLERQKIEGVKLESEGTYKILTIYSTVASSLVIAMGFAAFLFWAMSNGSCRCNICI

>QBK20924.1 hemagglutinin [Influenza A virus]
METVSLITILLVATVSNADKICIGYQSTNSTETVDTLTENNVPVTHAKELIHTEHNGMLCATSLGQPLIL
DTCTIEGLIYGNPSCDLSLEGREWSYIVERPSAVNGLCYPGNVENLEELRSLFSSARSYQRIQIFPDTIW
NVSYDGTSTACSGSFYRNMRWLTRKDGNYPTQDAQYTNNQGKNILFMWGINHPPTDTTQSGLYTRTDTTT
SVATEEINRIFKPLIGPRPLVNGLMGRIDYYWSVLKPGQTLRIKSDGNLIAPWFGHILLGESHGRILKTD
LKRGSCTVQCQTEKGGLNTTLPFQNVSKYAFGNCSKYIGIKSLKLAVGLRNVPSRSSRGLFGAIAGFIEG
GWSGLVAGWYGFQHSNDQGVGMAADRDSTQKAIDKITSKVNNIVDKMNKQYEIIDHEFSEVETRLNMINN
KIDDQIQDIWAYNAELLVLLENQKTLDEHDANVNNLYNKVKRALGSNAVEDGKGCFELYHKCDDQCMETI

RNGTYNRRKYQEESKLERQKIEGVKLESEGTYKILTIYSTVASSLVIAMGFAAFLFWAMSNGSCRCNICI

>QBK20923.1 hemagglutinin [Influenza A virus]
METVSLITILLVATVSNADKICIGYQSTNSTETVDTLTENNVPVTHAKELIHTEHNGMLCATSLGQPLIL
DTCTIEGLIYGNPSCDLSLEGREWSYIVERPSAVNGLCYPGNVENLEELRSLFSSARSYQRIQIFPDTIW
NVSYDGTSTACSGSFYRNMRWLTRKDGNYPTQDAQYTNNQGKNILFMWGINHPPTDTTQSGLYTRTDTTT
SVATEEINRIFKPLIGPRPLVNGLMGRIDYYWSVLKPGQTLRIKSDGNLIAPWFGHILSGESHGRILKTD
LKRGSCTVQCQTEKGGLNTTLPFQNVSKYAFGNCSKYIGIKSLKLAVGLRNVPSRSSRGLFGAIAGFIEG
GWSGLVAGWYGFQHSNDQGVGMAADRDSTQKAIDKITSKVNNIVDKMNKQYEIIDHEFSEVETRLNMINN
KIDDQIQDIWAYNAELLVLLENQITLDEHDANVNNLYNKVKRALGSNAVEDGKGCFELYHKCDDQCMETI
RNGTYNRRKYQEESKLERQKIEGVKLESEGTYKILTIYSTVASSLVIAMGFAAFLFWAMSNGSCRCNICI

>QBK20922.1 hemagglutinin [Influenza A virus]
METVSLITILLVATVSNADKICIGYQSTNSTETVDTLTENNVPVTHAKELIHTEHNGMLCATSLGQPLIL
DTCTIEGLIYGNPSCDLSLEGREWSYIVERPSAVNGLCYPGNVENLEELRSLFSSARSYQRIQIFPDTIW
NVSYDGTSTACSGSFYRNMRWLTRKDGNYPTQDAQYTNNQGKNILFMWGINHPPTDTTQSGLYTRTDTTT
SVATEEINRIFKPLIGPRPLVNGLMGRIDYYWSVLKPGQTLRIKSDGNLIAPWFGHILSGESHGRILKTD
LKRGSCTVQCQTEKGGLNTTLPFQNVSKYAFGNCSKYIGIKSLKLAVGLRNVPSRSSRGLFGAIAGFIEG
GWSGLVAGWYGFQHSNDQGVGMAADRDSTQKAIDKITSKVNNIVDKMNKQYEIIDHEFSEVETRLNMINN
KIDDQIQDIWAYNAELLVLLENQKTLDEHDANVNNLYNKVKRALCSNAVEDGKGCFELYHKCDDQCMETI
RNGTYNRRKYQEESKLERQKIEGVKLESEGTYKILSIYSTVASSLVIAMGSAAFLFWAMSNGSCRCNICI

>QBK20921.1 hemagglutinin [Influenza A virus]
METVSLITILLVATVSNADKICIGYQSTNSTETVDTLTENNVPVTHAKELIHTEHNGMLCATSLGQPLIL
DTCTIEGLIYGNPSCDLSLEGREWSYIVERPSAVNGLCYPGNVENLEELRSLFSSARSYQRIQIFPDTIW
NVSYDGTSTACSGSFYRNMRWLTRKDGNYPTQDAQYTNNQGKNILFMWGINHPPTDTTQSGLYTRTDTTT
SVATEEINRIFKPLIGPRPLVNGLMGRIDYYWSVLKPGQTLRIKSDGNLIAPWFGHILSGESHGRILKTD
LKRGSCTVQCQTEKGGLNTTLPFQNVSKYAFGNCSKYIGIKSLKLAVGLRNVPSRSSRGLFGAIAGFIEG
GWSGLVAGWYGFQHSNDQGVGMAADRDSTQKAIDKITSKVNNIVDKMNKQYEIIDHEFSEVETRLNMINN
KIDDQIQDIWAYNAELLVLLENQKTLDEHDANVNNLYNKVKRALGSNAVEDGKGCFELYHKCDDQCMETI
RNGTYNRRKYQEESKLERQKIEGVKLESEGTYKILTIYSTVASSLVIAMGFAAFLFWAMSNGSCRCNICI

>QBK20920.1 hemagglutinin [Influenza A virus]
METVSLITILIVATVSNADKICIGYQSTNSTETVNTLTENNVPVTHAKELLHTEHNGMLCATSLGQPLIL

DTCTIEGLIYGNPSCDLSLEGREWSYIVERPSAVNGLCYPGNVENLEELRSLFSSARSYQRIQIFPDTIW
NVSYDGTSTACSNSFYRSMRWLTRKNGDYPTQDAQYTNNQGKNILFMWGINHPPTDDTQRNLYTRTETTT
SVATEEINRIFKPLIGPRPLVNGLMGRIDYYWSVLKPGQTLRIKSDGNLIAPWYGHILSGESHGRILKTD
LKRGSCTVQCQTEKGGLNTTLPFQNVSKYAFGNCSKYIGIKSLKLAVGLRNVPSRSSRGLFGAIAGFIEG
GWSGLVAGWYGFQHSNDQGVGMAADRDSTQKAIDKITSKVNNIVDKMNKQYEIIDHEFSEVETRLNMINN
KIDDQIQDIWAYNAELLVLLENQKTLDEHDANVNNLYNKVKRALGSNAVEDGKGCFELYHKCNDQCMETI
RNGTYNRKKYQEESRLERQKIEGVKLESEGTYKILTIYSTVASSLVIAMGFAAFLFWAMSNGSCRCNICI

>QBK20919.1 hemagglutinin [Influenza A virus]
METVSLITILLVATVSNADKICIGYQSTNSTETVDTLTENNVPVTHAKELLHTEHNGMLCATSLGQPLIL
DTCTIEGLIYGNPSCDLSLEGREWSYIVERPSAVNGLCYPGNVENLEELRSLFSSARSYQRIQIFPDTIW
NVSYDGTSTACSNSFYRSMRWLTRKDGNYPTQDAQYTNNQGKNILFMWGINHPPTDDTQRNLYTRTDTTT
SVATEEINRIFKPLIGPRPLVNGLMGRIDYYWSVLKPGQTLRIKSDGNLIAPWYGYILSGESHGRILKTD
LKRGSCTVQCQTEKGGLNTTLPFQNVSKYAFGNCSKYIGIKSLKLAVGLRNVPSRSSRGLFGAIAGFIEG
GWSGLVAGWYGFQHSNDQGVGMAADRDSTQKAIDKITSKVNNIVDKMNKQYEIIDHEFSEVETRLNMINN
KIDDQIQDIWAYNAELLVLLENQKTLDEHDANVNNLYNKVKRALGSNAVEDGKGCFELYHKCNDQCMETI
RNGTYNRRKYQEESKLERQKIEGVKLESEGTYKILTIYSTVASSLVIAMGFAAFLFWAMSNGSCRCNICI

>QBK20918.1 hemagglutinin [Influenza A virus]
METVSLITILLVATVSNADKICIGYQSTNSTETVDTLTENNVPVTHAKELLHTEHNGMLCATSLGQPLIL
DTCTIEGLIYGNPSCDLSLEGREWSYIVERPSAVNGLCYPGNVENLEELRSLFSSARSYQRIQIFPDTIW
NVSYDGTSTACSNSFYRSMRWLTRKDGNYPTQDAQYTNNQGKNILFMWGINHPPTDDTQRNLYTRTDTTT
SVATEEINRIFKPLIGPRPLVNGLMGRIDYYWSVLKPGQTLRIKSDGNLIAPWYGYILSGESHGRILKTD
LKRGSCTVQCQTEKGGLNTTLPFQNVSKYAFGNCSKYIGIKSLKLAVGLRNVPSSSSRGLFGAIAGFIEG
GWSGLVAGWYGFQHSNDQGVGMAADRDSTQKAIDKITSKVNNIVDKMNKQYEIIDHEFSEVETRLNMINN
KIDDQIQDIWAYNAELLVLLENQKTLDEHDANVNNLYNKVKRALGSNAVEDGKGCFELYHKCNDQCMETI
RNGTYNRRKYQEESKLERQKIEGVKLESEGTYKILTIYSTVASSLVIAMGFAAFLFWAMSNGSCRCNICI

>QBK20917.1 hemagglutinin [Influenza A virus]
METISLITILLVATVSYADKICIGYQSTNSTETVDTLTENNVPVTHAKELLHTEHNGMLCATSLGQPLIL
DTCTIEGLIYGNPSCDLSLEGREWSYIVERPSAVNGLCYPGNVENLEELRSLFSSARSYQRVQIFPDTIW
NVSYDGTSTACSGSFYRSMRWLTRKDGNYPTQDAQYTNNQGKNILFMWGINHPPTDDTQRSLYTRTDTTT
SVATEEINRIFKPLIGPRPLVNGLMGRIDYYWSVLKPGQTLRIKSDGNLIAPWYGYILSGESHGRILKTD
LKRGSCTVQCQTEKGGLNTTLPFQNVSKYAFGNCSKYIGIKSLKLAVGLRNVPSRSSRGLFGAIAGFIEG

GWSGLVAGWYGFQHSNDQGVGMAADRDSTQKAIDKITSKVNNIVDKMNKQYEIIDHEFSEVETRLNMINN
KIDDQIQDIWAYNAELLVLLENQKTLDEHDANVNNLYNKVKRALGSNAAEDGKGCFELYHKCNDQCMETI
RNGTYNRRKYQEESKLERQKIEGVKLESEGTYKILTIYSTVASSLVIAMGFAAFLFWAMSNGSCRCNICI

>QBK20916.1 hemagglutinin [Influenza A virus]
METISLITILLVATVSYADKICIGYQSTNSTETVDTLTENNVPVTHAKELLHTEHNGMLCATSLGQPLIL
DTCTIEGLIYGNPSCDLSLEGREWSYIVERPSAVNGLCYPGNVENLEELRSLFSSARSYQRVQIFPDTIW
NVSYDGTSTACSGSFYRSMRWLTRKDGNYPTQDAQYTNNQGKNILFMWGINHPPTDDTQRSLYTRTDTTT
SVATEEINRIFKPLIGPRPLVNGLMGRIDYYWSVLKPGQTLRIKSDGNLIAPWYGYILSGESHGRILKTD
LKRGSCTVQCQTEKGGLNTTLPFQNVSKYAFGNCSKYIGIKSLKLAVGLRNVPSRSSRGLFGAIAGFIEG
GWSGLVAGWYGFQHSNDQGVGMAADRDSTQKAIDKITSKVNNIVDKMNKQYEIIDHEFSEVETRLNMINN
KIDDQIQDIWAYNAELLVLLENQKTLDEHDANVNNLYNKVKRALGSNAAEDGKGCFELYHKCNDQCMETI
RNGTYNRRKYQEESKLERQKIEGVKLESEGTYKILTIYSTVASSLVIAMGFAAFLFWAMSNGSCRCNICI

>QBK20915.1 hemagglutinin [Influenza A virus]
METVSLMTILLVATVSNADKICIGYQSTNSTETVDTLTENNVPVTHAKELLHTEHNGMLCATSLGNPLIL
DTCTIEGLIYGNPSCDPLLGGREWSYIVERPSAVNGLCYPGSVENLEELRSLFSSARSYQRIQIFPDTIW
NVSYSGTSKACSDSFYRSMRWLTQKNNAYPIQDAQYTNNQEKNILFMWGINHPPTETAQTNLYTRTDTTT
SVATEEINRIFKPLIGPRPLVNGLMGRINYYWSVLKPGQTLRIKSDGNLIAPWYGHILSGESHGRILKTD
LKRGSCTVQCQTEKGGLNTTLPFQNVSKYAFGNCSKYIGIKSLKLAVGLRNVPSRSSRGLFGAIAGFIEG
GWSGLVAGWYGFQHSNDQGVGMAADRDSTQKAIDKITSKVNNIVDKMNKQYEIIDHEFSEVETRLNMINN
KVDDQIQDIWAYNAELLVLLENQKTLDEHDSNVNNLYNKVKRALGSNAVEDGKGCFELYHKCDNQCMETI
RNGTYNRRKYQEESKLERQKIEGVKLESEGTYKILTIYSTVASSLVIAMGFAAFLFWAMSNGSCRCNICI

>QBK20914.1 hemagglutinin [Influenza A virus]
METVSLITILIVATVSNADKICIGYQSTNSTETVDTLTENNVPVTHAKELLHTEHNGMLCATSLGQPLIL
DTCTIEGLIYGNPSCDLSLEGREWSYIVERPSAVNGLCYPGNVENLEELRSLFSSARSYQRIQIFPDTIW
NVSYDGTSTACSNSFYRSMRWLTRKNGDYPTQDAQYTNNQGKNILFMWGINHPPTDDTQRNLYTRTETTT
SVATEEINRIFKPLIGPRPLVNGLMGRIDYYWSVLKPGQTLRIKSDGNLIAPWYGHILSGESHGRILKTD
LKRGSCTVQCQTEKGGLNTTLPFQNVSKYAFGNCSKYIGIKSLKLAVGLRNVPSRSSRGLFGAIAGFIEG
GWSGLVAGWYGFQHSNDQGVGMAADRDSTQKAIDKITSKVNNIVDKMNKQYEIIDHEFSEVETRLNMINN
KIDDQIQDIWAYNAELLVLLENQKTLDEHDANVNNLYNKVKRALGSNAVEDGKGCFELYHKCNDQCMETI
RNGTYNRKKYQEESRLERQKIEGVKLESEGTYKILTIYSTVASSLVIAMGFAAFLFWAMSNGSCRCNICI

>QBK20913.1 hemagglutinin [Influenza A virus]
METVSLITILIVATVSNADKICIGYQSTNSTETVDTLTENNVPVTHAKELLHTEHNGMLCATSLGQPLIL
DTCTIEGLIYGNPSCDLSLEGREWSYIVERPSAVNGLCYPGNVENLEELRSLFSSARSYQRIQIFPDTIW
NVSYDGTSTACSNSFYRSMRWLTRKNGDYPTQDAQYTNNQGKNILFMWGINHPPTDDTQRNLYTRTETTT
SVATEEINRIFKPLIGPRPLVNGLMGRIDYYWSVLKPGQTLRIKSDGNLIAPWYGHILSGESHGRILKTD
LKRGSCTVQCQTEKGGLNTTLPFQNVSKYAFGNCSKYIGIKSLKLAVGLRNVPSRSSRGLFGAIAGFIEG
GWSGLVAGWYGFQHSNDQGVGMAADRDSTQKAIDKITSKVNNIVDKMNKQYEIIDHEFSEVETRLNMINN
KIDDQIQDIWAYNAELLVLLENQKTLDEHDANVNNLYNKVKRALGSNAVEDGKGCFELYHKCNDQCMETI
RNGTYNRKKYQEESRLERQKIEGVKLESEGTYKILTIYSTVASSLVIAMGFAAFLFWAMSNGSCRCNICI

>QBK20912.1 hemagglutinin [Influenza A virus]
METISLMTILLVATVSNADKICIGYQSTNSTETVDTLTENNVPVTHAKELLHTEHNGMLCATSLGNPLIL
DTCTIEGLIYGNPSCDPLLGGREWSYIVERPSAVNGLCYPGSVENLEELRSLFSSARSYQRIQIFPDTIW
NVSYSGTSKACSDSFYRSMRWLTQKNNAYPIQDAQYTNNQEKNILFMWGINHPPTETAQTNLYTRTDTTT
SVATEEINRIFKPLIGPRPLVNGLMGRINYYWSVLKPGQTLRIKSDGNLIAPWYGHILSGESHGRILKTD
LKRGSCTVQCQTEKGGLNTTLPFQNVSKYAFGNCSKYIGIKSLKLAVGLRNVPSRSSRGLFGAIAGFIEG
GWSGLVAGWYGFQHSNDQGVGMAADRDSTQKAIDKITSKVNNIVDKMNKQYEIIDHEFSEVETRLNMINN
KVDDQIQDIWAYNAELLVLLENQKTLDEHDSNVNNLYNKVKRALGSNAVEDGKGCFELYHKCDNQCMETI
RNGTYNRRKYQEESKLERQKIEGVKLESEGTYKILTIYSTVASSLVIAMGFAAFLFWAMSNGSCRCNICI

>QBK20910.1 hemagglutinin [Influenza A virus]
METISLMTILLVATVSNADKICIGYQSTNSTETVDTLTENNVPVTHAKELLHTEHNGMLCATSLGNPLIL
DTCTIEGLIYGNPSCDPLLGGREWSYIVERPSAVNGLCYPGSVENLEELRSLFSSARSYQRIQIFPDTIW
NVSYSGTSKACSDSFYRSMRWLTQKNNAYPIQDAQYTNNQEKNILFMWGINHPPTETVQTNLYTRTDTTT
SVATEEINRIFKPLIGPRPLVNGLMGRINYYWSVLKPGQTLRIKSDGNLIAPWYGHILSGESHGRILKTD
LKRGSCTVQCQTEKGGLNTTLPFQNVSKYAFGNCSKYIGIKSLKLAVGLRNVPSRSSRGLFGAIAGFIEG
GWSGLVAGWYGFQHSNDQGVGMAADRDSTQKAIDKITSKVNNIVDKMNKQYEIIDHEFSEVETRLNMINN
KVDDQIQDIWAYNAELLVLLENQKTLDEHDSNVNNLYNKVKRALGSNAVEDGKGCFELYHKCDNQCMETI
RNGTYNRRKYQEESKLERQKIEGVKLESEGTYKILTIYSTVASSLVIAMGFAAFLFWAMSNGSCRCNICI

>QBK20909.1 hemagglutinin [Influenza A virus]
METVSLMTILLVATVSNADKICIGYQSTNSTETVDTLTENNVPVTHAKELLHTEHNGMLCATSLGNPLIL
DTCTIEGLIYGNPSCDPLLGGREWSYIVERPSAVNGLCYPGSVENLEELRSLFSSARSYQRIQIFPDTIW
NVSYSGTSKACSDSFYRSMRWLTQKNNAYPIQDAQYTNNQEKNILFMWGINHPPTETAQTNLYTRTDTTT

SVATEEINRIFKPLIGPRPLVNGLMGRINYYWSVLKPGQTLRIKSDGNLIAPWYGHILSGESHGRILKTD
LKRGSCTVQCQTEKGGLNTTLPFQNVSKYAFGNCSKYIGIKSLKLAVGLRNVPSRSSRGLFGAIAGFIEG
GWSGLVAGWYGFQHSNDQGVGMAADRDSTQKAIDKITSKVNNIVDKMNKQYEIIDHEFSEVETRLNMINN
KVDDQIQDIWAYNAELLVLLENQKTLDEHDSNVNNLYNKVKRALGSNAVEDGKGCFELYHXCDNQCMETI
RNGTYNRRKYQEESKLERQKIEGVKLESEGTYKILTIYSTVASSLVIAMGFAAFLFWAMSNGSCRCNICI

>QBK20908.1 hemagglutinin [Influenza A virus]
METASLITILLVATVSNADKICIGYQSTNSTETVDTLTENNVPVTHAKELLHTEHNGMLCATSLGQPIIL
DTCTIEGLIYGNPSCDLSLEGREWSYIVERPSAVNGLCYPGNVENLEELRSLFSSARSYQRIQIFPDTIW
NVSYDGTSTACSGSFYRNMRWLTRKNGDYPIQDAQYTNNQGKNILFMWGINHPPTDTTQRELYTRTDTTT
SVATEEINRIFKPLIGPRPLVNGLMGRIDYYWSVLKPGQTLRIKSDGNLIAPWYGHILSGESHGRILKTD
LKRGSCTVQCQTEKGGLNTTLPFQNVSKYAFGNCSKYIGIKSLKLAVGLRNVPSRSSRGLFGAIAGFIEG
GWSGLVAGWYGFQHSNDQGVGMAADRDSTQKAVDKITSKVNNIVDKMNKQYEIIDHEFSEVETRLNMINN
KIDDQIQDIWAYNAELLVLLENQKTLDEHDANVNNLYNKVKRALGSNAVEDGKGCFELYHKCDDQCMETI
RNGTYNRRKYQEESKLERQKIEGVKLESEGTYKILTIYSTVASSLVIAMGFAAFLFWAMSNGSCRCNICI

>QBK20907.1 hemagglutinin [Influenza A virus]
METVSLITILLVATVSYADKICIGYQSTNSTETVDTLTENNVPVTHAKELLHTEHNGMLCATSLGQPLIL
DTCTIEGLIYGNPSCDLSLEGREWSYIVERPSAVNGLCYPGNVENLEELRSLFSSARSYQRVQIFPDTIW
NVSYDGTSTACSSSFYRSMRWLTRKNGDYPTQDAQYTNNQGKNILFMWGINHPPTDDTQRNLYTRTDTTT
SVATEEINRIFKPLIGPRPLVNGLMGRIDYYWSVLKPGQTLRIKSDGNLIAPWYGYILSGESHGRILKTD
LKRGSCTVQCQTEKGGLNTTLPFQNVSKYAFGNCSKYIGIKSLKLAVGLRNVPSRSSRGLFGAIAGFIEG
GWSGLVAGWYGFQHSNDQGVGMAADRDSTQKAIDKITSKVNNIVDKMNKQYEIIDHEFSEVETRLNMINN
KIDDQIQDIWAYNAELLVLLENQKTLDEHDANVNNLYNKVKRALGSNAVEDGKGCFELYHKCNDQCMETI
RNGTYNRRKYQEESKLERQKIEGVKLESEGTYKILTIYSTVASSLVIAMGFAAFLFWAMSNGSCRCNICI

>QBK20906.1 hemagglutinin [Influenza A virus]
METASLITILLVATVSNADKICIGYQSTNSTETVDTLTENNVPVTHAKELLHTEHNGMLCATSLGQPIIL
DTCTIEGLIYGNPSCDLSLEGREWSYIVERPSAVNGLCYPGNVENLEELRSLFSSARSYQRIQIFPDTIW
NVSYDGTSTACSGSFYRNMRWLTRKNGDYPIQDAQYTNNQGKNILFMWGINHPPTDTTQRELYTRTDTTT
SVATEEINRIFKPLIGPRPLVNGLMGRIDYYWSVLKPGQTLRIKSDGNLIAPWYGHILSGESHGRILKTD
LKRGSCTVQCQTEKGGLNTTLPFQNVSKYAFGNCSKYIGIKSLKLAVGLRNVPSRSSRGLFGAIAGFIEG
GWSGLVAGWYGFQHSNDQGVGMAADRDSTQKAVDKITSKVNNIVDKMNKQYEIIDHEFSEVETRLNMINN
KIDDQIQDIWAYNAELLVLLENQKTLDEHDANVNNLYNKVKRALGSNAVEDGKGCFELYHKCDDQCMETI

RNGTYNRRKYQEESKLERQKIEGVKLESEGTYKILTIYSTVASSLVIAMGFAAFLFWAMSNGSCRCNICI

>QBK20903.1 hemagglutinin [Influenza A virus]
METVSLITILLIATVSNADKICIGYQSTNSTETVDTLTENNVPVTHAKELLHTEHNGMLCATSLGQPIIL
DTCTIEGLIYGNPSCDLSLEGREWSYIVERPSAVNGLCYPGNVENLEELRSLFSSARSYQRIQIFPDTIW
NVSYDGTSTACSGSFYRNMRWLTRKNGEYPIQDAQYTNNQGKNILFMWGINHPPADTTQRDLYTRTDTTT
SVATEEINRIFKPLIGPRPLVNGLMGRIDYYWSVLKPGQTLRIKSDGNLIAPWYGHILSGESHGRILKTD
LKRGSCTVQCQTEKGGLNTTLPFQNVSKYAFGNCSKYIGIKSLKLAVGLRNVPSRSSRGLFGAIAGFIEG
GWSGLVAGWYGFQHSNDQGVGMAADRESTQKAVDKITSKVNNIVDKMNKQYEIIDHEFSEVETRLNMINN
KIDDQIQDIWAYNAELLVLLENQKTLDEHDANVNNLYNKVKRALGSNAMEDGKGCFELYHKCDDQCMETI
RNGTYNRRKYQEESKLERQKIEGVKLESEGTYKILTIYSTVASSLVIAMGFAAFLFWAMSNGSCRCNICI

>QBK20902.1 hemagglutinin [Influenza A virus]
METVSLITILLIATVSNADKICIGYQSTNSTETVDTLTENNVPVTHAKELLHTEHNGMLCATSLGQPIIL
DTCTIEGLIYGNPSCDLSLEGREWSYIVERPSAVNGLCYPGNVENLEELRSLFSSARSYQRIQIFPDTIW
NVSYDGTSTACSGSFYRNMRWLTRKNGEYPIQDAQYTNNQGKNILFMWGINHPPADTTQRNLYTRTDTTT
SVATEEINRIFKPLIGPRPLVNGLMGRIDYYWSVLKPGQTLRIKSDGNLIAPWYGHILSGESHGRILKTD
LKRGSCTVQCQTEKGGLNTTLPFQNVSKYAFGNCSKYIGIKSLKLAVGLRNVPSRSSRGLFGAIAGFIEG
GWSGLVAGWYGFQHSNDQGVGMAADRESTQKAVDKITSKVNNIVDKMNKQYEIIDHEFSEVETRLNMINN
KIDDQIQDIWAYNAELLVLLENQKTLDEHDANVNNLYNKVKRALGSNAMEDGKGCFELYHKCDDQCMETI
RNGTYNRRKYQEESKLERQKIEGVKLESEGTYKILTIYSTVASSLVIAMGFAAFLFWAMSNGSCRCNICI

>QBK20900.1 hemagglutinin [Influenza A virus]
METVSLITILLVATVSNADKICIGYQSTNSTETVDTLTENNVPVTHAKELLHTEHNGMLCATSLGQPIIL
DTCTIEGLIYGNPSCDLSLEGREWSYIVERPSAVNGLCYPGNVENLEELRSLFSSARSYQRIQIFPDTIW
NVSYDGTSTACSGSFYRNMRWLTRKNGEYPIQDAQYTNNQGKNILFMWGINHPPADTTQRDLYTRTDTTT
SVATEEINRIFKPLIGPRPLVNGLMGRIDYYWSVLKPGQTLRIKSDGNLIAPWYGHILSGESHGRILKTD
LKRGSCTVQCQTEKGGLNTTLPFQNVSKYAFGNCSKYIGIKSLKLAVGLRNVPSRSSRGLFGAIAGFIEG
GWSGLVAGWYGFQHSNDQGVGMAADRESTQKAVDKITSKVNNIVDKMNKQYEIIDHEFSEVETRLHMINN
KIDDQIQDIWAYNAELLVLLENQKTLDEHDANVNNLYNKVKRALGSNAMEDGKGCFELYHKCDDQCMETI
RNGTYNRRKYQEESKLERQKIEGVKLESEGTYKILTIYSTVASSLVIAMGFAAFLFWAMSNGSCRCNICI

>QBK20899.1 hemagglutinin [Influenza A virus]
METVSLITILLVATVSNADKICIGYQSTNSTETVDTLTENNVPVTHAKELLHTEHNGMLCATSLGQPIIL

DTCTIEGLIYGNPSCDLSLEGREWSYIVERPSAVNGLCYPGNVENLEELRSLFSSARSYQRIQIFPDTIW
NVSYDGTSTACSGSFYRNMRWLTRKNGEYPIQDAQYTNNQGKNILFMWGINHPPADTTQRDLYTRTDTTT
SVATEEINRIFKPLIGPRPLVNGLMGRIDYYWSVLKPGQTLRIKSDGNLIAPWYGHILSGESHGRILKTD
LKRGSCTVQCQTEKGGLNTTLPFQNVSKYAFGNCSKYIGIKSLKLAVGLRNVPSRSSRGLFGAIAGFIEG
GWSGLVAGWYGFQHSNDQGVGMAADRESTQKAVDKITSKVNNIVDKMNKQYEIIDHEFSEVETRLNMINN
KIDDQIQDIWAYNAELLVLLENQKTLDEHDANVNNLYNKVKRALGSNAMEDGKGCFELYHKCDDQCMETI
RNGTYNRRKYQEESKLERQKIEGVKLESEGTYKILTIYSTVASSLVIAMGFAAFLFWAMSNGSCRCNICI

>QBK20898.1 hemagglutinin [Influenza A virus]
METVSLITILIVATVSNADKICIGYQSTNSTETVDTLTENNVPVTHAKELLHTEHNGMLCATSLGQPLIL
DTCTIEGLIYGNPSCDLSLEGREWSYIVERPSAVNGLCYPGNVENLEELRSLFSSARSYQRIQIFPDTIW
NVSYDGTSTACSNSFYRSMRWLTRKNGDYPTQDAQYTNNQGKNILFMWGINHPPTDDTQRNLYTRTDTTT
SVATEEINRIFKPLIGPRPLVNGLMGRIDYYWSVLKPGQTLRIKSDGNLIAPWYGHILSGESHGRILKTD
LKRGSCTVQCQTEKGGLNTTLPFQNVSKYAFGNCSKYIGIKSLKLAVGLRNVPSRSSRGLFGAIAGFIEG
GWSGLVAGWYGFQHSNDQGVGMAADRDSTQKAIDKITSKVNNIVDKMNKQYEIIDHEFSEVETRLNMINN
KIDDQIQDIWAYNAELLVLLENQKTLDEHDANVNNLYNKVKRALGSNAVEDGKGCFELYHKCNDQCMETI
RNGTYNRKKYQEESRLERQKIEGVKLESEGTYKILTIYSTVASSLVIAMGFAAFLFWAMSNGSCRCNICI

>QBK20897.1 hemagglutinin [Influenza A virus]
METVSLITILIVATVSNADKICIGYQSTNSTETVDTLTENNVPVTHAKELLHTEHNGMLCATSLGQPLIL
DTCTIEGLIYGNPSCDLSLEGREWSYIVERPSAVNGLCYPGNVENLEELRSLFSSARSYQRIQIFPDTIW
NVSYDGTSTACSNSFYRSMRWLTRKNGDYPTQDAQYTNNQGKNILFMWGINHPPTDDTQRNLYTRTETTT
SVATEEINRIFKPLIGPRPLVNGLMGRIDYYWSVLKPGQTLRIKSDGNLIAPWYGHILSGESHGRILKTD
LKRGSCTVQCQTEKGGLNTTLPFQNVSKYAFGNCSKYIGIKSLKLAVGLRNVPSRSSRGLFGAIAGFIEG
GWSGLVAGWYGFQHSNDQGVGMAADRDSTQKAIDKITSKVNNIVDKMNKQYEIIDHEFSEVETRLNMINN
KIDDQIQDIWAYNAELLVLLENQKTLDEHDANVNNLYNKVKRALGSNAVEDGKGCFELYHKCNDQCMETI
RNGTYNRKKYQEESRLERQKIEGVKLESEGTYKILTIYSTVASSLVIAMGFAAFLFWAMSNGSCRCNICI

>QBK20896.1 hemagglutinin [Influenza A virus]
METVSLITILIVATVSNADKICIGYQSTNSTETVDTLTENNVPVTHAKELLHTEHNGMLCATSLGQPLIL
DTCTIEGLIYGNPSCDLSLEGREWSYIVERPSAVNGLCYPGNVENLEELRSLFSSARSYQRIQIFPDTIW
NVSYDGTSTACSNSFYRSMRWLTRKNGDYPTQDAQYTNNQGKNILFMWGINHPPTDDTQRNLYTRTDTTT
SVATEEINRIFKPLIGPRPLVNGLMGRIDYYWSVLKPGQTLRIKSDGNLIAPWYGHILSGESHGRILKTD
LKRGSCTVQCQTEKGGLNTTLPFQNVSKYAFGNCSKYIGIKSLKLAVGLRNVPSRSSRGLFGAIAGFIEG

GWSGLVAGWYGFQHSNDQGVGMAADRDSTQKAIDKITSKVNNIVDKMNKQYEIIDHEFSEVETRLNMINN
KIDDQIQDIWAYNAELLVLLENQKTLDEHDANVNNLYNKVKRALGSNAVEDGKGCFELYHKCNDQCMETI
RNGTYNRKKYQEESRLERQKIEGVKLESEGTYKILTIYSTVASSLVIAMGFAAFLFWAMSNGSCRCNICI

>QBK20895.1 hemagglutinin [Influenza A virus]
METVSLITILLVATVSNADKICIGYQSTNSTETVDTLTENNVPVTHAKELLHTEHNGMLCATSLGQPLIL
DTCTIEGLIYGNPSCDLSLEGREWSYIVERPSAVNGLCYPGNVENLEELRSLFSSARSYQRIQIFPDTIW
NVSYDGTSTACSNSFYRSMRWLTRKDGNYPTQDAQYTNNQGKNILFMWGINHPPTDDTQRNLYTRTDTTT
SVATEEINRIFKPLIGPRPLVNGLMGRIDYYWSVLKPGQTLRIKSDGNLIAPWYGYILSGESHGRILKTD
LKRGSCTVQCQTEKGGLNTTLPFQNVSKYAFGNCSKYIGIKSLKLAVGLRNVPSRSSRGLFGAIAGFIEG
GWSGLVAGWYGFQHSNDQGVGMAADRDSTQKAIDKITSKVNNIVDKMNKQYEIIDHEFSEVETRLNMINN
KIDDQIQDIWAYNAELLVLLENQKTLDEHDANVNNLYNKVKRALGSNAVEDGKGCFELYHKCNDQCMETI
RNGTYNRRKYQEESKLERQKIEGVKLESEGTYKILTIYSTVASSLVIAMGFAAFLFWAMSNGSCRCNICI

>QBK20894.1 hemagglutinin [Influenza A virus]
METVSLITILIVATVSNADKICIGYQSTNSTETVDTLTENNVPVTHAKELLHTEHNGMLCATSLGQPLIL
DTCTIEGLIYGNPSCDLSLEGREWSYIVERPSAVNGLCYPGNVENLEELRSLFSSARSYQRIQIFPDTIW
NVSYDGTSTACSNSFYRSMRWLTRKNGDYPTQDAQYTNNQGKNILFMWGINHPPTDDTQRNLYTRTDTTT
SVATEEINRIFKPLIGPRPLVNGLMGRIDYYWSVLKPGQTLRIKSDGNLIAPWYGHILSGESHGRILKTD
LKRGSCTVQCQTEKGGLNTTLPFQNVSKYAFGNCSKYIGIKSLKLAVGLRNVPSRSSRGLFGAIAGFIEG
GWSGLVAGWYGFQHSNDQGVGMAADRDSTQKAIDKITSKVNNIVDKMNKQYEIIDHEFSEVETRLNMINN
KIDDQIQDIWAYNAELLVLLENQKTLDEHDANVNNLYNKVKRALGSNAVEDGKGCFELYHKCNDQCMETI
RNGTYNRKKYQEESRLERQKIEGVKLESEGTYKILTIYSTVASSLVIAMGFAAFLFWAMSNGSCRCNICI

>QBK20893.1 hemagglutinin [Influenza A virus]
METVSLITILLVATVSNADKICIGYQSTNSTETVDTLTENNVPVTHAKELLHTEHNGMLCATSLGQPLIL
DTCTIEGLIYGNPSCDLSLEGREWSYIVERPSAVNGLCYPGNVENLEELRSLFSSARSYQRIQIFPDTIW
NVSYDGTSTACSNSFYRSMRWLTRKDGDYPTQDAQYTNNQGKNILFMWGINHPPTDDTQRNLYTRTDTTT
SVATEEINRIFKPLIGPRPLVNGLMGRIDYYWSVLKPGQTLRIKSDGNLIAPWYGYILSGESHGRILKTD
LKRGSCTVQCQTEKGGLNTTLPFQNVSKYAFGNCSKYIGIKSLKLAVGLRNVPSRSSRGLFGAIAGFIEG
GWSGLVAGWYGFQHSNDQGVGMAADRDSTQKAIDKITSKVNNIVDKMNKQYEIIDHEFSEVETRLNMINN
KIDDQIQDIWAYNAELLVLLENQKTLDEHDANVNNLYNKVKRALGSNAVEDGKGCFELYHKCNDQCMETI
RNGTYNRRKYQEESKLERQKIEGVKLESEGTYKILTIYSTVASSLVIAMGFAAFLFWAMSNGSCRCNICI

>QBK20892.1 hemagglutinin [Influenza A virus]
METVSLITILLVATVSNADKICIGYQSTNSTETVDTLTENNVPVTHAKELLHTEHNGMLCATSLGQPLIL
DTCTIEGLIYGNPSCDLSLEGREWSYIVERPSAVNGLCYPGNVENLEELRSLFSSARSYQRIQIFPDTIW
NVSYDGTSTACSNSFYRSMRWLTRKDGNYPTQDAQYTNNQGKNILFMWGINHPPTDDTQRNLYTRTDTTT
SVATEEINRIFKPLIGPRPLVNGLMGRIDYYWSVLKPGQTLRIKSDGNLIAPWYGYILSGESHGRILKTD
LKRGSCTVQCQTEKGGLNTTLPFQNVSKYAFGNCSKYIGIKSLKLAVGLRNVPSRSSRGLFGAIAGFIEG
GWSGLVAGWYGFQHSNDQGVGMAADRDSTQKAIDKITSKVNNIVDKMNKQYEIIDHEFSEVETRLNMINN
KIDDQIQDIWAYNAELLVLLENQKTLDEHDANVNNLYNKVKRALGSNAVEDGKGCFELYHKCNDQCMETI
RNGTYNRRKYQEESKLERQKIEGVKLESEGTYKILTIYSTVASSLVIAMGFAAFLFWAMSNGSCRCNICI

>QBK20891.1 hemagglutinin [Influenza A virus]
METVSLITILIVATVSNADKICIGYQSTNSTETVDTLTENNVPVTHAKELLHTEHNGMLCATSLGQPLIL
DTCTIEGLIYGNPSCDLSLEGREWSYIVERPSAVNGLCYPGNVENLEELRSLFSSARSYQRIQIFPDTIW
NVSYDGTSTACSNSFYRSMRWLTRKNGDYPTQDAQYTNNQGKNILFMWGINHPPTDDTQRNLYTRTETTT
SVATEEINRIFKPLIGPRPLVNGLMGRIDYYWSVLKPGQTLRIKSDGNLIAPWYGHILSGESHGRILKTD
LKRGSCTVQCQTEKGGLNTTLPFQNVSKYAFGNCSKYIGIKSLKLAVGLRNVPSRSSRGLFGAIAGFIEG
GWSGLVAGWYGFQHSNDQGVGMAADRDSTQKAIDKITSKVNNIVDKMNKQYEIIDHEFSEVETRLNMINN
KIDDQIQDIWAYNAELLVLLENQKTLDEHDANVNNLYNKVKRALGSNAVEDGKGCFELYHKCNDQCMETI
RNGTYNRKKYQEESRLERQKIEGVKLESEGTYKILTIYSTVASSLVIAMGFAAFLFWAMSNGSCRCNICI

>QBK20890.1 hemagglutinin [Influenza A virus]
METVSLITILIVATVSNADKICIGYQSTNSTETVDTLTENNVPVTHAKELLHTEHNGMLCATSLGQPLIL
DTCTIEGLIYGNPSCDLSLEGREWSYIVERPSAVNGLCYPGNVENLEELRSLFSSARSYQRIQIFPDTIW
NVSYDGTSTACSNSFYRSMRWLTRKNGDYPTQDAQYTNNQGKNILFMWGINHPPTDDTQRNLYTRTDTTT
SVATEEINRIFKPLIGPRPLVNGLMGRIDYYWSVLKPGQTLRIKSDGNLIAPWYGHILSGESHGRILKTD
LKRGSCTVQCQTEKGGLNTTLPFQNVSKYAFGNCSKYIGIKSLKLAVGLRNVPSRSSRGLFGAIAGFIEG
GWSGLVAGWYGFQHSNDQGVGMAADRDSTQKAIDKITSKVNNIVDKMNKQYEIIDHEFSEVETRLNMINN
KIDDQIQDIWAYNAELLVLLENQKTLDEHDANVNNLYNKVKRALGSNAVEDGKGCFELYHKCNDQCMETI
RNGTYNRKKYQEESRLERQKIEGVKLESEGTYKILTIYSTVASSLVIAMGFAAFLFWAMSNGSCRCNICI

>QBK20889.1 hemagglutinin [Influenza A virus]
METVSLITILLVATVSNADKICIGYQSTNSTETVDTLTENNVPVTHAKELIHTKHNGMLCATSLGQPLIL
DTCTIEGLIYGNPSCDLSLEGREWSYIVERPSAVNGLCYPGNVENLEELRSLFSSARSYQRIQIFPDTIW
NVSYDGTSTACSGSFYRNMRWLTRKDGNYPTQDAQYTNNQGKNILFMWGINHPPTDTTQSGLYTRTDTTT

SVATEEINRIFKPLIGPRPLVNGLMGRIDYYWSVLKPGQTLRIKSDGNLIAPWFGHILSGESHGRILKTD
LKRGSCTVQCQTEKGGLNTTLPFQNVSKYAFGNCSKYIGIKSLKLAVGLRNVPSRSSRGLFGAIAGFIEG
GWSGLIAGWYGFQHSNDQGVGMAADRDSTQKAIDKITSKVNNIVDKMNKQYEIIDHEFSEVETRLNMINN
KIDDQIQDIWAYNAELLVLLENQKTLDEHDANVNNLYNKVKRALGSNAVEDGKGCFELYHKCDDQCMETI
RNGTYNRRKYQEESKLERQKIEGVKLESEGTYKILTIYSTVASSLVIAMGFAAFLFWAMSNGSCRCNICI

>QBK20888.1 hemagglutinin [Influenza A virus]
METVSLITILLVATVSNADKICIGYQSTNSTETVDTLTENNVPVTHAKELIHTKHNGMLCATSLGQPLIL
DTCTIEGLIYGNPSCDLSLEGREWSYIVERPSAVNGLCYPGNVENLEELRSLFSSARSYQRIQIFPDTIW
NVSYDGTSTACSGSFYRNMRWLTRKDGNYPTQDAQYTNNQGKNILFMWGINHPPTDTTQSGLYTRTDTTT
SVATEEINRIFKPLIGPRPLVNGLMGRIDYYWSVLKPGQTLRIKSDGNLIAPWFGHILSGESHGRILKTD
LKRGSCTVQCQTEKGGLNTTLPFQNVSKYAFGNCSKYIGIKSLKLAVGLRNVPSRSSRGLFGAIAGFIEG
GWSGLIAGWYGFQHSNDQGVGMAADRDSTQKAIDKITSKVNNIVDKMNKQYEIIDHEFSEVETRLNMINN
KIDDQIQDIWAYNAELLVLLENQKTLDEHDANVNNLYNKVKRALGSNAVEDGKGCFELYHKCDDQCMETI
RNGTYNRRKYQEESKLERQKIEGVKLESEGTYKILTIYSTVASSLVIAMGFAAFLFWAMSNGSCRCNICI

>QBK20887.1 hemagglutinin [Influenza A virus]
METISLITILLVATVSNADKICIGYQSTNSTETVDTLTENNVPVTHAKELIHTKHNGMLCATSLGQPLIL
DTCTIEGLIYGNPSCDLSLEGREWSYIVERPSAVNGLCYPGNVENLEELRSLFSSARSYQRIQIFPDTIW
NVSYDGTSTACSGSFYRNMRWLTRKDGNYPTQDAQYTNNQGKNILFMWGINHPPTDTTQSGLYTRTDTTT
SVATEEINRIFKPLIGPRPLVNGLMGRIDYYWSVLKPGQTLRIKSDGNLIAPWFGHILSGESHGRILKTD
LKRGSCTVQCQTEKGGLNTTLPFQNVSKYAFGNCSKYIGIKSLKLAVGLRNVPSRSSRGLFGAIAGFIEG
GWSGLIAGWYGFQHSNDQGVGMAADRDSTQKAIDKITSKVNNIVDKMNKQYEIIDHEFSEVETRLNMINN
KIDDQIQDIWAYNAELLVLLENQKTLDEHDANVNNLYNKVKRALGSNAVEDGKGCFELYHKCDDQCMETI
RNGTYNRRKYQEESKLERQKIEGVKLESEGTYKILTIYSTVASSLVIAMGFAAFLFWAMSNGSCRCNICI

>QBK20886.1 hemagglutinin [Influenza A virus]
METVSLITILLVATVSNADKICIGYQSTNSTETVDTLTENNVPVTHAKELIHTKHNGMLCATSLGQPLIL
DTCTIEGLIYGNPSCDLSLEGREWSYIVERPSAVNGLCYPGNVENLEELRSLFSSARSYQRIQIFPDTIW
NVSYDGTSTACSGSFYRNMRWLTRKDGNYPTQDAQYTNNQGKNILFMWGINHPPTDTTQSGLYTRTDTTT
SVATEEINRIFKPLIGPRPLVNGLMGRIDYYWSVLKPGQTLRIKSDGNLIAPWFGHILSGESHGRILKTD
LKRGSCTVQCQTEKGGLNTTLPFQNVSKYAFGNCSKYIGIKSLKLAVGLRNVPSRSSRGLFGAIAGFIEG
GWSGLIAGWYGFQHSNDQGVGMAADRDSTQKAIDKITSKVNNIVDKMNKQYEIIDHEFSEVETRLNMINN
KIDDQIQDIWAYNAELLVLLENQKTLDEHDANVNNLYNKVKRALGSNAVEDGKGCFELYHKCDDQCMETI

RNGTYNRRKYQEESKLERQKIEGVKLESEGTYKILTIYSTVASSLVIAMGFAAFLFWAMSNGSCRCNICI

>QBK20885.1 hemagglutinin [Influenza A virus]
METISLITILLVATVSNADKICIGYQSTNSTETVDTLTENNVPVTHAKELIHTKHNGMLCATSLGQPLIL
DTCTIEGLIYGNPSCDLSLEGREWSYIVERPSAVNGLCYPGNVENLEELRSLFSSARSYQRIQIFPDTIW
NVSYDGTSTACSGSFYRNMRWLTRKDGNYPTQDAQYTNNQGKNILFMWGINHPPTDTTQSGLYTRTDTTT
SVATEEINRIFKPLIGPRPLVNGLMGRIDYYWSVLKPGQTLRIKSDGNLIAPWFGHILSGESHGRILKTD
LKRGSCTVQFQTEKGGLNTTLPFQNVSKYAFGNCSKYIGIKSLKLAVVLRNVPSRSSRGLFGAIAGFIEG
GWSGLIAGWYGFQHSNDQGVGMAADRDSTQKAIDKITSKVNNIVDKMTKQYEIIDHEFSEVETRLNMINN
KIDDQIQDIWAYNAELLVLLENQKTLDEHDANVNNLYNKVKRALGSNAVEDGKGCFELYHKCDDQCMETI
RNGTYNRRKYQEESKLERQKIEGVKLESEGTYKILTIYSTVASSLVIAMGFAAFLFWAMSNGSCRCNICI

>QBK20884.1 hemagglutinin [Influenza A virus]
METVSLITILLVATVSNADKICIGYQSTNSTETVDTLTENNVPVTHAKELIHTKHNGMLCATSLGQPLIL
DTCTIEGLIYGNPSCDLSLEGREWSYIVERPSAVNGLCYPGNVENLEELRSLFSSAKSYQRIQIFPDTIW
NVSYDGTSTACSGSFYRNMRWLTRKDGNYPTQDAQYTNNQGKNILFMWGINHPPTDTTQSALYTRTDTTT
SVATEEINRIFKPLIGPRPLVNGLMGRIDYYWSVLKPGQTLRIKSDGNLIAPWFGHILSGESHGRILKTD
LKRGSCTVQCQTEKGGLNTTLPFQNVSKYAFGNCSKYIGIKSLKLAVGLRNVPSRSSRGLFGAIAGFIEG
GWSGLIAGWYGFQHSNDQGVGMAADRDSTQKAIDKITSKVNNIVDKMNKQYEIIDHEFSEVETRLNMINN
KIDDQIQDIWAYNAELLVLLENQKTLDEHDANVNNLYNKVKRALGSNAVEDGKGCFELYHKCDDQCMETI
RNGTYNRRKYQEESKLERQKIEGVKLESEGTYKILTIYSTVASSLVIAMGFAAFLFWAMSNGSCRCNICI

>QBK20883.1 hemagglutinin [Influenza A virus]
METVSLITILLVATVSNADKICIGYQSTNSTETVDTLTENNVPVTHAKELLHTEHNGMLCATSLGQPLIL
DTCTIEGLIYGNPSCDLSLEGREWSYIVERPSAVNGLCYPGNVENLEELRSLFSSARSYQRIQIFPDTIW
NVSYDGTSTACSGSFYRSMRWLTRKNGEYPIQDAQYTNNQGKNILFMWGINHPPTDTTQRDLYTRTDTTT
SVATEEINRVFKPLIGPRPLVNGLMGRIDYYWSVLKPGQTLRIKSDGNLIAPWFGHILSGESHGRILKTD
LKRGSCTVQCQTEKGGLNTTLPFQNVSKYAFGNCSKYIGIKSLKLAVGLRNVPSRSSRGLFGAIAGFIEG
GWSGLVAGWYGFQHSNDQGVGMAADRDSTQKAIDKITSKVNNIVDKMNKQYEIIDHEFSEVETRLNMINN
KIDDQIQDIWAYNAELLVLLENQKTLDEHDANVNNLYNKVKRALGSNAVEDGKGCFELYHKCDDQCMETI
RNGTYNRRKYQEESKLERQKIEGVKLESEGTYKILTIYSTVASSLVIAMGFAAFLFWAMSNGSCRCNICI

>QBK20882.1 hemagglutinin [Influenza A virus]
METVSLITILLVATVSNADKICIGYQSTNSTETVDTLTENNVPVTHAKELLHTEHNGMLCATSLGQPLIL

DTCTIEGLIYGNPSCDLSLEGREWSYIVERPSAVNGLCYPGNVENLEELRSLFSSARSYQRIQIFPDTIW
NVSYDGTSTACSGSFYRSMRWLTRKNGEYPIQDAQYTNNQGKNILFMWGINHPPTDTTQRDLYTRTDTTT
SVATEEINRVFKPLIGPRPLVNGLMGRIDYYWSVLKPGQTLRIKSDGNLIAPWFGHILSGESHGRILKTD
LKRGSCTVQCQTEKGGLNTTLPFQNVSKYAFGNCSKYIGIKSLKLAVGLRNVPSRSSRGLFGAIAGFIEG
GWSGLVAGWYGFQHSNDQGVGMAADRDSTQKAIDKITSKVNNIVDKMNKQYEIIDHEFSEVETRLNMINN
KIDDQIQDIWAYNAELLVLLENQKTLDEHDANVNNLYNKVKRALGSNAVEDGKGCFELYHKCDDQCMETI
RNGTYNRRKYQEESKLERQKIEGVKLESEGTYKILTIYSTVASSLVIAMGFAAFLFWAMSNGSCRCNICI

>QBK20881.1 hemagglutinin [Influenza A virus]
METVSLITILLVATVSNADKICIGYQSTNSTETVDTLTENNVPVTHAKELLHTEHNGMLCATSLGQPLIL
DTCTIEGLIYGNPSCDLSLEGREWSYIVERPSAVNGLCYPGNVENLEELRSLFSSAKSYQRIQIFSDTIW
NVSYDGTSTACSGSFYRSMRWLTRKDGEYPIQDAQYTNNQGKNILFMWGINHPPTDATQIALYTRTDTTT
SVATEEINRIFKPLIGPRPLVNGLMGRIDYYWSVLKPGQTLRIKSDGNLIAPWFGHILSGESHGRILKTD
LKRGSCTVQCQTEKGGLNTTLPFQNVSKYAFGNCSKYIGIKSLKLAVGLRNVPSRSSRGLFGAIAGFIEG
GWSGLVAGWYGFQHSNDQGVGMAADRDSTQKAIDKITSKVNNIVDKMNKQYEIIDHEFSEVETRLNMINN
KIDDQIQDIWAYNAELLVLLENQKTLDEHDANVNNLYNKVKRALGSNAVEDGKGCFELYHKCDDQCMETI
RNGTYNRRKYQEESKLERQKIEGVKLESEGTYKILTIYSTVASSLVIAMGFAAFLFWAMSNGSCRCNICI

>QBK20880.1 hemagglutinin [Influenza A virus]
METISLITILLVATVSNADKICIGYQSTNSTETVDTLTENNVPVTHAKELIHTEHNGMLCATSLGQPLIL
DTCTIEGLIYGNPSCDLSLEGREWSYIVERPSAVNGLCYPGNVENLEELRSLFSSARSYQRIQIFPDTIW
NVSYDGTSTACSGSFYRNMRWLTRKDGNYPTQDAQYTNNQGKNILFMWGINHPPTDTTQSGLYTRTDTTT
SVATEEINRIFKPLIGPRPLVNGLMGRIDYYWSVLKPGQTLRIKSDGNLIAPWFGHILSGESHGRILKTD
LKRGSCTVQCQTEKGGLNTTLPFQNVSKYAFGNCSKYIGIKSLKLAVGLRNVPSRSSRGLFGAIAGFIEG
GWSGLIAGWYGFQHSNDQGVGMAADRDSTQKAIDKITSKVNNIVDKMNKQYEIIDHEFSEVETRLNMIND
KIDDQIQDIWAYNAELLVLLENQKTLDEHDANVNNLYNKVKRALGSNAVEDGKGCFELYHKCDDQCMETI
RNGTYNRRKYQEESKLERQKIEGVKLESEGTYKILTIYSTVASSLVIAMGFAAFLFWAMSNGSCRCNICI

>QBK20879.1 hemagglutinin [Influenza A virus]
METVSLITILLVATVSSADKICIGYQSTNSTETVDTLTENNVPVTHAKELIHTEHNGMLCATSLGQPLIL
DTCTIEGLIYGNPSCNLSLEGREWSYIVERPSAVNGLCYPGNVENLEELRSLFSSARSYQRIQIFPDTIW
NVSYDGTSTACSGSFYRNMRWLTRKDGNYPTQDAQYTNNQGKNILFMWGINHPPTETTQSGLYTRTDTTT
SVATEEINRIFKPLIGPRPLVNGLMGRIDYYWSVLKPGQTLRIKSDGNLIAPWFGHILSGESHGRILKTD
LKRGSCTVQCQTEKGGLNTTLPFQNVSKYAFGNCSKYIGIKSLKLAVGLRNVPSRSSRGLFGAIAGFIEG

GWSGLVAGWYGFQHSNDQGVGMAADRDSTQKAIDKITSKVNNIVDKMNKQYEIIDHEFSEVETRLNMINN
KIDDQIQDIWAYNAELLVMLENQKTLDEHDANVNNLYNKVKRALGSNAVEDGKGCFELYHKCDDQCMETI
RNGTYNRRKYQEESKLERQKIEGVKLESEGTYKILTIYSTVASSLVIAMGFAAFLFWAMSNGSCRCNICI

>QBK20878.1 hemagglutinin [Influenza A virus]
METVSLITILLVATVSSADKICIGYQSTNSTETVDTLTENNVPVTHAKELIHTEHNGMLCATSLGQPLIL
DTCTIEGLIYGNPSCDLSLEGREWSYIVERPSAVNGLCYPGNVENLEELRSLFSSARSYQRIQIFPDTIW
NVSYDGTSTACSGSFYRNMRWLTRKDGNYPTQDAQYTNNQGKNILFMWGINHPPTETTQSGLYTRTDTTT
SVATEEINRIFKPLIGPRPLVNGLMGRIDYYWSVLKPGQTLRIKSDGNLIAPWFGHILSGESHGRILKTD
LKRGSCTVQCQTEKGGLNTTLPFQNVSKYAFGNCSKYIGIKSLKLAVGLRNVPSRSSRGLFGAIAGFIEG
GWSGLVAGWYGFQHSNDQGVGMAADRDSTQKAIDKITSKVNNIVDKMNKQYEIIDHEFSEVETRLNMINN
KIDDQIQDIWAYNAELLVMLENQKTLDEHDANVNNLYNKVKRALGSNAVEDGKGCFELYHKCDDQCMETI
RNGTYNRRKYQEESKLERQKIEGVKLESEGTYKILTIYSTVASSLVIAMGFAAFLFWAMSNGSCRCNICI

>QBK20877.1 hemagglutinin [Influenza A virus]
METVSLITILLVATVSSADKICIGYQSTNSTETVDTLTENNVPVTHAKELIHTEHNGMLCATSLGQPLIL
DTCTIEGLIYGNPSCDLSLEGREWSYIVERPSAVNGLCYPGNVENLEELRSLFSSARSYQRIQIFPDTIW
NVSYDGTSTACSGSFYRNMRWLTRKDGNYPTQDAQYTNNQGKNILFMWGINHPPTETTQSGLYTRTDTTT
SVATEEINRIFKPLIGPRPLVNGLMGRIDYYWSVLKPGQTLRIKSDGNLIAPWFGHILSGESHGRILKTD
LKRGSCTVQCQTEKGGLNTTLPFQNVSKYAFGNCSKYIGIKSLKLAVGLRNVPSRSSRGLFGAIAGFIEG
GWSGLVAGWYGFQHSNDQGVGMAADRDSTQKAIDKITSKVNNIVDKMNKQYEIIDHEFSEVETRLNMINN
KIDDQIQDIWAYNAELLVMLENQKTLDEHDANVNNLYNKVKRALGSNAVEDGKGCFELYHKCDDQCMETI
RNGTYNRRKYQEESKLERQKIEGVKLESEGTYKILTIYSTVASSLVIAMGFAAFLFWAMSNGSCRCNICI

>QBK20876.1 hemagglutinin [Influenza A virus]
METVSLITILLVATVSSADKICIGYQSTNSTETVDTLTENNVPVTHAKELIHTEHNGMLCATSLGQPLIL
DTCTIEGLIYGNPSCDLSLEGREWSYIVERPSAVNGLCYPGNVENLEELRSLFSSARSYQRIQIFPDTIW
NVSYDGTSTACSGSFYRNMRWLTRKDGNYPTQDAQYTNNQGKNILFMWGINHPPTETTQSGLYTRTDTTT
SVATEEINRIFKPLIGPRPLVNGLMGRIDYYWSVLKPGQTLRIKSDGNLIAPWFGHILSGESHGRILKTD
LKRGSCTVQCQTEKGGLNTTLPFQNVSKYAFGNCSKYIGIKSLKLAVGLRNVPSRSSRGLFGAIAGFIEG
GWSGLVAGWYGFQHSNDQGVGMAADRDSTQKAIDKITSKVNNIVDKMNKQYEIIDHEFSEVETRLNMINN
KIDDQIQDIWAYNAELLVMLENQKTLDEHDANVNNLYNKVKRALGSNAVEDGKGCFELYHKCDDQCMETI
RNGTYNRRKYQEESKLERQKIEGVKLESEGTYKILTIYSTVASSLVIAMGFAAFLFWAMSNGSCRCNICI

>QBK20875.1 hemagglutinin [Influenza A virus]

METASLITILLVATVSNADKICIGYQSTNSTETVDTLTENNVPVTHAKELLHTEHNGMLCATSLGQPLIL
DTCTIEGLIYGNPSCDLSLEGREWSYIVERPSAVNGLCYPGKVENLEELRSLFSSARSYQRIQIFPDTIW
NVSYDGTSTACSGSFYRSLRWLTRKNGEYPIQDAQYTNNQGKNILFMWGINHPPTDTTQRDLYTRTDTTT
SVATEEINRIFKPLIGPRPLVNGLMGRIDYYWSVLKPGQTLRIKSDGNLIAPWFGHILSGESHGRILKTD
LKRGSCTVQCQTEKGGLNTTLPFQNVSKYAFGNCSKYIGIKSLKLAVGLRNVPSRSSRGLFGAIAGFIEG
GWSGLVAGWYGFQHSNDQGVGMAADRESTQKAIDKITSKVNNIVDKMNKQYEIIDHEFSEVETRLNMINN
KIDDQIQDIWAYNAELLVLLENQKTLDEHDANVNNLYNKVKRALGSNAVEDGKGCFELYHKCDDQCMETI
RNGTYNRRKYQEESKLERQKIEGVKLESEGTYKILTIYSTVASSLVIAMGFAAFLFWAMSNGSCRCNICI

>QBK20874.1 hemagglutinin [Influenza A virus]

METVSLITILLVATVSNADKICIGYQSTNSTETVDTLTENNVPVTHAKELLHTEHNGMLCATSLGQPLIL
DTCTIEGLIYGNPSCDLSLEGREWSYIVERPSAVNGLCYPGNVENLEELRSLFSSARSYQRIQIFPDTIW
NVSYDGTSTACSGSFYRSMRWLTRKNGEYPVQDAQYTNNQGKNILFMWGINHPPTDTTQRNLYTRTDTTT
SVATEEINRVFKPLIGPRPLVNGLMGRIDYYWSVLKPGQTLRIKSDGNLIAPWFGHILSGESHGRILKTD
LKRGSCTVQCQTEKGGLNTTLPFQNVSKYAFGNCSKYIGIKSLKLAVGLRNVPSRSSRGLFGAIAGFIEG
GWSGLVAGWYGFQHSNDQGVGMAADRDSTQKAIDKITSKVNNIVDKMNKQYEIIDHEFSEVETRLNMINN
KIDDQIQDIWAYNAELLVLLENQKTLDEHDANVNNLYNKVKRALGSNAVEDGKGCFELYHKCDDQCMETI
RNGTYNRRKYQEESKLERQKIEGVKLESEGTYKILTIYSTVASSLVIAMGFAAFLFWAMSNGSCRCNICI

>QBK20873.1 hemagglutinin [Influenza A virus]

METISLITILLIATVSNADKICIGYQSTNSTETVDTLTENNVPVTHAKELIHTEHNGMLCATSLGQPLIL
DTCTIEGLIYGNPSCDLSLEGREWSYIVERPSAVNGLCYPGNVENLEELRSLFSSARSYQRIQIFPDTIW
NVSYDGTSTACSGSFYRNMRWLTRKDGNYPTQDAQYTNNQGKNILFMWGINHPPTDTTQSGLYTRTDTTT
SVATEEINRIFKPLIGPRPLVNGLMGRIDYYWSVLKPGQTLRIKSDGNLIAPWFGHILSGESHGRILKTD
LKRGSCTVQCQTEKGGLNTTLPFQNVSKYAFGNCSKYIGIKSLKLAVGLRNVPSRSSRGLFGAIAGFIEG
GWSGLVAGWYGFQHSNDQGVGMAADRDSTQKAIDKITSKVNNIVDKMNKQYEIIDHEFSEVETRLNMINN
KIDDQIQDIWAYNAELLVLLENQKTLDEHDANVNNLYNKVKRALGSNAVEDGKGCFELYHKCDDQCMETI
RNGTYNRRKYQEESKLERQKIEGVKLESEGTYKILTIYSTVASSLVIAMGFAAFLFWAMSNGSCRCNICI

>QBK20871.1 hemagglutinin [Influenza A virus]

METVSLITILLVATVSNADKICIGYQSTNSTETVDTLTENNVPVTHAKELLHTEHNGMLCATSLGQPLIL
DTCTIEGLIYGNPSCDLSLEGREWSYIVERPSAVNGLCYPGNVENLEELRSLFSSARSYQRIQIFPDTIW
NVSYDGTSTACSGSFYRSMRWLTRKNGEYPIQDAQYTNNQGKNILFMWGINHPPTDTTQRDLYTRTDTTT

SVATEEINRVFKPLIGPRPLVNGLMGRIDYYWSVLKPGQTLRIKSDGNLIAPWFGHILSGESHGRILKTD
LKRGSCTVQCQTEKGGLNTTLPFQNVSKYAFGNCSKYIGIKSLKLAVGLRNVPSRSSRGLFGAIAGFIEG
GWSGLVAGWYGFQHSNDQGVGMAADRDSTQKAIDKITSKVNNIVDKMNKQYEIIDHEFSEVETRLNMINN
KIDDQIQDIWAYNAELLVLLENQKTLDEHDANVNNLYNKVKRALGSNAVEDGKGCFELYHKCDDQCMETI
RNGTYNRRKYQEESKLERQKIEGVKLESEGTYKILTIYSTVASSLVIAMGFAAFLFWAMSNGSCRCNICI

>QBK20870.1 hemagglutinin [Influenza A virus]
METVSLITILLVATVSNADKICIGYQSTNSTETVDTLTENNVPVTHAKELLHTEHNGMLCATSLGQPLIL
DTCTIEGLIYGNPSCDLSLEGREWSYIVERPSAVNGLCYPGNVENLEELRSLFSSARSYQRIQIFPDTIW
NVSYDGTSTACSGSFYRSMRWLTRKNGEYPIQDAQYTNNQGKNILFMWGINHPPTDTTQRDLYTRTDTTT
SVATEEINRVFKPLIGPRPLVNGLMGRIDYYWSVLKPGQTLRIKSDGNLIAPWFGHILSGESHGRILKTD
LKRGSCTVQCQTEKGGLNTTLPFQNVSKYAFGNCSKYIGIKSLKLAVGLRNVPSRSSRGLFGAIAGFIEG
GWSGLVAGWYGFQHSNDQGVGMAADRDSTQKAIDKITSKVNNIVDKMNKQYEIIDHEFSEVETRLNMINN
KIDDQIQDIWAYNAELLVLLENQKTLDEHDANVNNLYNKVKRALGSNAVEDGKGCFELYHKCDDQCMETI
RNGTYNRRKYQEESKLERQKIEGVKLESEGTYKILTIYSTVASSLVIAMGFAAFLFWAMSNGSCRCNICI

>QBK20869.1 hemagglutinin [Influenza A virus]
METVSLITILLVATVSNADKICIGYQSTNSTETVDTLTENNVPVTHAKELLHTEHNGMLCATSLGQPLIL
DTCTIEGLIYGNPSCDLSLEGREWSYIVERPSAVNGLCYPGNVENLEELRSLFSSARSYQRIQIFPDTIW
NVSYDGTSTACSGSFYRSMRWLTRKNGEYPIQDAQYTNNQGKNILFMWGINHPPTDTTQRDLYTRTDTTT
SVATEEINRVFKPLIGPRPLVNGLMGRIDYYWSVLKPGQTLRIKSDGNLIAPWFGHILSGESHGRILKTD
LKRGSCTVQCQTEKGGLNTTLPFQNVSKYAFGNCSKYIGIKSLKLAVGLRNVPSRSSRGLFGAIAGFIEG
GWSGLVAGWYGFQHSNDQGVGMAADRDSTQKAIDKITSKVNNIVDKMNKQYEIIDHEFSEVETRLNMINN
KIDDQIQDIWAYNAELLVLLENQKTLDEHDANVNNLYNKVKRALGSNAVEDGKGCFELYHKCDDQCMETI
RNGTYNRRKYQEESKLERQKIEGVKLESEGTYKILTIYSTVASSLVIAMGFAAFLFWAMSNGSCRCNICI

>QBK20867.1 hemagglutinin [Influenza A virus]
METISLITILLVATVSNADKICIGYQSTNSTETVDTLTENNVPVTHAKELIHTEHNGMLCATSLGQPLIL
DTCTIEGLIYGNPSCDLSLEGREWSYIVERPSAVNGLCYPGNVENLEELRSLFSSARSYQRIQIFPDTIW
NVSYDGTSTACSGSFYRNMRWLTRKDGNYPTQDAQYTNNQGKNILFMWGINHPPTDTTQSGLYTRTDTTT
SVATEEINRIFKPLIGPRPLVNGLMGRIDYYWSVLKPGQTLRIKSDGNLIAPWFGHILSGESHGRILKTD
LKRGSCTVQCQTEKGGLNTTLPFQNVSKYAFGNCSKYIGIKSLKLAVGLRNVPSRSSRGLFGAIAGFIEG
GWSGLVAGWYGFQHSNDQGVGMAADRDSTQKAIDKITSKVNNIVDKMNKQYEIIDHEFSEVETRLNMINN
KIDDQIQDIWAYNAELLVLLENQKTLDEHDANVNNLYNKVKRALGSNAVEDGKGCFELYHKCDDQCMETI

RNGTYNRRKYQEESKLERQKIEGVKLESEGTYKILTIYSTVASSLVIAMGFAAFLFWAMSNGSCRCNICI

>QBK20864.1 hemagglutinin [Influenza A virus]
METISLITILLIATVSNADKICIGYQSTNSTETVDTLTENNVPVTHAKELIHTEHNGMLCATSLGQPLIL
DTCTIEGLIYGNPSCDLSLEGREWSYIVERPSAVNGLCYPGNVENLEELRSLFSSARSYQRIQIFPDTIW
NVSYDGTSTACSGSFYRNMRWLTRKDGNYPTQDAQYTNNQGKNILFMWGINHPPTDTTQSGLYTRTDTTT
SVATEEINRIFKPLIGPRPLVNGLMGRIDYYWSVLKPGQTLRIKSDGNLIAPWFGHILSGESHGRILKTD
LKRGSCTVQCQTEKGGLNTTLPFQNVSKYAFGNCSKYIGIKSLKLAVGLRNVPSRSSRGLFGAIAGFIEG
GWSGLVAGWYGFQHSNDQGIGMAADRDSTQKAIDKITSKVNNIVDKMNKQYEIIDHEFSEVETRLNMINN
KIDDQIQDIWAYNAELLVLLENQKTLDEHDANVNNLYNKVKRALGSNAVEDGKGCFELYHKCDDQCMETI
RNGTYNRRKYQEESKLERQKIEGVKLESEGTYKILTIYSTVASSLVIAMGFAAFLFWAMSNGSCRCNICI

>QBK20863.1 hemagglutinin [Influenza A virus]
METVSLITILLIATVSNADKICIGYQSTNSTETVDTLTENNVPVTHAKELIHTEHNGMLCATSLGQPLIL
DTCTIEGLIYGNPSCDLSLEGREWSYIVERPSAVNGLCYPGNVENLEELRSLFSSARSYQRIQIFPDTIW
NVSYDGTSTACSGSFYRNMRWLTRKDGNYPTQDAQYTNNQGKNILFMWGINHPPTDTTQSGLYTRTDTTT
SVATEEINRIFKPLIGPRPLVNGLMGRIDYYWSVLKPGQTLRIKSDGNLIAPWFGHILSGESHGRILKTD
LKRGSCTVQCQTEKGGLNTTLPFQNVSKYAFGNCSKYIGIKSLKLAVGLRNVPSRSSRGLFGAIAGFIEG
GWSGLVAGWYGFQHSNDQGVGMAADRDSTQKAIDKITSKVNNIVDKMNKQYEIIDHEFSEVETRLNMINN
KIDDQIQDIWAYNAELLVLLENQKTLDEHDANVNNLYNKVKRALGSNAVEDGKGCFELYHKCDDQCMETI
RNGTYNRRKYQEESKLERQKIEGVKLESEGTYKILTIYSTVASSLVIAMGFAAFLFWAMSNGSCRCNICI

>QBK20860.1 hemagglutinin [Influenza A virus]
METVSLITILLVATVSNADKICIGYQSTNSTETVDTLTENNVPVTHAKELLHTEHNGMLCATSLGQPLIL
DTCTIEGLIYGNPSCDLSLEGREWSYIVERPSAVNGLCYPGNVENLEELRSLFSSARSYQRIQIFPDTIW
NVSYDGTSTACSGSFYRSMRWLTRKNGEYPIQDAQYTNNQGKNILFMWGINHPPTDTTQRDLYTRTDTTT
SVATEEINRVFKPLIGPRPLVNGLMGRIDYYWSVLKPGQTLRIKSDGNLIAPWFGHILSGESHGRILKTD
LKRGSCTVQCQTEKGGLNTTLPFQNVSKYAFGNCSKYIGIKSLKLAVGLRNVPSRSSRGLFGAIAGFIEG
GWSGLVAGWYGFQHSNDQGVGMAADRDSTQKAIDKITSKVNNIVDKMNKQYEIIDHEFSEVETRLNMINN
KIDDQIQDIWAYNAELLVLLENQKTLDEHDANVNNLYNKVKRALGSNAVEDGKGCFELYHKCDDQCMETI
RNGTYNRRKYQEESKLERQKIEGVKLESEGTYKILTIYSTVASSLVIAMGFAAFLFWAMSNGSCRCNICI

>QBK20859.1 hemagglutinin [Influenza A virus]
METVSLITILLVATVSNADKICIGYQSTNSTETVDTLTENNVPVTHAKELLHTEHNGMLCATSLGQPLIL

DTCTIEGLIYGNPSCDLSLEGREWSYIVERPSAVNGLCYPGNVENLEELRSLFSSARSYQRIQIFPDTIW
NVSYDGTSTACSGSFYRSMRWLTRKNGEYPIQDAQYTNNQGKNILFMWGINHPPTDTTQRDLYTRTDTTT
SVATEEINRVFKPLIGPRPLVNGLMGRIDYYWSVLKPGQTLRIKSDGNLIAPWFGHILSGESHGRILKTD
LKRGSCTVQCQTEKGGLNTTLPFQNVSKYAFGNCSKYIGIKSLKLAVGLRNVPSRSSRGLFGAIAGFIEG
GWSGLVAGWYGFQHSNDQGVGMAADRDSTQKAIDKITSKVNNIVDKMNKQYEIIDHEFSEVETRLNMINN
KIDDQIQDIWAYNAELLVLLENQKTLDEHDANVNNLYNKVKRALGSNAVEDGKGCFELYHKCDDQCMETI
RNGTYNRRKYQEESKLERQKIEGVKLESEGTYKILTIYSTVASSLVIAMGFAAFLFWAMSNGSCRCNICI

>QBK20858.1 hemagglutinin [Influenza A virus]
METVSLITILLVATVSNADKICIGYQSTNSTETVDTLTENNVPVTHAKELLHTEHNGMLCATSLGQPLIL
DTCTIEGLIYGNPSCDLSLEGREWSYIVERPSAVNGLCYPGNVENLEELRSLFSSARSYQRIQIFPDTIW
NVSYDGTSTACSGSFYRSMRWLTRKNGEYPIQDAQYTNNQGKNILFMWGINHPPTDTTQRDLYTRTDTTT
SVATEEINRVFKPLIGPRPLVNGLMGRIDYYWSVLKPGQTLRIKSDGNLIAPWFGHILSGESHGRILKTD
LKRGSCTVQCQTEKGGLNTTLPFQNVSKYAFGNCSKYIGIKSLKLAVGLRNVPSRSSRGLFGAIAGFIEG
GWSGLVAGWYGFQHSNDQGVGMAADRDSTQKAIDKITSKVNNIVDKMNKQYEIIDHEFSEVETRLNMINN
KIDDQIQDIWAYNAELLVLLENQKTLDEHDANVNNLYNKVKRALGSNAVEDGKGCFELYHKCDDQCMETI
RNGTYNRRKYQEESKLERQKIEGVKLESEGTYKILTIYSTVASSLVIAMGFAAFLFWAMSNGSCRCNICI

>QBK20857.1 hemagglutinin [Influenza A virus]
METVSLITILLVATVSNADKICIGYQSTNSTETVDTLTENNVPVTHAKELIHTEHNGMLCATSLGQPLIL
DTCTIEGLIYGNPSCDLSLEGREWSYIVERPSAVNGLCYPGNVENLEELRSLFSSARSYQRIQIFPDTIW
NVSYDGTSTACSGSFYRNMRWLTRKDGNYPTQDAQYTNNQGKNILFMWGINHPPTDTTQSGLYTRTDTTT
SVATEEINRIFKPLIGPRPLVNGLMGRIDYYWSVLKPGQTLRIKSDGNLIAPWFGHILSGESHGRILKTD
LKRGSCTVQCQTEKGGLNTTLPFQNVSKYAFGNCSKYIGIKSLKLAVGLRNVPSRSSRGLFGAIAGFIEG
GWSGLVAGWYGFQHSNDQGVGMAADRDSTQKAIDKITSKVNNIVDKMNKQYEIIDHEFSEVETRLNMINN
KIDDQIQDIWAYNAELLVLLENQKTLDEHDANVNNLYNKVKRALGSNAVEDGKGCFELYHKCDDQCMETI
RNGTYNRRKYQEESKLERQKIEGVKLESEGTYKILTIYSTVASSLVIAMGFAAFLFWAMSNGSCRCNICI

>QBK20856.1 hemagglutinin [Influenza A virus]
METISLITILLIATVSNADKICIGYQSTNSTETVDTLTENNVPVTHAKELIHTEHNGMLCATSLGQPLIL
DTCTIEGLIYGNPSCDLSLEGREWSYIVERPSAVNGLCYPGNVENLEELRSLFSSARSYQRIQIFPDTIW
NVSYDGTSTACSGSFYRNMRWLTRKDGNYPTQDAQYTNNQGKNILFMWGINHPPTDTTQSGLYTRTDTTT
SVATEEINRIFKPLIGPRPLVNGLMGRIDYYWSVLKPGQTLRIKSDGNLIAPWFGHILSGESHGRILKTD
LKRGSCTVQCQTEKGGLNTTLPFQNVSKYAFGNCSKYIGIKSLKLAVGLRNVPSRSSRGLFGAIAGFIEG

GWSGLVAGWYGFQHSNDQGVGMAADRDSTQKAIDKITSKVNNIVDKMNKQYEIIDHEFSEVETRLNMINN
KIDDQIQDIWAYNAELLVLLENQKTLDEHDANVNNLYNKVKRALGSNAVEDGKGCFELYHKCDDQCMETI
RNGTYNRRKYQEESKLERQKIEGVKLESEGTYKILTIYSTVASSLVIAMGFAAFLFWAMSNGSCRCNICI

>QBK20855.1 hemagglutinin [Influenza A virus]
METVSLITILLVATVSNADKICIGYQSTNSTETVDTLTENNVPVTHAKELIHTEHNGMLCATSLGQPLIL
DTCTIEGLIYGNPSCDLSLEGREWSYIVERPSAVNGLCYPGNVENLEELRSLFSSARSYQRIQIFPDTIW
NVSYDGTSTACSGSFYRNMRWLTRKDGNYPTQDAQYTNNQGKNILFMWGINHPPTDTTQSGLYTRTDTTT
SVATEEINRIFKPLIGPRPLVNGLMGRIDYYWSVLKPGQTLRIKSDGNLIAPWFGHILSGESHGRILKTD
LKRGNCTVQCQTEKGGLNTTLPFQNVSKYAFGNCSKYIGIKSLKLAVGLRNVPSRSSRGLFGAIAGFIEG
GWSGLVAGWYGFQHSNDQGVGMAADRDSTQKAIDKITSKVNNIVDKMNKQYEIIDHEFSEVETRLNMINN
KIDDQIQDIWAYNAELLVLLENQKTLDEHDANVNNLYNKVKRALGSNAVEDGKGCFELYHKCDDQCMETI
RNGTYNRRKYQEESKLERQKIEGVKLESEGTYKILTIYSTVASSLVIAMGFAAFLFWAMSNGSCRCNICI

>QBK20854.1 hemagglutinin [Influenza A virus]
METISLITILLVATVSNADKICIGYQSTNSTETVDTLTENNVPVTHAKELIHTEHNGMLCATSLGQPLIL
DTCTIEGLIYGNPSCDLSPEGREWSYIVERPSAVNGLCYPGNVENLEELRSLFSSARSYQRIQIFPDTIW
NVSYDGTSTACSGSFYRNMRWLTRKDGNYPTQDAQYTNNQGKNILFMWGINHPPTDTTQSSLYTRTDTTT
SVATEEINRIFKPLIGPRPLVNGLMGRIDYYWSVLKPGQTLRIKSDGNLIAPWFGHILSGESHGRILKTD
LKRGSCTVQCQTEKGGLNTTLPFQNVSKYAFGNCSKYIGIKSLKLAVGLRNVPSRSSRGLFGAIAGFIEG
GWSGLVAGWYGFQHSNDQGVGMAADRDSTQKAIDKITSKVNNIVDKMNKQYEIIDHEFSEVETRLNMINN
KIDDQIQDIWAYNAELLVLLENQKTLDEHDANVNNLYNKVKRALGSNAVEDGKGCFELYHKCDDQCMETI
RNGTYNRRKYQEESKLERQKIEGVKLESEGTYKILTIYSTVASSLVIAMGFAAFLFWAMSNGSCRCNICI

>QBK20853.1 hemagglutinin [Influenza A virus]
METVSLITILLVATVSNADKICIGYQSTNSTETVDTLTENNVPVTHAKELLHTEHNGMLCATSLGQPLIL
DTCTIEGLIYGNPSCDPLLDGREWSYIVERPSAVNGLCYPGHVENLEELRSLFSSARSYQRIQIFPDTIW
NVSYDGTSTACSGSFYRSMRWLTRKNGDYPIQDAQYTNNQGKNILFMWGINQPPTDTTQRELYTRIDTTT
SVATEEINRIFKPLIGPRPLVNGLMGRIDYYWSVLRPGQTLRIRSDGNLIAPWYGHILSGESHGRILKTD
LKRGSCTVQCQTEKGGLNTTLPFQNVSKYAFGNCSKFIGIKSLKLAVGLRNVPSRSSRGLFGAIAGFIEG
GWSGLVAGWYGFQHSNDQGVGMAADRESTQKAIDKITSKVNNIVDKMNKQYEIIDHEFSEIETRLNMINN
KIDDQIQDIWAYNAELLVLLENQKTLDEHDANVNNLYNKVKRALGSNAVEDGKGCFELYHKCDDQCMETI
RNGTYNRRKYQEESKLERQKIEGVKLESEGTYKILTIYSTVASSLVIAMGFAAFLFWAMSNGSCRCNICI

>QBK20852.1 hemagglutinin [Influenza A virus]

METVSLITILLVATVSNADKICIGYQSTNSTETVDTLTENNVPVTHAKELIHTEHNGMLCATSLGQPLIL

DTCTIEGLIYGNPSCDLSLEGREWSYIVERPSAVNGLCYPGNVENLEELRSLFSSARSYQRIQIFPDTIW

NVSYDGTSTACSGSFYRSMRWLTRKNGEYPIQDAQYTNNQGKNILFMWGINHPPTDTTQRDLYTRTDTTT

SVATEEINRIFKPLIGPRPLVNGLMGRIDYYWSVLKPGQTLRIKSDGNLIAPWFGHILSGESHGRILKTD

LKRGSCTVQCQTEKGGLNTTLPFQNVSKYAFGNCSKYIGIKSLKLAVGLRNVPSKSSRGLFGAIAGFIEG

GWSGLVAGWYGFQHSNDQGVGMAADRDSTQKAIDKITSKVNNIVDKMNKQYEIIDHEFSEVETRLNMINN

KIDDQIQDIWAYNAELLVLLENQKTLDEHDANVNNLYNKVKRALGSNAVEDGKGCFELYHKCDDQCMETI

RNGTYNRRKYQEESKLERQKIEGVKLESEGTYKILTIYSTVASSLVIAMGFAAFLFWAMSNGSCRCNICI

>QBK20851.1 hemagglutinin [Influenza A virus]

METVSLITILLVATVSNADKICIGYQSTNSTDTVDTLTENNVPVTHAKELLHTEHNGMLCATSLGQPLIL

DTCTIEGLIYGNPSCDLSLEGREWSYIVERPSAVNGLCYPGNVENLEELRSLFSSARSYQRVQIFPDTIW

NVSYDGTSTACSGSFYRSMRWLTRKNGEYPIQDAQYTNSQGKNILFMWGINHPPTDTTQEALYTRTDTTT

SVATEEINRIFKPLIGPRPLVNGLMGRIDYYWSVLKPGQTLRIKSDGNLIAPWYGHVLSGESHGRILKTD

LKRGSCTVQCQTEKGGLNTTLPFQNVSKYAFGNCSKYIGIKSLKLAVGLRNVPSRSSRGLFGAIAGFIEG

GWPGLVAGWYGFQHSNDQGVGMAADRDSTQKAIDKITSKVNNIVDKMNKQYEIIDHEFSEVETRLNMINN

KIDDQIQDIWAYNAELLVLLENQKTLDEHDANVNNLYNKVKRALGSNAVEDGKGCFELYHKCDDQCMDTI

RNGTYNRRKYQEESKLERQKIEGVKLESEGTYKILTIYSTVASSLVIAMGFAAFLFWAMSNGSCRCNICI

>QBK20850.1 hemagglutinin [Influenza A virus]

METVSLITILLVATVSNADKICIGYQSTNSTETVDTLTENNVPVTHAKELLHTEHNGMLCATSLGQPLVL

DTCTIEGLIYGNPSCDLSLEGREWSYIVERPSAVHGLCYPGNVEDLEELRSLFSSARSYQRIQIFPDTIW

NVSYDGTSTACSGSFYRSMRWLTRKNGDYPIQDAQYTNNQGKNILFMWGINHPPTDETQRGLYTRIDTTT

SVATEEINRIFKPLIGPRPLVNGLMGRINYYWSVLKPGQTLRIKSDGNLIAPWYGHILSGESHGRILKTD

LKRGSCTVQCQTEKGGLNTTLPFQNVSKYAFGNCSKYIGIKSLKLAVGLRNVPSRSSRGLFGAIAGFIEG

GWSGLVAGWYGFQHSNDQGVGMAADRDSTQKAIDKITSKVNNIVDKMNKQYEIIDHEFSEVETRLNMINN

KIDDQIQDIWAYNAELLVLLENQKTLDEHDANVNNLYNKVKRALGSNAVEDGKGCFELYHKCDDQCMETI

RNGTYNRRKYQEESKLERQKIEGVKLESEGTYKILTIYSTVASSLVIAMGFAAFLFWAMSNGSCRCNICI

>QBK20849.1 hemagglutinin [Influenza A virus]

METVSLITILLVATVSNADKICIGYQSTNSTETVDTLTENNVPVTHAKELLHTEHNGMLCATSLGHPLIL

DTCTIEGLIYGNPSCDPLLGGREWSYIVERPSAVNGLCYPGNVENLEELRSLFSSSRSYQRIQIFPDTIW

NVSYSGTSKACSDSFYRSMRWLTQKNNAYPTQDAQYTNNQEKNILFMWGINHPPTDTAQTNLYTRTDTTT

SVATEEMNRIFKPLIGPRPLVNGLMGRINYYWSVLKPGQTLRIKSDGNLIAPWYGHILSGESHGRILKTD
LKSGSCTVQCQTEKGGLNTTLPFQNVSKYAFGNCSKYIGVKSLKLAVGLRNVPSRSSRGLFGAIAGFIEG
GWSGLVAGWYGFQHSNDQGVGMAADRDSTQKAIDKITSKVNNIVDKMNKQYEIIDHEFSEVETRLNMINN
KVDDQIQDIWAYNAELLVLLENQKTLDEHDANVNNLYNKVKRALGSNAVEDGKGCFELYHKCDDHCMETI
RNGTYNRRKYQEESKLERQKIEGVKLESEETYKILTIYSTVASSLVIAMGFAAFLFWAMSNGSCRCNICI

>QBK20848.1 hemagglutinin [Influenza A virus]
METVSLITILLVATVSNADKICIGYQSTNSTETVDTLTENNVPVTHAKELLHTEHNGMLCATSLGQPLVL
DTCTIEGLIYGNPSCDLSLEGREWSYIVERPSAVHGLCYPGNVEDLEELRSLFSSARSYQRIQIFPDTIW
NVSYDGTSTACSGSFYRSMRWLTRKNGDYPIQDAQYTNNQGKNILFMWGINHPPTDETQRGLYTRIDTTT
SVATEEINRIFKPLIGPRPLVNGLMGRINYYWSVLKPGQTLRIKSDGNLIAPWYGHILSGESHGRILKTD
LKRGSCTVQCQTEKGGLNTTLPFQNVSKYAFGNCSKYIGIKSLKLAVGLRNVPSRSSRGLFGAIAGFIEG
GWSGLVAGWYGFQHSNDQGVGMAADRDSTQKAIDKITSKVNNIVDKMNKQYEIIDHEFSEVETRLNMINN
KIDDQIQDIWAYNAELLVLLENQKTLDEHDANVNNLYNKVKRALGSNAVEDGKGCFELYHKCDDQCMETI
RNGTYNRRKYQEESKLERQKIEGVKLESEGTYKILTIYSTVASSLVIAMGFAAFLFWAMSNGSCRCNICI

>QBK20847.1 hemagglutinin [Influenza A virus]
METVSLITILLVATVSNADKICIGYQSTNSTETVDTLTENNVPVTHAKELLHTEHNGMLCATSLGQPLVL
DTCTIEGLIYGNPSCDLSLEGREWSYIVERPSAVHGLCYPGNVEDLEELRSLFSSARSYQRIQIFPDTIW
NVSYDGTSTACSGSFYRSMRWLTRKNGDYPIQDAQYTNNQGKNILFMWGINHPPTDETQRGLYTRIDTTT
SVATEEINRIFKPLIGPRPLVNGLMGRINYYWSVLKPGQTLRIKSDGNLIAPWYGHILSGESHGRILKTD
LKRGSCTVQCQTEKGGLNTTLPFQNVSKYAFGNCSKYIGIKSLKLAVGLRNVPSRSSRGLFGAIAGFIEG
GWSGLVAGWYGFQHSNDQGVGMAADRDSTQKAIDKITSKVNNIVDKMNKQYEIIDHEFSEVETRLNMINN
KIDDQIQDIWAYNAELLVLLENQKTLDEHDANVNNLYNKVKRALGSNAVEDGKGCFELYHKCDDQCMETI
RNGTYNRRKYQEESKLERQKIEGVKLESEGTYKILTIYSTVASSLVIAMGFAAFLFWAMSNGSCRCNICI

>QBK20846.1 hemagglutinin [Influenza A virus]
METVSLITILLVATVSNADKICIGYQSTNSTETVDTLTENNVPVTHAKELLHTEHNGMLCATSLGQPLVL
DTCTIEGLIYGNPSCDLSLEGREWSYIVERPSAVHGLCYPGNVEDLEELRSLFSSARSYQRIQIFPDTIW
NVSYDGTSTACSGSFYRSMRWLTRKNGDYPIQDAQYTNNQGKNILFMWGINHPPTDETQRGLYTRIDTTT
SVATEEINRIFKPLIGPRPLVNGLMGRINYYWSVLKPGQTLRIKSDGNLIAPWYGHILSGESHGRILKTD
LKRGSCTVQCQTEKGGLNTTLPFQNVSKYAFGNCSKYIGIKSLKLAVGLRNVPSRSSRGLFGAIAGFIEG
GWSGLVAGWYGFQHSNDQGVGMAADRDSTQKAIDKITSKVNNIVDKMNKQYEIIDHEFSEVETRLNMINN
KIDDQIQDIWAYNAELLVLLENQKTLDEHDANVNNLYNKVKRALGSNAVEDGKGCFELYHKCDDQCMETI

RNGTYNRRKYQEESKLERQKIEGVKLESEGTYKILTIYSTVASSLVIAMGFAAFLFWAMSNGSCRCNICI

>QBK20845.1 hemagglutinin [Influenza A virus]
METISLITILLVATVSNADKICIGYQSTNSTETVDTLTENNVPVTHAKELIHTEHNGMLCATSLGQPLIL
DTCTIEGLIYGNPSCDLSLEGREWSYIVERPSAVNGLCYPGIVENLEELRSLFSSARSYQRIQIFPDTIW
NVSYDGTSTACSGSFYRNMRWLTRKDGNYPTQDAQYTNNQGKNILFMWGINHPPTDATQSGLYTRTDTTT
SVATEEINRIFKPLIGPRPLVNGLMGRIDYYWSVLKPGQTLRIKSDGNLIAPWFGHILSGESHGRILKTD
LKRGSCTVQCQTEKGGLNTTLPFQNVSKYAFGNCSKYIGIKSLKLAVGLRNVPSRSSRGLFGAIAGFIEG
GWSGLVAGWYGFQHSNDQGVGIAADRDSTQKAIDKITSKVNNIVDKMNKQYEIIDHEFSEVETRLNMINN
KIDDQIQDIWAYNAELLVLLENQKTLDEHDANVNNLYNKVKRALGSNAVEDGKGCFELYHKCDDQCMETI
RNGTYNRRKYQEESKLERQKIEGVKLESEGTYKILTIYSTVASSLVIAMGFAAFLFWAMSNGSCRCNICI

>QBK20844.1 hemagglutinin [Influenza A virus]
METISLITILLVATVSNADKICIGYQSTNSTETVDTLTENNVPVTHAKELIHTEHNGMLCATSLGQPLIL
DTCTIEGLIYGNPSCDLSLEGREWSYIVERPSAVNGLCYPGNVENLEELRSLFSSARSYQRIQIFPDTIW
NVSYDGTSTACSGSFYRNMRWLTRKDGNYPTQDAQYTNNQGKNILFMWGINHPPTDTTQSGLYTRTDTTT
SVATEEINRIFKPLIGPRPLVNGLMGRIDYYWSVLKPGQTLRIKSDGNLIAPWFGHILSGESHGRILKTD
LKRGSCTVQCQTEKGGLNTTLPFQNVSKYAFGNCSKYIGIKSLKLAVGLRNVPSRSSRGLFGAIAGFIEG
GWSGLVAGWYGFQHSNDQGVGMAADRDSTQKAIDKITSKVNNIVDKMNKQYEIIDHEFSEVETRLNMINN
KIDDQIQDIWAYNAELLVLLENQKTLDEHDANVNNLYNKVKRALGSNAVEDGKGCFELYHKCDDQCMETI
RNGTYNRRKYQEESKLERQKIEGVKLESEGTYKILTIYSTVASSLVIAMGFAAFLFWAMSNGSCRCNICI

>QBK20843.1 hemagglutinin [Influenza A virus]
METISLITILLVATVSNADKICIGYQSTNSTETVDTLTENNVPVTHAKELIHTEHNGMLCATSLGQPLIL
DTCTIEGLIYGNPSCDLSLEGREWSYIVERPSAVNGLCYPGNVENLEELRSLFSSARSYQRIQIFPDTIW
NVSYDGTSTACSGSFYRNMRWLTRKDGNYPTQDAQYTNNQGKNILFMWGINHPPTDTTQSGLYTRTDTTT
SVATEEINRIFKPLIGPRPLVNGLMGRIDYYWSVLKPGQTLRIKSDGNLIAPWFGHILSGESHGRILKTD
LKRGSCTVQCQTEKGGLNTTLPFQNVSKYAFGNCSKYIGIKSLKLAVGLRNVPSRSSRGLFGAIAGFIEG
GWSGLVAGWYGFQHSNDQGVGMAADRDSTQKAIDKITSKVNNIVDKMNKQYEIIDHEFSEVETRLNMINN
KIDDQIQDIWAYNAELLVLLENQKTLDEHDANVNNLYNKVKRALGSNAVEDGKGCFELYHKCDDQCMETI
RNGTYNRRKYQEESKLERQKIEGVKLESEGTYKILTIYSTVASSLVIAMGFAAFLFWAMSNGSCRCNICI

>QBK20842.1 hemagglutinin [Influenza A virus]
METISLITILLVATVSNADKICIGYQSTNSTETVDTLTENNVPVTHAKELIHTEHNGMLCATSLGQPLIL

DTCTIEGLIYGNPSCDLSLEGREWSYIVERPSAVNGLCYPGNVENLEELRSLFSSARSYQRIQIFPDTIW
NVSYDGTSTACSGSFYRNMRWLTRKDGNYPTQDAQYTNNQGKNILFMWGINHPPTDTTQSGLYTRTDTTT
SVATEEINRIFKPLIGPRPLVNGLMGRIDYYWSVLKPGQTLRIKSDGNLIAPWFGHILSGESHGRILKTD
LKRGSCTVQCQTEKGGLNTTLPFQNVSKYAFGNCSKYIGIKSLKLAVGLRNVPSRSSRGLFGAIAGFIEG
GWSGLVAGWYGFQHSNDQGVGMAADRDSTQKAIDKITSKVNNIVDKMNKQYEIIDHEFSEVETRLNMINN
KIDDQIQDIWAYNAELLVLLENQKTLDEHDANVNNLYNKVKRALGSNAVEDGKGCFELYHKCDDQCMETI
RNGTYNRRKYQEESKLERQKIEGVKLESEGTYKILTIYSTVASSLVIAMGFAAFLFWAMSNGSCRCNICI

>QBK20841.1 hemagglutinin [Influenza A virus]
METVSLITILLVATVSNADKICIGYQSTNSTETVDTLTENNVPVTHAKELLHTEHNGMLCATSLGQPLIL
DTCTIEGLIYGNPSCDLSLEGREWSYIVERPSAVHGLCYPGNVEDLEELRSLFSSARSYQRIQIFPDTIW
NVSYDGTSTACSGSFYRSMRWLTRKNGDYPIQDAQYTNNQGKNILFMWGINHPPTDETQRGLYTRTDTTT
SVATEEINRIFKPLIGPRPLVNGLMGRINYYWSVLKPGQTLRIKSDGNLIAPWYGHILSGESHGRILKTD
LKRGSCTVQCQTEKGGLNTTLPFQNVSKYAFGNCSKYIGIKSLKLAVGLRNVPSRSSRGLFGAIAGFIEG
GWSGLVAGWYGFQHSNDQGVGMAADRDSTQKAIDKITSKVNNIVDKMNKQYEIIDHEFSEVETRLNMINN
KIDDQIQDIWAYNAELLVLLENQKTLDEHDANVNNLYNKVKRALGSNAVEDGKGCFELYHKCDDQCMETI
RNGTYNRRKYQEESKLERQKIEGVKLESEGTYKILTIYSTVASSLVIAMGFAAFLFWAMSNGSCRCNICI

>QBK20840.1 hemagglutinin [Influenza A virus]
METISLITILLVATVSNADKICIGYQSTNSTETVDTLTENNVPVTHAKELIHTEHNGMLCATSLGQPLIL
DTCTIEGLIYGNPSCDLSLEGREWSYIVERPSAVNGLCYPGNVENLEELRSLFSSARSYQRIQIFPDTIW
NVSYDGTSTACSGSFYRNMRWLTRKDGNYPTQDAQYTNNQGKNILFMWGINHPPTDTTQSGLYTRTDTTT
SVATEEINRIFKPLIGPRPLVNGLMGRIDYYWSVLKPGQTLRIKSDGNLIAPWFGHILSGESHGRILKTD
LKRGSCTVQCQTEKGGLNTTLPFQNVSKYAFGNCSKYIGIKSLKLAVGLRNVPSRSSRGLFGAIAGFIEG
GWSGLVAGWYGFQHSNDQGVGMAADRDSTQKAIDKITSKVNNIVDKMNKQYEIIDHEFSEVETRLNMINN
KIDDQIQDIWAYNAELLVLLENQKTLDEHDANVNNLYNKVKRALGSNAVEDGKGCFELYHKCDDQCMETI
RNGTYNRRKYQEESKLERQKIEGVKLESEGTYKILTIYSTVASSLVIAIGFAAFLFWAMSNGSCRCNICI

>QBK20839.1 hemagglutinin [Influenza A virus]
METVSLITILLVATVSNADKICIGYQSTNSTETVDTLTENNVPVTHAKELIHTEHNGMLCATSLGQPLIL
DTCTIEGLIYGNPSCDLSLEGREWSYIVERPSAVNGLCYPGNVENLEELRSLFSSARSYQRIQIFPDTIW
NVSYDGTSTACSGSFYRNMRWLTRKDGNYPTQDAQYTNNQGKNILFMWGINHPPTDTTQSGLYTRTDTTT
SVATEEINRIFKPLIGPRPLVNGLMGRIDYYWSVLKPGQTLRIKSDGNLIAPWFGHILSGESHGRILKTD
LKRGSCTVQCQTEKGGLNTTLPFQNVSKYAFGNCSKYIGIKSLKLAVGLRNVPSRSSRGLFGAIAGFIEG

GWSGLVAGWYGFQHSNDQGVGIAADRDSTQKAIDKITSKVNNIVDKMNKQYEIIDHEFSEVETRLSMINN
KIDDQIQDIWAYNAELLVLLENQKTLDEHDANVNNLYNKVKRALGSNAVEDGKGCFELYHKCDDQCMETI
RNGTYNRRKYQEESKLERQKIEGVKLESEGTYKILTIYSTVASSLVIAMGFAAFLFWAMSNGSCRCNICI

>QBK20838.1 hemagglutinin [Influenza A virus]
METISLITILLVATVSNADKICIGYQSTNSTETVDTLTENNVPVTHAKELIHTEHNGMLCATSLGQPLIL
DTCTIEGLIYGNPSCDLSLEGREWSYIVERPSAVNGLCYPGNVENLEELRSLFSSARSYQRIQIFPDTIW
NVSYDGTSTACSGSFYRNMRWLTRKDGNYPTQDAQYTNNQGKNILFMWGINHPPTDTTQSGLYTRTDTTT
SVATEEINRIFKPLIGPRPLVNGLMGRIDYYWSVLKPGQTLRIKSDGNLIAPWFGHILSGESHGRILKTD
LKRGSCTVQCQTEKGGLNTTLPFQNVSKYAFGNCSKYIGIKSLKLAVGLRNVPSRSSRGLFGAIAGFIEG
GWSGLVAGWYGFQHSNDQGVGMAADRDSTQKAIDKITSKVNNIVDKMNKQYEIIDHEFSEVETRLNMINN
KIDDQIQDIWAYNAELLVLLENQKTLDEHDANVNNLYNKVKRALGSNAVEDGKGCFELYHKCDDQCMETI
RNGTYNRRKYQEESKLERQKIEGVKLESEGTYKILTIYSTVASSLVIAMGFAAFLFWAMSNGSCRCNICI

>QBK20837.1 hemagglutinin [Influenza A virus]
METVSLITILLVATVSNADKICIGYQSTNSTETVDTLTENNVPVTHAKELLHTEHNGMLCATSLGQPLIL
DTCTIEGLIYGNPSCDLSLEGREWSYIVERPSAVHGLCYPGNVEDLEELRSLFSSARSYQRIQIFPDTIW
NVSYDGTSTACSGSFYRSMRWLTRKNGDYPIQDAQYTNNQGKNILFMWGINHPPTDATQRGLYTRTDTTT
SVATEEINRIFKPLIGPRPLVNGLMGRINYYWSVLKPGQTLRIKSDGNLIAPWYGHILSGESHGRILKTD
LKRGSCTVQCQTEKGGLNTTLPFQNVSKYAFGNCSKYIGIKSLKLAVGLRNVPSRSSRGLFGAIAGFIEG
GWSGLVAGWYGFQHSNDQGVGMAADRDSTQKAIDKITSKVNNIVDKMNKQYEIIDHEFSEVETRLNMINN
KIDDQIQDIWAYNAELLVLLENQKTLDEHDANVNNLYNKVKRALGSNAVEDGKGCFELYHKCDDQCMETI
RNGTYNRRKYQEESKLERQKIEGVKLESEGTYKILTIYSTVASSLVIAMGFAAFLFWAMSNGSCRCNICI

>QBK20836.1 hemagglutinin [Influenza A virus]
METVSLITILLVATVSNADKICIGYQSTNSTETVDTLTENNVPVTHAKELLHTEHNGMLCATSLGHPLIL
DTCTIEGLIYGNPSCDPLLGGREWSYIVERPSAVNGLCYPGNVENLEELRSLFSSSRSYQRIQIFPDTIW
NVSYSGTSKACSDSFYRSMRWLTQKNNAYPTQDAQYTNNQEKNILFMWGINHPPTDTAQTNLYTRTDTTT
SVATEEMNRIFKPLIGPRPLVNGLMGRINYYWSVLKPGQTLRIKSDGNLIAPWYGHILSGESHGRILKTD
LKSGSCTVQCQTEKGGLNTTLPFQNVSKYAFGNCSKYIGVKSLKLAVGLRNVPSRSSRGLFGAIAGFIEG
GWSGLVAGWYGFQHSNDQGVGMAADRDSTQKAIDKITSKVNNIVDKMNKQYEIIDHEFSEVETRLNMINN
KVDDQIQDIWAYNAELLVLLENQKTLDEHDANVNNLYNKVKRALGSNAVEDGKGCFELYHKCDDHCMETI
RNGTYNRRKYQEESKLERQKIEGVKLESEETYKILTIYSTVASSLVIAMGFAAFLFWAMSNGSCRCNICI

>QBK20835.1 hemagglutinin [Influenza A virus]
METVSLITILLVATVSNADKICIGYQSTNSTETVDTLTENNVPVTHAKELLHTEHNGMLCATSLGHPLIL
DTCTIEGLIYGNPSCDPLLGGREWSYIVERPSAVNGLCYPGNVENLEELRSLFSSSRSYQRIQIFPDTIW
NVSYSGTSKACSDSFYRSMRWLTQKNNAYPTQDAQYTNNQEKNILFMWGINHPPTDTAQTNLYTRTDTTT
SVATEEMNRIFKPLIGPRPLVNGLMGRINYYWSVLKPGQTLRIKSDGNLIAPWYGHILSGESHGRILKTD
LKSGSCTVQCQTEKGGLNTTLPFQNVSKYAFGNCSKYIGVKSLKLAVGLRNVPSRSSRGLFGAIAGFIEG
GWSGLVAGWYGFQHSNDQGVGMAADRDSTQKAIDKITSKVNNIVDKMNKQYEIIDHEFSEVETRLNMINN
KVDDQIQDIWAYNAELLVLLENQKTLDEHDANVNNLYNKVKRALGSNAVEDGKGCFELYHKCDDHCMETI
RNGTYNRRKYQEESKLERQKIEGVKLESEETYKILTIYSTVASSLVIAMGFAAFLFWAMSNGSCRCNICI

>QBK20834.1 hemagglutinin [Influenza A virus]
METVSLITILLVATVSNADKICIGYQSTNSTETVDTLTENNVPVTHAKELLHTEHNGMLCATSLGHPLIL
DTCTIEGLIYGNPSCDPLLGGREWSYIVERPSAVNGLCYPGNVENLEELRSLFSSSRSYQRIQIFPDTIW
NVSYSGTSKACSDSFYRSMRWLTQKNNAYPTQDAQYTNNQEKNILFMWGINHPPTDTAQTNLYTRTDTTT
SVATEEMNRIFKPLIGPRPLVNGLMGRINYYWSVLKPGQTLRIKSDGNLIAPWYGHILSGESHGRILKTD
LKSGSCTVQCQTEKGGLNTTLPFQNVSKYAFGNCSKYIGVKSLKLAVGLRNVPSRSSRGLFGAIAGFIEG
GWSGLVAGWYGFQHSNDQGVGMAADRDSTQKAIDKITSKVNNIVDKMNKQYEIIDHEFSEVETRLNMINN
KVDDQIQDIWAYNAELLVLLENQKTLDEHDANVNNLYNKVKRALGSNAVEDGKGCFELYHKCDDHCMETI
RNGTYNRRKYQEESKLERQKIEGVKLESEETYKILTIYSTVASSLVIAMGFAAFLFWAMSNGSCRCNICI

>QBK20833.1 hemagglutinin [Influenza A virus]
METVSLITILLVATVSNADKICIGYQSTNSTETVDTLTENNVPVTHAKELLHTEHNGMLCATSLGQPLIL
DTCTIEGLIYGNPSCDLSLEGREWSYIVERPSAVHGLCYPGNVEDLEELRSLFSSARSYQRIQIFPDTIW
NVSYDGTSTACSGSFYRSMRWLTRKNGDYPIQDAQYTNNQGKNILFMWGINHPPTDETQRGLYTRTDTTT
SVATEEINRIFKPLIGPRPLVNGLMGRINYYWSVLKPGQTLRIKSDGNLIAPWYGHILSGESHGRILKTD
LKRGSCTVQCQTEKGGLNTTLPFQNVSKYAFGNCSKYIGIKSLKLAVGLRNVPSRSSRGLFGAIAGFIEG
GWSGLVAGWYGFQHSNDQGVGMAADRDSTQKAIDKITSKVNNIVDKMNKQYEIIDHEFSEVETRLNMINN
KIDDQIQDIWAYNAELLVLLENQKTLDEHDANVNNLYNKVKRALGSNAVEDGKGCFELYHKCDDQCMETI
RNGTYNRRKYQEESKLERQKIEGVKLESEGTYKILTIYSTVASSLVIAMGFAAFLFWAMSNGSCRCNICI

>QBK20832.1 hemagglutinin [Influenza A virus]
METVSLITILLVATVSNADKICIGYQSTNSTETVDTLTENNVPVTHAKELLHTEHNGMLCATSLGQPLIL
DTCTIEGLIYGNPSCDLSLEGREWSYIVERPSAVHGLCYPGNVEDLEELRSLFSSARSYQRIQIFPDTIW
NVSYDGTSTACSGSFYRSMRWLTRKNGDYPIQDAQYTNNQGKNILFMWGINHPPTDETQRGLYTRTDTTT

SVATEEINRIFKPLIGPRPLVNGLMGRINYYWSVLKPGQTLRIKSDGNLIAPWYGHILSGESHGRILKTD
LKRGSCTVQCQTEKGGLNTTLPFQNVSKYAFGNCSKYIGIKSLKLAVGLRNVPSRSSRGLFGAIAGFIEG
GWSGLVAGWYGFQHSNDQGVGMAADRDSTQKAIDKITSKVNNIVDKMNKQYEIIDHEFSEVETRLNMINN
KIDDQIQDIWAYNAELLVLLENQKTLDEHDANVNNLYNKVKRALGSNAVEDGKGCFELYHKCDDQCMETI
RNGTYNRRKYQEESKLERQKIEGVKLESEGTYKILTIYSTVASSLVIAMGFAAFLFWAMSNGSCRCNICI

>QBK20831.1 hemagglutinin [Influenza A virus]
METVSLITILLVATVSNADKICIGYQSTNSTETVDTLTENNVPVTHAKELLHTEHNGMLCATSLGQPLIL
DTCTIEGLIYGNPSCDLSLEGREWSYIVERPSAVHGLCYPGNVEDLEELRSLFSSARSYQRIQIFPDTIW
NVSYDGTSTACSGSFYRSMRWLTRKNGDYPIQDAQYTNNQGKNILFMWGINHPPTDETQRGLYTRTDTTT
SVATEEINRIFKPLIGPRPLVNGLMGRINYYWSVLKPGQTLRIKSDGNLIAPWYGHILSGESHGRILKTD
LKRGSCTVQCQTEKGGLNTTLPFQNVSKYAFGNCSKYIGIKSLKLAVGLRNVPSRSSRGLFGAIAGFIEG
GWSGLVAGWYGFQHSNDQGVGMAADRDSTQKAIDKITSKVNNIVDKMNKQYEIIDHEFSEVETRLNMINN
KIDDQIQDIWAYNAELLVLLENQKTLDEHDANVNNLYNKVKRALGSNAVEDGKGCFELYHKCDDQCMETI
RNGTYNRRKYQEESKLERQKIEGVKLESEGTYKILTIYSTVASSLVIAMGFAAFLFWAMSNGSCRCNICI

>QBK20830.1 hemagglutinin [Influenza A virus]
METVSLITILLVATVSNADKICIGYQSTNSTETVDTLTENNVPVTHAKELLHTEHNGMLCATSLGQPLIL
DTCTIEGLIYGNPSCDLSLEGREWSYIVERPSAVHGLCYPGNVEDLEELRSLFSSARSYQRIQIFPDTIW
NVSYDGTSTACSGSFYRSMRWLTRKNGDYPIQDAQYTNNQGKNILFMWGINHPPTDETQRGLYTRTDTTT
SVATEEINRIFKPLIGPRPLVNGLMGRINYYWSVLKPGQTLRIKSDGNLIAPWYGHILSGESHGRILKTD
LKRGSCTVQCQTEKGGLNTTLPFQNVSKYAFGNCSKYIGIKSLKLAVGLRNVPSRSSRGLFGAIAGFIEG
GWSGLVAGWYGFQHSNDQGVGMAADRDSTQKAIDKITSKVNNIVDKMNKQYEIIDHEFSEVETRLNMINN
KIDDQIQDIWAYNAELLVLLENQKTLDEHDANVNNLYNKVKRALGSNAVEDGKGCFELYHKCDDQCMETI
RNGTYNRRKYQEESKLERQKIEGVKLESEGTYKILTIYSTVASSLVIAMGFAAFLFWAMSNGSCRCNICI

>QBK20829.1 hemagglutinin [Influenza A virus]
METVSLITILLVATVSNADKICIGYQSTNSTETVDTLTENNVPVTHAKELLHTEHNGMLCATSLGQPLIL
DTCTIEGLIYGNPSCDLSLEGREWSYIVERPSAVHGLCYPGNVEDLEELRSLFSSARSYQRIQIFPDTIW
NVSYDGTSTACSGSFYRSMRWLTRKNGDYPIQDAQYTNNQGKNILFMWGINHPPTDETQRGLYTRTDTTT
SVATEEINRIFKPLIGPRPLVNGLMGRINYYWSVLKPGQTLRIKSDGNLIAPWYGHILSGESHGRILKTD
LKRGSCTVQCQTEKGGLNTTLPFQNVSKYAFGNCSKYIGIKSLKLAVGLRNVPSRSSRGLFGAIAGFIEG
GWSGLVAGWYGFQHSNDQGVGMAADRDSTQKAIDKITSKVNNIVDKMNKQYEIIDHEFSEVETRLNMINN
KIDDQIQDIWAYNAELLVLLENQKTLDEHDANVNNLYNKVKRALGSNAVEDGKGCFELYHKCDDQCMETI

RNGTYNRRKYQEESKLERQKIEGVKLESEGTYKILTIYSTVASSLVIAMGFAAFLFWAMSNGSCRCNICI

>QBK20828.1 hemagglutinin [Influenza A virus]
METVSLITILLVATVSNADKICIGYQSTNSTETVDTLTENNVPVTHAKELLHTEHNGMLCATSLGQPLIL
DTCTIEGLIYGNPSCDLSLEGREWSYIVERPSAVHGLCYPGNVEDLEELRSLFSSARSYQRIQIFPDTIW
NVSYDGTSTACSGSFYRSMRWLTRKNGDYPIQDAQYTNNQGKNILFMWGINHPPTDETQRGLYTRTDTTT
SVATEEINRIFKPLIGPRPLVNGLMGRINYYWSVLKPGQTLRIKSDGNLIAPWYGHILSGESHGRILKTD
LKRGSCTVQCQTEKGGLNTTLPFQNVSKYAFGNCSKYIGIKSLKLAVGLRNVPSRSSRGLFGAIAGFIEG
GWSGLVAGWYGFQHSNDQGVGMAADRDSTQKAIDKITSKVNNIVDKMNKQYEIIDHEFSEVETRLNMINN
KIDDQIQDIWAYNAELLVLLENQKTLDEHDANVNNLYNKVKRALGSNAVEDGKGCFELYHKCDDQCMETI
RNGTYNRRKYQEESKLERQKIEGVKLESEGTYKILTIYSTVASSLVIAMGFAAFLFWAMSNGSCRCNICI

>QBK20827.1 hemagglutinin [Influenza A virus]
METVSLITILLVATVSNADKICIGYQSTNSTETVDTLTENNVPVTHAKELLHTEHNGMLCATSLGQPLIL
DTCTIEGLIYGNPSCDLSLEGREWSYIVERPSAVHGLCYPGNVEDLEELRSLFSSARSYQRIQIFPDTIW
NVSYDGTSTACSGSFYRSMRWLTRKNGDYPIQDAQYTNNQGKNILFMWGINHPPTDETQRGLYTRTDTTT
SVATEEINRIFKPLIGPRPLVNGLMGRINYYWSVLKPGQTLRIKSDGNLIAPWYGHILSGESHGRILKTD
LKRGSCTVQCQTEKGGLNTTLPFQNVSKYAFGNCSKYIGIKSLKLAVGLRNVPSRSSRGLFGAIAGFIEG
GWSGLVAGWYGFQHSNDQGVGMAADRDSTQKAIDKITSKVNNIVDKMNKQYEIIDHEFSEVETRLNMINN
KIDDQIQDIWAYNAELLVLLENQKTLDEHDANVNNLYNKVKRALGSNAVEDGKGCFELYHKCDDQCMETI
RNGTYNRRKYQEESKLERQKIEGVKLESEGTYKILTIYSTVASSLVIAMGFAAFLFWAMSNGSCRCNICI

>QBK20826.1 hemagglutinin [Influenza A virus]
METVSLITILLVATVSNADKICIGYQSTNSTETVDTLTENNVPVTHAKELLHTEHNGMLCATSLGQPLIL
DTCTIEGLIYGNPSCDLSLEGREWSYIVERPSAVHGLCYPGNVEDLEELRSLFSSARSYQRIQIFPDTIW
NVSYDGTSTACSGSFYRSMRWLTRKNGDYPIQDAQYTNNQGKNILFMWGINHPPTDETQRGLYTRTDTTT
SVATEEINRIFKPLIGPRPLVNGLMGRINYYWSVLKPGQTLRIKSDGNLIAPWYGHILSGESHGRILKTD
LKRGSCTVQCQTEKGGLNTTLPFQNVSKYAFGNCSKYIGIKSLKLAVGLRNVPSRSSRGLFGAIAGFIEG
GWSGLVAGWYGFQHSNDQGVGMAADRDSTQKAIDKITSKVNNIVDKMNKQYEIIDHEFSEVETRLNMINN
KIDDQIQDIWAYNAELLVLLENQKTLDEHDANVNNLYNKVKRALGSNAVEDGKGCFELYHKCDDQCMETI
RNGTYNRRKYQEESKLERQKIEGVKLESEGTYKILTIYSTVASSLVIAMGFAAFLFWAMSNGSCRCNICI

>QBK20825.1 hemagglutinin [Influenza A virus]
METVSLITILLVATVSNADKICIGYQSTNSTETVDTLTENNVPVTHAKELLHTEHNGMLCATSLGQPLIL

DTCTIEGLIYGNPSCDLSLEGREWSYIVERPSAVHGLCYPGNVEDLEELRSLFSSARSYQRIQIFPDTIW
NVSYDGTSTACSGSFYRSMRWLTRKNGDYPIQDAQYTNNQGKNILFMWGINHPPTDETQRGLYTRTDTTT
SVATEEINRIFKPLIGPRPLVNGLMGRINYYWSVLKPGQTLRIKSDGNLIAPWYGHILSGESHGRILKTD
LKRGSCTVQCQTEKGGLNTTLPFQNVSKYAFGNCSKYIGIKSLKLAVGLRNVPSRSSRGLFGAIAGFIEG
GWSGLVAGWYGFQHSNDQGVGMAADRDSTQKAIDKITSKVNNIVDKMNKQYEIIDHEFSEVETRLNMINN
KIDDQIQDIWAYNAELLVLLENQKTLDEHDANVNNLYNKVKRALGSNAVEDGKGCFELYHKCDDQCMETI
RNGTYNRRKYQEESKLERQKIEGVKLESEGTYKILTIYSTVASSLVIAMGFAAFLFWAMSNGSCRCNICI

>QBK20824.1 hemagglutinin [Influenza A virus]
METVSLITILLVATVSNADKICIGYQSTNSTETVDTLTENNVPVTHAKELLHTEHNGMLCATSLGHPLIL
DTCTIEGLIYGNPSCDPLLGGREWSYIVERPSAVNGLCYPGNVENLEELRSLFSSSRSYQRIQIFPDTIW
NVSYSGTSKACSDSFYRSMRWLTQKNNAYPTQDAQYTNNQEKNILFMWGINHPPTDTVQTNLYTRTDTTT
SVATEEMNRIFKPLIGPRPLVNGLMGRINYYWSVLKPGQTLRIKSDGNLIAPWYGHILSGESHGRILKTD
LKSGSCTVQCQTEKGGLNTTLPFQNVSKYAFGNCSKYIGVKSLKLAVGLRNVPSRSSRGLFGAIAGFIEG
GWSGLVAGWYGFQHSNDQGVGMAADRDSTQKAIDKITSKVNNIVDKMNKQYEIIDHEFSEVETRLNMINN
KVDDQIQDIWAYNAELLVLLENQKTLDEHDANVNNLYNKVKRALGSNAVEDGKGCFELYHKCDDHCMETI
RNGTYNRRKYQEESKLERQKIEGVKLESEETYKILTIYSTVASSLVIAMGFAAFLFWAMSNGSCRCNICI

>QBK20823.1 hemagglutinin [Influenza A virus]
METVSLITILLVATVSNADKICIGYQSTNSTETVDTLTENNVPVTHAKELLHTEHNGMLCATSLGQPLIL
DTCTIEGLIYGNPSCDLSLEGREWSYIVERPSAVHGLCYPGNVEDLEELRSLFSSARSYQRIQIFPDTIW
NVSYDGTSTACSGSFYRSMRWLTRKNGDYPIQDAQYTNNQGKNILFMWGINHPPTDETQRGLYTRTDTTT
SVATEEINRIFKPLIGPRPLVNGLMGRINYYWSVLKPGQTLRIKSDGNLIAPWYGHILSGESHGRILKTD
LKRGSCTVQCQTEKGGLNTTLPFQNVSKYAFGNCSKYIGIKSLKLAVGLRNVPSRSSRGLFGAIAGFIEG
GWSGLVAGWYGFQHSNDQGVGMAADRDSTQKAIDKITSKVNNIVDKMNKQYEIIDHEFSEVETRLNMINN
KIDDQIQDIWAYNAELLVLLENQKTLDEHDANVNNLYNKVKRALGSNAVEDGKGCFELYHKCDDQCMETI
RNGTYNRRKYQEESKLERQKIEGVKLESEGTYKILTIYSTVASSLVIAMGFAAFLFWAMSNGSCRCNICI

>QBK20822.1 hemagglutinin [Influenza A virus]
METVSLITILLVATVSNADKICIGYQSTNSTETVDTLTENNVPVTHAKELLHTEHNGMLCATSLGQPLIL
DTCTIEGLIYGNPSCDLSLEGREWSYIVERPSAVHGLCYPGNVEDLEELRSLFSSARSYQRIQIFPDTIW
NVSYDGTSTACSGSFYRSMRWLTRKNGDYPIQDAQYTNNQGKNILFMWGINHPPTDETQRGLYTRTDTTT
SVATEEINRIFKPLIGPRPLVNGLMGRINYYWSVLKPGQTLRIKSDGNLIAPWYGHILSGESHGRILKTD
LKRGSCTVQCQTEKGGLNTTLPFQNVSKYAFGNCSKYIGIKSLKLAVGLRNVPSRSSRGLFGAIAGFIEG

GWSGLVAGWYGFQHSNDQGVGMAADRDSTQKAIDKITSKVNNIVDKMNKQYEIIDHEFSEVETRLNMINN
KIDDQIQDIWAYNAELLVLLENQKTLDEHDANVNNLYNKVKRALGSNAVEDGKGCFELYHKCDDQCMETI
RNGTYNRRKYQEESKLERQKIEGVKLESEGTYKILTIYSTVASSLVIAMGFAAFLFWAMSNGSCRCNICI

>QBK20821.1 hemagglutinin [Influenza A virus]
METVSLITILLVATVSNADKICIGYQSTNSTETVDTLTENNVPVTHAKELLHTEHNGMLCATSLGQPLIL
DTCTIEGLIYGNPSCDLSLEGREWSYIVERPSAVHGLCYPGNVEDLEELRSLFSSARSYQRIQIFPDTIW
NVSYDGTSTACSGSFYRSMRWLTRKNGDYPIQDAQYTNNQGKNILFMWGINHPPTDETQRGLYTRTDTTT
SVATEEINRIFKPLIGPRPLVNGLMGRINYYWSVLKPGQTLRIKSDGNLIAPWYGHILSGESHGRILKTD
LKRGSCTVQCQTEKGGLNTTLPFQNVSKYAFGNCSKYIGIKSLKLAVGLRNVPSRSSRGLFGAIAGFIEG
GWSGLVAGWYGFQHSNDQGVGMAADRDSTQKAIDKITSKVNNIVDKMNKQYEIIDHEFSEVETRLNMINN
KIDDQIQDIWAYNAELLVLLENQKTLDEHDANVNNLYNKVKRALGSNAVEDGKGCFELYHKCDDQCMETI
RNGTYNRRKYQEESKLERQKIEGVKLESEGTYKILTIYSTVASSLVIAMGFAAFLFWAMSNGSCRCNICI

>QBK20820.1 hemagglutinin [Influenza A virus]
METVSLITILLVATVSNADKICIGYQSTNSTETVDTLTENNVPVTHAKELLHTEHNGMLCATSLGHPLIL
DTCTIEGLIYGNPSCDPLLGGREWSYIVERPSAVNGLCYPGNVENLEELRSLFSSSRSYQRIQIFPDTIW
NVSYSGTSKACSDSFYRSMRWLTQKNNAYPTQDAQYTNNQEKNILFMWGINHPPTDTAQTNLYTRTDTTT
SVATEEMNRIFKPLIGPRPLVNGLMGRINYYWSVLKPGQTLRIKSDGNLIAPWYGHILSGESHGRILKTD
LKSGSCTVQCQTEKGGLNTTLPFQNVSKYAFGNCSKYIGVKSLKLAVGLRNVPSRSSRGLFGAIAGFIEG
GWSGLVAGWYGFQHSNDQGVGMAADRDSTQKAIDKITSKVNNIVDKMNKQYEIIDHEFSEVETRLNMINN
KVDDQIQDIWAYNAELLVLLENQKTLDEHDANVNNLYNKVKRALGSNAVEDGKGCFELYHKCDDHCMETI
RNGTYNRRKYQEESKLERQKIEGVKLESEETYKILTIYSTVASSLVIAMGFAAFLFWAMSNGSCRCNICI

>QBK20819.1 hemagglutinin [Influenza A virus]
METVSLITILLVATVSNADKICIGYQSTNSTETVDTLTENNVPVTHAKELLHTEHNGMLCATSLGQPLIL
DTCTIEGLIYGNPSCDLSLEGREWSYIVERPSAVHGLCYPGNVEDLEELRSLFSSARSYQRIQIFPDTIW
NVSYDGTSTACSGSFYRSMRWLTRKNGDYPTQDAQYTNNQGKNILFMWGINHPPTDETQRGLYTRTDTTT
SVATEEINRIFKPLIGPRPLVNGLMGRINYYWSVLKPGQTLRIKSDGNLIAPWYGHILSGESHGRILKTD
LKRGSCTVQCQTEKGGLNTTLPFQNVSKYAFGNCSKYIGIKSLKLAVGLRNVPSRSSRGLFGAIAGFIEG
GWSGLVAGWYGFQHSNDQGVGMAADRDSTQKAIDKITSKVNNIVDKMNKQYEIIDHEFSEVETRLNMINN
KIDDQIQDIWAYNAELLVLLENQKTLDEHDANVNNLYNKVKRALGSNAVEDGKGCFELYHKCDDQCMETI
RNGTYNRRKYQEESNLERQKIEGVKLESEGTYKILTIYSTVASSLVIAMGFAAFLFWAMSNGSCRCNICI

>QBK20818.1 hemagglutinin [Influenza A virus]

METVSLITILLVATVSNADKICIGYQSTNSTETVDTLTENNVPVTHAKELLHTEHNGMLCATSLGQPLIL

DTCTIEGLIYGNPSCDLSLEGREWSYIVERPSAVNGLCYPGNVENLEELRSLFSSARSYQRIQIFPDTIW

NVSYDGTSTACSGSFYKSMRWLTRKNGEYPIQDAQYTNNQGKNILFIWGINHPPTDDTQRNLYTRTDTTT

SVATEEINRIFKPLIGPRPLVNGLMGRIDYYWSVLKPGQTLRIKSDGNLIAPWFGHILSGESHGRILKTD

LKRGSCTVQCQTEKGGLNTTLPFQNVSKYAFGNCSKYIGIKSLKLAVGLRNVPSRSSRGLFGAIAGFIEG

GWSGLVAGWYGFQHSNDQGVGMAADRDSTQKAIDKITSKVNNIVDKMNKQYEIINHEFSEVETRLNMINN

KIDDQIQDIWAYNAELLVLLENQKTLDEHDANVNNLYNKVKRALGSNAVEDGRGCFELYHKCDDQCMETI

RNGTYNRRKYQEESKLERQKIEGVKLESEGTYKILTIYSTVASSLVIAMGFAAFLFWAMSNGSCRCNICI

>QBK20817.1 hemagglutinin [Influenza A virus]

METVSLITILLVATVSNADKICIGYQSTNSTETVDTLTENNVPVTHAKELLHTEHNGMLCATSLGHPLIL

DTCTIEGLIYGNPSCDPLLGGREWSYIVERPSAVNGLCYPGNVENLEELRSLFSSSRSYQRIQIFPDTIW

NVSYSGTSKACSDSFYRSMRWLTQKNNAYPTQDAQYTNNQEKNILFMWGINHPPTDTAQTNLYTRTDTTT

SVATEEMNRIFKPLIGPRPLVNGLMGRINYYWSVLKPGQTLRIKSDGNLIAPWYGHILSGESHGRILKTD

LKSGSCTVQCQTEKGGLNTTLPFQNVSKYAFGNCSKYIGVKSLKLAVGLRNVPSRSSRGLFGAIAGFIEG

GWSGLVAGWYGFQHSNDQGVGMAADRDSTQKAIDKITSKVNNIVDKMNKQYEIIDHEFSEVETRLNMINN

KVDDQIQDIWAYNAELLVLLENQKTLDEHDANVNNLYNKVKRALGSNAVEDGKGCFELYHKCDDHCMETI

RNGTYNRRKYQEESKLERQKIEGVKLESEETYKILTIYSTVASSLVIAMGFAAFLFWAMSNGSCRCNICI

>QBK20813.1 hemagglutinin [Influenza A virus]

METVSLITILLVATISNADKICIGYQSTNSTETVDTLTENNVPVTHAKELLHTEHNGMLCATSLGQPLIL

DTCTIEGLIYGNPSCDLSLEGREWSYIVERPSAVNGLCYPGNVENLEELRSLFSSARSYQRIQIFPDTIW

NVSYDGTSTACSGSFYRSMRWLTRKNGDYPTQDAQYTNNQGKNILFMWGINHPPTDDTQRNLYTRIDTTT

SVATEEINRIFKPLIGPRPLVNGLMGRIDYYWSVLKPGQTLRIKSDGNLVAPWYGHILSGESHGRILKTD

LKRGSCTVQCQTEKGGLNTTLPFQNVSKYAFGNCSKYIGIKSLKLAVGLRNVPSRSSRGLFGAIAGFIEG

GWSGLVAGWYGFQHSNDQGVGMAADRDSTQKAIDKITSKVNNIVDKMNKQYEIIDHEFSEVETRLNMINN

KIDDQIQDIWAYNAELLVLLENQKTLDEHDANVNNLYNKVKRALGSNAVEDGKGCFELYHKCNDQCMETI

RNGTYNRRKYQEESKLERQKIEGVKLESEGTYKILTIYSTVASSLVIAMGFAAFLFWAMSNGSCRCNICI

>QBK20812.1 hemagglutinin [Influenza A virus]

METVSLITILLVATISNADKICIGYQSTNSTETVDTLTENNVPVTHAKELLHTEHNGMLCATSLGQPLIL

DTCTIEGLIYGNPSCDLSLEGREWSYIVERPSAVNGLCYPGNVENLEELRSLFSSARSYQRIQIFPDTIW

NVSYDGTSTACSGSFYRSMRWLTRKNGDYPTQDAQYTNNQGKNILFMWGINHPPTDDTQRNLYTRTDTTT

SVATEEINRIFKPLIGPRPLVNGLMGRIDYYWSVLKPGQTLRIKSDGNLVAPWYGHILSGESHGRILKTD
LKRGSCTVQCQTEKGGLNTTLPFQNVSKYAFGNCSKYIGIKSLKLAVGLRNVPSRSSRGLFGAIAGFIEG
GWSGLVAGWYGFQHSNDQGVGMAADRDSTQKAIDKITSKVNNIVDKMNKQYEIIDHEFSEVETRLNMINN
KIDDQIQDIWAYNAELLVLLENQKTLDEHDANVNNLYNKVKRALGSNAVEDGKGCFELYHKCNDQCMETI
RNGTYNRRKYQEESKLERQKIEGVKLESEGTYKILTIYSTVASSLVIAMGFAAFLFWAMSNGSCRCNICI

>QBK20811.1 hemagglutinin [Influenza A virus]
METVSLITILLVATISNADKICIGYQSTNSTETVDTLTENNVPVTHAKELLHTEHNGMLCATSLGQPLIL
DTCTIEGLIYGNPSCDLSLEGREWSYIVERPSAVNGLCYPGNVENLEELRSLFSSARSYQRIQIFPDTIW
NVSYDGTSTACSGSFYRSMRWLTRKNGDYPTQDAQYTNNQGKNILFMWGINHPPTDDTQRNLYTRTDTTT
SVATEEINRIFKPLIGPRPLVNGLMGRIDYYWSVLKPGQTLRIKSDGNLVAPWYGHILSGESHGRILKTD
LKRGSCTVQCQTEKGGLNTTLPFQNVSKYAFGNCSKYIGIKSLKLAVGLRNVPSRSSRGLFGAIAGFIEG
GWSGLVAGWYGFQHSNDQGVGMAADRDSTQRAIDKITSKVNNIVDKMNKQYEIIDHEFSEVETRLNMINN
KIDDQIQDIWAYNAELLVLLENQKTLDEHDANVNNLYNKVKRALGSNAVEDGKGCFELYHKCDDQCMETI
RNGTYNRRKYQEESKLERQKIEGVKLESEGTYKILTIYSTVASSLVIAMGFAAFLFWAMSNGSCRCNICI

>QBK20810.1 hemagglutinin [Influenza A virus]
METVSLITILLVATISNADKICIGYQSTNSTETVDTLTENNVPVTHAKELLHTEHNGMLCATSLGQPLIL
DTCTIEGLIYGNPSCDLSLEGREWSYIVERPSAVNGLCYPGNVENLEELRSLFSSARSYQRIQIFPDTIW
NVSYDGTSTACSGSFYRSMRWLTRKNGDYPTQDAQYTNNQGKNILFMWGINHPPTDDTQRNLYTRTDTTT
SVATEEINRIFKPLIGPRPLVNGLMGRIDYYWSVLKPGQTLRIKSDGNLVAPWDGHILSGESHGRILKTD
LKRGSCTVQCQTEKGGLNTTLPFQNVSKYAFGNCSKYIGIKSLKLAVGLRNVPSRSSRGLFGAIAGFIEG
GWSGLVAGWYGFQHSNDQGVGMAADRDSTQKAIDKITSKVNNIVDKMNKQYEIIDHEFSEVETRLNMINN
KIDDQXQDIWAYNAELLVLLENQKTLDEHDANVNNLYNKVKRALGSNAVEDGKGCFELYHKCNDQCMETI
RNGTYNRRKYQEESKLERQKIEGVKLESEGTYKILTIYSTVASSLVIAMGFAAFLFWAMSNGSCRCNICI

>QBK20809.1 hemagglutinin [Influenza A virus]
METVSLITILLVATISNADKICIGYQSTNSTETVDTLTENNVPVTHAKELLHTEHNGMLCATSLGQPLIL
DTCTIEGLIYGNPSCDLSLEGREWSYIVERPSAVNGLCYPGNVENLEELRSLFSSARSYQRIQIFPDTIW
NVSYDGTSTACSGSFYRSMRWLTRKNGDYPTQDAQYTNNQGKNILFMWGINHPPTDDTQRNLYTRTDTTT
SVATEEINRIFKPLIGPRPLVNGLMGRIDYYWSVLKPGQTLRIKSDGNLVAPWYGHILSGESHGRILKTD
LKRGSCTVQCQTEKGGLNTTLPFQNVSKYAFGNCSKYIGIKSLKLAVGLRNVPSRSSRGLFGAIAGFIEG
GWSGLVAGWYGFQHSNDQGVGMAADRDSTQKAIDKITSKVNNIVDKMNKQYEIIDHEFSEVETRLNMINN
KIDDQIQDIWAYNAELLVLLENQKTLDEHDANVNNLYNKVKRALGSNAVEDGKGCFELYHKCNDQCMETI

RNGTYNRRKYQEESKLERQKIEGVKLESEGTYKILTIYSTVASSLVIAMGFAAFLFWAMSNGSCRCNICI

>QBK20808.1 hemagglutinin [Influenza A virus]

METVSLITILLVATISNADKICIGYQSTNSTETVDTLTENNVPVTHAKELLHTEHNGMLCATSLGQPLIL

DTCTIEGLIYGNPSCDLSLEGREWSYIVERPSAVNGLCYPGNVENLEELRSLFSSARSYQRIQIFPDTIW

NVSYDGTSTACSGSFYRSMRWLTRKNGDYPTQDAQYTNNQGKNILFMWGINHPPTDDTQRNLYTRTDTTT

SVATEEINRIFKPLIGPRPLVNGLMGRIDYYWSVLKPGQTLRIKSDGNLVAPWYGHILSGKSHGRILKTD

LKRGSCTVQCQTEKGGLNTTLPFQNVSKYAFGNCSKYIGIKSLKLAVGLRNVPSRSSRGLFGAIAGFIEG

GWSGLVAGWYGFQHSNDQGVGMAADRDSTQRAIDKITSKVNNIVDKMNKQYEIIDHEFNEVETRLNMINN

KIDDQIQDIWAYNAELLVLLENQKTLDEHDANVNNLYNKVKRALGSNAVEDGKGCFELYHKCDDQCMETI

RNGTYNRRKYQEESKLERQKIEGVKLESEGTYKILTIYSTVASSLVIAMGFAAFLFWAMSNGSCRCNICI

>QBK20807.1 hemagglutinin [Influenza A virus]

METVSLITILLVATISNADKICIGYQSTNSTETVDTLTENNVPVTHAKELLHTEHNGMLCATSLGQPLIL

DTCTIEGLIYGNPSCDLSLEGREWSYIVERPSAVNGLCYPGNVENLEELRSLFSSARSYQRIQIFPDTIW

NVSYDGTSTACSGSFYRSMRWLTRKNGDYPTQDAQYTNNQGKNILFMWGINHPPTDDTQRNLYTRTDTTT

SVATEEINRIFKPLIGPRPLVNGLMGRIDYYWSVLKPGQTLRIKSDGNLVAPWYGHILSGESHGRILKTD

LKRGSCTVQCQTEKGGLNTTLPFQNVSKYAFGNCSKYIGIKSLKLAVGLRNVPSRSSRGLFGAIAGFIEG

GWSGLVAGWYGFQHSNDQGVGMAADRDSTQKAIDKITSKVNNIVDKMNKQYEIIDHEFSEVETRLNMINN

KIDDQIQDIWAYNAELLVLLENQKTLDEHDANVNNLYNKVKRALGSNAVEDGKGCFELYHKCNDQCMETI

RNGTYNRRKYQEESKLERQKIEGVKLESEGTYKILTIYSTVASSLVIAMGFAAFLFWAMSNGSCRCNICI

>QBK20806.1 hemagglutinin [Influenza A virus]

METVSLITILLVATISNADKICIGYQSTNSTETVDTLTENNVPVTHAKELLHTEHNGMLCATSLGQPLIL

DTCTIEGLIYGNPSCDLSLEGREWSYIVERPSAVNGLCYPGNVENLEELRSLFSSARSYQRIQIFPDTIW

NVSYDGTSTACSGSFYRSMRWLTRKNGDYPTQDAQYTNNQGKNILFMWGINHPPTDDTQRNLYTRTDTTT

SVATEEINRIFKPLIGPRPLVNGLMGRIDYYWSVLKPGQTLRIKSDGNLVAPWYGHILSGESHGRILKTD

LKRGSCTVQCQTEKGGLNTTLPFQNVSKYAFGNCSKYIGIKSLKLAVGLRNVPSRSSRGLFGAIAGFIEG

GWSGLVAGWYGFQHSNDQGVGMAADRDSTQKAIDKITSKVNNIVDKMNKQYEIIDHEFSEVETRLNMINN

KIDDQIQDIWAYNAELLVLLENQKTLDEHDANVNNLYNKVKRALGSNAVEDGKGCFELYHKCNDQCMETI

RNGTYNRRKYQEESKLERQKIEGVKLESEGTYKILTIYSTVASSLVIAMGFAAFLFWAMSNGSCRCNICI

>QBK20805.1 hemagglutinin [Influenza A virus]

METVSLITILLVATISNADKICIGYQSTNSTETVDTLTENNVPVTHAKELLHTEHNGMLCATSLGQPLIL

DTCTIEGLIYGNPSCDLSLEGREWSYIVERPSAVNGLCYPGNVENLEELRSLFSSARSYQRIQIFPDTIW
NVSYDGTSTACSGSFYRSMRWLTRKNGDYPTQDAQYTNNQGKNILFMWGINHPPTDDTQRNLYTRTDTTT
SVATEEINRIFKPLIGPRPLVNGLMGRIDYYWSVLKPGQTLRIKSDGNLVAPWYGHILSGESHGRILKTD
LKRGSCTVQCQTEKGGLNTTLPFQNVSKYAFGNCSKYIGIKSLKLAVGLRNVPSRSSRGLFGAIAGFIEG
GWSGLVAGWYGFQHSNDQGVGMAADRDSTQKAIDKITSKVNNIVDKMNKQYEIIDHEFSEVETRLNMINN
KIDDQIQDIWAYNAELLVLLENQKTLDEHDANVNNLYNKVKRALGSNAVEDGKGCFELYHKCNDQCMETI
RNGTYNRRKYQEESKLERQKIEGVKLESEGTYKILTIYSTVASSLVIAMGFAAFLFWAMSNGSCRCNICI

>QBK20804.1 hemagglutinin [Influenza A virus]
METVSLITILLVATISNADKICIGYQSTNSTETVDTLTENNVPVTHAKELLHTEHNGMLCATSLGQPLIL
DTCTIEGLIYGNPSCDLSLEGREWSYIVERPSAVNGLCYPGNVENLEELRSLFSSARSYQRIQIFPDTIW
NVSYDGTSTACSGSFYRSMRWLTRKNGDYPTQDAQYTNNQGKNILFMWGINHPPTDDTQRNLYTRTDTTT
SVATEEINRIFKPLIGPRPLVNGLMGRIDYYWSVLKPGQTLRIKSDGNLVAPWYGHILSGESHGRILKTD
LKRGSCTVQCQTEKGGLNTTLPFQNVSKYAFGNCSKYIGIKSLKLAVGLRNVPSRSSRGLFGAIAGFIEG
GWSGLVAGWYGFQHSNDQGVGMAADRDSTQKAIDKITSKVNNIVDKMNKQYEIIDHEFSEVETRLNMINN
KIDDQIQDIWAYNAELLVLLENQKTLDEHDANVNNLYNKVKRALGSNAVEDGKGCFELYHKCNDQCMETI
RNGTYNRRKYQEESKLERQKIEGVKLESEGTYKILTIYSTVASSLVIAMGFAAFLFWAMSNGSCRCNICI

>QBK20803.1 hemagglutinin [Influenza A virus]
METVSLITILLVATISNADKICIGYQSTNSTETVDTLTENNVPVTHAKELLHTEHNGMLCATSLGQPLIL
DTCTIEGLIYGNPSCDLSLEGREWSYIVERPSAVNGLCYPGNVENLEELRSLFSSARSYQRIQIFPDTIW
NVSYDGTSTACSGSFYRSMRWLTRKNGDYPTQDAQYTNNQGKNILFMWGINHPPTDDTQRNLYTRTDTTT
SVATEEINRIFKPLIGPRPLVNGLMGRIDYYWSVLKPGQTLRIKSDGNLVAPWYGHILSGESHGRILKTD
LKRGSCTVQCQTEKGGLNTTLPFQNVSKYAFGNCSKYIGIKSLKLAVGLRNVPSRSSRGLFGAIAGFIEG
GWSGLVAGWYGFQHSNDQGVGMAADRDSTQKAIDKITSKVNNIVDKMNKQYEIIDHEFSEVETRLNMINN
KIDDQIQDIWAYNAELLVLLENQKTLDEHDANVNNLYNKVKRALGSNAVEDGKGCFELYHKCNDQCMETI
RNGTYNRRKYQEESKLERQKIEGVKLESEGTYKILTIYSTVASSLVIAMGFAAFLFWAMSNGSCRCNICI

>QBK20802.1 hemagglutinin [Influenza A virus]
METVSLITILLVATISNADKICIGYQSTNSTETVDTLTENNVPVTHAKELLHTEHNGMLCATSLGQPLIL
DTCTIEGLIYGNPSCDLSLEGREWSYIVERPSAVNGLCYPGNVENLEELRSLFSSARSYQRIQIFPDTIW
NVSYDGTSTACSGSFYRSMRWLTRKNGDYPTQDAQYTNNQGKNILFMWGINHPPTDDTQRNLYTRTDTTT
SVATEEINRIFKPLIGPRPLVNGLMGRIDYYWSVLKPGQTLRIKSDGNLVAPWYGHILSGESHGRILKTD
LKRGSCTVQCQTEKGGLNTTLPFQNVSKYAFGNCSKYIGIKSLKLAVGLRNVPSRSSRGLFGAIAGFIEG

GWSGLVAGWYGFQHSNDQGVGMAADRDSTQKAIDKITSKVNNIVDKMNKQYEIIDHEFSEVETRLNMINN
KIDDQIQDIWAYNAELLVLLENQKTLDEHDANVNNLYNKVKRALGSNAVEDGKGCFELYHKCNDQCMETI
RNGTYNRRKYQEESKLERQKIEGVKLESEGTYKILTIYSTVASSLVIAMGFAAFLFWAMSNGSCRCNICI

>QBK20801.1 hemagglutinin [Influenza A virus]
METVSLITILLVATISNADKICIGYQSTNSTETVDTLTENNVPVTHAKELLHTEHNGMLCATSLGQPLIL
DTCTIEGLIYGNPSCDLSLEGREWSYIVERPSAVNGLCYPGNVENLEELRSLFSSARSYQRIQIFPDTIW
NVSYDGTSTACSGSFYRSMRWLTRKNGEYPTQDAQYTNNQGKNILFMWGINHPPTDDTQRNLYTRTDTTT
SVATEEINRIFKPLIGPRPLVNGLMGRIDYYWSVLKPGQTLRIKSDGNLVAPWYGHILSGESHGRILKTD
LKRGSCTVQCQTEKGGLNTTLPFQNVSKYAFGNCSKYIGIKSLKLAVGLRNVPSRSSRGLFGAIAGFIEG
GWSGLVAGWYGFQHSNDQGVGMAADRDSTQKAIDKITSKVNNIVDKMNKQYEIIDHEFSEVETRLNMINN
KIDDQIQDIWAYNAELLVLLENQKTLDEHDANVNNLYNKVKRALGSNAVEDGKGCFELYHKCNDQCMETI
RNGTYNRRKYQEESKLERQKIEGVKLESEGTYKILTIYSTVASSLVIAMGFAAFLFWAMSNGSCRCNICI

>QBK20800.1 hemagglutinin [Influenza A virus]
METVSLITILLVATISNADKICIGYQSTNSTETVDTLTENNVPVTHAKELLHTEHNGMLCATSLGQPLIL
DTCTIEGLIYGNPSCDLSLEGREWSYIVERPSAVNGLCYPGNVENLEELRSLFSSARSYQRIQIFPDTIW
NVSYDGTSTACSGSFYRSMRWLTRKNGEYPTQDAQYTNNQGKNILFMWGINHPPTDDTQRNLYTRTDTTT
SVATEEINRIFKPLIGPRPLVNGLMGRIDYYWSVLKPGQTLRIKSDGNLVAPWYGHILSGESHGRILKTD
LKRGSCTVQCQTEKGGLNTTLPFQNVSKYAFGNCSKYIGIKSLKLAVGLRNVPSRSSRGLFGAIAGFIEG
GWSGLVAGWYGFQHSNDQGVGMAADRDSTQKAIDKITSKVNNIVDKMNKQYEIIDHEFSEVETRLNMINN
KIDDQIQDIWAYNAELLVLLENQKTLDEHDANVNNLYNKVKRALGSNAVEDGKGCFELYHKCNDQCMETI
RNGTYNRRKYQEESKLERQKIEGVKLESEGTYKILTIYSTVASSLVIAMGFAAFLFWAMSNGSCRCNICI

>QBK20799.1 hemagglutinin [Influenza A virus]
METVSLITILIVATISNADKICIGYQSTNSTETVDTLTENNVPVTHAKELLHTEHNGMLCATSLGQPLIL
DTCTIEGLIYGNPSCDLSLEGREWSYIVERPSAVNGLCYPGNVENLEELRSLFSSARSYQRIQIFPDTIW
NVSYDGTSTACSGSFYRSMRWLTRKNGDYPTQDAQYTNNQGKNILFMWGINHPPTDDTQRNLYTRTDTTT
SVATEEINRIFKPLIGPRPLVNGLMGRIDYYWSVLKPGQTLRIKSDGNLVAPWYGHILSGESHGRILKTD
LKRGSCTVQCQTEKGGLNTTLPFQNVSKYAFGNCSKYIGIKSLKLAVGLRNVPSRSSRGLFGAIAGFIEG
GWSGLVAGWYGFQHSNDQGVGMAADRDSTQKAIDKITSKVNNIVDKMNKQYEIIDHEFSEVETRLNMINN
KIDDQIQDIWAYNAELLVLLENQKTLDEHDANVNNLYNKVKRALGSNAVEDGKGCFELYHKCNDQCMETI
RNGTYNRKKYQEESKLERQKIEGVKLESEGTYKILTIYSTVASSLVIAMGFAAFLFWAMSNGSCRCNICI

>QBK20798.1 hemagglutinin [Influenza A virus]

METVSLITILLVATISNADKICIGYQSTNSTETVDTLTENNVPVTHAKELLHTEHNGMLCATSLGQPLIL

DTCTIEGLIYGNPSCDLSLEGREWSYIVERPSAVNGLCYPGNVENLEELRSLFSSARSYQRIQIFPDTIW

NVSYDGTSTACSGSFYRSMRWLTRKNGEYPTQDAQYTNNQGKNILFMWGINHPPTDDTQRNLYTRTDTTT

SVATEEINRIFKPLIGPRPLVNGLMGRIDYYWSVLKPGQTLRIKSDGNLVAPWYGHILSGESHGRILKTD

LKRGSCTVQCQTEKGGLNTTLPFQNVSKYAFGNCSKYIGIKSLKLAVGLRNVPSRSSRGLFGAIAGFIEG

GWSGLVAGWYGFQHSNDQGVGMAADRDSTQKAIDKITSKVNNIVDKMNKQYEIIDHEFSEVETRLNMINN

KIDDQIQDIWAYNAELLVLLENQKTLDEHDANVNNLYNKVKRALGSNAVEDGKGCFELYHKCNDQCMETI

RNGTYNRRKYQEESKLERQKIEGVKLESEGTYKILTIYSTVASSLVIAMGFAAFLFWAMSNGSCRCNICI

>QBK20797.1 hemagglutinin [Influenza A virus]

METVSLITILIVATISNADKICIGYQSTNSTETVDTLTENNVPVTHAKELLHTEHNGMLCATSLGQPLIL

DTCTIEGLIYGNPSCDLSLEGREWSYIVERPSAVNGLCYPGNVENLEELRSLFSSARSYQRIQIFPDTIW

NVSYDGTSTACSGSFYRSMRWLTRKNGDYPTQDAQYTNNQGKNILFMWGINHPPTDDTQRNLYTRTDTTT

SVATEEINRIFKPLIGPRPLVNGLMGRIDYYWSVLKPGQTLRIKSDGNLVAPWYGHILSGESHGRILKTD

LKRGSCTVQCQTEKGGLNTTLPFQNVSKYAFGNCSKYIGIKSLKLAVGLRNVPSRSSRGLFGAIAGFIEG

GWSGLVAGWYGFQHSNDQGVGMAADRDSTQKAIDKITSKVNNIVDKMNKQYEIIDHEFSEVETRLNMINN

KIDDQIQDIWAYNAELLVLLENQKTLDEHDANVNNLYNKVKRALGSNAVEDGKGCFELYHKCNDQCMETI

RNGTYNRKKYQEESKLERQKIEGVKLESEGTYKILTIYSTVASSLVIAMGFAAFLFWAMSNGSCRCNICI

>QBK20796.1 hemagglutinin [Influenza A virus]

METVSLITILIVATISNADKICIGYQSTNSTETVDTLTENNVPVTHAKELLHTEHNGMLCATSLGQPLIL

DTCTIEGLIYGNPSCDLSLEGREWSYIVERPSAVNGLCYPGNVENLEELRSLFSSARSYQRIQIFPDTIW

NVSYDGTSTACSGSFYRSMRWLTRKNGDYPTQDAQYTNNQGKNILFMWGINHPPTDDTQRNLYTRTDTTT

SVATEEINRIFKPLIGPRPLVNGLMGRIDYYWSVLKPGQTLRIKSDGNLVAPWYGHILSGESHGRILKTD

LKRGSCTVQCQTEKGGLNTTLPFQNVSKYAFGNCSKYIGIKSLKLAVGLRNVPSRSSRGLFGAIAGFIEG

GWSGLVAGWYGFQHSNDQGVGMAADRDSTQKAIDKITSKVNNIVDKMNKQYEIIDHEFSEVETRLNMINN

KIDDQIQDIWAYNAELLVLLENQKTLDEHDANVNNLYNKVKRALGSNAVEDGKGCFELYHKCNDQCMETI

RNGTYNRKKYQEESKLERQKIEGVKLESEGTYKILTIYSTVASSLVIAMGFAAFLFWAMSNGSCRCNICI

>QBK20793.1 hemagglutinin [Influenza A virus]

METVSLITILLVATISNADKICIGYQSTNSTETVDTLTENNVPVTHAKELLHTEHNGMLCATSLGQPLIL

DTCTIEGLIYGNPSCDLSLEGREWSYIVERPSAVNGLCYPGNVENLEELRSLFSSARSYQRIQIFPDTIW

NVSYDGTSTACSGSFYRSMRWLTRKNGDYPTQDAQYTNNQGKNILFMWGINHPPTDDTQRNLYTRIDTTT

SVATEEINRIFKPLIGPRPLVNGLMGRIDYYWSVLKPGQTLRIKSDGNLVAPWYGHILSGESHGRILKTD
LKRGSCTVQCQTEKGGLNTTLPFQNVSKYAFGNCSKYIGIKSLKLAVGLRNVPSRSSRGLFGAIAGFIEG
GWSGLVAGWYGFQHSNDQGVGMAADRDSTQKAIDKITSKVNNIVDKMNKQYEIIDHEFSEVETRLNMINN
KIDDQIQDIWAYNAELLVLLENQKTLDEHDANVNNLYNKVKRALGSNAVEDGKGCFELYHKCNDQCMETI
RNGTYNRRKYQEESKLERQKIEGVKLESEGTYKILTIYSTVASSLVIAMGFAAFLFWAMSNGSCRCNICI

>QBK20792.1 hemagglutinin [Influenza A virus]
METVSLITILLVATISNADKICIGYQSTNSTETVDTLTENNVPVTHAKELLHTEHNGMLCATSLGQPLIL
DTCTIEGLIYGNPSCDLSLEGREWSYIVERPSAVNGLCYPGNVENLEELRSLFSSARSYQRIQIFPDTIW
NVSYDGTSTACSGSFYRSMRWLTRKNGDYPTQDAQYTNNQGKNILFMWGINHPPTDDTQRNLYTRTDTTT
SVATEEINRIFKPLIGPRPLVNGLMGRIDYYWSVLKPGQTLRIKSDGNLVAPWYGHILSGESHGRILKTD
LKRGSCTVQCQTEKGGLNTTLPFQNVSKYAFGNCSKYIGIKSLKLAVGLRNVPSRSSRGLFGAIAGFIEG
GWSGLVAGWYGFQHSNDQGVGMAADRDSTQKAIDKITSKVNNIVDKMNKQYEIIDHEFSEVETRLNMINN
KIDDQIQDIWAYNAELLVLLENQKTLDEHDANVNNLYNKVKRALGSNAVEDGKGCFELYHKCNDQCMETI
RNGTYNRRKYQEESKLERQKIEGVKLESEGTYKILTIYSTVASSLVIAMGFAAFLFWAMSNGSCRCNICI

>QBK20791.1 hemagglutinin [Influenza A virus]
METVSLITILLVATISNADKICIGYQSTNSTETVDTLTENNVPVTHAKELLHTEHNGMLCATSLGQPLIL
DTCTIEGLIYGNPSCDLSLEGREWSYIVERPSAVNGLCYPGNVENLEELRSLFSSARSYQRIQIFPDTIW
NVSYDGTSTACSGSFYRSMRWLTRKNGDYPTQDAQYTNNQGKNILFMWGINHPPTDDTQRNLYTRTDTTT
SVATEEINRIFKPLIGPRPLVNGLMGRIDYYWSVLKPGQTLRIKSDGNLVAPWYGHILSGESHGRILKTD
LKRGSCTVQCQTEKGGLNTTLPFQNVSKYAFGNCSKYIGIKSLKLAVGLRNVPSRSSRGLFGAIAGFIEG
GWSGLVAGWYGFQHSNDQGVGMAADRDSTQKAIDKITSKVNNIVDKMNKQYEIIDHEFSEVETRLNMINN
KIDDQIQDIWAYNAELLVLLENQKTLDEHDANVNNLYNKVKRALGSNAVEDGKGCFELYHKCNDQCMETI
RNGTYNRRKYQEESKLERQKIEGVKLESEGTYKILTIYSTVASSLVIAMGFAAFLFWAMSNGSCRCNICI

>QBK20790.1 hemagglutinin [Influenza A virus]
METVSLITILLVATISNADKICIGYQSTNSTETVDTLTENNVPVTHAKELLHTEHNGMLCATSLGQPLIL
DTCTIEGLIYGNPSCDLSLEGREWSYIVERPSAVNGLCYPGNVENLEELRSLFSSARSYQRIQIFPDTIW
NVSYDGTSTACSGSFYRSMRWLTRKNGDYPTQDAQYTNNQGKNILFMWGINHPPTDDTQRNLYTRTDTTT
SVATEEINRIFKPLIGPRPLVNGLMGRIDYYWSVLKPGQTLRIKSDGNLVAPWYGHILSGESHGRILKTD
LKRGSCTVQCQTEKGGLNTTLPFQNVSKYAFGNCSKYIGIKSLKLAVGLRNVPSRSSRGLFGAIAGFIEG
GWSGLVAGWYGFQHSNDQGVGMAADRDSTQKAIDKITSKVNNIVDKMNKQYEIIDHEFSEVETRLNMINN
KIDDQIQDIWAYNAELLVLLENQKTLDEHDANVNNLYNKVKRALGSNAVEDGKGCFELYHKCNDQCMETI

RNGTYNRKKYQEESKLERQKIEGVKLESEGTYKILTIYSTVASSLVIAMGFAAFLFWAMSNGSCRCNICI

>QBK20789.1 hemagglutinin [Influenza A virus]
METVSLITILIVATVSNADKICIGYQSTNSTETVDTLTENNVPVTHAKELLHTEHNGMLCATSLGQPLIL
DTCTIEGLIYGNPSCDLSLEGREWSYIVERPSAINGLCYPGNVENLEELRSLFSSARSYQRIQIFPDTIW
NVSYDGTSTACSNSFYRSMRWLTRKDGNYPTQDAQYTNNQGKNILFMWGINHPPTDDTQRNLYTRTDTTT
SVATEEINRIFKPLIGPRPLVNGLMGRIDYYWSVLKPGQTLRIKSDGNLIAPWYGHILSGESHGRILKTD
LKRGSCTVQCQTEKGGLNTTLPFQNVSKYAFGNCSKYIGIKSLKLAVGLRNVPSRSSRGLFGAIAGFIEG
GWSGLVAGWYGFQHSNDQGVGMAADRDSTQKAIDKITSKVNNIVDKMNKQYEIIDHEFSEVETRLNMINN
KIDDQIQDIWAYNAELLVLLENQKTLDEHDANVNNLYNKVKRALGSNAVEDGKGCFELYHKCNDQCMETI
RNGTYNRRKYQEESKLERQKIEGVKLESEGTYKILTIYSTVASSLVIAMGFAAFLFWAMSNGSCRCNICI

>QBK20788.1 hemagglutinin [Influenza A virus]
METVSLITILIVATVSNADKICIGYQSTNSTETVDTLTENNVPVTHAKELLHTEHNGMLCATSLGQPLIL
DTCTIEGLIYGNPSCDLSLEGREWSYIVERPSAINGLCYPGNVENLEELRSLFSSARSYQRIQIFPDTIW
NVSYDGTSTACSNSFYRSMRWLTRKDGNYPTQDAQYTNNQGKNILFMWGINHPPTDDTQRNLYTRTDTTT
SVATEEINRIFKPLIGPRPLVNGLMGRIDYYWSVLKPGQTLRIKSDGNLIAPWYGHILSGESHGRILKTD
LKRGSCTVQCQTEKGGLNTTLPFQNVSKYAFGNCSKYIGIKSLKLAVGLRNVPSRSSRGLFGAIAGFIEG
GWSGLVAGWYGFQHSNDQGVGMAADRDSTQKAIDKITSKVNNIVDKMNKQYEIIDHEFSEVETRLNMINN
KIDDQIQDIWAYNAELLVLLENQKTLDEHDANVNNLYNKVKRALGSNAVEDGKGCFELYHKCNDQCMETI
RNGTYNRRKYQEESKLERQKIEGVKLESEGTYKILTIYSTVASSLVIAMGFAAFLFWAMSNGSCRCNICI

>QBK20787.1 hemagglutinin [Influenza A virus]
METISLMTILLVATVSNADKICIGYQSTNSTETVDTLTENNVPVTHAKELLHTEHNGMLCATSLGNPLVL
DTCTIEGLIYGNPSCDPLLGGREWSYIVERPSAVNGLCYPGSVENLEELRSLFSSARSYQRIQIFPDTIW
NVSYSGTSKACSDSFYRSMRWLTQKNNAYPIQDAQYTNNQEKNILFMWGINHPPTETAQTNLYTRTDTTT
SVATEEINRIFKPLIGPRPLVNGLMGRINYYWSVLKPGQTLRIKSDGNLIAPWYGHILSGESHGRILKTD
LKRGSCTVQCQTEKGGLNTTLPFQNVSKYAFGNCSKYIGIKSLKLAVGLRNVPSRSSRGLFGAIAGFIEG
GWSGLVAGWYGFQHSNDQGVGMAADRDSTQKAIDKITSKVNNIVDKMNKQYEIIDHEFSEVETRLNMINN
KVDDQIQDIWAYNAELLVLLENQKTLDEHDSNVNNLYNKVKRALGSNAVEDGKGCFELYHKCDNQCMETI
RNGTYNRRKYQEESKLERQKIEGVKLESEGTYKILTIYSTVASSLVIAMGFAAFLFWAMSNGSCRCNICI

>QBK20786.1 hemagglutinin [Influenza A virus]
METVSLITILIVATVSNADKICIGYQSTNSTETVDTLTENNVPVTHAKELLHTEHNGMLCATSLGQPLIL

DTCTIEGLIYGNPSCDLSLEGREWSYMVERPSAINGLCYPGNVENLEELRSLFSSARSYQRIQIFPDTIW
NVSYDGTSTACSNSFYRSMRWLTRKDGNYPTQDAQYTNNQGKNILFMWGINHPPTDDTQRNLYTRTDTTT
SVATEEINRIFKPLIGPRPLVNGLMGRIDYYWSVLKPGQTLRIKSDGNLIAPWYGHILSGESHGRILKTD
LKRGSCTVQCQTEKGGLNTTLPFQNVSKYAFGNCSKYIGIKSLKLAVGLRNVPSRSSRGLFGAIAGFIEG
GWSGLVAGWYGFQHSNDQGVGMAADRDSTQKAIDKITSKVNNIVDKMNKQYEIIDHEFSEVETRLNMINN
KIDDQIQDIWAYNAELLVLLENQKTLDEHDANVNNLYNKVKRALGSNAVEDGKGCFELYHKCNDQCMETI
RNGTYNRRKYQEESKLERQKIEGVKLESEGTYKILTIYSTVASSLVIAMGFAAFLFWAMSNGSCRCNICI

>QBK20785.1 hemagglutinin [Influenza A virus]
METVSLITILIVATVSNADKICIGYQSTNSTETVDTLTENNVPVTHAKELLHTEHNGMLCATSLGQPLIL
DTCTIEGLIYGNPSCDLSLEGREWSYIVERPSAINGLCYPGNVENLEELRTLFSSARSYQRIQIFPDTIW
NVSYDGTSTACSNSFYRSMRWLTRKDGNYPTQDAQYTNNQGKNILFMWGINHPPTDDTQRNLYTRTDTTT
SVATEEINRIFKPLIGPRPLVNGLMGRIDYYWSVLKPGQTLRIKSDGNLIAPWYGHILSGESHGRILKTD
LKRGSCTVQCQTEKGGLNTTLPFQNVSKYAFGNCSKYIGIKSLKLAVGLRNVPSRSSRGLFGAIAGFIEG
GWSGLVAGWYGFQHSNDQGVGMAADRDSTQKAIDKITSKVNNIVDKMNKQYEIIDHEFSEVETRLNMINN
KIDDQIQDIWAYNAELLVLLENQKTLDEHDANVNNLYNKVKRALGSNAVEDGKGCFELYHKCNDQCMETI
RNGTYNRRKYQEESKLERQKIEGVKLESEGTYKILTIYSTVASSLVIAMGFAAFLFWAMSNGSCRCNICI

>QBK20784.1 hemagglutinin [Influenza A virus]
METVSLITILLVATVSNADKICIGYQSTNSTETVDTLTENNVPVTHAKELIHTEHNGMLCATSLGQPLIL
DTCTIEGLIYGNPSCDLSLEGREWSYIVERPSVVNGLCYPGNVENLEELRSLFSSARSYQRIQIFPDTIW
NVSYDGTSTACSGSFYRNMRWLTRKDGNYPTQDAQYTNNQGKNILFMWGINHPPTDTTQSGLYTRTDTTT
SVATEEINRIFKPLIGPRPLVNGLMGRIDYYWSVLKPGQTLRIKSDGNLIAPWFGHILSGESHGRILKTD
LKRGSCTVQCQTEKGGLNTTLPFQNVSKYAFGNCSKYIGIKSLKLAVGLRNVPSRSSRGLFGAIAGFIEG
GWSGLVAGWYGFQHSNDQGVGMAADRDSTQKAIDKITSKVNNIVDKMNKQYEIIDHEFSEVETRLNMINN
KIDDQIQDIWAYNAELLVLLENQKTLDEHDANVNNLYNKVKRALGSNAVEDGKGCFELYHKCDDQCMETI
RNGTYNRRKYQEESKLERQKIEGVKLESEGTYKILTIYSTVASSLVIAMGFAAFLFWAMSNGSCRCNICI

>QBK20781.1 hemagglutinin [Influenza A virus]
METVSLITILLAATVSNADKICIGYQSTNSTETVDTLTENNVPVTHAKELLHTEHNGMLCATSLGQPLIL
DTCTIEGLIYGNPSCDPLPEEREWSYIVERPSAVNGLCYPGNVENLEELRSLFSSARSYQRIQIFPDTIW
NVSYDGTSNTCSGSFYRNMRWLTRKDGNYPIQDAQYTNNQGKNILFMWGINNPPTDDTQRNLYTRTDTTT
SVATEEINRIFKPLIGPRPLVNGLMGRINYYWSVLKPGQTLRIKSDGNLVAPWYGSILSGESHGRILRTD
LKRGSCTVQCQTEKGGLNTTLPFQNVSKYAFGNCSKYIGIKSLKLAVGLRNVPSRSSRGLFGAIAGFIEG

GWSGLVAGWYGFQHSNDQGVGMAADRESTQKAVDKITSKVNNIVDKMNKQYEIIDHEFSEVETRLNMINN
KIDDQIQDIWAYNAELLVLLENQKTLDEHDANVNNLYNKVKRALGSNAVEDGKGCFELYHKCDDQCMETI
RNGTYNRRKYQEESKLERQKIEGVKLESEGTYKILTIYSTVASSLVIAMGFAAFLFWAMSNGSCRCNICI

>QBK20780.1 hemagglutinin [Influenza A virus]
METVSLITILLTATVSNADKICIGYQSTNSTETVDTLTENNVPVTHAKELLHTEHNGMLCATSLGQPLIL
DTCTIEGLIYGNPSCDPLPEEREWSYIVERPSAVNGLCYPGNVENLEELRSLFSSARSYQRIQIFPDTIW
NVSYDGTSNTCSGSFYRNLRWLTRKDGNYPIQDAQYTNNQGKNILFMWGINNPPTDDTQRNLYTRTDTTT
SVATEEINRIFKPLIGPRPLVNGLMGRINYYWSVLKPGQTLRIKSDGNLVAPWYGYILSGESHGRILRTD
LKRGSCTVQCQTEKGGLNTTLPFQNVSKYAFGNCSKYIGIKSLKLAVGLRNVPSRSSRGLFGAIAGFIEG
GWSGLVAGWYGFQHSNDQGVGMAADRESTQKAVDKITSKVNNIVDKMNKQYEIIDHEFSEVETRLNMINN
KIDDQIQDIWAYNAELLVLLENQKTLDEHDANVNNLYNKVKRALGSNAVEDGKGCFELYHKCDDQCMETI
RNGTYNRRKYQEESKLERQKIEGVKLESEGTYKILTIYSTVASSLVIAMGFAAFLFWAMSNGSCRCNICI

>QBK20779.1 hemagglutinin [Influenza A virus]
METVSLITILIVATVSNADKICIGYQSTNSTETVDTLTENNVPVTHAKELLHTEHNGMLCATSLGQPLIL
DTCTIEGLIYGNPSCDLSLEGREWSYIVERPSAVNGLCYPGNVENLEELRSLFSSARSYQRIQIFPDTIW
NVSYDGTSTACSNSFYRNMRWLTRKNGDYPAQDAQYTNNQGKNILFMWGINHPPTDDTQRNLYTRTDTTT
SVATEEINRIFKPLIGPRPLVNGLMGRIDYYWSVLKPGQTLRIKSDGNLIAPWYGHILSGKSHGRILKTD
LKRGSCTVQCQTEKGGLNTTLPFQNVSKYAFGNCSKYIGIKSLKLAVGLRNVPSRSSRGLFGAIAGFIEG
GWSGLVAGWYGFQHSNDQGVGMAADRDSTQKAIDKITSKVNNIVDKMNKQYEIIDHEFSEVETRLNMINN
KIDDQIQDIWAYNAELLVLLENQKTLDEHDANVNNLYNKVKRALGSNAVEDGKGCFELYHKCNDQCMETI
RNGTYNRRKYQEESKLERQKIEGVKLESEGTYKILTIYSTVASSLVIAMGFAAFLFWAMSNGSCRCNICI

>QBK20778.1 hemagglutinin [Influenza A virus]
METVSLITILLAATVSNADKICIGYQSTNSTETVDTLTENNVPVTHAKELLHTEHNGMLCATSLGQPLIL
DTCTIEGLIYGNPSCDPLPEEREWSYIVERPSAVNGLCYPGNVENLEELRSLFSSARSYQRIQIFPDTIW
NVSYDGTSNTCSGSFYRNMRWLTRKDGNYPIQDAQYTNNQGKNILFMWGINNPPTDDTQRNLYTRTDTTT
SVATEEINRIFKPLIGPRPLVNGLMGRINYYWSVLKPGQTLRIKSDGNLVAPWYGSILSGESPGRILRTD
LKRGSCTVQCQTEKGGLNTTLPFQNVSKYAFGNCSKYIGIKSLKLAVGLRNVPSRSSRGLFGAIAGFIEG
GWSGLVAGWYGFQHSNDQGVGMAADRESTQKAVDKITSKVNNIVDKMNKQYEIIDHEFSEVETRLNMINN
KIDDQIQDIWAYNAELLVLLENQKTLDEHDANVNNLYNKVKRALGSNAVEDGKGCFELYHKCDDQCMETI
RNGTYNRRKYQEESKLERQKIEGVKLESEGTYKILTIYSTVASSLVIAMGFAAFLFWAMSNGSCRCNICI

>QBK20777.1 hemagglutinin [Influenza A virus]

METVSLITILLAATVSNADKICIGYQSTNSTETVDTLTENNVPVTHAKELLHTEHNGMLCATSLGQPLIL

DTCTIEGLIYGNPSCDPLPEEREWSYIVERPSAVNGLCYPGNVENLEELRSLFSSARSYQRIQIFPDTIW

NVSYDGTSNTCSGSFYRNMRWLTRKDGNYPIQDAQYTNNQGKNILFMWGINNPPTDDTQRNLYTKTDTTT

SVATEEINRIFKPLIGPRPLVNGLMGRINYYWSVLKPGQTLRIKSDGNLVAPWYGYILSGESHGRILRTD

LKRGSCTVQCQTEKGGLNTTLPFQNVSKYAFGNCSKYIGIKSLKLAVGLRNVPSRSSRGLFGAIAGFIEG

GWSGLVAGWYGFQHSNDQGVGMAADRESTQKAVDKITSKVNNIVDKMNKQYEIIDHEFSEVETRLNMINN

KIDDQIQDIWAYNAELLVLLENQKTLDEHDANVNNLYNKVKRALGSNAVEDGKGCFELYHKCDDQCMETI

RNGTYNRRKYQEESKLERQKIEGVKLESEGTYKILTIYSTVASSLVIAMGFAAFLFWAMSNGSCRCNICI

>QBK20775.1 hemagglutinin [Influenza A virus]

METVSLITILLVATVSYADKICIGYQSTNSTETVDTLTENNVPVTHAKELLHTEHNGMLCATSLGQPLIL

DTCTIEGLIYGNPSCDLSLEGREWSYIVERPSAVNGLCYPGNVENLEELRSLFSSARSYQRVQIFPDTIW

NVSYDGTSTACSGSFYRSMRWLTRKNGDYPTQDAQYTNNQGKNILFMWGINHPPTDDTQRNLYTRTDTTT

SVATEEINRIFKPLIGPRPLVNGLMGRIDYYWSVLKPGQTLRIKSDGNLIAPWYGYILSGESHGRILKTD

LKRGSCTVQCQTEKGGLNTTLPFQNVSKYAFGNCSKYIGIKSLKLAVGLRNVPSRSSRGLFGAIAGFIEG

GWSGLVAGWYGFQHSNDQGVGMAADRDSTQKAIDKITSKVNNIVDKMNKQYEIIDHEFSEIETRLNMINN

KIDDQIQDIWAYNAELLVLLENQKTLDEHDANVNNLYNKVKRALGSNAVEDGKGCFELYHKCNDQCMETI

RNGTYNRRKYQEESKLERQKIEGVKLESEGTYKILTIYSTVASSLVIAMGFAAFLFWAMSNGSCRCNICI

>QBK20774.1 hemagglutinin [Influenza A virus]

METVSLITILLVATVSNADKICIGYQSTNSTETVDTLTENNVPVTHAKELIHTEHNGMLCATSLGQPLIL

DTCTIEGLIYGNPSCDLSLEGREWSYIVERPSAVNGLCYPGNVENLEELRSLFSSARSYQRIQIFPDTIW

NVSYDGTSTACSGSFYRNMRWLTRKDGNYPTQDAQYTNNQGKNILFMWGINHPPTDTTQSGLYTRTDTTT

SVATEEINRIFKPLIGPRPLVNGLMGRIDYYWSVLKPGQTLRIKSDGNLIAPWFGHILSGESHGRILKTD

LKRGSCTVQCQTEKGGLNTTLPFQNVSKYAFGNCSKYIGIKSLKLAVGLRNVPSRSSRGLFGAIAGFIEG

GWSGLVAGWYGFQHSNDQGVGMAADRDSTQKAIDKITSKVNNIVDKMNKQYEIIDHEFSEVETRLNMINN

KIDDQIQDIWAYNAELLVLLENQKTLDEHDANVNNLYNKVKRALGSNAVEDGKGCFELYHKCDDQCMETI

RNGTYNRRKYQEESKLERQKIEGVKLESEGTYKILTIYSTVASSLVIAMGFAAFLFWAMSNGSCRCNICI

>QBK20773.1 hemagglutinin [Influenza A virus]

METVSLITILLVATVSNADKICIGYQSTNSTETVDTLTENNVPVTHAKELIHTEHNGMLCATSLGQPLIL

DTCTIEGLIYGNPSCDLSLEGREWSYIVERPSAVNGLCYPGNVENLEELRSLFSSARSYQRIQIFPDTIW

NVSYDGTSTACSGSFYRNMRWLTRKDGNYPTQDAQYTNNQGKNILFMWGINHPPTDTTQSGLYTRTDTTT

SVATEEINRIFKPLIGPRPLVNGLMGRIDYYWSVLKPGQTLRIKSDGNLIAPWFGHILSGESHGRILKTD
LKRGSCTVQCQTEKGGLNTTLPFQNVSKYAFGNCSKYIGIKSLKLAVGLRNVPSRSSRGLFGAIAGFIEG
GWSGLVAGWYGFQHSNDQGVGMAADRDSTQKAIDKITSKVNNIVDKMNKQYEIIDHEFSEVETRLNMINN
KIDDQIQDIWAYNAELLVLLENQKTLDEHDANVNNLYNKVKRALGSNAVEDGKGCFELYHKCDDQCMETI
RNGTYNRRKYQEESKLERQKIEGVKLESEGTYKILTIYSTVASSLVIAMGFAAFLFWAMSNGSCRCNICI

>QBK20772.1 hemagglutinin [Influenza A virus]
METVSLITILLVATVSNADKICIGYQSTNSTETVDTLTENNVPVTHAKELIHTEHNGMLCATSLGQPLIL
DTCTIEGLIYGNPSCDLSLEGREWSYIVERPSAVNGLCYPGNVENLEELRSLFSSARSYQRIQIFPDTIW
NVSYDGTSTACSGSFYRNMRWLTRKDGNYPTQDAQYTNNQGKNILFMWGINHPPTDTTQSGLYTRTDTTT
SVATEEINRIFKPLIGPRPLVNGLMGRIDYYWSVLKPGQTLRIKSDGNLIAPWFGHILSGESHGRILKTD
LKRGSCTVQCQTEKGGLNTTLPFQNVSKYAFGNCSKYIGIKSLKLAVGLRNVPSRSSRGLFGAIAGFIEG
GWSGLVAGWYGFQHSNDQGVGMAADRDSTQKAIDKITSKVNNIVDKMNKQYEIIDHEFSEVETRLNMINN
KIDDQIQDIWAYNAELLVLLENQKTLDEHDANVNNLYNKVKRALGSNAVEDGKGCFELYHKCDDQCMETI
RNGTYNRRKYQEESKLERQKIEGVKLESEGTYKILTIYSTVASSLVIAMGFAAFLFWAMSNGSCRCNICI

>QBK20771.1 hemagglutinin [Influenza A virus]
METVSLITILLVATVSNADKICIGYQSTNSTETVDTLTENNVPVTHAKELIHTEHNGMLCATSLGQPLIL
DTCTIEGLIYGNPSCDLSLEGREWSYIVERPSAVNGLCYPGNVENLEELRSLFSSARSYQRIQIFPDTIW
NVSYDGTSTACSGSFYRNMRWLTRKDGNYPTQDAQYTNNQGKNILFMWGINHPPTDTTQSGLYTRTDTTT
SVATEEINRIFKPLIGPRPLVNGLMGRIDYYWSVLKPGQTLRIKSDGNLIAPWFGHILSGESHGRILKTD
LKRGSCTVQCQTEKGGLNTTLPFQNVSKYAFGNCSKYIGIKSLKLAVGLRNVPSRSSRGLFGAIAGFIEG
GWSGLVAGWYGFQHSNDQGVGMAADRDSTQKAIDKITSKVNNIVDKMNKQYEIIDHEFSEVETRLNMINN
KIDDQIQDIWAYNAELLVLLENQKTLDEHDANVNNLYNKVKRALGSNAVEDGKGCFELYHKCDDQCMETI
RNGTYNRRKYQEESKLERQKIEGVKLESEGTYKILTIYSTVASSLVIAMGFAAFLFWAMSNGSCRCNICI

>QBK20770.1 hemagglutinin [Influenza A virus]
MEAVSLITILLVATVSNADKICIGYQSTNSTETVDTLTENNVPVTHAKELIHTEHNGMLCATSLGQPLIL
DTCTIEGLIYGNPSCDLSLEGREWSYIVERPSAVNGLCYPGNVENLEELRSLFSSARSYQRIQIFPDTIW
NVSYDGTSTACSGSFYRNMRWLTRKDGNYPTQDAQYTNNQGKNILFMWGINHPPTDTTQSGLYTRTDTTT
SVATEEINRVFKPLIGPRPLVNGLMGRIDYYWSVLKPGQTLRIKSDGNLIAPWFGHILSGESHGRILKTD
LKRGSCTVQCQTEKGGLNTTLPFQNVSKYAFGNCSKYIGIKSLKLAVGLRNVPSRSSRGLFGAIAGFIEG
GWSGLVAGWYGFQHSNDQGVGMAADRDSTQKAIDKITSKVNNIVDKMNKQYEIIDHEFSEVETRLNMINN
KIDDQIQDIWAYNAELLVLLENQKTLDEHDANVNNLYNKVKRALGSNAVEDGKGCFELYHKCDDQCMETI

RNGTYNRRKYQEESKLERQKIEGVKLESEGTYKILTIYSTVASSLVIAMGFAAFLFWAMSTGSCRCNICI

>QBK20769.1 hemagglutinin [Influenza A virus]
MKTVSLITILLVATVSNADKICIGYQSTNSTETVDTLTENNVPVTHAKELIHTEHNGMLCATSLGQPLIL
DTCTIEGLIYGNPSCDLSLEGREWSYIVERPSAVNGLCYPGNVENLEELRSLFSSARSYQRIQIFPDTIW
NVSYDGTSTACSGSFYRNMRWLTRKDGNYPTQDAQYTNNQGKNILFMWGINHPPTDTTQSGLYTRTDTTT
SVATEEINRIFKPLIGPRPLVNGLMGRIDYYWSVLKPGQTLRIKSDGNLIAPWFGHILSGESHGRILKTD
LKRGSCTVQCQTEKGGLNTTLPFQNVSKYAFGNCSKYIGIKSLKLAVGLRNVPSRSSRGLFGAIAGFIEG
GWSGLVAGWYGFQHSNDQGVGMAADRDSTQKAIDKITSKVNNIVDKMNKQYEIIDHEFSEVETRLNMINN
KIDDQIQDIWAYNAELLVLLENQKTLDEHDANVNNLYNKVKRALGSNAVEDGKGCFELYHKCDDQCMETI
RNGTYNRRKYQEESKLERQKIEGVKLESEGTYKILTIYSTVASSLVIAMGFAAFLFWAMSNGSCRCNICI

>QBK20768.1 hemagglutinin [Influenza A virus]
METVSLITILLVATVSNADKICIGYQSTNSTETVDTLTENNVPVTHAKELIHTEHNGMLCATSLGQPLIL
DTCTIEGLIYGNPSCDLSLEGREWSYIVERPSAVNGLCYPGNVENLEELRSLFSSARSYQRIQIFPDTIW
NVSYDGTSTACSGSFYRNMRWLTRKDGNYPTQDAQYTNNQGKNILFMWGINHPPTDTTQSGLYTRTDTTT
SVATEEINRIFKPLIGPRPLVNGLMGRIDYYWSVLKPGQTLRIKSDGNLIAPWFGHILSGESHGRILKTD
LKRGSCTVQCQTEKGGLNTTLPFQNVSKYAFGNCSKYIGIKSLKLAVGLRNVPSRSSRGLFGAIAGFIEG
GWSGLVAGWYGFQHSNDQGVGMAADRDSTQKAIDKITSKVNNIVDKMNKQYEIIDHEFSEVETRLNMINN
KIDDQIQDIWAYNAELLVLLENQKTLDEHDANVNNLYNKVKRALGSNAVEDGKGCFELYHKCDDQCMETI
RNGTYNRRKYQEESKLERQKIEGVKLESEGTYKILTIYSTVASSLVIAMGFAAFLFWAMSNGSCRCNICI

>QBK20767.1 hemagglutinin [Influenza A virus]
METVSLITILLVATVSNADKICIGYQSTNSTETVDTLTENNVPVTHAKELIHTEHNGMLCATSLGQPLIL
DTCTIEGLIYGNPSCDLSLGGREWSYIVERPSAVNGLCYPGNVENLEELRSLFSSARSYQRIQIFPDTIW
NVSYDGTSTACSGSFYRNMRWLTRKDGNYPTQDAQYTNNQGKNILFMWGINHPPTDTTQSGLYTRTDTTT
SVATEEINRIFKPLIGPRPLVNGLMGRIDYYWSVLKPGQTLRIKSDGNLIAPWFGHILSGESHGRILKTD
LKRGSCTVQCQTEKGGLNTTLPFQNVSKYAFGNCSKYIGIKSLKLAVGLRNVPSRSSRGLFGAIAGFIEG
GWSGLVAGWYGFQHSNDQGVGMAADRDSTQKAIDKITSKVNNIVDKMNKQYEIIDHEFSEVETRLNMINN
KIDDQIQDIWAYNAELLVLLENQKTLDEHDANVNNLYNKVKRALGSNAVEDGKGCFELYHKCDDQCMETI
RNGTYNRRKYQEESKLERQKIEGVKLESEGTYKILTIYSTVASSLVIAMGFAAFLFWAMSNGSCRCNICI

>QBK20766.1 hemagglutinin [Influenza A virus]
METISLITILLVATVSNADKICIGYQSTNSTETVDTLTENNVPVTHAKELIHTEHNGMLCATSLGQPLIL

DTCTIEGLIYGNPSCDLSLEGREWSYIVERPSAVNGLCYPGNVENLEELRSLFSSARSYQRIQIFPDTIW

NVSYDGTSTACSGSFYRNMRWLTRKDGNYPTQDAQYTNNQGKNILFMWGINHPPTDTTQSSLYTRTDTTT

SVATEEINRIFKPLIGPRPLVNGLMGRIDYYWSVLKPGQTLRIKSDGNLIAPWFGHILSGESHGRILKTD

LKRGSCTVQCQTEKGGLNTTLPFQNVSKYAFGNCSKYIGIKSLKLAVGLRNVPSRSSRGLFGAIAGFIEG

GWSGLVAGWYGFQHSNDQGVGMAADRDSTQKAIDKITSKVNNIVDKMNKQYEIIDHEFSEVETRLNMINN

KIDDQIQDIWAYNAELLVLLENQKTLDEHDANVNNLYNKVKRALGSNAVEDGKGCFELYHKCDDQCMETI

RNGTYNRRKYQEESKLERQKIEGVKLESEGTYKILTIYSTVASSLVIAMGFAAFLFWAMSNGSCRCNICI

>QBK20765.1 hemagglutinin [Influenza A virus]

METVSLITILLVATVSNADKICIGYQSTNSTETVDTLTENNVPVTHAKELLHTEHNGMLCATSLGQPLIL

DTCTIEGLIYGNPSCDLSLEGREWSYIVERPSAVHGLCYPGNVEDLEELRSLFSSARSYQRIQIFPDTIW

NVSYDGTSTACSGSFYRSMRWLTRKNGEYPIQDAQYTNNQGKNILFMWGINHPPTDDTQRGLYTRTDTTT

SVATEEINRIFKPLIGPRPLVNGLMGRINYYWSVLKPGQTLRIKSDGNLIAPWYGHILSGESHGRILKTD

LKRGSCTVQCQTEKGGLNTTLPFQNVSKYAFGNCSKYIGIKSLKLAVGLRNVPSRSSRGLFGAIAGFIEG

GWSGLVAGWYGFQHSNDQGVGMAADRDSTQKAIDKITSKVNNIVDKMNKQYEIIDHEFSEVETRLNMINN

KIDDQIQDIWAYNAELLVLLENQKTLDEHDANVNNLYNKVKRALGSNAVEDGKGCFELYHKCDDQCMETI

RNGTYNRRKYQEESKLERQRIEGVKLESEGTYKILTIYSTVASSLVIAMGFAAFLFWAMSNGSCRCNICI

>QBK20764.1 hemagglutinin [Influenza A virus]

METVSLITILLVATVSNADKICIGYQSTNSTETVDTLTENNVPVTHAKELIHTEHNGMLCATSLGQPLIL

DTCTIEGLIYGNPSCNLSLEGREWSYIVERPSAVNGLCYPGNVENLEELRSLFSSARSYQRIQIFPDTIW

NVSYDGTSTACSGSFYRNMRWLTRKDGNYPTQDAQYTNNQRKNILFMWGINHPPTDTTQNGLYTRTDTTT

SVATEEINRIFKPLIGPRPLVNGLMGRIDYYWSVLKPGQTLRIKSNGNLIAPWFGHILSGESHGRILKTD

LKRGSCTVQCQTEKGGLNTTLPFQNVSKYAFGNCSKYIGIKSLKLAVGLRNVPSRSSRGLFGAIAGFIEG

GWSGLVAGWYGFQHSNDQGVGMAADRDSTQKAIDKITSKVNNIVDKMNKQYEIIDHEFSEVETRLNMINN

KIDDQIQDIWAYNAELLVLLENQKTLDEHDANVNNLYNKVKRALGSNAVEDGKGCFELYHKCDDQCMETI

RNGTYNRRKYQEESKLERQKIEGVKLESEGTYKILTIYSTVASSLVIAMGFAAFLFWAMSNGSCRCNICI

>QBK20763.1 hemagglutinin [Influenza A virus]

METVSLITILLVATVSNADKICIGYQSTNSTETVDTLTENNVPVTHAKELIHTEHNGMLCATSLGQPLIL

DTCTIEGLIYGNPSCNLSLEGREWSYIVERPSAVNGLCYPGNVENLEELRSLFSSARSYQRIQIFPDTIW

NVSYDGTSTACSGSFYRNMRWLTRKDGNYPTQDAQYTNNQRKNILFMWGINHPPTDTTQNGLYTRTDTTT

SVATEEINRIFKPLIGPRPLVNGLMGRIDYYWSVLKPGQTLRIKSNGNLIAPWFGHILSGESHGRILKTD

LKRGSCTVQCQTEKGGLNTTLPFQNVSKYAFGNCSKYIGIKSLKLAVGLRNVPSRSSRGLFGAIAGFIEG

GWSGLVAGWYGFQHSNDQGVGMAADRDSTQKAIDKITSKVNNIVDKMNKQYEIIDHEFSEVETRLNMINN
KIDDQIQDIWAYNAELLVLLENQKTLDEHDANVNNLYNKVKRALGSNAVEDGKGCFELYHKCDDQCMETI
RNGTYNRRKYQEESKLERQKIEGVKLESEGTYKILTIYSTVASSLVIAMGFAAFLFWAMSNGSCRCNICI

>QBK20762.1 hemagglutinin [Influenza A virus]
METISLITILLVATVSNADKICIGYQSTNSTETVDTLTENNVPVTHAKELIHTEHNGMLCATSLGQPLIL
DTCTIEGLIYGNPSCNLSLEGREWSYIVERPSAVNGLCYPGNVENLEELRSLFSSARSYQRIQIFPDTIW
NVSYDGTSTACSGSFYRNMRWLTRKDGNYPTQDAQYTNNQRKNILFMWGINHPPTDTTQNGLYTRTDTTT
SVATEEINRIFKPLIGPRPLVNGLMGRIDYYWSVLKPGQTLRIKSNGNLIAPWFGHILSGESHGRILKTD
LKRGSCTVQCQTEKGGLNTTLPFQNVSKYAFGNCSKYIGIKSLKLAVGLRNVPSRSSRGLFGAIAGFIEG
GWSGLVAGWYGFQHSNDQGVGMAADRDSTQKAIDKITSKVNNIVDKMNKQYEIIDHEFSEVETRLNMINN
KIDDQIQDIWAYNAELLVLLENQKTLDEHDANVNNLYNKVKRALGSNAVEDGKGCFELYHKCDDQCMETI
RNGTYNRRKYQEESKLERQKIEGVKLESEGTYKILTIYSTVASSLVIAMGFAAFLFWAMSNGSCRCNICI

>QBK20761.1 hemagglutinin [Influenza A virus]
METISLITILLVATVSNADKICIGYQSTNSTETVDTLTENNVPVTHAKELIHTEHNGMLCATSLGQPLIL
DTCTIEGLIYGNPSCNLSLEGREWSYIVERPSAVNGLCYPGNVENLEELRSLFSSARSYQRIQIFPDTIW
NVSYDGTSTACSGSFYRNMRWLTRKDGNYPTQDAQYTNNQRKNILFMWGINHPPTDTTQNGLYTRTDTTT
SVATEEINRIFKPLIGPRPLVNGLMGRIDYYWSVLKPGQTLRIKSNGNLIAPWFGHILSGESHGRILKTD
LKRGSCTVQCQTEKGGLNTTLPFQNVSKYAFGNCSKYIGIKSLKLAVGLRNVPSRSSRGLFGAIAGFIEG
GWSGLVAGWYGFQHSNDQGVGMAADRDSTQKAIDKITSKVNNIVDKMNKQYEIIDHEFSEVETRLNMINN
KIDDQIQDIWAYNAELLVLLENQKTLDEHDANVNNLYNKVKRALGSNAVEDGKGCFELYHKCDDQCMETI
RNGTYNRRKYQEESKLERQKIEGVKLESEGTYKILTIYSTVASSLVIAMGFAAFLFWAMSNGSCRCNICI

>QBK20760.1 hemagglutinin [Influenza A virus]
METVSLITILLVATVSNADKICIGYQSTNSTETVDTLTENNVPVTHAKELIHTEHNGMLCATSLGQPLIL
DTCTIEGLIYGNPSCNLSLEGREWSYIVERPSAVNGLCYPGNVENLEELRSLFSSARSYQRIQIFPDTIW
NVSYDGTSTACSGSFYRNMRWLTRKDGNYPTQDAQYTNNQRKNILFMWGINHPPTDTTQNGLYTRTDTTT
SVATEEINRIFKPLIGPRPLVNGLMGRIDYYWSVLKPGQTLRIKSNGNLIAPWFGHILSGESHGRILKTD
LKRGSCTVQCQTEKGGLNTTLPFQNVSKYAFGNCSKYIGIKSLKLAVGLRNVPSRSSRGLFGAIAGFIEG
GWSGLVAGWYGFQHSNDQGVGMAADRDSTQKAIDKITSKVNNIVDKMNKQYEIIDHEFSEVETRLNMINN
KIDDQIQDIWAYNAELLVLLENQKTLDEHDANVNNLYNKVKRALGSNAVEDGKGCFELYHKCDDQCMETI
RNGTYNRRKYQEESKLERQKIEGVKLESEGTYKILTIYSTVASSLVIAMGFAAFLFWAMSNGSCRCNICI

>QBK20759.1 hemagglutinin [Influenza A virus]

METISLITILLVATVSNADKICIGYQSTNSTETVDTLTENNVPVTHAKELIHTEHNGMLCATSLGQPLIL

DTCTIEGLIYGNPSCDLSLEGREWSYIVERPSAVNGLCYPGNVENLEELRSLFSSARSYQRIQIFPDTIW

NVSYDGTSTACSGSFYRNMRWLTRKDGNYPTQDAQYTNNQGKNILFMWGINHPPTDTTQSGLYTRTDTTT

SVATEEINRIFKPLIGPRPLVNGLMGRIDYYWSVLKPGQTLRIKSDGNLIAPWFGHILSGESHGRILKTD

LKRGSCTVQCQTEKGGLNTTLPFQNVSKYAFGNCSKYIGIKSLKLAVGLRNVPSRSSRGLFGAIAGFIEG

GWSGLVAGWYGFQHSNDQGVGMAADRDSTQKAIDKITSKVNNIVDKMNKQYEIIDHEFSEVETRLNMINN

KIDDQIQDIWAYNAELLVLLENQKTLDEHDANVNNLYNKVKRALGSNAVEDGKGCFELYHKCDDQCMETI

RNGTYNRRKYQEESKLERQKIEGVKLESEGTYKILTIYSTVASSLVIAMGFAAFLFWAMSNGSCRCNICI

>QBK20758.1 hemagglutinin [Influenza A virus]

METVSLITILLVATVSNADKICIGYQSTNSTETVDTLTENNVPVTHAKELIHTEHNGMLCATSLGQPLIL

DTCTIEGLIYGNPSCDLSLEGREWSYIVERPSAVNGLCYPGNVENLEELRSLFSSARSYQRIQIFPDTIW

NVSYDGTSTACSGSFYRNMRWLTRKDGNYPTQDAQYTNNQGKNILFMWGINHPPTDTTQSGLYTRTDTTT

SVATEEINRIFKPLIGPRPLVNGLMGRIDYYWSVLKPGQTLRIKSDGNLIAPWFGHILSGESHGRILKTD

LKRGSCTVQCQTEKGGLNTTLPFQNVSKYAFGNCSKYIGIKSLKLAVGLRNVPSRSSRGLFGAIAGFIEG

GWSGLVAGWYGFQHSNDQGVGMAADRDSTQKAIDKITSKVNNIVDKMNKQYEIIDHEFSEVETRLNMINN

KIDDQIQDIWAYNAELLVLLENQKTLDEHDANVNNLYNKVKRALGSNAVEDGKGCFELYHKCDDQCMETI

RNGTYNRRKYQEESKLERQKIEGVKLESEGTYKILTIYSTVASSLVIAMGFAAFLFWAMSNGSCRCNICI

>QBK20757.1 hemagglutinin [Influenza A virus]

METVSLITILLVATVSNADKICIGYQSTNSTETVDTLTENNVPVTHAKELIHTEHNGMLCATSLGQPLIL

DTCTIEGLIYGNPSCDLSLEGREWSYIVERPSAVNGLCYPGNVENLEELRSLFSSARSYQRIQIFPDTIW

NVSYDGTSTACSGSFYRNMRWLTRKDGNYPTQDAQYTNNQGKNILFMWGINHPPTDTTQSGLYTRTDTTT

SVATEEINRIFKPLIGPRPLVNGLMGRIDYYWSVLKPGQTLRIKSDGNLIAPWFGHILSGESHGRILKTD

LKRGSCTVQCQTEKGGLNTTLPFQNVSKYAFGNCSKYIGIKSLKLAVGLRNVPSRSSRGLFGAIAGFIEG

GWSGLVAGWYGFQHSNDQGVGMAADRDSTQKAIDKITSKVNNIVDKMNKQYEIIDHEFSEVETRLNMINN

KIDDQIQDIWAYNAELLVLLENQKTLDEHDANVNNLYNKVKRALGSNAVEDGKGCFELYHKCDDQCMETI

RNGTYNRRKYQEESKLERQKIEGVKLESEGTYKILTIYSTVASSLVIAMGFAAFLFWAMSNGSCRCNICI

>QBK20756.1 hemagglutinin [Influenza A virus]

METISLITILLVATVSNADKICIGYQSTNSTETVDTLTENNVPVTHAKELIHTEHNGMLCATSLGQPLIL

DTCTIEGLIYGNPSCDLSLEGREWSYIVERPSAVNGLCYPGNVENLEELRSLFSSARSYQRIQIFPDTIW

NVSYDGTSTACSGSFYRNMRWLTRKDGNYPTQDAQYTNNQGKNILFMWGINHPPTDTTQSGLYTRTDTTT

SVATEEINRIFKPLIGPRPLVNGLMGRIDYYWSVLKPGQTLRIKSDGNLIAPWFGHILSGESHGRILKTD
LKRGSCTVQCQTEKGGLNTTLPFQNVSKYAFGNCSKYIGIKSLKLAVGLRNVPSRSSRGLFGAIAGFIEG
GWSGLVAGWYGFQHSNDQGVGMAADRDSTQKAIDKITSKVNNIVDKMNKQYEIIDHEFSEVETRLNMINN
KIDDQIQDIWAYNAELLVLLENQKTLDEHDANVNNLYNKVKRALGSNAVEDGKGCFELYHKCDDQCMETI
RNGTYNRRKYQEESKLERQKIEGVKLESEGTYKILTIYSTVASSLVIAMGFAAFLFWAMSNGSCRCNICI

>QBK20755.1 hemagglutinin [Influenza A virus]
METVSLITILLAATVSNADKICIGYQSTNSTETVDTLTENNVPVTHAKELLHTEHNGMLCATSLGQPLIL
DTCTIEGLIYGNPSCDPLLEEREWSYIIERPSAVNGLCYPGNVENLEELRSLFSSARSYQRIQIFPDTIW
NVSYDGTSNTCSGSFYRNMRWLTRKNGNYPIQDAQYTNNQGKNILFMWGINHPPTDDTQRNLYTRTDTTT
SVATEEINRIFKPLIGPRPLVNGLMGRINYYWSVLKPGQTLRIKSDGNLIAPWYGYILSGESHGRILRTD
LKRGSCTVQCQTEKGGLNTTLPFQNVSKYAFGNCSKYIGIKSLKLAVGLRNVPSRSSRGLFGAIAGFIEG
GWSGLVAGWYGFQHSNDQGVGMAADRESTQKAIDKITSKVNNIVDKMNKQYEIIDHEFSEVETRLNMINN
KIDDQIQDIWAYNAELLVLLENQKTLDEHDANVNNLYNKVKRALGSNAVEDGKGCFELYHKCDDQCMETI
RNGTYNRRKYQEESKLERQKIEGVKLESEGTYKILTIYSTVASSLVIAMGFAAFLFWAMSNGSCRCNICI

>QBK20754.1 hemagglutinin [Influenza A virus]
METVSLITILLVATVSNADKICIGYQSTNSTETVDTLTENNVPVTHAKELLHTEHNGMLCATSLGQPLIL
DTCTIEGLIYGNPSCDLSLEGREWSYIVERPSAVHGLCYPGNVEDLEELRSLFSSARSYQRIQIFPDTIW
NVSYDGTSTACSGSFYRSMRWLTRKNGEYPIQDAQYTNNQGKNILFMWGINHPPTDDTQRGLYTRTDTTT
SVATEEINRIFKPLIGPRPLVNGLMGRINYYWSVLKPGQTLRIKSDGNLIAPWYGHILSGESHGRILKTD
LKRGSCTVQCQTEKGGLNTTLPFQNVSKYAFGNCSKYIGIKSLKLAVGLRNVPSRSSRGLFGAIAGFIEG
GWSGLVAGWYGFQHSNDQGVGMAADRDSTQKAIDKITSKVNNIVDKMNKQYEIIDHEFSEVETRLNMINN
KIDDQIQDIWAYNAELLVLLENQKTLDEHDANVNNLYNKVKRALGSNAVEDGKGCFELYHKCDDQCMETI
RNGTYNRRKYQEESKLERQRIEGVKLESEGTYKILTIYSTVASSLVIAMGFAAFLFWAMSNGSCRCNICI

>QBK20752.1 hemagglutinin [Influenza A virus]
METVSLITILLVATVSNADKICIGYQSTNSTETVDTLTENNVPVTHAKELLHTEHNGMLCATSLGQPLIL
DTCTIEGLIYGNPSCDLSLEGREWSYIVERPSAVHGLCYPGNVEDLEELRSLFSSARSYQRIQIFPDTIW
NVSYDGTSTACSGSFYRSMRWLTRKNGEYPIQDAQYTNNQGKNILFMWGINHPPTDDTQRGLYTRTDTTT
SVATEEINRIFKPLIGPRPLVNGLMGRINYYWSVLKPGQTLRIKSDGNLIAPWYGHILSGESHGRILKTD
LKRGSCTVQCQTEKGGLNTTLPFQNVSKYAFGNCSKYIGIKSLKLAVGLRNVPSRSSRGLFGAIAGFIEG
GWSGLVAGWYGFQHSNDQGVGMAADRDSTQKAIDKITSKVNNIVDKMNKQYEIIDHEFSEVETRLNMINN
KIDDQIQDIWAYNAELLVLLENQKTLDEHDANVNNLYNKVKRALGSNAVEDGKGCFELYHKCDDQCMETI

RNGTYNRRKYQEESKLERQRIEGVKLESEGTYKILTIYSTVASSLVIAMGFAAFLFWAMSNGSCRCNICI

>QBK20751.1 hemagglutinin [Influenza A virus]
METVSLITILLVATVSNADKICIGYQSTNSTETVDTLTENNVPVTHAKELLHTEHNGMLCATSLGQPLIL
DTCTIEGLIYGNPSCDLSLEGREWSYIVERPSAVHGLCYPGNVEDLEELRSLFSSARSYQRIQIFPDTIW
NVSYDGTSTACSGSFYRSMRWLTRKNGEYPIQDAQYTNNQGKNILFMWGINHPPTDDTQRGLYTRTDTTT
SVATEEINRIFKPLIGPRPLVNGLMGRINYYWSVLKPGQTLRIKSDGNLIAPWYGHILSGESHGRILKTD
LKRGSCTVQCQTEKGGLNTTLPFQNVSKYAFGNCSKYIGIKSLKLAVGLRNVPSRSSRGLFGAIAGFIEG
GWSGLVAGWYGFQHSNDQGVGMAADRDSTQKAIDKITSKVNNIVDKMNKQYEIIDHEFSEVETRLNMINN
KIDDQIQDIWAYNAELLVLLENQKTLDEHDANVNNLYNKVKRALGSNAVEDGKGCFELYHKCDDQCMETI
RNGTYNRRKYQEESKLERQRIEGVKLESEGTYKILTIYSTVASSLVIAMGFAAFLFWAMSNGSCRCNICI

>QBK20750.1 hemagglutinin [Influenza A virus]
METVSLITILLVATVSNADKICIGYQSTNSTETVDTLTENNVPVTHAKELLHTEHNGMLCATSLGQPLIL
DTCTIEGLIYGNPSCDLSLEGREWSYIVERPSAVHGLCYPGNVEDLEELRSLFSSARSYQRIQIFPDTIW
NVSYDGTSTACSGSFYRSMRWLTRKNGEYPIQDAQYTNNQGKNILFMWGINHPPTDDTQRGLYTRTDTTT
SVATEEINRIFKPLIGPRPLVNGLMGRINYYWSVLKPGQTLRIKSDGNLIAPWYGHILSGESHGRILKTD
LKRGSCTVQCQTEKGGLNTTLPFQNVSKYAFGNCSKYIGIKSLKLAVGLRNVPSRSSRGLFGAIAGFIEG
GWSGLVAGWYGFQHSNDQGVGMAADRDSTQKAIDKITSKVNNIVDKMNKQYEIIDHEFSEVETRLNMINN
KIDDQIQDIWAYNAELLVLLENQKTLDEHDANVNNLYNKVKRALGSNAVEDGKGCFELYHKCDDQCMETI
RNGTYNRRKYQEESKLERQRIEGVKLESEGTYKILTIYSTVASSLVIAMGFAAFLFWAMSNGSCRCNICI

>QBK20749.1 hemagglutinin [Influenza A virus]
METASLITILLVATVGNADKICIGYQSTNSTETVDTLTENNVPVTHAKELLHTEHNGMLCATSLGHPLIL
DTCTIEGLIYGNPSCDPLLGGREWSYIVERPSAVNGLCYPGNVENLEELRSLFSSSRSYQRIQIFPDTIW
NVSYSGTSKACSDSFYRSMRWLTQKNNAYPTQDAQYTNNQEKNILFMWGINHPPTDTAQTNLYTRTDTTT
SVATEEMNRIFKPLIGPRPLVNGLMGRINYYWSVLKPGQTLRIKSDGNLIAPWYGHILSGESHGRILKTD
LKRGSCTVQCQTEKGGLNTTLPFQNVSKYAFGNCSKYIGVKSLKLAVGLRNVPSKSSRGLFGAIAGFIEG
GWSGLVAGWYGFQHSNDQGVGMAADRDSTQKAIDKITSKVNNIVDKMNKQYEIIDHEFSEVETRLNMINN
KVDDQIQDIWAYNAELLVLLENQKTLDEHDANVNNLYNKVKRALGSNAVEDGKGCFELYHKCDDHCMETI
RNGTYNRRKYQEESKLERQKIEGVKLESEETYKILTIYSTVASSLVIAMGFAAFLFWAMSNGSCRCNICI

>QBK20748.1 hemagglutinin [Influenza A virus]
METVSLITILLVATVSNADKICIGYQSTNSTETVDTLTENNVPVTHAKELLHTEHNGMLCATSLGQPLIL

DTCTIEGLIYGNPSCDLSLEGREWSYIVERPSAVNGLCYPGNVEDLEELRSLFSSARSYQRVQIFPDTIW
NVSYDGTSTACSGSFYRSMRWLTRKNGEYPIQDAQYTNNQRKNILFMWGINHPPTDTTQRELYTRTDTTT
SVATEEINRIFKPLIGPRPLVNGLMGRINYYWSVLKPGQTLRIKSDGNLIAPWYGYILSGESHGRILKTD
LKRGSCTVQCQTEKGGLNTTLPFQNVSKYAFGNCSKYIGIKSLKLAVGLRNVPSRSSRGLFGAIAGFIEG
GWSGLVAGWYGFQHSNDQGVGMAADRDSTQKAIDKITSKVNNIVDKMNKQYEIIDHEFSEVETRLNMINN
KIDDQIQDIWAYNAELLVLLENQKTLDEHDANVNNLYNKVKRALGSNAVEDGKGCFELYHKCDDQCMETI
RNGTYNRRKYQEESKLERQKIEGVKLESEGTYKILTIYSTVASSLVIAMGFAAFLFWAMSNGSCRCNICI

>QBK20747.1 hemagglutinin [Influenza A virus]
METVSLITILLVATVSNADKICIGYQSTNSTETVDTLTENNVPVTHAKELLHTEHNGMLCATSLGQPLIL
DTCTIEGLIYGNPSCDLSLEGREWSYIVERPSAVHGLCYPGNVEDLEELRSLFSSARSYQRIQIFPDTIW
NVSYDGTSTACSGSFYRSMRWLTRKNGEYPIQDAQYTNNQGKNILFMWGINHPPTDDTQRGLYTRTDTTT
SVATEEINRIFKPLIGPRPLVNGLMGRINYYWSVLKPGQTLRIKSDGNLIAPWYGHILSGESHGRILKTD
LKRGSCTVQCQTEKGGLNTTLPFQNVSKYAFGNCSKYIGIKSLKLAVGLRNVPSRSSRGLFGAIAGFIEG
GWSGLVAGWYGFQHSNDQGVGMAADRDSTQKAIDKITSKVNNIVDKMNKQYEIIDHEFSEVETRLNMINN
KIDDQIQDIWAYNAELLVLLENQKTLDEHDANVNNLYNKVKRALGSNAVEDGKGCFELYHKCDDQCMETI
RNGTYNRRKYQEESKLERQRIEGVKLESEGTYKILTIYSTVASSLVIAMGFAAFLFWAMSNGSCRCNICI

>QBK20746.1 hemagglutinin [Influenza A virus]
METVSLITILLVATVSNADKICIGYQSTNSTETVDTLTENNVPVTHAKELLHTEHNGMLCATSLGQPLIL
DTCTIEGLIYGNPSCDLSLEGREWSYIVERPSAVNGLCYPGNVEDLEELRSLFSSARSYQRVQIFPDTIW
NVSYDGTSTACSGSFYRSMRWLTRKNGEYPIQDAQYTNNQRKNILFMWGINHPPTDTTQRELYTRTDTTT
SVATEEINRTFKPLIGPRPLVNGLMGRINYYWSVLKPGQTLRIKSDGNLIAPWYGYILSGESHGRILKTD
LKRGSCTVQCQTEKGGLNTTLPFQNVSKYAFGNCSKYIGIKSLKLAVGLRNVPSRSSRGLFGAIAGFIEG
GWSGLVAGWYGFQHSNDQGVGMAADRDSTQKAIDKITSKVNNIVDKMNKQYEIIDHEFSEVETRLNMINN
KIDDQIQDIWAYNAELLVLLENQKTLDEHDANVNNLYNKVKRALGSNAVEDGKGCFELYHKCDDQCMETI
RNGTYNRRKYQEESKLERQKIEGVKLESEGTYKILTIYSTVASSLVIAMGFAAFLFWAMSNGSCRCNICI

>QBK20745.1 hemagglutinin [Influenza A virus]
METVSLITILLVATVSNADKICIGYQSTNSTETVDTLTENNVPVTHAKELLHTEHNGMLCATSLGQPLIL
DTCTIEGLIYGNPSCDLSLEGREWSYIVERPSAVNGLCYPGNVEDLEELRSLFSSARSYQRVQIFPDTIW
NVSYDGTSTACSGSFYRSMRWLTRKNGEYPIQDAQYTNNQRKNILFMWGINHPPTDTTQRELYTRTDTTT
SVATEEINRTFKPLIGPRPLVNGLMGRINYYWSVLKPGQTLRIKSDGNLIAPWYGYILSGESHGRILKTD
LKRGSCTVQCQTEKGGLNTTLPFQNVSKYAFGNCSKYIGIKSLKLAVGLRNVPSRSSRGLFGAIAGFIEG

GWSGLVAGWYGFQHSNDQGVGMAADRDSTQKAIDKITSKVNNIVDKMNKQYEIIDHEFSEVETRLNMINN
KIDDQIQDIWAYNAELLVLLENQKTLDEHDANVNNLYNKVKRALGSNAVEDGKGCFELYHKCDDQCMETI
RNGTYNRRKYQEESKLERQKIEGVKLESEGTYKILTIYSTVASSLVIAMGFAAFLFWAMSNGSCRCNICI

>QBK20744.1 hemagglutinin [Influenza A virus]
METVSLITILLVATVSNADKICIGYQSTNSTETVDTLTENNVPVTHAKELLHTEHNGMLCATSLGQPLIL
DTCTIEGLIYGNPSCDLSLEGREWSYIVERPSAVHGLCYPGNVEDLEELRSLFSSARSYQRIQIFPDTIW
NVSYDGTSTACSGSFYRSMRWLTRKNGEYPIQDAQYTNNQGKNILFMWGINHPPTDDTQRGLYTRTDTTT
SVATEEINRIFKPLIGPRPLVNGLMGRINYYWSVLKPGQTLRIKSDGNLIAPWYGHILSGESHGRILKTD
LKRGSCTVQCQTEKGGLNTTLPFQNVSKYAFGNCSKYIGIKSLKLAVGLRNVPSRSSRGLFGAIAGFIEG
GWSGLVAGWYGFQHSNDQGVGMAADRDSTQKAIDKITSKVNNIVDKMNKQYEIIDHEFSEVETRLNMINN
KIDDQIQDIWAYNAELLVLLENQKTLDEHDANVNNLYNKVKRALGSNAVEDGKGCFELYHKCDDQCMETI
RNGTYNRRKYQEESKLERQRIEGVKLESEGTYKILTIYSTVASSLVIAMGFAAFLFWAMSNGSCRCNICI

>QBK20743.1 hemagglutinin [Influenza A virus]
METVSLITILLVATVSNADKICIGYQSTNSTETVDTLTENNVPVTHAKELLHTEHNGMLCATSLGQPLIL
DTCTIEGLIYGNPSCDLSLEGREWSYIVERPSAVHGLCYPGNVEDLEELRSLFSSARSYQRIQIFPDTIW
NVSYDGTSTACSGSFYRSMRWLTRKNGEYPIQDAQYTNNQGKNILFMWGINHPPTDDTQRGLYTRTDTTT
SVATEDINRIFKPLIGPRPLVNGLMGRINYYWSVLKPGQTLRIKSDGNLIAPWYGHILSGESHGRILKTD
LKRGSCTVQCQTEKGGLNTTLPFQNVSKYAFGNCSKYIGIKSLKLAVGLRNVPTRSSRGLFGAIAGFIEG
GWSGLVAGWYGFQHSNDQGVGMAADRDSTQKAIDKITSKVNNIVDKMNKQYEIIDHEFSEVETRLNMINN
KIDDQIQDIWAYNAELLVLLENQKTLDEHDANVNNLYNKVKRALGYNAVEDGKGCFELYHKCDDQCMETI
RNGTYNRRKYQEESKLERQRIEGVKLESEGTYKILTIYSTVASSLVIAMGFAAFLFWAMSNGSCRCNICI

>QBK20742.1 hemagglutinin [Influenza A virus]
METVSLITILLVATVSNADKICIGYQSTNSTETVDTLTENNVPVTHAKELLHTEHNGMLCATSLGQPLIL
DTCTIEGLIYGNPSCDLSLEGREWSYIVERPSAVNGLCYPGNVEDLEELRSLFSSARSYQRVQIFPDTIW
NVSYDGTSTACSGSFYRSMRWLTRKNGEYPIQDAQYTNNQRKNILFMWGINHPPTDTTQRELYTRTDTTT
SVATEEINRTFKPLIGPRPLVNGLMGRINYYWSVLKPGQTLRIKSDGNLIAPWYGYILSGESHGRILKTD
LKRGSCTVQCQTEKGGLNTTLPFQNVSKYAFGNCSKYIGIKSLKLAVGLRNVPSRSSRGLFGAIAGFIEG
GWSGLVAGWYGFQHSNDQGVGMAADRDSTQKAIDKITSKVNNIVDKMNKQYEIIDHEFSEVETRLNMINN
KIDDQIQDIWAYNAELLVLLENQKTLDEHDANVNNLYNKVKRALGSNAVEDGKGCFELYHKCDDQCMETI
RNGTYNRRKYQEESKLERQKIEGVKLESEGTYKILTIYSTVASSLVIAMGFAAFLFWAMSNGSCRCNICI

>QBK20741.1 hemagglutinin [Influenza A virus]

METVSLITILLVATVSNADKICIGYQSTNSTETVDTLTENNVPVTHAKELLHTEHNGMLCATSLGQPLIL
DTCTIEGLIYGNPSCDLSLEGREWSYIVERPSAVHGLCYPGNVEDLEELRSLFSSARSYQRIQIFPDTIW
NVSYDGTSTACSGSFYRSMRWLTRKNGEYPIQDAQYTNNQGKNILFMWGINHPPTDDTQRGLYTRTDTTT
SVATEEINRIFKPLIGPRPLVNGLMGRINYYWSVLKPGQTLRIKSDGNLIAPWYGHILSGESHGRILKTD
LKRGSCTVQCQTEKGGLNTTLPFQNVSKYAFGNCSKYIGIKSLKLAVGLRNVPTRSSRGLFGAIAGFIEG
GWSGLVAGWYGFQHSNDQGVGMAADRDSTQKAIDKITSKVNNIVDKMNKQYEIIDHEFSEVETRLNMINN
KIDDQIQDIWAYNAELLVLLENQKTLDEHDANVNNLYNKVKRALGYNAVEDGKGCFELYHKCDDQCMETI
RNGTYNRRKYQEESKLERQRIEGVKLESEGTYKILTIYSTVASSLVIAMGFAAFLFWAMSNGSCRCNICI

>QBK20740.1 hemagglutinin [Influenza A virus]

METVSLITILLVATVSNADKICIGYQSTNSTETVDTLTENNVPVTHAKELLHTEHNGMLCATSLGQPLIL
DTCTIEGLIYGNPSCDLSLEGREWSYIVERPSAVHGLCYPGNVEDLEELRSLFSSARSYQRIQIFPDTIW
NVSYDGTSTACSGSFYKSMRWLTRKNGEYPTQDAQYTNNQGKNILFMWGINHPPTDDTQRGLYTRTDTTT
SVATEEINRIFKPLIGPRPLVNGLMGRINYYWSVLKPGQTLRIKSDGNLIAPWYGHILSGESHGRILKTD
LKRGSCTVQCQTEKGGLNTTLPFQNVSKYAFGNCSKYIGIKSLKLAVGLRNVPSRSSRGLFGAIAGFIEG
GWSGLVAGWYGFQHSNDQGVGMAADRDSTQKAIDKITSKVNNIVDKMNKQYEIIDHEFSEVETRLNMINN
KIDDQIQDIWAYNAELLVLLENQKTLDEHDANVNNLYNKVKRALGSNAVEDGKGCFELYHKCDDQCMETI
RNGTYNRRKYQEESKLERQRIEGVKLESEGTYKILTIYSTVASSLVIAMGFAAFLFWAMSNGSCRCNICI

>QBK20738.1 hemagglutinin [Influenza A virus]

METVSLITILLVATVSNADKICIGYQSTNSTETVDTLTENNVPVTHAKELLHTEHNGMLCATSLGQPLIL
DTCTIEGLIYGNPSCDLSLEGREWSYIVERPSAVNGLCYPGNVEDLEELRSLFSSARSYQRVQIFPDTIW
NVSYDGTSTACSGSFYRSMRWLTRKNGEYPIQDAQYTNNQRKNILFMWGINHPPTDTTQRELYTRTDTTT
SVATEEINRIFKPLIGPRPLVNGLMGRINYYWSVLKPGQTLRIKSDGNLIAPWYGYILSGESHGRILKTD
LKRGSCTVQCQTEKGGLNTTLPFQNVSKYAFGNCSKYIGIKSLKLAVGLRNVPSRSSRGLFGAIAGFIEG
GWSGLVAGWYGFQHSNDQGVGMAADRDSTQKAIDKITSKVNNIVDKMNKQYEIIDHEFSEVETRLNMINN
KIDDQIQDIWAYNAELLVLLENQKTLDEHDANVNNLYNKVKRALGSNAVEDGKGCFELYHKCDDQCMETI
RNGTYNRRKYQEESKLERQKIEGVKLESEGTYKILTIYSTVASSLVIAMGFAAFLFWAMSNGSCRCNICI

>QBK20737.1 hemagglutinin [Influenza A virus]

METVSLITILLVATVSNADKICIGYQSTNSTETVDTLTENNVPVTHAKELLHTEHNGMLCATSLGQPLIL
DTCTIEGLIYGNPSCDLSLEGREWSYIVERPSAVHGLCYPGNVEDLEELRSLFSSARSYQRIQIFPDTIW
NVSYDGTSTACSGSFYRSMRWLTRKNGEYPIQDAQYTNNQGKNILFMWGINHPPTDDTQRGLYTRTDTTT

SVATEEINRIFKPLIGPRPLVNGLMGRINYYWSVLKPGQTLRIKSDGNLIAPWYGHILSGESHGRILKTD
LKRGSCTVQCQTEKGGLNTTLPFQNISKYAFGNCSKYIGIKSLKLAVGLRNVPSRSSRGLFGAIAGFIEG
GWSGLVAGWYGFQHSNDQGVGMAADRDSTQKAIDKITSKVNNIVDKMNKQYEIIDHEFSEVETRLNMINN
KIDDQIQDIWAYNAELLVLLENQKTLDEHDANVNNLYSKVKRALGSNAVEDGKGCFELYHKCDDQCMETI
RNGTYNRRKYQEESKLERQRIEGVKLESEGTYKILTIYSTVASSLVIAMGFAAFLFWAMSNGSCRCNICI

>QBK20736.1 hemagglutinin [Influenza A virus]
METVSLITILLVATVSNADKICIGYQSTNSTETVDTLTENNVPVTHAKELLHTEHNGMLCATSLGQPLIL
DTCTIEGLIYGNPSCDLSLEGREWSYIVERPSAVNGLCYPGNVEDLEELRSLFSSARSYQRVQIFPDTIW
NVSYDGTSTACSGSFYRSMRWLTRKNGEYPIQDAQYTNNQRKNILFMWGINHPPTDTTQRELYTRTDTTT
SVATEEINRIFKPLIGPRPLVNGLMGRINYYWSVLKPGQTLRIKSDGNLIAPWYGYILSGESHGRILKTD
LKRGSCTVQCQTEKGGLNTTLPFQNVSKYAFGNCSKYIGIKSLKLAVGLRNVPSRSSRGLFGAIAGFIEG
GWSGLVAGWYGFQHSNDQGVGMAADRDSTQKAIDKITSKVNNIVDKMNKQYEIIDHEFSEVETRLNMINN
KIDDQIQDIWAYNAELLVLLENQKTLDEHDANVNNLYNKVKRALGSNAVEDGKGCFELYHKCDDQCMETI
RNGTYNRRKYQEESKLERQKIEGVKLESEGTYKILTIYSTVASSLVIAMGFAAFLFWAMSNGSCRCNICI

>QBK20735.1 hemagglutinin [Influenza A virus]
METVSLITILLVATVSNADKICIGYQSTNSTETVDTLTENNVPVTHAKELLHTEHNGMLCATSLGQPLIL
DTCTIEGLIYGNPSCDLSLEGREWSYIVERPSAVNGLCYPGNVEDLEELRSLFSSARSYQRVQIFPDTIW
NVSYDGTSTACSGSFYRSMRWLTRKNGEYPIQDAQYTNNQRKNILFMWGINHPPTDTTQRELYTRTDTTT
SVATEEINRIFKPLIGPRPLVNGLMGRINYYWSVLKPGQTLRIKSDGNLIAPWYGYILSGESHGRILKTD
LKRGSCTVQCQTEKGGLNTTLPFQNVSKYAFGNCSKYIGIKSLKLAVGLRNVPSRSSRGLFGAIAGFIEG
GWSGLVAGWYGFQHSNDQGVGMAADRDSTQKAIDKITSKVNNIVDKMNKQYEIIDHEFSEVETRLNMINN
KIDDQIQDIWAYNAELLVLLENQKTLDEHDANVNNLYNKVKRALGSNAVEDGKGCFELYHKCDDQCMETI
RNGTYNRRKYQEESKLERQKIEGVKLESEGTYKILTIYSTVASSLVIAMGFAAFLFWAMSNGSCRCNICI

>QBK20734.1 hemagglutinin [Influenza A virus]
METVSLITILLVATVSNADKICIGYQSTNSTETVDTLTENNVPVTHAKELLHTEHNGMLCATSLGQPLIL
DTCTIEGLIYGNPSCDLSLEGREWSYIVERPSAVNGLCYPGNVEDLEELRSLFSSARSYQRVQIFPDTIW
NVSYDGTSTACSGSFYRSMRWLTRKNGEYPIQDAQYTNNQRKNILFMWGINHPPTDTTQRELYTRTDTTT
SVATEEINRIFKPLIGPRPLVNGLMGRINYYWSVLKPGQTLRIKSDGNLIAPWYGYILSGESHGRILKTD
LKRGSCTVQCQTEKGGLNTTLPFQNVSKYAFGNCSKYIGIKSLKLAVGLRNVPSRSSRGLFGAIAGFIEG
GWSGLVAGWYGFQHSNDQGVGMAADRDSTQKAIDKITSKVNNIVDKMNKQYEIIDHEFSEVETRLNMINN
KIDDQIQDIWAYNAELLVLLENQKTLDEHDANVNNLYNKVKRALGSNAVEDGKGCFELYHKCDDQCMETI

RNGTYNRRKYQEESKLERQKIEGVKLESEGTYKILTIYSTVASSLVIAMGFAAFLFWAMSNGSCRCNICI

>QBK20729.1 hemagglutinin [Influenza A virus]
METVSLITILLVATVSNADKICIGYQSTNSTETVDTLTENNVPVTHAKELLHTEHNGMLCATSLGQPLIL
DTCTIEGLIYGNPSCDLSLEGREWSYIVERPSAVHGLCYPGNVEDLEELRSLFSSARSYQRIQIFPDTIW
NVSYDGTSTACSGSFYRSMRWLTRKNGEYPIQDAQYTNNQGKNILFMWGINHPPTDDTQRGLYTRTDTTT
SVATEEINRIFKPLIGPRPLVNGLMGRINYYWSVLKPGQTLRIKSDGNLIAPWYGHILSGESHGRILKTD
LKRGSCTVQCQTEKGGLNTTLPFQNVSKYAFGNCSKYIGIKSLKLAVGLRNVPSRSSRGLFGAIAGFIEG
GWSGLVAGWYGFQHSNDQGVGMAADRDSTQKAIDKITSKVNNIVDKMNKQYEIIDHEFSEVETRLNMINN
KIDDQIQDIWAYNAELLVLLENQKTLDEHDANVNNLYNKVKRALGSNAVEDGKGCFELYHKCDDQCMETI
RNGTYNRRKYQEESKLERQRIEGVKLESEGTYKILTIYSTVASSLVIAMGFAAFLFWAMSNGSCRCNICI

>QBK20728.1 hemagglutinin [Influenza A virus]
METVSLITILLVATVSNADKICIGYQSTNSTETVDTLTENNVPVTHAKELLHTEHNGMLCATSLGQPLIL
DTCTIEGLIYGNPSCDLSLEGREWSYIVERPSAVHGLCYPGNVEDLEELRSLFSSARSYQRIQIFPDTIW
NVSYDGTSTACSGSFYRSMRWLTRKNGEYPIQDAQYTNNQGKNILFMWGINHPPTDDTQRGLYTRTDTTT
SVATEEINRIFKPLIGPRPLVNGLMGRINYYWSVLKPGQTLRIKSDGNLIAPWYGHILSGESHGRILKTD
LKRGSCTVQCQTEKGGLNTTLPFQNVSKYAFGNCSKYIGIKSLKLAVGLRNVPSRSSRGLFGAIAGFIEG
GWSGLVAGWYGFQHSNDQGVGMAADRDSTQKAIDKITSKVNNIVDKMNKQYEIIDHEFSEVETRLNMINN
KIDDQIQDIWAYNAELLVLLENQKTLDEHDANVNNLYNKVKRALGSNAVEDGKGCFELYHKCDDQCMETI
RNGTYNRRKYQEESKLERQRIEGVKLESEGTYKILTIYSTVASSLVIAMGFAAFLFWAMSNGSCRCNICI

>QBK20727.1 hemagglutinin [Influenza A virus]
METVSLITILLVATVSNADKICIGYQSTNSTETVDTLTENNVPVTHAKELLHTEHNGMLCATSLGQPLIL
DTCTIEGLIYGNPSCDLSLEGREWSYIVERPSAVHGLCYPGNVEDLEELRSLFSSARSYQRIQIFPDTIW
NVSYDGTSTACSGSFYRSMRWLTRKNGEYPIQDAQYTNNQGKNILFMWGINHPPTDDTQRGLYTRTDTTT
SVATEEINRIFKPLIGPRPLVNGLMGRINYYWSVLKPGQTLRIKSDGNLIAPWYGHILSGESHGRILKTD
LKRGSCTVQCQTEKGGLNTTLPFQNVSKYAFGNCSKYIGIKSLKLAVGLRNVPSRSSRGLFGAIAGFIEG
GWSGLVAGWYGFQHSNDQGVGMAADRDSTQKAIDKITSKVNNIVDKMNKQYEIIDHEFSEVETRLNMINN
KIDDQIQDIWAYNAELLVLLENQKTLDEHDANVNNLYNKVKRALGSNAVEDGKGCFELYHKCDDQCMETI
RNGTYNRRKYQEESKLERQRIEGVKLESEGTYKILTIYSTVASSLVIAMGFAAFLFWAMSNGSCRCNICI

>QBK20726.1 hemagglutinin [Influenza A virus]
METVSLITILLVATVSNADKICIGYQSTNSTETVDTLTENNVPVTHAKELLHTEHNGMLCATSLGQPLIL

DTCTIEGLIYGNPSCDLSLEGREWSYIVERPSAVHGLCYPGNVEDLEELRSLFSSARSYQRIQIFPDTIW
NVSYDGTSTACSGSFYKSMRWLTRKNGEYPTQDAQYTNNQGKNILFMWGINHPPTDDTQRGLYTRTDTTT
SVATEEINRIFKPLIGPRPLVNGLMGRINYYWSVLKPGQTLRIKSDGNLIAPWYGHILSGESHGRILKTD
LKRGSCTVQCQTEKGGLNTTLPFQNVSKYAFGNCSKYIGIKSLKLAVGLRNVPSRSSRGLFGAIAGFIEG
GWSGLVAGWYGFQHSNDQGVGMAADRDSTQKAIDKITSKVNNIVDKMNKQYEIIDHEFSEVETRLNMINN
KIDDQIQDIWAYNAELLVLLENQKTLDEHDANVNNLYNKVKRALGSNAVEDGKGCFELYHKCDDQCMETI
RNGTYNRRKYQEESKLERQRIEGVKLESEGTYKILTIYSTVASSLVIAMGFAAFLFWAMSNGSCRCNICI

>QBK20725.1 hemagglutinin [Influenza A virus]
METVSLITILLVATVSNADKICIGYQSTNSTETVDTLTENNVPVTHAKELLHTEHNGMLCATSLGQPLIL
DTCTIEGLIYGNPSCDLSLEGREWSYIVERPSAVNGLCYPGNVEDLEELRSLFSSARSYQRVQIFPDTIW
NVSYDGTSTACSGSFYRSMRWLTRKNGEYPIQDAQYTNNQRKNILFMWGINHPPTDTTQRALYTRTDTTT
SVATEEINRIFKPLIGPRPLVNGLMGRINYYWSVLKPGQTLRIKSDGNLIAPWYGYILSGESHGRILKTD
LKRGSCTVQCQTEKGGLNTTLPFQNVSKYAFGNCSKYIGIKSLKLAVGLRNVPSRSSRGLFGAIAGFIEG
GWSGLVAGWYGFQHSNDQGVGMAADRDSTQKAIDKITSKVNNIVDKMNKQYEIIDHEFSEVETRLNMINN
KIDDQIQDIWAYNAELLVLLENQKTLDEHDANVNNLYNKVKRALGSNAVEDGKGCFELYHKCDDQCMETI
RNGTYNRRKYQEESKLERQKIEGVKLESEGTYKILTIYSTVASSLVIAMGFAAFLFWAMSNGSCRCNICI

>QBK20724.1 hemagglutinin [Influenza A virus]
METVSLITILLVATVSNADKICIGYQSTNSTETVDTLTENNVPVTHAKELLHTEHNGMLCATSLGQPLIL
DTCTIEGLIYGNPSCDLSLEGREWSYLVERPSAVNGLCYPGNVEDLEELRSLFSSARSYQRVQIFPDTIW
NVSYDGTSTACSGSFYRSMRWLTRKNGEYPIQDAQYTNNQRKNILFMWGINHPPTDTTQRELYTRTDTTT
SVATEEINRTFKPLIGPRPLVNGLMGRINYYWSVLKPGQTLRIKSDGNLIAPWYGYILSGESHGRILKTD
LKRGSCTVQCQTEKGGLNTTLPFQNVSKYAFGNCSKYIGIKSLKLAVGLRNVPSRSSRGLFGAIAGFIEG
GWSGLVAGWYGFQHSNDQGVGMAADRDSTQKAIDKITSKVNNIVDKMNKQYEIIDHEFSEVETRLNMINN
KIDDQIQDIWAYNAELLVLLENQKTLDEHDANVNNLYNKVKRALGSNAVEDGKGCFELYHKCDDQCMETI
RNGTYNRRKYQEESKLERQKIEGVKLESEGTYKILTIYSTVASSLVIAMGFAAFLFWAMSNGSCRCNICI

>QBK20723.1 hemagglutinin [Influenza A virus]
METVSLITILLVATVSNADKICIGYQSTNSTETVDTLTENNVPVTHAKELLHTEHNGMLCATSLGQPLIL
DTCTIEGLIYGNPSCDLSLEGREWSYIVERPSAVHGLCYPGNVEDLEELRSLFSSARSYQRIQIFPDTIW
NVSYDGTSTACSGSFYKSMRWLTRKNGEYPTQDAQYTNNQGKNILFMWGINHPPTDDTQRGLYTRTDTTT
SVATEEINRIFKPLIGPRPLVNGLMGRINYYWSVLKPGQTLRIKSDGNLIAPWYGHILSGESHGRILKTD
LKRGSCTVQCQTEKGGLNTTLPFQNVSKYAFGNCSKYIGIKSLKLAVGLRNVPSRSSRGLFGAIAGFIEG

GWSGLVAGWYGFQHSNDQGVGMAADRDSTQKAIDKITSKVNNIVDKMNKQYEIIDHEFSEVETRLNMINN
KIDDQIQDIWAYNAELLVLLENQKTLDEHDANVNNLYNKVKRALGSNAVEDGKGCFELYHKCDDQCMETI
RNGTYNRRKYQEESKLERQRIEGVKLESEGTYKILTIYSTVASSLVIAMGFAAFLFWAMSNGSCRCNICI

>QBK20722.1 hemagglutinin [Influenza A virus]
METVSLITILLVATVSNADKICIGYQSTNSTETVDTLTENNVPVTHAKELLHTEHNGMLCATSLGQPLIL
DTCTIEGLIYGNPSCDLSLEGREWSYLVERPSAVNGLCYPGNVEDLEELRSLFSSARSYQRVQIFPDTIW
NVSYDGTSTACSGSFYRSMRWLTRKNGEYPIQDAQYTNNQRKNILFMWGINHPPTDTTQRELYTRTDTTT
SVATEEINRTFKPLIGPRPLVNGLMGRINYYWSVLKPGQTLRIKSDGNLIAPWYGYILSGESHGRILKTD
LKRGSCTVQCQTEKGGLNTTLPFQNVSKYAFGNCSKYIGIKSLKLAVGLRNVPSRSSRGLFGAIAGFIEG
GWSGLVAGWYGFQHSNDQGVGMAADRDSTQKAIDKITSKVNNIVDKMNKQYEIIDHEFSEVETRLNMINN
KIDDQIQDIWAYNAELLVLLENQKTLDEHDANVNNLYNKVKRALGSNAVEDGKGCFELYHKCDDQCMETI
RNGTYNRRKYQEESKLERQKIEGVKLESEGTYKILTIYSTVASSLVIAMGFAAFLFWAMSNGSCRCNICI

>QBK20721.1 hemagglutinin [Influenza A virus]
METVSLITILLVATVSNADKICIGYQSTNSTETVDTLTENNVPVTHAKELLHTEHNGMLCATSLGQPLIL
DTCTIEGLIYGNPSCDLSLEGREWSYIVERPSAVHGLCYPGNVEDLEELRSLFSSARSYQRIQIFPDTIW
NVSYDGTSTACSGSFYRSMRWLTRKNGEYPIQDAQYTNNQGKNILFMWGINHPPTDDTQRGLYTRTDTTT
SVATEEINRIFKPLIGPRPLVNGLMGRINYYWSVLKPGQTLRIKSDGNLIAPWYGHILSGESHGRILKTD
LKRGSCTVQCQTEKGGLNTTLPFQNVSKYAFGNCSKYIGIKSLKLAVGLRNVPSRSSRGLFGAIAGFIEG
GWSGLVAGWYGFQHSNDQGVGMAADRDSTQKAIDKITSKVNNIVDKMNKQYEIIDHEFSEVETRLNMINN
KIDDQIQDIWAYNAELLVLLENQKTLDEHDANVNNLYNKVKRALGSNAVEDGKGCFELYHKCDDQCMETI
RNGTYNRRKYQEESKLERQRIEGVKLESEGTYKILTIYSTVASSLVIAMGFAAFLFWAMSNGSCRCNICI

>QBK20720.1 hemagglutinin [Influenza A virus]
METVSLITILLVATVSNADKICIGYQSTNSTETVDTLTENNVPVTHAKELLHTEHNGMLCATSLGQPLIL
DTCTIEGLIYGNPSCDLSLEGREWSYIVERPSAVHGLCYPGNVEDLEELRSLFSSARSYQRIQIFPDTIW
NVSYDGTSTACSGSFYRSMRWLTRKNGEYPIQDAQYTNNQGKNILFMWGINHPPTDDTQRGLYTRTDTTT
SVATEEINRIFKPLIGPRPLVNGLMGRINYYWSVLKPGQTLRIKSDGNLIAPWYGHILSGESHGRILKTD
LKRGSCTVQCQTEKGGLNTTLPFQNVSKYAFGNCSKYIGIKSLKLAVGLRNVPSRSSRGLFGAIAGFIEG
GWSGLVAGWYGFQHSNDQGVGMAADRDSTQKAIDKITSKVNNIVDKMNKQYEIIDHEFSEVETRLNMINN
KIDDQIQDIWAYNAELLVLLENQKTLDEHDANVNNLYNKVKRALGSNAVEDGKGCFELYHKCDDQCMETI
RNGTYNRRKYQEESKLERQRIEGVKLESEGTYKILTIYSTVASSLVIAMGFAAFLFWAMSNGSCRCNICI

>QBK20719.1 hemagglutinin [Influenza A virus]

MGTVSLITILLVATVSNADKICIGYQSTNSTETVDTLTENNVPVTHAKELLHTEHNGMLCATSLGQPLIL
DTCTIEGLIYGNPSCDLSLEGREWSYIVERPSAVHGLCYPGNVEDLEELRSLFSSARSYQRIQIFPDTIW
NVSYDGTSTACSGSFYRSMRWLTRKNGEYPIQDAQYTNNQGKNILFMWGINHPPTDETQRGLYTRTDTTT
SVATEEINRIFKPLIGPRPLVNGLMGRINYYWSVLKPGQTLRIKSDGNLIAPWYGHILSGESHGRILKTD
LKRGSCTVQCQTEKGGLNTTLPFQNVSKYAFGNCSKYIGIKSLKLAVGLRNVPSRSSRGLFGAIAGFIEG
GWSGLVAGWYGFQHSNDQGVGMAADRDSTQKAIDKITSKVNNIVDKMNKQYEIIDHEFSEVETRLNMINN
KIDDQIQDIWAYNAELLVLLENQKTLDEHDANVNNLYNKVKRALGSNAVEDGKGCFELYHKCDDQCMETI
RNGTYNRRKYQEESKLERQRIEGVKLESEGTYKILTIYSTVASSLVIAMGFAAFLFWAMSNGSCRCNICI

>QBK20714.1 hemagglutinin [Influenza A virus]

METVSLITILLVATVSNADKICIGYQSTNSTETVDTLTENNVPVTHAKELLHTEHNGMLCATSLGQPLIL
DTCTIEGLIYGNPSCDLSLEGREWSYIVERPSAVHGLCYPGNVEDLEELRSLFSSARSYQRIQIFPDTIW
NVSYDGTSTACSGSFYRSMRWLTRKNGDYPIQDAQYTNNQGKNILFMWGINHPPTDETQRGLYTRTDTTT
SVATEEINRIFKPLIGPRPLVNGLMGRINYYWSVLKPGQTLRIKSDGNLIAPWYGHILSGESHGRILKTD
LKRGSCTVQCQTEKGGLNTTLPFQNVSKYAFGNCSKYIGIKSLKLAVGLRNVPSRSSRGLFGAIAGFIEG
GWSGLVAGWYGFQHSNDQGVGMAADRDSTQKAIDKITSKVNNIVDKMNKQYEIIDHEFSEVETRLNMINN
KIDDQIQDIWAYNAELLVLLENQKTLDEHDANVNNLYNKVKRALGSNAVEDGKGCFELYHKCDDQCMETI
RNGTYNRRKYQEESKLERQKIEGVKLESEGTYKILTIYSTVASSLVIAMGFAAFLFWAMSNGSCRCNICI

>QBK20713.1 hemagglutinin [Influenza A virus]

METVSLITILLVATVSNADKICIGYQSTNSTETVDTLTENNVPVTHAKELLHTEHNGMLCATSLGQPLIL
DTCTIEGLIYGNPSCDLSLEGREWSYIVERPSAVHGLCYPGNVEDLEELRSLFSSARSYQRIQIFPDTIW
NVSYDGTSTACSGSFYKSMRWLTRKNGEYPTQDAQYTNNQGKNILFMWGINHPPTDDTQRGLYTRTDTTT
SVATEEINRIFKPLIGPRPLVNGLMGRINYYWSVLKPGQTLRIKSDGNLIAPWYGHILSGESHGRILKTD
LKRGSCTVQCQTEKGGLNTTLPFQNVSKYAFGNCSKYIGIKSLKLAVGLRNVPSRSSRGLFGAIAGFIEG
GWSGLVAGWYGFQHSNDQGVGMAADRDSTQKAIDKITSKVNNIVDKMNKQYEIIDHEFSEVETRLNMINN
KIDDQIQDIWAYNAELLVLLENQKTLDEHDANVNNLYNKVKRALGSNAVEDGKGCFELYHKCDDQCMETI
RNGTYNRRKYQEESKLERQRIEGVKLESEGTYKILTIYSTVASSLVIAMGFAAFLFWAMSNGSCRCNICI

>QBK20712.1 hemagglutinin [Influenza A virus]

MGTVSLITILLIATVSNADKICIGYQSTNSTETVDTLTENNVPVTHAKELLHTEHNGMLCATSLGQPLIL
DTCTIEGLIYGNPSCDLSLEGREWSYIVERPSAVHGLCYPGNVEDLEELRSLFSSARSYQRIQIFPDTIW
NVSYDGTSTACSGSFYRSMRWLTRKNGEYPIQDAQYTNNQGKNILFMWGINHPPTDETQRGLYTRTDTTT

SVATEEINRIFKPLIGPRPLVNGLMGRINYYWSVLKPGQTLRIKSDGNLIAPWYGHILSGESHGRILKTD
LKRGSCTVQCQTEKGGLNTTLPFQNVSKYAFGNCSKYIGIKSLKLAVGLRNVPSRSSRGLFGAIAGFIEG
GWSGLVAGWYGFQHSNDQGVGMAADRDSTQKAIDKITSKVNNIVDKMNKQYEIIDHEFSEVETRLNMINN
KIDDQIQDIWAYNAELLVLLENQKTLDEHDANVNNLYNKVKRALGSNAVEDGKGCFELYHKCDDQCMETI
RNGTYNRRKYQEESKLERQRIEGVKLESEGTYKILTIYSTVASSLVIAMGFAAFLFWAMSNGSCRCNICI

>QBK20711.1 hemagglutinin [Influenza A virus]
METASLITILLVATVSNADKICIGYQSTNSTETVDTLTENNVPVTHAKELLHTEHNGMLCATSLGHPLIL
DTCTIEGLIYGNPSCDPLLGGREWSYIVERPSAVNGLCYPGNVENLEELRSLFSSSRSYQRIQIFPDTIW
NVSYSGTSKACSDSFYRSMRWLTQKNNAYPTQDAQYTNNQEKNILFMWGINHPPTDTAQTNLYTRTDTTT
SVATEEMNRIFKPLIGPRPLVNGLMGRINYYWSVLKPGQTLRIKSDGNLIAPWYGHILSGESHGRILKTD
LKRGSCTVQCQTEKGGLNTTLPFQNVSKYAFGNCSKYIGVKSLKIAVGLRNVPSKSSRGLFGAIAGFIEG
GWSGLVAGWYGFQHSNDQGVGMAADRDSTQKAIDKITSKVNNIVDKMNKQYEIIDHEFSEVETRLNMINN
KVDDQIQDIWAYNAELLVLLENQKTLDEHDANVNNLYNKVKRTLGSNAVEDGKGCFELYHKCDDHCMETI
RNGTYNRRKYQEESKLERQKIEGVKLESEETYKILTIYSTVASSLVIAMGFAAFLFWAMSNGSCRCNICI

>QBK20710.1 hemagglutinin [Influenza A virus]
METVSLITILLVATVSNADKICIGYQSTNSTETVDTLTENNVPVTHAKELLHTEHNGMLCATSLGQPLIL
DTCTIEGLIYGNPSCDLSLEGREWSYIVERPSAVHGLCYPGNVEDLEELRSLFSSARSYQRIQIFPDTIW
NVSYDGTSTACSGSFYRSMRWLTRKNGEYPIQDAQYTNNQGKNILFMWGINHPPTDDTQRGLYTRTDTTT
SVATEEINRIFKPLIGPRPLVNGLMGRINYYWSVLKPGQTLRIKSDGNLIAPWYGHILSGESHGRILKTD
LKRGSCTVQCQTEKGGLNTTLPFQNISKYAFGNCSKYIGIKSLKLAVGLRNVPSRSSRGLFGAIAGFIEG
GWPGLVAGWYGFQHSNDQGVGMAADRDSTQKAIDKITSKVNNIVDKMNKQYEIIDHEFSEVETRLNMINN
KIDDQIQDIWAYNAELLVLLENQKTLDEHDANVNNLYSKVKRALGSNAVEDGKGCFELYHKCDDQCMETI
RNGTYNRRKYQEESKLERQRIEGVKLESEGTYKILTIYSTVASSLVIAMGFAAFLFWAMSNGSCRCNICI

>QBK20709.1 hemagglutinin [Influenza A virus]
METVSLITILLVATVSNADKICIGYQSTNSTETVDTLTENNVPVTHAKELLHTEHNGMLCATSLGQPLIL
DTCTIEGLIYGNPSCDLSLEGREWSYIVERPSAVHGLCYPGNVEDLEELRSLFSSARSYQRIQIFPDTIW
NVSYDGTSTACSGSFYRSMRWLTRKNGEYPIQDAQYTNNQGKNILFMWGINHPPTDDTQRGLYTRTDTTT
SVATEEINRIFKPLIGPRPLVNGLMGRINYYWSVLKPGQTLRIKSDGNLIAPWYGHILSGESHGRILKTD
LKRGSCTVQCQTEKGGLNTTLPFQNVSKYAFGNCSKYIGIKSLKLAVGLRNVPSRSSRGLFGAIAGFIEG
GWSGLVAGWYGFQHSNDQGVGMAADRDSTQKAIDKITSKVNNIVDKMNKQYEIIDHEFSEVETRLNMINN
KIDDQIQDIWAYNAELLVLLENQKTLDEHDANVNNLYNKVKRALGSNAVEDGKGCFELYHKCDDQCMETI

RNGTYNRRKYQEESKLERQRIEGVKLESEGTYKILTIYSTVASSLVIAMGFAAFLFWAMSNGSCRCNICI

>QBK20708.1 hemagglutinin [Influenza A virus]
METVSLITILLVATVSNADKICIGYQSTNSTETVDTLTENNVPVTHAKELLHTEHNGMLCATSLGQPLIL
DTCTIEGLIYGNPSCDLSLEGREWSYIVERPSAVHGLCYPGNVEDLEELRSLFSSARSYQRIQIFPDTIW
NVSYDGTSTACSGSFYKSMRWLTRKNGEYPTQDAQYTNNQGKNILFMWGINHPPTDDTQRGLYTRTDTTT
SVATEEINRIFKPLIGPRPLVNGLMGRINYYWSVLKPGQTLRIKSDGNLIAPWYGHILSGESHGRILKTD
LKRGSCTVQCQTEKGGLNTTLPFQNVSKYAFGNCSKYIGIKSLKLAVGLRNVPSRSSRGLFGAIAGFIEG
GWSGLVAGWYGFQHSNDQGVGMAADRDSTQKAIDKITSKVNNIVDKMNKQYEIIDHEFSEVETRLNMINN
KIDDQIQDIWAYNAELLVLLENQKTLDEHDANVNNLYNKVKRALGSNAVEDGKGCFELYHKCDDQCMETI
RNGTYNRRKYQEESKLERQRIEGVKLESEGTYKILTIYSTVASSLVIAMGFAAFLFWAMSNGSCRCNICI

>QBK20707.1 hemagglutinin [Influenza A virus]
METVSLITILLVATVSNADKICIGYQSTNSTETVDTLTENNVPVTHAKELLHTEHNGMLCATSLGQPLIL
DTCTIEGLIYGNPSCDLSLEGREWSYIVERPSAVHGLCYPGNVEDLEELRSLFSSARSYQRIQIFPDTIW
NVSYDGTSTACSGSFYRSMRWLTRKNGEYPIQDAQYTNNQGKNILFMWGINHPPTDDTQRGLYTRTDTTT
SVATEEINRIFKPLIGPRPLVNGLMGRINYYWSVLKPGQTLRIKSDGNLIAPWYGHILSGESHGRILKTD
LKRGSCTVQCQTEKGGLNTTLPFQNVSKYAFGNCSKYIGIKSLKLAVGLRNVPSRSSRGLFGAIAGFIEG
GWSGLVAGWYGFQHSNDQGVGMAADRDSTQKAIDKITSKVNNIVDKMNKQYEIIDHEFSEVETRLNMINN
KIDDQIQDIWAYNAELLVLLENQKTLDEHDANVNNLYNKVKRALGSNAVEDGKGCFELYHKCDDQCMETI
RNGTYNRRKYQEESKLERQRIEGVKLESEGTYKILTIYSTVASSLVIAMGFAAFLFWAMSNGSCRCNICI

>QBK20706.1 hemagglutinin [Influenza A virus]
METVSLITILLVATVSNADKICIGYQSTNSTETVDTLTENNVPVTHAKELLHTEHNGMLCATSLGQPLIL
DTCTIEGLIYGNPSCDLSLEGREWSYIVERPSAVHGLCYPGNVEDLEELRSLFSSARSYQRIQIFPDTIW
NVSYDGTSTACSGSFYRSMRWLTRKNGEYPIQDAQYTNNQGKNILFMWGINHPPTDDTQRGLYTRTDTTT
SVATEEINRIFKPLIGPRPLVNGLMGRINYYWSVLKPGQTLRIKSDGNLIAPWYGHILSGESHGRILKTD
LKRGSCTVQCQTEKGGLNTTLPFQNVSKYAFGNCSKYIGIKSLKLAVGLRNVPSRSSRGLFGAIAGFIEG
GWSGLVAGWYGFQHSNDQGVGMAADRDSTQKAIDKITSKVNNIVDKMNKQYEIIDHEFSEVETRLNMINN
KIDDQIQDIWAYNAELLVLLENQKTLDEHDANVNNLYNKVKRALGSNAVEDGKGCFELYHKCDDQCMETI
RNGTYNRRKYQEESKLERQRIEGVKLESEGTYKILTIYSTVASSLVIAMGFAAFLFWAMSNGSCRCNICI

>QBK20705.1 hemagglutinin [Influenza A virus]
METVSLITILLVATVSNADKICIGYQSTNSTETVDTLTENNVPVTHAKELLHTEHNGMLCATSLGQPLIL

DTCTIEGLIYGNPSCDLSLEGREWSYIVERPSAVHGLCYPGNVEDLEELRSLFSSARSYQRIQIFPDTIW
NVSYDGTSTACSGSFYRSMRWLTRKNGEYPIQDAQYTNNQGKNILFMWGINHPPTDDTQRGLYTRTDTTT
SVATEEINRIFKPLIGPRPLVNGLMGRINYYWSVLKPGQTLRIKSDGNLIAPWYGHILSGESHGRILKTD
LKRGSCTVQCQTEKGGLNTTLPFQNVSKYAFGNCSKYIGIKSLKLAVGLRNVPSRSSRGLFGAIAGFIEG
GWSGLVAGWYGFQHSNDQGVGMAADRDSTQKAIDKITSKVNNIVDKMNKQYEIIDHEFSEVETRLNMINN
KIDDQIQDIWAYNAELLVLLENQKTLDEHDANVNNLYNKVKRALGSNAVEDGKGCFELYHKCDDQCMETI
RNGTYNRRKYQEESKLERQRIEGVKLESEGTYKILTIYSTVASSLVIAMGFAAFLFWAMSNGSCRCNICI

>QBK20704.1 hemagglutinin [Influenza A virus]
METVSLITILLVATVSNADKICIGYQSTNSTETVDTLTENNVPVTHAKELLHTEHNGMLCATSLGQPLIL
DTCTIEGLIYGNPSCDLSLEGREWSYIVERPSAVHGLCYPGNVEDLEELRSLFSSARSYQRIQIFPDTIW
NVSYDGTSTACSGSFYRSMRWLTRKNGEYPIQDAQYTNNQGKNILFMWGINHPPTDDTQRGLYTRTDTTT
SVATEEINRIFKPLIGPRPLVNGLMGRINYYWSVLKPGQTLRIKSDGNLIAPWYGHILSGESHGRILKTD
LKRGSCTVQCQTEKGGLNTTLPFQNVSKYAFGNCSKYIGIKSLKLAVGLRNVPSRSSRGLFGAIAGFIEG
GWSGLVAGWYGFQHSNDQGVGMAADRDSTQKAIDKITSKVNNIVDKMNKQYEIIDHEFSEVETRLNMINN
KIDDQIQDIWAYNAELLVLLENQKTLDEHDANVNNLYNKVKRALGSNAVEDGKGCFELYHKCDDQCMETI
RNGTYNRRKYQEESKLERQRIEGVKLESEGTYKILTIYSTVASSLVIAMGFAAFLFWAMSNGSCRCNICI

>QBK20703.1 hemagglutinin [Influenza A virus]
METVSLITILLVATVSNADKICIGYQSTNSTETVDTLTENNVPVTHAKELLHTEHNGMLCATSLGQPLIL
DTCTIEGLIYGNPSCDLSLEGREWSYIVERPSAVHGLCYPGNVEDLEELRSLFSSARSYQRIQIFPDTIW
NVSYDGTSTACSGSFYRSMRWLTRKNGEYPIQDAQYTNNQGKNILFMWGINHPPTDDTQRGLYTRTDTTT
SVATEEINRIFKPLIGPRPLVNGLMGRINYYWSVLKPGQTLRIKSDGNLIAPWYGHILSGESHGRILKTD
LKRGSCTVQCQTEKGGLNTTLPFQNVSKYAFGNCSKYIGIKSLKLAVGLRNVPSRSSRGLFGAIAGFIEG
GWSGLVAGWYGFQHSNDQGVGMAADRDSTQKAIDKITSKVNNIVDKMNKQYEIIDHEFSEVETRLNMINN
KIDDQIQDIWAYNAELLVLLENQKTLDEHDANVNNLYNKVKRALGSNAVEDGKGCFELYHKCDDQCMETI
RNGTYNRRKYQEESKLERQRIEGVKLESEGTYKILTIYSTVASSLVIAMGFAAFLFWAMSNGSCRCNICI

>QBK20702.1 hemagglutinin [Influenza A virus]
METVSLITILLVATVSNADKICIGYQSTNSTETVDTLTENNVPVTHAKELLHTEHNGMLCATSLGQPLIL
DTCTIEGLIYGNPSCDLSLEGREWSYIVERPSAVHGLCYPGNVEDLEELRSLFSSARSYQRIQIFPDTIW
NVSYDGTSTACSGSFYRSMRWLTRKNGEYPIQDAQYTNNQGKNILFMWGINHPPTDDTQRGLYTRTDTTT
SVATEEINRIFKPLIGPRPLVNGLMGRINYYWSVLKPGQTLRIKSDGNLIAPWYGHILSGESHGRILKTD
LKRGSCTVQCQTEKGGLNTTLPFQNVSKYAFGNCSKYIGIKSLKLAVGLRNVPTRSSRGLFGAIAGFIEG

GWSGLVAGWYGFQHSNDQGVGMAADRDSTQKAIDKITSKVNNIVDKMNKQYEIIDHEFSEVETRLNMINN
KIDDQIQDIWAYNAELLVLLENQKTLDEHDANVNNLYNKVKRALGYNAVEDGKGCFELYHKCDDQCMETI
RNGTYNRRKYQEESKLERQRIEGVKLESEGTYKILTIYSTVASSLVIAMGFAAFLFWAMSNGSCRCNICI

>QBK20701.1 hemagglutinin [Influenza A virus]
METVSLITILLVATVSNADKICIGYQSTNSTETVDTLTENNVPVTHAKELLHTEHNGMLCATSLGQPLIL
DTCTIEGLIYGNPSCDLSLEGREWSYIVERPSAVHGLCYPGNVEDLEELRSLFSSARSYQRIQIFPDTIW
NVSYDGTSTACSGSFYRSMRWLTRKNGEYPIQDAQYTNNQGKNILFMWGINHPPTDDTQRGLYTRTDTTT
SVATEEINRIFKPLIGPRPLVNGLMGRINYYWSVLKPGQTLRIKSDGNLIAPWYGHILSGESHGRILKTD
LKRGSCTVQCQTEKGGLNTTLPFQNVSKYAFGNCSKYIGIKSLKLAVGLRNVPTRSSRGLFGAIAGFIEG
GWSGLVAGWYGFQHSNDQGVGMAADRDSTQKAIDKITSKVNNIVDKMNKQYEIIDHEFSEVETRLNMINN
KIDDQIQDIWAYNAELLVLLENQKTLDEHDANVNNLYNKVKRALGYNAVEDGKGCFELYHKCDDQCMETI
RNGTYNRRKYQEESKLERQRIEGVKLESEGTYKILTIYSTVASSLVIAMGFAAFLFWAMSNGSCRCNICI

>QBK20700.1 hemagglutinin [Influenza A virus]
METVSLITILLVATVSNADKICIGYQSTNSTETVDTLTENNVPVTHAKELLHTEHNGMLCATSLGQPLIL
DTCTIEGLIYGNPSCDLSLEGREWSYIVERPSAVHGLCYPGNVEDLEELRSLFSSARSYQRIQIFPDTIW
NVSYDGTSTACSGSFYRSMRWLTRKNGEYPIQDAQYTNNQGKNILFMWGINHPPTDDTQRGLYTRTDTTT
SVATEEINRIFKPLIGPRPLVNGLMGRINYYWSVLKPGQTLRIKSDGNLIAPWYGHILSGESHGRILKTD
LKRGSCTVQCQTEKGGLNTTLPFQNVSKYAFGNCSKYIGIKSLKLAVGLRNVPTRSSRGLFGAIAGFIEG
GWSGLVAGWYGFQHSNDQGVGMAADRDSTQKAIDKITSKVNNIVDKMNKQYEIIDHEFSEVETRLNMINN
KIDDQIQDIWAYNAELLVLLENQKTLDEHDANVNNLYNKVKRALGYNAVEDGKGCFELYHKCDDQCMETI
RNGTYNRRKYQEESKLERQRIEGVKLESEGTYKILTIYSTVASSLVIAMGFAAFLFWAMSNGSCRCNICI

>QBK20699.1 hemagglutinin [Influenza A virus]
METVSLITILLVATVSNADKICIGYQSTNSTETVDTLTENNVPVTHAKELLHTEHNGMLCATSLGQPLIL
DTCTIEGLIYGNPSCDLSLEGREWSYIVERPSAVHGLCYPGNVEDLEELRSLFSSARSYQRIQIFPDTIW
NVSYDGTSTACSGSFYRSMRWLTRKNGEYPIQDAQYTNNQGKNILFMWGINHPPTDDTQRGLYTRTDTTT
SVATEEINRIFKPLIGPRPLVNGLMGRINYYWSVLKPGQTLRIKSDGNLIAPWYGHILSGESHGRILKTD
LKRGSCTVQCQTEKGGLNTTLPFQNVSKYAFGNCSKYIGIKSLKLAVGLRNVPTRSSRGLFGAIAGFIEG
GWSGLVAGWYGFQHSNDQGVGMAADRDSTQKAIDKITSKVNNIVDKMNKQYEIIDHEFSEVETRLNMINN
KIDDQIQDIWAYNAELLVLLENQKTLDEHDANVNNLYNKVKRALGYNAVEDGKGCFELYHKCDDQCMETI
RNGTYNRRKYQEESKLERQRIEGVKLESEGTYKILTIYSTVASSLVIAMGFAAFLFWAMSNGSCRCNICI

>QBK20698.1 hemagglutinin [Influenza A virus]

METVSLITILLVATVSNADKICIGYQSTNSTETVDTLTENNVPVTHAKELLHTEHNGMLCATSLGQPLIL
DTCTIEGLIYGNPSCDLSLEGREWSYIVERPSAVHGLCYPGNVEDLEELRSLFSSARSYQRIQIFPDTIW
NVSYDGTSTACSGSFYRSMRWLTRKNGEYPIQDAQYTNNQGKNILFMWGINHPPTDDTQRGLYTRTDTTT
SVATEEINRIFKPLIGPRPLVNGLMGRINYYWSVLKPGQTLRIKSDGNLIAPWYGHILSGESHGRILKTD
LKRGSCTVQCQTEKGGLNTTLPFQNVSKYAFGNCSKYIGIKSLKLAVGLRNVPTRSSRGLFGAIAGFIEG
GWSGLVAGWYGFQHSNDQGVGMAADRDSTQKAIDKITSKVNNIVDKMNKQYEIIDHEFSEVETRLNMINN
KIDDQIQDIWAYNAELLVLLENQKTLDEHDANVNNLYNKVKRALGYNAVEDGKGCFELYHKCDDQCMETI
RNGTYNRRKYQEESKLERQRIEGVKLESEGTYKILTIYSTVASSLVIAMGFAAFLFWAMSNGSCRCNICI

>QBK20697.1 hemagglutinin [Influenza A virus]

METVSLITILLVATVSNADKICIGYQSTNSTETVDTLTENNVPVTHAKELLHTEHNGMLCATSLGQPLIL
DTCTIEGLIYGDPSCDLSLEGREWSYIVERPSAVHGLCYPGNVEDLEELRSLFSSARSYQRIQIFPDTIW
NVSYDGTSTACSGSFYRSMRWLTRKNGEYPIQDAQYTNNQGKNILFMWGINHPPTDDTQRGLYTRTDTTT
SVATEEINRIFKPLIGPRPLVNGLMGRINYYWSVLKPGQTLRIKSDGNLIAPWYGHILSGESHGRILKTD
LKRGSCTVQCQTEKGGLNTTLPFQNVSKYAFGNCSKYIGIKSLKLAVGLRNVPTRSSRGLFGAIAGFIEG
GWSGLVAGWYGFQHSNDQGVGMAADRDSTQKAIDKITSKVNNIVDKMNKQYEIIDHEFSEVETRLNMINN
KIDDQIQDIWAYNAELLVLLENQKTLDEHDANVNNLYNKVKRALGSNAVEDGKGCFELYHKCDDQCMETI
RNGTYNRRKYQEESKLERQRIEGVKLESEGTYKILTIYSTVASSLVIAMGFAAFLFWAMSNGSCRCNICI

>QBK20696.1 hemagglutinin [Influenza A virus]

METASLITILLVATVSNADKICIGYQSTNSTETVDTLTENNVPVTHAKELLHTEHNGMLCATSLGHPLIL
DTCTIEGLIYGNPSCDPLLGGREWSYIVERPSAVNGLCYPGNVENLEELRSLFSSSRSYQRIQIFPDTIW
NVSYSGTSKACSDSFYRSMRWLTQKNNAYPTQDAQYTNNQEKNILFMWGINHPPTDTAQTNLYTRTDTTT
SVATEEMNRIFKPLIGPRPLVNGLMGRINYYWSVLKPGQTLRIKSDGNLIAPWYGHILSGESHGRILKTD
LKRGSCTVQCQTEKGGLNTTLPFQNVSKYAFGNCSKYIGVKSLKIAVGLRNVPSKSSRGLFGAIAGFIEG
GWSGLVAGWYGFQHSNDQGVGMAADRDSTQKAIDKITSKVNNIVDKMNKQYEIIDHEFSEVETRLNMINN
KVDDQIQDIWAYNAELLVLLENQKTLDEHDANVNNLYNKVKRTLGSNAVEDGKGCFELYHKCDDHCMETI
RNGTYNRRKYQEESKLERQKIEGVKLESEETYKILTIYSTVASSLVIAMGFAAFLFWAMSNGSCRCNICI

>QBK20695.1 hemagglutinin [Influenza A virus]

METVSLITILLVATVSNADKICIGYQSTNSTETVDTLTENNVPVTHAKELLHTEHNGMLCATSLGQPLIL
DTCTIEGLIYGDPSCDLSLEGREWSYIVERPSAVHGLCYPGNVEDLEELRSLFSSARSYQRIQIFPDTIW
NVSYDGTSTACSGSFYRSMRWLTRKNGEYPIQDAQYTNNQGKNILFMWGINHPPTDDTQRGLYTRTDTTT

SVATEEINRIFKPLIGPRPLVNGLMGRINYYWSVLKPGQTLRIKSDGNLIAPWYGHILSGESHGRILKTD
LKRGSCTVQCQTEKGGLNTTLPFQNVSKYAFGNCSKYIGIKSLKLAVGLRNVPTRSSRGLFGAIAGFIEG
GWSGLVAGWYGFQHSNDQGVGMAADRDSTQKAIDKITSKVNNIVDKMNKQYEIIDHEFSEVETRLNMINN
KIDDQIQDIWAYNAELLVLLENQKTLDEHDANVNNLYNKVKRALGSNAVEDGKGCFELYHKCDDQCMETI
RNGTYNRRKYQEESKLERQRIEGVKLESEGTYKILTIYSTVASSLVIAMGFAAFLFWAMSNGSCRCNICI

>QBK20694.1 hemagglutinin [Influenza A virus]
METASLITILLVATVSNADKICIGYQSTNSTETVDTLTENNVPVTHAKELLHTEHNGMLCATSLGHPLIL
DTCTIEGLIYGNPSCDPLLGGREWSYIVERPSAVNGLCYPGNVENLEELRSLFSSSRSYQRIQIFPDTIW
NVSYSGTSKACSDSFYRSMRWLTQKNNAYPTQDAQYTNNQEKNILFMWGINHPPTDTAQTNLYTRTDTTT
SVATEEMNRIFKPLIGPRPLVNGLMGRINYYWSVLKPGQTLRIKSDGNLIAPWYGHILSGESHGRILKTD
LKRGSCTVQCQTEKGGLNTTLPFQNVSKYAFGNCSKYIGVKSLKIAVGLRNVPSKSSRGLFGAIAGFIEG
GWSGLVAGWYGFQHSNDQGVGMAADRDSTQKAIDKITSKVNNIVDKMNKQYEIIDHEFSEVETRLNMINN
KVDDQIQDIWAYNAELLVLLENQKTLDEHDANVNNLYNKVKRTLGSNAVEDGKGCFELYHKCDDHCMETI
RNGTYNRRKYQEESKLERQKIEGVKLESEETYKILTIYSTVASSLVIAMGFAAFLFWAMSNGSCRCNICI

>QBK20693.1 hemagglutinin [Influenza A virus]
METVSLITILLVATVSNADKICIGYQSTNSTETVDTLTENNVPVTHAKELLHTEHNGMLCATSLGQPLIL
DTCTIEGLIYGDPSCDLSLEGREWSYIVERPSAVHGLCYPGNVEDLEELRSLFSSARSYQRIQIFPDTIW
NVSYDGTSTACSGSFYRSMRWLTRKNGEYPIQDAQYTNNQGKNILFMWGINHPPTDDTQRGLYTRTDTTT
SVATEEINRIFKPLIGPRPLVNGLMGRINYYWSVLKPGQTLRIKSDGNLIAPWYGHILSGESHGRILKTD
LKRGSCTVQCQTEKGGLNTTLPFQNVSKYAFGNCSKYIGIKSLKLAVGLRNVPTRSSRGLFGAIAGFIEG
GWSGLVAGWYGFQHSNDQGVGMAADRDSTQKAIDKITSKVNNIVDKMNKQYEIIDHEFSEVETRLNMINN
KIDDQIQDIWAYNAELLVLLENQKTLDEHDANVNNLYNKVKRALGSNAVEDGKGCFELYHKCDDQCMETI
RNGTYNRRKYQEESKLERQRIEGVKLESEGTYKILTIYSTVASSLVIAMGFAAFLFWAMSNGSCRCNICI

>QBK20692.1 hemagglutinin [Influenza A virus]
METVSLITILLVATVSNADKICIGYQSTNSTETVDTLTENNVPVTHAKELLHTEHNGMLCATSLGQPLIL
DTCTIEGLIYGDPSCDLSLEGREWSYIVERPSAVHGLCYPGNVEDLEELRSLFSSARSYQRIQIFPDTIW
NVSYDGTSTACSGSFYRSMRWLTRKNGEYPIQDAQYTNNQGKNILFMWGINHPPTDDTQRGLYTRTDTTT
SVATEEINRIFKPLIGPRPLVNGLMGRINYYWSVLKPGQTLRIKSDGNLIAPWYGHILSGESHGRILKTD
LKRGSCTVQCQTEKGGLNTTLPFQNVSKYAFGNCSKYIGIKSLKLAVGLRNVPTRSSRGLFGAIAGFIEG
GWSGLVAGWYGFQHSNDQGVGMAADRDSTQKAIDKITSKVNNIVDKMNKQYEIIDHEFSEVETRLNMINN
KIDDQIQDIWAYNAELLVLLENQKTLDEHDANVNNLYNKVKRALGSNAVEDGKGCFELYHKCDDQCMETI

RNGTYNRRKYQEESKLERQRIEGVKLESEGTYKILTIYSTVASSLVIAMGFAAFLFWAMSNGSCRCNICI

>QBK20691.1 hemagglutinin [Influenza A virus]

METVSLITILLVATVSNADKICIGYQSTNSTETVDTLTENNVPVTHAKELLHTEHNGMLCATSLGQPLIL
DTCTIEGLIYGDPSCDLSLEGREWSYIVERPSAVHGLCYPGNVEDLEELRSLFSSARSYQRIQIFPDTIW
NVSYDGTSTACSGSFYRSMRWLTRKNGEYPIQDAQYTNNQGKNILFMWGINHPPTDDTQRGLYTRTDTTT
SVATEEINRIFKPLIGPRPLVNGLMGRINYYWSVLKPGQTLRIKSDGNLIAPWYGHILSGESHGRILKTD
LKRGSCTVQCQTEKGGLNTTLPFQNVSKYAFGNCSKYIGIKSLKLAVGLRNVPTRSSRGLFGAIAGFIEG
GWSGLVAGWYGFQHSNDQGVGMAADRDSTQKAIDKITSKVNNIVDKMNKQYEIIDHEFSEVETRLNMINN
KIDDQIQDIWAYNAELLVLLENQKTLDEHDANVNNLYNKVKRALGSNAVEDGKGCFELYHKCDDQCMETI
RNGTYNRRKYQEESKLERQRIEGVKLESEGTYKILTIYSTVASSLVIAMGFAAFLFWAMSNGSCRCNICI

>QBK20690.1 hemagglutinin [Influenza A virus]

METASLITILLVATVSNADKICIGYQSTNSTETVDTLTENNVPVTHAKELLHTEHNGMLCATSLGHPLIL
DTCTIEGLIYGNPSCDPLLGGREWSYIVERPSAVNGLCYPGNVENLEELRSLFSSSRSYQRIQIFPDTIW
NVSYSGTSKACSDSFYRSMRWLTQKNNAYPTQDAQYTNNQEKNILFMWGINHPPTDTAQTNLYTRTDTTT
SVATEEMNRIFKPLIGPRPLVNGLMGRINYYWSVLKPGQTLRIKSDGNLIAPWYGHILSGESHGRILKTD
LKRGSCTVQCQTEKGGLNTTLPFQNVSKYAFGNCSKYIGVKSLKIAVGLRNVPSKSSRGLFGAIAGFIEG
GWSGLVAGWYGFQHSNDQGVGMAADRDSTQKAIDKITSKVNNIVDKMNKQYEIIDHEFSEVETRLNMINN
KVDDQIQDIWAYNAELLVLLENQKTLDEHDANVNNLYNKVKRTLGSNAVEDGKGCFELYHKCDDHCMETI
RNGTYNRRKYQEESKLERQKIEGVKLESEETYKILTIYSTVASSLVIAMGFAAFLFWAMSNGSCRCNICI

>QBK20689.1 hemagglutinin [Influenza A virus]

METASLITILLVATVSNADKICIGYQSTNSTETVDTLTENNVPVTHAKELLHTEHNGMLCATSLGHPLIL
DTCTIEGLIYGNPSCDPLLGGREWSYIVERPSAVNGLCYPGNVENLEELRSLFSSSRSYQRIQIFPDTIW
NVSYSGTSKACSDSFYRSMRWLTQKNNAYPTQDAQYTNNQEKNILFMWGINHPPTDTAQTNLYTRTDTTT
SVATEEMNRIFKPLIGPRPLVNGLMGRINYYWSVLKPGQTLRIKSDGNLIAPWYGHILSGESHGRILKTD
LKRGSCTVQCQTEKGGLNTTLPFQNVSKYAFGNCSKYIGVKSLKIAVGLRNVPSKSSRGLFGAIAGFIEG
GWSGLVAGWYGFQHSNDQGVGMAADRDSTQKAIDKITSKVNNIVDKMNKQYEIIDHEFSEVETRLNMINN
KVDDQIQDIWAYNAELLVLLENQKTLDEHDANVNNLYNKVKRTLGSNAVEDGKGCFELYHKCDDHCMETI
RNGTYNRRKYQEESKLERQKIEGVKLESEETYKILTIYSTVASSLVIAMGFAAFLFWAMSNGSCRCNICI

>QBK20688.1 hemagglutinin [Influenza A virus]

METVSLITILLVATVSNADKICIGYQSTNSTDTVDTLTENNVPVTHAKELLHTEHNGMLCATSLGQPLIL

DTCTIEGLIYGNPSCDLSLEGREWSYIVERPSAVNGLCYPGNVENLEELRSLFSSARSYQRVQIFPDTIW
NVSYDGTSTACSGSFYRSMRWLTRKNGEYPIQDAQYTNSQGKNILFMWGINHPPTDTTQEALYTRTDTTT
SVATEEINRIFKPLIGPRPLVNGLMGRIDYYWSVLKPGQTLRIKSDGNLIAPWYGHVLSGESHGRILKTD
LKRGSCTVQCQTEKGGLNTTLPFQNVSKYAFGNCSKYIGIKSLKLAVGLRNVPSRSSRGLFGAIAGFIEG
GWPGLVAGWYGFQHSNDQGVGMAADRDSTQKAIDKITSKVNNIVDKMNKQYEIIDHEFSEVETRLNMINN
KIDDQIQDIWAYNAELLVLLENQKTLDEHDANVNNLYNKVKRALGSNAVEDGKGCFELYHKCDDQCMDTI
RNGTYNRRKYQEESKLERQKIEGVKLESEGTYKILTIYSTVASSLVIAMGFAAFLFWAMSNGSCRCNICI

>QBK20687.1 hemagglutinin [Influenza A virus]
METVSLITILLVATVSNADKICIGYQSTNSTDTVDTLTENNVPVTHAKELLHTEHNGMLCATSLGQPLIL
DTCTIEGLIYGNPSCDLSLEGREWSYIVERPSAVNGLCYPGNVENLEELRSLFSSARSYQRVPIFPDTIW
NVSYDGTSTACSGSFYRSMRWLTRKNGEYPIQDAQYTNSQGKNILFMWGINHPPTDTTQEALYTRTDTTT
SVATEEINRIFKPLIGPRPLVNGLMGRIDYYWSVLKPGQTLRIKSDGNLIAPWYGHVLSGESHGRILKTD
LKRGSCTVQCQTEKGGLNTTLPFQNVSKYAFGNCSKYIGIKSLKLAVGLRNVPSRSSRGLFGAIAGFIEG
GWPGLVAGWYGFQHSNDQGVGMAADRDSTQKAIDKITSKVNNIVDKMNKQYEIIDHEFSEVETRLNMINN
KIDDQIQDIWAYNAELLVLLENQKTLDEHDANVNNLYNKVKRALGSNAVEDGKGCFELYHKCDDQCMDTI
RNGTYNRRKYQEESKLERQKIEGVKLESEGTYKILTIYSTVASSLVIAMGFAAFLFWAMSNGSCRCNICI

>QBK20686.1 hemagglutinin [Influenza A virus]
METVSLITILLVATVSNADKICIGYQSTNSTDTVDTLTENNVPVTHAKELLHTEHNGMLCATSLGQPLIL
DTCTIEGLIYGNPSCDLSLEGREWSYIVERPSAVNGLCYPGNVENLEELRSLFSSARSYQRVPIFPDTIW
NVSYDGTSTACSGSFYRSMRWLTRKNGEYPIQDAQYTNSQGKNILFMWGINHPPTDTTQEALYTRTDTTT
SVATEEINRIFKPLIGPRPLVNGLMGRIDYYWSVLKPGQTLRIKSDGNLIAPWYGHVLSGESHGRILKTD
LKRGSCTVQCQTEKGGLNTTLPFQNVSKYAFGNCSKYIGIKSLKLAVGLRNVPSRSSRGLFGAIAGFIEG
GWPGLVAGWYGFQHSNDQGVGMAADRDSTQKAIDKITSKVNNIVDKMNKQYEIIDHEFSEVETRLNMINN
KIDDQIQDIWAYNAELLVLLENQKTLDEHDANVNNLYNKVKRALGSNAVEDGKGCFELYHKCDDQCMDTI
RNGTYNRRKYQEESKLERQKIEGVKLESEGTYKILTIYSTVASSLVIAMGFAAFLFWAMSNGSCRCNICI

>QBK20685.1 hemagglutinin [Influenza A virus]
METISLITILLVATVSNADKICIGYQSTNSTETVDTLTENNVPVTHAKELIHTEHNGMLCATSLGQPLIL
DTCTIEGLIYGNPSCDLSLEGREWSYIVERPSAVNGLCYPGNVENLEELRSLFSSARSYQRIQIFPDTIW
NVSYDGTSTACSGSFYRNMRWLTRKDGNYPTQDAQYTNNQGKNILFMWGINHPPTDTTQSGLYTRTDTTT
SVATEEINRIFKPLIGPRPLVNGLMGRIDYYWSVLKPGQTLRIKSDGNLIAPWFGHILSGESHGRILKTD
LKRGSCTVQCQTEKGGLNTTLPFQNVSKYAFGNCSKYIGIKSLKLAVGLRNVPSRSSRGLFGAIAGFIEG

GWSGLVAGWYGFQHSNDQGVGMAADRDSTQKAIDKITSKVNNIVDKMNKQYEIIDHEFSEVETRLNMINN
KIDDQIQDIWAYNAELLVLLENQKTLDEHDANVNNLYNKVKRALGSNAVEDGKGCFELYHKCDDQCMETI
RNGTYNRRKYQEESKLERQKIEGVKLESEGTYKILTIYSTVASSLVIAMGFAAFLFWAMSNGSCRCNICI

>QBK20684.1 hemagglutinin [Influenza A virus]
METVSLITILLVATVSNADKICIGYQSTNSTDTVDTLTENNVPVTHAKELLHTEHNGMLCATSLGQPLIL
DTCTIEGLIYGNPSCDLSLEGREWSYIVERPSAVNGLCYPGNVENLEELRSLFSSARSYQRVQIFPDTIW
NVSYDGTSTACSGSFYRSMRWLTRKNGEYPIQDAQYTNSQGKNILFMWGINHPPTDTTQEALYTRTDTTT
SVATEEINRIFKPLIGPRPLVNGLMGRIDYYWSVLKPGQTLRIKSDGNLIAPWYGHILSGESHGRILKTD
LKRGSCIVQCQTEKGGLNTTLPFQNVSKYAFGNCSKYIGIKSLKLAVGLRNVPSRSSRGLFGAIAGFIEG
GWPGLVAGWYGFQHSNDQGVGMAADRDSTQKAIDKITSKVNNIVDKMNKQYEIIDHEFSEVETRLNMINN
KIDDQIQDIWAYNAELLVLLENQKTLDEHDANVNNLYNKVKRALGSNAVEDGKGCFELYHKCDDQCMDTI
RNGTYNRRKYQEESKLERQKIEGVKLESEGTYKILTIYSTVASSLVIAMGFAAFLFWAMSNGSCRCNICI

>QBK20683.1 hemagglutinin [Influenza A virus]
METISLITILLVATVSNADKICIGYQSTNSTETVDTLTENNVPVTHAKELIHTEHNGMLCATSLGQPLIL
DTCTIEGLIYGNPSCDLSLEGREWSYIVERPSAVNGLCYPGNVENLEELRSLFSSARSYQRIQIFPDTIW
NVSYDGTSTACSGSFYRNMRWLTRKDGNYPTQDAQYTNNQGKNILFMWGINHPPTDTTQSGLYTRTDTTT
SVATEEINRIFKPLIGPRPLVNGLMGRIDYYWSVLKPGQTLRIKSDGNLIAPWFGHILSGESHGRILKTD
LKRGSCTVQCQTEKGGLNTTLPFQNVSKYAFGNCSKYIGIKSLKLAVGLRNVPSRSSRGLFGAIAGFIEG
GWSGLVAGWYGFQHSNDQGVGMAADRDSTQKAIDKITSKVNNIVDKMNKQYEIIDHEFSEVETRLNMINN
KIDDQIQDIWAYNAELLVLLENQKTLDEHDANVNNLYNKVKRALGSNAVEDGKGCFELYHKCDDQCMETI
RNGTYNRRKYQEESKLERQKIEGVKLESEGTYKILTIYSTVASSLVIAMGFAAFLFWAMSNGSCRCNICI

>QBK20682.1 hemagglutinin [Influenza A virus]
MKTISLITILLVATVSNADKICIGYQSTNSTETVDTLTENNVPVTHAKELIHTEHNGMLCATSLGQPLIL
DTCTIEGLIYGNPSCDLSLEGREWSYIVERPSAVNGLCYPGNVENLEELRSLFSSARSYQRIQIFPDTIW
NVSYDGTSTACSGSFYRNMRWLTRKDGNYPTQDAQYTNNQGKNILFMWGINHPPTDTTQSGLYTRTDTTT
SVATEEINRIFKPLIGPRPLVNGLMGRIDYYWSVLKPGQTLRIKSDGNLIAPWFGHILSGESHGRILKTD
LKRGSCTVQCQTEKGGLNTTLPFQNVSKYAFGNCSKYIGIKSLKLAVGLRNVPSRSSRGLFGAIAGFIEG
GWSGLVAGWYGFQHSNDQGVGIAADRDSTQKAIDKITSKVNNIVDKMNKQYEIIDHEFSEVETRLNMINN
KIDDQIQDIWAYNAELLVLLENQKTLDEHDANVNNLYNKVKRALGSNAVEDGKGCFELYHKCDDQCMETI
RNGTYNRRKYQEESKLERQKIEGVKLESEGTYKILTIYSTVASSLVIAMGFAAFLFWAMSNGSCRCNICI

>QBK20681.1 hemagglutinin [Influenza A virus]

MKTISLITILLVATVSNADKICIGYQSTNSTETVDTLTENNVPVTHAKELIHTEHNGMLCATSLGQPLIL

DTCTIEGLIYGNPSCDLSLEGREWSYIVERPSAVNGLCYPGNVENLEELRSLFSSARSYQRIQIFPDTIW

NVSYDGTSTACSGSFYRNMRWLTRKDGNYPTQDAQYTNNQGKNILFMWGINHPPTDTTQSGLYTRTDTTT

SVATEEINRIFKPLIGPRPLVNGLMGRIDYYWSVLKPGQTLRIKSDGNLIAPWFGHILSGESHGRILKTD

LKRGSCTVQCQTEKGGLNTTLPFQNVSKYAFGNCSKYIGIKSLKLAVGLRNVPSRSSRGLFGAIAGFIEG

GWSGLVAGWYGFQHSNDQGVGIAADRDSTQKAIDKITSKVNNIVDKMNKQYEIIDHEFSEVETRLNMINN

KIDDQIQDIWAYNAELLVLLENQKTLDEHDANVNNLYNKVKRALGSNAVEDGKGCFELYHKCDDQCMETI

RNGTYNRRKYQEESKLERQKIEGVKLESEGTYKILTIYSTVASSLVIAMGFAAFLFWAMSNGSCRCNICI

>QBK20680.1 hemagglutinin [Influenza A virus]

MKTISLITILLVATVSNADKICIGYQSTNSTETVDTLTENNVPVTHAKELIHTEHNGMLCATSLGQPLIL

DTCTIEGLIYGNPSCDLSLEGREWSYIVERPSAVNGLCYPGNVENLEELRSLFSSARSYQRIQIFPDTIW

NVSYDGTSTACSGSFYRNMRWLTRKDGNYPTQDAQYTNNQGKNILFMWGINHPPTDTTQSGLYTRTDTTT

SVATEEINRIFKPLIGPRPLVNGLMGRIDYYWSVLKPGQTLRIKSDGNLIAPWFGHILSGESHGRILKTD

LKRGSCTVQCQTEKGGLNTTLPFQNVSKYAFGNCSKYIGIKSLKLAVGLRNVPSRSSRGLFGAIAGFIEG

GWSGLVAGWYGFQHSNDQGVGIAADRDSTQKAIDKITSKVNNIVDKMNKQYEIIDHEFSEVETRLNMINN

KIDDQIQDIWAYNAELLVLLENQKTLDEHDANVNNLYNKVKRALGSNAVEDGKGCFELYHKCDDQCMETI

RNGTYNRRKYQEESKLERQKIEGVKLESEGTYKILTIYSTVASSLVIAMGFAAFLFWAMSNGSCRCNICI

>QBK20679.1 hemagglutinin [Influenza A virus]

MKTISLITILLVATVSNADKICIGYQSTNSTETVDTLTENNVPVTHAKELIHTEHNGMLCATSLGQPLIL

DTCTIEGLIYGNPSCDLSLEGREWSYIVERPSAVNGLCYPGNVENLEELRSLFSSARSYQRIQIFPDTIW

NVSYDGTSTACSGSFYRNMRWLTRKDGNYPTQDAQYTNNQGKNILFMWGINHPPTDTTQSGLYTRTDTTT

SVATEEINRIFKPLIGPRPLVNGLMGRIDYYWSVLKPGQTLRIKSDGNLIAPWFGHILSGESHGRILKTD

LKRGSCTVQCQTEKGGLNTTLPFQNVSKYAFGNCSKYIGIKSLKLAVGLRNVPSRSSRGLFGAIAGFIEG

GWSGLVAGWYGFQHSNDQGVGIAADRDSTQKAIDKITSKVNNIVDKMNKQYEIIDHEFSEVETRLNMINN

KIDDQIQDIWAYNAELLVLLENQKTLDEHDANVNNLYNKVKRALGSNAVEDGKGCFELYHKCDDQCMETI

RNGTYNRRKYQEESKLERQKIEGVKLESEGTYKILTIYSTVASSLVIAMGFAAFLFWAMSNGSCRCNICI

>QBK20678.1 hemagglutinin [Influenza A virus]

MKTISLITILLVATVSNADKICIGYQSTNSTETVDTLTENNVPVTHAKELIHTEHNGMLCATSLGQPLIL

DTCTIEGLIYGNPSCDLSLEGREWSYIVERPSAVNGLCYPGNVENLEELRSLFSSARSYQRIQIFPDTIW

NVSYDGTSTACSGSFYRNMRWLTRKDGNYPTQDAQYTNNQGKNILFMWGINHPPTDTTQSGLYTRTDTTT

SVATEEINRIFKPLIGPRPLVNGLMGRIDYYWSVLKPGQTLRIKSDGNLIAPWFGHILSGESHGRILKTD

LKRGSCTVQCQTEKGGLNTTLPFQNVSKYAFGNCSKYIGIKSLKLAVGLRNVPSRSSRGLFGAIAGFIEG

GWSGLVAGWYGFQHSNDQGVGIAADRDSTQKAIDKITSKVNNIVDKMNKQYEIIDHEFSEVETRLNMINN

KIDDQIQDIWAYNAELLVLLENQKTLDEHDANVNNLYNKVKRALGSNAVEDGKGCFELYHKCDDQCMETI

RNGTYNRRKYQEESKLERQKIEGVKLESEGTYKILTIYSTVASSLVIAMGFAAFLFWAMSNGSCRCNICI

>QBK20677.1 hemagglutinin [Influenza A virus]

METVSLITILLVATVSNADKICIGYQSTNSTETVDTLTENNVPVTHAKELLHTEHNGMLCATSLGQPLIL

DTCTIEGLIYGNPSCDLSLEGREWSYIVERPSAIHGLCYPGNVEDLEELRSLFSSARSYQRIQIFPDTIW

NVSYDGTSTACSGSFYRSMRWLTRKNGDYPIQDAQYTNNQGKNILFMWGINHPPTDETQRGLYTRTDTTT

SVATEEINRIFKPLIGPRPLVNGLMGRINYYWSVLKPGQTLRIKSDGNLIAPWYGHILSGESHGRILKTD

LKRGSCTVQCQTEKGGLNTTLPFQNVSKYAFGNCSKYIGIKSLKLAVGLRNVPSRSSRGLFGAIAGFIEG

GWSGLVAGWYGFQHSNDQGVGMAADRDSTQKAIDKITSKVNNIVDKMNKQYEIIDHEFSEVETRLNMINN

KIDDQIQDIWAYNAELLVLLENQKTLDEHDANVNNLYNKVKRALGSNAVEDGKGCFELYHKCDDQCMETI

RNGTYNRRKYQEESKLERQKIEGVKLESEGTYKILTIYSTVASSLVIAMGFAAFLFWAMSNGSCRCNICI

>QBK20676.1 hemagglutinin [Influenza A virus]

METASLITILLVATVSNADKICIGYQSTNSTETVDTLTENNVPVTHAKELLHTEHNGMLCATSLGQPLIL

DTCTIEGLIYGNPSCDLSLEGREWSYIVERPSAVNGLCYPGKVENLEELRSLFSSARSYQRIQIFPDTIW

NVSYDGTSTACSGSFYRSMRWLTRKNGEYPIQDAQYTNNQGKNILFMWGINHPPTDTTQRDLYTRTDTTT

SVATEEINRIFKPLIGPRPLVNGLMGRIDYYWSVLKPGQTLRIKSDGNLIAPWFGHIFSGESHGRILKTD

LKRGSCTVQCQTEKGGLNTTLPFQNVSKYAFGNCSKYIGIKSLKLAVGLRNVPSRSSRGLFGAIAGFIEG

GWSGLVAGWYGFQHSNDQGVGMAADRESTQKAIDKITSKVNNIVDKMNKQYEIIDHEFSEVETRLNMINN

KIDDQIQDIWAYNAELLVLLENQKTLDEHDANVNNLYNKVKRALGSNAVEDGKGCFELYHKCDDQCMETI

RNGTYNRRKYQEESKLERQKIEGVKLESEGTYKILTIYSTVASSLVIAMGFAAFLFWAMSNGSCRCNICI

>QBK20675.1 hemagglutinin [Influenza A virus]

METVSLITILLVATVSNADKICIGYQSTNSTETVDTLTENNVPVTHAKELLHTEHNGMLCATSLGQPLIL

DTCTIEGLIYGNPSCDLSLEGREWSYIVERPSAIHGLCYPGNVEDLEELRSLFSSARSYQRIQIFPDTIW

NVSYDGTSTACSGSFYRSMRWLTRKNGDYPIQDAQYTNNQGKNILFMWGINHPPTDETQRGLYTRTDTTT

SVATEEINRIFKPLIGPRPLVNGLMGRINYYWSVLKPGQTLRIKSDGNLIAPWYGHILSGESHGRILKTD

LKRGSCTVQCQTEKGGLNTTLPFQNVSKYAFGNCSKYIGIKSLKLAVGLRNVPSRSSRGLFGAIAGFIEG

GWSGLVAGWYGFQHSNDQGVGMAADRDSTQKAIDKITSKVNNIVDKMNKQYEIIDHEFSEVETRLNMINN

KIDDQIQDIWAYNAELLVLLENQKTLDEHDANVNNLYNKVKRALGSNAVEDGKGCFELYHKCDDQCMETI

RNGTYNRRKYQEESKLERQKIEGVKLESEGTYKILTIYSTVASSLVIAMGFAAFLFWAMSNGSCRCNICI

>QBK20674.1 hemagglutinin [Influenza A virus]

METVSLITILLVATVSNADKICIGYQSTNSTETVDTLTENNVPVTHAKELLHTEHNGMLCATSLGQPLVL
DTCTIEGLIYGNPSCDLSLEGREWSYIVERPSAVHGLCYPGSVEDLEELRSLFSSARSYQRIQIFPDTIW
NVSYDGTSTACSGSFYRSMRWLTRKNGEYPIQDAQYTNNQGKNILFMWGINHPPTDETQRGLYTRIDTTT
SVATEEINRIFKPLIGPRPLVNGLMGRINYYWSVLKPGQTLRIKSDGNLIAPWYGHILSGESHGRILKTD
LKRGSCTVQCQTEKGGLNTTLPFQNVSKYAFGNCSKYIGIKSLKLAVGLRNVPSRSSRGLFGAIAGFIEG
GWSGLVAGWYGFQHSNDQGVGMAADRDSTQKAIDKITSKVNNIVDKMNKQYEIIDHEFSEVETRLNMINN
KIDDQIQDIWAYNAELLVLLENQKTLDEHDANVNNLYNKVKRALGSNAVEDGKGCFELYHKCDDQCMETI
RNGTYNRRKYQEESKLERQKIEGVKLESEGTYKILTIYSTVASSLVIAMGFAAFLFWAMSNGSCRCNICI

>QBK20673.1 hemagglutinin [Influenza A virus]

METISLITILLVATVSNADKICIGYQSTNSTETVDTLTENNVPVTHAKELIHTEHNGMLCATSLGQPLIL
DTCTIEGLIYGNPSCDLSLEGREWSYIVERPSAVNGLCYPGNVENLEELRSLFSSARSYQRIQIFPDTIW
NVSYDGTSTACSGSFYRNMRWLTRKDGNYPTQDAQYTNNQGKNILFMWGINHPPTDTTQSGLYTRTDTTT
SVATEEINRIFKPLIGPRPLVNGLMGRIDYYWSVLKPGQTLRIKSDGNLIAPWFGHILSGESHGRILKTD
LKRGSCTVQCQTEKGGLNTTLPFQNVSKYAFGNCSRYIGIKSLKLAVGLRNVPSRSSRGLFGAIAGFIEG
GWSGLVAGWYGFQHSNDQGVGMAADRDSTQKAIDKITSKVNNIVDKMNKQYEIIDHEFSEVETRLNMINN
KIDDQIQDIWAYNAELLVLLENQKTLDEHDANVNNLYNKVKRALGSNAVEDGKGCFELYHKCDDQCMEAI
RNGTYNRRKYQEESKLERQKIEGVKLESEGTYKILTIYSTVASSLVIAMGFAAFLFWAMSNGSCRCNICI

>QBK20672.1 hemagglutinin [Influenza A virus]

METISLITILLVATVSNADKICIGYQSTNSTETVDTLTENNVPVTHAKELIHTEHNGMLCATSLGQPLIL
DTCTIEGLIYGNPSCDLSLEGREWSYIVERPSAVNGLCYPGNVENLEELRSLFSSARSYQRIQIFPDTIW
NVSYDGTSTACSGSFYRNMRWLTRKDGNYPTQDAQYTNNQGKNILFMWGINHPPTDTTQSGLYTRTDTTT
SVATEEINRIFKPLIGPRPLVNGLMGRIDYYWSVLKPGQTLRIKSDGNLIAPWFGHILSGESHGRILKTD
LKRGSCTVQCQTEKGGLNTTLPFQNVSKYAFGNCSKYIGIKSLKLAVGLRNVPSRSSRGLFGAIAGFIEG
GWSGLVAGWYGFQHSNDQGVGMAADRDSTQKAIDKITSKVNNIVDKMNKQYEIIDHEFSEVETRLNMINN
KIDDQIQDIWAYNAELLVLLENQKTLDEHDANVNNLYNKVKRTLGSNAVEDGKGCFELYHKCDDQCMETI
RNGTYNRRKYQEESKLERQKIEGVKLESEGTYKILTIYSTVASSLVIAMGFAAFLFWAMSNGSCRCNICI

>QBK20671.1 hemagglutinin [Influenza A virus]

METISLITILLVATVSNADKICIGYQSTNSTETVDTLTENNVPVTHAKELIHTEHNGMLCATSLGQPLIL

DTCTIEGLIYGNPSCDLSLEGREWSYIVERPSAVNGLCYPGNVENLEELRSLFSSARSYQRIQIFPDTIW
NVSYDGTSTACSGSFYRNMRWLTRKDGNYPTQDAQYTNNQGKNILFMWGINHPPTDTTQSGLYTRTDTTT
SVATEEINRIFKPLIGPRPLVNGLMGRIDYYWSVLKPGQTLRIKSDGNLIAPWFGHILSGESHGRILKTD
LKRGSCTVQCQTEKGGLNTTLPFQNVSKYAFGNCSRYIGIKSLKLAVGLRNVPSRSSRGLFGAIAGFIEG
GWSGLVAGWYGFQHSNDQGVGMAADRDSTQKAIDKITSKVNNIVDKMNKQYEIIDHEFSEVETRLNMINN
KIDDQIQDIWAYNAELLVLLENQKTLDEHDANVNNLYNKVKRALGSNAVEDGKGCFELYHKCDDQCMEAI
RNGTYNRRKYQEESKLERQKIEGVKLESEGTYKILTIYSTVASSLVIAMGFAAFLFWAMSNGSCRCNICI

>QBK20670.1 hemagglutinin [Influenza A virus]
METVSLITILLVATVSNADKICIGYQSTNSTDTVDTLTENNVPVTHAKELLHTEHNGMLCATSLGQPLIL
DTCTIEGLIYGNPSCDLSLEGREWSYIVERPSAVNGLCYPGNVENLEELRSLFSSARSYQRVQIFPDTIW
NVSYDGTSTACSGSFYRSMRWLTRKNGEYPIQDAQYTNSQGKNILFMWGINHPPTDTTQEALYTRTDTTT
SVATEEINRIFKPLIGPRPLVNGLMGRIDYYWSVLKPGQTLRIKSDGNLIAPWYGHVLSGESHGRILKTD
LKRGSCTVQCQTEKGGLNTTLPFQNVSKYAFGNCSKYIGIKSLKLAVGLRNVPSRSSRGLFGAIAGFIEG
GWPGLVAGWYGFQHSNDQGVGMAADRDSTQKAIDKITSKVNNIVDKMNKQYEIIDHEFSEVETRLNMINN
KIDDQIQDIWAYNAELLVLLENQKTLDEHDANVNNLYNKVKRALGSNAVEDGKGCFELYHKCDDQCMDTI
RNGTYNRRKYQEESKLERQKIEGVKLESEGTYKILTIYSTVASSLVIAMGFAAFLFWAMSNGSCRCNICI

>QBK20669.1 hemagglutinin [Influenza A virus]
METISLITILLVATVSNADKICIGYQSTNSTETVDTLTENNVPVTHAKELIHTEHNGMLCATSLGQPLIL
DTCTIEGLIYGNPSCDLSLEGREWSYIVERPSAVNGLCYPGNVENLEELRSLFSSARSYQRIQIFPDTIW
NVSYDGTSTACSGSFYRNMRWLTRKDGNYPTQDAQYTNNQGKNILFMWGINHPPTDTTQSGLYTRTDTTT
SVATEEINRIFKPLIGPRPLVNGLMGRIDYYWSVLKPGQTLRIKSDGNLIAPWFGHILSGESHGRILKTD
LKRGSCTVQCQTEKGGLNTTLPFQNVSKYAFGNCSKYIGIKSLKLAVGLRNVPSRSSRGLFGAIAGFIEG
GWSGLVAGWYGFQHSNDQGVGMAADRDSTQKAIDKITSKVNNIVDKMNKQYEIIDHEFSEVETRLNMINN
KIDDQIQDIWAYNAELLVLLENQKTLDEHDANVNNLYNKVKRALGSNAVEDGKGCFELYHKCDDQCMETI
RNGTYNRRKYQEESKLERQKIEGVKLESEGTYKILTIYSTVASSLVIAMGFAAFLFWAMSNGSCRCNICI

>QBK20668.1 hemagglutinin [Influenza A virus]
METVSLITILLVATVSNADKICIGYQSTNSTETVDTLTENNVPVTHAKELIHTEHNGMLCATSLGQPLIL
DTCTIEGLIYGNPSCDLSLEGREWSYIVERPSAVNGLCYPGNVENLEELRSLFSSARSYQRIQIFPDTIW
NVSYDGTSTACSGSFYRNMRWLTRKDGNYPTQDAQYTNNQGKNILFMWGINHPPTDTTQSGLYTRTDTTT
SVATEEINRIFKPLIGPRPLVNGLMGRIDYYWSVLKPGQTLRIKSDGNLIAPWFGHILSGESHGRILKTD
LKRGSCTVQCQTEKGGLNTTLPFQNVSKYAFGNCSKYIGIKSLKLAVGLRNVPSRSSRGLFGAIAGFIEG

GWSGLVAGWYGFQHSNDQGVGMAADRDSTQKAIDKITSKVNNIVDKMNKQYEIIDHEFSEVETRLNMINN
KIDDQIQDIWAYNAELLVLLENQKTLDEHDANVNNLYNKVKRALGSNAVEDGKGCFELYHKCDDQCMETI
RNGTYNRRKYQEESKLERQKIEGVKLESEGTYKILTIYSTVASSLVIAMGFAAFLFWAMSNGSCRCNICI

>QBK20667.1 hemagglutinin [Influenza A virus]
METISLITILLVATVSNADKICIGYQSTNSTETVDTLTENNVPVTHAKELIHTEHNGMLCATSLGQPLIL
DTCTIEGLIYGNPSCDLSLEGREWSYIVERPSAVNGLCYPGNVENLEELRSLFSSARSYQRIQIFPDTIW
NVSYDGTSTACSGSFYRNMRWLTRKDGNYPTQDAQYTNNQGKNILFMWGINHPPTDTTQSGLYTRTDTTT
SVATEEINRIFKPLIGPRPLVNGLMGRIDYYWSVLKPGQTLRIKSDGNLIAPWFGHILSGESHGRILKTD
LKRGSCTVQCQTEKGGLNTTLPFQNVSKYAFGNCSKYIGIKSLKLAVGLRNVPSRSSRGLFGAIAGFIEG
GWSGLVAGWYGFQHSNDQGVGMAADRDSTQKAIDKITSKVNNIVDKMNKQYEIIDHEFSEVETRLNMINN
KIDDQIQDIWAYNAELLVLLENQKTLDEHDANVNNLYNKVKRALGSNAVEDGKGCFELYHKCDDQCMETI
RNGTYNRRKYQEESKLERQKIEGVKLESEGTYKILTIYSTVASSLVIAMGFAAFLFWAMSNGSCRCNICI

>QBK20666.1 hemagglutinin [Influenza A virus]
METVSLITILLVATVSSADKICIGYQSTNSTETVDTLTENNVPVTHAKELIHTEHNGMLCATSLGQPLIL
DTCTIEGLIYGNPSCDLSLEGREWSYIVERPSAVNGLCYPGNVENLEELRSLFSSARSYQRIQIFPDTIW
NVSYDGTSTACSGSFYRNMRWLTRKDGNYPTQDAQYTNNQGKNILFMWGINHPPTETTQSGLYTRTDTTT
SVATEEINRIFKPLIGPRPLVNGLMGRIDYYWSVLKPGQTLRIKSDGNLIAPWFGHILSGESHGRILKTD
LKRGSCTVQCQTEKGGLNTTLPFQNVSKYAFGNCSKYIGIKSLKLAVGLRNVPSRSSRGLFGAIAGFIEG
GWSGLVAGWYGFQHSNDQGVGMAADRDSTQKAIDKITSKVNNIVDKMNKQYEIIDHEFSEVETRLNMINN
KIDDQIQDIWAYNAELLVLLENQKTLDEHDANVNNLYNKVKRALGSNAVEDGKGCFELYHKCDDQCMETI
RNGTYNRRKYQEESKLERQKIEGVKLESEGTYKILTIYSTVASSLVIAMGFAAFLFWAMSNGSCRCNICI

>QBK20665.1 hemagglutinin [Influenza A virus]
METVSLITILLVATVSSADKICIGYQSTNSTETVDTLTENNVPVTHAKELIHTEHNGMLCATSLGQPLIL
DTCTIEGLIYGNPSCDLSLEGREWSYIVERPSAVNGLCYPGNVENLEELRSLFSSARSYQRIQIFPDTIW
NVSYDGTSTACSGSFYRNMRWLTRKDGNYPTQDAQYTNNQGKNILFMWGINHPPTETTQSGLYTRTDTTT
SVATEEINRIFKPLIGPRPLVNGLMGRIDYYWSVLKPGQTLRIKSDGNLIAPWFGHILSGESHGRILKTD
LKRGSCTVQCQTEKGGLNTTLPFQNVSKYAFGNCSKYIGIKSLKLAVGLRNVPSRSSRGLFGAIAGFIEG
GWSGLVAGWYGFQHSNDQGVGMAADRDSTQKAIDKITSKVNNIVDKMNKQYEIIDHEFSEVETRLNMINN
KIDDQIQDIWAYNAELLVLLENQKTLDEHDANVNNLYNKVKRALGSNAVEDGKGCFELYHKCDDQCMETI
RNGTYNRRKYQEESKLERQKIEGVKLESEGTYKILTIYSTVASSLVIAMGFAAFLFWAMSNGSCRCNICI

>QBK20664.1 hemagglutinin [Influenza A virus]

METVSLITILLVATVSSADKICIGYQSTNSTETVDTLTENNVPVTHAKELIHTEHNGMLCATSLGQPLIL
DTCTIEGLIYGNPSCDLSLEGREWSYIVERPSAVNGLCYPGNVENLEELRSLFSSARSYQRIQIFPDTIW
NVSYDGTSTACSGSFYRNMRWLTRKDGNYPTQDAQYTNNQGKNILFMWGINHPPTETTQSGLYTRTDTTT
SVATEEINRIFKPLIGPRPLVNGLMGRIDYYWSVLKPGQTLRIKSDGNLIAPWFGHILSGESHGRILKTD
LKRGSCTVQCQTEKGGLNTTLPFQNVSKYAFGNCSKYIGIKSLKLAVGLRNVPSRSSRGLFGAIAGFIEG
GWSGLVAGWYGFQHSNDQGVGMAADRDSTQKAIDKITSKVNNIVDKMNKQYEIIDHEFSEVETRLNMINN
KIDDQIQDIWAYNAELLVLLENQKTLDEHDANVNNLYNKVKRALGSNAVEDGKGCFELYHKCDDQCMETI
RNGTYNRRKYQEESKLERQKIEGVKLESEGTYKILTIYSTVASSLVIAMGFAAFLFWAMSNGSCRCNICI

>QBK20663.1 hemagglutinin [Influenza A virus]

METVSLITILLVATVSNADKICIGYQSTNSTETVDTLTENNVPVTHAKELLHTEHNGMLCATSLGQPLIL
DTCTIEGLIYGNPSCDLSLEGREWSYIVERPSAVHGLCYPGNVEDLEELRSLFSSARSYQRIQIFPDTIW
NVSYDGTSTACSGSFYRSMRWLTRKNGEYPIQDAQYTNNQGKNILFMWGINHPPTDDTQRGLYTRTDTTT
SVATEEINRIFKPLIGPRPLVNGLMGRINYYWSVLKPGQTLRIKSDGNLIAPWYGHILSGESHGRILKTD
LKRGSCTVQCQTEKGGLNTTLPFQNISKYAFGNCSKYIGIKSLKLAVGLRNVPSRSSRGLFGAIAGFIEG
GWPGLVAGWYGFQHSNDQGVGMAADRDSTQKAIDKITSKVNNIVDKMNKQYEIIDHEFSEVETRLNMINN
KIDDQIQDIWAYNAELLVLLENQKTLDEHDANVNNLYSKVKRALGSNAVEDGKGCFELYHKCDDQCMETI
RNGTYNRRKYQEESKLERQRIEGVKLESEGTYKILTIYSTVASSLVIAMGFAAFLFWAMSNGSCRCNICI

>QBK20662.1 hemagglutinin [Influenza A virus]

METVSLITILLVATVSNADKICIGYQSTNSTETVDTLTENNVPVTHAKELLHTEHNGMLCATSLGQPLIL
DTCTIEGLIYGNPSCDLSLEGREWSYIVERPSAVHGLCYPGNVEDLEELRSLFSSARSYQRIQIFPDTIW
NVSYDGTSTACSGSFYRSMRWLTRKNGEYPIQDAQYTNNQGKNILFMWGINHPPTDDTQRGLYTRTDTTT
SVATEEINRIFKPLIGPRPLVNGLMGRINYYWSVLKPGQTLRIKSDGNLIAPWYGHILSGESHGRILKTD
LKRGSCTVQCQTEKGGLNTTLPFQNISKYAFGNCSKYIGIKSLKLAVGLRNVPSRSSRGLFGAIAGFIEG
GWPGLVAGWYGFQHSNDQGVGMAADRDSTQKAIDKITSKVNNIVDKMNKQYEIIDHEFSEVETRLNMINN
KIDDQIQDIWAYNAELLVLLENQKTLDEHDANVNNLYSKVKRALGSNAVEDGKGCFELYHKCDDQCMETI
RNGTYNRRKYQEESKLERQRIEGVKLESEGTYKILTIYSTVASSLVIAMGFAAFLFWAMSNGSCRCNICI

>QBK20661.1 hemagglutinin [Influenza A virus]

METVSLITILLVATVSNADKICIGYQSTNSTETVDTLTENNVPVTHAKELLHTEHNGMLCATSLGQPLIL
DTCTIEGLIYGNPSCDLSLEGREWSYIVERPSAVHGLCYPGNVEDLEELRSLFSSARSYQRIQIFPDTIW
NVSYDGTSTACSGSFYRSMRWLTRKNGEYPIQDAQYTNNQGKNILFMWGINHPPTDDTQRGLYTRTDTTT

SVATEEINRIFKPLIGPRPLVNGLMGRINYYWSVLKPGQTLRIKSDGNLIAPWYGHILSGESHGRILKTD
LKRGSCTVQCQTEKGGLNTTLPFQNVSKYAFGNCSKYIGIKSLKLAVGLRNVPTRSSRGLFGAIAGFIEG
GWSGLVAGWYGFQHSNDQGVGMAADRDSTQKAIDKITSKVNNIVDKMNKQYEIIDHEFSEVETRLNMINN
KIDDQIQDIWAYNAELLVLLENQKTLDEHDANVNNLYNKVKRALGSNAVEDGKGCFELYHKCDDQCMETI
RNGTYNRRKYQEESKLERQRIEGVKLESEGTYKILTIYSTVASSLVIAMGFAAFLFWAMSNGSCRCNICI

>QBK20660.1 hemagglutinin [Influenza A virus]
METVSLITILLVATVSNADKICIGYQSTNSTETVDTLTENNVPVTHAKELLHTEHNGMLCATSLGQPLIL
DTCTIEGLIYGNPSCDLSLEGREWSYIVERPSAVHGLCYPGNVEDLEELRSLFSSARSYQRIQIFPDTIW
NVSYDGTSTACSGSFYRSMRWLTRKNGEYPIQDAQYTNNQGKNILFMWGINHPPTDDTQRGLYTRTDTTT
SVATEEINRIFKPLIGPRPLVNGLMGRINYYWSVLKPGQTLRIKSDGNLIAPWYGHILSGESHGRILKTD
LKRGSCTVQCQTEKGGLNTTLPFQNISKYAFGNCSKYIGIKSLKLAVGLRNVPSRSSRGLFGAIAGFIEG
GWPGLVAGWYGFQHSNDQGVGMAADRDSTQKAIDKITSKVNNIVDKMNKQYEIIDHEFSEVETRLNMINN
KIDDQIQDIWAYNAELLVLLENQKTLDEHDANVNNLYSKVKRALGSNAVEDGKGCFELYHKCDDQCMETI
RNGTYNRRKYQEESKLERQRIEGVKLESEGTYKILTIYSTVASSLVIAMGFAAFLFWAMSNGSCRCNICI

>QBK20659.1 hemagglutinin [Influenza A virus]
METVSLITILLVATVSNADKICIGYQSTNSTETVDTLTENNVPVTHAKELLHTEHNGMLCATSLGQPLIL
DTCTIEGLIYGNPSCDLSLEGREWSYIVERPSAVHGLCYPGNVEDLEELRSLFSSARSYQRIQIFPDTIW
NVSYDGTSTACSGSFYRSMRWLTRKNGEYPIQDAQYTNNQGKNILFMWGINHPPTDDTQRGLYTRTDTTT
SVATEEINRIFKPLIGPRPLVNGLMGRINYYWSVLKPGQTLRIKSDGNLIAPWYGHILSGESHGRILKTD
LKRGSCTVQCQTEKGGLNTTLPFQNVSKYAFGNCSKYIGIKSLKLAVGLRNVPTRSSRGLFGAIAGFIEG
GWSGLVAGWYGFQHSNDQGVGMAADRDSTQKAIDKITSKVNNIVDKMNKQYEIIDHEFSEVETRLNMINN
KIDDQIQDIWAYNAELLVMLENQKTLDEHDANVNNLYNKVKRALGSNAVEDGKGCFELYHKCDDQCMETI
RNGTYNRRKYQEESKLERQRIEGVKLESEGTYKILTIYSTVASSLVIAMGFAAFLFWAMSNGSCRCNICI

>QBK20658.1 hemagglutinin [Influenza A virus]
METVSLITILLVATVSNADKICIGYQSTNSTETVDTLTENNVPVTHAKELLHTEHNGMLCATSLGQPLIL
DTCTIEGLIYGNPSCDLSLEGREWSYIVERPSAVHGLCYPGNVEDLEELRSLFSSARSYQRIQIFPDTIW
NVSYDGTSTACSGSFYRSMRWLTRKNGEYPIQDAQYTNNQGKNILFMWGINHPPTDDTQRGLYTRTDTTT
SVATEEINRIFKPLIGPRPLVNGLMGRINYYWSVLKPGQTLRIKSDGNLIAPWYGHILSGESHGRILKTD
LKRGSCTVQCQTEKGGLNTTLPFQNISKYAFGNCSKYIGIKSLKLAVGLRNVPSRSSRGLFGAIAGFIEG
GWPGLVAGWYGFQHSNDQGVGMAADRDSTQKAIDKITSKVNNIVDKMNKQYEIIDHEFSEVETRLNMINN
KIDDQIQDIWAYNAELLVLLENQKTLDEHDANVNNLYSKVKRALGSNAVEDGKGCFELYHKCDDQCMETI

RNGTYNRRKYQEESKLERQRIEGVKLESEGTYKILTIYSTVASSLVIAMGFAAFLFWAMSNGSCRCNICI

>QBK20657.1 hemagglutinin [Influenza A virus]

METVSLITILLVATVSNADKICIGYQSTNSTETVDTLTENNVPVTHAKELLHTEHNGMLCATSLGQPLIL

DTCTIEGLIYGNPSCDLSLEGREWSYIVERPSAVHGLCYPGNVEDLEELRSLFSSARSYQRIQIFPDTIW

NVSYDGTSTACSGSFYRSMRWLTRKNGEYPIQDAQYTNNQGKNILFMWGINHPPTDDTQRGLYTRTDTTT

SVATEEINRIFKPLIGPRPLVNGLMGRINYYWSVLKPGQTLRIKSDGNLIAPWYGHILSGESHGRILKTD

LKRGSCTVQCQTEKGGLNTTLPFQNISKYAFGNCSKYIGIKSLKLAIGLRNVPSRSSRGLFGAIAGFIEG

GWPGLVAGWYGFQHSNDQGVGMAADRDSTQKAIDKITSKVNNIVDKMNKQYEIIDHEFSEVETRLNMINN

KIDDQIQDIWAYNAELLVLLENQKTLDEHDANVNNLYSKVKRALGSNAVEDGKGCFELYHKCDDQCMETI

RNGTYNRRKYQEESKLERQRIEGVKLESEGTYKILTIYSTVASSLVIAMGFAAFLFWAMSNGSCRCNICI

>QBK20656.1 hemagglutinin [Influenza A virus]

METASLITILLVATVSNADKICIGYQSTNSTETVDTLTENNVPVTHAKELLHTEHNGMLCATSLGHPLIL

DTCTIEGLIYGNPSCDPLLGGREWSYIVERPSAVNGLCYPGNVENLEELRSLFSSSRSYQRIQIFPDTIW

NVSYSGTSKACSDSFYRSMRWLTQKNNAYPTQDAQYTNNQEKNILFMWGINHPPTDTAQTNLYTRTDTTT

SVATEEMNRIFKPLIGPRPLVNGLMGRINYYWSVLKPGQTLRIKSDGNLIAPWYGHILSGESHGRILKTD

LKRGSCTVQCQTEKGGLNTTLPFQNVSKYAFGNCSKYIGVKSLKLAVGLRNVPSKSSRGLFGAIAGFIEG

GWSGLVAGWYGFQHSNDQGVGMAADRDSTQKAIDKITSKVNNIVDKMNKQYEIIDHEFSEVETRLNMINN

KVDDQIQDIWAYNAELLVLLENQKTLDEHDANVNNLYNKVKRALGSNAVEDGKGCFELYHKCDDHCMETI

RNGTYNRRKYQEESKLERQKIEGVKLESEETYKILTIYSTVASSLVIAMGFAAFLFWAMSNGSCRCNICI

>QBK20655.1 hemagglutinin [Influenza A virus]

METVSLITILLVATVSNADKICIGYQSTNSTETVDTLTENNVPVTHAKELLHTEHNGMLCATSLGQPLIL

DTCTIEGLIYGNPSCDLSLEGREWSYIVERPSAVHGLCYPGNVEDLEELRSLFSSARSYQRIQIFPDTIW

NVSYDGTSTACSGSFYRSMRWLTRKNGEYPIQDAQYTNNQGKNILFMWGINHPPTDDTQRGLYTRTDTTT

SVATEXINRIFKPLIGPRPLVNGLMGRINYYWSVLKPGQTLRIKSDGNLIAPWYGHILSGESHGRILKTD

XKRGSCTVQCQTEKGGLNTTLPFQNISKYAFGNCSKYIGIKSLKLAVGLRNVPSRSSRGLFGAIAGFIEG

GWSGLVAGWYGFQHSNDQGVGMAADRDSTQKAIDKITSKVNNIVDKMNKQYEIIDHEFSEVETRLNMINN

KIDDQIQDIWAYNAELLVLLENQKTLDEHDANVNNLYNKVKRALGSNAVEDGKGCFELYHKCDDQCMETI

RNGTYNRRKYQEESKLERQRIEGVKLESEGTYKILTIYSTVASSLVIAMGFAAFLFWAMSNGSCRCNICI

>QBK20654.1 hemagglutinin [Influenza A virus]

METVSLITILLVATVSNADKICIGYQSTNSTETVDTLTENNVPVTHAKELLHTEHNGMLCATSLGQPLIL

DTCTIEGLIYGNPSCDLSLEGREWSYIVERPSAVHGLCYPGNVEDLEELRSLFSSARSYQRIQIFPDTIW
NVSYDGTSTACSGSFYRSMRWLTRKNGEYPIQDAQYTNNQGKNILFMWGINHPPTDDTQRGLYTRTDTTT
SVATEEINRIFKPLIGPRPLVNGLMGRINYYWSVLKPGQTLRIKSDGNLIAPWYGPILSGESHGRILKTD
LKRGSCTVQCQTEKGGLNTTLPFQNISKYAFGNCSKYIGIKSLKLAVGLRNVPSRSSRGLFGAIAGFIEG
GWPGLVAGWYGFQHSNDQGVGMAADRDSTQKAIDKITSKVNNIVDKMNKQYEIIDHEFSEVETRLNMINN
KIDDQIQDIWAYNAELLVLLENQKTLDEHDANVNNLYSKVKRALGSNAVEDGKGCFELYHKCDDQCMETI
RNGTYNRRKYQEESKLERQRIEGVKLESEGTYKILTIYSTVASSLVIAMGFAAFLFWAMSNGSCRCNICI

>QBK20653.1 hemagglutinin [Influenza A virus]
METASLITILLVATVSNADKICIGYQSTNSTETVDTLTENNVPVTHAKELLHTEHNGMLCATSLGHPLIL
DTCTIEGLIYGNPSCDPLLGGREWSYIVERPSAVNGLCYPGNVENLEELRSLFSSSRSYQRIQIFPDTIW
NVSYSGTSKACSDSFYRSMRWLTQKNNAYPTQDAQYTNNQEKNILFMWGINHPPTDTAQTNLYTRTDTTT
SVATEEMNRIFKPLIGPRPLVNGLMGRINYYWSVLKPGQTLRIKSDGNLIAPWYGHILSGESHGRILKTD
LKRGSCTVQCQTEKGGLNTTLPFQNVSKYAFGNCSKYIGVKSLKLAVGLRNVPSKSSRGLFGAIAGFIEG
GWSGLVAGWYGFQHSNDQGVGMAADRDSTQKAIDKITSKVNNIVDKMNKQYEIIDHEFSEVETRLNMINN
KVDDQIQDIWAYNAELLVLLENQKTLDEHDANVNNLYNKVKRALGSNAVEDGKGCFELYHKCDDHCMETI
RNGTYNRRKYQEESKLERQKIEGVKLESEETYKILTIYSTVASSLVIAMGFAAFLFWAMSNGSCRCNICI

>QBK20652.1 hemagglutinin [Influenza A virus]
METVSLITILLVATVSNADKICIGYQSTNSTETVDTLTENNVPVTHAKELLHTEHNGMLCATSLGQPLIL
DTCTIEGLIYGNPSCDLSLEGREWSYIVERPSAVHGLCYPGNVEDLEELRSLFSSARSYQRIQIFPDTIW
NVSYDGTSTACSGSFYRSMRWLTRKNGEYPIQDAQYTNNQGKNILFMWGINHPPTDDTQRGLYTRTDTTT
SVATEEINRIFKPLIGPRPLVNGLMGRINYYWSVLKPGQTLRIKSDGNLIAPWYGHILSGESHGRILKTD
LKRGSCTVQCQTEKGGLNTTLPFQNISKYAFGNCSKYIGIKSLKLAVGLRNVPSRSSRGLFGAIAGFIEG
GWPGLVAGWYGFQHSNDQGVGMAADRDSTQKAIDKITSKVNNIVDKMNKQYEIIDHEFSEVETRLNMINN
KIDDQIQDIWAYNAELLVLLENQKTLDEHDANVNNLYSKVKRALGSNAVEDGKGCFELYHKCDDQCMETI
RNGTYNRRKYQEESKLERQRIEGVKLESEGTYKILTIYSTVASSLVIAMGFAAFLFWAMSNGSCRCNICI

>QBK20651.1 hemagglutinin [Influenza A virus]
METVSLITILLVATVSNADKICIGYQSTNSTETVDTLTENNVPVTHAKELLHTEHNGMLCATSLGQPLIL
DTCTIEGLIYGNPSCDLSLEGREWSYIVERPSAVHGLCYPGNVEDLEELRSLFSSARSYQRIQIFPDTIW
NVSYDGTSTACSGSFYRSMRWLTRKNGEYPIQDAQYTNNQGKNILFMWGINHPPTDDTQRGLYTRTDTTT
SVATEEINRIFKPLIGPRPLVNGLMGRINYYWSVLKPGQTLRIKSDGNLIAPWYGHILSGESHGRILKTD
LKRGSCTVQCQTEKGGLNTTLPFQNISKYAFGNCSKYIGIKSLKLAVGLRNVPSRSSRGLFGAIAGFIEG

GWPGLVAGWYGFQHSNDQGVGMAADRDSTQKAIDKITSKVNNIVDKMNKQYEIIDHEFSEVETRLNMINN
KIDDQIQDIWAYNAELLVLLENQKTLDEHDANVNNLYSKVKRALGSNAVEDGKGCFELYHKCDDQCMETI
RNGTYNRRKYQEESKLERQRIEGVKLESEGTYKILTIYSTVASSLVIAMGFAAFLFWAMSNGSCRCNICI

>QBK20650.1 hemagglutinin [Influenza A virus]
METVSLITILLVATVSNADKICIGYQSTNSTETVDTLTENNVPVTHAKELLHTEHNGMLCATSLGQPLIL
DTCTIEGLIYGNPSCDLSLEGREWSYIVERPSAVHGLCYPGNVEDLEELRSLFSSARSYQRIQIFPDTIW
NVSYDGTSTACSGSFYRSMRWLTRKNGEYPIQDAQYTNNQGKNILFMWGINHPPTDDTQRGLYTRTDTTT
SVATEEINRIFKPLIGPRPLVNGLMGRINYYWSVLKPGQTLRIKSDGNLIAPWYGHILSGESPGRILKTD
LKRGSCTVQCQTEKGGLNTTLPFQNVSKYAFGNCSKYIGIKSLKLAVGLRNVPTRSSRGLFGAIAGFIEG
GWSGLVAGWYGFQHSNDQGVGMAADRDSTQKAIDKITSKVNNIVDKMNKQYEIIDHEFSEVETRLNMINN
KIDDQIQDIWAYNAELLVLLENQKTLDEHDANVNNLYNKVKRALGSNAVEDGKGCFELYHKCDDQCMETI
RNGTYNRRKYQEESKLERQRIEGVKLESEGTYKILTIYSTVASSLVIAMGFAAFLFWAMSNGSCRCNICI

>QBK20649.1 hemagglutinin [Influenza A virus]
METASLITILLVATVSNADKICIGYQSTNSTETVDTLTENNVPVTHAKELLHTEHNGMLCATSLGHPLIL
DTCTIEGLIYGNPSCDPLLGGREWSYIVERPSAVNGLCYPGNVENLEELRSLFSSSRSYQRIQIFPDTIW
NVSYSGTSKACSDSFYRSMRWLTQKNNAYPTQDAQYTNNQEKNILFMWGINHPPTDTAQTNLYTRTDTTT
SVATEEMNRIFKPLIGPRPLVNGLMGRINYYWSVLKPGQTLRIKSDGNLIAPWYGHILSGESHGRILKTD
LKRGSCTVQCQTEKGGLNTTLPFQNVSKYAFGNCSKYIGVKSLKLAVGLRNVPSKSSRGLFGAIAGFIEG
GWSGLVAGWYGFQHSNDQGVGMAADRDSTQKAIDKITSKVNNIVDKMNKQYEIIDHEFSEVETRLNMINN
KVDDQIQDIWAYNAELLVLLENQKTLDEHDANVNNLYNKVKRALGSNAVEDGKGCFELYHKCDDHCMETI
RNGTYNRRKYQEESKLERQKIEGVKLESEETYKILTIYSTVASSLVIAMGFAAFLFWAMSNGSCRCNICI

>QBK20648.1 hemagglutinin [Influenza A virus]
METVSLITILLVATVSNADKICIGYQSTNSTETVDTLTENNVPVTHAKELLHTEHNGMLCATSLGQPLIL
DTCTIEGLIYGNPSCDLSLEGREWSYIVERPSAVHGLCYPGNVEDLEELRSLFSSARSYQRIQIFPDTIW
NVSYDGTSTACSGSFYRSMRWLTRKNGEYPIQDAQYTNNQGKNILFMWGINHPPTDDTQRGLYTRTDTTT
SVATEEINRIFKPLIGPRPLVNGLMGRINYYWSVLKPGQTLRIKSDGNLIAPWYGHILSGESHGRILKTD
LKRGSCTVQCQTEKGGLNTTLPFQNISKYAFGNCSKYIGIKSLKLAVGLRNVPSRSSRGLFGAIAGFIEG
GWPGLVAGWYGFQHSNDQGVGMAADRDSTQKAIDKITSKVNNIVDKMNKQYEIIDHEFSEVETRLNMINN
KIDDQIQDIWAYNAELLVLLENQKTLDEHDANVNNLYSKVKRALGSNAVEDGKGCFELYHKCDDQCMETI
RNGTYNRRKYQEESKLERQRIEGVKLESEGTYKILTIYSTVASSLVIAMGFAAFLFWAMSNGSCRCNICI

>QBK20647.1 hemagglutinin [Influenza A virus]

METVSLITILLVATVSNADKICIGYQSTNSTETVDTLTENNVPVTHAKELLHTEHNGMLCATSLGQPLIL

DTCTIEGLIYGNPSCDLSLEGREWSYIVERPSAVHGLCYPGNVEDLEELRSLFSSARSYQRIQIFPDTIW

NVSYDGTSTACSGSFYRSMRWLTRKNGEYPIQDAQYTNNQGKNILFMWGINHPPTDDTQRGLYTRTDTTT

SVATEEINRIFKPLIGPRPLVNGLMGRINYYWSVLKPGQTLRIKSDGNLIAPWYGHILSGESHGRILKTD

LKRGSCTVQCQTEKGGLNTTLPFQNVSKYAFGNCSKYIGIKSLKLAVGLRNVPTRSSRGLFGAIAGFIEG

GWSGLVAGWYGFQHSNGQGVGMAADRDSTQKAIDKITSKVNNIVDKMNKQYEIIDHEFSEVETRLNMINN

KIDDQIQDIWAYNAELLVLLENQKTLDEHDANVNNLYNKVKRALGSNAVEDGKGCFELYHKCDDQCMETI

RNGTYNRRKYQEESKLERQRIEGVKLESEGTYKILTIYSTVASSLVIAMGFAAFLFWAMSNGSCRCNICI

>QBK20646.1 hemagglutinin [Influenza A virus]

METASLITILLVATVSNADKICIGYQSTNSTETVDTLTENNVPVTHAKELLHTEHNGMLCATSLGHPLIL

DTCTIEGLIYGNPSCDPLLGGREWSYIVERPSAVNGLCYPGNVENLEELRSLFSSSRSYQRIQIFPDTIW

NVSYSGTSKACSDSFYRSMRWLTQKNNAYPTQDAQYTNNQEKNILFMWGINHPPTDTAQTNLYTRTDTTT

SVATEEMNRIFKPLIGPRPLVNGLMGRINYYWSVLKPGQTLRIKSDGNLIAPWYGHILSGESHGRILKTD

LKRGSCTVQCQTEKGGLNTTLPFQNVSKYAFGNCSKYIGVKSLKLAVGLRNVPSKSSRGLFGAIAGFIEG

GWSGLVAGWYGFQHSNDQGVGMAADRDSTQKAIDKITSKVNNIVDKMNKQYEIIDHEFSEVETRLNMINN

KVDDQIQDIWAYNAELLVLLENQKTLDEHDANVNNLYNKVKRALGSNAVEDGKGCFELYHKCDDHCMETI

RNGTYNRRKYQEESKLERQKIEGVKLESEETYKILTIYSTVASSLVIAMGFAAFLFWAMSNGSCRCNICI

>QBK20645.1 hemagglutinin [Influenza A virus]

METVSLITILLVATVSNADKICIGYQSTNSTETVDTLTENNVPVTHAKELLHTEHNGMLCATSLGQPLIL

DTCTIEGLIYGNPSCDLSLEGREWSYIVERPSAVHGLCYPGNVEDLEELRSLFSSARSYQRIQIFPDTIW

NVSYDGTSTACSGSFYRSMRWLTRKNGEYPIQDAQYTNNQGKNILFMWGINHPPTDDTQRGLYTRTDTTT

SVATEEINRIFKPLIGPRPLVNGLMGRINYYWSVLKPGQTLRIKSDGNLIAPWYGHILSGESHGRILKTD

LKRGSCTVQCQTEKGGLNTTLPFQNVSKYAFGNCSKYIGIKSLKLAVGLRNVPSRSSRGLFGAIAGFIEG

GWSGLVAGWYGFQHSNDQGVGMAADRDSTQKAIDKITSKVNNIVDKMNKQYEIIDHEFSEVETRLNMINN

KIDDQIQDIWAYNAELLVLLENQKTLDEHDANVNNLYSKVKRALGSNAVEDGKGCFELYHKCDDQCMETI

RNGTYNRRKYQEESKLERQRIEGVKLESEGTYKILTIYSTVASSLVIAMGFAAFLFWAMSNGSCRCNICI

>QBK20644.1 hemagglutinin [Influenza A virus]

METVSLITILLVATVSNADKICIGYQSTNSTETVDTLTENNVPVTHAKELLHTEHNGMLCATSLGQPLIL

DTCTIEGLIYGNPSCDLSLEGREWSYIVERPSAVHGLCYPGNVEDLEELRSLFSSARSYQRIQIFPDTIW

NVSYDGTSTACSGSFYRSMRWLTRKNGEYPIQDAQYTNNQGKNILFMWGINHPPTDDTQRGLYTRTDTTT

SVATEEINRIFKPLIGPRPLVNGLMGRINYYWSVLKPGQTLRIKSDGNLIAPWYGHILSGESHGRILKTD
LKRGSCTVQCQTEKGGLNTTLPFQNISKYAFGNCSKYIGIKSLKLAVGLRNVPSRSSRGLFGAIAGFIEG
GWPGLVAGWYGFQHSNDQGVGMAADRDSTQKAIDKITSKVNNIVDKMNKQYEIIDHEFSEVETRLNMINN
KIDDQIQDIWAYNAELLVLLENQKTLDEHDANVNNLYSKVKRALGSNAVEDGKGCFELYHKCDDQCMETI
RNGTYNRRKYQEESKLERQRIEGVKLESEGTYKILTIYSTVASSLVIAMGFAAFLFWAMSNGSCRCNICI

>QBK20643.1 hemagglutinin [Influenza A virus]
METVSLITILLVATVSNADKICIGYQSTNSTETVDTLTENNVPVTHAKELLHTEHNGMLCATSLGQPLIL
DTCTIEGLIYGNPSCDLSLEGREWSYIVERPSAVHGLCYPGNVEDLEELRSLFSSARSYQRIQIFPDTIW
NVSYDGTSTACSGSFYRSMRWLTRKNGEYPIQDAQYTNNQGKNILFMWGINHPPTDDTQRGLYTRTDTTT
SVATEEINRIFKPLIGPRPLVNGLMGRINYYWSVLKPGQTLRIKSDGNLIAPWYGHILSGESHGRILKTD
LKRGSCTVQCQTEKGGLNTTLPFQNVSKYAFGNCSKYIGIKSLKLAVGLRNVPTRSSRGLFGAIAGFIEG
GWSGLVAGWYGFQHSNDQGVGMAADRDSTQKAIDKITSKVNNIVDKMNKQYEIIDHEFSEVETRLNMINN
KIDDQIQDIWAYNAELLVLLENQKTLDEHDANVNNLYNKVKRALGSNAVEDGKGCFELYHKCDDQCMETI
RNGTYNRRKYQEESKLERQRIEGVKLESEGTYKILTIYSTVASSLVIAMGFAAFLFWAMSNGSCRCNICI

>QBK20642.1 hemagglutinin [Influenza A virus]
METVSLITILLVATVSNADKICIGYQSTNSTETVDTLTENNVPVTHAKELLHTEHNGMLCATSLGQPLIL
DTCTIEGLIYGNPSCDLSLEGREWSYIVERPSAVHGLCYPGNVEDLEELRSLFSSARSYQRIQIFPDTIW
NVSYDGTSTACSGSFYRSMRWLTRKNGEYPIQDAQYTNNQGKNILFMWGINHPPTDDTQRGLYTRTDTTT
SVATEEINRIFKPLIGPRPLVNGLMGRINYYWSVLKPGQTLRIKSDGNLIAPWYGHILSGESHGRILKTD
LKRGSCTVQCQTEKGGLNTTLPFQNISKYAFGNCSKYIGIKSLKLAVGLRNVPSRSSRGLFGAIAGFIEG
GWPGLVAGWYGFQHSNDQGVGMAADRDSTQKAIDKITSKVNNIVDKMNKQYEIIDHEFSEVETRLNMINN
KIDDQIQDIWAYNAELLVLLENQKTLDEHDANVNNLYSKVKRALGSNAVEDGKGCFELYHKCDDQCMETI
RNGTYNRRKYQEESKLERQRIEGVKLESEGTYKILTIYSTVASSLVIAMGFAAFLFWAMSNGSCRCNICI

>QBK20641.1 hemagglutinin [Influenza A virus]
METVSLITILLVATVSNADKICIGYQSTNSTETVDTLTENNVPVTHAKELLHTEHNGMLCATSLGQPLIL
DTCTIEGLIYGNPSCDLSLEGREWSYIVERPSAVHGLCYPGNVEDLEELRSLFSSARSYQRIQIFPDTIW
NVSYDGTSTACSGSFYRSMRWLTRKNGEYPIQDAQYTNNQGKNILFMWGINHPPTDDTQRGLYTRTDTTT
SVATEEINRIFKPLIGPRPLVNGLMGRINYYWSVLKPGQTLRIKSDGNLIAPWYGHILSGESHGRILKTD
LKRGSCTVQCQTEKGGLNTTLPFQNISKYAFGNCSKYIGIKSLKLAVGLRNVPSRSSRGLFGAIAGFIEG
GWPGLVAGWYGFQHSNDQGVGMAADRDSTQKAIDKITSKVNNIVDKMNKQYEIIDHEFSEVETRLNMINN
KIDDQIQDIWAYNAELLVLLENQKTLDEHDANVNNLYSKVKRALGSNAVEDGKGCFELYHKCDDQCMETI

RNGTYNRRKYQEESKLERQRIEGVKLESEGTYKILTIYSTVASSLVIAMGFAAFLFWAMSNGSCRCNICI

>QBK20640.1 hemagglutinin [Influenza A virus]
METVSLITILLVATVSNADKICIGYQSTNSTETVDTLTENNVPVTHAKELLHTEHNGMLCATSLGQPLIL
DTCTIEGLIYGNPSCDLSLEGREWSYIVERPSAVHGLCYPGNVEDLEELRSLFSSARSYQRIQIFPDTIW
NVSYDGTSTACSGSFYRSMRWLTRKNGEYPIQDAQYTNNQGKNILFMWGINHPPTDDTQRGLYTRTDTTT
SVATEEINRIFKPLIGPRPLVNGLMGRINYYWSVLKPGQTLRIKSDGNLIAPWYGHILSGESHGRILKTD
LKRGSCTVQCQTEKGGLNTTLPFQNISKYAFGNCSKYIGIKSLKLAVGLRNVPSRSSRGLFGAIAGFIEG
GWPGLVAGWYGFQHSNDQGVGMAADRDSTQKAIDKITSKVNNIVDKMNKQYEIIDHEFSEVETRLNMINN
KIDDQIQDIWAYNAELLVLLENQKTLDEHDANVNNLYSKVKRALGSNAVEDGKGCFELYHKCDDQCMETI
RNGTYNRRKYQEESKLERQRIEGVKLESEGTYKILTIYSTVASSLVIAMGFAAFLFWAMSNGSCRCNICI

>QBK20639.1 hemagglutinin [Influenza A virus]
METASLITILLVATVSNADKICIGYQSTNSTETVDTLTENNVPVTHAKELLHTEHNGMLCATSLGHPLIL
DTCTIEGLIYGNPSCDPLLGGREWSYIVERPSAVNGLCYPGNVENLEELRSLFSSSRSYQRIQIFPDTIW
NVSYSGTSKACSDSFYRSMRWLTQKNNAYPTQDAQYTNNQEKNILFMWGINHPPTDTVQTNLYTRTDTTT
SVATEEMNRIFKPLIGPRPLVNGLMGRINYYWSVLKPGQTLRIKSDGNLIAPWYGHILSGESHGRILKTD
LKRGSCTVQCQTEKGGLNTTLPFQNVSKYAFGNCSKYIGVKSLKLAVGLRNVPSKSSRGLFGAIAGFIEG
GWSGLVAGWYGFQHSNDQGVGMAADRDSTQKAIDKITSKVNNIVDKMNKQYEIIDHEFSEVETRLNMINN
KVDDQIQDIWAYNAELLVLLENQKTLDEHDANVNNLYNKVKRALGSNAVEDGKGCFELYHKCDDHCMETI
RNGTYNRRKYQEESKLERQKIEGVKLESEETYKILTIYSTVASSLVIAMGFAAFLFWAMSNGSCRCNICI

>QBK20638.1 hemagglutinin [Influenza A virus]
METVSLITILLVATVSNADKICIGYQSTNSTETVDTLTENNVPVTHATELLHTEHNGMLCATSLGQPLIL
DTCTIEGLIYGNPSCDLSLEGREWSYIVERPSAVHGLCYPGNVEDLEELRSLFSSARSYQRIQIFPDTIW
NVSYDGTSTACSGSFYRSMRWLTRKNGEYPIQDAQYTNNQGKNILFMWGINHPPTDDTQRGLYTRTDTTT
SVATEEINRIFKPLIGPRPLVNGLMGRINYYWSVLKPGQTLRIKSDGNLIAPWYGHILSGESHGRILKTD
LKRGSCTVQCQTEKGGLNTTLPFQNISKYAFGNCSKYIGIKSLKLAVGLRNVPSRSSRGLFGAIAGFIEG
GWPGLVAGWYGFQHSNDQGVGMAADRDSTQKAIDKITSKVNNIVDKMNKQYEIIDHEFSEVETRLNMINN
KIDDQIQDIWAYNAELLVLLENQKTLDEHDANVNNLYSKVKRALGSNAVEDGKGCFELYHKCDDQCMETI
RNGTYNRRKYQEESKLERQRIEGVKLESEGTYKILTIYSTVASSLVIAMGFAAFLFWAMSNGSCRCNICI

>QBK20637.1 hemagglutinin [Influenza A virus]
METVSLITILLVATVSNADKICIGYQSTNSTETVDTLTENNVPVTHAKELLHTEHNGMLCATSLGQPLIL

DTCTIEGLIYGNPSCDLSLEGREWSYIVERPSAVHGLCYPGNVEDLEELRSLFSSARSYQRIQIFPDTIW
NVSYDGTSTACSGSFYRSMRWLTRKNGEYPIQDAQYTNNQGKNILFMWGINHPPTDDTQRGLYTRTDTTT
SVATEEINRIFKPLIGPRPLVNGLMGRINYYWSVLKPGQTLRIKSDGNLIAPWYGHILSGESHGRILKTD
LKRGSCTVQCQTEKGGLNTTLPFQNVSKYAFGNCSKYIGIKSLKLAVGLRNVPTRSSRGLFGAIAGFIEG
GWSGLVAGWYGFQHSNDQGVGMAADRDSTQKAIDKITSKVNNIIDKMNKQYEIIDHEFSEVETRLNMINN
KIDDQIQDIWAYNAELLVLLENQKTLDEHDANVNNLYNKVKRALGSNAVEDGKGCFELYHKCDDQCMETI
RNGTYNRRKYQEESKLERQRIEGVKLESEGTYKILTIYSTVASSLVIAMGFAAFLFWAMSNGSCRCNICI

>QBK20636.1 hemagglutinin [Influenza A virus]
METVSLITILLVATVSNADKICIGYQSTNSTETVDTLTENNVPVTHAKELLHTEHNGMLCATSLGQPLIL
DTCTIEGLIYGNPSCDLSLEGREWSYIVERPSAVHGLCYPGNVEDLEELRSLFSSARSYQRIQIFPDTIW
NVSYDGTSTACSGSFYRSMRWLTRKNGEYPIQDAQYTNNQGKNILFMWGINHPPTDDTQRGLYTRTDTTT
SVATEEINRIFKPLIGPRPLVNGLMGRINYYWSVLKPGQTLRIKSDGNLIAPWYGHILSGESHGRILKTD
LKRGSCTVQCQTEKGGLNTTLPFQNVSKYAFGNCSKYIGIKSLKLAVGLRNVPTRSSRGLFGAIAGFIEG
GWSGLVAGWYGFQHSNDQGVGMAADRDSTQKAIDKITSKVNNIVDKMNKQYEIIDHEFSEVETRLNMINN
KIDDQIQDIWAYNAELLVLLENQKTLDEHDANVNNLYNKVKRALGSNAVEDGKGCFELYHKCDDQCMETI
RNGTYNRRKYQEESKLERQRIEGVKLESEGTYKILTIYSTVASSLVIAMGFAAFLFWAMSNGSCRCNICI

>QBK20635.1 hemagglutinin [Influenza A virus]
METVSLITILLVATVSNADKICIGYQSTNSTETVDTLTENNVPVTHAKELLHTEHNGMLCATSLGQPLIL
DTCTIEGLIYGNPSCDLSLEGREWSYIVERPSAVHGLCYPGNVEDLEELRSLFSSARSYQRIQIFPDTIW
NVSYDGTSTACSGSFYRSMRWLTRKNGEYPIQDAQYTNNQGKNILFMWGINHPPTDDTQRGLYTRTDTTT
SVATEEINRIFKPLIGPRPLVNGLMGRINYYWSVLKPGQTLRIKSDGNLIAPWYGHILSGESHGRILKTD
LKRGSCTVQCQTEKGGLNTTLPFQNVSKYAFGNCSKYIGIKSLKLAVGLRNVPTRSSRGLFGAIAGFIEG
GWSGLVAGWYGFQHSNDQGVGMAADRDSTQKAIDKITSKVNNIVDKMNKQYEIIDHEFSEVETRLNMINN
KIDDQIQDIWAYNAELLVLLENQKTLDEHDANVNNLYNKVKRALGSNAVEDGKGCFELYHKCDDQCMETI
RNGTYNRRKYQEESKLERQRIEGVKLESEGTYKILTIYSTVASSLVIAMGFAAFLFWAMSNGSCRCNICI

>QBK20634.1 hemagglutinin [Influenza A virus]
METVSLITILLVATVSNADKICIGYQSTNSTETVDTLTENNVPVTHAKELLHTEHNGMLCATSLGQPLIL
DTCTIEGLIYGNPSCDLSLEGREWSYIVERPSAVHGLCYPGNVEDLEELRSLFSSARSYQRIQIFPDTIW
NVSYDGTSTACSGSFYRSMRWLTRKNGEYPIQDAQYTNNQGKNILFMWGINHPPTDDTQRGLYTRTDTTT
SVATEEINRIFKPLIGPRPLVNGLMGRINYYWSVLKPGQTLRIKSDGNLIAPWYGHILSGESHGRILKTD
LKRGSCTVQCQTEKGGLNTTLPFQNVSKYAFGNCSKYIGIKSLKLAVGLRNVPTRSSRGLFGAIAGFIEG

GWSGLVAGWYGFQHSNDQGVGMAADRDSTQKAIDKITSKVNNIVDKMNKQYEIIDHEFSEVETRLNMINN
KIDDQIQDIWAYNAELLVLLENQKTLDEHDANVNNLYNKVKRALGSNAVEDGKGCFELYHKCDDQCMETI
RNGTYNRRKYQEESKLERQRIEGVKLESEGTYKILTIYSTVASSLVIAMGFAAFLFWAMSNGSCRCNICI

>QBK20633.1 hemagglutinin [Influenza A virus]
METVSLITILLVATVSNADKICIGYQSTNSTETVDTLTENNVPVTHAKELLHTEHNGMLCATSLGQPLIL
DTCTIEGLIYGNPSCELSLEGREWSYIVERPSAVHGLCYPGNVEDLEELRSLFSSARSYQRIQIFPDTIW
NVSYDGTSTACSGSFYRSMRWLTRKNGEYPIQDAQYTNNQGKNILFMWGINHPPTDDTQRGLYTRTDTTT
SVATEEINRIFKPLIGPRPLVNGLMGRINYYWAVLKPGQTLRIKSDGNLIAPWYGHILSGESHGRILKTD
LKRGSCTVQCQTEKGGLNTTLPFQNVSKYAFGNCSKYIGIKSLKLAVGLRNVPTRSSRGLFGAIAGFIEG
GWSGLVAGWYGFQHSNDQGVGMAADRDSTQKAIDKITSKVNNIVDKMNKQYEIIDHEFSEVETRLNMINN
KIDDQIQDIWAYNAELLVLLENQKTLDEHDANVNNLYNKVKRALGSNAVEDGKGCFELYHKCDDQCMETI
RNGTYNRRKYQEESKLERQRIEGVKLESEGTYKILTIYSTVASSLVIAMGFAAFLFWAMSNGSCRCNICI

>QBK20632.1 hemagglutinin [Influenza A virus]
METVSLITILLVATVSNADKICIGYQSTNSTETVDTLTENNVPVTHAKELLHTEHNGMLCATSLGQPLIL
DTCTIEGLIYGNPSCDLSLEGREWSYIVERPSAVHGLCYPGNVEDLEELRSLFSSARSYQRIQIFPDTIW
NVSYDGTSTACSGSFYRSMRWLTRKNGEYPIQDAQYTNNQGKNILFMWGINHPPTDDTQRGLYTRTDTTT
SVATEEINRIFKPLIGPRPLVNGLMGRINYYWSVLKPGQTLRIKSDGNLIAPWYGHILSGESHGRILKTD
LKRGSCTVQCQTEKGGLNTTLPFQNVSKYAFGNCSKYIGIKSLKLAVGLRNVPTRSSRGLFGAIAGFIEG
GWSGLVAGWYGFQHSNDQGVGMAADRDSTQKAIDKITSKVNNIVDKMNKQYEIIDHEFSEVETRLNMINN
KIDDQIQDIWAYNAELLVLLENQKTLDEHDANVNNLYNKVKRALGSNAVEDGKGCFELYHKCDDQCMETI
RNGTYNRRKYQEESKLERQRIEGVKLESEGTYKILTIYSTVASSLVIAMGFAAFLFWAMSNGSCRCNICI

>QBK20631.1 hemagglutinin [Influenza A virus]
METVSLITILLVATVSNADKICIGYQSTNSTETVDTLTENNVPVTHAKELLHTEHNGMLCATSLGQPLIL
DTCTIEGLIYGNPSCDLSLEGREWSYIVERPSAVHGLCYPGNVEDLEELRSLFSSARSYQRIQIFPDTIW
NVSYDGTSTACSGSFYRSMRWLTRKNGEYPIQDAQYTNNQGKNILFMWGINHPPTDDTQRGLYTRTDTTT
SVATEEINRIFKPLIGPRPLVNGLMGRINYYWSVLKPGQTLRIKSDGNLIAPWYGHILSGESHGRILKTD
LKRGSCTVQCQTEKGGLNTTLPFQNVSKYAFGNCSKYIGIKSLKLAVGLRNVPTRSSRGLFGAIAGFIEG
GWSGLVAGWYGFQHSNDQGVGMAADRDSTQKAIDKITSKVNNIVDKMNKQYEIIDHEFSEVETRLNMINN
KIDDQIQDIWAYNAELLVLLENQKTLDEHDANVNNLYNKVKRALGSNAVEDGKGCFELYHKCDDQCMETI
RNGTYNRRKYQEESKLERQRIEGVKLESEGTYKILTIYSTVASSLVIAMGFAAFLFWAMSNGSCRCNICI

>QBK20630.1 hemagglutinin [Influenza A virus]
METVSLITILLVATVSNADKICIGYQSTNSTETVDTLTENNVPVTHAKELLHTEHNGMLCATSLGQPLIL
DTCTIEGLIYGNPSCDLSLEGREWSYIVERPSAVHGLCYPGNVEDLEELRSLFSSARSYQRIQIFPDTIW
NVSYDGTSTACSGSFYRSMRWLTRKNGEYPIQDAQYTNNQGKNILFMWGINHPPTDDTQRGLYTRTDTTT
SVATEEINRIFKPLIGPRPLVNGLMGRINYYWSVLKPGQTLRIKSDGNLIAPWYGHILSGESHGRILKTD
LKRGSCTVQCQTEKGGLNTTLPFQNVSKYAFGNCSKYIGIKSLKLAVGLRNVPTRSSRGLFGAIAGFIEG
GWSGLVAGWYGFQHSNDQGVGMAADRDSTQKAIDKITSKVNNIVDKMNKQYEIIDHEFSEVETRLNMINN
KIDDQIQDIWAYNAELLVLLENQKTLDEHDANVNNLYNKVKRALGSNAVEDGKGCFELYHKCDDQCMETI
RNGTYNRRKYQEESKLERQRIEGVKLESEGTYKILTIYSTVASSLVIAMGFAAFLFWAMSNGSCRCNICI

>QBK20629.1 hemagglutinin [Influenza A virus]
METVSLITILLVATVSNADKICIGYQSTNSTETVDTLTENNVPVTHAKELLHTEHNGMLCATSLGQPLIL
DTCTIEGLIYGNPSCDLSLEGREWSYIVERPSAVHGLCYPGNVEDLEELRSLFSSARSYQRIQIFPDTIW
NVSYDGTSTACSGSFYRSMRWLTRKNGEYPIQDAQYTNNQGKNILFMWGINHPPTDDTQRGLYTRTDTTT
SVATEEINRIFKPLIGPRPLVNGLMGRINYYWSVLKPGQTLRIKSDGNLIAPWYGHILSGESHGRILKTD
LKRGSCTVQCQTEKGGLNTTLPFQNVSKYAFGNCSKYIGIKSLKLAVGLRNVPTRSSRGLFGAIAGFIEG
GWSGLVAGWYGFQHSNDQGVGMAADRDSTQKAIDKITSKVNNIVDKMNKQYEIIDHEFSEVETRLNMINN
KIDDQIQDIWAYNAELLVLLENQKTLDEHDANVNNLYNKVKRALGSNAVEDGKGCFELYHKCDDQCMETI
RNGTYNRRKYQEESKLERQRIEGVKLESEGTYKILTIYSTVASSLVIAMGFAAFLFWAMSNGSCRCNICI

>QBK20628.1 hemagglutinin [Influenza A virus]
METVSLITILLVATVSNADKICIGYQSTNSTETVDTLTENNVPVTHAKELLHTEHNGMLCATSLGQPLIL
DTCTIEGLIYGNPSCDLSLEGREWSYIVERPSAVHGLCYPGNVEDLEELRSLFSSARSYQRIQIFPDTIW
NVSYDGTSTACSGSFYRSMRWLTRKNGEYPIQDAQYTNNQGKNILFMWGINHPPTDDTQRGLYTRTDTTT
SVATEEINRIFKPLIGPRPLVNGLMGRINYYWSVLKPGQTLRIKSDGNLIAPWYGHILSGESHGRILKTD
LKRGSCTVQCQTEKGGLNTTLPFQNVSKYAFGNCSKYIGIKSLKLAVGLRNVPTRSSRGLFGAIAGFIEG
GWSGLVAGWYGFQHSNDQGVGMAADRDSTQKAIDKITSKVNNIVDKMNKQYEIIDHEFSEVETRLNMINN
KIDDQIQDIWAYNAELLVLLENQKTLDEHDANVNNLYNKVKRALGSNAVEDGKGCFELYHKCDDQCMETI
RNGTYNRRKYQEESKLERQRIEGVKLESEGTYKILTIYSTVASSLVIAMGFAAFLFWAMSNGSCRCNICI

>QBK20627.1 hemagglutinin [Influenza A virus]
METVSLITILLVATGSNADKICIGYQSTNSTETVDTLTENNVPVTHAKELLHTEHNGMLCATSLGQPLIL
DTCTIEGLIYGNPSCDLSLEGREWSYIVERPSAVHGLCYPGNVEDLEELRSLFSSARSYQRIQIFPDTIW
NVSYDGTSTACSGSFYRSMRWLTRKNGEYPIQDAQYTNNQGKNILFMWGINHPPTDDTQRGLYTRTDTTT

SVATEEINRIFKPLIGPRPLVNGLMGRINYYWSVLKPGQTLRIKSDGNLIAPWYGHILSGESHGRILKTD
LKRGSCTVQCQTEKGGLNTTLPFQNVSKYAFGNCSKYIGIKSLKLAVGLRNVPTRSSRGLFGAIAGFIEG
GWSGLVAGWYGFQHSNDQGVGMAADRDSTQKAIDKITSKVNNIVDKMNKQYEIIDHEFSEVETRLNMINN
KIDDQIQDIWAYNAELLVLLENQKTLDEHDANVNNLYNKVKRALGSNAVEDGKGCFELYHKCDDQCMETI
RNGTYNRRKYQEESKLERQRIEGVKLESEGTYKILTIYSTVASSLVIAMGFAAFLFWAMSNGSCRCNICI

>QBK20626.1 hemagglutinin [Influenza A virus]
METVSLITILLVATVSNADKICIGYQSTNSTETVDTLTENNVPVTHAKELLHTEHNGMLCATSLGQPLIL
DTCTIEGLIYGNPSCDLSLEGREWSYIVERPSAVHGLCYPGNVEDLEELRSLFSSARSYQRIQIFPDTIW
NVSYDGTSTACSGSFYRSMRWLTRKNGEYPIQDAQYTNNQGKNILFMWGINHPPTDDTQRGLYTRTDTTT
SVATEEINRIFKPLIGPRPLVNGLMGRINYYWSVLKPGQTLRIKSDGNLIAPWYGHILSGESHGRILKTD
LKRGSCTVQCQTEKGGLNTTLPFQNVSKYAFGNCSKYIGIKSLKLAVGLRNVPTRSSRGLFGAIAGFIEG
GWSGLVAGWYGFQHSNDQGVGMAADRDSTQKAIDKITSKVNNIVDKMNKQYEIIDHEFSEVETRLNMINN
KIDDQIQDIWAYNAELLVLLENQKTLDEHDANVNNLYNKVKRALGSNAVEDGKGCFELYHKCDDQCMETI
RNGTYNRRKYQEESKLERQRIEGVKLESEGTYKILTIYSTVASSLVIAMGFAAFLFWAMSNGSCRCNICI

>QBK20625.1 hemagglutinin [Influenza A virus]
METVSLITILLVATVSNADKICIGYQSTNSTETVDTLTENNVPVTHAKELLHTEHNGMLCATSLGQPLIL
DTCTIEGLIYGNPSCDLSLEGREWSYIVERPSAVHGLCYPGNVEDLEELRSLFSSARSYQRIQIFPDTIW
NVSYDGTSTACSGSFYRSMRWLTRKNGEYPIQDAQYTNNQGKNILFMWGINHPPTDDTQRGLYTRTDTTT
SVATEEINRIFKPLIGPRPLVNGLMGRINYYWSVLKPGQTLRIKSDGNLIAPWYGHILSGESHGRILKTD
LKRGSCTVQCQTEKGGLNTTLPFQNVSKYAFGNCSKYIGIKSLKLAVGLRNVPTRSSRGLFGAIAGFIEG
GWSGLVAGWYGFQHSNDQGVGMAADRDSTQKAIDKITSKVNNIVDKMNKQYEIIDHEFSEVETRLNMINN
KIDDQIQDIWAYNAELLVLLENQKTLDEHDANVNNLYNKVKRALGSNAVEDGKGCFELYHKCDDQCMETI
RNGTYNRRKYQEESKLERQRIEGVKLESEGTYKILTIYSTVASSLVIAMGFAAFLFWAMSNGSCRCNICI

>QBK20624.1 hemagglutinin [Influenza A virus]
METVSLITILLVATVSNADKICIGYQSTNSTETVDTLTENNVPVTHAKELLHTEHNGMLCATSLGQPLIL
DTCTIEGLIYGNPSCDLSLEGREWSYIVERPSAVHGLCYPGNVEDLEELRSLFSSARSYQRIQIFPDTIW
NVSYDGTSTACSGSFYRSMRWLTRKNGEYPIQDAQYTNNQGKNILFMWGINHPPTDDTQRGLYTRTDTTT
SVATEEINRIFKPLIGPRPLVNGLMGRINYYWSVLKPGQTLRIKSDGNLIAPWYGHILSGESHGRILKTD
LKRGSCTVQCQTEKGGLNTTLPFQNVSKYAFGNCSKYIGIKSLKLAVGLRNVPTRSSRGLFGAIAGFIEG
GWSGLVAGWYGFQHSNDQGVGMAADRDSTQKAIDKITSKVNNIVDKMNKQYEIIDHEFSEVETRLNMINN
KIDDQIQDIWAYNAELLVLLENQKTLDEHDANVNNLYNKVKRALGSNAVEDGKGCFELYHKCDDQCMETI

RNGTYNRRKYQEESKLERQRIEGVKLESEGTYKILTIYSTVASSLVIAMGFAAFLFWAMSNGSCRCNICI

>QBK20623.1 hemagglutinin [Influenza A virus]
METVSLITILLVATVSNADKICIGYQSTNSTETVDTLTENNVPVTHAKELLHTEHNGMLCATSLGQPLIL
DTCTIEGLIYGNPSCDLSLEGREWSYIVERPSAVHGLCYPGNVEDLEELRSLFSSARSYQRIQIFPDTIW
NVSYDGTSTACSGSFYRSMRWLTRKNGEYPIQDAQYTNNQGKNILFMWGINHPPTDDTQRGLYTRTDTTT
SVATEEINRIFKPLIGPRPLVNGLMGRINYYWSVLKPGQTLRIKSDGNLIAPWYGHILSGESHGRILKTD
LKRGSCTVQCQTEKGGLNTTLPFQNVSKYAFGNCSKYIGIKSLKLAVGLRNVPTRSSRGLFGAIAGFIEG
GWSGLVAGWYGFQHSNDQGVGMAADRDSTQKAIDKITSKVNNIVDKMNKQYEIIDHEFSEVETRLNMINN
KIDDQIQDIWAYNAELLVLLENQKTLDEHDANVNNLYNKVKRALGSNAVEDGKGCFELYHKCDDQCMETI
RNGTYNRRKYQEESKLERQRIEGVKLESEGTYKILTIYSTVASSLVIAMGFAAFLFWAMSNGSCRCNICI

>QBK20622.1 hemagglutinin [Influenza A virus]
METVSLITILLVATVSNADKICIGYQSTNSTETVDTLTENNVPVTHAKELLHTEHNGMLCATSLGQPLIL
DTCTIEGLIYGNPSCDLSLEGREWSYIVERPSAVHGLCYPGNVEDLEELRSLFSSARSYQRIQIFPDTIW
NVSYDGTSTACSGSFYRSMRWLTRKNGEYPIQDAQYTNNQGKNILFMWGINHPPTDDTQRGLYTRTDTTT
SVATEEINRIFKPLIGPRPLVNGLMGRINYYWSVLKPGQTLRIKSDGNLIAPWYGHILSGESHGRILKTD
LKRGSCTVQCQTEKGGLNTTLPFQNVSKYAFGNCSKYIGIKSLKLAVGLRNVPTRSSRGLFGAIAGFIEG
GWSGLVAGWYGFQHSNDQGVGMAADRDSTQKAIDKITSKVNNIVDKMNKQYEIIDHEFSEVETRLNMINN
KIDDQIQDIWAYNAELLVLLENQKTLDEHDANVNNLYNKVKRALGSNAVEDGKGCFELYHKCDDQCMETI
RNGTYNRRKYQEESKLERQRIEGVKLESEGTYKILTIYSTVASSLVIAMGFAAFLFWAMSNGSCRCNICI

>QBK20621.1 hemagglutinin [Influenza A virus]
METVSLITILLVATVSNADKICIGYQSTNSTETVDTLTENNVPVTHAKELLHTEHNGMLCATSLGQPLIL
DTCTIEGLIYGNPSCDLSLEGREWSYIVERPSAVHGLCYPGNVEDLEELRSLFSSARSYQRIQIFPDTIW
NVSYDGTSTACSGSFYRSMRWLTRKNGEYPIQDAQYTNNQGKNILFMWGINHPPTDDTQRGLYTRTDTTT
SVATEEINRIFKPLIGPRPLVNGLMGRINYYWSVLKPGQTLRIKSDGNLIAPWYGHILSGESHGRILKTD
LKRGSCTVQCQTEKGGLNTTLPFQNVSKYAFGNCSKYIGIKSLKLAVGLRNVPTRSSRGLFGAIAGFIEG
GWSGLVAGWYGFQHSNDQGVGMAADRDSTQKAIDKITSKVNNIVDKMNKQYEIIDHEFSEVETRLNMINN
KIDDQIQDIWAYNAELLVLLENQKTLDEHDANVNNLYNKVKRALGSNAVEDGKGCFELYHKCDDQCMETI
RNGTYNRRKYQEESKLERQRIEGVKLESEGTYKILTIYSTVASSLVIAMGFAAFLFWAMSNGSCRCNICI

>QBK20620.1 hemagglutinin [Influenza A virus]
METVSLITILLVATVSNADKICIGYQSTNSTETVDTLTENNVPVTHAKELLHTEHNGMLCATSLGQPLIL

DTCTIEGLIYGNPSCDLSLEGREWSYIVERPSAVHGLCYPGNVEDLEELRSLFSSARSYQRIQIFPDTIW
NVSYDGTSTACSGSFYRSMRWLTRKNGEYPIQDAQYTNNQGKNILFMWGINHPPTDDTQRGLYTRTDTTT
SVATEEINRIFKPLIGPRPLVNGLMGRINYYWSVLKPGQTLRIKSDGNLIAPWYGHILSGESHGRILKTD
LKRGSCTVQCQTEKGGLNTTLPFQNVSKYAFGNCSKYIGIKSLKLAVGLRNVPTRSSRGLFGAIAGFIEG
GWSGLVAGWYGFQHSNDQGVGMAADRDSTQKAIDKITSKVNNIVDKMNKQYEIIDHEFSEVETRLNMINN
KIDDQIQDIWAYNAELLVLLENQKTLDEHDANVNNLYNKVKRALGSNAVEDGKGCFELYHKCDDQCMETI
RNGTYNRRKYQEESKLERQRIEGVKLESEGTYKILTIYSTVASSLVIAMGFAAFLFWAMSNGSCRCNICI

>QBK20619.1 hemagglutinin [Influenza A virus]
METVSLITILLVATVSNADKICIGYQSTNSTETVDTLTENNVPVTHAKELLHTEHNGMLCATSLGQPLIL
DTCTIEGLIYGNPSCDLSLEGREWSYIVERPSAVHGLCYPGNVEDLEELRSLFSSARSYQRIQIFPDTIW
NVSYDGTSTACSGSFYRSMRWLTRKNGEYPIQDAQYTNNQGKNILFMWGINHPPTDDTQRGLYTRTDTTT
SVATEEINRIFKPLIGPRPLVNGLMGRINYYWSVLKPGQTLRIKSDGNLIAPWYGHILSGESHGRILKTD
LKRGSCTVQCQTEKGGLNTTLPFQNVSKYAFGNCSKYIGIKSLKLAVGLRNVPTRSSRGLFGAIAGFIEG
GWSGLVAGWYGFQHSNDQGVGMAADRDSTQKAIDKITSKVNNIVDKMNKQYEIIDHEFSEVETRLNMINN
KIDDQIQDIWAYNAELLVLLENQKTLDEHDANVNNLYNKVKRALGSNAVEDGKGCFELYHKCDDQCMETI
RNGTYNRRKYQEESKLERQRIEGVKLESEGTYKILTIYSTVASSLVIAMGFAAFLFWAMSNGSCRCNICI

>QBK20618.1 hemagglutinin [Influenza A virus]
METVSLITILLVATVSNADKICIGYQSTNSTETVDTLTENNVPVTHAKELLHTEHNGMLCATSLGQPLIL
DTCTIEGLIYGNPSCDLSLEGREWSYIVERPSAVHGLCYPGNVEDLEELRSLFSSARSYQRIQIFPDTIW
NVSYDGTSTACSGSFYRSMRWLTRKNGEYPIQDAQYTNNQGKNILFMWGINHPPTDDTQRGLYTRTDTTT
SVATEEINRIFKPLIGPRPLVNGLMGRINYYWSVLKPGQTLRIKSDGNLIAPWYGHILSGESHGRILKTD
LKRGSCTVQCQTEKGGLNTTLPFQNVSKYAFGNCSKYIGIKSLKLAVGLRNVPTRSSRGLFGAIAGFIEG
GWSGLVAGWYGFQHSNDQGVGMAADRDSTQKAIDKITSKVNNIVDKMNKQYEIIDHEFSEVETRLNMINN
KIDDQIQDIWAYNAELLVLLENQKTLDEHDANVNNLYNKVKRALGSNAVEDGKGCFELYHKCDDQCMETI
RNGTYNRRKYQEESKLERQRIEGVKLESEGTYKILTIYSTVASSLVIAMGFAAFLFWAMSNGSCRCNICI

>QBK20617.1 hemagglutinin [Influenza A virus]
METVSLITILLVATGSNADKICIGYQSTNSTEAVDTLTENNVPVTHARELLRTEQNGMLCATSLGQPLIL
DTCTIEGLIYGNPSCDLSLEGREWSYIVERPSAVHGLCYPGNVEDLEELRSLFSSARSYQRIQIFPDTIW
NVSYDGTSTACSGSFYRSMRWLTRKNGEYPIQDAQYTNNQGKNILFMWGINHPPTDDTQRGLYTRTDTTT
SVATEEINRIFKPLIGPRPLVNGLMGRINYYWSVLKPGQTLRIKSDGNLIAPWYGHILSGESHGRILKTD
LKRGSCTVQCQTEKGGLNTTLPFQNVSKYAFGNCSKYIGIKSLKLAVGLRNVPTRSSRGLFGAIAGFIEG

GWSGLVAGWYGFQHSNDQGVGMAADRDSTQKAIDKITSKVNNIVDKMNKQYEIIDHEFSEVETRLNMINN
KIDDQIQDIWAYNAELLVLLENQKTLDEHDANVNNLYNKVKRALGSNAVEDGKGCFELYHKCDDQCMETI
RNGTYNRRKYQEESKLERQRIEGVKLESEGTYKILTIYSTVASSLVIAMGFAAFLFWAMSNGSCRCNICI

>QBK20616.1 hemagglutinin [Influenza A virus]
METVSLITILLVAAVSNADKICIGYQSTNSTETVDTLTENNVPVTHAKELLHTAHNGMLCATSLGQPLIL
DTCTIEGLIYGNPSCDLSLEGREWSYIVERPSAVHGLCYPGNVEDLEELRSLFSSARSYQRIQIFPDTIW
NVSYDGTSTACSGSFYRSMRWLTRKNGEYPIQDAQYTNNQGKNILFMWGINHPPTDDTQRGLYTRTDTTT
SVATEEINRIFKPLIGPRPLVNGLMGRINYYWSVLKPGQTLRIKSDGNLIAPWYGHILSGESHGRILKTD
LKRGSCTVQCQTEKGGLNTTLPFQNVSKYAFGNCSKYIGIKSLKLAVGLRNVPSRSSRGLFGAIAGFIEG
GWSGLVAGWYGFQHSNDQGVGMAADRDSTQKAIDKITSKVNNIVDKMNKQYEIIDHEFSEVETRLNMINN
KIDDQIQDIWAYNAELLVLLENQKTLDEHDANVNNLYNKVKRALGSNAVEDGKGCFELYHKCDDQCMETI
RNGTYNRRKYQEESKLERQRIEGVKLESEGTYKILTIYSTVASSLVIAMGFAAFLFWAMSNGSCRCNICI

>QBK20615.1 hemagglutinin [Influenza A virus]
METVSLITILLVATVSNADKICIGYQSTNSTETVDTLTENNVPVTHAKELLHTEHNGMLCATSLGQPLIL
DTCTIEGLIYGNPSCDLSLEGREWSYIVERPSAVHGLCYPGNVEDLEELRSLFSSARSYQRIQIFPDTIW
NVSYDGTSTACSGSFYRSMRWLTRKNGEYPIQDAQYTNNQGKNILFMWGINHPPTDDTQRGLYTRTDTTT
SVATEEINRIFKPLIGPRPLVNGLMGRINYYWSVLKPGQTLRIKSDGNLIAPWYGHILSGESHGRILKTD
LKRGSCTVQCQTEKGGLNTTLPFQNVSKYAFGNCSKYIGIKSLKLAVGLRNVPTRSSRGLFGAIAGFIEG
GWSGLVAGWYGFQHSNDQGVGMAADRDSTQKAIDKITSKVNNIVDKMNKQYEIIDHEFSEVETRLNMINN
KIDDQIQDIWAYNAELLVLLENQKTLDEHDANVNNLYNKVKRALGSNAVEDGKGCFELYHKCDDQCMETI
RNGTYNRRKYQEESKLERQRIEGVKLESEGTYKILTIYSTVASSLVIAMGFAAFLFWAMSNGSCRCNICI

>QBK20614.1 hemagglutinin [Influenza A virus]
METVSLITILLVATVSNADKICIGYQSTNSTETVDTLTENNVPVTHAKELLHTEHNGMLCATSLGQPLIL
DTCTIEGLIYGNPSCDLSLEGREWSYIVERPSAVHGLCYPGNVEDLEELRSLFSSARSYQRIQIFPDTIW
NVSYDGTSTACSGSFYRSMRWLTRKNGEYPIQDAQYTNNQGKNILFMWGINHPPTDDTQRGLYTRTDTTT
SVATEEINRIFKPLIGPRPLVNGLMGRINYYWSVLKPGQTLRIKSDGNLIAPWYGHILSGESHGRILKTD
LKRGSCTVQCQTEKGGLNTTLPFQNVSKYAFGNCSKYIGIKSLKLAVGLRNVPTRSSRGLFGAIAGFIEG
GWSGLVAGWYGFQHSNDQGVGMAADRDSTQKAIDKITSKVNNIVDKMNKQYEIIDHEFSEVETRLNMINN
KIDDQIQDIWAYNAELLVLLENQKTLDEHDANVNNLYNKVKRALGSNAVEDGKGCFELYHKCDDQCMETI
RNGTYNRRKYQEESKLERQRIEGVKLESEGTYKILTIYSTVASSLVIAMGFAAFLFWAMSNGSCRCNICI

>QBK20613.1 hemagglutinin [Influenza A virus]
METVSLITILLVAAVSNADKICIGYQSTNSTETVDTLTENNVPVTHAKELLHTAHNGMLCATSLGQPLIL
DTCTIEGLIYGNPSCDLSLEGREWSYIVERPSAVHGLCYPGNVEDLEELRSLFSSARSYQRIQIFPDTIW
NVSYDGTSTACSGSFYRSMRWLTRKNGEYPIQDAQYTNNQGKNILFMWGINHPPTDDTQRGLYTRTDTTT
SVATEEINRIFKPLIGPRPLVNGLMGRINYYWSVLKPGQTLRIKSDGNLIAPWYGHILSGESHGRILKTD
LKRGSCTVQCQTEKGGLNTTLPFQNVSKYAFGNCSKYIGIKSLKLAVGLRNVPSRSSRGLFGAIAGFIEG
GWSGLVAGWYGFQHSNDQGVGMAADRDSTQKAIDKITSKVNNIVDKMNKQYEIIDHEFSEVETRLNMINN
KIDDQIQDIWAYNAELLVLLENQKTLDEHDANVNNLYNKVKRALGSNAVEDGKGCFELYHKCDDQCMETI
RNGTYNRRKYQEESKLERQRIEGVKLESEGTYKILTIYSTVASSLVIAMGFAAFLFWAMSNGSCRCNICI

>QBK20612.1 hemagglutinin [Influenza A virus]
METVSLITILLVAAVSNADKICIGYQSTNSTETVDTLTENNVPVTHAKELLHTAHNGMLCATSLGQPLIL
DTCTIEGLIYGNPSCDLSLEGREWSYIVERPSAVHGLCYPGNVEDLEELRSLFSSARSYQRIQIFPDTIW
NVSYDGTSIACSGSFYRSMRWLTRKNGEYPIQDAQYTNNQGKNILFMWGINHPPTDDTQRGLYTRTDTTT
SVATEEINRIFKPLIGPRPLVNGLMGRINYYWSVLKPGQTLRIKSDGNLIAPWYGHILSGESHGRILKTD
LKRGSCTVQCQTEKGGLNTTLPFQNVSKYAFGNCSKYIGIKSLKLAVGLRNVPSRSSRGLFGAIAGFIEG
GWSGLVAGWYGFQHSNDQGVGMAADRDSTQKAIDKITSKVNNIVDKMNKQYEIIDHEFSEVETRLNMINN
KIDDQIQDIWAYNAELLVLLENQKTLDEHDANVNNLYNKVKRALGSNAVEDGKGCFELYHKCDDQCMETI
RNGTYNRRKYQEESKLERQRIEGVKLESEGTYKILTIYSTVASSLVIAMGFAAFLFWAMSNGSCRCNICI

>QBK20611.1 hemagglutinin [Influenza A virus]
METVSLITILLVATVSNADKICIGYQSTNSTETVDTLTENNVPVTHAKELLHTEHNGMLCATSLGQPLIL
DTCTIEGLIYGNPSCDLSLEGREWSYIVERPSAVHGLCYPGNVEDLEELRSLFSSARSYQRIQIFPDTIW
NVSYDGTSTACSGSFYRSMRWLTRKNGEYPIQDAQYTNNQGKNILFMWGINHPPTDDTQRGLYTRTDTTT
SVATEEINRIFKPLIGPRPLVNGLMGRINYYWSVLKPGQTLRIKSDGNLIAPWYGHILSGESHGRILKTD
LKRGSCTVQCQTEKGGLNTTLPFQNVSKYAFGNCSKYIGIKSLKLAVGLRNVPTRSSRGLFGAIAGFIEG
GWSGLVAGWYGFQHSNDQGVGMAADRDSTQKAIDKITSKVNNIVDKMNKQYEIIDHEFSEVETRLNMINN
KIDDQIQDIWAYNAELLVLLENQKTLDEHDANVNNLYNKVKRALGSNAVEDGKGCFELYHKCDDQCMETI
RNGTYNRRKYQEESKLERQRIEGVKLESEGTYKILTIYSTVASSLVIAMGFAAFLFWAMSNGSCRCNICI

>QBK20610.1 hemagglutinin [Influenza A virus]
METVSLITILLVATVSNADKICIGYQSTNSTETVDTLTENNVPVTHAKELLHTEHNGMLCATSLGQPLIL
DTCTIEGLIYGNPSCDLSLEGREWSYIVERPSAVHGLCYPGNVEDLEELRSLFSSARSYQRIQIFPDTIW
NVSYDGTSTACSGSFYRSMRWLTRKNGEYPIQDAQYTNNQGKNILFMWGINHPPTDDTQRGLYTRTDTTT

SVATEEINRIFKPLIGPRPLVNGLMGRINYYWSVLKPGQTLRIKSDGNLIAPWYGHILSGESHGRILKTD
LKRGSCTVQCQTEKGGLNTTLPFQNVSKYAFGNCSKYIGIKSLKLAVGLRNVPTRSSRGLFGAIAGFIEG
GWSGLVAGWYGFQHSNDQGVGMAADRDSTQKAIDKITSKVNNIIDKMNKQYEIIDHEFSEVETRLNMINN
KIDDQIQDIWAYNAELLVLLENQKTLDEHDANVNNLYNKVKRALGSNAVEDGKGCFELYHKCDDQCMETI
RNGTYNRRKYQEESKLERQRIEGVKLESEGTYKILTIYSTVASSLVIAMGFAAFLFWAMSNGSCRCNICI

>QBK20609.1 hemagglutinin [Influenza A virus]
METVSLITILLVATVSNADKICIGYQSTNSTETVDTLTENNVPVTHAKELLHTEHNGMLCATSLGQPLIL
DTCTIEGLIYGNPSCDLSLEGREWSYIVERPSAVHGLCYPGNVEDLEELRSLFSSARSYQRIQIFPDTIW
NVSYDGTSTACSGSFYRSMRWLTRKNGEYPIQDAQYTNNQGKNILFMWGINHPPTDDTQRGLYTRTDTTT
SVATEEINRIFKPLIGPRPLVNGLMGRINYYWSVLKPGQTLRIKSDGNLIAPWYGHILSGESHGRILKTD
LKRGSCTVQCQTEKGGLNTTLPFQNISKYAFGNCSKYIGIKSLKLAVGLRNVPSRSSRGLFGAIAGFIEG
GWPGLVAGWYGFQHSNDQGVGMAADRDSTQKAIDKITSKVNNIVDKMNKQYEIIDHEFSEVETRLNMINN
KIDDQIQDIWAYNAELLVLLENQKTLDEHDANVNNLYSKVKRALGSNAVEDGKGCFELYHKCDDQCMETI
RNGTYNRRKYQEESKLERQRIEGVKLESEGTYKILTIYSTVASSLVIAMGFAAFLFWAMSNGSCRCNICI

>QBK20608.1 hemagglutinin [Influenza A virus]
METVSLITILLVAAVSNADKICIGYQSTNSTETVDTLTENNVPVTHAKELLHTAHNGMLCATSLGQPLIL
DTCTIEGLIYGNPSCDLSLEGREWSYIVERPSAVHGLCYPGNVEDLEELRSLFSSARSYQRIQIFPDTIW
NVSYDGTSTACSGSFYRSMRWLTRKNGEYPIQDAQYTNNQGKNILFMWGINHPPTDDTQRGLYTRTDTTT
SVATEEINRIFKPLIGPRPLVNGLMGRINYYWSVLKPGQTLRIKSDGNLIAPWYGHILSGESHGRILKTD
LKRGSCTVQCQTEKGGLNTTLPFQNVSKYAFGNCSKYIGIKSLKLAVGLRNVPSRSSRGLFGAIAGFIEG
GWSGLVAGWYGFQHSNDQGVGMAADRDSTQKAIDKITSKVNNIVDKMNKQYEIIDHEFSEVETRLNMINN
KIDDQIQDIWAYNAELLVLLENQKTLDEHDANVNNLYNKVKRALGSNAVEDGKGCFELYHKCDDQCMETI
RNGTYNRRKYQEESKLERQRIEGVKLESEGTYKILTIYSTVASSLVIAMGFAAFLFWAMSNGSCRCNICI

>QBK20607.1 hemagglutinin [Influenza A virus]
METVSLITILLVATVSNADKICIGYQSTNSTETVDTLTENNVPVTHAKELLHTAHNGMLCATSLGQPLIL
DTCTIEGLIYGNPSCDLSLEGREWSYIVERPSAVHGLCYPGNVEDLEELRSLFSSARSYQRIQIFPDTIW
NVSYDGTSTACSGSFYRSMRWLTRKNGEYPIQDAQYTNNQGKNILFMWGINHPPTDDTQRGLYTRTDTTT
SVATEEINRIFKPLIGPRPLVNGLMGRINYYWSVLKPGQTLRIKSDGNLIAPWYGHILSGESHGRILKTD
LKRGSCTVQCQTEKGGLNTTLPFQNVSKYAFGNCSKYIGIKSLKLAVGLRNVPSRSSRGLFGAIAGFIEG
GWSGLVAGWYGFQHSNDQGVGMAADRDSTQKAIDKITSKVNNIVDKMNKQYEIIDHEFSEVETRLNMINN
KIDDQIQDIWAYNAELLVLLENQKTLDEHDANVNNLYNKVKRALGSNAVEDGKGCFELYHKCDDQCMETI

RNGTYNRRKYQEESKLERQRIEGVKLESEGTYKILTIYSTVASSLVIAMGFAAFLFWAMSNGSCRCNICI

>QBK20606.1 hemagglutinin [Influenza A virus]
METASLITILLVATVSNADKICIGYQSTNSTETVDTLTENNVPVTHAKELLHTEHNGMLCATSLGHPLIL
DTCTIEGLIYGNPSCDPLLGGREWSYIVERPSAVNGLCYPGNVENLEELRSLFSSSRSYQRIQIFPDTIW
NVSYSGTSKACSDSFYRSMRWLTQKNNAYPTQDAQYTNNQEKNILFMWGINHPPTDTAQTNLYTRTDTTT
SVATEEMNRIFKPLIGPRPLVNGLMGRINYYWSVLKPGQTLRIKSDGNLIAPWYGHILSGESHGRILKTD
LKRGSCTVQCQTEKGGLNTTLPFQNVSKYAFGNCSKYIGVKSLKLAVGLRNVPSKSSRGLFGAIAGFIEG
GWSGLVAGWYGFQHSNDQGVGMAADRDSTQKAIDKITSKVNNIVDKMNKQYEIIDHEFSEVETRLNMINN
KVDDQIQDIWAYNAELLVLLENQKTLDEHDANVNNLYNKVKRALGSNAVEDGKGCFELYHKCDDHCMETI
RNGTYNRRKYQEESKLERQKIEGVKLESEETYKILTIYSTVASSLVIAMGFAAFLFWAMSNGSCRCNICI

>QBK20605.1 hemagglutinin [Influenza A virus]
METVSLITILLVATVSNADKICIGYQSTNSTETVDTLTENNVPVTHAKELLHTEHNGMLCATSLGQPLIL
DTCTIEGLIYGNPSCDLSLEGREWSYIVERPSAVHGLCYPGNVEDLEELRSLFSSARSYQRIQIFPDTIW
NVSYDGTSTACSGSFYRSMRWLTRKNGEYPIQDAQYTNNQGKNILFMWGINHPPTDDTQRGLYTRTDTTT
SVATEEINRIFKPLIGPRPLVNGLMGRINYYWSVLKPGQTLRIKSDGNLIAPWYGHILSGESHGRILKTD
LKRGSCTVQCQTEKGGLNTTLPFQNVSKYAFGNCSKYIGIKSLKLAVGLRNVPTRSSRGLFGAIAGFIEG
GWSGLVAGWYGFQHSNDQGVGMAADRDSTQKAIDKITSKVNNIVDKMNKQYEIIDHEFSEVETRLNMINN
KIDDQIQDIWAYNAELLVLLENQKTLDEHDANVNNLYNKVKRALGSNAVEDGKGCFELYHKCDDQCMETI
RNGTYNRRKYQEESKLERQRIEGVKLESEGTYKILTIYSTVASSLVIAMGFAAFLFWAMSNGSCRCNICI

>QBK20604.1 hemagglutinin [Influenza A virus]
METVSLITILLVATVSNADKICIGYQSTNSTETVDTLTENNVPVTHAKELLHTEHNGMLCATSLGQPLIL
DTCTIEGLIYGNPSCDLSLEGREWSYIVERPSAVHGLCYPGNVEDLEELRSLFSSARSYQRIQIFPDTIW
NVSYDGTSTACSGSFYRSMRWLTRKNGEYPIQDAQYTNNQGKNILFMWGINHPPTDDTQRGLYTRTDTTT
SVATEEINRIFKPLIGPRPLVNGLMGRINYYWSVLKPGQTLRIKSDGNLIAPWYGHILSGESHGRILKTD
LKRGSCTVQCQTEKGGLNTTLPFQNISKYAFGNCSKYIGIKSLKLAVGLRNVPSRSSRGLFGAIAGFIEG
GWPGLVAGWYGFQHSNDQGVGMAADRDSTQKAIDKITSKVNNIVDKMNKQYEIIDHEFSEVETRLNMINN
KIDDQIQDIWAYNAELLVLLENQKTLDEHDANVNNLYSKVKRALGSNAVEDGKGCFELYHKCDDQCMETI
RNGTYNRRKYQEESKLERQRIEGVKLESEGTYKILTIYSTVASSLVIAMGFAAFLFWAMSNGSCRCNICI

>QBK20603.1 hemagglutinin [Influenza A virus]
METVSLITILLVAAVSNADKICIGYQSTNSTETVDTLTENNVPVTHAKELLHTAHNGMLCATSLGQPLIL

DTCTIEGLIYGNPSCDLSLEGREWSYIVERPSAVHGLCYPGNVEDLEELRSLFSSARSYQRIQIFPDTIW
NVSYDGTSTACSGSFYRSMRWLTRKNGEYPIQDAQYTNNQGKNILFMWGINHPPTDDTQRGLYTRTDTTT
SVATEEINRIFKPLIGPRPLVNGLMGRINYYWSVLKPGQTLRIKSDGNLIAPWYGHILSGESHGRILKTD
LKRGSCTVQCQTEKGGLNTTLPFQNVSKYAFGNCSKYIGIKSLKLAVGLRNVPSRSSRGLFGAIAGFIEG
GWSGLVAGWYGFQHSNDQGVGMAADRDSTQKAIDKITSKVNNIVDKMNKQYEIIDHEFSEVETRLNMINN
KIDDQIQDIWAYNAELLVLLENQKTLDEHDANVNNLYNKVKRALGSNAVEDGKGCFELYHKCDDQCMETI
RNGTYNRRKYQEESKLERQRIEGVKLESEGTYKILTIYSTVASSLVIAMGFAAFLFWAMSNGSCRCNICI

>QBK20602.1 hemagglutinin [Influenza A virus]
METVSLITILLVATVSNADKICIGYQSTNSTETVDTLTENNVPVTHAKELLHTAHNGMLCATSLGQPLIL
DTCTIEGLIYGNPSCDLSLEGREWSYIVERPSAVHGLCYPGNVEDLEELRSLFSSARSYQRIQIFPDTIW
NVSYDGTSTACSGSFYRSMRWLTRKNGEYPIQDAQYTNNQGKNILFMWGINHPPTDDTQRGLYTRTDTTT
SVATEEINRIFKPLIGPRPLVNGLMGRINYYWSVLKPGQTLRIKSDGNLIAPWYGHILSGESHGRILKTD
LKRGSCTVQCQTEKGGLNTTLPFQNVSKYAFGNCSKYIGIKSLKLAVGLRNVPSRSSRGLFGAIAGFIEG
GWSGLVAGWYGFQHSNDQGVGMAADRDSTQKAIDKITSKVNNIVDKMNKQYEIIDHEFSEVETRLNMINN
KIDDQIQDIWAYNAELLVLLENQKTLDEHDANVNNLYNKVKRALGSNAVEDGKGCFELYHKCDDQCMETI
RNGTYNRRKYQEESKLERQRIEGVKLESEGTYKILTIYSTVASSLVIAMGFAAFLFWAMSNGSCRCNICI

>QBK20601.1 hemagglutinin [Influenza A virus]
METVSLITILLVATVSNADKICIGYQSTNSTETVDTLTENNVPVTHAKELLHTEHNGMLCATSLGQPLIL
DTCTIEGLIYGNPSCDLSLEGREWSYIVERPSAVHGLCYPGNVEDLEELRSLFSSARSYQRIQIFPDTIW
NVSYDGTSTACSGSFYRSMRWLTRKNGEYPIQDAQYTNNQGKNILFMWGINHPPTDDTQRGLYTRTDTTT
SVATEEIIRIFIPLIGPRPLVNGLMGRINYYWSVLKPGQTLRIKSDGNLIAPWYGHILSGESHGRILKTD
LKRGSCTVQCQTEKGGLNTTLPFQNISKYAFGNCSKYIGIKSLKLAVGLRNVPSRSSRGLFGAIAGFIEG
GWPGLVAGWYGFQHSNDQGVGMAADRDSTQKAIDKITSKVNNIVDKMNKQYEIIDHEFSEVETRLNMINN
KIDDQIQDIWAYNAELLVLLENQKTLDEHDANVNNLYSKVKRALGSNAVEDGKGCFELYHKCDDQCMETI
RNGTYNRRKYQEESKLERQRIEGVKLESEGTYKILTIYSTVASSLVIAMGFAAFLFWAMSNGSCRCNICI

>QBK20600.1 hemagglutinin [Influenza A virus]
METVSLITILLVATVSNADKICIGYQSTNSTETVDTLTENNVPVTHAKELLHTAHNGMLCATSLGQPLIL
DTCTIEGLIYGNPSCDLSLEGREWSYIVERPSAVHGLCYPGNVEDLEELRSLFSSARSYQRIQIFPDTIW
NVSYDGTSTACSGSFYRSMRWLTRKNGEYPIQDAQYTNNQGKNILFMWGINHPPTDDTQRGLYTRTDTTT
SVATEEINRIFKPLIGPRPLVNGLMGRINYYWSVLKPGQTLRIKSDGNLIAPWYGHILSGESHGRILKTD
LKRGSCTVQCQTEKGGLNTTLPFQNVSKYAFGNCSKYIGIKSLKLAVGLRNVPSRSSRGLFGAIAGFIEG

GWSGLVAGWYGFQHSNDQGVGMAADRDSTQKAIDKITSKVNNIVDKMNKQYEIIDHEFSEVETRLNMINN
KIDDQIQDIWAYNAELLVLLENQKTLDEHDANVNNLYNKVKRALGSNAVEDGKGCFELYHKCDDQCMETI
RNGTYNRRKYQEESKLERQRIEGVKLESEGTYKILTIYSTVASSLVIAMGFAAFLFWAMSNGSCRCNICI

>QBK20598.1 hemagglutinin [Influenza A virus]
METVSLITILLVATVSNADKICIGYQSTNSTETVDTLTENNVPVTHAKELLHTEHNGMLCATSLGQPLIL
DTCTIEGLIYGNPSCDLSLEGREWSYIVERPSAVHGLCYPGNVEDLEELRSLFSSARSYQRIQIFPDTIW
NVSYDGTSTACSGSFYRSMRWLTRKNGEYPIQDAQYTNNQGKNILFMWGINHPPTDDTQRGLYTRTDTTT
SVATEEINRIFKPLIGPRPLVNGLMGRINYYWSVLKPGQTLRIKSDGNLIAPWYGHILSGESHGRILKTD
LKRGSCTVQCQTEKGGLNTTLPFQNISKYAFGNCSKYIGIKSLKLAVGLRNVPSRSSRGLFGAIAGFIEG
GWPGLVAGWYGFQHSNDQGVGMAADRDSTQKAIDKITSKVNNIVDKMNKQYEIIDHEFSEVETRLNMINN
KIDDQIQDIWAYNAELLVLLENQKTLDEHDANVNNLYSKVKRALGSNAVEDGKGCFELYHKCDDQCMETI
RNGTYNRRKYQEESKLERQRIEGVKLESEGTYKILTIYSTVASSLVIAMGFAAFLFWAMSNGSCRCNICI

>QBK20596.1 hemagglutinin [Influenza A virus]
METVSLIAILLVATVSNADKICIGYQSTNSTETVDTLTENNVPVTHAKELLHTAHNGMLCATSLGQPLIL
DTCTIEGLIYGNPSCDLSLEGREWSYIVERPSAVHGLCYPGNVEDLEELRSLFSSARSYQRIQIFPDTIW
NVSYDGTSTACSGSFYRSMRWLTRKNGEYPIQDAQYTNNQGKNILFMWGINHPPTDDTQRGLYTRTDTTT
SVATEEINRIFKPLIGPRPLVNGLMGRINYYWSVLKPGQTLRIKSDGNLIAPWYGHILSGESHGRILKTD
LKRGSCTVQCQTEKGGLNTTLPFQNVSKYAFGNCSKYIGIKSLKLAVGLRNVPSRSSRGLFGAIAGFIEG
GWSGLVAGWYGFQHSNDQGVGMAADRDSTQKAIDKITSKVNNIVDKMNKQYEIIDHEFSEVETRLNMINN
KIDDQIQDIWAYNAELLVLLENQKTLDEHDANVNNLYNKVKRALGSNAVEDGKGCFELYHKCDDQCMETI
RNGTYNRRKYQEESKLERQRIEGVKLESEGTYKILTIYSTVASSLVIAMGFAAFLFWAMSNGSCRCNICI

>QBK20595.1 hemagglutinin [Influenza A virus]
METVSLIAILLVATVSNADKICIGYQSTNSTETVDTLTENNVPVTHAKELLHTAHNGMLCATSLGQPLIL
DTCTIEGLIYGNPSCDLSLEGREWSYIVERPSAVHGLCYPGNVEDLEELRSLFSSARSYQRIQIFPDTIW
NVSYDGTSTACSGSFYRSMRWLTRKNGEYPIQDAQYTNNQGKNILFMWGINHPPTDDTQRGLYTRTDTTT
SVATEEINRIFKPLIGPRPLVNGLMGRINYYWSVLKPGQTLRIKSDGNLIAPWYGHILSGESHGRILKTD
LKRGSCTVQCQTEKGGLNTTLPFQNVSKYAFGNCSKYIGIKSLKLAVGLRNVPSRSSRGLFGAIAGFIEG
GWSGLVAGWYGFQHSNDQGVGMAADRDSTQKAIDKITSKVNNIVDKMNKQYEIIDHEFSEVETRLNMINN
KIDDQIQDIWAYNAELLVLLENQKTLDEHDANVNNLYNKVKRALGSNAVEDGKGCFELYHKCDDQCMETI
RNGTYNRRKYQEESKLERQRIEGVKLESEGTYKILTIYSTVASSLVIAMGFAAFLFWAMSNGSCRCNICI

>QBK20594.1 hemagglutinin [Influenza A virus]

METASLITILLVATVSNADKICIGYQSTNSTETVDTLTENNVPVTHAKELLHTEHNGMLCATSLGHPLIL
DTCTIEGLIYGNPSCDPLLGGREWSYIVERPSAVNGLCYPGNVENLEELRSLFSSSRSYQRIQIFPDTIW
NVSYSGTSKACSDSFYRSMRWLTQKNNAYPTQDAQYTNNQEKNILFMWGINHPPTDTAQTNLYTRTDTTT
SVATEEMNRIFKPLIGPRPLVNGLMGRINYYWSVLKPGQTLRIKSDGNLIAPWYGHILSGESHGRILKTD
LKRGSCTVQCQTEKGGLNTTLPFQNVSKYAFGNCSKYIGVKSLKLAVGLRNVPSKSSRGLFGAIAGFIEG
GWSGLVAGWYGFQHSNDQGVGMAADRDSTQKAIDKITSKVNNIVDKMNKQYEIIDHEFSEVETRLNMINN
KVDDQIQDIWAYNAELLVLLENQKTLDEHDANVNNLYNKVKRALGSNAVEDGKGCFELYHKCDDHCMETI
RNGTYNRRKYQEESKLERQKIEGVKLESEETYKILTIYSTVASSLVIAMGFAAFLFWAMSNGSCRCNICI

>QBK20593.1 hemagglutinin [Influenza A virus]

METASLITILLVATVSNADKICIGYQSTNSTETVDTLTENNVPVTHAKELLHTEHNGMLCATSLGHPLIL
DTCTIEGLIYGNPSCDPLLGGREWSYIVERPSAVNGLCYPGNVENLEELRSLFSSSRSYQRIQIFPDTIW
NVSYSGTSKACSDSFYRSMRWLTQKNNAYPTQDAQYTNNQEKNILFMWGINHPPTDTAQTNLYTRTDTTT
SVATEEMNRIFKPLIGPRPLVNGLMGRINYYWSVLKPGQTLRIKSDGNLIAPWYGHILSGESHGRILKTD
LKRGSCTVQCQTEKGGLNTTLPFQNVSKYAFGNCSKYIGVKSLKLAVGLRNVPSKSSRGLFGAIAGFIEG
GWSGLVAGWYGFQHSNDQGVGMAADRDSTQKAIDKITSKVNNIVDKMNKQYEIIDHEFSEVETRLNMINN
KVDDQIQDIWAYNAELLVLLENQKTLDEHDANVNNLYNKVKRALGSNAVEDGKGCFELYHKCDDHCMETI
RNGTYNRRKYQEESKLERQKIEGVKLDSEETYKILTIYSTVASSLVIAMGFAAFLFWAMSNGSCRCNICI

>QBK20592.1 hemagglutinin [Influenza A virus]

METVSLITILLVATVSNADKICIGYQSTNSTETVDTLTENNVPVTHAKELLHTEHNGMLCATSLGQPLIL
DTCTIEGLIYGNPSCDLSLEGREWSYIVERPSAVHGLCYPGNVEDLEELRSLFSSARSYQRIQIFPDTIW
NVSYDGTSTACSGSFYRSMRWLTRKNGEYPIQDAQYTNNQGKNILFMWGINHPPTDDTQRGLYTRTDTTT
SVATEEINRIFKPLIGPRPLVNGLMGRINYYWSVLKPGQTLRIKSDGNLIAPWYGHILSGESHGRILKTD
LKRGSCTVQCQTEKGGLNTTLPFQNISKYAFGNCSKYIGIKSLKLAVGLRNVPSRSSRGLFGAIAGFIEG
GWPGLVAGWYGFQHSNDQGVGMAADRDSTQKAIDKITSKVNNIVDKMNKQYEIIDHEFSEVETRLNMINN
KIDDQIQDIWAYNAELLVLLENQKTLDEHDANVNNLYSKVKRALGSNAVEDGKGCFELYHKCDDQCMETI
RNGTYNRRKYQEESKLERQRIEGVKLESEGTYKILTIYSTVASSLVIAMGFAAFLFWAMSNGSCRCNICI

>QBK20591.1 hemagglutinin [Influenza A virus]

METVSLITILLVATVSNADKICIGYQSTNSTETVDTLTENNVPVTHAKELLHTEHNGMLCATSLGQPLIL
DTCTIEGLIYGNPSCDLSLEGREWSYIVERPSAVHGLCYPGNVEDLEELRSLFSSARSYQRIQIFPDTIW
NVSYDGTSTACSGSFYRSMRWLTRKNGEYPIQDAQYTNNQGKNILFMWGINHPPTDDTQRGLYTRTDTTT

SVATEEINRIFKPLIGPRPLVNGLMGRINYYWSVLKPGQTLRIKSDGNLIAPWYGHILSGESHGRILKTD
LKRGSCTVQCQTEKGGLNTTLPFQNVSKYAFGNCSKYIGIKSLKLAVGLRNVPTRSSRGLFGAIAGFIEG
GWSGLVAGWYGFQHSNDQGVGMAADRDSTQKAIDKITSKVNNIVDKMNKQYEIIDHEFSEVETRLNMINN
KIDDQIQDIWAYNAELLVLLENQKTLDEHDANVNNLYNKVKRALGSNAVEDGKGCFELYHKCDDQCMETI
RNGTYNRRKYQEESKLERQRIEGVKLESEGTYKILTIYSTVASSLVIAMGFAAFLFWAMSNGSCRCNICI

>QBK20590.1 hemagglutinin [Influenza A virus]
METVSLITILLVATVSNADKICIGYQSTNSTETVDTLTENNVPVTHAKELLHTEHNGMLCATSLGQPLIL
DTCTIEGLIYGNPSCDLSLEGREWSYIVERPSAVHGLCYPGNVEDLEELRSLFSSARSYQRIQIFPDTIW
NVSYDGTSTACSGSFYRSMRWLTRKNGEYPIQDAQYTNNQGKNILFMWGINHPPTDDTQRGLYTRTDTTT
SVATEEINRIFKPLIGPRPLVNGLMGRINYYWSVLKPGQTLRIKSDGNLIAPWYGHILSGESHGRILKTD
LKRGSCTVQCQTEKGGLNTTLPFQNISKYAFGNCSKYIGIKSLKLAVGLRNVPSRSSRGLFGAIAGFIEG
GWPGLVAGWYGFQHSNDQGVGMAADRDSTQKAIDKITSKVNNIVDKMNKQYEIIDHEFSEVETRLNMINN
KIDDQIQDIWAYNAELLVLLENQKTLDEHDANVNNLYSKVKRALGSNAVEDGKGCFELYHKCDDQCMETI
RNGTYNRRKYQEESKLERQRIEGVKLESEGTYKILTIYSTVASSLVIAMGFAAFLFWAMSNGSCRCNICI

>QBK20589.1 hemagglutinin [Influenza A virus]
METASLITILLVATVSNADKICIGYQSTNSTETVDTLTENNVPVTHAKELLHTEHNGMLCATSLGHPLIL
DTCTIEGLIYGNPSCDPLLGGREWSYIVERPSAVNGLCYPGNVENLEELRSLFSSSRSYQRIQIFPDTIW
NVSYSGTSKACSDSFYRSMRWLTQKNNAYPTQDAQYTNNQEKNILFMWGINHPPTDTVQTNLYTRTDTTT
SVATEEMNRIFKPLIGPRPLVNGLMGRINYYWSVLKPGQTLRIKSDGNLIAPWYGHILSGESHGRILKTD
LKRGSCTVQCQTEKGGLNTTLPFQNVSKYAFGNCSKYIGVKSLKLAVGLRNVPSKSSRGLFGAIAGFIEG
GWSGLVAGWYGFQHSNDQGVGMAADRDSTQKAIDKITSKVNNIVDKMNKQYEIIDHEFSEVETRLNMINN
KVDDQIQDIWAYNAELLVLLENQKTLDEHDANVNNLYNKVKRALGSNAVEDGKGCFELYHKCDDHCMETI
RNGTYNRRKYQEESKLERQKIEGVKLESEETYKILTIYSTVASSLVIAMGFAAFLFWAMSNGSCRCNICI

>QBK20588.1 hemagglutinin [Influenza A virus]
METASLITILLVATVSNADKICIGYQSTNSTETVDTLTENNVPVTHAKELLHTEHNGMLCATSLGHPLIL
DTCTIEGLIYGNPSCDPLLGGREWSYIVERPSAVNGLCYPGNVENLEELRSLFSSSRSYQRIQIFPDTIW
NVSYSGTSKACSDSFYRSMRWLTQKNNAYPTQDAQYTNNQEKNILFMWGINHPPTDTAQTNLYTRTDTTT
SVATEEMNRIFKPLIGPRPLVNGLMGRINYYWSVLKPGQTLRIKSDGNLIAPWYGHILSGESHGRILKTD
LKRGSCTVQCQTEKGGLNTTLPFQNVSKYAFGNCSKYIGVKSLKLAVGLRNVPSKSSRGLFGAIAGFIEG
GWSGLVAGWYGFQHSNDQGVGMAADRDSTQKAIDKITSKVNNIVDKMNKQYEIIDHEFSEVETRLNMINN
KVDDQIQDIWAYNAELLVLLENQKTLDEHDANVNNLYNKVKRALGSNAVEDGKGCFELYHKCDDHCMETI

RNGTYNRRKYQEESKLERQKIEGVKLESEETYKILTIYSTVASSLVIAMGFAAFLFWAMSNGSCRCNICI

>QBK20587.1 hemagglutinin [Influenza A virus]
METASLITILLVATVSNADKICIGYQSTNSTETVDTLTENNVPVTHAKELLHTEHNGMLCATSLGHPLIL
DTCTIEGLIYGNPSCDPLLGGREWSYIVERPSAVNGLCYPGNVENLEELRSLFSSSRSYQRIQIFPDTIW
NVSYSGTSKACSDSFYRSMRWLTQKNNAYPTQDAQYTNNQEKNILFMWGINHPPTDTAQTNLYTRTDTTT
SVATEEMNRIFKPLIGPRPLVNGLMGRINYYWSVLKPGQTLRIKSDGNLIAPWYGHILSGESHGRILKTD
LKRGSCTVQCQTEKGGLNTTLPFQNVSKYAFGNCSKYIGVKSLKLAVGLRNVPSKSSRGLFGAIAGFIEG
GWSGLVAGWYGFQHSNDQGVGMAADRDSTQKAIDKITSKVNNIVDKMNKQYEIIDHEFSEVETRLNMINN
KVDDQIQDIWAYNAELLVLLENQKTLDEHDANVNNLYNKVKRALGSNAVEDGKGCFELYHKCDDHCMETI
RNGTYNRRKYQEESKLERQKIEGVKLESEETYKILTIYSTVASSLVIAMGFAAFLFWAMSNGSCRCNICI

>QBK20586.1 hemagglutinin [Influenza A virus]
METASLITILLVATVSNADKICIGYQSTNSTETVDTLTENNVPVTHAKELLHTEHNGMLCATSLGHPLIL
DTCTIEGLIYGNPSCDPLLGGREWSYIVERPSAVNGLCYPGNVENLEELRSLFSSSRSYQRIQIFPDTIW
NVSYSGTSKACSDSFYRSMRWLTQKNNAYPTQDAQYTNNQEKNILFMWGINHPPTDTAQTNLYTRTDTTT
SVATEEMNRIFKPLIGPRPLVNGLMGRINYYWSVLKPGQTLRIKSDGNLIAPWYGHILSGESHGRILKTD
LKRGSCTVQCQTEKGGLNTTLPFQNVSKYAFGNCSKYIGVKSLKLAVGLRNVPSKSSRGLFGAIAGFIEG
GWSGLVAGWYGFQHSNDQGVGMAADRDSTQKAIDKITSKVNNIVDKMNKQYEIIDHEFSEVETRLNMINN
KVDDQIQDIWAYNAELLVLLENQKTLDEHDANVNNLYNKVKRALGSNAVEDGKGCFELYHKCDDHCMETI
RNGTYNRRKYQEESKLERQKIEGVKLESEETYKILTIYSTVASSLVIAMGFAAFLFWAMSNGSCRCNICI

>QBK20585.1 hemagglutinin [Influenza A virus]
METASLITILLVATVSNADKICIGYQSTNSTETVDTLTENNVPVTHAKELLHTEHNGMLCATSLGHPLIL
DTCTIEGLIYGNPSCDPLLGGREWSYIVERPSAVNGLCYPGNVENLEELRSLFSSSRSYQRIQIFPDTIW
NVSYSGTSKACSDSFYRSMRWLTQKNNAYPTQDAQYTNNQEKNILFMWGINHPPTDTAQTNLYTRTDTTT
SVATEEMNRIFKPLIGPRPLVNGLMGRINYYWSVLKPGQTLRIKSDGNLIAPWYGHILSGESHGRILKTD
LKRGSCTVQCQTEKGGLNTTLPFQNVSKYAFGNCSKYIGVKSLKLAVGLRNVPSKSSRGLFGAIAGFIEG
GWSGLVAGWYGFQHSNDQGVGMAADRDSTQKAIDKITSKVNNIVDKMNKQYEIIDHEFSEVETRLNMINN
KVDDQIQDIWAYNAELLVLLENQKTLDEHDANVNNLYNKVKRALGSNAVEDGKGCFELYHKCDDHCMETI
RNGTYNRRKYQEESKLERQKIEGVKLESEETYKILTIYSTVASSLVIAMGFAAFLFWAMSNGSCRCNICI

>QBK20584.1 hemagglutinin [Influenza A virus]
METVSLITILLVATVSNADKICIGYQSTNSTETVDTLTENNVPVTHAKELLHTEHNGMLCATSLGQPLIL

DTCTIEGLIYGNPSCDLSLEGREWSYIVERPSAVHGLCYPGNVEDLEELRSLFSSARSYQRIQIFPDTIW
NVSYDGTSTACSGSFYRSMRWLTRKNGEYPIQDAQYTNNQGKNILFMWGINHPPTDDTQRGLYTRTDTTT
SVATEEINRIFKPLIGPRPLVNGLMGRINYYWSVLKPGQTLRIKSDGNLIAPWYGHILSGESHGRILKTD
LKRGSCTVQCQTEKGGLNTTLPFQNISKYAFGNCSKYIGIKSLKLAVGLRNVPSRSSRGLFGAIAGFIEG
GWPGLVAGWYGFQHSNDQGVGMAADRDSTQKAIDKITSKVNNIVDKMNKQYEIIDHEFSEVETRLNMINN
KIDDQIQDIWAYNAELLVLLENQKTLDEHDANVNNLYSKVKRALGSNAVEDGKGCFELYHKCDDQCMETI
RNGTYNRRKYQEESKLERQRIEGVKLESEGTYKILTIYSTVASSLVIAMGFAAFLFWAMSNGSCRCNICI

>QBK20583.1 hemagglutinin [Influenza A virus]
METVSLITILLVATVSNADKICIGYQSTNSTETVDTLTENNVPVTHAKELLHTEHNGMLCATSLGQPLIL
DTCTIEGLIYGNPSCDLSLEGREWSYIVERPSAVHGLCYPGNVEDLEELRSLFSSARSYQRIQIFPDTIW
NVSYDGTSTACSGSFYRSMRWLTRKNGEYPIQDAQYTNNQGKNILFMWGINHPPTDDTQRGLYTRTDTTT
SVATEEINRIFKPLIGPRPLVNGLMGRINYYWSVLKPGQTLRIKSDGNLIAPWYGHILSGESHGRILKTD
LKRGSCTVQCQTEKGGLNTTLPFQNVSKYAFGNCSKYIGIKSLKLAVGLRNVPTRSSRGLFGAIAGFIEG
GWSGLVAGWYGFQHSNDQGVGMAADRDSTQKAIDKITSKVNNIVDKMNKQYEIIDHEFSEVETRLNMINN
KIDDQIQDIWAYNAELLVLLENQKTLDEHDANVNNLYNKVKRALGSNAVEDGKGCFELYHKCDDQCMETI
RNGTYNRRKYQEEFIFVREQKQGVKLESEGTYKILTIYSTVASSLVIAMGFAAFLFWAMSNGSCRCNICI

>QBK20582.1 hemagglutinin [Influenza A virus]
METASLITILLVATVSNADKICIGYQSTNSTETVDTLTENNVPVTHAKELLHTEHNGMLCATSLGHPLIL
DTCTIEGLIYGNPSCDPLLGGREWSYIVERPSAVNGLCYPGNVENLEELRSLFSSSRSYQRIQIFPDTIW
NVSYSGTSKACSDSFYRSMRWLTQKNNAYPTQDAQYTNNQEKNILFMWGINHPPTDTAQTNLYTRTDTTT
SVATEEMNRIFKPLIGPRPLVNGLMGRINYYWSVLKPGQTLRIKSDGNLIAPWYGHILSGESHGRILKTD
LKRGSCTVQCQTEKGGLNTTLPFQNVSKYAFGNCSKYIGVKSLKLAVGLRNVPSKSSRGLFGAIAGFIEG
GWSGLVAGWYGFQHSNDQGVGMAADRDSTQKAIDKITSKVNNIVDKMNKQYEIIDHEFSEVETRLNMINN
KVDDQIQDIWAYNAELLVLLENQKTLDEHDANVNNLYNKVKRALGSNAVEDGKGCFELYHKCDDHCMETI
RNGTYNRRKYQEESKLERQKIEGVKLESEETYKILTIYSTVASSLVIAMGFAAFLFWAMSNGSCRCNICI

>QBK20581.1 hemagglutinin [Influenza A virus]
METVSLITILLVATVSNADKICIGYQSTNSTETVDTLTENNVPVTHAKELLHTEHNGMLCATSLGQPLIL
DTCTIEGLIYGNPSCDLSLEGREWSYIVERPSAVHGLCYPGNVEDLEELRSLFSSARSYQRIQIFPDTIW
NVSYDGTSTACSGSFYRSMRWLTRKNGEYPIQDAQYTNNQGKNILFMWGINHPPTDDTQRGLYTRTDTTT
SVATEEINRIFKPLIGPRPLVNGLMGRINYYWSVLKPGQTLRIKSDGNLIAPWYGHILSGESHGRILKTD
LKRGSCTVQCQTEKGGLNTTLPFQNISKYAFGNCSKYIGIKSLKLAVGLRNVPSRSSRGLFGAIAGFIEG

GWSGLVAGWYGFQHSNDQGVGMAADRDSTQKAIDKITSKVNNIVDKMNKQYEIIDHEFSEVETRLNMINN
KIDDQIQDIWAYNAELLVLLENQKTLDEHDANVNNLYSKVKRALGSNAVEDGKGCFELYHKCDDQCMETI
RNGTYNRRKYQEESKLERQRIEGVKLESEGTYKILTIYSTVASSLVIAMGFAAFLFWAMSNGSCRCNICI

>QBK20580.1 hemagglutinin [Influenza A virus]
METVSLITILLVAAVSNADKICIGYQSTNSTETVDTLTENNVPVTHAKELLHTAHNGMLCATSLGQPLIL
DTCTIEGLIYGNPSCDLSLEGREWSYIVERPSAVHGLCYPGNVEDLEELRSLFSSARSYQRIQIFPDTIW
NVSYDGTSTACSGSFYRSMRWLTRKNGEYPIQDAQYTNNQGKNILFMWGINHPPTDDTQRGLYTRTDTTT
SVATEEINRIFKPLIGPRPLVNGLMGRINYYWSVLKPGQTLRIKSDGNLIAPWYGHILSGESHGRILKTD
LKRGSCTVQCQTEKGGLNTTLPFQNVSKYAFGNCSKYIGIKSLKLAVGLRNVPSRSSRGLFGAIAGFIEG
GWSGLVAGWYGFQHSNDQGVGMAADRDSTQKAIDKITSKVNNIVDKMNKQYEIIDHEFSEVETRLNMINN
KIDDQIQDIWAYNAELLVLLENQKTLDEHDANVNNLYNKVKRALGSNAVEDGKGCFELYHKCDDQCMETI
RNGTYNRRKYQEESKLERQRIEGVKLESEGTYKILTIYSTVASSLVIAMGFAAFLFWAMSNGSCRCNICI

>QBK20579.1 hemagglutinin [Influenza A virus]
METVSLITILLVATVSNADKICIGYQSTNSTETVDTLTENNVPVTHAKELLHTEHNGVLCATSLGQPLIL
DTCTIEGLIYGNPSCDLSLEGREWSYIVERPSAVHGLCYPGNVEDLEELRSLFSSARSYQRIQIFPDTIW
NVSYDGTSTACSGSFYRSMRWLTRKNGEYPIQDAQYTNNQGKNILFMWGINHPPTDDTQRGLYTRTDTTT
SVATEEINRIFKPLIGPRPRVNGLMGRINYYWSVLKPGQTLRIKSDGNLIAPWYGHILSGESHGRILKTD
LKRGSCTVQCQTEKGGLNTTLPFQNISKYAFGNCSKYIGIKSLKLAVGLRNVPSRSSRGLFGAIAGFIEG
GWPGLVAGWYGFQHSNDQGVGMAADRDSTQKAIDKITSKVNNIVDKMNKQYEIIDHEFSEVETRLNMINN
KIDDQIQDIWAYNAELLVLLENQKTLDEHDANVNNLYSKVKRALGSNAVEDGKGCFELYHKCDDQCMETI
RNGTYNRRKYQEESKLERQRIEGVKLESEGTYKILTIYSTVASSLVIAMGFAAFLFWAMSNGSCRCNICI

>QBK20578.1 hemagglutinin [Influenza A virus]
METASLITILLVATVSNADKICIGYQSTNSTETVDTLTENNVPVTHAKELLHTEHNGMLCATSLGHPLIL
DTCTIEGLIYGNPSCDPLLGGREWSYIVERPSAVNGLCYPGNVENLEELRSLFSSSRSYQRIQIFPDTIW
NVSYSGTSKACSDSFYRSMRWLTQKNNAYPTQDAQYTNNQEKNILFMWGINHPPTDTAQTNLYTRTDTTT
SVATEEMNRIFKPLIGPRPLVNGLMGRINYYWSVLKPGQTLRIKSDGNLIAPWYGHILSGESHGRILKTD
LKRGSCTVQCQTEKGGLNTTLPFQNVSKYAFGNCSKYIGVKSLKLAVGLRNVPSKSSRGLFGAIAGFIEG
GWSGLVAGWYGFQHSNDQGVGMAADRDSTQKAIDKITSKVNNIVDKMNKQYEIIDHEFSEVETRLNMINN
KVDDQIQDIWAYNAELLVLLENQKTLDEHDANVNNLYNKVKRALGSNAVEDGKGCFELYHKCDDHCMETI
RNGTYNRRKYQEESKLERQKIEGVKLESEETYKILTIYSTVASSLVIAMGFAAFLFWAMSNGSCRCNICI

>QBK20577.1 hemagglutinin [Influenza A virus]
METVSLITILLVATVSNADKICIGYQSTNSTETVDTLTENNVPVTHAKELLHTEHNGMLCATSLGQPLIL
DTCTIEGLIYGNPSCDLSLEGREWSYIVERPSAVHGLCYPGNVEDLEELRSLFSSARSYQRIQIFPDTIW
NVSYDGTSTACSGSFYRSMRWLTRKNGEYPIQDAQYTNNQGKNILFMWGINHPPTDDTQRGLYTRTDTTT
SVATEEINRIFKPLIGPRPLVNGLMGRINYYWSVLKPGQTLRIKSDGNLIAPWYGHILSGESHGRILKTD
LKRGSCTVQCQTEKGGLNTTLPFQNISKYAFGNCSKYIGIKSLKLAVGLRNVPSRSSRGLFGAIAGFIEG
GWPGLVAGWYGFQHSNDQGVGMAADRDSTQKAIDKITSKVNNIVDKMNKQYEIIDHEFSEVETRLNMINN
KIDDQIQDIWAYNAELLVLLENQKTLDEHDANVNNLYSKVKRALGSNAVEDGKGCFELYHKCDDQCMETI
RNGTYNRRKYQEESKLERQRIEGVKLESEGTYKILTIYSTVASSLVIAMGFAAFLFWAMSNGSCRCNICI

>QBK20576.1 hemagglutinin [Influenza A virus]
METVSLITILLVATVSNADKICIGYQSTNSTETVDTLTENNVPVTHAKELLHTEHNGMLCATSLGQPLIL
DTCTIEGLIYGNPSCDLSLEGREWSYIVERPSAVHGLCYPGNVEDLEELRSLFSSARSYQRIQIFPDTIW
NVSYDGTSTACSGSFYKSMRWLTRKNGEYPTQDAQYTNNQGKNILFMWGINHPPTDDTQRGLYTRTDTTT
SVATEEINRIFKPLIGPRPLVNGLMGRINYYWSVLKPGQTLRIKSDGNLIAPWYGHILSGESHGRILKTD
LKRGSCTVQCQTEKGGLNTTLPFQNVSKYAFGNCSKYIGIKSLKLAVGLRNVPSRSSRGLFGAIAGFIEG
GWSGLVAGWYGFQHSNDQGVGMAADRDSTQKAIDKITSKVNNIVDKMNKQYEIIDHEFSEVETRLNMINN
KIDDQIQDIWAYNAELLVLLENQKTLDEHDANVNNLYNKVKRALGSNAVEDGKGCFELYHKCDDQCMETI
RNGTYNRRKYQEESKLERQRIEGVKLESEGTYKILTIYSTVASSLVIAMGFAAFLFWAMSNGSCRCNICI

>QBK20575.1 hemagglutinin [Influenza A virus]
METVSLITILLVATVSNADKICIGYQSTNSTETVDTLTENNVPVTHAKELLHTEHNGMLCATSLGQPLIL
DTCTIEGLIYGNPSCDLSLEGREWSYIVERPSAVHGLCYPGNVEDLEELRSLFSSARSYQRIQIFPDTIW
NVSYDGTSTACSGSFYRSMRWLTRKNGEYPIQDAQYTNNQGKNILFMWGINHPPTDDTQRGLYTRTDTTT
SVATEEINRIFKPLIGPRPLVNGLMGRINYYWSVLKPGQTLRIKSDGNLIAPWYGHILSGESHGRILKTD
LKRGSCTVQCQTEKGGLNTTLPFQNISKYAFGNCSKYIGIKSLKLAIGLRNVPSRSSRGLFGAIAGFIEG
GWPGLVAGWYGFQHSNDQGVGMAADRDSTQKAIDKITSKVNNIVDKMNKQYEIIDHEFSEVETRLNMINN
KIDDQIQDIWAYNAELLVLLENQKTLDEHDANVNNLYSKVKRALGSNAVEDGKGCFELYHKCDDQCMETI
RNGTYNRRKYQEESKLERQRIEGVKLESEGTYKILTIYSTVASSLVIAMGFAAFLFWAMSNGSCRCNICI

>QBK20574.1 hemagglutinin [Influenza A virus]
METVSLITILLVATVSNADKICIGYQSTNSTETVDTLTENNVPVTHAKELLHTEHNGMLCATSLGQPLIL
DTCTIEGLIYGNPSCDLSLEGREWSYIVERPSAVHGLCYPGNVEDLEELRSLFSSARSYQRIQIFPDTIW
NVSYDGTSTACSGSFYRSMRWLTRKNGEYPIQDAQYTNNQGKNILFMWGINHPPTDDTQRGLYTRTDTTT

SVATEEINRIFKPLIGPRPLVNGLMGRINYYWSVLKPGQTLRIKSDGNLIAPWYGHILSGESHGRILKTD
LKRGSCTVQCQTEKGGLNTTLPFQNISKYAFGNCSKYIGIKSLKLAVGLRNVPSRSSRGLFGAIAGFIEG
GWSGLVAGWYGFQHSNDQGVGMAADRDSTQKAIDKITSKVNNIVDKMNKQYEIIDHEFSEVETRLNMINN
KIDDQIQDIWAYNAELLVLLENQKTLDEHDANVNNLYSKVKRALGSNAVEDGKGCFELYHKCDDQCMETI
RNGTYNRRKYQEESKLERQRIEGVKLESEGTYKILTIYSTVASSLVIAMGFAAFLFWAMSNGSCRCNICI

>QBK20573.1 hemagglutinin [Influenza A virus]
METVSLITILLVATVSNADKICIGYQSTNSTETVDTLTENNVPVTHAKELLHTEHNGMLCATSLGQPLIL
DTCTIEGLIYGNPSCDLSLEGREWSYIVERPSAVHGLCYPGNVEDLEELRSLFSSARSYQRIQIFPDTIW
NVSYDGTSTACSGSFYRSMRWLTRKNGEYPIQDAQYTNNQGKNILFMWGINHPPTDDTQRGLYTRTDTTT
SVATEEINRIFKPLIGPRPLVNGLMGRINYYWSVLKPGQTLRIKSDGNLIAPWYGHILSGESHGRILKTD
LKRGSCTVQCQTEKGGLNTTLPFQNISKYAFGNCSKYIGIKSLKLAVGLRNVPSRSSRGLFGAIAGFIEG
GWPGLVAGWYGFQHSNDQGVGMAADRDSTQKAIDKITSKVNNIVDKMNKQYEIIDHEFSEVETRLNMINN
KIDDQIQDIWAYNAELLVLLENQKTLDEHDANVNNLYSKVKRALGSNAVEDGKGCFELYHKCDDQCMETI
RNGTYNRRKYQEESKLERQRIEGVKLESEGTYKILTIYSTVASSLVIAMGFAAFLFWAMSNGSCRCNICI

>QBK20572.1 hemagglutinin [Influenza A virus]
METVSLITILLVATVSNADKICIGYQSTNSTETVDTLTENNVPVTHAKELLHTEHNGMLCATSLGQPLIL
DTCTIEGLIYGNPSCDLSLEGREWSYIVERPSAVHGLCYPGNVEDLEELRSLFSSARSYQRIQIFPDTIW
NVSYDGTSTACSGSFYRSMRWLTRKNGEYPIQDAQYTNNQGKNILFMWGINHPPTDDTQRGLYTRTDTTT
SVATEEINRIFKPLIGPRPLVNGLMGRINYYWSVLKPGQTLRIKSDGNLIAPWYGHILSGESHGRILKTD
LKRGSCTVQCQTEKGGLNTTLPFQNISKYAFGNCSKYIGIKSLKLAVGLRNVPSRSSRGLFGAIAGFIEG
GWPGLVAGWYGFQHSNDQGVGMAADRDSTQKAIDKITSKVNNIVDKMNKQYEIIDHEFSEVETRLNMINN
KIDDQIQDIWAYNAELLVLLENQKTLDEHDANVNNLYSKVKRALGSNAVEDGKGCFELYHKCDDQCMETI
RNGTYNRRKYQEESKLERQRIEGVKLESEGTYKILTIYSTVASSLVIAMGFAAFLFWAMSNGSCRCNICI

>QBK20571.1 hemagglutinin [Influenza A virus]
METVSLITILLVATVSNADKICIGYQSTNSTETVDTLTENNVPVTHAKELLHTEHNGMLCATSLGQPLIL
DTCTIEGLIYGNPSCDLSLEGREWSYIVERPSAVHGLCYPGNVEDLEELRSLFSSARSYQRIQIFPDTIW
NVSYDGTSTACSGSFYRSMRWLTRKNGEYPIQDAQYTNNQGKNILFMWGINHPPTDDTQRGLYTRTDTTT
SVATEEINRIFKPLIGPRPLVNGLMGRINYYWSVLKPGQTLRIKSDGNLIAPWYGHILSGESHGRILKTD
LKRGSCTVQCQTEKGGLNTTLPFQNISKYAFGNCSKYIGIKSLKLAVGLRNVPSRSSRGLFGAIAGFIEG
GWPGLVAGWYGFQHSNDQGVGMAADRDSTQKAIDKITSKVNNIVDKMNKQYEIIDHEFSEVETRLNMINN
KIDDQIQDIWAYNAELLVLLENQKTLDEHDANVNNLYSKVKRALGSNAVEDGKGCFELYHKCDDQCMETI

RNGTYNRRKYQEESKLERQRIEGVKLESEGTYKILTIYSTVASSLVIAMGFAAFLFWAMSNGSCRCNICI

>QBK20570.1 hemagglutinin [Influenza A virus]

METVSLITILLVATVSNADKICIGYQSTNSTETVDTLTENNVPVTHAKELLHTEHNGMLCATSLGQPLIL

DTCTIEGLIYGNPSCDLSLEGREWSYIVERPSAVHGLCYPGNVEDLEELRSLFSSARSYQRIQIFPDTIW

NVSYDGTSTACSGSFYRSMRWLTRKNGEYPIQDAQYTNNQGKNILFMWGINHPPTDDTQRGLYTRTDTTT

SVATEEINRIFKPLIGPRPLVNGLMGRINYYWSVLKPGQTLRIKSDGNLIAPWYGHILSGESHGRILKTD

LKRGSCPVQCQTEKGGLNTTLPFQNISKYAFGNCSKYIGIKSLKLAVGLRNVPSRSSRGLFGAIAGFIEG

GWPGLVAGWYGFQHSNDQGVGMAADRDSTQKAIDKITSKVNNIVDKMNKQYEIIDHEFSEVETRLNMINN

KIDDQIQDIWAYNAELLVLLENQKTLDEHDANVNNLYSKVKRALGSNAVEDGKGCFELYHKCDDQCMETI

RNGTYNRRKYQEESKLERQRIEGVKLESEGTYKILTIYSTVASSLVIAMGFAAFLFWAMSNGSCRCNICI

>QBK20569.1 hemagglutinin [Influenza A virus]

METVSLITILLVATVSNADKICIGYQSTNSTETVDTLTENNVPVTHAKELLHTEHNGMLCATSLGQPLIL

DTCTIEGLIYGNPSCDLSLEGREWSYIVERPSAVHGLCYPGNVEDLEELRSLFSSARSYQRIQIFPDTIW

NVSYDGTSTACSGSFYRSMRWLTRKNGEYPIQDAQYTNNQGKNILFMWGINHPPTDDTQRGLYTRTDTTT

SVATEEINRIFKPLIGPRPLVNGLMGRINYYWSVLKPGQTLRIKSDGNLIAPWYGHILSGESHGRILKTD

LKRGSCTVQCQTEKGGLNTTLPFQNISKYAFGNCSKYIGIKSLKLAVGLRNVPSRSSRGLFGAIAGFIEG

GWPGLVAGWYGFQHSNDQGVGMAADRDSTQKAIDKITSKVNNIVDKMNKQYEIIDHEFSEVETRLNMINN

KIDDQIQDIWAYNAELLVLLENQKTLDEHDANVNNLYSKVKRALGSNAVEDGKGCFELYHKCDDQCMETI

RNGTYNRRKYQEESKLERQRIEGVKLESEGTYKILTIYSTVASSLVIAMGFAAFLFWAMSNGSCRCNICI

>QBK20568.1 hemagglutinin [Influenza A virus]

METVSLITILLVATVSNADKICIGYQSTNSTETVDTLTENNVPVTHAKELLHTEHNGMLCATSLGQPLIL

DTCTIEGLIYGNPSCDLSLEGREWSYIVERPSAVHGLCYPGNVEDLEELRSLFSSARSYQRIQIFPDTIW

NVSYDGTSTACSGSFYRSMRWLTRKNGEYPIQDAQYTNNQGKNILFMWGINHPPTDDTQRGLYTRTDTTT

SVATEEINRIFKPLIGPRPLVNGLMGRINYYWSVLKPGQTLRIKSDGNLIAPWYGHILSGESHGRILKTD

LKRGSCTVQCQTEKGGLNTTLPFQNISKYAFGNCSKYIGIKSLKLAIGLRNVPSRSSRGLFGAIAGFIEG

GWPGLVAGWYGFQHSNDQGVGMAADRDSTQKAIDKITSKVNNIVDKMNKQYEIIDHEFSEVETRLNMINN

KIDDQIQDIWAYNAELLVLLENQKTLDEHDANVNNLYSKVKRALGSNAVEDGKGCFELYHKCDDQCMETI

RNGTYNRRKYQEESKLERQRIEGVKLESEGTYKILTIYSTVASSLVIAMGFAAFLFWAMSNGSCRCNICI

>QBK20567.1 hemagglutinin [Influenza A virus]

METVSLITILLVATVSNADKICIGYQSTNSTETVDTLTENNVPVTHAKELLHTEHNGMLCATSLGQPLIL

DTCTIEGLIYGNPSCDLSLEGREWSYIVERPSAVHGLCYPGNVEDLEELRSLFSSARSYQRIQIFPDTIW
NVSYDGTSTACSGSFYRSMRWLTRKNGEYPIQDAQYTNNQGKNILFMWGINHPPTDDTQRGLYTRTDTTT
SVATEEINRIFKPLIGPRPLVNGLMGRINYYWSVLKPGQTLRIKSDGNLIAPWYGHILSGESHGRILKTD
LKRGSCTVQCQTEKGGLNTTLPFQNISKYAFGNCSKYIGIKSLKLAIGLRNVPSRSSRGLFGAIAGFIEG
GWPGLVAGWYGFQHSNDQGVGMAADRDSTQKAIDKITSKVNNIVDKMNKQYEIIDHEFSEVETRLNMINN
KIDDQIQDIWAYNAELLVLLENQKTLDEHDANVNNLYSKVKRALGSNAVEDGKGCFELYHKCDDQCMETI
RNGTYNRRKYQEESKLERQRIEGVKLESEGTYKILTIYSTVASSLVIAMGFAAFLFWAMSNGSCRCNICI

>QBK20566.1 hemagglutinin [Influenza A virus]
METVSLITILLVATVSNADKICIGYQSTNSTETVDTLTENNVPVTHAKELLHTEHNGMLCATSLGQPLIL
DTCTIEGLIYGNPSCDLSLEGREWSYIVERPSAVHGLCYPGNVEDLEELRSLFSSARSYQRIQIFPDTIW
NVSYDGTSTACSGSFYKSMRWLTRKNGEYPTQDAQYTNNQGKNILFMWGINHPPTDDTQRGLYTRTDTTT
SVATEEINRIFKPLIGPRPLVNGLMGRINYYWSVLKPGQTLRIKSDGNLIAPWYGHILSGESHGRILKTD
LKRGSCTVQCQTEKGGLNTTLPFQNVSKYAFGNCSKYIGIKSLKLAVGLRNVPSRSSRGLFGAIAGFIEG
GWSGLVAGWYGFQHSNDQGVGMAADRDSTQKAIDKITSKVNNIVDKMNKQYEIIDHEFSEVETRLNMINN
KIDDQIQDIWAYNAELLVLLENQKTLDEHDANVNNLYNKVKRALGSNAVEDGKGCFELYHKCDDQCMETI
RNGTYNRRKYQEESKLERQRIEGVKLESEGTYKILTIYSTVASSLVIAMGFAAFLFWAMSNGSCRCNICI

>QBK20564.1 hemagglutinin [Influenza A virus]
METVSLITILLVATVSNADKICIGYQSTNSTETVDTLTENNVPVTHAKELLHTEHNGMLCATSLGQPLIL
DTCTIEGLIYGNPSCDLSLEGREWSYIVERPSAVHGLCYPGNVEDLEELRSLFSSARSYQRIQIFPDTIW
NVSYDGTSTACSGSFYKSMRWLTRKNGEYPTQDAQYTNNQGKNILFMWGINHPPTDDTQRGLYTRTDTTT
SVATEEINRIFKPLIGPRPLVNGLMGRINYYWSVLKPGQTLRIKSDGNLIAPWYGHILSGESHGRILKTD
LKRGSCTVQCQTEKGGLNTTLPFQNVSKYAFGNCSKYIGIKSLKLAVGLRNVPSRSSRGLFGAIAGFIEG
GWSGLVAGWYGFQHSNDQGVGMAADRDSTQKAIDKITSKVNNIVDKMNKQYEIIDHEFSEVETRLNMINN
KIDDQIQDIWAYNAELLVLLENQKTLDEHDANVNNLYNKVKRALGSNAVEDGKGCFELYHKCDDQCMETI
RNGTYNRRKYQEESKLERQRIEGVKLESEGTYKILTIYSTVASSLVIAMGFAAFLFWAMSNGSCRCNICI

>QBK20563.1 hemagglutinin [Influenza A virus]
METVSLITILLVATVSNADKICIGYQSTNSTETVDTLTENNVPVTHAKELLHTEHNGMLCATSLGQPLIL
DTCTIEGLIYGNPSCDLSLEGREWSYIVERPSAVHGLCYPGNVEDLEELRSLFSSARSYQRIQIFPDTIW
NVSYDGTSTACSGSFYRSMRWLTRKNGEYPIQDAQYTNNQGKNILFMWGINHPPTDDTQRGLYTRTDTTT
SVATEEINRIFKPLIGPRPLVNGLMGRINYYWSVLKPGQTLRIKSDGNLIAPWYGPILSGESHGRILKTD
LKRGSCTVQCQTEKGGLNTTLPFQNISKYAFGNCSKYIGIKSLKLAVGLRNVPSRSSRGLFGAIAGFIEG

GWPGLVAGWYGFQHSNDQGVGMAADRDSTQKAIDKITSKVNNIVDKMNKQYEIIDHEFSEVETRLNMINN
KIDDQIQDIWAYNAELLVLLENQKTLDEHDANVNNLYSKVKRALGSNAVEDGKGCFELYHKCDDQCMETI
RNGTYNRRKYQEESKLERQRIEGVKLESEGTYKILTIYSTVASSLVIAMGFAAFLFWAMSNGSCRCNICI

>QBK20562.1 hemagglutinin [Influenza A virus]
METVSLITILLVATVSNADKICIGYQSTNSTETVDTLTENNVPVTHAKELLHTEHNGMLCATSLGQPIIL
DTCTIEGLIYGNPSCDLSLEGREWSYIVERPSAVNGLCYPGNVENLEELRSLFSSARSYQRIQIFPDTIW
NVSYDGTSTACSGSFYRNMRWLTRKNGEYPIQDAQYTNNQGKNILFMWGINHPPADTTQRDLYTRTDTTT
SVATEEINRIFKPLIGPRPLVNGLMGRIDYYWSVLKPGQTLRIKSDGNLIAPWYGHILSGESHGRILKTD
LKRGSCTVQCQTEKGGLNTTLPFQNVSKYAFGNCSKYIGIKSLKLAVGLRNVPSRSSRGLFGAIAGFIEG
GWSGLVAGWYGFQHSNDQGVGMAADRESTQKAVDKITSKVNNIVDKMNKQYEIIDHEFSEVETRLNMINN
KIDDQIQDIWAYNAELLVLLENQKTLDEHDANVNNLYNKVKRALGSNAMEDGKGCFELYHKCDDQCMETI
RNGTYNRRKYQEESKLERQKIEGVKLESEGTYKILTIYSTVASSLVIAMGFAAFLFWAMSNGSCRCNICI

>QBK20559.1 hemagglutinin [Influenza A virus]
METVSLITILLVATVSNADKICIGYQSTNSTETVDTLTENNVPVTHAKELLHTEHNGMLCATSLGQPLIL
DTCTIEGLIYGNPSCDLSLEGREWSYIVERPSAVNGMCYPGNVENLEELRSLFSSARSYQRIQIFPDTIW
NVSYDGTSTACSGSFYRSMRWLTRKNGEYPIQDAQYTNNQGKNILFMWGINHPPTDTTQRDLYTRTDTTT
SVATEEINRVFKPLIGPRPLVNGLMGRIDYYWSVLKPGQTLRIKSDGNLIAPWFGHILSGESHGRILKTD
LKRGSCTVQCQTEKGGLNTTLPFQNVSKYAFGNCSKYIGIKSLKLAVGLRNVPSRSSRGLFGAIAGFIEG
GWSGLVAGWYGFQHSNDQGVGMAADRDSTQKAIDKITSKVNNIVDKMNKQYEIIDHEFSEVEARLNMINN
KIDDQIQDIWAYNAELLVLLENQKTLDEHDANVNNLYNKVKRALGSNAVEDGKGCFELYHKCDDQCMETI
RNGTYNRRKYQEESKLERQKIEGVKLESEGTYKILTIYSTVASSLVIAMGFAAFLFWAMSNGSCRCNICI

>QBK20557.1 hemagglutinin [Influenza A virus]
METASLITILLAVTVSNADKICIGYQSTNSTETVDTLTENNVPVTHAKELLHTEHNGMLCATSLGHPLIL
DTCTIEGLIYGNPSCDLLLGGREWSYIVERPSAVNGLCYPGNVENLEELRSLFSSARSYQRIQIFPDTIW
NVSYSGTSKACSDSFYRSMRWLTQKDNAYPVQDAQYTNNQERNILFMWGINHPPTDTAQTNLYTRTDTTT
SVATEEINRTFKPLIGPRPLVNGLMGRINYYWSVLKPGQTLRIKSNGNLIAPWYGHILSGESHGRILKTD
LKRGSCTVQCQTEKGGLNTTLPFQNVSKYAFGNCSKYIGVKSLKLAVGLRNVPSRSSRGLFGAIAGFIEG
GWSGLVAGWYGFQHSNDQGVGMAADRDSTQKAIDKITSKVNNIVDKMNKQYEIIDHEFSEVETRLNMINN
KIDDQIQDIWAYNAELLVLLENQKTLDEHDANVNNLYNKVKRALGSNAVEDGKGCFELYHKCDDQCMETI
RNGTYNRRKYQEESKLERQKIEGVKLESEGTYKILTIYSTVASSLVIAVGFAAFLFWAMSNGSCRCNICI

>QBK20556.1 hemagglutinin [Influenza A virus]

METASLITILLAVTVSNADKICIGYQSTNSTETVDTLTENNVPVTHAKELLHTEHNGMLCATSLGHPLIL
DTCTIEGLIYGNPSCDLLLGGREWSYIVERPSAVNGLCYPGNVENLEELRSLFSSARSYQRIQIFPDTIW
NVSYSGTSKACSDSFYRSMRWLTQKDNAYPVQDAQYTNNQERNILFMWGINHPPTDTAQTNLYTRTDTTT
SVATEEINRTFKPLIGPRPLVNGLMGRINYYWSVLKPGQTLRIKSNGNLIAPWYGHILSGESHGRILKTD
LKRGSCTVQCQTEKGGLNTTLPFQNVSKYAFGNCSKYIGVKSLKLAVGLRNVPSRSSRGLFGAIAGFIEG
GWSGLVAGWYGFQHSNDQGVGMAADRDSTQKAIDKITSKVNNIVDKMNKQYEIIDHEFSEVETRLNMINN
KIDDQIQDIWAYNAELLVLLENQKTLDEHDANVNNLYNKVKRALGSNAVEDGKGCFELYHKCDDQCMETI
RNGTYNRRKYQEESKLERQKIEGVKLESEGTYKILTIYSTVASSLVIAVGFAAFLFWAMSNGSCRCNICI

>QBK20555.1 hemagglutinin [Influenza A virus]

METASLITILLAVTVSNADKICIGYQSTNSTETVDTLTENNVPVTHAKELLHTEHNGMLCATSLGHPLIL
DTCTIEGLIYGNPSCDLLLGGREWSYIVERPSAVNGLCYPGNVENLEELRSLFSSARSYQRIQIFPDTIW
NVSYSGTSKACSDSFYRSMRWLTQKDNAYPVQDAQYTNNQERNILFMWGINHPPTDTAQTNLYTRTDTTT
SVATEEINRTFKPLIGPRPLVNGLMGRINYYWSVLKPGQTLRIKSNGNLIAPWYGHILSGESHGRILKTD
LKRGSCTVQCQTEKGGLNTTLPFQNVSKYAFGNCSKYIGVKSLKLAVGLRNVPSRSSRGLFGAIAGFIEG
GWSGLVAGWYGFQHSNDQGVGMAADRDSTQKAIDKITSKVNNIVDKMNKQYEIIDHEFSEVETRLNMINN
KIDDQIQDIWAYNAELLVLLENQKTLDEHDANVNNLYNKVKRALGSNAVEDGKGCFELYHKCDDQCMETI
RNGTYNRRKYQEESKLERQKIEGVKLESEGTYKILTIYSTVASSLVIAVGFAAFLFWAMSNGSCRCNICI

>QBK20554.1 hemagglutinin [Influenza A virus]

METVSLITILLVATVSNADKICIGYQSTNSTETVDTLTENNVPVTHAKELLHTEHNGMLCATSLGQPLIL
DTCTIEGLIYGNPSCDLSLEGREWSYIVERPSAVNGLCYPGNVENLEELRSLFSSARSYQRLQIFPDTIW
NVSYDGTSTACSGSFYRSMRWLTRKNGEYPTQDAQYTNNQGKNILFMWGINHPPTDTTQRDLYTRTDTTT
SVATEEINRIFKPLIGPRPLVNGLMGRIDYYWSILKPGQTLRIKSDGNLIAPWYGHILSGESHGRILKTD
LKRGSCTVQCQTEKGGLNTTLPFQNVSKYAFGNCSKYIGIKSLKLAVGLRNVPSRSSRGLFGAIAGFIEG
GWSGLVAGWYGFQHSNDQGVGMAADRDSTQKAIDKITSKVNNIVDKMNKQYEIIDHEFSEVETRLNMINN
KIDDQIQDIWAYNAELLVLLENQKTLDEHDANVNNLYNKVKRALGSNAVEDGKGCFELYHKCDNQCMETI
RNGTYNRRKYQEESKLERQKIEGVKLESEGTYKILTIYSTVASSLVIAMGFAAFLFWAMSNGSCRCNICI

>QBK20553.1 hemagglutinin [Influenza A virus]

METVSLITILLVATVSYADKICIGYQSTNSTETVDTLTENNVPVTHAKELLHTEHNGMLCATSLGQPLIL
DTCTIEGLIYGNPSCDLSLEGREWSYIVERPSAVNGLCYPGNVENLEELRSLFSSARSYQRVQIFPDTIW
NVSYDGTSTACSGSFYRSMRWLTRKNGDYPTQDAQYTNNQGKNILFMWGINHPPTDDTQRNLYTRTDTTT

SVATEEINRIFKPLIGPRPLVNGLMGRIDYYWSVLKPGQTLRIKSDGNLIAPWYGYILSGESHGRILKTD
LKRGSCTVQCQTEKGGLNTTLPFQNVSKYAFGNCSKYIGIKSLKLAVGLRNVPSRSSRGLFGAIAGFIEG
GWSGLVAGWYGFQHSNDQGVGMAADRDSTQKAIDKITSKVNNIVDKMNKQYEIIDHEFSEVETRLNMINN
KIDDQIQDIWAYNAELLVLLENQKTLDEHDANVNNLYNKVKRALGSNAVEDGKGCFELYHKCNDQCMETI
RNGTYNRRRYQEESKLERQKIEGVKLESEGTYKILTIYSTVASSLVIAMGFAAFLFWAMSNGSCRCNICI

>QBK20550.1 hemagglutinin [Influenza A virus]
METVSLITILLVATVSNADKICIGYQSTNSTETVDTLTENNVPVTHAKELLHTEHNGMLCATSLGQPLIL
DTCTIEGLIYGNPSCDLSLEGREWSYIVERPSAVNGLCYPGNVENLEELRSLFSSARSYQRIQIFPDTIW
NVSYDGTSTACSGSFYRSMRWLTRKNGEYPIQDAQYTNNQGKNILFMWGINHPPTDTTQRDLYTRTDTTT
SVATEEINRVFKPLIGPRPLVNGLMGRIDYYWSVLKPGQTLRIKSDGNLIAPWFGHILSGESHGRILKTD
LKRGSCTVQCQTEKGGLNTTLPFQNVSKYAFGNCSKYIGIKSLKLAVGLRNVPSRSSRGLFGAIAGFIEG
GWSGLVAGWYGFQHSNDQGVGMAADRDSTQKAIDKITSKVNNIVDKMNKQYEIIDHEFSEVEARLNMINN
KIDDQIQDIWAYNAELLVLLENQKTLDEHDANVNNLYNKVKRALGSNAVEDGKGCFELYHKCDDQCMETI
RNGTYNRRKYQEESKLERQKIEGVKLESEGTYKILTIYSTVASSLVIAMGFAAFLFWAMSNGSCRCNICI

>QBK20549.1 hemagglutinin [Influenza A virus]
METISLITILLVATVSYADKICIGYQSTNSTETVDTLTENNVPVTHAKELLHTEHNGMLCATSLGQPLIL
DTCTIEGLIYGNPSCDLSLEGREWSYIVERPSAVNGLCYPGNVENLEELRSLFSSARSYQRVKIFPDTIW
NVSYDGTSTACSGSFYRSMRWLTRKNGDYPTQDAQYTNNQGKNILFMWGINHPPTDDTQRNLYTRTDTTT
SVATEEINRIFKPLIGPRPLVNGLMGRIDYYWSVLKPGQTLRIKSDGNLIAPWYGYILSGESHGRILKTD
LKRGSCTVQCQTEKGGLNTTLPFQNVSKYAFGNCSKYIGIKSLKLAVGLRNVPSRSSRGLFGAIAGFIEG
GWSGLVAGWYGFQHSNDQGVGMAADRDSTQKAIDKITSKVNNIVDKMNKQYEIIDHEFSEVETRLNMINN
KIDDQIQDIWAYNAELLVLLENQKTLDEHDANVNNLYNKVKRALGSNAAEDGKGCFELYHKCNDQCMETI
RNGTYNRRKYQEESKLERQKIEGVKLESEGTYKILTIYSTVASSLVIAMGFAAFLFWAMSNGSCRCNICI

>QBK20548.1 hemagglutinin [Influenza A virus]
METVSLITILLVTTVSYADKICIGYQSTNSTETVDTLTENNVPVTHAKELLHTEHNGMLCATSLGQPLIL
DTCTIEGLIYGNPSCDLSLEGREWSYIVERPSAVNGLCYPGNVENLEELRSLFSSARSYQRVKIFPDTIW
NVSYDGTSTACSGSFYRSMRWLTRKNGDYPTQDAQYTNNQGKNILFMWGINHPPTDDTQRNLYTRTDTTT
SVATEEINRIFKPLIGPRPLVNGLMGRIDYYWSVLKPGQTLRIKSDGNLIAPWYGYILSGESHGRILKTD
LKRGSCTVQCQTEKGGLNTTLPFQNVSKYAFGNCSKYIGIKSLKLAVGLRNVPSRSSRGLFGAIAGFIEG
GWSGLVAGWYGFQHSNDQGVGMAADRDSTQKAIDKITSKVNNIVDKMNKQYEIIDHEFSEVETRLNMINN
KIDDQIQDIWAYNAELLVLLENQKTLDEHDANVNNLYNKVKRALGSNAAEDGKGCFELYHKCNDQCMETI

RNGTYNRRKYQEESKLERQKIEGVKLESEGTYKILTIYSTVASSLVIAMGFAAFLFWAMSNGSCRCNICI

>QBK20547.1 hemagglutinin [Influenza A virus]
METVSLITILLVAPVSNADKICIGYQSTTSTETVDTLTENNVPVTHAKELLHTEHNGMLCATSLGQPIIL
DTCTIEGLIYGNPSCDLSLEGREWSYIVERPSAVNGLCYPGNVENLEELRSLFSSARSYQRIQIFPDTIW
NVSYDGTSTACSGSFYRNMRWLTRKNGEYPIQDAQYTNNQGKNILFMWGINHPPADTTQRDLYTRTDTTT
SVATEEINRIFKPLIGPRPLVNGLMGRIDYYWSVLKPGQTLRIKSDGNLIAPWYGHILSGESHGRILKTD
LKRGSCTVQCQTEKGGLNTTLPFQNVSKYAFGNCSKYIGIKSLKLAVGLRNVPSRSSRGLFGAIAGFIEG
GWSGLVAGWYGFQHSNDQGVGMAADRESTQKAVDKITSKVNNIVDKMNKQYEIIDHEFSEVETRLNMINN
KIDDQIQDIWAYNAELLVLLENQKTLDEHDANVNNLYNKVKRALGSNAMEDGKGCFELYHKCDDQCMETI
RNGTYNRRKYQEESKLERQKIEGVKLESEGTYKILTIYSTVASSLVIAMGFAAFLFWAMSNGSCRCNICI

>QBK20546.1 hemagglutinin [Influenza A virus]
METVSLITILLVATVSYADKICIGYQSTNSTETVDTLTENNVPVTHAKELLHTEHNGMLCATSLGQPLIL
DTCTIEGLIYGNPSCDLSLEGREWSYIVERPSAVNGLCYPGNVENLEELRSLFSSARSYQRVKIFPDTIW
NVSYDGTSTACSGSFYRSMRWLTRKNGDYPTQDAQYTNNQGKNILFMWGINHPPTDDTQRNLYTRTDTTT
SVATEEINRIFKPLIGPRPLVNGLMGRIDYYWSVLKPGQTLRIKSDGNLIAPWYGYILSGESHGRILKTD
LKRGSCTVQCQTEKGGLNTTLPFQNVSKYAFGNCSKYIGIKSLKLAVGLRNVPSRSSRGLFGAIAGFIEG
GWSGLVAGWYGFQHSNDQGVGMAADRDSTQKAIDKITSKVNNIVDKMNKQYEIIDHEFSEVETRLNMINN
KIDDQIQDIWAYNAELLVLLENQKTLDEHDANVNNLYNKVKRALGSNAAEDGKGCFELYHKCNDQCMETI
RNGTYNRRKYQEESKLERQKIEGVKLESEGTYKILTIYSTVASSLVIAMGFAAFLFWAMSNGSCRCNICI

>QBK20545.1 hemagglutinin [Influenza A virus]
METVSLITILLVATVSNADKICIGYQSTNSTETVDTLTENNVPVTHAKELLHTEHNGMLCATSLGQPLIL
DTCTIEGLIYGNPSCDLSLEGREWSYIVERPSAVNGLCYPGNVENLEELRSLFSSARSYQRIQIFPDTIW
NVSYDGTSTACSNSFYRSMRWLTRKNGDYPTQDAQYTNNKGKNILFMWGINHPPTDDTQRNLYTRTDTTT
SVATEEINRIFKPLIGPRPLVNGLMGRIDYYWSVLKPGQTLRIKSDGNLIAPWYGHILSGESHGRILKTD
LKRGSCTVQCQTEKGGLNTTLPFQNVSKYAFGNCSKYIGIKSLKLAVGLRNVPSRSSRGLFGAIAGFIEG
GWSGLVAGWYGFQHSNDQGVGMAADRDSTQKAIDKITSKVNNIVDKMNKQYEIIDHEFSEVETRLNMINN
KIDDQIQDIWAYNAELLVLLENQKTLDEHDANVNNLYNKVKRALGSNAVEDGKGCFELYHKCNDQCMETI
RNGTYNRRKYQEESKLERQKIEGVKLESEGTYKILTIYSTVASSLVIAMGFAAFLFWAMSNGSCRCNICI

>QBK20543.1 hemagglutinin [Influenza A virus]
METVSLITILLVATVSNADKICIGYQSTNSTETVDTLTENNVPVTHAKELLHTEHNGMLCATSLGQPIIL

DTCTIEGLIYGNPSCDLSLEGREWSYIVERPSAVNGLCYPGNVENLEELRSLFSSARSYQRIQIFPDTIW
NVSYDGTSTACSGSFYRNMRWLTRKNGEYPIQDAQYTNNQGKNILFMWGINHPPADTTQRNLYTRTDTTT
SVATEEINRIFKPLIGPRPLVNGLMGRIDYYWSVLKPGQTLRIKSDGNLIAPWYGHILSGESHGRILKTD
LKRGSCTVQCQTEKGGLNTTLPFQNVSKYAFGNCSKYIGIKSLKLAVGLRNVPSRSSRGLFGAIAGFIEG
GWSGLVAGWYGFQHSNDQGVGMAADRDSTQKAVDKITSKVNTIVDKMNKQYEIIDHEFSEVETRLNMINN
KIDDQIQDIWAYNAELLVLLENQKTLDEHDANVNNLYNKVKRALGSNAVEDGKGCFELYHKCDDQCMETI
RNGTYNRRKYQEESKLERQKIEGVKLESEGAYKILTIYSTVASSLVIAMGFAAFLFWAMSNGSCRCNICI

>QBK20542.1 hemagglutinin [Influenza A virus]
METVSLITILLVATVSNADKICIGYQSTNSTETVDTLTENNVPVTHAKELLHTEHNGMLCATSLGQPLIL
DTCTIEGLIYGNPSCDLSLEGREWSYIVERPSAVNGLCYPGNVENLEELRSLFSSARSYQRIQIFPDTIW
NVSYDGTSTACSNSFYRSMRWLTRKNGDYPTQDAQYTNNQGKNILFMWGINHPPTDDTQRNLYTRTDTTT
SVATEEINRIFKPLIGPRPLVNGLMGRIDYYWSVLKPGQTLRIKSDGNLIAPWYGHILSGESHGRILKTD
LKRGSCTVQCQTEKGGLNTTLPFQNVSKYAFGNCSKYIGIKSLKLAVGLRNVPSRSSRGLFGAIAGFIEG
GWSGLVAGWYGFQHSNDQGVGMAADRDSTQKAIDKITSKVNNIVDKMNKQYEIIDHEFSEVETRLNMINN
KIDDQIQDIWAYNAELLILLENQKTLDEHDANVNNLYNKVKRALGSNAVEDGKGCFELYHKCNDQCMETI
RNGTYNRRKYQEESKLERQKIEGVKLESEGTYKILTIYSTVASSLVIAMGFAAFLFWAMSNGSCRCNICI

>QBK20539.1 hemagglutinin [Influenza A virus]
METVSLITILLVAAVSNADKICIGYQSTNSTETVDTLTENNVPVTHAKELLHTEHNGMLCATSLGQPIIL
DTCTIEGLIYGNPSCDLSLEGREWSYIVERPSAVNGLCYPGNVENLEELRSLFSSARSYQRIQIFPDTIW
NVSYDGTSTACSGSFYRNMRWLTRKNGEYPIQDAQYTNNQGKNILFMWGINHPPADTTQRNLYTRTDTTT
SVATEEINRIFKPLIGPRPLVNGLMGRIDYYWSVLKPGQTLRIKSDGNLIAPWYGHILSGESHGRILKTD
LKRGSCTVQCQTEKGGLNTTLPFQNVSKYAFGNCSKYIGIKSLKLAVGLRNVPSRSSRGLFGAIAGFIEG
GWSGLVAGWYGFQHSNDQGVGMAADRDSTQKAVDKITSKVNTIVDKMNKQYEIIDHEFSEVETRLNMINN
KIDDQIQDIWAYNAELLVLLENQKTLDEHDANVNNLYNKVKRALGSNAVEDGKGCFELYHKCDDQCMETI
RNGTYNRRKYQEESKLERQKIEGVKLESEGTYKILTIYSTVASSLVIAMGFAAFLFWAMSNGSCRCNICI

>QBK20538.1 hemagglutinin [Influenza A virus]
MGTVSLITILLVAAVSNADKICIGYQSTNSTETVDTLTENNVPVTHAKELLHTEHNGMLCATSLGQPIIL
DTCTIEGLIYGNPSCDLSLEGREWSYIVERPSAVNGLCYPGNVENLEELRSLFSSARSYQRIQIFPDTIW
NVSYDGTSTACSGSFYRNMRWLTRKNGEYPIQDAQYTNNQGKNILFMWGINHPPADTTQRNLYTRTDTTT
SVATEEINRIFKPLIGPRPLVNGLMGRIDYYWSVLKPGQTLRIKSDGNLIAPWYGHILSGESHGRILKTD
LKRGSCTVQCQTEKGGLNTTLPFQNVSKYAFGNCSKYIGIKSLKLAVGLRNVPSRSSRGLFGAIAGFIEG

GWSGLVAGWYGFQHSNDQGVGMAADRDSTQKAVDKITSKVNTIVDKMNKQYEIIDHEFNEVETRLNMINN
KIDDQIQDIWAYNAELLVLLENQKTLDEHDANVNNLYNKVKRALGSNAVEDGKGCFELYHKCDDQCMETI
RNGTYNRRKYQEESKLERQKIEGVKLESEGTYKILTIYSTVASSLVIAMGFAAFLFWAMSNGSCRCNICI

>QBK20537.1 hemagglutinin [Influenza A virus]
METVSLITILLVAAVSNADKICIGYQSTNSTETVDTLTENNVPVTHAKELLHTEHNGMLCATSLGQPIIL
DTCTIEGLIYGNPSCDLSLEGREWSYIVERPSAVNGLCYPGNVENLEELRSLFSSARSYQRIQIFPDTIW
NVSYDGTSTACSGSFYRNMRWLTRKNGEYPIQDAQYTNNQGKNILFMWGINHPPADTTQRNLYTRTDTTT
SVATEEINRIFKPLIGPRPLVNGLMGRIDYYWSVLKPGQTLRIKSDGNLIAPWYGHILSGESHGRILKTD
LKRGSCTVQCQTEKGGLNTTLPFQNVSKYAFGNCSKYIGIKSLKLAVGLRNVPSRSSRGLFGAIAGFIEG
GWSGLVAGWYGFQHSNDQGVGMAADRDSTQKAVDKITSKVNTIVDKMNKQYEIIDHEFSEVETRLNMINN
KIDDQIQDIWAYNAELLVLLENQKTLDEHDANVNNLYNKVKRALGSNAVEDGKGCFELYHKCDDQCMETI
RNGTYNRRKYQEESKLERQKIEGVKLESEGTYKILTIYSTVASSLVIAMGFAAFLFWAMSNGSCRCNICI

>QBK20536.1 hemagglutinin [Influenza A virus]
METVSLITILLVATVSNADKICIGYQSTNSTETVDTLTENNVPVTHAKELLHTEHNGMLCATSLGQPLIL
DTCTIEGLIYGNPSCDLSLEGREWSYIVERPSAVNGLCYPGNVENLEELRSLFSSARSYQRVQIFPDTIW
NVSYDGTSTACSGSFYRSMRWLTRKNGDYPTQDAQYTNNQGKNILFMWGINHPPTDDTQRNLYTRTDTTT
SVATEEINRIFKPLIGPRPLVNGLMGRIDYYWSVLRPGQTLRIKSNGNLIAPWYGHILSGESHGRILKTD
LKRGSCTVQCQTEKGGLNTTLPFQNVSKYAFGNCSKYIGIKSLKLAVGLRNVPSRSNRGLFGAIAGFIEG
GWSGLVAGWYGFQHSNDQGVGMAADRDSTQKAIDKITSKVNNIVDKMNKQYEIIDHEFSEVETRLNMINN
KIDDQIQDIWAYNAELLVLLENQKTLDEHDANVNNLYNKVKRALGSNAVEDGKGCFELYHKCNDQCMETI
RNGTYNRRKYQEESKLERQKIEGVKLESEGTYKILTIYSTVASSLVIAMGFAAFLFWAMSNGSCRCNICI

>QBK20534.1 hemagglutinin [Influenza A virus]
METVSLITILLVATVSNADKICIGYQSTNSTETVDTLTENNVPVTHAKELLHTEHNGMLCATSLGQPLIL
DTCTIEGLIYGNPSCDLSLEGREWSYIVERPSAVNGLCYPGNVENLEELRSLFSSARSYQRIQIFPDTIW
NVSYDGTSTACSGSFYRSMRWLTRKNGEYPIQDAQYTNNQGKNILFMWGINHPPTDTTQRDLYTRTDTTT
SVATEEINRIFKPLIGPRPLVNGLMGRIDYYWSVLKPGQTLRIKSDGNLIAPWFGHILSGESHGRILKTD
LKRGSCTVQCQTEKGGLNTTLPFQNVSKYAFGNCSKYIGIKSLKLAVGLRNVPSRSSRGLFGAIAGFIEG
GWSGLVAGWYGFQHSNDQGVGMAADRDSTQKAIDKITSKVNNIVDKMNKQYEIIDHEFSEVETRLNMINN
KIDDQIQDIWAYNAELLVLLENQKTLDEHDANVNNLYNKVKRALGSNAVEDGKGCFELYHKCDDQCMETI
RNGTYNGRKYQEESKLERQKIEGVKLESEGTYKILTIYSTVASSLVIAMGFAAFLFWAMSNGSCRCNICI

>QBK20533.1 hemagglutinin [Influenza A virus]

METVSLITILLVATVSNADKICIGYQSTNSTETVDTLTENNVPVTHAKELLHTEHNGMLCATSLGQPLIL

DTCTIEGLIYGNPSCDLSLEGREWSYIVERPSAVHGLCYPGNIENLEELRSLFSSARSYQRIQIFPDTIW

NVSYDGTSTACSGSFYRNMRWLTRKNGDYPIQDAQYTNNQGKNILFMWGINHPPTDTTQGNLYTRTDTTT

SVATEEINRIFKPLIGPRPLVNGLMGRIDYYWSVLKPGQTLRIKSDGNLIAPWYGHILSGESHGRILKTD

LKRGSCTVQCQTEKGGLNTTLPFQNVSRYAFGNCSKYIGIKSLKLAVGLRNVPSRSSRGLFGAIAGFIEG

GWSGLVAGWYGFQHSNDQGVGMAADRDTTQKAIDKITSKVNNIVDKMNKQYEIIDHEFSEVETRLNMINN

KIDDQIQDIWAYNAELLVLLENQKTLDEHDANVNNLYNKVKRALGSNAVEDGKGCFELYHKCDDQCMETI

RNGTYNRRKYQEESKLERQKIEGVKLESEGTYKILTIYSTVASSLVIAMGFAAFLFWAMSNGSCRCNICI

>QBK20532.1 hemagglutinin [Influenza A virus]

METVSLITILLVATVSNADKICIGYQSTNSTETVDTLTENNVPVTHAKELLHTEHNGMLCATSLGHPLIL

DTCTIEGIIYGNPSCDPLLGGREWSYIVERPSAVNGLCYPGNVENLEELRSLFSSSRSYQRIQIFPDTIW

NVSYSGTSKACSDSFYRSMRWLTQKNNAYPTQDAQYTNNQEKNILFMWGINHPPTDTAQTNLYTRTDTTT

SVATEEINRIFKPLIGPRPLVNGLMGRINYYWSVLKPGQTLRIKSNGNLIAPWYGHILSGESHGRILKTD

LKSGSCTVQCQTEKGGLNTTLPFQNVSKYAFGNCSKYIGVKSLKLAVGLRNVPSRSSRGLFGAIAGFIEG

GWSGLVAGWYGFQHSNDQGVGMAADRDSTQKAIDKITSKVNNIVDKMNKQYEIIDHEFSEVETRLNMINN

KVDDQIQDIWAYNAELLVLLENQKTLDEHDANVNNLYNKVKRALGSNAVEDGKGCFELYHKCDDHCMETI

RNGTYNRRKYQEESKLERQKIEGVKLESEETYKILTIYSTVASSLVIAMGFAAFLFWAMSNGSCRCNICI

>QBK20531.1 hemagglutinin [Influenza A virus]

METVSLITILLVATVSNADKICIGYQSTNSTETVDTLTENNVPVTHAKELLHTEHNGMLCATGLGQPLIL

DTCTIEGLIYGNPSCDLSMEGREWSYIVERPSAVNGLCYPGNVENLEELRSLFSSARSYQRIQIFPDTTW

NVSYDGTSTACSGSFYRNMRWLTRKNGEYPIQDAQYTNNQGKNILFMWGINHPPTDTTQRNLYTRTDTTT

SVATEEINRVFKPLIGPRPLVNGLMGRIDYYWSVLKPGQTLRIKSDGNLIAPWYGHILSGESHGRILKTD

LKKGSCTVQCQTEKGGLNTTLPFQNVSKYAFGNCSKYIGIKSLKLAVGLRNVPSRSSRGLFGAIAGFIEG

GWSGLVAGWYGFQHSNDQGVGMAADRDSTQRAIDKITSKVNNIVDKMNKQYEIIDHEFSEVETRLNMINN

KIDDQIQDIWAYNAELLVLLENQKTLDEHDANVNNLYNKVKRALGSNAVEDGKGCFELYHKCDDQCMETI

RNGTYNRRKYQEESKLERQKIEGVKLESEGTYKILTIYSTVASSLVIAMGFAAFLFWAMSNGSCRCNICI

>QBK20530.1 hemagglutinin [Influenza A virus]

METVSLITILLVATVSNADKICIGYQSTNSTETVDTLTENNVPVTHAKELLHTEHNGMLCATSLGQPLIL

DTCTIEGIIYGNPSCDLSLEGREWSYIVERPSAVNGLCYPGNVENLEELRSLFSSARSYQRIQIFPDTIW

NVSYDGTSTACSGSFYKSMRWLTRKNGDYPIQDAQYTNNQGKNILFMWGINHPPTDTTQRDLYTRTDTTT

SVATEEINRVFKPLIGPRPLVNGLMGRIDYYWSVLKPGQTLRIKSDGNLIAPWFGHILSGESHGRILKTD

LKRGSCTVQCQTEKGGLNTTLPFQNVSKYAFGNCSKYIGIKSLKLAVGLRNVPSRSSRGLFGAIAGFIEG

GWSGLVAGWYGFQHSNDQGVGMAADRDSTQKAIDKITSKVNNIVDKMNKQYEIIDHEFSEVETRLNMINN

KIDDQIQDIWAYNAELLVLLENQKTLDEHDANVNNLYNKVKRALGSNAVEDGKGCFELYHKCDDQCMETI

RNGTYNRRKYQEESKLERQKIEGVKLESEGTYKILTIYSTVASSLVIAMGFAAFLFWAMSNGSCRCNICI

>QBK20529.1 hemagglutinin [Influenza A virus]

METVSLITILLVATVSNADKICIGYQSTNSTETVDTLTENNVPVTHAKELLHTEHNGMLCATSLGQPLIL

DTCTIEGIIYGNPSCDLSLEGREWSYIVERPSAVNGLCYPGNVENLEELRSLFSSARSYQRIQIFPDTIW

NVSYDGTSAACSGSFYKSMRWLTRKNGDYPIQDAQYTNNQGKNILFMWGINHPPTDTTQRDLYTRTDTTT

SVATEEINRVFKPLIGPRPLVNGLMGRIDYYWSVLKPGQTLRIKSDGNLIAPWFGHILSGESHGRILKTD

LKRGSCTVQCQTEKGGLNTTLPFQNVSKYAFGNCSKYIGIKSLKLAVGLRNVPSRSSRGLFGAIAGFIEG

GWSGLVAGWYGFQHSNDQGVGMAADRDSTQKAIDKITSKVNNIVDKMNKQYEIIDHEFSEVETRLNMINN

KIDDQIQDIWAYNAELLVLLENQKTLDEHDANVNNLYNKVKRALGSNAVEDGKGCFELYHKCDDQCMETI

RNGTYNRRKYQEESKLERQKIEGVKLESEGTYKILTIYSTVASSLVIAMGFAAFLFWAMSNGSCRCNICI

>QBK20528.1 hemagglutinin [Influenza A virus]

METVSLITILLVATVSNADKICIGYQSTNSTETVDTLTENNVPVTHAKELLHTEHNGMLCATSLGQPLIL

DTCTIEGIIYGNPSCDLSLEGREWSYIVERPSAVNGLCYPGNVENLEELRSLFSSARSYQRIQIFPDTIW

NVSYDGTSAACSGSFYKSMRWLTRKNGDYPIQDAQYTNNQGKNILFMWGINHPPTDTTQRDLYTRTDTTT

SVATEEINRVFKPLIGPRPLVNGLMGRIDYYWSVLKPGQTLRIKSDGNLIAPWFGHILSGESHGRILKTD

LKRGSCTVQCQTEKGGLNTTLPFQNVSKYAFGNCSKYIGIKSLKLAVGLRNVPSRSSRGLFGAIAGFIEG

GWSGLVAGWYGFQHSNDQGVGMAADRDSTQKAIDKITSKVNNIVDKMNKQYEIIDHEFSEVETRLNMINN

KIDDQIQDIWAYNAELLVLLENQKTLDEHDANVNNLYNKVKRALGSNAVEDGKGCFELYHKCDDQCMETI

RNGTYNRRKYQEESKLERQKIEGVKLESEGTYKILTIYSTVASSLVIAMGFAAFLFWAMSNGSCRCNICI

>QBK20527.1 hemagglutinin [Influenza A virus]

METVSLITILLVATVSNADKICIGYQSTNSTETVDTLTENNVPVTHAKELLHTEHNGMLCATSLGQPLIL

DTCTIEGIIYGNPSCDLSLEGREWSYIVERPSAVNGLCYPGNVENLEELRSLFSSARSYQRIQIFPDTIW

NVSYDGTSAACSGSFYKSMRWLTRKNGDYPIQDAQYTNNQGKNILFMWGINHPPTDTTQRDLYTRTDTTT

SVATEEINRVFKPLIGPRPLVNGLMGRIDYYWSVLKPGQTLRIKSDGNLIAPWFGHILSGESHGRILKTD

LKRGSCTVQCQTEKGGLNTTLPFQNVSKYAFGNCSKYIGIKSLKLAVGLRNVPSRSSRGLFGAIAGFIEG

GWSGLVAGWYGFQHSNDQGVGMAADRDSTQKAIDKITSKVNNIVDKMNKQYEIIDHEFSEVETRLNMINN

KIDDQIQDIWAYNAELLVLLENQKTLDEHDANVNNLYNKVKRALGSNAVEDGKGCFELYHKCDDQCMETI

RNGTYNRRKYQEESKLERQKIEGVKLESEGTYKILTIYSTVASSLVIAMGFAAFLFWAMSNGSCRCNICI

>QBK20526.1 hemagglutinin [Influenza A virus]
METVSLITILLVATVSNADKICIGYQSTNSTETVDTLTENNVPVTHAKELLHTEHNGMLCATSLGQPLIL
DTCTIEGIIYGNPSCDLSLEGREWSYIVERPSAVNGLCYPGNVENLEELRSLFSSARSYQRIQIFPDTIW
NVSYDGTSAACSGSFYKSMRWLTRKNGDYPIQDAQYTNNQGKNILFMWGINHPPTDTTQRDLYTRTDTTT
SVATEEINRVFKPLIGPRPLVNGLMGRIDYYWSVLKPGQTLRIKSDGNLIAPWFGHILSGESHGRILKTD
LKRGSCTVQCQTEKGGLNTTLPFQNVSKYAFGNCSKYIGIKSLKLAVGLRNVPSRSSRGLFGAIAGFIEG
GWSGLVAGWYGFQHSNDQGVGMAADRDSTQKAIDKITSKVNNIVDKMNKQYEIIDHEFSEVETRLNMINN
KIDDQIQDIWAYNAELLVLLENQKTLDEHDANVNNLYNKVKRALGSNAVEDGKGCFELYHKCDDQCMETI
RNGTYNRRKYQEESKLERQKIEGVKLESEGTYKILTIYSTVASSLVIAMGFAAFLFWAMSNGSCRCNICI

>QBK20525.1 hemagglutinin [Influenza A virus]
METVSLITILLVATVSNADKICIGYQSTNSTETVDTLTENNVPVTHAKELLHTEHNGMLCATSLGQPLIL
DTCTIEGIIYGNPSCDLSLEGREWSYIVERPSAVHGLCYPGNIENLEELRSLFSSARSYQRIQIFPDTIW
NVSYDGTSTACSGSFYRNMRWLTRKNGDYPIQDAQYTNNQGKNILFMWGINHPPTDTTQGNLYTRTDTTT
SVATEEINRIFKPLIGPRPLVNGLMGRIDYYWSVLKPGQTLRIKSDGNLIAPWYGHILSGESHGRILKTD
LKRGSCTVQCQTEKGGLNTTLPFQNVSRYAFGNCSKYIGIKSLKLAVGLRNVPSRSSRGLFGAIAGFIEG
GWSGLVAGWYGFQHSNDQGVGMAADRDTTQKAIDKITSKVNNIVDKMNKQYEIIDHEFSEVETRLNMINN
KIDDQIQDIWAYNAELLVLLENQKTLDEHDANVNNLYNKVKRALGSNAVEDGKGCFELYHKCDDQCMETI
RNGTYNRRKYQEESKLERQKIEGVKLESEGTYKILTIYSTVASSLVIAMGFAAFLFWAMSNGSCRCNICI

>QBK20523.1 hemagglutinin [Influenza A virus]
METVSLITILLVATVSNADKICIGYQSTNSTETVDTLTENNVPVTHAKELLHTEHNGMLCATSLGHPLIL
DTCTIEGIIYGNPSCDPLLGGREWSYIVERPSAVNGLCYPGNVENLEELRSLFSSSRSYQRIQIFPDTIW
NVSYSGTSKACSDSFYRSMRWLTQKNNAYPTQDAQYTNNQEKNILFMWGINHPPTDTAQTNLYTRTDTTT
SVATEEINRIFKPLIGPRPLVNGLMGRINYYWSVLKPGQTLRIKSNGNLIAPWYGHILSGESHGRILKTD
LKSGSCTVQCQTEKGGLNTTLPFQNVSKYAFGNCSKYIGVKSLKLAVGLRNVPSRSSRGLFGAIAGFIEG
GWSGLVAGWYGFQHSNDQGVGMAADRDSTQKAIDKITSKVNNIVDKMNKQYEIIDHEFSEVETRLNMINN
KVDDQIQDIWAYNAELLVLLENQKTLDEHDANVNNLYNKVKRALGSNAVEDGKGCFELYHKCDDHCMETI
RNGTYNRRKYQEESKLERQKIEGVKLESEETYKILTIYSTVASSLVIAMGFAAFLFWAMSNGSCRCNICI

>QBK20522.1 hemagglutinin [Influenza A virus]
METVSLITILLVATVSNADKICIGYQSTNSTETVDTLTENNVPVTHAKELLHTEHNGMLCATSLGHPLIL

DTCTIEGIIYGNPSCDPLLGGREWSYIVERPSAVNGLCYPGNVENLEELRSLFSSSRSYQRIQIFPDTIW
NVSYSGTSKACSDSFYRSMRWLTQKNNAYPTQDAQYTNNQEKNILFMWGINHPPTDTAQTNLYTRTDTTT
SVATEEINRIFKPLIGPRPLVNGLMGRINYYWSVLKPGQTLRIKSNGNLIAPWYGHILSGESHGRILKTD
LKSGSCTVQCQTEKGGLNTTLPFQNVSKYAFGNCSKYIGVKSLKLAVGLRNVPSRSSRGLFGAIAGFIEG
GWSGLVAGWYGFQHSNDQGVGMAADRDSTQKAIDKITSKVNNIVDKMNKQYEIIDHEFSEVETRLNMINN
KVDDQIQDIWAYNAELLVLLENQKTLDEHDANVNNLYNKVKRALGSNAVEDGKGCFELYHKCDDHCMETI
RNGTYNRRKYQEESKLERQKIEGVKLESEETYKILTIYSTVASSLVIAMGFAAFLFWAMSNGSCRCNICI

>QBK20520.1 hemagglutinin [Influenza A virus]
METVSLITILLVATVSNADKICIGYQSTNSTETVDTLTENNVPVTHAKELLHTEHNGMLCATSLGQPLIL
DTCTIEGLIYGNPSCDLSLEGREWSYIVERPSAVHGLCYPGNIENLEELRSLFSSARSYQRIQIFPDTIW
NVSYDGTSTACSGSFYRNMRWLTRKNGDYPIQDAQYTNNQGKNILFMWGINHPPTDTTQGNLYTRTDTTT
SVATEEINRIFKPLIGPRPLVNGLMGRIDYYWSVLKPGQTLRIKSDGNLIAPWYGHILSGESHGRILKTD
LKRGSCTVQCQTEKGGLNTTLPFQNVSRYAFGNCSKYIGIKSLKLAVGLRNVPSRSSRGLFGAIAGFIEG
GWSGLVAGWYGFQHSNDQGVGMAADRDTTQKAIDKITSKVNNIVDKMNKQYEIIDHEFSEVETRLNMINN
KIDDQIQDIWAYNAELLVLLENQKTLDEHDANVNNLYNKVKRALGSNAVEDGKGCFELYHKCDDQCMETI
RNGTYNRRKYQEESKLERQKIEGVKLESEGTYKILTIYSTVASSLVIAMGFAAFLFWAMSNGSCRCNICI

>QBK20519.1 hemagglutinin [Influenza A virus]
METVSLITILLVATVSNADKICIGYQSTNSTETVDTLTENNVPVTHAKELLHTEHNGMLCATSLGQPLIL
DTCTIEGLIYGNPSCDLSLEGREWSYIVERPSAVHGLCYPGNIENLEELRSLFSSARSYQRIQIFPDTIW
NVSYDGTSTACSGSFYRNMRWLTRKNGDYPIQDAQYTNNQGKNILFMWGINHPPTDTTQGNLYTRTDTTT
SVATEEINRIFKPLIGPRPLVNGLMGRIDYYWSVLKPGQTLRIKSDGNLIAPWYGHILSGESHGRILKTD
LKRGSCTVQCQTEKGGLNTTLPFQNVSRYAFGNCSKYIGIKSLKLAVGLRNVPSRSSRGLFGAIAGFIEG
GWSGLVAGWYGFQHSNDQGVGMAADRDTTQKAIDKITSKVNNIVDKMNKQYEIIDHEFSEVETRLNMINN
KIDDQIQDIWAYNAELLVLLENQKTLDEHDANVNNLYNKVKRALGSNAVEDGKGCFELYHKCDDQCMETI
RNGTYNRRKYQEESKLERQKIEGVKLESEGTYKILTIYSTVASSLVIAMGFAAFLFWAMSNGSCRCNICI

>QBK20518.1 hemagglutinin [Influenza A virus]
METVSLITILLVATVSNADKICIGYQSTNSTETVDTLTENNVPVTHAKELLHTEHNGMLCATSLGQPLIL
DTCTIEGLIYGNPSCDLSLEGREWSYIVERPSAVHGLCYPGNIENLEELRSLFSSARSYQRIQIFPDTIW
NVSYDGTSTACSGSFYRNMRWLTRKNGDYPIQDAQYTNNQGKNILFMWGINHPPTDTTQGNLYTRTDTTT
SVATEEINRIFKPLIGPRPLVNGLMGRIDYYWSVLKPGQTLRIKSDGNLIAPWYGHILSGESHGRILKTD
LKRGSCTVQCQTEKGGLNTTLPFQNVSRYAFGNCSKYIGIKSLKLAVGLRNVPSRSSRGLFGAIAGFIEG

GWSGLVAGWYGFQHSNDQGVGMAADRDTTQKAIDKITSKVNNIVDKMNKQYEIIDHEFSEVETRLNMINN
KIDDQIQDIWAYNAELLVLLENQKTLDEHDANVNNLYNKVKRALGSNAVEDGKGCFELYHKCDDQCMETI
RNGTYNRRKYQEESKLERQKIEGVKLESEGTYKILTIYSTVASSLVIAMGFAAFLFWAMSNGSCRCNICI

>QBK20517.1 hemagglutinin [Influenza A virus]
METVSLITILLVATVSNADKICIGYQSTNSTETVDTLTENNVPVTHAKELLHTEHNGMLCATSLGQPLIL
DTCTIEGLIYGNPSCDLSLEGREWSYIVERPSAVNGLCYPGNVENLEELRSLFSSARSYQRIQIFPDTIW
NVSYDGTSTACSGSFYRSMRWLTRKNGEYPIQDAQYTNNQGKNILFMWGINHPPTDTTQRDLYTRTDTTT
SVATEEINRVFKPLIGPRPLVNGLMGRIDYYWSVLKPGQTLRIKSDGNLIAPWFGHILSGESHGRILKTD
LKRGSCTVQCQTEKGGLNTTLPFQNVSKYAFGNCSKYIGIKSLKLAVGLRNVPSRSSRGLFGAIAGFIEG
GWSGLVAGWYGFQHSNDQGVGMAADRDSTQKAIDKITSKVNNIVDKMNKQYEIIDHEFSEVEARLNMINN
KIDDQIQDIWAYNAELLVLLENQKTLDEHDANVNNLYNKVKRALGSNAVEDGKGCFELYHKCDDQCMETI
RNGTYNRRKYQEESKLERQKIEGVKLESEGTYKILTIYSTVASSLVIAMGFAAFLFWAMSNGSCRCNICI

>QBK20516.1 hemagglutinin [Influenza A virus]
METVSLITILLVATVSNADKICIGYQSTNSTETVDTLTENNVPVTHAKELLHTEHNGMLCATSLGQPLIL
DTCTIEGLIYGNPSCDLSLEGREWSYIVERPSAVNGLCYPGNVENLEELRSLFSSARSYQRIQIFPDTIW
NVSYDGTSAACSGSFYRSMRWLTRKNGEYPIQDAQYTNNQGKNILFMWGINHPPTDTTQRDLYTRTDTTT
SVATEEINRVFKPLIGPRPLVNGLMGRIDYYWSVLKPGQTLRIKSDGNLIAPWFGHILSGESHGRILKTD
LKRGSCTVQCQTEKGGLNTTLPFQNVSKYAFGNCSKYIGIKSLKLAVGLRNVPSRSSRGLFGAIAGFIEG
GWSGLVAGWYGFQHSNDQGVGMAADRDSTQKAIDKITSKVNNIVDKMNKQYEIIDHEFSEVEARLNMINN
KIDDQIQDIWAYNAELLVLLENQKTLDEHDANVNNLYNKVKRALGSNAVEDGKGCFELYHKCDDQCMETI
RNGTYNRRKYQEESKLERQKIEGVKLESEGIYKILTIYSTVASSLVIAMGFAAFLFWAMSNGSCRCNICI

>QBK20515.1 hemagglutinin [Influenza A virus]
METVSLITILLVATVSNADKICIGYQSTNSTETVDTLTENNVPVTHAKELLHTEHNGMLCATSLGQPLIL
DTCTIEGLIYGNPSCDLSLEGREWSYIVERPSAVNGLCYPGNVENLEELRSLFSSARSYQRIQIFPDTIW
NVSYDGTSTACSGSFYRSMRWLTRKNGEYPIQDAQYTNNQGKNILFMWGINHPPTDTTQRDLYTRTDTTT
SVATEEINRVFKPLIGPRPLVNGLMGRIDYYWSVLKPGQTLRIKSDGNLIAPWFGHILSGESHGRILKTD
LKRGSCTVQCQTEKGGLNTTLPFQNVSKYAFGNCSKYIGIKSLKLAVGLRNVPSRSSRGLFGAIAGFIEG
GWSGLVAGWYGFQHSNDQGVGMAADRDSTQKAIDKITSKVNNIVDKMNKQYEIIDHEFSEVEARLKMINN
KIDDQIQDIWAYNAELLVLLENQKTLDEHDANVNNLYNKVKRALGSNAVEDGKGCFELYHKCDDQCMETI
RNGTYNRRKYQEESKLERQKIEGVKLESEGTYKILTIYSTVASSLVIAMGFAAFLFWAMSNGSCRCNICI

>QBK20514.1 hemagglutinin [Influenza A virus]
METVSLITILLVATVSNADKICIGYQSTNSTETVDTLTENNVPVTHAKELLHTEHNGMLCATSLGQPLIL
DTCTIEGLIYGNPSCDLSLEGREWSYIVERPSAVNGLCYPGNVENLEELRSLFSSARSYQRIQIFPDTIW
NVSYDGTSTACSGSFYRSMRWLTRKNGEYPIQDAQYTNNQGKNILFMWGINHPPTDTTQRDLYTRTDTTT
SVATEEINRVFKPLIGPRPLVNGLMGRIDYYWSVLKPGQTLRIKSDGNLIAPWFGHILSGESHGRILKTD
LKRGSCTVQCQTEKGGLNTTLPFQNVSKYAFGNCSKYIGIKSLKLAVGLRNVPSRSSRGLFGAIAGFIEG
GWSGLVAGWYGFQHSNDQGVGMAADRDSTQKAIDKITSKVNNIVDKMNKQYEIIDHEFSEVEARLNMINN
KIDDQIQDIWAYNAELLVLLENQKTLDEHDANVNNLYNKVKRALGSNAVEDGKGCFELYHKCDDQCMETI
RNGTYNRRKYQEESKLERQKIEGVKLESEGTYKILTIYSTVASSLVIAMGFAAFLFWAMSNGSCRCNICI

>QBK20513.1 hemagglutinin [Influenza A virus]
METVSLITILLVATVSNADKICIGYQSTNSTETVDTLTENNVPVTHAKELLHTEHNGMLCATSLGQPLIL
DTCTIEGLIYGNPSCDLSLEGREWSYIVERPSAVNGLCYPGNVENLEELRSLFSSARSYQRIQIFPDTIW
NVSYDGTSTACSGSFYRSMRWLTRKNGEYPIQDAQYTNNQGKNILFMWGINHPPTDTTQRDLYTRTDTTT
SVATEEINRVFKPLIGPRPLVNGLMGRIDYYWSVLKPGQTLRIKSDGNLIAPWFGHILSGESHGRILKTD
LKRGSCTVQCQTEKGGLNTTLPFQNVSKYAFGNCSKYIGIKSLKLAVGLRNVPSRSSRGLFGAIAGFIEG
GWSGLVAGWYGFQHSNDQGVGMAADRDSTQKAIDKITSKVNNIVDKMNKQYEIIDHEFSEVEARLNMINN
KIDDQIQDIWAYNAELLVLLENQKTLDEHDANVNNLYNKVKRALGSNAVEDGKGCFELYHKCDDQCMETI
RNGTYNRRKYQEESKLERQKIEGVKLESEGTYKILTIYSTVASSLVIAMGFAAFLFWAMSNGSCRCNICI

>QBK20512.1 hemagglutinin [Influenza A virus]
METVSLITILLVATVSNADKICIGYQSTNSTETVDTLTENNVPVTHAKELLHTEHNGMLCATSLGQPLIL
DTCTIEGLIYGNPSCDLSLEGREWSYIVERPSAVNGLCYPGNVENLEELRSLFSSARSYQRIQIFPDTIW
NVSYDGTSTACSGSFYRSMRWLTRKNGEYPIQDAQYTNNQGKNILFMWGINHPPTDTTQRDLYTRTDTTT
SVATEEINRVFKPLIGPRPLVNGLMGRIDYYWSVLKPGQTLRIKSDGNLIAPWFGHILSGESHGRILKTD
LKRGSCTVQCQTEKGGLNTTLPFQNVSKYAFGNCSKYIGIKSLKLAVGLRNVPSRSSRGLFGAIAGFIEG
GWSGLVAGWYGFQHSNDQGVGMAADRDSTQKAIDKITSKVNNIVDKMNKQYEIIDHEFSEVEARLNMINN
KIDDQIQDIWAYNAELLVLLENQKTLDEHDANVNNLYNKVKRALGSNAVEDGKGCFELYHKCDDQCMETI
RNGTYNRRKYQEESKLERQKIEGVKLESEGTYKILTIYSTVASSLVIAMGFAAFLFWAMSNGSCRCNICI

>QBK20511.1 hemagglutinin [Influenza A virus]
METVSLITILLVATVSNADKICIGYQSTNSTETVDTLTENNVPVTHAKELLHTEHNGMLCATSLGQPLIL
DTCTIEGLIYGNPSCDLSLEGREWSYIVERPSAVNGLCYPGNVENLEELRSLFSSARSYQRIQIFPDTIW
NVSYDGTSTACSGSFYRSMRWLTRKNGEYPIQDAQYTNNQGKNILFMWGINHPPTDTTQRDLYTRTDTTT

SVATEEINRVFKPLIGPRPLVNGLMGRIDYYWSVLKPGQTLRIKSDGNLIAPWFGHILSGESHGRILKTD
LKRGSCTVQCQTEKGGLNTTLPFQNVSKYAFGNCSKYIGIKSLKLAVGLRNVPSRSSRGLFGAIAGFIEG
GWSGLVAGWYGFQHSNDQGVGMAADRDSTQKAIDKITSKVNNIVDKMNKQYEIIDHEFSEVEARLNMINN
KIDDQIQDIWAYNAELLVLLENQKTLDEHDANVNNLYNKVKRALGSNAVEDGKGCFELYHKCDDQCMETI
RNGTYNRRKYQEESKLERQKIEGVKLESEGTYKILTIYSTVASSLVIAMGFAAFLFWAMSNGSCRCNICI

>QBK20510.1 hemagglutinin [Influenza A virus]
METVSLITILLVATVSNADKICIGYQSTNSTETVDTLTENNVPVTHAKELLHTEHNGMLCATSLGQPLIL
DTCTIEGLIYGNPSCDLSLEGREWSYIVERPSAVNGLCYPGNVENLEELRSLFSSARSYQRIQIFPDTIW
NVSYDGTSTACSGSFYRSMRWLTRKNGKYPIQDAQYTNNQGKNILFMWGINHPPTDTTQRDLYTRTDTTT
SVATEEINRVFKPLIGPRPLVNGLMGRIDYYWSVLKPGQTLRIKSDGNLIAPWFGHILSGESHGRILKTD
LKRGSCTVQCQTEKGGLNTTLPFQNVSKYAFGNCSKYIGIKSLKLAVGLRNVPSRSSRGLFGAIAGFIEG
GWSGLVAGWYGFQHSNDQGVGMAADRDSTQKAIDKITSKVNNIVDKMNKQYEIIDHEFSEVEARLNMINN
KIDDQIQDIWAYNAELLVLLENQKTLDEHDANVNNLYNKVKRALGSNAVEDGKGCFELYHKCDDQCMETI
RNGTYNRRKYQEESKLERQKIEGVKLESEGTYKILTIYSTVASSLVIAMGFAAFLFWAMSNGSCRCNICI

>QBK20509.1 hemagglutinin [Influenza A virus]
METVSLITILLVATVSNADKICIGYQSTNSTETVDTLTENNVPVTHAKELLHTEHNGMLCATSLGQPLIL
DTCTIEGLIYGNPSCDLSLEGMEWSYIVERPSAVNGLCYPGNVENLEELRSLFSSARSYQRIQIFPDTIW
NVSYDGTSTACSDSFYRSMRWLTRKDGNYPTQDAQYTNNQGKNILFMWGINHPPTDDTQRKLYTRTDTTT
SVATEEINRIFKPLIGPRPLVNGLMGRIDYYWSVLKPGQTLRIKSDGNLIAPWYGHILSGESHGRILKTD
LKRGSCTVQCQTEKGGLNTTLPFQNVSKYAFGNCSKYIGIKSLKLAVGLRNVPSRSSRGLFGAIAGFIEG
GWSGLVAGWYGFQHSNDQGVGMAADRDSTQKAIDKITSKVNNIVDKMNKQYEIIDHEFSEVETRLNMINN
KIDDQIQDIWAYNAELLVLLENQKTLDEHDANVNNLYNKVKRALGSNAVEDGKGCFELYHKCNDQCMETI
RNGTYNRRKYQEESKLERQKIEGVNLESEGTYKILTIYSTVASSLVIAMGFAAFLFWAMSNGSCRCNICI

>QBK20508.1 hemagglutinin [Influenza A virus]
METVSLITILLVATVSNADKICIGYQSTNSTETVDTLTENNVPVTHAKELLHTEHNGMLCATSLGQPLIL
DTCTIEGLIYGNPSCDLSLEGMEWSYIVERPSAVNGLCYPGNVENLEELRSLFSSARSYQRIQIFPDTIW
NVSYDGTSTACSDSFYRSMRWLTRKDGNYPTQDAQYTNNQGKNILFMWGINHPPTDDTQRKLYTRTDTTT
SVATEEINRIFKPLIGPRPLVNGLMGRIDYYWSVLKPGQTLRIKSDGNLIAPWYGHILSGESHGRILKTD
LKRGSCTVQCQTEKGGLNTTLPFQNVSKYAFGNCSKYIGIKSLKLAVGLRNVPSRSSRGLFGAIAGFIEG
GWSGLVAGWYGFQHSNDQGVGMAADRDSTQKAIDKITSKVNNIVDKMNKQYEIIDHEFSEVETRLNMINN
KIDDQIQDIWAYNAELLVLLENQKTLDEHDANVNNLYNKVKRALGSNAVEDGKGCFELYHKCNDQCMETI

RNGTYNRRKYQEESKLERQKIEGVNLESEGTYKILTIYSTVASSLVIAMGFAAFLFWAMSNGSCRCNICI

>QBK20507.1 hemagglutinin [Influenza A virus]
METVSLITILLVATVSNADKICIGYQSTNSTETVDTLTENNVPVTHAKELLHTEHNGMLCATSLGQPLIL
DTCTIEGLIYGNPSCDLSLEGMEWSYIVERPSAVNGLCYPGNVENLEELRSLFSSARSYQRIQIFPDTIW
NVSYDGTSTACSDSFYRSMRWLTRKDGNYPTQDAQYTNNQGKNILFMWGINHPPTDDTQRKLYTRTDTTT
SVATEEINRIFKPLIGPRPLVNGLMGRIDYYWSVLKPGQTLRIKSDGNLIAPWYGHILSGESHGRILKTD
LKRGSCTVQCQTEKGGLNTTLPFQNVSKYAFGNCSKYIGIKSLKLAVGLRNVPSRSSRGLFGAIAGFIEG
GWSGLVAGWYGFQHSNDQGVGMAADRDSTQKAIDKITSKVNNIVDKMNKQYEIIDHEFSEVETRLNMINN
KIDDQIQDIWAYNAELLVLLENQKTLDEHDANVNNLYNKVKRALGSNAVEDGKGCFELYHKCNDQCMETI
RNGTYNRRKYQEESKLERQKIEGVNLESEGTYKILTIYSTVASSLVIAMGFAAFLFWAMSNGSCRCNICI

>QBK20506.1 hemagglutinin [Influenza A virus]
METVSLITILLVATVSNADKICIGYQSTNSTETVDTLTENNVPVTHAKELLHTEHNGMLCATSLGQPLIL
DTCTIEGLIYGNPSCDLSLEGMEWSYIVERPSAVNGLCYPGNVENLEELRSLFSSARSYQRIQIFPDTIW
NVSYDGTSTACSDSFYRSMRWLTRKDGNYPTQDAQYTNNQGKNILFMWGINHPPTDDTQRKLYTRTDTTT
SVATEEINRIFKPLIGPRPLVNGLMGRIDYYWSVLKPGQTLRIKSDGNLIAPWYGHILSGESHGRILKTD
LKRGSCTVQCQTEKGGLNTTLPFQNVSKYAFGNCSKYIGIKSLKLAVGLRNVPSRSSRGLFGAIAGFIEG
GWSGLVAGWYGFQHSNDQGVGMAADRDSTQKAIDKITSKVNNIVDKMNKQYEIIDHEFSEVETRLNMINN
KIDDQIQDIWAYNAELLVLLENQKTLDEHDANVNNLYNKVKRALGSNAVEDGKGCFELYHKCNDQCMETI
RNGTYNRRKYQEESKLERQKIEGVNLESEGTYKILTIYSTVASSLVIAMGFAAFLFWAMSNGSCRCNICI

>QBK20505.1 hemagglutinin [Influenza A virus]
METVSLITILLVATVSNADKICIGYQSTNSTETVDTLTENNVPVTHAKELLHTEHNGMLCATSLGQPLIL
DTCTIEGLIYGNPSCDLSLEGMEWSYIVERPSAVNGLCYPGNVENLEELRSLFSSARSYQRIQIFPDTIW
NVSYDGTSTACSDSFYRSMRWLTRKDGNYPTQDAQYTNNQGKNILFMWGINHPPTDDTQRKLYTRTDTTT
SVATEEINRIFKPLIGPRPLVNGLMGRIDYYWSVLKPGQTLRIKSDGNLIAPWYGHILSGESHGRILKTD
LKRGSCTVQCQTEKGGLNTTLPFQNVSKYAFGNCSKYIGIKSLKLAVGLRNVPSRSSRGLFGAIAGFIEG
GWSGLVAGWYGFQHSNDQGVGMAADRDSTQKAIDKITSKVNNIVDKMNKQYEIIDHEFSEVETRLNMINN
KIDDQIQDIWAYNAELLVLLENQKTLDEHDANVNNLYNKVKRALGSNAVEDGKGCFELYHKCNDQCMETI
RNGTYNRRKYQEESKLERQKIEGVNLESEGTYKILTIYSTVASSLVIAMGFAAFLFWAMSNGSCRCNICI

>QBK20504.1 hemagglutinin [Influenza A virus]
METVSLITILLVATVSNADKICIGYQSTNSTETVDTLTENNVPVTHAKELLHTEHNGMLCATSLGQPLIL

DTCTIEGLIYGNPSCDLSLEGREWSYIVERPSAVNGLCYPGNVENLEELRSLFSSARSYQRIQIFPDTIW
NVSYDGTSTACSGSFYRSMRWLTRKNGEYPIQDAQYTNNQGKNILFMWGINHPPTDTTQRDLYTRTDTTT
SVATEEINRVFKPLIGPRPLVNGLMGRIDYYWSVLKPGQTLRIKSDGNLIAPWFGHILSGESHGRILKTD
LKRGSCTVQCQTEKGGLNTTLPFQNVSKYAFGNCSKYIGIKSLKLAVGLRNVPSRSSRGLFGAIAGFIEG
GWSGLVAGWYGFQHSNDQGVGMAADRDSTQKAIDKITSKVNNIVDKMNKQYEIIDHEFSEVEARLNMINN
KIDDQIQDIWAYNAELLVLLENQKTLDEHDANVNNLYNKVKRALGSNAVEDGKGCFELYHKCDDQCMETI
RNGTYNRRKYQEESKLERQKIEGVKLESEGTYKILTIYSTVASSLVIAMGFAAFLFWAMSNGSCRCNICI

>QBK20503.1 hemagglutinin [Influenza A virus]
METVSLITILLVATVSNADKICIGYQSTNSTETVDTLTENNVPVTHAKELLHTEHNGMLCATSLGQPLIL
DTCTIEGLIYGNPSCDLSLEGREWSYIVERPSAVNGLCYPGNVENLEELRSLFSSARSYQRIQIFPDTIW
NVSYDGTSTACSGSFYRSMRWLTRKNGEYPIQDAQYTNNQGKNILFMWGINHPPTDTTQRDLYTRTDTTT
SVATEEINRVFKPLIGPRPLVNGLMGRIDYYWSVLKPGQTLRIKSDGNLIAPWFGHILSGESHGRILKTD
LKRGSCTVQCQTEKGGLNTTLPFQNVSKYAFGNCSKYIGIKSLKLAVGLRNVPSRSSRGLFGAIAGFIEG
GWSGLVAGWYGFQHSNDQGVGMAADRDSTQKAIDKITSKVNNIVDKMNKQYEIIDHEFSEVEARLNMINN
KIDDQIQDIWAYNAELLVLLENQKTLDEHDANVNNLYNKVKRALGSNAVEDGKGCFELYHKCDDQCMETI
RNGTYNRRKYQEESKLERQKIEGVKLESEGTYKILTIYSTVASSLVIAMGFAAFLFWAMSNGSCRCNICI

>QBK20502.1 hemagglutinin [Influenza A virus]
METVSLITILLVATVSNADKICIGYQSTNSTETVDTLTENNVPVTHAKELLHTEHNGMLCATSLGQPLIL
DTCTIEGLIYGNPSCDLSLEGMEWSYIVERPSAVNGLCYPGNVENLEELRSLFSSARSYQRIQIFPDTIW
NVSYDGTSTACSDSFYRSMRWLTRKDGNYPTQDAQYTNNQGKNILFMWGINHPPTDDTQRKLYTRTDTTT
SVATEEINRIFKPLIGPRPLVNGLMGRIDYYWSVLKPGQTLRIKSDGNLIAPWYGHILSGESHGRILKTD
LKRGSCTVQCQTEKGGLNTTLPFQNVSKYAFGNCSKYIGIKSLKLAVGLRNVPSRSSRGLFGAIAGFIEG
GWSGLVAGWYGFQHSNDQGVGMAADRDSTQKAIDKITSKVNNIVDKMNKQYEIIDHEFSEVETRLNMINN
KIDDQIQDIWAYNAELLVLLENQKTLDEHDANVNNLYNKVKRALGSNAVEDGKGCFELYHKCNDQCMETI
RNGTYNRRKYQEESKLERQKIEGVNLESEGTYKILTIYSTVASSLVIAMGFAAFLFWAMSNGSCRCNICI

>QBK20501.1 hemagglutinin [Influenza A virus]
METVSLITILLVATVSNADKICIGYQSTNSTETVDTLTENNVPVTHAKELLHTEHNGKLCATSLGQPLIL
DTCTIEGLIYGNPSCDLSLEGMEWSYIVERPSAVNGLCYPGNVENLEELRSLFSSARSYQRIQIFPDTIW
NVSYDGTSTACSDSFYRSMRWLTRKDGNYPTQDAQYTNNQGKNILFMWGINHPPTDDTQRKLYTRTDTTT
SVATEEINRIFKPLIGPRPLVNGLMGRIDYYWSVLKPGQTLRIKSDGNLIAPWYGHILSGESHGRILKTD
LKRGSCTVQCQTEKGGLNTTLPFQNVSKYAFGNCSKYIGIKSLKLAVGLRNVPSRSSRGLFGAIAGFIEG

GWSGLVAGWYGFQHSNDQGVGMAADRDSTQKAIDKITSKVNNIVDKMNKQYEIIDHEFSEVETRLNMINN
KIDDQIQDIWAYNAELLVLLENQKTLDEHDANVNNLYNKVKRALGSNAVEDGKGCFELYHKCNDQCMETI
RNGTYNRRKYQEESKLERQKIEGVNLESEGTYKILTIYSTVASSLVIAMGFAAFLFWAMSNGSCRCNICI

>QBK20500.1 hemagglutinin [Influenza A virus]
METVSLITILLVATVSNADKICIGYQSTNSTETVDTLTENNVPVTHAKELLHTEHNGMLCATSLGQPLIL
DTCTIEGLIYGNPSCDLSLEGREWSYIVERPSAVNGLCYPGNVENLEELRSLFSSARSYQRIQIFPDTIW
NVSYDGTSTACSGSFYRSMRWLTRKNGEYPIQDAQYTNNQGKNILFMWGINHPPTDTTQRDLYTRTDTTT
SVATEEINRVFKPLIGPRPLVNGLMGRIDYYWSVLKPGQTLRIKSDGNLIAPWFGHILSGESHGRILKTD
LKRGSCTVQCQTEKGGLNTTLPFQNVSKYAFGNCSKYIGIKSLKLAVGLRNVPSRSSRGLFGAIAGFIEG
GWSGLVAGWYGFQHSNDQGVGMAADRDSTQKAIDKITSKVNNIVDKMNKQYEIIDHEFSEVEARLNMINN
KIDDQIQDIWAYNAELLVLLENQKTLDEHDANVNNLYNKVKRALGSNAVEDGKGCFELYHKCDDQCMETI
RNGTYNRRKYQEESKLERQKIEGVKLESEGTYKILTIYSTVASSLVIAMGFAAFLFWAMSNGSCRCNICI

>QBK20499.1 hemagglutinin [Influenza A virus]
METVSLITILLVATVSNADKICIGYQSTNSTETVDTLTENNVPVTHAKELLHTEHNGMLCATSLGQPLIL
DTCTIEGLIYGNPSCDLSLEGREWSYIVERPSAVNGLCYPGNVENLEELRSLFSSARSYQRIQIFPDTIW
NVSYDGTSTACSDSFYRSMRWLTRKDGNYPTQDAQYTNNQGKNILFMWGINHPPTDDTQRKLYTRTDTTT
SVATEEINRIFKPLIGPRPLVNGLMGRIDYYWSVLKPGQTLRIKSDGNLIAPWYGHILSGESHGRILKTD
LKRGSCTVQCQTEKGGLNTTLPFQNVSKYAFGNCSKYIGIKSLKLAVGLRNVPSRSSRGLFGAIAGFIEG
GWSGLVAGWYGFQHSNDQGVGMAADRDSTQKAIDKITSKVNNIVDKMNKQYEIIDHEFSEVETRLNMINN
KIDDQIQDIWAYNAELLVLLENQKTLDEHDANVNNLYNKVKRALGSNAVEDGKGCFELYHKCNDQCMETI
RNGTYNRRKYQEESKLERQKIEGVNLESEGTYKILTIYSTVASSLVIAMGFAAFLFWAMSNGSCRCNICI

>QBK20498.1 hemagglutinin [Influenza A virus]
METVSLITILLVATVSNADKICIGYQSTNSTETVDTLTENNVPVTHAKELLHTEHNGMLCATSLGQPLIL
DTCTIEGLIYGNPSCDLSLEGMEWSYIVERPSAVNGLCYPGNVENLEELRSLFSSARSYQRIQIFPDTIW
NVSYDGTSTACSDSFYRSMRWLTRKDGNYPTQDAQYTNNQGKNILFMWGINHPPTDDTQRKLYTRTDTTT
SVATEEINRIFKPLIGPRPLVNGLMGRIDYYWSVLKPGQTLRIKSDGNLIAPWYGHILSGESHGRILKTD
LKRGSCTVQCQTEKGGLNTTLPFQNVSKYAFGNCSKYIGIKSLKLAVGLRNVPSRSSRGLFGAIAGFIEG
GWSGLVAGWYGFQHSNDQGVGMAADRDSTQKAIDKITSKVNNIVDKMNKQYEIIDHEFSEVETRLNMINN
KIDDQIQDIWAYNAELLVLLENQKTLDEHDANVNNLYNKVKRALGSNAVEDGKGCFELYHKCNDQCMETI
RNGTYNRRKYQEESKLERQKIEGVNLESEGTYKILTIYSTVASSLVIAMGFAAFLFWAMSNGSCRCNICI

>QBK20497.1 hemagglutinin [Influenza A virus]

METVSLITILLVATVSNADKICIGYQSTNSTETVDTLTENNVPVTHAKELLHTEHNGMLCATSLGQPLIL
DTCTIEGLIYGNPSCDLSLEGREWSYIVERPSAVNGLCYPGNVENLEELRSLFSSARSYQRIQIFPDTIW
NVSYDGTSTACSGSFYRSMRWLTRKNGEYPIQDAQYTNNQGKNILFMWGINHPPTDTTQRDLYTRTDTTT
SVATEEINRVFKPLIGPRPLVNGLMGRIDYYWSVLKPGQTLRIKSDGNLIAPWFGHILSGESHGRILKTD
LKRGSCTVQCQTEKGGLNTTLPFQNVSKYAFGNCSKYIGIKSLKLAVGLRNVPSRSSRGLFGAIAGFIEG
GWSGLVAGWYGFQHSNDQGVGMAADRDSTQKAIDKITSKVNNIVDKMNKQYEIIDHEFSEVEARLNMINN
KIDDQIQDIWAYNAELLVLLENQKTLDEHDANVNNLYNKVKRALGSNAVEDGKGCFELYHKCDDQCMETI
RNGTYNRRKYQEESKLERQKIEGVKLESEGTYKILTIYSTVASSLVIAMGFAAFLFWAMSNGSCRCNICI

>QBK20496.1 hemagglutinin [Influenza A virus]

METVSLITILLVATVSNADKICIGYQSTNSTETVDTLTENNVPVTHAKELLHTEHNGMLCATSLGQPLIL
DTCTIEGLIYGNPSCDLSLEGREWSYIVERPSAVNGLCYPGNVENLEELRSLFSSARSYQRIQIFPDTIW
NVSYDGTSTACSGSFYRSMRWLTRKNGEYPIQDAQYTNNQGKNILFMWGINHPPTDTTQRDLYTRTDTTT
SVATEEINRVFKPLIGPRPLVNGLMGRIDYYWSVLKPGQTLRIKSDGNLIAPWFGHILSGESHGRILKTD
LKRGSCTVQCQTEKGGLNTTLPFQNVSKYAFGNCSKYIGIKSLKLAVGLRNVPSRSSRGLFGAIAGFIEG
GWSGLVAGWYGFQHSNDQGVGMAADRDSTQKAIDKITSKVNNIVDKMNKQYEIIDHEFSEVEARLNMINN
KIDDQIQDIWAYNAELLVLLENQKTLDEHDANVNNLYNKVKRALGSNAVEDGKGCFELYHKCDDQCMETI
RNGTYNRRKYQEESKLERQKIEGVKLESEGTYKILTIYSTVASSLVIAMGFAAFLFWAMSNGSCRCNICI

>QBK20495.1 hemagglutinin [Influenza A virus]

METVSLITILLVATVSNADKICIGYQSTNSTETVDTLTENNVPVTHAKELLHTEHNGMLCATSLGQPLIL
DTCTIEGLIYGNPSCDLSLEGMEWSYIVERPSAVNGLCYPGNVENLEELRSLFSSARSYQRIQIFPDTIW
NVSYDGTSTACSDSFYRSMRWLTRKDGNYPTQDAQYTNNQGKNILFMWGINHPPTDDTQRKLYTRTDTTT
SVATEEINRIFKPLIGPRPLVNGLMGRIDYYWSVLKPGQTLRIKSDGNLIAPWYGHILSGESHGRILKTD
LKRGSCTVQCQTEKGGLNTTLPFQNVSKYAFGNCSKYIGIKSLKLAVGLRNVPSRSSRGLFGAIAGFIEG
GWSGLVAGWYGFQHSNDQGVGMAADRDSTQKAIDKITSKVNNIVDKMNKQYEIIDHEFSEVETRLNMINN
KIDDQIQDIWAYNAELLVLLENQKTLDEHDANVNNLYNKVKRALGSNAVEDGKGCFELYHKCNDQCMETI
RNGTYNRRKYQEESKLERQKIEGVNLESEGTYKILTIYSTVASSLVIAMGFAAFLFWAMSNGSCRCNICI

>QBK20494.1 hemagglutinin [Influenza A virus]

METVSLITILLVATVSNADKICIGYQSTNSTETVDTLTENNVPVTHAKELLHTEHNGMLCATSLGQPLIL
DTCTIEGLIYGNPSCDLSPEGREWSYIVERPSAVNGLCYPGNVENLEELRSLFSSARSYQRIQIFPDTIW
NVSYDGTSTACSGSFYRSMRWLTRKNGEYPIQDAQYTNNQGKNILFMWGINHPPTDTTQRDLYTRTDTTT

SVATEEINRVFKPLIGPRPLVNGLMGRIDYYWSVLKPGQTLRIKSDGNLIAPWFGHILSGESHGRILKTD
LKRGSCTVQCQTEKGGLNTTLPFQNVSKYAFGNCSKYIGIKSLKLAVGLRNVPSRSSRGLFGAIAGFIEG
GWSGLVAGWYGFQHSNDQGVGMAADRDSTQKAIDKITSKVNNIVDKMNKQYEIIDHEFSEVEARLNMINN
KIDDQIQDIWAYNAELLVLLENQKTLDEHDANVNNLYNKVKRALGSNAVEDGKGCFELYHKCDDQCMETI
RNGTYNRRKYQEESKLERQKIEGVKLESEGTYKILTIYSTVASSLVIAMGFAAFLFWAMSNGSCRCNICI

>QBK20493.1 hemagglutinin [Influenza A virus]
METVSLITILLVATVSNADKICIGYQSTNSTETVDTLTENNVPVTHAKELLHTEHNGMLCATSLGQPLIL
DTCTIEGLIYGNPSCDLSLEGREWSYIVERPSAVNGLCYPGNVENLEELRSLFSSARSYQRIQIFPDTIW
NVSYDGTSTACSGSFYRSMRWLTRKNGEYPIQDAQYTNNQGKNILFMWGINHPPTDTTQRDLYTRTDTTT
SVATEEINRVFKPLIGPRPLVNGLMGRIDYYWSVLKPGQTLRIKSDGNLIAPWFGHILSGESHGRILKTD
LKRGSCTVQCQTEKGGLNTTLPFQNVSKYAFGNCSKYIGIKSLKLAVGLRNVPSRSSRGLFGAIAGFIEG
GWSGLVAGWYGFQHSNDQGVGMAADRDSTQKAIDKITSKVNNIVDKMNKQYEIIDHEFSEVEARLNMINN
KIDDQIQDIWAYNAELLVLLENQKTLDEHDANVNNLYNKVKRALGSNAVEDGKGCFELYHKCDDQCMETI
RNGTYNRRKYQEESKLERQKIEGVKLESEGTYKILTIYSTVASSLVIAMGFAAFLFWAMSNGSCRCNICI

>QBK20492.1 hemagglutinin [Influenza A virus]
METVSLITILLVATVSNADKICIGYQSTNSTETVDTLTENNVPVTHAKELLHTEHNGMLCATSLGQPLIL
DTCTIEGLIYGNPSCDLSLEGREWSYIVERPSAVNGLCYPGNVENLEELRSLFSSARSYQRIQIFPDTIW
NVSYDGTSTACSGSFYRSMRWLTRKNGEYPIQDAQYTNNQGKNILFMWGINHPPTDTTQRDLYTRTDTTT
SVATEEINRVFKPLIGPRPLVNGLMGRIDYYWSVLKPGQTLRIKSDGNLIAPWFGHILSGESHGRILKTD
LKRGSCTVQCQTEKGGLNTTLPFQNVSKYAFGNCSKYIGIKSLKLAVGLRNVPSRSSRGLFGAIAGFIEG
GWSGLVAGWYGFQHSNDQGVGMAADRDSTQKAIDKITSKVNNIVDKMNKQYEIIDHEFSEVEARLNMINN
KIDDQIQDIWAYNAELLVLLENQKTLDEHDANVNNLYNKVKRALGSNAVEDGKGCFELYHKCDDQCMETI
RNGTYNRRKYQEESKLERQKIEGVKLESEGTYKILTIYSTVASSLVIAMGFAAFLFWAMSNGSCRCNICI

>QBK20489.1 hemagglutinin [Influenza A virus]
METVSLITILLVATVSNADKICIGYQSTNSTETVDTLTENNVPVTHAKELLHTEHNGMLCATSLGQPLIL
DTCTIEGLIYGNPSCDLSLEGREWSYIVERPSAVNGLCYPGNVENLEELRSLFSSARSYQRIQIFPDTTW
NVSYDGTSTACSGSFYRSMRWLTRKNGDYPIQDAQYTNNQGKSILFMWGINHPPTDTTQRDLYTRTDTTT
SVATEEINRIFKPLIGPRPLVNGLMGRINYYWSVLKPGQTLRIKSDGNLIAPWYGHILSGESHGRILKTD
LKRGSCTVQCQTEKGGLNTTLPFQNVSKYAFGNCSKYIGIKSLKLAVGLRNVPSRSSRGLFGAIAGFIEG
GWSGLVAGWYGFQHSNDQGVGMAADRDSTQKAIDKITSKVNNIVDKMNKQYEIIDHEFSEVETRLNMINN
KIDDQIQDIWAYNAELLVLLENQKTLDEHDANVNNLYNKIKRALGSNAVEDGKGCFELYHKCDDQCMETI

RNGTYNRRKYQEESKLERQKIEGVKLESEGTYKILTIYSTVASSLVIAMGFAAFLFWAMSNGSCRCNICI

>QBK20487.1 hemagglutinin [Influenza A virus]
METVSLMTILLVATVSNADKICIGYQSTNSTETVDTLTENNVPVTHAKELLHTEHNGMLCATSLGQPLIL
DTCTIEGLIYGNPSCDQLLEGREWSYIVERPSAVNGLCYPGNVENLEELRSLFSSARSYQRIQIFPDTIW
NVSYDGTSTACSGSFYRSMRWLTRKNGNYPIQDAQYTNNQGKNILFMWGINHPPTDTTQIDLYTRTDTTT
SVATEEINRIFKPLIGPRPRVNGLMGRINYYWSVLKPGQTLRIKSDGNLIAPWYGHILSGESHGRILKTD
LKKGSCTVQCQTEKGGLNTTLPFQNVSKYAFGNCSKYIGIKSLKLAVGLRNVPSRSSRGLFGAIAGFIEG
GWSGLVAGWYGFQHSNDQGVGMAADRGSTQKAIDKITSKVNNIVDKMNKQYEIIDHEFSEVETRLNMINN
KIDDQIQDIWAYNAELLVLLENQKTLDEHDANVNNLYNKVKRALGSNAVEDGRGCFELYHKCDDQCMETI
RNGTYNRSKYQEESKLERQKIEGVKLESEGTYKILTIYSTVASSLVIAMGFAAFLFWAMSNGSCRCNICI

>QBK20486.1 hemagglutinin [Influenza A virus]
METVSLITILLVATAGNADKICIGYQSTNSTETVDTLTENNVPVTHAKELLHTEHNGMLCATSLGQPLIL
DTCTIEGLIYGNPSCDLSLEGREWSYIVERPSAVNGLCYPGNVENLEELRSLFSSARSYQRIQIFPDTIW
NVSYDGTSTACSGSFYRSMRWLTRKNGDYPIQDAQYTNNQRKNILFMWGINHPPTDTTQRNLYTRNDTTT
SVATEEINRIFKPLIGPRPLVNGLMGRINYYWSVLKPGQTLRIKSDGNLIAPWYGHILSGESHGRILKTD
LKRGSCTVQCQTEKGGLNTTLPFQNVSKYAFGNCSKYIGIKSLKLAVGLRNVPSRSSRGLFGAIAGFIEG
GWSGLVAGWYGFQHSNDQGVGMAADRDSTQKAIDKITSKVNNIVDKMNKQYEIIDHEFSEVETRLNMINN
KIDDQIQDIWAYNAELLVLLENQKTLDEHDANVNNLYNKVKRALGSNAVEDGKGCFELYHKCDDQCMETI
RNGTYNRRKYQEESKLERQKIEGVKLESEGTYKILTIYSTVASSLVIAMGFAAFLFWAMSNGSCRCNICI

>QBK20485.1 hemagglutinin [Influenza A virus]
METVSLITILLVATVSNADKICIGYQSTNSTETVDTLTENNVPVTHAKELLHTEHNGMLCATSLGQPLIL
DTCTIEGLIYGNPSCDLSLEGREWSYIVERPSAVNGLCYPGNVENLEELRSLFSSARSYQRIQIFPDTIW
NVSYDGTSTACSGSFYRSMRWLTQKNGDYPIQDAQYTNNQGKNILFMWGINHPPADTTQRNLYTRIDTTT
SVATEEINRIFKPLIGPRPLVNGLMGRINYYWSVLKPGQTLRIKSDGNLIAPWYGHILSGESHGRILKTD
LKRGSCTVQCQTEKGGLNTTLPFQNVSKYAFGNCSKYIGTKSLKLAVGLRNVPSRSSRGLFGAIAGFIEG
GWSGLVAGWYGFQHSNDQGVGMAADRDSTQKAIDKITSKVNNIVDKMNKQYEIIDHEFSEVETRLNMINN
KIDDQIQDIWAYNAELLVLLENQKTLDEHDANVNNLYNKVKRALGSNAMEDGKGCFELYHKCDDQCMETI
RNGTYNRRKYQEESKLERQKIEGVKLESEGIYKILTIYSTVASSLVIAMGFAAFLFWAMSNGSCRCNICI

>QBK20484.1 hemagglutinin [Influenza A virus]
METVSLITILLVATVSNADKICIGYQSTNSTETVDTLTENNVPVTHAKELLHTEHNGMLCATSLGQPLIL

DTCTIEGLIYGNPSCDLSLEGREWSYIVERPSAVNGLCYPGNVENLEELRSLFSSVRSYQRIQIFPDTIW
NVSYDGTSTACSGSFYRSMRWLTRKNGEYPIQDAQYTNNQGKNILFMWGINHPPTDTTQRDLYTRTDTTT
SVATEEINRIFKPLIGPRPLVNGLMGRIDYYWSVLKPGQTLRIKSDGNLIAPWFGHILSGESHGRILKTD
LKRGSCTVQCQTEKGGLNTTLPFQNVSKYAFGNCSKYIGIKSLKLAVGLRNVPSRSSRGLFGAIAGFIEG
GWSGLVAGWYGFQHSNDQGVGMAADRDSTQKAIDKITSKVNNIVDKMNKQYEIIDHEFSEVETRLNMINN
KIDDQIQDIWAYNAELLVLLENQKTLDEHDANVNNLYNKVKRALGSNAVEDGKGCFELYHKCDDQCMETI
RNGTYNRRKYQKESKLERQKIEGVKLESEGTYKILTIYSTVASSLVIAMGFAAFLFWAMSNGSCRCNICI

>QBK20483.1 hemagglutinin [Influenza A virus]
METVSLITILLIATVSNADKICIGYQSTNSTETVDTLTENNVPVTHAKELLHTEHNGMLCATSLGQPLIL
DTCTIEGLIYGNPSCDLSLEGREWSYIVERPSAVNGLCYPGSVENLEELRSLFSSARSYQRIQIFPDTIW
NVSYDGTSTACSGSFYRSMRWLTRKNGDYPIQDAQYTNNQGKNILFMWGINHPPTDTTQRDLYTRTDTTT
SVATEEMNRIFKPLIGPRPLVNGLMGRINYYWSVLKPGQTLRIKSDGNLIAPWYGHILSGESHGRILKTD
LKRGSCTVQCQTEKGGLNTTLPFQNVSKYAFGNCSKYIGIKSLKLAVGLRNVPSRSSRGLFGAIAGFIEG
GWSGLVAGWYGFQHSNDQGVGMAADRDSTQKAIDKITSKVNNIVDKMNKQYEIIDHEFSEVETRLNMINN
KIDDQIQDIWAYNAELLVLLENQKTLDEHDANVNNLYNKVKRALGSNAVEDGKGCFELYHKCDDQCMETI
RNGTYNRRKYQEESKLERQKIEGVKLESEGTYKILTIYSTVASSLVIAMGFAAFLFWAMSNGSCRCNICI

>QBK20481.1 hemagglutinin [Influenza A virus]
METVSLITILLVATVSNADKICIGYQSTNSTETVDTLTENNVPVTHAKELLHTEHNGMLCATSLGHPLIL
DTCTIEGLIYGNPSCDPLLGGREWSYIVERPSAVNGLCYPGNVENLEELRSLFSSARSYQRIQIFPDTIW
NVSYSGTSKACSDSFYRSMRWLTQKNNAYPIQDAQYTNNQEKNILFMWGINHPPTDTAQTNLYTRTDTTT
SVATEEINRIFKPLIGPRPLVNGLMGRINYYWSVLKPGQTLRIKSDGNLIAPWYGHILSGESHGRILKTD
LTRGSCTVQCQTEKGGLNTTLPFQNVSKYAFGNCSKYIGIKSLKLAVGLRNVPSRSSRGLFGAIAGFIEG
GWSGLVAGWYGFQHSNDQGVGMAADRDSTQKAIDKITSKVNNIVDKMNKQYEIIDHEFSEVETRLNMINN
KVDDQIQDIWAYNAELLVLLENQKTLDEHDANVNNLYNKVKRALGSNAVEDGKGCFELYHKCDDQCMETI
RNGTYNRRKYQEESKLERQKIEGVKLESEETYKILTIYSTVASSLVIAMGFAAFLFWAMSNGSCRCNICI

>QBK20480.1 hemagglutinin [Influenza A virus]
METVSLITILLVATVSNADKICIGYQSTNSTETVDTLTENNVPVTHAKELLHTEHNGMLCATSLGHPLIL
DTCTIEGLIYGNPSCDPLLGGREWSYIVERPSAVNGLCYPGNVENLEELRSLFSSARSYQRIQIFPDTIW
NVSYSGTSKACSDSFYRSMRWLTQKNNAYPIQDAQYTNNQEKNILFMWGINHPPTDTAQTNLYTRTDTTT
SVATEEINRIFKPLIGPRPLVNGLMGRINYYWSVLKPGQTLRIKSDGNLIAPWYGHILSGESHGRILKTD
LTRGSCTVQCQTEKGGLNTTLPFQNVSKYAFGNCSKYIGIKSLKLAVGLRNVPSRSSRGLFGAIAGFIEG

GWSGLVAGWYGFQHSNDQGVGMAADRDSTQKAIDKITSKVNNIVDKMNKQYEIIDHEFSEVETRLNMINN
KVDDQIQDIWAYNAELLVLLENQKTLDEHDANVNNLYNKVKRALGSNAVEDGKGCFELYHKCDDQCMETI
RNGTYNRRKYQEESKLERQKIEGVKLESEETYKILTIYSTVASSLVIAMGFAAFLFWAMSNGSCRCNICI

>QBK20479.1 hemagglutinin [Influenza A virus]
METVSLITILLVATVSNADKICIGYQSTNSTETVDTITENNVPVTHAKELLHTEHNGMLCATSLGQPLIL
DTCTIEGLIYGNPSCDLSLEGREWSYIVERPSAVNGLCYPGNVENLEELRSLFSSARSYQRIQIFPDTIW
NVSYDGTSTACSGSFYRSMRWLTRKNGDYPIQDAQYTNNQGKNILFMWGINHPPTDTTQRDLYTRTDTTT
SVATEEINRIFKPLIGPRPLVNGLMGRINYYWSVLKPGQTLRIKSDGNLIAPWYGHILSGESHGRILKTD
LKRGSCTVQCQTEKGGLNTTLPFQNVSKYAFGNCSKYIGIKSLKLAVGLRNVPSRSSRGLFGAIAGFIEG
GWSGLVAGWYGFQHSNDQGVGMAADRDSTQKAIDKITSKVNNIVDKMNKQYEIIDHEFSEVETRLNMINN
KIDDQIQDIWAYNAELLVLLENQKTLDEHDANVNNLYNKVKRALGSNAVEDGKGCFELYHKCDDQCMETI
RNGTYNRRKYQEESKLERQKIEGVKLESEGTYKILTIYSTVASSLVIAMGFAAFLFWAMSNGSCRCNICI

>QBK20478.1 hemagglutinin [Influenza A virus]
METVSLITILLVATAGNADKICIGYQSTNSTETVDTLTENNVPVTHAKELLHTEHNGMLCATSLGQPLIL
DTCTIEGLIYGNPSCDLSLEGREWSYIVERPSAVNGLCYPGNVENLEELRSLFSSARSYQRIQIFPDTIW
NVSYDGTSTACSGSFYRSMRWLTRKNGDYPIQDAQYTNNQRKNILFMWGINHPPTDTTQRNLYTRNDTTT
SVATEEINRIFKPLIGPRPLVNGLMGRINYYWSVLKPGQTLRIKSDGNLIAPWYGHILSGESHGRILKTD
LKRGSCTVQCQTEKGGLNTTLPFQNVSKYAFGNCSKYIGIKSLKLAVGLRNVPSRSSRGLFGAIAGFIEG
GWSGLVAGWYGFQHSNDQGVGMAADRDSTQKAIDKITSKVNNIVDKMNKQYEIIDHEFSEVETRLNMINN
KIDDQIQDIWAYNAELLVLLENQKTLDEHDANVNNLYNKVKRALGSNAVEDGKGCFELYHKCDDQCMETI
RNGTYNRRKYQEESKLERQKIEGVKLESEGTYKILTIYSTVASSLVIAMGFAAFLFWAMSNGSCRCNICI

>QBK20477.1 hemagglutinin [Influenza A virus]
METVSLITILLVATVSNADKICIGYQSTNSTETVDTLTENNVPVTHAKELLHTEHNGMLCATSLGQPLIL
DTCTIEGLIYGNPSCDLSLEGREWSYIVERPSAVNGLCYPGNVENLEELRSLFSSARSYQRIQIFPDTIW
NVSYDGTSTACSGSFYRSMRWLTRKNGDYPIQDAQYTNNQGKNILFMWGINHPPTDTTQRDLYTRTDTTT
SVATEEINRIFKPLIGPRPLVNGLMGRINYYWSVLKPGQTLRIKSDGNLIAPWYGHILSGESHGRILKTD
LKRGSCTVQCQTEKGGLNTTLPFQNVSKYAFGNCSKYIGIKSLKLAVGLRNVPSRSSRGLFGAIAGFIEG
GWSGLVAGWYGFQHSNDQGVGMAADRDSTQKAIDKITSKVNNIVDKMNKQYEIIDHEFSEVETRLNMINN
KIDDQIQDIWAYNAELLVLLENQKTLDEHDANVNNLYNKVKRALGSNAVEDGKGCFELYHKCDDQCMETI
RNGTYNRRKYQEESKLERQKIEGVKLESEGTYKILTIYSTVASSLVIAMGFAAFLFWAMSNGSCRCNICI

>QBK20476.1 hemagglutinin [Influenza A virus]

METVSLITILLVATVSNADKICIGYQSTNSTETVDTLTENNVPVTHAKELLHTEHNGMLCATSLGQPLIL
DTCTIEGLIYGNPSCDLSLEGREWSYIVERPSAVNGLCYPGNVENLEELRSLFSSARSYQRIQIFPDTIW
NVSYDGTSTACSGSFYRSMRWLTRKNGDYPIQDAQYTNNQGKNILFMWGINHPPTDTTQRDLYTRTDTTT
SVATEEINRIFKPLIGPRPLVNGLMGRINYYWSVLKPGQTLRIKSDGNLIAPWYGHILSGESHGRILKTD
LKRGSCTVQCQTEKGGLNTTLPFQNVSKYAFGNCSKYIGIKSLKLAVGLRNVPSRSSRGLFGAIAGFIEG
GWSGLVAGWYGFQHSNDQGVGMAADRDSTQKAIDKITSKVNNIVDKMNKQYEIIDHEFSEVETRLTMINN
KIDDQIQDIWAYNAELLVLLENQKTLDEHDANVNNLYNKVKRALGSNAVEDGKGCFELYHKCDDQCMETI
RNGTYNRRKYQEESKLERQKIEGVKLESEGTYKILTIYSTVASSLVIAMGFAAFLFWAMSNGSCRCNICI

>QBK20475.1 hemagglutinin [Influenza A virus]

METISLITILLVATVSNADKICIGYQSTNSTETVDTLTENNVPVTHAKELLHTEHNGMLCATSLGQPLIL
DTCTIEGLIYGNPSCDLSLEGREWSYIVERPSAVNGLCYPGKVENLEELRSLFSSARSYQRIQIFPDTIW
NVSYDGTSTACSGSFYRSMRWLTRKNGEYPIQDAQYTNNQGKNILFMWGINHPPTDTTQRNLYTRTDTTT
SVATEEINRIFKPLIGPRPLVNGLMGRIDYYWSVLKPGQTLRINSDGNLIAPWFGHILSGESHGRILKTD
LKRGSCTVQCQTEKGGLNTTLPFQNVSKYAFGNCSKYIGIKSLKLAVGLRNVPSRSSRGLFGAIAGFIEG
GWSGLVAGWYGFQHSNDQGVGMAADRDSTQKAIDKITSKVNNIVDKMNKQYEIIDHEFSEVETRLNMINN
KIDDQIQDIWAYNAELLVLLENQKTLDEHDANVNNLYNKVKRALGSNAVEDGKGCFELYHKCDDQCMETI
RNGTYNRRKYQEESKLERQKIEGVKLESEGTYKILTIYSTVASSLVIAMGFAAFLFWAMSNGSCRCNICI

>QBK20474.1 hemagglutinin [Influenza A virus]

METISLITILLVATVSNADKICIGYQSTNSTETVDTLTENNVPVTHAKELLHTEHNGMLCATSLGQPLIL
DTCTIEGLIYGNPSCDLSLEGREWSYIVERPSAVNGLCYPGKVENLEELRSLFSSARSYQRIQIFPDTIW
NVSYDGTSTACSGSFYRSMRWLTRKNGEYPIQDAQYTNNQGKNILFMWGINHPPTDTTQRNLYTRTDTTT
SVATEEINRIFKPLIGPRPLVNGLMGRIDYYWSVLKPGQTLRINSDGNLIAPWFGHILSGESHGRILKTD
LKRGSCTVQCQTEKGGLNTTLPFQNVSKYAFGNCSKYIGIKSLKLAVGLRNVPSRSSRGLFGAIAGFIEG
GWSGLVAGWYGFQHSNDQGVGMAADRDSTQKAIDKITSKVNNIVDKMNKQYEIIDHEFSEVETRLNMINN
KIDDQIQDIWAYNAELLVLLENQKTLDEHDANVNNLYNKVKRALGSNAVEDGKGCFELYHKCDDQCMETI
RNGTYNRRKYQEESKLERQKIEGVKLESEGTYKILTIYSTVASSLVIAMGFAAFLFWAMSNGSCRCNICI

>QBK20473.1 hemagglutinin [Influenza A virus]

METVSLITILLVATVSNADKICIGYQSTNSTETVDTLTENNVPVTHAKELLHTEHNGMLCATSLGQPLIL
DTCTIEGLIYGNPSCDLSLEGREWSYIVERPSAVNGLCYPGNVENLEELRSLFSSARSYQRIQIFPDTIW
NVSYDGTSTACSGSFYRSMRWLTRKNGDYPTQDAQYTNNQGKNILFMWGINHPPTDTTQRDLYTRTDTTT

SVATEEINRIFKPLIGPRPLVNGLMGRINYYWSVLKPGQTLRIKSDGNLIAPWYGHILSGESHGRILKTD
LKRGSCTVQCQTEKGGLNTTLPFQNVSKYAFGNCSKYIGIKSLKLAVGLRNVPSRSSRGLFGAIAGFIEG
GWSGLVAGWYGFQHSNDQGVGMAADRDSTQKAIDKITSKVNNIVDKMNKQYEIIDHEFSEVETRLNMINN
KIDDQIQDIWAYNAELLVLLENQKTLDEHDANVNNLYNKVKRALGSNAVEDGKGCFELYHKCDDQCMETI
RNGTYNRRKYQEESKLERQKIEGVKLESEGTYKILTIYSTVASSLVIAMGFAAFLFWAMSNGSCRCNICI

>QBK20472.1 hemagglutinin [Influenza A virus]
METVSLITILLVATVSNADKICIGYQSTNSTETVDTLTENNVPVTHAKELLRTEHNGMLCATSLGQPLIL
DTCTIEGLIYGNPSCDLSLEGREWSYIVERPSAVNGLCYPGNVENLEELRSLFSSARSYQRIQIFPDTIW
NVSYDGTSTACSGSFYRSMRWLTRKNGDYPTQDAQYTNNQGKNILFMWGINHPPTDTTQRDLYTRTDTTT
SVATEEINRIFKPLIGPRPLVNGLMGRINYYWSVLKPGQTLRIKSDGNLIAPWYGHILSGESHGRILKTD
LKRGSCTVQCQTEKGGLNTTLPFQNVSKYAFGNCSKYIGIKSLKLAVGLRNVPSRSSRGLFGAIAGFIEG
GWSGLVAGWYGFQHSNDQGVGMAADRDSTQKAIDKITSKVNNIVDKMNKQYEIIDHEFSEVETRLNMINN
KIDDQIQDIWAYNAELLVLLENQKTLDEHDANVNNLYNKVKRALGPNAVEDGKGCFELYHKCDDQCMETI
RNGTYNRRKYQEESKLERQKIEGVKLESEGTYKILTIYSTVASSLVIAMGFAAFLFWAMSNGSCRCNICI

>QBK20471.1 hemagglutinin [Influenza A virus]
METVSLITILLVATVSNADKICIGYQSTNSTETVDTLTENNVPVTHAKELLHTEHNGMLCATSLGQPLIL
DTCTIEGLIYGNPSCDLSLEGREWSYIVERPSAVNGLCYPGNVENLEELRSLFSSARSYQRIQIFPDTIW
NVSYDGTSTACSSSFYRSMRWLTRKNGDYPTQDAQYTNNQGKNILFMWGINHPPTDDTQRNLYTRTDTTT
SVATEEINRIFKPLIGPRPLVNGLMGRIDYYWSVLKPGQTLRIKSDGNLIAPWYGHILSGESHGRILKTD
LKRGSCTVQCQTEKGGLNTTLPFQNVSKYAFGNCSKYIGIKSLKLAVGLRNVPSRSSRGLFGAIAGFIEG
GWSGLVAGWYGFQHSNDQGVGMAADRDSTQKAIDKITSKVNNIVDKMNKQYEIIDHEFSEVETRLNMINN
KIDDQIQDIWAYNAELLVLLENQKTLDEHDANVNNLYNKVKRALGSNAVEDGKGCFELYHKCNDQCMETI
RNGTYNRKKYQEESKLERQKIEGVKLESEGTYKILTIYSTVASSLVIAMGFAAFLFWAMSNGSCRCNICI

>QBK20470.1 hemagglutinin [Influenza A virus]
METVSLITILIVATVSNADKICIGYQSTNSTETVDTLTENNVPVTHAKELLHTEHNGMLCATSLGQPLIL
ETCTIEGLIYGNPSCDLSLEGREWSYIVERPSAVNGLCYPGNVENLEELRSLFSSARSYQRIQIFPDTIW
NVSYDGTSTACSNSFYRSMRWLTRKNGDYPTQDAQYTNNQGKNILFMWGINHPPTDDTQRNLYTRTDTTT
SVATEEINRIFKPLIGPRPLVNGLMGRIDYYWSVLKPGQTLRIKSDGNLIAPWYGHILSGESHGRILKTD
LKRGSCTVQCQTEKGGLNTTLPFQNVSKYAFGNCSKYIGIKSLKLAVGLRNVPSRSSRGLFGAIAGFIEG
GWSGLVAGWYGFQHSNDQGVGMAADRDSTQKAIDKITSKVNNIVDKMNKQYEIIDHEFSEVETRLNMINN
KIDDQIQDIWAYNAELLVLLENQKTLDEHDANVNNLYNKVKRALGSNAVEDGKGCFELYHKCNDQCMETI

RNGTYNRKKYQEESKLERQKIEGVKLESEGTYKILTIYSTVASSLVIAMGFAAFLFWAMSNGSCRCNICI

>QBK20469.1 hemagglutinin [Influenza A virus]
METVSLITILIVATVSNADKICIGYQSTNSTETVDTLTENNVPVTHAKELLHTEHNGMLCATSLGQPLIL
ETCTIEGLIYGNPSCDLSLEGREWSYIVERPSAVNGLCYPGNVENLEELRSLFSSARSYQRIQIFPDTIW
NVSYDGTSTACSNSFYRSMRWLTRKNGDYPTQDAQYTNNQGKNILFMWGINHPPTDDTQRNLYTRTDTTT
SVATEEINRIFKPLIGPRPLVNGLMGRIDYYWSVLKPGQTLRIKSDGNLIAPWYGHILSGESHGRILKTD
LKRGSCTVQCQTEKGGLNTTLPFQNVSKYAFGNCSKYIGIKSLKLAVGLRNVPSRSSRGLFGAIAGFIEG
GWSGLVAGWYGFQHSNDQGVGMAADRDSTQKAIDKITSKVNNIVDKMNKQYEIIDHEFSEVETRLNMINN
KIDDQIQDIWAYNAELLVLLENQKTLDEHDANVNNLYNKVKRALGSNAVEDGKGCFELYHKCNDQCMETI
RNGTYNRKKYQEESKLERQKIEGVKLESEGTYKILTIYSTVASSLVIAMGFAAFLFWAMSNGSCRCNICI

>QBK20468.1 hemagglutinin [Influenza A virus]
METVSLITILIVATVSNADKICIGYQSTNSTETVDTLTENNVPVTHAKELLHTEHNGMLCATSLGQPLIL
ETCTIEGLIYGNPSCDLSLEGREWSYIVERPSAVNGLCYPGNVENLEELRSLFSSARSYQRIQIFPDTIW
NVSYDGTSTACSNSFYRSMRWLTRKNGDYPTQDAQYTNNQGKNILFMWGINHPPTDDTQRNLYTRTDTTT
SVATEEINRIFKPLIGPRPLVNGLMGRIDYYWSVLKPGQTLRIKSDGNLIAPWYGHILSGESHGRILKTD
LKRGSCTVQCQTEKGGLNTTLPFQNVSKYAFGNCSKYIGIKSLKLAVGLRNVPSRSSRGLFGAIAGFIEG
GWSGLVAGWYGFQHSNDQGVGMAADRDSTQKAIDKITSKVNNIVDKMNKQYEIIDHEFSEVETRLNMINN
KIDDQIQDIWAYNAELLVLLENQKTLDEHDANVNNLYNKVKRALGSNAVEDGKGCFELYHKCNDQCMETI
RNGTYNRKKYQEESKLERQKIEGVKLESEGTYKILTIYSTVASSLVIAMGFAAFLFWAMSNGSCRCNICI

>QBK20467.1 hemagglutinin [Influenza A virus]
METVSLITILIVATVSNADKICIGYQSTNSTETVDTLTENNVPVTHAKELLHTEHNGMLCATSLGQPLIL
ETCTIEGLIYGNPSCDLSLEGREWSYIVERPSAVNGLCYPGNVENLEELRSLFSSARSYQRIQIFPDTIW
NVSYDGTSTACSNSFYRSMRWLTRKNGDYPTQDAQYTNNQGKNILFMWGINHPPTDDTQRNLYTRTDTTT
SVATEEINRIFKPLIGPRPLVNGLMGRIDYYWSVLKPGQTLRIKSDGNLIAPWYGHILSGESHGRILKTD
LKRGSCTVQCQTEKGGLNTTLPFQNVSKYAFGNCSKYIGIKSLKLAVGLRNVPSRSSRGLFGAIAGFIEG
GWSGLVAGWYGFQHSNDQGVGMAADRDSTQKAIDKITSKVNNIVDKMNKQYEIIDHEFSEVETRLNMINN
KIDDQIQDIWAYNAELLVLLENQKTLDEHDANVNNLYNKVKRALGSNAVEDGKGCFELYHKCNDQCMETI
RNGTYNRKKYQEESKLERQKIEGVKLESEGTYKILTIYSTVASSLVIAMGFAAFLFWAMSNGSCRCNICI

>QBK20466.1 hemagglutinin [Influenza A virus]
METVSLITILIVATVSNADKICIGYQSTNSTETVDTLTENNVPVTHAKELLHTEHNGMLCATSLGQPLIL

ETCTIEGLIYGNPSCDLSLEGREWSYIVERPSAVNGLCYPGNVENLEELRSLFSSARSYQRIQIFPDTIW

NVSYDGTSTACSNSFYRSMRWLTRKNGDYPTQDAQYTNNQGKNILFMWGINHPPTDDTQRNLYTRTDTTT

SVATEEINRIFKPLIGPRPLVNGLMGRIDYYWSVLKPGQTLRIKSDGNLIAPWYGHILSGESHGRILKTD

LKRGSCTVQCQTEKGGLNTTLPFQNVSKYAFGNCSKYIGIKSLKLAVGLRNVPSRSSRGLFGAIAGFIEG

GWSGLVAGWYGFQHSNDQGVGMAADRDSTQKAIDKITSKVNNIVDKMNKQYEIIDHEFSEVETRLNMINN

KIDDQIQDIWAYNAELLVLLENQKTLDEHDANVNNLYNKVKRALGSNAVEDGKGCFELYHKCNDQCMETI

RNGTYNRKKYQEESKLERQKIEGVKLESEGTYKILTIYSTVASSLVIAMGFAAFLFWAMSNGSCRCNICI

>QBK20465.1 hemagglutinin [Influenza A virus]

METVSLITILIVATVSNADKICIGYQSTNSTETVDTLTENNVPVTHAKELLHTEHNGMLCATSLGQPLIL

ETCTIEGLIYGNPSCDLSLEGREWSYIVERPSAVNGLCYPGNVENLEELRSLFSSARSYQRIQIFPDTIW

NVSYDGTSTACSNSFYRSMRWLTRKNGDYPTQDAQYTNNQGKNILFMWGINHPPTDDTQRNLYTRTDTTT

SVATEEINRIFKPLIGPRPLVNGLMGRIDYYWSVLKPGQTLRIKSDGNLIAPWYGHILSGESHGRILKTD

LKRGSCTVQCQTEKGGLNTTLPFQNVSKYAFGNCSKYIGIKSLKLAVGLRNVPSRSSRGLFGAIAGFIEG

GWSGLVAGWYGFQHSNDQGVGMAADRDSTQKAIDKITSKVNNIVDKMNKQYEIIDHEFSEVETRLNMINN

KIDDQIQDIWAYNAELLVLLENQKTLDEHDANVNNLYNKVKRALGSNAVEDGKGCFELYHKCNDQCMETI

RNGTYNRKKYQEESKLERQKIEGVKLESEGTYKILTIYSTVASSLVIAMGFAAFLFWAMSNGSCRCNICI

>QBK20464.1 hemagglutinin [Influenza A virus]

METVSLITILIVATVSNADKICIGYQSTNSTETVDTLTENNVPVTHAKELLHTEHNGMLCATNLGQPLIL

ETCTIEGLIYGNPSCDLSLEGREWSYIVERPSAVNGLCYPGNVENLEELRSLFSSARSYQRIQIFPDTIW

NVSYDGTSTACSNSFYRSMRWLTRKNGDYPTQDAQYTNNQGKNILFMWGINHPPTDDTQRNLYTRTDTTT

SVATEEINRIFKPLIGPRPLVNGLMGRIDYYWSVLKPGQTLRIKSDGNLIAPWYGHILSGESHGRILKTD

LKRGSCTVQCQTEKGGLNTTLPFQNVSKYAFGNCSKYIGIKSLKLAVGLRNVPSRSSRGLFGAIAGFIEG

GWSGLVAGWYGFQHSNDQGVGMAADRDSTQKAIDKITSKVNNIVDKMNKQYEIIDHEFSEVETRLNMINN

KIDDQIQDIWAYNAELLVLLENQKTLDEHDANVNNLYNKVKRALGSNAVEDGKGCFELYHKCNDQCMETI

RNGTYNRKKYQEESKLERQKIEGVKLESEGTYKILTIYSTVASSLVIAMGFAAFLFWAMSNGSCRCNICI

>QBK20463.1 hemagglutinin [Influenza A virus]

METVSLITILIVATVSNADKICIGYQSTNSTETVDTLTENNVPVTHAKELLHTEHNGMLCATSLGQPLIL

ETCTIEGLIYGNPSCDLSLEGREWSYIVERPSAVNGLCYPGNVENLEELRSLFSSARSYQRIQIFPDTIW

NVSYDGTSTACSNSFYRSMRWLTRKNGDYPTQDAQYTNNQGKNILFMWGINHPPTDDTQRNLYTRTDTTT

SVATEEINRIFKPLIGPRPLVNGLMGRIDYYWSVLKPGQTLRIKSDGNLIAPWYGHILSGESHGRILKTD

LKRGSCTVQCQTEKGGLNTTLPFQNVSKYAFGNCSKYIGIKSLKLAVGLRNVPSRSSRGLFGAIAGFIEG

GWSGLVAGWYGFQHSNDQGVGMAADRDSTQKAIDKITSKVNNIVDKMNKQYEIIDHEFSEVETRLNMINN
KIDDQIQDIWAYNAELLVLLENQKTLDEHDANVNNLYNKVKRALGSNAVEDGKGCFELYHKCNDQCMETI
RNGTYNRKKYQEESKLERQKIEGVKLESEGTYKILTIYSTVASSLVIAMGFAAFLFWAMSNGSCRCNICI

>QBK20462.1 hemagglutinin [Influenza A virus]
METVSLITILIVATVSNADKICIGYQSTNSTETVDTLTENNVPVTHAKELLHTEHNGMLCATNLGQPLIL
ETCTIEGLIYGNPSCDLSLEGREWSYIVERPSAVNGLCYPGNVENLEELRSLFSSARSYQRIQIFPDTIW
NVSYDGTSTACSNSFYRSMRWLTRKNGDYPTQDAQYTNNQGKNILFMWGINHPPTDDTQRNLYTRTDTTT
SVATEEINRIFKPLIGPRPLVNGLMGRIDYYWSVLKPGQTLRIKSDGNLIAPWYGHILSGESHGRILKTD
LKRGSCTVQCQTEKGGLNTTLPFQNVSKYAFGNCSKYIGIKSLKLAVGLRNVPSRSSRGLFGAIAGFIEG
GWSGLVAGWYGFQHSNDQGVGMAADRDSTQKAIDKITSKVNNIVDKMNKQYEIIDHEFSEVETRLNMINN
KIDDQIQDIWAYNAELLVLLENQKTLDEHDANVNNLYNKVKRALGSNAVEDGKGCFELYHKCNDQCMETI
RNGTYNRKKYQEESKLERQKIEGVKLESEGTYKILTIYSTVASSLVIAMGFAAFLFWAMSNGSCRCNICI

>QBK20461.1 hemagglutinin [Influenza A virus]
METVSLITILIVATVSNADKICIGYQSTNSTETVDTLTENNVPVTHAKELLHTEHNGMLCATSLGQPLIL
ETCTIEGLIYGNPSCDLSLEGREWSYIVERPSAVNGLCYPGNVENLEELRSLFSSARSYQRIQIFPDTIW
NVSYDGTSTACSNSFYRSMRWLTRKNGDYPTQDAQYTNNQGKNILFMWGINHPPTDDTQRNLYTRTDTTT
SVATEEINRIFKPLIGPRPLVNGLMGRIDYYWSVLKPGQTLRIKSDGNLIAPWYGHILSGESHGRILKTD
LKRGSCTVQCQTEKGGLNTTLPFQNVSKYAFGNCSKYIGIKSLKLAVGLRNVPSRSSRGLFGAIAGFIEG
GWSGLVAGWYGFQHSNDQGVGMAADRDSTQKAIDKITSKVNNIVDKMNKQYEIIDHEFSEVETRLNMINN
KIDDQIQDIWAYNAELLVLLENQKTLDEHDANVNNLYNKVKRALGSNAVEDGKGCFELYHKCNDQCMETI
RNGTYNRKKYQEESKLERQKIEGVKLESEGTYKILTIYSTVASSLVIAMGFAAFLFWAMSNGSCRCNICI

>QBK20450.1 hemagglutinin [Influenza A virus]
METVSLMTILLVAAVSNADKICIGYQSTNSTETVDTLTENNVPVTHAKELLHTEHNGMLCATSLGQPIIL
DTCTIEGLIYGNPSCDLSLEGREWSYIVERPSAVNGLCYPGNVENLEELRSLFSSARSYQRIQIFPDTIW
NVSYDGTSTACSGSFYRNMRWLTRKNGEYPIQDAQYTNNQGKNILFMWGINHPPSDTTQRDLYTRTDTTT
SVATEEINRIFKPLIGPRPLVNGLMGRIDYYWSVLKPGQTLRIKSDGNLIAPWYGHILSGESHGRILKTD
LKRGSCTVQCQTEKGGLNTTLPFQNVSKYAFGNCSKYIGIKSLKLAVGLRNVPSRSSRGLFGAIAGFIEG
GWSGLVAGWYGFQHSNDQGVGMAADRDSTQKAVDKITSKVNNIVDKMNKQYEIIDHEFSEVETRLNMINN
KIDDQIQDIWAYNAELLVLLENQKTLDEHDANVNNLYNKVKRALGSNAVEDGKGCFELYHKCDDQCMETI
RNGTYNRRKYQEESKLERQKIEGVKLESEGTYKILTIYSTVASSLVIAMGFAAFLFWAMSNGSCRCNICI

>QBK20449.1 hemagglutinin [Influenza A virus]

METVSLITILLVAAVSNADKICIGYQSTNSTETVDTLTENNVPVTHAKELLHTEHNGMLCATSLGQPIIL
DTCTIEGLIYGNPSCDLSLEGREWSYIVERPSAVNGLCYPGNVENLEELRSLFSSARSYQRIQIFPDTIW
NVSYDGTSTACSGSFYRNMRWLTRKNGEYPIQDAQYTNNQGKNILFMWGINHPPADTTQRDLYTRTDTTT
SVATEEINRIFKPLIGPRPLVNGLMGRIDYYWSVLKPGQTLRIKSDGNLIAPWYGHILSGESHGRILKTD
LRRGSCTVQCQTEKGGLNTTLPFQNVSKYAFGNCSKYIGIKSLKLAVGLRNVPSRSSRGLFGAIAGFIEG
GWSGLVAGWYGFQHSNDQGVGMAADRDSTQKAVDKITSKVNTIVDKMNKQYEIIDHEFSEVETRLNMINN
KIDDQIQDIWAYNAELLVLLENQKTLDEHDANVNNLYNKVKRALGSNAVEDGKGCFELYHKCDDQCMETI
RNGTYNRRKYQEESKLERQKIEGVKLESEGTYKILTIYSTVASSLVIAMGFAAFLFWAMSNGSCRCNICI

>QBK20448.1 hemagglutinin [Influenza A virus]

METVSLITILLVAAVSNADKICIGYQSTNSTETVDTLTENNVPVTHAKELLHTEHNGMLCATSLGQPIIL
DTCTIEGLIYGNPSCDLSLEGREWSYIVERPSAVNGLCYPGNVENLEELRSLFSSARSYQRIQIFPDTIW
NVSYDGTSTACSGSFYRNMRWLTRKNGEYPIQDAQYTNNQGKNILFMWGINHPPADTTQRDLYTRTDTTT
SVATEEINRIFKPLIGPRPLVNGLMGRIDYYWSVLKPGQTLRIKSDGNLIAPWYGHILSGESHGRILKTD
LKRGSCTVQCQTEKGGLNTTLPFQNVSKYAFGNCSKYIGIKSLKLAVGLRNVPSRSSRGLFGAIAGFIEG
GWSGLVAGWYGFQHSNDQGVGMAADRDSTQKAVDKITSKVNTIVDKMNKQYEIIDHEFSEVETRLNMINN
KIDDQIQDIWAYNAELLVLLENQKTLDEHDANVNNLYNKVKRALGSNAVEDGKGCFELYHKCDDQCMETI
RNGTYNRRKYQEESKLERQKIEGVKLESEGTYKILTIYSTVASSLVIAMGFAAFLFWAMSNGSCRCNICI

>QBK20447.1 hemagglutinin [Influenza A virus]

METVSLITILLVAAVSNADKICIGYQSTNSTETVDTLTENNVPVTHAKELLHTEHNGMLCATSLGQPIIL
DTCTIEGLIYGNPSCDLSLEGREWSYIVERPSAVNGLCYPGNVENLEELRSLFSSARSYQRIQIFPDTIW
NVSYDGTSTACSGSFYRNMRWLTRKNGEYPIQDAQYTNNQGKNILFMWGINHPPADTTQRDLYTRTDTTT
SVATEEINRIFKPLIGPRPLVNGLMGRIDYYWSVLKPGQTLRIKSDGNLIAPWYGHILSGESHGRILKTD
LKRGSCTVQCQTEKGGLNTTLPFQNVSKYAFGNCSKYIGIKSLKLAVGLRNVPSRSSRGLFGAIAGFIEG
GWSGLVAGWYGFQHSNDQGVGMAADRDSTQKAVDKITSKVNTIVDKMNKQYEIIDHEFSEVETRLNMINN
KIDDQIQDIWAYNAELLVLLENQKTLDEHDANVNNLYNKVKRALGSNAVEDGKGCFELYHKCDDQCMETI
RNGTYNRRKYQEESKLERQKIEGVKLESEGTYKILTIYSTVASSLVIAMGFAAFLFWAMSNGSCRCNICI

>QBK20446.1 hemagglutinin [Influenza A virus]

METVSLITILLVAAVSNADKICIGYQSTNSTETVDTLTENNVPVTHAKELLHTEHNGMLCATSLGQPIIL
DTCTIEGLIYGNPSCDLSLEGREWSYIVERPSAVNGLCYPGNVENLEELRSLFSSARSYQRIQIFPDTIW
NVSYDGTSTACSGSFYRNMRWLTRKNGEYPIQDAQYTNNQGKNILFMWGINHPPADTTQRDLYTRTDTTT

SVATEEINRIFKPLIGPRPLVNGLMGRIDYYWSVLKPGQTLRIKSDGNLIAPWYGHILSGESHGRILKTD
LKRGSCTVQCQTEKGGLNTTLPFQNVSKYAFGNCSKYIGIKSLKLAVGLRNVPSRSSRGLFGAIAGFIEG
GWSGLVAGWYGFQHSNDQGVGMAADRDSTQKAVDKITSKVNTIVDKMNKQYEIIDHEFSEVETRLNMINN
KIDDQIQDIWAYNAELLVLLENQKTLDEHDANVNNLYNKVKRALGSNAVEDGKGCFELYHKCDDQCMETI
RNGTYNRRKYQEESKLERQKIEGVKLESEGTYKILTIYSTVASSLVIAMGFAAFLFWAMSNGSCRCNICI

>QBK20445.1 hemagglutinin [Influenza A virus]
METVSLITILLVAAVSNADKICIGYQSTNSTETVDTLTENNVPVTHAKELLHTEHNGMLCATSLGQPIIL
DTCTIEGLIYGNPSCDLSLEGREWSYIVERPSAVNGLCYPGNVENLEELRSLFSSARSYQRIQIFPDTIW
NVSYDGTSTACSGSFYRNMRWLTRKNGEYPIQDAQYTNNQGKNILFMWGINHPPADTTQRDLYTRTDTTT
SVATEEINRIFKPLIGPRPLVNGLMGRIDYYWSVLKPGQTLRIKSDGNLIAPWYGHILSGESHGRILKTD
LKRGSCTVQCQTEKGGLNTTLPFQNVSKYAFGNCSKYIGIKSLKLAVGLRNVPSRSSRGLFGAIAGFIEG
GWSGLVAGWYGFQHSNDQGVGMAADRDSTQKAVDKITSKVNTIVDKMNKQYEIIDHEFSEVETRLNMINN
KIDDQIQDIWAYNAELLVLLENQKTLDEHDANVNNLYNKVKRALGSNAVEDGKGCFELYHKCDDQCMETI
RNGTYNRRKYQEESKLERQKIEGVKLESEGTYKILTIYSTVASSLVIAMGFAAFLFWAMSNGSCRCNICI

>QBK20444.1 hemagglutinin [Influenza A virus]
METVSLITILLVAAVSNADKICIGYQSTNSTETVDTLTENNVPVTHAKELLHTEHNGMLCATSLGQPIIL
DTCTIEGLIYGNPSCDLSLEGREWSYIVERPSAVNGLCYPGNVENLEELRSLFSSARSYQRIQIFPDTIW
NVSYDGTSTACSGSFYRNMRWLTRKNGEYPIQDAQYTNNQGKNILFMWGINHPPADTTQRDLYTRTDTTT
SVATEEINRIFKPLIGPRPLVNGLMGRIDYYWSVLKPGQTLRIKSDGNLIAPWYGHILSGESHGRILKTD
LKRGSCTVQCQTEKGGLNTTLPFQNVSKYAFGNCSKYIGIKSLKLAVGLRNVPSRSSRGLFGAIAGFIEG
GWSGLVAGWYGFQHSNDQGVGMAADRDSTQKAVDKITSKVNTIVDKMNKQYEIIDHEFSEVETRLNMINN
KIDDQIQDIWAYNAELLVLLENQKTLDEHDANVNNLYNKVKRALGSNAVEDGKGCFELYHKCDDQCMETI
RNGTYNRRKYQEESKLERQKIEGVKLESEGTYKILTIYSTVASSLVIAMGFAAFLFWAMSNGSCRCNICI

>QBK20443.1 hemagglutinin [Influenza A virus]
METVSLITILLVAAVSNADKICIGYQSTNSTETVDTLTENNVPVTHAKELLHTEHNGMLCATSLGQPIIL
DTCTIEGLIYGNPSCDLSLEGREWSYIVERPSAVNGLCYPGNVENLEELRSLFSSARSYQRIQIFPDTIW
NVSYDGTSTACSGSFYRNMRWLTRKNGEYPIQDAQYTNNQGKNILFMWGINHPPADTTQRDLYTRTDTTT
SVATEEINRIFKPLIGPRPLVNGLMGRIDYYWSVLKPGQTLRIKSDGNLIAPWYGHILSGESHGRILKTD
LKRGSCTVQCQTEKGGLNTTLPFQNVSKYAFGNCSKYIGIKSLKLAVGLRNVPSRSSRGLFGAIAGFIEG
GWSGLVAGWYGFQHSNDQGVGMAADRDSTQKAVDKITSKVNTIVDKMNKQYEIIDHEFSEVETRLNMINN
KIDDQIQDIWAYNAELLVLLENQKTLDEHDANVNNLYNKVKRALGSNAVEDGKGCFELYHKCDDQCMETI

RNGTYNRRKYQEESKLERQKIEGVKLESEGTYKILTIYSTVASSLVIAMGFAAFLFWAMSNGSCRCNICI

>QBK20442.1 hemagglutinin [Influenza A virus]
METVSLITILLVAAVSNADKICIGYQSTNSTETVDTLTENNVPVTHAKELLHTEHNGMLCATSLGQPIIL
DTCTIEGLIYGNPSCDLSLEGREWSYIVERPSAVNGLCYPGNVENLEELRSLFSSARSYQRIQIFPDTIW
NVSYDGTSTACSGSFYRNMRWLTRKNGEYPIQDAQYTNNQGKNILFMWGINHPPADTTQRDLYTRTDTTT
SVATEEINRIFKPLIGPRPLVNGLMGRIDYYWSVLKPGQTLRIKSDGNLIAPWYGHILSGESHGRILKTD
LKRGSCTVQCQTEKGGLNTTLPFQNVSKYAFGNCSKYIGIKSLKLAVGLRNVPSRSSRGLFGAIAGFIEG
GWSGLVAGWYGFQHSNDQGVGMAADRDSTQKAVDKITSKVNTIVDKMNKQYEIIDHEFSEVETRLNMINN
KIDDQIQDIWAYNAELLVLLENQKTLDEHDANVNNLYNKVKRALGSNAVEDGKGCFELYHKCDDQCMETI
RNGTYNRRKYQEESKLERQKIEGVKLESEGTYKILTIYSTVASSLVIAMGFAAFLFWAMSNGSCRCNICI

>QBK20441.1 hemagglutinin [Influenza A virus]
METVSLITILLVAAVSNADKICIGYQSTNSTETVDTLTENNVPVTHAKELLHTEHNGMLCATSLGQPIIL
DTCTIEGLIYGNPSCDLSLEGREWSYIVERPSAVNGLCYPGNVENLEELRSLFSSARSYQRIQIFPDTIW
NVSYDGTSTACSGSFYRNMRWLTRKNGEYPIQDAQYTNNQGKNILFMWGINHPPADTTQRDLYTRTDTTT
SVATEEINRIFKPLIGPRPLVNGLMGRIDYYWSVLKPGQTLRIKSDGNLIAPWYGHILSGESHGRILKTD
LKRGSCTVQCQTEKGGLNTTLPFQNVSKYAFGNCSKYIGIKSLKLAVGLRNVPSRSSRGLFGAIAGFIEG
GWSGLVAGWYGFQHSNDQGVGMAADRDSTQKAVDKITSKVNTIVDKMNKQYEIIDHEFSEVETRLNMINN
KIDDQIQDIWAYNAELLVLLENQKTLDEHDANVNNLYNKVKRALGSNAVEDGKGCFELYHKCDDQCMETI
RNGTYNRRKYQEESKLERQKIEGVKLESEGTYKILTIYSTVASSLVIAMGFAAFLFWAMSNGSCRCNICI

>QBK20440.1 hemagglutinin [Influenza A virus]
METVSLITILLVAAVSNADKICIGYQSTNSTETVDTLTENNVPVTHAKELLHTEHNGMLCATSLGQPIIL
DTCTIEGLIYGNPSCDLSLEGREWSYIVERPSAVNGLCYPGNVENLEELRSLFSSARSYQRIQIFPDTIW
NVSYDGTSTACSGSFYRNMRWLTRKNGEYPIQDAQYTNNQGKNILFMWGINHPPADTTQRDLYTRTDTTT
SVATEEINRIFKPLIGPRPLVNGLMGRIDYYWSVLKPGQTLRIKSDGNLIAPWYGHILSGESHGRILKTD
LKRGSCTVQCQTEKGGLNTTLPFQNVSKYAFGNCSKYIGIKSLKLAVGLRNVPSRSSRGLFGAIAGFIEG
GWSGLVAGWYGFQHSNDQGVGMAADRDSTQKAVDKITSKVNTIVDKMNKQYEIIDHEFSEVETRLNMINN
KIDDQIQDIWAYNAELLVLLENQKTLDEHDANVNNLYNKVKRALGSNAVEDGKGCFELYHKCDDQCMETI
RNGTYNRRKYQEESKLERQKIEGVKLESEGTYKILTIYSTVASSLVIAMGFAAFLFWAMSNGSCRCNICI

>QBK20439.1 hemagglutinin [Influenza A virus]
METVSLITILLVAAVSNADKICIGYQSTNSTETVDTLTENNVPVTHAKELLHTEHNGMLCATSLGQPIIL

DTCTIEGLIYGNPSCDLSLEGREWSYIVERPSAVNGLCYPGNVENLEELRSLFSSARSYQRIQIFPDTIW

NVSYDGTSTACSGSFYRNMRWLTRKNGEYPIQDAQYTNNQGKNILFMWGINHPPADTTQRDLYTRTDTTT

SVATEEINRIFKPLIGPRPLVNGLMGRIDYYWSVLKPGQTLRIKSDGNLIAPWYGHILSGESHGRILKTD

LKRGSCTVQCQTEKGGLNTTLPFQNVSKYAFGNCSKYIGIKSLKLAVGLRNVPSRSSRGLFGAIAGFIEG

GWSGLVAGWYGFQHSNDQGVGMAADRDSTQKAVDKITSKVNTIVDKMNKQYEIIDHEFSEVETRLNMINN

KIDDQIQDIWAYNAELLVLLENQKTLDEHDANVNNLYNKVKRALGSNAVEDGKGCFELYHKCDDQCMETI

RNGTYNRRKYQEESKLERQKIEGVKLESEGTYKILTIYSTVASSLVIAMGFAAFLFWAMSNGSCRCNICI

>QBK20438.1 hemagglutinin [Influenza A virus]

METVSLITILLVAAVSNADKICIGYQSTNSTETVDTLTENNVPVTHAKELLHTEHNGMLCATSLGQPIIL

DTCTIEGLIYGNPSCDLSLEGREWSYIVERPSAVNGLCYPGNVENLEELRSLFSSARSYQRIQIFPDTIW

NVSYDGTSTACSGSFYRNMRWLTRKNGEYPIQDAQYTNNQGKNILFMWGINHPPADTTQRDLYTRTDTTT

SVATEEINRIFKPLIGPRPLVNGLMGRIDYYWSVLKPGQTLRIKSDGNLIAPWYGHILSGESHGRILKTD

LKRGSCTVQCQTEKGGLNTTLPFQNVSKYAFGNCSKYIGIKSLKLAVGLRNVPSRSSRGLFGAIAGFIEG

GWSGLVAGWYGFQHSNDQGVGMAADRDSTQKAVDKITSKVNTIVDKMNKQYEIIDHEFSEVETRLNMINN

KIDDQIQDIWAYNAELLVLLENQKTLDEHDANVNNLYNKVKRALGSNAVEDGKGCFELYHKCDDQCMETI

RNGTYNRRKYQEESKLERQKIEGVKLESEGTYKILTIYSTVASSLVIAMGFAAFLFWAMSNGSCRCNICI

>QBK20437.1 hemagglutinin [Influenza A virus]

METVSLITILLVAAVSNADKICIGYQSTNSTETVDTLTENNVPVTHAKELLHTEHNGMLCATSLGQPIIL

DTCTIEGLIYGNPSCDLSLEGREWSYIVERPSAVNGLCYPGNVENLEELRSLFSSARSYQRIQIFPDTIW

NVSYDGTSTACSGSFYRNMRWLTRKNGEYPIQDAQYTNNQGKNILFMWGINHPPADTTQRDLYTRTDTTT

SVATEEINRIFKPLIGPRPLVNGLMGRIDYYWSVLKPGQTLRIKSDGNLIAPWYGHILSGESHGRILKTD

LKRGRCTVQCQTEKGGLNTTLPFQNVSKYAFGNCSKYIGIKSLKLAVGLRNVPSRSSRGLFGAIAGFIEG

GWSGLVAGWYGFQHSNDQGVGMAADRDSTQKAVDKITSKVNTIVDKMNKQYEIIDHEFSEVETRLNMINN

KIDDQIQDIWAYNAELLVLLENQKTLDEHDANVNNLYNKVKRALGSNAVEDGKGCFELYHKCDDQCMETI

RNGTYNRRKYQEESKLERQKIEGVKLESEGTYKILTIYSTVASSLVIAMGFAAFLFWAMSNGSCRCNICI

>QBK20436.1 hemagglutinin [Influenza A virus]

METVSLITILLVAAVSNADKICIGYQSTNSTETVDTLTENNVPVTHAKELLHTEHNGMLCATSLGQPIIL

DTCTIEGLIYGNPSCDLSLEGREWSYIVERPSAVNGLCYPGNVENLEELRSLFSSARSYQRIQIFPDTIW

NVSYDGTSTACSGSFYRNMRWLTRKNGEYPIQDAQYTNNQGKNILFMWGINHPPADTTQRDLYTRTDTTT

SVATEEINRIFKPLIGPRPLVNGLMGRIDYYWSVLKPGQTLRIKSDGNLIAPWYGHILSGESHGRILKTD

LKRGRCTVQCQTEKGGLNTTLPFQNVSKYAFGNCSKYIGIKSLKLAVGLRNVPSRSSRGLFGAIAGFIEG

GWSGLVAGWYGFQHSNDQGVGMAADRDSTQKAVDKITSKVNTIVDKMNKQYEIIDHEFSEVETRLNMINN
KIDDQIQDIWAYNAELLVLLENQKTLDEHDANVNNLYNKVKRALGSNAVEDGKGCFELYHKCDDQCMETI
RNGTYNRRKYQEESKLERQKIEGVKLESEGTYKILTIYSTVASSLVIAMGFAAFLFWAMSNGSCRCNICI

>QBK20432.1 hemagglutinin [Influenza A virus]
METVSLITILLVAAVSNADKICIGYQSTNSTETVDTLTENNVPVTHAKELLHTEHNGMLCATSLGQPIIL
DTCTIEGLIYGNPSCDLSLEGREWSYIVERPSAVNGLCYPGNVENLEELRSLFSSARSYQRIQIFPDTIW
NVSYDGTSTACSGSFYRNMRWLTRKNGEYPIQDAQYTNNQGKNILFMWGINHPPADTTQRDLYTRTDTTT
SVATEEINRIFKPLIGPRPLVNGLMGRIDYYWSVLKPGQTLRIKSDGNLIAPWYGHILSGESHGRILKTD
LKRGSCTVQCQTEKGGLNTTLPFQNVSKYAFGNCSKYIGIKSLKLAVGLRNVPSRSSRGLFGAIAGFIEG
GWSGLVAGWYGFQHSNDQGVGMAADRDSTQKAVDKITSKVNTIVDKMNKQYEIIDHEFSEVETRLNMINN
KIDDQIQDIWAYNAELLVLLENQKTLDEHDANVNNLYNKVKRALGSNAVEDGKGCFELYHKCDDQCMETI
RNGTYNRRKYQEESKLERQKIEGVKLESEGTYKILTIYSTVASSLVIAMGFAAFLFWAMSNGSCRCNICI

>QBK20431.1 hemagglutinin [Influenza A virus]
METVSLITILLVAAVSNADKICIGYQSTNSTETVDTLTENNVPVTHAKELLHTEHNGMLCATSLGQPIIL
DTCTIEGLIYGNPSCDLSLEGREWSYIVERPSAVNGLCYPGNVENLEELRSLFSSARSYQRIQIFPDTIW
NVSYDGTSTACSGSFYRNMRWLTRKNGEYPIQDAQYTNNQGKNILFMWGINHPPADTTQRDLYTRTDTTT
SVATEEINRIFKPLIGPRPLVNGLMGRIDYYWSVLKPGQTLRIKSDGNLIAPWYGHILSGESHGRILKTD
LKRGSCTVQCQTEKGGLNTTLPFQNVSKYAFGNCSKYIGIKSLKLAVGLRNVPSRSSRGLFGAIAGFIEG
GWSGLVAGWYGFQHSNDQGVGMAADRDSTQKAVDKITSKVNTIVDKMNKQYEIIDHEFSEVETRLNMINN
KIDDQIQDIWAYNAELLVLLENQKTLDEHDANVNNLYNKVKRALGSNAVEDGKGCFELYHKCDDQCMETI
RNGTYNRRKYQEESKLERQKIEGVKLESEGTYKILTIYSTVASSLVIAMGFAAFLFWAMSNGSCRCNICI

>QBK20430.1 hemagglutinin [Influenza A virus]
METVSLITILLVAAVSNADKICIGYQSTNSTETVDTLTENNVPVTHAKELLHTEHNGMLCATSLGQPIIL
DTCTIEGLIYGNPSCDLSLEGREWSYIVERPSAVNGLCYPGNVENLEELRSLFSSARSYQRIQIFPDTIW
NVSYDGTSTACSGSFYRNMRWLTRKNGEYPIQDAQYTNNQGKNILFMWGINHPPADTTQRDLYTRTDTTT
SVATEEINRIFKPLIGPRPLVNGLMGRIDYYWSVLKPGQTLRIKSDGNLIAPWYGHILSGESHGRILKTD
LKRGSCTVQCQTEKGGLNTTLPFQNVSKYAFGNCSKYIGIKSLKLAVGLRNVPSRSSRGLFGAIAGFIEG
GWSGLVAGWYGFQHSNDQGVGMAADRDSTQKAVDKITSKVNTIVDKMNKQYEIIDHEFSEVETRLNMINN
KIDDQIQDIWAYNAELLVLLENQKTLDEHDANVNNLYNKVKRALGSNAVEDGKGCFELYHKCDDQCMETI
RNGTYNRRKYQEESKLERQKIEGVKLESEGTYKILTIYSTVASSLVIAMGFAAFLFWAMSNGSCRCNICI

>QBK20429.1 hemagglutinin [Influenza A virus]

METVSLITILLVAAVSNADKICIGYQSTNSTETVDTLTENNVPVTHAKELLHTEHNGMLCATSLGQPIIL

DTCTIEGLIYGNPSCDLSLEGREWSYIVERPSAVNGLCYPGNVENLEELRSLFSSARSYQRIQIFPDTIW

NVSYDGTSTACSGSFYRNMRWLTRKNGEYPIQDAQYTNNQGKNILFMWGINHPPADTTQRDLYTRTDTTT

SVATEEINRIFKPLIGPRPLVNGLMGRIDYYWSVLKPGQTLRIKSDGNLIAPWYGHILSGESHGRILKTD

LKRGSCTVQCQTEKGGLNTTLPFQNVSKYAFGNCSKYIGIKSLKLAVGLRNVPSRSSRGLFGAIAGFIEG

GWSGLVAGWYGFQHSNDQGVGMAADRDSTQKAVDKITSKVNTIVDKMNKQYEIIDHEFSEVETRLNMINN

KIDDQIQDIWAYNAELLVLLENQKTLDEHDANVNNLYNKVKRALGSNAVEDGKGCFELYHKCDDQCMETI

RNGTYNRRKYQEESKLERQKIEGVKLESEGTYKILTIYSTVASSLVIAMGFAAFLFWAMSNGSCRCNICI

>QBK20428.1 hemagglutinin [Influenza A virus]

METVSLITILLVAAVSNADKICIGYQSTNSTETVDTLTENNVPVTHAKELLHTEHNGMLCATSLGQPIIL

DTCTIEGLIYGNPSCDLSLEGREWSYIVERPSAVNGLCYPGNVENLEELRSLFSSARSYQRIQIFPDTIW

NVSYDGTSTACSGSFYRNMRWLTRKNGEYPIQDAQYTNNQGKNILFMWGINHPPADTTQRDLYTRTDTTT

SVATEEINRIFKPLIGPRPLVNGLMGRIDYYWSVLKPGQTLRIKSDGNLIAPWYGHILSGESHGRILKTD

LKRGSCTVQCQTEKGGLNTTLPFQNVSKYAFGNCSKYIGIKSLKLAVGLRNVPSRSSRGLFGAIAGFIEG

GWSGLVAGWYGFQHSNDQGVGMAADRDSTQKAVDKITSKVNTIVDKMNKQYEIIDHEFSEVETRLNMINN

KIDDQIQDIWAYNAELLVLLENQKTLDEHDANVNNLYNKVKRALGSNAVEDGKGCFELYHKCDDQCMETI

RNGTYNRRKYQEESKLERQKIEGVKLESEGTYKILTIYSTVASSLVIAMGFAAFLFWAMSNGSCRCNICI

>QBK20427.1 hemagglutinin [Influenza A virus]

METVSLITILLVAAVSNADKICIGYQSTNSTETVDTLTENNVPVTHAKELLHTEHNGMLCATSLGQPIIL

DTCTIEGLIYGNPSCDLSLEGREWSYIVERPSAVNGLCYPGNVENLEELRSLFSSARSYQRIQIFPDTIW

NVSYDGTSTACSGSFYRNMRWLTRKNGEYPIQDAQYTNNQGKNILFMWGINHPPADTTQRDLYTRTDTTT

SVATEEINRIFKPLIGPRPLVNGLMGRIDYYWSVLKPGQTLRIKSDGNLIAPWYGHILSGESHGRILKTD

LKRGSCTVQCQTEKGGLNTTLPFQNVSKYAFGNCSKYIGIKSLKLAVGLRNVPSRSSRGLFGAIAGFIEG

GWSGLVAGWYGFQHSNDQGVGMAADRDSTQKAVDKITSKVNTIVDKMNKQYEIIDHEFSEVETRLNMINN

KIDDQIQDIWAYNAELLVLLENQKTLDEHDANVNNLYNKVKRALGSNAVEDGKGCFELYHKCDDQCMETI

RNGTYNRRKYQEESKLERQKIEGVKLESEGTYKILTIYSTVASSLVIAMGFAAFLFWAMSNGSCRCNICI

>QBK20426.1 hemagglutinin [Influenza A virus]

METVSLITILLVAAVSNADKICIGYQSTNSTETVDTLTENNVPVTHAKELLHTEHNGMLCATSLGQPIIL

DTCTIEGLIYGNPSCDLSLEGREWSYIVERPSAVNGLCYPGNVENLEELRSLFSSARSYQRIQIFPDTIW

NVSYDGTSTACSGSFYRNMRWLTRKNGEYPIQDAQYTNNQGKNILFMWGINHPPADTTQRDLYTRTDTTT

SVATEEINRIFKPLIGPRPLVNGLMGRIDYYWSVLKPGQTLRIKSDGNLIAPWYGHILSGESHGRILKTD
LKRGSCTVQCQTEKGGLNTTLPFQNVSKYAFGNCSKYIGIKSLKLAVGLRNVPSRSSRGLFGAIAGFIEG
GWSGLVAGWYGFQHSNDQGVGMAADRDSTQKAVDKITSKVNTIVDKMNKQYEIIDHEFSEVETRLNMINN
KIDDQIQDIWAYNAELLVLLENQKTLDEHDANVNNLYNKVKRALGSNAVEDGKGCFELYHKCDDQCMETI
RNGTYNRRKYQEESKLERQKIEGVKLESEGTYKILTIYSTVASSLVIAMGFAAFLFWAMSNGSCRCNICI

>QBK20425.1 hemagglutinin [Influenza A virus]
METVSLITILLVAAVSNADKICIGYQSTNSTETVDTLTENNVPVTHAKELLHTEHNGMLCATSLGQPIIL
DTCTIEGLIYGNPSCDLSLEGREWSYIVERPSAVNGLCYPGNVENLEELRSLFSSARSYQRIQIFPDTIW
NVSYDGTSTACSGSFYRNMRWLTRKNGEYPIQDAQYTNNQGKNILFMWGINHPPADTTQRDLYTRTDTTT
SVATEEINRIFKPLIGPRPLVNGLMGRIDYYWSVLKPGQTLRIKSDGNLIAPWYGHILSGESHGRILKTD
LKRGSCTVQCQTEKGGLNTTLPFQNVSKYAFGNCSKYIGIKSLKLAVGLRNVPSRSSRGLFGAIAGFIEG
GWSGLVAGWYGFQHSNDQGVGMAADRDSTQKAVDKITSKVNTIVDKMNKQYEIIDHEFSEVETRLNMINN
KIDDQIQDIWAYNAELLVLLENQKTLDEHDANVNNLYNKVKRALGSNAVEDGKGCFELYHKCDDQCMETI
RNGTYNRRKYQEESKLERQKIEGVKLESEGTYKILTIYSTVASSLVIAMGFAAFLFWAMSNGSCRCNICI

>QBK20423.1 hemagglutinin [Influenza A virus]
METVSLITILLVAAVSNADKICIGYQSTNSTETVDTLTENNVPVTHAKELLHTEHNGMLCATSLGQPIIL
DTCTIEGLIYGNPSCDLSLEGREWSYIVERPSAVNGLCYPGNVENLEELRSLFSSARSYQRIQIFPDTIW
NVSYDGTSTACSGSFYRNMRWLTRKNGEYPIQDAQYTNNQGKNILFMWGINHPPADTTQRDLYTRTDTTT
SVATEEINRIFKPLIGPRPLVNGLMGRIDYYWSVLKPGQTLRIKSDGNLIAPWYGHILSGESHGRILKTD
LKRGSCTVQCQTEKGGLNTTLPFQNVSKYAFGNCSKYIGIKSLKLAVGLRNVPSRSSRGLFGAIAGFIEG
GWSGLVAGWYGFQHSNDQGVGMAADRDSTQKAVDKITSKVNTIVDKMNKQYEIIDHEFSEVETRLNMINN
KIDDQIQDIWAYNAELLVLLENQKTLDEHDANVNNLYNKVKRALGSNAVEDGKGCFELYHKCDDQCMETI
RNGTYNRRKYQEESKLERQKIEGVKLESEGTYKILTIYSTVASSLVIAMGFAAFLFWAMSNGSCRCNICI

>QBK20422.1 hemagglutinin [Influenza A virus]
METVSLITILLVAAVSNADKICIGYQSTNSTETVDTLTENNVPVTHAKELLHTEHNGMLCATSLGQPIIL
DTCTIEGLIYGNPSCDLSLEGREWSYIVERPSAVNGLCYPGNVENLEELRSLFSSARSYQRIQIFPDTIW
NVSYDGTSTACSGSFYRNMRWLTRKNGEYPIQDAQYTNNQGKNILFMWGINHPPADTTQRDLYTRTDTTT
SVATEEINRIFKPLIGPRPLVNGLMGRIDYYWSVLKPGQTLRIKSDGNLIAPWYGHILSGESHGRILKTD
LKRGSCTVQCQTEKGGLNITLPFQNVSKYAFGNCSKYIGIKSLKLAVGLRNVPSRSSRGLFGAIAGFIEG
GWSGLVAGWYGFQHSNDQGVGMAADRDSTQKAVDKITSKVNTIVDKMNKQYEIIDHEFSEVETRLNMINN
KIDDQIQDIWAYNAELLVLLENQKTLDEHDANVNNLYNKVKRALGSNAVEDGKGCFELYHKCDDQCMETI

RNGTYNRRKYQEESKLERQKIEGVKLESEGTYKILTIYSTVASSLVIAMGFAAFLFWAMSNGSCRCNICI

>QBK20421.1 hemagglutinin [Influenza A virus]
METVSLITILLVAAVSNADKICIGYQSTNSTETVDTLTENNVPVTHAKELLHTEHNGMLCATSLGQPIIL
DTCTIEGLIYGNPSCDLSLEGREWSYIVERPSAVNGLCYPGNVENLEELRSLFSSARSYQRIQIFPDTIW
NVSYDGTSTACSGSFYRNMRWLTRKNGEYPIQDAQYTNNQGKNILFMWGINHPPADTTQRDLYTRTDTTT
SVATEEINRIFKPLIGPRPLVNGLMGRIDYYWSVLKPGQTLRIKSDGNLIAPWYGHILSGESHGRILKTD
LKRGSCTVQCQTEKGGLNTTLPFQNVSKYAFGNCSKYIGIKSLKLAVGLRNVPSRSSRGLFGAIAGFIEG
GWSGLVAGWYGFQHSNDQGVGMAADRDSTQKAVDKITSKVNTIVDKMNKQYEIIDHEFSEVETRLNMINN
KIDDQIQDIWAYNAELLVLLENQKTLDEHDANVNNLYNKVKRALGSNAVEDGKGCFELYHKCDDQCMETI
RNGTYNRRKYQEESKLERQKIEGVKLESEGTYKILTIYSTVASSLVIAMGFAAFLFWAMSNGSCRCNICI

>QBK20420.1 hemagglutinin [Influenza A virus]
METVSLITILLVAAVSNADKICIGYQSTNSTETVDTLTENNVPVTHAKELLHTEHNGMLCATSLGQPIIL
DTCTIEGLIYGNPSCDLSLEGREWSYIVERPSAVNGLCYPGNVENLEELRSLFSSARSYQRIQIFPDTIW
NVSYDGTSTACSGSFYRNMRWLTRKNGEYPIQDAQYTNNQGKNILFMWGINHPPADTTQRDLYTRTDTTT
SVATEEINRIFKPLIGPRPLVNGLMGRIDYYWSVLKPGQTLRIKSDGNLIAPWYGHILSGESHGRILKTD
LKRGSCTVQCQTEKGGLNTTLPFQNVSKYAFGNCSKYIGIKSLKLAVGLRNVPSRSSRGLFGAIAGFIEG
GWSGLVAGWYGFQHSNDQGVGMAADRDSTQKAVDKITSKVNTIVDKMNKQYEIIDHEFSEVETRLNMINN
KIDDQIQDIWAYNAELLVLLENQKTLDEHDANVNNLYNKVKRALGSNAVEDGKGCFELYHKCDDQCMETI
RNGTYNRRKYQEESKLERQKIEGVKLESEGTYKILTIYSTVASSLVIAMGFAAFLFWAMSNGSCRCNICI

>QBK20419.1 hemagglutinin [Influenza A virus]
METVSLITILLVAAVSNADKICIGYQSTNSTETVDTLTENNVPVTHAKELLHTEHNGMLCATSLGQPIIL
DTCTIEGLIYGNPSCDLSLEGREWSYIVERPSAVNGLCYPGNVENLEELRSLFSSARSYQRIQIFPDTIW
NVSYDGTSTACSGSFYRNMRWLTRKNGEYPIQDAQYTNNQGKNILFMWGINHPPADTTQRDLYTRTDTTT
SVATEEINRIFKPLIGPRPLVNGLMGRIDYYWSVLKPGQTLRIKSDGNLIAPWYGHILSGESHGRILKTD
LKRGSCTVQCQTEKGGLNTTLPFQNVSKYAFGNCSKYIGIKSLKLAVGLRNVPSRSSRGLFGAIAGFIEG
GWSGLVAGWYGFQHSNDQGVGMAADRDSTQKAVDKITSKVNTIVDKMNKQYEIIDHEFSEVETRLNMINN
KIDDQIQDIWAYNAELLVLLENQKTLDEHDANVNNLYNKVKRALGSNAVEDGKGCFELYHKCDDQCMETI
RNGTYNRRKYQEESKLERQKIEGVKLESEGTYKILTIYSTVASSLVIAMGFAAFLFWAMSNGSCRCNICI

>QBK20418.1 hemagglutinin [Influenza A virus]
METVSLITILLVAAVSNADKICIGYQSTNSTETVDTLTENNVPVTHAKELLHTEHNGMLCATSLGQPIIL

DTCTIEGLIYGNPSCDLSLEGREWSYIVERPSAVNGLCYPGNVENLEELRSLFSSARSYQRIQIFPDTIW
NVSYDGTSTACSGSFYRNMRWLTRKNGEYPIQDAQYTNNQGKNILFMWGINHPPADTTQRDLYTRTDTTT
SVATEEINRIFKPLIGPRPLVNGLMGRIDYYWSVLKPGQTLRIKSDGNLIAPWYGHILSGESHGRILKTD
LKRGSCTVQCQTEKGGLNTTLPFQNVSKYAFGNCSKYIGIKSLKLAVGLRNVPSRSSRGLFGAIAGFIEG
GWSGLVAGWYGFQHSNDQGVGMAADRDSTQKAVDKITSKVNTIVDKMNKQYEIIDHEFSEVETRLNMINN
KIDDQIQDIWAYNAELLVLLENQKTLDEHDANVNNLYNKVKRALGSNAVEDGKGCFELYHKCDDQCMETI
RNGTYNRRKYQEESKLERQKIEGVKLESEGTYKILTIYSTVASSLVIAMGFAAFLFWAMSNGSCRCNICI

>QBK20417.1 hemagglutinin [Influenza A virus]
METVSLITILLVAAVSNADKICIGYQSTNSTETVDTLTENNVPVTHAKELLHTEHNGMLCATSLGQPIIL
DTCTIEGLIYGNPSCDLSLEGREWSYIVERPSAVNGLCYPGNVENLEELRSLFSSARSYQRIQIFPDTIW
NVSYDGTSTACSGSFYRNMRWLTRKNGEYPIQDAQYTNNQGKNILFMWGINHPPADTTQRDLYTRTDTTT
SVATEEINRIFKPLIGPRPLVNGLMGRIDYYWSVLKPGQTLRIKSDGNLIAPWYGHILSGESHGRILKTD
LKRGSCTVQCQTEKGGLNTTLPFQNVSKYAFGNCSKYIGIKSLKLAVGLRNVPSRSSRGLFGAIAGFIEG
GWSGLVAGWYGFQHSNDQGVGMAADRDSTQKAVDKITSKVNTIVDKMNKQYEIIDHEFSEVETRLNMINN
KIDDQIQDIWAYNAELLVLLENQKTLDEHDANVNNLYNKVKRALGSNAVEDGKGCFELYHKCDDQCMETI
RNGTYNRRKYQEESKLERQKIEGVKLESEGTYKILTIYSTVASSLVIAMGFAAFLFWAMSNGSCRCNICI

>QBK20416.1 hemagglutinin [Influenza A virus]
METVSLITILLVAAVSNADKICIGYQSTNSTETVDTLTENNVPVTHAKELLHTEHNGMLCATSLGQPIIL
DTCTIEGLIYGNPSCDLSLEGREWSYIVERPSAVNGLCYPGNVENLEELRSLFSSARSYQRIQIFPDTIW
NVSYDGTSTACSGSFYRNMRWLTRKNGEYPIQDAQYTNNQGKNILFMWGINHPPADTTQRDLYTRTDTTT
SVATEEINRIFKPLIGPRPLVNGLMGRIDYYWSVLKPGQTLRIKSDGNLIAPWYGHILSGESHGRILKTD
LKRGSCTVQCQTEKGGLNTTLPFQNVSKYAFGNCSKYIGIKSLKLAVGLRNVPSRSSRGLFGAIAGFIEG
GWSGLVAGWYGFQHSNDQGVGMAADRDSTQKAVDKITSKVNTIVDKMNKQYEIIDHEFSEVETRLNMINN
KIDDQIQDIWAYNAELLVLLENQKTLDEHDANVNNLYNKVKRALGSNAVEDGKGCFELYHKCDDQCMETI
RNGTYNRRKYQEESKLERQKIEGVKLESEGTYKILTIYSTVASSLVIAMGFAAFLFWAMSNGSCRCNICI

>QBK20414.1 hemagglutinin [Influenza A virus]
METVSLITILLVATISNADKICIGYQSTNSTETVDTLTENNVPVTHAKELLHTEHNGMLCATSLGQPLIL
DTCTIEGLIYGNPSCDLSLEGREWSYIVERPSAVNGLCYPGNVENLEELRSLFSSARSYQRIQIFPDTIW
NVSYDGTSTACSGSFYRSMRWLTRKNGDYPTQDAQYTNNQGKNILFMWGINHPPTDDTQRNLYTRTDTTT
SVATEEINRIFKPLIGPRPLVNGLMGRIDYYWSVLKPGQTLRIKSDGNLVAPWYGHILSGESHGRILKTD
LKRGSCTVQCQTEKGGLNTTLPFQNVSKYAFGNCSKYIGIKSLKLAVGLRNVPSRSSRGLFGAIAGFIEG

GWSGLVAGWYGFQHSNDQGVGMAADRDSTQKAIDKITSKVNNIVDKMNKQYEIIDHEFSEVETRLNMINN
KIDDQIQDIWAYNAELLVLLENQKTLDEHDANVNNLYNKVKRALGSNAVEDGKGCFELYHKCNDQCMETI
RNGTYNRRKYQEESKVERQKIEGVKLESEGTYKILTIYSTVASSLVIAMGFAAFLFWAMSNGSCRCNICI

>QBK20413.1 hemagglutinin [Influenza A virus]
METVSLITILLVAAVSNADKICIGYQSTNSTETVDTLTENNVPVTHAKELLHTEHNGMLCATSLGQPIIL
DTCTIEGLIYGNPSCDLSLEGREWSYIVERPSAVNGLCYPGNVENLEELRSLFSSARSYQRIQIFPDTIW
NVSYDGTSTACSGSFYRNMRWLTRKNGEYPIQDAQYTNNQGKNILFMWGINHPPADTTQRDLYTRTDTTT
SVATEEINRIFKPLIGPRPLVNGLMGRIDYYWSVLKPGQTLRIKSDGNLIAPWYGHILSGESHGRILKTD
LKRGSCTVQCQTEKGGLNTTLPFQNVSKYAFGNCSKYIGIKSLKLAVGLRNVPSRSSRGLFGAIAGFIEG
GWSGLVAGWYGFQHSNDQGVGMAADRDSTQKAVDKITSKVNTIVDKMNKQYEIIDHEFSEVETRLNMINN
KIDDQIQDIWAYNAELLVLLENQKTLDEHDANVNNLYNKVKRALGSNAVEDGKGCFELYHKCDDQCMETI
RNGTYNRRKYQEESKLERQKIEGVKLESEGTYKILTIYSTVASSLVIAMGFAAFLFWAMSNGSCRCNICI

>QBK20412.1 hemagglutinin [Influenza A virus]
METVSLITILLVAAVSNADKICIGYQSTNSTETVDTLTENNVPVTHAKELLHTEHNGMLCATSLGQPIIL
DTCTIEGLIYGNPSCDLSLEGREWSYIVERPSAVNGLCYPGNVENLEELRSLFSSARSYQRIQIFPDTIW
NVSYDGTSTACSGSFYRNMRWLTRKNGEYPIQDAQYTNNQGKNILFMWGINHPPADTTQRDLYTRTDTTT
SVATEEINRIFKPLIGPRPLVNGLMGRIDYYWSVLKPGQTLRIKSDGNLIAPWYGHILSGESHGRILKTD
LKRGSCTVQCQTEKGGLNTTLPFQNVSKYAFGNCSKYIGIKSLKLAVGLRNVPSRSSRGLFGAIAGFIEG
GWSGLVAGWYGFQHSNDQGVGMAADRDSTQKAVDKITSKVNTIVDKMNKQYEIIDHEFSEVETRLNMINN
KIDDQIQDIWAYNAELLVLLENQKTLDEHDANVNNLYNKVKRALGSNAVEDGKGCFELYHKCDDQCMETI
RNGTYNRRKYQEESKLERQKIEGVKLESEGTYKILTIYSTVASSLVIAMGFAAFLFWAMSNGSCRCNICI

>QBK20411.1 hemagglutinin [Influenza A virus]
METVSLITILLVAAVSNADKICIGYQSTNSTETVDTLTENNVPVTHAKELLHTEHNGMLCATSLGQPIIL
DTCTIEGLIYGNPSCDLSLEGREWSYIVERPSAVNGLCYPGNVENLEELRSLFSSARSYQRIQIFPDTIW
NVSYDGTSTACSGSFYRNMRWLTRKNGEYPIQDAQYTNNQGKNILFMWGINHPPADTTQRDLYTRTDTTT
SVATEEINRIFKPLIGPRPLVNGLMGRIDYYWSVLKPGQTLRIKSDGNLIAPWYGHILSGESHGRILKTD
LKRGSCTVQCQTEKGGLNTTLPFQNVSKYAFGNCSKYIGIKSLKLAVGLRNVPSRSSRGLFGAIAGFIEG
GWSGLVAGWYGFQHSNDQGVGMAADRDSTQKAVDKITSKVNTIVDKMNKQYEIIDHEFSEVETRLNMINN
KIDDQIQDIWAYNAELLVLLENQKTLDEHDANVNNLYNKVKRALGSNAVEDGKGCFELYHKCDDQCMETI
RNGTYNRRKYQEESKLERQKIEGVKLESEGTYKILTIYSTVASSLVIAMGFAAFLFWAMSNGSCRCNICI

>QBK20410.1 hemagglutinin [Influenza A virus]

METVSLITILLVAAVSNADKICIGYQSTNSTETVDTLTENNVPVTHAKELLHTEHNGMLCATSLGQPIIL

DTCTIEGLIYGNPSCDLSLEGREWSYIVERPSAVNGLCYPGNVENLEELRSLFSSARSYQRIQIFPDTIW

NVSYDGTSTACSGSFYRNMRWLTRKNGEYPIQDAQYTNNQGKNILFMWGINHPPADTTQRDLYTRTDTTT

SVATEEINRIFKPLIGPRPLVNGLMGRIDYYWSVLKPGQTLRIKSDGNLIAPWYGHILSGESHGRILKTD

LKRGSCTVQCQTEKGGLNTTLPFQNVSKYAFGNCSKYIGIKSLKLAVGLRNVPSRSSRGLFGAIAGFIEG

GWSGLVAGWYGFQHSNDQGVGMAADRDSTQKAVDKITSKVNTIVDKMNKQYEIIDHEFSEVETRLNMINN

KIDDQIQDIWAYNAELLVLLENQKTLDEHDANVNNLYNKVKRALGSNAVEDGKGCFELYHKCDDQCMETI

RNGTYNRRKYQEESKLERQKIEGVKLESEGTYKILTIYSTVASSLVIAMGFAAFLFWAMSNGSCRCNICI

>QBK20409.1 hemagglutinin [Influenza A virus]

METVSLITILLVAAVSNADKICIGYQSTNSTETVDTLTENNVPVTHAKELLHTEHNGMLCATSLGQPIIL

DTCTIEGLIYGNPSCDLSLEGREWSYIVERPSAVNGLCYPGNVENLEELRSLFSSARSYQRIQIFPDTIW

NVSYDGTSTACSGSFYRNMRWLTRKNGEYPIQDAQYTNNQGKNILFMWGINHPPADTTQRDLYTRTDTTT

SVATEEINRIFKPLIGPRPLVNGLMGRIDYYWSVLKPGQTLRIKSDGNLIAPWYGHILSGESHGRILKTD

LKRGSCTVQCQTEKGGLNTTLPFQNVSKYAFGNCSKYIGIKSLKLAVGLRNVPSRSSRGLFGAIAGFIEG

GWSGLVAGWYGFQHSNDQGVGMAADRDSTQKAVDKITSKVNTIVDKMNKQYEIIDHEFSEVETRLNMINN

KIDDQIQDIWAYNAELLVLLENQKTLDEHDANVNNLYNKVKRALGSNAVEDGKGCFELYHKCDDQCMETI

RNGTYNRRKYQEESKLERQKIEGVKLESEGTYKILTIYSTVASSLVIAMGFAAFLFWAMSNGSCRCNICI

>QBK20408.1 hemagglutinin [Influenza A virus]

METVSLITILLVAAVSNADKICIGYQSTNSTETVDTLTENNVPVTHAKELLHTEHNGMLCATSLGQPIIL

DTCTIEGLIYGNPSCDLSLEGREWSYIVERPSAVNGLCYPGNVENLEELRSLFSSARSYQRIQIFPDTIW

NVSYDGTSTACSGSFYRNMRWLTRKNGEYPIQDAQYTNNQGKNILFMWGINHPPADTTQRDLYTRTDTTT

SVATEEINRIFKPLIGPRPLVNGLMGRIDYYWSVLKPGQTLRIKSDGNLIAPWYGHILSGESHGRILKTD

LKRGSCTVQCQTEKGGLNTTLPFQNVSKYAFGNCSKYIGIKSLKLAVGLRNVPSRSSRGLFGAIAGFIEG

GWSGLVAGWYGFQHSNDQGVGMAADRDSTQKAVDKITSKVNTIVDKMNKQYEIIDHEFSEVETRLNMINN

KIDDQIQDIWAYNAELLVLLENQKTLDEHDANVNNLYNKVKRALGSNAVEDGKGCFELYHKCDDQCMETI

RNGTYNRRKYQEESKLERQKIEGVKLESEGTYKILTIYSTVASSLVIAMGFAAFLFWAMSNGSCRCNICI

>QBK20407.1 hemagglutinin [Influenza A virus]

METVSLITILLVAAVSNADKICIGYQSTNSTETVDTLTENNVPVTHAKELLHTEHNGMLCATSLGQPIIL

DTCTIEGLIYGNPSCDLSLEGREWSYIVERPSAVNGLCYPGNVENLEELRSLFSSARSYQRIQIFPDTIW

NVSYDGTSTACSGSFYRNMRWLTRKNGEYPIQDAQYTNNQGKNILFMWGINHPPADTTQRDLYTRTDTTT

SVATEEINRIFKPLIGPRPLVNGLMGRIDYYWSVLKPGQTLRIKSDGNLIAPWYGHILSGESHGRILKTD
LKRGSCTVQCQTEKGGLNTTLPFQNVSKYAFGNCSKYIGIKSLKLAVGLRNVPSRSSRGLFGAIAGFIEG
GWSGLVAGWYGFQHSNDQGVGMAADRDSTQKAVDKITSKVNTIVDKMNKQYEIIDHEFSEVETRLNMINN
KIDDQIQDIWAYNAELLVLLENQKTLDEHDANVNNLYNKVKRALGSNAVEDGKGCFELYHKCDDQCMETI
RNGTYNRRKYQEESKLERQKIEGVKLESEGTYKILTIYSTVASSLVIAMGFAAFLFWAMSNGSCRCNICI

>QBK20406.1 hemagglutinin [Influenza A virus]
METVSLITILLVAAVSNADKICIGYQSTNSTETVDTLTENNVPVTHAKELLHTEHNGMLCATSLGQPIIL
DTCTIEGLIYGNPSCDLSLEGREWSYIVERPSAVNGLCYPGNVENLEELRSLFSSARSYQRIQIFPDTIW
NVSYDGTSTACSGSFYRNMRWLTRKNGEYPIQDAQYTNNQGKNILFMWGINHPPADTTQRDLYTRTDTTT
SVATEEINRIFKPLIGPRPLVNGLMGRIDYYWSVLKPGQTLRIKSDGNLIAPWYGHILSGESHGRILKTD
LKRGSCTVQCQTEKGGLNTTLPFQNVSKYAFGNCSKYIGIKSLKLAVGLRNVPSRSSRGLFGAIAGFIEG
GWSGLVAGWYGFQHSNDQGVGMAADRDSTQKAVDKITSKVNTIVDKMNKQYEIIDHEFSEVETRLNMINN
KIDDQIQDIWAYNAELLVLLENQKTLDEHDANVNNLYNKVKRALGSNAVEDGKGCFELYHKCDDQCMETI
RNGTYNRRKYQEESKLERQKIEGVKLESEGTYKILTIYSTVASSLVIAMGFAAFLFWAMSNGSCRCNICI

>QBK20405.1 hemagglutinin [Influenza A virus]
METVSLITILLVATVSNADKICIGYQSTNSTETVDTLTENNVPVTHAKELLHTEHNGMLCATSLGQPLIL
NTCTIEGLIYGNPSCDLSLEGREWSYIVERPSAVNGLCYPGNVENLEELRSLFSSARSYQRLQIFPDTIW
NVSYDGTSTACSGSFYRSMRWLTRKNGEYPTQDAQYTNNQGKNILFMWGINHPPTDTTQRNLYTRTDTTT
SVATEEINRIFKPLIGPRPLVNGLMGRIDYYWSILKPGQTLRIKSDGNLIAPWYGHILSGESHGRILKTD
LKRGSCTVQCQTEKGGLNTTLPFQNVSKYAFGNCSKYIGIKSLKLAVGLRNVPSRSSRGLFGAIAGFIEG
GWSGLVAGWYGFQHSNDQGVGMAADRDSTQKAIDKITSKVNNIVDKMNKQYEIIDHEFSEVETRLNMINN
KIDDQIQDIWAYNAELLVLLENQKTLDEHDANVNNLYNKVKRALGSNAVEDGKGCFELYHKCDNQCMETI
RNGTYNRRKYQEESKLERQKIEGVKLESEGTYKILTIYSTVASSLVIAMGFAAFLFWAMSNGSCRCNICI

>QBK20404.1 hemagglutinin [Influenza A virus]
METASLITILLVATVSNADKICIGYQSTNSTETVDTLTENNVPVTHAKELLHTEHNGMLCATSLGQPIIL
DTCTIEGLIYGNPSCDLSLEGREWSYIVERPSAVNGLCYPGNVENLEELRSLFSSARSYQRIQIFPDTIW
NVSYDGTSKACSGSFYRNMRWLTRKNGDYPIQDAQYTNNQGKNILFMWGINHPPADTTQRELYTRTDTTT
SVATEEINRIFKPLIGPRPLVNGLMGRIDYYWSVLKPGQTLRIKSDGNLIAPWYGHILSGESHGRILKTD
LKRGSCTVQCQTEKGGLNTTLPFQNVSKYAFGNCSKYIGIKSLKLAVGLRNVPSRSSRGLFGAIAGFIEG
GWSGLVAGWYGFQHSNDQGVGMAADRDSTQKAVDKITSKVNNIVDKMNKQYEIIDHEFSEVETRLNMINN
KIDDQIQDIWAYNAELLVLLENQKTLDEHDANVNNLYNKVKRALGSNAVEDGKGCFELYHKCDDQCMETI

RNGTYNRRKYQEESKLERQKIEGVKLESEGTYKILTIYSTVASSLVIAMGFAAFLFWAMSNGSCRCNICI

>QBK20403.1 hemagglutinin [Influenza A virus]
METVSLITILLVATVSNADKICIGYQSTNSTETVDTLTENNVPVTHAKELLHTEHNGMLCATSLGQPLIL
DTCTIEGLIYGNPSCDLSLEGREWSYIVERPSAVKGLCYPGNVENLEELRSLFSSARSYQRLQIFPDTIW
NVSYDGTSTACSGSFYRSMRWLTRKNGEYPTQDAQYTNNQGKNILFMWGINHPPTDTTQRDLYTRTDTTT
SVATEEINRIFKPLIGPRPLVNGLMGRIDYYWSILKPGQTLRIKSDGNLIAPWYGHILSGESHGRILKTD
LKRGSCTVQCQTEKGGLNTTLPFQNVSKYAFGNCSKYIGIKSLKLAVGLRNVPSRSSRGLFGAIAGFIEG
GWSGLVAGWYGFQHSNDQGVGMAADRDSTQKAIDKITSKVNNIVDKMNKQYEIIDHEFSEVETRLNMINN
KIDDQIQDIWAYNAELLVLLENQKTLDEHDANVNNLYNKVKRALGSNAVEDGKGCFELYHKCDNQCMETI
RNGTYNRRKYQEESKLERQKIEGVKLESEGTYKILTIYSTVASSLVIAMGFAAFLFWAMSNGSCRCNICI

>QBK20402.1 hemagglutinin [Influenza A virus]
METVSLITILIVATVSNADKICIGYQSTNSTETVDTLTENNVPVTHAKELLHTEHNGMLCATSLGQPLIL
DTCTIEGLIYGNPSCDLSLEGREWSYIVERPSAVNGLCYPGNVENLEELRSLFSSARSYQRIQIFPDTIW
NVSYDGTSTACSNSFYRSMRWLTRKNGNYPTQDAQYTNNQGKNILFMWGINHPPTDDTQRNLYTRTDTTT
SVATEEINRIFKPLIGPRPLVNGLMGRIDYYWSVLKPGQTLRIKSNGNLIAPWYGHILSGESHGRILKTD
LKRGSCTVQCQTEKGGLNTTLPFQNVSKYAFGNCSKYIGIKSLKLAVGLRNVPSRSSRGLFGAIAGFIEG
GWSGLVAGWYGFQHSNDQGVGMAADRDSTQKAIDKITSKVNNIVDKMNKQYEIIDHEFSEVETRLNMINN
KIDDQIQDIWAYNAELLVLLENQKTLDEHDANVNNLYNKVKRALGSNAVEDGKGCFELYHKCNDQCMETI
RNGTYNRKKYQEESKLERQKIDGVKLESEGTYKILTIYSTVASSLVIAMGFAAFLFWAMSNGSCRCNICI

>QBK20401.1 hemagglutinin [Influenza A virus]
METVSLITILLVATVSNADKICIGYQSTNSTETVDTLTENNVPVTHAKELLHTEHNGMLCATSLGQPIIL
DTCTVEGLIYGNPSCDLSLEGREWSYIVERPSAVNGLCYPGNVENLEELRSLFSSARSYQRIQIFPDTIW
NVSYDGTSTACSGSFYRNMRWLTRKNGDYPIQDAQYTNNQGKNILFMWGINHPPADTTQRELYTRTDTTT
SVATEEINRIFKPLIGPRPLVNGLMGRIDYYWSVLKPGQTLRIKSDGNLIAPWYGHILSGESHGRILKTD
LKRGSCTVQCQTEKGGLNTTLPFQNVSKYAFGNCSKYIGIKSLKLAVGLRNVPSRSSRGLFGAIAGFIEG
GWSGLVAGWYGFQHSNDQGVGMAADRDSTQKAVDKITSKVNNIVDKMNKQYEIIDHEFSEVETRLNMINN
KIDDQIQDIWAYNAELLVLLENQKTLDEHDANVNNLYNKVKRALGSNAVEDGKGCFELYHKCDDQCMETI
RNGTYNRRKYQEESKLERQKIEGVKLESEGTYKILTIYSTVASSLVIAMGFAAFLFWAMSNGSCRCNICI

>QBK20400.1 hemagglutinin [Influenza A virus]
METVSLITILLVATVSNADKICIGYQSTNSTETVDTLTENNVPVTHAKELLHTEHNGMLCATSLGQPIIL

DTCTVEGLIYGNPSCDLSLEGREWSYIVERPSAVNGLCYPGNVENLEELRSLFSSARSYQRIQIFPDTIW
NVSYDGTSTACSGSFYRNMRWLTRKNGDYPIQDAQYTNNQGKNILFMWGINHPPADTTQRELYTRTDTTT
SVATEEINRIFKPLIGPRPLVNGLMGRIDYYWSVLKPGQTLRIKSDGNLIAPWYGHILSGESHGRILKTD
LKRGSCTVQCQTEKGGLNTTLPFQNVSKYAFGNCSKYIGIKSLKLAVGLRNVPSRSSRGLFGAIAGFIEG
GWSGLVAGWYGFQHSNDQGVGMAADRDSTQKAVDKITSKVNNIVDKMNKQYEIIDHEFSEVETRLNMINN
KIDDQIQDIWAYNAELLVLLENQKTLDEHDANVNNLYNKVKRALGSNAVEDGKGCFELYHKCDDQCMETI
RNGTYNRRKYQEESKLERQKIEGVKLESEGTYKILTIYSTVASSLVIAMGFAAFLFWAMSNGSCRCNICI

>QBK20399.1 hemagglutinin [Influenza A virus]
METVSLITILLVATVSNADKICIGYQSTNSTETVDTLTENNVPVTHAKELLHTEHNGMLCATSLGQPIIL
DTCTIEGLIYGNPSCDLSLEGREWSYIVERPSAVNGLCYPGNVENLEELRSLFSSARSYQRIQIFPDTIW
NVSYDGTSTACSGSFYRNMRWLTRKNGDYPIQDAQYTNNQGKNILFMWGINHPPADTTQRDLYTRTDTTT
SVATEEINRIFKPLIGPRPLVNGLMGRIDYYWSVLKPGQTLRIKSDGNLIAPWYGHILSGESHGRILKTD
LKKGSCTVQCQTEKGGLNTTLPFQNISKYAFGNCSKYIGIKSLKLAVGLRNVPSRSSRGLFGAIAGFIEG
GWSGLVAGWYGFQHSNDQGVGMAADRDSTQKAVDKITSKVNNIVDKMNKQYEIIDHEFSEVETRLNMINN
KIDDQIQDIWAYNAELLVLLENQKTLDEHDANVNNLYNKVKRALGFNAVEDGKGCFELYHKCDDQCMETI
RNGTYNRRKYQEESKLERQKIEGVKLESEGTYKILTIYSTVASSLVIAMGFAAFLFWAMSNGSCRCNICI

>QBK20398.1 hemagglutinin [Influenza A virus]
METVSLITILLVATVSNADKICIGYQSTNSTETVDTLTENNVPVTHAKELLHTEHNGMLCATSLGQPLIL
DTCTIEGLIYGNPSCDLSLEGREWSYIVERPSAVKGLCYPGNVENLEELRSLFSSARSYQRLQIFPDTIW
NVSYDGTSTACSGSFYRSMRWLTRKNGEYPTQDAQYTNNQGKNILFMWGINHPPTDTTQRDLYTRTDTTT
SVATEEINRIFKPLIGPRPLVNGLMGRIDYYWSILKPGQTLRIKSDGNLIAPWYGHILSGESHGRILKTD
LKRGSCTVQCQTEKGGLNTTLPFQNVSKYAFGNCSKYIGIKSLKLAVGLRNVPSRSSRGLFGAIAGFIEG
GWSGLVAGWYGFQHSNDQGVGMAADRDSTQKAIDKITSKVNNIVDKMNKQYEIIDHEFSEVETRLNMINN
KIDDQIQDIWAYNAELLVLLENQKTLDEHDANVNNLYNKVKRALGSNAVEDGKGCFELYHKCDNQCMETI
RNGTYNRRKYQEESKLERQKIEGVKLESEGTYKILTIYSTVASSLVIAMGFAAFLFWAMSNGSCRCNICI

>QBK20397.1 hemagglutinin [Influenza A virus]
METVSLITILLVAAVSNADKICIGYQSTNSTETVDTLTENNVPVTHAKELLHTEHNGMLCATSLGQPIIL
DTCTIEGLIYGNPSCDLSLEGREWSYIVERPSAVNGLCYPGNVENLEELRSLFSSARSYQRIQIFPDAIW
NVSYDGTSTACSGSFYRNMRWLTRKNGEYPIQDAQYTNNQGKNILFMWGINHPPADTTQRNLYTRIDTTT
SVATEEINRIFKPLIGPRPLVNGLMGRIDYYWSVLKPGQTLRIKSDGNLIAPWYGHILSGESHGRILKTD
LKRGSCTVQCQTEKGGLNTTLPFQNVSKYAFGNCSKYIGIKSLKLAVGLRNVPSRSSRGLFGAIAGFIEG

GWSGLVAGWYGFQHSNDQGVGMAADRDSTQKAVDKITSKVNTIVDKMNKQYEIIDHEFSEVETRLNMINN
KIDDQIQDIWAYNAELLVLLENQKTLDEHDANVNNLYNKVKRALGSNAVEDGKGCFELYHKCDDQCMETI
RNGTYNRRKYQEESKLERQKIEGVKLESGGTYKILTIYSTVASSLVIAMGFAAFLFWAMSNGSCRCNICI

>QBK20396.1 hemagglutinin [Influenza A virus]
METVSLITILLVAAVSNADKICIGYQSTNSTETVDTLTENNVPVTHAKELLHTEHNGMLCATSLGQPIIL
DTCTIEGLIYGNPSCDLSLEGREWSYIVERPSAVNGLCYPGNVENLEELRSLFSSARSYQRIQIFPDAIW
NVSYDGTSTACSGSFYRNMRWLTRKNGEYPIQDAQYTNNQGKNILFMWGINHPPADTTQRNLYTRIDTTT
SVATEEINRIFKPLIGPRPLVNGLMGRIDYYWSVLKPGQTLRIKSDGNLIAPWYGHILSGESHGRILKTD
LKRGSCTVQCQTEKGGLNTTLPFQNVSKYAFGNCSKYIGIKSLKLAVGLRNVPSRSSRGLFGAIAGFIEG
GWSGLVAGWYGFQHSNDQGVGMAADRDSTQKAVDKITSKVNTIVDKMNKQYEIIDHEFSEVETRLNMINN
KIDDQIQDIWAYNAELLVLLENQKTLDEHDANVNNLYNKVKRALGSNAVEDGKGCFELYHKCDDQCMETI
RNGTYNRRKYQEESKLERQKIEGVKLESEGTYKILTIYSTVASSLVIAMGFAAFLFWAMSNGSCRCNICI

>QBK20395.1 hemagglutinin [Influenza A virus]
METVSLITILLVAAVSNADKICIGYQSTNSTETVDTLTENNVPVTHAKELLHTEHNGMLCATSLGQPIIL
DTCTIEGLIYGNPSCDLSLEGREWSYIVERPSAVNGLCYPGNVENLEELRSLFSSARSYQRIQIFPDAIW
NVSYDGTSTACSGSFYRNMRWLTRKNGEYPIQDAQYTNNQGKNILFMWGINHPPADTTQRNLYTRIDTTT
SVATEEINRIFKPLIGPRPLVNGLMGRIDYYWSVLKPGQTLRIKSDGNLIAPWYGHILSGESHGRILKTD
LKRGSCTVQCQTEKGGLNTTLPFQNVSKYAFGNCSKYIGIKSLKLAVGLRNVPSRSSRGLFGAIAGFIEG
GWSGLVAGWYGFQHSNDQGVGMAADRDSTQKAVDKITSKVNTIVDKMNKQYEIIDHEFSEVETRLNMINN
KIDDQIQDIWAYNAELLVLLENQKTLDEHDANVNNLYNKVKRALGSNAVEDGKGCFELYHKCDDQCMETI
RNGTYNRRKYQEESKLERQKIEGVKLESEGTYKILTIYSTVASSLVIAMGFAAFLFWAMSNGSCRCNICI

>QBK20394.1 hemagglutinin [Influenza A virus]
METVSLITILLVATISNADKICIGYQSTNSTETVDTLTENNVPVTHAKELLHTEHNGMLCATSLGQPLIL
DTCTIEGLIYGNPSCDLSLEGREWSYIVERPSAVNGLCYPGNVENLEELRSLFSSARSYQRIQIFPDTIW
NVSYDGTSTACSGSFYRSMRWLTRKNGNYPTQDAQYTNNQGKNILFMWGINHPPTDDTQRNLYTRTDTTT
SVATEEINRIFKPLIGPRPLVNGLMGRIDYYWSVLKPGQTLRIKSDGNLVAPWYGHILSGESHGRILKTD
LKRGSCTVQCQTEKGGLNTTLPFQNVSKYAFGNCSKYIGIKSLKLAVGLRNVPSRSSRGLFGAIAGFIEG
GWSGLVAGWYGFQHSNDQGVGMAADRDSTQKAIDKITSKVNNIVEKMNKQYEIIDHEFSEVETRLNMINN
KIDDQIQDIWAYNAELLVLLENQKTLDEHDANVNNLYNKVKRALGSNAVEDGKGCFELYHKCNDQCMETI
RNGTYNRRKYQEESKLERQKIEGVKLESEGTYKILTIYSTVASSLVIAMGFAAFLFWAMSNGSCRCNICI

>QBK20393.1 hemagglutinin [Influenza A virus]

METVSLITILLVATISNADKICIGYQSTNSTETVDTLTENNVPVTHAKELLHTEHNGMLCATSLGQPLIL
DTCTIEGLIYGNPSCDLSLEGREWSYIVERPSAVNGLCYPGNVENLEELRSLFSSARSYQRIQIFPDTIW
NVSYDGTSTACSGSFYRSMRWLTRKNGNYPTQDAQYTNNQGKNILFMWGINHPPTDDTQRNLYTRTDTTT
SVATEEINRIFKPLIGPRPLVNGLMGRIDYYWSVLKPGQTLRIKSDGNLVAPWYGHILSGESHGRILKTD
LKRGSCTVQCQTEKGGLNTTLPFQNVSKYAFGNCSKYIGIKSLKLAVGLRNVPSRSSRGLFGAIAGFIEG
GWSGLVAGWYGFQHSNDQGVGMAADRDSTQKAIDKITSKVNNIVEKMNKQYEIIDHEFSEVETRLNMINN
KIDDQIQDIWAYNAELLVLLENQKTLDEHDANVNNLYNKVKRALGSNAVEDGKGCFELYHKCNDQCMETI
RNGTYNRRKYQEESKLERQKIEGVKLESEGTYKILTIYSTVASSLVIAMGFAAFLFWAMSNGSCRCNICI

>QBK20392.1 hemagglutinin [Influenza A virus]

METVSLITILLVATISNADKICIGYQSTNSTETVDTLTENNVPVTHAKELLHTEHNGMLCATSLGQPLIL
DTCTIEGLIYGNPSCDLSLEGREWSYIVERPSAVNGLCYPGNVENLEELRSLFSSARSYQRIQIFPDTIW
NVSYDGTSTACSGSFYRSMRWLTRKNGNYPTQDAQYTNNQGKNILFMWGINHPPTDDTQRNLYTRTDTTT
SVATEEINRIFKPLIGPRPLVNGLMGRIDYYWSVLKPGQTLRIKSDGNLVAPWYGHILSGESHGRILKTD
LKRGSCTVQCQTEKGGLNTTLPFQNVSKYAFGNCSKYIGIKSLKLAVGLRNVPSRSSRGLFGAIAGFIEG
GWSGLVAGWYGFQHSNDQGVGMAADRDSTQKAIDKITSKVNNIVEKMNKQYEIIDHEFSEVETRLNMINN
KIDDQIQDIWAYNAELLVLLENQKTLDEHDANVNNLYNKVKRALGSNAVEDGKGCFELYHKCNDQCMETI
RNGTYNRRKYQEESKLERQKIEGVKLESEGTYKILTIYSTVASSLVIAMGFAAFLFWAMSNGSCRCNICI

>QBK20391.1 hemagglutinin [Influenza A virus]

METVSLITILLVATISNADKICIGYQSTNSTETVDTLTENNVPVTHAKELLHTEHNGMLCATSLGQPLIL
DTCTIEGLIYGNPSCDLSLEGREWSYIVERPSAVNGLCYPGNVENLEELRSLFSSARSYQRIQIFPDTIW
NVSYDGTSTACSGSFYRSMRWLTRKNGNYPTQDAQYTNNQGKNILFMWGINHPPTDDTQRNLYTRTDTTT
SVATEEINRIFKPLIGPRPLVNGLMGRIDYYWSVLKPGQTLRIKSDGNLVAPWYGHILSGESHGRILKTD
LKRGSCTVQCQTEKGGLNTTLPFQNVSKYAFGNCSKYIGIKSLKLAVGLRNVPSRSSRGLFGAIAGFIEG
GWSGLVAGWYGFQHSNDQGVGMAADRDSTQKAIDKITSKVNNIVEKMNKQYEIIDHEFSEVETRLNMINN
KIDDQIQDIWAYNAELLVLLENQKTLDEHDANVNNLYNKVKRALGSNAVEDGKGCFELYHKCNDQCMETI
RNGTYNRRKYQEESKLERQKIEGVKLESEGTYKILTIYSTVASSLVIAMGFAAFLFWAMSNGSCRCNICI

>QBK20390.1 hemagglutinin [Influenza A virus]

METVSLITILLVATISNADKICIGYQSTNSTETVDTLTENNVPVTHAKELLHTEHNGMLCATSLGQPLIL
DTCTIEGLIYGNPSCDLSLEGREWSYIVERPSAVNGLCYPGNVENLEELRSLFSSARSYQRIQIFPDTIW
NVSYDGTSTACSGSFYRSMRWLTRKNGNYPTQDAQYTNNQGKNILFMWGINHPPTDDTQRNLYTRTDTTT

SVATEEINRIFKPLIGPRPLVNGLMGRIDYYWSVLKPGQTLRIKSDGNLVAPWYGHILSGESHGRILKTD
LKRGSCTVQCQTEKGGLNTTLPFQNVSKYAFGNCSKYIGIKSLKLAVGLRNVPSRSSRGLFGAIAGFIEG
GWSGLVAGWYGFQHSNDQGVGMAADRDSTQKAIDKITSKVNNIVEKMNKQYEIIDHEFSEVETRLNMINN
KIDDQIQDIWAYNAELLVLLENQKTLDEHDANVNNLYNKVKRALGSNAVEDGKGCFELYHKCNDQCMETI
RNGTYNRRKYQEESKLERQKIEGVKLESEGTYKILTIYSTVASSLVIAMGFAAFLFWAMSNGSCRCNICI

>QBK20389.1 hemagglutinin [Influenza A virus]
METVSLITILLVATISNADKICIGYQSTNSTETVDTLTENNVPVTHAKELLHTEHNGMLCATSLGQPLIL
DTCTIEGLIYGNPSCDLSLEGREWSYIVERPSAVNGLCYPGNVENLEELRSLFSSARSYQRIQIFPDTIW
NVSYDGTSTACSGSFYRSMRWLTRKNGNYPTQDAQYTNNQGKNILFMWGINHPPTDDTQRNLYTRTDTTT
SVATEEINRIFKPLIGPRPLVNGLMGRIDYYWSVLKPGQTLRIKSDGNLVAPWYGHILSGESHGRILKTD
LKRGSCTVQCQTEKGGLNTTLPFQNVSKYAFGNCSKYIGIKSLKLAVGLRNVPSRSSRGLFGAIAGFIEG
GWSGLVAGWYGFQHSNDQGVGMAADRDSTQKAIDKITSKVNNIVEKMNKQYEIIDHEFSEVETRLNMINN
KIDDQIQDIWAYNAELLVLLENQKTLDEHDANVNNLYNKVKRALGSNAVEDGKGCFELYHKCNDQCMETI
RNGTYNRRKYQEESKLERQKIEGVKLESEGTYKILTIYSTVASSLVIAMGFAAFLFWAMSNGSCRCNICI

>QBK20388.1 hemagglutinin [Influenza A virus]
METVSLITILLVATISNADKICIGYQSTNSTETVDTLTENNVPVTHAKELLHTEHNGMLCATSLGQPLIL
DTCTIEGLIYGNPSCDLSLEGREWSYIVERPSAVNGLCYPGNVENLEELRSLFSSARSYQRIQIFPDTIW
NVSYDGTSTACSGSFYRSMRWLTRKNGNYPTQDAQYTNNQGKNILFMWGINHPPTDDTQRNLYTRTDTTT
SVATEEINRIFKPLIGPRPLVNGLMGRIDYYWSVLKPGQTLRIKSDGNLVAPWYGHILSGESHGRILKTD
LKRGSCTVQCQTEKGGLNTTLPFQNVSKYAFGNCSKYIGIKSLKLAVGLRNVPSRSSRGLFGAIAGFIEG
GWSGLVAGWYGFQHSNDQGVGMAADRDSTQKAIDKITSKVNNIVEKMNKQYEIIDHEFSEVETRLNMINN
KIDDQIQDIWAYNAELLVLLENQKTLDEHDANVNNLYNKVKRALGSNAVEDGKGCFELYHKCNDQCMETI
RNGTYNRRKYQEESKLERQKIEGVKLESEGTYKILTIYSTVASSLVIAMGFAAFLFWAMSNGSCRCNICI

>QBK20387.1 hemagglutinin [Influenza A virus]
METVSLITILLVATISNADKICIGYQSTNSTETVDTLTENNVPVTHAKELLHTEHNGMLCATSLGQPLIL
DTCTIEGLIYGNPSCDLSLEGREWSYIVERPSAVNGLCYPGNVENLEELRSLFSSARSYQRIQIFPDTIW
NVSYDGTSTACSGSFYRSMRWLTRKNGNYPTQDAQYTNNQGKNILFMWGINHPPADDTQRNLYTRTDTTT
SVATEEINRIFKPLIGPRPLVNGLMGRIDYYWSVLKPGQTLRIKSDGNLVAPWYGHILSGESHGRILKTD
LKRGSCTVQCQTEKGGLNTTLPFQNVSKYAFGNCSKYIGIKSLKLAVGLRNVPSRSSRGLFGAIAGFIEG
GWSGLVAGWYGFQHSNDQGVGMAADRDSTQKAIDKITSKVNNIVEKMNKQYEIIDHEFSEVETRLNMINN
KIDDQIQDIWAYNAELLVLLENQKTLDEHDANVNNLYNKVKRALGSNAVEDGKGCFELYHKCNDQCMETI

RNGTYNRRKYQEESKLERQKIEGVKLESEGTYKILTIYSTVASSLVIAMGFAAFLFWAMSNGSCRCNICI

>QBK20386.1 hemagglutinin [Influenza A virus]
METVSLITILLVATISNADKICIGYQSTNSTETVDTLTENNVPVTHAKELLHTEHNGMLCATSLGQPLIL
DTCTIEGLIYGNPSCDLSLEGREWSYIVERPSAVNGLCYPGNVENLEELRSLFSSARSYQRIQIFPDTIW
NVSYDGTSTACSGSFYRSMRWLTRKNGNYPTQDAQYTNNQGKNILFMWGINHPPTDDTQRNLYTRTDTTT
SVATEEINRIFKPLIGPRPLVNGLMGRIDYYWSVLKPGQTLRIKSDGNLVAPWYGHILSGESHGRILKTD
LKRGSCTVQCQTEKGGLNTTLPFQNVSKYAFGNCSKYIGIKSLKLAVGLRNVPSRSSRGLFGAIAGFIEG
GWSGLVAGWYGFQHSNDQGVGMAADRDSTQKAIDKITSKVNNIVEKMNKQYEIIDHEFSEVETRLNMINN
KIDDQIQDIWAYNAELLVLLENQKTLDEHDANVNNLYNKVKRALGSNAVEDGKGCFELYHKCNDQCMETI
RNGTYNRRKYQEESKLERQKIEGVKLESEGTYKILTIYSTVASSLVIAMGFAAFLFWAMSNGSCRCNICI

>QBK20385.1 hemagglutinin [Influenza A virus]
METVSLITILLVATISNADKICIGYQSTNSTETVDTLTENNVPVTHAKELLHTEHNGMLCATSLGQPLIL
DTCTIEGLIYGNPSCDLSLEGREWSYIVERPSAVNGLCYPGNVENLEELRSLFSSARSYQRIQIFPDTIW
NVSYDGTSTACSGSFYRSMRWLTRKNGNYPTQDAQYTNNQGKNILFMWGINHPPTDDTQRNLYTRTDTTT
SVATEEINRIFKPLIGPRPLVNGLMGRIDYYWSVLKPGQTLRIKSDGNLVAPWYGHILSGESHGRILKTD
LKRGSCTVQCQTEKGGLNTTLPFQNVSKYAFGNCSKYIGIKSLKLAVGLRNVPSRSSRGLFGAIAGFIEG
GWSGLVAGWYGFQHSNDQGVGMAADRDSTQKAIDKITSKVNNIVEKMNKQYEIIDHEFSEVETRLNMINN
KIDDQIQDIWAYNAELLVLLENQKTLDEHDANVNNLYNKVKRALGSNAVEDGKGCFELYHKCNDQCMETI
RNGTYNRRKYQEESKLERQKIEGVKLESEGTYKILTIYSTVASSLVIAMGFAAFLFWAMSNGSCRCNICI

>QBK20384.1 hemagglutinin [Influenza A virus]
METVSLITILLVATISNADKICIGYQSTNSTETVDTLTENNVPVTHAKELLHTEHNGMLCATSLGQPLIL
DTCTIEGLIYGNPSCDLSLEGREWSYIVERPSAVNGLCYPGNVENLEELRSLFSSARSYQRIQIFPDTIW
NVSYDGTSTACSGSFYRSMRWLTRKNGNYPTQDAQYTNNQGKNILFMWGINHPPTDDTQRNLYTRTDTTT
SVATEEINRIFKPLIGPRPLVNGLMGRIDYYWSVLKPGQTLRIKSDGNLVAPWYGHILSGESHGRILKTD
LKRGSCTVQCQTEKGGLNTTLPFQNVSKYAFGNCSKYIGIKSLKLAVGLRNVPSRSSRGLFGAIAGFIEG
GWSGLVAGWYGFQHSNDQGVGMAADRDSTQKAIDKITSKVNNIVEKMNKQYEIIDHEFSEVETRLNMINN
KIDDQIQDIWAYNAELLVLLENQKTLDEHDANVNNLYNKVKRALGSNAVEDGKGCFELYHKCNDQCMETI
RNGTYNRRKYQEESKLERQKIEGVKLESEGTYKILTIYSTVASSLVIAMGFAAFLFWAMSNGSCRCNICI

>QBK20383.1 hemagglutinin [Influenza A virus]
METVSLITILLVATISNADKICIGYQSTNSTETVDTLTENNVPVTHAKELLHTEHNGMLCATSLGQPLIL

DTCTIEGLIYGNPSCDLSLEGREWSYIVERPSAVNGLCYPGNVENLEELRSLFSSARSYQRIQIFPDTIW

NVSYDGTSTACSGSFYRSMRWLTRKNGNYPTQDAQYTNNQGKNILFMWGINHPPTDDTQRNLYTRTDTTT

SVATEEINRIFKPLIGPRPLVNGLMGRIDYYWSVLKPGQTLRIKSDGNLVAPWYGHILSGESHGRILKTD

LKRGSCTVQCQTEKGGLNTTLPFQNVSKYAFGNCSKYIGIKSLKLAVGLRNVPSRSSRGLFGAIAGFIEG

GWSGLVAGWYGFQHSNDQGVGMAADRDSTQKAIDKITSKVNNIVEKMNKQYEIIDHEFSEVETRLNMINN

KIDDQIQDIWAYNAELLVLLENQKTLDEHDANVNNLYNKVKRALGSNAVEDGKGCFELYHKCNDQCMETI

RNGTYNRRKYQEESKLERQKIEGVKLESEGTYKILTIYSTVASSLVIAMGFAAFLFWAMSNGSCRCNICI

>QBK20382.1 hemagglutinin [Influenza A virus]

METVSLITILLVATISNADKICIGYQSTNSTETVDTLTENNVPVTHAKELLHTEHNGMLCATSLGQPLIL

DTCTIEGLIYGNPSCDLSLEGREWSYIVERPSAVNGLCYPGNVENLEELRSLFSSARSYQRIQIFPDTIW

NVSYDGTSTACSGSFYRSMRWLTRKNGNYPTQDAQYTNNQGKNILFMWGINHPPTDDTQRNLYTRTDTTT

SVATEEINRIFKPLIGPRPLVNGLMGRIDYYWSVLKPGQTLRIKSDGNLVAPWYGHILSGESHGRILKTD

LKRGSCTVQCQTEKGGLNTTLPFQNVSKYAFGNCSKYIGIKSLKLAVGLRNVPSRSSRGLFGAIAGFIEG

GWSGLVAGWYGFQHSNDQGVGMAADRDSTQKAIDKITSKVNNIVEKMNKQYEIIDHEFSEVETRLNMINN

KIDDQIQDIWAYNAELLVLLENQKTLDEHDANVNNLYNKVKRALGSNAVEDGKGCFELYHKCNDQCMETI

RNGTYNRRKYQEESKLERQKIEGVKLESEGTYKILTIYSTVASSLVIAMGFAAFLFWAMSNGSCRCNICI

>QBK20381.1 hemagglutinin [Influenza A virus]

METVSLITILLVATISNADKICIGYQSTNSTETVDTLTENNVPVTHAKELLHTEHNGMLCATSLGQPLIL

DTCTIEGLIYGNPSCDLSLEGREWSYIVERPSAVNGLCYPGNVENLEELRSLFSSARSYQRIQIFPDTIW

NVSYDGTSTACSGSFYRSMRWLTRKNGNYPTQDAQYTNNQGKNILFMWGINHPPTDDTQRNLYTRTDTTT

SVATEEINRIFKPLIGPRPLVNGLMGRIDYYWSVLKPGQTLRIKSDGNLVAPWYGHILSGESHGRILKTD

LKRGSCTVQCQTEKGGLNTTLPFQNVSKYAFGNCSKYIGIKSLKLAVGLRNVPSRSSRGLFGAIAGFIEG

GWSGLVAGWYGFQHSNDQGVGMAADRDSTQKAIDKITSKVNNIVEKMNKQYEIIDHEFSEVETRLNMINN

KIDDQIQDIWAYNAELLVLLENQKTLDEHDANVNNLYNKVKRALGSNAVEDGKGCFELYHKCNDQCMETI

RNGTYNRRKYQEESKLERQKIEGVKLESEGTYKILTIYSTVASSLVIAMGFAAFLFWAMSNGSCRCNICI

>QBK20380.1 hemagglutinin [Influenza A virus]

METVSLITILLVATVSNADKICIGYQSTNSTETVDTLTENNVPVTHAKELLHTEHNGMLCATSLGQPIIL

DTCTIEGLIYGNPSCDLSLEGREWSYIVERPSAVNGLCYPGNVENLEELRSLFSSARSYQRIQIFPDTIW

NVSYDGTSTACSGSFYRNMRWLTRKNGDYPIQDAQFTNNQGKNILFMWGINHPPADTTQRDLYTRTDTTT

SVATEEINRIFKPLIGPRPLVNGLMGRIDYYWSVLKPGQTLRIKSDGNLIAPWYGHILSGESHGRILKTD

LKRGSCTVQCQTEKGGLNTTLPFQNVSKYAFGNCSKYIGIKSLKLAVGLRNVPSRSSRGLFGAIAGFIEG

GWSGLVAGWYGFQHSNDQGVGMAADRDSTQKAVDKITSKVNNIVDKMNKQYEIIDHEFSEVETRLNMINN

KIDDQIQDIWAYNAELLVLLENQKTLDEHDANVNNLYNKVKRALGSNAVEDGKGCFELYHKCDDQCMETI

RNGTYNRRKYQEESKLERQKIEGVKLESEGTYKILTIYSTVASSLVIAMGFAAFLFWAMSNGSCRCNICI

>QBK20378.1 hemagglutinin [Influenza A virus]

METVSLITILLVATVSNADKICIGYQSTNSTETVDTLTENNVPVTHAKELLHTEHNGMLCATSLGQPLIL

DTCTIEGLIYGNPSCDLSLEGREWSYIVERPSAVNGLCYPGNVENLEELRSLFSSARSYQRIQIFPDTIW

NVSYDGTSTACSGSFYRSMRWLTRKNGEYPIQDAQYTNNQGKNILFMWGINHPPTDTTQRDLYTRTDTTT

SVATEEINRVFKPLIGPRPLVNGLMGRIDYYWSVLKPGQTLRIKSDGNLIAPWFGHILSGESHGRILKTD

LKRGSCTVQCQTEKGGLNTTLPFQNVSKYAFGNCSKYIGIKSLKLAVGLRNVPSRSSRGLFGAIAGFIEG

GWSGLVAGWYGFQHSNDQGVGMAADRDSTQKAIDKITSKVNNIVDKMNKQYEIIDHEFSEVEARLNMINN

KIDDQIQDIWAYNAELLVLLENQKTLDEHDANVNNLYNKVKRALGSNAVEDGKGCFELYHKCDDQCMETI

RNGTYNRRKYQEESKLERQKIEGVKLESEGTYKILTIYSTVASSLVIAMGFAAFLFWAMSNGSCRCNICI

>QBK20377.1 hemagglutinin [Influenza A virus]

METVSLITILLVATVSNADKICIGYQSTNSTETVDTLTENNVPVTHAKELLHTEHNGMLCATSLGQPLIL

DTCTIEGLIYGNPSCDLSLEGREWSYIVERPSAVNGLCYPGNVENLEELRSLFSSARSYQRIQIFPDTIW

NVSYDGTSTACSGSFYRSMRWLTRKNGEYPIQDAQYTNNQGKNILFMWGINHPPTDTTQRDLYTRTDTTT

SVATEEINRVFKPLIGPRPLVNGLMGRIDYYWSVLKPGQTLRIKSDGNLIAPWFGHILSGESHGRILKTD

LKRGSCTVQCQTEKGGLNTTLPFQNVSKYAFGNCSKYIGIKSLKLAVGLRNVPSRSSRGLFGAIAGFIEG

GWSGLVAGWYGFQHSNDQGVGMAADRDSTQKAIDKITSKVNNIVDKMNKQYEIIDHEFSEVEARLNMINN

KIDDQIQDIWAYNAELLVLLENQKTLDEHDANVNNLYNKVKRALGSNAVEDGKGCFELYHKCDDQCMETI

RNGTYNRRKYQEESKLERQKIEGVKLESEGTYKILTIYSTVASSLVIAMGFAAFLFWAMSNGSCRCNICI

>QBK20376.1 hemagglutinin [Influenza A virus]

METVSLITILLVATVSNADKICIGYQSTNSTETVDTLTENNVPVTHAKELLHTEHNGMLCATSLGQPLIL

DTCTIEGLIYGNPSCDLSLEGREWSYIVERPSAVNGLCYPGNVENLEELRSLFSSARSYQRIQIFPDTIW

NVSYDGTSTACSGSFYRSMRWLTRKNGEYPIQDAQYTNNQGKNILFMWGINHPPTDTTQRDLYTRTDTTT

SVATEEINRVFKPLIGPRPLVNGLMGRIDYYWSVLKPGQTLRIKSDGNLIAPWFGHILSGESHGRILKTD

LKRGSCTVQCQTEKGGLNTTLPFQNVSKYAFGNCSKYIGIKSLKLAVGLRNVPSRSSRGLFGAIAGFIEG

GWSGLVAGWYGFQHSNDQGVGMAADRDSTQKAIDKITSKVNNIVDKMNKQYEIIDHEFSEVEARLNMINN

KIDDQIQDIWAYNAELLVLLENQKTLDEHDANVNNLYNKVKRALGSNAVEDGKGCFELYHKCDDQCMETI

RNGTYNRRKYQEESKLERQKIEGVKLESEGTYKILTIYSTVASSLVIAMGFAAFLFWAMSNGSCRCNICI

>QBK20375.1 hemagglutinin [Influenza A virus]

METVSLITILLVATVSNADKICIGYQSTNSTETVDTLTENNVPVTHAKELLHTEHNGMLCATSLGQPLIL
DTCTIEGLIYGNPSCDLSLEGREWSYIVERPSAVNGLCYPGNVENLEELRSLFSSARSYQRIQIFPDTIW
NVSYDGTSTACSGSFYRSMRWLTRKNGEYPIQDAQYTNNQGKNILFMWGINHPPTDTAQRNLYTRTDTTT
SVATEEINRIFKPLIGPRPLVNGLMGRIDYYWSVLKPGQTLRIKSDGNLIAPWFGHILSGESHGRILKTD
LKRGSCTVQCQTEKGGLNTTLPFQNVSKYAFGNCSKYIGIKSLKLAVGLRNVPSRSSRGLFGAIAGFIEG
GWSGLVAGWYGFQHSNDQGVGMAADRDSTQKAIDKITSKVNNIVDKMNKQYEIIDHEFSEVETRLNMINN
KIDDQIQDIWAYNAELLVLLENQKTLDEHDANVNNLYNKVKRALGSNAVEDGKGCFELYHKCDDQCMETI
RNGTYSRRKYQEESKLERQKIEGVKLESEGTYKILTIYSTVASSLVIAMGFAAFLFWAMSNGSCRCNICI

>QBK20374.1 hemagglutinin [Influenza A virus]

METVSLITILLVATVSNADKICIGYQSTNSTETVDTLTENNVPVTHAKELLHTEHNGMLCATSLGQPLIL
DTCTIEGLIYGNPSCDLSLEGREWSYIVERPSAVNGLCYPGNVENLEELRSLFSSARSYQRIQIFPDTIW
NVSYDGTSTACSGSFYRSMRWLTRKNGEYPIQDAQYTNNQGKNILFMWGINHPPTDTAQRNLYTRTDTTT
SVATEEINRIFKPLIGPRPLVNGLMGRIDYYWSVLKPGQTLRIKSDGNLIAPWFGHILSGESHGRILKTD
LKRGSCTVQCQTEKGGLNTTLPFQNVSKYAFGNCSKYIGIKSLKLAVGLRNVPSRSSRGLFGAIAGFIEG
GWSGLVAGWYGFQHSNDQGVGMAADRDSTQKAIDKITSKVNNIVDKMNKQYEIIDHEFSEVETRLNMINN
KIDDQIQDIWAYNAELLVLLENQKTLDEHDANVNNLYNKVKRALGSNAVEDGKGCFELYHKCDDQCMETI
RNGTYSRRKYQEESKLERQKIEGVKLESEGTYKILTIYSTVASSLVIAMGFAAFLFWAMSNGSCRCNICI

>QBK20373.1 hemagglutinin [Influenza A virus]

METVSLITILLVATVSNADKICIGYQSTNSTETVDTLTENNVPVTHAKELLHTEHNGMLCATSLGQPLIL
DTCTIEGLIYGNPSCDLSLEGREWSYIVERPSAVNGLCYPGNVENLEELRSLFSSARSYQRIQIFPDTIW
NVSYDGTSTACSGSFYRSMRWLTRKNGEYPIQDAQYTNNQGKNILFMWGINHPPTDTAQRNLYTRTDTTT
SVATEEINRIFKPLIGPRPLVNGLMGRIDYYWSVLKPGQTLRIKSDGNLIAPWFGHILSGESHGRILKTD
LKRGSCTVQCQTEKGGLNTTLPFQNVSKYAFGNCSKYIGIKSLKLAVGLRNVPSRSSRGLFGAIAGFIEG
GWSGLVAGWYGFQHSNDQGVGMAADRDSTQKAIDKITSKVNNIVDKMNKQYEIIDHEFSEVETRLNMINN
KIDDQIQDIWAYNAELLVLLENQKTLDEHDANVNNLYNKVKRALGSNAVEDGKGCFELYHKCDDQCMETI
RNGTYSRRKYQEESKLERQKIEGVKLESEGTYKILTIYSTVASSLVIAMGFAAFLFWAMSNGSCRCNICI

>QBK20372.1 hemagglutinin [Influenza A virus]

METVSLITILLVATVSNADKICIGYQSTNSTETVDTLTENNVPVTHAKELLHTEHNGMLCATSLGQPLIL
DTCTIEGLIYGNPSCDLSLEGREWSYIVERPSAVNGLCYPGNVENLEELRSLFSSARSYQRIQIFPDTIW
NVSYDGTSTACSGSFYRSMRWLTRKNGEYPIQDAQYTNNQGKNILFMWGINHPPTDTAQRNLYTRTDTTT

SVATEEINRIFKPLIGPRPRVNGLMGRIDYYWSVLKPGQTLRIKSDGNLIAPWFGHILSGESHGRILKTD
LKRGSCTVQCQTEKGGLNTTLPFQNVSKYAFGNCSKYIGIKSLKLAVGLRNVPSRSSRGLFGAIAGFIEG
GWSGLVAGWYGFQHSNDQGVGMAADRDSTQKAIDKITSKVNNIVDKMNKQYEIIDHEFSEVETRLNMINN
KIDDQIQDIWAYNAELLVLLENQKTLDEHDANVNNLYNKVKRALGSNAVEDGKGCFELYHKCDDQCMETI
RNGTYSRRKYQEESKLERQKIEGVKLESEGTYKILTIYSTVASSLVIAMGFAAFLFWAMSNGSCRCNICI

>QBK20371.1 hemagglutinin [Influenza A virus]
METVSLITILLVATVSNADKICIGYQSTNSTETVDTLTENNVPVTHAKELLHTEHNGMLCATSLGQPLIL
DTCTIEGLIYGNPSCDLSLEGREWSYIVERPSAVNGLCYPGNVENLEELRSLFSSARSYQRIQIFPDTIW
NVSYDGTSTACSGSFYRSMRWLTRKDGNYPTQDAQYTNNQGKNILFMWGINHPPTDETQRSLYTRTDTTT
SVATEEMNRIFKPLIGPRPLVNGLMGRIDYYWSVLKPGQTLRIKSDGNLIAPWYGHILSGESHGRILKTD
LKRGSCTVQCQTEKGGLNTTLPFQNVSKYAFGNCSKYIGIKSLKLAVGLRNVPSRSSRGLFGAIAGFIEG
GWSGLVAGWYGFQHSNDQGVGMAADRDSTQKAIDKITSKVNNIVDKMNKQYEIIDHEFSEVETRLNMINN
KIDDQIQDIWAYNAELLVLLENQKTLDEHDANVNNLYNKVKRALGSNAVEDGKGCFELYHKCNDQCMETI
RNGTYNRRKYQEESKLERQKIEGVKLESEGTYKILTIYSTVASSLVIAMGFAAFLFWAMSNGSCRCNICI

>QBK20370.1 hemagglutinin [Influenza A virus]
METVSLITILLVATVSNADKICIGYQSTNSTETVDTLTENNVPVTHAKELLHTEHNGMLCATSLGQPIIL
DTCTIEGLIYGNPSCDLSLEGREWSYIVERPSAVNGLCYPGNVENLEELRSLFSSARSYQRIQIFPDTIW
NVSYDGTSTACSGSFYRNMRWLTRKNGEYPIQDAQYTNNQGKNILFMWGINHPPADTTQRELYTRTDTTT
SVATEEINRIFKPLIGPRPLVNGLMGRIDYYWSVLKPGQTLRIKSDGNLIAPWYGHILSGESHGRILKTD
LKRGSCTVQCQTEKGGLNTTLPFQNVSKYAFGNCSKYIGIKSLKLAVGLRNVPSRSSRGLFGAIAGFIEG
GWSGLVAGWYGFQHSNDQGVGMAADRDSTQKAVDKITSKVNNIVDKMNKQYEIIDHEFSEVETRLNMINN
KIDDQIQDIWAYNAELLVLLENQKTLDEHDANVNNLYNKVKRALGSNAVEDGKGCFELYHKCDDQCMETI
RNGTYNRRKYQEESKLERQKIEGVKLESEGTYKILTIYSTVASSLVIAMGFAAFLFWAMSNGSCRCNICI

>QBK20369.1 hemagglutinin [Influenza A virus]
METVSLITILLVATVSNADKICIGYQSTNSTETVDTLTENNVPVTHAKELLHTEHNGMLCATSLGQPIIL
DTCTIEGLIYGNPSCDLSLEGREWSYIVERPSAVNGLCYPGNVENLEELRSLFSSARSYQRIQIFPDTIW
NVSYDGTSTACSGSFYRNMRWLTRKNGEYPIQDAQYTNNQGKNILFMWGINHPPADTTQRELYTRTDTTT
SVATEEINRIFKPLIGPRPLVNGLMGRIDYYWSVLKPGQTLRIKSDGNLIAPWYGHILSGESHGRILKTD
LKRGSCTVQCQTEKGGLNTTLPFQNVSKYAFGNCSKYIGIKSLKLAVGLRNVPSRSSRGLFGAIAGFIEG
GWSGLVAGWYGFQHSNDQGVGMAADRDSTQKAVDKITSKVNNIVDKMNKQYEIIDHEFSEVETRLNMINN
KIDDQIQDIWAYNAELLVLLENQKTLDEHDANVNNLYNKVKRALGSNAVEDGKGCFELYHKCDDQCMETI

RNGTYNRRKYQEESKLERQKIEGVKLESEGTYKILTIYSTVASSLVIAMGFAAFLFWAMSNGSCRCNICI

>QBK20366.1 hemagglutinin [Influenza A virus]
METVSLITILLVATVSNADKICIGYQSTNSTETVDTLTENNVPVTHAKELLHTEHNGMLCATSLGQPLIL
DTCTIEGLIYGNPSCDLSLEGREWSYIVERPSAVHGLCYPGNVEDLEELRSLFSSARSYQRIQIFPDTIW
NVSYDGTSTACSGSFYRSMRWLTRKNGEYPIQDAQYTNNQGKNILFMWGINHPPTDDTQRGLYTRTDTTT
SVATEEINRIFKPLIGPRPLVNGLMGRINYYWSVLKPGQTLRIKSDGNLIAPWYGHILSGESHGRILKTD
LKRGSCTVQCQTEKGGLNTTLPFQNVSKYAFGNCSKYIGIKSLKLAVGLRNVPSRSSRGLFGAIAGFIEG
GWSGLVAGWYGFQHSNDQGVGMAADRDSTQKAIDKITSKVNNIVDKMNKQYEIIDHEFSEVETRLNMINN
KIDDQIQDIWAYNAELLVLLENQKTLDEHDANVNNLYSKVKRALGSNAVEDGKGCFELYHKCDDQCMETI
RNGTYNRRKYQEESKLERQRIEGVKLESEGTYKILTIYSTVASSLVIAMGFAAFLFWAMSNGSCRCNICI

>QBK20365.1 hemagglutinin [Influenza A virus]
METVSLITILLVATASNADKICIGYQSTNSTETVDTLTENNVPVTHAKELLHTEHNGMLCATSLGQPLIL
DTCTIEGLIYGNPSCDLSLEGREWSYIVERPSAVNGLCYPGNVENLEELRSLFSSARSYQRIQIFPDTIW
NVSYDGTSTACSGSFYRSMRWLTRKNGDYPTQDAQYTNNQGKNILFMWGINHPPTDDTQRNLYTRTDTTT
SVATEEINRIFKPLIGPRPLVNGLMGRIDYYWSVLKPGQTLRIKSDGNLIAPWYGHILSGESHGRILKTD
LKRGSCTVQCQTEKGGLNTTLPFQNVSKYAFGNCSKYIGIKSLKLAVGLRNVPSRSSRGLFGAIAGFIEG
GWSGLVAGWYGFQHSNDQGVGMAADRDSTQKAIDKITSKVNNIVDKMNKQYEIIDHEFSEVETRLNMINN
KIDDQIQDIWAYNAELLVLLENQKTLDEHDANVNNLYNKVKRALGSNAVEDGKGCFELYHKCNDQCMETI
RNGTYNRRKYQEESKLERQKIEGVKLESEGTYKILTIYSTVASSLVIAMGFAAFLFWAMSNGSCRCNICI

>QBK20364.1 hemagglutinin [Influenza A virus]
METVSLITILLVATVSNADKICIGYQSTNSTETVDTLTENNVPVTHAKELLHTEHNGMLCATSLGQPLIL
DTCTIEGLIYGNPSCDLSLEGREWSYIVERPSAVHGLCYPGNVEDLEELRSLFSSARSYQRIQIFPDTIW
NVSYDGTSTACSGSFYRSMRWLTRKNGEYPIQDAQYTNNQGKNILFMWGINHPPTDDTQRGLYTRTDTTT
SVATEEINRIFKPLIGPRPXVNGLMGRINYYWSVLKPGQTLRIKSDGNLIAPWYGHILSGESHGRILKTD
LKRGSCTVQCQTEKGGLNTTLPFQNVSKYAFGNCSKYIGIKSLKLAVGLRNVPSRSSRGLFGAIAGFIEG
GWSGLVAGWYGFQHSNDQGVGMAADRDSTQKAIDKITSKVNNIVDKMNKQYEIIDHEFSEVETRLNMINN
KIDDQIQDIWAYNAELLILLENQKTLDEHDANVNNLYNKVKRALGSNAVEDGKGCFELYHKCDDQCMETI
RNGTYNRRKYQEESKLERQRIEGVKLESEGTYKILTIYSTVASSLVIAMGFAAFLFWAMSNGSCRCNICI

>QBK20363.1 hemagglutinin [Influenza A virus]
METVSLITILLVATVSNADKICIGYQSTNSTETVDTLTENNVPVTHAKELLHTEHNGMLCATSLGQPLIL

DTCTIEGLIYGNPSCDLSLEGREWSYIVERPSAVHGLCYPGNVEDLEELRSLFSSARSYQRIQIFPDTIW
NVSYDGTSTACSGSFYRSMRWLTRKNGEYPIQDAQYTNNQGKNILFMWGINHPPTDDTQRGLYTRTDTTT
SVATEEINRIFKPLIGPRPLVNGLMGRINYYWSVLKPGQTLRIKSDGNLIAPWYGHILSGESHGRILKTD
LKRGSCTVQCQTEKGGLNTTLPFQNVSKYAFGNCSKYIGIKSLKLAVGLRNVPSRSSRGLFGAIAGFIEG
GWSGLVAGWYGFQHSNDQGVGMAADRDSTQKAIDKITSKVNNIVDKMNKQYEIIDHEFSEVETRLNMINN
KIDDQIQDIWAYNAELLVLLENQKTLDEHDANVNNLYNKVKRALGSNAVEDGKGCFELYHKCDDQCMETI
RNGTYNRRKYQEESKLERQRIEGVKLESEGTYKILTIYSTVASSLVIAMGFAAFLFWAMSNGSCRCNICI

>QBK20362.1 hemagglutinin [Influenza A virus]
METVSLITILLVATVSNADKICIGYQSTNSTETVDTLTENNVPVTHAKELLHTEHNGMLCATSLGQPLIL
DTCTIEGLIYGNPSCDLSLEGREWSYIVERPSAVHGLCYPGNVEDLEELRSLFSSARSYQRIQIFPDTIW
NVSYDGTSTACSGSFYRSMRWLTRKNGEYPIQDAQYTNNQGKNILFMWGINHPPTDDTQRGLYTRTDTTT
SVATEEINRIFKPLIGPRPLVNGLMGRINYYWSVLKPGQTLRIKSDGNLIAPWYGHILSGESHGRILKTD
LKRGSCTVQCQTEKGGLNTTLPFQNVSKYAFGNCSKYIGIKSLKLAVGLRNVPSRSSRGLFGAIAGFIEG
GWSGLVAGWYGFQHSNDQGVGMAADRDSTQKAIDKITSKVNNIVDKMNKQYEIIDHEFSEVETRLNMINN
KIDDQIQDIWAYNAELLVLLENQKTLDEHDANVNNLYSKVKRALGSNAVEDGKGCFELYHKCDDQCMETI
RNGTYNRRKYQEESKLERQRIEGVKLESEGTYKILTIYSTVASSLVIAMGFAAFLFWAMSNGSCRCNICI

>QBK20361.1 hemagglutinin [Influenza A virus]
METVSLITILLVATVSNADKICIGYQSTNSTETVDTLTENNVPVTHAKELLHTEHNGMLCATSLGQPLIL
DTCTIEGLIYGNPSCDLSLEGREWSYIVERPSAVHGLCYPGNVEDLEELRSLFSSARSYQRIQIFPDTIW
NVSYDGTSTACSGSFYRSMRWLTRKNGEYPIQDAQYTNNQGKNILFMWGINHPPTDDTQRGLYTRTDTTT
SVATEEINRIFKPLIGPRPLVNGLMGRINYYWSVLKPGQTLRIKSDGNLIAPWYGHILSGESHGRILKTD
LKRGSCTVQCQTEKGGLNTTLPFQNVSKYAFGNCSKYIGIKSLKLAVGLRNVPSRSSRGLFGAIAGFIEG
GWSGLVAGWYGFQHSNDQGVGMAADRDSTQKAIDKITSKVNNIVDKMNKQYEIIDHEFSEVETRLNMINN
KIDDQIQDIWAYNAELLVLLENQKTLDEHDANVNNLYSKVKRALGSNAVEDGKGCFELYHKCDDQCMETI
RNGTYNRRKYQEESKLERQRIEGVKLESEGTYKILTIYSTVASSLVIAMGFAAFLFWAMSNGSCRCNICI

>QBK20360.1 hemagglutinin [Influenza A virus]
METVSLITILLVATVSNADKICIGYQSTNSTETVDTLTENNVPVTHAKELLHTEHNGMLCATSLGQPLIL
DTCTIEGLIYGNPSCDLSLEGREWSYIVERPSAVHGLCYPGNVEDLEELRSLFSSARSYQRIQIFPDTIW
NVSYDGTSTACSGSFYRSMRWLTRKNGEYPIQDAQYTNNQGKNILFMWGINHPPTDDTQRGLYTRTDTTT
SVATEEINRIFKPLIGPRPLVNGLMGRINYYWSVLKPGQTLRIKSDGNLIAPWYGHILSGESHGRILKTD
LKRGSCTVQCQTEKGGLNTTLPFQNVSKYAFGNCSKYIGIKSLKLAVGLRNVPSRSSRGLFGAIAGFIEG

GWSGLVAGWYGFQHSNDQGVGMAADRDSTQKAIDKITSKVNNIVDKMNKQYEIIDHEFSEVETRLNMINN
KIDDQIQDIWAYNAELLILLENQKTLDEHDANVNNLYNKVKRALGSNAVEDGKGCFELYHKCDDQCMETI
RNGTYNRRKYQEESKLERQRIEGVKLESEGTYKILTIYSTVASSLVIAMGFAAFLFWAMSNGSCRCNICI

>QBK20359.1 hemagglutinin [Influenza A virus]
METVSLITILLVATVSNADKICIGYQSTNSTETVDTLTENNVPVTHAKELLHTEHNGMLCATSLGQPLIL
DTCTIEGLIYGNPSCDLSLEGREWSYIVERPSAVHGLCYPGNVEDLEELRSLFSSARSYQRIQIFPDTIW
NVSYDGTSTACSGSFYRSMRWLTRKNGEYPIQDAQYTNNQGKNILFMWGINHPPTDDTQRGLYTRTDTTT
SVATEEINRIFKPLIGPRPLVNGLMGRINYYWSVLKPGQTLRIKSDGNLIAPWYGHILSGESHGRILKTD
LKRGSCTVQCQTEKGGLNTTLPFQNVSKYAFGNCSKYIGIKSLKLAVGLRNVPSRSSRGLFGAIAGFIEG
GWSGLVAGWYGFQHSNDQGVGMAADRDSTQKAIDKITSKVNNIVDKMNKQYEIIDHEFSEVETRLNMINN
KIDDQIQDIWAYNAELLILLENQKTLDEHDANVNNLYNKVKRALGSNAVEDGKGCFELYHKCDDQCMETI
RNGTYNRRKYQEESKLERQRIEGVKLESEGTYKILTIYSTVASSLVIAMGFAAFLFWAMSNGSCRCNICI

>QBK20358.1 hemagglutinin [Influenza A virus]
METVSLITILLVATVSNADKICIGYQSTNSTETVDTLTENNVPVTHAKELLHTEHNGMLCATSLGQPLIL
DTCTIEGLIYGNPSCDLSLEGREWSYIVERPSAVNGLCYPGNVEDLEELRSLFSSARSYQRVQIFPDTIW
NVSYDGTSTACSGSFYRSMRWLTRKNGEYPIQDAQYTNNQGKNILFMWGINHPPTDTTQRELYTRTDTTT
SVATEEINRIFKPLIGPRPLVNGLMGRINYYWSVLKPGQTLRIKSGGNLIAPWYGYILSGESHGRILKTN
LKRGSCTVQCQTEKGGLNTTLPFQNVSKYAFGNCSKYIGIKSLKLAVGLRNVPSRSSRGLFGAIAGFIEG
GWSGLVAGWYGFQHSNDQGVGMAADRDSTQKAIDKITSKVNNIVDKMNKQYEIIDHEFSEVETRLNMINN
KIDDQIQDIWAYNAELLVLLENQKTLDEHDANVNNLYNKVKRALGSNAVEDGKGCFELYHKCDDQCMETI
RNGTYNRRKYQEESKLERQKIEGVKLESEGTYKILTIYSTVASSLVIAMGFAAFLFWAMSNGSCRCNICI

>QBK20357.1 hemagglutinin [Influenza A virus]
METVSLITILLVATVSNADKICIGYQSTNSTETVDTLTENNVPVTHAKELLHTEHNGMLCATSLGQPLIL
DTCTIEGLIYGNPSCDLSLEGREWSYIVERPSAVNGLCYPGNVEDLEELRSLFSSARSYQRVQIFPDTIW
NVSYDGTSTACSGSFYRSMRWLTRKNGEYPIQDAQYTNNQGKNILFMWGINHPPTDTTQKELYTRTDTTT
SVATEEINRIFKPLIGPRPLVNGLMGRINYYWSVLKPGQTLRIKSGGNLIAPWYGYILSGESHGRILKTN
LKRGSCTVQCQTEKGGLNTTLPFQNVSKYAFGNCSKYIGIKSLKLAVGLRNVPSRSSRGLFGAIAGFIEG
GWSGLVAGWYGFQHSNDQGVGMAADRDSTQKAIDKITSKVNNIVDKMNKQYEIIDHEFSEVETRLNMINN
KIDDQIQDIWAYNAELLVLLENQKTLDEHDANVNNLYNKVKRALGSNAVEDGKGCFELYHKCDDQCMETI
RNGTYNRRKYQEESKLERQKIEGVKLESEGTYKILTIYSTVASSLVIAMGFAAFLFWAMSNGSCRCNICI

>QBK20356.1 hemagglutinin [Influenza A virus]

METVSLMTILLVATVSNADKICIGYQSTNSTETVDTLTENNVPVTHAKELLHTEHNGMLCATSLGNPLIL
DTCTIEGLIYGNPSCDPLLGGREWSYIVERPSAVNGLCYPGSVENLEELRSLFSSARSYQRIQIFPDTIW
NVSYSGTSKACSDSFYRSMRWLTQKNNAYPIQDAQYTNNQEKNILFMWGINHPPTETTQTNLYTRTDTTT
SVATEEINRIFKPLIGPRPLVNGLMGRINYYWSVLKPGQTLRIKSDGNLIAPWYGHILSGESHGRILKTD
LKRGSCTVQCQTEKGGLNTTLPFQNVSKYAFGNCSKYIGIKSLKLAVGLRNVPSRSSRGLFGAIAGFIEG
GWSGLVAGWYGFQHSNDQGVGMAADRDSTQKAIDKITSKVNNIVDKMNKQYEIIDHEFSEVETRLNMINN
KVDDQIQDIWAYNAELLVLLENQKTLDEHDSNVNNLYNKVKRALGSNAVEDGKGCFELYHKCDNQCMETI
RNGTYNRRKYQEESKLERQKIEGVKLESEGTYKILTIYSTVASSLVIAMGFAAFLFWAMSNGSCRCNICI

>QBK20355.1 hemagglutinin [Influenza A virus]

METVSLITILLVATVSNADKICIGYQSTNSTETVDTLTENNVPVTHAKELIHTEHNGMLCATSLGQPLIL
DTCTIEGLIYGNPSCDLSLEGREWSYIVERPSAVNGLCYPGNVENLEELRSFFSSARSYQRIQIFPDTIW
NVSYDGTSTACSGSFYRNMRWLTRKDGNYPTQDAQYTNNQGKNILFMWGINHPPTDTTQSGLYTRTDTTT
SVATEEINRIFKPLIGPRPLVNGLMGRIDYYWSVLKPGQTLRIKSDGNLIAPWFGHILSGESHGRILKTD
LKRGSCTVQCQTEKGGLNTTLPFQNVSKYAFGNCSKYIGIKSLKLAVGLRNVPSRSSRGLFGAIAGFIEG
GWSGLVAGWYGFQHSNDQGVGMAADRDSTQKAIDKITSKVNNIVDKMNKQYEIIDHEFSEVETRLNMINN
KIDDQIQDIWAYNAELLVLLENQKTLDEHDANVNNLYNKVKRALGSNAVEDGKGCFELYHKCDDQCMETI
RNGTYNRRKYQEESKLERQKIEGVKLESEGTYKILTIYSTVASSLVIAMGFAAFLFWAMSNGSCRCNICI

>QBK20354.1 hemagglutinin [Influenza A virus]

METVSLITILLVATVSNADKICIGYQSTNSTETVDTLTENNVPVTHAKELIHTEHNGMLCATSLGQPLIL
DTCTIEGLIYGNPSCDLSLEGREWSYIVERPSAVNGLCYPGNVENLEELRSLFSSARSYQRIQIFPDTIW
NVSYDGTSTACSGSFYRNMRWLTRKDGNYPTQDAQYTNNQGKNILFMWGINHPPTDTTQSGLYTRTDTTT
SVATEEINRIFKPLIGPRPLVNGLMGRIDYYWSVLKPGQTLRIKSDGNLIAPWFGHILSGESHGRILKTD
LKRGSCTVQCQTEKGGLNTTLPFQNVSKYAFGNCSKYIGIKSLKLAVGLRNVPSRSSRGLFGAIAGFIEG
GWSGLVAGWYGFQHSNDQGVGMAADRDSTQKAIDKITSKVNNIVDKMNKQYEIIDHEFSEVETRLNMINN
KIDDQIQDIWAYNAELLVLLENQKTLDEHDANVNNLYNKVKRALGSNAVEDGKGCFELYHKCDDKCMETI
RNGTYNRRKYQEESKLERQKIEGVKLESEGTYKILTIYSTVASSLVIAMGFAAFLFWAMSNGSCRCNICI

>QBK20353.1 hemagglutinin [Influenza A virus]

METVSLITILLVATVSNADKICIGYQSTNSTETVDTLTENNVPVTHAKELLHTEHNGMLCATSLGQPLIL
DTCTIEGLIYGNPSCDLSLEGREWSYIVERPSAVNGLCYPGKVENLEELRSLFSSARSYQRIQIFPDTIW
NVSYDGTSTACSGSFYRSMRWLTRKNGDYPTQDAQYTNNQGKNILFMWGINHPPTDDTQRNLYTRTDTTT

SVATEEINRIFKPLIGPRPLVNGLMGRIDYYWSVLKPGQTLGIKFDGNLIAPWYGHIFSGESHGRILKTD
LKRGSCTVQCQTEKGGLNTTLPFQNVSKYAFGNCSKYIGIKSLKLAVGLRNVPSRSSRGLFGAIAGFIEG
GWSGLVAGWYGFQHSNDQGVGMAADRDSTQKAIDKITSKVNNIVDKMNKQYEIIDHEFSEVETRLNMINN
KIDDQIQDIWAYNAELLVLLENQKTLDEHDANVNNLYNKVKRALGSNAVEDGKGCFELYHKCNDQCMETI
RNGTYNRRKYQEESKLERQKIEGVKLESEGTYKILTIYSTVASSLVIAMGFAAFLFWAMSNGSCRCNICI

>QBK20352.1 hemagglutinin [Influenza A virus]
METVSLITILLVATASNADKICIGYQSTNSTETVDTLTENNVPVTHAKELLHTEHNGMLCATSLGQPLIL
DTCTIEGLIYGNPSCDLSLEGREWSYIVERPSAVNGLCYPGNVENLEELRSLFSSARSYQRIQIFPDTIW
NVSYDGTSTACSGSFYRSMRWLTRKNGDYPTQDAQYTNNQGKNILFMWGINHPPTDDTQRNLYTRTDTTT
SVATEEINRIFKPLIGPRPLVNGLMGRIDYYWSVLKPSQTLRIKSDGNLIAPWYGHILSGESHGRILKTD
LKRGSCTVQCQTEKGGLNTTLPFQNVSKYAFGNCSKYIGIKSLKLAVGLRNVPSRSSRGLFGAIAGFIEG
GWSGLVAGWYGFQHSNDQGVGMAADRDSTQKAIDKITSKVNNIVDKMNKQYEIIDHEFSEVETRLNMINN
KIDDQIQDIWAYNAELLVLLENQKTLDEHDANVNNLYNKVKRALGSNAVEDGKGCFELYHKCNDQCMETI
RNGTYNRRKYQEESKLERQKIEGVKLESEGTYKILTIYSTVASSLVIAMGFAAFLFWAMSNGSCRCNICI

>QBK20351.1 hemagglutinin [Influenza A virus]
METVSLITILLVATVSNADKICIGYQSTNSTETVDTLTENNVPVTHAKELIHTEHNGMLCATSLGQPLIL
DTCTIEGLIYGNPSCDLSLEGREWSYIVERPSAVNGLCYPGNVENLEELRSLFSSARSYQRIQIFPDTIW
NVSYDGTSTACSGSFYRNMRWLTRKDGNYPTQDAQYTNNQGKNILFMWGINHPPTDTTQSGLYTRTDTTT
SVATEEINRIFKPLIGPRPLVNGLMGRIDYYWSVLKPGQTLRIKSDGNLIAPWFGHILSGESHGRILKTD
LKRGSCTVQCQTEKGGLNTTLPFQNVSKYAFGNCSKYIGIKSLKLAVGLRNVPSRSSRGLFGAIAGFIEG
GWSGLVAGWYGFQHSNDQGVGMAADRDSTQKAIDKITSKVNNIVDKMNKQYEIIDHEFSEVETRLNMINN
KIDDQIQDIWAYNAELLVLLENQKTLDEHDANVNNLYNKVKRALGSNAVEDGKGCFELYHKCDDKCMETI
RNGTYNRRKYQEESKLERQKIEGVKLESEGTYKILTIYSTVASSLVIAMGFAAFLFWAMSNGSCRCNICI

>QBK20350.1 hemagglutinin [Influenza A virus]
METVSLITILLVATVSNADKICIGYQSTNSTETVDTLTENNVPVTHAKELIHTEHNGMLCATSLGQPLIL
DTCTIEGLIYGNPSCDLSLEGREWSYIVERPSAVNGLCYPGNVENLEELRSLFSSARSYQRIQIFPDTIW
NVSYDGTSKACSGSFYRNMRWLTRKDGNYPTQDAQYTNNQGKNILFMWGINHPPTDTTQSGLYTRTDTTT
SVATEEINRIFKPLIGPRPLVNGLMGRIDYYWSVLKPGQTLRIKSDGNLIAPWFGHILSGESHGRILKTD
LKRGSCTVQCQTEKGGLNTTLPFQNVSKYAFGNCSKYIGIKSLKLAVGLRNVPSRSSRGLFGAIAGFIEG
GWSGLVAGWYGFQHSNDQGVGMAADRDSTQKAIDKITSKVNNIVDKMNKQYEIIDHEFSEVETRLNMINN
KIDDQIQDIWAYNAELLVLLENQKTLDEHDANVNNLYNKVKRALGSNAVEDGKGCFELYHKCDDKCMETI

RNGTYNRRKYQEESKLERQKIEGVKLESEGTYKILTIYSTVASSLVIAMGFAAFLFWAMSNGSCRCNICI

>QBK20349.1 hemagglutinin [Influenza A virus]
METVSLITILLVATVSNADKICIGYQSTNSTETVDTLTENNVPVTHAKELIHTEHNGMLCATSLGQPLIL
DTCTIEGLIYGNPSCDLSLEGREWSYIVERPSAVNGLCYPGNVENLEELRSLFSSARSYQRIQIFPDTIW
NVSYDGTSTACSGSFYRNMRWLTRKDGNYPTQDAQYTNNQGKNILFMWGINHPPTDTTQSGLYTRTDTTT
SVATEEINRIFKPLIGPRPLVNGLMGRIDYYWSVLKPGPTLGIKFDGNLIAPWFGHLLSGESHGRILKTD
LKRGSCTVQCQTEKGGLNTTLPFQNVSKYAFGNCSKYIGIKSLKLAVGLRNVPSRSSRGLFGAIAGFIEG
GWSGLVAGWYGFQHSNDQGVGMAADRDSTQKAIDKITSKVNNIVDKMNKQYEIIDHEFSEVETRLNMINN
KIDDQIQDIWAYNAELLVLLENQKTLDEHDANVNNLYNKVKRALGSNAVEDGKGCFELYHKCDDKCMETI
RNGTYNRRKYQEESKLERQKIEGVKLESEGTYKILTIYSTVASSLVIAMGFAAFLFWAMSNGSCRCNICI

>QBK20348.1 hemagglutinin [Influenza A virus]
METVSLITILLVATVSNADKICIGYQSTNSTETVDTLTENNVPVTHAKELLHTEHNGMLCATSLGQPLIL
DTCTIEGLIYGNPSCDLSLEGREWSYIVERPSAVNGLCYPGNVENLEELRSLFSSARSYQRIQIFPDTIW
NVSYDGTSTACSGSFYRSMRWLTRKNGDYPTQDAQYTNNQGKNILFMWGINHPPTDDTQRNLYTRTDTTT
SVATEEINRIFKPLIGPRPLVNGLMGRIDYYWSVLKPGQTLRIKSDGNLIAPWYGHILSGESHGRILKTD
LKRGSCTVQCQTEKGGLNTTLPFQNVSKYAFGNCSKYIGIKSLKLAVGLRNVPSRSNRGLFGAIAGFIEG
GWSGLVAGWYGFQHSNDQGVGMAADRDSTQKAIDKITSKVNNIVDKMNKQYEIIDHEFSEVETRLNMINN
KIDDQIQDIWAYNAELLVLLENQKTLDEHDANVNNLYNKVKRALGSNAVEDGKGCFELYHKCNDQCMETI
RNGTYNRRKYQEESKLERQKIEGVKLESEGTYKILTIYSTVASSLVIAMGFAAFLFWAMSNGSCRCNICI

>QBK20347.1 hemagglutinin [Influenza A virus]
METVSLITILLVATVSNADKICIGYQSTNSTETVDTLTENNVPVTHAKELLHTEHNGMLCATSLGQPLIL
DTCTIEGLIYGNPSCDLSLEGREWSYIVERPSAVHGLCYPGNVEDLEELRSLFSSARSYQRIQIFPDTIW
NVSYDGTSTACSGSFYRSMRWLTRKNGEYPIQDAQYTNNQGKNILFMWGINHPPTDDTQRGLYTRTDTTT
SVATEEINRIFKPLIGPRPLVNGLMGRINYYWSVLKPGQTLRIKSDGNLIAPWYGHILSGESHGRILKTD
LKRGSCTVQCQTEKGGLNTTLPFQNVSKYAFGNCSKYIGIKSLKLAVGLRNVPSRSSRGLFGAIAGFIEG
GWSGLVAGWYGFQHSNDQGVGMAADRDSTQKAIDKITSKVNNIVDKMNKQYEIIDHEFSEVETRLNMINN
KIDDQIQDIWAYNAELLVLLENQKTLDEHDANVNNLYSKVKRALGSNAVEDGKGCFELYHKCDDQCMETI
RNGTYNRRKYQEESKLERQRIEGVKLESEGTYKILTIYSTVASSLVIAMGFAAFLFWAMSNGSCRCNICI

>QBK20346.1 hemagglutinin [Influenza A virus]
METVSLITILLVATVSNADKICIGYQSTNSTETVDTLTENNVPVTHAKELLHTEHNGMLCATSLGQPLIL

DTCTIEGLIYGNPSCDLSLEGREWSYIVERPSAVHGLCYPGNVEDLEELRSLFSSARSYQRIQIFPDTIW
NVSYDGTSTACSGSFYRSMRWLTRKNGEYPIQDAQYTNNQGKNILFMWGINHPPTDDTQRGLYTRTDTTT
SVATEEINRIFKPLIGPRPLVNGLMGRINYYWSVLKPGQTLRIKSDGNLIAPWYGHILSGESHGRILKTD
LKRGSCTVQCQTEKGGLNTTLPFQNVSKYAFGNCSKYIGIKSLKLAVGLRNVPSRSSRGLFGAIAGFIEG
GWSGLVAGWYGFQHSNDQGVGMAADRDSTQKAIDKITSKVNNIVDKMNKQYEIIDHEFSEVETRLNMINN
KIDDQIQDIWAYNAELLVLLENQKTLDEHDANVNNLYSKVKRALGSNAVEDGKGCFELYHKCDDQCMETI
RNGTYNRRKYQEESKLERQRIEGVKLESEGTYKILTIYSTVASSLVIAMGFAAFLFWAMSNGSCRCNICI

>QBK20345.1 hemagglutinin [Influenza A virus]
METVSLITILLVATVSNADKICIGYQSTNSTETVDTLTENNVPVTHAKELLHTEHNGMLCATSLGQPLIL
DTCTIEGLIYGNPSCDLSLEGREWSYIVERPSAVHGLCYPGNVEDLEELRSLFSSARSYQRIQIFPDTIW
NVSYDGTSTACSGSFYRSMRWLTRKNGEYPIQDAQYTNNQGKNILFMWGINHPPTDDTQRGLYTRTDTTT
SVATEEINRIFKPLIGPRPLVNGLMGRINYYWSVLKPGQTLRIKSDGNLIAPWYGHILSGESHGRILKTD
LKRGSCTVQCQTEKGGLNTTLPFQNVSKYAFGNCSKYVGIKSLKLAVGLRNVPSRSSRGLFGAIAGFIEG
GWSGLVAGWYGFQHSNDQGVGMAADRDSTQKAIDKITSKVNNIVDKMNKQYEIIDHEFSEVETRLNMINN
KIDDQIQDIWAYNAELLVLLENQKTLDEHDANVNNLYSKVKRALGSNAVEDGKGCFELYHKCDDQCMETI
RNGTYNRRKYQEESKLERQRIEGVKLESEGTYKILTIYSTVASSLVIAMGFAAFLFWAMSNGSCRCNICI

>QBK20344.1 hemagglutinin [Influenza A virus]
METVSLITILLVATVSNADKICIGYQSTNSTETVDTLTENNVPVTHAKELLHTEHNGMLCATSLGQPLIL
DTCTIEGLIYGNPSCDLSLEGREWSYIVERPSAVHGLCYPGNVEDLEELRSLFSSARSYQRIQIFPDTIW
NVSYDGTSTACSGSFYRSMRWLTRKNGEYPIQDAQYTNNQGKNILFMWGINHPPTDDTQRGLYTRTDTTT
SVATEEINRIFKPLIGPRPLVNGLMGRINYYWSVLKPGQTLRIKSDGNLIAPWYGHILSGESHGRILKTD
LKRGSCTVQCQTEKGGLNTTLPFQNVSKYAFGNCSKYIGIKSLKLAVGLRNVPSRSSRGLFGAIAGFIEG
GWSGLVAGWYGFQHSNDQGVGMAADRDSTQKAIDKITSKVNNIVDKMNKQYEIIDHEFSEVETRLNMINN
KIDDQIQDIWAYNAELLVLLENQKTLDEHDANVNNLYSKVKRALGSNAVEDGKGCFELYHKCDDQCMETI
RNGTYNRRKYQEESKLERQRIEGVKLESEGTYKILTIYSTVASSLVIAMGFAAFLFWAMSNGSCRCNICI

>QBK20343.1 hemagglutinin [Influenza A virus]
METVSLITILLVATVSNADKICIGYQSTNSTETVDTLTENNVPVTHAKELLHTEHNGMLCATSLGQPLIL
DTCTIEGLIYGNPSCDLSLEGREWSYIVERPSAVHGLCYPGNVEDLEELRSLFSSARSYQRIQIFPDTIW
NVSYDGTSTACSGSFYRSMRWLTRKNGEYPIQDAQYTNNQGKNILFMWGINHPPTDDTQRGLYTRTDTTT
SVATEEINRIFKPLIGPRPLVNGLMGRINYYWSVLKPGQTLRIKSDGNLIAPWYGHILSGESHGRILKTD
LKRGSCTVQCQTEKGGLNTTLPFQNVSKYAFGNCSKYIGIKSLKLAVGLRNVPSRSSRGLFGAIAGFIEG

GWSGLVAGWYGFQHSNDQGVGMAADRDSTQKAIDKITSKVNNIVDKMNKQYEIIDHEFSEVETRLNMINN
KIDDQIQDIWAYNAELLVLLENQKTLDEHDANVNNLYSKVKRALGSNAVEDGKGCFELYHKCDDQCMETI
RNGTYNRRKYQEESKLERQRIEGVKLESEGTYKILTIYSTVASSLVIAMGFAAFLFWAMSNGSCRCNICI

>QBK20342.1 hemagglutinin [Influenza A virus]
METVSLITILLVATVSNADKICIGYQSTNSTETVDTLTENNVPVTHAKELLHTEHNGMLCATSLGQPLIL
DTCTIEGLIYGNPSCDLSLEGREWSYIVERPSAVHGLCYPGNVEDLEELRSLFSSARSYQRIQIFPDTIW
NVSYDGTSTACSGSFYRSMRWLTRKNGEYPIQDAQYTNNQGKNILFMWGINHPPTDDTQRGLYTRTDTTT
SVATEEINRIFKPLIGPRPLVNGLMGRINYYWSVLKPGQTLRIKSDGNLIAPWYGHILSGESHGRILKTD
LKRGSCTVQCQTEKGGLNTTLPFQNVSKYAFGNCSKYIGIKSLKLAVGLRNVPSRSSRGLFGAIAGFIEG
GWSGLVAGWYGFQHSNDQGVGMAADRDSTQKAIDKITSKVNNIVDKMNKQYEIIDHEFSEVETRLNMINN
KIDDQIQDIWAYNAELLVLLENQKTLDEHDANVNNLYSKVKRALGSNAVEDGKGCFELYHKCDDQCMETI
RNGTYNRRKYQEESKLERQRIEGVKLESEGTYKILTIYSTVASSLVIAMGFAAFLFWAMSNGSCRCNICI

>QBK20341.1 hemagglutinin [Influenza A virus]
METVSLITILLVATVSNADKICIGYQSTNSTETVDTLTENNVPVTHAKELLHTEHNGMLCATSLGQPLIL
DTCTIEGLIYGNPSCDLSLEGREWSYIVERPSAVHGLCYPGNVEDLEELRSLFSSARSYQRIQIFPDTIW
NVSYDGTSTACSGSFYRSMRWLTRKNGEYPIQDAQYTNNQGKNILFMWGINHPPTDDTQRGLYTRTDTTT
SVATEEINRIFKPLIGPRPLVNGLMGRINYYWSVLKPGQTLRIKSDGNLIAPWYGHILSGESHGRILKTD
LKRGSCTVQCQTEKGGLNTTLPFQNVSKYAFGNCSKYIGIKSLKLAVGLRNVPTRSSRGLFGAIAGFIEG
GWSGLVAGWYGFQHSNDQGVGMAADRDSTQKAIDKITSKVNNIVDKMNKQYEIIDHEFSEVETRLNMINN
KIDDQIQDIWAYNAELLVLLENQKTLDEHDANVNNLYNKVKRALGSNAVEDGKGCFELYHKCDDQCMETI
RNGTYNRRKYQEESKLERQRIEGVKLESEGTYKILTIYSTVASSLVIAMGFAAFLFWAMSNGSCRCNICI

>QBK20340.1 hemagglutinin [Influenza A virus]
METVSLITILLVATVSNADKICIGYQSTNSTETVDTLTENNVPVTHAKELLHTEHNGMLCATSLGQPLIL
DTCTIEGLIYGNPSCDLSLEGREWSYIVERPSAVHGLCYPGNVEDLEELRSLFSSARSYQRIQIFPDTIW
NVSYDGTSTACSGSFYRSMRWLTRKNGEYPIQDAQYTNNQGKNILFMWGINHPPTDDTQRGLYTRTDTTT
SVATEEINRIFKPLIGPRPLVNGLMGRINYYWSVLKPGQTLRIKSDGNLIAPWYGHILSGESHGRILKTD
LKRGSCTVQCQTEKGGLNTTLPFQNVSKYAFGNCSKYIGIKSLKLAVGLRNVPSRSSRGLFGAIAGFIEG
GWSGLVAGWYGFQHSNDQGVGMAADRDSTQKAIDKITSKVNNIVDKMNKQYEIIDHEFSEVETRLNMINN
KIDDQIQDIWAYNAELLVLLENQKTLDEHDANVNNLYSKVKRALGSNAVEDGKGCFELYHKCDDQCMETI
RNGTYNRRKYQEESKLERQRIEGVKLESEGTYKILTIYSTVASSLVIAMGFAAFLFWAMSNGSCRCNICI

>QBK20338.1 hemagglutinin [Influenza A virus]

METVSLITILLVATVSNADKICIGYQSTNSTETVDTLTENNVPVTHAKELLHTEHNGMLCATSLGQPLIL
DTCTIEGLIYGNPSCDLSLEGREWSYIVERPSAVHGLCYPGNVEDLEELRSLFSSARSYQRIQIFPDTIW
NVSYDGTSTACSGSFYRSMRWLTRKNGEYPIQDAQYTNNQGKNILFMWGINHPPTDDTQRGLYTRTDTTT
SVATEEINRIFKPLIGPRPLVNGLMGRINYYWSVLKPGQTLRIKSDGNLIAPWYGHILSGESHGRILKTD
LKRGSCTVQCQTEKGGLNTTLPFQNVSKYAFGNCSKYIGIKSLKLAVGLRNVPSRSSRGLFGAIAGFIEG
GWSGLVAGWYGFQHSNDQGVGMAADRDSTQKAIDKITSKVNNIVDKMNKQYEIIDHEFSEVETRLNMINN
KIDDQIQDIWAYNAELLVLLENQKTLDEHDANVNNLYNKVKRALGSNAVEDGKGCFELYHKCDDQCMETI
RNGTYNRRKYLEESKLERQRIEGVKLESEGTYKILTIYSTVASSLVIAMGFAAFLFWAMSNGSCRCNICI

>QBK20336.1 hemagglutinin [Influenza A virus]

METVSLITILLVATVSNADKICIGYQSTNSTETVDTLTENNVPVTHAKELLHTEHNGMLCATSLGQPLIL
DTCTIEGLIYGNPSCDLSLEGREWSYIVERPSAVHGLCYPGNVEDLEELRSLFSSARSYQRIQIFPDTIW
NVSYDGTSTACSGSFYRSMRWLTRKNGEYPIQDAQYTNNQGKNILFMWGINHPPTDDTQRGLYTRTDTTT
SVATEEINRIFKPLIGPRPLVNGLMGRINYYWSVLKPGQTLRIKSDGNLIAPWYGHILSGESHGRILKTD
LKRGSCTVQCQTEKGGLNTTLPFQNVSKYAFGNCSKYIGIKSLKLAVGLRNVPSRSSRGLFGAIAGFIEG
GWSGLVAGWYGFQHSNDQGVGMAADRDSTQKAIDKITSKVNNIVDKMNKQYEIIDHEFSEVETRLNMINN
KIDDQIQDIWAYNAELLILLENQKTLDEHDANVNNLYNKVKRALGSNAVEDGKGCFELYHKCDDQCMETI
RNGTYNRRKYQEESKLERQRIEGVKLESEGTYKILTIYSTVASSLVIAMGFAAFLFWAMSNGSCRCNICI

>QBK20335.1 hemagglutinin [Influenza A virus]

METVSLITILLVATVSNADKICIGYQSTNSTETVDTLTENNVPVTHAKELLHTEHNGMLCATSLGQPLIL
DTCTIEGLIYGNPSCDLSLEGREWSYIVERPSAVHGLCYPGNVEDLEELRSLFSSARSYQRIQIFPDTIW
NVSYDGTSTACSGSFYRSMRWLTRKNGEYPIQDAQYTNNQGKNILFMWGINHPPTDDTQRGLYTRTDTTT
SVATEEINRIFKPLIGPRPLVNGLMGRINYYWSVLKPGQTLRIKSDGNLIAPWYGHILSGESHGRILKTD
LKRGSCTVQCQTEKGGLNTTLPFQNVSKYAFGNCSKYIGIKSLKLAVGLRNVPSRSSRGLFGAIAGFIEG
GWSGLVAGWYGFQHSNDQGVGMAADRDSTQKAIDKITSKVNNIVDKMNKQYEIIDHEFSEVETRLNMINN
KIDDQIQDIWAYNAELLVLLENQKTLDEHDANVNNLYNKVKRALGSNAVEDGKGCFELYHKCDDQCMETI
RNGTYNRRKYQEESKLERQRIEGVKLESEGTYKILTIYSTVASSLVIAMGFAAFLFWAMSNGSCRCNICI

>QBK20334.1 hemagglutinin [Influenza A virus]

METVSLITILLVATVSNADKICIGYQSTNSTETVDTLTENNVPVTHAKELLHTEHNGMLCATSLGQPLIL
DTCTIEGLIYGNPSCDLSLEGREWSYIVERPSAVHGLCYPGNVEDLEELRSLFSSARSYQRIQIFPDTIW
NVSYDGTSTACSGSFYRSMRWLTRKNGEYPIQDAQYTNNQGKNILFMWGINHPPTDDTQRGLYTRTDTTT

SVATEEINRIFKPLIGPRPLVNGLMGRINYYWSVLKPGQTLRIKSDGNLIAPWYGHILSGESHGRILKTD
LKRGSCTVQCQTEKGGLNTTLPFQNVSKYAFGNCSKYIGIKSLKLAVGLRNVPSRSSRGLFGAIAGFIEG
GWSGLVAGWYGFQHSNDQGVGMAADRDSTQKAVDKITSKVNNIVDKMNKQYEIIDHEFSEVETRLNMINN
KIDDQIQDIWAYNAELLVLLENQKTLDEHDANVNNLYNKVKRALGSNAVEDGKGCFELYHKCDDQCMETI
RNGTYNRRKYQEESKLERQRIEGVKLESEGTYKILTIYSTVASSLVIAMGFAAFLFWAMSNGSCRCNICI

>QBK20333.1 hemagglutinin [Influenza A virus]
METVSLITILLVATVSNADKICIGYQSTNSTETVDTLTENNVPVTHAKELLHTEHNGMLCATSLGQPLIL
DTCTIEGLIYGNPSCDLSLEGREWSYIVERPSAVHGLCYPGNVEDLEELRSLFSSARSYQRIQIFPDTIW
NVSYDGTSTACSGSFYRSMRWLTRKNGEYPIQDAQYTNNQGKNILFMWGINHPPTDDTQRGLYTRTDTTT
SVATEEINRIFKPLIGPRPLVNGLMGRINYYWSVLKPGQTLRIKSDGNLIAPWYGHILSGESHGRILKTD
LKRGSCTVQCQTEKGGLNTTLPFQNVSKYAFGNCSKYIGIKSLKLAVGLRNVPSRSSRGLFGAIAGFIEG
GWSGLVAGWYGFQHSNDQGVGMAADRDSTQKAIDKITSKVNNIVDKMNKQYEIIDHEFSEVETRLNMINN
KIDDQIQDIWAYNAELLVLLENQKTLDEHDANVNNLYNKVKRALGSNAVEDGKGCFELYHKCDDQCMETI
RNGTYNRRKYQEESKLERQRIEGVKLESEGTYKILTIYSTVASSLVIAMGFAAFLFWAMSNGSCRCNICI

>QBK20332.1 hemagglutinin [Influenza A virus]
METVSLITILLVATVSNADKICIGYQSTNSTETVDTLTENNVPVTHAKELLHTEHNGMLCATSLGQPLIL
DTCTIEGLIYGNPSCDLSLEGREWSYIVERPSAVNGLCYPGNVEDLEELRSLFSSARSYQRVQIFPDTIW
NVSYDGTSTACSGSFYRSMRWLTRKNGEYPIQDAQYTNNQGKNILFMWGINHPPTDTTQRELYTRTDTTT
SVATEEINRIFKPLIGPRPLVNGLMGRINYYWSVLKPGQTLRIKSGGNLIAPWYGYILSGESHGRILKTN
LKRGSCTVQCQTEKGGLNTTLPFQNVSKYAFGNCSKYIGIKSLKLAVGLRNVPSRSSRGLFGAIAGFIEG
GWSGLVAGWYGFQHSNDQGVGMAADRDSTQKAIDKITSKVNNIVDKMNKQYEIIDHEFSEVETRLNMINN
KIDDQIQDIWAYNAELLVLLENQKTLDEHDANVNNLYNKVKRALGSNAVEDGKGCFELYHKCDDQCMETI
RNGTYNRRKYQEESKLERQKIEGVKLESEGTYKILTIYSTVASSLVIAMGFAAFLFWAMSNGSCRCNICI

>QBK20331.1 hemagglutinin [Influenza A virus]
METVSLITILLVATVSNADKICIGYQSTNSTETVDTLTENNVPVTHAKELLHTEHNGMLCATSLGQPLIL
DTCTIEGLIYGNPSCDLSLEGREWSYIVERPSAVHGLCYPGNVEDLEELRSLFSSARSYQRIQIFPDTIW
NVSYDGTSTACSGSFYRSMRWLTRKNGEYPIQDAQYTNNQGKNILFMWGINHPPTDDTQRGLYTRTDTTT
SVATEEINRIFKPLIGPRPLVNGLMGRINYYWSVLKPGQTLRIKSDGNLIAPWYGHILSGESHGRILKTD
LKRGSCTVQCQTEKGGLNTTLPFQNVSKYAFGNCSKYIGIKSLKLAVGLRNVPTRSSRGLFGAIAGFIEG
GWSGLVAGWYGFQHSNDQGVGMAADRDSTQKAIDKITSKVNNIVDKMNKQYEIIDHEFSEVETRLNMINN
KIDDQIQDIWAYNAELLVLLENQKTLDEHDANVNNLYNKVKRALGSNAVEDGKGCFELYHKCDDQCMETI

RNGTYNRRKYQEESKLERQRIEGVKLESEGTYKILTIYSTVASSLVIAMGFAAFLFWAMSNGSCRCNICI

>QBK20330.1 hemagglutinin [Influenza A virus]
METVSLITILLVATVSNADKICIGYQSTNSTETVDTLTENNVPVTHAKELLHTEHNGMLCATSLGQPLIL
DTCTIEGLIYGNPSCDLSLEGREWSYIVERPSAVNGLCYPGNVEDLEELRSLFSSARSYQRVQIFPDTIW
NVSYDGTSTACSGSFYRSMRWLTRKNGEYPIQDAQYTNNQGKNILFMWGINHPPTDTTQRELYTRTDTTT
SVATEEINRIFKPLIGPRPLVNGLMGRINYYWSVLKPGQTLRIKSGGNLIAPWYGYILSGESHGRILKTN
LKRGRCTVQCQTEKGGLNTTLPFQNVSKYAFGNCSKYIGIKSLKLAVGLRNVPSRSSRGLFGAIAGFIEG
GWSGLVAGWYGFQHSNDQGVGMAADRDSTQKAIDKITSKVNNIVDKMNKQYEIIDHEFSEVETRLNMINN
KIDDQIQDIWAYNAELLVLLENQKTLDEHDANVNNLYNKVKRALGSNAVEDGKGCFELYHKCDDQCMETI
RNGTYNRRKYQEESKLERQKIEGVKLESEGTYKILTIYSTVASSLVIAMGFAAFLFWAMSNGSCRCNICI

>QBK20329.1 hemagglutinin [Influenza A virus]
METVSLITILLVATVSNADKICIGYQSTNSTETVDTLTENNVPVTHAKELLHTEHNGMLCATSLGHPLIL
DTCTIEGLIYGNPSCDPLLGGREWSYIVERPSAVNGLCYPGNVENLEELRSLFSSARSYQRIQIFPDTIW
NVSYSGTSKACSDSFYRSMRWLTQKNNAYPIQDAQYTNNQEKNILFMWGINHPPTDTAQTNLYTRTDTTT
SVATEEINRIFKPLIGPRPLVNGLMGRINYYWSVLKPGQTLRIKSDGNLIAPWYGHILSGESHGRILKTD
LTRGSCTVQCQTEKGGLNTTLPFQNVSKYAFGNCSKYIGIKSLKLAVGLRNVPSRSSRGLFGAIAGFIEG
GWSGLVAGWYGFQHSNDQGVGMAADRDSTQKAIDKITSKVNNIVDKMNKQYEIIDHEFSEVETRLNMINN
KVDDQIQDIWAYNAELLVLLENQKTLDEHDANVNNLYNKVKRALGSNAVEDGKGCFELYHKCDDQCMETI
RNGTYNRRKYQEESKLERQKIEGVKLESEETYKILTIYSTVASSLVIAMGFAAFLFWAMSNGSCRCNICI

>QBK20328.1 hemagglutinin [Influenza A virus]
METVSLITILLVATVSNADKICIGYQSTNSTETVDTLTENNVPVTHAKELLHTEHNGMLCATSLGHPLIL
DTCTIEGLIYGNPSCDPLLGGREWSYIVERPSAVNGLCYPGNVENLEELRSLFSSARSYQRIQIFPDTIW
NVSYSGTSKACSDSFYRSMRWLTQKNNAYPIQDAQYTNNQEKNILFMWGINHPPTDTAQTNLYTRTDTTT
SVATEEINRIFKPLIGPRPLVNGLMGRINYYWSVLKPGQTLRIKSDGNLIAPWYGHILSGESHGRILKTD
LTRGSCTVQCQTEKGGLNTTLPFQNVSKYAFGNCSKYIGIKSLKLAVGLRNVPSRSSRGLFGAIAGFIEG
GWSGLVAGWYGFQHSNDQGVGMAADRDSTQKAIDKITSKVNNIVDKMSKQYEIIDHEFSEVETRLNMINN
KVDDQIQDIWAYNAELLVLLENQKTLDEHDANVNNLYNKVKRALGSNAVEDGKGCFELYHKCDDQCMETI
RNGTYNRRKYQEESKLERQKIEGVKLESEETYKILTIYSTVASSLVIAMGFAAFLFWAMSNGSCRCNICI

>QBK20327.1 hemagglutinin [Influenza A virus]
METVSLITILLVATVSNADKICIGYQSTNSTETVDTLTENNVPVTHAKELLHTEHNGMLCATSLGQPLIL

DTCTIEGLIYGNPSCDLSLEGREWSYIVERPSAVNGLCYPGNVENLEELRSLFSSARSYQRIQIFPDTIW
NVSYDGTSTACSGSFYRSMRWLTRKNGDYPTQDAQYTNNQGKNILFMWGINHPPTDDTQRNLYTRTDTTT
SVATEEINRIFKPLIGPRPLVNGLMGRIDYYWSVLKPGQTLRIKSDGNLIAPWYGHILSGESHGRILKTD
LKRGSCTVQCQTEKGGLNTTLPFQNVSKYAFGNCSKYIGIKSLKLAVGLRNVPSRSSRGLFGAIAGFIEG
GWSGLVAGWYGFQHSNDQGVGMAADRDSTQKAIDKITSKVNNIVDKMNKQYEIIDHEFSEVETRLNMINN
KIDDQIQDIWAYNAELLVLLENQKTLDEHDANVNNLYNKVKRALGSNAVEDGKGCFELYHKCNDQCMETI
RNGTYNRRKYQEESKLERQKIEGVKLESEGTYKILTIYSTVASSLVIAMGFAAFLFWAMSNGSCRCNICI

>QBK20326.1 hemagglutinin [Influenza A virus]
METVSLITILLVATVSNADKICIGYQSTNSTETVDTLTENNVPVTHAKELIHTEHNGMLCATSLGQPLIL
DTCTIEGLIYGNPSCDLSLEGREWSYIVERPSAVNGLCYPGNVENLEELRSLFSSARSYQRIQIFPDTIW
NVSYDGTSTACSGSFYRNMRWLTRKDGNYPTQDAQYTNNQGKNILFMWGINHPPTDTTQSGLYTRTDTTT
SVATEEINRIFKPLIGPRPLVNGLMGRIDYYWSVLKPGQTLRIKSDGNLIAPWFGHILSGESHGRILKTD
LKRGSCTVQCQTEKGGLNTTLPFQNVSKYAFGNCSKYIGIKSLKLAVGLRNVPSRSSRGLFGAIAGFIEG
GWSGLVAGWYGFQHSNDQGVGMAADRDSTQKAIDKITSKVNNIVDKMNKQYEIIDHEFSEVETRLNMINN
KIDDQIQDIWAYNAELLVLLENQKTLDEHDANVNNLYNKVKRALGSNAVEDGKGCFELYHKCDDQCMETI
RNGTYNRRKYQEESKLERQKIEGVKLESEGTYKILTIYSTVASSLVIAMGFAAFLFWAMSNGSCRCNICI

>QBK20325.1 hemagglutinin [Influenza A virus]
METVSLITILLVATVSNADKICIGYQSTNSTETVDTLTENNVPVTHAKELIHTEHNGMLCATSLGQPLIL
DTCTIEGLIYGNPSCDLSLEGREWSYIVERPSAVNGLCYPGNVENLEELRSLFSSARSYQRIQIFPDTIW
NVSYDGTSTACSGSFYRNMRWLTRKDGNYPTQDAQYTNNQGKNILFMWGINHPPTDTTQSGLYTRTDTTT
SVATEEINRIFKPLIGPRPLVNGLMGRIDYYWSVLKPGQTLRIKSDGNLIAPWFGHILSGESHGRILKTD
LKRGSCTVQCQTEKGGLNTTLPFQNVSKYAFGNCSKYIGIKSLKLAVGLRNVPSRSSRGLFGAIAGFIEG
GWSGLVAGWYGFQHSNDQGVGMAADRDSTQKAIDKITSKVNNIVDKMNKQYEIIDHEFSEVETRLNMINN
KIDDQIQDIWAYNAELLVLLENQKTLDEHDANVNNLYNKVKRALGSNAVEDGKGCFELYHKCDDQCMETI
RNGTYNRRKYQEESKLERQKIEGVKLESEGTYKILTIYSTVASSLVIAMGFAAFLFWAMSNGSCRCNICI

>QBK20324.1 hemagglutinin [Influenza A virus]
METVSLITILLVATVSNADKICIGYQSTNSTETVDTLTENNVPVTHAKELLHTEHNGMLCATSLGQPIIL
DTCTIEGLIYGNPSCDLSLEGREWSYIVERPSAVNGLCYPGNVENLEELRSLFSSARSYKRIQIFPDTIW
NVSYDGTSTACSGSFYRNMRWLTRKNGDYPIQDAQYTNNQGKNILFMWGINHPPADTKQRELYTRTDTTT
SVATEEINRIFKPLIGPRPLVNGLMGRIDYYWSVLKPGQTLRIKSDGNLIAPWYGHILSGESHGRILKTD
LKRGSCTVQCQTEKGGLNTTLPFQNVSKYAFGNCSKYIGIKSLKLAVGLRNVPSRSSRGLFGAIAGFIEG

GWSGLVAGWYGFQHSNDQGVGMAADRDSTQKAVDKITSKVNNIVDKMNKQYEIIDHEFSEVETRLNMINN
KIDDQIQDIWAYNAELLVLLENQKTLDEHDANVNNLYNKVKRALGSNAVEDGKGCFELYHKCDDQCMETI
RNGTYNRRKYQEESKLERQKIEGVKLESEGTYKILTIYSTVASSLVIAMGFAAFLFWAMSNGSCRCNICI

>QBK20323.1 hemagglutinin [Influenza A virus]
METVSLITILLVATASNADKICIGYQSTNSTETVDTLTENNVPVTHAKELIHTEHNGMLCATSLGQPLIL
DTCTIEGLIYGNPSCDLSLEGREWSYIVERPSAVNGLCYPGNVENLEELRSLFSSARSYQRIQIFPDTIW
NVSYDGTSTACSGSFYRNMRWLTRKNGNYPTQDVQYTNNQGKNILFMWGINHPPTDTTQSGLYTRTDTTT
SVATEEINRIFKPLIGPRPLVNGLMGRIDYYWSVLKPGQTLRIKSDGNLIAPWFGHILSGESHGRILKTD
LKRGSCTVQCQTEKGGLNTTLPFQNVSKYAFGNCSKYIGIKSLKLAVGLRNVPSRSSRGLFGAIAGFIEG
GWSGLVAGWYGFQHSNDQGVGMAADRDSTQKAIDKITSKVNNIVDKMNKQYEIIDHEFSEVETRLNMINN
KIDDQIQDIWAYNAELLVLLENQKTLDEHDANVNNLYNKVKRALGSNAVEDGRGCFELYHKCDDQCMETI
RNGTYNRRKYQEESKLERQKIEGVKLESEGTYKILTIYSTVASSLVIAMGFAAFLFWAMSNGSCRCNICI

>QBK20322.1 hemagglutinin [Influenza A virus]
METVSLITILLVATVSNADKICIGYQSTNSTETVDTLTENNVPVTHAKELIHTEHNGMLCATSLGQPLIL
DTCTIEGLIYGNPSCDLSLEGREWSYIVERPSAVNGLCYPGNVENLEELRSLFSSARSYQRIQIFPDTIW
NVSYDGTSTACSGSFYRNMRWLTRKDGNYPTQDAQYTNNQGKNILFMWGINHPPTDTTQSGLYTRTDTTT
SVATEEINRIFKPLIGPRPLVNGLMGRIDYYWSVLKPGQTLRIKSDGNLIAPWFGHILSGESHGRILKTD
LKRGSCTVQCQTEKGGLNTTLPFQNVSKYAFGNCSKYIGIKSLKLAVGLRNVPSRSSRGLFGAIAGFIEG
GWSGLVAGWYGFQHSNDQGVGMAADRDSTQKAIDKITSKVNNIVDKMNKQYEIIDHEFSEVETRLNMINN
KIDDQIQDIWAYNAELLVLLENQKTLDEHDANVNNLYNKVKRALGSNAVEDGKGCFELYHKCDDQCMETI
RNGTYNRRKYQEESKLERQKIEGVKLESEGTYKILTIYSTVASSLVIAMGFAAFLFWAMSNGSCRCNICI

>QBK20321.1 hemagglutinin [Influenza A virus]
METVSLITILLVATVSNADKICIGYQSTNSTETVDTLTENNVPVTHAKELINTEHNGMLCATSLGQPLIL
DTCTIEGLIYGNPSCDLSLEGREWSYIVERPSAVNGLCYPGNVENLEELRSLFSSARSYQRIQIFPDTIW
NVSYDGTSTACSGSFYRNMRWLTRKDGNYPTQDAQYTNNQGKNILFMWGINHPPTDTTQSGLYTRTDTTT
SVATEEINRIFKPLIGPRPLVNGLMGRIDYYWSVLKPGQTLRIKSDGNLIAPWFGHILSGESHGRILKTD
LKRGSCTVQCQTEKGGLNTTLPFQNVSKYAFGNCSKYIGIKSLKLAVGLRNVPSRSSRGLFGAIAGFIEG
GWSGLVAGWYGFQHSNDQGVGMAADRDSTQKAIDKITSKVNNIVDKMNKQYEIIDHEFSEVETRLNMINN
KIDDQIQDIWAYNAELLVLLENQKTLDEHDANVNNLYNKVKRALGSNAVEDGKGCFELYHKCDDQCMETI
RNGTYNRRKYQEESKLERQKIEGVKLESEGTYKILTIYSTVASSLVIAMGFAAFLFWAMSNGSCRCNICI

>QBK20320.1 hemagglutinin [Influenza A virus]

METVSLITILLVTTVSNADKICIGYQSTNSTETVDTLTENNVPVTHAKELIHTEHNGMLCATSLGQPLIL
DTCTIEGLIYGNPSCDLSLEGREWSYIVERPSAVNGLCYPGNVENLEELRSLFSSARSYQRIQIFPDTIW
NVSYDGTSTACSGSFYRNMRWLTRKDGNYPTQDAQYTNNQGKNILFMWGINHPPTDTTQSGLYTRTDTTT
SVATEEINRIFKPLIGPRPLVNGLMGRIDYYWSVLKPGQTLRIKSDGNLIAPWFGHILSGESHGRILKTD
LKRGSCTVQCQTEKGGLNTTLPFQNVSKYAFGNCSKYIGIKSLKLAVGLRNVPSRSSRGLFGAIAGFIEG
GWSGLVAGWYGFQHSNDQGVGMAADRDSTQKAIDKITSKVNNIVDKMNKQYEIIDHEFSEVETRLNMINN
KIDDQIQDIWAYNAELLVLLENQKTLDEHDANVNNLYNKVKRALGSNAVEDGKGCFELYHKCDDQCMETI
RNGTYNKRKYQEESKLERQKIEGVKLESEGTYKILTIYSTVASSLVIAMGFAAFLFWAMSNGSCRCNICI

>QBK20319.1 hemagglutinin [Influenza A virus]

METVSLITILLVTTVSNADKICIGYQSTNSTETVDTLTENNVPVTHAKELIHTEHNGMLCATSLGQPLIL
DTCTIEGLIYGNPSCDLSLEGREWSYIVERPSAVNGLCYPGNVENLEELRSLFSSARSYQRIQIFPDTIW
NVSYDGTSTACSGSFYRNMRWLTRKDGNYPTQDAQYTNNQGKNILFMWGINHPPTDTTQSGLYTRTDTTT
SVATEEINRIFKPLIGPRPLVNGLMGRIDYYWSVLKPGQTLRIKSDGNLIAPWFGHILSGESHGRILKTD
LKRGSCTVQCQTEKGGLNTTLPFQNVSKYAFGNCSKYIGIKSLKLAVGLRNVPSRSSRGLFGAIAGFIEG
GWSGLVAGWYGFQHSNDQGVGMAADRDSTQKAIDKITSKVNNIVDKINKQYEIIDHEFSEVETRLNMINN
KIDDQIQDIWAYNAELLVLLENQKTLDEHDANVNNLYNKVKRALGSNAVEDGKGCFELYHKCDDQCMETI
RNGTYNKRKYQEESKLERQKIEGVKLESEGTYKILTIYSTVASSLVIAMGFAAFLFWAMSNGSCRCNICI

>QBK20318.1 hemagglutinin [Influenza A virus]

METVSLITILLVATVSNADKICIGYQSTNSTETVDTLTENNVPVTHAKELIHTEHNGMLCATSLGQPLIL
DTCTIEGLIYGNPSCDLSLEGREWSYIVERPSAVNGLCYPGNVENLEELRSLFSSARSYQRIQIFPDTIW
NVSYDGTSTACSGSFYRNMRWLTRKDGNYPTQDAQYTNNQGKNILFMWGINHPPTDTTQSGLYTRTDTTT
SVATEEINRIFKPLIGPRPLVNGLMGRIDYYWSVLKPGQTLRIKSDGNLIAPWFGHILSGESHGRILKTD
LKRGSCTVQCQTEKGGLNTTLPFQNVSKYAFGNCSKYIGIKSLKLAVGLRNVPSRSSRGLFGAIAGFIEG
GWSGLVAGWYGFQHSNDQGVGMAADRDSTQKAIDKITSKVNNIVDKMNKQYEIIDHEFSEVETRLNMINN
KIDDQIQDIWAYNAELLVLLENQKTLDEHDANVNNLYNKVKRALGSNAVEDGKGCFELYHKCDDQCMETI
RNGTYNRRKYQEESKLERQKIEGVKLESEGTYKILTIYSTVASSLVIAMGFAAFLFWAMSNGSCRCNICI

>QBK20317.1 hemagglutinin [Influenza A virus]

METVSLITILLVATVSNADKICIGYQSTNSTETVDTLTENNVPVTHAKELIHTEHNGMLCATSLGQPLIL
DTCTIEGLIYGNPSCDLSLEGREWSYIVERPSAVNGLCYPGNVENLEELRSLFSSARSYQRIQIFPDTIW
NVSYDGTSTACSGSFYRNMRWLTRKDGNYPTQDAQYTNNQGKNILFMWGINHPPTDTTQSGLYTRTDTTT

SVATEEINRIFKPLIGPRPLVNGLMGRIDYYWSVLKPGQTLRIKSDGNLIAPWFGHILSGESHGRILKTD
LKRGSCTVQCQTEKGGLNTTLPFQNVSKYAFGNCSKYIGIKSLKLAVGLRNVPSRSSRGLFGAIAGFIEG
GWSGLVAGWYGFQHSNDQGVGMAADRDSTQKAIDKITSKVNNIVDKMNKQYEIIDHEFSEVETRLNMINN
KIDDQIQDIWAYNAELLVLLENQKTLDEHDANVNNLYNKVKRALGSNAVEDGKGCFELYHKCDDQCMETI
RNGTYNRRKYQEESKLERQKIEGVKLESEGTYKILTIYSTVASSLVIAMGFAAFLFWAMSNGSCRCNICI

>QBK20316.1 hemagglutinin [Influenza A virus]
METVSLITILLVATVSNADKICIGYQSTNSTETVDTLTENNVPVTHAKELIHTEHNGMLCATSLGQPLIL
DTCTIEGLIYGNPSCDLSLEGREWSYIVERPSAVNGLCYPGNVENLEELRSLFSSARSYQRIQIFPDTIW
NVSYDGTSTACSGSFYRNMRWLTRKDGNYPTQDAQYTNNQGKNILFMWGINHPPTDTTQSGLYTRTDTTT
SVATEEINRIFKPLIGPRPLVNGLMGRIDYYWSVLKPGQTLRIKSDGNLIAPWFGHILSGESHGRILKTD
LKRGSCTVQCQTEKGGLNTTLPFQNVSKYAFGNCSKYIGIKSLKLAVGLRNVPSRSSRGLFGAIAGFIEG
GWSGLVAGWYGFQHSNDQGVGMAADRDSTQKAIDKITSKVNNIVDKMNKQYEIIDHEFSEVETRLNMINN
KIDDQIQDIWAYNAELLVLLENQKTLDEHDANVNNLYNKVKRALGSNAVEDGKGCFELYHKCDDQCMETI
RNGTYNRRKYQEESKLERQKIEGVKLESEGTYKILTIYSTVASSLVIAMGFAAFLFWAMSNGSCRCNICI

>QBK20314.1 hemagglutinin [Influenza A virus]
METVSLITILLVATVSNADKICIGYQSTNSTETVDTLTENNVPVTHAKELIHTEHNGMLCATSLGQPLIL
DTCTIEGLIYGNPSCDLSLEGREWSYIVERPSAVNGLCYPGNVENLEELRSLFSSARSYQRIQIFPDTIW
NVSYDGTSTACSGSFYRNMRWLTRKDGNYPTQDAQYTNNQGKNILFMWGINHPPTDTTQSGLYTRTDTTT
SVATEEINRIFKPLIGPRPLVNGLMGRIDYYWSVLKPGQTLRIKSDGNLIAPWFGHILSGESHGRILKTD
LKRGSCTVQCQTEKGGLNTTLPFQNVSKYAFGNCSKYIGIKSLKLAVGLRNVPSRSSRGLFGAIAGFIEG
GWSGLVAGWYGFQHSNDQGVGMAADRDSTQKAIDKITSKVNNIVDKMNKQYEIIDHEFSEVETRLNMINN
KIDDQIQDIWAYNAELLVLLENQKTLDEHDANVNNLYNKVKRALGSNAVEDGKGCFELYHKCDDQCMETI
RNGTYNRRKYQEESKLERQKIEGVKLESEGTYKILTIYSTVASSLVIAMGFAAFLFWAMSNGSCRCNICI

>QBK20313.1 hemagglutinin [Influenza A virus]
METVSLITILLVATASNADKICIGYQSTNSTETVDTLTENNVPVTHAKELIHTEHNGMLCATSLGQPLIL
DTCTIEGLIYGNPSCDLSLEGREWSYIVERPSAVNGLCYPGNVENLEELRSLFSSARSYQRIQIFPDTIW
NVSYDGTSTACSGSFYRNMRWLTRKNGNYPTQDVQYTNNQGKNILFMWGINHPPTDTTQSGLYTRTDTTT
SVATEEINRIFKPLIGPRPLVNGLMGRIDYYWSVLKPGQTLRIKSDGNLIAPWFGHILSGESHGRILKTD
LKRGSCTVQCQTEKGGLNTTLPFQNVSKYAFGNCSKYIGIKSLKLAVGLRNVPSRSSRGLFGAIAGFIEG
GWSGLVAGWYGFQHSNDQGVGMAADRDSTQKAIDKITSKVNNIVDKMNKQYEIIDHEFSEVETRLNMINN
KIDDQIQDIWAYNAELLVLLENQKTLDEHDANVNNLYNKVKRALGSNAMEDGKGCFELYHKCDDQCMETI

RNGTYNRRKYQEESKLERQKIEGVKLESEGTYKILTIYSTVASSLVIAMGFAAFLFWAMSNGSCRCNICI

>QBK20312.1 hemagglutinin [Influenza A virus]
METVSLITILLVATASNADKICIGYQSTNSTETVDTLTENNVPVTHAKELIHTEHNGMLCATSLGQPLIL
DTCTIEGLIYGNPSCDLSLEGREWSYIVERPSAVNGLCYPGNVENLEELRSLFSSARSYQRIQIFPDTIW
NVSYDGTSTACSGSFYRNMRWLTRKNGNYPTQDVQYTNNQGKNILFMWGINHPPTDTTQSGLYTRTDTTT
SVATEEINRIFKPLIGPRPLVNGLMGRIDYYWSVLKPGQTLRIKSDGNLIAPWFGHILSGESHGRILKTD
LKRGSCTVQCQTEKGGLNTTLPFQNVSKYAFGNCSKYIGIKSLKLAVGLRNVPSRSSRGLFGAIAGFIEG
GWSGLVAGWYGFQHSNDQGVGMAADRDSTQKAIDKITSKVNNIVDKMNKQYEIIDHEFSEVETRLNMINN
KIDDQIQDIWAYNAELLVLLENQKTLDEHDANVNNLYNKVKRALGSNAMEDGKGCFELYHKCDDQCMETI
RNGTYNRRKYQEESKLERQKIEGVKLESEGTYKILTIYSTVASSLVIAMGFAAFLFWAMSNGSCRCNICI

>QBK20311.1 hemagglutinin [Influenza A virus]
METVSLITILLVATVSNADKICIGYQSTNSTETVDTLTENNVPVTHAKELLHTEHNGMLCATSLGQPLIL
DTCTIEGLIYGNPSCDLSLEGREWSYIVERPSAVNGLCYPGNVENLEELRSLFSSARSYQRIQIFPDTIW
NVSYDGTSTACSGSFYRSMRWLTRKNGDYPTQDAQYTNNQGKNILFMWGINHPPTDDTQRNLYTRTDTTT
SVATEEINRIFKPLIGPRPLVNGLMGRIDYYWSVLKPGQTLRIKSDGNLIAPWYGHILSGESHGRILKTD
LKRGSCTVQCQTEKGGLNTTLPFQNVSKYAFGNCSKYIGIKSLKLAVGLRNVPSRSSRGLFGAIAGFIEG
GWSGLVAGWYGFQHSNDQGVGMAADRDSTQKAIDKITSKVNNIVDKMNKQYEIIDHEFSEVETRLNMINN
KIDDQIQDIWAYNAELLVLLENQKTLDEHDANVNNLYNKVKRALGSNAVEDGKGCFELYHKCNDQCMETI
RNGTYNRRKYQEESKLERQKIEGVKLESEGTYKILTIYSTVASSLVIAMGFAAFLFWAMSNGSCRCNICI

>QBK20310.1 hemagglutinin [Influenza A virus]
METVSLITILLVATVSNADKICIGYQSTNSTETVDTLTENNVPVTHAKELLHTEHNGMLCATSLGQPLIL
DTCTIEGLIYGNPSCDLSLEGREWSYIVERPSAVNGLCYPGNVENLEELRSLFSSARSYQRIQIFPDTIW
NVSYDGTSTACSGSFYRSMRWLTRKNGDYPTQDAQYTNNQGKNILFMWGINHPPTDDTQRNLYTRTDTTT
SVATEEINRIFKPLIGPRPLVNGLMGRIDYYWSVLKPGQTLRIKSDGNLIAPWYGHILSGESHGRILKTD
LKRGSCTVQCQTEKGGLNTTLPFQNVSKYAFGNCSKYIGIKSLKLAVGLRNVPSRSSRGLFGAIAGFIEG
GWSGLVAGWYGFQHSNDQGVGMAADRDSTQKAIDKITSKVNNIVDKMNKQYEIIDHEFSEVETRLNMINN
KIDDQIQDIWAYNAELLVLLENQKTLDEHDANVNNLYNKVKRALGSNAVEDGKGCFELYHKCNDQCMETI
RNGTYNRRKYQEESKLERQKIEGVKLESEGTYKILTIYSTVASSLVIAMGFAAFLFWAMSNGSCRCNICI

>QBK20309.1 hemagglutinin [Influenza A virus]
METVSLITILLVATVSNADKICIGYQSTNSTETVDTLTENNVPVTHAKELIHTEHNGMLCATSLGQPLIL

DTCTIEGLIYGNPSCDLSLEGREWSYIVERPSAVNGLCYPGNVENLEELRSLFSSARSYQRIQIFPDTIW
NVSYDGTSTACSGSFYRNMRWLTRKDGNYPTQDAQYTNNQGKNILFMWGINHPPTDTTQSGLYTRTDTTT
SVATEEINRIFKPLIGPRPLVNGLMGRIDYYWSVLKPGQTLRIKSDGNLIAPWFGHILSGESHGRILKTD
LKRGSCTVQCQTEKGGLNTTLPFQNVSKYAFGNCSKYIGIKSLKLAVGLRNVPSRSSRGLFGAIAGFIEG
GWSGLVAGWYGFQHSNDQGVGMAADRDSTQKAIDKITSKVNNIVDKMNKQYEIIDHEFSEVETRLNMINN
KIDDQIQDIWAYNAELLVLLENQKTLDEHDANVNNLYNKVKRALGSNAVEDGKGCFELYHKCNDQCMETI
RNGTYNRRKYQEESKLERQKIEGVKLESEGTYKILTIYSTVASSLVIAMGFAAFLFWAMSNGSCRCNICI

>QBK20308.1 hemagglutinin [Influenza A virus]
METVSLITILLVATVSNADKICIGYQSTNSTETVDTLTENNVPVTHAKELIHTEHNGMLCATSLGQPLIL
DTCTIEGLIYGNPSCDLSLEGREWSYIVERPSAVNGLCYPGNVENLEELRSLFSSARSYQRIQIFPDTIW
NVSYDGTSTACSGSFYRNMRWLTRKDGNYPTQDAQYTNNQGKNILFMWGINHPPTDTTQSGLYTRTDTTT
SVATEEINRIFKPLIGPRPLVNGLMGRIDYYWSVLKPGQTLRIKSDGNLIAPWFGHILSGESHGRILKTD
LKRGSCTVQCQTEKGGLNTTLPFQNVSKYAFGNCSKYIGIKSLKLAVGLRNVPSRSSRGLFGAIAGFIEG
GWSGLVAGWYGFQHSNDQGVGMAADRDSTQKAIDKITSKVNNIVDKMNKQYEIIDHEFSEVETRLNMINN
KIDDQIQDIWAYNAELLVLLENQKTLDEHDANVNNLYNKVKRALGSNAVEDGKGCFELYHKCDDQCMETI
RNGTYNRRKYQEESKLERQKIEGVKLESEGTYKILTIYSTVASSLVIAMGFAAFLFWAMSNGSCRCNICI

>QBK20307.1 hemagglutinin [Influenza A virus]
METVSLITILLVATVSNADKICIGYQSTNSTETVDTLTENNVPVTHAKELIHTEHNGMLCATSLGQPLIL
DTCTIEGLIYGNPSCDLSLEGREWSYIVERPSAVNGLCYPGNVENLEELRSLFSSARSYQRIQIFPDTIW
NVSYDGTSTACSGSFYRNMRWLTRKDGNYPTQDAQYTNNQGKNILFMWGINHPPTDTTQSGLYTRTDTTT
SVATEEINRIFKPLIGPRPLVNGLMGRIDYYWSVLKPGQTLRIKSDGNLIAPWFGHILSGESHGRILKTD
LKRGSCTVQCQTEKGGLNTTLPFQNVSKYAFGNCSKYIGIKSLKLAVGLRNVPSRSSRGLFGAIAGFIEG
GWSGLVAGWYGFQHSNDQGVGMAADRDSTQKAIDKITSKVNNIVDKMNKQYEIIDHEFSEVETRLNMINN
KIDDQIQDIWAYNAELLVLLENQKTLDEHDANVNNLYNKVKRALGSNAVEDGKGCFELYHKCDDQCMETI
RNGTYNRRKYQEESKLERQKIEGVKLESEGTYKILTIYSTVASSLVIAMGFAAFLFWAMSNGSCRCNICI

>QBK20306.1 hemagglutinin [Influenza A virus]
METVSLITILLVATVSNADKICIGYQSTNSTETVDTLTENNVPVTHAKELIHTEHNGMLCATSLGQPLIL
DTCTVEGLIYGNPSCDLSLEGREWSYIVERPSAVNGLCYPGNVENLEELRSLFSSARSYQRIQIFPDTIW
NVSYDGTSTACSGSFYRNMRWLTRKNGNYPTQDVQYTNNQGKNILFMWGINHPPTDSTQSGLYTRTDTTT
SVATEEINRIFKPLIGPRPLVNGLMGRIDYYWSVLKPGQTLRIKSDGNLIAPWFGHILSGESHGRILKTD
LKRGSCTVQCQTEKGGLNTTLPFQNVSKYAFGNCSKYIGIKSLKLAVGLRNVPSRSSRGLFGAIAGFIEG

GWSGLVAGWYGFQHSNDQGVGMAADRDSTQKAIDKITSKVNNIVDKMNKQYEIIDHEFSEVETRLNMINN
KIDDQIQDIWAYNAELLVLLENQKTLDEHDANVNNLYNKVKRTLGSNAVEDGKGCFELYHKCDDQCMETI
RNGTYNRRKYQEESKLERQKIEGVKLESEGTYKILTIYSTVASSLVIAIGFAAFLFWAMSNGSCRCNICI

>QBK20305.1 hemagglutinin [Influenza A virus]
METVSLITILLVATVSNADKICIGYQSTNSTETVNTLTENDVPVTHAQELLHTEHNGMLCATSLGQPLVL
DTCTIEGLIYGNPSCDLSLEGREWSYIVERPSAVNGLCYPGNVENLEELRSLFSSARSYQRIQIFPDTIW
NVSYDGTSNACSGSFYRSMRWLTRKNGNYPIQDAQYTNNQGKNILFMWGINHPPTNTAQTNLYTRNDTTT
SVATEEINRIFKPLIGPRPLVNGLMGRIDYYWSVLKPGHTLRIKSDGNLIAPWYGHILSGESHGRILKTD
LKRGSCTMQCQTEKGGFNTTLPFQNVSKYAFGNCSKYIGIKSLKLAVGLRNVPSRSSRGLFGAIAGFIEG
GWPGLVAGWYGFQHSNDQGVGMAADRDSTQKAIDKITSKVNNIVDKMNKQYEIIDHEFSEVETRLNMINN
KIDDQIQDIWAYNAELLVLLENQKTLDEHDANVNNLYNKVKRALGSNAVEDGKGCFELYHKCDDQCMETI
RNGTYNRRKYQEESKLERQKIEGVKLESEGTYKILTIYSTVASSLVIAMGFAAFLFWAMSNGSCRCNICI

>QBK20304.1 hemagglutinin [Influenza A virus]
METVSLITILLVATVSNADKICIGYQSTNSTETVDTLTENNVPVTHAKELIHTEHNGMLCATSLGQPLIL
DTCTIEGLIYGNPSCDLSLEGREWSYIVERPSAVNGLCYPGNVENLEELRSLFSSARSYQRIQIFPDTIW
NVSYDGTSTACSGSFYRNMRWLTRKDGNYPTQDAQYTNNQGKNILFMWGINHPPTDTTQSGLYTRTDTTT
SVATEEINRIFNPLIGPRPLVNGLMGRIDYYWSVLKPGQTLRIKSDGNLIAPWYGHILSGESHGRILKTD
LKRGSCTVQCQTEKGGLNTTLPFQNVSKYAFGNCSKYIGIKSLKLAVGLRNVPSRSSRGLFGAIAGFIEG
GWSGLVAGWYGFQHSNDQGVGMAADRDSTQKAIDKITSKVNNIVDKMNKQYEIIDHEFSEVETRLNMINN
KIDDQIQDIWAYNAELLVLLENQKTLDEHDANVNNLYNKVKRALGSNAVEDGKGCFELYHKCDDQCMETI
RNGTYNRRKYQEESKLERQKIEGVKLESEGTYKILTIYSTVASSLVIAMGFAAFLFWAMSNGSCRCNICI

>QBK20303.1 hemagglutinin [Influenza A virus]
METVSLITILLVATASNADKICIGYQSTNSTETVDTLTENNVPVTHAKELLHTEHNGMLCATSLGQPLIL
DTCTIEGLIYGNPSCDLSLEGREWSYIVERPSAVNGLCYPGNVENLEELRSLFSSARSYQRIQIFPDTIW
NVSYDGTSTACSGSFYRSMRWLTRKNGNYPTQDAQYTNNQGKNILFMWGINHPPTDDTQRNLYTRTDTTT
SVATEEINRIFKPLIGPRPLVNGLMGRIDYYWSVLKPGQTLRIKSDGNLIAPWYGHILSGESHGRILKTD
LKRGSCTVQCQTEKGGLNTTLPFQNVSKYAFGNCSKYIGIKSLKLAVGLRNVPSRSSRGLFGAIAGFIEG
GWSGLVAGWYGFQHSNDQGVGMAADRDSTQKAIDKITSKVNNIVDKMNKQYEIIDHEFSEVETRLNMINN
KIDDQIQDIWAYNAELLVLLENQKTLDEHDANVNNLYNKVKRALGSNAVEDGKGCFELYHKCNDQCMETI
RNGTYNRRKYQEESKLERQKIEGVKLESEGTYKILTIYSTVASSLVIAMGFAAFLFWAMSNGSCRCNICI

>QBK20302.1 hemagglutinin [Influenza A virus]

METVSLITILLVATASNADKICIGYQSTNSTETVDTLTENNVPVTHAKELLHTEHNGMLCATSLGQPLIL

DTCTIEGLIYGNPSCDLSLEGREWSYIVERPSAVNGLCYPGNVENLEELRSLFSSARSYQRIQIFPDTIW

NVSYDGTSIACSGSFYRSMRWLTRKNGDYPTQDAQYTNNQGKNILFMWGINHPPTDDTQRNLYTRTDTTT

SVATEEINRIFKPLIGPRPLVNGLMGRIDYYWSVLKPGQTLRIKSDGNLIAPWYGHILSGESHGRILKTD

LKRGSCTVQCQTEKGGLNTTLPFQNVSKYAFGNCSKYIGIKSLKLAVGLRNVPSRSSRGLFGAIAGFIEG

GWSGLVAGWYGFQHSNDQGVGMAADRDSTQKAIDKITSKVNNIVDKMNKQYEIIDHEFSEVETRLNMINN

KIDDQIQDIWAYNAELLVLLENQKTLDEHDANVNNLYNKVKRALGSNAVEDGKGCFELYHKCNDQCMETI

RNGTYNRRKYQEESKLERQKIEGVKLESEGTYKILTIYSTVASSLVIAMGFAAFLFWAMSNGSCRCNICI

>QBK20299.1 hemagglutinin [Influenza A virus]

METVSLITILLVATVSNADKICIGYQSTNSTETVDTLTENNVPVTHAKELIHTEHNGMLCATSLGQPLIL

DTCTIEGLIYGNPSCDLSLEGREWSYIVERPSAVNGLCYPGNVENLEELRSLFSSARSYQRIQIFPDTIW

NVSYDGTSTACSGSFYRNMRWLTRKDGNYPTQDAQYTNNQGKNILFMWGINHPPTDTTQSGLYTRTDTTT

SVATEEINRIFKPLIGPRPLVNGLMGRIDYYWSVLKPGQTLRIKSDGNLIAPWFGHILSGESHGRILKTD

LKRGSCTVQCQTEKGGLNTTLPFQNVSKYAFGNCSKYIGIKSLKLAVGLRNVPSRSSRGLFGAIAGFIEG

GWSGLVAGWYGFQHSNDQGVGMAADRDSTQKAIDKITSKVNNIVDKMNKQYEIIDHEFSEVETRLNMINN

KIDDQIQDIWAYNAELLVLLENQKTLDEHDANVNNLYNKVKRALGSNAVEDGKGCFELYHKCDDKCMETI

RNGTYNRRKYQEESKLERQKIEGVKLESEGTYKILTIYSTVASSLVIAMGFAAFLFWAMSNGSCRCNICI

>QBK20298.1 hemagglutinin [Influenza A virus]

METVSLITILLVATVSNADKICIGYQSTNSTETVDTLTENNVPVTHAKELIHTEHNGMLCATSLGQPLIL

DTCSIEGLIYGNPSCDLSLEGREWSYIVERPSAVNGLCYPGNVENLEELRSLFSSARSYQRIQIFPDTIW

NVSYDGTSTACSGSFYRNMRWLTRKDGNYPTQDAQYTNNQGKNILFMWGINHPPTDTTQSGLYTRTDTTT

SVATEEINRIFKPLIGPRPLVNGLMGRIDYYWSVLKPGQTLRIKSDGNLIAPWFGHILSGESHGRILKTD

LKRGSCTVQCQTEKGGLNTTLPFQNVSKYAFGNCSKYIGIKSLKLAVGLRNVPSRSSRGLFGAIAGFIEG

GWSGLVAGWYGFQHSNDQGVGMAADRDSTQKAIDKITSKVNNIVDKMNKQYEIIDHEFSEVETRLNMINN

KIDDQIQDIWAYNAELLVLLENQKTLDEHDANVNNLYNKVKRALGSNAVEDGKGCFELYHKCDDQCMETI

RNGTYNRRKYQEESKLERQKIEGVKLESEGTYKILTIYSTVASSLVIAMGFAAFLFWAMSNGSCRCNICI

>QBK20296.1 hemagglutinin [Influenza A virus]

METVSLITILLVATVSNADKICIGYQSTNSTETVDTLTENNVPVTHAKELLHTEHNGMLCATSLGQPLIL

DTCTIEGLIYGNPSCDLSLEGREWSYIVERPSAVNGLCYPGNVENLEELRSLFSSARSYQRIQIFPDTIW

NVSYDGTSTACSGSFYRSMRWLTRKNGDYPVQDAQYTNNQGKNILFMWGINHPPTDTTQRDLYTRNDTTT

SVATEEINRIFKPLIGPRPLVNGLMGRIDYYWSVLKPGQTLRIKSDGNLIAPWYGHILSRESHGRILKTD
LKRGSCTVQCQTEKGGLNTTLPFQNVSKYAFGKCSKYIGIKSLKLAVGLRNVPSRSSRGLFGAIAGFIEG
GWSGLVAGWYGFQHSNDQGVGMAADRDSTQKAIDKITSKVNNIVDKMNKQYEIIDHEFSEVETRLNMINN
KIDDQIQDIWAYNAELLVLLENQKTLDEHDANVNNLYNKVKRALGSNAVEDGKGCFELYHKCDDQCMETI
RNGTYNRRKYQEESKLERQKIEGVKLESEGTYKILSIYSTVASSLVIAMGFAAFLFWALSNWSCRCNICI

>QBK20295.1 hemagglutinin [Influenza A virus]
METVSLITILLVATVSNADKICIGYQSTNSTETVDTLTENNVPVTHAKELLHTEHNGMLCATSLGQPLIL
DTCTIEGLIYGNPSCDLSLEGREWSYIVERPSAVNGLCYPGNVENLEELRSLFSSARSYQRIQIFPDTIW
NVSFDGTSTACSGSFYKSMRWLTRKNGDYPIQDAQYTNNQGKNILFMWGINHPPTDTTQRDLYTRIDTTT
SVATEEINRVFKPLIGPRPLVNGLMGRIDYYWSVLKPGQTLRIKSDGNLIAPWFGHILSGESHGRILKTD
LKRGSCTVQCQTEKGGLNTTLPFQNVSKYAFGNCSKYIGIKSLKLAVGLRNVPSRSSRGLFGAIAGFIEG
GWSGLVAGWYGFQHSNDQGVGMAADRDSTQKAIDKITSKVNNIVDKMNKQYEIIDHEFSEVETRLNMINN
KIDDQIQDIWAYNAELLVLLENQKTLDEHDANVNNLYNKVKRALGSNAVEDGKGCFELYHKCDDQCMETI
RNGTYNRRKYQEESKLERQKIEGVKLESEGTYKILSIYSTVASSLVIAMGFAAFLFWAMSNGSCRCNICI

>QBK20294.1 hemagglutinin [Influenza A virus]
METVSLITILLVATVSNADKICIGYQSTNSTETVDTLTENNVPVTHAKELLHTEHNGMLCATSLGQPLIL
DTCTIEGLIYGNPSCDLSLEGREWSYIVERPSAVNGLCYPGNVENLEELRSLFSSARSYQRIQIFPDTIW
NVSFDGTSTACSGSFYKSMRWLTRKNGDYPIQDAQYTNNQGKNILFMWGINHPPTDTTQRDLYTRIDTTT
SVATEEINRVFKPLIGPRPLVNGLMGRIDYYWSVLKPGQTLRIKSDGNLIAPWFGHILSGESHGRILKTD
LKRGSCTVQCQTEKGGLNTTLPFQNVSKYAFGNCSKYIGIKSLKLAVGLRNVPSRSSRGLFGAIAGFIEG
GWSGLVAGWYGFQHSNDQGVGMAADRDSTQKAIDKITSKVNNIVDKMNKQYEIIDHEFSEVETRLNMINN
KIDDQIQDIWAYNAELLVLLENQKTLDEHDANVNNLYNKVKRALGSNAVEDGKGCFELYHKCDDQCMETI
RNGTYNRRKYQEESKLERQKIEGVKLESEGTYKILTIYSTVASSLVIAMGFAAFLFWAMSNGSCRCNICI

>QBK20293.1 hemagglutinin [Influenza A virus]
METVSLITILLIATVSNADKICIGYQSTNSTETVDTLTENNVPVTHAKELLHTEHNGMLCATSLGQPLIL
DTCTIEGLIYGNPSCDLSLEGREWSYIVERPSAVNGLCYPGNVENLEELRSLFSSARSYQRIQIFPDTIW
NVSFDGTSTACSGSFYKSMRWLTRKNGDYPIQDAQYTNNQGKNILFMWGINHPPTDTTQRDLYTRIDTTT
SVATEEINRVFKPLIGPRPLVNGLMGRIDYYWSVLKPGQTLRIKSDGNLIAPWFGHILSRESHGRILTTD
LKRGSCTVQCQTEKGGLNTTLPFQNVSKYAFGNCSKYIGIKSLKLAVGLRNVPSRSSRGLFGAIAGFIEG
GWSGLVAGWYGFQHSNDQGVGMAADRDSTQKAIDKITSKVNNIVDKMNKQYEIIDHEFSEVETRLNMINN
KIDDQIQDIWAYNAELLVLLENQKTLDEHDANVNNLYNKVKRALGSNAVEDGKGCFELYHKCDDQCMETI

GNGTYNRRKYQEESKLERQKIEGVKLESEGTYKILSIYSTVASSLVIAMGFAAFLFWAMSNWSCRSNICI

>QBK20292.1 hemagglutinin [Influenza A virus]
METVSLITILLVATVSNADKICIGYQSTNSTETVDTLTENNVPVTHAKELLHTEHNGMLCATSLGQPLIL
DTCTIEGLIYGNPSCDLSLEGREWSYIVERPSAVNGLCYPGNVENLEELRSLFSSARSYQRIQIFPDTIW
NVSFDGTSTACSGSFYKSMRWLTRKNGDYPIQDAQYTNNQGKNILFMWGINHPPTDTTQRDLYTRIDTTT
SVATEEINRVFKPLIGPRPLVNGLMGRIDYYWSVLKPGQTLRIKSDGNLIAPWFGHILSGESHGRILKTD
LKRGSCTVQCQTEKGGLNTTLPFQNVSKYAFGNCSKYIGIKSLKLAVGLRNVPSRSSRGLFGAIAGFIEG
GWSGLVAGWYGFQHSNDQGVGMAADRDSTQKAIDKITSKVNNIVDKMNKQYEIIDHEFSEVETRLNMINN
KIDDQIQDIWAYNAELLVLLENQKTLDEHDANVNNLYNKVKRALGSNAVEDGKGCFELYHKCDDQCMETI
RNGTYNRRKYQEESKLERQKIEGVKLESEGTYKILSIYSTVASSLVIAMGFAAFLFWAMSNGSCRCNISI

>QBK20291.1 hemagglutinin [Influenza A virus]
METVSLITILLVATVSNADKICIGYQSTNSTETVDTLTENNVPVTHAKELLHTEHNGMLCATSLGQPLIL
DTCTIEGLIYGNPSCDLSLEGREWSYIVERPSAVNGLCYPGNVENLEELRSLFSSARSYQRIQIFPDTIW
NVSFDGTSTACSGSFYKSMRWLTRKNGDYPIQDAQYTNNQGKNILFMWGINHPPTDTTQRDLYTRIDTTT
SVATEEINRVFKPLIGPRPLVNGLMGRIDYYWSVLKPGQTLRIKSDGNLIAPWFGHILSRESHGRILKTD
LKRGSCTVQCQTEKGGLNTTLPFQNVSKYAFGNCSKYIGIKSLKLAVGLRNVPSRSSRGLFGAIAGFIEG
GWSGLVAGWYGFQHSNDQGVGMAADRDSTQKAIDKITSKVNNIVDKMNKQYEIIDHEFSEVETRLNMINN
KIDDQIQDIWAYNAELLVLLENQKTLDEHDANVNNLYNKVKRALGSNAVEDGKGCFELYHKCDDQCMETI
RNGTYNRRKYQEESKLERQKIEGVKLESEGTYKILTIYSTVASSLVIAMGFAAFLFWAMSNGSCRCNICI

>QBK20290.1 hemagglutinin [Influenza A virus]
METVSLITILLVATVSNADKICIGYQSTNSTETVDTLTENNVPVTHAKELLHTEHNGMLCATSLGQPLIL
DTCTIEGLIYGNPSCDLSLEGREWSYIVERPSAVNGLCYPGNVENLEELRSLFSSARSYQRIQIFPDTIW
NVSFDGTSTACSGSFYKSMRWLTRKNGDYPIQDAQYTNNQGKNILFMWGINHPPTDTTQRDLYTRIDTTT
SVATEEINRVFKPLIGPRPLVNGLMGRIDYYWSVLKPGQTLRIKSDGNLIAPWFGHILSGESHGRILKTD
LKRGSCTVQCQTEKGGLNTTLPFQNVSKYAFGNCSKYIGIKSLKLAVGLRNVPSRSSRGLFGAIAGFIEG
GWSGLVAGWYGFQHSNDQGVGMAADRDSTQKAIDKITSKVNNIVDKMNKQYEIIDHEFSEVETRLNMINN
KIDDQIQDIWAYNAELLVLLENQKTLDEHDANVNNLYNKVKRALGSNAVEDGKGCFELYHKCDDQCMETI
RNGTYNRRKYQEESKLERQKIEGVKLESEGTYKILSIYSTVASSLVIAMGFAAFLFWAMSNGSCRCNICI

>QBK20289.1 hemagglutinin [Influenza A virus]
METVSLITILLVATVSNADKICIGYQSTNSTETVDTLTENNVPVTHAKELLHTEHNGMLCATSLGQPLIL

DTCTIEGLIYGNPSCDLSLEGREWSYIVERPSAVNGLCYPGNVENLEELRSLFSSARSYQRIQIFPDTIW
NVSFDGTSTACSGSFYKSMRWLTRKNGDYPIQDAQYTNNQGKNILFMWGINHPPTDTTQRDLYTRIDTTT
SVATEEINRVFKPLIGPRPLVNGLMGRIDYYWSVLKPGQTLRIKSDGNLIAPWFGHILSRESHGRILKTD
LKRGSCTVQCQTEKGGLNTTLPLQNVSKYAFGNCSKYIGIKSLKLAVGLRNVPSRSSRGLFGAIAGFIEG
GWSGLVAGWYGFQHSNDQGVGMAADRDSTQKAIDKITSKVNNIVDKMNKQYEIIDHEFSEVETRLNMINN
KIDDQIQDIWAYNAELLVLLENQKTLDEHDANVNNLYNKVKRALGSNAVEDGKGCFELYHKCDDQCMETI
RNGTYNRRKYQEESKLERQKIEGVKLESEGTYKILSIYSTVASSLVIAMGFAAFLFWAMSNGSCRCNISI

>QBK20288.1 hemagglutinin [Influenza A virus]
METVSLITILLVATVSNADKICIGYQSTNSTETVDTLTENNVPVTHAKELLHTEHNGMLCATSLGQPLIL
DTCTIEGLIYGNPSCDLSLEGREWSYIVERPSAVNGLCYPGNVENLEELRSLFSSARSYQRIQIFPDTIW
NVSFDGTSTACSGSFYKSMRWLTRKNGDYPIQDAQYTNNQGKNILFMWGINHPPTDTTQRDLYTRIDTTT
SVATEEINRVFKPLIGPRPLVNGLMGRIDYYWSVLKPGQTLRIKSDGNLIAPWFGHILSRESHGRILKTD
LKRGSCTVQCQTEKGGLNTTLPFQNVSKYAFGNCSKYIGIKSLKLAVGLRNVPSRSSRGLFGAIAGFIEG
GWSGLVAGWYGFQHSNDQGVGMAADRDSTQKAIDKITSKVNNIVDKMNKQYEIIDHEFSEVETRLNMINN
KIDDQIQDIWAYNAELLVLLENQKTLDEHDANVNNLYNKVKRALGSNAVEDGKGCFELYHKCDDQCMETI
RNGTYNRRKYQEESKLERQKIEGVKLESEGTYKILTIYSTVASSLVIAMGFAAFLFWAMSNGSCRCNICI

>QBK20287.1 hemagglutinin [Influenza A virus]
METVSLITILLIATVSNADKICIGYQSTNSTETVDTLTENNVPVTHAKELLHTEHNGMLCATSLGQPLIL
DTCTIEGLIYGNPSCDLSLEGREWSYIVERPSAVNGLCYPGNVENLEELRSLFSSARSYQRIQIFPDTIW
NVSFDGTSTACSGSFYKSMRWLTRKNGDYPIQDAQYTNNQGKNILFMWGINHPPTDTTQRDLYTRIDTTT
SVATEEINRVFKPLIGPRPLVNGLMGRIDYYWSVLKPGQTLRIKSDGNLIAPWFGHILSRESHGRILKTD
LKRGSCTVQCQTEKGGLNTTLPFQNVSKYAFGNCSKYIGIKSLKLAVGLRNVPSRSSRGLFGAIAGFIEG
GWSGLVAGWYGFQHSNDQGVGMAADRDSTQKAIDKITSKVNNIVDKMNKQYEIIDHEFSEVETRLNMINN
KIDDQIQDIWAYNAELLVLLENQKTLDEHDANVNNLYNKVKRALGSNAVEDGKGCFELYHKCDDQCMETI
RNGTYNRRKYQEESKLERQKIEGVKLESEGTYKILSIYSTVASSLVIAMGFAAFLFWAMSNGSCRCNISI

>QBK20286.1 hemagglutinin [Influenza A virus]
METVSLITILLVATVSNADKICIGYQSTNSTETVDTLTENNVPVTHAKELLHTEHNGMLCATSLGQPLIL
DTCTIEGLIYGNPSCDLSLEGREWSYIVERPSAVNGLCYPGNVENLEELRSLFSSARSYQRIQIFPDTIW
NVSFDGTSTACSGSFYKSMRWLTRKNGDYPIQDAQYTNNQGKNILFMWGINHPPTDTTQRDLYTRIDTTT
SVATEEINRVFKPLIGPRPLVNGLMGRIDYYWSVLKPGQTLRIKSDGNLIAPWFGHILSGESHGRILKTD
LKRGSCTVQCQTEKGGLNTTLPFQNVSKYAFGNCSKYIGIKSLKLAVGLRNVPSRSSRGLFGAIAGFIEG

GWSGLVAGWYGFQHSNDQGVGMAADRDSTQKAIDKITSKVNNIVDKMNKQYEIIDHEFSEVETRLNMINN
KIDDQIQDIWAYNAELLVLLENQKTLDEHDANVNNLYNKVKRALGSNAVEDGKGCFELYHKCDDQCMETI
RNGTYNRRKYQEESKLERQKIEGVKLESEGTYKILTIYSTVASSLVIAMGFAAFLFWAMSNGSCRCNICI

>QBK20285.1 hemagglutinin [Influenza A virus]
METVSLITILLIATVSNADKICIGYQSTNSTETVDTLTENNVPVTHAKELLHTEHNGMLCATSLGQPLIL
DTCTIEGLIYGNPSCDLSLEGREWSYIVERPSAVNGLCYPGNVENLEELRSLFSSARSYQRIQIFPDTIW
NVSFDGTSTACSGSFYKSMRWLTRKNGDYPIQDAQYTNNQGKNILFMWGINHPPTDTTQRDLYTRIDTTT
SVATEEINRVFKPLIGPRPLVNGLMGRIDYYWSVLKPGQTLRIKSDGNLIAPWFGHILSRESHGRILKTD
LKRGSCTVQCQTEKGGLNTTLPFQNVSKYAFGNCSKYIGIKSLKLAVGLRNVPSRSSRGLFGAIAGFIEG
GWSGLVAGWYGFQHSNDQGVGMAADRDSTQKAIDKITSKVNNIVDKMNKQYEIIDHEFSEVETRLNMINN
KIDDQIQDIWAYNAELLVLLENQKTLDEHDANVNNLYNKVKRALGSNAVEDGKGCFELYHKCDDQCMETI
RNGTYNRRKYQEESKLERQKIEGVKLESEGTYKILSIYSTVASSLVIAMGFAAFLFWAMSNGSCRCNISI

>QBK20284.1 hemagglutinin [Influenza A virus]
METVSLITILLVATVSNADKICIGYQSTNSTETVDTLTENNVPVTHAKELLHTEHNGMLCATSLGQPLIL
DTCTIEGLIYGNPSCDLSLEGREWSYIVERPSAVNGLCYPGNVENLEELRSLFSSARSYQRIQIFPDTIW
NVSFDGTSTACSGSFYKSMRWLTRKNGDYPIQDAQYTNNQGKNILFMWGINHPPTDTTQRDLYTRIDTTT
SVATEEINRVFKPLIGPRPLVNGLMGRIDYYWSVLKPGQTLRIKSDGNLIAPWFGHILSRESHGRILKTD
LKRGSCTVQCQTEKGGLNTTLPFQNVSKYAFGNCSKYIGIKSLKLAVGLRNVPSRSSRGLFGAIAGFIEG
GWSGLVAGWYGFQHSNDQGVGMAADRDSTQKAIDKITSKVNNIVDKMNKQYEIIDHEFSEVETRLNMINN
KIDDQIQDIWAYNAELLVLLENQKTLDEHDANVNNLYNKVKRALGSNAVEDGKGCFELYHKCDDQCMETI
RNGTYNRRKYQEESKLERQKIEGVKLESEGTYKILTIYSTVASSLVIAMGFAAFLFWAMSNGSCRCNICI

>QBK20283.1 hemagglutinin [Influenza A virus]
METVSLITILLVATVSNADKICIGYQSTNSTETVDTLTENNVPVTHAKELLHTEHNGMLCATSLGQPLIL
DTCTIEGLIYGNPSCDLSLEGREWSYIVERPSAVNGLCYPGNVENLEELRSLFSSARSYQRIQIFPDTIW
NVSFDGTSTACSGSFYKSMRWLTRKNGDYPIQDAQYTNNQGKNILFMWGINHPPTDTTQRDLYTRIDTTT
SVATEEINRVFKPLIGPRPLVNGLMGRIDYYWSVLKPGQTLRIKSDGNLIAPWFGHILSRESHGRILKTD
LKRGSCTVQCQTEKGGLNTTLPFQNVSKYAFGNCSKYIGIKSLKLAVGLRNVPSRSSRGLFGAIAGFIEG
GWSGLVAGWYGFQHSNDQGVGMAADRDSTQKAIDKITSKVNNIVDKMNKQYEIIDHEFSEVETRLNMINN
KIDDQIQDIWAYNAELLVLLENQKTLDEHDANVNNLYNKVKRALGSNAVEDGKGCFELYHKCDDQCMETI
RNGTYNRRKYQEESKLERQKIEGVKLESEGTYKILTIYSTVASSLVIAMGFAAFLFWAMSNGSCRCNICI

>QBK20282.1 hemagglutinin [Influenza A virus]

METVSLITILLVATVSNADKICIGYQSTNSTETVDTLTENNVPVTHAKELLHTEHNGMLCATSLGQPLIL
DTCTIEGLIYGNPSCDLSLEGREWSYIVERPSAVNGLCYPGNVENLEELRSLFSSARSYQRIQIFPDTIW
NVSFDGTSTACSGSFYKSMRWLTRKNGDYPIQDAQYTNNQGKNILFMWGINHPPTDTTQRDLYTRIDTTT
SVATEEINRVFKPLIGPRPLVNGLMGRIDYYWSVLKPGQTLRIKSDGNLIAPWFGHILSGESHGRILKTD
LKRGSCTVQCQTEKGGLNTTLPFQNVSKYAFGNCSKYIGIKSLKLAVGLRNVPSRSSRGLFGAIAGFIEG
GWSGLVAGWYGFQHSNDQGVGMAADRDSTQKAIDKITSKVNNIVDKMNKQYEIIDHEFSEVETRLNMINN
KIDDQIQDIWAYNAELLVLLENQKTLDEHDANVNNLYNKVKRALGSNAVEDGKGCFELYHKCDDQCMETI
RNGTYNRRKYQEESKLERQKIEGVKLESEGTYKILTIYSTVASSLVIAMGFAAFLFWAMSNGSCRCNICI

>QBK20281.1 hemagglutinin [Influenza A virus]

METVSLITILLVATVSNADKICIGYQSTNSTETVDTLTENNVPVTHAKELLHTEHNGMLCATSLGQPLIL
DTCTIEGLIYGNPSCDLSLEGREWSYIVERPSAVNGLCYPGNVQNLEELRSLFSSARSYQRIQIFPDTIW
NVSFDGTSTACSGSFYKSMRWLTRKNGDYPIQDAQYTNNQGKNILFMWGINHPPTDTTQRDLYTRIDTTT
SVATEEINRVFKPLIGPRPLVNGLMGRIDYYWSVLKPGQTLRIKSDGNLIAPWFGHILSRESHGRILKTD
LKRGSCTVQCQTEKGGLNTALPLQNVSKYAFGNCSKYIGIKSLKLAVGLRNVPSRYSRGLFGAIAGFIEG
GWSGLVAGWYGFQHSNDQGVGMAADRDSTQKAIDKITSKVNNIVAKMNKPYEIIDHEFSVVETRINMINN
KFDDQIQDIWAYNAESLVLLENQKTLDEQDANVNNLYNKVKRAAGSNAVEDGKGCFELCHKCDDQCMETI
GNGTYNRRKYQEESKLERQKMEGVKLESDRTYKILSIYSRVASYRVIAMGSVALLVWALYNGSYRQNSYT

>QBK20280.1 hemagglutinin [Influenza A virus]

MDTVSLITILLISTVSNADKICIGYQSTNSTETVDTLTENNVPVTHAKELLHTEHNGMLCATSLGQPLIL
DTCTIEGLIYGNPSCDLSLEGREWSYIVERPSAVNGLCYPGNVENLEELRSLFSSARSYQRIQIFPDTIW
NVSFYGTSTACSGSFYKSMRWLTRKNGDYPIQDAQYTNNQGKNILFMWGINHPPTDTTQRDLYTRIDTTT
SVATEEINRVFKPLIGPRPLVNGLMGRIDYYWSVLKPGQTLRIKSDGNLIAPWFGHILSRESHGRILKTD
LKRGSCTVQCQTEKGGLNTTLPLQNVSKYAFGNCSKYIGIKSLKLAVGLRNVPSRSSRGLFGAIAGFIEG
GWSGLVAGWYGFQHSNDQGVGMAADRDSTQKAIDKITSKVNNIVDKMNKQYEIIDHEFSEVETRINMINN
KIDDQIQDIWAYNAELLVLLENQKTLDEHDANVNNLYNKVKRALGSNAVEDGKGCFELYHKCDDQCMETI
GNGTYNRRKYQEESKLERQKIEGVKLESEGTYKILSIYSTVASSRVIAMGFAALLFWAMYNGSCRCNSSI

>QBK20279.1 hemagglutinin [Influenza A virus]

METVSLITILLVATVSNADKICIGYQSTNSTETVDTLTENNVPVTHAKELLHTEHNGMLCATSLGQPLIL
DTCTIEGLIYGNPSCDLSLEGREWSYIVERPSAVNGLCYPGNVENLEELRSLFSSARSYQRIQIFPDTIW
NVSFDGTSTACSGSFYKSMRWLTRKNGDYPIQDAQYTNNQGKNILFMWGINHPPTDTTQRDLYTRIDTTT

SVATEEINRVFKPLIGPRPLVNGLMGRIDYYWSVLKPGQTLRIKSDGNLIAPWFGHILSGESHGRILKTD
LKRGSCTVQCQTEKGGLNTTLPFQNVSKYAFGNCSKYIGIKSLKLAVGLRNVPSRYSRGLFGAIAGFIEG
GWSGLVAGWYGFQHSNDQGVGMAADRDSTQKAIDKITSKVNNIVDKMNKQYEIIDHEFSEVETRLNMINN
KIDDQIQDIWAYNAELLVLLENQKTLDEHDANVNNLYNKVKRALGSNAVEDGKGCFELYHKCDDQCMETI
GNGTYNRRKYQEESKLERQKIEGVKLESEGTYKILSIYSTVASSLVIAMGFVAFLFWAMYNGSCRCNISI

>QBK20278.1 hemagglutinin [Influenza A virus]
METVSLITILLVATVSNADKICIGYQSTNSTETVDTLTENNVPVTHAKELLHTEHNGMLCATSLGQPLIL
DTCTIEGLIYGNPSCDLSLEGREWSYIVERPSAVNGLCYPGNVENLEELRSLFSSARSYQRIQIFPDTIW
NVSYDGTSTACSGSFYKSMRWLTRKNGDYPVQDAQYTNNQGKNILFMWGINHPPTDTTQRDLYTRIDTTT
SVATEEINRIFKPLIGPRPLVNGLMGRIDYYWSVLKPGQTLRIKSDGNLIAPWYGHILSGESHGRILKTD
LKRGSCTVQCQTEKGGLNTTLPFQNVSKYAFGNCSKYIGIKSLKLAVGLRNVRSRSSRGLFGAIAGFIEG
GWSGLVAGWYGFQHSNDQGVGMAADRDSTQKAIDKITSKVNNIVDKMNKQYEIIDHEFSEVETRLNMINN
KIDDQIQDIWAYNAELLVLLENQKTLDEHDANVNNLYNKVKRALGSNAVEDGKGCFELYHKCDDQCMETI
RNGTYNRRKYQEESKLERQKIEGVKLESEGTYKILSIYSTVASSLVIAMGSAAFLFWAMSNGSCRCNICI

>QBK20277.1 hemagglutinin [Influenza A virus]
METVSLITILLVATVSNADKICIGYQSTNSTETVDTLTENNVPVTHAKELLHTEHNGMLCATSLGQPLIL
DTCTIEGLIYGNPSCDLSLEGREWSYIVERPSAVNGLCYPGNVENLEELRSLFSSARSYQRIQIFPDTIW
NVSFDGTSTACSGSFYKSMRWLTRKNGDYPIQDAQYTNNQGKNILFMWGINHPPTDTTQRDLYTRIDTTT
SVATEEINRVFKPLIGPRPLVNGLMGRIDYYWSVLKPGQTLRIKSDGNLIAPWFGHILSRESHGRILKTD
LKRGSCTVQCQTEKGGLNTTLPFQNVSKYAFGNCSKYIGIKSLKLAVGLRNVPSRSSRGLFGAIAGFIEG
GWSGLVAGWYGFQHSNDQGVGMAADRDSTQKAIDKITSKVNNIVDKMNKQYEIIDHEFSEVETRLNMINN
KIDDQIQDIWAYNAELLVLLENQKTLDEHDANVNNLYNKVKRALGSNAVEDGKGCFELYHKCDDQCMETI
RNGTYNRRKYQEESKLERQKIEGVKLESEGTYKILTIYSTVASSLVIAMGFAAFLFWAMSNGSCRCNICI

>QBK20276.1 hemagglutinin [Influenza A virus]
METVSLITILLIATVSNADKICIGYQSTNSTETVDTLTENNVPVTHAKELLHTEHNGMLCATSLGQPLIL
DTCTIEGLIYGNPSCDLSLEGREWSYIVERPSAVNGLCYPGNVENLEELRSLFSSARSYQRIQIFPDTIW
NVSFDGTSTACSGSFYKSMRWLTRKNGDYPIQDAQYTNNQGKNILFMWGINHPPTDTTQRDLYTRIDTTT
SVATEEINRVFKPLIGPRPLVNGLMGRIDYYWSVLKPGQTLRIKSDGNLIAPWFGHILSRESHGRILKTD
LKRGSCTVQCQTEKGGLNTTLPFQNVSKYAFGNCSKYIGIKSLKLAVGLRNVPSRYSRGLFGAIAGFIEG
GWSGLVAGWYGFQHSNDQGVGMAADRDSTQKAIDKITSKVNNIVDKMNKQYEIIDHEFSEVETRINMINN
KIDDQIQDIWAYNAELLVLLENQKTLDEHDANVNNLYNKVKRALGSNAVEDGKGCFELYHKCDDQCMETI

RNGTYNRRKYQEESKLERQKIEGVKLESEGTYKILSIYSTVASSRVIAMGFAAFLFWAMSNWSCRQNICI

>QBK20275.1 hemagglutinin [Influenza A virus]
METVSLITILLVATVSNADKICIGYQSTNSTETVDTLTENNVPVTHAKELLHTEHNGMLCATSLGQPLIL
DTCTIEGLIYGNPSCDLSLEGREWSYIVERPSAVNGLCYPGNVENLEELRSLFSSARSYQRIQIFPDTIW
NVSFDGTSTACSGSFYKSMRWLTRKNGDYPIQDAQYTNNQGKNILFMWGINHPPTDTTQRDLYTRIDTTT
SVATEEINRVFKPLIGPRPLVNGLMGRIDYYWSVLKPGQTLRIKSDGNLIAPWFGHILSGESHGRILKTD
LKRGSCTVQCQTEKGGLNTTLPFQNVSKYAFGNCSKYIGIKSLKLAVGLRNVPSRSSRGLFGAIAGFIEG
GWSGLVAGWYGFQHSNDQGVGMAADRDSTQKAIDKITSKVNNIVDKMNKQYEIIDHEFSEVETRLNMINN
KIDDQIQDIWAYNAELLVLLENQKTLDEHDANVNNLYNKVKRALGSNAVEDGKGCFELYHKCDDQCMETI
RNGTYNRRKYQEESKLERQKIEGVKLESEGTYKILTIYSTVASSLVIAMGFAAFLFWAMSNGSCRCNICI

>QBK20274.1 hemagglutinin [Influenza A virus]
METVSLITILLVATVSNADKICIGYQSTNSTETVDTLTENNVPVTHAKELLHTEHNGMLCATSLGQPLIL
DTCTIEGLIYGNPSCDLSLEGREWSYIVERPSAVNGLCYPGNVENLEELRSLFSSARSYQRIQIFPDTIW
NVSYYGTSTACSGSFYRSMRWLTRKNGDYPVQDAQYTNNQGKNILFMWGINHPPSDTTQRNLYTRNDTTT
SVATEEINRIFKPLIGPRPLVNGLMGRIDYYWSVLKPGQTLRIKSDGNLIAPWYGHILSRESHGRILKTD
LKRGSCTVQCQTEKGGLNTTLPFQNVSKYAFGNCSKYIGLKSLKLAVGLRNVPSRSSRGLFGAIAGFIEG
GWSGLVAGWYGFQHSNDQGVGMAADRDSTQKAIDKITSKVNNIVDKMNKQYEIIDHEFSEVETRLNMINN
KIDDQIQDIWAYNAELLVLLENQKTLDEHDANVNNLYNKVKRALGSNAVEDGKGCFELYHKCDDQCMETI
RNGTYNRRKYQEESKLERQKIEGVKLESEGTYKILSIYSTVASYLVIAMGFAAFLFWAMSNGSCRCNICI

>QBK20273.1 hemagglutinin [Influenza A virus]
METVSLITILLVATVSNADKICIGYQSTNSTETVDTLTENNVPVTHAKELLHTEHNGMLCATSLGQPLIL
DTCTIEGLIYGNPSCDLSLEGREWSYIVERPSAVNGLCYPGNVKNLEELRSLFSSARSYQRIQIFPDTIW
NVSYYGTSTACSGSFYISMRWLTRKNGDYPVQDAQYTNNQGKNILFMWGINHPPSDTTQRNLYTRNDTTT
SVATEEINRIFKPLIGPRPLVNGLMGRIDYYWSVLKPGQTLRIKSDGNLIAPWYGHILSRESHGRILKTD
LKRGSCTVQCQTEKGGLNTTLSLRNVRKYAFGKCSKYIGLKSLKLAVGLRNVPSRSSRGLFGAIAGSIEG
GWSGLVAGWYGFQHSNDQGVGMAADRDSTQKAIDKITSKVNNIVDKMNKQYEIIDHEFSEVETRLNVINN
KIDDQIQDIWAYNAELLVLLENQKTLDEHDANVNNLYNKVKRALGSNAVEDGKGCFELYHKCDDQCMETI
RNGTYNRRKYQEESKLERQKIEGVKLESEGTYKILSIYSTVASSLVIAMGFAAFLFWAMSNGSCRCNICI

>QBK20272.1 hemagglutinin [Influenza A virus]
METVSLITILLVATVSNADKICIGYQSTNSTETVDTLTENNVPVTHAKELLHTEHNGMLCATSLGQPLIL

DTCTIEGLIYGNPSCDLSLEGREWSYIVERPSAVNGLCYPGNVENLEELRSLFSSARSYQRIQIFPDTIW
NVSYDGTSTACSGSFYRSMRWLTRKNGDYPVQDAQYTNNQGKNILFMWGINHPPSDTTQRNLYTRNDTTT
SVATEEINRIFKPLIGPRPLVNGLMGRIDYYWSVLKPGQTLRIKSDGNLIAPWYGHILSGESHGRILKTD
LKRGSCTVQCQTEKGGLNTTLPFQNVSKYAFGNCSKYIGIKSLKLAVGLRNVPSRSSRGLFGAIAGFIEG
GWSGLVAGWYGFQHSNDQGVGMAADRDSTQKAIDKITSKVNNIVDKMNKQYEIIDHEFSEVETRLNMINN
KIDDQIQDIWAYNAELLVLLENQKTLDEHDANVNNLYNKVKRALGSNAVEDGKGCFELYHKCDDQCMETI
RNGTYNRRKYQEESKLERQKIEGVKLESEGTYKILTIYSTVASSLVIAMGFAAFLFWAMSNGSCRCNICI

>QBK20271.1 hemagglutinin [Influenza A virus]
METVSLITILLVATVSNADKICIGYQSTNSTETVDTLTENNVPVTHAKELLHTEHNGMLCATSLGQPLIL
DTCTIEGLIYGNPSCDLSLEGREWSYIVERPSAVNGLCYPGNVENLEELRSLFSSARSYQRIQIFPDTIW
NVSYDGTSTACSGSFYRSMRWLTRKNGDYPVQDAQYTNNQGKNILFMWGINHPPSDTTQRNLYTRNDTTT
SVATEEINRIFKPLIGPRPLVNGLMGRIDYYWSVLKPGQTLRIKSDGNLIAPWYGHILSGESHGRILKTD
LKRGSCTVQCQTEKGGLNTTLPFQNVSKYAFGNCSKYIGLKSLKLAVGLRNVPSRSSRGLFGAIAGFIEG
GWSGLVAGWYGFQHSNDQGVGMAADRDSTQKAIDKITSKVNNIVDKMNKQYEIIDHEFSEVETRLNMINN
KIDDQIQDIWAYNAELLVLLENQKTLDEHDANVNNLYNKVKRALGSNAVEDGKGCFELYHKCDDQCMETI
RNGTYNRRKYQEESKLERQKIEGVKLESEGTYKILTIYSTVASSLVIAMGFAAFLFWAMSNGSCRCNICI

>QBK20270.1 hemagglutinin [Influenza A virus]
METVLLITILLVSTVSNADKICIGYHSTNSTETVDTLTENNVPVTHAKELLHTEHNGMLCATSLGQPLIL
DTCTIEGLIYGNPSCDLSLEGREWSYIVERPSAVNGLCYPGNVENLEELRSLFSSARSYQRIQIFPDTIW
NVSYYGTSTACSGSFYITMRWLTRKNGDYPTQDAQYTNNQGKNILFMWGINHPPTDDTQRNLYTRTDTTT
SVATEDINRIFKPLIGPRPLVNGLMGRIDYYWSVLKPGQTLRIKSDGNLISPWYGHILSRESHGRILKTD
LKRGSCAVQCQTEKGGFNTALSWQNVRMYAFGQCSQYIGIKSLKLAGGLKNMSSRCSRGIFEGIARSIEG
GWSGLVAGWYGFQHSNDQGVGMAADSDSTQKAIDKITYKVNNIVDKMNKQYEIIDHEFSEVETRINVIGN
MIVDQIQDIWADNAELVVSLENQKTLDEHDANVNNLYNKVKRAVGCNAVEDGKGCFEIYHKCNDQCMETI
WNGSSNRRKYQEESKLERQKIEGVKLEFEGTYKILSIYSTVASSLVIAMGSVAFLFWAMSNGSCRCNSCI

>QBK20269.1 hemagglutinin [Influenza A virus]
METVSLITILLIATVSNADKICIGYQSTNSTETVDTLTENNVPVTHAKELLHTEHNGMLCATSLGQPLIL
DTCTIEGLIYGNPSCDLSLEGREWSYIVERPSAVNGLCYPGNVENLEELRSLFSSARSYQRIQIFPDTIW
NVSYYGTSAACSGSFYITMRWLTRKNGDYPVQDAQYTNNQGKNILFMWGINHPPSDTTQRNLYMRNDTTT
SVATEDINRIFKPLIGPRPLVNGLMGRIDYYWSVLKPGQTLRIKSDGNLISPWYGHIISRESHGRILKTD
LQSGSCTVQCQTEKGDFNTTLSWRNVSKYAFGKCSQYIGIKSLKLAVGMRNVRSRCSRGIFEAIAGSIEG

GWSGLVAGWYGFQHSNDQGVGMAADSDSTQKAIDKITSKVNNIVDKMNKQYEIIDHEFIEVETRINVISN
KIDDQIQDIWAYNAELLVSLENQKTLDEHDANVNNLYNKVKRAVGSNAVEDGKGCFEIYHKCDDQCMETI
WNGTYNRRKYQEESKLERQKIEGVKLESEGTYKILTIYSTVASSLVIAMGFAAFLFWAMSNGSCRCNICI

>QBK20268.1 hemagglutinin [Influenza A virus]
METVSLITILLIATVSNADKICIGYQSTNSTETVDTLTENNVPVTHAKELLHTEHNGMLCATSLGQPLIL
DTCTIEGLIYGNPSCDLSLEGREWSYIVERPSAVNGLCYPGNVENLEELRSLFSSARSYQRIQIFPDTIW
NVSYDGTSTACSGSFYRSMRWLTRKNGDYPVQDAQYTNNQGKNILFMWGINHPPSDTTQRNLYTRNDTTT
SVATEEINRIFKPLIGPRPLVNGLMGRIDYYWSVLKPGQTLRIKSDGNLIAPWYGHILSRESHGRILKTD
LKRGSCTVQCQTEKGGLNTTLPFQNVSKYAFGNCSKYIGIKSLKLAVGLRNVPSRSSRGLFGAIAGFIEG
GWSGLVAGWYGFQHSNDQGVGMAADRDSTQKAIDKITSKVNNIVDKMNKQYEIIDHEFSEVETRLNMINN
KIDDQIQDIWAYNAELLVLLENQKTLDEHDANVNNLYNKVKRALGSNAVEDGKGCFELYHKCDDQCMETI
RNGTYNRRKYQEESKLERQKIEGVKLESEGTYKILTIYSTVASSLVIAMGFAAFLFWAMSNGSCRCNICI

>QBK20267.1 hemagglutinin [Influenza A virus]
METVSLITILLVATVSNADKICIGYQSTNSTETVDTLTENNVPVTHAKELLHTEHNGMLCATSLGQPLIL
DTCTIEGLIYGNPSCDLSLEGREWSYIVERPSAVNGLCYPGNVENLEELRSLFSSARSYQRIQIFPDTIW
NVSYDGTSTACSGSFYRSMRWLTRKNGDYPVQDAQYTNNQGKNILFMWGINHPPSDTTQRNLYMRNDTTT
SVATEEINRIFKPLIGPRPLVNGLMGRIDYYWSVLKPGQTLRIKSDGNLIAPWYGHILSRESHGRILKTD
LKRGSCTVQCQTEKGGLNTTLPFQNVSKYAFGNCSKYIGIKSLKLAVGLRNVPSRSSRGLFGAIAGFIEG
GWSGLVAGWYGFQHSNDQGVGMAADRDSTQKAIDKITSKVNNIVDKMNKQYEIIDHEFSEVETRLNMINN
KIDDQIQDIWAYNAELLVLLENQKTLDEHDANVNNLYNKVKRALGSNAVEDGKGCFELYHKCDDQCMETI
RNGTYNRRKYQEESKLERQKIEGVKLESEGTYKILTIYSTVASSLVIAMGFAAFLFWAMSNGSCRCNICI

>QBK20266.1 hemagglutinin [Influenza A virus]
METVSLITILLVATVSNADKICIGYQSTNSTETVDTLTENNVPVTHAKELLHTEHNGMLCATSLGQPLIL
DTCTIEGLIYGNPSCDLSLEGREWSYIVERPSAVNGLCYPGNVENLEELRSLFSSARSYQRIQIFPDTIW
NVSYDGTSTACSGSFYRSMRWLTRKNGDYPVQDAQYTNNQGKNILFMWGINHPPSDTTQRNLYTRNDTTT
SVATEEINRIFKPLIGPRPLVNGLMGRIDYYWSVLKPGQTLRIKSDGNLIAPWYGHILSRESHGRILKTD
LKRGSCTVQCQTEKGGLNTTLPFQNVSKYAFGNCSKYIGIKSLKLAVGLRNVPSRSSRGLFGAIAGFIEG
GWSGLVAGWYGFQHSNDQGVGMAADRDSTQKAIDKITSKVNNIVDKMNKQYEIIDHEFSEVETRINMINN
KIDDQIQDIWAYNAELLVLLENQKTLDEHDANVNNLYNKVKRALGSNAVEDGKGCFELYHKCDDQCMETI
RNGTYNRRKYQEESKLERQKIEGVKLESEGTYKILTIYSTVASSLVIAMGFAAFLFWAMSNGSCRCNICI

>QBK20265.1 hemagglutinin [Influenza A virus]

METVSLITILLVVTVSNADKICIGYQSTNSTETVDTLTENNVPVTHAKELLHTEHNGMLCATSLGQPLIL

DTCTIEGLIYGNPSCDLSLEGREWSYIVERPSAVNGLCYPGNVENLEELRSLFSSARSYQRIQIFPDTIW

NVSYDGTSTACSGSFYRSMRWLTRKNGDYPVQDAQYTNNQGKNILFTWGINHPPSDTTQRNLYTRNDTTT

SVATEEINRIFKPLIGPRPLVNGLMGRIDYYWSVLKPGQTLRIKSDGNLIAPWYGHILSRESHGRILKTD

LKRGSCTVQCQTEKGGLNTTLPFQNVSKYAFGNCSKYIGIKSLKLAVGLRNVPSRYSRGLFGAIAGFIEG

GWSGLVAGWYGFQHSNDQGVGMAADRDSTQKAIDKITSKVNNIVDKMNKQYEIIDHEFSEVETRLNMINN

KIDDQIQDIWAYNAELLVLLENQKTLDEHDANVNNLYNKVKRALGSNAVEDGKGCFELYHKCDDQCMETI

RNGTYNRRKYQEESKLERQKIEGVKLESEGTYKILSIYSTVASSLVIAMGFAAFLFWAMSNGSCRCNICI

>QBK20264.1 hemagglutinin [Influenza A virus]

METVSLITILLAATVSNADKICIGYQSTNSTETVDTLTENNVPVTHAKELLHTEHNGMLCATSLGQPLIL

DTCTIEGLIYGNPSCDLSLEGREWSYIVERPSAVNGLCYPGNVENLEELRSLFSSARSYQRIQIFSDTIW

NVSYDGTSTACSGSFYKSMRWLTRKNGDYPTQDAQYTNNQGKNILFMWGINHPPTDTTQIDLYTRTDTTT

SVATEEINRIFKPLIGPRPLVNGLMGRIDYYWSVLKPGQTLRIKSDGNLIAPWYGHILSGESHGRILKTD

LKRGSCTVQCQTEKGGLNTTLPFQNVSKYAFGNCSKYIGIKSLKLAVGLRNVPSRSSRGLFGAIAGFIEG

GWSGLVAGWYGFQHSNNQGVGMAADRDSTQKAIDKITSKVNNIVDKMNKQYEIIDHEFNEVETRLNMINN

KIDDQIQDIWAYNAELLVLLENQKTLDEHDANVNNLYNKVKRALGSNAVEDGKGCFELYHKCDDQCMETI

RNGTYNRRKYQEESKLERQKIEGVKLESEGTYKILTIYSTVASSLVIAMGFAAFLFWAMSNGSCRCNICI

>QBK20263.1 hemagglutinin [Influenza A virus]

METVPLITILLVATVSNADKICIGYQSTNSTETVDTLTENNVPVTHAKELLHTEHNGMLCATSLGHPLIL

DTCTIEGLIYGNPSCDPLLGGREWSYIVERPSAVNGLCYPGNVENLEELRSLFSSARSYQRIQIFPDTIW

NVSYSGTSKACSDSFYRSMRWLTQKNNAYPIQDAQYTNNQEKNILFMWGINHPPTETAQTNLYTRTDTTT

SVATEEINRIFKPLIGPRPLVNGLMGRINYYWSVLKPGQTLRIKSDGNLIAPWYGHILSRESHGRILMTD

LKRGSCTVQRQTEKGGLNTTLPFQNVSKYAFGKCSKYIGIKSLKLAVGLRNVRSRCSRGLFGTIAGFIEG

GWSGLVAGWYGFQHSNDQGVGMAADRDSTQKAIDKITSKVNNIVDKMNRQYEIIDHEFSEVETRLNMINN

KVDDQIQDIWAYNAELLVLLENQKTLDEHDSNVNNLYNKVKRALGSNAVEDGKGCFELYHKCDDQCMETI

RNGTYNRRKYQEESKLERQKIEGVKLESEGTYKILSIYSTVASSLVIAMGFVAFLFWAMSNGSCRCNICI

>QBK20262.1 hemagglutinin [Influenza A virus]

METVSLITILLVVTVSNADKICIGYQSTNSTETVDTLTENNVPVTHAKELLHTEHNGMLCATSLGQPLIL

DTCTIEGLIYGNPSCDLSLEGREWSYIVERPSAVNGLCYPGNVENLEELRSLFSSARSYQRIQIFPDTIW

NVSYDGTSTACSGSFYRSMRWLTRKNGDYPVQDAQYTNNQGKNILFTWGINHPPSDTTQRNLYTRNDTTT

SVATEEINRIFKPLIGPRPLVNGLMGRIDYYWSVLKPGQTLRIKSDGNLIAPWYGHILSRESHGRILTTD
LKRGSCTVQCQTEKGGLNTTLPFQNVSKYAFGNCSKYIGIKSLKLAVGLRNVPSRSSRGLFGAIAGFIEG
GWSGLVAGWYGFQHSNDQGVGMAADRDSTQKAIDKITSKVNNIVDKMNKQYEIIDHEFSEVETRLNMINN
KIDDQIQDIWAYNAELLVLLENQKTLDEHDANVNNLYNKVKRALGSNAVEDGKGCFELYHKCDDQCMETI
RNGTYNRRKYQEESKLERQKIEGVKLESEGTYKILTIYSTVASSLVIAMGFAAFLFWAMSNGSCRCNICI

>QBK20261.1 hemagglutinin [Influenza A virus]
METVSLITILLVVTVSNADKICIGYQSTNSTETVDTLTENNVPVTHAKELLHTEHNGMLCATSLGQPLIL
DTCTIEGLIYGNPSCDLSLEGREWSYIVERPSAVNGLCYPGNVENLEELRSLFSSARSYQRIQIFPDTIW
NVSYDGTSTACSGSFYRSMRWLTRKNGDYPVQDAQYTNNQGKNILFTWGINHPPSDTTQRNLYTRNDTTT
SVATEEINRIFKPLIGPRPLVNGLMGRIDYYWSVLKPGQTLRIKSDGNLIAPWYGHILSRESHGRILKTD
LKRGSCTVQCQTEKGGLNTTLPFQNVSKYAFGNCSKYIGIKSLKLAVGLRNVPSRSSRGLFGAIAGFIEG
GWSGLVAGWYGFQHSNDQGVGMAADRDSTQKAIDKITSKVNNIVDKMNKQYEIIDHEFSEVETRLNMINN
KIDDQIQDIWAYNAELLVLLENQKTLDEHDANVNNLYNKVKRALGSNAVEDGKGCFELYHKCDDQCMETI
RNGTYNRRKYQEESKLERQKIEGVKLESEGTYKILTIYSTVASSLVIAMGFAAFLFWAMSNGSCRCNICI

>QBK20260.1 hemagglutinin [Influenza A virus]
METVSLITILLVVTVSNADKICIGYQSTNSTETVDTLTENNVPVTHAKELLHTEHNGMLCATSLGQPLIL
DTCTIEGLIYGNPSCDLSLEGREWSYIVERPSAVNGLCYPGNVENLEELRSLFSSARSYQRIQIFPDTIW
NVSYDGTSTACSGSFYRSMRWLTRKNGDYPVQDAQYTNNQGKNILFTWGINHPPSDTTQRNLYTRNDTTT
SVATEEINRIFKPLIGPRPLVNGLMGRIDYYWSVLKPGQTLRIKSDGNLIAPWYGHILSRESHGRILKTD
LKRGSCTVQCQTEKGGLNTTLPFQNVSKYAFGNCSKYIGIKSLKLAVGLRNVPSRSSRGLFGAIAGFIEG
GWSGLVAGWYGFQHSNDQGVGMAADRDSTQKAIDKITSKVNNIVDKMNKQYEIIDHEFSEVETRLNMINN
KIDDQIQDIWAYNAELLVLLENQKTLDEHDANVNNLYNKVKRALGSNAVEDGKGCFELYHKCDDQCMETI
RNGTYNRRKYQEESKLERQKIEGVKLESEGTYKILTIYSTVASSLVIAMGFAAFLFWAMSNGSCRCNICI

>QBK20259.1 hemagglutinin [Influenza A virus]
METVSLITILLVVTVSNADKICIGYQSTNSTETVDTLTENNVPVTHAKELLHTEHNGMLCATSLGQPLIL
DTCTIEGLIYGNPSCDLSLEGREWSYIVERPSAVNGLCYPGNVENLEELRSLFSSARSYQRIQIFPDTIW
NVSYDGTSTACSGSFYRSMRWLTRKNGDYPVQDAQYTNNQGKNILFTWGINHPPSDTTQRNLYTRNDTTT
SVATEEINRIFKPLIGPRPLVNGLMGRIDYYWSVLKPGQTLRIKSDGNLIAPWYGHILSRESHGRILKTD
LKRGSCTVQCQTEKGGLNTTLPFQNVSKYAFGNCSKYIGIKSLKLAVGLRNVPSRSSRGLFGAIAGFIEG
GWSGLVAGWYGFQHSNDQGVGMAADRDSTQKAIDKITSKVNNIVDKMNKQYEIIDHEFSEVETRLNMINN
KIDDQIQDIWAYNAELLVLLENQKTLDEHDANVNNLYNKVKRALGSNAVEDGKGCFELYHKCDDQCMETI

RNGTYNRRKYQEESKLERQKIEGVKLESEGTYKILSIYSTVASSLVIAMGFAAFLFWAMSNGSCRCNICI

>QBK20258.1 hemagglutinin [Influenza A virus]
METVSLITILLVATVSNADKICIGYQSTNSTETVDTLTENNVPVTHAKELLQTEHNGMLCATSLGQPLIL
DTCTIEGLIYGNPSCDLSLEGREWSYIVERPSAVNGLCYPGNVENLEELRSLFSSARSYQRIQIFPDTIW
NVSYDGTSTACSGSFYKSMRWLTRKNGDYPIQDAQYTNNQGKNILFMWGINHPPTDTTQRDLYTRTDTTT
SVATEEINRVFKPLIGPRPLVNGLMGRIDYYWSVLKPGQTLRIKSDGNLIAPWFGHILSGESHGRILKTD
LKRGSCTVQCQTEKGGLNTTLPFQNVSKYAFGNCSKYIGIKSLKLAVGLRNVPSRSSRGLFGAIAGFIEG
GWSGLVAGWYGFQHSNDQGVGMAADRDSTQKAIDKITSKVNNIVDKMNKQYEIIDHEFSEVETRLNMINN
KIDDQIQDIWAYNAELLVLLENQKTLDEHDANVNNLYNKVRRALGSNAVEDGKGCFELYHKCDDQCMETI
RNGTYNRRKYQEESKLGRQKIEGVKLESEGTYKILTIYSTVASSLVIAMGFAAFLFWAMSNGSCRCNICI

>QBK20257.1 hemagglutinin [Influenza A virus]
METVSLITILLVATVSNADKICIGYQSTNSTETVDTLTENNVPVTHAKELLQTEHNGMLCATSLGQPLIL
DTCTIEGLIYGNPSCDLSLEGREWSYIVERPSAVNGLCYPGNVENLEELRSLFSSARSYQRIQIFPDTIW
NVSYDGTSTACSGSFYKSMRWLTRKNGDYPIQDAQYTNNQGKNILFMWGINHPPTDTTQRDLYTRTDTTT
SVATEEINRVFKPLIGPRPLVNGLMGRIDYYWSVLKPGQTLRIKSDGNLIAPWFGHILSRESHGRILKTD
LKRGSCTVQCQTEKGGLNTTLPFQNVSKYAFGNCSKYIGIKSLKLAVGLRNVPSRSSRGLFGAIAGFIEG
GWSGLVAGWYGFQHSNDQGVGMAADRDSTQKAIDKITSKVNNIVDKMNKQYEIIDHEFSEVETRLNMINN
KIDDQIQDIWAYNAELLVLLENQKTLDEHDANVNNLYNKVRRALGSNAVEDGKGCFELYHKCDDQCMETI
RNGTYNRRKYQEESKLGRQKIEGVKLESEGTYKILTIYSTVASSLVIAMGFAAFLFWAMSNGSCRCNICI

>QBK20256.1 hemagglutinin [Influenza A virus]
METVSLITILLIATVSNADKICIGYQSTNSTETVDTLTENNVPVTHAKELLHTEHNGMLCATSLGQPLIL
DTCTIEGLIYGNPSCDLSLEGREWSYIVERPSAVNGLCYPGNVENLEELRSLFSSARSYQRIQIFPDTIW
NVSYYGTSTACSGSFYRSMRWLTRKNGDYPVQDAQYTNNQGKNILFMWGINHPPSDTTQRNLYTRNDTTT
SVATEEINRIFKPLIGPRPLVNGLMGRIDYYWSVLKPGQTLRIKSDGNLIAPWYGHILSRESHGRILKTD
LKRGSCSMRCQTENVGFHTALSLQNVSKYAFGNCSKYIGIKSLKLAVGLRNVPSRYSRGLFGAIAGFIEG
GWSGLVAGWYGFQHSNDQGVGMAADRDSTQKAIDKITSKVNNIVDKMNKQYEIIDHEFSEVETRINMINN
KIDDQIQDIWAYNAELLVLLENQKTLDEHDANVNNLYNKVKRALGSNAVEDGKGCFELYHKCDDQCMETI
RNGTYNRRKYQAESKLERQKIEGVKLESEGTYKILSIYSTVTSYLVIAMGFVAFLFWAMSNGSCRCEQCI

>QBK20255.1 hemagglutinin [Influenza A virus]
METVSLITILLVATVSNADKICIGYQSTNSTETVDTLTENNVPVTHAKELLHTEHNGMLCATSLGQPLIL

DACTIEGLIYGNPSCDLSLEGREWSYIVERPSAVNGLCYPGNVENLEELRSLFSSARSYQRIQIFPDTIW
NVSYDGTSTACSGSFYRSMRWLTRKNGEYPIQDAQYTNNQGKNILFMWGINHPPTDTTQRDLYTRTDTTT
SVATEEINRVFKPLIGPRPLVNGLMGRIDYYWSVLKPGQTLRIKSDGNLIAPWFGHILSRESHGRILKTD
LKRGSCTVQCQTEKGGLNTTLPCQNVSKYAFGNCSKYIGIKSLKLAVGLRNVPSRSSRGLFGAIAGFIEG
GWSGLVAGWYGFQHSNDQGVGMAADRDSTQKAIDKITSKVNNIVDKMNKQYEIIDHEFSEVEARLNMINN
KIDDQIQDIWAYNAELLVLLENQKTLDEHDANVNNLYNKVKRALGSNAVEDGKGCFELYHKCDDQCMETI
RNGTYNRRKYQEESKLERQKIEGVKLESEGTYKILTIYSTVASSLVIAMGFAAFLFWAMSNGSCRCNICI

>QBK20254.1 hemagglutinin [Influenza A virus]
METVPLITILLVATVSNADKICIGYQSTNSTETVDTLTENNVPVTHAKELLHTEHNGMLCATSLGHPLIL
DTCTIEGLIYGNPSCDPLLGGREWSYIVERPSAVNGLCYPGNVENLEELRSLFSSARSYQRIQIFPDTIW
NVSYSGTSKACSDSFYRSMRWLTQKNNAYPIQDAQYTNNQEKNILFMWGINHPPTETAQTNLYTRTDTTT
SVATEEINRIFKPLIGPRPLVNGLMGRINYYWSVLKPGQTLRIKSDGNLIAPWYGHILSGESHGRILKTD
LKRGSCTVQCQTEKGGLNTTLPFQNVSKYAFGNCSKYIGIKSLKLAVGLRNVPSRSSRGLFGAIAGFIEG
GWSGLVAGWYGFQHSNDQGVGMAADRDSTQKAIDKITSKVNNIVDKMNRQYEIIDHEFSEVETRLNMINN
KVDDQIQDIWAYNAELLVLLENQKTLDEHDSNVNNLYNKVKRALGSNAVEDGKGCFELYHKCDDQCMETI
RNGTYNRRKYQEESKLERQKIEGVKLESEGTYKILSIYSTVASSLVIAMGFAAFLFWAMSNGSCRCNICI

>QBK20253.1 hemagglutinin [Influenza A virus]
METVSLITILLIATVSNADKICIGYQSTNSTETVDTLTENNVPVTHAKELLHTEHNGMLCATSLGQPLIL
DTCTIEGLIYGNPSCDLSLEGREWSYIVERPSAVNGLCYPGNVQNLEELRSLFSSARSYQRIQIFPDTIW
NVSYDGTSTACSGSFYRSMRWLTRKNGDYPVQDAQYTNNQGKNILFMWGINHPPSDTTQRNLYTRNDTTT
SVATEEINRIFKPLIGPRPLVNGLMGRIDYYWSVLKPGQTLRIKSDGNLIAPWYGHILSRESHGRILKTD
LKRGSCTVQCQTEKGGLNTTLPFQNVSKYAFGNCSKYIGIKSLKLAVGLRNVRSRYSRGLFGAIAGFIER
GWSGLVAGWYGFQHSNDQGVGMAADRDSTQKAIDKITSKVNNIVDKMNKQYEIIDHEFSEVETRLNMINN
KIDDQIQDIWAYNAELLVLLENQKTLDEHDANVNNLYNKVKRALGSNAVEDGKGCFELYHKCDDQCMETI
RNGTYNRRKYQEESKLERQKIEGVKLESEGTYKILSIYSTVASSLVIAMGSAAFLFWAMSNGSCRCEQCI

>QBK20252.1 hemagglutinin [Influenza A virus]
METVSLITILLVATVSNADKICIGYQSTNSTETVDTLTENNVPVTHAKELLHTEHNGMLCATSLGQPLIL
DACTIEGLIYGNPSCDLSLEGREWSYIVERPSAVNGLCYPGNVENLEELRSLFSSARSYQRIQIFPDTIW
NVSYDGTSTACSGSFYRSMRWLTRKNGEYPIQDAQYTNNQGKNILFMWGINHPPTDTTQRDLYTRTDTTT
SVATEEINRVFKPLIGPRPLVNGLMGRIDYYWSVLKPGQTLRIKSDGNLIAPWFGHILSRESHGRILKTD
LKRGSCTVQCQTEKGGLNTTLPFQNVSKYAFGNCSKYIGIKSLKLAVGLRNVPSRSSRGLFGAIAGFIEG

GWSGLVAGWYGFQHSNDQGVGMAADRDSTQKAIDKITSKVNNIVDKMNKQYEIIDHEFSEVEARLNMINN
KIDDQIQDIWAYNAELLVLLENQKTLDEHDANVNNLYNKVKRALGSNAVEDGKGCFELYHKCDDQCMETI
RNGTYNRRKYQEESKLERQKIEGVKLESEGTYKILTIYSTVASSLVIAMGFAAFLFWAMSNGSCRCNICI

>QBK20251.1 hemagglutinin [Influenza A virus]
MDTVLLITILLIATVSNADKICIGYQSTNSTETVDTLTENNVPVTHAKELLHTEHNGMLCATSLGQPLIL
DACTIEGLIYGNPSCDLSLEGREWSYIVERPSAVNGLCYPGNVENLEELRSLFSSARSYQRIQIFPDTIW
NVSYYGTSTACSGSFYRSMRWLTRKNGEYPIQDAQYTNNQGKNILFMWGINHPPTDTTQRDLYTRTDTTT
SVATEEINRVFKPLIGPRPLVNGLMGRIDYYWSVLKPGQTLRIKSDGNLIAPWFGHILSRESHGRILKTD
LKRGSCTVQCQTEKGGLHTTLPLQNVSKYAFGKCSKYIGIKSLKLAVGLRNVPSRSSRGLFGAIAGFIEG
GWSGLVAGWYGFQHSNDQGVGMAADRDSTQKAIDKITSKVNNIVAKMNKQYEIIDHEFSEVEARINMINN
KIDDQIQDIWAYNAELIVLLENQKTLDEHDANVNNLYNKVKRALGSNAVEDGKGCFELYHKCDDQCMETI
GNGTYNRRKYQEESKLERQKIEGVKLESEGTYKILSIYSTVASSLVIAMGFAAFLFWAMSNWSCRQNSCI

>QBK20250.1 hemagglutinin [Influenza A virus]
METVSLITILLVATVSNADKICIGYQSTNSTETVDTLTENNVPVTHAKELLHTEHNGMLCATSLGQPLIL
DTCTIEGLIYGNPSCDLSLEGREWSYIVERPSAVNGLCYPGNVENLEELRSLFSSARSYQRIQIFPDTIW
NVSYDGTSTACSGSFYRSMRWLTRKNGDYPVQDAQYTNNQGKNILFMWGINHPPSDTTQRNLYTRNDTTT
SVATEEINRIFKPLIGPRPLVNGLMGRIDYYWSVLKPGQTLRIKSDGNLIAPWYGHILSRESHGRILKTD
LKRGSCTVQCQTEKGGLHTTLPFQNVSKYAFGKCSKYIGIKSLKLAVGLRNVPSRSSRGLFGAIAGFIEG
GWSGLVAGWYGFQHSNDQGVGMAADRDSTQKAIDKITSKVNNIVDKMYKQYEIIDHEFSEVETRLNMINN
KIDDQIQDIWAYNAELLVLLENQKTLDEHDANVNNLYNKVKRALGSNAVEDGKGCFELYHKCDDQCMETI
RNGTYNRRKYQEESKLERQKIEGVKLESEGTYKILSIYSTVASSLVIAMGSAAFLFWAMSNGSCRCNICI

>QBK20249.1 hemagglutinin [Influenza A virus]
METVLLITILLVATVSNADKICIGYQSTNSTETVDTLTENNVPVTHAKELLHTEHNGMLCATSLGHPLIL
DTCTIEGLIYGNPSCDPLLGGREWSYIVERPSAVNGLCYPGNVENLEELRSLFSSARSYQRIQIFPDTIW
NVSYSGTSKACSDSFYRSMRWLTQKNNAYPIQDAQYTNNQEKNILFMWGINHPPTETAQTNLYTRTDTTT
SVATEEINRIFKPLIGPRPLVNGLMGRINYYWSVLKPGQTLRIKSDGNLIAPWYGHILSRESHGRILKTD
LKRGSCTVQCQTEKGGLNTTLPLQNVSKYAFGKCSKYIGIKSLKLAVGMRNVRSRCSRGLFGAIAGFIEG
GWSGLVAGWYGFQHSNDQGVGMAADRDSTQKAIDKITSKVNNIVDKMNRQYEIIDHEFSEVETRLNMINN
KVDDQIQDIWAYNAELLVMLENQKTLDEHDSNVNNLYNKVKRALGSNAVEDGKGCFELYHKCDDQCMETI
RNGTYNRRKYQEESKLERQKIEGVKLEFEGTYKILTIYSTVASSLVIAMGFAAFLFWAMSNGSCRCNICI

>QBK20248.1 hemagglutinin [Influenza A virus]

METVSLITILLVATVSNADKICIGYQSTNSTETVDTLTENNVPVTHAKELLHTEHNGMLCATSLGQPLIL

DTCTIEGLIYGNPSCDLSLEGREWSYIVERPSAVNGLCYPGNVENLEELRSLFSSARSYQRIQIFPDTIW

NVSYDGTSTACSGSFYRSMRWLTRKNGDYPVQDAQYTNNQGKNILFMWGINHPPSDTTQRNLYTRNDTTT

SVATEEINRIFKPLIGPRPLVNGLMGRIDYYWSVLKPGQTLRIKSDGNLIAPWYGHILSGESHGRILKTD

LKRGSCTVQCQTEKGGLNTTLPFQNVSKYAFGNCSKYIGIKSLKLAVGLRNVRSRSSRGLFGAIAGFIER

GWSGLVAGWYGFQHSNDQGVGMAADRDSTQKAIDKITSKVNNIVDKMNKQYEIIDHEFSEVETRLNMINN

KIDDQIQDIWAYNAELLVLLENQKTLDEHDANVNNLYNKVKRALGSNAVEDGKGCFELYHKCDDQCMETI

RNGTYNRRKYQEESKLERQKIEGVKLESEGTYKILSIYSTVASSLVIAMGFAAFLFWAMSNGSCRCNICI

>QBK20247.1 hemagglutinin [Influenza A virus]

METVSLITILLVATVSNADKICIGYQSTNSTETVDTLTENNVPVTHAKELLHTEHNGMLCATSLGQPLIL

DACTIEGLIYGNPSCDLSLEGREWSYIVERPSAVNGLCYPGNVENLEELRSLFSSARSYQRIQIFPDTIW

NVSYDGTSTACSGSFYRSMRWLTRKNGEYPIQDAQYTNNQGKNILFMWGINHPPTDTTQRDLYTRTDTTT

SVATEEINRVFKPLIGPRPLVNGLMGRIDYYWSVLKPGQTLRIKSDGNLIAPWFGHILSRESHGRILKTD

LKRGSCTVQCQTEKGGLNTTLPFQNVSKYAFGNCSKYIGIKSLKLAVGLRNVPSRSSRGLFGAIAGFIEG

GWSGLVAGWYGFQHSNDQGVGMAADRDSTQKAIDKITSKVNNIVDKMNKQYEIIDHEFSEVEARLNMINN

KIDDQIQDIWAYNAELLVLLENQKTLDEHDANVNNLYNKVKRALGSNAVEDGKGCFELYHKCDDQCMETI

RNGTYNRRKYQEESKLERQKIEGVKLESEGTYKILTIYSTVASSLVIAMGFAAFLFWAMSNGSCRCNICI

>QBK20246.1 hemagglutinin [Influenza A virus]

METVSLITILLVATVSNADKICIGYQSTNSTETVDTLTENNVPVTHAKELLHTEHNGMLCATSLGQPLIL

DACTIEGLIYGNPSCDLSLEGREWSYIVERPSAVNGLCYPGNVENLEELRSLFSSARSYQRIQIFPDTIW

NVSYDGTSTACSGSFYRSMRWLTRKNGEYPIQDAQYTNNQGKNILFMWGINHPPTDTTQRDLYTRTDTTT

SVATEEINRVFKPLIGPRPLVNGLMGRIDYYWSVLKPGQTLRIKSDGNLIAPWFGHILSRESHGRILKTD

LKRGSCTVQCQTEKGGLNTTLPFQNVSKYAFGNCSKYIGIKSLKLAVGLRNVPSRSSRGLFGAIAGFIEG

GWSGLVAGWYGFQHSNDQGVGMAADRDSTQKAIDKITSKVNNIVDKMNKQYEIIDHEFSEVEARLNMINN

KIDDQIQDIWAYNAELLVLLENQKTLDEHDANVNNLYNKVKRALGSNAVEDGKGCFELYHKCDDQCMETI

RNGTYNRRKYQEESKLERQKIEGVKLESEGTYKILTIYSTVASSLVIAMGFAAFLFWAMSNGSCRCNICI

>QBK20245.1 hemagglutinin [Influenza A virus]

METVSLITILLVATVSNADKICIGYQSTNSTETVDTLTENNVPVTHAKELLHTEHNGMLCATSLGQPLIL

DACTIEGLIYGNPSCDLSLEGREWSYIVERPSAVNGLCYPGNVENLEELRSLFSSARSYQRIQIFPDTIW

NVSYDGTSTACSGSFYRSMRWLTRKNGEYPIQDAQYTNNQGKNILFMWGINHPPTDTTQRDLYTRTDTTT

SVATEEINRVFKPLIGPRPLVNGLMGRIDYYWSVLKPGQTLRIKSDGNLIAPWFGHILSRESHGRILKTD
LKRGSCTVQCQTEKGGLNTTLPFQNVSKYAFGNCSKYIGIKSLKLAVGLRNVPSRSSRGLFGAIAGFIEG
GWSGLVAGWYGFQHSNDQGVGMAADRDSTQKAIDKITSKVNNIVDKMNKQYEIIDHEFSEVEARLNMINN
KIDDQIQDIWAYNAELLVLLENQKTLDEHDANVNNLYNKVKRALGSNAVEDGKGCFELYHKCDDQCMETI
RNGTYNRRKYQEESKLERQKIEGVKLESEGTYKILSIYSTVASSLVIAMGFAAFLFWAMSNGSCRCNICI

>QBK20244.1 hemagglutinin [Influenza A virus]
METVSLITILLVATVSNADKICIGYQSTNSTETVDTLTENNVPVTHAKELLHTEHNGMLCATSLGHPLIL
DTCTIEGLIYGNPSCDPLLGGREWSYIVERPSAVNGLCYPGNVENLEELRSLFSSARSYQRIQIFPDTIW
NVSYSGTSKACSDSFYRSMRWLTQKNNAYPIQDAQYTNNQEKNILFMWGINHPPTETVQTNLYTRTDTTT
SVATEEINRIFKPLIGPRPLVNGLMGRINYYWSVLKPGQTLRIKSDGNLIAPWYGHILSGESHGRLLKTD
LKRGSCTVQCQTEKGGLNTTLPFQNVSKYAFGNCSKYIGIKSLKLAVGLRNVPSRSSRGLFGAIAGFIEG
GWSGLVAGWYGFQHSNDQGVGMAADRDSTQKAIDKITSKVNNIVDKMNRQYEIIDHEFSEVETRLNMINN
KVDDQIQDIWAYNAELLVLLENQKTLDEHDSNVNNLYNKVKRALGSNAVEDGKGCFELYHKCDDQCMETI
RNGTYNRRKYQEESKLERQKIEGVKLESEGTYKILTIYSTVASSLVIAMGFAAFLFWAMSNGSCRCNICI

>QBK20243.1 hemagglutinin [Influenza A virus]
METVSLITILLVATVSNADKICIGYQSTNSTETVDTLTENNVPVTHAKELLHTEHNGMLCATSLGQPLIL
DTCTIEGLIYGNPSCDLSLEGREWSYIVERPSAVNGLCYPGNVENLEELRSLFSSARSYQRIQIFPDTIW
NVSYDGTSTACSGSFYRSMRWLTRKNGDYPVQDAQYTNNQGKNILFMWGINHPPSDTTQRNLYTRNDTTT
SVATEEINRIFKPLIGPRPLVNGLMGRIDYYWSVLKPGQTLRIKSDGNLIAPWYGHILSRESHGRILTTD
LKRGSCTVQCQTEKGGLNTTLPFQNVSKYAFGNCSKYIGIKSLKLAVGLRNVPSRSSRGLFGAIAGFIEG
GWSGLVAGWYGFQHSNDQGVGMAADRDSTQKAIDKITSKVNNIVDKMNKQYEIIDHEFSEVETRLNMINN
KIDDQIQDIWAYNAELLVLLENQKTLDEHDANVNNLYNKVKRALGSNAVEDGKGCFELYHKCDDQCMETI
RNGTYNRRKYQEESKLERQKIEGVKLESEGTYKILSIYSTVASSLVIAMGFAAFLFWAMSNGSCRCNICI

>QBK20242.1 hemagglutinin [Influenza A virus]
METVPLITILLVATVSNADKICIGYQSTNSTETVDTLTENNVPVTHAKELLHTEHNGMLCATSLGHPLIL
DTCTIEGLIYGNPSCDPLLGGREWSYIVERPSAVNGLCYPGNVENLEELRSLFSSARSYQRIQIFPDTIW
NVSYSGTSKACSDSFYRSMRWLTQKNNAYPIQDAQYTNNQEKNILFMWGINHPPTETVQTNLYTRTDTTT
SVATEEINRIFKPLIGPRPLVNGLMGRINYYWSVLKPGQTLRIKSDGNLIAPWYGHILSGESHGRILKTD
LKRGSCTVQCQTEKGGLNTTLPFQNVSKYAFGNCSKYIGIKSLKLAVGLRNVPSRSSRGLFGAIAGFIEG
GWSGLVAGWYGFQHSNDQGVGMAADRDSTQKAIDKITSKVNNIVDKMNRQYEIIDHEFSEVETRLNMINN
KVDDQIQDIWAYNAELLVLLENQKTLDEHDSNVNNLYNKVKRALGSNAVEDGKGCFELYHKCDDQCMETI

RNGTYNRRKYQEESKLERQKIEGVKLESEGTYKILTIYSTVASSLVIAMGFAAFLFWAMSNGSCRCNICI

>QBK20241.1 hemagglutinin [Influenza A virus]

METVSLITILLIATVSNADKICIGYQSTNSTETVDTLTENNVPVTHAKELLHTEHNGMLCATSLGQPLIL
DACTIEGLIYGNPSCDLSLEGREWSYIVERPSAVNGLCYPGNVENLEELRSLFSSARSYQRIQIFPDTIW
NVSYDGTSTACSGSFYRSMRWLTRKNGEYPIQDAQYTNNQGKNILFMWGINHPPTDTTQRDLYTRTDTTT
SVATEEINRVFKPLIGPRPLVNGLMGRIDYYWSVLKPGQTLRIKSDGNLIAPWFGHILSRESHGRILKTD
LKRGSCTVQCQTEKGGLNTTLPFQNVSKYAFGKCSKYIGIKSLKLAVGLRNVPSRSSRGLFGAIAGFIEG
GWSGLVAGWYGFQHSNDQGVGMAADRDSTQKAIDKITSKVNNIVDKMNKQYEIIDHEFSEVEARINMINN
KIDDQIQDIWAYNAELLVLLENQKTLDEHDANVNNLYNKVKRALGSNAVEDGKGCFELYHKCDDQCMETI
RNGTYNRRKYQEESKLERQKIEGVKLESEGTYKILSIYSTVASSLVIAMGFAAFLFWAMSNGSCRCNICI

>QBK20240.1 hemagglutinin [Influenza A virus]

METVPLITILLIATVSNADKICIGYQSTNSTETVDTLTENNVPVTHAKELLHTEHNGMLCATSLGHPLIL
DTCTIEGLIYGNPSCDPLLGGREWSYIVERPSAVNGLCYPGNVENLEELRSLFSSARSYQRIQIFPDTIW
NVSYSGTSKACSDSFYRSMRWLTQKNNAYPIQDAQYTNNQEKNILFMWGINHPPTETAQTNLYTRTDTTT
SVATEEINRIFKPLIGPRPLVNGLMGRINYYWSVLKPGQTLRIKSDGNLIAPWYGHILSGESHGRILKTD
LKRGSCTVQCQTEKGGLNTTLPFQNVSKYAFGNCSKYIGIKSLKLAVGLRNVPSRSSRGLFGAIAGFIEG
GWSGLVAGWYGFQHSNDQGVGMAADRDSTQKAIDKITSKVNNIVDKMNRQYEIIDHEFSEVETRLNMINN
KVDDQIQDIWAYNAELLVLLENQKTLDEHDSNVNNLYNKVKRALGSNAVEDGKGCFELYHKCDDQCMETI
RNGTYNRRKYQEESKLERQKIEGVKLESEGTYKILTIYSTVASSLVIAMGFAAFLFWAMSNGSCRCNICI

>QBK20239.1 hemagglutinin [Influenza A virus]

METVSLITILLIATVSNADKICIGYQSTNSAETVDTLTENNVPVTHAKELLHTEHNGMLCATSLGQPLIL
DTCTIEGLIYGNPSCDLSLEGREWSYIVERPSAVNGLCYPGNVQNLEELRSLFSSARSYQRIQIFPDTIW
NVSYYGTSTACSGSFYRSMRWLTRKNGDYPVQDAQYTNNQGKNILFMWGINHPPSDTTQRNLYTRNDTTT
SVATEEINRIFKPLIGPRPLVNGLMGRIDYYWSVLKPGQTLRIKSDGNLIAPWYGHILSRESHGRILTTD
LKRGSCTVQCQTEKGGLNTALPFQNVSKYAFGKCSKYIGIKSLKLAVGLRNVRCRYSRGLFGAIAGFIER
GWSGLVAGWYGFQHSNDQGVGMAADRDSTQKAIDKITSKVNNIVAKMYKPYEIIDHEFSEVGTRINTSNN
KIDDQIQDIWAYNAESLVLLENQKTLDEHDANVNNLYNKVKRALGSNAVEDGKGCFEQYHKCDDQCMETI
RNGTYNRRKYQEESKLERQKIEGVKLESEGTYKIVSIYSRVASYRVIAMGSAALLFGALSNWSCRRNSCI

>QBK20238.1 hemagglutinin [Influenza A virus]

METVSLITILLVATVSNADKICIGYQSTNSTETVDTLTENNVPVTHAKELLHTEHNGMLCATSLGQPLIL

DACTIEGLIYGNPSCDLSLEGREWSYIVERPSAVNGLCYPGNVENLEELRSLFSSARSYQRIQIFPDTIW
NVSYDGTSTACSGSFYRSMRWLTRKNGEYPIQDAQYTNNQGKNILFMWGINHPPTDTTQRDLYTRTDTTT
SVATEEINRVFKPLIGPRPLVNGLMGRIDYYWSVLKPGQTLRIKSDGNLIAPWFGHILSRESHGRILTTD
LKRGSCTVQCQTEKGGLNTTLPFQNVSKYAFGNCSKYIGIKSLKLAVGLRNVPSRSSRGLFGAIAGFIEG
GWSGLVAGWYGFQHSNDQGVGMAADRDSTQKAIDKITSKVNNIVDKMNKQYEIIDHEFSEVEARLNMINN
KIDDQIQDIWAYNAELLVLLENQKTLDEHDANVNNLYNKVKRALGSNAVEDGKGCFELYHKCDDQCMETI
RNGTYNRRKYQEESKLERQKIEGVKLESEGTYKILTIYSTVASSLVIAMGFAAFLFWAMSNGSCRCNICI

>QBK20237.1 hemagglutinin [Influenza A virus]
METVSLITILLVATVSNADKICIGYQSTNSTETVDTLTENNVPVTHAKELLHTEHNGMLCATSLGQPLIL
DACTIEGLIYGNPSCDLSLEGREWSYIVERPSAVNGLCYPGNVENLEELRSLFSSARSYQRIQIFPDTIW
NVSYDGTSTACSGSFYRSMRWLTRKNGEYPIQDAQYTNNQGKNILFMWGINHPPTDTTQRDLYTRTDTTT
SVATEEINRVFKPLIGPRPLVNGLMGRIDYYWSVLKPGQTLRIKSDGNLIAPWFGHILSGESHGRILKTD
LKRGSCTVQCQTEKGGLNTTLPFQNVSKYAFGNCSKYIGIKSLKLAVGLRNVPSRSSRGLFGAIAGFIEG
GWSGLVAGWYGFQHSNDQGVGMAADRDSTQKAIDKITSKVNNIVDKMNKQYEIIDHEFSEVEARLNMINN
KIDDQIQDIWAYNAELLVLLENQKTLDEHDANVNNLYNKVKRALGSNAVEDGKGCFELYHKCDDQCMETI
RNGTYNRRKYQEESKLERQKIEGVKLESEGTYKILSIYSTVASSLVIAMGFAAFLFWAMSNGSCRCNICI

>QBK20236.1 hemagglutinin [Influenza A virus]
METVSLITILLIATVSNADKICIGYQSTNSTETVDTLTENNVPVTHAKELLHTEHNGMLCATSLGQPLIL
DACTIEGLIYGNPSCDLSLEGREWSYIVERPSAVNGLCYPGNVENLEELRSLFSSARSYQRIQIFPDTIW
NVSYDGTSTACSGSFYRSMRWLTRKNGEYPIQDAQYTNNQGKNILFMWGINHPPTDTTQRDLYTRTDTTT
SVATEEINRVFKPLIGPRPLVNGLMGRIDYYWSVLKPGQTLRIKSDGNLIAPWFGHILSRESHGRILKTD
LKRGSCTVQCQTEKGGLNTTLPFQNVSKYAFGKCSKYIGIKSLKLAVGLRNVPSRSSRGLFGAIAGFIEG
GWSGLVAGWYGFQHSNDQGVGMAADRDSTQKAIDKITSKVNNIVDKMNKQYEIIDHEFSEVEARINMINN
KIDDQIQDIWAYNAELLVLLENQKTLDEHDANVNNLYNKVKRALGSNAVEDGKGCFELYHKCDDQCMETI
RNGTYNRRKYQEESKLERQKIEGVKLESEGTYKILSIYSTVASYLVIAMGFAAFLFWAMSNGSCRCNICI

>QBK20235.1 hemagglutinin [Influenza A virus]
METVSLITILLVATVSNADKICIGYQSTNSAETVDTLTENNVPVTHAKELLHTEHNGMLCATSLGQPLIL
DACTIEGLIYGNPSCDLSLEGREWSYIVERPSAVNGLCYPGNVQNLEELRSLFSSARSYQRIQIFPDTIW
NVSYDGTSTACSGSFYRSMRWLTRKNGEYPIQDAQYTNNQGKNILFMWGINHPPTDTTQRDLYTRTDTTT
SVATEEINRVFKPLIGPRPLVNGLMGRIDYYWSVLKPGQTLRIKSDGNLIAPWFGHILSRESHGRILKTD
LKRGSCTVQCQTEKGGLNTTLPLLNVSKYAFGKCSKYIGIKSLKLAVGLRNVHSRSSRGLFGAIAGFIEG

GWSGLVAGWYGFQHSNDQGVGMAADRDSTQKAIDKITSKVNNIVAKMNKQYEIIDHEFSEVEARINMINN
KIDDQIQDIWAYNAESLVLLENQKTLDEHDANVNNLYNKVKRALGSNAVEDGKGCFELYHKCDDQCMETI
RNGTYNRRKYQEESKLERQKIEGAKLESEGTYKILSIYSTVASYRVIAMGSAAFLFWAMSNGSCRCNSCI

>QBK20233.1 hemagglutinin [Influenza A virus]
METVSLITILLVATVSNADKICIGYQSTNSTETVDTLTENNVPVTHAKELLHTEHNGMLCATSLGQPLIL
DTCTIEGLIYGNPSCDLSLEGREWSYIVERPSAVNGLCYPGNVENLEELRSLFSSARSYQRIQIFPDTIW
NVSYDGTSTACSGSFYRSMRWLTRKNGDYPVQDAQYTNNQGKNILFMWGINHPPSDTTQRNLYTRNDTTT
SVATEEINRIFKPLIGPRPLVNGLMGRIDYYWSVLKPGQTLRIKSDGNLIAPWYGHILSRESHGRILKTD
LKRGSCTVQCQTEKGGLNTTLPFQNVSKYAFGNCSKYIGIKSLKLAVGLRNVPSRSSRGLFGAIAGFIEG
GWSGLVAGWYGFQHSNDQGVGMAADRDSTQKAIDKITSKVNNIVDKMNKQYEIIDHEFSEVETRLNMINN
KIDDQIQDIWAYNAELLVLLENQKTLDEHDANVNNLYNKVKRALGSNAVEDGKGCFELYHKCDDQCMETI
RNGTYNRRKYQEESKLERQKIEGVKLESEGTYKILTIYSTVASSLVIAMGFAAFLFWAMSNGSCRCNICI

>QBK20232.1 hemagglutinin [Influenza A virus]
METVSLITILLIATVSNADKICIGYQSTNSTETVDTLTENNVPVTHAKELLHTEHNGMLCATSLGQPLIL
DTCTIEGLIYGNPSCDLSLEGREWSYIVERPSAVNGLCYPGNVENLEELRSLFSSARSYQRIQIFPDTIW
NVSYYGTSTACSGSFYRSMRWLTRKNGDYPVQDAQYTNNQGKNILFMWGINHPPTDTTQRDLYTRTDTTT
SVATEEINRIFKPLIGPRPLVNGLMGRIDYYWSVLKPGQTLRIKSDGNLIAPWYGHILSRESHGRILKTD
LKRGSCTVQCQTEKGGLNTTLSLQNVSKYAFGKCSKYIGIKSLKLAVGLRNVPSRSSRGLFGAIAGFIEG
GWSGLVAGWYGFQHSNDQGVGMAADRDSTQKAIDKITSKVNNIVDKMNKQYEIIDHEFSEVETRINMINN
MIDDQIQDIWAYNAELLVLLDNQKTLDEHDANVNNLYNKVKRALGSNAVEDGKGCFELYHKCDDQCMETI
RNGTYNRRKYQEESKLERQKIEGVKLESDGTYKILSIYSTVASSLVIAMGSAAFLFWALSNGSCRCKSCI

>QBK20231.1 hemagglutinin [Influenza A virus]
METVSLITILLVATVSNADKICIGYQSTNSTETVDTLTENNVPVTHAKELLHTEHNGMLCATSLGQPLIL
DTCTIEGLIYGNPSCDLSLEGREWSYIVERPSAVNGLCYPGNVENLEELRSLFSSARSYQRIQIFPDTIW
NVSYDGTSTACSGSFYRSMRWLTRKNGDYPVQDAQYTNNQGKNILFMWGINHPPSDTTQRNLYTRNDTTT
SVATEEINRIFKPLIGPRPLVNGLMGRIDYYWSVLKPGQTLRIKSDGNLIAPWYGHILSRESHGRILKTD
LKRGSCTVQCQTEKGGLNTTLPFQNVSKYAFGNCSKYIGIKSLKLAVGLRNVPSRSSRGLFGAIAGFIEG
GWSGLVAGWYGFQHSNDQGVGMAADRDSTQKAIDKITSKVNNIVDKMNKQYEIIDHEFSEVETRLNMINN
KIDDQIQDIWAYNAELLVLLENQKTLDEHDANVNNLYNKVKRALGSNAVEDGKGCFELYHKCDDQCMETI
RNGTYNRRKYQEESKLERQKIEGVKLESEGTYKILTIYSTVASSLVIAMGFAAFLFWAMSNGSCRCNICI

>QBK20230.1 hemagglutinin [Influenza A virus]

METVSLITILLVATVSNADKICIGYQSTNSTETVDTLTENNVPVTHAKELLHTEHNGMLCATSLGQPLIL

DTCTIEGLIYGNPSCDLSLEGREWSYIVERPSAVNGLCYPGNVENLEELRSLFSSARSYQRIQIFPDTIW

NVSYDGTSTACSGSFYRSMRWLTRKNGDYPVQDAQYTNNQGKNILFMWGINHPPTDTTQRDLYTRTDTTT

SVATEEINRIFKPLIGPRPLVNGLMGRIDYYWSVLKPGQTLRIKSDGNLIAPWYGHILSGESHGRILKTD

LKRGSCTVQCQTEKGGLNTTLPFQNVSKYAFGNCSKYIGIKSLKLAVGLRNVPSRSSRGLFGAIAGFIEG

GWSGLVAGWYGFQHSNDQGVGMAADRDSTQKAIDKITSKVNNIVDKMNKQYEIIDHEFSEVETRLNMINN

KIDDQIQDIWAYNAELLVLLENQKTLDEHDANVNNLYNKVKRALGSNAVEDGKGCFELYHKCDDQCMETI

RNGTYNRRKYQEESKLERQKIEGVKLESEGTYKILSIYSTVASSLVIAMGFAAFLFWAMSNGSCRCNICI

>QBK20229.1 hemagglutinin [Influenza A virus]

METVSLITILLIATVSNADKICIGYQSTNSTETVDTLTENNVPVTHAKELLHTEHNGMLCATSLGQPLIL

DTCTIEGLIYGNPSCDLSLEGREWSYIVERPSAVNGLCYPGNVENLEELRSLFSSARSYQRIQIFPDTIW

NVSFDGTSTACSGSFYKSMRWLTRKNGDYPIQDAQYTNNQGKNILFMWGINHPPTDTTQRDLYTRIDTTT

SVATEEINRVFKPLIGPRPLVNGLMGRIDYYWSVLKPGQTLRIKSDGNLIAPWFGHILSRESHGRILTTD

LKRGSCTVQCQTEKGGLHTALPLQNVSKYAFGKCSKYIGIKSLKLAVGLRNVHSRSSRGLFGAIAGFIEG

GWSGLVAGWYGFQHSNDQGVGMAADRDSTQKAIDKITSKVNNIVDKMYKQYEIIDHEFSEVETRINMINN

KIDDQIQDIWAYNAESLVLLENQKTLDEHDANVNNLYNKVKRALGSNAVEDGKGCFELYHKCDDQCMETI

RNGTYNRRKYQEESKLERQKIEGVKLESEGTYKILSIYSTVASSLVIAMGSAAFLFWALSNWSCRCNSCI

>QBK20228.1 hemagglutinin [Influenza A virus]

METVSLITILLIATVSNADKICIGYQSTNSTETVDTLTENNVPVTHAKELLHTEHNGMLCATSLGQPLIL

DTCTIEGLIYGNPSCDLSLEGREWSYIVERPSAVNGLCYPGNVENLEELRSLFSSARSYQRIQIFPDTIW

NVSYYGTSTACSGSFYRSMRWLTRKNGDYPVQDAQYTNNQGKNILFMWGINHPPTDTTQRDLYTRTDTTT

SVATEEINRIFKPLIGPRPLVNGLMGRIDYYWSVLKPGQTLRIKSDGNLIALWYGHILSRESHGRILKTD

LKRGSCTVQCQTEKGGLNTALPFQNVSKYAFGKCSKYIGIKSLKLAVGLRNVPSRSSRGLFGAIAGFIEG

GWSGLVAGWYGFQHSNDQGVGMAADRDSTQKAIDKITSKVNNIVDKMNKQYEIIDHEFSEVETRINMINN

KIDDQIQDIWAYNAELLVLLENQKTLDEHDANVNNLYNKVKRALGSNAVEDGKGCFELYHKCDDQCMETI

RNGTYNRRKYQAESKLERQKIEGVKLESEGTYKIVSIYSTVASSLVIAMGSAAFLFWAMSNGECRCNSCI

>QBK20227.1 hemagglutinin [Influenza A virus]

METVSLITILLVATVSNADKICIGYQSTNSTETVDTLTENNVPVTHAKELLHTEHNGMLCATSLGQPLIL

DTCTIEGLIYGNPSCDLSLEGREWSYIVERPSAVNGLCYPGNVENLEELRSLFSSARSYQRIQIFPDTIW

NVSYDGTSTACSGSFYRSMRWLTRKNGDYPVQDAQYTNNQGKNILFMWGINHPPTDTTQRDLYTRTDTTT

SVATEEINRIFKPLIGPRPLVNGLMGRIDYYWSVLKPGQTLRIKSDGNLIAPWYGHILSRESHGRILKTD
LKRGSCTVQCQTEKGGLNTTLPFQNVSKYAFGKCSKYIGIKSLKLAVGLRNVPSRSSRGLFGAIAGFIEG
GWSGLVAGWYGFQHSNDQGVGMAADRDSTQKAIDKITSKVNNIVDKMNKQYEIIDHEFSEVETRINMINN
KIDDQIQDIWAYNAELLVLLENQKTLDEHDANVNNLYNKVKRALGSNAVEDGKGCFELYHKCDDQCMETI
RNGTYNRRKYQEESKLERQKIEGVKLESEGTYKILSIYSTVASYLVIAMGSAAFLFWAMSNGSCRCNICI

>QBK20226.1 hemagglutinin [Influenza A virus]
METVSLITILLVATVSNADKICIGYQSTNSTETVDTLTENNVPVTHAKELLHTEHNGMLCATSLGQPLIL
DTCTIEGLIYGNPSCDLSLEGREWSYIVERPSAVNGLCYPGNVENLEELRSLFSSARSYQRIQIFPDTIW
NVSFDGTSTACSGSFYKSMRWLTRKNGDYPIQDAQYTNNQGKNILFMWGINHPPTDTTQRDLYTRIDTTT
SVATEEINRVFKPLIGPRPLVNGLMGRIDYYWSVLKPGQTLRIKSDGNLIAPWFGHILSRESHGRILKTD
LKRGSCTVQCQTEKGGLNTTLPFQNVSKYAFGNCSKYIGIKSLKLAVGLRNVPSRSSRGLFGAIAGFIEG
GWSGLVAGWYGFQHSNDQGVGMAADRDSTQKAIDKITSKVNNIVDKMNKQYEIIDHEFSEVETRINMINN
KIDDQIQDIWAYNAELLVLLENQKTLDEHDANVNNLYNKVKRALGSNAVEDGKGCFELYHKCDDQCMETI
GNGTYNRRKYQEESKLERQKIEGVKLESEGTYKILSIYSTVASSLVIAMGFAAFLFWALSNGECRCNSCI

>QBK20225.1 hemagglutinin [Influenza A virus]
METVSLITILLVATVSNADKICIGYQSTNSTETVDTLTENNVPVTHAKELLHTEHNGMLCATSLGQPLIL
DTCTIEGLIYGNPSCDLSLEGREWSYIVERPSAVNGLCYPGNVENLEELRSLFSSARSYQRIQIFPDTIW
NVSYDGTSTACSGSFYRSMRWLTRKNGDYPVQDAQYTNNQGKNILFMWGINHPPTDTTQRDLYTRTDTTT
SVATEEINRIFKPLIGPRPLVNGLMGRIDYYWSVLKPGQTLRIKSDGNLIAPWYGHILSRESHGRILKTD
LKRGSCTVQCQTEKGGLNTTLPFQNVSKYAFGKCSKYIGIKSLKLAVGLRNVPSRSSRGLFGAIAGFIEG
GWSGLVAGWYGFQHSNDQGVGMAADRDSTQKAIDKITSKVNNIVDKMNKQYEIIDHEFSEVETRINMINN
KIDDQIQDIWAYNAELLVLLENQKTLDEHDANVNNLYNKVKRALGSNAVEDGKGCFELYHKCDDQCMETI
RNGTYNRRKYQEESKLERQKIEGVKLESEGTYKILSIYSTVASSLVIAMGFAAFLFWAMSNGSCRCNICI

>QBK20224.1 hemagglutinin [Influenza A virus]
METVSLITILLVATVSNADKICIGYQSTNSTETVDTLTENNVPVTHAKELLHTEHNGMLCATSLGQPLIL
DTCTIEGLIYGNPSCDLSLEGREWSYIVERPSAVNGLCYPGNVENLEELRSLFSSARSYQRIQIFPDTIW
NVSYDGTSTACSGSFYRSMRWLTRKNGDYPVQDAQYTNNQGKNILFMWGINHPPTDTTQRDLYTRTDTTT
SVATEEINRIFKPLIGPRPLVNGLMGRIDYYWSVLKPGQTLRIKSDGNLIAPWYGHILSRESHGRILTTD
LKRGSCTVQCQTEKGGLNTTLPFQNVSKYAFGNCSKYIGIKSLKLAVGLRNVPSRSSRGLFGAIAGFIEG
GWSGLVAGWYGFQHSNDQGVGMAADRDSTQKAIDKITSKVNNIVDKMNKQYEIIDHEFSEVETRLNMINN
KIDDQIQDIWAYNAELLVLLENQKTLDEHDANVNNLYNKVKRALGSNAVEDGKGCFELYHKCDDQCMETI

RNGTYNRRKYQEESKLERQKIEGVKLESEGTYKILTIYSTVASSLVIAMGFAAFLFWAMSNGSCRCNICI

>QBK20223.1 hemagglutinin [Influenza A virus]

METVSLITILLVATVSNADKICIGYQSTNSTETVDTLTENNVPVTHAKELLHTEHNGMLCATSLGQPLIL

DTCTIEGLIYGNPSCDLSLEGREWSYIVERPSAVNGLCYPGNVENLEELRSLFSSARSYQRIQIFPDTIW

NVSFDGTSTACSGSFYKSMRWLTRKNGDYPIQDAQYTNNQGKNILFMWGINHPPTDTTQRDLYTRIDTTT

SVATEEINRVFKPLIGPRPLVNGLMGRIDYYWSVLKPGQTLRIKSDGNLIAPWFGHILSGESHGRILKTD

LKRGSCTVQCQTEKGGLNTTLPFQNVSKYAFGNCSKYIGIKSLKLAVGLRNVPSRSSRGLFGAIAGFIEG

GWSGLVAGWYGFQHSNDQGVGMAADRDSTQKAIDKITSKVNNIVDKMNKQYEIIDHEFSEVETRLNMINN

KIDDQIQDIWAYNAELLVLLENQKTLDEHDANVNNLYNKVKRALGSNAVEDGKGCFELYHKCDDQCMETI

RNGTYNRRKYQEESKLERQKIEGVKLESEGTYKILTIYSTVASSLVIAMGFAAFLFWAMSNGSCRCNICI

>QBK20222.1 hemagglutinin [Influenza A virus]

METVSLITILLVATVSNADKICIGYQSTNSTETVDTLTENNVPVTHAKELLHTEHNGMLCATSLGQPLIL

DTCTIEGLIYGNPSCDLSLEGREWSYIVERPSAVNGLCYPGNVENLEELRSLFSSARSYQRIQIFPDTIW

NVSFDGTSTACSGSFYKSMRWLTRKNGDYPIQDAQYTNNQGKNILFMWGINHPPTDTTQRDLYTRIDTTT

SVATEDINRVFKPLIGPRPLVNGLMGRIDYYWSVLKPGQTLRIKSDGNLIAPWFGHILSGESHGRILKTD

LKRGSCTVQCQTEKGGLNTTLPFQNVSKYAFGNCSKYIGIKSLKLAVGLRNVPSRSSRGLFGAIAGFIEG

GWSGLVAGWYGFQHSNDQGVGMAADRDSTQKAIDKITSKVNNIVDKMNKQYEIIDHEFSEVETRLNMINN

KIDDQIQDIWAYNAELLVLLENQKTLDEHDANVNNLYNKVKRALGSNAVEDGKGCFELYHKCDDQCMETI

RNGTYNRRKYQEESKLERQKIEGVKLESEGTYKILTIYSTVASSLVIAMGFAAFLFWAMSNGSCRCNICI

>QBK20221.1 hemagglutinin [Influenza A virus]

METVSLITILLVATVSNADKICIGYQSTNSTETVDTLTENNVPVTHAKELLHTEHNGMLCATSLGQPLIL

DTCTIEGLIYGNPSCDLSLEGREWSYIVERPSAVNGLCYPGNVENLEELRSLFSSARSYQRIQIFPDTIW

NVSYDGTSTACSGSFYRSMRWLTRKNGDYPVQDAQYTNNQGKNILFMWGINHPPTDTTQRDLYTRTDTTT

SVATEEINRIFKPLIGPRPLVNGLMGRIDYYWSVLKPGQTLRIKSDGNLIAPWYGHILSGESHGRILKTD

LKRGSCTVQCQTEKGGLNTTLPFQNVSKYAFGNCSKYIGIKSLKLAVGLRNVPSRSSRGLFGAIAGFIEG

GWSGLVAGWYGFQHSNDQGVGMAADRDSTQKAIDKITSKVNNIVDKMNKQYEIIDHEFSEVETRLNMINN

KIDDQIQDIWAYNAELLVLLENQKTLDEHDANVNNLYNKVKRALGSNAVEDGKGCFELYHKCDDQCMETI

RNGTYNRRKYQEESKLERQKIEGVKLESEGTYKILTIYSTVASSLVIAMGFAAFLFWAMSNGSCRCNICI

>QBK20220.1 hemagglutinin [Influenza A virus]

METVSLITILLIATVSNADKICIGYQSTNSTETVDTLTENNVPVTHAKELLHTEHNGMLCATSLGQPLIL

DTCTIEGLIYGNPSCDLSLEGREWSYIVERPSAVNGLCYPGNVENLEELRSLFSSARSYQRIQIFPDTIW
NVSYDGTSTACSGSFYRSMRWLTRKNGDYPVQDAQYTNNQGKNILFMWGINHPPSDTTQRNLYTRNDTTT
SVATEEINRIFKPLIGPRPLVNGLMGRIDYYWSVLKPGQTLRIKSDGNLIAPWYGHILSGESHGRILKTD
LKRGSCTVQCQTEKGGLNTTLPFQNVSKYAFGNCSKYIGIKSLKLAVGLRNVPSRSSRGLFGAIAGFIEG
GWSGLVAGWYGFQHSNDQGVGMAADRDSTQKAIDKITSKVNNIVDKMNKQYEIIDHEFSEVETRLNMINN
KIDDQIQDIWAYNAELLVLLENQKTLDEHDANVNNLYNKVKRALGSNAVEDGKGCFELYHKCDDQCMETI
RNGTYNRRKYQEESKLERQKIEGVKLESEGTYKILSIYSTVASSLVIAMGSAAFLFWAMSNGSCRCNICI

>QBK20219.1 hemagglutinin [Influenza A virus]
METVPLITILLVATVSNADKICIGYQSTNSTETVDTLTENNVPVTHAKELLHTEHNGMLCATSLGHPLIL
DTCTIEGLIYGNPSCDPLLGGREWSYIVERPSAVNGLCYPGNVQNLEELRSLFSSARSYQRIQIFPDTIW
NVSYSGTSKACSDSFYRSMRWLTQKNNAYPIQDAQYTNNQEKNILFMWGINHPPTETAQTNLYTRTDTTT
SVATEEINRIFKPLIGPRPLVNGLMGRINYYWSVLKPGQTLRIKSDGNLIAPWYGHILSGESHGRILKTD
LKRGSCTVQCQTEKGGLNTTLPFQNVSKYAFGKCSKYIGIKSLKLAVGLRNVRSRYSRGLFGAIAGFIEG
GWSGLVDGWYGFQHSNDQGVGMAADRDSTQKAIDKITSKVNNIVDKMNRQYEIIDHEFSEVETRLNMINN
KVDDQIQDIWAYNAELIVLLENQKTLDEHDSNVNNLYNKVKRALGSNAVEDGKGCFELYHKCDDQCMETI
RNGTYNRRKYQEESKLERQKIEGVKLESEGTYKILSIYSTVASYRVIAMGFAAFLFWAMSNGSCRCNSCI

>QBK20218.1 hemagglutinin [Influenza A virus]
METVSLITILLVATVSNADKICIGYQSTNSTETVDTLTENNVPVTHAKELLHTEHNGMLCATSLGQPLIL
DTCTIEGLIYGNPSCDLSLEGREWSYIVERPSAVNGLCYPGNVENLEELRSLFSSARSYQRIQIFPDTIW
NVSYDGTSTACSGSFYRSMRWLTRKNGDYPVQDAQYTNNQGKNILFMWGINHPPTDTTQRDLYTRTDTTT
SVATEEINRIFKPLIGPRPLVNGLMGRIDYYWSVLKPGQTLRIKSDGNLIAPWYGHILSGESHGRILKTD
LKRGSCTVQCQTEKGGLNTTLPFQNVSKYAFGKCSKYIGIKSLKLAVGLRNVPSRSSRGLFGAIAGFIEG
GWSGLVAGWYGFQHSNDQGVGMAADRDSTQKAIDKITSKVNNIVDKMNKQYEIIDHEFSEVETRINMINN
KIDDQIQDIWAYNAELLVLLENQKTLDEHDANVNNLYNKVKRALGSNAVEDGKGCFELYHKCDDQCMETI
RNGTYNRRKYQEESKLERQKIEGVKLESEGTYKILSIYSTVASSLVIAMGFAAFLFWAMSNGSCRCNICI

>QBK20217.1 hemagglutinin [Influenza A virus]
METVSLITILLVATVSNADKICIGYQSTNSTETVDTLTENNVPVTHAKELLHTEHNGMLCATSLGQPLIL
DTCTIEGLIYGNPSCDLSLEGREWSYIVERPSAVNGLCYPGNVENLEELRSLFSSARSYQRIQIFPDTIW
NVSYDGTSTACSGSFYRSMRWLTRKNGDYPVQDAQYTNNQGKNILFMWGINHPPSDTTQRNLYTRNDTTT
SVATEEINRIFKPLIGPRPLVNGLMGRIDYYWSVLKPGQTLRIKSDGNLIAPWYGHILSRESHGRILKTD
LKRGSCTVQCQTEKGGLNTTLPFQNVSKYAFGNCSKYIGIKSLKLAVGLRNVPSRSSRGLFGAIAGFIEG

GWSGLVAGWYGFQHSNDQGVGMAADRDSTQKAIDKITSKVNNIVDKMNKQYEIIDHEFSEVETRLNMINN
KIDDQIQDIWAYNAELLVLLENQKTLDEHDANVNNLYNKVKRALGSNAVEDGKGCFELYHKCDDQCMETI
RNGTYNRRKYQEESKLERQKIEGVKLESEGTYKILSIYSTVASSLVIAMGFAAFLFWAMSNGSCRCNISI

>QBK20216.1 hemagglutinin [Influenza A virus]
METVSLITILLVATVSNADKICIGYQSTNSTETVDTLTENNVPVTHAKELLHTEHNGMLCATSLGQPLIL
DTCTIEGLIYGNPSCDLSLEGREWSYIVERPSAVNGLCYPGNVENLEELRSLFSSARSYQRIQIFPDTIW
NVSYDGTSTACSGSFYRSMRWLTRKNGDYPVQDAQYTNNQGKNILFMWGINHPPSDTTQRNLYTRNDTTT
SVATEEINRIFKPLIGPRPLVNGLMGRIDYYWSVLKPGQTLRIKSDGNLIAPWYGHILSGESHGRILKTD
LKRGSCTVQCQTEKGGLNTTLPFQNVSKYAFGNCSKYIGIKSLKLAVGLRNVPSRSSRGLFGAIAGFIEG
GWSGLVAGWYGFQHSNDQGVGMAADRDSTQKAIDKITSKVNNIVDKMNKQYEIIDHEFSEVETRLNMINN
KIDDQIQDIWAYNAELLVLLENQKTLDEHDANVNNLYNKVKRALGSNAVEDGKGCFELYHKCDDQCMETI
RNGTYNRRKYQEESKLERQKIEGVKLESEGTYKILSIYSTVASSLVIAMGFAAFLFWAMSNGSCRCNICI

>QBK20215.1 hemagglutinin [Influenza A virus]
METVSLITILLVATVSNADKICIGYQSTNSTETVDTLTENNVPVTHAKELLHTEHNGMLCATSLGQPLIL
DTCTIEGLIYGNPSCDLSLEGREWSYIVERPSAVNGLCYPGNVENLEELRSLFSSARSYQRIQIFPDTIW
NVSYDGTSTACSGSFYRSMRWLTRKNGDYPVQDAQYTNNQGKNILFMWGINHPPTDTTQRDLYTRTDTTT
SVATEEINRIFKPLIGPRPLVNGLMGRIDYYWSVLKPGQTLRIKSDGNLIAPWYGHILSGESHGRILKTD
LKRGSCTVQCQTEKGGLNTTLPFQNVSKYAFGKCSKYIGIKSLKLAVGLRNVPSRSSRGLFGAIAGFIEG
GWSGLVAGWYGFQHSNDQGVGMAADRDSTQKAIDKITSKVNNIVDKMNKQYEIIDHEFSEVETRLNMINN
KIDDQIQDIWAYNAELLVLLENQKTLDEHDANVNNLYNKVKRALGSNAVEDGKGCFELYHKCDDQCMETI
RNGTYNRRKYQEESKLERQKIEGVKLESEGTYKILSIYSTVASSLVIAMGFAAFLFWAMSNGSCRCNICI

>QBK20209.1 hemagglutinin [Influenza A virus]
METVSLITILLVATVSNADKICIGYQSTNSTETVDTLTENNVPVTHAKELLHTEHNGMLCATSLGQPLIL
DACTIEGLIYGNPSCDLSLEGREWSYIVERPSAVNGLCYPGNVENLEELRSLFSSARSYQRIQIFPDTIW
NVSYDGTSTACSGSFYRSMRWLTRKNGEYPIQDAQYTNNQGKNILFMWGINHPPTDTTQRDLYTRTDTTT
SVATEEINRVFKPLIGPRPLVNGLMGRIDYYWSVLKPGQTLRIKSDGNLIAPWFGHILSRESHGRILKTD
LKRGSCTVQCQTEKGGLNTTLPFQNVSKYAFGNCSKYIGIKSLKLAVGLRNVPSRSSRGLFGAIAGFIEG
GWSGLVAGWYGFQHSNDQGVGMAADRDSTQKAIDKITSKVNNIVDKMNKQYEIIDHEFSEVEARLNMINN
KIDDQIQDIWAYNAELLVLLENQKTLDEHDANVNNLYNKVKRALGSNAVEDGKGCFELYHKCDDQCMETI
RNGTYNRRKYQEESKLERQKIEGVKLESEGTYKILTIYSTVASSLVIAMGFAAFLFWAMSNGSCRCNICI

>QBK20208.1 hemagglutinin [Influenza A virus]

METVSLITILLVATVSNADKICIGYQSTNSTETVNTLTENNVPVTHAKELLHTEHNGMLCATSLGQPLIL
DTCTIEGLIYGNPSCDLSLEGREWSYIVERPSAVNGLCYPGNVENLEELRSLFSSARSYQRIQIFPDTIW
NVSYDGTSTACSGSFYRSMRWLTRKNGDYPVQDAQYTNNQGKNILFMWGINHPPSDTTQRNLYTRNDTTT
SVATEEINRIFKPLIGPRPLVNGLMGRIDYYWSVLKPGQTLRIKSDGNLIAPWYGHILSGESHGRILKTD
LKRGSCTVQCQTEKGGLNTTLPFQNVSKYAFGNCSKYIGIKSLKLAVGLRNVPSRSSRGLFGAIAGFIEG
GWSGLVAGWYGFQHSNDQGVGMAADRDSTQKAIDKITSKVNNIVDKMNKQYEIIDHEFSEVETRLNMINN
KIDDQIQDIWAYNAELLVLLENQKTLDEHDANVNNLYNKVKRALGSNAVEDGKGCFELYHKCDDQCMETI
RNGTYNRRKYQEESKLERQKIEGVKLESEGTYKILTIYSTVASSLVIAMGFAAFLFWAMSNGSCRCNICI

>QBK20207.1 hemagglutinin [Influenza A virus]

METVSLITILLVAAVSNADKICIGYQSTNSTETVDTLTENNVPVTHAKELLHTEHNGMLCATSLGQPLIL
DTCTIEGLIYGNPSCDLSLEGREWSYIVERPSAVNGLCYPGNVENLEELRSLFSSARSYQRIQIFPDTIW
NVSYDGTSTACSGSFYKSMRWLTRKNGDYPTQDAQYTNNQGKNILFMWGINHPPTDTTQRDLYTRTDTTT
SVATEEINRVFKPLIGPRPLVNGLMGRIDYYWSVLKPGQTLRIKSDGNLIAPWFGHILSRESHGRILKTD
LKRGSCTVQCQTEKGGLNTTLPFQNVSKYAFGNCSKYIGIKSLKLAVGLRNVPSRSSRGLFGAIAGFIEG
GWSGLVAGWYGFQHSNDQGVGMAADRDSTQKAIDKITSKVNNIVDKMNKQYEIIDHEFSEVETRLNMINN
KIDDQIQDIWAYNAELLVLLENQKTLDEHDANVNNLYNKVKRALGSNAVEDGKGCFELYHKCDDQCMETI
RNGTYNRRKYQEESKLERQKIEGVKLESEGTYKILSIYSTVASSLVIAMGFAAFLFWAMSNGSCRCNICI

>QBK20206.1 hemagglutinin [Influenza A virus]

METVSLITILLVATVSNADKICIGYQSTNSTETVDTLTENNVPVTHAKELLHTEHNGMLCATSLGQPLIL
DTCTIEGLIYGNPSCDLSLEGREWSYIVERPSAVNGLCYPGNVENLEELRSLFSSARSYQRIQIFPDTIW
NVSYDGTSTACSGSFYRSMRWLTRKNGDYPVQDAQYTNNQGKNILFMWGINHPPSDTTQRNLYTRNDTTT
SVATEEINRIFKPLIGPRPLVNGLMGRIDYYWSVLKPGQTLRIKSDGNLIAPWYGHILSRESHGRILKTD
LKRGSCTVQCQTEKGGLNTTLPFQNVSKYAFGNCSKYIGIKSLKLAVGLRNVPSRSSRGLFGAIAGFIEG
GWSGLVAGWYGFQHSNDQGVGMAADRDSTQKAIDKITSKVNNIVDKMNKQYEIIDHEFSEVETRLNMINN
KIDDQIQDIWAYNAELLVLLENQKTLDEHDANVNNLYNKVKRALGSNAVEDGKGCFELYHKCDDQCMETI
RNGTYNRRKYQEESKLERQKIEGVKLESEGTYKILTIYSTVASSLVIAMGFAAFLFWAMSNGSCRCNICI

>QBK20205.1 hemagglutinin [Influenza A virus]

METVSLITILLVATVSNADKICIGYQSTNSTETVDTLTENNVPVTHAKELLHTEHNGMLCATSLGQPLIL
DACTIEGLIYGNPSCDLSLEGREWSYIVERPSAVNGLCYPGNVENLEELRSLFSSARSYQRIQIFPDTIW
NVSYDGTSTACSGSFYRSMRWLTRKNGEYPIQDAQYTNNQGKNILFMWGINHPPTDTTQRDLYTRTDTTT

SVATEEINRIFKPLIGPRPLVNGLMGRIDYYWSVLKPGQTLRIKSDGNLIAPWFGHILSRESHGRILKTD
LKRGSCTVQCQTEKGGLNTTLPFQNVSKYAFGNCSKYIGIKSLKLAVGLRNVPSRSSRGLFGAIAGFIEG
GWSGLVAGWYGFQHSNDQGVGMAADRDSTQKAIDKITSKVNNIVDKMNKQYEIIDHEFSEVEARLNMINN
KIDDQIQDIWAYNAELLVLLENQKTLDEHDANVNNLYNKVKRALGSNAVEDGKGCFELYHKCDDQCMETI
RNGTYNRRKYQEESKLERQKIEGVKLESEGTYKILTIYSTVASSLVIAMGFAAFLFWAMSNGSCRCNICI

>QBK20204.1 hemagglutinin [Influenza A virus]
METVSLITILLVATVSNADKICIGYQSTNSTETVDTLTENNVPVTHAKELLHTEHNGMLCATSLGQPLIL
DTCTIEGLIYGNPSCDLSLEGREWSYIVERPSAVNGLCYPGNVENLEELRSLFSSARSYQRIQIFPDTIW
NVSYYGTSTACSGSFYRSMRWLTRKNGDYPVQDAQYTNNQGKNILFMWGINHPPTDTTQRDLYTRTDTTT
SVATEEINRIFKPLIGPRPLVNGLMGRIDYYWSVLKPGQTLRIKSDGNLIAPWYGHILSRESHGRILKTD
LKRGSCTVQCQTEKGGLNTTLPFQNVSKYAFGKCSKYIGIKSLKLAVGLRNVPSRSSRGLFGAIAGFIEG
GWSGLVAGWYGFQHSNDQGVGMAADRDSTQKAIDKITSKVNNIVDKMNKQYEIIDHEFSEVETRINMINN
KIDDQIQDIWAYNAELLVLLENQKTLDEHDANVNNLYNKVKRALGSNAVEDGKGCFELYHKCDDQCMETI
RNGTYNRRKYQEESKLERQKIEGVKLESEGTYKILSIYSTVASSLVIAMGSAAFLFWALSNGSCRCNSCI

>QBK20203.1 hemagglutinin [Influenza A virus]
METVSLITILLVATVSNADKICIGYQSTNSTETVDTLTENNVPVTHAKELLHTEHNGMLCATSLGQPLIL
DTCTIEGLIYGNPSCDLSLEGREWSYIVERPSAVNGLCYPGNVENLEELRSLFSSARSYQRIQIFPDTIW
NVSYDGTSTACSGSFYRSMRWLTRKNGDYPVQDAQYTNNQGKNILFMWGINHPPSDTTQRNLYTRNDTTT
SVATEEINRIFKPLIGPRPLVNGLMGRIDYYWSVLKPGQTLRIKSDGNLIAPWYGHILSGESHGRILTTD
LKRGSCTVQCQTEKGGLNTTLPFQNVSKYAFGNCSKYIGIKSLKLAVGLRNVPSRSSRGLFGAIAGFIEG
GWSGLVAGWYGFQHSNDQGVGMAADRDSTQKAIDKITSKVNNIVDKMNKQYEIIDHEFSEVETRLNMINN
KIDDQIQDIWAYNAELLVLLENQKTLDEHDANVNNLYNKVKRALGSNAVEDGKGCFELYHKCDDQCMETI
RNGTYNRRKYQEESKLERQKIEGVKLESEGTYKILTIYSTVASSLVIAMGFAAFLFWAMSNGSCRCNICI

>QBK20202.1 hemagglutinin [Influenza A virus]
METVSLITILLIATVSNADKICIGYQSTNSTETVDTLTENNVPVTHAKELLHTEHNGMLCATSLGQPLIL
DTCTIEGLIYGNPSCDLSLEGREWSYIVERPSAVNGLCYPGNVENLEELRSLFSSARSYQRIQIFPDTIW
NVSYDGTSTACSGSFYRSMRWLTRKNGDYPVQDAQYTNNQGKNILFMWGINHPPSDTTQRNLYTRNDTTT
SVATEEINRIFKPLIGPRPLVNGLMGRIDYYWSVLKPGQTLRIKSDGNLIAPWYGHILSRESHGRILTTD
LKRGSCTVQCQTEKGGLNTTLPFQNVSKYAFGNCSKYIGIKSLKLAVGLRNVPSRSSRGLFGAIAGFIEG
GWSGLVAGWYGFQHSNDQGVGMAADRDSTQKAIDKITSKVNNIVDKMNKQYEIIDHEFSEVETRLNMINN
KIDDQIQDIWAYNAELLVLLENQKTLDEHDANVNNLYNKVKRALGSNAVEDGKGCFELYHKCDDQCMETI

RNGTYNRRKYQEESKLERQKIEGVKLESEGTYKILSIYSTVASSLVIAMGFAAFLFWAMSNGSCRCNICI

>QBK20201.1 hemagglutinin [Influenza A virus]
METVSLITILLVATVSNADKICIGYQSTNSTETVDTLTENNVPVTHAKELLHTEHNGMLCATSLGQPLIL
DACTIEGLIYGNPSCDLSLEGREWSYIVERPSAVNGLCYPGNVENLEELRSLFSSARSYQRIQIFPDTIW
NVSYDGTSTACSGSFYRSMRWLTRKNGEYPIQDAQYTNNQGKNILFMWGINHPPTDTTQRDLYTRTDTTT
SVATEEINRVFKPLIGPRPLVNGLMGRIDYYWSVLKPGQTLRIKSDGNLIAPWFGHILSRESHGRLLTTD
LKRGSCTVQCQTEKGGLNTTLPFQNVSKYAFGNCSKYIGIKSLKLAVGLRNVPSRSSRGLFGAIAGFIEG
GWSGLVAGWYGFQHSNDQGVGMAADRDSTQKAIDKITSKVNNIVDKMNKQYEIIDHEFSEVEARLNMINN
KIDDQIQDIWAYNAELLVLLENQKTLDEHDANVNNLYNKVKRALGSNAVEDGKGCFELYHKCDDQCMETI
RNGTYNRRKYQEESKLERQKIEGVKLESEGTYKILSIYSTVASYLVIAMGFAAFLFWAMSNGSCRCNICI

>QBK20200.1 hemagglutinin [Influenza A virus]
METVFLITILLLSTVINADLICIGYHSTDSTETVDTLTENNVPVTHAKELLHTEHNGMLCATSLGQPLIL
DACTIEGLIYGNPSCDLSLKGRKWSYIVKRPSTVDGLCYPGNVKNLKELRSLFSSARSYQTIQIFPDPIW
NVSYYGTSTACSGSFYLSMRWLTQKNGEYPIQDAQYTNNQGKNILFMWGINHPPTDTTQRDLYTRTDTTT
SVATEEINRVFKPLIGPRPLVNGLMGRIDYYWSGLKPAQTLRIKSDGNLISPWFGHILSRESHGIILNTD
LKRGSCAVQCQTEKGGYTTSLSLQNLRKYAFGQCSKYIGIKSLSLAVGMRNVPSRSSRGIFDTIAGSIEG
GWSVLVAGWHGLQHSIDQGVGMPADSDSTQKAIDKITSKVNNIVDKMSKQYEIIDHEFSEVEARINVINI
KIVDQIQDIWAFIAEMVVTLEIQKSIDEHDANVINLHNKVKTTVGSNGVEDVNRYFEIYRKCGVRSMESI
WNGSSDGRRYQEESKLERQKIEGVKLEFEGTYKILTIYLAVASFLVVAMGWVAFMLWALCNGSCRCNICI

>QBK20198.1 hemagglutinin [Influenza A virus]
METVSLITILLVATVSNADKICIGYQSTNSTETVDTLTENNVPVTHAKELLHTEHNGMLCATSLGQPLIL
DTCTIEGLIYGNPSCDLSLEGREWSYIVERPSAVNGLCYPGNVENLEELRSLFSSARSYQRIQIFPDTIW
NVSYDGTSTACSGSFYRSMRWLTRKNGDYPVQDAQYTNNQGKNILFMWGINHPPTDTTQRDLYTRTDTTT
SVATEEINRIFKPLIGPRPLVNGLMGRIDYYWSVLKPGQTLRIKSDGNLIAPWYGHILSRESHGRILKTD
LKRGSCTVQCQTEKGGLNTTLPFQNVSKYAFGNCSKYIGIKSLKLAVGLRNVPSRSSRGLFGAIAGFIEG
GWSGLVAGWYGFQHSNDQGVGMAADRDSTQKAIDKITSKVNNIVDKMNKQYEIIDHEFSEVETRLNMINN
KIDDQIQDIWAYNAELLVLLENQKTLDEHDANVNNLYNKVKRALGSNAVEDGKGCFELYHKCDDQCMETI
RNGTYNRRKYQEESKLERQKIEGVKLESEGTYKILSIYSTVASSLVIAMGFAAFLFWAMSNGSCRCNICI

>QBK20197.1 hemagglutinin [Influenza A virus]
METVSLITMLLVATVSNADKICIGYQSTNSAETVDTLTENNVPVTHAKELLHTEHNGMLCATSLGQPLIL

DTCTIEGLIYGNPSCDLSLEGREWSYIVERPSAVNGLCYPGNVENLEELRSLFSSARSYQRIQIFPDTIW
NVSYYGTSTACSGSFYRSMRWLTRKNGDYPVQDAQYTNNQGKNILFMWGINHPPTDTTQRDLYTRTDTTT
SVATEEINRIFKPLIGPRPLVNGLMGRIDYYWSVLKPGQTLRIKSDGNLIAPWYGHILSRESHGRILNTD
LERGRCTVQCQAETGGLNTALPLQNVSKYAFGKCSKYIGIKSLKLAVGLRNVRSRYSRGLFGAIAGFIEG
GWSGLVAGWYGFQHSNDQGVGMAADRDSTQKAIDKITSKVNNIVAKLYKQYEIIDLEFSEVGTRIDPSSN
KFDDQIQDIWAYNAESIVFLDNQKTLDEHDANVNNLYNKVKRALGSNAVEDGKGCSELYHKCDDQCMETI
RNGTYNRRKYQEESKLERQKIEGAKLESDRTNKILSIYSRVASSLVIAMGSVAFLFWALSNWSCRCNSCI

>QBK20196.1 hemagglutinin [Influenza A virus]
METVSLITILLVATVSNADKICIGYQSTNSTETVDTLTENNVPVTHAKELLHTEHNGMLCATSLGQPLVL
DTCTIEGLIYGNPSCDLSLEGREWSYIVERPSAVNGLCYPGNVENLEELRSLFSSARSYQRIQIFPDTIW
NVSYDGTSSACSGSFYKSMRWLTRKNGDYPIQDAQYTNNQGKNILFMWGINHPPTDTTQRDLYTRTDTTT
SVATEEINRIFKPLIGPRPLVNGLMGRIDYYWSVLKPSQTLRIKSDGNLIAPWYGHILSRESHGRILKTD
LKRGSCTVQCQTEKGGLNTTLPFQNVSKYAFGNCSKYIGIKSLKLAVGLRNVPSRSSRGLFGAIAGFIEG
GWSGLVAGWYGFQHSNDQGVGMAADRDSTQKAIDKITSKVNNIVDKMNKQYEIIDHEFSEVETRLNMINN
KIDDQIQDIWAYNAELLVLLENQKTLDEHDANVNNLYNKVKRALGSNAVEDGKGCFELYHKCDDQCMETI
RNGTYNRRKYQEESKLERQKIEGVKLEYEGTYKILTIYSTVASSLVIAMGFAAFLFWAMSNGSCRCNICI

>QBK20195.1 hemagglutinin [Influenza A virus]
METVSLITILLVATVSNADKICIGYQSTNSTETVDTLTENNVPVTHAKELLHTEHNGMLCATSLGQPLIL
DACTIEGLIYGNPSCDLSLEGREWSYIVERPSAVNGLCYPGNVENLEELRSLFSSARSYQRIQIFPDTIW
NVSYDGTSTACSGSFYRSMRWLTRKNGEYPIQDAQYTNNQGKNILFMWGINHPPTDTTQRDLYTRTDTTT
SVATEEINRVFKPLIGPRPLVNGLMGRIDYYWSVLKPGQTLRIKSDGNLIAPWFGHILSRESHGRILKTD
LKRGSCTVQCQTEKGGLNTTLSLQNVSKYAFGKCSKYIGIKSLKLAVGLRNVRSRSSRGLFGAIAGFIEG
GWSGLVAGWYGFQHSNDQGVGMAADRDSTQKAIDKITSKVNNIVDKMNKQYEIIDHEFSEVEARINMINN
KIDDQIQDIWAYNAELLVLLENQKTLDEHDANVNNLYNKVKRALGSNAVEDGKGCFELYHKCDDQCMETI
RNGTYNRRKYQEESKLERQKIEGVKLEFEGTYKILSIYSTVASSLVIAMGFAAFLFWAMSNGSCRCNICI

>QBK20194.1 hemagglutinin [Influenza A virus]
METVSLITILLVATVSNADKICIGYQSTNSTETVDTLTENNVPVTHAKELLHTEHNGMLCATSLGQPLIL
DTCTIEGLIYGNPSCDLSLEGREWSYIVERPSAVNGLCYPGNVENLEELRSLFSSARSYQRIQIFPDTIW
NVSYDGTSTACSGSFYRSMRWLTRKNGDYPVQDAQYTNNQGKNILFMWGINHPPSDTTQRNLYTRNDTTT
SVATEEINRIFKPLIGPRPLVNGLMGRIDYYWSVLKPGQTLRIKSDGNLIAPWYGHILSRESHGRILKTD
LKRGSCTVQCQTEKGGLNTTLPFQNVSKYAFGNCSKYIGIKSLKLAVGLRNVPSRSSRGLFGAIAGFIEG

GWSGLVAGWYGFQHSNDQGVGMAADRDSTQKAIDKITSKVNNIVDKMNKQYEIIDHEFSEVETRLNMINN
KIDDQIQDIWAYNAELLVLLENQKTLDEHDANVNNLYNKVKRALGSNAVEDGKGCFELYHKCDDQCMETI
RNGTYNRRKYQEESKLERQKIEGVKLESEGTYKILSIYSTVASSLVIAMGSAAFLFWAMSNGSCRCNICI

>QBK20193.1 hemagglutinin [Influenza A virus]
METVSLITILLVATVSNADKICIGYQSTNSTETVDTLTENNVPVTHAKELLHTEHNGMLCATSLGQPLIL
DACTIEGLIYGNPSCDLSLEGREWSYIVERPSAVNGLCYPGNVENLEELRSLFSSARSYQRIQIFPDTIW
NVSYDGTSTACSGSFYRSMRWLTRKNGEYPIQDAQYTNNQGKNILFMWGINHPPTDTTQRDLYTRTDTTT
SVATEEINRVFKPLIGPRPLVNGLMGRIDYYWSVLKPGQTLRIKSDGNLIAPWFGHILSRESHGRILKTD
LKRGSCTVQCQTEKGGLNTTLPCQNVSKYAFGNCSKYIGIKSLKLAVGLRNVPSRSSRGLFGAIAGFIEG
GWSGLVAGWYGFQHSNDQGVGMAADRDSTQKAIDKITSKVNNIVDKMNKQYEIIDHEFSEVETRLNMINN
KIDDQIQDIWAYNAELLVLLENQKTLDEHDANVNNLYNKVKRALGSNAVEDGKGCFELYHKCDDQCMETI
RNGTYNRRKYQEESKLERQKIEGAKLESEGTYKILSIYSTVASSLVIAMGSAAFLFWAMSNGSCRCNICI

>QBK20192.1 hemagglutinin [Influenza A virus]
METVSLITILLAATVSNADKICIGYQSTNSTETVDTLTENNVPVTHAKELLHTEHNGMLCATSLGQPLIL
DTCTIEGLIYGNPSCDLSLEGREWSYIVERPSAVNGLCYPGNVENLEELRSLFSSARSYQRIQIFSDTIW
NVSYDGTSTACSGSFYKSMRWLTRKNGDYPTQDAQYTNNQGKNILFMWGINHPPTDTTQINLYTRTDTTT
SVATEEINRVFKPLIGPRPLVNGLMGRIDYYWSVLKPGQTLRIKSDGNLIAPWFGHILSGESHGRILKTD
LKRGSCTVQCQTEKGGLNTTLPFQNVSKYAFGNCSKYIGIKSLKLAVGLRNVPSRSSRGLFGAIAGFIEG
GWSGLVAGWYGFQHSNNQGVGMAADRDSTQKAIDKITSKVNNIVDKMNKQYEIIDHEFNEVETRLNMINN
KIDDQIQDIWAYNAELLVLLENQKTLDEHDANVNNLYNKVKRALGSNAVEDGKGCFELYHKCDDQCMETI
RNGTYNRRKYQEESKLERQKIEGVKLESEGTYKILSIYSTVASSLVIAMGFAAFLFWAMSNGSCRCNICI

>QBK20191.1 hemagglutinin [Influenza A virus]
MDTVSLITILLVATVSNADKSGIGYQSTNSTETVDTLTENNVPVTHAKELLHTEHNGMLCATSLGQPLVL
DTCTIEGLIYGNPSCDLSLEGREWSYIVERPSAVNGLCYPGNVENLEELRSLFSSARSYQRIQIFPDTIW
NVSYDGTSSACSGSFYKSMRWLTRKNGDYPIQDAQYTNNQGKNILFMWGINHPPTDTTQRDLYTRTDTTT
SVATEEINRIFKPLIGPRPLVNGLMGRIDYYWSVLKPGQTLPIKSDGNLIAPWYGHILSRESHGRILTTD
LKRGSCTVQCQTEKGGLNTTLPFQNVSKYAFGKCSKYIGIKSLKLAVGLRNVRSRYSRGLFGAIAGFIEG
GWSGLVDGWYGFQHSNDHGVGMAADRDSTQKAIDKITSKVNNIVAKMNKQYEIIDHEFSEVETRLNMINN
KIDDQIQDIWAYNAELLVLLENQKTLDEHDANVNNVYNKVKRALGSNAVEDGKGCFELYHKCDDQCMETI
RNGTFNRRKYQEESKLERQKIEGVKLEYEGTYKILSIYSTVASYLVIAMGFAAFLFWAMSNGSCRCNICI

>QBK20190.1 hemagglutinin [Influenza A virus]

MDTVSLITILLIATVSNADKICIGYQSTNSTETVDTLTENNVPVTHAKELLHTEHNGMLCATSLGQPLVL

DTCTIEGLIYGNPSCDLSLEGREWSYIVERPSAVNGLCYPGNVENLEELRSLFSSARSYQRIQIFPDTIW

NVSYYGTSSACSGSFYKSMRWLTRKNGDYPIQDAQYTNNQGKNILFMWGINHPPTDTTQRDLYTRTDTTT

SVATEEINRIFKPLIGPRPLVNGLMGRIDYYWSVLKPGQTLRIKSDGNLIAPWYGHILSRESHGRILTTD

LKRGSCTVQCQTEKGGLNTTLPFQNVSEYAFGKCSKYIGIKSLKLAVGLRNVRSRYSRGLFGAIAGFIEG

GWSGLVDGWYGFQHSNDQGVGMAADRDSTQKAIDKITSKVNNIVAKMNKQYEIIDHEFSEVETRINMINN

KIDDQIQDIWAYNAELLVLLENQKTLDEHDANVNNLYNKVKRALGSNAVEDGKGCFEQYHKCDDQCMETI

RNGTYNRRKYQAESKKERQKIEGAKLEYERTYKILSIYSTVASSLVIAMGSAAFLFWAMSNGSCRCNICI

>QBK20189.1 hemagglutinin [Influenza A virus]

METVSLITILLVSAVSNADKICIGYQSTNSTETVDTLTENNVPVTHAKELLHTEHNGMLCATSLGQPLIL

DTCTIEGLIYGNPSCDLSLEGREWSYIVERPSAVNGLCYPGNVQNLEELRSLFSSARSYQRIQIFPHTIW

NVSYYGTSTACSGSFYKSMRWLTRKSGDYPIQDAQYTNNQGKNILFTWGINHPPTDTTQRDLYTRTDTTT

SVATEEINRVFKPLIGPRPLVNGLMGRIDYYWSVLKPGQTLRIKSDGNLIAPWFGHILSRESHGRILTTD

LKRGSCTAQCQREKGGLHTAQSLQNVSKYAFGECSKYIGIKSLKLAVGLRNVPSRYSRGLFGAIAGFIEG

GWSGLVGGWYGFQHSNDQGVGMAADRDSTQKAMDKITSNVNNIVAKMNKPYEIIDLEFIVVGTRIDTSSH

KSDDQIQDIWAYNAESIVLLENQKTLDEQDVNVSNGYNKVKRAACSNAVEDGKGCLEQCHKCDDQCMETI

RNGTYNRRKYQAESKLERQKMEGLKLESERTNKILSMCSRVAWYRVIAMGSVALLIWAMYNWSCRCNICI

>QBK20188.1 hemagglutinin [Influenza A virus]

MDTVSLITILLIATVSNADKICIGYQSTNSTETVDTLTENNVPVTHAKELLHTEHNGMLCATSLGQPLIL

DTCTIEGLIYGNPSCDLSLEGREWSYIVERPSAVNGLCYPGNVENLEELRSLFSSARSYQRIQIFPDTIW

NVSYYGTSTACSGSFYRSMRWLTRKNGDYPVQDAQYTNNQGKNILFMWGINHPPTDTTQRDLYTRTDTTT

SVATEEINRIFKPLIGPRPLVNGLMGRIDYYWSVLKPGQTLRIKSDGNLIAPWYGHILSRESHGRILTTD

LKRGSCTVQCQTEKGGLHTALPFQNVSKYAFGKCSKYIGIKSLKLAVGLRNVRCRSSRGLFGAIAGFIEG

GWSGLVAGWYGFQHSNDQGVGMAADRDSTQKAIDKITSKVNNIVDKMNKQYEIIDHEFSVVETRINMINN

KIDDQIQDIWAYNAELLVLLENQKTLDEHDANVNNLYNKVKRALGSNAVEDGKGCFELYHKCDDQCMETI

GNGTYNRRKYQEESKLERQKIEGVKLESEGTNKILSIYSTVASSLVIAMGSAAFLFWAMSNWSCRCNICI

>QBK20186.1 hemagglutinin [Influenza A virus]

MDTVSLITMLLVSSVSNANKICIGYQSTNSAETVDTLTQNNVPVTHAVELLHTQHNGMLCATSLGQPLIL

DTCTIEGLIYGNPSCDLSLEGREWSYIVERPSAVNGLCYPGNVQNLEELRSLFSSARSYQRLQIFPDTIW

NVSYYGTSTACSGSFYRSMRWLTQKNGEYPTQDAQYTNNQGKNILFMWGINHPPTDTTQRDLYTRTDTTT

SVATEEINRIFKPLIGPRPLVNGLMGRIDYYWSILKPGQTLRIKSDGNLIAPWDGHILSRESHGRILTTD
LERGSCTVQCQRENGELHTALPLLNVSKYAFGRCSKYIGIKSLKRAVGLRNVPSRYIRGLFGAIAGFIEG
GWSGLVAGWYGFQHSNDQGVGMAADRDSTQKAIDKITSKVINIVAKMYKQYEIIDHAFSEVETRINMINN
KIDDQIQDIWAYNAEAIVFNDNQKTSEEHDAKPDNEYNKVKRALGSNAVEDGKGCSEQYHKCEKECMETI
GNGTFNRRECQAESKKERQKIEGVDLESDRTWKILSIYSRVTSYRVIAVGSFALMIEALYNWSCRCNICI

>QBK20185.1 hemagglutinin [Influenza A virus]
METVSLITILLIATVSNADKICIGYQSTNSTETVDTLTENNVPVTHAKELLHTEHNGMLCATSLGQPLIL
DTCTIEGLIYGNPSCDLSLEGREWSYIVERPSAVNGLCYPGNVENLEELRSLFSSARSYQRIQIFPDTIW
NVSYDGTSTACSGSFYRSMRWLTRKNGDYPVQDAQYTNNQGKNILFMWGINHPPTDTKQRDLYTRTDTTT
SVATEEINRIFKPLIGPRPLVNGLMGRIDYYWSVLKPGQTLRIKSDGNLIAPWYGHILSGESHGRILTTD
LKRGSCTVQCQTEKGGLNTALPFQNVSKYAFGNCSKYIGIKSLKLAVGLRNVRCRSSRGLFGAIAGFIEG
GWSGLVAGWYGFQHSNDQGVGMAADRDSTQKAIDKITSKVNNIVDKMNKQYEIIDHEFSEVETRLNMINN
KIDDQIQDIWAYNAESLVLLENQKTLDEHDANVNNLYNKVKRALGSNAVEDGKGCFELYHKCDDQCMETI
RNGTYNRRKYQAESKLERQKIEGVKLESEGTYKILSIYSTVASSLVIAMGFAAFLFWAMSNGSCRCNICI

>QBK20184.1 hemagglutinin [Influenza A virus]
METVSLITILLIATVSNADKICIGYQSTNSTETVDTLTENNVPVTHAKELLHTEHNGMLCATSLGQPLIL
DTCTIEGLIYGNPSCDLSLEGREWSYIVERPSAVNGLCYPGNVENLEELRSLFSSARSYQRIQIFPDTIW
NVSYYGTSTACSGSFYRSMRWLTRKNGDYPVQDAQYTNNQGKNILFMWGINHPPTDTTQRNLYTRTDTTT
SVATEEINRIFKPLIGPRPLVNGLMGRIDYYWSVLKPGQTLRIKSDGNLIAPWYGHILSRESHGRILKTD
LKRGSCTVQCQTEKGGLNTALPLQNVSKYAFGNCSKYIGIKSLKLAVGLRNVRCRSSRGLFGAIAGFIEG
GWSGLVGGWYGFQHSNDQGVGMAADRDSTQKAIDKITSKVNNIVDKMNKQYEIIDHEFSVVETRINMINN
KFDDQIQDIWAYNAESLVLLENQKTLDEHDANVNNLYNKVKRALGSNAVEDGKGCFEQYHKCDDQCMETI
RNGTYNRRKYQEESKLERQKIEGVKLESEGTYKILSIYSTVASSRVIAMGFAAFLFWAMSNGSCRCNICI

>QBK20182.1 hemagglutinin [Influenza A virus]
METVSLITILLIATVSNADKICIGYQSTNSAETVDTLTQNNVPVTHAKELLHTEHNGMLCATSLGQPLIL
DTCTIEGLIYGNPSCDLSLEGREWSYIVERPSPVNGLCYPGNVQNLEELRSLFSSARSYQKLQIFPDTIW
NVSYYGTSTACSGSFYRIMRWLTRKNGEYPTQDAQYTNNQGKNILFMWGINHPPTDTTQRDLYTRTDTTT
SVATEEINRIFKPLIGPRPLVNGLMGRIDYYWSILKPGQTLRIKSDGNLIAPWYGHILSRESHGRILTTD
SEGGSCTVQCQTEKGGLNTALSLQNVSKYAFGRCSKYIGIKSVKRAVGLRNVRSRYSRGLFGAIAGFIEG
GMSGLVGGWYGFQHSNDQGVGMAADRDSTQKAIDKIPSKVNNIVAKMYKQYEIIDHAFSEVETRLNMINN
KTDDQIQDIWAYNAESIVFLDNQKTLDEHDAKVNNLYNKVKRALGSNAVEDGKGCFEIYHKCDDQCMETI

GNGTYNRRKYQAESKLERQKIEGVKLESEGTYKILSICSRVAWYRVIAVGSAAFLFGAMYNGSCRCNICI

>QBK20181.1 hemagglutinin [Influenza A virus]
MDTVSLITILLISSVSNADKICIGYQSTNSAETVDTLTENNVPVTHAKELLHTEHNGMLCATSLGQPLIL
DTCTIEGLIYGNPSCDLSLEGREWSYIVERPSAVNGLCYPGNVENLEELRSLFSSARSYQRIQIFPHTIW
NVSYYGTSTACSGSFYRSMRWLTRKNGDYPVQDAQYTNNQGKNILFMWGINHPPTDTTQRDLYTRTDTTT
SVATEEINRIFKPLIGPRPLVNGLMGRIDYYWSVLKPGQTLRIKSDGNLIAPWYGHILSRESHGRILTTD
LKRGSCTVQCQTEKGGLHTALPLLNVSKYAFGKCSKYIGIKSLKLAVGLRNVRSRSSRGLFGAIAGFIEG
GWSGLVAGWYGFQHSNDQGVGMAADRDSTQKAMDKITSNVNNIVAKMYKQYEIIDLEFSVVGTRIIMINN
KFDDQIQDIWAYNAESIVSLENQKTLDEHDANVNNLGNKVKRGAGSNAVEDGKGCFEQYHKCDDQCMETI
RNGTYNRREYQEESKLEREKIEGLDLESEGTYEILSICSRVASYRVIAMGFVAFLFGALYNGSCRCNICI

>QBK20180.1 hemagglutinin [Influenza A virus]
MDTVSLITILLIATVSNADKICIGYQSTNSAETVDTLTENNVPVTHAKELLHTEHNGMLCATSLGQPLIL
DTCTIEGLIYGNPSCDLSLEGREWSYIVERPSAVNGLCYPGNVENLEELRSLFSSARSYQRIQIFPDTIW
NVSYYGTSTACSGSFYRSMRWLTRKNGDYPVQDAQYTNNQGKNILFMWGINHPPTDTTQRDLYTRTDTTT
SVATEEINRIFKPLIGPRPLVNGLMGRIDYYWSVLKPGQTLRIKSDGNLIAPWYGHILSRESHGRILTTD
LKRGSCTVQCQTEKGGLHTALPFQNVSKYASGKCSKYIGIKSLKLAVGLRNVRCRSSRGLFGAIAGFIEG
GWSGLVAGWYGFQHSNDQGVGMAADRDSTQKAIDKITSKVNNIVDKMNKQYEIIDHEFSVVGTRINTSNN
KFDDQIQDIWAYNAESLVLLENQKTLDEQDANVNNLYNKVKRAAGSNAVEDGKGCFEQYHKCDDQCMETI
RNGTYNRRKYQEESKLERQKMEGLKLESEGTWKILSIYSRVASYLVIAMGSVAFLFWAMSNWSCRCNICI

>QBK20179.1 hemagglutinin [Influenza A virus]
METVSLITILLIATVSNADKICIGYQSTNSTETVDTLTENNVPVTHAKELLHTEHNGMLCATSLGQPLIL
DTCTIEGLIYGNPSCDLSLEGREWSYIVERPSAVNGLCYPGNVQNLEELRSLFSSARSYQRIQIFPHTIW
NVSYYGTSTACSGSFYKSMRWLTRKNGDYPIQDAQYTNNQGKNILFMWGINHPPTDTTQRDLYTRIDTTT
SVATEEINRVFTPLIGPRPLVNGLMGRIDYYWSVLKPGQTLRIKSDGNLIAPWFGHILSGESHGRILTTD
LKRGSCTVQCQREKGGLHSTLSLLNVSKYAFGKCSKYIGIKSLKLAVGLRNVPSRYSRGLFGAIAGFIEG
GWSGLVAGWYGFQHSNDQGVGMAADRDSTQKAIDKITSKVNNIVDKMNKQYEIIDLEFSEVETRINTINN
KFDDQIQDIWAYNAESLVLLENQKTLDEHDANVNNLYNKVKRALGSNAVEDGKGCLELYHKCDDQCMETI
RNGTYNRRKYQAESKLERQKIEGVKQESEGTYKILSIYSTVASYRVIAMGFAAFLFWAMYNGSCRCNICI

>QBK20178.1 hemagglutinin [Influenza A virus]
MDTVSLITMLLLSTVSNADKICIGYQSTNSAETVDTLTENNVPVTHAKELLHTEHNGMLCATSLGQPLIL

DTCTIEGLIYGNPSCDLSLEGREWSYIVERPSAVNGLCYPGNVENLKELMSLFSSARSYQKNQIFPHTIW
NVSYYGTSTACSGSFYRSMRWLTRKNGDYPVQDAQYTNNQGKNILFMWGINHPPTDTNQIDLYTITDTTT
SVATEEINRIFKPLIGPRPLVNGLMGRIDYYWSVLKPGQTLRIKSDGNLISPWYGHILSRESHGRILTTD
LNRGSCTVQCQTEKGGLNTTLPFQNVSKYAFGKCSKYIGIKSLKLAVGLRNVPCRSSRGLFGAIAGFIER
GWSGLVGGWYGFQHSNDQGVGMAADRDSTQKAIDKITSKVNNIVDKMNKPYEIIDHEFSEVETRLNTINN
KIDDQIQDIWAYNAESLVLLENQKTLDEHDANVNNLYNKVKRALGSNAVEDGKGCLELYHKSDDQCMETI
GNGTYNRRKYQAESKLERQKMEGLKQESDRTYEILSIYSTVASYRVIAMGFAALRFGAMYNGSCRCNSSI

>QBK20177.1 hemagglutinin [Influenza A virus]
METVSLITILLIATVSNADKICIGYQSTNSTETVDTLTENNVPVTHAKELLHTEHNGMLCATSLGQPLIL
DTCTIEGLIYGNPSCDLSLEGREWSYIVERPSAVNGLCYPGNVENLEELRSLFSSARSYQRIQIFPDTIW
NVSYDGTSTACSGSFYRSMRWLTRKNGDYPVQDAQYTNNQGKNILFMWGINHPPTDTTQRDLYTRTDTTT
SVATEEINRIFKPLIGPRPLVNGLMGRIDYYWSVLKPGQTLRIKSDGNLIAPWYGHILSGESHGRILKTD
LKRGSCTVQCQTEKGGLNTTLPFQNVSKYAFGNCSKYIGIKSLKLAVGLRNVPSRSSRGLFGAIAGFIEG
GWSGLVAGWYGFQHSNDQGVGMAADRDSTQKAIDKITSKVNNIVDKMNKQYEIIDHEFSEVETRLNMINN
KIDDQIQDIWAYNAELLVLLENQKTLDEHDANVNNLYNKVKRALGSNAVEDGKGCFELYHKCDDQCMETI
RNGTYNRRKYQEESKLERQKIEGVKLESEGTYKILSIYSTVASSLVIAMGFAAFLFWAMSNGSCRCNICT

>QBK20176.1 hemagglutinin [Influenza A virus]
METVSLITILLIATVSNADKICIGYQSTNSTETVDTLTENNVPVTHAKELLHTEHNGMLCATSLGQPLIL
DTCTIEGLIYGNPSCDLSLEGREWSYIVERPSAVNGLCYPGNVENLEELRSLFSSARSYQRIQIFPDTIW
NVSYDGTSTACSGSFYRSMRWLTRKNGDYPVQDAQYTNNQGKNILFMWGINHPPTDTTQRDLYTRTDTTT
SVATEEINRIFKPLIGPRPLVNGLMGRIDYYWSVLKPGQTLRIKSDGNLIAPWYGHILSRESHGRILKTD
LKRGSCTVQCQTEKGGLNTTLPFQNVSKYAFGNCSKYIGIKSLKLAVGLRNVPSRSSRGLFGAIAGFIEG
GWSGLVAGWYGFQHSNDQGVGMAADRDSTQKAIDKITSKVNNIVDKMNKQYEIIDHEFSEVETRLNMINN
KIDDQIQDIWAYNAELLVLLENQKTLDEHDANVNNLYNKVKRALGSNAVEDGKGCFELYHKCDDQCMETI
RNGTYNRRKYQEESKLERQKIEGVKLESEGTYKILSIYSTVASSLVIAMGFAAFLFWAMSNGSCRCNSCI

>QBK20175.1 hemagglutinin [Influenza A virus]
METVSLITILLIATVSNADKICIGYQSTNSTETVDTLTENNVPVTHAKELLHTEHNGMLCATSLGQPLIL
DTCTIEGLIYGNPSCDLSLEGREWSYIVERPSAVNGLCYPGNVENLEELRSLFSSARSYQRIQIFPDTIW
NVSYDGTSTACSGSFYRSMRWLTRKNGDYPVQDAQYTNNQGKNILFMWGINHPPTDTTQRDLYTRTDTTT
SVATEEINRIFKPLIGPRPLVNGLMGRIDYYWSVLKPGQTLRIKSDGNLIAPWYGHILSRESHGRILKTD
LKRGSCTVQCQTEKGGLNTTLPFQNVSKYAFGNCSKYIGIKSLKLAVGLRNVPSRSSRGLFGAIAGFIEG

GWSGLVAGWYGFQHSNDQGVGMAADRDSTQKAIDKITSKVNNIVDKMNKQYEIIDHEFSEVETRLNMINN
KIDDQIQDIWAYNAELLVLLENQKTLDEHDANVNNLYNKVKRALGSNAVEDGKGCFELYHKCDDQCMETI
RNGTYNRRKYQEESKLERQKIEGVKLESEGTYKILSIYSTVASSLVIAMGFAAFLFWAMSNGSCRCNICI

>QBK20174.1 hemagglutinin [Influenza A virus]
MDTVSLITMLLVSTVSNADKICIGYQSTNSAETVDTLTENNVPVTHAKELLHTEHNGMLCATSLGQPLIL
DTCTIEGLIYGNPSCDLSLEGREWSYIVERPSAVNGLCYPGNVENLKELMSLFSSARSYQKIQIFPHTIW
NVSYYGTSTACSGSFYRSMRWLTRKNGDYPVQDAQYTNNQGKNILFMWGINHPPTDTTQRDLYTRTDTTT
SVATEEINRIFKPLIGPRPLVNGLMGRIDYYWSVLKPGQSLRIKCDGNLIATWYGHILSGESHGRIVKTD
LKRGSCTVQCQREKGGLNTTLPLQNVSKYAFGKCSKYIGIKSLKLAVGLRNVRCRSSRGLFGAIAGFIEG
GWSGLVAGWYGFQHSNDQGVGMAADRDSTQKAIDKITSKVNNIVDKMNKQYEIIDHEFSEVETRLNMINN
KIDDQIQDIWAYNAESIVLLENQKTLDEHDANVNNLYNKVKRALGSNAVEDGKGCFELYHKCDDQCMETI
GNRTFNRRKYQAESKLERQKIEGVKQESEGTYKILSIYSTVASYRVIAMGFAAFLFWAMSNGSGRRNSSI

>QBK20173.1 hemagglutinin [Influenza A virus]
METVSLITILLVSTVSNADKICIGYQSTNSTETVDTLTENNVPVTHAKELLHTEHNGMLCATSLGQPLIL
DTCTIEGLIYGNPSCDLSLEGREWSYIVERPSAVNGLCYPGNVENLEELRSLFSSARSYQRIQIFPDTIW
NVSYDGTSTACSGSFYRSMRWLTRKNGDYPVQDAQYTNNQGKNILFMWGINHPPTDTTQRDLYTRTDTTT
SVATEEINRIFKPLIGPRPLVNGLMGRIDYYWSVLKPGQTLRIKSDGNLIAPWYGHILSGESHGRILKTD
LKRGSCTVQCQTEKGGLNTTLPFQNVSKYAFGNCSKYIGIKSLKLAVGLRNVPSRSSRGLFGAIAGFIEG
GWSGLVAGWYGFQHSNDQGVGMAADRDSTQKAIDKITSKVNNIVDKMNKQYEIIDHEFSEVETRLNMINN
KIDDQIQDIWAYNAELLVLLENQKTLDEHDANVNNLYNKVKRALGSNAVEDGKGCFELYHKCDDQCMETI
RNGTYNRRKYQEESKLERQKIEGVKLEFEGTYKILSIYSTVASYLVIAMGFAAFLFWAMSNGSCRCNICI

>QBK20172.1 hemagglutinin [Influenza A virus]
METVSLITILLIATVSNADKICIGYQSTNSTETVDTLTENNVPVTHAKELLHTEHNGMLCATSLGQPLIL
DTCTIEGLIYGNPSCDLSLEGREWSYIVERPSAVNGLCYPGNVENLEELRSLFSSARSYQRIQIFPDTIW
NVSYDGTSTACSGSFYRSMRWLTRKNGDYPVQDAQYTNNQGKNILFMWGINHPPTDTTQRDLYTRTDTTT
SVATEEINRIFKPLIGPRPLVNGLMGRIDYYWSVLKPGQTLRIKSDGNLIAPWYGHILSGERHGRILKTD
SKRGSCTVQCQTDKGGLNKALRLLNVSKYAFGNCSKYIGIKSLKLAVGLRNVRSRSSRGLFGAIAGFIER
GWSGLVAGWYGFQHSNDQGVGMAADRDSTQKAIDKITSKVNNIVDKMNKQYEIIDHEFSEVETRLNMINN
KIDDQIQDIWAYNAELLVLLENQKTLDEHDANVNNLYNKVKRALGSNAVEDGKGCFELYHKCDDQCMETI
RNGTYNRRKYQEESKLERQKIEGVKLESEGTYKILSIYSTVASSLVIAMGSAAFLFWAMSNGSCRCNSST

>QBK20171.1 hemagglutinin [Influenza A virus]
METVSLITILLIATVSNADKSCIGYQSTNSTETVDTLTENNVPVTHAKELLHTEHNGMLCATSLGQPLIL
DTCTIEGLIYGNPSCDLSLEGREWSYIVERPSAVNGLCYPGNVENLEELRSLFSSARSYQRIQIFPDTIW
NVSYDGTSTACSGSFYRSMRWLTRKNGDYPVQDAQYTNNQGKNILFMWGINHPPTDTTQRDLYTRTDTTT
SVATEEINRIFKPLIGPRPLVNGLMGRIDYYWSVLKPGQTLRIKSDGNLIAPWYGHILSGESHGRILKTD
LKRGSCTVQCQTEKGGLNTALSWQNVSKYAFGNCSKYIGIKSLKLAVGLRSVRCRSSRGLFGAIAGFIEG
GWSGLVAGWYGFQHSNDQGVGMAADRDSTQKAIDKITSKVNNIVDKMNKQYEIIDHEFSEVETRINMINN
KIDDQIQDIWAYNAESLVLLENQKTLDEHDANVNNLYNKVKRALGSNAVEDGKGCFELYHKCDDQCMETI
RNGTYNRRKYQEESKLERQKIEGVKLESEGTYKILSIYSTVASSLVIAMGSAAFLFWAMSNGSRRCNIST

>QBK20170.1 hemagglutinin [Influenza A virus]
METVSLITILLIATVSNADKICIGYQSTNSTETVDTLTENNVPVTHAKELLHTEHNGMLCATSLGQPLIL
DTCTIEGLIYGNPSCDLSLEGREWSYIVERPSAVNGLCYPGNVENLEELRSLFSSARSYQRIQIFPDTIW
NVSYDGTSTACSGSFYRSMRWLTRKNGDYPVQDAQYTNNQGKNILFMWGINHPPTDTTQRDLYTRTDTTT
SVATEEINRIFKPLIGPRPLVNGLMGRIDYYWSVLKPGQTLRIKSDGNLIAPWYGHILSGESHGRILKTD
LKRGSCTVQCQTEKGGLNTTLPFQNVSKYAFGKCSKYIGIKSLKLAVGLRNVRCRSSRGLFGAIAGFIEG
GWSGLVAGWYGFQHSNDQGVGMAADRDSTQKAIDKITSKVNNIVDKMNKQYEIIDHEFSEVETRINMINN
KIDDQIQDIWAYNAELLVLLENQKTLDEHDANVNNLYNKVKRALGSNAVEDGKGCFELYHKCDDQCMETI
RNGTYNRRKYQEESKLERQKIEGVKLEFEGTYKILSIYSTVASSLVIAMGSAAFLFWAMSNGSRRCNISI

>QBK20169.1 hemagglutinin [Influenza A virus]
MDTVSLITILLISTVSNADKICIGYQSTNSTETVDTLTENNVPVTHAKELLHTEHNGMLCATSLGQPLIL
DTCTIEGLIYGNPSCDLSLEGREWSYIVERPSAVNGLCYPGNVENLEELRSLFSSARSYQKIQIFPDTIW
NVSYYGTSTACSGSFYRSMRWLTRKNGDYPVQDAQYTNNQGKNILFMWGINHPPTDTTQRDLYTITDTTT
SVATEEINRIFTPLIGPRPLVNGLMGRIDYYWSVLKPGQTLRIKSDGNLIAPWYGHILSGESHGRILTTD
LTRGSCTVSCQTEKGGLHTALPLQNVSKYAFGKCSKYIGIECVKLAVGLRNVRSRSSRGLFGAIAGFIER
GWSGLVAGWYGFQHSNDQGVGMAADRDSTQTAIDKITSKVNNIVDKMYKPYEIIDHEFSEVGTRINMINN
KIDDQIQDIWAYNAESLVLLENQKTLDEQDANVNNLYNKVKRALGSNAVEDGKGCFELYHKCDDQCMETI
RNGTYNRRKYQEESKLERQKIEGVKLEFEGTCKILSIYSTVASYRVIAMGSVAFLFGAMYNGSCRCNICI

>QBK20168.1 hemagglutinin [Influenza A virus]
METVSLITILLVATVSNADKICIGYQSTNSTETVDTLTENNVPVTHAKELLHTEHNGMLCATSLGQPLIL
DTCTIEGLIYGNPSCDLSLEGREWSYIVERPSAVNGLCYPGNVENLEELRSLFSSARSYQRIQIFPDTIW
NVSYYGTSTACSGSFYRSMRWLTRKNGDYPVQDAQYTNNQGKNILFMWGINHPPTDTTQRDLYTRTDTTT

SVATEEINRIFKPLIGPRPLVNGLMGRIDYYWSVLKPGQTLRIKSDGNLIAPWYGHILSGESHGRILKTD
LKRGSCTVQCQTEKGGLNTTLPLQNVSKYAFGNCSKYIGIKSLKLAVGLRNVRCRSSRGLFGAIAGFIEG
GWSGLVAGWYGFQHSNDQGVGMAADRDSTQKAIDKITSKVNNIVDKMYKQYEIIDHEFSEVETRINMINN
KIDDQIQDIWAYNAESLVLLENQKTLDEHDANVNNLYNKVKRALGSNAVEDGKGCFELYHKCDDQCMETI
RNGTYNRRKYQEESKLERQKIEGVKLESEGTYKILSIYSTVASYLVIAMGSAAFLFWAMYNGSCRCNICI

>QBK20167.1 hemagglutinin [Influenza A virus]
MHTVSLITILLISTVSNADKICIGYQSTNSAETVDTLTENNVPVTHAKELLHTEHNGMLCATSLGQPLIL
DTCTIEGLIYGNPSCDLSLEGREWSYIVERPSAVNGLCYPGNVENLEELRSLFSSARSYQRIQIFPHTIW
NVSYYGTSTACSGSFYRSMRWLTRKNGDYPVQDAQYTNNQGKNILFMWGINHPPTDTTQRDLYTRTDTTT
SVATEEINRIFKPLIGPRPLVNGLMGRIDYYWSVLKPGQTLRIKSDGNLIAPWYGHILSRESHGRILKTD
LKRGSCTVQCQTEKGGLNTTLPLQNVSKYAFGNCSKYIGIKSLKLAVGLRNVRCRSSRGLFGAIAGFIER
GWSGLVAGWYGFQHSNDQGVGMAADRDSTQKAIDKITSKVNNIVAKLYKQYEIIDHEFSEVETRINMINN
KFDDQIQDIWAYNAESIVLLENQKTLDEHDANVNNLYNKVKRALGSNAVEDGKGCFEQYHKCDDQCMETI
RNGTYNRRKYQAESKLERQKIEGLNLESEGTNEILSIYSRVASSLVIAMGFVAFLFGAMYNGSCRCNICI

>QBK20166.1 hemagglutinin [Influenza A virus]
MDTVSLITILLIATVSNADKICIGYQSTNSTETVDTLTENNVPVTHAKELLHTEHNGMLCATSLGQPLIL
DTCTIEGLIYGNPSCDLSLEGREWSYIVERPSAVNGLCYPGNVENLEELRSLFSSARSYQRIQIFPDTIW
NVSYYGTSTACSGSFYRSMRWLTRKNGDYPVQDAQYTNNQGKNILFMWGINHPPTDTTQRDLYTRTDTTT
SVATEEINRIFKPLIGPRPLVNGLMGRIDYYWSVLKPGQTLRIKSDGNLIAPWYGHILSRESHGRILTTD
LKRGSCTVQCQTEKGGLNTALSLQNVSKYAFGKCSKYIGIKSLKLAVGLRNVRCRSSRGLFGAIAGFIER
GWSGLVAGWYGFQHSNDQGVGMAADRDSTQKAIDKITSKVNNIVAKMNKPYEIIDHEFSVVETRINMINN
KFDDQIQDIWAYNAESLVLLENQKTLDEHDANVNNLYNKVKRAAGSNAVEVGKGCFELYHKCDDQCMETI
GNGTYNRRKYQEESKLERQKIEGLKLESEGTWKILSIYSTVASSRMIAMGSAAFLFGALSNWSCRCNICI

>QBK20165.1 hemagglutinin [Influenza A virus]
MDTVSLITMLLISTVSNADKICIGYQSTNSAETVDTLTENNVPVTHAKELLHTEHNGMLCATSLGQPLIL
DTCTIEGLIYGNPSCDLSLEGREWSYIVERPSAVNGLCYPGNVENLEELRSLFSSARSYQRIQIFPHTIW
NVSYYGTSTACSGSFYRSMRWLTRKNGDYPVQDAQYTNNQGKNILFMWGINHPPTDTTQRDLYTRTDTTS
SVATEEINRIFTPLIGPRPLVNGLMGRIDYYWSVLKPGQTLRIKSDGNLIAPWYGHILSGESHGTILTTD
LKRGSCTVQCQTEKGGLNTALPFQNVSKYAFGNCSKYIGIKSLKLAVGLRNVRCRSSRGLFGAIAGFIER
GWSGLVAGWYGFQHSNDQGVGMAADRDSTQKAIDKITSKVNNIVDKMNKQYEIIGHEFSEVETRINMINN
KFDDQIQDIWAYNAESIVLLENQKTLDEHDANVNNLYNKVKRALGSNAVEDGKGCFEQYHKCDDQCMETI

RNGTYNRREYQAESKLERQKIEGLKLELEGTSKSHSIYSTVASYRVIAMGSVAFLFWAMYNGSCRCNICI

>QBK20164.1 hemagglutinin [Influenza A virus]
METVSLITILLIATVSNADKICIGYQSTNSTETVDTLTENNVPVTHAKELLHTEHNGMLCATSLGQPLIL
DTCTIEGLIYGNPSCDLSLEGREWSYIVERPSAVNGLCYPGNVENLEELRSLFSSARSYQRIQIFPDTIW
NVSYYGTSTACSGSFYRSMRWLTRKNGDYPVQDAQYTNNQGKNILFMWGINHPPTDTTQRDLYTRTDTTT
SVATEEINRIFKPLIGPRPLVNGLMGRIDYYWSVLKPGQTLRIKSDGNLIAPWYGHILSRESHGRILTTD
LKRGSCTVQCQTEKGGLNTALPLQNVSKYAFGNCSKYIGIKSLKLAVGLRNVPCRSSRGLFGAIAGFIEG
GWSGLVAGWYGFQHSNDQGVGMAADRDSTQTAIDKITSKVNNIVDKMNKQYEIIDHEFSEIETRLNMINN
KIDDQIQDIWAYNAELLVLLENQKTLDEHDANVNNLYNKVKRALGSNAVEDGKGCFELYHKCDDQCMETI
RNGTYNRRKYQEESKLERQKIEGVKLESEGTYKILSIYSTVASSLVIAMGFAAFLFWAMSNGSCRCNICI

>QBK20163.1 hemagglutinin [Influenza A virus]
METVSLITILLVATVSNADKICIGYQSTNSTETVDTLTENNVPVTHAKELLHTEHNGMLCATSLGQPLIL
DTCTIEGLIYGNPSCDLSLEGREWSYIVERPSAVNGLCYPGNVENLEELRSLFSSARSYQRIQIFPDTIW
NVSYDGTSTACSGSFYRSMRWLTRKNGDYPVQDAQYTNNQGKNILFMWGINHPPTDTTQRDLYTRTDTTT
SVATEEINRIFKPLIGPRPLVNGLMGRIDYYWSVLKPGQTLRIKSDGNLIAPWYGHILSRESHGRILTTD
LKRGSCTVQCQTEKGGLNTTLPFQNVSKYAFGNCSKYIGIKSLKLAVGLRNVRCRSSRGLFGAIAGFIEG
GWSGLVAGWYGFQHSNDQGVGMAADRDSTQKAIDKITSKVNNIVDKMNKQYEIIDHEFSEVETRINMINN
KIDDQIQDIWAYNAESLVLLENQKTLDEHDANVNNLYNKVKRALGSNAVEDGKGCFELYHKCDDQCMETI
RNGTYNRRKYQEESKLERQKIEGVKLESEGTYKILSIYSTVASSLVIAMGFAAFLFWAMSNGSCRCNICI

>QBK20162.1 hemagglutinin [Influenza A virus]
METVSLITILLIATVSNADKICIGYQSTNSTETVDTLTENNVPVTHAKELLHTEHNGMLCATSLGQPLIL
DTCTIEGLIYGNPSCDLSLEGREWSYIVERPSAVNGLCYPGNVENLEELRSLFSSARSYQRIQIFPDTIW
NVSYDGTSTACSGSFYRSMRWLTRKNGDYPVQDAQYTNNQGKNILFMWGINHPPTDTTQRDLYTRTDTTT
SVATEEINRIFKPLIGPRPLVNGLMGRIDYYWSVLKPGQTLRIKSDGNLIAPWYGHILSGESHGRILKTD
LKRGSCTVQCQTEKGGLNTTLPFQNVSKYAFGNCSKYIGIKSLKLAVGLRNVPSRSSRGLFGAIAGFIEG
GWSGLVAGWYGFQHSNDQGVGMAADRDSTQKAIDKITSKVNNIVDKMNKQYEIIDHEFSEVETRLNMINN
KIDDQIQDIWAYNAELLVLLENQKTLDEHDANVNNLYNKVKRALGSNAVEDGKGCFELYHKCDDQCMETI
RNGTYNRRKYQEESKLERQKIEGVKLESEGTYKILSIYSTVASSLVIAMGFAAFLFWAMSNGSCRCNISI

>QBK20161.1 hemagglutinin [Influenza A virus]
MDTVSLITILLISTVSNADKICIGYQSTNSTETVDTLTENNVPVTHAKELLHTEHNGMLCATSLGQPLIL

DTCTIEGLIYGNPSCDLSLEGREWSYIVERPSAVNGLCYPGNVENLEELRSLFSSARSYQRIQIFPDTIW

NVSYYGTSTACSGSFYRSMRWLTRKNGDYPVQDAQYTNNQGKNILFMWGINHPPTDTTQRDLYTRTDTTS

SVATEEINRIFKPLIGPRPLVNGLMGRIDYYWSVLKPGQTLRIKSDGNLIAPWYGHILSGESHGRILTTD

LKRGSCTVQCQTEKGGLHTAQSFQNVSKYASGKCSKYIGIKSLKLAVGLRNVRCRSSRGLFGAIAGFIER

GWSGLVAGWYGFQHSNDQGVGMAADRDSTQKAIDKITSKVNNIVDKMNKQYEIIDHEFSVVETRINMINN

KFDDQIQDIWAYNAESLVLLENQKTLDEHDANVNNLYNKVKRALGSNAVEDGKGCFELYHKCDDQCMETI

RNGTYNRRKYQEESKLERQKIEGVKLESEGTYKILSIYSTVTSSLVIAMGSAAFLFWAMSNGSCRCNICI

>QBK20160.1 hemagglutinin [Influenza A virus]

MDTVSLITILLIATVSNADKICIGYQSTNSTETVDTLTENNVPVTHAKELLHTEHNGMLCATSLGQPLIL

DTCTIEGLIYGNPSCDLSLEGREWSYIVERPSAVNGLCYPGNVENLEELRSLFSSARSYQRIQIFPDTIW

NVSYDGTSTACSGSFYRSMRWLTRKNGDYPVQDAQYTNNQGKNILFMWGINHPPTDTTQRDLYTRTDTTT

SVATEEINRIFKPLIGPRPLVNGLMGRIDYYWSVLKPGQTLRIKSDGNLIAPWYGHILSGESHGRILTTD

LKRGSCTVQCQTEKGGLNTALPFQNVSKYAFGNCSKYIGIKSLKLAVGLRNVRCRSSRGLFGAIAGFIEG

GWSGLVAGWYGFQHSNDQGVGMAADRDSTQKAIDKITSKVNNIVDKMNKQYEIIDHEFSEVETRLNMINN

KIDDQIQDIWAYNAESLVLLENQKTLDEHDANVNNLYNKVKRALGSNAVEDGKGCFELYHKCDDQCMETI

RNGTYNRRKYQEESKLERQKIEGVKLESEGTYKILSIYSTVASSRVIAMGSAAFLFWAMSNGSCRCNICI

>QBK20159.1 hemagglutinin [Influenza A virus]

MHTVSLITILLIATVSNADKICIGYQSTNSTETVDTLTENNVPVTHAKELLHTEHNGMLCATSLGQPLIL

DTCTIEGLIYGNPSCDLSLEGREWSYIVERPSAVNGLCYPGNVENLEELRSLFSSARSYQKIQIFPHTIW

NVSYYGTSTACSGSFYRSMRWLTRKNGDYPVQDAQYTNNQGKNILFMWGINHPPTDTTQRDLYTRTDTTT

SVATEEINRIFKPLIGPRPLVNGLMGRIDYYWSVLKPGQTLRIKSDGNLIAPWYGHILSGESHGRILTTD

LKRGSCTVQCQTEKGGLNTALPLLNVSKYAFGKCSKYIGIKSLKLAVGLRNVRCRSSRGLFGAIAGFIER

GWSGLVAGWYGFQHSNDQGVGMAADRDSTQKAIDKITSKVNNIVAKMNKQYEIIDHEFSEVETRINMINN

KIDDQIQDIWAYNAESIVLLENQKTLDEHDANVNNLYNKVKRALGSNAVEDGKGCFELYHKCDDQCMETI

RNGTYNRRKYQEESKLERQKIEGVKLESEGTYKILSIYSTVASYLVIAMGFAAFLFWAYSNGSCRCNICI

>QBK20158.1 hemagglutinin [Influenza A virus]

METVSLITILLVATVSNADKICIGYQSTNSTETVDTLTENNVPVTHAKELLHTEHNGMLCATSLGQPIIL

DTCTIEGLIYGNPSCDLSLEGREWSYIVERPSAVNGLCYPGNVENLEELRSLFSSARSYQRIQIFPDTIW

NVSYDGTSTACSGSFYRNMRWLTRKNGDYPIQDAQYTNNQGKNILFMWGINHPPADTTQRDLYTRTDTTT

SVATEEINRIFKPLIGPRPLVNGLMGRIDYYWSVLKPGQTLRIKSDGNLIAPWYGHILSRESHGRILKTD

LKRGSCTVQCQTEKGGLNTTLPFQNVSKYAFGNCSKYIGIKSLKLAVGLRNVRCRYSRGLFGAIAGFIEG

GWSGLVAGWYGFQHSNDQGVGMAADRDSTQKAVDKITSKVNNIVAKMYKPYEIIDHEFSVVETRINTINN
KFDDQIQDIWAYNAESLVLLENQKTLDEQDANVNNLYNKVKRAAGSNAVEDGKGCFELYHKCDDQCMETI
GNGTYNRRKYQEESKLERQKIEGHKLESEGTYKILSIYSTVASSRVIAMGFAAFLFWAMSNGSCRCNICI

>QBK20157.1 hemagglutinin [Influenza A virus]
METVSLITILLIATVSNADKICIGYQSTNSTETVDTLTENNVPVTHAKELLHTEHNGMLCATSLGQPIIL
DTCTIEGLIYGNPSCDLSLEGREWSYIVERPSAVNGLCYPGNVENLEELRSLFSSARSYQRIQIFPDTIW
NVSYDGTSTACSGSFYRNMRWLTRKNGDYPIQDAQYTNNQGKNILFMWGINHPPADTTQRDLYTRTDTTT
SVATEEINRIFKPLIGPRPLVNGLMGRIDYYWSVLKPGQTLRIKSDGNLIAPWYGHILSGESHGRILKTD
LKRGSCTVQCQTEKGGLNTTLPFQNVSKYAFGNCSKYIGIKSLKLAVGLRNVRSRYSRGLFGAIAGFIEG
GWSGLVAGWYGFQHSNDQGVGMAADRDSTQKAVDKITSKVNNIVDKMYKQYEIIDHEFSEVETRLNMINN
KIDDQIQDIWAYNAELLVLLENQKTLDEHDANVNNLYNKVKRALGSNAVEDGKGCFELYHKCDDQCMETI
GNGTYNRRKYQEESKLERQKIEGVKLESEGTYKILSIYSRVTSSRVIAMGFAAFLFWALYNWSCRCNISI

>QBK20156.1 hemagglutinin [Influenza A virus]
METVSLITILLIATVSNADKICIGYQSTNSAETVDTLTENNVPVTHAKELLHTEHNGMLCATSLGQPLIL
DTCTIEGLIYGNPSCDLSLEGREWSYIVERPSAVNGLCYPGNVENLEELRSLFSSARSYQRIQIFPDTIW
NVSYYGTSTACSGSFYRSMRWLTRKNGDYPVQDAQYTNNQGKNILFMWGINHPPTDTTQRDLYTRTDTTT
SVATEEINRIFKPLIGPRPLVNGLMGRIDYYWSVLKPGQTLRIKSDGNLIAPWYGHILSRESHGRILTTD
LKRGSCTVQCQTEKGGLHTAQPLQNVSKYAFGKCSKYIGIKSLKLAVGLRNVRCRSSRGLFGAIAGFIEG
GWSGLVAGWYGFQHSNDQGVGMAADRDSTQKAIDKITSKVNNIVAKMNKQYEIIDLEFSVVGTRINMINN
KFDDQIQDIWAYNAESLVLLENQKTLDEHDANVNNLYNKVKRALGSNAVEDGKGCFELYHKCDDQCMETI
RNGTYNRRKYQEESKLERQKIEGVKLESEGTCKILSIYSTVASSLVIAMGFAAFLFGAMSNGSCRCNICI

>QBK20155.1 hemagglutinin [Influenza A virus]
MDTVSLITILLIATVSNADKICIGYQSTNSTETVDTLTENNVPVTHAKELLHTEHNGMLCATSLGQPLIL
DTCTIEGLIYGNPSCDLSLEGREWSYIVERPSAVNGLCYPGNVENLEELRSLFSSARSYQRIQIFPDTIW
NVSYYGTSTACSGSFYRSMRWLTRKNGDYPVQDAQYTNNQGKNILFMWGINHPPTDTTQRDLYTRTDTTT
SVATEEINRIFKPLIGPRPLVNGLMGRIDYYWSVLKPGQTLRIKSDGNLIALWYGHILSGESHGRILTTD
LKRGSCTVQCQTEKGGLNTALPLQNVSKYAFGKCSKYIGIKSLKLAVGLRNVRCRSSRGLFGAIAGFIER
GWSGLVAGWYGFQHSNDQGVGMAADRDSTQKAIDKITSKVNNIVDKMNKQYEIIDHEFSVVGTRINTINN
KFDDQIQDIWAYNAESLVLLENQKTLDEHDANVNNLYNKVKRAAGSNAVEDGKGCFELYHKCDDQCMETI
RNGTYNRRKYQAESKLERQKIEGLKLESERTYKILSIYSTVASSLVIAMGSAAFLFWAMYNGSCRCNSSI

>QBK20154.1 hemagglutinin [Influenza A virus]

METVSLITILLVATVSNADKICIGYQSTNSTETVDTLTENNVPVTHAKELLHTEHNGMLCATSLGQPLIL

DTCTIEGLIYGNPSCDLSLEGREWSYIVERPSAVNGLCYPGNVENLEELRSLFSSARSYQRIQIFPDTIW

NVSYDGTSTACSGSFYRSMRWLTRKNGDYPVQDAQYTNNQGKNILFMWGINHPPTDTTQRDLYTRTDTTT

SVATEEINRIFKPLIGPRPLVNGLMGRIDYYWSVLKPGQTLRIKSDGNLIAPWYGHILSGESHGRILKTD

LKRGSCTVQCQTEKGGLNTTLPFQNVSKYAFGNCSKYIGIKSLKLAVGLRNVPSRSSRGLFGAIAGFIEG

GWSGLVAGWYGFQHSNDQGVGMAADRDSTQKAIDKITSKVNNIVDKMNKQYEIIDHEFSEVETRLNMINN

KIDDQIQDIWAYNAELLVLLENQKTLDEHDANVNNLYNKVKRALGSNAVEDGKGCFELYHKCDDQCMETI

RNGTYNRRKYQEESKLERQKIEGVKLESEGTYKILTIYSTVASSLVIAMGFAAFLFWAMSNGSCRCNICI

>QBK20153.1 hemagglutinin [Influenza A virus]

MATVSLITILLIATVSNADKICIGYQSTNSAETVDTLTENNVPVTHAKELLHTEHNGMLCATSLGQPLIL

DTCTIEGLIYGNPSCDLSLEGREWSYIVERPSAVNGLCYPGNVQNLEELRSLFSSARSYQRIQIFPDTIW

NVSYDGTSTACSGSFYRSMRWLTRKNGDYPTQDAQYTNNQGKNILFMWGINHPPTDDTQRNLYTRTDTTS

SVATEEINRIFTPLIGPRPLVNGLMGRIDYYWSVLKPGQTLRIKSDGNLIAPWYGHVLSGESHGRILTTD

LKRGSCTVPCQTENGGLHTALPLLNVSKYAFGKFSKYIGIKSLKLAVGLRNVPSRSSRGLFGAIAGFIEG

GWSGLVGGWYGFQHSNDQGVGMAADRDSTQKAIDKIPSKVNNIVAKLYKQYEIIDHEFSEVGTRINMSNN

KFDDQIQDIWAYNAESVVFLENQKTLDEHDINPDNEYNKVKRALGSNAVEDGKGCFRQYHKCNEECMETI

RNRTFNRREYQAESKLERQKIEGVKLEFEGTCKIVSIYSTVASYRVIAMGFAAFLFGALSNGSCRCNICI

>QBK20152.1 hemagglutinin [Influenza A virus]

METVSLITILLIATVSNADKICIGYQSTNSAETVDTLTENNVPVTHAKELLHTEHNGMLCATSLGQPLIL

DTCTIEGLIYGNPSCDLSLEGREWSYIVERPSAVNGLCYPGNVENLEELRSLFSSARSYQRIQIFPDTIW

NVSYYGTSTACSGSFYRSMRWLTRKNGDYPVQDAQYTNNQGKNILFMWGINHPPTDTTQRDLYTRTDTTT

SVATEEINRIFKPLIGPRPLVNGLMGRIDYYWSVLKPGQTLRIKSDGNLIAPWYGHILSGESHGRILTTD

LKRGSCTVQCQTEKGGLNTALPFQNVSKYAFGNCSKYIGIKSLKLAVGLRNVRCRSSRGLFGAIAGFIEG

GWSGLVAGWYGFQHSNDQGVGMAADRDSTQKAIDKITSKVNNIVDKMNKQYEIIDHEFSVVETRINMINN

KFDDQIQDIWAYNAESLVLLENQKTLDEHDANVNNLYNKVKRALGSNAVEDGKGCFELYHKCDDQCMETI

RNGTYNRRKYQAESKLERQKIEGLKQESEGTYKILSIYSTVASSLVIAMGSAAFLFGAMSNGSCRCNICI

>QBK20151.1 hemagglutinin [Influenza A virus]

METVSLITILLVATVSNADKICIGYQSTNSTETVDTLTENNVPVTHAKELLHTEHNGMLCATSLGQPLIL

DTCTIEGLIYGNPSCDLSLEGREWSYIVERPSAVNGLCYPGNVENLEELRSLFSSARSYQRIQIFPDTIW

NVSYDGTSTACSGSFYRSMRWLTRKNGDYPVQDAQYTNNQGKNILFMWGINHPPTDTTQRDLYTRTDTTT

SVATEEINRIFKPLIGPRPLVNGLMGRIDYYWSVLKPGQTLRIKSDGNLIAPWYGHILSGESHGRILKTD
LKRGSCTVQCQTEKGGLNTTLPFQNVSKYAFGNCSKYIGIKSLKLAVGLRNVPSRSSRGLFGAIAGFIEG
GWSGLVAGWYGFQHSNDQGVGMAADRDSTQKAIDKITSKVNNIVDKMNKQYEIIDHEFSEVETRLNMINN
KIDDQIQDIWAYNAELLVLLENQKTLDEHDANVNNLYNKVKRALGSNAVEDGKGCFELYHKCDDQCMETI
RNGTYNRRKYQEESKLERQKIEGVKLESEGTYKILTIYSTVASSLVIAMGFAAFLFWAMSNGSCRCNICI

>QBK20150.1 hemagglutinin [Influenza A virus]
MDTVSLITILLISTVSNADKICIGYQSTNSAETVDTLTENNVPVTHAKELLHTEHNGMLCATSLGQPLIL
DTCTIEGLIYGNPSCDLSLEGREWSYIVERPSAVNGLCYPGNVENLEELRSLFSSARSYQRIQIFPHTIW
NVSYYGTSTACSGSFYRSMRWLTRKNGDYPVQDAQYTNNQGKNILFMWGINHPPTDTTQRDLYTRTDTTT
SVATEEINRIFKPLIGPRPLVNGLMGRIDYYWSVLKPGQTLRIKSDGNLIAPWYGHILSGESHGRILKTD
LKRGSCTVQCQTEKGGLHTALPFQNVSKYAFGNCSKYIGIKSLKLAVGLRNVRCRSSRGLFGAIAGFIER
GWSGLVAGWYGFQHSNDQGVGMAADRDSTQKAIDKITSKVNNIVDKMNKQYEIIDHEFSVVGTRINMINN
KFDDQIQDIWAYNAESLVSLENQKTLDEHDANVNNLYNKVKRALGSNAVEDGKGCFEQYHKCDDQCMETI
RNGTYNRRKYQAESKLERQKIEGLKLESDRTNKSLSIYSTVASYRVIAMGFAAFLFWAMSNGSCRCNICI

>QBK20149.1 hemagglutinin [Influenza A virus]
METVSLITILLIATVSNADKICIGYQSTNSTETVDTLTENNVPVTHAKELLHTEHNGMLCATSLGQPLIL
DTCTIEGLIYGNPSCDLSLEGREWSYIVERPSAVNGLCYPGNVENLEELRSLFSSARSYQRIQIFPDTIW
NVSYDGTSTACSGSFYRSMRWLTRKNGDYPVQDAQYTNNQGKNILFMWGINHPPTDTTQRDLYTRTDTTT
SVATEEINRIFKPLIGPRPLVNGLMGRIDYYWSVLKPGQTLRIKSDGNLIAPWYGHILSRESHGRILTTD
LKRGSCTVQCQTEKGGLNTTLPFQNVSKYAFGKCSKYIGIKSLKLAVGLRNVRCRSSRGLFGAIAGFIEG
GWSGLVAGWYGFQHSNDQGVGMAADRDSTQKAIDKITSKVNNIVDKMNKQYEIIDHEFSEVETRINMINN
KIDDQIQDIWAYNAELLVLLENQKTLDEHDANVNNLYNKVKRALGSNAVEDGKGCFELYHKCDDQCMETI
RNGTYNRRKYQEESKLERQKIEGVKLESEGTYKIVSIYSTVASSLVIAMGFAAFLFWAMSNGSCRCNICI

>QBK20148.1 hemagglutinin [Influenza A virus]
MEAVSLITILLIVTVSNADKICIGYQSTNSTETVDTLTENNVPVTHAKELLHTEHNGMLCATSLGQPLVL
DTCTIEGLIYGNPSCDLSLEGREWSYIVERPSAVNGLCYPGNVQNLEELRSLFSSARSYQRIQIFPDTIW
NVSYDGTSNACSGSFYRSMRWLTRKNGDYPTQDAQYTNNQGKNILFMWGINHPPTDTTQRDLYTRTDTTT
SVATEEINRIFKPLIGPRPLVNGLMGRIDYYWSVLKPGQTLRIKSDGNLIAPWYGHILSGESHGRIVKHD
LKRGSCTVPCQSEKGGLNTALRFQNVSKYAFGRCSKYIGIKSLKLAVGLRNVRSRYSRGLFGAIAGFIEG
GWSGLVDGWYGFQHSNDQGVGMAADKDSTQKAIDKITSKVNNIVAKMYKQYEIIDHEFSEVETRINMINN
KIDDQIQDIWAYNAESIVFLDNQKTLDEHDANVNNLYNKVKRALGSNAVEDGKGCSELCHKCDDQCMETI

RNGTFNRRKYQAESKLERQKIEGVNLEFEGTWKILSIYSRVASYLVIAMGSVALLFGALSNGSCRCNICI

>QBK20147.1 hemagglutinin [Influenza A virus]
METVSLITILLVATVSNADKICIGYQSTNSTETVDTLTENNVPVTHAKELLHTEHNGMLCATSLGQPLIL
DTCTIEGLIYGNPSCDLSLEGREWSYIVERPSAVNGLCYPGNVENLEELRSLFSSARSYQRIQIFPDTIW
NVSYDGTSTACSGSFYRSMRWLTRKNGDYPTQDAQYTNNQGKNILFMWGINHPPTDDTQRNLYTRTDTTT
SVATEEINRIFRPLIGPRPLVNGLMGRIDYYWSVLKPGQTLRIKSDGNLIAPWYGHILSRESHGRILKTD
LKRGSCTVQCQTEKGGLNTTLPFQNVSKYAFGNCSKYIGIKSLKLAVGLRNVPSRSSRGLFGAIAGFIEG
GWSGLVAGWYGFQHSNDQGVGMAADRDSTQKAIDKITSKVNNIVDKMNKQYEIIDHEFSEVETRLNMINN
KIDDQIQDIWAYNAELLVLLENQKTLDEHDANVNNLYNKVKRALGSNAVEDGKGCFELYHKCNDQCMETI
RNGTYNRRKYQEESKLERQKIEGVKLESEGTYKILTIYSTVASSLVIAMGFAAFLFWAMSNGSCRCNICI

>QBK20146.1 hemagglutinin [Influenza A virus]
METVSLITILLVATVSNADKICIGYQSTNSAETVDTLTENNVPVTHAKELLHTEHNGMLCATSLGQPLIL
DTCTIEGLIYGNPSCDLSLEGREWSYIVERPSAVNGLCYPGNVQNLEELRSLFSSARSYQRIQIFPHTIW
NVSYYGTSTACSGSFYRSMRWLTRKNGNYPTQDAQYTNNQGKNILFMWGINHPPTDDTQRNLYTRNDTTT
SVATEEINRIFRPLIGPRPLVNGLMGRIDYYWSVLKPGQTLPIKSDGNLIAPWYGHILSGESHGRILTTD
LKRGSCTVQCQTVKGGLNTTLPFQNVSKYAFGNCSKYIGIKSLKLAVGLRNVRSRSSRGLFGAIAGFIEG
GWSGLVAGWYGFQHSNDQGVGMAADRDSTQKAIDKITSKVNNIVDKMNKQYEIIDHEFSEVETRLNMINN
KIDDQIQDIWAYNAEMVVLLENQKTLDEHDANVNNLYNKVKRALGSNAVEDGKGCFELYHKCNDQCMETI
RNGTYNRRKYHEESKFERQKIEGVKEEFEGTCKVLTIYSTKASSLVIAMGFAAFLLWAMSNGSCRCNICI

>QBK20145.1 hemagglutinin [Influenza A virus]
METVSLITILLVATVSNADKICIGYQSTNSAETVDTLTENNVPVTHAKELLHTEHNGMLCASSLGQPLIL
DTCTIEGLIYGNPSCDLSLEGREWSYIVERPSAVNGLCYPGNVQNLEELRSLFSSARSYQRIQIFPDTIW
NVSYYGTSTACSGSFYRSMRWLTRKNGNYPTQDAQYTNNQGKNILFMWGINHPPTDDTQRNLYTRNDTTT
SVATEEINRIFRPLIGPRPLVNGLMGRIDYYWSVLKPGQTLRIKSDGNLIAPWYGHILSRESHGRILKTD
LKRGSCTVQCQTEKGGLHTALPFQNVSKYAFGNCSKYIGIKSLKLAVGLRNVRSRYSRGLFGAIAGFIEG
GWSGLVAGWYGFQHSNDQGVGMAADRDSTQKAIDKITSKVNNIVDKMNKQYEIIDHEFSEVETRLNMINN
KIDDQIQDIWAYNAELLVLLENQKTLDEHDANVNNLYNKVKRALGSNAVEDGKGCFELYHKCNDQCMETI
RNGTYNRRKYQEESKLERQKIEGVKLEFEGTCKILSIYSTVASSLVIAMGFAAFLFWAMSNGSCRCNICI

>QBK20144.1 hemagglutinin [Influenza A virus]
MDTVSLITILLIATVSNADKICIGYQSTNSTETVDTLTENNVPVTHAKELLHTEHNGMLCATSLGQPLIL

DTCTIEGLIYGNPSCDLSLEGREWSYIVERPSAVNGLCYPGNVQNLEELRSLFSSARSYQRIQIFPDTIW
NVSYDGTSTACSGSFYRSMRWLTRKNGDYPTQDAQYTNNQGKNILFMWGINHPPTDDTQRNLYTRTDTTT
SVATEEINRIFKPLIGPRPLVNGLMGRIDYYWSVLKPGQTLRIKSDGNLIAPWYGHILSRESHGRILKTD
LKRGSCTVQCQTENGGLHTTLPLLNVSKYAFGRCSKYIGIKSLKLAVGLRNVPSRFSRGLFGAIAGFIEG
GWSGLVGGWYGFQHSNDQGVGMAADRDSTRKAIDKITSKENNLVAKLYKQYEILDHEISEVGTRINMSNH
KSDGQIQDIWAYNAESVVFNDNQKTSREHDIKPDNEYNKVKRALGSNAVEDGKGCFEIYHKCNEECMETI
RNRTFIRREYQAESKLERQKIEGVKLELEGTCKVVSIYSTVASYRVIAIGSVAFLFGALYNWSCRCNICI

>QBK20143.1 hemagglutinin [Influenza A virus]
METVSLITILLVATVSNADKICIGYQSTNSTETVDTLTENNVPVTHAKELLHTEHNGMLCATSLGQPLIL
DTCTIEGLIYGNPSCDLSLEGREWSYIVERPSAVNGLCYPGNVENLEELRSLFSSARSYQRIQIFPDTIW
NVSYDGTSTACSGSFYRSMRWLTRKNGNYPTQDAQYTNNQGKNILFMWGINHPPTDDTQRNLYTRNDTTT
SVATEEINRIFRPLIGPRPLVNGLMGRIDYYWSVLKPGQTLRIKSDGNLIAPWYGHILSGESHGRILKTD
LKRGSCTVQCQTEKGGLNTTLPFQNVSKYAFGNCSKYIGIKSLKLAVGLRNVPSRSSRGLFGAIAGFIEG
GWSGLVAGWYGFQHSNDQGVGMAADRDSTQKAIDKITSKVNNIVDKMNKQYEIIDHEFSEVETRLNMINN
KIDDQIQDIWAYNAELLVLLENQKTLDEHDANVNNLYNKVKRALGSNAVEDGKGCFELYHKCNDQCMETI
RNGTYNRRKYQEESKLERQKIEGVKLEFEGTYKILTIYSTVASSLVIAMGFAAFLLWAMSNGSCRCNICI

>QBK20142.1 hemagglutinin [Influenza A virus]
METVSLITILLIATVSNADKICIGYQSTNSTETVDTLTENNVPVTHAKELLHTEHNGMLCATSLGQPLIL
DTCTIEGLIYGNPSCDLSLEGREWSYIVERPSAVNGLCYPGNVENLEELRSLFSSARSYQRIQIFPDTIW
NVSYDGTSTACSGSFYRSMRWLTRKNGDYPTQDAQYTNNQGKNILFMWGINHPPTDDTQRNLYTRTDTTT
SVATEEINRIFKPLIGPRPLVNGLMGRIDYYWSVLKPGQTLRIKSDGNLIAPWYGHILSGESHGRILTTD
LKRGSCTVQCQTEKGGLNTTLPFQNVSKYAFGKCSKYIGIKSLKLAVGLRNVPSRSSRGLFGAIAGFIEG
GWSGLVAGWYGFQHSNDQGVGMAADRDSTQKAIDKITSKVNNIVDKMNKQYEIIDHEFSEVETRLNMINN
KIDDQIQDIWAYNAELLVLLENQKTLDEHDANVNNLYNKVKRALGSNAVEDGKGCFELYHKCNDQCMETI
RNGTYNRRKYQEESKLERQKIEGVKLESEGTYKILSIYSTVASSLVIAMGFAAFLFWAMSNGSCRCNICI

>QBK20141.1 hemagglutinin [Influenza A virus]
MEAVSLIAILLVVTVSNANEICIGYQSTNSTETVDTLTENNVPVTHAKELLHTQHNGMLCATSLGQPLVL
DTCTIEGLIYGNPSCDLSLEGREWSYIVERPSAVNGLCYPGNVQNLEELRSLFSSARSYQRIQIFPDTIW
NVSYYGTSNACSGSFYRSMRWLTRKNGDYPTQDAQYTNNQGNNILFMWGINHPPTDTTQRDLYTRTDTTT
SVATEEINRIFKPLVGPRPLVNGLMGRIDYYWSVLKPGQTLRIKSDGNLIAPWYGHILSRESHGRILTHD
LKRGSCTVQCQSENGGLNTALSLLNVSKYAFGKCSKYIGIKSLKLAVGLRNVQSRYSRGLFGAIAGFIEG

GWSGLVAGWYGFQHSNDQGVGMAADKDSTQKAIDKITSKVNNIVAKMYKQYEIIDHEFSEVETRINMINN
KIDDQIQDIWAYNAELIVLLDNQKTLDEHDANVNNEYNKVKRALGSNAVEDGKGCSEQCHKCDDQCMETI
RNGTFNRREYQAESKLEIQKIEGVKLEFDGTCEILSICSRVASYRVIAMGSVAFLFGAMYNGSCRCNICI

>QBK20140.1 hemagglutinin [Influenza A virus]
MDTVSLIPILLISTVSNANEICIGYQSTNSTETVDTLSENYVPVTHAKELLHTQHNGMLGASSLGQPLIL
DTCTIEGLIYGNPSCDLSLEGREWSYIVQRPSAVNGLCYPGNVQNLEELRSLFSSARSYQKIQIFPHTIW
NVSYDGTSTACSGSFYRSMRWLTRKNGDYPTQDAQYTNNQGKNILFMWGINHPPTDDTQRNLYTRTDTTS
SVATEEINRIFKPLIGPRPLVNGLMGRIDYYWSVLKPGHTLRIKSDGNLIAPWYGHILSGESHGRILKTD
LKRGSCTVQCQRENGGLHTALSLLNVSKYAFGNCSKYIGIKSLKRAVGLRNVQCRSSRGLFGAIAGFIEG
GWSVLVGGWYGFQHSNDQGVGMAADRDSTQKAIDKITSKVINIVAKLYKQYEILDHEFSEVGTRINMSSN
KFDDQIQDIWAYNAESVVFIDNQKTSDEHDANVDNEYNKVKRRLGSDAVEDGKGCSEQYHKCNEECMETI
RNRTFNRREYQAESKLERQKIEGVKLEFDGTSEVVSIYSTVASYRVIAMGSVAFLFWAISNWSCRCNICI

>QBK20139.1 hemagglutinin [Influenza A virus]
METVSVITILLIATVSNADKICIGYQSTNSAETVDTLTENNVPVTHAKELLHTEHNGMLCATSLGQPLIL
DTCTIEGLIYGNPSCDLSLEGREWSYIVERPSAVNGLCYPGNVENLEELRSLFSSARSYQRIQIFPDTIW
NVSYYGTSTACSGSFYRSMRWLTRKNGDYPVQDAQYTNNQGKNILFMWGINHPPTDTTQRDLYTRTDTTS
SVATEEINRIFKPLIGPRPLVNGLMGRIDYYWSVLKPGQTLRIKSDGNLIAPWYGHILSGESHGRILTTD
LKRGSCTVQCQTEKGGLHTALSLLNVSKYAFGNCSKYIGIKSLKLAVGLRNVRCRSSRGLFGAIAGFIER
GWSGLVDGWYGFQHSNDQGVGMAADRDSTQKAIDKITSKVNNIVDKMYKQYEIIDHEFSVVGTRIITINN
KFDDQIQDIWAYNAESLVLLDNQKTLDEHDANVNNLYNKVKRAAGSNAVEDGKGCFEQYHKCDDQCMETI
RNGTYNRRKYQAESKLERQKIEGLKLESDRTNKILSIYSRVASYRVIAMGFVAFLFGALYNGSCRCNICI

>QBK20138.1 hemagglutinin [Influenza A virus]
MDTVSLITILLIATVSNADKICIGYQSTNSTETVDTLTENNVPVTHAKELLHTEHNGMLCATSLGQPLIL
DTCTIEGLIYGNPSCDLSLEGREWSYIVERPSAVNGLCYPGNVENLEELRSLFSSARSYQRIQIFPDTIW
NVSYYGTSTACSGSFYRSMRWLTRKNGDYPVQDAQYTNNQGKNILFMWGINHPPTDTTQRDLYTRTDTTT
SVATEEINRIFKPLIGPRPLVNGLMGRIDYYWSVLKPGQTLRIKSDGNLIAPWYGHILSGESHGRILKTD
LKRGSCTVQCQTEKGGLNTALPFQNVSKYAFGNCSKYIGIKSLKLAVGLRNVRCRSSRGLFGAIAGFIEG
GWSGLVAGWYGFQHSNDQGVGMAADRDSTQKAIDKITSKVNNIVDKMNKQYEIIDHEFSEVETRINMINN
KIDDQIQDIWAYNAESLVLLENQKTLDEHDANVNNLYNKVKRALGSNAVEDGKGCFELYHKCDDQCMETI
RNGTYNRRKYQEESKLERQKIEGLKLESEGTYKILSIYSTVASSLVIAMGFAAFLFWAMSNGSCRCNICI

>QBK20137.1 hemagglutinin [Influenza A virus]

METVSLITILLIATVSNADKICIGYQSTNSTETVDTLTENNVPVTHAKELLHTEHNGMLCATSLGQPLIL
DTCTIEGLIYGNPSCDLSLEGREWSYIVERPSAVNGLCYPGNVENLEELRSLFSSARSYQRIQIFPDTIW
NVSYDGTSTACSGSFYRSMRWLTRKNGDYPVQDAQYTNNQGKNILFMWGINHPPTDTTQRDLYTRTDTTT
SVATEEINRIFKPLIGPRPLVNGLMGRIDYYWSVLKPGQTLRIKSDGNLIAPWYGHILSGESQGRIVKPD
LKRGSCTVQCQTEKGGLNTALPFQNVSKYAFGNCSKYIGIKSLKLAVGLRNVRSRSSRGLFGAIAGFIEG
GWSGLVAGWYGFQHSNDQGVGMAADRDSTQKAIDKITSKVNNIVDKMNKQYEIIDHEFSEVETRLNMINN
KIDDQIQDIWAYNAESLVLLENQKTLDEHDANVNNLYNKVKRALGSNAVEDGKGCFEQYHKCDDQCMETI
RNGTYNRRKYQEESKLERQKIEGVKQESDRTNKILSIYSRVASSRVIAMGFAALLFWAMSNGSCRCNSSI

>QBK20136.1 hemagglutinin [Influenza A virus]

MDTVSLITILLISTVSNADKICIGYQSTNSAETVDTLTENNVPVTHAKELLHTEHNGMLCATSLGQPLIL
DTCTIEGLIYGNPSCDLSLEGREWSYIVERPSAVNGLCYPGNVENLEELRSLFSSARSYQKIQIFPDTIW
NVSYYGTSTACSGSFYRSMRWLTRKNGDYPVQDAQYTNNQGKNILFMWGINHPPTDTTQRDLYTRTDTTT
SVATEEINRIFKPLIGPRPLVNGLMGRIDYYWSVLKPGQTLRIKSDGNLIAPWYGHILSRESHGRILTTD
LKRGSCTVQCQTEKGGLHTALPLQNVSKYAFGKCSKYIGIKSLKLAVGLRNVRCRSSRGLFGAIAGFIEG
GWSGLVGGWYGFQHSNDQGVGMAADRDSTQKAIDKITSKVNNLVAKLYKQYEIIDLEFSVVGTRINMSNN
KFDDQIQDIWAYNAESIVLLENQKTLDEQDANTNNLYNKVKRAAGSNAVEDGKGCLEQYHKCDDQCMETI
RNGTYNRREYQAESKLERQKMEGLKLEFDGTCKILSIYSTVASYRVIAMGSVALLFGAMSNGSCRCNICI

>QBK20135.1 hemagglutinin [Influenza A virus]

MDTVSLITILLIATVSNADKICIGYQSTNSTETVDTLTENNVPVTHAKELLHTEHNGMLCATSLGQPLIL
DTCTIEGLIYGNPSCDLSLEGREWSYIVERPSAVNGLCYPGNVENLEELRSLFSSARSYQRIQIFPDTIW
NVSYYGTSTACSGSFYRSMRWLTRKNGDYPVQDAQYTNNQGKNILFMWGINHPPTDTTQRDLYTRTDTTT
SVATEEINRIFKPLIGPRPLVNGLMGRIDYYWSVLKPGQTLRIKSDGNLIAPWYGHILSRESHGRILKTD
LKRGSCTVQCQTEKGGLHTAQPLLNVSKYAFGKCSKYIGIKSLKLAVGLRNVRCRSSRGLFGAIAGFIER
GWSGLVAGWYGFQHSNDQGVGMAADRDSTQKAIDKITSKVNNIVAKLYKPYEIIDLEFIVVGTRIVTISN
KFADQIQDIWAYNAESLVLLENQKTLDERDANTRSGGNKVKRGAGSNAVEDGNGCLEQYHKRDDQCMETI
RNGTYNRRKYQAESKKERQKMEGVKLEFEGTWKRVSIYSRVASYRVIAMGSFALRFGALYNGSCRCNICI

>QBK20134.1 hemagglutinin [Influenza A virus]

MDTVSLITILLIATVSNADKICIGYQSTNSTETVDTLTENNVPVTHAKELLHTEHNGMLCATSLGQPLIL
DTCTIEGLIYGNPSCDLSLEGREWSYIVERPSAVNGLCYPGNVENLEELRSLFSSARSYQRIQIFPDTIW
NVSYYGTSTACSGSFYRSMRWLTRKNGDYPVQDAQYTNNQGKNILFMWGINHPPTDTTQRDLYTRTDTTT

SVATEEINRIFKPLIGPRPLVNGLMGRIDYYWSVLKPGQTLRIKSDGNLIAPWYGHILSGESHGRILKTD
LKRGSCTVQCQTEKGGLHTALPLQNVSKYAFGKCSKYIGIKSLKLAVGLRNVRCRSSRGLFGAIAGFIER
GWSGLVAGWYGFQHSNDQGVGMAADRDSTQKAIDKITSKVNNIVAKMNKPYEIIDHEFSVVETRINTINH
KSADQIQDIWAYNAESLVSLENQKTLDEHDANVNNLYNKVKRAAGSNAVEDGKGCFELYHKCDDQCMETI
RNGTYNRRKYQAESKLERQKIEGLKLESEGTWKIVSIYSTVASSLVIAMGSAAFLFWAMSNGSCRCNSSI

>QBK20133.1 hemagglutinin [Influenza A virus]
METVSLITILLIATVSNADKICIGYQSTNSTETVDTLTENNVPVTHAKELLHTEHNGMLCATSLGQPLIL
DTCTIEGLIYGNPSCDLSLEGREWSYIVERPSAVNGLCYPGNVENLEELRSLFSSARSYQRIQIFPDTIW
NVSYYGTSTACSGSFYRSMRWLTRKNGDYPVQDAQYTNNQGKNILFMWGINHPPTDTNQRDLYTRTDTTT
SVATEEINRIFKPLIGPRPLVNGLMGRIDYYWSVLKPGQTLRIKSDGNLIAPWYGHILSGESHGRILKTD
LKRGSCTVQCQTEKGGLNTALPFQNVSKYAFGKCSKYIGIKSLKLAVGLRNVPCRSSRGLFGAIAGFIEG
GWSGLVAGWYGFQHSNDQGVGMAADRDSTQKAIDKITSKVNNIVAKMYKQYEIIDHEFSEVETRINTINN
KIDDQIQDIWAYNAESIVLLENQKTLDEHDANVNNLYNKVKRALGSNAVEDGKGCFELYHKCDDQCMETI
RNGTYNRRKYQEESKLERQKIEGVKLESEGTYKILSIYSTVASYRVIAMGFAAFLFWALSNGSCRCNSSI

>QBK20132.1 hemagglutinin [Influenza A virus]
METVSLITILLVATVSNADKICIGYQSTNSTETVDTLTENNVPVTHAKELLHTEHNGMLCATSLGQPLIL
DTCTIEGLIYGNPSCDLSLEGREWSYIVERPSAVNGLCYPGNVENLEELRSLFSSARSYQRIQIFPDTIW
NVSYDGTSTACSGSFYRSMRWLTRKNGDYPVQDAQYTNNQGKNILFMWGINHPPTDTTQRDLYTRTDTTT
SVATEEINRIFKPLIGPRPLVNGLMGRIDYYWSVLKPGQTLRIKSDGNLIAPWYGHILSGESHGRILKTD
LKRGSCTVQCQTEKGGLNTTLPFQNVSKYAFGNCSKYIGIKSLKLAVGLRNVPSRSSRGLFGAIAGFIEG
GWSGLVAGWYGFQHSNDQGVGMAADRDSTQKAIDKITSKVNNIVDKMNKQYEIIDHEFSEVETRLNMINN
KIDDQIQDIWAYNAELLVLLENQKTLDEHDANVNNLYNKVKRALGSNAVEDGKGCFELYHKCDDQCMETI
RNGTYNRRKYQEESKLERQKIEGVKLESEGTYKILSIYSTVASSLVIAMGFAAFLFWAMSNGSCRCNICI

>QBK20131.1 hemagglutinin [Influenza A virus]
MDTVSLITILLISTVSNADKICIGYQSTNSAETVDTLTENNVPVTHAKELLHTEHNGMLCATSLGQPLIL
DTCTIEGLIYGNPSCDLSLEGREWSYIVERPSAVNGLCYPGNVENLEELRSLFSSARSYQRIQIFPDTIW
NVSYYGTSTACSGSFYRSMRWLTRKNGDYPVQDAQYTNNQGKNILFMWGINHPPTDTTQRDLYTRTDTTT
SVATEEINRIFKPLIGPRPLVNGLMGRIDYYWSVLKPGQTLRIKSDGNLIAPWYGHILSRESHGRILTTD
LKRGSCTVQCQTEKGGLHTALRLLNVSKYAFGKCSKYIGIKSLKLAVGLRNVRCRSSRGLFGAIAGFIEG
GWSGLVGGWYGFQHSNDQGVGMAADRDSTQKAIDKIPSKVNNLVAKLYKPYEILDLEFSVVGTRIITSNN
KFDDQIQDIWAYNAESIVFIENQKTLDEHDANTNNGGNKVKRGAGSNAVEDGKGCFEQYHKCDDQCMETI

GNGTYNRREYQAESKLERQKMEGLKLEFDRTWEVVSIYSTVAWYRVIAMGSFALRFGALYNGSCRCNICI

>QBK20130.1 hemagglutinin [Influenza A virus]

MDTVSLITILLIATVSNADKICIGYQSTNSTETVDTLTENNVPVTHAKELLHTEHNGMLCATSLGQPLIL

DTCTIEGLIYGNPSCDLSLEGREWSYIVERPSAVNGLCYPGNVENLEELRSLFSSARSYQRIQIFPDTIW

NVSYDGTSTACSGSFYRSMRWLTRKNGDYPAQDAQYTNNQGKNILFMWGINHPPTDDTQRNLYTRTDTTT

SVATEEINRIFKPLIGPRPLVNGLMGRIDYYWSVLKPGQTLRIKSDGNLIAPWYGHILSRESHGRILTTD

LKRGSCTVQCQTEKGGLHTALPFQNVSKYAFGKCSKYIGIKSLKLAVGLRNVPSRSSRGLFGAIAGFIEG

GWSGLVAGWYGFQHSNDQGVGMAADRDSTQKAIDKITSKVNNIVAKMYKQYEIIDHEFSEVETRINMINN

KIDDQIQDIWAYNAESLVLLENQKTLDEHDANVNNLYNKVKRALGSNAVEDGKGCFEIYHKCNDQCMETI

RNGTYNRRKYQAESKIERQKIEGVKLEFEGTYKILSIYSTVASSRVIAMGFVAFLFWAMSNGSCRCNSSI

>QBK20129.1 hemagglutinin [Influenza A virus]

MDTVSLITILLISTVSNADKICIGYQSTNSAETVDTLTENNVPVTHAKELLHTEHNGMLCATSLGQPLIL

DTCTIEGLIYGNPSCDLSLEGREWSYIVERPSAVNGLCYPGNVQNLEELRSLFSSARSYQRIQIFPDTIW

NVSYDGTSTACSGSFYRSMRWLTRKNGDYPTQDAQYTNNQGKNILFMWGINHPPTDDTQRNLYTRTDTTS

SVATEEINRIFKPLIGPRPLVNGLMGRIDYYWSVLKPGQTLRIKSDGNLIAPWYGHILSRESHGRILTTD

LTRGSCTVQCQTEKGGLHTALPLLNVSKYAFGKCSKYISIKSLKLAVGLRNVRCRFSRGLFGAIAGFIEG

GWSGLVGGWYGFQHSNDQGVGMAADRDSTRKAIDKITSKENNIVAKLYKQYEILDHAFSEVGTRINMSNN

MFDDQIQDIWAYNAESVVFLENQKTREEHDIKTDNEYNKVKRASGSNAVEDGKGCSEIYHKCKKECMETI

RNRTFIRREYQAEGKKERQKIEGVKLELEGTCEVVSIYSRMTSYRTIAMRAFAFLFEALYNGSCRCNICI

>QBK20128.1 hemagglutinin [Influenza A virus]

MDTVSLITILLIATVSNADKICIGYQSTNSTETVDTLTENNVPVTHAKELLHTEHNGMLCATSLGQPLIL

DTCTIEGLIYGNPSCDLSLEGREWSYIVERPSAVNGLCYPGNVENLEELRSLFSSARSYQRIQIFPDTIW

NVSYDGTSTACSGSFYRSMRWLTRKNGDYPVQDAQYTNNQGKNILFMWGINHPPTDTTQRDLYTRTDTTT

SVATEEINRIFKPLIGPRPLVNGLMGRIDYYWSVLKPGQTLRIKSDGNLIAPWYGHILSGESHGRILKTD

LKRGSCTVQCQTEKGGLNTTLPCQNVSKYAFGKCSKYIGIKSLKLAVGLRNVPCRSSRGLFGAIAGFIEG

GWSGLVAGWYGFQHSNDQGVGMAADRDSTQKAIDKITSKVNNIVDKMNKQYEIIDHEFSEVETRINMINN

KIDDQIQDIWAYNAESLVLLENQKTLDEQDANVNNLYNKVKRALGSNAVEDGKGCFELYHKCDDQCMETI

RNGTYNRRKYQEESKLERQKIEGVKLESEGTYKILSIYSTVASSLVIAMGSVAFLFWAMSNGSCRCNSSI

>QBK20127.1 hemagglutinin [Influenza A virus]

METVSLITILLVATVSNADKICIGYQSTNSTETVDTLTENNVPVTHAKELLHTEHNGMLCATSLGQPLIL

DTCTIEGLIYGNPSCDLSLEGREWSYIVERPSAVNGLCYPGNVENLEELRSLFSSARSYQRIQIFPDTIW
NVSYYGTSTACSGSFYRSMRWLTRKTGDYPVQDAQYTNNQGKNILFMWGINHPPTDTTQRDLYTRTDTTT
SVATEEINRIFKPLIGPRPLVNGLMGRIDYYWSVLKPGQTLRIKSDGNLIAPWYGHILSGESHGRILKTD
LKRGRCTVQCQTEKGGLNTAQPLQNVSKYASGKCSKYIGIKSLKLAVGLRNVRCRSSRGLFGAIAGFIER
GWSGLVAGWYGFQHSNDQGVGMAADRDSTQKAIDKITSKVNNIVDKMNKPYEIIGLEFSVVETRINTISH
KFDDQIQDIWAYNAESIVSLENQKTLDEHDANVNNLYNKVKRAAGSNAVEDGKGCFEQYHKCDDQCMETI
RNGTYNRRKYQAESKLERQKMEGLKQESEGTYKILSIYSTVASYRVIAMGFAAFLFGAMSNGSCRCNICI

>QBK20126.1 hemagglutinin [Influenza A virus]
METVSLITILLVATVSNADKICIGYQSTNSTETVDTLTENNVPVTHAKELLHTEHNGMLCATSLGQPLIL
DTCTIEGLIYGNPSCDLSLEGREWSYIVERPSAVNGLCYPGNVENLEELRSLFSSARSYQRIQIFPDTIW
NVSYDGTSTACSGSFYRSMRWLTRKTGDYPVQDAQYTNNQGKNILFMWGINHPPTDTTQRDLYTRTDTTT
SVATEEINRIFKPLIGPRPLVNGLMGRIDYYWSVLKPGQTLRIKSDGNLIAPWYGHILSGESHGRIVKTD
LKRGSCTVQCQTEKGGLNTTLPFQNVSKYAFGNCSKYIGIKSLKLAVGLRNVPSRSSRGLFGAIAGFIEG
GWSGLVAGWYGFQHSNDQGVGMAADRDSTQKAIDKITSKVNNIVDKMNKQYEIIDHEFSEVETRLNMINN
KIDDQIQDIWAYNAELLVLLENQKTLDEHDANVNNLYNKVKRALGSNAVEDGKGCFELYHKCDDQCMETI
RNGTYNRRKYQEESKLERQKIEGVKLESEGTYKILSIYSTVASSLVIAMGFAAFLFWAMSNGSCRCNISI

>QBK20125.1 hemagglutinin [Influenza A virus]
MDTVSLITMLLISTVSNADKICIGYQSTNSAETVDTLTENNVPVTHAKELLHTEHNGMLCATSLGQPLIL
DTCTIEGLIYGNPSCDLSLEGREWSYIVERPSAVNGLCYPGNVENLEELRSLFSSARSYQKIQIFPHTIW
NVSYYGTSTACSGSFYRSMRWLTRKNGDYPVQDAQYTNNQGKNILFMWGINHPPTDTTQRDLYTRTDTTS
SVATEEINRIFKPLIGPRPLVNGLMGRIDYYWSVLKPGQTLRIKSDGNLIAPWYGHILSRESHGRILTTD
LKRGSCTVQCQTEKGGLNTALPLQNVSKYASGNCSKYIGIKSLKVAVGLRNVRCRSSRGLFGAIAGFIER
GWSGLVAGWYGFQHSNDQGVGMAADRDSTQKAMDKITSNVNNIVAKLYKQYEIIGLEFSVVGTRIITSNN
KFDDQIQDIWAYNAESIVLLENQKTIDEHDANVNNLYNKVKRGAGSNAVEDGKGCFEQYHKCDDQCMETI
RNGTYNRRKYQAESKLERQKIEGDNLEFEGTYKILSIYSTVASSRVIAMGSAALRFGAMYNGSCRCNICI

>QBK20124.1 hemagglutinin [Influenza A virus]
METVSLITILLIATVSNADKICIGYQSTNSTETVDTLTENNVPVTHAKELLHTEHNGMLCATSLGQPLIL
DTCTIEGLIYGNPSCDLSLEGREWSYIVERPSAVNGLCYPGNVENLEELRSLFSSARSYQRIQIFPDTIW
NVSYYGTSTACSGSFYRSMRWLTRKNGDYPVQDAQYTNNQGKNILFMWGINHPPTDTTQRDLYTRTDTTT
SVATEEINRIFKPLIGPRPLVNGLMGRIDYYWSVLKPGQTLRIKSDGNLIAPWYGHTRSGERHGRNVEPD
LKRGSCTVQCQTEKGGLNTALPLQNVSKYAFGNCSKYIGIKSLKLAVGLRNVRCRSSRGLFGAIAGFIER

GWSGLVAGWYGFQHSNDQGVGMAADRDSTQKAIDKITSKVNNIVAKMNKQYEIIDHEFSEVETRINMINN
KIDDQIQDIWAYNAESLVLLENQKTLDEHDANVNNLYNKVKRALGSNAVEDGKGCFELYHKCDDQCMETI
RNGTYNRRKYQEESKLERQKIEGVKLESEGTYKILSIYSTVASSRVIAMGFAAFLFWAMSNGSCRCNICI

>QBK20123.1 hemagglutinin [Influenza A virus]
MATVSAITILLISTVSNADEICIGYQSTNSAETVDTLTENYVPVTHAKELLHTQHNGMLGASSLGQPLIL
DTCTIEGLIYGNPSCDLSLEGREWSYIVQRPSAVNGLCYPGNVQNLEELRSLFSSARSYQRIQIFPHTIW
NVSYDGTSTACSGSFYRSMRWLTRKNGDYPTQDAQYTNNQGKNILFMWGINHPPTDDTQRNLYTRTDTTS
SVATEEINRIFKPLIGPRPLVNGLMGRIDYYWSVLKPGQTLRIKSDGNLIAPWYGHILSRESHGRILTTD
LQRGSCTVQCQRENGELNTALPLLNVSKYAIGNCSKYIGIKSLKLAVGLRNVPSRSSRGLFGAIAGFIEG
GWSGLVGGWYGFQHSNDQGVGMAADRDSTQKAIDKITSKVNNIVAKMYKQYEIIDHEFSEVGTRINMINN
KFDDQIQDIWAYNAESVVFLDNQKTLDEHDANVNNVYNKVKRALGSNAVEDGKGCFEQYHKCNDQCMETI
RNRTFNRRKYQAESKLERQKIEGVKLEFEGTCKVLSIYSTVASYRVIAMGFVAFLFGALSNWSCRCNICI

>QBK20122.1 hemagglutinin [Influenza A virus]
METVSLITILLVATVSNADKICIGYQSTNSTETVDTLTENNVPVTHAKELLHTEHNGMLCATSLGQPLIL
DTCTIEGLIYGNPSCDLSLEGREWSYIVERPSAVNGLCYPGNVENLEELRSLFSSARSYQRIQIFPDTIW
NVSYDGTSTACSGSFYRSMRWLTRKNGDYPVQDAQYTNNQGKNILFMWGINHPPTDTTQRDLYTRTDTTT
SVATEEINRIFKPLIGPRPLVNGLMGRIDYYWSVLKPGQTLRIKSDGNLIAPWYGHILSGESHGRILKTD
LKRGSCTVQCQTEKGGLNTTLPFQNVSKYAFGNCSKYIGIKSLKLAVGLRNVPSRSSRGLFGAIAGFIEG
GWSGLVAGWYGFQHSNDQGVGMAADRDSTQKAIDKITSKVNNIVDKMNKQYEIIDHEFSEVETRLNMINN
KIDDQIQDIWAYNAELLVLLENQKTLDEHDANVNNLYNKVKRALGSNAVEDGKGCFELYHKCDDQCMETI
RNGTYNRRKYQEESKLERQKIEGVKLESEGTYKILSIYSTVASSLVIAMGFAAFLFWAMSNGSCRCNICI

>QBK20121.1 hemagglutinin [Influenza A virus]
MDTVSLITILLIATVSNADKICIGYQSTNSAETVDTLTENNVPVTHAKELLHTEHNGMLCATSLGQPLIL
DTCTIEGLIYGNPSCDLSLEGREWSYIVERPSAVNGLCYPGNVENLEELRSLFSSARSYQRIQIFPDTIW
NVSYYGTSTACSGSFYRSMRWLTRKNGDYPVQDAQYTNNQGKNILFMWGINHPPTDATQRNLYTRTDTTT
SVATEEINRIFKPLIGPRPLVNGLMGRIDYYWSVLKPGQTLRIKSDGNLIAPWYGHILSRESHGRILKTD
LKRGSCTVQCQTEKGGLNTAQSLLNVSKYAFGKCSKYIGIKSLKLAVGLRNVRCRSSRGLFGAIAGFIEG
GWSGLVGGWYGFQHSNDQGVGMAADRDSTQKAIDKITSKENNLVAKLYKQYEILDLAISVVGTRIITSNN
KSDDQIQDIWAYNAESIVLLENQKTLEEQDANTSNRGNKVKRAACSNRVEDGKGCFEQYHKCYKECMETI
RNRTSNRREYQAESKKSRQKMEGLNLELERTSEIVSIYSRMTSYRMIAMGPFALMIGALYNWSCRCNICI

>QBK20120.1 hemagglutinin [Influenza A virus]

METVSLITILLIATVSNADKICIGYQSTNSTETVDTLTENNVPVTHAKELLHTEHNGMLCATSLGQPLIL
DTCTIEGLIYGNPSCDLSLEGREWSYIVERPSAVNGLCYPGNVENLEELRSLFSSARSYQRIQIFPDTIW
NVSYDGTSTACSGSFYRSMRWLTRKTGDYPVQDAQYTNNQGKNILFMWGINHPPTDTTQRDLYTRTDTTT
SVATEEINRIFKPLIGPRPLVNGLMGRIDYYWSVLKPGQTLRIKSDGNLIAPWYGHILSGESHGRILTTD
LKRGSCTVQCQTEKGGLNTTLPFQNVSKYAFGNCSKYIGIKSLKLAVGLRNVRCRSSRGLFGAIAGFIEG
GWSGLVAGWYGFQHSNDQGVGMAADRDSTQKAIDKITSKVNNIVDKMNKQYEIIDHEFSEVETRINMINN
KIDDQIQDIWAYNAESIVLLENQKTLDEHDANVNNLYNKVKRALGSNAVEDGKGCFELYHKCDDQCMETI
RNGTFNRRKYQEESKLERQKIEGVKLESEGTNKILSIYSTVASSLVIAMGFAAFLFWAMSNGSCRCNICI

>QBK20119.1 hemagglutinin [Influenza A virus]

METVSLITILLIATVSNADKICIGYQSTNSTETVDTLTENNVPVTHAKELLHTEHNGMLCATSLGQPLIL
DTCTIEGLIYGNPSCDLSLEGREWSYIVERPSAVNGLCYPGNVENLEELRSLFSSARSYQRIQIFPDTIW
NVSYDGTSTACSGSFYRSMRWLTRKNGDYPVQDAQYTNNQGKNILFMWGINHPPTDTTQRDLYTRTDTTT
SVATEEINRIFKPLIGPRPLVNGLMGRIDYYWSVLKPGQTLRIKSDGNLIAPWYGHILSGESHGRILTTD
LKRGSCTVQCQTEKGGLNTTLPFQNVSKYAFGNCSKYIGIKSLKLAVGLRNVRSRSSRGLFGAIAGFIEG
GWSGLVAGWYGFQHSNDQGVGMAADRDSTQKAIDKITSKVNNIVDKMNKQYEIIDHEFSEVETRINMINN
KIDDQIQDIWAYNAELLVLLENQKTLDEHDANVNNLYNKVKRALGSNAVEDGKGCFELYHKCDDQCMETI
RNGTYNRRKYQEESKLERQKIEGVKLESEGTYKILSIYSTVASSLVIAMGFAAFLFWAMSNGSCRCNISI

>QBK20118.1 hemagglutinin [Influenza A virus]

METVSLITILLVATVSNADKICIGYQSTNSTETVDTLTENNVPVTHAKELLHTEHNGMLCATSLGQPLIL
DTCTIEGLIYGNPSCDLSLEGREWSYIVERPSAVNGLCYPGNVENLEELRSLFSSARSYQRIQIFPDTIW
NVSYDGTSTACSGSFYRSMRWLTRKNGDYPVQDAQYTNNQGKNILFMWGINHPPTDTTQRDLYTRTDTTT
SVATEEINRIFKPLIGPRPLVNGLMGRIDYYWSVLKPGQTLRIKSDGNLIAPWYGHILSGESHGRNLKPD
LKRGSCTVQCQTEKGGLNTTLPFQNVSKYAFGNCSKYIGIKSLKLAVGLRNVPCRSSRGLFGAIAGFIEG
GWSGLVAGWYGFQHSNDQGVGMAADRDSTQKAIDKITSKVNNIVDKMNKQYEIIDHEFSEVETRLNMINN
KIDDQIQDIWAYNAELLVLLENQKTLDEHDANVNNLYNKVKRALGSNAVEDGKGCFELYHKCDDQCMETI
RNGTYNRRKYQEESKLERQKIEGVKLESEGTYKILSIYSTVASSLVIAMGFAAFLFWAMSNGSCRCNICI

>QBK20117.1 hemagglutinin [Influenza A virus]

MDTVSLITILLIATVSNADKICIGYQSTNSAETVDTLTENNVPVTHAKELLHTEHNGMLCATSLGQPLIL
DTCTIEGLIYGNPSCDLSLEGREWSYIVERPSAVNGLCYPGNVENLEELRSLFSSARSYQRIQIFPDTIW
NVSYYGTSTACSGSFYRSMRWLTRKNGDYPVQDAQYTNNQGKNILFMWGINHPPTDTTQRDLYTRTDTTS

SVATEEINRIFKPLIGPRPLVNGLMGRIDYYWSVLKPGQTLRIKSDGNLIAPWYGHILSRESHGRILKTD
LKRGSCTVQCQTEKGGLNTAQPLQNVSKYAFGNCSKYIGIKSLKLAVGLRNVRCRSSRGLFGAIAGFIEG
GWSGLVGGWYGFQHSNDQGVGMAADRDSTQKAIDKITSKVNNIVDKMNKPYEIIDHEFSVVGTRINTSNN
KFDDQIQDIWAYNAESIVLLENQKTLDEHDANVNNLYNKVKRALGSNAVEDGKGCFELYHKCDDQCMETI
RNGTYNRRKYQAESKLERQKIEGLKLEFERTCKILSIYSTVASYLVIAMGSAAFLFWAMSNGSCRCNICI

>QBK20116.1 hemagglutinin [Influenza A virus]
METVSLITILLVATVSNADKICIGYQSTNSTETVDTLTENNVPVTHAKELLHTEHNGMLCATSLGQPLIL
DTCTIEGLIYGNPSCDLSLEGREWSYIVERPSAVNGLCYPGNVENLEELRSLFSSARSYQRIQIFPDTIW
NVSYDGTSTACSGSFYRSMRWLTRKNGDYPVQDAQYTNNQGKNILFMWGINHPPTDTTQRDLYTRTDTTT
SVATEEINRIFKPLIGPRPLVNGLMGRIDYYWSVLKPGQTLRIKSDGNLIAPWYGHILSGESHGRIVKPD
LKRGSCTVQCQTEKGGLNTTLPFQNVSKYAFGNCSKYIGIKSLKLAVGLRNVPSRSSRGLFGAIAGFIEG
GWSGLVAGWYGFQHSNDQGVGMAADRDSTQKAIDKITSKVNNIVDKMNKQYEIIDHEFSEVETRLNMINN
KIDDQIQDIWAYNAELLVLLENQKTLDEHDANVNNLYNKVKRALGSNAVEDGKGCFELYHKCDDQCMETI
RNGTYNRRKYQEESKLERQKIEGVKLESEGTYKILSIYSTVASSLVIAMGFAAFLFWAMSNGSCRCNICI

>QBK20115.1 hemagglutinin [Influenza A virus]
METVSLITILLIATVSNADKICIGYQSTNSTETVDTLTENNVPVTHAKELLHTEHNGMLCATSLGQPLIL
DTCTIEGLIYGNPSCDLSLEGREWSYIVERPSAVNGLCYPGNVENLEELRSLFSSARSYQRIQIFPDTIW
NVSYDGTSTACSGSFYRSMRWLTRKNGDYPVQDAQYTNNQGKNILFMWGINHPPTDTTQRDLYTRTDTTT
SVATEEINRIFKPLIGPRPLVNGLMGRIDYYWSVLKPGQTLRIKSDGNLIAPWYGHILSGESHGRILKTD
LKRGSCTVQCQTEKGGLNTTLPFQNVSKYAFGNCSKYIGIKSLKLAVGLRNVPSRSSRGLFGAIAGFIEG
GWSGLVAGWYGFQHSNDQGVGMAADRDSTQKAIDKITSKVNNIVDKMNKQYEIIDHEFSEVETRLNMINN
KIDDQIQDIWAYNAELLVLLENQKTLDEHDANVNNLYNKVKRALGSNAVEDGKGCFELYHKCDDQCMETI
RNGTYNRRKYQEESKLERQKIEGVKLESEGTYKILSIYSTVASSLVIAMGFAAFLFWAMSNGSCRCNICI

>QBK20114.1 hemagglutinin [Influenza A virus]
METVSLITILLVATVSNADKICIGYQSTNSTETVDTLTENNVPVTHAKELLHTEHNGMLCATSLGQPLIL
DTCTIEGLIYGNPSCDLSLEGREWSYIVERPSAVNGLCYPGNVENLEELRSLFSSARSYQRIQIFPDTIW
NVSYDGTSTACSGSFYRSMRWLTRKNGDYPVQDAQYTNNQGKNILFMWGINHPPTDTTQRDLYTRTDTTT
SVATEEINRIFKPLIGPRPLVNGLMGRIDYYWSVLKPGQTLRIKSDGNLIAPWYGHILSGESHGRILKTD
LKRGSCTVQCQTEKGGLNTTLPFQNVSKYAFGNCSKYIGIKSLKLAVGLRNVPSRSSRGLFGAIAGFIEG
GWSGLVAGWYGFQHSNDQGVGMAADRDSTQKAIDKITSKVNNIVDKMNKQYEIIDHEFSEVETRLNMINN
KIDDQIQDIWAYNAELLVLLENQKTLDEHDANVNNLYNKVKRALGSNAVEDGKGCFELYHKCDDQCMETI

RNGTYNRRKYQEESKLERQKIEGVKLESEGTYKILSIYSTVASSLVIAMGFAAFLFWAMSNGSCRCNICI

>QBK20113.1 hemagglutinin [Influenza A virus]
METVSLITILLIATVSNADKICIGYQSTNSTETVDTLTENNVPVTHAKELLHTEHNGMLCATSLGQPLIL
DTCTIEGLIYGNPSCDLSLEGREWSYIVERPSAVNGLCYPGNVENLEELRSLFSSARSYQRIQIFPDTIW
NVSYYGTSTACSGSFYRSMRWLTRKNGDYPVQDAQYTNNQGKNILFMWGINHPPTDTTQRDLYTRTDTTT
SVATEEINRIFKPLIGPRPLVNGLMGRIDYYWSVLKPGQTLRIKSDGNLIAPWYGHILSGESHGRILTTD
LNRGSCTVQCQTEKGGLNTALPFQNVSKYAFGNCSKYIGIKSLKLAVGLRNVRCRSSRGLFGAIAGFIEG
GWSGLVAGWYGFQHSNDQGVGMAADRDSTQKAIDKITSKVNNIVDKMNKQYEIIDHEFSEVETRINMINN
KFDDQIQDIWAYNAESLVLLENQKTLDEHDANVNNLYNKVKRALGSNAVEDGKGCFELYHKCDDQCMETI
RNGTYNRRKYQAESKLERQKIEGVKLESEGTYKILSIYSTVTSYLVIAMGFAAFLFWAMSNGSCRCNICI

>QBK20112.1 hemagglutinin [Influenza A virus]
METVSLITILLIATVSNADKICIGYQSTNSTETVDTLTENNVPVTHAKELLHTEHNGMLCATSLGQPLIL
DTCTIEGLIYGNPSCDLSLEGREWSYIVERPSAVNGLCYPGNVENLEELRSLFSSARSYQRIQIFPDTIW
NVSYDGTSTACSGSFYRSMRWLTRKNGDYPVQDAQYTNNQGKNILFMWGINHPPTDTTQRDLYTRTDTTT
SVATEEINRIFKPLIGPRPLVNGLMGRIDYYWSVLKPGQTLRIKSDGNLIAPWYGHILSGESHGRILKTD
LKRGSCTVQCQTEKGGLNTTLPFQNVSKYAFGNCSKYIGIKSLKLAVGLRNVPSRSSRGLFGAIAGFIEG
GWSGLVAGWYGFQHSNDQGVGMAADRDSTQKAIDKITSKVNNIVDKMNKQYEIIDHEFSEVETRINMINN
KIDDQIQDIWAYNAELLVLLENQKTLDEHDANVNNLYNKVKRALGSNAVEDGKGCFELYHKCDDQCMETI
RNGTYNRRKYQEESKLERQKIEGVKLESEGTYKILSIYSTVASSLVIAMGFAAFLFWAMSNGSCRCNICI

>QBK20111.1 hemagglutinin [Influenza A virus]
METVSLITILLIATVSNADKICIGYQSTNSTETVDTLTENNVPVTHAKELLHTEHNGMLCATSLGQPLIL
DTCTIEGLIYGNPSCDLSLEGREWSYIVERPSAVNGLCYPGNVENLEELRSLFSSARSYQRIQIFPDTIW
NVSYYGTSTACSGSFYRSMRWLTRKNGDYPVQDAQYTNNQGKNILFMWGINHPPTDTTQRDLYTRTDTTT
SVATEEINRIFKPLIGPRPLVNGLMGRIDYYWSVLKPGQTLRIKSDGNLIAPWYGHILSRESHGRILKTD
LKRGSCTVQCQTEKGGLNTAQPLQNVSKYAFGKCSKYIGIKSLKLAVGLRNVRSRSSRGLFGAIAGFIER
GWSGLVGGWYGFQHSNDQGVGMAADRDSTQKAIDKITSKVNNIVAKMNKPYEIIGLEFSVVGTRIDTSSH
KSADQIQDIWAYNAESIVLLENQKTLDEQDANVRSGGNKVKRAAGSNAVEDGKGCFEQYHKCDDQCMETI
RNGTYNRRKYQEESKLERQKIEGHNLESERTCKILSIYSRVASYRVIAMGSFALMFGAMYNWSCRCNICI

>QBK20110.1 hemagglutinin [Influenza A virus]
MDTVSLITMLLISTVSNADKICIGYQSTNSAKTVNTLSENNVPVTHAKELLHTEHNGMLCAISLGQPLIL

DTCTIEGLIYGNPSCDLSLEGREWSYIVERPSAVNGLCYPGNVQNLEELMSLFSSARSYQKIQIFPHTIW
NVSYYGTSTACSGSFYRSMRWLTRKNGDYPVQDAQYTNNQGKNILFMWGINHPPTDTTQRDLYTRTDTTS
SVATEEINTIFKPLIGPRPLVNGLMGRIDYYWSVLKPGQTLRIKSDGNLIAPWYGHILSRESLGRILTTD
LQRGSCTVQCQREKGGLHTALPFLNVSKYASGNCSKYIGIKSLKVAVGLRNVRSRSSRGLFGAIAGFIEG
GWSGLVGGWYGFQHSNDQGVGMAADRDSTQKAIDKITSNVNNIVAKLYKPYEIIGLEFIVVGTRIITSSH
KFADQIQDIWAYNAESIVLLENQKTLDEQDINVRSGGNKVKRGAGSNAVEDGKGCLVQYHKCDDQCMETI
RNGTYNRREYQEESKLERQKMEGLKLESERTWEILSIYSRVASYRVIAMGSVAFLFWALYNGSCRCNICI

>QBK20109.1 hemagglutinin [Influenza A virus]
METVSLITILLIATVSNADKICIGYQSTNSTETVDTLTENNVPVTHAKELLHTEHNGMLCATSLGQPLIL
DTCTIEGLIYGNPSCDLSLEGREWSYIVERPSAVNGLCYPGNVENLEELRSLFSSARSYQRIQIFPDTIW
NVSYYGTSTACSGSFYRSMRWLTRKNGDYPVQDAQYTNNQGKNILFMWGINHPPTDTTQRDLYTRTDTTT
SVATEEINRIFKPLIGPRPLVNGLMGRIDYYWSVLKPGQTLRIKSDGNLIAPWYGHILSRESHGRILKTD
LKRGSCTVQCQTEKGGLNTALSFQNVSKYASGNCSKYIGIKSLKLAVGLRNVRCRSSRGLFGAIAGFIER
GWSGLVAGWYGFQHSNDQGVGMAADRDSTQKAIDKITSKVNNIVDKMNKQYEIIDHEFSEVETRINMINN
KIDDQIQDIWAYNAELLVLLENQKTLDEHDANVNNLYNKVKRALGSNAVEDGKGCFEQYHKCDDQCMETI
RNGTYNRRKYQAESKLERQKIEGVKQESEGTYKILSIYSTVASYLVIAMGFAAFLFGAMSNGSCRCNICI

>QBK20108.1 hemagglutinin [Influenza A virus]
METVSLITILLVATVSNADKICIGYQSTNSTETVDTLTENNVPVTHAKELLHTEHNGMLCATSLGQPLIL
DTCTIEGLIYGNPSCDLSLEGREWSYIVERPSAVNGLCYPGNVENLEELRSLFSSARSYQRIQIFPDTIW
NVSYDGTSTACSGSFYRSMRWLTRKNGDYPVQDAQYTNNQGKNILFMWGINHPPTDTTQRDLYTRTDTTT
SVATEEINRIFKPLIGPRPLVNGLMGRIDYYWSVLKPGQTLRIKSDGNLIAPWYGHILSGESHGRILKTD
LKRGSCTVQCQTEKGGLNTTLPFQNVSKYAFGNCSKYIGIKSLKLAVGLRNVPSRSSRGLFGAIAGFIEG
GWSGLVAGWYGFQHSNDQGVGMAADRDSTQKAIDKITSKVNNIVDKMNKQYEIIDHEFSEVETRLNMINN
KIDDQIQDIWAYNAELLVLLENQKTLDEHDANVNNLYNKVKRALGSNAVEDGKGCFELYHKCDDQCMETI
RNGTYNRRKYQEESKLERQKIEGVKLESEGTYKILTIYSTVASSLVIAMGFAAFLFWAMSNGSCRCNICI

>QBK20107.1 hemagglutinin [Influenza A virus]
METVSLITILLVATVSNADKICIGYQSTNSTETVDTLTENNVPVTHAKELLHTEHNGMLCATSLGQPLIL
DTCTIEGLIYGNPSCDLSLEGREWSYIVERPSAVNGLCYPGNVENLEELRSLFSSARSYQRIQIFPDTIW
NVSYDGTSTACSGSFYRSMRWLTRKNGDYPVQDAQYTNNQGKNILFMWGINHPPTDTTQRDLYTRTDTTT
SVATEEINRIFKPLIGPRPLVNGLMGRIDYYWSVLKPGQTLRIKSDGNLIAPWYGHILSGESHGRILKTD
LKRGSCTVQCQTEKGGLNTTLPFQNVSKYAFGNCSKYIGIKSLKLAVGLRNVPSRSSRGLFGAIAGFIEG

GWSGLVAGWYGFQHSNDQGVGMAADRDSTQKAIDKITSKVNNIVDKMNKQYEIIDHEFSEVETRLNMINN
KIDDQIQDIWAYNAELLVLLENQKTLDEHDANVNNLYNKVKRALGSNAVEDGKGCFELYHKCDDQCMETI
RNGTYNRRKYQEESKLERQKIEGVKLESEGTYKILTIYSTVASSLVIAMGFAAFLFWAMSNGSCRCNICI

>QBK20106.1 hemagglutinin [Influenza A virus]
METVSLITILLIATVSNADKICIGYQSTNSTETVDTLTENNVPVTHAKELLHTEHNGMLCATSLGQPLIL
DTCTIEGLIYGNPSCDLSLEGREWSYIVERPSAVNGLCYPGNVENLEELRSLFSSARSYQRIQIFPDTIW
NVSYDGTSTACSGSFYRSMRWLTRKNGDYPVQDAQYTNNQGKNILFMWGINHPPTDTTQRDLYTRTDTTT
SVATEEINRIFKPLIGPRPLVNGLMGRIDYYWSVLKPGQTLRIKSDGNLIAPWYGHILSRESHGRILKTD
LKRGSCTVQCQTEKGGLNTALPCQNVSKYAFGNCSKYIGIKSLKLAVGLRNVPCRSSRGLFGAIAGFIEG
GWSGLVAGWYGFQHSNDQGVGMAADRDSTQKAIDKITSKVNNIVDKMNKQYEIIDHEFSEVETRINMINN
KIDDQIQDIWAYNAELLVLLENQKTLDEHDANVNNLYNKVKRALGSNAVEDGKGCFELYHKCDDQCMETI
RNGTYNRRKYQEESKLERQKIEGVKLESEGTYKILSIYSTVASSLVIAMGFAAFLFWAMSNGSCRCNISI

>QBK20105.1 hemagglutinin [Influenza A virus]
METVSLITILLIATVSNADKICIGYQSTNSTETVDTLTENNVPVTHAKELLHTEHNGMLCATSLGQPLIL
DTCTIEGLIYGNPSCDLSLEGREWSYIVERPSAVNGLCYPGNVQNLEELRSLFSSARSYQRIQIFPDTIW
NVSYDGTSTACSGSFYRSMRWLTRKNGDYPTQDAQYTNNQGKNILFMWGINHPPTDDTQRNLYTRTDTTT
SVATEEINRIFKPLIGPRPLVNGLMGRIDYYWSVLKPGQTLRIKSDGNLIAPWYGHILSGESHGRILKTD
LKRGSCTVQCQTDNGGLHTAQSLLNVSKSAFGRCSKYIGIKSLQGAVGLRNVRSRYSRGLFGAIAGFIEG
GWSGLVGGWYGFQHSNDQGVGMAADRDSTQKAIDKITSKENNIVAKLYKQYEIIDHEFSEVGTRINMSSN
KFDDRIQDIWAYNAESVVFLDNQKTSDEHDAKLDNEYNKVKRAPGSNAVEDGKGCSEMYHKCNEECMETI
RNRTFNRREYQAESKLEREKIEGVKLELEGTCKVVSIYSSVASYRVIAIGFVAFLIGALYNWSCRCNICI

>QBK20104.1 hemagglutinin [Influenza A virus]
MDTVSLITILLIATVSNADKICIGYQSTNSTETVDTLTENNVPVTHAKELLHTEHNGMLCATSLGQPLIL
DTCTIEGLIYGNPSCDLSLEGREWSYIVERPSAVNGLCYPGNVENLEELRSLFSSARSYQRIQIFPDTIW
NVSYYGTSTACSGSFYRSMRWLTRKNGDYPVQDAQYTNNQGKNILFMWGINHPPTDTTQRDLYTRTDTTT
SVATEEINRIFKPLIGPRPLVNGLMGRIDYYWSVLKPGQTLRIKSDGNLIAPWYGHILSRESHGRILKTD
LKGGSCTVQCQTEKGGLHTAQSLLNVSKYAFGKCSKYIGIKSLKLAVGLRNVPSRSSRGLFGAIAGFIER
GWSGLVGGWYGFQHSNDQGVGMAADRDSTQKAMDKIPSNVINIVAKLYKPYEIIGLEFIVVGTRIITSNH
KFADQIQDIWAYNAESIVSLDNQITLDEQDANVRSGGNKVKRGACSNAVEDGKGCLVQSHKRDDQCMETI
RNGTYNRRKYQAESKKERQKMEGLNQEFDRTNERVSIYSRVASYRVIAMGSFALRIGALYNWSCRCNICI

>QBK20103.1 hemagglutinin [Influenza A virus]

METVSLITILLVATVSNADKICIGYQSTNSTETVDTLTENNVPVTHAKELLHTEHNGMLCATSLGQPLIL

DTCTIEGLIYGNPSCDLSLEGREWSYIVERPSAVNGLCYPGNVENLEELRSLFSSARSYQRIQIFPDTIW

NVSYDGTSTACSGSFYRSMRWLTRKNGDYPVQDAQYTNNQGKNILFMWGINHPPTDTTQRDLYTRTDTTT

SVATEEINRIFKPLIGPRPLVNGLMGRIDYYWSVLKPGQTLRIKSDGNLIAPWYGHILSGESHGRILKTD

LKRGSCTVQCQTEKGGLNTTLPFQNVSKYAFGNCSKYIGIKSLKLAVGLRNVPSRSSRGLFGAIAGFIEG

GWSGLVAGWYGFQHSNDQGVGMAADRDSTQKAIDKITSKVNNIVDKMNKQYEIIDHEFSEVETRLNMINN

KIDDQIQDIWAYNAELLVLLENQKTLDEHDANVNNLYNKVKRALGSNAVEDGKGCFELYHKCDDQCMETI

RNGTYNRRKYQEESKLERQKIEGVKLESEGTYKILTIYSTVASSLVIAMGFAAFLFWAMSNGSCRCNICI

>QBK20102.1 hemagglutinin [Influenza A virus]

METVSLITILLVATVSNADKICIGYQSTNSTETVDTLTENNVPVTHAKELLHTEHNGMLCATSLGQPLIL

DTCTIEGLIYGNPSCDLSLEGREWSYIVERPSAVNGLCYPGNVENLEELRSLFSSARSYQRIQIFPDTIW

NVSYYGTSTACSGSFYRSMRWLTRKNGDYPVQDAQYTNNQGKNILFMWGINHPPTDTTQRDLYTRTDTTT

SVATEEINRIFKPLIGPRPLVNGLMGRIDYYWSVLKPGQTLRIKSDGNLIAPWYGHILSGESHGRILTTD

LKRGSCTVQCQTEKGGLHTALPLQNVSKYAFGKCSKYIGIKSLKLAVGLRNVRCRSSRGLFGAIAGFIER

GWSGLVAGWYGFQHSNDQGVGMAADRDSTQKAIDKITSKVNNIVAKMYKPYEIIDHEFSEVETRINMINN

KIDDQIQDIWAYNAESLVLLENQKTLDEHDANVNNLYNKVKRALGSNAVEDGKGCFELYHKCDDQCMETI

RNGTYNRRKYQEESKLERQKIEGVKLESEGTYKILSIYSTVASSLVIAMGFAAFLFWAMSNGSCRCNICI

>QBK20101.1 hemagglutinin [Influenza A virus]

MDTVSLITILLIATVSNADKICIGYQSTNSTETVDTLTENNVPVTHAKELLHTEHNGMLCATSLGQPLIL

DTCTIEGLIYGNPSCDLSLEGREWSYIVERPSAVNGLCYPGNVQNLEELRSLFSSARSYQKIQIFPDTIW

NVSYYGTSTACSGSFYRSMRWLTRKNGDYPTQDAQYTNNQGKNILFMWGINHPPTDDTQRNLYTRTDTTT

SVATEEINRIFKPLIGPRPLVNGLMGRIDYYWSVLKPGQTLRIKSDGNLIAPWYGHILSGESHGRILTTD

LQRGSCTVQCQTENGGLHTALPFQNVSKYAFGKCSKYIGIKSLKLAVGLRNVPSRSSRGLFGAIAGFIEG

GWSGLVAGWYGFQHSNDQGVGMAADRDSTQKAIDKITSKVNNIVAKLYKQYEILDHEFSEVGTRINMSSN

KFDDQIQDIWAYNAESLVFLENQKTLDEHDANVNNLYNKVKRALGSNAVEDGKGCFEQYHKCKKECMETI

RNGTYNRREYQAESKLERQKIEGVNLELERTCKIVSIYSTVASYRVIAMGSAALMFGALSNWSCRCNICI

>QBK20100.1 hemagglutinin [Influenza A virus]

METVSLITILLVATVSNADKICIGYQSTNSTETVDTLTENNVPVTHAKELLHTEHNGMLCATSLGQPLIL

DTCTIEGLIYGNPSCDLSLEGREWSYIVERPSAVNGLCYPGNVENLEELRSLFSSARSYQRIQIFPDTIW

NVSYDGTSTACSGSFYRSMRWLTRKNGDYPVQDAQYTNNQGKNILFMWGINHPPTDTTQRDLYTRTDTTT

SVATEEINRIFKPLIGPRPLVNGLMGRIDYYWSVLKPGQTLRIKSDGNLIAPWYGHILSRESHGRILKTD
LKRGSCTVQCQTEKGGLNTTLPFQNVSKYAFGNCSKYIGIKSLKLAVGLRNVPSRSSRGLFGAIAGFIEG
GWSGLVAGWYGFQHSNDQGVGMAADRDSTQKAIDKITSKVNNIVDKMNKQYEIIDHEFSEVETRLNMINN
KIDDQIQDIWAYNAELLVLLENQKTLDEHDANVNNLYNKVKRALGSNAVEDGKGCFELYHKCDDQCMETI
RNGTYNRRKYQEESKLERQKIEGVKLESEGTYKILSIYSTVASSLVIAMGFAAFLFWAMSNGSCRCNICI

>QBK20099.1 hemagglutinin [Influenza A virus]
METVSLIPILLIATVSNADKICIGYQSTNSTETVDTLTENYVPVTHAKELLHTEHNGMLCATSLGQPLIL
DTCTIEGLIYGNPSCDLSLEGREWSYIVERPSAVNGLCYPGNVQNLEELRSLFSSARSYQRIQIFPDTIW
NVSYDGTSTACSGSFYRSMRWLTRKNGDYPTQDAQYTNNQGKNILFMWGINHPPTDDTQRNLYTRTDTTT
SVATEEINRIFKPLIGPRPLVNGLMGRIDYYWSVLKPGQTLRIKSDGNLIAPWYGNILSRESHGRILTTD
LKRGSCTVHCQTENGGLHTTLPLQNVSKYAFGKCSKYIGIKSLKLAVGLRNVPSRSSRGLFGAIAGFIEG
GWSGLVAGWYGFQHSNDQGVGMAADRDSTQKAIDKITSKVNNIVAKLYKQYEIIDHEFSEVETRINMSNN
KFDDQIQDIWAYNAESVVFLDNQKTLDEHDANPDNVYNKVKRALGSNAVEDGKGCSEQYHKCNEECMETI
RNGTFNRREYQAEGKKEIEKIEGVKLELEGTSEVVSIYSTVASYRVIAMGFAAFLFGALSNWSCRCNICI

>QBK20098.1 hemagglutinin [Influenza A virus]
METVSLITILLIATVSNADKICIGYQSTNSTETVDTLTENNVPVTHAKELLHTEHNGMLCATSLGQPLIL
DTCTIEGLIYGNPSCDLSLEGREWSYIVERPSAVNGLCYPGNVENLEELRSLFSSARSYQRIQIFPDTIW
NVSYDGTSTACSGSFYRSMRWLTRKNGDYPVQDAQYTNNQGKNILFMWGINHPPTDTTQRDLYTRTDTTT
SVATEEINRIFKPLIGPRPLVNGLMGRIDYYWSVLKPGQTLRIKSDGNLIAPWYGHILSRESHGRILTTD
LKRGSCTVQCQTEKGGLNTALPFQNVSKYAFGNCSKYIGIKSLKLAVGLRNVRSRSSRGLFGAIAGFIEG
GWSGLVAGWYGFQHSNDQGVGMAADRDSTQKAIDKITSKVNNIVDKMNKQYEIIDHEFSEVETRLNMINN
KIDDQIQDIWAYNAELLVLLENQKTLDEHDANVNNLYNKVKRALGSNAVEDGKGCFELYHKCDDQCMETI
RNGTYNRRKYQEESKLERQKIEGVKLESEGTYKILSIYSTVASSLVIAMGFAAFLFWAMSNGSCRCNICI

>QBK20097.1 hemagglutinin [Influenza A virus]
METVSLITILLVATVSNADKICIGYQSTNSTETVDTLTENNVPVTHAKELLHTEHNGMLCATSLGQPLIL
DTCTIEGLIYGNPSCDLSLEGREWSYIVERPSAVNGLCYPGNVENLEELRSLFSSARSYQRIQIFPDTIW
NVSYDGTSTACSGSFYRSMRWLTRKNGDYPVQDAQYTNNQGKNILFMWGINHPPTDTTQRDLYTRTDTTT
SVATEEINRIFKPLIGPRPLVNGLMGRIDYYWSVLKPGQTLRIKSDGNLIAPWYGHILSGESHGRILKTD
LKRGSCTVQCQTEKGGLNTTLPFQNVSKYAFGNCSKYIGIKSLKLAVGLRNVPSRSSRGLFGAIAGFIEG
GWSGLVAGWYGFQHSNDQGVGMAADRDSTQKAIDKITSKVNNIVDKMNKQYEIIDHEFSEVETRLNMINN
KIDDQIQDIWAYNAELLVLLENQKTLDEHDANVNNLYNKVKRALGSNAVEDGKGCFELYHKCDDQCMETI

RNGTYNRRKYQEESKLERQKIEGVKLESEGTYKILSIYSTVASSLVIAMGFAAFLFWAMSNGSCRCNICI

>QBK20096.1 hemagglutinin [Influenza A virus]
METVSLITILLIATVSNADKICIGYQSTNSTETVDTLTENNVPVTHAKELLHTEHNGMLCATSLGQPLIL
DTCTIEGLIYGNPSCDLSLEGREWSYIVERPSAVNGLCYPGNVENLEELRSLFSSARSYQRIQIFPDTIW
NVSYDGTSTACSGSFYRSMRWLTRKNGDYPTQDAQYTNNQGKNILFMWGINHPPTDDTQRNLYTRTDTTT
SVATEEINRIFKPLIGPRPLVNGLMGRIDYYWSVLKPGQTLRIKSDGNLIAPWYGHILSGESHGRILKTD
LKRGRCTVQCQTEKGGLNTALPLQNVSKYAFGKCSKYIGIKSLKLAVGLRNVPSRSSRGLFGAIAGFIEG
GWSGLVAGWYGFQHSNDQGVGMAADRDSTQKAIDKITSKVNNIVAKMYKQYEIIDHEFSEVETRINMINN
KIDDQIQDIWAYNAESVVLLENQKTLDEHDANVNNLYNKVKRALGSNAVEDGKGCFELYHKCNDQCMETI
RNRTFNRRKYQAESKLERQKIEGVKLEFEGTYKILSIYSTVASSLVIAMGFAAFLFWALSNGSCRCNICI

>QBK20095.1 hemagglutinin [Influenza A virus]
MDTVSLITMLLVSTVSNADKICIGYQSTNSRETVDTLTENNVPVTHAKELLHTEHNGMLCATSLGQPLIL
DTCTIEGLIYGNPSCDLSLEGREWSYIVERPSAVNGLCYPGNVQNLEELRSLFSSARSYQRIQIFPHTIW
NVSYYGTSTACSGSFYRSMRWLTRKNGDYPVQDAQYTNNQGKNILFMWGINHPPTDTTQRDLYTRTDTTT
SVATEEINRIFKPLIGPRPLVNGLMGRIDYYWSVLKPGQTLRIKSDGNLIAPWYGHILSRESHGRILTTD
LNGGSCTVQCQREKGGLHTALPLLNVSKYAFGNCSKYIGIKSLKLAVGLRNVRCRSSRGVFGAIAGFIER
GWSGLVDGWYGFQHSNDQGVGMAADRDSTQKAMDKITSKENNIVAKLYKPYEIIGLEFIVVGTRIITINN
KFDDQIQDIWAYNAESIVFLDNQKTLDEHDANTNNGGNKVKRGAGSNAVEDGKGCLVQSHKCDEECMETI
RNGTFNRREYQAESKKEREKMEGLKLELDRTREIVSIYSRVAWYRVIAMGSFALRFWALYNWSCRCNICI

>QBK20094.1 hemagglutinin [Influenza A virus]
METVSLITILLIATVSNADKICIGYQSTNSTETVDTLTENNVPVTHAKELLHTEHNGMLCATSLGQPLIL
DTCTIEGLIYGNPSCDLSLEGREWSYIVERPSAVNGLCYPGNVENLEELRSLFSSARSYQRIQIFPDTIW
NVSYDGTSTACSGSFYRSMRWLTRKNGDYPTQDAQYTNNQGKNILFMWGINHPPTDDTQRNLYTRTDTTT
SVATEEINRIFKPLIGPRPLVNGLMGRIDYYWSVLKPGQTLRIKSDGNLIAPWYGHILSGESHGRILTTD
LKRGSCTVQCQTEKGGLNTALPLQNVSKYAFGKCSKYIGIKSLKLAVGLRNVPSRSSRGLFGAIAGFIEG
GWSGLVAGWYGFQHSNDQGVGMAADRDSTQKAIDKITSKVNNIVAKMYKQYEIIDHEFSEVETRINMINN
KFDDQIQDIWAYNAESLVFLDNQKTLDEHDANVNNLYNKVKRALGSNAVEDGKGCFEQYHKCNDQCMETI
RNGTYNRRKYQAESKLERQKIEGVNQESEGTCKILSIYSTVASYRVIAMGFAAFLIGAMSNWSCRCNICI

>QBK20093.1 hemagglutinin [Influenza A virus]
METVSLITILLVATVSNADKICIGYQSTNSTETVDTLTENNVPVTHAKELLHTEHNGMLCATSLGQPLIL

DTCTIEGLIYGNPSCDLSLEGREWSYIVERPSAVNGLCYPGNVQNLEELRSLFSSARSYQRIQIFPDTIW
NVSYDGTSTACSGSFYRSMRWLTRKNGDYPTQDAQYTNNQGKNILFMWGINHPPTDDTQRNLYTRTDTTT
SVATEEINRIFKPLIGPRPLVNGLMGRIDYYWSVLKPGQTLRIKSDGNLIAPWYGHILSRESHGRILTTD
LKRGSCTVQCQREKGGLHTALPLQNVSKYAIGNCSKYIGIKSLKLAVGLRNVPSRSSRGLFGAIAGFIEG
GWSGLVGGWYGFQHSNDQGVGMAADRDSTQKAIDKITSKVNNMVAKLYKQYEIIDHEFSEVGTRINMSSN
KFDGQIQDIWAYNAESVVLLDNQKTRDEHDANPDNVYNKVKRASGSNAVEDGKGCFEQSHKCNEECMETI
RNRTFNRRKYQAESKLERQKIEGVNQELDRTCKVVSIYSTVAWYRVIAMGFVAFLFGALYNGSCRCNICI

>QBK20092.1 hemagglutinin [Influenza A virus]
METVSLITILLVATVSNADKICIGYQSTNSTETVDTLTENNVPVTHAKELLHTEHNGMLCATSLGQPLIL
DTCTIEGLIYGNPSCDLSLEGREWSYIVERPSAVNGLCYPGNVENLEELRSLFSSARSYQRIQIFPDTIW
NVSYDGTSTACSGSFYRSMRWLTRKNGDYPTQDAQYTNNQGKNILFMWGINHPPTDDTQRNLYTRTDTTT
SVATEEINRIFKPLIGPRPLVNGLMGRIDYYWSVLKPGQTLRIKSDGNLIAPWYGHILSGESHGRILKTD
LKRGSCTVQCQTEKGGLNTTLPFQNVSKYAFGNCSKYIGIKSLKLAVGLRNVPSRSSRGLFGAIAGFIEG
GWSGLVAGWYGFQHSNDQGVGMAADRDSTQKAIDKITSKVNNIVDKMNKQYEIIDHEFSEVETRLNMINN
KIDDQIQDIWAYNAELLVLLENQKTLDEHDANVNNLYNKVKRALGSNAVEDGKGCFELYHKCNDQCMETI
RNGTYNRRKYQEESKLERQKIEGVKLESEGTYKILSIYSTVASSLVIAMGFAAFLFWAMSNGSCRCNICI

>QBK20091.1 hemagglutinin [Influenza A virus]
METVSLITILLIATVSNADKICIGYQSTNSTETVDTLTENNVPVTHAKELLHTEHNGMLCATSLGQPLIL
DTCTIEGLIYGNPSCDLSLEGREWSYIVERPSAVNGLCYPGNVENLEELRSLFSSARSYQRIQIFPDTIW
NVSYDGTSTACSGSFYRSMRWLTRKNGDYPVQDAQYTNNQGKNILFMWGINHPPTDTTQRDLYTRTDTTT
SVATEEINRIFKPLIGPRPLVNGLMGRIDYYWSVLKPGQTLRIKSDGNLIAPWYGHILSGESHGRILKTD
LKRGSCTVQCQTEKGGLNTALPFQNVSKYAFGNCSKYIGIKSLKLAVGLRNVRCRSSRGLFGAIAGFIEG
GWSGLVAGWYGFQHSNDQGVGMAADRDSTQKAIDKITSKVNNIVAKMNKQYEIIDHEFSEVETRLNMINN
KIDDQIQDIWAYNAESLVLLENQKTLDEHDANVNNLYNKVKRALGSNAVEDGKGCFELYHKCDDQCMETI
RNGTYNRRKYQAESKLERQKIEGVKLESEGTYKILSIYSTVASSRVIAMGSAAFLFGAMSNGSCRCNSSI

>QBK20090.1 hemagglutinin [Influenza A virus]
METVSLITILLIATVSNADKICIGYQSTNSTETVDTLTENNVPVTHAKELLHTEHNGMLCATSLGQPLIL
DTCTIEGLIYGNPSCDLSLEGREWSYIVERPSAVNGLCYPGNVENLEELRSLFSSARSYQRIQIFPDTIW
NVSYDGTSTACSGSFYRSMRWLTRKNGDYPVQDAQYTNNQGKNILFMWGINHPPTDTTQRDLYTRTDTTT
SVATEEINRIFKPLIGPRPLVNGLMGRIDYYWSVLKPGQTLRIKSDGNLIAPWYGHILSGESHGRILKTD
LKRGSCTVQCQTEKGGLNTTLPFQNVSKYAFGNCSKYIGIKSLKLAVGLRNVPSRSSRGLFGAIAGFIEG

GWSGLVAGWYGFQHSNDQGVGMAADRDSTQKAIDKITSKVNNIVDKMNKQYEIIDHEFSEVETRLNMINN
KIDDQIQDIWAYNAELLVLLENQKTLDEHDANVNNLYNKVKRALGSNAVEDGKGCFELYHKCDDQCMETI
RNGTYNRRKYQEESKLERQKIEGVKLESEGTYKILSIYSTVASSLVIAMGFAAFLFWAMSNGSCRCNISI

>QBK20089.1 hemagglutinin [Influenza A virus]
METVSLITILLIATVSNADKICIGYQSTNSTETVDTLTENNVPVTHAKELLHTEHNGMLCATSLGQPLIL
DTCTIEGLIYGNPSCDLSLEGREWSYIVERPSAVNGLCYPGNVENLEELRSLFSSARSYQRIQIFPDTIW
NVSYDGTSTACSGSFYRSMRWLTRKNGDYPVQDAQYTNNQGKNILFMWGINHPPTDTTQRDLYTRTDTTT
SVATEEINRIFKPLIGPRPLVNGLMGRIDYYWSVLKPGQTLRIKSDGNLIAPWYGHILSRESHGRILKTD
LKRGSCTVQCQTEKGGLNTTLPFQNVSKYAFGNCSKYIGIKSLKLAVGLRNVPCRSSRGLFGAIAGFIEG
GWSGLVAGWYGFQHSNDQGVGMAADRDSTQKAIDKITSKVNNIVDKMNKQYEIIDHEFSEVETRLNMINN
KIDDQIQDIWAYNAESLVLLENQKTLDEHDANVNNLYNKVKRALGSNAVEDGKGCFELYHKCDDQCMETI
RNGTYNRRKYQEESKLERQKIEGVKLESEGTYKILSIYSTVASSLVIAMGFAAFLFWAMSNGSCRCNISI

>QBK20088.1 hemagglutinin [Influenza A virus]
METVSLITILLVATVSNADKICIGYQSTNSTETVDTLTENNVPVTHAKELLHTEHNGMLCATSLGQPLIL
DTCTIEGLIYGNPSCDLSLEGREWSYIVERPSAVNGLCYPGNVENLEELRSLFSSARSYQRIQIFPDTIW
NVSYDGTSTACSGSFYRSMRWLTRKNGDYPVQDAQYTNNQGKNILFMWGINHPPTDTTQRDLYTRTDTTT
SVATEEINRIFKPLIGPRPLVNGLMGRIDYYWSVLKPGQTLRIKSDGNLIAPWYGHILSRESHGRILTTD
LKRGSCTVQCQTEKGGLNTTLPFQNVSKYAFGNCSKYIGIKSLKLAVGLRNVPCRSSRGLFGAIAGFIEG
GWSGLVAGWYGFQHSNDQGVGMAADRDSTQKAIDKITSKVNNIVDKMNKQYEIIDHEFSEVETRLNMINN
KIDDQIQDIWAYNAELLVLLENQKTLDEHDANVNNLYNKVKRALGSNAVEDGKGCFELYHKCDDQCMETI
RNGTYNRRKYQEESKLERQKIEGVKLESEGTYKILSIYSTVASSLVIAMGFAAFLFWAMSNGSCRCNSCI

>QBK20087.1 hemagglutinin [Influenza A virus]
METVSLITILLVATVSNADKICIGYQSTNSTETVDTLTENNVPVTHAKELLHTEHNGMLCATSLGQPLIL
DTCTIEGLIYGNPSCDLSLEGREWSYIVERPSAVNGLCYPGNVENLEELRSLFSSARSYQRIQIFPDTIW
NVSYDGTSTACSGSFYRSMRWLTRKNGDYPVQDAQYTNNQGKNILFMWGINHPPTDTTQRDLYTRTDTTT
SVATEEINRIFKPLIGPRPLVNGLMGRIDYYWSVLKPGQTLRIKSDGNLIAPWYGHILSGERHGRILKTD
LKRGSCTVQCQTEKGGLHTTLPFQNVSKYAFGNCSKYIGIKSLKLAVGLRNVPSRSSRGLFGAIAGFIEG
GWSGLVAGWYGFQHSNDQGVGMAADRDSTQKAIDKITSKVNNIVDKMNKQYEIIDHEFSEVETRLNMINN
KIDDQIQDIWAYNAELLVLLENQKTLDEHDANVNNLYNKVKRALGSNAVEDGKGCFELYHKCDDQCMETI
RNGTYNRRKYQEESKLERQKIEGVKLESEGTYKILSIYSTVASSLVIAMGFAAFLFWAMSNGSCRCNICI

>QBK20086.1 hemagglutinin [Influenza A virus]

METVSLITILLVATVSNADKICIGYQSTNSTETVDTLTENNVPVTHAKELLHTEHNGMLCATSLGQPLIL
DTCTIEGLIYGNPSCDLSLEGREWSYIVERPSAVNGLCYPGNVENLEELRSLFSSARSYQRIQIFPDTIW
NVSYDGTSTACSGSFYRSMRWLTRKNGDYPVQDAQYTNNQGKNILFMWGINHPPTDTTQRDLYTRTDTTT
SVATEEINRIFKPLIGPRPLVNGLMGRIDYYWSVLKPGQTLRIKSDGNLIAPWYGHILSGESHGRILKTD
LKRGSCTVQCQTEKGGLNTALPLQNVSKYAFGNCSKYIGIKSVKLAVGLRNVRCRSSRGLFGAIAGFIER
GWSGLVAGWYGFQHSNDQGVGMAADRDSTQKAIDKITSKVNNIVDKMYKPYEIIDHEFSVVETRINTINN
KFDDQIQDIWAYNAESIVLLENQKTLDEHDANVNNLYNKVKRALGSNAVEDGKGCFEQYHKCDDQCMETI
RNGTFNRRKYQAESKLERQKIEGVKQESEGTCKIVSIYSTVTSSLVIAMGFAAFLFWAMSNGSCRCNICI

>QBK20085.1 hemagglutinin [Influenza A virus]

METVSLITILLVATVSNADKICIGYQSTNSTETVDTLTENNVPVTHAKELLHTEHNGMLCATSLGQPLIL
DTCTIEGLIYGNPSCDLSLEGREWSYIVERPSAVNGLCYPGNVENLEELRSLFSSARSYQRIQIFPDTIW
NVSYDGTSTACSGSFYRSMRWLTRKNGDYPVQDAQYTNNQGKNILFMWGINHPPTDTTQRDLYTRTDTTT
SVATEEINRIFKPLIGPRPLVNGLMGRIDYYWSVLKPGQTLRIKSDGNLIAPWYGHILSGESHGRILKTD
LKRGSCTVQCQTEKGGLNTTLPFQNVSKYAFGNCSKYIGIKSLKLAVGLRNVPSRSSRGLFGAIAGFIEG
GWSGLVAGWYGFQHSNDQGVGMAADRDSTQKAIDKITSKVNNIVDKMNKQYEIIDHEFSEVETRLNMINN
KIDDQIQDIWAYNAELLVLLENQKTLDEHDANVNNLYNKVKRALGSNAVEDGKGCFELYHKCDDQCMETI
RNGTYNRRKYQEESKLERQKIEGVKLESEGTYKILTIYSTVASSLVIAMGFAAFLFWAMSNGSCRCNICI

>QBK20084.1 hemagglutinin [Influenza A virus]

MDTVSLITILLVSTVSNADKICIGYQSTNSTETVNTLTENNVPVTHAKELLHTEHNGMLCATSLGQPLIL
DTCTIEGLIYGNPSCDLSLEGREWSYIVERPSAVNGLCYPGNVENLEELRSLFSSARSYQKIQIFPHTIW
NVSYYGTSTACSGSFYRIMRWLTRKNGDYPVQDAQYTNNQGKNILFMWGINHPPTDTTQRDLYTRTDTTT
SVATEEINRIFKPLIGPRPLVNGLMGRIDYYWSELKPGQTLRIKSDGNLIATWYGHILSGESHGRIVKTD
LKRGSCTVQCQTEKGGLNTALPLQNVSKYAFGKCSKYIGIKSLKLAVGLRNVPCRSSRGLFGAIAGFIER
GWSGLVAGWYGFQHSNDQGVGMAADRDSTQKAIDKITSKVNNIVAKMYKPYEIIGHEFSVVETRINTISN
KFDDQIQDIWAYNAESIVLLDNQKTLDEHDANVNNLYNKVKRALGSNAVEDGKGCFEQYHKCDDQCMETI
RNGTYNRRKYQAESKLERQKMEGVKQESEGTWKILSIYSTVASYRVIAMGFAALRIGAMYNGSCRCNICI

>QBK20083.1 hemagglutinin [Influenza A virus]

METVSLITILLVATVSNADKICIGYQSTNSTETVDTLTENNVPVTHAKELLHTEHNGMLCATSLGQPLIL
DTCTIEGLIYGNPSCDLSLEGREWSYIVERPSAVNGLCYPGNVENLEELRSLFSSARSYQRIQIFPDTIW
NVSYYGTSTACSGSFYRSMRWLTRKNGDYPVQDAQYTNNQGKNILFMWGINHPPTDTTQRDLYTRTDTTT

SVATEEINRIFKPLIGPRPLVNGLMGRIDYYWSVLKPGQTLRIKSDGNLIAPWYGHILSGESHGRILTTD

LKRGSCTVQCQTEKGGLNTTLPFQNVSKYAFGKCSKYIGIKSLKLAVGLRNVRCRSSRGLFGAIAGFIER

GWSGLVAGWYGFQHSNDQGVGMAADRDSTQKAIDKITSKVNNIVAKMYKPYEIIDHEFSEVETRLNMINN

KIDDQIQDIWAYNAESLVLLENQKTLDEHDANVNNLYNKVKRALGSNAVEDGKGCFELYHKCDDQCMETI

RNGTYNRRKYQEESKLERQKIEGVKLESEGTYKILSIYSTVASSLVIAMGFAAFLFWAMSNGSADCNSSI

>QBK20082.1 hemagglutinin [Influenza A virus]

MDTVSLISMLLLSTVSKANKICIGYQSTNSAETVNTLTQNNVPVTHAKELLHTQHNGMLCATSLGQPLIL

DTCTIEGLIYGNPSCDLSLEGREWSYIVQRPSTVNGLCYPGNVQNLEELMSLFSSATSSQKIQIFPHTIW

NVSYYGTSAACSGSFYRIMRWLTRKNGDYPIQDAQYTNNQGNNILFMWGINHPPTDTTQIDLYTITDTTT

SLATEEINRIFKPLIGPRPLVNGLMGRIHYYWSVLKPGKTLPIKSDGNLIAPWYGHILSRESHGRILTTD

LKRGSCTVQCQTEKGGQHTTLSLQNVSKYAFGKCSKYIGIKSLKLAVGLRNVPSRSSRGLLGAIARFIEG

GWSGLVGGWYGFQHSNDQGVGMAADRDSTQKAIDKITSKVNNIVDKMNKQYEIIDHEFSVVETRIIMINN

KSDDQIQDIWAYNAELIVLLENQKTLDEQDANVSNLYNKVKRGACSNAVEDGNGCLELCHKCDDQCMETI

GNGTYNRREYQEEGKLARQKMEGLKLESDRTRERVSIYSTVTSYIVIAMGFVALRFGGMYNGSCRCNICI

>QBK20081.1 hemagglutinin [Influenza A virus]

METVSLITILLIATVSNADKICIGYQSTNSTETVDTLTENNVPVTHAKELLHTEHNGMLCATSLGQPLIL

DTCTIEGLIYGNPSCDLSLEGREWSYIVERPSAVNGLCYPGNVENLEELRSLFSSARSSQRIQIFPDTIW

NVSYYGTSTACSGSFYRSMRWLTRKNGDYPIQDAQYTNNQGKNILFMWGINHPPTDTTQRDLYTRTDTTT

SLATEEINRIFKPLIGPRPLVNGLMGRIHYYWSVLKPGQTLRIKSDGNLIAPWYGHILSRESHGRILKTD

LKRGSCTVQCQTEKGGLNTTLPFQNVSKYAFGNCSKYIGIKSLKLAVGLRNVPSRSSRGLFGAIAGFIEG

GWSGLVAGWYGFQHSNDQGVGMAADRDSTQKAIDKITSKVNNIVDKMNKQYEIIDHEFSEVETRLNMINN

KIDDQIQDIWAYNAELLVLLENQKTLDEHDANVNNLYNKVKRALGSNAVEDGKGCFELYHKCDDQCMETI

RNGTYNRRKYQEEAKLERQKIEGVKLESEGTYKILSIYSTVASSIVIAMGFAAFLFWAMSNGSCRCNICI

>QBK20080.1 hemagglutinin [Influenza A virus]

METVSLITILLVATVSNADKICIGYQSTNSTETVDTLTENNVPVTHAKELLHTEHNGMLCATSLGQPLIL

DTCTIEGLIYGNPSCDLSLEGREWSYIVERPSAVNGLCYPGNVQNLEELRSLFSSARSSQRIQIFPDTIW

NVSYDGTSTACSGSFYRSMRWLTRKNGDYPIQDAQYTNNQGKNILFMWGINHPPTDTTQRDLYTRTDTTT

SLATEEINRIFKPLIGPRPLVNGLMGRIHYYWSVLKPGQTVRIKSDGNLIATWYGHILSGESHGRIVKTD

LKRGSCTVQCKTEKGGLHTALPLQNVSKYAFGKCSKYIGIKSLKLAVGSRNVPSRSSRGLFGAIAGFIEG

GWSGLVGGWYGFQHSNDQGVGMAADRDSTQKAIDKITSKVNNIVDKMNKQYEIIDHEFSVVETRINMINN

KFDDQIQDIWAYNAELIVLLENQKTLDEQDANVSNLYNKVKRGACSNAVEDGKGCFELCHKCDDQCMETI

GNGTYNRRKYQEEGKLERQKIEGLKLESDGTNKILSICSRVASYIVIAMGFVALEFGAMYNWSCRCNICI

>QBK20079.1 hemagglutinin [Influenza A virus]

METVSLITILLIATVSNADKICIGYQSTNSTETVDTLTENNVPVTHAKELLHTEHNGMLCATSLGQPLIL

DTCTIEGLIYGNPSCDLSLEGREWSYIVERPSAVNGLCYPGNVQNLEELRSLFSSARSSQRIQIFPDTIW

NVSYYGTSTACSGSFYRSMRWLTRKNGDYPIQDAQYTNNQGKNILFMWGINHPPTDTTQRDLYTRTDTTT

SLATEEINRIFKPLIGPRPLVNGLMGRIHYYWSVLKPGQTLRIKSDGNLIAPWYGHILSRESHGRILKTD

LKRGSCTVQCQTEKGGLNTALPLQNVSKYAFGNCSKYIGIKSLKRAVGSRNVPSRSSRGLFGAIAGFIEG

GWSGLVGGWYGFQHSNDQGVGMAADRDSTQKAIDKITSKVNNIVDKMNKQYEIIDHEFSVVETRLIMINN

KFDDQIQDIWAYNAESIVLLENQKTLDEQDANVSNLYNKVKRAACSNAVEDGKGCFELCHKCDDQCMETI

GNGTYNRRKYQEEAKLERQKIEGLKLESDRTNKILSIYSTVASYIVIAMGFVALEFGALYNGSCRCNICI

>QBK20078.1 hemagglutinin [Influenza A virus]

MDTVSLITILLIATVSNADKICIGYQSTNSTETVDTLTENNVPVTHAKELLHTEHNGMLCATSLGQPLIL

DTCTIEGLIYGNPSCDLSLEGREWSYIVERPSTVNGLCYPGNVQNLEELRSLFSSARSSQRIQIFPDTIW

NVSYYGTSTACSGSFYRSMRWLTRKNGDYPIQDAQYTNNQGKNILFMWGINHPPTDTTQRDLYTRTDTTT

SLATEEINRIFKPLIGPRPLVNGLMGRIHYYWSVLKPGQTLRIKSDGNLIAPWYGHILSRESHGRILNTD

LKRGSCTVQCQTEKGGLNTTLPFQNVSKYAFGNCSKYIGIKSLKLAVGLRNVPSRSSRGLFGAIAGFIEG

GWSGLVGGWYGFQHSNDQGVGMAADRDSTQKAIDKITSKVNNIVDKMNKQYEIIDHEFSEVETRINMINN

KFDDQIQDIWAYNAELIVLLENQKTLDEQDANVNNLYNKVKRGACSNAVEDGKGCLELCHKCDDQCMETI

RNGTYNRRKYQEEGKKEREKMEGLKLESERTNKILSIYSRVASYIVIAMGSVALMIEALYNWSCRCNICI

>QBK20077.1 hemagglutinin [Influenza A virus]

METVSLITILLIATVSNADKICIGYQSTNSTETVDTLTENNVPVTHAKELLHTEHNGMLCATSLGQPLIL

DTCTIEGLIYGNPSCDLSLEGREWSYIVERPSAVNGLCYPGNVQNLEELRSLFSSARSSQRIQIFPDTIW

NVSYYGTSTACSGSFYRSMRWLTRKNGDYPIQDAQYTNNQGKNILFMWGINHPPTDTTQRDLYTRTDTTT

SLATEEINRIFKPLIGPRPLVNGLMGRIHYYWSVLKPGQTLRIKSDGNLIAPWYGHILSRESHGRLLKTD

LKRGSCTVQCQTEKGGLNTTLPLQNVSKYAFGNCSKYIGIKSLKLAVGLRNVPSRSSRGLFGAIAGFIEG

GWSGLVAGWYGFQHSNDQGVGMAADRDSTQKAIDKITSKVNNIVDKMNKQYEIIDHEFSEVETRLNMINN

KIDDQIQDIWAYNAELLVLLENQKTLDEHDANVNNLYNKVKRALGSNAVEDGKGCFELYHKCDDQCMETI

RNGTYNRRKYQEEAKLERQKIEGVKLESEGTYKILSIYSTVASYIVIAMGSAAFLFWAMSNGSCRCNICI

>QBK20076.1 hemagglutinin [Influenza A virus]

MDTVSLITILLIATVSNADKICIGYQSTNSTETVDTLTENNVPVTHAKELLHTEHNGMLCATSLGQPLIL

DTCTIEGLIYGNPSCDLSLEGREWSYIVERPSAVNGLCYPGNVQNLEELRSLFSSARSSQRIQIFPDTIW
NVSYYGTSTACSGSFYRSMRWLTRKNGDYPIQDAQYTNNQGKNILFMWGINHPPTDTTQRDLYTRTDTTT
SLATEEINRIFKPLIGPRPPVNGLMGRIHYYWSVLKPGQTLRIKSDGNLIAPWYGHILSRESHGRILNTD
LKRGSCTVQCQTEKGGLHTTQSLLNVDRAAFGKCSKYIVIKSLQGAVGSRNDLSRSSRGLFGAIAGFIEG
GWSGLVGGRYRIQHSNDQGVGMAADRDSTQKAIDKITSKVNNIVGKMNKQYEIIDLEFSVVETRIIMINH
KSDDQIQDIWAYNAEAIVSLENQKTLDEQDVNVSNGGNKVKRGACSNAMEDGNGCLEQRHKCDDQCMETI
RNGTYNRREYQEEGKKERQKMEGVDLESDRTWERDSICSRVAWYRVIAMGSFALSIEALYNWSCRCNICI

>QBK20075.1 hemagglutinin [Influenza A virus]
METVSLITILLVATVSNADKICIGYQSTNSTETVDTLTENNVPVTHAKELLHTEHNGMLCATSLGQPLIL
DTCTIEGLIYGNPSCDLSLEGREWSYIVERPSAVNGLCYPGNVQNLEELRSLFSSARSSQRIQIFPDTIW
NVSYYGTSTACSGSFYRSMRWLTRKNGDYPIQDAQYTNNQGKNILFMWGINHPPTDTTQRDLYTRTDTTT
SLATEEINRIFKPLIGPRPLVNGLMGRIHYYWSVLKPGQTLRIKSDGNLIAPWYGHILSRESHGRILKTD
LKRGSCTVQCQTEKGGLNTTLPLQNVSKYAFGKCSKYIGIKSLKLAVGLRNVPSRSSRGLFGAIAGFIEG
GWSGLVGGWYGFQHSNDQGVGMAADRDSTQKAIDKITSKVNNIVDKMNKQYEIIDHEFSEVETRLNTINN
KSDDQIQDIWAYNAELLVLLENQKTLDEHDANVSNLYNKVKRAACSNAVEDGKGCFELYHKCDDQCMETI
RNGTYNRRKYQEEAKLERQKMEGLKLESEGTYKILSIYSRVASYIVIAMGSVALRFGALYNGSCRQNSSI

>QBK20074.1 hemagglutinin [Influenza A virus]
MDTASLITMLLLLTVSIADGICLRYQSTNSTETVNTLTQNNVPVTHAKELLHTQHNGMLCATSLGQPLIL
DTCTIEGIIYGNPSCDILLGGREWSYIVERPSTVNGLCYPGNVQNLKELMSLFSSAKSYQKIQIFPHTLW
NVSYCGTSKACSDSFYRSMRWLTQKNNAYPIQDAQYTNNQENNILFMWGINHPPTDTVQTNLYTRTDTTT
SVATEEINRTFKPLIGPRPLVNGLMGRINYYWSVLKPGQTLRIKSNGNLIAPWYGHILSRESHGRILKTD
LERGSCTVQCQTENGGLNTTLPFQNVTMYAFGNCSKYIGIKSLKLAVGLRNVPSRSSRGLFGAIAGFIEG
GWSGLVDGWYGFQHSNDQGVGMAADRDSTQKAIDKITSKLNNIVDKTIKQYEILDHEFSEVETRINMISN
KIDDQIQDIWAYNAEFIVLLENQKTLDEHDANVNNLYNKVKRALGSNAVEDGKGCFELYHKCDDQCMETI
RNGTYNRRRYQEESREVIRKMEGVKPESEGTHKILVIYLTVASSMVIAMGFVAHELWAMSIVSCRCNICI

>QBK20073.1 hemagglutinin [Influenza A virus]
METASLITILLVVTVSIANKICIGYQSTNSTETVNTLTENNVPVTHAKELLHTEHNGMLCATSLGQPLIL
DTCTIEGIIYGNPSCDILLGGREWSYIVERPSAVNGLCYPGNVQNLEELRSLFSSAKSYQRIQIFPHTIW
NVSYSGTSKACSDSFYRSMRWLTQKNNAYPIQDAQYTNNQEKNILFMWGINHPPTDTAQTNLYTRTDTTT
SVATEEINRTFKPLIGPRPLVNGLMGRINYYWSVLKPGQTLRIKSNGNLIAPWYGHILSGESHGRILKTD
LKRGSCTVQCQTEKGGLNTTLPLQNVSKYAFGNCSKYIGIKSLKLAVGLRNVPSRSSRGLFGAIAGFIEG

GWSGLVAGWYGFQHSNDQGVGMAADRDSTQKAIDKITSKVNNIVDKMIKQYEILDHEFSEVETRINMISN
KIDDQIQDIWAYNAEFIVLLENQKTLDEHDANVNNLYNKVKRALGSNAVEDGKGCFELYHKCDDQCMETI
RNGTYNRRKYQEESRLERQKIEGAKPESEGTHKILVIYLTVASSLVIAMGFVAFLLWAMSIVSCRCNICI

>QBK20072.1 hemagglutinin [Influenza A virus]
METASLITILLLVTVSNANEICIGYQSTNSTETVNTLTENNVPVTHAKELLHTEHNGMLCATNLGHPLIL
DTCTIEGLIYGNPSCDPLLGGREWSYIVERPSTVNGLCYPGNVQNLEELRSLFSSARSYQRIQIFPDTIW
NVSYSGTSKACSDSFYRSMRWLTQKDNAYPIQDAQYTNNQEKNILFMWGINHPPTDTVQTNLYTRTDTTT
SVATEEINRTFKPLIGPRPLVNGLMGRINYYWSVLEPGQTLTIKSNGNLIAPWYGHILSGESHGRILKTN
LKRGNCIVQCQAEKGGLNTTLPLLNVSKYAFGNCSKYMGIKSLKLAVGSRNVPSRSSRGLLGAIAGFIEG
GWSGLVGGWYGFQHSNDQGVGMAADRDSTQKAIDKITSKVNNIVAKMNKQYEIIDHEFSEVETRINMINN
KFDDQIQDIWAYNAESLVLLENQKTLDEHDANVSNLYNKVKRALGSNAVEDGNGCFELYHKCDDQCMETI
RNGTFNRRKYQAESKLERQKIEGLKQESERTYKILSIYSTVASYLAIAMGSAAFLFGAMYNGSCRCNICI

>QBK20071.1 hemagglutinin [Influenza A virus]
METASLITILLVVTVSRADKICIGYQSTNSTETVNTLTENNVPVTHAKELLHTEHNGMLCATSLGQPLIL
DTCTIEGIIYGNPSCDILLGGREWSYIVERPSAVNGLCYPGNVQNLEELRSLFSSAKSYQRIQIFPDTIW
NVSYSGTSKACSDSFYRSMRWLTQKNNAYPIQDAQYTNNQEKNILFMWGINHPPTDTAQTNLYTRTDTTT
SVATEEINRTFKPLIGPRPLVNGLMGRINYYWSVLKPGQTLRIKSDGNLIAPWYGHVLSRESHGRILKTD
LKRGSCTVQCQTEKGGLNTTSPLQNVSKYAFGNCSKYIGIKSLKLAVGLRNVPSRSSRGLFGAIAGFIEG
GWSGLVAGWYGFQHSNDQGVGMAADRDSTQKAIDKITSKVNNIVDKMIKQYEILDHEFSEVETRINMISN
KIDDQIQDIWAYNAEFIVLLENQKTLDEHDANVNNLYNKVKRALGSNAVEDGKGCFELYHKCDDQCMETI
RNGTNNRRKYQEESRLERQKIEGVKPEYEGTHKILTIYLTVASSLVIAMGFVAFLLWAMSIVSCRCNICI

>QBK20070.1 hemagglutinin [Influenza A virus]
METASLITILLLVTVSNADKICIGYQSTNSTETVDTLTENNVPVTHAKELLHTEHNGMLCATNLGHPLIL
DTCTIEGLIYGNPSCDPLLGGREWSYIVERPSAVNGLCYPGNVENLEELRSLFSSARSYQRIQIFPDTIW
NVSYSGTSKACSDSFYRSMRWLTQKDNAYPIQDAQYTNNQEKNILFMWGINHPPTDTAQTNLYTRTDTTT
SVATEEINRTFKPLIGPRPLVNGLMGRINYYWSVLKPGQTLRIKSNGNLIAPWYGHILSRESHGRILKTN
LKRGNCIVQCQTEKGGLNTTLPFQNVSKYAFGNCSKYIGIKSLKLAVGLRNVPSRSSRGLFGAIAGFIEG
GWSGLIAGWYGFQHSNDQGVGMAADRDSTQKAIDKITSKVNNIVDKMNKQYEIIDHEFSEVETRLNMINN
KIDDQIQDIWAYNAELLVLLENQKTLDEHDANVNNLYNKVKRALGSNAVEDGKGCFELYHKCDDQCMETI
RNGTYNRRKYQEESKLERQKIEGVKLESEGTYKILSIYSTVASSLVIAMGFAAFLFWAMSNGSCRCNICI

>QBK20069.1 hemagglutinin [Influenza A virus]
MDTASLMTILLLLTVSNANDICLGYQSTNSTETVNTLTENNVPVTHAKELLHTQHNGMLCATNLGHPLIL
DTCTIEGLIYGNPSCDLLLGGREWSYIVQRPSAVNGLCYPGNVQNLEELISLFSSARSYQRIQIFPHTIW
NVSYSGTSKACSDSFYISMRWLTQKNNNYPIQDAQYTNNQEKNILFMWGINHPPTDTAQTNLYTRTDTTT
SVATEEINRTFKPLIGPRPLVNGLMGRINYYWSVLKPGQTLRIKSNVNLIAPWYGHILSGESHGRILKTD
LKRGSCTVQCQTEKGGLDTTLPLQNVSKYAFGNCSKYIGIKSLKLAVGLRNVPSRSSRGLFGAIAGFIEG
GWSGLVAGWYGFQHSNDQGVGMAADRDSTQKAIDKITSKLNNIVDKTIKQYEILDHEFSEVETRINMINN
KIDDQIQDIWAYNAEFIVLLQNQKTMDEHDANVNNLYNKVKRALGSNAVEDGKGCFELYHKCDDQCMESI
RNGTYNRRKYQEESKVVRQKMEGAKPESEGIHKILIFYLSVASSLVIARGFAALVLWAMSIVSCRCNICI

>QBK20068.1 hemagglutinin [Influenza A virus]
METASLITILLLVTVSNADKICIGYQSTNSTETVDTLTENNVPVTHAKELLHTEHNGMLCATNLGHPLIL
DTCTIEGLIYGNPSCDPLLGGREWSYIVERPSAVNGLCYPGNVQNLEELRSLFSSARSYQRIQIFPDTIW
NVSYSGTSKACSDSFYRSMRWLTQKDNAYPIQDAQYTNNQGKNILFMWGINHPPTDTAQTNLYTRTDTTT
SVATEEINRTFKPLIGPRPLVNGLMGRINYYWSVLKPGQTLRIKSNGNLIAPWYGHILSGESHGRILKTD
LKRGSCTVQCQTEKGGLNTTLPFQNVSKYAFGNCSKYMGIKSLKLAVGSRNVLSRSSRGLFGAIAGFIEG
GWSGLVAGWYGFQHSNDQGVGMAADRDSTQKAIDKITSKVNNIVAKMNKQYEIIDLEFSEVETRLNMINN
KFDDQIQDIWAYNAESIVLLENQKTLDEHDANVNNLYNKVKRALGSNAVEDGKGCLELYHKCDDQCMETI
GNGTYNRRKYQEESKLERQKMEGVKLESEGTYKILSIYSTVASYLVIAMGFVAFWFGAMSNGSCRCNICI

>QBK20067.1 hemagglutinin [Influenza A virus]
METASLMTILLVVTVSNANKICIGYQSTNSTETVNTLTENNVPVTHAKELLHTEHNGMLCATSLGHPLIL
DTCTIEGLIYGNPSCDLLLGGREWSYIVERPSAVNGLCYPGNVQNLEELRSLFSSARSYQRIQIFPDTIW
NVSYSGTSKACSDSFYRSMRWLTQKNNNYPIQDAQYTNNQEKNILFMWGINHPPTDTAQTNLYTRTDTTT
SVATEEINRTFKPLIGPRPLVNGLMGRINYYWSVLKPGQTLRIKSNGNLIAPWYGHILSRESHGRILKTD
LKRGSCTVPSQTEKGGLHPTLPFRNVSKYAVGKCSKYIGIKSLKLAGGLRNVLCRSSRGLFGAIAGIIEG
GWSGLVAGWYGFQHSNDQGVGMAADRDSTQRAIDKITSKGNNIVDKMHKQYEIVDQEFSEVESRLNVISN
QFYDQIQEIWANKAELAVVFENQKTLGEHDANVNNLYNKVKRARGSNAVEDGKGSFEVYHKCDDESMETI
GNGTYNRRKDQEESKLERQKIGGVQVQYEGHYKILAFYWSVASCLVIAMGFASFLFRDGSNWSCRCNICI

>QBK20066.1 hemagglutinin [Influenza A virus]
MDTVSLITMLLLSTLSNANKICIGYQSTNSAKTVNTLTQNNVPVTHAKELLHTEHNGMLCATSLGQPLIL
DTCTIEGLIYGNPSCDLSLEGREWSYIVERPSTVNGLCYPGNVQNLEELMSLFSSATSSQKIQIFPHTIW
NVSYYGTSTACSGSFYRIMRWLTRKNGDYPIQDAQYTNNQGNNILFMWGINHPPTDTTQRDLYTRTDTTT

SVATEEINRIFKPLIGPRPLVNGLMGRIHYYWSVLKPGQTLRIKSDGNLIAPWYGHILSGESHGRILKTD
LKRGSCTVQCQTEKGGLNTTLPLQNVSKYAFGNCSKYIGIKSLKLAVGLRNVPSRSSRGLFGAIAGFIEG
GWSGLVGGWYGFQHSNDQGVGMAADRDSTQKAIDKITSKVNNIVDKMNKQYEIIDHEFSVVETRINMINN
KIDDQIQDIWAYNAELIVLLENQKTLDEHDANVSNLYNKVKRALCSNAVEDGKGCFELYHKCDDQCMETI
GNGTYNRRKYQEEAKLERQKIEGLKQESEGTYKILSIYSTVASYIVIAMGSAALMFWAMSNGSCRCNICI

>QBK20065.1 hemagglutinin [Influenza A virus]
METASLMTILLVVTVSNADKICIGYQSTNSTETVDTLTENNVPVTHAKELLHTEHNGMLCATSLGHPLIL
DTCTIEGLIYGNPSCDLLLGGREWSYIVERPSAVNGLCYPGNVENLEELRSLFSSARSYQRIQIFPDTIW
NVSYSGTSKACSDSFYRSMRWLTQKNNNYPIQDAQYTNNQEKNILFMWGINHPPTDTAQTNLYTRTDTTT
SVATEEINRTFKPLIGPRPLVNGLMGRINYYWSVLKPGQTLRIKSNGNLIAPWYGHILSGESHGRILKTD
LKRGSCTVQCQTEKGGLNTTLPFQNVSKYAFGNCSKYIGIKSLKLAVGLRNVPSRSSRGLFGAIAGFIEG
GWSGLVAGWYGFQHSNDQGVGMAADRDSTQKAIDKITSKVNNIVDKMNKQYEIIDHEFSEVETRLNMINN
KIDDQIQDIWAYNAELLVLLENQKTLDEHDANVNNLYNKVKRALGSNAVEDGKGCFELYHKCDDQCMETI
RNGTYNRRKYQEESKLERQKIEGVKLESEGTYKILTIYLTVASSLVIAMGFAAFLFWAMSNGSCRCNICI

>QBK20064.1 hemagglutinin [Influenza A virus]
METVSLITILLVATVSNADKICIGYQSTNSTETVDTLTENNVPVTHAKELLHTEHNGMLCATSLGQPLIL
DTCTIEGLIYGNPSCDLSLEGREWSYIVERPSAVNGLCYPGNVENLEELRSLFSSARSSQRIQIFPDTIW
NVSYDGTSTACSGSFYRSMRWLTRKNGDYPIQDAQYTNNQGKNILFMWGINHPPTDTTQRDLYTRTDTTT
SVATEEINRIFKPLIGPRPLVNGLMGRIHYYWSVLKPGQTLRIKSDGNLIAPWYGHILSGESHGRILKTD
LKRGSCTVQCQTEKGGLNTTLPFQNVSKYAFGNCSKYIGIKSLKLAVGLRNVPSRSSRGLFGAIAGFIEG
GWSGLVAGWYGFQHSNDQGVGMAADRDSTQKAIDKITSKVNNIVDKMNKQYEIIDHEFSEVETRLNMINN
KIDDQIQDIWAYNAELLVLLENQKTLDEHDANVNNLYNKVKRALGSNAVEDGKGCFELYHKCDDQCMETI
RNGTYNRRKYQEEAKLERQKIEGVKLESEGTYKILSIYSTVASYIVIAMGFAAFLFWAMSNGSCRCEHCI

>QBK20063.1 hemagglutinin [Influenza A virus]
METVSLITILLIATVSNADKICIGYQSTNSTETVDTLTENNVPVTHAKELLHTEHNGMLCATSLGQPLIL
DTCTIEGLIYGNPSCDLSLEGREWSYIVERPSAVNGLCYPGNVQNLEELRSLFSSARSSQRIQIFPDTIW
NVSYYGTSTACSGSFYRSMRWLTRKNGDYPIQDAQYTNNQGKNILFMWGINHPPTDTTQRDLYTRTDTTT
SVATEEINRIFKPLIGPRPLVNGLMGRIHYYWSVLKPGQTLRIKSDGNLIAPWYGHILSRESHGRILKTD
LKRGSCTVQCQTEKGGLHTTLPLQNVSKYAFGNCSKYIGIKSLKLAVGSRNVPSRFSRGLFGAIAGFIEG
GWSGLVGGWYGFQHSNDQGVGMAADRDSTQKAIDKITSKVNNIVDKMNKQYEIIDLEFSVVETRINMINN
KFDDQIQDIWAYNAESIVSIENQKTLDEQDANVNNGYNKVKRGACSNAVEDGNGCLEQCHKCDDQCMETI

RNGTYNRRKYQAEGKKEREKMEGLKLEFERTYKILSIYSRVTSYRVIAMGSFALRIEALYNGSCRCNICI

>QBK20062.1 hemagglutinin [Influenza A virus]
METVSLITILLIATVSNADKICIGYQSTNSTETVDTLTENNVPVTHAKELLHTEHNGMLCATSLGQPLIL
DTCTIEGLIYGNPSCDLSLEGREWSYIVERPSAVNGLCYPGNVQNLEELRSLFSSARSSQRIQIFPDTIW
NVSYDGTSTACSGSFYRSMRWLTRKNGDYPIQDAQYTNNQGKNILFMWGINHPPTDTTQRDLYTRTDTTT
SVATEEINRIFKPLIGPRPLVNGLMGRIHYYWSVLKPGQTLRIKSDGNLIAPWYGHILSRESHGRILKTD
LKRGSCTVQCQTEKGGLNTTLPLQNVSKYAFGNCSKYIGIKSLKLAVGLRNVPSRSSRGLFGAIAGFIEG
GWSGLVAGWYGFQHSNDQGVGMAADRDSTQKAIDKITSKVNNIVDKMNKQYEIIDHEFSEVETRLNMINN
KIDDQIQDIWAYNAELLVLLENQKTLDEHDANVNNLYNKVKRALGSNAVEDGKGCFELYHKCDDQCMETI
RNGTYNRRKYQEEAKLERQKIEGVKLESEGTYKILSIYSTVASYIVIAMGFAAFLFWAMSNGSCRCNICI

>QBK20061.1 hemagglutinin [Influenza A virus]
METVSLITILLIATVSNADKICIGYQSTNSTETVDTLTENNVPVTHAKELLHTEHNGMLCATSLGQPLIL
DTCTIEGLIYGNPSCDLSLEGREWSYIVERPSAVNGLCYPGNVENLEELRSLFSSARSSQRIQIFPDTIW
NVSYDGTSTACSGSFYRSMRWLTRKNGDYPIQDAQYTNNQGKNILFMWGINHPPTDTTQRDLYTRTDTTT
SVATEEINRIFKPLIGPRPLVNGLMGRIHYYWSVLKPGQTLRIKSDGNLIAPWYGHILSRESHGRILKTD
LKRGSCTVQCQTEKGGLNTTLPLLNVSKYAFGNCSKYIGIKSLKLAVGSRNVPSRSSRGLFGAIAGFIEG
GWSGLVGGWYGFQHSNDQGVGMAADRDSTQKAIDKITSKVNNIVDKMNKQYEIIDHEFSEVETRLNMINN
KFDDQIQDIWAYNAELLVLLENQKTLDEHDANVNNLYNKVKRALGSNAVEDGKGCFELYHKCDDQCMETI
RNGTYNRRKYQEEAKLERQKIEGVKLESEGTNKILSIYSTVASYIVIAMGFAAFLFWAMSNGSCRCNICI

>QBK20060.1 hemagglutinin [Influenza A virus]
MDTVSLISMLLVSTVSNANKICIGYQSANSAKTVNTLTQNNVPVTHAKELLHTQHNGMLGATSLGQPLIL
DTCSIEGLIYGNPSCDLSLEGREWSYIVERPSAVNGLCYPGNVQNLEELMSLFSSARSSQKIQIFPHTIW
NVSYYGTSAACSGSFYRIMRWLTRKNGDYPIQDAQYTNNQGNNILFMWGINHPPTDTTQIDLYTITDTTT
SVATEEINRIFKPLIGPRPLVNGLMGRIHYYWSVLKPGQTLRIKSDGNLIAPWYGHILSGESHGRIVETD
LKRGSCTVQCQTEKGGVNTALPFLNVSRYAFGKCSKYMVIKSLQLAVGLRNVPSRSSRGLFGAIAGFIEG
GWSGLVGGWYGFQHSNDQGVGMAADRDSTQKAIDKITSKVNNIVDKMNKQYEIIDLEFSVVETRLVMINH
KFDDQIQDIWAYNAESLVLLENQKTLDEQDVNVSNLYNKVKRAACSNAVEDGNGCFELYHKCDDQCMETI
RNGTYNRRKYQAEAKLERQKIEGLKLESDRTWKILSIYSTVASYIAIAMGSAAFLFGAMSNGSCRCNICI

>QBK20059.1 hemagglutinin [Influenza A virus]
METVSLITILLIATVSNADKICIGYQSTNSTETVDTLTENNVPVTHAKELLHTEHNGMLCATSLGQPLIL

DTCTIEGLIYGNPSCDLSLEGREWSYIVERPSAVNGLCYPGNVENLEELRSLFSSARSSQRIQIFPDTIW
NVSYDGTSTACSGSFYRSMRWLTRKNGDYPIQDAQYTNNQGKNILFMWGINHPPTDTTQRDLYTRTDTTT
SVATEEINRIFKPLIGPRPLVNGLMGRIHYYWSVLKPGQTLRIKSDGNLIAPWYGHILSRESHGRILKTD
LKRGSCTVQCQTEKGGLNTTLPFQNVSKYAFGNCSKYIGIKSLKLAVGLRNVPSRSSRGLFGAIAGFIEG
GWSGLVAGWYGFQHSNDQGVGMAADRDSTQKAIDKITSKVNNIVDKMNKQYEIIDHEFSEVETRLNMINN
KIDDQIQDIWAYNAELLVLLENQKTLDEHDANVNNLYNKVKRALGSNAVEDGKGCFELYHKCDDQCMETI
GNGTYNRRKYQEEAKLERQKIEGVKLESEGTYKILSIYSTVASYIVIAMGFAAFLFWAMSNGSCRCNICI

>QBK20058.1 hemagglutinin [Influenza A virus]
METVSLITILLVATVSNANKICIGYQSTNSTETVDTLTENNVPVTHAKELLHTEHNGMLCATSLGQPLIL
DTCTIEGLIYGNPSCDLSLEGREWSYIVERPSAVNGLCYPGNVQNLEELRSLFSSARSSQRIQIFPDTIW
NVSYYGTSTACSGSFYRSMRWLTRKNGDYPIQDAQYTNNQGKNILFMWGINHPPTDTTQRDLYTRTDTTT
SVATEEINRIFKPLIGPRPLVNGLMGRIHYYWSVLKPGQTLRIKSDGNLIAPWYGHILSRESHGRILKTD
LKRGSCTVQCQTEKDGLHTTLPLQNVGKYAFGNCSKYIGIKSLKLAVGLRNVPSRSSRGLFGAIAGFIEG
GWSGLVGGWYGFQHSNDQGVGMAADRDSTQKAIDKITSKVNNIVDKMNKQYEIIDHEFSEVETRLNTINN
KFDDQIQDIWAYNAESLVLLENQKTLDEHDANVSNLYNKVKRAVGSNAVEDGKGCFELCHKCDDQCMETI
RNGTYNRRKYQEEGKLERQKIEGLKLESERTNKILSIYSTVASYIVIAMGSAALMIWAMYNGSCRCNICI

>QBK20057.1 hemagglutinin [Influenza A virus]
MDTVSLITILLISTVSNANKICIGYQSTNSTETVDTLTENNVPVTHAKELLHTEHNGMLCATSLGQPLIL
DTCTIEGLIYGNPSCDLSLEGREWSYIVERPSAVNGLCYPGNVQNLEELRSLFSSARSSQRIQIFPDTIW
NVSYYGTSTACSGSFYRSMRWLTRKNGDYPIQDAQYTNNQGKNILFMWGINHPPTDTTQRDLYTRTDTTT
SVATEEINRIFKPLIGPRPLVNGLMGRIHYYWSVLKPGQTLRIKSDGNLIAPWYGHILSRESHGRILNTD
LKRGSCTVQCQTEKGGLNTALPLQNVSKYAFGKCSKYIGIKSLKLAVGLRNVPSRSSRGLFGAIAGFIEG
GWSGLVGGWYGFQHSNDQGVGMAADRDSTQKAIDKITSKVNNIVDKMNKQYEIIDHEFSEVETRLNMINN
KFDDQIQDIWAYNAELIVLLENQKTLDEHDANVSNLYNKVKRAVCSNAVEDGKGCFELYHKCDDQCMETI
RNGTYNRRKYQEEAKLERQKIEGVKLESEGTYKIVSIYSRVASYIVIAMGSAALRIGAMYNGSCRCNICI

>QBK20056.1 hemagglutinin [Influenza A virus]
MDTVSLITILLVATVSNANKICIGYQSTNSTETVDTLTENNVPVTHAKELLHTEHNGMLCATSLGQPLIL
DTCTIEGLIYGNPSCDLSLEGREWSYIVERPSTVNGLCYPGNVQNLEELRSLFSSARSSQRIQIFPDTIW
NVSYYGTSTACSGSFYRSMRWLTRKNGDYPIQDAQYTNNQGKNILFMWGINHPPTDTTQRDLYTRTDTTT
SVATEEINRIFKPLIGPRPLVNGLMGRIHYYWSVLKPGQTLRIKSDGNLIAPWYGHILSRESHGRLLKTD
LKRGSCTVQCQTEKGGLNTALPLLNVSKYAFGKCSKYIGIKSLKLAVGLRNVLCRYSRGLFGAIAGFIEG

GWSGLVGGWYGFQHSNDQGVGMAADRDSTQKAMDKITSKVNNIVGKMNKQYEIIDHEFSVVETRMVTINN
KFDDQIQDIWADNAESIVFLENQKTLDEHDVNVSNLYNKVKRGACSNAVEDENGCLEQCHKCDDQCMETI
RNGTYNRRERQEEGKKEREKMEGHDQEIERTREILSIYSRVASYRVIAMGSFALRIEGLYNWSCRCNICI

>QBK20055.1 hemagglutinin [Influenza A virus]
METVSLITILLIATVSNADKICIGYQSTNSTETVDTLTENNVPVTHAKELLHTEHNGMLCATSLGQPLIL
DTCTIEGLIYGNPSCDLSLEGREWSYIVERPSAINGLCYPGNVENLEELRSLFSSARSSQRIQIFPDTIW
NVSYDGTSTACSGSFYRSMRWLTRKNGDYPIQDAQYTNNQGKNILFMWGINHPPTDTTQRDLYTRTDTTT
SVATEEINRIFKPLIGPRPLVNGLMGRIHYYWSVLKPGQTLRIKSDGNLIAPWYGHILSGESHGRILKTD
LKRGSCTVQCQTEKGGLNTALPFQNVSKYAFGNCSKYIGIKSLKLAVGLRNVPSRSSRGLFGAIAGFIEG
GWSGLVAGWYGFQHSNDQGVGMAADRDSTQKAIDKITSKVNNIVDKMNKQYEIIDLEFSVVETRLNMINN
KFDDQIQDIWAYNAELLVLLENQKTLDEQDANVNNLYNKVKRALGSNAVEDGKGCFELYHKCDDQCMETI
GNGTYNRRKYQEEAKLERQKIEGVKLESEGTYKILSIYSTVASYIVIAMGFVAFLFGAMSNGSCRRNICI

>QBK20054.1 hemagglutinin [Influenza A virus]
MDTASLMTILLLVTVSNANGICLRYHSTNSTETVNTLTENYVPVTHAKELLHTEHNGMLCATSLGHPLIL
DTCTIEGLIYGNPSCDLLLGGREWSYIVQRPSAVNGLCYPGNVQNLEELRSLFSSARSYQRIQIFPDTIW
NVSYSGTSKACSDSFYRSMRWLTQKNNNYPIQDAQYTNNQEKNILFMWGINHPPTDTAQTNLYTRTDTTT
SVATEEINRTFKPLIGPRPLVNGLMGRINYYWSVLKPGHTLRIKSNGNLIAPWYGHILSRESHGRILKTD
LKRGSCTVQCQTEKGGLNTTLPFQNVSKYAFGSSSKYIGIKSLKLAVGLRKDPCRSSRGLFGAIAGFLEA
GWSGLVAGWYGFQHSNDQGVGMAADRDSTQKASDKITSESNNIVGKTINRYESLDHEFSEVGNRNNIINT
KIHDQIQDIWAYNPEIIGLLQYQKTKDEHDANVNKRYNNQTTEGVCNAVEDSKRFFGTYHKCDGKSMESI
RNARNNRSRYQEEWKEERQKMEGARPEDESFQGILVFYSSVESAMIIARGFVALELWTMSIVSCRCNICI

>QBK20053.1 hemagglutinin [Influenza A virus]
METASLMTILLVVTVSNANKICIGYQSTNSTETVNTLTENNVPVTHAKELLHTEHNGMLCATSLGHPLIL
DTCTIEGLIYGNPSCDLLLGGREWSYIVERPSAVNGLCYPGNVQNLEELRSLFSSARSYQRIQIFPDTIW
NVSYSGTSKACSDSFYRSMRWLTQKNNNYPIQDAQYTNNQEKNILFMWGINHPPTDTAQTNLYTRTDTTT
SVATEEINRTFKPLIGPRPLVNGLMGRINYYWSVLKPGQTLRIKSNGNLIAPWYGHILSRESHGRILKTD
LKRGSCTVQCQTEKGGLTTTLPLQNVSKYAFGNSSKYIGIKSLKLAVGLRNVPSRSSRGLFGAIAGFIEG
GWSGLVAGWYGFQHSNDQGVGMAADRDSTQKAIDKITSKLNNIVDKTIKHYEILDHEFSEVENRINMINT
KIDDQIQDIWAYNPEIIVLLQNQKTLEEHDANVNNRYNKVKRAGVSNAVEDGKGCFELYHKCDDQCMESI
RNGTYNRRKYQEESKLERQKMEGAKPEDEAIHKILVFDLSVASSLIIARGFVALELWAMSIVSCRCNICI

>QBK20052.1 hemagglutinin [Influenza A virus]

MDTASLMTILLVVTVSNANEICLGYQSTNSTETVNTLTENNVPVTHAKELLHTQHNGMLCATSLGHPLIL
DTCTIEGLIYGNPSCDLLLGGREWSYIVQRPSAVNGLCYPGNVQNLEELISLFSSARSYQRIQIFPHTIW
NVSYSGTSKACSDSFYRSMRWLTHKNNNYPIQDAQYTNNQEKNILFMWGINHPPTDTAQTNLYTRTDTTT
SVATEEINRTFKPLIGPRPLVNGLMGRINYYWSVLKPGQTLRIKSNGNLIAPWYGHILSRESRGRIRKTD
LKRGRCTVLCQTEKGGLDPTLPLHKVSKYAFGNCSKYIGIKCLKLAVGLRNVPCRISRGLFGAIAGFLEA
GWSGLDAGWYGFQHSNDQGVGMAADRDSTQKASDKITSELNNIVGKTIKRFESLDHEFSEVGNRNDIINT
KIHDQIQDIWAYKPEIIGLLQNQKTMEEHDANVNNRYNKVKREGVCNAVEDWKGCFGTYHKCDGKSMESI
RNGGYNRRRYQEEWKLERQKMEGAKPEDEATQGILSFISTVASSMVIARGFVALELWSMSIVSCRCNICI

>QBK20051.1 hemagglutinin [Influenza A virus]

METASLMTILLVVTVSNADKICIGYQSTNSTETVNTLTENNVPVTHAKELLHTEHNGMLCATSLGHPLIL
DTCTIEGLIYGNPSCDLLLGGREWSYIVERPSAVNGLCYPGNVQNLEELRSLFSSARSYQRIQIFPDTIW
NVSYSGTSKACSDSFYRSMRWLTQKNNNYPIQDAQYTNNQEKNILFMWGINHPPTDTAQTNLYTRTDTTT
SVATEEINRTFKPLIGPRPLVNGLMGRINYYWSVLKPGQTLRIKSNGNLIAPWYGHILSRESHGRILKTD
LKRGSCTVLCQTEKGGLNTTSPLQNVSKYAFGNCSKYIGIKSLKLAVGLRNVPSRFSRGLFGAIAGFIEG
GWSGLVAGWYGFQHSNDQGVGMAADRDSTQKAIDKITSKLNNIVDKTIKQYEILDHEFSEVETRINMINT
KIHDQIQDIWAYNAEFIVLLQNQKTLDEHDANVNNLYNKVKTAGGSNAVEDGKGCFELYHKCDDQCMESI
RNGTYNRRKYQEESKVERQKMEGAKPESEATHKILIFDLSVASSLVIARGFVALELWAMSNVSCRCNICI

>QBK20050.1 hemagglutinin [Influenza A virus]

MDTASLMTILLLLTVSNANEICLRYQSTNSTETVNTLTENNVPVTHAKELLHTEHNGMLCATSLGHPLIL
DTCTIEGLIYGNPSCDLLLGGREWSYIVQRPSAVNGLCYPGNVQNLEELISLFSSARSYQRIQIFPHTIW
NVSYSGTSKACSDSFYRSMRWLTQKNNNYPIQDAQYTNNQENNILFMWGINHPPTDTAQTNLYTRTDPTT
SVATEEINRTFKPLIGPRPLVNGLMGRINYYWSVLKPGQTLPIKSNGNLIAPWYGHILSRESHGRILKTD
LKRGSCTVLCQTEKDGLDPTLPLQNVSKYAYGNCSKYIGIKSLKLAVGSRKVPCRSSRGLLGAIAGFLEA
GWSGLVAGWYGFQHSNDQGVGMAADRDSTQKASDKITSELNNIVGKTIKRYEILDHEFSEVENRINIINT
KIHDQIQDIWAYNPEFIVLLQNQKTLEEHDANVNNRYNKVKREGGCNAVEDSKRCFGTYHKCDGKSMGSI
RNGRNNNRSRYQEESKLERRKMEGAKPEDESFQGILVFDLSVESAMIIARGFVALELWSMSIVSCRCNICI

>QBK20049.1 hemagglutinin [Influenza A virus]

METASLMTILLVVTVSNANKICIGYQSTNSTETVNTLTENNVPVTHAKELLHTEHNGMLCATSLGHPLIL
DTCTIEGLIYGNPSCDLLLGGREWSYIVERPSAVNGLCYPGNVQNLEELRSLFSSARSYQRIQIFPDTIW
NVSYSGTSKACSDSFYRSMRWLTQKNNNYPIQDAQYTNNQEKNILFMWGINHPPTDTAQTNLYTRTDTTT

SVATEEINRTFKPLIGPRPLVNGLMGRINYYWSVLKPGQTLRIKSNGNLIAPWYGHILSGESHGRILKTD
LKRGSCTVQCQTEKGGLNTTLPFQNVSKYAFGNCSKYIGIKSLKLAVGLRNVPSRSSRGLFGAIAGFIEG
GWSGLVAGWYGFQHSNDQGVGMAADRDSTQKAIDKITSKLNNIVDKTIKQYEILDHEFSEVETRINMINN
KIDDQIQDIWAYNAEFIVLLENQKTLDEHDANVNNLYNKVKRALGSNAVEDGKGCFELYHKCDDQCMETI
RNGTYNRRRYQEEWKEERQKMEGAKPESEGIHKILAFYLTVASSIVIARGFVALVLWAMSNGSCRCNICI

>QBK20048.1 hemagglutinin [Influenza A virus]
METASLMTILLIVTVSNADKICIGYQSTNSTETVNTLTENNVPVTHAKELLHTEHNGMLCATSLGHPLIL
DTCTIEGLIYGNPSCDLLLGGREWSYIVERPSAVNGLCYPGNVQNLEELRSLFSSARSYQRIQIFPDTIW
NVSYSGTSKACSDSFYRSMRWLTQKNNNYPIQDAQYTNNQEKNILFMWGINHPPTDTAQTNLYTRTDTTT
SVATEEINRTFKPLIGPRPLVNGLMGRINYYWSVLKPGQTLRIKSNGNLIAPWYGHILSGESHGRILKTD
LKRGSCTVQCQTEKGGLNTTLPFQNVSKYAFGNCSKYIGIKSLKLAVGLRNVPSRSSRGLFGAIAGFIEG
GWSGLVAGWYGFQHSNDQGVGMAADRDSTQKAIDKITSKVNNIVDKMNKQYEIIDHEFSEVETRLNMINN
KIDDQIQDIWAYNAELLVLLENQKTLDEHDANVNNLYNKVKRALGSNAVEDGKGCFELYHKCDDQCMETI
RNGTYNRRKYQEESKLERQKIEGVKPESEGTYKILTIYLTVASSLVIAMGFAAFLLWAMSNGSCRCEHCI

>QBK20047.1 hemagglutinin [Influenza A virus]
MDTASLMTILLVVTVSNANKICIGYQSTNSTETVNTLTENNVPVTHAKELLHTEHNGMLCATSLGHPLIL
DTCTIEGLIYGNPSCDLLLGGREWSYIVERPSAVNGLCYPGNVQNLEELRSLFSSARSYQRIQIFPDTIW
NVSYSGTSKACSDSFYRSMRWLTQKNNNYPIQDAQYTNNQEKNILFMWGINHPPTDTAQTNLYTRTDTTT
SVATEEINRTFKPLIGPRPLVNGLMGRINYYWSVLKPGQTLRIKSNGNLIAPWYGHILSRESHGRILKTD
LKRGSCTVQCQTEKGGLNTTLPLQNVSKYAFGNCSKYIGIKSLKLAVGLRNVPSRSSRGLFGAIAGFIEA
GWSGLVAGWYGFQHSNDQGVGMAADRDSTQKAIDKITSKLNNIVDKMIKQYEILDHEFSEVETRINMINN
KIDDQIQDIWAYNAEFIVLLENQKTLDEHDANVNNLYNKVKRALGSNAVEDGKGCFELYHKCDDQSMESI
RNGTNNRRKYQEESKVERQKMEGAKPESEGIQKILIIYLTVASSLVIAMGFVAFLLWAMSNGCCRCRRYI

>QBK20046.1 hemagglutinin [Influenza A virus]
METASLMTILLVVTVSNADEICIGYQSTNSTETVNTLTENNVPVTHAKELLHTEHNGMLCATSLGHPLIL
DTCTIEGLIYGNPSCDLLLGGREWSYIVERPSAVNGLCYPGNVQNLEELRSLFSSARSYQRIQIFPDTIW
NVSYSGTSKACSDSFYRSMRWLTQKNNNYPIQDAQYTNNQEKNILFMWGINHPPTDTAQTNLYTRTDTTT
SVATEEINRTFKPLIGPRPLVNGLMGRINYYWSVLKPGQTLRIKSNGNLIAPWYGHILSRESHGRILRTD
LKRGSCTEQCQTEKGGLNPTSPLQNVSKYAFGNCSKYIGIKSLKLAVGLRKVPCRSSRGLFGAIAGFIEG
GWSGLVAGWYGFQHSNDQGVGMAADRDSTQKASDKITSKLNNIVGKTIKRYEILDHEFSEVGTRISMINT
KIDDQIQDIWAYNAEFIVLLENQKTMDEHDANVNNLYNNQTTAGGSNAVEDGKGCFELYHKCDDQCMESI

RNGTYNRRKYQEESKLERQKIEGAKPESEGTQEILIIYLSVASSMVIARGFVALELWAMSIVSCRCNICI

>QBK20045.1 hemagglutinin [Influenza A virus]
METASLMTILLVVTVSNADKICIGYQSTNSTETVDTLTENNVPVTHAKELLHTEHNGMLCATSLGHPLIL
DTCTIEGLIYGNPSCDLLLGGREWSYIVERPSAVNGLCYPGNVQNLEELRSLFSSARSYQRIQIFPDTIW
NVSYSGTSKACSDSFYRSMRWLTQKNNNYPIQDAQYTNNQEKNILFMWGINHPPTDTAQTNLYTRTDTTT
SVATEEINRTFKPLIGPRPLVNGLMGRINYYWSVLKPGQTLRIKSNGNLIAPWYGHILSRESHGRIRKTE
LKRGRCTVLCQTEKDGLNTTSPLQNVSKYAFGKCSKYIGIKSLKLAVGMRNVPCRSSRGLFGAIAGFIEG
GWSGLGESWYGFQQSNDQGVGMAADRDSTQKASDKITYELNNIVGKTIKHYEILDHEFSEVETRINIINT
KIDDQIQDIWAYNAEIIVLLENQKTMEEHDANVNNRYNKVKRAGGSNAVEDGKGCFELYHKCDDQCMESI
RNGTYNRRRYQEESKLERQKMEGAKPESEGIHKILIFYLSVASSMVIARGFVALELWGMSIVSCRCNVCI

>QBK20044.1 hemagglutinin [Influenza A virus]
MDTASLMTILLLLTVSNANDICIGYQSTNSTETVNTLTENYVPVTHAEELLHTQHNGMLCATSLGHPLIL
DTCTIEGLIYGNPSCDLLLGGREWSYIVQRPSAVNGLCYPGNVQNLEELISLFSSARSYQRIQIFPHTIW
NVSYSGTSKACSDSFYISMRWLTQKNNNYPIQDAQYTNNQEKNILFMWGINHPPTDTAQTNLYTITDTTT
SVATEEINRTFKPLIGPRPLVNGLMGRINYYWSVLKPGQTLRIKSNGNLIAPWYGHILSGESHGRILKTD
LKRGSCTVQCQTEKGGLNTTLPLQNVSKYAFGNCSKYIGIKSLKLAVGLRNVPCRSSRGLFGAIAGFIEG
GWSGLVAGWYGFQHSNDQGVGMAADRDSTQKAIDKITSKLNNIVDKTIKQYEILDHEFSEVETRINMINN
KIDDQIQDIWAYNAEFIVLLENQKTLDEHDANVNNLYNKVKRALGSNAVEDGKGCFELYHKCDDQCMETI
RNGTYNRRKYQEESKLERQKIEGAKPESEGTHKILIIYLSVASSMVIAMGFAALLLWAMSNVSCRCNICI

>QBK20043.1 hemagglutinin [Influenza A virus]
METASLMTILLVVTVSNADKICIGYQSTNSTETVDTLTENNVPVTHAKELLHTEHNGMLCATSLGHPLIL
DTCTIEGLIYGNPSCDLLLGGREWSYIVERPSAVNGLCYPGNVENLEELRSLFSSARSYQRIQIFPDTIW
NVSYSGTSKACSDSFYRSMRWLTQKNNNYPIQDAQYTNNQEKNILFMWGINHPPTDTAQTNLYTRTDTTT
SVATEEINRTFKPLIGPRPLVNGLMGRINYYWSVLKPGQTLRIKSNGNLIAPWYGHILSRESHGRILKTD
LKRGSCTVQCQTEKGGLNTTLPFQNVSKYAFGNCSKYIGIKSLKLAVGLRNVPSRSSRGLFGAIAGFIEG
GWSGLVAGWYGFQHSNDQGVGMAADRDSTQKAIDKITSKVNNIVDKMNKQYEIIDHEFSEVETRLNMINN
KIDDQIQDIWAYNAEMLVLLENQKTLDEHDANVNNLYNKVKRALGSNAVEDGKGCFELYHKCDDQCMETI
RNGTYNRRKYQEESKLERQKIEGVKLESEGTYKILTIYLTVASSLVIAMGFAAFLFWAMSNGSCRCNICI

>QBK20042.1 hemagglutinin [Influenza A virus]
MDTASLMTILLLLTVSNANDICIGYQSTNSTETVNTLTENNVPVTHAKELLHTQHNGMLCATNLGHPLIL

DTCTIEGLIYGNPSCDLLLGGREWSYIVQRPSAVNGLCYPGNVQNLEELISLFSSARSYQRIQIFPHTIW
NVSYSGTSKACSDSFYISMRWLTQKNNNYPIQDAQYTNNQEKNILFMWGINHPPTDTAQTNLYTRTDTTT
SVATEEINRTFKPLIGPRPLVNGLMGRINYYWSVLKPGQTLRIKSNGNLIAPWYGHILSRESHGRILKTD
LKRGSCTVQCQTEKGGLNTTLPLQNVSKYAFGNCSKYIGIKSLKLAVGLRNVPSRSSRGLFGAIAGFIEG
GWSGLVAGWYGFQHSNDQGVGMAADRDSTQKAIDKITSKLNNIVDKMIKQYEILDHEFSEVETRINMINN
KIDDQIQDIWAYNAEFIVLLQNQKTLDEHDANVNHLYNKVKRALGSNAVEDGKGCFELYHKCDDQCMETI
RNGTYNRRKYQEESKLERQKIEGAKPEYEGTHKILTFDLSVASSLVIARGFAALLLWAMSNVSCRCNICI

>QBK20041.1 hemagglutinin [Influenza A virus]
MDTASLMTILLVVTVSNADKICIGYQSTNSTETVDTLTENNVPVTHAKELLHTEHNGMLCATSLGHPLIL
DTCTIEGLIYGNPSCDLLLGGREWSYIVERPSAVNGLCYPGNVQNLEELRSLFSSARSYQRIQIFPDTIW
NVSYSGTSKACSDSFYRSMRWLTQKNNNYPIQDAQYTNNQEKNILFMWGINHPPTDTAQTNLYTRTDTTT
SVATEEINRTFKPLIGPRPLVNGLMGRINYYWSVLKPGQTLRIKSNGNLIAPWYGHILSGESHGRILKTD
LKRGSCTVQCQTEKGGLNTTLPLQNVSKYAFGNCSKYIGIKSLKLAVGLRNVPSRSSRGLFGAIAGFIEG
GWSGLVAGWYGFQHSNDQGVGMAADRDSTQKAIDKITSKVNNIVGKMIKQYEILDHEFSEVETRINMINN
KIDDQIQDIWAYNAEFIVLLENQKTLDEHDANVNNLYNKVKRALGSNAVEDGKGCFELYHKCDDQCMETI
RNGTYNRRKYQEESKLERQKIEGAKPEFEGIHKILIIYLSVASSLVIAMGFVALLLWAMSNGSCRCNICI

>QBK20040.1 hemagglutinin [Influenza A virus]
METASLMTILLVVTVSNADKICIGYQSTNSTETVDTLTENNVPVTHAKELLHTEHNGMLCATSLGHPLIL
DTCTIEGLIYGNPSCDLLLGGREWSYIVERPSAVNGLCYPGNVENLEELRSLFSSARSYQRIQIFPDTIW
NVSYSGTSKACSDSFYRSMRWLTQKNNNYPIQDAQYTNNQEKNILFMWGINHPPTDTAQTNLYTRTDTTT
SVATEEINRTFKPLIGPRPLVNGLMGKIHYYWSVLKPGQTLRIKSNGNLIAPWYGHILSGESHGRILKTD
LKRGSCTVQCQTEKGGLNTTLPFQNVSKYAFGNCSKYIGIKSLKLAVGLRNVPSRSSRGLFGAIAGFIEG
GWSGLVAGWYGFQHSNDQGVGMAADRDSTQKAIDKITSKVNNIVDKMIKQYEIIDHEFSEVETRINMINN
KIDDQIQDIWAYNAELIVLLENQKTLDEHDANVNNLYNKVKRALGSNAVEDGKGCFELYHKCDDQCMETI
RNGTYNRRKYQEESKLERQKIEGVKPEYEGTYKILIIYLSVASSLVIAMGFAALLLWAMSNGSCRCNVCI

>QBK20039.1 hemagglutinin [Influenza A virus]
METASLMTILLVVTVSNADKICIGYQSTNSTETVNTLTENNVPVTHAKELLHTEHNGMLCATSLGHPLIL
DTCTIEGLIYGNPSCDLLLGGREWSYIVERPSAVNGLCYPGNVQNLEELRSLFSSARSYQRIQIFPDTIW
NVSYSGTSKACSDSFYRSMRWLTQKNNNYPIQDAQYTNNQEKNILFMWGINHPPTDTAQTNLYTRTDTTT
SVATEEINRTFKPLIGPRPLVNGLMGRINYYWSVLKPGQTLRIKSNGNLIAPWYGHILSRESHGRILKTD
LKRGSCTVHCQTEKDGLNPTLPLQNVSKYAYRSCSKYIGIKSLKLAVGLRNVPCRSSRGLFGAIAGFIEG

GWSGLVAGWYGFQHSNDQGVGMAADRDSTQKAIDKITSKSNNIVGKTINHFEILDHEFSEVENRINMINN
KIDDQIQDIWAYNAEFIVLLQNQKTLDEHDANVNNRYNKVKREGGSNAVEDGKRCFDTYHKCDDQCMETI
RNGTYNRRRYQEEWKLERRKIEGAKPEDEAIYKILIFYLSVASSMEIARGFVALELWTMSIVSDRCNSCI

>QBK20038.1 hemagglutinin [Influenza A virus]
MDTASLMTILLILTVSNANEICLGYQSTNSTETVNTLTENYVPVTHAKELLHTQHNGMLGATSLGHPLIL
DTCTIEGLIYGNPSCDLLLGGREWSYIVERPSAVNGLCYPGNVQNLEELRSLFSSARSYQRIQIFPHTIW
NVSYSGTSKACSDSFYRSMRWLTQKNNNYPIQDAQYTNNQEKNILFMWGINHPPTDTAQTNLYTRTDTTT
SVATEEINRTFKPLIGPRPLVNGLMGRINYYWSVLKPGHTLRIKSNGNLIAPWDGHILSRESHGRILKTD
LKRESCSVLCQAELGGLDTTLLLQNVSKYAFRKCSKYIGIKSLKLAVGLRNVPCRFSRGLFGAIAGFIEA
GWSGLDESWYGFQHSNDQGVGMAADRDSTQKASDPITSELNNIVGKTINRFESLDHEFSEVGNRIDIIST
KIHDQIQNIWAYKPEIIVLLQNQKTMEEHDANVNNRRDKVKTEGGCNAVEDSKRFFDTYHKCDDQSMESI
RNGGNNRRRYQEESKEVRQKMEGVKPEDEGFQGILAFYLSVESSMETQGRFVALELWSMSNWSDRCNSCI

>QBK20037.1 hemagglutinin [Influenza A virus]
METASLMTILLVVTVSNADKICIGYQSTNSTETVDTLTENNVPVTHAKELLHTEHNGMLCATSLGHPLIL
DTCTIEGLIYGNPSCDLLLGGREWSYIVERPSAVNGLCYPGNVENLEELRSLFSSARSYQRIQIFPDTIW
NVSYNGTSKACSDSFYRSMRWLTQKNNNYPIQDAQYTNNQGKNILFMWGINHPPTDTAQTNLYTRTDTTT
SVATEEINRTFKPLIGPRPLVNGLMGRIHYYWSVLKPGQTLRIKSDGNLIAPWYGHILSGESHGRILKTD
LKRGSCTVQCQTEKGGLNTTLPLQNVSRYAFRNCSKYNGIKSLKLAGCSRNVPSRSSRRLIGAIAGFIEG
GWSRLVAGWYGFQQSNDPGVGMAADRDSTPKAIDKITSQVNNIVGKMNQPFEILDPEFSEVGNRINLINN
KIDDQIPDLWAYKAEIIGLLENPKTIDEPGAKVNHLYNKMKRAVGFNAVEGWKGCFGVYHKSDGPSKGTI
WNGTFNRRKNQEEWKLERPKIEGVKPEFEGNYKILLLYLAVAFSFVNAKGFVSLLFWGMSKGLDRCNICI

>QBK20036.1 hemagglutinin [Influenza A virus]
MDTVSLITILLVATVSNADKICIGYQSTNSTETVDTLTENNVPVTHAKELLHTEHNGMLCATSLGQPLIL
DTCTIEGLIYGNPSCDLSLEGREWSYIVERPSAINGLCYPGNVQNLEELRSLFSSARSSQRIQIFPDTIW
NVSYYGTSTACSGSFYRSMRWLTRKNGDYPIQDAQYTNNQGKNILFMWGINHPPTDTTQRDLYTRTDTTT
SVATEEINRIFKPLIGPRPLVNGLMGRIHYYWSVLKPGQTLRIKSDGNLIAPWYGHILSGESHGRILKTD
LKRGSCTVQCQTEKGGLNTALPFQNVSKYAFGNCSKYIGIKSLKLAVGLRNVPSRSSRGLFGAIAGFIEG
GWSGLVAGWYGFQHSNDQGVGMAADRDSTQKAIDKITSKVNNIVAKMNKQYEIIDHEFSEVETRINMINN
KFDDQIQDIWAYNAELLVLLENQKTLDEQDANVNNLYNKVKRGLGSNAVEDGKGCLELCHKCDDQCMETI
GNGTYNRRKYQEEAKLERQKMEGVKLESEGTYKIVSIYSTVTSYIVIAMGSVAFLFGALSNGSCRCNICI

>QBK20035.1 hemagglutinin [Influenza A virus]
MDTASLMTILLVVTVSNANEICIGYQSTNSTETVNTLTENNVPVTHAKELLHTEHNGMLCATSLGHPLIL
DTCTIEGLIYGNPSCDLLLGGREWSYIVERPSAVNGLCYPGNVQNLEELRSLFSSARSYQRIQIFPDTIW
NVSYSGTSKACSDSFYRSMRWLTQKNNNYPIQDAQYTNNQEKNILFMWGINHPPTDTTQTNLYTRTDTTT
SVATEEINRTFKPLIGPRPLVNGLMGRINYYWSVLKPGQTLRIKSNGNLIAPWYGHILSRESHGRILKTD
LKRGSCTVQCQSKKDGLNKTLPLHQVSKYAFGNCSKYKGIKSLKLADALRKVPCRSSRGLFGAIAGFIEG
GWSGLVAGWYGFQHSNDQGVCMAADRESTQKAIDQITSKLNNIVGKTIKQYEILDHEFSEVETRLNMINN
KIHDQIQDIWAYNPEIIVLLENQKTMEEHDANVNNLYNKVKRALGSNAVEDGKGCFELYHKCDDQCMETI
RNGTYNRRRYQEEWKLERQKIEGAKPESEGIYKILIIYLSVASSMVIARGFVALLLWTMSNGSCRCNICI

>QBK20034.1 hemagglutinin [Influenza A virus]
METASLMTILLIVTVSNANEICLRYQSTNSTETVNTLTENYVPVTHAKELLHTQHNGMLGATSLGHPLIL
DTCTIEGLIYGNPSCDLLLGGREWSYIVERPSAVNGLCYPGNVQNLEELRSLFSSARSYQRIQIFPDTIW
NVSYSGTSKACSDSFYRSMRWLTQKNNNYPIQDAQYTNNQEKNILFMWGINHPPTDTAQTNLYTRTDTTT
SVATEEINRTFKPLIGPRPLVNGLMGRINYYWSVLKPGQTLRIKSNVNLIAPWYGHILSRESHGRILKTD
VKRGRCTVHCQTEFRGLDTTSPLQNVSKYAYRKCSKYIGIKSLKLAVGLRNVPCRSSRGLFGAIAEFLEA
VWSGLVAGWYGFQHSNDQGVGMAADRDSTQKASDKITSESNNIVGKTINRFESLDHEFSEVENRNDIINT
KIHDQIQDIWACKPEIIVLLQYQKTLEEHDANVNNRYNNQTTEGISNAVEDSKRCFGTYHKCDGKSMESI
RNARNNRRRYQEEWKLVRQKMEGAKPESEGTHRILIIYLSVASSMVIARGFVALELWTMSNWSCRCNICI

>QBK20033.1 hemagglutinin [Influenza A virus]
METASLMTILLVVTVSNANEICIGYQSTNSTETVNTLTENNVPVTHAKELLHTEHNGMLGATSLGHPLIL
DTCTIEGLIYGNPSCDLLLGGREWSYIVERPSAVNGLCYPGNVQNLEELRSLFSSARSYQRIQIFPDTIW
NVSYSGTSKACSDSFYRSMRWLTQKNNNYPIQDAQYTNNQEKNILFMWGINHPPTDTAQTNLYTRTDTTT
SVATEEINRTFKPLIGPRPLVNGLMGRINYYWSVLKPGQTLRIKSNGNLIAPWYGHILSRESHGRILKTD
VKRDRCTVLCQTEKDGLDTTSPLQNVSKYAFGSCSKYIGIKSLKLAVGLRKDPCRSSRGLFGAIAGFLEA
GWSGLVAGWYGFQHSNDQGVGMAADRDSTQKASDKITSELNNIVGKTIKRFEILDHEFSEVENRINIINT
KIHDQIQDIWAYNPEIIVLLQNQKTMEEHDANVNNRYNKVKTEGGSNAVEDSKRCFELYHKCDDQCMETI
RNGTNNRRRYQEESKLVRQKMEGAKPEDEGFQGRGVFELSVASSLVIARGFVALELWSMSNWSCRCNICI

>QBK20032.1 hemagglutinin [Influenza A virus]
METASLMTILLVVTVSNANGICIGYQSTNSTETVDTLTENNVPVTHAKELLHTEHNGMLCATSLGHPLIL
DTCTIEGLIYGNPSCDLLLGGREWSYIVERPSAVNGLCYPGNVQNLEELRSLFSSARSYQRIQIFPDTIW
NVSYSGTSKACSDSFYRSMRWLTHKNNNYPIQDAQYTNNQEKNILFMWGINHPPTDTAQTNLYTRTDTTT

SVATEEINRTFKPLIGPRPLVNGLMGRINYYWSVLKPGQTLRIKSNGNLIAPWYGHILSRESHGRILKTD
LKRGSCTVHCQTEKGGLNPTLPLQKVSKYAFGSCSKYIGIKSLKLAVGLRNVPCRSSRGLFGAIAGFIEA
GWSGLVAGWYGFQHSNDQGVGMAADRDSTQKAIDKITSKLNNIVGKTIKHFEILDHEFSEVENRINMINT
KIHDQIQDIWAYNAEIIVLLQNQKTMEEHDANVNNLYNKVKRAGVSNAVEDGKRCFDTYHKCDDQCMESI
RNGTYNRRRYQEEWKLVIQKMEGARPEDEGFQGILIFYLTVASSLVNARGFVALELWSMSIVSCRCNICI

>QBK20031.1 hemagglutinin [Influenza A virus]
METASLMTILLVVTVSNADKICIGYQSTNSTETVDTLTENNVPVTHAKELLHTEHNGMLCATSLGHPLIL
DTCTIEGLIYGNPSCDLLLGGREWSYIVERPSAVNGLCYPGNVENLEELRSLFSSARSYQRIQIFPDTIW
NVSYSGTSKACSDSFYRSMRWLTQKNNNYPIQDAQYTNNQEKNILFMWGINHPPTDTAQTNLYTRTDTTT
SVATEEINRTFKPLIGPRPLVNGLMGRINYYWSVLKPGQTLRIKSNGNLIAPWYGHILSGESHGRILKTD
LKRGSCTVQCQTEKGGLNTTLPFQNVSKYAFGNCSKYIGIKSLKLAVGLRNVPSRSSRGLFGAIAGFIEG
GWSGLVAGWYGFQHSNDQGVGMAADRDSTQKAIDKITSKVNNIVDKMNKQYEIIDHEFSEVETRINMINN
KIDDQIQDIWAYNAELIVLLENQKTLDEHDANVNNLYNKVKRALGSNAVEDGKGCFELYHKCDDQCMETI
RNGTYNRRKYQEESKLERQKIEGVKPESEGTYKILIIYLTVASSMVIAMGFVAFLLWAMSNVSCRCNICI

>QBK20030.1 hemagglutinin [Influenza A virus]
METASLITILLLVTVSNANKICIGYQSTNSTETVNTLTENNVPVTHAKELLHTEHNGMLCATNLGHPLIL
DTCTIEGLIYGNPSCDPLLGGREWSYIVQRPSTVNGLCYPGNVQNLEELRSLFSSARSYQRIQIFPDTIW
NVSYSGTSKACSDSFYRSMRWLTQKDNAYPIQDAQYTNNQEKNILFMWGINHPPTDTTQTNLYTRTDTTT
SVATEEINRTFKPLIGPRPLVNGLMGRINYYWSVLEPGQTLRIKSNGNLIAPWYGHILSGESHGRILKTN
LKRGNCIVQCQTEKGGLNTTLPLQNVSKYAFGKCSKYIGIKSLKLAVGLRNVPSRSSRGLFGAIAGFIEG
GWSGLIGGWYGFQHSNDQGVGMAADRDSTQKAIDKITSKVNNIVAKMNKQYEIIDHEFSEVETRINMINN
KFDDQIQDIWAYNAESIVLLENQKTLDEHDANVSNLYNKVKRALGSNAVEDGKGCFELYHKCDDQCMETI
RNGTYNRRKYQEESKLERQKIEGVKQESEGTRKILSIYSTVASYLVIAMGFVALLFWAMYNGSCRCNICI

>QBK20029.1 hemagglutinin [Influenza A virus]
METVSLITILLVATVSNADKICIGYQSTNSTETVDTLTENNVPVTHAKELLHTEHNGMLCATSLGQPLIL
DTCTIEGLIYGNPSCDLSLEGREWSYIVQRPSTINGLCYPGNVQNLEELRSLFSSARSSQRIQIFPDTIW
NVSYYGTSTACSGSFYRSMRWLTRKNGDYPIQDAQYTNNQGKNILFMWGINHPPTDTTQRDLYTRTDTTT
SVATEEINRIFKPLIGPRPLVNGLMGRIHYYWSVLKPGQTLRIKSDGNLIATWDGHILSGESDGRIVKHD
LKRGSCTVQCQTEKGGLHTTLPLQNVGKYAFGNCSKYIGIKSLKLAVGLRNVPSRSSRGLFGAIAGFIEG
GWSGLVGGWYGFQHSNDQGVGMAADRDSTQKAIDKITSKVNNIVDKMNKQYEIIDHEFSEVETRINMINN
KFDDQIQDIWAYNAELLVLLENQKTLDEQDANVSNLYNKVKRGAGSNAVEDGKGCLELYHKCDDQCMETI

RNGTYNRREYQEEGKLERQKMEGLKLESEGTYKILSIYSRVASYRVIAMGSAAFLFGAMYNGSCRCNSCI

>QBK20028.1 hemagglutinin [Influenza A virus]
MDTASLMTILLVVTVSNANEICIGYQSTNSTETVNTLTENNVPVTHAKELLHTEHNGMLCATSLGHPLIL
DTCTIEGLIYGNPSCDLLLGGREWSYIVERPSAVNGLCYPGNVQNLEELRSLFSSARSYQRIQIFPDTIW
NVSYSGTSKACSDSFYRSMRWLTQKNNNYPIQDAQYTNNQEKNILFMWGINHPPTDTAQTNLYTRTDTTT
SVATEEINRTFKPLIGPRPLVNGLMGRINYYWSVLKPGQTLRIKSNGNLIAPWYGHILSGESHGRILKTD
LKRGSCTVHCQTEKDGLNTTLPLQNVSKYAFGNCSKYIGIKSLKLAVGSRNVPCRSSRGLLGAIAGFIEG
GWSGLVAGWYGFQHSNDQGVGMAADRDSTQKAIDKITSKSNNIVDKTIKQYEILDHEFSEVENRINMINT
KIDDQIQDIWAYKPEFIVLLENQKTMEEHDANVNNRSNKVKTEGGSNAVEDGKGCFELYHKCDDQCMESI
RNGTYNRRRYQEESKLVRQKMEGAKPESEAIHKILIIDLSVESSMEIARGFVALEFWAMSIVSCRCNICI

>QBK20027.1 hemagglutinin [Influenza A virus]
MDTVSLITILLIATVSNADKICIGYQSTNSTETVDTLTENNVPVTHAKELLHTEHNGMLCATSLGQPLIL
DTCTIEGLIYGNPSCDLSLEGREWSYIVERPSAVNGLCYPGNVQNLEELRSLFSSARSSQRIQIFPDTIW
NVSYDGTSTACSGSFYRSMRWLTRKNGDYPIQDAQYTNNQGKNILFMWGINHPPTDTTQRDLYTRTDTTT
SVATEEINRIFKPLIGPRPLVNGLMGRIHYYWSVLKPGQTLRIKSDGNLIAPWYGHILSRESHGRILKTD
LKRGSCTVQCQTEKGGLNTTLPLQNVSKYAFGNCSKYIGIKSLKLAVGSRNVPSRSSRGLFGAIAGFIEG
GWSGLVAGWYGFQHSNDQGVGMAADRDSTQKAIDKITSKVNNIVDKMNKQYEIIDHEFSEVETRLNMINN
KIDDQIQDIWAYNAELLVLLENQKTLDEHDANVNNLYNKVKRALGSNAVEDGKGCFELYHKCDDQCMETI
RNGTYNRRKYQEEAKLERQKIEGVKLESEGTYKILSIYSTVASSIVIAMGFAAFLFWAMSNGSCRCNICI

>QBK20026.1 hemagglutinin [Influenza A virus]
METVSLITILLVATVSNADKICIGYQSTNSTETVDTLTENNVPVTHAKELLHTEHNGMLCATSLGQPLIL
DTCTIEGLIYGNPSCDLSLEGREWSYIVERPSAINGLCYPGNVQNLEELRSLFSSARSSQRIQIFPDTIW
NVSYDGTSTACSGSFYRSMRWLTRKNGDYPIQDAQYTNNQGKNILFMWGINHPPTDTTQRDLYTRTDTTT
SVATEEINRIFKPLIGPRPLVNGLMGRIHYYWSVLKPGQTLRIKSDGNLIAPWYGHILSGESHGRILKTD
LKRGSCTVQCQTEKGGLNTTLPFQNVSKYAFGNCSKYIGIKSLKLAVGLRNVPSRSSRGLFGAIAGFIEG
GWSGLVAGWYGFQHSNDQGVGMAADRDSTQKAIDKITSKVNNIVDKMNKQYEIIDHEFSEVETRLNMINN
KIDDQIQDIWAYNAELLVLLENQKTLDEHDANVNNLYNKVKRALGSNAVEDGKGCFELYHKCDDQCMETI
RNGTYNRRKYQEEAKLERQKIEGVKLESEGTYKILSIYSTVASSIVIAMGFAAFLFWAMSGGSCRCNICI

>QBK20025.1 hemagglutinin [Influenza A virus]
METVSLITILLVATVSNADKICIGYQSTNSTETVDTLTENNVPVTHAKELLHTEHNGMLCATSLGQPLIL

DTCTIEGLIYGNPSCDLSLEGREWSYIVERPSAINGLCYPGNVQNLEELRSLFSSARSSQRIQIFPDTIW
NVSYYGTSTACSGSFYRSMRWLTRKNGDYPIQDAQYTNNQGKNILFMWGINHPPTDTTQRDLYTRTDTTT
SVATEEINRIFKPLIGPRPLVNGLMGRIHYYWSVLKPGQTLRIKSDGNLIAPWYGHILSRESHGRILKTD
LKRGSCTVQCQTEKGGLHTTLPLLNVSKYAFGDCSKYIGIKSLKLAVGLRNVPSRSSRGLFGAIAGFIEG
GWSGLVAGWYGFQHSNDQGVGMAADRDSTQKAIDKITSKVNNIVAKMNKQYEIIDHEFSEVETRLNMINN
KIDDQIQDIWAYNAELLVLLENQKTLDEQDANVNNLYNKVKRALGSNAVEDGKGCFELYHKCDDQCMETI
RNGTYNRREYQAEAKLERQKIEGVKQESEGTCKILSIYSTVASSIVIAMGFAAFLFWAMSGGSCRCNICI

>QBK20024.1 hemagglutinin [Influenza A virus]
METASLMTILLVVTVSNADEICIGYQSTNSTETVNTLTENNVPVTHAKELLHTEHNGMLCATSLGHPLIL
DTCTIEGLIYGNPSCDLLLGGREWSYIVERPSAVNGLCYPGNVQNLEELRSLFSSARSYQRIQIFPDTIW
NVSYSGTSKACSDSFYRSMRWLTQKNNNYPIQDAQYTNNQEKNILFMWGINHPPTDTTQTNLYTRTDTTT
SVATEEINRTFKPLIGPRPLVNGLMGRINYYWSVLKPGQTLRIKSNGNLIAPWYGHILSRESHGRILKTD
LTRGSCTVQCQTEKGGLNPTLPLQNVSKYAFGNCSKYIGIKSLKLAVGLRNVPSRSSRGLFGAIAGFIEG
GWSGLVAGWYGFQHSNDQGVGMAADRDSTQKAIDKITSKLNNIVDKTIKHYEILDHEFSEVETRINMINT
KIDDQIQDIWAYNAEFIVLLQNQKTLDEHDANVNNLYNKVKRALGSNAVEDGKGCFELYHKCDDQCMESI
RNGTYNRRRYQEESKLERQKIEGVKPESEGIYKILTFYLSVASSLVIARGFVALLFWAMSIVSCRCNICI

>QBK20023.1 hemagglutinin [Influenza A virus]
METVSLITILLVSTVSNANKICIGYQSTNSTETVDTLTENNVPVTHAKELLHTEHNGMLCATSLGQPLIL
DTCTIEGLIYGNPSCDLSLEGREWSYIVERPSTINGLCYPGNVQNLEELRSLFSSARSSQRIQIFPDTIW
NVSYYGTSTACSGSFYRSMRWLTRKNGDYPIQDAQYTNNQGKNILFMWGINHTPTDTTQRDLYTRTDTTT
SVATEEINRIFKPLIGPRPLVNGLMGRIHYYWSVLKPGQTLRIKSDGNLIAPWYGHILSGESHGRILKTD
LKRGSCTVQCQTEKGGLNTALPLQNVSRYAFGDCSKYIGIKSLKLAVGSRNVPSRSSRGLFGAIAGFIEG
GWSGLVAGWYGFQHSNDQGVGMAADRDSTQKAIDKITSKVNNIVDKMNKQYEIIDHEFSVVETRINMINN
KFDDQIQDIWAYNAELIVLLENQKTIDEQDANVNNLYNKVKRAAGSNAVEDGKGCLELYHKCDDQCMETI
RNGTYNRRKYQEEAKLERQKIEGLKLESDGTYKILSIYSTVASSIVIAMGFVAFLFGAMYNGSCRCNSCI

>QBK20022.1 hemagglutinin [Influenza A virus]
METASLMTILLVLTVSNANDICIGYQSTNSTETVNTLTENNVPVTHAKELLHTEHNGMLCATSLGHPLIL
DTCTIEGLIYGNPSCDLLLGGREWSYIVQRPSAVNGLCYPGNVQNLEELRSLFSSARSYQRIQIFPDTIW
NVSYSGTSKACSDSFYRSMRWLTQKNNNYPIQDAQYTNNQEKNILFMWGINHPPTDTAQTNLYTRTDTTT
SVATEEINRTFKPLIGPRPLVNGLMGRINYYWSVLKPGQTLRIKSNGNLIATWYGHILSGESHGRILKTD
LKRGSCTVQCQTEKGGLNTTLPFQNVSKYAFGNCSKYIGIKSLKLAVGLRNVPSRSSRGLFGAIAGFIEG

GWSGLVAGWYGFQHSNDQGVGMAADRDSTQKAIDKITSKVNNIVDKMIKQYEIIDHEFSEVETRLNMINN
KIDDQIQDIWAYNAEFIVLLENQKTLDEHDANVNNLYNKVKRALGSNAVEDGKGCFELYHKCDDQCMETI
RNGTYNRRKYQEESKLERQKIEGAKPEYEGTHKILIIYLSVASSMVIAMGFVALLFWAMSNVSCRCNICI

>QBK20021.1 hemagglutinin [Influenza A virus]
METASLMTILLVVTVSNANKICIGYQSTNSTETVNTLTENNVPVTHAKELLHTEHNGMLCATSLGHPLIL
DTCTIEGLIYGNPSCDLLLGGREWSYIVERPSAVNGLCYPGNVQNLEELRSLFSSARSYQRIQIFPDTIW
NVSYSGTSKACSDSFYRSMRWLTQKNNNYPIQDAQYTNNQEKNILFMWGINHPPTDTAQTNLYTRTDTTT
SVATEEINRTFKPLIGPRPLVNGLMGRINYYWSVLKPGQTLRIKSNGNLIAPWYGHILSGESHGRILKTD
LKRGSCTVQCQTEKGGLNTTLPLQNVSKYALGNCSKYIGIKSLKLAVGLRNVLSRFSRGLFGAIAGFIEG
GWSGLVAGWYGFQHSNDQGVGMAADRDSTQKAIDKITSKVNNIVDKTIKQYEILDHEFSEVETRINMINN
KIDDQIQDIWAYNAEFIVLLENQKTMDEHDANVNNLYNKVKRALGSNAVEDGKGCFELYHKCDDQCMETI
RNGTNNGRRYQEESKLERQKMEGAKPESEATHKILIIYLSVASSMVIARGFVALVFWAMSNVSCRCNICI

>QBK20020.1 hemagglutinin [Influenza A virus]
MDTASLMTILLLLTVSNANEICLGYQSTNSTETVNTLTENNVPVTHAKELLHTQHNGMLCATSLGHPLIL
DTCTIEGLIYGNPSCDLLLGGREWSYIVQRPSAVNGLCYPGNVQNLEELRSLFSSARSYQRIQIFPDTIW
NVSYSGTSKACSDSFYRSMRWLTQKNNNYPIQDAQYTNNQEKNILFMWGINHPPTDTAQTNLYTRTDTTT
SVATEEINRTFKPLIGPRPLVNGLMGRINYYWSVLKPGQTLRIKSNGNLIAPWYGHILSGESHGRILKTD
LKRGSCTVQCQTEKGGLNTTLPFQNVSKYAFGNCSKYIGIKSLKLAVGLRNVPSRSSRGLFGAIAGFIEG
GWSGLVAGWYGFQHSNDQGVGMAADRDSTQKAIDKITSKVNNIVDKMIKQYEILDHEFSEVETRINMINN
KIDDQIQDIWAYNAEFIVLLENQKTMEEHDANVNNLYNKVKRALGSNAVEDGKGCFELYHKCDDQCMESI
RNGRNNRRRYQEESKLERQKMEGAKPESEGTHKILIIYLSVASSMVIAMGFVALLFWAMSNGSCRCNICI

>QBK20019.1 hemagglutinin [Influenza A virus]
METVSLITILLVATVSNADKICIGYQSTNSTETVDTLTENNVPVTHAKELLHTEHNGMLCATSLGQPLIL
DTCTIEGLIYGNPSCDLSLEGREWSYIVERPSAINGLCYPGNVQNLEELRSLFSSARSSQRIQIFPDTIW
NVSYYGTSTACSGSFYRSMRWLTRKNGDYPIQDAQYTNNQGKNILFMWGINHPPTDTTQRDLYTRTDTTT
SVATEEINRIFKPLIGPRPLVNGLMGRIHYYWSVLKPGQTLRIKSDGNLIAPWYGHILSRESHGRILKTD
LKRGSCTVQCQTEKGGLNTALPLLNVSKYAFGNCSKYIVIKSLKLAVGLRNVPSRSSRGLFGAIAGFIEG
GWSGLVGGWYGFQHSNDQGVGMAADRDSTQKAIDKITSKVNNIVAKMNKQYEIIDHEFSVVETRLNTINN
KFDDQIQDIWAYNAESLVSLENQKTLDEHDANVSNLYNKVKRALGSNAVEDGKGCFELYHKCDDQCMETI
RNRTYNRRKYQEEAKLERQKIEGVKLEFEGTCKILSIYSTVASSIVIAMGFAAFLFWAMSNGSCRCNICI

>QBK20018.1 hemagglutinin [Influenza A virus]

MDTVSLITILLVATVSNADKICIGYQSTNSTETVDTLTENNVPVTHAKELLHTEHNGMLCATSLGQPLIL

DTCTIEGLIYGNPSCDLSLEGREWSYIVERPSTINGLCYPGNVQNLEELRSLFSSARSSQRIQIFPDTIW

NVSYDGTSTACSGSFYRSMRWLTRKNGDYPIQDAQYTNNQGKNILFMWGINHPPTDTTQRDLYTRTDTTT

SVATEEINRIFKPLIGPRPLVNGLMGRIHYYWSVLKPGQTLRIKSDGNLIAPWYGHILSGESHGRILKTD

LKRGSCTVQCQTEKGGLNTTLPFENVSKYAFGNCSKYIGIKSLKLAVGLRNVLSRYSRGLLGAIAGFIEG

GWSGLVGGWYGFQHSNDQGVGMAADRDSTQKAIDKITSKVNNIVDKMNKQYEIIDHEFSEVETRINMINN

KIDDQIQDIWAYNAELLVLLENQKTLDEQDANVNNLYNKVKRGVGSNAVEDGKGCLELYHKCDDQCMETI

GNGTYNRREYQEEGKKERQKMEGVKLESEGTYKIVSIYSRVASYIVIAMGFVALLFGAMYNGSSRCNSCI

>QBK20017.1 hemagglutinin [Influenza A virus]

METVSLITILLVATVSNADKICIGYQSTNSTETVDTLTENNVPVTHAKELLHTEHNGMLCATRLGQPLIL

DTCTIEGLIYGNPSCDLSLEGREWSYIVERPSAVNGLCYPGNVENLEELRSLFSSARSSQRIQIFPDTIW

NVSYDGTSTACSGSFYRSMRWLTRKNGDYPIQDAQYTNNQGKNILFMWGINHPPTDTTQRDLYTRTDTTT

SVATEEINRIFKPLIGPRPLVNGLMGRIHYYWSVLKPGQTLRIKSDGNLIAPWYGHILSRESHGRILKTD

LKRGSCTVQCQTEKGGLNTTLPLQNVSKYAFGNCSKYIGIKSLKLAVGLRNVPSRSSRGLFGAIAGFIEG

GWSGLVAGWYGFQHSNDQGVGMAADRDSTQKAIDKITSKVNNIVDKMNKQYEIIDHEFSEVETRLNMINN

KIDDQIQDIWAYNAELLVLLENQKTLDEHDANVNNLYNKVKRALGSNAVEDGKGCFELYHKCDDQCMETI

GNGTYNRRKYQEEAKLERQKIEGVKLESEGTYKILSMYSTVASYIMIAMGSVAFLFWALSNGSCRCNSCI

>QBK20016.1 hemagglutinin [Influenza A virus]

METVSLITILLVATVSNADKICIGYQSTNSTETVDTLTENNVPVTHAKELLHTEHNGMLCATSLGQPLIL

DTCTIEGLIYGNPSCDLSLEGREWSYIVERPSAVNGLCYPGNVENLEELRSLFSSARSSQRIQIFPDTIW

NVSYDGTSTACSGSFYRSMRWLTRKNGNYPIQDAQYTNNQGKNILFMWGINHPPTDTTQRDMYTRTDTTT

SVATEEINRIFKPLIGPRPLVNGLMGRIHYYWSVLKPGQTLRIKSDGNLIAPWYGHILSGESHGRILKTD

LKRGSCTVQCQTEKGGLNTTLPFQNVSKYAFGNCSKYIGIKSLKLAVGLRNVPSRSSRGLFGAIAGFIEG

GWSGLVAGWYGFQHSNDQGVGMAADRDSTQKAIDKITSKVNNIVDKMNKQYEIIDHEFSEVETRLNMINN

KIDDQIQDIWAYNAELLVLLENQKTLDEHDANVNNLYNKVKRALGSNAVEDGKGCFELYHKCDDQCMETI

RNGTYNRRKYQEEAKLERQKIEGVKLESEGTYKILSIYSTVASYIVIAMGFAAFLFWAMSNGSCRCNICI

>QBK20015.1 hemagglutinin [Influenza A virus]

MDTVSLITILLVATVSNADKICIGYQSTNSTETVDTLTENNVPVTHAKELLHTEHNGMLCATSLGQPLIL

DTCTIEGLIYGNPSCDLSLEGREWSYIVERPSAVNGLCYPGNVQNLEELRSLFSSARSSQRIQIFPDTIW

NVSYYGTSTACSGSFYRSMRWLTRKNGDYPIQDAQYTNNQGKNILFMWGINHPPTDTTQRDLYTRTDTTT

SVATEEINRIFKPLIGPRPLVNGLMGRIHYYWSVLKPGQTLRIKSDGNLIAPWYGHILSGESHGRIVKTD
LKRGSCTVQCQTEKGGLNTTLPLQNVSKYAFGNCSKYIGIKSLKLAVGLRNVPSRSSRGLFGAIAGFIEG
GWSGLVAGWYGFQHSNDQGVGMAADRDSTQKAIDKITSKVNNIVDKMNKQYEIIDHEFSEVETRINMINN
KIDDQIQDIWAYNAELLVLLENQKTLDEHDANVSNLYNKVKRALGSNAVEDGKGCFELYHKCDDQCMETI
GNGTYNRRKYQEEGKLERQKIEGVKLESEGTYKILSIYSTVASYSVIAMGFVAFLFWALYNGSCRCNSCI

>QBK20014.1 hemagglutinin [Influenza A virus]
MDTVSLITILLISTVSNANKICIGYQSTNSTETVDTLTENNVPVTHAKELLHTEHNGMLCATSLGQPLIL
DTCTIEGLIYGNPSCDLSLEGREWSYIVERPSAVNGLCYPGNVQNLEELRSLFSSARSSQRIQIFPDTIW
NVSYYGTSTACSGSFYRSMRWLTRKNGDYPIQDAQYTNNQGKNILFMWGINHPPTDTTQRDLYTRTDTTT
SVATEEINRIFKPLIGPRPLVNGLMGRIHYYWSVLKPGQTLRIKSDGNLIAPWYGHILSRESHGRILNTD
LKRGSCTVQCQTEKGRLHTTLPLLNVDKAAFGKCSKYIIIKSLQRAVGSRNDLSRSSRGLLGAIAGFIEG
GWSGLVRGLYGFQHSNDQGVGMAADRDSTQKAIDKITSKVNNIVGKMNKQYEIIDLEFSVVETRMVPINN
KSDDQIQDIWAYNAESIVSIENQKTSDEQDANVSNGGNKVKRGACSNAVEVGNGCLEQCHKCDDQCMETI
GNGTYNRREYQAEGKKERQKMEGLNLESEGTWKILSIYSRVASYRVIAMGSFALRFGALYNWSCRCNICI

>QBK20013.1 hemagglutinin [Influenza A virus]
MDTVSLITILLLSTLSIANKICIGYQSTNSAKTVNTLTQNNVPVTHAKELLHTQHNGMLCATSLGQPLIL
DTCTIEGLIYGNPSCDLSLEGREWSYIVERPSAVNGLCYPGNVQNLEELMSLFSSATSSQKIQIFPHTIW
NVSYYGTSTACSGSFYRIMRWLTRKNGDYPIQDAQYTNNQGNNILFMWGINHPPTDTTQRDLYTITDTTT
SVATEEINRIFKPLIGPRPLVNGLMGRIHYYWSVLKPGQTLRIKSDGNLIAPWYGHILSGESHGRILKTD
LKRGSCTVQCQTEKGGLNTTLPFQNVTKYAFGNCSKYIGIKSLKLAVGLRNVPSRSSRGLFGAIAGFIEG
GWSGLVGGWYGFQHSNDQGVGMAADRDSTQKAIDKITSKVNNIVDKMNKQYEIIDHEFSEVETRINMINN
KIDDQIQDIWAYNAELIVLLENQKTLDEHDANVSNLYNKVKRALGSNAVEDGKGCFELCHKCDDQCMETI
GNGTYNRRKYQEEAKLERQKMEGHKLESERTSKILSIYSRVASYIVIAMGFVALRFGAMYNGSCRCNICI

>QBK20012.1 hemagglutinin [Influenza A virus]
MDTVSLISILLVSTLSNADKICIGYQSTNSAESVNTLTQNNVPVTHAKELLHTEHNGMLCATSLGQPLIL
DTCTIEGLIYGNPSCDLSLEGREWSYIVERPSAVNGLCYPGNVQNLEELRSLFSSARSSQKIQIFPDTIW
NVSYYGTSTACSGSFYRSMRWLTQKNGDYPIQDAQYTNNQGNNILFMWGINHPPTDTTQRDLYTITDTTT
SVATEEINRIFKPLIGPRPLVNGLMGRIHYYWSVLKPGQTLRIKSDGNLIAPWYGHILSRESHGRILKTD
LKRGSCTVQCQTEKGGLNTTLPFQNVSKYAFGNCSKYIGIKSLKLAVGLRNVPSRSSRGLFGAIAGFIEG
GWSGLVGGWYGFQHSNDQGVGMAADRDSTQKAIDKITSKVNNIVDKMNKQYEIIDHEFSEVETRINMINN
KIDDQIQDIWAYNAELIVLLENQKTLDEHDANVNNLYNKVKRALGSNAVEDGKGCFELYHKCDDQCMETI

GNGTYNRRKYQEEAKLERQKIEGVKLESEGTYKILSIYSTVASYIVIAMGFAAFLFWAMSNGSCRCNSSI

>QBK20011.1 hemagglutinin [Influenza A virus]
MDTVSLITILLVATVSNADKICIGYQSTNSTETVDTLTENNVPVTHAKELLHTEHNGMLCATSLGQPLIL
DTCTIEGLIYGNPSCDLSLEGREWSYIVERPSAVNGLCYPGNVQNLEELRSLFSSARSSQRIQIFPDTIW
NVSYYGTSTACSGSFYRSMRWLTRKNGDYPIQDAQYTNNQGKNILFMWGINHPPTDTTQRDLYTRTDTTT
SVATEEINRIFKPLIGPRPLVNGLMGRIHYYWSVLKPGQTLRIKSDGNLIAPWYGHILSRESHGRILKTD
LKRGSCTVQCQTEKGGLNTTLPFQNVSKYAFGKCSKYIGIKSLKLAVGLRNVPSRFSRGLFGAIAGFIEG
GWSGLVGGWYGFQHSNDQGVGMAADRDSTQKAIDKITSKVNNIVDKMNKQYEIIDHEFSEVETRINMINN
KIDDQIQDIWAYNAELIVLLENQKTLDEHDANVSNLYNKVKRGVCSNAVEDGKGCLELCHKCDDQCMETI
RNGTSNRREYQEEGKLEREKMEGLKLESEGTYKRDSIYSRVASYIVIAMGSVALRFGALYNGSSRCNSCI

>QBK20010.1 hemagglutinin [Influenza A virus]
MDTVSLITILLVSTVSNADKICIGYQSTNSAETVNTLTENNVPVTHAVELLHTEHNGMLCATSLGQPLIL
DTCTIEGLIYGNPSCDLSLEGREWSYIVERPSAVNGLCYPGNVENLEELRSLFSSARSYQKIQIFPHTIW
NVSYYGTSTACSGSFYRSMRWLTRKNGDYPVQDAQYTNNQGKNILFMWGINHPPTDTTQRDLYTRTDTTT
SVATEEINRIFKPLIGPRPLVNGLMGRIDYYWSVLKPGQTLRIKSDGNLIAPWYGHILSGESHGRIVKTD
LKRGSCAVQCQTEKGGLNTTLPLQNVSKYAFGNCSKYIGIKSLKLAVGLRNVPSRYSRGLFGAIAGFIEG
GWSGLVAGWYGFQHSNDQGVGMAADRDSTQKAIDKITSKVNNIVDKMNKQYEIIDHEFSEVETRLNMINN
KIDDQIQDIWAYNAELIVLLENQKTLDEHDANVNNLYNKVKRALGSNAVEDGKGCFELYHKCDDQCMETI
GNGTYNRRKYQEESKLERQKIEGVKLESDRTWKILSIYSRVTSYLVIAMGFVALMFWALSNWSCRCNICI

>QBK20009.1 hemagglutinin [Influenza A virus]
METVSLITILLVATVSNADKICIGYQSTNSTETVDTLTENNVPVTHAKELLHTEHNGMLCATSLGQPLIL
DTCTIEGLIYGNPSCDLSLEGREWSYIVERPSAVNGLCYPGNVENLEELRSLFSSARSYQRIQIFPDTIW
NVSYDGTSTACSGSFYKSMRWLTRKNGDYPIQDAQYTNNQGKNILFMWGINHPPTDTTQRDLYTRIDTTT
SVATEEINRVFKPLIGPRPLVNGLMGRIDYYWSVLKPGQTLRIKSDGNLIAPWFGHILSGESHGRIMKTD
LKRGSCTVQCQTEKGGLNSTLPFQNVSKYAFGNCSKYIGIKSLKLAVGLRNVPSRSSRGLFGAIAGFIEG
GWSGLVAGWYGFQHSNDQGVGMAADRDSTQKAIDKITSKVNNIVDKMNKQYEIIDHEFSEVETRLNMINN
KIDDQIQDIWAYNAELLVLLENQKTLDEHDANVNNLYNKVKRALGSNAVEDGKGCFELYHKCDDQCMETI
RNGTYNRRKYQEESKLERQKIEGVKLESEGTYKILSIYSTVASSLVIAMGFAAFLFWAMSNGSCRCNICI

>QBK20008.1 hemagglutinin [Influenza A virus]
MDTVSLISMLLTITLSNADKICIGYQSTNSAETVDTLTQNNVPVTHAKELLHTQHNGMLCATSLGQPLIL

DTCTIEGLIYGNPSCDLSLEGREWSYIVERPSAVNGLCYPGNVQNLEELRSLFSSARSYQKIQIFPHTIW
NVSYYGTSTACSGSFYKSMRWLTRKNGDYPIQDAQYTNNQGKNILFMWGINHPPTDTTQRDLYTIIDTTT
SVATEEINRVFKPLIGPRPLVNGLMGRIDYYWSVLKPGQTLRIKSDGNLIAPWFGHILSGESHGRILKTD
LKRGSCTVQCQTEKGGLNSTLPFQNVSKYAFGNCSKYIGIKSLKLAVGLRNVPSRYSRGLFGAIAGFIEG
GWSGLVAGWYGFQHSNDQGVGMAADRDSTQKAIDKITSKVNNIVDKMNKQYEIIDHEFSEVETRINMINN
KIDDQIQDIWAYNAELLVLLENQKTLDEHDANVNNLYNKVKRALGSNAVEDGKGCFELYHKCDDQCMETI
GNGTYNRRKYQEESKLERQKIEGLKQESEGTNKILSIYSTVTSYRVIAMGFVALMFGALYNGSCRCNICI

>QBK20007.1 hemagglutinin [Influenza A virus]
MDTVSLISMLLTIPLSNADRISIGYRSSNSGNSVNTGSQNNVPVTHAVELLHTQHHGMLGDPSLGHPLIL
DTCSIEGLIDGNPSCDLSLEGREWSYIVERPSAVNGLCYPGNVQNLEELMSLFSSARSYQKIQIFPHTIW
NVSYYGTSTACSGSFYKSMRWLTPKNGDYPIQDAQYTNNQGNNILFMWGINHPPTDTTQIDLYTIIDTTT
SVATEEINRVFKPLIGPRPLVNGLMGRIDYYWSVLKPGQTLRIKSDGNLISPWFGHILSGESHGRILKTD
LKRGSCTVQCQTEKGGLNSALPLQNVSKYAFGNCSKYIGIKSLKLAVGLRNVPSRYSRGLFGAIAGFIEG
GWSGLVAGWYGFQHSNDQGVGMAADRDSTQKAIDKITSKVNNIVAKMYKQYEIIDHEFSVVETRINMINN
KFDDQIQDIWAYNAESIVLLENQKTLDEHDANVNNLYNKVKRAAGSNAVEDGKGCLELYHKCDDQCMETI
RNGTFNRREYQAESKLERQKMEGLKLESERTWGGVSMSSRVAWYRVIAMGSFALRIWALYNGSCRCNICI

>QBK20006.1 hemagglutinin [Influenza A virus]
MDTVSLISMLLTIPLTNADRISIGYRSSNSGNTVNTLSQNNVPVTTAVELLHTQHHGMLGATSLGQPLIL
DTCSIEGLIDGNPSCDLSLEGREWSYIVERPSAVNGLCYPGNVQNLEELRSLFSSARSYQKIQIFPHTIW
NVSYYGTSTACSGSFYKSMRWLTRKNGDYPIQDAQYTNNQGKNILFMWGINHPPTDTTQIDLYTIIDTTT
SVATEEINRVFKPLIGPRPLVNGLMGRIDYYWSVLKPGQTLRIKSDGNLIAPWFGHILSGESHGRILKTD
LKRGSCTVQCQTEKGGLNSTLPLQNVSKYAFGNCSKYIGIKSLKLAVGLRNVPSRYSRGLFGAIAGFIEG
GWSGLVAGWYGFQHSNDQGVGMAADRDSTQKAIDKITSKVNNIVAKMNKQYEIIDHEFSEVETRINMINN
KIDDQIQDIWAYNAESIVLLENQKTLDEQDANVNNVYNKVKRALGSNAVEDQKGCLELYHKRDDQCMETI
RNGTFNRRKYQAESKLERQKMEGLKLELDGTCEIVSIYSRVASYRMIAMGSFALRIWAMYNGSCRCNICI

>QBK20005.1 hemagglutinin [Influenza A virus]
MDTVSLITILLIATVSNADKICIGYQSTNSAETVDTLTENNVPVTHAKELLHTEHNGMLGATSLGQPLIL
DTCTIEGLIYGNPSCDLSLEGREWSYIVERPSAVNGLCYPGNVQNLEELRSLFSSARSYQRIQIFPHTIW
NVSYYGTSTACSGSFYKSMRWLTRKNGDYPIQDAQYTNNQGKNILFMWGINHPPTDTTQRDLYTRIDTTT
SVATEEINRVFKPLIGPRPLVNGLMGRIDYYWSVLKPGQTLRIKSDGNLIAPWFGHILSGESHGRILTTD
LKRGSCTVQCQTEKGGLNSAQPLQNVSKYAFGNCSKYIGIKSLKLAVGLRNVRSRYSRGLFGAIAGFIEG

GWSGLVAGWYGFQHSNDQGVGMAADRDSTQKAVDKITSKVNNIVAKLYKPYEIIDLEFSVVGTRIIMSNN

KFDDQIQDIWAYNAESLVLLENQKTREEQDIKPDNEGNKVKRAACSNAVEDGKGCLELYHKCDDQCMETI

RNGTYNRRECQAESKLERQKMEGVKLESERTCEILSIYSTVASYRVIAMGSVAFLIGAMSNGSCRCNICI

>QBK20004.1 hemagglutinin [Influenza A virus]

MDTVSLVSMLLVSTLSNADKICIGYQSTNSAETVDTLTQNNVPVTHAKELLHTEHNGMLCATSLGQPLIL

DTCTIEGLIYGNPSCDLSLEGREWSYIVERPSAVNGLCYPGNVQNLEELRSLFSSARSYQKIQIFPDTIW

NVSYDGTSTACSGSFYKSMRWLTRKNGDYPIQDAQYTNNQGKNILFMWGINHPPTDTTQRDLYTRIDTTT

SVATEEINRVFKPLIGPRPLVNGLMGRIDYYWSVLKPGQTLRIKSDGNLIAPWFGHILSGESHGRILKTD

LKRGSCTVQCQTEKGGLNSTLPFQNVSKYAFGNCSKYIGIKSLKLAVGLRNVPSRYSRGLFGAIAGFIEG

GWSGLVAGWYGFQHSNDQGVGMAADRDSTQKAIDKITSKVNNIVDKMNKQYEIIDHEFSEVETRLNMINN

KIDDQIQDIWAYNAELLVLLENQKTLDEHDANVNNLYNKVKRALGSNAVEDGKGCLELYHKCDDQCMETI

RNGTFNRRKYQEESKLERQKIEGVKLESEGTWKILSIYSRVASYLVIAMGFAALRFWAMYNGSCRQNSSI

>QBK20003.1 hemagglutinin [Influenza A virus]

MDTVSLISMLLVSTLSNADKICIGYQSTNSADTVDTLTQNNVPVTHAVELLHTQHNGMLCATSLGQPLIL

DTCTIEGLIYGNPSCDLSLEGREWSYIVERPSAVNGLCYPGNVQNLEELRSLFSSARSYQKIQIFPHTIW

NVSYYGTSTACSGSFYKSMRWLTRKNGDYPIQDAQYTNNQGKNILFMWGINHPPTDTTQRDLYTIIDTTT

SVATEEINRVFKPLIGPRPLVNGLMGRIDYYWSVLKPGQTLRIKSDGNLIAPWFGHILSGESHGRILKTD

LKRGSCTVQCQTEKGGLNSTLPLQNVSKYAFGKCSKYIGIKSLKLAVGLRNVPSRYSRGLFGAIAGFIEG

GWSGLVGGWYGFQHSNDQGVGMAADRDSTQKAIDKITSKVNNIVDKMNKQYEIIDHEFSEVETRINMINN

KIDDQIQDIWAYNAELIVLLENQKTLDEHDANVNNLYNKVKRALGSNAVEDGKGCLELYHKCDDQCMETI

RNGTYNRRKYQAESKLERQKIEGVKLEFEGTWKVVSIYSRVAWYLVIAMGFVALRFWAMYNGSCRCNICI

>QBK20002.1 hemagglutinin [Influenza A virus]

MDTVSLISMLLTIPLTNADRSSIGYRSTNSGNCVNTLSQNNVPVTTAVELLHTQHHGMLGDPSLGHPLIL

DTCSIEGLIDGNPSCDLSLEGREWSYIVERPSAVNGLCYPGNVQNLEELRSLFSSARSYQKIQIFPHTIW

NVSYYGTSTACSGSFYKSMRWLTRKNGDYPIQDAQYTNNQGKNILFMWGINHPPTDTTQIDLYTIIDTTS

SVATEEINTVFKPLIGPRPLVNGLMGRIDYYWSVLKPGQTLRIKSDGNLISPWFGHILSGESHGRILTTD

LKRGSCTVQCQTEKGGLNSTLPLQNVSKYAFGNCSKYIGIKSLKLAVGLRNVPSRYSRGLFGAIAGFIEG

GWSGLVGGWYGFQHSNDQGVGMAADRDSTQKAIDKITSKVNNIVDKMNKPYEIIGLEFSVVETRINTINN

KFDDQIQDIWAYNAEAIVLLDNQKTLDEQDANVNNGGNKVKRAAGSNAVEDGKGCLELYHKCDDQCMETI

RNRTFNRRKCQAESKLERQKMEGLKQESDRTCEGDSMYSRVKWYRAMAMGSFALRIGALYNWSCRCNICI

>QBK20001.1 hemagglutinin [Influenza A virus]
METVSLITILLIATVSNADKICIGYQSTNSTETVDTLTENNVPVTHAKELLHTEHNGMLCATSLGQPIIL
DTCTIEGLIYGNPSCDLSLEGREWSYIVERPSAVNGLCYPGNVQNLEELRSLFSSARSYQRIQIFPDTIW
NVSYYGTSKACSGSFYKNMRWLTRKNGDYPIQDAQYTNNQGKNILFMWGINHPPADTTQRDLYTRTDTTT
SVATEEINRIFKPLIGPRPLVNGLMGRIDYYWSVLKPGQTLRIKSDGNLIAPWYGHILSRESHGRILKTD
LKRGSCTVQCQTEKGGLNTTLPFQNVSKYAFGNCSKYIGIKSLKLAVGLRNVRSRYSRGLFGAIAGFIER
GWSGLVDGWYGFQHSNDQGVGMAADRDSTQKAVDKITSKVNNIVAKMYKQYEIIDHEFSEVETRINMINN
KIDDQIQDIWAYNAELLVLLENQKTLDEHDANVNNGGNKVKRAAGSNAVEDGKGCLELYHKCDDQCMETI
GNGTYNRREYQEESKLERQKMEGLKLESEGTYKILSIYSRVASSRVIAMGSVAFLIGALYNWSCRCNICI

>QBK20000.1 hemagglutinin [Influenza A virus]
METVSLITILLISTVSNADKICIGYQSTNSTETVDTLTENNVPVTHAKELLHTEHNGMLCATSLGQPLIL
DTCTIEGLIYGNPSCDLSLEGREWSYIVERPSAVNGLCYPGNVQNLEELRSLFSSARSYQRIQIFPDTIW
NVSYYGTSTACSGSFYKSMRWLTRKNGDYPIQDAQYTNNQGKNILFMWGINHPPTDTTQRDLYTRIDTTT
SVATEEINRVFKPLIGPRPLVNGLMGRIDYYWSVLKPGQTLRIKSDGNLIAPWFGHILSGESHGRIVKHD
LKRGSCTVQCQTEKGGLNSALPLQNVSKYAFGNCSKYIGIKSLKLAVGLRNVPSRYSRGLFGAIAGFIEG
GWSGLVAGWYGFQHSNDQGVGMAADRDSTQKAIDKITSKVNNIVAKMNKQYEIIDHEFSEVETRINMINN
KIDDQIQDIWAYNAESIVLLENQKTLDEHDANVNNLYNKVKRALGSNAVEDGKGCLELYHKCDDQCMETI
RNGTYNRRKYQAESKLERQKIEGVKLESEGTNKILSIYSRVASYRVIAMGFVAFLFGAMYNGSCRCNSSI

>QBK19999.1 hemagglutinin [Influenza A virus]
METVSLITILLIATVSNADKICIGYQSTNSTETVDTLTENNVPVTHAKELLHTEHNGMLCATSLGQPLIL
DTCTIEGLIYGNPSCDLSLEGREWSYIVERPSAVNGLCYPGNVQNLEELRSLFSSARSYQRIQIFPDTIW
NVSYYGTSTACSGSFYKSMRWLTRKNGDYPIQDAQYTNNQGKNILFMWGINHPPTDTTQRDLYTRIDTTS
SVATEEINRVFKPLIGPRPLVNGLMGRIDYYWSVLKPGQTLRIKSDGNLIAPWFGHILSGESHGRIVKND
LKRGSCTVQCQTEKGGLNSTLPFQNVSKYAFGNCSKYIGIKSLKLAVGLRNVPSRYSRGLFGAIAGFIEG
GWSGLVAGWYGFQHSNDQGVGMAADRDSTQKAIDKITSKVNNIVAKMNKQYEIIDHEFSEVETRINMINN
KIDDQIQDIWAYNAESLVLLENQKTLDEHDANVNNLYNKVKRALGSNAVEDGKGCLELYHKCDDQCMETI
RNGTYNRRKYQAESKLERQKIEGVKLELEGTYKILSIYSTVASSRVIAMGFAAFLFWAMYNGSCRCNICI

>QBK19998.1 hemagglutinin [Influenza A virus]
METVSLITILLIATVSNADKICIGYQSTNSTETVDTLTENNVPVTHAKELLHTEHNGMLCATSLGQPLIL
DTCTIEGLIYGNPSCDLSLEGREWSYIVERPSAVNGLCYPGNVQNLEELRSLFSSARSYQRIQIFPDTIW
NVSYDGTSTACSGSFYRSMRWLTRKNGDYPIQDAQYTNNQGKNILFMWGINHPPTDTTQRNLYTRNDTTT

SVATEEINRIFKPLIGPRPLVNGLMGRIDYYWSILKPGQTLRIKSDGNLIAPWYGHILSRESHGRILKTD
LKKGSCIVQCQTEKGGLNTTLPFQNVSKYAFGNCSKYIGIKSLKLAVGLRNVPSRYSRGLFGAIAGFIEG
GWSGLVAGWYGFQHSNEQGVGMAADRDSTQKAIDKITSKVNNIVDKMNKQYEIIDHEFSEVETRLNMINN
KIDDQIQDIWAYNAELLVLLENQKTLDEHDANVNNLYNKVKRALGSNAVEDGKGCFELYHKCDDQCMETI
RNGTYNRRKYQEESKLERQKIEGVKLESEGTYKILSIYSTVASSLVIAMGFAAFLFWAMSNGSCRCNICI

>QBK19997.1 hemagglutinin [Influenza A virus]
METVSLITILLVATVSNADKICIGYQSTNSTETVDTLTENNVPVTHAKELLHTEHNGMLCATSLGQPIIL
DTCTIEGLIYGNPSCDLSLEGREWSYIVERPSAVNGLCYPGNVQNLEELRSLFSSARSYQRIQIFPDTIW
NVSYYGTSTACSGSFYRNMRWLTRKNGDYPIQDAQYTNNQGKNILFMWGINHPPADTTQRDLYTRTDTTT
SVATEEINRIFKPLIGPRPLVNGLMGRIDYYWSVLKPGQTLRIKSDGNLIAPWYGHILSRESHGRILTTD
LKRGSCTVQCQTEKGGLNTALPLQNVSKYAFGKCSKYIGIKSLKLAVGLRNVRCRYSRGLFGAIAGFIER
GWSGLVAGWYGFQHSNDQGVGMAADRDSTQKAVDKITSKVNNIVAKMYKPYEIIDHEFSVVETRINTINN
KFDDQIQDIWAYNAESLVLLENQKTLDEQDANVNNGYNKVKRAAGSNAVEDGKGCFELYHKCDDQCMETI
RNGTYNRRKYQEESKLERQKIEGLKLESEGTYKILSMCSRVASSRVIAMGSAALMIGALSNWSCRCNICI

>QBK19996.1 hemagglutinin [Influenza A virus]
METVSLITILLIATVSNADKICIGYQSTNSTETVDTLTENNVPVTHAKELLHTEHNGMLCATSLGQPLIL
DTCTIEGLIYGNPSCDLSLEGREWSYIVERPSAVNGLCYPGNVQNLEELRSLFSSARSYQRIQIFPDTIW
NVSYDGTSTACSGSFYKSMRWLTRKNGDYPIQDAQYTNNQGKNILFMWGINHPPTDTTQRDLYTRIDTTT
SVATEEINRVFKPLIGPRPLVNGLMGRIDYYWSVLKPGQTLRIKSDGNLIAPWFGHILSGESDGRIVKHD
LKRGSCTVQCQTEKGGLNSTLPLQNVSKYAFGNCSKYIGIKSLKLAVGLRNVPSRSSRGLFGAIAGFIEG
GWSGLVAGWYGFQHSNDQGVGMAADRDSTQKAIDKITSKVNNIVDKMNKQYEIIDHEFSEVETRLNMINN
KIDDQIQDIWAYNAELLVLLENQKTLDEHDANVNNLYNKVKRALGSNAVEDGKGCFELYHKCDDQCMETI
RNGTYNRRKYQEESKLERQKIEGVKLESEGTYKILSIYSTVASSLVIAMGSAAFLFWAMSNGSCRCNICI

>QBK19995.1 hemagglutinin [Influenza A virus]
MDTVSLISMLLISTVSNADKICIGYQSRNSAETVNTLTQNNVPVTHAKELLHTQHNGMLCATSLGQPLIL
DTCTIEGLIYGNPSCDLSLEGREWSYIVERPSAVNGLCYPGNVQNLEELRSLFSSARSYQKNQIFPHTIW
NVSYYGTSTACSGSFYKSMRWLTRKNGDYPIQDAQYTNNQGKNILFMWGINHPPTDTTQIDLYTRIDTTT
SVATEEINRVFKPLIGPRPLVNGLMGRIDYYWSVLKPGQTLLIKSDGNLISPWFGHILSGESHGRILTTD
LQRGSCTAQCQREKDGYNSALPLQNVSKYAFGNCSKYIGIKSLKLAVGLRNVRSRYSRGLFGAIAGFIEG
GWSGLVDGWYGFQHSNDQGVGMAADRDSTQKAIDKITSKVNNIVAKMNKPYEIIDLEFSVVETRINTINN
KFDDQIQDIWAYNAESIVLLDNQKTLDEQDANVNNLYNKVKRAACSNAVEDGKGCLELYHKCDDQCMETI

RNGTYNRRKYQEESKLERQKMEGVKLESEGTWKRVSMSSRVAWYRVIAMGFFAFFFGALYNWSCRCNICI

>QBK19994.1 hemagglutinin [Influenza A virus]
METVSLITILLIATVSNADKICIGYQSTNSTETVDTLTENNVPVTHAKELLHTEHNGMLCATSLGQPLIL
DTCTIEGLIYGNPSCDLSLEGREWSYIVERPSAVNGLCYPGNVENLEELRSLFSSARSYQRIQIFPDTIW
NVSYDGTSTACSGSFYKSMRWLTRKNGDYPIQDAQYTNNQGKNILFMWGINHPPTDTTQRDLYTRIDTTT
SVATEEINRVFKPLIGPRPLVNGLMGRIDYYWSVLKPGQTLRIKSDGNLIAPWFGHILSGESHGRILKTD
LKRGSCTVQCQTEKDGLNSTLPFQNVSKYAFGNCSKYIGIKSLKLAVGLRNVPSRSSRGLFGAIAGFIEG
GWSGLVAGWYGFQHSNDQGVGMAADRDSTQKAIDKITSKVNNIVDKMNKQYEIIDHEFSEVETRLNMINN
KIDDQIQDIWAYNAELLVLLENQKTLDEHDANVNNLYNKVKRALGSNAVEDGKGCFELYHKCDDQCMETI
RNGTYNRRKYQEESKLERQKIEGVKLESEGTYKILSIYSTVASSLVIAMGFAAFLFWAMSNGSCRCNICI

>QBK19993.1 hemagglutinin [Influenza A virus]
METVSLITILLIATVSNADKSCIGYQSTNSTETVDTLTENNVPVTHAKELLHTEHNGMLCATSLGQPLIL
DTCTIEGLIYGNPSCDLSLEGREWSYIVERPSAVNGLCYPGNVQNLEELRSLFSSARSYQRIQIFPDTIW
NVSYDGTSTACSGSFYKSMRWLTRKNGDYPIQDAQYTNNQGKNILFMWGINHPPTDTTQRDLYTRIDTTT
SVATEEINRVFKPLIGPRPLVNGLMGRIDYYWSVLKPGQTLRIKSDGNLIAPWFGHILSGESHGRILKTD
LKRGSCTVQCQTEKGGLNSTLPLQNVSKYAFGNCSKYIGIKSLKLAVGLRNVPSRYSRGLFGAIAGFIEG
GWSGLVAGWYGFQHSNDQGVGMAADRDSTQKAIDKITSKVNNIVDKMNKQYEIIDHEFSEVETRLNMINN
KIDDQIQDIWAYNAELLVLLENQKTLDEHDANVNNLYNKVKRALGSNAVEDGKGCFELYHKCDDQCMETI
RNGTYNRRKYQEESKLERQKIEGVKLESEGTYKILSIYSTVASSLVIAMGFAAFLFWAMSNGSCRCNICI

>QBK19992.1 hemagglutinin [Influenza A virus]
METVSLITILLIATVSNADKICIGYQSTNSTETVDTLTENNVPVTHAKELLHTEHNGMLCATSLGQPLIL
DTCTIEGLIYGNPSCDLSLEGREWSYIVERPSAVNGLCYPGNVENLEELRSLFSSARSYQRIQIFPDTIW
NVSYDGTSTACSGSFYKSMRWLTRKNGDYPIQDAQYTNNQGKNILFMWGINHPPTDTTQRDLYTRIDTTT
SVATEEINRVFKPLIGPRPLVNGLMGRIDYYWSVLKPGQTLRIKSDGNLIAPWFGHILSGESHGRILKTD
LKRGSCTVQCQTEKGGLNSTLPFQNVSKYAFGNCSKYIGIKSLKLAVGLRNVPSRSSRGLFGAIAGFIEG
GWSGLVAGWYGFQHSNDQGVGMAADRDSTQKAIDKITSKVNNIVDKMNKQYEIIDHEFSEVETRLNMINN
KIDDQIQDIWAYNAELLVLLENQKTLDEHDANVNNLYNKVKRALGSNAVEDGKGCFELYHKCDDQCMETI
RNGTYNRRKYQEESKLERQKIEGVKLESEGTYKILSIYSTVASSLVIAMGFAAFLFWAMSNGSCRCNICI

>QBK19991.1 hemagglutinin [Influenza A virus]
METVSLITILLIATVSNADKICIGYQSTNSTETVDTLTENNVPVTHAKELLHTEHNGMLCATSLGQPIIL

DTCTIEGLIYGNPSCDLSLEGREWSYIVERPSAVNGLCYPGNVQNLEELRSLFSSARSYQKIQIFPHTIW
NVSYYGTSTACSGSFYRNMRWLTRKNGDYPIQDAQYTNNQGKNILFMWGINHPPADTTQRDLYTRTDTTT
SVATEEINRIFKPLIGPRPLVNGLMGRIDYYWSVLKPGQTLRIKSDGNLIAPWFGHILSGESHGRILKTD
LKRGSCTVQCQTEKGGLNTALSFQNVSKYAFGNCSKYIGIKSLKLAVGLRNVRCRYSRGLFGAIAGFIEG
GWSGLVAGWYGFQHSNDQGVGMAADRDSTQKAVDKITSKVNNIVAKMYKQYEIIDHEFSVVETRINMINN
KFDDQIQDIWAYNAESLVLLENQKTLDEQDANVNNLYNKVKRAAGSNAVEDGKGCFELYHKCDDQCMETI
RNGTYNRRKYQEESKLERQKIEGLKLESEGTYKILSIYSTVASSLVIAMGSAAFLFWAMSNGSCRCNICI

>QBK19990.1 hemagglutinin [Influenza A virus]
METVSLITILLIATVSNADKICIGYQSTNSTETVDTLTENNVPVTHAKELLHTEHNGMLCATSLGQPLIL
DTCTIEGLIYGNPSCDLSLEGREWSYIVERPSAVNGLCYPGNVQNLEELRSLFSSARSYQRIQIFPDTIW
NVSYYGTSTACSGSFYKSMRWLTRKNGDYPIQDAQYTNNQGKNILFMWGINHPPTDTTQRDLYTRIDTTT
SVATEEINRVFKPLIGPRPLVNGLMGRIDYYWSVLKPGQTLRIKSDGNLIAPWFGHILSGESQGRIVKPD
LKRGSCTVQCQTEKGGLNSTLPLQNVSKYAFGNCSKYIGIKSLKLAVGLRNVPSRYSRGLFGAIAGFIEG
GWSGLVAGWYGFQHSNDQGVGMAADRDSTQKAIDKITSKVNNIVDKMNKQYEIIDHEFSEVETRINMINN
KIDDQIQDIWAYNAESLVLLENQKTLDEHDANVNNLYNKVKRALGSNAVEDGKGCFELYHKCDDQCMETI
RNGTYNRRKYQEESKLERQKIEGVKLESEGTYKILSIYSTVASSLVIAMGFAAFLFWALSNGSCRCNICI

>QBK19989.1 hemagglutinin [Influenza A virus]
METVSLITILLIATVSNADKICIGYQSTNSTETVDTLTENNVPVTHAKELLHTEHNGMLCATSLGQPIIL
DTCTIEGLIYGNPSCDLSLEGREWSYIVERPSAVNGLCYPGNVQNLEELRSLFSSARSYQRIQIFPDTIW
NVSYYGTSTACSGSFYRNMRWLTRKNGDYPIQDAQYTNNQGKNILFMWGINHPPADTTQRDLYTRTDTTT
SVATEEINRIFKPLIGPRPLVNGLMGRIDYYWSVLEPGQTLRIKSDGNLIDTWYGHILSGERHGRILEQD
LKRGSCTVQCQTEKGGLNTALSFQNVSKYASGKCSKYIGIKSLKLAVGLRSVRCRYSRGLFGAIAGFIER
GWSGLVAGWYGFQHSNDQGVGMAADRDSTQKAVDKITSKVNNIVAKLYKQYEIIDHEFSVVGTRINMINN
KFDDQIQDIWAYNAESLVLLENQKTLDEQDANVNNLSNKVKRAAGSNAVEDGKGCFELYHKCDDQCMETI
RNGTYNRRKYQEESKLERQKIEGVKLESEGTYKILSIYSTVASSLVIAMGSAAFLFGAMSNWSCRCNICI

>QBK19988.1 hemagglutinin [Influenza A virus]
METVSLITILLIATVSNADKICIGYQSTNSTETVDTLTENNVPVTHAKELLHTEHNGMLCATSLGQPLIL
DTCTIEGLIYGNPSCDLSLEGREWSYIVERPSAVNGLCYPGNVQNLEELRSLFSSARSYQRIQIFPDTIW
NVSYDGTSTACSGSFYKSMRWLTRKNGDYPIQDAQYTNNQGKNILFMWGINHPPTDTTQRDLYTRIDTTT
SVATEEINRVFKPLIGPRPLVNGLMGRIDYYWSVLKPGQTLRIKSDGNLIATWFGHILSGESHGRILKND
LKRGSCTVQCQTEKGGLNSTLPFQNVSRYAFGNCSKYIGIKSLKLAVGLRNVPSRYSRGLFGAIAGFIEG

GWSGLVAGWYGFQHSNDQGVGMAADRDSTQKAIDKITSKVNNIVDKMNKQYEIIDHEFSEVETRLNMINN
KIDDQIQDIWAYNAELLVLLENQKTLDEHDANVNNLYNKVKRALGSNAVEDGKGCFELYHKCDDQCMETI
RNGTYNRRKYQEESKLERQKIEGVKLESEGTYKILSIYSTVASSLVIAMGFAAFLFWAMSNGSCRCNICI

>QBK19987.1 hemagglutinin [Influenza A virus]
MDAVSLITILLILTVSNANKICIGYQSTNSAETVDTLTENNVPVTHAKELLHTQHNGMLCATSLGQPLVL
DTCTIEGLIYGNPSCDLSLEGREWSYIVERPSTVNGLCYPGNVQNLEELRSLFSSARSYQKIQIFPHTLW
NVSYYGTSNACSGSFYISMRWLTRKNGDYPTQDAQYTNNQGKNILFMWGINHPPTDTTQRDLYTRTDTTS
SVATEEINRIFTPLIGPRPLVNGLMGRIDYYWSVLKPGQTLRIKSDGNLIATWYGHIRSGERHGRILKTD
LKRGSCTVQCQTEKGGLNTALSLQNVSKYAFGNCSKYIGIKSLKLAVGLRNVRCRYSRGLFGAIAGFIEG
GWSGLVAGWYGFQHSNDQGVGMAADKDSTQKAIDKITSKVNNLVAKLYKQYEILDHEISEVGTRINMINN
KIDDQIQDIWAYNAESIVFLENQKTLDEHDANVNNLYNKVKRALGSNAVEDGKGCFELYHKCDDQCMETI
RNGTFNRRKYQAESKLERQKIEGLNLESEGTCKRVSIYSTVASSRVIAMGSAAFLFWAMSNGSCRCNICI

>QBK19986.1 hemagglutinin [Influenza A virus]
MDTVSLITILLIATVSNADKICIGYQSTNSTETVDTLTENNVPVTHAKELLHTEHNGMLCATSLGQPLIL
DTCTIEGLIYGNPSCDLSLEGREWSYIVERPSAVNGLCYPGNVQNLEELRSLFSSARSYQRIQIFPDTIW
NVSYDGTSTACSGSFYKSMRWLTRKNGDYPIQDAQYTNNQGKNILFMWGINHPPTDTTQRDLYTRIDTTT
SVATEEINRVFKPLIGPRPLVNGLMGRIDYYWSVLKPGQTLRIKSDGNLIAPWFGHILSGESHGRIQKHD
LNRGSCAVQCQTEKGRLHSALPFLNVSKYAFGNCSKYIGIKSLKLAVGLRNVRSRYSRGLFGAIAGFIEG
GWSGLVAGWYGFQHSNDQGVGMAADRDSTQKAMDKITSKVNNIVAKMNKQYEIIDHEFSEVETRINMINN
KIDDQIQDIWAYNAELLVLLENQKTLDEHDANVNNLYNKVKRALGSNAVEDGKGCFELYHKCDDQCMETI
RNGTYNRRKYQEESKLERQKIEGVKLESEGTYKILSIYSTVASSLVIAMGFAAFLFWAMSNGSCRCNICI

>QBK19985.1 hemagglutinin [Influenza A virus]
METVSLITILLIATVSNADKKGIGYQSTNSTETVDTLTENNVPVTHAKELLHTEHNGMLCATSLGQPLIL
DTCTIEGLIYGNPSCDLSLEGREWSYIVERPSAVNGLCYPGNVENLEELRSLFSSARSYQRIQIFPDTIW
NVSYDGTSTACSGSFYKSMRWLTRKNGDYPIQDAQYTNNQGKNILFMWGINHPPTDTTQRDLYTRIDTTT
SVATEEINRVFKPLIGPRPLVNGLMGRIDYYWSVLKPGQTLRIKSDGNLIATWFGHILSGESHGRILKND
LQRGSCTVQCQTEKGGLNSTLPLQNVSKYAFGNCSKYIGIKSLKLAVGLRNVPSRYSRGLFGAIAGFIEG
GWSGLVDGWYGFQHSNDQGVGMAADSDSTQKAIDKITSKVNNIVAKMNKQYEIIDHEFSEVETRINMINN
KIDDQIQDIWAYNAELLVLLENQKTLDEQDANVNNLYNKVKRALGSNAVEDGKGCFELYHKCDDQCMETI
RNGTYNRRKYQEESKLERQKIEGVKLESEGTYKILSIYSTVASSLVIAMGSAAFLFWAMYNGSCRCNICI

>QBK19984.1 hemagglutinin [Influenza A virus]

MDTVSLISMLLVSTLSNADKICIGYQSTNSAETVDTLTQNNVPVTHAKELLHTQHNGMLCATSLGQPLIL
DTCTIEGLIYGNPSCDLSLEGREWSYIVERPSAVNGLCYPGNVQNLEELRSLFSSARSYQKIQIFPHTIW
NVSYYGTSTACSGSFYKSMRWLTRKNGDYPIQDAQYTNNQGNNILFMWGINHPPTDTTQIDLYTIIDTTS
SVATEEINRVFTPLIRPRPLVNGLMGRIDYYWSVLKPGQTLRIKSDGNLIAPWFGHILSGESHGRIVKHD
LKRGRCTVQCQTEKGGLHSTLPLQNVSKYAFGKCSKYIGIKSLKLAVGLRNVPSRYSRGLFGAIAGFIEG
GWSGLVGGWYGFQHSNDQGVGMAADRDSTQKAMDKITSKVNNIVAKLNKPYEIIDHEFSVVETRINTINN
KFDDQIQDIWAYNAESLVLLENQKTLDEHDANVNNLYNKVKRALGSNAVEDGKGCLELYHKCDDQCMETI
RNGTYNRRKYQAESKLERQKIEGVKQESEGTYKILSIYSRVASYRVIAMGFAAFRIGAMSNGSCRCNICI

>QBK19983.1 hemagglutinin [Influenza A virus]

MDTVSLITMLLISHVSNADKICIGYQSTNSAETVDTLTENNVPVTHAKELLHTEHNGMLCATSLGQPLIL
DTCTIEGLIYGNPSCDLSLEGREWSYIVERPSAVNGLCYPGNVQNLEELRSLFSSARSYQKIQIFPHTLW
NVSYYGTSTACSGSFYKSMRWLTRKNGDYPIQDAQYTNNQGNNILFMWGINHPPTDTTQRDLYTRIDTTS
SVATEEINRVFKPLIGPRPLVSGLMGRIDYYWSVLKPGHTLRIKSDGNLIATWFGHTRTGERQGRIVKHD
LHRGSCAAQCQRENGRLNSTQPLLNVSKHAFGKCSKYMGIKCVKLAVGLRNVPCRYSRGLFGAIAGFIER
GWSGLVAGWYGFQHSNDQGVGMAADRDSTQKAIDKITSNENNIVAKLYKPYEVIDLEFSVGETRINTSNH
MFDDQIQDIWAYNAESIVFIENQKTIEEQDINVRNLYNKVKRGACSNAVEDGKGCSELYHKCDERCMETI
RNGTFNRRECQAESKKERQRMEGVELESDGTWGGVSIYSRVTWYRMIAMGSFALRIEGLYNWSCRCNICI

>QBK19982.1 hemagglutinin [Influenza A virus]

MDTVSLITILLIATVSNADKICIGYQSTNSTETVDTLTENNVPVTHAKELLHTEHNGMLCATSLGQPLIL
DTCTIEGLIYGNPSCDLSLEGREWSYIVERPSAVNGLCYPGNVENLEELRSLFSSARSYQRIQIFPDTIW
NVSYDGTSTACSGSFYKSMRWLTRKNGDYPIQDAQYTNNQGKNILFMWGINHPPTDTTQRDLYTRIDTTT
SVATEEINRVFKPLIGPRPLVNGLMGRIDYYWSVLKPGQTLRIKSDGNLIAPWFGHILFGVSHGRNLKTD
LKRGSCTVQCQTEKGGLNSTLPLQNVSKYAFGNCSKYIGIKSLKLAVGLRNVPSRYSRGLFGAIAGFIEG
GWSGLVAGWYGFQHSNDQGVGMAADRDSTQKAIDKITSKVNNIVDKMNKQYEIIDHEFSEVETRLNMINN
KIDDQIQDIWAYNAELLVLLENQKTLDEHDANVNNLYNKVKRALGSNAVEDGKGCFELYHKCDDQCMETI
RNGTYNRRKYQEESKLERQKIEGVKLESEGTYKILSIYSTVASSLVIAMGFAAFLFWAMSNGSCRCNICI

>QBK19981.1 hemagglutinin [Influenza A virus]

MDTVSLISMLLVSTLSNADKICIGYRSTNSADTVDTVTENNVPVTHAVELLHTQHNGMLCATSLGQPLIL
DTCTIEGLIYGNPSCDLSLEGREWSYIVERPSAVNGLCYPGNVQNLEELRSLFSSARSYQKIQIFPHTIW
NVSYYGTSTACSGSFYKSMRWLTRKNGDYPIQDAQYTNNQGKNILFMWGINHPPTDTTQIDLYTRIDTTT

SVATEEINRVFKPLIGPRPLVNGLMGRIDYYWSVLKPGQTLRIKSDGNLIAPWFGHILSGESHGRILKTD
LKRGSCTVQCQTEKGGLNSTLPFQNVSKYAFGNCSKYIGIKSLKLAVGLRNVPSRSSRGLFGAIAGFIEG
GWSGLVAGWYGFQHSNDQGVGMAADRDSTQKAIDKITSKVNNIVDKMNKQYEIIDHEFSEVETRINMINN
KIDDQIQDIWAYNAELLVLLENQKTLDEHDANVNNLYNKVKRALGSNAVEDGKGCLELYHKCDDQCMETI
RNGTFNRRKYQAESKLERQKIEGDKQESDRTNEIVSIYSTVASYLVIAMGSVALMFGALYNGSCRCNICI

>QBK19980.1 hemagglutinin [Influenza A virus]
MEAVSLITILLVLTVSNADKICIGYQSTNSAETVNTLTENNVPVTHAKELLHTQHNGMLCATSLGQPLVL
DTCTIEGLIYGNPSCDLSLEGREWSYIVERPSAVNGLCYPGNVQNLEELRSLFSSARSYQRIQIFPDTIW
NVSYDGTSNACSGSFYRSMRWLTRKNGDYPTQDAQYTNNQGKNILFMWGINHPPTDTTQRDLYTRTDTTT
SVATEEINRIFKPLIGPRPLVNGLMGRIDYYWSVLKPGQTLRIKSDGNLIAPWYGHILSGESHGRILKTD
LKRGSCTVQCQTEKGGLNTTLPFQNVSKYAFGNCSKYIGIKSLKLAVGLRNVPSRSSRGLFGAIAGFIEG
GWSGLVAGWYGFQHSNDQGVGMAADKDSTQKAIDKITSKVNNIVDKMNKQYEIIDHEFSEVETRLNMINN
KIDDQIQDIWAYNAELLVLLENQKTLDEHDANVNNLYNKVKRALGSNAVEDGKGCFELYHKCDDQCMETI
RNGTYNRRKYQEESKLERQKIEGVKLESEGTYKILSIYSTVASYLVIAMGFAAFLFWAMSNGSCRCNICI

>QBK19979.1 hemagglutinin [Influenza A virus]
METVSLITMLLVATVSNADKICIGYQSTNSTETVDTLTENNVPVTHAKELLHTEHNGMLCATSLGQPLIL
DTCTIEGLIYGNPSCDLSLEGREWSYIVERPSAVNGLCYPGNVQNLEELRSLFSSARSYQRIQIFPHTIW
NVSYYGTSTACSGSFYKSMRWLTRKNGDYPIQDAQYTNNQGKNILFMWGINHPPTDTTQRDLYTRIDTTT
SVATEEINTVFKPLIGPRPLVNGLMGRIDYYWSVLKPGQTLPIKSDGNLIAPWFGHILSGESHGRIVKHD
LKRGRCTVQCQTEKGGLNSALSLQNVSKYAFGKCSKYIGIKSLKLAVGLRNVPCRYSRGLFGAIAGFIEG
GLSGLVGGWYGFQHSNDQGVGMAADRDSTQKAMDTTTSKENNIVAKLNKPYEIIDLEFSEVETRINTINH
KSDDQIQDIWAYNAESIVLLENQITLDEHDANVNNLYNKVKRAAGSNAVEDGKGCLELCHKCDDQCMETI
GNRTYNRRKCQAESKLERQKMEGLEQESERTREILSIYSTVASYRVIAMGFVALRFWALYNWSCRCNICI

>QBK19978.1 hemagglutinin [Influenza A virus]
MDTVSLITILLVSTLSNADKICIGYQSTNSAETVNTLTENNVPVTHAKELLHTEHNGMLCATSLGQPIIL
DTCTIEGLIYGNPSCDLSLEGREWSYIVERPSAVNGLCYPGNVQNLEELMSLFSSARSYQKIQIFPHTIW
NVSYYGTSTACSGSFYRNMRWLTRKNGDYPIQDAQYTNNQGKNILFMWGINHPPTDTTQRDLYTITDTTT
SVATEEINRIFKPLIGPRPLVNGLMGRIDYYWSVLKPGQTLRIKSDGNLIAPWYGHILSGESHGRILKTD
LKRGSCTVQCQTEKGGLHTALPFQNVSKYAFGNCSKYIGIKSLKLAVGLRNVRSRYSRGLFGAIAGFIER
GWSGLVAGWYGFQHSNDQGVGMAADRDSTQKAVDKITSKVNNIVDKMNKQYEIIDHEFSEVETRLNMINN
KIDDQIQDIWAYNAELLVLLENQKTLDEHDANVNNLYNKVKRALGSNAVEDGKGCFELYHKCDDQCMETI

GNGTYNRRKYQEESKLERQKIEGLKLESDRTWKILSMYSTVASYLVIAMGFVAFLFWAMYNWSCRCNICI

>QBK19977.1 hemagglutinin [Influenza A virus]

MDTVSLISMLLVSTLTHADKISIGYRSTNSGNTVNTLTQNNVPVTHAVELLHTQHNGMLCAPSLGQPLIL
DTCTIEGLIYGNPSCDLSLERREWSYIIERPSAVNGLCYPGNVQNLKELMSLFSSATSYQKNPIFPHTLW
NVSYYGTSTACSGSFYNLMRWLTRKNGDYPIQDAQYTNNQGKNILFMWGINHPPTDTTQINLYTRIDPTS
SVATDEINTVFAPLIGPRPLVSGLMGRIDYYWSELKPGQTLLIKSDGNLIVPWFGLILSGESHGTILTTD
LIGGSCTVQCQTEKGGLHSALPFQNVSKYAFGKCSKYIGIKSLKLAVGLRNVPCRYSRGLFGAIAGFIER
GWSGLVGGWYGFQHSNDQGVGMAADRDSTQKAMDKITSKVNNIVAKMYKPYEIIDLEFSVVETRIITINN
KFDDQIQDIWAYNAESIVLLDNQKTLDEQDANVNNLYNKVKRAACSNAVEDGKGCLELCHKRDDQCMETI
RNRTYNRREYQAESKLERQKMEGLNLESDRTWEILSMYSRVAWYRVIAMGSAALRIWALYNWSCRCNICI

>QBK19976.1 hemagglutinin [Influenza A virus]

MDAVSLITMLLISTVSIANRFCIGYALTNSAETVNTVTQNNVPVTHAVELLHTQHNGMLGATSLGQPLVL
DTCSIEGLIYGNPSCDLSLEGREWSYIVERPSAVNGLCYPGNVQNLEELRSLFSSARSYQKIQIFPHTIW
NVSYDGTSNACSGSFYISMRWLTRKNGDYPTQDAQYTNNQGKNILFMWGINHPPTDTTQRDLYTRTDTTT
SVATEEINRIFTPLIGPRPLVNGLMGRIDYYWSVLKPGQTLRIKSDGNLIAPWYGHILSGESHGRIVKTD
LKRGSCTVQCQTEKGGLNTTLPFQNVSKYAFGNCSKYIGIKSLKLAVGLRNVPSRSSRGLFGAIAGFIEG
GWSGLVAGWYGFQHSNDQGVGMAADKDSTQKAIDKITSKVNNIVAKMYKQYEIIDHEFSEVETRLNMINN
KIDDQIQDIWAYNAESIVFLENQKTLDEHDANVNNLYNKVKRALGSNAVEDGKGCFELYHKCDDQCMETI
RNGTYNRRKYQEESKLERQKIEGVKLESEGTYKILSIYSTVASSLVIAMGFAAFLFWALSNWSCRCNICI

>QBK19975.1 hemagglutinin [Influenza A virus]

MDTVSLITILLIATVSNADKICIGYQSTNSTETVDTLTENNVPVTHAKELLHTEHNGMLCATSLGQPLIL
DTCTIEGLIYGNPSCDLSLEGREWSYIVERPSAVNGLCYPGNVQNLEELRSLFSSARSYQRIQIFPDTIW
NVSYYGTSTACSGSFYKSMRWLTRKNGDYPIQDAQYTNNQGKNILFMWGINHPPTDTTQRDLYTRIDTTT
SVATEEINRVFKPLIGPRPLVNGLMGRIDYYWSVLKPGQTLRIKSDGNLIAPWFGHILSGESHGRIVKPD
LKRGSCTVQCQTEKGGLNSTLPLQNVSKYAFGNCSKYIGIKSLKLAVGLRNVPSRYSRGLFGAIAGFIEG
GWSGLVAGWYGFQHSNDQGVGMAADRDSTQKAIDKITSKVNNIVDKMNKQYEIIDHEFSEVETRINMINN
KIDDQIQDIWAYNAESLVLLENQKTLDEHDANVNNLYNKVKRALGSNAVEDGKGCFELYHKCDDQCMETI
RNGTYNRRKYQEESKLERQKIEGVKLESEGTYKILSIYSTVASSLVIAMGFAAFLFWAMYNGSCRCNICI

>QBK19974.1 hemagglutinin [Influenza A virus]

MDTVSLISMLLVSTLSNADKISIGYRSTNSAETVDTLTQNNVPVTHAKELLHTQHNGMLCATSLGQPLIL

DTCTIEGLIYGNPSCDLSLEGREWSYIVERPSAVNGLCYPGNVQNLKELRSLFSSARSYQKIQIFPHTIW
NVSYYGTSTACSGSFYKSMRWLTRKNGDYPIQDAQYTNNQGNNILFMWGINHPPTDTTQIDLYTIIDTTT
SVATEEINRVFTPLIGPRPLVNGLMGRIDYYWSVLKPGHTLRIKSDGNLIAPWFGHILSGESHGRILTTD
LNRGSCTVQCQTEKGGLHSTLPLQNVSKYAFGNCSKYIGIKSLKLAVGLRNVPCRYSRGLFGAIAGFIEG
GWSGLVAGWYGFQHSNDQGVGMAADRDSTQKAIDKITSKVNNIVAKMYKQYEIIDLEFSVVETRINTINN
KFDDQIQDIWAYNAESIVLLENQKTLDEHDANVNNLYNKVKRALGSNAVEDGKGCFELYHKCDDQCMETI
RNGTYNRRKYQAESKLERQKIEGVKLESEGTYKILSIYSRVASYRVIAMGFAAFLFWAMSNWSCRCNICI

>QBK19973.1 hemagglutinin [Influenza A virus]
MHTVSLISMLLIATLSNADKICIGYQSTNSAETVDTLTENNVPVTHAKELLHTEHNGMLCATSLGQPLIL
DTCTIEGIIYGNPSCDLSLEGREWSYIVERPSAVNGLCYPGNVQNLEELRSLFSSARSYQKNQIFPHTIW
NVSYYGTSTACSGSFYKSMRWLTRKNGDYPIQDAQYTNNQGKNILFMWGINHPPTDTTQIDLYTIIDTTT
SVATEEINRVFKPLIGPRPLVSGLMGRIDYYWSVLKPGQTLRIKSDGNLIAPWFGHIPSGESHGRVVEHD
LHRGSCTAQCQTENGRLHSALPLLNVSKYAFGKCSKYIGIKSLKLAVGLRNVPSRYSRGLFGAIAGFIEG
GWSGLVGGWYGFQHSNDQGVGMAADRDSTQKAVDKIPSNVNNIVAKLYKPYEIIGLEFSVVGTRIITSNN
KFDDQIQDIWAYNAESIVFIENQKTREEHDIKPNKEGNKVKRAAGSNAVEDGNGCSELYHKRDKECMETI
RNGTYNRRECQAESKKERQKMEGLKQELDRTREIVSFYSRVTSYRVIAMGSFALRIEAMYNGSCRCNICI

>QBK19972.1 hemagglutinin [Influenza A virus]
METVSLITILLVATVSNADKICIGYQSTNSTETVDTLTENNVPVTHAKELLHTEHNGMLCATSLGQPLIL
DTCTIEGLIYGNPSCDLSLEGREWSYIVERPSAVNGLCYPGNVQNLEELRSLFSSARSYQRIQIFPDTIW
NVSYDGTSTACSGSFYKSMRWLTRKNGDYPIQDAQYTNNQGKNILFMWGINHPPTDTTQRDLYTRIDTTT
SVATEEINRVFKPLIGPRPLVNGLMGRIDYYWSVLKPGQTLRIKSDGNLIAPWFGHILSGESHGRILKTD
LKRGSCTVQCQTEKGGLNSTLPLQNVSKYAFGNCSKYIGIKSLKLAVGLRNVPSRYSRGLFGAIAGFIEG
GWSGLVAGWYGFQHSNDQGVGMAADRDSTQKAIDKITSKVNNIVDKMNKQYEIIDHEFSEVETRINMINN
KIDDQIQDIWAYNAELLVLLENQKTLDEHDANVNNLYNKVKRALGSNAVEDGKGCFELYHKCDDQCMETI
RNGTYNRRKYQEESKLERQKIEGVKLESEGTYKILSIYSTVASSLVIAMGFAAFLFWAMSNGSCRCNICI

>QBK19971.1 hemagglutinin [Influenza A virus]
MDTVSLITMLLIATVSIADKICIGYQSTNSAETVDTLTENNVPVTHAKELLHTEHNGMLGATSLGQPLIL
DTCTIEGLIDGNPSCDLSLEGREWSYIVERPSAVNGLCYPGNVQNLEELRSLFSSARSYQRIQIFPHTIW
NVSYYGTSTACSGSFYKSMRWLTRKNGDYPIQDAQYTNNQGKNILFMWGINHPPTDTTQRDLYTRIDTTT
SVATEEINRVFTPLIGPRPLVNGLMGRIDYYWSVLKPGHTLRIKSDGNLIAPWGGHILSGESQGRNVKHD
LKRGSCTVQCQTEKGGLNSAQPLQNVSKHAIGNCSKYIGIKSVKLAVGLRNVRCRYSRGLFGAIAGFIEG

GWSGLVAGWYGFQHSNDQGVGMAADRDSTQKAMDKTTSNVNNIVDKMYKPYEIIDLEFSVVETRIITSNN
MFDDQIQDIWAYNAESLVLLENQKTLDEQDAKPANLYNKVKRGAGSNAVEDGKGCLELYHKCDDQCMETI
RNGTYNRRECQAESKKERQKIEGLKLELEGTCEILSIYSTVASSRVIAMGSVAFLFGAMYNGSCRCNICI

>QBK19970.1 hemagglutinin [Influenza A virus]
MDTVSLITILLISTLSNADKICIGYQSTNSAETVDTLTENNVPVTHAKELLHTEHNGMLCATSLGQPLIL
DTCTIEGLIYGNPSCDLSLEGREWSYIVERPSAVNGLCYPGNVQNLEELRSLFSSARSYQKIQIFPDTIW
NVSYYGTSTACSGSFYKSMRWLTRKNGDYPIQDAQYTNNQGKNILFMWGINHPPTDTTQRDLYTRIDTTT
SVATEEINRIFKPLIGPRPLVNGLMGRIDYYWSVLKPGQTLRIKSDGNLIAPWFGHILSGESHGRIVKTD
LKRGSCTVQCQTEKGGLNSTLPFQNVSKYAFGNCSKYIGIKSLKLAVGLRNVPSRSSRGLFGAIAGFIEG
GWSGLVAGWYGFQHSNDQGVGMAADRDSTQKAVDKITSKVNNIVDKMNKQYEIIDHEFSEVETRINMINN
KIDDQIQDIWAYNAELLVLLENQKTLDEHDANVNNLYNKVKRALGSNAVEDGKGCFELYHKCDDQCMETI
RNGTYNRRKYQEESKLERQKIEGLKLESEGTYKILSIYSTVASSRVIAMGFAAFLFWAMSNGSCRCNICI

>QBK19969.1 hemagglutinin [Influenza A virus]
METVSLITILLISTVSNADKICIGYQSTNSAETVDTLTENNVPVTHAKELLHTEHNGMLCATSLGQPLIL
DTCTIEGLIYGNPSCDLSLEGREWSYIVERPSAVNGLCYPGNVENLEELRSLFSSARSYQRIQIFPDTIW
NVSYDGTSTACSGSFYKSMRWLTRKNGDYPIQDAQYTNNQGKNILFMWGINHPPTDTTQRDLYTRIDTTT
SVATEEINRVFKPLIGPRPLVNGLMGRIDYYWSVLKPGQTLRIKSDGNLIAPWFGHILSGESHGRILKTD
LKRGSCTVQCQTEKGGLNSTLPFQNVSKYAFGNCSKYIGIKSLKLAVGLRNVPSRSSRGLFGAIAGFIEG
GWSGLVAGWYGFQHSNDQGVGMAADRDSTQKAIDKITSKVNNIVDKMNKQYEIIDHEFSEVETRLNMINN
KIDDQIQDIWAYNAELLVLLENQKTLDEHDANVNNLYNKVKRALGSNAVEDGKGCFELYHKCDDQCMETI
RNGTYNRRKYQEESKLERQKIEGVKQESEGTNKILSIYSTVASYLVIAMGFAAFLFWAMSNGSCRCNICI

>QBK19968.1 hemagglutinin [Influenza A virus]
METVSLITILLIATVSNADKICIGYQSTNSTETVDTLTENNVPVTHAKELLHTEHNGMLCATSLGQPLIL
DTCTIEGLIYGNPSCDLSLEGREWSYIVERPSAVNGLCYPGNVQNLEELRSLFSSARSYQRIQIFPDTIW
NVSYYGTSTACSGSFYKSMRWLTRKNGDYPIQDAQYTNNQGKNILFMWGINHPPTDTTQRDLYTRIDTTT
SVATEEINRVFKPLIGPRPLVNGLMGRIDYYWSVLKPGQTLRIKSDGNLIATWFGHIRSGESQGRIVEHD
LHRGSCTVPCQTEKGGLHSAQPLLNVSKYAFGNCSKYIGIKSLKLAVGLRNVRSRYSRGLFGAIAGFIEG
GWSGLVAGWYGFQHSNDQGVGMAADRDSTQKAVDKITSKVNNIVAKMYKQYEIIDHEFSEVETRINMINN
KFDDQIQDIWAYNAESLVLLENQKTLDEHDANVNNLYNKVKRALGSNAVEDGKGCLELYHKCDDQCMETI
RNGTYNRRKYQEESKLERQKIEGVKLESEGTYKILSIYSTVASSLVIAMGSAAFLFWAMSNGSCRCNICI

>QBK19967.1 hemagglutinin [Influenza A virus]

METVSLITILLISTVSNADKICIGYQSTNSTETVDTLTENNVPVTHAKELLHTEHNGMLCATSLGQPLIL
DTCTIEGLIYGNPSCDLSLEGREWSYIVERPSAVNGLCYPGNVENLEELRSLFSSARSYQRIQIFPHTIW
NVSYYGTSTACSGSFYRSMRWLTRKNGDYPVQDAQYTNNQGKNILFMWGINHPPTDTTQRDLYTRTDTTT
SVATEEINRIFKPLIGPRPLVNGLMGRIDYYWSVLKPGQTLRIKSDGNLIAPWYGHILSGESHGRILKTD
LKRGSCTVQCQTEKGGLHTTLPFQNVSKYAFGKCSKYIGIKSLKLAVGLRNVPSRSSRGLFGAIAGFIEG
GWSGLVGGWYGFQHSNDQGVGMAADRDSTQKAIDKITSKVNNIVDKMNKQYEIIDHEFSVVETRINMINN
KFDDQIQDIWAYNAESLVLLENQKTLDEQDANVNNLYNKVKRAAGSNAVEDGKGCLEQYHKCDDQCMETI
RNGTYNRRKYQEESKLERQKIEGLKLESEGTYKILSIYSTVASSLVIAMGFAAFLFWAMSNGSCRCNICI

>QBK19966.1 hemagglutinin [Influenza A virus]

METVSLITILLIATVSNADKICIGYQSTNSTETVDTLTENNVPVTHAKELLHTEHNGMLCATSLGQPLVL
DTCTIEGLIYGNPSCDLSLEGREWSYIVERPSAVNGLCYPGNVQNLEELRSLFSSARSYQRIQIFPDTIW
NVSYYGTSSACSGSFYKSMRWLTRKNGDYPIQDAQYTNNQGKNILFMWGINHPPTDTTQRDLYTRTDTTT
SVATEEINRIFKPLIGPRPLVNGLMGRIDCYWSVLKPGQTLRIKSDGNLIAPWYGHILSGESHGRILKTD
LKRGSCTVQCQTEKGGLHTALPFQNVSKYAFGNCSKYIGIKSLKLAVGLRNVRSRYSRGLFGAIAGFIEG
GWSGLVAGWYGFQHSNDQGVGMAADRDSTQKAIDKITSKVNNIVAKMYKQYEIIDHEFSEVETRINMINN
KIDDQIQDIWAYNAESIVLLENQKTLDEHDANVNNGDNKVKRAAGSNAVEDGKGCLELCHKCDDQCMETI
RNGTYNRRKYQAESKLERQKIEGVKQESEGTYKIDSMYSTVASYLVIAMGFVAFLFWALSNGSCRCNICI

>QBK19965.1 hemagglutinin [Influenza A virus]

METVSLITILLVATVSNADKICIGYQSTNSAETVDTLTENNVPVTHAKELLHTEHNGMLCATSLGQPLIL
DTCTIEGLIYGNPSCDLSLEGREWSYIVERPSAVNGLCYPGNVENLEELRSLFSSARSYQRIQIFPDTIW
NVSYDGTSTACSGSFYRSMRWLTRKNGDYPTQDAQYTNNQGKNILFMWGINHPPTDDTQRNLYTRTDTTT
SVATEEINRIFKPLIGPRPLVNGLMGRIDYYWSVLKPGQTLRIKSDGNLIAPWYGHILSGESHGRIVKTD
LKRGSCTVQCQTEKGGLNTTLPFQNVSKYAFGNCSKYIGIKSLKLAVGLRNVPSRSSRGLFGAIAGFIEG
GWSGLVAGWYGFQHSNDQGVGMAADRDSTQKAIDKITSKVNNIVDKMNKQYEIIDHEFSEVETRLYMINN
KIDDQIQDIWAYNAELLVLLENQKTLDEHDANVNNVYNKVKRALGSNAVEDGKGCFELYHKCNDQCMETI
RNGTYDRRKYQEESKLERQKIEGVKLEFEGTYKILSIYSTVASYLVIAMGFAAFLFWAMSNGSCRCNICI

>QBK19964.1 hemagglutinin [Influenza A virus]

MPTVSLITMLLIATVSNADKICIGYQSTNSAETVDTLTENNVPVTHAKELLHTEHNGMLCASSLGQPIIL
DTCTIEGLIYGNPSCDLSLEGREWSYIVERPSAVNGLCYPGNVQNLEELRSLFSSARSYQKIQIFPDTLW
NVSYYGTSTACSGSFYRNMRWLTRKNGDYPIQDAQYTNNQGKNILFMWGINHPAADTTQRDLYTRTDTTT

SVATEEINRIFKPLIGPRPLVNGLMGRIDYYWSVLKPGQTLPIKSDGNLIAPWYGHILSRESHGTILKTD
LKRGSCTVPCQTEKGGLHTALPLLNVSKHASGKCSKCIGIKCVKLAVGLRNVRCRYSRGVFGGIAGFIER
GWSGLVDGWYGFQHSNDQGVGMAADSDSTQQAVDKITSNVNNMMAKLYKPYEILDHAISEVGTRIDMSNN
MFDDQIQDIWAYNAESLVLLENQKTLDEQDIKPNNGGNKVKRAAGSNAMEDGKGCLELCHKCDDQCMETI
GNGTYNRREYQEESKKERQKMEGVKLESDRTWEILSFYSRVASSLVIAMGSVAFLFGAMYYWSCRCNSSI

>QBK19963.1 hemagglutinin [Influenza A virus]
METVSLITILLVATVSNADKICIGYQSTNSTETVDTLTENNVPVTHAKELLHTEHNGMLCATSLGQPLIL
DTCTIEGLIYGNPSCDLSLEGREWSYIVERPSAVNGLCYPGNVENLEELRSLFSSARSYQRIQIFPDTIW
NVSYDGTSTACSGSFYKSMRWLTRKNGDYPIQDAQYTNNQGKNILFMWGINHPPTDTTQRDLYTRIDTTT
SVATEEINRVFKPLIGPRPLVNGLMGRIDYYWSVLKPGQTLRIKSDGNLIAPWFGHILSGESHGRILKTD
LKRGSCTVQCQTEKGGLHSTLPFQNVSKYAFGNCSKYIGIKSLKLAVGLRNVPSRSSRGLFGAIAGFIEG
GWSGLVAGWYGFQHSNDQGVGMAADRDSTQKAIDKITSKVNNIVDKMNKQYEIIDHEFSEVETRLNMINN
KIDDQIQDIWAYNAELLVLLENQKTLDEHDANVNNLYNKVKRALGSNAVEDGKGCFELYHKCDDQCMETI
RNGTYNRRKYQEESKLERQKIEGVKLESEGTYKILSIYSTVASSLVIAMGFAAFLFWAMSNGSGRCNICI

>QBK19962.1 hemagglutinin [Influenza A virus]
METVSLITILLIATVSNADKICIGYQSTNSTETVDTLTENNVPVTHAKELLHTEHNGMLCATSLGQPLIL
DTCTIEGLIYGNPSCDLSLEGREWSYIVERPSAVNGLCYPGNVQNLEELRSLFSSARSYQKIQIFPDTIW
NVSYYGTSTACSGSFYKSMRWLTRKNGDYPIQDAQYTNNQGKNILFMWGINHPPTDTTQRDLYTRIDTTT
SVATEEINRVFKPLIGPRPLVNGLMGRIDYYWSVLKPGHTLRIKSDGNLIAPWFGHILSRESHGRILTTD
LKRGSCTVQCQTEKGGLHSALPLQNVSKYAFGNFSKYIGIKSLKLAVGLRNVPSRYSRGLFGAIAGFIEG
GWSGLVAGWYGFQHSNDQGVGMAADRDSTQKAIDKITSKVNNIVAKMYKQYEIIDHEFSEVETRINMINN
KFDDQIQDIWAYNAESLVFLENQKTLDEHDANVNNLYNKVKRALGSNAVEDGKGCFELYHKCDDQCMETI
RNGTYNRRKYQAESKLERQKIEGLKLESERTYKILSIYSTVASSRVIAMGSAAFLFGAMSYGSCRCNICI

>QBK19961.1 hemagglutinin [Influenza A virus]
METVSLITILLIATVSNADKICIGYQSTNSAETVDTLTENNVPVTHAKELLHTEHNGMLGATSLGQPLIL
DTCTIEGLIYGNPSCDLSLEGREWSYIVERPSAVNGLCYPGNVQNLEELRSLFSSARSYQRIQIFPDTIW
NVSYYGTSTACSGSFYKSMRWLTRKNGDYPIQDAQYTNNQGKNILFMWGINHPPTDTTQRDLYTRIDTTS
SVATEEINRIFKPLIGPRPLVNGLMGRIDYYWSVLKPGQTLRIKSDGNLIAPWFGHILSGESHGRILKTD
LKRGSCTVQCQTDKGGLHSALPLLNVSKYAFGKCSKYIGIKSLKLAVGLRNVPSRYSRGLFGAIAGFIEG
GWSGLVAGWYGFQHSNDQGVGMAADRDSTQKAVDKITSKVNNIVDKMNKQYEIIDHEFSEVETRINMINN
KIDDQIQDIWAYNAESLVLLENQKTLDEQDANVNNLYNKVKRALGSNAVEDGKGCLELYHKCDDQCMETI

RNGTYNRREYQAESKKERQKIEGLKLEFERTWEILSMYSTVTSSLVIAMGSVAFLFWALYNGSCRCNICI

>QBK19960.1 hemagglutinin [Influenza A virus]
MDTVSLITILLISTVSNADKICIGYQSTNSAETVDTLTENNVPVTHAKELLHTEHNGMLCATSLGQPLIL
DTCTIEGLIYGNPSCDLSLEGREWSYIVERPSAVNGLCYPGNVQNLEELRSLFSSARSYQKIQIFPHTIW
NVSYYGTSTACSGSFYKSMRWLTRKNGDYPIQDAQYTNNQGKNILFMWGINHPPTDTTQIDLYTRIDTTT
SVATEEINRVFKPLIGPRPLVNGLMGRIDYYWSVLKPGQTLRIKSDGNLIAPWFGHIRSGERHGRNEKND
LHRGSCTVQCQTEKDGLNSALSLQNVSKYAFGNCSKYIGIKSVKLAVGLRNVRSRYSRGLFGAIAGFIER
GWSGLVAGWYGFQHSNDQGVGMAADRDSTQKAMDKITSKVNNIVAKMNKQYEIIGLEFSVVETRINMINN
KFDDQIQDIWAYNAESIVLLENQKTLDEHDAKPNNLYNKVKRGACSNAVEDGKGCSELYHKCEKECMETI
RNRTFNRREYQAESKKERQKMEGLKQELERTWEGVSIYSRVTSYRVIAMGSFALLFWAMYNGSCRCNICI

>QBK19959.1 hemagglutinin [Influenza A virus]
METVSLITILLVATVSNADKICIGYQSTNSAETVDTLTENNVPVTHAKELLHTEHNGMLCATSLGQPLIL
DTCTIEGLIYGNPSCDLSLEGREWSYIVERPSAVNGLCYPGNVQNLEELRSLFSSARSYQRIQIFPDTIW
NVSYDGTSTACSGSFYKSMRWLTRKNGDYPIQDAQYTNNQGKNILFMWGINHPPTDTTQRDLYTRIDTTT
SVATEEINRVFKPLIGPRPLVNGLMGRIDYYWSVLKPGQTLRIKSDGNLIAPWFGHILSGESHGRILTTD
LKRGSCTVQCQREKGGLHSALPLQNVSKYAFGKCSKYIGIKSLKLAVGLRNVPSRYSRGLFGAIAGFIER
GWSGLVAGWYGFQHSNDQGVGMAADRDSTQKAIDKITSKVNNIVDKMNKPYEIIDHEFSVVETRINTINN
KFDDQIQDIWAYNAESIVLLENQKTLDEHDANVNNLYNKVKRALGSNAVEDGKGCLELYHKCDDQCMETI
RNRTYNRRKYQAESKLERQKIEGVKQESEGTWKILSIYSRVAWYRVIAMGFAAFLFGAMYNGSCRCNISI

>QBK19958.1 hemagglutinin [Influenza A virus]
METVSLITILLVATVSNADKICIGYQSTNSTETVDTLTENNVPVTHAKELLHTEHNGMLCATSLGQPLIL
DTCTIEGLIYGNPSCDLSLEGREWSYIVERPSAVNGLCYPGNVQNLEELRSLFSSARSYQRIQIFPDTIW
NVSYDGTSTACSGSFYKSMRWLTRKNGDYPIQDAQYTNNQGKNILFMWGINHPPTDTTQRDLYTRIDTTT
SVATEEINRVFKPLIGPRPLVNGLMGRIDYYWSVLKPGQTLRIKSDGNLIAPWFGHILSGESHGRIVEHD
LKRGSCTVQCQTEKGGLHSTLPFQNVSKYAFGNCSKYIGIKSLKLAVGLRNVPSRYSRGLFGAIAGFIEG
GWSGLVAGWYGFQHSNDQGVGMAADRDSTQKAIDKITSKVNNIVAKMNKQYEIIDHEFSEVETRLNMINN
KIDDQIQDIWAYNAELLVLLENQKTLDEHDANVNNLYNKVKRALGSNAVEDGKGCFELYHKCDDQCMETI
RNGTYNRRKYQEESKLERQKIEGVKLESEGTYKILSIYSTVASSLVIAMGFAAFLFWAMSNGSCRCNICI

>QBK19957.1 hemagglutinin [Influenza A virus]
MDTVSLITILLIAPVSNADKICIGYQSTNSAETVDTLTENNVPVTHAVELLHTEHNGMLCATSLGQPLIL

DTCTIEGLIDGNPSCDLSLEGREWSYIVERPSAVNGLCYPGNVQNLEELRSLFSSARSYQRIQIFPHTIW
NVSYDGTSTACSGSFYKSMRWLTRKNGDYPIQDAQYTNNQGKNILFMWGINHPPTDTTQRDLYTRIDTTT
SVATEEINRVFKPLIGPRPLVNGLMGRIDYYWSVLKPGQTLRIKSDGNLIAPWFGHILSGESHGTILTTD
LQRGSCAVQCQTETGGLHSAQSLLNVSKSAFGKCSKYIGIKSVKLAVGLRNVRSRYSRGLFGAIAGFIEG
GWSGLVAGWYGFQHSNDQGVGMAADRDSTQKAIDKITSNVNNIVAKMYKPYEIIGLEFSVVGTRINTSNN
KFDDQIQDIWAYNAEAIVFLENQKTLDEQDAKPGAEGNKVKRAAGSNAVEDGKGCSELYHKCDDQCMETI
GNGTFNRREYQAESKLERQKIEGLKQEFDRTCEIVSMSSRVASSRVIAMGSAAFLFWAMYNGSCRCNICI

>QBK19956.1 hemagglutinin [Influenza A virus]
METASLITILLVATVSNADKICIGYQSTNSTETVDTLTENNVPVTHAKELLHTEHNGMLCATSLGQPIIL
DTCTIEGLIYGNPSCDLSLEGREWSYIVERPSAVNGLCYPGNVENLEELRSLFSSARSYQRIQIFPDTIW
NVSYDGTSTACSGSFYRNMRWLTRKNGDYPIQDAQYTNNQGKNILFMWGINHPPADTTQRDLYTRTDTTT
SVATEEINRIFKPLIGPRPLVNGLMGRIDYYWSVLKPGQTLRIKSDGNLIAPWYGHILSGESHGRNQKHD
LKRGSCTVQCQTEKGGLNTALPFQNVSKYAFGKCSKYIGIKSLKLAVGLRNVPSRYSRGLFGAIAGFIEG
GWSGLVAGWYGFQHSNDQGVGMAADRDSTQKAVDKITSKVNNIVDKMYKQYEIIDHEFSEVETRINMINN
KIDDQIQDIWAYNAELLVLLENQKTLDEHDANVNNLYNKVKRALGSNAVEDGKGCFELYHKCDDQCMETI
RNGTYNRRKYQEESKLERQKIEGVKLESEGTYKILSIYSTVASSLVIAMGSVAFLFWAMSNGSCRCNMCI

>QBK19955.1 hemagglutinin [Influenza A virus]
METVLLITILLIATVSNADKICIGYQSTNSTETVDTLTENNVPVTHAKELLHTEHNGMLCATSLGQPLIL
DTCTIEGLIYGNPSCDLSLEGREWSYIVERPSAVNGLCYPGNVQNLEELRSLFSSARSYQRIQIFPDTIW
NVSYDGTSTACSGSFYKSMRWLTRKNGDYPIQDAQYTNNQGKNILFMWGINHPPTDTTQRDLYTRIDTTT
SVATEEINRVFKPLIGPRPLVNGLMGRIDYYWSVLKPGQTLRIKSDGNLIAPWFGHILSGESHGRILKTD
LKRGSCTVQCQTEKGGLNSTLPFQNVSKYAFGNCSKYIGIKSLKLAVGLRNVPSRYSRGLFGAIAGFIEG
GWSGLVDGWYGFQHSNDQGVGMAADSDSTQKAIDKITSKVNNIVDKMNKQYEIIDHEFSEVETRINMINN
KIDDQIQDIWAYNAELLVLLENQKTLDEHDANVNNLYNKVKRALGSNAVEDGKGCFELYHKCDDQCMETI
RNGTYNRRKYQEESKLERQKIEGVKLESEGTYKILSIYSTVASSLVIAMGSAAFLFWAYSNGSCRCNICI

>QBK19954.1 hemagglutinin [Influenza A virus]
METVSLITILLIATVSNADKICIGYQSTNSTETVDTLTENNVPVTHAKELLHTEHNGMLCATSLGQPLIL
DTCTIEGLIYGNPSCDLSLEGREWSYIVERPSAVNGLCYPGNVQNLEELRSLFSSARSYQRIQIFPDTIW
NVSYYGTSTACSGSFYKIMRWLTRKNGDYPIQDAQYTNNQGKNILFMWGINHPPTDTTQRDLYTRIDTTT
SVATEEINRIFKPLIGPRPLVNGLMGRIDYYWSVLKPGQTLRIKSDGNLIAPWFGHILSGESHGRILTTD
LKRGSCTVQCQTENGGLHSALSLQNVSKYAFGNCSKYIGIKSVKLAVGLRNVRCRYSRGLFGAIAGFIER

GWSGLVAGWYGFQHSNDQGVGMAADRDSTQKAVDKITSKVNNIVAKMYKQYEIIDHEFSVVETRINMINN
KFDDQIQDIWAYNAESLVLLENQKTLDEQDANVNNLYNKVKRAAGSNAVEDGKGCLELYHKCDDQCMETI
RNGTYNRRKYQAESKLEREKIEGVKQESEGTYKILSIYSTVASSLVIAMGSAAFLFWAMYNGSCRCNICI

>QBK19953.1 hemagglutinin [Influenza A virus]
METVSLITILLIATVSNADKICIGYQSTNSTETVDTLTENNVPVTHAKELLHTEHNGMLCATSLGQPLIL
DTCTIEGLIYGNPSCDLSLEGREWSYIVERPSAVNGLCYPGNVENLEELRSLFSSARSYQRIQIFPDTIW
NVSYDGTSTACSGSFYKSMRWLTRKNGDYPIQDAQYTNNQGKNILFMWGINHPPTDTTQRDLYTRIDTTT
SVATEEINRVFKPLIGPRPLVNGLMGRIDYYWSVLKPGQTLRIKSDGNLIAPWFGHILSGESHGRILKTD
LKRGSCTVQCQTEKGGLNSTLPFQNVSKYAFGNCSKYIGIKSLKLAVGLRNVPSRYSRGLFGAIAGFIEG
GWSGLVAGWYGFQHSNDQGVGMAADRDSTQKAIDKITSKVNNIVDKMNKQYEIIDHEFSEVETRLNMINN
KIDDQIQDIWAYNAELLVLLENQKTLDEHDANVNNLYNKVKRALGSNAVEDGKGCFELYHKCDDQCMETI
RNGTYNRRKYQEESKLERQKIEGVKLESEGTYKILSIYSTVASSLVIAMGFAAFLFWAMSNGSGRCNICI

>QBK19952.1 hemagglutinin [Influenza A virus]
METVSLITILLIATVSNADKICIGYQSTNSTETVDTLTENNVPVTHAKELLHTEHNGMLCATSLGQPLIL
DTCTIEGLIYGNPSCDLSLEGREWSYIVERPSAVNGLCYPGNVQNLEELRSLFSSARSYQRIQIFPDTIW
NVSYYGTSTACSGSFYKSMRWLTRKNGDYPIQDAQYTNNQGKNILFMWGINHPPTDTTQRDLYTRIDTTT
SVATEEINRIFKPLIGPRPLVNGLMGRIDYYWSVLKPGQTLRIKSDGNLIAPWFGHILSGESHGRILTTD
LKRGSCTVQCQTEKGGLNSALPLQNVSKYAFGNCSKYIGIKSLKLAVGLRNVPSRYSRGLFGAIAGFIEG
GWSGLVAGWYGFQHSNDQGVGMAADRDSTQKAVDKITSKVNNIVDKMNKQYEIIDHEFSEVETRINMINN
KIDDQIQDIWAYNAESLVLLENQKTLDEHDANVNNLYNKVKRALGSNAVEDGKGCFELYHKCDDQCMETI
RNGTYNRRKYQEESKLERQKIEGVKLESEGTYKILSIYSTVASSLVIAMGSAAFLFWAMSNGSDRCNICI

>QBK19951.1 hemagglutinin [Influenza A virus]
MQTVSLVSMLLIATVSNADKICIGYQSTNSAETVDTVTQNNVPVTHAKELLHTEHNGMLCATSLGQPLIL
DTCTIEGLIYGNPSCDLSLEGREWSYIVERPSPVNGLCYPGNVQNLEELRSLFSSARSYQKIQIFPDTIW
NVSYNGTSTACSGSFYKIMRWLTRKNGDYPIQDAQYTNNQGKNILFMWGINHPPTDTTQIDLYTRIDTTT
SVATEEINRVFTPLIGPRPLVNGLMGRIDYYWSVLKPGQTLRIKSDGNLIAPWFGHILSGESHGRILTTD
LSRGSCTVQCQTEKGRLHSTLPFQNVSTYAFGNFSKYIGIKSLKRAVGLRNVPCRYSRGVFGAIAGFIEG
GWSGLVGRLYGFQHSNDQGVGMAADRDSTQKAIDKITSKVNNIVAKMYKQYEIIDLEFSVVETRINMINN
KFDDQIQDIWAYNAELLVFIENQKTLDEHDAKVNNLYNKVKRALGSNAVEDGKGCFELYHKCDDQCMETI
RNGTYNRRKYQAESKLERQKIEGLKLESERTCKVLSIYSRVTSYRVIAMGSVAFLFWALSNGSCRCNICI

>QBK19950.1 hemagglutinin [Influenza A virus]

METVSLITILLIATVSNADKICIGYQSTNSTETVDTLTENNVPVTHAKELLHTEHNGMLCATSLGQPIIL
DTCTIEGLIYGNPSCDLSLEGREWSYIVERPSAVNGLCYPGNVQNLEELRSLFSSARSYQRIQIFPDTIW
NVSYYGTSTACSGSFYKNMRWLTRKNGDYPIQDAQYTNNQGKNILFMWGINHPPTDTTQRDLYTRTDTTT
SVATEEINRIFKPLIGPRPLVNGLMGRIDYYWSVLKPGQTLRIKSDGNLIAPWYGHILSGESHGRILKTD
LKRGSCTVQCQTEKGGLNTALSLQNVSKYAFGKCSKYIGIKSVKLAVGLRNVRCRYSRGLFGAIAGFIER
GWSGLVAGWYGFQHSNDQGVGMAADRDSTQKAVDKITSKVNNIVAKLYKQYEIIDHEISVVGTRINTSNN
KFDDQIQDIWAYNAESLVLLENQKTLDEQDAKPNNGGNKVKRGACSNAVEDGKGCLELYHKCDDQCMETI
RNGTYNRRKYQEESKKEREKMEGLKLESERTYKILSIYSTVASSLVIAMGSAAFLFWALYNGSDRCEHCI

>QBK19949.1 hemagglutinin [Influenza A virus]

MDTVSLITILLIATVSNADKICIGYQSTNSAETVDTLTENNVPVTHAKELLHTEHNGMLCATSLGQPLIL
DTCTIEGLIYGNPSCDLSLEGREWSYIVERPSAVNGLCYPGNVQNLEELRSLFSSARSYQRIQIFPHTIW
NVSYYGTSTACSGSFYKSMRWLTRKNGDYPIQDAQYTNNQGKNILFMWGINHPPTDTTQRDLYTRIDTTT
SVATEEINRVFKPLIGPRPLVNGLMGRIDYYWSVLKPGQTLRIKSDGNLIAPWFGHILSGESHGRILKTD
LKRGSCTVQCQTEKGGLNSALPLLNVSKYAFGNCSKYIGIKSVKLAVGLRNVPSRYSRGLFGAIAGFIEE
GWSGLVAGWYGFQHSNDQGVGMAADRDSTQKAIDKITSKVNNIVAKMNKPYEIIDLEFSVVETRINTSNN
KFDDQIQDIWAYNAESLVLLENQKTLDEHDANVNNLYNKVKRAAGSNAVEDGKGCLELYHKCDDQCMETI
RNGTYNRRKYQEESKLERQKIEGLKLESEGTCKIVSIYSTVASYRVIAMGSAAFLFWAMYNGSCRCEHCI

>QBK19948.1 hemagglutinin [Influenza A virus]

METVSLITILLVATVSNADKICIGYQSTNSTETVDTLTENNVPVTHAKELLHTEHNGMLCATSLGQPIIL
DTCTIEGLIYGNPSCDLSLEGREWSYIVERPSAVNGLCYPGNVENLEELRSLFSSARSYQRIQIFPDTIW
NVSYDGTSTACSGSFYRNMRWLTRKNGDYPIQDAQYTNNQGKNILFMWGINHPPADTTQRDLYTRTDTTT
SVATEEINRIFKPLIGPRPLVNGLMGRIDYYWSVLKPGQTLRIKSDGNLIAPWYGHILSGESHGRILKND
LKRGSCTVQCQTEKGGLNTTLPFQNVSKYAFGNCSKYIGIKSLKLAVGLRNVPCRYSRGVFGAIAGFIEG
GWSGLVAGWYGFQHSNDQGVGMAADRDSTQKAVDKITSKVNNIVAKMYKQYEIIDHEFSVVETRINMINN
KIDDQIQDIWAYNAELLVLLENQKTLDEHDANVNNLYNKVKRALGSNAVEDGKGCFELYHKCDDQCMETI
GNGTYNRRKYQEESKLERQKIEGVKLESEGTYKILSIYSRVASSLVIAMGSAAFLFWAMYNGSGRCEHCI

>QBK19947.1 hemagglutinin [Influenza A virus]

METVSLISMLLISPLSNADKICIGYQSTNSAETVDTLTENNVPVTHAKELLHTEHNGMLCATSLGQPLIL
DTCTIEGLIYGNPSCDLSLEGREWSYIVERPSAVNGLCYPGNVQNLEELRSLFSSARSYQRIQIFPHTIW
NVSYYGTSTACSGSFYKSMRWLTRKNGDYPIQDAQYTNNQGKNILFMWGINHPPTDTTQRDLYTRIDTTT

SVATEEINRVFKPLIGPRPLVNGLMGRIDYYWSVLKPGQTLRIKSDGNLIALWFGHILSGESHGRILKTD
LKRGSCTVQCQTEKGGLHSAQPLLNVSKYAFGNCSKYIGIKSVKLAVGLRNVPSRYSRGLFGAIAGFIEG
GWSGLVGGWYGFQHSNDQGVGMAADRDSTQKAMDKITSNVNNIVAKMYKPYEIIDLEFSVVETRINTINH
KFDDQIQDIWAYNAEAIVLLENQKTLEEHDANVNNEGNKVKRAAGSNAVEDGKGCLELYHKPDDQCMETI
RNGTYNRREYQAESKLERQKMEGVNLEFDRTWEIVSIYSRVTWYRVIAMGSVALRFGALYNGSCRCEQCI

>QBK19946.1 hemagglutinin [Influenza A virus]
METVSLITILLVATVSNADKICIGYQSTNSTETVDTLTENNVPVTHAKELLHTEHNGMLCATSLGQPIIL
DTCTIEGLIYGNPSCDLSLEGREWSYIVERPSAVNGLCYPGNVENLEELRSLFSSARSYQRIQIFPDTIW
NVSYYGTSTACSGSFYRNMRWLTRKNGDYPIQDAQYTNNQGKNILFMWGINHPPADTTQRDLYTRTDTTT
SVATEEINRIFKPLIGPRPLVNGLMGRIDYYWSVLKPGQTLRIKSDGNLIAPWYGHILSRESHGRILKHD
LKRGGCTVPCQTEKGGLHTALSLQNVSKYAFGRCSKYIGIKSLKLAVGLRNVRSRYSRGLFGAIAGFIEE
GRSGLVAGWYGFQHSNDQGVGMAADRDSTQKAVDKITSKVNNMVAKLYKQYEIIDHEISEVGTRINTSSN
KFDDQIQDIWAYNAEAIVLLENQKTLDEQDANVSNGGNKVKRAAGSNAVEDGNGCSELCHKCDDQCMETI
RNGTYNRRKYQAESKKERQKMEGAKLESDGTYKILSIYSRVASSLVIAMGSAAFLIGAMYYGSCRCNICI

>QBK19945.1 hemagglutinin [Influenza A virus]
METASLITILLVATVSNADKICIGYQSTNSTETVDTLTENNVPVTHAKELLHTEHNGMLCATSLGQPIIL
DTCTIEGLIYGNPSCDLSLEGREWSYIVERPSAVNGLCYPGNVENLEELRSLFSSARSYQRIQIFPDTIW
NVSYYGTSTACSGSFYRNMRWLTRKNGDYPIQDAQYTNNQGKNILFMWGINHPPADTTQRDLYTRTDTTT
SVATEEINRIFKPLIGPRPLVNGLMGRIDYYWSVLKPGQTLRIKSDGNLIAPWYGHILSGESHGRILKHD
LKRGSCTVQCQTEKGGLNTTLPFQNVSKYAFGNCSKYIGIKSLKLAVGLRNVPSRYSRGLFGAIAGFIEG
GWSGLVAGWYGFQHSNDQGVGMAADRDSTQKAVDKITSKVNNIVAKMNKQYEIIDHEFSEVETRINMINN
KIDDQIQDIWAYNAELLVLLENQKTLDEHDANVNNLYNKVKRALGSNAVEDGKGCFELYHKCDDQCMETI
RNGTYNRRKYQEESKLERQKIEGVKLESEGTYKILSIYSRVASSLVIAMGFAAFLFWAMYNGSCRCNICI

>QBK19944.1 hemagglutinin [Influenza A virus]
MEAVSLITMLLVVTVSNADGICIGYQSTNSAETVDTLTENNVPVTHAKELLHTEHNGMLCATSLGQPLVL
DTCTIEGLIYGNPSCDLSLEGREWSYIVERPSAVNGLCYPGNVQNLEELRSLFSSARSYQRIQIFPDTIW
NVSYYGTSTACSGSFYKSMRWLTRKNGDYPTQDAQYTNNQGKNILFMWGINHPPTDTTQRDLYTRTDTTT
SVATEEINRIFKPLIGPRPLVNGLMGRIDYYWSVLKPGQTLRIKSDGNLIAPWYGHILSGESHGRILTTD
FERGSCTVQCQTEKGGLNTAQPLQNVSKYAFGKCSKYIGIKSLKLAVGLRNVRSRYSRGLFGAIAGFIEG
GWSGLVAGWYGFQHSNDQGVGMAADKDSTQKAIDKITSKVNNIVAKMYKPYEIIDHEFSEVETRINMINN
KFDDQIQDIWAYNAESIVFLENQKTLDEHDANVNNLYNKVKRALGSNAVEDGKGCLELYHKCDDQCMETI

RNGTYNRRKYQAESKLERQKIEGLNLESEGTWEILSICSRVAWYRVIAMGSAALRIGAMYNGSCRCNICI

>QBK19943.1 hemagglutinin [Influenza A virus]
METVSLITILLVATLSNADKICIGYQSTNSAETVDTLTENNVPVTHAKELLHTEHNGMLCATSLGQPLIL
DTCTIEGLIYGNPSCDLSLEGREWSYIVERPSAVNGLCYPGNVQNLEELRSLFSSARSYQRIQIFPHTIW
NVSYYGTSTACSGSFYKSMRWLTRKNGDYPIQDAQYTNNQGKNILFMWGINHPPTDTTQRDLYTRIDTTS
SVATEEINRVFKPLIGPRPLVNGLMGRIDYYWSVLKPGQTLRIKSDGNLIAPWFGHILSGESHGRILTTD
LKRGSCTVQCQTEKGGLHSTLPFQNVSKYAFGKCSKYIGIKSLKLAVGLRNVRSRYSRGLFGAIAGFIEG
GWSGLVAGWYGFQHSNDQGVGMAADRDSTQKAIDKITSKVNNIVAKMYKQYEIIDHEFSVVETRINMINN
KFDDQIQDIWAYNAESIVLLENQKTLDEHDANVNNLYNKVKRALGSNAVEDGKGCLELYHKCDDQCMETI
RNRTYNRRKYQAESKLERQKIEGVKQESDRTRKILSMYSTVTSYRVIAMGSAAFLFWAMSNGSCRCNICI

>QBK19942.1 hemagglutinin [Influenza A virus]
METVSLITILLIATVSNADKICIGYQSTNSTETVDTLTENNVPVTHAKELLHTEHNGMLCATSLGQPLIL
DTCTIEGLIYGNPSCDLSLEGREWSYIVERPSAVNGLCYPGNVQNLEELRSLFSSARSYQRIQIFPDTIW
NVSYDGTSTACSGSFYKSMRWLTRKNGDYPIQDAQYTNNQGKNILFMWGINHPPTDTTQRDLYTRIDTTT
SVATEEINRVFKPLIGPRPLVNGLMGRIDYYWSVLKPGQTLRIKSDGNLIAPWFGHILSGESHGRILKTD
LKRGSCTVQCQTEKGGLNSTLPFQNVSKYAFGNCSKYIGIKSLKLAVGLRNVPSRSSRGLFGAIAGFIEG
GWSGLVAGWYGFQHSNDQGVGMAADRDSTQKAIDKITSKVNNIVDKMNKQYEIIDHEFSEVETRLNMINN
KIDDQIQDIWAYNAELLVLLENQKTLDEHDANVNNLYNKVKRALGSNAVEDGKGCFELYHKCDDQCMETI
RNGTYNRRKYQEESKLERQKIEGVKLESEGTYKILSIYSTVASSLVIAMGFAAFLFWAMYNGSCRCEHCI

>QBK19941.1 hemagglutinin [Influenza A virus]
MDTVSLITILLIATVSNADKICIGYQSTNSTETVDTLTENNVPVTHAKELLHTEHNGMLCATSLGQPLIL
DTCTIEGLIYGNPSCDLSLEGREWSYIVERPSAVNGLCYPGNVQNLEELRSLFSSARSYQRIQIFPDTIW
NVSYYGTSTACSGSFYKSMRWLTRKNGDYPIQDAQYTNNQGKNILFMWGINHPPTDTTQRDLYTRIDTTT
SVATEEINRVFKPLIGPRPLVNGLMGRIDYYWSVLKPGQSLRIKSDGNLIATWLGHIRSGERQGRIEEHD
LQRGSCTVQCQTENGGLNSALPLLNVSKYAFGNCSKYIGIKSLKLAVGLRNVPSRYSRGLFGAIAGFIEG
GWSGLVAGWYGFQHSNDQGVGMAADRDSTQKAIDKITSKVNNIVDKLYKPYEIIDHEFSVVETRINTINN
KFDDQIQDIWAYNAESLVLLENQKTLDEHDANVNNLYNKVKRALGSNAVEDGKGCLELYHKCDDQCMETI
RNGTYNRRKYQAESKLERQKMEGVKLESERTCKILSIYSTVASSLVIAMGSAAFLFGAMSNGSSRCRACI

>QBK19940.1 hemagglutinin [Influenza A virus]
METVSLITILLVATVSNADKICIGYQSTNSTETVDTLTENNVPVTHAKELLHTEHNGMLCATSLGQPLIL

DTCTIEGLIYGNPSCDLSLEGREWSYIVERPSAVNGLCYPGNVENLEELRSLFSSARSYQRIQIFPDTIW
NVSYDGTSTACSGSFYKSMRWLTRKNGDYPIQDAQYTNNQGKNILFMWGINHPPTDTTQRDLYTRIDTTT
SVATEEINRVFKPLIGPRPLVNGLMGRIDYYWSVLKPGQTLRIKSDGNLIAPWFGHILSGESHGRILKTD
LKRGSCTVHCQTEKGGLNSTLPFQNVSKYAFGNCSKYIGIKSLKLAVGLRNVPSRYSRGLFGAIAGFIEG
GWSGLVAGWYGFQHSNDQGVGMAADRDSTQKAIDKITSKVNNIVDKMNKQYEIIDHEFSEVETRLNMINN
KIDDQIQDIWAYNAELIVLLENQKTLDEHDANVNNLYNKVKRALGSNAVEDGKGCFELYHKCDDQCMETI
RNGTYNRRKYQEESKLERQKIEGVKLESEGTYKILTIYSTVASSLVIAMGFAAFLFWAMSNGSGRCEHCI

>QBK19939.1 hemagglutinin [Influenza A virus]
METVSLITILLIATVSNADKICIGYQSTNSTETVDTLTENNVPVTHAKELLHTEHNGMLCATSLGQPLIL
DTCTIEGLIYGNPSCDLSLEGREWSYIVERPSAVNGLCYPGNVQNLEELRSLFSSARSYQRIQIFPDTIW
NVSYYGTSTACSGSFYKSMRWLTRKNGDYPIQDAQYTNNQGKNILFMWGINHPPTDTTQRDLYTRIDTTS
SVATEEINRVFKPLIGPRPLVNGLMGRIDYYWSVLKPGQTLRIKSDGNLIAPWFGHILSGESHGRILKTD
LKRGSCTVQCQTEKGGLNSTLPFQNVSKYAFGNCSKYIGIKCVKLAVGLRNVPSRYSRGLFGAIAGFIEG
GWSGLVAGWYGFQHSNDQGVGMAADRDSTQKAIDKITSKVNNIVDKMNKQYEIIDHEFSEVETRINMINN
KIDDQIQDIWAYNAESLVLLENQKTLDEHDANVNNLYNKVKRALGSNAVEDGKGCFELYHKCDDQCMETI
RNGTYNRRKYQEESKLERQKIEGVKLESEGTYKILSIYSTVASSLVIAMGSAAFLFGAMSNGSCRCEHCI

>QBK19938.1 hemagglutinin [Influenza A virus]
MDTVSLITMLLIATVSNADKICIGYQSTNSTETVDTLTENNVPVTHAKELLHTEHNGMLCATSLGQPLIL
DTCTIEGLIYGNPSCDLSLEGREWSYIVERPSAVNGLCYPGNVQNLEELRSLFSSARSYQKIQIFPHTIW
NVSYYGTSTACSGSFYKIMRWLTRKNGDYPIQDAQYTNNQGKNILFMWGINHPPTDTTQRDLYTIIDTTT
SVATEEINRVFKPLIGPRPLVNGLMGRIDYYWSVLKPGQTLPIKSDGNLIAPWLGHTLSGESHGRIVKHD
LHRGSCTVPCQTEKGRLHSALPFLNVSKHAFGKCSKYIGIKSLKLAVGLRNVPCRYSRGLFGAIAGFIEG
GWSGLVAGWYGFQHSNDQGVGMAADRDSTQKAMDKITSKVNNIVAKMYKPYEIIGHEFSEVETRINMINN
KFDDQIQDIWAYNAESLVFIENQKTLDEHDANVNNLYNKVKRAAGSNAVEDGKGCSELYHKCDDQCMETI
RNGTFNRRKYQAESKLERQKIEGLKLELDGTWEVVSIYSRVASYRVIAMGSAAFLIWALYNWSGRSEQYI

>QBK19937.1 hemagglutinin [Influenza A virus]
MDTVSLITMLLISTLSNADKICIGYQSTNSAETVDTLTENNVPVTHAKELLHTEHNGMLCATSLGQPLIL
DTCTIEGLIYGNPSCDLSLEGREWSYIVERPSAVNGLCYPGNVQNLEELRSLFSSARSYQRIQIFPHTIW
NVSYYGTSTACSGSFYKSMRWLTRKNGDYPIQDAQYTNNQGKNILFMWGINHPPTDTTQRDLYTRIDTTT
SVATEEINRVFKPLIGPRPLVNGLMGRIDYYWSVLKPGQTLRIKSDGNLIAPWFGHILSRESHGTILNTD
LKRGSCTVQCQTEKGGLHSAQSLLNVSKYAFGNCSKYIGIKSVKLAVGLRNVRSRYSRGLFGAIAGFIER

GWSGLVAGWYGFQHSNDQGVGMAADRDSTQKAMDKITSKVNNIVAKMNKQYEIIDHEFSVVETRINTINN
KFDDQIQDIWAYNAESLVLLENQKTLDEQDANVNNLYNKVKRAAGSNAVEDGKGCLELYHKCDDQCMETI
RNGTYNRREYQAESKKERQKMEGLKLEFEGTSKILSMNSRVTSYRVIAMGSVAFLFWAMYNGSCRCEHCI

>QBK19936.1 hemagglutinin [Influenza A virus]
METVSLITILLVATVSNADKICIGYQSTNSTETVDTLTENNVPVTHAKELLHTEHNGMLCATSLGQPLIL
DTCTIEGLIYGNPSCDLSLEGREWSYIVERPSAVNGLCYPGNVENLEELRSLFSSARSYQRIQIFPDTIW
NVSYDGTSTACSGSFYKSMRWLTRKNGDYPIQDAQYTNNQGKNILFMWGINHPPTDTTQRDLYTRIDTTT
SVATEEINRVFKPLIGPRPLVNGLMGRIDYYWSVLKPGQTLRIKSDGNLIAPWFGHILSGESHGRILKTD
LKRGSCTVQCQTEKGGLNSTLPFQNVSKYAFGNCSKYIGIKSLKLAVGLRNVPSRSSRGLFGAIAGFIEG
GWSGLVAGWYGFQHSNDQGVGMAADRDSTQKAIDKITSKVNNIVDKMNKQYEIIDHEFSEVETRLNMINN
KIDDQIQDIWAYNAELLVLLENQKTLDEHDANVNNLYNKVKRALGSNAVEDGKGCFELYHKCDDQCMETI
RNGTYNRRKYQEESKLERQKIEGVKLESEGTYKILTIYSTVASSLVIAMGFAAFLFWAMSNGSCRCEHCI

>QBK19935.1 hemagglutinin [Influenza A virus]
METVSLITILLVATVSNADKICIGYQSTNSTETVDTLTENNVPVTHAKELLHTEHNGMLCATSLGQPLIL
DTCTIEGLIYGNPSCDLSLEGREWSYIVERPSAVNGLCYPGNVQNLEELRSLFSSARSYQRIQIFPDTIW
NVSYYGTSTACSGSFYKSMRWLTRKNGDYPIQDAQYTNNQGKNILFMWGINHPPTDTTQRDLYTRIDTTT
SVATEEINRVFKPLIGPRPLVNGLMGRIDYYWSVLKPGQTLRIKSDGNLIAPWCGHIVSGESHGRIVKPD
LHRGSCTVPCQTEKGGLHSTLPLLNVSKYAFGNCSKYIGIKSLKLAVGLRNVRSRYSRGLFGAIAGFIEG
GWSGLVGGWYGFQHSNDQGVGMAADRDSTQKAMDKITSKVNNIVAKMNKQYEIIDHEFSEVETRINMINN
KIDDQIQDIWAYNAESIVFLENQKTLDEHDANVNNLYNKVKRALGSNAVEDGKGCLELYHKCDDQCMETI
RNGTFNRRKYQAESKLERQKIEGLNLESEGTWKILSINSRVASSLVIAMGSAAFLFWAMYNGSCRCEHCI

>QBK19934.1 hemagglutinin [Influenza A virus]
METVSLITMLLVSTVSNADKICIGYQSTNSAETVDTLTENNVPVTHAKELLHTQHNGMLGATSLGQPLIL
DTCTIEGLIYGNPSCDLSLEGREWSYIVERPSAVNGLCYPGNVQNLEELRSLFSSARSYQRIQIFPHTLW
NVSYYGTSTACSGSFYKSMRWLTRKNGDYPIQDAQYTNNQGKNILFMWGINHPPTDTTQRDLYTRIDTTS
SVATEEINRVFKPLIGPRPLVNGLMGRIDYYWSVLKPGHTLRIKSDGNLIAPWFGHILSGESHGRILTTD
LKWGSCTVQCQTEKGGLHSAQSLQNVSKHAFGKCSKYIGIKSLKVAVRLRNVPSRYSRGLFGAIAGFIER
GWSGLVGRWYGFQHSNDQGVGMAADRDSTQKAMDKTPSNVNNIVAKLYKPYEVIGLEFSVGGTRIIPSNH
KSADQIQDIWAYNAESIVSLENQKTLDEHDANVNNLYNKVKRAACSNAVEDGNTCLELYHKCDDQCMETI
RNGTYNRRKYQAESKLERQKMEGHNQESDRTNEIVSICSTVTSSRVIAMGSAAFLFWAMSNGSCRCNICI

>QBK19933.1 hemagglutinin [Influenza A virus]

MDTVSLITMLLVATVSNADKICIGYQSTNSTETVDTLTENNVPVTHAKELLHTEHNGMLCATSLGQPLIL

DTCTIEGLIYGNPSCDLSLEGREWSYIVERPSAVNGLCYPGNVQNLEELRSLFSSARSYQRIQIFPDTIW

NVSYYGTSTACSGSFYKSMRWLTRKNGDYPIQDAQYTNNQGKNILFMWGINHPPTDTTQRDLYTRIDTTT

SVATEEINRVFKPLIGPRPLVNGLMGRIDYYWSVLKPGQTLRIKSDGNLIASWFGHFLSGESHGRILKTD

LNGGSCTVQCQTEKGGLNSAQPLQNVSKYAFGKCSKYIGIKSVKLAVGLRNVRSRYSRGLFGAIAGFIEG

GWSGLVAGWYGFQHSNDQGVGMAADRDSTQKAMDKITSKVNNIVAKMYKPYEIIDLEFSVVETRINTINH

KFDDQIQDIWAYNAESLVLLENQKTLDEQDANVNNLYNKVKRAAGSNAVEDGKGCLELYHKCDDQCMETI

GNGTYNRREYQEESKLERQKMEGLKLESDGTREILSLCSRVAWYRVIAMGSAALRIWALYNGSCRCNKCI

>QBK19932.1 hemagglutinin [Influenza A virus]

MDTVSLISMLLVSTVSNADKICIGYQSTNSAETVDTLTQNNVPVTHAKELLHTQHNGMLCATSLGQPLIL

DTCTIEGLIYGNPSCDLSLEGREWSYIVERPSAVNGLCYPGNVQNLEELRSLFSSARSYQKIQIFPDTIW

NVSYYGTSTACSGSFYKSMRWLTRKNGDYPIQDAQYTNNQGKNILFMWGINHPPTDTTQRDLYTRIDTTT

SVATEEINRVFKPLIGPRPLVNGLMGRIDYYWSVLKPGQTLRIKSDGNLIAPWFGHILSGESHGRILKTD

LNRGSCTVQCQTEKGGLNSTLPFQNVSKYAFGNCSKYIGIKSLKLAVGLRNVPCRYSRGLFGAIAGFIEG

GWSGLVAGWYGFQHSNDQGVGMAADRDSTQKAIDKITSKVNNIVDKMNKQYEIIDHEFSEVETRINMINN

KIDDQIQDIWAYNAESIVLLENQKTLDEHDANVNNLYNKVKRALGSNAVEDGKGCLELYHKCDDQCMETI

RNGTYNRRKYQEESKLERQKIEGVKLELEGTWKILSIYSRVASYLVIAMGFVALLFWAMYNGSCRCNICI

>QBK19931.1 hemagglutinin [Influenza A virus]

MDTVSLITILLVATVSNADKICIGYQSTNSTETVDTLTENNVPVTHAKELLHTEHNGMLCATSLGQPLIL

DTCTIEGLIYGNPSCDLSLEGREWSYIVERPSAVNGLCYPGNVENLEELRSLFSSARSYQRIQIFPDTIW

NVSYDGTSTACSGSFYKSMRWLTRKNGDYPIQDAQYTNNQGKNILFMWGINHPPTDTTQRDLYTRIDTTT

SVATEEINRVFKPLIGPRPLVNGLMGRIDYYWSVLKPGQTLRIKSDGNLIVPWVGHILSGESHGRILKTD

LKRGSCTVQCQTEKGGLNSTLPFQNVSKYAFGNCSKYIGIKSLKLAVGLRNVPSRSSRGLFGAIAGFIEG

GWSGLVAGWYGFQHSNDQGVGMAADRDSTQKAVDKITSKVNNIVDKMNKQYEIIDHEFSEVETRLNMINN

KIDDQIQDIWAYNAELLVLLENQKTLDEHDANVNNLYNKVKRALGSNAVEDGKGCFELYHKCDDQCMETI

RNGTYNRRKYQEESKLERQKIEGVKLESEGTYKILSIYSTVASSLVIAMGFAAFLFWAMSNGSCRCNICI

>QBK19930.1 hemagglutinin [Influenza A virus]

MDTVSLITILLVSTLSNADKICIGYQSTNSTETVDTLTENNVPVTHAKELLHTEHNGMLCATSLGQPLIL

DTCTIEGLIYGNPSCDLSLEGREWSYIVERPSAVNGLCYPGNVQNLEELRSLFSSARSYQKIQIFPHTIW

NVSYYGTSTACSGSFYKSMRWLTRKNGDYPIQDAQYTNNQGKNILFMWGINHPPTDTTQRDLYTRIDTTT

SVATEEINRVFKPLIGPRPLVNGLMGRIDYYWSELKPGQTLRIKSDGNLIAPWFGHILSGESHGRIVKHD
LKRGSCTVQCQREKGGLNSTLPLQNVSKYAFGKCSKYIGIKSLKLAVGLRNVRSRYSRGLFGAIAGFIEG
GWSGLVGGWYGFQHSNDQGVGMAADRDSTQKAMDKITSKVNNIVDKMNKPYEIIGHEFSEVETRINTINN
KFDDQIQDIWAYNAESIVLLENQKTLDEHDANVNNLYNKVKRALGSNAVEDGKGCLELYHKCDDQCMETI
RNGTFNRREYQAESKLERQKIEGVKQESDRTREILSIYSRVASYLAIAMGSAALRFGALYNGSCRCNICI

>QBK19929.1 hemagglutinin [Influenza A virus]
MDTVSLITMLLVSTLSNADKICIGYRSTNSAETVDTLTQNNVPVTHAKELLHTEHNGMLCATSLGQPLIL
DTCTIEGLIYGNPSCDLSLEGREWSYIVERPSAVNGLCYPGNVQNLEELRSLFSSARSYQRIQIFPDTIW
NVSYDGTSTACSGSFYKSMRWLTRKNGDYPIQDAQYTNNQGKNILFMWGINHPPTDTTQRDLYTRIDTTT
SVATEEINRVFKPLIGPRPLVNGLMGRIDYYWSVLKPGQTLRIKSDGNLIAPWFGHILSGESHGRILTTD
LKRGSCTEQCQTEKGGLNSALPLQNVSKYAFGNCSKYMGIKSLKLAVGLKNVPSRYSRGLFGAIAGFIEG
GWSGLVAGWYGFQHSNDQGVGMAADRDSTQKAMDKITSKVNNIVDKMNKQYEIIDHEFSEVETRLNMINN
KIDDQIQDIWAYNAELLVLLENQKTLDEHDANVNNLYNKVKRALGSNAVEDGKGCFELYHKCDDQCMETI
RNGTFNRRKYQEESKLERQKIEGVKLESEGTCKILSIYSTVASSLVIAMGFAAFLFWAMYNGSCRCNICI

>QBK19928.1 hemagglutinin [Influenza A virus]
MDTVSLISMLLVSTLTHADKICIGYQSTNSGKTVDTLTQNNVPVTHAKELLHTQHNGMLCATSLGQPLIL
DTCTIEGLIYGNPSCDLSLEGREWSYIVERPSAVNGLCYPGNVQNLEELRSLFSSARSYQKIQIFPDTIW
NVSYDGTSTACSGSFYKSMRWLTRKNGDYPIQDAQYTNNQGKNILFMWGINHPPTDTTQRDLYTRIDTTT
SVATEEINRVFKPLIGPRPLVNGLMGRIDYYWSVLKPGQTLRIKSDGNLIAPWFGHILSGESHGRILKTD
LKRGSCTVQCQTEKGRLNSTLPFQNVSKYAFGNCSKYIGIKSLKLAVGLRNVPSRYSRGLFGAIAGFIEG
GWSGLVAGWYGFQHSNDQGVGMAADRDSTQKAIDKITSNVNNIVAKMNKPYEIIDHEFSEVETRINTINN
KFDDQIQDIWAYNAESLVLLENQKTLDEQDANVNNLYNKVKRALGSNAVEDGKGCLELYHKRDDQCMETI
RNGTFNRRECQAESKKERQKMEGVKQEFDRTRGGVSIYSRVASYRVIAMGSVALRIWALYNGSCRCNICI

>QBK19927.1 hemagglutinin [Influenza A virus]
MDTVSLISMLLISTLSNADKICIGYRSTNSAETVDTLTQNNVPVTHAKELLHTQHNGMLCATSLGQPLIL
DTCTIEGLIYGNPSCDLSLEGREWSYIVERPSAVNGLCYPGNVQNLEELRSLFSSARSYQKIQIFPHTIW
NVSYDGTSTACSGSFYKSMRWLTRKNGDYPIQDAQYTNNQGKNILFMWGINHPPTDTTQRDLYTRIDTTT
SVATEEINRVFTPLIGPRPLVNGLMGRIDYYWSVLKPGQTLRIKSDGNLIAPWFGHILSGESHGRILKND
LKRGSCTVQCQTEKGGLNSTLPFQNVSKYAFGNCSKYIGIKSLKLAVGLRNVPSRSSRGLFGAIAGFIEG
GWSGLVAGWYGFQHSNDQGVGMAADRDSTQKAIDKITSKVNNIVDKMNKQYEIIDHEFSEVETRINMINN
KIDDQIQDIWAYNAELLVLLENQKTLDEHDANVNNLYNKVKRALGSNAVEDGKGCLELYHKCDDQCMETI

RNGTYNRRKYQAESKLERQKIEGVKLESEGTWKIVSIYSRVASYLVIAMGFVALLFGAMYNGSCRCNICI

>QBK19926.1 hemagglutinin [Influenza A virus]

MDTVSLITILLIATVSNADKICIGYQSTNSTETVDTLTENNVPVTHAKELLHTEHNGMLCATSLGHPLIL
DTCTIEGLIYGNPSCDPLLGGREWSYIVERPSAVNGLCYPGNVQNLEELRSLFSSARSYKRIQIFPDTIW
NVSYSGTSRACSDSFYRSMRWLTQKNNAYPIQDAQYTNDQEKNILFMWGINHPPTETAQTNLYTRTDTTT
SVATEEINRIFKPLIGPRPLVNGLMGRINYYWSVLKPGQTLRIKSDGNLIAPWYGHILSRESHGRILNTD
LKRGSCTVQCQTEKGGLHTALPFLNVSKYAFGKCSKYIGIKSLKLAVGLRNVPCRYSRGLFGAIAGFIEG
GWSGLVAGWYGFQHSNDQGVGMAADRDSTQKAIDKITSKVNNIVDKMYKQYEIIDHEFSEVGTRINMINN
KVDDQIQDIWAYNAESLVLLENQKTLDEHDSNVNNLYNKVKRALGSNAMEDGKGCSELYHKCDDQCMETI
RNGTYNRRKYQAESKLEREKMEGVKLESEGTNKILSIYSTVASSLVIAMGFAAFLFWALSNGSCRCNICI

>QBK19925.1 hemagglutinin [Influenza A virus]

MDTVSLITILLIATVSNADKICIGYQSTNSTETVDTLTENNVPVTHAKELLHTEHNGMLCATSLGHPLIL
DTCTIEGLIYGNPSCDPLLGGREWSYIVERPSAVNGLCYPGNVQNLEELRSLFSSARSYKRIQIFPDTIW
NVSYSGTSRACSDSFYRSMRWLTQKNNAYPIQDAQYTNDQEKNILFMWGINHPPTETAQTNLYTRTDTTT
SVATEEINRIFKPLIGPRPLVNGLMGRINYYWSVLKPGQTLRIKSDGNLIAPWYGHILSRESHGRILKTD
LKRGSCTVQCQTEKGGLNTALPFLNVSKYAFGKCSKYIGIKSLKLAVGLRNVPSRSSRGLFGAIAGFIEG
GWSGLVAGWYGFQHSNDQGVGMAADRDSTQKAIDKITSKVNNIVDKMYKQYEIIDHEFSEVGTRLNMINN
KVDDQIQDIWAYNAESLVFLENQKTLDEHDSNVNNLYNKVKRALGSNAMEDGKGCFELCHKCDDQCMETI
RNGTYNRRKYQEESKLERQKMEGVKLESEGTNKILSIYSTVASSLVIAMGSAAVLFWALSNGSCRCNICI

>QBK19924.1 hemagglutinin [Influenza A virus]

MDTVSLITILLIATVSNADKICIGYQSTNSTETVDTLTENNVPVTHAKELLHTEHNGMLCATSLGHPLIL
DTCTIEGLIYGNPSCDPLLGGREWSYIVERPSAVNGLCYPGNVENLEELRSLFSSARSYKRIQIFPDTIW
NVSYSGTSRACSDSFYRSMRWLTQKNNAYPIQDAQYTNDQEKNILFMWGINHPPTETAQTNLYTKTDTTT
SVATEEINRIFKPLIGPRPLVNGLMGRINYYWSVLKPGQTLRIKSDGNLIAPWYGHILSGEKHGRILKHD
LKRGSCTVQCQTEKGGLNTTLPFQNVSKYAFGKCSKYIGIKSLKLAVGLRNVRSRFSRGLFGAIAGFIEG
GWSGLVAGWYGFQHSNDQGVGMAADRDSTQKAIDKITSKVNNIVDKMNKQYEIIDHEFSEVETRLNMINN
KVDDQIQDIWAYNAELLVLLENQKTLDEHDSNVNNLYNKVKRALGSNAMEDGKGCFELYHKCDDQCMETI
RNGTYNRRKYQEESKLERQKIEGVKLESEGTYKILSIYSTVASSLVIAMGSVAFLFWAMSNGSCRCNICI

>QBK19923.1 hemagglutinin [Influenza A virus]

MHTVSAITMLLIATVSNADKICIGYQSTNSTETVDTLTENNVPVTHAKELLHTEHNGMLCATSLGHPLIL

DTCTIEGLIYGNPSCDPLLGGREWSYIVERPSAVNGLCYPGNVQNLEELRSLFSSARSYKRIQIFPDTIW
NVSYCGTSRACSDSFYRSMRWLTQKNNAYPIQDAQYTNDQENNILFMWGINHPPTETAQTNLYTRTDTTT
SVATEEINRIFKPLIGPRPLVNGLMGRINYYWSVLKPGQTLRIKSDGNLIAPWYGHIRSWESQGRIVKHD
LKRGSCTVQCQTEKGGLNTTLPFQNVSKYAFGKCSKYIGIKSLKLAVGLRNVRSRYSRGLFGAIAGFIEG
GWSGLVAGWYGFQHSNDQGVGMAADRDSTQKAIDKIPSKVNNIVAKLYKQYEILDHAIIEVGTRINPINH
KVDDQIQDIWAYNAESIVFLDNQKTLDEHDSNTNNLYNKVKRAAGSNAMEDGKGCSELCHKCDDQCMETI
RNGTYNRREYQAEGKLERQKMEGAKLESEGTYKILSMYSTVASYLVIAMGSFAFLFWALYNGSCRCNICI

>QBK19922.1 hemagglutinin [Influenza A virus]
MDTVSLITILLIATVSNADKICIGYQSTNSAETVDTLTENNVPVTHAKELLHTEHNGMLCATSLGHPLIL
DTCTIEGLIYGNPSCDPLLGGREWSYIVERPSAVNGLCYPGNVQNLEELRSLFSSARSYKRIQIFPHTIW
NVSYSGTSRACSDSFYRSMRWLTQKNNAYPIQDAQYTNDQENNILFMWGINHPPTETAQTNLYTRTDTTT
SVATEEINRIFKPLIGPRPLVNGLMGRINYYWSVLKPGQTLPIKSDGNLIAPWYGHILSRESHGRILNTD
LKRGSCTVQCQTEKGGLNTARPFQNVSKYASGKCSKYIGIKSVKLAVGLRNVPCRYSRGLFGAIAGFIEG
GWSGLVDGWYGFQHSNDQGVGMAADRDSTQKAIDTITSKVNNIVAKMYKQYEILDHEFSEVGTRINTINN
KVDDQIQDIWAYNAESIVFHDNQKTLDEHDSNVNNLYNKVKRALGSNAMEDGKGCSELCHKCYDQCMETI
RNGTFNRREYQAESKLERQKMEGVKQESDGTWKILSIYSRVASYRVIAMGSVAFLFWALSNGSCRCNICI

>QBK19921.1 hemagglutinin [Influenza A virus]
METVSLITILLIATISNADKICIGYQSTNSTETVDTLTENNVPVTHAKELLHTEHNGMLCATSLGQPLIL
DTCTIEGLIYGNPSCDLSLEGREWSYIVERPSAVNGLCYPGNVQNLEELRSLFSSARSYQRIQIFPDTIW
NVSYDGTSTACSGSFYRSMRWLTRKNGDYPIQDAQYTNNQGKNILFMWGINHPPTDTTQRDLYTRTDTTS
SVATEEINRVFKPLIGPRPLVNGLMGRIDYYWSVLKPGQTLRIKSDGNLIAPWFGHILSGESHGRILTTD
LKRGSCTVQCQREKGGLNTALPLLNVSKYAFGNCSKYIGIKSLKLAVGLRNVRSRYSRGLFGAIAGFIER
GWSGLVAGWYGFQHSNDQGVGMAADRDSTQKAIDKITSKVNNIVAKMYKQYEIIDHEFSEVETRINMINH
KIDDQIQDIWAYNAESLVLLENQKTLDEHDANVNNLYNKVKRALGSNAVEDGKGCFELYHKCDDQCMETI
RNGTYNRRKYQAESQLERQKIEGVKQESERTYKILSIYSTVASYRVIAMGFAAFLFWALSNGSCRCNICI

>QBK19920.1 hemagglutinin [Influenza A virus]
MDTVSLITMLLISTVSNANKICIGYQSTNSAETVDTLTENNVPVTHAKELLHTEHNGMLCATSLGHPLIL
DTCTIEGLIYGNPSCDPLLGGREWSYIVERPSAVNGLCYPGNVQNLEELRSLFSSARSYKRIQIFPDTIW
NVSYSGTSRACSDSFYRSMRWLTQKNNAYPIQDAQYTNDQENNILFMWGINHPPTETAQTNLYTRTDTTT
SVATEEINRIFKPLIGPRPLVNGLMRRINYYWSVLKPGHTLPIKSDGNLIAPWYGHILFRESHGRILNTD
LKGGSCTVQCQTEKGGLNSALPLQNVSKYAFGKCSKYIGIKSLKLAVGLRNVPCRYSRGLFGAIAGFIEG

GWPGLVDGWYGFQHSNDQGVGMAADRDSTQKAIDKITSKVNNIVAKMYKQYEIIDHEFSEVGTRINMINN
KVDDQIQDIWAYNAESIVLNENQKTLDEHDSNVNNEGNKVKRALGSNAMEDGKGCSELCHKCDDQCMETI
RNGTYNRRKYQAESKLERQKMEGAKLESDGTYKILSICSRVAWYLVIAMGSVAFLIWAMSNWSCRCNICI

>QBK19919.1 hemagglutinin [Influenza A virus]
METVSLITILLVATVSNADKICIGYQSTNSTETVDTLTENNVPVTHAKELLHTEHNGMLCATSLGHPLIL
DTCTIEGLIYGNPSCDPLLGGREWSYIVERPSAVNGLCYPGNVENLEELRSLFSSARSYKRIQIFPDTIW
NVSYSGTSRACSDSFYRSMRWLTQKNNAYPIQDAQYTNDQEKNILFMWGINHPPTETAQTNLYTRTDTTT
SVATEEINRIFKPLIGPRPLVNGLMGRINYYWSVLKPGQTLRIKSDGNLIAPWYGHILSGESHGRILKTD
LKRGSCTVQCQTEKGGLNTTLPFQNVSKYAFGNCSKYIGIKSLKLAVGLRNVPSRSSRGLFGAIAGFIEG
GWSGLVAGWYGFQHSNDQGVGMAADRDSTQKAIDKITSKVNNIVDKMNKQYEIIDHEFSEVETRLNMINN
KVDDQIQDIWAYNAELLVLLENQKTLDEHDSNVNNLYNKVKRTLGSNAMEDGKGCFELYHKCDDQCMETI
RNGTYNRRKYQEESKLERQKIEGVKLESEGTYKILSIYSTVASSLVIAMGSAAFLFWAMSNGSCRCNICI

>QBK19918.1 hemagglutinin [Influenza A virus]
MDTVSLITILLIATVSNADKICIGYQSTNSTETVDTLTENNVPVTHAKELLHTEHNGMLCATSLGHPLIL
DTCTIEGLIYGNPSCDPLLGGREWSYIVERPSAVNGLCYPGNVQNLEELRSLFSSARSYKRIQIFPDTIW
NVSYCGTSRACSDSFYRSMRWLTQKNNAYPIQDAQYTNDQENNILFMWGINHPPTETAQTNLYTKTDTTT
SVATEEINRIFKPLIGPRPLVNGLMGRINYYWSVLKPGQTLRIKSDGNLIAPWYGHILSRESHGRILKTD
LKRGSCTVQCQTEKGGLNTALPFQNVSKYAFGNCSKYIGIKSLKLAVGLRNVRCRYSRGLFGAIAGFIER
GWSGLVAGWYGFQHSNDQGVGMAADRDSTQKAIDKITSKVNNIVAKMYKQYEIIDHEFSEVGTRLNMINN
KVDDQIQDIWAYNAESLVLLENQKTLDEHDSNVNNLYNKVKRAAGSNAMEDGKGCFELYHKCDDQCMETI
RNGTYNRRKYQEESKLERQKIEGLKLESEGTWKILSIYSTVASSLVIAMGFAAFLFWALSNGSCRCNICI

>QBK19917.1 hemagglutinin [Influenza A virus]
METVSLITILLVSTVSNADKICIGYQSTNSAETVDTLTENNVPVTHAKELLHTEHNGMLCATSLGHPLIL
DTCTIEGLIYGNPSCDPLLGGREWSYIVERPSAVNGLCYPGNVQNLEELRSLFSSARSYKRIQIFPDTIW
NVSYSGTSRACSDSFYRSMRWLTQKNNAYPIQDAQYTNDQENNILFMWGINHPPTETAQTNLYTRTDTTT
SVATEEINRIFKPLIGPRPLVNGLMGRINYYWSVLKPGQTLRIKSDGNLIAPWYGHILSGESHGRIVKTD
LKRGSCTVQCQTEKGGLNTTLPFQNVSKYAFGNCSKYIGIKSLKLAVGLRNVPSRSSRGLFGAIAGFIEG
GWSGLVAGWYGFQHSNDQGVGMAADRDSTQKAIDKITSKVNNIVDKMNKQYEIIDHEFSEVETRLNMINN
KVDDQIQDIWAYNAELLVLLENQKTLDEHDSNVNNLYNKVKRALGSNAMEDGKGCFELYHKCDDQCMETI
GNGTFNRRKYQEESKLERQKIEGVKLESEGTYKILSIYSTVASYLVIAMGFAAFLFWAMSNGSCRCNICI

>QBK19916.1 hemagglutinin [Influenza A virus]

MDTVSLISMLLLSHVSIADKICIGYQSTNSAKTVDTVTQNNVPVTHAKELLHTQHNGMLCATSLGHPLIL
DTCTIEGLIYGNPSCDPLLGGREWSYIVERSSAVNGLCYPGNVQNLKELMSLFSSARSYKKIQIFPHTLW
NVSYSGTMIACSNSFYLSMRWLTLPNNAYPIQDALYTNESENTILFMWGINHPPTEPAQTNLYTITDTTS
SVATDEINRIFKPLIGPRPLVNGLMARINYYWSVLKPGHTLRIKSDGNHIALWYGHILSGESHGRIVKHD
LKRGRCTAQCQTEKGGLHTTLPLQNVSKYAFGKCSKYIGIKSLKLAVGLRNVPSRYSRGLFGAIAGFIEG
GWSGLVAGWYGFQHSNDQGVGMAADRDSTQKAIDKITSKVNNIVAKLYKQYEIIDLEFSEVETRLNTINN
KVDDQIQDIWAYNAESIVFLDNQKTLDEHDSNVSNLYNKVKRAAGSNAMEDGNGCSELCHKCDDQCMETI
RNGTYNRRKYQAESKLERQKMEGAKQESERTREILSIYSTVASYRVIAMGSAAFLFWALSNGSCRCNICI

>QBK19915.1 hemagglutinin [Influenza A virus]

MDTVSLITILLIATVSNADKICIGYQSTNSTETVDTLTENNVPVTHAKELLHTEHNGMLCATSLGHPLIL
DTCTIEGLIYGNPSCDPLLGGREWSYIVERPSAVNGLCYPGNVQNLEELRSLFSSARSYKRIQIFPDTIW
NVSYSGTSRACSDSFYRSMRWLTQKNNAYPIQDAQYTNDQEKNILFMWGINHPPTETVQTNLYTKTDTTT
SVATEEINRIFKPLIGPRPLVNGLMGRINYYWSVLKPGQTLRIKSDGNLIAPWYGHILSRESHGRILKTD
LKRGSCTVQCQTEKGGLNTALPFQNVSKYAFGNCSKYIGIKSLKLAVGLRNVRSRYSRGLFGAIAGFIEG
GWSGLVAGWYGFQHSNDQGVGMAADRDSTQKAIDKITSKVNNIVAKMYKQYEIIDHEFSEVGTRLNMINH
KVDDQIQDIWAYNAESLVFLENQKTIDEHDSNVNNLYNKVKRALGSNAMEDGKGCFELYHKCDDQCMETI
RNGTSNRRKYQAESKLERQKIEGAKLESDGTNKILSIYSRVASSLVIAMGSAAFLFGALSNGSCRCNICI

>QBK19914.1 hemagglutinin [Influenza A virus]

METVSLITILLVATVSNADKICIGYQSTNSTETVDTLTESNVPVTHAKELLHTEHNGMLCATSLGQPLVL
DTCTIEGLIYGNPSCDLSLEGREWSYIVERPSAVNGLCYPGNVENLEELRSLFSSARSYQRIQIFPDTIW
NVSYDGTSNACSGSFYRSMRWLTQKNGDYPTQDAQYTNNQGKNILFMWGINHPPTDTTQINLYTRTDTTT
SVATEEINRIFKPLIGPRPLVNGLMGRIDYYWSVLKPGQTLRIKSDGNLIAPWYGHILSGESHGRILKTD
LKRGSCTVQCQTEKGGLNTTLPFQNVSKYAFGNCSKYIGIKSLKLAVGLRNVPSRSSRGLFGAIAGFIEG
GWPGLVAGWYGFQHSNDQGVGMAADRDSTQKAIDKITSKVNNIVDKMNKQYEIIDHEFSEVETRLNMINN
KIDDQIQDIWAYNAELLVLLENQKTLDEHDANVNNLYNKVKRALGSNAVEDGKGCFELYHKCDDQCMETI
RNGTYNRRKYQEESKLERQKIEGVKLESEGTYKILSIYSTVASYLVIAMGFAAFLFWAMSNGSCRCNICI

>QBK19913.1 hemagglutinin [Influenza A virus]

METVSLITILLVATVSNADKICIGYQSTNSTETVDTLTESNVPVTHAKELLHTEHNGMLCATSLGQPLVL
DTCTIEGLIYGNPSCDLSLEGREWSYIVERPSAVNGLCYPGNVENLEELRSLFSSARSYQRIQIFSDTIW
NVSYDGTSNACSGSFYRSMRWLTQKNGDYPTQDAQYTNNQGKNILFMWGINHPPTDTTQINLYTRTDTTT

SVATEEINRIFKPLIGPRPLVNGLMGRIDYYWSVLKPGQTLRIKSDGNLIAPWYGHILSRESHGRILKTD
LKRGSCTVQCQTEKGGLNTALPFQNVSEYAFGKCSKYICIKSVKLAVDLRNVRGRYSRGVFGGIAGFIER
GRPGLVDGWYGFQHSNDHGVGMAADRDSTQKAIDKITSKVNNIVAKMNKQYEIIDHEFSEVETRLNMINN
KIDDQIQDIWAYNAELLVLLENQKTLDEHDANVNNVYNKVKRALGSNAVEDGKGCSEQYHKCDDQCMETI
RNGTYNRRKYQAESQLERQKIEGAKLESEGTREILSMYSTVASSRVIAMGSVALRFGALYNWSCRCNICI

>QBK19912.1 hemagglutinin [Influenza A virus]
METVSLITILLVATVSNADKICIGYQSTNSTETVDTLTESNVPVTHAKELLHTEHNGMLCATSLGQPLVL
DTCTIEGLIYGNPSCDLSLEGREWSYIVERPSAVNGLCYPGNVENLEELRSLFSSARSYQRIQIFPDTIW
NVSYDGTSNACSGSFYRSMRWLTQKNGDYPTQDAQYTNNQGKNILFMWGINHPPTDTTQINLYTRTDTTT
SVATEEINRIFKPLIGPRPLVNGLMGRIDYYWSVLKPGQTLRIKSDGNLIAPWYGHILSRESHGRILKTD
LKRGSCTVQCQTEKGGLNTTLPFQNVSKYAFGNCSKYIGIKSLKLAVGLRNVPSRSSRGLFGAIAGFIEG
GWPGLVAGWYGFQHSNDQGVGMAADRDSTQKAIDKITSKVNNIVDKMNKQYEIIDHEFSEVETRLNMINN
KIDDQIQDIWAYNAELLVLLENQKTLDEHDANVNNLYNKVKRALGSNAVEDGKGCFELYHKCDDQCMETI
RNGTYNRRKYQEESKLERQKIEGVKLESEGTYKILSIYSTVASSLVIAMGFAAFLFWAMSNGSCRCNICI

>QBK19911.1 hemagglutinin [Influenza A virus]
METASLITILLIVTVSNADKICIGYQSTNSTETVNTLTENNVPVTHAKELLHTEHNGMLCATSLGQPLIL
DTCTIEGLIYGNPSCDLLLGGREWSYIVERPSAVNGLCYPGNVQNLEELRSLFSSARSYQRIQIFPDTIW
NVSYSGTSKACSDSFYRSMRWLTQKDNAYPTQDAQYTNNQEKNILFMWGINHPPTDTVQTNLYTRTDTTT
SVATEEINRTFKPLIGPRPLVNGLMGRINYYWSVLKPGQTLRIKSNGNLIAPWYGHVLSRESHGRILNTD
LNRGSCTVQCQTEKSGLHTTLPFQNVSKYAFGKCSKYIGIKSLKLAVGLRNVRSRYSRGVCGAIAGFIEG
GWSGLVDGWYGFQHSNDHGVGMAADRDSTQKAIDKITSKVNNIVAKMNKQYEIIDHEFSEVETRLNMINN
KIDDQIQDIWAYNAELLVSNENQKTLDEHDANVNNLYNKVKRALGSNAVEDGKGCSEQYQKCDDQCMETI
RNGTYNRRKYQAESKLEREKMEGAKQESEGTNKILSMYSRVASYRVIAMGSVAFLFGALYNGSCRCNICI

>QBK19910.1 hemagglutinin [Influenza A virus]
METVSLITILLVATVSNADKICIGYQSTNSTETVDTLTESNVPVTHAKELLHTEHNGMLCATSLGQPLVL
DTCTIEGLIYGNPSCDLSLEGREWSYIVERPSAVNGLCYPGNVENLEELRSLFSSARSYQRIQIFPDTIW
NVSYDGTSNACSGSFYRSMRWLTQKNGDYPTQDAQYTNNQGKNILFMWGINHPPTDTTQINLYTRTDTTT
SVATEEINRIFKPLIGPRPLVNGLMGRIDYYWSVLKPGQTLRIKSDGNLIAPWYGHILSRESHGRILKTD
LKRGSCTVQCQTEKGGLNTTLPFQNVSKYAFGNCSKYIGIKSLKLAVGLRNVPSRSSRGLFGAIAGFIEG
GWPGLVAGWYGFQHSNDQGVGMAADRDSTQKAIDKITSKVNNIVDKMNKQYEIIDHEFSEVETRLNMINN
KIDDQIQDIWAYNAELLVLLENQKTLDEHDANVNNLYNKVKRALGSNAVEDGKGCFELYHKCDDQCMETI

RNGTYNRRKYQEESKLERQKIEGVKLESEGTYKILSIYSTVASSLVIAMGFAAFLFWAMSNGSCRCNICI

>QBK19908.1 hemagglutinin [Influenza A virus]

MDTASLITILLVITVSNADKICIGYQSTNSTETVDTLTENNVPVTHAKELLHTEHNGMLCATSLGHPLIL
DTCTIEGLIYGNPSCDLFLGGREWSYIVERPSAVNGLCYPGNVENLEELRSLFSSARSYQRIQIFPDTIW
NVSYSGTSKACSDSFYRSMRWLTQKNNAYPIQDAQYTNNQEKNILFMWGINHPPTDTAQTNLYTRTDTTT
SVATEEINRTFKPLIGPRPLVNGLMGRINYYWSVLKPGQTLRIKSNGNLIAPWYGHILSGESHGRILKTD
LKRGSCTVQCQTEKGGLNTTLPFQNVSKYAFGNCSKYIGIKSLKLAVGLRNVPSRSSRGLFGAIAGFIEG
GWSGLVAGWYGFQHSNDQGVGMAADRDSTQKAIDKITSKVNNIVDKMNKQYKIIDHEFSEVETRLNMINN
KIDDQIQDIWAYNAELLVLLENQKTLDEHDANVNNLYNKVKRALGSNAVEDGKGCFELYHKCDDQCMETI
RNGTYNRRKYQEESKLERQKIEGVKLESEGTYKILTIYSTVASSLVIAMGFAAFLFWAMSNGSCRCNICI

>QBK19907.1 hemagglutinin [Influenza A virus]

METVSLITMLLVATVSNADGICIGYQSTNSAETVDTLTESNVPVTHAKELLHTEHNGMLGATSLGQPLVL
DTCTIEGLIYGNPSCDLSLEGREWSYIVERPSAVNGLCYPGNVQNLEELRSLFSSARSYQKIQIFPDTIW
NVSYYGTSNACSGSFYRSMRWLTQKNGDYPTQDAQYTNNQGNNILFMWGINHPPTDTTQINLYTRTDTTT
SVATEEINRIFRPLIGPRPLVNGLMGRIDYYWSVLKPGQTLRIKSDGNLIAPWYGHILSRESHGRILNTD
LKRGSCTVQCQTENGGLHTALPLLNVSKYASGKCSKYIGIKSLKLAVGLRNVQSRYSRGLFGAIAGFIEG
GWPGLVDGWYGFQHSNDQGVGMAADRDSTQKAIDKITSKVNNIVAKMYKQYEIIDHEFSEVETRINMINN
KIDDQIQDIWAYNAELIVLLENQKTLDEHDAKVNNLYNKVKRALGSNTVEDGKGCSELYHKCDDQCMETI
RNGTSNRRKYQAESKKERQKMEGAKLESEGTWKILSIYSTVTSYRVIAMGSVAFLIGAMYNGSCRCNICI

>QBK19906.1 hemagglutinin [Influenza A virus]

METVSLITILLVATVSNADKICIGYQSTNSTETVDTLTESNVPVTHAKELLHTEHNGMLCATSLGQPLVL
DTCTIEGLIYGNPSCDLSLEGREWSYIVERPSAVNGLCYPGNVENLEELRSLFSSARSYQRIQIFPDTIW
NVSYDGTSNACSGSFYRSMRWLTQKDGDYPTQDAQYTNNQGKNILFMWGINHPPTDTTQINLYTRTDTTT
SVATEEINRIFKPLIGPRPLVNGLMGRIDYYWSVLKPGQTLRIKSDGNLIAPWYGHILSGESHGRILKTD
LKRGSCTVQCQTEKGGLNTTLPFQNVSKYAFGNCSKYIGIKSLKLAVGLRNVPSRSSRGLFGAIAGFIEG
GWPGLVAGWYGFQHSNDQGVGMAADRDSTQKAIDKITSKVNNIVDKMNKQYEIIDHEFSEVETRLNMINN
KIDDQIQDIWAYNAELLVLLENQKTLDEHDANVNNLYNKVKRALGSNAVEDGKGCFELYHKCDDQCMETI
RNGTYNRRKYQEESKLERQKIEGVKLESEGTYKILSIYSTVASSLVIAMGFAAFLFWAMSNGSCRCNICI

>QBK19899.1 hemagglutinin [Influenza A virus]

METVSLITILLVATVSNADKICIGYQSTNSTETVDTLTESNVPVTHAKELLHTEHNGMLCATSLGQPLVL

DTCTIEGLIYGNPSCDLSLEGREWSYIVERPSAVNGLCYPGNVENLEELRSLFSSARSYQKIQIFPDTIW
NVSYDGTSNACSGSFYRSMRWLTQKNGDYPTQDAQYTNNQGKNILFMWGINHPPTDTTQINLYTRTDTTT
SVATEEINRIFKPLIGPRPLVNGLMGRIDYYWSVLKPGQTLRIKSDGNLIAPWYGHILSGESHGRILEAD
LKRGSCTVQCQTEKGGLNTTLPLQNVSKYAFGKCSKYIGIKSLKLAVGLRNVPSRSSRGLFGAIAGFIEG
GWPGLVAGWYGFQHSNDQGVGMAADRDSTQKAIDKITSKVNNIVAKMNKQYEIIDHEFSEVETRINMINN
KIDDQIQDIWAYNAELLVLLENQKTLDEHDANVNNLYNKVKRALGSNAVEDGKGCFELYHKCDDQCMETI
RNGTFNRRKYQEESKLERQKIEGVKLESEGTWKILSIYSTVASYRVIAMGFVAFFFGAMYNGSCRCNICI

>QBK19898.1 hemagglutinin [Influenza A virus]
MDTASLITILLIITVSNADKICIGYQSTNSTETVNTLTENNVPVTHAKELLHTEHNGMLCATSLGHPLIL
DTCTIEGLIYGNPSCDLFLGGREWSYIVERPSAVNGLCYPGNVQNLEELRSLFSSARSYQRIQIFPDTIW
NVSYSGTSKACSDSFYRSMRWLTQKNNAYPIQDAQYTNNQEKNILFMWGINHPPTDTAQTNLYTRTDTTT
SVATEEINRTFKPLIGPRPLVNGLMGRINYYWSVLKPGQTLRIKSNGNLIAPWYGHILSRESHGRILKTD
LKRGSCTVQCQTEKGGLNTTLPLQNVSKYAFGNCSKYIGIKSLKLAVGLRNVPSRYSRGLFGAIAGFIEG
GWSGLVAGWYGFQHSNDQGVGMAADRDSTQKAIDKITSKVNNIVAKMYKPYEIIDHEFSEVETRINMINN
KIDDQIQDIWAYNAELLVLLENQKTLDEHDANVNNVYNKVKRALGSNAVEDGKGCSELYHKCDDQCMETI
GNGTYNRRKYQEESKLERQKIEGVKLESEGTYKILSIYSRVASSRVIAMGSAAFLFWAMSNGSCRCNSSI

>QBK19897.1 hemagglutinin [Influenza A virus]
METASLITILLIVTVSNADKICIGYQSTNSAETVNTLTENNVPVTHAKELLHTEHNGMLCATSLGQPLIL
DTCTIEGLIYGNPSCDLLLGGREWSYIVERPSAVNGLCYPGNVQNLKELRSLFSSARSYQRIQIFPHTIW
NVSYSGTSKACSDSFYRSMRWLTQKDNAYPTQDAQYTNNQENNILFMWGINHPPTDTAQTNLYTRTDTTT
SVATEEINRTFKPLIGPRPLVNGLMGRINYYWSVLKPGQTLRIKSNGNLIAPWYGHVLSRESHGRILKTD
LKRGSCTVQCQTEKGGLNNTLPLQNVSKYAFGKCSKYIGIKSLKLAVGLRNVPSRYSRGLFGAIAGFIEG
GWSGLVDGWYGFQHSNDQGVGMAADRDSTQKAIDKITSKVNNIVAKMNKQYEIIDHEFSEVETRLNMINN
KIDDQIQDIWAYNAELLVLLENQKTLDEHDANVNNLYNKVKRALGSNAVEDGKGCFELYHKCDDQCMETI
RNGTFNRRKYQEESKLERQKMEGAKLESEGTYKILSIYSTVASYRVIAMGFFAFFFWALSNGSGRQNICI

>QBK19896.1 hemagglutinin [Influenza A virus]
METASLITILLVVTVSNADKICIGYQSTNSTETVDTLTENNVPVTHAKELLHTEHNGMLCATSLGQPLIL
DTCTIEGLIYGNPSCDLLLGGREWSYIVERPSAVNGLCYPGNVQNLEELRSLFSSARSYQRIQIFPDTIW
NVSYSGTSKACSDSFYRSMRWLTQKDNAYPTQDAQYTNNQEKNILFMWGINHPPTDTAQTNLYTRTDTTT
SVATEEINRTFKPLIGPRPLVNGLMGRINYYWSVLKPGQTLRIKSNGNLIAPWYGHVLSRESHGRILKTD
LNRGSCTVPCQTEKGGLHTALPFQNVSKYAFGNCSKYIGIKSLKLAVGLRNVPSRSSRGLFGAIAGFIEG

GWSGLVDGWYGFQHSNDHGVGMAADRDSTQKAIDKITSKVNNIVAKMNKQYEIIDHEFSEVETRLNMINN
KIDDQIQDIWAYNAELLVLLENQKTLDEHDANVNNLYNKVKRALGSNAVEDGKGCFELYHKCDDQCMETI
RNGTYNRRKYQEESKLERQKIEGVKLESEGTYKILSIYSTVASSRVIAMGFAAFLFWAMSNGSCRCNICI

>QBK19895.1 hemagglutinin [Influenza A virus]
MDTVLLITILLISTVSNADKICIGYQSTNSTETVDTLTENNVPVTHAKELLHTEHNGMLCATSLGQPLIL
DTCTIEGLIYGNPSCDLSLEGREWSYIVERPSAVNGLCYPGNVENLEELRSLFSSARSYQRIQIFPDTIW
NVSYYGTSTACSGSFYRSMRWLTRKNGDYPVQDAQYTNNQGKNILFMWGINHPPTDTTQRDLYTRTDTTT
SVATEEINRIFKPLIGPRPLVNGLMGRIDYYWSVLKPGQTLRIKSDGNLIAPWYGHILSRESHGRILKTD
LKRGSCTVQCQTEKGGLHTTLSLQNVSKYAFGKCSKYIGIKSLKLAVGLRNVPSRSSRGLFGAIAGFIEG
GWSGLVAGWYGFQHSNDQGVGMAADRDSTQKAIDKITSKVNNIVAKMNKQYEIIDHEFSEVETRINMINN
MIDDQIQDIWAYNAELLVLLENQKTLDEHDANVNNLYNKVKRALGSNAVEDGKGCFELYHKCDDQCMETI
RNGTYNRRKYQEESKLERQKIEGVKLEFEGTYKIVSIYSTVASYRVIAMGSVAFLFWALSNGSCRCNICI

>QBK19892.1 hemagglutinin [Influenza A virus]
METVSLITILLAATVSNADKICIGYQSTNSTETVDTLTENNVPVTHAKELLHTEHNGMLCATSLGQPLIL
NTCTIEGLIYGNPSCDLSLEGREWSYIVERPSAVNGLCYPGNVENLEELRSLFSSARSYQRIQIFPDTIW
NVSYDGTSNACSGSFYRSMRWLTRKNGDYPIQDAQYTNNQGKNILFMWGINHPPTDTTQRDLYTRTDTTT
SVATEEINRIFKPLIGPRPLVNGLMGRIDYYWSILKPGQTLRIKSDGNLIAPWYGYILSGESHGRILKTD
LKRGSCTVQCQTEKGGLNTTLPFQNVSKYAFGNCSKYIGIKSLKLAVGLRNVPSRSSRGLFGAIAGFIEG
GWSGLVAGWYGFQHSNDQGVGMAADRDSTQKAIDKITSKVNNIVDKMNKQYEIIDHEFSEVETRLSMINN
KIDDQIQDIWAYNAELLVLLENQKTLDEHDANVNNLYNKVKRALGSNAVEDGKGCFELYHKCDDQCMETI
RNGTYNRRKYQEESKLERQKIEGVKLESEGTYKILTIYSTVASSLVIAMGFAAFLFWAMSNGSCRCNICI

>QBK19891.1 hemagglutinin [Influenza A virus]
METVSLITILLAATVSNADKICIGYQSTNSTETVDTLTENNVPVTHAKELLHTEHNGMLCATSLGQPLIL
NTCTIEGLIYGNPSCDLSLEGREWSYIVERPSAVNGLCYPGNVENLEELRSLFSSARSYQRIQIFPDTIW
NVSYDGTSNACSGSFYRSMRWLTRKNGDYPIQDAQYTNNQGKNILFMWGINHPPTDTTQRDLYTRTDTTT
SVATEEINRIFKPLIGPRPLVNGLMGRIDYYWSILKPGQTLRIKSDGNLIAPWYGYILSRESHGRILKTD
LKRGSCTVQCQTEKGGLNTTLPFQNVSKYAFGNCSKYIGIKSLKLAVGLRNVPSRYSRGLFGAIAGFIEG
GWSGLVDGWYGFQHSNDQGVGMAADRDSTQKAIDKITSKVNNIVDKMNKQYEIIDHEFSEVETRLSMINN
KIDDQIQDIWAYNAELLVLLENQKTLDEHDANVNNLYNKVKRALGSNAVEDGKGCFELYHKCDDQCMETI
RNGTYNRRKYQEESKLERQKIEGVKLESEGTYKILSIYSTVASSLVIAMGFAAFLFWAMSNGSCRCNICI

>QBK19890.1 hemagglutinin [Influenza A virus]

METVSLITILLVATVSNADKICIGYQSTNSTETVDTLTENNVPVTHAKELLHTEHNGMLCATSLGQPLIL
DTCTIEGLIYGNPSCELLLEGREWSYIVERPSAINGLCYPGNVENLEELRSLFSSARSYQRVQIFPVTIW
NVSYNGTSKACSGSFYRSMRWLTRKNGDYPIQDAQYTNNQGKNILFMWGINHPPTDTKQRDLYTRIDTTT
SVATEEINRVFKPLIGPRPLVNGLMGRIDYYWSVLKPGQTLRIKSDGNLIAPWYGYILSGESHGRILKTD
LKRGSCTVQCQTEKGGLNTTLPFQNVSKYAFGNCSKFIGIKSLKLAVGLRNVPSRYSRGLFGAIAGFIEG
GWSGLVDGWYGFQHSNDQGVGMAADRDSTQKAIDKITSKVNNIVDKMNKQYEIIDHEFSEVEARINMINN
KIDDQIQDIWAYNAELLVLLENQKTLDEHDANVNNLYNKVKRALGSNAVEDGKGCFELYHKCDDQCMETI
RNGTYNRRKYQEESKLKRQKIEGVKLESEGTYKILSIYSTVASSLVIAMGFAAFLFWAMSNGSCRCNICI

>QBK19889.1 hemagglutinin [Influenza A virus]

METVSLITILLAATVSNADKICIGYQSTNSTETVDTLTENNVPVTHAKELLHTEHNGMLCATSLGQPLIL
NTCTIEGLIYGNPSCDLSLEGREWSYIVERPSAVNGLCYPGNVENLEELRSLFSSARSYQRIQIFPDTIW
NVSYDGTSNACSGSFYRSMRWLTRKNGDYPIQDAQYTNNQGKNILFMWGINHPPTDTTQRDLYTRTDTTT
SVATEEINRIFKPLIGPRPLVNGLMGRIDYYWSILKPGQTLRIKSDGNLIAPWYGYILSGESHGRILKTD
LKRGSCTVQCQTEKGGLNTTLPFQNVSKYAFGNCSKYIGIKSLKLAVGLRNVPSRSSRGLFGAIAGFIEG
GWSGLVDGWYGFQHSNDQGVGMAADRDSTQKAIDKITSKVNNIVDKMNKQYEIIDHEFSEVETRLSMINN
KIDDQIQDIWAYNAELLVLLENQKTLDEHDANVNNLYNKVKRALGSNAVEDGKGCFEIYHKCDDQCMETI
RNGTYNRRKYQEESKLERQKIEGVKLEFEGTYKILSIYSTVASSLVIAMGFAAFLFWAMYNGSCRCNICI

>QBK19888.1 hemagglutinin [Influenza A virus]

METVSLITILLASTVSNADKICIGYQSTNSTETVNTLTENNVPVTHAKELLHTEHNGMLCATSLGQPLIL
DTCTIEGLIYGNPSCDLSLEGREWSYIVERPSAVNGLCYPGNVENLEELRSLFSSARSYQRIQIFPDTIW
NVSYDGTSNACSGSFYRSMRWLTRKNGDYPIQDAQYTNNQGKNILFMWGINHPPTDTTQRDLYTRTDTTT
SVATEEINRIFKPLIGPRPLVNGLMGRIDYYWSILKPGQTLRIKSDGNLIAPWYGYILSRESHGRILKTD
LKRGSCTVQCQTEKGGLNTTLPFQNVSKYAFGNCSKYIGIKSLKLAVGLRNVPSRSSRGLFGAIAGFIEG
GWSGLVAGWYGFQHSNDQGVGMAADRDSTQKAIDKITSKVNNIVDKMNKQYEIIDHEFSEVETRLSMINN
KIDDQIQDIWAYNAELLVLLENQKTLDEHDANVNNLYNKVKRALGSNAVEDGKGCFELYHKCDDQCMETI
RNGTYNRRKYQEESKLERQKIEGVKLESEGTYKILSIYSTVASSLVIAMGFAAFLFWAMSNGSGRCNICI

>QBK19887.1 hemagglutinin [Influenza A virus]

METVSLITILLVATVSNADKICIGYQSTNSTETVDTLTENNVPVTHAKELIHTEHNGMLCATSLGQPLIL
DTCTIEGLIYGNPSCDLMLEGREWSYIVERPSAVNGLCYPGHVQNLEELRSLFSSARSYQRVQIFPDTIW
NVSYYGTSTACSGSFYRSMRWLTRKNGDYPIQDAQYTNNQGKNILFMWGINHPPADTTQRDLYTRTDTTT

SVATEEINRIFKPLIGPRPLVNGLMGRIDYYWSVLKPGQTLRIKSDGNIIAPWYGHILSGESHGRILTTD
LKRGSCTVQCQTEKGGLNTTLPFQNVSKYAFGKCSKYIGMKSLKLAVGLRNVPSRSSRGLFGAIAGFIEG
GWSGLVAGWYGFQHSNDQGVGMAADRDSTQKAIDKITSNVNNIVAKLYKPYEIIDLAFSEVETRIITSSN
KFDDQIQDIWAYNAESIVLLENQKTLDEQDANVNNLYNNVKRGACSNAVEDGNGCLDLCHKCDDQGTETI
RNGTYNRRKYQEESKKEREKMEGAKLESDGSRERVSSYSTVASYRVIAMGSVALMIEALYNWSDRCNICI

>QBK19886.1 hemagglutinin [Influenza A virus]
METVSLITILLAATVSNADKICIGYQSTNSTETVDTLTENNVPVTHAKELLHTEHNGMLCATSLGQPLIL
NTCTIEGLIYGNPSCDLSLEGREWSYIVERPSAVNGLCYPGNVENLEELRSLFSSARSYQRIQIFPDTIW
NVSYDGTSNACSGSFYRSMRWLTRKNGDYPIQDAQYTNNQGKNILFMWGINHPPTDTTQRDLYTRTDTTT
SVATEEINRIFKPLIGPRPLVNGLMGRIDYYWSILKPGQTLRIKSDGNLIAPWYGYILSGESHGRILKTD
LKRGSCTVQCQTEKGGLNTTLPFQNVSKYAFGNCSKYIGIKSLKLAVGLRNVPSRSSRGLFGAIAGFIEG
GWSGLVAGWYGFQHSNDQGVGMAADRDSTQKAIDKITSKVNNIVDKMNKQYEIIDHEFSEVETRLSMINN
KIDDQIQDIWAYNAELLVLLENQKTLDEHDANVNNLYNKVKRALGSNAVEDGKGCFELYHKCDDQCMETI
RNGTYNRRKYQEESKLERQKIEGVKLESEGTYKILTIYSTVASSLVIAMGFAAFLFWAMSNGSCRCNICI

>QBK19885.1 hemagglutinin [Influenza A virus]
METVSLITILLAATVSNADKICIGYQSTNSTETVDTLTENNVPVTHAKELLHTEHNGMLCATSLGQPLIL
NTCTIEGLIYGNPSCDLSLEGREWSYIVERPSAVNGLCYPGNVENLEELRSLFSSARSYQRIQIFPDTIW
NVSYDGTSNACSGSFYRSMRWLTRKNGDYPIQDAQYTNNQGKNILFMWGINHPPTDTTQRDLYTRTDTTT
SVAAEEINRIFKPLIGPRPLVNGLMGRIDYYWSILKPGQSLRIKSDGNLIATWYGYILSGERHGRILKTD
LKRGSCTVQCQTEKGGLNTTLPFQNVSKYAFGNCSKYIGIKSLKLAVGLRNVPSRYSRGLFGAIAGFIEG
GWSGLVDGWYGFQHSNDQGVGMAADRDSTQKAIDKITSKVNNIVDKMNKQYEIIDHEFSEVETRISMINN
KIDDQIQDIWAYNAELLVLLENQKTLDEHDANVNNLYNKVKRALGSNAVEDGKGCFELYHKCDDQCMETI
GNGTYNRRKYQEESKKERQKIEGLKLESEGTYKILSINSTVASSLVIAMGSFAFLFWALYNGSDRCEHCI

>QBK19884.1 hemagglutinin [Influenza A virus]
METVSLITILLAATVSNADKICIGYQSTNSTETVDTLTENNVPVTHAKELLHTEHNGMLCATSLGQPLIL
NTCTIEGLIYGNPSCDLSLEGREWSYIVERPSAVNGLCYPGNVENLEELRSLFSSARSYQRIQIFPDTIW
NVSYDGTSNACSGSFYRSMRWLTRKNGDYPIQDAQYTNNQGKNILFMWGINHPPTDTKQRDLYTRTDTTT
SVATEEINRIFKPLIGPRPLVNGLMGRIDYYWSILKPGQTLRIKSDGNLIAPWYGYILSGESHGRILKTD
LKRGSCTVQCQTEKGGLNTTLPCQNVSKYAFGNCSKYIGIKSLKLAVGLRNVPSRYSRGLFGAIAGFIEG
GWSGLVDGWYGFQHSNDQGVGMAADRDSTQKAIDKITSKVNNIVDKMNKQYEIIDHEFSEVETRLSMINN
KIDDQIQDIWAYNAELLVLLENQKTLDEHDANVNNLYNKVKRALGSNAVEDGKGCFELYHKCDDQCMETI

RNGTYNRRKYQEESKLERQKIEGVKLESEGTYKILSIYSTVASSLVIAMGSAAFLFWAMSNGSCRCEHCI

>QBK19883.1 hemagglutinin [Influenza A virus]
METVSLITILLAATVSNADKICIGYQSTNSTETVDTLTENNVPVTHAKELLHTEHNGMLCATSLGQPLIL
NTCTIEGLIYGNPSCDLSLEGREWSYIVERPSAVNGLCYPGNVENLEELRSLFSSARSYQRIQIFPDTIW
NVSYDGTSNACSGSFYRSMRWLTRKNGDYPIQDAQYTNNQGKNILFMWGINHPPTDTTQRDLYTRTDTTT
SVATEEINRIFKPLIGPRPLVNGLMGRIDYYWSILKPGQTLRIKSDGNLIAPWYGYILSRESHGRILKTD
LKRGSCTVQCQTEKGGLNTTLPFQNVSKYAFGNCSKYIGIKSLKLAVGLRNVPSRYSRGLFGAIAGFIEG
GWSGLVDGWYGFQHSNDQGVGMAADRDSTQKAIDKITSKVNNIVDKMNKQYEIIDHEFSEVETRISMINN
KIDDQIQDIWAYNAELLVLLENQKTLDEHDANVNNLYNKVKRALGSNAVEDGKGCFEIYHKCDDQCMETI
RNGTYNRRKYQEESKLERQKIEGVKLESEGTYKILSIYSTVASSLVIAMGSAAFLFWAMSNGSGRCEHCI

>QBK19882.1 hemagglutinin [Influenza A virus]
METVSLITILLVATVSNADKICIGYQSTNSTETVDTLTENNVPVTHAKELIHTEHNGMLCATSLGQPLIL
DTCTIEGLIYGNPSCDLMLEGREWSYIVERPSAVNGLCYPGHVQNLEELRSLFSSARSYQRVQIFPDTIW
NVSYDGTSTACSGSFYRSMRWLTRKNGDYPIQDAQYTNNQGKNILFMWGINHPPADTTQRDLYTRTDTTT
SVATEEINRIFKPLIGPRPLVNGLMGRIDYYWSVLKPGQTLRIKSDGNIIAPWYGHILSRESHGRILTTD
LKRGSCTVQCQTEKGGLNTALSLQNVSKYAFGKCSKYIGMKSLKLAVGLRNVPSRSSRGLFGAIAGFIEG
GWSGLVAGWYGFQHSNDQGVGMAADRDSTQKAIDKITSKVNNIVDKMNKQYEIIDHEFSEVETRLNMINN
KIDDQIQDIWAYNAESIVLLENQKTLDEHDANVNNLYNKVKRALGSNAVEDGKGCFDLYHKCDDQCIETI
RNGTYNRRKYQEESKLERQKIEGAKLESEGTYKILSIYSTVASSLVIAMGSAAFLFGAMSNGSCRCEQCI

>QBK19881.1 hemagglutinin [Influenza A virus]
METVSLITILLAATVSNADKICIGYQSTNSTETVDTLTENNVPVTHAKELLHTEHNGMLCATSLGQPLIL
NTCTIEGLIYGNPSCDLSLEGREWSYIVERPSAVNGLCYPGNVENLEELRSLFSSARSYQRIQIFPDTIW
NVSYDGTSNACSGSFYRSMRWLTRKNGDYPIQDAQYTNNQGKNILFMWGINHPPTDTTQRDLYTRTDTTT
SVATEEINRIFKPLIGPRPLVNGLMGRIDYYWSILKPGQTLRIKSDGNLIAPWYGYILSGESHGRILKTD
LKRGSCTVQCQTEKGGLNTTLPFQNVSKYAFGNCSKYIGIKSLKLAVGLRNVPSRSSRGLFGAIAGFIEG
GWSGLVAGWYGFQHSNDQGVGMAADRDSTQKAIDKITSKVNNIVDKMNKQYEIIDHEFSEVETRLSMINN
KIDDQIQDIWAYNAELLVLLENQKTLDEHDANVNNLYNKVKRALGSNAVEDGKGCFELYHKCDDQCMETI
RNGTYNRRKYQEESKLERQKIEGVKLESEGTYKILTIYSTVASSLVIAMGFAAFLFWAMSNGSCRCNICI

>QBK19880.1 hemagglutinin [Influenza A virus]
METVSLITILLAATVSNADKICIGYQSTNSTETVDTLTENNVPVTHAKELLHTEHNGMLCATSLGQPLIL

NTCTIEGLIYGNPSCDLSLEGREWSYIVERPSAVNGLCYPGNVENLEELRSLFSSARSYQRIQIFPDTIW
NVSYDGTSNACSGSFYRSMRWLTRKNGDYPIQDAQYTNNQGKNILFMWGINHPPTDTTQRDLYTRTDTTT
SVATEEINRIFKPLIGPRPLVNGLMGRIDYYWSILKPGQTLRIKSDGNLIAPWYGYILSGESHGRILKTD
LKRGSCTVQCQTEKGGLNTTLPFQNVSKYAFGNCSKYIGIKSLKLAVGLRNVPSRSSRGLFGAIAGFIEG
GWSGLVAGWYGFQHSNDQGVGMAADRDSTQKAIDKITSKVNNIVDKMNKQYEIIDHEFSEVETRLSMINN
KIDDQIQDIWAYNAELLVLLENQKTLDEHDANVNNLYNKVKRALGSNAVEDGKGCFELYHKCDDQCMETI
RNGTYNRRKYQEESKLERQKIEGVKLESEGTYKILTIYSTVASSLVIAMGFAAFLFWAMSNGSCRCNICI

>QBK19879.1 hemagglutinin [Influenza A virus]
METVSLITILLAATVSNADKICIGYQSTNSTETVDTLTENNVPVTHAKELLHTEHNGMLCATSLGQPLIL
NTCTIEGLIYGNPSCDLSLEGREWSYIVERPSAVNGLCYPGNVENLEELRSLFSSARSYQRIQIFPDTIW
NVSYDGTSNACSGSFYRSMRWLTRKNGDYPIQDAQYTNNQGKNILFMWGINHPPTDTTQRDLYTRTDTTT
SVATEEINRIFKPLIGPRPLVNGLMGRIDYYWSILKPGQTLRIKSDGNLIAPWYGYILSGESHGRILKTD
LKRGSCTVQCQTEKGGLNTTLPFQNVSKYAFGNCSKYIGIKSLKLAVGLRNVPSRSSRGLFGAIAGFIEG
GWSGLVAGWYGFQHSNDQGVGMAADRDSTQKAIDKITSKVNNIVDKMNKQYEIIDHEFSEVETRLSMINN
KIDDQIQDIWAYNAELLVLLENQKTLDEHDANVNNLYNKVKRALGSNAVEDGKGCFELYHKCDDQCMETI
RNGTYNRRKYQEESKLERQKIEGVKLESEGTYKILTIYSTVASSLVIAMGFAAFLFWAMSNGSCRCEHCI

>QBK19878.1 hemagglutinin [Influenza A virus]
METVSLITILLAATVSNADKICIGYQSTNSTETVDTLTENNVPVTHAKELLHTEHNGMLCATSLGQPLIL
NTCTIEGLIYGNPSCDLSLEGREWSYIVERPSAVNGLCYPGNVENLEELRSLFSSARSYQRIQIFPDTIW
NVSYDGTSNACSGSFYRSMRWLTRKNGDYPIQDAQYTNNQGKNILFMWGINHPPTDTTQRDLYTRTDTTT
SVATEEINRIFKPLIGPRPLVNGLMGRIDYYWSILKPGQTLRIKSDGNLIAPWYGYILSGESHGRILKTD
LKRGSCTVQCQTEKGGFNTTLPLQNVSKYAFGNCSKYIGIKSLKLADGLRNVRSRYSRGLFGAIAGFIEG
GWSGLVAGWYGFQHSNDQGVGMAADRDSTQKAIDKITSKVNNIVDKMNKQYEIIDHEFSEVETRLSMINN
KIDDQIQDIWAYNAELLVLLENQKTIDEHDANVNNLYNKVKRGLGSNAVEDGKGCFELYHKCDDQCMETI
RNGTYNRRKYQEESKLERQKIEGVKLECEGTYKILSIYSTVASYLVIAMGFVAFLFWAMFNGSSRCRACI

>QBK19877.1 hemagglutinin [Influenza A virus]
METVSLITILLAATVSNADKICIGYQSTNSTETVDTLTENNVPVTHAKELLHTEHNGMLCATSLGQPLIL
NTCTIEGLIYGNPSCDLSLEGREWSYIVERPSAVNGLCYPGNVENLEELRSLFSSARSYQRIQIFPDTIW
NVSYDGTSNACSGSFYRSMRWLTRKNGDYPIQDAQYTNNQGKNILFMWGINHPPTDTTQRDLYTRTDTTT
SVATEEINRIFKPLIGPRPLVNGLMGRIDYYWSILKPGQTLRIKSDGNLIAPWYGYILSGESHGRILKTD
LKRGSCTVQCQTEKGGLNTTLPFQNVSKYAFGNCSKYIGIKSLKLAVGLRNVPSRYSRGLFGAIAGFIEG

GWSGLVDGWYGFQHSNDQGVGMAADRDSTQKAIDKITSKVNNIVDKMNKQYEIIDHEFSEVETRISMINN
KIDDQIQDIWAYNAELLVLLENQKTLDEHDANVNNLYNKVKRALGSNAVEDGKGCFELYHKCDDQCMETI
RNGTYNRRKYQEESKLERQKIEGVKLESEGTYKILSIYSTVASSLVIAMGSAAFLFGAMSNGSGRCEHCI

>QBK19876.1 hemagglutinin [Influenza A virus]
METVSLITILLAATVSNADKICIGYQSTNSTETVDTLTENNVPVTHAKELLHTEHNGMLCATSLGQPLIL
NTCTIEGLIYGNPSCDLSLEGREWSYIVERPSAVNGLCYPGNVENLEELRSLFSSARSYQRIQIFPDTIW
NVSYDGTSNACSGSFYRSMRWLTRKNGDYPIQDAQYTNNQGKNILFMWGINHPPTDTTQRDLYTRTDTTT
SVATEEINRIFKPLIGPRPLVNGLMGRIDYYWSILKPGQTLRIKSDGNLIAPWYGYILSGESHGRILKTD
LKRGSCTVQCQTEKGGLNTTLPFQNVSKYAFGNCSKYIGIKSLKLAVGLRNVPSRSSRGLFGAIAGFIEG
GWSGLVDGWYGFQHSNDQGVGMAADRDSTQKAIDKITSKVNNIVDKMNKQYEIIDHEFSEVETRLSMINN
KIDDQIQDIWAYNAELLVLLENQKTLDEHDANVNNLYNKVKRALGSNAVEDGKGCFEIYHKCDDQCMETI
RNGTYNRRKYQEESKLERQKIEGVKLESEGTYKILSIYSTVASSLVIAMGSAAFLFWAMSNGSCRCEHCI

>QBK19875.1 hemagglutinin [Influenza A virus]
METVSVITILLIATVSNADKICIGYQSTNSAETVDTLTENNVPVTHAKELLHTEHNGMLCATSLGQPLIL
DTCTIEGLIYGNPSCDLSLEGREWSYIVERPSAVNGLCYPGNVENLEELRSLFSSARSYQRIQIFPDTIW
NVSYYGTSTACSGSFYRSMRWLTRKNGDYPVQDAQYTNNQGKNILFMWGINHPPTDTTQRDLYTRTDTTS
SVATEEINRIFKPLIGPRPLVNGLMGRIDYYWSVLKPGQTLRIKSDGNLIAPWYGHILSGESHGRILTTD
LKRGSCTVQCQTEKGGLHTALSLLNVSKYAFGNCSKYIGIKSLKLAVGLRNVRCRSSRGLFGAIAGFIER
GWSGLVAGWYGFQHSNDQGVGMAADRDSTQKAIDKITSKVNNIVDKMYKQYEIIDHEFSVVGTRIITINN
KFDDQIQDIWAYNAESLVLLDNQKTLDEHDANVNNLYNKVKRAAGSNAVEDGKGCFEQYHKCDDQCMETI
RNGTYNRRKYQAESKLERQKIEGLKLESDRTNKILSIYSRVASYRVIAMGFVAFLFGALYNGSGRSEQYI

>QBK19874.1 hemagglutinin [Influenza A virus]
METVSLITILLASTLSNADKICIGYRSTNSAETVNTLTENNVPVTHAKELLHTEHNGMLCATSLGQPLIL
NTCTIEGLIYGNPSCDLSLEGREWSYIVERPSAVNGLCYPGNVENLEELRSLFSSARSYQRIQIFPDTIW
NVSYDGTSNACSGSFYRSMRWLTQKNGDYPIQDAQYTNNQGKNILFMWGINHPPTDTTQRDLYTRTDTTT
SVATEEINRIFKPLIGPRPLVNGLMGRIDYYWSILKPGQTLRIKSDGNLIAPWYGYILSRESHGRILKTD
LKRGSCTVQCQTEKGGLNTTLPFQNVSKYAFGNCSKYIGIKSLKLAVGLRNVPSRYSRGLFGAIAGFIEG
GWSGLVDGWYGFQHSNDQGVGMAADRDSTQKAIDKITSKVNNIVDKMNKQYEIIDHEFSEVETRLSMINN
KIDDQIQDIWAYNAELLVLLENQKTLDEHDANVNNVYNKVKRALGSNAVEDGKGCFELYHKCDDQCMETI
RNGTYNRRKYQEESKLERQKIEGVKLESEGTYKILSIYSTVASSLVIAMGFAAFLFWAMSNGSCRCEHCI

>QBK19873.1 hemagglutinin [Influenza A virus]

METVSLITILLAATVSNADKICIGYQSTNSTETVDTLTENNVPVTHAKELLHTEHNGMLCATSLGQPLIL
NTCTIEGLIYGNPSCDLSLEGREWSYIVERPSAVNGLCYPGNVENLEELRSLFSSARSYQRIQIFPDTIW
NVSYDGTSNACSGSFYRSMRWLTRKNGDYPIQDAQYTNNQGKNILFMWGINHPPTDTTQRDLYTRTDTTT
SVATEEINRIFKPLIGPRPLVNGLMGRIDYYWSILKPGQTLRIKSDGNLIAPWYGYILSGESHGRILKTD
LKRGSCTVQCQTEKGGLNTTLPFQNVSKYAFGNCSKYIGIKSLKLAVGLRNVPSRYSRGLFGAIAGFIEG
GWSGLVDGWYGFQHSNDQGVGMAADRDSTQKAIDKITSKVNNIVDKMNKQYEIIDHEFSEVETRLSMINN
KIDDQIQDIWAYNAELLVLLENQKTLDEHDANVNNLYNKVKRALGSNAVEDGKGCFELYHKCDDQCMETI
RNGTYNRRKYQEESKLERQKIEGVKLESEGTYKILSIYSTVASYLVIAMGSAAFLFWAMSNGSCRCEHCI

>QBK19872.1 hemagglutinin [Influenza A virus]

METVSLITILLAATVSNADKICIGYQSTNSTETVDTLTENNVPVTHAKELLHTEHNGMLCATSLGQPLIL
NTCTIEGLIYGNPSCDLSLEGREWSYIVERPSAVNGLCYPGNVENLEELRSLFSSARSYQRIQIFPDTIW
NVSYDGTSNACSGSFYRSMRWLTRKNGDYPIQDAQYTNNQGKNILFMWGINHPPTDTTQRDLYTRTDTTT
SVATEEINRIFKPLIGPRPLVNGLMGRIDYYWSILKPGQTLRIKSDGNLIAPWYGYILSGESHGRILKTD
LKRGSCTVQCQTEKGGLNTTLPFQNVSKYAFGNCSKYIGIKSLKLAVGLRNVPSRSSRGLFGAIAGFIEG
GWSGLVAGWYGFQHSNDQGVGMAADRDSTQKAIDKITSKVNNIVDKMNKQYEIIDHEFSEVETRLSMINN
KIDDQIQDIWAYNAELLVLLENQKTLDEHDANVNNLYNKVKRALGSNAVEDGKGCFELYHKCDDQCMETI
RNGTYNRRKYQEESKLERQKIEGVKLESEGTYKILTIYSTVASSLVIAMGFAAFLFWAMSNGSCRCEHCI

>QBK19871.1 hemagglutinin [Influenza A virus]

METVSLITILLAATVSNADKICIGYQSTNSTETVDTLTENNVPVTHAKELLHTEHNGMLCATSLGQPLIL
NTCTIEGLIYGNPSCDLSLEGREWSYIVERPSAVNGLCYPGNVENLEELRSLFSSARSYQRIQIFPDTIW
NVSYDGTSNACSGSFYRSMRWLTRKNGDYPIQDAQYTNNQGKNILFMWGINHPPTDTTQRDLYTRTDTTT
SVATEEINRIFKPLIGPRPLVNGLMGRIDYYWSILKPGQTLRIKSDGNLIAPWYGYILSGESHGRILKTD
LKRGSCTVQCQTEKGGLNTTLPFQNVSKYAFGNCSKYIGIKSLKLAVGLRNVPSRSSRGLFGAIAGFIEG
GWSGLVAGWYGFQHSNDQGVGMAADRDSTQKAIDKITSKVNNIVDKMNKQYEIIDHEFSEVETRLSMINN
KIDDQIQDIWAYNAELLVLLENQKTLDEHDANVNNLYNKVKRALGSNAVEDGKGCFELYHKCDDQCMETI
RNGTYNRRKYQEESKLERQKIEGVKLESEGTYKILTIYSTVASSLVIAMGFAAFLFWAMSNGSCRCNICI

>QBF57703.1 hemagglutinin [Influenza A virus]

METVSLITILLVATVSNADKICIGYQSTNSTETVDTLTENNVPVTHAKELLHTEHNGMLCATSLGQPLIL
DTCTIEGLIYGNPSCDLSLEGREWSYIVERPSAVNGLCYPGNVENLEELRSLFSSARSYQRIQIFPDTIW
NVSYDGTSTACSNSFYRSMRWLTRKDGNYPTQDAQYTNNQGKNILFMWGINHPPTDETQRTLYTRTDTTT

SVATEEINRIFKPLIGPRPLVNGLMGRIDYYWSVLKPGQTLRIKSDGNLIAPWYGHILSGESHGRILKTD

LKRGSCTVQCQTEKGGLNTTLPFQNVSKYAFGNCSKYIGIKSLKLAVGLRNVPSRSSRGLFGAIAGFIEG

GWSGLVAGWYGFQHSNDQGVGMAADRDSTQKAIDKITSKVNNIVDKMNKQYEIIDHEFSEVETRLNMINN

KIDDQIQDIWAYNAELLVLLENQKTLDEHDANVNNLYNKVKRALGSNAVEDGKGCFELYHKCNDQCMETI

RNGTYNRKKYQEESKLERQRIEGVKLESEGTYKILTIYSTVASSLVIAMGFAAFLFWAMSNGSCRCIIFK

>QBF57702.1 hemagglutinin [Influenza A virus]

METVSLITILLIATVSNADKICIGYQSTNSTETVDTLTENNVPVTHAKELIHTEHNGMLCATSLGQPLIL

DTCTIEGLIYGNPSCDLPLEGKEWSYIVERPSAVNGLCYPGNVENLEELRSLFSSARSYQRIQIFPDTIW

NVSYDGTSRACSDSFYRNMRWLTRKDGNYPTQDAQYTNNQGKNILFMWGINHPPTDTTQNDLYTRTDTTT

SVATEEINRIFKPLIGPRPLVNGLMGRIDYYWSVLKPGQTLRIKSDGNLIAPWFGHILSGESHGRILKTD

LKRGSCTVQCQTEKGGLNTTLPFQNVSKYAFGNCSKYIGIKSLKLAVGLRNVPSRSSRGLFGAIAGFIEG

GWSGLVAGWYGFQHSNDQGVGMAADRDSTQRAIDKITSKVNNIVDKMNKQYEIIDHEFSEVETRLNMINN

KIDDQIQDIWAYNAELLVLLENQKTLDEHDANVNNLYNKVKRALGSNAVEDGKGCFELYHKCDDQCMETI

RNGTYNRRKYQEESKLERQKIEGVKLESEGTYKILTIYSTVASSLVIAMGFAAFLFWAMSNGSCRCNICI

>QBF57701.1 hemagglutinin [Influenza A virus]

METVSLMTILLVATVSNADKICIGYQSTNSTETVDTLTENNVPVTHAKELLHTEHNGMLCATSLGQPLIL

DTCTIEGLIYGNPSCDLSLEGREWSYIVERPSAVHGLCYPGNVEDLEELRSLFSSARSYQKIQIFPDTIW

NVSYDGTSTACSGSFYKSMRWLTRKNGEYPTQDAQYTNNQGKNILFMWGINHPPTDDTQRGLYTRTDTTT

SVATEEINRIFKPLIGPRPLVNGLMGRINYYWSVLKPGQTLRIKSDVNLIAPWYGHILSRESQGRILKTD

LKRGSCTVQCQTEKGGLNTTLPFQNVSKYAFGNCSKYIGIKSLKLAVGLRNVPSRSSRGLFGAIAGFIEG

GWSGLVAGWYGFQHSNDQGVGMAADRDSTQKAIDKITSKVNNIVDKMNKQYEIIDHEFSEVETRLNMINN

KIDDQIQDIWAYNAELLVLLENQKTLDEHDANVNNLYNKVKRALGSNAVEDGKGCFELYHKCDDQCMETI

RNGTYNRRKYQEESKLERQKIEGVKLESEGTYKILSIYSTVASSLVIAMGFAAFLFWAMSNGSCRCNICI

>QBF57700.1 hemagglutinin [Influenza A virus]

METVSLITILLVATVSNADKICIGYQSTNSTETVDTLTENNVPVTHAKELLHTEHNGMLCATSLGQPLIL

DTCTIEGLIYGNPSCDLSLEGREWSYIVERPSAVHGLCYPGNVEYLEELRSLFSSARSYQKIQIFPHTIW

NVSYDGTSTACSGSFYRSMRWLTRKNGEYPIQDAQYTNNQGKNILFMWGINHPPTDDTQIGLYTITDTTT

SVATEEINRIFKPLIGPRPLVNGLMGRINYYWSVLKPGQTLRIKSDGNLIAPWYGHILSRESYGSLLTTD

LKRGSCTVQCQTGKGGLNTALPFQNVSKYAFGNCSKYIGIKSLKLAVGLRNVRSRYSRGLFGAIAGFIEG

GWSGLVAGWYGFQHSNDQGVGMAADRDSTQKAIDKITSKVNNIVDKMNKQYEIIDHEFSEVETRINMINN

KIDDQIQDIWAYNAELLVLLENQKTLDEHDANVNNLYNKVKRALGSNAVEDGKGCFELYHKCDDQCMETI

RNGTYNRRKYQEESKLERQKIEGVKLESEGTYKILSIYSTVASSLVIAMGSAAFLFWAMSNGSCRCNICI

>QBF57697.1 hemagglutinin [Influenza A virus]
METVSLMTILLVATVSNADKICIGYQSTNSTETVDTLTENNVPVTHAKELLHTEHNGMLCATSLGQPLIL
DTCTIEGLIYGNPSCDLSLEGREWSYIVERPSAVHGLCYPGNVEDLEELRSLFSSARSYQRIQIFPDTIW
NVSYDGTSTACSGSFYKSMRWLTRKNGEYPTQDAQYTNNQGKNILFMWGINHPPTDDTQRGLYTRTDTTT
SVATEEINRIFKPLIGPRPLVNGLMGRINYYWSVLKPGQTLRIKSDGNLIAPWYGHILSGESHGRILKTD
LKRGSCTVQCQTEKGGLNTTLPFQNVSKYAFGNCSKYIGIKSLKLAVGLRNVPSRSSRGLFGAIAGFIEG
GWSGLVAGWYGFQHSNDQGVGMAADRDSTQKAIDKITSKVNNIVDKMNKQYEIIDHEFSEVETRLNMINN
KIDDQIQDIWAYNAELLVLLENQKTLDEHDANVNNLYNKVKRALGSNAVEDGKGCFELYHKCDDQCMETI
RNGTYNRRKYQEESKLERQKIEGVKLESEGTYKILTIYSTVASSLVIAMGFAAFLFWAMSNGSCRCNICI

>QBF57696.1 hemagglutinin [Influenza A virus]
METVSLITILLVATVSNADKICIGYQSTNSTETVDTLTENNVPVTHAKELLHTEHNGMLCATSLGQPLIL
DTCTIEGLIYGNPSCDLSLEGREWSYIVERPSAVHGLCYPGNVEDLEELRSLFSSARSYQRIQIFPDTIW
NVSYDGTSTACSGSFYRSMRWLTRKNGEYPIQDAQYTNNQGKNILFMWGINHPPTDDTQRGLYTRTDTTT
SVATEEINRIFKPLIGPRPLVNGLMGRINYYWSVLKPGQTLRIKSDGNLIAPWYGHILSGESHGRILKTD
LKRGSCTVQCQTEKGGLNTTLPFQNVSKYAFGNCSRYIGIKSLKLAVGLRNVPSRSSRGLFGAIAGFIEG
GWSGLVAGWYGFQHSNDQGVGMAADRDSTQKAIDKITSKVNNIVDKMNKQYEIIDHEFSEVETRLNMINN
KIDDQIQDIWAYNAELLVLLENQKTLDEHDANVNNLYNKVKRALGSNAVEDGKGCFELYHKCDDQCMETI
RNGTYNRRKYQEESKLERQKIEGVKLESEGTYKILTIYSTVASSLVIAMGFAAFLFWAMSNGSCRCNICI

>QBF57684.1 hemagglutinin [Influenza A virus]
METVSLMTILLVATVSNADKICIGYQSTNSTETVDTLTENNVPVTHAKELLHTEHNGMLCATSLGQPLIL
DTCTIEGLIYGNPSCDLSLEGREWSYIVERPSAVHGLCYPGNVEDLEELRSLFSSARSYQRIQIFPDTIW
NVSYDGTSTACSGSFYKSMRWLTRKNGEYPTQDAQYTNNQGKNILFMWGINHPPTDDTQRGLYTRTDTTT
SVATEEINRIFKPLIGPRPLVNGLMGRINYYWSVLKPGQTLRIKSDGNLIAPWYGHILSGESHGRILKTD
LKRGSCTVQCQTEKGGLNTTLPFQNVSKYAFGNCSKYIGIKSLKLAVGLRNVPSRSSRGLFGAIAGFIEG
GWSGLVAGWYGFQHSNDQGVGMAADRDSTQKAIDKITSKVNNIVDKMNKQYEIIDHEFSEVETRLNMINN
KIDDQIQDIWAYNAELLVLLENQKTLDEHDANVNNLYNKVKRALGSNAVEDGKGCFELYHKCDDQCMETI
RNGTYNRRKYQEESKLERQKIEGVKLESEGTYKILTIYSTVASSLVIAMGFAAFLFWAMSNGSCRCNICI

>QBF57676.1 hemagglutinin [Influenza A virus]
METVSLMTILLIATVSNADKICIGYQSTNSTETVDTLTENNVPVTHAKELLHTEHNGMLCATSLGQPLIL

DTCTIEGLIYGNPSCDLSLEGREWSYIVERPSAVHGLCYPGNVEDLEELRSLFSSARSYQRIQIFPDTIW
NVSYDGTSTACSGSFYKSMRWLTRKNGEYPTQDAQYTNNQGKNILFMWGINHPPTDDTQRGLYTRTDTTT
SVATEEINRIFKPLIGPRPLVNGLMARINYYWSVLKPDQTLRIKSDGNLIAPWYGHILSEESHGRILKTE
LKRGRCTGQCQTEKGGLNTTQSFQNVSKYAFGNCSKYIGIKSLKLAAGLRNVPSRYSRGLFGAIAGFIEG
GWSGLVAGWYGFQHSNDQGVGMAADRDSTQKAIDKITSKVNNIVDKMNKQYEIIDHEFSEVETRINMINN
KIDDQIQDIWAYNAELIVLLENQKTLDEHDANVNNLYNKVKRALGSNAVEDGKGCFELYHKCDDQCMETI
RNGTYNRRKYQEESKLERQKIEGVKLESEGTYKILSIYSTVASYLVIAMGSAALLFWAMSNGSCRCNNCI

>QBF57675.1 hemagglutinin [Influenza A virus]
METVSLMTILLVATVSNADKICIGYQSTNSTETVDTLTENNVPVTHAKELLHTEHNGMLCATSLGQPLIL
DTCTIEGLIYGNPSCDLSLEGREWSYIVERPSAVHGLCYPGNVEDLEELRSLFSSARSYQRIQIFPDTIW
NVSYDGTSTACSGSFYKSMRWLTRKNGEYPTQDAQYTNNQGKNILFMWGINHPPTDDTQRGLYTRTDTTT
SVATEEINRIFKPLIGPRPLVNGLMGRINYYWSVLKPGQTLRIKSDGNLIAPWYGHILSGESHGRILKTD
LKRGSCTVQCQTEKGGLNTTLPFQNVSKYAFGNCSKYIGIKSLKLAVGLRNVPSRSSRGLFGAIAGFIEG
GWSGLVAGWYGFQHSNDQGVGMAADRDSTQKAIDKITSKVNNIVDKMNKQYEIIDHEFSEVETRLNMINN
KIDDQIQDIWAYNAELLVLLENQKTLDEHDANVNNLYNKVKRALGSNAVEDGKGCFELYHKCDDQCMETI
RNGTYNRRKYQEESKLERQKIEGVKLESEGTYKILTIYSTVASSLVIAMGFAAFLFWAMSNGSCRCNICI

>QBF57674.1 hemagglutinin [Influenza A virus]
METVSLMTILLVATVSNADKICIGYQSTNSTETVDTLTENSVPVTHAKELLHTEHNGMLCATSLGQPLIL
DTCTIEGLIYGNPSCDLSLEGREWSYIVERPSAVHGLCYPGNVEDLEELRSLFSSARSYQRIQIFPDTIW
NVSYDGTSTACSGSFYKSMRWLTRKNGEYPTQDAQYTNNQGKNILFMWGINHPPTDDTQRGLYTRTDTTT
SVATEEINRIFKPLIGPRPLVNGLMGRINYYWSVLKPGQTLPIKSDGNLIAPWYGHILSEEPHGRILKTD
LKRGSCTVQCQTEKDGLHTTLSLQNVSKYASGNCSKYSGIKSLKLAVGLRNVPSRYSRGVFGAIAGFIER
GWSGLVAGWYGFQHSNDQGVGMAADRDSTQKAIDKITSKVNNIVDKLNKQYEIIDHEFSEVETRINMINN
KIDDQIQDIWAYNAELLVLLENQKTLDEHDANVNNLYNKVKRALGSNAVEDGKGCFELYHKCDDQCMETI
RNGTYNRRKYQEESKLERQKIEGVKLESEGTYKILSIYSTVASSLVIAMGFAAFLFWAMSNGSCRCNDGI

>QBF57673.1 hemagglutinin [Influenza A virus]
METVSLITILLVATVSNADKICIGYQSTNSTETVDTLTENNVPVTHAKELLHTEHNGMLCATSLGQPLIL
DTCTIEGLIYGNPSCDLSLEGREWSYIVERPSAVHGLCYPGNVQDLEELRSLFSSARSYQRIQIFPDTIW
NVSYDGTSTACSGSFYRSMRWLTRKNGEYPIQDAQYTNNQGKNILFMWGINHPPTDDTQRGLYTRTDTTT
SVATEEINRIFKPLIGPRPLVNGLMGRINYYWSVLKPGQTLRIKSDGNLIAPWYGHILSGESHGRILETD
LKRGSCTVQCQTEKGGLNTTLPFQNVSKYAFGNCSKYIGIKSLKLAVGLRNVPSRSSRGLFGAIAGFIEG

GWSGLVAGWYGFQHSNDQGVGMAADRDSTQKAIDKITSKVNNIVDKMNKQYEIIDHEFSEVETRLNMINN
KIDDQIQDIWAYNAELLVLLENQKTLDEHDANVNNLYNKVKRALGSNAVEDGKGCFELYHKCDDQCMETI
RNGTYNRRKYQEESKLERQKIEGVKLESEGTYKILTIYSTVASSLVIAMGFAAFLFWAMSNGSCRCNICI

>QBF57672.1 hemagglutinin [Influenza A virus]
METVSLITILLVATVSNADKICIGYQSTNSTETVDTLTENNVPVTHAKELLHTEHNGMLCATSLGQPLIL
DTCTIEGLIYGNPSCDLSLEGREWSYIVQRPSTVHGLCYPGNVEYLEELRSLFSSARSYQRIQIFPDTIW
NVSYDGTSTACSGSFYKSMRWLTRKNGEYPIQDAQYTNNLGKNILFMWGINHPPTDDTQIGLYTITDTTS
SVATDEINTIFKQLIGPRPLVNGLMGRINYYWSVLKPGQTLRIKSDGNLIAPWYGHILSRESHGRILKTD
LKRGSCTVQCQTEKGGLNTTLRLQNVSKYALENCSKYIGIKSLKLAVGLRNVPSRSSRGLCGAIAGFIEG
GWSGLVAGWYGFQHSNDQGVGMAADRDSTQKAIDKITSKVNNIVDKMNKQYEIIDHEFSEVETRINMINN
KIDDQIQDIWAYNAELLVLLENQKTLDEHDANVNNLYNKVKRALGSNAVEDGKGCFELYHKCDDQCMETI
RNGTYNRRKYQEESKLERQKIEGVKLESEGTYKILTIYSTVASSLVIAMGFAAFLFWAMSNGSCRCNICI

>QBF57671.1 hemagglutinin [Influenza A virus]
METVSLMTILLVATVSNADKICIGYQSTNSTETVDTLTENNVPVTHAKELLHTEHNGMLCATSLGQPLIL
DTCTIEGLIYGNPSCDLSLEGREWSYIVERPSAVHGLCYPGNVEDLEELRSLFSSARSYQRIQIFPDTIW
NVSYDGTSTACSGSFYKSMRWLTRKNGEYPTQDAQYTNNQGKNILFMWGINHPPTDDTQRGLYTRTDTTT
SVATEEINRIFKPLIGPRPLVNGLMGRINYYWSVLKPGQTLRIKSDGNLIAPWYGHILSGESHGRILKTD
LKRGSCTVQCQTEKGGLNTTLPFQNVSKYAFGNCSKYIGIKSLKLAVGLRNVPSRSSRGLFGAIAGFIEG
GWSGLVAGWYGFQHSNDQGVGMAADRDSTQKAIDKITSKVNNIVDKMNKQYEIIDHEFSEVETRLNMINN
KIDDQIQDIWAYNAELLVLLENQKTLDEHDANVNNLYNKVKRALGSNAVEDGKGCFELYHKCDDQCMETI
RNGTYNRRKYQEESKLERQKIEGVKLESEGTYKILTIYSTVASSLVIAMGFAAFLFWAMSNGSCRCNICI

>QBF57670.1 hemagglutinin [Influenza A virus]
METVSLITILLVATVSNADKICIGYQSTNSTETVDTLTENNVPVTHAKELLHTQHNGMLCATSLGQPLIL
DTCTIEGLIYGNPSCDLSLEGREWSYIVQRPSTVHGLCYPGNVQYLEKLRSLFSSATSYQKIQIFPHTIW
NVSYDGTSTACSGSFYRTMRWLTPINGEYPIQDAQYTNNQGKNILFMWGINHPPTDDTQIGLYTITDTTT
SVATDEINTIFKQLIEPRPLVNGLMGRINYYRSVLKPGQTLPIKSDGNLIASWYGHVLSRESHGVLLKTD
LKRGICTVQCQTDKGGLNTTQSCQNVSKYAFGNCSKYIGIKSVKLAAGLRNVRSRYSRGLFGAIAGFIEG
GWSGLVAGWYGFQHSNDQGVGMAADRDSTQKAIDKITSKVNNIVDKMNKQYEIIDHEFSEVETRINMINN
KIDDQIQDIWAYNAELLVLLENQKTLDEHDANVNNLYNKVKRALGSNAVEDGKGCFELYHKCDDQCMETI
RNGTYNRRKYQEESKLERQKIEGVKLESEGTYKILTIYSTVASSLVIAMGFAAFLFWAMSNGSCRCNICI

>QBF57669.1 hemagglutinin [Influenza A virus]

METVSRITILLVATVINAYNFCIGYQSANSTETVDTLTENNVPVTHAKELLHTEHNGMLCATSLGQPLIL

DTCTIEGLIYGNPSCDLSLEGREWSYIVQRPSAVHGLCYPGNVQDLEELRSLFSSARSYQRIQIFPHTIW

NVSYDGTSTACSGSFYRSMRWLTRKNGEYPIQDAQYTNNQGKNILFMWGINHPPTDDTQIGLYTRTDTTS

SVATEEINRIFKPLIGPRPLVNGLMGRINYYWSVLKPGQTLRIKSDGNLIALWYGHILFMKSHGSTLTTD

LDMGSCTVQCQTEKGGLNTTLSFQNVSKYAFGNCSKYIGIKSLKLAVGLRNVPSRSSRGLFGAIAGFIEG

GWSGLVAGWYGFQHSNDQGVGMAADRDSTQKAIDKITSKVNNIVDKMNKQYEIIDHEFSEVETRLNMINN

KIDDQIQDIWAYNAELLVLLENQKTLDEHDANVNNLYNKVKRALGSNAVEDGKGCFELYHKCDDQCMETI

RNGTYNRRKYQEESKLERQKIEGVKLESEGTYKILTIYSTVASSLVIAMGFAAFLFWAMSNGSCRCNICI

>QBF57668.1 hemagglutinin [Influenza A virus]

METVSLITILLVATVSNADKICIGYQSTNSTETVDTLTENNVPVTHAKELLHTEHNGMLCATSLGQPLIL

DTCTIEGLIYGNPSCDLSLEGREWSYIVERPSAVHGLCYPGNVEDLEELRSLFSSARSYQRIQIFPDTIW

NVSYDGTSTACSGSFYRSMRWLTRKNGEYPIQDAQYTNNQGKNILFMWGINHPPTDDTQRGLYTRTDTTT

SVATEEINRIFKPLIGPRPLVNGLMGRINYYWSVLKPGQTLRIKSDGNLIAPWYGHILSGESHGRILKTD

LKRGSCTVQCQTEKGGLNTTLPFQNVSKYAFGNWSKYIGIKSLKLAVGLRNVPSRSSRGLFGAIAGFIEG

GWSGLVAGWYGFQHSNDQGVGMAADRDSTQKAIDKITSKVNNIVDKMNKQYEIIDHEFSEVETRLNMINN

KIDDQIQDIWAYNAELLVLLENQKTLDEHDANVNNLYNKVKRALGSNAVEDGKGCFELYHKCDDQCMETI

RNGTYNRRKYQEESKLERQKIEGVKLESEGTYKILTIYSTVASSLVIAMGFAAFLFWAMSNGSCRCSLSK

>QBF57667.1 hemagglutinin [Influenza A virus]

METVSLITILLVSTVSNANKICLGYQSTNSTETVDTLTENNVPVTHAKELLHTEHNGMLCATSLGQPLIL

DTCTIEGLIYGNPSCDLSLEGREWSYIVQRPSAVHGLCYPGNVQYLEELRSLFSSARSYQKIQIFPHTIW

NVSYDGTSTACSGSFYRSMRWLTRKNGEYPIQDAQYTNNQGKNILFMWGINHPPTDDTQRGLYTITDTTT

SVATEEINRIFKPLIGPRPLVNGLMGRINYYWSVLKPGQTLRIKSDGNLIAPWYGHILHEESHGSILNTD

LKRGSCTVQCQTDKGGLNTTLPLQNVSKYAFGNCSKYIGIKSLKLAVGLRNVPSRSSRGLFGAIAGFIER

GWSGLVAGWYGFQHSNDQGVGMAADRDSTQKAIDKITSKVNNIVDKMNKQYEIIDHEFSEVETRLNMINN

KIDDQIQDIWAYNAELLVLLENQKTLDEHDANVNNLYNKVKRALGSNAVEDGKGCFELYHKCDDQCIETI

RNGTYNRRKYQEESKLERQKIEGVKLESEGTYKILTIYSTVASSLVIAMGSAAFLFWAMSNGSCRCNICI

>QBF57666.1 hemagglutinin [Influenza A virus]

METVSLITILLVATVSNADKICIGYQSTNSTETVDTLTENNVPVTHAKELLHTEHNGMLCATSLGQPLIL

DTCTIEGLIYGNPSCDLSLEGREWSYIVERPSAVHGLCYPGNVEDLEELRSLFSSARSYQRIQIFPDTIW

NVSYDGTSTACSGSFYRSMRWLTRKNGEYPIQDAQYTNNQGKNILFMWGINHPPTDDTQRGLYTRTDTTT

SVATEEINRIFKPLIGPRPLVNGLMGRINYYWSVLKPGQTLRIKSDGNLIAPWYGHILSGESHGRILKTD
LKRGSCTVQCQTEKGGLNTTLPFQNVSKYAFGNCSKYIGIKSLKLAVGLRNVPSRSSRGLFGAIAGFIEG
GWSGLVAGWYGFQHSNDQGVGMAADRDSTQKAIDKITSKVNNIVDKMNKQYEIIDHEFSEVETRLNMINN
KIDDQIQDIWAYNAELLVLLENQKTLDEHDANVNNLYNKVKRALGSNAVEDGKGCFELYHKCDDQCMETI
RNGTYNRRKYQEESKLERQKIEGVKLESEGTYKILTIYSTVASSLVIAMGFAAFLFWAMSNGSCRCNICI

>QBF57665.1 hemagglutinin [Influenza A virus]
METVSLITILLVATVSNADKICIGYQSTNSTETVDTLTENNVPVTHAKELLHTEHNGMLCATSLGQPLIL
DTCTIEGLIYGNPSCDLSLEGREWSYIVERPSAVHGLCYPGNVEDLEELRSLFSSARSYQRIQIFPDTIW
NVSYDGTSTACSGSFYRSMRWLTRKNGEYPIQDAQYTNNQGKNILFMWGINHPPTDDTQRGLYTRTDTTT
SVATEEINRIFKPLIGPRPLVNGLMGRINYYWSVLKPGQTLRIKSDGNLIAPWYGHILSGESHGRILKTD
LKRGSCTVQCQTEKGGLNTTLPFQNVSKYAFGNCSKYIGLKSLKLAVGLRNVPSRSSRGLFGAIAGFIEG
GWSGLVAGWYGFQHSNDQGVGMAADRDSTQKAIDKITSKVNNIVDKMNKQYEIIDHEFSEVETRLNMINN
KIDDQIQDIWAYNAELLVLLENQKTLDEHDANVNNLYNKVKRALGSNAVEDGKGCFELYHKCDDQCMETI
RNGTYNRRKYQEESKLERQKIEGVKLESEGTYKILTIYSTVASSLVIAMGFAAFLFWAMSNGSCRCNICL

>QBF57664.1 hemagglutinin [Influenza A virus]
METVSLITILLVATVSNADKICIGYQSTNSTETVDTLTENNVPVTHAKELLHTEHNGMLCATSLGQPLIL
DTCTIEGLIYGNPSCDLSLEGREWSYIVERPSAVHGLCYPGNVEDLEELRSLFSSARSYQRIQIFPDTIW
NVSYDGTSTACSGSFYRSMRWLTRKNGEYPIQDAQYTNNQGKNILFMWGINHPPTDDTQRGLYTRTDTTT
SVATEEINRIFKPLIGPRPLVNGLMGRINYYWSVLKPGQTLRIKSDGNLIAPWYGHILFRESHGAFLKTD
LKRGSCTVQCQTEKGGLNTTLSFQNVSKYAFGNCSKYIGIKSLKLAVGLRNVPSRSSRGLFGAIAGFIEG
GWSGLVAGWYGFQHSNDQGVGMAADRDSTQKAIDKITSKVNNIVDKMNKQYEIIDHEFSEVETRLNMINN
KIDDQIQDIWAYNAELLVLLENQKTLDEHDANVNNLYNKVKRALGSNAVEDGKGCFELYHKCDDQCMETI
RNGTYNRRKYQEESKLERQKIEGVKLESEGTYKILTIYSTVASSLVIAMGFAAFLFWAMSNGSCRCNICI

>QBF57663.1 hemagglutinin [Influenza A virus]
METVSLITILLVATVSNADKICIGYQSTNSTETVDTLTENNVPVTHAKELLHTEHNGMLCATSLGQPLIL
DTCTIEGLIYGNPSCDLSLEGREWSYIVERPSAVHGLCYPGNVEDLEELRSLFSSARSYQRIQIFPDTIW
NVSYDGTSTACSGSFYRSMRWLTRKNGEYPIQDAQYTNNQGKNILFMWGINHPPTDDTQRGLYTRTDTTT
SVATEEINRIFKPLIGPRPLVNGLMGRINYYWSVLKPGQTLRIKSDGNLIALWYGHILSVESHGRILKTD
LKRGSCTVQVRTEKGGLNTTLPFQNVSKYAFGNCSKYIGIKSLKLAVGLRNVPSRSSRGLFGAIAGFIEG
GWSGLVAGWYGFQHSNDQGVGMAADRDSTQKAIDKITSKVNNIVDKMNKQYEIIDHEFSEVETRLNMINN
KIDDQIQDIWAYNAELLVLLENQKTLDEHDANVNNLYNKVKRALGSNAVEDGKGCFELYHKCDDQCMETI

RNGTYNRRKYQEESKLERQKIEGVKLESEGTYKILTIYSTVASSLVIAMGFAAFLFWAMSNGSCRCNICI

>QBF57662.1 hemagglutinin [Influenza A virus]
METVSLITILLVATVSNANEICIGYQSTNSTETVDTLTENNVPVTHAKELLHTEHNGMLCATNLGQPLIL
DTCTIEGLIYGNPSCDLSLEGREWSYIVQRPSTVHGLCYPGNVQYLEELMSLFSSARSYQKIQIFPHTIW
NVSYDGTSTACSGSFYRSMRWLTRKNGEYPIQDAQYTNNQGKNILFMWGINHTPTDDTQIGLYTITDTTT
SVATEEINKIFKPLIGPRPLVSGLMGRINYYWSVLEPGQTLRIKSDGNLIAPWYGHILSRESHGGILKAD
LKRGRCTGQCQTEKDGLNTTQSFQNVSKYASGNCSKYIGIKSLKLAAGLRNVPSRYSRGLFGAIAGFIEG
GWSGLVAGWYGFQHSNDQGVGMAADRDSTQKAIDKITSKVNNIVDKMNKQYEIIDHEFSEVETRLNMINN
KIDDQIQDIWAYNAELLVLLENQKTLDEHDANVNNLYNKVKRALGSNAVEDGKGCFELYHKCNDQCMETI
RNGTYNRRKYQEESKLERQKIEGVKLESEGTYKILSIYSTVASSLVIAMGSAALLFWAMSNGSCRCNDCM

>QBF57661.1 hemagglutinin [Influenza A virus]
METVSVITMLLGSTESNANEICLGYQSANSAETVDTLTQHYVPVTHAKELLHTQRNGMLCAPNLGQPLIL
DTCSIEGLIYGYPSCDLSLEGREWSYIVQIPSTVHGLCYPGNVQYQEELRSLFSSATSYQKIQIFPHTIW
NVSYDGTSMPCSGSFYISLIWLTPLNCDHPIQDPPYPNNLELNILFMWGINHTPTDDTQIRLYTITDTTF
CVATDEINTIFKQLIEPRPLVNELMTRINYYRSVLMPGQTLQVKSDVNLLASWYGPILSRESYGALLTTD
LKRGNCTVQCQTEKGGLNTTQSCQNVSKQASGNCSKYIGIKSVKLAVGLRKVRCRYIRGVFGAVAGFIER
GWSGLVGGWYGFQHSNDQGVGMAADRDSTQKAMDKITSKVNNIVDKMNKPYEIIGLEFIVVGTRMDTSGH
KFDDQIQDIWAYNAESIVLLENQKTLDEQDANVSNGGNKVKRALGSNEVEDENGCFELYHKCDDQCIETI
RNGTYNRRKYQEESQLERQKMEGVKLESEGTREILSLYSRVASYLVIAMRSAALLIGAMSNGYCRCNECI

>QBF57660.1 hemagglutinin [Influenza A virus]
METVSLITILLVATVSNADKICIGYQSTNSTETVDTLTENNVPVTHAKELLHTEHNGMLCATSLGQPLIL
DTCTIEGLIYGNPSCDLSLEGREWSYIVERPSAVHGLCYPGNVEDLEELRSLFSSARSYQRIQIFPDTIW
NVSYDGTSTACSGSFYRSMRWLTRKNGEYPIQDAQYTNNQGKNILFMWGINHPPTDDTQRGLYTRTDTTT
SVATEEINRIFKPLIGPRPLVNGLMGRINYYWSVLNPGQTLRIKSDGNLIAPWYGHILLRESHGRILKTD
LKRGSCTVQCQTEKGGLNTTLSFQNVSKYAFGNCSRYIGIKSLKLAVGLRNVPSRSSRGLFGAIAGFIEG
GWSGLVAGWYGFQHSNDQGVGMAADRDSTQKAIDKITSKVNNIVDKMNKQYEIIDHEFSEVETRLNMINN
KIDDQIQDIWAYNAELLVLLENQKTLDEHDANVNNLYNKVKRALGSNAVEDGKGCFELYHKCNDQCMETI
RNGTYNRRKYQEESKLERQKIEGVKLESEGTYKILTIYSTVASSLVIAMGFAAFLFWAMSNGSCRCNICI

>QBF57659.1 hemagglutinin [Influenza A virus]
METVSLITILLVATVSNADKICIGYQSTNSTETVDTLTENNVPVTHAKELLHTEHNGMLCATSLGQPLIL

DTCTIEGLIYGNPSCDLSLEGREWSYIVERPSAVHGLCYPGNVEDLEELRSLFSSARSYQRIQIFPDTIW
NVSYDGTSTACSGSFYRSMRWLTRKNGEYPIQDAQYTNNQGKNILFMWGINHPPTDDTQRGLYTRTDTTT
SVATEEINRIFKPLIGPRPLVNGLMGRINYYWSVLKPGQTLRIKSDGNLIAPWYGHISLRESYGRILKTD
LKRGSCTVQCQTEKGGLNTTLSFQNVSKYAFGNCSRYIGIKSLKLAVGLRNVPSRSSRGLFGAIAGFIEG
GWSGLVAGWYGFQHSNDQGVGMAADRDSTQKAIDKITSKVNNIVDKMNKQYEIIDHEFSEVETRLNMINN
KIDDQIQDIWAYNAELLVLLENQKTLDEHDANVNNLYNKVKRALGSNAVEDGKGCFELYHKCNDQCMETI
RNGTYNRRKYQEESKLERQKIEGVKLESEGTYKILTIYSTVASSLVIAMGFAAFLFWAMSNGSCRCNICI

>QBF57658.1 hemagglutinin [Influenza A virus]
METVSLITILLVATESNANEICIGYQSTNSTETVDTLTENNVPVTHAKELLHTEHNGMLCATSLGQPLIL
DTCTIEGLIYGNPSCDLSLEGREWSYIVERPSTVHGLCYPGNVQYLEELRSLFSSARSYQKIQIFPHTIW
NVSYDGTSTACSGSFYRSMRWLTRKNGEYPIQDAQYTNNQGKNILFMWGINHPPTDDTQIRLYTITDTTT
SVATEEINRIFKQLIGPRPLVNGLMGRINYYWSVLKPGQTLQVKSDGNLLAPWYGHVLSGERHGRILNTD
LKRGSCTASCQTEKGGLHTTLSFQNVSKYASGNCSRYIGIKSLKLAVGLRNVPSRYSRGLFGAIAGFIEG
GWSGLVGGWYGFQHSNDQGVGMAADRDSTQKAIDKITSKVNNIVDKMNKQYEIIDLEFSEVETRINMSNN
KIDDQIQDIWAYNAELLVLLENQKTLDEHDANVNNLYNKVKRALGSNAVEDGKGCFELYHKCNDQCMETI
RNGTYNRRKYQEESKLERQKIEGVKLESEGTYKILSIYSTVASSLVIAMGSAALLFWAMSNGSCRCNICI

>QBF57657.1 hemagglutinin [Influenza A virus]
MDTVSLITILLLSTERIANEICIGYHSANSAETVDTLTENCVPVTNAIELLHTQRNGMLGATNLGQPLIL
DTCSIGWLIYGNPSCDLSLEGREWSYIVQIPSTVHGLCYPGNVQYQKELMSLFSSARSYQKIQIFPHTLW
NVSYDGTSMPCSGSFYLSMIWLTPKNSDYPIQDPPYTNNQELNILFMWGINPTATDDTQIVLYTITDTTS
CVATDEINTIFKQLIGPSLLVNGLMTTVNYYWSVLNPGQTLQVKSDGNLIALWYGHVLSRESYGRILKTD
LKGGNCTVQCQTEKGGLNTIRSGQNVGKYASGKCLRYIGIKSVKLSVGLRNVRCRYSRGLFGAIAGFIEG
GWSGLVAGWYGFQHSNDQGVGMAADRDSTQKAVDKITSKVNNMVAKMNKPYEIIDLEFIVVGTRIDTSGH
KFDDQIQDIWAYNAESIVLLENQKTLDEQDANVNNLYNKVKRAACSNAVEDGKGCLELCHKCNDQCTETI
RNGTYNRRKYQEESKKERQKIEGAKLESEGTYKILSIYSTIASSRVIAMGSVALLFGALSNGYCRCNICV

>QBF57656.1 hemagglutinin [Influenza A virus]
METVSLITILLVATVSNADKICIGYQSTNSTETVDTLTENNVPVTHAKELLHTEHNGMLCATSLGQPLIL
DTCTIEGLIYGNPSCDLSLEGREWSYIVERPSAVHGLCYPGNVEDLEELRSLFSSARSYQRIQIFPDTIW
NVSYDGTSTACSGSFYRSMRWLTRKNGEYPIQDAQYTNNQGKNILFMWGINHPPTDDTQRGLYTRTDTTT
SVATEEINRIFKPLIGPRPLVNGLMGRINYYWSVLKPGQTLRIKSDGNLIAPWYGHILSGESHGRILKTD
LKRGSCTVQCQTEKGGLNTTLPFQNGSKYAFGNCSKYIGIKSLKLAVGLRNVPSRSSRGLFGAIAGFIEG

GWSGLVAGWYGFQHSNDQGVGMAADRDSTQKAIDKITSKVNNIVDKMNKQYEIIDHEFSEVETRLNMINN
KIDDQIQDIWAYNAELLVLLENQKTLDEHDANVNNLYNKVKRALGSNAVEDGKGCFELYHKCDDQCMETI
RNGTYNRRKYQEESKLERQKIEGVKLESEGTYKILTIYSTVASSLVIAMGFAAFLFWAMSNGSCRCNKRL

>QBF57655.1 hemagglutinin [Influenza A virus]
METVSLMTILLVATVSNADYFCIGYQSTNSTETVDTLTENNVPVTHAKELLHTEHNGMLCATSLGQPLIL
DTCTIEGLIYGNPSCDLSLEGREWSYIVERPSAVHGLCYPGNVEDLEELRSLFSSARSYQRIQIFPDTIW
NVSYDGTSTACSGSFYKSMRWLTLKNGEYPTQDAQYTNNQGKNILFMWGINHPPTDDTQRGLYTRTDTTT
SVATEEINRIFKPLIGPRPLVNCLMGRINYYWSVLNPGQTLRIKSDGNLIAPWYGHILSAESHGRILKTD
LKGGSCTVQCQTEKGGLNTTLSFQNVSKYAFGNCSKYIGIKSLKLAVGLRNVPSRSSRGLFGAIAGFIEG
GWSGLVAGWYGFQHSNDQGVGMAADRDSTQKAIDKITSKVNNIVDKMNKQYEIIDHEFSEVETRLNMINN
KIDDQIQDIWAYNAELLVLLENQKTLDEHDANVNNLYNKVKRALGSNAVEDGKGCFELYHKCDDQCMETI
RNGTYNRRKYQEESKLERQKIEGVKLESEGTYKILTIYSTVASSLVIAMGFAAFLFWAMSNGSCRCNICI

>QBF57654.1 hemagglutinin [Influenza A virus]
METVSLITILLVATVSNADKICIGYQSTNSTEIVDTLTENNVPVTHAKELLHTEHNGMLCATRLGQPLIL
DTCTIEGLIYGNPSCDLSLEGREWSYIVERPSAVHGLCYPGNVEDLEELRSLFSSARSYQRIQIFPDTIW
NVSYDGTSTACSGSFYRSMRWLTRKNGEYPIQDAQYTNNQGKNILFMWGINHPPTDDTQRGLYTITDTTT
SVATEEINRIFKPLIGPRPLVNGLMGRINYYWSVLKPGQTLRIKSDGNLIAPWNGHILSGESHGRILKTD
LKRGSCTVQCQTEKGGLHTTLSFQNVSKYAFGNCPKYIGIKSLKLAVGLRNVPSRSSRGLFGAIAGFIEG
GWSGLVAGWYGFQHSNDQGVGMAADRDSTQKAIDKITSKVNNIVDKMNKQYEIIDHEFSEVETRLNMINN
KIDDQIQDIWAYNAELLVLLENQKTLDEHDANVNNLYNKVKRALGSNAVEDGKGCFELYHKCDDQCMETI
RNGTYNRRKYQEESKLERQKIEGVKLESEGTYKILSIYSTVASSLVIAMGFAAFLFWAMSNGSCRCNICI

>QBF57653.1 hemagglutinin [Influenza A virus]
METVSLITILLVATVSNADKICIGYQSTNSTEIVDTLTENNVPVTHAKELLHTEHNGMLCATRLGQPLIL
DTCTIEGLIYGNPSCDLSLEGREWSYIVERPSAVHGLCYPGNVEDLEELRSLFSSARSYQRIQIFPDTIW
NVSYDGTSTACSGSFYRSMRWLTRKNGEYPIQDAQYTNNQGKNILFMWGINHPPTDDTQRGLYTRTDTTT
SVATEEINRIFKPLIGPRPLVNGLMGRINYYWSVLKPGQTLRIKSDGNLIAPWYGHILSGESHGRILKTD
LKRGSCTVQCQTEKGGLNTTLPFQNVSKYAFGNCPKYIGIKSLKLAVGLRNVPSRSSRGLFGAIAGFIEG
GWSGLVAGWYGFQHSNDQGVGMAADRDSTQKAIDKITSKVNNIVDKMNKQYEIIDHEFSEVETRLNMINN
KIDDQIQDIWAYNAELLVLLENQKTLDEHDANVNNLYNKVKRALGSNAVEDGKGCFELYHKCDDQCMETI
RNGTYNRRKYQEESKLERQKIEGVKLESEGTYKILTIYSTVASSLVIAMGFAAFLFWAMSNGSCRCNICI

>QBF57652.1 hemagglutinin [Influenza A virus]
METVSLITMLLVSTVSNANEICIGYQSTNSSEIVDTLTENNVPVTHAKELLHTQHNGMLGATKLGQPLIL
DTCTIEGLIYGNPSCDLSLERREWSYIVQIPSTVHGLCYPGNVQYLEELRSLFSSARSYQKIQIFPHTIW
NVSYDGTSTACSGSFYRSMRWLTRKNGEYPIQDAQYTNNQGNNILFMWGINPTPTDDTQIGLYTITDTTS
CVATDEINKIFKPLIGPRPLVNGLMTRINYYWSVLKPGQTLRIKSDGNLIASWYGHVLSRESHGRHLTTD
LNWGNCTVRRAINKGRLNTTQPFPNVSKYAFGNCPKYIGIKSVKLAAGLRNVRCRYSRGLFGAIAGFIER
GRSGLVAGWYGFQHSNDQGVGMAADRDSTQKAMNKITSKVNNIVAKLYKPYEIIGLEFSEVETRINTSNH
KIDDQIQDIWAYNAESIVLLENQKTLDEQDANVNNLYNKVKRALGSNAVEDGKGCFELCHKCDDQCMETI
GNGTYNRRKYQEESKLERQKIEGAKQESEGTYKILSIYSTVASSLVIAMGSAALLIGAMFNGSCRCDECI

>QBF57651.1 hemagglutinin [Influenza A virus]
METVSLITMLLVSTVSNANKICIGYQSTNSTEIVDTLTENNVPVTHAKELLHTEHNGMLCATRLGQPLIL
DTCTIEGLIYGNPSCDLSLEGREWSYIVQRPSAVHGLCYPGNVQYLEELMSLFSSARSYQKIQIFPHTIW
NVSYDGTSTACSGSFYRSMRWLTPKNGEYPIQDAQYTNNQGKNILFMWGINHPPTDDTQRGLYTITDTTT
SVATEEINTIFKPLIGPSPLVNSLMGRINYYWSVLKPGHTLRIKSDGNLIASWYGHALSRESHGRILKTD
LKRGSCTVQCQTDKGGLNTTLSFPNVSEYAFGKCLKYIGIKSVKLAVGLRNVRSRYIRGLFGAIAGFIEG
GWSGLVDGWYGFQHSNDQGVGMAADRDSTQKAIDKITSKVNNIVDKMNKQYEIIDHEFSEVETRLNMINN
KIDDQIQDIWAYNAELLVLLENQKTLDEHDANVNNLYNKVKRALGSNAVEDGKGCFELYHKCDDQCMETI
RNGTYNRRKYQEESKLERQKIEGVKLESEGTYKILSIYSTVASSLVIAMGSAALLFWAMSNGSCRCDDSI

>QBF57650.1 hemagglutinin [Influenza A virus]
METVSLITILLVATVSNADKICIGYQSTNSTEIVDTLTENNVPVTHAKELLHTEHNGMLCATRLGQPLIL
DTCTIEGLIYGNPSCDLSLEGREWSYIVERPSAVHGLCYPGNVEDLEELRSLFSSARSYQRIQIFPDTIW
NVSYDGTSTACSGSFYRSMRWLTRKNGEYPIQDAQYTNNQGNNILFMWGINHPPTDDTQRGLYTITDTTT
SVATEEINRIFKPLIGPRPLVNGLMGRINYYWSVLKPGQTLRIKSDGNLIAPWYGHSLSRESHGKILETD
LKRGSCTVQCQTEKGGLNTTLPFQNVSKYAFGNCPKYIGIKSLKLAVGLRNVPSRSSRGLFGAIAGFIEG
GWSGLVAGWYGFQHSNDQGVGMAADRDSTQKAIDKITSKVNNIVDKMNKQYEIIDHEFSEVETRLNMINN
KIDDQIQDIWAYNAELLVLLENQKTLDEHDANVNNLYNKVKRALGSNAVEDGKGCFELYHKCDDQCMETI
RNGTYNRRKYQEESKLERQKIEGVKLESEGTYKILTIYSTVASSLVIAMGFAAFLFWAMSNGSCRCNICI

>QBF57649.1 hemagglutinin [Influenza A virus]
MDTVSLMTILLVATVSNADEICIGYESTNSTETVDTLTENYVPVTHAKELLHTQHNGMLGATSLGQPLIL
DTCTIEGLIYGNPSCDLSLEGREWSYIVERPSTVHGLCYPGNVQYLEELMSLFSSARSYQKIQIFPHTIW
NVSYYGTSTACSGSFYKSMRWLTRKNGEYPTQDAQYTNNQGNNILFMWGINHPPTDDTQIGLYTITDTTF

CVATEEINRIFKPLIGPRPLVNGLMRRITYYWSVLKPGQTLPIYADGNLYASWYGHILTRESHGRILKTD
LKRGSCTVHRQTDNSGLNTTQSIENVSEYAFGNCSKYIGIKSVKLAAGLRNVRCRYSRGLFGAIAGFIER
GWSGLVAGWYGFQHSNDQGVGMAADRDSTQKAIDKITSKVNNIVDKMNKQYEIIDHEFSEVETRINMSNN
KIDDQIQDIWAYNAESIVLLENQKTLDEHDANVNNLYNKVKRALGSNAVEDGKGCFELYHKCDDQCMETI
RNGTYNRRKYQEESKLERQKIEGVKLESEGTYKILSIYSTVASSLVIAMGSAALLFWAMSNGSCRCDDCI

>QBF57648.1 hemagglutinin [Influenza A virus]
METVSLMTILLVSTVSNADKICIGYQSTNSTETVDTLTENNVPVTHAKELLHTEHNGMLCATSLGQPLIL
DTCTIEGLIYGNPSCDLSLEGREWSYIVERPSAVHGLCYPGNVQDLEELRSLFSSARSYQKIQIFPHTIW
NVSYDGTSTACSGSFYKSMRWLTRKNGEYPTQDAQYTNNQGNNILFMWGINHPPTDDTQRGLYTRTDTTT
SVATDEINRIFKPLIGPRPLVNGLMGRINYYWSVLKPGQTMRIKSDVNLIAPWYGHILSRESLGRLLKTD
LKRGRCTVQCQTEKGGLNTTLPFQNVSKYASGNCSKYIGIKSLKLAVGLRNVRSRYSRGLFGAIAGFIER
GWSGLVAGWYGFQHSNDQGVGMAADRDSTQKAIDKITSKVNNIVDKMNKQYEIIDHEFSEVETRLNMINN
KIDDQIQDIWAYNAELLVLLENQKTLDEHDANVNNLYNKVKRALGSNAVEDGKGCFELYHKCDDQCMETI
RNGTYNRRKYQEESKLERQKIEGVKLESEGTYKILSIYSTVASSLVIAMGSAAFLFWALFNGACRCKICI

>QBF57647.1 hemagglutinin [Influenza A virus]
METVSLMTILLVATVSNADEICIGYESANSSETVDTLTENYVPVTHAKELLHTQRNGMLGATNLGQPLIL
DTCTIEGLIYGNPSCDLSLEGREWSYIVERPSTVHGLCYPGNVQYLEELMSLFSSARSYQKIQIFPHTIW
NVSYDGTSTACSGSFYKSMRWLTRKNSEYPTQDAQYTNNHGNNILFMWGINHPPTDDTQIGLYTITDTTF
CVATEEINRIFKPLIGPQPLVNGLMGRINYYWSVLKPDQTLPVKSDMNLLASLYGHILSVESYGRLLITD
LKRGSCTEQCHTEKGGLNTTLACQNVSEYAFGNCSKYIGIKSVKLAVGLRNVRCRYSRGVFGAIAGFIER
GWSGLVAGWYGFQHSNDQGVGMAADRDSTQKAIDKITSKVNNIVDKMNKQYEIIDLEFSEVETRINMSNN
KFDDQIQDIWAYNAESIVLLENQKTLDEHDANVNNLYNKVKRALGSNAVEDGKGCFELYHKCDDQCMETI
RNGTYNRRKYQEESKLERQKIEGVKLESEGTNKILSINSRVASSLVIAMGSVALLFWAMSNGSGRPNICI

>QBF57646.1 hemagglutinin [Influenza A virus]
METVSLITILLVATVSNADEICIGYQSTNSTETVDTLTENNVPVTHAKELLHTEHNGMLGATSLGQPLIL
DTCTIEGLIYGNPSCDLSLEGREWSYIVQRPSTVHGLCYPGNVQYLEELMSLFSSARSYQKIQIFPHTIW
NVSYDGTSTACSGSFYISMRWLTRKNGEYPIQDAQYTNNQGKNILFMWGINHPPTDDTQIGLYTITDTTS
SVATEEINRIFKQLIGPRPLVNGLMGRINYYWSVLKPGQTLRIKSDGNLLASWYGHILSRESHGRILKHD
LKRGSCTVQCQTEKGGLNTTQSFQNVSKYAFGNCSRYIGIKSLKLAVGLRNVPSRSSRGLFGAIAGFIEG
GWSGLVAGWYGFQHSNDQGVGMAADRDSTQKAIDKITSKVNNIVDKMNKQYEIIDHEFSEVETRLNMINN
KIDDQIQDIWAYNAELLVLLENQKTLDEHDANVNNLYNKVKRALGSNAVEDGKGCFELYHKCNDQCMETI

RNGTYNRRKYQEESKLERQKIEGVKLESEGTYKILSIYSTVASSLVIAMGSAAFLFWAMSNGSCRCIYCI

>QBF57644.1 hemagglutinin [Influenza A virus]
METVSLITILLVATVSNADKICIGYQSTNSTETVDTLTENNVPVTHAKELLHTEHNGMLCATSLGQPLIL
DTCTIEGLIYGNPSCDLSLEGREWSYIVERPSAVHGLCYPGNVEDLEELRSLFSSARSYQRIQIFPDTIW
NVSYDGTSTACSGSFYRSMRWLTRKNGEYPIQDAQYTNNQGKNILFMWGINHPPTDDTQRGLYTRTDTTT
SVATEEINRIFKPLIGPRPLVNGLMGRINYYWSVLKPGQTLRIKSDGNLIAPWYGHILSGESQRRILKTD
LKRGSCTVQCQTEKGGLNTTLSFQNVSKYAFGNCSKYIGIKSLKLAVGLRNVPSRSSRGLFGAIAGFIEG
GWSGLVAGWYGFQHSNDQGVGMAADRDSTQKAIDKITSKVNNIVDKMNKQYEIIDHEFSEVETRLNMINN
KIDDQIQDIWAYNAELLVLLENQKTLDEHDANVNNLYNKVKRALGSNAVEDGKGCFELYHKCDDQCMETI
RNGTYNRRKYQEESKLERQKIEGVKLESEGTYKILTIYSTVASSLVIAMGFAAFLFWAMSNGSCRCNICI

>QBF57643.1 hemagglutinin [Influenza A virus]
METVSLITILLVSTVSNADKICIGYQSTNSTETVDTLTENNVPVTHAKELLHTEHNGMLCATSLGQPLIL
DTCTIEGLIYGNPSCDLSLEGREWSYIVERPSAVHGLCYPGNVEYLEELRSLFSSARSYQRIQIFPHTIW
NVSYDGTSTACSGSFYKSMRWLTRKNGDYPIQDAQYTNNQGKNILFMWGINHPPTDDTQRGLYTITDTTT
SVATDEINTIFKQLIGPMPLVNGLMGRINYYWSVLKPGQTLRIKSDVNLIAPWYGHVLSGESHGSTLKTD
LKWGSCTVQCQTEKGGLHTTLSFQNVSEYAFGNCSKYIGIKSLKLAVGLRNVRSRYSRGLFGAIAGFIEG
GWSGLVAGWYGFQHSNDQGVGMAADRDSTQKAIDKITSKVNNIVDKMNKQYEIIDHEFSEVETRLNMINN
KIDDQIQDIWAYNAELLVLLENQKTLDEHDANVNNLYNKVKRALGSNAVEDGKGCFELYHKCDDQCMETI
RNGTYNRRKYQEESKLERQKIEGVKLESEGTYKILTIYSTVASSLVIAMGSAAFLFWAMSNGSCRCNICI

>QBF57641.1 hemagglutinin [Influenza A virus]
METVSLITILLVATVSNADKICIGYQSTNSTETVDTLTENNVPVTHAKELLHTEHNGMLCATSLGQPLIL
DTCTIEGLIYGNPSCDLSLEGREWSYIVERPSAVHGLCYPGNVEDLEELRSLFSSARSYQRIQIFPDTIW
NVSYDGTSTACSGSFYKSMRWLTRKNGEYPTQDAQYTNNQGKNILFMWGINHPPTDDTQRGLYTRTDTTT
SVATEEINRIFKPLIGPRPLVNGLMGRINYYWSVLKPGQTLRIKSDGNLIAPWYGHILSGESHGRILKTD
LKRGSCTVQCQTEKGGLNTTLPFQNVSKYAFGNCSKYIGIKSLKLAVGLRNVPSRSSRGLFGAIAGFIEG
GWSGLVAGWYGFQHSNDQGVGMAADRDSTQKAIDKITSKVNNIVDKMNKQYEIIDHEFSEVETRLNMINN
KIDDQIQDIWAYNAELLVLLENQKTLDEHDANVNNLYNKVKRALGSNAVEDGKGCFELYHKCDDQCMETI
RNGTYNRRKYQEESKLERQRIEGVKLESEGTYKILTIYSTVASSLVIAMGFAAFLFWAMSNGSCRCNICI

>QBF57640.1 hemagglutinin [Influenza A virus]
METVSLITILLVATVSNADKICIGYQSTNSTETVDTLTENNVPVTHAKELLHTEHNGMLCATSLGQPLIL

DTCTIEGLIYGNPSCDLSLEGREWSYIVERPSAVHGLCYPGNVEDLEELRSLFSSARSYQRIQIFPDTIW
NVSYDGTSTACSGSFYKSMRWLTRKNGEYPTQDAQYTNNQGKNILFMWGINHPPTDDTQRGLYTRTDTTT
SVATEEINRIFKPLIGPRPLVNGLMGRINYYWSVLKPGQTLRIKSDGNLIAPWYGHILSGESHGRILKTD
LKRGSCTVQCQTEKGGLNTTLPFQNVSKYAFGNCSKYIGIKSLKLAVGLRNVPSRSSRGLFGAIAGFIEG
GWSGLVAGWYGFQHSNDQGVGMAADRDSTQKAIDKITSKVNNIVDKMNKQYEIIDHEFSEVETRLNMINN
KIDDQIQDIWAYNAELLVLLENQKTLDEHDANVNNLYNKIKRALGSNAVEDGKGCFELYHKCDDQCMETI
RNGTYNRRKYQEESKLERQRIEGVKLESEGTYKILTIYSTVASSLVIAMGFAAFLFWAMSNGSCRCNICI

>QBF57639.1 hemagglutinin [Influenza A virus]
METVSLITILLVATVSNADKICIGYQSTNSTETVDTLTENNVPVTHAKELLHTEHNGMLCATSLGQPLIL
DTCTIEGLIYGNPSCDLSLEGREWSYIVERPSAVHGLCYPGNVEDLEELRSLFSSARSYQRIQIFPDTIW
NVSYDGTSTACSGSFYKSMRWLTRKNGEYPTQDAQYTNNQGKNILFMWGINHPPTDDTQRGLYTRTDTTT
SVATEEINRIFKPLIGPRPLVNGLMGRINYYWSVLKPGQTLRIKSDGNLIAPWYGHILSGESHGRILKTD
LKEGSCTVQCQTEKGGLNTTLPFQNVSKYAFGNCSKYIGIKSLKLAVGLRNVPSRSSRGLFGAIAGFIEG
GWSGLVAGWYGFQHSNDQGVGMAADRDSTQKAIDKITSKVNNIVDKMNKQYEIIDHEFSEVETRLNMINN
KIDDQIQDIWAYNAELLVLLENQKTLDEHDANVNNLYNKVKRALGSNAVEDGKGCFELYHKCDDQCMETI
RNGTYNRRKYQEESKLERQRIEGVKLESEGTYKILTIYSTVASSLVIAMGFAAFLFWAMSNGSCRCNICI

>QBF57638.1 hemagglutinin [Influenza A virus]
METVSLITILLVATVSNADKICIGYQSTNSTETVDTLTENNVPVTHAKELLHTEHNGMLCATSLGQPLIL
DTCTIEGLIYGNPSCDLSLEGREWSYIVERPSAVHGLCYPGNVEDLEELRSLFSSARSYQRIQIFPDTIW
NVSYDGTSTACSGSFYKSMRWLTRKNGEYPTQDAQYTNNQGKNILFMWGINHPPTDDTQRGLYTRTDTTT
SVATEEINRIFKPLIGPRPLVNGLMGRINYYWSVLKPGQTLRIKSDGNLIAPWYGHILSGESHGRILKTD
LKKGSCTVQCQTEKGGLNTTLPFQNVSKYAFGNCSKYIGIKSLKLAVGLRNVPSRSSRGLFGAIAGFIEG
GWSGLVAGWYGFQHSNDQGVGMAADRDSTQKAIDKITSKVNNIVDKMNKQYEIIDHEFSEVETRLNMINN
KIDDQIQDIWAYNAELLVLLENQKTLDEHDANVNNLYNKVKRALGSNAVEDGKGCFELYHKCDDQCMETI
RNGTYNRRKYQEESKLERQRIEGVKLESEGTYKILTIYSTVASSLVIAMGFAAFLFWAMSNGSCRCSSSR

>QBF57637.1 hemagglutinin [Influenza A virus]
METVSLITILLVATVSNADKICIGYQSTNSTETVDTLTENNVPVTHAKELLHTEHNGMLCATSLGQPLIL
DTCTIEGLIYGNPSCDLSLEGREWSYIVERPSAVHGLCYPGNVEDLEELRSLFSSARSYQRIQIFPDTIW
NVSYDGTSTACSGSFYKSMRWLTRKNGEYPTQDAQYTNNQGKNILFMWGINHPPTDDTQRGLYTRTDTTT
SVATEEINRIFKPLIGPRPLVNGLMGRINYYWSVLKPGQTLRIKSDGNLIAPWYGHILSGESHGRILKTD
LKRGSCTVQCQTEKGGLNTTLPFQNVSKYAFGNCSKYIGIKSLKLAVGLRNVPSRSSRGLFGAIAGFIEG

GWSGLVAGWYGFQHSNDQGVGMAADRDSTQKAIDKITSKVNNIVDKMNKQYEIIDHEFSEVETRLNMINN
KIDDQIQDIWAYNAELLVLLENQKTLDEHDANVNNLYNKVKRALGSNAVEDGKGCFELYHKCDDQCMETI
RNGTYNRRKYQEESKLERQRIEGVKLESEGTYKILTIYSTVASSLVIAMGFAAFLFWAMSNGSCRCNICI

>QBF57635.1 hemagglutinin [Influenza A virus]
METVSLITILIVATVSNADKICIGYQSTNSTETVDTLTENNVPVTHAKELLHTEHNGMLCATSLGHPLIL
NTCTIEGLIYGNPSCDPLLGGREWSYIVERPSAVNGLCYPGNVENLEELRSLFSSSRSYQRIQIFPDTIW
NVSYSGTSKACSDSFYRSMRWLTQKNNAYPTQDAQYTNNQGKNILFMWGINHPPTDTAQTNLYTRTDTTT
SVATEEMNRIFKPLIGPRPLVNGLMGRINYYWSVLKPGQTLRIKSDGNLIAPWYGHILSGESHGRILKTD
LKRGSCTVQCQTEKGGLNTTLPFQNISKYAFGNCSKYIGVKSLKLAVGLRNVPSRSSRGLFGAIAGFIEG
GWSGLVAGWYGFQHSNDQGVGMAADRDSTQKAIDKITSKVNNIVDKMNKQYEIIDHEFSEVETRLNMINN
KVDDQIQDIWAYNAELLVLLENQKTLDEHDANVNNLYNKVKRALGSNAVEDGKGCFELYHKCDDHCMETI
RNGTYNRRKYQEESKLERQKIEGVKLESEETYKILTIYSTVASSLVIAMGFAAFLFWAMSNGSCRCNICI

>QBF57634.1 hemagglutinin [Influenza A virus]
METVSLITILIVATVSNADKICIGYQSTNSTETVDTLTENNVPVTHAKELLHTEHNGMLCATSLGQPLIL
DTCTIEGLIYGNPSCDLSLEGREWSYIVERPSAVNGLCYPGNVENLEELRSLFSSARSYQRIQIFPDTIW
NVSYDGTSTACSNSFYRSMRWLTRKDGNYPTQDAQYTNNQGKNILFMWGINHPPTDETQRNLYTRTDTTT
SVATEEINRIFKPLIGPRPLVNGLMGRIDYYWSVLKPGQTLRIKSDGNLIAPWYGHILSGESHGRILKTD
LKRGSCTVQCQTEKGGLNTTLPFQNVSKYAFGNCSKYIGIKSLKLAVGLRNVPSRSSRGLFGAIAGFIEG
GWSGLVAGWYGFQHSNDQGVGMAADRDSTQKAIDKITSKVNNIVDKMNKQYEIIDHEFSEVETRLNMINN
KIDDQIQDIWAYNAELLVLLENQKTLDEHDANVNNLYNKVKRALGSNAVEDGKGCFELYHKCNDQCMETI
RNGTYNRKKYQEESKLERQRIEGVKLESEGTYKILTIYSTVASSLVIAMGFAAFLFWAMSNGSCRCNICI

>QBF57633.1 hemagglutinin [Influenza A virus]
METVSLITILLVATASNADKICIGYQSTNSTETVDTLTENNVPVTHAKELLHTEHNGMLCATSLGQPLIL
DTCTVEGLIYGNPSCDLSLEGREWSYIVERPSAVSGLCYPGNVENLEELRSLFSSARSYQRIQIFPDTIW
NVSYDGTSTACSNSFYRSMRWLTRKDGNYPTQDAQYTNNQGKNILFMWGINHPPTDDTQRNLYTRTDTTT
SVATEEINRIFKPLIGPRPLVNGLMGRIDYYWSVLKPGQTLRIKSDGNLIAPWYGHILSGESHGRILKTD
LKRGSCTVQCQTKKGGLNPTLPFQNVSKYAFGNCSKYIGIKSLKLAVGLRNVPSRSSRGLFGAIAGFIEG
GWSGLVAGWYGFQHSNDQGVGMAADRDSTQKAIDKITSKVNNIVDKMNKQYEIIDHEFSEVETRLNMINN
KIDDQIQDIWAYNAELLVLLENQKTLDEHDANVNNLYNKVKRALGSNAVEDGKGCFELYHKCNDQCMETI
RNGTYNRRKYQEESKLERQKIEGVKLESEGTYKILTIYSTVASSLVIAMGFAAFLFWAMSNGSCRCIICM

>QBF57632.1 hemagglutinin [Influenza A virus]

METVSLITILLVATASNADKICIGYQSTNSTETVDTLTENNVPVTHAKELLHTEHNGMLCATSLGQPLIL

DTCTVEGLIYGNPSCDLSLEGREWSYIVERPSAVNGLCYPGNVENLEELRSLFSSARSYQRIQIFPDTIW

NVSYDGTSTACSNSFYRSMRWLTRKDGNYPTQDAQYTNNQGKNILFMWGINHPPTDDTQRNLYTRTDTTT

SVATEEINRIFKPLIGPRPLVNGLMGRIDYYWSVLKPGQTLRIKSDGNLIAPWYGHILSGESHGRILKTD

LKRGSCTVQCQTEKGGLNTTLPFQNVSKYAFGNCSKYIGIKSLKLAVGLRNVPSRSSRGLFGAIAGFIEG

GWSGLVAGWYGFQHSNDQGVGMAADRDSTQKAIDKITSKVNNIVDKMNKQYEIIDHEFSEVETRLNMINN

KIDDQIQDIWAYNAELLVLLENQKTLDEHDANVNNLYNKVKRALGSNAVEDGKGCFELYHKCNDQCMETI

RNGTYNRRKYQEESKLERQKIEGVKLESEGTYKILTIYSTVASSLVIAMGFAAFLFWAMSNGSCRCNICI

>QBF57631.1 hemagglutinin [Influenza A virus]

MDTVSLITILIVSTVSNADKICIGYQSTNSTETVDTLTENNVPVTHAKELLHTEHNGMLCATSLGQPLIL

DTCTIEGLIYGNPSCDLSLEGREWSYIVERPSAVNGLCYPGNVENLEELRSLFSSARSYQRIQIFPDTIW

NVSYDGTSTACSNSFYRSMRWLTRKDGNYPTQDAQYTNNQGNNILFMWGINHPPTDETQRNLYTITDTTT

SVATEEINRIFKPLIGPRPLVNGLMGRIDYYWSVLKPGQTLRIKSDGNLIAPWYGHILSGESHGSILTTD

LKRGSCTVQCQTEKGGLNTTLPFQNVSKYAFGNCSKYIGIKSLKLAVGLRNVPSRSSRGLFGAIAGFIEG

GWSGLVAGWYGFQHSNDQGVGMAADRDSTQKAIDKITSKVNNIVDKMNKQYEIIDHEFSEVETRLNMINN

KIDDQIQDIWAYNAELLVLLENQKTLDEHDANVNNLYNKVKRALGSNAVEDGKGCFELYHKCNDQCMETI

RNGTYNRKKYQEESKLERQRIEGVKLESEGTYKILTIYSTVASSLVIAMGFAAFLFWAMSNGSCRCIICI

>QBF57629.1 hemagglutinin [Influenza A virus]

METVSLITILIVATVSNADKICIGYQSTNSTETVDTLTENNVPVTHAKELLHTEHNGMLCATSLGQPLIL

DTCTIEGLIYGNPSCDLSLEGREWSYIVERPSAVNGLCYPGNVENLEELRSLFSSARSYQRIQIFPDTIW

NVSYDGTSTACSNSFYRSMRWLTRKDGNYPTQDAQYTNNQGKNILFMWGINHPPTDETQRNLYTRTDTTT

SVATEEINRIFKPLIGPRPLVNGLMGRIDYYWSVLKPGQTLRIKSDGNLIAPWYGHILSGESHGRILKTD

LKRGSCTVQCQTEKGGLNTTLPFQNVSKYAFGNCSKYIGIKSLKLAVGLRNVPSRSSRGLFGAIAGFIEG

GWSGLVAGWYGFQHSNDQGVGMAADRDSTQKAIDKITSKVNNIVDKMNKQYEIIDHEFSEVETRLNMINN

KIDDQIQDIWAYNAELLVLLENQKTLDEHDANVNNLYNKVKRALGSNAVEDGKGCFELYHKCNDQCMETI

RNGTYNRKKYQEESKLERQRIEGVKLESEGTYKILTIYSTVASSLVIAMGFAAFLFWAMSNGSCRCNYCI

>QBF57628.1 hemagglutinin [Influenza A virus]

MDTVSLITILLVATAGNADKICIGYQSANSTETVDTLTENNVPVTHAKELLHTEHNGMLCATSLGQPLLL

DTCTVEGLIYGNPSCDLSLEGREWSYIVKRPSAVNGLCYPGNVENLEKLRSLFSSARSYQRIQIFPDTIW

NVSYDGTSTACSNSFYKSMRWLTRKDGNYPTQDAQYTNNQGKNILFMWGINHPPTDDTQINLYTITDTTT

SVATEEINRIFKPLIGPRPLVSGLMGRIHYYWSVLNQGQTLRIISDGNLIAPWHGHILCRESHGRILKTD
LKRGSCTVPCQTEKGGLNTTLPLQNVSKYAFGNCSKYIGIKSVKLAVGMRNVRSRFSRGLFEAIAGFIEG
GWSGLVDGWYGFQHSNDQGVGMAADRDSTQKAIDKITSKVNNIVDKMNKQYEIIDHEFSEVETRINMINN
KIDDQIQDIWAYNAELLVLLENQKTLDEHDANVNNLYNKVKRALGSNAVEDGKGCFELYHKCNDQCMETI
RNGTYNRRKYQEESKLERQKIEGVKLESEGTYKILTIYSTVASSLVIAMGFAAFLFWAMSNGSCRCNICI

>QBF57627.1 hemagglutinin [Influenza A virus]
METVSLITILLVATVSNADKICIGYQSTNSTETVDTLTENNVPVTHAKELLHTEHNGMLCATSLGQPLIL
DTCTIEGLIYGNPSCDLSLEGREWSYIVERPSAVHGLCYPGNVEDLEELRSLFSSARSYQRIQIFPDTIW
NVSYDGTSTACSGSFYKSMRWLTRKNGEYPTQDAQYTNNQGKNILFMWGINHPPTDDTQRGLYTRTDTTT
SVATEEINRVFKPLIGPRPLVNGLMGRINYYWSVLKPGQTLRIKSDGNLIAPWYGHILSGESHGRILKTD
LKRGSCTVQCQTEKGGLNTTLPFQNVSKYAFGNCSKYIGIKSLKLAVGLRNVPSRSSRGLFGAIAGFIEG
GWSGLVAGWYGFQHSNDQGVGMAADRDSTQKAIDKITSKVNNIVDKMNKQYEIIDHEFSEVETRLNMINN
KIDDQIQDIWAYNAELLVLLENQKTLDEHDANVNNLYNKVKRALGSNAVEDGKGCFELYHKCDDQCMETI
RNGTYNRRKYQEESKLERQRIEGVKLESEGTYKILTIYSTVASSLVIAMGFAAFLFWAMSNGSCRCNICI

>QBF57625.1 hemagglutinin [Influenza A virus]
METVSLITILLVATVSNADKICIGYQSTNSTETVDTLTENNVPVTHAKELLHTEHNGMLCATSLGQPLIL
DTCTIEGLIYGNPSCDLSLEGREWSYIVERPSAVHGLCYPGNVEDLEELRSLFSSARSYQRIQIFPDTIW
NVSYDGTSTACSGSFYKSMRWLTRKNGEYPTQDAQYTNNQGKNILFMWGINHPPTDDTQRGLYTRTDTTT
SVATEEINRIFKPLIGPRPLVNGLMGRINYYWSVLKPGQTLRIKSDGNLIAPWYGHILSGESHGRILKTD
LKRGSCTVQCQTEKGGLNTTLPFQNVSKYAFGNCSKYIGIKSLKLAVGLRNVPSRSSRGLFGAIAGFIEG
GWSGLVAGWYGFQHSNDQGVGMAADRDSTQKAIDKITSKVNNIVDKMNKQYEIIDHEFSEVETRLNMINN
KIDDQIQDIWAYNAELLVLLENQKTLDEHDANVNNLYNKVKRALGSNAVEDGKGCFELYHKCDDQCMETI
RNGTYNRRKYQEESKLERQRIEGVKLESEGTYKILTIYSTVASSLVIAMGFAAFLFWAMSNGSCRCNICI

>QBF57624.1 hemagglutinin [Influenza A virus]
METVSLITILLVATVSNADKICIGYQSTNSTETVDTLTENNVPVTHAKELLHTEHNGMLCATSLGQPLIL
DTCTIEGLIYGNPSCDLSLEGREWSYIVERPSAVHGLCYPGNVEYLEELRSLFSSARSYQRIQIFPDTIW
NVSYDGTSTACSGSFYKSMRWLTRKNGEYPTQDAQYTNNQGKNILFMWGINHPPTDDTQRGLYTRTDTTT
SVATEEINRIFKPLIGPRPLVNGLMGRINYYWSVLKPGQTLRIKSDGNLIAPWYGHILSGEIHGRILRTD
LKRGSCTVQCQTEKGGLNTTLPFQNVSKYAFGNCSKYIGIKSLKLAVGLRNVPSRSSRGLFGAIAGFIEG
GWSGLVAGWYGFQHSNDQGVGMAADRDSTQKAIDKITSKVNNIVDKMNKQYEIIDHEFSEVETRLNMINN
KIDDQIQDIWAYNAELLVLLENQKTLDEHDANVNNLYNKVKRALGSNAVEDGKGCFELYHKCDDQCMETI

RNGTYNRRKYQEESKLERQRIEGVKLESEGTYKILTIYSTVASSLVIAMGFAAFLFWAMSNGSCRCNISI

>QBF57622.1 hemagglutinin [Influenza A virus]
METVSLITILLVATVSNADKICIGYQSTNSTETVDTLTENNVPVTHAKELLHTEHNGMLCATSLGQPLIL
DTCTIEGLIYGNPSCDLSLEGREWSYIVERPSAVHGLCYPGNVEDLEELRSLFSSARSYQRIQIFPDTIW
NVSYDGTSTACSGSFYKSMRWLTRKNGEYPTQDAQYTNNQGKNILFMWGINHPPTDDTQRGLYTRTDTTT
SVATEEINRIFKPLIGPRPLVNGLMGRINYYWSVLKPGQTLRIKSDGNLIAPWYGHILSGESHWRILKTD
LKRGSCTVQCQTEKGGLNTTLPFQNVSKYAFGNCSKYIGIKSLKLAVGLRNVPSRSSRGLFGAIAGFIEG
GWPGLVAGWYGFQHSNDQGVGMAADRDSTQKAIDKITSKVNNIVDKMNKQYEIIDHEFSEVETRLNMINN
KIDDQIQDIWAYNAELLVLLENQKTLDEHDANVNNLYNKVKRALGSNAVEDGKGCFELYHKCDDQCMETI
RNGTYNRRKYQEESKLERQRIEGVKLESEGTYKILTIYSTVASSLVIAMGFAAFLFWAMSNGSCRCNIGI

>QBF57618.1 hemagglutinin [Influenza A virus]
METVSLITILLVSTVSNADKICIGYQSTNSTETVDTLTENNVPVTHAKELLHTEHNGMLCATSLGQPLIL
DTCTIEGLIYGNPSCDLSLEGREWSYIVERPSAVNGLCYPGNVENLEELRSLFSSARSYQKIQIFPDTIW
NVSYDGTSTACSNSFYKSMRWLTRKDGNYPTQDAQYTNNQGKNILFMWGINHPPTDDTQRSLYTRTDTTT
SVATEEINRIFKPLIGPRPLVNGLMGRIDYYWSVLKPGQTLRIKSDGNLIAPWYGHILSGESHGRILKTD
LKRGSCTVQCQTEKGGLNTTLPFQNVSKYAFGNCSKYIGIKSLKLAVGLRNVPSRSSRGLFGAIAGFIEG
GWSGLVAGWYGFQHSNDQGVGMAADRDSTQKAIDKITSKVNNIVDKMYKQYEIIDHEFSEVETRLNMINN
KIDDQIQDIWAYNAELLVLLENQKTLDEHDANVNNLYNKVKRALGSNAVEDGKGCFELYHKCNDQCMDTI
RNGTYNRRKYQEESKLERQKIEGVKLESEGTYKILSIYSTVASSLVIAMGFAAFLFWAMSNGSCRCNICV

>QBF57617.1 hemagglutinin [Influenza A virus]
METVSLITILLVATVSNADKICIGYQSTNSTETVDTLTENNVPVTHAKELLHTEHNGMLCATSLGQPLIL
DTCTIEGLIYGNPSCDLSLEGREWSYIVERPSAVHGLCYPGNVEDLEELRSLFSSARSYQRIQIFPDTIW
NVSYDGTSTACSGSFYKSMRWLTRKNGEYPTQDAQYTNNQGKNILFMWGINHPPTDDTQRGLYTRTDTTT
SVGTEEINRIFKPLIRPRPLVHGLMGRINYYWSVLKPGQTLRIKSDGNLIAPWYGHILSGESHGRIVKTD
LKKGSCTVQCQTEKGGLNTTLPFQNVSKYAFGNCSKYIGIKSLKLAVGLRNVPSRYSRGLFGAIAGFIEG
GWSGLVAGWYGFQHSNDQGVGMAADRDSTQKAIDKITSKVNNIVDKMNKQYEIIDHEFSEVETRLNMINN
KIDDQIQDIWAYNAELLVLLENQKTLDEHDANVNNLYNKVKRALGSNAVEDGKGCFELYHKCDDQCMETI
RNGTYNRRKYQEESKLERQRIEGVKLESEGTYKILTIYSTVASSLVIAMGFAAFLFWAMYNGSCRCNICI

>QBF57615.1 hemagglutinin [Influenza A virus]
METVSLITILIVATVSNADKICIGYQSTNSTETVDTLTENNVPVTHAKELLHTEHNGMLCATSLGQPLIL

DTCTIEGLIYGNPSCDLSLEGREWSYIVERPSAVNGLCYPGNVENLEELRSLFSSARSYQRIQIFPDTIW
NVSYDGTSTACSNSFYRSMRWLTRKDGNYPTQDAQYTNNQGKNILFMWGINHPPTDETQRNLYTRTDTTT
SVATEEINRIFKPLIGPRPLVNGLMGRIDYYWSVLKPGQTLRIKSDGNLIAPWYGHILSGESHGRILKTD
LKRGSCTVQCQTEKGGLNTTLPFQNVSKYAFGNCSKYIGIKSLKLAVGLRNVPSRSSRGLFGAIAGFIEG
GWSGLVAGWYGFQHSNDQGVGMAADRDSTQKAIDKITSKVNNIVDKMNKQYEIIDHEFSEVETRLNMINN
KIDDQIQDIWAYNAELLVLLENQKTLDEHDANVNNLYNKVKRALGSNAVEDGKGCFELYHKCNDQCMETI
RNGTYNRKKYQEESKLERQRIEGVKLESEGTYKILTIYSTVASSLVIAMGFAAFLFWAMSNGSCRCNICI

>QBF57614.1 hemagglutinin [Influenza A virus]
METVSLITILIVATVSNADKICIGYQSTNSTETVDTLTENNVPVTHAKELLHTEHNGMLCATSLGQPLIL
DTCTIEGLIYGNPSCDLSLEGREWSYIVERPSAVNGLCYPGNVENLEELRSLFSSARSYQRIQIFPDTIW
NVSYDGTSTACSNSFYRSMRWLTRKDGNYPTQDAQYTNNQGKNILFMWGINHPPTDETQRNLYTRTDTTT
SVATEEINRIFKPLIGPRPLVNGLMGRIDYYWSVLKPGQTLRIKSDGNLIAPWYGHILSGESHGRILKTD
LKRGSCTVQCQTEKGGLNTTLPFQNVSKYAFGNCSKYIGIKSLKLAVGLRNVPSRSSRGLFGAIAGFIEG
GWSGLVAGWYGFQHSNDQGVGMAADRDSTQKAIDKITSKVNNIVDKMNKQYEIIDHEFSEVETRLNMINN
KIDDQIQDIWAYNAELLVLLENQKTLDEHDANVNNLYNKVKRALGSNAVEDGKGCFELYHKCNDQCMETI
RNGTYNRKKYQEESKLERQRIEGVKLESEGTYKILTIYSTVASSLVIAMGFAAFLFWAMSNGSCRCNICI

>QBF57613.1 hemagglutinin [Influenza A virus]
METVSLITILLVATASNADKICIGYQSTNSTETVDTLTENNVPVTHAKELLHTEHNGMLCATSLGQPLIL
DTCTVEGLIYGNPSCDLSLEGREWSYIVERPSAVNGLCYPGNVENLEELRSLFSSARSYQRIQIFPDTIW
NVSYDGTSTACSNSFYRSMRWLTRKDGNYPTQDAQYTNNQGKNILFMWGINHPPTDDTQRNLYTRTDTTT
SVATEEINRIFKPLIGPRPLVNGLMGRIDYYWSVLKPGQTLRIKSDGNLIAPWYGHILSGESHGRILKTD
LKRGSCTVQCQTEKGGLNTTLPFQNVSKYAFGNCSKYIGIKSLKLAVGLRNVPSRSSRGLFGAIAGFIEG
GWSGLVAGWYGFQHSNDQGVGMAADRDSTQKAIDKITSKVNNIVDKMNKQYEIIDHEFSEVETRLNMINN
KIDDQIQDIWAYNAELLVLLENQKTLDEHDANVNNLYNKVKRALGSNAVEDGKGCFELYHKCNDQCMETI
RNGTYNRRKYQEESKLERQKIEGVKLESEGTYKILTIYSTVASSLVIAMGFAAFLFWAMSNGSCRCNICI

>QBF57612.1 hemagglutinin [Influenza A virus]
METVSLITILIVATVSNADKICIGYQSTNSTETVDTLTENNVPVTHAKELLHTEHNGMLCATSLGQPLIL
DTCTIEGLIYGNPSCDLSLEGREWSYIVERPSAVNGLCYPGNVENLEELRSLFSSARSYQRIQIFPDTIW
NVSYDGTSTACSNSFYRSMRWLTRKDGNYPTQDAQYTNNQGKNILFMWGINHPPTDETQRNLYTRTDTTT
SVATEEINRIFKPLIGPRPLVNGLMGRIDYYWSVLKPGQTLRIKSDGNLIAPWYGHILSGESHGRILKTD
LKRGSCTVQCQTEKCGLNTTLPFQNVSKYAFGNCSKYIGIKSLKLAVGLRNVPSRSSRGLFGAIAGFIEG

GWSGLVAGWYGFQHSNDQGVGMAADRDSTQKAIDKITSKVNNIVDKMNKQYEIIDHEFSEVETRLNMINN
KIDDQIQDIWAYNAELLVLLENQKTLDEHDANVNNLYNKVKRALGSNAVEDGKGCFELYHKCNDQCMETI
RNGTYNRKKYQEESKLERQRIEGVKLESEGTYKILTIYSTVASSLVIAMGFAAFLFWAMSNGSCRCNICI

>QBF57611.1 hemagglutinin [Influenza A virus]
METVSLITILLVATASNADKICIGYQSTNSTETVDTLTENNVPVTHAKELLHTEHNGMLCATSLGQPLIL
DTCTVEGLIYGNPSCDLSLEGREWSYIVERPSAVNGLCYPGNVENLEELRSLFSSARSYQRIQIFPDTIW
NVSYDGTSTACSNSFYRSMRWLTRKDGNYPTQDAQYTNNQGKNILFMWGINHPPTDDTQRNLYTRTDTTT
SVATEEINRIFKPLIGPRPLVNGLMGRIDYYWSVLKPGQTLRIKSDGNLIAPWYGHILSGESHGRILKTD
LKRGSCTVQCQTEKGGLNTTLPFQNVSKYAFGNCSKYIGIKSLKLAVGLRNVPSRSSKGLFGAIPGFIEG
GWSGLVAGWYGFQHSNDQGVGMAADRDSTQKAIDKITSKVNNIVDKMNKQYEIIDHEFSEVETRLNMINN
KIDDQIQDIWAYNAELLVLLENQKTLDEHDANVNNLYNKVKRALGSNAVEDGKGCFELYHKCNDQCMETI
RNGTYNRRKYQEESKLERQKIEGVKLESEGTYKILTIYSTVASSLVIAMGFAAFLFWAMSNGSCRCEHCI

>QBF57610.1 hemagglutinin [Influenza A virus]
METVSLITILLVATVSNADKICIGYQSTNSTETVDTLTENNVPVTHAKELLHTEHNGMLCATSLGQPLIL
DTCTIEGLIYGNPSCDLSLEGREWSYIVERPSAVHGLCYPGNVQDLEELRSLFSSARSYQRIQIFPDTIW
NVSYDGTSTACSGSFYKSMRWLTRKNGEYPTQDAQYTNNQGKNILFMWGINHPPTDDTQRGLYTRTDTTT
SVATEEINRIFKPLIGPRPLVNGLMGRINYYWSVLKPGQTLRIKSDGNLIAPWYGHILSGESHGRIVKTD
LERGRCTVQCQTEKGGLNTTLPFQNVSKYAFGNCSKYIGIKSLKLAVGLRNVPSRYSRGLFGAIAGFIEG
GWSGLVDGWYGFQHSNDQGVGMAADRDSTQKAIDKITSKVNNIVDKMNKPYEIIDHEFSEVETRLNMINN
KIDDQIQDIWAYNAELIVLLENQKTLDEHDANVNNVYNKVKRALGSNAVEDGKGCLELYHKCDDQCMETI
RNGTYNRRKYQEESKLERQRIEGVKLESEGTYKILSIYSTVASYLVIAMGFAAFLFWAMYNGSCRCEHCI

>QBF57609.1 hemagglutinin [Influenza A virus]
METVSLITILIVATVSNADKICIGYQSTNSTETVDTLTENNVPVTHAKELLHTEHNGMLCATSLGQPLIL
DTCTIEGLIYGNPSCDLSLEGREWSYIVERPSAVNGLCYPGNVENLEELRSLFSSARSYQRIQIFPDTIW
NVSYDGTSTACSNSFYRSMRWLTRKDGNYPTQDAQYTNNQGKNILFMWGINHPPTDETQRNLYTRTDTTT
SVATEEINRIFKPLIGPRPLVNGLMGRIDYYWSVLKPGQTLRIKSDGNLIAPWYGHILSGESHGRILKTD
LKRGSCTVQCQTEKGGLNTTLPFQNVSKYAFGNCSKYIGIKSLKLAVGLRNVPSRSSRGLFGAIAGFIEG
GWSGLVAGWYGFQHSNDQGVGMAADRDSTQKAIDKITSKVNNIVDKMNKQYEIIDHEFSEVETRLNMINN
KIDDQIQDIWAYNAELLVLLENQKTLDEHDANVNNLYNKVKRALGSNAVEDGKGCFELYHKCNDQCMETI
RNGTYNRKKYQEESKLERQRIEGVKLESEGTYKILTIYSTVASSLVIAMGFAAFLFWAMSNGSCRCNICI

>QBF57607.1 hemagglutinin [Influenza A virus]

METVSLITILLVATASNADKICIGYQSTNSTETVDTLTENNVPVTHAKELLHTEHNGMLCATSLGQPLIL

DTCTVEGLIYGNPSCDLSLEGREWSYIVERPSAVNGLCYPGNVENLEELRSLFSSARSYQRIQIFPDTIW

NVSYDGTSTACSNSFYRSMRWLTRKDGNYPTQDAQYTNNQGKNILFMWGINHPPTDDTQRNLYTRTDTTT

SVATEEINRIFKPLIGPRPLVNGLMGRIDYYWSVLKPGQTLRIKSDGNLIAPWYGHILSGESHGRILKTD

LKRGSCTVQCQTEKGGLNTTLPFQNVSKYAFGNCSKYIGIKSLKLAVGLRNVPSKSSRGLFGAIAGFIEG

GWSGLVAGWYGFQHSNDQGVGMAADRDSTQKAIDKITSKVNNIVDKMNKQYEIIDHEFSEVETRLNMINN

KIDDQIQDIWAYNAELLVLLENQKTLDEHDANVNNLYNKVKRALGSNAVEDGKGCFELYHKCNDQCMETI

RNGTYNRRKYQEESKLERQKIEGVKLESEGTYKILTIYSTVASSLVIAMGFAAFLFWAMSNGSCRCNICI

>QBF57606.1 hemagglutinin [Influenza A virus]

MDTVSLITILLVATASNADKICIGYQSTNSTETVDTLTENNVPVTHAKELLHTEHNGMLCATSLGQPLIL

DTCTVEGLIYGNPSCDLSLEGREWSYIVERPSAVNGLCYPGNVENLEKLRSLFSSARSYQRIQIFPDTIW

NVSYDGTSTACSNSFYRSMRWLTQKDGNYPTQDAQYTNNQGKNILFMWGINHPPTDDTQRNLYTRTDTTT

SVATEEINRIFDPLIGPRPLVNDLMRRIDYYWSVMKPGQTLRIKSDGNLIAPWYGHILSGESHGRILKTD

LKRGSCTVQCQTEKGGLNTTLPFQNVSKYAFGNCSKYIGIKSLKLAVGLRNVPSRCSRGLFGAIAGFIEG

GWSGLVAGWYGFQHSNDQGVGMAADRDSTQKAIDKITSKVNNIVDKMNKQYEIIDHEFSEVETRINMINN

KIDDQIQDIWAYNAELLVLLENQKTLDEHDANVNNLYNKVKRALGSNAVEDGKGCFELYHKCNDQCMETI

RNGTYNRRKYQEESKLERQKIEGVKLESEGTYKILSIYSTVASSLVIAMGFAAFLFWAMSNGSCRCNICI

>QBF57605.1 hemagglutinin [Influenza A virus]

METVLLITILLVATASNADKICIGYQSTNSTETVDTLTENNVPVTHAKELLHTEHNGMLCATSLGQPLIL

DTCTVEGLIYGNPSCDLSLEGREWSYIVERPSAVNGLCYPGNVENLEELRSLFSSARSYQRIQIFPDTIW

NVSYDGTSTACSNSFYRSMRWLTRKDGNYPTQDAQYTNNQGKNILFMWGINHPPTDDTQRNLYTRTDTTT

SVATEEINRIFKPLIGPRPLVNGLMGRIDYYWSVLKPGQTLRIKSDGNLIAPWYGHILSGESHGRIVKTD

LKRGSCTVQCQTEKGGLNTTLPFQNVSKYAFGNCSKYIGIKSLKLAVGLRNVPSRSSRGLFGAIAGFIEG

GWSGLVAGWYGFQHSNDQGVGMAADRDSTQKAIDKTTSKVNNIVDKMNKQYEIIDHEFSEVETRINMINN

KIDDQIQDIWAYNAELLVLLENQKTLDEHDANVNNLYNKVKRALGSNAVEDGKGCFELYHKCNDQCMETI

RNGTYNRRKYQEESKLERQKIEGVKLESEGTYKILTIYSTVASSLVIAMGFAAFLFWAMYNGSCRCEHCI

>QBF57604.1 hemagglutinin [Influenza A virus]

METVSLITILIVATVSNADKICIGYQSTNSTETVDTLTENNVPVTHAKELLHTEHNGMLCATSLGQPLIL

DTCTIEGLIYGNPSCDLSLEGREWSYIVERPSAVNGLCYPGNVENLEELRSLFSSARSYQRIQIFPDTIW

NVSYDGTSTACSNSFYRSMRWLTRKDGNYPTQDAQYTNNQGKNILFMWGINHPPTDETQRNLYTRTDTTT

SVATEEINRIFKPLIGPRPLVNGLMGRIDYYWSVLKPGQTLRIKSDGNLIAPWYGHILSGESHGRILKTD
LKRGSCTVQCQTEKGGLNTTLPFQNVSKYAFGNCSKYIGIKSLKLAVGLRNVPSRSSRGLFGAIAGFIEG
GWSGLVAGWYGFQHSNDQGVGMAADRDSTQKAIDKITSKVNNIVDKMNKQYEIIDHEFSEVETRINMINN
KIDDQIQDIWAYNAEVLVLLENQKTLDEHDANVNNLYNKVKRALGSNAVEDGKGCFELYHKCNDQCMETI
RNGTYNRKKYQEESKLERQRIEGVKLEFEGTYKILTIYSTVASSLVIAMGFAAFLFWAMSNGSCRCEHCI

>QBF57601.1 hemagglutinin [Influenza A virus]
METVSLITILLVATVSNADKICIGYQSTNSTETVDTLTENNVPVTHAKELLHTEHNGMLCATSLGQPLIL
DTCTVEGLIYGNPSCDLSLEGREWSYIVERPSAVNGLCYPGHVQNLEELRSLFSSARSYQKIQIFPDTIW
NVSYDGTSTACSNSFYRSMRWLTRKDGNYPTQDAQYTNNQGKNILFMWGINHPPTDDTQRNLYTRTDTTT
SVATEEINRIFKPLIGPRPLVNGLMGRIDYYWSVLKPGQTLRIKSDGNLIAPWYGHILSGESHGSILTTD
LKRGSCTVQCQTEKGGFNTTLPFQNVSKYAFGNCSKYIGIKSLKLAVGLRNVPSRSSRGLFGAIAGFIEG
GWSGLVAGWYGFQHSNDQGVGMAADRDSTQKAIDKITSKVNNIVDKMNKQYEIIDHEFSEVETRLNMINN
KIDDQIQDIWAYNAELLVLLENQKTLDEHDANVNNLYNKVKRALGSNAVEDGKGCFELYHKCNDQCMETI
RNGTYNRKKYQEESKLERQRIEGVKLESEGTYKILSIYSTVASSLVIAMGFAAFLFWAMSNGSCRCNYCI

>QBF57600.1 hemagglutinin [Influenza A virus]
METVSLITILLVATVSNADKICIGYQSTNSTETVDTLTENNVPVTHAKELLHTEHNGMLCATSLGQPLIL
DTCTVEGLIYGNPSCDLSLEGREWSYIVERPSAVNGLCYPGNVQNLEELRSLFSSARSYHKIQIFPDTIW
NVSYDGTSTACSNSFYRSMRWLTRKDGNYPTQDAQYTNNQGKNILFMWGINHPPTDETQRNLYTRTDTTT
SVATEEINRIFKPLIGPRPLVNGLMGRIDYYWSVLKPGQTLRIKSDGNLIAPWYGHILSGESHGSILTTD
LKRGSCTVQCQTEKGGLNTTLPLQNVSKYAFGNCSKYIGIKSLKLAVGLRNVPSRSSRGLFGAIAGFIEG
GWSGLVAGWYGFQHSNDQGVGMAADRDSTQKAIDKITSKVNNIVDKMNKQYEIIDHEFSEVETRLNMINN
KIDDQIQDIWAYNAELLVLLENQKTLDEHDANVNNLYNKVKRALGSNAVEDGKGCFELYHKCNDQCMETI
RNGTYNRKKYQEESKLERQRIEGVKLESEGTYKILTIYSTVASSLVIAMGFAAFLFWAMSNGSCRCNICI

>QBF57599.1 hemagglutinin [Influenza A virus]
METVSLITILLVATVSNADKICIGYQSTNSTETVDTLTENNVPVTHAKELLHTEHNGMLCATSLGQPLIL
DTCTIEGLIYGNPSCDLSLEGREWSYIVERPSAVNGLCYPGNVENLEELRSLFSSARSYQRIQIFPDTIW
NVSYDGTSTACSNSFYRSMRWLTRKDGNYPTQDAQYTNNQGKNILFMWGINHPPTDDTQRNLYTRTDTTT
SVATEEINRIFKPLIGPRPLVNGLMGRIDYYWSVLKPGQTLRIKSDGNLIAPWYGHILSGESHGRILKTD
LKRGSCTVQCQTEKGGLNTTLPFQNVSKYAFGNCSKYIGIKSLKLAVGLRNVPSRSSRGLFGAIAGFIEG
GWSGLVAGWYGFQHSNDQGVGMAADRDSTQKAIDKITSKVNNIVDKMNKQYEIIDHEFSEVETRLNMINN
KIDDQIQDIWAYNAELLVLLENQKTLDEHDANVNNLYNKVKRALGSNAVEDGKGCFELYHKCNDQCMDTI

RNGTYNRRKYQEESKLERQKIEGVKLESEGTYKILSIYSTVASSLVIAMGFAAFLFWAMSNGSCRCNICI

>QBF57598.1 hemagglutinin [Influenza A virus]
METVSLITILLVATVSNADKICIGYQSTNSTETVDTLTENNVPVTHAKELLHTEHNGMLCATSLGQPLIL
DTCTIEGLIYGNPSCDLSLEGREWSYIVERPSAVHGLCYPGNVEDLEELRSLFSSARSYQRIQIFPDTIW
NVSYDGTSTACSGSFYKSMRWLTRKNGEYPTQDAQYTNNQGKNILFMWGINHPPTDDTQRGLYTRTDTTT
SVATEEINRIFKPLIGPRPLVNGLMGRINYYWSVLKPGQTLRIKSDGNLIAPWYGHILSGESHGRILKTD
LKRGSCTVQCQTEKGGLNTTLPFQNVSKYAFGNCSKYIGIKSLKLAVGLRNVPSRSSRGLFGAIAGFIEG
GWSGLVAGWYGFQHSNDQGVGMAADRDSTQKAIDKITSKVNNIVDKMNKQYEIIDHEFSEVETRLNMINN
KIDDQIQDIWAYNAELLVLLENQKTLDEHDANVNNLYNKVKRALGSNAVEDGKGCFELYHKCDDQCMETI
RNGTYNRRKYQEESKLERQRIEGVKLESEGTYKILTIYSTVASSLVIAMGFAAFLFWAMSNGSCRCNICI

>QBF57595.1 hemagglutinin [Influenza A virus]
METVSLITILLVATVSNADKICIGYQSTNSTETVDTLTENNVPVTHAKELLHTEHNGMLCATSLGQPLIL
DTCTVEGLIYGNPSCDLSLEGREWSYIVERPSAVNGLCYPGNVENLEELRSLFSSARSYQRIQIFPDTIW
NVSYDGTSTACSDSFYRSMRWLTRKDGNYPTQDAQYTNNQGKNILFMWGINHPPTDDTQRNLYTRTDTTT
SVATEEINRIFKPLIGPRPLVNGLMGRIDYYWSVLKPGQTLRIKSDGNLIAPWYGHILSGESHGRILKTD
LKRGSCTVQCQTEKGGLNPTLPFQNGSKYAFGNCSKYIGIKILKLAVGLRNVPSRSSRGLFGAIAGFIEG
GWSGLVAGWYGFQHSNDQGVGMAADRDSTQKAIDKITSKVNNIVDKMNKQYEIIDHEFSEVETRLNMINN
KIDDQIQDIWAYNAELLVLLENQKTLDEHDANVNNLYNKVKRALGSNAVEDGKGCFELYHKCNDQCMETI
RNGTYNRRKYQEESKLERQKIEGVKLESEGTYKILTIYSTVASSLVIAMGFAAFLFWAMSNGSCRCNICT

>QBF57594.1 hemagglutinin [Influenza A virus]
METVSLITILLVATVSNADKICIGYQSTNSTETVDTLTENNVPVTHAKELLHTEHNGMLCATSLGQPLIL
DTCTIEGLIYGNPSCDLSLEGREWSYIVERPSAVHGLCYPGNVEDLEELRSLFSSARSYQRIQIFPDTIW
NVSYDGTSTACSGSFYKSMRWLTRKNGEYPTQDAQYTNNQGKNILFMWGINHPPTDDTQRGLYTRTDTTT
SVATEEINRIFKPLIGPRPLVNGLMGRINYYWSVLKPGQTLRIKSDGNLIAPWYGHILSGESTGSLLTTD
LKRGSCTVQCQTEKGGLNTTLPFQNVSKYAFGNCSKYIGIKSLKLAVGLRNVPSRSSRGLFGAIAGFIEG
GWSGLVAGWYGFQHSNDQGVGIAADRDSTQKAIDKITSKVNNIVDKMNKQYEIIDHEFSEVETRLNMINN
KIDDQIQDIWAYNAELLVLLENQKTLDEHDANVNNLYNKVKRALGSNAVEDGKGCFELYHKCDDQCMETI
RNGTYNRRKYQEESKLERQRIEGVKLESEGTYKILSIYSTVASSLVIAMGFAAFLFWAMSNGSCRCKNCI

>QBF57593.1 hemagglutinin [Influenza A virus]
METVSLITILLVATVSNADKICIGYQSTNSTETVDTLTENNVPVTHAKELLHTEHNGMLCATSLGQPLIL

DTCTIEGLIYGNPSCDLSLEGREWSYIVERPSAVHGLCYPGNVEDLEELRSLFSSARSYQRIQIFPDTIW
NVSYDGTSTACSGSFYKSMRWLTRKNGEYPTQDAQYTNNQGKNILFMWGINHPPTDDTQRGLYTRTDTTT
SVATEEINRIFKPLIGPRPLVNGLMGRINYYWSVLKPGQTLRIKSDGNLIAPWYGHILSGESHGRILKTD
LKRGSCTVQCQTEKGGLNTTLPFQNVSKYAFGNCSKYIGIKSLKLAVGLRNVPSRSSKRLFGAIAGFIEG
GWSGLVAGWYGFQHSNDQGVGMAADRDSTQKAIDKITSKVNNIVDKMNKQYEIIDHEFSEVETRLNMINN
KIDDQIQDIWAYNAELLVLLENQKTLDEHDANVNNLYNKVKRALGSNAVEDGKGCFELYHKCDDQCMETI
RNGTYNRRKYQEESKLERQRIEGVKLESEGTYKILTIYSTVASSLVIAMGFAAFLFWAMSNGSCRCNICI

>QBF57592.1 hemagglutinin [Influenza A virus]
MKTVSLITILLVATVSNADKICIGYQSTNSTETVDTLTENNVPVTHAKELLHTEHNGMLCATSLGQPLIL
DTCTIEGLIYGNPSCDLSLEGREWSYIVERPSAVHGLCYPGNVEDLEELRSLFSSARSYQRIQIFPDTIW
NVSYDGTSTACSGSFYKSMRWLTRKDGDYPTQDAQYTNNQGKNILFMWGINHPPTDDTQRSLYTRTDTTT
SVATEEINRIFKPLIGPRPLVNGLMGRINYYWSVLKPGQTLRIKSDGNLIAPWYGHILSGESPGRILKTD
LKRGSCTVQCQTDKGGLNTTLPFQNVSKYAFGNCSKYIGIKSLKLAVGLRNVPSRSSRGLFGAIAGFIEG
GWSGLVAGWYGFQHSNDQGVGMAADRDSTQKAIDKITSKVNNIVDKMNKQYEIIDHEFSEVETRLNMINN
KIDDQIQDIWAYNAELLVLLENQKTLDEHDANVNNLYNKVKRALGSNAVEDGKGCFELYHKCDDQCMETI
RNGTYNRRKYQEESKLERQRIEGVKLESEGTYKILTIYSTVASSLVIAMGFAAFLFWAMSNGSCRCNICI

>QBF57590.1 hemagglutinin [Influenza A virus]
METVSLITILLVATVSNADKICIGYQSTNSTETVDTLTENNVPVTHAKELLHTEHNGMLCATSLGQPLIL
DTCTIEGLIYGNPSCDLSLEGREWSYIVERPSAVHGLCYPGNVEDLEELRSLFSSARSYQRIQIFPDTIW
NVSYDGTSTACSGSFYKSMRWLTRKNGEYPTQDAQYTNNQGKNILFMWGINHPPTDDTQRGLYTRTDTTT
SVATEEINRIFKPLIGPRPLVNGLMGRINYYWSVLKPGQTLRIKSDGNLIAPWYGHILSGESHGSILKTD
LKKSSCTVQCQTEKGGLNTTLPLQNVSKYAFGNCSKYIGIKSLKLAVGLRNVPSRSSRGLFGAIAGFIEG
GWSGLVAGWYGFQHSNDQGVGMAADRDSTQKAIDKITSKVNNIVDKMNKQYEIIDHEFSEVETRLNMINN
KIDDQIQDIWAYNAELLVLLENQKTLDEHDANVNNLYNKVKRALGSNAVEDGKGCFELYHKCDDQCMETI
RNGTYNRRKYQEESKLERQRIEGVKLESEGTYKILTIYSTVASSLVIAMGFAAFLFWAMSNGSCRCNICI

>QBF57589.1 hemagglutinin [Influenza A virus]
METVSLITILLVATVSNADKICIGYQSTNSTETVDTLTENNVPVTHAKELLHTEHNGMLCATSLGQPLIL
DTCTIEGLIYGNPSCDLSLEGREWSYIVERPSAVHGLCYPGNVEDLEELRSLFSSARSYQRIQIFPDTIW
NVSYDGTSTACSGSFYKSMRWLTRKNGEYPTQDAQYTNNQGKNILFMWGINHPPTDDTQRGLYTRTDTTT
SVATEEINRIFKPLIGPRPLVNGLMGRINYYWSVLKPGQTLRIKSDGNLIAPWYGHILSGESHGRILKTD
LKRGSCTVQCQTEKGGLNTTLPFQNVSKYAFGNCSKYIGIKSLKLAVGLKNVPSKSNKRLFGVKTGFIEG

GWSGLVAGWYGFQHSNDHGVGMAADRDSTQKAIDKITSKVNNIVDKMYKQYEIIDHEFSEVETRLNMINN
KIDDQIQDIWAYNAELLVLLENQKTLDEHDANVNNLYNKVKRALGSNAVEDGKGCFELYHKSDDQCIETI
RNGTYNRRKYQEESKLERQRIEGVKLESEGTYKILSIYSTVASSLVIAMGFAAFLFWAMYNGSCRCNICI

>QBF57588.1 hemagglutinin [Influenza A virus]
MVTVSLITILLVATVSNADKICIGYQSTNSTETVDTLTENNVPVTHAKELLHTEHNGMLCATSLGQPLIL
DTCTVEGLIYGNPSCDLSLEGREWSYIVERPSAVNGLCYPGNVENLEELRSLFSSARSYQRIQIFPDTIW
NVSYDGTSTACSDSFYRSMRWLTRKDGNYPTQDAQYTNNQGKNILFMWGINHPPTDDTQRNLYTRTDTTT
SVATEEINRIFKPLIGPRPLVNGLMGRIDYYWSVLKPGQTLRIKSDGNLIAPWYGHILSGESHGRILKTD
LKRGSCTVQCQTEKGGLNTTLPFQNVSKYAFGNCSKYIGIKSLKLAVGLRNVPSRSSRGLFGAIAGFIEG
GWSGLVAGWYGFQHSNDQGVGMAADRDSTQKAIDKITSKVNNIVDKMYKQYEIIDHEFSEVETRLNMINN
KIDDQIQDIWAYNAELLVLLENQKTLDEHDANVNNLYNKVKRALGSNAVEDGKGCFELYHKRNDQCMETI
RNGTYNRRKYQEESKLERQKIEGVKLESEGTYKILSIYSTVASSLVIAMGFAAFLFWAMSNGSCRCNICI

>QBF57587.1 hemagglutinin [Influenza A virus]
METVSLITILLVATVSNADKICIGYQSTNSTETVDTLTENNVPVTHAKELLHTEHNGMLCATSLGQPLIL
DTCTIEGLIYGNPSCDLSLEGREWSYIVERPSAVHGLCYPGNVQDLEELRSLFSSARSYQRIQIFPDTIW
NVSYDGTSTACSGSFYKSMRWLTRKNGEYPTQDAQYTNNQGKNILFMWGINHPPTDDTQRGLYTRTDTTT
SVATEEINRIFKPLIGPRPLVNGLMGRINYYWSVLKPGQTLRIKSDGNLIAPWYGHILSGESHGEVLKTD
LKRGSCTVQCQTKRGGLNTTLPFQNVSKYASGNCSKYIGIKCLKLAVGLRNVRSRSSRGLFGAIAGFIEG
GWSGLVDGWYGFQHSNDQGVGMAADRDSTQKAIDKITSKVNNIVDKMNKQYEIIDHEFSEVETRLNMINN
KIDDQIQDIWAYNAELLVLLENQKTLDEHDANVNNLYNKVKRALGSNAVEDGKGCFELYHKCDDQCMETI
RNGTYNRRKYQEESKLERQRIEGVKLESEGTYKILSIYSTVASSLVIAMGFAAFLFWAMSNGSCRCNICI

>QBF57586.1 hemagglutinin [Influenza A virus]
METVSLMTILLVATVSNADKICIGYQSTNSTETVDTLTENNVPVTHAKELLHTEHNGMLCATSLGQPLIL
DTCTIEGLIYGNPSCDLSLEGREWSYIVERPSAVHGLCYPGNVEDLEELRSLFSSARSYQRIQIFPDTIW
NVSYDGTSTACSGSFYRSMRWLTRKNGEYPIQDAQYTNNQGKNILFMWGINHPPTDDTQRGLYTRTDTTT
SVATEEINRIFKPLIGPRPLVDGLMGRINYYWSVLKPGQTLRIKSDGNLIAPWYGHILSGESHGRILKTD
LKRGSCTVQCQTEKCGINTTLPFQNVSKYAFGKCSKYIGIKSLKLAVGLRNVPSRSSRGLFGAIAGFIEG
GWSGLVAGWYGFQHSNDHGVGMAADRDSTQKAIDKITSKVNNIVDKMYKQYEIIDHEFSEVETRLNMINN
KIDDQIQDIWAYNAELLVLLENQKTLDEHDANVNNLYNKVKRALGSNAVEDGKGCFELYHKCDDQCMETI
RNGTYNRRKYQEESKLERQRIEGVKLESEGTYKILSIYSTVASSLVIAMGFAAFLFWAMYNGSCRCNICI

>QBF57585.1 hemagglutinin [Influenza A virus]

MDTVSLMTILLVATVSNANEICIGYQSTNSTKSVNTLTENNVPVTHAVELLHTQHNGMLCAPSLGQPLIL

DTCSIEWLIYGIPSCDLSLEGREWSYIVERPSTVHGLCYPGNVQYLKELMSLFSSATSYEKNQIFPHTLW

NVSYDGTSMPCSGSFYRTMIWLTPKNGEHPIQDAPYTNNLGKNILFMWGINHPPTDDTQIRLYTRTDTTS

SVATDEIKTIFTPLIRPRPLVNRLMGRINYYWSELKPGQTLRIKSDENLISPWYGHILSGESHGRILKTD

LERGSCTELRKTEKGGLTTTLPFQNVSKYASGNCSKYNGIKSVKLAAGLRNVRSRYSRGLFGAIAGFIEG

GWSGVGDGWYGFQHSNDHGVGMAADRDLIQKAIDKITSKVNNIVAKMYKQYEIIDHEFSEVGTRINMSNN

KIDDQIQDIWAYNAETIVLLDNQKTLDEHDANVNNLYNKVKRALGSNAVEDGKGCFELYHKCDDQCMETI

RNGTYNRRKYQEESKLERQRMEGVKLESERTYKILSIYSRVASYRVIAMGSVALRFGAMYYGSCRCDSCI

>QBF57583.1 hemagglutinin [Influenza A virus]

MDTVSLITILIVSTVSIANEFCIGYQSTNSTETVNTLSQNCVPVTHAVELLHTQHNGMLCATSLGHPLIL

DTCTIEWLIYGNPSCDPLLGGREWSYIVERPSTVNGLCYPGNVQNLEELISLFSSSRSYQRIQIFPDTIW

NVSYSGTSTACSDSFYRSMRWLTPKNSAYPTQDAHYTNNQGKNILFMWGINHPPTDTTQTILYTITDTTT

SVATDEMNRIFKPLIGPRPLVNGLMGRINYYWSVLKPGPTLRVKSDGNLIAPWYGWIRSGERPGTILKTD

LKKGSCTVQCQTEKGGLHAPLPVQNMSKYTFGNCSKYIGVKSVKLAVGLRNVPSRYSRGLFGAIAGFIEG

GWPGVVDGWYGFRHSNDHGVGMAADRDSTQKAIDKITSKVNNIVAKMNKQYEIIDHEFSEVETRINMINN

KVDDQIQDIWAYNAETIVFQDNQKTLDEHDANVNNVYNKVKRALGSNAVEDGKGCFELYHKRDDHCMETI

RNRTYNRRKYQAESKLERQRIEGVNQELEETCKIVSIYSTVASSRVIAMGFAAILFWAMYNGSARQNSCT

>QBF57582.1 hemagglutinin [Influenza A virus]

METVSLITILLVATASNADKICIGYQSTNSTETVDTLTENNVPVTHAKELLHTEHNGMLCATSLGQPLIL

DTCTVEGLIYGNPSCDLSLEGREWSYIVERPSAVNGLCYPGNVENLEELRSLFSSARSYQRIQIFPDTIW

NVSYDGTSTACSNSFYRSMRWLTRKDGNYPTQDAQYTNNQGKNILFMWGINHPPTDDTQRNLYTRTDTTT

SVATEEINRIFKPLIGPRPLVNGLMGRIDYYWSVLKPGQTLRIKSDGNLIAPWYGHILSGESHGRILKTD

LKRGSCTVQCQTEKGGLNTTLPFQNVSKYAFGNCSKYIGIKSLKLAVGLRNVPSRSSRVLFGAIAGFIEG

GWSGLVAGWYGFQHSNDQGVGMAADRDSTQKAIDKITSKVNNIVDKMYKQYEIIDHEFSEVETRLNMINN

KIDDQIQDIWAYNAELLVLLENQKTLDEHDANVNNLYNKVKRALGSNAVEDGKGCFELYHKSNDQCIETI

RNGTYNRRKYQAESKLERQKIEGVKLESEGTYKILSIYSTVASSLVIAMGFAAFLFWAMSNGSCRCNICI

>QBF57581.1 hemagglutinin [Influenza A virus]

MDTVSLMTILLVATVSNADKICIGYQSTNSTKTVNTLTENNVPVTHAEELLHTQHNGMLCATSLGQPLIL

DTCTIEGLIYGNPSCDLSLEGREWSYIVERPSAVHGLCYPGNVQDLEELMSLFSSARSYQKIQIFPHTIW

NVSYDGTSTACSGSFYRSMRWLTRKNGEYPIQDAQYTNNQGKNILFMWGINHPPTDDTQIGLYTRTDTTT

SVATEEINTIFEPLIRPRPLVDGLMGRINYYWSVLKPGQTLRIKSDGNLIAPWYGHVLSGESQGRILKTD
LKRGSCTVQRQTDKGGINTTLPFQNVSKYASGNCSKYIGIKSLKLAVGLRNVPSRYSRGLFGAIAGFIEG
GWSGVVDGWYGFQHSNDHGVGMAADRDSIQKAIDKITSKVNNIVAKMYKQYEIIDHEFSEVETRINMINN
KIDDQIQDIWAYNAEPIVLLDNQKTLDEHDANVNNVYNKVKRALGSNAVEDGKGCFELYHKCDDQCMETI
RNGTYNRRKYQEESKLERQRIEGVKLESEGTYKILSIYSTVASSLVIAMGFAALLFWAMYNGSCRCNICI

>QBF57580.1 hemagglutinin [Influenza A virus]
METVSLMTILLVATVSNADKICIGYQSTNSTETVDTLTENNVPVTHAEELLHTEHNGMLCATSLGQPLIL
DTCTIEGLIYGNPSCDLSLEGREWSYIVERPSAVHGLCYPGNVEDLEELRSLFSSARSYQRIQIFPDTIW
NVSYDGTSTACSGSFYRSMRWLTRKNGEYPIQDAQYTNNQGKNILFMWGINHPPTDDTQRGLYTRTDTTT
SVATEEINRIFKPLIGPRPLVNGLMGRINYYWSVLRPGQTLRIKSDGNLIAPWYGHILSGEGHGRILKTD
LKRGSCTVQCRTEKRGINTTLPFQNVSKYAFGNCSKYIGIKSLKLAVGLRNVPSRSSRGLFGAIAGFIEG
GWSGLVAGWYGFQHSNDHGVGMAADRDSTQKAIDKITSKVNNIVDKMYKQYEIIDHEFSEVETRLNMINH
KIDDQIQDIWAYNAELLVLLDNQKTLDEHDANVNNLYNKVKRALGSNAVEDGKGCFELYHKCDDQCIETI
RNGTYNRRKYQEESKLERQRIEGVKLESEGTYKILSIYSTVASYLVIAMGFAAFLFWAMYNGSCRCNICI

>QBF57579.1 hemagglutinin [Influenza A virus]
METVSLMTILLVATVSNADKICIGYQSTNSTETVNTLTENNVPVTHAKELLHTEHNGMLCATSLGQPLIL
DTCTIEGLIYGNPSCDLSLEGREWSYIVQRPSAVHGLCYPGNVEDLEELRSLFSSARSYQRIQIFPDTIW
NVSYDGTSTACSGSFYRSMRWLTRKNGEYPIQDAQYTNNQGKNILFMWGINHPPTDDTQRGLYTRTDTTT
SVATEEINTIFEPLIRPRPLVDGLMGRINYYWSVLKPDQTLRIKSDGNLIAPWYGHILSRERHGSNLTTD
LKRGSCPVQCQTQKSGINTTLPFQNVSKYAFGNCSKYIGIKSVKLAVGLRNVPSRSSRGLFGAIAGFIEG
GWSGVVDGWYGFQHSNDQGVGMAADRDSTQKAIDKITSKVNNIVDKMNKQYEIIDHEFSEVETRINMINN
KIDDQIQDIWAYNAELIVLLDNQKTLDEHDANVNNVYNKVKRALGSNAVEDGKGCFELYHKCDDQCMETI
RNGTYNRRKYQEESKLERQRIEGVKLESEGTYKILSIYSTVASSLVIAMGFAAFLFWAMYNGSCRCNICI

>QBF57578.1 hemagglutinin [Influenza A virus]
METVSLMTILLVATVSNADKICIGYQSTNSTETVDTLTENNVPVTHAKELLHTEHNGMLCATSLGQPLIL
DTCTIEGLIYGNPSCDLSLEGREWSYIVERPSAVHGLCYPGNVEDLEELRSLFSSARSYQRIQIFPDTIW
NVSYDGTSTACSGSFYRSMRWLTRKNGEYPIQDAQYTNNQGKNILFMWGINHPPTDDTQRGLYTRTDTTT
SVATEEINRIFKPLIGPRPLVNGLMGRINYYWSVLKPGQTLRIKSDGNLIAPWYGHILSGESHGRILKTD
LKRGSCTVQCQTEKGGINTTLPFQNVSKYAFGNCSKYIGIKSLKLAVGLRNVPSRSSRGLFGAIAGFIEG
GWSGLVAGWYGFQHSNDQGVGMAADRDSTQKAIDKITSKVNNIVDKMNKQYEIIDHEFSEVETRLNMINN
KIDDQIQDIWAYNAELLVLLENQKTLDEHDANVNNLYNKVKRALGSNAVEDGKGCFELYHKCDDQCMETI

RNGTYNRRKYQEESKLERQRIEGVKLESEGTYKILSIYSTVASSLVIAMGFAAFLFWAMSNGSCRCNICI

>QBF57576.1 hemagglutinin [Influenza A virus]
MDTVSLITILLLSTVSIADKICIGYQSTNSAETVNTLTQNNVPVTHAEELLHTQHNGMLCATSLGQPLIL
DTCSVEWLIYGNPSCDLSLEGREWSYIVQRPSTVNGLCYPGNVQNLKELMSLFSSARSYQKIQIFPHTIW
NVSYDGTSTPCSNSFYRSMRWLTPKDGNYPTQDAQYTNNQGKNILFMWGINHPPTDDTQINLYTITDTTS
SVATEEISTIFTPLIRPRPLVNSLMGRIDYYWSVLKPDQTLPIKSDENLIALWYGHILSGESHERIMKTN
LKGGSCTVQCQTEKGGLNTAQSMENVSKYEFGKCSKYRCIKCVKLAVGLRKVRCRYSRGVFGAIAGFIER
GWSGLVGSWYGFQHSNDQGVGMAADRDSTRKAMDKITSNVNNMVAKMYKPYEVIGLEFIVVGTRIVTINN
KFDDQIQDIWAYNAESIVLIDNQKTLGEHDANVNNLWNKVKRGACSNAVEDGKGCLVQYHKCNDQCMETI
RNGTYNRRKYQAESKKERQKIEGVKLELDRTCKIVSIYSTVASYRVIAMGFVAILFGAMYNGSCRQNICI

>QBF57575.1 hemagglutinin [Influenza A virus]
METVSLITILLVATVSNADKICIGYQSTNSTETVDTLTENNVPVTHAKELLHTEHNGMLCATSLGQPLIL
DTCTVEGLIYGNPSCDLSLEGREWSYIVQRPSAVNGLCYPGNVENLEELRSLFSSARSYHRIQIFPDTIW
NVSYDGTSTACSNSFYRSMRWLTRKDGNYPTQDAQYTNNQGKNILFMWGINHPPTDETQRNLYTRTDTTT
SVATEEINRIFKPLIGPRPLVNGLMGRIDYYWSVLKPGQTLRIKSDGNLIAPWYGHILSGESHGRILKTD
LKRGSCTVQCQTEKGGLNTTLPFQNVSKFAFGNCSKYIGIKSLKLAVGLRNVPSRSSRGLFGAIAGFIEG
GWSGLVAGWYGFQHSNDQGVGMAADRDSTQKAIDKITSKVNNIVDKMYKQYEIIDHEFSEIETRLNMINN
KIDDQIQDIWAYNAELLVLLENQKTLDEHDANVNNLYNKVKRALGSNAVEDGKGCFELYHKRNDQCMETI
RNGTYNRKKYQEESKLERQRIEGVKLEFEGTYKILTIYSTVASSLVIAMGFAAFLFWAMSNGSCRCNICI

>QBF57574.1 hemagglutinin [Influenza A virus]
METVSLITILVVATVSNADKICIGYQSTNSTETVDTLTENNVPVTHAKELLHTEHNGMLCATSLGHPLIL
DTCTIEGLIYGNPSCDPLLGGREWSYIVERPSAVNGLCYPGNVENLEELRSLFSSSRSYQRIQIFPDTIW
NVSYSGTSKACSDSFYRSMRWLTQKNNAYPTQDAQYTNNQGKNILFMWGINHPPTDTVQTNLYTRTDTTT
SVATEEMNRIFKPLIGPRPLVNGLMGRINYYWSVLKPGQTLRIKSDGNLIAPWYGHILSGESHGRILKTD
LKRGSCTVQCQTEKGGLNTTLPFQNISKYAFGNCSKYIGVKSLKLAVGLKNVPSRSSKGLFGAIAGFIEG
GWPGLVAGWYGFQHSNDQGVGMAADRDSTQKAIDKITSKVNNIVDKMNKQYEIIDHEFSEVETRLNMINN
KVDDQIQDIWAYNAELLVLLENQKTLDEHDANVNNLYNKVKRALGSNAVEDGKGCFELYHKCDDHCMETI
RNGTYNRRKYQEESKLERQKIEGVKLESEETYKILTIYSTVASSLVIAMGFAAFLFWAMSNGSCRCNICI

>QBF57573.1 hemagglutinin [Influenza A virus]
METVSLITILLVATVSNADKICIGYQSTNSTETVDTLTENNVPVTHAKELLHTEHNGMLCATSLGQPLIL

DTCTVEGLIYGNPSCDLSLEGREWSYIVERPSAVNGLCYPGNVENLEELRSLFSSARSYHRIQIFPDTIW
NVSYDGTSTACSNSFYRSMRWLTRKDGNYPTQDAQYTNNQGKNILFMWGINHPPTDETQRNLYTRTDTTT
SVATEEINRIFKPLIGPRPLVNGLMGRIDYYWSVLKPGQTLRIKSDGNLIAPWYGHILSGESHGRILKTD
LKRGSCTVQCQTEKGGLNTTLPFQNVSKFAFGNCSKYIGIKSLKLAVGLRNVPSRSSRGLFGAIAGFIEG
GWSGLVAGWYGFQHSNDQGVGMAADRDSTQKAIDKITSKVNNIVDKMNKQYEIIDHEFSEIETRLNMINN
KIDDQIQDIWAYNAELLVLLENQKTLDEHDANVNNLYNKVKRALGSNAVEDGKGCFELYHKCNDQCMETI
RNGTYNRKKYQEESKLERQRIEGVKLESEGTYKILTIYSTVASSLVIAMGFAAFLFWAMSNGSCRCNICI

>QBF57572.1 hemagglutinin [Influenza A virus]
METVSLITILLVATASNADKICIGYQSTNSTETVDTLTENNVPVTHAKELLHTEHNGMLCATSLGQPLIL
DTCTVEGLIYGNPSCDLSLEGREWSYIVERPSAVNGLCYPGNVENLEELRSLFSSARSYQRIQIFPDTIW
NVSYDGTSTACSNSFYRSMRWLTRKDGNYPTQDAQYTNNQGKNILFMWGINHPPTDDTQRNLYTRTDTTT
SVATEEINRIFKPLIGPRPLVNGLMGRIDYYWSVLKPGQTLRIKSDGNLIAPWYGHILSGESHGRILKTD
LNRGSCTVQCQAEKGGLNTTLPFQNVSKYAFGNCSKYIGIKSLKLAVGLRNVPSRSSRGLFGAIAGFIEG
GWSGLVAGWYGFQHSNDQGVGMAADRDSTQKAIDKITSKVNNIVDKMNKQYEIIDHEFSEIETRLNMINN
KIDDQIQDIWAYNAELLVLLDNQKTLDEHDANVNNLYNKVKRALGSNAVEDGKGCFELYHKCNDQCMETI
RNGTYNRRKYQEESKLERQRIEGVKLESEGTYKILTIYSTVASSLVIAMGFAAFLFWAMSNGSCRCNICI

>QBF57571.1 hemagglutinin [Influenza A virus]
MDTVSLITILLVATVSNADKICIGYQSTNSTETVNTLTENNVPVTHAEELLHTQHNGMLCATSLGQPLIL
DTCTIEGLIYGNPSCDLSLEGREWSYIVQRPSTVHGLCYPGNVQYLEELISLFSSARSYQKIQIFPHTIW
NVSYDGTSTACSSSFYKSMRWLTPKNGEYPTQDAQYTNNRGKNILFMWGINHPPTDDTQRRLYTRTDTTT
SVATEEINTIFTPLIRPRPLVNSLMGTIHYYWSVLKPGQTLRIKADGNLIAPWYGHILSGESHGAILKTE
LDRGSCTEQRQTDKDGLNNTQALQNVSKYASGKCSKYIGIKCVKLAAGLRNVRSRYSRGLFGAIAGFIEG
GWSGLVDGWYGFQHSNDQGVGMAADRDSIRKAIDKITSKVNNIVAKMYKQYEIIDHEFSEVGTRINMSNN
KIDDQIQDIWAYNAESIVLLDNQKTLDEHDANVNNLYNKVKRALGSNAVEDGKGCFELYHKCDDQCMETI
RNGTYNRRKYQEESKLERQRMEGLNQESERTNKILSIYSRVASYRVIAMGSAALRFGAMYNGECRCEHCT

>QBF57570.1 hemagglutinin [Influenza A virus]
MDTVSLITILLVSTVSIANEICIGYQSTNSTKTVNTLTQNNVPVTHAEELLHTQRNGMLGDPSLGQPLIL
DTCTIEGLIYGNPSCDLSLEGREWSYIVQRPSTVHGLCYPGNVQYLKELRSLFSSARSYQKIQIFPHTIW
NVSYDGTSTPCSGSFYITMIWLTPKNGDYPTQDPLYTNNLGKNILFMWGINHPPTDDTQRGLYTITDTTT
SVATEEINTIFTPLIRPRPLVNRLMGRINYYWSVLKPDQTLPIKSDGNLIALWYGHIPLRESHGRILKTD
LKRGSCTEQRQTDKGGLNTTMAFQNVSKYASGKCSKSIGIKCVKLAAGLRNVRCTYSRGLFGAIAGFIEG

GRSGLVDGWYGFQHSNDHGVGMAADRDSIQKAIDKITSKVNNIVAKMYKQYEIIDHEFSEVETRINMSNN
KIDDQIQDIWAYNAESIVLLDNQKTLDEHDANVNNVYNKVKRALGSNAVEDGKGCFELYHKRDDQCTETI
RNGTYNRRKYQAESKLERQRMEGLNLESERTYKILSTYSRVASYRVIAMGSAALRFGAMYYWDCRCEQCI

>QBF57569.1 hemagglutinin [Influenza A virus]
MDTVSLITILLLSTASIADKICIGYQSTNSAETVDTLTENNVPVTHAEELLHTEHNGMLCATSLGQPLIL
DTCTVEGLIYGNPSCDLSLEGREWSYIVERPSTVNGLCYPGNVQNLEELRSLFSSARSYQKIQIFPHTIW
NVSYDGTSTACSNSFYRSMRWLTRKDGNYPTQDAQYTNNQGKNILFMWGINHPPTDDTQRNLYTRTDTTS
SVATEEINRIFKPLIRPRPLVNGLMGRIDYYWSVLKPGQTLRIKSDGNLISLWYGHIVLGESHGRMLKTD
LNRGRCTVQCQTEKGGLNTTLPFLNVSKYAFGKCSKYICIKSLKLAVGLRNVRSRYSRGLFGAIAGFIER
GWSGLVAGWYGFQHSNDQGVGMAADRDSTRKAIDKITSNVNNIVDKLYKQYEIIDHEFSEVETRLIMINN
KFDDQIQDIWAYNAELLVLLENQKTLDEHDANVNNLYNKVKRALGSNAVEDGKGCFELYHKCNDQCMETI
RNGTYNRRKYQEESKLERQKIEGVKLESEGTYKIVTIYSTVASSLVIAMGFAAFLFWAMSNGSCRCNICI

>QBF57568.1 hemagglutinin [Influenza A virus]
METVSLITILLVATVSNADKICIGYQSTNSTETVDTLTESNVPVTHAKELLHTEHNGMLCATSLGQPLIL
DTCTIEGLIYGNPSCDLSLEGREWSYIVERPSAVHGLCYPGNVEDLEELRSLFSSARSYQRIQIFPDTIW
NVSYDGTSTACSGSFYKSMRWLTRKNGEYPTQDAQYTNNQGKNILFMWGINHPPTDDTQRGLYTRTDTTT
SVATEEINRIFKPLIGPRPLVNGLMGRINYYWSVLKPGQTLRIKSDGNLIAPWYGHILSGESHGRILKTD
LKRGSCPVQCQTEKGGLNTTLPFQNVSKYAFGNCSKYIGIKSLKLAVGLRNVPSRSSRGLFGAIAGFIEG
GWSGLVDGWYGFQHSNDQGVGMAADRDSTQKAIDKITSKVNNIVDKMNKQYEIIDHEFSEVETRLNMINN
KIDDQIQDIWAYNAELLVLLENQKTLDEHDANVNNLYNKVKRALGSNAVEDGKGCFELYHKCDDQCMETI
RNGTYNRRKYQEESKLERQRIEGVKLESEGTYKILTIYSTVASSLVIAMGFAAFLFWAMYNGSCRCNICI

>QBF57567.1 hemagglutinin [Influenza A virus]
METVSLITILLVATVSNADKICIGYQSTNSTETVNTLTENNVPVTHAKELLHTEHNGMLCATSLGQPLIL
DTCTIEGLIYGNPSCDLSLEGREWSYIVQRPSAVHGLCYPGNVQDLEELRSLFSSARSYQRIQIFPDTIW
NVSYDGTSTACSGSFYKSMRWLTRKNGEYPTQDAQYTNNQGKNILFMWGINHPPTDDTQIGLYTRTDTTT
SVATDEINRIFKPLIGPRPLVNGLMGRINYYWSVLKPGQTLRIKSDGNLIAPWYGHILSGESHGRILKTD
LKKGSCTVQCQTDKGGLNTTLPFQNVSKYASGNCSKYIGIKSLKLAVGLRNVPSRSSRGLFGAIAGFIEG
GWSGLVDGWYGFQHSNDQGVGMAADRDSTQKAIDKITSKVNNIVAKMYKQYEIIDHEFSEVETRINMINN
KIDDQIQDIWAYNAELIVLLENQKTLDEHDANVNNLYNKVKRALGSNAVEDGKGCFELYHKCDDQCMETI
RNGTYNRRKYQEESKLERQRIEGVKLESEGTYKIVSIYSTVASYLVIAMGFAAFLFWAMYNGSCRCNSCI

>QBF57566.1 hemagglutinin [Influenza A virus]

METVSLITILLVATVSNADKICIGYQSTNSTETVDTLTENNVPVTHAKELLHTEHNGMLCATSLGQPLIL
DTCTIEGLIYGNPSCDLSLEGREWSYIVERPSAVHGLCYPGNVEDLEELRSLFSSARSYQRIQIFPDTIW
NVSYDGTSTACSGSFYKSMRWLTRKNGEYPTQDAQYTNNQGKNILFMWGINHPPTDDTQRGLYTRTDTTT
SVATEEINRIFKPLIGPRPLVNGLMGRINYYWSVLKPGQTLRIKSDGNLIAPWYGHILSGESHGRILKTD
LKRGRCTVQCQTEKGGLNTTLPFQNVSKYAFGNCSKYIGIKSLKLAVGLRNVPSRSSRGLFGAIAGFIEG
GWSGLVAGWYGFQHSNDQGVGMAADRDSTQKAIDKITSKVNNIVDKMNKQYEIIDHEFSEVETRLNMINN
KIDDQIQDIWAYNAELLVLLENQKTLDEHDANVNNLYNKVKRALGSNAVEDGKGCFELYHKCDDQCMETI
RNGTYNRRKYQEESKLERQRIEGVKLESEGTYKILTIYSTVASSLVIAMGFAAFLFWAMYNGSCRCNYCI

>QBF57565.1 hemagglutinin [Influenza A virus]

METVSLITILLVATVSNADKICIGYQSTNSTETVDTLTENNVPVTHAKELLHTEHNGMLCATSLGQPLIL
DTCTVEGLIYGNPSCDLSLEGKEWSYIVERPSAVNGLCYPGNVENLEELRSLFSSARSYQRIQIFPDTIW
NVSYDGTSTACSDSFYRSMRWLTRKDGNYPTQDAQYTNNQGKNILFMWGINHPPTDDTQRNLYTRTDTTT
SVATEEINRIFKPLIGPRPLVNGLMGRIDYYWSVLKPGQTLRIKSDGNLIAPWYGHILSGESHGRILKTD
LKRGSCTVQCQTEKGGLNTTLPFQNVSKYAFGNCSKYIGIKSLKLAVGLRNVPSRSSRGLFGAIAGFIEG
GWSGLVAGWYGFQHSNDQGVGMAADRDSTQKAIDKITSKVNNIVDKMNKQYEIIDHEFSEVETRLNMINN
KIDDQIQDIWAYNAELLVLLENQKTLDEHDANVNNLYNKVKRALGSNAVEDGKGCFELYHKCNDQCMETI
RNGTYNRRKYQEESKLERQKIEGVRLESEGTYKILTIYSTVASSLVIAMGFAAFLFWAMSNGSCRCNICI

>QBF57564.1 hemagglutinin [Influenza A virus]

METVSLITILLVATVSNADKICIGYQSTNSTETVDTLTENNVPVTHAKELLHTEHNGMLCATSLGQPLIL
DTCTIEGLIYGNPSCDLSLEGREWSYIVERPSAVHGLCYPGNVEDLEELRSLFSSARSYQRIQIFPDTIW
NVSYDGTSTACSGSFYKSMRWLTRKNGEYPTQDAQYTNNQGKNILFMWGINHPPTDDTQRGLYTRTDTTT
SVATEEINRIFKPLIGPRPLVNGLMGRINYYWSVLKPGQTLRIKSDGNLIAPWYGHILSGESHGRILKTD
LKRGSCTVQCQTEKGGLNTTLPFQNVSKYAFGNCSKYIGIKSLKLAVGLRNVPSRSSRGLFGAIAGFIEG
GWSGLVAGWYGFQHSNDQGVGMAADRDSTQKAIDKITSKVNNIVDKMNKQYEIIDHEFSEVETRLNMINN
KIDDQIQDIWAYNAELLVLLENQKTLDEHDANVNNLYNKVKRALGSNAVEDGKGCFELYHKCDDQCMETI
RNGTYNRRKYQEESKLERQRIEGVKLESEGTYKILTIYSTVASSLVIAMGFAAFLFWAMSNGSCRCNICI

>QBF57563.1 hemagglutinin [Influenza A virus]

METVSLITILLVATVSNADKICIGYQSTNSTETVDTLTENNVPVTHAKELLHTEHNGMLCATSLGQPLIL
DTCTVEGLIYGNPSCDLSLEGKEWSYIVERPSAVNGLCYPGNVENLEELRSLFSSARSYQRIQIFPDTIW
NVSYDGTSTACSDSFYRSMRWLTRKDGNYPTQDAQYTNNQGKNILFMWGINHPPTDDTQRNLYTRTDTTT

SVATEEINRIFKPLIGPRPLVNGLMGRIDYYWSVLKPGQTLRIKSDGNLIAPWYGHIPSGESHGSILTTD
LKRGSCHCQCQTEKGGSNTTLPFQNLSIYASGNCSKYIGIKSLKLAVGLRNVHSRSSRGLFGAIAGFIEG
GWSGLVAGWYGFQHSNDQGVGMAADRDSTQKAIDKITSKVNNIVDKMNKQYEIIDHEFSEVETRLNMINN
KIDDQIQDIWAYNAELLVLLENQKTLDEHDANVNNLYNKVKRALGSNAVEDGKGCFELYHKCNDQCMETI
RNGTYNRRKYQEESKLERQKIEGVRLEFEGTYKILTIYSTVASSLVIAMGFAAFLFWAMSNGSCRCNICI

>QBF57562.1 hemagglutinin [Influenza A virus]
METVSLITILLVATVSNADKICIGYQSTNSTETVDTLTENNVPVTHAKELLHTEHNGMLCATSLGQPLIL
DTCTVEGLIYGNPSCDLSLEGKEWSYIVERPSAVNGLCYPGNVENLEELRSLFSSARSYQRIQIFPDTIW
NVSYDGTSTACSDSFYRSMRWLTRKDGNYPTQDAQYTNNQGKNILFMWGINHPPTDDTQRNLYTRTDTTT
SVATEEINRIFKPLIGPRPLVNGLMGRIDYYWSVLKPGQTLRIKSDGNLIAPWYGHILSGESHGRILKTD
LKRGSCTVQCQTEKGGLNTTLPFQNVSKYAFGNCSKYIGIKSLKLAVGLRNVPSRSSRGLFGAIAGFIEG
GWSGLVAGWYGFQHSNDQGVGMAADRDSTQKAIDKITSKVNNIVDKMNKQYEIIDHEFSEVETRLNMINN
KIDDQIQDIWAYNAELLVLLENQKTLDEHDANVNNLYNKVKRALGSNAVEDGKGCFELYHKCNDQCMETI
RNGTYNRRKYQEESKLERQKIEGVRLESEGTYKILTIYSTVASSLVIAMGFAAFLFWAMSNGSCRCNICI

>QBF57561.1 hemagglutinin [Influenza A virus]
METVSLITILLVATVSNADKICIGYQSTNSTETVDTLTENNVPVTHAKELLHTEHNGMLCATSLGQPLIL
DTCTIEGLIYGNPSCDLSLEGREWSYIVERPSAVHGLCYPGNVEDLEELRSIFSSARSYQRIQIFPDTIW
NVSYDGTSTACSGSFYKSMRWLTRKNGEYPTQDAQYTNNQGKNILFMWGINHPPTDDTQRGLYTRTDTTT
SVATEEINRIFKPLIGPRPLVNGLMGRINYYWSVLKPGQTLRIKSDGNLIAPWYGHILSGESHGGILKTD
LKRGSCTVQCQTEKGGLNTTLAIQNVSKYASGNCSKYIGIKSLKLAVGLRNVPSRSSRGLFGAIAGFIEG
GWSGLVAGWYGFQHSNDQGVGMAADRDSTQKAIDKITSKVNNIVDKMNKQYEIIDHEFSEVETRLNMINN
KIDDQIQDIWAYNAELLVLLENQKTLDEHDANVNNLYNKVKRALGSNAVEDGKGCFELYHKCDDQCMETI
RNGTYNRRKYQEESKLERQRIEGVKLESEGTYKILTIYSTVASSLVIAMGFAAFLFWAMSNGSCRCNICI

>QBF57560.1 hemagglutinin [Influenza A virus]
METVSLITILLVATVSNADKICIGYQSTNSTETVDTLTENNVPVTHAKELLHTEHNGMLCATSLGQPLIL
DTCTVEGLIYGNPSCDLSLEGKEWSYIVERPSAVNGLCYPGNVENLEELRSLFSSARSYQRIQIFPDTIW
NVSYDGTSTACSDSFYRSMRWLTRKDGNYPTQDAQYTNNQGKNILFMWGINHPPTDDTQRNLYTRTDTTT
SVATEEINRIFKPLIGPRPLVNGLMGRIDYYWSVLKPGQTLRIKSDGNLIAPWYGHIPSGESQGRILKTD
LKRGSCTVQCQTEKGGLNTTLPFQNVSKYAFGKCSKYIGIKSLKLAVGLRNVPSRSSRGLFGAIAGFIEG
GWSGLVAGWYGFQHSNDQGVGMAADRDSTQKAIDKITSKVNNIVDKMNKQYEIIDHEFSEVETRLNMINN
KIDDQIQDIWAYNAELLVLLENQKTLDEHDANVNNLYNKVKRALGSNAVEDGKGCFELYHKCNDQCMETI

RNGTYNRRKYQEESKLERQKIEGVRLESEGTYKILSIYSTVASSLVIAMGFAAFLFWAMSNGSCRCNICI

>QBF57559.1 hemagglutinin [Influenza A virus]
METVSLITILLVATVSNADKICIGYQSTNSTETVDTLTENNVPVTHAKELLHTEHNGMLCATSLGQPLIL
DTCTIEGLIYGNPSCDLSLEGREWSYIVERPSAVHGLCYPGNVEDLEELRSLFSSARSYQRIQIFPDTIW
NVSYDGTSTACSGSFYKSMRWLTRKNGEYPTQDAQYTNNQGKNILFMWGINHPPTDDTQRGLYTRTDTTT
SVATEEINRIFKPLIGPRPLVNGLMGRINYYWSVLKPGQTLRIKSDGNLIAPWYGHILSGESHGRILKTD
LNRGSCTVQCQTEKGGLNTTLPFQNVSKYAFGNCSKYIGIKSLKLAVGLRNVPSRSSRGLFGAIAGFIEG
GWSGLVAGWYGFQHSNDQGVGIAADRDSTQKAIDKITSKVNNIVDKMYKQYEIIDHEFSEVETRLNMINN
KIDDQIQDIWAYNAESLVLLENQKTLDEHDANVNNLYNKVKRALGSNAVEDGKGCFELYHKCDDQCIETI
RNGTYNRRKYQEESKLERQRIEGVKLESEGTYKILSIYSTVASSLVIAMGFAAFLFWAMYNGSCRCNICI

>QBF57558.1 hemagglutinin [Influenza A virus]
MDTVSLITILLVSTVSNADKICIGYQSTNSAETVDTLTENNVPVTHAKELLHTEHNGMLCATSLGQPLIL
DTCTIEGLIYGNPSCDLSLEGREWSYIVQRPSAVHGLCYPGNVQDLEELRSLFSSATSYQKIQIFPDTIW
NVSYDGTSTACSGSFYKSMRWLTRKNGEYPTQDAHYTNNQGKNILFMWGINHPPTDDTQIGLYTRTDTTT
SVATDEINRIFEPLIGPRPLVNGLMGRIHYYWSVLKPDQTLPIKSDGNLIAPWYGPILSGESHGRILKTR
IRRHSCTVQCQTEKDRLNTEQRCQNVSKYAFGKCSKYSGIKSVKLAAGLKNVPSRYSRGLFGCIGGFIEG
GWSGLVAGWYGFQHSNDQGVGIAADRDSTQKAMDKITSKVNNIVAKMNKQYEIIDLEFSVVGTRIITINN
KFDDQIQDIWAYNAESIVLLENQKTLDEHDANVNNLYNKVKRAAGSNAVEDGNGCLELYHKCDDQCMETI
RNGTYNRRKYQAESKLERQRMEGLKQESDRTSEIVSIYSRVASYRVIAMGFVAMLIGGMYNGSCRQNIYI

>QBF57557.1 hemagglutinin [Influenza A virus]
METVSLITILLVATVSNADKICIGYQSTNSTETVDTLTENNVPVTHAKELLHTEHNGMLCATSLGQPLIL
DTCTVEGLIYGNPSCDLSLEGKEWSYIVERPSAVNGLCYPGNVENLEELRSLFSSARSYQRIQIFPDTIW
NVSYDGTSTACSDSFYRSMRWLTRKDGNYPTQDAQYTNNQGKNILFMWGINHPPTDDTQRNLYTRTDTTT
SVATEEINRIFKPLIGPRPLVNGLMGRIDYYWSVLKPGQTLRIKSDGNLIAPWYGHILSGESHGRILKTD
LKRGCCTVQCQTEKGGLNTTLPFQNVSKYASGNCSKYIGIKSLKLAVGLRNVPSRSSRGLFGAIAGFIER
GWSGLVAGWYGFQHSNDQGVGMAADRDSTQKAIDKITSKVNNIVDKLYKQYEIIDHEFSEVETRLNMINN
KIDDQIQDIWAYNAELLVLLENQKTLDEHDANVNNLYNKVKRALGSNAVEDGKGCFELYHKCNDQCIETI
RNGTYNRRKYQEESKLERQKIEGVRLEFEGTYKILSIYSTVASSLVIAMGFAAFLFWAMSNGSCRCNICI

>QBF57556.1 hemagglutinin [Influenza A virus]
METVSLITILLVATVSNANKICIGYQSTNSTETVDTLTENNVPVTHAKELLHTEHNGMLCATSLGQPLIL

DTCTIEGLIYGNPSCDLSLEGREWSYIVERPSAVHGLCYPGNVEDLEELRSLFSSARSYQRIQIFPDTIW
NVSYDGTSTACSGSFYKSMRWLTRKNGEYPTQDAQYTNNQGKNILFMWGINHPPTDDTQRGLYTRTDTTT
SVATEEINRIFKPLIGPRPLVNGLMGRINYYWSVLKPGQTLRIKSDGNLIAPWYGHILSGESHGRILKTD
LKRGSCTVQCQTEKGGLNTTLPFQNVSKYAFGNCSKYSGIKSLKLAVGLRNVPSRSSRGLFGAIAGFIEG
GWSGLVAGWYGFQHSNDQGVGIAADRDSTQKAIDKITSKVNNIVDKMNKQYEIIDHEFSEVETRLNMINN
KIDDQIQDIWAYNAELLVLLENQKTLDEHDANVNNLYNKVKRALGSNAVEDGKGCFELYHKCDDQCMETI
RNGTYNRRKYQEESKLERQRIEGVKLESEGTYKILSIYSTVASSLVIAMGFAAFLFWAMYNGSCRCNICI

>QBF57555.1 hemagglutinin [Influenza A virus]
METVSLITILLVATVSNADKICIGYQSTNSTETVDTLTENNVPVTHAKELLHTEHNGMLCATSLGQPLIL
DTCTIEGLIYGNPSCDLSLEGREWSYIVERPSAVHGLCYPGNVEDLEELRSLFSSARSYQRIQIFPDTIW
NVSYDGTSTACSGSFYKSMRWLTRKNGEYPTQDAQYTNNQGKNILFMWGINHPPTDDTQRGLYTRTDTTT
SVATEEINRIFKPLIGPRPLVNGLMGRINYYWSVLKPGQTLRIKSDGNLIAPWYGPILSGESHGRIVRTD
LKRGSCTVQCQAEKGGLNTTLPFQNVSKYAFGNCSKYIGIKSLKLAVGLRNVPSRSSRGLFGAIAGFIEG
GWSGLVAGWYGFQHSNDQGVGIAADRDSTQKAIDKITSKVNNIVDKMNKQYEIIDHEFSEVETRLNMINN
KIDDQIQDIWAYNAELLVLLENQKTLDEHDANVNNLYNKVKRALGSNAVEDGKGCFELYHKCDDQCMETI
RNGTYNRRKYQEESKLERQRIEGVKLESEGTYKILSIYSTVASSLVIAMGFAAFLFWAMYNGSCRCNRVI

>QBF57554.1 hemagglutinin [Influenza A virus]
METVSLITILLVSTVSNADKICIGYQSTNSTETVDTLTENNVPVTHAKELLHTEHNGMLCATSLGQPLIL
DTCTVEGLIYGNPSCDLSLEGKEWSYIVERPSAVNGLCYPGNVENLEELRSLFSSARSYQRIQIFPDTIW
NVSYDGTSTACSDSFYRSMRWLTRKDGNYPTQDAQYTNNQGKNILFMWGINHPPTDDTQRNLYTRTDTTT
SVATEEINRIFKPLIGPRPLVNGLMGRIDYYWSVLKPGQTLLIKSDGNLIAPWYGHILSGESHGTILKTD
LKRGSCTVQCQTEKGGLNTTLAIQNVSKYAFGNCSKYIGIKSLKLAVGLRNVPSRSSRGLFGAIAGFIEG
GWSGLVAGWYGFQHSNDQGVGMAADRDSTQKAIDKITSKVNNIVDKMNKQYEIIDHEFSEVETRLNMINN
KIDDQIQDIWAYNAELLVLLENQKTLDEHDANVNNLYNKVKRALGSNAVEDGKGCFELYHKCNDQCMETI
RNGTFNRRKYQEESKLERQKIEGVRQEFEGTYKIVTIYSTVASSLVIAMGFAAFLFWAMSNGSCRCNICI

>QBF57553.1 hemagglutinin [Influenza A virus]
METVSLITILLVATVSNADKICIGYQSTNSTETVDTLTENNVPVTHAKELLHTEHNGMLCATSLGQPLIL
DTCTVEGLIYGNPSCDLSLEGKEWSYIVERPSAVNGLCYPGNVENLEELRSLFSSARSYQRIQIFPDTIW
[truncated: 1,663,593 more chars]
